# Molecular evolutionary dynamics of cytochrome P450 monooxygenases across kingdoms: Special focus on mycobacterial P450s

**DOI:** 10.1038/srep33099

**Published:** 2016-09-12

**Authors:** Mohammad Parvez, Lehlohonolo Benedict Qhanya, Ntsane Trevor Mthakathi, Ipeleng Kopano Rosinah Kgosiemang, Hans Denis Bamal, Nataraj Sekhar Pagadala, Ting Xie, Haoran Yang, Hengye Chen, Chrispian William Theron, Richie Monyaki, Seiso Caiphus Raselemane, Vuyani Salewe, Bogadi Lorato Mongale, Retshedisitswe Godfrey Matowane, Sara Mohamed Hasaan Abdalla, Wool Isaac Booi, Mari van Wyk, Dedré Olivier, Charlotte E. Boucher, David R. Nelson, Jack A. Tuszynski, Jonathan Michael Blackburn, Jae-Hyuk Yu, Samson Sitheni Mashele, Wanping Chen, Khajamohiddin Syed

**Affiliations:** 1 Unit for Drug Discovery Research, Department of Health Sciences, Faculty of Health and Environmental Sciences, Central University of Technology, Bloemfontein 9300, Free State, South Africa; 2 Department of Medical Microbiology and Immunology, 6-020 Katz Group Centre, University of Alberta, Edmonton, Alberta T6G 2E1, Canada; 3 College of Informatics, Huazhong Agricultural University, Wuhan, Hubei Province, China; 4 College of Food Science and Technology, Huazhong Agricultural University, Wuhan, Hubei Province, China; 5 Faculty of Natural and Agricultural Science, Department of Microbial, Biochemical and Food Biotechnology, University of the Free State, P.O. Box 339, Bloemfontein, 9300, South Africa; 6 Department of Microbiology, Immunology and Biochemistry, University of Tennessee Health Science Center, Memphis, TN 38163, USA; 7 Department of Physics, University of Alberta, Edmonton, Alberta T6G 2J1, Canada; 8 Institute of Infectious Disease & Molecular Medicine; Department of Integrative Biomedical Sciences, Faculty of Health Sciences, University of Cape Town, Cape Town 7925, South Africa; 9 Department of Bacteriology, University of Wisconsin-Madison, 3155 MSB, 1550 Linden Drive, Madison WI 53706, USA

## Abstract

Since the initial identification of cytochrome P450 monooxygenases (CYPs/P450s), great progress has been made in understanding their structure-function relationship, diversity and application in producing compounds beneficial to humans. However, the molecular evolution of P450s in terms of their dynamics both at protein and DNA levels and functional conservation across kingdoms still needs investigation. In this study, we analyzed 17 598 P450s belonging to 113 P450 families (bacteria −42; fungi −19; plant −28; animal −22; plant and animal −1 and common P450 family −1) and found highly conserved and rapidly evolving P450 families. Results suggested that bacterial P450s, particularly P450s belonging to mycobacteria, are highly conserved both at protein and DNA levels. Mycobacteria possess the highest P450 diversity percentage compared to other microbes and have a high coverage of P450s (≥1%) in their genomes, as found in fungi and plants. Phylogenetic and functional analyses revealed the functional conservation of P450s despite belonging to different biological kingdoms, suggesting the adherence of P450s to their innate function such as their involvement in either generation or oxidation of steroids and structurally related molecules, fatty acids and terpenoids. This study’s results offer new understanding of the dynamic structural nature of P450s.

Cytochrome P450 monooxygenases, also known as CYPs/P450s, are heme-thiolate enzymes playing key roles in nature, particularly in the evolution of organisms, including the dawn of multicellular life^1^. P450s are well known for their capabilities for stereo- and regio-specific oxidation of substrates, which makes these enzymes essential in the primary and secondary metabolism of organisms. Since their identification five decades ago, quite a large number of P450s have been identified in species across biological kingdoms, especially due to the current genome sequencing rush^2^. Studies on P450 enzymes have been reported from animals owing to their role in drug metabolism (particularly from mammals)^3^ or analysis of diversity^1^,^4^; from fungi owing to their role as drug targets^5^,^6^ and for evolutionary analysis^7^,^8^,^9^; from bacteria owing to P450 structure-functional analysis^10^ and generation of products valuable to humans^11^; and from plants owing to their roles in key cellular processes and defense mechanisms^12^,^13^,^14^. Irrespective of their origins, P450s from all organisms have been exploited for their biotechnological potential^15^. Although quite a large number of P450s have been identified to date^2^, genome annotation of P450s in recently elucidated organisms genomes has led to the discovery of a novel P450 family containing a novel P450 fusion protein (CYP5619 family) with an N-terminal P450 domain fused to a heme peroxidase/dioxygenase domain^16^, suggesting that much remains to be explored and understood about the evolution of these enzymes.

The origin of P450s in organisms was ascribed to CYP51^17^,^18^. CYP51 is regarded as an ancient P450 despite confusion on its origin, either in prokaryotes or in eukaryotes^17^,^18^. It has been postulated that the evolution of present-day P450s is due to the divergence and duplication of this ancient P450^17^,^18^. The proposed hypothesis of “descendance of P450s from CYP51” is strongly supported by the fact that CYP51 is the only P450 that is conserved across species belonging to different biological kingdoms^19^. Recent studies showing the conservation of amino acid patterns in EXXR and CXG motifs in CYP51 P450s collected from species across biological kingdoms^20^ further supported the idea that the origin of CYP51 predates the divergence of biological kingdoms, as proposed in earlier studies^21^.

Since CYP51 P450 was established to be the origin of all P450s, further studies have focused on unravelling the P450 complements from different organisms, including analysis of P450 diversity and their evolution. To date, a large number of P450 families have been reported in species from different biological kingdoms, with fungi reported to have more diverse P450 families than plants, animals or bacteria^22^. Thorough studies on the origins of P450 diversity have been conducted using animal and plant P450s as model P450s^4^. Eukaryotic microbes such as fungi and oomycete P450s have also been extensively studied with respect to their diversity and duplications^7^,^8^,^9^,^16^,^23^. However, bacterial P450s have been virtually unexplored in the above context, considering the general assumption that bacterial species have a low number of P450s, with many bacterial species lacking P450s.

Although a plethora of information on P450s is available, to date two key research gaps have not been addressed in P450 research. The first gap is that, since the origin and divergence of P450s from CYP51 into different biological kingdoms^21^, the molecular dynamics of P450s in terms of their primary structure both at protein and DNA level have not been studied. Studies on understanding the molecular dynamics of P450s’ primary structure are limited to the P450 family CYP51^24^ (in 1997) and the biosynthetic-type (710 P450s) and detoxification-type P450s (543 P450s) from vertebrate (14) and invertebrate (6) genomes^25^. Moreover, comprehensive analysis of P450 families from different biological kingdoms and their dynamics in terms of their evolutionary rates, both at protein and DNA levels, have not been reported. Identification of P450 families with the highest evolutionary rate will provide important answers, such as P450 families that are possibly adapting to different substrates or emerging into new P450s, thereby creating new P450 families. Quite the opposite is true for the P450s with lower evolutionary rates, which would indicate their conservation by the organisms owing to their critical role in the organisms’ physiology or probable strict substrate specificity. The second gap is that comparative genomic analyses of P450s in bacteria have not been reported despite the availability of comprehensive bacterial P450 functional data^11^. As mentioned above, compared to fungal, animal and plant P450s, bacterial P450s have not been explored in terms of their diversity and evolutionary analysis.

In this study, we address these research gaps *per se* by performing genome-wide identification, annotation and phylogenetic analysis of P450s in 60 mycobacterial species belonging to six different categories. The species belonging to the genus *Mycobacterium* are selected because of the public availability of quite a large number of mycobacterial species genomes and the presence of diverse species^26^, thus potentially representing diverse P450 complements. Furthermore, the highest G + C content compared to other bacterial species and high genome level conservation among mycobacterial species make them interesting candidates for observation of the distribution, diversity and evolution of P450s in these organisms. The results from the mycobacterial P450 analysis allowed us to carry out comprehensive evolutionary analysis (both at protein and DNA level) and phylogenetic analysis of 17 598 P450s belonging to 113 P450s families from species across biological domains: bacteria, fungi, plants and animals.

## Methods

### Mycobacterial species

A total of 60 mycobacterial species belonging to six different categories were used in this study (Supplementary Table S1). The six categories include *Mycobacterium tuberculosis* complex (MTBC) (27 species), *M. chelonae-abscessus* complex (MCAC) (6 species), *M. avium* complex (MAC) (8 species), Mycobacteria causing leprosy (MCL) (2 species), nontuberculous mycobacteria (NTM) (6 species) and Saprophytes (SAP) (11 species). The criteria for separation of mycobacterial species into six different groups is based on their characteristic features, including ecological niches, as well as the nature and site of infection as described elsewhere^26^. Also taxonomical grouping of mycobacterial species is taken into consideration as described elsewhere^27^. Detailed information on species and their category is listed in the supplementary Table S1.

### P450 mining in mycobacterial genomes

Mycobacterial genomes that are publicly available at different genome databases as listed in Supplementary Table S2 were mined for P450s as described elsewhere^16^,^20^,^23^,^28^. Briefly, the whole proteome of mycobacterial species was downloaded from the respective data bases listed in Supplementary Table S2, and subjected to the NCBI *Batch* Web CD-Search Tool (http://www.ncbi.nlm.nih.gov/Structure/bwrpsb/bwrpsb.cgi). Proteins that belong to a P450 superfamily were selected and further subjected to BLAST analysis against bacterial P450s at the Cytochrome P450 Homepage^2^. Based on the International P450 Nomenclature Committee rule, proteins with >40% identity and >55% identity were grouped under the same family and subfamily, respectively. For each species of P450s different databases were consulted; all databases gave the same results and hence P450s from one of the databases were selected. Some mycobacterial P450s were annotated and made available at the Cytochrome P450 Homepage^2^. In this case, the same nomenclature for P450s was continued. The same procedure was followed for mycobacterial P450s documented in the literature. P450s that showed less than 40% identity to known P450s at the Cytochrome P450 Homepage^2^ were assigned to new P450 families and subfamilies as per International P450 Nomenclature Committee rules.

### Protein and cDNA sequence collection

Protein sequences and the respective cDNAs for 113 P450 family members (17 598 P450s) were collected from different databases and published resources. Some of the bacterial P450 family members’ protein sequences and cDNAs, particularly P450 families present in mycobacterial species, were collected from the KEGG database (http://www.genome.jp/kegg/catalog/org_list.html). These P450 families include: CYP108, CYP121, CYP123-CYP126, CYP128, CYP130, CYP132, CYP135-CYP144, CYP150, CYP164, CYP185, CYP187-CYP191, CYP268, CYP279 and CYP291. Identification of P450 family members was carried out using the methodology described elsewhere^16^,^20^,^23^,^28^. Briefly, a representative P450 for each of the families was taken from mycobacterial species from the Cytochrome P450 Homepage^2^ and protein BLAST was performed at KEGG against prokaryote genomes. The resulting hit proteins were sorted into P450 families according to the International P450 Nomenclature Criteria i.e. >40% identity grouped as a family. The hit proteins were subjected to BLAST analysis against named bacterial P450s at the Cytochrome P450 Homepage^2^. Based on the percentage identity to the homolog P450 (>40% identity), the hit proteins were sorted into different P450 families.

Fungal P450 sequences and corresponding cDNAs belonging to P450 families CYP61, CYP63, CYP512, CYP5035, CYP5037, CYP5136, CYP5139, CYP5141, CYP5144, CYP5150 and CYP5152 were retrieved from published work^8^,^9^,^20^ and their corresponding cDNAs were retrieved from each of the species’ genome databases at MycoCosm^29^. The remaining P450 family members’ protein sequences, i.e. CYP1-9, CYP11, CYP12, CYP17, CYP19, CYP21, CYP24, CYP26-CYP28, CYP33, CYP39, CYP46, CYP51-CYP53, CYP55, CYP58, CYP65, CYP71-CYP76, CYP78, CYP79, CYP81, CYP82, CYP84, CYP86, CYP87, CYP89, CYP90, CYP92-CYP94, CYP96-CYP98, CYP102, CYP105-CYP107, CYP110, CYP116, CYP147, CYP152, CYP153, CYP157, CYP195, CYP202, CYP325, CYP501, CYP505, CYP584, CYP620, CYP704-CYP707, CYP709, CYP714 and CYP716 were retrieved from the Cytochrome P450 Engineering Database (https://cyped.biocatnet.de/)^30^ and their cDNAs were collected from NCBI using the respective P450 family member protein IDs. The P450 protein sequences (17 598 P450 sequences) belonging to 113 P450 families used in this study are listed in Supplementary Dataset 1.

### Analysis of amino acid conservation

Analysis of the number of amino acids conserved across different mycobacterial P450 families was carried out using PROfile Multiple Alignment with predicted Local Structures and 3D constraints (PROMALS3D)^31^. PROMALS3D aligns multiple protein sequences based on their secondary structure prediction using the available homolog crystal structures. The output alignment assigns numbers as conservation index from 4 to 9, where number 9 is the invariantly conserved amino acid across the analyzed protein sequences^31^. The majority of mycobacterial P450 families contain fewer than 10 members. Hence in this study, mycobacterial P450 families with more than 15 members were selected for amino acid conservation analysis, considering that this number of member P450s will provide sufficient information on amino acid conservation. This cut-off value is also set taking into account that bacteria possess a low number of P450s in their genomes compared to species from other biological kingdoms^22^. Based on the cut-off value, a total of 32 mycobacterial P450 families (CYP51, CYP105, CYP108, CYP121, CYP123, CYP124, CYP125, CYP126, CYP128, CYP130, CYP132, CYP135, CYP136, CYP137, CYP138, CYP139, CYP140, CYP141, CYP142, CYP143, CYP144, CYP150, CYP164, CYP185, CYP187, CYP188, CYP189, CYP190, CYP191, CYP268, CYP279 and CYP291) qualified for this analysis. The P450 families from fungi, plants and animals were selected based on criteria described elsewhere^20^. The P450 families used from different biological kingdoms were listed in the Supplementary Dataset 1. CYP51 members from bacteria and fungi individually were included in the analysis to see their ranking of conservation compared to CYP51 members across biological kingdoms.

### Construction of the P450s phylogenetic trees

The phylogenetic analysis of P450s was conducted as described elsewhere^8^. Briefly, the P450 protein sequences were aligned by HMMER package 3.1 (http://hmmer.janelia.org/) through adjusting them to the P450 profile hidden Markov model PF00067 downloaded from the Pfam protein families database (http://pfam.xfam.org/)^32^,^33^. Then, the phylogenetic trees from alignments were inferred by FastTree version 2.1.4 with the maximum-likelihood method (http://www.microbesonline.org/fasttree/)^34^. In this study, the phylogenetic tree of 1 772 mycobacterial P450s was viewed by iTOL (http://itol.embl.de/upload.cgi)^35^, and the phylogenetic tree of 17 598 P450s from 113 families was viewed by Hypertree 1.2.2^36^.

### Analysis of P450 diversity

The P450 diversity percentage is the percentage contribution of the number of P450 families in the total number of P450s in an organism^16^. The P450 diversity percentage in mycobacterial species was calculated using the methodology described elsewhere^16^. For comparative analysis of the P450 diversity percentage between different microbial populations, the P450 diversity percentage data from lower eukaryote microbes such as fungi and oomycetes was retrieved from published literature^2^,^9^,^16^,^23^,^37^,^38^.

### Evolutionary rate analysis

The above-mentioned cDNA sequences of 113 P450 family members were used for evaluating their evolutionary rates. Evolutionary rate for all P450s were calculated using full-length cDNA sequences. Firstly, the cDNA sequences of each P450 family member were aligned by Muscle in the codons module^39^. All positions containing gaps and missing data were eliminated. Then, the aligned sequences were subjected to examination in order to estimate their evolutionary rates under the Tamura-Nei model^40^. A discrete Gamma distribution was used to model evolutionary rate differences among sites. The rate of substitution for each site was drawn from a Gamma distribution with shape parameter α. If α was <1, the distribution implied that there was a relatively large amount of rate variation, with many sites evolving very slowly but some sites evolving at a high rate. For values of α > 1, the shape of the distribution changed qualitatively, with less variation and most sites having roughly similar rates^41^. The evolutionary rate analyses were conducted in MEGA7^42^.

## Results and Discussion

### Mycobacterial P450s

**Figure 1:**
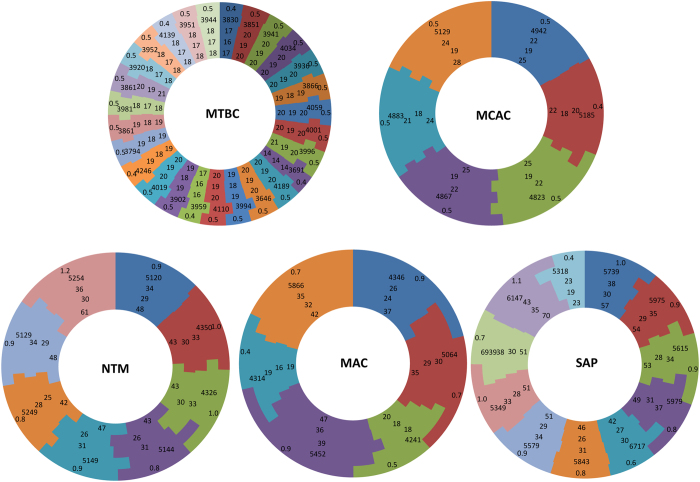
Comparative analysis of P450s in mycobacteria. Mycobacterial species were grouped into different groups such as MTBC (*Mycobacterium tuberculosis* complex), MCAC (*Mycobacterium chelonae-abscessus* complex), MAC (*Mycobacterium avium* complex), NTM (Nontuberculous mycobacteria) and SAP (Saprophytes). Each color in the circle represents a mycobacterial species in that group. The numerical order from the inside to the outside of the circle is as follows: number of P450s, number of P450 families, number of P450 subfamilies, number of ORFs in an organism and percentage of P450s compared to ORFs of an organism. Considering the presence of a single P450 in MCL (Mycobacteria causing leprosy) species, this group is omitted from comparative analysis. Details on species and their P450s were listed in Supplementary Table S3.

Genome data-mining and annotation of P450s in 60 mycobacterial species revealed the presence of 1 784 P450s (Fig. 1 and Supplementary Table S3 and Datasets 2 and 3). Among the species, *M. leprae* species showed a single P450, whereas the highest number of P450s (70 P450s) was found in *M. rhodesiae* NBB3, followed by *M. indicus pranii* MTCC 9506 (61 P450s). An interesting pattern was observed when comparing the P450 pattern between mycobacterial species belonging to different categories (Fig. 1). Comparison of the number of P450s and average number of P450s among different categories revealed a gradual loss of P450s from SAP to MTBC species (Fig. 1 and Supplementary Table S3). The order is as follows, where the minimum and maximum number of P450s, and after the semicolon the average number of P450s, are shown in parenthesis: SAP (23-70:50) > MAC (42-61:47) > NTM (19-47:33) > MCAC (22-28:25) > MTBC (14-21:19) (Fig. 1 and Supplementary Table S3). This clearly indicates that the progression from soil mycobacteria (SAP) into human pathogens such as those living in human blood and ultimately adapted as a lung pathogen (MTBC), resulted in gradual loss of a considerable number of P450s. Furthermore, the reduction in P450 count followed the pattern of genome reduction as the overall number of Open Reading Frames (ORFs) in organisms was reduced from SAP to MTBC (see ORF data in Supplementary Table S3). The average number of P450s in mycobacterial species, especially belonging to NTM, MCAC and SAP, is in fact higher than some human fungal pathogens^9^,^38^. The percentage analysis of P450s in mycobacterial species genomes reflected the same pattern and some of the species belonging to MAC and SAP showed ≥1% of P450s in their genomes, indicating the important role of this large number of P450s in their physiology (Fig. 1 and Supplementary Table S3). It is noteworthy that some fungi and plants also have ≥1% P450s in their genomes^2^, similar to SAP and MAC, highlighting the important roles P450s play in both prokaryotic and eukaryotic organisms. Considering the presence of a single P450 in MCL species, this group was omitted from further comparative analysis.

### P450 family and subfamily analysis in mycobacteria

All 1 784 P450s were grouped into 77 P450 families and 132 subfamilies (see Supplementary Table S4 and Dataset 2). P450 family annotation revealed the presence of 17 new P450 families in mycobacterial species. The new P450 families included: CYP1067 and CYP1119- CYP1134. Among subfamilies, 43 new subfamilies were identified in mycobacterial species used in this study. The lowest number of P450 families was found in *M. tuberculosis* RGTB327 (14 P450 families) and the highest number of P450 families was found in *M. marinum* (36 P450 families), followed by *M. rhodesiae* NBB3 (35 P450 families) (see Supplementary Table S3).

Analysis of P450 families revealed that the CYP125 family is the dominant P450 family with 114 members (6.4% of total P450s found in 60 mycobacterial species), followed by CYP189 with 86 members (4.8%), CYP150 with 84 members (4.7%) and CYP136 with 82 members (4.6%) (see Supplementary Table S4). CYP150 and CYP189 P450 families were present with a high copy number in mycobacteria, with one to six copies, followed by CYP125 (one to five copies), CYP187 and CYP279 (one to four copies) (see Supplementary Table S4). The presence of a high number of member P450s in the above P450 families suggests possible blooming of these P450 families. This implies that the bloomed P450 families play a key role(s) in the physiology of mycobacteria; hence they are present in high copy numbers, as suggested for other organisms^9^. Ten P450 families, CYP51, CYP123, CYP125, CYP130, CYP135, CYP136, CYP138, CYP140, CYP144 and CYP1128, were conserved across the different mycobacterial categories, suggesting their important role in mycobacteria (see Table Supplementary S4). The P450 families CYP124, CYP126, CYP128, CYP142 and CYP143 were missing only in MCAC (see Supplementary Table S4). Interestingly, two P450 families, CYP121 and CYP141, are only present in MTBC. Among 27 MTBC species, CYP121 is present in 23 species and CYP141 is present in 19 species. The presence of CYP121 and CYP141 only in MTBC species suggests that these P450 families can serve as diagnostic markers in the detection of MTBC species. The use of CYP141 as a diagnostic marker in the detection of *M. tuberculosis* was reported earlier^43^. The results from this study however suggest that the use of CYP141 as a diagnostic marker is not limited to the detection of *M. tuberculosis,* but can also be employed in detecting other MTBC species. Furthermore, this study suggests that CYP121 can serve as a better diagnostic marker compared to CYP141, as 23 species out of 27 contain this P450 (see Supplementary Dataset 2). The retention/evolution of these two P450 families by MTBC species suggest the key/essential role of these P450 families, and this was strengthened further by experimental data as CYP121 was found to be essential for the survival of *M. tuberculosis*^44^. The new P450 family CYP1131 is present only in MCAC species. All six MCAC species contain this P450, suggesting that CYP1131 can serve as a diagnostic marker in the detection of MCAC species. Five P450 families, CYP1016, CYP1017, CYP1018, CYP1019 and CYP1067 (new P450 family), are present only in MAC species. Ten P450 families (CYP183, CYP226, CYP269, CYP271, CYP274, CYP276, CYP1120, CYP1123, CYP1127 and CYP1133) were unique to NTM and 11 P450 families (CYP110, CYP145, CYP151, CYP186, CYP289, CYP292, CYP1119, CYP1121, CYP1122, CYP1125, and CYP1126) were present only in SAP species. Among these unique P450 families four and five P450 families were new P450 families that were present in NTM and SAP species, respectively. One of the most studied self-sufficient P450s, CYP102, involved in fatty acid hydroxylation^45^, was found in both MCAC and SAPs. A detailed analysis on the comparison of different P450 families across different mycobacterial categories is presented in Supplementary Table S4.

Despite different geographical locations, all analyzed *M. bovis* species showed a truncated CYP142 in their genome (see Supplementary Dataset 3). This suggests that the event of the CYP142 P450 truncation occurred before speciation and thus resulted in a non-functional CYP142 in *M. bovis* species. Further evidence for the non-functionality of CYP142 P450 in these species can be obtained from earlier studies where CYP125 gene-knockout strains of *M. bovis* and *M. bovis* BCG were unable to grow on cholesterol as a carbon source compared to *M. tuberculosis* H37Rv, where CYP142 normally complements CYP125^46^. The CYP51 P450 family that is highly conserved across different living organisms^19^ is absent from *M. tuberculosis* UT205, *M. canetii* CIPT 140060008, *M. canetii* CIPT 140710010, *M. liflandii* 128FXT and *M. massiliense,* further strengthening the argument that CYP51 is not an essential gene in mycobacteria and it is therefore unlikely to be an azole drug target in mycobacteria, as identified earlier^47^. Interestingly, *M. intracellulare* MOTT-02 contains two copies of CYP51 in its genome (see Supplementary Dataset 2).

### Phylogenetic analysis of mycobacterial P450s

**Figure 2:**
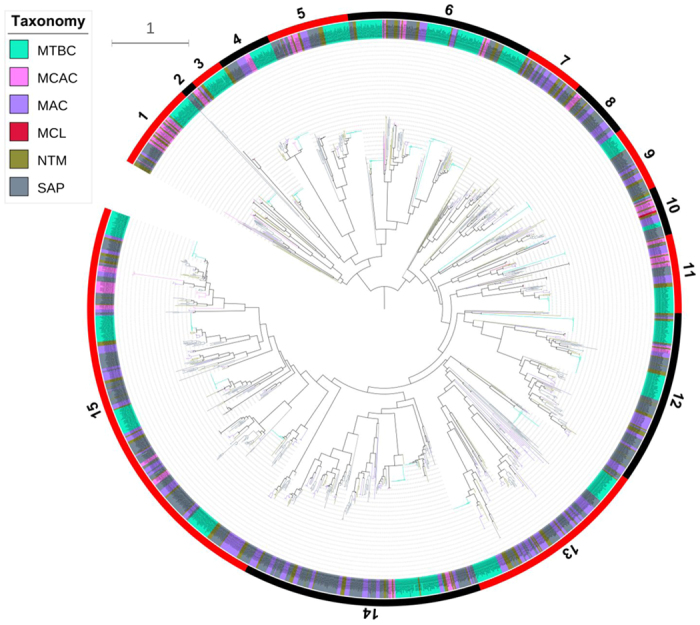
Phylogenetic analysis of P450s in mycobacteria. A phylogenetic tree was constructed with 1772 mycobacterial P450s. The inner circle is the phylogenetic tree based on the consensus sequences of the mycobacterial P450s against the Pfam seed PF00067. The branches with different colors show their taxonomic groups: MTBC (*Mycobacterium tuberculosis* complex), MCAC (*Mycobacterium chelonae-abscessus* complex), MAC (*Mycobacterium avium* complex), MCL (Mycobacteria causing leprosy), NTM (Nontuberculous mycobacteria) and SAP (Saprophytes). Ancestral branches with children that had identical colors were assigned the same color as the children. The middle circle shows the corresponding CYPs, which are covered by different colors to show their taxonomic groups. Each taxon links the branch with a dotted line. The outermost numbers indicate the 15 clades based on this study, and their ranges are marked by alternating red and black. Distribution of P450s families into different clans was listed in Supplementary Table S5. A high-resolution phylogenetic tree is provided in the supplementary Fig. S1.

Phylogenetic analysis of mycobacterial P450s revealed that P450s within the same family are clustered together (Fig. 2 and Supplementary Fig. S1 and Table S5). This suggests that the annotation (assigning the family and subfamilies) of P450s in mycobacterial species in this study is correct. Moreover, the phylogenetic relationship of the mycobacterial P450s is also related to species taxonomy. It is evident that the mycobacterial P450s from the same taxonomic group are generally clustered together in the tree. Particularly, P450s in the same family from the group MTBC are clustered together in the branches, suggesting their conserved evolution after speciation into different groups.

In order to understand the evolution of mycobacterial P450s and their relation to their groups, higher-level P450 classifications, i.e. clan level^48^, have been carried out. Clan-level classification of P450s will give an idea of P450 families that probably diverged from a single common ancestor irrespective of their parent organism and possibly with overlapping functions. This type of classification has been reported for P450s belonging to different biological kingdoms^8^,^16^,^48^. In this study, based on their phylogenetic relationships, 77 mycobacterial P450 families were grouped into 15 clans (Fig. 2 and Supplementary Fig. S1 and Table S5). Clan 13 has most P450 families (16 families), followed by clan 1 (11 families), clan 15 (10 families) and clan 12 (8 families). Clans 3–5 contain a single P450 family, suggesting the independent divergence of these P450 families. Analysis of P450 families with respect to mycobacterial groups suggested that most clans consist of members from wide mycobacterial groups, thus these P450 families probably diverged from a common ancestor. The grouping of CYP124, CYP125 and CYP142 P450 families in the same clan (clan 15) strongly authenticates our clan-level classification of mycobacterial P450s, being consistent with the argument that these P450 family members have overlapping functions, as it is evident from experimental data that these P450 families are shown to be involved in cholesterol catabolism in *M. tuberculosis*^46^,^49^,^50^,^51^,^52^,^53^. In addition to cholesterol catabolism, CYP124 and CYP153 P450s are involved in oxidation of aliphatic hydrocarbons^54^,^55^ and were also grouped in the same clan, indicating divergence in function for CYP124 that was possibly acquired during the course of evolution. Considering that most mycobacterial P450s are orphans, future functional characterization of mycobacterial P450s will provide more information on clan-level grouping of P450 families with respect to their functional relationship.

### Mycobacterial species have the highest P450 diversity

P450 diversity is a good indication of the diversity of P450 families that organisms harbor. Low P450 diversity suggests blooming of certain P450 families, possibly *via* duplication of the same P450 in an organism^9^,^16^,^56^. Analysis of P450s in microbes, particularly lower eukaryotes such as fungi and oomycetes, suggested that fungal species have the highest P450 diversity in their genomes^16^.

**Figure 3:**
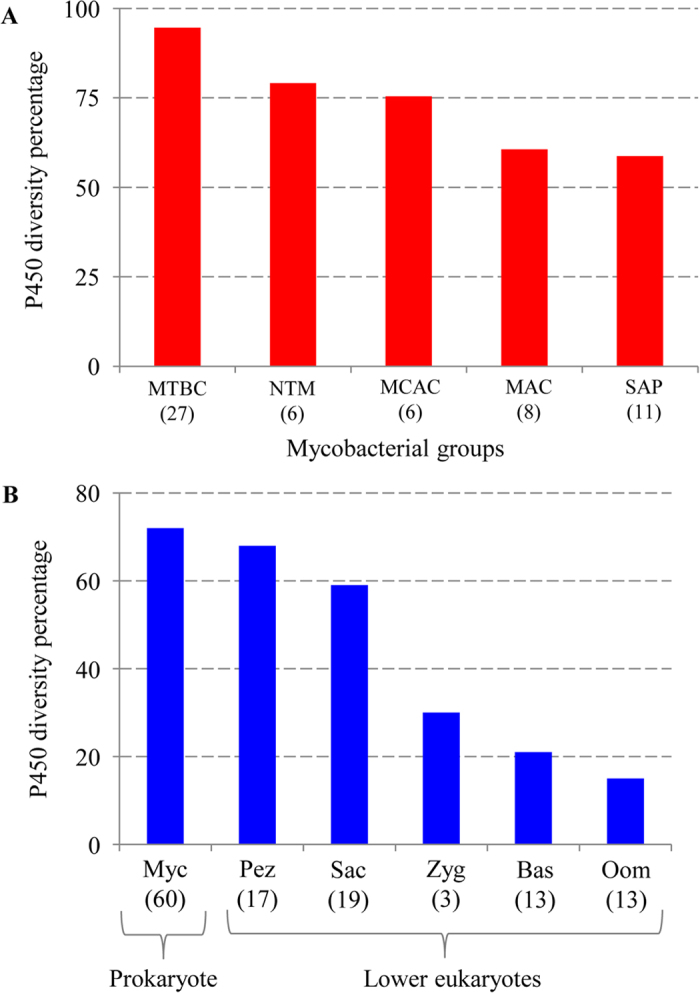
P450 diversity percentage analysis. (**A**) Comparative analysis of P450 diversity percentage between different mycobacterial categories. (**B**) Comparative analysis of P450 diversity percentage between microbes such as prokaryote mycobacterial species and lower eukaryote fungi and oomycetes. The number of species in each of the groups (**A**) or microbe (**B**) used for this analysis is shown in parenthesis. Detailed analysis of the P450 diversity percentage values were presented in Supplementary Table S6. Abbreviations: MTBC, *Mycobacterium tuberculosis* complex; MCAC, *Mycobacterium chelonae-abscessus* complex; MAC, *Mycobacterium avium* complex; MCL, Mycobacteria causing leprosy; NTM, nontuberculous mycobacteria; SAP, Saprophytes; Myc, Mycobacterial species; Sac, Saccharomycetes; Pez, Pezizomycetes; Bas, Basidiomycetes; Zyg, Zygomycetes and Oom, Oomycetes.

In this study, analysis of P450 diversity between different mycobacterial categories revealed that MTBC species have the highest P450 diversity percentage in their genomes (Fig. 3 and Supplementary Table S6). The P450 diversity percentage between different mycobacterial groups is as follows: MTBC > NTM > MCAC > MAC > SAP (Fig. 3A). The low P450 diversity percentage observed in MAC and SAP group species is due to the fact that certain P450 family members are populated in their genomes (see Supplementary Table S6 and Dataset 2). For example, five and six copies of CYP189 P450s are present in MAC and SAP; up to four copies of CYP125 and CYP187 are present in both groups; six and five copies of CYP150 P450s are present in MAC and SAP respectively (see Supplementary Table S4). Overall, the 60 mycobacterial species showed an average P450 diversity percentage of 72%.

Comparative analysis of the P450 diversity percentage between different microbial populations such as mycobacterial species and other lower eukaryote microbes (fungi and oomycetes) revealed that the mycobacterial species have the highest P450 diversity percentage in their genomes (Fig. 3B and Supplementary Table S6). The P450 diversity percentage observed in mycobacterial species (average 72%) is slightly higher compared to Pezizomycetes (68%) and is clearly the highest compared to other fungal categories and oomycetes. Future analysis of P450s in a greater number of bacterial species will provide a clear answer on whether the observed highest P450 diversity percentage in mycobacterial species is unique or a general characteristic of prokaryotes, and thus provide information of the P450 diversity percentage of prokaryotic versus eukaryotic microbes.

### Some P450s show the highest amino acid conservation in Mycobacteria

**Figure 4:**
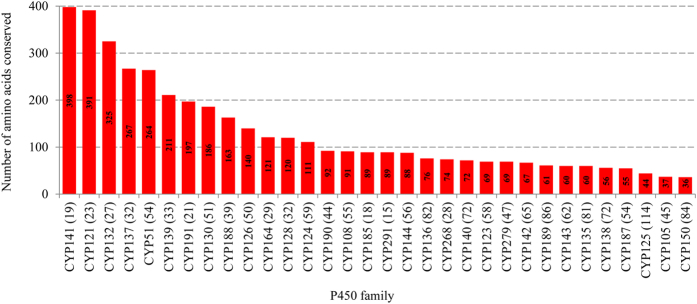
Conserved amino acid analysis in mycobacterial P450 families. Numbers of amino acids that are conserved in member P450s were determined using PROMALS3D^31^ and presented in the figure with conservation index where the number “9” indicates conserved amino acid in P450 family members. Number of member P450s analyzed for each P450 family is presented in parenthesis next to P450 family. A PROMALS3D analysis of member P450s and the conservation index scores for each mycobacterial P450 family were presented in Supplementary Table S7.

During the annotation of mycobacterial P450s (this study) we observed that the mycobacterial P450s belonging to the same family showed the highest percentage identity, suggesting the highest conservation in member P450s’ primary structure, i.e. amino acid level. This phenomenon, the highest number of amino acids conserved in the primary structure of P450, was recently reported for CYP53 family members in fungi^6^. In order to understand the nature and extent of conserved amino acids, mycobacterial P450 families were subjected to PROMALS3D analysis^31^. PROMALS3D analysis of 32 mycobacterial P450 families revealed the highest number of amino acids conserved in some of the P450 families (Fig. 4 and Supplementary Table S7). As shown in Fig. 4, the CYP141 P450 family has the highest number of conserved amino acids (389 amino acids) in its members, followed by CYP121, CYP132, CYP137 and CYP51. It is noteworthy that CYP141 and CYP121 P450 families are present only in MTBC species, and both families have the highest conservation in their primary structure, indicating that these P450 families were protected from any changes in their primary structure. Changes in the primary structure might be deleterious, as CYP121 is known to be essential for *M. tuberculosis* survival^44^. The highest conservation of amino acids in CYP121 and CYP141 is quite understandable, considering these families exist only in MTBC species. The lowest number of conserved amino acids was observed for P450 families CYP150 (36 amino acids), CYP105 (37 amino acids) and CYP125 (44 amino acids). This might suggest that P450 families that are populated across mycobacterial species (see Supplementary Table S4) are subjected to changes in their primary structure. This is however not true, as P450 families such as CYP51, CYP137, CYP132, CYP191 and CYP140 that are present across different mycobacterial categories (see Supplementary Table S4) displayed among the highest number of conserved amino acids (Fig. 4). This clearly suggests that irrespective of the widespread nature of a P450 family, some of the P450 families have been highly conserved despite their divergence into different mycobacterial species.

### Bacterial P450s show highest amino acid conservation across biological kingdoms

The highest conservation of mycobacterial P450s at primary structure level (as shown above) led us to assess the amino acid conservation in the P450 families across biological kingdoms to see the conservation pattern with respect to biological kingdoms, if any, and also to assess where bacterial P450s, particularly P450 families present in mycobacterial species, rank among other P450 families.

**Figure 5:**
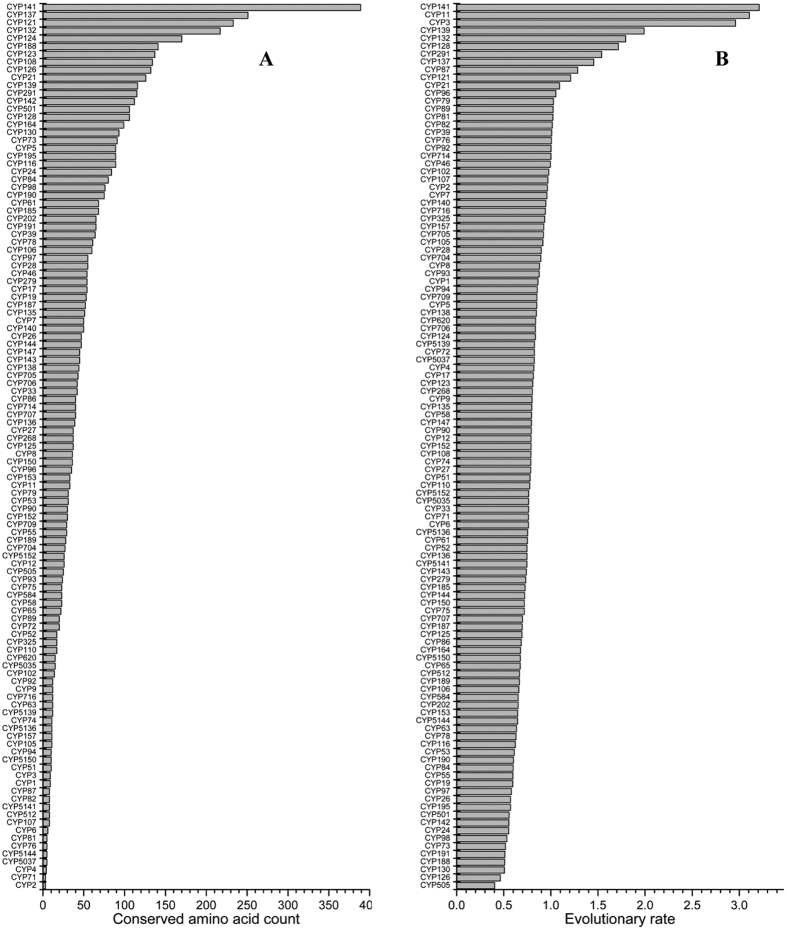
Protein-level (A) and DNA-level (B) P450s structural dynamic analysis. Structural dynamics for 17 598 P450s belonging to 113 P450 families from different biological kingdoms were analyzed. (**A**) Protein-level structure dynamics were assessed based on the number of conserved amino acids present in each P450 family. The P450 families in the graph are presented such that the P450 family CYP141 that has the highest number of conserved amino acids is on top of the graph and the lowest number of conserved amino acids observed for the CYP2 family is on the bottom of the graph. A detailed analysis of the number of conserved amino acids and number of member P450s used and their hosts (biological kingdoms) and conservation ranking for each member of the family is presented in Supplementary Table S8. (**B**) Evolutionary rate analysis of P450s. Evolutionary rates were estimated based on their cDNA sequences under the Tamura-Nei model^40^. A discrete Gamma distribution was used to model the evolutionary rate differences, as X axis of Fig. 5B presents, and more details are provided in Methods section. A discrete Gamma distribution was used to model the evolutionary rate differences. The P450 families in the graph are presented such that the P450 family CYP141 showing the lowest evolutionary rate is on top of the graph and the highest evolutionary rate observed for the CYP505 family is on the bottom of the graph. A detailed analysis of evolutionary rates for each of the P450 family and their ranking is presented in Supplementary Table S9.

To this end, 17 598 P450 sequences belonging to 113 P450 families (see Supplementary Dataset 1) were collected, as described in the Methods. Among the P450 families, 42 belong to bacteria, 28 to plants, 22 to animals and 19 to fungi (excluding bacteria and fungi specific CYP51 members). CYP74 is common between plants and animals and CYP51 is common among all biological kingdoms. Analysis of conserved amino acids in 113 P450 families revealed that among the top 20 conservation ranked P450 families, 18 P450 families belonged to bacteria, three belonged to animals and one family each belonged to fungi and plants (Fig. 5A and Supplementary Table S8). Among the 18 bacterial P450s, seven P450 families were exclusively present in mycobacterial species (CYP108, CYP121, CYP123, CYP124, CYP132, CYP137 and CYP141). The animal CYP21 P450 family was ranked 11 and CYP501 and CYP73 from fungi and plants were ranked 15 and 18, respectively. The animal CYP5 family ranked 19. Interestingly, bacterial CYP51 members ranked 2 compared to fungal CYP51 members (rank 37) and in general CYP51 members from all biological kingdoms (rank 62), suggesting that after speciation, CYP51 in each biological kingdom had been subjected to speciation/kingdom-specific primary structure conservation, as observed in a study described elsewhere^8^.

The amino acid conservation ranking is independent of the number of member P450s, as some of the P450 families, although they contain a low number of members, for example CYP325 from animals (53 members), CYP706 and CYP87 from plants (51 and 78 members, respectively) and CYP147 from bacteria (52 members) were ranked 57, 39 and 64, and 35, respectively (see Supplementary Table S8). In contrast to the CYP5 P450 family (ranked 19), animal P450 families such as human P450 families CYP1 and 3, CYP2 and CYP4 ranked 63, 67 and 66, respectively. Interestingly, fungal P450 families CYP5141, CYP512, CYP5144 and CYP5037 that ranked above 63 are shown to have bloomed in fungi *via* duplication of their member P450s^9^, hence their variation in primary structure. Overall, based on the conserved amino acid analysis (see Supplementary Table S8), it can be concluded that after the origin and divergence of P450 families into different biological kingdoms, some P450 families ranked in the top 20 (see Supplementary Table S8) have been conserved in organisms. The reason for the conservation of these P450 families is clearly that these families play key roles in the physiology of the organisms. For example, two P450 families, CYP121 and CYP128, are known to be critical for the survival of *M. tuberculosis*^44^,^47^, suggesting that any changes in their primary structure might create a problem for the organism’s survival. The P450 families that are lowest ranked are the ones prone to diversifying their substrate range, thus possibly serving to generate new P450s. The broad substrate specificity of P450 families such as CYP1-3 (from animals)^3^ and CYP5141, CYP512, CYP5144 and CYP5037 (from fungi)^9^ strongly supports this argument.

### Evolutionary rate analysis of P450 families

In order to assess whether the conservation of amino acids observed for the top 20 ranked P450 families (as discussed in the above section) will align at the DNA level, we performed evolutionary rate analysis using the cDNAs of P450 family members (Fig. 5B and Supplementary Table S9). Evolutionary rate analysis revealed that the CYP505 family showed the highest evolutionary rate at the DNA level. It is noteworthy that this family showed the low amino acid conservation, suggesting substitutions were nonsynonymous. Among the top 20 ranked P450 families for the highest evolutionary rate, eight P450 families belonged to bacteria, three P450 families to animals, four P450 families to fungi and six P450 families to plants (Fig. 5B and Supplementary Table S9). Evolutionary rate analysis of P450 families revealed that among the top 20 conserved bacterial P450 families at the protein level only eight P450 families, the CYP21, CYP121, CYP128, CYP132, CYP137, CYP139, CYP141 and CYP291, showed the lowest evolutionary rate at the DNA level. This indicates these P450 families are conserved both at the DNA and the protein levels. In the P450 families such as CYP24, CYP73, CYP116, CYP126, CYP130, CYP142, CYP188, CYP195 and CYP501, despite showing the highest conservation at the protein level, DNA-level analysis revealed the highest evolutionary rate (ranking below 20) for these families, suggesting that nucleotide substitutions are synonymous (Fig. 5B and Supplementary Table S9). This suggests that changes occurring at the DNA level in these P450 families are neutral, since they do not alter the protein sequence. Some of the other top 20 conserved P450 families at protein level, such as CYP5, CYP108, CYP123, CYP124 and CYP164, also showed the highest evolutionary rate at the DNA level, suggesting most of the changes were synonymous. It is quite interesting to observe that the CYP1-3 families that showed the lowest protein-level conservation surprisingly showed the lowest evolutionary rate at the DNA-level, as CYP1-3 ranked 76, 88 and 109, respectively, suggesting the DNA-level changes were nonsynonymous substitutions and led to a change in the amino acid sequence, thus altering protein function. It is noteworthy that these P450s are known for their catalytic versatility and the single nucleotide polymorphism of CYP1-3 is well known to change the response to xenobiotics such as drugs etc.^3^. The fungal P450 families with the lowest conserved amino acids, such as CYP5144, CYP512, and CYP5141, also showed a moderate evolutionary rate as they ranked 22, 27 and 41, respectively, suggesting DNA-level changes were nonsynonymous substitutions.

### P450 family dynamics of divergence

From the above results it is clear that irrespective of their parent organism (bacteria, fungi, plants and animals) P450s follow an independent evolutionary route either for their conservation or diversity at the protein or DNA level. This poses the question on how these P450 families diverged into different organisms since their origin.

**Figure 6:**
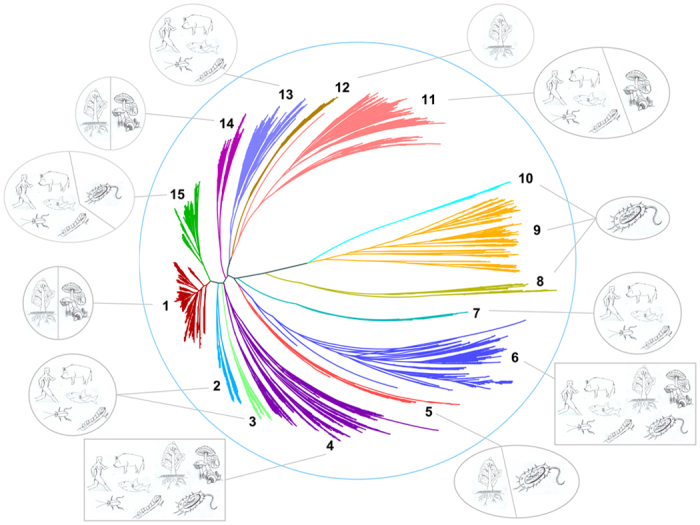
Phylogenetic analysis of 17 598 P450s belonging to 113 families from different biological kingdoms such as bacteria, fungi, animals and plants. All 113 P450 families were grouped under 15 clans based on their phylogenetic relationships and following the methods described elsewhere^8^. Clans were presented in different colors and the host containing the respective P450 families that grouped under each clan is shown in schematic diagrams. Schematic diagrams are representative only and the P450 family is not necessarily confined to the depicted animals. Cartoon figures for plants, bacteria and fungi are shown as representative of their kingdoms. The tree was viewed by Hypertree in fisheye pattern. Detailed information on clan-level grouping of 113 P450 families is presented in Supplementary Table S10 and functional data on P450s at family level is presented in Supplementary Table S11.

In order to understand the dynamics of divergence of P450 families, we carried out evolutionary analysis of 17 598 P450 sequences from 113 P450 families covering the biological kingdoms animals, bacteria, fungi and plants. Based on their phylogenetic relationships, these 113 families were grouped into 15 clans (Fig. 6 and Supplementary Table S10). As indicated earlier, the P450 families grouped into a clan possibly diverged from a common ancestor irrespective of their host^17^. P450 families grouped into clans 4 and 6 are distributed among all biological kingdoms, suggesting the ancestral P450 of these P450 families was evolved before the evolution of different biological kingdoms. Clan 11 and clan 15 contain P450 families that are distributed between animals and fungi and animals and bacteria, respectively. Clans 1 and 14 contain P450 families of plants and fungi, whereas clan 5 contains P450 families of plants and bacteria. The presence of P450 families belonging to two different biological kingdoms in the same clan suggests that these P450 families originated from a common ancestral P450 that evolved before the divergence of biological kingdoms. Clans 2, 3, 7 and 13 contain animal P450 families, suggesting an independent origin of these P450 families. The same applies to P450 families distributed among clans 8, 9 and 10 that contain exclusively bacterial P450s and clan 12 that contains plant P450s (Fig. 6 and Supplementary Table S10). Considering the presence of these P450 families in a single biological kingdom, it appears that these P450 families originated after the divergence of biological kingdoms.

### Functional conservation of P450s

**Figure 7:**
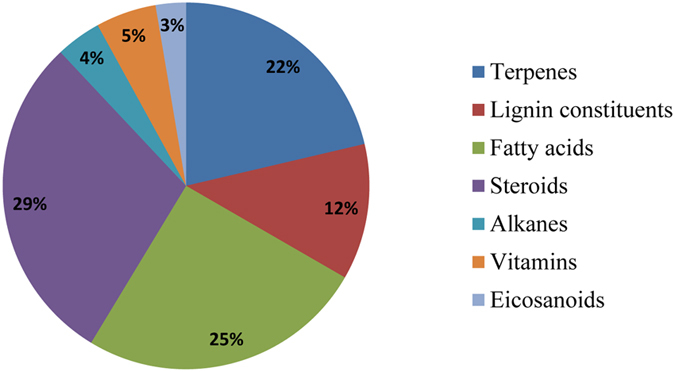
Classification of P450s based on their main substrate class. Percentage of P450s involved in oxidation of particular substrate class is shown in the figure. The functional classification is presented in a broader terms of substrates as described elsewhere^3^. Detailed information on classification of P450 family members into different substrate class is presented in Supplementary Table S12.

To assess the relationship, if any, at the clan-level grouping of P450 families and their functional conservation we assessed each of the P450 family functions at the family level (see Supplementary Table S11). Overall analysis of P450s’ function revealed that the majority of the P450s in all biological kingdoms are involved either in generation or oxidation steroids and structurally related molecules, fatty acids and terpenoids (Fig. 7 and Supplementary Tables S11 and S12). The generation/oxidation of these molecules is critical in the generation of different molecules of biological significance, indicating that P450s are primarily evolved to serve organisms’ physiological process. The best example of functional conservation of P450s can be obtained from Clan 1, where both plant P450s (CYP73, CYP75, CYP84, CYP98) and fungal P450s (CYP5037, CYP5144 and CYP5152) are involved in oxidation of different molecules involved either in biosynthesis of lignin (in plants) or oxidation of lignin-derived molecules generated during fungal-mediated degradation of wood, particularly the lignin component (see Supplementary Tables S11 and S12). It is noteworthy that the molecules involved in biosynthesis of lignin were also found during the fungal-mediated degradation of lignin^57^,^58^. This indicates some overlap in substrate specificity in plant and fungal P450s grouped in this clan, with the key exception that the plant P450s reactions are directed towards lignin biosynthesis whereas fungal P450s reactions are directed towards de-lignification. Oxidation of different plant chemicals by fungal P450s^9^,^59^,^60^ that are part of the lignin biosynthesis pathway further strongly supports this hypothesis (see Supplementary Tables S11 & S12). Furthermore, plant P450s (CYP86, CYP94, CYP96, CYP704) and fungal P450s (CYP52 and CYP63) grouped under Clan 14 show perfect functional conservation, as these P450s are involved in the oxidation of aliphatic hydrocarbons such as alkanes and fatty acids. Clan 4 harbors P450s from all biological kingdoms that possess oxidation activity towards steroids, terpenoids and other structurally related aromatic compounds (see Supplementary Table S11). The same phenomenon can be found in Clan 6, where fungal (CYP505), bacterial (CYP102 and CYP153) and plant/animal (CYP74) P450s are involved in oxidation of aliphatic hydrocarbons, including fatty acids. Functional conservation can also be found between animal and fungal P450 families grouped under Clan 11 where these P450s perform diverse catalytic reactions, including oxidation of different xenobiotics (carcinogenic and endocrine-disrupting chemicals) and plant-related compounds (see Supplementary Table S11). Functional conservation of different P450s belonging to different biological kingdoms grouped under the same clans indicate that these P450s potentially have a common parental ancestor, and hence our analysis of grouping different P450 families into clans makes sense.

Animal P450s that are grouped into clans such as 2, 3, 7 and 13 are involved in steroid hydroxylation. However, based on functional data, a functional divergence was observed for CYP4, CYP12 and CYP325 P450s, as these P450s are known for their role in insecticide resistance. CYP55, grouped under clan 6 that performs denitrification, shows functional divergence compared to other members that are involved in oxidation of aliphatic hydrocarbons (see Supplementary Table S11). Functional divergence of P450s in terms of their function can be observed for P450s grouped across clans, as they involved in oxidation of different xenobiotics. Clan 8 harbors a single P450 CYP157 that is known to lack the EXXR motif. One of the P450 CYP121 belonging to bacteria that are exclusively confined to the MTBC complex has been found to be highly specific towards its known substrate^11^,^44^.

Based on the above data we conclude that functional conservation of P450s is quite common, considering P450s’ prime evolution is to serve organisms through involvement in different biological reactions of physiological importance and during evolution, in response to constant pressure, these P450s evolved to acquire new capabilities such as enhancing their substrate specificity and performing diverse catalytic reactions. Functional conservation of P450s from different biological kingdoms grouped into the same clan further strengthens the hypothesis of common ancestral origin of these P450s. The results presented in this article enhance our understanding of the molecular evolutionary analysis of P450s in terms of their dynamic nature (both at protein and gene level) across biological kingdoms.

## Additional Information

**How to cite this article**: Parvez, M. *et al*. Molecular evolutionary dynamics of cytochrome P450 monooxygenases across kingdoms: Special focus on mycobacterial P450s. *Sci. Rep.* **6**, 33099; doi: 10.1038/srep33099 (2016).

## Supplementary Material

Supplementary Information

Supplementary Dataset S1

Supplementary Dataset S2

Supplementary Dataset S3
