# Supplementary material for: Molecular evolutionary dynamics of cytochrome P450 monooxygenases across kingdoms: Special focus on mycobacterial P450s
Source: Sci Rep. 2016 Sep 12;6:33099. doi: 10.1038/srep33099 (PMC5018878; doi:10.1038/srep33099)
Supplement: Supplementary Information [file srep33099-s1.pdf]

Molecular evolutionary dynamics of cytochrome P450 monooxygenases across kingdoms: Special focus on mycobacterial P450s

Mohammad Parvez[1], Lehlohonolo Benedict Qhanya[1], Ntsane Trevor Mthakathi[1], Ipeleng Kopano Rosinah Kgosiemang[1], Hans Denis Bamal[1], Nataraj Sekhar Pagadala[2], Ting Xie[3], Haoran Yang[4], Hengye Chen[4], Chrispian William Theron[5], Richie Monyaki[1], Seiso Caiphus Raselemane[1], Vuyani Salewe[1], Bogadi Lorato Mongale[1], Retshedisitswe Godfrey Matowane[1], Sara Mohamed Hasaan Abdalla[1], Wool Isaac Booi[1], Mari van Wyk[1], Dedré Olivier[1], Charlotte E Boucher[5], David R Nelson[6], Jack A Tuszynski[7], Jonathan Michael Blackburn[8], Jae-Hyuk Yu[9], Samson Sitheni Mashele[1*], Wanping Chen[4*], Khajamohiddin Syed[1*]

[1] Unit for Drug Discovery Research, Department of Health Sciences, Faculty of Health and Environmental Sciences, Central University of Technology, Bloemfontein 9300, Free State, South Africa

[2] Department of Medical Microbiology and Immunology, 6-020 Katz Group Centre, University of Alberta, Edmonton, Alberta T6G 2E1, Canada

[3] College of Informatics, Huazhong Agricultural University, Wuhan, Hubei Province, China

[4] College of Food Science and Technology, Huazhong Agricultural University, Wuhan, Hubei Province, China

[5] Faculty of Natural and Agricultural Science, Department of Microbial, Biochemical and Food Biotechnology, University of the Free State, P.O Box 339, Bloemfontein, 9300, South Africa.

[6] Department of Microbiology, Immunology and Biochemistry, University of Tennessee Health Science Center, Memphis, TN 38163, USA

[7] Department of Physics, University of Alberta, Edmonton, Alberta T6G 2J1, Canada

[8] Institute of Infectious Disease & Molecular Medicine; Department of Integrative Biomedical Sciences, Faculty of Health Sciences, University of Cape Town, Cape Town 7925, South Africa

[9] Department of Bacteriology, University of Wisconsin-Madison, 3155 MSB, 1550 Linden Drive, Madison WI 53706, USA

Keywords: Mycobacteria, P450, Evolution, Conservation, Divergence, Bacteria, Plant, Fungi, Animal, *Mycobacterium tuberculosis*, Diagnostic-markers, Evolutionary rates

* Corresponding authors' email: khajamohiddinsyed@gmail.com & chenwanping@mail.hzau.edu.cn & smashele@cut.ac.za

Fig. S1. A high-resolution phylogenetic tree of Mycobacterial P450s.

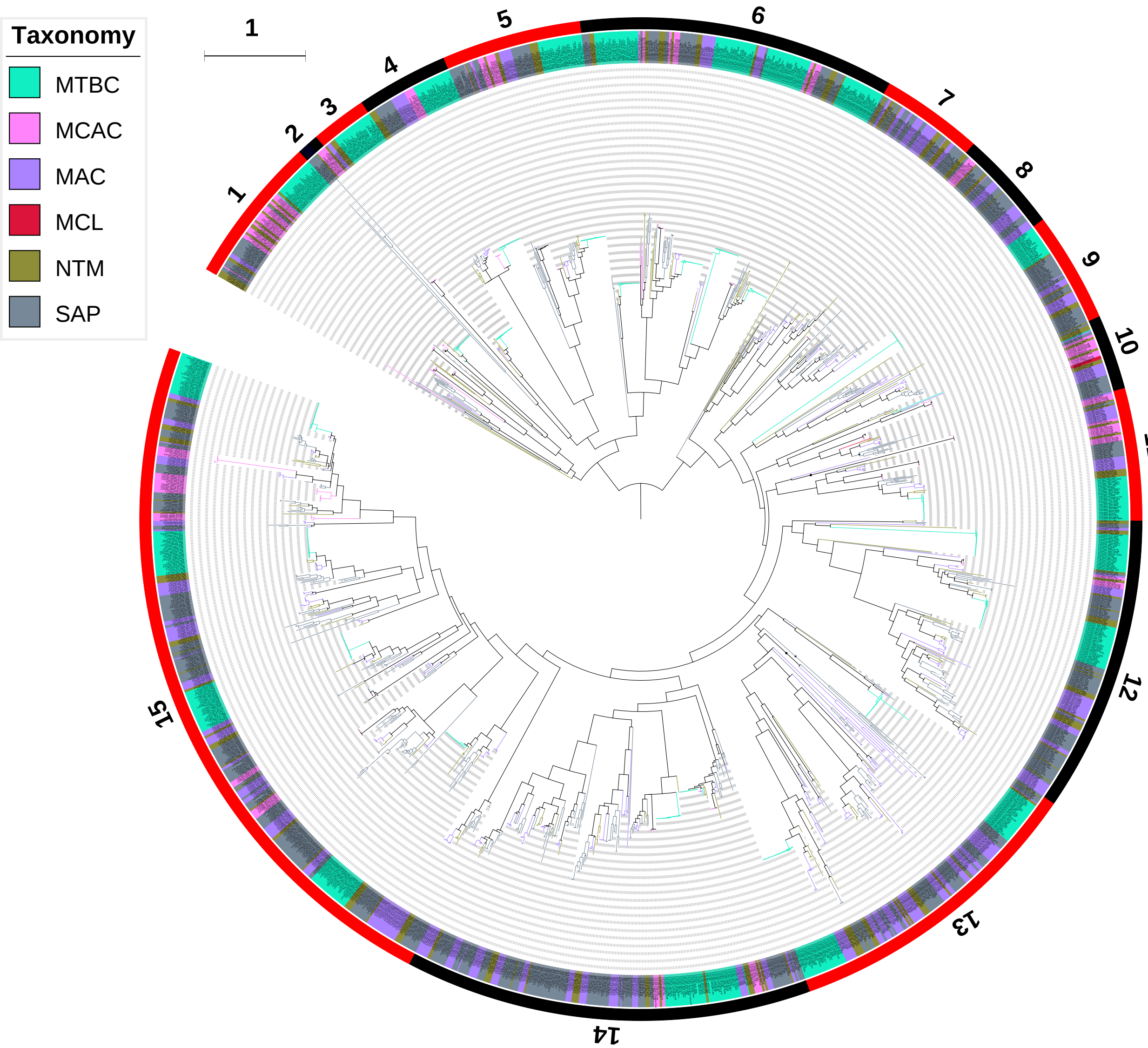

Supplementary Table S1. List of mycobacterial species used in the study. As listed in the table, 60 mycobacterial species were grouped under six different categories based on their characteristic features, including ecological niches, nature of infection and site of infection as described elsewhere (1-5). Also taxonomical grouping of mycobacterial is taken into consideration as described elsewhere (6).

***Mycobacterium tuberculosis* complex (MTBC)**	
1	*Mycobacterium africanum* GM041182
2	*Mycobacterium tuberculosis* C
3	*Mycobacterium tuberculosis* F11
4	*Mycobacterium tuberculosis* H37Ra
5	*Mycobacterium tuberculosis* H37Rv
6	*Mycobacterium tuberculosis* Haarlem
7	*Mycobacterium tuberculosis* KZN 1435
8	*Mycobacterium tuberculosis* KZN 605
9	*Mycobacterium tuberculosis* KZN 4207
10	*Mycobacterium tuberculosis* RGTB327
11	*Mycobacterium tuberculosis* CDC1551
12	*Mycobacterium tuberculosis* strains CCDC5079
13	*Mycobacterium tuberculosis* 7199-99
14	*Mycobacterium tuberculosis* Beijing/NITR203
15	*Mycobacterium tuberculosis* CAS/NITR204
16	*Mycobacterium tuberculosis* EAI5
17	*Mycobacterium tuberculosis* EAI5/NITR206
18	*Mycobacterium tuberculosis* Erdman= ATCC 35801
19	*Mycobacterium tuberculosis* UT205
20	*Mycobacterium canetii* CIPT 140010059
21	*Mycobacterium canetii* CIPT 140060008
22	*Mycobacterium canetii* CIPT 140710010
23	*Mycobacterium bovis* AF 2122/97
24	*Mycobacterium bovis* BCG Pasteur 1173P2
25	*Mycobacterium bovis* BCG Korea 1168P
26	*Mycobacterium bovis* BCG Mexico
27	*Mycobacterium bovis* BCG Tokyo 172
***Mycobacterium chelonae-abscessus* complex (MCAC)**	
28	*Mycobacterium abscessus* ATCC 19977
29	*Mycobacterium abscessus* subsp. *bolletii* 50594
30	*Mycobacterium abscessus* 47J26
31	*Mycobacterium abscessus*103
32	*Mycobacterium abscessus* subsp. *bolletii* MA 1948
33	*Mycobacterium abscessus* VO6705
***Mycobacterium avium* complex (MAC)**	
34	*Mycobacterium Avium* 104
35	*Mycobacterium Avium* subsp. *paratuberculosis* K10

36	*Mycobacterium avium* subsp. *paratuberculosis* MAP4
37	*Mycobacterium intracellulare* ATCC 13950
38	*Mycobacterium intracellulare* MOTT-02
39	*Mycobacterium Intracellulare* MOTT-64
40	*Mycobacterium intracellulare* MOTT-36Y
41	*Mycobacterium Indicus pranii* MTCC 9506
**Mycobacteria causing leprosy (MCL)**	
42	*Mycobacterium leprae* Br4923
43	*Mycobacterium Leprae* TN
**Nontuberculous mycobacteria (NTM)**	
44	*Mycobacterium* sp. JDM601
45	*Mycobacterium liflandii* 128FXT
46	*Mycobacterium ulcerans* Agy99
47	*Mycobacterium Marinum*
48	*Mycobacterium massiliense*
49	*Mycobacterium kansassii* ATCC 12478
**Saprophytes (SAP)**	
50	*Mycobacterium* sp. JLS
51	*Mycobacterium* sp. KMS
52	*Mycobacterium* sp. MCS
53	*Mycobacterium vanbaalenii* PYR-1
54	*Mycobacterium smegmatis* MC2 155
55	*Mycobacterium chubuense* NBB4
56	*Mycobacterium gilvum* PYR-GCK
57	*Mycobacterium gilvum* Spyr1
58	*Mycobacterium smegmatis* JS623
59	*Mycobacterium rhodesiae* NBB3
60	*Mycobacterium neoaurum* VKM Ac-1815D

Supplementary Table S2. Information on mycobacterial species genome databases and their web links used in the study.

Database	Weblink	Reference
TB Database	http://genome.tbdb.org/tbdb_sysbio/GenomesIndex.html	1, 2
KEGG	http://www.genome.jp/kegg-bin/show_organism?category=Mycobacterium	3,4
UniProt	http://www.uniprot.org/taxonomy/36809	5

Supplementary Table S3. Genome data-mining, identification and annotation of cytochrome P450 monooxygenases in 60 mycobacterial species. Open reading frames (ORFs) for each species were obtained from KEGG database[3,4].

**Name of the species**	**P450 count**	**Families**	**Sub-families**	**ORFs**	**% of P450s**
**Mycobacterium tuberculosis complex (MTBC)**					
Mycobacterium africanum GM041182	17	16	17	3830	0.4
Mycobacterium tuberculosis C	20	19	20	3851	0.5
Mycobacterium tuberculosis F11	20	19	20	3941	0.5
Mycobacterium tuberculosis H37Ra	20	19	20	4034	0.5
Mycobacterium tuberculosis H37Rv	20	19	20	3936	0.5
Mycobacterium tuberculosis Haarlem	19	18	19	3866	0.5
Mycobacterium tuberculosis KZN 1435	20	19	20	4059	0.5
Mycobacterium tuberculosis KZN 605	20	19	20	4001	0.5
Mycobacterium tuberculosis KZN 4207	21	19	20	3996	0.5
Mycobacterium tuberculosis RGTB327	14	14	14	3691	0.4
Mycobacterium tuberculosis CDC1551	20	19	20	4189	0.5
Mycobacterium tuberculosis strains CCDC5079	20	19	20	3646	0.5
Mycobacterium tuberculosis 7199-99	19	18	19	3994	0.5
Mycobacterium tuberculosis Beijing/NITR203	20	19	20	4110	0.5
Mycobacterium tuberculosis CAS/NITR204	17	16	16	3959	0.4
Mycobacterium tuberculosis EAI5	19	18	19	3902	0.5
Mycobacterium tuberculosis EAI5/NITR206	20	19	20	4019	0.5
Mycobacterium tuberculosis Erdman= ATCC 35801	19	18	19	4246	0.4
Mycobacterium tuberculosis UT205	19	18	19	3794	0.5
Mycobacterium canetii CIPT 140010059	19	18	19	3861	0.5
Mycobacterium canetii CIPT 140060008	18	17	18	3981	0.5
Mycobacterium canetii CIPT 140710010	21	19	20	3861	0.5
Mycobacterium bovis AF 2122/97	18	17	18	3920	0.5
Mycobacterium bovis BCG Pasteur 1173P2	18	17	18	3952	0.5
Mycobacterium bovis BCG Korea 1168P	18	17	18	4139	0.4
Mycobacterium bovis BCG Mexico	18	17	18	3951	0.5
Mycobacterium bovis BCG Tokyo 172	18	17	18	3944	0.5
**Mycobacterium chelonae-abscessus complex (MCAC)**					
Mycobacterium abscessus ATCC 19977	25	19	22	4942	0.5
Mycobacterium abscessus subsp. bolletii 50594	22	18	20	5185	0.4
Mycobacterium abscessus 47J26	25	19	22	4823	0.5
*Mycobacterium abscessus*103	25	19	22	4867	0.5
Mycobacterium abscessus subsp. bolletii MA 1948	24	18	21	4883	0.5

Mycobacterium abscessus VO6705	28	19	24	5129	0.5
**Mycobacterium avium complex (MAC)**					
Mycobacterium Avium 104	48	29	34	5120	0.9
Mycobacterium Avium subsp. paratuberculosis K10	43	30	33	4350	1.0
Mycobacterium avium subsp. paratuberculosis MAP4	43	30	33	4326	1.0
Mycobacterium intracellulare ATCC 13950	43	26	31	5144	0.8
Mycobacterium intracellulare MOTT-02	47	26	31	5149	0.9
Mycobacterium Intracellulare MOTT-64	42	25	28	5249	0.8
Mycobacterium intracellulare MOTT-36Y	48	29	34	5129	0.9
Mycobacterium Indicus pranii MTCC 9506	61	30	36	5254	1.2
**Mycobacteria causing leprosy (MCL)**					
Mycobacterium leprae Br4923	1	1	1	1604	0.1
Mycobacterium Leprae TN	1	1	1	1605	0.1
**Nontuberculous mycobacteria (NTM)**					
Mycobacterium sp. JDM601	37	24	26	4346	0.9
Mycobacterium liflandii 128FXT	35	29	30	5064	0.7
Mycobacterium ulcerans Agy99	20	18	18	4241	0.5
Mycobacterium Marinum	47	36	39	5452	0.9
Mycobacterium massiliense	19	16	19	4314	0.4
Mycobacterium kansassii ATCC 12478	42	32	35	5866	0.7
**Saprophytes (SAP)**					
Mycobacterium sp. JLS	56	30	38	5739	1.0
Mycobacterium sp. KMS	54	29	35	5975	0.9
Mycobacterium sp. MCS	53	28	34	5615	0.9
Mycobacterium vanbaalenii PYR-1	49	31	37	5979	0.8
Mycobacterium smegmatis MC2 155	42	27	30	6717	0.6
Mycobacterium chubuense NBB4	46	26	31	5843	0.8
Mycobacterium gilvum PYR-GCK	51	29	34	5579	0.9
Mycobacterium gilvum Spyr1	51	28	33	5349	1.0
Mycobacterium smegmatis JS623	51	30	38	6939	0.7
Mycobacterium rhodesiae NBB3	70	35	43	6147	1.1
Mycobacterium neoaurum VKM Ac-1815D	23	19	23	5318	0.4

Supplementary Table S4. Comparative analysis of P450 families between different mycobacterial categories. For each P450 family, the number of member P450s (P450 count) and their percentage in the total number of P450s (1 786) found in mycobacterial species were shown. This includes CYP164 P450s from the Mycobacterium causing leprosy (MCL). However, the MCL category is not presented in the table, considering the presence of a single P450 CYP164 in their genome. The number of species belonging to different categories (MTBC, MCAC, MAC, NTM and SAP) used in the analysis is shown in parenthesis next to the category name. The distribution of member P450s in the category is as follows: min-max number of P450s or number of member P450s in the category members/number of category members possessing the member P450. The empty boxes with green fluorescent color indicate the absence of P450 family members in the category. Abbreviations: MTBC, *Mycobacterium tuberculosis* complex; MCAC, *Mycobacterium chelonae-abscessus* complex; MAC, *Mycobacterium avium* complex; NTM, Nontuberculous mycobacteria; SAP, Saprophytes.

P450 family	P450 count	Percentage	MTBC (27)	MCAC (6)	MAC (8)	NTM (6)	SAP (11)
CYP51	54	3.0	**1/24**	**1/6**	**1-2/8**	**1/4**	**1/11**
CYP123	63	3.5	**1-2/26**	**1/6**	**1-2/5**	**1/5**	**1-2/11**
CYP125	114	6.4	**1/25**	**3-4/6**	**1-3/8**	**1-5/5**	**1-4/10**
CYP130	51	2.9	**1/21**	**1/6**	**1/8**	**1/5**	**1/11**
CYP135	81	4.5	**1/27**	**1/6**	**1/4**	**1-3/4**	**1/10**
CYP136	82	4.6	**1/27**	**1/6**	**1/8**	**1-2/6**	**1-2/11**
CYP138	72	4.0	**1/26**	**1-2/6**	**1/8**	**1-3/5**	**1-2/11**
CYP140	72	4.0	**1/27**	**1/6**	**1/8**	**1/6**	**1/9**
CYP144	58	3.2	**1/27**	**1/6**	**1/6**	**1-2/6**	**1-2/11**

CYP1128	10	0.6	**1/1**	**1/6**	**1/1**	**1/1**	**1/1**
CYP108	56	3.1		**1/6**	**1-2/8**	**1/4**	**1-3/11**
CYP164	29	1.6		**1/6**	**1/8**	**1/3**	**1/10**
CYP124	59	3.3	**1/27**		**1-2/7**	**1/4**	**1-2/11**
CYP126	50	2.8	**1/26**		**1/8**	**1/5**	**1/11**
CYP128	32	1.8	**1-2/26**		**1/2**	**1/1**	**1/3**
CYP142	59	3.3	**1/26**		**1-2/8**	**1/5**	**1/11**
CYP143	62	3.5	**1/27**		**1-2/7**	**1-2/5**	**1-2/6**
CYP105	45	2.5			**1-2/5**	**1/5**	**1-2/11**
CYP185	18	1.0			**1/3**	**1/2**	**1-2/10**
CYP187	54	3.0			**2-4/8**	**1-2/5**	**1-4/10**
CYP188	39	2.2			**2/8**	**1/5**	**1-3/10**
CYP189	86	4.8			**3-5/8**	**1-2/5**	**1-6/10**
CYP190	44	2.5			**1-3/8**	**1/4**	**1-3/10**
CYP191	21	1.2			**1/8**	**1/4**	**1/10**
CYP243	6	0.3			**1/4**	**1/1**	**1/1**
CYP268	28	1.6			**1/8**	**1/4**	**1-3/10**
CYP279	47	2.6			**1-4/8**	**1/3**	**1-4/10**
CYP150	84	4.7			**2-6/8**	**1-5/5**	**3-5/10**
CYP1027	6	0.3			**1/4**	**1/1**	**1/1**
CYP137	32	1.8	**1-2/27**			**1/3**	**1/1**
CYP139	33	1.8	**1/26**		**1/3**	**1/4**	
CYP1134	7	0.4		**1/6**	**1/1**	**1/1**	
CYP286	8	0.4		**1/6**		**1/1**	**1/1**
CYP153	11	0.6		**1/4**		**1/1**	**1/6**
CYP161	8	0.4		**1/6**		**1/1**	**1/1**

CYP132	27	1.5	**1/26**			**1/1**	
CYP102	5	0.3		**1/1**			**1/4**
CYP1110	5	0.3		**1/4**		**1/1**	
CYP1130	16	0.9		**1/6**		**1/2**	
CYP1132	7	0.4		**1/6**		**1/1**	
CYP272	4	0.2			**1/3**	**1/1**	
CYP291	15	0.8			**1/8**		**1-2/5**
CYP1034	3	0.2			**1/1**		**1/1**
CYP1124	6	0.3			**1/4**		**1/2**
CYP1129	6	0.3			**1-2/4**		**1/1**
CYP107	2	0.1				**1/1**	**1/1**
CYP147	5	0.3				**1/3**	**1/1**
CYP278	4	0.2				**1/2**	**1/2**
CYP121	23	1.3	**1/23**				
CYP141	19	1.1	**1/19**				
CYP1131	1	0.1		**1/6**			
CYP1016	2	0.1			**1/2**		
CYP1017	2	0.1			**1/2**		
CYP1018	6	0.3			**1-2/2**		
CYP1019	4	0.2			**2/2**		
CYP1067	1	0.1			**1/1**		
CYP183	2	0.1				**1/2**	
CYP226	2	0.1				**1/2**	
CYP269	2	0.1				**1/2**	
CYP271	1	0.1				**1/1**	
CYP274	2	0.1				**1/2**	

CYP276	2	0.1				**1/2**	
CYP1120	1	0.1				**1/1**	
CYP1123	1	0.1				**1/1**	
CYP1133	1	0.1				**1/1**	
CYP1127	1	0.1				**1/1**	
CYP110	3	0.2					**1/3**
CYP145	4	0.2					**1/3**
CYP151	2	0.1					**1/2**
CYP186	4	0.2					**1/4**
CYP289	2	0.1					**1/2**
CYP292	5	0.3					**1/5**
CYP1119	1	0.1					**1/1**
CYP1121	1	0.1					**1/1**
CYP1122	1	0.1					**1/1**
CYP1125	1	0.1					**1/1**
CYP1126	1	0.1					**1/1**

Supplementary Table S5. Clan-level classification of mycobacterial P450 families. Abbreviations: MTBC, *Mycobacterium tuberculosis* complex; MCAC, *Mycobacterium chelonae-abscessus* complex; MAC, *Mycobacterium avium* complex; NTM, Nontuberculous mycobacteria; SAP, Saprophytes.

Clan	CYP family	Group
1	CYP183, CYP274, CYP102, CYP185, CYP1131, CYP1132, CYP1130, CYP1121, CYP1133, CYP1134, CYP132	MTBC, MCAC, MAC, NTM, SAP
2	CYP1119, CYP186, CYP286	SAP, MCAC, NTM, SAP
3	CYP139	MTBC, MAC, NTM
4	CYP51	MTBC, MCAC, MAC, NTM, SAP
5	CYP136	MTBC, MCAC, MAC, NTM, SAP
6	CYP110, CYP137, CYP138, CYP135	MTBC, MCAC, MAC, NTM, SAP
7	CYP1125, CYP226, CYP1034, CYP187	MAC, SAP, NTM, MTBC
8	CYP276, CYP161, CYP151, CYP279	NTM, MCAC, MAC, SAP
9	CYP141, CYP1127, CYP105	MTBC, NTM, MAC, SAP
10	CYP147, CYP1128, CYP164	MTBC, MCAC, MAC, NTM, SAP
11	CYP1067, CYP1110, CYP140	MTBC, MCAC, MAC, NTM, SAP
12	CYP278, CYP1016, CYP269, CYP121, CYP1120, CYP1126, CYP144, CYP150	MTBC, MCAC, MAC, NTM, SAP
13	CYP1122, CYP1123, CYP128, CYP145, CYP289, CYP1018, CYP1019, CYP292, CYP1017, CYP1129, CYP243, CYP188, CYP271, CYP272, CYP1027, CYP143	MTBC, MCAC, MAC, NTM, SAP

14	CYP123, CYP130, CYP190, CYP189	MTBC, MCAC, MAC, NTM, SAP
15	CYP191, CYP142, CYP108, CYP291, CYP153, CYP125, CYP126, CYP268, CYP124, CYP1124	MTBC, MCAC, MAC, NTM, SAP

Supplementary Table S6. P450 diversity percentage analysis in mycobacterial species. Comparative analysis of P450 diversity percentage between mycobacterial species with other lower eukaryote microbes such as fungi and oomycetes are also shown in the table. The P450 diversity percentage data for lower eukaryotes such as fungi and oomycetes were retrieved from published literature[7-12].

**Group**	**Number of species**	**P450 diversity percentage**		
MTBC	27	95		
NTM	6	79		
MCAC	6	75		
MAC	8	61		
SAP	11	59		
	**Number of species**	**Average number of P450s**	**Average number of P450 families**	**Average P450 diversity percentage**
Mycobacterial species	60	29.75	21.5	72
Saccharomycetes	19	8.11	4.79	59
Pezizomycetes	17	87.35	59.71	68
Basidiomycetes	13	157.00	32.85	21
Zygomycetes	3	50.33	15.00	30
Oomycetes	13	27.38	4.00	15

Supplementary Table S7. Conserved amino acid analysis in mycobacterial P450s. The conservation score (5-9) obtained *via* PROMALS3D is shown in the table, where the number "9" indicates conserved amino acid in P450 members.

P450 family	Number of member P450s	PROMALS3D conservation score				
		5	6	7	8	9
CYP141	19	0	0	0	0	398
CYP51	54	11	102	0	0	264
CYP137	32	146	0	0	0	267
CYP132	27	0	0	0	0	325
CYP121	23	0	0	0	0	391
CYP191	21	56	51	54	0	197
CYP140	72	65	24	57	0	72
CYP139	33	130	0	0	0	211
CYP124	59	60	23	86	0	111
CYP126	50	54	89	36	0	140
CYP123	58	86	9	76	0	69
CYP108	55	76	9	90	0	91
CYP130	51	48	35	66	0	186
CYP190	44	54	14	82	0	92
CYP144	56	33	7	88	0	88
CYP185	18	84	23	82	0	89
CYP291	15	0	128	0	0	89
CYP188	39	66	34	76	0	163
CYP136	82	61	20	76	0	76
CYP268	28	61	29	62	0	74
CYP142	65	99	55	48	0	67
CYP143	62	66	12	73	0	60
CYP135	81	51	30	73	0	60
CYP138	72	44	38	31	30	56
CYP128	32	0	147	0	0	120
CYP189	86	42	36	37	21	61
CYP164	29	58	30	60		121
CYP187	54	37	29	60		55
CYP150	84	43	50	23	30	36
CYP279	47	42	31	74	1	69
CYP105	45	41	18	34	23	37
CYP125	114	43	34	41	18	44

Supplementary Table S8. Amino acid conservation analysis in P450 families from different biological kingdoms. The conservation score (5-9) obtained *via* PROMALS3D is shown in the table where the number "9" indicates conserved amino acid in P450 members. P450 families were arranged in order of the highest to the lowest number of amino acids conserved.

**P450 family**	**Number of member P450s**	**Kingdom**	**PROMALS3D conservation Index**					**Rank (highest to lowest conservation)**
			**5**	**6**	**7**	**8**	**9**	
CYP141	29	Bacteria	0	0	0	0	389	1
CYP51	50	Bacteria	11	102	0	0	264	2
CYP137	38	Bacteria	145	0	0	0	251	3
CYP121	34	Bacteria	0	0	0	0	233	4
CYP132	39	Bacteria	175	0	0	0	217	5
CYP124	71	Bacteria	52	35	59	0	170	6
CYP188	67	Bacteria	62	0	100	0	141	7
CYP123	74	Bacteria	62	0	82	0	137	8
CYP108	67	Bacteria	52	12	92	0	134	9
CYP126	78	Bacteria	65	16	98	0	132	10
CYP21	84	Animal	84	30	104	0	126	11
CYP139	54	Bacteria	126	0	0	0	116	12
CYP291	23	Bacteria	0	111	0	0	115	13
CYP142	90	Bacteria	60	6	83	0	112	14
CYP501	106	Fungi	44	60	113	0	106	15
CYP128	49	Bacteria	0	132	0	0	106	15
CYP164	50	Bacteria	49	9	94	0	99	16
CYP130	98	Bacteria	31	31	91	0	93	17
CYP73	155	Plant	52	69	57	71	91	18
CYP5	71	Animal	37	48	117	0	89	19
CYP116	93	Bacteria	98	92	39	55	89	19
CYP195	52	Bacteria	35	38	54	21	89	19
CYP24	65	Animal	87	11	96	0	84	20
CYP84	62	Plant	68	64	48	44	80	21
CYP98	77	Plant	68	83	44	48	76	22
CYP190	76	Bacteria	34	19	87	0	75	23
CYP185	42	Bacteria	65	20	72	0	68	24
CYP61	70	Fungi	54	63	38	25	68	24
CYP191	36	Bacteria	92	29	92	0	65	25
CYP202	84	Bacteria	34	34	27	23	65	25
CYP39	51	Animal	51	47	79	0	64	26
CYP78	112	Plant	40	51	36	40	61	27
CYP106	93	Bacteria	38	27	64	9	60	28

CYP28	51	Animal	84	59	44	28	55	29
CYP97	100	Plant	37	49	29	25	55	29
CYP17	99	Animal	56	68	44	29	54	30
CYP46	53	Animal	53	42	99	0	54	30
CYP279	71	Bacteria	38	49	26	37	54	30
CYP19	176	Animal	53	57	44	60	53	31
CYP187	103	Bacteria	35	33	42	13	52	32
CYP135	124	Bacteria	48	46	22	23	51	33
CYP140	113	Bacteria	38	32	21	26	50	34
CYP7	89	Animal	69	43	34	26	50	34
CYP144	107	Bacteria	45	33	34	35	47	35
CYP26	131	Animal	69	63	30	46	47	35
CYP143	103	Bacteria	43	43	28	30	45	35
CYP147	52	Bacteria	45	29	35	32	45	35
CYP138	114	Bacteria	42	44	24	32	44	36
CYP51	82	Fungi	64	42	39	33	43	37
CYP705	50	Plant	66	49	41	28	43	37
CYP33	67	Animal	61	49	34	40	42	38
CYP706	51	Plant	53	49	34	32	42	39
CYP86	139	Plant	64	39	34	29	40	40
CYP707	100	Plant	59	54	47	57	40	40
CYP714	58	Plant	58	43	25	26	40	40
CYP136	171	Bacteria	48	34	25	28	39	41
CYP268	73	Bacteria	43	43	27	33	37	42
CYP125	103	Bacteria	42	39	29	17	37	42
CYP27	116	Animal	66	46	25	25	37	42
CYP150	164	Bacteria	59	45	20	35	36	43
CYP8	91	Animal	65	34	36	37	36	43
CYP96	61	Plant	57	40	29	30	35	44
CYP11	171	Animal	46	47	27	29	33	45
CYP153	164	Bacteria	41	34	37	28	33	45
CYP53	102	Fungi	64	45	48	33	31	46
CYP79	102	Plant	67	55	30	31	31	46
CYP90	117	Plant	63	44	44	30	30	47
CYP152	90	Bacteria	46	38	19	25	30	47
CYP55	60	Fungi	34	56	51	21	29	48
CYP709	113	Plant	50	42	18	26	29	48
CYP189	188	Bacteria	54	37	17	27	28	49
CYP704	109	Plant	45	43	33	43	27	50
CYP5152	66	Fungi	46	28	25	15	26	51
CYP12	119	Animal	59	37	23	20	26	51
CYP505	165	Fungi	95	76	48	31	25	52
CYP93	151	Plant	68	42	26	20	24	53
CYP58	106	Fungi	52	43	29	21	23	54

CYP75	251	Plant	74	48	31	29	23	54
CYP584	96	Fungi	56	35	30	21	23	54
CYP65	203	Fungi	38	25	13	11	22	55
CYP72	208	Plant	57	27	28	27	20	56
CYP89	134	Plant	48	38	28	25	20	56
CYP52	161	Fungi	51	26	24	19	17	57
CYP110	113	Bacteria	50	27	17	18	17	57
CYP325	53	Animal	50	31	19	10	17	57
CYP5035	129	Fungi	37	30	17	9	15	58
CYP620	178	Fungi	33	21	12	17	15	58
CYP102	333	Bacteria	45	43	16	19	14	59
CYP63	133	Fungi	51	39	23	13	12	60
CYP5139	181	Fungi	35	12	3	9	12	60
CYP9	312	Animal	45	24	9	10	12	60
CYP92	167	Plant	51	32	33	17	12	60
CYP716	103	Plant	53	30	17	23	12	60
CYP5136	68	Fungi	44	42	20	15	11	61
CYP74	159	Plant/Animal	40	29	23	19	11	61
CYP105	329	Bacteria	28	14	19	6	11	61
CYP157	115	Bacteria	44	26	26	18	11	61
CYP5150	336	Fungi	48	26	8	7	10	62
CYP51	409	Bacteria/fungi /animal/plant	36	19	11	13	10	62
CYP94	170	Plant	39	44	34	26	10	62
CYP1	289	Animal	67	28	26	24	9	63
CYP3	248	Animal	59	44	27	20	9	63
CYP5141	86	Fungi	40	21	13	11	8	64
CYP512	247	Fungi	25	12	8	5	8	64
CYP82	174	Plant	68	31	20	21	8	64
CYP87	78	Plant	58	29	23	20	8	64
CYP107	217	Bacteria	40	25	8	12	8	64
CYP6	921	Animal	21	9	7	8	6	65
CYP5037	261	Fungi	19	6	7	5	5	65
CYP5144	514	Fungi	10	4	5	3	5	65
CYP76	206	Plant	56	31	21	10	5	65
CYP81	235	Plant	40	30	29	14	5	65
CYP4	1076	Animal	27	11	5	6	4	66
CYP2	857	Animal	48	18	10	9	3	67
CYP71	780	Plant	22	7	6	5	3	67

Supplementary Table S9. Evolutionary rate analysis of P450 families. The maximum likelihood estimates for P450 evolutionary rates. Substitution pattern and rates were estimated under the Tamura-Nei model (+G)[13]. A discrete Gamma distribution was used to model evolutionary rate differences among sites. The rate of substitution for each site is drawn from a Gamma distribution with shape parameter α. If α is <1, the distribution implies that there is a relatively large amount of rate variation, with many sites evolving very slowly but some sites evolving at a high rate. For values of α >1, the shape of the distribution changes qualitatively, with less variation and most sites having roughly similar rates[14].

CYP family	Biological kingdom	Shape parameter (α)	Ranking (highest to lowest rate of evolution)
CYP505	Fungi	0.4034	1
CYP126	Bacteria	0.4646	2
CYP130	Bacteria	0.5066	3
CYP188	Bacteria	0.5089	4
CYP191	Bacteria	0.5125	5
CYP73	Plant	0.5186	6
CYP98	Plant	0.5341	7
CYP24	Animal	0.5525	8
CYP142	Bacteria	0.555	9
CYP501	Fungi	0.557	10
CYP195	Bacteria	0.5709	11
CYP26	Animal	0.5711	12
CYP97	Plant	0.5816	13
CYP19	Animal	0.5959	14
CYP55	Fungi	0.5982	15
CYP84	Plant	0.6002	16
CYP190	Bacteria	0.6028	17
CYP53	Fungi	0.6119	18
CYP116	Bacteria	0.6236	19
CYP78	Plant	0.6308	20
CYP63	Fungi	0.6371	21
CYP5144	Fungi	0.647	22
CYP153	Bacteria	0.6485	23
CYP202	Bacteria	0.654	24
CYP584	Fungi	0.654	24
CYP106	Bacteria	0.6603	25
CYP189	Bacteria	0.6673	26
CYP512	Fungi	0.6681	27

CYP65	Fungi	0.6746	28
CYP5150	Fungi	0.6756	29
CYP164	Bacteria	0.6781	30
CYP86	Plant	0.6854	31
CYP125	Bacteria	0.6934	32
CYP187	Bacteria	0.695	33
CYP707	Plant	0.6989	34
CYP75	Plant	0.7185	35
CYP150	Bacteria	0.7188	36
CYP144	Bacteria	0.7228	37
CYP185	Bacteria	0.726	38
CYP279	Bacteria	0.7342	39
CYP143	Bacteria	0.7398	40
CYP5141	Fungi	0.7465	41
CYP136	Bacteria	0.7472	42
CYP52	Fungi	0.7476	43
CYP61	Fungi	0.7514	44
CYP5136	Fungi	0.7543	45
CYP6	Animal	0.7618	46
CYP71	Plant	0.7618	46
CYP33	Animal	0.763	47
CYP5035	Fungi	0.7634	48
CYP5152	Fungi	0.7641	49
CYP110	Bacteria	0.7748	50
CYP51	Animal/Plant/ Bacteria/Fungi	0.7794	51
CYP27	Animal	0.7858	52
CYP74	Plant/Animal	0.7879	53
CYP108	Bacteria	0.7896	54
CYP152	Bacteria	0.7904	55
CYP12	Animal	0.7908	56
CYP90	Plant	0.7922	57
CYP147	Bacteria	0.794	58
CYP58	Fungi	0.7948	59
CYP135	Bacteria	0.7983	60
CYP9	Animal	0.8016	61
CYP268	Bacteria	0.8037	62
CYP123	Bacteria	0.8084	63
CYP17	Animal	0.8148	64
CYP4	Animal	0.8196	65
CYP5037	Fungi	0.8245	66
CYP72	Plant	0.8262	67
CYP5139	Fungi	0.8267	68
CYP124	Bacteria	0.8361	69
CYP706	Plant	0.8369	70

CYP620	Fungi	0.8391	71
CYP138	Bacteria	0.8462	72
CYP5	Animal	0.8516	73
CYP709	Plant	0.8529	74
CYP94	Plant	0.8562	75
CYP1	Animal	0.8636	76
CYP93	Plant	0.8768	77
CYP8	Animal	0.8791	78
CYP704	Plant	0.8941	79
CYP28	Animal	0.8978	80
CYP105	Bacteria	0.915	81
CYP705	Plant	0.9213	82
CYP157	Bacteria	0.9259	83
CYP325	Animal	0.9344	84
CYP716	Plant	0.9416	85
CYP140	Bacteria	0.9464	86
CYP7	Animal	0.9584	87
CYP2	Animal	0.9639	88
CYP107	Bacteria	0.969	89
CYP102	Bacteria	0.9767	90
CYP46	Animal	0.995	91
CYP714	Plant	1.0019	92
CYP92	Plant	1.0027	93
CYP76	Plant	1.0065	94
CYP39	Animal	1.0091	95
CYP82	Plant	1.0158	96
CYP81	Plant	1.0219	97
CYP89	Plant	1.0247	98
CYP79	Plant	1.0276	99
CYP96	Plant	1.053	100
CYP21	Animal	1.0927	101
CYP121	Bacteria	1.2102	102
CYP87	Plant	1.2841	103
CYP137	Bacteria	1.4553	104
CYP291	Bacteria	1.5395	105
CYP128	Bacteria	1.7168	106
CYP132	Bacteria	1.7932	107
CYP139	Bacteria	1.9896	108
CYP3	Animal	2.9595	109
CYP11	Animal	3.1065	110
CYP141	Bacteria	3.2104	111

Supplementary Table S10. Clan-level classification of 113 P450 families belonging to different biological kingdoms such as bacteria, fungi, animal and plant. A hundred and thirteen P450 families were classified into different clans based on their phylogenetic relationships, as shown in Fig. 6, and following the procedure described elsewhere[12].

Clan	CYP family	Biological kingdoms
1	CYP71, CYP73, CYP75, CYP76, CYP78, CYP79, CYP81, CYP82, CYP84, CYP89, CYP92, CYP93, CYP98, CYP620, CYP705, CYP706, CYP5037, CYP5144, CYP5152	Plant, Fungi
2	CYP7, CYP8, CYP19, CYP39	Animal
3	CYP11, CYP12, CYP24, CYP27	Animal
4	CYP26, CYP51, CYP53, CYP58, CYP61, CYP65, CYP87, CYP90, CYP110, CYP116, CYP135, CYP136, CYP137, CYP138, CYP501, CYP512, CYP707, CYP716, CYP5035	Animal, Fungi, Plant, Bacteria
5	CYP97, CYP132, CYP185	Plant, Bacteria
6	CYP55, CYP74, CYP102, CYP105, CYP106, CYP141, CYP153, CYP505	Fungi, Plant, Animal, Bacteria
7	CYP46	Animal
8	CYP157	Bacteria
9	CYP101, CYP107, CYP108, CYP121, CYP123, CYP124, CYP125, CYP126, CYP128, CYP130, CYP140, CYP142, CYP144, CYP147, CYP150, CYP164, CYP187, CYP188, CYP189, CYP190, CYP191, CYP195, CYP202, CYP268, CYP279, CYP291	Bacteria
10	CYP143	Bacteria
11	CYP3, CYP5, CYP6, CYP9, CYP28, CYP5136, CYP5139, CYP5141, CYP5150	Animal, Fungi
12	CYP72, CYP709, CYP714	Plant

13	CYP4, CYP325	Animal
14	CYP52, CYP63, CYP86, CYP94, CYP96, CYP584, CYP704	Plant, Fungi
15	CYP1, CYP2, CYP17, CYP21, CYP33, CYP139, CYP152	Animal, Bacteria

Supplementary Table S11. Family-level functional analysis of P450s. P450s known for their major role (majority of member P450s function) is shown in the table.

Clan	CYP family	Broader biological/functional role	Biological kingdoms	References
**1**	CYP71	Synthesis of indole aldoxime (hormones) -terpenoid metabolism	Plant, Fungi	15
	CYP73	Biosynthesis of lignin (Cinnamic acid hydroxylation)		16-18
	CYP75	Biosynthesis of lignin (Flavonoid hydroxylation)		19
	CYP76	Terpenoid metabolism; biosynthesis of secoiridoids and terpene indole alkaloids; herbicide metabolism; 7-ethoxycoumarin O-de-ethylation (hydroxylation of geraniol and its derivatives, monoterpenes and diterpenes and phenylurea)		20,21
	CYP78	Fatty acid hydroxylation; growth and gametophore formation		22-24
	CYP79	Synthesis of indole aldoxime (hormones)		25,26
	CYP81	Indole glucosinolates hydroxylation		27
	CYP82	Terpenoic metabolism		27
	CYP84	Biosynthesis of lignin (coniferaldehyde/coniferyl alcohol 5-hydroxylase)		29, 30
	CYP89	Formation of major chlorophyll catabolites during leaf senescence		31
	CYP92	Brassinosteroid synthesis		32
	CYP93	Flavonoid biosynthesis		33
	CYP98	Biosynthesis of lignin (3’-hydroxylation of phenolic esters ρ-coumaroyltyramine meta-hydroxylation)		16
	CYP620	Orphan		
	CYP705	Sterols desaturation and triterpenoid metabolism		16, 30
	CYP706	Sesquiterpenoid synthesis (nootkatol and nootkatone)		34
	CYP5037	Plant defence chemicals and degradation of compounds derived from plant components (lignin, cellulose or hemicellulose; coumarin)		10, 35, 36
	CYP5144	Polycyclic aromatic hydrocarbons and plant defence chemicals and degradation of compounds derived from plant components (lignin, cellulose or hemicellulose)		10, 35, 36
	CYP5152	Plant defence chemicals and degradation of compounds derived from plant components (lignin, cellulose or hemicellulose)		10, 35, 36

**2**	CYP7	Steroids hydroxylation (bile acid biosynthesis -cholesterol 7α-hydroxylation)	Animal	37,38
	CYP8	Steroids hydroxylation (Prostacyclin and bile acid biosynthesis)		38,39
	CYP19	Steroids (aromatase: aromatization of androgens into estrogens)		38, 40
	CYP39	Steroids hydroxylation (cholesterol hydroxylation; Steroid 7-alpha hydroxylase)		38
**3**	CYP11	Steroids hydroxylation (steroids biosynthesis)	Animal	38
	CYP12	Insecticide resistance		41
	CYP24	Steroids hydroxylation (vitamin D)		38
	CYP27	Steroids hydroxylation (bile acid biosynthesis and viamin D)		38
**4**	CYP26	Retinoic acid hydroxylation (Vitamin A)	Animal, Fungi, Plant, Bacteria	38
	CYP51	Steroid metabolism (biosynthesis of membrane ergosterol)		42
	CYP53	Benzoate and its derivatives hydroxylation; plant material stilbene demethylation		10, 35, 36
	CYP58	Mycotoxin biosynthesis (Aflatoxin and trichothecene)		43
	CYP61	Steroid metabolism (biosynthesis of membrane ergosterol)		44
	CYP65	Mycotoxin biosynthesis (Trichothecene and fumonisin)		45-47
	CYP87	Triterpoind biosynthesis (Maesasaponins - C-16α oxidations of β-amyrin)		48
	CYP90	Steroid hydroxylation (Brassinosteroids)		49
	CYP110	Fatty acid ω-hydroxylase		50
	CYP116	Xenobiotics degradation		51, 52
	CYP135	Orphan		
	CYP136	Orphan (up-regulated by hydrocarbons)		
	CYP137	Orphan		
	CYP138	Orphan		
	CYP501	Orphan		
	CYP512	Steroids hydroxylation (progesterone and testosterone and dehydroabietic acid)		10, 35, 36
	CYP707	Hormones hydroxylaiton (Abscisic acid - plants)		53
	CYP716	Triterpoind biosynthesis (Maesasaponins - oxidation of b-amyrin to oleanolic acid)		48
	CYP5035	Plant chemicals, i.e. resin and flavonoids and the pharmaceutical chemical naproxen		10, 35, 36
**5**	CYP97	Tetraterpenoids hydroxylaiton (carotenoids)	Plant, Bacteria	54

	CYP132	Orphan		
	CYP185	Orphan (up-regulated by hydrocarbons)		
**6**	CYP55	Denitrification	Fungi, Plant, Animal, Bacteria	55
	CYP74	Conversions of fatty acid hydroperoxides to bioactive oxylipins		56, 57
	CYP102	Fatty acid hydroxylation		58
	CYP105	Diterpenoids oxidation (abietic acid, dehydroabietic acid, isopimaric acid); Biosynthesis of drugs-antibiotics		59
	CYP106	Steroids, di- and triterpene hydroxylation (progesterone, 11-deoxycortisol; dehydroepiandrosterone; 11-keto-β-boswellic acid)		60
	CYP141	Orphan		
	CYP153	Alkane hydroxylation		61
	CYP505	Fatty acid hydroxylation and mycotoxin biosynthesis (fumonisin)		62
**7**	CYP46	Steroids hydroxylation (cholesterol)	Animal	38, 63
**8**	CYP157	Orphan (no EXXR)	Bacteria	64
**9**	CYP101	Terpenoid hydroxylaiton (camphor)	Bacteria	65
	CYP107	Macrolide hydroxylation (antibiotics/drugs), fatty acids oxidative cleavage, steroid hydroxylation (vitamin D3)		66-68
	CYP108	Terpineol oxidation		69
	CYP121	Mycocyclosin production via intramolecular C-C bond formation of cyclo (L-tyr-L-tyr) (cYY)-highly specific		70
	CYP123	Orphan		
	CYP124	Steroids and fatty acids hydroxylation (Omega hydroxylation of methyl-branched fatty acids and cholesterol/cholest-4-en-3-one oxidation)		71, 72
	CYP125	Steroids hydroxylation (cholesterol and 4-cholesten-3-one oxidation)		73
	CYP126	Orphan		
	CYP128	Menaquinone hydroxylaiton (prediction)		74
	CYP130	Orphan		
	CYP140	Macrolide hydroxylation (mycolactone synthesis)		75
	CYP142	Steroid hydroxylation (cholesterol and 4-cholesten-3-one oxidation)		76

	CYP144	Orphan		
	CYP147	Omega-fatty acid hydroxylation		77
	CYP150	polycyclic aromatic hydrocarbon hydroxylation (pyrene, dibenzothiophene and 7-methylbenz(alpha)anthracene)		78
	CYP164	Fatty acid hydroxylation		79
	CYP187	Orphan		
	CYP188	Orphan		
	CYP189	Orphan		
	CYP190	Orphan		
	CYP191	Orphan		
	CYP195	Orphan		
	CYP202	Orphan		
	CYP268	Orphan		
	CYP279	Orphan		
	CYP291	Orphan		
**10**	CYP143	Orphan	Bacteria	
**11**	CYP3	Drug/Xenobiotic metabolism	Animal, Fungi	38
	CYP5	Eicosanoids hydroxylation (Thromboxane A2 synthesis)		38
	CYP6	Xenobiotic metabolism (fatty acids, insecticides), plant chemicals (furanocoumarins)		80
	CYP9	Monoterpene conversion into pheromones, xenobiotics		80
	CYP28	Orphan		
	CYP5136	Xenobiotics degradation (Polycyclic aromatic and alkylphenols hydroxylation)		10, 35, 36
	CYP5139	Xenobioitics degradation (7-ethoxycoumarin; carbazole and phenanthrene)		10, 35, 36
	CYP5141	Plant defense chemicals or degradation of compounds derived from plant components (lignin, cellulose or hemicellulose)		10, 35, 36
	CYP5150	Xenobiotics degradation (Polycyclic aromatic compounds)		10, 35, 36
**12**	CYP72	Steroid catabolism (Brassinosteroids)	Plant	81
	CYP709	Fatty acid hydroxylation		82
	CYP714	Diterpene hydroxylation (Gibberellins)		30

**13**	CYP4	Arachidonic acid and fatty acid hydroxylation; insecticide resistance	Animal	38
	CYP325	Insecticide resistance (based on up-regulation)		83,84
**14**	CYP52	Alkanes and fatty acid hydroxylation	Plant, Fungi	85,86
	CYP63	Xenobiotics degradation (alkanes, polycyclic aromatic compounds and alkylphenols)		87
	CYP86	Fatty acid hydroxylation		38
	CYP94	Fatty acid hydroxylation		38
	CYP96	Alkane hydroxylation		88
	CYP584	Orphan		
	CYP704	Fatty acid hydroxylation		89
**15**	CYP1	Drug/xenobiotic metabolism; Steroids hydroxylation	Animal, Bacteria	38,90
	CYP2	Drug/xenobiotic and steroid metabolism; Fatty acids hydroxylation		38,90
	CYP17	Steroid biosynthesis (testosterone and estrogen biosynthesis)		38,90
	CYP21	Steroid biosynthesis		38,90
	CYP33	Orphan		
	CYP139	Orphan		
	CYP152	Fatty acid hydroxylation		91

Supplementary Table S12. Classification of P450s based on their main substrate class as described elsewhere (38). Percent of P450s is calculated considering 75 P450s that are functionally characterized as 100% for each of the substrate class. Considering study is aimed to find common functions of all P450s and many P450s are involved in oxidation of different class of xenobiotics, xenobiotics as substrate class data is not represented. This table is derived from the functional data presented in Table S11.

Substrate class	P450s	Number of P450s	Percent of P450s
Terpenes	CYP714,CYP9,CYP108,CYP101,CYP106,CYP105,CYP97,CYP716,CYP87,CYP706,CYP705,CYP82,CYP81,CYP79,CYP76,CYP71	16	21
Lignin constituents	CYP5141,CYP5139,CYP5152,CYP5144,CYP5037,CYP98,CYP84,CYP76,CYP73	9	12
Fatty acids	CYP152,CYP2,CYP704,CYP94,CYP86,CYP63,CYP52,CYP4,CYP709,CYP6,CYP164,CYP147, CYP124,CYP107,CYP505,CYP102,CYP74,CYP110,CYP78	19	25
Steroids	CYP121,CYP17,CYP2,CYP72,CYP142,CYP125,CYP124,CYP46,CYP106,CYP512,CYP90, CYP61,CYP51,CYP27,CYP11,CYP39,CYP19,CYP8,CYP7,CYP705,CYP92,CYP76	22	29
Alkanes	CYP96,CYP52,CYP153	3	4
Vitamins	CYP107,CYP26,CYP27,CYP24	4	5
Eicosanoids	CYP4, CYP5	2	3

REFERENCES

1. Reddy, T. B. et al. TB database: an integrated platform for tuberculosis research. *Nucleic Acids Res.***37,** D499-508 (2009).
2. Galagan, J. E. et al. TB database 2010: overview and update. *Tuberculosis (Edinb)* **90,** 225-235 (2010).
3. Kanehisa, M. & Goto, S. KEGG: kyoto encyclopedia of genes and genomes. *Nucleic Acids Res.* **28,** 27-30 (2000).
4. Kanehisa, M., Sato, Y., Kawashima, M., Furumichi, M. & Tanabe, M. KEGG as a reference resource for gene and protein annotation. *Nucleic Acids Res.* **44,** D457-D462 (2016).
5. The UniProt Consortium. UniProt: a hub for protein information. *Nucleic Acids Res.* **43,** D204-D212 (2015).
6. Tortoli, E. Phylogeny of the genus *Mycobacterium*: Many doubts, few certainties. Infect Genet Evol **12,** 827-831.
7. Nelson, D. R. The cytochrome P450 homepage. *Human Genomics* **4,** 59–65 (2009).
8. Syed, K, Nelson, D. R., Riley, R. & Yadav. J. S. Genome-wide annotation and comparative genomics of cytochrome P450 monooxygenases (P450s) in the polypore species *Bjerkandera adusta*, *Ganoderma* sp. and *Phlebia brevispora*. *Mycologia* **105,** 1445-1455 (2013).
9. Kgosiemang, I. K. R., Mashele, S. S. & Syed, K. Comparative genomics and evolutionary analysis of cytochrome P450 monooxygenases in fungal subphylum Saccharomycotina. *J. Pure Appl. Microbiol.* **8,** 291-302 (2014).
10. Syed, K., Shale, K., Pagadala, N. S. & Tuszynski, J. Systematic identification and evolutionary analysis of catalytically versatile cytochrome P450 monooxygenase families enriched in model basidiomycete fungi. *PLoS ONE* **9(1),** e86683 (2014).
11. Sello, M. M. et al. Diversity and evolution of cytochrome P450 monooxygenases in Oomycetes. *Scientific Reports* **5,** 11572 (2015).
12. Chen, W. et al. Fungal cytochrome P450 monooxygenases: Their distribution, structure, functions, family expansion, and evolutionary origin. Genome Biol. Evol. **6,** 1620–34 (2014).
13. Tamura, K. & Nei, M. Estimation of the number of nucleotide substitutions in the control region of mitochondrial DNA in humans and chimpanzees. *Mol. Biol. Evol.* **10,** 512-526 (1993).
14. Liò, P. & Goldman, N. Models of molecular evolution and phylogeny. *Genome Res.* **8,** 1233-1244 (1998).
15. Nafisi, M. et al. *Arabidopsis* cytochrome P450 monooxygenase 71A13 catalyzes the conversion of indole-3-acetaldoxime in camalexin synthesis. *Plant Cell.* **19,** 2039-2052 (2007).
16. Hamberger, B. & Bak, S. Plant P450s as versatile drivers for evolution of species-specific chemical diversity. *Phil. Trans. R. Soc. B. Biol. Sci.* **368,** 20120426 (2013).

17. Teutsch, H. G. Isolation and sequence of a cDNA encoding the Jerusalem artichoke cinnamate 4-hydroxylase, a major plant cytochrome P450 involved in the general phenylpropanoid pathway. *Proc. Natl. Acad. Sci. U. S. A.* **90,** 4102-6 (1993).
18. Pierrel, M. A. et al. 1994. Catalytic properties of the plant cytochrome P450 CYP73 expressed in yeast. Substrate specificity of cinnamte hydroxylase. *Eur. J. Biochem.* **224,** 835-844.
19. Kaltenbach, M., Schröder, G., Schmelzer, E., Lutz, V. & Schröder J. Flavonoid hydroxylase from *Catharanthus roseus*: cDNA, heterologous expression, enzyme properties and cell-type specific expression in plants. *Plant J.* **19,** 183-193.
20. Höfer, R. et al. Dual function of the cytochrome P450 CYP76 family from *Arabidopsis thaliana* in the metabolism of monoterpenols and phenylurea herbicides. *Plant Physiol*. **166,** 1149-61 (2014).
21. Batard, Y. et al. Molecular cloning and functional expression in yeast of CYP76B1, a xenobiotic-inducible 7-ethoxycoumarin O-de-ethylase from *Helianthus tuberosus*. *Plant J.* **14,** 111-120 (1998).
22. Katsumata, T. et al. Involvement of the CYP78A subfamily of cytochrome P450 monooxygenases in protonema growth and gametophore formation in the moss *Physcomitrella patens. Biosci. Biotechnol. Biochem.* **75,** 331-336 (2011).
23. Imaishi, H., Matsuo, S., Swai, E. & Ohkawa, H. CYP78A1 preferentially expressed in developing inflorescences of *Zea mays* encoded a cytochrome P450-dependent lauric acid 12-monooxygenase. *Biosci. Biotechnol. Biochem.* **64,** 1696-1701 (2000).
24. Kai, K. et al. Metabolomics for the characterization of cytochromes P450-dependent fatty acid hydroxylation reactions in *Arabidopsis*. *Plant Biotechnol*. **26,** 175-182 (2009).
25. Sugawara, S. et al. Biochemical analyses of indole-3-acetaldoxime-dependent auxin biosynthesis in *Arabidopsis*. *Proc. Natl. Acad. Sci. U. S. A.* **106,** 5430-5435 (2009).
26. Irmisch, S., Zeltner, P., Handrick, V., Gershenzon, J. & Kollner, T. G. The maize cytochrome P450 CYP79A61 produces phenylacetaldoxime and indole-3-acetaldoxime in heterologous systems and might contribute to plant defense and auxin formation. BMC Plant Biology 15, 128 (2015).
27. Pfalz, M. et al. Metabolic engineering in *Nicotiana benthamiana* reveals key enzyme functions in *Arabidopsis* indole glucosinolate modification. *Plant Cell* **23,** 716-729.
28. Lee, S. et al. Herbivore-induced and floral homoterpene volatiles are biosynthesized by a single P450 enzyme (CYP82G1) in *Arabidopsis*. *Proc. Natl. Acad. Sci. U.S.A.* **107,** 21205–21210 (2010).
29. Ehlting, J., Hamberger, B., Million-Rousseau, R. & Werck-Reichhart, D. Cytochromes P450 in phenolic metabolism. Phytochem. Rev. 5, 239-270 (2006).
30. Mizutani, M. & Ohta, D. Diversification of P450 genes during land plant evolution. *Annu. Rev. Plant Biol*. **61,** 291-315 (2010).
31. Christ, B. et al. Cytochrome P450 CYP89A9 is Involved in the Formation of Major Chlorophyll Catabolites during Leaf Senescence in *Arabidopsis*. *Plant Cell* **25,** 1868-1880.
32. Kang, J.G. et al. (2001). Light and brassinosteroid signals are integrated via a dark-induced small G protein in etiolated seedling growth. *Cell* **105,** 625–636 (2011).

33. Du, Y., Chu, H., Chu, I. K. & Lo, C. CYP93G2 is a flavone 2-hydroxylase required for c-glycosylflavone biosynthesis in Rice. *Plant Physiol*. **154,** 324-333 (2010).
34. Cankar, K. et al. Valencene oxidase CYP706M1 from Alaska cedar (*Callitropsis nootkatensis*). FEBS Lett. 588, 1001-1007 (2014).
35. Ide, M., Ichinose, H. & Wariishi, H. Molecular identification and functional characterization of cytochrome P450 monooxygenases from the brown-rot basidiomycete *Postia placenta*. *Arch. Microbiol*. **194,** 243–53 (2012).
36. Hirosue, S. et al. Insight into functional diversity of cytochrome P450 in the white-rot basidiomycete *Phanerochaete chrysosporium*: Involvement of versatile monooxygenase. *Biochem. Biophys. Res. Commun*. **407,** 118–23 (2011).
37. Cohen, J. C. et al. Cloning of the human cholesterol 7 alpha-hydroxylase gene (CYP7) and localization to chromosome 8q11-q12. *Genomics* **14,** 153-61 (1992).
38. Guengerich, F. P. Human cytochrome P450 enzymes in *Cytochrome P450: Structure, mechanism, and biochemistry* 4th edn, (eds Oritz de Montellano, P. R.) Ch. 9, 523-786. (Springer International Publishing , 2015).
39. Yokoyama, C. et al. Human gene encoding prostacyclin synthase (PTGIS): genomic organization, chromosomal localization, and promoter activity. *Genomics* **36,** 296-304 (1996).
40. Corbin, C. J. et al. Isolation of a full-length cDNAinsert encoding human aromatase system cytochrome P-450 and itsexpression in nonsteroidogenic cells. Proc. Natl. Acad. Sci. U.S.A. 85, 8948-8952 (1988).
41. Feyereisen, R. Evolution of insect P450. *Biochem. Soc. Transc*. **34,** 6 (2006).
42. Kelly, S. L. & Kelly, D. E. Microbial cytochrome P450: Biodiversity and biotechnology, where do cytochrome P450 come from, what do they do and what can they do for us? *Phil. Trans. R. Soc. B. Biol. Sci.* **368,** 161220120430 (2013).
43. Ehrlich, K. C., Chang, P. K., Yu, J. & Cotty, P. J. Aflatoxin biosynthesis cluster gene *cypA* is required for G-aflatoxin formation. *Appl. Environ. Microbiol*. **70,** 6518-6524 (2004).
44. Kelly, S. L., Lamb, D. C., Baldwin, B. C., Corran, A. J. & Kelly, D. E. Characterization of *Saccharomyces cerevisiae* CYP61, sterol delta22-desaturase, and inhibition by azole antifungal agents. *J. Biol. Chem.* **272,** 9986-8 (1997).
45. Kimura, M., Tokai, T., Takahashi-Ando, N., Ohsato, S. & Fujimura, M. (2007). Molecular and genetic studies of trichothecene biosynthesis: pathways, genes, and evolution. *Biosci. Biotechnol. Biochem.* **71,** 2105– 2123 (2007).
46. Proctor, R. H., Plattner, R. D., Desjardins, A. E., Busman, M. & Butchko, R. A. (2006). Fumonisin production in the maize pathogen *Fusarium verticillioides*: genetic basis of naturally occurring chemical variation. *J. Agric. Food. Chem*. **54,** 2424-2430 (2006).
47. Bojja, R. S., Cerny, R. L., Proctor, R. H. & Du, L. Determining the biosynthetic sequence in the early steps of the fumonisin pathway by use of three gene-disruption mutants of *Fusarium verticillioides*. *J. Agric. Food. Chem.* **52,** 2855-2860 (2004).
48. Moses, T. et al. Unravelling the triterpenoid Saponin biosynthesis of the African shrub *Maesa lanceolate*. *Mol. Plant* **8,** 122-135 (2015).

49. Szekeres, M. Brassinosteroids rescue the deficiency of CYP90, a cytochrome P450, controlling cell elongation and de-etiolation in *Arabidopsis*. *Cell* **85,** 171-182 (1996).
50. Torres, S., Fjetland, C. R. & Lammers, P. J. Alkane-induced expression, substrate binding profile, and immunolocalization of a cytochrome P450 encoded on the *nifD* excision element of *Anabaena* 7120. *BMC Microbiol.* **5,** 16 (2005).
51. Nagy, I. Degradation of the thiocarbamate herbicide EPTC (S-ethyl dipropylcarbamothioate) and biosafening by *Rhodococcus* sp. strain NI86/21 involve an inducible cytochrome P-450 system and aldehyde dehydrogenase. *J. Bacteriol*. **177,** 676-687.
52. Warman, A. J. et al. Characterization of *Cupriavidus metallidurans* CYP116B1 – a thiocarbamate herbicide oxygenating P450-phthalate dioxygenase reductase fusion protein. *FEBS J.* **279,** 1675-1693 (2012).
53. Saito, S. et al. Arabidopsis *CYP707A*s encode (+)-abscisic acid 8'-hydroxylase, a key enzyme in the oxidative catabolism of abscisic acid. *Plant Physiology* **134,** 1439-1449 (2004).
54. Ruiz-Sola, M. A. & Rodriguez-Concepcion, M. Carotenoid biosynthesis in *Arabidopsis*: A colourful pathway. *Arabidopsis Book* **10,** e0158 (2012).
55. Tomura, D., Obika, K., Fukumizu, A. & Shoun, H. Nitric oxide reductase cytochrome P-450 gene, CYP 55, of the fungus *Fusarium oxysporum* containing a potential binding-site for FNR, the transcription factor involved in the regulation of anaerobic growth of *Escherichia coli*. *J Biochem*. **116,** 88-94.
56. Stumpe, M. & Feussner, I. Formation of oxylipins by CYP74 enzymes. *Phytochem. Rev.* **5,** 347-357 (2006).
57. Grechkin, A. N. Hydroperoxide lyase and divinyl ether synthase. *Prostaglandins Other Lipid Mediat*. **68-69,** 457-470 (2002).
58. Noble, M. A., Miles, C. S., Reid, G. A., Chapman, S. K. & Munro, A. W. Catalytic properties of key active site mutants of flavocytochrome P-450 BM3. *Biochem. Soc. Trans*. **27,** A44–A44 (1999).
59. Moody, S .C. & Loveridge, E. J. CYP105-diverse structures, functions and roles in an intriguing family of enzymes in *Streptomyces*. *J. Appl. Microbiol*. **117,** 1549-1563.
60. Kiss, F. M. et al. Comparison of CYP106A1 and CYP106A2 from *Bacillus megaterium*-identification of a novel 11-oxidase activity. *Appl. Microbiol. Biotechnol*. **99,** 8495-8514.
61. Maier, T., Foerster, H.-H., Asperger, O. & Hahn, U. Molecular characterization of the 56-kDa CYP153 from *Acinetobacter* sp. EB104. *Biochem. Biophys. Res. Commun*. **286,** 652-658 (2001).
62. Kitazume, T., Takaya, N., Nakayama, N. & Shoun, H. *Fusarium oxysporum* fatty-acid subterminal hydroxylase (CYP505) is a membrane-bound eukaryotic counterpart of *Bacillus megaterium* cytochrome P450BM3. *J. Biol. Chem*. **275,** 39734-40 (2000).
63. Lund, E. G., Guileyardo, J. M., Russell, D. W. cDNA cloning of cholesterol 24-hydroxylase, a mediator of cholesterol homeostasis in the brain. *Proc. Natl. Acad. Sci. U. S. A.* **96,** 7238-7243.

64. Rupasinghe, S. et al. The cytochrome P450 gene family CYP157 does not contain EXXR in the K-helix reducing the absolute conserved P450 residues to a single cysteine. *FEBS Lett.* **580,** 6338-6342 (2006).
65. Poulos, T. L., Finzel, B. C. & Howard, A. J. Crystal structure of substrate-free *Pseudomonas putida* cytochrome P450. *Biochemistry* **25,** 5314–5322 (1986).
66. Cupp–Vickery, J. R. & Poulos, T. L. Structure of cytochrome P450eryF involved in erythromycin biosynthesis. *Nat. Struct. Biol*. **2,** 144-153 (1995).
67. Sakaki, T. et al. Bioconversion of vitamin D to its active form by bacterial or mammalian cytochrome P450. *Biocheim. Biophys. Acta*. **1814,** 249-256 (2011).
68. Cryle, M. J. & De Voss, J. J. Carbon-carbon bond cleavage by cytochrome P450(boil) (CYP107H1). *Chem. Commun*. **1,** 86-87 (2004).
69. Fruetel, J. A., Mackman, R. L., Peterson, J. A. & Ortiz de Montellano, P. R. Relationship of active site topology to substrate specificity for cytochrome P450terp (CYP108). J. Biol. Chem. 269, 28815-28821 (1994).
70. McLean, K. J. et al. (2008). Characterization of active site structure in CYP121. A cytochrome P450 essential for viability of *Mycobacterium tuberculosis* H37Rv. *J. Biol. Chem.* **283,** 33406-33416 (2008).
71. Johnston, J. B., Kells, P. M., Podust, L. M. & Ortiz de Montellano, P. R. Biochemical and structural characterization of CYP124: a methyl-branched lipid *ω*-hydroxylase from *Mycobacterium tuberculosis*. *Proc. Natl. Acad. Sci. U.S.A.* **106,** 20687-20692 (2009).
72. Johnston, J.B., Ouellet, H. & Ortiz de Montellano, P. R. Functional redundancy of steroid C-26-monooxygenase activity in *Mycobacterium tuberculosis* revealed by biochemical and genetic analyses. *J. Biol. Chem*. **285,** 36352–36360 (2010).
73. McLean, K. J. et al. The structure of *Mycobacterium tuberculosis* CYP125 molecular basis for cholesterol binding in a P450 needed for host infection. *J. Biol. Chem.* **284,** 35524–35533 (2009).
74. Holsclaw, C. M. et al. Structural characterization of a novel sulfated menaquinone produced by stf3 from *Mycobacterium tuberculosis*. *ACS Chem. Biol.* **3,** 619-624 (2008).
75. Mve-Obiang, A. et al. A newly discovered Mycobacterial pathogen isolated from laboratory colonies of *Xenopus* species with lethal infections produces a novel form of mycolactone, the *Mycobacterium ulcerans* macrolide toxin. *Infect. Immun*. **73,** 3307-3312.
76. Driscoll, M. D. et al. Structural and biochemical characterization of *Mycobacterium tuberculosis* CYP142: evidence for multiple cholesterol 27-hydroxylase activities in a human pathogen. *J. Biol. Chem***. 285,** 38270–38282 (2010).
77. Bhattarai, S., Liou, K. & Oh, T. J. Hydroxylation of long chain fatty acids by CYP147F1, a new cytochromeP450 subfamily protein from *Streptomyces peucetius*. *Arch. Biochem. Biophys*. **539,** 63-69 (2013).
78. Brezna, B. et al. Molecular characterization of cytochrome P450 genes in the polycyclic aromatic hydrocarbon degrading *Mycobacterium vanbaalenii* PYR-1. *Appl. Microbiol. Biotechnol.* **71,** 522-352 (2006).

79. Agnew, C. R. et al. An enlarged, adaptable active site in CYP164 family P450 enzymes, the sole P450 in *Mycobacterium leprae*. *Antimicrob. Agents Chemother*. **56,** 391-402 (2012).
80. Schuler, M. A. P450s in plants, insects, and their fungal pathogens in Cytochrome P450: Structure, mechanism, and biochemistry 4th edn, (eds Oritz de Montellano, P. R.) Ch. 7, 409-449. (Springer International Publishing , 2015).
81. Neff, M. M. et al. BAS1: A gene regulating brassinosteroid levels and light responsiveness in *Arabidopsis*. *Proc. Natl. Acad. Sci. U.S.A.* **96,** 15316-15323 (1999).
82. Kandel, S. et al. (2005) Cloning, functional expression, and characterization of CYP709C1, the first sub-terminal hydroxylase of long chain fatty acid in plants. Induction by chemicals and methyl jasmonate. *J. Biol. Chem.* **280,** 35881-35889 (2005).
83. David, J-P. et al. The Anopheles gambiae detoxification chip: A highly specific microarray to study metabolic-based insecticide resistance in malaria vectors. *Proc. Natl. Acad. Sci. U.S.A.***102,** 4080-4084.
84. Reddy, B. P. N., Rao, B. P., Prasad, G. B. K. S. & Raghavendra, K. Identification and classification of detoxification enzymes from *Culex quinquefasciatus* (Diptera: Culicidae). *Bioinformation* 8, 430-436.
85. Kim, D., Cryle, M. J., Voss, J. J. & Ortiz de Montellano, P. R. (2007) Functional expression and characterization of cytochrome P450 52A21 from *Candida albicans*. *Arch. Biochem. Biophys*. **464,** 213-220 (2007).
86. van Bogaert, I. N., De Mey, M., Develter, D., Soetaert, W. & Vandamme, E. J. Importance of the cytochrome P450 monooxygenase CYP52 family for the sophorolipid-producing yeast *Candida bombicola*. *FEMS Yeast Res*. **9,** 87-94 (2009).
87. Syed, K., Porollo, A., Lam, Y. W., Grimmett, P. E. & Yadav, J. S. CYP63A2, a catalytically versatile fungal P450 monooxygenase capable of oxidizing higher molecular-weight polycyclic aromatic hydrocarbons, alkylphenols, and alkanes. *Appl. Environ. Microbiol.* **79,** 2692-2702.
88. Greer, S. et al. The cytochrome P450 enzyme CYP96A15 is the midchain alkane hydroxylase responsible for formation of secondary alcohols and ketones in stem cuticular wax of *Arabidopsis*. *Plant Physiol*. **145,**653-667 (2007).
89. Li, H. et al. Cytochrome P450 family member CYP704B2 catalyzes the ω-hydroxylation of fatty acids and is required for anther cutin biosynthesis and pollen exine formation in rice. *Plant Cell* **22,** 173-190 (2010).
90. McKinnon, R. A., Sorich, M. J. & Ward, M. B. Cytochrome P450 Part 1: multiplicity and function. *J. Pharm. Pract. Res.* **38,** 55-57 (2008).
91. Rude, M. A. et al. Terminal olefin (1-alkene) biosynthesis by a novel p450 fatty acid decarboxylase from Jeotgalicoccus species. *Appl. Environ. Microbiol.* **77,** 1718-27.
