# Supplementary material for: Molecular evolutionary dynamics of cytochrome P450 monooxygenases across kingdoms: Special focus on mycobacterial P450s
Source: Sci Rep. 2016 Sep 12;6:33099. doi: 10.1038/srep33099 (PMC5018878; doi:10.1038/srep33099)
Supplement: Supplementary Dataset S2 [file srep33099-s3.doc]

**Molecular evolutionary dynamics of cytochrome P450 monooxygenases across kingdoms: Special focus on mycobacterial P450s**

Mohammad Parvez^1^, Lehlohonolo Benedict Qhanya^1^, Ntsane Trevor Mthakathi^1^, Ipeleng Kopano Rosinah Kgosiemang^1^, Hans Denis Bamal^1^, Nataraj Sekhar Pagadala^2^, Ting Xie^3^, Haoran Yang^4^, Hengye Chen^4^, Chrispian William Theron^5^, Richie Monyaki^1^, Seiso Caiphus Raselemane^1^, Vuyani Salewe^1^, Bogadi Lorato Mongale^1^, Retshedisitswe Godfrey Matowane^1^, Sara Mohamed Hasaan Abdalla^1^, Wool Isaac Booi^1^, Mari van Wyk^1^, Dedré Olivier^1^, Charlotte E Boucher^5^, David R Nelson^6^, Jack A Tuszynski^7^, Jonathan Michael Blackburn^8^, Jae-Hyuk Yu^9^, Samson Sitheni Mashele^1*^, Wanping Chen^4*^, Khajamohiddin Syed^1*^

^1^ Unit for Drug Discovery Research, Department of Health Sciences, Faculty of Health and Environmental Sciences, Central University of Technology, Bloemfontein 9300, Free State, South Africa

^2^ Department of Medical Microbiology and Immunology, 6-020 Katz Group Centre, University of Alberta, Edmonton, Alberta T6G 2E1, Canada

^3^ College of Informatics, Huazhong Agricultural University, Wuhan, Hubei Province, China

^4^ College of Food Science and Technology, Huazhong Agricultural University, Wuhan, Hubei Province, China

^5^ Faculty of Natural and Agricultural Science, Department of Microbial, Biochemical and Food Biotechnology, University of the Free State, P.O Box 339, Bloemfontein, 9300, South Africa.

^6^ Department of Microbiology, Immunology and Biochemistry, University of Tennessee Health Science Center, Memphis, TN 38163, USA

^7^ Department of Physics, University of Alberta, Edmonton, Alberta T6G 2J1, Canada

^8^ Institute of Infectious Disease & Molecular Medicine; Department of Integrative Biomedical Sciences, Faculty of Health Sciences, University of Cape Town, Cape Town 7925, South Africa

^9^ Department of Bacteriology, University of Wisconsin-Madison, 3155 MSB, 1550 Linden Drive, Madison WI 53706, USA

**Keywords**: Mycobacteria, P450, Evolution, Conservation, Divergence, Bacteria, Plant, Fungi, Animal, *Mycobacterium tuberculosis*, Diagnostic-markers, Evolutionary rates

* Corresponding authors’ email: khajamohiddinsyed@gmail.com & chenwanping@mail.hzau.edu.cn & smashele@cut.ac.za

**Supplementary Dataset 3**. Mycobacterial P450 sequences.

| ***Mycobacterium tuberculosis* complex (MTBC)** |
| --- |
| ***Mycobacterium africanum*** **GM041182** |
| Database: KEGG; P450 count: 17; P450 families: 16; Subfamilies: 17 |
| >CYP138A1(MAF_01360)  MSEVVTAAPAPPVVRLPPAVRGPKLFQGLAFVVSRRRLLGRFVRRYGKAFTANILMYGRV VVVADPQLARQVFTSSPEELGNIQPNLSRMFGSGSVFALDGDDHRRRRRLLAPPFHGKSM KNYETIIEEETLRETANWPQGQAFATLPSMMHITLNAILRAIFGAGGSELDELRRLIPPW VTLGSRLAALPKPKRDYGRLSPWGRLAEWRRQYDTVIDKLIEAERADPNFADRTDVLALM LRSTYDDGSIMSRKDIGDELLTLLAAGHETTAATLGWAFERLSRHPDVLAALVEEVDNGG HELRQAAILEVQRARTVIDFAARRVNPPVYQLGEWVIPRGYSIIINIAQIHGDPDVFPQP DRFDPQRYIGSKPSPFAWIPFGGGTRRCVGAAFANMEMDVVLRTVLRHFTLETTTAAGER SHGRGVAFTPKDGGRVVMRRR  >CYP135A1(MAF_03290)  MASTLTTGLPPGPRLPRYLQSVLYLRFREWFLPAMHRKYGDVFSLRVPPYADNLVVYTRP EHIKEIFAADPRSLHAGEGNHILGFVMREHSVLMTDEAEHARMRSLLMPAFTRAALRGYR DMIASVAREHITRWRPHATINSLDHMNALTLDIILRVVFGVTDPKVKAELTSRLQQIINI HPAILAGVPYPSLKRMNPWKRFFHNQTKIDEILYREIASRRIDSDLTARTDVLSRLLQTK DTPTKPLTDAELRDQLITLLLAGHETTAAALSWTLWELAHAPEIQSQVVWAAVGGDDGFL EAVLKEGMRRHTVIASTARKVTAPAEIGGWRLPAGTVVNTSILLAHASEVSHPKPTEFRP SRFLDGSVAPNTWLPFGGGVRRCLGFGFALTEGAVILQEIFRRFTITAAGPSKGETPLVR NITTVPKHGAHLRLIPQRRLGGLGDSDPP  >CYP135B1(MAF_05750)  MSGTSSMGLPPGPRLSGSVQAVLMLRHGLRFLTACQRRYGSVFTLHVAGFGHMVYLSDPA AIKTVFAGNPSVFHAGEANSMLAGLLGDSSLLLIDDDVHRDRRRLMSPPFHRDAVARQAG PIAEIAAANIAGWPMAKAFAVAPKMSEITLEVILRTVIGASDPVRLAALRKVMPRLLNVG PWATLALANPSLLNNRLWSRLRRRIEEADALLYAEIADRRADPDLAARTDTLAMLVRAAD EDGRTMTERELRDQLITLLVAGHDTTATGLSWALERLTRHPVTLAKAVQAADASAAGDPA GDEYLDAVAKETLRIRPVVYDVGRVLTEAVEVAGYRLPAGVMVVPAIGLVHASAQLYPDP ERFDPDRMVGATLSPTTWLPFGGGNRRCLGATFAMVEMRVVLREILRRVELSTTTTSGER PKLKHVIMVPHRGARIRVRATRDVSATSQATAQGAGCPAARGGGPSRAVGSQ  >CYP51B1(MAF_07760)  MSAVALPRVSGGHDEHGHLEEFRTDPIGLMQRVRDECGDVGTFQLAGKQVVLLSGSHANE FFFRAGDDDLDQAKAYPFMTPIFGEGVVFDASPERRKEMLHNAALRGEQMKGHAATIEDQ VRRMIADWGEAGEIDLLDFFAELTIYTSSACLIGKKFRDQLDGRFAKLYHELERGTDPLA YVDPYLPIESFRRRDEARNGLVALVADIMNGRIANPPTDKSDRDMLDVLIAVKAETGTPR FSADEITGMFISMMFAGHHTSSGTASWTLIELMRHRDAYAAVIDELDELYGDGRSVSFHA LRQIPQLENVLKETLRLHPPLIILMRVAKGEFEVQGHRIHEGDLVAASPAISNRIPEDFP DPHDFVPARYEQPRQEDLLNRWTWIPFGAGRHRCVGAAFAIMQIKAIFSVLLREYEFEMA QPPESYRNDHSKMVVQLAQPACVRYRRRTGV  >CYP123A1(MAF_07780)  MTVRVGDPELVLDPYDYDFHEDPYPYYRRLRDEAPLYRNEERNFWAVSRHHDVLQGFRDS TALSNAYGVSLDPSSRTSEAYRVMSMLAMDDPAHLRMRTLVSKGFTPRRIRELEPQVLEL ARIHLDSALQTESFDFVAEFAGKLPMDVISELIGVPDTDRARIRALADAVLHREDGVADV PPPAMAASIELMRYYADLIAEFRRRPANNLTSALLAAELDGDRLSDQEIMAFLFLMVIAG NETTTKLLANAVYWAAHHPGQLARVFADHSRIPMWVEETLRYDTSSQILARTVAHDLTLY DTTIPEGEVLLLLPGSANRDDRVFDDPDDYRIGREIGCKLVSFGSGAHFCLGAHLARMEA RVALGALLRRIRNYEVDDDNVVRVHSSNVRGFAHLPISVQAR  >CYP126A1(MAF_07900)  MTTAAGLSGIDLTDLDNFADGFPHHLFAIHRREAPVYWHRPTEHTPDGEGFWSVATYAET LEVLRDPVTYSSVTGGQRRFGGTVLQDLPVAGQVLNMMDDPRHTRIRRLVSSGLTPRMIR RVEDDLRRRARGLLDGVEPGAPFDFVVEIAAELPMQMICILLGVPETDRHWLFEAVEPGF DFRGSRRATMPRLNVEDAGSRLYTYALELIAGKRAEPADDMLSVVANATIDDPDAPALSD AELYLFFHLLFSAGAETTRNSIAGGLLALAENPDQLQTLRSDFELLPTAIEEIVRWTSPS PSKRRTASRAVSLGGQPIEAGQKVVVWEGSANRDPSVFDRADEFDITRKPNPHLGFGQGV HYCLGANLARLELRVLFEELLSRFGSVRVVEPAEWTRSNRHTGIRHLVVELRGG  >CYP130A1(MAF_12750)  MTSVMSHEFQLATAETWPNPWPMYRALRDHDPVHHVVPPQRPEYDYYVLSRHADVWSAAR DHQTFSSAQGLTVNYGELEMIGLHDTPPMVMQDPPVHTEFRKLVSRGFTPRQVETVEPTV RKFVVERLEKLRANGGGDIVTELFKPLPSMVVAHYLGVPEEDWTQFDGWTQAIVAANAVD GATTGALDAVGSMMAYFTGLIERRRTEPADDAISHLVAAGVGADGDTAGTLSILAFTFTM VTGGNDTVTGMLGGSMPLLHRRPDQRRLLLDDPEGIPDAVEELLRLTSPVQGLARTTTRD VTIGDTTIPAGRRVLLLYGSANRDERQYGPDAAELDVTRCPRNILTFSHGAHHCLGAAAA RMQCRVALTELLARCPDFEVAESRIVWSGGSYVRRPLSVPFRVTS  >CYP132A1(MAF_14160)  MATATTQRPLKGPAKRMSTWTMTREAITIGFDAGDGFLGRLRGSDITRFRCAGRRFVSIS HPDYVDHVLHEARLKYVKSDEYGPIRATAGLNLLTDEGDSWARHRGALNSTFARRHLRGL VGLMIDPIADVTAALVPGAQFDMHQSMVETTLRVVANALFSQDFGPLVQSMHDLATRGLR RAEKLERLGLWGLMPRTVYDTLIWCIYSGVHLPPPLREMQEITLTLDRAINSVIDRRLAE PTNSADLLNVLLSADGGIWPRQRVRDEALTFMLAGHETTANAMSWFWYLMALNPQARDHM LTELDDVLGMRRPTADDLGKLAWTTACLQESQRYFSSVWIIAREAVDDDIIDGHRIRRGT TVVIPIHHIHHDPRWWPDPDRFDPGRFLRCPTDRPRCAYLPFGGGRRICIGQSFALMEMV LMAAIMSQHFTFDLAPGYHVELEATLTLRPKHGVHVIGRRR  >CYP139A1(MAF_16830)  MRYPLGEALLALYRWRGPLINAGVGGHGYTYLLGAEANRFVFANADAFSWSQTFESLVPV DGPTALIVSDGADHRRRRSVVAPGLRHHHVQRYVATMVSNIDTVIDGWQPGQRLDIYQEL RSAVRRSTAESLFGQRLAVHSDFLGEQLQPLLDLTRRPPQVMRLQQRVNSPGWRRAMAAR KRIDDLIDAQIADARTAPRPDDHMLTTLISGCSEEGTTLSDNEIRDSIVSLITAGYETTS GALAWAIYALLTVPGTWESAASEVARVLGGRVPAADDLSALTYLNGVVHETLRLYSPGVI SARRVLRDLWFDGHRIRAGRLLIFSAYVTHRLPEIWPEPTEFRPLRWDPNAADYRKPAPH EFIPFSGGLHRCIGAVMATTEMTVILARLVARAMLQLPAQRTHRIRAANFAALRPWPGLT VEIRKSAPAQ  >CYP144A1(MAF_17990)  MRRSPKGSPGAVLDLQRRVDQAVSADHAELMTIAKDANTFFGAESVQDPYPLYERMRAAG SVHRIANSDFYAVCGWDAVNEAIGRPEDFSSNLTATMTYTAEGTAKPFEMDPLGGPTHVL ATADDPAHAVHRKLVLRHLAAKRIRVMEQFTVQAADRLWVDGMQDGCIEWMGAMANRLPM MVVAELIGLPDPDIAQLVKWGYAATQLLEGLVENDQLVAAGVALMELSGYIFEQFDRAAA DPRDNLLGELATACASGELDTLTAQVMMVTLFAAGGESTAALLGSAVWILATRPDIQQQV RANPELLGAFIEETLRYEPPFRGHYRHVRNATTLDGTELPADSHLLLLWGAANRDPAQFE APGEFRLDRAGGKGHISFGKGAHFCVGAALARLEARIVLRLLLDRTSVIEAADVGGWLPS ILVRRIERLELAVQ  >CYP143A1(MAF_18070)  MTTPGEDHAGSFYLPRLEYSTLPMAVDRGVGWKTLRDAGPVVFMNGWYYLTRREDVLAAL RNPKVFSSRKALQPPGNPLPVVPLAFDPPEHTRYRRILQPYFSPAALSKALPSLRRHTVA MIDAIAGRGECEAMADLANLFPFQLFLVLYGLPLEDRDRLIGWKDAVIAMSDRPHPTEAD VAAARELLEYLTAMVAERRRNPGPDVLSQVQIGEDPLSEIEVLGLSHLLILAGLDTVTAA VGFSLLELARRPQLRAMLRDNPKQIRVFIEEIVRLEPSAPVAPRVTTEPVTVGGMTLPAG SPVRLCMAAVNRDGSDAMSTDELVMDGKVHRHWGFGGGPHRCLGSHLARLELTLLVGEWL NQIPDFELAPDYAPEIRFPSKSFALKNLPLRWS  >CYP140A1(MAF_19020)  MKDKLHWLAMHGVIRGIAAIGIRRGDLQARLIADPAVATDPVPFYDEVRSHGALVRNRAN YLTVDHRLAHDLLRSDDFRVVSFGENLPPPLRWLERRTRGDQLHPLREPSLLAVEPPDHT RYRKTVSAVFTSRAVSALRDLVEQTAINLLDRFAEQPGIVDVVGRYCSQLPIVVISEILG VPEHDRPRVLEFGELAAPSLDIGIPWRQYLRVQQGIRGFDCWLEGHLQQLRHAPGDDLMS QLIQIAESGDNETQLDETELRAIAGLVLVAGFETTVNLLGNGIRMLLDTPEHLATLRQHP ELWPNTVEEILRLDSPVQLTARVACRDVEVAGVRIKRGEVVVIYLAAANRDPAVFPDPHR FDIERPNAGRHLAFSTGRHFCLGAALARAEGEVGLRTFFDRFPDVRAAGAGSRRDTRVLR GWSTLPVTLGPARSMVSP  >CYP124A1(MAF_22760)  MGLNTAIATRVNGTPPPEVPIADIELGSLDFWALDDDVRDGAFATLRREAPISFWPTIEL PGFVAGNGHWALTKYDDVFYASRHPDIFSSYPNITINDQTPELAEYFGSMIVLDDPRHQR LRSIVSRAFTPKVVARIEAAVRDRAHRLVSSMIANNPDRQADLVSELAGPLPLQIICDMM GIPKADHQRIFHWTNVILGFGDPDLATDFDEFMQVSADIGAYATALAEDRRVNHHDDLTS SLVEAEVDGERLSSREIASFFILLVVAGNETTRNAITHGVLALSRYPEQRDRWWSDFDGL APTAVEEIVRWASPVVYMRRTLTQDIELRGTKMAAGDKVSLWYCSANRDESKFADPWTFD LARNPNPHLGFGGGGAHFCLGANLARREIRVAFDELRRQMPDVVATEEPARLLSQFIHGI KTLPVTWS  >CYP128A1(MAF_22780)  MTATQSPPEPAPDRVRLAGCPLAGTPDVGLTAQDATTALGVPTRRRASSGGIPVATSMWR DAQTVRTYGPAVAKALALRVAGKARSRLTGRHCRKFMQLTDFDPFDPAIAADPYPHYREL LAGERVQYNPKRDVYILSRYADVREAARNHDTLSSARGVTFSRGWLPFLPTSDPPAHTRM RKQLAPGMARGALETWRPMVDQLARELVGGLLTQTPADVVSTVAAPMPMRAITSVLGVDG PDEAAFCRLSNQAVRITDVALSASGLISLVQGFAGFRRLRALFTHRRDNGLLRECTVLGK LATHAEQGRLSDDELFFFAVLLLVAGYESTAHMISTLFLTLADYPDQLTLLAQQPDLIPS AIEEHLRFISPIQNICRTTRVDYSVGQAVIPAGSLVLLAWGAANRDPRQYEDPDVFRADR NPVGHLAFGSGIHLCPGTQLARMEGQAILREIVANIDRIEVVEPPTWTTNANLRGLTRLR VAVTPRVAP  >CYP136A1(MAF_30660)  MATIHPPAYLLDQAKRRFTPSFNNFPGMSLVEHMLLNTKFPEKKLAEPPPGSGLKPVVGD AGLPILGHMIEMLRGGPDYLMFLYKTKGPVVFGDSAVLPGVAALGPDAAQVIYSNRNKDY SQQGWVPVIGPFFHRGLMLLDFEEHMFHRRIMQEAFVRSRLAGYLEQMDRVVSRVVADDW VVNDARFLVYPAMKALTLDIASMVFMGHEPGTDHELVTKVNKAFTITTRAGNAVIRTSVP PFTWWRGLRARELLENYFTARVKERREASGNDLLTVLCQTEDDDGNRFSDADIVNHMIFL MMAAHDTSTSTATTMAYQLAAHPEWQQRCRDESDRHGDGPLDIESLEQLESLDLVMNESI RLVTPVQWAMRQTVRDTELLGYYLPKGTNVIAYPGMNHRLPEIWTDPLTFDPERFTEPRN EHKRHRYAFTPFGGGVHKCIGMVFGQLEIKTILHRLLRRYRLELSRPDYQPRWDYSAMPI PMDGMPIVLRPR  >CYP125A1(MAF_35570)  MSWNHQSVEIAVRRTTVPSPNLPPGFDFTDPAIYAERLPVAEFAELRSAAPIWWNGQDPG KGGGFHDGGFWAITKLNDVKEISRHSDVFSSYENGVIPRFKNDIAREDIEVQRFVMLNMD APHHTRLRKIISRGFTPRAVGRLHDELQERAQKIAAEAAAAGSGDFVEQVSCELPLQAIA GLLGVPQEDRGKLFHWSNEMTGNEDPEYAHIDPKASSAELIGYAMKMAEEKAKNPADDIV TQLIQADIDGEKLSDDEFGFFVVMLAVAGNETTRNSITQGMMAFAEHPDQWELYKKVRPE TAADEIVRWATPVTAFQRTALRDYELSGVQIKKGQRVVMFYRSANFDEEVFQDPFTFNIL RNPNPHVGFGGTGAHYCIGANLARMTINLIFNAVADHMPDLKPISAPERLRSGWLNGIKH WQVDYTGRCPVAH  >CYP137A1(MAF_36930)  MVLRSLASPAALTDPKRCASVVGVAAFAVRREHAPDALGGPPGLPAPRGFRAAFAAAYAV AYLAGGERRMLRLIRRYGPIMTMPILSLGDVAIVSDSALAKEVFTAPTDVLLGGEGVGPA AAIYGSGSMFVQEEPEHLRRRKLLTPPLHGAALDRYVPIIENSTRAAMHTWPVDRPFAML TVARSLMLDVIIKVIFGVDDPEEVRRLGRPFERLLNLGVSEQLTVRYALRRLGALRVWPA RARANTEIDDVVMALIAQRRADPRLGERHDVLSLLVSARGESGEQLSDSEIRDDLITLVL AGHETTATTLAWAFDLLLHHPDALRRVRAEAVGGGEAFTTAVINETLRVRPPAPLTARVA AQPLTIGGYRVEAGTRIVVHIIAINRSAEVYEHPHEFRPERFLGTRPQTYAWVPFGGGVK RCLGANFSMRELITVLHVLLREGEFTAVDDEPERIVRRSIMLVPRRGTRVRFRPAR |
| ***Mycobacterium tuberculosis* C** |
| Database: TB; P450 count: 20; Families: 19; Subfamilies:20 |
| >CYP125A1(TBCG_03474.1)  MSWNHQSVEIAVRRTTVPSPNLPPGFDFTDPAIYAERLPVAEFAELRSAAPIWWNGQDPG  KGGGFHDGGFWAITKLNDVKEISRHSDVFSSYENGVIPRFKNDIAREDIEVQRFVMLNMD  APHHTRLRKIISRGFTPRAVGRLHDELQERAQKIAAEAAAAGSGDFVEQVSCELPLQAIA  GLLGVPQEDRGKLFHWSNEMTGNEDPEYAHIDPKASSAELIGYAMKMAEEKAKNPADDIV  TQLIQADIDGEKLSDDEFGFFVVMLAVAGNETTRNSITQGMMAFAEHPDQWELYKKVRPE  TAADEIVRWATPVTAFQRTALRDYELSGVQIKKGQRVVMFYRSANFDEEVFQDPFTFNIL  RNPNPHVGFGGTGAHYCIGANLARMTINLIFNAVADHMPDLKPISAPERLRSGWLNGIKH  WQVDYTGRCPVAH*  >CYP142A1(TBCG_03448.1)  MTEAPDVDLADGNFYASREARAAYRWMRANQPVFRDRNGLAAASTYQAVIDAERQPELFS  NAGGIRPDQPALPMMIDMDDPAHLLRRKLVNAGFTRKRVKDKEASIAALCDTLIDAVCER  GECDFVRDLAAPLPMAVIGDMLGVRPEQRDMFLRWSDDLVTFLSSHVSQEDFQITMDAFA  AYNDFTRATIAARRADPTDDLVSVLVSSEVDGERLSDDELVMETLLILIGGDETTRHTLS  GGTEQLLRNRDQWDLLQRDPSLLPGAIEEMLRWTAPVKNMCRVLTADTEFHGTALCAGEK  MMLLFESANFDEAVFCEPEKFDVQRNPNSHLAFGFGTHFCLGNQLARLELSLMTERVLRR  LPDLRLVADDSVLPLRPANFVSGLESMPVVFTPSPPLG*  >CYP137A1(TBCG_03609.1)  MVLRSLASPAALTDPKRCASVVGVAAFAVRREHAPDALGGPPGLPAPRGFRAAFAAAYAV  AYLAGGERRMLRLIRRYGPIMTMPILSLGDVAIVSDSALAKEVFTAPTDVLLGGEGVGPA  AAIYGSGSMFVQEEPEHLRRRKLLTPPLHGAALDRYVPIIENSTRAAMHTWPVDRPFAML  TVARSLMLDVIVKVIFGVDDPEEVRRLGRPFERLLNLGVSEQLTVRYALRRLGALRVWPA  RARANTEIDDVVMALIAQRRADPRLGERHDVLSLLVSARGESGEQLSDSEIRDDLITLVL  AGHETTATTLAWAFDLLLHHPDALRRVRAEAVGGGEAFTTAVINETLRVRPPAPLTARVA  AQPLTIGGYRVEAGTRIVVHIIAINRSAEVYEHPHEFRPERFLGTRPQTYAWVPFGGGVK  RCLGANFSMRELITVLHVLLREGEFTAVDDEPERIVRRSIMLVPRRGTRVRFRPAR*  >CYP138A1(TBCG_00135.1)  MSEVVTAAPAPPVVRLPPAVRGPKLFQGLAFVVSRRRLLGRFVRRYGKAFTANILMYGRV  VVVADPQLARQVFTSSPEELGNIQPNLSRMFGSGSVFALDGDDHRRRRRLLAPPFHGKSM  KNYETIIEEETLRETANWPQGQAFATLPSMMHITLNAILRAIFGAGGSELDELRRLIPPW  VTLGSRLAALPKPKRDYGRLSPWGRLAEWRRQYDTVIDKLIEAERADPNFADRTDVLALM  LRSTYDDGSIMSRKDIGDELLTLLAAGHETTAATLGWAFERLSRHPDVLAALVEEVDNGG  HELRQAAILEVQRARTVIDFAARRVNPPVYQLGEWVIPRGYSIIINIAQIHGDPDVFPQP  DRFDPQRYIGSKPSPFAWIPFGGGTRRCVGAAFANMEMDVVLRTVLRHFTLETTTAAGER  SHGRGVAFTPKDGGRVVMRRR*  >CYP130A1(TBCG_01237.1)  MTSVMSHEFQLATAETWPNPWPMYRALRDHDPVHHVVPPQRPEYDYYVLSRHADVWSAAR  DHQTFSSAQGLTVNYGELEMIGLHDTPPMVMQDPPVHTEFRKLVSRGFTPRQVETVEPTV  RKFVVERLEKLRANGGGDIVTELFKPLPSMVVAHYLGVPEEDWTQFDGWTQAIVAANAVD  GATTGALDAVGSMMAYFTGLIERRRTEPADDAISHLVAAGVGADGDTAGTLSILAFTFTM  VTGGNDTVTGMLGGSMPLLHRRPDQRRLLLDDPEGIPDAVEELLRLTSPVQGLARTTTRD  VTIGDTTIPAGRRVLLLYGSANRDERQYGPDAAELDVTRCPRNILTFSHGAHHCLGAAAA  RMQCRVALTELLARCPDFEVAESRIVWSGGSYVRRPLSVPFRVTS*  >CYP126A1(TBCG_00769.1)  MTTAAGLSGIDLTDLDNFADGFPHHLFAIHRREAPVYWHRPTEHTPDGEGFWSVATYAET  LEVLRDPVTYSSVTGGQRRFGGTVLQDLPVAGQVLNMMDDPRHTRIRRLVSSGLTPRMIR  RVEDDLRRRARGLLDGVEPGAPFDFVVEIAAELPMQMICILLGVPETDRHWLFEAVEPGF  DFRGSRRATMPRLNVEDAGSRLYTYALELIAGKRAEPADDMLSVVANATIDDPDAPALSD  AELYLFFHLLFSAGAETTRNSIAGGLLALAENPDQLQTLRSDFELLPTAIEEIVRWTSPS  PSKRRTASRAVSLGGQPIEAGQKVVVWEGSANRDPSVFDRADEFDITRKPNPHLGFGQGV  HYCLGANLARLELRVLFEELLSRFGSVRVVEPAEWTRSNRHTGIRHLVVELRGG*  >CYP123A1(TBCG_00757.1)  MTVRVGDPELVLDPYDYDFHEDPYPYYRRLRDEAPLYRNEERNFWAVSRHHDVLQGFRDS  TALSNAYGVSLDPSSRTSEAYRVMSMLAMDDPAHLRMRTLVSKGFTPRRIRELEPQVLEL  ARIHLDSALQTESFDFVAEFAGKLPMDVISELIGVPDTDRARIRALADAVLHREDGVADV  PPPAMAASIELMRYYADLIAEFRRRPANNLTSALLAAELDGDRLSDQEIMAFLFLMVIAG  NETTTKLLANAVYWAAHHPGQLARVFADHSRIPMWVEETLRYDTSSQILARTVAHDLTLY  DTTIPEGEVLLLLPGSANRDDRVFDDPDDYRIGREIGCKLVSFGSGAHFCLGAHLARMEA  RVALGALLRRIRNYEVDDDNVVRVHSSNVRGFAHLPISVQAR*  >CYP51B1(TBCG_00755.1)  MSAVALPRVSGGHDEHGHLEEFRTDPIGLMQRVRDECGDVGTFQLAGKQVVLLSGSHANE  FFFRAGDDDLDQAKAYPFMTPIFGEGVVFDASPERRKEMLHNAALRGEQMKGHAATIEDQ  VRRMIADWGEAGEIDLLDFFAELTIYTSSACLIGKKFRDQLDGRFAKLYHELERGTDPLA  YVDPYLPIESFRRRDEARNGLVALVADIMNGRIANPPTDKSDRDMLDVLIAVKAETGTPR  FSADEITGMFISMMFAGHHTSSGTASWTLIELMRHRDAYAAVIDELDELYGDGRSVSFHA  LRQIPQLENVLKETLRLHPPLIILMRVAKGEFEVQGHRIHEGDLVAASPAISNRIPEDFP  DPHDFVPARYEQPRQEDLLNRWTWIPFGAGRHRCVGAAFAIMQIKAIFSVLLREYEFEMA  QPPESYRNDHSKMVVQLAQPACVRYRRRTGV*  >CYP140A1(TBCG_01832.1)  MKDKLHWLAMHGVIRGIAAIGIRRGDLQARLIADPAVATDPVPFYDEVRSHGALVRNRAN  YLTVDHRLAHDLLRSDDFRVVSFGENLPPPLRWLERRTRGDQLHPLREPSLLAVEPPDHT  RYRKTVSAVFTSRAVSALRDLVEQTAINLLDRFAEQPGIVDVVGRYCSQLPIVVISEILG  VPEHDRPRVLEFGELAAPSLDIGIPWRQYLRVQQGIRGFDCWLEGHLQQLRHAPGDDLMS  QLIQIAESGDNETQLDETELRAIAGLVLVAGFETTVNLLGNGIRMLLDTPEHLATLRQHP  ELWPNTVEEILRLDSPVQLTARVACRDVEVAGVRIKRGEVVVIYLAAANRDPAVFPDPHR  FDIERPNAGRHLAFSTGRHFCLGAALARAEGEVGLRTFFDRFPDVRAAGAGSRRDTRVLR  GWSTLPVTLGPARSMVSP*  >CYP121A1(TBCG_02221.1)  MTATVLLEVPFSARGDRIPDAVAELRTREPIRKVRTITGAEAWLVSSYALCTQVLEDRRF  SMKETAAAGAPRLNALTVPPEVVNNMGNIADAGLRKAVMKAITPKAPGLEQFLRDTANSL  LDNLITEGAPADLRNDFADPLATALHCKVLGIPQEDGPKLFRSLSIAFMSSADPIPAAKI  NWDRDIEYMAGILENPNITTGLMGELSRLRKDPAYSHVSDELFATIGVTFFGAGVISTGS  FLTTALISLIQRPQLRNLLHEKPELIPAGVEELLRINLSFADGLPRLATADIQVGDVLVR  KGELVLVLLEGANFDPEHFPNPGSIELDRPNPTSHLAFGRGQHFCPGSALGRRHAQIGIE  ALLKKMPGVDLAVPIDQLVWRTRFQRRIPERLPVLW*  >CYP128A1(TBCG_02215.1)  MTATQSPPEPAPDRVRLAGCPLAGTPDVGLTAQDATTALGVPTRRRASSGGIPVATSMWR  DAQTVRTYGPAVAKALALRVAGKARSRLTGRHCRKFMQLTDFDPFDPAIAADPYPHYREL  LAGERVQYNPKRDVYILSRYADVREAARNHDTLSSARGVTFSRGWLPFLPTSDPPAHTRM  RKQLAPGMARGALETWRPMVDQLARELVGGLLTQTPADVVSTVAAPMPMRAITSVLGVDG  PDEAAFCRLSNQAVRITDVALSASGLISLVQGFAGFRRLRALFTHRRDNGLLRECTVLGK  LATHAEQGRLSDDELFFFAVLLLVAGYESTAHMISTLFLTLADYPDQLTLLAQQPDLIPS  AIEEHLRFISPIQNICRTTRVDYSVGQAVIPAGSLVLLAWGAANRDPRQYEDPDVFRADR  NPVGHLAFGSGIHLCPGTQLARMEGQAILREIVANIDRIEVVEPPTWTTNANLRGLTRLR  VAVTPRVAP*  >CYP124A1(TBCG_02213.1)  MGLNTAIATRVNGTPPPEVPIADIELGSLDFWALDDDVRDGAFATLRREAPISFWPTIEL  PGFVAGNGHWALTKYDDVFYASRHPDIFSSYPNITINDQTPELAEYFGSMIVLDDPRHQR  LRSIVSRAFTPKVVARIEAAVRDRAHRLVSSMIANNPDRQADLVSELAGPLPLQIICDMM  GIPKADHQRIFHWTNVILGFGDPDLATDFDEFMQVSADIGAYATALAEDRRVNHHDDLTS  SLVEAEVDGERLSSREIASFFILLVVAGNETTRNAITHGVLALSRYPEQRDRWWSDFDGL  APTAVEEIVRWASPVVYMRRTLTQDIELRGTKMAAGDKVSLWYCSANRDESKFADPWTFD  LARNPNPHLGFGGGGAHFCLGANLARREIRVAFDELRRQMPDVVATEEPARLLSQFIHGI  KTLPVTWS*  >CYP143A1(TBCG_01740.1)  MVGARPRAIMARSAPLGYVCDLRQERRHERLERMTTPGEDHAGSFYLPRLEYSTLPMAVD  RGVGWKTLRDAGPVVFMNGWYYLTRREDVLAALRNPKVFSSRKALQPPGNPLPVVPLAFD  PPEHTRYRRILQPYFSPAALSKALPSLRRHTVAMIDAIAGRGECEAMADLANLFPFQLFL  VLYGLPLEDRDRLIGWKDAVIAMSDRPHPTEADVAAARELLEYLTAMVAERRRNPGPDVL  SQVQIGEDPLSEIEVLGLSHLLILAGLDTVTAAVGFSLLELARRPQLRAMLRDNPKQIRV  FIEEIVRLEPSAPVAPRVTTEPVTVGGMTLPAGSPVRLCMAAVNRDGSDAMSTDELVMDG  KVHRHWGFGGGPHRCLGSHLARLELTLLVGEWLNQIPDFELAPDYAPEIRFPSKSFALKN  LPLRWS*  >CYP144A1(TBCG_01733.1)(truncated)  MRRSPKGSPGAVLDLQRRVTRRYPPITLN**QLPRMPTRSLVPNPCRTPTRCMSACAPQA  RSTGSLTRTSMPCAVGTLSMRPSVVRRTSPRI*PPR*PIRPRAPLNRSRWTHSADPHTCW  PPPTILPTPCTASSCCVTWRPSGSALWSSSPYRLPTGCGSTACRMGASNGWAPWPIAYR*  WS*LSSSACPTPTSPSW*SGDTRPLSYSKGWSKTISSSPRVWR*WSSAVTSSSSLTVPRP  IRGTICSVSLPPPAHRGSWTLSPPRS*WSPCSPPAASPRRRCWAARYGYWRHVPISSNRC  ARTPSCWERLSKRRCVTSRHFAATTATCETPPPWTARNCPRIRTCCCCGARPTAIQPSSR  HPASSVLTVQEAKATSVSEKGPTSVSALHWHAWRLESSCVCCSIAPR*LRQPMSAGGCPV  SWCAASSG*S*LYN  >CYP135B1(TBCG_00563.1)  MSGTSSMGLPPGPRLSGSVQAVLMLRHGLRFLTACQRRYGSVFTLHVAGFGHMVYLSDPA  AIKTVFAGNPSVFHAGEANSMLAGLLGDSSLLLIDDDVHRDRRRLMSPPFHRDAVARQAG  PIAEIAAANIAGWPMAKAFAVAPKMSEITLEVILRTVIGASDPVRLAALRKVMPRLLNVG  PWATLALANPSLLNNRLWSRLRRRIEEADALLYAEIADRRADPDLAARTDTLAMLVRAAD  EDGRTMTERELRDQLITLLVAGHDTTATGLSWALERLTRHPVTLAKAVQAADASAAGDPA  GDEYLDAVAKETLRIRPVVYDVGRVLTEAVEVAGYRLPAGVMVVPAIGLVHASAQLYPDP  ERFDPDRMVGATLSPTTWLPFGGGNRRCLGATFAMVEMRVVLREILRRVELSTTTTSGER  PKLKHVIMVPHRGARIRVRATRDVSATSQATAQGAGCPAARGGGPSRAVGSQ*  >CYP139A1(TBCG_01620.1)  MRTYRTVRYPLGEALLALYRWRGPLINAGVGGHGYTYLLGAEANRFVFANADAFSWSQTF  ESLVPVDGPTALIVSDGADHRRRRSVVAPGLRHHHVQRYVATMVSNIDTVIDGWQPGQRL  DIYQELRSAVRRSTAESLFGQRLAVHSDFLGEQLQPLLDLTRRPPQVMRLQQRVNSPGWR  RAMAARKRIDDLIDAQIADARTAPRPDDHMLTTLISGCSEEGTTLSDNEIRDSIVSLITA  GYETTSGALAWAIYALLTVPGTWESAASEVARVLGGRVPAADDLSALTYLNGVVHETLRL  YSPGVISARRVLRDLWFDGHRIRAGRLLIFSAYVTHRLPEIWPEPTEFRPLRWDPNAADY  RKPAPHEFIPFSGGLHRCIGAVMATTEMTVILARLVARAMLQLPAQRTHRIRAANFAALR  PWPGLTVEIRKSAPAQ*  >CYP135A1(TBCG_00322.1)  MASTLTTGLPPGPRLPRYLQSVLYLRFREWFLPAMHRKYGDVFSLRVPPYADNLVVYTRP  EHIKEIFAADPRSLHAGEGNHILGFVMGEHSVLMTDEAEHARMRSLLMPAFTRAALRGYR  DMIASVAREHITRWRPHATINSLDHMNALTLDIILRVVFGVTDPKVKAELTSRLQQIINI  HPAILAGVPYPSLKRMNPWKRFFHNQTKIDEILYREIASRRIDSDLTARTDVLSRLLQTK  DTPTKPLTDAELRDQLITLLLAGHETTAAALSWTLWELAHAPEIQSQVVWAAVGGDDGFL  EAVLKEGMRRHTVIASTARKVTAPAEIGGWRLPAGTVVNTSILLAHASEVSHPKPTEFRP  SRFLDGSVAPNTWLPFGGGVRRCLGFGFALTEGAVILQEIFRRFTITAAGPSKGETPLVR  NITTVPKHGAHLRLIPQRRLGGLGDSDPP*  >CYP132A1(TBCG_01374.1)  MATATTQRPLKGPAKRMSTWTMTREAITIGFDAGDGFLGRLRGSDITRFRCAGRRFVSIS  HPDYVDHVLHEARLKYVKSDEYGPIRATAGLNLLTDEGDSWARHRGALNSTFARRHLRGL  VGLMIDPIADVTAALVPGAQFDMHQSMVETTLRVVANALFSQDFGPLVQSMHDLATRGLR  RAEKLERLGLWGLMPRTVYDTLIWCIYSGVHLPPPLREMQEITLTLDRAINSVIDRRLAE  PTNSADLLNVLLSADGGIWPRQRVRDEALTFMLAGHETTANAMSWFWYLMALNPQARDHM  LTELDDVLGMRRPTADDLGKLAWTTACLQESQRYFSSVWIIAREAVDDDIIDGHRIRRGT  TVVIPIHHIHHDPRWWPDPDRFDPGRFLRCPTDRPRCAYLPFGGGRRICIGQSFALMEMV  LMAAIMSQHFTFDLAPGYHVELEATLTLRPKHGVHVIGRRR*  >CYP141A1(TBCG_03058.1)  MTSTSIPTFPFDRPVPTEPSPMLSELRNSCPVAPIELPSGHTAWLVTRFDDVKGVLSDKR  FSCRAAAHPSSPPFVPFVQLCPSLLSIDGPQHTAARRLLAQGLNPGFIARMRPVVQQIVD  NALDDLAAAEPPVDFQEIVSVPIGEQLMAKLLGVEPETVHELAAHVDAAMSVCEIGDEEV  SRRWSALCTMVIDILHRKLAEPGDDLLSTIAQANRQQSTMTDEQVVGMLLTVVIGGVDTP  IAVITNGLASLLHHRDQYERLVEDPGRVARAVEEIVRFNPATEIEHLRVVTEDVVIAGTA  LSAGSPAFTSITSANRDSDQFLDPDEFDVERNPNEHIAFGYGPHACPASAYSRMCLTTFF  TSLTQRFPQLQLARPFEDLERRGKGLHSVGIKELLVTWPT*  >CYP136A1(TBCG_02995.1)  MATIHPPAYLLDQAKRRFTPSFNNFPGMSLVEHMLLNTKFPEKKLAEPPPGSGLKPVVGD  AGLPILGHMIEMLRGGPDYLMFLYKTKGPVVFGDSAVLPGVAALGPDAAQVIYSNRNKDY  SQQGWVPVIGPFFHRGLMLLDFEEHMFHRRIMQEAFVRSRLAGYLEQMDRVVSRVVADDW  VVNDARFLVYPAMKALTLDIASMVFMGHEPGTDHELVTKVNKAFTITTRAGNAVIRTSVP  PFTWWRGLRARELLENYFTARVKERREASGNDLLTVLCQTEDDDGNRFSDADIVNHMIFL  MMAAHDTSTSTATTMAYQLAAHPEWQQRCRDESDRHGDGPLDIESLEQLESLDLVMNESI  RLVTPVQWAMRQTVRDTELLGYYLPKGTNVIAYPGMNHRLPEIWTDPLTFDPERFTEPRN  EHKRHRYAFTPFGGGVHKCIGMVFDQLEIKTILHRLLRRYRLELSRPDYQPRWDYSAMPI  PMDGMPIVLRPR* |
| ***Mycobacterium tuberculosis* F11** |
| Database: TB; P450 count:20; Families: 19; Subfamilies: 20 |
| >CYP138A1(TBFG_10137.4)  MSEVVTAAPAPPVVRLPPAVRGPKLFQGLAFVVSRRRLLGRFVRRYGKAFTANILMYGRV  VVVADPQLARQVFTSSPEELGNIQPNLSRMFGSGSVFALDGDDHRRRRRLLAPPFHGKSM  KNYETIIEEETLRETANWPQGQAFATLPSMMHITLNAILRAIFGAGGSELDELRRLIPPW  VTLGSRLAALPKPKRDYGRLSPWGRLAEWRRQYDTVIDKLIEAERADPNFADRTDVLALM  LRSTYDDGSIMSRKDIGDELLTLLAAGHETTAATLGWAFERLSRHPDVLAALVEEVDNGG  HELRQAAILEVQRARTVIDFAARRVNPPVYQLGEWVIPRGYSIIINIAQIHGDPDVFPQP  DRFDPQRYIGSKPSPFAWIPFGGGTRRCVGAAFANMEMDVVLRTVLRHFTLETTTAAGER  SHGRGVAFTPKDGGRVVMRRR*  >CYP135A1(TBFG_10332.4)  MASTLTTGLPPGPRLPRYLQSVLYLRFREWFLPAMHRKYGDVFSLRVPPYADNLVVYTRP  EHIKEIFAADPRSLHAGEGNHILGFVMGEHSVLMTDEAEHARMRSLLMPAFTRAALRGYR  DMIASVAREHITRWRPHATINSLDHMNALTLDIILRVVFGVTDPKVKAELTSRLQQIINI  HPAILAGVPYPSLKRMNPWKRFFHNQTKIDEILYREIASRRIDSDLTARTDVLSRLLQTK  DTPTKPLTDAELRDQLITLLLAGHETTAAALSWTLWELAHAPEIQSQVVWAAVGGDDGFL  EAVLKEGMRRHTVIASTARKVTAPAEIGGWRLPAGTVVNTSILLAHASEVSHPKPTEFRP  SRFLDGSVAPNTWLPFGSGVRRCLGFGFALTEGAVILQEIFRRFTITAAGPSKGETPLVR  NITTVPKHGAHLRLIPQRRLGGLGDSDPP*  >CYP135B1(TBFG_10578.4)  MSGTSSMGLPPGPRLSGSVQAVLMLRHGLRFLTACQRRYGSVFTLHVAGFGHMVYLSDPA  AIKTVFAGNPSVFHAGEANSMLAGLLGDSSLLLIDDDVHRDRRRLMSPPFHRDAVARQAG  PIAEIAAANIAGWPMAKAFAVAPKMSEITLEVILRTVIGASDPVRLAALRKVMPRLLNVG  PWATLALANPSLLNNRLWSRLRRRIEEADALLYAEIADRRADPDLAARTDTLAMLVRAAD  EDGRTMTERELRDQLITLLVAGHDTTATGLSWALERLTRHPVTLAKAVQAADASAAGDPA  GDEYLDAVAKETLRIRPVVYDVGRVLTEAVEVAGYRLPAGVMVVPAIGLVHASAQLYPDP  ERFDPDRMVGATLSPTTWLPFGGGNRRCLGATFAMVEMRVVLREILRRVELSTTTTSGER  PKLKHVIMVPHRGARIRVRATRDVSATSQATAQGAGCPAARGGGPSRAVGSQ*  >CYP51B1(TBFG_10779.4)  MSAVALPRVSGGHDEHGHLEEFRTDPIGLMQRVRDECGDVGTFQLAGKQVVLLSGSHANE  FFFRAGDDDLDQAKAYPFMTPIFGEGVVFDASPERRKEMLHNAALRGEQMKGHAATIEDQ  VRRMIADWGEAGEIDLLDFFAELTIYTSSACLIGKKFRDQLDGRFAKLYHELERGTDPLA  YVDPYLPIESFRRRDEARNGLVALVADIMNGRIANPPTDKSDRDMLDVLIAVKAETGTPR  FSADEITGMFISMMFAGHHTSSGTASWTLIELMRHRDAYAAVIDELDELYGDGRSVSFHA  LRQIPQLENVLKETLRLHPPLIILMRVAKGEFEVQGHRIHEGDLVAASPAISNRIPEDFP  DPHDFVPARYEQPRQEDLLNRWTWIPFGAGRHRCVGAAFAIMQIKAIFSVLLREYEFEMA  QPPESYRNDHSKMVVQLAQPACVRYRRRTGV*  >CYP123A1(TBFG_10781.4)  MTVRVGDPELVLDPYDYDFHEDPYPYYRRLRDEAPLYRNEERNFWAVSRHHDVLQGFRDS  TALSNAYGVSLDPSSRTSEAYRVMSMLAMDDPAHLRMRTLVSKGFTPRRIRELEPQVLEL  ARIHLDSALQTESFDFVAEFAGKLPMDVISELIGVPDTDRARIRALADAVLHREDGVADV  PPPAMAASIELMRYYADLIAEFRRRPANNLTSALLAAELDGDRLSDQEIMAFLFLMVIAG  NETTTKLLANAVYWAAHHPGQLARVFADHSRIPMWVEETLRYDTSSQILARTVAHDLTLY  DTTIPEGEVLLLLPGSANRDDRVFDDPDDYRIGREIGCKLVSFGSGAHFCLGAHLARMEA  RVALGALLRRIRNYEVDDDNVVRVHSSNVRGFAHLPISVQAR*  >CYP126A1(TBFG_10793.4)  MTTAAGLSGIDLTDLDNFADGFPHHLFAIHRREAPVYWHRPTEHTPDGEGFWSVATYAET  LEVLRDPVTYSSVTGGQRRFGGTVLQDLPVAGQVLNMMDDPRHTRIRRLVSSGLTPRMIR  RVEDDLRRRARGLLDGVEPGAPFDFVVEIAAELPMQMICILLGVPETDRHWLFEAVEPGF  DFRGSRRATMPRLNVEDAGSRLYTYALELIAGKRAEPADDMLSVVANATIDDPDAPALSD  AELYLFFHLLFSAGAETTRNSIAGGLLALAENPDQLQTLRSDFELLPTAIEEIVRWTSPS  PSKRRTASRAVSLGGQPIEAGQKVVVWEGSANRDPSVFDRADEFDITRKPNPHLGFGQGV  HYCLGANLARLELRVLFEELLSRFGSVRVVEPAEWTRSNRHTGIRHLVVELRGG*  >CYP130A1(TBFG_11282.4)  MTSVMSHEFQLATAETWPNPWPMYRALRDHDPVHHVVPPQRPEYDYYVLSRHADVWSAAR  DHQTFSSAQGLTVNYGELEMIGLHDTPPMVMQDPPVHTEFRKLVSRGFTPRQVETVEPTV  RKFVVERLEKLRANGGGDIVTELFNPLPSMVVAHYLGVPEEDWTQFDGWTQAIVAANAVD  GATTGALDAVGSMMAYFTGLIERRRTEPADDAISHLVAAGVGADGDTAGTLSILAFTFTM  VTGGNDTVTGMLGGSMPLLHRRPDQRRLLLDDPEGIPDAVEELLRLTSPVQGLARTTTRD  VTIGDTTIPAGRRVLLLYGSANRDERQYGPDAAELDVTRCPRNILTFSHGAHHCLGAAAA  RMQCRVALTELLARCPDFEVAESRIVWSGGSYVRRPLSVPFRVTS*  >CYP132A1(TBFG_11423.4)  MATATTQRPLKGPAKRMSTWTMTREAITIGFDAGDGFLGRLRGSDITRFRCAGRRFVSIS  HPDYVDHVLHEARLKYVKSDEYGPIRATAGLNLLTDEGDSWARHRGALNSTFARRHLRGL  VGLMIDPIADVTAALVPGAQFDMHQSMVETTLRVVANALFSQDFGPLVQSMHDLATRGLR  RAEKLERLGLWGLMPRTVYDTLIWCIYSGVHLPPPLREMQEITLTLDRAINSVIDRRLAE  PTNSADLLNVLLSADGGIWPRQRVRDEALTFMLAGHETTANAMSWFWYLMALNPQARDHM  LTELDDVLGMRRPTADDLGKLAWTTACLQESQRYFSSVWIIAREAVDDDIIDGHRIRRGT  TVVIPIHHIHHDPRWWPDPDRFDPGRFLRCPTDRPRCAYLPFGGGRRICIGQSFALMEMV  LMAAIMSQHFTFDLAPGYHVELEATLTLRPKHGVHVIGRRR*  >CYP139A1(TBFG_11684.4)  MRYPLGEALLALYRWRGPLINAGVGGHGYTYLLGAEANRFVFANADAFSWSQTFESLVPV  DGPTALIVSDGADHRRRRSVVAPGLRHHHVQRYVATMVSNIDTVIDGWQPGQRLDIYQEL  RSAVRRSTAESLFGQRLAVHSDFLGEQLQPLLDLTRRPPQVMRLQQRVNSPGWRRAMAAR  KRIDDLIDAQIADARTAPRPDDHMLTTLISGCSEEGTTLSDNEIRDSIVSLITAGYETTS  GALAWAIYALLTVPGTWESAASEVARVLGGRVPAADDLSALTYLNGVVHETLRLYSPGVI  SARRVLRDLWFDGHRIRAGRLLIFSAYVTHRLPEIWPEPTEFRPLRWDPNAADYRKPAPH  EFIPFSGGLHRCIGAVMATTEMTVILARLVARAMLQLPAQRTHRIRAANFAALRPWPGLT  VEIRKSAPAQ*  >CYP144A1(TBFG_11808.4)  MTPSFPRTGVSGHAGAVHSSNLTATMTYTAEGTAKPFEMDPLGGPTHVLATADDPAHAVH  RKLVLRHLAAKRIRVMEQFTVQAADRLWVDGMQDGCIEWMGAMANRLPMMVVAELIGLPD  PDIAQLVKWGYAATQLLEGLVENDQLVAAGVALMELSGYIFEQFDRAAADPRDNLLGELA  TACASGELDTLTAQVMMVTLFAAGGESTAALLGSAVWILATRPDIQQQVRANPELLGAFI  EETLRYEPPFRGHYRHVRNATTLDGTELPADSHLLLLWGAANRDPAQFEAPGEFRLDRAG  GKGHISFGKGAHFCVGAALARLEARIVLRLLLDRTSVIEAADVGGWLPSILVRRIERLEL  AVQ*  >CYP143A1(TBFG_11815.4)  MTTPGEDHAGSFYLPRLEYSTLPMAVDRGVGWKTLRDAGPVVFMNGWYYLTRREDVLAAL  RNPKVFSSRKALQPPGNPLPVVPLAFDPPEHTRYRRILQPYFSPAALSKALPSLRRHTVA  MIDAIAGRGECEAMADLANLFPFQLFLVLYGLPLEDRDRLIGWKDAVIAMSDRPHPTEAD  VAAARELLEYLTAMVAERRRNPGPDVLSQVQIGEDPLSEIEVLGLSHLLILAGLDTVTAA  VGFSLLELARRPQLRAMLRDNPKQIRVFIEEIVRLEPSAPVAPRVTTEPVTVGGMTLPAG  SPVRLCMAAVNRDGSDAMSTDELVMDGKVHRHWGFGGGPHRCLGSHLARLELTLLVGEWL  NQIPDFELAPDYAPEIRFPSKSFALKNLPLRWS*  >CYP140A1(TBFG_11908.4)  MKDKLHWLAMHGVIRGIAAIGIRRGDLQARLIADPAVATDPVPFYDEVRSHGALVRNRAN  YLTVDHRLAHDLLRSDDFRVVSFGENLPPPLRWLERRTRGDQLHPLREPSLLAVEPPDHT  RYRKTVSAVFTSRAVSALRDLVEQTAINLLDRFAEQPGIVDVVGRYCSQLPIVVISEILG  VPEHDRPRVLEFGELAAPSLDIGIPWRQYLRVQQGIRGFDCWLEGHLQQLRHAPGDDLMS  QLIQIAESGDNETQLDETELRAIAGLVLVAGFETTVNLLGNGIRMLLDTPEHLATLRQHP  ELWPNTVEEILRLDSPVQLTARVACRDVEVAGVRIKRGEVVVIYLAAANRDPAVFPDPHR  FDIERPNAGRHLAFSTGRHFCLGAALARAEGEVGLRTFFDRFPDVRAAGAGSRRDTRVLR  GWSTLPVTLGPARSMVSP*  >CYP124A1(TBFG_12293.4)  MGLNTAIATRVNGTPPPEVPIADIELGSLDFWALDDDVRDGAFATLRREAPISFWPTIEL  PGFVAGNGHWALTKYDDVFYASRHPDIFSSYPNITINDQTPELAEYFGSMIVLDDPRHQR  LRSIVSRAFTPKVVARIEAAVRDRAHRLVSSMIANNPDRQADLVSELAGPLPLQIICDMM  GIPKADHQRIFHWTNVILGFGDPDLATDFDEFMQVSADIGAYATALAEDRRVNHHDDLTS  SLVEAEVDGERLSSREIASFFILLVVAGNETTRNAITHGVLALSRYPEQRDRWWSDFDGL  APTAVEEIVRWASPVVYMRRTLTQDIELRGTKMAAGDKVSLWYCSANRDESKFADPWTFD  LARNPNPHLGFGGGGAHFCLGANLARREIRVAFDELRRQMPDVVATEEPARLLSQFIHGI  KTLPVTWS*  >CYP128A1(TBFG_12295.4)  MTATQSPPEPAPDRVRLAGCPLAGTPDVGLTAQDATTALGVPTRRRASSGGIPVATSMWR  DAQTVRTYGPAVAKALALRVAGKARSRLTGRHCRKFMQLTDFDPFDPAIAADPYPHYREL  LAGERVQYNPKRDVYILSRYADVREAARNHDTLSSARGVTFSRGWLPFLPTSDPPAHTRM  RKQLAPGMARGALETWRPMVDQLARELVGGLLTQTPADVVSTVAAPMPMRAITSVLGVDG  PDEAAFCRLSNQAVRITDVALSASGLISLVQGFAGFRRLRALFTHRRDNGLLRECTVLGK  LATHAEQGRLSDDELFFFAVLLLVAGYESTAHMISTLFLTLADYPDQLTLLAQQPDLIPS  AIEEHLRFISPIQNICRTTRVDYSVGQAVIPAGSLVLLAWGAANRDPRQYEDPDVFRADR  NPVGHLAFGSGIHLCPGTQLARMEGQAILREIVANIDRIEVVEPPTWTTNANLRGLTRLR  VAVTPRVAP*  >CYP121A1(TBFG_12301.4)  MTATVLLEVPFSARGDRIPDAVAELRTREPIRKVRTITGAEAWLVSSYALCTQVLEDRRF  SMKETAAAGAPRLNALTVPPEVVNNMGNIADAGLRKAVMKAITPKAPGLEQFLRDTANSL  LDNLITEGAPADLRNDFADPLATALHCKVLGIPQEDGPKLFRSLSIAFMSSADPIPAAKI  NWDRDIEYMAGILENPNITTGLMGELSRLRKDPAYSHVSDELFATIGVTFFGAGVISTGS  FLTTALISLIQRPQLRNLLHEKPELIPAGVEELLRINLSFADGLPRLATADIQVGDVLVR  KGELVLVLLEGANFDPEHFPNPGSIELDRPNPTSHLAFGRGQHFCPGSALGRRHAQIGIE  ALLKKMPGVDLAVPIDQLVWRTRFQRRIPERLPVLW*  >CYP136A1(TBFG_13076.4)  MATIHPPAYLLDQAKRRFTPSFNNFPGMSLVEHMLLNTKFPEKKLAEPPPGSGLKPVVGD  AGLPILGHMIEMLRGGPDYLMFLYKTKGPVVFGDSAVLPGVAALGPDAAQVIYSNRNKDY  SQQGWVPVIGPFFHRGLMLLDFEEHMFHRRIMQEAFVRSRLAGYLEQMDRVVSRVVADDW  VVNDARFLVYPAMKALTLDIASMVFMGHEPGTDHELVTKVNKAFTITTRAGNAVIRTSVP  PFTWWRGLRARELLENYFTARVKERREASGNDLLTVLCQTEDDDGNRFSDADIVNHMIFL  MMAAHDTSTSTATTMAYQLAAHPEWQQRCRDESDRHGDGPLDIESLEQLESLDLVMNESI  RLVTPVQWAMRQTVRDTELLGYYLPKGTNVIAYPGMNHRLPEIWTDPLTFDPERFTEPRN  EHKRHRYAFTPFGGGVHKCIGMVFDQLEIKTILHRLLRRYRLELSRPDYQPRWDYSAMPI  PMDGMPIVLRPR*  >CYP141A1(TBFG_13141.4)  MTSTSIPTFPFDRPVPTEPSPMLSELRNSCPVAPIELPSGHTAWLVTRFDDVKGVLSDKR  FSCRAAAHPSSPPFVPFVQLCPSLLSIDGPQHTAARRLLAQGLNPGFIARMRPVVQQIVD  NALDDLAAAEPPVDFQEIVSVPIGEQLMAKLLGVEPETVHELAAHVDAAMSVCEIGDEEV  SRRWSALCTMVIDILHRKLAEPGDDLLSTIAQANRQQSTMTDEQVVGMLLTVVIGGVDTP  IAVITNGLASLLHHRDQYERLVEDPGRVARAVEEIVRFNPATEIEHLRVVTEDVVIAGTA  LSAGSPAFTSITSANRDSDQFLDPDEFDVERNPNEHIAFGYGPHACPASAYSRMCLTTFF  TSLTQRFPQLQLARPFEDLERRGKGLHSVGIKELLVTWPT*  >CYP142A1(TBFG_13551.4)  MTEAPDVDLADGNFYASREARAAYRWMRANQPVFRDRNGLAAASTYQAVIDAERQPELFS  NAGGIRPDQPALPMMIDMDDPAHLLRRKLVNAGFTRKRVKDKEASIAALCDTLIDAVCER  GECDFVRDLAAPLPMAVIGDMLGVRPEQRDMFLRWSDDLVTFLSSHVSQEDFQITMDAFA  AYNDFTRATIAARRADPTDDLVSVLVSSEVDGERLSDDELVMETLLILIGGDETTRHTLS  GGTEQLLRNRDQWDLLQRDPSLLPGAIEEMLRWTAPVKNMCRVLTADTEFHGTALCAGEK  MMLLFESANFDEAVFCEPEKFDVQRNPNSHLAFGFGTHFCLGNQLARLELSLMTERVLRR  LPDLRLVADDSVLPLRPANFVSGLESMPVVFTPSPPLG*  >CYP125A1(TBFG_13578.4)  MSWNHQSVEIAVRRTTVPSPNLPPGFDFTDPAIYAERLPVAEFAELRSAAPIWWNGQDPG  KGGGFHDGGFWAITKLNDVKEISRHSDVFSSYENGVIPRFKNDIAREDIEVQRFVMLNMD  APHHTRLRKIISRGFTPRAVGRLHDELQERAQKIAAEAAAAGSGDFVEQVSCELPLQAIA  GLLGVPQEDRGKLFHWSNEMTGNEDPEYAHIDPKASSAELIGYAMKMAEEKAKNPADDIV  TQLIQADIDGEKLSDDEFGFFVVMLAVAGNETTRNSITQGMMAFAEHPDQWELYKKVRPE  TAADEIVRWATPVTAFQRTALRDYELSGVQIKKGQRVVMFYRSANFDEEVFQDPFTFNIL  RNPNPHVGFGGTGAHYCIGANLARMTINLIFNAVADHMPDLKPISAPERLRSGWLNGIKH  WQVDYTGRCPVAH*  >CYP137A1(TBFG_13716.4)  MVLRSLASPAALTDPKRCASVVGVAAFAVRREHAPDALGGPPGLPAPRGFRAAFAAAYAV  AYLAGGERRMLRLIRRYGPIMTMPILSLGDVAIVSDSALAKEVFTAPTDVLLGGEGVGPA  AAIYGSGSMFVQEEPEHLRRRKLLTPPLHGAALDRYVPIIENSTRAAMHTWPVDRPFAML  TVARSLMLDVIVKVIFGVDDPEEVRRLGRPFERLLNLGVSEQLTVRYALRRLGALRVWPA  RARANTEIDDVVMALIAQRRADPRLGERHDVLSLLVSARGESGEQLSDSEIRDDLITLVL  AGHETTATTLAWAFDLLLHHPDALRRVRAEAVGGGEAFTTAVINETLRVRPPAPLTARVA  AQPLTIGGYRVEAGTRIVVHIIAINRSAEVYEHPHEFRPERFLGTRPQTYAWVPFGGGVK  RCLGANFSMRELITVLHVLLREGEFTAVDDEPERIVRRSIMLVPRRGTRVRFRPAR* |
| ***Mycobacterium tuberculosis* H37Ra** |
| Database: TB; P450 count: 20; Families: 19; Subfamilies: 20 |
| >CYP140A1(MRA_1891)  MKDKLHWLAMHGVIRGIAAIGIRRGDLQARLIADPAVATDPVPFYDEVRSHGALVRNRAN  YLTVDHRLAHDLLRSDDFRVVSFGENLPPPLRWLERRTRGDQLHPLREPSLLAVEPPDHT  RYRKTVSAVFTSRAVSALRDLVEQTAINLLDRFAEQPGIVDVVGRYCSQLPIVVISEILG  VPEHDRPRVLEFGELAAPSLDIGIPWRQYLRVQQGIRGFDCWLEGHLQQLRHAPGDDLMS  QLIQIAESGDNETQLDETELRAIAGLVLVAGFETTVNLLGNGIRMLLDTPEHLATLRQHP  ELWPNTVEEILRLDSPVQLTARVACRDVEVAGVRIKRGEVVVIYLAAANRDPAVFPDPHR  FDIERPNAGRHLAFSTGRHFCLGAALARAEGEVGLRTFFDRFPDVRAAGAGSRRDTRVLR  GWSTLPVTLGPARSMVSP*  >CYP130A1(MRA_1264)  MTSVMSHEFQLATAETWPNPWPMYRALRDHDPVHHVVPPQRPEYDYYVLSRHADVWSAAR  DHQTFSSAQGLTVNYGELEMIGLHDTPPMVMQDPPVHTEFRKLVSRGFTPRQVETVEPTV  RKFVVERLEKLRANGGGDIVTELFKPLPSMVVAHYLGVPEEDWTQFDGWTQAIVAANAVD  GATTGALDAVGSMMAYFTGLIERRRTEPADDAISHLVAAGVGADGDTAGTLSILAFTFTM  VTGGNDTVTGMLGGSMPLLHRRPDQRRLLLDDPEGIPDAVEELLRLTSPVQGLARTTTRD  VTIGDTTIPAGRRVLLLYGSANRDERQYGPDAAELDVTRCPRNILTFSHGAHHCLGAAAA  RMQCRVALTELLARCPDFEVAESRIVWSGGSYVRRPLSVPFRVTS*  >CYP142A1(MRA_3557)  MTEAPDVDLADGNFYASREARAAYRWMRANQPVFRDRNGLAAASTYQAVIDAERQPELFS  NAGGIRPDQPALPMMIDMDDPAHLLRRKLVNAGFTRKRVKDKEASIAALCDTLIDAVCER  GECDFVRDLAAPLPMAVIGDMLGVRPEQRDMFLRWSDDLVTFLSSHVSQEDFQITMDAFA  AYNDFTRATIAARRADPTDDLVSVLVSSEVDGERLSDDELVMETLLILIGGDETTRHTLS  GGTEQLLRNRDQWDLLQRDPSLLPGAIEEMLRWTAPVKNMCRVLTADTEFHGTALCAGEK  MMLLFESANFDEAVFCEPEKFDVQRNPNSHLAFGFGTHFCLGNQLARLELSLMTERVLRR  LPDLRLVADDSVLPLRPANFVSGLESMPVVFTPSPPLG*  >CYP51B1(MRA_0773)  MSAVALPRVSGGHDEHGHLEEFRTDPIGLMQRVRDECGDVGTFQLAGKQVVLLSGSHANE  FFFRAGDDDLDQAKAYPFMTPIFGEGVVFDASPERRKEMLHNAALRGEQMKGHAATIEDQ  VRRMIADWGEAGEIDLLDFFAELTIYTSSACLIGKKFRDQLDGRFAKLYHELERGTDPLA  YVDPYLPIESFRRRDEARNGLVALVADIMNGRIANPPTDKSDRDMLDVLIAVKAETGTPR  FSADEITGMFISMMFAGHHTSSGTASWTLIELMRHRDAYAAVIDELDELYGDGRSVSFHA  LRQIPQLENVLKETLRLHPPLIILMRVAKGEFEVQGHRIHEGDLVAASPAISNRIPEDFP  DPHDFVPARYEQPRQEDLLNRWTWIPFGAGRHRCVGAAFAIMQIKAIFSVLLREYEFEMA  QPPESYRNDHSKMVVQLAQPACVRYRRRTGV*  >CYP123A1(MRA_0775)  MTVRVGDPELVLDPYDYDFHEDPYPYYRRLRDEAPLYRNEERNFWAVSRHHDVLQGFRDS  TALSNAYGVSLDPSSRTSEAYRVMSMLAMDDPAHLRMRTLVSKGFTPRRIRELEPQVLEL  ARIHLDSALQTESFDFVAEFAGKLPMDVISELIGVPDTDRARIRALADAVLHREDGVADV  PPPAMAASIELMRYYADLIAEFRRRPANNLTSALLAAELDGDRLSDQEIMAFLFLMVIAG  NETTTKLLANAVYWAAHHPGQLARVFADHSRIPMWVEETLRYDTSSQILARTVAHDLTLY  DTTIPEGEVLLLLPGSANRDDRVFDDPDDYRIGREIGCKLVSFGSGAHFCLGAHLARMEA  RVALGALLRRIRNYEVDDDNVVRVHSSNVRGFAHLPISVQAR*  >CYP126A1(MRA_0787)  MTTAAGLSGIDLTDLDNFADGFPHHLFAIHRREAPVYWHRPTEHTPDGEGFWSVATYAET  LEVLRDPVTYSSVTGGQRRFGGTVLQDLPVAGQVLNMMDDPRHTRIRRLVSSGLTPRMIR  RVEDDLRRRARGLLDGVEPGAPFDFVVEIAAELPMQMICILLGVPETDRHWLFEAVEPGF  DFRGSRRATMPRLNVEDAGSRLYTYALELIAGKRAEPADDMLSVVANATIDDPDAPALSD  AELYLFFHLLFSAGAETTRNSIAGGLLALAENPDQLQTLRSDFELLPTAIEEIVRWTSPS  PSKRRTASRAVSLGGQPIEAGQKVVVWEGSANRDPSVFDRADEFDITRKPNPHLGFGQGV  HYCLGANLARLELRVLFEELLSRFGSVRVVEPAEWTRSNRHTGIRHLVVELRGG*  >CYP125A1(MRA_3584)  MSWNHQSVEIAVRRTTVPSPNLPPGFDFTDPAIYAERLPVAEFAELRSAAPIWWNGQDPG  KGGGFHDGGFWAITKLNDVKEISRHSDVFSSYENGVIPRFKNDIAREDIEVQRFVMLNMD  APHHTRLRKIISRGFTPRAVGRLHDELQERAQKIAAEAAAAGSGDFVEQVSCELPLQAIA  GLLGVPQEDRGKLFHWSNEMTGNEDPEYAHIDPKASSAELIGYAMKMAEEKAKNPADDIV  TQLIQADIDGEKLSDDEFGFFVVMLAVAGNETTRNSITQGMMAFAEHPDQWELYKKVRPE  TAADEIVRWATPVTAFQRTALRDYELSGVQIKKGQRVVMFYRSANFDEEVFQDPFTFNIL  RNPNPHVGFGGTGAHYCIGANLARMTINLIFNAVADHMPDLKPISAPERLRSGWLNGIKH  WQVDYTGRCPVAH*  >CYP124A1(MRA_2286)  MGLNTAIATRVNGTPPPEVPIADIELGSLDFWALDDDVRDGAFATLRREAPISFWPTIEL  PGFVAGNGHWALTKYDDVFYASRHPDIFSSYPNITINDQTPELAEYFGSMIVLDDPRHQR  LRSIVSRAFTPKVVARIEAAVRDRAHRLVSSMIANNPDRQADLVSELAGPLPLQIICDMM  GIPKADHQRIFHWTNVILGFGDPDLATDFDEFMQVSADIGAYATALAEDRRVNHHDDLTS  SLVEAEVDGERLSSREIASFFILLVVAGNETTRNAITHGVLALSRYPEQRDRWWSDFDGL  APTAVEEIVRWASPVVYMRRTLTQDIELRGTKMAAGDKVSLWYCSANRDESKFADPWTFD  LARNPNPHLGFGGGGAHFCLGANLARREIRVAFDELRRQMPDVVATEEPARLLSQFIHGI  KTLPVTWS*  >CYP128A1(MRA_2288)  MTATQSPPEPAPDRVRLAGCPLAGTPDVGLTAQDATTALGVPTRRRASSGGIPVATSMWR  DAQTVRTYGPAVAKALALRVAGKARSRLTGRHCRKFMQLTDFDPFDPAIAADPYPHYREL  LAGERVQYNPKRDVYILSRYADVREAARNHDTLSSARGVTFSRGWLPFLPTSDPPAHTRM  RKQLAPGMARGALETWRPMVDQLARELVGGLLTQTPADVVSTVAAPMPMRAITSVLGVDG  PDEAAFCRLSNQAVRITDVALSASGLISLVQGFAGFRRLRALFTHRRDNGLLRECTVLGK  LATHAEQGRLSDDELFFFAVLLLVAGYESTAHMISTLFLTLADYPDQLTLLAQQPDLIPS  AIEEHLRFISPIQNICRTTRVDYSVGQAVIPAGSLVLLAWGAANRDPRQYEDPDVFRADR  NPVGHLAFGSGIHLCPGTQLARMEGQAILREIVANIDRIEVVEPPTWTTNANLRGLTRLR  VAVTPRVAP*  >CYP121A1(MRA_2295)  MTATVLLEVPFSARGDRIPDAVAELRTREPIRKVRTITGAEAWLVSSYALCTQVLEDRRF  SMKETAAAGAPRLNALTVPPEVVNNMGNIADAGLRKAVMKAITPKAPGLEQFLRDTANSL  LDNLITEGAPADLRNDFADPLATALHCKVLGIPQEDGPKLFRSLSIAFMSSADPIPAAKI  NWDRDIEYMAGILENPNITTGLMGELSRLRKDPAYSHVSDELFATIGVTFFGAGVISTGS  FLTTALISLIQRPQLRNLLHEKPELIPAGVEELLRINLSFADGLPRLATADIQVGDVLVR  KGELVLVLLEGANFDPEHFPNPGSIELDRPNPTSHLAFGRGQHFCPGSALGRRHAQIGIE  ALLKKMPGVDLAVPIDQLVWRTRFQRRIPERLPVLW*  >CYP138A1(MRA_0143)  MSEVVTAAPAPPVVRLPPAVRGPKLFQGLAFVVSRRRLLGRFVRRYGKAFTANILMYGRV  VVVADPQLARQVFTSSPEELGNIQPNLSRMFGSGSVFALDGDDHRRRRRLLAPPFHGKSM  KNYETIIEEETLRETANWPQGQAFATLPSMMHITLNAILRAIFGAGGSELDELRRLIPPW  VTLGSRLAALPKPKRDYGRLSPWGRLAEWRRQYDTVIDKLIEAERADPNFADRTDVLALM  LRSTYDDGSIMSRKDIGDELLTLLAAGHETTAATLGWAFERLSRHPDVLAALVEEVDNGG  HELRQAAILEVQRARTVIDFAARRVNPPVYQLGEWVIPRGYSIIINIAQIHGDPDVFPQP  DRFDPQRYIGSKPSPFAWIPFGGGTRRCVGAAFANMEMDVVLRTVLRHFTLETTTAAGER  SHGRGVAFTPKDGGRVVMRRR*  >CYP139A1(MRA_1677)  MRYPLGEALLALYRWRGPLINAGVGGHGYTYLLGAEANRFVFANADAFSWSQTFESLVPV  DGPTALIVSDGADHRRRRSVVAPGLRHHHVQRYVATMVSNIDTVIDGWQPGQRLDIYQEL  RSAVRRSTAESLFGQRLAVHSDFLGEQLQPLLDLTRRPPQVMRLQQRVNSPGWRRAMAAR  KRIDDLIDAQIADARTAPRPDDHMLTTLISGCSEEGTTLSDNEIRDSIVSLITAGYETTS  GALAWAIYALLTVPGTWESAASEVARVLGGRVPAADDLSALTYLNGVVHETLRLYSPGVI  SARRVLRDLWFDGHRIRAGRLLIFSAYVTHRLPEIWPEPTEFRPLRWDPNAADYRKPAPH  EFIPFSGGLHRCIGAVMATTEMTVILARLVARAMLQLPAQRTHRIRAANFAALRPWPGLT  VEIRKSAPAQ*  >CYP144A1(MRA_1793)  MRRSPKGSPGAVLDLQRRVDQAVSADHAELMTIAKDANTFFGAESVQDPYPLYERMRAAG  SVHRIANSDFYAVCGWDAVNEAIGRPEDFSSNLTATMTYTAEGTAKPFEMDPLGGPTHVL  ATADDPAHAVHRKLVLRHLAAKRIRVMEQFTVQAADRLWVDGMQDGCIEWMGAMANRLPM  MVVAELIGLPDPDIAQLVKWGYAATQLLEGLVENDQLVAAGVALMELSGYIFEQFDRAAA  DPRDNLLGELATACASGELDTLTAQVMMVTLFAAGGESTAALLGSAVWILATRPDIQQQV  RANPELLGAFIEETLRYEPPFRGHYRHVRNATTLDGTELPADSHLLLLWGAANRDPAQFE  APGEFRLDRAGGKGHISFGKGAHFCVGAALARLEARIVLRLLLDRTSVIEAADVGGWLPS  ILVRRIERLELAVQ*  >CYP135A1(MRA_0336)  MASTLTTGLPPGPRLPRYLQSVLYLRFREWFLPAMHRKYGDVFSLRVPPYADNLVVYTRP  EHIKEIFAADPRSLHAGEGNHILGFVMGEHSVLMTDEAEHARMRSLLMPAFTRAALRGYR  DMIASVAREHITRWRPHATINSLDHMNALTLDIILRVVFGVTDPKVKAELTSRLQQIINI  HPAILAGVPYPSLKRMNPWKRFFHNQTKIDEILYREIASRRIDSDLTARTDVLSRLLQTK  DTPTKPLTDAELRDQLITLLLAGHETTAAALSWTLWELAHAPEIQSQVVWAAVGGDDGFL  EAVLKEGMRRHTVIASTARKVTAPAEIGGWRLPAGTVVNTSILLAHASEVSHPKPTEFRP  SRFLDGSVAPNTWLPFGGGVRRCLGFGFALTEGAVILQEIFRRFTITAAGPSKGETPLVR  NITTVPKHGAHLRLIPQRRLGGLGDSDPP*  >CYP136A1(MRA_3091)  MATIHPPAYLLDQAKRRFTPSFNNFPGMSLVEHMLLNTKFPEKKLAEPPPGSGLKPVVGD  AGLPILGHMIEMLRGGPDYLMFLYKTKGPVVFGDSAVLPGVAALGPDAAQVIYSNRNKDY  SQQGWVPVIGPFFHRGLMLLDFEEHMFHRRIMQEAFVRSRLAGYLEQMDRVVSRVVADDW  VVNDARFLVYPAMKALTLDIASMVFMGHEPGTDHELVTKVNKAFTITTRAGNAVIRTSVP  PFTWWRGLRARELLENYFTARVKERREASGNDLLTVLCQTEDDDGNRFSDADIVNHMIFL  MMAAHDTSTSTATTMAYQLAAHPEWQQRCRDESDRHGDGPLDIESLEQLESLDLVMNESI  RLVTPVQWAMRQTVRDTELLGYYLPKGTNVIAYPGMNHRLPEIWTDPLTFDPERFTEPRN  EHKRHRYAFTPFGGGVHKCIGMVFDQLEIKTILHRLLRRYRLELSRPDYQPRWDYSAMPI  PMDGMPIVLRPR*  >CYP137A1(MRA_3720)  MVLRSLASPAALTDPKRCASVVGVAAFAVRREHAPDALGGPPGLPAPRGFRAAFAAAYAV  AYLAGGERRMLRLIRRYGPIMTMPILSLGDVAIVSDSALAKEVFTAPTDVLLGGEGVGPA  AAIYGSGSMFVQEEPEHLRRRKLLTPPLHGAALDRYVPIIENSTRAAMHTWPVDRPFAML  TVARSLMLDVIVKVIFGVDDPEEVRRLGRPFERLLNLGVSEQLTVRYALRRLGALRVWPA  RARANTEIDDVVMALIAQRRADPRLGERHDVLSLLVSARGESGEQLSDSEIRDDLITLVL  AGHETTATTLAWAFDLLLHHPDALRRVRAEAVGGGEAFTTAVINETLRVRPPAPLTARVA  AQPLTIGGYRVEAGTRIVVHIIAINRSAEVYEHPHEFRPERFLGTRPQTYAWVPFGGGVK  RCLGANFSMRELITVLHVLLREGEFTAVDDEPERIVRRSIMLVPRRGTRVRFRPAR*  >CYP135B1(MRA_0575)  MSGTSSMGLPPGPRLSGSVQAVLMLRHGLRFLTACQRRYGSVFTLHVAGFGHMVYLSDPA  AIKTVFAGNPSVFHAGEANSMLAGLLGDSSLLLIDDDVHRDRRRLMSPPFHRDAVARQAG  PIAEIAAANIAGWPMAKAFAVAPKMSEITLEVILRTVIGASDPVRLAALRKVMPRLLNVG  PWATLALANPSLLNNRLWSRLRRRIEEADALLYAEIADRRADPDLAARTDTLAMLVRAAD  EDGRTMTERELRDQLITLLVAGHDTTATGLSWALERLTRHPVTLAKAVQAADASAAGDPA  GDEYLDAVAKETLRIRPVVYDVGRVLTEAVEVAGYRLPAGVMVVPAIGLVHASAQLYPDP  ERFDPDRMVGATLSPTTWLPFGGGNRRCLGATFAMVEMRVVLREILRRVELSTTTTSGER  PKLKHVIMVPHRGARIRVRATRDVSATSQATAQGAGCPAARGGGPSRAVGSQ*  >CYP143A1(MRA_1799)  MTTPGEDHAGSFYLPRLEYSTLPMAVDRGVGWKTLRDAGPVVFMNGWYYLTRREDVLAAL  RNPKVFSSRKALQPPGNPLPVVPLAFDPPEHTRYRRILQPYFSPAALSKALPSLRRHTVA  MIDAIAGRGECEAMADLANLFPFQLFLVLYGLPLEDRDRLIGWKDAVIAMSDRPHPTEAD  VAAARELLEYLTAMVAERRRNPGPDVLSQVQIGEDPLSEIEVLGLSHLLILAGLDTVTAA  VGFSLLELARRPQLRAMLRDNPKQIRVFIEEIVRLEPSAPVAPRVTTEPVTVGGMTLPAG  SPVRLCMAAVNRDGSDAMSTDELVMDGKVHRHWGFGGGPHRCLGSHLARLELTLLVGEWL  NQIPDFELAPDYAPEIRFPSKSFALKNLPLRWS*  >CYP132A1(MRA_1403)  MATATTQRPLKGPAKRMSTWTMTREAITIGFDAGDGFLGRLRGSDITRFRCAGRRFVSIS  HPDYVDHVLHEARLKYVKSDEYGPIRATAGLNLLTDEGDSWARHRGALNSTFARRHLRGL  VGLMIDPIADVTAARVPGAQFDMHQSMVETTLRVVANALFSQDFGPLVQSMHDLATRGLR  RAEKLERLGLWGLMPRTVYDTLIWCIYSGVHLPPPLREMQEITLTLDRAINSVIDRRLAE  PTNSADLLNVLLSADGGIWPRQRVRDEALTFMLAGHETTANAMSWFWYLMALNPQARDHM  LTELDDVLGMRRPTADDLGKLAWTTACLQESQRYFSSVWIIAREAVDDDIIDGHRIRRGT  TVVIPIHHIHHDPRWWPDPDRFDPGRFLRCPTDRPRCAYLPFGGGRRICIGQSFALMEMV  LMAAIMSQHFTFDLAPGYHVELEATLTLRPKHGVHVIGRRR*  >CYP141A1(MRA_3153)  MTSTSIPTFPFDRPVPTEPSPMLSELRNSCPVAPIELPSGHTAWLVTRFDDVKGVLSDKR  FSCRAAAHPSSPPFVPFVQLCPSLLSIDGPQHTAARRLLAQGLNPGFIARMRPVVQQIVD  NALDDLAAAEPPVDFQEIVSVPIGEQLMAKLLGVEPKTVHELAAHVDAAMSVCEIGDEEV  SRRWSALCTMVIDILHRKLAEPGDDLLSTIAQANRQQSTMTDEQVVGMLLTVVIGGVDTP  IAVITNGLASLLHHRDQYERLVEDPGRVARAVEEIVRFNPATEIEHLRVVTEDVVIAGTA  LSAGSPAFTSITSANRDSDQFLDPDEFDVERNPNEHIAFGYGPHACPASAYSRMCLTTFF  TSLTQRFPQLQLARPFEDLERRGKGLHSVGIKELLVTWPT* |
| ***Mycobacterium tuberculosis* H37Rv** |
| Database: TB; P450 count:20; P450 families: 19; Subfamilies: 20 |
| >CYP123A1(Rv0766c)  MTVRVGDPELVLDPYDYDFHEDPYPYYRRLRDEAPLYRNEERNFWAVSRHHDVLQGFRDS  TALSNAYGVSLDPSSRTSEAYRVMSMLAMDDPAHLRMRTLVSKGFTPRRIRELEPQVLEL  ARIHLDSALQTESFDFVAEFAGKLPMDVISELIGVPDTDRARIRALADAVLHREDGVADV  PPPAMAASIELMRYYADLIAEFRRRPANNLTSALLAAELDGDRLSDQEIMAFLFLMVIAG  NETTTKLLANAVYWAAHHPGQLARVFADHSRIPMWVEETLRYDTSSQILARTVAHDLTLY  DTTIPEGEVLLLLPGSANRDDRVFDDPDDYRIGREIGCKLVSFGSGAHFCLGAHLARMEA  RVALGALLRRIRNYEVDDDNVVRVHSSNVRGFAHLPISVQAR*  >CYP125A1(Rv3545c)  MSWNHQSVEIAVRRTTVPSPNLPPGFDFTDPAIYAERLPVAEFAELRSAAPIWWNGQDPG  KGGGFHDGGFWAITKLNDVKEISRHSDVFSSYENGVIPRFKNDIAREDIEVQRFVMLNMD  APHHTRLRKIISRGFTPRAVGRLHDELQERAQKIAAEAAAAGSGDFVEQVSCELPLQAIA  GLLGVPQEDRGKLFHWSNEMTGNEDPEYAHIDPKASSAELIGYAMKMAEEKAKNPADDIV  TQLIQADIDGEKLSDDEFGFFVVMLAVAGNETTRNSITQGMMAFAEHPDQWELYKKVRPE  TAADEIVRWATPVTAFQRTALRDYELSGVQIKKGQRVVMFYRSANFDEEVFQDPFTFNIL  RNPNPHVGFGGTGAHYCIGANLARMTINLIFNAVADHMPDLKPISAPERLRSGWLNGIKH  WQVDYTGRCPVAH*  >CYP51B1(Rv0764c)  MSAVALPRVSGGHDEHGHLEEFRTDPIGLMQRVRDECGDVGTFQLAGKQVVLLSGSHANE  FFFRAGDDDLDQAKAYPFMTPIFGEGVVFDASPERRKEMLHNAALRGEQMKGHAATIEDQ  VRRMIADWGEAGEIDLLDFFAELTIYTSSACLIGKKFRDQLDGRFAKLYHELERGTDPLA  YVDPYLPIESFRRRDEARNGLVALVADIMNGRIANPPTDKSDRDMLDVLIAVKAETGTPR  FSADEITGMFISMMFAGHHTSSGTASWTLIELMRHRDAYAAVIDELDELYGDGRSVSFHA  LRQIPQLENVLKETLRLHPPLIILMRVAKGEFEVQGHRIHEGDLVAASPAISNRIPEDFP  DPHDFVPARYEQPRQEDLLNRWTWIPFGAGRHRCVGAAFAIMQIKAIFSVLLREYEFEMA  QPPESYRNDHSKMVVQLAQPACVRYRRRTGV*  >CYP138A1(Rv0136)  MSEVVTAAPAPPVVRLPPAVRGPKLFQGLAFVVSRRRLLGRFVRRYGKAFTANILMYGRV  VVVADPQLARQVFTSSPEELGNIQPNLSRMFGSGSVFALDGDDHRRRRRLLAPPFHGKSM  KNYETIIEEETLRETANWPQGQAFATLPSMMHITLNAILRAIFGAGGSELDELRRLIPPW  VTLGSRLAALPKPKRDYGRLSPWGRLAEWRRQYDTVIDKLIEAERADPNFADRTDVLALM  LRSTYDDGSIMSRKDIGDELLTLLAAGHETTAATLGWAFERLSRHPDVLAALVEEVDNGG  HELRQAAILEVQRARTVIDFAARRVNPPVYQLGEWVIPRGYSIIINIAQIHGDPDVFPQP  DRFDPQRYIGSKPSPFAWIPFGGGTRRCVGAAFANMEMDVVLRTVLRHFTLETTTAAGER  SHGRGVAFTPKDGGRVVMRRR*  >CYP135A1(Rv0327c)  MASTLTTGLPPGPRLPRYLQSVLYLRFREWFLPAMHRKYGDVFSLRVPPYADNLVVYTRP  EHIKEIFAADPRSLHAGEGNHILGFVMGEHSVLMTDEAEHARMRSLLMPAFTRAALRGYR  DMIASVAREHITRWRPHATINSLDHMNALTLDIILRVVFGVTDPKVKAELTSRLQQIINI  HPAILAGVPYPSLKRMNPWKRFFHNQTKIDEILYREIASRRIDSDLTARTDVLSRLLQTK  DTPTKPLTDAELRDQLITLLLAGHETTAAALSWTLWELAHAPEIQSQVVWAAVGGDDGFL  EAVLKEGMRRHTVIASTARKVTAPAEIGGWRLPAGTVVNTSILLAHASEVSHPKPTEFRP  SRFLDGSVAPNTWLPFGGGVRRCLGFGFALTEGAVILQEIFRRFTITAAGPSKGETPLVR  NITTVPKHGAHLRLIPQRRLGGLGDSDPP*  >CYP126A1(Rv0778)  MTTAAGLSGIDLTDLDNFADGFPHHLFAIHRREAPVYWHRPTEHTPDGEGFWSVATYAET  LEVLRDPVTYSSVTGGQRRFGGTVLQDLPVAGQVLNMMDDPRHTRIRRLVSSGLTPRMIR  RVEDDLRRRARGLLDGVEPGAPFDFVVEIAAELPMQMICILLGVPETDRHWLFEAVEPGF  DFRGSRRATMPRLNVEDAGSRLYTYALELIAGKRAEPADDMLSVVANATIDDPDAPALSD  AELYLFFHLLFSAGAETTRNSIAGGLLALAENPDQLQTLRSDFELLPTAIEEIVRWTSPS  PSKRRTASRAVSLGGQPIEAGQKVVVWEGSANRDPSVFDRADEFDITRKPNPHLGFGQGV  HYCLGANLARLELRVLFEELLSRFGSVRVVEPAEWTRSNRHTGIRHLVVELRGG*  >CYP130A1(Rv1256c)  MTSVMSHEFQLATAETWPNPWPMYRALRDHDPVHHVVPPQRPEYDYYVLSRHADVWSAAR  DHQTFSSAQGLTVNYGELEMIGLHDTPPMVMQDPPVHTEFRKLVSRGFTPRQVETVEPTV  RKFVVERLEKLRANGGGDIVTELFKPLPSMVVAHYLGVPEEDWTQFDGWTQAIVAANAVD  GATTGALDAVGSMMAYFTGLIERRRTEPADDAISHLVAAGVGADGDTAGTLSILAFTFTM  VTGGNDTVTGMLGGSMPLLHRRPDQRRLLLDDPEGIPDAVEELLRLTSPVQGLARTTTRD  VTIGDTTIPAGRRVLLLYGSANRDERQYGPDAAELDVTRCPRNILTFSHGAHHCLGAAAA  RMQCRVALTELLARCPDFEVAESRIVWSGGSYVRRPLSVPFRVTS*  >CYP121A1(Rv2276)  MTATVLLEVPFSARGDRIPDAVAELRTREPIRKVRTITGAEAWLVSSYALCTQVLEDRRF  SMKETAAAGAPRLNALTVPPEVVNNMGNIADAGLRKAVMKAITPKAPGLEQFLRDTANSL  LDNLITEGAPADLRNDFADPLATALHCKVLGIPQEDGPKLFRSLSIAFMSSADPIPAAKI  NWDRDIEYMAGILENPNITTGLMGELSRLRKDPAYSHVSDELFATIGVTFFGAGVISTGS  FLTTALISLIQRPQLRNLLHEKPELIPAGVEELLRINLSFADGLPRLATADIQVGDVLVR  KGELVLVLLEGANFDPEHFPNPGSIELDRPNPTSHLAFGRGQHFCPGSALGRRHAQIGIE  ALLKKMPGVDLAVPIDQLVWRTRFQRRIPERLPVLW*  >CYP124A1(Rv2266)  MGLNTAIATRVNGTPPPEVPIADIELGSLDFWALDDDVRDGAFATLRREAPISFWPTIEL  PGFVAGNGHWALTKYDDVFYASRHPDIFSSYPNITINDQTPELAEYFGSMIVLDDPRHQR  LRSIVSRAFTPKVVARIEAAVRDRAHRLVSSMIANNPDRQADLVSELAGPLPLQIICDMM  GIPKADHQRIFHWTNVILGFGDPDLATDFDEFMQVSADIGAYATALAEDRRVNHHDDLTS  SLVEAEVDGERLSSREIASFFILLVVAGNETTRNAITHGVLALSRYPEQRDRWWSDFDGL  APTAVEEIVRWASPVVYMRRTLTQDIELRGTKMAAGDKVSLWYCSANRDESKFADPWTFD  LARNPNPHLGFGGGGAHFCLGANLARREIRVAFDELRRQMPDVVATEEPARLLSQFIHGI  KTLPVTWS*  >CYP144A1(Rv1777)  MRRSPKGSPGAVLDLQRRVDQAVSADHAELMTIAKDANTFFGAESVQDPYPLYERMRAAG  SVHRIANSDFYAVCGWDAVNEAIGRPEDFSSNLTATMTYTAEGTAKPFEMDPLGGPTHVL  ATADDPAHAVHRKLVLRHLAAKRIRVMEQFTVQAADRLWVDGMQDGCIEWMGAMANRLPM  MVVAELIGLPDPDIAQLVKWGYAATQLLEGLVENDQLVAAGVALMELSGYIFEQFDRAAA  DPRDNLLGELATACASGELDTLTAQVMMVTLFAAGGESTAALLGSAVWILATRPDIQQQV  RANPELLGAFIEETLRYEPPFRGHYRHVRNATTLDGTELPADSHLLLLWGAANRDPAQFE  APGEFRLDRAGGKGHISFGKGAHFCVGAALARLEARIVLRLLLDRTSVIEAADVGGWLPS  ILVRRIERLELAVQ*  >CYP143A1(Rv1785c)  MTTPGEDHAGSFYLPRLEYSTLPMAVDRGVGWKTLRDAGPVVFMNGWYYLTRREDVLAAL  RNPKVFSSRKALQPPGNPLPVVPLAFDPPEHTRYRRILQPYFSPAALSKALPSLRRHTVA  MIDAIAGRGECEAMADLANLFPFQLFLVLYGLPLEDRDRLIGWKDAVIAMSDRPHPTEAD  VAAARELLEYLTAMVAERRRNPGPDVLSQVQIGEDPLSEIEVLGLSHLLILAGLDTVTAA  VGFSLLELARRPQLRAMLRDNPKQIRVFIEEIVRLEPSAPVAPRVTTEPVTVGGMTLPAG  SPVRLCMAAVNRDGSDAMSTDELVMDGKVHRHWGFGGGPHRCLGSHLARLELTLLVGEWL  NQIPDFELAPDYAPEIRFPSKSFALKNLPLRWS*  >CYP135B1(Rv0568)  MSGTSSMGLPPGPRLSGSVQAVLMLRHGLRFLTACQRRYGSVFTLHVAGFGHMVYLSDPA  AIKTVFAGNPSVFHAGEANSMLAGLLGDSSLLLIDDDVHRDRRRLMSPPFHRDAVARQAG  PIAEIAAANIAGWPMAKAFAVAPKMSEITLEVILRTVIGASDPVRLAALRKVMPRLLNVG  PWATLALANPSLLNNRLWSRLRRRIEEADALLYAEIADRRADPDLAARTDTLAMLVRAAD  EDGRTMTERELRDQLITLLVAGHDTTATGLSWALERLTRHPVTLAKAVQAADASAAGDPA  GDEYLDAVAKETLRIRPVVYDVGRVLTEAVEVAGYRLPAGVMVVPAIGLVHASAQLYPDP  ERFDPDRMVGATLSPTTWLPFGGGNRRCLGATFAMVEMRVVLREILRRVELSTTTTSGER  PKLKHVIMVPHRGARIRVRATRDVSATSQATAQGAGCPAARGGGPSRAVGSQ*  >CYP142A1(Rv3518c)  MTEAPDVDLADGNFYASREARAAYRWMRANQPVFRDRNGLAAASTYQAVIDAERQPELFS  NAGGIRPDQPALPMMIDMDDPAHLLRRKLVNAGFTRKRVKDKEASIAALCDTLIDAVCER  GECDFVRDLAAPLPMAVIGDMLGVRPEQRDMFLRWSDDLVTFLSSHVSQEDFQITMDAFA  AYNDFTRATIAARRADPTDDLVSVLVSSEVDGERLSDDELVMETLLILIGGDETTRHTLS  GGTEQLLRNRDQWDLLQRDPSLLPGAIEEMLRWTAPVKNMCRVLTADTEFHGTALCAGEK  MMLLFESANFDEAVFCEPEKFDVQRNPNSHLAFGFGTHFCLGNQLARLELSLMTERVLRR  LPDLRLVADDSVLPLRPANFVSGLESMPVVFTPSPPLG*  >CYP141A1(Rv3121)  MTSTSIPTFPFDRPVPTEPSPMLSELRNSCPVAPIELPSGHTAWLVTRFDDVKGVLSDKR  FSCRAAAHPSSPPFVPFVQLCPSLLSIDGPQHTAARRLLAQGLNPGFIARMRPVVQQIVD  NALDDLAAAEPPVDFQEIVSVPIGEQLMAKLLGVEPKTVHELAAHVDAAMSVCEIGDEEV  SRRWSALCTMVIDILHRKLAEPGDDLLSTIAQANRQQSTMTDEQVVGMLLTVVIGGVDTP  IAVITNGLASLLHHRDQYERLVEDPGRVARAVEEIVRFNPATEIEHLRVVTEDVVIAGTA  LSAGSPAFTSITSANRDSDQFLDPDEFDVERNPNEHIAFGYGPHACPASAYSRMCLTTFF  TSLTQRFPQLQLARPFEDLERRGKGLHSVGIKELLVTWPT*  >CYP139A1(Rv1666c)  MRYPLGEALLALYRWRGPLINAGVGGHGYTYLLGAEANRFVFANADAFSWSQTFESLVPV  DGPTALIVSDGADHRRRRSVVAPGLRHHHVQRYVATMVSNIDTVIDGWQPGQRLDIYQEL  RSAVRRSTAESLFGQRLAVHSDFLGEQLQPLLDLTRRPPQVMRLQQRVNSPGWRRAMAAR  KRIDDLIDAQIADARTAPRPDDHMLTTLISGCSEEGTTLSDNEIRDSIVSLITAGYETTS  GALAWAIYALLTVPGTWESAASEVARVLGGRVPAADDLSALTYLNGVVHETLRLYSPGVI  SARRVLRDLWFDGHRIRAGRLLIFSAYVTHRLPEIWPEPTEFRPLRWDPNAADYRKPAPH  EFIPFSGGLHRCIGAVMATTEMTVILARLVARAMLQLPAQRTHRIRAANFAALRPWPGLT  VEIRKSAPAQ*  >CYP137A1(Rv3685c)  MVLRSLASPAALTDPKRCASVVGVAAFAVRREHAPDALGGPPGLPAPRGFRAAFAAAYAV  AYLAGGERRMLRLIRRYGPIMTMPILSLGDVAIVSDSALAKEVFTAPTDVLLGGEGVGPA  AAIYGSGSMFVQEEPEHLRRRKLLTPPLHGAALDRYVPIIENSTRAAMHTWPVDRPFAML  TVARSLMLDVIVKVIFGVDDPEEVRRLGRPFERLLNLGVSEQLTVRYALRRLGALRVWPA  RARANTEIDDVVMALIAQRRADPRLGERHDVLSLLVSARGESGEQLSDSEIRDDLITLVL  AGHETTATTLAWAFDLLLHHPDALRRVRAEAVGGGEAFTTAVINETLRVRPPAPLTARVA  AQPLTIGGYRVEAGTRIVVHIIAINRSAEVYEHPHEFRPERFLGTRPQTYAWVPFGGGVK  RCLGANFSMRELITVLHVLLREGEFTAVDDEPERIVRRSIMLVPRRGTRVRFRPAR*  >CYP140A1(Rv1880c)  MKDKLHWLAMHGVIRGIAAIGIRRGDLQARLIADPAVATDPVPFYDEVRSHGALVRNRAN  YLTVDHRLAHDLLRSDDFRVVSFGENLPPPLRWLERRTRGDQLHPLREPSLLAVEPPDHT  RYRKTVSAVFTSRAVSALRDLVEQTAINLLDRFAEQPGIVDVVGRYCSQLPIVVISEILG  VPEHDRPRVLEFGELAAPSLDIGIPWRQYLRVQQGIRGFDCWLEGHLQQLRHAPGDDLMS  QLIQIAESGDNETQLDETELRAIAGLVLVAGFETTVNLLGNGIRMLLDTPEHLATLRQHP  ELWPNTVEEILRLDSPVQLTARVACRDVEVAGVRIKRGEVVVIYLAAANRDPAVFPDPHR  FDIERPNAGRHLAFSTGRHFCLGAALARAEGEVGLRTFFDRFPDVRAAGAGSRRDTRVLR  GWSTLPVTLGPARSMVSP*  >CYP128A1(Rv2268c)  MTATQSPPEPAPDRVRLAGCPLAGTPDVGLTAQDATTALGVPTRRRASSGGIPVATSMWR  DAQTVRTYGPAVAKALALRVAGKARSRLTGRHCRKFMQLTDFDPFDPAIAADPYPHYREL  LAGERVQYNPKRDVYILSRYADVREAARNHDTLSSARGVTFSRGWLPFLPTSDPPAHTRM  RKQLAPGMARGALETWRPMVDQLARELVGGLLTQTPADVVSTVAAPMPMRAITSVLGVDG  PDEAAFCRLSNQAVRITDVALSASGLISLVQGFAGFRRLRALFTHRRDNGLLRECTVLGK  LATHAEQGRLSDDELFFFAVLLLVAGYESTAHMISTLFLTLADYPDQLTLLAQQPDLIPS  AIEEHLRFISPIQNICRTTRVDYSVGQAVIPAGSLVLLAWGAANRDPRQYEDPDVFRADR  NPVGHLAFGSGIHLCPGTQLARMEGQAILREIVANIDRIEVVEPPTWTTNANLRGLTRLR  VAVTPRVAP*  >CYP132A1(Rv1394c)  MATATTQRPLKGPAKRMSTWTMTREAITIGFDAGDGFLGRLRGSDITRFRCAGRRFVSIS  HPDYVDHVLHEARLKYVKSDEYGPIRATAGLNLLTDEGDSWARHRGALNSTFARRHLRGL  VGLMIDPIADVTAARVPGAQFDMHQSMVETTLRVVANALFSQDFGPLVQSMHDLATRGLR  RAEKLERLGLWGLMPRTVYDTLIWCIYSGVHLPPPLREMQEITLTLDRAINSVIDRRLAE  PTNSADLLNVLLSADGGIWPRQRVRDEALTFMLAGHETTANAMSWFWYLMALNPQARDHM  LTELDDVLGMRRPTADDLGKLAWTTACLQESQRYFSSVWIIAREAVDDDIIDGHRIRRGT  TVVIPIHHIHHDPRWWPDPDRFDPGRFLRCPTDRPRCAYLPFGGGRRICIGQSFALMEMV  LMAAIMSQHFTFDLAPGYHVELEATLTLRPKHGVHVIGRRR*  >CYP136A1(Rv3059)  MATIHPPAYLLDQAKRRFTPSFNNFPGMSLVEHMLLNTKFPEKKLAEPPPGSGLKPVVGD  AGLPILGHMIEMLRGGPDYLMFLYKTKGPVVFGDSAVLPGVAALGPDAAQVIYSNRNKDY  SQQGWVPVIGPFFHRGLMLLDFEEHMFHRRIMQEAFVRSRLAGYLEQMDRVVSRVVADDW  VVNDARFLVYPAMKALTLDIASMVFMGHEPGTDHELVTKVNKAFTITTRAGNAVIRTSVP  PFTWWRGLRARELLENYFTARVKERREASGNDLLTVLCQTEDDDGNRFSDADIVNHMIFL  MMAAHDTSTSTATTMAYQLAAHPEWQQRCRDESDRHGDGPLDIESLEQLESLDLVMNESI  RLVTPVQWAMRQTVRDTELLGYYLPKGTNVIAYPGMNHRLPEIWTDPLTFDPERFTEPRN  EHKRHRYAFTPFGGGVHKCIGMVFDQLEIKTILHRLLRRYRLELSRPDYQPRWDYSAMPI  PMDGMPIVLRPR* |
| ***Mycobacterium tuberculosis* Haarlem** |
| Database: TB; P450 count:19; P450 families: 18; Subfamilies: 19 |
| >CYP136A1(TBHG_02989.1)  MATIHPPAYLLDQAKRRFTPSFNNFPGMSLVEHMLLNTKFPEKKLAEPPPGSGLKPVVGD  AGLPILGHMIEMLRGGPDYLMFLYKTKGPVVFGDSAVLPGVAALGPDAAQVIYSNRNKDY  SQQGWVPVIGPFFHRGLMLLDFEEHMFHRRIMQEAFVRSRLAGYLEQMDRVVSRVVADDW  VVNDARFLVYPAMKALTLDIASMVFMGHEPGTDHELVTKVNKAFTITTRAGNAVIRTSVP  PFTWWRGLRARELLENYFTARVKERREASGNDLLTVLCQTEDDDGNRFSDADIVNHMIFL  MMAAHDTSTSTATTMAYQLAAHPEWQQRCRDESDRHGDGPLDIESLEQLESLDLVMNESI  RLVTPVQWAMRQTVRDTELLGYYLPKGTNVIAYPGMNHRLPEIWTDPLTFDPERFTEPRN  EHKRHRYAFTPFGGGVHKCIGMVFDQLEIKTILHRLLRRYRLELSRPDYQPRWDYSAMPI  PMDGMPIVLRPR*  >CYP51B1(TBHG_00756.1)  MSAVALPRVSGGHDEHGHLEEFRTDPIGLMQRVRDECGDVGTFQLAGKQVVLLSGSHANE  FFFRAGDDDLDQAKAYPFMTPIFGEGVVFDASPERRKEMLHNAALRGEQMKGHAATIEDQ  VRRMIADWGEAGEIDLLDFFAELTIYTSSACLIGKKFRDQLDGRFAKLYHELERGTDPLA  YVDPYLPIESFRRRDEARNGLVALVADIMNGRIANPPTDKSDRDMLDVLIAVKAETGTPR  FSADEITGMFISMMFAGHHTSSGTASWTLIELMRHRDAYAAVIDELDELYGDGRSVSFHA  LRQIPQLENVLKETLRLHPPLIILMRVAKGEFEVQGHRIHEGDLVAASPAISNRIPEDFP  DPHDFVPARYEQPRQEDLLNRWTWIPFGAGRHRCVGAAFAIMQIKAIFSVLLREYEFEMA  QPPESYRNDHSKMVVQLAQPACVRYRRRTGV*  >CYP123A1(TBHG_00758.1)  MTVRVGDPELVLDPYDYDFHEDPYPYYRRLRDEAPLYRNEERNFWAVSRHHDVLQGFRDS  TALSNAYGVSLDPSSRTSEAYRVMSMLAMDDPAHLRMRTLVSKGFTPRRIRELEPQVLEL  ARIHLDSALQTESFDFVAEFAGKLPMDVISELIGVPDTDRARIRALADAVLHREDGVADV  PPPAMAASIELMRYYADLIAEFRRRPANNLTSALLAAELDGDRLSDQEIMAFLFLMVIAG  NETTTKLLANAVYWAAHHPGQLARVFADHSRIPMWVEETLRYDTSSQILARTVAHDLTLY  DTTIPEGEVLLLLPGSANRDDRVFDDPDDYRIGREIGCKLVSFGSGAHFCLGAHLARMEA  RVALGALLRRIRNYEVDDDNVVRVHSSNVRGFAHLPISVQAR*  >CYP142A1(TBHG_03458.1)  MTEAPDVDLADGNFYASREARAAYRWMRANQPVFRDRNGLAAASTYQAVIDAERQPELFS  NAGGIRPDQPALPMMIDMDDPAHLLRRKLVNAGFTRKRVKDKEASIAALCDTLIDAVCER  GECDFVRDLAAPLPMAVIGDMLGVRPEQRDMFLRWSDDLVTFLSSHVSQEDFQITMDAFA  AYNDFTRATIAARRADPTDDLVSVLVSSEVDGERLSDDELVMETLLILIGGDETTRHTLS  GGTEQLLRNRDQWDLLQRDPSLLPGAIEEMLRWTAPVKNMCRVLTADTEFHGTALCAGEK  MMLLFESANFDEAVFCEPEKFDVQRNPNSHLAFGFGTHFCLGNQLARLELSLMTERVLRR  LPDLRLVADDSVLPLRPANFVSGLESMPVVFTPSPPLG*  >CYP126A1(TBHG_00770.1)  MTTAAGLSGIDLTDLDNFADGFPHHLFAIHRREAPVYWHRPTEHTPDGEGFWSVATYAET  LEVLRDPVTYSSVTGGQRRFGGTVLQDLPVAGQVLNMMDDPRHTRIRRLVSSGLTPRMIR  RVEDDLRRRARGLLDGVEPGAPFDFVVEIAAELPMQMICILLGVPETDRHWLFEAVEPGF  DFRGSRRATMPRLNVEDAGSRLYTYALELIAGKRAEPADDMLSVVANATIDDPDAPALSD  AELYLFFHLLFSAGAETTRNSIAGGLLALAENPDQLQTLRSDFELLPTAIEEIVRWTSPS  PSKRRTASRAVSLGGQPIEAGQKVVVWEGSANRDPSVFDRADEFDITRKPNPHLGFGQGV  HYCLGANLARLELRVLFEELLSRFGSVRVVEPAEWTRSNRHTGIRHLVVELRGG*  >CYP140A1(TBHG_01835.1)  MKDKLHWLAMHGVIRGIAAIGIRRGDLQARLIADPAVATDPVPFYDEVRSHGALVRNRAN  YLTVDHRLAHDLLRSDDFRVVSFGENLPPPLRWLERRTRGDQLHPLREPSLLAVEPPDHT  RYRKTVSAVFTSRAVSALRDLVEQTAINLLDRFAEQPGIVDVVGRYCSQLPIVVISEILG  VPEHDRPRVLEFGELAAPSLDIGIPWRQYLRVQQGIRGFDCWLEGHLQQLRHAPGDDLMS  QLIQIAESGDNETQLDETELRAIAGLVLVAGFETTVNLLGNGIRMLLDTPEHLATLRQHP  ELWPNTVEEILRLDSPVQLTARVACRDVEVAGVRIKRGEVVVIYLAAANRDPAVFPDPHR  FDIERPNAGRHLAFSTGRHFCLGAALARAEGEVGLRTFFDRFPDVRAAGAGSRRDTRVLR  GWSTLPVTLGPARSMVSP*  >CYP124A1(TBHG_02216.1)  MGLNTAIATRVNGTPPPEVPIADIELGSLDFWALDDDVRDGAFATLRREAPISFWPTIEL  PGFVAGNGHWALTKYDDVFYASRHPDIFSSYPNITINDQTPELAEYFGSMIVLDDPRHQR  LRSIVSRAFTPKVVARIEAAVRDRAHRLVSSMIANNPDRQADLVSELAGPLPLQIICDMM  GIPKADHQRIFHWTNVILGFGDPDLATDFDEFMQVSADIGAYATALAEDRRVNHHDDLTS  SLVEAEVDGERLSSREIASFFILLVVAGNETTRNAITHGVLALSRYPEQRDRWWSDFDGL  APTAVEEIVRWASPVVYMRRTLTQDIELRGTKMAAGDKVSLWYCSANRDESKFADPWTFD  LARNPNPHLGFGGGGAHFCLGANLARREIRVAFDELRRQMPDVVATEEPARLLSQFIHGI  KTLPVTWS*  >CYP128A1(TBHG_02218.1)  MTATQSPPEPAPDRVRLAGCPLAGTPDVGLTAQDATTALGVPTRRRASSGGIPVATSMWR  DAQTVRTYGPAVAKALALRVAGKARSRLTGRHCRKFMQLTDFDPFDPAIAADPYPHYREL  LAGERVQYNPKRDVYILSRYADVREAARNHDTLSSARGVTFSRGWLPFLPTSDPPAHTRM  RKQLAPGMARGALETWRPMVDQLARELVGGLLTQTPADVVSTVAAPMPMRAITSVLGVDG  PDEAAFCRLSNQAVRITDVALSASGLISLVQGFAGFRRLRALFTHRRDNGLLRECTVLGK  LATHAEQGRLSDDELFFFAVLLLVAGYESTAHMISTLFLTLADYPDQLTLLAQQPDLIPS  AIEEHLRFISPIQNICRTTRVDYSVGQAGIPAGSLVLLAWGAANRDPRQYEDPDVFRADR  NPVGHLAFGSGIHLCPGPSWRAWRVRRSCARSSPISTE*RWSSRRRGRQTPTFAA*PGYG  SPLPPASHH  >CYP141A1(TBHG_03052.1)  MTSTSIPTFPFDRPVPTEPSPMLSELRNSCPVAPIELPSGHTAWLVTRFDDVKGVLSDKR  FSCRAAAHPSSPPFVPFVQLCPSLLSIDGPQHTAARRLLAQGLNPGFIARMRPVVQQIVD  NALDDLAAAEPPVDFQEIVSVPIGEQLMAKLLGVEPETVHELAAHVDAAMSVCEIGDEEV  SRRWSALCTMVIDILHRKLAEPGDDLLSTIAQANRQQSTMTDEQVVGMLLTVVIGGVDTP  IAVITNGLASLLHHRDQYERLVEDPGRVARAVEEIVRFNPATEIEHLRVVTEDVVIAGTA  LSAGSPAFTSITSANRDSDQFLDPDEFDVERNPNEHIAFGYGPHACPASAYSRMCLTTFF  TSLTQRFPQLQLARPFEDLERRGKGLHSVGIKELLVTWPT*  >CYP138A1(TBHG_00136.1)  MSEVVTAAPAPPVVRLPPAVRGPKLFQGLAFVVSRRRLLGRFVRRYGKAFTANILMYGRV  VVVADPQLARQVFTSSPEELGNIQPNLSRMFGSGSVFALDGDDHRRRRRLLAPPFHGKSM  KNYETIIEEETLRETANWPQGQAFATLPSMMHITLNAILRAIFGAGGSELDELRRLIPPW  VTLGSRLAALPKPKRDYGRLSPWGRLAEWRRQYDTVIDKLIEAERADPNFADRTDVLALM  LRSTYDDGSIMSRKDIGDELLTLLAAGHETTAATLGWAFERLSRHPDVLAALVEEVDNGG  HELRQAAILEVQRARTVIDFAARRVNPPVYQLGEWVIPRGYSIIINIAQIHGDPDVFPQP  DRFDPQRYIGSKPSPFAWIPFGGGTRRCVGAAFANMEMDVVLRTVLRHFTLETTTAAGER  SHGRGVAFTPKDGGRVVMRRR*  >CYP130A1(TBHG_01240.1)  MSHEFQLATAETWPNPWPMYRALRDHDPVHHVVPPQRPEYDYYVLSRHADVWSAARDHQT  FSSAQGLTVNYGELEMIGLHDTPPMVMQDPPVHTEFRKLVSRGFTPRQVETVEPTVRKFV  VERLEKLRANGGGDIVTELFKPLPSMVVAHYLGVPEEDWTQFDGWTQAIVAANAVDGATT  GALDAVGSMMAYFTGLIERRRTEPADDAISHLVAAGVGADGDTAGTLSILAFTFTMVTGG  NDTVTGMLGGSMPLLHRRPDQRRLLLDDPEGIPDAVEELLRLTSPVQGLARTTTRDVTIG  DTTIPAGRRVLLLYGSANRDERQYGPDAAELDVTRCPRNILTFSHGAHHCLGAAAARMQC  RVALTELLARCPDFEVAESRIVWSGGSYVRRPLSVPFRVTS*  >CYP139A1(TBHG_01628.1)  MRTYRTVRYPLGEALLALYRWRGPLINAGVGGHGYTYLLGAEANRFVFANADAFSWSQTF  ESLVPVDGPTALIVSDGADHRRRRSVVAPGLRHHHVQRYVATMVSNIDTVIDGWQPGQRL  DIYQELRSAVRRSTAESLFGQRLAVHSDFLGEQLQPLLDLTRRPPQVMRLQQRVNSPGWR  RAMAARKRIDDLIDAQIADARTAPRPDDHMLTTLISGCSEEGTTLSDNEIRDSIVSLITA  GYETTSGALAWAIYALLTVPGTWESAASEVARVLGGRVPAADDLSALTYLNGVVHETLRL  YSPGVISARRVLRDLWFDGHRIRAGRLLIFSAYVTHRLPEIWPEPTEFRPLRWDPNAADY  RKPAPHEFIPFSGGLHRCIGAVMATTEMTVILARLVARAMLQLPAQRTHRIRAANFAALR  PWPGLTVEIRKSAPAQ*  >CYP135B1(TBHG_00565.1)  MSGTSSMGLPPGPRLSGSVQAVLMLRHGLRFLTACQRRYGSVFTLHVAGFGHMVYLSDPA  AIKTVFAGNPSVFHAGEANSMLAGLLGDSSLLLIDDDVHRDRRRLMSPPFHRDAVARQAG  PIAEIAAANIAGWPMAKAFAVAPKMSEITLEVILRTVIGASDPVRLAALRKVMPRLLNVG  PWATLALANPSLLNNRLWSRLRRRIEEADALLYAEIADRRADPDLAARTDTLAMLVRAAD  EDGRTMTERELRDQLITLLVAGHDTTATGLSWALERLTRHPVTLAKAVQAADASAAGDPA  GDEYLDAVAKETLRIRPVVYDVGRVLTEAVEVAGYRLPAGVMVVPAIGLVHASAQLYPDP  ERFDPDRMVGATLSPTTWLPFGGGNRRCLGATFAMVEMRVVLREILRRVELSTTTTSGER  PKLKHVIMVPHRGARIRVRATRDVSATSQATAQGAGCPAARGGGPSRAVGSQ*  >CYP125A1(TBHG_03485.1)  MSGNHQSVEIAVRRTTVPSPNLPPGFDFTDPAIYAERLPVAEFAELRSAAPIWWNGQDPG  KGGGFHDGGFWAITKLNDVKEISRHSDVFSSYENGVIPRFKNDIAREDIEVQRFVMLNMD  APHHTRLRKIISRGFTPRAVGRLHDELQERAQKIAAEAAAAGSGDFVEQVSCELPLQAIA  GLLGVPQEDRGKLFHWSNEMTGNEDPEYAHIDPKASSAELIGYAMKMAEEKAKNPADDIV  TQLIQADIDGEKLSDDEFGFFVVMLAVAGNETTRNSITQGMMAFAEHPDQWELYKKVRPE  TAADEIVRWATPVTAFQRTALRDYELSGVQIKKGQRVVMFYRSANFDEEVFQDPFTFNIL  RNPNPHVGFGGTGAHYCIGANLARMTINLIFNAVADHMPDLKPISAPERLRSGWLNGIKH  WQVDYTGRCPVAH*  >CYP137A1(TBHG_03620.1)  MVLRSLASPAALTDPKRCASVVGVAAFAVRREHAPDALGGPPGLPAPRGFRAAFAAAYAV  AYLAGGERRMLRLIRRYGPIMTMPILSLGDVAIVSDSALAKEVFTAPTDVLLGGEGVGPA  AAIYGSGSMFVQEEPEHLRRRKLLTPPLHGAALDRYVPIIENSTRAAMHTWPVDRPFAML  TVARSLMLDVIVKVIFGVDDPEEVRRLGRPFERLLNLGVSEQLTVRYALRRLGALRVWPA  RARANTEIDDVVMALIAQRRADPRLGERHDVLSLLVSARGESGEQLSDSEIRDDLITLVL  AGHETTATTLAWAFDLLLHHPDALRRVRAEAVGGGEAFTTAVINETLRVRPPAPLTARVA  AQPLTIGGYRVEAGTRIVVHIIAINRSAEVYEHPHEFRPERFLGTRPQTYAWVPFGGGVK  RCLGANFSMRELITVLHVLLREGEFTAVDDEPERIVRRSIMLVPRRGTRVRFRPAR*  >CYP132A1(TBHG_01374.1)  MATATTQRPLKGPAKRMSTWTMTREAITIGFDAGDGFLGRLRGSDITRFRCAGRRFVSIS  HPDYVDHVLHEARLKYVKSDEYGPIRATAGLNLLTDEGDSWARHRGALNSTFARRHLRGL  VGLMIDPIADVTAALVPGAQFDMHQSMVETTLRVVANALFSQDFGPLVQSMHDLATRGLR  RAEKLERLGLWGLMPRTVYDTLIWCIYSGVHLPPPLREMQEITLTLDRAINSVIDRRLAE  PTNSADLLNVLLSADGGIWPRQRVRDEALTFMLAGHETTANAMSWFWYLMALNPQARDHM  LTELDDVLGMRRPTADDLGKLAWTTACLQESQRYFSSVWIIAREAVDDDIIDGHRIRRGT  TVVIPIHHIHHDPRWWPDPDRFDPGRFLRCPTDRPRCAYLPFGGGRRICIGQSFALMEMV  LMAAIMSQHFTFDLAPGYHVELEATLTLRPKHGVHVIGRRR*  >CYP144A1(TBHG_01736.1)  MRRSPKGSPGAVLDLQRRVDQAVSADHAELMTIAKDANTFFGAESVQDPYPLYERMRAAG  SVHRIANSDFYAVCGWDAVNEAIGRPEDFSSNLTATMTYTAEGTAKPFEMDPLGGPTHVL  ATADDPAHAVHRKLVLRHLAAKRIRVMEQFTVQAADRLWVDGMQDGCIEWMGAMANRLPM  MVVAELIGLPDPDIAQLVKWGYAATQLLEGLVENDQLVAAGVALMELSGYIFEQFDRAAA  DPRDNLLGELATACASGELDTLTAQVMMVTLFAAGGESTAALLGSAVWILATRPDIQQQV  RANPELLGAFIEETLRYEPPFRGHYRHVRNATTLDGTELPADSHLLLLWGAANRDPAQFE  APGEFRLDRAGGKGHISFGKGAHFCVGAALARLEARIVLRLLLDRTSVIEAADVGGWLPS  ILVRRIERLELAVQ*  >CYP143A1(TBHG_01743.1)  MTTPGEDHAGSFYLPRLEYSTLPMAVDRGVGWKTLRDAGPVVFMNGWYYLTRREDVLAAL  RNPKVFSSRKALQPPGNPLPVVPLAFDPPEHTRYRRILQPYFSPAALSKALPSLRRHTVA  MIDAIAGRGECEAMADLANLFPFQLFLVLYGLPLEDRDRLIGWKDAVIAMSDRPHPTEAD  VAAARELLEYLTAMVAERRRNPGPDVLSQVQIGEDPLSEIEVLGLSHLLILAGLDTVTAA  VGFSLLELARRPQLRAMLRDNPKQIRVFIEEIVRLEPSAPVAPRVTTEPVTVGGMTLPAG  SPVRLCMAAVNRDGSDAMSTDELVMDGKVHRHWGFGGGPHRCLGSHLARLELTLLVGEWL  NQIPDFELAPDYAPEIRFPSKSFALKNLPLRWS*  >CYP135A1(TBHG_00322.1)  MASTLTTGLPPGPRLPRYLQSVLYLRFREWFLPAMHRKYGDVFSLRVPPYADNLVVYTRP  EHIKEIFAADPRSLHAGEGNHILGFVMGEHSVLMTDEAEHARMRSLLMPAFTRAALRGYR  DMIASVAREHITRWRPHATINSLDHMNALTLDIILRVVFGVTDPKVKAELTSRLQQIINI  HPAILAGVPYPSLKRMNPWKRFFHNQTKIDEILYREIASRRIDSDLTARTDVLSRLLQTK  DTPTKPLTDAELRDQLITLLLAGHETTAAALSWTLWELAHAPEIQSQVVWAAVGGDDGFL  EAVLKEGMRRHTVIASTARKVTAPAEIGGWRLPAGTVVNTSILLAHASEVSHPKPTEFRP  SRFLDGSVAPNTWLPFGGGVRRCLGFGFALTEGAVILQEIFRRFTITAAGPSKGETPLVR  NITTVPKHGAHLRLIPQRRLGGLGDSDPP* |
| ***Mycobacterium tuberculosis* KZN 1435** |
| Database: TB; P450 count:20; families: 19; Subfamilies: 20 |
| >CYP138A1(TBMG_00137)  MSEVVTAAPAPPVVRLPPAVRGPKLFQGLAFVVSRRRLLGRFVRRYGKAFTANILMYGRV VVVADPQLARQVFTSSPEELGNIQPNLSRMFGSGSVFALDGDDHRRRRRLLAPPFHGKSM KNYETIIEEETLRETANWPQGQAFATLPSMMHITLNAILRAIFGAGGSELDELRRLIPPW VTLGSRLAALPKPKRDYGRLSPWGRLAEWRRQYDTVIDKLIEAERADPNFADRTDVLALM LRSTYDDGSIMSRKDIGDELLTLLAAGHETTAATLGWAFERLSRHPDVLAALVEEVDNGG HELRQAAILEVQRARTVIDFAARRVNPPVYQLGEWVIPRGYSIIINIAQIHGDPDVFPQP DRFDPQRYIGSKPSPFAWIPFGGGTRRCVGAAFANMEMDVVLRTVLRHFTLETTTAAGER SHGRGVAFTPKDGGRVVMRRR  >CYP135A1(TBMG_00331)  MASTLTTGLPPGPRLPRYLQSVLYLRFREWFLPAMHRKYGDVFSLRVPPYADNLVVYTRP EHIKEIFAADPRSLHAGEGNHILGFVMGEHSVLMTDEAEHARMRSLLMPAFTRAALRGYR DMIASVAREHITRWRPHATINSLDHMNALTLDIILRVVFGVTDPKVKAELTSRLQQIINI HPAILAGVPYPSLKRMNPWKRFFHNQTKIDEILYREIASRRIDSDLTARTDVLSRLLQTK DTPTKPLTDAELRDQLITLLLAGHETTAAALSWTLWELAHAPEIQSQVVWAAVGGDDGFL EAVLKEGMRRHTVIASTARKVTAPAEIGGWRLPAGTVVNTSILLAHASEVSHPKPTEFRP SRFLDGSVAPNTWLPFGGGVRRCLGFGFALTEGAVILQEIFRRFTITAAGPSKGETPLVR NITTVPKHGAHLRLIPQRRLGGLGDSDPP  >CYP135B1(TBMG_00573)  MSGTSSMGLPPGPRLSGSVQAVLMLRHGLRFLTACQRRYGSVFTLHVAGFGHMVYLSDPA AIKTVFAGNPSVFHAGEANSMLAGLLGDSSLLLIDDDVHRDRRRLMSPPFHRDAVARQAG PIAEIAAANIAGWPMAKAFAVAPKMSEITLEVILRTVIGASDPVRLAALRKVMPRLLNVG PWATLALANPSLLNNRLWSRLRRRIEEADALLYAEIADRRADPDLAARTDTLAMLVRAAD EDGRTMTERELRDQLITLLVAGHDTTATGLSWALERLTRHPVTLAKAVQAADASAAGDPA GDEYLDAVAKETLRIRPVVYDVGRVLTEAVEVAGYRLPAGVMVVPAIGLVHASAQLYPDP ERFDPDRMVGATLSPTTWLPFGGGNRRCLGATFAMVEMRVVLREILRRVELSTTTTSGER PKLKHVIMVPHRGARIRVRATRDVSATSQATAQGAGCPAARGGGPSRAVGSQ  >CYP51B1(TBMG_00779)  MSAVALPRVSGGHDEHGHLEEFRTDPIGLMQRVRDECGDVGTFQLAGKQVVLLSGSHANE FFFRAGDDDLDQAKAYPFMTPIFGEGVVFDASPERRKEMLHNAALRGEQMKGHAATIEDQ VRRMIADWGEAGEIDLLDFFAELTIYTSSACLIGKKFRDQLDGRFAKLYHELERGTDPLA YVDPYLPIESFRRRDEARNGLVALVADIMNGRIANPPTDKSDRDMLDVLIAVKAETGTPR FSADEITGMFISMMFAGHHTSSGTASWTLIELMRHRDAYAAVIDELDELYGDGRSVSFHA LRQIPQLENVLKETLRLHPPLIILMRVAKGEFEVQGHRIHEGDLVAASPAISNRIPEDFP DPHDFVPARYEQPRQEDLLNRWTWIPFGAGRHRCVGAAFAIMQIKAIFSVLLREYEFEMA QPPESYRNDHSKMVVQLAQPACVRYRRRTGV  >CYP123A1(TBMG_00781)  MTVRVGDPELVLDPYDYDFHEDPYPYYRRLRDEAPLYRNEERNFWAVSRHHDVLQGFRDS TALSNAYGVSLDPSSRTSEAYRVMSMLAMDDPAHLRMRTLVSKGFTPRRIRELEPQVLEL ARIHLDSALQTESFDFVAEFAGKLPMDVISELIGVPDTDRARIRALADAVLHREDGVADV PPPAMAASIELMRYYADLIAEFRRRPANNLTSALLAAELDGDRLSDQEIMAFLFLMVIAG NETTTKLLANAVYWAAHHPGQLARVFADHSRIPMWVEETLRYDTSSQILARTVAHDLTLY DTTIPEGEVLLLLPGIGQP  >CYP126A1(TBMG_00793)  MTTAAGLSGIDLTDLDNFADGFPHHLFAIHRREAPVYWHRPTEHTPDGEGFWSVATYAET LEVLRDPVTYSSVTGGQRRFGGTVLQDLPVAGQVLNMMDDPRHTRIRRLVSSGLTPRMIR RVEDDLRRRARGLLDGVEPGAPFDFVVEIAAELPMQMICILLGVPETDRHWLFEAVEPGF DFRGSRRATMPRLNVEDAGSRLYTYALELIAGKRAEPADDMLSVVANATIDDPDAPALSD AELYLFFHLLFSAGAETTRNSIAGGLLALAENPDQLQTLRSDFELLPTAIEEIVRWTSPS PSKRRTASRAVSLGGQPIEAGQKVVVWEGSANRDPSVFDRADEFDITRKPNPHLGFGQGV HYCLGANLARLELRVLFEELLSRFGSVRVVEPAEWTRSNRHTGIRHLVVELRGG  >CYP136A1(TBMG_00908)  MATIHPPAYLLDQAKRRFTPSFNNFPGMSLVEHMLLNTKFPEKKLAEPPPGSGLKPVVGD AGLPILGHMIEMLRGGPDYLMFLYKTKGPVVFGDSAVLPGVAALGPDAAQVIYSNRNKDY SQQGWVPVIGPFFHRGLMLLDFEEHMFHRRIMQEAFVRSRLAGYLEQMDRVVSRVVADDW VVNDARFLIYPAMKALTLDIASMVFMGHEPGTDHELVTKVNKAFTITTRAGNAVIRTSVP PFTWWRGLRARELLENYFTARVKERREASGNDLLTVLCQTEDDDGNRFSDADIVNHMIFL MMAAHDTSTSTATTMAYQLAAHPEWQQRCRDESDRHGDGPLDIESLEQLESLDLVMNESI RLVTPVQWAMRQTVRDTELLGYYLPKGTNVIAYPGMNHRLPEIWTDPLTFDPERFTEPRN EHKRHRYAFTPFGGGVHKCIGMVFDQLEIKTILHRLLRRYRLELSRPDYQPRWDYSAMPI PMDGMPIVLRPR  >CYP121A1(TBMG_01706)  MTATVLLEVPFSARGDRIPDAVAELRTREPIRKVRTITGAEAWLVSSYALCTQVLEDRRF SMKETAAAGAPRLNALTVPPEVVNNMGNIADAGLRKAVMKAITPKAPGLEQFLRDTANSL LDNLITEGAPADLRNDFADPLATALHCKVLGIPQEDGPKLFRSLSIAFMSSADPIPAAKI NWDRDIEYMAGILENPNITTGLMGELSRLRKDPAYSHVSDELFATIGVTFFGAGVISTGS FLTTALISLIQRPQLRNLLHEKPELIPAGVEELLRINLSFADGLPRLATADIQVGDVLVR KGELVLVLLEGANFDPEHFPNPGSIELDRPNPTSHLAFGRGQHFCPGSALGRRHAQIGIE ALLKKMPGVDLAVPIDQLVWRTRFQRRIPERLPVLW  >CYP128A1(TBMG_01714)  MTATQSPPEPAPDRVRLAGCPLAGTPDVGLTAQDATTALGVPTRRRASSGGIPVATSMWR DAQTVRTYGPAVAKALALRVAGKARSRLTGRHCRKFMQLTDFDPFDPAIAADPYPHYREL LAGERVQYNPKRDVYILSRYADVREAARNHDTLSSARGVTFSRGWLPFLPTSDPPAHTRM RKQLAPGMARGALETWRPMVDQLARELVGGLLTQTPADVVSTVAAPMPMRAITSVLGVDG PDEAAFCRLSNQAVRITDVALSASGLISLVQGFAGFRRLRALFTHRRDNGLLRECTVLGK LATHAEQGRLSDDELFFFAVLLLVAGYESTAHMISTLFLTLADYPDQLTLLAQQPDLIPS AIEEHLRFISPIQNICRTTRVDYSVGQAVIPAGSLVLLAWGAANRDPRQYEDPDVFRADR NPVGHLAFGSGIHLCPGTQLARMEGQAILREIVANIDRIEVVEPPTWTTNANLRGLTRLR VAVTPRVAP  >CYP124A1(TBMG_01716)  MGLNTAIATRVNGTPPPEVPIADIELGSLDFWALDDDVRDGAFATLRREAPISFWPTIEL PGFVAGNGHWALTKYDDVFYASRHPDIFSSYPNITINDQTPELAEYFGSMIVLDDPRHQR LRSIVSRAFTPKVVARIEAAVRDRAHRLVSSMIANNPDRQADLVSELAGPLPLQIICDMM GIPKADHQRIFHWTNVILGFGDPDLATDFDEFMQVSADIGAYATALAEDRRVNHHDDLTS SLVEAEVDGERLSSREIASFFILLVVAGNETTRNAITHGVLALSRYPEQRDRWWSDFDGL APTAVEEIVRWASPVVYMRRTLTQDIELRGTKMAAGDKVSLWYCSANRDESKFADPWTFD LARNPNPHLGFGGGGAHFCLGANLARREIRVAFDELRRQMPDVVATEEPARLLSQFIHGI KTLPVTWS    >CYP140A1(TBMG_02114)  MKDKLHWLAMHGVIRGIAAIGIRRGDLQARLIADPAVATDPVPFYDEVRSHGALVRNRAN YLTVDHRLAHDLLRSDDFRVVSFGENLPPPLRWLERRTRGDQLHPLREPSLLAVEPPDHT RYRKTVSAVFTSRAVSALRDLVEQTAINLLDRFAEQPGIVDVVGRYCSQLPIVVISEILG VPEHDRPRVLEFGELAAPSLDIGIPWRQYLRVQQGIRGFDCWLEGHLQQLRHAPGDDLMS QLIQIAESGDNETQLDETELRAIAGLVLVAGFETTVNLLGNGIRMLLDTPEHLATLRQHP ELWPNTVEEILRLDSPVQLTARVACRDVEVAGVRIKRGEVVVIYLAAANRDPAVFPDPHR FDIERPNAGRHLAFSTGRHFCLGAALARAEGEVGLRTFFDRFPDVRAAGAGSRRDTRVLR GWSTLPVTLGPARSMVSP  >CYP143A1(TBMG_02212)  MTTPGEDHAGSFYLPRLEYSTLPMAVDRGVGWKTLRDAGPVVFMNGWYYLTRREDVLAAL RNPKVFSSRKALQPPGNPLPVVPLAFDPPEHTRYRRILQPYFSPAALSKALPSLRRHTVA MIDAIAGRGECEAMADLANLFPFQLFLVLYGLPLEDRDRLIGWKDAVIAMSDRPHPTEAD VAAARELLEYLTAMVAERRRNPGPDVLSQVQIGEDPLSEIEVLGLSHLLILAGLDTVTAA VGFSLLELARRPQLRAMLRDNPKQIRVFIEEIVRLEPSAPVAPRVTTEPVTVGGMTLPAG SPVRLCMAAVNRDGSDAMSTDELVMDGKVHRHWGFGGGPHRCLGSHLARLELTLLVGEWL NQIPDFELAPDYAPEIRFPSKSFALKNLPLRWS  >CYP144A1(TBMG_02218)  MRRSPKGSPGAVLDLQRRVDQAVSADHAELMTIAKDANTFFGAESVQDPYPLYERMRAAG SVHRIANSDFYAVCGWDAVNEAIGRPEDFSSNLTATMTYTAEGTAKPFEMDPLGGPTHVL ATADDPAHAVHRKLVLRHLAAKRIRVMEQFTVQAADRLWVDGMQDGCIEWMGAMANRLPM MVVAELIGLPDPDIAQLVKWGYAATQLLEGLVENDQLVAAGVALMELSGYIFEQFDRAAA DPRDNLLGELATACASGELDTLTAQVMMVTLFAAGGESTAALLGSAVWILATRPDIQQQV RANPELLGAFIEETLRYEPPFRGHYRHVRNATTLDGTELPADSHLLLLWGAANRDPAQFE APGEFRLDRAGGKGHISFGKGAHFCVGAALARLEARIVLRLLLDRTSVIEAADVGGWLPS ILVRRIERLELAVQ  >CYP139A1(TBMG_02327)  MRYPLGEALLALYRWRGPLINAGVGGHGYTYLLGAEANRFVFANADAFSWSQTFESLVPV DGPTALIVSDGADHRRRRSVVAPGLRHHHVQRYVATMVSNIDTVIDGWQPGQRLDIYQEL RSAVRRSTAESLFGQRLAVHSDFLGEQLQPLLDLTRRPPQVMRLQQRVNSPGWRRAMAAR KRIDDLIDAQIADARTAPRPDDHMLTTLISGCSEEGTTLSDNEIRDSIVSLITAGYETTS GALAWAIYALLTVPGTWESAASEVARVLGGRVPAADDLSALTYLNGVVHETLRLYSPGVI SARRVLRDLWFDGHRIRAGRLLIFSAYVTHRLPEIWPEPTEFRPLRWDPNAADYRKPAPH EFIPFSGGLHRCIGAVMATTEMTVILARLVARAMLQLPAQRTHRIRAANFAALRPWPGLT VEIRKSAPAQ  >CYP132A1(TBMG_02586)  MATATTQRPLKGPAKRMSTWTMTREAITIGFDAGDGFLGRLRGSDITRFRCAGRRFVSIS HPDYVDHVLHEARLKYVKSDEYGPIRATAGLNLLTDEGDSWARHRGALNSTFARRHLRGL VGLMIDPIADVTAALVPGAQFDMHQSMVETTLRVVANALFSQDFGPLVQSMHDLATRGLR RAEKLERLGLWGLMPRTVYDTLIWCIYSGVHLPPPLREMQEITLTLDRAINSVIDRRLAE PTNSADLLNVLLSADGGIWPRQRVRDEALTFMLAGHETTANAMSWFWYLMALNPQARDHM LTELDDVLGMRRPTADDLGKLAWTTACLQESQRYFSSVWIIAREAVDDDIIDGHRIRRGT TVVIPIHHIHHDPRWWPDPDRFDPGRFLRCPTDRPRCAYLPFGGGRRICIGQSFALMEMV LMAAIMSQHFTFDLAPGYHVELEATLTLRPKHGVHVIGRRR  >CYP130A1(TBMG_02725)  MSHEFQLATAETWPNPWPMYRALRDHDPVHHVVPPQRPEYDYYVLSRHADVWSAARDHQT FSSAQGLTVNYGELEMIGLHDTPPMVMQDPPVHTEFRKLVSRGFTPRQVETVEPTVRKFV VERLEKLRANGGGDIVTELFNPLPSMVVAHYLGVPEEDWTQFDGWTQAIVAANAVDGATT GALDAVGSMMAYFTGLIERRRTEPADDAISHLVAAGVGADGDTAGTLSILAFTFTMVTGG NDTVTGMLGGSMPLLHRRPDQRRLLLDDPEGIPDAVEELLRLTSPVQGLARTTTRDVTIG DTTIPAGRRVLLLYGSANRDERQYGPDAAELDVTRCPRNILTFSHGAHHCLGAAAARMQC RVALTELLARCPDFEVAESRIVWSGGSYVRRPLSVPFRVTS  >CYP141A1(TBMG_03165)  MTSTSIPTFPFDRPVPTEPSPMLSELRNSCPVAPIELPSGHTAWLVTRFDDVKGVLSDKR FSCRAAAHPSSPPFVPFVQLCPSLLSIDGPQHTAARRLLAQGLNPGFIARMRPVVQQIVD NALDDLAAAEPPVDFQEIVSVPIGEQLMAKLLGVEPETVHELAAHVDAAMSVCEIGDEEV SRRWSALCTMVIDILHRKLAEPGDDLLSTIAQANRQQSTMTDEQVVGMLLTVVIGGVDTP IAVITNGLASLLHHRDQYERLVEDPGRVARAVEEIVRFNPATEIEHLRVVTEDVVIAGTA LSAGSPAFTSITSANRDSDQFLDPDEFDVERNPNEHIAFGYGPHACPASAYSRMCLTTFF TSLTQRFPQLQLARPFEDLERRGKGLHSVGIKELLVTWPT  >CYP142A1(TBMG_03557)  MTEAPDVDLADGNFYASREARAAYRWMRANQPVFRDRNGLAAASTYQAVIDAERQPELFS NAGGIRPDQPALPMMIDMDDPAHLLRRKLVNAGFTRKRVKDKEASIAALCDTLIDAVCER GECDFVRDLAAPLPMAVIGDMLGVRPEQRDMFLRWSDDLVTFLSSHVSQEDFQITMDAFA AYNDFTRATIAARRADPTDDLVSVLVSSEVDGERLSDDELVMETLLILIGGDETTRHTLS GGTEQLLRNRDQWDLLQRDPSLLPGAIEEMLRWTAPVKNMCRVLTADTEFHGTALCAGEK MMLLFESANFDEAVFCEPEKFDVQRNPNSHLAFGFGTHFCLGNQLARLELSLMTERVLRR LPDLRLVADDSVLPLRPANFVSGLESMPVVFTPSPPLG  >CYP125A1(TBMG_03584)  MSWNHQSVEIAVRRTTVPSPNLPPGFDFTDPAIYAERLPVAEFAELRSAAPIWWNGQDPG KGGGFHDGGFWAITKLNDVKEISRHSDVFSSYENGVIPRFKNDIAREDIEVQRFVMLNMD APHHTRLRKIISRGFTPRAVGRLHDELQERAQKIAAEAAAAGSGDFVEQVSCELPLQAIA GLLGVPQEDRGKLFHWSNEMTGNEDPEYAHIDPKASSAELIGYAMKMAEEKAKNPADDIV TQLIQADIDGEKLSDDEFGFFVVMLAVAGNETTRNSITQGMMAFAEHPDQWELYKKVRPE TAADEIVRWATPVTAFQRTALRDYELSGVQIKKGQRVVMFYRSANFDEEVFQDPFTFNTL RNPNPHVGFGGTGAHYCIGANLARMTINLIFNAVADHMPDLKPISAPERLRSGWLNGIKH WQVDYTGRCPVAH  >CYP137A1(TBMG_03727)  MVLRSLASPAALTDPKRCASVVGVAAFAVRREHAPDALGGPPGLPAPRGFRAAFAAAYAV AYLAGGERRMLRLIRRYGPIMTMPILSLGDVAIVSDSALAKEVFTAPTDVLLGGEGVGPA AAIYGSGSMFVQEEPEHLRRRKLLTPPLHGAALDRYVPIIENSTRAAMHTWPVDRPFAML TVARSLMLDVIVKVIFGVDDPEEVRRLGRPFERLLNLGVSEQLTVRYALRRLGALRVWPA RARANTEIDDVVMALIAQRRADPRLGERHDVLSLLVSARGESGEQLSDSEIRDDLITLVL AGHETTATTLAWAFDLLLHHPDALRRVRAEAVGGGEAFTTAVINETLRVRPPAPLTARVA AQPLTIGGYRVEAGTRIVVHIIAINRSAEVYEHPHEFRPERFLGTRPQTYAWVPFGGGVK RCLGANFSMRELITVLHVLLREGEFTAVDDEPERIVRRSIMLVPRRGTRVRFRPAR |
| ***Mycobacterium tuberculosis* KZN 605** |
| Database: TB; P450 count:20; Families: 19; Subfamilies: 20 |
| >CYP138A1(TBXG_000137)  MSEVVTAAPAPPVVRLPPAVRGPKLFQGLAFVVSRRRLLGRFVRRYGKAFTANILMYGRV VVVADPQLARQVFTSSPEELGNIQPNLSRMFGSGSVFALDGDDHRRRRRLLAPPFHGKSM KNYETIIEEETLRETANWPQGQAFATLPSMMHITLNAILRAIFGAGGSELDELRRLIPPW VTLGSRLAALPKPKRDYGRLSPWGRLAEWRRQYDTVIDKLIEAERADPNFADRTDVLALM LRSTYDDGSIMSRKDIGDELLTLLAAGHETTAATLGWAFERLSRHPDVLAALVEEVDNGG HELRQAAILEVQRARTVIDFAARRVNPPVYQLGEWVIPRGYSIIINIAQIHGDPDVFPQP DRFDPQRYIGSKPSPFAWIPFGGGTRRCVGAAFANMEMDVVLRTVLRHFTLETTTAAGER SHGRGVAFTPKDGGRVVMRRR  >CYP135A1(TBXG_000329)  MASTLTTGLPPGPRLPRYLQSVLYLRFREWFLPAMHRKYGDVFSLRVPPYADNLVVYTRP EHIKEIFAADPRSLHAGEGNHILGFVMGEHSVLMTDEAEHARMRSLLMPAFTRAALRGYR DMIASVAREHITRWRPHATINSLDHMNALTLDIILRVVFGVTDPKVKAELTSRLQQIINI HPAILAGVPYPSLKRMNPWKRFFHNQTKIDEILYREIASRRIDSDLTARTDVLSRLLQTK DTPTKPLTDAELRDQLITLLLAGHETTAAALSWTLWELAHAPEIQSQVVWAAVGGDDGFL EAVLKEGMRRHTVIASTARKVTAPAEIGGWRLPAGTVVNTSILLAHASEVSHPKPTEFRP SRFLDGSVAPNTWLPFGGGVRRCLGFGFALTEGAVILQEIFRRFTITAAGPSKGETPLVR NITTVPKHGAHLRLIPQRRLGGLGDSDPP  >CYP135B1(TBXG_000570)  MSGTSSMGLPPGPRLSGSVQAVLMLRHGLRFLTACQRRYGSVFTLHVAGFGHMVYLSDPA AIKTVFAGNPSVFHAGEANSMLAGLLGDSSLLLIDDDVHRDRRRLMSPPFHRDAVARQAG PIAEIAAANIAGWPMAKAFAVAPKMSEITLEVILRTVIGASDPVRLAALRKVMPRLLNVG PWATLALANPSLLNNRLWSRLRRRIEEADALLYAEIADRRADPDLAARTDTLAMLVRAAD EDGRTMTERELRDQLITLLVAGHDTTATGLSWALERLTRHPVTLAKAVQAADASAAGDPA GDEYLDAVAKETLRIRPVVYDVGRVLTEAVEVAGYRLPAGVMVVPAIGLVHASAQLYPDP ERFDPDRMVGATLSPTTWLPFGGGNRRCLGATFAMVEMRVVLREILRRVELSTTTTSGER PKLKHVIMVPHRGARIRVRATRDVSATSQATAQGAGCPAARGGGPSRAVGSQ  >CYP51B1(TBXG_000772)  MSAVALPRVSGGHDEHGHLEEFRTDPIGLMQRVRDECGDVGTFQLAGKQVVLLSGSHANE FFFRAGDDDLDQAKAYPFMTPIFGEGVVFDASPERRKEMLHNAALRGEQMKGHAATIEDQ VRRMIADWGEAGEIDLLDFFAELTIYTSSACLIGKKFRDQLDGRFAKLYHELERGTDPLA YVDPYLPIESFRRRDEARNGLVALVADIMNGRIANPPTDKSDRDMLDVLIAVKAETGTPR FSADEITGMFISMMFAGHHTSSGTASWTLIELMRHRDAYAAVIDELDELYGDGRSVSFHA LRQIPQLENVLKETLRLHPPLIILMRVAKGEFEVQGHRIHEGDLVAASPAISNRIPEDFP DPHDFVPARYEQPRQEDLLNRWTWIPFGAGRHRCVGAAFAIMQIKAIFSVLLREYEFEMA QPPESYRNDHSKMVVQLAQPACVRYRRRTGV  >CYP126A1(TBXG_000786)  MTTAAGLSGIDLTDLDNFADGFPHHLFAIHRREAPVYWHRPTEHTPDGEGFWSVATYAET LEVLRDPVTYSSVTGGQRRFGGTVLQDLPVAGQVLNMMDDPRHTRIRRLVSSGLTPRMIR RVEDDLRRRARGLLDGVEPGAPFDFVVEIAAELPMQMICILLGVPETDRHWLFEAVEPGF DFRGSRRATMPRLNVEDAGSRLYTYALELIAGKRAEPADDMLSVVANATIDDPDAPALSD AELYLFFHLLFSAGAETTRNSIAGGLLALAENPDQLQTLRSDFELLPTAIEEIVRWTSPS PSKRRTASRAVSLGGQPIEAGQKVVVWEGSANRDPSVFDRADEFDITRKPNPHLGFGQGV HYCLGANLARLELRVLFEELLSRFGSVRVVEPAEWTRSNRHTGIRHLVVELRGG  >CYP136A1(TBXG_000900)  MATIHPPAYLLDQAKRRFTPSFNNFPGMSLVEHMLLNTKFPEKKLAEPPPGSGLKPVVGD AGLPILGHMIEMLRGGPDYLMFLYKTKGPVVFGDSAVLPGVAALGPDAAQVIYSNRNKDY SQQGWVPVIGPFFHRGLMLLDFEEHMFHRRIMQEAFVRSRLAGYLEQMDRVVSRVVADDW VVNDARFLIYPAMKALTLDIASMVFMGHEPGTDHELVTKVNKAFTITTRAGNAVIRTSVP PFTWWRGLRARELLENYFTARVKERREASGNDLLTVLCQTEDDDGNRFSDADIVNHMIFL MMAAHDTSTSTATTMAYQLAAHPEWQQRCRDESDRHGDGPLDIESLEQLESLDLVMNESI RLVTPVQWAMRQTVRDTELLGYYLPKGTNVIAYPGMNHRLPEIWTDPLTFDPERFTEPRN EHKRHRYAFTPFGGGVHKCIGMVFDQLEIKTILHRLLRRYRLELSRPDYQPRWDYSAMPI PMDGMPIVLRPR  >CYP121A1(TBXG_001689)  MTATVLLEVPFSARGDRIPDAVAELRTREPIRKVRTITGAEAWLVSSYALCTQVLEDRRF SMKETAAAGAPRLNALTVPPEVVNNMGNIADAGLRKAVMKAITPKAPGLEQFLRDTANSL LDNLITEGAPADLRNDFADPLATALHCKVLGIPQEDGPKLFRSLSIAFMSSADPIPAAKI NWDRDIEYMAGILENPNITTGLMGELSRLRKDPAYSHVSDELFATIGVTFFGAGVISTGS FLTTALISLIQRPQLRNLLHEKPELIPAGVEELLRINLSFADGLPRLATADIQVGDVLVR KGELVLVLLEGANFDPEHFPNPGSIELDRPNPTSHLAFGRGQHFCPGSALGRRHAQIGIE ALLKKMPGVDLAVPIDQLVWRTRFQRRIPERLPVLW  >CYP128A1(TBXG_001696)  MTATQSPPEPAPDRVRLAGCPLAGTPDVGLTAQDATTALGVPTRRRASSGGIPVATSMWR DAQTVRTYGPAVAKALALRVAGKARSRLTGRHCRKFMQLTDFDPFDPAIAADPYPHYREL LAGERVQYNPKRDVYILSRYADVREAARNHDTLSSARGVTFSRGWLPFLPTSDPPAHTRM RKQLAPGMARGALETWRPMVDQLARELVGGLLTQTPADVVSTVAAPMPMRAITSVLGVDG PDEAAFCRLSNQAVRITDVALSASGLISLVQGFAGFRRLRALFTHRRDNGLLRECTVLGK LATHAEQGRLSDDELFFFAVLLLVAGYESTAHMISTLFLTLADYPDQLTLLAQQPDLIPS AIEEHLRFISPIQNICRTTRVDYSVGQAVIPAGSLVLLAWGAANRDPRQYEDPDVFRADR NPVGHLAFGSGIHLCPGTQLARMEGQAILREIVANIDRIEVVEPPTWTTNANLRGLTRLR VAVTPRVAP  >CYP124A1(TBXG_001698)  MGLNTAIATRVNGTPPPEVPIADIELGSLDFWALDDDVRDGAFATLRREAPISFWPTIEL PGFVAGNGHWALTKYDDVFYASRHPDIFSSYPNITINDQTPELAEYFGSMIVLDDPRHQR LRSIVSRAFTPKVVARIEAAVRDRAHRLVSSMIANNPDRQADLVSELAGPLPLQIICDMM GIPKADHQRIFHWTNVILGFGDPDLATDFDEFMQVSADIGAYATALAEDRRVNHHDDLTS SLVEAEVDGERLSSREIASFFILLVVAGNETTRNAITHGVLALSRYPEQRDRWWSDFDGL APTAVEEIVRWASPVVYMRRTLTQDIELRGTKMAAGDKVSLWYCSANRDESKFADPWTFD LARNPNPHLGFGGGGAHFCLGANLARREIRVAFDELRRQMPDVVATEEPARLLSQFIHGI KTLPVTWS  >CYP140A1(TBXG_002096)  MKDKLHWLAMHGVIRGIAAIGIRRGDLQARLIADPAVATDPVPFYDEVRSHGALVRNRAN YLTVDHRLAHDLLRSDDFRVVSFGENLPPPLRWLERRTRGDQLHPLREPSLLAVEPPDHT RYRKTVSAVFTSRAVSALRDLVEQTAINLLDRFAEQPGIVDVVGRYCSQLPIVVISEILG VPEHDRPRVLEFGELAAPSLDIGIPWRQYLRVQQGIRGFDCWLEGHLQQLRHAPGDDLMS QLIQIAESGDNETQLDETELRAIAGLVLVAGFETTVNLLGNGIRMLLDTPEHLATLRQHP ELWPNTVEEILRLDSPVQLTARVACRDVEVAGVRIKRGEVVVIYLAAANRDPAVFPDPHR FDIERPNAGRHLAFSTGRHFCLGAALARAEGEVGLRTFFDRFPDVRAAGAGSRRDTRVLR GWSTLPVTLGPARSMVSP  >CYP143A1(TBXG_002193)  MTTPGEDHAGSFYLPRLEYSTLPMAVDRGVGWKTLRDAGPVVFMNGWYYLTRREDVLAAL RNPKVFSSRKALQPPGNPLPVVPLAFDPPEHTRYRRILQPYFSPAALSKALPSLRRHTVA MIDAIAGRGECEAMADLANLFPFQLFLVLYGLPLEDRDRLIGWKDAVIAMSDRPHPTEAD VAAARELLEYLTAMVAERRRNPGPDVLSQVQIGEDPLSEIEVLGLSHLLILAGLDTVTAA VGFSLLELARRPQLRAMLRDNPKQIRVFIEEIVRLEPSAPVAPRVTTEPVTVGGMTLPAG SPVRLCMAAVNRDGSDAMSTDELVMDGKVHRHWGFGGGPHRCLGSHLARLELTLLVGEWL NQIPDFELAPDYAPEIRFPSKSFALKNLPLRWS  >CYP144A1(TBXG_002200)  MRRSPKGSPGAVLDLQRRVDQAVSADHAELMTIAKDANTFFGAESVQDPYPLYERMRAAG SVHRIANSDFYAVCGWDAVNEAIGRPEDFSSNLTATMTYTAEGTAKPFEMDPLGGPTHVL ATADDPAHAVHRKLVLRHLAAKRIRVMEQFTVQAADRLWVDGMQDGCIEWMGAMANRLPM MVVAELIGLPDPDIAQLVKWGYAATQLLEGLVENDQLVAAGVALMELSGYIFEQFDRAAA DPRDNLLGELATACASGELDTLTAQVMMVTLFAAGGESTAALLGSAVWILATRPDIQQQV RANPELLGAFIEETLRYEPPFRGHYRHVRNATTLDGTELPADSHLLLLWGAANRDPAQFE APGEFRLDRAGGKGHISFGKGAHFCVGAALARLEARIVLRLLLDRTSVIEAADVGGWLPS ILVRRIERLELAVQ  >CYP139A1(TBXG_002309)  MRYPLGEALLALYRWRGPLINAGVGGHGYTYLLGAEANRFVFANADAFSWSQTFESLVPV DGPTALIVSDGADHRRRRSVVAPGLRHHHVQRYVATMVSNIDTVIDGWQPGQRLDIYQEL RSAVRRSTAESLFGQRLAVHSDFLGEQLQPLLDLTRRPPQVMRLQQRVNSPGWRRAMAAR KRIDDLIDAQIADARTAPRPDDHMLTTLISGCSEEGTTLSDNEIRDSIVSLITAGYETTS GALAWAIYALLTVPGTWESAASEVARVLGGRVPAADDLSALTYLNGVVHETLRLYSPGVI SARRVLRDLWFDGHRIRAGRLLIFSAYVTHRLPEIWPEPTEFRPLRWDPNAADYRKPAPH EFIPFSGGLHRCIGAVMATTEMTVILARLVARAMLQLPAQRTHRIRAANFAALRPWPGLT VEIRKSAPAQ  >CYP132A1(TBXG_002566)  MATATTQRPLKGPAKRMSTWTMTREAITIGFDAGDGFLGRLRGSDITRFRCAGRRFVSIS HPDYVDHVLHEARLKYVKSDEYGPIRATAGLNLLTDEGDSWARHRGALNSTFARRHLRGL VGLMIDPIADVTAALVPGAQFDMHQSMVETTLRVVANALFSQDFGPLVQSMHDLATRGLR RAEKLERLGLWGLMPRTVYDTLIWCIYSGVHLPPPLREMQEITLTLDRAINSVIDRRLAE PTNSADLLNVLLSADGGIWPRQRVRDEALTFMLAGHETTANAMSWFWYLMALNPQARDHM LTELDDVLGMRRPTADDLGKLAWTTACLQESQRYFSSVWIIAREAVDDDIIDGHRIRRGT TVVIPIHHIHHDPRWWPDPDRFDPGRFLRCPTDRPRCAYLPFGGGRRICIGQSFALMEMV LMAAIMSQHFTFDLAPGYHVELEATLTLRPKHGVHVIGRRR  >CYP130A1(TBXG_002705)  MTSVMSHEFQLATAETWPNPWPMYRALRDHDPVHHVVPPQRPEYDYYVLSRHADVWSAAR DHQTFSSAQGLTVNYGELEMIGLHDTPPMVMQDPPVHTEFRKLVSRGFTPRQVETVEPTV RKFVVERLEKLRANGGGDIVTELFNPLPSMVVAHYLGVPEEDWTQFDGWTQAIVAANAVD GATTGALDAVGSMMAYFTGLIERRRTEPADDAISHLVAAGVGADGDTAGTLSILAFTFTM VTGGNDTVTGMLGGSMPLLHRRPDQRRLLLDDPEGIPDAVEELLRLTSPVQGLARTTTRD VTIGDTTIPAGRRVLLLYGSANRDERQYGPDAAELDVTRCPRNILTFSHGAHHCLGAAAA RMQCRVALTELLARCPDFEVAESRIVWSGGSYVRRPLSVPFRVTS  >CYP141A1(TBXG_003143)  MTSTSIPTFPFDRPVPTEPSPMLSELRNSCPVAPIELPSGHTAWLVTRFDDVKGVLSDKR FSCRAAAHPSSPPFVPFVQLCPSLLSIDGPQHTAARRLLAQGLNPGFIARMRPVVQQIVD NALDDLAAAEPPVDFQEIVSVPIGEQLMAKLLGVEPETVHELAAHVDAAMSVCEIGDEEV SRRWSALCTMVIDILHRKLAEPGDDLLSTIAQANRQQSTMTDEQVVGMLLTVVIGGVDTP IAVITNGLASLLHHRDQYERLVEDPGRVARAVEEIVRFNPATEIEHLRVVTEDVVIAGTA LSAGSPAFTSITSANRDSDQFLDPDEFDVERNPNEHIAFGYGPHACPASAYSRMCLTTFF TSLTQRFPQLQLARPFEDLERRGKGLHSVGIKELLVTWPT  >CYP142A1(TBXG_003533)  MTEAPDVDLADGNFYASREARAAYRWMRANQPVFRDRNGLAAASTYQAVIDAERQPELFS NAGGIRPDQPALPMMIDMDDPAHLLRRKLVNAGFTRKRVKDKEASIAALCDTLIDAVCER GECDFVRDLAAPLPMAVIGDMLGVRPEQRDMFLRWSDDLVTFLSSHVSQEDFQITMDAFA AYNDFTRATIAARRADPTDDLVSVLVSSEVDGERLSDDELVMETLLILIGGDETTRHTLS GGTEQLLRNRDQWDLLQRDPSLLPGAIEEMLRWTAPVKNMCRVLTADTEFHGTALCAGEK MMLLFESANFDEAVFCEPEKFDVQRNPNSHLAFGFGTHFCLGNQLARLELSLMTERVLRR LPDLRLVADDSVLPLRPANFVSGLESMPVVFTPSPPLG  >CYP125A1(TBXG_003560)  MSWNHQSVEIAVRRTTVPSPNLPPGFDFTDPAIYAERLPVAEFAELRSAAPIWWNGQDPG KGGGFHDGGFWAITKLNDVKEISRHSDVFSSYENGVIPRFKNDIAREDIEVQRFVMLNMD APHHTRLRKIISRGFTPRAVGRLHDELQERAQKIAAEAAAAGSGDFVEQVSCELPLQAIA GLLGVPQEDRGKLFHWSNEMTGNEDPEYAHIDPKASSAELIGYAMKMAEEKAKNPADDIV TQLIQADIDGEKLSDDEFGFFVVMLAVAGNETTRNSITQGMMAFAEHPDQWELYKKVRPE TAADEIVRWATPVTAFQRTALRDYELSGVQIKKGQRVVMFYRSANFDEEVFQDPFTFNTL RNPNPHVGFGGTGAHYCIGANLARMTINLIFNAVADHMPDLKPISAPERLRSGWLNGIKH WQVDYTGRCPVAH  >CYP137A1(TBXG_003698)  MVLRSLASPAALTDPKRCASVVGVAAFAVRREHAPDALGGPPGLPAPRGFRAAFAAAYAV AYLAGGERRMLRLIRRYGPIMTMPILSLGDVAIVSDSALAKEVFTAPTDVLLGGEGVGPA AAIYGSGSMFVQEEPEHLRRRKLLTPPLHGAALDRYVPIIENSTRAAMHTWPVDRPFAML TVARSLMLDVIVKVIFGVDDPEEVRRLGRPFERLLNLGVSEQLTVRYALRRLGALRVWPA RARANTEIDDVVMALIAQRRADPRLGERHDVLSLLVSARGESGEQLSDSEIRDDLITLVL AGHETTATTLAWAFDLLLHHPDALRRVRAEAVGGGEAFTTAVINETLRVRPPAPLTARVA AQPLTIGGYRVEAGTRIVVHIIAINRSAEVYEHPHEFRPERFLGTRPQTYAWVPFGGGVK RCLGANFSMRELITVLHVLLREGEFTAVDDEPERIVRRSIMLVPRRGTRVRFRPAR  >CYP123A1(TBXG_000774)(truncated)  MTVRVGDPELVLDPYDYDFHEDPYPYYRRLRDEAPLYRNEERNFWAVSRHHDVLQGFRDS  TALSNAYGVSLDPSSRTSEAYRVMSMLAMDDPAHLRMRTLVSKGFTPRRIRELEPQVLEL  ARIHLDSALQTESFDFVAEFAGKLPMDVISELIGVPDTDRARIRALADAVLHREDGVADV  PPPAMAASIELMRYYADLIAEFRRRPANNLTSALLAAELDGDRLSDQEIMAFLFLMVIAG  NETTTKLLANAVYWAAHHPGQLARVFADHSRIPMWVEETLRYDTSSQILARTVAHDLTLY  DTTIPEGEVLLLLPGIGQP-RPGVRRPGRLSHRPRNRLQTSQFRQRCPLLSGGSPGPDGS  PGGPGRAAASDPQLRSRRRQRRARPFQQRARICPSADQRAGQV |
| ***Mycobacterium tuberculosis* KZN 4207** |
| Database: TB; P450 count:21; Families: 19; Subfamilies: 20 |
| >CYP138A1(TBSG_00138)  MSEVVTAAPAPPVVRLPPAVRGPKLFQGLAFVVSRRRLLGRFVRRYGKAFTANILMYGRV VVVADPQLARQVFTSSPEELGNIQPNLSRMFGSGSVFALDGDDHRRRRRLLAPPFHGKSM KNYETIIEEETLRETANWPQGQAFATLPSMMHITLNAILRAIFGAGGSELDELRRLIPPW VTLGSRLAALPKPKRDYGRLSPWGRLAEWRRQYDTVIDKLIEAERADPNFADRTDVLALM LRSTYDDGSIMSRKDIGDELLTLLAAGHETTAATLGWAFERLSRHPDVLAALVEEVDNGG HELRQAAILEVQRARTVIDFAARRVNPPVYQLGEWVIPRGYSIIINIAQIHGDPDVFPQP DRFDPQRYIGSKPSPFAWIPFGGGTRRCVGAAFANMEMDVVLRTVLRHFTLETTTAAGER SHGRGVAFTPKDGGRVVMRRR  >CYP135A1(TBSG_00334)  MASTLTTGLPPGPRLPRYLQSVLYLRFREWFLPAMHRKYGDVFSLRVPPYADNLVVYTRP EHIKEIFAADPRSLHAGEGNHILGFVMGEHSVLMTDEAEHARMRSLLMPAFTRAALRGYR DMIASVAREHITRWRPHATINSLDHMNALTLDIILRVVFGVTDPKVKAELTSRLQQIINI HPAILAGVPYPSLKRMNPWKRFFHNQTKIDEILYREIASRRIDSDLTARTDVLSRLLQTK DTPTKPLTDAELRDQLITLLLAGHETTAAALSWTLWELAHAPEIQSQVVWAAVGGDDGFL EAVLKEGMRRHTVIASTARKVTAPAEIGGWRLPAGTVVNTSILLAHASEVSHPKPTEFRP SRFLDGSVAPNTWLPFGGGVRRCLGFGFALTEGAVILQEIFRRFTITAAGPSKGETPLVR NITTVPKHGAHLRLIPQRRLGGLGDSDPP  >CYP135B1(TBSG_00579)  MSGTSSMGLPPGPRLSGSVQAVLMLRHGLRFLTACQRRYGSVFTLHVAGFGHMVYLSDPA AIKTVFAGNPSVFHAGEANSMLAGLLGDSSLLLIDDDVHRDRRRLMSPPFHRDAVARQAG PIAEIAAANIAGWPMAKAFAVAPKMSEITLEVILRTVIGASDPVRLAALRKVMPRLLNVG PWATLALANPSLLNNRLWSRLRRRIEEADALLYAEIADRRADPDLAARTDTLAMLVRAAD EDGRTMTERELRDQLITLLVAGHDTTATGLSWALERLTRHPVTLAKAVQAADASAAGDPA GDEYLDAVAKETLRIRPVVYDVGRVLTEAVEVAGYRLPAGVMVVPAIGLVHASAQLYPDP ERFDPDRMVGATLSPTTWLPFGGGNRRCLGATFAMVEMRVVLREILRRVELSTTTTSGER PKLKHVIMVPHRGARIRVRATRDVSATSQATAQGAGCPAARGGGPSRAVGSQ  >CYP51B1(TBSG_00783)  MSAVALPRVSGGHDEHGHLEEFRTDPIGLMQRVRDECGDVGTFQLAGKQVVLLSGSHANE FFFRAGDDDLDQAKAYPFMTPIFGEGVVFDASPERRKEMLHNAALRGEQMKGHAATIEDQ VRRMIADWGEAGEIDLLDFFAELTIYTSSACLIGKKFRDQLDGRFAKLYHELERGTDPLA YVDPYLPIESFRRRDEARNGLVALVADIMNGRIANPPTDKSDRDMLDVLIAVKAETGTPR FSADEITGMFISMMFAGHHTSSGTASWTLIELMRHRDAYAAVIDELDELYGDGRSVSFHA LRQIPQLENVLKETLRLHPPLIILMRVAKGEFEVQGHRIHEGDLVAASPAISNRIPEDFP DPHDFVPARYEQPRQEDLLNRWTWIPFGAGRHRCVGAAFAIMQIKAIFSVLLREYEFEMA QPPESYRNDHSKMVVQLAQPACVRYRRRTGV  >CYP123A1(TBSG_00786)  MTVRVGDPELVLDPYDYDFHEDPYPYYRRLRDEAPLYRNEERNFWAVSRHHDVLQGFRDS TALSNAYGVSLDPSSRTSEAYRVMSMLAMDDPAHLRMRTLVSKGFTPRRIRELEPQVLEL ARIHLDSALQTESFDFVAEFAGKLPMDVISELIGVPDTDRARIRALADAVLHREDGVADV PPPAMAASIELMRYYADLIAEFRRRPANNLTSALLAAELDGDRLSDQEIMAFLFLMVIAG NETTTKLLANAVYWAAHHPGQLARVFADHSRIPMWVEETLRYDTSSQILARTVAHDLTLY DTTIPEGEVLLLLPGIGQP  >CYP126A1(TBSG_00798)  MTTAAGLSGIDLTDLDNFADGFPHHLFAIHRREAPVYWHRPTEHTPDGEGFWSVATYAET LEVLRDPVTYSSVTGGQRRFGGTVLQDLPVAGQVLNMMDDPRHTRIRRLVSSGLTPRMIR RVEDDLRRRARGLLDGVEPGAPFDFVVEIAAELPMQMICILLGVPETDRHWLFEAVEPGF DFRGSRRATMPRLNVEDAGSRLYTYALELIAGKRAEPADDMLSVVANATIDDPDAPALSD AELYLFFHLLFSAGAETTRNSIAGGLLALAENPDQLQTLRSDFELLPTAIEEIVRWTSPS PSKRRTASRAVSLGGQPIEAGQKVVVWEGSANRDPSVFDRADEFDITRKPNPHLGFGQGV HYCLGANLARLELRVLFEELLSRFGSVRVVEPAEWTRSNRHTGIRHLVVELRGG  >CYP136A1(TBSG_00914)  MATIHPPAYLLDQAKRRFTPSFNNFPGMSLVEHMLLNTKFPEKKLAEPPPGSGLKPVVGD AGLPILGHMIEMLRGGPDYLMFLYKTKGPVVFGDSAVLPGVAALGPDAAQVIYSNRNKDY SQQGWVPVIGPFFHRGLMLLDFEEHMFHRRIMQEAFVRSRLAGYLEQMDRVVSRVVADDW VVNDARFLIYPAMKALTLDIASMVFMGHEPGTDHELVTKVNKAFTITTRAGNAVIRTSVP PFTWWRGLRARELLENYFTARVKERREASGNDLLTVLCQTEDDDGNRFSDADIVNHMIFL MMAAHDTSTSTATTMAYQLAAHPEWQQRCRDESDRHGDGPLDIESLEQLESLDLVMNESI RLVTPVQWAMRQTVRDTELLGYYLPKGTNVIAYPGMNHRLPEIWTDPLTFDPERFTEPRN EHKRHRYAFTPFGGGVHKCIGMVFDQLEIKTILHRLLRRYRLELSRPDYQPRWDYSAMPI PMDGMPIVLRPR  >CYP121A1(TBSG_01716)  MTATVLLEVPFSARGDRIPDAVAELRTREPIRKVRTITGAEAWLVSSYALCTQVLEDRRF SMKETAAAGAPRLNALTVPPEVVNNMGNIADAGLRKAVMKAITPKAPGLEQFLRDTANSL LDNLITEGAPADLRNDFADPLATALHCKVLGIPQEDGPKLFRSLSIAFMSSADPIPAAKI NWDRDIEYMAGILENPNITTGLMGELSRLRKDPAYSHVSDELFATIGVTFFGAGVISTGS FLTTALISLIQRPQLRNLLHEKPELIPAGVEELLRINLSFADGLPRLATADIQVGDVLVR KGELVLVLLEGANFDPEHFPNPGSIELDRPNPTSHLAFGRGQHFCPGSALGRRHAQIGIE ALLKKMPGVDLAVPIDQLVWRTRFQRRIPERLPVLW  >CYP128A1(TBSG_01724)  MTATQSPPEPAPDRVRLAGCPLAGTPDVGLTAQDATTALGVPTRRRASSGGIPVATSMWR DAQTVRTYGPAVAKALALRVAGKARSRLTGRHCRKFMQLTDFDPFDPAIAADPYPHYREL LAGERVQYNPKRDVYILSRYADVREAARNHDTLSSARGVTFSRGWLPFLPTSDPPAHTRM RKQLAPGMARGALETWRPMVDQLARELVGGLLTQTPADVVSTVAAPMPMRAITSVLGVDG PDEAAFCRLSNQAVRITDVALSASGLISLVQGFAGFRRLRALFTHRRDNGLLRECTVLGK LATHAEQGRLSDDELFFFAVLLLVAGYESTAHMISTLFLTLADYPDQLTLLAQQPDLIPS AIEEHLRFISPIQNICRTTRVDYSVGQAVIPAGSLVLLAWGAANRDPRQYEDPDVFRADR NPVGHLAFGSGIHLCPGTQLARMEGQAILREIVANIDRIEVVEPPTWTTNANLRGLTRLR VAVTPRVAP  >CYP124A1(TBSG_01726)  MGLNTAIATRVNGTPPPEVPIADIELGSLDFWALDDDVRDGAFATLRREAPISFWPTIEL PGFVAGNGHWALTKYDDVFYASRHPDIFSSYPNITINDQTPELAEYFGSMIVLDDPRHQR LRSIVSRAFTPKVVARIEAAVRDRAHRLVSSMIANNPDRQADLVSELAGPLPLQIICDMM GIPKADHQRIFHWTNVILGFGDPDLATDFDEFMQVSADIGAYATALAEDRRVNHHDDLTS SLVEAEVDGERLSSREIASFFILLVVAGNETTRNAITHGVLALSRYPEQRDRWWSDFDGL APTAVEEIVRWASPVVYMRRTLTQDIELRGTKMAAGDKVSLWYCSANRDESKFADPWTFD LARNPNPHLGFGGGGAHFCLGANLARREIRVAFDELRRQMPDVVATEEPARLLSQFIHGI KTLPVTWS  >CYP140A1(TBSG_02125)  MKDKLHWLAMHGVIRGIAAIGIRRGDLQARLIADPAVATDPVPFYDEVRSHGALVRNRAN YLTVDHRLAHDLLRSDDFRVVSFGENLPPPLRWLERRTRGDQLHPLREPSLLAVEPPDHT RYRKTVSAVFTSRAVSALRDLVEQTAINLLDRFAEQPGIVDVVGRYCSQLPIVVISEILG VPEHDRPRVLEFGELAAPSLDIGIPWRQYLRVQQGIRGFDCWLEGHLQQLRHAPGDDLMS QLIQIAESGDNETQLDETELRAIAGLVLVAGFETTVNLLGNGIRMLLDTPEHLATLRQHP ELWPNTVEEILRLDSPVQLTARVACRDVEVAGVRIKRGEVVVIYLAAANRDPAVFPDPHR FDIERPNAGRHLAFSTGRHFCLGAALARAEGEVGLRTFFDRFPDVRAAGAGSRRDTRVLR GWSTLPVTLGPARSMVSP  >CYP143A1(TBSG_02224)  MTTPGEDHAGSFYLPRLEYSTLPMAVDRGVGWKTLRDAGPVVFMNGWYYLTRREDVLAAL RNPKVFSSRKALQPPGNPLPVVPLAFDPPEHTRYRRILQPYFSPAALSKALPSLRRHTVA MIDAIAGRGECEAMADLANLFPFQLFLVLYGLPLEDRDRLIGWKDAVIAMSDRPHPTEAD VAAARELLEYLTAMVAERRRNPGPDVLSQVQIGEDPLSEIEVLGLSHLLILAGLDTVTAA VGFSLLELARRPQLRAMLRDNPKQIRVFIEEIVRLEPSAPVAPRVTTEPVTVGGMTLPAG SPVRLCMAAVNRDGSDAMSTDELVMDGKVHRHWGFGGGPHRCLGSHLARLELTLLVGEWL NQIPDFELAPDYAPEIRFPSKSFALKNLPLRWS  >CYP144A1(TBSG_02231)  MRRSPKGSPGAVLDLQRRVDQAVSADHAELMTIAKDANTFFGAESVQDPYPLYERMRAAG SVHRIANSDFYAVCGWDAVNEAIGRPEDFSSNLTATMTYTAEGTAKPFEMDPLGGPTHVL ATADDPAHAVHRKLVLRHLAAKRIRVMEQFTVQAADRLWVDGMQDGCIEWMGAMANRLPM MVVAELIGLPDPDIAQLVKWGYAATQLLEGLVENDQLVAAGVALMELSGYIFEQFDRAAA DPRDNLLGELATACASGELDTLTAQVMMVTLFAAGGESTAALLGSAVWILATRPDIQQQV RANPELLGAFIEETLRYEPPFRGHYRHVRNATTLDGTELPADSHLLLLWGAANRDPAQFE APGEFRLDRAGGKGHISFGKGAHFCVGAALARLEARIVLRLLLDRTSVIEAADVGGWLPS ILVRRIERLELAVQ  >CYP139A1(TBSG_02339)  MRYPLGEALLALYRWRGPLINAGVGGHGYTYLLGAEANRFVFANADAFSWSQTFESLVPV DGPTALIVSDGADHRRRRSVVAPGLRHHHVQRYVATMVSNIDTVIDGWQPGQRLDIYQEL RSAVRRSTAESLFGQRLAVHSDFLGEQLQPLLDLTRRPPQVMRLQQRVNSPGWRRAMAAR KRIDDLIDAQIADARTAPRPDDHMLTTLISGCSEEGTTLSDNEIRDSIVSLITAGYETTS GALAWAIYALLTVPGTWESAASEVARVLGGRVPAADDLSALTYLNGVVHETLRLYSPGVI SARRVLRDLWFDGHRIRAGRLLIFSAYVTHRLPEIWPEPTEFRPLRWDPNAADYRKPAPH EFIPFSGGLHRCIGAVMATTEMTVILARLVARAMLQLPAQRTHRIRAANFAALRPWPGLT VEIRKSAPAQ  >CYP132A1(TBSG_02599)  MATATTQRPLKGPAKRMSTWTMTREAITIGFDAGDGFLGRLRGSDITRFRCAGRRFVSIS HPDYVDHVLHEARLKYVKSDEYGPIRATAGLNLLTDEGDSWARHRGALNSTFARRHLRGL VGLMIDPIADVTAALVPGAQFDMHQSMVETTLRVVANALFSQDFGPLVQSMHDLATRGLR RAEKLERLGLWGLMPRTVYDTLIWCIYSGVHLPPPLREMQEITLTLDRAINSVIDRRLAE PTNSADLLNVLLSADGGIWPRQRVRDEALTFMLAGHETTANAMSWFWYLMALNPQARDHM LTELDDVLGMRRPTADDLGKLAWTTACLQESQRYFSSVWIIAREAVDDDIIDGHRIRRGT TVVIPIHHIHHDPRWWPDPDRFDPGRFLRCPTDRPRCAYLPFGGGRRICIGQSFALMEMV LMAAIMSQHFTFDLAPGYHVELEATLTLRPKHGVHVIGRRR  >CYP130A1(TBSG_02739)  MTSVMSHEFQLATAETWPNPWPMYRALRDHDPVHHVVPPQRPEYDYYVLSRHADVWSAAR DHQTFSSAQGLTVNYGELEMIGLHDTPPMVMQDPPVHTEFRKLVSRGFTPRQVETVEPTV RKFVVERLEKLRANGGGDIVTELFNPLPSMVVAHYLGVPEEDWTQFDGWTQAIVAANAVD GATTGALDAVGSMMAYFTGLIERRRTEPADDAISHLVAAGVGADGDTAGTLSILAFTFTM VTGGNDTVTGMLGGSMPLLHRRPDQRRLLLDDPEGIPDAVEELLRLTSPVQGLARTTTRD VTIGDTTIPAGRRVLLLYGSANRDERQYGPDAAELDVTRCPRNILTFSHGAHHCLGAAAA RMQCRVALTELLARCPDFEVAESRIVWSGGSYVRRPLSVPFRVTS  >CYP141A1(TBSG_03185)  MTSTSIPTFPFDRPVPTEPSPMLSELRNSCPVAPIELPSGHTAWLVTRFDDVKGVLSDKR FSCRAAAHPSSPPFVPFVQLCPSLLSIDGPQHTAARRLLAQGLNPGFIARMRPVVQQIVD NALDDLAAAEPPVDFQEIVSVPIGEQLMAKLLGVEPETVHELAAHVDAAMSVCEIGDEEV SRRWSALCTMVIDILHRKLAEPGDDLLSTIAQANRQQSTMTDEQVVGMLLTVVIGGVDTP IAVITNGLASLLHHRDQYERLVEDPGRVARAVEEIVRFNPATEIEHLRVVTEDVVIAGTA LSAGSPAFTSITSANRDSDQFLDPDEFDVERNPNEHIAFGYGPHACPASAYSRMCLTTFF TSLTQRFPQLQLARPFEDLERRGKGLHSVGIKELLVTWPT  >CYP142A1(TBSG_03584)  MTEAPDVDLADGNFYASREARAAYRWMRANQPVFRDRNGLAAASTYQAVIDAERQPELFS NAGGIRPDQPALPMMIDMDDPAHLLRRKLVNAGFTRKRVKDKEASIAALCDTLIDAVCER GECDFVRDLAAPLPMAVIGDMLGVRPEQRDMFLRWSDDLVTFLSSHVSQEDFQITMDAFA AYNDFTRATIAARRADPTDDLVSVLVSSEVDGERLSDDELVMETLLILIGGDETTRHTLS GGTEQLLRNRDQWDLLQRDPSLLPGAIEEMLRWTAPVKNMCRVLTADTEFHGTALCAGEK MMLLFESANFDEAVFCEPEKFDVQRNPNSHLAFGFGTHFCLGNQLARLELSLMTERVLRR LPDLRLVADDSVLPLRPANFVSGLESMPVVFTPSPPLG  >CYP125A1(TBSG_03611)  MSWNHQSVEIAVRRTTVPSPNLPPGFDFTDPAIYAERLPVAEFAELRSAAPIWWNGQDPG KGGGFHDGGFWAITKLNDVKEISRHSDVFSSYENGVIPRFKNDIAREDIEVQRFVMLNMD APHHTRLRKIISRGFTPRAVGRLHDELQERAQKIAAEAAAAGSGDFVEQVSCELPLQAIA GLLGVPQEDRGKLFHWSNEMTGNEDPEYAHIDPKASSAELIGYAMKMAEEKAKNPADDIV TQLIQADIDGEKLSDDEFGFFVVMLAVAGNETTRNSITQGMMAFAEHPDQWELYKKVRPE TAADEIVRWATPVTAFQRTALRDYELSGVQIKKGQRVVMFYRSANFDEEVFQDPFTFNTL RNPNPHVGFGGTGAHYCIGANLARMTINLIFNAVADHMPDLKPISAPERLRSGWLNGIKH WQVDYTGRCPVAH  >CYP137A1(TBSG_03751)  MVLRSLASPAALTDPKRCASVVGVAAFAVRREHAPDALGGPPGLPAPRGFRAAFAAAYAV AYLAGGERRMLRLIRRYGPIMTMPILSLGDVAIVSDSALAKEVFTAPTDVLLGGEGVGPA AAIYGSGSMFVQEEPEHLRRRKLLTPPLHGAALDRYVPIIENSTRAAMHTWPVDRPFAML TVARSLMLDVIVKVIFGVDDPEEVRRLGRPFERLLNLGVSEQLTVRYALRRLGALRVWPA RARANTEIDDVVMALIAQRRADPRLGERHDVLSLLVSARGESGEQLSDSEIRDDLITLVL AGHETTATTLAWAFDLLLHHPDALRRVRAEAVGGGEAFTTAVINETLRVRPPAPLTARVA AQPLTIGGYRVEAGTRIVVHIIAINRSAEVYEHPHEFRPERFLGTRPQTYAWVPFGGGVK RCLGANFSMRELITVLHVLLREGEFTAVDDEPERIVRRSIMLVPRRGTRVRFRPAR  >CYP123A1-fragment(TBSG_00785)  MRCCCCYLGSANRDDRVFDDPDDYRIGREIGCKLVSFGSGAHFCLGAHLARMEARVALGA LLRRIRNYEVDDDNVVRVHSSNVRGFAHLPISVQAR |
| ***Mycobacterium tuberculosis* RGTB327** |
| Database: TB; P450 count: 14; Families: 14; Subfamilies: 14 |
| >CYP138A1(MRGA327_00875)  MSEVVTAAPAPPVVRLPPAVRGPKLFQGLAFVVSRRRLLGRFVRRYGKAFTANILMYGRV VVVADPQLARQVFTSSPEELGNIQPNLSRMFGSGSVFALDGDDHRRRRRLLAPPFHGKSM KNYETIIEEETLRETANWPQGQAFATLPSMMHITLNAILRAIFGAGGSELDELRRLIPPW VTLGSRLAALPKPKRDYGRLSPWGRLAEWRRQYDTVIDKLIEAERADPNFADRTDVLALM LRSTYDDGSIMSRKDIGDELLTLLAAGHETTAATLGWAFERLSRHPDVLAALVEEVDNGG HELRQAAILEVQRARTVIDFAARRVNPPVYQLGEWVIPRGYSIIINIAQIHGDPDVFPQP DRFDPQRYIGSKPSPFAWIPFGGGTRRCVGAAFANMEMDVVLRTVLRHFTLETTTAAGER SHGRGVAFTPKDGGRVVMRRR  >CYP135A1(MRGA327_02080)  MASTLTTGLPPGPRLPRYLQSVLYLRFREWFLPAMHRKYGDVFSLRVPPYADNLVVYTRP EHIKEIFAADPRSLHAGEGNHILGFVMGEHSVLMTDEAEHARMRSLLMPAFTRAALRGYR DMIASVAREHITRWRPHATINSLDHMNALTLDIILRVVFGVTDPKVKAELTSRLQQIINI HPAILAGVPYPSLKRMNPWKRFFHNQTKIDEILYREIASRRIDSDLTARTDVLSRLLQTK DTPTKPLTDAELRDQLITLLLAGHETTAAALSWTLWELAHAPEIQSQVVWAAVGGDDGFL EAVLKEGMRRHTVIASTARKVTAPAEIGGWRLPAGTVVNTSILLAHASEVSHPKPTEFRP SRFLDGSVAPNTWLPFGGGVRRCLGFGFAPGTEGAVILQEIFRRFTITAAGPSKGETPLV RNITTVPKHGAHLRLIPQRRLGGLGDSDPP  >CYP51B1(MRGA327_04755)  MSAVALPRVSGGHDEHGHLEEFRTDPIGLMQRVRDECGDVGTFQLAGKQVVLLSGSHANE FFFRAGDDDLDQAKAYPFMTPIFGEGVVFDASPERRKEMLHNAALRGEQMKGHAATIEDQ VRRMIADWGEAGEIDLLDFFAELTIYTSSACLIGKKFRDQLDGRFAKLYHELERGTDPLA YVDPYLPIESFRRRDEARNGLVALVADIMNGRIANPPTDKSDRDMLDVLIAVKAETGTPR FSADEITGMFISMMFAGHHTSSGTASWTLIELMRHRDAYAAVIDELDELYGDGRSVSFHA LRQIPQLENVLKETLRLHPPLIILMRVAKGEFEVQGHRIHEGDLVAASPAISNRIPEDFP DPHDFVPARYEQPRQEDLLNRWTWIPFGAGRHRCVGAAFAIMQIKAIFSVLLREYEFEMA QPPESYRNDHSKMVVQLAQPACVRYRRRTGV  >CYP130A1(MRGA327_07880)  MSHEFQLATAETWPNPWPMYRALRDHDPVHHVVPPQRPEYDYYVLSRHADVWSAARDHQT FSSAQGLTVNYGELEMIGLHDTPPMVMQDPPVHTEFRKLVSRGFTPRQVETVEPTVRKFV VERLEKLRANGGGDIVTELFNPLPSMVVAHYLGVPEEDWTQFDGWTQAIVAANAVDGATT GALDAVGSMMAYFTGLIERRRTEPADDAISHLVAAGVGADGDTAGTLSILAFTFTMVTGG NDTVTGMLGGSMPLLHRRPDQRRLLLDDPEGIPDAVEELLRLTSPVQGLARTTTRDVTIG DTTIPAGRRVLLLYGSANRDERQYGPDAAELDVTRCPRNILTFSHGAHHCLGAAAARMQC RVALTELLARCPDFEVAESRIVWSGGSYVRRPLSVPFRVTS  >CYP132A1(MRGA327_08730)  MATATTQRPLKGPAKRMSTWTMTREAITIGFDAGDGFLGRLRGSDITRFRCAGRRFVSIS HPDYVDHVLHEARLKYVKSDEYGPIRATAGLNLLTDEGDSWARHRGALNSTFARRHLRGL VGLMIDPIADVTAALVPGAQFDMHQSMVETTLRVVANALFSQDFGPLVQSMHDLATRGLR RAEKLERLGLWGLMPRTVYDTLIWCIYSGVHLPPPLREMQEITLTLDRAINSVIDRRLAE PTNSADLLNVLLSADGGIWPRQRVRDEALTFMLAGHETTANAMSWFLVSDGAEPAGPRPH AHRAGRRAGHAPPDRRRPGQAGLDHRVPAGIATLLLVGVDNRARGRR  >CYP144A1(MRGA327_11030)  MRRSPKGSPGAVLDLQRRVDQAVSADHAELMTIAKDANTFFGAESVQDPYPLYERMRAAG SVHRIANSDFYAVCGWDAVNEAIGRPEDFSSNLTATMTYTAEGTAKPFEMDPLGGPTHVL ATADDPAHAVHRKLVLRHLAAKRIRVMEQFTVQAADRLWVDGMQDGCIEWMGAMANRLPM MVVAELIGLPDPDIAQLVKWGYAATQLLEGLVENDQLVAAGVALMELSGYIFEQFDRAAA DPRDNLLGELATACASGELDTLTAQVMMVTLFAAGGESTAALLGSAVWILATRPDIQQQV RANPELLGAFIEETLRYEPPFRGHYRHVRNATTLDGTELPADSHLLLLWGAANRDPAQFE APGEFRLDRAGGKGHISFGKGAHFCVGAALARLEARIVLRLLLDRTSVIEAADVGGWLPS ILVRRIERLELAVQ  >CYP143A1(MRGA327_11070)  MTTPGEDHAGSFYLPRLEYSTLPMAVDRGVGWKTLRDAGPVVFMNGWYYLTRREDVLAAL RNPKVFSSRKALQPPGNPLPVVPLAFDPPEHTRYRRILQPYFSPAALSKALPSLRRHTVA MIDAIAGRGECEAMADLANLFPFQLFLVLYGLPLEDRDRLIGWKDAVIAMSDRPHPTEAD VAAARELLEYLTAMVAERRRNPGPDVLSQVQIGEDPLSEIEVLGLSHLLILAGLDTVTAA VGFSLLELARRPQLRAMLRDNPKQIRVFIEEIVRLEPSAPVAPRVTTEPVTVGGMTLPAG SPVRLCMAAVNRDGSDAMSTDELVMDGKVHRHWGFGGGPHRCLGSHLARLELTLLVGEWL NQIPDFELAPDYAPEIRFPSKSFALKNLPLRWS  >CYP140A1(MRGA327_11610)  MKDKLHWLAMHGVIRGIAAIGIRRGDLQARLIADPAVATDPVPFYDEVRSHGALVRNRAN YLTVDHRLAHDLLRSDDFRVVSFGENLPPPLRWLERRTRGDQLHPLREPSLLAVEPPDHT RYRKTVSAVFTSRAVSALRDLVEQTAINLLDRFAEQPGIVDVVGRYCSQLPIVVISEILG VPEHDRPRVLEFGELAAPSLDIGIPWRQYLRVQQGIRGFDCWLEGHLQQLRHAPGDDLMS QLIQIAESGDNETQLDETELRAIAGLVLVAGFETTVNLLGNGIRMLLDTPEHLATLRQHP ELWPNTVEEILRLDSPVQLTARVACRDVEVAGVRIKRGEVVVIYLAAANRDPAVFPDPHP L  >CYP124A1(MRGA327_13975)  MGLNTAIATRVNGTPPPEVPIADIELGSLDFWALDDDVRDGAFATLRREAPISFWPTIEL PGFVAGNGHWALTKYDDVFYASRHPDIFSSYPNITINDQTPELAEYFGSMIVLDDPRHQR LRSIVSRAFTPKVVARIEAAVRDRAHRLVSSMIANNPDRQADLVSELAGPLPLQIICDMM GIPKADHQRIFHWTNVILGFGDPDLATDFDEFMQVSADIGAYATALAEDRRVNHHDDLTS SLVEAEVDGERLSSREIASFFILLVVAGNETTRNAITHGVLALSRYPEQRDRWWSDFDGL APTAVEEIVRWASPVVYMRRTLTQDIELRGTKMAAGDKVSLWYCSANRDESKFADPWTFD LARNPNPHLGFGGGGAHFCLGANLARREIRVAFDELRRQMPDVVATEEPARLLSQFIHGI KTLPVTWS  >CYP121A1(MRGA327_14025)  MTATVLLEVPFSARGDRIPDAVAELRTREPIRKVRTITGAEAWLVSSYALCTQVLEDRRF SMKETAAAGAPRLNALTVPPEVVNNMGNIADAGLRKAVMKAITPKAPGLEQFLRDTANSL LDNLITEGAPADLRNDFADPLATALHCKVLGIPQEDGPKLFRSLSIAFMSSADPIPAAKI NWDRDIEYMAGILENPNITTGLMGELSRLRKDPAYSHVSDELFATIGVTFFGAGVISTGS FLTTALISLIQRPQLRNLLHEKPELIPAGVEELLRINLSFADGLPRLATADIQVGDVLVR KGELVLVLLEGANFDPEHFPNPGSIELDRPNPTSHLAFGRGQHFCPGSALGRRHAQIGIE ALLKKMPGVDLAVPIDQLVWRTRFQRRIPERLPVLW  >CYP136A1(MRGA327_18795)  MATIHPPAYLLDQAKRRFTPSFNNFPGMSLVEHMLLNTKFPEKKLAEPPPGSGLKPVVGD AGLPILGHMIEMLRGGPDYLMFLYKTKGPVVFGDSAVLPGVAALGPDAAQVIYSNRNKDY SQQGWVPVIGPFFHRGLMLLDFEEHMFHRRIMQEAFVRSRLAGYLEQMDRVVSRVVADDW VVNDARFLVYPAMKALTLDIASMVFMGHEPGTDHELVTKVNKAFTITTRAGNAVIRTSVP PFTWWRGLRARELLENYFTARVKERREASGNDLLTVLCQTEDDDGNRFSDADIVNHMIFL MMAAHDTSTSTATTMAYQLAAHPEWQQRCRDESDRHGDGPLDIESLEQLESLDLVMNESI RLVTPVQWAMRQTVRDTELLGYYLPKGTNVIAYPGMNHRLPEIWTDPLTFDPERFTEPRN EHKRHRYAFTPFGGGVHKCIGMVFDQLEIKTILHRLLRRYRLELSRPDYQPRWDYSAMPI PMDGMPIVLRPR  >CYP141A1(MRGA327_19190)  MTSTSIPTFPFDRPVPTEPSPMLSELRNSCPVAPIELPSGHTAWLVTRFDDVKGVLSDKR FSCRAAAHPSSPPFVPFVQLCPSLLSIDGPQHTAARRLLAQGLNPGFIARMRPVVQQIVD NALDDLAAAEPPVDFQEIVSVPIGEQLMAKLLGVEPETVHELAAHVDAAMSVCEIGDEEV SRRWSALCTMVIDILHRKLAEPGDDLLSTIAQANRQQSTMTDEQVVGMLLTVVIGGVDTP IAVITNGLASLLHHRDQYERLVEDPGRVARAVEEIVRFNPATEIEHLRVVTEDVVIAGTA LSAGSPAFTSITSANRDSDQFLDPDEFDVERNPNEHIAFGYGPHACPASAYSRMCLTTFF TSLTQRFPQLQLARPFEDLERRGKGLHSVGIKELLVTWPT  >CYP142A1(MRGA327_21740)  MDLADGNFYASREARAAYRWMRANQPVFRDRNGLAAASTYQAVIDAERQPELFSNAGGIR PDQPALPMMIDMDDPAHLLRRKLVNAGFTRKRVKDKEASIAALCDTLIDAVCERGECDFV RDLAAPLPMAVIGDMLGVRPEQRDMFLRWSDDLVTFLSSHVSQEDFQITMDAFAAYNDFT RATIAARRADPTDDLVSVLVSSEVDGERLSDDELVMETLLILIGGDETTRHTLSGGTEQL LRNRDQWDLLQRDPSLLPGAIEEMLRWTAPVKNMCRVLTADTEFHGTALCAGEKMMLLFE SANFDEAVFCEPEKFDVQRNPNSHLAFGFGTHFCLGNQLARLELSLMTERVLRRLPDLRL VADDSVLPLRPANFVSGLESMPVVFTPSPPLG  >CYP137A1(MRGA327_22695)  MGRPFERLLNLGVSEQLTVRYALRRLGALRVWPARARANTEIDDVVMALIAQRRADPRLG ERHDVLSLLVSARGESGEQLSDSEIRDDLITLVLAGHETTATTLAWAFDLLLHHPDALRR VRAEAVGGGEAFTTAVINETLRVRPPAPLTARVAAQPLTIGGYRVEAGTRIVVHIIAINR SAEVYEHPHEFRPERFLGTRPQTYAWVPFGGGVKRCLGANFSMRELITVLHVLLREGEFT AVDDEPERIVRRSIMLVPRRGTRVRFRPAR |
| ***Mycobacterium tuberculosis* CDC1551** |
| Database: KEGG; P450 count: 20; Families: 19; Subfamilies: 20 |
| >CYP138A1(MT0144)  MSEVVTAAPAPPVVRLPPAVRGPKLFQGLAFVVSRRRLLGRFVRRYGKAFTANILMYGRV  VVVADPQLARQVFTSSPEELGNIQPNLSRMFGSGSVFALDGDDHRRRRRLLAPPFHGKSM  KNYETIIEEETLRETANWPQGQAFATLPSMMHITLNAILRAIFGAGGSELDELRRLIPPW  VTLGSRLAALPKPKRDYGRLSPWGRLAEWRRQYDTVIDKLIEAERADPNFADRTDVLALM  LRSTYDDGSIMSRKDIGDELLTLLAAGHETTAATLGWAFERLSRHPDVLAALVEEVDNGG  HELRQAAILEVQRARTVIDFAARRVNPPVYQLGEWVIPRGYSIIINIAQIHGDPDVFPQP  DRFDPQRYIGSKPSPFAWIPFGGGTRRCVGAAFANMEMDVVLRTVLRHFTLETTTAAGER  SHGRGVAFTPKDGGRVVMRRR  >CYP135A1(MT0342)  MASTLTTGLPPGPRLPRYLQSVLYLRFREWFLPAMHRKYGDVFSLRVPPYADNLVVYTRP  EHIKEIFAADPRSLHAGEGNHILGFVMGEHSVLMTDEAEHARMRSLLMPAFTRAALRGYR  DMIASVAREHITRWRPHATINSLDHMNALTLDIILRVVFGVTDPKVKAELTSRLQQIINI  HPAILAGVPYPSLKRMNPWKRFFHNQTKIDEILYREIASRRIDSDLTARTDVLSRLLQTK  DTPTKPLTDAELRDQLITLLLAGHETTAAALSWTLWELAHAPEIQSQVVWAAVGGDDGFL  EAVLKEGMRRHTVIASTARKVTAPAEIGGWRLPAGTVVNTSILLAHASEVSHPKPTEFRP  SRFLDGSVAPNTWLPFGGGVRRCLGFGFALTEGAVILQEIFRRFTITAAGPSKGETPLVR  NITTVPKHGAHLRLIPQRRLGGLGDSDPP  >CYP135B1(MT0594)  MSGTSSMGLPPGPRLSGSVQAVLMLRHGLRFLTACQRRYGSVFTLHVAGFGHMVYLSDPA  AIKTVFAGNPSVFHAGEANSMLAGLLGDSSLLLIDDDVHRDRRRLMSPPFHRDAVARQAG  PIAEIAAANIAGWPMAKAFAVAPKMSEITLEVILRTVIGASDPVRLAALRKVMPRLLNVG  PWATLALANPSLLNNRLWSRLRRRIEEADALLYAEIADRRADPDLAARTDTLAMLVRAAD  EDGRTMTERELRDQLITLLVAGHDTTATGLSWALERLTRHPVTLAKAVQAADASAAGDPA  GDEYLDAVAKETLRIRPVVYDVGRVLTEAVEVAGYRLPAGVMVVPAIGLVHASAQLYPDP  ERFDPDRMVGATLSPTTWLPFGGGNRRCLGATFAMVEMRVVLREILRRVELSTTTTSGER  PKLKHVIMVPHRGARIRVRATRDVSATSQATAQGAGCPAARGGGPSRAVGSQ  >CYP51B1(MT0788)  MSAVALPRVSGGHDEHGHLEEFRTDPIGLMQRVRDECGDVGTFQLAGKQVVLLSGSHANE  FFFRAGDDDLDQAKAYPFMTPIFGEGVVFDASPERRKEMLHNAALRGEQMKGHAATIEDQ  VRRMIADWGEAGEIDLLDFFAELTIYTSSACLIGKKFRDQLDGRFAKLYHELERGTDPLA  YVDPYLPIESFRRRDEARNGLVALVADIMNGRIANPPTDKSDRDMLDVLIAVKAETGTPR  FSADEITGMFISMMFAGHHTSSGTASWTLIELMRHRDAYAAVIDELDELYGDGRSVSFHA  LRQIPQLENVLKETLRLHPPLIILMRVAKGEFEVQGHRIHEGDLVAASPAISNRIPEDFP  DPHDFVPARYEQPRQEDLLNRWTWIPFGAGRHRCVGAAFAIMQIKAIFSVLLREYEFEMA  QPPESYRNDHSKMVVQLAQPACVRYRRRTGV  >CYP123A1(MT0790)  MTVRVGDPELVLDPYDYDFHEDPYPYYRRLRDEAPLYRNEERNFWAVSRHHDVLQGFRDS  TALSNAYGVSLDPSSRTSEAYRVMSMLAMDDPAHLRMRTLVSKGFTPRRIRELEPQVLEL  ARIHLDSALQTESFDFVAEFAGKLPMDVISELIGVPDTDRARIRALADAVLHREDGVADV  PPPAMAASIELMRYYADLIAEFRRRPANNLTSALLAAELDGDRLSDQEIMAFLFLMVIAG  NETTTKLLANAVYWAAHHPGQLARVFADHSRIPMWVEETLRYDTSSQILARTVAHDLTLY  DTTIPEGEVLLLLPGSANRDDRVFDDPDDYRIGREIGCKLVSFGSGAHFCLGAHLARMEA  RVALGALLRRIRNYEVDDDNVVRVHSSNVRGFAHLPISVQAR  >CYP126A1(MT0802)  MTTAAGLSGIDLTDLDNFADGFPHHLFAIHRREAPVYWHRPTEHTPDGEGFWSVATYAET  LEVLRDPVTYSSVTGGQRRFGGTVLQDLPVAGQVLNMMDDPRHTRIRRLVSSGLTPRMIR  RVEDDLRRRARGLLDGVEPGAPFDFVVEIAAELPMQMICILLGVPETDRHWLFEAVEPGF  DFRGSRRATMPRLNVEDAGSRLYTYALELIAGKRAEPADDMLSVVANATIDDPDAPALSD  AELYLFFHLLFSAGAETTRNSIAGGLLALAENPDQLQTLRSDFELLPTAIEEIVRWTSPS  PSKRRTASRAVSLGGQPIEAGQKVVVWEGSANRDPSVFDRADEFDITRKPNPHLGFGQGV  HYCLGANLARLELRVLFEELLSRFGSVRVVEPAEWTRSNRHTGIRHLVVELRGG  >CYP130A1(MT1295)  MTSVMSHEFQLATAETWPNPWPMYRALRDHDPVHHVVPPQRPEYDYYVLSRHADVWSAAR  DHQTFSSAQGLTVNYGELEMIGLHDTPPMVMQDPPVHTEFRKLVSRGFTPRQVETVEPTV  RKFVVERLEKLRANGGGDIVTELFKPLPSMVVAHYLGVPEEDWTQFDGWTQAIVAANAVD  GATTGALDAVGSMMAYFTGLIERRRTEPADDAISHLVAAGVGADGDTAGTLSILAFTFTM  VTGGNDTVTGMLGGSMPLLHRRPDQRRLLLDDPEGIPDAVEELLRLTSPVQGLARTTTRD  VTIGDTTIPAGRRVLLLYGSANRDERQYGPDAAELDVTRCPRNILTFSHGAHHCLGAAAA  RMQCRVALTELLARCPDFEVAESRIVWSGGSYVRRPLSVPFRVTS  >CYP132A1(MT1439)  MATATTQRPLKGPAKRMSTWTMTREAITIGFDAGDGFLGRLRGSDITRFRCAGRRFVSIS  HPDYVDHVLHEARLKYVKSDEYGPIRATAGLNLLTDEGDSWARHRGALNSTFARRHLRGL  VGLMIDPIADVTAALVPGAQFDMHQSMVETTLRVVANALFSQDFGPLVQSMHDLATRGLR  RAEKLERLGLWGLMPRTVYDTLIWCIYSGVHLPPPLREMQEITLTLDRAINSVIDRRLAE  PTNSADLLNVLLSADGGIWPRQRVRDEALTFMLAGHETTANAMSWFWYLMALNPQARDHM  LTELDDVLGMRRPTADDLGKLAWTTACLQESQRYFSSVWIIAREAVDDDIIDGHRIRRGT  TVVIPIHHIHHDPRWWPDPDRFDPGRFLRCPTDRPRCAYLPFGGGRRICIGQSFALMEMV  LMAAIMSQHFTFDLAPGYHVELEATLTLRPKHGVHVIGRRR  >CYP139A1(MT1706)  MRTYRTVRYPLGEALLALYRWRGPLINAGVGGHGYTYLLGAEANRFVFANADAFSWSQTF  ESLVPVDGPTALIVSDGADHRRRRSVVAPGLRHHHVQRYVATMVSNIDTVIDGWQPGQRL  DIYQELRSAVRRSTAESLFGQRLAVHSDFLGEQLQPLLDLTRRPPQVMRLQQRVNSPGWR  RAMAARKRIDDLIDAQIADARTAPRPDDHMLTTLISGCSEEGTTLSDNEIRDSIVSLITA  GYETTSGALAWAIYALLTVPGTWESAASEVARVLGGRVPAADDLSALTYLNGVVHETLRL  YSPGVISARRVLRDLWFDGHRIRAGRLLIFSAYVTHRLPEIWPEPTEFRPLRWDPNAADY  RKPAPHEFIPFSGGLHRCIGAVMATTEMTVILARLVARAMLQLPAQRTHRIRAANFAALR  PWPGLTVEIRKSAPAQ  >CYP144A1(MT1827)  MRRSPKGSPGAVLDLQRRVDQAVSADHAELMTIAKDANTFFGAESVQDPYPLYERMRAAG  SVHRIANSDFYAVCGWDAVNEAIGRPEDFSSNLTATMTYTAEGTAKPFEMDPLGGPTHVL  ATADDPAHAVHRKLVLRHLAAKRIRVMEQFTVQAADRLWVDGMQDGCIEWMGAMANRLPM  MVVAELIGLPDPDIAQLVKWGYAATQLLEGLVENDQLVAAGVALMELSGYIFEQFDRAAA  DPRDNLLGELATACASGELDTLTAQVMMVTLFAAGGESTAALLGSAVWILATRPDIQQQV  RANPELLGAFIEETLRYEPPFRGHYRHVRNATTLDGTELPADSHLLLLWGAANRDPAQFE  APGEFRLDRAGGKGHISFGKGAHFCVGAALARLEARIVLRLLLDRTSVIEAADVGGWLPS  ILVRRIERLELAVQ  >CYP143A1(MT1834)  MVGARPRAILARSAPLGYVCDLRQERRHERLERMTTPGEDHAGSFYLPRLEYSTLPMAVD  RGVGWKTLRDAGPVVFMNGWYYLTRREDVLAALRNPKVFSSRKALQPPGNPLPVVPLAFD  PPEHTRYRRILQPYFSPAALSKALPSLRRHTVAMIDAIAGRGECEAMADLANLFPFQLFL  VLYGLPLEDRDRLIGWKDAVIAMSDRPHPTEADVAAARELLEYLTAMVAERRRNPGPDVL  SQVQIGEDPLSEIEVLGLSHLLILAGLDTVTAAVGFSLLELARRPQLRAMLRDNPKQIRV  FIEEIVRLEPSAPVAPRVTTEPVTVGGMTLPAGSPVRLCMAAVNRDGSDAMSTDELVMDG  KVHRHWGFGGGPHRCLGSHLARLELTLLVGEWLNQIPDFELAPDYAPEIRFPSKSFALKN  LPLRWS  >CYP140A1(MT1929)  MHGVIRGIAAIGIRRGDLQARLIADPAVATDPVPFYDEVRSHGALVRNRANYLTVDHRLA  HDLLRSDDFRVVSFGENLPPPLRWLERRTRGDQLHPLREPSLLAVEPPDHTRYRKTVSAV  FTSRAVSALRDLVEQTAINLLDRFAEQPGIVDVVGRYCSQLPIVVISEILGVPEHDRPRV  LEFGELAAPSLDIGIPWRQYLRVQQGIRGFDCWLEGHLQQLRHAPGDDLMSQLIQIAESG  DNETQLDETELRAIAGLVLVAGFETTVNLLGNGIRMLLDTPEHLATLRQHPELWPNTVEE  ILRLDSPVQLTARVACRDVEVAGVRIKRGEVVVIYLAAANRDPAVFPDPHRFDIERPNAG  RHLAFSTGRHFCLGAALARAEGEVGLRTFFDRFPDVRAAGAGSRRDTRVLRGWSTLPVTL  GPARSMVSP  >CYP124A1(MT2328)  MGLNTAIATRVNGTPPPEVPIAGIELGSLDFWALDDDVRDGAFATLRREAPISFWPTIEL  PGFVAGNGHWALTKNDDVFYASRHPDIFSSYPNITINDQTPELAEYFGSMIVLDDPRHQR  LRSIVSRAFTPKVVARIEAAVRDRAHRLVSSMIANNPDRQADLVSELAGPLPLQIICDMM  GIPKADHQRIFHWTNVILGFGDPDLATDFDEFMQVSADIGAYATALAEDRRVNHHDDLTS  SLVEAEVDGERLSSREIASFFILLVVAGNETTRNAITHGVLALSRYPEQRDRWWSDFDGL  APTAVEEIVRWASPVVYMRRTLTQDIELRGTKMAAGDKVSLWYCSANRDESKFADPWTFD  LARNPNPHLGFGGGGAHFCLGANLARREIRVAFDELRRQMPDVVATEEPARLLSQFIHGI  KTLPVTWS  >CYP128A1(MT2330)  MTATQSPPEPAPDRVRLAGCPLAGTPDVGLTAQDATTALGVPTRRRASSGGIPVATSMWR  DAQTVRTYGPAVAKALALRVAGKARSRLTGRHCRKFMQLTDFDPFDPAIAADPYPHYREL  LAGERVQYNPKRDVYILSRYADVREAARNHDTLSSARGVTFSRGWLPFLPTSDPPAHTRM  RKQLAPGMARGALETWRPMVDQLARELVGGLLTQTPADVVSTVAAPMPMRAITSVLGVDG  PDEAAFCRLSNQAVRITDVALSASGLISLVQGFAGFRRLRALFTHRRDNGLLRECTVLGK  LATHAEQGRLSDDELFFFAVLLLVAGYESTAHMISTLFLTLADYPDQLTLLAQQPDLIPS  AIEEHLRFISPIQNICRTTRVDYSVGQAVIPAGSLVLLAWGAANRDPRQYEDPDVFRADR  NPVGHLAFGSGIHLCPGTQLARMEGQAILREIVANIDRIEVVEPPTWTTNANLRGLTRLR  VAVTPRVAP  >CYP121A1(MT2336)  MTATVLLEVPFSARGDRIPDAVAELRTREPIRKVRTITGAEAWLVSSYALCTQVLEDRRF  SMKETAAAGAPRLNALTVPPEVVNNMGNIADAGLRKAVMKAITPKAPGLEQFLRDTANSL  LDNLITEGAPADLRNDFADPLATALHCKVLGIPQEDGPKLFRSLSIAFMSSADPIPAAKI  NWDRDIEYMAGILENPNITTGLMGELSRLRKDPAYSHVSDELFATIGVTFFGAGVISTGS  FLTTALISLIQRPQLRNLLHEKPELIPAGVEELLRINLSFADGLPRLATADIQVGDVLVR  KGELVLVLLEGANFDPEHFPNPGSIELDRPNPTSHLAFGRGQHFCPGSALGRRHAQIGIE  ALLKKMPGVDLAVPIDQLVWRTRFQRRIPERLPVLW  >CYP136A1(MT3145)  MATIHPPAYLLDQAKRRFTPSFNNFPGMSLVEHMLLNTKFPEKKLAEPPPGSGLKPVVGD  AGLPILGHMIEMLRGGPDYLMFLYKTKGPVVFGDSAVLPGVAALGPDAAQVIYSNRNKDY  SQQGWVPVIGPFFHRGLMLLDFEEHMFHRRIMQEAFVRSRLAGYLEQMDRVVSRVVADDW  VVNDARFLVYPAMKALTLDIASMVFMGHEPGTDHELVTKVNKAFTITTRAGNAVIRTSVP  PFTWWRGLRARELLENYFTARVKERREASGNDLLTVLCQTEDDDGNRFSDADIVNHMIFL  MMAAHDTSTSTATTMAYQLAAHPEWQQRCRDESDRHGDGPLDIESLEQLESLDLVMNESI  RLVTPVQWAMRQTVRDTELLGYYLPKGTNVIAYPGMNHRLPEIWTDPLTFDPERFTEPRN  EHKRHRYAFTPFGGGVHKCIGMVFDQLEIKTILHRLLRRYRLELSRPDYQPRWDYSAMPI  PMDGMPIVLRPR  >CYP141A1(MT3203)  MTSTSIPTFPFDRPVPTEPSPMLSELRNSCPVAPIELPSGHTAWLVTRFDDVKGVLSDKR  FSCRAAAHPSSPPFVPFVQLCPSLLSIDGPQHTAARRLLAQGLNPGFIARMRPVVQQIVD  NALDDLAAAEPPVDFQEIVSVPIGEQLMAKLLGVEPETVHELAAHVDAAMSVCEIGDEEV  SRRWSALCTMVIDILHRKLAEPGDDLLSTIAQANRQQSTMTDEQVVGMLLTVVIGGVDTP  IAVITNGLASLLHHRDQYERLVEDPGRVARAVEEIVRFNPATEIEHLRVVTEDVVIAGTA  LSAGSPAFTSITSANRDSDQFLDPDEFDVERNPNEHIAFGYGPHACPASAYSRMCLTTFF  TSLTQRFPQLQLARPFEDLERRGKGLHSVGIKELLVTWPT  >CYP142A1(MT3619)  MRANQPVFRDRNGLAAASTYQAVIDAERQPELFSNAGGIRPDQPALPMMIDMDDPAHLLR  RKLVNAGFTRKRVKDKEASIAALCDTLIDAVCERGECDFVRDLAAPLPMAVIGDMLGVRP  EQRDMFLRWSDDLVTFLSSHVSQEDFQITMDAFAAYNDFTRATIAARRADPTDDLVSVLV  SSEVDGERLSDDELVMETLLILIGGDETTRHTLSGGTEQLLRNRDQWDLLQRDPSLLPGA  IEEMLRWTAPVKNMCRVLTADTEFHGTALCAGEKMMLLFESANFDEAVFCEPEKFDVQRN  PNSHLAFGFGTHFCLGNQLARLELSLMTERVLRRLPDLRLVADDSVLPLRPANFVSGLES  MPVVFTPSPPLG  >CYP125A1(MT3649)  MSWNHQSVEIAVRRTTVPSPNLPPGFDFTDPAIYAERLPVAEFAELRSAAPIWWNGQDPG  KGGGFHDGGFWAITKLNDVKEISRHSDVFSSYENGVIPRFKNDIAREDIEVQRFVMLNMD  APHHTRLRKIISRGFTPRAVGRLHDELQERAQKIAAEAAAAGSGDFVEQVSCELPLQAIA  GLLGVPQEDRGKLFHWSNEMTGNEDPEYAHIDPKASSAELIGYAMKMAEEKAKNPADDIV  TQLIQADIDGEKLSDDEFGFFVVMLAVAGNETTRNSITQGMMAFAEHPDQWELYKKVRPE  TAADEIVRWATPVTAFQRTALRDYELSGVQIKKGQRVVMFYRSANFDEEVFQDPFTFNIL  RNPNPHVGFGGTGAHYCIGANLARMTINLIFNAVADHMPDLKPISAPERLRSGWLNGIKH  WQVDYTGRCPVAH  >CYP137A(MT3787)  MVLRSLASPAALTDPKRCASVVGVAAFAVRREHAPDALGGPPGLPAPRGFRAAFAAAYAV  AYLAGGERRMLRLIRRYGPIMTMPILSLGDVAIVSDSALAKEVFTAPTDVLLGGEGVGPA  AAIYGSGSMFVQEEPEHLRRRKLLTPPLHGAALDRYVPIIENSTRAAMHTWPVDRPFAML  TVARSLMLDVIVKVIFGVDDPEEVRRLGRPFERLLNLGVSEQLTVRYALRRLGALRVWPA  RARANTEIDDVVMALIAQRRADPRLGERHDVLSLLVSARGESGEQLSDSEIRDDLITLVL  AGHETTATTLAWAFDLLLHHPDALRRVRAEAVGGGEAFTTAVINETLRVRPPAPLTARVA  AQPLTIGGYRVEAGTRIVVHIIAINRSAEVYEHPHEFRPERFLGTRPQTYAWVPFGGGVK  RCLGANFSMRELITVLHVLLREGEFTAVDDEPERIVRRSIMLVPRRGTRVRFRPAR |
| ***Mycobacterium tuberculosis* strains CCDC5079** |
| Database: TB; P450 count: 20; Families: 19; Subfamilies: 20 |
| >CYP138A1(CCDC5079_0122)  MSEVVTAAPAPPVVRLPPAVRGPKLFQGLAFVVSRRRLLGRFVRRYGKAFTANILMYGRV VVVADPQLARQVFTSSPEELGNIQPNLSRMFGSGSVFALDGDDHRRRRRLLAPFFHGKSM KNYETIIEEETLRETANWPQGQAFATLPSMMHITLNAILRAIFGAGGSELDELRRLIPPW VTLGSRLAALPKPKRDYGRLSPWGRLAEWRRQYDTVIDKLIEAERADPNFADRTDVLALM LRSTYDDGSIMSRKDIGDELLTLLAAGHETTAATLGWAFERLSRHPDVLAALVEEVDNGG HELRQAAILEVQRARTVIDFAARRVNPPVYQLGEWVIPRGYSIIINIAQIHGDPDVFPQP DRFDPQRYIGSKPSPFAWIPFGGGTRRCVGAAFANMEMDVVLRTVLRHFTLETTTAAGER SHGRGVAFTPKDGGRVVMRRR  >CYP135A1(CCDC5079_0304)  MASTLTTGLPPGPRLPRYLQSVLYLRFREWFLPAMHRKYGDVFSLRVPPYADNLVVYTRP EHIKEIFAADPRSLHAGEGNHILGFVMGEHSVLMTDEAEHARMRSLLMPAFTRAALRGYR DMIASVAREHITRWRPHATINSLDHMNALTLDIILRVVFGVTDPKVKAELTSRLQQIINI HPAILAGVPYPSLKRMNPWKRFFHNQTKIDEILYREIASRRIDSDLTARTDVLSRLLQTK DTPTKPLTDAELRDQLITLLLAGHETTAAALSWTLWELAHAPEIQSQVVWAAVGGDDGFL EAVLKEGMRRHTVIASTARKVTAPAEIGGWRLPAGTVVNTSILLAHASEVSHPKPTEFRP SRFLDGSVAPNTWLPFGGGVRRCLGFGFALTEGAVILQEIFRRFTITAAGPSKGETPLVR NITTVPKHGAHLRLIPQRRLGGLGDSDPP  >CYP135B1(CCDC5079_0534)  MSGTSSMGLPPGPRLSGSVQAVLMLRHGLRFLTACQRRYGSVFTLHVAGFGHMVYLSDPA AIKTVFAGNPSVFHAGEANSMLAGLLGDSSLLLIDDDVHRDRRRLMSPPFHRDAVARQAG PIAEIAAANIAGWPMAKAFAVAPKMSEITLEVILRTVIGASDPVRLAALRKVMPRLLNVG PWATLALANPSLLNNRLWSRLRRRIEEADALLYAEIADRRADPDLAARTDTLAMLVRAAD EDGRTMTERELRDQLITLLVAGHDTTATGLSWALERLTRHPVTLAKAVQAADASAAGDPA GDEYLDAVAKETLRIRPVVYDVGRVLTEAVEVAGYRLPAGVMVVPAIGLVHASAQLYPDP ERFDPDRMVGATLSPTTWLPFGGGNRRCLGATFAMVEMRVVLREILRRVELSTTTTSGER PKLKHVIMVPHRGARIRVRATRDVSATSQATAQGAGCPAARGGGPSRAVGSQ  >CYP51B1(CCDC5079_0706)  MSAVALPRVSGGHDEHGHLEEFRTDPIGLMQRVRDECGDVGTFQLAGKQVVLLSGSHANE FFFRAGDDDLDQAKAYPFMTPIFGEGVVFDASPERRKEMLHNAALRGEQMKGHAATIEDQ VRRMIADWGEAGEIDLLDFFAELTIYTSSACLIGKKFRDQLDGRFAKLYHELERGTDPLA YVDPYLPIESFRRRDEARNGLVALVADIMNGRIANPPTDKSDRDMLDVLIAVKAETGTPR FSADEITGMFISMMFAGHHTSSGTASWTLIELMRHRDAYAAVIDELDELYGDGRSVSFHA LRQIPQLENVLKETLRLHPPLIILMRVAKGEFEVQGHRIHEGDLVAASPAISNRIPEDFP DPHDFVPARYEQPRQEDLLNRWTWIPFGAGRHRCVGAAFAIMQIKAIFSVLLREYEFEMA QPPESYRNDHSKMVVQLAQPACVRYRRRTGV  >CYP123A1(CCDC5079_0708)  MTVRVGDPELVLDPYDYDFHEDPYPYYRRLRDEAPLYRNEERNFWAVSRHHDVLQGFRDS TALSNAYGVSLDPSSRTSEAYRVMSMLAMDDPAHLRMRTLVSKGFTPRRIRELEPQVLEL ARIHLDSALQTESFDFVAEFAGKLPMDVISELIGVPDTDRARIRALADAVLHREDGVADV PPPAMAASIELMRYYADLIAEFRRRPANNLTSALLAAELDGDRLSDQEIMAFLFLMVIAG NETTTKLLANAVYWAAHHPGQLARVFADHSRIPMWVEETLRYDTSSQILARTVAHDLTLY DTTIPEGEVLLLLPGSANRDDRVFDDPDDYRIGREIGCKLVSFGSGAHFCLGAHLARMEA RVALGALLRRIRNYEVDDDNVVRVHSSNVRGFAHLPISVQAR  >CYP126A1(CCDC5079_0720)  MQFFSVMTTAAGLSGIDLTDLDNFADGFPHHLFAIHRREAPVYWHRPTEHTPDGEGFWSV ATYAETLEVLRDPVTYSSVTGGQRRFGGTVLQDLPVAGQVLNMMDDPRHTRIRRLVSSGL TPRMIRRVEDDLRRRARGLLDGVEPGAPFDFVVEIAAELPMQMICILLGVPETDRHWLFE AVEPGFDFRGSRRATMPRLNVEDAGSRLYTYALELIAGKRAEPADDMLSVVANATIDDPD APALSDAELYLFFHLLFSAGAETTRNSIAGGLLALAENPDQLQTLRSDFELLPTAIEEIV RWTSPSPSKRRTASRAVSLGGQPIEAGQKVVVWEGSANRDPSVFDRADEFDITRKPNPHL GFGQGVHYCLGANLARLELRVLFEELLSRFGSVRVVEPAEWTRSNRHTGIRHLVVELRGG  >CYP130A1(CCDC5079_1159)  MSHEFQLATAETWPNPWPMYRALRDHDPVHHVVPPQRPEYDYYVLSRHADVWSAARDHQT FSSAQGLTVNYGELEMIGLHDTPPMVMQDPPVHTEFRKLVSRGFTPRQVETVEPTVRKFV VERLEKLRANGGGDIVTELFKPLPSMVVAHYLGVPEEDWTQFDGWTQAIVAANAVDGATT GALDAVGSMMAYFTGLIERRRTEPADDAISHLVAAGVGADGDTAGTLSILAFTFTMVTGG NDTVTGMLGGSMPLLHRRPDQRRLLLDDPEGIPDAVEELLRLTSPVQGLARTTTRDVTIG DTTIPAGRRVLLLYGSANRDERQYGPDAAELDVTRCPRNILTFSHGAHHCLGAAAARMQC RVALTELLARCPDFEVAESRIVWSGGSYVRRPLSVPFRVTS  >CYP132A1(CCDC5079_1294)  MATATTQRPLKGPAKRMSTWTMTREAITIGFDAGDGFLGRLRGSDITRFRCAGRRFVSIS HPDYVDHVLHEARLKYVKSDEYGPIRATAGLNLLTDEGDSWARHRGALNSTFARRHLRGL VGLMIDPIADVTAALVPGAQFDMHQSMVETTLRVVANALFSQDFGPLVQSMHDLATRGLR RAEKLERLGLWGLMPRTVYDTLIWCIYSGVHLPPPLREMQEITLTLDRAINSVIDRRLAE PTNSADLLNVLLSADGGIWPRQRVRDEALTFMLAGHETTANAMSWFWYLMALNPQARDHM LTELDDVLGMRRPTADDLGKLAWTTACLQESQRYFSSVWIIAREAVDDDIIDGHRIRRGT TVVIPIHHIHHDPRWWPDPDRFDPGRFLRCPTDRPRCAYLPFGGGRRICIGQSFALMEMV LMAAIMSQHFTFDLAPGYHVELEATLTLRPKHGVHVIGRRR  >CYP139A1(CCDC5079_1545)  MRTYRTVRYPLGEALLALYRWRGPLINAGVGGHGYTYLLGAEANRFVFANADAFSWSQTF ESLVPVDGPTALIVSDGADHRRRRSVVAPGLRHHHVQRYVATMVSNIDTVIDGWQPGQRL DIYQELRSAVRRSTAESLFGQRLAVHSDFLGEQLQPLLDLTRRPPQVMRLQQRVNSPGWR RAMAARKRIDDLIDAQIADARTAPRPDDHMLTTLISGCSEEGTTLSDNEIRDSIVSLITA GYETTSGALAWAIYALLTVPGTWESAASEVARVLGGRVPAADDLSALTYLNGVVHETLRL YSPGVISARRVLRDLWFDGHRIRAGRLLIFSAYVTHRLPEIWPEPTEFRPLRWDPNAADY RKPAPHEFIPFSGGLHRCIGAVMATTEMTVILARLVARAMLQLPAQRTHRIRAANFAALR PWPGLTVEIRKSAPAQ  >CYP144A1(CCDC5079_1639)  MTIAKDANTFFGAESVQDPYPLYERMRAAGSVHRIANSDFYAVCGWDAVNEAIGRPEDFS SNLTATMTYTAEGTAKPFEMDPLGGPTHVLATADDPAHAVHRKLVLRHLAAKRIRVMEQF TVQAADRLWVDGMQDGCIEWMGAMANRLPMMVVAELIGLPDPDIAQLVKWGYAATQLLEG LVENDQLVAAGVALMELSGYIFEQFDRAAADPRDNLLGELATACASGELDTLTAQVMMVT LFAAGGESTAALLGSAVWILATRPDIQQQVRANPELLGAFIEETLRYEPPFRGHYRHVRN ATTLDGTELPADSHLLLLWGAANRDPAQFEAPGEFRLDRAGGKGHISFGKGAHFCVGAAL ARLEARIVLRLLLDRTSVIEAADVGGWLPSILVRRIERLELAVQ  >CYP143A1(CCDC5079_1646)  MTTPGEDHAGSFYLPRLEYSTLPMAVDRGVGWKTLRDAGPVVFMNGWYYLTRREDVLAAL RNPKVFSSRKALQPPGNPLPVVPLAFDPPEHTRYRRILQPYFSPAALSKALPSLRRHTVA MIDAIAGRGECEAMADLANLFPFQLFLVLYGLPLEDRDRLIGWKDAVIAMSDRPHPTEAD VAAARELLEYLTAMVAERRRNPGPDVLSQVQIGEDPLSEIEVLGLSHLLILAGLDTVTAA VGFSLLELARRPQLRAMLRDNPKQIRVFIEEIVRLEPSAPVAPRVTTEPVTVGGMTLPAG SPVRLCMAAVNRDGSDAMSTDELVMDGKVHRHWAFGGGPHRCLGSHLARLELTLLVGEWL NQIPDFELAPDYAPEIRFPSKSFALKNLPLRWS  >CYP140A1(CCDC5079_1737)  MKDKLHWLAMHGVIRGIAAIGIRRGDLQARLIADPAVATDPVPFYDEVRSHGALVRNRAN YLTVDHRLAHDLLRSDDFRVVSFGENLPPPLRWLERRTRGDQLHPLREPSLLAVEPPDHT RYRKTVSAVFTSRAVSALRDLVEQTAINLLDRFAEQPGIVDVVGRYCSQLPIVVISEILG VPEHDRPRVLEFGELAAPSLDIGIPWRQYLRVQQGIRGFDCWLEGHLQQLRHAPGDDLMS QLIQIAESGDNETQLDETELRAIAGLVLVAGFETTVNLLGNGIRMLLDTPEHLATLRQHP ELWPNTVEEILRLDSPVQLTARVACRDVEVAGVRIKRGEVVVIYLAAANRDPAVFPDPHR FDIERPNAGRHLAFSTGRHFCLGAALARAEGEVGLRTFFDRFPDVRAAGAGSRRDTRVLR GWSTLPVTLGPARSMVSP  >CYP124A1(CCDC5079_2102)  MGLNTAIATRVNGTPPPEVPIADIELGSLDFWALDDDVRDGAFATLRREAPISFWPTIEL PGFVAGNGHWALTKYDDVFYASRHPDIFSSYPNITINDQTPELAEYFGSMIVLDDPRHQR LRSIVSRAFTPKVVARIEAAVRDRAHRLVSSMIANNPDRQADLVSELAGPLPLQIICDMM GIPKADHQRIFHWTNVILGFGDPDLATDFDEFMQVSADIGAYATALAEDRRVNHHDDLTS SLVEAEVDGERLSSREIASFFILLVVAGNETTRNAITHGVLALSRYPEQRDRWWSDFDGL APTAVEEIVRWASPVVYMRRTLTQDIELRGTKMAAGDKVSLWYCSANRDESKFADPWTFD LARNPNPHLGFGGGGAHFCLGANLARREIRVAFDELRRQMPDVVATEEPARLLSQFIHGI KTLPVTWS  >CYP128A1(CCDC5079_2104)  MQLTDFDPFDPAIAADPYPHYRELLAGERVQYNPKRDVYILSRYADVREAARNHDTLSSA RGVTFSRGWLPFLPTSDPPAHTRMRKQLAPGMARGALETWRPMVDQLARELVGGLLTQTP ADVVSTVAAPMPMRAITSVLGVDGPDEAAFCRLSNQAVRITDVALSASGLISLVQGFAGF RRLRALFTHRRDNGLLRECTVLGKLATHAEQGRLSDDELFFFAVLLLVAGYESTAHMIST LFLTLADYPDQLTLLAQQPDLIPSAIEEHLRFISPIQNICRTTRVDYSVGQAVIPAGSLV LLAWGAANRDPRQYEDPDVFRADRNPVGHLAFGSGIHLCPGTQLARMEGQAILREIVANI DRIEVVEPPTWTTNANLRGLTRLRVAVTPRVAP  >CYP121A1(CCDC5079_2109)  MTATVLLEVPFSARGDRIPDAVAELRTREPIRKVRTITGAEAWLVSSYALCTQVLEDRRF SMKETAAAGAPRLNALTVPPEVVNNMGNIADAGLRKAVMKAITPKAPGLEQFLRDTANSL LDNLITEGAPADLRNDFADPLATALHCKVLGIPQEDGPKLFRSLSIAFMSSADPIPAAKI NWDRDIEYMAGILENPNITTGLMGELSRLRKDPAYSHVSDELFATIGVTFFGAGVISTGS FLTTALISLIQRPQLRNLLHEKPELIPAGVEELLRINLSFADGLPRLATADIQVGDVLVR KGELVLVLLEGANFDPEYFPNPGSIELDRPNPTSHLAFGRGQHFCPGSALGRRHAQIGIE ALLKKMPGVDLAVPIDQLVWRTRFQRRIPERLPVLW  >CYP136A1(CCDC5079_2815)  MATIHPPAYLLDQAKRRFTPSFNNFPGMSLVEHMLLNTKFPEKKLAEPPPGSGLKPVVGD AGLPILGHMIEMLRGGPDYLMFLYKTKGPVVFGDSAVLPGVAALGPDAAQVIYSNRNKDY SQQGWVPVIGPFFHRGLMLLDFEEHMFHRRIMQEAFVRSRLAGYLEQMDRVVSRVVADDW VVNDARFLVYPAMKALTLDIASMVFMGHEPGTDHELVTKVNKAFTITTRAGNAVIRTSVP PFTWWRGLRARELLENYFTARVKERREASGNDLLTVLCQTEDDDGNRFSDADIVNHMIFL MMAAHDTSTSTATTMAYQLAAHPEWQQRCRDESDRHGDGPLDIESLEQLESLDLVMNESI RLVTPVQWAMRQTVRDTELLGYYLPKGTNVIAYPGMNHRLPEIWTDPLTFDPERFTEPRN EHKRHRYAFTPFGGGVHKCIGMVFGQLEIKTILHRLLRRYRLELSRPDYQPRWDYSAMPI PMDGMPIVLRPR  >CYP141A1(CCDC5079_2874)  MLSDKRFSCRAAAHPSSPPFVPFVQLCPSLLSIDGPQHTAARRLLAQGLNPGFIARMRPV VQQIVDNALDDLAAAEPPVDFQEIVSVPIGEQLMAKLLGVEPETVHELAAHVDAAMSVCE IGDEEVSRRWSALCTMVIDILHRKLAEPGDDLLSTIAQANRQQSTMTDEQVVGMLLTVVI GGVDTPIAVITNGLASLLHHRDQYERLVEDPGRVARAVEEIVRFNPATEIEHLRVVTEDV VIAGTALSAGSPAFTSITSANRDSDQFLDPDEFDVERNPNEHIAFGYGPHACPASAYSRM CLTTFFTSLTQRFPQLQLARPFEDLERRGKGLHSVGIKELLVTWPT  >CYP142A1(CCDC5079_3259)  MTEAPDVDLADGNFYASREARAAYRWMRANQPVFRDRNGLAAASTYQAVIDAERQPELFS NAGGIRPDQPALPMMIDMDDPAHLLRRKLVNAGFTRKRVKDKEASIAALCDTLIDAVCER GECDFVRDLAAPLPMAVIGDMLGVRPEQRDMFLRWSDDLVTFLSSHVSQEDFQITMDAFA AYNDFTRATIAARRADPTDDLVSVLVSSEVDGERLSDDELVMETLLILIGGDETTRHTLS GGTEQLLRNRDQWDLLQRDPSLLPGAIEEMLRWTAPVKNMCRVLTADTEFHGTALCAGEK MMLLFESANFDEAVFCEPEKFDVQRNPNSHLAFGFGTHFCLGNQLARLELSLMTERVLRR LPDLRLVADDSVLPLRPANFVSGLESMPVVFTPSPPLG  >CYP125A1(CCDC5079_3286)  MPSPNLPPGFDFTDPAIYAERLPVAEFAELRSAAPIWWNGQDPGKGGGFHDGGFWAITKL NDVKEISRHSDVFSSYENGVIPRFKNDIAREDIEVQRFVMLNMDAPHHTRLRKIISRGFT PRAVGRLHDELQERAQKIAAEAAAAGSGDFVEQVSCELPLQAIAGLLGVPQEDRGKLFHW SNEMTGNEDPEYAHIDPKASSAELIGYAMKMAEEKAKNPADDIVTQLIQADIDGEKLSDD EFGFFVVMLAVAGNETTRNSITQGMMAFAEHPDQWELYKKVRPETAADEIVRWATPVTAF QRTALRDYELSGVQIKKGQRVVMFYRSANFDEEVFQDPFTFNILRNPNPHVGFGGTGAHY CIGANLARMTINLIFNAVADHMPDLKPISAPERLRSGWLNGIKHWQVDYTGRCPVAH  >CYP137A1(CCDC5079_3416)  MAAFAVRREHAPDALGGPPGLPAPRGFRAAFAAAYAVAYLAGGERRMLRLIRRYGPIMTM PILSLGDVAIVSDSALAKEVFTAPTDVLLGGEGVGPAAAIYGSGSMFVQEEPEHLRRRKL LTPPLHGAALDRYVPIIENSTRAAMHTWPVDRPFAMLTVARSLMLDVIVKVIFGVDDPEE VRRLGRPFERLLNLGVSEQLTVRYALRRLGALRVWPARARANTEIDDVVMALIAQRRADP RLGERHDVLSLLVSARGESGEQLSDSEIRDDLITLVLAGHETTATTLAWAFDLLLHHPDA LRRVRAEAVGGGEAFTTAVINETLRVRPPAPLTARVAAQPLTIGGYRVEAGTRIVVHIIA INRSAEVYEHPHEFRPERFLGTRPQTYAWVPFGGGVKRCLGANFSMRELITVLHVLLREG EFTAVDDEPERIVRRSIMLVPRRGTRVRFRPAR |
| ***Mycobacterium tuberculosis* 7199-99** |
| Database: TB; P450 count: 19; Families: 18; Subfamilies: 19 |
| >CYP138A1(MT7199_0138)  MSEVVTAAPAPPVVRLPPAVRGPKLFQGLAFVVSRRRLLGRFVRRYGKAFTANILMYGRV VVVADPQLARQVFTSSPEELGNIQPNLSRMFGSGSVFALDGDDHRRRRRLLAPPFHGKSM KNYETIIEEETLRETANWPQGQAFATLPSMMHITLNAILRAIFGAGGSELDELRRLIPPW VTLGSRLAALPKPKRDYGRLSPWGRLAEWRRQYDTVIDKLIEAERADPNFADRTDVLALM LRSTYDDGSIMSRKDIGDELLTLLAAGHETTAATLGWAFERLSRHPDVLAALVEEVDNGG HELRQAAILEVQRARTVIDFAARRVNPPVYQLGEWVIPRGYSIIINIAQIHGDPDVFPQP DRFDPQRYIGSKPSPFAWIPFGGGTRRCVGAAFANMEMDVVLRTVLRHFTLETTTAAGER SHGRGVAFTPKDGGRVVMRRR  >CYP135A1(MT7199_0333)  MASTLTTGLPPGPRLPRYLQSVLYLRFREWFLPAMHRKYGDVFSLRVPPYADNLVVYTRP EHIKEIFAADPRSLHAGEGNHILGFVMGEHSVLMTDEAEHARMRSLLMPAFTRAALRGYR DMIASVAREHITRWRPHATINSLDHMNALTLDIILRVVFGVTDPKVKAELTSRLQQIINI HPAILAGVPYPSLKRMNPWKRFFHNQTKIDEILYREIASRRIDSDLTARTDVLSRLLQTK DTPTKPLTDAELRDQLITLLLAGHETTAAALSWTLWELAHAPEIQSQVVWAAVGGDDGFL EAVLKEGMRRHTVIASTARKVTAPAEIGGWRLPAGTVVNTSILLAHASEVSHPKPTEFRP SRFLDGSVAPNTWLPFGGGVRRCLGFGFALTEGAVILQEIFRRFTITAAGPSKGETPLVR NITTVPKHGAHLRLIPQRRLGGLGDSDPP  >CYP135B1(MT7199_0582)  MSGTSSMGLPPGPRLSGSVQAVLMLRHGLRFLTACQRRYGSVFTLHVAGFGHMVYLSDPA AIKTVFAGNPSVFHAGEANSMLAGLLGDSSLLLIDDDVHRDRRRLMSPPFHRDAVARQAG PIAEIAAANIAGWPMAKAFAVAPKMSEITLEVILRTVIGASDPVRLAALRKVMPRLLNVG PWATLALANPSLLNNRLWSRLRRRIEEADALLYAEIADRRADPDLAARTDTLAMLVRAAD EDGRTMTERELRDQLITLLVAGHDTTATGLSWALERLTRHPVTLAKAVQAADASAAGDPA GDEYLDAVAKETLRIRPVVYDVGRVLTEAVEVAGYRLPAGVMVVPAIGLVHASAQLYPDP ERFDPDRMVGATLSPTTWLPFGGGNRRCLGATFAMVEMRVVLREILRRVELSTTTTSGER PKLKHVIMVPHRGARIRVRATRDVSATSQATAQGAGCPAARGGGPSRAVGSQ  >CYP51B1(MT7199_0785)  MSAVALPRVSGGHDEHGHLEEFRTDPIGLMQRVRDECGDVGTFQLAGKQVVLLSGSHANE FFFRAGDDDLDQAKAYPFMTPIFGEGVVFDASPERRKEMLHNAALRGEQMKGHAATIEDQ VRRMIADWGEAGEIDLLDFFAELTIYTSSACLIGKKFRDQLDGRFAKLYHELERGTDPLA YVDPYLPIESFRRRDEARNGLVALVADIMNGRIANPPTDKSDRDMLDVLIAVKAETGTPR FSADEITGMFISMMFAGHHTSSGTASWTLIELMRHRDAYAAVIDELDELYGDGRSVSFHA LRQIPQLENVLKETLRLHPPLIILMRVAKGEFEVQGHRIHEGDLVAASPAISNRIPEDFP DPHDFVPARYEQPRQEDLLNRWTWIPFGAGRHRCVGAAFAIMQIKAIFSVLLREYEFEMA QPPESYRNDHSKMVVQLAQPACVRYRRRTGV  >CYP123A1(MT7199_0787)  MTVRVGDPELVLDPYDYDFHEDPYPYYRRLRDEAPLYRNEERNFWAVSRHHDVLQGFRDS TALSNAYGVSLDPSSRTSEAYRVMSMLAMDDPAHLRMRTLVSKGFTPRRIRELEPQVLEL ARIHLDSALQTESFDFVAEFAGKLPMDVISELIGVPDTDRARIRALADAVLHREDGVADV PPPAMAASIELMRYYADLIAEFRRRPANNLTSALLAAELDGDRLSDQEIMAFLFLMVIAG NETTTKLLANAVYWAAHHPGQLARVFADHSRIPMWVEETLRYDTSSQILARTVAHDLTLY DTTIPEGEVLLLLPGSANRDDRVFDDPDDYRIGREIGCKLVSFGSGAHFCLGAHLARMEA RVALGALLRRIRNYEVDDDNVVRVHSSNVRGFAHLPISVQAR  >CYP126A1(MT7199_0799)  MTTAAGLSGIDLTDLDNFADGFPHHLFAIHRREAPVYWHRPTEHTPDGEGFWSVATYAET LEVLRDPVTYSSVTGGQRRFGGTVLQDLPVAGQVLNMMDDPRHTRIRRLVSSGLTPRMIR RVEDDLRRRARGLLDGVEPGAPFDFVVEIAAELPMQMICILLGVPETDRHWLFEAVEPGF DFRGSRRATMPRLNVEDAGSRLYTYALELIAGKRAEPADDMLSVVANATIDDPDAPALSD AELYLFFHLLFSAGAETTRNSIAGGLLALAENPDQLQTLRSDFELLPTAIEEIVRWTSPS PSKRRTASRAVSLGGQPIEAGQKVVVWEGSANRDPSVFDRADEFDITRKPNPHLGFGQGV HYCLGANLARLELRVLFEELLSRFGSVRVVEPAEWTRSNRHTGIRHLVVELRGG  >CYP130A1(MT7199_1285)  MTSVMSHEFQLATAETWPNPWPMYRALRDHDPVHHVVPPQRPEYDYYVLSRHADVWSAAR DHQTFSSAQGLTVNYGELEMIGLHDTPPMVMQDPPVHTEFRKLVSRGFTPRQVETVEPTV RKFVVERLEKLRANGGGDIVTELFKPLPSMVVAHYLGVPEEDWTQFDGWTQAIVAANAVD GATTGALDAVGSMMAYFTGLIERRRTEPADDAISHLVAAGVGADGDTAGTLSILAFTFTM VTGGNDTVTGMLGGSMPLLHRRPDQRRLLLDDPEGIPDAVEELLRLTSPVQGLARTTTRD VTIGDTTIPAGRRVLLLYGSANRDERQYGPDAAELDVTRCPRNILTFSHGAHHCLGAAAA RMQCRVALTELLARCPDFEVAESRIVWSGGSYVRRPLSVPFRVTS  >CYP132A1(MT7199_1424)  MATATTQRPLKGPAKRMSTWTMTREAITIGFDAGDGFLGRLRGSDITRFRCAGRRFVSIS HPDYVDHVLHEARLKYVKSDEYGPIRATAGLNLLTDEGDSWARHRGALNSTFARRHLRGL VGLMIDPIADVTAALVPGAQFDMHQSMVETTLRVVANALFSQDFGPLVQSMHDLATRGLR RAEKLERLGLWGLMPRTVYDTLIWCIYSGVHLPPPLREMQEITLTLDRAINSVIDRRLAE PTNSADLLNVLLSADGGIWPRQRVRDEALTFMLAGHETTANAMSWFWYLMALNPQARDHM LTELDDVLGMRRPTADDLGKLAWTTACLQESQRYFSSVWIIAREAVDDDIIDGHRIRRGT TVVIPIHHIHHDPRWWPDPDRFDPGRFLRCPTDRPRCAYLPFGGGRRICIGQSFALMEMV LMAAIMSQHFTFDLAPGYHVELEATLTLRPKHGVHVIGRRR  >CYP139A1(MT7199_1689)  MRYPLGEALLALYRWRGPLINAGVGGHGYTYLLGAEANRFVFANADAFSWSQTFESLVPV DGPTALIVSDGADHRRRRSVVAPGLRHHHVQRYVATMVSNIDTVIDGWQPGQRLDIYQEL RSAVRRSTAESLFGQRLAVHSDFLGEQLQPLLDLTRRPPQVMRLQQRVNSPGWRRAMAAR KRIDDLIDAQIADARTAPRPDDHMLTTLISGCSEEGTTLSDNEIRDSIVSLITAGYETTS GALAWAIYALLTVPGTWESAASEVARVLGGRVPAADDLSALTYLNGVVHETLRLYSPGVI SARRVLRDLWFDGHRIRAGRLLIFSAYVTHRLPEIWPEPTEFRPLRWDPNAADYRKPAPH EFIPFSGGLHRCIGAVMATTEMTVILARLVARAMLQLPAQRTHRIRAANFAALRPWPGLT VEIRKSAPAQ  >CYP144A1(MT7199_1804)  MRRSPKGSPGAVLDLQRRVDQAVSADHAELMTIAKDANTFFGAESVQDPYPLYERMRAAG SVHRIANSDFYAVCGWDAVNEAIGRPEDFSSNLTATMTYTAEGTAKPFEMDPLGGPTHVL ATADDPAHAVHRKLVLRHLAAKRIRVMEQFTVQAADRLWVDGMQDGCIEWMGAMANRLPM MVVAELIGLPDPDIAQLVKWGYAATQLLEGLVENDQLVAAGVALMELSGYIFEQFDRAAA DPRDNLLGELATACASGELDTLTAQVMMVTLFAAGGESTAALLGSAVWILATRPDIQQQV RANPELLGAFIEETLRYEPPFRGHYRHVRNATTLDGTELPADSHLLLLWGAANRDPAQFE APGEFRLDRAGGKGHISFGKGAHFCVGAALARLEARIVLRLLLDRTSVIEAADVGGWLPS ILVRRIERLELAVQ  >CYP143A1(MT7199_1811)  MTTPGEDHAGSFYLPRLEYSTLPMAVDRGVGWKTLRDAGPVVFMNGWYYLTRREDVLAAL RNPKVFSSRKALQPPGNPLPVVPLAFDPPEHTRYRRILQPYFSPAALSKALPSLRRHTVA MIDAIAGRGECEAMADLANLFPFQLFLVLYGLPLEDRDRLIGWKDAVIAMSDRPHPTEAD VAAARELLEYLTAMVAERRRNPGPDVLSQVQIGEDPLSEIEVLGLSHLLILAGLDTVTAA VGFSLLELARRPQLRAMLRDNPKQIRVFIEEIVRLEPSAPVAPRVTTEPVTVGGMTLPAG SPVRLCMAAVNRDGSDAMSTDELVMDGKVHRHWGFGGGPHRCLGSHLARLELTLLVGEWL NQIPDFELAPDYAPEIRFPSKSFALKNLPLRWS  >CYP140A1(MT7199_1906)  MKDKLHWLAMHGVIRGIAAIGIRRGDLQARLIADPAVATDPVPFYDEVRSHGALVRNRAN YLTVDHRLAHDLLRSDDFRVVSFGENLPPPLRWLERRTRGDQLHPLREPSLLAVEPPDHT RYRKTVSAVFTSRAVSALRDLVEQTAINLLDRFAEQPGIVDVVGRYCSQLPIVVISEILG VPEHDRPRVLEFGELAAPSLDIGIPWRQYLRVQQGIRGFDCWLEGHLQQLRHAPGDDLMS QLIQIAESGDNETQLDETELRAIAGLVLVAGFETTVNLLGNGIRMLLDTPEHLATLRQHP ELWPNTVEEILRLDSPVQLTARVACRDVEVAGVRIKRGEVVVIYLAAANRDPAVFPDPHR FDIERPNAGRHLAFSTGRHFCLGAALARAEGEVGLRTFFDRFPDVRAAGAGSRRDTRVLR GWSTLPVTLGPARSMVSP  >CYP124A1(MT7199_2297)  MGLNTAIATRVNGTPPPEVPIADIELGSLDFWALDDDVRDGAFATLRREAPISFWPTIEL PGFVAGNGHWALTKYDDVFYASRHPDIFSSYPNITINDQTPELAEYFGSMIVLDDPRHQR LRSIVSRAFTPKVVARIEAAVRDRAHRLVSSMIANNPDRQADLVSELAGPLPLQIICDMM GIPKADHQRIFHWTNVILGFGDPDLATDFDEFMQVSADIGAYATALAEDRRVNHHDDLTS SLVEAEVDGERLSSREIASFFILLVVAGNETTRNAITHGVLALSRYPEQRDRWWSDFDGL APTAVEEIVRWASPVVYMRRTLTQDIELRGTKMAAGDKVSLWYCSANRDESKFADPWTFD LARNPNPHLGFGGGGAHFCLGANLARREIRVAFDELRRQMPDVVATEEPARLLSQFIHGI KTLPVTWS  >CYP128A1(MT7199_2299)  MTATQSPPEPAPDRVRLAGCPLAGTPDVGLTAQDATTALGVPTRRRASSGGIPVATSMWR DAQTVRTYGPAVAKALALRVAGKARSRLTGRHCRKFMQLTDFDPFDPAIAADPYPHYREL LAGERVQYNPKRDVYILSRYADVREAARNHDTLSSARGVTFSRGWLPFLPTSDPPAHTRM RKQLAPGMARGALETWRPMVDQLARELVGGLLTQTPADVVSTVAAPMPMRAITSVLGVDG PDEAAFCRLSNQAVRITDVALSASGLISLVQGFAGFRRLRALFTHRRDNGLLRECTVLGK LATHAEQGRLSDDELFFFAVLLLVAGYESTAHMISTLFLTLADYPDQLTLLAQQPDLIPS AIEEHLRFISPIQNICRTTRVDYSVGQAVIPAGSLVLLAWGAANRDPRQYEDPDVFRADR NPVGHLAFGSGIHLCPGPSWRAWRVRRSCARSSPISTE  >CYP136A1(MT7199_3093)  MATIHPPAYLLDQAKRRFTPSFNNFPGMSLVEHMLLNTKFPEKKLAEPPPGSGLKPVVGD AGLPILGHMIEMLRGGPDYLMFLYKTKGPVVFGDSAVLPGVAALGPDAAQVIYSNRNKDY SQQGWVPVIGPFFHRGLMLLDFEEHMFHRRIMQEAFVRSRLAGYLEQMDRVVSRVVADDW VVNDARFLVYPAMKALTLDIASMVFMGHEPGTDHELVTKVNKAFTITTRAGNAVIRTSVP PFTWWRGLRARELLENYFTARVKERREASGNDLLTVLCQTEDDDGNRFSDADIVNHMIFL MMAAHDTSTSTATTMAYQLAAHPEWQQRCRDESDRHGDGPLDIESLEQLESLDLVMNESI RLVTPVQWAMRQTVRDTELLGYYLPKGTNVIAYPGMNHRLPEIWTDPLTFDPERFTEPRN EHKRHRYAFTPFGGGVHKCIGMVFDQLEIKTILHRLLRRYRLELSRPDYQPRWDYSAMPI PMDGMPIVLRPR  >CYP141A1(MT7199_3154)  MTSTSIPTFPFDRPVPTEPSPMLSELRNSCPVAPIELPSGHTAWLVTRFDDVKGVLSDKR FSCRAAAHPSSPPFVPFVQLCPSLLSIDGPQHTAARRLLAQGLNPGFIARMRPVVQQIVD NALDDLAAAEPPVDFQEIVSVPIGEQLMAKLLGVEPETVHELAAHVDAAMSVCEIGDEEV SRRWSALCTMVIDILHRKLAEPGDDLLSTIAQANRQQSTMTDEQVVGMLLTVVIGGVDTP IAVITNGLASLLHHRDQYERLVEDPGRVARAVEEIVRFNPATEIEHLRVVTEDVVIAGTA LSAGSPAFTSITSANRDSDQFLDPDEFDVERNPNEHIAFGYGPHACPASAYSRMCLTTFF TSLTQRFPQLQLARPFEDLERRGKGLHSVGIKELLVTWPT  >CYP142A1(MT7199_3578)  MTEAPDVDLADGNFYASREARAAYRWMRANQPVFRDRNGLAAASTYQAVIDAERQPELFS NAGGIRPDQPALPMMIDMDDPAHLLRRKLVNAGFTRKRVKDKEASIAALCDTLIDAVCER GECDFVRDLAAPLPMAVIGDMLGVRPEQRDMFLRWSDDLVTFLSSHVSQEDFQITMDAFA AYNDFTRATIAARRADPTDDLVSVLVSSEVDGERLSDDELVMETLLILIGGDETTRHTLS GGTEQLLRNRDQWDLLQRDPSLLPGAIEEMLRWTAPVKNMCRVLTADTEFHGTALCAGEK MMLLFESANFDEAVFCEPEKFDVQRNPNSHLAFGFGTHFCLGNQLARLELSLMTERVLRR LPDLRLVADDSVLPLRPANFVSGLESMPVVFTPSPPLG  >CYP125A1(MT7199_3607)  MSGNHQSVEIAVRRTTVPSPNLPPGFDFTDPAIYAERLPVAEFAELRSAAPIWWNGQDPG KGGGFHDGGFWAITKLNDVKEISRHSDVFSSYENGVIPRFKNDIAREDIEVQRFVMLNMD APHHTRLRKIISRGFTPRAVGRLHDELQERAQKIAAEAAAAGSGDFVEQVSCELPLQAIA GLLGVPQEDRGKLFHWSNEMTGNEDPEYAHIDPKASSAELIGYAMKMAEEKAKNPADDIV TQLIQADIDGEKLSDDEFGFFVVMLAVAGNETTRNSITQGMMAFAEHPDQWELYKKVRPE TAADEIVRWATPVTAFQRTALRDYELSGVQIKKGQRVVMFYRSANFDEEVFQDPFTFNIL RNPNPHVGFGGTGAHYCIGANLARMTINLIFNAVADHMPDLKPISAPERLRSGWLNGIKH WQVDYTGRCPVAH  >CYP137A1(MT7199_3749)  MVLRSLASPAALTDPKRCASVVGVAAFAVRREHAPDALGGPPGLPAPRGFRAAFAAAYAV AYLAGGERRMLRLIRRYGPIMTMPILSLGDVAIVSDSALAKEVFTAPTDVLLGGEGVGPA AAIYGSGSMFVQEEPEHLRRRKLLTPPLHGAALDRYVPIIENSTRAAMHTWPVDRPFAML TVARSLMLDVIVKVIFGVDDPEEVRRLGRPFERLLNLGVSEQLTVRYALRRLGALRVWPA RARANTEIDDVVMALIAQRRADPRLGERHDVLSLLVSARGESGEQLSDSEIRDDLITLVL AGHETTATTLAWAFDLLLHHPDALRRVRAEAVGGGEAFTTAVINETLRVRPPAPLTARVA AQPLTIGGYRVEAGTRIVVHIIAINRSAEVYEHPHEFRPERFLGTRPQTYAWVPFGGGVK RCLGANFSMRELITVLHVLLREGEFTAVDDEPERIVRRSIMLVPRRGTRVRFRPAR |
| ***Mycobacterium tuberculosis* Beijing/NITR203** |
| Database: TB; P450 count: 20; Families: 19; Subfamilies: 20 |
| >CYP138A1(J112_00750)  MSEVVTAAPAPPVVRLPPAVRGPKLFQGLAFVVSRRRLLGRFVRRYGKAFTANILMYGRV VVVADPQLARQVFTSSPEELGNIQPNLSRMFGSGSVFALDGDDHRRRRRLLAPFFHGKSM KNYETIIEEETLRETANWPQGQAFATLPSMMHITLNAILRAIFGAGGSELDELRRLIPPW VTLGSRLAALPKPKRDYGRLSPWGRLAEWRGQYDTVIDKLIEAERADPNFADRTDVLALM LRSTYDDGSIMSRKDIGDELLTLLAAGHETTAATLGWAFERLSRHPDVLAALVEEVDNGG HELRQAAILEVQRARTVIDFAAPRVNPPVYQLGEWVIPRGYSIIINIAQIHGDPDVFPQP DRFDPQRYIGSKPSPFAWIPFGGGPRRCVGAAFANMEMDVVLRTVLRHFTLETTTAAGER SHGRGVAFTPKDGGRVVMRRR  >CYP135A1(J112_01765)  MASTLTTGLPPGPRLPRYLQSVLYLRFREWFLPAMHRKYGDVFSLRVPPYADNLVVYTRP EHIKEIFAADPRSLHAGEGNHILGFVMGEHSVLMTDEAEHARMRSLLMPAFTRAALRGYR DMIASVAREHITRWRPHATINSLDHMNALTLDIILRVVFGVTDPKVKAELTSRLQQIINI HPAILAGVPYPSLKRMNPWKRFFHNQTKIDEILYREIASRRIDSDLTARTDVLSRLLQTK DTPTKPLTDAELRDQLITLLLAGHETTAAALSWTLWELAHAPEIQSQVVWAAVGGDDGFL EAVLKEGMRRHTVIASTARKVTAPAEIGGWRLPAGTVVNTSILLAHASEVSHPKPTEFRP SRFLDGSVAPNTWLPFGGGVRRCLGFGFALTEGAVILQEIFRRFTITAAGPSKGETPLVR NITTVPKHGAHLRLIPQRRLGGLGDSDPP  >CYP135B1(J112_03045)  MSGTSSMGLPPGPRLSGSVQAVLMLRHGLRFLTACQRRYGSVFTLHVAGFGHMVYLSDPA AIKTVFAGNPSVFHAGEANSMLAGLLGDSSLLLIDDDVHRDRRRLMSPPFHRDAVARQAG PIAEIAAANIAGWPMAKAFAVAPKMSEITLEVILRTVIGASDPVRLAALRKVMPRLLNVG PWATLALANPSLLNNRLWSRLRRRIEEADALLYAEIADRRADPDLAARTDTLAMLVRAAD EDGRTMTERELRDQLITLLVAGHDTTATGLSWALERLTRHPVTLAKAVQAADASAAGDPA GDEYLDAVAKETLRIRPVVYDVGRVLTEAVEVAGYRLPAGGMVVPAIGLVHASAQLYPDP ERFDPDRMVGATLSPTTWLPFGGGTRRCLGATFAMVEMRVVLREILRRVELSTTTTSGER PKLKHVIMVPHRGARIRVRATRDVSATSQATAQGAGCPAARGGGPSRAVGSQ  >CYP51B1(J112_04110)  MSAVALPRVSGGHDEHGHLEEFRTDPIGLMQRVRDECGDVGTFQLAGKQVVLLSGSHANE FFFRAGDDDLDQAKAYPFMTPIFGEGVVFDASPERRKEMLHNAALRGEQMKGHAATIEDQ VRRMIADWGEAGEIDLLDFFAELTIYTSSACLIGKKFRDQLDGRFAKLYHELERGTDPLA YVDPYLPIESFRRRDEARNGLVALVADIMNGRIANPPTDKSDRDMLDVLIAVKAETGTPR FSADEITGMFISMMFAGHHTSSGTASWTLIELMRHRDAYAAVIDELDELYGDGRSVSFHA LRQIPQLENVLKETLRLHPPLIILMRVAKGEFEVQGHRIHEGDLVAASPAISNRIPEDFP DPHDFVPARYEQPRQEDLLNRWTWIPFGAGRHRCVGAAFAIMQIKAIFSVLLREYEFEMA QPPESYRNDHSKMVVQLAQPACVRYRRRTGV  >CYP123A1(J112_04120)  MTVRVGDPELVLDPYDYDFHEDPYPYYRRLRDEAPLYRNEERNFWAVSRHHDVLQGFRDS TALSNAYGVSLDPSSRTSEAYRVMSMLAMDDPAHLRMRTLVSKGFTPRRIRELEPQVLEL ARIHLDSALQTESFDFVAEFAGKLPMDVISELIGVPDTDRARIRALADAVLHREDGVADV PPPAMAASIELMRYYADLIAEFRRRPANNLTSALLAAELDGDRLSDQEIMAFLFLMVIAG NETTTKLLANAVYWAAHHPGQLARVFADHSRIPMWVEETLRYDTSSQILARTVAHDLTLY DTTIPEGEVLLLLPGSANRDDRVFDDPDDYRIGREIGCKLVSFGSGAHFCLGAHLARMEA RVALGALLRRIRNYEVDDDNVVRVHSSNVRGFAHLPISVQAR  >CYP126A1(J112_04180)  MTTAAGLSGIDLTDLDNFADGFPHHLFAIHRREAPVYWHRPTEHTPDGEGFWSVATYAET LEVLRDPVTYSSVTGGQRRFGGTVLQDLPVAGQVLNMMDDPRHTRIRRLVSSGLTPRMIR RVEDDLRRRARGLLDGVEPGAPFDFVVEIAAELPMQMICILLGVPETDRHWLFEAVEPGF DFRGSRRATMPRLNVEDAGSRLYTYALELIAGKRAEPADDMLSVVANATIDDPDAPALSD AELYLFFHLLFSAGAETTRNSIAGGLLALAENPDQLQTLRSDFELLPTAIEEIVRWTSPS PSKRRTASRAVSLGGQPIEAGQKVVVWEGSANRDPSVFDRADEFDITRKPNPHLGFGQGV HYCLGANLARLELRVLFEELLSRFGSVRVVEPAEWTRSNRHTGIRHLVVELRGG  >CYP130A1(J112_06760)  MTSVMSHEFQLATAETWPNPWPMYRALRDHDPVHHVVPPQRPEYDYYVLSRHADVWSAAR DHQTFSSAQGLTVNYGELEMIGLHDTPPMVMQDPPVHTEFRKLVSRGFTPRQVETVEPTV RKFVVERLEKLRANGGGDIVTELFKPLPSMVVAHYLGVPEEDWTQFDGWTQAIVAANAVD GATTGALDAVGSMMAYFTGLIERRRTEPADDAISHLVAAGVGADGDTAGTLSILAFTFTM VTGGNDTVTGMLGGSMPLLHRRPDQRRLLLDDPEGIPDAVEELLRLTSPVQGLARTTTRD VTIGDTTIPAGRRVLLLYGSANRDERQYGPDAAELDVTRCPRNILTFSHGAHHCLGAAAA RMQCRVALTELLARCPDFEVAESRIVWSGGSYVRRPLSVPFRVTS  >CYP132A1(J112_07510)  MATATTQRPLKGPAKRMSTWTMTREAITIGFDAGDGFLGRLRGSDITRFRCAGRRFVSIS HPDYVDHVLHEARLKYVKSDEYGPIRATAGLNLLTDEGDSWARHRGALNSTFARRHLRGL VGLMIDPIADVTAALVPGAQFDMHQSMVETTLRVVANALFSQDFGPLVQSMHDLATRGLR RAEKLERLGLWGLMPRTVYDTLIWCIYSGVHLPPPLREMQEITLTLDRAINSVIDRRLAE PTNSADLLNVLLSADGGIWPRQRVRDEALTFMLAGHETTANAMSWFWYLMALNPQARDHM LTELDDVLGMRRPTADDLGKLAWTTACLQESQRYFSSVWIIAREAVDDDIIDGHRIRRGT TVVIPIHHIHHDPRWWPDPDRFDPGRFLRCPTDRPRCAYLPFGGGRRICIGQSFALMEMV LMAAIMSQHFTFDLAPGYHVELEATLTLRPKHGVHVIGRRR  >CYP139A1(J112_08910)  MRYPLGEALLALYRWRGPLINAGVGGHGYTYLLGAEANRFVFANADAFSWSQTFESLVPV DGPTALIVSDGADHRRRRSVVAPGLRHHHVQRYVATMVSNIDTVIDGWQPGQRLDIYQEL RSAVRRSTAESLFGQRLAVHSDFLGEQLQPLLDLTRRPPQVMRLQQRVNSPGWRRAMAAR KRIDDLIDAQIADARTAPRPDDHMLTTLISGCSEEGTTLSDNEIRDSIVSLITAGYETTS GALAWAIYALLTVPGTWESAASEVARVLGGRVPAADDLSALTYLNGVVHETLRLYSPGVI SARRVLRDLWFDGHRIRAGRLLIFSAYVTHRLPEIWPEPTEFRPLRWDPNAADYRKPAPH EFIPFSGGLHRCIGAVMATTEMTVILARLVARAMLQLPAQRTHRIRAANFAALRPWPGLT VEIRKSAPAQ  >CYP144A1(J112_09505)  MRRSPKGSPGAVLDLQRRVDQAVSADHAELMTIAKDANTFFGAESVQDPYPLYERMRAAG SVHRIANSDFYAVCGWDAVNEAIGRPEDFSSNLTATMTYTAEGTAKPFEMDPLGGPTHVL ATADDPAHAVHRKLVLRHLAAKRIRVMEQFTVQAADRLWVDGMQDGCIEWMGAMANRLPM MVVAELIGLPDPDIAQLVKWGYAATQLLEGLVENDQLVAAGVALMELSGYIFEQFDRAAA DPRDNLLGELATACASGELDTLTAQVMMVTLFAAGGESTAALLGSAVWILATRPDIQQQV RANPELLGAFIEETLRYEPPFRGHYRHVRNATTLDGTELPADSHLLLLWGAANRDPAQFE APGEFRLDRAGGKGHISFGKGAHFCVGAALARLEARIVLRLLLDRTSVIEAADVGGWLPS ILVRRIERLELAVQ  >CYP143A1(J112_09545)  MTTPGEDHAGSFYLPRLEYSTLPMAVDRGVGWKTLRDAGPVVFMNGWYYLTRREDVLAAL RNPKVFSSRKALQPPGNPLPVVPLAFDPPEHTRYRRILQPYFSPAALSKALPSLRRHTVA MIDAIAGRGECEAMADLANLFPFQLFLVLYGLPLEDRDRLIGWKDAVIAMSDRPHPTEAD VAAARELLEYLTAMVAERRRNPGPDVLSQVQIGEDPLSEIEVLGLSHLLILAGLDTVTAA VGFSLLELARRPQLRAMLRDNPKQIRVFIEEIVRLEPSAPVAPRVTTEPVTVGGMTLPAG SPVRLCMAAVNRDGSDAMSTDELVMDGKVHRHWAFGGGPHRCLGSHLARLELTLLVGEWL NQIPDFELAPDYAPEIRFPSKSFALKNLPLRWS  >CYP140A1(J112_10020)  MKDKLHWLAMHGVIRGIAAIGIRRGDLQARLIADPAVATDPVPFYDEVRSHGALVRNRAN YLTVDHRLAHDLLRSDDFRVVSFGENLPPPLRWLERRTRGDQLHPLREPSLLAVEPPDHT RYRKTVSAVFTSRAVSALRDLVEQTAINLLDRFAEQPGIVDVVGRYCSQLPIVVISEILG VPEHDRPRVLEFGELAAPSLDIGIPWRQYLRVQQGIRGFDCWLEGHLQQLRHAPGDDLMS QLIQIAESGDNETQLDETELRAIAGLVLVAGFETTVNLLGNGIRMLLDTPEHLATLRQHP ELWPNTVEEILRLDSPVQLTARVACRDVEVAGVRIKRGEVVVIYLAAANRDPAVFPDPHR FDIERPNAGRHLAFSTGRHFCLGAALARAEGEVGLRTFFDRFPDVRAAGAGSRRDTRVLR GWSTLPVTLGPARSMVSP  >CYP124A1(J112_12165)  MGLNTAIATRVNGTPPPEVPIADIELGSLDFWALDDDVRDGAFATLRREAPISFWPTIEL PGFVAGNGHWALTKYDDVFYASRHPDIFSSYPNIMINDQTPELAEYFGSMIVLDDPRHQR LRSIVSRAFTPKVVARIEAAVRDRAHRLVSSMIANNPDRQADLVSELAGPLPLQIICDMM GIPKADHQRIFHWTNVILGFGDPDLATDFDEFMQVSADIGAYATALAEDRRVNHHDDLTS SLVEAEVDGERLSSREIASFFILLVVAGNETTRNAITHGVLALSRYPEQRDRWWSDFDGL APTAVEEIVRWASPVVYMRRTLTQDIELRGTKMAAGDKVSLWYCSANRDESKFADPWTFD LARNPNPHLGFGGGGAHFCLGANLARREIRVAFDELRRQMPDVVATEEPARLLSQFIHGI KTLPVTWS  >CYP128A1(J112_12175)  MTATQSPPEPAPDRVRLAGCPLAGTPDVGLTAQDATTALGVPTRRRASSGGIPVATSMWR DAQTVRTYGPAVAKALALRVAGKARSRLTGRHCRKFMQLTDFDPFDPAIAADPYPHYREL LAGERVQYNPKRDVYILSRYADVREAARNHDTLSSARGVTFSRGWLPFLPTSDPPAHTRM RKQLAPGMARGALETWRPMVDQLARELVGGLLTQTPADVVSTVAAPMPMRAITSVLGVDG PDEAAFCRLSNQAVRITDVALSASGLISLVQGFAGFRRLRALFTHRRDNGLLRECTVLGK LATHAEQGRLSDDELFFFAVLLLVAGYESTAHMISTLFLTLADYPDQLTLLAQQPDLIPS AIEEHLRFISPIQNICRTTRVDYSVGQAVIPAGSLVLLAWGAANRDPRQYEDPDVFRADR NPVGHLAFGSGIHLCPGTQLARMEGQAILREIVANIDRIEVVEPPTWTTNANLRGLTRLR VAVTPRVAP  >CYP121A1(J112_12205)  MTATVLLEVPFSARGDRIPDAVAELRTREPIRKVRTITGAEAWLVSSYALCTQVLEDRRF SMKETAAAGAPRLNALTVPPEVVNNMGNIADAGLRKAVMKAITPKAPGLEQFLRDTANSL LDNLITEGAPADLRNDFADPLATALHCKVLGIPQEDGPKLFRSLSIAFMSSADPIPAAKI NWDRDIEYMAGILENPNITTGLMGELSRLRKDPAYSHVSDELFATIGVTFFGAGVISTGS FLTTALISLIQRPQLRNLLHEKPELIPAGVEELLRINLSFADGLPRLATADIQVGDVLVR KGELVLVLLEGANFDPEYFPNPGSIELDRPNPTSHLAFGRGQHFCPGSALGRRHAQIGIE ALLKKMPGVDLAVPIDQLVWRTRFQRRIPERLPVLW  >CYP136A1(J112_16385)  MATIHPPAYLLDQAKRRFTPSFNNFPGMSLVEHMLLNTKFPEKKLAEPPPGSGLKPVVGD AGLPILGHMIEMLRGGPDYLMFLYKTKGPVVFGDSAVLPGVAALGPDAAQVIYSNRNKDY SQQGWVPVIGPFFHRGLMLLDFEEHMFHRRIMQEAFVRSRLAGYLEQMDRVVSRVVADDW VVNDARFLVYPAMKALTLDIASMVFMGHEPGTDHELVTKVNKAFTITTRAGNAVIRTSVP PFTWWRGLRARELLENYFTARVKERREASGNDLLTVLCQTEDDDGNRFSDADIVNHMIFL MMAAHDTSTSTATTMAYQLAAHPEWQQRCRDESDRHGDGPLDIESLEQLESLDLVMNESI RLVTPVQWAMRQTVRDTELLGYYLPKGTNVIAYPGMNHRLPEIWTDPLTFDPERFTEPRN EHKRHRYAFTPFGGGVHKCIGMVFGQLEIKTILHRLLRRYRLELSRPDYQPRWDYSAMPI PMDGMPIVLRPR  >CYP141A1(J112_16725)  MTSTSIPTFPFDRPVPTEPSPMLSELRNSCPVAPIELPSGHTAWLVTRFDDVKGVLSDKR FSCRAAAHPSSPPFVPFVQLCPSLLSIDGPQHTAARRLLAQGLNPGFIARMRPVVQQIVD NALDDLAAAEPPVDFQEIVSVPIGEQLMAKLLGVEPETVHELAAHVDAAMSVCEIGDEEV SRRWSALCTMVIDILHRKLAEPGDDLLSTIAQANRQQSTMTDEQVVGMLLTVVIGGVDTP IAVITNGLASLLHHRDQYERLVEDPGRVARAVEEIVRFNPATEIEHLRVVTEDVVIAGTA LSAGSPAFTSITSANRDSDQFLDPDEFDVERNPNEHIAFGYGPHACPASAYSRMCLTTFF TSLTQRFPQLQLARPFEDLERRGKGLHSVGIKELLVTWPT  >CYP142A1(J112_18940)  MTEAPDVDLADGNFYASREARAAYRWMRANQPVFRDRNGLAAASTYQAVIDAERQPELFS NAGGIRPDQPALPMMIDMDDPAHLLRRKLVNAGFTRKRVKDKEASIAALCDTLIDAVCER GECDFVRDLAAPLPMAVIGDMLGVRPEQRDMFLRWSDDLVTFLSSHVSQEDFQITMDAFA AYNDFTRATIAARRADPTDDLVSVLVSSEVDGERLSDDELVMETLLILIGGDETTRHTLS GGTEQLLRNRDQWDLLQRDPSLLPGAIEEMLRWTAPVKNMCRVLTADTEFHGTALCAGEK MMLLFESANFDEAVFCEPEKFDVQRNPNSHLAFGFGTHFCLGNQLARLELSLMTERVLRR LPDLRLVADDSVLPLRPANFVSGLESMPVVFTPSPPLG  >CYP125A1(J112_19085)  MSWNHQSVEIAVRRTTVPSPNLPPGFDFTDPAIYAERLPVAEFAELRSAAPIWWNGQDPG KGGGFHDGGFWAITKLNDVKEISRHSDVFSSYENGVIPRFKNDIAREDIEVQRFVMLNMD APHHTRLRKIISRGFTPRAVGRLHDELQERAQKIAAEAAAAGSGDFVEQVSCELPLQAIA GLLGVPQEDRGKLFHWSNEMTGNEDPEYAHIDPKASSAELIGYAMKMAEEKAKNPADDIV TQLIQADIDGEKLSDDEFGFFVVMLAVAGNETTRNSITQGMMAFAEHPDQWELYKKVRPE TAADEIVRWATPVTAFQRTALRDYELSGVQIKKGQRVVMFYRSANFDEEVFQDPFTFNIL RNPNPHVGFGGTGAHYCIGANLARMTINLIFNAVADHMPDLKPISAPERLRSGWLNGIKH WQVDYTGRCPVAH  >CYP137A1(J112_19805)  MVLRSLASPAALTDPKRCASVVGVAAFAVRREHAPDALGGPPGLPAPRGFRAAFAAAYAV AYLAGGERRMLRLIRRYGPIMTMPILSLGDVAIVSDSALAKEVFTAPTDVLLGGEGVGPA AAIYGSGSMFVQEEPEHLRRRKLLTPPLHGAALDRYVPIIENSTRAAMHTWPVDRPFAML TVARSLMLDVIVKVIFGVDDPEEVRRLGRPFERLLNLGVSEQLTVRYALRRLGALRVWPA RARANTEIDDVVMALIAQRRADPRLGERHDVLSLLVSARGESGEQLSDSEIRDDLITLVL AGHETTATTLAWAFDLLLHHPDALRRVRAEAVGGGEAFTTAVINETLRVRPPAPLTARVA AQPLTIGGYRVEAGTRIVVHIIAINRSAEVYEHPHEFRPERFLGTRPQTYAWVPFGGGVK RCLGANFSMRELITVLHVLLREGEFTAVDDEPERIVRRSIMLVPRRGTRVRFRPAR |
| ***Mycobacterium tuberculosis* CAS/NITR204** |
| Database: TB; P450 count: 17; Families: 16; Subfamilies: 16 |
| >CYP135A1(J113_02365)  MASTLTTGLPPGPRLPRYLQSVLYLRFREWFLPAMHRKYGDVFSLRVPPYADNLVVYTRP EHIKEIFAADPRSLHAGEGNHILGFVMGEHSVLMTDEAEHARMRSLLMPAFTRAALRGYR DMIASVAREHITRWRPHATINSLDHMNALTLDIILRVVFGVTDPKVKAELTSRLQQIINI HPAILAGVPYPSLKRMNPWKRFFHNQTKIDEILYREIASRRIDSDLTARTDVLSRLLQTK DTPTKPLTDAELRDQLITLLLAGHETTAAALSWTLWELAHAPEIQSQVVWAAVGGDDGFL EAVLKEGMRRHTVIASTARKVTAPAEIGGWRLPAGTVVNTSILLAHASEVSHPKPTEFRP SRFLDGSVAPNTWLPFGGGVRRCLGFGFALTEGAVILQEIFRRFTITAAGPSKGETPLVR NITTVPKHGAHLRLIPQRRLGGLGDSDPP  >CYP51B1(J113_05390)  MSAVALPRVSGGHDEHGHLEEFRTDPIGLMQRVRDECGDVGTFQLAGKQVVLLSGSHANE FFFRAGDDDLDQAKAYPFMTPIFGEGVVFDASPERRKEMLHNAALRGEQMKGHAATIEDQ VRRMIADWGEAAEIDLLDFFAELTIYTSSACLIGKKFRDQLDGRFAKLYHELERGTDPLA YVDPYLPIESFRRRDEARNGLVALVADIMNGRIANPPTDKSDRDMLDVLIAVKAETGTPR FSADEITGMFISMMFAGHHTSSGTASWTLIELMRHRDAYAAVIDELDELYGDGRSVSFHA LRQIPQLENVLKETLRLHPPLIILMRVAKGEFEVQGHRIHEGDLVAASPAISNRIPEDFP DPHDFVPARYEQPRQEDLLNRWTWIPFGAGRHRCVGAAFAIMQIKAIFSVLLREYEFEMA QPPESYRNDHSKMVVQLAQPACVRYRRRTGV  >CYP126A1(J113_05480)  MTTAAGLSGIDLTDLDNFADGFPHHLFAIHRREAPVYWHRPTEHTPDGEGFWSVATYAET LEVLRDPVTYSSVTGGQRRFGGTVLQDLPVAGQVLNMMDDPRHTRIRRLVSSGLTPRMIR RVEDDLRRRARGLLDGVEPGAPFDFVVEIAAELPMQMICILLGVPETDRHWLFEAVEPGF DFRGSRRATMPRLNVEDAGSRLYTYALELIAGKRAEPADDMLSVVANATIDDPDAPALSD AELYLFFHLLFSAGAETTRNSIAGGLLALAENPDQLQTLRSDFELLPTAIEEIVRWTSPS PSKRRTASRAVSLGGQPIEAGQKVVVWEGSANRDPSVFDRADEFDITRKPNPHLGFGQGV HYCLGANLARLELRVLFEELLSRFGSVRVVEPAEWTRSNRHTGIRHLVVELRGG  >CYP139A1(J113_11610)  MRYPLGEALLALYRWRGPLINAGVGGHGYTYLLGAEANRFVFANADAFSWSQTFESLVPV DGPTALIVSDGADHRRRRSVVAPGLRHHHVQRYVATMVSNIDTVIDGWQPGQRLDIYQEL RSAVRRSTAESLFGQRLAVHSDFLGEQLQPLLDLTRRPPQVMRLQQRVNSPGWRRAMAAR KRIDDLIDAQIADARTAPRPDDHMLTTLISGCSEEGTTLSDNEIRDSIVSLITAGYETTS GALAWAIYALLTVPGTWESAASEVARVLGGRVPAADDLSALTYLNGVVHETLRLYSPGVI SARRVLRDLWFDGHRIRAGRLLIFSAYVTHRLPEIWPEPTEFRPLRWDPNAADYRKPAPH EFIPFSGGLHRCIGAVMATTEMTVILARLVARAMLQLPAQRTHRIRAANFAALRPWPGLT VEIRKSAPAQ  >CYP144A1(J113_12350)  MRRSPKGSPGAVLDLQRRVDQAVSADHAELMTIAKDANTFFGAESVQDPYPLYERMRAAG SVHRIANSDFYAVCGWDAVNEAIGRPEDFSSNLTATMTYTAEGTAKPFEMDPLGGPTHVL ATADDPAHAVHRKLVLRHLAAKRIRVMEQFTVQAADRLWVDGMQDGCIEWMGAMANRLPM MVVAELIGLPDPDIAQLVKWGYAATQLLEGLVENDQLVAAGVALMELSGYIFEQFDRAAA DPRDNLLGELATACASGELDTLTAQVMMVTLFAAGGESTAALLGSAVWILATRPDIQQQV RANPQLLGAFIEETLRYEPPFRGHYRHVRNATTLDGTELPADSHLLLLWGAANRDPAQFE APGEFRLDRAGGKGHISFGKGAHFCVGAALARLEARIVLRLLLDRTSVIEAADVGGWLPS ILVRRIERLELAVQ  >CYP143A1(J113_12420)  MTTPGEDHAGSFYLPRLEYSTLPMAVDRGVGWKTLRDAGPVVFMNGWYYLTRREDVLAAL RNPKVFSSRKALQPPGNPLPVVPLAFDPPEHTRYRRILQPYFSPAALSKALPSLRRHTVA MIDAIAGRGECEAMADLANLFPFQLFLVLYGLPLEDRDRLIGWKDAVIAMSDRPHPTEAD VAAARELLEYLTAMVAERRRNPGPDVLSQVQIGEDPLSEIEVLGLSHLLILAGLDTVTAA VGFSLLELARRPQLRAMLRDNPKQIRVFIEEIVRLEPSAPVAPRVTTEPVTVGGMTLPAG SPVRLCMAAVNRDGSDAMSTDELVMDGKVHRHWAFGGGPHRCLGSHLARLELTLLVGEWL NQIPDFELAPDYAPEIRFPSKSFALKNLPLRWS  >CYP140A1(J113_13030)  MKDKLHWLAMHGVIRGIAAIGIRRGDLQARLIADPAVATDPVPFYDEVRSHGALVRNRAN YLTVDHRLAHDLLRSDDFRVVSFGENLPPPLRWLERRTRGDQLHPLREPSLLAVEPPDHT RYRKTVSAVFTSRAVSALRDLVEQTAINLLDRFAEQPGIVDVVGRYCSQLPIVVISEILG VPEHDRPRVLEFGELAAPSLDIGIPWRQYLRVQQGIRGFDCWLEGHLQQLRHAPGDDLMS QLIQIAESGDNETQLDETELRAIAGLVLVAGFETTVNLLGNGIRMLLDTPEHLATLRQHP ELWPNTVEEILRLDSPVQLTARVACRDVEVAGVRIKRGEVVVIYLAAANRDPAVFPDPHR FDIERPNAGRHLAFSTGRHFCLGAALARAEGEVGLRTFFDRFPDVRAAGAGSRRDTRVLR GWSTLPVTLGPARSMVSP  >CYP124A1(J113_15800)  MGLNTAIATRVNGTPPPEVPIADIELGSLDFWALDDDVRDGAFATLRREAPISFWPTIEL PGFVAGNGHWALTKYDDVFYASRHPDIFSSYPNITINDQTPELAEYFGSMIVLDDPRHQR LRSIVSRAFTPKVVARIEAAVRDRAHRLVSPMIANNPDRQADLVSELAGPLPLQIICDMM GIPKADHQRIFHWTNVILGFGDPDLATDFDEFMQVSADIGAYATALAEDRRVNHHDDLTS SLVEAEVDGERLSSREIASFFILLVVAGNETTRNAITHGVLALSRYPEQRDRWWSDFDGL APTAVEEIVRWASPVVYMRRTLTQDIELRGTKMAAGDKVSLWYCSANRDESKFADPWTFD LARNPNPHLGFGGGGAHFCLGANLARREIRVAFDELRRQMPDVVATEEPARLLSQFIHGI KTLPVTWS  >CYP128A1(J113_15810)  MQLTDFDPFDPAIAADPYPHYRELLAGERVQYNPKRDVYILSRYADVREAARNHDTLSSA RGVTFSRGWLPFLPTSDPPAHTRMRKQLAPGMARGALETWRPMVDQLARELVGGLLTQTP ADVVSTVAAPMPMRAITSVLGVDGPDEAAFCRLSNQAVRITDVALSASGLISLVQGFAGF RRLRALFTHRRDNGLLRECTVLGKLATHAEQGRLSDDELFFFAVLLLVAGYESTAHMIST LFLTLADYPDQLTLLAQQPDLIPSAIEEHLRFISPIQNICRTTRVDYSVGQAVIPAGSLV LLAWGAANRDPRQYEDPDVFRADRNPVGHLAFGSGIHLCPGTQLARMEGQAILREIVANI DRIEVVEPPTWTTNANLRGLTRLRVAVTPRVAP  >CYP121A1(J113_15840)  MTATVLLEVPFSARGDRIPDAVAELRTREPIRKVRTITGAEAWLVSSYALCTQVLEDRRF SMKETAAAGAPRLNALTVPPEVVNNMGNIADAGLRKAVMKAITPKAPGLEQFLRDTANSL LDNLITEGAPADLRNDFADPLATALHCKVLGIPQEDGPKLFRSLSIAFMSSADPIPAAKI NWDRDIEYMAGILENPNITTGLMGELSRLRKDPAYSHVSDELFATIGVTFFGAGVISTGS FLTTALISLIQRPQLRNLLHEKPELIPAGVEELLRINLSFADGLPRLATADIQVGDVLVR KGELVLVLLEGANFDPEHFPNPGSIELDRPNPTSHLAFGRGQHFCPGSALGRRHAQIGIE ALLKKMPGVDLAVPIDQLVWRTRFQRRIPERLPVLW  >CYP136A1(J113_21300)  MATIHPPAYLLDQAKRRFTPSFNNFPGMSLVEHMLLNTKFPEKKLAEPPPGSGLKPVVGD AGLPILGHMIEMLRGGPDYLMFLYKTKGPVVFGDSAVLPGVAALGPDAAQVIYSNRNKDY SQQGWVPVIGPFFHRGLMLLDFEEHMFHRRIMQEAFVRSRLAGYLEQMDRVVSRVVADDW VVNDARFLVYPAMKALTLDIASMVFMGHEPGTDHELVTKVNKAFTITTRAGNAVIRTSVP PFTWWRGLRARELLENYFTARVKERREASGNDLLTVLCQTEDDDGNRFSDADIVNHMIFL MMAAHDTSTSTATTMAYQLAAHPEWQQRCRDESDRHGDGPLDIESLEQLESLDLVMNESI RLVTPVQWAMRQTVRDTELLGYYLPKGTNVIAYPGMNHRLPEIWTDPLTFDPERFTEPRN EHKRHRYAFTPFGGGVHKCIGMVFGQLEIKTILHRLLRRYRLELSRPDYQPRWDYSAMPI PMDGMPIVLRPR  >CYP141A1(J113_21750)  MTSTSIPTFPFDRPVPTEPSPMLSELRNSCPVAPIELPSGHTAWLVTRFDDVKGVLSDKR FSCRAAAHPSSPPFVPFVQLCPSLLSIDGPQHTAARRLLAQGLNPGFIARMRPVVQQIVD NALDDLAAAEPPVDFQEIVSVPIGEQLMAKLLGVEPETVHELAAHVDAAMSVCEIGDEEV SRRWSALCTMVIDILHRKLAEPGDDLLSTIAQANRQQSTMTDEQVVGMLLTVVIGGVDTP IAVITNGLASLLHHRDQYERLVEDPGRVARAVEEIVRFNPATEIEHLRVVTEDVVIAGTA LSAGSPAFTSITSANRDSDQFLDPDEFDVERNPNEHIAFGYGPHACPASAYSRMCLTTFF TSLTQRFPQLQLARPFEDLERRGKGLHSVGIKELLVTWPT  >CYP142A1(J113_24605)  MTEAPDVDLADGNFYASREARAAYRWMRANQPVFRDRNGLAAASTYQAVIDAERQPELFS NAGGIRPDQPALPMMIDMDDPAHLLRRKLVNAGFTRKRVKDKEASIAALCDTLIDAVCER GECDFVRDLAAPLPMAVIGDMLGVRPEQRDMFLRWSDDLVTFLSSHVSQEDFQITMDAFA AYNDFTRATIAARRADPTDDLVSVLVSSEVDGERLSDDELVMETLLILIGGDETTRHTLS GGTEQLLRNRDQWDLLQRDPSLLPGAIEEMLRWTAPVKNMCRVLTADTEFHGTALCAGEK MMLLFESANFDEAVFCEPEKFDVQRNPNSHLAFGFGTHFCLGNQLARLELSLMTERVLRR LPDLRLVADDSVLPLRPANFVSGLESMPVVFTPSPPLG  >CYP125A1(J113_24790)  MSWNHQSVEIAVRRTTVPSPNLPPGFDFTDPAIYAERLPVAEFAELRSAAPIWWNGQDPG KGGGFHDGGFWAITKLNDVKEISRHSDVFSSYENGVIPRFKNDIAREDIEVQRFVMLNMD APHHTRLRKIISRGFTPRAVGRLHDELQERAQKIAAEAAAAGSGDFVEQVSCELPLQAIA GLLGVPQEDRGKLFHWSNEMTGNEDPEYAHIDPKASSAELIGYAMKMAEEKAKNPADDIV TQLIQADIDGEKLSDDEFGFFVVMLAVAGNETTRNSITQGMMAFAEHPDQWELYKKVRPE TAADEIVRWATPVTAFQRTALRDYELSGVQIKKGQRVVMFYRSANFDEEVFQDPFTFNIL RNPNPHVGFGGTGAHYCIGANLARMTINLIFNAVADHMPDLKPISAPERLRSGWLNGIKH WQVDYTGRCPVAH  >CYP137A(J113_25740)  MAAFAVRREHAPDALGGPPGLPAPRGFRAAFAAAYAVAYLAGGERRMLRLIRRYGPIMTM PILSLGDVAIVSDSALAKEVFTAPTDVLLGGEGVGPAAAIYGSGSMFVQEEPEHLRRRKL LTPPLHGAALDRYVPIIENSTRAAMHTWPVDRPFAMLTVARSLMLDVIVKVIFGVDDPEE VRRLGRPFERLLNLGVSEQLTVRYALRRLGALRVWPARARANTEIDDVVMALIAQRRSDP RLGDQTAFQRRGESGEQLSDSEIRDDLITLVLAGHETTATTLAWAFDLLLHHPDALRRVR AEAVGGGGLHDGGDQRDVAGASARAVDGSCRRATTNHRRLPCGGWHTNRGPHHRDQPQRR GV  >CYP123A1-fragment(J113_05400)  MAASIELMRYYADLIAEFRRRPANNLTSALLAAELDGDRLSDQEIMAFLFLMVIAGNETT TKLLANAVYWAAHHPGQLARVFADHSRIPMWVEETLRYDTSSQILARTVAHDLTLYDTTI PEGEVLLLLPGSANRDDRVFDDPDDYRIGREIGCKLVSFGSGAHFCLGAHLARMEARVAL GALLRRIRNYEVDDDNVVRVHSSNVRGFAHLPISVQAR  >CYP137A1-fragment(J113_25735)  MVHIIAINRSAEVYEHPHEFRPERFLGTRPQTYAWVPFGGGVKRCLGANFSMRELITVLH VLLREGEFTAVDDEPERIVRRSIMLVPRRGTRVRFRPAR |
| ***Mycobacterium tuberculosis* EAI5** |
| Database: TB; P450 count: 19; Families: 18; Subfamilies: 19 |
| >CYP138A1(M943_00750)  MSEVVTAAPAPPVVRLPPAVRGPKLFQGLAFVVSRRRLLGRFVRRYGKAFTANILMYGRV VVVADPQLARQVFTSSPEELGNIQPNLSRMFGSGSVFALDGDDHRRRRRLLAPPFHGKSM KNYETIIEEETLRETANWPQGQAFATLPSMMHITLNAILRAIFGAGGSELDELRRLIPPW VTLGSRLAALPKPKRDYGRLSPWGRLAEWRRQYDTVIDKLIEAERADPNFADRTDVLALM LRSTYDDGSIMSRKDIGDELLTLLAAGHETTAATLGWAFERLSRHPDVLAALVEEVDNGG HELRQAAILEVQRARTVIDFAAPRVNPPVYQLGEWVIPRGYSIIINIAQIHGDPDVFPQP DRFDPQRYIGSKPSPFAWIPFGGGTRRCVGAAFANMEMDVVLRTVLRHFTLETTTAAGER SHGRGVAFTPKDGGRVVMRRR  >CYP135A1(M943_01720)  MASTLTTGLPPGPRLPRYLQSVLYLRFREWFLPAIHRKYGDVFSLRVPPYADNLVVYTRP EHIKEIFAADPRSLHAGEGNHILGFVMGEHSVLMTDEAEHARMRSLLMPAFTRAALRGYR DMIASVAREHITRWRPHATINSLDHMNALTLDIILRVVFGVTDPKVKAELTSRLQQIINI HPAILAGVPYPSLKRMNPWKRFFHNQTKIDEILYREIASRRIDSDLTARTDVLSRLLQTK DTPTKPLTDAELRDQLITLLLAGHETTAAALSWTLWELAHAPEIQSQVVWAAVGGDDGFL EAVLKEGMRRHTVIASTARKVTAPAEIGGWRLPAGTVVNTSILLAHASEVSHPKPTEFRP SRFLDGSVAPNTWLPFGGGVRRCLGFGFALTEGAVILQEIFRRFTITAAGPSKGETPLVR NITTVPKHGAHLRLIPQRRLGGLGDSDPP  >CYP135B1(M943_02945)  MSGTSSMGLPPGPRLSGSVQAVLMLRHGLRFLTACQRRYGSVFTLHVAGFGHMVYLSDPA AIKTVFAGNPSVFHAGEANSMLAGLLGDSSLLLIDDDVHRDRRRLMSPPFHRDAVARQAG PIAEIAAANIAGWPMAKAFAVAPKMSEITLEVILRTVIGASDPVRLAALRKVMPRLLNVG PWATLALANPSLLNNRLWSRLRRRIEEADALLYAEIADRRADPDLAARTDTLAMLVRAAD EDGRTMTERELRDQLITLLVAGHDTTATGLSWALERLTRHPVTLAKAVQAADASAAGDPA GDEYLDAVAKETLRIRPVVYDVGRVLTEAVEVAGYRLPAGVMVVPAIGLVHASAQLYPDP ERFDPDRMVGATLSPTTWLPFGGGNRRCLGATFAMVEMRVVLREILRRVELSTTTTSGER PKLKHVIMVPHRGARIRVRATRDVSATSQATAQGAGCPAARGGGPSRAVGSQ  >CYP51B1(M943_04000)  MSAVALPRVSGGHDEHGHLEEFRTDPIGLMQRVRDECGDVGTFQLAGKQVVLLSGSHANE FFFRAGDDDLDQAKAYPFMTPIFGEGVVFDASPERRKEMLHNAALRGEQMKGHAATIEDQ VRRMIADWGEAGEIDLLDFFAELTIYTSSACLIGKKFRDQLDGRFAKLYHELERGTDPLA YVDPYLPIESFRRRDEARNGLVALVADIMNGRIANPPTDKSDRDMLDVLIAVKAETGTPR FSADEITGMFISMMFAGHHTSSGTASWTLIELMRHRDAYAAVIDELDELYGDGRSVSFHA LRQIPQLENVLKETLRLHPPLIILMRVAKGEFEVQGHRIHEGDLVAASPAISNRIPEDFP DPHDFVPARYEQPRQEDLLNRWTWIPFGAGRHRCVGAAFAIMQIKAIFSVLLREYEFEMA QPPESYRNDHSKMVVQLAQPACVRYRRRTGV  >CYP123A1(M943_04010)  MTVRVGDPELVLDPYDYDFHEDPYPYYRRLRDEAPLYRNEERNFWAVSRHHDVLQGFRDS TALSNAYGVSLDPSSRTSEAYRVMSMLAMDDPAHLRMRTLVSKGFTPRRIRELEPQVLEL ARIHLDSALQTESFDFVAEFAGKLPMDVISELIGVPDTDRARIRALADAVLHREDGVADV PPPAMAASIELMRYYADLIAEFRRRPANNLTSALLAAELDGDRLSDQEIMAFLFLMVIAG NETTTKLLANAVYWAAHHPGQLARVFADHSRIPMWVEETLRYDTSSQILARTVAHDLTLY DTTIPEGEVLLLLPGSANRDDRVFDDPDDYRIGREIGCKLVSFGSGAHFCLGAHLARMEA RVALGALLRRIRNYEVDDDNVVRVHSSNVRGFAHLPISVQAR  >CYP126A1(M943_04070)  MTTAAGLSGIDLTDLDNFADGFPHHLFAIHRREAPVYWHRPTEHTPDGEGFWSVATYAET LEVLRDPVTYSSVTGGQRRFGGTVLQDLPVAGQVLNMMDDPRHTRIRRLVSSGLTPRMIR RVEDDLRRRARGLLDGVEPGAPFDFVVEIAAELPMQMICILLGVPETDRHWLFEAVEPGF DFRGSRRATMPRLNVEDAGSRLYTYALELIAGKRAEPADDMLSVVANATIDDPDAPALSD AELYLFFHLLFSAGAETTRNSIAGGLLALAENPDQLQTLRSDFELLPTAIEEIVRWTSPS PSKRRTASRAVSLGGQPIEAGQKVVVWEGSANRDPSVFDRADEFDITRKPNPHLGFGQGV HYCLGANLARLELRVLFEELLSRFGSVRVVEPAEWTRSNRHTGIRHLVVELRGG  >CYP130A1(M943_06565)  MTSVMSHEFQLATAETWPNPWPMYRALRDHDPVHHVVPPQRPEYDYYVLSRHADVWSAAR DHQTFSSAQGLTVNYGELEMIGLHDTPPMVMQDPPVHTEFRKLVSRGFTPRQVETVEPTV RKFVVERLEKLRANGGGDIVTELFKPLPSMVVAHYLGVPEEDWTQFDGWTQAIVAANAVD GATTGALDAVGSMMAYFTGLIERRRTEPADDAISHLVAAGVGADGDTAGTLSILAFTFTM VTGGNDTVTGMLGGSMPLLHRRPDQRRLLLDDPEGIPDAVEELLRLTSPVQGLARTTTRD VTIGDTTIPAGRRVLLLYGSANRDERQYGPDAAELDVTRCPRNILTFSHGAHHCLGAAAA RMQCRVALTELLARCPDFEVAESRIVWSGGSYVRRPLSVPFRVTS  >CYP132A1(M943_07315)  MATATTQRPLKGPAKRMSTWTMTREAITIGFDAGDGFLGRLRGSDITRFRCAGRRFVSIS HPDYVDHVLHEARLKYVKSDEYGPIRATAGLNLLTDEGDSWARHRGALNSTFARRHLRGL VGLMIDPIADVTAALVPGAQFDMHQSMVETTLRVVANALFSQDFGPLVQSMHDLATRGLR RAEKLERLGLWGLMPRTVYDTLIWCIYSGVHLPPPLREMQEITLTLDRAINSVIDRRLAE PTNSADLLNVLLSADGGIWPRQRVRDEALTFMLAGHETTANAMSWFWYLMALNPQARDHM LTELDDVLGMRRPTADDLGKLAWTTACLQESQRYFSSVWIIAREAVDDDIIDGHRIRRGT TVVIPIHHIHHDPRWWPDPDRFDPGRFLRCPTDRPRCAYLPFGGGRRICIGQSFALMEMV LMAAIMSQHFTFDLAPGYHVELEATLTLRPKHGVHVIGRRR  >CYP139A1(M943_08690)  MRTYRTVRYPLGEALLALYRWRGPLINAGVGGHGYTYLLGAEANRFVFANADAFSWSQTF ESLVPVDGPTALIVSDGADHRRRRSVVAPGLRHHHVQRYVATMVSNIDTVIDGWQPGQRL DIYQELRSAVRRSTAESLFGQRLAVHSDFLGEQLQPLLDLTRRPPQVMRLQQRVNSPGWR RAMAARKRIDDLIDAQIADARTAPRPDDHMLTTLISGCSEEGTTLSDNEIRDSIVSLITA GYETTSGALAWAIYALLTVPGTWESAASEVARVLGGRVPAADDLSALTYLNGVVHETLRL YSPGVISARRVLRDLWFDGHRIRAGRLLIFSAYVTHRLPEIWPEPTEFRPLRWDPNAADY RKPAPHEFIPFSGGLHRCIGAVMATTEMTVILARLVARAMLQLPAQRTHRIRAANFAALR PWPGLTVEIRKSAPAQ  >CYP144A1(M943_09255)  MRRSPKGSPGAVLDLQRRVDQAVSADHAELMTIAKDANTFFGAESVQDPYPLYERMRAAG SVHRIANSDFYAVCGWDAVNEAIGRPEDFSSNLTATMTYTAEGTAKPFEMDPLGGPTHVL ATADDPAHAVHRKLVLRHLAAKRIRVMEQFTVQAADRLWVDGMQDGCIEWMGAMANRLPM MVVAELIGLPDPDIAQLVKWGYAATQLLEGLVENDQLVAAGVALMELSGYIFEQFDRAAA DPRDNLLGELATACASGELDTLTAQVMMVTLFAAGGESTAALLGSAVWILATRPDIQQQV RANPELLGAFIEETLRYEPPFRGHYRHVRNATTLDGTELPADSHLLLLWGAANRDPAQFE APGEFRLDRAGGKGHISFGKGAHFCVGAALARLEARIVLRLLLDRTSVIEAADVGGWLPS ILVRRIERLELAVQ  >CYP143A1(M943_09295)  MTTPGEDHAGSFYLPRLEYSTLPMAVDRGVGWKTLRDAGPVVFMNGWYYLTRREDVLAAL RNPKVFSSRKALQPPGNPLPVVPLAFDPPEHTRYRRILQPYFSPAALSKALPSLRRHTVA MIDAIAGRGECEAMADLANLFPFQLFLVLYGLPLEDRDRLIGWKDAVIAMSDRPHPTEAD VAAARELLEYLTAMVAERRRNPGPDVLSQVQIGEDPLSEIEVLGLSHLLILAGLDTVTAA VGFSLLELARRPQLRAMLRDNPKQIRVFIEEIVRLEPSAPVAPRVTTEPVTVGGMTLPAG SPVRLCMAAVNRDGSDAMSTDELVMDGKVHRHWGFGGGPHRCLGSHLARLELTLLVGEWL NQIPDFELAPDYAPEIRFPSKSFALKNLPLRWS  >CYP140A1(M943_09765)  MKDKLHWLAMHGVIRGIAAIGIRRGDLQARLIADPAVATDPVPFYDEVRSHGALVRNRAN YLTVDHRLAHDLLRSDDFRVVSFGENLPPPLRWLERRTRGDQLHPLREPSLLAVEPPDHT RYRKTVSAVFTSRAVSALRDLVEQTAINLLDRFAEQPGIVDVVGRYCSQLPIVVISEILG VPEHDRPRVLEFGELAAPSLDIGIPWRQYLRVQQGIRGFDCWLEGHLQQLRHAPGDDLMS QLIQIAESGDNETQLDETELRAIAGLVLVAGFETTVNLLGNGIRMLLDTPEHLATLRQHP ELWPNTVEEILRLDSPVQLTARVACRDVEVAGVRIKRGEVVVIYLAAANRDPAVFPDPHR FDIERPNAGRHLAFSTGRHFCLGAALARAEGEVGLRTFFDRFPDVRAAGAGSRRDTRVLR GWSTLPVTLGPARSMVSP  >CYP124A1(M943_11730)  MGLNTAIATRVNGTPPPEVPIADIELGSLDFWALDDDVRDGAFATLRREAPISFWPTIEL PGFVAGNGHWALTKYDDVFYASRHPDIFSSYPNITINDQTPELAEYFGSMIVLDDPRHQR LRSIVSRAFTPKVVARIEAAVRDRAHRLVSSMIANNPDRQADLVSELAGPLPLQIICDMM GIPKADHQRIFHWTNVILGFGDPDLATDFDEFMQVSADIGAYATALAEDRRVNHHDDLTS SLVEAEVDGERLSSREIASFFILLVVAGNETTRNAITHGVLALSRYPEQRDRWWSDFDGL APTAVEEIVRWASPVVYMRRTLTQDIELRGTKMAAGDKVSLWYCSANRDESKFADPWTFD LARNPNPHLGFGGGGAHFCLGANLARREIRVAFDELRRQMPDVVATEEPARLLSQFIHGI KTLPVTWS  >CYP128A1(M943_11740)  MTATQSPPEPAPDRVRLAGCPLAGTPDVGLTAQDATTALGVPTRRRASSGGIPVATSMWR DAQTVRTYGPAVAKALALRVAGKARSRLTGRHCRKFMQLTDFDPFDPAIAADPYPHYREL LAGERVQYNPKRDVYILSRYADVREAARNHDTLSSARGVTFSRGWLPFLPTSDPPAHTRM RKQLAPGMARGALETWRPMVDQLARELVGGLLTQTPADVVSTVAAPMPMRAITSVLGVDG PDEAAFCRLSNQAVRITDVALSASGLISLVQGFAGFRRLRALFTHRRDNGLLRECTVLGK LATHAEQGRLSDDELFFFAVLLLVAGYESTAHMISTLFLTLADYPDQLTLLAQQPDLIPS AIEEHLRFISPIQNICRTTRVDYSVGQAVIPAGSLVLLAWGAANRDPRQYEDPDVFRADR NPVGHLAFGSGIHLCPGTQLARMEGQAILREIVANIDRIEVVEPPTWTTNANLRGLTRLR VAVTPRVAP  >CYP121A1(M943_11770)  MTATVLLEVPFSARGDRIPDAVAELRTREPIRKVRTITGAEAWLVSSYALCTQVLEDRRF SMKETAAAGAPRLNALTVPPEVVNNMGNIADAGLRKAVMKAITPKAPGLEQFLRDTANSL LDNLITEGAPADLRNDFADPLATALHCKVLGIPQEDGPKLFRSLSIAFMSSADPIPAAKI NWDRDIEYMAGILENPNITTGLMGELSRLRKDPAYSHVSDELFATIGVTFFGAGVISTGS FLTTALISLIQRPQLRNLLHEKPELIPAGVEELLRINLSFADGLPRLATADIQVGDVLVR KGELVLVLLEGANFDPEHFPNPGSIELDRPNPTSHLAFGRGQHFCPGSALGRRHAQIGIE ALLKKMPGVDLAVPIDQLVWRTRFQRRIPERLPVLW  >CYP136A1(M943_15785)  MATIHPPAYLLDQAKRRFTPSFNNFPGMSLVEHMLLNTKFPEKKLAEPPPGSGLKPVVGD AGLPILGHMIEMLRGGPDYLMFLYKTKGPVVFGDSAVLPGVAALGPDAAQVIYSNRNKDY SQQGWVPVIGPFFHRGLMLLDFEEHMFHRRIMQEAFVRSRLAGYLEQMDRVVSRVVADDW VVNDARFLVYPAMKALTLDIASMVFMGHEPGTDHELVTKVNKAFTITTRAGNAVIRTSVP PFTWWRGLRARELLENYFTARVKERREASGNDLLTVLCQTEDDDGNRFSDADIVNHMIFL MMAAHDTSTSTATTMAYQLAAHPEWQQRCRDESDRHGDGPLDIESLEQLESLDLVMNESI RLVTPVQWAMRQTVRDTELLGYYLPKGTNVIAYPGMNHRLPEIWTDPLTFDPERFTEPRN EHKRHRYAFTPFGGGVHKCIGMVFGQLEIKTILHRLLRRYRLELSRPDYQPRWDYSAMPI PMDGMPIVLRPR  >CYP141A1(M943_16110)  MTSTSIPTFPFDRPVPTEPSPMLSELRNSCPVAPIELPSGHTAWLVTRFDDVKGVLSDKR FSCRAAAHPSSPPFVPFVQLCPSLLSIDGPQHTAARRLLAQGLNPGFIARMRPVVQQIVD NALDDLAAAEPPVDFQEIVSVPIGEQLMAKLLGVEPETVHELAAHVDAAMSVCEIGDEEV SRRWSALCTMVIDILHRKLAEPGDDLLSTIAQANRQQSTMTDEQVVGMLLTVVIGGVDTP IAVITNGLASLLHHRDQYERLVEDPGRVARAVEEIVRFNPATEIEHLRVVTEDVVIAGTA LSAGSPAFTSITSANRDSDQFLDPDEFDVERNPNEHIAFGYGPHACPASAYSRMCLTTFF TSLTQRFPQLQLARPFEDLERRGKGLHSVGIKELLVTWPT  >CYP142A1(M943_18090)  MTEAPDVDLADGNFYASREARAAYRWMRANQPVFRDRNGLAAASTYQAVIDAERQPELFS NAGGIRPDQPALPMMIDMDDPAHLLRRKLVNAGFTRKRVKDKEASIAALCDTLIDAVCER GECDFVRDLAAPLPMAVIGDMLGVRPEQRDMFLRWSDDLVTFLSSHVSQEDFQITMDAFA AYNDFTRATIAARRADPTDDLVSVLVSSEVDGERLSDDELVMETLLILIGGDETTRHTLS GGTEQLLRNRDQWDLLQRDPSLLPGAIEEMLRWTAPVKNMCRVLTADTEFHGTALCAGEK MMLLFESANFDEAVFCEPEKFDVQRNPNSHLAFGFGTHFCLGNQLARLELSLMTERVLRR LPDLRLVADDSVLPLRPANFVSGLESMPVVFTPSPPLG  >CYP137A1(M943_18930)  MVLRSLASPAALTDPKRCASVVGVAAFAVRREHAPDALGGPPGLPAPRGFRAAFAAAYAV AYLAGGERRMLRLIRRYGPIMTMPILSLGDVAIVSDSALAKEVFTAPTDVLLGGEGVGPA AAIYGSGSMFVQEEPEHLRRRKLLTPPLHGAALDRYVPIIENSTRAAMHTWPVDRPFAML TVARSLMLDVIVKVIFGVDDPEEVRRLGRPFERLLNLGVSEQLTVRYALRRLGALRVWPA RARANTEIDDVVMALIAQRRADPRLGERHDVLSLLVSARGESGEQLSDSEIRDDLITLVL AGHETTATTLAWAFDLLLHHPDALRRVRAEAVGGGEAFTTAVINETLRVRPPAPLTARVA AQPLTIGGYRVEAGTRIVVHIIAINRSAEVYEHPHEFRPERFLGTRPQTYAWVPFGGGVK RCLGANFSMRELITVLHVLLREGEFTAVDDEPERIVRRSIMLVPRRGTRVRFRPAR |
| ***Mycobacterium tuberculosis* EAI5/NITR206** |
| Database: TB; P450 count:20; Families: 19; Subfamilies: 20 |
| >CYP138A1(J114_00750)  MSEVVTAAPAPPVVRLPPAVRGPKLFQGLAFVVSRRRLLGRFVRRYGKAFTANILMYGRV VVVADPQLARQVFTSSPEELGNIQPNLSRMFGSGSVFALDGDDHRRRRRLLAPPFHGKSM KNYETIIEEETLRETANWPQGQAFATLPSMMHITLNAILRAIFGAGGSELDELRRLIPPW VTLGSRLAALPKPKRDYGRLSPWGRLAEWRGQYDTVIDKLIEAERADPNFADRTDVLALM LRSTYDDGSIMSRKDIGDELLTLLAAGHETTAATLGWAFERLSRHPDVLAALVEEVDNGG HELRQAAILEVQRARTVIDFAAPRVNPPVYQLGEWVIPRGYSIIINIAQIHGDPDVFPQP DRFDPQRYIGSKPSPFAWIPFGGGPRRCVGAAFANMEMDVVLRTVLRHFTLETTTAAGER SHGRGVAFTPKDGGRVVMRRR  >CYP135A1(J114_01765)  MASTLTTGLPPGPRLPRYLQSVLYLRFREWFLPAIHRKYGDVFSLRVPPYADNLVVYTRP EHIKEIFAADPRSLHAGEGNHILGFVMGEHSVLMTDEAEHARMRSLLMPAFTRAALRGYR DMIASVAREHITRWRPHATINSLDHMNALTLDIILRVVFGVTDPKVKAELTSRLQQIINI HPAILAGVPYPSLKRMNPWKRFFHNQTKIDEILYREIASRRIDSDLTARTDVLSRLLQTK DTPTKPLTDAELRDQLITLLLAGHETTAAALSWTLWELAHAPEIQSQVVWAAVGGDDGFL EAVLKEGMRRHTVIASTARKVTAPAEIGGWRLPAGTVVNTSILLAHASEVSHPKPTEFRP SRFLDGSVAPNTWLPFGGGVRRCLGFGFALTEGAVILQEIFRRFTITAAGPSKGETPLVR NITTVPKHGAHLRLIPQRRLGGLGDSDPP  >CYP135B1(J114_03035)  MSGTSSMGLPPGPRLSGSVQAVLMLRHGLRFLTACQRRYGSVFTLHVAGFGHMVYLSDPA AIKTVFAGNPSVFHAGEANSMLAGLLGDSSLLLIDDDVHRDRRRLMSPPFHRDAVARQAG PIAEIAAANIAGWPMAKAFAVAPKMSEITLEVILRTVIGASDPVRLAALRKVMPRLLNVG PWATLALANPSLLNNRLWSRLRRRIEEADALLYAEIADRRADPDLAARTDTLAMLVRAAD EDGRTMTERELRDQLITLLVAGHDTTATGLSWALERLTRHPVTLAKAVQAADASAAGDPA GDEYLDAVAKETLRIRPVVYDVGRVLTEAVEVAGYRLPAGVMVVPAIGLVHASAQLYPDP ERFDPDRMVGATLSPTTWLPFGGGNRRCLGATFAMVEMRVVLREILRRVELSTTTTSGER PKLKHVIMVPHRGARIRVRATRDVSATSQATAQGAGCPAARGGGPSRAVGSQ  >CYP51B1(J114_04080)  MSAVALPRVSGGHDEHGHLEEFRTDPIGLMQRVRDECGDVGTFQLAGKQVVLLSGSHANE FFFRAGDDDLDQAKAYPFMTPIFGEGVVFDASPERRKEMLHNAALRGEQMKGHAATIEDQ VRRMIADWGEAGEIDLLDFFAELTIYTSSACLIGKKFRDQLDGRFAKLYHELERGTDPLA YVDPYLPIESFRRRDEARNGLVALVADIMNGRIANPPTDKSDRDMLDVLIAVKAETGTPR FSADEITGMFISMMFAGHHTSSGTASWTLIELMRHRDAYAAVIDELDELYGDGRSVSFHA LRQIPQLENVLKETLRLHPPLIILMRVAKGEFEVQGHRIHEGDLVAASPAISNRIPEDFP DPHDFVPARYEQPRQEDLLNRWTWIPFGAGRHRCVGAAFAIMQIKAIFSVLLREYEFEMA QPPESYRNDHSKMVVQLAQPACVRYRRRTGV  >CYP123A1(J114_04090)  MTVRVGDPELVLDPYDYDFHEDPYPYYRRLRDEAPLYRNEERNFWAVSRHHDVLQGFRDS TALSNAYGVSLDPSSRTSEAYRVMSMLAMDDPAHLRMRTLVSKGFTPRRIRELEPQVLEL ARIHLDSALQTESFDFVAEFAGKLPMDVISELIGVPDTDRARIRALADAVLHREDGVADV PPPAMAASIELMRYYADLIAEFRRRPANNLTSALLAAELDGDRLSDQEIMAFLFLMVIAG NETTTKLLANAVYWAAHHPGQLARVFADHSRIPMWVEETLRYDTSSQILARTVAHDLTLY DTTIPEGEVLLLLPGSANRDDRVFDDPDDYRIGREIGCKLVSFGSGAHFCLGAHLARMEA RVALGALLRRIRNYEVDDDNVVRVHSSNVRGFAHLPISVQAR  >CYP126A1(J114_04150)  MTTAAGLSGIDLTDLDNFADGFPHHLFAIHRREAPVYWHRPTEHTPDGEGFWSVATYAET LEVLRDPVTYSSVTGGQRRFGGTVLQDLPVAGQVLNMMDDPRHTRIRRLVSSGLTPRMIR RVEDDLRRRARGLLDGVEPGAPFDFVVEIAAELPMQMICILLGVPETDRHWLFEAVEPGF DFRGSRRATMPRLNVEDAGSRLYTYALELIAGKRAEPADDMLSVVANATIDDPDAPALSD AELYLFFHLLFSAGAETTRNSIAGGLLALAENPDQLQTLRSDFELLPTAIEEIVRWTSPS PSKRRTASRAVSLGGQPIEAGQKVVVWEGSANRDPSVFDRADEFDITRKPNPHLGFGQGV HYCLGANLARLELRVLFEELLSRFGSVRVVEPAEWTRSNRHTGIRHLVVELRGG  >CYP130A1(J114_06770)  MTSVMSHEFQLATAETWPNPWPMYRALRDHDPVHHVVPPQRPEYDYYVLSRHADVWSAAR DHQTFSSAQGLTVNYGELEMIGLHDTPPMVMQDPPVHTEFRKLVSRGFTPRQVETVEPTV RKFVVERLEKLRANGGGDIVTELFKPLPSMVVAHYLGVPEEDWTQFDGWTQAIVAANAVD GATTGALDAVGSMMAYFTGLIERRRTEPADDAISHLVAAGVGADGDTAGTLSILAFTFTM VTGGNDTVTGMLGGSMPLLHRRPDQRRLLLDDPEGIPDAVEELLRLTSPVQGLARTTTRD VTIGDTTIPAGRRVLLLYGSANRDERQYGPDAAELDVTRCPRNILTFSHGAHHCLGAAAA RMQCRVALTELLARCPDFEVAESRIVWSGGSYVRRPLSVPFRVTS  >CYP132A1(J114_07480)  MATATTQRPLKGPAKRMSTWTMTREAITIGFDAGDGFLGRLRGSDITRFRCAGRRFVSIS HPDYVDHVLHEARLKYVKSDEYGPIRATAGLNLLTDEGDSWARHRGALNSTFARRHLRGL VGLMIDPIADVTAARVPGAQFDMHQSMVETTLRVVANALFSQDFGPLVQSMHDLATRGLR RGEKLERLGLWGLMPRTVYDTLIWCIYSGVHLPPPLREMQEITLTLDRAINSVIDRRLAE PTNSADLLNVLLSADGGIWPRQRVRDEALTFMLAGHETTANAMSWFWYLMALNPQARDHM LTELDDVLGMRRPTADDLGKLAWTTACLQESQRYFSSVWIIAREAVDDDIIDGHRIRRGT TVVIPIHHIHHDPRWWPDPDRFDPGRFLRCPTDRPRCASLPFGGGRRICIGQSFALMEMV LMAAIMSQHFTFDLAPGYHVELEATLTLRPKHGVHVIGRRR  >CYP139A1(J114_08910)  MRYPLGEALLALYRWRGPLINAGVGGHGYTYLLGAEANRFVFANADAFSWSQTFESLVPV DGPTALIVSDGADHRRRRSVVAPGLRHHHVQRYVATMVSNIDTVIDGWQPGQRLDIYQEL RSAVRRSTAESLFGQRLAVHSDFLGEQLQPLLDLTRRPPQVMRLQQRVNSPGWRRAMAAR KRIDDLIDAQIADARTAPRPDDHMLTTLISGCSEEGTTLSDNEIRDSIVSLITAGYETTS GALAWAIYALLTVPGTWESAASEVARVLGGRVPAADDLSALTYLNGVVHETLRLYSPGVI SARRVLRDLWFDGHRIRAGRLLIFSAYVTHRLPEIWPEPTEFRPLRWDPNAADYRKPAPH EFIPFSGGLHRCIGAVMATTEMTVILARLVARAMLQLPAQRTHRIRAANFAALRPWPGLT VEIRKSAPAQ  >CYP144A1(J114_09500)  MRRSPKGSPGAVLDLQRRVDQAVSADHAELMTIAKDANTFFGAESVQDPYPLYERMRAAG SVHRIANSDFYAVCGWDAVNEAIGRPEDFSSNLTATMTYTAEGTAKPFEMDPLGGPTHVL ATADDPAHAVHRKLVLRHLAAKRIRVMEQFTVQAADRLWVDGMQDGCIEWMGAMANRLPM MVVAELIGLPDPDIAQLVKWGYAATQLLEGLVENDQLVAAGVALMELSGYIFEQFDRAAA DPRDNLLGELATACASGELDTLTAQVMMVTLFAAGGESTAALLGSAVWIMATRPDIQQQV RANPELLGAFIEETRRYEPPFRGHYRHVRNATTLDGTELPADSHLLLLWGAANRDPAQFE APGEFRLDRAGGKGHISFGKGAHFCVGAALARLEARIVLRLLLDRTSVIEAADVGGWLPS ILVRRIERLELAVQ  >CYP143A1(J114_09540)  MTTPGEDHAGSFYLPRLEYSTLPMAVDRGVGWKTLRDAGPVVFMNGWYYLTRREDVLAAL RNPKVFSSRKALQPPGNPLPVVPLAFDPPEHTRYRRILQPYFSPAALSKALPSLRRHTVA MIDAIAGRGECEAMADLANLFPFQLFLVLYGLPLEDRDRLIGWKDAVIAMSDRPHPTEAD VAAARELLEYLTAMVAERRRNPGPDVLSQVQIGEDPLSEIEVLGLSHLLILAGLDTVTAA VGFSLLELARRPQLRAMLRDNPKQIRVFIEEIVRLEPSAPVAPRVTTEPVTVGGMTLPAG SPVRLCMAAVNRDGSDAMSTDELVMDGKVHRHWGFGGGPHRCLGSHLARLELTLLVGEWL NQIPDFELAPDYAPEIRFPSKSFALKNLPLRWS  >CYP140A1(J114_10020)  MKDKLHWLAMHGVIRGIAAIGIRRGDLQARLIADPAVATDPVPFYDEVRSHGALVRNRAN YLTVDHRLAHDLLRSDDFRVVSFGENLPPPLRWLERRTRGDQLHPLREPSLLAVEPPDHT RYRKTVSAVFTSRAVSALRDLVEQTAINLLDRFAEQPGIVDVVGRYCSQLPIVVISEILG VPEHDRPRVLEFGELAAPSLDIGIPWRQYLRVQQGIRGFDCWLEGHLQQLRHAPGDDLMS QLIQIAESGDNETQLDETELRAIAGLVLVAGFETTVNLLGNGIRMLLDTPEHLATLRQHP ELWPNTVEEILRLDSPVQLTARVACRDVEVAGVRIKRGEVVVIYLAAANRDPAVFPDPHR FDIERPNAGRHLAFSTGRHFCLGAALARAEGEVGLRTFFDRFPDVRAAGAGSRRDTRVLR GWSTLPVTLGPARSMVSP  >CYP124A1(J114_12155)  MGLNTAIATRVNGTPPPEVPIADIELGSLDFWALDDDVRDGAFATLRREAPISFWPTIEL PGFVAGNGHWALTKYDDVFYASRHPDIFSSYPNITINDQTPELAEYFGSMIVLDDPRHQR LRSIVSRAFTPKVVARIEAAVRDRAHRLVSSMIANNPDRQADLVSELAGPLPLQIICDMM GIPKADHQRIFHWTNVILGFGDPDLATDFDEFMQVSADIGAYATALAEDRRVNHHDDLTS SLVEAEVDGERLSSREIASFFILLVVAGNETTRNAITHGVLALSRYPEQRDRWWSDFDGL APTAVEEIVRWASPVVYMRRTLTQDIELRGTKMAAGDKVSLWYCSANRDESKFADPWTFD LARNPNPHLGFGGGGAHFCLGANLARREIRVAFDELRRQMPDVVATEEPARLLSQFIHGI KTLPVTWS  >CYP128A1(J114_12165)  MTATQSPPEPAPDRVRLAGCPLAGTPDVGLTAQDATTALGVPTRRRASSGGIPVATSMWR DAQTVRTYGPAVAKALALRVAGKARSRLTGRHCRKFMQLTDFDPFDPAIAADPYPHYREL LAGERVQYNPKRDVYILSRYADVREAARNHDTLSSARGVTFSRGWLPFLPTSDPPAHTRM RKQLAPGMARGALETWRPMVDQLARELVGGLLTQTPADVVSTVAAPMPMRAITSVLGVDG PDEAAFCRLSNQAVRITDVALSASGLISLVQGFAGFRRLRALFTHRRDNGLLRECTVLGK LATHAEQGRLSDDELFFFAVLLLVAGYESTAHMISTLFLTLADYPDQLTLLAQQPDLIPS AIEEHLRFISPIQNICRTTRVDYSVGQAVIPAGSLVLLAWGAANRDPRQYEDPDVFRADR NPVGHLAFGSGIHLCPGTQLARMEGQAILREIVANIDRIEVVEPPTWTTNANLRGLTRLR VAVTPRVAP  >CYP121A1(J114_12200)  MTATVLLEVPFSARGDRIPDAVAELRTREPIRKVRTITGAEAWLVSSYALCTQVLEDRRF SMKETAAAGAPRLNALTVPPEVVNNMGNIADAGLRKAVMKAITPKAPGLEQFLRDTANSL LDNLITEGAPADLRNDFADPLATALHCKVLGIPQEDGPKLFRSLSIAFMSSADPIPAAKI NWDRDIEYMAGILENPNITTGLMGELSRLRKDPAYSHVSDELFATIGVTFFGAGVISTGS FLTTALISLIQRPQLRNLLHEKPELIPAGVEELLRINLSFADGLPRLATADIQVGDVLVR KGELVLVLLEGANFDPEHFPNPGSIELDRPNPTSHLAFGRGQHFCPGSALGRRHAQIGIE ALLKKMPGVDLAVPIDQLVWRTRFQRRIPERLPVLW  >CYP136A1(J114_16360)  MATIHPPAYLLDQAKRRFTPSFNNFPGMSLVEHMLLNTKFPEKKLAEPPPGSGLKPVVGD AGLPILGHMIEMLRGGPDYLMFLYKTKGPVVFGDSAVLPGVAALGPDAAQVIYSNRNKDY SQQGWVPVIGPFFHRGLMLLDFEEHMFHRRIMQEAFVRSRLAGYLEQMDRVVSRVVADDW VVNDARFLVYPAMKALTLDIASMVFMGHEPGTDHELVTKVNKAFTITTRAGNAVIRTSVP PFTWWRGLRARELLENYFTARVKERREASGNDLLTVLCQTEDDDGNRFSDADIVNHMIFL MMAAHDTSTSTATTMAYQLAAHPEWQQRCRDESDRHGDGPLDIESLEQLESLDLVMNESI RLVTPVQWAMRQTVRDTELLGYYLPKGTNVIAYPGMNHRLPEIWTDPLTFDPERFTEPRN EHKRHRYAFTPFGGGVHKCIGMVFGQLEIKTILHRLLRRYRLELSRPDYQPRWDYSAMPI PMDGMPIVLRPR  >CYP141A1(J114_16690)  MTSTSIPTFPFDRPVPTEPSPMLSELRNSCPVAPIELPSGHTAWLVTRFDDVKGVLSDKR FSCRAAAHPSSPPFVPFVQLCPSLLSIDGPQHTAARRLLAQGLNPGFIARMRPVVQQIVD NALDDLAAAEPPVDFQEIVSVPIGEQLMAKLLGVEPETVHELAAHVDAAMSVCEIGDEEV SRRWSALCTMVIDILHRKLAEPGDDLLSTIAQANRQQSTMTDEQVVGMLLTVVIGGVDTP IAVITNGLASLLHHRDQYERLVEDPGRVARAVEEIVRFNPATEIEHLRVVTEDVVIAGTA LSAGSPAFTSITSANRDSDQFLDPDEFDVERNPNEHIAFGYGPHACPASAYSRMCLTTFF TSLTQRFPQLQLARPFEDLERRGKGLHSVGIKELLVTWPT  >CYP142A1(J114_18810)  MTEAPDVDLADGNFYASREARAAYRWMRANQPVFRDRNGLAAASTYQAVIDAERQPELFS NAGGIRPDQPALPMMIDMDDPAHLLRRKLVNAGFTRKRVKDKEASIAALCDTLIDAVCER GECDFVRDLAAPLPMAVIGDMLGVRPEQRDMFLRWSDDLVTFLSSHVSQEDFQITMDAFA AYNDFTRATIAARRADPTDDLVSVLVSSEVDGERLSDDELVMETLLILIGGDETTRHTLS GGTEQLLRNRDQWDLLQRDPSLLPGAIEEMLRWTAPVKNMCRVLTADTEFHGTALCAGEK MMLLFESANFDEAVFCEPEKFDVQRNPNSHLAFGFGTHFCLGNQLARLELSLMTERVLRR LPDLRLVADDSVLPLRPANFVSGLESMPVVFTPSPPLG  >CYP125A1(J114_18960)  MSWNHQSVEIAVRRTTVPSPNLPPGFDFTDPAIYAERLPVAEFAELRSAAPIWWNGQDPG KGGGFHDGGFWAITKLNDVKEISRHSDVFSSYENGVIPRFKNDIAREDIEVQRFVMLNMD APHHTRLRKIISRGFTPRAVGRLHDELQERAQKIAAEAAAAGSGDFVEQVSCELPLQAIA GLLGVPQEDRGKLFHWSNEMTGNEDPEYAHIDPKASSAELIGYAMKMAEEKAKNPADDIV TQLIQADIDGEKLSDDEFGFFVVMLAVAGNETTRNSITQGMMAFAEHPDQWELYKKVRPE TAADEIVRWATPVTAFQRTALRDYELSGVQIKKGQRVVMFYRSANFDEEVFQDPFTFNIL RNPNPHVGFGGTGAHYCIGANLARMTINLIFNAVADHMPDLKPISAPERLRSGWLNGIKH WQVDYTGRCPVAH  >CYP137A1(J114_19685)  MVLRSLASPAALTDPKRCASVVGVAAFAVRREHAPDALGGPPGLPAPRGFRAAFAAAYAV AYLAGGERRMLRLIRRYGPIMTMPILSLGDVAIVSDSALAKEVFTAPTDVLLGGEGVGPA AAIYGSGSMFVQEEPEHLRRRKLLTPPLHGAALDRYVPIIENSTRAAMHTWPVDRPFAML TVARSLMLDVIVKVIFGVDDPEEVRRLGRPFERLLNLGVSEQLTVRYALRRLGALRVWPA RARANTEIDDVVMALIAQRRADPRLGERHDVLSLLVSARGESGEQLSDSEIRDDLITLVL AGHETTATTLAWAFDLLLHHPDALRRVRAEAVGGGEAFTTAVINETLRVRPPAPLTARVA AQPLTIGGYRVEAGTRIVVHIIAINRSAEVYEHPHEFRPERFLGTRPQTYAWVPFGGGVK RCLGANFSMRELITVLHVLLREGEFTAVDDEPERIVRRSIMLVPRRGTRVRFRPAR |
| ***Mycobacterium tuberculosis* Erdman= ATCC 35801** |
| Database: TB; P450 count: 19; Families: 18; Subfamilies: 19 |
| >CYP138A1(ERDMAN_0158)  MSEVVTAAPAPPVVRLPPAVRGPKLFQGLAFVVSRRRLLGRFVRRYGKAFTANILMYGRV VVVADPQLARQVFTSSPEELGNIQPNLSRMFGSGSVFALDGDDHRRRRRLLAPPFHGKSM KNYETIIEEETLRETANWPQGQAFATLPSMMHITLNAILRAIFGAGGSELDELRRLIPPW VTLGSRLAALPKPKRDYGRLSPWGRLAEWRRQYDTVIDKLIEAERADPNFADRTDVLALM LRSTYDDGSIMSRKDIGDELLTLLAAGHETTAATLGWAFERLSRHPDVLAALVEEVDNGG HELRQAAILEVQRARTVIDFAARRVNPPVYQLGEWVIPRGYSIIINIAQIHGDPDVFPQP DRFDPQRYIGSKPSPFAWIPFGGGTRRCVGAAFANMEMDVVLRTVLRHFTLETTTAAGER SHGRGVAFTPKDGGRVVMRRR  >CYP135A1(ERDMAN_0362)  MASTLTTGLPPGPRLPRYLQSVLYLRFREWFLPAMHRKYGDVFSLRVPPYADNLVVYTRP EHIKEIFAADPRSLHAGEGNHILGFVMGEHSVLMTDEAEHARMRSLLMPAFTRAALRGYR DMIASVAREHITRWRPHATINSLDHMNALTLDIILRVVFGVTDPKVKAELTSRLQQIINI HPAILAGVPYPSLKRMNPWKRFFHNQTKIDEILYREIASRRIDSDLTARTDVLSRLLQTK DTPTKPLTDAELRDQLITLLLAGHETTAAALSWTLWELAHAPEIQSQVVWAAVGGDDGFL EAVLKEGMRRHTVIASTARKVTAPAEIGGWRLPAGTVVNTSILLAHASEVSHPKPTEFRP SRFLDGSVAPNTWLPFGGGVRRCLGFGFALTEGAVILQEIFRRFTITAAGPSKGETPLVR NITTVPKHGAHLRLIPQRRLGGLGDSDPP  >CYP135B1(ERDMAN_0622)  MSGTSSMGLPPGPRLSGSVQAVLMLRHGLRFLTACQRRYGSVFTLHVAGFGHMVYLSDPA AIKTVFAGNPSVFHAGEANSMLAGLLGDSSLLLIDDDVHRDRRRLMSPPFHRDAVARQAG PIAEIAAANIAGWPMAKAFAVAPKMSEITLEVILRTVIGASDPVRLAALRKVMPRLLNVG PWATLALANPSLLNNRLWSRLRRRIEEADALLYAEIADRRADPDLAARTDTLAMLVRAAD EDGRTMTERELRDQLITLLVAGHDTTATGLSWALERLTRHPVTLAKAVQAADASAAGDPA GDEYLDAVAKETLRIRPVVYDVGRVLTEAVEVAGYRLPAGVMVVPAIGLVHASAQLYPDP ERFDPDRMVGATLSPTTWLPFGGGNRRCLGATFAMVEMRVVLREILRRVELSTTTTSGER PKLKHVIMVPHRGARIRVRATRDVSATSQATAQGAGCPAARGGGPSRAVGSQ  >CYP51B1(ERDMAN_0846)  MSAVALPRVSGGHDEHGHLEEFRTDPIGLMQRVRDECGDVGTFQLAGKQVVLLSGSHANE FFFRAGDDDLDQAKAYPFMTPIFGEGVVFDASPERRKEMLHNAALRGEQMKGHAATIEDQ VRRMIADWGEAGEIDLLDFFAELTIYTSSACLIGKKFRDQLDGRFAKLYHELERGTDPLA YVDPYLPIESFRRRDEARNGLVALVADIMNGRIANPPTDKSDRDMLDVLIAVKAETGTPR FSADEITGMFISMMFAGHHTSSGTASWTLIELMRHRDAYAAVIDELDELYGDGRSVSFHA LRQIPQLENVLKETLRLHPPLIILMRVAKGEFEVQGHRIHEGDLVAASPAISNRIPEDFP DPHDFVPARYEQPRQEDLLNRWTWIPFGAGRHRCVGAAFAIMQIKAIFSVLLREYEFEMA QPPESYRNDHSKMVVQLAQPACVRYRRRTGV  >CYP123A1(ERDMAN_0848)  MTVRVGDPELVLDPYDYDFHEDPYPYYRRLRDEAPLYRNEERNFWAVSRHHDVLQGFRDS TALSNAYGVSLDPSSRTSEAYRVMSMLAMDDPAHLRMRTLVSKGFTPRRIRELEPQVLEL ARIHLDSALQTESFDFVAEFAGKLPMDVISELIGVPDTDRARIRALADAVLHREDGVADV PPPAMAASIELMRYYADLIAEFRRRPANNLTSALLAAELDGDRLSDQEIMAFLFLMVIAG NETTTKLLANAVYWAAHHPGQLARVFADHSRIPMWVEETLRYDTSSQILARTVAHDLTLY DTTIPEGEVLLLLPGSANRDDRVFDDPDDYRIGREIGCKLVSFGSGAHFCLGAHLARMEA RVALGALLRRIRNYEVDDDNVVRVHSSNVRGFAHLPISVQAR  >CYP126A1(ERDMAN_0860)  MQFFSVMTTAAGLSGIDLTDLDNFADGFPHHLFAIHRREAPVYWHRPTEHTPDGEGFWSV ATYAETLEVLRDPVTYSSVTGGQRRFGGTVLQDLPVAGQVLNMMDDPRHTRIRRLVSSGL TPRMIRRVEDDLRRRARGLLDGVEPGAPFDFVVEIAAELPMQMICILLGVPETDRHWLFE AVEPGFDFRGSRRATMPRLNVEDAGSRLYTYALELIAGKRAEPADDMLSVVANATIDDPD APALSDAELYLFFHLLFSAGAETTRNSIAGGLLALAENPDQLQTLRSDFELLPTAIEEIV RWTSPSPSKRRTASRAVSLGGQPIEAGQKVVVWEGSANRDPSVFDRADEFDITRKPNPHL GFGQGVHYCLGANLARLELRVLFEELLSRFGSVRVVEPAEWTRSNRHTGIRHLVVELRGG  >CYP130A1(ERDMAN_1403)  MSHEFQLATAETWPNPWPMYRALRDHDPVHHVVPPQRPEYDYYVLSRHADVWSAARDHQT FSSAQGLTVNYGELEMIGLHDTPPMVMQDPPVHTEFRKLVSRGFTPRQVETVEPTVRKFV VERLEKLRANGGGDIVTELFKPLPSMVVAHYLGVPEEDWTQFDGWTQAIVAANAVDGATT GALDAVGSMMAYFTGLIERRRTEPADDAISHLVAAGVGADGDTAGTLSILAFTFTMVTGG NDTVTGMLGGSMPLLHRRPDQRRLLLDDPEGIPDAVEELLRLTSPVQGLARTTTRDVTIG DTTIPAGRRVLLLYGSANRDERQYGPDAAELDVTRCPRNILTFSHGAHHCLGAAAARMQC RVALTELLARCPDFEVAESRIVWSGGSYVRRPLSVPFRVTS  >CYP132A1(ERDMAN_1552)  MATATTQRPLKGPAKRMSTWTMTREAITIGFDAGDGFLGRLRGSDITRFRCAGRRFVSIS HPDYVDHVLHEARLKYVKSDEYGPIRATAGLNLLTDEGDSWARHRGALNSTFARRHLRGL VGLMIDPIADVTAALVPGAQFDMHQSMVETTLRVVANALFSQDFGPLVQSMHDLATRGLR RAEKLERLGLWGLMPRTVYDTLIWCIYSGVHLPPPLREMQEITLTLDRAINSVIDRRLAE PTNSADLLNVLLSADGGIWPRQRVRDEALTFMLAGHETTANAMSWFWYLMALNPQARDHM LTELDDVLGMRRPTADDLGKLAWTTACLQESQRYFSSVWIIAREAVDDDIIDGHRIRRGT TVVIPIHHIHHDPRWWPDPDRFDPGRFLRCPTDRPRCAYLPFGGGRRICIGQSFALMEMV LMAAIMSQHFTFDLAPGYHVELEATLTLRPKHGVHVIGRRR  >CYP139A1(ERDMAN_1833)  MRTYRTVRYPLGEALLALYRWRGPLINAGVGGHGYTYLLGAEANRFVFANADAFSWSQTF ESLVPVDGPTALIVSDGADHRRRRSVVAPGLRHHHVQRYVATMVSNIDTVIDGWQPGQRL DIYQELRSAVRRSTAESLFGQRLAVHSDFLGEQLQPLLDLTRRPPQVMRLQQRVNSPGWR RAMAARKRIDDLIDAQIADARTAPRPDDHMLTTLISGCSEEGTTLSDNEIRDSIVSLITA GYETTSGALAWAIYALLTVPGTWESAASEVARVLGGRVPAADDLSALTYLNGVVHETLRL YSPGVISARRVLRDLWFDGHRIRAGRLLIFSAYVTHRLPEIWPEPTEFRPLRWDPNAADY RKPAPHEFIPFSGGLHRCIGAVMATTEMTVILARLVARAMLQLPAQRTHRIRAANFAALR PWPGLTVEIRKSAPAQ  >CYP144A1(ERDMAN_1966)  MQRRVDQAVSADHAELMTIAKDANTFFGAESVQDPYPLYERMRAAGSVHRIANSDFYAVC GWDAVNEAIGRPEDFSSNLTATMTYTAEGTAKPFEMDPLGGPTHVLATADDPAHAVHRKL VLRHLAAKRIRVMEQFTVQAADRLWVDGMQDGCIEWMGAMANRLPMMVVAELIGLPDPDI AQLVKWGYAATQLLEGLVENDQLVAAGVALMELSGYIFEQFDRAAADPRDNLLGELATAC ASGELDTLTAQVMMVTLFAAGGESTAALLGSAVWILATRPDIQQQVRANPELLGAFIEET LRYEPPFRGHYRHVRNATTLDGTELPADSHLLLLWGAANRDPAQFEAPGEFRLDRAGGKG HISFGKGAHFCVGAALARLEARIVLRLLLDRTSVIEAADVGGWLPSILVRRIERLELAVQ  >CYP143A1(ERDMAN_1974)  MTTPGEDHAGSFYLPRLEYSTLPMAVDRGVGWKTLRDAGPVVFMNGWYYLTRREDVLAAL RNPKVFSSRKALQPPGNPLPVVPLAFDPPEHTRYRRILQPYFSPAALSKALPSLRRHTVA MIDAIAGRGECEAMADLANLFPFQLFLVLYGLPLEDRDRLIGWKDAVIAMSDRPHPTEAD VAAARELLEYLTAMVAERRRNPGPDVLSQVQIGEDPLSEIEVLGLSHLLILAGLDTVTAA VGFSLLELARRPQLRAMLRDNPKQIRVFIEEIVRLEPSAPVAPRVTTEPVTVGGMTLPAG SPVRLCMAAVNRDGSDAMSTDELVMDGKVHRHWGFGGGPHRCLGSHLARLELTLLVGEWL NQIPDFELAPDYAPEIRFPSKSFALKNLPLRWS  >CYP140A1(ERDMAN_2072)  MKDKLHWLAMHGVIRGIAAIGIRRGDLQARLIADPAVATDPVPFYDEVRSHGALVRNRAN YLTVDHRLAHDLLRSDDFRVVSFGENLPPPLRWLERRTRGDQLHPLREPSLLAVEPPDHT RYRKTVSAVFTSRAVSALRDLVEQTAINLLDRFAEQPGIVDVVGRYCSQLPIVVISEILG VPEHDRPRVLEFGELAAPSLDIGIPWRQYLRVQQGIRGFDCWLEGHLQQLRHAPGDDLMS QLIQIAESGDNETQLDETELRAIAGLVLVAGFETTVNLLGNGIRMLLDTPEHLATLRQHP ELWPNTVEEILRLDSPVQLTARVACRDVEVAGVRIKRGEVVVIYLAAANRDPAVFPDPHR FDIERPNAGRHLAFSTGRHFCLGAALARAEGEVGLRTFFDRFPDVRAAGAGSRRDTRVLR GWSTLPVTLGPARSMVSP  >CYP124A1(ERDMAN_2490)  MGLNTAIATRVNGTPPPEVPIADIELGSLDFWALDDDVRDGAFATLRREAPISFWPTIEL PGFVAGNGHWALTKYDDVFYASRHPDIFSSYPNITINDQTPELAEYFGSMIVLDDPRHQR LRSIVSRAFTPKVVARIEAAVRDRAHRLVSSMIANNPDRQADLVSELAGPLPLQIICDMM GIPKADHQRIFHWTNVILGFGDPDLATDFDEFMQVSADIGAYATALAEDRRVNHHDDLTS SLVEAEVDGERLSSREIASFFILLVVAGNETTRNAITHGVLALSRYPEQRDRWWSDFDGL APTAVEEIVRWASPVVYMRRTLTQDIELRGTKMAAGDKVSLWYCSANRDESKFADPWTFD LARNPNPHLGFGGGGAHFCLGANLARREIRVAFDELRRQMPDVVATEEPARLLSQFIHGI KTLPVTWS  >CYP128A1(ERDMAN_2492)  MQLTDFDPFDPAIAADPYPHYRELLAGERVQYNPKRDVYILSRYADVREAARNHDTLSSA RGVTFSRGWLPFLPTSDPPAHTRMRKQLAPGMARGALETWRPMVDQLARELVGGLLTQTP ADVVSTVAAPMPMRAITSVLGVDGPDEAAFCRLSNQAVRITDVALSASGLISLVQGFAGF RRLRALFTHRRDNGLLRECTVLGKLATHAEQGRLSDDELFFFAVLLLVAGYESTAHMIST LFLTLADYPDQLTLLAQQPDLIPSAIEEHLRFISPIQNICRTTRVDYSVGQAVIPAGSLV LLAWGAANRDPRQYEDPDVFRADRNPVGHLAFGSGIHLCPGPSWRAWRVRRSCARSSPIS TE  >CYP136A1(ERDMAN_3348)  MATIHPPAYLLDQAKRRFTPSFNNFPGMSLVEHMLLNTKFPEKKLAEPPPGSGLKPVVGD AGLPILGHMIEMLRGGPDYLMFLYKTKGPVVFGDSAVLPGVAALGPDAAQVIYSNRNKDY SQQGWVPVIGPFFHRGLMLLDFEEHMFHRRIMQEAFVRSRLAGYLEQMDRVVSRVVADDW VVNDARFLVYPAMKALTLDIASMVFMGHEPGTDHELVTKVNKAFTITTRAGNAVIRTSVP PFTWWRGLRARELLENYFTARVKERREASGNDLLTVLCQTEDDDGNRFSDADIVNHMIFL MMAAHDTSTSTATTMAYQLAAHPEWQQRCRDESDRHGDGPLDIESLEQLESLDLVMNESI RLVTPVQWAMRQTVRDTELLGYYLPKGTNVIAYPGMNHRLPEIWTDPLTFDPERFTEPRN EHKRHRYAFTPFGGGVHKCIGMVFDQLEIKTILHRLLRRYRLELSRPDYQPRWDYSAMPI PMDGMPIVLRPR  >CYP141A1(ERDMAN_3416)  MLSDKRFSCRAAAHPSSPPFVPFVQLCPSLLSIDGPQHTAARRLLAQGLNPGFIARMRPV VQQIVDNALDDLAAAEPPVDFQEIVSVPIGEQLMAKLLGVEPETVHELAAHVDAAMSVCE IGDEEVSRRWSALCTMVIDILHRKLAEPGDDLLSTIAQANRQQSTMTDEQVVGMLLTVVI GGVDTPIAVITNGLASLLHHRDQYERLVEDPGRVARAVEEIVRFNPATEIEHLRVVTEDV VIAGTALSAGSPAFTSITSANRDSDQFLDPDEFDVERNPNEHIAFGYGPHACPASAYSRM CLTTFFTSLTQRFPQLQLARPFEDLERRGKGLHSVGIKELLVTWPT  >CYP142A1(ERDMAN_3857)  MDLADGNFYASREARAAYRWMRANQPVFRDRNGLAAASTYQAVIDAERQPELFSNAGGIR PDQPALPMMIDMDDPAHLLRRKLVNAGFTRKRVKDKEASIAALCDTLIDAVCERGECDFV RDLAAPLPMAVIGDMLGVRPEQRDMFLRWSDDLVTFLSSHVSQEDFQITMDAFAAYNDFT RATIAARRADPTDDLVSVLVSSEVDGERLSDDELVMETLLILIGGDETTRHTLSGGTEQL LRNRDQWDLLQRDPSLLPGAIEEMLRWTAPVKNMCRVLTADTEFHGTALCAGEKMMLLFE SANFDEAVFCEPEKFDVQRNPNSHLAFGFGTHFCLGNQLARLELSLMTERVLRRLPDLRL VADDSVLPLRPANFVSGLESMPVVFTPSPPLG  >CYP125A1(ERDMAN_3890)  MPSPNLPPGFDFTDPAIYAERLPVAEFAELRSAAPIWWNGQDPGKGGGFHDGGFWAITKL NDVKEISRHSDVFSSYENGVIPRFKNDIAREDIEVQRFVMLNMDAPHHTRLRKIISRGFT PRAVGRLHDELQERAQKIAAEAAAAGSGDFVEQVSCELPLQAIAGLLGVPQEDRGKLFHW SNEMTGNEDPEYAHIDPKASSAELIGYAMKMAEEKAKNPADDIVTQLIQADIDGEKLSDD EFGFFVVMLAVAGNETTRNSITQGMMAFAEHPDQWELYKKVRPETAADEIVRWATPVTAF QRTALRDYELSGVQIKKGQRVVMFYRSANFDEEVFQDPFTFNILRNPNPHVGFGGTGAHY CIGANLARMTINLIFNAVADHMPDLKPISAPERLRSGWLNGIKHWQVDYTGRCPVAH  >CYP137A1(ERDMAN_4036)  MVLRSLASPAALTDPKRCASVVGVAAFAVRREHAPDALGGPPGLPAPRGFRAAFAAAYAV AYLAGGERRMLRLIRRYGPIMTMPILSLGDVAIVSDSALAKEVFTAPTDVLLGGEGVGPA AAIYGSGSMFVQEEPEHLRRRKLLTPPLHGAALDRYVPIIENSTRAAMHTWPVDRPFAML TVARSLMLDVIVKVIFGVDDPEEVRRLGRPFERLLNLGVSEQLTVRYALRRLGALRVWPA RARANTEIDDVVMALIAQRRADPRLGERHDVLSLLVSARGESGEQLSDSEIRDDLITLVL AGHETTATTLAWAFDLLLHHPDALRRVRAEAVGGGEAFTTAVINETLRVRPPAPLTARVA AQPLTIGGYRVEAGTRIVVHIIAINRSAEVYEHPHEFRPERFLGTRPQTYAWVPFGGGVK RCLGANFSMRELITVLHVLLREGEFTAVDDEPERIVRRSIMLVPRRGTRVRFRPAR |
| ***Mycobacterium tuberculosis* UT205** |
| Database: KEGG; P450 count 19; Families: 18; Subfamilies: 19 |
| >CYP135A1(UDA_0327c)  MASTLTTGLPPGPRLPRYLQSVLYLRFREWFLPAMHRKYGDVFSLRVPPYADNLVVYTRP EHIKEIFAADPRSLHAGEGNHILGFVMGEHSVLMTDEAEHARMRSLLMPAFTRAALRGYR DMIASVAREHITRWRPHATINSLDHMNALTLDIILRVVFGVTDPKVKAELTSRLQQIINI HPAILAGVPYPSLKRMNPWKRFFHNQTKIDEILYREIASRRIDSDLTARTDVLSRLLQTK DTPTKPLTDAELRDQLITLLLAGHETTAAALSWTLWELAHAPEIQSQVVWAAVGGDDGFL EAVLKEGMRRHTVIASTARKVTAPAEIGGWRLPAGTVVNTSILLAHASEVSHPKPTEFRP SRFLDGSVAPNTWLPFGGGVRRCLGFGFALTEGAVILQEIFRRFTITAAGPSKGETPLVR NITTVPKHGAHLRLIPQRRLGGLGDSDPP  >CYP135B1(UDA_0568)  MSGTSSMGLPPGPRLSGSVQAVLMLRHGLRFLTACQRRYGSVFTLHVAGFGHMVYLSDPA AIKTVFAGNPSVFHAGEANSMLAGLLGDSSLLLIDDDVHRDRRRLMSPPFHRDAVARQAG PIAEIAAANIAGWPMAKAFAVAPKMSEITLEVILRTVIGASDPVRLAALRKVMPRLLNVG PWATLALANPSLLNNRLWSRLRRRIEEADALLYAEIADRRADPDLAARTDTLAMLVRAAD EDGRTMTERELRDQLITLLVAGHDTTATGLSWALERLTRHPVTLAKAVQAADASAAGDPA GDEYLDAVAKETLRIRPVVYDVGRVLTEAVEVAGYRLPAGVMVVPAIGLVHASAQLYPDP ERFDPDRMVGATLSPTTWLPFGGGNRRCLGATFAMVEMRVVLREILRRVELSTTTTSGER PKLKHVIMVPHRGARIRVRATRDVSATSQATAQGAGCPAARGGGPSRAVGSQ  >CYP123A1 (UDA_0766c)  MTVRVGDPELVLDPYDYDFHEDPYPYYRRLRDEAPLYRNEERNFWAVSRHHDVLQGFRDS TALSNAYGVSLDPSSRTSEAYRVMSMLAMDDPAHLRMRTLVSKGFTPRRIRELEPQVLEL ARIHLDSALQTESFDFVAEFAGKLPMDVISELIGVPDTDRARIRALADAVLHREDGVADV PPPAMAASIELMRYYADLIAEFRRRPANNLTSALLAAELDGDRLSDQEIMAFLFLMVIAG NETTTKLLANAVYWAAHHPGQLARVFADHSRIPMWVEETLRYDTSSQILARTVAHDLTLY DTTIPEGEVLLLLPGIGQP  >CYP126A1(UDA_0778)  MTTAAGLSGIDLTDLDNFADGFPHHLFAIHRREAPVYWHRPTEHTPDGEGFWSVATYAET LEVLRDPVTYSSVTGGQRRFGGTVLQDLPVAGQVLNMMDDPRHTRIRRLVSSGLTPRMIR RVEDDLRRRARGLLDGVEPGAPFDFVVEIAAELPMQMICILLGVPETDRHWLFEAVEPGF DFRGSRRATMPRLNVEDAGSRLYTYALELIAGKRAEPADDMLSVVANATIDDPDAPALSD AELYLFFHLLFSAGAETTRNSIAGGLLALAENPDQLQTLRSDFELLPTAIEEIVRWTSPS PSKRRTASRAVSLGGQPIEAGQKVVVWEGSANRDPSVFDRADEFDITRKPNPHLGFGQGV HYCLGANLARLELRVLFEELLSRFGSVRVVEPAEWTRSNRHTGIRHLVVELRGG  >CYP130A (UDA_1256c)  MTSVMSHEFQLATAETWPNPWPMYRALRDHDPVHHVVPPQRPEYDYYVLSRHADVWSAAR DHQTFSSAQGLTVNYGELEMIGLHDTPPMVMQDPPVHTEFRKLVSRGFTPRQVETVEPTV RKFVVERLEKLRANGGGDIVTELFNPLPSMVVAHYLGVPEEDWTQFDGWTQAIVAANAVD GATTGALDAVGSMMAYFTGLIERRRTEPADDAISHLVAAGVGADGDTAGTLSILAFTFTM VTGGNDTVTGMLGGSMPLLHRRPDQRRLLLDDPEGIPDAVEELLRLTSPVQGLARTTTRD VTIGDTTIPAGRRVLLLYGSANRDERQYGPDAAELDVTRCPRNILTFSHGAHHCLGAAAA RMQCRVALTELLARCPDFEVAESRIVWSGGSYVRRPLSVPFRVTS  >CYP132A1(UDA_1394c)  MATATTQRPLKGPAKRMSTWTMTREAITIGFDAGDGFLGRLRGSDITRFRCAGRRFVSIS HPDYVDHVLHEARLKYVKSDEYGPIRATAGLNLLTDEGDSWARHRGALNSTFARRHLRGL VGLMIDPIADVTAALVPGAQFDMHQSMVETTLRVVANALFSQDFGPLVQSMHDLATRGLR RAEKLERLGLWGLMPRTVYDTLIWCIYSGVHLPPPLREMQEITLTLDRAINSVIDRRLAE PTNSADLLNVLLSADGGIWPRQRVRDEALTFMLAGHETTANAMSWFWYLMALNPQARDHM LTELDDVLGMRRPTADDLGKLAWTTACLQESQRYFSSVWIIAREAVDDDIIDGHRIRRGT TVVIPIHHIHHDPRWWPDPDRFDPGRFLRCPTDRPRCAYLPFGGGRRICIGQSFALMEMV LMAAIMSQHFTFDLAPGYHVELEATLTLRPKHGVHVIGRRR  >CYP139A1 (UDA_1666c)  MRYPLGEALLALYRWRGPLINAGVGGHGYTYLLGAEANRFVFANADAFSWSQTFESLVPV DGPTALIVSDGADHRRRRSVVAPGLRHHHVQRYVATMVSNIDTVIDGWQPGQRLDIYQEL RSAVRRSTAESLFGQRLAVHSDFLGEQLQPLLDLTRRPPQVMRLQQRVNSPGWRRAMAAR KRIDDLIDAQIADARTAPRPDDHMLTTLISGCSEEGTTLSDNEIRDSIVSLITAGYETTS GALAWAIYALLTVPGTWESAASEVARVLGGRVPAADDLSALTYLNGVVHETLRLYSPGVI SARRVLRDLWFDGHRIRAGRLLIFSAYVTHRLPEIWPEPTEFRPLRWDPNAADYRKPAPH EFIPFSGGLHRCIGAVMATTEMTVILARLVARAMLQLPAQRTHRIRAANFAALRPWPGLT VEIRKSAPAQ  >CYP144A1(UDA_1777)  MRRSPKGSPGAVLDLQRRVDQAVSADHAELMTIAKDANTFFGAESVQDPYPLYERMRAAG SVHRIANSDFYAVCGWDAVNEAIGRPEDFSSNLTATMTYTAEGTAKPFEMDPLGGPTHVL ATADDPAHAVHRKLVLRHLAAKRIRVMEQFTVQAADRLWVDGMQDGCIEWMGAMANRLPM MVVAELIGLPDPDIAQLVKWGYAATQLLEGLVENDQLVAAGVALMELSGYIFEQFDRAAA DPRDNLLGELATACASGELDTLTAQVMMVTLFAAGGESTAALLGSAVWILATRPDIQQQV RADPELLGAFIEETLRYEPPFRGHYRHVRNATTLDGTELPADSHLLLLWGAANRDPAQFE APGEFRLDRAGGKGHISFGKGAHFCVGAALARLEARIVLRLLLDRTSVIEAADVGGWLPS ILVRRIERLELAVQ  >CYP143A1(UDA_1785c)  MTTPGEDHAGSFYLPRLEYSTLPMAVDRGVGWKTLRDAGPVVFMNGWYYLTRREDVLAAL RNPKVFSSRKALQPPGNPLPVVPLAFDPPEHTRYRRILQPYFSPAALSKALPSLRRHTVA MIDAIAGRGECEAMADLANLFPFQLFLVLYGLPLEDRDRLIGWKDAVIAMSDRPHPTEAD VAAARELLEYLTAMVAERRRNPGPDVLSQVQIGEDPLSEIEVLGLSHLLILAGLDTVTAA VGFSLLELARRPQLRAMLRDNPKQIRVFIEEIVRLEPSAPVAPRVTTEPVTVGGMTLPAG SPVRLCMAAVNRDGSDAMSTDELVMDGKVHRHWGFGGGPHRCLGSHLARLELTLLVGEWL NQIPDFELAPDYAPEIRFPSKSFALKNLPLRWS  >CYP140A1(UDA_1880c)  MKDKLHWLAMHGVIRGIAAIGIRRGDLQARLIADPAVATDPVPFYDEVRSHGALVRNRAN YLTVDHRLAHDLLRSDDFRVVSFGENLPPPLRWLERRTRGDQLHPLREPSLLAVEPPDHT RYRKTVSAVFTSRAVSALRDLVEQTAINLLDRFAEQPGIVDVVGRYCSQLPIVVISEILG VPEHDRPRVLEFGELAAPSLDIGIPWRQYLRVQQGIRGFDCWLEGHLQQLRHAPGDDLMS QLIQIAESGDNETQLDETELRAIAGLVLVAGFETTVNLLGNGIRMLLDTPEHLATLRQHP ELWPNTVEEILRLDSPVQLTARVACRDVEVAGVRIKRGEVVVIYLAAANRDPAVFPDPHR FDIERPNAGRHLAFSTGRHFCLGAALARAEGEVGLRTFFDRFPDVRAAGAGSRRDTRVLR GWSTLPVTLGPARSMVSP  >CYP124A1(UDA_2266)  MGLNTAIATRVNGTPPPEVPIADIELGSLDFWALDDDVRDGAFATLRREAPISFWPTIEL PGFVAGNGHWALTKYDDVFYASRHPDIFSSYPNITINDQTPELAEYFGSMIVLDDPRHQR LRSIVSRAFTPKVVARIEAAVRDRAHRLVSSMIANNPDRQADLVSELAGPLPLQIICDMM GIPKADHQRIFHWTNVILGFGDPDLATDFDEFMQVSADIGAYATALAEDRRVNHHDDLTS SLVEAEVDGERLSSREIASFFILLVVAGNETTRNAITHGVLALSRYPEQRDRWWSDFDGL APTAVEEIVRWASPVVYMRRTLTQDIELRGTKMAAGDKVSLWYCSANRDESKFADPWTFD LARNPNPHLGFGGGGAHFCLGANLARREIRVAFDELRRQMPDVVATEEPARLLSQFIHGI KTLPVTWS  >CYP128A1(UDA_2268c)  MTATQSPPEPAPDRVRLAGCPLAGTPDVGLTAQDATTALGVPTRRRASSGGIPVATSMWR DAQTVRTYGPAVAKALALRVAGKARSRLTGRHCRKFMQLTDFDPFDPAIAADPYPHYREL LAGERVQYNPKRDVYILSRYADVREAARNHDTLSSARGVTFSRGWLPFLPTSDPPAHTRM RKQLAPGMARGALETWRPMVDQLARELVGGLLTQTPADVVSTVAAPMPMRAITSVLGVDG PDEAAFCRLSNQAVRITDVALSASGLISLVQGFAGFRRLRALFTHRRDNGLLRECTVLGK LATHAEQGRLSDDELFFFAVLLLVAGYESTAHMISTLFLTLADYPDQLTLLAQQPDLIPS AIEEHLRFISPIQNICRTTRVDYSVGQAVIPAGSLVLLAWGAANRDPRQYEDPDVFRADR NPVGHLAFGSGIHLCPGTQLARMEGQAILREIVANIDRIEVVEPPTWTTNANLRGLTRLR VAVTPRVAP  >CYP121A1(UDA_2276)  MTATVLLEVPFSARGDRIPDAVAELRTREPIRKVRTITGAEAWLVSSYALCTQVLEDRRF SMKETAAAGAPRLNALTVPPEVVNNMGNIADAGLRKAVMKAITPKAPGLEQFLRDTANSL LDNLITEGAPADLRNDFADPLATALHCKVLGIPQEDGPKLFRSLSIAFMSSADPIPAAKI NWDRDIEYMAGILENPNITTGLMGELSRLRKDPAYSHVSDELFATIGVTFFGAGVISTGS FLTTALISLIQRPQLRNLLHEKPELIPAGVEELLRINLSFADGLPRLATADIQVGDVLVR KGELVLVLLEGANFDPEHFPNPGSIELDRPNPTSHLAFGRGQHFCPGSALGRRHAQIGIE ALLKKMPGVDLAVPIDQLVWRTRFQRRIPERLPVLW  >CYP136A1(UDA_3059)  MATIHPPAYLLDQAKRRFTPSFNNFPGMSLVEHMLLNTKFPEKKLAEPPPGSGLKPVVGD AGLPILGHMIEMLRGGPDYLMFLYKTKGPVVFGDSAVLPGVAALGPDAAQVIYSNRNKDY SQQGWVPVIGPFFHRGLMLLDFEEHMFHRRIMQEAFVRSRLAGYLEQMDRVVSRVVADDW VVNDARFLVYPAMKALTLDIASMVFMGHEPGTDHELVTKVNKAFTITTRAGNAVIRTSVP PFTWWRGLRARELLENYFTARVKERREASGNDLLTVLCQTEDDDGNRFSDADIVNHMIFL MMAAHDTSTSTATTMAYQLAAHPEWQQRCRDESDRHGDGPLDIESLEQLESLDLVMNESI RLVTPVQWAMRQTVRDTELLGYYLPKGTNVIAYPGMNHRLPEIWTDPLTFDPERFTEPRN EHKRHRYAFTPFGGGVHKCIGMVFDQLEIKTILHRLLRRYRLELSRPDYQPRWDYSAMPI PMDGMPIVLRPR  >CYP141A1(UDA_3121)  MTSTSIPTFPFDRPVPTEPSPMLSELRNSCPVAPIELPSGHTAWLVTRFDDVKGVLSDKR FSCRAAAHPSSPPFVPFVQLCPSLLSIDGPQHTAARRLLAQGLNPGFIARMRPVVQQIVD NALDDLAAAEPPVDFQEIVSVPIGEQLMAKLLGVEPETVHELAAHVDAAMSVCEIGDEEV SRRWSALCTMVIDILHRKLAEPGDDLLSTIAQANRQQSTMTDEQVVGMLLTVVIGGVDTP IAVITNGLASLLHHRDQYERLVEDPGRVARAVEEIVRFNPATEIEHLRVVTEDVVIAGTA LSAGSPAFTSITSANRDSDQFLDPDEFDVERNPNEHIAFGYGPHACPASAYSRMCLTTFF TSLTQRFPQLQLARPFEDLERRGKGLHSVGIKELLVTWPT  >CYP142A1(UDA_3518c)  MTEAPDVDLADGNFYASREARAAYRWMRANQPVFRDRNGLAAASTYQAVIDAERQPELFS NAGGIRPDQPALPMMIDMDDPAHLLRRKLVNAGFTRKRVKDKEASIAALCDTLIDAVCER GECDFVRDLAAPLPMAVIGDMLGVRPEQRDMFLRWSDDLVTFLSSHVSQEDFQITMDAFA AYNDFTRATIAARRADPTDDLVSVLVSSEVDGERLSDDELVMETLLILIGGDETTRHTLS GGTEQLLRNRDQWDLLQRDPSLLPGAIEEMLRWTAPVKNMCRVLTADTEFHGTALCAGEK MMLLFESANFDEAVFCEPEKFDVQRNPNSHLAFGFGTHFCLGNQLARLELSLMTERVLRR LPDLRLVADDSVLPLRPANFVSGLESMPVVFTPSPPLG  >CYP125A1(UDA_3545c)  MSWNHQSVEIAVRRTTVPSPNLPPGFDFTDPAIYAERLPVAEFAELRSAAPIWWNGQDPG KGGGFHDGGFWAITKLNDVKEISRHSDVFSSYENGVIPRFKNDIAREDIEVQRFVMLNMD APHHTRLRKIISRGFTPRAVGRLHDELQERAQKIAAEAAAAGSGDFVEQVSCELPLQAIA GLLGVPQEDRGKLFHWSNEMTGNEDPEYAHIDPKASSAELIGYAMKMAEEKAKNPADDIV TQLIQADIDGEKLSDDEFGFFVVMLAVAGNETTRNSITQGMMAFAEHPDQWELYKKVRPE TAADEIVRWATPVTAFQRTALRDYELSGVQIKKGQRVVMFYRSANFDEEVFQDPFTFNIL RNPNPHVGFGGTGAHYCIGANLARMTINLIFNAVADHMPDLKPISAPERLRSGWLNGIKH WQVDYTGRCPVAH  >CYP137A1(UDA_3685c)  MVLRSLASPAALTDPKRCASVVGVAAFAVRREHAPDALGGPPGLPAPRGFRAAFAAAYAV AYLAGGERRMLRLIRRYGPIMTMPILSLGDVAIVSDSALAKEVFTAPTDVLLGGEGVGPA AAIYGSGSMFVQEEPEHLRRRKLLTPPLHGAALDRYVPIIENSTRAAMHTWPVDRPFAML TVARSLMLDVIVKVIFGVDDPEEVRRLGRPFERLLNLGVSEQLTVRYALRRLGALRVWPA RARANTEIDDVVMALIAQRRADPRLGERHDVLSLLVSARGESGEQLSDSEIRDDLITLVL AGHETTATTLAWAFDLLLHHPDALRRVRAEAVGGGEAFTTAVINETLRVRPPAPLTARVA AQPLTIGGYRVEAGTRIVVHIIAINRSAEVYEHPHEFRPERFLGTRPQTYAWVPFGGGVK RCLGANFSMRELITVLHVLLREGEFTAVDDEPERIVRRSIMLVPRRGTRVRFRPAR  >CYP138A-fragment (UDA_0136)  MSEVVTAAPAPPVVRLPPAVRGPKLFQGLAFVVSRRRLLGRFVRRYGKAFTANILMYGRV VVVAARS |
| ***Mycobacterium canetii* CIPT 140010059** |
| Database: KEGG; P450 count 19; Families 18; Subfamilies: 19 |
| >CYP138A1(MCAN_01391)  MSEVVTAAPAPPVVRLPPAVRGPKLFQGLAFVVSRRRLLGRFVRRYGKAFTANILMYGRV VVVADPQLARQVFTSSPEELGNIQPNLSRMFGSGSVFALDGDDHRRRRRLLAPPFHGKSM KNYETIIEEETLRETANWPQGQAFATLPSMMHITLNAILRAIFGAGGSELDELRRLIPPW VTLGSRLAALPKPKRDYGRLSPWGRLAEWRRQYDTVIDKLIEAERADPNFADRTDVLALM LRSTYDDGSIMSRKDIGDELLTLLAAGHETTAATLGWAFERLSRHPDVLAALVEEVDNGG HELRQAAILEVQRARTVIDFAARRVNPPVYQLGEWVIPRGYSIIINIAQIHGDPDVFPQP DRFDPQRYIGSKPSPFAWIPFGGGTRRCVGAAFANMEMDVVLRTVLRHFTLETTTAAGER SHGRGVAFTPKDGGRVVMRRR  >CYP135A1(MCAN_03291)  MASTLTTGLPPGPRLPRYLQSVLYLRFREWFLPAMHRKYGDVFSLRVPPYADNLVVYTRP EHIKEIFAADPRSLHAGEGNHILGFVMGEHSVLMTDEAEHARMRSLLMPAFTRAALRGYR DMIASVAREHITRWRPHATINSLDHMNALTLDIILRVVFGVTDPKVKAELTSRLQQIINI HPAILAGVPYPSLKRMNPWKRFFHNQTKIDEILYREIASRRIDSDLTARTDVLSRLLQTK DTPTKPLTDAELRDQLITLLLAGHETTAAALSWTLWELAHAPEIQSQVVWAAVGGDDGFL EAVLKEGMRRHTVIASTARKVTAPAEIGGWRLPAGTVVNTSILLAHASEVSHPKPTEFRP SRFLDGSVAPNTWLPFGGGVRRCLGFGFALTEGAVILQEIFRRFTITAAGPSKGETPLVR NITTVPKHGAHLRLIPQRRLGGLGDSDPP  >CYP135B1(MCAN_05711)  MSGTSSMGLPPGPRLSGSVQAVLMLRHGLRFLTACQRRYGSVFTLHVAGFGHMVYLSDPA AIKTVFAGNPRVFHAGEANSMLAGLLGDSSLLLIDDDVHRDRRRLMLPPFHRDAVARQAG LMAEIAAANIAGWPMAKQFAVAPKMSEITLEVILRTVIGASDPARLAALRKVMPRLLNVG PWATLALANPSLLNNRLWSRLRRRIEEADALLYAEIADRRADPDLAARTDTLAMLVRAAD EDGRTMTERELRDQLITLLVAGHDTTATGLSWALERLTRHPVTLAKAVQAADASAAGDPA GDEYLDAVAKETLRIRPVVYDVGRVLTEAVEVAGYRLPAGVMVVPAIGLVHASSQLYPDP ERFDPDRMVGATLSPTTWLPFGGGNRRCLGATFAMVEMRVVLREILRRVELSTTTTSGER PKLKHVIMVPHRGARIRVRATRDVSATSQATAQGAGCPAARGGGPSRAVGSQ  >CYP51B1(MCAN_07681)  MSAVALPRVSGGHDEHGHLEEFRTDPIGLMQRVRDECGDVGTFQLAGKQVVLLSGAQANE FFFRAGDDDLDQAKAYPFMKPIFGEGVVFDASPERRKEMLHNAALRGEQMKGHAATIEDQ VRRMIADWGEAGEIDLLDFFAELTIYTSSACLIGKKFRDQLDRRFAKLYHELERGTDPLA YVDPYLPIESFRRRDEARKGLVALVADIMNGRIANPPTDKSDRDMLDVLIAVKDETGTPR FSADEITGMFISMMFAGHHTSSGTASWTLIELMRHRDAYAAVINELDELYGDGRSVSFHA LRQIPRLENVLKETLRLHPPLIILMRVAKGEFEVQGHRIHEGDLVAASPAISNRIPEDFP DPDDFVPARYEQPRQEDLLNRWTWIPFGAGRHRCVGAAFAIMQIKAIFSVLLREYEFEMA QPPESYRNDHSKMVVQLAQPACVRYRLRSGTGG  >CYP123A1(MCAN_07701)  MTVRIGDPELVLDPYDYDFHEDPYSYYRRLRDEAPLYRNEERNFWAVSRHHDVLQGFRDS TALSNAYGVSLDPSSRTSEAYRVMSMLAMDDPAHLRMRTLVSKGFTPRRIRELEPQVLEL ARIHLDSALQTESFDFVAEFAGKLPMDVISELMGVPDTDRARIRALADAVLHREDGVADV PPSAMAASIELMRYYADLIAEFRRRPADNLTSALLAAELDGDRLSDQEIMAFLFLMVIAG NETTTKLLANAVYWAAHHPGQLARVFADHSRIPMWVEETLRYDTSSQILARTVAHDLTLY DTTIPEGEVLLLLPGSANRDDRVFDDPDDYRIGREIGCKLVSFGSGAHFCLGAHLARMEA RVALGELLRRIRNYEVDDDNAVRVHSSNVRGFAHLPISVQAR  >CYP126A1(MCAN_07821)  MTTAAGLSGIDLTDLDNFADGFPHHLFAIHRREAPVFWHRPTEHTPDGEGFWSVATYAQT LEVLRDPVTYSSVTGGQRRFGGTVLQDLPVAGQVLNMMDDPRHTRIRRLVSSGLTPRMIR RVEDDLRRRARGLLDGVEPGAPFDFVVEIAAELPMQMICILLGVPETDRHWLFEAVEPGF DFRGSRRATMPKLNVEDAGSRLYTYALELIAGKRAEPADDMLSVVANATIDDPDAPALSD AELYLFFHLLFSAGAETTRNSIAGGLLALAENPDQLQTLRSDFELLPTAIEEIVRWTSPS PSKRRTASRAVSLGGQPIEAGQKVVVWEGSANRDPSVFDRADEFDITRKPNPHLGFGQGV HYCLGANLARLELRVLFEELLSRFGSVRVVEPAEWTRSNRHTGIRHLVVELRGG  >CYP130A1(MCAN_12701)  MTSVMSHEFQLATAETWPNPWPMYRALRDHDPVHHVVPPQRPEYDYYVLSRHADVWSAAR DHQTFSSAQGLTVNYGELEMIGLHDTPPMVMQDPPVHTEFRKLVSRGFTPRQVETVEPTV RKFVVERLEKLRANGGGDIVTELFKPLPSMVVAHYLGVPEEDWTQFDGWTQAIVAANAVD GATTGALDAVGSMMAYFTGLIERRRTEPADDAISHLVAAGVGADGDTAGTLSILAFTFTM VTGGNDTVTGMLGGSMPLLHRRPDQRRLLLDDPEGIPDAVEELLRLTSPVQGLARTTTRD VTIGDTTIPAGRRVLLLYGSANRDERQYGPDAAELDVTRCPRNILTFSHGAHHCLGAAAA RMQCRVALTELLARCPDFEVAESRIVWSGGSYVRRPLSVPFRVTS  >CYP132A1(MCAN_14101)  MATATTQRPLKGPAKRMSTWTMTREAITIGFDAGDGFLGRLRGSDITRFRCAGRRFVSIS HPDYVDHVLYEARLKYVKSDEYGPIRATAGLNLLTDEGDSWARHRGALNPTFARRHLRGL VGLMIDPIADVTAALVPGAQFDMHQSMVETTLRVVANALFSQDFGPLVQSMHDLATRGLR RAEKLERLGLWGLMPRTVYDTLIWCIYSGVHLPPPLRETQEITLTLDRAVNSLIDRRLAE PTNSADLLNVLLSADGGIWPRQRVRDEALTFMLAGHETTANAMSWFWYLMALNPQARDHM LTELDDVLGTRRPTADDLGKLAWTTACLQESQRYFSSVWIIAREAVDDDIIDGHRIRRGT TVVIPIHHIHHDPRWWPDPDRFDPGRFLRCPTDRPRCAYLPFGGGRRICIGQSFALMEMV LMAAIMSQHFTFDLAPGYHVELEATLTLRPKHGVHVIGRRR  >CYP139A1(MCAN_16741)  MRYPLGEALLALYRWRGPLINAGVGGHGYTYLLGAEANRFVFANADAFSWSQTFESLVPV DGPTALIVSDGADHRRRRSVVAPGLRHHHVQRYVATMVSNIDTVIDGWQPGQRLDIYQEL RSAVRRSTAESLFGQRLAVHSDFLGEQLQPLLDLTRRPPQVMRLQQRVNSPGWRRAMAAR KRIDDLIDAQIADARTAPRPDDHMLTTLISGCSEEGTTLSDNEIRDSIVSLITAGYETTS GALAWAIYALLTVPGTWESAASEVARVLGGRVPAADDLSALTYLNGVVHETLRLYSPGVI SARRVLRDLWFDGHRIRAGRLLIFSAYVTHRLPEIWPEPTEFRPLRWDPNAADYRKPAPH EFIPFSGGLHRCIGAVMATTEMTVILARLVARAMLQLPAQRTHRIRAANFAALRPWPGLT VEIRKSAPAQ  >CYP144A1(MCAN_17951)  MRRSPKGSPGAVLDLQRRVDQAVSADHAELMTIAKDANTFFGAESVQDPYPLYERMRAAG SVHRIANSDFYAVCGWDAVNEAIGRPEDFSSNLTATMTYTAEGTVKPFEMDPLGGPTHVL ATADDPAHAVHRKLVLRHLAAKRIRVMEQFTVQAADRLWVDGMQDGCIEWMGAMANRLPM MVVAELIGLPDPDIAQLVKWGYAATQLLEGLVENDQLVAAGVALMELSGYIFEQFDRAAA DPRDNLLGELATACASGELDTLTAQVMMVTLFAAGGESTAALLGSAVWILATRPDIQQQV RANPELLGAFIEETLRYEPPFRGHYRHVRNATTLDGTELPADSHLLLLWGAANRDPAQFE APGEFRLDRAGGKGHISFGKGAHFCVGAALARLEARIVLRLLLDRTSVIEAADVGGWLPS ILVRRIERLELAVQ  >CYP143A1(MCAN_18031)  MTTPGEDHAGSFYLPRLEYSTLPMAVDRGVGWKTLRDAGPVVFMNGWYYLTRREDVLAAL RNPKVFSSRKALQPPGNPLPVVPLAFDPPEHTRYRRILQPYFSPAALSKALPSLRRHTVA MIDAIAGRGECEAMADLANLFPFQLFLVLYGLPLEDRDRLIGWKDAVIAMSDRPHPTEAD VAAARELLEYLTAMVAERRRNPGPDVLSQVQIGEDPLSEIEVLGLSHLLILAGLDTVTAA VGFSLLELARRPQLRAMLRDNPKQIRVFIEEIVRLEPSAPVAPRVTTELVTVGGMTLPAG SPVRLCMAAVNRDGSDAMSTDELVMDGKVHRHWGFGGGPHRCLGSHLARLELTLLVGEWL NQIPDFELAPDYAPEIRFPSKSFALKNLPLRWS  >CYP140A1(MCAN_18951)  MKDKLHWLAMHGVIRGIAAIGIRRGDLQARLIADPAVATDPVPFYDEVRSHGALVRNRAN YLTVDHRLAHDLLRSDDFRVVSFGENLPPPLRWLERRTRGDQLHPLREPSLLAVEPPDHT RYRKTVSAVFTSRAVSALRDLVEQTAINLLDRFAEQPGIVDVVGRYCSQLPIVVISEILG VPEHDRPRVLEFGELAAPSLDIGIPWRQYLRVQQGIRGFDCWLEGHLQQLRHAPGDDLMS QLIQIAESGDNETQLDETELRAIAGLVLVAGFETTVNLLGNGIRMLLDTPEHLATLRQHP ELWPNTVEEILRLDSPVQLTARVACRDVEVAGVRIKRGEVVVIYLAAANRDPAVFPDPHR FDIERPNAGRHLAFSTGRHFCLGAALARAEGEVGLRTFFDRFPDVRAAGAGSRRDTRVLR GWSTLPVTLGPARSMVSP  >CYP124A1(MCAN_22901)  MGLNTAIATRVNGTPPPEVPIADIELGSLDFWALDDDVRDGAFATLRREAPISFWPTIEL PGFVAGNGHWALTKYDDVFYASRHPDIFSSYPNITINDQTPELAEYFGSMIVLDDPRHQR LRSIVSRAFTPKVVARIEAAVRDRAHRLVSSMIANNPDRQADLVSELAGPLPLQIICDMM GIPKADHQRIFHWTNVILGFGDPDLATDFDEFMQVSADIGAYATALAEDRRVNHHDDLTS SLVEAEVDGERLSSREIASFFILLVVAGNETTRNAITHGVLALSRYPEQRDRWWSDFDGL APTAVEEIVRWASPVVYMRRTLTQDIELRGTKMAAGDKVSLWYCSANRDESKFADPWTFD LARNPNPHLGFGGGGAHFCLGANLARREIRVAFDELRRQMPDVVATEEPARLLSQFIHGI KTLPVTWS  >CYP128A1(MCAN_22921)  MTATQSPPEPAPDRVQLAGCPLAGTPDVGLTAQDATTALGVPTRRRASSGGIPVATSMWR DAQTVRTYGPAVAKALALRVAGKARSRLAGRHCRKFMQLTDFDPFDPAIAADPYPHYREL LAGERVQYNPKRDVYILSRYADVREAARNHDTLSSARGVTFSRGWLPFLPTSDPPAHTRM RKQLAPGMARGALETWRPMVDQLARELVGGLLTQTPADVVSTVAAPMPMRAITSVLGVDG PDEAAFCRLSNQAVRITDVALSASGLISLVQGFAGFRRLRALFTHRRDNGLLRECTVLGK LATHAEQGRLSDDELFFFAVLLLVAGYESTAHMISTLFLTLADYPDQLTLLAQQPDLIPS AIEEHLRFISPIQNICRTTRVDYSVGQAVIPAGSLVLLAWGAANRDPRQYEDPDVFRADR NPVGHLAFGSGIHLCPGTQLARMEGQAILREIVANIDRIEVVEPPTWTTNANLRGLTRLR VAVTPRVAP  >CYP121A1(MCAN_23001)  MTATVLLEVPFSARGDRIPDAVAELRTREPIRKVRTITGAEAWLVSSYALCTQVLEDRRF SMKETAAAGAPRLNALTVPPEVVNNMGNIADAGLRKAVMKAITPKAPGLEQFLRDTANSL LDNLITEGAPADLRNDFADPLATALHCKVLGIPQEDGPKLFRSLSIAFMSSADPIPAAKI NWDRDIEYMAGILENPNITTGLMGELGRLRKDPAYSHVSDELFATIGVTFFGAGVISTGS FLTTALISLIQRPQLRNLLHEKPELIPAGVEELLRINLSFADGLPRLATADIQVGDVLVR KGELVLVLLEGANFDPEHFPNPGSIELDRPNPTSHLAFGRGQHFCPGSALGRRHAQIGIE ALLKKMPDVDLAVPIDQLVWRTRFQRRIPERLPVLW  >CYP136A1(MCAN_30841)  MATIHPPAYLLDQAKRRFTPSFNNFPGMSLVEHMLLNTKFPEKKLAEPPPGSGLKPVVGD AGLPILGHMIEMLRGGPDYLMFLYKTKGPVVFGDSAVLPGVAALGPDAAQVIYSNRNKDY SQQGWVPVIGPFFHRGLMLLDFEEHMFHRRIMQEAFVRSRLAGYLEQMDRVVSRVVADDW VVNDARFLVYPAMKALTLDIASMVFMGHEPGTDHELVTKVNKAFTITTRAGNAVIRTSVP PFTWWRGLRARELLENYFTARVKERREASGNDLLTVLCQTEDDDGNRFSDADIVNHMIFL MMAAHDTSTSTATTMAYQLAAHPEWQQRCRDESDRHGDGPLDIESLEQLESLDLVMNESI RLVTPVQWAMRQTVRDTELLGYYLPKGTNVIAYPGMNHRLPEIWTDPLTFDPERFTEPRN EHKRHRYAFTPFGGGVHKCIGMVFGQLEIKTILHRLLRRYRLELSRPDYQPRWDYSAMPI PMDGMPIVLRPR  >CYP142A1(MCAN_35291)  MTEAPDVDLADGNFYASREARAAYRWMRANQPVFRDRNGLAAASTYQAVIDAERQPELFS NAGGIRPDQPALPMMIDMDDPAHLLRRKLVNAGFTRKRVKDKEASIAALCDTLIDAVCER GECDFVRDLAAPLPMAVIGDMLGVRPEQRDMFLRWSDDLVTFLSSHVSQEDFQITMDAFA AYNDFTRATIAARRADPTDDLVSVLVSSEVDGERLSDDELVMETLLILIGGDETTRHTLS GGTEQLLRNRDQWDLLQRDPSLLPGAIEEMLRWTAPVKNMCRVLTADTEFHGTALCAGEK MMLLFESANFDEAVFCEPEKFDVQRNPNSHLAFGFGTHFCLGNQLARLELSLMTERVLRR LPDLRLVADDSVLPLRPANFVSGLESMPVVFTPSPPLG  >CYP125A1(MCAN_35561)  MSWNHQSVEIAVRRTTVPSPNLPPGFDFTDPAIYAERLPVAEFAELRSAAPIWWNGQDPG KGGGFHDGGFWAITKLNDVKEISRHSDVFSSYENGVIPRFKNDIAREDIEVQRFVMLNMD APHHTRLRKIISRGFTPRAVGRLHDELQERAQKIAAEAAAAGSGDFVEQVSCELPLQAIA GLLGVPQEDRGKLFHWSNEMTGNEDPEYAHIDPKASSAELIGYAMKMAEEKAKNPADDIV TQLIQADIDGEKLSDDEFGFFVVMLAVAGNETTRNSITQGMMAFAEHPDQWELYKKVRPE TAADEIVRWATPVTAFQRTALRDYELSGVQIKKGQRVVMFYRSANFDEEVFQDPFTFNIL RSPNPHVGFGGTGAHYCIGANLARMTINLIFNAVADHMPDLKPISAPERLRSGWLNGIKH WQVDYTGRCPVAH  >CYP137A1(MCAN_37061)  MVLRSLASPAALTDPKRCASVVGVAAFAVRREHAPDALGGPPGLPAPRGFRAAFAAAYAV AYLAGGERRMLRLIRRYGPIMTMPILSLGDVAIVSDPALAKEVFTAPTDVLLGGEGVGPA AAIYGSGSMFVQEEPEHLRRRKLLTPPLHGAALDRYVPIIENSTRAAMHTWPVDRPFAML TVARSLMLDVIVKVIFGVDDPEEARRLGRPFERLLNLGVSEQLTVRYVLRRLGALRVWPA RARANTEIDDVVMALIAQRRADPRLGERHDVLSLLVSARGESGEQLSDSEIRDDLITLVL AGHETTATTLAWAFDLLLHHPDALRRVRAEAVGGGEAFTTAVINETLRVRPPAPLTARVA AQPLTIGGYRVEAGTRIVVHIIAINRSAEVYEHPHEFRPERFLGTRPQTYAWVPFGGGVK RCLGANFSMRELITVLHVLLREGEFTAVDDEPERIVRRSIMLVPRRGTRVRFRPAR |
| ***Mycobacterium canetii* CIPT 140060008** |
| Database: KEGG; P450 count: 18; Families 17; Subfamilies: 18 |
| >CYP138A1(BN44_10164)  MSEVVTAAPAPPVVRLPPAVRGPKLFQGLAFVVSRRRLLGRFVRRYGKAFTANILMYGRV VVVADPQLARQVFTSSPEELGNIQPNLSRMFGSGSVFALDGDDHRRRRRLLAPPFHGKSM KNYETIIEEETLRETANWPQGQAFATLPSMMHITLNAILRAIFGAGGSELDELRRLIPPW VTLGSRLAALPKPKRDYGRLSPWGRLAEWRRQYDTVIDKLIEAERADPNFADRTDVLALM LRSTYDDGSIMSRKDIGDELLTLLAAGHETTAATLGWAFERLSRHPDVLAALVEEVDNGG HELRQAAILEVQRARTVIDFAARRVNPPVYQLGEWVIPRGYSIIINIAQIHGDPDVFPQP DRFDPQRYIGSKPSPFAWIPFGGGTRRCVGAAFANMEMDVVLRTVLRHFTLETTTAAGER SHGRGVAFTPKDGGRVVMRRR  >CYP135A1(BN44_10367)  MASTLTTGLPPGPRLPRYLQSVLYLRFREWFLPAMHRKYGDVFSLRVPPYADNLVVYTRP EHIKEIFAADPRSLHAGEGNHILGFVMGEHSVLMTDEAEHARMRSLLMPAFTRAALRGYR DMIASVAREHITRWRPHATINSLDHMNALTLDIILRVVFGVTDPKVKAELTSRLQQIINI HPAILAGVPYPSLKRMNPWKRFFHNQTKIDEILYREIASRRIDSDLTARTDVLSRLLQTK DTPTKPLTDAELRDQLITLLLAGHETTAAALSWTLWELAHAPEIQSQVVWAAVGGDDGFL EAVLKEGMRRHTVIASTARKVTAPAEIGGWRLPAGTVVNTSILLAHASEVSHPKPTEFRP SRFLDGSVAPNTWLPFGGGVRRCLGFGFALTEGAVILQEIFRRFTITAAGPSKGETPLVR NITTVPKHGAHLRLIPQRRLGGLGDSDPP  >CYP135B1(BN44_10629)  MSGTSSMGLPPGPRLSGSVQAVLMLRHGLRFLTACQRRYGSVFTLHVAGFGHMVYLSDPA AIKTVFAGNPSVFHAGEANSMLAGLLGDSSLLLIDDDVHRDRRRLMSPPFHRDAVARQAG PIAEIAAANIAGWPMAKAFAVAPKMSEITLEVILRTVIGASDPVRLAALRKVMPRLLNVG PWATLALANPSLLNNRLWSRLRRRIEEADALLYAEIADRRADPDLAARTDTLAMLVRAAD EDGRTMTERELRDQLITLLVAGHDTTATGLSWALERLTRHPVTLAKAVQAADASAAGDPA GDEYLDAVAKETLRIRPVVYDVGRVLTEAVEVAGYRLPAGVMVVPAIGLVHASAQLYPDP ERFDPDRMVGATLSPTTWLPFGGGNRRCLGATFAMVEMRVVLREILRRVELSTTTTSGER PKLKHVIMVPHRGARIRVRATRDVSATSQATAQGAGCPAARGGGPSRAVGSQ  >CYP123A1(BN44_10836)  MTVRVGDPELVLDPYDYDFHEDPYPYYRRLRDEAPLYRNEERNFWAVSRHHDVLQGFRDS TALSNAYGVSLDPSSRTSEAYRVMSMLAMDDPAHLRMRTLVSKGFTPRRIRELEPQVLEL ARIHLDSALQTESFDFVAEFAGKLPMDVISELIGVPDTDRARIRALADAVLHREDGVADV PPPAMAASIELTRYYADLIAEFRRRPANNLTSALLAAELDGDRLSDQEIMAFLFLMVIAG NETTTKLLANAVYWAAHHPGQLARVFADHSRIPMWVEETLRYDTSSQILARTVAHDLTLY DTTIPEGEVLLLLPGSANRDDRVFDDPDDYRIGREIGCKLVSFGSGAHFCLGAHLARMEA RVALGALLRRIRNYEVDDDNVVRVHSSNVRGFAHLPISVQAR  >CYP126A1(BN44_10850)  MTTAAGLSGIDLTDLDNFADGFPHHLFAIHRREAPVYWHRPTEHTPDGEGFWSVATYAET LEVLRDPVTYSSVTGGQRRFGGTVLQDLPVAGQVLNMMDDPRHTRIRRLVSSGLTPRMIR RVEDDLRRRARGLLDGVEPGAPFDFVVEIAAELPMQMICILLGVPETDRHWLFEAVEPGF DFRGSRRATMPRLNVEDAGSRLYTYALELIAGKRAEPADDMLSVVANATIDDPDAPALSD AELYLFFHLLFSAGAETTRNSIAGGLLALAENPDQLQTLRSDFELLPTAIEEIVRWTSPS PSKRRTASRAVSLGGQPIEAGQKVVVWEGSANRDPSVFDRADEFDITRKPNPHLGFGQGV HYCLGANLARLELRVLFEELLSRFGSVRVVEPAEWTRSNRHTGIRHLVVELRGG  >CYP130A1(BN44_11404)  MYRALRDHDPVHHVVPPQRPEYDYYVLSRHADVWSAARDHQTFSSAQGLTVNYGELEMIG LHDTPPMVMQDPPVHTEFRKLVSRGFTPRQVETVEPTVRKFVVERLEKLRANGGGDIVTE LFKPLPSMVVAHYLGVPEEDWTQFDGWTQAIVAANAVDGATTGALDAVGSMMAYFTGLIE RRRTEPADDAISHLVAAGVGADGDTAGTLSILAFTFTMVTGGNDTVTGMLGGSMPLLHRR PDQRRLLLDDPEGIPDAVEELLRLTSPVQGLARTTTRDVTIGDTTIPAGRRVLLLYGSAN RDERQYGPDAAELDVTRCPRNILTFSHGAHHCLGAAAARMQCRVALTELLARCPDFEVAE SRIVWSGGSYVRRPLSVPFRVTS  >CYP132A1(BN44_11570)  MATATTQRPLKGPAKRMSTWTMTREAITIGFDAGDGFLGRLRGSDITRFRCAGRRFVSIS HPDYVDHVLYEARLKYVKSDEYGPIRATAGLNLLTDEGDSWARHRGALNPTFARRHLRGL VGLMIDPIADVTAALVPGAQFDMHQSMVETTLRVVANALFSQDFGPLVQSMHDLATRGLR RAEKLERLGLWGLMPRTVYDTLIWCIYSGVHLPPPLRETQEITLTLDRAVNSLIDRRLAE PTNSADLLNVLLSADGGIWPRQRVRDEALTFMLAGHETTANAMSWFWYLMALNPQARDHM LTELDDVLGTRRPTADDLGKLAWTTACLQESQRYFSSVWIIAREAVDDDIIDGHRIRRGT TVVIPIHHIHHDPRWWPDPDRFDPGRFLRCPTDRPRCAYLPFGGGRRICIGQSFALMEMV LMAAIMSQHFTFDLAPGYHVELEATLTLRPKHGVHVIGRRR  >CYP139A1(BN44_20227)  MRYPLGEALLALYRWRGPLINAGVGGHGYTYLLGAEANRFVFANADAFSWSQTFESLVPV DGPTALIVSDGADHRRRRSVVAPGLRHHHVQRYVATMVSNIDTVIDGWQPGQRLDIYQEL RSAVRRSTAESLFGQRLAVHSDFLGEQLQPLLDLTRRPPQVMRLQQRVNSPGWRRAMAAR KRIDDLIDAQIADARTAPRPDDHMLTTLISGCSEEGTTLSDNEIRDSIVSLITAGYETTS GALAWAIYALLTVPGTWESAASEVARVLGGRVPAADDLSALTYLNGVVHETLRLYSPGVI SARRVLRDLWFDGHRIRAGRLLIFSAYVTHRLPEIWPEPTEFRPLRWDPNAADYRKPAPH EFIPFSGGLHRCIGAVMATTEMTVILARLVARAMLQLPAQRTHRIRAANFAALRPWPGLT VEIRKSAPAQ  >CYP144A1(BN44_40033)  MRRSPKGSPGAVLDLQRRVDQAVSADHAELMTIAKDANTFFGAESVQDPYPLYERMRAAG SVHRIANSDFYAVCGWDAVNEAIGRPEDFSSNLTATMTYTAEGTAKPFEMDPLGGPTHVL ATADDPAHAVHRKLVLRHLAAKRIRVMEQFTVQAADRLWVDGMQDGCIEWMGAMANRLPM MVVAELIGLPDPDIAQLVKWGYAATQLLEGLVENDQLVAAGVALMELSGYIFEQFDRAAA DPRDNLLGELATACASGELDTLTAQVMMVTLFAAGGESTAALLGSAVWILATRPDIQQQV RANPELLGAFIEETLRYEPPFRGHYRHVRNATTLDGTELPADSHLLLLWGAANRDPAQFE APGEFRLDRAGGKGHISFGKGAHFCVGAALARLEARIVLRLLLDRTSVIEAADVGGWLPS ILVRRIERLELAVQ  >CYP143A1(BN44_40042)  MTTPGEDHAGSFYLPRLEYSTLPMAVDRGVGWKTLRDAGPVVFMNGWYYLTRREDVLAAL RNPKVFSSRKALQPPGNPLPVVPLAFDPPEHTRYRRILQPYFSPAALSKALPSLRRHTVA MIDAIAGRGECEAMADLANLFPFQLFLVLYGLPLEDRDRLIGWKDAVIAMSDRPHPTEAD VAAARELLEYLTAMVAERRRNPGPDVLSQVQIGEDPLSEIEVLGLSHLLILAGLDTVTAA VGFSLLELARRPQLRAMLRDNPKQIRVFIEEIVRLEPSAPVAPRVTTELVTVGGMTLPAG SPVRLCMAAVNRDGSDAMSTDELVMDGKVHRHWGFGGGPHRCLGSHLARLELTLLVGEWL NQIPDFELAPDYAPEIRFPSKSFALKNLPLRWS  >CYP140A1(BN44_40143)  MKDKLHWLAMHGVIRGIAAIGIRRGDLQARLIADPAVATDPVPFYDEVRSHGALVRNRAN YLTVDHRLAHDLLRSDDFRVVSFGENLPPPLRWLERRTRGDQLHPLREPSLLAVEPPDHT RYRKTVSAVFTSRAVSALRDLVEQTAINLLDRFAEQPGIVDVVGRYCSQLPIVVISEILG VPEHDRPRVLEFGELAAPSLDIGIPWRQYLRVQQGIRGFDCWLEGHLQQLRHAPGDDLMS QLIQIAESGDNETQLDETELRAIAGLVLVAGFETTVNLLGNGIRMLLDTPEHLATLRQHP ELWPNTVEEILRLDSPVQLTARVACRDVEVAGVRIKRGEVVVIYLAAANRDPAVFPDPHR FDIERPNAGRHLAFSTGRHFCLGAALARAEGEVGLRTFFDRFPDVRAAGAGSRRDTRVLR GWSTLPVTLGPARSMVSP  >CYP124A1(BN44_50212)  MGLNTAIATRVNGTPPPEVPIADIELGSLDFWALDDDVRDGAFATLRREAPISFWPTIEL PGFVAGNGHWALTKYDDVFYASRHPDIFSSYPNITINDQTPELAEYFGSMIVLDDPRHQR LRSIVSRAFTPKVVARIEAAVRDRAHRLVSSMIANNPDRQADLVSELAGPLPLQIICDMM GIPKADHQRIFHWTNVILGFGDPDLATDFDEFMQVSADIGAYATALAEDRRVNHHDDLTS SLVEAEVDGERLSSREIASFFILLVVAGNETTRNAITHGVLALSRYPEQRDRWWSDFDGL APTAVEEIVRWASPVVYMRRTLTQDIELRGTKMAAGDKVSLWYCSANRDESKFADPWTFD LARNPNPHLGFGGGGAHFCLGANLARREIRVAFDELRRQMPDVVATEEPARLLSQFIHGI KTLPVTWS  >CYP128A1(BN44_50214)  MTATQSPPEPAPDRVQLAGCPLAGTPDVGLTAQDATTALGVPTRRRASSGGIPVATSMWR DAQTVRTYGPAVAKALALRVAGKARSRLAGRHCRKFMQLTDFDPFDPAIAADPYPHYREL LAGERVQYNPKRDVYILSRYADVREAARNHDTLSSARGVTFSRGWLPFLPTSDPPAHTRM RKQLAPGMARGALETWRPMVDQLARELVGGLLTQTPADVVSTVAAPMPMRAITSVLGVDG PDEAAFCRLSNQAVRITDVALSASGLISLVQGFAGFRRLRALFTHRRDNGLLRECTVLGK LATHAEQGRLSDDELFFFAVLLLVAGYESTAHMISTLFLTLADYPDQLTLLAQQPDLIPS AIEEHLRFISPIQNICRTTRVDYSVGQAVIPAGSLVLLAWGAANRDPRQYEDPDVFRADR NPVGHLAFGSGIHLCPGTQLARMEGQAILREIVANIDRIEVVEPPTWTTNANLRGLTRLR VAVTPRVAP  >CYP121A1(BN44_50222)  MTATVLLEVPFSARGDRIPDAVAELRTREPIRKVRTVTGAEAWLVSSYALCTQVLEDRRF SMKETAAAGAPRLNALTVPPEVVNNMGNIADAGLRKAVMKAITPKAPGLEQFLRDTANSL LDNLITEGAPADLRNDFADPLATALHCKVLGIPQEDGPKLFRSLSIAFMSSADPIPAAKI NWDRDIEYMAGILENPNITTGLMGELGRLRKDPAYSHVSDELFATIGVTFFGAGVISTGS FLTTALISLIQRPQLRNLLHEKPELIPAGVEELLRINLSFADGLPRLATADIQVGDVLVR KGELVLVLLEGANFDPEHFPNPGSIELDRPNPTSHLAFGRGQHFCPGSALGRRHAQIGIE ALLKKMPGVDLAVPIDQLVWRTRFQRRIPERLPVLW  >CYP136A1(BN44_60570)  MATIHPPAYLLDQAKRRFTPSFNNFPGMSLVEHMLLNTKFPEKKLAEPPPGSGLKPVVGD AGLPILGHMIEMLRGGPDYLMFLYKTKGPVVFGDSAVLPGVAALGPDAAQVIYSNRNKDY SQQGWVPVIGPFFHRGLMLLDFEEHMFHRRIMQEAFVRSRLAGYLEQMDRVVSRVVADDW VVNDARFLVYPAMKALTLDIASMVFMGHEPGTDHELVTKVNKAFTITTRAGNAVIRTSVP PFTWWRGLRARELLENYFTARVKERREASGNDLLTVLCQTEDDDGNRFSDADIVNHMIFL MMAAHDTSTSTATTMAYQLAAHPEWQQRCRDESDRHGDGPLDIESLEQLESLDLVMNESI RLVTPVQWAMRQTVRDTELLGYYLPKGTNVIAYPGMNHRLPEIWTDPLTFDPERFTEPRN EHKRHRYAFTPFGGGVHKCIGMVFGQLEIKTILHRLLRRYRLELSRPDYQPRWDYSAMPI PMDGMPIVLRPR  >CYP142A1(BN44_110006)  MTEAPDVDLADGNFYASREARAAYRWMRANQPVFRDRNGLAAASTYQAVIDAERQPELFS NAGGIRPDQPALPMMIDMDDPAHLLRRKLVNAGFTRKRVKDKEASIAALCDTLIDAVCER GECDFVRDLAAPLPMAVIGDMLGVRPEQRDMFLRWSDDLVTFLSSHVSQEDFQITMDAFA AYNDFTRATIAARRADPTDDLVSVLVSSEVDGERLSDDELVMETLLILIGGDETTRHTLS GGTEQLLRNRDQWDLLQRDPSLLPGAIEEMLRWTAPVKNMCRVLTADTEFHGTALCAGEK MMLLFESANFDEAVFCEPEKFDVQRNPNSHLAFGFGTHFCLGNQLARLELSLMTERVLRR LPDLRLVADDSVLPLRPANFVSGLESMPVVFTPSPPLG  >CYP125A1(BN44_110037)  MSWNHQSVEIAVRRTTVPSPNLPPGFDFTDPAIYAERLPVAEFAELRSAAPIWWNGQDPG KGGGFHDGGFWAITKLNDVKEISRHSDVFSSYENGVIPRFKNDIAREDIEVQRFVMLNMD APHHTRLRKIISRGFTPRAVGRLHDELQERAQKIAAEAAAAGSGDFVEQVSCELPLQAIA GLLGVPQEDRGKLFHWSNEMTGNEDPEYAHIDPKASSAELIGYAMKMAEEKAKNPADDIV TQLIQADIDGEKLSDDEFGFFVVMLAVAGNETTRNSITQGMMAFAEHPDQWELYKKVRPE TAADEIVRWATPVTAFQRTALRDYELSGVQIKKGQRVVMFYRSANFDEEVFQDPFTFNIL RSPNPHVGFGGTGAHYCIGANLARMTINLIFNAVADHMPDLKPISAPERLRSGWLNGIKH WQVDYTGRCPVAH  >CYP137A1(BN44_120082)  MTDPKRCASVVGVAAFAVRREHAPDALGGPPGLPAPRGFRAAFAAAYAVAYLAGGERRML RLIRRYGPIMTMPILSLGDVAIVSDSALAKEVFTAPTDVLLGGEGVGPAAAIYGSGSMFV QEEPEHLRRRKLLTPPLHGAALDRYVPIIENSTRAAMHTWPVDRPFAMLTVARSLMLDVI VKVIFGVDDPEEVRRLGRPFERLLNLGVSEQLTVRYALRRLGALRVWPARARANTEIDDV VMALIAQRRADPRLGERHDVLSLLVSARGESGEQLSDSEIRDDLITLVLAGHETTATTLA WAFDLLLHHPDALRRVRAEAVGGGEAFTTAVINETLRVRPPAPLTARVAAQPLTIGGYRV EAGTRIVVDIIAINRSAEVYEHPHEFRPERFLGTRPQTYAWVPFGGGVKRCLGANFSMRE LITVLHVLLREGEFTAVDDEPERIVRRSIMLVPRRGTRVRFRPAR |
| ***Mycobacterium canetii* CIPT 140710010** |
| Database: KEGG; P450 count: 21; Families: 19; Subfamilies: 20 |
| >CYP138A1(BN42_10175)  MSEVVTAAPAAPVVRLPPAVRGPKLFQGLAFVLSRRRLLGRFVRRYGKAFTANILMYGRV VVVADPQLARQVFTSSPEELGNIQPNLSRMFGSGSVFGLDGEDHRRRRRLLAPPFHGKSM KNYETIIEEETLRETANWPQRQPFATLPSMMHITLNAILRAIFGAGGSELDELRRLIPPW VTLGSRLAALPKPKRDYGRLSPWGRLAEWRRQYDIVIDKLIEAERADPNFADRTDVLALM LRSTYDDGSIMSRKDIGDELLTLLAAGHETTAATLGWAFERLSRHPDVLAALVEEVDNGG HELRQAAILEVQRARTVIDFAARRVNPPVYQLGEWVIPRGYSIIINIAQIHGDPDVFPEP DRFDPQRYIGSKPSPFAWIPFGGGTRRCVGAAFANMEMDVVLRTVLRHFTLETTTAAGER SHGRGVAFTPKDGGRVVMRRR  >CYP135A1(BN42_20056)  MASTLTTGLPPGPRLPRYLQSVLYLRFREWFLPAMHRKYGDVFSLRVPPYADNLVVYTRP EHIKEIFAADPRSLHAGEGNHILGFVMGEHSVLMTDEAEHARMRSLLMPAFTRAALRGYR DMIASVAREHITRWRPHATINSLDHMNALTLDIILRVVFGVTDPKVKAELTSRLQQIINI HPAILAGVPYPSLKRMNPWKRFFHNQTKIDEILYREIASRRIDSDLTARTDVLSRLLQTK DTPTKPLTDAELRDQLITLLLAGHETTAAALSWTLWELAHAPEIQSQVVWAAVGGDDGFL EAVLKEGMRRHTVIASTARKVTAPAEIGGWRLPAGTVVNTSILLAHASEVSHPKPTEFRP SRFLDGSVAPNTWLPFGGGVRRCLGFGFALTEGAVILQEIFRRFTITAAGPSKGETPLVR NITTVPKHGAHLRLIPQRRLGGLGDSDPP  >CYP135B1(BN42_20307)  MSGTSSMGLPPGPRLSGSVQAVLMLRHGLRFLTACQRRYGSVFTLHVAGFGHMVYLSDPA AIKTVFAGNPSVFHAGEANSMLAGLLGDSSLLLIDDDVHRDRRRLMLPPFHRDAVARQAG LMAEIAAANIAGWPMAKQFAVAPKMSEITLEVILRTVIGASDPARLAALRKVMPRLLNVG PWATLALANPSLLNNRLWSRLRRRIEEADALLYAEIADRRADPDLAARTDTLAMLVRAAD EDGRTMTERELRDQLITLLVAGHDTTATGLSWALERLTRHPVTLAKAVQAADASAAGDPA GDEYLDAVAKETLRIRPVVYDVGRVLTEAVEVAGYRLPAGVMVVPAIGLVHASAQLYPDP ERFDPDRMVGATLSPTTWLPFGGGNRRCLGATFAMVEMRVVLREILRRVELSTTTTSGER PKLKHVIMVPHRGARIRVRATRDVPATSQATAQGAGCPAARGGGPSRAVGSQ  >CYP1128A1(BN42_20317)  MTIRSATGPRLPWDAADPYPFYERHRGVADPVWDDTAQAWLVFGYHAAQQVLGGPGWAID PLANPNAGLAVDAASSELFKRSMLFADGTEHLRLRGAVRDVFTPSFITGLTAGVEAIAAA VIDHPRTATVFDFMTEIALPLPLAVMGEWLGLDVESSRLFRTLSSATIRILVPLATAEEI QTGAAALTRLVAHILPLAADRRSNPSDDLLSFIAADPDLQLDEVVMTALNTAVGGHETTA YLLGTAAIRLFTPRADGNRMLDELDPADPSLITELLRLESPIQATARTATQIQRIGDTEI AQGQQVIVVIAAANRDPAVFDEPDRLRLGRRGPAPLAFGHGPHYCLGAALARLQISVALR RIVARNPVVCGAASWDRSPALRGPVSLPMRFIGP  >CYP128A(BN42_20320)  MTATRSSADHPSAEVAQGGCPFDRGVRPALALKGAAKPGESVRWRASSRVSLPVSARLRR DVQLARLYAPMLAKTLALTLARKARSKLVVEHDPKHVAITDFDPFDPTVARDPYPHYRAL LAGPRVHYNPKRDVYILSRYADVRAAARNHDVLSSAGGVTYSRLQLPFLPTSDPPEHTRM RKQLRPAFTRSALESWRPTIDQLAQELIARLMTQAGADVVSTVAAPIPMRTITHILGVSG PDQAAFRDWSSQAARMTNINLSASGLFSLARTFNGFRHLHAFFTQRLRHDGPLRVETVLE RLAAQADDGALSDEELFFFAVLLLVAGYESTANLLSTLFLTLASWPDQLRLLAQRPELIP SAIEEQLRIASPIQNICRTTRVDYPVGRAVIPKGSLVLLAWGAANRDPRQFDDPDVFRAD RNPTAHVAFGSGIHSCPGAQLARMEGQAVLREIVENIERIEVVEPPRWSTNANLRGLTRL RVSVTRRTPASGPSQI  >CYP123A1(BN42_20524)  MTVRVGDPELVLDPYDYDFHEDPYPYYRRLRDEAPLYRNEERNFWAVSRHHDVLQGFRDS TALSNAYGVSLDPSSRTSEAYRVMSMLAMDDPAHLRMRTLVSKGFTPRRIRELEPQVLEL ARIHLDSALQTESFDFVAEFAGKLPMDVISELMGVPDTDRARIRALADAVLHRQDGVADV PPSAMAASIELMRYYADLIAEFRRRPANNLTSALLAAELDGDRLSDQEIMAFLFLMVIAG NETTTKLLANAVYWAAHHPGQLARVFADHSRIPMWVEETLRYDTSSQILARTVAHDLTLY DTTIPEGEVLLLLPGSANRDDRVFDDPDDYRIGREIGCKLVSFGSGAHFCLGAHLARMEA RVALGELLRRIRNYEVDDDNAVRVHSSNVRGFAHLPISVQAR  >CYP126A1(BN42_20537)  MTTAAGLSGIDLTDLDNFADGFPHHLFAIHRREAPVYWHRPTEHTPDGEGFWSVATYAET LEVLRDPVTYSSVTGGQRRFGGTVLQDLPVAGQVLNMMDDPRHTRIRRLVSSGLTPRMIR RVEDDLRRRARGLLDGVEPGAPFDFVVEIAAELPMQMICILLGVPETDRHWLFEAVEPGF DFRGSRRATMPRLNVEDAGSRLYTYALELIAGKRAEPADDMLSVVANATIDDPDAPALSD AELYLFFHLLFSAGAETTRNSIAGGLLALAENPDQLQTLRSDFELLPTAIEEIVRWTSPS PSKRRTASRAVSLGGQPIEAGQKVVVWEGSANRDPSVFDRADEFDITRKPNPHLGFGQGV HYCLGANLARLELRVLFEELLSRFGSVRVVEPAEWTRSNRHTGIRHLVVELRGG  >CYP130A1(BN42_21130)  MYRALRDHDPVHHVVPPQRPEHDYYVLSRHADVWSAARDHQTFSSAQGLTVNYGELEMIG LHDTPPMVMQDPPVHTEFRKLVSRGFTPRQVETVEPTVRKFVVERLEKLRANGGGDIVTE LFKPLPSMVVAHYLGVPEEDWTQFDGWTQAIVAANAVDGATTGALDAVGSMMAYFTGLIE RRRTEPADDAISHLVAAGVGADGDTAGTLSILAFTFTMVTGGNDTVTGMLGGSMPLLHRR PDQRRLLLDDPEGIPDAVEELLRLTSPVQGLARTTTREVTIGDTTIPTGRKVLLLYGSAN RDERRYGPDAAELDVIRRPRNILTFSHGAHHCLGAAAARMQSRVTLTELLARCPNFEVDE SRIVWSGGSYVRRPLSVPFRVTS  >CYP132A(BN42_21311)  MATATTQRPLKGPAKRMSTWTMTREAITIGFDAGDGFLGRLRGSDITRFRCAGRRFVSIS HPDYVDHVLHEARLKYVKSDEYGPIRATAGLNLLTDEGDSWARHRGALNPTFARRHLSGL VGLMIDPIADVTATLVPGARFDMHQSMVEATLRVVANALFSQDFGPLVQSMNDLATRGLR RAEKLERLGLWGLMPRTVYDALIWFIYSGVHLPPPLREMQEITLTLDHAVNSVIDRRLAE PTDCADLLNVLLSADGGIWPRQRVRDEALTFMLAGHETTANAMSWFWYLMARNPQARDHM LTEVDDVLGTRRPSADDLGKLAWTTACLQESQRYFSSVWIIAREAIDDDIIDGHRIRRGT TVVIPIHHIHHDPRWWPDPDRFDPGRFLGTCATDRPRCAYLPFGGGRRICIGQSFALMEM VLIAAIMSQHFTFDLAPGYHVELEATLTLRPKHGVHVIGRRR  >CYP139A1(BN42_21593)  MRYPLGEALLALYRWRGPLINAGVGGHGYTYLLGAEANRFVFANAAAFSWRQTFESLVPV DGPTALIVSDGADHRRRRSVVAPGLRHHHVQRYVATMVSNIDTVIDGWQPGQRLDIYQEL RSAVRRSTAESLFGQRLAVHSDFLGEQLQPLLDLTRRPPQVMRLQQRVNSPGWRRAMAAR KRIDDLIDAQIADARTAPRPDDHMLTTLISGCPKEGTTLSDNEIRDSIVSLITAGYETTS GALAWAIYALLTVPGAWESAASEVARVLGGRVPAADDLSALTYLNGVVHETLRLYSPGVI SARRVLRDLWFDGHRIRAGRLLIFSAYVTHRLPEIWPEPTEFRPLRWDPNAADYRKPAPH EFIPFSGGLHRCIGAVMATTEMTVMLARLVARAMLQLPAQRAHRIRAANFAALRPWPGLT VEIRKSAPAQ  >CYP144A1(BN42_30031)  MRRSPKDSPGAVLDLQRRVDLAVSSDHAELMTIAKDANTFFGAESVQDPYPLYERMRAAG SVHRIANSDFYAVCGWDAVNEAIGRPEDFSSNLTATMTYTAEGTVKPFEMDPLGGPTHVL ATADDPAHAVHRKLVLRHLAAKRIRVMEQFTVQAADRLWVDGMQDGCIEWMGAMANRLPM MVVAELIGLPDPDIAQLVKWGYAATQLLEGLVENDQLVAAGVALMELSGYIFEQFDRAAA DPRDNLLGELATACASGELDTLTAQVMMVTLFAAGGESTAALLGSAVWILATRPDIQQQV RANPELLGAFIEETLRYEPPFRGHYRHVRNATTLDGTELPADSHLLLLWGAANRDPAQFE APGEFRLDRAGGKGHISFGKGAHFCVGAALARLEARIVLRLLLDRTSVIEAADVGGWLPS ILVRRIERLELAVQ  >CYP143A1(BN42_30040)  MRTPGEDHAGSFYLPRLEYSTLPMAVDRGVGWKTLRDAGPVVFMNGWYYLTRREDVLAAL RNPKVFSSRKALQPPGNPLPVVPLAFDPPEHTRYRRILQPYFSPAALSKALPSLRRHTVA MIDAIAGRGECEAMADLANLFPFQLFLVLYGLPLEDRDRLIGWKDAVIAMSDRPHPTEAD VAAARELLEYLTAMVAERRRNPGPDVLSQVQIGEDPLSEIEVLGLSHLLILAGLDTVTAA VGFSLLELARRPQLRAMLRDNPKQIRVFIEEIVRLEPSAPVAPRVTTETVTVGGMTLPAG SPVRLCMAAVNRDGSDAMSTDELVMDGKVHRHWGFGGGPHRCLGSHLARLELTLLVGEWL NQIPDFELAPDYAPEIRFPSKSFALKNLPLRWS  >CYP140A1(BN42_3014)  MKDKLHWLAMHGVIRGIAAIGIRRGDLQARLIADPAVATDPVSFYDEVRSHGALVRNRAN YLTVDHRLAHDLLRSDDFRVVSFGENLPAPLRWLERRTRGDQLHPLREPSLLAVEPPDHT RYRKTVSAVFTSRAVSALRDLVEQTAINLLDRFAEQPGIVDVAGRYCSQLPIVVISEILG VPEHDRPRVLEFGELAAPSLDIGIPWRQYLRVQQGIRGFNCWLEGHLQQLRRAPGDDLMS QLIQIAESGDNDTQLDETELRAIAGLVLVAGFETTVNLLGNGIRMLLDTPEHLATLRQHP ELWPNTVEEILRLDSPVQLTARVACRDVEVAGVRIKRGEVVVIYLAAANRDPAVFPDPHR FDIERPNAGRHLAFSTGRHFCLGAALARAEGEVGLRTFFDRFPDVRAAGAGSRRDTRVLR GWSTLPVTLGPARSMVSP  >CYP124A1(BN42_40188)  MGLNTAIATRVNGTAPPEVPIADIELGSLDFWGLDDDVRDGAFATLRREAPISFWPTIEL PGFVAGNGHWALTKYDDVFYASRHPDIFSSYPNITINDQTAELAEYFGSMIVLDDPRHQR LRSIVSRAFTPKVVARIEAAVRDRAHRLVSSMIANNPERQADLVSELAGPLPLQIICDMM GIPEADHQRIFHWTNVILGFGDPDLATDFDEFMQVSADIGAYATALAEDRRVNHHDDLTS SLVEAEVDGERLSSREIASFFILLVVAGNETTRNAITHGVLALSRYPEQRDRWWSDFDGL APTAVEEIVRWASPVVYMRRTLTQDIELRGTKMAAGDKVSLWYCSANRDESKFADPWTFD LARNPNPHLGFGGGGAHFCLGANLARREIRVAFDELRREMPDVVATEEPARLLSQFIHGI KTLPVTWS  >CYP128A(BN42_40190)  MTAAQSPPEPVPDRVQLAGCPLAGTPDVGLTTQDATAALGGPMRRRGSSGGIPVATSMWR DARTVRTYGPAVAKALALRVAGKARSRLAGRHCRKSMQLTDFDPFDPAIAADPYPHYREL LTGERVQYNRKRDVYILSRYADVREAARNHDTLSSARGVTFSRGWLPFLPTSDPPAHTRM RKQLAPGMARGALETWRPMVDQLARELVGGLLTQTPADVVSTVAAPMPMRAITSVLGVDG PDEAAFCRLSNQAVRITDVALSASGLISLVQGFAGFRRLRALFTHRRDNGLLRECTVLGK LATHAEQGRLSDDELFFFAVLLLVAGYESTAHMISTLFLTLADYPDQLTLLAQQPDLIPS AIEEHLRFVSPIQNICRTTRVDYSVGQAVIPAGSLVLLAWGAANRDPRQYDDPDVFRADR NPVGHLAFGSGIHLCPGTQLVRMEGQAILREIVANFDRIEVVEPPTWTTNANLRGLTRLR VAVTPRVAP  >CYP121A1(BN42_40197)  MTATVLLEVPFSARGDRIPDAVAELRTREPIRKVRTVTGAEAWLVSSYALCTQVLEDRRF SMKETAAAGAPRLNALTVPPEVVNNMGNIADAGLRKAVMKAITPKAPGLEQFLRDTANSL LDNLITEGAPADLRNDFADPLATALHCKVLGIPQEDGPKLFRSLSIAFMSSADPIPAAKI NWDRDIEYMAGILENPNITTGLMGELGRLRKDPAYSHVSDELFATIGVTFFGAGVISTGS FLTTALISLIQRPQLRNLLHEKPQLIPAGVEELLRINLSFADGLPRLATADIQVGDVLVR KGELVLVLLEGANFDPEHFPNPGSIELDRPNPTSHLAFGRGQHFCPGSALGRRHAQIGIE ALLKKMPGVDLAVPIDQLVWRTRFQRRIPERLPVLW  >CYP136A1(BN42_41067)  MATISPPAYLLEQAKRRFTPSFNNFPGMNLVEHMLLNTKFPEKKLADPPPGSGLKPVVGD AGLPILGHMIEMLRGGPDYLMFLYKTKGPVVFGDSAVLPGVAALGPDAAQVIYSNRNKDY SQQGWVPVIGPFFHRGLMLLDFEEHMFHRRIMQEAFVRSRLAGYLEQMDRVVSRVVADDW VVNDARFLVYPAMKALTLDIASMVFMGHEPGTDHELVTKVNKAFTITTRAGNAVIRTSVP PFTWWRGLRARELLENYFTARVKERREASGNDLLTVLCQTEDDDGNRFSDADIVNHMIFL MMAAHDTSTSTATTMAYQLAAHPEWQQRCRDESDRHGDGPLDIESLEQLESLDLVMNESI RLVTPVQWAMRQTVRDTELLGYYLPKGTNVIAYPGMNHRLPEIWTDPLTFDPERFTEPRN EHKRHRYAFTPFGGGAHKCIGMVFGQLEIKTILHRLLRRYRLELSRPDYQPRWDYSAMPI PMDGMPIVLRPR  >CYP141A1(BN42_41156)  MTSTSIPTFPFDRPVPTEPSPMLSELRNSCPVAPIELPSGHTAWLVTRFDDVKGVLSDKR FSCRAAAHPSSPQFVPFVQLCPSLLSIDGPQHTAARRLLAQGLNPGFIARMRPVVQQIVD NALDDLAAAEPPVDFQEIVSVPIGEQLMAKLLGVEPETVHELAAHVDAAMSVCEIGDEEV SRRWSALCTMVIDILHRKLAEPGDDLLSTIAQANRQQSTMTDEQVVGMLLTVVIGGVDTP IAVITNGLASLLHHRDQYERLVEDPGRVARAVEEIVRFNPATEIEHLRVVTEDVVIAGTA LSAGSPAFTSITSANRDSDQFLDPDEFDVERNPNEHIAFGYGPHACPASAYSRMCLTTFF TSLTQRFPQLQLARPFEDLERRGKGLHSVGIKELLVTWPT  >CYP142A1(BN42_90009)  MTEAPDVDLADGNFYASREARAAYRWMRANQPVFRDRNGLAAASTYQAVIDAERQPELFS NAGGIRPDQPALPMMIDMDDPAHLLRRKLVNAGFTRKRVKDKEASIAALCDTLIDAVCER GECDFVRDLAAPLPMAVIGDMLGVRPEQRDMFLRWSDDLVTFLSSHVSQEDFQITMDAFA AYNDFTRATIAARRAEPTDDLVSVLVSSEVDGERLSDDELVMETLLILIGGDETTRHTLS GGTEQLLCNRDQWDLLQRDPSLLPGAIEEMLRWTAPVKNMCRVLTADTEFHGTALCAGEK MMLLFESANFDEAVFTEPEKFDILRNPNSHLAFGFGTHFCLGNQLARLELSLMTERVLRR LPDLRLVADSSELPLRPANFVSGLESMPVVFTPSPPLG  >CYP125A1(BN42_90040)  MSWNHQSVEIAVRRTTVPSPNLPPGFDFTDPAIYAERLPVAEFAELRSAAPIWWNGQDPG KGGGFHDGGFWAITKLNDVKEISRHSDVFSSYENGVIPRFKNDIAREDIEVQRFVMLNMD APHHTRLRKIISRGFTPRAVGRLHDELQERAQKIAAEAAAAGSGDFVEQVSCELPLQAIA GLLGVPQEDRGKLFHWSNEMTGNEDPEYAHIDPKASSAELIGYAMKMAEEKAKNPADDIV TQLIQADIDGEKLSDDEFGFFVVMLAVAGNETTRNSITQGMMAFAEHPDQWELYKKERPE TAADEIVRWATPVTAFQRTALRDYELSGVQIKKGQRVVLFYRSANFDEEVFQDPFTFNIL RNPNPHVGFGGTGAHYCIGANLARMTINLIFNAVADHMPDLKPISEPERLRSGWLNGIKH WQVDYTGRCPVAH  >CYP137A1(BN42_90200)  MTDPKRCASVVGVAAFAVRREHAPDALGGPPGLPAPRGFRAAFAAAYAVAYLAGGERRML RLIRRYGPIMTMPILSLGDVAIVSDPALAKEVFTAPTDVLLGGEGVGPAAAIYGSGSMFV QEEPEHLRRRKLLTPPLHGAALDSYVPIIENSTRAAMHTWPVDRPFAMLTVARSLMLDVI VKVIFGVDDPKEVRRLGRPFERLLILGVSEQLTVRYALRRLGALRVWPARARANTEIDDV VMALIAQRRADPRLGERHDVLSLLVSARGESGEQLSDSEIRDDLITLVLAGHETTATTLA WAFDLLLHHPDALRRVRAEAVGGGEAFTTAVINETLRVRPPAPLTARVAAQPLTIGGYRV EAGTRIVVHIIAINRSAEVYEHPHEFRPERFLGTRPQTYAWVPFGGGVKRCLGANFSMRE LITVLHVLLREGEFTAVDDEPERIVRRSIMLVPRRGTRVRFRPAR |
| ***Mycobacterium bovis* AF 2122/97** |
| Database: KEGG; P450 count: 18; P450 families: 17; Subfamilies: 18 |
| >CYP138A1(Mb0141)  MSEVVTAAPAPPVVRLPPAVRGPKLFQGLAFVVSRRRLLGRFVRRYGKAFTANILMYGRV  VVVADPQLARQVFTSSPEELGNIQPNLSRMFGSGSVFALDGDDHRRRRRLLAPPFHGKSM  KNYETIIEEETLRETANWPQGQAFATLPSMMHITLNAILRAIFGAGGSELDELRRLIPPW  VTLGSRLAALPKPKRDYGRLSPWGRLAEWRRQYDTVIDKLIEAERADPNFADRTDVLALM  LRSTYDDGSIMSRKDIGDELLTLLAAGHETTAATLGWAFERLSRHPDVLAALVEEVDNGG  HELRQAAILEVQRARTVIDFAARRVNPPVYQLGEWVIPRGYSIIINIAQIHGDPDVFPQP  DRFDPQRYIGSKPSPFAWIPFGGGTRRCVGAAFANMEMDVVLRTVLRHFTLETTTAAGER  SHGRGVAFTPKDGGRVVMRRR  >CYP135A1(Mb0334c)  MASTLTTGLPPGPRLPRYLQSVLYLRFREWFLPAMHRKYGDVFSLRVPPYADNLVVYTRP  EHIKEIFAADPRSLHAGEGNHILGFVMGEHSVLMTDEAEYARMRSLLMPAFTRAALRGYR  DMIASVAREHITRWRPHATINSLDHMNALTLDIILRVVFGVTDPKVKAELTSRLQQIINI  HPAILAGVPYPSLKRMNPWKRFFHNQTKIDEILYREIASRRIDSDLTARTDVLSRLLQTK  DTPTKPLTDAELRDQLITLLLAGHETTAAALSWTLWELAHAPEIQSQVVWAAVGGDDGFL  EAVLKEGMRRHTVIASTARKVTAPAEIGGWRLPAGTVVNTSILLAHASEVSHPKPTEFRP  SRFLDGSVAPNTWLPFGGGVRRCLGFGFALTEGAVILQEIFRRFTITAAGPSKGETPLVR  NITTVPKHGAHLRLIPQRRLGGLGDSDPP  >CYP135B1(Mb0583)  MSGTSSMGLPPGPRLSGSVQAVLMLRHGLRFLTACQRRYGSVFTLHVAGFGHMVYLSDPA  AIKTVFAGNPSVFHAGEANSMLAGLLGDSSLLLIDDDVHRDRRRLMSPPFHRDAVARQAG  PIAEIAAANIAGWPMAKAFAVAPKMSEITLEVILRTVIGASDPVRLAALRKVMPRLLNVG  PWATLALANPSLLNNRLWSRLRRRIEEADALLYAEIADRRADPDLAARTDTLAMLVRAAD  EDGRTMTERELRDQLITLLVAGHDTTATGLSWALERLTRHPVTLAKAVQAADASAAGDPA  GDEYLDAVAKETLRIRPVVYDVGRVLTEAVEVAGYRLPAGVMVVPAIGLVHASAQLYPDP  ERFDPDRMVGATLSPTTWLPFGGGNRRCLGATFAMVEMRVVLREILRRVELSTTTTSGER  PKLKHVIMVPHRGARIRVRATRDVSATSQATAQGAGCPAARGGGPSRAVGSQ  >CYP51B1(Mb0787c)  MSAVALPRVSGGHDEHGHLEEFRTDPIGLMQRVRDECGDVGTFQLAGKQVVLLSGSHANE  FFFRAGDDDLDQAKAYPFMTPIFGEGVVFDASPERRKEMLHNAALRGEQMKGHAATIEDQ  VRRMIADWGEAGEIDLLDFFAELTIYTSSACLIGKKFRDQLDGRFAKLYHELERGTDPLA  YVDPYLPIESFRRRDEARNGLVALVADIMNGRIANPPTDKSDRDMLDVLIAVKAETGTPR  FSADEITGMFISMMFAGHHTSSGTASWTLIELMRHRDAYAAVIDELDELYGDGRSVSFHA  LRQIPQLENVLKETLRLHPPLIILMRVAKGEFEVQGHRIHEGDLVAASPAISNRIPEDFP  DPHDFVPARYEQPRQEDLLNRWTWIPFGAGRHRCVGAAFAIMQIKAIFSVLLREYEFEMA  QPPESYRNDHSKMVVQLAQPACVRYRRRTGV  >CYP123A1(Mb0789c)  MTVRVGDPELVLDPYDYDFHEDPYPYYRRLRDEAPLYRNEERNFWAVSRHHDVLQGFRDS  TALSNAYGVSLDPSSRTSEAYRVMSMLAMDDPAHLRMRTLVSKGFTPRRIRELEPQVLEL  ARIHLDSALQTESFDFVAEFAGKLPMDVISELIGVPDTDRARIRALADAVLHREDGVADV  PPPAMAASIELMRYYADLIAEFRRRPANNLTSALLAAELDGDRLSDQEIMAFLFLMVIAG  NETTTKLLANAVYWAAHHPGQLARVFADHSRIPMWVEETLRYDTSSQILARTVAHDLTLY  DTTIPEGEVLLLLPGSANRDDRVFDDPDDYRIGREIGCKLVSFGSGAHFCLGAHLARMEA  RVALGALLRRIRNYEVDDDNVVRVHSSNVRGFAHLPISVQAR  >CYP126A1(Mb0801)  MTTAAGLSGIDLTDLDNFADGFPHHLFAIHRREAPVYWHRPTEHTPDGEGFWSVATYAET  LEVLRDPVTYSSVTGGQRRFGGTVLQDLPVAGQVLNMMDDPRHTRIRRLVSSGLTPRMIR  RVEDDLRRRARGLLDGVEPGAPFDFVVEIAAELPMQMICILLGVPETDRHWLFEAVEPGF  DFRGSRRATMPRLNVEDAGSRLYTYALELIAGKRAEPADDMLSVVANATIDDPDAPALSD  AELYLFFHLLFSAGAETTRNSIAGGLLALAENPDQLQTLRSDFELLPTAIEEIVRWTSPS  PSKRRTASRAVSLGGQPIEAGQKVVVWEGSANRDPSVFDRADEFDITRKPNPHLGFGQGV  HYCLGANLARLELRVLFEELLSRFGSVRVVEPAEWTRSNRHTGIRHLVVELRGG  >CYP132A1(Mb1429c)  MATATTQRPLKGPAKRMSTWTMTREAITIGFDAGDGFLGRLRGSDITRFRCAGRRFVSIS  HPDYVDHVLHEARLKYVKSDEYGPIRATAGLNLLTDEGDSWARHRGALNSTFARRHLRGL  VGLMIDPIADVTAALVPGAQFDMHQSMVETTLRVVANALFSQDFGPLVQSMHDLATRGLR  RAEKLERLGLWGLMPRTVYDTLIWCIYSGVHLPPPLREMQEITLTLDRAINSVIDRRLAE  PTNSADLLNVLLSADGGIWPRQRVRDEALTFMLAGHETTANAMSWFWYLMALNPQARDHM  LTELDDVLGMRRPTADDLGKLAWTTACLQESQRYFSSVWIIAREAVDDDIIDGHRIRRGT  TVVIPIHHIHHDPRWWPDPDRFDPGRFLRCPTDRPRCAYLPFGGGRRICIGQSFALMEMV  LMAAIMSQHFTFDLAPGYHVELEATLTLRPKHGVHVIGRRR  >CYP139A1(Mb1694c)  MRYPLGEALLALYRWRGPLINAGVGGHGYTYLLGAEANRFVFANADAFSWSQTFESLVPV  DGPTALIVSDGADHRRRRSVVAPGLRHHHVQRYVATMVSNIDTVIDGWQPGQRLDIYQEL  RSAVRRSTAESLFGQRLAVHSDFLGEQLQPLLDLTRRPPQVMRLQQRVNSPGWRRAMAAR  KRIDDLIDAQIADARTAPRPDDHMLTTLISGCSEEGTTLSDNEIRDSIVSLITAGYETTS  GALAWAIYALLTVPGTWESAASEVARVLGGRVPAADDLSALTYLNGVVHETLRLYSPGVI  SARRVLRDLWFDGHRIRAGRLLIFSAYVTHRLPEIWPEPTEFRPLRWDPNAADYRKPAPH  EFIPFSGGLHRCIGAVMATTEMTVILARLVARAMLQLPAQRTHRIRAANFAALRPWPGLT  VEIRKSAPAQ  >CYP144A1(Mb1806)  MRRSPKGSPGAVLDLQRRVDQAVSADHAELMTIAKDANTFFGAESVQDPYPLYERMRAAG  SVHRIANSDFYAVCGWDAVNEAIGRPEDFSSNLTATMTYTAEGTAKPFEMDPLGGPTHVL  ATADDPAHAVHRKLVLRHLAAKRIRVMEQFTVQAADRLWVDGMQDGCIEWMGAMANRLPM  MVVAELIGLPDPDIAQLVKWGYAATQLLEGLVENDQLVAAGVALMELSGYIFEQFDRAAA  DPRDNLLGELATACASGELDTLTAQVMMVTLFAAGGESTAALLGSAVWILATRPDIQQQV  RANPELLGAFIEETLRYEPPFRGQYRHVRNATTLDGTELPADSHLLLLWGAANRDPAQFE  APGEFRLDRAGGKGHISFGKGAHFCVGAALARLEARIVLRLLLDRTSVIEAADVGGWLPS  ILVRRIERLELAVQ  >CYP143A1(Mb1813c)  MTTPGEDHAGSFYLPRLEYSTLPMAVDRGVGWKTLRDAGPVVFMNGWYYLTRREDVLAAL  RNPKVFSSRKALQPPGNPLPVVPLAFDPPEHTRYRRILQPYFSPAALSKALPSLRRHTVA  MIDAIAGRGECEAMADLANLFPFQLFLVLYGLPLEDRDRLIGWKDAVIAMSDRPHPTEAD  VAAARELLEYLTAMVAERRRNPGPDVLSQVQIGEDPLSEIEVLGLSHLLILAGLDTVTAA  VGFSLLELARRPQLRAMLRDNPKQIRVFIEEIVRLEPSAPVAPRVTTEPVTVGGMTLPAG  SPVRLCMAAVNRDGSDAMSTDELVMDGKVHRHWGFGGGPHRCLGSHLARLELTLLVGEWL  NQIPDFELAPDYAPEIRFPSKSFALKNLPLRWS  >CYP140A1(Mb1912c) MKDKLHWLAMHGVIRGIAAIGIRRGDLQARLIADPAVATDPVPFYDEVRSHGALVRNRAN  YLTVDHRLAHDLLRSDDFRVVSFGENLPPPLRWLERRTRGDQLHPLREPSLLAVEPPDHT  RYRKTVSAVFTSRAVSALRDLVEQTAINLLDRFAEQPGIVDVVGRYCSQLPIVVISEILG  VPEHDRPRVLEFGELAAPSLDIGIPWRQYLRVQQGIRGFDCWLEGHLQQLRHAPGDDLMS  QLIQIAESGDNETQLDETELRAIAGLVLVAGFETTVNLLGNGIRMLLDTPEHLATLRQHP  ELWPNTVEEILRLDSPVQLTARVACRDVEVAGVRIKRGEVVVIYLAAANRDPAVFPDPHR  FDIERPNAGRHLAFSTGRHFCLGAALARAEGEVGLRTFFDRFPDVRAAGAGSRRDTRVLR  GWSTLPVTLGPARSMVSP  >CYP124A1(Mb2289)  MGLNTAIATRVNGTPPPEVPIADIELGSLDFWALDDDVRDGAFATLRREAPISFWPTIEL  PGFVAGNGHWALTKYDDVFYASRHPDIFSSYPNITINDQTPELAEYFGSMIVLDDPRHQR  LRSIVSRAFTPKVVARIEAAVRDRAHRLVSSMIANNPDRQADLVSELAGPLPLQIICDMM  GIPKADHQRIFHWTNVILGFGDPDLATDFDEFMQVSADIGAYATALAEDRRVNHHDDLTS  SLVEAEVDGERLSSREIASFFILLVVAGNETTRNAITHGVLALSRYPEQRDRWWSDFDGL  APTAVEEIVRWASPVVYMRRTLTQDIELRGTKMAAGDKVSLWYCSANRDESKFADPWTFD  LARNPNPHLGFGGGGAHFCLGANLARREIRVAFDELRRQMPDVVATEEPARLLSQFIHGI  KTLPVTWS  >CYP128A1(Mb2291c)  MTATQSPPEPAPDRVRLAGCPLAGTPDVGLTAQDATTALGVPTRRRASSGGIPVATSMWR  DAQTVRTYGPAVAKALALRVAGKARSRLTGRHCRKFMQLTDFDPFDPAIAADPYPHYREL  LAGERVQYNPKRDVYILSRYADVREAARNHDTLSSARGVTFSRGWLPFLPTSDPPAHTRM  RKQLAPGMARGALETWRPMVDQLARELVGGLLTQTPADVVSTVAAPMPMRAITSVLGVDG  PDEAAFCRLSNQAVRITDVALSASGLISLVQGFAGFRRLRALFTHRRDNGLLRECTVLGK  LATHAEQGRLSDDELFFFAVLLLVAGYESTAHMISTLFLTLADYPDQLTLLAQQPDLIPS  AIEEHLRFISPIQNICRTTRVDYSVGQAVIPAGSLVLLAWGAANRDPRQYEDPDVFRADR  NPVGHLAFGSGIHLCPGTQLARMEGQAILREIVANIDRIEVVEPPTWTTNANLRGLTRLR  VAVTPRVAP  >CYP121A1(Mb2299)  MTATVLLEVPFSARGDRIPDAVAELRTREPIRKVRTITGAEAWLVSSYALCTQVLEDRRF  SMKETAAAGAPRLNALTVPPEVVNNMGNIADAGLRKAVMKAITPKAPGLEQFLRDTANSL  LDNLITEGAPADLRNDFADPLATALHCKVLGIPQEDGPKLFRSLSIAFMSSADPIPAAKI  NWDRDIEYMAGILENPNITTGLMGELSRLRKDPAYSHVSDELFATIGVTFFGAGVISTGS  FLTTALISLIQRPQLRNLLHEKPELIPAGVEELLRINLSFADGLPRLATADIQVGDVLVR  KGELVLVLLEGANFDPEHFPNPGSIELDRPNPTSHLAFGRGQHFCPGSALGRRHAQIGIE  ALLKKMPGVDLAVPIDQLVWRTRFQRRIPERLPVLW  >CYP136A1(Mb3085)  MATIHPPAYLLDQAKRRFTPSFNNFPGMSLVEHMLLNTKFPEKKLAEPPPGSGLKPVVGD  AGLPILGHMIEMLRGGPDYLMFLYKTKGPVVFGDSAVLPGVAALGPDAAQVIYSNRNKDY  SQQGWVPVIGPFFHRGLMLLDFEEHMFHRRIMQEAFVRSRLAGYLEQMDRVVSRVVADDW  VVNDARFLVYPAMKALTLDIASMVFMGHEPGTDHELVTKVNKAFTITTRAGNAVIRTSVP  PFTWWRGLRARELLENYFTARVKERREASGNDLLTVLCQTEDDDGNRFSDADIVNHMIFL  MMAAHDTSTSTATTMAYQLAAHPEWQQRCRDESDRHGDGPLDIESLEQLESLDLVMNESI  RLVTPVQWAMRQTVRDTELLGYYLPKGTNVIAYPGMNHRLPEIWTDPLTFDPERFTEPRN  EHKRHRYAFTPFGGGVHKCIGMVFGQLEIKTILHRLLRRYRLELSRPDYQPRWDYSAMPI  PMDGMPIVLRPR  >CYP142A1bP(Mb3547c)  MSSEVDGERLSDDELVMETLLILIGGDETTRHTLSGGTEQLLRNRDQWDLLQRDPSLLPG  AIEEMLRWTAPVKNMCRVLTADTEFHGTALCAGEKMMLLFESANFDEAVFCEPEKFDVQR  NPNSHLAFGFGTHFCLGNQLARLELSLMTERVLRRLPDLRLVADDSVLPLRPANFVSGLE  SMPVVFTPSPPLG  >CYP142A1aP(Mb3548c)  MTEAPDVDLADGNFYASREARAAYRWMRANQPVFRDRNGLAAASTYQAVIDAERQPELFS  NAGGIRPDQPALPMMIDMDDPAHLLRRKLVNAGFTRKRVKDKEASIAALCDTLIDAVCER  GECDFVRDLAAPLPMAVIGDMLGVRPEQRDMFLRWSDDLVTFLSSHVSQEDFQITMDAFA  AYNDFTRATIAARRADPPTTWSACW  >CYP125A1(Mb3575c)  MSWNHQSVEIAVRRTTVPSPNLPPGFDFTDPAIYAERLPVAEFAELRSAAPIWWNGQDPG  KGGGFHDGGFWAITKLNDVKEISRHSDVFSSYENGVIPRFKNDIAREDIEVQRFVMLNMD  APHHTRLRKIISRGFTPRAVGRLHDELQERAQKIAAEAAAAGSGDFVEQVSCELPLQAIA  GLLGVPQEDRGKLFHWSNEMTGNEDPEYAHIDPKASSAELIGYAMKMAEEKAKNPADDIV  TQLIQADIDGEKLSDDEFGFFVVMLAVAGNETTRNSITQGMMAFAEHPDQWELYKKVRPE  TAADEIVRWATPVTAFQRTALRDYELSGVQIKKGQRVVMFYRSANFDEEVFQDPFTFNIL  RNPNPHVGFGGTGAHYCIGANLARMTINLIFNAVADHMPDLKPISAPERLRSGWLNGIKH  WQVDYTGRCPVAH  >CYP137A1(Mb3710c)  MVLRSLASPAALTDPKRCASVVGVAAFAVRREHAPDALGGPPGLPAPRGFRAAFAAAYAV  AYLAGGERRMLRLIRRYGPIMTMPILSLGDVAIVSDSALAKEVFTAPTDVLLGGEGVGPA  AAIYGSGSMFVQEEPQHLRRRKLLTPPLHGAALDRYVPIIENSTRAAMHTWPVDRPFAML  TVARSLMLDVIVKVIFGVDDPEEVRRLGRPFERLLNLGVSEQLTVRYALRRLGALRVWPA  RARANTEIDDVVMALIAQRRADPRLGERHDVLSLLVSARGESGEQLSDSEIRDDLITLVL  AGHETTATTLAWAFDLLLHHPDALRRVRAEAVGGGEAFTTAVINETLRVRPPAPLTARVA  AQPLTIGGYRVEAGTRIVVHIIAINRSAEVYEHPHEFRPERFLGTRPQTYAWVPFGGGVK  RCLGANFSMRELITVLHVLLREGEFTAVDDEPERIVRRSIMLVPRRGTRVRFRPAR |
| ***Mycobacterium bovis* BCG Pasteur 1173P2** |
| Database: KEGG; P450 count:18; Families: 17; Subfamilies: 18 |
| >CYP137A1(BCG_3744c)  MVLRSLASPAALTDPKRCASVVGVAAFAVRREHAPDALGGPPGLPAPRGFRAAFAAAYAV  AYLAGGERRMLRLIRRYGPIMTMPILSLGDVAIVSDSALAKEVFTAPTDVLLGGEGVGPA  AAIYGSGSMFVQEEPQHLRRRKLLTPPLHGAALDRYVPIIENSTRAAMHTWPVDRPFAML  TVARSLMLDVIVKVIFGVDDPEEVRRLGRPFERLLNLGVSEQLTVRYALRRLGALRVWPA  RARANTEIDDVVMALIAQRRADPRLGERHDVLSLLVSARGESGEQLSDSEIRDDLITLVL  AGHETTATTLAWAFDLLLHHPDALRRVRAEAVGGGEAFTTAVINETLRVRPPAPLTARVA  AQPLTIGGYRVEAGTRIVVHIIAINRSAEVYEHPHEFRPERFLGTRPQTYAWVPFGGGVK  RCLGANFSMRELITVLHVLLREGEFTAVDDEPERIVRRSIMLVPRRGTRVRFRPAR  >CYP142A1bP(BCG_3581c)  MSSEVDGERLSDDELVMETLLILIGGDETTRHTLSGGTEQLLRNRDQWDLLQRDPSLLPG  AIEEMLRWTAPVKNMCRVLTADTEFHGTALCAGEKMMLLFESANFDEAVFCEPEKFDVQR  NPNSHLAFGFGTHFCLGNQLARLELSLMTERVLRRLPDLRLVADDSVLPLRPANFVSGLE  SMPVVFTPSPPLG  >CYP142A1aP(BCG_3582c)  MTEAPDVDLADGNFYASREARAAYRWMRANQPVFRDRNGLAAASTYQAVIDAERQPELFS  NAGGIRPDQPALPMMIDMDDPAHLLRRKLVNAGFTRKRVKDKEASIAALCDTLIDAVCER  GECDFVRDLAAPLPMAVIGDMLGVRPEQRDMFLRWSDDLVTFLSSHVSQEDFQITMDAFA  AYNDFTRATIAARRADPPTTWSACW  >CYP126A1(BCG_0830)  MTTAAGLSGIDLTDLDNFADGFPHHLFAIHRREAPVYWHRPTEHTPDGEGFWSVATYAET  LEVLRDPVTYSSVTGGQRRFGGTVLQDLPVAGQVLNMMDDPRHTRIRRLVSSGLTPRMIR  RVEDDLRRRARGLLDGVEPGAPFDFVVEIAAELPMQMICILLGVPETDRHWLFEAVEPGF  DFRGSRRATMPRLNVEDAGSRLYTYALELIAGKRAEPADDMLSVVANATIDDPDAPALSD  AELYLFFHLLFSAGAETTRNSIAGGLLALAENPDQLQTLRSDFELLPTAIEEIVRWTSPS  PSKRRTASRAVSLGGQPIEAGQKVVVWEGSANRGPSVFDRADEFDITRKPNPHLGFGQGV  HYCLGANLARLELRVLFEELLSRFGSVRVVEPAEWTRSNRHTGIRHLVVELRGG  >CYP123A1(BCG_0818c)  MTVRVGDPELVLDPYDYDFHEDPYPYYRRLRDEAPLYRNEERNFWAVSRHHDVLQGFRDS  TALSNAYGVSLDPSSRTSEAYRVMSMLAMDDPAHLRMRTLVSKGFTPRRIRELEPQVLEL  ARIHLDSALQTESFDFVAEFAGKLPMDVISELIGVPDTDRARIRALADAVLHREDGVADV  PPPAMAASIELMRYYADLIAEFRRRPANNLTSALLAAELDGDRLSDQEIMAFLFLMVIAG  NETTTKLLANAVYWAAHHPGQLARVFADHSRIPMWVEETLRYDTSSQILARTVAHDLTLY  DTTIPEGEVLLLLPGSANRDDRVFDDPDDYRIGREIGCKLVSFGSGAHFCLGLTWPGWKP  GWPWARCCVGSATTKSTTTTSCASIPATCADLPICRSACRPGNATLRTSPRPADHRRRRR  IVGHRRGHRNRTRRPRVSGRLGGPPHGQVGRAGRQNPRRRWRGGSLPPRRDRSRVGEIVC  GANGRGTRRGRTAGVQCRRHAPGTAARGQHRGLCRAGSDTPGRCQPAGHGRATGHGGTPA  R  >CYP138A1(BCG_0172)  MSEVVTAAPAPPVVRLPPAVRGPKLFQGLAFVVSRRRLLGRFVRRYGKAFTANILMYGRV  VVVADPQLARQVFTSSPEELGNIQPNLSRMFGSGSVFALDGDDHRRRRRLLAPPFHGKSM  KNYETIIEEETLRETANWPQGQAFATLPSMMHITLNAILRAIFGAGGSELDELRRLIPPW  VTLGSRLAALPKPKRDYGRLSPWGRLAEWRRQYDTVIDKLIEAERADPNFADRTDVLALM  LRSTYDDGSIMSRKDIGDELLTLLAAGHETTAATLGWAFERLSRHPDVLAALVEEVDNGG  HELRQAAILEVQRARTVIDFAARRVNPPVYQLGEWVIPRGYSIIINIAQIHGDPDVFPQP  DRFDPQRYIGSKPSPFAWIPFGGGTRRCVGAAFANMEMDVVLRTVLRHFTLETTTAAGER  SHGRGVAFTPKDGGRVVMRRR  >CYP135A1(BCG_0366c)  MASTLTTGLPPGPRLPRYLQSVLYLRFREWFLPAMHRKYGDVFSLRVPPYADNLVVYTRP  EHIKEIFAADPRSLHAGEGNHILGFVMGEHSVLMTDEAEYARMRSLLMPAFTRAALRGYR  DMIASVAREHITRWRPHATINSLDHMNALTLDIILRVVFGVTDPKVKAELTSRLQQIINI  HPAILAGVPYPSLKRMNPWKRFFHNQTKIDEILYREIASRRIDSDLTARTDVLSRLLQTK  DTPTKPLTDAELRDQLITLLLAGHETTAAALSWTLWELAHAPEIQSQVVWAAVGGDDGFL  EAVLKEGMRRHTVIASTARKVTAPAEIGGWRLPAGTVVNTSILLAHASEVSHPKPTEFRP  SRFLDGSVAPNTWLPFGGGVRRCLGFGFALTEGAVILQEIFRRFTITAAGPSKGETPLVR  NITTVPKHGAHLRLIPQRRLGGLGDSDPP  >CYP135B1(BCG_0613)  MSGTSSMGLPPGPRLSGSVQAVLMLRHGLRFLTACQRRYGSVFTLHVAGFGHMVYLSDPA  AIKTVFAGNPSVFHAGEANSMLAGLLGDSSLLLIDDDVHRDRRRLMSPPFHRDAVARQAE  PIAEIAAANIAGWPMAKAFAVAPKMSEITLEVILRTVIGASDPVRLAALRKVMPRLLNVG  PWATLALANPSLLNNRLWSRLRRRIEEADALLYAEIADRRADPDLAARTDTLAMLVRAAD  EDGRTMTERELRDQLITLLVAGHDTTATGLSWALERLTRHPVTLAKAVQAADASAAGDPA  GDEYLDAVAKETLRIRPVVYDVGRVLTEAVEVAGYRLPAGVMVVPAIGLVHASAQLYPDP  ERFDPDRMVGATLSPTTWLPFGGGNRRCLGATFAMVEMRVVLREILRRVELSTTTTSGER  PKLKHVIMVPHRGARIRVRATRDVSATSQATAQGAGCPAARGGGPSRAVGSQ  >CYP51B(BCG_0816c)  MSAVALPRVSGGHDEHGHLEEFRTDPIGLMQRVRDECGDVGTFQLAGKQVVLLSGSHANE  FFFRAGDDDLDQAKAYPFMTPIFGEGVVFDASPERRKEMLHNAALRGEQMKGHAATIEDQ  VRRMIADWGEAGEIDLLDFFAELTIYTSSACLIGKKFRDQLDGRFAKLYHELERGTDPLA  YVDPYLPIESFRRRDEARNGLVALVADIMNGRIANPPTDKSDRDMLDVLIAVKAETGTPR  FSADEITGMFISMMFAGHHTSSGTASWTLIELMRHRDAYAAVIDELDELYGDGRSVSFHA  LRQIPQLENVLKETLRLHPPLIILMRVAKGEFEVQGHRIHEGDLVAASPAISNRIPEDFP  DPHDFVPARYEQPRQEDLLNRWTWIPFGAGRHRCVGAAFAIMQIKAIFSVLLREYEFEMA  QPPESYRNDHSKMVVQLAQPACVRYRRRTGV  >CYP132A1(BCG_1455c)  MATATTQRPLKGPAKRMSTWTMTREAITIGFDAGDGFLGRLRGSDITRFRCAGRRFVSIS  HPDYVDHVLHEARLKYVKSDEYGPIRATAGLNLLTDEGDSWARHRGALNSTFARRHLRGL  VGLMIDPIADVTAALVPGAQFDMHQSMVETTLRVVANALFSQDFGPLVQSMHDLATRGLR  RAEKLERLGLWGLMPRTVYDTLIWCIYSGVHLPPPLREMQEITLTLDRAINSVIDRRLAE  PTNSADLLNVLLSADGGIWPRQRVRDEALTFMLAGHETTANAMSWFWYLMALNPQARDHM  LTELDDVLGMRRPTADDLGKLAWTTACLQESQRYFSSVWIIAREAVDDDIIDGHRIRRGT  TVVIPIHHIHHDPRWWPDPDRFDPGRFLRCPTDRPRCAYLPFGGGRRICIGQSFALMEMV  LMAAIMSQHFTFDLAPGYHVELEATLTLRPKHGVHVIGRRR  >CYP139A1(BCG_1705c)  MRYPLGEALLALYRWRGPLINAGVGGHGYTYLLGAEANRFVFANADAFSWSQTFESLVPV  DGPTALIVSDGADHRRRRSVVAPGLRHHHVQRYVATMVSNIDTVIDGWQPGQRLDIYQEL  RSAVRRSTAESLFGQRLAVHSDFLGEQLQPLLDLTRRPPQVMRLQQRVNSPGWRRAMAAR  KRIDDLIDAQIADARTAPRPDDHMLTTLISGCSEEGTTLSDNEIRDSIVSLITAGYETTS  GALAWAIYALLTVPGTWESAASEVARVLGGRVPAADDLSALTYLNGVVHETLRLYSPGVI  SARRVLRDLWFDGHRIRAGRLLIFSAYVTHRLPEIWPEPTEFRPLRWDPNAADYRKPAPH  EFIPFSGGLHRCIGAVMATTEMTVILARLVARAMLQLPAQRTHRIRAANFAALRPWPGLT  VEIRKSAPAQ  >CYP144A1(BCG_1810)  MRRSPKGSPGAVLDLQRRVDQAVSADHAELMTIAKDANTFFGAESVQDPYPLYERMRAAG  SVHRIANSDFYAVCGWDAVNEAIGRPEDFSSNLTATMTYTAEGTAKPFEMDPLGGPTHVL  ATADDPAHAVHRKLVLRHLAAKRIRVMEQFTVQAADRLWVDGMQDGCIEWMGAMANRLPM  MVVAELIGLPDPDIAQLVKWGYAATQLLEGLVENDQLVAAGVALMELSGYIFEQFDRAAA  DPRDNLLGELATACASGELDTLTAQVMMVTLFAAGGESTAALLGSAVWILATRPDIQQQV  RANPELLGAFIEETLRYEPPFRGQYRHVRNATTLDGTELPADSHLLLLWGAANRDPAQFE  APGEFRLDRAGGKGHISFGKGAHFCVGAALARLEARIVLRLLLDRTSVIEAADVGGWLPS  ILVRRIERLELAVQ  >CYP143A1(BCG_1817c)  MTTPGEDHAGSFYLPRLEYSTLPMAVDRGVGWKTLRDAGPVVFMNGWYYLTRREDVLAAL  RNPKVFSSRKALQPPGNPLPVVPLAFDPPEHTRYRRILQPYFSPAALSKALPSLRRHTVA  MIDAIAGRGECEAMADLANLFPFQLFLVLYGLPLEDRDRLIGWKDAVIAMSDRPHPTEAD  VAAARELLEYLTAMVAERRRNPGPDVLSQVQIGEDPLSEIEVLGLSHLLILAGLDTVTAA  VGFSLLELARRPQLRAMLRDNPKQIRVFIEEIVRLEPSAPVAPRVTTEPVTVGGMTLPAG  SPVRLCMAAVNRDGSDAMSTDELVMDGKVHRHWGFGGGPHRCLGSHLARLELTLLVGEWL  NQIPDFELAPDYAPEIRFPSKSFALKNLPLRWS  >CYP140A1(BCG_1917c)  MKDKLHWLAMHGVIRGIAAIGIRRGDLQARLIADPAVATDPVPFYDEVRSHGALVRNRAN  YLTVDHRLAHDLLRSDDFRVVSFGENLPPPLRWLERRTRGDQLHPLREPSLLAVEPPDHT  RYRKTVSAVFTSRAVSALRDLVEQTAINLLDRFAEQPGIVDVVGRYCSQLPIVVISEILG  VPEHDRPRVLEFGELAAPSLDIGIPWRQYLRVQQGIRGFDCWLEGHLQQLRHAPGDDLMS  QLIQIAESGDNETQLDETELRAIAGLVLVAGFETTVNLLGNGIRMLLDTPEHLATLRQHP  ELWPNTVEEILRLDSPVQLTARVACRDVEVAGVRIKRGEVVVIYLAAANRDPAVFPDPHR  FDIERPNAGRHLAFSTGRHFCLGAALARAEGEVGLRTFFDRFPDVRAAGAGSRRDTRVLR  GWSTLPVTLGPARSMVSP  >CYP124A1(BCG_2283)  MGLNTAIATRVNGTPPPEVPIADIELGSLDFWALDDDVRDGAFATLRREAPISFWPTIEL  PGFVAGNGHWALTKYDDVFYASRHPDIFSSYPNITINDQTPELAEYFGSMIVLDDPRHQR  LRSIVSRAFTPKVVARIEAAVRDRAHRLVSSMIANNPDRQADLVSELAGPLPLQIICDMM  GIPKADHQRIFHWTNVILGFGDPDLATDFDEFMQVSADIGAYATALAEDRRVNHHDDLTS  SLVEAEVDGERLSSREIASFFILLVVAGNETTRNAITHGVLALSRYPEQRDRWWSDFDGL  APTAVEEIVRWASPVVYMRRTLTQDIELRGTKMAAGDKVSLWYCSANRDESKFADPWTFD  LARNPNPHLGFGGGGAHFCLGANLARREIRVAFDELRRQMPDVVATEEPARLLSQFIHGI  KTLPVTWS  >CYP128A1(BCG_2285c)  MTATQSPPEPAPDRVRLAGCPLAGTPDVGLTAQDATTALGVPTRRRASSGGIPVATSMWR  DAQTVRTYGPAVAKALALRVAGKARSRLTGRHCRKFMQLTDFDPFDPAIAADPYPHYREL  LAGERVQYNPKRDVYILSRYADVREAARNHDTLSSARGVTFSRGWLPFLPTSDPPAHTRM  RKQLAPGMARGALETWRPMVDQFARELVGGLLTQTPADVVSTVAAPMPMRAITSVLGVDG  PDEAAFCRLSNQAVRITDVALSASGLISLVQGFAGFRRLRALFTHRRDNGLLRECTVLGK  LATHAEQGRLSDDELFFFAVLLLVAGYESTAHMISTLFLTLADYPDQLTLLAQQPDLIPS  AIEEHLRFISPIQNICRTTRVDYSVGQAVIPAGSLVLLAWGAANRDPRQYEDPDVFRADR  NPVGHLAFGSGIHLCPGTQLARMEGQAILREIVANIDRIEVVEPPTWTTNANLRGLTRLR  VAVTPRVAP  >CYP121A1(BCG_2293)  MTATVLLEVPFSARGDRIPDAVAELRTREPIRKVRTITGAEAWLVSSYALCTQVLEDRRF  SMKETAAAGAPRLNALTVPPEVVNNMGNIADAGLRKAVMKAITPKAPGLEQFLRDTANSL  LDNLITEGAPADLRNDFADPLATALHCKVLGIPQEDGPKLFRSLSIAFMSSADPIPAAKI  NWDRDIEYMAGILENPNITTGLMGELSRLRKDPAYSHVSDELFATIGVTFFGAGVISTGS  FLTTALISLIQRPQLRNLLHEKPELIPAGVEELLRINLSFADGLPRLATADIQVGDVLVR  KGELVLVLLEGANFDPEHFPNPGSIELDRPNPTSHLAFGRGQHFCPGSALGRRHAQIGIE  ALLKKMPGVDLAVPIDQLVWRTRFQRRIPERLPVLW  >CYP136A1(BCG_3084)  MATIHPPAYLLDQAKRRFTPSFNNFPGMSLVEHMLLNTKFPEKELAEPPPGSGLKPVVGD  AGLPILGHMIEMLRGGPDYLMFLYKTKGPVVFGDSAVLPGVAALGPDAAQVIYSNRNKDY  SQQGWVPVIGPFFHRGLMLLDFEEHMFHRRIMQEAFVRSRLAGYLEQMDRVVSRVVADDW  VVNDARFLVYPAMKALTLDIASMVFMGHEPGTDHELVTKVNKAFTITTRAGNAVIRTSVP  PFTWWRGLRARELLENYFTARVKERREASGNDLLTVLCQTEDDDGNRFSDADIVNHMIFL  MMAAHDTSTSTATTMAYQLAAHPEWQQRCRDESDRHGDGPLDIESLEQLESLDLVMNESI  RLVTPVQWAMRQTVRDTELLGYYLPKGTNVIAYPGMNHRLPEIWTDPLTFDPERFTEPRN  EHKRHRYAFTPFGGGVHKCIGMVFGQLEIKTILHRLLRRYRLELSRPDYQPRWDYSAMPI  PMDGMPIVLRPR  >CYP125A1(BCG_3609c)  MSWNHQSVEIAVRRTTVPSPNLPPGFDFTDPAIYAERLPVAEFAELRSAAPIWWNGQDPG  KGGGFHDGGFWAITKLNDVKEISRHSDVFSSYENGVIPRFKNDIAREDIEVQRFVMLNMD  APHHTRLRKIISRGFTPRAVGRLHDELQERAQKIAAEAAAAGSGDFVEQVSCELPLQAIA  GLLGVPQEDRGKLFHWSNEMTGNEDPEYAHIDPKASSAELIGYAMKMAEEKAKNPADDIV  TQLIQADIDGEKLSDDEFGFFVVMLAVAGNETTRNSITQGMMAFAEHPDQWELYKKVRPE  TAADEIVRWATPVTAFQRTALRDYELSGVQIKKGQRVVMFYRSANFDEEVFQDPFTFNIL  RNPNPHVGFGGTGAHYCIGANLARMTINLIFNAVADHMPDLKPISAPERLRSGWLNGIKH  WQVDYTGRCPVAH |
| ***Mycobacterium bovis* BCG Korea 1168P** |
| DATABASE: KEGG; P450 count: 18; Families:17; Subfamilies: 18 |
| >CYP138A1(K60_001520)  MSEVVTAAPAPPVVRLPPAVRGPKLFQGLAFVVSRRRLLGRFVRRYGKAFTANILMYGRV VVVADPQLARQVFTSSPEELGNIQPNLSRMFGSGSVFALDGDDHRRRRRLLAPPFHGKSM KNYETIIEEETLRETANWPQGQAFATLPSMMHITLNAILRAIFGAGGSELDELRRLIPPW VTLGSRLAALPKPKRDYGRLSPWGRLAEWRRQYDTVIDKLIEAERADPNFADRTDVLALM LRSTYDDGSIMSRKDIGDELLTLLAAGHETTAATLGWAFERLSRHPDVLAALVEEVDNGG HELRQAAILEVQRARTVIDFAARRVNPPVYQLGEWVIPRGYSIIINIAQIHGDPDVFPQP DRFDPQRYIGSKPSPFAWIPFGGGTRRCVGAAFANMEMDVVLRTVLRHFTLETTTAAGER SHGRGVAFTPKDGGRVVMRRR  >CYP135A1(K60_003470)  MASTLTTGLPPGPRLPRYLQSVLYLRFREWFLPAMHRKYGDVFSLRVPPYADNLVVYTRP EHIKEIFAADPRSLHAGEGNHILGFVMGEHSVLMTDEAEYARMRSLLMPAFTRAALRGYR DMIASVAREHITRWRPHATINSLDHMNALTLDIILRVVFGVTDPKVKAELTSRLQQIINI HPAILAGVPYPSLKRMNPWKRFFHNQTKIDEILYREIASRRIDSDLTARTDVLSRLLQTK DTPTKPLTDAELRDQLITLLLAGHETTAAALSWTLWELAHAPEIQSQVVWAAVGGDDGFL EAVLKEGMRRHTVIASTARKVTAPAEIGGWRLPAGTVVNTSILLAHASEVSHPKPTEFRP SRFLDGSVAPNTWLPFGGGVRRCLGFGFALTEGAVILQEIFRRFTITAAGPSKGETPLVR NITTVPKHGAHLRLIPQRRLGGLGDSDPP  >CYP135B1(K60_006020)  MSGTSSMGLPPGPRLSGSVQAVLMLRHGLRFLTACQRRYGSVFTLHVAGFGHMVYLSDPA AIKTVFAGNPSVFHAGEANSMLAGLLGDSSLLLIDDDVHRDRRRLMSPPFHRDAVARQAE PIAEIAAANIAGWPMAKAFAVAPKMSEITLEVILRTVIGASDPVRLAALRKVMPRLLNVG PWATLALANPSLLNNRLWSRLRRRIEEADALLYAEIADRRADPDLAARTDTLAMLVRAAD EDGRTMTERELRDQLITLLVAGHDTTATGLSWALERLTRHPVTLAKAVQAADASAAGDPA GDEYLDAVAKETLRIRPVVYDVGRVLTEAVEVAGYRLPAGVMVVPAIGLVHASAQLYPDP ERFDPDRMVGATLSPTTWLPFGGGNRRCLGATFAMVEMRVVLREILRRVELSTTTTSGER PKLKHVIMVPHRGARIRVRATRDVSATSQATAQGAGCPAARGGGPSRAVGSQ  >CYP51B1(K60_008170)  MSAVALPRVSGGHDEHGHLEEFRTDPIGLMQRVRDECGDVGTFQLAGKQVVLLSGSHANE FFFRAGDDDLDQAKAYPFMTPIFGEGVVFDASPERRKEMLHNAALRGEQMKGHAATIEDQ VRRMIADWGEAGEIDLLDFFAELTIYTSSACLIGKKFRDQLDGRFAKLYHELERGTDPLA YVDPYLPIESFRRRDEARNGLVALVADIMNGRIANPPTDKSDRDMLDVLIAVKAETGTPR FSADEITGMFISMMFAGHHTSSGTASWTLIELMRHRDAYAAVIDELDELYGDGRSVSFHA LRQIPQLENVLKETLRLHPPLIILMRVAKGEFEVQGHRIHEGDLVAASPAISNRIPEDFP DPHDFVPARYEQPRQEDLLNRWTWIPFGAGRHRCVGAAFAIMQIKAIFSVLLREYEFEMA QPPESYRNDHSKMVVQLAQPACVRYRRRTGV  >CYP123A1(K60_008190)  MTVRVGDPELVLDPYDYDFHEDPYPYYRRLRDEAPLYRNEERNFWAVSRHHDVLQGFRDS TALSNAYGVSLDPSSRTSEAYRVMSMLAMDDPAHLRMRTLVSKGFTPRRIRELEPQVLEL ARIHLDSALQTESFDFVAEFAGKLPMDVISELIGVPDTDRARIRALADAVLHREDGVADV PPPAMAASIELMRYYADLIAEFRRRPANNLTSALLAAELDGDRLSDQEIMAFLFLMVIAG NETTTKLLANAVYWAAHHPGQLARVFADHSRIPMWVEETLRYDTSSQILARTVAHDLTLY DTTIPEGEVLLLLPGSANRDDRVFDDPDDYRIGREIGCKLVSFGSGAHFCLGLTWPGWKP GWPWARCCVGSATTKSTTTTSCASIPATCADLPICRSACRPGNATLRTSPRPADHRRRRR IVGHRRGHRNRTRRPRVSGRLGGPPHGQVGRAGRQNPRRRWRGGSLPPRRDRSRVGEIVC GANGRGTRRGRTAGVQCRRHAPGTAARGQHRGLCRAGSDTPGRCQPAGHGRATGHGGTPA R  >CYP126A1(K60_008310)  MTTAAGLSGIDLTDLDNFADGFPHHLFAIHRREAPVYWHRPTEHTPDGEGFWSVATYAET LEVLRDPVTYSSVTGGQRRFGGTVLQDLPVAGQVLNMMDDPRHTRIRRLVSSGLTPRMIR RVEDDLRRRARGLLDGVEPGAPFDFVVEIAAELPMQMICILLGVPETDRHWLFEAVEPGF DFRGSRRATMPRLNVEDAGSRLYTYALELIAGKRAEPADDMLSVVANATIDDPDAPALSD AELYLFFHLLFSAGAETTRNSIAGGLLALAENPDQLQTLRSDFELLPTAIEEIVRWTSPS PSKRRTASRAVSLGGQPIEAGQKVVVWEGSANRGPSVFDRADEFDITRKPNPHLGFGQGV HYCLGANLARLELRVLFEELLSRFGSVRVVEPAEWTRSNRHTGIRHLVVELRGG  >CYP132A1(K60_014970)  MATATTQRPLKGPAKRMSTWTMTREAITIGFDAGDGFLGRLRGSDITRFRCAGRRFVSIS HPDYVDHVLHEARLKYVKSDEYGPIRATAGLNLLTDEGDSWARHRGALNSTFARRHLRGL VGLMIDPIADVTAALVPGAQFDMHQSMVETTLRVVANALFSQDFGPLVQSMHDLATRGLR RAEKLERLGLWGLMPRTVYDTLIWCIYSGVHLPPPLREMQEITLTLDRAINSVIDRRLAE PTNSADLLNVLLSADGGIWPRQRVRDEALTFMLAGHETTANAMSWFWYLMALNPQARDHM LTELDDVLGMRRPTADDLGKLAWTTACLQESQRYFSSVWIIAREAVDDDIIDGHRIRRGT TVVIPIHHIHHDPRWWPDPDRFDPGRFLRCPTDRPRCAYLPFGGGRRICIGQSFALMEMV LMAAIMSQHFTFDLAPGYHVELEATLTLRPKHGVHVIGRRR  >CYP139A1(K60_017510)  MRTYRTVRYPLGEALLALYRWRGPLINAGVGGHGYTYLLGAEANRFVFANADAFSWSQTF ESLVPVDGPTALIVSDGADHRRRRSVVAPGLRHHHVQRYVATMVSNIDTVIDGWQPGQRL DIYQELRSAVRRSTAESLFGQRLAVHSDFLGEQLQPLLDLTRRPPQVMRLQQRVNSPGWR RAMAARKRIDDLIDAQIADARTAPRPDDHMLTTLISGCSEEGTTLSDNEIRDSIVSLITA GYETTSGALAWAIYALLTVPGTWESAASEVARVLGGRVPAADDLSALTYLNGVVHETLRL YSPGVISARRVLRDLWFDGHRIRAGRLLIFSAYVTHRLPEIWPEPTEFRPLRWDPNAADY RKPAPHEFIPFSGGLHRCIGAVMATTEMTVILARLVARAMLQLPAQRTHRIRAANFAALR PWPGLTVEIRKSAPAQ  >CYP144A1(K60_018620)  MRRSPKGSPGAVLDLQRRVDQAVSADHAELMTIAKDANTFFGAESVQDPYPLYERMRAAG SVHRIANSDFYAVCGWDAVNEAIGRPEDFSSNLTATMTYTAEGTAKPFEMDPLGGPTHVL ATADDPAHAVHRKLVLRHLAAKRIRVMEQFTVQAADRLWVDGMQDGCIEWMGAMANRLPM MVVAELIGLPDPDIAQLVKWGYAATQLLEGLVENDQLVAAGVALMELSGYIFEQFDRAAA DPRDNLLGELATACASGELDTLTAQVMMVTLFAAGGESTAALLGSAVWILATRPDIQQQV RANPELLGAFIEETLRYEPPFRGQYRHVRNATTLDGTELPADSHLLLLWGAANRDPAQFE APGEFRLDRAGGKGHISFGKGAHFCVGAALARLEARIVLRLLLDRTSVIEAADVGGWLPS ILVRRIERLELAVQ  >CYP143A1(K60_018700)  MVGARPRAILARSAPLGYVCDLRQERRHERLERMTTPGEDHAGSFYLPRLEYSTLPMAVD RGVGWKTLRDAGPVVFMNGWYYLTRREDVLAALRNPKVFSSRKALQPPGNPLPVVPLAFD PPEHTRYRRILQPYFSPAALSKALPSLRRHTVAMIDAIAGRGECEAMADLANLFPFQLFL VLYGLPLEDRDRLIGWKDAVIAMSDRPHPTEADVAAARELLEYLTAMVAERRRNPGPDVL SQVQIGEDPLSEIEVLGLSHLLILAGLDTVTAAVGFSLLELARRPQLRAMLRDNPKQIRV FIEEIVRLEPSAPVAPRVTTEPVTVGGMTLPAGSPVRLCMAAVNRDGSDAMSTDELVMDG KVHRHWGFGGGPHRCLGSHLARLELTLLVGEWLNQIPDFELAPDYAPEIRFPSKSFALKN LPLRWS  >CYP140A1(K60_019690)  MKDKLHWLAMHGVIRGIAAIGIRRGDLQARLIADPAVATDPVPFYDEVRSHGALVRNRAN YLTVDHRLAHDLLRSDDFRVVSFGENLPPPLRWLERRTRGDQLHPLREPSLLAVEPPDHT RYRKTVSAVFTSRAVSALRDLVEQTAINLLDRFAEQPGIVDVVGRYCSQLPIVVISEILG VPEHDRPRVLEFGELAAPSLDIGIPWRQYLRVQQGIRGFDCWLEGHLQQLRHAPGDDLMS QLIQIAESGDNETQLDETELRAIAGLVLVAGFETTVNLLGNGIRMLLDTPEHLATLRQHP ELWPNTVEEILRLDSPVQLTARVACRDVEVAGVRIKRGEVVVIYLAAANRDPAVFPDPHR FDIERPNAGRHLAFSTGRHFCLGAALARAEGEVGLRTFFDRFPDVRAAGAGSRRDTRVLR GWSTLPVTLGPARSMVSP  >CYP124A1(K60_023510)  MGLNTAIATRVNGTPPPEVPIADIELGSLDFWALDDDVRDGAFATLRREAPISFWPTIEL PGFVAGNGHWALTKYDDVFYASRHPDIFSSYPNITINDQTPELAEYFGSMIVLDDPRHQR LRSIVSRAFTPKVVARIEAAVRDRAHRLVSSMIANNPDRQADLVSELAGPLPLQIICDMM GIPKADHQRIFHWTNVILGFGDPDLATDFDEFMQVSADIGAYATALAEDRRVNHHDDLTS SLVEAEVDGERLSSREIASFFILLVVAGNETTRNAITHGVLALSRYPEQRDRWWSDFDGL APTAVEEIVRWASPVVYMRRTLTQDIELRGTKMAAGDKVSLWYCSANRDESKFADPWTFD LARNPNPHLGFGGGGAHFCLGANLARREIRVAFDELRRQMPDVVATEEPARLLSQFIHGI KTLPVTWS  >CYP128A1(K60_023530)  MTATQSPPEPAPDRVRLAGCPLAGTPDVGLTAQDATTALGVPTRRRASSGGIPVATSMWR DAQTVRTYGPAVAKALALRVAGKARSRLTGRHCRKFMQLTDFDPFDPAIAADPYPHYREL LAGERVQYNPKRDVYILSRYADVREAARNHDTLSSARGVTFSRGWLPFLPTSDPPAHTRM RKQLAPGMARGALETWRPMVDQFARELVGGLLTQTPADVVSTVAAPMPMRAITSVLGVDG PDEAAFCRLSNQAVRITDVALSASGLISLVQGFAGFRRLRALFTHRRDNGLLRECTVLGK LATHAEQGRLSDDELFFFAVLLLVAGYESTAHMISTLFLTLADYPDQLTLLAQQPDLIPS AIEEHLRFISPIQNICRTTRVDYSVGQAVIPAGSLVLLAWGAANRDPRQYEDPDVFRADR NPVGHLAFGSGIHLCPGTQLARMEGQAILREIVANIDRIEVVEPPTWTTNANLRGLTRLR VAVTPRVAP  >CYP121A1(K60_023610)  MTATVLLEVPFSARGDRIPDAVAELRTREPIRKVRTITGAEAWLVSSYALCTQVLEDRRF SMKETAAAGAPRLNALTVPPEVVNNMGNIADAGLRKAVMKAITPKAPGLEQFLRDTANSL LDNLITEGAPADLRNDFADPLATALHCKVLGIPQEDGPKLFRSLSIAFMSSADPIPAAKI NWDRDIEYMAGILENPNITTGLMGELSRLRKDPAYSHVSDELFATIGVTFFGAGVISTGS FLTTALISLIQRPQLRNLLHEKPELIPAGVEELLRINLSFADGLPRLATADIQVGDVLVR KGELVLVLLEGANFDPEHFPNPGSIELDRPNPTSHLAFGRGQHFCPGSALGRRHAQIGIE ALLKKMPGVDLAVPIDQLVWRTRFQRRIPERLPVLW  >CYP136A1(K60_031730)  MATIHPPAYLLDQAKRRFTPSFNNFPGMSLVEHMLLNTKFPEKELAEPPPGSGLKPVVGD AGLPILGHMIEMLRGGPDYLMFLYKTKGPVVFGDSAVLPGVAALGPDAAQVIYSNRNKDY SQQGWVPVIGPFFHRGLMLLDFEEHMFHRRIMQEAFVRSRLAGYLEQMDRVVSRVVADDW VVNDARFLVYPAMKALTLDIASMVFMGHEPGTDHELVTKVNKAFTITTRAGNAVIRTSVP PFTWWRGLRARELLENYFTARVKERREASGNDLLTVLCQTEDDDGNRFSDADIVNHMIFL MMAAHDTSTSTATTMAYQLAAHPEWQQRCRDESDRHGDGPLDIESLEQLESLDLVMNESI RLVTPVQWAMRQTVRDTELLGYYLPKGTNVIAYPGMNHRLPEIWTDPLTFDPERFTEPRN EHKRHRYAFTPFGGGVHKCIGMVFGQLEIKTILHRLLRRYRLELSRPDYQPRWDYSAMPI PMDGMPIVLRPR  >CYP142A1bP(K60_036520)  MSSEVDGERLSDDELVMETLLILIGGDETTRHTLSGGTEQLLRNRDQWDLLQRDPSLLPG AIEEMLRWTAPVKNMCRVLTADTEFHGTALCAGEKMMLLFESANFDEAVFCEPEKFDVQR NPNSHLAFGFGTHFCLGNQLARLELSLMTERVLRRLPDLRLVADDSVLPLRPANFVSGLE SMPVVFTPSPPLG  >CYP142A1aP(K60_036530)  MTEAPDVDLADGNFYASREARAAYRWMRANQPVFRDRNGLAAASTYQAVIDAERQPELFS NAGGIRPDQPALPMMIDMDDPAHLLRRKLVNAGFTRKRVKDKEASIAALCDTLIDAVCER GECDFVRDLAAPLPMAVIGDMLGVRPEQRDMFLRWSDDLVTFLSSHVSQEDFQITMDAFA AYNDFTRATIAARRADPPTTWSACW  >CYP125A1(K60_036830)  MSWNHQSVEIAVRRTTVPSPNLPPGFDFTDPAIYAERLPVAEFAELRSAAPIWWNGQDPG KGGGFHDGGFWAITKLNDVKEISRHSDVFSSYENGVIPRFKNDIAREDIEVQRFVMLNMD APHHTRLRKIISRGFTPRAVGRLHDELQERAQKIAAEAAAAGSGDFVEQVSCELPLQAIA GLLGVPQEDRGKLFHWSNEMTGNEDPEYAHIDPKASSAELIGYAMKMAEEKAKNPADDIV TQLIQADIDGEKLSDDEFGFFVVMLAVAGNETTRNSITQGMMAFAEHPDQWELYKKVRPE TAADEIVRWATPVTAFQRTALRDYELSGVQIKKGQRVVMFYRSANFDEEVFQDPFTFNIL RNPNPHVGFGGTGAHYCIGANLARMTINLIFNAVADHMPDLKPISAPERLRSGWLNGIKH WQVDYTGRCPVAH  >CYP137A1(K60_038230)  MVLRSLASPAALTDPKRCASVVGVAAFAVRREHAPDALGGPPGLPAPRGFRAAFAAAYAV AYLAGGERRMLRLIRRYGPIMTMPILSLGDVAIVSDSALAKEVFTAPTDVLLGGEGVGPA AAIYGSGSMFVQEEPQHLRRRKLLTPPLHGAALDRYVPIIENSTRAAMHTWPVDRPFAML TVARSLMLDVIVKVIFGVDDPEEVRRLGRPFERLLNLGVSEQLTVRYALRRLGALRVWPA RARANTEIDDVVMALIAQRRADPRLGERHDVLSLLVSARGESGEQLSDSEIRDDLITLVL AGHETTATTLAWAFDLLLHHPDALRRVRAEAVGGGEAFTTAVINETLRVRPPAPLTARVA AQPLTIGGYRVEAGTRIVVHIIAINRSAEVYEHPHEFRPERFLGTRPQTYAWVPFGGGVK RCLGANFSMRELITVLHVLLREGEFTAVDDEPERIVRRSIMLVPRRGTRVRFRPAR |
| ***Mycobacterium bovis* BCG Mexico** |
| DATABASE: KEGG; P450 count: 18; Families: 17; Subfamilies: 18 |
| >CYP138A1(BCGMEX_0142)  MSEVVTAAPAPPVVRLPPAVRGPKLFQGLAFVVSRRRLLGRFVRRYGKAFTANILMYGRV VVVADPQLARQVFTSSPEELGNIQPNLSRMFGSGSVFALDGDDHRRRRRLLAPPFHGKSM KNYETIIEEETLRETANWPQGQAFATLPSMMHITLNAILRAIFGAGGSELDELRRLIPPW VTLGSRLAALPKPKRDYGRLSPWGRLAEWRRQYDTVIDKLIEAERADPNFADRTDVLALM LRSTYDDGSIMSRKDIGDELLTLLAAGHETTAATLGWAFERLSRHPDVLAALVEEVDNGG HELRQAAILEVQRARTVIDFAARRVNPPVYQLGEWVIPRGYSIIINIAQIHGDPDVFPQP DRFDPQRYIGSKPSPFAWIPFGGGTRRCVGAAFANMEMDVVLRTVLRHFTLETTTAAGER SHGRGVAFTPKDGGRVVMRRR  >CYP135A1(BCGMEX_0336c)  MASTLTTGLPPGPRLPRYLQSVLYLRFREWFLPAMHRKYGDVFSLRVPPYADNLVVYTRP EHIKEIFAADPRSLHAGEGNHILGFVMGEHSVLMTDEAEYARMRSLLMPAFTRAALRGYR DMIASVAREHITRWRPHATINSLDHMNALTLDIILRVVFGVTDPKVKAELTSRLQQIINI HPAILAGVPYPSLKRMNPWKRFFHNQTKIDEILYREIASRRIDSDLTARTDVLSRLLQTK DTPTKPLTDAELRDQLITLLLAGHETTAAALSWTLWELAHAPEIQSQVVWAAVGGDDGFL EAVLKEGMRRHTVIASTARKVTAPAEIGGWRLPAGTVVNTSILLAHASEVSHPKPTEFRP SRFLDGSVAPNTWLPFGGGVRRCLGFGFALTEGAVILQEIFRRFTITAAGPSKGETPLVR NITTVPKHGAHLRLIPQRRLGGLGDSDPP  >CYP135B1(BCGMEX_0584)  MSGTSSMGLPPGPRLSGSVQAVLMLRHGLRFLTACQRRYGSVFTLHVAGFGHMVYLSDPA AIKTVFAGNPSVFHAGEANSMLAGLLGDSSLLLIDDDVHRDRRRLMSPPFHRDAVARQAE PIAEIAAANIAGWPMAKAFAVAPKMSEITLEVILRTVIGASDPVRLAALRKVMPRLLNVG PWATLALANPSLLNNRLWSRLRRRIEEADALLYAEIADRRADPDLAARTDTLAMLVRAAD EDGRTMTERELRDQLITLLVAGHDTTATGLSWALERLTRHPVTLAKAVQAADASAAGDPA GDEYLDAVAKETLRIRPVVYDVGRVLTEAVEVAGYRLPAGVMVVPAIGLVHASAQLYPDP ERFDPDRMVGATLSPTTWLPFGGGNRRCLGATFAMVEMRVVLREILRRVELSTTTTSGER PKLKHVIMVPHRGARIRVRATRDVSATSQATAQGAGCPAARGGGPSRAVGSQ  >CYP51B1(BCGMEX_0787c)  MSAVALPRVSGGHDEHGHLEEFRTDPIGLMQRVRDECGDVGTFQLAGKQVVLLSGSHANE FFFRAGDDDLDQAKAYPFMTPIFGEGVVFDASPERRKEMLHNAALRGEQMKGHAATIEDQ VRRMIADWGEAGEIDLLDFFAELTIYTSSACLIGKKFRDQLDGRFAKLYHELERGTDPLA YVDPYLPIESFRRRDEARNGLVALVADIMNGRIANPPTDKSDRDMLDVLIAVKAETGTPR FSADEITGMFISMMFAGHHTSSGTASWTLIELMRHRDAYAAVIDELDELYGDGRSVSFHA LRQIPQLENVLKETLRLHPPLIILMRVAKGEFEVQGHRIHEGDLVAASPAISNRIPEDFP DPHDFVPARYEQPRQEDLLNRWTWIPFGAGRHRCVGAAFAIMQIKAIFSVLLREYEFEMA QPPESYRNDHSKMVVQLAQPACVRYRRRTGV  >CYP123A1(BCGMEX_0789c)  MTVRVGDPELVLDPYDYDFHEDPYPYYRRLRDEAPLYRNEERNFWAVSRHHDVLQGFRDS TALSNAYGVSLDPSSRTSEAYRVMSMLAMDDPAHLRMRTLVSKGFTPRRIRELEPQVLEL ARIHLDSALQTESFDFVAEFAGKLPMDVISELIGVPDTDRARIRALADAVLHREDGVADV PPPAMAASIELMRYYADLIAEFRRRPANNLTSALLAAELDGDRLSDQEIMAFLFLMVIAG NETTTKLLANAVYWAAHHPGQLARVFADHSRIPMWVEETLRYDTSSQILARTVAHDLTLY DTTIPEGEVLLLLPGSANRDDRVFDDPDDYRIGREIGCKLVSFGSGAHFCLGLTWPGWKP GWPWARCCVGSATTKSTTTTSCASIPATCADLPICRSACRPGNATLRTSPRPADHRRRRR IVGHRRGHRNRTRRPRVSGRLGGPPHGQVGRAGRQNPRRRWRGGSLPPRRDRSRVGEIVC GANGRGTRRGRTAGVQCRRHAPGTAARGQHRGLCRAGSDTPGRCQPAGHGRATGHGGTPA R  >CYP126A1(BCGMEX_0801)  MTTAAGLSGIDLTDLDNFADGFPHHLFAIHRREAPVYWHRPTEHTPDGEGFWSVATYAET LEVLRDPVTYSSVTGGQRRFGGTVLQDLPVAGQVLNMMDDPRHTRIRRLVSSGLTPRMIR RVEDDLRRRARGLLDGVEPGAPFDFVVEIAAELPMQMICILLGVPETDRHWLFEAVEPGF DFRGSRRATMPRLNVEDAGSRLYTYALELIAGKRAEPADDMLSVVANATIDDPDAPALSD AELYLFFHLLFSAGAETTRNSIAGGLLALAENPDQLQTLRSDFELLPTAIEEIVRWTSPS PSKRRTASRAVSLGGQPIEAGQKVVVWEGSANRGPSVFDRADEFDITRKPNPHLGFGQGV HYCLGANLARLELRVLFEELLSRFGSVRVVEPAEWTRSNRHTGIRHLVVELRGG  >CYP132A1(BCGMEX_1427c)  MATATTQRPLKGPAKRMSTWTMTREAITIGFDAGDGFLGRLRGSDITRFRCAGRRFVSIS HPDYVDHVLHEARLKYVKSDEYGPIRATAGLNLLTDEGDSWARHRGALNSTFARRHLRGL VGLMIDPIADVTAALVPGAQFDMHQSMVETTLRVVANALFSQDFGPLVQSMHDLATRGLR RAEKLERLGLWGLMPRTVYDTLIWCIYSGVHLPPPLREMQEITLTLDRAINSVIDRRLAE PTNSADLLNVLLSADGGIWPRQRVRDEALTFMLAGHETTANAMSWFWYLMALNPQARDHM LTELDDVLGMRRPTADDLGKLAWTTACLQESQRYFSSVWIIAREAVDDDIIDGHRIRRGT TVVIPIHHIHHDPRWWPDPDRFDPGRFLRCPTDRPRCAYLPFGGGRRICIGQSFALMEMV LMAAIMSQHFTFDLAPGYHVELEATLTLRPKHGVHVIGRRR  >CYP139A1(BCGMEX_1677c)  MRYPLGEALLALYRWRGPLINAGVGGHGYTYLLGAEANRFVFANADAFSWSQTFESLVPV DGPTALIVSDGADHRRRRSVVAPGLRHHHVQRYVATMVSNIDTVIDGWQPGQRLDIYQEL RSAVRRSTAESLFGQRLAVHSDFLGEQLQPLLDLTRRPPQVMRLQQRVNSPGWRRAMAAR KRIDDLIDAQIADARTAPRPDDHMLTTLISGCSEEGTTLSDNEIRDSIVSLITAGYETTS GALAWAIYALLTVPGTWESAASEVARVLGGRVPAADDLSALTYLNGVVHETLRLYSPGVI SARRVLRDLWFDGHRIRAGRLLIFSAYVTHRLPEIWPEPTEFRPLRWDPNAADYRKPAPH EFIPFSGGLHRCIGAVMATTEMTVILARLVARAMLQLPAQRTHRIRAANFAALRPWPGLT VEIRKSAPAQ  >CYP144A1(BCGMEX_1791)  MRRSPKGSPGAVLDLQRRVDQAVSADHAELMTIAKDANTFFGAESVQDPYPLYERMRAAG SVHRIANSDFYAVCGWDAVNEAIGRPEDFSSNLTATMTYTAEGTAKPFEMDPLGGPTHVL ATADDPAHAVHRKLVLRHLAAKRIRVMEQFTVQAADRLWVDGMQDGCIEWMGAMANRLPM MVVAELIGLPDPDIAQLVKWGYAATQLLEGLVENDQLVAAGVALMELSGYIFEQFDRAAA DPRDNLLGELATACASGELDTLTAQVMMVTLFAAGGESTAALLGSAVWILATRPDIQQQV RANPELLGAFIEETLRYEPPFRGQYRHVRNATTLDGTELPADSHLLLLWGAANRDPAQFE APGEFRLDRAGGKGHISFGKGAHFCVGAALARLEARIVLRLLLDRTSVIEAADVGGWLPS ILVRRIERLELAVQ  >CYP143A1(BCGMEX_1798c)  MTTPGEDHAGSFYLPRLEYSTLPMAVDRGVGWKTLRDAGPVVFMNGWYYLTRREDVLAAL RNPKVFSSRKALQPPGNPLPVVPLAFDPPEHTRYRRILQPYFSPAALSKALPSLRRHTVA MIDAIAGRGECEAMADLANLFPFQLFLVLYGLPLEDRDRLIGWKDAVIAMSDRPHPTEAD VAAARELLEYLTAMVAERRRNPGPDVLSQVQIGEDPLSEIEVLGLSHLLILAGLDTVTAA VGFSLLELARRPQLRAMLRDNPKQIRVFIEEIVRLEPSAPVAPRVTTEPVTVGGMTLPAG SPVRLCMAAVNRDGSDAMSTDELVMDGKVHRHWGFGGGPHRCLGSHLARLELTLLVGEWL NQIPDFELAPDYAPEIRFPSKSFALKNLPLRWS  >CYP140A1(BCGMEX_1898c)  MKDKLHWLAMHGVIRGIAAIGIRRGDLQARLIADPAVATDPVPFYDEVRSHGALVRNRAN YLTVDHRLAHDLLRSDDFRVVSFGENLPPPLRWLERRTRGDQLHPLREPSLLAVEPPDHT RYRKTVSAVFTSRAVSALRDLVEQTAINLLDRFAEQPGIVDVVGRYCSQLPIVVISEILG VPEHDRPRVLEFGELAAPSLDIGIPWRQYLRVQQGIRGFDCWLEGHLQQLRHAPGDDLMS QLIQIAESGDNETQLDETELRAIAGLVLVAGFETTVNLLGNGIRMLLDTPEHLATLRQHP ELWPNTVEEILRLDSPVQLTARVACRDVEVAGVRIKRGEVVVIYLAAANRDPAVFPDPHR FDIERPNAGRHLAFSTGRHFCLGAALARAEGEVGLRTFFDRFPDVRAAGAGSRRDTRVLR GWSTLPVTLGPARSMVSP  >CYP124A1(BCGMEX_2271)  MGLNTAIATRVNGTPPPEVPIADIELGSLDFWALDDDVRDGAFATLRREAPISFWPTIEL PGFVAGNGHWALTKYDDVFYASRHPDIFSSYPNITINDQTPELAEYFGSMIVLDDPRHQR LRSIVSRAFTPKVVARIEAAVRDRAHRLVSSMIANNPDRQADLVSELAGPLPLQIICDMM GIPKADHQRIFHWTNVILGFGDPDLATDFDEFMQVSADIGAYATALAEDRRVNHHDDLTS SLVEAEVDGERLSSREIASFFILLVVAGNETTRNAITHGVLALSRYPEQRDRWWSDFDGL APTAVEEIVRWASPVVYMRRTLTQDIELRGTKMAAGDKVSLWYCSANRDESKFADPWTFD LARNPNPHLGFGGGGAHFCLGANLARREIRVAFDELRRQMPDVVATEEPARLLSQFIHGI KTLPVTWS  >CYP128A1(BCGMEX_2273c)  MTATQSPPEPAPDRVRLAGCPLAGTPDVGLTAQDATTALGVPTRRRASSGGIPVATSMWR DAQTVRTYGPAVAKALALRVAGKARSRLTGRHCRKFMQLTDFDPFDPAIAADPYPHYREL LAGERVQYNPKRDVYILSRYADVREAARNHDTLSSARGVTFSRGWLPFLPTSDPPAHTRM RKQLAPGMARGALETWRPMVDQFARELVGGLLTQTPADVVSTVAAPMPMRAITSVLGVDG PDEAAFCRLSNQAVRITDVALSASGLISLVQGFAGFRRLRALFTHRRDNGLLRECTVLGK LATHAEQGRLSDDELFFFAVLLLVAGYESTAHMISTLFLTLADYPDQLTLLAQQPDLIPS AIEEHLRFISPIQNICRTTRVDYSVGQAVIPAGSLVLLAWGAANRDPRQYEDPDVFRADR NPVGHLAFGSGIHLCPGTQLARMEGQAILREIVANIDRIEVVEPPTWTTNANLRGLTRLR VAVTPRVAP  >CYP121A1(BCGMEX_2281)  MTATVLLEVPFSARGDRIPDAVAELRTREPIRKVRTITGAEAWLVSSYALCTQVLEDRRF SMKETAAAGAPRLNALTVPPEVVNNMGNIADAGLRKAVMKAITPKAPGLEQFLRDTANSL LDNLITEGAPADLRNDFADPLATALHCKVLGIPQEDGPKLFRSLSIAFMSSADPIPAAKI NWDRDIEYMAGILENPNITTGLMGELSRLRKDPAYSHVSDELFATIGVTFFGAGVISTGS FLTTALISLIQRPQLRNLLHEKPELIPAGVEELLRINLSFADGLPRLATADIQVGDVLVR KGELVLVLLEGANFDPEHFPNPGSIELDRPNPTSHLAFGRGQHFCPGSALGRRHAQIGIE ALLKKMPGVDLAVPIDQLVWRTRFQRRIPERLPVLW  >CYP136A1(BCGMEX_3081)  MATIHPPAYLLDQAKRRFTPSFNNFPGMSLVEHMLLNTKFPEKELAEPPPGSGLKPVVGD AGLPILGHMIEMLRGGPDYLMFLYKTKGPVVFGDSAVLPGVAALGPDAAQVIYSNRNKDY SQQGWVPVIGPFFHRGLMLLDFEEHMFHRRIMQEAFVRSRLAGYLEQMDRVVSRVVADDW VVNDARFLVYPAMKALTLDIASMVFMGHEPGTDHELVTKVNKAFTITTRAGNAVIRTSVP PFTWWRGLRARELLENYFTARVKERREASGNDLLTVLCQTEDDDGNRFSDADIVNHMIFL MMAAHDTSTSTATTMAYQLAAHPEWQQRCRDESDRHGDGPLDIESLEQLESLDLVMNESI RLVTPVQWAMRQTVRDTELLGYYLPKGTNVIAYPGMNHRLPEIWTDPLTFDPERFTEPRN EHKRHRYAFTPFGGGVHKCIGMVFGQLEIKTILHRLLRRYRLELSRPDYQPRWDYSAMPI PMDGMPIVLRPR  >CYP142A1bP(BCGMEX_3579c)  MSSEVDGERLSDDELVMETLLILIGGDETTRHTLSGGTEQLLRNRDQWDLLQRDPSLLPG AIEEMLRWTAPVKNMCRVLTADTEFHGTALCAGEKMMLLFESANFDEAVFCEPEKFDVQR NPNSHLAFGFGTHFCLGNQLARLELSLMTERVLRRLPDLRLVADDSVLPLRPANFVSGLE SMPVVFTPSPPLG  >CYP142A1aP(BCGMEX_3580c)  MTEAPDVDLADGNFYASREARAAYRWMRANQPVFRDRNGLAAASTYQAVIDAERQPELFS NAGGIRPDQPALPMMIDMDDPAHLLRRKLVNAGFTRKRVKDKEASIAALCDTLIDAVCER GECDFVRDLAAPLPMAVIGDMLGVRPEQRDMFLRWSDDLVTFLSSHVSQEDFQITMDAFA AYNDFTRATIAARRADPPTTWSACW  >CYP125A1(BCGMEX_3607c)  MSWNHQSVEIAVRRTTVPSPNLPPGFDFTDPAIYAERLPVAEFAELRSAAPIWWNGQDPG KGGGFHDGGFWAITKLNDVKEISRHSDVFSSYENGVIPRFKNDIAREDIEVQRFVMLNMD APHHTRLRKIISRGFTPRAVGRLHDELQERAQKIAAEAAAAGSGDFVEQVSCELPLQAIA GLLGVPQEDRGKLFHWSNEMTGNEDPEYAHIDPKASSAELIGYAMKMAEEKAKNPADDIV TQLIQADIDGEKLSDDEFGFFVVMLAVAGNETTRNSITQGMMAFAEHPDQWELYKKVRPE TAADEIVRWATPVTAFQRTALRDYELSGVQIKKGQRVVMFYRSANFDEEVFQDPFTFNIL RNPNPHVGFGGTGAHYCIGANLARMTINLIFNAVADHMPDLKPISAPERLRSGWLNGIKH WQVDYTGRCPVAH  >CYP137A1(BCGMEX_3744c)  MVLRSLASPAALTDPKRCASVVGVAAFAVRREHAPDALGGPPGLPAPRGFRAAFAAAYAV AYLAGGERRMLRLIRRYGPIMTMPILSLGDVAIVSDSALAKEVFTAPTDVLLGGEGVGPA AAIYGSGSMFVQEEPQHLRRRKLLTPPLHGAALDRYVPIIENSTRAAMHTWPVDRPFAML TVARSLMLDVIVKVIFGVDDPEEVRRLGRPFERLLNLGVSEQLTVRYALRRLGALRVWPA RARANTEIDDVVMALIAQRRADPRLGERHDVLSLLVSARGESGEQLSDSEIRDDLITLVL AGHETTATTLAWAFDLLLHHPDALRRVRAEAVGGGEAFTTAVINETLRVRPPAPLTARVA AQPLTIGGYRVEAGTRIVVHIIAINRSAEVYEHPHEFRPERFLGTRPQTYAWVPFGGGVK RCLGANFSMRELITVLHVLLREGEFTAVDDEPERIVRRSIMLVPRRGTRVRFRPAR |
| ***Mycobacterium bovis* BCG Tokyo 172** |
| DATABASE: KEGG; P450 count: 18; Families: 17; Subfamilies: 18 |
| >CYP138A1(JTY_0142)  MSEVVTAAPAPPVVRLPPAVRGPKLFQGLAFVVSRRRLLGRFVRRYGKAFTANILMYGRV VVVADPQLARQVFTSSPEELGNIQPNLSRMFGSGSVFALDGDDHRRRRRLLAPPFHGKSM KNYETIIEEETLRETANWPQGQAFATLPSMMHITLNAILRAIFGAGGSELDELRRLIPPW VTLGSRLAALPKPKRDYGRLSPWGRLAEWRRQYDTVIDKLIEAERADPNFADRTDVLALM LRSTYDDGSIMSRKDIGDELLTLLAAGHETTAATLGWAFERLSRHPDVLAALVEEVDNGG HELRQAAILEVQRARTVIDFAARRVNPPVYQLGEWVIPRGYSIIINIAQIHGDPDVFPQP DRFDPQRYIGSKPSPFAWIPFGGGTRRCVGAAFANMEMDVVLRTVLRHFTLETTTAAGER SHGRGVAFTPKDGGRVVMRRR  >CYP135A1(JTY_0336)  MASTLTTGLPPGPRLPRYLQSVLYLRFREWFLPAMHRKYGDVFSLRVPPYADNLVVYTRP EHIKEIFAADPRSLHAGEGNHILGFVMGEHSVLMTDEAEYARMRSLLMPAFTRAALRGYR DMIASVAREHITRWRPHATINSLDHMNALTLDIILRVVFGVTDPKVKAELTSRLQQIINI HPAILAGVPYPSLKRMNPWKRFFHNQTKIDEILYREIASRRIDSDLTARTDVLSRLLQTK DTPTKPLTDAELRDQLITLLLAGHETTAAALSWTLWELAHAPEIQSQVVWAAVGGDDGFL EAVLKEGMRRHTVIASTARKVTAPAEIGGWRLPAGTVVNTSILLAHASEVSHPKPTEFRP SRFLDGSVAPNTWLPFGGGVRRCLGFGFALTEGAVILQEIFRRFTITAAGPSKGETPLVR NITTVPKHGAHLRLIPQRRLGGLGDSDPP  >CYP135B1(JTY_0583)  MSGTSSMGLPPGPRLSGSVQAVLMLRHGLRFLTACQRRYGSVFTLHVAGFGHMVYLSDPA AIKTVFAGNPSVFHAGEANSMLAGLLGDSSLLLIDDDVHRDRRRLMSPPFHRDAVARQAE PIAEIAAANIAGWPMAKAFAVAPKMSEITLEVILRTVIGASDPVRLAALRKVMPRLLNVG PWATLALANPSLLNNRLWSRLRRRIEEADALLYAEIADRRADPDLAARTDTLAMLVRAAD EDGRTMTERELRDQLITLLVAGHDTTATGLSWALERLTRHPVTLAKAVQAADASAAGDPA GDEYLDAVAKETLRIRPVVYDVGRVLTEAVEVAGYRLPAGVMVVPAIGLVHASAQLYPDP ERFDPDRMVGATLSPTTWLPFGGGNRRCLGATFAMVEMRVVLREILRRVELSTTTTSGER PKLKHVIMVPHRGARIRVRATRDVSATSQATAQGAGCPAARGGGPSRAVGSQ  >CYP51B1(JTY_0786)  MSAVALPRVSGGHDEHGHLEEFRTDPIGLMQRVRDECGDVGTFQLAGKQVVLLSGSHANE FFFRAGDDDLDQAKAYPFMTPIFGEGVVFDASPERRKEMLHNAALRGEQMKGHAATIEDQ VRRMIADWGEAGEIDLLDFFAELTIYTSSACLIGKKFRDQLDGRFAKLYHELERGTDPLA YVDPYLPIESFRRRDEARNGLVALVADIMNGRIANPPTDKSDRDMLDVLIAVKAETGTPR FSADEITGMFISMMFAGHHTSSGTASWTLIELMRHRDAYAAVIDELDELYGDGRSVSFHA LRQIPQLENVLKETLRLHPPLIILMRVAKGEFEVQGHRIHEGDLVAASPAISNRIPEDFP DPHDFVPARYEQPRQEDLLNRWTWIPFGAGRHRCVGAAFAIMQIKAIFSVLLREYEFEMA QPPESYRNDHSKMVVQLAQPACVRYRRRTGV  >CYP123A1(JTY_0788)  MTVRVGDPELVLDPYDYDFHEDPYPYYRRLRDEAPLYRNEERNFWAVSRHHDVLQGFRDS TALSNAYGVSLDPSSRTSEAYRVMSMLAMDDPAHLRMRTLVSKGFTPRRIRELEPQVLEL ARIHLDSALQTESFDFVAEFAGKLPMDVISELIGVPDTDRARIRALADAVLHREDGVADV PPPAMAASIELMRYYADLIAEFRRRPANNLTSALLAAELDGDRLSDQEIMAFLFLMVIAG NETTTKLLANAVYWAAHHPGQLARVFADHSRIPMWVEETLRYDTSSQILARTVAHDLTLY DTTIPEGEVLLLLPGSANRDDRVFDDPDDYRIGREIGCKLVSFGSGAHFCLGLTWPGWKP GWPWARCCVGSATTKSTTTTSCASIPATCADLPICRSACRPGNATLRTSPRPADHRRRRR IVGHRRGHRNRTRRPRVSGRLGGPPHGQVGRAGRQNPRRRWRGGSLPPRRDRSRVGEIVC GANGRGTRRGRTAGVQCRRHAPGTAARGQHRGLCRAGSDTPGRCQPAGHGRATGHGGTPA R  >CYP126A1(JTY_0800)  MTTAAGLSGIDLTDLDNFADGFPHHLFAIHRREAPVYWHRPTEHTPDGEGFWSVATYAET LEVLRDPVTYSSVTGGQRRFGGTVLQDLPVAGQVLNMMDDPRHTRIRRLVSSGLTPRMIR RVEDDLRRRARGLLDGVEPGAPFDFVVEIAAELPMQMICILLGVPETDRHWLFEAVEPGF DFRGSRRATMPRLNVEDAGSRLYTYALELIAGKRAEPADDMLSVVANATIDDPDAPALSD AELYLFFHLLFSAGAETTRNSIAGGLLALAENPDQLQTLRSDFELLPTAIEEIVRWTSPS PSKRRTASRAVSLGGQPIEAGQKVVVWEGSANRGPSVFDRADEFDITRKPNPHLGFGQGV HYCLGANLARLELRVLFEELLSRFGSVRVVEPAEWTRSNRHTGIRHLVVELRGG  >CYP132A1(JTY_1430)  MATATTQRPLKGPAKRMSTWTMTREAITIGFDAGDGFLGRLRGSDITRFRCAGRRFVSIS HPDYVDHVLHEARLKYVKSDEYGPIRATAGLNLLTDEGDSWARHRGALNSTFARRHLRGL VGLMIDPIADVTAALVPGAQFDMHQSMVETTLRVVANALFSQDFGPLVQSMHDLATRGLR RAEKLERLGLWGLMPRTVYDTLIWCIYSGVHLPPPLREMQEITLTLDRAINSVIDRRLAE PTNSADLLNVLLSADGGIWPRQRVRDEALTFMLAGHETTANAMSWFWYLMALNPQARDHM LTELDDVLGMRRPTADDLGKLAWTTACLQESQRYFSSVWIIAREAVDDDIIDGHRIRRGT TVVIPIHHIHHDPRWWPDPDRFDPGRFLRCPTDRPRCAYLPFGGGRRICIGQSFALMEMV LMAAIMSQHFTFDLAPGYHVELEATLTLRPKHGVHVIGRRR  >CYP139A1(JTY_1680)  MRYPLGEALLALYRWRGPLINAGVGGHGYTYLLGAEANRFVFANADAFSWSQTFESLVPV DGPTALIVSDGADHRRRRSVVAPGLRHHHVQRYVATMVSNIDTVIDGWQPGQRLDIYQEL RSAVRRSTAESLFGQRLAVHSDFLGEQLQPLLDLTRRPPQVMRLQQRVNSPGWRRAMAAR KRIDDLIDAQIADARTAPRPDDHMLTTLISGCSEEGTTLSDNEIRDSIVSLITAGYETTS GALAWAIYALLTVPGTWESAASEVARVLGGRVPAADDLSALTYLNGVVHETLRLYSPGVI SARRVLRDLWFDGHRIRAGRLLIFSAYVTHRLPEIWPEPTEFRPLRWDPNAADYRKPAPH EFIPFSGGLHRCIGAVMATTEMTVILARLVARAMLQLPAQRTHRIRAANFAALRPWPGLT VEIRKSAPAQ  >CYP144A1(JTY_1794)  MRRSPKGSPGAVLDLQRRVDQAVSADHAELMTIAKDANTFFGAESVQDPYPLYERMRAAG SVHRIANSDFYAVCGWDAVNEAIGRPEDFSSNLTATMTYTAEGTAKPFEMDPLGGPTHVL ATADDPAHAVHRKLVLRHLAAKRIRVMEQFTVQAADRLWVDGMQDGCIEWMGAMANRLPM MVVAELIGLPDPDIAQLVKWGYAATQLLEGLVENDQLVAAGVALMELSGYIFEQFDRAAA DPRDNLLGELATACASGELDTLTAQVMMVTLFAAGGESTAALLGSAVWILATRPDIQQQV RANPELLGAFIEETLRYEPPFRGQYRHVRNATTLDGTELPADSHLLLLWGAANRDPAQFE APGEFRLDRAGGKGHISFGKGAHFCVGAALARLEARIVLRLLLDRTSVIEAADVGGWLPS ILVRRIERLELAVQ  >CYP143A1(JTY_1801)  MTTPGEDHAGSFYLPRLEYSTLPMAVDRGVGWKTLRDAGPVVFMNGWYYLTRREDVLAAL RNPKVFSSRKALQPPGNPLPVVPLAFDPPEHTRYRRILQPYFSPAALSKALPSLRRHTVA MIDAIAGRGECEAMADLANLFPFQLFLVLYGLPLEDRDRLIGWKDAVIAMSDRPHPTEAD VAAARELLEYLTAMVAERRRNPGPDVLSQVQIGEDPLSEIEVLGLSHLLILAGLDTVTAA VGFSLLELARRPQLRAMLRDNPKQIRVFIEEIVRLEPSAPVAPRVTTEPVTVGGMTLPAG SPVRLCMAAVNRDGSDAMSTDELVMDGKVHRHWGFGGGPHRCLGSHLARLELTLLVGEWL NQIPDFELAPDYAPEIRFPSKSFALKNLPLRWS  >CYP140A1(JTY_1901)  MKDKLHWLAMHGVIRGIAAIGIRRGDLQARLIADPAVATDPVPFYDEVRSHGALVRNRAN YLTVDHRLAHDLLRSDDFRVVSFGENLPPPLRWLERRTRGDQLHPLREPSLLAVEPPDHT RYRKTVSAVFTSRAVSALRDLVEQTAINLLDRFAEQPGIVDVVGRYCSQLPIVVISEILG VPEHDRPRVLEFGELAAPSLDIGIPWRQYLRVQQGIRGFDCWLEGHLQQLRHAPGDDLMS QLIQIAESGDNETQLDETELRAIAGLVLVAGFETTVNLLGNGIRMLLDTPEHLATLRQHP ELWPNTVEEILRLDSPVQLTARVACRDVEVAGVRIKRGEVVVIYLAAANRDPAVFPDPHR FDIERPNAGRHLAFSTGRHFCLGAALARAEGEVGLRTFFDRFPDVRAAGAGSRRDTRVLR GWSTLPVTLGPARSMVSP  >CYP124A1(JTY_2277)  MGLNTAIATRVNGTPPPEVPIADIELGSLDFWALDDDVRDGAFATLRREAPISFWPTIEL PGFVAGNGHWALTKYDDVFYASRHPDIFSSYPNITINDQTPELAEYFGSMIVLDDPRHQR LRSIVSRAFTPKVVARIEAAVRDRAHRLVSSMIANNPDRQADLVSELAGPLPLQIICDMM GIPKADHQRIFHWTNVILGFGDPDLATDFDEFMQVSADIGAYATALAEDRRVNHHDDLTS SLVEAEVDGERLSSREIASFFILLVVAGNETTRNAITHGVLALSRYPEQRDRWWSDFDGL APTAVEEIVRWASPVVYMRRTLTQDIELRGTKMAAGDKVSLWYCSANRDESKFADPWTFD LARNPNPHLGFGGGGAHFCLGANLARREIRVAFDELRRQMPDVVATEEPARLLSQFIHGI KTLPVTWS  >CYP128A1(JTY_2279)  MTATQSPPEPAPDRVRLAGCPLAGTPDVGLTAQDATTALGVPTRRRASSGGIPVATSMWR DAQTVRTYGPAVAKALALRVAGKARSRLTGRHCRKFMQLTDFDPFDPAIAADPYPHYREL LAGERVQYNPKRDVYILSRYADVREAARNHDTLSSARGVTFSRGWLPFLPTSDPPAHTRM RKQLAPGMARGALETWRPMVDQFARELVGGLLTQTPADVVSTVAAPMPMRAITSVLGVDG PDEAAFCRLSNQAVRITDVALSASGLISLVQGFAGFRRLRALFTHRRDNGLLRECTVLGK LATHAEQGRLSDDELFFFAVLLLVAGYESTAHMISTLFLTLADYPDQLTLLAQQPDLIPS AIEEHLRFISPIQNICRTTRVDYSVGQAVIPAGSLVLLAWGAANRDPRQYEDPDVFRADR NPVGHLAFGSGIHLCPGTQLARMEGQAILREIVANIDRIEVVEPPTWTTNANLRGLTRLR VAVTPRVAP  >CYP121A1(JTY_2287)  MTATVLLEVPFSARGDRIPDAVAELRTREPIRKVRTITGAEAWLVSSYALCTQVLEDRRF SMKETAAAGAPRLNALTVPPEVVNNMGNIADAGLRKAVMKAITPKAPGLEQFLRDTANSL LDNLITEGAPADLRNDFADPLATALHCKVLGIPQEDGPKLFRSLSIAFMSSADPIPAAKI NWDRDIEYMAGILENPNITTGLMGELSRLRKDPAYSHVSDELFATIGVTFFGAGVISTGS FLTTALISLIQRPQLRNLLHEKPELIPAGVEELLRINLSFADGLPRLATADIQVGDVLVR KGELVLVLLEGANFDPEHFPNPGSIELDRPNPTSHLAFGRGQHFCPGSALGRRHAQIGIE ALLKKMPGVDLAVPIDQLVWRTRFQRRIPERLPVLW  >CYP136A1(JTY_3079)  MATIHPPAYLLDQAKRRFTPSFNNFPGMSLVEHMLLNTKFPEKELAEPPPGSGLKPVVGD AGLPILGHMIEMLRGGPDYLMFLYKTKGPVVFGDSAVLPGVAALGPDAAQVIYSNRNKDY SQQGWVPVIGPFFHRGLMLLDFEEHMFHRRIMQEAFVRSRLAGYLEQMDRVVSRVVADDW VVNDARFLVYPAMKALTLDIASMVFMGHEPGTDHELVTKVNKAFTITTRAGNAVIRTSVP PFTWWRGLRARELLENYFTARVKERREASGNDLLTVLCQTEDDDGNRFSDADIVNHMIFL MMAAHDTSTSTATTMAYQLAAHPEWQQRCRDESDRHGDGPLDIESLEQLESLDLVMNESI RLVTPVQWAMRQTVRDTELLGYYLPKGTNVIAYPGMNHRLPEIWTDPLTFDPERFTEPRN EHKRHRYAFTPFGGGVHKCIGMVFGQLEIKTILHRLLRRYRLELSRPDYQPRWDYSAMPI PMDGMPIVLRPR  >CYP142A1bP(JTY_3582)  MSSEVDGERLSDDELVMETLLILIGGDETTRHTLSGGTEQLLRNRDQWDLLQRDPSLLPG AIEEMLRWTAPVKNMCRVLTADTEFHGTALCAGEKMMLLFESANFDEAVFCEPEKFDVQR NPNSHLAFGFGTHFCLGNQLARLELSLMTERVLRRLPDLRLVADDSVLPLRPANFVSGLE SMPVVFTPSPPLG  >CYP142A1aP(JTY_3583)  MTEAPDVDLADGNFYASREARAAYRWMRANQPVFRDRNGLAAASTYQAVIDAERQPELFS NAGGIRPDQPALPMMIDMDDPAHLLRRKLVNAGFTRKRVKDKEASIAALCDTLIDAVCER GECDFVRDLAAPLPMAVIGDMLGVRPEQRDMFLRWSDDLVTFLSSHVSQEDFQITMDAFA AYNDFTRATIAARRADPPTTWSACW  >CYP125A1(JTY_3610)  MSWNHQSVEIAVRRTTVPSPNLPPGFDFTDPAIYAERLPVAEFAELRSAAPIWWNGQDPG KGGGFHDGGFWAITKLNDVKEISRHSDVFSSYENGVIPRFKNDIAREDIEVQRFVMLNMD APHHTRLRKIISRGFTPRAVGRLHDELQERAQKIAAEAAAAGSGDFVEQVSCELPLQAIA GLLGVPQEDRGKLFHWSNEMTGNEDPEYAHIDPKASSAELIGYAMKMAEEKAKNPADDIV TQLIQADIDGEKLSDDEFGFFVVMLAVAGNETTRNSITQGMMAFAEHPDQWELYKKVRPE TAADEIVRWATPVTAFQRTALRDYELSGVQIKKGQRVVMFYRSANFDEEVFQDPFTFNIL RNPNPHVGFGGTGAHYCIGANLARMTINLIFNAVADHMPDLKPISAPERLRSGWLNGIKH WQVDYTGRCPVAH  >CYP137A1(JTY_3745)  MVLRSLASPAALTDPKRCASVVGVAAFAVRREHAPDALGGPPGLPAPRGFRAAFAAAYAV AYLAGGERRMLRLIRRYGPIMTMPILSLGDVAIVSDSALAKEVFTAPTDVLLGGEGVGPA AAIYGSGSMFVQEEPQHLRRRKLLTPPLHGAALDRYVPIIENSTRAAMHTWPVDRPFAML TVARSLMLDVIVKVIFGVDDPEEVRRLGRPFERLLNLGVSEQLTVRYALRRLGALRVWPA RARANTEIDDVVMALIAQRRADPRLGERHDVLSLLVSARGESGEQLSDSEIRDDLITLVL AGHETTATTLAWAFDLLLHHPDALRRVRAEAVGGGEAFTTAVINETLRVRPPAPLTARVA AQPLTIGGYRVEAGTRIVVHIIAINRSAEVYEHPHEFRPERFLGTRPQTYAWVPFGGGVK RCLGANFSMRELITVLHVLLREGEFTAVDDEPERIVRRSIMLVPRRGTRVRFRPAR |
| ***Mycobacterium chelonae-abscessus* complex (MCAC)** |
| ***Mycobacterium abscessus* ATCC 19977** |
| Database: TB; P450 count: 25; Families: 19; Subfamilies: 22 |
| >CYP125A(MAB_1211c)  MTAMKTAAELGLPEGFDFTDPELYGNRMPHEEFATLRREAPVWWNPQPRTVGGFADEGYW  VISKHRDVREVSLHTDTFSSGRKGAIPRLEDHISPEEFQATLSVLINKDAPEHTQLRGLV  SRMFTPRSIAALRITLEERAERIVRAALEGGHGEFVREVASELPMQAIAELIGVPEEDRV  KLFEWSNQMTGYDEADVEIDPRVGAAQILGYSYQLAEQRRDCPGNDVVSRLLTGTVDGEQ  LTPEQFGFFVVMLSVAGNETTRNATTMGMMAFLEHPGQWELFKSARPSTTVDEIVRYTSP  LISQQRTALQDTVISDVRIRAGERVVMLYPSANFDEEVFENPHTFDITRDPNPHLGFGGT  GAHYCLGANLAKAELEIIFNKIADRMPDISRIGDAPRFHSGWINGIKKFDTAYCPVTH*  >CYP51B(MAB_1214c)  MTTPTVPRVSGGEDQYGHLEEFRTDPIALMKRIRAECGNVGTFQLADKQVVFLSGAEANE  FFFRSSDEDLDQAEAYPFMTPIFGKGVVFDADPERRKEMLHNAALRGEQMKGHAATIENE  VRQMISRWGESGEVDLLDFFAELTIYTSSACLIGKKFRDELDGRFAHLYHQLEQGTDPLC  YVDPYLEIESFRQRDEARTGLVALVQEIINGRVQRSADSPTDKSQRDMLDVLIMIKDEHG  NPRFTADEITGMFISMMFAGHHTSSGTASWVLIELLRHPDIQSQVIDELDELYADGSEVS  FHALRQIPKLENVLKETLRLHPPLIILMRVAKGEFEVGGFPIHDGDMVAASPAVSNRIAE  DFPNPDGFVPDRYEKPRQEDIVNRWTWIPFGAGRHRCVGAAFATMQIKAIFSVLLREYEF  EMAQPADSYHNDHSKMVVQLAQPAKVRYRKRSA*  >CYP123A(MAB_1216c)  MTVAETSAVPLVFDPYDYDFHEDPYPYYRRLRDEAPLYRNDDLKFWALSRHHDVLQGFRN  SEALSNANGVSMDKASFGPHAKLVMSFLAMDDPEHLRLRALVSKGFTPRRIRELEGQVVA  LARTHLDRALANASDRSFDFIAEYAGKLPMDVISELMGVPESDRARIRELADGVMHREDG  LADVPPEAIQASFDLMTYYIEMVRERRRRPTEDLTSALLQAEIDGDRLTDEEVLAFLFLM  VIAGNETTTKLLANAVYWGHRNPDQLATVHADHDRIPLWVEESLRYDTSSQILARTVAED  ITVYDTKIPAGDILLLLPGSANRDDRVFDDADQYRIGREIGAKLVSFGSGAHFCLGAHLA  RMEAKVALTELFTRISGYEIDERNSVRVHSSNVRGFAHLPMTVQLREGR*  >CYP164A(MAB_0276)  MPITTDPGTLLLQVLDPANRANPYPVYGQLVEQTQAGPVHPPGMDVSVLATFADCNAVLR  HPQASSDRRKSNIVQRQLAQNPNLIARPSFLGLDAPDHTRLRKLASKAFAPRVINAMAED  IQTFVDKMLDDIALRGTFNLVTEFAYPLPVAVICRMLGVPIEDEPEFGRASALLGQGLDP  VYALTGQSPANMDERFQAARWMWDYFIDLIASRRRNLGDDLLSALIQVEEAGDQLTEEEI  ISTCTLLLIAGHETTVNLIANASLAMLRHPEQWKLLGQNADRAPLVVEETLRYDPPVHMV  ARVADGEMAIRDFILPDGEWVLLMLAAAQRDPDVGPDLDVFNPDRSEIKHLAFGHGPHFC  LGAPLARLEARLALSSITARFPGARLMDEPTYKPNVTLRGLAELPVSIA*  >CYP1110B1(MAB_0917c)  MTLSSTELDDLVGAVDRYPVYKDLRDNQPVLPVEVNGHEAYLLTRYADVSRVLKTASARV  QPRAGEFPAHIGTGPASEFYRFSLPSMDAPSHTRLRKLAAAAFSPRAVAAMRSWVEEIIG  AGIDRLMDFDGEFDFVGEFASRVPAEIACRLLHAPMSDAHSVLERMPDLNPILSHGGITA  EQLAAADAAAQFYIDYIGDLVDTLQGKLDSDDAVGALLEAEADGSKMTRTELIITLVGLF  IASYHTTMVALTNAVYGFSSHPAQMRTLADNPDLAPKAWEESLRYRSPVHVVHRYAGEDM  VLHDQSIPEGAQLLLGLASANRDERFFDSPDTLDITRGTNRHLAFTGGGHYCLGAPLSRL  EGDYFMRVLPQRLPNIRVTSDRPDWGTDLSFAFMRSMMVSSGR*  >CYP1132A(MAB_2151c)  MRSIDTDSVAPAVPKPPVPRLPPRLPIHKAALLGLSCLGDPTRLPYLLLPKIANRYGDIV  RLFTGPTPALTLTLINHPDYVDHVFTRHHDRYVKHEATIELVSGEPVALPLLEGREWKRV  RSAFNPYFGERALAQATPLMMEGITERVDAWSRHVHSGELVDLEHELGAVVMDGLMRSMF  KVRLRPAEIDHAVDGARRYGVYVISRVAMHFLPRWLPNPLRRSGEEAKAELFGILDRFVQ  ERAGCPSTGTPDLVDTLLALEFDGCPQIRERRRRSEAAGLVFAGFETTAAALAWTIALLC  RNPIALGKAYAEVDALGGKTLAYDDLENLKYLRACFDEAQRFQAAPANVRTAIEDDEVGG  YFIPRGSQVIITQYALQRDPRFWNEPERFNPDRFLTDRINRNTFLPFSIGPRKCMGTRMA  YIEGTLVLGAILQRYAFQIRDGWTPRHRVRVSTGLAGGLPARLFAR*  >CYP125A(MAB_0611)  MVQAQHPHLPDGIDFTDPELFVHGIPERELAELRHTEPIWWNHTERGVAGFDDDGFWVVS  KHKDVKEVSLRCEVFSSEQNTAIPRYLPTTPRERIDATRLIMLNMDPPRHSRLRHIISRG  FTPRAISRLRDDLNARAQGIARAAAQLRHGDFVEQVACELPLQAIAGLMGTPLDEREQLF  DWSNRLVGSSDGEDDSAVASAELLMYAMGVAARKTAEPGADICTDLVNADIDGQKLSDDE  FGFFVMLLAVAGNETTRNSITHGMHAFTQFPEQWELYKKTRPETAADEIVRWATPVTSFQ  RTALEDTELGGVRIKKGQRVVMMYRSANFDEEVFENPFTFDIMRDPNPHVGFGGNGEHHC  VGANLARMTINLMFNAIADHMPDLASAGEPDRLRSGWLNGVKHWEVDFCPAGYGRAS*  >CYP125A(MAB_0613)  MVHPSLPAGFDFTDPEIYAERLPVEELKELRKTAPIWWQEQPDGVGGFNDGGYWVVTKHK  DVKEVSLRSDVFSSWENTAIPRFQDDITREAIELQRYVMLNMDAPHHTRLRKIISRGFTP  RAIGRLRDELNERAQEIAKAAAASGTGDFVEQVSCELPLQAIAGLLGVPIEDRGKLFNWS  NEMTSYDDPEYADIDPAASSMEILAYSMEMAKQKAENPGEDIVTTLINAEVEGEGKLSDD  EFGFFVIMLAVAGNETSRNSITQGMMAFTQFPEQWELYKKERPETAADEIVRWATPVTSF  QRTALEDTELDGVKIKKGQRVVMMYRSANFDEDVFEDPFSFNIMRNPNPHMGFGGSGAHY  CIGANLARLTINLMFNAIADHMPNLAPAGDPKRLQSGWLNGIKHWQVDFTGASGCPVLQ*  >CYP136A(MAB_3423)  MATISSTDYLIDQAKRRLPTMNTLPGMGYIENYLNNREWPMTELAAPPPGSGLKPVMGDQ  GLPMLGHMVEMFRGGIDWVLNMYQERGPVSWTQTPIGKIVAALGPDATQAVFSNANKDFS  QQGWVPVIGPFFNRGLMLLDFQEHREHRLIMQQAFLRSRLAGYVEQIDAVASEIVAQWPT  NDNRFLFYPAIKELTLDVASVVFMGNEPHAQHERLEKVNKAFVATTRAGGAIFRFGLPPF  KWWQGLQGRKLLEEYFAERVGIARQKTEGADMLTALCHAETDEGDAFTDTDIVNHMIFLM  MAAHDTTTSTTTTMAYYLAANPEWQERVRDESDRLGDGPLDIDSLEKLESLDLVMNEALR  LVTPLPFNIRSTVRDTDLLGFHIPAGTMINIWPGMNHRLPELWTEPDKFDPDRFSEPRNE  HKRHRYAFSPFGGGAHKCIGMVFGQLEIKAVMHRLLRRYRFELAHPHYQPKWDYAGMPLP  IDGMPIILRPLH*  >CYP144NSF1(MAB_1939)  MNTDELFDPQVLEDPYPFYRRLRETAPVWPVGDSGFYFVSRWDLVVEATERAEDFSSNLT  AALMKSAEGLAVAPMGPPADPTHVLATGDDPLHHAHRRLVLPTLVARRITALEPVMAQTG  SCLWERGVNGDGIDWMAAMGDALPMTMVARLIGLPAGDVPQLVQWGYSSTEMLGGLNTPQ  RQAQVVTDTMHLVLYLREHLEEELAAPGDDLLGYLAQACNRGDISLDIGVMILVQLVGAG  GESTAGLMGNAVRILGENPRLQQRIRENRALLPTFLEEALRLESPFRGHHRHVLTDTTLG  GVQLPAGSHLTLLWGAANRDPAIFEDPDVLRLDRPSPRGHITFGKGLHFCVGAALARLEA  RTAINLLLDRTREFAIKPDGAHWVPSIMVRRHQKLELEVSAA*  >CYP125A(MAB_0101)  MTTCPFTPGFDFTDPDLIQHRIPAEEFAYLRKTEPIWWNAQPRGVAGFDDDGYWVVTKHA  DVKEVSRLNEVFSNSVNTTVVRYNEDITAEQLEIQRENLLIDMDEPKHRILRRIVSPLFT  PKAVNGLHARLVERAHGIVEEAAEKSSGNFVSDIASVLPMHAIADLVGIPESDRQQVLDW  TNQMFAYDDPAIGRDTATTATVSMLGYAYAMAEERQLNPQDDILTGLVRGAYDDRPLTPL  EFAYFVIQLMVAGNETSRNAITHGVLAFADNPAQWRLYRERRPSTAADEIIRWASPIIAF  QRTALQDVELGGVQIRKDQRVGMFYASANFDEDVFDDPFAFNIERDPNPHLAFGGHGIHY  CLGANLARLEIGIMFDALADRLPDLMPTGAPTRFRSGWINGVVALPANYHGSGPRG*  >CYP108B(MAB_3825)  MATALSEIEEAGRVLADPAAYADELRLHAAMTLLRREQPVTKVITDDYRPFWAVTKHDDI  MAVERDNALWINEPRPLLMNLEQEAELDKQAAMGIELKTLVHIDDPKHRVLRAIGADWFR  PKAMRDMKLRTDELANRYVNKLLEAGGSCDFAQDIAVHFPLYVIMSLLGIPESDFDRMLK  LTQELFGGDDSEFQRGTTPEEQLMALLDFFGYFSGLTASRRQHPTDDLASTIANARVDGE  LLSDVDTASYYTIIATAGHDTTSATIAGGLEALLEHPDQLARLRENPGLMPLAVDEMIRW  VTPVKEFMRTATADTEIRGVPITEGESVLLSYPSGNRDEDIFTDPFTFDIARDPNKHLAF  GFGVHFCLGAALARMEVNSFFSALLPRLESIEVDGNIERTSTIFVGGIKHLPIRYTLR*  >CYP130A(MAB_1406c)  MSVDVSHTVAPRYVPGTADNWTNPWPMYAALRDHDPVHHVVPEYTPDQDYYVLTRHADVY  EAARDWETFSSARGLTVTYGDLEKTGMGDNPPMVMQDPPTHTEFRKLVSRGFTPRQVTAV  EPKVREFVVQRLERLKERGEGDIVVELFKPLPSMVVAHYLGVPEEDRTQFDAWTDGIVAA  ASGGAIDIEAMQGEVAQTIGELMMYFTGLIERRRAEPEDDTVSHLVAAGVGADGDISGTL  QILGFAFTMVTGGNDTTTGMLGGAIQLLHQNPAQRQKLIDDPSLIPGAVEEFLRLTSPVQ  GLARTATRDVIIGDTTIPAGRRALLLYGSANRDEREYGNDAAELDVLRKPRNILTFSHGN  HHCLGAAAARMQSRIALEELLTRIPDFEVDLDGVTWADGSYVRRPLTVPIRVR*  >CYP1130A(MAB_3275)  MIASLSDDPFWPTRKPLRASFSLLTGWGRDRPIPPGPIGLPFIGSAIPMARNPYKFLQDC  HRRYGDIYRVPLPIHPLVLANHPDLVSEFMENTELKYSMSAPIQGKRLQKAVASVGCPVQ  VLEGQALRDRRKRLMPMFGKRHLSVVSDKFVEVFTDRIDRWLLVADTGKEVNLQAELPKV  VLPAFMYAMFSTRLSDDEVLHADTATRSVMRAIASGLFLASPPNIFPLRGRENLPVSGLR  LMRTIRQMIRDRRANPTDDADLLNILLAARGDSSRPLTEIDVYSEIMSAIGGGYETIVAS  MSWTLALLLQHPEHLDRLYDEISILHGNAPTPDDLPRLPWARACFDEGQRLQGAPINPRY  AMEDTELGGYPIPKYTLVASSLYVVHRDPRWWGENAETYDPMQFFDQDRVNARPRLAFQA  FGAGPHHCMGTGMAYMMAQYLLAIIFQRYRLHLRPGWQPRQFFSLSTLVKGGVPATITKA  *  >CYP1134A1(MAB_1426)  MSASALQPEPLSAPVEVAAGECPVNHDLYPGGPTFLRWHHTRVGYLKSWRLALGAVWSTY  RHTISEYLADLPGQDDVIVARAPMRKAVIVRNPELARHVLVANQDNYIKSAEYDLLAVGF  GRGLVTDLNEGLWNRNRRLVQPIFAKRQVDLFAPQMAEAAARTISRWDELYAEGKPVDIT  AEMNYLTMDIVAQTMFGIDLSGDMAERMRIYFARLLKLFGVGFIVGAAPPLRWVVDKLAA  HGPDELSSHTPRLAIRALRIGASVAAPRTMKGLRWVERTIDQLIADHRSGRIARQDNLLA  LLMAAEDPETGAKYTDLEIRDELMTFLGAGFETTAAALAWTWYLLSRNPDARAKLGQEVD  RVLGGRQPTAADVDNLPWTAAVLNEAMRVYPPILGLARTAKADDVLGDYPISAGTTVMVL  IDSIHHNERVWDDAKTFDPARFLKENLQPEQRKAHMPFGAGKRMCVASGFANLEAIIGIA  ALAQNYELDLLPGQQPRREVTFTGGPEGEILMRLRKRHP*  >CYP138A(MAB_1427c)  MEGDVTEISTIDRAADVPKPIAPPLVTLPKLAQGIAFVASRRWTTSRLAKKYGKVYTINI  PKFGYTVVVADPDLTREVFTTSTEILGNIQPNLSQQLGPGSVFALERNEHRHRRKLLAPK  FHGKAMVQYEQIIEEETLRECATWPEGQQFETMEPMMRITLNAILRAVFGADGAELDVLR  RLIPPWVVLASLTTRIPQPKRNYGRFSPWGRLKTYRAHYDAVIGSLIEKALAAPDFEDRT  DILALLLRSTYDDGTTMSRSDVSDELLTLLAAGHETTATTLAWAFERITRHPEVLSRLAE  EAQGDSNEYRQATILEVQRSRPVIDFAGRHVLAPHVDIGPYRIPQGHSVVVSINLMHDDP  VAFPCPERFDPERFMGAKPGNSWVPYGGGTRRCVGAAFANMEMDIVLRTVLRHFTIETTT  APDEKWHSRGVASCPKNNGRVTVRRR*  >CYP153A(MAB_2048c)  MSIPAAVAAKAQSAVPLELQIRGAHLYDKTRRWVTGTNGKKIFTETPIPPVEDVDIADID  LSNPFLYRQGRWKSYFERVRNEAPVHYQARSPFGPFWSVTRHADIIAVDKNHEAFSAEPF  IIIGRPPRFMDIAMFIAMDPPQHDLQRASVQGVVAPKNLREMEGLIRSRVQEVLDDLPVD  QPFNWVHHVSIELTARMLATLLDFPYEQRHKLVEWSDLATSMEQANGGPSDNDRVFRGMV  DMAKGLSALWHDKAARTANGERPGFDLITMLQANEDTKDLIDRPMEFLGNLVLLIVGGND  TTRNSMSGGVLALNQFPDQFEKLKANPDLIPNMVSEIIRWQTPLAYMRRVAKKDIMLNGQ  FIRKGDKVVMWYASGNRDERVFERADELIIDRSNARNHISFGFGVHRCMGNRLAELQLRI  LWEELLPRFENIEVVGEPEYVQSNFVRGISKLMVRLTPRPSA*  >CYP1128A(MAB_3316)  MTTANPVTPRIPWDSRDPYVYLEDLRAHGDVVLDENAGTWVVLGYQPAREVLGGSGWSSD  PLASPQLRAQAPEFLDSSGFGRNMLFADEPDHTELRGTVRDVFTPGFIAGLREGVHTIAS  HVVEYPSTGEYFDFMADIALPLPIAIIGEWLGLDDSSAAVLRQESPAIIQMLGAFADVDT  VMAGTAAGVTLATELLPLAADRRAHPGDDLLSLIASNTDLPLEDVVTTALIIAIAGHETT  ANLLGASMVRLLTPRPDGTRLADDIDPDDPTVITELLRLDGPVLATARVATRAHTLAGNT  IEGGQTVLIAIAAANRDPQVFVDPAVFRRDRTSPPLAFGYGTHHCLGSALARLETTLVLR  EVLARQPVITGPVSWRDTPAIRGPRSIPMRFQT*  >CYP136D1(MAB_2078)  MTNHLLAPAHHVKERLSSVIMVPAPHAVDDRWRRWSRDWPVRELAPAPAGSGLKAVRGDA  GLPFVGHTLDYIRFGSDFSRERYDRLGSVSWMGAFGTKMVVIAGPDATREAFTSEAKAFS  QDGWSFLIDAFFHRGLMLMSFDEHLMHRRIMQEAFTRPRLTGYVEQVTPCVRSAVPAWPV  GPSVRIYPLLKELTLDIATDVFMGGRGKDESDAVNKAFVATVRAASSLVRAPLPGTRFRA  GVQGRRVLEDYFFRHLPAARAGETEDLFAALCQATTEDGERFSDEDVVNHMIFLMMAAHD  TSTITTTAVTYFLAKYPQWQEAAAAEAAAIGDGLPDIEALEKMTVIDRVIKEALRLLAPV  PLVMRKTVRDVAIDGYHIPSNTLCAITPAVNHFDRTIWNDPERFDPSRFDEPRREDQHHR  FAWVPFGGGAHKCIGMQFGTLEVKAILHRMLRSFTWKVPENYHVRWDNTSLPIPVDGLPL  EMKRR*  >CYP161NSF1(MAB_3961c)  MPAAQQRELQRAGTVHRVTTAVGDPAWLITGYATVRQLFDDERVGRSHPEPDTAARSGDS  AFFGGPIGSYETEREDHARGRRLMQPHFTPKQMRTLAGRVHELADELITAMIDRGSPADF  YTAVAVPLPMMVLCELLGVPYADRDEFREWTVAASNTRDRSRSEWGMGQLFVYGMQLVGR  KRQQPGDDVISRLCTIDGVADHEIASQSMALLLGGHETTVVQLGLATLLLLANPEQWALL  VSQPDLVPNAVEETLRASRTGGGEIPRYAREDLEIDGVHIAAGELLLLDVGAANHDPAVF  GCPDQLDVARKHLAHVIFGYGSRYCVGAPLARMQLTEVLGQLVERLPGLHLRRDISELTM  RGDLLTGGLREVPVGW*  >CYP286D1(MAB_3962)  MARITSVPCDGETTTYVSPPKLRVARALARLPRLGDTVGLMLDPTFYLFEKYLRHGPVFT  VKMPYQTYTVLAGSEAATFMSSKDGRECLTVGSSWRFVEEQFGGKDSLVAVDGPQHKQWR  TLLQRGYSREAIADRYSEAIDVIDDAVDKHWRPGESVPVLPAMQKLSIAQVGTFLGGVRP  TDDDIEDIALVTREILKIAPVQHIPKFMLQLPRYVNARSRVARVAQNALALAGHSSASGR  RGQSTLLEDILESCRDDPNAANQRNMLFHSVLPYFAGVETTSATATYALYLILRHPDVLC  RIQDEVAVMFERGGITHDSLFHSTPVLHGAIMEAMRLRPIASFIIRVAAKDFEFHGHQIR  SGEGVFIGTTVPHFLSEFYENPMKFDVDRYGDSAGPNRTPGAYSPFGRGPHMCVGKGLAE  SLMQLMLARIIHRRTLELSSRSYRLSNRVSSSSPSPKFAVRVVSSRR*  >CYP140NSF1(MAB_4456)  MRTRDQIKYWSRWATMHGISRAALLTQVRTLPLAALFLGPDRAEKHYRYIEEIRASGSVT  PSRAGGLIFTDLALTREILRDNRFITMAPTNIPSPILPLAFSRWVFARTEPGLPNPVEPP  AMLAVDPPEHTRFRKLVSKAFTPRAVSKLEDRVREVTTELLDNLERHERADLLHDYASQL  PVAIIAEMLGVPRADAPFLLEWGNHGAALLDIGMTWSAYRDATQALIEIDRYFDAHLVRL  RGELAEDPTVDGILASIVRDGDLNDRELKATMALLLGAGFETTVNLIGNGIAALLRHPQQ  LAHLRENPDGWPNAVEEILRYDSPVQITGRVATQACEFEGHTLAAGSMAILLLGGANRDP  AVFDQPDVFDVLRANAREHVAFGSGIHVCLGASLARMEGVVALQSLFERFPELALAADPT  PGKHVNLHGFGSLPVNLGRARVPASS*  >CYP140B(MAB_4457)  MQHRIKQRTHWAVTHGIGRAYLKVLARRGEPVAQLGIDVGQAPDIYRIIDKIRERGRLSR  AGDGWITADAQIVRTIFRDNRFVTFKPEHRSASPIIQRLAAWSDPQLLNPAEPPSILITD  PPDHGRLRRLVAAPFTPRAIEGLRDRIQEVTNGHLDVLQQRQSPDLIADFTAKIPIAVIG  EMIAVPPPDYSRLYTAMNRAIQLIATTAPSWSEYQDGTAALREIDEYLEKHVARLRREGT  ESELATALLDSDLSHFELKMFFAVFLGAGFVTTTHLMGKAIVTLLRHPEQLALLQADPSL  WPNAVEELMRYDTSNQWSARVATETVEIEGHTIEAGQSALLLLGGANRDPAAFENPDVFD  ITRPNARENITLGTGIHVCLGQVLARAELHTALQTLFERFPRLSLAGEPEYLNGMGIHGL  RLLPVTLG*  >CYP1130B1(MAB_4944c)  MTELVGKEVTRGCPAEPHQDGPTGFGDFPRELAGQWGPLPLNPSVHEVNEIARARQRFEY  SRKDPWWLTRASFPLASSIVRGVGSPALTPPGPVGHPVVGSVPEMRREPFEFFRRCAREF  GDVYRVPFPLGGSIVVVNHPDYASQVMDDPVGRYSMIGPGQAAMGVIGAAIPMLEGDKFR  QRRRMLMPMFGRRHLARVAEVIADEFVRRVDRWACWADTGQVVDLQHAIAQVTLPAFLRA  MFSSTITEQEISETDVDLRTFMSLMASVTMMSPLPSVLPLPGRESAPRSMWRLWRLTRRL  IQQRRKNPIETPDLLSLLLEASYDDGSQLSERDLSMELMILMAGGYETVVASLSWTLALL  LGHPEHLRRLYAEVDGLAGALPTPDDLPKLAWAKACFDEGQRLQGHPLNPRFAMDDDVIG  GYFIPKHTIVGPSLYSIHRDPRWWADPDTYDPNRFMDEVAARSRPRLAFMPFGSGRHHCL  GTGMAYMNAQFLLAIIFQRYRLALPEGWKPKHHFNFSVTLEGGLPVTLTTV*  >CYP135B(MAB_4693)  MSKTAKIALPPGPPLPLAVQSILMASYGLRFLAGCQRRYGNMFTLRIPLSGKVVYLADPA  DIKTVYAGDPHVFHSGEAHWFFRGLLGDSSLFVLDEDEHHHQRRLLMPAFHRDAVARQAT  QMAQIAAANIAEWPVGESFPVAPRTTDITLEVILRTVIGASDPVRLAALRKVVPRLLYMK  PWETPAITNPGLRRYWPWTAVGRRMAETDALLYAEIAERRADPNLTERTDVLAMLVRATD  DDGRTMSDQELRDHLLTSIAAGHETTATALSWVLERLTRHPDALLKAAQAAEASAAGDPA  GDEYLDAVVKETLRIRPVIFSSGRVLKESVEVGGYQLPVGIMVDPAIGLVHASGAVYPDP  ARFDPDRMVGTTLSPTTWLPFGGGNRRCLGATFAMVEIRVVLREILRRVELNTTTAPDEK  QQAKHVTFVPHRGGSISVRAIRTCAPAVTPTCPADVHGTGQARDPQR* |
| ***Mycobacterium abscessus* subsp. *bolletii* 50594** |
| DATABASE: KEGG; P450 count: 22; Families: 18; Subfamilies : 20 |
| >CYP125A(MASS_0104)  MTACPFTPGFDFTDPDLIQHRIPAEEFAYLRKTEPIWWNAQPRGVAGFDDDGYWVVTKHA DVKEVSRLNEVFSNSVNTTVVRYNEDITAEQLEIQRENLLIDMDEPKHRILRRIVSPLFT PKAINGLHSRLVERAHSIVEEAAEKSSGNFVSDIASVLPMHAIADLVGIPESDRQQVLDW TNQMFAYDDPAIGRDTATAATVSMLGYAYAMAEERQLNPQDDILTGLVRGAYDDRPLTPL EFAYFVIQLMVAGNETSRNAITHGVLAFADNPAQWRLYRERRPATAADEIIRWASPIIAF QRTALQDVELGGVQICKDQRVGMFYASANFDEDVFDDPFTFNIERDPNPHLAFGGHGIHY CLGANLARLEIGIMFDALADRLPDLMPTGAPTRFRSGWINGVVALPANYLGAGPRG  >CYP164A(MASS_0278)  MRLLHLRALHPGLYASPVQGQGDPCLDAGHHPQRAVHHRLGRPHSRQHRGWLLPGAARRH PGPLLPLSHEAQVHLSISAHTGHMPITTDPGTLLLQVLDPANRANPYPVYGQLVEQTQAG PVHPPGMDVSVLATFADCNAVLRHPQASSDRRKSSIVQRQLAQNPNLIARPSFLGLDAPD HTRLRKLASKAFAPRVINAMAEDIQTFVDKMLDDIALRGTFNLVTEFAYPLPVAVICRML GVPIEDEPEFGRASALLGQGLDPVYALTGQSPANMDERFQAARWMWDYFIDLIASRRRNL GDDLLSALIQVEEAGDQLTEEEIISTCTLLLIAGHETTVNLIANASLAMLRDPEQWKLLG RNADRAPLVVEETLRYDPPVHMVARVADGEMAIRDFVLPRGEWVLLMLAAAQRDPDVGPD LDVFNPDRTEIKHLAFGHGPHFCLGAPLARLETRLALSSLTARFPGARLMDEPTYKPNVT LRGLAELPVSIA  >CYP125A(MASS_0581)  MVQAQHPHLPDGIDFTDPELFVHGIPERELAELRHTEPIWWNHTERGVAGFDDDGFWVVS KHKDVKEVSLRCEVFSSEQNTAIPRYLPTTPRERIDATRLIMLNMDPPRHSRLRHIISRG FTPRAISRLRDDLNARAQGIAKAAAQLRHGDFVEQVACELPLQAIAGLMGTPLDEREQLF DWSNRLVGSSDGEDDSAVASAELLMYAMGVAARKTAEPGADICTDLVNADIDGQKLSDDE FGFFVMLLAVAGNETTRNSITHGMHAFTQFPEQWELYKKTRPETAADEIVRWATPVTSFQ RTALEDTELGGVRIKKGQRVVMMYRSANFDEEVFENPFTFDIMRDPNPHVGFGGNGEHHC VGANLARMTINLMFNAIADHMPDLASAGEPDRLRSGWLNGVKHWEVDFCPAGYGRAS  >CYP125A(MASS_0583)  MVHPSLPAGFDFTDPEIYAERLPVEELKELRKTAPIWWQEQPDGVGGFNDGGYWVVTKHK DVKEVSLRSDVFSSWENTAIPRFQDDITREAIELQRYVMLNMDAPHHTRLRKIISRGFTP RAIGRLRDELNERAQEIAKAAAASGTGDFVEQVSCELPLQAIAGLLGVPIEDRGKLFNWS NEMTSYDDPEYADIDPAASSMEILAYSMEMAKQKAENPGEDIVTTLINAEVEGEGKLSDD EFGFFVIMLAVAGNETSRNSITQGMMAFTQFPEQWELYKKERPETAADEIVRWATPVTSF QRTALEDTELDGVKIKKGQRVVMMYRSANFDEDVFEDPFSFNIMRNPNPHMGFGGSGAHY CIGANLARLTINLMFNAIADHMPNLAPAGDPKRLQSGWLNGIKHWQVDFTGASGCPVLQ  >CYP51B(MASS_1212)  MTTPTVPRVSGGEDQYGHLEEFRTDPIALMKRIRAECGNVGTFQLADKQVVFLSGAEANE FFFRSSDEDLDQAEAYPFMTPIFGKGVVFDADPERRKEMLHNAALRGEQMKGHAATIENE VRQMISRWGESGEVDLLDFFAELTIYTSSACLIGKKFRDELDGRFAHLYHQLEQGTDPLC YVDPYLEIESFRQRDEARTGLVALVQEIINGRVQRSADSPTDKSQRDMLDVLIMIKDEHG NPRFTADEITGMFISMMFAGHHTSSGTASWVLIELLRHPDIRSQVIDELDELYADGGEVS FHALRQIPKLENVLKETLRLHPPLIILMRVAKGEFEVGGFPIHDGDMVAASPAVSNRIAE DFPNPDGFVPDRYEKPRQEDIVNRWTWIPFGAGRHRCVGAAFATMQIKAIFSVLLREYEF EMAQPADSYHNDHSKMVVQLAQPAKVRYRKRSA  >CYP123A(MASS_1214)  MTAAETSAAPLVFDPYDYDFHEDPYPYYRRLRDEAPLYRNDDLKFWALSRHHDVLQGFRN SEALSNANGVSMDKASFGPHAKLVMSFLAMDDPEHLRLRTLVSKGFTPRRIRELEGQVVT LARTHLDRALTNASDRSFDFIAEYAGKLPMDVISELMGVPESDRTRIRELADGVMHREDG LADVPPEAIQASFDLMTYYIEMVRERRRRPTEDLTSALLQAEIDGDRLTDEEVLAFLFLM VIAGNETTTKLLANAVYWGHRNPDQLATVHADHDRIPLWVEESLRYDTSSQILARTVAED MTLYDTKIPAGDILLLLPGSANRDDRVFDDADQYRIGREIGAKLVSFGSGAHFCLGAHLA RMEAKVALTELFTRISGYEIDERNSVRVHSSNVRGFAHLPMTVQLREGH  >CYP130A(MASS_1397)  MSADVSHAVAPRYVPGTADNWTNPWPMYAALRDHDPVHHVVPEYTPDQDYYVLTRHADVY EAARDWETFSSARGLTVTYGDLEKTGMGDNPPMVMQDPPTHTEFRKLVSRGFTPRQVTAV EPKVREFVVQRLERLKERGEGDIVVELFKPLPSMVVAHYLGVPEEDRTQFDAWTDGIVAA ASGGAIDIEAMQGEVAQTIGELMMYFTGLIERRRAEPEDDTVSHLVAAGVGADGDISGTL QILGFAFTMVTGGNDTTTGMLGGAIQLLHQNPAQRQKLIDDPSLIPGAVEEFLRLTSPVQ GLARTATRDVIIGDTTIPAGRRALLLYGSANRDEREYGNDAAELDVLRKPRNILTFSHGN HHCLGAAAARMQSRIALEELLTRIPDFEVDLDGVTWADGSYVRRPLTVPIRVR  >CYP1134A(MASS_1417)  MSASALQPEPLSAPVEVAAGECPVNHDLYPGGPTFLRWHHTRVGYLKSWRLALGAVWSTY RHTISEYLADLPGQDDVIVARAPMRKAVIVRNPELARHVLVANQDNYIKSAEYDLLAVGF GRGLVTDLNEGLWNRNRRLVQPIFAKRQVDLFAPQMAEAAARTVSRWDELYAEGKPVDIT AEMNYLTMDIVAQTMFGIDLSGDMAERMRIYFARLLKLFGVGFIVGAAPPLRWVVDKLAA HGPHELSSHTPRLAIRALRIGASVAAPRTMKGLRWVERTIDQLIADHRSGRIARQDNLLA LLMAAEDPETGAKYTDLEIRDELMTFLGAGFETTAAALAWTWYLLSRNPDARAKLGQEVD RVLGGRQPTAADVDNLPWTAAVLNEAMRVYPPILGLARTAKADDVLGDYPISAGTTVMVL IDSIHHNERVWDDAKTFDPARFLKENLQPEQRKAHMPFGAGKRMCIASGFANLEAIIGIA ALAQNYELDLLPGQQPRREVTFTGGPEGEILMRLRKRDR  >CYP138A(MASS_1419)  MEGDVTEISTIDRAADVPEPIAPPLVTLPKLAQGIAFVASRRWTTSRLAKKYGKVYTINI PKFGYTVVIADPGLTREVFTTSTEILGNIQPNLSQQLGPGSVFALERNEHRHRRKLLAPK FHGKAMVQYEQIIEEETLRECASWPEGRQFETMEPMMRITLNAILRAVFGADGAELDVLR RLIPPWVVLASLTTRIPQPKRDYGRFSPWGRLKTYRAHYDAVIGSLIDKALAAPDFEDRT DILALLLRSTYDDGTTMSRSDVSDELLTLLAAGHETTATTLAWAFERITRHPRVLSRLAE EAQGDSNEYRQATILEVQRSRPVIDFAGRHVLAPHVDIGPYRIPQGHSVVVSINLMHDDP VAFPRPECFDPERFMGAKPGNSWVPYGGGTRRCVGAAFANMEMDIVLRTVLRHFTIETTT APDEKWHSRGVASCPKKNGRVTVRRR  >CYP102NSF3(MASS_1645)  MTNTRASTHGTAPLPHPRFRLPILGDLASVDFAQPIQGLAREARRHHGIFEQRIGDFPVV VVDGPELIEEINNEELWEKNVGPTLHKLRSVAGDGLFTAYNTEDNWRKAHDILMPAFTKQ AMTNYHASIVDTVHELVTVWNSHAKNKSWVDVPFDLNRLTIEIISRSGFGYSFSSLADPR ENPFLSAVLRELQYANRRTDSIPLYEQFLGGARRREHVADKRAIRAQIDKIIDARRAESR TGQSPDILDIMLTSSDPLTGEKLDNNNIGNQILTFLVAGSETSANAIAFALHFLSTHPDI AAKARAEVDASWPARAFPAFAFDQIAKLRYLRLVVDETLRLWPVAPGYFRQAKQDTTIGE GRYSFKEKDWVFVNLLAAQRHPSWGPDADQFNPDRFLTENRRKLPTHVHRPFGVGARACI GRQFAQHEILIALAAILHQYELTPRPGYKLKVSETLTLKPSALELGLTKRT  >CYP144NSF1(MASS_1928)  MAMNTDELFDPQVLEDPYPFYRRLREKAPVWPVGDSGFYFVSRWDLVVEATERAEDFSSN LTAALMKSADGLTVAPMGPPADPTHVLATGDDPVHHAHRRLVLPTLVAKRITALEPVMAQ TGSRLWERGVNGDGIDWMAAMGDALPMTMVARLIGLPVGDVPQLVQWGYSSTEMLGGLNT PQRQAQVVTDTMYLVLYLREHLEKELAAPGDDLLGYLAQACNRGDISLDIGVMILVQLVG AGGESTAGLMGNAVRILGENPRLQQHIRENRALLPTFLEEALRLESPFRGHHRHVLTDTT LGGVELPAGSHLTLLWGAANRDPAIFEDPDVLRLDRRSPRGHITFGKGLHFCVGAALARL EARTAINLLLDRTREFAIKRDGAHWVPSIMVRRHQKLELEVSAA  >CYP1132A(MASS_2078)  MRSIDTDSMAPAVPKPRGHRLPARLPIHKAALLGLSCMGDPTRLPYLLLPKIANRYGDIV RLFTGPTPALTLTLINHPDYVDHVFTRHHGRYVKHEATIELVSGEPVALPLLEGQEWKRV RSAFNPYFGERALAQATPLMMEGITDRVDAWSRHVNSGELVDLEHELGAVVMDGLMRSMF KVRLSPAEIDHAVDGARRYGVYVISRVAMHFLPRWLPNPLRRSGEEAKAELFGILDRFVQ ERAGCPSTGTPDLVDTLLALEFDGCPQTRERRRRSEAAGLVFAGFETTAAALAWTIALLC RNPIALGKAYAEVDALGGKALAYDDLENLNYLRACFDEAQRFQAAPANVRTAIEDDEVGG YFIPRGSQVIITQYALQRDPRFWNEPERFNPDRFLTDKINRNTFLPFSIGPRKCMGTRMA YIEGTLVLGAILQRYAFQIRDGWTPRHRVRVSTGLAGGLPARLFAR  >CYP1130A(MASS_3220)  MIASLSDDPFWPTRKPLRASFSLLTGWGRDRPIPPGPIGLPFIGSAIPMARNPYKFLQDC HRRYGDIYRVPLPIHPLVLANHPDLVSEFMENTELKYSMSAPIQGRRLQKAVTSVGCPVQ VLEGQALRDRRKRLMPMFGKRHLSVVSDKFVEVFTDRIDRWLLVADTGKEVNLQAELPKV VLPAFMYAMFSTRLSDDEVLHADTATRSVMRAIASGLFLASPPNIFPLRGRENLPVSGLR LMRTIRQMIRDRRANPTDDADLLNILLAARGDSSRPLTEIDVYSEIMSAIGGGYETIVAS MSWTLALLLQHPEHLERLYDEIAILNGNAPTPDDLPRLPWARACFDEGQRLQGAPINPRY AMEDTELGGYPIPKYTLVASSLYVVHRDPRWWGENAETYDPMQFFDQDRVNARPRLAFQA FGAGPHHCMGTGMAYMMAQYLLAIIFQRYRLHLRPGWQPRQFFSLSTLVKGGVPATITKA  >CYP1128A(MASS_3254)  MNTANPVIPRIPWDSRDPYAYLEDLRAHGDVVLDDNAGTWVVLGYQAAREVLSGSGWSSD PLASPQLQAQAPEFLDSSGFGRNMLFADEPDHTELRATVRDVFTPGFIAGLREGIHTIAS YVVEYPATGENFDFMADIALPLPIAIIGEWLGLDNSSAAVLRQESPAIIQMLGAFADVDT VMAGTAAGAALATELLPLAADRRVHPGDDLLSLIASNTDLPLEDVVATALIIAIAGHETT ANLLGASMIRILAPRPDGTRLADDIDPDDPTVITELLRLDGPVLATARVATSAHLLAGNT IEEGQTVLIAIAAANRDPQVFVDPAVFRRDRTSPPLAFGYGTHHCLGSALARLETTVVLR EVLARQPVVTGPVSWRETPAIRGPRSIPMRFQT  >CYP136A(MASS_3368)  MATISSTDYLIDQAKRRLPTMNTLPGMGYIENYLNNREWPMTELAAPPPGSGLKPVMGDQ GLPMLGHMVEMFRGGIDWVLNMYQERGPVSWTQTPIGKIVAALGPDATQAVFSNANKDFS QQGWVPVIGPFFNRGLMLLDFQEHREHRLIMQQAFLRSRLAGYVEQIDAVASEIVAQWPT NDNRFLFYPAIKELTLDVASVVFMGNEPHAQHERLEKVNKAFVATTRAGGAIFRFGLPPF KWWQGLQGRKLLEEYFAERVGIARQKTEGADMLTALCHAETDEGDAFTDTDIVNHMIFLM MAAHDTTTSTTTTMAYYLAANPEWQERVRDESDRLGDGPLDIDSLEKLESLDLVMNEALR LVTPLPFNIRSTVRDTDLLGFHIPAGTMINIWPGMNHRLPELWTEPDKFDPDRFSEPRNE HKRHRYAFSPFGGGAHKCIGMVFGQLEIKAVMHRLLRRYRFELAHPHYQPKWDYAGMPLP IDGMPIILRPLH  >CYP108B(MASS_3837)  MATALSEIEEAGQVLADPTAYADEPRLHAAMTLLRREQPVTKVITDDYRPFWAVTKHDDI MAVERDNALWINEPRPLLMNLEQEAEMDKQAAMGIELKTLVHIDDPKHRVLRAIGADWFR PKAMRDMKLRTDELANRYVNKLLEAGGSCDFAQDIAVHFPLYVIMSLLGIPESDFDRMLK LTQKLFGGDDSEFQRGTTPEEQLMALLDFFGYFSGLTASRRQHPTDDLASTIANARVDGE LLSDVDTASYYTIIATAGHDTTSATIAGGLEALLEHPDQLARLRENPGLMPLAVDEMIRW VTPVKEFMRTATADTEIRGVPITEGESVLLSYPSGNRDEDIFTDPFTFDIARDPNKHLAF GFGVHFCLGAALARMEVNSFFSALLPRLESIEVDGNIERTSTIFVGGIKHLPIRYTLR  >CYP161NSF1(MASS_3974)  MNAVQPPQLPFPREGLFVPAAKQRELQRAGTVHRVTTAVGDPAWLITGYATVRRLFDDER VGRSHPEPDTAARSGDSAFFGGPIGSYETEREDHARARRLMQPHFTPKQMRTLAGRVHEL ADELITAMIDQGSPADFYTAVAVPLPMMVLCELLGVPYADRDEFREWTVAASNTRDRSRS EWGMGQLFVYGMQLVGRKRKQPGDDVISRLCAIDGVADHEIASQSMALLLGGHETTVVQL GLATLLLLANPEQWALLVSQPDLVPNAVEETLRASRTGGGEIPRYAREDLEIDGVHIAAG ELLLLDVGAANHDPAVFGCPDQLDVARKHLAHVIFGYGSRYCVGAPLARMQLTEVLGQLV ERVPGLHLRRDISELTMRGDLLTGGLREVPVGW  >CYP286D1(MASS_3976)  MGVEMARITSVPCDGETTTYVSPPKLRVARALARLPRLGDTVGLMLDPTFYLFEKYLRHG PVFTVKMPYQTYTVLAGSEAATFMSSKDGRECLTVGSSWRFVEEQFGGKDSLVAVDGPQH KQWRTLLQRGYSREAIADRYSEAIDVIDDAVDKHWRPGESVPVLSAMQKLSIAQVGTFLG GVRPTDDDIEDIALVTREILKIAPVQHIPKFMLQLPRYVNARSRVARVAQNALALAGHSS ASGHRGQSTLLEDILESCRDDPNAANQRNMLFHSVLPYFAGVETTSATATYALYLILRHP DVLCRIQDEVAVMFERGDITHDSLFHSTPVLHGAIMEAMRLRPIASFIIRVAAKDFEFHG HQIRSGEGVFIGTTVPHFLSEFYENPMKFDVDRYGDSVGPNRTPGAYSPFGRGPHMCVGK GLAESLMQLMLARIIHRRTLELSSRSYRLSNRVSSSSPSPKFAVRVVSSRR  >CYP140NSF1(MASS_4490)  MRTRDQIKYWSRWATMHGISRAALLTQVRTLPLAALFLGPDRAEKHYRYIEEIRASGSVT PSRAGGLIFTDLALTREILRDNRFITMAPTNIPSPILPLAFSRWVFARTEPGLPNPVEPP AMLAVDPPEHTRFRKLVSKAFTPRAVSKLEDRVREVTTELLDNLERQERADLLHDYASQL PVAIIAEMLGVPRADAPFLLEWGNHGAALLDIGMTWSAYRDAIQALIEIDHYFDAHLVRL RGELAEDPTVDGILASIVRDGDLNARELKATMALLLGAGFETTVNLIGNGIAALLRHPQQ LAHLRENPDGWPNAVEEILRYDSPVQITGRVATQTCEFEGHTLAAGSMAILLLGGANRDP AVFDQPDVFDVLRANAREHVAFGSGIHVCLGASLARMEGVVALQSLFERFPELALAADPT PGKHVNLHGFGSLPVNLGRARVPASS  >CYP140B(MASS_4491)  MQDRIKQRTHWAVTHGIGRAYLKVLARRGEPVAQLGIDVGQAPDIYRIIDKIRERGRLSR AGGGWITADAQIVRTIFRDNRFVTFKPEHRSTSPIIQRLAAWSDPQLLNPAEPPSILITD PPDHGRLRRLVAAPFTPRAIEGLRDRIQEVTNGHLDVLQQRQSPDLIADFTAKIPIAVIG EMIAVPPPDYSRLYTAMNRAIQLIATTAPSWSEYQDGTAALREIDEYLEKHVARLRREGT ESELATALLGSDLSHFELKMFFAVFLGAGFVTTTHLMGKAIVTLLRHPEQLALLQADPSL WPNAVEELMRYDTSNQWSARVATETVEIEGHTIEAGQSALLLLGGANRDPAAFENPDVFD ITRPNARENITLGTGIHVCLGQVLARAELHTALQTLFERFPRLSLAGEPEYLNGMGIHGL RRLPVTLG  >CYP135B(MASS_4723)  MSKTAKIALPPGPPLPLAVQSILMASYGLRFLAGCQRRYGNLFTLRIPLSGKVVYLADPG DIKTVYAGDPHVFHSGEAHWFFRGLLGDSSLFVLDEDEHHHQRRLLMPAFHRDAVARQVS QMAQIAAANIAEWPVGESFPVAPRTTDITLEVILRTVIGASDPVRLAALRKVVPRLLYMK PWETPAITNPGLRRYWPWRAVGRRMAETDALLYAEIAERRADPNLTERTDVLAMLVRATD DDGRTMSDRELRDHLLTSIAAGHETTATALSWVLERLTRHPDALLKAAQAAEASAAGDPA GDEYLDAVVKETLRIRPVIFSSGRVLKESVEVGGYQLPAGIMVDPAIGLVHASDAVYPDP ARFDPDRMVGTTLSPTTWLPFGGGNRRCLGATFAMVEIRVVLREILRRVELNTTTAPDEK QQAKHVTFVPHRGGSISVRAIRTCAPAVTPTCPADVHGTSQARDPQR  >CYP1130B(MASS_4945)  MTELVGKEATRGCPAEPHQAGPTGFSDFPRELAGRWGPLPLNPSVHEVNEIARARQRFEY SRKDPWWLTRAGIPLASSIVRGVGSPALTPPGPVGHPVVGSVPEMRREPFEFFRRCAREF GDVYRVPFPLGGSIVVVNHPDYASQVMDDPVGRYSMIGPGQAAMGVIGAAIPMLEGDKFR QRRRMLMPMFGRRHLARVAEVIADEFVRRVDRWACWADTGRVVDLQHAIAQVTLPAFLRA MFSSTITEQEIRETDVDLRTFMSLMASVTMMSPLPSVLPLPGRESAPRSMWRLWRLTRRL IKQRRKTPIETPDLLSLLLEASYDDGSQLSERDLSMELMILMAGGYETVVASLSWTLALL LGHPEHLRRLYAEVDSLAGALPTPDDLPKLAWAKACFDEGQRLQGHPLNPRFAMDDDVIG GYFIPKHTIVGPSLYSIHRDPRWWTDPDTYDPNRFMDEVAARSRPRLAFMPFGSGRHHCL GTGMAYMNAQFLLAIIFQRYRLALPEGWKPKHHFNFSVTLEGGLPVTLTAV |
| ***Mycobacterium abscessus* 47J26** |
| UniProtKB; P450 count :25; Families: 19; Subfamilies: 22 |
| >CYP108B6(G6X178_MYCAB)  MATALSEIEEAGQVLADPTAYADEPRLHAAMTLLRREQPVTKVITDDYRPFWAVTKHDDI  MAVERDNALWINEPRPLLMNLEQEAEMDKQAAMGIELKTLVHIDDPKHRVLRAIGADWFR  PKAMRDMKLRTDELANRYVNKLLEAGGSCDFAQDIAVHFPLYVIMSLLGIPESDFDRMLK  LTQELFGGDDSEFQRGTTPEEQLMALLDFFGYFSGLTASRRQHPTDDLASTIANARVDGE  LLSDVDTASYYTIIATAGHDTTSATIAGGLEALLEHPDQLARLRENPGLMPLAVDEMIRW  VTPVKEFMRTATADTEIRGVPITEGESVLLSYPSGNRDEDIFTDPFTFDIARDPNKHLAF  GFGVHFCLGAALARMEVNSFFSALLPRLESIEVDGNIERTSTIFVGGIKHLPIRYTLR  >CYP1131A1(G6X451_MYCAB)  MSSVTFSPADIAGLPTAPGPSGIAGAGALVRLIRDPWLYPLTLTEKYGDVVSIPTPFVKT  VYIGHPDWVDHVLVKHPDRYRRSDMVAKQMVPPKVGHNFFAFADDQEWQRGRSLYRPSFT  QKNLAELGDLFTESVTGQVDSWAQMPCSADGYLDIEEMTRKLALIVLFNAMFDEYLSPRM  LEASVTKNAISFGMLATTVRVAMYSLPAWVPRPLQRRMDGLLEIIMGGADQLIEMRRRHP  TERTDILNLLIGATYDDGRPLENDKIAVEIAGMIIGGHETTAAALAWTFALVSGHSDIER  RLIDEVDALGGKPVTVADMARLPLARACFDEAQRLQGGLVINPKTALVDDEMGGYRIAAG  TTILYSSLAMQRDPRFWPEPDRYNPDRFLNNEADLRAFVPFGRGQRLCLGMRMAYIEAVL  TIATAYQRYRFELPKGYEPRHQYRMSMGLKGGLPARIVRR  >CYP135B(G6XE26_MYCAB)  MSKTAKIALPPGPPLPLAVQSILMASYGLRFLAGCQRRYGNLFTLRIPLSGKVVYLADPG  DIKTVYAGDPHVFHSGEAHWFFRGLLGDSSLFVLDEDEHHHQRRLLMPAFHRDAVARQVS  QMAQIAAANIAEWPVGESFPVAPRTTDITLEVILRTVIGASDPVRLAALRKVVPRLLYMK  PWETPAITNPGLRRYWPWRAVGRRMAETDALLYAEIAERRADPNLTERTDVLAMLVRATD  DDGRTMSDRELRDHLLTSIAAGHETTATALSWVLERLTRHPDALLKAAQAAEASAAGDPA  GDEYLDAVVKETLRIRPVIFSSGRVLKESVEVGGYQLPAGIMVDPAIGLVHASDAVYPDP  ARFDPDRMVGTTLSPTTWLPFGGGNRRCLGATFAMVEIRVVLREILRRVELNTTTAPDEK  QQAKHVTFVPHRGGSISVRAIRTCAPAVTPTCPADVHGTSQARDPQR  >CYP1130B(G6XDJ4_MYCAB)  MPEMRREPFEFFRRCAREFGDVYRVPFPLGGSIVVVNHPDYASQVMDDPVGRYSMIGPGQ  AAMGVIGAAIPMLEGDKFRQRRRMLMPMFGRRHLARVAEVIADEFVRRVDRWACWADTGR  VVDLQHAIAQVTLPAFLRAMFSSTITEQEIRETDVDLRTFMSLMASVTMMSPLPSVLPLP  GRESAPRSMWRLWRLTRRLIKQRRKNPIETPDLLSLLLETSYDDGSQLSERDLSMELMIL  MAGGYETVVASLSWTLALLLGHPEHLRRLYAEVDGLAGALPTPDDLPKLAWAKACFDEGQ  RLQGHPLNPRFAMDDDVIGGYFIPKHTIVGPSLYSIHRDPRWWTDPDTYDPNRFMDEVAA  RSRPRLAFMPFGSGRHHCLGTGMAYMNAQFLLAIIFQRYRLALPEGWKPKHHFNFSVTLE  GGLPVTLTAV  >CYP136D1(G6X706_MYCAB)  MTNHLLAPAHHVKERLSSVIMVPAPHAVDDRWRRWSRDWPVRELAPAPAGSGLKAVRGDA  GLPFVGHTLDYIRFGSDFSRERYDRLGSVSWMGAFGTKMVVIAGPDATREAFTSEAKAFS  QDGWSFLIDAFFHRGLMLMSFDEHLMHRRIMQEAFTRPRLTGYVEQVTPCVRSAVPAWPV  GPSVRIYPLLKELTLDIATDVFMGGRGKDESDAVNKAFVATVRAASSLVRAPLPGTRFRA  GVQGRRVLEDYFFRHLPAARAGETEDLFAALCQATTEDGERFSDEDVVNHMIFLMMAAHD  TSTITTTAVTYFLAKYPQWQEAAAAEAAAIGDGLPDIEALEKMTVIDRVIKEALRLLAPV  PLVMRKTVRDVAIDGYHIPSNTLCAITPAVNHFDRTIWNDPERFDPSRFDEPRREDQHHR  FAWVPFGGGAHKCIGMQFGTLEVKAILHRMLRSFTWKVPENYHVRWDNTSLPIPVDGLPL  EMKRR  >CYP1132A(G6X772_MYCAB)    MGDPTRLPYLLLPKIANRYGDIVRLFTGPTPALTLTLINHPDYVDHVFTRHHDRYVKHEA  TIELVSGEPVALPLLEGQEWKRVRSAFNPYFGERALAQATPLMMEGITDRVDAWSRHVNS  GELVDLEHELGAVVMDGLMRSMFKVRLSPAEIDHAVDGARRYGVYVISRVAMHFLPRWLP  NPLRRSGEEAKAELFGILDRFVQERAGCPSTGTPDLVDTLLALEFDGCPQTRERRRRSEA  AGLVFAGFETTAAALAWTIALLCRNPIALGKAYAEVDALGGKALAYDDLENLKYLRACFD  EAQRFQAAPANVRTAIEDDEVGGYFIPRGSQVIITQYALQRDPRFWKEPERFNPDRFLTD  KINRNTFLPFSIGPRKCMGTRMAYIEGTLVLGAILQRYAFQIRDGWTPRHRVRVSTGLAG  GLPARLFAR  >CYP136A(G6X3G3_MYCAB)    MATISSTDYLIDQAKRRLPTMNTLPGMGYIENYLNNREWPMTELAAPPPGSGLKPVMGDQ  GLPMLGHMVEMFRGGIDWVLNMYQERGPVSWTQTPIGKIVAALGPDATQAVFSNANKDFS  QQGWVPVIGPFFNRGLMLLDFQEHREHRLIMQQAFLRSRLAGYVEQIDAVASEIVAQWPT  NDNRFLFYPAIKELTLDVASVVFMGNEPHAQHERLEKVNKAFVATTRAGGAIFRFGLPPF  KWWQGLQGRKLLEEYFAERVGIARQKTEGADMLTALCHAETDEGDAFTDTDIVNHMIFLM  MAAHDTTTSTTTTMAYYLAANPEWQERVRDESDRLGDGPLDIDSLEKLESLDLVMNEALR  LVTPLPFNIRSTVRDTDLLGFHIPAGTMINIWPGMNHRLPELWTEPDKFDPDRFSEPRNE  HKRHRYAFSPFGGGAHKCIGMVFGQLEIKAVMHRLLRRYRFELAHPHYQPKWDYAGMPLP  IDGMPIILRPLH  >CYP161NSF1(G6X9D1_MYCAB)  MPAAKQRELQRAGTVHRVTTAVGDPAWLITGYATVRRLFDDERVGRSHPEPDTAARSGDS  AFFGGPIGSYETEREDHARARRLMQPHFTPKQMRTLAGRVHELADELITAMIDQGSPADF  YTAVAVPLPMMVLCELLGVPYADRDEFREWTVAASNTRDRSRSEWGMGQLFVYGMQLVGR  KRKQPGDDVISRLCAIDGVADHEIASQSMALLLGGHETTVVQLGLATLLLLANPEQWALL  VSQPDLVPNAVEETLRASRTGGGEIPRYAREDLEIDGVHIAAGELLLLDVGAANHDPAVF  GCPDQLDVARKHLAHVIFGYGSRYCVGAPLARMQLTEVLGQLVERVPGLHLRRDISELTM  RGDLLTGGLREVPVGW  >CYP164A(G6XAU3_MYCAB)  MPITTDPGTLLLQVLDPANRANPYPVYGQLVEQTQAGPVHPPGMDVSVLATFADCNAVLR  HPQASSDRRKSSIVQRQLAQNPNLIARPSFLGLDAPDHTRLRKLASKAFAPRVINAMAED  IQTFVDKMLDDIALRGTFNLVTEFAYPLPVAVICRMLGVPIEDEPEFGRASALLGQGLDP  VYALTGQSPANMDERFQAARWMWDYFIDLIASRRRNLGDDLLSALIQVEEAGDQLTEEEI  ISTCTLLLIAGHETTVNLIANASLAMLRDPEQWKLLGRNADRAPLVVEETLRYDPPVHMV  ARVADGEMAIRDFVLPHGEWVLLMLAAAQRDPDVGPDLDVFNPDRTEIKHLAFGHGPHFC  LGAPLARLETRLALSSLTARFPGARLMDEPTYKPNVTLRGLAELPVSIA  >CYP1128A(G6X3T9_MYCAB)  MVLDENAGTWVVLGYQPAREVLGGSGWSSDPLASPQLRAQAPEFLDSSGFGRNMLFADEP  DHTELRGTVRDVFTPGFIAGLREGVHTIASHVVEYPSTGEYFDFMADIALPLPIAIIGEW  LGLDDSSAAVLRQESPAIIQMLGAFADVDTVMAGTAAGVTLATELLPLAADRRAHPGDDL  LSLIASNTDLPLEDVVTTALIIAIAGHETTANLLGASMVRLLTPRLDGTRLADDIDPDDP  TVITELLRLDGPVLATARVATRAHTLAGNTIEGGQTVLIAIAAANRDPQVFVDPAVFRRD  RTSPPLAFGYGTHHCLGSALARLETTLVLREVLARQPVITGPVSWRDTPAIRGPRSIPMR  FQT  >CYP1134A(G6X8I8_MYCAB)    MSASALQPEPLSAPVEVAAGECPVNHDLYPGGPTFLRWHHTRVGYLKSWRLALGAVWSTY  RHTISEYLADLPGQDDVIVARAPMRKAAIVRNPELARHVLVANQDNYIKSAEYDLLAVGF  GRGLVTDLNEGLWNRNRRLVQPIFAKRQVDLFAPQMAEAAARTVSRWDELYAEGKPVDIT  AEMNYLTMDIVAQTMFGIDLSGDMAERMRIYFARLLKLFGVGFIVGAAPPLRWVVDKLAA  HGPHELSSHTPRLAIRALRIGASVAAPRTMKGLRWVERTIDQLIADHRSGRIARQDNLLA  LLMAAEDPETGAKYTDLEIRDELMTFLGAGFETTAAALAWTWYLLSRNPDARAKLGQEVD  RVLGGRQPTAADVDNLPWTAAVLNEAMRVYPPILGLARTAKADDVLGDYPISAGTTVMVL  IDSIHHNERVWDDAKTFDPARFLKENLQPEQRKAHMPFGAGKRMCIASGFANLEAIIGIA  ALAQNYELDLLPGQQPRREVTFTGGPEGEILMRLRKRDR  >CYP286D(G6X9D2_MYCAB)    MARALARLPRLGDTVGLMLDPTFYLFEKYLRHGPVFTVKMPYQTYTVLAGSEAATFMSSK  DGRECLTVGSSWRFVEEQFGGKDSLVAVDGPQHKQWRTLLQRGYSREAIADRYSEAIDVI  DDAVDKHWRPGESVPVLSAMQKLSIAQVGTFLGGVRPTDDDIEDIALVTREILKIAPVQH  IPKFMLQLPRYVNARSRVARVAQNALALAGHSSASGHRGQSTLLEDILESCRDDPNAANQ  RNMLFHSVLPYFAGVETTSATATYALYLILRHPDVLCRIQDEVAVMFERGDITHDSLFHS  TPVLHGAIMEAMRLRPIASFIIRVAAKDFEFHGHQIRSGEGVFIGTTVPHFLSEFYENPM  KFDVDRYGDSVGPNRTPGAYSPFGRGPHMCVGKGLAESLMQLMLARIIHRRTLELSSRSY  RLSNRVSSSSPSPKFAVRVVSSRR  >CYP51B(G6X7Y9_MYCAB)    MTTPTVPRVSGGEDQYGHLEEFRTDPIALMKRIRAECGNVGTFQLADKQVVFLSGAEANE  FFFRSSDEDLDQAEAYPFMTPIFGKGVVFDADPERRKEMLHNAALRGEQMKGHAATIENE  VRQMISRWGESGEVDLLDFFAELTIYTSSACLIGKKFRDELDGRFAHLYHQLEQGTDPLC  YVDPYLEIESFRQRDEARTGLVALVQEIINGRVQRSADSPADKSQRDMLDVLIMIKDEHG  NPRFTADEITGMFISMMFAGHHTSSGTASWVLIELLRHPDIRSQVIDELDELYADGGEVS  FHALRQIPKLENVLKETLRLHPPLIILMRVAKGEFEVGGFPIHDGDMVAASPAVSNRIAE  DFPNPDGFVPDRYEKPRQEDIVNRWTWIPFGAGRHRCVGAAFATMQIKAIFSVLLREYEF  EMAQPADSYHNDHSKMVVQLAQPAKVRYRKRSA  >CYP144NSF1(G6X6M1_MYCAB)    MNTDELFDPQVLEDPYPFYRRLREKAPVWPVGDSGFYFVSRWDLVVEATERAEDFSSNLT  AALMKSADGLTVAPMGPPADPTHVLATGDDPVHHAHRRLVLPTLVAKRITALEPVMAQTG  SRLWERGVNGDGIDWMAAMGDALPMTMVARLIGLPVGDVPQLVQWGYSSTEMLGGLNTPQ  RQAQVVTDTMYLVLYLREHLEKELAAPGDDLLGYLAQACNRGDISLDIGVMILVQLVGAG  GESTAGLMGNAVRILGENPRLQQHIRENRALLPTFLEEALRLESPFRGHHRHVLTDTTLG  GVELPAGSHLTLLWGAANRDPAIFEDPDVLRLDRRSPRGHITFGKGLHFCVGAALARLEA  RTAINLLLDRTREFAIKRDGAHWVPSIMVRRHQKLELEVSAA  >CYP140NSF1(G6XCK4_MYCAB)  MRTRDQIKYWSRWATMHGISRAALLTQVRTLPLAALFLGPDRAEKHYRYIEEIRASGSVT  PSRAGGLIFTDLALTREILRDNRFITMAPTNIPSPILPLAFSRWVFARTEPGLPNPVEPP  AMLAVDPPEHTRFRKLVSKAFTPRAVSKLEDRVREVTTELLDNLERHERADLLHDYASQL  PVAIIAEMLGVPRADAPFLLEWGNHGAALLDIGMTWSAYRDAIQALIEIDRYFDAHLVRL  RGELAEDPTVDGILASIVRDGDLNDRELKATMALLLGAGFETTVNLIGNGIAALLRHPQQ  LAHLRENPDGWPNAVEEILRYDSPVQITGRVATQTCEFEGHTLAAGSMAILLLGGANRDP  AVFDQPDVFDVLRANAREHVAFGSGIHVCLGASLARMEGVVALQSLFERFPELVLAADPI  PGKHVNLHGFGSLPVNLGRARVPASS  >CYP1130A(G6X3Y6_MYCAB)  MIASLSDDPFWPTRKPLRASFSLLTGWGRDRPIPPGPIGLPFIGSAIPMARNPYKFLQDC  HRRYGDIYRVPLPIHPLVLANHPDLVSEFMENTELKYSMSAPIQGRRLQKAVTSVGCPVQ  VLEGQALRDRRKRLMPMFGKRHLSVVSDKFVEVFTDRIDRWLLVADTGKEVNLQAELPKV  VLPAFMYAMFSTRLSDDEVLHADTATRSVMRAIASGLFLASPPNIFPLRGRENLPVSGLR  LMRTIRQMIRDRRANPTDDADLLNILLAARGDSSRPLTEIDVYSEIMSAIGGGYETIVAS  MSWTLALLLQHPEHLERLYDEIAILNGNAPTPDDLPRLPWARACFDEGQRLQGAPINPRY  AMEDTELGGYPIPKYTLVASSLYVVHRDPRWWGENAETYDPMQFFDQDRVNARPRLAFQA  FGAGPHHCMGTGMAYMMAQYLLAIIFQRYRLHLRPGWQPRQFFSLSTLVKDGVSATITKA  >CYP123A(G6X7Z1_MYCAB)    MTAAETSAAPLVFDPYDYDFHEDPYPYYRRLRDEAPLYRNDDLKFWALSRHHDVLQGFRN  SEALSNANGVSMDKASFGPHAKLVMSFLAMDDPEHLRLRTLVSKGFTPRRIRELEGQVVT  LARTHLDRALTNASDRSFDFIAEYAGKLPMDVISELMGVPEADRTRIRELADGVMHREDG  LADVPPEAIQASFDLMTYYIEMVRERRRRPTEDLTSALLQAEIDGDRLTDEEVLAFLFLM  VIAGNETTTKLLANAVYWGHRNPDQLATVHADHDRIPLWVEESLRYDTSSQILARTVAED  MTLYDTKIPAGDILLLLPGSANRDDRVFDDADQYRIGREIGAKLVSFGSGAHFCLGAHLA  RMEAKVALTELFTRISGYEIDERNSVRVHSSNVRGFAHLPMTVQLREGH  >CYP153A(G6X6X6_MYCAB)    MSIPAAVAAKAQSAVPLELQIRGAHLYDKTRRWVTGTNGKKIFTETPIPPVEDVDIADID  LSNPFLYRQGRWKSYFERVRNEAPVHYQARSPFGPFWSVTRHADIIAVDKNHEAFSAEPF  IIIGRPPRFMDIAMFIAMDPPQHDLQRASVQGVVAPKNLREMEGLIRSRVQEVLDDLPVD  QPFNWVHHVSIELTARMLATLLDYPYEQRHKLVEWSDLATSMEQANGGPSDNDRVFRGMV  DMAKGLSALWHDKAARTANGERPGFDLITMLQANEDTKDLIDRPMEFLGNLVLLIVGGND  TTRNSMSGGVLALNQFPDQFEKLKANPDLIPNMVSEIIRWQTPLAYMRRVAKKDIMLNGQ  FIRKGDKVVMWYASGNRDERVFERADELIIDRSNARNHISFGFGVHRCMGNRLAELQLRI  LWEELLPRFENIEVVGEPEYVQSNFVRGISKLMVRLTPRPSA  >CYP130A(G6X8G7_MYCAB)    MSVDVSHAVAPRYVPGTADNWTNPWPMYAALRDHDPVHHVVPEYTPDQDYYVLTRHADVY  EAARDWETFSSARGLTVTYGDLEKTGMGDNPPMVMQDPPTHTEFRKLVSRGFTPRQVTAV  EPKVRKFVVQRLERLKERGEGDIVVELFKPLPSMVVAHYLGVPEEDRTQFDAWTDGIVAA  ASGGAIDIEAMQGEVAQTIGELMMYFTGLIERRRAEPEDDTVSHLVAAGVGADGDISGTL  QILGFAFTMVTGGNDTTTGMLGGAIQLLHQNPAQRQKLIDDPSLIPGAVEEFLRLTSPVQ  GLARTATRDVIIGDTTIPAGRRALLLYGSANRDEREYGNDAAELDVLRKPRNILTFSHGN  HHCLGAAAARMQSRIALEELLTRIPDFEVDLDGVTWADGSYVRRPLTVPIRVR  >CYP138A(G6X8J0_MYCAB)  MTEISTIDRAADVPKPIAPPLVTLPKLAQGIAFVASRRWTTSRLAKKYGKVYTINIPKFG  YTVVVADPDLTREVFTTSTEILGNIQPNLSQQLGPGSVFALERNEHRHRRKLLAPKFHGK  AMVQYEQIIEEETLRECATWPEGQQFETMEPMMRITLNAILRAVFGADGAELDVLRRLIP  PWVVLASLTTRIPQPKRNYGRFSPWGRLKTYRAHYDAVIGSLIEKALAAPDFEDRTDILA  LLLRSTYDDGTTMSRSDVSDELLTLLAAGHETTATTLAWAFERITRHPEVLSRLAAEAQG  DSNEYRQATILEVQRSRPVIDFAGRHVLAPHVDIGPYRIPQGHSVVVSINLMHDDPVAFP  CPERFDPERFMGAKPGNSWVPYGGGTRRCVGAAFANMEMDIVLRTVLRHFTIETTTAPDE  KWHSRGVASCPKKNGRVTVRRR  >CYP125A(G6XE67_MYCAB)  MVQAQHPHLPDGIDFTDPELFVHGIPERELAELRHTEPIWWNHTERGVAGFDDDGFWVVS  KHKDVKEVSLRCEVFSSEQNTAIPRYLPTTPRERIDATRLIMLNMDPPRHSRLRHIISRG  FTPRAISRLRDDLNARAQGIAKAAAQLRHGDFVEQVACELPLQAIAGLMGTPLDEREQLF  DWSNRLVGSSDGEDDSAVASAELLMYAMGVAARKTAEPGADICTDLVNADIDGQKLSDDE  FGFFVMLLAVAGNETTRNSITHGMHAFTQFPEQWELYKKTRPETAADEIVRWATPVTSFQ  RTALEDTELGGVRIKKGQRVVMMYRSANFDEEVFENPFTFDIMRDPNPHVGFGGNGEHHC  VGANLARMTINLMFNAIADHMPDLASAGEPDRLRSGWLNGVKHWEVDFCPAGYGRAS  >CYP125A(G6XE69_MYCAB)    MVHPSLPAGFDFTDPEIYAERLPVEELKELRKTAPIWWQEQPDGVGGFNDGGYWVVTKHK  DVKEVSLRSDVFSSWENTAIPRFQDDITREAIELQRYVMLNMDAPHHTRLRKIISRGFTP  RAIGRLRDELNERAQEIAKAAAASGTGDFVEQVSCELPLQAIAGLLGVPIEDRGKLFNWS  NEMTSYDDPEYADIDPAASSMEILAYSMEMAKQKAENPGEDIVTTLINAEVEGEGKLSDD  EFGFFVIMLAVAGNETSRNSITQGMMAFTQFPEQWELYKKERPETAADEIVRWATPVTSF  QRTALEDTELDGVKIKKGQRVVMMYRSANFDEDVFEDPFSFNIMRNPNPHMGFGGSGAHY  CIGANLARLTINLMFNAIADHMPNLAPAGDPKRLQSGWLNGIKHWQVDFTGASGCPVLQ  >CYP125A(G6XAE6_MYCAB)  MTACPFTPGFDFTDPDLIQHRIPAEEFAYLRKTEPIWWNAQPRGVAGFDDDGYWVVTKHA  DVKEVSRLNEVFSNSVNTTVVRYNEDITAEQLEIQRENLLIDMDEPKHRILRRIVSPLFT  PKAVNGLHSRLVERAHSIVEEAAEKSSGNFVSDIASVLPMHAIADLVGIPESDRQQVLDW  TNQMFAYDDPAIGRDTATAATVSMLGYAYAMAEERQLNPQDDILTGLVRGAYDDRPLTPL  EFAYFVIQLMVAGNETSRNAITHGVLAFADNPAQWRLYRERRPATAADEIIRWASPIIAF  QRTALQDVELGGVQICKDQRVGMFYASANFDEDVFDDPFTFNIERDPNPHLAFGGHGIHY  CLGANLARLEIGIMFDALADRLPDLMPTGAPTRFRSGWINGVVALPANYLGAGPRG  >CYP125A(G6X7Y6_MYCAB)    MTAMKTAAELGLPEGFDFTDPELYGNRMPHEEFATLRREAPVWWNPQPRTVGGFADEGYW  VISKHRDVREVSLHTDIFSSGRKGAIPRLEDHISPEEFQATLSVLINKDAPEHTQLRGLV  SRMFTPRSIAALRITLEERAERIVRAALEGGHGEFVREVASELPMQAIAELIGVPEEDRV  KLFEWSNQMTGYDEADVEIDPRIGAAQILGYSYQLAEQRRDCPGNDVVSRLLTGTVDGEQ  LTPEQFGFFVVMLSVAGNETTRNATTMGMMAFLEHPDQWELFKSARPSTTVDEIVRYTSP  LISQQRTALQDTVIGDVRIRAGERVVMLYPSANFDEEVFENPHAFDITRDPNPHLGFGGT  GAHYCLGANLAKAELEIIFNKIADRMPDISRIGDAPRFHSGWINGIKKFDTAYCPVTH  >CYP140B(G6XCK3_MYCAB)  MQHRIKQRTHWAVTHGIGRAYLKVLARRGEPVAQLGIDVGQAPDIYRIIDKIRERGRLSR  AGDGWITADAQIVRTIFRDNRFVTFKPEHRSASPIIQRLAAWSDPQLLNPAEPPSILITD  PPDHGRLRRLVAAPFTPRAIEGLRDRIQEVTNGHLDVLQQRQSPDLIADFTAKIPIAVIG  EMIAVPPPDHSRLYTAMNRAIQLIATTAPSWSEYQDGTAALREIDEYLEKHVARLRREGT  ESELATALLDSDLSHFELKMFFAVFLGAGFVTTTHLMGKAIVTLLRHPEQLALLQADPSL  WPNAVEELMRYDTSNQWSARVATETVEIEGHTIEAGQSALLLLGGANRDPAAFENPDVFD  ITRPNARENITLGTGIHVCLGQVLARAELHTALQTLFERFPRLSLAGEPEYLNGMGIHGL  RRLPVTLG |
| ***Mycobacterium abscessus* 103** |
| UniProtKB;P450count:25;Families 19;Subfamilies:22 |
| >CYP286D(X8EK13_MYCAB)  MARALARLPRLGDTVGLMLDPTFYLFEKYLRHGPVFTVKMPYQTYTVLAGSEAATFMSSK  DGRECLTVGSSWRFVEEQFGGKDSLVAVDGPQHKQWRTLLQRGYSREAIADRYSEAIDVI  DDAVDKHWRPGESVPVLPAMQKLSIAQVGTFLGGVRPTDDDIEDIALVTREILKIAPVQH  IPKFMLQLPRYVNARSRVARVAQNALALAGHSSASGRRGQSTLLEDILESCRDDPNAANQ  RNMLFHSVLPYFAGVETTSATATYALYLILRHPDVLCRIQDEVAVMFERGGITHDSLFHS  TPVLHGAIMEAMRLRPIASFIIRVAAKDFEFHGHQIRSGEGVFIGTTVPHFLSEFYENPM  KFDVDRYGDSAGPNRTPGAYSPFGRGPHMCVGKGLAESLMQLMLARIIHRRTLELSSRSY  RLSNRVSSSSPSPKFAVRVVSSRR  >CYP1130B1(X8EN95_MYCAB)  MTELVGKEVTRGCPAEPHQDGPTGFGDFPRELAGQWGPLPLNPSVHEVNEIARARQRFEY  SRKDPWWLTRASFPLASSIVRGVGSPALTPPGPVGHPVVGSVPEMRREPFEFFRRCAREF  GDVYRVPFPLGGSIVVVNHPDYASQVMDDPVGRYSMIGPGQAAMGVIGAAIPMLEGDKFR  QRRRMLMPMFGRRHLARVAEVIADEFVRRVDRWACWADTGQVVDLQHAIAQVTLPAFLRA  MFSSTITEQEISETDVDLRTFMSLMASVTMMSPLPSVLPLPGRESAPRSMWRLWRLTRRL  IQQRRKNPIETPDLLSLLLEASYDDGSQLSERDLSMELMILMAGGYETVVASLSWTLALL  LGHPEHLRRLYAEVDGLAGALPTPDDLPKLAWAKACFDEGQRLQGHPLNPRFAMDDDVIG  GYFIPKHTIVGPSLYSIHRDPRWWADPDTYDPNRFMDEVAARSRPRLAFMPFGSGRHHCL  GTGMAYMNAQFLLAIIFQRYRLALPEGWKPKHHFNFSVTLEGGLPVTLTTV  >CYP164A(X8EN37_MYCAB)  MYGQLVEQTQAGPVHPPGMDVSVLATFADCNAVLRHPQASSDRRKSNIVQRQLAQNPNLI  ARPSFLGLDAPDHTRLRKLASKAFAPRVINAMAEDIQTFVDKMLDDIALRGTFNLVTEFA  YPLPVAVICRMLGVPIEDEPEFGRASALLGQGLDPVYALTGQSPANMDERFQAARWMWDY  FIDLIASRRRNLGDDLLSALIQVEEAGDQLTEEEIISTCTLLLIAGHETTVNLIANASLA  MLRHPEQWKLLGQNADRAPLVVEETLRYDPPVHMVARVADGEMAIRDFILPDGEWVLLML  AAAQRDPDVGPDLDVFNPDRSEIKHLAFGHGPHFCLGAPLARLEARLALSSITARFPGAR  LMDEPTYKPNVTLRGLAELPVSIA  >CYP1128A(X8EHZ8_MYCAB)  MVLGYQPAREVLGGSGWSSDPLASPQLRAQAPEFLDSSGFGRNMLFADEPDHTELRGTVR  DVFTPGFIAGLREGVHTIASHVVEYPSTGEYFDFMADIALPLPIAIIGEWLGLDDSSAAV  LRQESPAIIQMLGAFADVDTVMAGTAAGVTLATELLPLAADRRAHPGDDLLSLIASNTDL  PLEDVVTTALIIAIAGHETTANLLGASMVRLLTPRPDGTRLADDIDPDDPTVITELLRLD  GPVLATARVATRAHTLAGNTIEGGQTVLIAIAAANRDPQVFVDPAVFRRDRTSPPLAFGY  GTHHCLGSALARLETTLVLREVLARQPVITGPVSWRDTPAIRGPRSIPMRFQT  >CYP135B1(X8EM19_MYCAB)  MASYGLRFLAGCQRRYGNMFTLRIPLSGKVVYLADPADIKTVYAGDPHVFHSGEAHWFFR  GLLGDSSLFVLDEDEHHHQRRLLMPAFHRDAVARQATQMAQIAAANIAEWPVGESFPVAP  RTTDITLEVILRTVIGASDPVRLAALRKVVPRLLYMKPWETPAITNPGLRRYWPWTAVGR  RMAETDALLYAEIAERRADPNLTERTDVLAMLVRATDDDGRTMSDQELRDHLLTSIAAGH  ETTATALSWVLERLTRHPDALLKAAQAAEASAAGDPAGDEYLDAVVKETLRIRPVIFSSG  RVLKESVEVGGYQLPVGIMVDPAIGLVHASGAVYPDPARFDPDRMVGTTLSPTTWLPFGG  GNRRCLGATFAMVEIRVVLREILRRVELNTTTAPDEKQQAKHVTFVPHRGGSISVRAIRT  CAPAVTPTCPADVHGTGQARDPQR  >CYP161NSF1(X8EJI8_MYCAB)  MPAAQQRELQRAGTVHRVTTAVGDPAWLITGYATVRQLFDDERVGRSHPEPDTAARSGDS  AFFGGPIGSYETEREDHARGRRLMQPHFTPKQMRTLAGRVHELADELITAMIDRGSPADF  YTAVAVPLPMMVLCELLGVPYADRDEFREWTVAASNTRDRSRSEWGMGQLFVYGMQLVGR  KRQQPGDDVISRLCTIDGVADHEIASQSMALLLGGHETTVVQLGLATLLLLANPEQWALL  VSQPDLVPNAVEETLRASRTGGGEIPRYAREDLEIDGVHIAAGELLLLDVGAANHDPAVF  GCPDQLDVARKHLAHVIFGYGSRYCVGAPLARMQLTEVLGQLVERLPGLHLRRDISELTM  RGDLLTGGLREVPVGW  >CYP1132A(X8E9K1_MYCAB)    MRSIDTDSVAPAVPKPPVPRLPPRLPIHKAALLGLSCLGDPTRLPYLLLPKIANRYGDIV  RLFTGPTPALTLTLINHPDYVDHVFTRHHDRYVKHEATIELVSGEPVALPLLEGREWKRV  RSAFNPYFGERALAQATPLMMEGITERVDAWSRHVHSGELVDLEHELGAVVMDGLMRSMF  KVRLRPAEIDHAVDGARRYGVYVISRVAMHFLPRWLPNPLRRSGEEAKAELFGILDRFVQ  ERAGCPSTGTPDLVDTLLALEFDGCPQIRERRRRSEAAGLVFAGFETTAAALAWTIALLC  RNPIALGKAYAEVDALGGKTLAYDDLENLKYLRACFDEAQRFQAAPANVRTAIEDDEVGG  YFIPRGSQVIITQYALQRDPRFWNEPERFNPDRFLTDRINRNTFLPFSIGPRKCMGTRMA  YIEGTLVLGAILQRYAFQIRDGWTPRHRVRVSTGLAGGLPARLFAR  >CYP1134A1(X8ES41_MYCAB)  MSASALQPEPLSAPVEVAAGECPVNHDLYPGGPTFLRWHHTRVGYLKSWRLALGAVWSTY  RHTISEYLADLPGQDDVIVARAPMRKAVIVRNPELARHVLVANQDNYIKSAEYDLLAVGF  GRGLVTDLNEGLWNRNRRLVQPIFAKRQVDLFAPQMAEAAARTISRWDELYAEGKPVDIT  AEMNYLTMDIVAQTMFGIDLSGDMAERMRIYFARLLKLFGVGFIVGAAPPLRWVVDKLAA  HGPDELSSHTPRLAIRALRIGASVAAPRTMKGLRWVERTIDQLIADHRSGRIARQDNLLA  LLMAAEDPETGAKYTDLEIRDELMTFLGAGFETTAAALAWTWYLLSRNPDARAKLGQEVD  RVLGGRQPTAADVDNLPWTAAVLNEAMRVYPPILGLARTAKADDVLGDYPISAGTTVMVL  IDSIHHNERVWDDAKTFDPARFLKENLQPEQRKAHMPFGAGKRMCVASGFANLEAIIGIA  ALAQNYELDLLPGQQPRREVTFTGGPEGEILMRLRKRHP  >CYP136A(X8EF95_MYCAB)  MATISSTDYLIDQAKRRLPTMNTLPGMGYIENYLNNREWPMTELAAPPPGSGLKPVMGDQ  GLPMLGHMVEMFRGGIDWVLNMYQERGPVSWTQTPIGKIVAALGPDATQAVFSNANKDFS  QQGWVPVIGPFFNRGLMLLDFQEHREHRLIMQQAFLRSRLAGYVEQIDAVASEIVAQWPT  NDNRFLFYPAIKELTLDVASVVFMGNEPHAQHERLEKVNKAFVATTRAGGAIFRFGLPPF  KWWQGLQGRKLLEEYFAERVGIARQKTEGADMLTALCHAETDEGDAFTDTDIVNHMIFLM  MAAHDTTTSTTTTMAYYLAANPEWQERVRDESDRLGDGPLDIDSLEKLESLDLVMNEALR  LVTPLPFNIRSTVRDTDLLGFHIPAGTMINIWPGMNHRLPELWTEPDKFDPDRFSEPRNE  HKRHRYAFSPFGGGAHKCIGMVFGQLEIKAVMHRLLRRYRFELAHPHYQPKWDYAGMPLP  IDGMPIILRPLH  >CYP136D1(X8E7C2_MYCAB)  MKERLSSVIMVPAPHAVDDRWRRWSRDWPVRELAPAPAGSGLKAVRGDAGLPFVGHTLDY  IRFGSDFSRERYDRLGSVSWMGAFGTKMVVIAGPDATREAFTSEAKAFSQDGWSFLIDAF  FHRGLMLMSFDEHLMHRRIMQEAFTRPRLTGYVEQVTPCVRSAVPAWPVGPSVRIYPLLK  ELTLDIATDVFMGGRGKDESDAVNKAFVATVRAASSLVRAPLPGTRFRAGVQGRRVLEDY  FFRHLPAARAGETEDLFAALCQATTEDGERFSDEDVVNHMIFLMMAAHDTSTITTTAVTY  FLAKYPQWQEAAAAEAAAIGDGLPDIEALEKMTVIDRVIKEALRLLAPVPLVMRKTVRDV  AIDGYHIPSNTLCAITPAVNHFDRTIWNDPERFDPSRFDEPRREDQHHRFAWVPFGGGAH  KCIGMQFGTLEVKAILHRMLRSFTWKVPENYHVRWDNTSLPIPVDGLPLEMKRR  >CYP140NSF1(X8ELS8_MYCAB)  MRTRDQIKYWSRWATMHGISRAALLTQVRTLPLAALFLGPDRAEKHYRYIEEIRASGSVT  PSRAGGLIFTDLALTREILRDNRFITMAPTNIPSPILPLAFSRWVFARTEPGLPNPVEPP  AMLAVDPPEHTRFRKLVSKAFTPRAVSKLEDRVREVTTELLDNLERHERADLLHDYASQL  PVAIIAEMLGVPRADAPFLLEWGNHGAALLDIGMTWSAYRDATQALIEIDRYFDAHLVRL  RGELAEDPTVDGILASIVRDGDLNDRELKATMALLLGAGFETTVNLIGNGIAALLRHPQQ  LAHLRENPDGWPNAVEEILRYDSPVQITGRVATQACEFEGHTLAAGSMAILLLGGANRDP  AVFDQPDVFDVLRANAREHVAFGSGIHVCLGASLARMEGVVALQSLFERFPELALAADPT  PGKHVNLHGFGSLPVNLGRARVPASS  >CYP130A(X8EQI7_MYCAB)  MSHTVAPRYVPGTADNWTNPWPMYAALRDHDPVHHVVPEYTPDQDYYVLTRHADVYEAAR  DWETFSSARGLTVTYGDLEKTGMGDNPPMVMQDPPTHTEFRKLVSRGFTPRQVTAVEPKV  REFVVQRLERLKERGEGDIVVELFKPLPSMVVAHYLGVPEEDRTQFDAWTDGIVAAASGG  AIDIEAMQGEVAQTIGELMMYFTGLIERRRAEPEDDTVSHLVAAGVGADGDISGTLQILG  FAFTMVTGGNDTTTGMLGGAIQLLHQNPAQRQKLIDDPSLIPGAVEEFLRLTSPVQGLAR  TATRDVIIGDTTIPAGRRALLLYGSANRDEREYGNDAAELDVLRKPRNILTFSHGNHHCL  GAAAARMQSRIALEELLTRIPDFEVDLDGVTWADGSYVRRPLTVPIRVR  >CYP123A(X8ER38_MYCAB)  MTVAETSAVPLVFDPYDYDFHEDPYPYYRRLRDEAPLYRNDDLKFWALSRHHDVLQGFRN  SEALSNANGVSMDKASFGPHAKLVMSFLAMDDPEHLRLRALVSKGFTPRRIRELEGQVVA  LARTHLDRALANASDRSFDFIAEYAGKLPMDVISELMGVPESDRARIRELADGVMHREDG  LADVPPEAIQASFDLMTYYIEMVRERRRRPTEDLTSALLQAEIDGDRLTDEEVLAFLFLM  VIAGNETTTKLLANAVYWGHRNPDQLATVHADHDRIPLWVEESLRYDTSSQILARTVAED  ITVYDTKIPAGDILLLLPGSANRDDRVFDDADQYRIGREIGAKLVSFGSGAHFCLGAHLA  RMEAKVALTELFTRISGYEIDERNSVRVHSSNVRGFAHLPMTVQLREGR  >CYP1130A(X8EEV4_MYCAB)  MARNPYKFLQDCHRRYGDIYRVPLPIHPLVLANHPDLVSEFMENTELKYSMSAPIQGKRL  QKAVASVGCPVQVLEGQALRDRRKRLMPMFGKRHLSVVSDKFVEVFTDRIDRWLLVADTG  KEVNLQAELPKVVLPAFMYAMFSTRLSDDEVLHADTATRSVMRAIASGLFLASPPNIFPL  RGRENLPVSGLRLMRTIRQMIRDRRANPTDDADLLNILLAARGDSSRPLTEIDVYSEIMS  AIGGGYETIVASMSWTLALLLQHPEHLDRLYDEISILHGNAPTPDDLPRLPWARACFDEG  QRLQGAPINPRYAMEDTELGGYPIPKYTLVASSLYVVHRDPRWWGENAETYDPMQFFDQD  RVNARPRLAFQAFGAGPHHCMGTGMAYMMAQYLLAIIFQRYRLHLRPGWQPRQFFSLSTL  VKGGVPATITKA  >CYP153A(X8E851_MYCAB)  MSIPAAVAAKAQSAVPLELQIRGAHLYDKTRRWVTGTNGKKIFTETPIPPVEDVDIADID  LSNPFLYRQGRWKSYFERVRNEAPVHYQARSPFGPFWSVTRHADIIAVDKNHEAFSAEPF  IIIGRPPRFMDIAMFIAMDPPQHDLQRASVQGVVAPKNLREMEGLIRSRVQEVLDDLPVD  QPFNWVHHVSIELTARMLATLLDFPYEQRHKLVEWSDLATSMEQANGGPSDNDRVFRGMV  DMAKGLSALWHDKAARTANGERPGFDLITMLQANEDTKDLIDRPMEFLGNLVLLIVGGND  TTRNSMSGGVLALNQFPDQFEKLKANPDLIPNMVSEIIRWQTPLAYMRRVAKKDIMLNGQ  FIRKGDKVVMWYASGNRDERVFERADELIIDRSNARNHISFGFGVHRCMGNRLAELQLRI  LWEELLPRFENIEVVGEPEYVQSNFVRGISKLMVRLTPRPSA  >CYP144NSF1(X8E7R6_MYCAB)    MNTDELFDPQVLEDPYPFYRRLRETAPVWPVGDSGFYFVSRWDLVVEATERAEDFSSNLT  AALMKSAEGLAVAPMGPPADPTHVLATGDDPLHHAHRRLVLPTLVARRITALEPVMAQTG  SCLWERGVNGDGIDWMAAMGDALPMTMVARLIGLPAGDVPQLVQWGYSSTEMLGGLNTPQ  RQAQVVTDTMHLVLYLREHLEEELAAPGDDLLGYLAQACNRGDISLDIGVMILVQLVGAG  GESTAGLMGNAVRILGENPRLQQRIRENRALLPTFLEEALRLESPFRGHHRHVLTDTTLG  GVQLPAGSHLTLLWGAANRDPAIFEDPDVLRLDRPSPRGHITFGKGLHFCVGAALARLEA  RTAINLLLDRTREFAIKPDGAHWVPSIMVRRHQKLELEVSAA  >CYP138A(X8EQJ9_MYCAB)  MEGDVTEISTIDRAADVPKPIAPPLVTLPKLAQGIAFVASRRWTTSRLAKKYGKVYTINI  PKFGYTVVVADPDLTREVFTTSTEILGNIQPNLSQQLGPGSVFALERNEHRHRRKLLAPK  FHGKAMVQYEQIIEEETLRECATWPEGQQFETMEPMMRITLNAILRAVFGADGAELDVLR  RLIPPWVVLASLTTRIPQPKRNYGRFSPWGRLKTYRAHYDAVIGSLIEKALAAPDFEDRT  DILALLLRSTYDDGTTMSRSDVSDELLTLLAAGHETTATTLAWAFERITRHPEVLSRLAE  EAQGDSNEYRQATILEVQRSRPVIDFAGRHVLAPHVDIGPYRIPQGHSVVVSINLMHDDP  VAFPCPERFDPERFMGAKPGNSWVPYGGGTRRCVGAAFANMEMDIVLRTVLRHFTIETTT  APDEKWHSRGVASCPKNNGRVTVRRR  >CYP1110B1(X8EN24_MYCAB)  MTLSSTELDDLVGAVDRYPVYKDLRDNQPVLPVEVNGHEAYLLTRYADVSRVLKTASARV  QPRAGEFPAHIGTGPASEFYRFSLPSMDAPSHTRLRKLAAAAFSPRAVAAMRSWVEEIIG  AGIDRLMDFDGEFDFVGEFASRVPAEIACRLLHAPMSDAHSVLERMPDLNPILSHGGITA  EQLAAADAAAQFYIDYIGDLVDTLQGKLDSDDAVGALLEAEADGSKMTRTELIITLVGLF  IASYHTTMVALTNAVYGFSSHPAQMRTLADNPDLAPKAWEESLRYRSPVHVVHRYAGEDM  VLHDQSIPEGAQLLLGLASANRDERFFDSPDTLDITRGTNRHLAFTGGGHYCLGAPLSRL  EGDYFMRVLPQRLPNIRVTSDRPDWGTDLSFAFMRSMMVSSGR  >CYP140B(X8EL16_MYCAB)  MQHRIKQRTHWAVTHGIGRAYLKVLARRGEPVAQLGIDVGQAPDIYRIIDKIRERGRLSR  AGDGWITADAQIVRTIFRDNRFVTFKPEHRSASPIIQRLAAWSDPQLLNPAEPPSILITD  PPDHGRLRRLVAAPFTPRAIEGLRDRIQEVTNGHLDVLQQRQSPDLIADFTAKIPIAVIG  EMIAVPPPDYSRLYTAMNRAIQLIATTAPSWSEYQDGTAALREIDEYLEKHVARLRREGT  ESELATALLDSDLSHFELKMFFAVFLGAGFVTTTHLMGKAIVTLLRHPEQLALLQADPSL  WPNAVEELMRYDTSNQWSARVATETVEIEGHTIEAGQSALLLLGGANRDPAAFENPDVFD  ITRPNARENITLGTGIHVCLGQVLARAELHTALQTLFERFPRLSLAGEPEYLNGMGIHGL  RLLPVTLG  >CYP108B(X8EJN0_MYCAB)  MATALSEIEEAGRVLADPAAYADELRLHAAMTLLRREQPVTKVITDDYRPFWAVTKHDDI  MAVERDNALWINEPRPLLMNLEQEAELDKQAAMGIELKTLVHIDDPKHRVLRAIGADWFR  PKAMRDMKLRTDELANRYVNKLLEAGGSCDFAQDIAVHFPLYVIMSLLGIPESDFDRMLK  LTQELFGGDDSEFQRGTTPEEQLMALLDFFGYFSGLTASRRQHPTDDLASTIANARVDGE  LLSDVDTASYYTIIATAGHDTTSATIAGGLEALLEHPDQLARLRENPGLMPLAVDEMIRW  VTPVKEFMRTATADTEIRGVPITEGESVLLSYPSGNRDEDIFTDPFTFDIARDPNKHLAF  GFGVHFCLGAALARMEVNSFFSALLPRLESIEVDGNIERTSTIFVGGIKHLPIRYTLR  >CYP51B(X8EPW6_MYCAB)  MTTPTVPRVSGGEDQYGHLEEFRTDPIALMKRIRAECGNVGTFQLADKQVVFLSGAEANE  FFFRSSDEDLDQAEAYPFMTPIFGKGVVFDADPERRKEMLHNAALRGEQMKGHAATIENE  VRQMISRWGESGEVDLLDFFAELTIYTSSACLIGKKFRDELDGRFAHLYHQLEQGTDPLC  YVDPYLEIESFRQRDEARTGLVALVQEIINGRVQRSADSPTDKSQRDMLDVLIMIKDEHG  NPRFTADEITGMFISMMFAGHHTSSGTASWVLIELLRHPDIQSQVIDELDELYADGSEVS  FHALRQIPKLENVLKETLRLHPPLIILMRVAKGEFEVGGFPIHDGDMVAASPAVSNRIAE  DFPNPDGFVPDRYEKPRQEDIVNRWTWIPFGAGRHRCVGAAFATMQIKAIFSVLLREYEF  EMAQPADSYHNDHSKMVVQLAQPAKVRYRKRSA  >CYP125A(X8ER31_MYCAB)    MKTAAELGLPEGFDFTDPELYGNRMPHEEFATLRREAPVWWNPQPRTVGGFADEGYWVIS  KHRDVREVSLHTDTFSSGRKGAIPRLEDHISPEEFQATLSVLINKDAPEHTQLRGLVSRM  FTPRSIAALRITLEERAERIVRAALEGGHGEFVREVASELPMQAIAELIGVPEEDRVKLF  EWSNQMTGYDEADVEIDPRVGAAQILGYSYQLAEQRRDCPGNDVVSRLLTGTVDGEQLTP  EQFGFFVVMLSVAGNETTRNATTMGMMAFLEHPGQWELFKSARPSTTVDEIVRYTSPLIS  QQRTALQDTVISDVRIRAGERVVMLYPSANFDEEVFENPHTFDITRDPNPHLGFGGTGAH  YCLGANLAKAELEIIFNKIADRMPDISRIGDAPRFHSGWINGIKKFDTAYCPVTH  >CYP125A(X8EQ35_MYCAB)    MVQAQHPHLPDGIDFTDPELFVHGIPERELAELRHTEPIWWNHTERGVAGFDDDGFWVVS  KHKDVKEVSLRCEVFSSEQNTAIPRYLPTTPRERIDATRLIMLNMDPPRHSRLRHIISRG  FTPRAISRLRDDLNARAQGIARAAAQLRHGDFVEQVACELPLQAIAGLMGTPLDEREQLF  DWSNRLVGSSDGEDDSAVASAELLMYAMGVAARKTAEPGADICTDLVNADIDGQKLSDDE  FGFFVMLLAVAGNETTRNSITHGMHAFTQFPEQWELYKKTRPETAADEIVRWATPVTSFQ  RTALEDTELGGVRIKKGQRVVMMYRSANFDEEVFENPFTFDIMRDPNPHVGFGGNGEHHC  VGANLARMTINLMFNAIADHMPDLASAGEPDRLRSGWLNGVKHWEVDFCPAGYGRAS  >CYP125A(X8EN49_MYCAB)    MTTCPFTPGFDFTDPDLIQHRIPAEEFAYLRKTEPIWWNAQPRGVAGFDDDGYWVVTKHA  DVKEVSRLNEVFSNSVNTTVVRYNEDITAEQLEIQRENLLIDMDEPKHRILRRIVSPLFT  PKAVNGLHARLVERAHGIVEEAAEKSSGNFVSDIASVLPMHAIADLVGIPESDRQQVLDW  TNQMFAYDDPAIGRDTATTATVSMLGYAYAMAEERQLNPQDDILTGLVRGAYDDRPLTPL  EFAYFVIQLMVAGNETSRNAITHGVLAFADNPAQWRLYRERRPSTAADEIIRWASPIIAF  QRTALQDVELGGVQIRKDQRVGMFYASANFDEDVFDDPFAFNIERDPNPHLAFGGHGIHY  CLGANLARLEIGIMFDALADRLPDLMPTGAPTRFRSGWINGVVALPANYHGSGPRG  >CYP125A(X8EM53_MYCAB)  MVHPSLPAGFDFTDPEIYAERLPVEELKELRKTAPIWWQEQPDGVGGFNDGGYWVVTKHK  DVKEVSLRSDVFSSWENTAIPRFQDDITREAIELQRYVMLNMDAPHHTRLRKIISRGFTP  RAIGRLRDELNERAQEIAKAAAASGTGDFVEQVSCELPLQAIAGLLGVPIEDRGKLFNWS  NEMTSYDDPEYADIDPAASSMEILAYSMEMAKQKAENPGEDIVTTLINAEVEGEGKLSDD  EFGFFVIMLAVAGNETSRNSITQGMMAFTQFPEQWELYKKERPETAADEIVRWATPVTSF  QRTALEDTELDGVKIKKGQRVVMMYRSANFDEDVFEDPFSFNIMRNPNPHMGFGGSGAHY  CIGANLARLTINLMFNAIADHMPNLAPAGDPKRLQSGWLNGIKHWQVDFTGASGCPVLQ |
| ***Mycobacterium abscessus* subsp. *bolletii* MA1948** |
| UniProtKB;P450count:24;Families:18;Subfamilies:21 |
| >CYP164A(X8D8F5_MYCAB)  MHLSISAHTGHMPITTDPGTLLLQVLDPANRANPYPVYGQLVEQTQAGPVHPPGMDVSVL  ATFADCNAVLRHPQASSDRRKSNIVQRQLAQNPNLIARPSFLGLDAPDHTRLRKLASKAF  APRVINAMAEDIQTFVDKMLDDIALRGTFNLVTEFAYPLPVAVICRMLGVPIEDEPEFGR  ASALLGQGLDPVYALTGQSPANMDERFQAARWMWDYFIDLIASRRRNLGDDLLSALIQVE  EAGDQLTEEEIISTCTLLLIAGHETTVNLIANASLAMLRHPEQWKLLGQNADRAPLVVEE  TLRYDPPVHMVARVADGEMAIRDFILPDGEWVLLMLAAAQRDPDVGPDLDVFNPDRSEIK  HLAFGHGPHFCLGAPLARLEARLALSSITARFPGARLMDEPTYKPNVTLRGLAELPVSIA  >CYP161NSF1(X8D212_MYCAB)  MPAAQQRELQRAGTVHRVTTAVGDPAWLITGYATVRQLFDDERVGRSHPEPDTAARSGDS  AFFGGPIGSYETEREDHARGRRLMQPHFTPKQMRTLAGRVHELADELITAMIDRGSPADF  YTAVAVPLPMMVLCELLGVPYADRDEFREWTVAASNTRDRSRSEWGMGQLFVYGMQLVGR  KRQQPGDDVISRLCTIDGVADHEIASQSMALLLGGHETTVVQLGLATLLLLANPEQWALL  VSQPDLVPNAVEETLRASRTGGGEIPRYAREDLEIDGVHIAAGELLLLDVGAANHDPAVF  GCPDQLDVARKHLAHVIFGYGSRYCVGAPLARMQLTEVLGQLVERLPGLHLRRDISELTM  RGDLLTGGLREVPVGW  >CYP286D1(X8D2S5_MYCAB)    MEMARITSVPCDGETTTYVSPPKLRVARALARLPRLGDTVGLMLDPTFYLFEKYLRHGPV  FTVKMPYQTYTVLAGSEAATFMSSKDGRECLTVGSSWRFVEEQFGGKDSLVAVDGPQHKQ  WRTLLQRGYSREAIADRYSEAIDVIDDAVDKHWRPGESVPVLPAMQKLSIAQVGTFLGGV  RPTDDDIEDIALVTREILKIAPVQHIPKFMLQLPRYVNARSRVARVAQNALALAGHSSAS  GRRGQSTLLEDILESCRDDPNAANQRNMLFHSVLPYFAGVETTSATATYALYLILRHPDV  LCRIQDEVAVMFERGGITHDSLFHSTPVLHGAIMEAMRLRPIASFIIRVAAKDFEFHGHQ  IRSGEGVFIGTTVPHFLSEFYENPMKFDVDRYGDSAGPNRTPGAYSPFGRGPHMCVGKGL  AESLMQLMLARIIHRRTLELSSRSYRLSNRVSSSSPSPKFAVRVVSSRR  >CYP136A(X8D2T7_MYCAB)  MATISSTDYLIDQAKRRLPTMNTLPGMGYIENYLNNREWPMTELAAPPPGSGLKPVMGDQ  GLPMLGHMVEMFRGGIDWVLNMYQERGPVSWTQTPIGKIVAALGPDATQAVFSNANKDFS  QQGWVPVIGPFFNRGLMLLDFQEHREHRLIMQQAFLRSRLAGYVEQIDAVASEIVAQWPT  NDNRFLFYPAIKELTLDVASVVFMGNEPHAQHERLEKVNKAFVATTRAGGAIFRFGLPPF  KWWQGLQGRKLLEEYFAERVGIARQKTEGADMLTALCHAETDEGDAFTDTDIVNHMIFLM  MAAHDTTTSTTTTMAYYLAANPEWQERVRDESDRLGDGPLDIDSLEKLESLDLVMNEALR  LVTPLPFNIRSTVRDTDLLGFHIPAGTMINIWPGMNHRLPELWTEPDKFDPDRFSEPRNE  HKRHRYAFSPFGGGAHKCIGMVFGQLEIKAVMHRLLRRYRFELAHPHYQPKWDYAGMPLP  IDGMPIILRPLH  >CYP135B(X8D4A2_MYCAB)    MQSILMASYGLRFLAGCQRRYGNMFTLRIPLSGKVVYLADPADIKTVYAGDPHVFHSGEA  HWFFRGLLGDSSLFVLDEDEHHHQRRLLMPAFHRDAVARQATQMAQIAAANIAEWPVGES  FPVAPRTTDITLEVILRTVIGASDPVRLAALRKVVPRLLYMKPWETPAITNPGLRRYWPW  TAVGRRMAETDALLYAEIAERRADPNLTERTDVLAMLVRATDDDGRTMSDQELRDHLLTS  IAAGHETTATALSWVLERLTRHPDALLKAAQAAEASAAGDPAGDEYLDAVVKETLRIRPV  IFSSGRVLKESVEVGGYQLPVGIMVDPAIGLVHASGAVYPDPARFDPDRMVGTTLSPTTW  LPFGGGNRRCLGATFAMVEIRVVLREILRRVELNTTTAPDEKQQAKHVTFVPHRGGSISV  RAIRTCAPAVTPTCPADVHGTGQARDPQR  >CYP1134A1(X8CV07_MYCAB)    MSASALQPEPLSAPVEVAAGECPVNHDLYPGGPTFLRWHHTRVGYLKSWRLALGAVWSTY  RHTISEYLADLPGQDDVIVARAPMRKAVIVRNPELARHVLVANQDNYIKSAEYDLLAVGF  GRGLVTDLNEGLWNRNRRLVQPIFAKRQVDLFAPQMAEAAARTISRWDELYAEGKPVDIT  AEMNYLTMDIVAQTMFGIDLSGDMAERMRIYFARLLKLFGVGFIVGAAPPLRWVVDKLAA  HGPDELSSHTPRLAIRALRIGASVAAPRTMKGLRWVERTIDQLIADHRSGRIARQDNLLA  LLMAAEDPETGAKYTDLEIRDELMTFLGAGFETTAAALAWTWYLLSRNPDARAKLGQEVD  RVLGGRQPTAADVDNLPWTAAVLNEAMRVYPPILGLARTAKADDVLGDYPISAGTTVMVL  IDSIHHNERVWDDAKTFDPARFLKENLQPEQRKAHMPFGAGKRMCVASGFANLEAIIGIA  ALAQNYELDLLPGQQPRREVTFTGGPEGEILMRLRKRHP  >CYP1128A(X8D0U2_MYCAB)    MVLGYQPAREVLGGSGWSSDPLASPQLRAQAPEFLDSSGFGRNMLFADEPDHTELRGTVR  DVFTPGFIAGLREGVHTIASHVVEYPSTGEYFDFMADIALPLPIAIIGEWLGLDDSSAAV  LRQESPAIIQMLGAFADVDTVMAGTAAGVTLATELLPLAADRRAHPGDDLLSLIASNTDL  PLEDVVTTALIIAIAGHETTANLLGASMVRLLTPRPDGTRLADDIDPDDPTVITELLRLD  GPVLATARVATRAHTLAGNTIEGGQTVLIAIAAANRDPQVFVDPAVFRRDRTSPPLAFGY  GTHHCLGSALARLETTLVLREVLARQPVITGPVSWRDTPAIRGPRSIPMRFQT  >CYP1130B1(X8D7N2_MYCAB)  MRREPFEFFRRCAREFGDVYRVPFPLGGSIVVVNHPDYASQVMDDPVGRYSMIGPGQAAM  GVIGAAIPMLEGDKFRQRRRMLMPMFGRRHLARVAEVIADEFVRRVDRWACWADTGQVVD  LQHAIAQVTLPAFLRAMFSSTITEQEISETDVDLRTFMSLMASVTMMSPLPSVLPLPGRE  SAPRSMWRLWRLTRRLIQQRRKNPIETPDLLSLLLEASYDDGSQLSERDLSMELMILMAG  GYETVVASLSWTLALLLGHPEHLRRLYAEVDGLAGALPTPDDLPKLAWAKACFDEGQRLQ  GHPLNPRFAMDDDVIGGYFIPKHTIVGPSLYSIHRDPRWWADPDTYDPNRFMDEVAARSR  PRLAFMPFGSGRHHCLGTGMAYMNAQFLLAIIFQRYRLALPEGWKPKHHFNFSVTLEGGL  PVTLTTV  >CYP136D1(X8CWY5_MYCAB)  MGHTLDYIRFGSDFSRERYDRLGSVSWMGAFGTKMVVIAGPDATREAFTSEAKAFSQDGW  SFLIDAFFHRGLMLMSFDEHLMHRRIMQEAFTRPRLTGYVEQVTPCVRSAVPAWPVGPSV  RIYPLLKELTLDIATDVFMGGRGKDESDAVNKAFVATVRAASSLVRAPLPGTRFRAGVQG  RRVLEDYFFRHLPAARAGETEDLFAALCQATTEDGERFSDEDVVNHMIFLMMAAHDTSTI  TTTAVTYFLAKYPQWQEAAAAEAAAIGDGLPDIEALEKMTVIDRVIKEALRLLAPVPLVM  RKTVRDVAIDGYHIPSNTLCAITPAVNHFDRTIWNDPERFDPSRFDEPRREDQHHRFAWV  PFGGGAHKCIGMQFGTLEVKAILHRMLRSFTWKVPENYHVRWDNTSLPIPVDGLPLEMKR  R  >CYP130A(X8DCC0_MYCAB)    MYAALRDHDPVHHVVPEYTPDQDYYVLTRHADVYEAARDWETFSSARGLTVTYGDLEKTG  MGDNPPMVMQDPPTHTEFRKLVSRGFTPRQVTAVEPKVREFVVQRLERLKERGEGDIVVE  LFKPLPSMVVAHYLGVPEEDRTQFDAWTDGIVAAASGGAIDIEAMQGEVAQTIGELMMYF  TGLIERRRAEPEDDTVSHLVAAGVGADGDISGTLQILGFAFTMVTGGNDTTTGMLGGAIQ  LLHQNPAQRQKLIDDPSLIPGAVEEFLRLTSPVQGLARTATRDVIIGDTTIPAGRRALLL  YGSANRDEREYGNDAAELDVLRKPRNILTFSHGNHHCLGAAAARMQSRIALEELLTRIPD  FEVDLDGVTWADGSYVRRPLTVPIRVR  >CYP1130A(X8D2E4_MYCAB)    MARNPYKFLQDCHRRYGDIYRVPLPIHPLVLANHPDLVSEFMENTELKYSMSAPIQGKRL  QKAVASVGCPVQVLEGQALRDRRKRLMPMFGKRHLSVVSDKFVEVFTDRIDRWLLVADTG  KEVNLQAELPKVVLPAFMYAMFSTRLSDDEVLHADTATRSVMRAIASGLFLASPPNIFPL  RGRENLPVSGLRLMRTIRQMIRDRRANPTDDADLLNILLAARGDSSRPLTEIDVYSEIMS  AIGGGYETIVASMSWTLALLLQHPEHLDRLYDEISILHGNAPTPDDLPRLPWARACFDEG  QRLQGAPINPRYAMEDTELGGYPIPKYTLVASSLYVVHRDPRWWGENAETYDPMQFFDQD  RVNARPRLAFQAFGAGPHHCMGTGMAYMMAQYLLAIIFQRYRLHLRPGWQPRQFFSLSTL  VKGGVPATITKA  >CYP144NSF1(X8CXA6_MYCAB)    MNTDELFDPQVLEDPYPFYRRLRETAPVWPVGDSGFYFVSRWDLVVEATERAEDFSSNLT  AALMKSAEGLAVAPMGPPADPTHVLATGDDPLHHAHRRLVLPTLVARRITALEPVMAQTG  SCLWERGVNGDGIDWMAAMGDALPMTMVARLIGLPAGDVPQLVQWGYSSTEMLGGLNTPQ  RQAQVVTDTMHLVLYLREHLEEELAAPGDDLLGYLAQACNRGDISLDIGVMILVQLVGAG  GESTAGLMGNAVRILGENPRLQQRIRENRALLPTFLEEALRLESPFRGHHRHVLTDTTLG  GVQLPAGSHLTLLWGAANRDPAIFEDPDVLRLDRPSPRGHITFGKGLHFCVGAALARLEA  RTAINLLLDRTREFAIKPDGAHWVPSIMVRRHQKLELEVSAA  >CYP140NSF1(X8D5A1_MYCAB)  MRTRDQIKYWSRWATMHGISRAALLTQVRTLPLAALFLGPDRAEKHYRYIEEIRASGSVT  PSRAGGLIFTDLALTREILRDNRFITMAPTNIPSPILPLAFSRWVFARTEPGLPNPVEPP  AMLAVDPPEHTRFRKLVSKAFTPRAVSKLEDRVREVTTELLDNLERHERADLLHDYASQL  PVAIIAEMLGVPRADAPFLLEWGNHGAALLDIGMTWSAYRDATQALIEIDRYFDAHLVRL  RGELAEDPTVDGILASIVRDGDLNDRELKATMALLLGAGFETTVNLIGNGIAALLRHPQQ  LAHLRENPDGWPNAVEEILRYDSPVQITGRVATQACEFEGHTLAAGSMAILLLGGANRDP  AVFDQPDVFDVLRANAREHVAFGSGIHVCLGASLARMEGVVALQSLFERFPELALAADPT  PGKHVNLHGFGSLPVNLGRARVPASS  >CYP123A-fragment(X8D966_MYCAB)  MDVISELMGVPESDRARIRELADGVMHREDGLADVPPEAIQASFDLMTYYIEMVRERRRR  PTEDLTSALLQAEIDGDRLTDEEVLAFLFLMVIAGNETTTKLLANAVYWGHRNPDQLATV  HADHDRIPLWVEESLRYDTSSQILARTVAEDITVYDTKIPAGDILLLLPGSANRDDRVFD  DADQYRIGREIGAKLVSFGSGAHFCLGAHLARMEAKVALTELFTRISGYEIDERNSVRVH  SSNVRGFAHLPMTVQLREGR  >CYP138A(X8CXE6_MYCAB)  MTEISTIDRAADVPKPIAPPLVTLPKLAQGIAFVASRRWTTSRLAKKYGKVYTINIPKFG  YTVVVADPDLTREVFTTSTEILGNIQPNLSQQLGPGSVFALERNEHRHRRKLLAPKFHGK  AMVQYEQIIEEETLRECATWPEGQQFETMEPMMRITLNAILRAVFGADGAELDVLRRLIP  PWVVLASLTTRIPQPKRNYGRFSPWGRLKTYRAHYDAVIGSLIEKALAAPDFEDRTDILA  LLLRSTYDDGTTMSRSDVSDELLTLLAAGHETTATTLAWAFERITRHPEVLSRLAEEAQG  DSNEYRQATILEVQRSRPVIDFAGRHVLAPHVDIGPYRIPQGHSVVVSINLMHDDPVAFP  CPERFDPERFMGAKPGNSWVPYGGGTRRCVGAAFANMEMDIVLRTVLRHFTIETTTAPDE  KWHSRGVASCPKNNGRVTVRRR  >CYP1132A1(X8CX78_MYCAB)  MRSIDTDSVAPAVPKPPVPRLPPRLPIHKAALLGLSCLGDPTRLPYLLLPKIANRYGDIVRLFTGPTPALTLTLINHPDYVDHVFTRHHDRYVKHEATIELVSGEPVALPLLEGREWKRVRSAFNPYFGERALAQATPLMMEGITERVDAWSRHVHSGELVDLEHELGAVVMDGLMRSMFKVRLRPAEIDHAVDGARRYGVYVISRVAMHFLPRWLPNPLRRSGEEAKAELFGILDRFVQERAGCPSTGTPDLVDTLLALEFDGCPQIRERRRRSEAAGLVFAGFETTAAALAWTIALLCRNPIALGKAYAEVDALGGKTLAYDDLENLKYLRACFDEAQRFQAAPANVRTAIEDDEVGGYFIPRGSQVIITQYALQRDPRFWNEPERFNPDRFLTDKINRNTFLPFSIGPRKCMGTRMAYIEGTLVLGAILQRYAFQIRDGWTPMHRVRVSTGLAGGLPARLFAR  >CYP1110B1(X8DBA1_MYCAB)  MTLSSTELDDLVGAVDRYPVYKDLRDNQPVLPVEVNGHEAYLLTRYADVSRVLKTASARV  QPRAGEFPAHIGTGPASEFYRFSLPSMDAPSHTRLRKLAAAAFSPRAVAAMRSWVEEIIG  AGIDRLMDFDGEFDFVGEFASRVPAEIACRLLHAPMSDAHSVLERMPDLNPILSHGGITA  EQLAAADAAAQFYIDYIGDLVDTLQGKLDSDDAVGALLEAEADGSKMTRTELIITLVGLF  IASYHTTMVALTNAVYGFSSHPAQMRTLADNPDLAPKAWEESLRYRSPVHVVHRYAGEDM  VLHDQSIPEGAQLLLGLASANRDERFFDSPDTLDITRGTNRHLAFTGGGHYCLGAPLSRL  EGDYFMRVLPQRLPNIRVTSDRPDWGTDLSFAFMRSMMVSSGR  >CYP140B(X8D3J1_MYCAB)  MQHRIKQRTHWAVTHGIGRAYLKVLARRGEPVAQLGIDVGQAPDIYRIIDKIRERGRLSR  AGDGWITADAQIVRTIFRDNRFVTFKPEHRSASPIIQRLAAWSDPQLLNPAEPPSILITD  PPDHGRLRRLVAAPFTPRAIEGLRDRIQEVTNGHLDVLQQRQSPDLIADFTAKIPIAVIG  EMIAVPPPDYSRLYTAMNRAIQLIATTAPSWSEYQDGTAALREIDEYLEKHVARLRREGT  ESELATALLDSDLSHFELKMFFAVFLGAGFVTTTHLMGKAIVTLLRHPEQLALLQADPSL  WPNAVEELMRYDTSNQWSARVATETVEIEGHTIEAGQSALLLLGGANRDPAAFENPDVFD  ITRPNARENITLGTGIHVCLGQVLARAELHTALQTLFERFPRLSLAGEPEYLNGMGIHGL  RLLPVTLG  >CYP108B(X8D4S2_MYCAB)    MATALSEIEEAAGRVLADPAAYADELRLHAAMTLLRREQPVTKVITDDYRPFWAVTKHDD  IMAVERDNALWINEPRPLLMNLEQEAELDKQAAMGIELKTLVHIDDPKHRVLRAIGADWF  RPKAMRDMKLRTDELANRYVNKLLEAGGSCDFAQDIAVHFPLYVIMSLLGIPESDFDRML  KLTQELFGGDDSEFQRGTTPEEQLMALLDFFGYFSGLTASRRQHPTDDLASTIANARVDG  ELLSDVDTASYYTIIATAGHDTTSATIAGGLEALLEHPDQLARLRENPGLMPLAVDEMIR  WVTPVKEFMRTATADTEIRGVPITEGESVLLSYPSGNRDEDIFTDPFTFDIARDPNKHLA  FGFGVHFCLGAALARMEVNSFFSALLPRLESIEVDGNIERTSTIFVGGIKHLPIRYTLR  >CYP51B(X8DCD3_MYCAB)    MTTPTVPRVSGGEDQYGHLEEFRTDPIALMKRIRAECGNVGTFQLADKQVVFLSGAEANE  FFFRSSDEDLDQAEAYPFMTPIFGKGVVFDADPERRKEMLHNAALRGEQMKGHAATIENE  VRQMISRWGESGEVDLLDFFAELTIYTSSACLIGKKFRDELDGRFAHLYHQLEQGTDPLC  YVDPYLEIESFRQRDEARTGLVALVQEIINGRVQRSADSPTDKSQRDMLDVLIMIKDEHG  NPRFTADEITGMFISMMFAGHHTSSGTASWVLIELLRHPDIQSQVIDELDELYADGSEVS  FHALRQIPKLENVLKETLRLHPPLIILMRVAKGEFEVGGFPIHDGDMVAASPAVSNRIAE  DFPNPDGFVPDRYEKPRQEDIVNRWTWIPFGAGRHRCVGAAFATMQIKAIFSVLLREYEF  EMAQPADSYHNDHSKMVVQLAQPAKVRYRKRSA  >CYP125A(X8D963_MYCAB)  MTAMKTAAELGLPEGFDFTDPELYGNRMPHEEFATLRREAPVWWNPQPRTVGGFADEGYW  VISKHRDVREVSLHTDTFSSGRKGAIPRLEDHISPEEFQATLSVLINKDAPEHTQLRGLV  SRMFTPRSIAALRITLEERAERIVRAALEGGHGEFVREVASELPMQAIAELIGVPEEDRV  KLFEWSNQMTGYDEADVEIDPRVGAAQILGYSYQLAEQRRDCPGNDVVSRLLTGTVDGEQ  LTPEQFGFFVVMLSVAGNETTRNATTMGMMAFLEHPGQWELFKSARPSTTVDEIVRYTSP  LISQQRTALQDTVISDVRIRAGERVVMLYPSANFDEEVFENPHTFDITRDPNPHLGFGGT  GAHYCLGANLAKAELEIIFNKIADRMPDISRIGDAPRFHSGWINGIKKFDTAYCPVTH  >CYP125A(X8D810_MYCAB)  MTTCPFTPGFDFTDPDLIQHRIPAEEFAYLRKTEPIWWNAQPRGVAGFDDDGYWVVTKHA  DVKEVSRLNEVFSNSVNTTVVRYNEDITAEQLEIQRENLLIDMDEPKHRILRRIVSPLFT  PKAVNGLHARLVERAHGIVEEAAEKSSGNFVSDIASVLPMHAIADLVGIPESDRQQVLDW  TNQMFAYDDPAIGRDTATTATVSMLGYAYAMAEERQLNPQDDILTGLVRGAYDDRPLTPL  EFAYFVIQLMVAGNETSRNAITHGVLAFADNPAQWRLYRERRPSTAADEIIRWASPIIAF  QRTALQDVELGGVQIRKDQRVGMFYASANFDEDVFDDPFAFNIERDPNPHLAFGGHGIHY  CLGANLARLEIGIMFDALADRLPDLMPTGAPTRFRSGWINGVVALPANYHGSGPRG  >CYP125A(X8D8Q9_MYCAB)  MVHPSLPAGFDFTDPEIYAERLPVEELKELRKTAPIWWQEQPDGVGGFNDGGYWVVTKHK  DVKEVSLRSDVFSSWENTAIPRFQDDITREAIELQRYVMLNMDAPHHTRLRKIISRGFTP  RAIGRLRDELNERAQEIAKAAAASGTGDFVEQVSCELPLQAIAGLLGVPIEDRGKLFNWS  NEMTSYDDPEYADIDPAASSMEILAYSMEMAKQKAENPGEDIVTTLINAEVEGEGKLSDD  EFGFFVIMLAVAGNETSRNSITQGMMAFTQFPEQWELYKKERPETAADEIVRWATPVTSF  QRTALEDTELDGVKIKKGQRVVMMYRSANFDEDVFEDPFSFNIMRNPNPHMGFGGSGAHY  CIGANLARLTINLMFNAIADHMPNLAPAGDPKRLQSGWLNGIKHWQVDFTGASGCPVLQ  >CYP125A(X8D7U8_MYCAB)  MVQAQHPHLPDGIDFTDPELFVHGIPERELAELRHTEPIWWNHTERGVAGFDDDGFWVVS  KHKDVKEVSLRCEVFSSEQNTAIPRYLPTTPRERIDATRLIMLNMDPPRHSRLRHIISRG  FTPRAISRLRDDLNARAQGIARAAAQLRHGDFVEQVACELPLQAIAGLMGTPLDEREQLF  DWSNRLVGSSDGEDDSAVASAELLMYAMGVAARKTAEPGADICTDLVNADIDGQKLSDDE  FGFFVMLLAVAGNETTRNSITHGMHAFTQFPEQWELYKKTRPETAADEIVRWATPVTSFQ  RTALEDTELGGVRIKKGQRVVMMYRSANFDEEVFENPFTFDIMRDPNPHVGFGGNGEHHC  VGANLARMTINLMFNAIADHMPDLASAGEPDRLRSGWLNGVKHWEVDFCPAGYGRAS |
| ***Mycobacterium abscessus* VO6705** |
| UniProtKB;P450count:28;Families:19;Subfamilies:24 |
| >CYP51B(T2R7D7_MYCAB)  MTTPTVPRVSGGEDQYGHLEEFRTDPIALMKRIRAECGNVGTFQLADKQVVFLSGAEANE  FFFRSSDEDLDQAEAYPFMTPIFGKGVVFDADPERRKEMLHNAALRGEQMKGHAATIENE  VRQMISRWGESGEVDLLDFFAELTIYTSSACLIGKKFRDELDGRFAHLYHQLEQGTDPLC  YVDPYLEIESFRQRDEARTGLVALVQEIINGRVQRSADSPTDKSQRDMLDVLIMIKDEHG  NPRFTADEITGMFISMMFAGHHTSSGTASWVLIELLRHPDIQSQVIDELDELYADGSEVS  FHALRQIPKLENVLKETLRLHPPLIILMRVAKGEFEVGGFPIHDGDMVAASPAVSNRIAE  DFPNPDGFVPDRYEKPRQEDIVNRWTWIPFGAGRHRCVGAAFATMQIKAIFSVLLREYEF  EMAQPADSYHNDHSKMVVQLAQPAKVRYRKRSA  >CYP138C2(T0AZ73_MYCAB)  MVTAAAKRYGSPFKVHLPILGTTVIVGDPVMIKEVFTASSELVGQASNLGVVFGPGSTFS  LEGEAHRARRKLLVPPFHGKRMHGYEDIVEQEAMHEFAKWKQGVSFPVMPSMMRITVNAI  LRAVFGAQGSEVEELSKLLPSMVELGSKIVVIPLAQKNLGRLSPWVRLMRMRARYDAIIA  EMIERARSDSALDQRADVLALLLQATYEDGTRITDGHIADELLTLLAAGHETTATSLAWT  VERLRRHPWLLARLTEEVESGDSALRQATIWEVQRTRPVIGGALRRAQTRIRMGEWVIPE  GCGIVTSATLAHQQAALYGQPDDFNPDRFLNGRPDTYGWIPFGGGTHRCIGAAFANMEMD  VVLRTMLREFELTSTYTAGERMLSRGVAVAPSDGGAVVVYKRTPAHMPAVPVAAVGAV  >CYP108B6(T2RAT4_MYCAB)  MATALSEIEEAGRVLADPAAYADELRLHAAMTLLRREQPVTKVITDDYRPFWAVTKHDDI  MAVERDNALWINEPRPLLMNLEQEAELDKQAAMGIELKTLVHIDDPKHRVLRAIGADWFR  PKAMRDMKLRTDELANRYVNKLLEAGGSCDFAQDIAVHFPLYVIMSLLGIPESDFDRMLK  LTQELFGGDDSEFQRGTTPEEQLMALLDFFGYFSGLTASRRQHPTDDLASTIANARVDGE  LLSDVDTASYYTIIATAGHDTTSATIAGGLEALLEHPDQLARLRENPGLMPLAVDEMIRW  VTPVKEFMRTATADTEIRGVPITEGESVLLSYPSGNRDEDIFTDPFTFDIARDPNKHLAF  GFGVHFCLGAALARMEVNSFFSALLPRLESIEVDGNIERTSTIFVGGIKHLPIRYTLR  >CYP136D1(T2R8E9_MYCAB)  MDAVKAAQRPGGTMTNHLLAPAHHVKERLSSVIMVPAPHAVDDRWRRWSRDWPVRELAPA  PAGSGLKAVRGDAGLPFVGHTLDYIRFGSDFSRERYDRLGSVSWMGAFGTKMVVIAGPDA  TREAFTSEAKAFSQDGWSFLIDAFFHRGLMLMSFDEHLMHRRIMQEAFTRPRLTGYVEQV  TPCVRSAVPAWPVGPSVRIYPLLKELTLDIATDVFMGGRGKDESDAVNKAFVATVRAASS  LVRAPLPGTRFRAGVQGRRVLEDYFFRHLPAARAGETEDLFAALCQATTEDGERFSDEDV  VNHMIFLMMAAHDTSTITTTAVTYFLAKYPQWQEAAAAEAAAIGDGLPDIEALEKMTVID  LVIKEALRLLAPVPLVMRKTVRDVAIDGYHIPSNTLCAITPAVNHFDRTIWSDPGRFDPS  RFDEPRREDQHHRFAWVPFGGGAHKCIGMQFGTLEVKAILHRMLRSLTWTVPENYHVRWD  NTSLPIPVDGLPLQLKRR  >CYP164A3(T2R940_MYCAB)    MPITTDPGTLLLQVLDPANRANPYPVYGQLVEQTQAGPVHPPGMDVSVLATFADCNAVLR  HPQASSDRRKSNIVQRQLAQNPNLIARPSFLGLDAPDHTRLRKLASKAFAPRVINAMAED  IQTFVDKMLDDIALRGTFNLVTEFAYPLPVAVICRMLGVPIEDEPEFGRASALLGQGLDP  VYALTGQSPANMDERFQAARWMWDYFIDLIASRRRNLGDDLLSALIQVEEAGDQLTEEEI  ISTCTLLLIAGHETTVNLIANASLAMLRHPEQWKLLGQNADRAPLVVEETLRYDPPVHMV  ARVADGEMAIRDFILPDGEWVLLMLAAAQRDPDVGPDLDVFNPDRSEIKHLAFGHGPHFC  LGAPLARLEARLALSSITARFPGARLMDEPTYKPNVTLRGLAELPVSIA  >CYP1128A(T2R7A1_MYCAB)  MTTANPVTPRIPWDSRDPYVYLEDLRAHGDVVLDENAGTWVVLGYQPAREVLGGSGWSSD  PLASPQLRAQAPEFLDSSGFGRNMLFADEPDHTELRGTVRDVFTPGFIAGLREGVHTIAS  HVVEYPSTGEYFDFMADIALPLPIAIIGEWLGLDDSSAAVLRQESPAIIQMLGAFADVDT  VMAGTAAGVTLATELLPLAADRRAHPGDDLLSLIASNTDLPLEDVVTTALIIAIAGHETT  ANLLGASMVRLLTPRPDGTRLADDIDPDDPTVITELLRLDGPVLATARVATRAHTLAGNT  IEGGQTVLIAIAAANRDPQVFVDPAVFRRDRTSPPLAFGYGTHHCLGSALARLETTLVLR  EVLARQPVITGPVSWRDTPAIRGPRSIPMRFQT  >CYP1130B1(T2R992_MYCAB)  MTELVGKEVTRGCPAEPHQDGPTGFGDFPRELAGQWGPLPLNPSVHEVNEIARARQRFEY  SRKDPWWLTRASFPLASSIVRGVGSPALTPPGPVGHPVVGSVPEMRREPFEFFRRCAREF  GDVYRVPFPLGGSIVVVNHPDYASQVMDDPVGRYSMIGPGQAAMGVIGAAIPMLEGDKFR  QRRRMLMPMFGRRHLARVAEVIADEFVRRVDRWACWADTGQVVDLQHAIAQVTLPAFLRA  MFSSTITEQEISETDVDLRTFMSLMASVTMMSPLPSVLPLPGRESAPRSMWRLWRLTRRL  IQQRRKNPIETPDLLSLLLEASYDDGSQLSERDLSMELMILMAGGYETVVASLSWTLALL  LGHPEHLRRLYAEVDGLAGALPTPDDLPKLAWAKACFDEGQRLQGHPLNPRFAMDDDVIG  GYFIPKHTIVGPSLYSIHRDPRWWADPDTYDPNRFMDEVAARSRPRLAFMPFGSGRHHCL  GTGMAYMNAQFLLAIIFQRYRLALPEGWKPKHHFNFSVTLEGGLPVTLTTV  >CYP1134A1(S9ZPJ2_MYCAB)  MSASALQPEPLSAPVEVAAGECPVNHDLYPGGPTFLRWHHTRVGYLKSWRLALGAVWSTY  RHTISEYLADLPGQDDVIVARAPMRKAVIVRNPELARHVLVANQDNYIKSAEYDLLAVGF  GRGLVTDLNEGLWNRNRRLVQPIFAKRQVDLFAPQMAEAAARTISRWDELYAEGKPVDIT  AEMNYLTMDIVAQTMFGIDLSGDMAERMRIYFARLLKLFGVGFIVGAAPPLRWVVDKLAA  HGPDELSSHTPRLAIRALRIGASVAAPRTMKGLRWVERTIDQLIADHRSGRIARQDNLLA  LLMAAEDPETGAKYTDLEIRDELMTFLGAGFETTAAALAWTWYLLSRNPDARAKLGQEVD  RVLGGRQPTAADVDNLPWTAAVLNEAMRVYPPILGLARTAKADDVLGDYPISAGTTVMVL  IDSIHHNERVWDDAKTFDPARFLKENLQPEQRKAHMPFGAGKRMCVASGFANLEAIIGIA  ALAQNYELDLLPGQQPRREVTFTGGPEGEILMRLRKRHP  >CYP135B(S9ZVW4_MYCAB)  MSKTAKIALPPGPPLPLAVQSILMASYGLRFLAGCQRRYGNMFTLRIPLSGKVVYLADPA  DIKTVYAGDPHVFHSGEAHWFFRGLLGDSSLFVLDEDEHHHQRRLLMPAFHRDAVARQAT  QMAQIAAANIAEWPVGESFPVAPRTTDITLEVILRTVIGASDPVRLAALRKVVPRLLYMK  PWETPAITNPGLRRYWPWTAVGRRMAETDALLYAEIAERRADPNLTERTDVLAMLVRATD  DDGRTMSDQELRDHLLTSIAAGHETTATALSWVLERLTRHPDALLKAAQAAEASAAGDPA  GDEYLDAVVKETLRIRPVIFSSGRVLKESVEVGGYQLPVGIMVDPAIGLVHASGAVYPDP  ARFDPDRMVGTTLSPTTWLPFGGGNRRCLGATFAMVEIRVVLREILRRVELNTTTAPDEK  QQAKHVTFVPHRGGSISVRAIRTCAPAVTPTCPADVHGTGQARDPQR  >CYP136A(T2RCD9_MYCAB)  MATISSTDYLIDQAKRRLPTMNTLPGMGYIENYLNNREWPMTELAAPPPGSGLKPVMGDQ  GLPMLGHMVEMFRGGIDWVLNMYQERGPVSWTQTPIGKIVAALGPDATQAVFSNANKDFS  QQGWVPVIGPFFNRGLMLLDFQEHREHRLIMQQAFLRSRLAGYVEQIDAVASEIVAQWPT  NDNRFLFYPAIKELTLDVASVVFMGNEPHAQHERLEKVNKAFVATTRAGGAIFRFGLPPF  KWWQGLQGRKLLEEYFAERVGIARQKTEGADMLTALCHAETDEGDAFTDTDIVNHMIFLM  MAAHDTTTSTTTTMAYYLAANPEWQERVRDESDRLGDGPLDIDSLEKLESLDLVMNEALR  LVTPLPFNIRSTVRDTDLLGFHIPAGTMINIWPGMNHRLPELWTEPDKFDPDRFSEPRNE  HKRHRYAFSPFGGGAHKCIGMVFGQLEIKAVMHRLLRRYRFELAHPHYQPKWDYAGMPLP  IDGMPIILRPLH  >CYP138A(T0B5W1_MYCAB)  MYQGVVMIENALQLKAKLPPGPRWPALVQAVAYGTARQQVLRYADRRFGSAFTARLPVFG  HTVVISDPALIKQLFQTSTDVAQGVRPNLDTVLGPGSTFGLQGEEHHRRRKLLIPPFHGK  RMRAYEDLIEHETLREISTWSQGREFSVMPSTMRITLNAILRAVFGAHGAEFEVLRDLMP  KMVHLGSPLVLVPWMHRDLGRWSPWGKFMAMRRQFDHTVGVLIERAQSDPHFNQREDVLS  LMLQARYDDGTSMSHSDIGDELLTLLAAGHETTATSLAWAVERLRRHPEILAQLVEEVDS  GGTGLLQATVYEVQRVRPVIDGALRHIVAPMMLGPWVIPAGTTVIVHVSQIHNSESVFPD  AETFNPQRFLGGIPDTYSWVPFGGGTRRCLGAAFANMEMNTVLRTILREFTLAPTTARGE  RWHNRGVAAAPSGGGRALVYRRSLSAAGVSQQQQSPAV  >CYP161NSF1(T0B3R5_MYCAB)  MNAVQPPQLPFPREGLFVPAAQQRELQRAGTVHRVTTAVGDPAWLITGYATVRQLFDDER  VGRSHPEPDTAARSGDSAFFGGPIGSYETEREDHARGRRLMQPHFTPKQMRTLAGRVHEL  ADELITAMIDRGSPADFYTAVAVPLPMMVLCELLGVPYADRDEFREWTVAASNTRDRSRS  EWGMGQLFVYGMQLVGRKRQQPGDDVISRLCTIDGVADHEIASQSMALLLGGHETTVVQL  GLATLLLLANPEQWALLVSQPDLVPNAVEETLRASRTGGGEIPRYAREDLEIDGVHIAAG  ELLLLDVGAANHDPAVFGCPDQLDVARKHLAHVIFGYGSRYCVGAPLARMQLTEVLGQLV  ERLPGLHLRRDISELTMRGDLLTGGLREVPVGW  >CYP138NSF1(S9ZL76_MYCAB)  MFAYQNRLEASMTTVESKTAVVLPPGPSLPKTIQGAAALINRRLALRAMRHRFGSAFTVD  LPIFGRAVVLSEPALIRQLFKSTPDEVDIVDANLGRVMGPNSMFALTGERHRAHRKLMTP  PFHGRRLSAYETIVENESTREFATWPIDQPFATMPSMMRITLNVILRAVFGAEGEEFEQL  RILLPKMVELGSKLALVPLPQWDWGSWSPWGRFSQYRREYDAIVGRLFDKGAADPGLDER  NDVLALMMQSRYEDGTSMTRSEIADELITLLSAGHETTATTLAWAVERLQRHPRLLDRLV  ADIDVGSDQLLEATITEVQRTRPVIDTAFRSVRAPTLTIGPWTLPQGQTIIAGIGLLHAD  DAVYADAKRFDPDRFIGTHPDTSQWIPFGGGLRRCIGAAFATMEMRVVLRTLLRDFTITS  STAAGERWHSRGVAIAPSRGGRVRLRLRNQIASAPVKQC  >CYP286D(T0BAJ3_MYCAB)  MGVEMARITSVPCDGETTTYVSPPKLRVARALARLPRLGDTVGLMLDPTFYLFEKYLRHG  PVFTVKMPYQTYTVLAGSEAATFMSSKDGRECLTVGSSWRFVEEQFGGKDSLVAVDGPQH  KQWRTLLQRGYSREAIADRYSEAIDVIDDAVDKHWRPGESVPVLPAMQKLSIAQVGTFLG  GVRPTDDDIEDIALVTREILKIAPVQHIPKFMLQLPRYVNARSRVARVAQNALALAGHSS  ASGRRGQSTLLEDILESCRDDPNAANQRNMLFHSVLPYFAGVETTSATATYALYLILRHP  DVLCRIQDEVAVMFERGGITHDSLFHSTPVLHGAIMEAMRLRPIASFIIRVAAKDFEFHG  HQIRSGEGVFIGTTVPHFLSEFYENPMKFDVDRYGDSAGPNRTPGAYSPFGRGPHMCVGK  GLAESLMQLMLARIIHRRTLELSSRSYRLSNRVSSSSPSPKFAVRVVSSRR  >CYP1132A(T2R807_MYCAB)  MRSIDTDSVAPAVPKPPVPRLPPRLPIHKAALLGLSCLGDPTRLPYLLLPKIANRYGDIV  RLFTGPTPALTLTLINHPDYVDHVFTRHHDRYVKHEATIELVSGEPVALPLLEGREWKRV  RSAFNPYFGERALAQATPLMMEGITERVDAWSRHVHSGELVDLEHELGAVVMDGLMRSMF  KVRLRPAEIDHAVDGARRYGVYVISRVAMHFLPRWLPNPLRRSGEEAKAELFGILDRFVQ  ERAGCPSTGTPDLVDTLLALEFDGCPQIRERRRRSEAAGLVFAGFETTAAALAWTIALLC  RNPIALGKAYAEVDALGGKTLAYDDLENLKYLRACFDEAQRFQAAPANVRTAIEDDEVGG  YFIPRGSQVIITQYALQRDPRFWNEPERFNPDRFLTDRINRNTFLPFSIGPRKCMGTRMA  YIEGTLVLGAILQRYAFQIRDGWTPRHRVRVSTGLAGGLPARLFAR  >CYP140NSF1(T0B316_MYCAB)  MRTRDQIKYWSRWATMHGISRAALLTQVRTLPLAALFLGPDRAEKHYRYIEEIRASGSVT  PSRAGGLIFTDLALTREILRDNRFITMAPTNIPSPILPLAFSRWVFARTEPGLPNPVEPP  AMLAVDPPEHTRFRKLVSKAFTPRAVSKLEDRVREVTTELLDNLERHERADLLHDYASQL  PVAIIAEMLGVPRADAPFLLEWGNHGAALLDIGMTWSAYRDATQALIEIDRYFDAHLVRL  RGELAEDPTVDGILASIVRDGDLNDRELKATMALLLGAGFETTVNLIGNGIAALLRHPQQ  LAHLRENPDGWPNAVEEILRYDSPVQITGRVATQACEFEGHTLAAGSMAILLLGGANRDP  AVFDQPDVFDVLRANAREHVAFGSGIHVCLGASLARMEGVVALQSLFERFPELALAADPT  PGKHVNLHGFGSLPVNLGRARVPASS  >CYP123A(T2R8E2_MYCAB)  MTVAETSAVPLVFDPYDYDFHEDPYPYYRRLRDEAPLYRNDDLKFWALSRHHDVLQGFRN  SEALSNANGVSMDKASFGPHAKLVMSFLAMDDPEHLRLRALVSKGFTPRRIRELEGQVVA  LARTHLDRALANASDRSFDFIAEYAGKLPMDVISELMGVPESDRARIRELADGVMHREDG  LADVPPEAIQASFDLMTYYIEMVRERRRRPTEDLTSALLQAEIDGDRLTDEEVLAFLFLM  VIAGNETTTKLLANAVYWGHRNPDQLATVHADHDRIPLWVEESLRYDTSSQILARTVAED  ITVYDTKIPAGDILLLLPGSANRDDRVFDDADQYRIGREIGAKLVSFGSGAHFCLGAHLA  RMEAKVALTELFTRISGYEIDERNSVRVHSSNVRGFAHLPMTVQLREGR  >CYP130A(S9ZXM6_MYCAB)  MSVDVSHTVAPRYVPGTADNWTNPWPMYAALRDHDPVHHVVPEYTPDQDYYVLTRHADVY  EAARDWETFSSARGLTVTYGDLEKTGMGDNPPMVMQDPPTHTEFRKLVSRGFTPRQVTAV  EPKVREFVVQRLERLKERGEGDIVVELFKPLPSMVVAHYLGVPEEDRTQFDAWTDGIVAA  ASGGAIDIEAMQGEVAQTIGELMMYFTGLIERRRAEPEDDTVSHLVAAGVGADGDISGTL  QILGFAFTMVTGGNDTTTGMLGGAIQLLHQNPAQRQKLIDDPSLIPGAVEEFLRLTSPVQ  GLARTATRDVIIGDTTIPAGRRALLLYGSANRDEREYGNDAAELDVLRKPRNILTFSHGN  HHCLGAAAARMQSRIALEELLTRIPDFEVDLDGVTWADGSYVRRPLTVPIRVR  >CYP144NSF1(T2R751_MYCAB)  MNTDELFDPQVLEDPYPFYRRLRETAPVWPVGDSGFYFVSRWDLVVEATERAEDFSSNLT  AALMKSAEGLAVAPMGPPADPTHVLATGDDPLHHAHRRLVLPTLVARRITALEPVMAQTG  SCLWERGVNGDGIDWMAAMGDALPMTMVARLIGLPAGDVPQLVQWGYSSTEMLGGLNTPQ  RQAQVVTDTMHLVLYLREHLEEELAAPGDDLLGYLAQACNRGDISLDIGVMILVQLVGAG  GESTAGLMGNAVRILGENPRLQQRIRENRALLPTFLEEALRLESPFRGHHRHVLTDTTLG  GVQLPAGSHLTLLWGAANRDPAIFEDPDVLRLDRPSPRGHITFGKGLHFCVGAALARLEA  RTAINLLLDRTREFAIKPDGAHWVPSIMVRRHQKLELEVSAA  >CYP1130A1(S9ZUJ6_MYCAB)    MIASLSDDPFWPTRKPLRASFSLLTGWGRDRPIPPGPIGLPFIGSAIPMARNPYKFLQDC  HRRYGDIYRVPLPIHPLVLANHPDLVSEFMENTELKYSMSAPIQGKRLQKAVASVGCPVQ  VLEGQALRDRRKRLMPMFGKRHLSVVSDKFVEVFTDRIDRWLLVADTGKEVNLQAELPKV  VLPAFMYAMFSTRLSDDEVLHADTATRSVMRAIASGLFLASPPNIFPLRGRENLPVSGLR  LMRTIRQMIRDRRANPTDDADLLNILLAARGDSSRPLTEIDVYSEIMSAIGGGYETIVAS  MSWTLALLLQHPEHLDRLYDEISILHGNAPTPDDLPRLPWARACFDEGQRLQGAPINPRY  AMEDTELGGYPIPKYTLVASSLYVVHRDPRWWGENAETYDPMQFFDQDRVNARPRLAFQA  FGAGPHHCMGTGMAYMMAQYLLAIIFQRYRLHLRPGWQPRQFFSLSTLVKGGVPATITKA  >CYP153A(T0B128_MYCAB)  MSIPAAVAAKAQSAVPLELQIRGAHLYDKTRRWVTGTNGKKIFTETPIPPVEDVDIADID  LSNPFLYRQGRWKSYFERVRNEAPVHYQARSPFGPFWSVTRHADIIAVDKNHEAFSAEPF  IIIGRPPRFMDIAMFIAMDPPQHDLQRASVQGVVAPKNLREMEGLIRSRVQEVLDDLPVD  QPFNWVHHVSIELTARMLATLLDFPYEQRHKLVEWSDLATSMEQANGGPSDNDRVFRGMV  DMAKGLSALWHDKAARTANGERPGFDLITMLQANEDTKDLIDRPMEFLGNLVLLIVGGND  TTRNSMSGGVLALNQFPDQFEKLKANPDLIPNMVSEIIRWQTPLAYMRRVAKKDIMLNGQ  FIRKGDKVVMWYASGNRDERVFERADELIIDRSNARNHISFGFGVHRCMGNRLAELQLRI  LWEELLPRFENIEVVGEPEYVQSNFVRGISKLMVRLTPRPSA  >CYP138A(S9ZXP9_MYCAB)  MEGDVTEISTIDRAADVPKPIAPPLVTLPKLAQGIAFVASRRWTTSRLAKKYGKVYTINI  PKFGYTVVVADPDLTREVFTTSTEILGNIQPNLSQQLGPGSVFALERNEHRHRRKLLAPK  FHGKAMVQYEQIIEEETLRECATWPEGQQFETMEPMMRITLNAILRAVFGADGAELDVLR  RLIPPWVVLASLTTRIPQPKRNYGRFSPWGRLKTYRAHYDAVIGSLIEKALAAPDFEDRT  DILALLLRSTYDDGTTMSRSDVSDELLTLLAAGHETTATTLAWAFERITRHPEVLSRLAE  EAQGDSNEYRQATILEVQRSRPVIDFAGRHVLAPHVDIGPYRIPQGHSVVVSINLMHDDP  VAFPCPERFDPERFMGAKPGNSWVPYGGGTRRCVGAAFANMEMDIVLRTVLRHFTIETTT  APDEKWHSRGVASCPKNNGRVTVRRR  >CYP140B(T0B9X2_MYCAB)  MQHRIKQRTHWAVTHGIGRAYLKVLARRGEPVAQLGIDVGQAPDIYRIIDKIRERGRLSR  AGDGWITADAQIVRTIFRDNRFVTFKPEHRSASPIIQRLAAWSDPQLLNPAEPPSILITD  PPDHGRLRRLVAAPFTPRAIEGLRDRIQEVTNGHLDVLQQRQSPDLIADFTAKIPIAVIG  EMIAVPPPDYSRLYTAMNRAIQLIATTAPSWSEYQDGTAALREIDEYLEKHVARLRREGT  ESELATALLDSDLSHFELKMFFAVFLGAGFVTTTHLMGKAIVTLLRHPEQLALLQADPSL  WPNAVEELMRYDTSNQWSARVATETVEIEGHTIEAGQSALLLLGGANRDPAAFENPDVFD  ITRPNARENITLGTGIHVCLGQVLARAELHTALQTLFERFPRLSLAGEPEYLNGMGIHGL  RLLPVTLG  >CYP1110B1(S9ZWG3_MYCAB)  MTLSSTELDDLVGAVDRYPVYKDLRDNQPVLPVEVNGHEAYLLTRYADVSRVLKTASARV  QPRAGEFPAHIGTGPASEFYRFSLPSMDAPSHTRLRKLAAAAFSPRAVAAMRSWVEEIIG  AGIDRLMDFDGEFDFVGEFASRVPAEIACRLLHAPMSDAHSVLERMPDLNPILSHGGITA  EQLAAADAAAQFYIDYIGDLVDTLQGKLDSDDAVGALLEAEADGSKMTRTELIITLVGLF  IASYHTTMVALTNAVYGFSSHPAQMRTLADNPDLAPKAWEESLRYRSPVHVVHRYAGEDM  VLHDQSIPEGAQLLLGLASANRDERFFDSPDTLDITRGTNRHLAFTGGGHYCLGAPLSRL  EGDYFMRVLPQRLPNIRVTSDRPDWGTDLSFAFMRSMMVSSGR  >CYP125A(T0B0P1_MYCAB)  MVQAQHPHLPDGIDFTDPELFVHGIPERELAELRHTEPIWWNHTERGVAGFDDDGFWVVS  KHKDVKEVSLRCEVFSSEQNTAIPRYLPTTPRERIDATRLIMLNMDPPRHSRLRHIISRG  FTPRAISRLRDDLNARAQGIARAAAQLRHGDFVEQVACELPLQAIAGLMGTPLDEREQLF  DWSNRLVGSSDGEDDSAVASAELLMYAMGVAARKTAEPGADICTDLVNADIDGQKLSDDE  FGFFVMLLAVAGNETTRNSITHGMHAFTQFPEQWELYKKTRPETAADEIVRWATPVTSFQ  RTALEDTELGGVRIKKGQRVVMMYRSANFDEEVFENPFTFDIMRDPNPHVGFGGNGEHHC  VGANLARMTINLMFNAIADHMPDLASAGEPDRLRSGWLNGVKHWEVDFCPAGYGRAS  >CYP125A(T2R8E5_MYCAB)  MTAMKTAAELGLPEGFDFTDPELYGNRMPHEEFATLRREAPVWWNPQPRTVGGFADEGYW  VISKHRDVREVSLHTDTFSSGRKGAIPRLEDHISPEEFQATLSVLINKDAPEHTQLRGLV  SRMFTPRSIAALRITLEERAERIVRAALEGGHGEFVREVASELPMQAIAELIGVPEEDRV  KLFEWSNQMTGYDEADVEIDPRVGAAQILGYSYQLAEQRRDCPGNDVVSRLLTGTVDGEQ  LTPEQFGFFVVMLSVAGNETTRNATTMGMMAFLEHPGQWELFKSARPSTTVDEIVRYTSP  LISQQRTALQDTVISDVRIRAGERVVMLYPSANFDEEVFENPHTFDITRDPNPHLGFGGT  GAHYCLGANLAKAELEIIFNKIADRMPDISRIGDAPRFHSGWINGIKKFDTAYCPVTH  >CYP125A(T2R8J5_MYCAB)  MTTCPFTPGFDFTDPDLIQHRIPAEEFAYLRKTEPIWWNAQPRGVAGFDDDGYWVVTKHA  DVKEVSRLNEVFSNSVNTTVVRYNEDITAEQLEIQRENLLIDMDEPKHRILRRIVSPLFT  PKAVNGLHARLVERAHGIVEEAAEKSSGNFVSDIASVLPMHAIADLVGIPESDRQQVLDW  TNQMFAYDDPAIGRDTATTATVSMLGYAYAMAEERQLNPQDDILTGLVRGAYDDRPLTPL  EFAYFVIQLMVAGNETSRNAITHGVLAFADNPAQWRLYRERRPSTAADEIIRWASPIIAF  QRTALQDVELGGVQIRKDQRVGMFYASANFDEDVFDDPFAFNIERDPNPHLAFGGHGIHY  CLGANLARLEIGIMFDALADRLPDLMPTGAPTRFRSGWINGVVALPANYHGSGPRG  >CYP125A(S9ZVJ7_MYCAB)  MVHPSLPAGFDFTDPEIYAERLPVEELKELRKTAPIWWQEQPDGVGGFNDGGYWVVTKHK  DVKEVSLRSDVFSSWENTAIPRFQDDITREAIELQRYVMLNMDAPHHTRLRKIISRGFTP  RAIGRLRDELNERAQEIAKAAAASGTGDFVEQVSCELPLQAIAGLLGVPIEDRGKLFNWS  NEMTSYDDPEYADIDPAASSMEILAYSMEMAKQKAENPGEDIVTTLINAEVEGEGKLSDD  EFGFFVIMLAVAGNETSRNSITQGMMAFTQFPEQWELYKKERPETAADEIVRWATPVTSF  QRTALEDTELDGVKIKKGQRVVMMYRSANFDEDVFEDPFSFNIMRNPNPHMGFGGSGAHY  CIGANLARLTINLMFNAIADHMPNLAPAGDPKRLQSGWLNGIKHWQVDFTGASGCPVLQ |
| ***Mycobacterium avium* complex (MAC)** |
| ***Mycobacterium Avium* 104** |
| Database: TB; P450 count: 48; Families: 29; Subfamilies: 34 |
| >CYP105Q(MAV_2968)  MTQPSTDADTDIPDFPMTRAPGCPFAPPPKVLQLNADKQLSRVRIWDGSTPWLVHGYQAI  RALFADARTSVDDRLPGYPHWNEGMLATVHKRPRSVFTSDAEEHTRFRRMLSKPFTFKRV  EALRPAVQKITDDHIDALLRGPNPGDIVSTVSLPVPSLVISELLGVPYEDAEFFQTQAQR  GMGRYATEEDTAQGAASLAKYLANLVRAKMQSPSEDLVSDLAERVNAEEISVREAAQLAT  GVLIAGHETTANMISLSVAALLEHPDQRALLCDTDDPKVIATAVEELMRYLSIIQTGQRR  IAIEDIEIGGETIRAGEGIILDVAPANWDARQFPNPDRLDLRRQDGPHVGFGYGRHQCVG  QQLARMELQIVLPTLLRRVPTLRLAAPLDELPFKHDALAYGLYELPVTW*  >CYP150A(MAV_0849)  MTDLAQVDYFTDADVAQDPYDYWDYLREQGPVFREPHYGVVAVTGYQEVQAAFKDVESFS  AVNAIGGPFPPLPFTPEGDDISELIEAHRHEFPIFEHMVVMDPPEHDKARSLLGRLLTPR  RLQENKDYIWQLADRQFDEFIANGHCEFLSEYAKPFATLAIADLLGVPDEDRPQIRRNLG  AGNAPGARVGALDHEPVGSNPLQYLDDLFSGYIADRRERPRDDVLTGLATATYPDGSTPP  LLEVVRPATFLFAAGQETVTKLLSAAVQVLGDQPELQAQLRADRGLIGPFIEEALRMQSP  TKVDFRLARKTTTLGGVHIPAGTVIMLCLGAANRDPRKFENPNEFRIDRKNVREHIAFGR  GIHTCAGAPLARVEGQITINRLLDRTSELRINEAKHGPASSRQYRYESTFLLRGLTELHI  EFTQAG*  >CYP1027B(MAV_2634)  MKNIEMNTPESPALAHFHLLDECQDEARPVFRNTEAGIDYWVFTDNSVILDGLQHPELWS  SSVIVPTEPEPPYKWIPIMIDPPDHAKWRHVLAEYFSPGRVKGLRDAQQKLAAELIDQVA  GEGGCDFVARISRVFPSTVFLTIMGMPVEDLEKFLAWEDMILHQSGVGEEVNAARLEGMT  HVMGYFSGLIQQRRENRDPDADDIVSKAIDWTIDDEPINDLELLNCLLLLFMAGLDTVSN  QLSYAMLHLATHPADRARIVAEPQLIPRAVEELLRVYPIVQTARKATRDMDFHGCPVKAG  DMASFSLAFAGRDESAYPNARTVDFDRGVTRHLSFGGGPHRCLGSHLARQELAVVLEEWH  KRIPEYEVSGQPIEHGGQVFGVDSLNLTWG*  >CYP124NSF1(MAV_2642)  MSAIDTDEQQFAPLIDLRWWQERPAERSELYRRLREAGGPVFVRTNRPDSPRPRGFWAIG  AHRDVVDISRRPGDFSSGQGTQIFDQTAEMREYRGSIIDMDDPEHMRLRKIVSRGFTPRI  LSELRGLVEETTAEILEEMPRTGECDFVSSFATLLPLRIIDTMLGVPREHEQFILRATNI  VLGASDPEYVPDQTVAGIGAAVTKISEQLIELLKGIAEDRIAHPRDDVISKLVNSDEENL  TPQELAKFFILLIGAGNETTRNALTHGLLILSAHPEQRDRLLANYDDMASTAVEEILRYA  SPVIHFRRTVTRDGVTLTDAHGEVTHTFNAGDKVVVWYPAANRDPAVFDQPERFDIARKP  NNHIAFGGPGPHYCLGAHLARLELNVAFQMLYARYPDITAAGEPVMLRSNFVNGIKHLKA  TYTP*  >CYP189A(MAV_2984)  MTASTDSDVRFDPYDVGLIADPYPMFARLREEAPLYYNAEYDFYAVSRYADVSKALVDHE  TYSSARGAILELIKANLDIPSGMLIFEDPPIHDVHRKLLSRMFTPRRIAALEPMIRDFCA  QCLDPLVGTGHFDFVTDLGAQMPMKVISMLLGIPEDDQEYIRDRGNAQLRTEAGKPMKAA  EQGLSVGEQFEAYIDWRAEHPSDDIMTELLNVEFVDETGTTRRLTREEILVYLNVVAGAG  NETTTRLIGWTGKVLAEHPDQRRDLVENPALIPQAIEELLRYEPPAPHVSRYVTRDVVVH  DQTVPEGSVMMMLIGSACRDEAQFGSDAGEFNIHRAVRPHLTFSMGTHFCLGSALARLEG  RVALEEILKRFPEWEVDLSRATLSPTSTVRGWECMPALVP*  >CYP187A2(MAV_1598) MTTNVPTAAGGETVSLRDPYPFFARKRREAGVFAGTVMDYSKTPESLMPKQEYSAVSFDA  VNTVFRDGRVFSSKPYDKTIGLFMGPTILAMEGKKHRDHRNLVSAAFKSKALARWEPTIV  RPICNALIDDFIDAGTADLVRQFTFEFPTRVIARLLGLPDEDLPMFHTRAVQLISYHVDY  ERAFEASAALKDYFLEQIEQRKSKPTEDIIGDLVTAEIDGEKLSDEAIYSFLRLLLPAGL  ETTYRSSGNLLYLLLTHPDQFAALQADRELLAPAIEEGLRFETPLTTVQRFTTEDTELHG  VRIPARSVIGVCIGSANRDERRWERSEEFDIFRKHVPHISFAAGEHTCLGLHLARLETRV  AMECLLNRLTNVTLLSDGDPHIHGQPFRSPTALPVTFDAK*  >CYP124NSF1(MAV_1940)  MSATDIDEQQFAPLIDLKWWQKRPGERTELYRRLRDAGGPVFVRTNRPDSPRVRGFWAVG  THRDVTDISRRPAEFCSGHGTQIFDQTAEMREYRGSIIDMDDPEHQRLRKIVSRGFSPRM  VSELRGLVQETTAEILEEMPRSGSCDFVSSFATLLPLRIIDDMLGVPREHEQFIVRATNV  VLGASDPEYVPDQTVTGIERAVSQTSEQLIELLRGIAEDRIANPRDDVISKLVTSNEENL  TPQELAKFFILLIGAGNETTRNALTHGLLILSEHPEQRNRLIANYDELAPTAVEEILRYA  SPVIHMRRTVTRDGVTLTDGDGQVTHTFNEGDKVVMWYPAANRDPAVFDDPECFDIARKP  NNHIAFGGPGPHYCLGAHLARLELNVAFQMLYARYPDITAAGEPVMLRSNFVNGIKHLKA  TYTP*  >CYP143B(MAV_1950)  MTTDALPKVCTDDLPMPEARDDAWHQLDKHPLVEVEDGYAVTRRDVVELVLKNPSLFSSK  RAFDVLASPVPLVPIAFDPPEQTRYRRILQPFFSPRMIKPLEPDLRRQVVDLIEPIARRG  QCDFIAEVAAVFPIQVFLTLFGLPLDMRDQFIEWKDAVLNLSAAAGQTSVDEAAQEGMQK  ALELFMYLTDLIQKRRGAPGDDVLSQVLSIEPPDALSDEEAVGLCFLFVLAGLDTVMDAL  GFGMQRLAENPDKRQEIVNDPSLIPAAMEELLRLDPPAPFVPRVSTQEVTVAGHTLPEGT  RITNYLAVANRDERQFPNPYDIDFHRTENRHVSFGMGVHRCLGSHLARLEMAVLYEEWHK  RIPNYHITPGTTPRVHWPRGTVGLDSLHLTIEAGDRA*  >CYP143NSF1(MAV_1953)  MNGTANINDLPFAEDRSRAWRELREAGEAVLSGEEIVLTSAEAVEFAAKRPDIFSSAKAF  DRLGSPVPLIPIAIDPPDHTRFRRMLDPFFSPKKMAEREPELRKQAGELIDAILAKGHSD  VVADLATPFPSQVFLTLFGLPMADRDRLVQWKDSILEFTDPSSTEATPEVLAHAMELFTY  LTEHIAARRADTTGSDMLTQLMQNSEEGGMDDNEILGLCFMFVLAGLDTVTSAVGFALAK  LAGDPELRRRISNDYSLIPAFIEELLRVDGPVPFAPRVTTQEVEVAGRVVPKDTTVMLSY  GSADRDPLRYEDADNVHLDSKAVHFAFGRGPHRCLGSHLARLELRIILEEWHKRIPEYSL  ADGKDPQMPWPTGTMGLKSVPLTFPT*  >CYP143NSF1(MAV_2658)  MTELGITGTLNINDLPFAEDRTRAWRELREAGEAVSSGEEVVLTSAEAVEFAAKKPEIFS  SARAFDRLGSPVPLVPIAIDPPDHTRFRRMLDPFFGPKKMAEREPELRRQAGELIDAIVA  SGECDVVPDLATPFPSQVFLTLFGLPMADRDRLVKWKDAILQFTDPSSTEATPEVMAHAL  ELFSYLTEHIAERRADATGNDMLTQLIQDTDEGGMSDNEILGLCFMFVLAGLDTVTSAVG  FSLARLAADDDMRRRVAHDFTLIPAFIEEILRVDGPVPFAPRVTTEGVEVAGRFVPKDTT  VMLSYGSADRDPRRYQDADQIHLDSKVVHFAFGRGPHRCLGSHLARLELRLILEEWHSRI  PEYTLALGKPPQMPWPTGTMSLQSVPLNIKPSSRGQ*  >CYP108B5(MAV_1609)  MSTTTMDEAAKLLADPMAYTDEQRLHAALTHLRANAPVSWVEVPNYKPFWAITKHADVMD  IERENMLFTNWPRPVLTTAEGDEMQAAAGVRTLIHMDDPQHRVVRAIGSDWFRPKAMRAL  KVRVDELAKIYVDKMLAAGPECDFVQEVAVNYPLYVIMSLLGLPEADFPRMLKLTQELFG  SDDSEFKRGSSNEDQLPALLDMFGYFNGVTAARREHPTEDLASAIANARVDGEPLSDIDT  VSYYLIVATAGHDTTSATISGGLQALIENPDQLQRLRDNLDLMPLATEEMIRWVTPVKEF  MRTAAKDTVVRGVPIAAGESVLLSYVSANRDEDVFDEPFRFDVGRDPNKHLAFGYGVHFC  MGAALARMEVNSFFTELLPRLKSIELTGDPELVATTFVGGLKHLPVRYSLA*  >CYP187A3(MAV_1615) MPLSTGPTGQPVVPTLDFTGETSPYPFFEHMRRTDPVWHGSLADASQLPEELRPEDEWVL  FDYESVSQAFRDDRIFSSHKYDETIGLVMGHTILAMGGREHHDHRNLVAKAFRATALERW  EPSVIGPVCEQLVDEIKNDGHADLVKAVTFEFPTRIISTLLGLPAEDLDLFRRLSLDLIS  IPTDIEAGLNAATELYDYFLKQVEQRRRKPTDDIIGDLVAAEIDGEKLTDEAIIAFLRLL  LPAGLETTYRSSGNLLYLLLTHPEQLAMVYRDRSLIPMAIEEGLRFETPLTMVTRTTTEE  VEIGGKTIPANAQIDMCMGSANRDETRWTDPNAFDIRRPRQAHIAFAGGIHMCLGMHLAR  LETRVMLNSLFDRVRDLAFVPDDGTGEESKIVGLTFRSPNKLPVTFAPAA*  >CYP279A1(MAV_4569)  MTVGAAPPSVFDSDLPTLHYHSDETPAQVYPRLREAQRRAAVAIGPHGPEVLSYHLVRSV  LRDPRFQIPPGINLLAQGIDSGPLWDKVANSLLCLEGDAHHRLRSLCSKAFTPRTVARLH  DTMAAVMNELVDRVAAAGRCDVVTDIARPYPVPIICALLGAPREDWRRFSSWADDVFKAF  SFTVDLREVEPVVMRAWRELDDYVDEMVARRRHSLTDDLLSDLIRAGDEGDRLDAAELRM  LAGGLLLAGTDTTRNQVAASVQVLCEHPHQWELLRQRPELAMRAVEETMRHSPIACGTLR  LVVEDAELDGHLFPAGTAVLVNTFAANRDPAVYDDPDRVDITREAAPPILTFGGGVHYCL  GANLARREIAEALNVLANRLRNPRLAGPAPWKPMVSLSGPTSLPIEFDR*  >CYP150A(MAV_0912)  MTNEFSELDFFRGSELIENPYPYYEALRQRCPVTKESHHNVTMITGWDEACAVLNDAETW  SSCISVTGPFPGFPVPLEGDDVTELIERHRDELPFSDQLPTLDPPTHTNHRSLLMRLITP  KRLKENEDAMWVLADQALDTFLAPGHGEFIKGFAGPFTLLVIADLLGVPEEDRDKFVKGI  RQHSGGGVGGTGEETLAHSPLEFLYGLFSDYVRDRRRQPREDVLTGLATATYPDGSIPEV  EDVARVASNVFSAGQETTVRLLGAALQTLGERPDIQAQLRKDRSLIPNFIEESLRHESPV  KGDFRLNRRPVTVGGVDLPAGTTVMVVQAAANRDPRRFDDPATFDPARKNARQHISFGRG  IHSCPGAPLARAETRVAIERLLDRTTDIRINENIHGPANDRRYQYVPTYILRGLTELHLE  FTLA*  >CYP190A2(MAV_0916)  MTKPKLVFDPYSEDYFNNPYEIYRRMREEAPLYYDEKEDFYALTRHVDVAAAFKDYETYS  SARGCDLAMVRRGISPEQKSIIFMDPPEHRHMRSLLNKAFTPRAIQSQRETIIEVVDKYL  SAADPDNFDVVQDFSGPFPVEVITRMAGVPEEYRQQVRHWIDTSLHHEPGQIEVSEAGMQ  ANIDTAMYYFGLVQERRQNPQDDMISRLIAAEIPGENGQMRKLDDIEITGFATLLGGAGA  ETVTKLLGNAAVIFARHPDQWQKLQDDRDKIPGAVEELLRYEGPVQYNVRYTLKEAHVSG  GVIPAGKPVFLCGAAANRDPEAFTDADTFDIERDQTEAQHLGLGYGIHSCLGAALARLES  RIALERLLDFMPRYEVDWAGCRRVTMQNVAGWKNVPVKVLR*  >CYP187A(MAV_1274)  MEQLFDDLEDFGAFDDAVSGDVRDPYTELARLRREEPIQRLDTSGMPHEESKPVFIVYRH  EDAQQMLRDNETFSSAAVIAAFGPVLGERVMLGMDEPVHGRLRSLVSKAFSQKALARWED  ELVGRVGNSLIDRFAGNGKADLVKEFTFDYPSRIIAGLLGLPEQDYPQFQRWSISLLSWI  LNPQRGLAASAALCDYFAPILAARRTEPKDDLISGLAQAEIDGEKLEDEEIYSFLRLLLP  AGVETTYRALGSLLLALLSDPEQLDAIRGDRSLLPQAIEEGVRWEPPLLTITRVATRDTE  LGGVPIPAGSTVMPMLGAANRQEDRYPDPDRFDIFRAPKSHLGWGHGVHVCLGMHLARLE  MRTAVNLLLDRLPNLRLDPDADDPHIRGQVFRSPTSVPVLFDPQ*  >CYP1124A(MAV_1637)  MNVNAATAACGDDPAERGLAMTTAAVDLSDFSLWCNGFPDELFTELRRTRPLFHHDLTPG  VAATVHRDFWVATKHRHAVRLHRDTESFTAADGPLIQPVAMFSSSPTIITMDPPELNKRR  KLISNAFNPRAIAKLEDGIRARAARMIDNLLAHGGGDWIEDVADALPMTVIGDILGIPER  DRPRIFDLFDRILKALAPDAHPRGGVELELFASVFDYAMQLTADKRRNPTGDIWSTLATA  VITGEDGEEFRLPANELEFFFFVLAFAGSDTTKNALAIGLQAFLANPGQVERYRADEALR  PTAVEEVLRWASPVAYWTRTAKVDVEMDGQRIAKGERVVSMLRSANRDEEVFDAPFTFDI  GRQPNPHVAFGGGGPHHCLGAMLARAELRAVFDELLLRCDDIEIGPAKAAYPNLITNMSI  YDEMPISLRRR*  >CYP191A2(MAV_5123)  MDFAYDPFDAEVMANPLPYYRILRDHHPVYYMPQWDTFALSRFDDIWRVLEVNNGTFVAS  EGTLPPASVLAQHNDGPVDDPPLHPLPFHAMFDADLYGEIRRTHSRPFRPRAVTDLEGRI  RTLANERLDELLARGSFDLTQEYGGVVVATIVCELLGIPTDLAPQVLAAVNAGSLAEPGV  GVDTGQARPNYFEFLLPAVQRRRADPSGPPLEVVDGLLGYQLPDGSALDDLEVATQMLCI  FIGGTETVPKIVAQGLWELSRHPDQLAAVRADPQHNIPVAREEMIRYCAPAQWFARTVRK  PFDIHGQTLNPGQRVITLLASANRDEREYPDPDDFVWDRPIRRSLAFGRGQHFCIGYHLA  RLEVAVLLQEWLRRVPDYAIRADAATRLPSSFQWGWNKIPVEV*  >CYP188A2(MAV_0940)  MSVDDGVNDSDRKKNRYHFDRHSPDYRSRFKAITEEMHAKCPMAWTDTYGGHWVAAGSHE  VFELARCPAVSNDHDINGERRGYKGISIPTASRVSAVRGGILEMDDPEHRIYRTVLNPYL  SPAAVKRWEPFIDEVTRAALDEKIEEGSIDFVDDLANIVPAVLTLAMLGIPLKKWKMYSE  PVHAAVYTPEHSPDIERVTAMHREMGLDMVNNMLEIRENPRPGIVNALLQMRIDGEPAPD  LEILGNLGLVIGGGFDTTTALTAHSLEWLSEHPEQRQLLSDERKTLLDPATEEFLRYFTP  APGDGRTFSEDFELDGTVFKEGERLWISWAMANRDPAVFHDPDEVILDRKGNRHFSFGLG  IHRCIGSNVARTVFKSMLIAVLDRMPDYRCDPEGTVHYETIGVIQGMRKLPATFTPGRRI  GAGLDETLEKLQRICDEQELARPITERKEAAVID*  >CYP150A(MAV_2371)  MSAPENRDFFTDKSVVDDPYDYYDAIRRCPVWREPAHGVVMVSGYDEALAVQRDTDHALS  VCNIVSGPWSGIPANTGTDDISELIERYRKKVTFGDYFITFDPPMHTAHRSLLSRLFTPK  QLKNNEDFLWRLADEQLNRFIANGKCEMVIDYNFPFTLDAITDLLDVPEADRERFRRAAI  ASRLEGDRSGFVGVKEEWFVEYVEERRRNPRDDVLTELALAKFPDGTTPEPIDVARVATF  MFAAGHGTTIDLLSLSMLTLAERPDLQDLLREDNSKIPAFIEEMLRIESPIKSNFRLARR  TTRIGDVEVQAGTSILVMNGAANRDPRRFDEPNEFRLDRPNILHHMAFGRGIHTCPGAPI  ARAEVRVSLERILSRMADIRLSEAKHGPAGARRLRWDPTLLFRRLKELHLEFTPIR*  >CYP150A(MAV_0964)  MSNFDSIDFFTDPSLVPDPHPYFDYLRSQNPVLRLPHYGVVAITGYEEATEVYKDPETFS  NIVALGGPFPPLPFTPEGDDISAQIDAHRSSFPMFEHMVTMDPPEHTKARSVLAKLLTPS  RLKQNEEFMWRLADRQLDEFLHNGECEFIAEYSKPFATLVIADLLGVPEEDHTQFRTVLG  ADRPGARVGALDHETVGINPLEWLDDKFCNYIEERRREPRADVLTFLAEAKYPDGSTPPV  IEVVRSATFLFAAGQETTAKLLSAALQVLGDRPDIQQQLREDRSLIPAFIEESLRMESPV  KSDSRLARRATTIGGVDIPAGTVVMILPGAANRDPRRFENPHEFDLRRKNVREHMAFARG  VHSCPGGPLARVEGRVSIERILDRMPHIEINETHHGPAGDRRYTYEPTYILRGLSELHLT  FTTADAVAPVG*  >CYP139A(MAV_3106)  MRYRPGEALLALYRRRGPVIDAGAGRRGYTLLLGAEANKFVFANADAFSWRATFENLALV  DGPTALIVSDGDDHRRRRSVVAPGLRHRQIQDYVTTMVSCIDRVIDGWRPGQRLDVYQHC  RAAVRRSTAESLFGRRLAVHSDALGEYLQPLLDLTHQPPQLVGLQRRINAPAWRRAMAAR  QRINNLVDTLIAEARAAPNPDDHMLTMLIDGRGDEGYALNDNEIRDAIVSLVTAGYETTS  GALAWAVYLLLSQPGAWATAAGEVRRVLAGRLPAAADLSGLTYLNGVVHETLRLYPPGVI  SARRVMRDLRFEGRRIRSGRLLIFSPYVTHRLPEIWPEPMRFAPERWNPDAPGYRRPAPH  EFIPFSAGLHRCVGAAMATTEMTVMLARLLARTRLRLPAQRLRAANVAALRPTPGLTVEV  IDSVPAQ*  >CYP138A(MAV_5160)  MSEVATAPLAAVHLPPAVRGPKLLQGIGFAVSRRTMMRRLSRRYGNVFTLRLPMWGPVVM  VSDPQLAKQIFTTSPDELGNIQPNLSRLFGSGSVFGLEGDDHRRRRRLLAPPFHGKSMKN  YESIIEEETLRETAGWPEGESFPTLPPMMRITLNAILRAVFGAEGAELDELRRLIPPWVT  LGSRLAALPKPQRYPRFGPWGQLDRWRRQYDGVIERLIAAEQADPNFAERTDVLALLLRS  TYDDGAAMSHKEIGDELLTLLAAGHETTASTLAWAFERISRHPELLARLVEEADNGGNEL  RQAAILEVQRARTVIDFAGRHVYPDVYRLGEWVIPRGYSIIVGIAQIHDNPDVFPDPRRF  DPQRFIDNKPSALSWIPFGGGTRRCVGAAFANMEMDVVLRTVLRHFTIETTDAPDEPWHC  RGVAFTPKHGGRIVVHRR*  >CYP125A(MAV_0616)  MPSPNLPPGFDLLDPDVCVKGLPVAELAELRKSAPIYWMDVPGGTGGFGDKGYWAITKHK  DVKEISVRSDIFSSQQDCAIPVWPKEMTREQIDLQRNVMLNMDAPHHTRLRKIISRGFTP  RAVGRLRDELDARAQNIAKTAAAAGAGDFVEQVSCELPLQAIAGLLGVPQEDRDKIFRWS  NEMTGNEDPEYAHIDPAMSSAELIMYAMKMAEERAKNPGDDIVTQLIQADLDGEKLSDDE  FGFFVVMLAVAGNETTRNSITHGMIAFADNPDQWELFKKERPETAPDEIVRWATPVTAFQ  RTALEDYELSGVQIKKGQRVVMFYRSANFDEEVFEDPHRFNILRNPNPHVGFGGTGAHYC  IGANLARMTISLIFNAVADHMPDLKPLSAPERLRSGWLNGIKHWQVDYTGKCPVAH*  >CYP291A3(MAV_1339)  MQTHPPLRSPSFPLHSPDFYAGNPYPAYRELRATAPVCWNDVTNFWALLKYEDIRFVSSN  PALFTSTRGITIPDPQLPNPVQQGSLIFTDPPRHRQLRKLINSGFTRRRVSVLEPKIREI  VRGILDGIERGAVHEFAEQIAAPLPTRMIAELIGAPPDDWEQFRAWSDAATGTADPEIEL  DPAVAAGQLYEYFQRLIAARRARPRADLLSVLAEAEIDEHRLTDEDLLNFAFLLLVAGNE  TTRNLIALGTLALIAHPDQYRLLVEEPARIPLAVEEMLRWNSPVVHMARTATADVEIRGQ  RIRAGEVVVMLYGSANRDEDVFGPDSEEFDVTRHPNPHIAFGCGEHSCVGAQLARLEATV  FFEELLRRYPRIELVGEVDRMRATMVPGVKRMPVRMGA*  >CYP142A(MAV_0641)  MTSTIPEAIANIDLADGNFYADRRASREAYRWMRANQPVFRDRNGLAGATTYQAIQDAER  NPELFSSTGGIRPDQPGMPYMIDMDDPAHLLRRKLVNAGFTRKRVKEKEPSIGTLCDTLI  DAVCERGECDFVRDIAAPLPMAVIGDMLGVLPTERGMLLKWSDDLVCGLSSHIDPTSAEF  QTVMDAFAAYTAFTMDIIAKRRAEPTDDLFSILVNAEVEGQRMSDDEIVMETLLILIGGD  ETTRHTLSGGTEQLLRHRDQWDALVRDPSLLPGAIEEMLRWTSPVKNMCRTLTADTEFHG  TELRAGEKIMLLFESGNFDESVFDDPDSFDIRRNPNSHMAFGFGTHFCLGNQLARLELSM  MTERVLKRLPDLRLADDGDLPLRPANFVSGLEAMPVVFTPSAPLLR*  >CYP268A(MAV_1734)  MAVLTGESGTDRSRRAYDEIDLSSRAFWSGTAAERERSFAALRAERPVSWHPPVEDSLLP  DPTDPGFWAVTRRADIVTVSRNNDVFLSGHGVMFESIPAELLEASQSFLAMDPPRHTKLR  KLAHAALSPRQVRRIEDSIKANAKAIVEELRAAGSGCDFVDHCAKELPIRTLSDMMGIPE  SERERMAHATDALVSWADPEFLNGRPALEVLLENQMYLHQVVGDLATQRRERPGDDLISS  LVTAEVDGDRLEDAEVAAFFVLLSVAGNDTTRQTISHTLRALTVFPDEKSWLLEDFDHRI  GTAVEEFIRWASPVMTFRRTAAADVELGGQTILAGEKVVMFYPSGNWDTEAFDHPERLNL  GRDPNPHVGFGGGGLHFCLGAHVARAQLRAIFSELFRQLPGIQAGEPTYLAGNFVHAIRA  MPCTF*  >CYP189A(MAV_1389)  MTGLYYDPWNREIDADPYPIYKMLRNEAPLYYNERHDFWGLSRYDDVDAALRDPLRLSSA  KGDILDVVKADPVMPPGVFINEDPPLHTIHRALVARAFTPKKMRTLEDKIRAFCVASLDM  VADSDRFDFVEDLGAELPMRTIGMLLGIPDADQPSVREHARATLHNDTGGPMPIRKDHYF  DGDMFSDYVEWRKQNPSDDLITELLSVDFEDQSGTVRRLTTPELLIFLAVIAGAGVETTG  RLFGWMGKVLADHPDQRKELAEDRSLIPAAVEELLRYEPPGPSVARYVTEDVEFHGQTVP  SGSAMLLMLSSANRDERRFDHPDRFDIHRKPGRHLTFGRGVHFCLGAPLARLEGRIGLEE  VLNRWPEWEIDMDNARRSRTSTVRGWDSMPAIVT*  >CYP188A(MAV_1397)  MSVEVAGSDSRKALQFHFDRHAPEYRERFLDVTQEMHQRCPIAWTDTYGGHWVAAGGDAV  FELARCPHVSNDHDVNNERRGYRGVTIPLTTESDQIRGGMLEMDDPEHRIYRSLLNPYLS  PAAVSRWQPFIDDVVRACLDERIESGRIDFVDDLANVVPAVLTLAMLGVPLRKWTMYNEP  VHAMVYTPPDSPEAAKVHDMWVSVVVDLFANLTEIREHPRPGIINALAQLRIDGEPAPDM  EIIGMLTLLIGGGFDTTTALTAHALEWLSEHPDQRARLHGELDVLLNPATEEFLRYFTPA  PGDARTISADMELDGIRFAEGERLWLSWAMANRDPSLFDNPNELMLARKANRHFSFGIGV  HRCIGSNVARTVFKSMLTAVLERMPDYRCDRVNTVHYDTIGIIQGMRNLPADFTPGKRRG  PGLDETLDRLQSVCDSQGLARPITEYKEQARIHE*  >CYP105NSF1(MAV_2464)  MSISFETSESRADAELPVLPMPRAAHCPLAPPPEFVDWRQQPGLRRALFQGNPVWVVSRY  HDIRAALVDPRLSAKTIPDSIMPTDADNKVPVMFARTDDPEHHRLRRMLTGNFTFRRCES  MRPQIQDTVDHYLDRMVDGGVPADLVREFALPVPSLVIALLLGVPPEDLELFQFNTTKGL  DQKSSDEEKGKAFGAMYAYIEELVQRKAREPGDDLISRLITEYVATGQLDHATTAMNSVI  MMQAGHETTANMISLGTVALLGHPEIYARLGQTDDSAVVANIVEELMRYLSIVHSQVDRV  ATEGLTIAGQLIRAGEFVVMNLPAGNWDTEFVDNPESFDADRNPRGHLGFGYGVHQCIGA  NLARVEMQVAFATLARRLPGLRLAVPPEQLKFKDANIYGMKELPVSW*  >CYP125A(MAV_2811)  MATVEPTTKPVPNLPPGFDFTDPDIYAERLPVEELAEMRRVAPIWWNEQPIGAGGFDDGG  FWVVTKHKDVKEVSLRSDVFSSLQKTALPRYKDGTVAEQVERGKFVLLNMDAPQHTRLRK  IISRAFTPRAVERLRDDLRERARRIVEAAAAEGSGDFVEQVSCELPLQAIASLMGVPQED  RKKLFHWSNEMVGDQDPEFASNDAITASVELIMYGMQMAADRAKNPGEDLVTKLVQADID  GHKLSDDEFGFFVILLAVAGNETTRNSITQGMMAFTDFPDQWELFKRERPATAADEIVRW  ATPVTSFQRTALQDYELSGVKIKKGQRVVMFYRSANFDEDVFDDPFTFNILRDPNPHVGF  GGTGAHYCIGANLARMTIDLMFNAIADAMPDLESIGKPERLRSGWLNGIKHWQVDYHTNG  SSKCPVAH*  >CYP140A(MAV_2822)  MKERLHWFAMHGFIRGAAALGARRGDVHARLIADPAVAADPARFYDEARARGTLVKGRVA  YLTADHALAHELLRSEDFRVLVFGSNLPAPLRWLERRTRDDLLHPLRAPSLLAVEPPEHT  RYRKTVSAVFTPRAVAALRDRVERTAAELLDQLTGGPGVVDIVGRYCSQLPVAIISEILG  VPEQDRSRVLEFGELAAPSLDIGLPWRQYRSVQRGIAGFSSWLAGHLQQLRSNPSDNLMS  QLIQTAESGSAETYLDETELAAIAGLVLAAGFETTVNLLGNGIRMLLDAPEHLDTLRRRP  ELWPNAVEEILRLESPVQLTARMALNDVEVAGRQLHRGDLVLVYLAAANRDPAVFGDPHR  FDIERPNAGRHLAFSGGRHFCLGAALARAEGEVGLRTFFERFPEARAAGAGSRRETRVLR  GWSSLPVRLGPARSLAAAEAGGRPDEPTAG*  >CYP130A(MAV_1404)  MSHNVPVAFELPNADTWADPWPMYRALRDHDPVHHVVPPKRPEHDYYVLSRHADVWAAAR  DHETFSSAKGLTVNYDDLELIGLQDNPPFVMQDPPVHTEFRKLVSRGFTPRQVEAVEPKV  RDFVVERIERLRAAGGGDIVAELFKPLPSMVVAHYLGVPEEDRAQFDGWTEAIVAANTAD  GGVAGALGSAGDAVTSMMAYFTGLIERRRTDPEDDTISHLVSAGVGADGDIAGTLSVLAF  TFTMVTGGNDTTTGMLGGSMPLLHQRPDQRQRLVDEPELIPDAVEELLRLTSPVQGLART  TTRDVTIGRTTIPAGRRVLLLYGSANRDERQYGPDAGELDVARCPRNILTFSHGAHHCLG  AAAARMQSRVALTELLARCPDFEVDESGIVWAGGNYVRRPLSVPIRVKS*  >CYP189A5(MAV_2138)  MTGTSAIELYYDPFDSDIDDNPYPVWQRMREEAPLYYNEKYNFYALSRYEDVARELPNWQ  TYQSGRGTTADILFSNVEVPPGILLFEDPPLHDLHRRLLSRVFTPRRMLAVEDLVRGFCV  RELDPLVGAGGFDFIRDLGAMMPMRTIGYLLGIPEEDQEKIRDRSVANIELSRDSDPAAV  DANVFANSIALFAEYIEWRADHPSDDLMTELLRAEIDEPDGTRRPLSRTEVLAYTAMIAG  AGNETTARLIGFMGQLLSDHPDQRRELAADPSLIPGAVEETLRFEPPSPVQARYVARDAE  HYGRVVPEGSFMLLLNGSANRDPRRFTDPDRYDIHRQGGGHLSFGQGLHFCLGSALARME  ARVAFEEVLKRWRDWEVDYANAERARTASVRGWARLPVVTGG*  >CYP51B1(MAV_0708)  MTTSTVVPRVSGGEEEHGHLEEFRTDPIGLMQRVRDECGDVGWFQLVDKHVILLSGAQAN  EFFFRSADEDLDQAEAYPFMTPIFGKGVVFDASPERRKEMLHNSALRGEQMKGHASTIEG  EVKKMIADWGDEGEIELLDFFAELTIYTSTACLIGLKFREQLDHRFAEYYHDLERGTDPL  CYVDPYLPIESFKRRDEARVKLVALVQEIMDQRLANPPKDKADRDMLDVLVSIKDEDGKP  RFSADEITGMFISLMFAGHHTSSGTSAWTLIELIRHPDVYAEVLAELEELYADGQEVSFH  ALRSIPKLDNVVKETLRLHPPLIILMRVAKGEFEVEGFPIHEGDYVAASPAISNRIPEDF  PDPDAFKPDRYNKPEQADIVNRWTWIPFGAGRHRCVGAAFAQMQIKAIFSVLLREYDFEM  AQPADSYRNDHSKMVVQLARPAKVRYRKRNA*  >CYP140B(MAV_2156)  MRVRLGARWMAMHGLPRAYFAVQARRGDPLARLLRSGTTGEDRYALMEQIRARGPLMRAP  FVWASVDHAVCRQVLRDKRFGVTSPTEMELPRPVRALIARTDPGVANPVAPPAMVIVDPP  DHTRYRQLVAQSFTPRAIEALNTRVAQVTMELIERIAATPQPDLIADFATRLPVAIIAEI  LGMPPDSYPRMLAWGRSGSPLLDLGIDWKTYRDAIAGLRGVDEYLLAHFHQLRADPHSDN  PFGRMAADGSLTDRELTANAALIVGAGFETTVNLIGNGIVLLLRHPEQLALLHDNPDLWP  SAVEEILRIASPVQMTARTPACDVDIAGAHIGAGEMVGLFLGGANRDPKVFSDPTTFDVT  RPNAREHLAFASGIHACLGAALARIEGATALRALFENFPDLRLTAAPQRRSLINLHGYTR  LPAQLGGRRTTSTTIPV*  >CYP243A(MAV_2159)  MSSASRATVPSAYLHPRFQGQEPPGFPGRFTVAWNENYGGFWFLSSYDAVSQTARNGDTF  AHKYEPNAADGVDYQGEMGVPRPEGQPALGLGEVDGPYHQALRHALAPFFSPGAVEKLKP  FMEQSAHWFLDQQITTGQMDLVLDYASPVPAILTMKLMGLPYDNWHLYANLFHSVMAVSQ  DSDEYATAIAKVPAMMHEVLDYAATRRAKPEEDLTSFLIRFEFDGHRLTDEQLLNILWNL  IGGGVDTTTSQTALTLLHLGTHPDLRQQLIDHPELYRTATDEFLRYFSVNQTLSRTVTHD  VVLAGQRLRKNDRVVISWLSANHDENEFDRPDEIILDRSPNRHVAFGLGPHRCIGSHLAR  LMSEVMVRAVLDRIPDYQVDVENVHQYLGNPSMTGLGQLPVTFAPGKSRKTLLPW*  >CYP136A(MAV_3927)  MTATISTPQYLLDQARRRFTPTLNTIPGMGAIEKRLLAHEWQTKVLAEPPAGSGLKPVLG  DAGLPILGHIIELFRGGPDYALFLYRNHGPLIYLDSPIMPAVTALGPDATQAVFSNRNKD  YSQKGWHPVIGPFFNRGLMMLDFDEHMYHRRIMQEAFTRSRLTGYVEHIDRVATDIVADW  PTNDARFLFHPAMKELTLDIASLVFMGHEPGTDHDLVTKVNQAFTTTTRAGGAIIRQPIP  PFKWWRGLRARQLLEDYFSERVKERRNATGNDMLTVLCHTEDDDGNSFTDDDIVNHMIFL  MMAAHDTSTSTTTTMVYNMAAHPEWQERAREESTRLGDGPLDIEALEKLETLELIMNESL  RMVTPLPFNMRQAVRDTELLGHYIPAGTNITIWPGMNHRLPELWTDPDKFDPERFAEPRA  EHKKHRYAFAPFGGGAHKCIGMVFGQLEVKTVVHRLLRRYRLELARPGYQPRWDYGGMPI  PMDGMPIVLRPL*  >CYP144A(MAV_0011)  MAVLDSATRFFGSEAIQDPYPLYERMHAEAPVHRIGDSVFYAVCGWDAVHEAIERVEDFS  SNLTATMVFHEDGTVTPFDMGAPGAPMHALATADDPVHAVHRKILLPHLSAKRIRIIEEF  ATQTADRLWDENLSDGRIEWMSAIANRLPMMVVCRLLGLPDDDVDKLIRLGYATTTLLDG  IVAPEQLEQAGMAAIELSGYVLEHFEKASEKPESSLMADLAARCAAGELEQLPALGIMLT  LFSAAGESTASLLGSAAWILADRPAIQRQLRENPELLSTFIEETLRFEAPFRGHYRHVWR  DTTLGGIELPEGAHLLLMWGAANRDPTHFKDPNEFRLDRAAAKSHLSFGKGVHFCVGAAL  ARLEAHIVLRRLLERTSWIDATDVGDWLPSILVRRRERLGLAVR*  >CYP164A(MAV_0358)  MTTAPTESAESQGLLLQLLDPANRADPYRLYAQFRERGALQLPEANLAVFLSYRDCDEVL  RHPSSSSDNVNSTVAKRQAAAGTAPVRQGPPGFLFLDPPDHTRLRRLVSKAFAPRVVAAL  EPDIRSLVDGLLDRAADRGELEIVEDFAYPLPVAVICRLLGVPLDDEPQFSRASALLAQA  LDPFSTITGVPAEVASERQQAGTWLRDYFHQLIEARRSRPGDDLLSGLIAVEESGDQLTE  EEIVSTCNLLLIAGHETTVNLIGNAVLAMLRDPGQWAALGADPGRAPAIVEETLRYDPPV  QLAGQIALDDMVIGGVEVPAGDVMMLLLAAANRDPAEFDRPDTFDPDRKSLRHLGFGRGV  HYCLGAPLARLEAGVALSAVTARFPRARLDGEPQYKTNVTLRGLSRLVVAV*  >CYP189A(MAV_0366)  MTVANDTAVYYDPYDIGIITDPYPTYARLREEAPIYYNERYDFWALSRHSDVERALANWQ  VFSNRRSDILELIQSKFDMPGGVMMFQDPPEHTVLRGLMSRVFTPRRMAALEDQIRQYCI  RCLDPLVGSSSFDIIAELASMMPMRVIGMLLGIPESEQVSVRDANDANLRTKPGAPLRVA  DADSIADGRIYADYVEWRSKNPSDDLMTTLLNVEFDDEDGVRRKLTRKEVLHYTQVVAGA  GNETTGRLIGWLAKVLAEHPDQRREVYRDRSLLTRTVDETLRFEPTGPHVARWMAADFEC  YGTTVPAGSAMLLLFGAANRDPRRYTDPDTFNIHRDNISHITFGKGVHYCLGANLARLEG  RVALDELLNRWPEWDIDYDTAQLASTSTVRGWERLRIVVP*  >CYP126A(MAV_0723)  MTLAGTLSQIDFTDLDNFANGFPHHLFAVHRREAPVYWHEPTDNTPDGEGFWSVASYAET  LEVLKDPATYSSVTGGERPYGGTLLQDLAIAGQVLNMMDDPRHSQIRRLVSSGLTPRMIR  LVEDDLRARARRLLDAVVPGEPFDFLVDIAAELPMQMICILLGVPESERHWLFQAIEPQF  DFGGSRKAALSQLSEAEAGSRMYEYGQQLIAAKRAEPTDDMLSVVANATLDDAAAPALSD  LELYLFFSLLFSAGAETTRNAVAGGLLALAEHPEQLRWLRDDLGALPRAVEEMVRWTSPS  PSKRRTATRDATLGGQSIKAGQKVQIWEGSANRDASVFDRADEFDVTRKPNPHLGFGQGV  HYCLGANLARLELRVLFEELLSRFGAVRVVRPVEWARSNRHTGIRHLVVELREEQ*  >CYP124A1(MAV_2174)  MSLKTRPKKGLATRINGAPPPRVPLADIHLESLDFWGYDDDFRDGAFATLRREAPISFWP  AIEMDGFVAGNGYWALTKHEDVHFASRHPEIFSSVPNITINDQTPELAEYFGSMIVLDDP  RHQRLRSIVSRAFTPKVVARIEASVRERARRLVSSLVANHPNGEAELVSELAGPLPLQVI  CDMMGIPEEDHQRVFHWTNVILGFGDPDLATDFEEFLQVSMDIGAYATALAEDRRVNHHD  DLTTSLVEAEVDGERLTSSEIASFFILLVVAGNETTRNAISHGVLALSRYPDERDKWFSD  FDRLTPTAVEEVVRWASPVVYMRRTLTRDVKLRGTKMKAGDKVALWYNSANRDESTFDNP  WLFDVARTPNPHLGFGGGGAHFCLGANLARREIRVVFDELRHEIPDIVATEEPARLLSQF  IHGIKRLPVAWTPPR*  >CYP1129A(MAV_1841)  MTETRTEAQARAKVDLDHHSPEFREDPYGKFREMRESGCPVAHSDHYEGFWALVDYASVF  EAARDDDLFNSFPSVGVPASELPLPILPIESDPPETQQLREVTLKRFSPGSAERFRDAAI  EMTNEAIDAFIERGECDLVTELTTPLPARLILRLLKFDESRHMDWVHWVHSTVHDRAHDP  ERAGAAGMEMFGEIVKHMEERRAEGLGDDLFSDILRGTLNGKPLDDGQITMYTVLMMLGG  MDTTSGFTGNVLLRLCQDLELRQQFIDDPSLVKKGTDELLRLYTPTLGLARTVSRDAEFH  GQRLCKGDRAILMWAAANRDPAMFEDPDTLDLSRPNAKKQMAFGVGMHRCLGSHYAKMMF  DVMLTQVLKRLPDFELAGEPELFEDAGEVYAVRKLPVKFTPGPRVG*  >CYP143A(MAV_2548)  METLGAPELSFASLPMAADRGVGWKVLRDAGRVVSVDGIFYLTHREDVLAALRDPELFSS  KKAFDVLGSPLPLVPISFDPPEHTRFRKILQPFFSPHTLKEMLPSLQQQAIEIIERVAAQ  GECEVVADVAIPYPSQVFLTLFGLPLEDRDKLVAWKTSVIAISEAPSLEDADLTPALELV  AYLTEAINARRADPGPDILSQLLNGEEPLDDAEIMGLSFLFVLAGLDTVTAAMSSALLEL  ARNPELRATLHDDPDQIDVFVEEIVRLEPPAPMLPRVTTTEVTIGDVTLPADTMVRLCVA  AINRDDSDEISTNDVVMDGKVHRHWGFGGGPHRCLGSHLARLELKLIVGEWLRRIPEFSV  KVGEEPQIVFPASTFSLERVPLKLG*  >CYP272A1(MAV_2213)  MTEAGGFVDQTVTGVAEPQPMYKALRESNPVFRSPQAVVLSRLADIEMALKHTELFSSNM  DAVDLGNVRPLIPLQIDPPEHAKYRRILDPLFTPREMARREPLVTELVNEMIDRFAARGE  CDFHAEFAVPLPCTVFLQLLGLPLEDLDRFLLWKDGVIRPAGDSGFDRRHESSAGVAQQI  YEYFDKAIDEHIAVPRDDVLSAMIAADVGGQPLSREELLDICFLFLIAGLDTVTDSLDCF  FVYLARHPQHRRQLVERPDVLPGAVEELLRWETPVPGVARVATQDVEVGGCPISKGERVS  PLLGAANTDPAEFPDPETVDFTRSPNRHRAFGGGPHRCLGSHLARMELRVALREFHRRIP  DYEIKPGTQLTYTAALRSVESLPLVFPVR*  >CYP1129A(MAV_2572)  MTDTQAESRPRAEVDLDHHSPEFRDDPYGRFREMRESGCPVAHSEHYEGFWALVDYASVF  EAARDDDLFNSFPSVGVPASELPLPILPIESDPPETQELREVTLKRFSPGSAERFRESAI  EMTNEAIDAFIERGGCDLVGELTTPLPARLILRLLNFDESRFMDWVGWVHTTVHDRAHDP  EKAGIAGMEMFGEIVKHMEQRRAEGLGDDLFSDILRGTLNGEPLDDGQITMYTVLMMLGG  MDTTSGFTANVLLRLCKDAELRAKFTADPSLVKKSTDELLRLYTPTLGLARTVSRDADFH  GQHLCQGDRAILMWAAANRDPAMFENPDELDLDRPNAKKHMAFGVGMHRCLGSHYAKLMF  DVMITQVLKRLPDFELAGEAKLFEDAGEVYAVRELPVSFTPGPRVG*  >CYP143A(MAV_2931)  MSEGQYGTFHLPRLDFATLPMSVDRGLGWKTLRDAGPVVFMNGHYYLTRREDVLAALRNP  KVFSSTVLQPPGHPLPVLPLAFDPPQHTRYRKILQPYFSPHALGKSRPVLERHAAEMIAA  LADRGECEVMADFAHLYPFQVFMDLYGLPLQDRDRLLDWKNAVVGEKPFVTESDVEKSEQ  LLAYLADAIAQRRQHPGTDMLSQVMTGEGNFTDIELLGMSHLLILAGLDTVTAAIGFSLF  ELARRPQLRKELRDNPKQTRVFIEEIVRLEPSAPVAPRITTEYVEVGGMTLPPGTSVRLC  MAAVNRDDSDSMSTNELNMDGKVHRHWGFGGGPHRCLGSHLARIELTVVVAEWLTQIPDF  ELPDGYEPVINYPSKSFALKELPLHWG* |
| ***Mycobacterium avium* subsp. *paratuberculosis* K10** |
| Database: TB: P450 count: 43; Families: 30; Subfamilies: 33 |
| >CYP140A(MAP1603c)  MKERLHWFAMHGFIRGAAALGARRGDVHARLIADPAVAADPARFYDEARARGTLVKGRVA  YLTADHALAHELLRSEDFRVLVFGSNLPAPLRWLERRTRDDLLHPLRAPSLLAVEPPEHT  RYRKTVSAVFTPRAVAALRDRVERTAAELLDQLTGGPGVVDIVGRYCSQLPVAIISEILG  VPEQDRSRVLEFGELAAPSLDIGLPWRQYRSVQRGIAGFSSWLAGHLQQLRSNPSDNLMS  QLIQTAESGSAETYLDETELAAIAGLVLAAGFETTVNLLGKGIRMLLDAPEHLDTLRRRP  ELWPNAVEEILRLESPVQLTARMALNDVEVAGRQLHRGDLVLVYLAAANRDPAVFGDPHR  FDIERPNAGRHLAFSGGRHFCLGAALARAEGEVGLRTFFERFPEARAAGAGSRRETRVLR  GWSSLPVRLGPARSLAAAEAGGRPDEPTAG*  >CYP125A(MAP1614c)  MATVEPTTKPVPNLPPGFDFTDPDIYAERLPVEELAEMRRVAPIWWNEQPIGAGGFDDGG  FWVVTKHKDVKEVSLRSDVFSSLQKTALPRYKDGTVAEQVERGKFVLLNMDAPQHTRLRK  IISRAFTPRAVERLRDDLRERARRIVEAAAAEGSGDFVEQVSCELPLQAIAGLMGVPQED  RKKLFHWSNEMVGDQDPEFASNDAITASVELIMYGMQMAADRAKNPGEDLVTKLVQADID  GHKLSDDEFGFFVILLAVAGNETTRNSITQGMMAFTDFPDQWELFKRERPATAADEIVRW  ATPVTSFQRTALQDYELSGVKIRKGQRVVMFYRSANFDEDVFDDPFTFNILRDPNPHVGF  GGTGAHYCIGANLARMTIDLMFNAIADAMPDLESIGKPERLRSGWLNGIKHWQVDYHTNG  SSKCPVAH*  >CYP189A(MAP0336c)  MTVTNDTAVYYDPYDIGIITDPYPTYARLREEAPIYYNERYDFWALSRHSDVERALANWQ  VFSNRRSDILELIQSKFDMPGGVMMFQDPPEHTVLRGLMSRVFTPRRMAALEDQIRQYCI  RCLDPLVGSSSFDIIAELASMMPMRVIGMLLGIPESEPVSVRDANDANLRTKPGAPLRVA  DADSIADGRIYADYVEWRSKNPSDDLMTTLLNVEFDDEDGVRRKLTRKEVLHYTQVVAGA  GNETTGRLIGWLAKVLAEHPDQRREVYRDRSLLTRTVDETLRFEPTGPHVARWMAADFEC  YGTTVPAGSAMLLLFGAANRDPRRYTDPDTFNIHRDNISHITFGKGVHYCLGANLARLEE  RVALDELLNRWPEWDIDYDTAQLASTSTVRGWERLRIVVP*  >CYP164A(MAP0344c)  MTTAPTESAESQGLLLQLLDPANRADPYRLYAQFRERGALQLPEANLAVFLSYRDCDEVL  RHPSSSSDNVNSTVAKRQAAAGTAPVRQGPPGFLFLDPPDHTRLRRLVSKAFAPRVVAAL  EPDIRSLVDGLLDRAADKGELEIVEDFAYPLPVAVICRLLGVPLDDEPQFSRASALLAQA  LDPFSTITGVPAEVASERQRAGTWLRDYFHQLIEARRSRPGDDLLSGLIAVEESGDQLTE  EEIVSTCNLLLIAGHETTVNLIGNAVLAMLRDPGQWAALGADPGRAPAIVEETLRYDPPV  QLAGRIALDDMVIGGVEVPAGDVMMLLLAAANRDPAEFDRPDTFDPDRKCLRHLGFGRGV  HYCLGAPLARLEAGVALSAVTARFPRARLDGEPQYKTNVTLRGLSRLVVAV*  >CYP105NSF1(MAP1782c)  METWVMSISFETSESRADAELPVLPMPRAAHCPLAPPPEFVDWRQQPGLRRALFQGNPVW  VVSRYHDIRAALVDPRLSAKTIPDSIMPTDADNKVPVMFARTDDPEHHRLRRMLTGNFTF  RRCESMRPQIQDTVDHYLDRMLDGGAPADLVREFALPVPSLVIALLLGVPPEDLELFQFN  TSKGLDQKSSDEEKGKAFGAMYAYIEELVQRKAREPGDDLISRLITEYVATGQLDHATTA  MNSVIMMQAGHETTANMISLGTVALLGNPEIYARLGQTDDSAVVANIVEELMRYLSIVHS  QVDRVATEDLTIAGQLIRAGEFVVMNLPAGNWDTEFVDNPESFDADRNTRGHLGFGYGVH  QCIGANLARVEMQVAFATLARRLPGLRLAVPPEQLKFKDANIYGMKELPVSW*  >CYP136A(MAP3109)  MTATISTPQYLLDQARRRFTPTLNTIPGMGAIEKRLLAHEWQTKVLAEPPAGSGLKPVLG  DAGLPILGHIIELFRGGPDYALFLYRNHGPLIYLDSPIMPAVTALGPDATQAVFSNRNKD  YSQKGWHPVIGPFFNRGLMMLDFDEHMYHRRIMQEAFTRSRLTGYVEHIDRVATAIVADW  PTNDARFLFHPAMKELTLDIASLVFMGHEPGTDHDLVTTVNQAFTTTTRAGGAIIRQPIP  PFKWWRGLRARQLLEDYFSERVKERRNATGNDMLTVLCHTEDDDGNSFTDDDIVNHMIFL  MMAAHDTSTSTTTTMVYNMAAHPEWQERAREESARLGDGPLDIEALEKLETLELIMNESL  RMVTPLPFNMRQAVRDTELLGHYIPAGTNITIWPGMNHRLPELWTDPDKFDPERFAEPRA  EHKKHRYAFAPFGGGAHKCIGMVFGQLEVKTVVHRLLRRYRLELARPGYQPRWDYGGMPI  PMDGMPIVLRPL*  >CYP150A(MAP1866c)  MRHKGGEVSAPENLDFFTDKSVVDDPYDYYDAIRRCPVWREPAHGVVMVSGYDEALAVQR  DTDHALSVCNIVSGPWSGIPVNTGSDDISELIERYRKKVTFGDYFITFDPPMHTAHRSLL  SRLFTPKQLKNNEDFLWRLADEQLNRFIANGKCEMVIDYNFPFTLDAITDLLDVPEADRE  RFRRAAIASRLEGDRSGFVGVKEEWFVEYVEERRRNPRDDVLTELALAKVPDGTTPEPID  VARVATFMFAAGHGTTIDLLSLSMLTLAERPDLQDLLREDNSKIPAFIEEMLRIESPIKS  NFRLARRTTRIGDVEVQAGTSILVMNGAANRDPRRFDEPNEFRLDRPNILHHMAFGRGIH  TCPGAPIARAEVRVSLERILSRMADIRLSEAKHGPAGARRLRWDPTLLFRRLKELHLEFT  PIR*  >CYP125A(MAP0522)  MPSPNLPPGFDLLDPDVCVKGLPVAELAELRKSAPIYWVDVPGGTGGFGDKGYWAITKHK  DVKEISVRSDIFSSQQDCAIPVWPKEMTREQIDLQRNVMLNMDAPHHTRLRKIISRGFTP  RAVGRLRDELDARAQNIAKTAAAAGAGDFVEQVSCELPLQAIAGLLGVPQEDRDKIFRWS  NEMTGNEDPEYAHIDPAMSSAELIMYAMKMAEERAKNPGDDIVTQLIQADLDGEKLSDDE  FGFFVVMLAVAGNETTRNSITHGMIAFADNPDQWELFKKERPETAPDEIVRWATPVTAFQ  RTALEDYELSGVQIKKGQRVVMFYRSANFDEEVFEDPHRFNILRNPNPHVGFGGTGAHYC  IGANLARMTISLIFNAVADHMPDLKPLSAPERLRSGWLNGIKHWQVDYTGKCPVAH*  >CYP142A(MAP0547)  MTSTIPEAIANIDLADGNFYADRRASREAYRWMRANQPVFRDRNGLAGATTYQAIQDAER  NPELFSSTGGIRPDQPGMPYMIDMDDPAHLLRRKLVNAGFTRKRVKEKEPSIGTLCDTLI  DAVCERGECDFVRDIAAPLPMAVIGDMLGVLPTERGMLLKWSDDLVCGLSSHIDPTSAEF  QTVMDAFAAYTAFTMDIIAKRRAEPTDDLFSILVNAEVEGQRMSDDEIVMETLLILIGGD  ETTRHTLSGGTEQLLRHRDQWDALVRDPSLLPGAIEEMLRWTSPVKNMCRTLTADTEFHG  TELRAGEKIMLLFESGNFDESVFDDPDSFDIRRNPNSHMAFGFGTHFCLGNQLARLELSM  MTERVLKRLPDLRLADDGDLPLRPANFVSGLEAMPVVFTPSAPLLR*  >CYP51B1(MAP0598c)  MTTSTVVPRISGGEEEHGHLEEFRTDPIGLMQRVRDECGDVGWFQLVDKHVILLSGAQAN  EFFFRSADEDLDQAEAYPFMTPIFGKGVVFDASPERRKEMLHNSALRGEQMKGHASTIEG  EVKKMIADWGDEGEIELLDFFAELTIYTSTACLIGLKFREQLDHRFAEYYHDLERGTDPL  CYVDPYLPIESFKRRDEARVKLVALVQEIMDQRLANPPKDKADRDMLDVLVSIKDEDGKP  RFSADEITGMFISLMFAGHHTSSGTSAWTLIELIRHPDVYAEVLAELEELYADGQEVSFH  ALRSIPKLDNVVKETLRLHPPLIILMRVAKGEFEVEGFPIHEGDYVAASPAISNRIPEDF  PDPDAFKPDRYNKPEQADIVNRWTWIPFGAGRHRCVGAAFAQMQIKAIFSVLLREYDFEM  AQPADSYRNDHSKMVVQLARPAKVRYRKRNA*  >CYP123A(MAP0600c)  MTVHVGDHELVLDPYDYDFHEDPYPYYKRLRDEAPLYRNDELKFWALSRHQDVLQGFRNS  TTLSNKYGVSLDPASRGPHASKTMSFLAMDDPAHLRLRTLVSKGFTPRRIRELEPRVTEI  ATQHLDTMLDKAGSAAGGAVDYVDEFAGKLPMDVISELMGVPQADRVQVRAWADGVMHRE  EGVTDVPPEAVEASLNLIVYYQGMVEERRKKPTGDLTSALLEAEIDGDRLTDDEVLGFMF  LMVIAGNETTTKLLANAAFWGHKNPDQLTPVYDDLSRVPLWVEETLRYDTSSQILARTVS  GPLTLYDTTIPEGDVLLLLPGSGHRDERVFDNPDDYLIGREIGPKLLSFGSGAHFCLGAH  LARMEARVALTELFKRIRGYEVDEANAVRVHSSNVRGFAHLPMSVEVR*  >CYP126A(MAP0612)  MTLAGTLSQIDFTDLDNFANGFPHHLFAVHRREAPVYWHEPTDNTPDGEGFWSVASYAET  LEVLKDPATYSSVTGGERPYGGTLLQDLAIAGQVLNMMDDPRHSQIRRLVSSGLTPRMIR  LVEDDLRARARRLLDAVVPGEPFDFLVDIAAELPMQMICILLGVPESERHWLFQAIEPQF  DFGGSRKAALSQLSEAEAGSRMYEYGQQLIAAKRAEPTDDMLSVVANATLDDAAAPALSD  LELYLFFSLLFSAGAETTRNAVAGGLLALAEHPEQLRWLRDDLGALPTAVEEMVRWTSPS  PSKRRTATRDATLGGQSIKAGQKVQIWEGSANRDASVFDRADEFDVTRKPNPHLGFGQGV  HYCLGANLARLELRVLFEELLSRFGAVRVVRPVEWTRSNRHTGIRHLVVELRAEQ*  >CYP272A1(MAP1979)  MTEAGGFVDQTVTGVAEPQPMYKALRESNPVFRSTQAVVLSRLADIEMALKHTELFSSNM  DAVDLGNVRPLIPLQIDPPDHAKYRRILDPLFTPREMARREPLVTELVNEMIDRFAPRGE  CDFHAEFAVPLPCTVFLQLLGLPLEDLDRFLLWKDGVIRPAGDSGFDRRHESSAGVAQQI  YEYFDKAIDEHIAVPRDDVLSAMIAADVGGQPLSREELLDICFLFLIAGLDTVTDSLDCF  FVYLARHPQHRRQLVERPDVLPGAVEELLRWETPVPGVARVATQDVEVGGCPISKGERVS  PLLGAANTDPAEFPDPEIVDFTRSPNRHRAFGGGPHRCLGSHLARMELRVALREFHRRIP  DYEIRPGTQLTYTAALRSVESLPLVFPVR*  >CYP124A1(MAP2015)  MSLKTRPKKGLATRINGAPPPRVPLADIHLESLDFWGYDDDFRDGAFATLRREAPISFWP  AIEMDGFVAGNGYWALTKHEDVHFASRHPEIFSSVPNITINDQTPELAEYFGSMIVLDDP  RHQRLRSIVSRAFTPKVVARIEASVRERARRLVSSLVANHPNGEAELVSELAGPLPLQVI  CDMMGIPEEDHQRVFHWTNVILGFGDPDLATDFEEFLQVSMDIGAYATALAEDRRVNHHD  DLTTSLVEAEVDGERLTSSEIASFFILLVVAGNETTRNAISHGVLALSRYPDERDKWFSD  FDRLTPTAVEEVVRWASPVVYMRRTLTRDVKLRGTKMKAGDKVALWYNSANRDESTFGNP  WLFDVARTPNPHLGFGGGGAHFCLGANLARREIRVVFDELRHEIPDIVATEEPARLLSQF  IHGIKRLPVAWTPPR*  >CYP243A(MAP2030)  MATRPNVSPDAIVDFDHHSDAFNLNELAVNAELRQRCPVAWNENYGGFWFLSSYDAVSQT  ARDGDTFAHKYEPNAADGVDYQGEMGVPRPEGQPALGLGEVDGPYHQALRHALAPFFSPG  AVEKLKPFMEHSAHWFLDQQITTGQMDLVLDYASPVPAILTMKLMGLPYDNWHLYANLFH  SVMAVSQDSDEYAAAIAKVPAMMQEVLDYAATRRAKPEEDLTSFLIRFEFDGHRLTDEQL  LNILWNLIGGGVDTTTSQTALTLLHLGTHPDLRQQLIDHPELYRTATDEFLRYFSVNQTL  SRTVTHDVVLAGQRLRKNDRVVISWLSANHDENEFHRPDEIILDRAPNRHVAFGLGPHRC  IGSHLARLMSEVMVRAVLVRIPDYQVDVDNVHQYLGNPSMTGLGQLPVTFAPGRSRKALR  PW*  >CYP140B(MAP2033)  MRVRLGARWMAMHGLPRAYFAVQARRGDPLARLLRSGTTGEDRYALMEQIRARGPLMRAP  FVWASVDHALCRQVLRDKRFGVTSPTEMELPRPVRALIARTDPGVANPVEPPAMVIVDPP  DHTRYRQLVAQSFTPRAIEALNTRVAQVTLELIERIATIPQPDLIADFATRLPVAIIAEI  LGMPPDSYPRMLAWGRSGSPLLDLGIDWRTYRDAIDGLRGVDEYLLAHFHQLRADPHSDN  PFGRMAADGSLTDRELTANAALIVGAGFETTVNLIGNGIVLLLRHPEQLALLHDNPDLWP  SAVEEILRIASPVQMTARTPACDVDIAGAHIGAGEMVGLFLGGANRDPKVFSDPTTFDVT  RPNAREHLAFASGIHACLGAALARIEGATALRALFENFPDLRLTAAPQRRSLINLHGYTR  LPAQLGGRRTTSATIPV*  >CYP150A(MAP0668)  MTDLAQVDYFTDADVAQDPYDYWDYLREQGPVFREPHYGVVAVTGYQEVQAAFKDVESFS  AVNAIGGPFPPLPFTPEGDDISELIEAHRHEFPIFEHMVVMDPPEHDKARSLLGRLLTPR  RLQENKDYIWQLADRQFDEFIANGHCEFLSEYAKPFATLAIADLLGVPDEDRPQIRRNLG  AGNAPGARVGALDHEPVGSNPLQYLDDLFSGYIADRRERPRDDVLTGLATATYPDGSTPP  LLEVVRPATFLFAAGQETVTKLLSAAVQVLGDQPELQARLRADRGLIGPFIEEALRMQSP  TKVDFRLARKTTTLGGVHIPAGTVIMLCLGAANRDPRKFENPNEFRIDRKNVREHIAFGR  GIHTCAGAPLARVEGQITINRLLDRTSELRINKAKHGPASSRQYRYESTFLLRGLTELHI  EFTRAG*  >CYP105Q(MAP0704)  MKNDDTARNNTMSDTLTSTATEQTADIPDYPMPRQAGCPFAPPPDVMALAHDKPLSRVRI  WDGSTPWLITGYEQVRELFSDSRVSVDDRLPGFPHWNAGMLSTVHKRPRSVFTADGEEHT  RFRRMLSKPFTFKRVEGLRPTIQQITDEHIDAILAGPKPADIVSALALPVPSLVISQLLG  VPYEDADMFQHHANVGLARYATGEDTVKGAMSLNKYLAQLVEAKMENPAEDAVSDLAERV  KAGELSVKEAAQLGTGLLIAGHETTSNMIGLGVLALLENPDQLAVIRDAEDPKVIASAVE  ELLRYLSIIQNGQRRVALEDIHIAGETIRAGEGIIIDLAPANWDARVFPEPDRLYLHRSG  ADRNVAFGYGRHQCVGQQLARAELQIVFHSLVRRIPTLQLAIPIEEVPFKDDRLAYGVYE  LPVTW*  >CYP150A(MAP0727)  MTNEFSELDFFRGSELIENPYPYYEALRQRCPVTKESHHNVTMITGWDEACAVLNDAETW  SSCISVTGPFPGFPVPLEGDDVTELIERHRDELPFSDQLPTLDPPTHTNHRSLLMRLITP  KRLKENEDAMWVLADQALDTFLAPGHGEFIKGFAGPFTLLVIADLLGVPEEDRDKFVKGI  RQHSGGGVGGTGEETLAHSPLEFLYGLFFDYVRDRRRQPREDVLTGLATATYPDGSIPEV  EDVARVASNVFSAGQETTVRLLGAALQTLGERPDIQAQLRKDRSLIPNFIEESLRHESPV  KGDFRLNRRPVTVGGVDLPAGTTVMVVQAAANRDPRRFDDPATFDPARKNARQHISFGRG  IHSCPGAPLARAETRVAIERLLDRTTDIRINENIHGPANDRRYQYVPTYILRGLTELHLE  FTLA*  >CYP190A2(MAP0730c)  MTKPKLVFDPYSEDYFNNPYEIYRRMREEAPLYYDEKEDFYALTRHVDVAAAFKDYETYS  SARGCDLAMVRRGISPEQKSIIFMDPPEHRHMRSLLNKAFTPRAIQSQRETIIEVVDKYL  SAADPDNFDVVQDFSGPFPVEVITRMAGVPEEYRQQVRHWIDTSLHHEPGQIEVSEAGMQ  ANIDTAMYYFGLVQERRQDPQDDMISRLIAAEIPGENGQMRKLDDIEITGFATLLGGAGA  ETVTKLLGNAAVIFARHPDQWQKLQEDRDKIPGAVEELLRYEGPVQYNVRYTLKEAHVSG  GVIPAGKPVFLCGAAANRDPEAFTDADTFDIERDQTEAQHLGLGYGIHSCLGAALARLES  RIALERLLDFMPRYDVDWAGCRRVTMQNVAGWKNVPVKVLR*  >CYP189A5(MAP2051c)  MVMTGTSAIELYYDPFDSGIDDNPYPVWQRMREEAPLYYNEKYNFYALSRYEDVARELPN  WQTYRSGRGTTADILFSNVEVPPGILLFEDPPLHDLHRRLLSRVFTPRRMLAVEDLVRGF  CVRELDPLVGAGGFDFIRDLGAMMPMRTIGYLLGIPEEDQEKIRDRSVANIELSRDSDPA  AVDANVFANSIALFADYIEWRADHPSDDLMTELLRAEIDEPDGTRRPLSRTEVLAYTAMI  AGAGNETTARLIGFMGQLLSDHPDQRRELAADPSLIPGAVEETLRFEPPSPVQARYVARD  AEHYGRVVPEGSFMLLLNGSANRDPRRFTDPDRYDIHRQGGGHLSFGQGLHFCLGSALAR  MEARVAFEEVLKRWPDWEVDYANAERARTASVRGWARLPVVTGG*  >CYP188A2(MAP0752c)  MPDAWTDTYGGHRVAAGSHEVFELARCPAVSNDHDINGERRGYKGISIPTASRVSAVRGG  ILEMDDPEHRIYRTVLNPYLSPAAVKRWEPFIDEVTRAALDEKIEEGSIDFVDDLANIVP  AVLTLAMLGIPLKKWKMYSEPVHAAVYTPEHSPDIERVTAMHREMGLDMVNNMLEIRENP  RPGIVNALLQMRIDGEPAPDLEILGNLGLVIGGGFDTTTALTAHSLEWLSEHPEQRQLLS  DERKTLLDPATEEFLRYFTPAPGDGRTFSEDFELDGTVFKEGERLWISWAMANRDPAVFH  DPDEVILDRKGNRHFSFGLGIHRCIGSNVARTVFKSMLIAVLDRMPDYRCDPEGTVHYET  IGVIQGMRKLPATFTPGRRIGAGLDETLEKLQRICDEQELARPITERKEAAVID*  >CYP150A(MAP0774c)  MLSTFSISQSGGGTACRRTPPVKPSPGGRTPMSNFDSIDFFTDPSLVPDPHPYFDYLRSQ  NPVLRLPHYGVVAITGYEEATEVYKDPETFSNIVALGGPFPPLPVTPEGDDISAQIDAHR  SSFPMFEHMVTMDPPEHTKARSVLAKLLTPSRLKQNEEFMWRLADRQLDEFLHNGECEFI  AEYSKPFATLVIADLLGVPEEDHTQFRTVLGADRPGARVGALDHETVGINPLEWLDDKFC  NYIEERRREPRADVLTFLAEAKYPDGSTPPVIEVVRSATFLFAAGQETTAKLLSAALQVL  GDRPDIQQQLREDRSLIPAFIEESLRMESPVKSDSRLARRATTIGGVDIPAGTVVMILPG  AANRDPRRFENPHEFDLRRKNVREHMAFARGVHSCPGGPLARVEGRVSIERILDRMPHIE  INETHHGPAGDRRYTYEPTYILRGLSELHLTFTTADAVAPVG*  >CYP1034A(MAP2183c)  MTAPALDRDRLRELFDLRSSYNAWAGGAYEDDPYPVWHRLREKGPVLPGVLHELTGSTDT  MFFHGLPYPDCPHFTVFDYDSCMIAYRNPEVFASSPEPVDLEHGPLGLTNSMLSMNGEQH  KRYRALVQPSFLPANGKWWIDNWISETVDLLIDGLVHEGRAELNVDFCAAIPVLTITGSF  GVPVEQALDIREALARDPQKVVDLLKPVIAARPEEPRDDLISVLVQAELTDEDGAKDRLT  DREIDSFVLLLLGAGSGTTWKQMGTTLTTLLQRPELLEAVRADRSLLRPAIEEAIRWMPT  DPMFSRWVMADTELAGVSIPAGSVVHLALGAANRDPARWDRPDEYDITRKFKPSLGFGQG  SHICLGMHVARAEMTIAISALLDRLPNLRLDPDAEPPRFVGMYERGATAIPVVFDV*  >CYP138A(MAP3553)  MHERSSHRPAGRGPPASRGPSPKLLQGIGFAVSRRTMMRRLSRRYGNVFTLRLPMWGPVV  MVSDPQLAKQIFTTTPDELGNIQPNLSRLFGSGSVFGLEGDDHRRRRRLLAPPFHGKSMK  NYESIIEEETLRETAGWPEGESFPTLPPMMRITLNAILRAVFGAEGAELDELRRLIPPWV  TLGSRLAALPKPQRYPRFGPWGQLDRWRRHYDGVIERLIAAEQADPNFAERTDVLALLLR  STYDDGAAMSHKEIGDELLTLLAAGHETTASTLAWAFERISRHPELLARLVEEADNGGNE  LRQATILEVQRARTVIDFAGRHVYPDVYRLGEWVIPRGYSIIVGIAQIHDNPDVFPDPRR  FDPQRFIDNKPSALSWIPFGGGTRRCVGAAFANMEMDVVLRTVLRHFTIETTDAPDEPWH  CRGVAFTPKHGGRIVVHRR*  >CYP191A2(MAP3590)  MDFAYDPFDAEVMANPLPYYRILRDHHPVYYMPQWDTFALSRFDDIWRVLEVNNGTFVAS  EGTLPPASVLAQHNDGPVDDPPLHPLPFHAMFDADLYGEIRRTHSRPFRPRAVTDLEGRI  RTLANERLDELLARGSFDLTQEYGGVVVATIVCELLGIPTDLAPQVLAAVNAGSLAEPGV  GVDTGQARPNYFEFLLPAVQRRRADPSGPPLEVVDGLLGYQLPDGSALDDLEVATQMLCI  FIGGTETVPKIVAHGLWELSRHPDQLAAVRADPQHNIPVAREEMIRYCAPAQWFARTVRK  PFDIHGQTPNPGQRVITLLASANRDEREYPDPDDFVWDRPIRRSLAFGRGQHFCIGYHLA  RLEVAVLLQEWLRRVPDYAIRADAATRLPSSFQWGWNKIPVEV*  >CYP268A1(MAP2261c)  MTGVIAERIGIMAVLTGESGTDRSGRPYDEIDLSSRAFWSGTAAERERSFAVLRAERPVS  WHPPVEDSLLPDPTDPGFWAVTRRADIVTVSRNNDVFLSGHGVMFESIPAELLEASQSFL  AMDPPRHTKLRKLAHAALSPRQVRRIEDSIKANAKAIVEELRSAGSGCDFVDHCAKELPI  RTLSDMMGIPESERERMAHATDALVSWADPEFLNGRPALEVLLENQMYLHQVVGDLATQR  RERPGDDLISSLVTAEVDGDRLEDAEVAAFFVLLSVAGNDTTRQTISHTLRALTVFPDEK  FWLLEDFGHRIGTAVEEFIRWASPVMTFRRTAAADVELGGQTILAGEKVVMFYPSGNWDT  EAFDHPERLNLGRDPNPHVGFGGGGLHFCLGAHVARAQLRAIFSELFRQLPGIQAGEPTY  LAGNFVHAIRAMPCTF*  >CYP1124A1(MAP2344)  MNVNAATAACGDDPAERGSAMTTAAVDLSDFSLWCNGFPDELFAELRRTRPLFHHDLTPG  VAATVHRDFWVATKHRHAVRLHRDTESFTAADGPLIQPVAMFSSSPTIITMDPPELNKRR  KLISNAFNPRAIAKLEDGIRARAARMIDSLLAHGGGDWIEDVADALPMTVIGDILGIPER  DRPRIFDLFDRILKALAPEAHPRGGVELELFASVFDYAMQLTADKRRNPTGDIWSTLATA  VITGEDGEEFRLPANELEFFFFVLAFAGSDTTKNALAIGLQAFLANPEQVERYCADEALR  PTAVEEVLRWASPVAYWTRTAKVDVEMDGQRIAKGERVVSMLRSANRDEEVFDAPFTFDI  GRQPNPHVAFGGGGPHHCLGAMLARAELRAVFDELLLRCDDIEIGPAKAAYPNLITNMSI  YDEMPISLRRR*  >CYP187A3(MAP2365)  MPLSTGPTGQPVVPTLDFTGETSPYPFFEHMRRTDPVWHGSLADASQLPEELRPEDEWVL  FDYESVSQAFRDDRIFSSHKYDETIGLVMGHTILAMGGREHHDHRNLVAKAFRATALERW  EPSVIGPVCEQLVDEIKNDGHADLVKAVTFEFPTRIISTLLGLPAEDLDLFRRLSLDLIS  IPTDIEAGLNAATELYDYFLKQVEQRRRKPTDDIIGDLVAAEIDGEKLTDEAIIAFLRLL  LPGGLETTYRSSGNLLYLLLTHPEQLAMVYRDRSLIPMAIEEGLRFETPLTMVTRTTTEE  VEIGGKTIPANAQIDMCMGSANRDETRWTDPNAFDIRRPRQAHIAFAGGIHMCLGMHLAR  LETRVMLNSLFDRVRDLAFVPDDGTGEESKIVGLTFRSPNKLPVTFAPAA*  >CYP108B5(MAP2371c)  MSTTTMDEAAKLLADPMAYTDEQRLHAALTHLRANAPVSWVEVPNYKPFWAITKHADVMD  IERENMLFTNWPRPVLTTAEGDEMQAAAGVRTLIHMDDPQHRVVRAIGSDWFRPKAMRAL  KVRVDELAKIYVDKMLAAGPECDFVQEVAVNYPLYVIMSLLGLPEADFPRMLKLTQELFG  SDDSEFKRGSSNEDQLPALLDMFGYFNGVTAARREHPTEDLASAIANARVDGEPLSDIDT  VSYYLIVATAGHDTTSATISGGLQALIENPDQLQRLRDNLDLMPLATEEMIRWVTPVKEF  MRTAAKDTVVRGVPIAAGESVLLSYVSANRDEDVFDEPFRFDVGRDPNKHLAFGYGVHFC  MGAALARMEVNSFFTELLPRLKSIELTGDPELVATTFVGGLKHLPVRYSLA*  >CYP187A2(MAP2382)  MMTNVPTAAGDETVSLRDPYPFFARKRREAGVFAGTVMDYSKTPESLMPKQEYSAVSFDA  VNTVFRDGRVFSSKPYDKTIGLFMGPTILAMEGKKHRDHRNLVSAAFKSKALARWEPTIV  RPICNALIDDFIDAGTADLVRQFTFEFPTRVIARLLGLPDEDLPMFHTRAVQLISYHVDY  ERAFEASAALKDYFLEQIEQRKSKPTEDIIGDLVTAEIDGEKLSDEAIYSFLRLLLPAGL  ETTYRSSGNLLYLLLTHPDQFAALQADRELLAPAIEEGLRFETPLTTVQRFTTEDTELQG  VRIPARSVIGVCIGSANRDERRWERSEEFDIFRKHVPHISFAAGEHTCLGLHLARLETRV  AMECLLNRLTNVTLLSDGDPHIHGQPFRSPTALPVTFDAK*  >CYP125F1(MAP3818)  MRTPVTVGQHRHPFGRDIYVGRSGYVTEDAISIGGVNLADPDTYRAGMPYGAFRKLRERA  PVAWHPQKDGSGFWALTGYEEIHAVSRDSATWSSQINGAMFDAPPPGEVPPVMIFMDPPQ  HTALRKLINKGFTPRQVTRLNEHIVEMAKQIVDDVIERGECEFADDVAGALPSYVIAEML  GIPLEDGRRLYQITEILHTGSVGDSDDERQQAMVEMFQYGVELAVRKRAEPGDDIATSLL  HAEVDGQSLSDLEFNLFFMLLIDAGGDTTRNLVAAGILALLEHPQELQRLKADPSLMPTA  IEEMLRYTSPVTAFLRTATKDTELRGVPVKAGERVAMFYPSGNRDDSHFADPDRLDVGRA  PNPHLAFGGGGTHFCLGANLARVEASAMVPEVLSRMNDLELAGPVERLRSDLINGIRSMP  VRFTPGKRLGTA*  >CYP130A(MAP2518)  MSHNVPVAFELPNADTWADPWPMYRALRDHDPVHHVVPPKRPEHDYYVLSRHADVWAAAR  DHETFSSAKGLTVNYDDLELIGLQDNPPFVMQDPPVHTEFRKLVSRSFTPRQVEAVEPKV  RDFVVERIERLRAAGGGDIVAELFKPLPSMVVAHYLGVPEEDRAQFDGWTEAIVAANTAD  GGVAGALGSAGDAVTSMMAYFTGLIERRRTDPEDDTISHLVSAGVGADGDIAGTLSVLAF  TFTMVTGGNDTTTGMLGGSMPLLHQRPDQRQRLVDEPELIPDAVEELLRLTSPVQGLART  TTRDVTIGRTTIPAGRRVLLLYGSANRDERQYGPDAGELDVARCPRNILTFSHGAHHCLG  AAAARMQSRVALTELLARCPDFEVDESGIVWAGGNYVRRPLSVPIRVKS*  >CYP188A(MAP2525c)  MSVEVAGSGSRKAPQFHFDRHTPEYRERFLDVTQEMHQRCPIAWTDTYGGHWVAAGAGAV  FELARCPHVSNDHDVNNERRGYRGVTIPLTTESDQIRGGMLEMDDPEHRIYRSLLNPYLS  PAAVSRWQPFIDDVVRACLDERIESGRIDFVDDLANVVPAVLTLAMLGVPLRKWTMYNEP  VHAMVYTPPGSPEAAKVHDMWVSVVVDLFANLTEIREHPRPGIINALAQLRIDGEPAPDM  EIIGMLTLLIGGGFDTTTALTAHALKWLSEHPDQRARLHGELDVLLNPATEEFLRYFTPA  PGDARTISADMELDGIRFAEGERVWLSWAMANRDPSLFDNPNELMLARKANRHFSFGIGV  HRCIGSNVARTVFKSMLTAVLERMPDYRCDRVNTVHYDTIGIIQGMRNLPADFTPGKRLG  PGLDETLDRLQSVCDSQGLARPITEYKEQARLPG*  >CYP189A-fragment(MAP2532)  MTGLYYDPWNREIDADPYPIYQRLRNEAPLYYNERHDFWGLSRYDDVDAALRDPLRLSSA  KGDILDVVKADPVMPPGVFINEDPPLHTIHRALVARAFTPKKMRTLEDKIRAFCVASLDM  VADSDRFDFVEDLGAELPMRTIGMLLGIPDADQPSVREHARATLQNDTGGPMPIRKDHYF  DGDMFSDYVEWRKRPEWEIDMDNARRSRTSTVRGWDSMPAIVT*  >CYP291A3(MAP2584)  MQTHPPLRSPSFPLHSPDFYAGNPYPAYRELRATAPVCWNDVTNFWALLKYEDIRFVSSN  PALFTSTRGITIPDPQLPNPVQQGSLIFTDPPRHRQLRKLINSGFTRRRVSVLEPKIRKI  VRGILDGIERGAVHEFAEQIAAPLPTRMIAELIGAPPDDWEQFRAWSDAATGTADPEIEL  DPAVAAGQLYEYFQRLIAARRARPRADLLSVLAEAEIDEHRLTDEDLLNFAFLLLVAGNE  TTRNLIALGTLALIAHPDQYRLLVEEPARIPLAVEEMLRWNSPVVHMARTATADVEIRGQ  RIRAGEVVVMLYGSANRDEDVFGPDSEEFDVTRHPNPHIAFGCGEHSCVGAQLARLEATV  FFEELLRRYPRIELVGEVDRMRATMVPGVKRMPVRMGA*  >CYP187A(MAP2649c)  MEQLFDDLEDFGAFDDAVSGDVRDPYTELARLRREEPIQRLDTSGMPHEESKPVFIVYRH  EDAQQMLRDNETFSSAAVIAAFGPVLGERVMLGIDEPVHGRLRSLVSKAFSQKALARWED  ELVGRVGNSLIDRFAGNGKADLVKEFTFDYPSRIIAGLLGLPEQDYPQFQRWSISLLSWI  LNPERGLAASAALCDYFAPILAARRAEPKDDLISGLAQAEIDGEKLEDEEIYSFLRLLLP  AGVETTYRALGSLLLALLSDPEQLDAIRGDRSLLPQAIEEGVRWEPPLLTITRVATRDTE  LGGVPIPAGSTVMPMLGAANRQEDRYPDPDRFDIFRAPKSHLGWGHGVHVCLGMHLARLE  MRTAVNLLLDRLPNLRLDPDADDPHIRGQVFRSPTSVPVLFDPQ*  >CYP185A3(MAP1344)  MSVAEPKRDIAGLPLAPKNPLSYRERLRAIKEFHTGTNKLRDAGGPVTRVTLGPRWLISP  IVLATSPQGIRDIVSVRDGSIDKTSTVATELRRLLGPNLFVLPHTEWLPRRRTLQPVFTR  QRVREFGGHMAEAAESVCAGWPEDTEIDLDAQCRTLTLRALGRSVLGLDLDERSDAIAEP  LRVATSYAVRRALRPLRAPEWLPTPSRRRARAAAGAIRALADEILQACRADPGREAPLVH  ALIAATDPETGQALSDKEIRDEMIIFLFAGHDTTATTLTYALWALGRHPEYQARVAAEVA  ELPDRHLTPDDVARLGFTVRVLQEALRLCPPGPTGTRMATRDVEVAGYRVEAGTMLAFGR  MAVQTDPSLWDAPLRFDPDRFDPRRAGDRDRWQYLPFGGGPRSCIGDHFAMLEATLALAT  IVRRVEIESLSDDFPLAVPFTMVAAAPIRAMVRRRR*  >CYP279A1(MAP4068c)  MTVGAAPPSVFDSDLPTLHYHSDETPAQVYPRLREAQRRAAVAIGPHGPEVLSYHLVRSV  LRDPRFQIPPGINLLAQGIDSGPLWDKVANSLLCLEGDAHHRLRSLCSKAFTPRTVARLH  DTMAAVMNELVDRVAAAGRCDVVTDIARPYPVPIICALLGAPREDWRRFSSWADDVFKAF  SFTVDLREVEPVVMRAWRELDDYVDEMVARRRHNLTDDLLSDLIRVGDEGDRLDAAELRM  LAGGLLLAGTDTTRNQVAASVQVLCEHPDQWELLRQRPELAMRAVEETMRHSPIACGTLR  LVVEDAELDGHLFPAGTAVLVNTFAANRDPVVYNDPDRVDITREAAPPILTFGGGVHYCL  GANLARREIAEALNVLANRLRNPRLAGPAPWKPMVSLSGPTSLPIEFDR*  >CYP144A(MAP0009)  MLATGHPHPQAPSPAVVAPQLRRGPLTPRDGASSLKRMAVLDSATRFFGSEAIQDPYPLY  ERMHAEAPMHRIGDSVFYAVCGWDAVHEAIERVEDFSSNLTATMVFHEDGTVTPFDMGAP  GAPMHALATADDPVHAVHRKILLPHLSAKRIRIIEEFATQTADRLWDENLSDGRIEWMSA  IANRLPMMVVCRLLGLPDDDVDKLIRLGYATTTLLDGIVAPEQLEQAGMAAIELSGYVLE  HFEKASEKPESSLMADLAARCAAGELEQLPALGIMLTLFSAAGESTASLLGSAAWILADR  PAIQRQLRENPELLSTFIEETLRFEAPFRGHYRHVWRDTTLGGIELPEGAHLLLMWGAAN  RDPTHFKDPNEFRLDRAAAKSHLSFGKGVHFCVGAALARLEAHIVLRRLLERTSWIDATD  VGDWLPSILVRRRERLGLAVR*  >CYP139A(MAP1373c)  MRYRPGEALLALYRRRGPVIDAGAGRHGYTLLLGAEANKFVFANADAFSWRATFENLALV  DGPTALIVSDGDDHRRRRSVVAPGLRHRQIQDYVTTMVSCIDRVIDGWRPGQRLDVYQHC  RAAVRRSTAESLFGPRLAVHSDALGEHLQPLLDLTHQPPQLVGLQRRINAPAWRRAMAAR  QRINNLVDTLIADARAAPNPNDHMLTMLIDGRGDEGYTLSDNEIRDAIVSLVTAGYETTS  GALAWAVYLLLSQPGAWATAAGEVRRVLAGLPPAAADLSGLTYLNGVVHETLRLYPPGVI  SARRVMRDLRFKGRRIRSGRLLIFSPYVTHRLHEIWPEPRRFAPERWNPDAPGYRRAAPH  EFIPFSAGLHRCVGAAMATTEMTVMLARLLARTRLRLPAQRLRAANVAALRPTPGLTVEV  IDSVPAQ*  >CYP105Q(MAP1469c)  MTQPSTDADTDIPDFPMTRAPGCPFAPPPKVLQLNADKQLSRVRIWDGSTPWLVHGYQAI  RALFADARTSVDDRLPGYPHWNEGMLATVHKRPRSVFTSDAEEHTRFRRMLSKPFTFKRV  EALRPAVQKITDDHIDALLRGPNPGDIVSTVSLPVPSLVISELLGVPYEDAEFFQTQAQR  GMGRYATEEDTAQGAASLAKYLANLVRAKMQSPSEDLVSDLAERVNAEEISVREAAQLAT  GVLIAGHETTANMISLSVAALLEHPDQRALLCDTDDPKVIATAVEELMRYLSIIQTGQRR  IAIEDIEIGGETIRAGEGIILDVAPANWDARQFPNPDRLDLRREDGPHVGFGYGRHQCVG  QQLARMELQIVLPTLLRRVPTLRLAAPLDELPFKHDALAYGLYELPVTW*  >CYP143A(MAP1503c)  MSEGQYGTFHLPRLDFATLPMSVDRGLGWKTLRDAGPVVFMNGHYYLTRREDVLAALRNP  KVFSSTVLQPPGHPLPVLPLAFDPPQHTRYRKILQPYFSPHALGKSRPVLERHAAEMIAA  LADRGECEVMADFAHLYPFQVFMDLYGLPLQDRDRLLDWKNAVVGEKPFVTESDVEKSEQ  LLAYLADAIAQRRQHPGTDMLSQVMTGEGNFTDIELLGMSHLLILAGLDTVTAAIGFSLF  ELARRPQLRKELRDNPKQTRVFIEEIVRLEPSAPVAPRITTEYVEVGGMTLPPGTSVRLC  MAAVNRDDSDSMSTNELNMDGKVHRHWGFGGGPHRCLGSHLARIELTVVVAEWLTQIPDF  ELPDGLRAGDQLPLKEFRAQGAAAALGLKDPPRCSGTVPACRTTCGIRSRRPERTRRCPR  PSAGHGRGAADRPSSCRG* |
| ***Mycobacterium avium* subsp. *paratuberculosis* MAP4** |
| DATABASE: KEGG; P450 count: 43; Families: 30; Subfamilies: 33 |
| >CYP191A2(MAP4_0183)  MDFAYDPFDAEVMANPLPYYRILRDHHPVYYMPQWDTFALSRFDDIWRVLEVNNGTFVAS EGTLPPASVLAQHNDGPVDDPPLHPLPFHAMFDADLYGEIRRTHSRPFRPRAVTDLEGRI RTLANERLDELLARGSFDLTQEYGGVVVATIVCELLGIPTDLAPQVLAAVNAGSLAEPGV GVDTGQARPNYFEFLLPAVQRRRADPSGPPLEVVDGLLGYQLPDGSALDDLEVATQMLCI FIGGTETVPKIVAHGLWELSRHPDQLAAVRADPQHNIPVAREEMIRYCAPAQWFARTVRK PFDIHGQTPNPGQRVITLLASANRDEREYPDPDDFVWDRPIRRSLAFGRGQHFCIGYHLA RLEVAVLLQEWLRRVPDYAIRADAATRLPSSFQWGWNKIPVEV  >CYP138A(MAP4_0221)  MHERSSHRPAGRGPPASRGPSPKLLQGIGFAVSRRTMMRRLSRRYGNVFTLRLPMWGPVV MVSDPQLAKQIFTTTPDELGNIQPNLSRLFGSGSVFGLEGDDHRRRRRLLAPPFHGKSMK NYESIIEEETLRETAGWPEGESFPTLPPMMRITLNAILRAVFGAEGAELDELRRLIPPWV TLGSRLAALPKPQRYPRFGPWGQLDRWRRHYDGVIERLIAAEQADPNFAERTDVLALLLR STYDDGAAMSHKEIGDELLTLLAAGHETTASTLAWAFERISRHPELLARLVEEADNGGNE LRQATILEVQRARTVIDFAGRHVYPDVYRLGEWVIPRGYSIIVGIAQIHDNPDVFPDPRR FDPQRFIDNKPSALSWIPFGGGTRRCVGAAFANMEMDVVLRTVLRHFTIETTDAPDEPWH CRGVAFTPKHGGRIVVHRR  >CYP136A(MAP4_0689)  MTATISTPQYLLDQARRRFTPTLNTIPGMGAIEKRLLAHEWQTKVLAEPPAGSGLKPVLG DAGLPILGHIIELFRGGPDYALFLYRNHGPLIYLDSPIMPAVTALGPDATQAVFSNRNKD YSQKGWHPVIGPFFNRGLMMLDFDEHMYHRRIMQEAFTRSRLTGYVEHIDRVATAIVADW PTNDARFLFHPAMKELTLDIASLVFMGHEPGTDHDLVTTVNQAFTTTTRAGGAIIRQPIP PFKWWRGLRARQLLEDYFSERVKERRNATGNDMLTVLCHTEDDDGNSFTDDDIVNHMIFL MMAAHDTSTSTTTTMVYNMAAHPEWQERAREESARLGDGPLDIEALEKLETLELIMNESL RMVTPLPFNMRQAVRDTELLGHYIPAGTNITIWPGMNHRLPELWTDPDKFDPERFAEPRA EHKKHRYAFAPFGGGAHKCIGMVFGQLEVKTVVHRLLRRYRLELARPGYQPRWDYGGMPI PMDGMPIVLRPL  >CYP187A(MAP4_1169)  MEQLFDDLEDFGAFDDAVSGDVRDPYTELARLRREEPIQRLDTSGMPHEESKPVFIVYRH EDAQQMLRDNETFSSAAVIAAFGPVLGERVMLGMDEPVHGRLRSLVSKAFSQKALARWED ELVGRVGNSLIDRFAGNGKADLVKEFTFDYPSRIIAGLLGLPEQDYPQFQRWSISLLSWI LNPERGLAASAALCDYFAPILAARRAEPKDDLISGLAQAEIDGEKLEDEEIYSFLRLLLP AGVETTYRALGSLLLALLSDPEQLDAIRGDRSLLPQAIEEGVRWEPPLLTITRVATRDTE LGGVPIPAGSTVMPMLGAANRQEDRYPDPDRFDIFRAPKSHLGWGHGVHVCLGMHLARLE MRTAVNLLLDRLPNLRLDPDADDPHIRGQVFRSPTSVPVLFDPQ  >CYP291A3(MAP4_1235)  MQTHPPLRSPSFPLHSPDFYAGNPYPAYRELRATAPVCWNDVTNFWALLKYEDIRFVSSN PALFTSTRGITIPDPQLPNPVQQGSLIFTDPPRHRQLRKLINSGFTRRRVSVLEPKIRKI VRGILDGIERGAVHEFAEQIAAPLPTRMIAELIGAPPDDWEQFRAWSDAATGTADPEIEL DPAVAAGQLYEYFQRLIAARRARPRADLLSVLAEAEIDEHRLTDEDLLNFAFLLLVAGNE TTRNLIALGTLALIAHPDQYRLLVEEPARIPLAVEEMLRWNSPVVHMARTATADVEIRGQ RIRAGEVVVMLYGSANRDEDVFGPDSEEFDVTRHPNPHIAFGCGEHSCVGAQLARLEATV FFEELLRRYPRIELVGEVDRMRATMVPGVKRMPVRMGA  >CYP188A(MAP4_1297)  MSVEVAGSGSRKAPQFHFDRHTPEYRERFLDVTQEMHQRCPIAWTDTYGGHWVAAGAGAV FELARCPHVSNDHDVNNERRGYRGVTIPLTTESDQIRGGMLEMDDPEHRIYRSLLNPYLS PAAVSRWQPFIDDVVRACLDERIESGRIDFVDDLANVVPAVLTLAMLGVPLRKWTMYNEP VHAMVYTPPGSPEAAKVHDMWVSVVVDLFANLTEIREHPRPGIINALAQLRIDGEPAPDM EIIGMLTLLIGGGFDTTTALTAHALKWLSEHPDQRARLHGELDVLLNPATEEFLRYFTPA PGDARTISADMELDGIRFAEGERVWLSWAMANRDPSLFDNPNELMLARKANRHFSFGIGV HRCIGSNVARTVFKSMLTAVLERMPDYRCDRVNTVHYDTIGIIQGMRNLPADFTPGKRLG PGLDETLDRLQSVCDSQGLARPITEYKEQARLPG  >CYP130A(MAP4_1304)  MSHNVPVAFELPNADTWADPWPMYRALRDHDPVHHVVPPKRPEHDYYVLSRHADVWAAAR DHETFSSAKGLTVNYDDLELIGLQDNPPFVMQDPPVHTEFRKLVSRSFTPRQVEAVEPKV RDFVVERIERLRAAGGGDIVAELFKPLPSMVVAHYLGVPEEDRAQFDGWTEAIVAANTAD GGVAGALGSAGDAVTSMMAYFTGLIERRRTDPEDDTISHLVSAGVGADGDIAGTLSVLAF TFTMVTGGNDTTTGMLGGSMPLLHQRPDQRQRLVDEPELIPDAVEELLRLTSPVQGLART TTRDVTIGRTTIPAGRRVLLLYGSANRDERQYGPDAGELDVARCPRNILTFSHGAHHCLG AAAARMQSRVALTELLARCPDFEVDESGIVWAGGNYVRRPLSVPIRVKS  >CYP187A2(MAP4_1441)  MMTNVPTAAGDETVSLRDPYPFFARKRREAGVFAGTVMDYSKTPESLMPKQEYSAVSFDA VNTVFRDGRVFSSKPYDKTIGLFMGPTILAMEGKKHRDHRNLVSAAFKSKALARWEPTIV RPICNALIDDFIDAGTADLVRQFTFEFPTRVIARLLGLPDEDLPMFHTRAVQLISYHVDY ERAFEASAALKDYFLEQIEQRKSKPTEDIIGDLVTAEIDGEKLSDEAIYSFLRLLLPAGL ETTYRSSGNLLYLLLTHPDQFAALQADRELLAPAIEEGLRFETPLTTVQRFTTEDTELQG VRIPARSVIGVCIGSANRDERRWERSEEFDIFRKHVPHISFAAGEHTCLGLHLARLETRV AMECLLNRLTNVTLLSDGDPHIHGQPFRSPTALPVTFDAK  >CYP108B5(MAP4_1452)  MSTTTMDEAAKLLADPMAYTDEQRLHAALTHLRANAPVSWVEVPNYKPFWAITKHADVMD IERENMLFTNWPRPVLTTAEGDEMQAAAGVRTLIHMDDPQHRVVRAIGSDWFRPKAMRAL KVRVDELAKIYVDKMLAAGPECDFVQEVAVNYPLYVIMSLLGLPEADFPRMLKLTQELFG SDDSEFKRGSSNEDQLPALLDMFGYFNGVTAARREHPTEDLASAIANARVDGEPLSDIDT VSYYLIVATAGHDTTSATISGGLQALIENPDQLQRLRDNLDLMPLATEEMIRWVTPVKEF MRTAAKDTVVRGVPIAAGESVLLSYVSANRDEDVFDEPFRFDVGRDPNKHLAFGYGVHFC MGAALARMEVNSFFTELLPRLKSIELTGDPELVATTFVGGLKHLPVRYSLA  >CYP187A3(MAP4_1458)  MPLSTGPTGQPVVPTLDFTGETSPYPFFEHMRRTDPVWHGSLADASQLPEELRPEDEWVL FDYESVSQAFRDDRIFSSHKYDETIGLVMGHTILAMGGREHHDHRNLVAKAFRATALERW EPSVIGPVCEQLVDEIKNDGHADLVKAVTFEFPTRIISTLLGLPAEDLDLFRRLSLDLIS IPTDIEAGLNAATELYDYFLKQVEQRRRKPTDDIIGDLVAAEIDGEKLTDEAIIAFLRLL LPGGLETTYRSSGNLLYLLLTHPEQLAMVYRDRSLIPMAIEEGLRFETPLTMVTRTTTEE VEIGGKTIPANAQIDMCMGSANRDETRWTDPNAFDIRRPRQAHIAFAGGIHMCLGMHLAR LETRVMLNSLFDRVRDLAFVPDDGTGEESKIVGLTFRSPNKLPVTFAPAA  >CYP1124A(MAP4_1479)  MNVNAATAACGDDPAERGSAMTTAAVDLSDFSLWCNGFPDELFAELRRTRPLFHHDLTPG VAATVHRDFWVATKHRHAVRLHRDTESFTAADGPLIQPVAMFSSSPTIITMDPPELNKRR KLISNAFNPRAIAKLEDGIRARAARMIDSLLAHGGGDWIEDVADALPMTVIGDILGIPER DRPRIFDLFDRILKALAPEAHPRGGVELELFASVFDYAMQLTADKRRNPTGDIWSTLATA VITGEDGEEFRLPANELEFFFFVLAFAGSDTTKNALAIGLQAFLANPEQVERYCADEALR PTAVEEVLRWASPVAYWTRTAKVDVEMDGQRIAKGERVVSMLRSANRDEEVFDAPFTFDI GRQPNPHVAFGGGGPHHCLGAMLARAELRAVFDELLLRCDDIEIGPAKAAYPNLITNMSI YDEMPISLRRR  >CYP268A1(MAP4_1563)  MTGVIAERIGIMAVLTGESGTDRSGRPYDEIDLSSRAFWSGTAAERERSFAVLRAERPVS WHPPVEDSLLPDPTDPGFWAVTRRADIVTVSRNNDVFLSGHGVMFESIPAELLEASQSFL AMDPPRHTKLRKLAHAALSPRQVRRIEDSIKANAKAIVEELRSAGSGCDFVDHCAKELPI RTLSDMMGIPESERERMAHATDALVSWADPEFLNGRPALEVLLENQMYLHQVVGDLATQR RERPGDDLISSLVTAEVDGDRLEDAEVAAFFVLLSVAGNDTTRQTISHTLRALTVFPDEK FWLLEDFGHRIGTAVEEFIRWASPVMTFRRTAAADVELGGQTILAGEKVVMFYPSGNWDT EAFDHPERLNLGRDPNPHVGFGGGGLHFCLGAHVARAQLRAIFSELFRQLPGIQAGEPTY LAGNFVHAIRAMPCTF  >CYP1034A(MAP4_1639)  MTAPALDRDRLRELFDLRSSYNAWAGGAYEDDPYPVWHRLREKGPVLPGVLHELTGSTDT MFFHGLPYPDCPHFTVFDYDSCMIAYRNPEVFASSPEPVDLEHGPLGLTNSMLSMNGEQH KRYRALVQPSFLPANGKWWIDNWISETVDLLIDGLVHEGRAELNVDFCAAIPVLTITGSF GVPVEQALDIREALARDPQKVVDLLKPVIAARPEEPRDDLISVLVQAELTDEDGAKDRLT DREIDSFVLLLLGAGSGTTWKQMGTTLTTLLQRPELLEAVRADRSLLRPAIEEAIRWMPT DPMFSRWVMADTELAGVSIPAGSVVHLALGAANRDPARWDRPDEYDITRKFKPSLGFGQG SHICLGMHVARAEMTIAISALLDRLPNLRLDPDAEPPRFVGMYERGATAIPVVFDV  >CYP189A5(MAP4_1774)  MTGTSAIELYYDPFDSGIDDNPYPVWQRMREEAPLYYNEKYNFYALSRYEDVARELPNWQ TYRSGRGTTADILFSNVEVPPGILLFEDPPLHDLHRRLLSRVFTPRRMLAVEDLVRGFCV RELDPLVGAGGFDFIRDLGAMMPMRTIGYLLGIPEEDQEKIRDRSVANIELSRDSDPAAV DANVFANSIALFADYIEWRADHPSDDLMTELLRAEIDEPDGTRRPLSRTEVLAYTAMIAG AGNETTARLIGFMGQLLSDHPDQRRELAADPSLIPGAVEETLRFEPPSPVQARYVARDAE HYGRVVPEGSFMLLLNGSANRDPRRFTDPDRYDIHRQGGGHLSFGQGLHFCLGSALARME ARVAFEEVLKRWPDWEVDYANAERARTASVRGWARLPVVTGG  >CYP140B(MAP4_1792)  MRVRLGARWMAMHGLPRAYFAVQARRGDPLARLLRSGTTGEDRYALMEQIRARGPLMRAP FVWASVDHALCRQVLRDKRFGVTSPTEMELPRPVRALIARTDPGVANPVEPPAMVIVDPP DHTRYRQLVAQSFTPRAIEALNTRVAQVTLELIERIATIPQPDLIADFATRLPVAIIAEI LGMPPDSYPRMLAWGRSGSPLLDLGIDWRTYRDAIDGLRGVDEYLLAHFHQLRADPHSDN PFGRMAADGSLTDRELTANAALIVGAGFETTVNLIGNGIVLLLRHPEQLALLHDNPDLWP SAVEEILRIASPVQMTARTPACDVDIAGAHIGAGEMVGLFLGGANRDPKVFSDPTTFDVT RPNAREHLAFASGIHACLGAALARIEGATALRALFENFPDLRLTAAPQRRSLINLHGYTR LPAQLGGRRTTSATIPV  >CYP243A(MAP4_1795)  MATRPNVSPDAIVDFDHHSDAFNLNELAVNAELRQRCPVAWNENYGGFWFLSSYDAVSQT ARDGDTFAHKYEPNAADGVDYQGEMGVPRPEGQPALGLGEVDGPYHQALRHALAPFFSPG AVEKLKPFMEHSAHWFLDQQITTGQMDLVLDYASPVPAILTMKLMGLPYDNWHLYANLFH SVMAVSQDSDEYAAAIAKVPAMMQEVLDYAATRRAKPEEDLTSFLIRFEFDGHRLTDEQL LNILWNLIGGGVDTTTSQTALTLLHLGTHPDLRQQLIDHPELYRTATDEFLRYFSVNQTL SRTVTHDVVLAGQRLRKNDRVVISWLSANHDENEFHRPDEIILDRAPNRHVAFGLGPHRC IGSHLARLMSEVMVRAVLVRIPDYQVDVDNVHQYLGNPSMTGLGQLPVTFAPGRSRKALR PW  >CYP124A1(MAP4_1810)  MSLKTRPKKGLATRINGAPPPRVPLADIHLESLDFWGYDDDFRDGAFATLRREAPISFWP AIEMDGFVAGNGYWALTKHEDVHFASRHPEIFSSVPNITINDQTPELAEYFGSMIVLDDP RHQRLRSIVSRAFTPKVVARIEASVRERARRLVSSLVANHPNGEAELVSELAGPLPLQVI CDMMGIPEEDHQRVFHWTNVILGFGDPDLATDFEEFLQVSMDIGAYATALAEDRRVNHHD DLTTSLVEAEVDGERLTSSEIASFFILLVVAGNETTRNAISHGVLALSRYPDERDKWFSD FDRLTPTAVEEVVRWASPVVYMRRTLTRDVKLRGTKMKAGDKVALWYNSANRDESTFGNP WLFDVARTPNPHLGFGGGGAHFCLGANLARREIRVVFDELRHEIPDIVATEEPARLLSQF IHGIKRLPVAWTPPR  >CYP272A1(MAP4_1847)  MTEAGGFVDQTVTGVAEPQPMYKALRESNPVFRSTQAVVLSRLADIEMALKHTELFSSNM DAVDLGNVRPLIPLQIDPPDHAKYRRILDPLFTPREMARREPLVTELVNEMIDRFAPRGE CDFHAEFAVPLPCTVFLQLLGLPLEDLDRFLLWKDGVIRPAGDSGFDRRHESSAGVAQQI YEYFDKAIDEHIAVPRDDVLSAMIAADVGGQPLSREELLDICFLFLIAGLDTVTDSLDCF FVYLARHPQHRRQLVERPDVLPGAVEELLRWETPVPGVARVATQDVEVGGCPISKGERVS PLLGAANTDPAEFPDPEIVDFTRSPNRHRAFGGGPHRCLGSHLARMELRVALREFHRRIP DYEIRPGTQLTYTAALRSVESLPLVFPVR  >CYP150A(MAP4_1962)  MRHKGGEVSAPENLDFFTDKSVVDDPYDYYDAIRRCPVWREPAHGVVMVSGYDEALAVQR DTDHALSVCNIVSGPWSGIPVNTGSDDISELIERYRKKVTFGDYFITFDPPMHTAHRSLL SRLFTPKQLKNNEDFLWRLADEQLNRFIANGKCEMVIDYNFPFTLDAITDLLDVPEADRE RFRRAAIASRLEGDRSGFVGVKEEWFVEYVEERRRNPRDDVLTELALAKVPDGTTPEPID VARVATFMFAAGHGTTIDLLSLSMLTLAERPDLQDLLREDNSKIPAFIEEMLRIESPIKS NFRLARRTTRIGDVEVQAGTSILVMNGAANRDPRRFDEPNEFRLDRPNILHHMAFGRGIH TCPGAPIARAEVRVSLERILSRMADIRLSEAKHGPAGARRLRWDPTLLFRRLKELHLEFT PIR  >CYP105NSF1(MAP4_2048)  MSISFETSESRADAELPVLPMPRAAHCPLAPPPEFVDWRQQPGLRRALFQGNPVWVVSRY HDIRAALVDPRLSAKTIPDSIMPTDADNKVPVMFARTDDPEHHRLRRMLTGNFTFRRCES MRPQIQDTVDHYLDRMLDGGAPADLVREFALPVPSLVIALLLGVPPEDLELFQFNTSKGL DQKSSDEEKGKAFGAMYAYIEELVQRKAREPGDDLISRLITEYVATGQLDHATTAMNSVI MMQAGHETTANMISLGTVALLGNPEIYARLGQTDDSAVVANIVEELMRYLSIVHSQVDRV ATEDLTIAGQLIRAGEFVVMNLPAGNWDTEFVDNPESFDADRNTRGHLGFGYGVHQCIGA NLARVEMQVAFATLARRLPGLRLAVPPEQLKFKDANIYGMKELPVSW  >CYP125A(MAP4_2225)  MATVEPTTKPVPNLPPGFDFTDPDIYAERLPVEELAEMRRVAPIWWNEQPIGAGGFDDGG FWVVTKHKDVKEVSLRSDVFSSLQKTALPRYKDGTVAEQVERGKFVLLNMDAPQHTRLRK IISRAFTPRAVERLRDDLRERARRIVEAAAAEGSGDFVEQVSCELPLQAIAGLMGVPQED RKKLFHWSNEMVGDQDPEFASNDAITASVELIMYGMQMAADRAKNPGEDLVTKLVQADID GHKLSDDEFGFFVILLAVAGNETTRNSITQGMMAFTDFPDQWELFKRERPATAADEIVRW ATPVTSFQRTALQDYELSGVKIRKGQRVVMFYRSANFDEDVFDDPFTFNILRDPNPHVGF GGTGAHYCIGANLARMTIDLMFNAIADAMPDLESIGKPERLRSGWLNGIKHWQVDYHTNG SSKCPVAH  >CYP140A(MAP4_2236)  MKERLHWFAMHGFIRGAAALGARRGDVHARLIADPAVAADPARFYDEARARGTLVKGRVA YLTADHALAHELLRSEDFRVLVFGSNLPAPLRWLERRTRDDLLHPLRAPSLLAVEPPEHT RYRKTVSAVFTPRAVAALRDRVERTAAELLDQLTGGPGVVDIVGRYCSQLPVAIISEILG VPEQDRSRVLEFGELAAPSLDIGLPWRQYRSVQRGIAGFSSWLAGHLQQLRSNPSDNLMS QLIQTAESGSAETYLDETELAAIAGLVLAAGFETTVNLLGKGIRMLLDAPEHLDTLRRRP ELWPNAVEEILRLESPVQLTARMALNDVEVAGRQLHRGDLVLVYLAAANRDPAVFGDPHR FDIERPNAGRHLAFSGGRHFCLGAALARAEGEVGLRTFFERFPEARAAGAGSRRETRVLR GWSSLPVRLGPARSLAAAEAGGRPDEPTAG  >CYP143A(MAP4_2338)  MSEGQYGTFHLPRLDFATLPMSVDRGLGWKTLRDAGPVVFMNGHYYLTRREDVLAALRNP KVFSSTVLQPPGHPLPVLPLAFDPPQHTRYRKILQPYFSPHALGKSRPVLERHAAEMIAA LADRGECEVMADFAHLYPFQVFMDLYGLPLQDRDRLLDWKNAVVGEKPFVTESDVEKSEQ LLAYLADAIAQRRQHPGTDMLSQVMTGEGNFTDIELLGMSHLLILAGLDTVTAAIGFSLF ELARRPQLRKELRDNPKQTRVFIEEIVRLEPSAPVAPRITTEYVEVGGMTLPPGTSVRLC MAAVNRDDSDSMSTNELNMDGKVHRHWGFGGGPHRCLGSHLARIELTVVVAEWLTQIPDF ELPDGYEPVINYPSKSFALKELPLHWG  >CYP105Q(MAP4_2373)  MTQPSTDADTDIPDFPMTRAPGCPFAPPPKVLQLNADKQLSRVRIWDGSTPWLVHGYQAI RALFADARTSVDDRLPGYPHWNEGMLATVHKRPRSVFTSDAEEHTRFRRMLSKPFTFKRV EALRPAVQKITDDHIDALLRGPNPGDIVSTVSLPVPSLVISELLGVPYEDAEFFQTQAQR GMGRYATEEDTAQGAASLAKYLANLVRAKMQSPSEDLVSDLAERVNAEEISVREAAQLAT GVLIAGHETTANMISLSVAALLEHPDQRALLCDTDDPKVIATAVEELMRYLSIIQTGQRR IAIEDIEIGGETIRAGEGIILDVAPANWDARQFPNPDRLDLRREDGPHVGFGYGRHQCVG QQLARMELQIVLPTLLRRVPTLRLAAPLDELPFKHDALAYGLYELPVTW  >CYP139A(MAP4_2472)  MRYRPGEALLALYRRRGPVIDAGAGRHGYTLLLGAEANKFVFANADAFSWRATFENLALV DGPTALIVSDGDDHRRRRSVVAPGLRHRQIQDYVTTMVSCIDRVIDGWRPGQRLDVYQHC RAAVRRSTAESLFGPRLAVHSDALGEHLQPLLDLTHQPPQLVGLQRRINAPAWRRAMAAR QRINNLVDTLIADARAAPNPNDHMLTMLIDGRGDEGYTLSDNEIRDAIVSLVTAGYETTS GALAWAVYLLLSQPGAWATAAGEVRRVLAGLPPAAADLSGLTYLNGVVHETLRLYPPGVI SARRVMRDLRFKGRRIRSGRLLIFSPYVTHRLHEIWPEPRRFAPERWNPDAPGYRRAAPH EFIPFSAGLHRCVGAAMATTEMTVMLARLLARTRLRLPAQRLRAANVAALRPTPGLTVEV IDSVPAQ  >CYP185A3(MAP4_2502)  MSVAEPKRDIAGLPLAPKNPLSYRERLRAIKEFHTGTNKLRDAGGPVTRVTLGPRWLISP IVLATSPQGIRDIVSVRDGSIDKTSTVATELRRLLGPNLFVLPHTEWLPRRRTLQPVFTR QRVREFGGHMAEAAESVCAGWPEDTEIDLDAQCRTLTLRALGRSVLGLDLDERSDAIAEP LRVATSYAVRRALRPLRAPEWLPTPSRRRARAAAGAIRALADEILQACRADPGREAPLVH ALIAATDPETGQALSDKEIRDEMIIFLFAGHDTTATTLTYALWALGRHPEYQARVAAEVA ELPDRHLTPDDVARLGFTVRVLQEALRLCPPGPTGTRMATRDVEVAGYRVEAGTMLAFGR MAVQTDPSLWDAPLRFDPDRFDPRRAGDRDRWQYLPFGGGPRSCIGDHFAMLEATLALAT IVRRVEIESLSDDFPLAVPFTMVAAAPIRAMVRRRR  >CYP150A(MAP4_3085)  MSNFDSIDFFTDPSLVPDPHPYFDYLRSQNPVLRLPHYGVVAITGYEEATEVYKDPETFS NIVALGGPFPPLPVTPEGDDISAQIDAHRSSFPMFEHMVTMDPPEHTKARSVLAKLLTPS RLKQNEEFMWRLADRQLDEFLHNGECEFIAEYSKPFATLVIADLLGVPEEDHTQFRTVLG ADRPGARVGALDHETVGINPLEWLDDKFCNYIEERRREPRADVLTFLAEAKYPDGSTPPV IEVVRSATFLFAAGQETTAKLLSAALQVLGDRPDIQQQLREDRSLIPAFIEESLRMESPV KSDSRLARRATTIGGVDIPAGTVVMILPGAANRDPRRFENPHEFDLRRKNVREHMAFARG VHSCPGGPLARVEGRVSIERILDRMPHIEINETHHGPAGDRRYTYEPTYILRGLSELHLT FTTADAVAPVG  >CYP188A2(MAP4_3108)  MPDAWTDTYGGHRVAAGSHEVFELARCPAVSNDHDINGERRGYKGISIPTASRVSAVRGG ILEMDDPEHRIYRTVLNPYLSPAAVKRWEPFIDEVTRAALDEKIEEGSIDFVDDLANIVP AVLTLAMLGIPLKKWKMYSEPVHAAVYTPEHSPDIERVTAMHREMGLDMVNNMLEIRENP RPGIVNALLQMRIDGEPAPDLEILGNLGLVIGGGFDTTTALTAHSLEWLSEHPEQRQLLS DERKTLLDPATEEFLRYFTPAPGDGRTFSEDFELDGTVFKEGERLWISWAMANRDPAVFH DPDEVILDRKGNRHFSFGLGIHRCIGSNVARTVFKSMLIAVLDRMPDYRCDPEGTVHYET IGVIQGMRKLPATFTPGRRIGAGLDETLEKLQRICDEQELARPITERKEAAVID  >CYP190A2(MAP4_3132)  MTKPKLVFDPYSEDYFNNPYEIYRRMREEAPLYYDEKEDFYALTRHVDVAAAFKDYETYS SARGCDLAMVRRGISPEQKSIIFMDPPEHRHMRSLLNKAFTPRAIQSQRETIIEVVDKYL SAADPDNFDVVQDFSGPFPVEVITRMAGVPEEYRQQVRHWIDTSLHHEPGQIEVSEAGMQ ANIDTAMYYFGLVQERRQDPQDDMISRLIAAEIPGENGQMRKLDDIEITGFATLLGGAGA ETVTKLLGNAAVIFARHPDQWQKLQEDRDKIPGAVEELLRYEGPVQYNVRYTLKEAHVSG GVIPAGKPVFLCGAAANRDPEAFTDADTFDIERDQTEAQHLGLGYGIHSCLGAALARLES RIALERLLDFMPRYDVDWAGCRRVTMQNVAGWKNVPVKVLR  >CYP150A(MAP4_3136)  MTNEFSELDFFRGSELIENPYPYYEALRQRCPVTKESHHNVTMITGWDEACAVLNDAETW SSCISVTGPFPGFPVPLEGDDVTELIERHRDELPFSDQLPTLDPPTHTNHRSLLMRLITP KRLKENEDAMWVLADQALDTFLAPGHGEFIKGFAGPFTLLVIADLLGVPEEDRDKFVKGI RQHSGGGVGGTGEETLAHSPLEFLYGLFFDYVRDRRRQPREDVLTGLATATYPDGSIPEV EDVARVASNVFSAGQETTVRLLGAALQTLGERPDIQAQLRKDRSLIPNFIEESLRHESPV KGDFRLNRRPVTVGGVDLPAGTTVMVVQAAANRDPRRFDDPATFDPARKNARQHISFGRG IHSCPGAPLARAETRVAIERLLDRTTDIRINENIHGPANDRRYQYVPTYILRGLTELHLE FTLA  >CYP105Q(MAP4_3159)  MSDTLTSTATEQTADIPDYPMPRQAGCPFAPPPDVMALAHDKPLSRVRIWDGSTPWLITG YEQVRELFSDSRVSVDDRLPGFPHWNAGMLSTVHKRPRSVFTADGEEHTRFRRMLSKPFT FKRVEGLRPTIQQITDEHIDAILAGPKPADIVSALALPVPSLVISQLLGVPYEDADMFQH HANVGLARYATGEDTVKGAMSLNKYLAQLVEAKMENPAEDAVSDLAERVKAGELSVKEAA QLGTGLLIAGHETTSNMIGLGVLALLENPDQLAVIRDAEDPKVIASAVEELLRYLSIIQN GQRRVALEDIHIAGETIRAGEGIIIDLAPANWDARVFPEPDRLYLHRSGADRNVAFGYGR HQCVGQQLARAELQIVFHSLVRRIPTLQLAIPIEEVPFKDDRLAYGVYELPVTW  >CYP150A(MAP4_3197)  MTDLAQVDYFTDADVAQDPYDYWDYLREQGPVFREPHYGVVAVTGYQEVQAAFKDVESFS AVNAIGGPFPPLPFTPEGDDISELIEAHRHEFPIFEHMVVMDPPEHDKARSLLGRLLTPR RLQENKDYIWQLADRQFDEFIANGHCEFLSEYAKPFATLAIADLLGVPDEDRPQIRRNLG AGNAPGARVGALDHEPVGSNPLQYLDDLFSGYIADRRERPRDDVLTGLATATYPDGSTPP LLEVVRPATFLFAAGQETVTKLLSAAVQVLGDQPELQARLRADRGLIGPFIEEALRMQSP TKVDFRLARKTTTLGGVHIPAGTVIMLCLGAANRDPRKFENPNEFRIDRKNVREHIAFGR GIHTCAGAPLARVEGQITINRLLDRTSELRINKAKHGPASSRQYRYESTFLLRGLTELHI EFTRAG  >CYP126A(MAP4_3253)  MTLAGTLSQIDFTDLDNFANGFPHHLFAVHRREAPVYWHEPTDNTPDGEGFWSVASYAET LEVLKDPATYSSVTGGERPYGGTLLQDLAIAGQVLNMMDDPRHSQIRRLVSSGLTPRMIR LVEDDLRARARRLLDAVVPGEPFDFLVDIAAELPMQMICILLGVPESERHWLFQAIEPQF DFGGSRKAALSQLSEAEAGSRMYEYGQQLIAAKRAEPTDDMLSVVANATLDDAAAPALSD LELYLFFSLLFSAGAETTRNAVAGGLLALAEHPEQLRWLRDDLGALPTAVEEMVRWTSPS PSKRRTATRDATLGGQSIKAGQKVQIWEGSANRDASVFDRADEFDVTRKPNPHLGFGQGV HYCLGANLARLELRVLFEELLSRFGAVRVVRPVEWTRSNRHTGIRHLVVELRAEQ  >CYP123A(MAP4_3265)  MTVHVGDHELVLDPYDYDFHEDPYPYYKRLRDEAPLYRNDELKFWALSRHQDVLQGFRNS TTLSNKYGVSLDPASRGPHASKTMSFLAMDDPAHLRLRTLVSKGFTPRRIRELEPRVTEI ATQHLDTMLDKAGSAAGGAVDYVDEFAGKLPMDVISELMGVPQADRVQVRAWADGVMHRE EGVTDVPPEAVEASLNLIVYYQGMVEERRKKPTGDLTSALLEAEIDGDRLTDDEVLGFMF LMVIAGNETTTKLLANAAFWGHKNPDQLTPVYDDLSRVPLWVEETLRYDTSSQILARTVS GPLTLYDTTIPEGDVLLLLPGSGHRDERVFDNPDDYLIGREIGPKLLSFGSGAHFCLGAH LARMEARVALTELFKRIRGYEVDEANAVRVHSSNVRGFAHLPMSVEVR  >CYP51B1(MAP4_3267)  MTTSTVVPRISGGEEEHGHLEEFRTDPIGLMQRVRDECGDVGWFQLVDKHVILLSGAQAN EFFFRSADEDLDQAEAYPFMTPIFGKGVVFDASPERRKEMLHNSALRGEQMKGHASTIEG EVKKMIADWGDEGEIELLDFFAELTIYTSTACLIGLKFREQLDHRFAEYYHDLERGTDPL CYVDPYLPIESFKRRDEARVKLVALVQEIMDQRLANPPKDKADRDMLDVLVSIKDEDGKP RFSADEITGMFISLMFAGHHTSSGTSAWTLIELIRHPDVYAEVLAELEELYADGQEVSFH ALRSIPKLDNVVKETLRLHPPLIILMRVAKGEFEVEGFPIHEGDYVAASPAISNRIPEDF PDPDAFKPDRYNKPEQADIVNRWTWIPFGAGRHRCVGAAFAQMQIKAIFSVLLREYDFEM AQPADSYRNDHSKMVVQLARPAKVRYRKRNA  >CYP142A(MAP4_3320)  MTSTIPEAIANIDLADGNFYADRRASREAYRWMRANQPVFRDRNGLAGATTYQAIQDAER NPELFSSTGGIRPDQPGMPYMIDMDDPAHLLRRKLVNAGFTRKRVKEKEPSIGTLCDTLI DAVCERGECDFVRDIAAPLPMAVIGDMLGVLPTERGMLLKWSDDLVCGLSSHIDPTSAEF QTVMDAFAAYTAFTMDIIAKRRAEPTDDLFSILVNAEVEGQRMSDDEIVMETLLILIGGD ETTRHTLSGGTEQLLRHRDQWDALVRDPSLLPGAIEEMLRWTSPVKNMCRTLTADTEFHG TELRAGEKIMLLFESGNFDESVFDDPDSFDIRRNPNSHMAFGFGTHFCLGNQLARLELSM MTERVLKRLPDLRLADDGDLPLRPANFVSGLEAMPVVFTPSAPLLR  >CYP125A(MAP4_3345)  MPSPNLPPGFDLLDPDVCVKGLPVAELAELRKSAPIYWVDVPGGTGGFGDKGYWAITKHK DVKEISVRSDIFSSQQDCAIPVWPKEMTREQIDLQRNVMLNMDAPHHTRLRKIISRGFTP RAVGRLRDELDARAQNIAKTAAAAGAGDFVEQVSCELPLQAIAGLLGVPQEDRDKIFRWS NEMTGNEDPEYAHIDPAMSSAELIMYAMKMAEERAKNPGDDIVTQLIQADLDGEKLSDDE FGFFVVMLAVAGNETTRNSITHGMIAFADNPDQWELFKKERPETAPDEIVRWATPVTAFQ RTALEDYELSGVQIKKGQRVVMFYRSANFDEEVFEDPHRFNILRNPNPHVGFGGTGAHYC IGANLARMTISLIFNAVADHMPDLKPLSAPERLRSGWLNGIKHWQVDYTGKCPVAH  >CYP164A(MAP4_3526)  MTTAPTESAESQGLLLQLLDPANRADPYRLYAQFRERGALQLPEANLAVFLSYRDCDEVL RHPSSSSDNVNSTVAKRQAAAGTAPVRQGPPGFLFLDPPDHTRLRRLVSKAFAPRVVAAL EPDIRSLVDGLLDRAADKGELEIVEDFAYPLPVAVICRLLGVPLDDEPQFSRASALLAQA LDPFSTITGVPAEVASERQRAGTWLRDYFHQLIEARRSRPGDDLLSGLIAVEESGDQLTE EEIVSTCNLLLIAGHETTVNLIGNAVLAMLRDPGQWAALGADPGRAPAIVEETLRYDPPV QLAGRIALDDMVIGGVEVPAGDVMMLLLAAANRDPAEFDRPDTFDPDRKCLRHLGFGRGV HYCLGAPLARLEAGVALSAVTARFPRARLDGEPQYKTNVTLRGLSRLVVAV  >CYP189A(MAP4_3534)  MTVTNDTAVYYDPYDIGIITDPYPTYARLREEAPIYYNERYDFWALSRHSDVERALANWQ VFSNRRSDILELIQSKFDMPGGVMMFQDPPEHTVLRGLMSRVFTPRRMAALEDQIRQYCI RCLDPLVGSSSFDIIAELASMMPMRVIGMLLGIPESEPVSVRDANDANLRTKPGAPLRVA DADSIADGRIYADYVEWRSKNPSDDLMTTLLNVEFDDEDGVRRKLTRKEVLHYTQVVAGA GNETTGRLIGWLAKVLAEHPDQRREVYRDRSLLTRTVDETLRFEPTGPHVARWMAADFEC YGTTVPAGSAMLLLFGAANRDPRRYTDPDTFNIHRDNISHITFGKGVHYCLGANLARLEE RVALDELLNRWPEWDIDYDTAQLASTSTVRGWERLRIVVP  >CYP144A(MAP4_3866)  MLATGHPHPQAPSPAVVAPQLRRGPLTPRDGASSLKRMAVLDSATRFFGSEAIQDPYPLY ERMHAEAPMHRIGDSVFYAVCGWDAVHEAIERVEDFSSNLTATMVFHEDGTVTPFDMGAP GAPMHALATADDPVHAVHRKILLPHLSAKRIRIIEEFATQTADRLWDENLSDGRIEWMSA IANRLPMMVVCRLLGLPDDDVDKLIRLGYATTTLLDGIVAPEQLEQAGMAAIELSGYVLE HFEKASEKPESSLMADLAARCAAGELEQLPALGIMLTLFSAAGESTASLLGSAAWILADR PAIQRQLRENPELLSTFIEETLRFEAPFRGHYRHVWRDTTLGGIELPEGAHLLLMWGAAN RDPTHFKDPNEFRLDRAAAKSHLSFGKGVHFCVGAALARLEAHIVLRRLLERTSWIDATD VGDWLPSILVRRRERLGLAVR  >CYP125F1(MAP4_3931)  MRTPVTVGQHRHPFGRDIYVGRSGYVTEDAISIGGVNLADPDTYRAGMPYGAFRKLRERA PVAWHPQKDGSGFWALTGYEEIHAVSRDSATWSSQINGAMFDAPPPGEVPPVMIFMDPPQ HTALRKLINKGFTPRQVTRLNEHIVEMAKQIVDDVIERGECEFADDVAGALPSYVIAEML GIPLEDGRRLYQITEILHTGSVGDSDDERQQAMVEMFQYGVELAVRKRAEPGDDIATSLL HAEVDGQSLSDLEFNLFFMLLIDAGGDTTRNLVAAGILALLEHPQELQRLKADPSLMPTA IEEMLRYTSPVTAFLRTATKDTELRGVPVKAGERVAMFYPSGNRDDSHFADPDRLDVGRA PNPHLAFGGGGTHFCLGANLARVEASAMVPEVLSRMNDLELAGPVERLRSDLINGIRSMP VRFTPGKRLGTA  >CYP279A1(MAP4_4192)  MTVGAAPPSVFDSDLPTLHYHSDETPAQVYPRLREAQRRAAVAIGPHGPEVLSYHLVRSV LRDPRFQIPPGINLLAQGIDSGPLWDKVANSLLCLEGDAHHRLRSLCSKAFTPRTVARLH DTMAAVMNELVDRVAAAGRCDVVTDIARPYPVPIICALLGAPREDWRRFSSWADDVFKAF SFTVDLREVEPVVMRAWRELDDYVDEMVARRRHNLTDDLLSDLIRVGDEGDRLDAAELRM LAGGLLLAGTDTTRNQVAASVQVLCEHPDQWELLRQRPELAMRAVEETMRHSPIACGTLR LVVEDAELDGHLFPAGTAVLVNTFAANRDPVVYNDPDRVDITREAAPPILTFGGGVHYCL GANLARREIAEALNVLANRLRNPRLAGPAPWKPMVSLSGPTSLPIEFDR  >CYP189A-fragment(MAP4_1289)  MTGLYYDPWNREIDADPYPIYQRLRNEAPLYYNERHDFWGLSRYDDVDAALRDPLRLSSA KGDILDVVKADPVMPPGVFINEDPPLHTIHRALVARAFTPKKMRTLEDKIRAFCVASLDM VADSDRFDFVEDLGAELPMRTIGMLLGIPDADQPSVREHARATLQNDTGGPMPIRKDHYF DGDMFSDYVEWRKRPEWEIDMDNARRSRTSTVRGWDSMPAIVT |
| ***Mycobacterium intracellulare* ATCC 13950** |
| DATABASE: KEGG; P450 count: 43; Families: 26; Subfamilies: 31 |
| >CYP189A(OCU_00890)  MDDLYYDPWDVEIDLDPYPTYRRLRDESPLYYNERHNFWGISRYADVDAALKDTTRLSSA KGDILEVVLTDPVIPAGVFINEDPPVHTIHRAIVSRAFTPKKMRAIEDKVRAFCTACLDP LVGGERFDFVQDLGAELPMKTIGMLAGIPDSEQPAVRAHANAVLRNEAGKPMHVDKEHYF TGEMFGEYVEWREKHPSEDLITELLNVEFDDETGTTRKLTKQELVIFLAVVAGAGVETTG RLFGWMGKVLAEHPDQRKELAEDHSLIPTAIEELLRFEPPGPHVARYVATEDVAFQEQIV PAGSAILLMLASANRDERRFEDPDRFDIRRKPGGHLTFGRGAHFCVGSPLARLEGRVALE EVLKRFPEWTIDMDNARRSRTSTVRGWDSMPAIVG  >CYP142A(OCU_01340)  MPSTVDARNRPRPDVDLTDGAFYAGDSRPVYRWMRENEPVFRDRNGLAAAATYQAVIDAE RAPELFSNAGGIRPDQDAPPMMIAMDDPAHLLRRKLVNAGFTRKRVKDREASIGALCDAL IDNVCERGECDFVWDLAAPLPMAVIGDMLGVLPEERQMFLRWSDDMVTMLSSTTAQEDFQ VSIDAFAAYTEYMTSMIAARKAEPTDDLVSVLVHAEVDGEKLADHEIVTEVLLLLIGGDE TTRHTLSGGTAQILRHPRQHRQLVDDMALLPNAIEEMLRWTAPVKNMARTMTADLEFHGT QLARGEKIILLFESANFDEAVFGDPESFRIDRYPNNHLAFGFGTHFCLGNQLARLELSMM LERLLKRLPDMELASQDPLPLRPANFVSGLERMPVTFTPSAPSGG  >CYP164A(OCU_03180)  MTTAPTEATESQGLLLQLLDPANRADPYRLCAQFRDRGPLQLPDANLVVFLSYRDCDEVL RHPSSSSENANSTVAKRQAEAGTAPPRQGPPGFLFLDPPDHTRLRKLVSKAFAPRVVSAL QPDIRSLVDGLLDRVAEKGQFEVVEDFAYPLPVAVICRLLGVPLEDEPQFSRASALLAQA LDPFSTITGVPAEVANERQQAGTWLRDYFHGLIDKRRSRPGEDLLSGLIAVEESGDQLTE EEIVSTCNLLLIAGHETTVNLIGNAVLAMLRNPGQWAALGADADRAPAIVEETLRYDPPV QLAGRIALADMVIGGVEVPAGDVMMLLLAGANRDPAEYDRPDVFDPDRKNLRHLGFGRGA HYCLGAPLARLEASVALAAVAARFPNARLDGEPQYKTNVTLRGLSQLTLAI  >CYP189A(OCU_03300)  MTVTNDTDVYYDPYDVGINADPYPTYARLRDEAPIYYNEKYDFWALSRHSDVERGLANWE TFSNKRSDILELVQSKFEMPGGVMMFQDPPEHTRLRGLMSRVFTPRRMAALEDQIRQYCV RCLDPLVGSGGFDIIAELASMMPMRVIGMLLGIPESEQVSVRDANDANLRTKPGAPLKVA NADSIADGRIYADYVEWRSKNPSDDLMTTLLNVEFDDDQGVHRKLTRKEVLHYTQVVAGA GNETTGRLIGWLAKVLAEHPDQRREVYRDRSLLTRTVDETLRFEPTGPHVARWMARDFEC YGTTVPAGSAMLLLFGAANRDPRRYTDPDTYNIRRDNISHITFGKGVHYCLGANLARLEG RVALDEILNRWPEWDIDYDTAQLASTSTVRGWERLRVVLP  >CYP142A(OCU_05550)  MTSTIPEAISNIDLTDGNFYADRHTSREAYRWMRANQPVFRDRNGLAGATTYQAILDAER NPELFSSTGGIRPDQPGMPYMIDMDDPAHLLRRKLVNAGFTRKRVKEKEPSIAKLCDTLI DAVCERGECDFVRDIAAPLPMAVIGDMLGVLPTERSMLLKWSDDLVCGLSSHIDPTSETA QAVMAAFAAYTEFTTSIITKRRAEPTDDLFSILVNAEVEGQRMSDDEIVMETLLILIGGD ETTRHTLSGGTEQLVRHRDQWDALVRDPSLLPGAIEEMLRWTSPVKNMCRTLTADTEFHG TSLREGEKIMLLFESGNFDESVFEDPDSFDIRRNPNSHMAFGFGTHFCLGNQLARLELSM MTERVLKRLPDLRLADDDSTLPLRPANFVSGLEAMPVVFTPSAPLLG  >CYP51B(OCU_06050)  MTTAIVPRVSGGEEEHGHLEEFRTDPIGLMKRVRDECGDVGWFQLAGKHVILLSGAGANE FFFRSADEDLDQAEAYPFMTPIFGKGVVFDASPERRKEMLHNSALRGEQMKGHASTIEGE VKKMIADWGDEGEIELLDFFAELTIYTSTACLIGLKFREQLDHRFAEYYHELERGTDPLC YVDPYLPIDSFRRRDEARVKLVAVVQEIMDQRLANPPKDKADRDMLDVLVSIKDEDGNLR FSADEVTGMFISLMFAGHHTSSGTSAWTLIELIRHPEVYAEVLAELEELYADGQEVSFHA LRSIPKLDNVVKETLRLHPPLIILMRVAKGEFEVEGFPIHNGDYVAASPAISNRIPEDFP DPDAFRPDRYNKPEQADIVNRWTWIPFGAGRHRCVGAAFAQMQIKAIFSVLLREYDFEMA QPADSYHNDHSKMVVQLARPAKVRYRKRNA  >CYP126A(OCU_06210)  MTLAGALAEIDFTDLDNFADGFPHDLFAIHRREAPVYWHPPTDNTPDNEGFWSVATYAET LEVLKDPVTYSSVTGGERPYGGTLLQDLAIAGQVLNMMDDPRHSQIRRLVSSGLTPRMIR LVEDDLRTRARRLLDAVVPGEPFDFLVDIAAELPMQMICILLGVPESERHWLFDAIEPQF DFGGSRKASLSQLSVEEAGSRMYDYGRQLIASKRENPTDDMLSVVANAMLDDSDASALSD LELYLFFSLLFSAGAETTRNAVAGGLLALANHPSQLRSLREDLGALPTAVEEMVRWTSPS PSKRRTATRDVTLGGRPIEAGQKIQIWEGSANRDASVFDHADTFDIARKPNPHLGFGQGV HYCLGANLARLELRVLFEELLSRFGAVQVVRPVEWARSNRHTGIRHLVVELREDQ  >CYP150A(OCU_07020)  MTDLANVDYFTDADVAQDPYDYWDYLRNQGPVFREPHYGVVAVTGYQEVQAAFKDVDSFS AVNAIGGPFPPLPFTPEGDDISEQIEAHRHEFPIFEHMVVMDPPEHDKARSLLGRLLTPR RLQENKDYIWQLADRQFDEFIANGHCEFLSEYAKPFATLAIADLLGVPDEDRPEIRRNLG AGNAPGARVGALDHEPVGSNPLQYLDDLFSAYITDRRRQPREDVLTGLATATYPDGSVPP LLEVVRPATFLFAAGQETVTKLLSSAVQVLGDDPELQERLRSDRSLIGTFIEEALRMQSP TKVDFRLARKTTTLGGVHIPAGTVLMLCLGAANRDPRKFDNPNEFRPDRKNVREHIAFGR GIHTCAGAPLARVEGQITINRLLDRTRKFRISEAKHGSPSGRQYHYEPTFLLRGLTELHI DFIPAD  >CYP150A(OCU_07780)  MTKEFSELDFFRGSELIADPYPYYEALRQRCPVTRESHHDVTMITGWDEACAVLNDAETW SSCISVTGPFPGFPVPLEGDDVTELIERHRDELPFSDQLPTLDPPTHTNHRSLLMRLITP KRLKENEDAMWALADQALDEFLAPGHGEWIKGFAGPFTLLVIADLLGVPMEDRDKFVKGI REHSGGGVGGTGKESLAHSPLEFLYGLFSDYVRDRRREPRDDVLTGLATATFPDGSIPEV EDVARVAANVFSAGQETTVRLLGAALQTLGERPDVQAQLRKDRSLIPNFIEESLRHESPV KGDFRMNRVPANVGGVDLPAGSTVMIVQAAANRDPRRFEDPATFDPARKNARQHISFGRG IHSCPGAPLARAETRVALERLLDRTTDIRINEDKHGPANNRRYQYVPTYILRGLTELHLE FTLA  >CYP188A(OCU_08160)  MSVDDVVNDGDRKKNRYHFDRHSSEYRSQFKAITEEMHAKCPMAWTDTYGGHWVAAGSHE VFELARCPAVSNDHDIHGERRGYKGISIPTASRVSAVRGGILEMDDPEHRTYRTVLNPYL SPAAIKRWEPFIDDVTRACLDEKIEGGSIDFVDDLANIVPAVFTLAMLGIPLKKWNMYSE PVHAAVYTPEHSPDIERVTAMHREMGLDMVNNMIEIRANPRPGIVNGLLQMRIDGEPAPE LEILGNLGLVIGGGFDTTTALTAHSLEWLSEHPEQRQLLSDERKTLLDPATEEFLRYFTP APGDGRTFAEDTELDGTRFKEGERLWISWAMANRDPAVFHDPDEIILDRKGNRHFSFGLG VHRCIGSNVARTVFKSMLTAVLDRMPDYKCDPEGTVHYETIGVIQGMRKLPATFTPGRRI GAGLDETLEKLQRICDEQELARPITERKEAAVID  >CYP135B(OCU_09850)  MDRAAARYGDTFTMKLVGEGPIVMVSHPDAVRQIFTSPPELMLAGEANRILQPVVGQNSV LLLDRDAHREQRRLLMPAFRGNQLQSYMSTMTAIAQAEIARWPRGVPVRLHPRMQALTLE VILHLVFGLERGQSMDRLRQALQRTLVLTTNGAAQFFLLLVGPRKMRTALIHKLLADVDR LLHEEIATPRRQADLDTRTDVLSMLLKARHEDGNPMSDQEIRDELITLLLAGHETTASGL AWAVERIIRHPDAHSRLIEEAHADDGQRYVDAVVKETLRMRPVLALVSRRLTEPMEVDGF SLPAGVKVAPSIYLMHRRPDIYPDPKQFRPERFLDNRAGTYTWIPFGGGVRRCLGATFAE CEMRIVLGAMFTTMRVRPVDAKSEPIHRRSITQVPGRGTTVVLE  >CYP279A(OCU_10400)  MTTLISGCPNVFDAGLPVIAYDHLTDPDAAHRVIDDARELSPIAVGPHGPEVLSYELVRT VLRDSRFVTAHGLGLGLQGVTSGPLWDRAISNILGLDGAAHHRLRRLVSKAFAPRGAERL RALVIQIIDGLVDPLAEAGHCDVVADIARRYPTPVICALLGAPPEDWHLFSDWTDDIKKI FDWNVAEDGPAILAAWDQLDAYLEELIARRRASLSDDLISELIRSEDDGDRLTHDELLML CATLLGAGTDTTRNQLAAAVQVLADQPDQWALLAQHPELAANAVHELMRYYPIVFGTIRE TVEDVELAGVCIPAGTLVLANTAAANRDASVYDRPDRVDITREDPPAMLTFGGGVHFCLG AHLARLELTEALRVITRRMPNPRRTGPAPWKAISGITGPTTLPLAFDVGTVQP  >CYP130A(OCU_13000)  MSQNAPVAFQLATAATWSNPWPMYRALRDHDPVHHVVPANSPEHDYYVLSRHADVWAAAR DHETFSSAKGLTVNYDDLELIGLQDNPPFVMQDPPVHTQFRKLVSRGFTPRQVEAVEPKV REFVVERIEGLRAAGGGDIVTELFKPLPSMVVAHYLGVPEEDWVQFDGWTQAIVAANTAE GGVAGALETVGDAVGSMMAYFTGLIERRRTDPEDDTISHLVTAGVGADGDVAGTLSILAF TFTMVTGGNDTVTGMLGGSMPLLHERPDQRKLLVDNPELITDAVEELLRMTSPVQGLART TTREVTIGDTTIPAGRRALLLYGSANRDERQYGPDAAELDVTRCPRNILTFSHGAHHCLG AAAARMQSRVALTELLARCPDFEVDESGIAWAGGNYVRRPLSVPIRVGS  >CYP190NSF1(OCU_13290)  MSTDHGAGSVTKQREDGSPMEFDPYSDSFFDDPYDTYRWMRDEAPVYYSERWDFYALTRN EDVVAAHRDWETFSSAYGVTLDALSMRHRFDELKMLILMDPPEHELLRKLVRQVFTKAAI ANLEPLVTDVVTSYADALSGRDDFDIVADFAALFPVEIISSMLGVPPGERQQIRHWTDGF LHREPNNPFATESGVADSMAMNAYFLALSKEKRRQPDDLIISRLVDATYEDETGVTHRLT DEDIATFAVLIAAAGSETVTKLIGNGTMAFHHNPDQWELVLADPGLVPGAIEEMLRLNPP SQYQGRFATRDVVLDGGTIPAGSPTLLVTGAATRDPRAYDQPDAFDIRRGGSTTLAFGYG AHSCLGSWLARLETRVALHAIRERWPRFTVDTEGLRRVTMSNVAGYSHIPVHVA  >CYP1067A1(OCU_13460)  MLEFDPFKPGPADELPAVYAALRERFPVYRTGTNIWVISRFDDVKAVQSNPAVFSSRPNP YEGDSAPADAEMKPEVIERLMALTAGIPVDMNEMASAKTIAAADPPQHTRIRRIVSRGFT PPRIKEMADAIAKIVDRCLSGIDELPSFDLVERLAIPLPVEMICHILGIDRSEYGRAKCW SDAFAAAAGGNFSSAAERNELMLTTIKEFSTYFVPLIEARRVEPRNDLVSAMVAAIDNES LSNVETLMVAITIMVAGNETTTNLIGNAVVELLANPDQLKLLLDDPSLLPNAVDEANRLT TPIQFAFREATEDTVVAGTTIPKGAIIALHMAAANRDPRRFDEPDRFLITRPPGKSLAFG HGIHFCLGAHLAGQEVRAAIGGLLPYLDRLKLTDAPLERNPTALLNGWQRVELAWVS  >CYP144B1(OCU_13470)  MLTASMLLDPAVLQNPYAFYAMLREQAPVWMVPGTDVVAISTFALLSEAVARPEDFSSIM RCLLYRDQDGLPARLDFGEAAMPTLATADPPVHTTHRRAVFPELVARRMRTLEDDIRALS AAAVKQALDDGSFDFMAKIGNMVPITVVARLIGFRDIDPMQLLEAAFDSTTMVGGTMSMD ELSTFVARTETIEAWIADQIAGTGEGGEEGILLAVRRALDAGTLRIGEATIILHTMLSAG GESTTSLLGNAVGMLAENPGLQQRLREKPQDIDVFVEEALRLESPFRQMMRSVPRDTTLG GVEIASGSTVLLLFAAGNRDPAQFKHPDRIDLTRESPKRHLAFGHGIHFCVGAALARIEA RAVLGAILEQTRCFTLDEDRAPRWVDSLLVRRHAELHVCAAPR  >CYP143A(OCU_13750)  METLGAPELSFASLPMAADRGVGWKVLRDAGRVVSVDGIFYLTHREDVLAALRDPELFSS KKAFDVLGSPLPLVPISFDPPEHTRFRKILQPFFSPHTLKEMLPSLQQQAIEIIERVAAQ GECEVVADVAIPYPSQVFLTLFGLPLEDRDKLVAWKTSVIAISEAPSLEDADLTPALELV AYLTEAINARRADPGPDILSQLLNGEEPLDDAEIMGLSFLFVLAGLDTVTAAMSSALLEL ARNPELRATLHDDPDQIDVFVEEIVRLEPPAPMLPRVTTTEVTIGDVTLPADTMVRLCVA AINRDDSDEISTNDVVMDGKVHRHWGFGGGPHRCLGSHLARLELKLIVGEWLRRIPEFSV KVGEEPQIVFPASTFSLERVPLKLG  >CYP1129A(OCU_13990)  MTDTQAESRPRAEVDLDHHSPEFRDDPYGRFREMRESGCPVAHSEHYEGFWALVDYASVF EAARDDDLFNSFPSVGVPASELPLPILPIESDPPETQELREVTLKRFSPGSAERFRESAI EMTNEAIDAFIERGECDLVGELTTPLPARLILRLLNFDESRFMDWVGWVHTTVHDRAHDP EKAGIAGMEMFGEIVKHMEQRRAEGLGDDLFSDILRGTLNGEPLDDGQITMYTVLMMLGG MDTTSGFTANVLLRLCKDAELRAKFTADPSLVKKSTDELLRLYTPTLGLARTVSRDADFH GQHLCQGDRAILMWAAANRDPAMFENPDELDLDRPNAKKHMAFGVGMHRCLGSHYAKLMF DVMITQVLKRLPDFELAGEAKLFEDAGEVYAVRELPVSFTPGPRVG  >CYP1027B(OCU_14610)  MKNIEMNTPESPALAHFHLLDECQDEARPVFRNTEAGIGYWVFTDNSVILDGLQHPELWS SSVIVPTEPEPPYKWIPIMIDPPDHAKWRHVLAEYFSPGRVKGLRDAQQKLAAELIDQVA GEGGCDFVARISRVFPSTVFLTIMGMPVEDLEKFLAWEDMILHQSGVGEEVNAARLEGMT HVMGYFSGLIQQRRENRDPDADDIVSKAIDWTIDDEPINDLELLNCLLLLFMAGLDTVSN QLSYAMLHLATHPADRARIVAEPQLIPRAVEELLRVYPIVQTARKATRDMDFHGCPVKAG DMASFSLAFAGRDESAYPNARTVDFDRGVTRHLSFGGGPHRCLGSHLARQELAVVLEEWH KRIPEYEVSGQPIEHGGQVFGVDSLNLTWG  >CYP124NSF1(OCU_14690)  MSAIDTDEQQFAPLIDLRWWQERPAERSELYRRLREAGGPVFVRTNRPDSPRPRGFWAIG AHRDVVDISRRPGDFSSGQGTQIFDQTAEMREYRGSIIDMDDPEHMRLRKIVSRGFTPRI LSELRGLVEETTAEILEEMPRTGECDFVSSFATLLPLRIIDTMLGVPREHEQFILRATNI VLGASDPEYVPDQTVAGIGAAVTEISEQLIELLKGIAEDRIAHPRDDVISKLVNSDEENL TPQELAKFFILLIGAGNETTRNALTHGLLILSAHPEQRDRLLANYDDMVSTAVEEILRYA SPVIHFRRTVTRDGVTLTDAHGEVTHTFNAGDKVVVWYPAANRDPAVFDQPERFDIARKP NNHIAFGGPGPHYCLGAHLARLELNVAFQMLYARYPDITAAGEPVMLRSNFVNGIKHLKA TYTP  >CYP143NSF1(OCU_14850)  MTELGMTGTLNINDLPFAEDRTRAWRELREAGEAVSSGEEVVLTSAEAVEFAAKKPEIFS SARAFDRLGSPVPLVPIAIDPPDHTRFRRMLDPFFGPKKMAEREPELRRQAGELIDAIVA SGECDVVPDLATPFPSQVFLTLFGLPMADRDRLVKWKDAILQFTDPSSTEATPEVMAHAL ELFSYLTEHIAERRADATGNDMLTQLIQDTDEGGMSDNEILGLCFMFVLAGLDTVTSAVG FSLARLAADDDMRRRIAHDFTLIPAFIEEILRVDGPVPFAPRVTTEGVEVAGRFVPKDTT VMLSYGSADRDPRRYQDADQIHLDSKVVHFAFGRGPHRCLGSHLARLELRLILEEWHSRI PEYTLALGKPPQMPWPTGTMSLQSVPLNIKPSSRGQ  >CYP190A(OCU_16860)  MTVAELEFDPFSEDFFNGPYETYRRMREEAPVYYSERYDFYALSRHVDVAAAYKNFETYS SARGVDLAAVRSEEPMGHKSIIMMDPPEHRHMRGLVNKVFTPRAIEAMRPLVTGLVDRYL DATDPAGFDVVQDFSALFPVEVITTMLGVPEEDRQQIRLWLDTFLHREPGEVEMSEAGVQ AATQMAIAYYQLVKERRAAPRDDMISALVAARVERENGELTALDNIEIAQFAVLLGGAGA ETVTKLVANAVVLFARHPEQWQKLLDDRSKVPAAVEEVLRYESPNQYNVRCSREDVTLHG VTIPAGKPVFLLLGSANRDPDAWTKPEVFDIERDRTEAQNLGFGYGIHSCLGAALARMES SVALQKLLDHMPRYEVDWAGCRRVNMQNVAGWNNVPVRVLP  >CYP108B(OCU_16990)  MDEAANLLADPLAYTDEQGLHAALTHLRANAPVSWVKVPNYRPFWAITKHADIMDIEREN MLFTNWPRPVLTTAEGDEMQAAAGVRTLIHMDDPQHRVVRAIGSDWFRPKAMRALKVRVD ELAKIYVDKMMAAGPECDFVQEVAVNYPLYVIMSLLGLPEADFPRMLKLTQELFGSDDSE FKRGSSNEDQLPALFDMFQYFNGVTASRREHPTEDLASAIANARVDGEPLSDIDTVSYYL IVATAGHDTTSATISGGLQALIENPDQLQRLRDNLDLMPLATDEMIRWVTPVKEFMRTAS SDTVVRGVPIAAGESVLLSYVSANRDEEVFDDPFRFDVGRDPNKHLAFGYGVHFCMGAAL ARMEVSSFFSELLPRLESIELTGDPELVATTFVGGLKHLPVRYSLRR  >CYP187A(OCU_17050)  MVPPLDFTGETSPYPFFEYMRRTDPVWHGSLADSGQLPEELRPDDEWVLFDYDAVSQAFR DDRVFTSAAYDKTIGLVMGHTILAMGGREHHDHRNLVAKAFRATALARWEPSVIGPVCDQ LVDEIKNDGQADLVKAVTFEFPTRIISTLLGLPAEDLDLFRRLSLDLISIPTDIEAGLNA ANELYDYFLKQVEQRRRKLTDDIIGDLVAAEIDGEKLTDEAIIAFLRLLLPAGLETTYRS SGNLLYLLLTHPEQLAMVRRDRSLLPMAIEEGLRFETPLTTVMRTTTEEVEIGGKTIPAN AQIDMCMGSANRDETRWTDADTFDICRPRQAHIAFAGGIHMCLGMHLARLETRVMLNSLF DRVEDLAFVPDDGTGVESKIVGLTFRSPNKLPVTFAPAA  >CYP188A(OCU_17400)  MSTEDTLTGITRTKAGKTHRYHFDRHTPEYRERFTAITEEMHAKCPVAWTDTYDGHWVAA GSREVFELARCPHVSNDHDVTGEGTGYKGITIPTIPQATGVRGGMLEMDEPEHSAYRSIL NPYLSPAAIKRWKPFVDEIVRASIDEKIETGRIDFVDDLANVVPAVLTLALMGVPLKKWQ LYCEPAHANVYTPADSPDAPRVLEQTMAMGADLFNHLLEIREHPRPGIVDALARLRIDGE PAPDAELLGMLGLIIGGGFDTTTALTAHALEWLGENPEQRELLRRESDTLLASATEEFLR FFTPAPGDGRTIAADLEIAGTQFKEGDRLWLSWAMANRDPAVFPDPNRLDLARKNNRHFS FGLGIHRCIGSNVARVVFKSMLTAVLDRMPDYRCDPEGTVHYPTIGVIQGMQHLPATFTP GRRLGPGIDETLEKLQRVCDEQGIARPITEYAEAAVISD  >CYP268A(OCU_18140)  MAVMTGTPGAEKSGRAYDEIDLSSRAFWSTTAADRERSFAVLRAERPVSWHPPVEDSLMP DPTDPGFWAVTRRADIVAVSRNNDAFLSGQGVMFESIPVELLEASQSFLAMDPPRHTKLR KLAHAALSPRQVRRIEDSIKANAKTIVEELRTAGSGADFVDHCAKELPIRTLSDMMGIPE SERERMAHATDALVSWADPEFLNGRPAMEVLVENQLYLHQVVGTLATERRERPGDDLISS LVTAEVDGDRLEDAEVAAFFVLLSVAGNDTTRQTISHTLKALTDFPDEKAWLLHDFDNRI GTAVEEFIRWATPVMTFRRTAATDIELGGQTILAGEKVVMFYPSGNWDTDAFDHPERLNL ARDPNPHVGFGGGGLHFCLGAHVARAQLRAIFHELFRQLPGIQAGEPTYLAGNFVHAVRS MPCTF  >CYP187A(OCU_18970)  MTEPAVAGDIEQRIRDAQEKFNAGMGADGAATPYPLLRELRRKAAVHPGWPEMGVPENGP DGTKTFTAYSFDAVKAVFTDNITFSTRIYEDMVRPLQGPTILEMQEPEHATYRRLHEFAF ARSSMKRWDTELVGPLVDRTIAKFRDNKRADVVNAVFMPIPVRIIAALLGLPESDVGEFH RLAIDLLGFRADMETAMKASAQMKEYFVGVLADRRKSPKDDMVTILSRAEIDGVKMSDEQ IYGFMRNLLPAGAETTSRSTASLALGLLTHTDQLDALRADRSLLPQAIEEGIRWETPLLN FIREVTADIDFFGLHIPKGSTMMVNLGSANHDETRWEDAESFDIFRERKPHIGFGHGAHV CLGMHLARLESTKIFDALLDELPGLRLDPDAPPPYVTGTMFRSPSRLDVVWD  >CYP190A(OCU_19120)  MSATPSQIASENPVDFDPFSDDFFNGAFDTYRRLRDEAPVYYNAKWDFWALTRYDDVAPA TKDYETFSSAKGATLDMVQAHDDALPVPKVIISMDPPEHQKMRRLVSNVFTPRAIAALED MVREKVYERIHALDPSSFDVVADFSALFPNEVITTMLGVPKQDRDQIRLWLDLLLERRPG EIATTAEGFEASTKTGIYYYNLVQKRRAEPCDDMISRLIETEIERDGQVEKLTDVDITGF ATMLGGAGAETVTKLIGNAMVAFADFPDQWQKLQKDRSKIPAAIEELLRYEAPSQYQVRT ATRDVTLHGKTIPEGSAVLLVTGSATRDERMFPDPDRLDIDRERKMGFNLAFGYGIHSCL GAALARMESRIALGALLDLLPEYEVDRSGLRRVAMSNVCGWSNVPVARRAG  >CYP291A(OCU_19200)  MHSPDFYAGDPYPVYRELRNTTPVVWNDVTNFWALLKYEDVRYVSGHPLTFSSTKGITIP DPSQPEPVQEGNLIFTDPPRHRQLRKLINSGFSRRQVQLLEPKVRQIVKGIVDSVEPSRE YEFAEEIAAPLPTRMIAEMLGAPPEDWEQFRAWSDAAVGTADPDIELDSIVALGELYEYF TKLIAARRSGEVSGQDDLLSILAAAEVDGERLTDADLLNFSFLLLVAGNETTRNLIALGT LALIDHPDQFALLRSDPSLLPSAVEEMLRFTSPVTHMARRATEDVEIRGQQIRAGDTVVM LYGSANRDEEIFGPTCEEFDITRNPNPHIAFGAGEHACLGAQLARLEARVMFEVLLGAFP TIELTGDVTRLRATMVPGVKRMPIRLGAGN  >CYP189A(OCU_19650)  MTVPTAPTESDLYYDPYNIDLNMNPYPVFARIREEAPLYYNEQHDFYALSRYDDVNKAVI DHETFISGRGALLEIIKSGMEIPPGTLIFEDPPIHNIHRNLLSRVFTPRKVLALEPQIRE FTARCLDPLVGSGRFDFVNDLGEQMPMRVIGMLLGLPEDRQRQITDHGEETLQGKTVDAL ATGEVFAEFVDWRAEHPSDDIMTDLLNAEFEDETGTVRKLRRDELLMYLTVIATAGSETT TRLIGWAGKTLADYPDQRRDLVENPGLIPQAIEEILRWEPPALQIARYVTRDVEYYGQTV PEGSAMLMLVGAANRDHRRFPPDGDVFDIYREQRSHMTFGAGTHFCMGNALARLEGRIAL EEILKRFPAWEVDWANAVPSETAAVRGWAAMPTLVSRS  >CYP125D(OCU_21370)  MSIAKPTLVKSLVPQNLDTAADRDAAAVLDPDTFVTGAPYDAMTRLRATSPVHPVQLPGL PRSWLLTKHADVRLVSRDTDTFTSSKGNTLVEAEAGPNSAMLPGIDPPRHVHFRKLINQG FTVRNVQRLEPRMRLVTRDIVDTIIDKGEFDAVTDISAEMSLQVIADVLGVPAEDRMNVF RWSNAIGSLGIEDPDYAPTPEALGQAAAEMFAYCGELVEHRRKHGLTDDILSALLAAEVD GEKLNRDQLNEFFLLLAIAGNETTRNTLSHGILALAEHPEQQAQLARDPAAIKPAVEELL RWATPVMHFRRTVVRDVEIRGQRIPCGDWVLMHYLSANRDEEVFDRPDQFDVTRPDAGHA AFGGGGVHFCLGAQLARLELRVMLEELYANVPGLAVTGPPDRLRSSFFHGIKRLPCTT    >CYP140B(OCU_21520)  MHGVPRAYFAVQARRGDPLARLLRSGTADDRYALMEQIRARGPVVRSPYVWASVDHAVCR QILRDKRFGVTAPSEMELPRPLKALIAKTDPGVANPVEPPAMVIVNPPDHTRYRQLVAQS FTPRAIDSLDTRVAEVTMELIERIAATPPPDLIADFAMQLPVAIIAEILDLPPDSYPRML GWGRSGSPLLDIGIDWKTYRDAIDGLRGADDYLLEHLHRLRAGQKSDNPFGRMAADGSLT DRELTANAALIVGAGFETTVNLIGNGIVLLLQHPEQLALLHDNPDLWPSAVEEILRFASP VQMTARTPNCDVEIAGAHIASGDMVGLFLGGANRDPKVFIDPTTFDITRANARDHLAFAS GIHACLGAALARIEGTTALRALFESFPDLRLTAPPQPRRLINLHGYTRLPAQLGGRRTTS AKLPV  >CYP124A(OCU_21760)  MSLKTGPKKGLAAQTNGAPPPEIPLADIHLESLDFWALDDDVRDGTFATLRREAPISFWP AVEYEGFEPGNGHWALTKHDDVYFASRHPDIFSSSPNITINDNTPEISEYFGSMIVLDDP RHQRLRSIVSRAFTPKVVARIEASVRERARRLVASLIANHPDGEAELVGELAGPLPLQVI CDMMGIPEEDHQRIFHWTNVILGFGDPDLATDFGEFLQVSMDIGAYASALAEDRRSNHHD DLTTSLVEAEVDGERLTSAEIASFFILLVVAGNETTRNAISHGVLALSRYPAERDKWWSN FDRLTPTAVEEIVRWASPVIYMRRTLTRDFKLSGTKMKAGDKVTLWYNSANRDESTFDNP WLFDVARTPNPHFGFGGGGAHFCLGANLARREIRVVFDELRREIPDIVATDEPARLLSQF IHGIKTLPVAWTPPR  >CYP279A(OCU_24320)  MTVDTAAPSVFDAGLPTLHYDITDTVHQVAPRIHEARKRSPIALGPLGPEVLGYELARGI LRDPRFVFPPGMHMTARGITSGPLYDRVLGTILGMEGEEHRRLRGLVSRAFTPRASARME DTIDRVINELIDQVAGAGRCDFVADIARPYPIPIICALLGAPPEDWEQFSLWAEDIFKIV RFDSDLANEQHIVMRAWDEFDAYIDDMIAERRSRLTDDLISELIRAQDDGDRLSNAEMRM LAFSILSAGTDTTRNQLAACMHALCDHPEQLAMLRKNPNLAMPAVEECMRHSPAVCTTLR TVLDDVTFADYTFPAGTFISVNTFAANCDPEVYAHPGRFDITREDPPPILTFGGGAHYCL GANLARTELAGALKVLARRMTNPRRVATAQWKPMLGLSGPVGLEMEFD  >CYP140A(OCU_26420)  MKERLHWFAMHGFIRGAAAYGARRGDVQARLIADPAVAADPVRFYDEVRARGKLVKGRVA YLTADHALAHELLRSDDFRVVIFGSNLPAPLRWLERRTRDDLLHPLRAPSLLAVEPPDHT RYRKTVSAVFTPRAVAALRERVEQTAADLLERLTGESGVVDIVGRYCAQLPVSIISEILG VPESDRPRVLEFGELAAPSLDIGLPWRQYRSVQDGIAGFSSWLAEHLQRLRREPSDNVMS QLIQTAESGSAETYLNENELQAIAGLVLAAGFETTVNLLGNGIRTLLDAPEHLDALRRRP ELWPNAVEEILRLESPVQLTARMARGDVEIAGHQVARGDLVLVYLAAANRDPAVFSDPHR FDIERPNAGRHLAFSGGRHFCLGAALARAEGEVGLRTFFDRFPEVRAAGAGSRRETRVLR GWSSLPAALGPARSMAAAES  >CYP143A(OCU_27490)  MSDGQYGSFHLPRLDVDKLPMSADRGLGWKTLRDAGPVVFMNGHYYLTRREDVLAALRNP KVFSSTVLQPPGHPLPVLPLAFDPPQHTRYRKILQPYFSPHALSKSRPALERHARDMIGA LAGRGKCEVMADLASLYPFQVFLDLYGLPLEDRDRLIDWKDSVIADKPFLTQADMAKGQQ LLQYLVDAIAQRRQNPGSDMLSQVMTGGGDFSDIELLGMSHLLILAGLDTVTAAIGFSLF ELARRPQLRAELRDNPKQIRVFIEEIVRLEPSAPVAPRITTEFVEVGGMTLPPGTSVRLC MAAVNRDDSDPMSTNELNMDGKVHRHWGFGGGPHRCLGSHLARIELTVIVAEWLNQIPDF ELPADYSPVINYPSKSFALKELPLRWG  >CYP189A(OCU_28020)  MTASTDSHVRFDPYDVGLIADPYPMFARLRQEAPLYYNAEFDFYAVSRYADVSKALVDHE TYSSARGAILELIKANLEIPSGMLIFEDPPIHDVHRKLLSRMFTPRRIAALEPMIRDFCA QLLDPLVGSGHFDFVTDLGAQMPMKVISMLLGIPEDDQEYIRDRGNAQLRTEAGKPMDAA QHGLSVGEQFEAYIDWRAEHPSDDIMTELLNVEFVDETGVNRHLSREEILVYLNVVAGAG NETTTRLIGWSGKVLAEHPDQRRDLLENPALIPQAIEELLRYEPPAPHVSRYVTRDVTVH DQTVPEGSVMMMLIGSACRDEAQFGPDAGEFNIHRAVRPHLTFSMGTHFCLGSALARLEG RVALEEILKRFPEWEVDLSKATLSPTSTVRGWECMPALVSR  >CYP136A(OCU_37620)  MTATISTPHYLLDQARRRFTPTLNTIPGMGAIEKRLLAHEWDTKVLAEPPAGSDLKPVMG DAGLPILGHIIELFRGGPDYALYLYRNKGPLIYLDSPIMPAVTALGPDATQAVFSNRNKD FSQKGWHPVIGPFFNRGLMMLDFDEHMYHRRIMQEAFTRSRLTGYVEHIDRVATDIVAGW PTNDARFLFHPAMKELTLDIASLVFMGHEPGSDHDLVTKVNQAFTTTTRAGGAIIRQPVP PFKWWRGLRARQLLEDYFNERVKERRNATGNDMLTVLCHTEDDDGNSFTDEDIVNHMIFL MMAAHDTSTSTTTTMVYNMAANPEWQERAREESARLGDGPLDIESLEKLETLELIMNESL RMVTPLPFNMRMAVRDTELLGHYIPAGTNITIWPGMNHRLPELWTEPDKFDPERFAEPRS EHKNHRYAFAPFGGGAHKCIGMVFGQLEVKTVVHRLLRRYRIELARPGYQPRWDYGGMPI PMDGMPIVLRPL  >CYP108B(OCU_43390)  MAGAEAKTSAMSTPTVDPETAQAVKVFADPIAYADEPRLHAALAHLRAHAPVSLVDCPPY RPFWAITKHADVMEIERANDLFLNEPRPLLAPAEADDFARAQLDAGMGLRTLIHMDDPRH RVVRAIGADWFRPKAMRALKVRVDELAKSYVDKMMAAGGECDFVQEVAVNYPLYVILSLL GLPESDFPRMLRLTQELFGGDDEEFKRGTTPEEQMQILLDFFGYFSDLTAARRAHPTDDL ASAIANARVDGEPLSDVDTASYYVIIATAGHDTTSATIAGGLQALIENPDQRARLTADPA LMPLATEEMIRWVTPVKEFMRTAVADTAVRGVPIAAGESVYLSYVSANRDEDVFDDPFRF DVGRDPNKHLAFGYGVHFCLGAALARMEVNSFFTELLPRLESVECAGEPELVATTFVGGL KHLPIRYSLR  >CYP125A(OCU_44240)  MATPNLPPGFDFTDPDIYAHRLPVREFAELRATEPVWWNEQAPDKGGFGDGGYWAVTKHR DIRDVSLRSDVFSSAAKSIVPRYREDLAAGQIEAGRASMIMMDDPEHSRLRRIVSRAFTP RAVERLRAELSERARCIVTEAAAAGSGDFVRQVACELPLQAISALLGVPHEDYDKLFDWT NNMIGSDDPEFAGNDALTSAGELMWYAMQLAARKAEEPGDDIVTTLIQADADGQRLSEAE FGMFVVTLAVAGNETTRNSITQGMMAFTDYPVQWELFKARRPKTAADEIIRWATPITAFQ RTAREDTELGGVAIREGQRVVLFYRSANFDEEVFDDPFTFDILRSPNPHLGFGGTGAHYC IGANLARMTIDVMFNALADRLPDLAPLGNPERLRSSFINGIKHWPVDYRGGHPVAS  >CYP279A(OCU_44280)  MNEETVMTVGAAPPSVFDADLPTLSYRDDETPAEVYPRLREAQRHAPVALGPHGPEVLGY HMVRSVLRDTRFQIPPGLNLLVQGITSGPLWDKVVNSLLCLEGDAHHRLRSLTSKAFTPK ATLRLHDTMVGVLNELVDRVAGAGRCDVVTDIARPYPVPIICALLGAPREDWQRFSLWAD DVFKAFSFTADLTEVEPVVMRAWRQLDAYVDEMVAGRRRSLTDDLLSDLIRAEDEGDRLD AAELRMLAGGLLLAGTDTTRNQVAASVHLLCEHPEQWELLRRRPELAMRAVEETMRHSPI VCGTLRLVAEDAEVDGYVFPAGTMVLLNTGAANRDPTVYDDPDRVDITREGAPPILTFGG GAHYCLGANLARREIAEALTVLTARLRNPRIAGPAPWKPMGTLGGPLSLPLEFDR  >CYP191A(OCU_49410)  MDHTDFRYDPFDAAVMANPLPYYRILRDQHPVYYMPQWDTFALSRFEDIWKVLEVNNGTF VASEGTLPAASVLAQHNSGPVDDPPLHPLPFHAMFDADLYGEIRRTHSRPFRPRSVTDLE GRIRELANERLDLLLPRGSFDLTQDYGGVVVAAIVCELLGIPTDLAPQVLAAVNAGSLAQ PGVGVDTGQARPNYFEFLLPAVARRRADRSGPPLDVVDGLLGYHLPDGSALDDMEVATQM LCIFIGGTETVPKIVAHGLWELSRRPDQLAAVRADPEGNVPVAREEMIRYCAPAQWFART ARKPFDIHGQTINPGQRVITLLASASRDEREYPEPDEFIWDRPIRRSLAFGRGQHFCIGY HLARLEIDVLLAEWLRRVPDYAIQTHAATRLPSSFQWGWNNIPVEV  >CYP138A(OCU_49810)  MSELATVPPATVHLPPAVRSPKLVQGIGFAVSRRMMMRRLSRRYGNVFTLRLPMWGRVIM VSDPQLAKQIFTTSPDELGNIQPNLSRLFGPGSVFGLEGDDHRRRRRLLAPPFHGKSMKN YESIIEEETLREMAGWPEGSPFATLPPMMRITLNAILRAVFGAEGAELDELRRLIPPWVT LGSRLAALPKPKRYPRFGPWGRLDKWRRQYDIVIEKLIAAERADPDFAERTDVLALLLRS TYDDGSTMSHKDIGDELLALLAAGHETTASTLAWAFERISRHPELLARLVEEADGAGEGG NELRQATILEVQRARTVIDFAGRHVYPDAYRLGEWAIPRGYSIIVGIAQIHDNPDVFPDP RRFDPQRFTDTKPSALSWIPFGGGTRRCVGAAFANMEMDVVLRTVLRHLTIETTDAPGER WHCRGVAFTPKDGGRITVRRR |
| ***Mycobacterium intracellulare* MOTT-02** |
| DATABASE: KEGG; P450 count: 47; Families: 26; Subfamilies: 31 |
[truncated: 433,104 more chars]
