# Supplementary material for: Molecular evolutionary dynamics of cytochrome P450 monooxygenases across kingdoms: Special focus on mycobacterial P450s
Source: Sci Rep. 2016 Sep 12;6:33099. doi: 10.1038/srep33099 (PMC5018878; doi:10.1038/srep33099)
Supplement: Supplementary Dataset S1 [file srep33099-s2.doc]

**Molecular evolutionary dynamics of cytochrome P450 monooxygenases across kingdoms: Special focus on mycobacterial P450s**

Mohammad Parvez^1^, Lehlohonolo Benedict Qhanya^1^, Ntsane Trevor Mthakathi^1^, Ipeleng Kopano Rosinah Kgosiemang^1^, Hans Denis Bamal^1^, Nataraj Sekhar Pagadala^2^, Ting Xie^3^, Haoran Yang^4^, Hengye Chen^4^, Chrispian William Theron^5^, Richie Monyaki^1^, Seiso Caiphus Raselemane^1^, Vuyani Salewe^1^, Bogadi Lorato Mongale^1^, Retshedisitswe Godfrey Matowane^1^, Sara Mohamed Hasaan Abdalla^1^, Wool Isaac Booi^1^, Mari van Wyk^1^, Dedré Olivier^1^, Charlotte E Boucher^5^, David R Nelson^6^, Jack A Tuszynski^7^, Jonathan Michael Blackburn^8^, Jae-Hyuk Yu^9^, Samson Sitheni Mashele^1*^, Wanping Chen^4*^, Khajamohiddin Syed^1*^

^1^ Unit for Drug Discovery Research, Department of Health Sciences, Faculty of Health and Environmental Sciences, Central University of Technology, Bloemfontein 9300, Free State, South Africa

^2^ Department of Medical Microbiology and Immunology, 6-020 Katz Group Centre, University of Alberta, Edmonton, Alberta T6G 2E1, Canada

^3^ College of Informatics, Huazhong Agricultural University, Wuhan, Hubei Province, China

^4^ College of Food Science and Technology, Huazhong Agricultural University, Wuhan, Hubei Province, China

^5^ Faculty of Natural and Agricultural Science, Department of Microbial, Biochemical and Food Biotechnology, University of the Free State, P.O Box 339, Bloemfontein, 9300, South Africa.

^6^ Department of Microbiology, Immunology and Biochemistry, University of Tennessee Health Science Center, Memphis, TN 38163, USA

^7^ Department of Physics, University of Alberta, Edmonton, Alberta T6G 2J1, Canada

^8^ Institute of Infectious Disease & Molecular Medicine; Department of Integrative Biomedical Sciences, Faculty of Health Sciences, University of Cape Town, Cape Town 7925, South Africa

^9^ Department of Bacteriology, University of Wisconsin-Madison, 3155 MSB, 1550 Linden Drive, Madison WI 53706, USA

**Keywords**: Mycobacteria, P450, Evolution, Conservation, Divergence, Bacteria, Plant, Fungi, Animal, *Mycobacterium tuberculosis*, Diagnostic-markers, Evolutionary rates

* Corresponding authors’ email: khajamohiddinsyed@gmail.com & chenwanping@mail.hzau.edu.cn & smashele@cut.ac.za

**Supplementary Dataset 1.** P450 sequences of 113 P450 families used in this study.

**CYP1**

>BAD96828.1

MLFPISMSATEFLLASVIFCLVFWVIRASRPQVPKGLKNPPGPWDWPLIGHMLTLGKNPH

LALSRMSQQYGDVLQIRIGSTPVVVLSGLDTIRQALVRQGDDFKGRPDLYTFTLISNGQS

MSFSPDSGPVWAARRRLAQNGLKSFSIASDPASSTSCYLEEHVSKGAEVLISTLQELMAG

PGHFNPYRYVVVSVTNVICAICFGRRYDHNHQELLSLVNLNNNFGEVVGSGNPADFIPIL

RYLPNPSLNAFKDLNEKFYSFMQKMVKEHYKTFEKGHIRDITDSLIEHCQEKQLDENANV

QLSDEKIINIVLDLFGAGFDTVTTATSWSLMYLVMNPRVQRKIQEELDTVIGRSRRPRLS

DRSHLPYMEAFILETFRHSSFVPFTIPHSTTRDTSLKGFYIPKGRCVFVNQWQIDHDQKL

WVNPSEFLPERFLTPDGAIDKVLSEKVIIFGMGKRKCIGETIARWEVFLFLAILLQRVEF

SVPLGVKVDMTPIYGLTVKHACCEHFQMQLRS

>AAV38804.1

MGTSLSPNDPWPLNPLSIQQTTLLLLLSVLATVHVGQRLLRQRRRQLRSAPPGPFAWPLI

GNAAAVGQAAHLSFARLARRYGDVFQIRLGSCPIVVLNGERAIHQALVQQGSAFADRPAF

ASFRVVSGGRSMAFGHYSEHWKVQRRAAHSMMRNFFTRQPRSRQVLEGHVLSEARELVAL

LVRGSADGAFLDPRPLTVVAMANVMSAVCFGCRYSHDDPEFRELLSHNEEFGRTVGAGSL

VDVMPWLQYFPNPVRTVFREFEQLNRNFSSFILDKFLRHCESLRPGAAPRDMMDAFILSA

EKKAAGDSHGGGARLDLENVPATITDIFGASQDTLSTALQWLLLLFTRYPDVQTRVQAEL

DQVVGRDRLPCMGDQPNLPYVLAFLYEAMRFSSFVPVTIPHATTANTSVLGYHIPKDTVV

FVNQWSVNHDPVKWPNPENFDPARFLDKDGLINKDLTSRVMIFSVGKRRCIGEELSKMQL

FLFISILAHQCDFRANPNEPAKMNFSYGLTIKPKSFKVNVTLRESMELLDSAVQNLQAKE

TCQ

>AAR15082.1

MALWDSEFGVKGSSIIREWSGQVQPALVASFIFLFCLEACLWVRNLRHKRRLPGPFPWPV

VGNAMQLGQMPHITFSKLSKKYGNVYQIRLGCNDIVVLNGDTAIRQALIQHSTAFAGRPN

FLSFQMISGGKSMTFSNYSTQWKMHRRIAQSTIRAFSSANSQTKKAFEQHIVGEALDLVQ

VFLRHSADGRYFNPSHELTVAAANVICALCFGKRYGHEDLEFKTLLSRVDKFGETVGAGS

LVDVMPWLQSFPNPVRSIYQNFKELNEEFFAFVKDKVIQHRETYNPEVTRDMSDAIISVI

EHGKDNGLTKDFVEGTVTDLIGAGQDTVSTLLQWILLLLVKYPHIQAKLQEQIDKVVGRD

XLPCMEDKSSLAYLDAFVYETMRFTSFVPVTIPHSTTSDVTIEGVHIPRDTVVFINQWSV

NHDPQKWKDPHVFNPSRFLDENGVLDKDLTSSVMIFSIGKRRCIGDQIAKVEVFLFFAIL

LHQCSFESNPSQEISLDCSYGLTLKPLNHSISAKLRGKFLGLVSPA

>ACJ71248.1

MNLENISHTATSEVTLILCALALLLLALHGRRRAPGVPVPPGPRPWPIVGNFLQMEEQVH

LSLTNLRVQYGDVFQVKMGSLVVVVLSGYSTIKEALVRQGDAFAGRPDLYTFSAVANGTS

MTFSEKYGGAWVLHKKICKNALRTFSQTEPKDSNASCLLEERICVEAIDMVETLKTQGEE

FGDSGIDPVQLLVTSVANVVCTLCFGKRYSHNDKEFLTIVHINNEVLRLFAAGNLADFFP

IFRYLPSPSLRKMVEFINRMNNFMERNIMEHLVNFDTNCIRDITDALIAMCEDRQEDKES

AVLSNSQIVHSVIDIFGAGFDTIITGLQWSLLYLIKFPNIQDKIVQEIDNQVGMDRLPQF

KDRPNMPYTEAFINEVFRHASYMPFTIPHCTTENVTLNGYFIPKDTCVFINQYQVNHDIE

IWDDPESFRPERFLTLSGHLNKSLTEKVMIFGMGIRRCLGDNIARLEMFVFLTTLLHRLH

IENVPGQELDLSSTFGLTMKPRPYRIKIIPRN

>ABV54638.1

MMITAAILLDAGRSFAVPVAFTAVSVLTLYVCLRKRQGIPPGPTAWPLIGNLFSMGRQSH

LVLESMRKTYGDVFSVYFGSTLVVVVNGKAVEECLSTHSARYSMRPELHTAQYILEGKSF

AFSHIEVSEHKRYRTLAVAVVKQLVNGGGEKTDDAVKHGLQNGTRHTSIEDRIYMEATCM

CDKLLDTSDSPDLKDEILQLVTKELCRSAADMLLGNTLSEYELDEISRVVENLRNSNEAI

MLVNFIPVVRMLWRNGLRKYIQLTQSLNRFFERCIRNRKAQLATVSNGHTEDNGVLLTNG

VDCTVKFWQKLKNDPQYEESRVMKVVADLFGARVDTMTVALAWMIVYWATYQATQEKAQK

EIDHFVKNEKRLPRYSERNQLPYTMALITEVERHCSFVPFTLPHAPVQDTMLNGYLIPKG

TMMLISMRSINYDTALWDSPAQFRPERFLLDQSGGFNSALAEQVMLFGAGRRRCAGEALG

RMQIFLYSVLILRKCTFRRSDKDSHVLPESLAGISLIPQTMCVSISRREADGSKNTEP

>ABV54639.1

MDSLVFVLVDTVLVMKYEILLLLVILYVVKLFATSQSRRLNIPGPYPWPVIGNVIEMGGQ

PQFSLTNMAKKYGPVYLMKLGTADVLVLNNYEVIKEALLRQRRIFGGRPIFDSFKKISQG

RGVVFNSTTTQGDEWMKLKMTVVKHVHRFVSSEETKGYVAHHVQMEAVELVRILTEKCRS

SPNEVIVPIEQINLAIANVVCAIMFGHRYQHGNKEFQDLISLNEQFGDVIGSGSQVDVIP

WMKIFPKFRNALKVFDFLTNRLNDWMRLRTKEHRLTYKHGVIRDIVDSFIAESIDNPEQS

ALNDDVIMALTTDVFGAGQDTMSTTVQWVFVYMMHFKECQRKIHAELDSVIGPGELPHIS

DRRRLPYLEAVMHEIFRHSTFTSTTIPHVTTQDTVLDGHFIPKGILVFINQFGANHDPNH

WVDPDKFIPERFLDGKGNLISRPHDRYLLFSTGARKCPGDELSRMLVLHFMATMFALCEV

SSDPKKPATLDAVYNLSMRPKELRAIVRSRNMPLLKNSVAQMSEAESHVLTVPGETTSFL

LSHAERTDQENQESQFSDNDFEKVATKMPKRKAFSRSTLTHDDINGNDVRKRGNLHQSAM

YRIQ

>P04798.1

MLFPISMSATEFLLASVIFCLVFWVIRASRPQVPKGLKNPPGPWGWPLIGHMLTLGKNPH

LALSRMSQQYGDVLQIRIGSTPVVVLSGLDTIRQALVRQGDDFKGRPDLYTFTLISNGQS

MSFSPDSGPVWAARRRLAQNGLKSFSIASDPASSTSCYLEEHVSKEAEVLISTLQELMAG

PGHFNPYRYVVVSVTNVICAICFGRRYDHNHQELLSLVNLNNNFGEVVGSGNPADFIPIL

RYLPNPSLNAFKDLNEKFYSFMQKMVKEHYKTFEKGHIRDITDSLIEHCQEKQLDENANV

QLSDEKIINIVLDLFGAGFDTVTTAISWSLMYLVMNPRVQRKIQEELDTVIGRSRRPRLS

DRSHLPYMEAFILETFRHSSFVPFTIPHSTTRDTSLKGFYIPKGRCVFVNQWQINHDQKL

WVNPSEFLPERFLTPDGAIDKVLSEKVIIFGMGKRKCIGETIARWEVFLFLAILLQRVEF

SVPLGVKVDMTPIYGLTMKHACCEHFQMQLRS

>114658093

MLFPISMSATEFLLASVIFCLVFWVIRASRPRVPKGLKNPPGPWGWPLIGHMLTLGKNPH

LALSRMSQQYGDVLQIRIGSTPVVVLSGLDTIRQALVRQGDDFKGRPDLYTFTLISNGQS

MSFSPDSGPVWAARRRLAQNGLKSFSIASDPASSTSCYLEEHVSKEAEVLISTLQELMAG

PGHFNPYRYVVVSVTNVICAICFGRRYDHNHQELLSLVNLNNNFGEVVGSGNPADFIPIL

RYLPNPSLNAFKDLNEKFYSFMQKMVKEHYKTFEKGHIRDITDSLIEHCQEKQLDENANV

QLSDEKIINIVLDLFGAGFDTVTTAISWSLMYLVMNPRVQRKIQEELDTVIGRSRRPRLS

DRSHLPYMEAFILETFRHSSFVPFTIPHSTTRDTSLKGFYIPKGRCVFVNQWQINHDQKL

WVNPSEFLPERFLTPDGAIDKVLSEKVIIFGMGKRKCIGETIARWEVFLFLAILLQRVEF

SVPLGVKVDMTPIYGLTMKHACCEHFQMQLRS

>397479702

MLFPISMSATEFLLASVIFCLVFWVIRASRPRVPKGLKNPPGPWGWPLIGHMLTLGKNPH

LALSRMSQQYGDVLQIRIGSTPVVVLSGLDTIRQALVRQGDDFKGRPDLYTFTLISNGQS

MSFSPDSGPVWAARRRLAQNGLKSFSIASDPASSTSCYLEEHVSKEAEVLISTLQELMAG

PGHFNPYRYVVVSVTNVICAICFGRRYDHNHQELLSLVNLNNNFGEVVGSGNPADFIPIL

RYLPNPSLNAFKDLNEKFYSFMQKMVKEHYKTFEKGHIRDITDSLIEHCQEKQLDENANV

QLSDEKIINIVLDLFGAGFDTVTTAISWSLMYLVMNPRVQRKIQEELDTVIGRSRRPRLS

DRSHLPYMEAFILETFRHSSFVPFTIPHSTTRDTSLKGFYIPKGRCVFVNQWQINHDQKL

WVNPSEFLPERFLTPDGAIDKVLSEKVIIFGMGKRKCIGETIARWEVFLFLAILLQRVEF

SVPLGVKVDMTPIYGLTMKHACCEHFQMQLRS

>CAA26458.1

MLFPISMSATEFLLASVIFCLVFWVIRASRPQVPKGLKNPPGPWGWPLIGHMLTLGKNPH

LALSRMSQQYGDVLQIRIGSTPVVVLSGLDTIRQALVRQGDDFKGRPDLYTFTLISNGQS

MSFSPDSGPVWAARRRLAQNGLKSFSIASDPASSTSCYLEEHVSKEAEVLISTLQELMAG

PGHFNPYRYVVVSVTNVICAICFGRRYDHNHQELLSLVNLNNNFGEVVGSGNPADFIPIL

RYLPNPSLNAFKDLNEKFYSFMQKMVKEHYKTFEKGHIRDITDSLIEHCQEKQLDENANV

QLSDEKIINIVLDLFGAGFDTVTTAISWSLMYLVMNPRVQRKIQEELDTVIGRSRRPRLS

DRSHLPYMEAFILETFRHSSLVPFTIPHSTTRDTSLKGFYIPKGRCVFVNQWQINHDQKL

WVNPSEFLPERFLTPDGAIDKVLSEKVIIFGMGKRKCIGETVARWEVFLFLAILLQRVEF

SVPLGVKVDMTPIYGLTMKHACCEHFQMQLRS

>AAA52139.1

MLFPISMSATEFLLASVIFCLVFWVMRASRPQVPKGLKNPPGPWGWPLIGHMLTLGKNPH

LALSRMSQQYGDVLQIRIGSTPVVVLSGLDTIRQALVRQGDDFKGRPDLYTFTLISNGQS

MSFSPDSGPVWAARRRLAQNGLKSFSIASDPASSTSCYLEEHVSKEAEVLISTLQELMAG

PGHFNPYRYVVVSVTNVICAICFGRRYDHNHQELLSLVNLNNNFGEVVGSGNPAEFIPIL

RYLPNPSLNAFKDLNEKFYSFMQKMVKEHYKTFEKGHIRDITDSLIEHCQEKQLDENANV

QLSDEKIINIVLDLFGAGFDTVTTAISWSLMYLVMNPRVQRKIQEELDTVIGRSRRPRLS

DRSHLPYMEAFILETFRHSSFVPFTIPHSTTRDTSLKGFYIPKGRCVFVNQWQINHDQKL

WVNPSEFLPERFLTPDGAIDKVLSEKVIIFGMGKRKCIGETVARWEVFLFLAILLQRVEF

SVPLGVKVDMTPIYGLTMKHACCEHFQMQLRS

>AAD56646.1

MLFPISMSATEFLLASVIFCLVFWVIRASRPQVPKGLKNLLGPWGWPLIGHMLTLGKNPH

LALSRMSQQYGDVLQIRIGSTPVVVLSGLDTIRQALVRQGDDFKGRPDLYTFTLISNGQS

MSFSPDSGPVWAARRRLAQNGLKSFSIASDPASSTSCYLEEHVSKEAEVLISTLQELMAG

PGHFNPYRYVVVSVTNVICAICFGRRYDHNHQELLSLVNLNNNFGEVVGSGNLADFIPIL

RYLPNPSLNAFKDLNEKFYSFMQKMVKEHYKTFEKGHIRDITDSLIEHCQEKQLDENANV

QLSDEKIINIVLDLFGAGFDTVTTAISWSLMYLVMNPRVQRKIQEELDTVIGRSRRPRLS

DRSHLPYMEAFILETFRHSSFVLFTIPHSTTRDTSLKGFYIPKGRCVFVNQWQINHDQKL

WVNPSEFLPERFLTPDGAIDKVLSEKVIIFGMGKRKCIGETIARWEVFLFLAILLQRVEF

SVPLGVKVDMTPIYGLTMKHACCEHFQMQLRS

>AAF07051.1

MLFPISMSATEFLLASVIFCLVFWVIRASRPQVPKGLKNLLGPWGWPLIGHMLTLGKNPH

LALSRMSQQYGDVLQIRIGSTPVVVLSGLDTIRQALVRQGDDFKGRPDLYTFTLISNGQS

MSFSPDSGPVWAARRRLAQNGLKSFSIASDPASSTSCYLEEHVSKEAEVLISTLQELMAG

PGHFNPYRYVVVSVTNVICAICFGRRYDHNHQELLSLVNLNNNFGEVVGSGNLADFIPIL

RYLPNPSLNAFKDLNEKFYSFMQKMVKEHYKTFEKGHIRDITDSLIEHCQEKQLDENANV

QLSDEKIINIVLDLFGAGFDTVTTAISWSLMYLVMNPRVQRKIQEELDTVIGRSRRPRLS

DRSHLPYMEAFILETFRHSSFVLFTIPHSTTRDTSLKGFYIPKGRCVFVNQWQINHDQKL

WVNPSEFLPERFLTPDGAIDKVLSEKVIIFGMGKRKCIGETIARWEVFLFLAILLQRVEF

SVPLGVKVDMTPIYGLTMKHACCEHFQMQLRS

>332236145

MLFPISMSATEFLLASVIFCLVFWVIRALRPRVPKGLKNPPGPWGWPLIGHMLTLGKNPH

LALSRMSQQYGDVLQIRIGSTPVLVLSGLDTIQQALVRQGDDFKGRPDLYTFTLISNGQS

MSFSPDSGPVWAARRRLAQNGLKSFSIASDPASSSSCYLEEHVSKEAEVLISTLQELMAG

PGHFNPYRYVVVSVTNVICAICFGQRYDHNHQELLSLVNLSNNFAEVVGSGNPADFIPIL

RYLPNPSLNAFKDLNEKFYSFMQKMIKEHYKTFEKGHIRDITDSLIEHCQEKQLDENANI

QLSDEKIINIVLDLFGAGFDTVTTAISWSLSYLVMNPRVQRKIQEELDTVIGRSRRPRLS

DRSHLPYMEAFILETFRHSSFVPFTIPHSTTRDTSLKGFYIPKGRCVFVNQWQINHDQKL

WVNPSEFLPERFLTPDGAIDKVLSEKVIIFGMGKRKCIGETIARWEVFLFLAILLQRVEF

SVPLGVKVDMTPIYGLTMKHACCEHFQMQLRS

>402874884

MLFRISMSATEFLLASVIFCLVFWVIRASRPRVPKGLKNPPGPWGWPLIGHMLTLGKNPH

LALSRMSQRYGDVLQIRIGSTPVLVLSGLDTIRQALVQQGDDFKGRPNLYSFTLISNGQS

MSFGPDSGPVWAARRRLAQNGLKSFSIASDPASSSSCYLEEHVSKEAEVLISKLQEQMAG

PGHFNPYRYVVVSVANVICAICFGQRYDHNHQELLSLVNLSNNFGEVVGSGNPADFIPIL

RYLPNRSLNGFKDLNEKFHSFMQKMIKEHYKTFEKGHIRDITDSLIEHCQEKQLDENANI

QLSDEKIVNVVSDLFGAGFDTVTTAISWSLMYLVTNPRVQRKIQEELDTVIGRSRRPRLS

DRSHLPYMEAFILETFRHSSFVPFTIPHSTTRDTSLKGFYIPKGRCVFVNQWQINHDQKL

WVNPSEFLPERFITPDGAIDKVLSEKVILFGLGKRKCIGETIARWEVFLFLAILLQRVEF

SVPPGVKVDMTPIYGLTMKHACCEHFQMQLRS

>Q6GUR1.1

MLFRISMSATEFLLASLIFCLVFWVIRASRPRVPKGLKNPPGPWGWPLIGHILTLGKNPH

LALSRMSQRYGDVLQIRIGSTPVLVLSGLDTIRQALVQQGDDFKGRPNLYSFTLISNGQS

MSFGPDSGPVWAARRRLAQNGLKSFSIASDPASSSSCYLEEHVSKEAEVLISKLQEQMAG

PGHFNPYRYVVISVANVICAICFGQRYDHNHQELLSLVNLSNNFGEVVGSGNPADFIPIL

RYLPNRSLNGFKDLNEKFHSFMQKMIKEHYKTFEKGHIRDITDSLIEHCQEKQLDENANI

QLSDEKIVNVVLDLFGAGFDTVTTAISWSLMYLVTNPRVQRKIQEELDTVIGRSRRPRLS

DRSHLPYMEAFILETFRHSSFVPFTIPHSTTRDTSLKGFYIPKGRCVFVNQWQINHDQKL

WVNPSEFLPERFITPDGAIDKVLSEKVILFGLGKRKCIGETIARWEVFLFLAILLQRVEF

SVPPGVKVDMTPIYGLTMKHACCEHFQMQLRS

>EHH63218.1

MLFRISMSATEFLLASLIFCLVFWVIRASRPRVPKGLKNPPGPWGWPLIGHILTLGKNPH

LALSRMSQRYGDVLQIRIGSTPVLVLSGLDTIRQALVQQGDDFKGRPNLYSFTLISNGQS

MSFGPDSGPVWAARRRLAQNGLKSFSIASDPASSSSCYLEEHVSKEAEVLISKLQEQMAG

PGHFNPYRYVVISVANVICAICFGQRYDHNHQELLSLVNLSNNFGEVVGSGNPADFIPIL

RYLPNRSLNGFKDLNEKFHSFMQKMIKEHYKTFEKGHIRDITDSLIEHCQEKQLDENANI

QLSDEKIVNVVLDLFGAGFDTVTTAISWSLMYLVTNPRVQRKIQEELDTVIGRSRRPRLS

DRSHLPYMEAFILETFRHSSFVPFTIPHSTTRDTSLKGFYIPKGRCVFVNQWQINHDQKL

WVNPSEFLPERFITPDGAIDKVLSEKVILFGLGKRKCIGETIARWEVFLFLAILLQRVEF

SVPPGVKVDMTPIYGLTMKHACCEHFQMQLRS

>EHH27476.1

MLFRISMSATEFLLASLIFCLVFWVIRASRPRVPKGLKNPPGPWGWPLIGHILTLGKNPH

LALSRMSQRYGDVLQIRIGSTPVLVLSGLDTIRQALVQQGDDFKGRPNLYSFTLISNGQS

MSFGPDSGPVWAARRRLAQNGLKSFSIASDPASSSSCYLEEHVSKEAEVLISKLQEQMAG

PGHFNPYRYVVISVANVICAICFGQRYDHNHQELLSLVNLSNNFGEVVGSGNPADFIPIL

RYLPNRSLNGFKDLNEKFHSFMQKMIKEHYKTFEKGHIRDITDSLIEHCQEKQLDENANI

QLSDEKIVNVVLDLFGAGFDTVTTAISWSLMYLVTNPRIQRKIQEELDTVIGRSRRPRLS

DRSHLPYMEAFILETFRHSSFVPFTIPHSTTRDTSLKGFYIPKGRCVFVNQWQINHDQKL

WVNPSEFLPERFITPDGAIDKVLSEKVILFGLGKRKCIGETIARWEVFLFLAILLQRVEF

SVPPGVKVDMTPIYGLTMKHACCEHFQMQLRS

>BAA04500.1

MLFRISMSATEFLLASLIFCLVFWVIRASRPRVPKGLKNPPGPWGWPLIGHILTLGKNPH

LALSRMSQRYGDVLQIRIGSTPVLVLSGLDTIRQALVQQGDDFKGRPNLYSFTLISNGQS

MSFGPDSGPVWAARRRLAQNGLKSFSIASDPASSSSCYLEEHVSKEAEVLISKLQEQMAG

PGHFNPYRYVVISVANVICAICFGQRYDHNHQELLSLVNLSNNFGEVVGSGNPADFIPIL

RYLPNRSLNGFKDLNEKFHSFMQKMIKEHYKTFEKGHIRDITDSLIEHCQEKQLDENANI

QLSDEKIVNVVLDLFGAGFDTVTTAISWSLMYLVTNPRVQRKIQEELDTVIGRSRRPRLS

DRSHLPYMEAFILETFRHSSFVPFTIPHSTTRDTSLKGFYIPKGRCVFVNQWQINHDQKL

WVNPSEFLPERFITPDGAIDKVLSEKVILFGLGKRKCIGETIARWEVFLFLAILLQRVEF

SVPPGVKVDVTPIYGLTMKHACCEHFQMQLRS

>P33616.1

MLFRISMSATEFLLASLIFCLVFWVIRASRPRVPKGLKNPPGPWGWPLIGHILTLGKNPH

LALSRMSQRYGDVLQIRIGSTPVLVLSGLDTIRQALVQQGDDFKGRPNLYSFTLISNGQS

MSFGPDSGPVWAARRRLAQNGLKSFSIASDPASSSSCYLEEHVSKEAEVLISKLQEQMAG

PGHFNPYRYVVISVANVICAICFGQRYDHNHQELLSLVNLSNNFGEVVGSGNPADFIPIL

RYLPNRSLNGFKDLNEKFHSFMQKMIKEHYKTFEKGYIRDITDSLIEHCQEKQLDENANI

QLSDEKIVNVVLDLFGAGFDTVTTAISWSLMYLVTNPRVQRKIQEELDTVIGRSRRPRLS

DRSHLPYMEAFILETFRHSSFVPFTIPHSTTRDTSLKGFYIPKGRCVFVNQWQINHDQKL

WVNPSEFLPERFITPDGAIDKVLSEKVILFGLGKRKCIGETIARWEVFLFLAILLQRVEF

SVPPGVKVDMTPIYGLTMKHACCEHFQMQLRS

>395746969

MLFPISMSATEFLLASVIFCLVFWVIRTSRPRVPKGLKNPPGPMGGPLIGHMLTLGKNPH

LALSRMSQQYGECVQIRIGSTPVVVLSGVDTIRQALVRQGDDFKGRPDLYTFTLISNGQS

MSFSPDSGPVWAARRRLAQNGLKSFSIASDPASSSSCYLEEHVSKEAEVLISTLQELMAG

PGHFNPYTMVVVSVANVIXCPFALARRYDHNHQELLSVVNLNNNFGRWTGSGNPADFIPI

LRYLPNPSLNGFKDLNEKFYSFMQKMIKEHYKTFEKGHIRDITDSLIEHCQEKQLDENAN

IQLSDEKIINIVLDLFGAGFDTVTTAISWSLMYLVMNPRVQRKIQEELDTVIGRSRQPRL

SDRSHLPYMEAFILETFRHSSLLPFTIPHSTTRDTSLKGFYIPKGRCVFVNQWQINHDQK

LWVNPSEFLPERFLTPDGAIDKVLSEKVIIFGMGKRKCIGETIARWEVFLFLAILLQRVE

FSVPLGVKVDMTPIYGLTMKHACCEHFQMQLRS

>AAQ76692.1

MLFPISMSATEFLLASVIFCLVFWVIRASRPQVPKGLKNPPGPWGWPLIGHMLTLGKNPH

LALSRMSQQYGDVLQIRIGSTPVVVLSGLDTIRQALVRQGDDFKGRPDLYTFTLISNGQS

MSFSPDSGPVWAARRRLAQNGLKSFSIASDPASSTSCYLEEHVSKEAEVLISTLQELMAG

PGHFNPYRYVVVSVTNVICAICFGRRYDHNHQELLSLVNLNAFKDLNEKFYSFMQKMVKE

HYKTFEKGHIRDITDSLIEHCQEKQLDENANVQLSDEKIINIVLDLFGAGFDTVTTAISW

SLMYLVMNPRVQRKIQEELDTVIGRSRRPRLSDRSHLPYMEAFILETFRHSSFVPFTIPH

STTRDTSLKGFYIPKGRCVFVNQWQINHDQKLWVNPSEFLPERFLTPDGAIDKVLSEKVI

IFGMGKRKCIGETIARWEVFLFLAILLQRVEFSVPLGVKVDMTPIYGLTMKHACCEHFQM

QLRS

>296213692

MLFPISVSVTELLLASVIFCLVFWVIKASRPRVPKGLKSPPEPWRWPLLGHMLTLGKNPH

LALTKMSQRYGDVLQIHIGSTPVVVLSGLDTIRQALVRQGDDFKGRPDLYSFTLITGGQS

MSFSPDSGPVWAARRRLAQNSLKSFSIASDPASSSSCYLEKHVSKEAEALISKLQELMAG

PGRFNPYRYVMVSVANVICAICFGQLYDHNHQELLSIVNLSDNFAEVVGSGNPADFIPIL

RYLPNSSLNTFKDLNEKFYNFLQKTVKEHYKTFEKGHIRDITDSLIEHCQEKQLDENANI

QLSDEKIINVVLDLFGAGFDTVTTAISWSLMYLVTNPRVQRKIQEELDTVIGRERQPRLS

DRPHLPYMEAFILETFRHSSFVPFTIPHSTTRDTSLKGFYIPKGRCVFVNQWQINHDQKL

WVNPSEFLPERFLTRDGGINKVLSEKVILFGMGKRKCIGETIARWEVFLFLAILLHQVEF

SVPPGVKVDMTPIYGLTMKHTCCEHFQMQLRS

>403308781

MLFPISVSVTELLLASVIFCLVFWVIKASRPQVPKGLKSPPEPWGWPLLGHVLTLGKNPH

LALTRMSQRYGDVLQIRIGCTPVVVLSGLDTIRQALVRQGDDFKGRPDLYSFTLISGGQS

MSFSPDSGPVWAARRRLAQNGLKSFSIASDPASSSSYLEKHVSKEAETLISRLQELMAGP

GRFNPYRHVMVSVANVICAICFGQLYDHNHQELLSIVNLSNHFAEVAGSGNPADFIPILR

YLPNSSLNAFKDLNEKFYNFLQKMIKEHYKTFEKGRIRDITDSLIEHCQEKQLDENANIQ

LSDEKIINVVLDLFGAGFDTVTTAISWSLMYLVTNPRVQRKIQEELDTVIGRERRPRLSD

RFHLPYMEAFILETFRHSSFVPFTIPHSTTRDTSLKGFYIPKGRCVFVNQWQINHDQKLW

ITPSEFLPERFLTPDGGINKVLSEKVVLFGMGKRRCIGEAIARWEVFLFLAILLQQVEFS

VPSGVKVDMTPIYGLTMKHTCCEHFQMQLRS

>332236149

MLFPISMSATEFLLASVIFCLVFWVIRALRPRVPKGLKNPPGPWGWPLIGHMLTLGKNPH

LALSRMSQQYGDVLQIRIGSTPVLVLSGLDTIQQALVRQGDDFKGRPDLYTFTLISNGQS

MSFSPDSGPVWAARRRLAQNGLKSFSIASDPASSSSCYLEEHVSKEAEVLISTLQELMAG

PGHFNPYRYVVVSVTNVICAICFGQRYDHNHQELLSLVNLSNNFAEVVGSGNPADFIPIL

RYLPNPSLNAFKDLNEKFYSFMQKMIKEHYKTFEKGHIRDITDSLIEHCQEKQLDENANI

QLSDEKIINIVLDLFGAGFDTVTTAISWSLSYLVMNPRVQRKIQEELDTVIGRSRRPRLS

DRSHLPYMEAFILETFRHSSFVPFTIPHRKLWVNPSEFLPERFLTPDGAIDKVLSEKVII

FGMGKRKCIGETIARWEVFLFLAILLQRVEFSVPLGVKVDMTPIYGLTMKHACCEHFQMQ

LRS

>332844347

MLFPISMSATEFLLASVIFCLVFWVIRASRPRVPKGLKNPPGPWGWPLIGHMLTLGKNPH

LALSRMSQQYGDVLQIRIGSTPVVVLSGLDTIRQALVRQGDDFKGRPDLYTFTLISNGQS

MSFSPDSGPVWAARRRLAQNGLKSFSIASDPASSTSCYLEEHVSKEAEVLISTLQELMAG

PGHFNPYRYVVVSVTNVICAICFGRRYDHNHQELLSLVNLNNNFGEVVGSGNPADFIPIL

RYLPNPSLNAFKDLNEKFYSFMQKMVKEHYKTFEKGHIRDITDSLIEHCQEKQLDENANV

QLSDEKIINIVLDLFGAGFDTVTTAISWSLMYLVMNPRVQRKIQEELDTVIGRSRRPRLS

DRSHLPYMEAFILETFRHSSFVPFTIPHRKLWVNPSEFLPERFLTPDGAIDKVLSEKVII

FGMGKRKCIGETIARWEVFLFLAILLQRVEFSVPLGVKVDMTPIYGLTMKHACCEHFQMQ

LRS

>397479704

MLFPISMSATEFLLASVIFCLVFWVIRASRPRVPKGLKNPPGPWGWPLIGHMLTLGKNPH

LALSRMSQQYGDVLQIRIGSTPVVVLSGLDTIRQALVRQGDDFKGRPDLYTFTLISNGQS

MSFSPDSGPVWAARRRLAQNGLKSFSIASDPASSTSCYLEEHVSKEAEVLISTLQELMAG

PGHFNPYRYVVVSVTNVICAICFGRRYDHNHQELLSLVNLNNNFGEVVGSGNPADFIPIL

RYLPNPSLNAFKDLNEKFYSFMQKMVKEHYKTFEKGHIRDITDSLIEHCQEKQLDENANV

QLSDEKIINIVLDLFGAGFDTVTTAISWSLMYLVMNPRVQRKIQEELDTVIGRSRRPRLS

DRSHLPYMEAFILETFRHSSFVPFTIPHRKLWVNPSEFLPERFLTPDGAIDKVLSEKVII

FGMGKRKCIGETIARWEVFLFLAILLQRVEFSVPLGVKVDMTPIYGLTMKHACCEHFQMQ

LRS

>AAX62803.1

MLFPISMSATKFLLASVIFCLVFWVIRASRPQVPKGLENPPGPWGWPLIGHMLTLGKNPH

LALSRMSQQYGDVLQIRIGSTPVVVLSGLDTIRQALVRQGDDFKGRPDLYTFTLISNGQS

MSFSPDSGPVWAARRRLAQNGLKSFSIASDPASSTSCYLEEHVSKEAEVLISTLQELMAG

PGHFNPYRYVVVSVTNVICAICFGRRYDHNHQELLSLVNLNNNFGEVVGSGNPADFIPIL

RYLPNPSLNAFKDLNEKFYSFMQKMVKEHYKTFEKGHIRDITDSLIEHCQEKQLDENANV

QLSDEKIINIVLDLFGAGFDTVTTAISWSLMYLVMNPRVQRKIQEELDTVIGRSRRPRLS

DRSHPPYMEAFILETFRHSSFVPFTIPHKKLWVNPSEFLPERFLTPDGAIDKVLSEKVII

FGMGKRKCIGETIARWEVFLFLAILLQRVEFSVPLGVKVDMTPIYGLTMKHACCEHFQMQ

LRS

>402874886

MLFRISMSATEFLLASVIFCLVFWVIRASRPRVPKGLKNPPGPWGWPLIGHMLTLGKNPH

LALSRMSQRYGDVLQIRIGSTPVLVLSGLDTIRQALVQQGDDFKGRPNLYSFTLISNGQS

MSFGPDSGPVWAARRRLAQNGLKSFSIASDPASSSSCYLEEHVSKEAEVLISKLQEQMAG

PGHFNPYRYVVVSVANVICAICFGQRYDHNHQELLSLVNLSNNFGEVVGSGNPADFIPIL

RYLPNRSLNGFKDLNEKFHSFMQKMIKEHYKTFEKGHIRDITDSLIEHCQEKQLDENANI

QLSDEKIVNVVSDLFGAGFDTVTTAISWSLMYLVTNPRVQRKIQEELDTVIGRSRRPRLS

DRSHLPYMEAFILETFRHSSFVPFTIPHRKLWVNPSEFLPERFITPDGAIDKVLSEKVIL

FGLGKRKCIGETIARWEVFLFLAILLQRVEFSVPPGVKVDMTPIYGLTMKHACCEHFQMQ

LRS

>395822516

MLSVFGLLIPISATELLLASAIFCLVFWVVSASKSQVPKGLKSPPGPWGWPLIGHVLTLG

KNPHLALSKMSQQYGDVLQIRIGSTPVLVLSGLDTVRQALVRQGDDFKGRPDFYSFTLIT

EGQSMTFSPDSGPVWAARRRLAQNGLKTFSIASDPASSSSCYLEEHVSKEAEALISKFQE

LMAGAGHFDPYRYVVVSVANVICAICFGQRYDHNNQELLSIVNLNNEFGEVAASGNPADF

FPILRYLPNPALDAFKDLNKKFCSFMEKMVKEHYKTFEKGHIRDITDSLIEHCQDKKLDE

NANIQLSDEKIVNIVSDLFGAGFDTVTTAISWSLMYLVTNPKIQKKIQEELDTVIGRARR

PRLSDRLQLPYLEAFILETFRHSSFVPFTIPHCTTRNTSINGFYIPKGHCVFVNQWQINH

DQKLWGDPFEFRPTRFLTADGIINKALSEKVILFGLGKRKCIGETIARMEVFLFLAILLQ

QVEFSVLPGVKVDMTPIYGLSMKHAHCEHFQVQMRY

>344284460

MKFLPREGATVSGTVCEAASQACRAALGLRVLPQRTRGAPARPGSAGSAADLPSPHGPRG

TGPLRAPPRLPSPSRDCTGTRAGFGCWAALWALLTQPPGTAPPSSPFPCPAEGFSLTLSI

ATRREVAAQLPELPIPLPCQRASKPVRQKGAAKSGGSGGGSPRLQQASGGPRGGAEPLVS

AQRDCVSAMHSSPSIQEHELLSGARAPGAEAAPPPVPSTRFREGGVATSHLKVFELIMLS

VFELPISATGFLLASAIFCLMFWVIRAWKPQVPRGLKSPPGPWGWPLIGHVLTLGKNPHL

ALTRLSQQYGDVMQVRIGSTPVLVLSGLDTIRQALVRQGDDFKGRPDLYSFTLIMDGQSM

TFNPDSGPVWAARRRLAQNALKNFSMASDPASSSSCYLEEHVSKEAEYLISKFQELMAEV

GHFDPYRYVVVSVTNVICAVCFGQRYDHDHQELLGIIDLNNEFGEVVGSGNPADFIPILR

YLPNSTLNDFKELNEKFYSFTQKIVKEHYKTFEKNRIRDITDSLIKHCQDKKLDENANIQ

LPDKKIVNIVMDLFGAGFDTVTTAISWSLMYLVTNPSVQRKIQEELDTVIGRARRPRLSD

RSQLPYLEAFILETFRHSSFLPFTIPHSTTRDTSLSGFYIPKGRYVFVNQWQINHDQKLW

GDPSEFRPERFLTSDGAINRALSEKVILFGMGKRKCIGETMGRLEVFLFLAILLQQVEFS

VSPGVKVDMTPIYGLTMKHARCQHFQVKLRS

>296213694

MLFPISVSVTELLLASVIFCLVFWVIKASRPRVPKGLKSPPEPWRWPLLGHMLTLGKNPH

LALTKMSQRYGDVLQIHIGSTPVVVLSGLDTIRQALVRQGDDFKGRPDLYSFTLITGGQS

MSFSPDSGPVWAARRRLAQNSLKSFSIASDPASSSSCYLEKHVSKEAEALISKLQELMAG

PGRFNPYRYVMVSVANVICAICFGQLYDHNHQELLSIVNLSDNFAEVVGSGNPADFIPIL

RYLPNSSLNTFKDLNEKFYNFLQKTVKEHYKTFEKGHIRDITDSLIEHCQEKQLDENANI

QLSDEKIINVVLDLFGAGFDTVTTAISWSLMYLVTNPRVQRKIQEELDTVIGRERQPRLS

DRPHLPYMEAFILETFRHSSFVPFTIPHRKLWVNPSEFLPERFLTRDGGINKVLSEKVIL

FGMGKRKCIGETIARWEVFLFLAILLHQVEFSVPPGVKVDMTPIYGLTMKHTCCEHFQMQ

LRS

>345794793

MMSMFRLSIPISASELLLASTVFCLVLWVVKAWQPRLPKGLKSPPGPWGWPLLGNVLTLG

KSPHLALSRLSQRYGDVLQIRIGSTPVLVLSGLDTIRQALVRQGDDFKGRPDLYSFSLVT

DGQSLTFSPDSGPVWAARRRLAQNALKSFSIASDPASSCSCYLEEHVSKEAEVLLSRLQE

QMAEVGRFDPYRYIVVSVANVICAMCFSKRYDHDDQELLSLVNLSNEFGEGVASANPLDF

FPILRYLPNPALDFFKDLNKRFYSFMQKMVKEHYKTFEKGQIRDVTDSLIEHCQDKRLDE

NANIQLSDEKIVNVVLDLFGAGFDTVTTAISWSLLYLVTNPNVQKKIQKELDTVIGRARQ

PRLSDRPQLPYMEAFILETFRHASFVPFTIPHSTTRDTSLSGFYIPKGRCVFVNQWQINH

DQKLWGNPSEFQPERFLTLDGTINKALSEKVILFGLGKRKCIGETIARLEVFLFLAILLQ

QVEFSVPEGTKVDMTPIYGLTMKHARCEHFQVRVRTEGAESSAA

>BAB85660.1

MFSVFGLPIPISATELLLASAIFCLVFWVVRTWQPQVPKGLKSPPGPWGWPLLGHVLTLG

KSPHLALARLSQRYGDVLQIRIGCTPVLVLSGLDTIRQALVRQGDDFKGRPDLYSFTLIS

NGQSMTFNPDSGPVWAARRRLAQKALNTFSIASDPASSSSCYLEDHVSKEAECLLGKFQE

LMAGPGHFDPYKYVLVSVANVICAICFGQRYDHDNPELLSLINLSNEFGEVTAAGNPADF

IPILRYLPNTSLDLFKDLNQKFYIFMQKMVREHYKIFEKGRIRDITDSLIEHCQDKRMDE

NANIQLSDEKIVNIVIDLFGAGFDTVTTAISWSLMYLVTNPSIQRKIQEELDTVIGQARR

PRLSDRPQLPYMEAFILELFRHTSFVPFTIPHSTTRDTSLNGFYIPKGRCVFVNQWQINH

DQKLWDDPSVFRPERFLTADGTINKALGEKVILFGLGKRKCIGETIARLEVFLFLAILLQ

QVEFRVTPGVKVDMTPIYGLTMKHAHCEHVQMHVRS

>DAA17555.1

MFSVFGLPIPISATELLLASAVFCLVFWVVRTWRPRVPQGLKSPPEPWGWPLLGHMLMLG

KNPHVVLSQLSQRYGDVLQIRIGCTPVLVLSGLDTVRQALVRQGDDFKGRPDLYSFTLIT

NGQSMTFNPDSGPVWAARRRLAQNALKSFSTASDPASSSSCYLEEHVNKEAKYLLGKFQE

LMSGPGRFDPYRYIVVSVANVICAICFGRRYDHNDQEFLSLVNLSNEFGEITASGNPSDF

IPVLRYLPNTALDLFKDLNQRFYVFVQKIVKEHYKTFEKGHIRDITDSLIEHCQDKRLDE

NANIQLSDEKIINVVIDLFGAGFDTVTTALSWSLLYLVTSPRVQKKIQEELDTVIGRARR

PRLSDRPQLPYLEAFILETFRHSSFVPFTIPHSTTRDSNLNGFYIPKGRCVFVNQWQINH

DQKLWEDPSEFRPERFLTADGTINKVLSEKVIIFGLGKRKCIGETIARLEVFLFLAILLH

QVEFCVTPGVKVDMTPVYGLTMKYARCEHFQAHMRS

>403308783

MLFPISVSVTELLLASVIFCLVFWVIKASRPQVPKGLKSPPEPWGWPLLGHVLTLGKNPH

LALTRMSQRYGDVLQIRIGCTPVVVLSGLDTIRQALVRQGDDFKGRPDLYSFTLISGGQS

MSFSPDSGPVWAARRRLAQNGLKSFSIASDPASSSSYLEKHVSKEAETLISRLQELMAGP

GRFNPYRHVMVSVANVICAICFGQLYDHNHQELLSIVNLSNHFAEVAGSGNPADFIPILR

YLPNSSLNAFKDLNEKFYNFLQKMIKEHYKTFEKGRIRDITDSLIEHCQEKQLDENANIQ

LSDEKIINVVLDLFGAGFDTVTTAISWSLMYLVTNPRVQRKIQEELDTVIGRERRPRLSD

RFHLPYMEAFILETFRHSSFVPFTIPHRKLWITPSEFLPERFLTPDGGINKVLSEKVVLF

GMGKRRCIGEAIARWEVFLFLAILLQQVEFSVPSGVKVDMTPIYGLTMKHTCCEHFQMQL

RS

>P56590.1

MMSMFRLSIPISASELLLASTVFCLVLWVVKAWQPRLPKGLKSPPGPWGWPVLGNVLTLG

KSPHLALSRLSQRYGDVLQIRIGSTPVLVLSGLDTIRQALVRQGDDFKGRPDLYSFSLVT

DGQSLTFSPDSGPVWAARRRLAQNALKSFSIASDPASSCSCYLEEHVSKEAEVLLSRLQE

QMAEVGRFDPYRYIVVSVANVICAMCFSKRYDHDDQELLSLVNLSNEFGEGVASANPLDF

FPILRYLPNPALDFFKDLNKRFYSFMQKMVKEHYKTFEKGQIRDVTDSLIEHCQDKRLDE

NANIQLSDEKIVNVVLDLFGAGFDTVTTAISWSLLYLVTNPNVQKKIQKELDTVIGRARQ

PRLSDRPQLPYMEAFILETFRHASFVPFTIPHSTTRDTSLSGFYIPKGRCVFVNQWQINH

DQKLWGNPSEFQPERFLTLDGTINKALSEKVILFGLGKRKCIGETIARLEVFLFLAILLQ

QVEFSVPEGTKVDMTPIYGLTMKHARCEHFQVRVRTEGAESPAA

>AAV34440.1

MFSVFGLSIPISATELLLASATFCLVFWVVRAWQPRVPKGLKSPPGPWSWPLIGHMLTLG

KSPHLALSRLSQRYGDVLQIRIGCTPVLVLSGLDTIRQALVRQGDDFKGRPDLYSFTLVA

DGQSMTFNPDSGPVWAARRRLAQNALNSFSIASDPASSSSCYLEEHVSKEAKHLISKFQE

LMAESGRFDPYRYVVVSVANVICAMCFGRRYDHESQELLSILTLSNEFGEVTASGNPADF

IPILRYLPNTALDVFKDLNQRFYIFMQKMLKEHYKTFEKGHIRDITDSLIEHCQDKRLDE

NANIQVSDEKIVNVVMDLFGAGFDTVTTAISWSLMYLVTSPRVQKKIQEELDTVIGSARQ

PRLSDRPQLPYLEAFILETFRHSSFMPFTIPHSTTRDTSLNGFYIPKGRCVFVNQWQSNH

DQKLWDNPSAFWPERFLTAGGTINKALSEKVILFGLGKRKCIGETIARGEVFLFLAILLQ

QVEFRVTPGVKVDMTPIYGLTMKHAPCEHFQVHMRS

>Q3LFU0.1

MFSVFGLSIPISATELLLASATFCLVFWVVRAWQPRVPKGLKSPPGPWSWPLIGHVLTLG

KSPHLALSRLSQRYGDVLQIRIGCTPVLVLSGLDTIRQALVRQGDDFKGRPDLYSFTLVA

DGQSMTFNPDSGPVWAARRRLAQNALKSFSIASDPASSSSCYLEEHVSKESEYLIGKFQE

LMAGSGRFDPYRYVVVSVANVICAMCFGRRYDHESQVLLSVVGLSNEFGAVAASGNPADF

IPILRYLPNTALDDFKDLNRRFYIFMQKMLKEHYKTFEKGRIRDITDSLIEHCQGKRLDE

NANIQLSDEKIVNVVMDLFGAGFDTVTTAISWSLMYLVTSPSVQKKIQEELDTVIGSARQ

PRLSDRPRLPYLEAFILETFRHSSFLPFTIPHSTTRDTSLNGFYIPKGRCVFVNQWQINH

DQKLWDDPSAFWPERFLTADGTINKALSEKVILFGLGKRKCIGETIARWEVFLFLAILLQ

QVEFRVTPGVKVDMTPVYGLTMKHAHCEHFQAHMRS

>Q5KQT7.1

MMLSVFGLSVPISATELLLASFVFCLVFWVVRAWQPRVPKGLKSPPGPWGWPLLGHVLTL

GKNPHLVLARLSQHYGDVLQIRIGSTPVLVLSGLDTIRQALVRQGDDFKGRPNLYSFTLI

SEGQSMSFSPDSGPVWAARRRLAQNALKSFSIASDPASSSSCYLEDHVSKEAEYLIGKFQ

ELMAKVGHFDPYRYVVVSVANVICAMCFGRRYDHDDQELLSIVNLSNEFGDGTASGNPVD

FFPILRYLPNPALDFFKDVNEKFSIFIHKMVKEHYKTFEKGHIRDITDSLIEHCQDKRLD

ENANIQLSDEKIVNVVSDLFGAGFDTVTTAISWCLMYLVTSPNVQEKIQKELDTVIGRER

QPRLSDRLQLPYMEAFILEMFRHTSFVPFTIPHSTTKDTSLSGFYIPKERCVFVNQWQIN

HDQKLWGDPSEFRPERFLTPDGTINKALSEKVILFGLGKRKCIGETIARLEVFLFLAILL

QQVEFSVPQGTKVDMTPIYGLTMKHARCEHFQVRMRT

>ABS87659.1

MMSSVSRLSIPISATEFLLASAVFCLVLWVVRAWQPRVPKGLKSPPGPWGWPLLGNVLTL

GKTPHLALTRLSQRYGDVLQIHIGSTPVLVLSGLDTIRQALVRQGDDFKGRPDLYSFTLV

TDGQSMTFNPDSGPAWAARRRLAQNALKSFSTASDPASSCTCYLEEHVSKEAEALLGRLQ

QRMAEAGCFEPYRYVVVSVANVICAMCFGKRYDHDDQELLSLVNLSNEFGEAVASGSPMD

FFPILRYLPNPTLDSFKDLNKKFYNFMQKLVKEHYRTFEKGHIRDITDSLIEHCQEKRLD

ENANIQLSDEKIVNVVLDLFGAGFDTVTTAISWSLLYLVTNPNVQKKIQEELDTVIGRAR

QPRLSDRPQLPYMEAFILEIFRHASFVPFTIPHSTTRDTSLSGFYIPKGRCVFVNQWKIN

HDQKLWGDPSKFRPERFLNLDGTINKALSEKVILFGMGKRKCIGETIARLEVFLFLAILL

QQVEFSVPQGTRVDMTPIYGLTMKHARCEHFQVRVRT

>ABR10295.1

MMFSAFGLSIPISATEFLLASSVFCLVLWVVRAWQPRVPKGLKSPPGPWGWPLLGNVLTL

GKTPHLALTRLSQRYGDVLQIHIGSTPVLVLSGLDTIRQALVQQGNDFKGRPDLYSFTLV

TDGQSMSFSPDSGPVWAARRRLAQSALKSFSTASDPASSCTCYLEEHVSKEAEALLGRLQ

EQMAEAGRFEPYRYVVVSVANVICAMCFGKRYDHDDQELLSLVNLSNEFGEAVASGSPTD

FFPILRYLPNPALDSFKDLNKKFYSFMQKLVKEHYRTFEKGHIRDITDSLIEHCQEKRLD

ENANILLSDEKIVNVVLDLFGAGFDTVTTAISWSLLYLVTNPSVQKKIQEELDTVIGRAR

QPRLSDRPQLPYMEAFILEIFRHASFVPFTIPHCTTKDTSLSGFYIPKGRCVFVNQWQID

HDQKLWGDPSEFRPERFLTLDGTINKALSEKVILFGMGKRKCIGETIARLEVFLFLAILL

QQVEFSVPQGTRVDMTPIYGLTMKHARCEHVQVRVRA

>AFJ20719.1

MFSVFGLSIPISATELLLASAIFCLIFWVVRAWQSRVPKGLKSPPGPWGWPLLGHVLTLG

KSPHVALSRLSQRYGDVLQIRIGCTPVLVLSGLDTIRQALVRQGDDFKGRPDFYSFTLVS

DGQSMTFNPDSGPVWAARRRLAQNALKSFSIASDPASSSSCYLEEHVTKEAEYLLVKFQE

LMAGPGHFDPYRYVVVSVANVICAICFGHRYDHDNQELLSLVNLGNEFGEVAASGNPADF

IPILRYLPNTALDLYKDLKDLNQRIYIFMQKLVKEHYKMFEEGHIRDITDSLIKHCQDKR

LDENANIKLLDKQIVNVVLDLFGAGFDTVTTAISWSLMYLVTSPNVQKKVQEELDTVIGR

VRWPRLSDRLQLPYLEAFILETFRHSSFVPFTIPHSTTRDTSLNGFYIPKGRCVFVNQWQ

INHDQKLWDDPSEFRPERFLTPDGTINKALSEKVILFGMGKRKCIGETIARLEVFLFLAI

LLQQVEFSVTPGVKVDMTPIYGLTMKHARCEHFQVHMRS

>BAF58167.1

MFSASRLSIPISATELLLASAVFCLMLWVVRAWQPRVPKGLKSPPGPWGWPLLGNVLTLG

KNPHLALSRLSQRYGDVLQIHIGSTPVLVLSGLDTVRQALVRQGEDFKGRPDLYSFTLIT

NGQSMSFSPDSGPVWAARRRLAQNALKSFSIASDPGSSSSCYLEEHVSKEAEALLSRLQE

QMAEVGHFDPYRYVVVSVANVVCAMCFGKRYDHDDQELLSLINLNNEFGEAVASGNPVDF

FPILRYLPNPALDFFKDLNKRFYSFMQKLVKEHYKTFEKGHIRDITDSLIKHCQDKRLDE

NANIQLSDEKIVNVVLDLFGAGFDTVTTAISWSLLYLVTSPSVQKKIQEELDTVIGRARQ

PRLSDRPQLPYLEAFILETFRHASFVPFTIPHSTTKDTSLSGFYIPKGRCVFVNQWQINH

DQELWGDPSEFRPERFLTLDGTINKALSEKVILFGMGKRKCIGETIARLEVFLFLAILLQ

QVEFSVPRGTKVDMTPIYGLTMKHARCEHVQVRVRA

>BAB20377.2

MLMMFSASRLSIPISATELLLASAVFCLMLWVVRAWQPRVPKGLKSPPGPWGWPLLGNVL

TLGKNPHLALSRLSQRYGDVLQIHIGSTPVLVLSGLDTVRQALVRQGEDFKGRPDLYSFT

LITNGQSMSFSPDSGPVWAARRRLAQNALKSFSIASDPGSSSSCYLEEHVSKEAEALLSR

LQEQMAEVGHFDPYRYVVVSVANVVCAMCFGKRYDHDDQELLSLINLNNEFGEAVASGNP

VDFFPILRYLPNPALDFFKDLNKRFYSFMQKLVKEHYKTFEKGHIRDITDSLIKHCQDKR

LDENANIQLSDEKIVNVVLDLFGAGFDTVTTAISWSLLYLVTSPSVQKKIQEELDTVIGR

ARQPRLSDKPQLPYLEAFILETFRHASFVPFTIPHSTTKDTSLSGFYIPKGRCVFVNQWQ

INHDQELWGDPSEFRPERFLTLDGTINKALSEKVILFGMGKRKCIGETIARLEVFLFLAI

LLQQVEFSVPRGTKVDMTPIYGLTMKHARCEHVQVRVRA

>EGW02098.1

MSSMYGILNFMSATELLLAITVFCLGFWVVRATRTQVPKGLKSPPGPWGWPLLGHILTLG

KNPHLSLTKLSKQYGDILQIRIGSTPVVVLSGLDTIRQALVRQGDDFKGRPDFYSFNFIG

NGQSMTFNPESGPVWAARRRLAQNALKSFSIASDPASASSCYLEEHVSKEADYLISKFQK

LMAEVGHFDPYRYLVVSVTNVICAMCFGQRYDHDNQELLSIVNLSNEFGKVTGSGYPADF

IPVLRYLPNSSLDVFKDLNKKFYSFMQKLIKEHYRTFEKGHIRDITDSLIERCQDKRLDE

NANVQLSDDKVISIIFDLFGAGFDTVTTAISWSLMYLVTNPEVQRKIQEELDTVIGRNRR

PRLCDRSQLPYLEAFILETFRHSSFLPFTIPHSTTRDTNLCGFYIPKGRCVFVSQWQINH

DQELWGDPNKFRPERFLTSSGTLDKVLSEKVTLFGLGKRKCIGETIGRLEVFLFLAILLQ

QIEFTVSPGEKVDMTPIYGLTLKHARCEHFQVQMRSSGPLHV

>P56591.1

MWIMFSVFGLPIPISATELLLASAVFCLVFWVVRTWRPRVPQGLKSPPEPWGWPCLGHVL

TLGKNPHVVLSQLSQRYGDVLQIRIGCTPVLVLSGLDTIRQALVRQGDDFKGRPDLYSFT

LVSDGQSMTFNPDSGPVWAARRRLAQNALKSFSTASDPASLSSCYLEEHVSKEAEYLLGK

FQELMSGPGRFDPYRYVVVSVANVICAICFGRRYDHDDQEFLSLINLSNEFGEITASGNP

ADFIPVLRYLPNTALDLFKDLNRRFYVFVQKIVKEHYKTFEKGHIRDITDSLIEHCQDKR

LDENANIQLSDEKIINVVMDLFGAGFDTVTTAISWSLLYLVTSPRVQKKIQEELDTVIGR

ARWPQLSDRPQLPYLEAFILETFRHSSFVPFTIPHSTTRDTNLNGFYIPKGXCVFVNQWQ

INHDQKLWEDPSEFRPERFLTTDGTVNKVLSEKVIIFGLGKRQCIGEIIARLEVFLFLAI

LLHQVEFHVTPGVKVDMTPLYGLTMKHARCEHFQVRIRS

>AAA37506.1

MYGLPAFVSATELLLAVTVFCLGFWVVRATRTWVPKGLKTPPGPWGLPFIGHMLTVGKNP

HLSLTRLSQQYGDVLQIRIGSTPVVVLSGLNTIKQALVRQGDDFKGRPDLYSFTLITNGK

SMTFNPDSGPVWAARRRLAQNALKSFSIASDPTSASSCYLEEHVSKEANYLVSKLQKVMA

EVGHFDPYKYLVVSVANVICAICFGQRYDHDDQELLSIVNLSNEFGEVTGSGYPADFIPV

LRYLPNSSLDAFKDLNDKFYSFMKKLIKEHYRTFEKGHIRDITDSLIEHCQDRKLDENAN

VQLSDDKVITIVLDLFGAGFDTVTTAISWSLMYLVTNPRVQRKIQEELDTVIGRDRQPRL

SDRPQLPYLEAFILETFRHSSFVPFTIPHSTTRDTSLNGFYIPKGCCVFVNQWQVNHDRE

LWGDPNEFRPERFLTPSGTLDKRLSEKVTLFGLGKRKCIGETIGRSEVFLFLAILLQQIE

FKVSPGEKVDMTPTYGLTLKHARCEHFQVQMRSSGPQHLQA

>EHB07660.1

MELLLAATIFCLVLWVVRASRPQVPKGLKRPPGPWGWPLIGHALTLGKNPHLSLARLSER

YGDVMQIRIGSTPVVVLSGLDTIRQALVRQADDFKGRPDLYSFTLISDGQSMTFNPDSGP

VWAARRRLAQDALKSFSVASDPASSSSCYLEEHVRKEAEYLINKFQELMAGPGHFDPYRY

VVVSVTNVICAMCFGQRYDHDNQELLDLVDLNNEFGEVTASGNPADFIPVLQYLPNSAMD

NFKELTRKFSVFTQKMVKEHYKTFEEGHIRDITDSLIEHCEDRKLDENANIQLSDHKIIS

IVLDLFGAGFDTVTTAISWSLLYLVTNPRVQRKIQEELDTVIGRERRPRLTDRSQLPYLE

AFIMEVFRHSSFLPFTIPHSTIRDTSLSGFYIPKGRCVFVNQWQINHDEKLWSDPSVFRP

ERFLCLDGTVDKALSEKVIIFGLGKRRCIGETIGRWEVFLFLAILVQQLEFSISPGMKVD

MTPIYGLTMKHPRCKHFQAELRPSRP

>149692248

MFSVFGFSVPISATELLLTSAIFCLVFWLVRAWQPQIPKGLKSPPGPWGWPLLGHVLTLG

KNPHLALSRLSQRYGDVMQIRIGSTPVLVLSGLDTVRQALVRQGDDFKGRPDLHSFTLIS

DGQSMTFSPDSGPVWAARRRLAQNALKSFSIASDPASMSSCYLEEHVSKEAEYLIRKFQE

LMAGVGHFDPYKYVVMSVANVICAMCFGRRYDHDDEELLNLINLNNEFGEVAASGNPADF

IPILRYLPNSALDTFKDLNKKFYIFMQKMIKEHNKTFEKGHIRDITDSLIEHCQDKRLDE

NANIQLSDEKIINVVLDLFGAGFDTVTTAISWSLLYLVTRPSMQKKIQEELDTVIGRARQ

PRLSDRPQLPYMEAFILETFRHSSFVPFTIPHCTTRNTSLSGFYIPKGHCVFVNQWQINH

DQKLWGDPSEFRPERFLNPNGTINKALSEKVVLFGLGKRKCIGETIGRLEVFLFLAILLQ

QVEFSVPPGVKVDMTPIYGLSMKHARCEHFQVQLQFAVNTEDEETR

>P00185.1

MPSVYGFPAFTSATELLLAVTTFCLGFWVVRVTRTWVPKGLKSPPGPWGLPFIGHVLTLG

KNPHLSLTKLSQQYGDVLQIRIGSTPVVVLSGLNTIKQALVKQGDDFKGRPDLYSFTLIA

NGQSMTFNPDSGPLWAARRRLAQNALKSFSIASDPTLASSCYLEEHVSKEAEYLISKFQK

LMAEVGHFDPFKYLVVSVANVICAICFGRRYDHDDQELLSIVNLSNEFGEVTGSGYPADF

IPILRYLPNSSLDAFKDLNKKFYSFMKKLIKEHYRTFEKGHIRDITDSLIEHCQDRRLDE

NANVQLSDDKVITIVFDLFGAGFDTITTAISWSLMYLVTNPRIQRKIQEELDTVIGRDRQ

PRLSDRPQLPYLEAFILETFRHSSFVPFTIPHSTIRDTSLNGFYIPKGHCVFVNQWQVNH

DQELWGDPNEFRPERFLTSSGTLDKHLSEKVILFGLGKRKCIGETIGRLEVFLFLAILLQ

QMEFNVSPGEKVDMTPAYGLTLKHARCEHFQVQMRSSGPQHLQA

>CAA25153.1

MPSVYGFPAFTSATELLLAVTTFCLGFWVVRVTRTWVPKGLKSPPGPWGLPFMGHVLTLG

KNPHLSLTKLSQQYGDVLQIRIGSTPVVVLSGLNTIKQALVKQGDDFKGRPDLYSFTLIA

NGQSMTFNPDSGPLWAARRRLAQNALKSFSIASDPTLASSCYLEEHVSKEAEYLISKFQK

LMAEVGHFDPFKYLVVSVANVICAICFGRRYDHDDQELLSIVNLSNEFGEVTGSGYPADF

IPILRYLPNSSLDAFKDLNKKFYSFMKKLIKEHYRTFEKGHIRDITDSLIEHCQDRRLDE

NANVQLSDDKVITIVFDLFGAGFDTITTAISWSLMYLVTNPRIQRKIQEELDTVIGRDRQ

PRLSDRPQLPYLEAFILETFRHSSFVPFTIPHSTIRDTSLNGFYIPKGHCVFVNQWQVNH

DQELWGDPNEFRPERFLTSSGTLDKHLSEKVILFGLGKRKCIGETIGRLEVFLFLAILLQ

QMEFNVSPGEKVDMTPAYGLTLKHARCEHFQVQMRSSGPQHLQA

>AAA41025.1

MPSVYGFPAFTSATELLLAVTTFCLGFWVVRVTRTWVPKGLKSPPGPWGLPFIGHVLTLG

KNPHLSLTKLSQQYGDVLQIRIGSTPVVVLSGLNTIKQALVKQGDDFKGRPDLYSFTLIA

NGQSMTFNPDSGPLWAARRRLAQNALKSFSIASDPTLASSCYLEEHVSKEAEYLISKFQK

LMAEVGHFDPFKYLVVSVANVICAICFGRRYDHDDQELLSIVNLSNEFGEVTGSGYPADF

IPILRYLPNSSLDAFKDLNKKFYSFMKKLIKEHYRTFEKGHIRDITDSLIEHCQDRRLDE

NANVQLSDDKVITIVFDLFGAGFDTITTAISWSLMYLVTNPRIQRKIQEELDTVIGRDRQ

PRLSDRPQLPYLEAFILETFRHSSFVPFTIPHSTIRDTSLNGFYIPKGHCVFVNQWQVNH

DQELWGDPNEFRPERFLTSSGTLDKHLSEKVILFGLGKRKCIGETIGRLEVFLFLAILLQ

QMEFNVSPGEKVDSTPAYGLTLKHARCEHFQVQMRSSGPQHLQA

>Q06367.1

MSTSAMELLLTATIFCLVLWVVRIFRPQVPKGLKSPPGPWGWPLIGHMLTLGKNPHLALT

RLSARYGDVLQIRIGSTPVVVLSGLDTIRQALVRQGDDFKGRPDLYSFTFISDGQSMTFN

PDSGPVWAARRRLAQSALKSFSVASDPASVSSCYLEEHVKKEAEYLIKKFQELMAGPGHF

DPYRYVVVSVANVISAICFGQRYSHDDQQLLELIDLNNEFGEVTGSGNPSDFIPILRYLP

SATMDTFKDLNRRFSVFIQKMIKEHYKTFEKGHIRDITDSLIEHCQDRKLDKNANIQISD

QKIIGIVLDLFGAGFDTITTAISWSLLYLVMNPRIQKKIQEELDTVIGRERQPQLADRPK

LPYMEAFISEVFRYSSFMPFTIPHSTTKDTSLNGFYIPKGCCIFVNQWQINHDQKLWGDP

SVFRPERFLSPDGTVDKALSEKVTIFGLGKRRCLGEVIGRWEVFLFLAILLQQLEFSTSP

GVKIDMTPIYGLTMKYSRCEHFQAQTRPFVLKCPEA

>BAJ24835.1

MPSRYGLPVFMSATELLLAVTVFCLGFWVVRATRTRVPKGLKSPPGPWGLPFIGHMLTLG

KNPHLSLTKLSQQYGDVLQIRIGSTPVVVLSGLNTIKQALVRQGDDFKGRPDLYSFTLIA

NGKSLTFNPDSGPVWTARQRLAQNALKSFSIASDPTSASSCYLEEHVSKEANHLISKFQK

LMAEVGHFDPCKYLVVSVANVICAICFGQRYDHDDQELLSIINLSNEFGEVTGSGYPADF

IPVLRYLPNSSLDAFKDLNKKFYSFMEKLIKEHYRTFEKGHIRDITDSLIEHCQDRRLDE

NANVQMSEDKVIATVFDLFGAGFDTVTSAISWSLMYLVTNPRVQRKIQEELDTVIGRARQ

PRFSDRPQLPYLEAFILETFRHSSFLPFTIPHSTTRDTSLNDFYIPKGRCVFVNQWQVNH

DRELWGDPNEFRPERFLTPRGVLDKCLTEKVILFGLGKRKCIGETIGRLEVFLFLAILLQ

QIEFKVSPGKKVDMTPTYGLILKHPRCEHFQVQMRSSDSQHLQA

>Q00557.2

MSSIYGLLNFMSATELLLAITVFCLGFWVVRALRTQVPKGLKTPPGPWGLPILGHVLTLG

KNPHLSLTKLSKQYGDVLQIRIGSTPVVVLSGLDTIRQALVRQGDDFKGRPDFYSFTLIT

NGKSMTFNPDCGPVWAARRRLAQDALKSFSIALDPASASSCYLEEYVIKEADYLISKFQK

LMAEVGHFDPDRYLVVSVTNVICAMCFGQRYDHDDQELLSIVNLSNEFGKVTGSGYPPDF

IPILRYLPNSSLDAFKDLNKKFYSFMQKSVKEHYRTFEKGHIRDITDSLIEHCQDKSLDE

NANVQLSDDRVINIIVDLFGAGFDTVTTAISWSLMYLVTNPGVQRKIQEELDTVIGRSRR

PRLCDRSQLPYLEAFILETFRHSSFLPFTIPHSTTRDTSLCGFYIPKGHCVFVNQWQINH

NQELWGDPNKFRPERFLTSSGTLDKVLSGKVTLFGLGKRKCIGETIGRLEVFLFLAILLQ

QIEFTVSPGEKVDMTPIYGLTLKHARCEYFQAQTRSSGPQHPQA

>JS0746

MSSIYGLLNFMSATELLLAITVFCLGFWVVRALRTQVPKGLKTPPGPWGLPILGHVLTLG

KNPHLSLTKLSKQYGDVLQIRIGSTPVVVLSGLDTIRQALVRQGDDFKGRPDFYSFTLIT

NGKSMTFNPDCGPVWAARRRLAQDALKSFSIALDPASASSCYLEEYVIKEADYLISKFQK

LMAEVGHFDPDRYLVVSVTNVICAMCFGQRYDHDDQELLSIVNLSNEFGKVTGSGYPPDF

IPILRYLPNSSLDAFKDLNKKFYSFMQKSVKEHYRTFEKGHIRDITDSLIEHCQDKSLDE

NANVQLSDDRVINIIVDLFGAGFDTVTTAISWSLMYLVTNPGVQRKIQEELDTVIGRSRR

PRLCDRSQLPYLEAFILETFRHSSFLPFTIPHSTTRDTSLCGFYIPKGHCVFVNQWQINH

NQELWGDPNKFRPERFLTSSGTLDKVLSGKVTLFGLGKRKCIGETIGRLEVFLFLAILLQ

QIEFTVSPGEKVDMTPIYGLTLKHARCEYFQAQTRSSGPQHPQA

>BAA01096.1

MSSIYGLLNFMSATELLVAITVFCLGFWVVRALRTQVPKGLKTPPGPWGLPILGHVLTLG

KNPHLSLTKLSKQYGDVLQIRIGSTPVVVLSGLDTIRQALVRQGDDFKGRPDFYSFTLIT

NGKSMTFNPDCGPVWAARRRLAQDALKSFSIALDPASASSCYLEEYVIKEADYLISKFQK

LMAEVGHFDPDRYLVVSVTNVICAMCFGQRYDHDDQELLSIVNLSNEFGKVTGSGYPPDF

IPILRYLPNSSLDAFKDLNKKFYSFMQKSVKEHYRTFEKGHIRDITDSLIEHCQDKSLDE

NANVQLSDDRVINIIVDLFGAGFDTVTTAISWSLMYLVTNPGVQRKIQEELDTVIGRSRR

PRLCDRSQLPYLEAFILETFRHSSFLPFTIPHSTTRDTSLCGFYIPKGHCVFVNQWQINH

NQELWGDPNKFRPERFLTSSGTLDKVLSGKVTLFGLGKRKCIGETIGRLEVFLFLAILLQ

QIEFTVSPGEKVDMTPIYGLTLKHARCEYFQAQTRSSGPQYPQA

>CAA35039.1

MPSVYGFPAFTSATELLLAVTTFCLGFWVVRVTRTWVPKGLKSPPGPWGLPFIGHVLTLG

KNPHLSLTKLSQQYGDVLQIRIGSTPVVVLSGLNTIKQALVKQGDDFKGRPDLYSFTLIA

NGQSMTFNPDSGPLWAARRRLAQNALKSFSIASDPTLASSCYLEEHVSKEAEYLISKFQK

LMAEVGLFDPFKYLVVSVANVICAICFGRRYDHDDQDLLSIVNLSNEFGEVTGSGYPADF

IPIPRYLPNSSLDAFKDLNKKFYSFSKKLIKEHYRTFEKGHIRDITDSLIEHCQDRRLDE

NANVQLSDDKVITIVFDLFGAGFDTITTAISWSLSYLVTNPRIQRKIQEELDTVIGRDRQ

PRLSDRPQLPYLEAFILETFRHSSFVPFTIPHSTIRDTSLNGFYIPKGHCVFVNQWQVNH

DQELWGDPNEFRPERFLTSSGTLDKHLSEKVLSEKVILFGLGKRKCIGETIGQLEVFLFL

AILLQQMEFNVSPGEKVDSTPAYGLTLKHARCEHFQVQMRSSGPQHLQA

>P05176.1

MVSDFGLPTFISATELLLASAVFCLVFWVAGASKPRVPKGLKRLPGPWGWPLLGHVLTLG

KNPHVALARLSRRYGDVFQIRLGSTPVVVLSGLDTIKQALVRQGDDFKGRPDLYSFSFVT

KGQSMIFGSDSGPVWAARRRLAQNALNSFSVASDPASSSSCYLEEHVSQEAENLISKFQE

LMAAVGHFDPYRYVVMSVANVICAMCFGRRYDHDDQELLSLVNLNDEFGKVAASGSPADF

FLILRYLPNPALDTFKDLNERFYSFTQERVKEHCRSFEKGHIRDITDSLIKHYRVDRLDE

NANVQVSDEKTVGIVLDLFGAGFDTVTTAISWSLMYLVTKPRIQRKIQEELDAVVGRARR

PRFSDRPQLPYLEAVIMETFRHTSFLPFTIPHSTTRDTSLGGFYIPKGRCVFVNQWQNNH

DPELWGDPEAFRPERFLTPSGAVDKALTEKVLLFGLGKRKCIGETIGRLEVFLFLATLLQ

QVEFSVSPGTTVDMTPIYGLTMKHARCEHFQAKLRFEA

>1307202A

MVSDFGLPTFISATELLLASAVFCLVFWVAGASKPRVPKGLKRLPGPWGWPLLGHVLTLG

KNPHVALARLSRRYGDVFQIRLGSTPVVVLSGLDTIKQALVRQGDDFKGRPDLYSFSFVT

KGQSMIFGSDSGPVWAARRRLAQNALNSFSVASDPASSSSCYLEEHVSQEAENLISKFQE

LMAAVGHFDPYRYVVMSVANVICAMCFGRRYDHDDQELLSLVNLNDEFGKVAASGSPADF

FLILRYLPNPALDTFKDLNERFYSFTQERVKEHCRSFEKGHIRDITDSLIKHYRVDRLDE

NANVQVSDEKTVGIVLDLFGAGFDTVTTAISWSLMYLVTKPSIQRKIQEELDAVVGRARR

PRFSDRPQLPYLEAVIMETFRHTSFLPFTIPHSTTRDTSLGGFYIPKGRCVFVNQWQNNH

DPELWGDPEPFRPERFLTPSGAVDKALTEKVLLFGLGKRKCIGETIGRLEVFLFLATLLQ

QVEFSVSPGTTVDMTPIYGLTMKHARCEHFQAKLRFEA

>126272482

MMTSILSLLGFSKSFTVTELLVVSAVFCLVFWIIDSYHQRVPKGFKSPPGPWAWPLIGNV

LTLGKNPHLVLTQMREKYGDVMQIQIGSTPVLVLSGLETIRHALVKQGDDFKGRPDLYSF

SLILDGESLSFGPDSGEVWAARRKLTQNALKAFSISSSPSSSFCYLEEHVIKEAEYLIQK

FQEQKGHFDPVRYIVVSVANVICAICFGQRYDHDDQELLNIVRLSNKFGEVAASGNPVDF

IPILRYLPNSKITAFRDLNEKIVAFTQKLVKEHYRKFEKGCIRDITDSLIEHCQEKKLDE

NANIMLSEKKVVNVVIDLFGAGFDTVTTAISWGLMYLVAKPEVQKKIHEELDTVIGRERL

PQLSDKTQLPYMEAFILETFRHSSFLPFTIPHSTTRDITLNGFYIPKGRCVFVNQWQINH

DPKIWGDPSVFRPERFLSVDGTINKALSEKVIMFGLGKRKCIGETIARWEVFLFLSILLH

RMEFSVPSGVKVDLTPVYGLTMKHIPCEHFQTKLRS

>395501301

MITSVLSTFTSLSTFSVTELLLASAVFGLAFWIIESYSQKIPKGLKSPPGPWAWPLIGNI

LTLGKNPHLALAQLREKYGDVMQIQIGSTPVLVLSGLETIRQALIRQGDDFKGRPDLYSF

SLIEDGQSMSFGPDTGEAWAARRKMAQNALKTFSVSSSPSSSSCYLEEHVRKEAECLIQK

FQEKKGPFDPFRYVMVSVANVICAICFGKRYNHEDQELLSIITLSTKFGEVTASGNPADF

IPLLQYLPSSKMTDFRDLNEKITNFVQKLVNEHYKKYEKGCIRDITDSLIEHCQERKLDE

NANVLLSEKKIVNIVIDLFGAGFDTITTGISWSLMYLVAKPEIQKKVHEELDTVIGRARW

PKLSDKPQLPYLEAFILETFRHSSFLPFTIPHSTTRATTLNNFYIPKGRCVFVNQWQINH

DPKIWGDPSEFRPERFLSADGTINKSLSEKVIMFGLGKRKCIGETIARWEVFLFLAILLH

QMEFSVPSGVKVDLTPIYGLTMKHASCEHFQVKLRS

>402874888

MALSQSVPFSATELLLASAIFCLVFWVLRGSRPQVPKGLKSPPEPWGWPLLGHVLTLGKN

PHLALSRMSQLYGDVLQIRIGSTPVLVLSGLDTIRQALVRQGDDFKGRPDLYSFTFITDG

QSMSFSPDSGPVWAARRRLAQNALNTFSIASDPASSSSCYLEEHVSKEAEALSSRLQELM

AGPGHFDPYNQVVVSVANVIGAMCFGQHYPESSDEMLSLVKNSHEFVESASSGNPVDFFP

ILRYLPNPALQRFKAFNQRFRQFLQKTVQEHYQDFDKNSVQDITGALFKHSKKGPRASGN

LIPQEKIVNLVNDIFGAGFDTIATAISWSLMYLVTKPEIQRKIQKELDAVIGRGRRPRLS

DRLQLPYLEAFILETFRHSSFVPFTIPHSTTRDTTLNGFYIPRECCVFINQWQVNHDPQL

WGDPSEFRPERFLTAEGTTINKPLSEKIMLFGLGKRRCIGEVLGKWEVFLFLAILLQQLE

FSVPPGVKVDLTPIYGLTMKHARCEHFQARLRFSIK

>397479700

MALSQSVPFSATELLLASAIFCLVFWVLKGLRPRVPKGLKSPPEPWGWPLLGHVLTLGKN

PHLALSRMSQRYGDVLQIRIGSTPVLVLSRLDTIRQALVRQGDDFKGRPDLYTSTLITDG

QSMTFSTDSGPVWAARRRLAQNALNTFSIASDPASSSSCYLEEHVSKEAEALISRLQELM

AGPGHFDPYNQVVMSVANVIGAMCFGQHFPESSDEMLSLVKNTHEFVETASSGNPLDFFP

ILRYLPNPALQRFKAFNQRFLRFLQKTVQEHYQDFDKNSVRDITGALFKHSKKGPRASGG

DLIPQEKIVNLVNDIFGAGFDTVTTAISWSLMYLVTKPEIQRKIQKELDTVIGRERRPRL

SDRPQLPYLEAFILETFRHSSFLPFTIPHSTTRDTTLNGFYIPKKCCVFVNQWQVNHDPE

LWEDPSEFRPERFLTADGTAINKPLSEKMMLFGMGKRRCIGEVLAKWEVFLFLAILLQQL

EFSVPPGVKVDLTPIYGLTMKHARCEHVQARLRFSIN

>O77809.3

MALSQSVPFLATELLLASAIFCLVFWVLRGSRPRVPKGLKSPPEPWGWPLLGHVLTLGKN

PHLALSRMSQLYGDVLQIRIGSTPVLVLSGLDTIRQALVRQGDDFKGRPDLYSFTFITDG

QSMSFSPDSGPVWAARRRLAQNALNTFSIASDPASSSSCYLEEHVSKEAEALISRLQELM

AGPGHFDPYNQVVVSVANVIGAMCFGQHFPESSDEMLSLVKNSHEFVESASSGNPVDFFP

ILRYLPNPALQRFKAFNQRFRRFLQKTVQEHYQDFDKNSVQDITGALFKHSKKGPRASGN

LIPQEKIVNLVNDIFGAGFDTIATAISWSLMYLVTKPEIQRKIQKELDAVIGRGRRPRLS

DRPQLPYLEAFILETFRHSSFVPFTIPHSTTRDTTLNGFYIPRECCVFINQWQVNHDPQL

WGDPSEFRPERFLTAEGTTINKPLSEKIMLFGLGKRRCIGEVLGKWEVFLFLAILLQQLE

FSVPPGVKVDLTPIYGLTMKHARCEHFQARLRFSIK

>332236152

MALSQSVPFSATELLLASAIFCLVFWVLKGSRPRVPKGLKSPPEPWGWPLLGHVLTLRKN

PHLALSKMSQRYGDVLQIRIGSTPVLVLSGLDTIRQALVRQGDDFKGRPDLYSSTFITDG

HSMSFSPDSGPVWAARRRLAQNALNAFSIASDPASSSSCYLEEHVSKEAEALISRLQELM

AGPGHFDPYNQVVVSVANVIGAMCFGQHFPESSDEMLSLVKNTHEFVEVASSGNPVDFFP

ILRYLPNPALQRFKAFNQRFLRFLQKTVQEHYQDFDKNSVQDITGALFKYSKKGPRASGD

LIPQEKIVNLVNDIFGAGFDTVTTAISWSLMYLVTKPEIQRKIQKELDTVIGRGRRPRLS

DRPQLPYLEAFILETFRHSSFLPFTIPHSTTRDTTLNGFYIPKECCVFVNQWQVNHDPEL

WEDPSEFRPERFLTADGTAIYKPLSEKMMLFGMGKRRCIGEVLAKWEVFLFLAILLQQLE

FSVPPGVKVDLTPIYGLTMKHARCEHVQARLRFSIK

>Q4H4C3.3

MALSQFVPFSATELLLASAIFCLVFWVLRGSRPRVPKGLKSPPEPWGWPLLGHVLTLGKN

PHLALSRMSQLYGDVLQIRIGSTPVLVLSGLDTIRQALVRQGDDFKGRPDLYSFTFITDG

QSMSFSPDSGPVWAARRRLAQNALNTFSIASDPASSSSCYLEEHVSKEAEALISRLQELM

AGPGHFDPYNQVVVSVANVIGAMCFGQHFPESSDEMLSLVKNSHEFVESASSGNPVDFFP

ILRYLPNPALQRFKAFNQRFRRFLQKTVQEHYQDFDKNSVQDITGALFKHSKKGPRASGN

LIPQEKIVNLVNDIFGAEFDTIATAISWSLMYLVTKPEIQRKIQKELDAVIGRGRRPRLS

DRPQLPYLEAFILETFRHSSFVPFTIPHSTTRDTTLNGFYIPRECCVFINQWQVNHDPQL

WGDPSEFRPERFLTAEGTTINKPLSEKIMLFGLGKRRCIGEVLGKWEVFLFLAILLQQLE

FSVPPGVKVDLTPIYGLTMKHARCEHFQARLRFSFQ

>338717778

MMLSQLSPFSATELLLASTIFCLVFWVVRAWQPQIPKGLKSPPGPWGWPFLGHVLTLGKN

PHLALSRLSQRYGDVMQIRIGSTPVLVLSGLDTIRQALVRQGDDFKGRPDLYSFTLITNG

QSMTFNPDSGPVWAARRRLAQNALNTFSIASDPASMSSCYLEEHVSKEAEALLSRLQKLM

SVAGRFDPSSQVVASVANVIGAMCFGQHFPHSSEEMISLLRSSHEFVQTASSGNPVDFFP

ILRYLPNPPLQRFKSFNQRFLRFLQKIIQEHYRDFDKNSIQDITGALFKHREKSSRASGV

LIPQEKIINIINDIFGAGFDTVTTAITWSLTYLVTNPKIQRKIQEELDTVVGRARQPRLS

DRPQLPYMEAFILETFRHSSFVPFTIPHSTVRDTTLNGFYIPKERCVFINQWHVNHDEEL

WENPFEFRPERFLSADGTTINKTLSEKVMLFGMGKRRCIGEVLAKWEVFLFLAILLQRLE

FSVPPGVKLDLTPIYGLTMKHASCEHVQARLRFSIK

>AAK25728.1

MALSQSVPFSATELLLASAIFCLVFWVLKGLRPRVPKGLKSPPEPWGWPLLGHVLTLGKN

PHLALSRMSQRYGDVLQIRIGSTPVLVLSRLDTIRQALVRQGDDFKGRPDLYTSTLITDG

QSLTFSTDSGPVWAARRRLAQNALNTFSIASDPASSSSCYLEEHVSKEAKALISRLQELM

AGPGHFDPYNQVVVSVANVIGAMCFGQHFPESSDEMLSLVKNTHEFVETASSGNPLDFFP

ILRYLPNPALQRFKAFNQRFLWFLQKTVQEHYQDFDKNSVRDITGALFKHSKKGPRASGN

LIPQEKIVNLVNDIFGAGFDTVTTAISWSLMYLVTKPEIQRKIQKELDTVIGRERRPRLS

DRPQLPYLEAFILETFRHSSFLPFTIPHSTTRDTTLNGFYIPKKCCVFVNQWQVNHDPEL

WEDPSEFRPERFLTADGTAINKPLSEKMMLFGMGKRRCIGEVLAKWEIFLFLAILLQQLE

FSVPPGVKVDLTPIYGLTMKHARCEHVQARLRFSIN

>AAH67429.1

MALSQSVPFSATELLLASAIFCLVFWVLKGLRPRVPKGLKSPPEPWGWPLLGHVLTLGKN

PHLALSRMSQRYGDVLQIRIGSTPVLVLSRLDTIRQALVRQGDDFKGRPDLYTSTLITDG

QSLTFSTDSGPVWAARRRLAQNALNTFSIASDPASSSSCYLEEHVSKEAKALISRLQELM

AGPGHFDPYNQVVVSVANVIGAMCFGQHFPESSDEMLSLVKNTHEFVETASSGNPLDFFP

ILRYLPNPALQRFKAFNQRFLWFLQKTVQEHYQDFDKNSVRDITGALFKHSKKGPRASGN

LIPQEKIVNLVNDIFGAGFDTVTTAISWSLMYLVTKPEIQRKIQKELDTVIGRERRPRLS

DRPQLPYLEAFILETFRHSPFLPFTIPHSTTRDTTLNGFYIPKKCCVFVNQWQVNHDPEL

WEDPSEFRPERFLTADGTAINKPLSEKMMLFGMGKRRCIGEVLAKWEIFLFLAILLQQLE

FSVPPGVKVDLTPIYGLTMKHARCEHVQARLRFSIN

>AAH67427.1

MALSQSVPFSATELLLASAIFCLVFWVLKGLRPRVPKGLKSPPEPWGWPLLGHVLTLGKN

PHLALSRMSQRYGDVLQIRIDSTPVLVLSRLDTIRQALVRQGDDFKGRPDLYTSTLITDG

QSLTFSTDSGPVWAARRRLAQNALNTFSIASDPASSSSCYLEEHVSKEAKALISRLQELM

AGPGHFDPYNQVVVSVANVIGAMCFGQHFPESSDEMLSLVKNTHEFVETASSGNPLDFFP

ILRYLPNPALQRFKAFNQRFLWFLQKTVQEHYQDFDKNSVRDITGALFKHSKKGPRASGN

LIPQEKIVNLVNDIFGAGFDTVTTAISWSLMYLVTKPEIQRKIQKELDTVIGRERRPRLS

DRPQLPYLEAFILETFRHSSFLPFTIPHSTTRDTTLNGFYIPKKCCVFVNQWQVNHDPEL

WEDPSEFRPERFLTADGTAINKPLSEKMMLFGMGKRRCIGEVLAKWEIFLFLAILLQQLE

FSVPPGVKVDLTPIYGLTMKHARCEHVQARLRFSIN

>AAF13599.1

MALSQSVPFSATELLLASAIFCLVFWVLKGLRPRVPKGLKSPPEPWGWPLLGHVLTLGKN

PHLALSRMSQRYGDVLQIRIGSTPVLVLSRLDTIRQALVRQGDDFKGRPDLYTSTLITDG

QSLTFSTDSGPVWAARRRLAQNALNTFSIASDPASSSSCYLEEHVSKEAMALISRLQELM

AGPGHFDPYNQVVVSVANVIGAMCFGQHFPESSDEMLSLVKNTHEFVETASSGNPLDFFP

ILRYLPNPALQRFKAFNQRFLWFLQKTVQEHYQDFDKNSVRDITGALFKHSKKGPRASGN

LIPQEKIVNLVNDVFGAGFDTVTTAISWSLMYLVTKPEIQRKIQKELDTVIGRERRPRLS

DRPQLPYLEAFILETFRHSSFLPFTIPHSTTRDTTLNGFYIPKKCCVFVNQWQVNHDPEL

WEDPSEFRPERFLTADGTAINKPLSEKMMLFGMGKRRCIGEVLAKWEIFLFLAILLQQLE

FSVPPGVKVDLIPIYGLTMKHARCEHVQARLRFSIN

>P05177.3

MALSQSVPFSATELLLASAIFCLVFWVLKGLRPRVPKGLKSPPEPWGWPLLGHVLTLGKN

PHLALSRMSQRYGDVLQIRIGSTPVLVLSRLDTIRQALVRQGDDFKGRPDLYTSTLITDG

QSLTFSTDSGPVWAARRRLAQNALNTFSIASDPASSSSCYLEEHVSKEAKALISRLQELM

AGPGHFDPYNQVVVSVANVIGAMCFGQHFPESSDEMLSLVKNTHEFVETASSGNPLDFFP

ILRYLPNPALQRFKAFNQRFLWFLQKTVQEHYQDFDKNSVRDITGALFKHSKKGPRASGN

LIPQEKIVNLVNDIFGAGFDTVTTAISWSLMYLVTKPEIQRKIQKELDTVIGRERRPRLS

DRPQLPYLEAFILETFRHSSFLPFTIPHSTTRDTTLNGFYIPKKCCVFVNQWQVNHDPEL

WEDPSEFRPERFLTADGTAINKPLSEKMMLFGMGKRRCIGEVLAKWEIFLFLAILLQQLE

FSVPPGVKVDLTPIYGLTMKHARCEHVQARRFSIN

>AAA35738.1

MALSQSVPFSATELLLASAIFCLVFWVLKGLRPRVPKGLKSPPEPWGWPLLGHVLTLGKN

PHLALSRMSQRYGDVLQISIGSTPVLVLSRLDTIRQALVRQGDDFKGRPDLYTSTLITDG

QSLTFSTDSGPVWAARRRLAQNALNTFSIASDPASSSSCYLEEHVSKEAKALISRLQELM

AGPGHFDPYNQVVVSVANVIGAMCFGQHFPESSDEMLSLVKNTHEFVETASSGNPLDFFP

ILRYLPNPALQRFKAFNQRFLWFLQKTVQEHYQDFDKNSVRDITGALFKHSKKGPRASGN

LIPQEKIVNLVNDIFGAGFDTVTTAISWSLMYLVTKPEIQRKIQKELDTVIGRERRPRLS

DRPQLPYLEAFILETFRHSSFLPFTIPHSTTRDTTLNGFYIPKKCCVFVNQWQVNHDPEL

WEDPSEFRPERFLTADGTAINKPLSEKMMLFGMGKRRCIGEVLAKWEIFLFLAILLQQLE

FSVPPGVKVDLTPIYGLTMKHARCEHVQARLPFSIN

>301775212

MASSQMATELLLASAVFCLVLWVVRAWQPRVPKGLKSPPGPWGWPLLGNVLTLGKSPHLA

LSRLSQRYGDVLQIRIGSTPVLVLSGLDTIRQALVQRGDDFKGRPDLYSFTLVTDGQSMT

FNPDSGPVWAARRRLAQNALKSFSTASDPASASSCYLEEHVSKEAEALLSRLQERMAEVG

HFNPYNQVLLSVANVIGAMCFGQHFPQSSEEMLTLVKHSNDFVEIASSGNPVDFFPILQY

MPSPALQRFKAFNQKLVRFLQKIVQEHYRDFDEGSVQDITGALLKHSEKGSRASGGHIPH

EKIVNLINDIFGAGFDTVTTALSWSLMYLVTNPEIQRKIQKELDTVIGRVRRPQLSDRPQ

LPYMEAFILEIFRHTSFVPFTIPHSTTRDTTLKGFYIPKERCVFINQWQVNHDQKVWGDP

SEFRPERFLTVDGTAINKTLSEKMMIFGMGKRRCIGEVLAKWEIFLFLAILLQQLEFSVP

AGVKVDLTPIFGLTMKHTHCEHVQARPRFSTK

>395822518

MALSQYVPFSATELLLASAIFCLVFWVVSASKSQVPKGLKSPPGPWGWPLIGHVLTLGKN

PHLALSKMSQQYGDVLQIRIGSTPVLVLSGLDTVRQALVRQGDDFKGRPDFYSFTLITDG

QSMTFNPDSGPVWAARRRLAQNALNTFSIASNPASSSSCYLEEHVSKEAEALISKFQELM

AEAGRFDPYNQVVGSVANVIGAMCFGQHFTQDSEEMLSLVRNSHEFVETASSANPVDFFP

FLRYLPSPTLQRFKDFNQRFVRFLQKIVQEHYQDFDKNSVQDITGALFKHSEKGSGTSGH

LIAREKIVNLVNDLFGAGFDTVTTAISWSLMYLVTKPEIQKKIQKELDTVIGRARQPRLS

DRPQLPYMEAFILETFRHSSFVPFTIPHSTTRDTTLNGFFIPKECCVFVNQWQVNHDQEL

WGDPFEFRPERFLTDNDSALNKPLSEKVLLFGLGKRRCIGEVLAKWEVFLFLAILLQQLE

FSVPPGLKVDLTPIYGLTMKHPRCEHVRARLRFSIK

>ABQ53137.1

MAMSQTATELLLASSVFCLVLWVVRAWQPRVPKGLKSPPGPWGWPLLGNVLTLGKTPHLA

LTRLSQRYGDVLQIHIGSTPVLVLSGLDTIRQALVQQGDDFKGRPDLYSFTLVTDGQSMS

FSPDSGPVWAARRRLAQNVLKSFSIASDPASSCTCYLEEHVSKEAEALLGRLQQRMAEAG

RFDPYNEVMLSVTGVIGAMCFGRRFPQSSEDMLSLISSTNDFVEMASSGNPVDFFPILRY

LPNPSLQKFKAFNQKFFRFLQNIVREHYRDFDESNIQDITGALLKHNEKGSRTSAGHIPH

EKIVNIINDIFGAGFDTITTAISWSIMYLVTNPEIQRKIQEELDRVIGRARQPRLSDRLQ

LPYLEAFILEIFRHTSFVPFTIPHSTTRDTTLKGFYIPKERCVFVNQWQVNHDRKVWGDP

SEFRPERLLTADGTAINKILSEKVILFSMGKRRCIGEVMAKSEIFLFLAILLQRLEFSVP

ACVKVDLTPIYGLTMKHPHCEHVQARPRFSTK

>BAB20378.2

MALSQMATELLLASAVFCLVLWVVRAWQPRVPKGLKSPPGPWGWPLLGNVLTLGKNPHLA

LSRLSQRYGDVLQIHIGSTPVLVLSGLDTVRQALVRQGEDFKGRPDLYSFTLITDGQSMS

FSPDSGPVWAARRRLAQNALKSFSIASDPGSLSSCYLEEHVSKEAEALLSRLQEQMAEVG

HFDPYNQVLLSVANVIGAMCFGQHFPQSNEEMLSLIKSSNDFVETASSGNPVDFFPILQY

MPNPALQRFKAFNQKLVQFLQKIVQEHYQDFDESSIQDVTGALLKHNEKGSRAGGGHIPH

EKIVSLINDIFGAGFEPITTAISWSLIYLVTNPEIQRKIQEELDTVTGRARQPRLSDRPQ

LPYMEAFILEIFRHTSFVPFTIPHSTTRDTTLKGFYIPKERCVFINQWHVNHDQKVWGDP

FEFRPERFLTADGTSINKILSEKVMIFGMGKRRCIGELLAKWEIFLFLAILLQRLEFSVP

DGVKVDLTPIYGLTMKHTRCEHVQARPRFSTK

>227430405

MTLFQPSLFSATELLLASAIFCLVFWVVRTWQPQVPKGLKSPPGPWGWPLLGHVLTLGKS

PHLALARLSQCYGDVLQIRIGCTPVLVLSGLDTIRQALVRQGDDFKGRPNLYSFTLVTDG

QSMTFNPDSGPVWAARRRLAQKALNTFSIASDPASSSSCYLEDHVSKEAECLLGKFQELM

AGPGHFDPYDHIVVSVGRVIGAMCFGKGFPQSSEEMFSIVKNSHEFVETASSGNPVDFFP

ILRYLPSPTLQRFKSFNQRLLQFLRKMVQERYRDFDKNCIQDITSALFKHSEENSSTSGG

LISQEKTINLVNDIFGAGFDTITTAISWSLLYLVTYPEIQKKIQKELDTVIGRARRPRLS

DRPQLPYMEAFILELFRHTSFVPFTIPHSTTRDTTLNGFYIPKERHVLVNQWQVNHDPKL

WGDPSEFRPERFLTADGTAIHKTMSEKVILFGMGKRRCIGEVLAKWEVFLFLAILLQQLE

FSVPPGVKVDLTPIYGLTMKHAHCKHFQAHLRFPIK

>O77810.3

MALSQFVPFSATELLLTSTVFCLVFWVFKGLRPRVPKGLKSPPEPWRWPLLGHVLTLGKN

PHLALTKMSQRYGDVLQIHIGSTPVVVLSGLDTIRQALVRQGDDFKGRPDLYSFTLITDG

QSMSFSPDSGPVWAARRRLAQNALNTFSIASDPASSSSCYLEEHVSKEAEALIGRLQELM

AGPGRFDPYNQIVESVVKVIGAMCFGQHFPESSDEMLSLMKNSHVFVENATSGNPVDFFP

ILRYLPNPALQRFKAFNQRFLRFLRETVQEHYQDSDKNSVQDITGALFKHCEKRSGASGD

LIPQEKIVNLVNDIFGAGFDTITTAISWSLMYLVTKPEIQRKIQKELDTVIGRGRRPRLS

DRPQLPYLEAFILETFRHSSFVPFTIPHSTTRDTTLKGFYIPKECCVFINQWQVNHDPQL

WGDPSEFRPERFLTAKGTALNKPLSEKILLFGLGKRRCIGEVLGKWEVFLFLAILLQQLE

FSVPPGVQIDLTPTYGLTMKHARCEHVQARLRFSFQ

>345327710

MAVSLSVLGLVGSVSVTEILLASAIFFLAFWIIGSLSEKVPHGMKSPPGPWAWPLIGNIL

ELRKDPHLALTRMSQVYGDVMQVQIGTTPVLVLSGLETLRQALVKQGEDFMGRPDLYTFQ

HIGDGESLSFSTDSGEVWVARRKLAQNALKTFSLASSPSSSCTYLEEHVSKEAVSLIGKF

QELMKKRGSFDPYRYVMVSVANVICAMCFGKRYDHNDQELLSVVSLSDKFGEFAASGNPA

DFIPILRYLPNHALRNFKDLNQKMVAFLQKMLMEHYRTYEKNQVRDITDSLIEHCQDKKL

DENANVQLSDKKIVNVVLDLFGAGFDTITTSLSWSLMYLVKYPEVQKKIQEELDMVIGRG

RMPRLSDRSLLPYTEAFILESFRHSSFIPFTIPHSTTRATTLNGYYIPKGRCVFVNQWQI

NHDPELWEDPSAFRPERFLTQEGTKINKALSERVTMFGLGKRKCIGETIARWEVFLFLTI

LLQQIEFSVQSGGKLDMSRIYGLAMKHPRCEHFQVHPRF

>AAF07049.1

MALSQSVPFSATELLLASAIFCLVFWVLKGLRPRVPKGLKSPPEPWGWPLLGHVLTLGKN

PHLALSRMSQRYGDVLQIRIGSTPVLVLSRLDTIRQALVRQGDDFKGRPDLYISTLITDG

QSLTFSTDSGPVWAARRRLAQNALNTFSIASDPASSSSCYLEEHVSKEAKALISRLQELM

AGPGHFDPYNQVVVAVANVIGAMCFGQHFPESSDEMLSLVKNTHEFVETASSGNPLDFFP

ILRYLPNPALQRFKAFNQRFLWFLQKTVQEHYQDFDKNSVRDITGALFKHSKKGPRASGN

LIPQEKIVNLVNDIFGAGFDTVTTAISWSLMYLVTKPEIQRKIQKELDTVIGRERRPRLS

DRPQLPYLEAFILETFRHSSFLPFTIPHSTTRDTTLNGFYIPKKCCVFVNQWQVNHDPEL

WEDPSEFRPERFLTADGTAINKPLSEKMMLFGMGKRRCIGEVLAKWEIFLFLAILLQQLE

FSVPPGVKVDLTPIYGLTMKHARCEHVQARRFSIN

>AAF09457.1

MALSQSVPFSATELLLASAIFCLVFWVLKGLRPRVPKGLKSPPEPWGWPLLGHVLTLGKN

PHLALSRMSQRYGDVLQIRIGSTPVLVLSRLDTIRQALVRQGDDFKGRPDLYISTLITDG

QSLTFSTDSGPVWAARRRLAQNALNTFSIASDPASSSSCYLEEHVSKEAKALISRLQELM

AGPGHFDPYNQVVVAVANVIGAMCFGQHFPESSDEMLSLVKNTHEFVETASSGNPLDFFP

ILRYLPNPALQRFKAFNQRFLWFLQKTVQEHYQDFDKNSVRDITGALFKHSKKGPRASGN

LIPQEKIVNLVNDIFGAGFDTVTTAISWSLMYLVTKPEIQRKIQKELDTVIGRERRPRLS

DRPQLPYLEAFILETFRHSSFLPFTIPHSTTRDTTLNGFYIPKKCCVFVNQWQVNHDPEL

WEDPSEFRPERFLTADGTAINKPLSEKMMLFGMGKRRCIGEVLAKWEIFLFLAILLQQLE

FSVPPGVKVDLTPIYGLTMKHARCEHVQARRFSIN

>AAF09460.1

MALSQSVPFSATELLLASAIFCLVFWVLKGLRPRVPKGLKSPPEPWGWPLLGHVLTLGKN

PHLALSRMSQRYGDVLQIRIGSTPVLVLSRLDTIRQALVRQGDDFKGRPDLYISTLITDG

QSLTFSTDSGPVWAARRRLAQNALNTFSIASDPASSSSCYLEEHVSKEAKALISRLQELM

AGPGHFDPYNQVVVAVANVIGAMCFGQHFPESSDEMLSLVKNTHEFVETASSGNPLDFFP

ILRYLPNPALQRFKAFNQRFLWFLQKTVQEHYQDFDKNSVRDITGALFKHSKKGPRASGN

LIPQEKIVNLVNDIFGAGFDTVTTAISWSLMYLVTKPEIQRKIQKELDTVIGRERRPRLS

DRPQLPYLEAFILETFRHSSFLPFTIPHSTTRDTTLNGFYIPKKCCVFVNQWQVNHDPEL

WEDPSEFRPERFLTADGTAINKPLSEKMMLFGMGKRRCIGEVLAKWEIFLFLAILLQQLE

FSVPPGVKVDLTPIYGLTMKHARCEHVQARRFSIN

>Q5RBQ1.3

MALSQSVPFSATELLLASAIFCLVFWVLKGLRPRVPKGLKSPPEPWGWPLLGHVLTLRKN

PHLALSRMSQRYGDVLQIRIGSTPVLVLSGLDTIRQALVRQGDDFKGRPDLYSSTLITDG

QSLTFSPDSGPVWAARRHLAQNALNTFSIASDPASSYSCYLEEHVSKEAEALISRLQELM

AGPGHFDPYNQVVVSVVNVIGAMCFGQHFPESSDEMLSLVKNTHEFVETASSGNPVDFFP

ILRYLPNPALQRFKAFNQRFLRFLRKTVQEHYQDFDKNSVQDIMGALFKYSKKGPRASGN

LIPQEKIVNLVNDIFGAGFDTVTTAISWSLMYLVTKPEIQRKIQKELDTMIGRGRRPRLS

DRPQLPYLKAFILETFRHSSFLPFTIPHSTTRDTTLNGFYIPKECCVFVNQWQVNHDPEL

WEDPSEFWPERFLTTDGTAINKPLSEKVMLFGMGKRRCIGEVLANWEVFLFLAILLQQLE

FSVPPGVKVDLTPIYGLTMKHARCEHVQARLRFSIK

>BAF58168.1

MALSQMATELLLASAVFCLMLWVVRAWQPRVPKGLKSPPGPWGWPLLGNVLTLRKNPHLA

LSRLSQRYGDVLQIHIGSTPVLVLSGLDTVRQALVRQGEDFKGRPNLYSFTLITNGQSMS

FSPDSGPVWAARRRLAQNALESFSIASDPGSSSSCYLEEHVSKEAEALLSRLQEQMAEVG

QFDPYNQVLLSVANVIGAMCFGQHFPQSNEEMLSLIKSSNDFVETASSGNPVDFFPILQY

MPNPALQRFKAFNQKLVQFLQKIVQEHYQDFDESSIQDITGALLKHNEKGSRAGGGHIPH

EKIVSLINDIFGAGFEPITMAISWSLIYLVTNPEIQRKIQEELDTVTGRARQPRLSDRPQ

LPYMEAFILEVFRHTSFVPFTIPHSTTRDTTLKGFYIPKERCVFINQWHVNHDQKVWGDP

FEFRPERFLTADGTSINKILSEKVMIFGMGKRRCIGELLAKWEIFLFLAILLQRLEFSVP

DGVKVDLTPIYGLIMKHTRCEHVQARPRFSTK

>344284462

MVLSQSVPFSATEFLLAFVIFCLMFWVIRAWKPQVPKGLKSPPGPWGWPLIGHVLTLGRN

PHLALTRLSQQYGDVMQVRIGSTPVLVLSGLDTIQQALVQQGDDFKGRPDLYSSTLINDG

QSMTFSPDSGPVWAARRRLAQKALNTFSMASDPASLSSCYLEEHVINEAEALLHRLQELM

AGAGGFDPYSQVVVSVTNIIGAMCFGQHFPQHSEELLSLIENTHEFVETATSGNPMDFFP

FLRYLPSPALQKFKVFNQRFLQFVQKTVQGHYRDFDKNSVQDITGALFKHSEEGSRASGG

LMPQKKIINLVNDIFGAGIDTVTTAISWSLMYLMTKPGIQKKIQKELDTVIGKARKPRLS

DRPQLPYMEAFILEIFRHSSFVPFTIPHSTTRDTTLNGLYIPKGRCVFVNQWHVNHDQKL

WGDPFEFRPERFLTAEGSTINKTLSEKVMLFGMGKRRCIGEVLAKCEIFLFLAILLQQLE

FSLPPGIKVDLTPTYGLTMKHARCKHVQAQLRFSVK

>ABS87660.1

MAWSPIATELLLVSAVFCLVLWVARAWQPRVPKGLKRPPGPWGWPLLGNVLTLGKTPHLA

LTRLSHQYGDVLQIHIGSTPVLVLTGMDTIRQALVQQGDDFKGRPDLYNFTLVTDGQSMT

FNPDSGPLWAARRRLAQNALKSFSIASDPASLCTCYLEEHVSKEAEALLGRLQQRMAEAG

RFDPYNEVLFSVASVIGAMCFGKRFPQSSEEMLSLISSTNDFVETASSGNPVDFFPILRY

LPNPSLQKFKAFNQKFFRFLQNIVQEHYQDFDESNIQDITGALLKHNEKGSRTSAGHIPH

EKIVNIINDIFGAGFDTITTAISWSIMYLVTNPEIQRKIQEELDRVIGRARQPRLSDRLQ

LPYLEAFILELFRHTSFVPFTIPHSTTRDTILKGFYIPKERCVFVNQWQVNHDPKVWGDP

SKFRPERFLTADGTAINKTLSEKVILFGMGKRRCIGEVMAKSEIFLFLAILLQRLEFSVP

AGVKVDLTPIYGLTMKHPHCEHVQARPRFSTK

>P00187.3

MAMSPAAPLSVTELLLVSAVFCLVFWAVRASRPKVPKGLKRLPGPWGWPLLGHLLTLGKN

PHVALARLSRRYGDVFQIRLGSTPVVVLSGLDTIKQALVRQGDDFKGRPDLYSSSFITEG

QSMTFSPDSGPVWAARRRLAQDSLKSFSIASNPASSSSCYLEEHVSQEAENLIGRFQELM

AAVGRFDPYSQLVVSAARVIGAMCFGRRFPQGSEEMLDVVRNSSKFVETASSGSPVDFFP

ILRYLPNRPLQRFKDFNQRFLRFLQKTVREHYEDFDRNSIQDITGALFKHSEKNSKANSG

LIPQEKIVNLVNDIFGAGFDTITTALSWSLMYLVTNPRRQRKIQEELDAVVGRARQPRLS

DRPQLPYLEAFILELFRHTSFVPFTIPHSTTRDTTLNGFHIPKECCIFINQWQINHDPQL

WGDPEEFRPERFLTADGAAINKPLSEKVTLFGLGKRRCIGETLARWEVFLFLAILLQRLE

FSVPPGVPVDLTPIYGLTMKHPRCEHVQARPRFSDQ

>Q3LFT9.3

MALSQATPFSATELLLASATFCLVFWVVKAWQPRVPKGLKSPPGPWSWPLIGHVLTLGKS

PHLALSRLSQRYGDVLQIRIGCTPVLVLSGLDTIRQALVRQGDDFKGRPDLYSFTLVADG

QSMTFNPDSGPVWAAQRRLAQNALNSFSVASDPASSSSCYLEMHVSKEAEALIGKFQELM

AGSGRFDPYDHVVVSVAKVIGAMCFGQHFPQSSGEMVSLVRNTHDFVETASSGSPVDFFP

ILKYLPNPALQKYKSFNRRFLQFLWKMVQEHHQDFDKNRVQDIVGALFKHYEDNSRASGG

LMPQKKTVNLVNDIFAAGFDPITTAISWSLLYLVTNPEIQRKIQQELDTVIGRARRPRLS

DRSQLPYLEAFILETFRHSSFVPFTIPHSTIRDTTLNGFYIPKELCVFINQWQVNHDPKL

WGDPSEFRPERFLTSHDTTISKTLSEKVMLFGMGKRRCIGEVLAKWEIFLFLAILLQQLE

FSVPPGVKVDLTPTYGLTMKPAPCEHVQARLRFPIK

>DAA17508.1

MALSQLSPFSAMELLLASAIFCLVFWVVRTWRPRVPQGLKSPPEPWGWPLLGHMLTLGKN

PHVVLSQLSQRYGDVLQIRIGCTPVLVLSGLDTVRQALVRQGDDFKGRPDLYSFTLVTDG

QSMTFNPDSGPVWAARRRLAQNALNTFSVASDPSSSSSCYLEDHVSKEAEALLGKFQELM

SGPGRFDPYGHVVASVANVIGAMCFGQHFPQSSKEMLSLVESSHDFVESASSGNPVDFFP

ILKYLPNPALQRFKSFNQRFLQFVRKTVQEHYQDFDKNSIQDIIGALFKHSEDNSRASSR

LISQEKTVNLVNDLFAAGFDTITTAISWSLMYLVTNPKIQRKIQEELDRVVGRARRPRLS

DRPQLPYLESFILETFRHSSFVPFTIPHSTTRDTTLNGFFIPKERCVFINQWQVNHDPKL

WGDPSVFRPERFLTSDGTTIDKTASEKVLLFGMGKRRCIGEVMARWEVFLFLAILLQRLE

FSVPPGVKVDLTPTYGLTMKHARCEHMQARLRFPIK

>Q5KQT6.1

MAISPTATELLLASFAFCLVFWVVRAWQPRVPKGLKSPPGPWGWPLLGHVLTLGKNPHLV

LARLSQRYGDVLQIRIGSTPVLVLSGLDTIRQALVQQGDDFKGRPNLYSFSLVTDGHSMS

FSPDSGPVWAARRRLAQSALNTFSIASDPASSSSCYLEDHVSKEAEALLSRLQEQMAEVG

SFDPHSQVVLSVANVIGAMCFGQHFPQDSEEKLSLIHSSNIFVENAYSGNPVDFFPILQY

IPTPGLQRFKAFNQKLVQFLQKIIQEHYQDFDENNIQDITGALLKHCKKGSRANGGRIPH

EKIVSLINDIFGAGFDTVTTAISWSLMYLVTNPEKQRKIQEELDTVVGRARRPRLSDRLQ

LPYLEASILEIFRHSSFIPFTVPHSTTRDTTLNGFYIPEKHLVFINQWQVNHDQKVWGDP

FEFRPERFLTADGTAINKILSEKVMIFGLGKRRCIGEVLAKWEVFLFLAILLQQLEFSVP

AGVKVDLTPIYGLTMRHVRCEHVQARPRFSIK

>AAI46112.1

MALSQLSPFSAMELLLASAIFCLVFWVVRTWRPRVPQGLKSPPEPWGWPLLGHMLTLGKN

PHVVLSQLSQRYGDVLQIRIGCTPVLVLSGLDTVRQALVRQGDDFKGRPDLYSFTLVTDG

QSMTFNPDSGPVWAARRRLAQNALNTFSVASDPSSSSSCYLEDHVSKEAEALLGKFQELM

SGPGRFDPYGHVVASVANVIGAMCFGQHFPQSSKEMLSLVESSHDFVESASSGNPVDFFP

ILKYLPNPALQRFKSFNQRFLQFVRKTVQEHYQDFDKNSIQDIIGALFKHSEDNSRASSR

LISQEKTVNLVNDLFAAGFDTITTAISWSLMYLVTNPKIQRKIQEELERVVGRARRPRLS

DRPQLPYLESFILETFRHSSFVPFTIPHSTTRDTTLNGFFIPKERCVFINQWQVNHDPKL

WGDPSVFRPERFLTSDGTTIDKTASEKVLLFGMGKRRCIGEVMARWEVFLFLAILLQRLE

FSVPPGVKVDLTPTYGLTMKHARCEHMQARLRFPIK

>CAA29171.1

MAMSPAAPLSVTELLLVSAVFCLVFWAVRASRPKVPKGLKRLPGPWGWPLLGHLLTLGKN

PHVALARLSRRYGDVFQIRLGSTPVVVLSGLDTIKQALVRQGDDFKGRPDLYSSSFITEG

HSMTFSPDSGPVWAARRRLAQDSLKSFSIASNPASSSSCYLEEHVSQEAENLIGRFQELM

AAVGRFDPYSQLVVSAARVIGAMCFGRRFPQGSEEMLDVVRNSSKFVETASSGSPVDFFP

ILRYLPNRPLQRFKDFNQRFLRFLQKTVREHYEDFDRNSIQDITGALFKHSEKNSKANSG

LIPQEKIVNLVNDIFGAGFDTITTALSWSLMYLVTNPRRQRKIQEELDAVVGRARQPRLS

DRPQLPYLEAFILELFRHTSFVPFTIPHSTTRDTTLNGFHIPKECCIFINQWQINHDPQL

WGDPEEFRPERFLTADGAAINKPLSEKVTLFGLGKRRCIGETLARWEVFLFLAILLQRLE

FSVPPGVPVDLTPIYGLTMKHPRCEHVQARPRFSDQ

>2HI4A

MAVLKGLRPRVPKGLKSPPEPWGWPLLGHVLTLGKNPHLALSRMSQRYGDVLQIRIGSTP

VLVLSRLDTIRQALVRQGDDFKGRPDLYTSTLITDGQSLTFSTDSGPVWAARRRLAQNAL

NTFSIASDPASSSSCYLEEHVSKEAKALISRLQELMAGPGHFDPYNQVVVSVANVIGAMC

FGQHFPESSDEMLSLVKNTHEFVETASSGNPLDFFPILRYLPNPALQRFKAFNQRFLWFL

QKTVQEHYQDFDKNSVRDITGALFKHSKKGPRASGNLIPQEKIVNLVNDIFGAGFDTVTT

AISWSLMYLVTKPEIQRKIQKELDTVIGRERRPRLSDRPQLPYLEAFILETFRHSSFLPF

TIPHSTTRDTTLNGFYIPKKCCVFVNQWQVNHDPELWEDPSEFRPERFLTADGTAINKPL

SEKMMLFGMGKRRCIGEVLAKWEIFLFLAILLQQLEFSVPPGVKVDLTPIYGLTMKHARC

EHVQARRFSINHHHH

>CAA32066.1

MAMSPAAPLSVTELLLVSAVFCLVFWAVRASRPKVPKGLKRLPGPWGWPLLGHLLTLGKN

PHVALARLSRRYGDVFQIRLGSTPVVVLSGLDTIKQALVRQGDDFKGRPDLYSSSFITEG

QSMTFSPDSGPVWAARRRLAQDSLKSFSIASNPASSSSCYLEEHVSQEAENLISRFQELM

AAVGRFDPYSQLVVSAARVIGAMCFGRHFPQGSEEMLDVVRNSSKFVETASSSSPVDFFP

ILRYLPNRPLQRFKDFNQRFLRFLQKTVREHYEDFDRNSIQDITGALFKHSEKNSKANGG

LIPQEKIVNLVNDIFGAGFDTITTALSWSLMYLVTNPRRQRKIQEELDAVVGRPGQPRLS

DRPQLPYLEAFILELFRHTSFVPFTIPHSTTRDTTLNGFHIPKECCIFINQWQINHDPQL

WGDPEEFRPERFLTADGAAINKPLSEKVTLFGLGKRRCIGETLARWEVFLFLAILLQRLE

FSVPPGVPVDLTPIYGLTMKHPRCEHVQARPRFSDQ

>P56592.2

MALSQMATGLLLASTIFCLILWVVKAWQPRLPKGLKSPPGPWGWPLLGNVLTLGKSPHLA

LSRLSQRYGDVLQIRIGSTPVLVLSGLDTIRQALVRQGDDFKGRPDLYSLSLITDSQSMS

FSPDSGPVWAAGRRLAQNALNTFSIASDPASSCSCYLEEHVSKEAEALLSRLQEQMAEVG

RFDPYNQVLLSVANVIGAMCFGHHFSQRSEEMLPLLMSSSDFVETVSSGNPLDFFPILQY

MPNSALQRFKNFNQTFVQSLQKIVQEHYQDFDERSVQDITGALLKHNEKSSRASDGHIPQ

EKIVNLINDIFGAGFDTVTTAISWSLMYLVANPEIQRQIQKELDTVIGRARQPRLSDRPQ

LPLMEAFILEIFRHTSFVPFTIPHSTTKDTTLKGFYIPKECCVFINQWQVNHDQQVWGDP

FAFRPERFLTADGTTINKTLSEKVMLFGMGKRRCIGEVLAKWEIFLFLAILLQRLEFSVP

AGVKVDLTPIYGLTMKHTRCEHVQARPRFSIK

>AAA31437.1

MAMSPAAPLSVTELLLVSAVFCLVFWAVRASRPKVPKGLKRLPGPWGWPLLGHLLTLGKN

PHVALAPLSRRYGDVFQIRLGSTPVVVLSGLDTIKQALVRQGDDFKGRPDLYSSSFITEG

QSMTFSPDSGPVWAARRRLAQDSLKSFSIASNPASSSSCYLEEHVSQEAENLISRFQELM

AAVGRFDPYSQLVVSAARVIGAMCFGRHFPQGSEEMLDVVRNSSKFVETASSSSPVDFFP

ILRYLPNRPLQRFKDFNQRFLRFLQKTVREHYEDFDRNSIQDITGALFKHSEKNSKANGG

LIPQEKIVNLVNDIFGAGFDTITTALSWSLMYLVTNPRRQRKIQEELDAVVGRPGQPRLS

DRPQLPYLEAFILELFRHTSFVPFTIPHSTTRDTTLNGFHIPKECCIFINQWQINHDPQL

WGDPEEFRPERFLTADGAAINKPLSEKVTLFGLGKRRCIGETLARWEVFLFLAILLQRLE

FSVPPGVPVDLTPIYGLTMKHPRCEHVQARPRFSDQ

>BAE93470.1

MPAAMKAAMSLVESQGIVSATEVLLTAAVFCLVFLLIQSLQQHVPQGLKSPPGPRGYPIL

GNALELRKDTHLALTRLSQKYGDVMEVRIGTRPVLVLSGLDTIRQALVKQGEDFMGRPDL

HSFHHVADGQSLAFSPDSGEVWKARRKLAQNALKTFSVAPSPTSSSTCLLEEHVSKEADY

LVIKFLQLMDEGKSFDPYRYIVVSVANVICAMCFGKRYDHNDQELLDIVNVSDQFGEVAA

SGNPADFIPLLRYLPSRTMSLFKDFNKRFLHFLQKIVKEHYRTYDKNNIRDITDSLIEQC

LEKKVEANTAMQIPKEKIVNLVNDLFGAGFDTVATALSWSLMYLVTYPNIQKRIQEELDQ

TIGQERRPRLSDRGMLPYTEAFILEMFRHSSFLPFTIPHSTTRDTVLNGYYIPKDRCVFV

NQWQVNHDEKLWKDPLTFDPERFLNAEGTEVNKVDGEKVLLFGLGKRKCIGEPIARWQVF

LFLSTLLQQLEFSVCNGKKVDMTPLYGLTLKHKRCEHFQAKQRSPMKSTN

>AAA37509.1

MAFSQYISLAPELLLATAIFCLVFWMVRASRTQVPKGLKNPPGPWGLPFIGHMLTVGKNP

HLSLTRLSQQYGDVLQIRIGSTPVVVLSGLNTIKQALVRQGDDFKGRPDLYSFTLITNGK

SMTFNPDSGPVWAARRRLAQDALKSFSIASDPTSASSCYLEEHVSKEANHLVSKLQKAMA

EVGHFEPVSQVVESVANVIGAMCFGKNFPRKSEEMLNIVNNSKDFVENVTSGNAVDFFPV

LRYLPNPALKRFKTFNDNFVLFLQKTVQEHYQDFNKNSIQDITSALFKHSENYKDNGGLI

PEEKIVNIVNDIFGAGFDTVTTAITWSILLLVTWPNVQRKIHEELDTVVGRDRQPRLSDR

PQLPYLEAFILEIYRYTSFVPFTIPHSTTRDTSLNGFYIPKGCCVFVNQWQVNHDRELWG

DPFVFRPERFLTNNNSAIDKTQSEKVMLFGLGKRRCIGEIPAKWEVFLFLAILLQHLEFS

VPPGVKVDLTPNYGLTMKPGTCEHVQAWPRFSK

>P00186.1

MAFSQYISLAPELLLATAIFCLVFWMVRASRTQVPKGLKNPPGPWGLPFIGHMLTVGKNP

HLSLTRLSQQYGDVLQIRIGSTPVVVLSGLNTIKQALVRQGDDFKGRPDLYSFTLITNGK

SMTFNPDSGPVWAARRRLAQDALKSFSIASDPTSASSCYLEEHVSKEANHLVSKLQKAMA

EVGHFEPVSQVVESVANVIGAMCFGKNFPRKSEEMLNIVNNSKDFVENVTSGNAVDFFPV

LRYLPNPALKRFKTFNDNFVLFLQKTVQEHYQDFNKNSIQDITSALFKHSENYKDNGGLI

PEEKIVNIVNDIFGAGFDTVTTAITWSILLLVTWPNVQRKIHEELDTVVGRDRQPRLSDR

PQLPYLEAFILEIYRYTSFVPFTIPHSTTRDTSLNGFHIPKERCIYINQWQVNHDEKQWK

DPFVFRPERFLTNNNSAIDKTQSEKVMLFGLGKRRCIGEIPAKWEVFLFLAILLQHLEFS

VPPGVKVDLTPNYGLTMKPGTCEHVQAWPRFSK

>CAA27832.1

MAFSQYISLAPELLLATAIFCLVFWMVRASRTQVPKGLKNPPGPWGLPFIGHMLTVGKNP

HLSLTRLSQQYGDVLQIRIGSTPVVVLSGLNTIKQALVRQGDDFKGRPDLYSFTLITNGK

SMTFNPDSGPVWAARRRLAQDALKSFSIASDPTSASSCYLEEHVSKEANHLVSKLQKAMA

EVGHFEPVSQVVESVANVIGAMCFGKNFPRKSEEMLNIVNNSKDFVENVTSGNAVDFFPV

LRYLPNPALKRFKTFNDNFVLFLQKTVQEHYQDFNKNSIQDITSALFKHSENYKDNGGLI

PEEKIVNIVNDIFGAGFDTVTTAITWSILLLVTWPNVQRKIHEELDTVVGRDRQPRLSDR

PQLPYLEAFILEIYRYTSFVPFTMPHSTTRDTSLNGFHIPKERCIYINQWQVNHDEKQWK

DPFVFRPERFLTNNNSAIDKTQSEKVMLFGLGKRRCIGEIPAKWEVFLFLAILLQHLEFS

VPPGVKVDLTPNYGLTMKPGTCEHVQAWPRFSK

>AAB70866.1

MPLSWLLPFSAMELLLTATIFYLVLWVVKAFRLQVPKGLKSPPGPWGWPLIGHVLTLGKN

PHLALTRLSARYGDVLQIRIGSTPVVVLSGLDTIRQALVRQSDDFKGRPDLYSSTFISDG

QSMIFNPDSGPVWAARRRLAQSALQSFSLASDPASVSSCYLEEHVSREAEHLVTKLLDLM

AGPGCFEPSSQIVGSVANVIGAMCFGKNFPQTSEEMLQIVNTSKEFTEFASSGNPVDFFP

ILRYLPNPMLQQFKDFNKRFLQFLQKTVQEHYQDFDKNHVQDIASALFKHSEESPHVNGD

LIPRKKIVNLVNDIFGAGFDTVTTAISWSLLYLVTKPEIQKKIHKELDAVIGRDRKPRLA

DRPQLPYMEAFILEVFRYSSFLPFTIPHCTTRDTILNGFYIPKDRCVFINQWQVNHDPKQ

WEDPFEFRPERFLLANNTAVDKTLSDKILLFGLGKRRCIGETLGRWEVFLFLAILLQQLE

FSVPPGVKVDLTPVYGLTMKPPHCQHVQARPRFSK

>Q64391.1

MPLSWLLPFSAMELLLTATIFYLVLWVVKAFRLQVPKGLKSPPGPWGWPLIGHVLTLGKN

PHLALTRLSARYGDVLQIRIGSTPVVVLSGLDTIRQALVRQSDDFKGRPDLYSSTFISDG

QSMIFNPDSGPVWAARRRLAQSALQSFSVASDPASVSSCYLEEHVSREAEHLVTKLLDLM

AGPGCFEPSSQIVGSVANVIGAMCFGKNFPQTSEEMLQIVNTSKEFTEFASSGNPVDFFP

ILRYLPNPMLQQFKDFNKRFLQFLQKTVQEHYQDFDKNHVQDIASALFKHSEESPHVNGD

LIPRKKIVNLVNDIFGAGFDTVTTAISWSLLYLVTKPEIQKKIHKELDAVIGRDRKPRLA

DRPQLPYMEAFILEVFRYSSFLPFTIPHCTTRDTILNGFYIPKDRCVFINQWQVNHDPKQ

WEDPFEFRPERFLLANNTAVDKTLSDKILLFGLGKRRCIGETLGRWEVFLFLAILLQQLE

FSVPPGVKVDLTPVYGLTMKPPHCQHVQARPRFSK

>AAA41053.1

MAFSQYISLAPELLLATAIFCLVFWVLRGTRTQVPKGLKSPPGPWGLPFIGHMLTLGKNP

HLSLTKLSQQYGDVLQIRIGSTPVVVLSGLNTIKQALVKQGDDFKGRPDLYSFTLITNGK

SMTFNPDSGPVWAARRHLAQDALKSFSIASDPTSVSSCYLEEHVSKEANHLISKFQKLMA

EVGHFEPVNQVVESVANVIGAMCFGKNFPRKSEEMLNLVKSSKDFVENVTSGNAVDFFPV

LRYLPNPALKRFKNFNDNFVLFLQKTVQEHYQDFNKNSIQDITGALFKHSENYKDNGGLI

PQEKIVNIVNDIFGAGFETVTTAIFWSILLLVTEPKVQRKIHEELDTVIGRDRQPRLSDR

PQLPYLEAFILEIYRYTSFVPFTIPHSTTRDTSLNGFHIPKERCIFINQWQVNHDEKQWK

DPFVFRPERFLTNDNTAIDKTLSEKVMLFGLGKRRCIGEIPAKWEVFLFLAILLHQLEFT

VPPGVKVDLTPSYGLTMKPRTCEHVQAWPRFSK

>P04799.2

MAFSQYISLAPELLLATAIFCLVFWVLRGTRTQVPKGLKSPPGPWGLPFIGHMLTLGKNP

HLSLTKLSQQYGDVLQIRIGSTPVVVLSGLNTIKQALVKQGDDFKGRPDLYSFTLITNGK

SMTFNPDSGPVWAARRRLAQDALKSFSIASDPTSVSSCYLEEHVSKEANHLISKFQKLMA

EVGHFEPVNQVVESVANVIGAMCFGKNFPRKSEEMLNLVKSSKDFVENVTSGNAVDFFPV

LRYLPNPALKRFKNFNDNFVLFLQKTVQEHYQDFNKNSIQDITGALFKHSENYKDNGGLI

PQEKIVNIVNDIFGAGFETVTTAIFWSILLLVTEPKVQRKIHEELDTVIGRDRQPRLSDR

PQLPYLEAFILEIYRYTSFVPFTIPHSTTRDTSLNGFHIPKECCIFINQWQVNHDEKQWK

DPFVFRPERFLTNDNTAIDKTLSEKVMLFGLGKRRCIGEIPAKWEVFLFLAILLHQLEFT

VPPGVKVDLTPSYGLTMKPRTCEHVQAWPRFSK

>BAE93469.1

MKAAMSLVESQGIVSATEVLLAAAVFCLVFLLIQSLQQHVPQGLKSPPGPRGYPILGNVL

ELRKDTHLALTRLSQKYGDVMEVRIGTRPVLVLSGLDTIRQALVKQGEDFMGRPDLHSFQ

YISNGQSLAFSPDSGEVWKARRKLAQNALKTFSVAPSPTSSSTCLLEEHVSKEADYLVIK

FLQLMDEGKSFDLNRYIVVSVANVICAMCFGKRYDHNDQELLSLVNLNNEFGEVAASGNP

ADFIPLLRYLPSRTMQVFKDINRRFSFFVQKIVQEHFISFDKEHIRDITDSLIEHCQEKS

VGEDAHVPVSNEKIISIVNDLFGAGFDTVATALSWSLMYAALYPDIQKRIQEELDQTIGQ

ERRPRLSDRGMLPYTEAFILEMFRHSSFLPFTIPHSTTKATVLNGYYIPKDTCVFINQWQ

VNHDEKLWKDPSTFNPERFLNATGTEISRTESDKVMAFGLGKRRCIGESIGRWEVFLFLA

TMLQQLEFSLRPGEEVDITPQYGLTMKYKQCECFAIKRRFPMKSSP

>AAA41028.1

MAFSQYISLAPELLLATAIFCLVFWVLRGTRTQVPKGLKSPPGPWGLPFIGHMLTLGKNP

HLSLTKLSQQYGDVLQIRIGSTPVVVLSGLNTIKQALVKQGDDFKGRPDLYSFTLITNGK

SMTFNPDSGPVWAARRRLAQDALKSFSIASDPTSVSSCYLEEHVSKEANHLISKFQKLMA

EVGHFEPVNQVVESVANVIGAMCFGKNFPRKSEEMLNLVKSSKDFVENVTSGNAVDFFPV

LRYLPNPALKRFKNFNDNFVLSLQKTVQEHYQDFNKNSIQDITGALFKHSENYKDNGGLI

PQEKIVNIVNDIFGAGFETVTTAIFWSILLLVTEPKVQRKIHEELDTVIGRDRQPRLSDR

PQLPYLEAFILEIYRYTSFVPFTIPHSTTRDTSLNGFHIPKECCIFINQWQVNHDEKQWK

DPFVFRPERFLTNDNTAIDKTLSEKVMLFGLGKRRCIGEIPAKWEVFLFLAILLHQLEFT

VPPGVKVDLTPSYGLTMKPRTCEHVQAWPRFSK

>BAE75841.1

MLKAAMSLAAGSHGAVSASEVLLVAAAFCLVFLLLQRLQQQQQPVPKGLQRPPGPRGYPM

VGNVLELRKDTHLALTRLSRQYGDVMEVRIGTRPVLVLSGLDTIRQALVKQGEDFMGRPD

LYSFRFVTDGQSLTFSPDSGEVWKARRKLAQSALKSFSIAPSPTSSSTCLLEEHVSKEAE

YLVTKFLQLMEEEKRFDPHRYLVVSVANVICAMCFGKRYEHDDQELLSLVNSAQDFTDVA

AAGNPADFIPLLRYLPSCSMKLFIDFNRYLLSFLQKRVKEHYETYDENNIRDITDSLIQQ

CLDKKVETNAATQIPKKKIVNLVNDLFGAGFDTVTTALSWSLMYLVTDPSIQKRIQEELD

QTIGRERRPRLSDRGTLPYTEAFILEVFRHSSFVPFAIPHSTTQDTVLNGFYIPKGRCVF

INQWQVNHDEKLWKDPETFNPERFLSADGTKVNKEDGEKVLMFGLGKRKCIGETIARWQV

FLFLATLLQQLEFSVCEGTEVDMTPLYGLALKHKRCEHFQVRQRFPVKSRS

>P24453.4

MALSQYTSLSTELVLATAIFCIVFWVARALRTQVPKGLKTPPGPWGLPILGHVLTLGKNP

HLSLTKLSKQYGDVLQIRIGSTPVVVLSGLDTIRQALVRQGDDFKGRPDLYSFTLITNGK

SMTFNPDCGPVWAARRRLAQDALKSFSIASDPTSASSCYLEDHVIKEANHLVSKLQKLTA

EVGHFEPVNQVVESVANVIGAMCFGKNFPRKSEEMLRIVKGSSDFVENVSSGNAVDFFPI

LRYLPNPDLKRFKNFNDNFVLFLQKTVQEHYQDFNKNSIQDITGALFKHSENSKDSGGLI

PQEKIVNIVNDLFGAGFDTVTTAITLSILLLVTWPNVQRKIHKELDTVIGRDRQPRLSDR

LQLPYMEAFILELYRYTSFVPFTIPHSTTRDTSLNGFYIPKDRCIFINQWQVNHDEKQWK

DPFVFRPERFLTDNDTVINKTLSEKVMLFGLGKRRCIGEIPAKWEVFLFLAILLQQLEFS

VPPGTKVDLTPTYGLTMKPQTCKYIQAWPRFSK

>126272484

MTGRASGNLRAGGCLEAGCSPRHRCSQQPGTVKMVSSLLASISISELLLASVIFCLVFWV

TRSSHQRVPKGLKSPPGPWAWPLFGNVWTLGKNPHLTLAQLSEKYGDVMKIHIGSTPVIV

LSGLETIRQALVKQGEDFKGRPDLYSSTFVADGYSLAFNPDSGEVWAVRRKLAQNALNTF

SVSSSPSSSSCYLEEHVNKEVKHLIQKFQELMEGVGCFDPYRHIVASVANVISAMCFSQR

YEDHKNPEFTTLINASHEFVESATSGNPVDFFPILRYIPNPQLQRFKEFNQRFLKFLQNT

IREHHKAFDENNIQDITGALYKHSQDKAFGNTSSSVPEMLIINLINDIFGAGFDTVTTAI

SWSLMYLVTNPKVQKKIQQELDTVIGRDRWPLLSDRPQLPFMEAFILEIFRHTSFVPFTI

PHSTTRATTLNNFYIPKGTCVFVNQWQTNHDPKLWEDPSVFRPERFLSADGTVNKALSEK

VILFGLGKRRCIGETIARWEVFLFLAILLHQIEFSVPSGVKVDMTPTYGLTMKHPRCEHF

QARPRFSRKG

>AAA37070.1

MALSQYTSLSTELVLATAIFCIVFWVARALRTQVPKGLKTPPGPWGLPFLGMCLTLGKNP

HLSLTKLSKQYGDVLQIRIGSTPVVVLSGLDTIRQALVRQGDDFKGRPDLYSFTLITNGK

SMTFNPDCGPVWAARRRLAQDALKSFSIASDPTSASSCYLEDHVIKEANHLVSKLQKLTA

EVGHFEPVNQVVESVANVIGAMCFGKNFPRKSEEMLRIVKGSSDFVENVSSGNAVDFFPI

LRYLPNPDLKRFGGFNDNFVLFLQKTVQEHYQDFNKNSIQDITGALFKHSENSKDSGGLI

PQEKIVNIVNDLFGAGFDTVTTAITWSILLLVTWPNVQRKIHKELDTVIGRDRQPLLSDR

LQLPYMEAFILELYRYTSFVPFTIPHSTTRDTSLNGFYIPKDRCIFINQWQVNHDEKQWK

DPFVFRPERFLTDNDTVINKTLSEKVMLFGLGKRRCIGEIPAKWEVFLFLAILLQQLEFS

VPPGQKVDLTPTYGLTMKPQTCKYIQAWPRFSK

>AAI35261.1

MTSWIGLIADMMDNSTTTEVLVASIVFAIVFLVIRSQRVKLPPGTKKLPGPMPYPVIGNL

LSLSKNPHLSLTKMSETYGDVFQIQIGTKPMLVLSGLETLRQALIRQSDEFAGRPDLFTF

RLVGDGQSMTFSSDSGEVWRARRRLAQNALKTFATSPSPTSSNSCLVEENIITEAEYLIR

KFKELIDDKGEFDPYRYVVVSVANVICGMCFGKRYNHDDEELLNVVNLTDEFGAAAASGN

PADFIPILQYLPNSSMKAFKEINQKFLAFMQKFTKEHYKTFDKNHIRDITDSLIQHSQEK

RVDENSNIQLSNEKIVNIVNDLFGAGFDTITTALSWSLMYLVAHPNIQQRIQDELDQVIG

RERRPRLSDRAQLPYTEAFILEMFRHSSFMPFTIPHCTTKDTMLNGYFIPKGICVLINQW

QVNHDPNLWQDPFKFCPERFLNNDGTMVNKTEMEKVMIFGLGKRRCVGEAIGRMEVFLFL

TTMLQQMQFFKQDGEKLDMSPQYGLTMKHKRCHLTAKLRFALLTN

>P79761.1

MGPEEVMVQASSPGLISATEVLVAAATFCLLLLLTQTRRQHAPKGLRSPPGPRGLPMLGS

VLELRKDPHLVLTRLSRKYGDVMEVTIGSRPVVVLSGLETIKQALVRQAEDFMGRPDLYS

FRHITDGQSLTFSTDTGEMWKARRKLAQNALKNFSIAASPTASSSCLLEEHVSTEASYLV

TKFLQLMEEKQSFDPYRYMVVSVANVICAICFGKRYDHDDQELLSVVNVVDEFVDVTAAG

NPADFIPLLRYLPSRNMDSFLDFNKRFMKLLQTAVEEHYQTFDKNNIRDVTDSLIEQCVE

KKAEANGATQIPNEKIINLVNDIFGAGFDTVTTALSWSLMYLVTYPHMQKKIQAELDQTI

GRERRPRLSDRGMLPYTEAFILEMFRHSSFMPFTIPHSTTRDTVLNGYYIPKDRCVFINQ

WQVNHDEKLWKDPQAFNPERFLNAEGTEVNKVDAEKVMTFGLGKRRCIGENIGKWEVFLF

LSTLLQQLEFSIQDGKKADMTPIYGLSMKHKRCEHFQVKKRFSMKSSN

>AAX73011.1

MGPEEVMVQVGSPGLISATEMLVAAATFCLLLLLTQTRRQHTPKGLRRPPGPRGLPLLGS

VLELRKDPHLVLTQMSRKYGDVMEVTIGSRPVVVLSGLETIKQALVRQAEDFMGRPDLYS

FRHVTDGQSLTFSTDTGEVWKARRKLAQNALKNFSIAASPTASSSCLLEEHVTNEASYLV

TKFLQLMEEKQSFDPYRYMVVSVANVICAICFGKRYDHDNQELLSVVNVVEEFGDVTAVG

NPTDFIPLLQYLPSRNMDLFLDFNKRFMKLLKTAVEEHYETFDKNNIRDVTDSLIEQCME

KKTEANSATQIPNEKIINLVNDIFGAGFDTVTTALSWSLMYLVTYPHIQKKIQAELDQTI

GRERRPRLSDRGTLPYTEAFILEMFRHSSFMPFTIPHSTTRDTVLNGYYIPKDRCVFINQ

WQVNHDEKLWKDPQAFNPERFLNAEGTEVNKVDAEKVMTFGLGKRRCIGENIGKWEVFLF

LSTLLQQLEFSIRDGKKADMTPIYGLSVKHKRCEHFQVKKRFSMKSSN

>AAI70421.1

MTNWIGTVAGMMANTTITEFLVASVVFAIVFLVIRSQRVKIPPGTKKLPGPMPYPVIGNL

LSLSKNPHLSLTRMSETYGDVFQIQIGTKPVLVLSGLETLKQALIRQGDEFAGRPDLFTF

RMVGDGQSMTFSSDSGEVWRARRRLAQNALKTFATSPSPTSSSSCLVEENIITEAEYLVK

KFMQLIDEKGEFDPYRYVVVSVANIICGMCFGKRYNHDDEELLNVVNLTDEFGAAAASGN

PADFIPILQYLPSSSMKAFKEINRKFIDFMQKFATEHYKTFDKNHIRDITDSLIQHSQEK

RVDENSNVQLSNQKIVNIVNDLFGAGFDTITTALSWSLMYLVAHPNIQEKIQDELDRVIG

KERRPRLSDRAQLPYTEAFIFEMFRHSSFMPFTIPHCTTKDTVLNGYFIPKGICVLVNQW

QVNHDPNLWKDPSKFYPERFLNTDGTMVNKTEMEKVMVFGLGKRRCVGEAIGRMEVFLFL

TTMLQQMQFFKQDGEKLDMSPQYGLTMKHKRCHVTAKLRFPLLTTD

>BAA37079.1

MTDWIGSIAGLMANTTITEFLLVSTVFAIVFLVLRSERVKIPPGTKKLPGPMPYPIIGNL

LSLSKNPHLSLTRMSKTYGDVFQIQIGTKPVLVLSGLETLKQALIRQGDEFAGRPDLFTF

RLVGDGKSLTFSSDSGEVWRARRRLAHNALKTFATSPSPTSSSSCLVEENIITEAEYLVR

KFKQLIDEKGEFDPYRYVVVSVANVICGMCFGKRYNHDDEELLNVVNLTDEFGAAAASGN

PADFIPILQYLPSSSMKAFKEINRKFLDFIQKLVKEHYKTFDKNHIRDITDSLIQHSQEK

RVDENSNVQLSNQKIVNIVNDLFGAGFDTITTALSWSLMYLVAHPNIQEKIQDELDQVIG

RERRPRLSDRAQLPYTEAFILEMFRHSSFVPFTIPHSSTTDTVLNGYFIPKGICVLINQW

QVNHDPNLWKDPFKFCPERFLNTDGTTLNKIEMEKVMIFGLGKRRCVGEVIGRMEVFLFL

TTMLQQMQFFKQDGEKLDMSPQYGLTMKHKRCHVTAKIRFPLLATH

>ACX54107.1

MGPEEVMVQVGSPGLISSTEVLVAAATFCLLLLLTQTRRQNVPKGLRSPPGPHGLPVLGS

ALELRKDTHLVLTEMSRKYGDVMEVTIGSRPVVVLSGLETIKQALVRQAEDFMGRPDLYS

FRHVTDGQSLTFSTDTGEMWKARRKLAQNALKNFSIAASPTASSSCLLEEHVTNEASYLV

TKFLQLMEEKQSFDPYRYTVVSVANVICAICFGKRYDHEDQELLNVVNVVDEFVNVTAVG

NLADFIPLLQYLPSRNMDLFLDFNKRLMKLLQAAVDEHYKTYDKNSIRDVTDSLIEQCME

KKAEGSGALQIPNEKIINLVNDIFGAGFDTVTTALSWSLMYLVTYPHIQKKIQAELDQTI

GRERRPRLSDRSMLPYTEAFILEMFRHSSFIPFTIPHSTTRDTVLNGYYIPKDRCVFINQ

WQVNHDEKLWKDPQTFNPERFLSAEGTELNKVDAEKVMTFGLGKRRCIGENIARWEVFLF

LSTLLQQLEFSIRDGEKADMTPIYGLTMKHKRCEHFQVKKRFSTKSSN

>326926364

MGPEEVMVQVGSPGLISATEMLVAAATFCLLLLLTQTRRQHTPKGLRRPPGPRGLPLLGS

VLELRKDPHLVLTQMSRKYGDVMEVTIGSRPVVVLSGLETIKQALVRQAEDFMGRPDLYS

FRHVTDGQSLTFSTDTGEVWKARRKLAQNALXXXXXXXXPTASSSCLLEEHVTNEASYLV

TKFLQLMEEKQSFDPYRYMVVSVANVICAICFGKRYDHDNQELLSVVNVVEEFGDVTAVG

NPTDFIPLLQYLPSRNMDLFLDFNKRFMKLLKTAVEEHYETFDKNNIRDVTDSLIEQCME

KKTEANSATQIPNEKIINLVNDIFGAGFDTVTTALSWSLMYLVTYPHIQKKIQAELDQTI

GRERRPRLSDRGTLPYTEAFILEMFRHSSFMPFTIPHSTTRDTVLNGYYIPKDRCVFINQ

WQVNHDEKLWKDPQAFNPERFLNAEGTEVNKVDAEKVMTFGLGKRRCIGENIGKWEVFLF

LSTLLQQLEFSIRDGKKADMTPIYGLSVKHKRCEHFQVKKRFSMKSSN

>AEN19340.2

MALISLPILGPILVSESPVAVATLCAVYLLLRLLRTEIPAGLRRPPGPTPLPLIGNVLEL

GGNPHLSLTKMSQRYGDVLQIQIGTRPVVVLSGNETVRQALIKQGDDFAGRPDLYSFQFI

SNGKSLTFSNDHAGVWRARRKLAQNALRSFSTVESPTSSYSCVLEEHISLEAEYLVKRLS

GIMEADGSFDPFRHIVVSVANVICAMCFGRRYSHDDQELVNLVNLSAEFGRVIASGNPAD

FIPALRILPNRNMRAFIGISNRFNTFIQNIVTDHYRSFDKSNIRDITDSLIEHCQDKKVD

ENANIQISDEKIVSIVNDLFGAGFDTISTALSWCLMYLVSHPDIQKKIHQVLDEHVGRER

TPRLSDRPSLPYVEAFILETFRHSSFLPFTIPHCMTKDRALNGFYIPKDTCIFVNQWQVN

HDPSLWKDPSTFSPERFLNAEGTGINKAESEKVMLFGLGKRRCIGESIGRSEVFLFLAVL

LQRLEFRSLPGQKLDLTPEYGLTMKHKRCQLSASLLGRGSGCFLS

>224061771

MPGAAVWPAGSPGAVWASEALLAAAAFFELLLALQRLRPPGAVPEGLRRPPGPRGFPVLG

NVLELRRDTHLALTRLGRRYGDVMEVRIGTRPVLVLSGLDTIRQALVRQGDDFMGRPDLY

SSRFVADGQSLTFSPDSGEVWKARRKLAQSALKSFSIAPSPTSSCSCLLEEHVSKEAEYL

VTKFLQLMEEEKSFEPCRYLVVSVANVICAICFGKRYEHEDQELLRLVNSSEKFTDVAAA

GNPADFIPLLRYLPSRSMKLFIDFNRYFVGFLQRRVKEHYETYDENNIRDITDSLIEQCL

DKKLGTNTAAQIPKEKIVNLVNDLFGAGFDTVTTALSWSLMYLVTNPNIQKKIHEELDRT

IGRERRPRLSDRGTLPYTEAFILEMFRHSSFLPFTIPHSTTKDTVLNGYFIPKDRCVFVN

QWQVNHDEKLWKDPETFNPERFLSADGTRVNKEDAEKVLVFGLGRRRCIGENIARSQVFL

FLVTLLQQLEFSVCEGGRVDMTPLYGLSLKHKRCEHFQVRQRFPVKGRS

>ACX54108.1

MAQVSSSGLISSTEVLVAAATFCLLLLLTQTRRQNVPKGLRSPPGPRGLPLLGNVLELRK

DPHLVLTEMSRKYGDVMEVTIGSRPVVVLSGLDTVRQALVRQAEDFMGRPDLPSFKYVSN

GHSLAFSYECGDAWKARRKLAQNALKTFSIAASPTASSSCLLEEHVSTEASYLVTKFLQL

MEEKQTFNPNNYLVVSVANVICAICFGKRYDHDDQELLNVVNMNTEFGDVAAAGNPSDFI

PLLRYLPNRAMATFKDVNTRFDAFIEKIVQNHYTTFDKEHIRDVTDSLIGQCQEKKTGGN

VRVQPSDKSIISIVNDLFGAGFDTVTTALSWCVMYAALYPHIQKKIQAELDQIIGRERRP

RLSDRSMLPYTEAFILEVFRHSSLLPFTIPHSTTKDTVLNGYFIPKNTCVFINQWQVNHD

EKIWKDPSSFNPERFLNAAGTEINRTEGDKVVIFGLGKRRCIGESIGRWEVFLFLTTILQ

QLEINLAPGQQVDITPQYGLTMKYKQCECFQMKKRFPTKSSA

>P79760.1

MAAGPQAAMEQASSPGLISATEVLVAAATFCLLLLLTQTRRQHAPKGLRSPPGPRGLPML

GNVLELRKDPHLVLTRLSRKYGDVMEVTIGSRPVVVLSGLETIKQALVRQAEDFMGRPDL

PSWQYVSNGHSLAFSYECGDAWKARRKLAQNALKTFSIAASPTASSSCLLEEHVSTEASY

LVTKFLQLMEEKQSFNPNSYLMVSVANVICAICFGKRYDHDDQELLSVVNMNTEFGDVAA

AGNPADFIPLLRYLPNRAMAAFKDVNARFSAFVQKIVQNHYSTFDKEHIRDVTDSLIGHC

QEKRTGEDVRVQPSDESIISIVNDLFGAGFDTVTTALSWCMMYAALYPHIQKKIQAELDQ

TIGRERRPRLSDRGMLPYTEAFILEAFRHSSLLPFTIPHCTTKDTVLNGYYIPKDTCVFI

NQWQANHDEKIWKDPPSFKPERFLNAAGTELSRTEADKVLIFGLGKRRCIGESIGRWEVF

LFLTTILQQLEISLAPGQRVDITPQYGLTMKYKQCECFQMKKRFPSKGSA

>327288210

MSSMESLSHITATEALIATAVFCLLFMIVKSFRNRVPHGLKKIPGPMGYPLIGNMLELGK

NPHLSLTRMSQKYGDVMMIHIGSTPVLVLSGLETIRKALVRQGAEFLGRPDLYSFRYVAD

GESLAFGHDSGEVWRTRRKLAQNALKSFAASPSPVSPSIYLLEEHLSKEVDYLIQKLQEV

MREKKSLDPYRYIVVSVANVICAMCFGKRYSHDNQEFLSIIDESEKFVEVAASGNLADFI

PLLQYLPMRSMKMFKQFNEKFTVFLLNMVKEHYESFSKDSIRDITDSLIEQSQEKFQISS

KKIVNLVNDIFGAGFDTVTTTLSWSLMYLVTHPEIQKKIHEEIDEVIGRERKPRLSDRLL

MPYTEAFTMEVFRHSSLLPFTIPHCTVKETSLNGYYIPKDLCVFVNQWQVNHDEKLWKDP

SSFNPERFLSADGKDVNKDESEKVLTFGLGKRRCIGEQIARWEVFLFLTFLLQELEFSVK

EGVEVDMTPRYGLSMKHKRCPHFLVKPRPPKNAS

>326926366

MAAGPQAAMAQVGSPGLISATEMLVAAATFCLLLLLTQTRRQHTPKGLRRPPGPRGLPLL

GSALELRKDPHLVLTQMSRKYGDVMEVTIGSRPVVVLSGLETIKQALVRQAEDFMGRPKL

PSFKYISNGHSLAFSYECGEMWKARRKLAQNALKTFSIAASPTASSSCLLEEHVTNEASY

LVTKFLQLMEEKQSFDPNSYLIVSVANVICAICFGKRYDHDDQELLSVVNMNTEFGDVVA

AGNPTDFIPLLRYLPNRAMAAFKDVNARFNAFIQKIVQNHYTTFDKEHIRDVTDSLIGHC

QEKKTGEDVRVQPSDESIISIVNDLFGAGFDTVTTTLSWCIMYAALYPHIQKKIQAELDQ

TIGCERRPRLSDRGILPYTEAFILEVFRHSSLLPFTIPHSTTKDTVLNGYYIPKDTCVFV

NQWQVNHDEKIWKDPSSFKPERFLNAAGTEINKTEGDKVVIFGLGKRRCIGESIGRWEVF

LFLSTILQQLEVSGXLAPGQQVDVTPQYGLTMKYKQCECFQMKKRFPTKSSA

>Q92116.1

MVLMILPVIGSVSVSEGLVAMITMCLAYLILRLFRTEIPEGLLQLPGPKPLPIIGNVLEV

GRNPYLSLTAMSKRYGDVFQIQIGMRPVVVLSGSETVRQALIKQGDXFAGRPDLYSFRFI

NDGKSLAFSTDQAGVWRARRKLAYSALRSFATLEGTTPEYSCALEEHVSKEAEYLVKQLH

TVMEADGSFDPFRHIVVSVANVICGMCFGRRYDHNHQELLNLVNLSDEFGQVVASGNPAD

FIPILQYLPSTTMKKFLNINDRFNTFVQKIVSEHYTTFDKDNIRDITDSLIDHCEDRKLD

ENSNVQMSDEKIVGIVNDLFGAGFDTISTALSWSVMYLVAYPEIQERLYQEMNETVGPDR

TPCLSDKPKLPFLEAFILETFRHSSFLPFTIPHCTSKDTSLNGYFIPKDTCVFINQWQIN

HDAELWKDPSSFNPDRFLNADGTEVNKLEGEKMMVFGMGKRRCIGEVIARSEVFLFLAIL

VQNLRFHSMPGEPLDMTPEYGLTMKHKRCQLRAAMRARNEE

>327288204

MGSQTVMSLTELLLAFVVFCLILVAVKSFWRQIPPGLKRLPGPKGYPLIGNILDLGKNPH

LSLNQMRQKYGDVMQIRIGTRPILVLSGLETIRQALIKQGEDFASRPNLYSFQFVGEGQS

LTFGSCPAEVWRSRRKVAQNALKVISIAANETLSTCPMEEFVSTEADSLVVKFQELMKEK

NSFEPYRYLVVSVANVICGMCFGKRYDHEDQELLSLVNINNEFGEAAASCNPADFIPLLQ

YLPNQTMKVFKDLNKRFGALVERIAKEHYTTFDKNNIRDITDSLIDYWQSKKVDVNANIQ

QLDQNIVHIVGDIFGAGFDTVSTGLSWCLMYLVTYPEIQKKIQDELDQNIGQERKARLSD

RNVLPYTEAFILEMFRHSSFIPFTIPHCTTKDTALNGFYIPKDTCVFVNQWQVNHDPKLW

KDPFAFNPERFLAEDGSGINRAEGEKILTFGLGRRRCIGENIGRSEIFLFLTTLVQKLEF

SLRPGKEVDFTPQYGLTMKFKKCEHFQIKTRF

>ADM47615.1

MLNAGTRVLQNLMAVTVTEAFVGIAVFSVIFLLIRAYRSKVPKGLKPLPGPPSVPIIGHT

LSLGESPHLALTSLGQQYGDIFQIQIGTRPVLVLSGLDTLKQALVKQGDDFAGRPSLYSF

KFINNGKSLTFSSDYSEVWRLRRKLAQNALRTFSTEEWKNSTHSCLLEEHVCSEARHLIQ

IFVEQMKRRGSFDPMRYIVVSVANVICALCFGKRYSHEDQELLNIVNVSDEFGKIVAAGN

PADFIPILRFLPNHSMDKFIAINKRFATFVENIVMEHYRTFDKDNIRDITDSLIGHCQDK

KVDENANIQISDEKIVGIVNDLFGAGFDTITTAVSWCVMYLVYYPDIQKKLHEEIDETIG

KDRSPRITDKPSLPYTEAFIMETFRHSSFLPFTIPHCTTKDTALNGYYISKNTCVFVNQW

QVNHDSSLWKDPFAFRPERFLNADGKTTNKVECEKVLLFGMGKRRCIGEAIGRIEVFLFL

TSLVQQLQFEKKPGQDIDMTPQYGLTMKPKRCEVQAMQRVSAKAAP

>Q9W683.1

MALMILPFIGALSVSESLVAVVTVCLVYLIIKSFQDKIPEGLSRLPGPKPLPIIGNVLEV

GSRPYLSLTEMGKRYGNVFQIQIGMRPVVVLSGNETVRQALIKQGDEFAGRPDLYSFRFI

SEGKSLAFSTDQAGVWRARRKLAYSALRSFSTLEGTTPEYSCVLEEHISKEAKYLIQQLD

TVMKADGSFDPFRYIVVSVANVICGMCFGRRYDHHDQELLSLVNLSDEFGQVVGSGNPAD

FIPILQYLPNKTMKKFVSINDRFISFVQKIVSEHYATFNKDNIRDITDSLIDHCEDRKLD

ENANVQMSDEKVVGIVNDLFGAGFDTISTALSWSVMYLVAYPEIQEXLYQELKENVGLDR

TPVLSDRNNLPLLESFILEIFRHSSFLPFTIPHCTTKDTSLNGYYIPKDTCVFINQWQIN

HDPELWKEPSSFNPDRFLSADGTEVNKVDGEKVMIFGMGKRRCIGEVIARNEVYLFLAIL

IQKLHFCNLPGEPLDMTPEYGLTMKHKRCQLRATARVRSDH

>O42231.1

MALMILPFIGALSVSESLVALVTVCLVYLIIKSFQANIPEGLSRLPGPKPLPIIGNVLEV

GSRPYLSLTEMSKRYGNVFQIQIGMRPVVVLSGNETVRQALIKQGDEFAGRPDLYSFRFI

SEGKSLAFSTDQAGVWRARRKLAYSALRSFSTLEGTTPEYSCVLEEHISKEAEYLIKQLD

TVMKADGSFDPFRYIVVSVANVICGMCFGRRYDHHDRELLSLVNLSDEFGQVVGSGNPAD

FIPILQYLPNKTMKKFVNINDRFISFVQKIVSEHYATFNKDNIRDITDSLIDHCEDRKLD

ENANVQMSDEKVVGIVNDLFGAGLDTISTALSWSVMYLVAYPEIQERLYQELKENVGLDR

TPVLSDRNNLPLLEAFILEIFRHSSFLPFTIPHCTTKDTSLNGYYIPKDTCVFINQWQIN

HDPELWKEPSSFNPDRFLSADGTEVNKVDGEKVMVFGLGKRRCIGEVIARNEVYMFLAIL

IQKLHFYNLPGEPLDMTPEYGLTMKHKRCHLRATVRVRSDH

>ACT64126.1

MVLMILPFIGSVSVSEGLVAITVLCLVYLALRFFHTEIPEGLRRLPGPKPFPLIGNVLEV

GSKPYLSLTAMSKRYGDVFQIQIGMRPVIVLSGSDTVRQALIKQGEEFAGRPDLYSFRFI

NDGKSLAFSTDQAGVWRARRKLAYSALRSFSSLDGTTPEYSCMLEEHICKEGEYLIKQLN

NVMKADGSFDPFRHIVVSVANVICGMCFGRRYDHNDQELLSLVNLSDEFGRVVGSGNPAD

FIPVLQYLPSSTMKSFMSINARFSAFVQKIVIEHYATYNKDNIRDITDSLIDHCEDRKLD

ENSNVQMSDEKIVGIVNDLFGAGFDTISTALSWSVMYLVAHPEMQERLHQELKDAVGLDR

TPRLSDRPNLPFLDAFILELFRHSSFLPFTIPHCTSKNTSLNGYFIPKDTCVFINQWQMN

HDPEIWKDPSSFNPDRFLSADGTAVNKMEGEKVMLFGMGKRRCIGEVIARNEVYLFLAIL

AQKLQFQAKPGQPLDMTPEYGLTMKHKRCHLRATMRVRNKQ

>O42457.1

MVLMILPFVGPVSVSESLVAIITMCLVYMILKFFRTEIPEGLCQLPGPKPLPIIGNVLEV

GRNPYLSLTAMSKRYGDVFQIQIGMRPVVVLSGSETVRQALIKQGDDFAGRPDLYSFRFI

NDGKSLAFSTDQAGVWRARRKLAYSALRSFSTLEGTTPEYSCALEEHVSKEAEYLVKQLN

TVMETDGSFDPFRHIVVSVANVICGMCFGRRYDHNNQELLNLVNLSDEFGQVVASGNPAD

FIPILQYLPSTSMKKFVSINDRFNAFVQKIVSEHYTTFDKDNIRDITDSLIDHCEDRKLD

ENSNVQMSDEKVVGIVNDLFGAGFDTISTALSWSVMYLVAYPEIQERLYQEMKESVGLDR

TPCLSDKPKLPFLEAFILEIFRHSSFLPFTIPHCSSKDTSLNGYFIPKDTCVFINQWQIN

HDPELWKDPSSFNPDRFLNTDGTELNKLEGEKMMVFGLGKRRCIGEVIARNEVFLFLAIL

VQNLRFHAKPGEPLDMTPEYGLTMKHKRCHLRAAMRSRNEE

>AFJ44751.1

MVLMILPFIGSVSVSESLVAITVLCVVYLALRCFHTEIPEGLRRLPGPKPFPLIGNVLEV

GSKPYLSLTAMSKRYGDVFQIQVGMRPVIVLSGTDTVRQALIKQGEEFAGRPDLYSFRFI

NDGKSLAFSTDQAGVWRARRKLAYSALRSFSSLEGTTPEYSCMLEEHICKEGEYLIKQLN

NVMKADGSFDPFRHIVVSVANVICGMCFGRRYDHNDQELLSLVNLSDEFGQVVGSGNPAD

FIPILQYLPSSTMKTFMDINVRFNTFVQKIVSEHYANYNKDNIRDITDSLIDHCEDRKLD

ENSNVQMSDEKVVGIVNDLFGAGFDTISTALSWSVMYLVAHPEMQERLYQELKDTVGLDR

SPRLSDRPSLPFLDAFILEIFRHSSFLPFTIPHCTSKDTSLNGYFIPKDTCVFINQWQIN

HDPEIWKDPSSFNPDRFLSADGTEVNKMEGEKISIFGMGKRRCIGEVIARNEVYLFLAII

VQKLQFHAMPGKPLDMTPEYGLTMKHKRCHLRATMRVRNEQ

>AAM00254.1

MVLMILPIIGSVSVSEGLVAMVTLCLVYMFMKYKHTEIPEGLKRLPGPKPLPIIGNVLEV

HNNPHLSLTAMSERYGSVFQIQIGMRPVVVLSGSETVRQALIKQGEDFAGRPDLYSFKFI

NDGKSLAFSTDKAGVWRARRKLAMSALRSFATLEGSTPEYSCALEEHVCKEGEYLVKQLT

SVMDVSGSFDPFRHIVVSVANVICGMCFGRRYSHDDQELLSLVNLSDEFGQVVGSGNPAD

FIPILRYLPNRTMKRFMDINDRFNAFVQKIVSEHYESYDKDNIRDITDSLIDHCEDRKLD

ENANIQVSDEKIVGIVNDLFGAGFDTISTALSWAVVYLVAYPEIQERLHQELTEKVGLNR

TPRLSDKTNLPLLEAFILEIFRHSSFLPFTIPHCTIKDTSLNGYFIPKDTCVFINQWQVN

HDPELWKEPSLFNPDRFLSADGTELNKLEGEKVLVFGMGKRRCIGEAIGRNEVYLFLAIL

LQRLRFQEKPGHPLDMTPEYGLTMKHKRCQLKASLRPWGQEE

>ACI33928.1

MVLMILPIIGSVSVSEGLVVMVTLCLVYMIMKYMHTEIPEGLKRLPGPKPLPIIGNVLEV

HNNPHLSLTAMSERYGSVFQIQIGMRPVVVLSGSETVRQALIKQGEDFAGRPDLYSFKFI

NDGKSLAFSTDKAGVWRARRKLAMSALRSFATLEGSTPEYSCALEEHVCKEGEYLVKQLT

SVMDVSGSFDPFRHIVVSVANVICGMCFGRRYSHDDQELLGLVNLSDEFGQVVGSGNPAD

FIPILRYLPNRTMKRFMDINDRFNTFVQKIVSEHYESYDKDNIRDITDSLIDHCEDRKLD

ENANVQVSDEKIVGIVNDLFGAGFDTISTALSWAVVYLVAYPEIQERLHQELKEKVGMTR

TPRLSDKTNLPLLEAFILEIFRHSSFLPFTIPHCTVKDTSLNGYFIPKDTCVFINQWQVN

HDPELWKEPSSFNPDRFLSADGTELNKLEGEKVLVFGMGKRRCIGEAIGRNEVYLFLAIL

LQRLCFQEKPGHPLDMTPEYGLTMKHKRCQLKASLRPWGQEE

>AAD14035.1

MVLMILPIIGSVSASEGLVAMVTLCLVYMIMKYMHTEIPEGLKRLPGPKPLPIIGNVLEV

HNNPHLSLTAMSERYGSVFQIQIGMRPVVVLSGSETVRQALIKQGEDFAGRPDLYSFKFI

NDGKSLAFSTDKAGVWRARRKLAMSALRSFATLEGSTPEYSCALEEHVCKEGEYLVKQLT

SVMDVSGSFDPFRHIVVSVANVICGMCFGRRYSHDDQELLGLVNMSDEFGQVVGSGNPAD

FIPILRYLPNRTMKRFMDINDRFNTFVQKIVSEHYDSYDKDNIRDITDSLIDHCEDRKLD

ENANVQVSDEKIVGIVNDLFGAGFDTISTALSWAVVYLVAYPEIQERLHQELKEKVGMIR

TPRLSDKINLPLLEAFILEIFRHSSFLPFTIPHCTVKDTSLNGYFIPKDTCVFINQWQVN

HDPELWKEPSSFNPDRFLSADGTELNKLEGEKVLVFGMGKRRCIGEAIGRNEVYLFLAIL

LQRLCFQEKPGHPLDMTPEYGLTMKHKRCQLKASLRHGGRKSEGHGHIYDSQHHYN

>ABO38813.1

MVLMILPFIGSVSVSESLVAMTTVCLVYLILKFFHTDIPKGLRRLPGPKPLPIIGNVLEV

GSKPYLSLTAMSKRYGHVFQIQIGMRPVVVLSGSETVRQALIKQGEEFSGRPDLYSFRFI

SDGKSLAFSTDQAGVWRARRKLAYSALRSFSTLEGTTPEYSCVLEEHICKEGEYLIKQLN

TVMKADGSFDPFRYIVVSVANVICGMCFGRRYDHDDQELVSLVNLSDEFGQVVGSGNPAD

FIPILQFLPSKSMKNFMSINERFISFVQKIVTEHYATFDKDNIRDITDSLIDHCEDRKLD

ENSNIQMSDEKIVGIVNDLFGAGFDTISTALSWSVMYLVAYPEIQERLYEEIKEKVGLDR

TPLLSDKPNLLFLEAFILEIFRHSSFLPFTIPHCTSKDTSLNGYFIPKDTCVFINQWQIN

HDEELWKDPSSFNPDRFLSADGSGIKKLEGEKVMVFGMGKRRCIGEVIARNEVYLFLAII

IQKLHFHTMPGELLDMTPEYGLTMKHKRCHLKATMRARNEH

>AAQ10900.1

MVLMILPIIGSVSVSEGLVAMVTLCLVYMIMKYMHTEIPEGLKRLPGPKPLPIIGNVLEV

HNNPHLSLTAMSERYGSVFQIQIGMRPVVVLSGSETVRQALIKQGEDFAGRPDLYSFKFI

NDGKSLAFSTDKAGVWRARRKLAMSALRSFATLEGSTPEYSCALEEHVCKEGEYLVKQLT

SVMDVSGSFDPFRHIVVSVANVICGMCFGRRYSHDDQELLGLVNLSDEFGQVVGSGNPAD

FIPILRYLPNRTMKRFMDINDRFNTFVQKIVSEHYESYDKDNIRDITDSLIDHCEDRKLD

ENANIQVSDEKIVGIVNDLFGAGFDTISTALSWAVVYLVAYPEIQERLHQELKEKVGMIR

TPRLSDKTNLPLLEAFILEIFRHSSFLPFTIPHCTVKDTSLNGYFIPKDTCVFINQWQVN

HDPELWKEPSSFNPDRFLSADGTELNKLEGEKVLVFGMGKRRCIGEAIGRNEVYLFLAIL

LQRLCFKEKPGHPLDMTPEYGLTMKHKRCQLKASLRPWGQEE

>AAD45966.1

MVLMILPIIGSVSASEGLVAMVTLCLVYMIMKYMHTEIPEGLKRLPGPKPLPIIGNVLEV

HNNPHLSLTAMSERYGSVFQIQIGMRPVVVLSGSETVRQALIKQGEDFAGRPDLYSFKFI

NDGKSLAFSTDKAGVWRARRKLAMSALRSFATLEGSTPEYSCALEEHVCKEGEYLVKQLT

SVMDVSGSFDPFRHIVVSVANVICGMCFGRRYSHDDQELLGLVNMSDEFGQVVGSGNPAD

FIPILRYLPNRTMKRFMDINDRFNTFVQKIVSEHYDSYDKDNIRDITDSLIDHCEDRKLD

ENANVQVSDEKIVGIVNDLFGAGFDTISTALSWAVVYLVAYPEIQERLHQELKEKVGMIR

TPRLSDKINLPLLEAFILEIFRHSSFLPFTIPHCTVKDTSLNGYFIPKDTCVFINQWQVN

HDPELWKEPSSFNPDRFLSADGTELNKLEGEKVLVFGMGKRRCIGEAIGRNEVYLFLAIL

LQRLCFQEKPGHPLDMTPEYGLTMKHKRCQLKASLRPWGQEE

>AAK69390.1

MVLMILPFIGPVSVSESLVAMITMCLVYMILKFFRTEIPEGLRQLPGPKPLPIIGNVLEV

GRNPYLSLTAMSKRYGDVFQIQIGMRPVVVLSGNETVRQALIKQGEEFAGRPDLYSFRFI

NDGKSLAFSTDQAGVWRARRKLAYSALRSFATLEGTTPEYSCALEEHVSKEAEYLVKQFN

TVMETDGSFDPFRHIVVSVANVICGMCFGRRYDHNNQELLNLVNLSDEFGQVVASGNPAD

FIPILQYLPSTTMKKFLNINDRFNEFVQKIVSEHYTTFDKDNIRDITDSLIDHCEDRKLD

ENSNVQMSDEKIVGIVNVLFGAGFDTISTALSWSVMYLVAYPEIQERLHQEMKETVGMDR

TPCLSDRLKLPFLEAFILEIFRHSSFLPFTIPHCSTKDTSLNGYFIPKDTCVFINQWQIN

HDPELWKDPSSFNPDRFLNADGTEVNKLEGEKMMVFGLGKRRCIGEVIARHEVFLFLAIL

VQNLRFHAKPGEPLDMTPEYGLTMKHKRCQLRAAMRARNEE

>CAB63650.1

MVLMILPFIGSVSVSESLVALTTVCLVYLILKFFRTEIPEGLHRLPGPKPLPLIGNVLEV

GNKPYLSLTAMSKRYGDVFQIQIGMRPVVVLSGSETVRQALIKQGDEFAGRPDLYSFRFI

NDGKSLAFSTDQAGVWRARRKLAYSALRSFSSLGGTTPEYSCVLEEHICKEGEYLIKQLN

TVMKADGSFDPFRHIVVSVANVICGMCFGRRYDHNDQELLSLVNLSDEFGQVVGSGNPAD

FIPVLQFLPSTTMKKFMDINARFNKFVQKIVSEHYTTYDKDNIRDITDSLIDHCEDRKLD

ENSNVQMSDEKIVGIVNDLFGAGFDTISTALSWSVMYLVAYPEIEERLYQELKENVGLDR

TPLLCDRPNLPFLEAFILEIFRHSSFLPFTIPHCTSKDTSLNGYFIPKDTCVFINQWQIN

HDPELWKDPSSFNPDRFLSTDGTELNKLEGEKVMVFGLGKRRCIGEVIARNEVFLFLAII

VQKLHFKTLPGEPLDMTPEYGLTMKHKRCHLRATMRASE

>AAQ10899.1

MVLMILPIIGSVSVSEGLVAIVSLCLVYMLMKYKHTEIPEGLKRLPGPKPLPIIGNVLEV

HNNPHLSLTAMSERYGSVFQIQIGMRPVVVLSGSETVRQALIKQGEDFAGRPDLYSFKFI

NDGKSLAFSTDKAGVWRARRKLAMSALRSFATLEGSTPEYSCALEEHVCKEGEYLVKQLT

SVMDVSGSFDPFRHIVVSVANVICGMCFGRRYSHDDEELLGLVNLSDEFGQVVGSGNPAD

FIPILRYLPNRTMKRFMDINDRFNTFVQKIVSEHYESYDKDNIRDITDSLIDHCEDRKLD

ENANIQVSDEKIVGIVNDLFGAGFDTISTALSWAVVYLVAYPEIQERLYQELTEKVGMNR

TPRLSDKTNLPLLEAFILEIFRHSSFLPFTIPHCTIKDTSLNGYFIPKDTCVFINQWQVN

HDPELWKEPSLFNPDRFLSADGTELNKLEGEKVLVFGMGKRRCIGEAIGRNEVYLFLAIL

LQRLRFQEKPGHPLDMTPEYGLTMKHKRCQLKASLRPWGQEE

>Q92109.2

MVLMILPIIGSVSASEGLVAMVTLCLVYMIMKYMHTEIPEGLKRLPGPKPLPIIGNMLEV

HNNPHLSLTAMSERYGSVFQIQIGMRPVVVLSGNETVRQALIKQGEDFAGRSDLYSFKFI

NDGKSLAFSTDKAGVWRARRKLAMSALRSFATLEGTTPEYSCALEEHVLKEGEYLVKQLT

SVMDVSGSFDPFRHIVVSVANVICGMCFGRRYSHDDQELLGLVNMSDEFGQVVGSGNPAD

FIPILRYLPNRTMKRFMDINDRFNNFVQKIVSEHYESYDKDNIRDITDSLIDHCEDRKLD

ENANIQVSDEKIVGIVNDLFGAGFDTISTALSWAVVYLVAYPETQERLHQELKEKVGMIR

TPRLSDKINLPLLEAFILEIFRHSSFLPFTIPHCTIKDTSLNGYFIPKDTCVFINQWQVN

HDPELWKEPSSFNPDRFLSADGTELNKLEGEKVLVFGMGKRRCIGEAIGRNEVYLFLAIL

LQRLRFQEKPGHPLDMTPEYGLTMKHKRCQLKASMRPWGQEE

>AAB40627.1

MVLMILPIIGSVSVSEGLVAIVTLCLVYMLMKYKHTEIPEGLKRLPGPKPLPIIGNVLEV

YNNPHLSLTAMSERYGSVFQIQIGMRPVVVLSGNETVRQALIKQGEDFAGRPDLYSFKFI

NDGKSLAFSTDKAGVWRARRKLAMSALRSFATLEGTTPEYSCALEEHVCKEGEYLVKQLT

SVMDVSGSFDPFRHIVVSVANVICGMCFGRRYSHDDQELLGLVNMSDEFGQVVGSGNPAD

FIPILRYLPNRTMKRFMDINDRFNNFVQKIVSEHYESYDKDNIRDITDSLIDHCEDRKLD

ENANIQVSDEKIVGIVNDLFGAGFDTISTALSWAVVYLVAYPEIQERLHQELKEKVGMIR

TPRLSDKINLPLLEAFILEIFRHSSFLPFTIPHCTIKDTSLNGYFIPKDTCVFINQWQVN

HDPELWKEPSSFNPDRFLSADGTELNKLEGEKVLVFGMGKRRCIGEAIGRNEVYLFLAIL

LQKLRFQEKPGHPLDMTPEYGLTMKHKRCQLKASMRPWGQEE

>ACL31529.1

MALMILPIIGSVSVTEGLLAITTVCLVYMILKFLQPEIPEGLCRLPGPKPLPLIGNVLEL

RHKPYQSLTAMSKRYGHVFQIHIGTRPVVVLSGSETVRQALIKQGEEFAGRPDLYSFQFI

NDGKSLAFSTDQSGVWRARRKLAYSALRSFSNLESKNSEYSCVLEEHVSKEAEYLIKRLC

TVMKADGSFDPVRHIVVSVANVICGICFGRRYSHDDQELLSLVTLADDFNQVAGSGNPAD

FIPILQYLPSRNMKNFMDLNARFNSFVQKIVREHYATYDKDNIRDITDSLIDHCEDRKLD

ENFNVQVSDEKIVGIVNDLFGAGFDTVTTALSWSVMYMVAYPEIQERLYDELKVNVGLER

SPRLSDKPNLPFLEAFILEMLRHSSFLPFTIPHCTTKDTSLNGYFIPKDTCVFINQWQIN

HDPELWKDPFSFNPDRFLSADSTEVNKVEGEKVVAFGLGKRRCIGEVIARNEVYLFLAIL

IQKLEFHQMPGVPLDMTPQYGLTMKHKPCHLRATMRAINEQ

>AAD14036.1

MVLMILPIIGSVSVSEGLVAIVTLCLVYMLMKYKHTEIPEGLKRLPGPKPLPIIGNVLEV

YNNPHLSLTAMSERYGSVFQIQIGMRPVVVLSGNETVRQALIKQGEDFAGRPDLYSFKFI

NDGKSLAFSTDKAGVWRARRKLAMSALRSFATLEGTTPEYSCALEEHVCKEGEYLVKQLT

SVMDVSGSFDPFRHIVVSVANVICGMCFGRRYSHDDQELLGLVNMSDEFGQVVGSGNPAD

FIPILRYLPNRTMKRFMDINDRFNNFVQKIVSEHYESYDKDNIRDITDSLIDHCEDRKLD

ENANIQVSDEKIVGIVNDLFGAGFDTISTALSWAVVYLVAYPEIQERLHQELKEKVGMIR

TPRLSDKINLPLLEAFILEIFRHSSFLPFTIPHCTIKDTSLNGYFIPKDTCVFINQWQVN

HDPELWKEPSSFNPDRFLSADGTELNKLEGEKVLVFGMDKRRCIGEAIGRNEVFLFLAIL

LQRLRFQEKPGHPLDMTPEYGLTMKHKRCQLKASMRPWGQEE

>ADO15701.1

MVLMMLPLVGPVSVSEGLVAMTTLCLVYLIYKFFHTEIPKGLRRLPGPKPLPLIGNVLEL

GSRPYLSLTAMSKRYGHIFQIQIGMRPVVVLSGNDTLRQALIKQGDDFAGRPDLYSFRLI

NDGKSLAFSTDQAGVWRARRKLAYSALRSFSTLEGTTPEYSCMLEEHILKEGKYLIQQLK

DVMASDGSFDPFRHIVVSVANVICGMCFGRRYSHDDQELLSLVNLSDEFSQVAGSGNPAD

FIPALRFLPNALMTKFKDINARFNIFVQKIVSEHYATYDKDHIRDITDSLIDHCEDRKLD

ENSNVQMSDEKIVGIVNDLFGAGFDTVSTTLSWSVMYMVAHPEIQERLHQELKDKVGLDR

SPLLSDKPNLPFLEAFILESLRHSSFLPFTIPHCTTKDTSLNGYFIPKDTCVFINQWQVN

QDPELWKDPSTFNPDRFLSADGTEINKLKGERVMAFGMGKRRCIGEVIARNEVYLFLAII

VQKLHFHAIPGEPLDMTPEYGLTMKHKRCHLRATMRARSEE

>348500086

MALMILPFIGALSVSHVLVAVTTACLVYLIIKNAQNKIPEGLQQLPGPKSLPIIGNLLEL

GKRPYLSLTSMSKRYGDVFQIQIGMRPVVVLSGNETVRQALIKQGDEFAGRPDLYSFRYI

NDGKSLSFSTDQAGIWRARRKLAYSALRSFSNLEGTTPEYSCALEEHISKEAEYLIKELN

TVMKTKGSFDPFRYVVVSVANVICGMCFGRRYDHHDDELVSLVNLSDDFVKVVGSGNPAD

FIPLLQYLPSTKMKKFVSLNARFSKFVQKLVTEHYATFDKDNIRDITDSLIDHCEDRKLD

ENANIQMSDEKIVGIVNDLFGAGFDTISTALSWSLMYFVAYPEIQNRLFEEMKEKVGLDR

MPVFSDRNNLPLLEAYILELFRHSSYLPFTIPHCTTKDTSLNGYFIPKDTCVFINQWQIN

HDPELWEDPFSFKPERFLNADGTEVNKVEGEKVMTFGLGKRRCIGEVIARNEVFLFLAIL

IQNLNFQALPGDQLDLTPEYGLTMKHKRYHLRATMRVKNEQ

>ABZ88704.1

MALMIPPFIGSVSVSEGLVAMTTVCLIYLILKFFHTGIPEGLRRLPGPKPLPIIGSVLEV

GSRPYLSLTAMSKRYGDVFQIQIGMRPVVVLSGSDTVRQALIKQGEEFAGRPDLYSFRFI

NDGKSLAFSTDQAGVWRARRKLAYSALRSFSTLDSKTPEYSCMLEEHICKEGDYLIRQLN

TVMKADGSFDPFRHIVVSVANVICGMCFGRRYDHNDEELLSLVNLSDEFGQVVGSGNPAD

FIPVLQFLPSSTMKKFVSINDRFNKFVQKIVSEHYVTYDKDNIRDITDSLIDHCEDRKLD

ENSNIQMSDEKIVGIVNDLFGAGFDTISTALSWSVMYLVAYPEIQERLYQELKDKVGLER

SPRLSDKSNLPXLEAFILEIFRHSSFLPFTIPHCTSKDTSLNGYFIPKDTCVFINQWQIN

HDPELWKDPSSFNPDRFLSADGTELKKLEGEKVMVFGMGKRRCIGEVIARNEVFLFLAII

VQKLHFHTMPGEPLDMTPEYGLTMKHKRCHLRATMRARDEQ

>Q92110.2

MVLMILPIIGSVSVSEGLVAIVTLCLVYMLMKYKHTEIPEGLKRLPGPKPLPIIGNVLEV

YNNPHLSLTAMSERYGSVFQIQIGMRPVVVLSGNETVRQALIKQGEDFAGRPDLYSFKFI

NDGKSLAFSTDKAGVWRARRKLAMSALRSFATLEGTTPEYSCALEEHVCKEGEYLVKQLT

SVMDVSGSFDPFRHIVVSVANVICGMCFGRRYSHDDQELLGLVNMSDEFGQVVGSGNPAD

FIPILRYLPNRTMKRFMDINDRFNNFVQKIVSEHYESYDKDNIRDITDSLIDHCEDRKLD

ENANIQVSDEKIVGIVNDLFGAGFDTISTALSWAVVYLVAYPEIQERLHQELKEKVGMIR

TPRLSDKINLPLLEAFILEIFRHSSFLPFTIPHCTIKDTSLNGYFIPKDTCVFINQWQVN

HDPELWKEPSSFNPDRFLSADGTELNKLEGEKVLVFGMDKRRCIGEAIGRNEVYLFLAIL

LQRLRFQEKPGHPLDMTPEYGLTMKHKRCQLKASMRPWGQEE

>BAC87834.1

MMLMMLPFIGSVSVSESLVAMTMMCLVYLILKYLQTEIPEGLRRLPGPKPLPIIGNVLEL

GSKPYLSLTAMSKRYGHVFQIQIGMRPVVVLSGSETVRQALIKQGDEFAGRPDLYSFRFI

NEGKSLAFSTDQAGVWRARRKLAYSALRSFSKLEGTTPEYSCVLEEHICKEGEYLIKRLN

TVMKADGSFDPFRHIVVSVANVICGMCFGRRYDHDDQELVGLVTLSDEFGRVVGSGNPAD

FIPILQYLPSATMKNFVRINARFTTFVQKIVTEHYTTFNKDNIRDITDSLIDHCEDRKLD

ENSNVQMSDEKIVGIVNDLFGAGFDTVSTAMSWSVMYLVAHPEIQERLYQEIEDKVGLDR

MPLLSDKPNLPFLEAFILEVFRHSSFLPFTIPHCTSKDTSLNGYFIPKDTCVFINQWQIN

HDPELWKDPSSFNPDRFLSADGSEVNKLDGEKVMAFGMGKRRCIGEVIARNEVYLFLAII

IQKLHFLPIPGEKLDMTPEYGLTMKHKRCHLKATMRARNQH

>ADV36120.1

MMLMMLPFIGSVSVSESLVAMTMMCLVYLILKYLQTEIPEGLRRLPGPKPLPIIGNVLEV

GSRPYLSLSAMSKRYGHVFQIQIGMRPVVVLSGSETVRQALIKQGDDFAGRPDLYSFRFI

NEGKSLAFSTDKAGVWRARRKLAYSALRSFSNLEGTTPEYSCVLEEHICKEGEYLIKQLN

TVMKADGSFDPFRHIVVSVANVICGMCFGRRYDHDDQGLVGLVTLSDEFGQVVGSGNPAD

FIPILQYLPSATMKNFARINVRFTKFVQKIVTEHYTTFNKDNIRDITDSLIDHCEDRKLD

ENSNVQMSDEKIVGIVNDLFGAGFDTVSTAMSWSVMYLVAHPEIQERLYQEIEDKVGLDR

MPLLSDRPNLPFLEAFILEILRHSSFLPFTIPHCTTKDTSLNGYFIPKDTCVFINQWQIN

HDPELWKDPSSFNPDRFLSADGSEVNKLDGEKVMAFGMGKRRCIGEVIARNEVYLFLAIL

IQKLHFLPIPGEKLDMTPEYGLTMKHKRCHLKATMRARNQH

>AAA49550.1

MVLMILPIIGSVSASEGLVAMVTLCLVYMIMKYMHTEIPEGLKRLPGPKPLPIIGNVLEV

HNNPHLSLTAMSERYGSVFQIQIGMRPVVVLSGSETVRQALIKQGEDFAGRPDLYSFKFI

NDGKSLAFSTDKAGVWRARRKLAMSALRSFATLEGTTPEYSCALEEHVCKEGEYLVKQLT

SVMDVSGSFDPFRHIVVSVANVICGMCFGRRYSHDDQELLGLVNMSDEFGQVVGSGNPAD

SIPILRYLPNRTMKRFMDINDRFNTFVQNIISEHYESYDKDNIRDITDSLIDHCEDRKLD

ENANIQVSDEKIVGIVNDLFGAGFDTISTALSWAVVYLVAYPEIQERLHQELKEKVGMIR

TPRLSDKINLPLLEAFILEIFRHSSFLPFTIPHCTIKDTSLNGYFIPKDTCVFINQWQVN

HDPDLWKEPSSFNPDRFLSADGTELNKLEGEKVLVFGMDKRRCIGEAIGRNEVFLFLAIL

LQRLRFQEKPGHPLDMTPEYGLTMKHKRCQLKASMRPWGQEE

>ABX10186.1

MILPFIGSVSVSESLVAMITMCLAYLILKFFRTEIPEGLRELPGPKPLPIIGNVLEVGRN

PYLSLTAMSKRYGDVFQIQIGMRPVVVLSGNETVRQAFIKQGEEFAGRPDLYSFKFINDG

KSLAFSTDQAGVWRARRKLAYSALRSFATLEGTTPEYSCALEEHVSKEAEYLVKQFNNVM

KADGSFDPFRHIVVSVANVICGMCFGRRYDYNNQELLDLVNLSDEFGQVVASGNPADFIP

ILQYLPSTTMKKFLNISRNARFNEFVQKIVSEHYTTFDKDNIRDITDSLIDHCEDRKLDE

NSNVQMSDEKIVGIVNDLFGAGFDTISTALSWSVMYLVAYPEIQERLYQEMKETVGPDRM

PCLSDKPNLPFLEAFILEILRHSSFLPFTIPHCTTKDTSLNGYFIPKDTCVFVNQWQINH

DPELWKDPSSFNPDRFLNADGTEVNKLDGEKMMVFGMGKRRCIGEVIARNEVYLFLAILV

QNLQFHAMPGEPLDMTPEYGLTMKHKRCHLRAAMRPRNEE

>ABV01348.1

MALTVLPVLGPISVSESLVAIITICVVYLLMRLMRVKIPEGLHKLPGPKPLPIIGNVLEV

GSNPHLSLTAMSKCYGPIFQIQIGTRPVVVLSGNDVIRQALLKQGEEFSGRPDLYSTRYI

SDGKSLAFSTDQVGVWRARRKLALSALRTFSTLQSQSSQYSCALEEHISKEGLYLIERLH

SVMKSDGSFDPFRHIVVSVANVICGICFGRRYSHDDDELVSLVNPSDEFGKIVGSGNPAD

FIPILRILPSCSMKKFLAINARFSAFMQKIVKDHYDSFSKDNIRDITDSLIDHCEDHKLD

ENSNVQVSDEKIVGIVNDLFGAGFDTISTALSWSVVYLVAYPEIQERLQRELNEKVGMER

TPRLSDRTELPLLEAFILEIFRHSSFLPFTIPHCTTKDTSLNGFFIPKDTCVFVNQWQVN

HDPELWKDPSCFNPDRFLTADGTDLNRMEGEKVLVFGLGKRRCIGESIGRAEVFLFLAIL

LQRLEFSGMPGELLDMTPEYGLTMKHKRCLLRVAPRPGYAS

>Q92100.1

MMLMMLPFIGSVSVSESLVAMTTMCLVYLILKFFQTEIPEGLRRLPGPKPLPIIGNVLGL

GSKPYLSLTAMSKRYGHVFQIQIGMRPVVVLSGTGTVRQALIKQGDEFAGRPDLYSFRFI

NAGKSLAFSTDQAGVWRARRKLAYSALRSFSTLEGTTPEYSCVLEEHICKEGEYLIKQLN

TVMKADGSFDPFRHIVVSVANVICGMCFGRRYDHDDQELVSLVTLSDEFGRVVGSGNPAD

FIPILQYLPSAEMKNFLRINEHFTEFVQKIVTEHYTTFNKDNIRDITDSLIDHCEDRKLD

ENSNVQMSDEKIVGIVNDLFGAGFDTVSTALSWSVMYLVAHPEIQERLYQEIEDKVGLDR

MPLLSDKPNLPFLEAFILEILRHSSFLPFTIPHCTTKDTSLNGYFIPKDTCVFINQWQIN

HDPELWKDPSSFNPDRFLSADGSEVNKLDGEKVMAFGMGKRRCIGEVIARNEVYLFLAII

IQKLHFLPIPGEKLDMTPEYGLTMKHKRCHLKATMRARNEH

>ACN38059.1

MALMILPFIGALSVSHVLVAVTTACLVYLIIKNAQNKIPEGLQQLPGPKPLPIIGNLLEL

GKRPYLSLTSMSKRYGDVFQIQIGMRPVVVLSGNETVRQALIKQGDEFAGRPDLYSFRYI

NDGKSLSFSTDQAGIWRARRKLAYSALRSFSNLEGTTPEYSCALEEHISKEAEYLIKELN

TVMKTKGSFDPFRYVVVSVANVICGTCFGRRYDHHDDELVSLVNLSDDFVKVVGSGNPAD

FIPLLQYLPSTKMKKFVSLNARFSKFVQKLVTEHYATFDKDNIRDITDSLIDHCEDRKLD

ENANIQMSNEKIVGIVNDLFGAGFDTISTALSWSLMYFVAYPEIQNRLFEEMKEKVGLDR

MPVFSDRNNLPLLEAYILELFRHSSYLPFTIPHCTTKDTSLNGYFIPKDTCVFINQWQIN

HDPELWEDPFSFKPERFLNADGTEVNKVEGEKVMTFGLGKRRCIGEVIARNELFLFLAIL

IQKLNFQALPGDQLDLTPEYGLTMKHKRYHLRATMRVKNEQ

>O42430.1

MMLMMLPFIGSVSVSESLVAMTTVCLVYLILKFFQTEIPEGLRRLPGPKPLPIIGNVLEM

GSRPYLSLTAMSKRYGNVFQIQIGMRPVVVLSGSETVRQALIKQGDDFAGRPDLYSFRFI

NEGKSLAFSTDKAGIWRARRKLAYSALRSFATLEGTTPEYSCVLEEHVCKEGEYLIKQLN

TAMTADGSFDPFRHIVVSVANVICGMCFGRRYDHDDQELVGLVTLSDEFGRVVGSGNPAD

FIPILQYLPSATMKNFLRINGRFTEFVQKIVTEHYTTFDKDNIRDITDSLIDHCEDRKLD

ENSNVQMSDEKIVGIVNDLFGAGFDTVSTALSWSVMYLVAHPEIQERLYQEIEDKVGLDR

MPLLSDKPNLPFLEAFILEILRHSSFLPFTIPHCTTKDTSLNGYFIPKDTCVFINQWQIN

HDPEMWKDPSSFNPDRFLSADGSEVNKLDGEKVMAFGMGKRRCIGEVIARNEVYLFLAIL

IQKLHFLPIPGEKLDMTPEYGLTMKHKRCHLKATMRARNEH

>AAD01809.1

MALMILPFIGALSVSEGLIALVTVCLVYLTLKHFRREIPEGLRRLPGPTPLPIIGNFLEL

GSKPYLSLTEMSKRFGDVFQIQLGMRPVVILSGYETVKQALTKQGDDFAGRPDLYSFRFI

NDGKSLAFSTDKAGVWRARRKLAYSALRSFSSLEGKLPEYSCVLEEHICKETEHLIKELH

NVMTAEGKFDPFRYIVVSVANVICGMCFGRRYDHHNQELLSLVNLAEDFVQVTGSGNPAD

FIPALQFLPNKSMKKFVNLNNRFNNFVQKIVSEHYSTFDKDNIRDITDSLIDHCEDRKLD

ENSNIQMSDEKIVGIVNDLFGAGFDTISTALSWAVMYLVAYPEVEERLYEEIKEKVGLDR

TPVMSDRSNLPLLESFILELFRHSSYLPFTIPHCSTKDTSLNGYFIPKDTCVFVNQWQIN

HDPELWKDPSMFIPDRFLSADGTEVNKQEGEKVLIFGLGRRRCIGEVIARNEVFLFLAII

IQKLHFYKLPGEPVDMTPEYGLTMKHKRCYLGVAMRAKDVQ

>ABZ88706.1

MALMILPFIGALSVSESLVAVLTVCLAYLILKSFQDKIPEGLSRLPGPKPLPIIGNVLEM

GSRPYLSLSEMSKRYGDVFQIQIGMRPVVVLSGNETVRQALIKQGDEFAGRPDLYSFRFI

NDGKSLAFSTDKAGVWRARRKLAYSALRTFATLEGTTPEYSCVLEEHICKEADYPIKQLD

SAMKADGSFDPFRYIVVSVANVICGMYFGRRYDHNDQELLGLVNLSDEFVQVVGSGNPAD

FIPILQYLPNQTMKKFVGINDRFINFVQKIVTEHYATYNKDNIRDITDSLIDHCEDRKLD

ENANVQISDEKVVGIVNDLFGAGFDTISTALSWSVMYLVTYPEIQERLYEELKEKVGVDR

TPVLADRNNLPLLEAFILEIFRHSSFLPFTIPHCTTKDTSLNGYFIPKDTCVFINQWQIN

HDPELWKDPSSFNPDRFLNADGTEVNRVEGEKVVIFGMGKRRCIGEVIARNEVYMFLAIL

IQNLNFYNLPGEPLDMTPEYGLTMKHKRCQLRATARVRDDY

>ADR30129.1

MVLAVLPILGPISVSESLVAMITICTVYLLLQFTRTKIPEGLQHLPGPKPLPLIGNILQL

AQNPHLTLTAMSKRYGPIFQVQIGMRPVVVLGGKEVIHQALIKQGDEFSGRPDLYSFKFI

NNGKSLAFSSDQVNVWRTRRKLAHSALRSFAILKDPSGQYSCTLEMHISKEALYLVKRLH

EVMEADGCFDPFRHIVVSVANVICGMCFGRRYSHDDQELLGFVNLSDEFGRVVGNGNPAD

FIPFLRMLPNQDITIFQDINRRFNVFVQRIVREHYDSYDKNNIRDITDSLIDHCEDRKLD

ENSNLQVSDEKIVGIVNDLFGAGFDTISTALSWSIVYLVVYPEIQERLHQELREKVGMDR

TPSLLDKSRLPYLEAFILEIFRHSSFLPFTIPHCTTKDTSLNGYFIPNNTCVFINQWQIN

HDPELWKEPSLFNPDRFLSADGTELNKMEAEKVMVFGLGKRRCVGEAIGRSEVFLFLAVL

IQRLKFSSPGGQMPDMTPEYGLTMKHKRCFLCATPRPGSEV

>Q9YH64.1

MMLMMLPFIGSVSVSESLVAMTTMCLVYLILKFFQTEIPEGLLRLPGPKPLPIIGNVLGL

GSKPYLSLTDMSKRYGHVFQIQIGMRPVVVLSGSETVRQALIKQGDDFAGRPDLYSFRFI

NAGKSLAFSTDQAGVWRARRKLAYSALRSFSNLEGTTPEYSCVLEEHICKEGEYLIKQLN

TVMKADGSFDPFRHIVVSVANVICGMCFGRRYDHDDQELVGLVTLSDEFGRVVGSGNPAD

FIPILQYLPSAAMKNFLRINSRFTEFVQKIVTEHYTTFDKDNIRDITDSLIDHCEDRKLD

ENSNVQMSDEKIVGIVNDLFGAGFDTVSTALSWSVMYLVAHPEIQERLYQEIEDKVGLDR

MPLLSDKPNLPFLEAFILEILRHSSFLPFTIPHCTTKDTSLNGYFIPKDTCVFINQWQIN

HDPELWKDPSSFNPDRFLSADGSEVNKLDGEKVMAFGMGKRRCIGEVIARNEVYLFLAII

IQKLHFLPIPGEKLDMTPEYGLTMKHKRCHLKATMRARNEH

>ACJ60906.2

MALMILPFIGALSVSHVLVAVTTACLVYLIIKNAQNKIPEGLQQLPGPKPLPIIGNLLEL

GKRPYLSLTSMSKRYGDVFQIQIGMRPVVVLSGNETVRQALIKQGDEFAGRPDLYSFRYI

NDGKSLSFSTDQAGIWRARRKLAYSALRSFSNLEGTTPEYSCALEEHISKEAEYLIKELN

TVMKTKGSFDPFRYVVVSVANVICGTCFGRRYDHHDDELVSLVNLSDDFVKVVGSGNPAD

FIPLLQYLPSTKMKKFVSLNARFSKFVQKLVTEHYATFDKDNIRDITDSLIDHCEDRKLD

ENANIQMSDEKIVGIVNDLFGAGFDTISTALSWSLMYFVAYPEIQNRLFEEMKEKVGLDR

MPVFSDRNNLPLLEAYILELFRHSSYLPFTIPHCTTKDTSLNGYFIPKDTCVFINQWQIN

HDPELWEDPFSFKPGRFLNADGTEVNKVEGEKVMTFGLGKRRCIGEVIARNELFLFLAIL

IQKLNFQALPGDQLDLTPEYGLTMKHKRYHLRATMRVKNEQ

>ABZ88705.1

MALMILPFVGALSVSESLVAVVTVCLVYLIMKSFQEKIPEGLSRLPGPKPLPIIGNVLEM

GSRPYLSLTEMSKRYGNVFQIQIGMRPVVVLSGNETVRQALIKQGDDFAGRPDLYSFRFI

SEGKSLAFSTDQAGVWRARRKLAYSALRSFSTLEGTTPEYSCVLEEHISKEAEYLVKQLN

TVMKADGSFDPFRYIVVSVANVICGMCFGRRYDHHDQELLSLVNLSDEFGQWWAVATQAD

FIPILQYLPNKTMKKFVNINGRFISFVQKIVNEHYATFNKDNIRDITDSLIDHCEDRKLD

ENANVQMSDEKVVGIVNDLFGVGFDTISTALSWSVMYLVAYPEIQERLYQELKENVGLDR

TPVLSDRNNLPLLEAFILEIFRHSSFLPFTIPHCTTKDTSLNGYHIPKDTCVFINQWQIN

HDPELWKEPSSFNPDRFLSADGTEVNRVDGEKVMIFGMGKRRCIGEVIARNEVYMFLAIL

IQKLHFHNLPGEPLDMTPEYGLTMKHKRCQLRATARVRSDH

>ADC35580.1

MALMILPFIGSVSVSESLVAMATVCLVYLILKFFHTEIPEGLRRLPGPKPLPIIGNVLEV

GSKPYLSLTAMSKRYGHIFQIQIGMRPVVVLSGSETVRQALIKQGEEFAGRPDLYSFRFI

NDGKSLAFSTDQAGVWRARRKLAYSALRSFSSLEGTTPEYSCVLEEHICREGEYLIKQLN

TVMKADGSFDPFRHIVVSVANVICGMCFGRRYDHNDQELLSLVNLSDEFGQVVGSGNPAD

FIPALQFLPSKTMKKFMNINARFNTFVQKIVSEHYATYDKDNIRDITDSLIDHCGDRKLD

ENSNVQMSDEKVVGIVNDLFGAGFDTISTALSWSVMYLVAYPEIQERLYQELKNNVGLDR

TPVLSDKPNLPFLEAFILEIFRHSSFLPFTIPHCTSKDTSLNGYFIPKDTCVFINQWQIN

HDPELWKDPSSFNPDRFLSADGTDVNKLEGEKVMVFGMGKRRCIGEVIARNEVYLFLAII

IQKLHFHTMPGEPLDMTPEYGLTMKHKRCHLRATMRARNE

>ABV24057.1

MVLMVLPLIGSVSVSEVLVALTTACLVYLMGRYFYTEIPAGLRRLPGPTPLPIIGNVLEI

GRRPYLSLTAMSKRYGDVFQIQIGTRPVIVLSGMETVRQALVEQGEEFSSRPDLYSFRFI

NEGKSLSFSTDQAGVWRARRKLAYNALRSFSTLQGTTPEYSCVLEEHICKEGEYLVNRLS

SVLQADGRFEPFPHIVVSVANVICGMCFGRRYDHDDQELVSLVNLSNEFGKVVSSGNPAD

FIPALRFLLSSTMKSFVDLNTRFTTFVQKIVNEHYATFDKENIRDITDSLIDHCEDRKLD

ENSNIQVSDEKIVGIVNDLFGAGFDTVSTALSWSIMYLVTYPDVQERLYQELKSNVDQNR

KPRLSDKPNLPLLEAFILELFRHSSFLPFTIPHCTSKDTSLNGYFIPKDTCVFINQWQIN

HDPEQWEDPSSFNPDRFLSADGTEVNKAEGEKVTTFGMGKRRCIGEIIARNEVYLFLAIL

IQRLQFLPIPGETVDMTPEYGLTMKHKDCRLKARMRTRDER

>AAL54873.2

MALTILPILGPISVSESLVAIITICLVYLLMRLNRTKIPDGLQKLPGPKPLPIIGNVLEI

GNNPHLSLTAMSKCYGPVFQIQIGMRPVVVLSGNDVIRQALLKQGEEFSGRPELYSTKFI

SDGKSLAFSTDQVGVWRARRKLALNALRTFSTVQGKSPKYSCALEEHISNEGLYLVQRLH

SVMKADGSFDPFRHIVVSVANVICGICFGRRHSHDDDELVRLVNMSDEFGKIVGSGNPAD

FIPFLRILPSTTMKKFLDINERFSKFMKRLVMEHYDTFDKDNIRDITDSLINHCEDRKLD

ENSNLQVSDEKIVGIVNDLFGAGFDTISTALSWAVVYLVHYPEVQERLQRELDEKIGKDR

TPLLSDRANLPLLESFILEIFRHSSFLPFTIPHCTSKDTSLNGYFIPKDTCVFVNQWQVN

HDPELWKDPSSFIPDRFLTADGTELNKLEGEKVLVFGLGKRRCIGESIGRAEVFLFLAIL

LQRLKFTGMPGEMLDMTPEYGLTMKHKRCLLRVTPQPGF

>327291832

MFVGNENIISVAEALIALVVFLLVLSITRSFRKKIPPGLKRLPGPVAYPLIGNIVQMGKN

PHLSFNRMRGKYGDVMQVHIGMRPVLVLSGLETIKQALVKQGEEFMARPDLYTFNMIADG

QSLTFGRDTEAVWRVRKKLAQNALKTFSSAPSLTSASSCIVEEHVSEEASYLVTKLLQVM

EEKGRFCPYRYVVISVANVICAVTFGKRYSHDDKELLDIIHLMDEAEKATGLGNLADFIP

VLQYLPNPLMKRFKALVMNFNAFLQKNINRHYESFNKDHIRDITDSLIAQSQDMKFLAKN

SIQLPEQKIVNLVNDLVGASFDTVTAALSWCIMYLVSFPEIQKKIQKELDQTIGKERTPR

LSDRALLPYAEAFILEVFRHSSYVPFTIPHCTTKDTSLNGFYIPKDLCVFVNQWQVNHDE

TLWEDPSSFNPDRFLSADGTEIDRAESEKVMLFGMGKRRCIGENLARWEVFLFLTTLLQR

LEFTVSDGERVNMTPEYGLVMKPKKCENLQVRERFPRQSSE

>AAQ97766.1

MALTILPILGPISVSESLVAIITICLVYLLMRLNRTKIPDGLQKLPGPKPLPIIGNVLEI

GNNPHLSLTAMSKCYGPVFQIQIGMRPVVVLSGNDVIRQALLKQGEEFSGRPELYSTKFI

SDGKSLAFSTDQVGVWRARRKLALNALRTFSTVQGKSPKYSCALEEHISNEGLYLVQRLH

SVMKADGSFDPFRHIVVSVANVICGICFGRRHSHDDDELVRLVNMSDEFGKIVGSGNPAD

FIPFLRILPSTTMKKFLDINERFSKFMKRLVMEHYDTFDKDNIRDITDSLINHCEDRKLD

ENSNLQVSDEKIVGIVNDLFGAGFDTISTALSWAVVYLVHYPEVQERLQRELDEKIGKDR

TPLLSDRANLPLLESFILEIFRHSSFLPFTIPHCTSKDTSLNGYFIPKDTCVFVNQWQVN

HDPELWKDPSSFIPDRFLTADGTELNKLEGEKVLVFGLGKRRCIGESIGRAEVFLFLAIL

LQRLKFTGMPGEMLDMTPEYGLTMKHKRCLLRVTPQPVF

>BAA88241.1

MALMILPLVGPVSVSESLLAMATVCIVYLLMRLLRSDIPEGLQRLPGPKPLPIIGNVLEV

GANPHLSLTAMSERFGQVYQIQIGMRPVVVLNGSETIRQALIKQGEEFASRPDLYSFQFI

NNGKSLAFSSDQAGVWRTRRKLAMNALRSFSTIKGSDLGYSCVLEEHVCKEGEYLVKQLS

NVTEASGSFDPFRHIVVSVANVICGMCFGRRYNHDDQELLSFVNLSDEFGQVAGSGNPAD

FIPFLRFLPSRTMKTFMDINARFNVFVQKIVTEHYRTFDKNNIRDITDSLIDHCEDRKLD

ENSNVQVSDEKIVGIVNDLFGAGFDTISTALSWAVVYLVAYPEIQEKLHQEMKDSVGLER

TPLLSDKANLPLLEAFILELFRHSSFLPFTIPHCTTKDTALNGYFIPKDTCVFINQWQVN

HDPALWKDPSSFSPERFLNADGVTVNKMDAEKVMVFGMGKRRCIGEAIGRTEVFLFLAIL

VQKLRFHELPGHPLDLTPEYGLTMKHKRCQLRASLRVQE

>BAA88248.1

MALMILPLVGPVSVSESLLAMATVCIVYLLMRLLRSDIPEGLQRLPGPKPLPIIGNVLEV

GANPHLSLTAMSERFGQVYQIQIGMRPVVVLNGSETIRQALIKQGEEFASRPDLYSFQFI

NNGKSLAFSSDQAGVWRTRRKLAMNALRSFSTIKGSDLGYSCVLEEHVCKEGEYLVKQLS

NVTEASGSFDPFRHIVVSVANVICGMCFGRRYNHDDQELLSFVNLSDEFGQVAGSGNPAD

FIPFLRFLPSRTMKTFMDINDRFNVFVQKIVTEHYRTFDKNNIRDITDSLIDHCEDRKLD

ENSNVQVSDEKIVGIVNDLFGAGFDTISTALSWAVVYLVAYPEIQEKLHQEMKDSVGLER

TPLLSDKANLPLLEAFILELFRHSSFLPFTIPHCTTKDTALNGYFIPKDTCVFINQWQVN

HDPALWKDPSSFSPERFLNADGVTVNKMDAEKVMVFGMGKRHCIGEAIGRTEVFLFLAIL

VQKLRFHELPGHPLDLTPEYGLTMKHKRCQLRASLRVQE

>AFN02446.1

MALMILPFIGALSVSEGLIALVTVCLVYLTLKHFHREIPEGLRQLPGPSAFPIIGNLIEL

GSKPYLSLTEMSKRFGDVFQIQIGMRPVVVLSGYDTVKQALTKQGDDFAGRPDLYSFRFI

NDGKSLAFSTDQAGIWRARRKLAYSALRSFSSLNGKLPEYSCVLEEHICKEAEHLIKELQ

NVMITEGKFEPFRYIVVSVANVICGMCFGRRYDHHDQELVGLVNLAEDFVQVTGSGNPAD

FIPAMQYLPNKSMKKFVNINNRFNNFVQKLVTEHYATFDKDNIRDITDSLIDHCEDRKLD

ENSNIQMSDEKVVGIVNDLFGAGFDTISTGLSWAVMYLLSYPEVQERLFQEIKEQIGLDR

TPTLADRNNLPLLESFILELFRHSSYLPFTIPHCSTKDTSLNGYFIPKDTCVFINQWQIN

HDPELWKDPSSFIPDRFLNDDGTEVNKLEGEKVLIFGLGRRRCIGEVIARNEVFLFLAII

IQKLHFYKLPGEPVDMTPEYGLTMKHKRCYLGVSMRAKDDQ

>327288212

MLTLEMPFMEVLSHITATEALLGVAVFCLFFMYVKSFQNRIPKGLKKIPGPTGFPLIGNA

LQMGKYPHLSLTRMSQKYGDVMMIHIGNTPVLVLSGLKTIHQALVRQATEFMGRPDLYSF

RCIANGESLGFGRDSGEVWRARRKMVQNALKAFATSPSSNSFSTYLVEEHVSKEANYLIE

KFQEVMLEKQSFDPYEHILVSTANIICAMCFSKSYHHDDEELLGIVNTSEKFVEVATSGN

LADFIPLLRYLPMNSMKMFHEFNRKFYTFMLKEIKEHYESFSKDSIRDITDSLIDQSQKM

FSISSENLFSFLNDLFGAAFDTVTTVMSWGLMYLVVHPEIQKKIQEEIDEVIGRARKPRL

SDRPLMPYTEAFILEVFRHSSLLPFTIPHCTTKETVLNGYYIPKDICVFINQWQVNHDEN

LWKDPSSFNPERFLSADGKDVNKDEREKVLIFGLGKRRCIGEPIARWEIFLFLTFLLQEL

EFSVKEGVKVDMTPRYGLSMKVKRCPHFQVKRRSLKNK

>AAP48792.1

MALMVLPFIGPLSVLEGLIALTTVCVVYLLLKHFNKEIPGGLRQLPGPTPLPIIGNLLEL

GSKPYLSLTEMSKRFGDVFQIQIGMRPVVVLSGNETVRQALIKQGDDFSGRPDLYSFQFI

NDGKSLAFSTDQAGVWRARRKLAYSALRSFSSLEGSNAEYSCMLEEHICKETEHLVKEIE

KVMKTEGKFDPYRYIVVSVANVICGMCFGRRYDHHDQELVGLVNLSEDFVQVTGSGNPAD

FIPALRYLPSKAMKKFVEINNRFQCFVQKIVNEHYATYDKDNIRDITDSLIDHCEDRKLD

ENSNIQMSDEKVVGIVNDLFGAGFDTISTALSWAVGYLVAYPDIEKRLFEEIKENIGLNR

NPTISDRSNLPLTEAFILEIFRHSSFLPFTIPHCTTRDTSLNGYYIPKDTCVFINQWQIN

HDPKLWQDPSSFNPDRFLSEDGSEVNRLDGEKVMVFGLGKRRCIGEVIARNEVFLFLAIM

IQKLCFEEMPGEPLDLTPEYGLTMKHKRCNVRASLRLKDGC

>AAH94977.1

MALTILPILGPISVSESLVAIITICLVYLLMRLNRTKIPDGLQKLPGPKPLPIIGNVLEI

GNNPHLSLTAMSKCYGPVFQIQIGMRPVVVLSGNDVIRQALLKQGEEFSGRPELYSTKFI

SDGKSLAFSTDQVGVWRARRKLALNALRTFSTVQGKSPKYSCALEEHISNEGLYLVQRLH

SVMKADGSFDPFRHIVVSVANVICGICFGRRHSHDDELVRLVNMSDEFGKIVGSGNPADF

IPFLRILPSTTMKKFLDINERFSKFMKRLVMEHYDTFDKDNIRDITDSLINHCEDRKLDE

NSNLQVSDEKIVGIVNDLFGAGFDTISTALSWAVVYLVHYPEVQERLQRELDEKIGKDRT

PLLSDRANLPLLESFILEIFRHSSFLPFTIPHCTPKDTSLNGYFIPKDTCVFVNQWQVNH

DPELWKDPSSFIPDRFLTADGTELNKLEGEKVLVFGLGKRRCIGESIGRAEVFLFLAILL

QRLKFTGMPGEMLDMTPEYGLTMKHKRCLLRVTPQPGF

>AFJ15525.1

MALMILPFIGALSVSEGLIALVTVCFVYLTLKQFRREIPEGLRQLPGPSAFPIIGNLIEL

GSKPYLSLTEMSKRFGDVFQIQIGMRPVVVLSGYETVKQALTKQGDDFAGRPDLYSFRFI

NDGKSLSFSTDQAGIWRARRKLAYSALRSFSSLDGKLPEYSCVLEEHICKESEHLIKELQ

NVMRVEGKFDPFRYIVVSVANVICGMCFGRRYDHHDQELLSLVNLAEDFVQVAGSGNPAD

FIPAMQYLPNKSMRKFVNLNNRFNNFVQKIVTEHYTTFDEDNIRDITDSLIDHCEDRKLD

ENSNIQMSDEKIVGIVNDLFGAGFDTISTGLSWAVMHLVSYPEVEERLFQEIKVQIGLDR

TPTLADRNNLPLLESFILELFRHSSYLPYTIPHCSTKDTSLNGYFIPKDTCVFINQWQIN

HDPELWKDPSSFIPDRFVNDDGTEVNKLEGEKVLIFGMGRRRCIGEVIARNEVFLFLAII

IQKLHFYKLPGEPVDMTPEYGLTMKNKRCYLGVSMRAKGDQ

>AAL99904.1

MALMVLPLVVPVSVSESLLAMATVCIVYLLMRLLRSDIPEGLQRLPGPKPLPIIGNVLDV

GANPHLSLTAMSERFGQVYQIQIGMRPVVVLNGSETIRQALIKQGEEFASRPDLYSFQFI

NNGKSLAFSSDQAGVWRTRRKLALNALRSFSTIKGSDLGYSCVLEEHVCKEGEYLVKQLS

NVTEASGSFDPFRHIVVSVANVICGMCFGRRYNHDDQELLSFVNLSDEFGQVAGSGNPAD

FIPFLRFLPSRTMKTFMEINARFNAFVQKIVTEHYRTFDKNNIRDITDSLIDHCEDRKLD

ENSNVQVSDEKIVGIVNDLFAAGFDTISTALSWAVVYLVAYPEIQEKLHQEMKDSVGLER

TPLLSDKANLPLLEAFILELFRHSSFLPFTIPHCTTKDTALNGYFIPKDTCVFINQWQVN

HDPALWKDPSSFSPERFLNADGITVNKMDAEKVMIFGMGKRRCIGEAIGRTEVFLFLAIL

VQKLRFHELPGHPLDLTPEYGLTMKHKRCQLRASLRVQEQN

>ADD14079.1

MALMILPFIGSVSVSESLVAMATVCLVYLILKFFHTEIPEGLRRLPGPKPLPIIGNVLEV

GSKPYLSLTAMSKRYGHIFQIQIGMRPVVVLSGSETVRQALIKQGEEFAGRPDLYSFRFI

NDGKSLAFSTDQAGVWRTRRKLAYSALRSFSSLEGTTPEYSCVLEERICKEGEYLIKRLN

TVMKADGSFDPFRHIVVSVANVICGMCLGRRYDHNDQELLSLVNLSDEFGQVVGSGNPAD

FIPAPQFLPSKTMKKFMNINARFNTFVQKIVSEHYATYDKDNIRDITDSLIDHCEDRKLD

ENSNVQMSDEKVVGIVNDLFGAGFDTISTALSWSVMYLVAYPEIQERLYQELKNNVGLDR

TPVLSDKPNLPFLEAFILEIFRHSSFLPFTIPHCTSKDTSLNGYFIPKDTCVFINQWQIN

HDPELWKDPSSFNPDRFLSADGTDVNKLEGEKVMVFGMGKRRCIGEVIARNEVYLFLAII

IQKLHFHTMPGEPLDMTPEYGLTMKHKRCHLRATMRARNE

>Q6JZS3.1

MALMVLPFIGPLSVLEGLIALTTVCVVYLLLKHFNKEIPGGLRQLPGPTPLPIIGNLLKL

GSKPYLSLTEMSKRFGDVFQIQIGMRPVVVLSGNETVRQALIKQGDDFSGRPDLYSFQFI

NDGKSLAFSTDQAGVWRARRKLAYSALRSFSSLEGSNAEYSCMLEEHICKETEHLVKEIE

KVMKTEGKFDPYRYIVVSVANVICGMCFGRRYDHHDQELVGLVNLSEDFVQVTGSGNPAD

FIPALRYLPSKAMKKFVEINNRFQSFVQKIVNEHSATYDKDNIRDITDSLIDHCEDRKLD

ENSNIQMSDEKVVGIVNDLFGAGFDTISTALSWAVGYLVAYPDIEKGLFEEIKENIGLNR

NPTISDRSNLPLTDAFILEIFRHSSFLPFTIPHCTTRDTSLNGYYIPKDTCVFINQWQIN

HDPKLWQDPSSFNPDRFLSEDGSEVNRLDGEKVMVFGLGKRRCIGEVIARNEVFLFLAIM

IQKLCFEEMPGEPLDMTPEYGLTMKHKRCNVRASLRLKDGC

>ABF60890.1

MPLTVLPILGPISVSESLVAIITICVVYLLMRLMRTKIPEGLQKLPGPKPLPIIGNVLEV

GNNPHLSLTAMSKFYGPVFQIQIGMRPVVVLSGNDVIRQALLKQGEEFAGRPDLHSSKFI

SDGKSLAFSTDQVGVWRARRKLALSALRTFSTVQAKSSEYSCALEEHISKEGLYLIERLH

SVMKADGSFDPFRHIVVSVANVICGICFGQRYSHDDDELVSLVNLSDEFGKIVGSGNPAD

FIPFLRILPSTMMKKFVAINARFSKLMKKMVNDHYDTFNKDNIRDITDSLINHCEDRKLD

ENSNVQVSDEKIVGIVNDLFGAGFDTISTALSWGVVYLVAYPEIQERLQRELREKIGMDR

TPRLSDRTDLPLLEAFILEIFRHSSFLPFTIPHCTSKDTSLNGYFIPKDTCVFVNQWQIN

HDPELWKDPSSFNLDRFLTADGTELNKIEGEKVLVFGLGKRRCIGESIGRAEVFLFMAIL

LQRLKFSGMPGEMLDMTPEYGLTMKHKRCMLRVTPQPGFELPGTAA

>Q92039.1

MALMILPFIGSVSVSESLVALTAVCLVYLILKFFRTEIPAGLRQLPGPKPLPIIGNVLEV

GSKPHLSLTAMSKRYGDVFQIQIGMRPVVVLSGSETVRQALIKQGDEFSGRPDLYSFTFI

NDGKSLAFSTDQAGVCGACRKLAYSALRSFSTLDGTTPEYSCMLEEHICKEGECLINQLN

TVMKADGSFDPFRHIVVSVANVICGMCFGRRYDHNDQDLLRLVNLSDEFGQVAGSGNPAD

FINILRFLPSTTMKKFMTINADFNTFVKKIVGEHYATFDKNNIRDITDSLIDHCEDRKLD

ENCNVQMSDEKIVGIVNDLFGAGFDTVSTALSWSVMYLVAYPDIQERLFQEIKDNVGLDR

TPLLSDRSKVPYLEAFILELFRHSSFLPFTIPHCSAKDTSLNGYFIPKDTCVFINQWQIN

RDPELWKDPSSFNPDRFLSCNGTEVNKQEGEKVMVFGMGKRRCIGEVIARNEVYRGLAIL

IQRLQFHEMPGELLDMTPEYGLTMKHKRCHLRATMRARNEQ

>Q92095.1

MALIILPFIGSLSVSESLVALITICVVYLILTYSHTKIPAGLQRLPGPKPLPIIGNVLEI

GRKPYQTLTALSKRYGPVFQIQIGMRPVVVLSGSETVRQALIKQGEDFSGRPDLYTFQFI

SDGKSLAFSTDKVGVWRARRKLAYSALRSFSSLESTNQEYSCMLEEHICKEGEYLVKQLN

TSMKANGSFDPFRNIVVSVANVICGMCFGRRYDHYDQELVSLVNLSEEFGQVVGTGNLAD

FIPVLRFLPSTAMKKFLSINDRFDKFVKKIVSEHYATYNKDNIRDITDSLIDHCEDRKLD

ENCNVQVSDEKIVGIVNDLFGAGFDTVSTGLSWSVMYLVAYPEIQERLYQEIKDSVGTER

MPLLSDRPSLPFLDAFILEIFRHSSFLPFTIPHCTTKNTSLNGYFIPKDTCVFINQWQIN

HDPELWKDPFSFNPERFLSADGTELNRLEGEKVMLFGLGKRRCIGEVIARNEVFLFLAII

IQRLQFHMLPGEPLDMTPEYGLTMKHKRCQLRATMREKNEQ

>P79716.1

MVLMILPFIGSVSVSESLVALTTVCLVYLILKFFRTEIPEGLHRLPGPKPLPLIGNVLEV

GNKPYLSLTAMSKRYGDVFRFRLGMRPSGCLSGSETVRKALIKQGDEFAGRPDLYSFRFI

NDGKSLAFSTDKAGVWRALRKLAYSALRSFSSLEESTPRDSCVLEEHSAKEGEYLYSNML

NAVMKVTGSFDPFRHIVVSVANVICGMCFGRRYGHNDQELLSLVNLSDPFGQVVGSGNPA

DFIPVLQFLPSTTMKNFMDINARFNKFVQKIVSEHYTTYDKDNIRDITDSLIDHCEDRKL

DENANVQMSDEKIVGIVNDLFGAGFDTISTALSWSVMYLVAYPEIEERLYQELKENVGLD

RTPLLCDRPNLPFLEAFILEIFRHSSFLPFTIPHCTSKDTSLNGYFIPKDTCVFINQWQI

NHDPELWKDPSSFNPDRFLSADGTELNKLEGEKVMVFGLGKRRCIGEVIARNGVFLFLAI

IVQKLHFKTLPGEPLDMTPEYGLTMKHKRCHLRATMRASE

>ADR30132.1

MVLAVLPILGPISVSESLVAMITICTVYLLLQFTRTKIPEGLQHLPGPKPLPLIGNILQL

AQNPHLTLTAMSKRYGPIFQVQIGMRPVVVLGGKEVIHQALIKQGDEFSGRPDLYSFKFI

NNGKSLAFSSDQVNVWRTRRKLAHSALRSFAILKDPSGQYSCTLEMHISKEALYLVKRLH

EVMEADGCFDPFRHIVVSVANVICGMCFGRRYSHDDQELLGFVNLSDEFGRVVGNGNPAD

FIPFLRMLPNQDITIFQDINRRFNVFVQRIVREHYDSYDKNNIRDITDSLIDHCEDRKLD

ENSNLQVSDEKIVGIVNDLFGAGFDTISTALSWSIVYLVVYPEIQERLHQELREKVGMDR

TPRLLDKSRLPYLEAFILEIFRHSSFLPFTIPHWYYIQLLTLDRYFIPNNTCVFINQWQI

NHDPELWKEPSLFNPDRFLSADGTELNKMEAEKVMVFGLGKRRCVGEAIGRSEVFLFLAV

LIQRLKFSSPGGQMPDMTPEYGLTMKHKRCFLCATPRPGSEV

>ADR30130.1

MVLAVLPMLGPISVSESLVAMITICIVYLLLRLTRTKIPEGLQPLPGPKPLPLIGNILEL

SQNPHLSLTAMSKHYGPIFQVQIGMRPVVVLGGNEVVRQALIKQGEEFAGRPDLYSFKFI

NNGKSLAFSSDQVNVWRTRRKLAHSALRSFAILKDPTGEYSCPLEMHISKEGLYLVKRLR

EVMKSDGSFDPFRHIVVSVANVICGMCFGRRYSHDDQELLSFVNLSDEFGRVVGNGNPAD

FIPFLRMLPNQDITKFQDINRRFNVFVQRIVREHYDSFDKNNIRDITDSLIDHCEDRKLD

ENSNLQVSDEKIVGIVNDLFGAGFDTISTALSWSVVYLVVYPEIQERLHQELREKVGTDR

TPTLLDKNLLPYLEAFILEILRHSSFLPFTIPHCTTKDTLLNGYFIPNNTCVFINQWQVN

HDPELWKEPSLFNPDRFLNADGTELNKMEAEKVMVFGLGKRRCVGEAIGRFEVFLFLAVL

IQRLKFSSPDGQMPDMTPEYGLTMKHKRCFLCATPRPGFEA

>AAQ16634.1

MALMILPIFGAVSVFEALLALVVLCLVYLALKRYSTKIPEGLRRLPGPRALPVIGNMLEL

GRKPYLSLTEMSKRYGEVFQVQLGSRPVVVLSGYETVKQALTKQGDDFSGRPDLYSFSFI

NDGKSLAFSTDKAGIWRTRRKLAYSALRSFSNLEGTTSEYSCVLEEHICKESGYLIKELN

NVMKTEGKFDPFRYIVVSVANVICGMCFGRRYDHHDQELLGLINLSEDFVKVTGSGNPAD

FIPALQYLPDKAMKKFVDINSRFCNFVQKIVSEHYITFDKDNIRDITDSLIDHCEDRKLD

ENSNVQISDEKIVGIVNDLFGAGFDTISTGLSWAVMYLVAYPEIEERLFTEIKEKIGLDR

TPTLSDRNNLPLLEAFILELFRHSSYLPFTIPHCATKDTSLNGYFIPKDTCVFINQWQIN

HDPELWEDPSSFIPDRFLSDDGTEVNKLDGEKVVIFGLGRRRCIGEVIARNEVFLFLAII

IQKLHFYQLPGEPLDLTPEYGLTMKHKRCHLGVKLRDENAQ

>BAA88242.1

MALMNLPVVGPISVCESLVAVVTVCVVYLLLRLLSTQIPEGLKRLPGPKPLPIIGNVLEL

SANPHLSLTAMSERYGPVFQIQIGMRPVVVLSGGETVRQALIKQGEEFSGRPDLHSFQYI

SDGKSLAFSTDQAGVWRGRRKLALSALRFFSTIEGSSSEYSCVLEEHISKEGMYLVEQLS

TVMKANGTFDPFRHIVVSVANVICGMCFGRRYSHDDQELLSIVNLSHEFSQVASTGNPAD

FIPFLRFLPSRTMKAFMDMNDRFTQFVQKIVREHYISYDKNNIRDITDSLIDHCEDRKLD

ENSNTQISDEKVVGIVNDLFGAGFDTVSTFLSWAVMYLVTYPEIQEKLHQELREKVGMDR

LPRLTDKSNLPFLEAFITEVFRHSSFVPFTIPHCTTKNTSLNGFFIPKDTCVFVNQWQVN

HDPTVWKEPTDFSIERFLNADGMSINKLEAEKVMMFGLGKRRCIGEAIARSEVFLFLAIL

LQRLRFYQKPGQAPDMTPEYGLTMKHKRCQLSASLRT

>AAB69383.1

MVLMILPIIGSVSVSEGLVAIVTLCLVYMLMKYKHTEIPEGLKRLPGPKPPPIIGNVLEV

YNNPHLSLTAMSERYGSVFQIQIGMRPVVVLSGNETDRQALIKQGEDFAGRPDLYSFKFI

NDGNSLAFSTDKAGVWRARRKLAMSALRSFATLEGTTPEYSCALEEHVCKEGEYLVKQLT

SVMDVSGSFDPFRHIVVSVANVICGMCFGRRYSHDDQELLGLVNMSDEFGQVVGSGNPAD

FIPILRYLPNRTMKRFMDINDRFNNFVQKIVSEHYESYDKDNIRDITDSLIDHCEDRKLD

ENANIHVSDEKIVGIVNDLFGAGFDTISTALSWAVVYLVAYPEIQERLHQELKEKVGMIR

TPRLSDKINLPLLEAFILEIFRHSSFLPFIIPHCTIKDTSLNGYFIPKDTCVFINQWQVN

HDPELWKEPSSFNPDRFLSADGTELNKLEGEKVLVFGMDKRRCIGEAIGHNEVYLFLAIL

LQRLRFQEKPGHPLDMTPEYGLTMKHKRCQLKASMRPWGQEE

>AAL99905.1

MALMILPVVGPISVCKSLVAVVTVCVVYLLLRLLSTQIPEGLKRLPGPKPLPIIGNVLEL

SANPHLSLTAMSERYGPVFQIQIGMRPVVVLSGGETVRQALIKQGEEFSGRPDLHSFQYI

SDGKSLAFSTDQAGVWRGRRKLALSALRFFSTIEGSSSEYSCVLEEHISKEGEYLVERLS

TVMKANGTFDPFRHIVVSVANVICGMCFGRRYSHDDQELLSIVNLSHEFSQVASTGNPAD

FIPFLRFQPSRTMKAFMEMNDRFTQFVQKIVREHYISYDKNNIRDITDSLIDHCEDRKLD

ENSNTQISDEKIVGIVNDLFGAGFDTVSTFLSWAVMYLVTYPEIQEKLHQELREKVVMDR

FPRLTDKSNLPFLEAFITEVFRHSSFVPFTIPHCTTKNTSLNGFFIPKDTCVFVNQWQVN

HDPTVWKEPTDFSIERFLDADGISINKLEAEKVMTFGLGKRRCIGEAIARSEVFLFLAIL

LQRLRFYQKPGQAPDMTPEYGLTMKHKRCQLSASLRT

>ADR30131.1

MALAVLPLLGPVSVSESLVAMITICTVYLLLRLMRTKIPEGLQPLPGPKPLPLIGNVLEL

TQNPHLSLTAMSKRYGPVFQIQIGTRPVVVLGGSEVVRQALIKQGEEFSGRPDLYSFRFI

NDGKSLAFSTDQVGVWRTRRKLALNALRSFAILKSQSGEYSCALEEHVSKEGLYLVKRLQ

GVMKASGGFDPFRQIVVSVANVICGMCFGRRYSHDDAELLSFVNLSHEFNQVAGSGNPAD

YIPFLRLLPSKTMAKFQDINRRFNTFVKKIVGEHYDTYDKDNIRDITDALIDHCKDRKLD

ENSNLNLSDEKIVGIVNDLFGAGFDTISTALSWSIVYLVAYPEIQERLHQEIIEKVGTDR

VPNLPDKSNLAYLEAFILEIFRHSSFLPFTIPHCTTKDTSLNGFFIPKDTCVFINQWQTN

HDPELWKEPSLFNPDRFLSADGTEINKIEGEKVLVFGFAKRRCVGEVIGRSEVFLFLAIL

IQRLKFSSLPGQVLDMTPEYGLTMKHKRIEFPPPL

>ABU62828.1

MVLADIPFFGPISVSESLVAIITICTVYLLLWVTRTKIPEGLLPLPGPKPLPLIGNVLEL

GRNPHLSLTAMSKQYGPVFQVQIGTRPVAVLGGSEVVRQALIKQGEEFAGRPDLYSFQFI

SEGKSLAFSTDQAGVWRTRRKLAHNALRSFANIEGQSVEYSCALEEHISKEGLCLIERLC

SVMESNGGFDPFRYIVVSVANVICGMCFGRRYSHDDNELLNLVNMSDEFNQVVASGNPAD

FIPFLRLLPSTNMKKFLAINKRFNTLMQKLVKEHYDTYNKDNIRDITDSLIDHCEDRKLD

ENSNLQVSDEKIVAIVNDLFGAGFDTISTALSWSVVYLVAYPEIQERLHEELKQKVGIDS

TPRLVDKTNLIYLEAFIMEIFRHSSFLPFTIPHCTTKNTSLNGYFIPKDTCVFINQWQVN

HDLEIWKDPSMFNPDRFLTADGTELNKMEAEKVMIFGLGKRRCVGEVIARSGVYLFLAIL

IQRLKFSSMPGQMPDMTPEYGLTMKHKRCLLRATLRPGFETPCTV

>AAK52513.3

MVLMILPIIGSVSVSEGLVVMVTLCLVYMIMKYMHTEIPEGLKRLPGPKPLPIIGNVLEV

HNNPHLSLTAMSERYGSVFQIQIGMRPVVVLSGSETVRKALIKQGEDFAGRPDLYSFKFI

NDGKSLAFSTDKAGVWRARRKLAMSALRSFATLEGSTPEYSCALEEHVCKEGEYLVKQLT

SVMDVSGSFDPFRHIVVSVANVICGMCFGRRYSHDDQELLSLVNLSDEFGQVVGSGNPAD

FIPILRYLPNRTMKRFMDINDRFNAFVQKIVSEHYESYDKDNIRDITDSLIDHCEDRKLD

ENANIQVSDEKIVGIVNDLFGAGFDTISTALSWAVVYLVAYPEIQERLHQELTEKVGLNR

TPRLSDKTNLPLLEAFILEIFRHSSFLPFTIPHCTIKDTSLNGYFHSQGHLCLHQPVAGQ

PPGAVEGAFFIQPYRFLSADGTELNKLEGEKVLVFGMGKRRCIGEAIGRNEVYLFLAILL

QRLCFQEKPGHPLDMTPEYGLTMKHKRCQLKASLRPWGQEE

>P98181.1

MVLMILPFIGSVSVSESLVAMITMCLAYLILKFFRTEIPEGLRELPGPKPLPIIGNVLEV

GRNPYLSLTAMTAPRTDVFQIQIGMRPVVVLSGNETVRQALIKQGQEFAGRPDLYSFKFI

NDGKSLAFSTDGPAVWRARRKLAYSALRSFATKEGTTPEYSVRLFEHVSKERYAAISMFM

KLMADFDPVRHIVVSVANVICGMCFGRRYDHNNRSWLNLVNPRRDVRKVVASGNPADFIP

ILQYLPSTTMKKFLNINARFNEFVQKIVSEHYTTFNKDNIRDITDSLIDHCEDEPMDPMP

DADDKIVGIVNDLFGAGFDTISTALSWSVMYLVAYPEIQERLYQEMETVMGPDRMPCLSD

RTQLTLPEAFILEIFRHSSFLPFTIPHCTTKDTSLNGYFIPKAPCIYIKQWPVIHDDDDV

LDPSSFNPDRFLKADGTEVNKLNGEKMMVFGMGKRRCIGEVIARNEVYLFLAILLVQLQF

HAMPGEPLDMTPEYGLTMKHKRCHLRAAMRRGTEE

>Q92148.1

MALMILPLIGSVSVSETLVAMITVCMIYMLMKFLHPDVPEAPPAPGPQALPIIGNVWSWE

NGLPEPHGHGQRYGDILQIQIGMRSVVVLSGHETVRQALIKQGHDLRPPDLYSFQFINDG

KSLAFSTDQAGVWSPPKAGHECPALLFHARGHHAAVLLHVGGACLQGGRLPRQAAVQRHG

TDASFDPFRHIVVSVANVICGMCFGRRYGHEDQELLSLVNLTDEFGKVVGSGNLADFIPL

LRFLPNATMKRFMAINERFMTFVQKIVTEHYNTYDKDNIRDITDSLIDHCEDRKLDENSN

IQMSDEKIVGIVNDLFGAGFDTVSTALSWSVMYLVAHPEIQERLHQEIKDKVGLSRSPVL

TDRHNLPILEAFIFEIFRHSSFLPFTIPHCATKDTSLDGYFIPKDTCVFINQWQINHDPE

LWKEPSTFNPDRFLSADASELNKLAGEKVMLFGMGKRRCIGEMVARNEVFLFLAILVQRL

TFHAVPGEPLDMTPEYGLTMKHKRCHLRATVRTTE

>ACO51073.1

MGLILSGYLSTKEDPSAALSSVTVALCLLTLLLMAIRAKQSQGFFFLYHHDSHLDRAKFP

SPPGPNPWALVGNLLQVGDQMHLSLTRLGLQYGDVFKMRFGSLTVVILSGYATIRQALVR

QGEDFAGRPELFTFSAVADGTSMTFSEKYGPAWLLHKKLCKNALRSFSQAEPRGSGATCL

LEEQVCAEAAEMVEVIREQAAKSNEMRGIDPAISLVTSVANVVCALCFGRRYDHNDQEFL

TIVNINNEVLKLFAAGTLADFFPVFRYFPSPSLRKMVQYIHRMNGFMERRIQEHINTFDK

NCIRDITDALIALCDGREEDGDTSLLSNSQIIHTVIDIFSAGFDTIIAGLQWSLLYLIKF

PDIQDEIQQEIDEHVGSARLPRFPDKPSMPFTEAFIFEVFRHSSYVPFTIPHCTTRDTIL

NGYFIPKDTCVFVNQYQVNHDVDLWGDPETFRPRRFLGPSGQLNKELTEKVLIFGLGKRR

CLGDGVARLEMFVFLTTLLHGLRIENVPGQKLDLSADFGLTMKPRPYRIMVSPRF

>ADR10206.1

MESAVKKTLMDMMPMLLKASISFLTVLLVMSILWKKRNSLPGPWAVPIVGNFFQLGDQIH

ITLTDMRNRYGDVFQIKLGLMPIVVVSGLETVKRVLLKEGENFADRPNFYSFSLFSNGSS

MTFSEKYGESWKIHKKIMKNALRNLSNESTNSSNCSCRLEEYVCAEASDLVQELTDLSAE

KVAFDPSSLIVITVANVVCALSFGKRYDHHDKEFLTLIDFNNDLRKAAGGGLLADFIPIL

RFIPSSSVKALKKFVQSFHSFIAKCVKDHFATFEENNIRDITDALIQLCKERKSEDKNQL

LSDDQIISTVNDIFGAGFDTITSALLWAIFYLLRYPEFQDKIHKEIEEKIGCNRAPRFND

RKDLHYTEAFINEVLRHSSFVPFGLPHCTTMDTKLNGYFLPKGTCVFTNLYQVNHDNTVW

KDADMFMPERFLDQNGQIIKSLTEKVLIFGMGVRKCLGEDVARNEMFVIMTIMMQRLKLV

KSTKHELDPIPVYGLTLKPKPYYLVAKVRT

>352962133

MESAVKKTLMDMMPMLLKASISFLTVLLVMSILWKKRNSLPGPWAVPIVGNFFQLGDQIH

ITLTDMRNRYGDVFQIKLGLMPIVVVSGLETVKRVLLKEGENFADRPNFYSFSLFSNGSS

MTFSEKYGESWKIHKKIMKNALRNLSNESTNSSNCSCRLEEYVCAEASDLVQELTDLSAE

KVAFDPSSLIVITVANVVCALSFGKRYDHHDKEFLTLIDFNNDLRKAAGGGLLADFIPIL

RFIPSSSVKALKKFVQSFHSFIAKCVKDHFATFEENNIRDITDALIQLCKERKSEDKNQL

LSDDQIISTVNDIFGAGFDTITSALLWAIFYLLRYPEFQDKIHKEIEEKIGCNRAPRFND

RKDLHYTEAFINEVLRHSSFVPFGLPHCTTMDTKLNGYFLPKGTCVFTNLYQVNHDNTVW

KDADMFMPERFLDQNGQIIKSLTEKVLVFGMGVRKCLGEDVARNEMFVIMTIMMQRLKLV

KSTKHELDPIPVYGLTLKPKPYYLVAKVRT

>ADM53744.1

MILNLAVTPGEVTTSLIILVMVFVFVRALRSKGRKQLSPPGPWSFPIIGNLLQLGEHPYL

TLMEMRKKYGDVFLLKLGMVPVLVVNGMEMVKQVLLKDGEHFAGRPNMHTFSFLAEGKSL

SFSVNYGESWKLHKKIASKALRTLSNAEAKSSTCSCLLEEHVTEEVSELVTVFVELSSKN

GGFDPRNAITCAVANVVCALCFGKRYDHSDEEFLKIVKTNDDLLKASRAANPADFIPCLR

YLPLQIINAPREFYRALNGFIALHVQDHLATYDKDHIRDITDALINVCHNKYAATKTDTL

NDSEIISTVNDLFGAGFETVSTCLYWSFLYLIHYPEIQAKIQEEIDGNIGLKPPRFEDRK

ILPYTEAFISEVFRHASFLPFTIPHCTTADTTLNGYFIPRKTCTFINMYQVNHDETIWDN

PSLFRPDRFLNENRELNKSLVEKVLIFGMGIRKCLGEDVARNEIFIFITAVLQQLKLKKC

PRVKLDLTPTYGLVMRPKPYELEAERRSSGSSSASILRLRGGFLTQFRKIDDLNLLN

>AAI60554.1

MPMLLKASISFLTVLLVMSILWKKRNSLPGPWAVPIVGNFFQLGDQIHITLTDMRNRYGD

VFQIKLGLMPIVVVSGLETVKRVLLKEGENFADRPNFYSFSLFSNGSSMTFSEKYGESWK

IHKKIMKNALRNLSNESTNSSNCSCRLEEYVCAEASDLVQELTDLSAEKVAFDPSSLIVI

TVANVVCALSFGKRYDHHDKEFLTLIDFNNDLRKAAGGGLLADFIPILRFIPSSSVKALK

KFVQSFHSFIAKCVKDHFATFEENNIRDITDALIQLCKERKSEDKNQLLSDDQIISTVND

IFGAGFDTITSALLWAIFYLLRYPEFQDKIHKEIEEKIGCNRAPRFNDRKDLHYTEAFIN

EVLRHSSFVPFGLPHCTTMDTKLNGYFLPKGTCVFTNLYQVNHDNTVWKDADMFMPERFL

DQNGQIIKSLTEKVLVFGMGVRKCLGEDVARNEMFVIMTIMMQRLKLVKSTKHELDPIPV

YGLTLKPKPYYLVAKVRT

>ADM53745.1

MILNLAVTPGEVTTSLTILVMVFVFVRALRSKGRKQLSPPGPWSFPIIGNLLQLGEHPYL

TLMEMRKKYGDVFLLKLGMVPVLVVNGMEMVKQVLLKDGEHFAGRPNMHTFSFLAEGKSL

SFSVNYGESWKLHKKIASKALRTLSNAEAKSSTCSCLLEEHVTEEVSELVTVFVELSSKN

GGFDPRNAITCAVANVVCALCFGKRYDHSDEEFLKIVKTNDDLLKASSAANPADFIPCLR

YLPLQIINAPREFYRALNGFIALHVQDHLATYDKDHIRDITDALINVCHNKYAAAKTDTL

NDSEIISTVNDLFGAGFETVSTCLYWSFLYLIHYPEIQAKIQEEIDGNIGLKPPRFEDRK

ILPYTEAFISEVFRHASFLPFTIPHCTTADTTLNGYFIPRKTCTFINMYQVNHDETIWDN

PSLFRPDRFLNENRELNKSLVEKVLIFGMGIRKCLGEDVARNEIFIFITAVLQQLKLKKC

PRAKLDLTPTYGLAMRPKPYQLEAERRSSGSSSASILRLRGGFLTQFRKIDDLNLLN

>402897645

MILNLAVTPGEVTTSLIILVMVFVFVRALRSKGRKQLSPPGPWSFPIIGNLLQLGEHPYL

TLMEMRKKYGDVFLLKLGMVPVLVVNGMEMVKQVLLKDGEHFAGRPNMYTFSFLAEGKSL

SFSVNYGESWKLHXKIASKALRTLSNAEAKSSTCSCLLEEHVTEEVSELVTVFVELSSKN

GGFDPRNAITCAVANVVCALCFGKRYDHSDEEFLKIVKTNDDLLKASSAANPADFIPCLR

YLPLQIINAPREFYRALNGFIALHVQDHLATYDKDHIRDITDALINVCHNKYAATKTDTL

NDSEIISTVNDLFGAGFETVSTCLYWSFLYLIHYPEIQAKIQGEIDGNIGLKPPRFEDRK

ILPYTEAFISEVFRHASFLPFTIPHCTTADTTLNGYFIPRKTCTFINMXQVNHDETIWDN

PSLFRPDRFLNENRELNKSLVEKVLIFGMGIRKCLGEDVARNEIFIFITAVLQQLKLKKC

PRAKLDLTPTYGLVMRPKPYQLQVELRSSGSASAXILRLRGGLLTQFRKIDDLNLLN

>344271214

MIFSLAVTPGEATTCLIVLVIVFVFVRALRNRDGKQVSLPGPWSFPIIGNLPQIGDHPYL

TFMEMRKKYGDVFLIRLGMVPVVVVNGMEMVKQVLLKDGEKFAGRPNMHTFSVLAEKKSL

SFSVNYGESWKLHKKIASNALRTFSKAEAKSSTCSCLLEEHVTEEVSELVKVFAELTSKN

GSFEPRSVITCSVANVVCALCFGKRYEHNDEEFLQIVKTNDELLKASSAANPADFIPCFR

YLPLGVINAPRKFYQALYQFIALHVQDHLTTYDKDHIRDITDALINTCHNKHAATKTATL

NDDEIINTVGDLFGAGFETVSTCLYWSFLYLIRYPEIQAKIQEEIDGNIGLKSPRFDDRK

ILPYTEAFVNEIFRHASFFPFTIPHCTTVDTSLNGYFIPRKTCTFINMYQVNHDETIWDD

PNLFQPERFLNQNGELNKSLVEKVLIFGMGIRKCLGEDVARNEIFIFLTTVLQQLKLQKC

PGAQLDLTPMCGLTMKPKPYQLKAELRPSGGSPD

>327263435

MFFSTEVSFSEVTITLFVVAAIFISIHMLMKTKRPHPPGPWSLPILGNLLQVEEHPYISF

QRMRKKYGDVFQIKLGMVPVVVVNGLDAVKQVLLRDGESFAGRPDMHTFSFFADGDSMSF

SVNYGESWKLQKKIAGRALKLLSKSEAKSSTCSCLLEEHVCDEASELVKILLELSKNGGF

DPAAVTTCTAANVVCALCFGKRYNHNDEEFLGVIKLNDDFVKASSAFNPADFIPCLRYLP

LPAAKVARTFYRKLNDFVSACVEYHCTTYDKNYVRDITDALINVGNEKKEDGKTAALSDK

KIISTVNDIFGAGFSTVSACLLWIYLYLISKPEIQTKIQEEIDEKIGLRPPRFDDRKYLH

YTEAFINEIFRHCSFLPFTIPHCTTRDAVLNGYYIPQSTCIFINMYQVNHDEDVWEDPYS

FKPERFLNESGELNKSLVEKVLIFGMGIRKCLGEELARNEVFVIITTILQQLRLEKPPED

KLDLTPMYGLTMSPKPYRLQAALRT

>345323214

MIPGELTTSLLMLVIVLISINVLRNRGQKPPSPPGPWALPVIGNLLQLGEHPYLSFIEMR

KKYGDVFLIKLGMVPVVVVNGMEPVKRVLFQDGENYAGRPNMHTFSFFANGKSLSFSTNY

GDSWKHHKKMAINALKSFSKAEAKSSTCSCLLEEHVCGEVSELVKIFTELTATQGNFDPR

GSLTCAVANVVCALCFGKRYEHTDEKFLKVIKINDDLLKASSAVNPADFIPCFRYLPLRV

VNAPREYYHMLNQFIMQHVQEHYVTYDEGYLRDITDALISICYDKNSTGKTPILPDDTII

STVNDIFGAGFDTVSTCLNWSFLYLINYPEIQTKIQAEIDGNIGLKPPRFEDRKNLPYTE

AFINEIFRHTTFLPFTIPHCTTADTILNGYFIPQKTCVFVNIYQVNHDETLWEKPDLFRP

ERFLNENGELNKGLVEKVLIFGLGIRKCLGEDVARNEIFIFITNVLQHLKLEKCSGAQLD

LTPVYGLSMKPKPYHIKAEPRF

>395514975

MSPPGPWPFPIIGNLLQLGDHPYLTFMEMRKKYGDVFLIKLGMVPVVVVNGAEMVKKGLL

KDGENFAGRPHMHTFSFFAEGKSLSFSVNYGESWKLHKKIAINALRSFSKAEAKSSTCSS

ILEEHVTEEVSELVKVFKKLSSKQGSFDPKNPITSAVANVVCALCFGKRYDHFDEEFLRV

IKTNEEFLKASNAANPADFIPCFRYLPLSIINAPREFYHQLNQFIKQHVQDHITTYDKNH

LRDITDALVSICHDKNATTKAASLSDNEIINTVCDIFGAGFETVSAFLNWSFLYLIYYPE

VQTKIHEEIDENIGLKPPRFEDRKNLPYTEAFINEIFRHTTFIPLTIPHCATTDTILNGY

FIPQKTCVFFNMYQVNHDETIWENPDSFQPERFLNENGKLNKSVVEKVLIFGMGIRKCLG

EDVARNEIFVFIASILQQLKLKKCPGAQLDLTPAYGLVMKNKPFQLTVEPRILENLNIVF

N

>395819443

MISHLAITPREVTISLVILVIVFVFLRVLRSKGRKQVSPPGPLSFPIIGNLLQLGEHPYL

TFMEMRRQYGDIFLLRLGTVPVVVVNGVEMVKQVLLKDGEYFAGRPNMHTFSFLAEGKSL

TFSVNYGESWKLHKKIASNALRTFSKAEAKSSTCSCLLEEHVTEEASELVKVFVELTSKN

GSFNPRSAITCAVANVVCALCFGKRYDHGDEEFLRIVKTNDDLLKASSAANPADFIPCFR

YLPLRIINAPREFYQALNRFIALQVQDHLTTYDKDHIRDITDALINTCHNKYAATKTATL

NDSEIISTVSDLFGAGFETVSTCLYWSFLYLIRYPDIQAKIQEEIGRNIGLKSPRFEDRK

ILPYTEAFINEIFRHTSFLPFTIPHCTTADTTLNGYFIPKKICVFINMYQVNHDETIWDT

PNLFRPERFLNKKRELNKTLVEKVMIFGMGIRKCLGEDVARNETFIFITTVLQQLKLRKC

PGAELDLTPMYGLTMKPKPYQLQAELHSTGSLSTXITVRGGFLISFKLSSSTTLSHENIL

>334333060

MSPPGPRPFPIIGNLLQLGDHPYLTFMEMKKKYGDVFLIKLGMVPVVVVNGTEMVKKGLL

KDGENFAGRPHMYTFSFFAEGKSLSFSVNYGESWKLHKKIAMNALRNFSKAEAKSSTCSC

VLEEHVTEEASELVKIFSKLSLKQGSFDPKSSITCAVANVVCALCFGKRYGHFDKEFLRI

IKTNEEFLKASSAANPADFIPCFRYLPLRIIHAPREFYCQLNHFIEQHVQDHITTFDKNH

LRDITDALVSICRDKSATIKTATLSDNEIISTVSDIFGAGFETVSGFLHWSFLYLIYYPE

IQAKIHEEIDGIIGFKPPRFKDRKNLPYTEAFINEIFRHTTFVPFTIPHCTTKDTTLNGY

FIPQKTCVFFNMYQVNHDETLWENPDSFQPERFLNEKGEMNKNLVEKVLIFGMGIRKCLG

EDVARNEVFIFIVSILQQLKLKKCPEVQLDLTPVYGLVMKPKPYQLIVEPRFHVNSST

>348516236

MVIWGRKSKRSFFHFHHDSQLDHTKYPPPPGPKPWPLVGNLLQVGEQIHLSLTGLRLQYG

DIFKLRLGSLTVVVLSGYNTIRQALVRHGEAFAGRPNLFIFSAIANGTSMTFNENYGPVW

LLHKKLCKNALRSFSQAEPRGFGATCLLEEHICAEAAGMVQVIREKAAKEDMEGIDPATT

LVTSVANVVCALCFGKRYDYSDKEFLTIVDVNNEVLKLFAAGNLADFFPVFRYFPSPSLR

KIVQYVRRMNRFMERNIEEHIDTFDKNRIRDITDALIALCEEREESKDTSMLSNDQIIHT

VIDIFGAGFDTIIAGLQWSLLYLIKFPDIQDKIQQEIDEHIGKPQLPRFSNKAKMPFTEA

FIYEVFRHASYVPFTIPHCTTRDITFNGYFIPKDTCVFINQYQVNHDIDLWGDPEKFRPQ

RFLNPCGLLNKELTEKVQIFGMGKRRCLGDEFARLEMFVFLTTLLHGLRIENVPGQELDL

STDFGLTMKPRPYRISLTSLARKEVLQPVIVQLQHIG

>AAH85648.1

MNLENISHTATSEVTLILCAFALLLLALHGRRRAPGVPVPPGPRPWPIVGNFLQMEEQVH

LSLTNLRVQYGDVFQVKMGSLVVVVLSGYTTIKEALVRQGDAFAGRPDLYTFSAVANGTS

MTFSEKYGEAWVLHKKICKNALRTFSQTEPKDSNASCLLEERICVEAIDMVETLKAQGEE

FGDSGIDPVQLLVTSVANVVCTLCFGKRYSHNDKEFLTIVHINNEVLRLFAAGNLADFFP

IFRYLPSPSLRKMVEFINRMNNFMERNIMEHLVNFDTNCIRDITDALIAMCEDRQEDKES

AVLSNSQIVHSVIDIFGAGFDTIITGLQWSLLYLIKFPNIQDKIVQEIDNQVGMDRLPQF

KDRPNMPYTEAFINEVFRHASYMPFTIPHCTTENITLNGYFIPKDTCVFINQYQVNHDIE

IWDDPESFRPERFLTLSGHLNKSLTEKVMIFGMGIRRCLGDNIARLEMFVFLTTLLHRLH

IENVPGQELDLSSTFGLTMKPRPYRIKIIPRN

>397503319

MILDLAVTPGEVTTSLIILVMVFVFVRALRSKGRKQVSPPGPXSFPIIENLLQLGDHPYL

TLMEMRKKYGDVFLLKLGMVPVLVVNGMEMVKQVLHKDGEHFAGRPNMHTFSFLAEGKSL

SFSVNYGESWKLHXKIASKGLXTFSNAEAKSSTCSCSLEEHVTEEISELATVFVELTSKN

GSFDPRNAITCVVANIVCALCFGKRXDHSDEEFLRIVKTNDDLLKASSAANPADFIPCLH

YLPLQIINAPLEFYQALNGFIALHVQDHLATYGKDHIRDITDALINVCHNKYAATKTDTL

NDSEIISTVTDLFGAGFETVSTCLYWSFLYLIHYPEIQARIQEEIDGNIGLRPPRFEDRK

ILPYTEAFVSEVFRHASFLPFTIPHCTTADTTLNGYFIPRKTCTFINMYQVNHDETIWDN

PSLFRPDRFLNENRELNKSLVEKVLIFGMGIRKCLGEDVARNEIFIFITTVLQQFKLKKC

PRAKLDLTPTYGLVMRPKPYQLQAELHPSGSSSAXILRLSGGFLTQFRKIDDLSLFN

>AFB82731.1

MTPMDTATPQAEWKDSVQPALVFSFVILICLEACIWLRNHGQKRSPPGPFPWPVVGNAMQ

LGQLPHLTFCKMAQKYGNVFQIRLGNQDIVVLNGDSTIREALVKHSKEFAGRPNFSSFQL

ISGGKSIAFGGYSTLWKAQKKIAHSTLRAFSTVNTKTQNLFEKHVAAEAQDLIRVFLRLT

SEEQYFDPTRECTVAAANVICALCFGKRYSHDDEEFKALIGRNDKFGQTVGAGSLVDIMP

WLLTFPNPVRSLYQSFKDLNWEFYDFVKEKISHHRQTYKPEITRDMSDAFISHIEQAEEA

GHGLSKDYVESIVNDILGAGQDTTATALTWILLLLIKYPDIQQKLRDEIDLVVGPNRLPS

ADDKVHLPYVQAFIYETLRFSSFVPVTIPHSTTSDVLIDGFYIPQDTVVFVNQWSVNHDG

SKWKNPEVFDPSRFLDEDGQMDRDAAFGVMIFSVGKRRCIGDQLSMLQIFLFTTIFLHQC

TFQGNPKEIPTMDCTSGLALKPLPYGMSVTARAGYTTTEEPA

>CAX13204.1

MMDVLLALRDLLQLSTRSVLLSLMVCLMLMFRRRQLVPGPFSWPVIGNAAQLGNTPHFYF

SRMAQKYGDVFQIKLGSRNVVVLNGDAIKEALVKKATDFAGRPDFASFRFVSNGKSMAFG

NYTPWWKLHRKVAQSTVRNFSTANIQTKQTFEKHIVSEIGELIRLFLNKSREQQFFQPHR

YLVVSVANTMSAVCFGNRYAYDDAEFQQVVGRNDQFTKTVGAGSMVDVMPWMQYFPNPIR

TLFDQFKELNKEFCAFIELKVSEHRKTISPSHVRDMTDAFIVALDKGLSGGSGVSLDKEF

VPPTISDIFGASQDTLSTALQWIILLLVRYPEIQKRLQEDVDRVVDRSRLPTIADQPHLP

YLMAFIYEVMRFTSFTPLTIPHSTTKDTSINGYPIPKDTVIFVNQWSLNHDPTKWDQPEV

FNPQRFLDEDGSLNKDLTTNVLIFSLGKRRCIGEDVSKIQLFLFTSVLVHQCSFKAESTP

NMDYEYGLTLKPKPFKVSVTARDSSDLLDSLVGTSQTPTEKRQKCD

>AAT07036.1

MMDVLLALRDLLQLSTRSVLLSLMVCLMLMFRRRQLVPGPFSWPVIGNAAQLGNTPHFYF

SRMAQKYGDVFQIKLGSRNVVVLNGDAIKEALVKKATDFAGRPDFASFRFVSNGKSMAFG

NYTPWWKLHRKVAQSTVRNFSTANIQTKQTFEKHIVSEIGELIRLFLNKSREQQFFQPHR

YLVVSVANTMSAVCFGNRYAYDDAEFQQVVGRNDQFTKTVGAGSMVDVMPWMQYFPNPIR

TLFDQFKELNKEFCAFIELKVSEHRKTISPSHVRDMTDAFIVALDKGLSGGSGVSLDKEF

VPPTISDIFGASQDTLSTALQWIILLLVRYPEIQKRLQEDVDRVVDRSRLPTIADQPHLP

YLMAFIYEVMRFTSFTPLTIPHSTTKDTSINGYPIPKDTVIFVNQWSLNHDPTKWDQPEV

FNPQRFLDEDGSLNKDLTTNVLIFSLGKRRCIGEDVSKIQLFLFTSVLVHQCSFKAESTP

NMDYEYGLTLKPKPFKVSVTARDSSDLLDSLVGTSQTPTEKRLKCD

>AAW52507.1

MMDVLLALRDLLQLSTRSVLLSLMVCLMLMFRRRQLVPGPFSWPVIGNAAQLGNTPHFYF

SRMAQKYGDVFQIKLGSRNVVVLNGDAIKEALVKKATDFAGRPDFASFRFVSNGKSMAFG

NYTPWWKLHRKVAQSTVRNFSTANIQTKQTFEKHIVSEIGELIRLFLNKSREQQFFQPHR

YLVVSVANTMSAVCFGNRYAYDDAEFQQVVGRNDQFTKTVGAGSMVDVMPWMQYFPNPIR

TLFDQFKELNKEFCAFIELKVSEHRKTISPSHVRDMTDAFIVALDKGLSGGSGVSLDKEF

VPPTISDIFGASQDTLSTALQWIILLLVRYPEIQKRLQEDVDRVVDRSRLPTIADQPHLP

YLMAFIYEVMRFTSFTPLTIPHSTTKDTSINGYPIPKDTVIFVNQWSLNHDPTKWDQPEV

FNPQRFLDEDGSLNKDLTTNVLIFSLGKRRCIGEDVSKIQLFLFTSVLVHQCNFKAESTP

NMDYEYGLTLKPKPFKVSVTARDSSDLLDSLVGTSQTPTEKRLKCD

>AAH95332.1

MREWSGQVQPALIASFVILFFLEACLWVRNLTFKKRLPGPFAWPLVGNAMQLGQMPHITF

SKLAKKYGNVYQIRLGCSDIVVLNGDAAIRKALVQHSTEFAGRPNFVSFQMISGGRSLTF

TNYSKQWKTHRKVAQSTLRAFSMANSQTRKTFEQHVVGEAMDLVQKFLRLSADGRHFNPA

HEATVAAANVICALCFGKRYGHDDPEFRTLLGRVNKFGETVGAGSLVDVMPWLQSFPNPV

RSVYQNFKTINKEFFNYVKDKVLQHRDTYDPDVTRDMSDAIIGVIEHGKESTLTKDFVES

TVTDLIGAGQDTVSTAMQWMLLLLVKYPSIQSKLQEQIDKVVGRDRLPSIEDRCNLAYLD

AFIYETMRFTSFVPVTIPHSTTSDVTIEGLHIPKDTVVFINQWSVNHDPQKWSDPHIFNP

SRFLDENGALNKDLTNSVMIFSTGKRRCIGEQIAKVEVFLFSAILLHQCKFERDPSQDLS

MDCSYGLALKPLHYTISAKLRGKLFGLVSPA

>AAY26142.1

MEAEFGLKSSSIMREWSGQVQPALIASFVILFFLEACLWVRNLTFKKRLPGPFAWPLVGN

AMQLGHMPHITFSKLAKKYGNVYQIRLGCSDIVVLNGDAAIRKALVQHSTEFAGRPNLVS

FQMISGGRSLTFTNYSKQWKTHRKVAQSTLRAFSMANSQTRKTFEQHVVGEAMDLVQKFL

RLSADGRHFNPAHEATVAAANVICALCFGKRYGHDDPEFRTLLGRVNKFGETVGAGSLVD

VMPWLQSFPNPVRSVYQNFKTINKEFFNYVKDKVLQHRDTYDPDVTRDISDAIIGVIEHG

KESTLTKDFVESTVTDLIGAGQDTVSTAMQWMLLLLVKYPSIQSKLQEQIDKVVGRDRLP

SIEDRCNLAYLDAFIYETMRFTSFVPVTIPHSTTSDVTIEGLHTPKDTVVFINQWSVNHD

PQKWSDPHIFNPSRFLDENGALNKDLTNSVMIFSTGKRRCIGEQIAKVEVFLFSAILLHQ

CKFERDPSQDLSMDCSYGLALKPLHYTISAKLRGKLFGLVSPA

>334313147

MATSRSSEETWLPGLLSTQQTTLLLLFSVLAVVHLGQWLLRRVRRTPWASCRPPGPFQWP

LIGNAVEVGSIPHLSFTRLARRYGDVFQIRLGNCPVVVLNGERAIRQALLQQGAAFASRP

PFASFQVVSNGRSLAFNKYSELWKVQRRVAHGTVRAFSTGQVRSRQVLEQHVLSETRELV

ELLVQGSAGGAFLNPGPLTVVAVANVMSAVCFGCRYSHSDEEFRELLSHNERFGRTVGAG

SLVDVLPWLQRFPNPVRTAFRDFQKLNQDFYSFVLDKFLKHKSSLQPGAPPRDMMDAFIH

TVGKEEENPGEVKPGLRLDTEYVPATVTDIFGASQDTLSTALQWLLILFIRYPKVQAQVQ

EELDRVVGRDRLPSLNDQPHLPYVMAFLYEAMRFSSFVPVTIPHATTIDTSVMGYHIPKD

TVVFINQWSVNHDPEKWQNPEDFNPARFLDKKGFIDKDLASGVMIFSMGKRRCIGEELSK

IQLFLFIAILAHQCNFLANPDEDPEMNFSYGLTIKPQSFTINVTLRESMELLDSTVQRLQ

EENNQ

>AAR87724.1

MALSDTEFGMKGNSILKEWSGQIQPALVASFIFLCCLEACFWVKNLTLKKRLPGPFAWPL

VGNAMQLGQMPHITFSKLAKRYGDVYQIRLGSSDIVVLNGESAIRKALLQHSTEFAGRPN

FVSFQSVSGGTGMTFINYSKQWKMHRKIAQSTIRAFSSANSQTKKSFEKHIAAEAQELIE

TFLRLSSNNQYFNPSHELTVTAANIICALCFGKRYGHDDFEFRTILTKVNKFGETVGAGS

LVDVMPWLQSFPNPVRSIFQSFKELNKDFFTFVKGKVVEHRQSYDPEVTRDMSDAFIGVI

EHADTETGLTEAHTEGTVSDLIGAGLDTVSTALHWMLLLLVKYPSIQTKLQEQIDKVVGR

GRLPSIDDKSNLAYLDAFIYETMRYTSFVPVTIPHSTTSDVTIEGLHIPKDTVVFINQWP

VNHNPQKWQDPHIFNPSRFLDETGALDKDLTNSVMIFSIGKRRCIGDQIAKVEVFLITAM

LIHQLTFENDPSQDLSLNCSYGLTLKPFDYKISAKPRGSVFNEE

>AAX37330.1

MEAEFGLKSSSIMREWSGQVQPALIASFIILFFLEACLWVRNLTFKKRLPGPFAWPLVGN

AMQLGQMPHITFSKLAKKYGNVYQIRLGCSDIVVLNGDAAIRKALVQHSTEFAGRPNFVS

FQMISGGRSSTFTNYSKQWKTHRKVAQSTLRAFSMANSQTRKTFEQHVVGEAMDLVQKFL

RLSADGRHFNPAHEATVAAANVVCALCFGKRYGHDDPEFRTLLGRVNKFGETVGAGSLVD

VMPWLQSFPNPVRSVYQNFKTINKEFFNYVKDKVLQHRDTYDPDVTRDMSDAIIGVIEHG

KESTLTKDFVESTVTDLIGAGQDTVSTAMQWMLLLLVKYPSIQSKLQEQIDKVVGRDRLP

SIEDRCNLAYLDAFIYETMRFTSFVPVTIPHSTTSDVTIEGLHIPKDTVVFINQWSVNHD

PQKWSDPHIFNPSRFLDENGALNKDLTNSVMIFSTGKRRCIGEQIAKVEVFLFSAILLHQ

CKFERDPSQDLSMDCSYGLALKPLHYTISAKLRGKLFGLVSPA

>ADD51162.1

MALLETDFEVKGSSIIREWSGQVQPALVASLVFLFCLEACLWVRNLRLKRRLPGPFAWPV

VGNAMQLGQMPHITFSKLAKKYGNVYQIRLGCNNIVVLNGDASIREALVQHSTEFAGRPN

FVSFQSVSGGNSMTFTNYSKQWRTHRKIAQTTIRAFSSANSQTKKAFEQHVVAEATELIE

AFLKLNAEGQFFNPAHELTVAAANVICALCFGKRYGHDDIEFRTLLGSVDKFGETVGAGS

LVDVMPWLQYFPNPVRRVYQSFKDLNKEFFTFVRDKVVEHRETFDPEVTRDMSDAIIGVI

DKADSDTGLTEAHTEGTVSDLIGAGLDTVSTCLHWMLLLLVKYPNIQTRLQEQIDKVVGR

DRLPCIEDKANLAYLDAVIYETMRYTSFVPLTIPHSTTSDVTIEGFHIPKDTVVFINQWS

VNHDPLQWKDPHLFDPSRFLDENGALDKDLTSSVMIFSAGKRRCIGDQIAKVEVFLFSAI

LIHQCTFENNPSQDLSLDCSYGLTLKPLNYKISAKLRGELLTGA

>ADR10205.1

MTPMDTAEPPAEWKDSVQPALVFSFLILICLEVCLWLRNNGQRRSPPGPFPWPVVGNAMQ

LGQLPHLTFCKMSQKYGNVFQIRLGTQDIVVLNGDSTIREALVKHSKEFAGRPNFSSFQL

ISGGKSIAFGGYSTLWKAQKKIAHSTLRAFSTVNSKTQKLFEKHVVAEAQDLIDVFLRLT

SEEEYFDPTRECTVAAANVICALCFGKRYSHDDEEFKALIGRNDKFGQTVGAGSLVDIMP

WLLTFPNPVRSLYQSFKDLNWEFYGFVKEKVSHHRQTYNPEIARDMSDAFISHIDNAEGI

EAGDGLSKDYVESIVNDILGAGQDTTATALTWILLLLIKYPDIQQKLQEEIDLVVGPNRL

PTADDKVQLPYVQAFIYEALRFSSFVPVTIPHSTTSDVVIDGFYIPKDTVVFVNQWSVNH

DESKWKNPDVFDPSRFLDEEGQLDRDAAFGVMIFSVGKRRCIGDQLSMLQIFLCTAIFLH

QCTLHGNPKEIPTMDCISGLSLKPLPYGMSVRARVGRTTMKEPV

>AAY90143.1

MSNKTRFCEILEANIESILFIVKHPRMELSGIITDFLKLSKENVLLVCLAVLVAVHLMTS

LVRNWSVRTLPGPFPWPIIGNAAQIGRYPHIYFTRMAQHYGDVFQIKLGNRPVVVLNGDA

IRQALVQKGVDFAGRPDFASFRFVSCGRSMAFGSYSEWWKVHRRVAHATVRKFTTGNPDT

KNAIENHVVSEVKELISLFMHETRTHGYFQPHRYLVVSMANITSALCFGKRYSYDDAEFQ

QVVGRNDKFTMTVGAGSIVDVMPWLQYFPNPMKTLFDQFKELNAEFYEFILTKVVEHRKT

KEPSVVRDMTDALIMSLDRGMTGPSGVLLDEKYVPPTIGDIFGASQDTTSSAMQWIILLL

VRYPDVQKRLQEEVDKVVGRNRLPSIQDQPHLPYLMAFIYEMMRFTSFIPVTIPHSTTTN

TSINGYPIPKGTVVFINQWSLNHDPKKWDQPEIFNPLRFLDEDGELNKDKTGNVLIYSMG

KRRCIGEELSKIQLFLFTTLLTHQCHFTVEKQPTMDCMYGLTLKPNPFKVAVTLRD

>348520548

MAQTDAEFGVKSSSSIIREWSGQVQPALVVSLLFLFCLEACLWVRNLKLKRRLPGPFAWP

VVGNALQLGQMPHITFTRLAKKYGEVYQIRLGCSDVVVLNGGQVIRQALIQHSTEFAGRP

NFVSFQFVSGGKSMTFTNYSKQWKMHRKIAQSTIRAFSSANSQTKKAFEQQIVAEATELV

ESFLKLSAQGQYFNPAQELTVAAANVICALCFGKRYGHDDMEFRALLHKVDQFGQTVGAG

SLVDVMPWLQSFPNPVRTVFRNFKSLNQDFFAFVKNKVEKHKETFDPEVTRDISDAIIGV

LNEVEKGEELTVGYTEATVADLIGAGLDTVSTALHWIVLLLAKHPEIQTRLHELIDKVVG

QNRLPSVEDRQHLAYLDAFIYETMRYTSFVPVTIPHSTTSDVTVSGLTIPKDTVVFINQW

SVNHDPLMWKDPHIFDPSRFIDENGSLNKDLTNNVMIFSAGKRRCIGDQIAKVEIFVFFA

ILLHQCSFEKCPNEDFSLNSTYGLTLKPLNYKITAKLRGESLKSQ

>AAR99332.1

MDIQEVMEEMSRPSPRSLLLASVSLLVAVHLWLWIQRCRNADGPPGPFPWPVIGNAAQLG

SAPHLYFSRMAQKYGEVFQIKLGSRAVVVLNGDAIRTALIKKGADFAGRPDFTSFQFVSN

GKSMAFGNYSDWWKAQRKIAQTTVRTFSTGNVHTKKVLENHVLCEGRELLHLFMKMTNER

KYFQPLTYLTVSTANIMSAVCFGKRYSYNDAEFQAVVGRNDQFTKTVGAGSIVDVMPWLQ

YFPNPIKTIFGNFKDLNREFYVFISQKVAEHRKTIQSRTVRDMTDAFILALDQGLTSSTG

VYLDKEYVPPTVSDIFGASQDTLSTALQWIILVLVRFPDIQKQLREEVDKVVDSSRLPSI

EDQPRLPYVMAFLYEVMRFTSFIPVTIPHSTTTDTAIQGYRIPKDTVVFINQWSVNHSPA

KWVRPEVFDPNRFLDKSGALDKDQTSSVLIFSLGKRRCIGEDLSKMQLFLYTTLLIHQCL

FKANPAEPLSLDYLYGLTLKPKAFSIAVEHRNSMALLDAYVQQHAKAEADGESTSSEQN

>395508195

MKPAPLSCPAKKLQKHLSLRLMGLAGDLATRQGLPGSFEFRGGRWLDGACACRAGDGGDG

RRGAAGSPSPTLLAEQPLRVRGCSAQTNPVRRSGLRRVKEDRARGQSLPRLRRERASERA

RVWSLFDAAKLSLYPQLRSIPQLPIHQILLEAAPTHSMATSRNSAEAWIPGLLSTQQTTL

LLFLSVLVAVHLSQCILRRCRRLPGAGCSPPGPFQWPLIGNAVEVGNNPHLSFTRLARRY

GDVFQIRLGSCPVVVLNGERAIRQALVQQGSTFASRPPFPSFHVVSNGRSLAFSKYSEIW

KMQRRXAHGTVRAFSTGHVRSRRVLEQHVLSEARELVRLLVQGSAGGAFLNPGPLAVVAV

ANVMSAVCFGCRYSHSDEEFRELLSHNERFGRTVGAGSLVDVLPWLQRFPNPVRTAYRDF

QKINHDFYSFVLDKFLKHRSSLKPGAPPRDMMDAFIHTVGKEEESTGKTTSGLRLDTDYV

PATVTDIFGASQDTLSTALQWLLILFTRYPEVQAQVQEELDRVVGRDRLPCLDDQPHLPY

VMAFLYEAMRFSSFVPVTIPHATTVDTSIMGYHIPKDTVVFVNQWSVNHDPVKWQNPEDF

NPARFLDNKGFIDKDMASGVMIFSVGKRRCIGEELSKVQLFLFIAILAHQCNFLANPDED

RKMNFSYGLTIKPQSFTINVTLRESMELLDNTVQRLQTEDNQ

>BAB39160.2

MMDFQAVLGDFIQLSARSVLLSLLVCLMLMFLRRSGPGRLPGPFPWPVIGNAAQLGTSPH

FYFTRMAQKYGDVFQIKLGSRNVVVLNGDTIKEALIKKGVDFAGRPDFASFRFVSNGKSL

AFGNYSQWWKLHRKIAHSTVRTFSAANIHTKETFEKHIVSEIGELIRLFLNKTREQQYFE

PHRYLVVSVANTMSAVCFGNRYAFDDEEFLQVVGRNDQFSKTVGAGSIVDVMPWLRYFPN

PIKTLFDQFKALNKEFNEFIHSKVVEHRKTLLLSLIRDMTDAFIVALDKGLSGGPGVYLD

KEYVPSTIADIFGASQDTLSTALQWIILLLVRYPEVQKRLQEDVDKVADRSRLPTIADQP

HLPYVMAFIYEVMRFTSFVPVTIPHSTTTDTSINGYPIPKDTVIFVNQWSLNHDPTKWDQ

PEVFNPQRFLDEDGTLNKDLTTNVLIFSVGKRRCIGEDLSKIQLFLFTSLLVHQCSFTAE

STPSMDYLYGLTLKPKAFKVSVTPLDRTELLQSLVGTSQTPTEKRERPQV

>AAI16464.1

MMDVLLALRDLLQLSTRSVLLSLMVCLMLMFRRRQLVPGPFSWPVIGNAAQLGNTPHFYF

SRMAQKYGDVFQIKLGSRNVVVLNGDAIKEALVKKATDFAGRPDFASFRFVSNGKSMAFG

NYTPWWKLLRKVAQSTVRNFSTANIQTKQTFEKHIVSEIGELIRLFLNKSREQQFFQPHR

YLVVSVANTMSAVCFGNRYAYDDAEFQQVVGRNDQFTKTVGAGSMVDVMPWMQYFPNPIR

TLFDQFKELNKEFCAFIELKVSEHRKTISPSHVRDMTDAFIVALDKGLSGGSGVSLDKEF

VPPTISDIFGASQDTLSTALQWIILLLVRYPEIQNRLQEDVGRVVDRSRLPTIADQPHLP

YLMAFIYEVMRFTSFTPLTIPHSTTKDTSINGYPIPKDTVIFVNQWSLNHAPAKWDQPEV

FNPQRFLDEDGSLNKDLTTNVLIFSLGKRRCIGEDVSKIQLFLFTSVLVHQCSFKAESTP

NMDYEYGLTLKPKPFKVSVTARDSSDLLDSLVGTSQTPTEKRLKCN

>CAB51367.1

MFLQDPPAMDVTLEGIDPVTLRAVLLACVTLLFSLHLWRWLGGQPSVPGPPGPLAWPLIG

NAAEMGKLPHLYLTRMAHKYGNVFQIKLGSRTVVVLNGDSIKQALVKQGTDFAGRPDFAS

FKYIFDGDSLAFGPFTDWWKVHRRVAQSTVRTFSTGNADTKKTFEHHVLCEFRELLQLFV

GKTEQQRFFQPMTYLVVSTANIMSAVCFGKRYAYEDEEFLQVVGRNDQFTQTVGAGSIVD

VMPWLQYFPNPIRTIFDNFKKLNLEFGQFIRDKVIEHRKTIQSSTTRDMTDALIVALDKL

GDKSELTGGKDYVSPTMGDIFGASQDTLSTALQWIVLILVKYPEMQLRIQQEVDKVVDRT

RLPSIEDQLQLPYIMAFVYEVMRFTSFVPLTIPHSTVTDTSIMGYTIPKNTVIFINQWSI

NHDPALWSHPETFDPQRFLDQNGALNKDLTSSVLIFSLGKRRCIGEELSKMQLFLFTALI

AHQCHISPDPARPPKLDYTYGLTLKPCAFSIAVALRGHDMSLLDEATRSSAEEVKGEPSS

DSQTKN

>AAL78297.1

MAMDTDYGVKGSSIIREWSGQVQPALVASFVFLFCLEACLWVRNLRLKRRLPGPFAWPLV

GNAMQLGQMPHITFARLAKKYGNVYQIRLGCSDIVVLNGDKAIREALIQHSTEFAGRPNF

VSFQMVSGGRSLTFNNYGKQWKAHRKIAQSSLRAFSSANSQTKKAFEQHITAEAMDLVQS

FLRQSADGRYFDPAHEFTVAAANIMCALCFGRRYGHEDLEFRTLLKKVEKFGETVGAGSL

VDVMPWLQSFPNPVRSVYENFKNLNEEFFAFVKDKVVQHRESFDPEVTRDMSDAIINVIE

HGKDSGLSKEFVEATVTDLIGAGQDTVSTVMQWIVLLLVKHPDMQAKLQELIDKVVGQDR

LPSIEDRGSLAYLDAFIYETMRFTSFVPVTIPHSTTSDVTIEGLHIPKDTVVFINQWSVN

HDHLKWKDPHTFDPSRFLDENGALDKDITNNVMIFSSGKRRCIGDQIAKVEVFLYAAILL

HQCSFESDPSKPLTLDCSYGLTLKPLRYCVSARLRGKLLGLVSPA

>AAL78299.1

MAQIDGEFGVKGSSIIREWSGQVQPALVASFVFLFCLEACLWVRNLRLKRRLPGPFAWPV

VGNAMQLGQMPHITFARLAKKYGNVYQIRLGCSDIVVLNGDKAIREALIQHSTEFAGRPD

FISFQMVSGGRSMTFTSYSKQWKMHRKIAQSTIRAFSSANSQTKKAFEQQIVAEATELVE

IFLKLSAQGQHFNPAHELTVAAANVICALCFGKRYGHDDVEFRTLLQRVDMFGQTVGAGS

LVDVMPWLQSFPNPVRSMFKSFKVLNQEFFGFVKHKVEEHRETFDPEVTRDISDAIIGVI

EKASGDNGLTKSHAEGTVSDLIGAGLDTVSTALHWSLLLLLKHPEIQTKLHELIDKVVGR

QRLPSIEDRGSLAYLDAFIYETMRFTSFVPVTIPHSTTSDVTIEGLHIPKDTVVFINQWS

VNHDPLKWKDPHIFDPSRFLDENGDLDKDITNNVMIFSSGKRRCIGDQIAKVEVFLFFAI

LLHQCSFEKCADENLSLNCTYGLTLKPLDYKITAKLRGELLTGQ

>AAR87723.1

MALTEAEFESGSIMKEWGGQVQPALIASFIILFFLEACLWVRNLTIKKRLPGPFAWPLVG

NAMQLGQMPHITFSKLAKKYGNVYQIRLGRSDIVVLNGDVAIRKALIEHSTEFAGRPNFL

SFQMISGGRSMVFSSYSKQWKVHRKVAQSTLRAFSMANTQTKQTFEQHVVGEAMDLVQKF

LRLSADRRDFNPSHEFTVAAANVICALCFGKRYGLDDPEFRTLLKRVEKFGETVGAGSLV

DVMPWLQLFPNPVRSVYQNFKLINEEFFAYVKDKVVQHRDTYDPEVTRDMSDAIIGVIEH

GKESMLTKDFVESTVTDLIGAGQDTVSTVMQWILLLLVRYPSIQTKLQEQIDKVVGRDRL

PSIEDKSNLAYLDAFIYETMRYTSFVPVTIPHSTTSDVTIEGLHIPKDTVVFINQWSVNH

DPQKWQDPHIFNPSRFLDETGALDKDLTNSVMIFSTGKRRCIGDQIAKVEVFLFSAILLH

QCSFESNPSQDLSMDCSYGLTLKPLHYTISAKLRGKLFGLVSPA

>CAG10688.1

MEEEFCVEGGSSSIREWSGHIQAALVAFFVFLFCLEARLWAKNLKRRLPGPFAWPVVGNA

MQLGQMPHITFSKLAKKYGNVYQIRLGCSDIVVLNGDRVIRQALIQHSTEFAGRPNFVSF

QTVSGGKGMTFSSYSKRWKMHRKIAQSTIRAFSSANSQTKENFEQQIAAEATELVEVFLK

LSARGQHFNPEHELTVAAANVICALCFGKRYGHDDAEFRELLHRVNMFGQTVGAGSLVDV

MPWLQSFPNPVRSMFKSFKTPQQAVLWLKLKEHRETFDPKVTRDMSDAIISVLDRSASEY

GLTKDNAEGTVSDLIGAGLDTVSTALHWMLLLLVKHPQLQHKLQQLIDQVVGRNRLPSVG

DRSSLAYLDAFIYETMRFTSFVPVTIPHSTTSDVTIEGLRIPKDTVVFINQWSVNHDSLM

WTDPHVFDPSRFLDEQGSLNRDLASNVMIFSAGKRRCIGTQIAKAEIFLFLAILLHQCSF

ERSAGEEPSLDCSYGLTLKPLDYRITAKLRGKLLKSP

>AAR87722.1

MMDLLAVLGDFIQLSARSVLLSLLVCLTLMLWLLSGRRQLPGPFSWPVIGNAAQLGTSPH

FYFSRMAQKYGDVFQIKLGSRNVVVLNGDAIKEALIKKGVDFAGRPDFASFRFVSHGKSM

AFGNYSQWWKLHRKIAHSTVRNFSTANIHTKQTFEKHIVCEIGELIRLFLNKTREQQYFE

PHRYLVVSVANTMSAVCFGNRYSFDDEEFQQVVGRNDQFTKTVGAGSIVDVMPWLQYFPN

PIKTLFDQFKALNKEFNEFIYSKVAEHRKTLLMSLVRDMTDAFIVALDKGLSGGPGVFLD

KEYVPPTIADIFGASQDTLSTALQWIILLIVRYPEVQKRLQEDVDKVADRSRLPTIADQP

HLPYVMAFIYEVMRFTSFVPVTIPHSTTTDTSINGYPIPKDTIIFVNQWSLNHDPTKWDQ

PEVFNPQRFLDEDGALNKDLTTNVLIFSMGKRRCIGEDVSKIQLFLFTSLLVHQCRFTAE

TTPSMDYVYGLTLKPRSFKVSVTLRDSTEPLEGLVGTSQTPTQKRQRPQV

>AEV12203.1

MSAMGTPNRAAMAPVLSPHSALLLIAVVLTAILLLARTRHKATRGQSPPGPFAWPLVGNV

LQMGRLPHLTFMRMACRYGAIFQLRLGRHWVVVLNGEAAIRRALVGLGTRFAGRPDFPSF

GLVSGGRSIAFGGCTPQWRARRRLAHAALRARSTVAEVERHVAAEAGDLVRLFLRHSQGG

AYFQPCPLLVVANANVLCALCFGRRYDHADGEFTALLGRNDRFGQTVGAGSLVDVLPWLL

RFPNPVRHVYRDFQALNRELHGFVQAKVAQHRQTFDWRAVRDISDVMIASVERGGGSPDG

LGPEDVEGAMTDIFGAGQDTTSTALSWIILLLLKHPQVQQDLQAELDRVVGRSRLPTAED

RPHLPLLEAFIYETLRYSSFVPITIPHATTADVELEGFRIPKGTVVFVNQWSVNHDCSKW

PEPQRFDPTRFLDKQQRLDRERAGSVMIFSAGQRRCIGDQLSKLQIFLFTAILLHQCSFH

ANPAEHLTMDCIHGLALKPLPFTVNVRPRIPLLIQP

>ADD51160.1

MALLDTEFEVKGSSSIIREWSGQVQPALVASLVFLFCLEACLWVRNLRLKRRLPGPFAWP

VVGNAMQLGQMPHITFSKLAKKYGNVYQIRLGCNNIVVLNGDTAIREALVQHSTEFAGRP

DFVSFRMISGGRSMTFTNYSKQWKMHRRIAQSTIRAFSSANSQTKKAFEQHVLGESMDLV

QVFLRMSADGRYFNPAHEFTVAAANVICALCFGKRYGHDDIEFRTLLGRMDKFGETVGAG

SLVDVMPWLQNFPNPVRSVYQNFKHINEEFFTFVKDKVVQHRETFNPDVTRDISDAIINV

IEHGKDSGLTKDFTEGTVTDLIGAGQDTMSTILQWILLLLIKYPIIQTKLQEQIDKVVGR

DRLPCIEDKANLAYLDAVIYETMRYTSFVPLTIPHSTTSDVTIEGFHIPKDTVVFINQWS

VNHDPLQWKDPHLFDPSRFLDESGALDKDLTNSVMIFSTGKRRCIGDQIAKVEIFLFTAI

LLHQCTFESNPSETLTLGCSYGLALKPLYYTITTKLRGKLLGLVSPA

>118091033

MGTPNGAAMAPVLSPHSALLLIAVVLTAILLLARTRHKATRGQSPPGPFASPLVGNVLQM

GRLPHLTFMRMACRYGAIFQLRLGRHRVVVLNGEAAIRRALVGLGTRFAGRPDFPSFGLV

SGGRSIAFGGCTPQWRARRRLAHAALRAHSTVAEVERHVVAEAGDLVRLFLRHSQGGAYF

QPCPLLVVANANVLCALCFGRRYDHADGEFTALLGRNDRFGQTVGAGSLVDVLPWLLRFP

NPVRHVYRDFQALNRELHGFVQAKVAQHRQTFDWRAVRDISDVMIASVERGGGSPDGLGP

EDVEGAMTDIFGAGQDTTSTALSWIILLLLKHPQVQQDLQAELDRVVGRSRLPTAEDRPH

LPLLEAFIYETLRYSSFVPITIPHATTADVELEGFRIPKGTVVFVNQWSVNHDCSKWPEP

QRFDPTRFLDKQQRLDRERAGSVMIFSAGQRRCIGDQLSKLQIFLFTAILLHQCSFHANP

AEHLTMDCIHGLALKPLPFTVNVRPRIPLLIQP

>AAA19567.1

MGTSLSPNDPWPLNPLSIQQTTLLLLLSVLATVHVGQRLLRQRRRQLRSAPPGPFAWPLI

GNAAAVGQAAHLSFARLARRYGDVFQIRLGSCPIVVLNGERAIHQALVQQGSAFADRPAF

ASFRVVSGGRSMAFGHYSEHWKVQRRAAHSMMRNFFTRQPRSRQVLEGHVLSEARELVAL

LVRGSADGAFLDPRPLTVVAVANVMSAVCFGCRYSHDDPEFRELLSHNEEFGRTVGAGSL

VDVMPWLQYFPNPVRTVFREFEQLNRNFSNFILDKFLRHCESLRPGAAPRDMMDAFILSA

EKKAAGDSHGGGARLDLENVPATITDIFGASQDTLSTALQWLLLLFTRYPDVQTRVQAEL

DQVVGRDRLPCMGDQPNLPYVLAFLYEAMRFSSFVPVTIPHATTANTSVLGYHIPKDTVV

FVNQWSVNHDPVKWPNPENFDPARFLDKDGLINKDLTSRVMIFSVGKRRCIGEELSKMQL

FLFISILAHQCDFRANPNEPAKMNFSYGLTIKPKSFKVNVTLRESMELLDSAVQNLQAKE

TCQ

>Q16678.2

MGTSLSPNDPWPLNPLSIQQTTLLLLLSVLATVHVGQRLLRQRRRQLRSAPPGPFAWPLI

GNAAAVGQAAHLSFARLARRYGDVFQIRLGSCPIVVLNGERAIHQALVQQGSAFADRPAF

ASFRVVSGGRSMAFGHYSEHWKVQRRAAHSMMRNFFTRQPRSRQVLEGHVLSEARELVAL

LVRGSADGAFLDPRPLTVVAVANVMSAVCFGCRYSHDDPEFRELLSHNEEFGRTVGAGSL

VDVMPWLQYFPNPVRTVFREFEQLNRNFSNFILDKFLRHCESLRPGAAPRDMMDAFILSA

EKKAAGDSHGGGARLDLENVPATITDIFGASQDTLSTALQWLLLLFTRYPDVQTRVQAEL

DQVVGRDRLPCMGDQPNLPYVLAFLYEAMRFSSFVPVTIPHATTANTSVLGYHIPKDTVV

FVNQWSVNHDPLKWPNPENFDPARFLDKDGLINKDLTSRVMIFSVGKRRCIGEELSKMQL

FLFISILAHQCDFRANPNEPAKMNFSYGLTIKPKSFKVNVTLRESMELLDSAVQNLQAKE

TCQ

>AAV38782.1

MGTSLSPNDPWPLNPLSIQQTTLLLLLSVLATVHVGQRLLRQRRRQLRSAPPGPFAWPLI

GNAAAVGQAAHLSFARLARRYGDVFQIRLGSCPIVVLNGERAIHQALVQQGSAFADRPAF

ASFRVVSGGRSMAFGHYSEHWKVQRRAAHSMMRNFFTRQPRSRQVLEGHVLSEARELVAL

LVRGSADGAFLDPRPLTVVAVANVMSAVCFGCRYSHDDPEFRELLSHNEEFGRTVGAGSL

VDVMPWLQYFPNPVRTVFREFEQLNRNFSNFILDKFLRHCESLRPGAAPRDMMDAFILSA

EKKAAGDSHGGGARLDLENVPATITDIFGASQDTLSTALQWLLLLFTRYPDVQTRVQAEL

DQVVGRDRLPCMGDQPNLPYVLAFLYEAMRFSSFVPVTIPHATTANTSVLGYHIPKDTVV

FVNQWSVNHDPLKWPNPENFDPARFLDKDGLISKDLTSRVMIFSVGKRRCIGEELSKMQL

FLFISILAHQCDFRANPNEPAKMNFSYGLTIKPKSFKVNVTLRESMELLDSAVQNLQAKE

TCQ

>114576990

MGTSLSPNDPWPLNPLSIQQTTLLLLLSVLATVHVGQRLLRQRRRQLGSAPPGPFAWPLI

GNAAAVGQAAHLSFARLARRYGDVFQIRLGSCPIVVLNGERAIHQALVQQGSAFADRPSF

ASFRVVSGGRSMAFGHYSEHWKVQRRAAHSMMRNFFTRQPRSRQVLEGHVLSEARELVAL

LVRGSADGAFLDPRPLTVVAVANVMSAVCFGCRYSHDDPEFRELLSHNEEFGRTVGAGSL

VDVMPWLQYFPNPVRTVFREFEQLNRNFSNFILDKFLRHCESLRPGAAPRDMMDAFILSA

EKKAAGDSDDGGARLDLENVPATVTDIFGASQDTLSTALQWLLLLFTRYPDVQTRVQAEL

DQVVGRDRLPCMGDQPNLPYVLAFLYEAMRFSSFVPVTIPHATTANTSVLGYHIPKDTVV

FVNQWSVNHDPVKWPNPENFDPARFLDKDGFINKDLTSRVMIFSVGKRRCIGEELSKMQL

FLFISILAHQCNFRANPNEPAKMNFSYGLTIKPKSFKVNVTLRESMELLDSAVQKLQAKE

TCQ

>332227226

MGTSLSPNDSWPLNPLSIQQTTLLLLLSVLATVHVGQRLLRQRRRQLGSAPPGPFAWPLI

GNAAAVGQAAHLSFARLARRYGDVFQIRLGSCPIVVLNGERAIHQALVQQGSAFADRPSF

ASFRVVSGGRSMAFGHYSEHWKVQRRAAHSMMRNFSTRQPRSRQVLEGHVLSEARELVAL

LVRGSADGAFLDPRPLTVVAVANVMSAVCFGCRYSHDDPEFRELLSHNEEFGRTVGAGSL

VDVMPWLQYFPNPVRTVFREFEQLNRNFSNFVLDKFLRHCESLRPGAGPRDMMDAFILSA

EKKAAGDSGDGGARLDLENVPATITDIFGASQDTLSTALQWLLLLFTRYPDVQARVQAEL

DQVVGRDRLPCMGDQPKLPYVLAFLYEAMRFSSFVPVTIPHATTANTSLLGYHIPKDTVI

FVNQWSVNHDPVKWPNPENFDPARFLDKDGLINKDLTSRVMIFSVGKRRCIGEELSKMQL

FLFISILAHQCNFRASPNEPAKMNFSYGLTIKPKSFKVNVTLRESMELLDSAVQKLQAEE

TCQ

>3PM0A

MAKKTSSKGKPPGPFAWPLIGNAAAVGQAAHLSFARLARRYGDVFQIRLGSCPIVVLNGE

RAIHQALVQQGSAFADRPSFASFRVVSGGRSMAFGHYSEHWKVQRRAAHSMMRNFFTRQP

RSRQVLEGHVLSEARELVALLVRGSADGAFLDPRPLTVVAVANVMSAVCFGCRYSHDDPE

FRELLSHNEEFGRTVGAGSLVDVMPWLQYFPNPVRTVFREFEQLNRNFSNFILDKFLRHC

ESLRPGAAPRDMMDAFILSAEKKAAGDSHGGGARLDLENVPATITDIFGASQDTLSTALQ

WLLLLFTRYPDVQTRVQAELDQVVGRDRLPCMGDQPNLPYVLAFLYEAMRFSSFVPVTIP

HATTANTSVLGYHIPKDTVVFVNQWSVNHDPLKWPNPENFDPARFLDKDGLINKDLTSRV

MIFSVGKRRCIGEELSKMQLFLFISILAHQCDFRANPNEPAKMNFSYGLTIKPKSFKVNV

TLRESMELLDSAVQNLQAKETCQHHHH

>AFH31263.1

MGTGLSPKDPWPLNLLSTQQTTLLLLLSVLVAVHVGQWLLRQRRRQLGSTPPGPFAWPLI

GNAAAVGQASHLSFARLARRYGDVFQIRLGSCPIVVLNGERAIHQALVQQGSAFADRPSF

ASFRVISGGRSMAFGHYSEHWKVQRRAAHSTMRNFSTRQLRSRQVLEGHVLSEARELVAL

LVRGSADGAFLDPRPLTVVAVANVMSAVCFGCRYSHDDPEFRELLSHNEEFGRTVGAGSL

VDVMPWLQYFPNPMRTAFREFEQLNRNFSNFVLDKFLRHCESLRPGAAPRDMMDAFILSA

EKKAARDSDDGGARLDLENVPATVTDIFGASQDTLSTALQWLLLLFIRYPDVQARVQAEL

DQVVGRDRLPCMDDQPNLPYVLAFLYEAMRFSSFVPVTIPHATNANTSVLGYHIPKDTVI

FVNQWSVNHDPVKWPNPENFDPARFLDKDGLINKDLTSRVMIFSVGKRRCIGEELSKMQL

FLFISILAHQCNFRANPNGPEMNFSYGLTIKPKSFKVNVTLRESMELLDSAVQKLQAEET

CQ

>EHH22047.1

MGTGLSPKDPWPLNLLSTQQTTLLLLLSVLVAVHVGQWLLRQRRRQLGSTPPGPFAWPLI

GNAAAVGQASHLSFARLARRYGDVFQIRLGSCPIVVLNGERAIHQALVQQGSAFADRPSF

ASFRVISGGRSMAFGHYSEHWKVQRRAAHSTMRNFSTRQLRSRQVLEGHVLSEARELVAL

LVRGSADGAFLDPRPLTIVAVANVMSAVCFGCRYSHDDPEFRELLSHNEEFGRTVGAGSL

VDVMPWLQYFPNPMRTAFREFEQLNRNFSNFVLDKFLRHCESLRPGAAPRDMMDAFILSA

EKKAARDSDDGGARLDLENVPATVTDIFGASQDTLSTALQWLLLLFIRYPDVQARVQAEL

DQVVGRDRLPCMDDQPNLPYVLAFLYEAMRFSSFVPVTIPHATNANTSVLGYHIPKDTVI

FVNQWSVNHDPVKWPNPENFDPARFLDKDGLINKDLTSRVMIFSVGKRRCIGEELSKMQL

FLFISILAHQCNFRANPNGPEMNFSYGLTIKPKSFKVNVTLRESMELLDSAVQKLQAEET

CQ

>402890595

MGTSLSPKDPWPLNLLSTQQTTLLLLLSVLAAVHVAQWLLRQRRRQLGSTPPGPFAWPLI

GNAAAVGQASHLSFARLARRYGDVFQIRLGSCPIVVLNGERAIHQALVQQGSAFADRPPF

ASFRVISGGRSMAFGHYSEHWKVQRRAAHSTMRNFSTRQLRSRQVLEGHVLSEARELVVL

LVRGSADGAFLDPRPLTVVAVANVMSAVCFGCRYSHDDPEFRELLSHNEQFGRTVGAGSL

VDVMPWLQYFPNPMRTAFREFEQLNRNFSNFVLDKFLRHCESLRPGAAPRDMMDAFILSA

EKKAARDSDDGGARLDLENVPATVTDIFGASQDTLSTALQWLLLLFIRYPDVQARVQAEL

DQVVGRDRLPCMDDQPNLPYVLAFLYEAMRFSSFVPVTIPHATNANTSVLGYHIPKDTVI

FVNQWSVNHDPVKWPNPENFDPARFLDKDGLINKDLTSRVMIFSVGKRRCIGEELSKMQL

FLFISILAHQCNFRANPNGPEMNFSYGLTIKPKSFKVNVTLRESMELLDSAVQKLQAEET

CQ

>390474567

MATSLSPNDPWPLNPLSVQQTTLLLLLSVLAFVHAGQWLLRQRRRPLGFAPPGPFAWPLI

GNAAAVGQAAHLSFARLARRYGDVFQIRLGSCPIVVLNGERAIHQALLQQGAAFADRPSF

ASFRVVSGGRSMAFGHYSEHWKVQRRAAHSTMRAFSTRQPRSRQVLEGHVLSEARELVAL

LVRGSADGAFLDPRPLTIVAVANVMSAVCFGCRYSHDDPEFRELLSHNDEFGRTVGAGSL

VDVMPWLQRFPNPVRTAFREFEQLNRNFSNFVLDKFLRHRESLRPGATPRDMMDAFILSA

EKKATGDSDDDGARLDLADVPATVTDIFGASQDTLSTALQWLLLLFTRYPDVQAQVQAEL

DQVVGRDRLPCMDDQPXLHYVMAFLYEAMRFSSFVPVTIPHATAANTSLLGYHIPKDTVI

FVNQWSVNHDPVKWPNPENFDPARFLDKDGFINKDLTSRVMIFSVGKRRCIGEELSKMQL

FLFISILAHQCNFRANPNEPAKMNFSYGLTIKPKSFKVNVTLRESMEILDSAVQKLQAEE

ICQ

>403269693

MATSLSSNDPWPLNQLSVQQTTLLLLLSVLAVVHAGQWLLRQRRRPLGFAPGPFAWPLIG

NAAAMGQAVHLSFARLARRYGDVFQIRLGSCPIVVLNGERAIHQALLQQGAVFADRPSFA

SFRVVSGGRSMAFGHYSEHWKVQRRAAHRTMRAFATRQPRSRQVLEGHVLSEARELVALL

VRGSADGAFLDPRPLTVVAVANVMSAVCFGCRYSHDDPEFRELLSHNEEFGRTVGAGSLV

DVMPWLQRFPNPVRTAFREFQQLNRKFSNFVLDKFLRHRESLRPGAAPRDMMDAFILSAE

KKAAGDSDDDGARLDLADVPATVTDIFGASQDTLSTALQWLLLLFTRYPDVQARVQAELD

QVVGRDRLPCMDDQPNLHYVMAFLYEAMRFSSFVPVTIPHATAANTSLLGYHIPKDTVIF

VNQWSVNHDPVKWPNPENFDPARFLDKDGFINKDLTSRVMIFSVGKRRCIGEELSKMQLF

LFISILAHQCNFRANPNEPVKMSFSYGLTIKPKSFKVNVTLRESMEILDSAVQKLQAEEI

CQ

>397493656

MGTSLSPNDPWPLNPLSIQQTTLLLLLSVLATVHVGQRLLRQRRRQLGSAPPGPFAWPLI

GNAAAVGQAAHLSFARLARRYGDVFQIRLGSCPIVVLNGERAIHQALVQQGSAFADRPSF

ASFRVVSGGGHVLSEARELVALLVRGSADGAFLDPRPLTVVAVANVMSAVCFGCRYSHDD

PEFRELLSHNEEFGRTVSAGSLVDVMPWLQYFPNPVRTVFREFEQLNRNFSNFILDKFLR

HCESLRPGAAPRDMMDAFILSAEKKAAGDSDDGGARLDLENVPATVTDIFGASQDTLSTA

LQWLLLLFTRYPDVQTRVQAELDQVVGRDRLPCMGDQPNLPYVLAFLYEAMRFSSFVPVT

IPHATTANTSVFGYHIPKDTVVFVNQWSVNHDPVKWPNPENFDPARFLDKDGFINKDLTS

RVMIFSVGKRRCIGEELSKMQLFLFISILAHQCNFRANPNEPAKMNFSYGLTIKPKSFKV

NVTLRESMELLDSAVQKLQAKETCQ

>ACO52509.1

MATSLGPDAPLQPSALSAQQTTLLLLLSVLAAVHAGQWLLRQRRRQPGSAPPGPFAWPLI

GNAAAMGPAPHLSFARLARRYGDVFQIRLGSCPVVVLNGERAIRQALVQQGAAFADRPRF

ASFRVVSGGRSLAFGQYSPRWKVQRRAAHSTMRAFSTRQPRSRRVLEGHVLAETRELVAL

LARGSAGGAFLDPRPLTVVAVANVMSAVCFGCRYSHDDAEFRELLSHNEEFGRTVGAGSL

VDVLPWLQRFPNPVRTAFREFEQLNRNFSNFVLRKFLRHRESLQPGAAPRDMMDAFILSA

GTEAAEGSGDGGARLDMEYVPATVTDIFGASQDTLSIALQWLLILFTRYPQVQARVQEEL

DQVVGRNRLPCLDDQPNLPYTMAFLYEGMRFSSFVPVTIPHATTTSACVLGYHIPKDTVV

FVNQWSVNHDPVKWPNPEDFDPARFLDKDGFIDKDLASSVMIFSVGKRRCIGEELSKMQL

FLFISILAHQCNFKANPDEPSKMDFNYGLTIKPKAFSINVTLRESMELLDSAVQKLQAEE

DCQ

>194220796

MATSLSLDDPLLPISLSTQQTTLLLFLSALAAVHVGQWLLRQRRRQPGCAPPGPFAWPLI

GNAAAMGPAPHLAFARLARRYGDVFQIRLGSCPVVVLNGERAIRQALVQQGAAFADRPPF

ASFRVVSGGHSLAFSQYSEHWKVHRRAAHSTMRAFSTRQPRSRRVLEGHVLGEARELVAL

LVRGSAGGAFLDPVPLTVVAVANVMSAVCFGCRYNHDDAEFLELLSHNEKFGRTVGAGSL

VDVLPWLQLFPNPVRTAFREFEQLNRNFSNFVLNKFLSHRESLRPGAAPRDMMDAFILSA

GKEAAEGSGDGGARLDMEYVPGTVTDIFGASQDTLSTALQWLLILFTRYPEVQARVQAEL

DQVVGRDRLPCLDDQPKLPYVMAFLYEAMRFSSFVPVTIPHATTANASVLGYHIPKDTVV

FVNQWSVNHDPVKWPNPEDFDPARFLDKDGSINRDLASSVMIFSVGKRRCIGEELSKMQL

FLFISILAHECNIKANPDELSKMDFHYGLTIKPKSFKINVTLRESMELLDSAVQKLQAEE

DSQ

>291386917

MVTSLSPDAPLLLNSLSSQQTMLLLVLSVLAAVHVGQWLLRQRRRQPLSAPPGPFQWPLI

GNAAAVGRAAHLSFARLARRYGDVFQIRLGSCPVVVLNGERAIHQALVQQGATFADRPPF

ASFRVVSGGRSLAFGHYSEHWKVQRRAAHGTMRAFSTRQPRSRSILEGHVLGEARELVAL

LVRGSAGGAFLDPRQPTIVAVANVMSAVCFGCRYSHDDAEFLELLSHNEEFGRTVGAGSL

VDVLPWLQRFPNPLRTTFRQFERVNRNFSNFILDKFLRHRESLRPGAAPRDMMDAFILSA

GKAEGDSGEGGARLDLESVPATVTDIFGASQDTLSTALQWLLLILTRYPDVQARMQAELD

QVVGRKRLPCMGDQPNLPYVMAFLYETMRFSSFVPVTIPHATTANAFILGYHIPKNTVIF

VNQWSVNHDPAKWPHPEDFNPARFLHKDGSFNKDLASSVMIFSTGKRRCIGEELSKMQLF

LFISILAHQCHFRANPDEPWQTSFSYGLTIKPKSFTVNVTLRDSMELLDSAVQKLEAEGV

CH

>395846044

MATSLCGNDPWPLSLLSVQQTTLLLLLSVLAAVHAGQWLLRQRRQQVGSAPPGPFAWPVI

GNVLTVGRAAHLSFARLAQRYGDVFQIHLGSCPIVVLNGERAIHEALVQQGAVFADRPPF

ASFRVVSGGRSIAFGRYSEYWKTQRRAAHGTMRAFSTRQPHSRRVLEGHVLGEARELVAL

LVRGSAGGTFLDPRPLTLVAVANVMSAVCFGCRYSHDDAEFRELLSHNEEFGRTVGAGSL

VDVLPWLQLFPNPVRTAFREFAQLNLSFSNFVYDKFLKHQESPGPRAAPRDLMDAFILSA

EKKVTGDSGAGGAQLDLENVPATLTDIFGASQDTLSTALQWLLLLLVRYPDVQARVQAEL

DQVVGRDRLPCMADQPNLPYVMAFLYEAMRFSSFVPVTIPHATTASTSILGYHIPKDTVV

FVNQWSVNHDPGKWLNPEDFDPTRFLDKDGFINKDLTSSVMIFSAGRRRCIGEELSKMQL

FLFTSILTHQCNFRANPKEPLTMNFTYGLSIKPKSFKINITLRGSMELLDGAVQKLQAEE

VCQ

>BAF58169.1

MATSLGAEAPLQPSALSSQQTTLLLLLSVLAAVHVGQWLLRQRRRQPGSAPPGPFAWPLI

GNAAAMGPAPHLSFARLARRYGDVFQIRLGNCPVVVLNGERAIRQALVQQGAAFADRPRF

ASFRVVSGGRSLAFGPYSQSWKVRRRAAHSTMRAFSTRQPRSRRVLEGHVLGEARELVAL

LVRGSAGGAFVDPRPLTVVAVANVMSAVCFGCRYSHDDAEFRELLSHNEEFGRTVGAGSL

VDVLPWLQRFPNPVRTAFREFEQLNRNFSNFVLDKFLRHRESLQPGAGPRDMMDAFIISA

GTEAAEGSEDGGARQDLEYVPATVTDIFGASQDTLSTALQWLLILFTRYPEVQARVQAEL

DQVVGRDRLPCLDDQPNLPYVVAFLYEAMRFSSFVPVTIPHATTTSTSVLGYHIPKDTVV

FVNQWSVNHDPAKWPNPEDFDPGRFLDKDGCIDKDLASSVMIFSMGKRRCIGEELSKMQL

FLFISILAHECNFKANPDEPSKMDFNYGLTIKPKSFRINVTLRESMELLDSAVQKFQAEE

DCQ

>CAA55205.1

MATSLSADSPQQLSSLSTQQTTLLLLFSVLAAVHLGQWLLRQWQRKPWSSPPGPFPWPLI

GNAAAVGQASHLYFARLARRYGDVFQIRLGSCPVVVLNGESAIHQALVQQGSIFADRPPF

ASFRVVSGGRSLAFGHYSEHWKTQRRSAYSTMRAFSTRHPRSRGLLEGHALAEARELVAV

LVRRCAGGAFLDPTPPVIVAVANVMSAVCFGCRYNHDDAEFLELLSHNEEFGRTVGAGSL

VDVLPWLQLFPNPVRTTFRKFEQLNRNFSNFVLDKFLRHRESLVPGAAPRDMTDAFILSA

EKKASGDPGDDSSGLDLEDVPATITDIGGASQDTLSTALLWLLILFTRYPDVQARVQAEL

DQVVGRDRLPCMSDQPNLPYVMAFLYESMRFSSFLPVTIPHATTANTFVLGYYIPKNTVV

FVNQWSVNHDPAKWPNPEDFDPARFLDKDGFINKALTSSVMIFSVGKRRCIGEELSKMLL

FLFISILAHQCNFKANQNESSNMSFSYGLTIKPKSVRIHVSLRESMELLDNAVKKLQTEE

GCK

>AAH50063.1

MATSLSADSPQQLSSLSTQQTTLLLLFSVLAAVHLGQWLLRQWQRKPWSSPPGPFPWPLI

GNAAAVGQASHLYFARLARRYGDVFQIRLGSCPVVVLNGESAIHQALVQQGSIFADRPPF

ASFRVVSGGRSLAFGHYSEHWKTQRRSAYSTMRAFSTRHPRSRGLLEGHALAEARELVAV

LVRRCAGGAFLDPTQPVIVAVANVMSAVCFGCRYNHDDAEFLELLSHNEEFGRTVGAGSL

VDVLPWLQLFPNPVRTTFRKFEQLNRNFSNFVLDKFLRHRESLVPGAAPRDMTDAFILSA

EKKASGAPGDDSSGLDLEDVPATITDIFGASQDTLSTALLWLLILFTRYPDVQARVQAEL

DQVVGRDRLPCMSDQPNLPYVMAFLYESMRFSSFLPVTIPHATTANTFVLGYYIPKNTVV

FVNQWSVNHDPTKWPNPEDFDPARFLDKDGIINKALASSVMIFSVGKRRCIGEELSKMLL

FLFISILAHQCNFKANQNESSNMSFSYGLTIKPKSFRIHVSLRESMELLDNAVKKLQTEE

GCK

>Q64429.3

MATSLSADSPQQLSSLSTQQTTLLLLFSVLAAVHLGQWLLRQWQRKPWSSPPGPFPWPLI

GNAAAVGQASHLYFARLARRYGDVFQIRLGSCPVVVLNGESAIHQALVQQGSIFADRPPF

ASFRVVSGGRSLAFGHYSEHWKTQRRSAYSTMRAFSTRHPRSRGLLEGHALAEARELVAV

LVRRCAGGAFLDPTQPVIVAVANVMSAVCFGCRYNHDDAEFLELLSHNEEFGRTVGAGSL

VDVLPWLQLFPNPVRTTFRKFEQLNRNFSNFVLDKFLRHRESLVPGAAPRDMTDAFILSA

EKKASGAPGDDSSGLDLEDVPATITDIFGASQDTLSTALLWLLILFTRYPDVQARVQAEL

DQVVGRDRLPCMSDQPNLPYVMAFLYESMRFSSFLPVTIPHATTANTFVLGYYIPKNTVV

FVNQWSVNHDPAKWPNPEDFDPARFLDKDGFINKALASSVMIFSVGKRRCIGEELSKMLL

FLFISILAHQCNFKANQNESSNMSFSYGLTIKPKSFRIHVSLRESMELLDNAVKKLQTEE

GCK

>Q64678.1

MATSLSADSPQQLSSLSTQQTILLLLVSVLAIVHLGQWLLRQWRRKPWSSPPGPFPWPLI

GNAASVGRASHLYFARLARRYGDVFQIRLGSCPVVVLNGESAIHQALVQQGGVFADRPPF

ASFRVVSGGRSLAFGHYSERWKERRRAAYGTMRAFSTRHPRSRGLLEGHALGEARELVAV

LVRRCAGGACLDPTQPIIVAVANVMSAVCFGCRYNHDDAEFLELLSHNEEFGRTVGAGSL

VDVMPWLQLFPNPVRTIFREFEQINRNFSNFVLDKFLRHRESLVPGAAPRDMMDAFILSA

EKKATGDPGDSPSGLDLEDVPATITDIFGASQDTLSTALLWLLILFTRYPDVQARVQAEL

DQVVGRDRLPCMSDQPNLPYVMAFLYESMRFTSFLPVTLPHATTANTFVLGYYIPKNTVV

FVNQWSVNHDPAKWSNPEDFDPARFLDKDGFINKALASSVMIFSVGKRRCIGEELSKTLL

FLFISILAHQCNFKANQNEPSNMSFSYGLSIKPKSFKIHVSLRESMKLLDSAVEKLQAEE

ACQ

>301758074

MATSLGPDAPLQPSALSSQQTTLLLLLSVLAAVHVGQWLLQQRRRQPGSAPPGPFAWPLI

GNAAAMGPAPHLSFARLARRYGDVFQIRLGNCPVVVLNGERAIRQALVQQGTAFADRPHF

ASFRWVSGGRSLAFGQYSQQWKVQRRAAQSTMRAFSXRQPRSRRVLEGHVLGEARELVAL

LVRGSAGGAFLDPRPLTVVAVANVMSAVCRYSHDDAEFRELLSHNEEFGRTVGAGSLVDV

LPWLQRFPNPVRTAFREFQQLNRDFSNFVLDKFLRHRESLQPGAGPRDMMDAFILSAGTD

AAEGSDDGGARLDLEYVPATVTDIFGASQDTLSTALQWLLVLFTRYPEVQARVQAELDRV

VGRARLPCLDDQPNLPYVMAFLYEAMRFSSFVPVTIPHATTTHASVLGYHIPKDTVVFVN

QWSVNHDPAKWPNPERFDPGRFLDKDGFIDKDVASGVMIFSVGKRRCIGEEISKMQLFLF

TSILAHQCNFKANPDEPSKMDFNYGLTIKPKSFRINVTLRESMELLDSAVQKLQAKEDCQ

>DAA24756.1

MATGLSPDDHLSPTLLSVQQTMLLLLLSVLAAVHVGQWLLRQRRRQPGSAPPGPFAWPLI

GNAASMGSAPHLLFARLARRYGDVFQIHLGSCRVVVLNGERAIRQALVHQSAAFADRPPF

ASFRLVSGGRSLAFGQYSESWKAQRRAAHSTMRAFSTRQPRGRRVLEGHVVGEVRELVEL

LVRRSAGGAFLDPRPLTLVAVANVMSALCFGCRYSHDDAEFLELLSHNEEFGRTVGAGSL

VDVLPWLQRFPNPVRTAFREFEQLNRNFSNFVLDKFLRHRESLRPGAAPRDMMDAFIHSA

GADSGDGGPRLDVDYVPATVTDIFGASQDTLSTALQWLLVLFTRYSEVQARVQAELDQVV

GRHRLPTLEDQPRLPYVMAFLYEAMRFSSFVPVTIPHATTANASVLGYHIPKDTVVFVNQ

WSVNHDPVKWSNPEDFDPTRFLDKDGLINKDLTGSVMVFSVGKRRCIGEEISKMQLFLFI

SILAHQCNFKANPDEPSKMDFNYGLTIKPKSFKINVTLRESMELLDSAVQKLQVEKECQ

>344288791

MATSLSPDDPRPASPLSTQQTTLLLFLSVLVAVHVGQWLLKQRRRQAASAPPGPFPWPLI

GNAAAVGPAPHLSFARLAQRYGDVFQIRLXNCPVVVLNGERAIHQALVQQGAAFADRPPF

ASFRVCRTAAACLCRYSENXRRARAANSXXXXXXXRQPRSRRVLEGHVLGEARELVALLV

RGSAGGTFVDPRPLTVVAVANVMSAVCFGCRYSHDDAEFRGLLSHNEDFGRTVGAGSLVD

VLPWLQRFPNPVRTAFRQFQQLNRDFSNFVLDKFVRHRESLRPGAAPRDMMDAFILLAGK

EETNGSGNGGVRLDLEYVPSTVTDIFGASQDTLSTALQWLLVLFTRYPEVQARVQAELDQ

VVGRDRLPCMDDQPNLPYIMAFLYEAMRFSSFVPVTIPHATTTDASVLGYHIPKDTVVFV

NQWSVNHDPGKWANPEDFNPARFLDKDGFINKNLASSVMIFSVGKRRCIGEELSKMQLFL

FFSILAHQCNFRANPDEPWKMDFTYGLTIKPKSFKINVTLRESMELLDHAVQKLQAQEDC

Q

>348574357

MALSADDPLQPSLLSTQQTTLLLLLSLLAAVHVGQWLLRQRRQPWSSPPGPFPWPLIGNV

SAVGPATHLSFARLARRYGDVFQIRLGSCPVVVLNSESAIHQALVQQGATFADRPNFASF

RVVSGGRSVAFGPYSEAWKAQRRAAFSIMRAFSTRQPRSRRILEGHVLGEARELVAILVR

GSAGGTCLDPMQPIIVAVANVMSAVCFGCRYNHGDAEFQELLSHNEEFGRTVGAGSLVDV

MPWLQLFPNPVRTTFREFQQLNRAFSNFVFDKFLRHRETLRPGAAPRDMMDAFILSAERK

AAGDSGGVGAQLDMENVPAAVSDIFGASQDTLSTALLWLLILFTRYPDVQARVQAELDQV

VGRDRLPCMSDQPNLPYVMAFLYEAMRFSSFLPVTIPHATTDNAFVLGYYIPKNTVIFVN

QWSVNHDPVKWHNPEDFDPARFLDKDGFINKDLASSVMIFSVGKRRCIGEELSKMLMFLF

ISILAHQCNFKVNQNEPSKMSFSYGLTIKPKSFKIHVSLRESMELLDSAVQKLQDEDACQ

>BAG59567.1

MAFGHYSEHWKVQRRAAHSMMRNFFTRQPRSRQVLEGHVLSEARELVALLVRGSADGAFL

DPRPLTVVAVANVMSAVCFGCRYSHDDPEFRELLSHNEEFGRTVGAGSLVDVMPWLQYFP

NPVRTVFREFEQLNRNFSNFILDKFLRHCESLRPGAAPRDMMDAFILSAEKKAAGDSHGG

GARLDLENVPATITDIFGASQDTLSTALQWLLLLFTRYPDVQTRVQAELDQVVGRDRLPC

MGDQPNLPYVLAFLYEAMRFSSFVPVTIPHATTANTSVLGYHIPKDTVVFVNQWSVNHDP

LKWPNPENFDPARFLDKDGLINKDLTSRVMIFSVGKRRCIGEELSKMQLFLFISILAHQC

DFRANPNEPAKMNFSYGLTIKPKSFKVNDTLRESMELLDSAVQNLQAKETCQ

>EDM02787.1

MATSLSADSPQQLSSLSTQQTILLLLVSVLAIVHLGQWLLRQWRRKPWSSPPGPFPWPLI

GNAASVGRASHLYFARLARRYGDVFQIRLGSCPVVVLNGESAIHQALVQQGGVFADRPPF

ASFRVVSGGRSLAFGHYSERWKERRRAAYGTMRAFSTRHPRSRGLLEGHALGEARELVAV

LVRRCAGGACLDPTQPIIVAVANVMSAVCFGCRYNHDDAEFLELLSHNEEFGRTVGAGSL

VDVMPWLQLFPNPVRTIFREFEQINRNFSNFVLDKFLRHRESLVPGAAPRDMMDAFILSA

EKKATGDPGDSPSGLDLEDVPATITDIFGASQDTLSTALLWLLILFTSCTSDQPNLPYVM

AFLYESMRFTSFLPVTLPHATTANTFVLGYYIPKNTVVFVNQWSVNHDPAKWSNPEDFDP

ARFLDKDGFINKALASSVMIFSVGKRRCIGEELSKTLLFLFISILAHQCNFKANQNEPSN

MSFSYGLSIKPKSFKIHVSLRESMKLLDSAVEKLQAEEACQ

>BAE23978.1

MATSLSADSPQQLSSLSTQQTTLLLLFSVLAAVHLGQWLLRQWQRKPWSSPPGPFPWPLI

GNAAAVGQASHLYFARLARRYGDVFQIRLGSCPVVVLNGESAIHQALVQQGSIFADRPPF

ASFRVVSGGRSLAFGHYSEHWKTQRRSAYSTMRAFSTRHPRSRGLLEGHALAEARELVAV

LVRRCAGGAFLDPTQPVIVAVANVMSAVCFGCRYNHDDAEFLELLSHNEEFGRTVGAGSL

VDVLPWLQLFPNPVRTTFRKFEQLNRNFSNFVLDKFLRHRESLVPGAAPRDMTDAFILSA

EKKASGAPGDDSSGLDLEDVPATITDIFGASQDTLSTALLWLLILFTRYPDVQARVQAEL

DQVVGRDRLPCMSDQPNLPYVMAFLYESMRFSSFLPVTIPHATTANTFVLGYYIPKNTVV

FVNQWSVNHDPAKWPNPEDFDPARFLDKDGFINKALASSVMIFSVGKRRCIGWHTTMSSH

FLEAERG

>EDM02788.1

MATSLSADSPQQLSSLSTQQTILLLLVSVLAIVHLGQWLLRQWRRKPWSSPPGPFPWPLI

GNAASVGRASHLYFARLARRYGDVFQIRLGSCPVVVLNGESAIHQALVQQGGVFADRPPF

ASFRVVSGGRSLAFGHYSERWKERRRAAYGTMRAFSTRHPRSRGLLEGHALGEARELVAV

LVRRCAGGACLDPTQPIIVAVANVMSAVCFGCRYNHDDAEFLELLSHNEEFGRTVGAGSL

VDVMPWLQLFPNPVRTIFREFEQINRNFSNFVLDKFLRHRESLVPGAAPRDMMDAFILSA

EKKATGDPGDSPSGLDLEDVPATITDIFGASQDTLSTALLWLLILFTSCTSDQPNLPYVM

AFLYESMRFTSFLPVTLPHATTANTFVLGYYIPKNTVVFVNQWSVNHDPAKWSNPEDFDP

ARFLDKDGFINKALASSVMIFSVGKRRMAD

>224047677

MALERLGEALRGMPPLQSSLLLLLCLLAAIHLGKLLLQHQQRRRQGQRRAPPGPFPWPLI

GNAAQLGSAPHLSFARLASTYGAVFQLRLGRWPVVVLNGERAIRQALVRQGAAFAGRPPF

PSFQLVSGGLSLAFGGYSELWKFQWRYKHTTHATVRAFFTGSPATRRMLERHLVSEARAL

MALLVRGSAGGAFLDPSRVLVVAVANVMSALCFGRRYSHGDGEFLRIVGRNEQFGRAVGA

GSLVDALPWLQRFPSPVRAAYRAFRDLNRDFYGFVRGKFLQHQRSLRPGAAPRDMMDAFI

RLQREQPWLQLEHVPATVTDIFGASQDTLSTALQWLLIFLIRYPKVQAKMQEEVDRIVGR

DRLPCVEDQPHLPYIMAFLYESMRFSSFVPVTIPHATTTNTFIMGYLIPKDTVIFVNQWS

VNHDPAKWSNPEDFDPTRFLDENGLINKDLTSSVMIFSLGKRRCIGEELSKVQLFLFTSI

LVHQCNFTANPNEDPKMDFTYGLTIKPKPFTLNVTLRDTMELLDQAVQRLQAEKAASESQ

LSANA

>BAE02060.1

MSAVCFGCRYSHDDPEFRELLSHNEEFGRTVGAGSLVDVMPWLQYFPNPMRTAFREFEQL

NRNFSNFVLDKFLRHCESLRPGAAPRDMMDAFILSAEKKAARDSDDGGARLDLENVPATV

TDIFGASQDTLSTALQWLLLLFIRYPDVQARVQAELDQVVGRDRLPCMDDQPNLPYVLAF

LYEAMRFSSFVPVTIPHATNANTSVLGYHIPKDTVIFVNQWSVNHDPVKWPNPENFDPAR

FLDKDGLINKDLTSRVMIFSVGKRRCIGEELSKMQLFLFISILAHQCNFRANPNGPEMNF

SYGLTIKPKSFKVNVTLRESMELLDSAVQKLQAEETCQ

>301608708

MNWKIWEDLGQSSVPKLLLSFLCALTVAHILKWIHEWIIPRWIRSSQPPGPFPWPLFGNA

LQMGSYPHLAFIDLAKRYGNIFQIKLGSQKIVVLNGDLVIRHALLHKGEDFAGRPKFTSY

QFVSGGRSLAFGCYTEKWKAHRKLAHSTVRAFSTGNPQTKRCLAENVLKEARDLIALFSE

LGQGGKYFYPGRHTVVSVANVMSAVCFGRRYQHGDLEFQSLLSNNDKFTRSVGAGSLVDV

MPWLQRFPNPVRSVFRSFQQVNYEFYDFVYKKFLLHRNTANQAVTRDMMDAFIHILITKE

GKVRADDADGGEEKGKNGQYFFHSLEAEHVPSTVTDIFGASQDTLSTALQWVIFFLVRYP

EIQTKLQDEMDRVIGKDRLPCIEDQPKLPYLMAFLYEFMRFSSFVPITIPHATTKNTTIM

GYQIPKDTVVFVNQWSVNHDPQKWSNPGEFNPSRFLDDNGLINKDLVSNIMIFSVGKRRC

IGEELSKIQLFMFSSILLHQCIFTALPADNLNPKGDYGLSIKPKPFRISMTLRHGSMDLL

NNSVLSGMAE

>AFB82730.1

MLWKTYEDLGQSSAPKLLLSFLCALATLHILKWIHEWIIPRWSGSSQPPGPFPWPVIGNA

LQIGGYPHLAFIDLANRFGNVFQIKLGSQKVVVLNGDLVIRHALLHKGADFAGRPKFTSF

KFVSGGRSLTFGCYNERWKAHRKLAHSTLRTFSTGNPQTKRCLAENVLNEAQDLIALFSE

LGEGGKYFYPERHIAVAVANVMSAVCFGKRYQHGDLEFQSLLSNNDKFTRSVGAGSLVDV

MPWLQCFPNPVRSVFKSFQQVNYEFYNFVYTKFLLHRSTVTNTVTRDMMDAFIHILITKD

QQCKTNADDDADGGEDEAKNGQHSFHLLEADHVPSIVTDIFGASQDTLSTALQWVIFYLI

RYPEIQSKIRDELDRIIGKDRLPCIEDQPNLPYLMAFLYELMRFSSFVPVTIPHATTKDT

NIMGYQIPKDTVVFVNQWSVNHDPQKWSKPGEFNPSRFLDDNGFLNKDLVSNVMIFSIGK

RRCIGEELSKIQLFMFTSILLHQCIFTASPVDDLNQKGHYGLSIRPKTFRMNMTLRNGSM

DLLNNSVRRGTDD

>AEI99246.1

MTLLVSLHLFRWLCRQRSHSCPPGPLAWPVIGNAPQLGNAPHLYFTRLVKKYGNVFQIKL

GSRTVVVLNGGSIKQALVKQGSDFSGRPDFTSFQYISNGESMAFGNSTDWWKVYRKVAQS

TVRMFSTGNPQTKKTFENHVLSEGKELLRLFLRKTKEDKYFQPLTYLVVSTANIMSAVCF

GKRYSYEDEEFQQVVGRNDQFTKTVGAGSIVDVMPWLQYFPNPIKTIFENFKKLNLEFSA

FVRDKVVEHRKTIDSSTIRDMTDAFIVALDQIRDKMGLPEKDYVSSTVGDVFGASQDTLS

TALQWIILILVKYPEIQVRLQQEVDKVVDRSRVPAIEDQQQLPYVMAFIYEVMRFTSFVP

LTIPHSTTTDTSIMGYTVPKNTVIFINQWSVNHDPSNWSQPDIFDPERFLDQSGMLNKDL

TGNVLLFSLGKRRCIGEELSKMELFLLTALIAHQCNITADPAVPVKLGYNYGLTLKPHAF

SVAVSVRDDMKLLEVDTSQPSSDSQTKE

>348507171

MTLLVSLHLFRWLCRQRSHSCPPGPLAWPVIGNAPQLGNAPHLYFTRMVKKYGNVFQIKL

GSRTVVVLNGGSIKQALVKQGSDFSGRPDFTSFQYISNGESMAFGNSTDWWKVHRKVAQS

TVRMFSTGNPQTKKTFENHVLSEGKELLRLFLRKTKEDKYFQPLTYLVVSTANIMSAVCF

GKRYSYEDEEFQQVVGRNDQFTKTVGAGSIVDVMPWLQYFPNPIKTIFENFKKLNLEFSE

FVRDKVVAHRKTIDSSTIRDMTDAFIVALDQIRDKMGLPEKDYVSSTVGDVFGASQDTLS

TALQWIILILVKYPEIQVRLQQEVDKVVDRSRVPAIEDQQQLPYVMAFIYEVMRFTSFVP

LTIPHSTTTDTSIMGYTVPKNTVIFINQWSVNHDPSNWSQPDIFDPERFLDQSGMLNKDL

TGNVLLFSLGKRRCIGEELSKMELFLLTALIAHQCNITADPAVPVKLGYNYGLTLKPHAF

SVAVSVRDDMKLLEADTSQPSSDSQTKE

>ADD51159.1

MDMPIIFEEMTWPSPRSILLASLTVVFAVHLWRRIRRNWDVCSPPGPFAWPVIGNALEVG

KTPHLYFSRMARKYGDVFRIKLGCRDVVVLNGDAIRQALIKNGYDFAGRPDFTSFQYVSN

GDGIAFGKFSEWWKVHRKVAQSTVRMFSTGNMNSKKTFENHVVCEVRELLRLFLGKTQEH

KYFQPMTYLVVSTANIMTAVCFGKRYSYDDAEFAQVVGRNDQFTQTVGAGSIVDVMPWLQ

YFPNPIKTIFDNFKELNREFSKFIVTKVVEHRKTIQPSTIRDMTDAFIMALDHSQDSSPG

VSPGKDYVPPTIGDIFGASQDTLSTALQWIILILVRFPHIQLRLQEEVDKVVYRSRLPTM

EDQSQLPYVMAFIYEVMRFTSFVPLTIPHSTITDTTIMGYTILKDTVIFINQWSSNHDPA

RWTQPETFDPLRFLDQDSSLNKDLASSVLIFSLGKRRCIGEELSKMQLFLFTALLAHQAH

FSPDPDKLPTIDYTYGLTLKPNNFSIAVNLRDTMDVLEEASQRPFYGETQEDTGNSRSD

>ACO51071.1

MEVTPEHIPAVNPFTPRAALVACLALLLSVWLRLRLRQRRALPGLPGPFAWPVIGNATQL

GNAPHLYFSRMVSKYGNVFQIQLGSRAVLVLNGDAIREALIKQGLNFAGRPDLTSFKHIS

AGRSMAFGTVTDWWKTHRKVAQSTVRMFSTGNPQTKRAFEQHVVGEFRELLRLFVEKTRG

ERHFQPGAYLVVSTANVMSAVCFGKRYAYEDAEFREVVGRNDKFTQTVGAGSIVDVMPWL

QYFPNPIKTIFDDFKKLNQDFVVFIQDKVTEHRKTMESGITRDMTDAFIKALDQIKETSG

LQGGTDYVTPTIGDIFGASQDTLSTALQWIILIFVKYPEMQVRLQLEVDRAVDRSRLPSI

EDQSRLPYVMAFIYEVMRFTSFVPLTIPHSTLTDTSLMGYAVPKDTVVFINQWSINHDPA

TWSNPESFDPERFLDAQGALNKDLTSNVLIFSVGRRRCIGEELSKMQLFLFTSLLAHQCN

ITGDPLRAPTLDYKYGLTLKPLDYSIAVSLREDMALLDAATAQPARDEQPAGVQATG

>348520546

MALDTEFGVKSSSSIIREWSGQVHPALVATFVFLFCVEACLWVRNLRLKRRLPGPFAWPV

VGNAMQLGQMPHITFARLAKKYGNVYQIRLGCSDVVVLNGERAIRQALIQHSTEFAGRPN

FLSFQMISGGRSLTFTNYSKQWKAHRKVAQSSLRAFSSANSHTKKAFEQHVTAEAMELVQ

ALLRQSTDRRYSDLVHEFTVASANIMCALCFGRRYGHDDLEFRTLLKRMDKFGETVGAGS

LVDVMPWLQSFPNPVRSVYENFKNINEEFFGFVKDKVMQHRESFNPQVTRDMSDAIISVI

EHGEDSRLTKEFVEATVTDLIGAGQDTISTFMQWILLLLVKYPDVQAKLQELIDKVVGRD

RLPSIEDRSSLAYLDAFIYETMRFISFVPLTIPHSTTSDVTIEGLHIPKDTVVFINQWSV

NHDPLKWKDPHVFDPSRFLDESGALDKDKANSVMIFSTGKRRCIGNQIAKVEVFIFTAVL

LHQCSFESNPSEPVTLDCSYGLALKPLPFRVSTKLRGKLLGLVSPA

>AEI99247.1

MALDTEFGVKSSSSIIREWSGQVHPALVATFVFLFCVEACLWVRNLRLKRRLPGPFAWPV

VGNAMQLGQMPHITFARLAKKYGNVYQIRLGCSDVVVLNGERAIRQALIQHSTEFAGRPN

FLSFQMISGGRSLTFTNYSKQWKAHRKVAQSSLRAFSSANSHTKKAFEQHVTAEAMELVQ

ALLRQSTDRRYSDLVHEFTVASANIMCALCFGRRYGHDDLEFRTLLKRMDKFGETVGAGS

LVDVMPWLQSFPNPVRSVYENFKNINEEFFGFVKDKVMQHRESFNPQVTRDMSDAIISVI

EHGEDSRLTKEFVEATVTDLIGAGQDTISTFMQWILLLLVKYPDVQAKLQELIDKVVGRD

RLPSIEDRSSLAYLDAFIYETMRFISLVPLTIPHSTTSDVTIEGLHIPKDTVVFINQWSV

NHDPLKWKDPHVFDPSRFLDESGALDKDKANSVMIFSTGKRRCIGNQIAKVEVFIFTAVL

LHQCSFESNPSEPVTLDCSYGLALKPLPFRVSTKLRRKLLGLVSPA

>ADO15703.1

MALDTEFGVKGSSIIREWSGQVQPALVASFVFLVCLEACLWVRNLRLKRRLPGPFAWPVV

GNAMQLGQMPHITFATLAKKYGNVYQIRLGCSDVVVLNGDRAIREALLQHGTEFAGRPNF

VSFQMVSGGRSMSFSNYSKQWKAHRKIAQSSLRAFTSGNSQTKKAFEQQITAEAMELVQV

FLRHSTDGRYFNPSQEFTEAAANIMCALCFGRRYAHEDQEFRNMLKKLDKFGETVGAGSL

VDVMPWLQRFPNPVRSGYENFKNLNEEFFAFVKDKVVQHRESFDPDVTRDMSDAFINVIE

HGQDSTLDNEYVEATVTDLIGAGQDTISTTLQWILLLLAKHPDIQAKLHELIDKAVGRDR

LPSSEDRSRLSYLDAFIYETMRFTSFVPLTIPHATTSGVTIDGLHIPKDTVVFVNQWSVN

HDPLKWKDPHTFDPARFLDENGALNKDATSGVMIFSTGKRRCIGNQIAMVEVFLFAAVLL

HQCSFESDPSEPLTLNCSYGLTLKPLQLSVRAKLRGKLLGLVSPA

>ADD51161.1

MALLDTEFGVNISSIIREWSGQVQPALVASFVFLFCLEACLWVRNLRLKRRLPGPFAWPV

VGNAMQLGQMPHITFSKLAKKYGNVYQIRLGCNNIVVLNGDTAIREALVQHSTEFAGRPN

FVSFQMISGGRSLTFTNYSNQWKMHRKIAQSTIRAFSSANSQTKKAFEHHVLGESMDLVQ

VFLRMSADGRYFNPSHEFTVAAANVICALCFGKRYGHDDIEFRTLLGRMDRFGETVGAGS

LVDVMPWLQNFPNPVRSIYQNFKHINEEFFAFVKDKVMQHRETFTPDVTRDMSDAIINAI

EHRKDSGLAKDFVEGTVTDLIGAGQETMSTIFQWILLLLVKYPNIQTRLQEQIDKVVGRD

RLPCIEDKASLAYLDAVIYETMRYTSFVPLTIPHSTTSDVTIEGFHIPKDTVVFVNQWSV

NHNPLKWKDPHLFDPSRFLDENGALDKDLTNSVMIFSTGKRRCIGDQIAKVETFLFTAVL

LHQCTFESNPSEALTLDCSYGLTLKPLHYTITTKLRGKLLGLVSPA

>ACI33284.1

MALLDTEFGVKGSSIIREWSGQVQPALVASFVFLFCLEACLWVRNLRLKRRLPGPFAWPV

VGNAMQLGQMPHITFSKLAKKYGNVYQIRLGCNNIVVLNGDTAIREALVQHSTEFAGRPN

FISFQMISGGRSLTFTNYSKQWKMHRKIAQSTIRAFSSANSQTKKAFEHHVLGECMDLVQ

VFLRRSADGRYFNPAHEFTVAAANVICALCFGKRYGHDDIEFRTLLGRMDRFGETVGAGS

LVDVMPWLQNFPNPVRSIYQNFKHINEEFFAFVKDKVMQHRETFTPDVTRDMSDAIINAI

DHRKDSGLAEDFVEGTVTDLIGAGQETMSTIFQWILLLLIKYPNIQTKLQEQIDKVVGRD

RLPSIEDKASLAYLDAVIYETMRYTSFVPLTIPHSTTSDVTIEGFHIPKDTVVFINQWSV

NHNPLKWKDPHLFDPSRFLDENGALDKDLTNSVMIFSTGKRRCIGNQIAKVETFLFTAVL

LHQCTFESNPSEALTLDCSYGLTLKPLHYTITTKLRGKLLGLVSPA

>ABA03137.1

MAITSEFGLKSSSIIKEWSGQVHPALVASFVFLFCLEACLWVRNLRLKRRLPGPFAWPVV

GNAMQLGQMPHITLAKLAKKYGNVYQIRLGCSDVVVLNGDQAIHQALIQHSTEFAGRPNF

VSFQMISGGRSLTFTNYSKQWKAHRKVAQSTLRAFSSANSQTKKNFEQHVLAEATELVQV

FLRQSANGQYFYPAYEFTVAAANIMCALCFGRRYGHDDQEFRTLLQSIDKFGETVGAGSL

VDVMPWLQSFPNPVRNIYETFKTINTEFFNYVKDKVVQHRESFNPEVTRDMSDAFIRVIE

HEESTLSREFVEATVTDLIGAGQDTMSTFMQWLVHLLVKYPDYQTKLQQLIDKVVGRDRL

PSVEDRSNLALLDAFIYETMRFTSFVPFTIPHSTTSDVTIESLHIPKDTVVFINQWSVNH

DPLKWKDPHVFDPMRFLDENGALDRDRTNSVMIFSTGKRRCIGSQIAKVQVFLFSAVLLH

QLTFESDSSLPPTLECSYGLTLRPLQFNVRAKLRGKLLDVVSPSINTLP

>EHB14600.1

MGWRWRGCAESEAEARLFPSAHRAWVSMALSPDDALQPSPLSIQQTALLLLLSVLAAVHV

GQWLLRQRQQPRSSPPGPFPWPLIGNASAVGPASHLSFARLARRYGDVFQIRLGSCPVVV

LNGESAIHQALVQQGATFAGRPPFASFREVSGGRSMAFGPYSEAWKAQRRAAFSITRAFT

TRQPRSRRLLFGRTVGAGSLVDVMPWLRLFPNPVRTTFREFQQLNRNFSNFVFEKFRRHR

ESLRPGADPRDMMDAFILSAEGTAAGGSGEAGARLDMESVAATVTDIFGASQDTLSTALL

WLLILFTRYPDVQARVQAELDRVVGRDRLPRMSDQPNLPYVMAFLYETMRFTSFLPVTIP

HATTASALVLGYYIPKNTVIFVNQWSVNHDPVKWPNPEDFDPARFLDEDGFINKDLANSV

MIFSVGRRRCIGEELSKMLMFLFISILAHQCDFKVNQNEPSKMSFSYGLTIKPKSFKIHV

SLRESMELLDSAVQKLQDEEACQ

>ACO51072.1

MAQMDAEFDLRSGSIIKGWSGHVQPALVAAVVFLFCLEACLWVRNLKLKRRLPGPFAWPV

VGNALQLGHMPHITFAELAKKYGDVYQIRLGCSDIVVLNGARVIREALVQHSTEFAGRPN

FVSFQNVSGGKSLSFNNYSKQWRMHRKIAQTTIRAFSSFNSRTKKAFEHQIVAEATELVE

IFLQLSTQGQYFNPGNELTVAAANVICALCFGKRYGHNDAEFRALLRHVDLFGRTVGAGS

LVDVMPWLQSFPNPVRSVFKTFKWSNQEFFNFVSSKVEEHRQTFDPHNIRDMSDAIIELI

DESDGDTEITKEYTEATVADLIGAGMDTVSTALHWIVLLLAKHPDIQTKLHELIDRVVGR

GRLPSVEDRVHMPYLDAFIYETMRFTSFVPVTIPHLTTSDVTVGDLSIPKDTVIFINQWS

VNHDPLRWKDPQAFDPSRFLDENXSLDKDLTNNVMIFSAGKRRCIGDQVAKVEIFLFFAI

LLHQCSFEKCPDEDFSLNYSYGLTLKPLDYKIAAKLRGELLKHK

>AFJ49832.1

MVQSEDRRGGEIAAPVQSVLQPALLLSCALLLYFWAARKRSAAPGKRLPGPVGWPLVGNA

LQLGRHPHLTFCELAKRYGDVFQFRLGSRSIVVLNGEATIRQALVQQGGPFAGRPDFPSF

RMVSDGKSVAFGRYTERWRAQRRVAHATLRAFSTGNAPNKRLFEEHVTAEAQELIDGLVK

LSEGGLYVNPLPLLAVANANVMCALCFGQRYSHADGEFRALLGRNDRFGQTVAAGSLVDV

LPWLLSFPNPVRSVFRDFQAINGEMLEFVRAKVAQHRLTFQPGTPPRHISDAILGRMEHG

PGGKEGPLGDYAESTLTDLFGAGQDTTSAALAWLLLLLLKHPQLREQLQTDLDRVVGRER

LPTADDRASLPRLEAFLYEMLRYSSLVPVTIPHATTADVFLDGFHIPEGTVVFVNQWSVN

HDGVCWKDPHIFDPTRFLDVRQETLDRDLARRVLIFSMGKRRCIGDQLAKLQLFLFTAIL

LHQCDLTANPAEEIRVDCEHGLVLRPLPYTLSVTRRSTSRAEEDRSRSNTDIACPS

>AAI59256.1

MQLGQMPHITFSKLAKKYGNVYQIRLGSSDIVVLNGESAIRSALLQHSTEFAGRPNFVSF

QYVSGGTSMTFASYSKQWKMHRKIAQSTIRAFSSANSRTKKSFEKHIVAEAVDLVETFLK

SQHFNPSHELTVAAANIICALCFGKRYGHDDLEFRTLLGNVNRFSETVGAGSLVDVMPWL

QTFPNPIRSIFQSFKDLNNDFFSFVKGKVVEHRLSYDPEVIRDMSDAFIGVMDHADEETG

LTEAHTEGTVSDLIGAGLDTVSTALNWMLLLLVKYPSIQSKLQEQIDKVVGRDRLPSIED

RCNLAYLDAFIYETMRFTSFVPVTIPHSTTSDVTIEGLHIPKDTVVFINQWSVNHDPQKW

SDPHIFNPSRFLDENGALDKDLTSSVMIFSIGRRRCIGDQIAKVEVFLISAILIHQLTFE

SDPSQDLTLNCSYGLTLKPFDYKISAKPRGSIVN

>363731574

MALERLGEALRGTPPLQSSLLLLLCLLAAFLRLVGRNEQFGRAVGAGSLVDALPWLRRFP

SPVRAAYRAFRDLNRDFYGFVRGKFLQHQRSLRPGAAPRDMMDAFIRLQREQPRLQLEHV

PATVTDIFGASQDTLSTALLWLLIFLIRYPKVQAKMQEEVDRIVGRDRLPCAEDQPHLPY

IVAFLYESMRFSSFVPVTIPHATTTNTFIMGYLIPKDTVIFVNQWSVNHDPAKWSNPEDF

DPTRFLDENGFINKDLTSSVMIFSMGKRRCIGEELSKVQLFLFTSILVHQCHFTANPNED

PKMDYTYGLTIKPKPFTLNVTLRDTMELLDKAVQRLQAEKTGNEN

>327262379

MNTNLEPVLALMTLLCSVDEEFRRLVGRNEQFGRAVGAGSLVDALPWLRRFPNPVRSAFR

AFRALNRDFYGFVRGKFLRRRRILRLRPGDRARDLMDACIRLQQDRPGLPLEHVPATLTD

IFGASQDTLSTALQWLLLCLVRYPEVQTKLQEEIDKVVGRDRLPCAEDQPHLPYVMAFLY

ETMRFSSFVPVTIPHFTTMDTTLMGYHIPKDTVIFVNQWSVNHDPVKWPSPEDFNPARFL

YENGSLNKDLTSSVMIFSVGKRRCIGEELSKAQLFLFIAILVHQCNFTANPKEDSKMDFT

YGLTTKPKPFTLHVKLRDNLDLLGKAVQRLQAEKDSENSLSDM

>EEN63759.1

MAAVATAALFGLSYLQVVLIAVLLVLVAAVVASSLRQNTPSLPPGPWGFPVVGIFPALGS

RPHHAFSRMAEKYGDVFRVKFGSRTVIILNGIDMVKDAFVKQSACFAGRPALYSFKQVKN

GITFKTYSPSWVARKKVTVGALKGFVNGRVGALTASAETMITEEAQELARVFLSKSGQPS

NPEEYAHTAVANVVCALCFGKRYEHGDQEFRQLLRNTEKFRQAIGAGNPADFMPWLRFFP

NKNMKLFKEAMESSTQLFDKHINAHLQTYDPSVIRDIADALIYNMRENKEAGLTDEFVLE

CVIDIFGAGQDTTSQMLHWAFLYMLVFPDVQARVQREIDGVVGRERAPTLADEASLPYTV

AVIQEIVRHTGVVPMSIPHLTTKDTQLHGYTLPKDTIVFANLFSVGHDRRIWGDPSSFRP

ERFLDPSGTTLDPAAVEKNLPFSAGKRRCPGEHLAKQEMFLFFSILLQQCSFERVNGTAS

PTLEGTFGLVMRPQPYSMIVRPR

>198433484

MMITAAILLDAGRSFAVPVAFTAVSVLTLYVCLRKRQGIPPGPTAWPLVGNLFSMGRQSH

LILESMRKTYGDVFSVYFGSTLVVVVNGKAVEECLSTHSARYSMRPELHTTQYILEGKSF

AFSHIAVSEHKRYRTLAVAVVKQLVNGGGEKTDVAVKHGLQNGTRHSSIEDRIYMEAACM

CDKLLETSDKPDLKDEILQVITKELCRSAADMLLGNTLSEYELDEISRVVENLRNSNEAI

MLVNFIPAVRMLWRNGLQKYIQLTQSLNRFFERCIRNRKAQLATVSNGHTEDNGVWLTNG

VDCTVKFWQKLKNDPQYEESRVMKVVADLFGARVDTMTVALAWMIVYWSTYQAAQERAQK

EIDHFVKNEKRLPRYSERNQLPYTMALIMEVERHCSFVPFTLPHAPVQDTMLNGYLIPKG

TMMLISMRSINHDTAVWDSPAQFRPERFLLDQSGGFNSALAEQVMLFGAGRRRCAGEALG

RMQIFLYSVLFLRKCTFRRSDKDGHVLPESLAGISLIPQTMCVSISRREADGSKNTEP

>198434495

MDSLVFVLVDTVLVMKYQILLFLVIVYAIKLLATSQSRRLNIPGPYPWPVIGNVIEMGGQ

PQFSLTNMAKKYGPVYLMKLGTADVLVLNNYEVIKEALLRQRRIFGGRPIFDSFKKISQG

RGVVFNSTMTQGDEWMKLKMTIVKHVHRFVSSEETKGYVAHHVQMEAVELVRILTEKCRS

SPNKVIFPIEQINLAIANVVCAIMFGHRYQHGNKEFQDLISLNEQFGDVIGSGSQVDVIP

WMKIFPKFRNALKVFDFLTNRLNDWMRLRTKEHRLTYKHGVIRDIVDSFIAESIDHPEQS

ALNDDVIMALTTDVFGAGQDTMSTTMQWVFVYMMHFKECQRKIHAELDSVIGPGELPHIS

DRRRLPYLEAVMHEIFRHSTFTSTTIPHVTTQDTVLDGHFIPKGILVFINQFGANHDPNH

WVDPDKFIPERFLDGKGNLISRPHDRYLLFSTGARKCPGDELSRMLILHFMATMFALCEV

SSDPKKPATLDAVYNLSMRPKELRTIVRSRNLPFLKNSVAQMSEADSHVLTVPGETTSFL

TSRVESTVPDNQESQFSDNDFEKVDTKIPKRKVFSRPTLTHDDINGNNVRKRGNLHQSAM

YRIQLAT

**CYP2**

>AAI43326.1

MLASGLLLVALLACLTVMVLMSVWQQRKSRGKLPPGPTPLPFIGNYLQLNTEHICDSIMK

FSECYGPVFTIHLGPRRVVVLCGHDAVREALVDQAEEFSGRGEQATFDWVFKGYGVAFSN

GERAKQLLRFAIATLRDFGVGKRGIEERIQEESGFLIEAIRSTHGANIDPTFFLSRTVSN

VISSIVFGDRFDYEDKEFLSLLSVMLGIFQFTSTSTGQLYEMFSSVMKHLPGPQQQAFKL

LQGLEDFIAKKVEHNQRTLDPNSPRDFIDSFLIRMQEEEKNPNTEFYLKNLMMSTLNLFI

AGTETVSTTLRYGFLLLMKHPEVEAKVHEEIDRVIGKNRQPKFEDRAKMPYMEAVIHEIQ

RFGDVIPMSLARRVKKDTKFRDFFLPKGTEVFPMLGSVLRDPSFFSNPQDFNPQHFLDDK

GQFKKSDAFVPFSIGKRYCFGEGLARMELFLFFTTVMQNFRLKSSQSPKDIDVSPKHVGF

ATIPRNYTMSFLPR

>AAX37318.1

MFTALLKLDLASVGLTLFLGLIILVLFEIFRIHSYKRRTPPGPTPLPFVGTIPHFLKNPL

GFIRSMSQYGDMSTMYLGRKPAIFLNTIQLAKETLVQDTFSGKPYLPVIEWISKGFGIAM

VTFNHSWRQQRRFALHTLKNFGLGKKSVEDRVLEESHYLISEMLKDEGKPVDPHHPIQNA

VSNIICSIVFGDRFEYNNKRFEYLLKMLNETIMLAGSASGRIFNLVPFIKHFPGPHQKIK

KNTDDLLIFLGDEVEEHRKTLDPGSPRDFIDAYLLEIEKQKFNKDSTFHEGNLLASAGDL

FMAGTDTTETTIRWGLLLLIQNPDVQERCHEEIVRVLGYDRLPSMNDRDRLPYTLATVHE

IQRYGNIIPILIHETILPTKLQGYSIPQGTTIVTNIQAIFSSKDHWKHPDSFNPENFLED

GHFIKPESFIMFSLGPRSCLGEMLARTELFLFITSLLQRIHFSWPPDAKPIDMDGIFGLV

HSPQTFNVICRSRDTK

>AAS83982.1

MILQFIYENFDFKSWILLGFGLLLLVDLIKYWNPSSFPPGPRPLPFLGNVFTEIQDFRNI

NKLAQVYGTIFSLRLGSEKLIIVSGYKMAKEALVTQNDSFIDRPNVPLFHKVFKGLGTIL

SNGYLWRMHRKFAVSHLRNFGVGNESLEVKIQQECIFLCDAFKAEKGPVDPMVTLNSAIS

NIISSLIFGQRFDYHDEYYQRILRLDTECIQLACSPRAQLYNACPWLFEYLPGPHQTIFS

NYKKIKDFLRGEIIKHREDWDPANPRDFIDNYLTEMERKKSDPEAGFNIEGLVVTCLDLI

EAGTETATTTLRWGLLFMIKFPEIQEKVQAEIDRVIGQSRQPCLNDRVNMPYTEAVIHEI

QRFGDVVPLGFPKRAVKDTQLGKYFIPKGTAITVNLSSVLHDPNEWETPDTFNPGHFLDE

IGQFRKRDAFMPFFAGKRACLGENLARQELFLYFTSLLQQFKISKCPGEEPSLEGEIWFT

YAPAPFHICVSSR

>AAH60973.1

MEPSVLLLLALLVGFLLLLARGHPKSRGNFPPGPRPLPLLGNLLQMDRGGLLKSFIQLRE

KYGDVFTVHLGPRPVVMLCGTDTIREALVGQAEAFSGRGTVAVVEPTFKEYGVIFANGER

WKTLRRFSLATMRDFGMGKRSVEERIQEEAQCLVEELRKSQGAPLDPTFLFQCITANIIC

SIVFGERFEYTDRQFLRLLELFYQTFSLISSFSSQMFELFSGFLKYFPGAHRQISKNLQE

LLDYIGHSVEKHRATLDPSVPRDFIDIYLLRMEKEKSNQHTEFHHQNLMMSVLSLFFAGT

ETSSTTLRYGFLLMLKYPHVAEKVQKEIDQVIGSHRLPTLDDRTKMPYTDAVIHEIQRFS

DLIPIGVPHRVTKDTMFRGYLLPKNTEVYPILSSALHDPQYFEQPDSFNPDHFLDANGAL

KKSEAFLPFSTGKRICLGESIARNELFLFFTSILQNFSVASHVAPKDIDLTPKESGIGKI

PPTYQICFLAR

>ADI87117.1

MELDALVPLAVTVAIFLLLVDLMHRRQRWAARYPPGPLPLPGLGNLLHVDFKNTPYCFDQ

LRRRFGDVFSLQLAWTPVVVLNGLAAVREALVTCGEDTADRPPVPIYQVLGIGPRSQGVF

LARYGPAWREQRRFSVSTLRNLGLGKKSLEQWVTEEAACLCAAFTDQAGRPFRPNSLLDK

AVSNVIASLTYGRRFEYDDPRFLRLFDLTHEALKEESGFLREVLNAVPLLLRIPGLAGKV

LRSQKAFLTQLDELLTEHRMTWDPAQPPRDLTEAFLAEMEKAKGNPESSFNEENLRIVVA

DLFSAGMVTTSTTLAWGLLLMILHPDVQRRVQQEIDNVIGQVRRPEMGDQARMPYTTAVI

HEVQRFGDIVPLGVTHMTSRDIELQGFLIPKGTTLFTNLSSVLKDEAVWEKPFRFHPEHF

LDAQGRFVKPEAFLPFSAGRHACLGEPLARMELFLFFTCLLQRFSFSVPAGQPRPSHHGV

FAFLVTPSPYELCAVPR

>P05181.1

MSALGVTVALLVWAAFLLLVSMWRQVHSSWNLPPGPFPLPIIGNLFQLELKNIPKSFTRL

AQRFGPVFTLYVGSQRMVVMHGYKAVKEALLDYKDEFSGRGDLPAFHAHRDRGIIFNNGP

TWKDIRRFSLTTLRNYGMGKQGNESRIQREAHFLLEALRKTQGQPFDPTFLIGCAPCNVI

ADILFRKHFDYNDEKFLRLMYLFNENFHLLSTPWLQLYNNFPSFLHYLPGSHRKVIKNVA

EVKEYVSERVKEHHQSLDPNCPRDLTDCLLVEMEKEKHSAERLYTMDGITVTVADLFFAG

TETTSTTLRYGLLILMKYPEIEEKLHEEIDRVIGPSRIPAIKDRQEMPYMDAVVHEIQRF

ITLVPSNLPHEATRDTIFRGYLIPKGTVVVPTLDSVLYDNQEFPDPEKFKPEHFLNENGK

FKYSDYFKPFSTGKRVCAGEGLARMELFLLLCAILQHFNLKPLVDPKDIDLSPIHIGFGC

IPPRYKLCVIPRS

>AAL69652.1

MDSISTAILLLLLALVCLLLTLSSRDKGKLPPGPRPLSILGNLLLLCSQDMLTSLTKLSK

EYGSMYTVHLGPRRVVVLSGYQAVKEALVDQGEEFSGRGDYPAFFNFTKGNGIAFSSGDR

WKVLRQFSIQILRNFGMGKRSIEERILEEGSFLLAELRKTEGEPFDPTFVLSRSVSNIIC

SVLFGSRFDYDDERLLTIIRLINDNFQIMSSPWGELYDILDPRFPSLLDWVPGPHQRIFQ

NFKCLRDLIAHSVHDHQASLDPRSPRDFIHCFLTKMAEEKEDPLSHFHMDTLLMTTHNLL

FGGTKTVSTTLHHAFLALMKYPKVQARVQEEIDLVVGRARLPALKDRAAMPYTDAVIHEV

QRFADIIPMNLPHRVTRDTAFRGFLIPKGTDVITLLNTVHYDPSQFLTPQEFNPEHFLDA

NQSFKKSPAFMPFSAGRRLCLGESLARMELFLYLTAILQSFSLQPLGAPEDIDLTPLSSG

LGNLPRPFQLCLRPR

>P24461.1

MELGGAFTIFLALCFSCLLILIAWKRVQKPGRLPPGPTPIPFLGNLLQVRTDATFQSFLK

LREKYGPVFTVYMGPRPVVILCGHEAVKEALVDRADEFSGRGELASVERNFQGHGVALAN

GERWRILRRFSLTILRDFGMGKRSIEERIQEEAGYLLEEFRKTKGAPIDPTFFLSRTVSN

VISSVVFGSRFDYEDKQFLSLLRMINESFIEMSTPWAQLYDMYSGVMQYLPGRHNRIYYL

IEELKDFIAARVKVNEASLDPQNPRDFIDCFLIKMHQDKNNPHTEFNLKNLVLTTLNLFF

AGTETVSSTLRYGFLLIMKHPEVQTKIYEEINQVIGPHRIPSVDDRVKMPFTDAVIHEIQ

RLTDIVPMGVPHNVIRDTHFRGYLLPKGTDVFPLLGSVLKDPKYFCHPDDFYPQHFLDEQ

GRFKKNEAFVPFSSGKRICLGEAMARMELFLYFTSILQNFSLHPLVPPVNIDITPKISGF

GNIPPTYELCLIAR

>P20678.1

MDFLGLPTILLLVCISCFLIAAWRSTSQRGKEPPGPTPIPIIGNVFQLNPWDLMESFKEL

SKKYGPIFTIHLGPKKVVVLYGYDVVKEALIDNGEAFSGRGNLPLFEKVFKGTGIVTSNG

ESWRQMRRFALTTLRDFGMGKKSIEERIQEEARFLVERIRNTHEKPFNPTVFLMHAVSNI

ICSTVFGDRFDYEDKKFLDLIEMLDENERYQNRIQTQLYNFFPTILDYLPGPHKTLIKSI

ETVDDFITEIIRAHQESFDASCPRDFIDAFINKMQQEKENSYFTVESLTRTTLDLFLAGT

GTTSTTLRYGLLILLKHPEIEEKMHKEIDRVVGRDRSPCMADRSQLPYTDAVIHEIQRFI

DFLPVNLPRAVIKDTKLRDYFIPKDTMIFPLLSPILQDCKEFPNPEKFDPGHFLNANGTF

RKSNYFMPFSAGKRICAGEGLARMELFLFLTSILQNFSLKPVKDRKDIDISPIVTSAANI

PRPYEVSFIPR

>P51589.2

MLAAMGSLAAALWAVVHPRTLLLGTVAFLLAADFLKRRRPKNYPPGPWRLPFLGNFFLVD

FEQSHLEVQLFVKKYGNLFSLELGDISAVLITGLPLIKEALIHMDQNFGNRPVTPMREHI

FKKNGLIMSSGQAWKEQRRFTLTALRNFGLGKKSLEERIQEEAQHLTEAIKEENGQPFDP

HFKINNAVSNIICSITFGERFEYQDSWFQQLLKLLDEVTYLEASKTCQLYNVFPWIMKFL

PGPHQTLFSNWKKLKLFVSHMIDKHRKDWNPAETRDFIDAYLKEMSKHTGNPTSSFHEEN

LICSTLDLFFAGTETTSTTLRWALLYMALYPEIQEKVQAEIDRVIGQGQQPSTAARESMP

YTNAVIHEVQRMGNIIPLNVPREVTVDTTLAGYHLPKGTMILTNLTALHRDPTEWATPDT

FNPDHFLENGQFKKREAFMPFSIGKRACLGEQLARTELFIFFTSLMQKFTFRPPNNEKLS

LKFRMGITISPVSHRLCAVPQV

>AAF72563.1

MSLMESLLLQAPSTVTLLGAVLFLLVLYLLSSSSSSEEHGKEPPGPRPLPLLGNMLQLDL

KRPYQTLCELSKKYGSVFTVHIGPRKVVVLAGYKTVKQALVNHAEEFGDRGISHLFTELN

KGHGIVFANGDSWKEMRRFALTNLRDFGMGKKASEEKIIEEVHYLIEVFEEHKGKAFDAT

QAMNYAVSNIICSIVYGSRFDYSDPRFRRSVDQANESIRLGGSISIQLYNMFPWLGPLLK

NKTLILKNIADNKGEMKELVRGLKETLNPQMCRGFVDSFLVRKQTLEESGNMNSHYHTEN

LIQTVANLFAAGTDTTGTTLRWGLLLMAKYPDIQDRVQEEISRVIGSRQALVEDRKNLPY

TDAVIHETQRLANIVPMSIPHTTSQDVTFQGYFIKKGTDVLPLLTSVLRDESEWKSPHTF

NPEHFLDEEGRFVKKDAFMPFSAGRRVCLGESLARMELFLFFTSLLQHFCFSPPPGVREE

DLDLTPSLGFTLSPSPHQLCAVSRV

>EDS41084.1

MWWFLLGIVLVSLTVFDSIKPADYPPGPRWIPWFGTTFVVQKLSKKLGGLHEVFKYLSQK

HQSPVVGLRFGKELTVGIRGYDLAKEALTGDDLLGRPDNFFIRLRTDGERLGLTFTDGKL

WEEHRSFIMRTLKEVGYGRSRMEELIQKEAIELKQTYSQRINSTSSTLPTAVAKCIERQR

IERDDNRLQMLLKLLRKRSGQFDLAGGLLNQMPWLRFLAPNWSGYNLVVDFNRQLMEFFA

ETIRNHYETYTDTKSSNDLIYAYIREFRNQGHNPGTTFTEKQMLMTILDLFLAGGLNTST

VTDLLLLMMVVRPDIQAKVHQEIDNHLNPIRWSDRSQLPFVEAVIMEVQRFFPTAAFGGP

RRAVHDFNIGGYRIPKNTTVFVDLESVHHDEAYWKDPEVFRPERFLDEGGHLVTCDRLLP

FSLGKRKCPGDGLAKTTIFLFFVELLRNFELSLPEGAEVPSTELVPGRIYMPKPYWIIFS

RRS

>Q92088.1

MDVLHILQTNFVSIIIGFVVIILLWMNRGKQSNSRLPPGPAPIPLLGNLLRMDVKAPYKL

YMELSKKYGSVFTVWLGSKPVVVISGYQAIKDAFVTQGEEFSGRANYPVIMTVSKGYGVL

VSSGKRSKDLRRFSLMTLKTFGMGRRSIEERVQEEAKMLVKAFGEYRDSVVNPKELLCNC

VGNVICSIVFGHRFENDDPMFQLIQKAVDAYFNVLSSPIGAMYNMFPRIVWCFPGNHHEM

FAIVNKAKVYIQEQAEIRLKTLNISEPQDFIEAFLVKMLEEKDDPNTEFNNGNMVMTAWS

LFAAGTETTSSTLRQSFLMMIKYPHIQESVQKEIDEVIGSRVPTVDDRVKMPYTDAVIHE

VQRYMDLSPTSVPHKVMRDTEFYNYHIPEGTMVLPLLSSVLVDPKLFKNPDEFDPENFLD

ENGVFKKNDGFFAFGVGKRACPGEALARVELFLFFTSVLQRFTFTGTKPPEEINIEPACS

SFGRLPRSYDCYIKLRTEK

>AAD54015.1

MWLYNFLLVLDLKAILLFIFSFLLIADFLRNRKPANFPPGPKALPFVGNMLNLDSQHPHI

FFSKLADIYGNVFSFRLGKESMVVVSGHKLVKEAIVTQGENFVDRPPNAIAERFYTEPSG

GLFFNNGEIWKRQRRFALSTLRTFGLGKNTLELSICEEIRHLQEEIENEKGKPFSPAGLF

NNAVSNIICQLVMGRRFDYHDQSFQTMLKYMSEALWLEGSIWGQLYQAFPQVMKYIPGPH

NKLFSNFTAIKELLQEEIEKHKKDLDHSNPRDYIDTFLIKMENQQEAELGFTERNLAFCS

LDLFLAGTETTATTLLWALLFLIKYPEVQEKVHAEIDRVIGQTRLPSMADRPNLPYTDAV

IHEIQRMSNIVPLNGLRVASKDTTLGGYFIPKGTAVMPMLTSVLFDKTEWETPDTFNPGH

FLDANGKFVKKEAFLPFSAGKRVCLGEGLAKMELFLFLVALLQKFSFSAPEGVELSTEGI

TGITLVPHPYKVSAKAR

>AAF21999.1

MEALYSLLGLEWLDTRSVLIFFCVFLLLSDILKNRKPKNFPPGPAALPFIGDLHHINPSR

IHLQITDFAEKYGNVFSLHLFGGKAVVINGYKHVKEALVEKGEDFMDRPTIPLFSDVFKN

KGIVMSNGYPWKVQRRFALHALRNFGLGKKTMERYIQQECQYLNEVFVDQQGKPFSGQTL

INNAVSNIICCLVFGNRFEYDDKEYHTILDNMNELLRLQGGFWVQVYNMFPSVMKWLPGP

HKKIFIHLQKIIDFLEIRIKEHRENLDPSSPRDYIDSFLIEMGDKEDKDSGFDLFNLSAC

TLDLFAAGTETTTTTLHWGLLYMIYYPDIQERVHAEINAVIGSSRQPAVADRENMPYTDA

VIHEIQRMGNILPLNVARMTSKDTTLDKYSIPKGTVIIATLHSVLHDESMWETPHSFNPQ

HFLDQDGKFRKRDAFMPFSAGKRVCLGEQLARMELFLFFTSLLQRFKFSPPPGEQPSLEY

KLGATHCPKPYRLCAVPR

>Q6VVW9.1

MLELPGARACAGALAGALLLLLFVLVVRQLLRQRRPAGFPPGPPRLPFVGNICSLALSAD

LPHVYMRKQSRVYGEIFSLDLGGISTVVLNGYDVVKECLVHQSEIFADRPCLPLFMKMTK

MGGLLNSRYGRGWIDHRRLAVNSFHYFGSGQKSFESKILEETWSLIDAIETYKGGPFDLK

QLITNAVSNITNLILFGERFTYEDTDFQHMIELFSENVELAASAPVFLYNAFPWIGILPF

GKHQRLFRNADVVYDFLSRLIEKAAVNRKPHLPHHFVDAYLDEMDQGQNDPLSTFSKENL

IFSVGELIIAGTETTTNVLRWAILFMALYPNIQGQVHKEIDLIVGHNRRPSWEYKCKMPY

TEAVLHEVLRFCNIVPLGIFHATSEDAVVRGYSIPKGTTVITNLYSVHFDEKYWKDPDMF

YPERFLDSNGYFTKKEALIPFSLGRRHCLGEQLARMEMFLFFTSLLQQFHLHFPHELVPN

LKPRLGMTLQPQPYLICAERR

>Q9DBX6.1

MEAASTWALLLALLLLLLLLSLTLFRTPARGYLPPGPTPLPLLGNLLQLRPGALYSGLLR

LSKKYGPVFTVYLGPWRRVVVLVGHDAVREALGGQAEEFSGRGTLATLDKTFDGHGVFFA

NGERWKQLRKFTLLALRDLGMGKREGEELIQAEVQSLVEAFQKTEGRPFNPSMLLAQATS

NVVCSLVFGIRLPYDDKEFQAVIQAASGTLLGISSPWGQAYEMFSWLLQPLPGPHTQLQH

HLGTLAAFTIQQVQKHQGRFQTSGPARDVVDAFLLKMAQEKQDPGTEFTEKNLLMTVTYL

LFAGTMTIGATIRYALLLLLRYPQVQQRVREELIQELGPGRAPSLSDRVRLPYTDAVLHE

AQRLLALVPMGMPHTITRTTCFRGYTLPKGTEVFPLIGSILHDPAVFQNPGEFHPGRFLD

EDGRLRKHEAFLPYSLGKRVCLGEGLARAELWLFFTSILQAFSLETPCPPGDLSLKPAIS

GLFNIPPDFQLRVWPTGDQSR

>AAK53421.1

MVTCEATLLLLLILTLMLMSWGWLAHQARARMQKDLPPGPAPLPLLGNLLQLQSGHLDRV

LMELSSRWGPVFTVWLGPRPAVVLSGYAALRDALVLQADAFSGPRVHGCLRTLTHGNGIV

FSNGPALATLRTLHLNTEELRGSSTIEERILEETACVLDEFQATMGAPFDPRRLLDNAVS

NVICTVVFGKRYNYGDPEFLRLLDLFSDNFRIMSSRWGETYNMFPSFMDWIPGPHHRIFK

NFQELRLFISEQIQWHRQSRQTGEPRDFIDCFLEQMDKEHQDPESHFQDETLVMTTHNLF

FGGTETTSTTLRYGLLIMLKYPEVAAKVQEELDATVGRTRAPSLADRAHLPYTNAVLHEI

QRFISVLPLGLPRALIRDVNLRNHFLHKGTFVIPLLVSAHRDPTQFKDPDHFNPTNFLDD

QGEFQNNDAFMPFAPGKRMCLGAGLARSEIFLFLTAILQKFSLLPVGSPADIDLTPQCTG

LGNVPPAFQLRLVAR

>Q4V8D1.1

MSSIGGLRPAAGEQPGVGPHLQAVGGALLLCGLAVLLDWVWLQRQRAGGIPPGPKPRPLV

GNFGYLLLPRFLRLHFWLGSGSQTDTVGRHVYLARLARVYGNIFSFFIGHRLVVVLSDFQ

SVREALVQQAEVFSDRPRMPLISILTKEKGIVFAHYGPIWKQQRRFSHSTLRHFGLGKLS

LEPRIIEEFAYVKAEMQKHGEAPFSPFPVISNAVSNIICSLCFGQRFDYTNKEFKKVLDF

MSRGLEICLHSQLFLINLCPWFYYLPFGPFKELRQIERDITCFLKNIIKEHQESLDANNP

QDFIDMYLLHTQEEKDKCKGTNFDEDYLFYIIGDLFIAGTDTTTNSLLWCLLYMSLNPGV

QKKVHEEIERVIGRDRAPSLTDKAQMPYTEATIMEVQRLSMVVPLAIPHMTSEKTVLQGY

SIPKGTVVLPNLWSIHRDPVIWEKPDDFCPHRFLDDQGQLLKRETFIPFGIGKRVCMGEQ

LAKMELFLMFVSLMQSFTFALPEGSEKPIMTGRFGLTLAPHPFNVTVSKR

>BAA92374.1

MALENILLHLNSKVWTDAGTILLLFILFLLVSVKLRNRNKPTKTFLLGPTPLPFIGNVFN

LDTSQPHICLTKMSDHYGNIFSLRLGSLNTVVVNTYSMVKKVLNDQGNSFMYRPSNDITE

RILSKCQGLTFNNGYSWKQHRRFTLSTLKFFGVGKRSLEFIIMEEYKFLHQSILDTNGLP

FNPHYIINNGVSNIICSMVFGRRFEYTDQRFLNMLSLISKALKLQTSVFIQLYAAFPRLM

DLLPGPHKELFSCFHQVRAFIKEEVDKHRADWDPSSPRDFIDCYLTEIEKMKDDLEAGFH

DEGLQYAVLDLFVAGTETTSTTLLWAFVYMMKYQKSKKVQAEIDKVVGRYRRPSMDDRPC

MPYTDAVIHEIQRMGNVVPLSVPRMTNEDTLLEGLFHSLRVLQIIPNLTSVLFDQTKWKT

QHSFDPQNFLNAQGKLKSLKLLSPFSLGKRSCPGESLARMELFLFFTSFLQSFSLSAPDE

TQTSLDFKCGMTLSPKPFKICFTPR

>AAH25761.1

MELSERYGPVFTVHLGRQKTVVLTGFEAVKEALAGPGQELADRPPIAIFQLIQRGGGIFF

SSGARWRAARQFTVRALHSLGVGREPVADKILQELKCLSGQLDGYRGRPFPLALLGWAPS

NITFALLFGRRFDYRDPVFVSLLGLIDEVMVLLGSPGLQLFNVYPWLGALLQLHRPVLRK

IEEVRAILRTLLEARRPHVCPGDPVCSYVDALIQQGQGDDPEGLFAEANAVACTLDMVMA

GTETTSATLQWAALLMGRHPDVQGRVQEELDRVLGPGRTPRLEDQQALPYTSAVLHEVQR

FITLLPHVPRCTAADTQLGGFLLPKGTPVIPLLTSVLLDETQWQTPGQFNPGHFLDANGH

FVKREAFLPFSAGRRVCVGERLARTELFLLFAGLLQRYRLLPPPGVSPASLDTTPARAFT

MRPRAQALCAVPRP

>189521688

MLTALVLLCLGAFLLYLQVRIRRPKDFPPGPAPVPFFGNLLQLNRINPIKDLDKFAQHYG

SIYGIYIGSKPAVVLTGQKMIKEALITQAAEFAGRPNHMMISHITRSKGVIMADYGESWR

EHRRFALTTLRNFGLGKKSMEQRILEEVKHICLLLEESAGKSIDPQHLYHQAASNIIASV

IFGSRFNYKDEYFQTLIQTMEKLTKIAIGTWAMLYEIAPVLRIFPLPFWKAFHYFEKITR

HSLKVVEEHKKSFVAGEPKDLIDCYLEEMKKVNQRTTFDEAQMVTLLFDLFLAGTETTSN

TLRTLTLYLMTHTHIQEQCQREIDEVLGARDHVTYEDRNDMHFVQAVIHEGQRVADIVPL

NVFHTARTDTQLRGYSIPKGTIIIPYLSSSLREESQWKFPHEFNPQNFLNEKGEFVKNEA

FMPFSAGPRVCLGENLARMELFLILVTVLRKFRLVWPKDAGEPDFTYIYGGTQSLKPYPM

IVELRTPGETHEYAN

>AAX37329.1

MELSSSLLLVLVLTVLMLIRWRRKENGLSLPPGPLALPLIGNLLTLDKSAPFKSFMKWRK

TYGSVMTVHLGPQRMVVLVGYETVKEALVDQAEDFAPRAPIAFMNRIVKGYGLAISNGER

WRQLRRFTLTTLRDFGMGRKQMEQWIQEESRYLLKSFEETKSKPVDPTFFFSRTVSNVIC

SLVFGQRFDYGDKNFLQLLQIISKLLRFLSSPWGQLYNIFPQVMERFSSRHHAILKDVEN

IRTFIRNKVKEHEQRLDFSDPSDFIDCFLIRLTQEKDKLDTEFHKDNLMATVLNLFIAGT

ETTSTTLRYALMLLIKHPQIQEQMQREIDRVIGQNRIPTMEDRKSLPFTDAVIHEVQRYM

DIVPLSLPHYAMKDITFRGYKIPKDTVIIPMLHSVLRDEGQWETPWTFNPEHFLDSNGNF

KKNPAFMPFSAGKRSCVGESLARMELFLFTVSLLQKFIFSSPNGPDGIDLSPELSSFANM

PRFYELIASPR

>AAI17166.1

MLASGLLLVALLACLTVMVLMSVWQQRKSRGKLPPGPTPLPFIGNYLQLNTEHICDSIMK

FSECYGPVFTIHLGPRRAVVLCGHDAVREALVDQAEEFSGRGEQATFDWVFKGYGVAFSN

GERAKQLLRFAIATLRDFGVGKRGIEERIQEESGFLIEAIRSTHGANIDPTFFLSRTVSN

VISSIVFGDRFDYEDKEFLSLLSVMLGIFQFTSTSTGQLYEMFSSVMKHLPGPQQQAFKL

LQGLEDFIAKKVEHNQRTLDPNSPRDFIDSFLIRMQEEEKNPNTEFYLKNLMMSTLNLFI

AGTETVSTTLRYGFLLLMKHPEVEAKVHEEIDRVIGKNRQPKFEDRAKMPYMEAVIHEIQ

RFGDVIPMSLARRVKKDTKFRDFFLPKGTEVFPMLGSVLRDPSFFSNPQDFNPQHFLDDK

GQFKKSDAFVPFSIGKRYCFGEGLARMELFLFFTTVMQNFRLKSSQSPKDIDVSPKHVGF

ATIPRNYTMSFLPR

>AAA52138.1

MLASGLLLVALLACLTVMVLMSVWQQRKSRGKLPPGPTPLPFIGNYLQLNTEHICDSIMK

FSECYGPVFTIHLGPRRVVVLCGHDAVREALVDQAEEFSGRGEQATFDWVFKGYGVAFSN

GERAKQLLRFAIATLRDFGVGKRGIEERIQEESGFLIEAIRSSHGANIDPTFFLSRTVSN

VISSIVFGDRFDYEDKEFLSLLSMMLGIFQFTSTSTGQLYEMFSSVMKHLPGPQQQAFKL

LQGLEDFIAKKVEHNQRTLDPNSPQDFIDSFLIHMQEEEKNPNTEFYLKNLMMSTLNLFI

AGTETVSTTLRYGFLLLMKHPEVEAKVHEEIDRVIGKNRQPKFEDRTKMPYMEAVIHEIQ

RFGDVIPMSLARRVKKDTKFRDFFLPKGTEVFPMLGSVLRDPSFFSNPQDFNPQHFLDDK

GQFKKSDAFVPFSIGKRYCFGEGLARMELFLFFTTVMQNFRLKSSQSPKDIDVSSKHVGF

ATIPRNYTMSFLPR

>P20853.2

MLASGLLLVALLACLTVMVLMSVWQQRKSRGKLPPGPTPLPFIGNYLQLNTEHICDSIMK

FSECYGPVFTIHLGPRRVVVLCGHDAVREALVDQAEEFSGRGEQATFDWVFKGYGVAFSN

GERAKQLLRFAIATLRDFGVGKRGIEERIQEESGFLIEAIRSTHGANIDPTFFLSRTVSN

VISSIVFGDRFDYEDKEFLSLLSMMLGIFQFTSTSTGQLYEMFSSVMKHLPGPQQQAFKL

LQGLEDFIAKKVEHNQRTLDPNSPQDFIDSFLIHMQEEEKNPNTEFYLKNLMMSTLNLFI

AGTETVSTTLRYGFLLLMKHPEVEAKVHEEIDRVIGKNRQPKFEDRTKMPYMEAVIHEIQ

RFGDVIPMSLARRVKKDTKFRDFFLPKGTEVFPMLGSVLRDPSFFSNPQDFNPQHFLDDK

GQFKKSDAFVPFSIGKRNCFGEGLARMELFLFFTTVMQNFRLKSSQSPKDIDVSPKHVVF

ATIPRNYTMSFLPR

>EAW57013.1

MLASGLLLVALLACLTVMVLMSVWQQRKSRGKLPPGPTPLPFIGNYLQLNTEHICDSIMK

ISERYGPVFTIHLGPRRVVVLCGHDAVREALVDQAEEFSGRGEQATFDWVFKGYGVAFSN

GERAKQLLRFAIATLRDFGVGKRGIEERIQEESGFLIEAIRSTHGANIEPTFFLSRTVSN

VISSIVFGDRFDYEDKEFLSLLSMMLGIFQFTSTSTGQLYEMFSSVMKHLPGPQQQAFKL

LQGLEDFIAKKVEHNQRTLDPNSPQDFIDSFLIHMQEEEKNPNTEFYLKNLMMSTLNLFI

AGTETVSTTLRYGFLLLMKHPEVEAKVHEEIDRVIGKNRQPKFEDRTKMPYMEAVIHEIQ

RFGDVIPMSLARRVKKDTKFRDFFLPKGTEVFPMLGSVLRDPSFFSNPQDFNPQHFLDDK

GQFKKSDAFVPFSIGKRNCFGEGLARMELFLFFTTVMQNFRLKSSQSPKDIDVSPKHVVF

ATIPRNYTMSFLPR

>AAB40518.1

MLASGMLLVALLACLTVMVLMSVWQQRKSKGKLPPGPTPLPFIGNYLQLNTEQMYNSLMK

ISERYGPVFTIHLGPRRVVVLCGHDAVREALVDQAEEFSGRGEQATFDWVFKGYGVVFSN

GERAKQLLRFAIATLRDFGVGKRGIEERIQEESGFLIEAIRSTHGANIDPTFFLSRTVSN

VISSIVFGDRFDYKDKEFLSLLRMMLGIFQFTSTSTGQLYEMFSSVMKHLPGPQQQAFQL

LQGLEDFIAKKVEHNQRTLDPNSPRDFIDSFLIRMQEEEKNPNTEFYLKNLMMSTLNLFI

AGTETVSTTLHYGFLLLMKHPEVEAKVHEEIDRVIGKNRQPKFEDRAKMPYMEAVIHEIQ

RFGDVIPMSLARRVKKDTKFRDFFLPKGIEVFPMLGSVLRDLRFFSNPRDFNPQHFLGEK

GQFKKRDAFVPFSIRKRNCFGEGLARMELFLFFTTVMQNFRLKSSQSPKDIDVSPKHVGF

ATIPRNYTMSFLPR

>P11509.3

MLASGMLLVALLVCLTVMVLMSVWQQRKSKGKLPPGPTPLPFIGNYLQLNTEQMYNSLMK

ISERYGPVFTIHLGPRRVVVLCGHDAVREALVDQAEEFSGRGEQATFDWVFKGYGVVFSN

GERAKQLRRFSIATLRDFGVGKRGIEERIQEEAGFLIDALRGTGGANIDPTFFLSRTVSN

VISSIVFGDRFDYKDKEFLSLLRMMLGIFQFTSTSTGQLYEMFSSVMKHLPGPQQQAFQL

LQGLEDFIAKKVEHNQRTLDPNSPRDFIDSFLIRMQEEEKNPNTEFYLKNLVMTTLNLFI

GGTETVSTTLRYGFLLLMKHPEVEAKVHEEIDRVIGKNRQPKFEDRAKMPYMEAVIHEIQ

RFGDVIPMSLARRVKKDTKFRDFFLPKGTEVFPMLGSVLRDPSFFSNPQDFNPQHFLNEK

GQFKKSDAFVPFSIGKRNCFGEGLARMELFLFFTTVMQNFRLKSSQSPKDIDVSPKHVGF

ATIPRNYTMSFLPR

>AAA52067.1

MLASGMLLVALLVCLTVMVLMSVWQQRKSKGKLPPGPTPLPFIGNYLQLNTEQMYNSLMK

ISERYGPVFTIHLGPRRVVVLCGHDAVREALVDQAEEFSGRGEQATFDWVFKGYGVVFSN

GERAKQLRRFSIATLRDFGVGKRGIEERIQEEAGFLIDALRGTGGANIDPTFFLSRTVSN

VISSIVFGDRFDYKDKEFLSLLRMMLGIFQFTSTSTGQLYEMFSSVMKHLPGPQQQAFQL

LQGLEDFIAKKVEHNQRTLDPNSPRDFIDSFLIRMQEEEKNPNTEFYLKNLVMTTLNLFI

GGTETVSTTLRYGFLLLMKHPEVEAKVHEEIDRVIGKNRQPKFEDRAKMPYMEAVIHEIQ

RFGDVIPMSLARRVKKDTKFRDFFLPKGTEVYPMLGSVLRDPSFFSNPQDFNPQHFLNEK

GQFKKSDAFVPFSIGKRNCFGEGLARMELFLFFTTVMQNFRLKSSQSPKDIDVSPKHVGF

ATIPRNYTMSFLPR

>AAH96256.1

MLASGMLLVALLVCLTVMVLMSVWQQRKSKGKLPPGPTPLPFIGNYLQLNTEQMYNSLMK

ISERYGPVFTIHLGPRRVVVLCGHDAVREALVDQAEEFSGRGEQATFDWVFKGYGVVFSN

GERAKQLRRFSIATLRDFGVGKRGIEERIQEEAGFLIDALRGTGGANIDPTFFLSRTVSN

VISSIVFGDRFDYKDKEFLSLLRMMLGIFQFTSTSTGQLYEMFSSVMKHLPGPQQQAFQL

LQGLEDFIAKKVEHNQRTLDPNSPRDFIDSFLIRMQEEEKNPNTEFYLKNLVMTTLNLFI

GGTETVSTTLRYGFLLLMKHPEVEAKVHEEIDRVIGKNRQPKFEDRAKMPYMEAVIHEIQ

RFGDVIPMSLARRVKKDTKFRDFFLPKGTEVYPMLGSVLRDPSFFSNPQDFNPQHFLNEK

GQFKKSDAFVPFSIGKRNCFGEGLARMELFLFFTTVMQNFRLKSSQSPKDIDVSPRHVGF

ATIPRNYTMSFLPR

>AAH96255.1

MLASGMLLVALLVCLTVMVLMSVWQQRKSKGKLPPGPTPLPFIGNYLQLNTEQMYNSLMK

ISERYGPVFTIHLGPRRVVVLCGHDAVREALVDQAEEFSGRGEQATFDWVFKGYGVVFSN

GERAKQLRRFSIATLRDFGVGKRGIEERIQEEAGFLIDAHRGTGGANIDPTFFLSRTVSN

VISSIVFGDRFDYKDKEFLSLLRMMLGIFQFTSTSTGQLYEMFSSVMKHLPGPQQQAFQL

LQGLEDFIAKKVEHNQRTLDPNSPRDFIDSFLIRMQEEEKNPNTEFYLKNLVMTTLNLFI

GGTETVSTTLRYGFLLLMKHPEVEAKVHEEIDRVIGKNRQPKFEDRAKMPYMEAVIHEIQ

RFGDVIPMSLARRVKKDTKFRDFFLPKGTEVYPMLGSVLRDPSFFSNPQDFNPQHFLNEK

GQFKKSDAFVPFSIGKRNCFGEGLARMELFLFFTTVMQNFRLKSSQSPKDIDVSPKHVGF

ATIPRNYTMSFLPR

>AAF13600.1

MLASGMLLVALLVCLTVMVLMSVWQQRKSKGKLPPGPTPLPFIGNYLQLNTEQMYNSLMK

ISERYGPVFTIHLGPRRVVVLCGHDAVREALVDQAEEFSGRGEQATFDWVFKGYGVVFSN

GERAKQLRRFSIATLRDFGVGKRGIEERIQEEAGFLIDALRGTGGANIDPTFFLSRTVSN

VISSIVFGDRFDYKDKEFLSLLRMMLGIFQFTSTSTGQLYEMFSSVMKHLPGPQQQAFQL

LQGLEDFIAKKVEHNQRTLDPNSPRDFIDSFLIRMQEEEKNPNTEFYLKNLVMTTLNLFI

GGTETVSTTLRYGFLLLMKHPEVEAKVHEEIDRVIGKNRQPKFEDRAKMPYMEAVIHEIQ

RFGDVIPMSLARRVKKDTKFRDFFLPKGTEVYPMLGSVLRDPSFFSNPQDFNPQHFLNEK

GQFKKSDAFVPFSIGKRNCFGEGLARMELFLFFTTVMQNFRLKSSQSPKDIDVSPKHVVF

ATIPRNYTMSFLPR

>1609083A

MLASGMLLVALLVCLTVMVLMSVWQQRKNKGKLPPGPTPLPFIGNYLQLNTEQMYNSLMK

ISERYGPVFTIHLGPRRVVVLCGHDAVREALVDQAEEFSGRGEQATFDWVFKGYGVVFSN

GERAKQLRRFSIATLRDFGVGKRGIEERIQEEAGFLIDALRGTGGANIDPTFFLSRTVSN

VISSIVFGDRFDYKDKEFLSLLRMMLGIFQFTSTSTGQLYEMFSSVMKHLPGPQQQAFQL

LQGLEDFIAKKVEHKQRTLDPNSPRDFIDSFLIRMQEEEKNPNTEFYLKNLVMTTLNLFI

GGTETVSTTLRYGFLLLMKHPEVEAQVHEEIDRVIGKNRQPKFEDRAKMPYMEAVIHEIQ

RFGDVIPMSLARRVKKDTKFRDFFLPKGTEVYPMLGSVLRDPSFFSNPQDFNPQHFLNEK

GQFKKSDAFVPFSIGKRNCFGEGLARMELFLFFTTVMQNFRLKSSQSPKDIDVSPKHVGF

ATIPRNYTMSFLPR

>AAB40520.1

MLASGLLLVALLACLTVMVLMSVWQQRKSRGKLPPGPTPLPFIGNYLQLNTEHICDSIMK

FSECYGPVFTIHLGPRRVVVLCGHDAVREALVDQAEEFSGRGEQATFEWVFKGYGVAFSN

GERAKQLLRFAIATLRDFGVGKRGIEERIQEESGFLIEAIRSTHGANIDPTFFLSRTVSN

VISSIVFGDRFDYEDKEFLSLLSMMLGIFQFTSTSTGQLYEMFSSLMKHLPGPQQQAFKL

LLGLEDFIAKKVEHNQRTLDPNSPQDFIDSFLIHMQEEEKNPNTEFYLKNLMMSTLNLFI

AGTETVSTTLRYGFLLLMKHPEVEAKVHEEIDRVIGKNRQPKFEDRTKMPYMEAVIHEIQ

RFGDVIPMSLARRVKKDTKFRDFFLPKGTEVFPMLGSVLRDLRFFSNPRDFNPQHFLGEK

GQFKKRDAFVPFSIRKRNCFGEGLARMELFLFFTTVMQNFRLKSSQSPKDIDVSPKHVVF

ATIPRNYTMSFLPR

>CAA32097.1

MLLVALLVCLTVMVLMSVWQQRKNKGKLPPGPTPLPFIGNYLQLNTEQMYNSLMKISERY

GPVFTIHLGPRRVVVLCGHDAVREALVDQAEEFSGRGEQATFDWVFKGYGVVFSNGERAK

QLRRFSIATLRDFGVGKRGIEERIQEEAGFLIDALRGTGGANIDPTFFLSRTVSNVISSI

VFGDRFDYKDKEFLSLLRMMLGIFQFTSTSTGQLYEMFSSVMKHLPGPQQQAFQLLQGLE

DFIAKKVEHKQRTLDPNSPRDFIDSFLIRMQEEEKNPNTEFYLKNLVMTTLNLFIGGTET

VSTTLRYGFLLLMKHPEVEAQVHEEIDRVIGKNRQPKFEDRAKMPYMEAVIHEIQRFGDV

IPMSLARRVKKDTKFRDFFLPKGTEVYPMLGSVLRDPSFFSNPQDFNPQHFLNEKGQFKK

SDAFVPFSIGKRNCFGEGLARMELFLFFTTVMQNFRLKSSQSPKDIDVSPKHVGFATIPR

NYTMSFLPR

>AAZ29438.1

MLASGLLLVALLACLTVMVLMSVWQQRNSKGKLPPGPTPLPFIGNYLQLNTEQMCNSIMK

ISERYGPVFTIHLGPRRVVVLCGYDAVKEALVDQAEEFSGRGEQATFDWVFKGYGVVFSN

GERAKQLRRFSIATLRDFGVGKRGIEERIQEEAGFLIEALRDTHGANIDPTFFLSRTVSN

VISSIVFGDRFDYKDKEFLSLLGMMLAIFQFTSTSTGQLYEMFSSVMKHLPGPQQQAFKE

LQGLEDFIAKKVEHNQRTLDPNSPRDFIDSFLIRMQEEEKNPNTEFYLKNLMMTTLNLFI

AGTETVSTTLRYGFLLLMKYPEVEAKVHEEIDRVIGKNRQPKFEDRVKMPYMEAVIHEIQ

RFGDVIPMSLARRVNKDTKFRDFFLPKGTEVFPMLGSVLRDPRFFSNPQDFNPQHFLDEK

GQFKKSDAFVPFSIGKRNCFGEGLARMELFLFFTTIMQNFRFKSPQSPKDIDVSPKHAGF

ATIPRNYTMSFLPR

>402905622

MLASGLLLVALLACLTVMVLMSVWQQRNSKGKLPPGPTPLPFIGNYLQLNTEQMCNSIMK

ISERYGPVFTIHLGPRRIVVLCGYDAVKEALVDHAEEFSGRGEQATFDWVFKGYGVVFSN

GERAKQLRRFSIATLRDFGVGKRGIEERIQEEAGFLIEALRDTHGANIDPTFFLSRTVSN

VISSIVFGDRFDYEDKEFLSLLGMMLAIFQFTSTSTGQLYEIFSSVMKHLPGPQQQAFKE

LQGLEDFIAKKVEHNQRTLDPNSPRDFIDSFLIRMQEEEKNPNTEFYLKNLMMTTLNLFI

AGTETVSTTLRYGFLLLMKHPEVEAKVHEEIDRVIGKNRQPKFEDRVKMPYMEAVIHEIQ

RFGDVIPMSLARRVNKDTKFQDFFLPKGTEVFPMLGSVLRDPRFFSNPQDFNPQHFLDEK

GQFKKSDAFVPFSIGKRNCFGEGLARMELFLFFTTIMQNFRFKSPQSPKDIDVSPKHAGF

ATIPRNYTMSFLPR

>AAR90939.1

MLASGLLLVTLLACLTVMVLMSVWRQRKSRGKLPPGPTPLPFIGNYLQLNTEQMYNSLMK

ISERYGPVFTIHLGPRRVVVLCGHDAVKEALVDQAEEFSGRGEQATFDWLFKGYGVAFSN

GERAKQLRRFSIATLRGFGVGKRGIEERIQEEAGFLIEALRGTHGANIDPTFFLSRTVSN

VISSIVFGDRFDYEDKEFLSLLRMMLGSFQFTATSTGQLYEMFSSVMKHLPGPQQQAFKE

LQGLEDFIAKKVEHNQRTLDPNSPRDFIDSFLIRMQEEEKNPNTEFYLKNLVMTTLNLFF

AGTETVSTTLRYGFLLLMKHPEVEAKVHEEIDRVIGKNRQPKFEDRAKMPYTEAVIHEIQ

RFGDMLPMGLAHRVNKDTKFRDFFLPKGTEVFPMLGSVLRDPRFFSNPRDFNPQHFLDKK

GQFKKSDAFVPFSIGKRYCFGEGLARMELFLFFTTIMQNFRFKSPQSPKDIDVSPKHVGF

ATIPRNYTMSFLPR

>Q16696.3

MLASGLLLVTLLACLTVMVLMSVWRQRKSRGKLPPGPTPLPFIGNYLQLNTEQMYNSLMK

ISERYGPVFTIHLGPRRVVVLCGHDAVKEALVDQAEEFSGRGEQATFDWLFKGYGVAFSN

GERAKQLRRFSIATLRGFGVGKRGIEERIQEEAGFLIDALRGTHGANIDPTFFLSRTVSN

VISSIVFGDRFDYEDKEFLSLLRMMLGSFQFTATSTGQLYEMFSSVMKHLPGPQQQAFKE

LQGLEDFIAKKVEHNQRTLDPNSPRDFIDSFLIRMQEEEKNPNTEFYLKNLVMTTLNLFF

AGTETVSTTLRYGFLLLMKHPEVEAKVHEEIDRVIGKNRQPKFEDRAKMPYTEAVIHEIQ

RFGDMLPMGLAHRVNKDTKFRDFFLPKGTEVFPMLGSVLRDPRFFSNPRDFNPQHFLDKK

GQFKKSDAFVPFSIGKRYCFGEGLARMELFLFFTTIMQNFRFKSPQSPKDIDVSPKHVGF

ATIPRNYTMSFLPR

>AAR90940.1

MLASGLLLVTLLACLTVMVLMSVWRQRKSRGKLPPGPTPLPFIGNYLQLNTEQMYNSLMK

ISERYGPVFTIHLGPRRVVVLCGHDAVKEALVDQAEEFSGRGEQATFDWLFKGYGVAFSN

GERAKQLRRFSIATLRGFGVGKRGIEERIQEEAGFLIDALRGTHGANIDPTFFLSRTVSN

VISSIVFGDRFDYEDKEFLSLLRMMLGSFQFTATSTGQLYEMFSSVMKHLPGPQQQAFKE

LQGLEDFIAKKVEHNQRTLDPNSPRDFIDSFLIRMQEEEKNPNTEFYLKNLVMTTLNLFF

AGTETVSTTLRYGFLLLMKHPELEAKVHEEIDRVIGKNRQPKFEDRAKMPYTEAVIHEIQ

RFGDMLPMGLAHRVNKDTKFRDFFLPKGTEVFPMLGSVLRDPRFFSNPRDFNPQHFLDKK

GQFKKSDAFVPFSIGKRYCFGEGLARMELFLFFTTIMQNFRFKSPQSPKDIDVSPKHVGF

ATIPRNYTMSFLPR

>AAT49264.1

MLASGLLLVALLACLTVMVLMSVWQQRNSKGKLPPGPTPLPFIGNYLQLNTEQMCNSLMK

ISERYGPVFTIHLGPRRVVVLCGYDAVKEALVDQAEEFSGRGEQATFDWVFKGYGVVFSN

GERAKQLRRFSIATLRDFGVGKRGIEERIQEEAGFLIEALRDTHGANIDPTFFLSRTVSN

VISSIVFGDRFDYEDKEFLSLLGMMLGSFQFTSTSTGQLYEMFSSVMKHLPGPQQQAFKE

LQGLEDFIAKKVEHNQHTLDPNSPRDFIDSFLIRMQEEEKNPNTEFYLKNLMMTTLNLFI

AGTETVSTTLRYGFLLLMKYPEVEAKVHEEIDRVIGKNRQPKFEDRVKMPYMEAVIHEIQ

RFGDVIPMSLARRVNKDTKFRDFFLPKGTEVFPMLGSVLRDPRFFSNPQDFNPQHFLDEK

GQFKKSDAFVPFSIGKRNCFGEGLARMELFLFFTTIMQNFRFKSPQLPKDIDVSPKHVGF

ATIPPNYTMSFLPR

>AAB40519.1

MLASGLLLVTLLACLTVMVLMSVWRQRKSRGKLPPGPTPLPFIGNYLQLNTEQMYNSLMK

ISERYGPVFTIHLGPRRVVVLCGHDAVKEALVDQAEEFSGRGEQATFDWLFKGYGVAFSN

GERAKQLRRFSIATLRGFGVGKRGIEERIQEEAGFLIDALRGTHGANIDPTFFLSRTVSN

VISSIVFGDRFDYEDKEFLSLLRMMLGRFQFTGTSTGQLYEMFSSVMKHLPGPQQQAFKE

LQGLEDFIAKKVEHNQRTLDPNSPRDFIDSFLIRMQEEEKNPNTEFYLKNLVMTTLNLFF

AGTETVSTTLRYGFLLLMKHPEVEAKVHEEIDRVIGKNRQPKFEDRAKMPYTEAVIHEIQ

RFGDMLPMGLAHRVNKDTKFRDFFLPKGTEVFPMLGSELRDPRFFSNPQDCSPQHFLDEK

GQFKKSDAFVPFSIGKRYCFGEGLARMELFLFFTTIMQNFRFKSPQSPKDIDVSPKHVGF

ATIPRNYTMSFLPR

>402905604

MLASGLLLVALLACLTVMVLMSVWQQRNSKGKLPPGPTPLPFIGNYLQLNTEQMYNSIMK

ISERYGPVFTIHLGPRRVVVLCGHDAVREALVDHAEEFSGRGEQATFNWVFKGYGVAFSN

GERAKQLRRFSIATLRDFGVGKRGIEERIQEEAGFLIEALRGTRGANIDPTFFLSRTVCN

VISSIVFGDRFDYEDKEFLSLLRMMLGSFQFTSTSMGQLHEMFSSVMKHLPGPQQQAFKE

LQGLEDFIAKKVEHNQRTLDPNSPRDFIDSFLIRMQEEEKNPNTEFYMQNLLMTALNLFI

AGTETVSTTLRYGFLLLMKHPEVEAKVHEEIDRVIGKNQQPKFEDRVKMPYMEAVIHEIQ

RFGNVIPMSLARRVNKDTKFRDFFLPKGTEVFPMLGSVLKDPRFFSNPQDFNPQHFLDEK

GQFKKSDAFVPFSIGKRNCFGEGLARMELFLFFTTIMQNFRFKSPQSPKDIDVSPKHVGF

ATIPRNYTMSFLPR

>397482609

MLASGLLLVTLLACLTVIVLMSIWRQRKSKGKLPPGPTPLPFIGNYLQLNTEQMYNSLMK

ISERYGPVFTIHLGPRRVVVLCGHDAVKEALVDQAEEFSGRGEQATFDWLFKGYGVAFSN

GERAKQLRRFSIPTLRGFGVGKRGIEERIQEEAGFLIEALRGTHGANIDPTFFLSRTVSS

VISSIVFGDRFDYEDKEFLSLLRMMLESFQFTATSTGQLYEMFSSVMKHLPGPQQQAFKE

LQRLEDFIAKKVEHNQRTLDPNSPRDFIDSFLIRMQEEEKNPNTEFYLKNLVMTTLNLFF

AGTETVSTTLRYGFLLLMKHPEVEAKVHEEIDRVIGKNRQPKFEDRAKMPYTEAVIHEIQ

RFGDMLPMGLAHRVNKDTKFRDFFLPKGTEVFPMLGSVLRDPRFFSNPQDFNPQHFLDKK

GQFKKSDAFVPFSIGKRYCFGEGLARMELFLFFTTIMQNFRFKSPQPPKDIDVSPKHVGF

ATIPRNYTMSFLPR

>1Z10A

MAKKTSSKGKLPPGPTPLPFIGNYLQLNTEQMYNSLMKISERYGPVFTIHLGPRRVVVLC

GHDAVREALVDQAEEFSGRGEQATFDWVFKGYGVVFSNGERAKQLRRFSIATLRDFGVGK

RGIEERIQEEAGFLIDALRGTGGANIDPTFFLSRTVSNVISSIVFGDRFDYKDKEFLSLL

RMMLGIFQFTSTSTGQLYEMFSSVMKHLPGPQQQAFQLLQGLEDFIAKKVEHNQRTLDPN

SPRDFIDSFLIRMQEEEKNPNTEFYLKNLVMTTLNLFIGGTETVSTTLRYGFLLLMKHPE

VEAKVHEEIDRVIGKNRQPKFEDRAKMPYMEAVIHEIQRFGDVIPMSLARRVKKDTKFRD

FFLPKGTEVYPMLGSVLRDPSFFSNPQDFNPQHFLNEKGQFKKSDAFVPFSIGKRNCFGE

GLARMELFLFFTTVMQNFRLKSSQSPKDIDVSPKHVGFATIPRNYTMSFLPRHHHH

>ADM53741.1

MLALGLLLVALLACLTVMVLMSVWQQRNSKGKLPPGPTPLPFIGNYLQLNTEQMYNSIMK

ISERYGPVFTIHLGPRRVVVLCGYDAVKEALVDQAEEFSGRGEQATFNWVFKGYGVAFSN

GERAKQLRRFSIATLRDFGVGKRGIEERIQEEADFLIKALRGTHGANIDPTFFLSRTVCN

VISSIVFGDRFDYEDKEFLSLLRMMLGSFQFTSTSMGQLHEMFSSVMKHLPGPQQQAFKE

LQGLEDFIAKKVEHNQRTLDPNSPRDFIDSFLIRMQEEEKNPNTEFYMQNLLMTALNLFI

AGTETVSTTLRYGFLLLMKHPEVEAKVHEEIDRVISKNRQPKFEDRVKMPYMEAVIHEIQ

RFGNVIPMSLARRVNKDTKFRDFFLPKSTEVFPMLGSVLKDPKFFSNPQDFNPQHFLDEK

GQFKKSDAFVPFSIGKRNCFGEGLARMELFLFFTTIMQNFRFKSPQSPKDIDVSPKHVGF

ATIPRNYTMSFLPR

>332267039

MLASGLLLGALLACLTVMVLMSVWQQRKSRGKLPPGPTPLPFIGNYLQLNTEQMYNSLMK

ISERYGPVFTIHLGPRRVVVLCGHDAVKEALVDQAEEFSGRGEQATFDWLFKGYGVVFSN

GERAKQLRRFSITILRDFGVGKRGIEERIQEEAGFLIQALRGTRGASIDPTFFLSRTVSN

VISSIVFGDRFDYEDKEFLSLLRMMLGSFQFTATSTGQLYEMFSSVMKHLPGPQQQAFKL

LKGLEDFIAKKVEHNQRTLDPSSPRDFIDSFLIRMQEEEKNPNTEFCLKNLVLTALNLFF

AGTETVSTTLRYGFLLLMKHPEVEAKVHEEIDRVIGKNRQPKFEDRAKMPYMEAVIHEIQ

RFGDMLPMGVARRVNKDTKFRDFFLPKGTEVFPMLGSVLKDPKFFSNPWDFNPQHFLDEK

GQFKKSDAFVPFSIGKRFCFGEGLARMELFLFFTTIMQNFRFKSSQSPKDIDVSPKHVGF

ATIPRNYTMSFLPR

>2PG5A

MAKKTSSKGKLPPGPTPLPFIGNYLQLNTEQMYNSLMKISERYGPVFTIHLGPRRVVVLC

GHDAVREALVDQAEEFSGRGEQATFDWVFKGYGVVFSNGERAKQLRRFSIATLRDFGVGK

RGIEERIQEEAGFLIDALRGTGGANIDPTFFLSRTVSNVISSIVFGDRFDYKDKEFLSLL

RMMLGIFQFTSTSTGQLYEMFSSVMKHLPGPQQQAFQLLQGLEDFIAKKVEHNQRTLDPN

SPRDFIDSFLIRMQEEEKNPNTEFYLKNLVMTTLQLFIGGTETVSTTLRYGFLLLMKHPE

VEAKVHEEIDRVIGKNRQPKFEDRAKMPYMEAVIHEIQRFGDVIPMSLARRVKKDTKFRD

FFLPKGTEVYPMLGSVLRDPSFFSNPQDFNPQHFLNEKGQFKKSDAFVPFSIGKRNCFGE

GLARMELFLFFTTVMQNFRLKSSQSPKDIDVSPKHVGFATIPRNYTMSFLPRHHHH

>2PG7A

MAKKTSSKGKLPPGPTPLPFIGNYLQLNTEQMYNSLMKISERYGPVFTIHLGPRRVVVLC

GHDAVREALVDQAEEFSGRGEQATFDWVFKGYGVVFSNGERAKQLRRFSIATLRDFGVGK

RGIEERIQEEAGFLIDALRGTGGANIDPTFFLSRTVSNVISSIVFGDRFDYKDKEFLSLL

RMMLGIFQFTSTSTGQLYEMFSSVMKHLPGPQQQAFQLLQGLEDFIAKKVEHNQRTLDPN

SPRDFIDSFLIRMQEEEKNPNTEFYLKNLVMTTLQLFVGGTETVSTTLRYGFLLLMKHPE

VEAKVHEEIDRVIGKNRQPKFEDRAKMPYMEAVIHEIQRFGDVIPMSLARRVKKDTKFRD

FFLPKGTEVYPMLGSVLRDPSFFSNPQDFNPQHFLNEKGQFKKSDAFVPFSIGKRNCFGE

GLARMELFLFFTTVMQNFRLKSSQSPKDIDVSPKHVGFATIPRNYTMSFLPRHHHH

>395859637

MLASGMLLVALLACLTVMVLMSVWRQRKIWGRLPPGPTPLPFIGNYLQLNTEQMYNSLMK

LSERYGPVFTIHLGPRRIVVLCGYDAVKEALVDQAEEFSGRGKQATFDWLFKGYGVAFSN

GERAKQLRRFSITTLRDFGVGKRGIEERIQEEAGYLIEAFRDTRGAVIDPTFFLSRTVSN

VISSIVFGDRFDYENKEFLSLLRMMLGSFQFTATSTGQLYEMFYSVMKHLPGPQQQAFKE

LQGLEDFIAKKVEHNQRTLDPNSPRNFIDSFLIRLQEEQKNPNTEFYMKNLVLTTLNLFF

AGTETVSTTLRYGFLLLMKHPDVEAKVCEEIDRVIGKNRQPKFEDRAKMPYTEAVIHEIQ

RFGDMIPMGLARRVTKDTKFRDFFLPKGTEVFPMLGSVLRDPKFFSKPQDFNPQHFLDEK

GQFKKSDAFVPFSIGKRYCFGEGLARMELFLFLTTIMQNFRFKSPQSPQDIDVSPRLVGF

ATIPPNYTMSFLPR

>402905630

MLASGLLLVALLACLTVMVLMSVWQQRNSKGKLPPGPTPLPFIGNYLQLNTEQMCTSLMK

ISEHYGPVFTIHLGPRRVVVLCGHDAVREALVDQAEEFSGRGEQATFDWLFKGYGVAFSN

GERAKQLRRFSIATLRDFGVGKRGIEERIQEEAGFLIEALRGTRGANIDPTFFLSRTVCN

VISSIVFGDRFDYEDKEFLSLLRMMLGSFQFTATSTGQLYEMFSSVMKHLPGPQQQAFKE

LQGLEDFIAKKVEHNQRTLDPNSPRDFIDSFLIRMQEEEKNPNTEFHLKNLVMTSLNLFF

AGTETVSTTLRYGFLLLMKHPEVEAKVHEEIDRVIGKNRQPKFEDRVKMPYTEAVIHEIQ

RFGNVIPMSLAHRVIRDTKFRDFFLPKGTEVFPMLGSVLRDPRFFSNPQDFNPQHFLDEK

GQFKKSDAFVPFSIGKRNCFGEGLARMELFLFLTTIMQNFGFESPQLPKDIDVSPKHVGF

ATIPRNYTMSFLPR

>ADM53742.1

MLASGLLLVALLACLTVMVLMSVWQQRNSKGKLPPGPTPLPFIGNYLQLNTEQMYNSIMK

ISERYGPVFTIHLGPRRVVVLCGYDAVKEALVDQAEEFSGRGEQATFNWVFKGYGVAFSN

GERAKQLRRFSIATLRDFGVGKRGIEERIQEEADFLIKALRGTHGANIDPTFFLSRTVCN

VISSIVFGDRFDYEDKEFLSLLRMMLGSFQFTSTSMGQLHEMFSSVMKHLPGPQQQAFKE

LQGLEDFIAKKVEHNQRTLDPNSPRDFIDSFLIRMQEEEKNPNTEFYMQNLLMTALNLFI

AGTETVSTTLRYGFLLLMKHPEVEAKVHEEIDRVISKNRQPKFEDRVKMPYMEAVIHEIQ

RFGNVIPMSLARRVNKDTKFRDFFLPKSTEVFPMLGSVLKDPKFFSNPQDFNPQHFLDEK

GQFKKSDAFVPFSIGKRNCFGEGLARMELFLFFTTIMQNFRFKSPQSPKDIDVSPKHVGF

ATIPPNYTMSFLPR

>2PG6A

MAKKTSSKGKLPPGPTPLPFIGNYLQLNTEQMYNSLMKISERYGPVFTIHLGPRRVVVLC

GHDAVREALVDQAEEFSGRGEQATFDWVFKGYGVVFSNGERAKQLRRFSIATLRDFGVGK

RGIEERIQEEAGFLIDALRGTGGANIDPTFFLSRTVSNVISSIVFGDRFDYKDKEFLSLL

RMMLGIFQFTSTSTGQLYEMFSSVMKHLPGPQQQAFQCLQGLEDFIAKKVEHNQRTLDPN

SPRDFIDSFLIRMQEEEKNPNTEFYLKNLVMTTLQLFIGGTETVSTTLRYGFLLLMKHPE

VEAKVHEEIDRVIGKNRQPKFEDRAKMPYMEAVIHEIQRFGDVIPMSLARRVKKDTKFRD

FFLPKGTEVYPMLGSVLRDPSFFSNPQDFNPQHFLNEKGQFKKSDAFVPFSIGKRNCFGE

GLARMELFLFFTTVMQNFRLKSSQSPKDIDVSPKHVGFATIPRNYTMSFLPRHHHH

>3EBSA

MAKKTSSKGKLPPGPTPLPFIGNYLQLNTEQMYNSLMKISERYGPVFTIHLGPRRVVVLC

GHDAVREALVDQAEEFSGRGEQATFDWVFKGYGVVFSNGERAKQLRRFSIATLRDFGVGK

RGIEERIQEEAGFLIDALRGTGGANIDPTFFLSRTVSNVISSIVFGDRFDYKDKEFLSLL

RMMLGSFQFTSTSTGQLYEMFSSVMKHLPGPQQQAFQLLQGLEDFIAKKVEHNQRTLDPN

SPRDFIDSFLIRMQEEEKNPNTEFYLKNLVMTTLNLFFAGTETVSTTLRYGFLLLMKHPE

VEAKVHEEIDRVIGKNRQPKFEDRAKMPYMEAVIHEIQRFGDVIPMGLARRVKKDTKFRD

FFLPKGTEVYPMLGSVLRDPSFFSNPQDFNPQHFLNEKGQFKKSDAFVPFSIGKRNCFGE

GLARMELFLFFTTVMQNFRLKSSQSPKDIDVSPKHVGFATIPRNYTMSFLPRHHHH

>194215480

MLASGLLLVAFLACLTLMVLMSVWRQGKFLGKLPPGPTPLPFIGNYLQLNTEQMYNSLMK

ISKRYGPVFTVHLGPRRIVVLCGYEAVKEALLDQAEEFSGRGEQATFDWLFKGYGVAFSN

GERAKQLRRFSITTLRDFGVGKRGIEERIQEEAGFLIEAFRSTRGTLIDPTFFLSRAVSN

VISSIVFGDRFDYEDKEFLSLLRMMLGSFQFTATSTGQLYEMFYSVMKHLPGPQQQAFKE

LQGLEDFIAKKVEQNQRTLDPNSPRNFIDSFLIRMQQEQKNPNTEFYLKNLVLTTLNVFF

AGTETVSTTLRYGFLLLMKHPDVEAKVHEEVDRVIGKNRQPKFEDRAKMPYTEAVIHEIQ

RFGDMIPMGVARRVTKDTKFRDFLLPKGTEVFPMLGSVLRDPRFFSNPHDFNPQHFLDEK

GQFKKSDGFVPFSIGKRYCFGEGLARMELFLFLTTIMQNFHFKSPQSPQDIDVSPKHVGF

ATIPRNYTMSFQPR

>BAE66633.1

MLASGLLLVALLVCLTVMVLMSVWQQRNSKGKLPPGPTPLPFIGNYLQLNTEQMYNSIMK

ISERYGPVFTIHLGPRRIVVLCGYDAVKEALVDQAEEFSGRGEQATFDWLFKGYGVVFSN

GERAKQLRRFAIATLRDFGVGKRGIEERIQEEAGFLIEALRDTQGANIDPTFFLSRTVSN

VISSIVFGDRFDYEDKEFLSLLRMMLGSFQFTATSTGQLYEMFSSVMKHLPGPQQQAFKE

LQGLEDFIAKKVEHNRRTLDPNSPRDFIDSFLIRMQEEEKNPNTEFHLKNLVLTSLNFFF

GGTETVSTTLRYGFLLLMKHPEVEAKVHEEIDRVIGKNRQPKFEDQARMPYMEAVIHEIQ

RFGDMLPLGVAHRVIKDTKFRDFFLPKGTEVFPMLGSVLKDPKFFSNPQDFNPQHFLDEK

GQFKKSDAFVPFSIGKRNCFGEGLARMELFLFFTTIMQNFRFKSPQSPKDIDVSPKHVGF

ATIPPNYTMSFLPR

>AAT49263.1

MLASGLLLVALLACLTVMVLMSVWQQRNSKGKLPPGPTPLPFIGNYLQLNTEQMYNSIMK

ISERYGPVFTIHLGPRRIVVLCGYDAVKEALVDQAEEFSGRGEQATFDWLFKGYGVVFSN

GERAKQLRRFSIATLRDFGVGKRGIEERIQEEAGFLIEALRDTQGANIDPTFFLSRTVSN

VISSIVFGDRFDYEDKEFLSLLRMMLGSFQFTATSAGQLYEMFSSVMKHLPGPQQQAFKE

LQGLEDFIAKKVEHNRRTLDPNSPRDFIDSFLIRMQEEEKNPNTEFHLKNLVLTSLNLFF

GGTETVSTTLRYGFLLLMKHPEVEAKVHEEIDRVIGKNRQPKFEDQARMPYMEAVIHEIQ

RFGDMLPLGVAHRVIKDTKFRDFFLPKGTEVFPMLGSVLKDPKFFSNPQDFNPQHFLDEK

GQFKKSDAFVPFSIGKRNCFGEGLARMELFLFFTTIMQNFRFKSPQSPKDIDVSPKHVGF

ATIPPNYTMSFLPR

>403305346

MLASGLLLVALLTCLTVMLLMSVWRQRKSKGKLPPGPTPLPFIGNYLQLNTEQMYNSLMK

ISERYGPVFTIHLGSRRVVVLCGYDAIKEALLDQAEEFSGRGEQATFDWLFKGYGVAFSN

GERAKQLRRFSITTLRDFGVGKRGIEERIQEEAGFLIEVLRGTHGASIDPTFFLSRTVSN

VISSIVFGDRFDYEDKEFLSLLRMMLGSFQFTATSMGQLYEMFSSVMKHLPGPQQQAFKE

LQGLEDFIAKKVEHNQRTLDPNSPRDFIDSFLIRMREEEKNPNTEFYLKNLVLTTLNLFF

AGTETVSTTLRYGFLLLMKHPEVEAKVREEIDRVIGKNRQPKFEDRAKMPYTEAVIHEMQ

RFGDMLPMGVARRVTKDTEFRDFMLPKGTEVFPLLGSVLRDSRFFSNPRDFNPQHFLDEK

GQFKKSDAFVPFSIGKRYCFGEGLARMELFLFLTTIMQNFRFKSPQSPKDIDVSPKHVGF

ATIPPNYTMSFLPR

>2P85A

MAKKTSSKGKLPPGPTPLPFIGNYLQLNTEQMYNSLMKISERYGPVFTIHLGPRRVVVLC

GHDAVKEALVDQAEEFSGRGEQATFDWLFKGYGVAFSNGERAKQLRRFSIATLRGFGVGK

RGIEERIQEEAGFLIDALRGTHGANIDPTFFLSRTVSNVISSIVFGDRFDYEDKEFLSLL

RMMLGSFQFTATSTGQLYEMFSSVMKHLPGPQQQAFKELQGLEDFIAKKVEHNQRTLDPN

SPRDFIDSFLIRMQEEEKNPNTEFYLKNLVMTTLNLFFAGTETVSTTLRYGFLLLMKHPE

VEAKVHEEIDRVIGKNRQPKFEDRAKMPYTEAVIHEIQRFGDMLPMGLAHRVNKDTKFRD

FFLPKGTEVFPMLGSVLRDPRFFSNPRDFNPQHFLDKKGQFKKSDAFVPFSIGKRYCFGE

GLARMELFLFFTTIMQNFRFKSPQSPKDIDVSPKHVGFATIPRNYTMSFLPRHHHH

>AAI63376.1

MFTALLKLDLASVGLTLFLGLIILVLFEIFRIHSYKRRTPPGPTPLPFVGTIPHFLKNPL

GFIRSMSQYGDMSTMYLGRKPAIFLNTIQLAKETLVQDTFSGKPYLPVIEWISKGFGIAM

VTFNHSWRQQRRFALHTLKNFGLGKKSVEDRVLEESRYLIAEMLKDEGKPVDPHHPIQNA

VSNIICSIVFGDRFEYNNKRFEYLLKMLNETIMLAGSASGRIFNLVPFIKHFPGPHQKIK

KNTDDLLIFLGDEVEEHRKTLDPGSPRDFIDAYLLEIEKQKFNKDSTFHEGNLLASAGDL

FMAGTDTTETTIRWGLLFLIQNPDVQERCHEEIVQVLGYDRLPSMDDRDRLPYTLATVHE

IQRYGNIIPILIHETILPTKLQGYSIPQGTTIVTNIQAIFSSKDHWKHPDSFNPENFLED

RHFIKPESFIMFSLGPRSCLGEILARTELFLFITSLLQRIHFSWPPDAKPIDMDGIFGLV

HSPQAFNVICRSRDTK

>AAI34007.1

MLVGLVKLDLASVGLTLFLGLIFLVLFEIFRIHSYKGRFPPGPTPLPFVGNLPHFLKSPM

EFIRFMPQYGEMTTIFFGRKPVIMLNTIQLAKEAYVQDVFSGRPAIPLFDWITNGLGIVM

VTFNNSWRQQRRFALHTLRNFGLGKKTVEDRVLEESRYLIAEMLKEEGKSMNPQHALQNA

ISNIICSIVFGDRFEYDNKRFEYLLKTLNENIMLAGSAAGQIFNLVPFIKHFPGPHQKIK

QNADELLGFIRDEAKEHRQTLDPDSPRDFIDAYLLEIEKQKSSKDSTFHEENLVVSASDL

FLAGTDTTETTIRWGLINLIQNPDVQERCHEEIVRVLGYDRLPSMDDRDKLPYTLATVYE

IQRCANIAPNVMHQTILPTKLHGYNIPQGTIILTNLAAIFSNKEHWKHPDAFNPENFLDE

NGHFSKPESFIPFSLGPRVCLGETLARTELFLFITALLQRIRFSWPPDAKPIDMDGIMGL

VRSPQTFNVVCHSRDNVK

>AAM90905.1

MLVGLVKLDLASVGLTLFLGLIFLVLFEIFRINSYKFRFPPGPTPLPFVGNLPHFLKSPM

EFIRSMPQYGEMTTIFFGRKPVIMLNTIQLAKEAYVQDAFSGRPAIPLFDWITNGLGIVM

VTFNNSWRQQRRFALHTLRNFGLGKKTVEDRVLEESRYLIAEMLKEEGKSMNPQHALQNA

ISNIICSIVFGDRFEYDNKRFEYLLKTLNENIMLAGSAAGQIFNLVPFIKHFPGPHQKIK

QNADELLGFIRDEAKEHKQTLDPDSPRDFIDAYLLEIEKQKSSKDSTFHEENLVVSASDL

FLAGTDTTETTIRWGLINLIQNPDVQERCHEEIVRVLGYDRLPSMDDRDKLPYTLATVYE

IQRCANIAPNVMHQTILPTRLHGYDIPQGTIILTNLAAIFSNKDHWKHPDAFNPENFLDE

NGHFSKPESFIPFSLGPRVCLGETLARTELFLFITALLQRIRFSWPPDAKPIDMDGIMGL

VRSPQTFNVVCHSRDNVK

>AAI52009.1

MFAALLKLDLASVGLTLFLGLIFLVLFEIFRINSYKGRFPPGPTPLPFVGNLPHSLKNPM

EFIRSMPQYGEMTTMYLGRRPAIVLNTIQLAKEAFVQDAFSGRPFLPVMDWVANGLGIIM

VTFNHSWRQQRRFALHTLRNFGLGKKSVEDRVLEESRYLIAEILKGEGKPMSPHHPIQNA

VSNIICSIVFGDRFDYDNKRFEYLLELLNENFVLTGSAAGQIFNLAPFIKHFPGPHQKIK

QNANELLAFIQGEVKEHKKTLDPDSPRDFIDAYLLEIEKQKSNKDSTFHEGNLAISTADL

FLAGTDTTSTTIRWGLLFLTQNPDVQERCHEEIVQVLGYDRLPSMDDRDKLPYTLATVHE

IQRCANLVPFGVIHETIQPTKLRGYDIPQGTVVMTNLAAILSDKEHWKHPDTFNPENFLD

ENRQFSKPESFIPFSLGLRFCLGETLAKMELFLFITSLLQRIHFSSPPDAKPIDMDGVVG

IVRYPQPFSIICCSRDTTK

>AAI24397.1

MLAALLKLDLATVGLTLFLGLIFLVLFEIFRINSYKGRFPPGPTPLPFVGNLPHLLKNPM

GFKRSLSEYGGLATVFIGRQPAISINTIQLAKEALVQDVFSGRPPLPIFDWISHGLGIIM

VTFNHSWRQQRRFALHTLRNFGLGKKTVEDRVLEESQYLIAEMLKDEGKSMNPQHALQNA

VSNIICSIVFGDRFEYDNKRFEYLLKILNENIMLTGSAAGQIFNLVPFIKHFPGPHQKVK

QNADDLFNFIRDEAKEHKQTLDPDSPRDFIDAYLLEIEKQKSSKESTFHEEHLVVSTSDL

FLAGTDTTETTIRWGLIYLIQNPDVQERCHEEIVQVLGYDRLPSMDDRDKLPYTLATVHE

IQRCGNIAPKLLHETIRRTKLHGYDIPQGTTIIANFTAMFSDKELWKHPDAFNPENFLDE

NGQFSKPEYFFPFSLGPRACLGEALARTELFLFITSLLQRIRFSWPPNAKPIDMDGIVGI

VRSPEPFNIICHSRDTKD

>AAX37317.1

MFTALLKVDLASVGLTLFLGLIFLVVFEIFRIYSYKCRFPPGPTPLPFVGNLPHLLKKPM

EFIRSLSQYGEMTTMYLGRKPAIVLNTYQVAKEALVQEAFAGRPHLPIIEWITKGLGIVM

VTFNNSWKQQRRFAQHTLRNFGLGKKSLESRVLEESQYLIAEMLKDEGRPMNPQHAVQNA

LSNIICSIVFGDRFDYNNKRFEYLLKILNESIILTGSAAGQIFNFAPIIKHFPGPHQMIN

ENANEVYSFVRHEVEEHRKTLDPGSPRDFIDGYLLEIEKQKSNKDSTFHEDNLITTTVDL

FLAGSDSTSSSIRWGLLFLIQNPDVQERCHEEIVQVLGYDRLPCMDDRDRLPYTLATVHE

IQRCGNIAPFGLFHETAQPTKLRGYNIPQGTIIMTNYTAIFSNKEHWKHPDTFNPENFLD

ENGHFSKPKCFIAFGVGPRICLGDTLAKTALFLFITSLLQRIRFSLPPDAKPMDMDGILS

IIRYPETFCIICCSRDTKE

>AAI62826.1

MFTALLKVDLASVGLTLFLGLIFLVVFEISRIYSYKCRFPPGPTPLPFVGNLPHLLKKPM

EFIRSLSQYGEMTTMYLGRKPAIVLNTYQVAKEALVQEAFAGRPHLPIIEWITKGLGIVM

VTFNNSWKQQRRFAQHTLRNFGLGKKSLESRVLEESQYLIAEMLKDEGRPMNPQHAVQNA

LSNIICSIVFGDRFDYNNKRFEYLLKILNESIILTGSAAGQIFNFAPIIKHFPGPHQMIN

ENANEVYSFVRHEVEEHRKTLDPGSPRDFIDGYLLEIEKQKSNKDSTFHEDNLITTTVDL

FLAGSDSTSSSIRWGLLFLIQNPDVQERCHEEIVQVLGYDRLPCMDDRDRLPYTLATVHE

IQRCGNIAPFGLFHETVQPTKLRGYNIPQGTIIMTNYTAIFSNKEHWKHPDTFNPENFLD

ENGHFSKPKCFIAFGVGPRICLGDTLAKTALFLFITSLLQRIRFSLPPDAKPMDMDGILS

IIRYPETFSIICCSRDTKE

>AAH55136.1

MVTFNHSWRQQRRFALHTLKNFGLGKKSVEDRVLEESRYLIAEMLKDEGKPVDPHHPIQN

AVSNIICSIVFGDRFEYNNKRFEYLLKMLNETIMLAGSASGRIFNLVPFIKHFPGPHQKI

KKNTDDLLIFLGDEVEEHRKTLDPGSPRDFIDAYLLEIEKQKFNKDSTFHEGNLLASAGD

LFMAGTDTTETTIRWGLLLLIQNPDVQERCHEEIVRVLGYDRLPSMNDRDRLPYTLATVH

EIQRYGNIIPILIHETILPTKLQGYSIPQGTTIVTNIQAIFSSKDHWKHPDTFNPENFLE

DGHFIKPESFIMFSLGPRSCLGEMLARTELFLFITSLLQRIHFSWPPDAKPIDMDGIFGL

VHSPQTFNVICRSRDTK

>BAF62160.1

MFGALVKLDLSSVGLTLLLGLILLVLFEIFRIHSYKSRFPPGPTPLPFVGNLPHLLRDPM

GFNRLMAQYGEMSTMYLGKKPAIVLNTIQVAKEALVQEAFAGRPCLPVIDWTSNGCGIIM

ATFNNSWKQQRRFALHTLRNFGLGKKSIESRVLEESQYLFAELLKDEGEPVNPHHALQNA

VSNIFCSIMFGERFDYDNKRLGYLLKILNENMMLTGSAIGQIFNLAPFIKHFPGPHQKIK

KNSNELYSFIEDEVEEHRKTLDPVSPRDFIDAYLLEIEKQKSNKDSTFQEENLIGSAIDL

FFAGTDSTATSIRWGLLFLIQNPDVQERCHEEIVQVLGYDRLPCMDDCDRLPYTHATVHE

IQRFAKTVPFGVFHETIWPTKLHGFDIPQGTMIMTNLAAIFSSKDHWKHPDTFNPENFLD

ENGHFSKPESYIPFSLGLRACIGESLVRTELFLFATVLLQRIHFSWPPDAKPLDMDGIVG

IVRYPQTFSIICCSRDSKK

>AAI52007.1

MFASLLKLDLATVGLTLFLGLIFLVVFEIFRIRSYSGRFPPGPTPLPFVGTIPHFLKDSM

GFIRSLSQYGEMTTVYLGRKPAMVLNTLQVIKEAIVQNGTSSSGRPSIPILTWITEGYGI

VLATFGHSWRQQRRFALHTLRNFGLGKKSVEERVTEESGYLVPEMLKMEGKPFDPQHAIQ

NAVSNIICSIVFGDRFEYDNKRFEYLLEIIKENINQAGSLIGQVFNLIPFIKHFPGPHQK

IYQNAEELKAFIRESTKSHRETLDPDSPRDFIDAYLLEMEKQKSSQDSSFHEDNMVMSVA

DLFLAGSDTTATTIRWGLIYLTQNPDVQERCHEEIVRVLGYDRLPCMDDRDRLPYTLATV

HELQRCGNIVPSSVPHETTQPMKLRGYDIPQGTQMLINLSDILANKEHWKHPNTFNPENF

LDDKGNFYRPEAFLPFSLGPRVCLGETLAKTELFLFITSLLQRIRFSWPTGEKWPNMDGI

VSVVRSPEPFKIICHSRGSKE

>316983117

MLAALLKLDLSSVGLSLFLGLIFLVLFEIFRIHSCKGRFPPGPTPLPFVGSIPHFLNNPM

GFIKSLSQYGEMTTVYPGRKPAIILNTLQLMKEALVQNGSSFSGRPPVPVFNWVTDGYGI

VMATFGHSWRQQRRFALHTLRNFGLGKKSVEERVTEESGYLISEFLKAEGKPFNPQHAIH

NAAANIICSIVFGDRFDYDNKSFTYLLEIIKENIIQAGSLVGQVFNLVPIIKHVPGPHQK

IYQNGQAFKSFIRESVKEHRQTLDPDSPRDFIDAYLLEMEKQKSTQDSTFHEDNMVMAVG

DLFLAGSDTTATTIRWGLIYLTQNPDVQERCHEEIVRVLGFDRLPSMDDRDRLPYTYATV

HEFQRCANLVPTGVPHETTQATKLRGYDIPQGTQILINLTEILTNKEHWKHPDTFNPENF

LDDKGHFFKPEAFLPFSLGPRACLGETLAKAELFLFVTSLLQRIRFSWPTGEKLPDMNGI

FGIVRSPKPFNIICHSRGSKH

>AAH71533.1

MGFNRLMAQYGEMSTMYLGKKPAIVLNTIQVAKEALVQEAFAGRPCLPVIDWTSNGCGII

MATFNNSWKQQRRFALHTLRNFGLGKKSIESRVLEESQYLFAELLKDEGEPVNPHHALQN

AVSNIFCSIMFGERFDYDNKRLGYLLKILNENMMLTGSAIGQIFNLAPFIKHFPGPHQKI

KKNSNELYSFIEDEVEEHRKTLDPVSPRDFIDAYLLEIEKQKSNKDSTFQEENLIGSAID

LFFAGTDSTATSIRWGLLFLIQNPDVQERCHEEIVQVLGYDRLPCMDDCDRLPYTHATVH

EIQRFAKTVPFGVFHETIWPTKLHGFDIPQGTMIMTNLAAIFSSKEHWKHPDTFNPENFL

DENGHFSKPESYIPFSLGLRACIGESLVRTELFLFATVLLQRIHFSWPPDAKPLDMDGIV

GIVRYPQTFSIICCSRDSKK

>AAH77115.1

MFNTVQVAKEALVQDAFAGRPHLPIVDWITNGLGIVAVTFNHSWRQQRRFALHTLRNFGL

GKKSIESRVLEESQYLIAEMLKEKGRSVNPHHIIQNALSNIICSIMFGDRFDYDDKRFEY

FLKLLNENILLIGSAAVQIFNFAPFIKHFPGPHQKFKQNVNELSGFVRHEVEEHKKTLDP

DSPRDFIDAYLLEIEKQKSNKDSTFHDENLVRSAADLFEAGSDSTATTIRWGLLFLIQNP

DVQERCHEEIVQVLGYDRLPCMDDRDRLPYTLATVHEIQRCGNIVPFGLIHETIQPTNLH

GYDIPQGTVVMANFTAILSNKENWKHPDTFNPENFLDENGHFSKPESFIPFSLGPRSCLG

ETLAKTELFLFITSLLQRIHFSWPPDAQPIDMDGIMGIVRYPQPFSIICCSRDTKK

>AAH83240.1

MLNTVQLAKEVLIQDAFAGKPSLPVLDWVSNGLGIVMVTFNHSWRQQRRFALHTLRNFGL

GRKSVESRVLEESQYLIAELLKKKGKSVNPQHALQNAFSNVICSIVFGDRFDYDDKRFEH

FLEILGKSMILTGSTAGQIFNFAPIIKHFPGPHQKIKKNADELSGFFQHEVKEHKKTLDP

GSPRDYIDAYLLEMEKQKSNKDSTFHDENLIGSTTDLFVAGSDSTATTFRWGLLFLIQNP

DVQERCHKEIVQVLGYDRLPSMEDRDRLPYTLATVYEIQRCANLAPFGLIHETIQPTKLQ

GYDLPRGTTIIVNLTAIFSNKENWKHPDTFNPENFLDESGQFSKHESFIPFSLGVRVCLG

ETLARTELFLFITALLQRIRFSLPPDAKPMDMDGILSVLRYPQNFSFICCSRDTKE

>AAI65796.1

MLNTVQLAKEVLIQDAFAGKPSLPVLDWVSNGLGIVMVTFNHSWRQQRRFALHTLRNFGL

GRKSVESRVLEESQYLIAELLKKKGKSVNPQHALQNAFSNVICSIVFGDRFDYDDKRFEH

FLEILGKSMILTGSTAGQIFNFAPIIKHFPGPHQKIKKNADELSGFFQHEVKEHKKTLDP

GSPRDYIDAYLQEMEKQKSNKDSTFHDENLIGSTTDLFVAGSDSTATTFRWGLLFLIQNP

DVQERCHKEIVQVLGYDRLPSMEDRDRLPYTLATVYEIQRCANLAPFGLIHETIQPTKLQ

GYDLPRGTTIIVNLTAIFSNKENWKHPDTFNPENFLDESGQFSKHESFIPFSLGVRVCLG

ETLARTELFLFITALLQRIRFSLPPDAKPMDMDGILSVLRYPQNFSFICCSRDTKE

>73956380

MLAAVGSLAATLWAVLHLRTLLLGAVAFLFFADFLKRRRPKNYPPGPVPLPFVGNFFHLD

FEQSHLKLQRFVKKYGNVFSVQMGDMPLVVVTGLPLIKEVLVDQNQVFVNRPITPIRERV

FKNSGLIMSSGQIWKEQRRFTLATLKNFGLGRKSIEERIQEEAHHLIQAIEEENGQPFNP

HFKINNAVSNIICSITFGKRFEYQDEQFQELLRLLDEVTCLETSMRCQLYNVFPWIIKFL

PGPHQKLFNDWEKLKLFIAHMTENHRRDWNPAEPRDFIDAYLKEMEKNRGNATSSFHEEN

LIYSTLDLFFAGTETTSTTLRWGLLYLALNPEIQEKVQAEIDRVIGQSQLPGLAVRESMP

YTNAFIHEVQRMGNIVPLNVPREVTGDTTLAGYYLPKGTVIVTNLTALHRDPAEWATPDT

FNPEHFLENGQFKKREAFLPFSIGKRVCIGEQLARSELFIFFTSLVQRFTFRPPDNEKLS

LEFRTGLTISPVSHRLRAIPRS

>350586158

MPLALGSLAEALWTALRPSTILLGAVAFLFFADFLKKRRPKNYPPGPPRLPFIGNLFHLD

LDKGHLSLQRFVKKYGNVFSLDFGALSSVVITGLPFIKEAFVHQDKNFSNRPIVPIQQRV

FKDKGVVMSNGQVWKEQRRFALTTLRNFGLGKKSLEERIQEEAQYLIQAIGEENGQPFNP

HFKINNAVSNIICSITFGERFDYQDNQFQELLKLLDEVMCLQTSVWCQIYNIIPWIMKFL

PGPHQTLFSNWEKLKMFVAHVIENHRRDWNPAEARDFIEAYLQEIEKHKGDATSSFQEEN

LICSTLDLFVAGTETTSTTLRWGLLYMALYPEIQEKVQAEIDRVLGQLQQPSTAARESMP

YTNAVIHEVQRMGNIIPLNVPREVAEDTTLAGYHLPKGTMVIANLTALHSDPTEWATPDT

FNPEHFLENGKFKKREAFLPFSVGKRACLGEQLARTELFVFFTSLLQKFSFRPPDNEKLS

LKFRMGLTLSPVTYRICAVPRA

>301763659

MLAAMGSLAAAVWAMGHLRTLLLGAVAFLFFADCLKRRRPKNYPPGPLALPFVGNFFHLD

FERSHLTIQRFVKKYGNIMSMQLGDLPLVLVTGLPLIKEILVDQNQIFVNRPITPIRERI

FKNNGLIMSSGQTWKEQRRFTLATLRNFGLGRKSLEERIQEEAHHLIQAIEEENGQPFNP

HFKMNNAVSNIICSLTFGKRFEYEDEQFQELLRLLDEVMYLETSMRCQLYNVFPRIMKFL

PGPHQTLFSHWEKLKSFIAHMTENHRKDWNPAEPRDFIDAYLKEIEKNRDNAASSFHGEN

LICSTLDLFFAGTETTSTTLRWGLLYLALNPEIQEKVQAEIDRVIGRSQLPSIAARESLP

YTNAVIHEVQRMGNIIPLNVPREVTGDTTLAGYYLPKGTMILTNLTALHRDPAEWATPDT

FNPEHFLENGQFKKRETFLPFSVGKRVCLGEQLAKSELFIFFTSLVQKFTFRPPNNEQLS

LKFRTGLTISPVNHRLCAVPRA

>DAA31250.1

MLEALSSLATALWAALRPDTXLLGTLAFLLFVDFLKRRHPKNYPPGPPGLPFVGNLFQLD

PEKVPLVLHQFVKKYGNVFSLDFGTVPSVLITGLPLIKEVLVHQGQIFSNRPIVPLQEHI

INNKGLIMSSGQLWKEQRRFALTTLRNFGLGKKSLEERIQEEASYLIQTIREENGQPFDP

HLTINNAVSNIICSITFGERFDYQDDQFQELLRMLDEILNLQTSMCCQLYNVFPRIMNFL

PGPHQALFSNMEKMKMFVARMIENHKRDWNPAEARDFIDAYLQEIEKHKGDATSSFQEEN

LIYNTLDLFLAGTETTSTSLRWGLLFMALNPEIQEKVQAEIDRVLGQSQQPSMAARESMP

YTNAVIHEVLRMGNIIPLNVPREVAVDTTLAGYHLPKGTMVMTNLTALHRDPTEWATPDT

FNPEHFLENGQFKKRESFLPFSIGKRMCLGEQLARTELFIFFTSLLQKFTFRPPENEQLS

LKFRVSLTLAPVSHRLCAVPRG

>358411529

MLEALSSLATALWAALRPDTVLLGTLAFLLFVDFLKRRHPKNYPPGPPGLPFVGNLFQLD

PEKVPLVLHQVGAGENGRAWNPAESSRHRPHRREPDVAVMLEALSSLATALWAALRPDTV

LLGTLAFLLFVDFLKRRHPKNYPPGPPGLPFVGNLFQLDPEKVPLVLHQFVKKYGNVFSL

DFGTVPSVLITGLPLIKEVLVHQGQIFSNRPIVPLQEHIINNKGLIMSSGQLWKEQRRFA

LTTLRNFGLGKKSLEERIQEEASYLIQTIREENGQPFDPHLTINNAVSNIICSITFGERF

DYQDDQFQELLRMLDEILNLQTSMCCQLYNVFPRIMNFLPGPHQALFSNMEKMKMFVARM

IENHKRDWNPAEARDFIDAYLQEIEKHKGDATSSFQEENLIYNTLDLFLAGTETTSTSLR

WGLLFMALNPEIQEKVQAEIDRVLGQSQQPSMAARESMPYTNAVIHEVLRMGNIIPLNVP

REVAVDTTLAGYHLPKGTMVMTNLTALHRDPTEWATPDTFNPEHFLENGQFKKRESFLPF

SIGKRMCLGEQLARTELFIFFTSLLQKFTFRPPENEQLSLKFRVSLTLAPVSHRLCAVPR

G

>AAZ29440.1

MLAALGSLAAALWAVVHPRTLLLGTVAFLLVADFLKRRRPKNYPPGPWPLPFVGNFFHVN

FEQSHLEIQQFVKKYGNLFSLELGDISAVLITGLPLIKEALIHMDQNFGNRPMTPMRERT

FKKNGLIMSSGQIWKEQRRFTLTALRNFGLGKKSLEERIQEEAQHLTEAIKEENGQPFDP

HFKINNAVSNIICSITFGERFEYQDSQFQELLKLLDEVTYLEASKTCQLYNIFPWLMKFL

PGPHQTLFSNWEKLKLFVSHMIEKHRKDWNPAETRDFIDAYLKEMSKHTGNSTSSFHEEN

LICSTLDLFFAGTETTSTTLRWALLYMALYPEIQEKVQAEIDRVIGQGQQPSTAARESMP

YTNAVIHEVQRMGNIVPLNVPREATVDTTLAGYHLPKGTMILTNLTALHRDPTEWATPDT

FNPEHFLENGQFKKREAFLPFSIGKRACLGEQLARTELFIFFTSLVQKFTFRPPNNEKLS

LKFRMGITISPVSHHLCAVPRV

>EHH14839.1

MLAALGSLAAALWAVVHPRTLLLGTVAFLLVADFLKRRRPKNYPPGPWPLPFVGNFFHVN

FEQSHLEIQQFVKKYGNLFSLELGDISAVLITGLPLIKEALIHMDQNFGNRPMTPMRERT

FKKNGLIMSSGQIWKEQRRFTLTALRNFGLGKKSLEERIQEEAQHLTEAIKEENGQPFDP

HFKINNAVSNIICSITFGERFEYQDSQFQELLKLLDEVTYLEASKTCQLYNIFPWLMKFL

PGPHQTLFSNWEKLKLFVSHMIEKHRKDWNPAETRDFIDAYLKEMSKHTGNSTSSFHEEN

LICSTLDLFFAGTETTSTTLRWALLYMALYPEIQEKVQAEIDRVIGQGQQPSTAARESMP

YTNAVIHEVQRMGNIVPLNVPREATVDTTLAGYHLPKGTMILTNLTALHRDPTEWATPDT

FNPEHFLENGQFKKREAFLPFSIGKRACLGEQLARTELFIFFTSLVQKFTFRPPNNEKLS

LKFRMGITISPVSHHLCAVPRV

>301611288

MELAVAPRFPQQLVNKTRLDMELGIALLLGLVILVIFCCKKQQPVGFPPGPAAMPVLGNI

LQMDFSNPLKDLQKFSEVYGPVYSIYLGFTPAVVVRGLKSIKEVLVNKGTDFAGRPQNRI

TDVISKSKGLVVAPYGQAWKEHRRFTLSTLRSFGLGKKSMEDRIQEENMHLIQAFKNSKD

VLFDPHFVLENAVSNIICSIVFGRRYDYNDTSFQNILNLVHENMKLATGFWAQLYNAFGF

IQFLPLPHKKIFQNVDVVFAFLGKVVEEHKETLLPGEPRDYIDCYLEELKKEEDGNKMTF

DEENLFSCVADLFVAGTETTTSSLEWCLLYMLKFPEIQEKCHEEIDRVFGNKNCLEYEDR

ERMPYTQAVLQEVQRHASVVPLGVSHSPIRDVHLNGFFIPKGTVIITDLSSVHYDKTHWK

YPHEFNPENFLNENGELIKTEAFLPFSAGPRVCLGENLARMEIFLFFTAMLQHFEFHWPD

PTSPPDLRPVFGLSQAPKHYKMRIVSRKKK

>EHH49955.1

MLAALGSLAXXXXXXXXPRTLLLGTVAFLLVADFLKRRRPKNYPPGPWPLPFVGNFFHVN

FEQSHLEIQQFVKKYGNLFSLELGDISAVLITGLPLIKEALIHMDQNFGNRPMTPMRERT

FKKNGLIMSSGQIWKEQRRFTLTALRNFGLGKKSLEERIQEEAQHLTEAIKEENGQPFDP

HFKINNAVSNIICSITFGERFDYQDSQFQELLKLLDEVTYLEASKTCQLYNIFPWLMKFL

PGPHQTLFSNWEKLKLFVSHMIEKHRKDWNPAETRDFIDAYLKEMSKHTGNSTSSFHEEN

LICSTLDLFFAGTETTSTTLRWALLYMALYPEIQEKVQAEIDRVIGQGQQPSTAARESMP

YTNAVIHEVQRMGNIVPLNVPREATVDTTLAGYHLPKGTMILTNLTALHRDPTEWATPDT

FNPEHFLENGQFKKREAFLPFSIGKRACLGEQLARTELFIFFTSLVQKFTFRPPNNEKLS

LKFRMGITISPVSHHLCAVPRV

>395840736

MQLTPENGKQRKEWATEELAVIPGLLLAMGFLQAALGAVLHPQTLLLGAVAFLFFADFLK

SRRPKNYPPGPWRLPFLGNFFPLNIEQAHLTLQRIVKKYGNIVSLEFGSISSVVITGLPL

IKEALIDLHQNFVNRPVFPIQERIFQGNGLFGSKGQVWKEQRRFALTTLRNFGLGKKSLE

ERIQEEAHHLVEAIKEENGQPFDPHFKINNAVSNIICSITFGERFEYQDGEFQELLRLLD

EAANMETSFTSQLYNFLPRIMKFLPGPHQTVFRNWEKLKLFVSHIVEKHKRDWNPDETRD

FIDAYLKEIAKNASNATSSFHKDNLIGCTLDLFFAGTETTSATLRWGLLYMALYPDIQEK

VHAEIDRVIGQEQLPSVAARESLPYTNAVIHEVQRMGNIVPLNVPREVAADTTLAGYHLP

KGTMVFTNLTALHRDPAEWTSPDTFNPENFLENGQFKKRESFLPFSIGKRACPGEQLART

ELFIFFTCLMQKFTFRPPDNEKLSLKFRMGITVSPVKHRLCAVPRGRRC

>402854744

MLAALGSLAAALWAVVHPRTLLLGTVAFLLVADFLKRRRPKNYPPGPWPLPFVGNFFHVN

FEQSHLEIQQFVKKYGNLFSLELGDISAVLITGLPLIKEALIHMDQNFGNRPMTPMRERT

FKKNGLIMSSGQIWKEQRRFTLTALRNFGLGKKSLEERIQEEAQHLTEAIKEENGQPFDP

HFKINNAVSNIICSITFGERFEYQDSQFQELLKLLDEVTYLEASKTCQLYNIFPWLMKFL

PGPHQTLFSNWEKLKLFVSHMIEKHRKDWNPAETRDFIDAYLKEMSKHTGNSTSSFHEEN

LICSTLDLFFAGTETTSTTLRWALLYMALYPEIQEKVQAEIDRVIGQGQQPSTAARESMP

YTNAVIHEVQRMGNIIPLNVPREATVDTTLAGYHLPKGTMILTNLTALHRDPTEWATPDT

FNPEHFLENGQFKKREAFLPFSIGKRACLGEQLARTELFIFFTSLVQKFTFRPPNNEKLS

PKFRMGITISPVSHHLCAVPRV

>395840738

MTTRGRAQPAASRNSASAQRFNQISTELFKQLHSRRLACLWECRFSEINGALWVFTKEDV

GCPRSNSNLSFSFQTTQTTFPGVDSASMDRPCKHHLLSKFIQEAETHTLHVIDKRNKSPR

GPWPEKARARVIPGLLLAMGFLGAVLWAVLHPQTLLLGAVTFLLFADFLKSRRPKNYPPG

PWRLPFLGNFFHLGFEQSHLRVQRFVKKYGNIVNFEFRNVSSVVITGLPLIREAFIHKDQ

NFANRPVFPVRERIIKGHGLIMSNGQVWKEQRRFALTALRNFGLGRKSLEERIQEEAYHL

VEAVKEENGQPFDPHFKINNAVSNVICSITFGKRFEYQDGEFQELLRLLDEATYMEASVA

SQLYNIFPWIMKFLPGSHTTVFRNWEKLKLFVAHIIENHKRDWNPDETRDFIDAYLKEIS

KNASNANSSFHEENLIWSTLDLFFAGTETTSTTLRWGLLYMALYPDIQEKVHAEIDRVIG

QEQLPSVAARESLPYTNAVIHEVQRMGNIIPLNVPREVAADTTLAGYHLPKGTMVLTNLT

ALHRDPAEWATPDTFNPEHFLENGQFKKREAFLPFSIGKRACLGEQLARTELFIFFISFM

QKFTFKAPDNEKLSLEFREGLTLTPVKHQLCAVPRG

>109005667

MLAALGSLAAALWAVVHPRTLLLGTVAFLLVADFLKRRRPKNYPPGPWPLPFVGNFFHVN

FEQSHLEIQQFVKKYGNLFSLELGDISAVLITGLPLIKEALIHMDQNFGNRPMTPMRERT

FKKNGLIMSSGQIWKEQRRFTLTALRNFGLGKKSLEERIQEEAQHLTEAIKEENGQPFDP

HFKINNAVSNIICSITFGERFDYQDSQFQELLKLLDEVTYLEASKTCQLYNIFPWLMKFL

PGPHQTLFSNWEKLKLFVSHMIEKHRKDWNPAETRDFIDAYLKEMSKHTGNSTSSFHEEN

LICSTLDLFFAGTETTSTTLRWALLYMALYPEIQEKVQAEIDRVIGQGQQPSTAARESMP

YTNAVIHEVQRMGNIVPLNVPREVTVDTTLAGYHLPKGTMILTNLTALHRDPTEWATPDT

FNPEHFLENGQFKKREAFLPFSIGKRACLGEQLARTELFIFFTSLVQKFTFRPPNNEKLS

LKFRMGITISPVSHHLCAVPRV

>395840732

MAKPGTKRGSWLGIARVIPDLLLAMGFLQAALWAVLHPQTLLLGAVTFLFFADFLKSRRP

KNYPPGPWRLPFVGNFFLLGFEQSHLTLQRFVKKYGNILSLDLGNISSVVITGLPLIREA

LTNMGQNFVNRPVSPMRERIFKGNGLIMSNGQVWKEQRRFALAALRNFGLGRKSLEERIQ

EEAHHLVEAVKEENGQPFDPHFKINNAVSNIICSITFGKRFEYQDGEFQELLRLLDEATY

MEASVASQLYTIFPWIMKFLPGSHQTVFRNWEKLRLFVAHIIEKHKRDWNPDETRDFIDT

YLKEIAKNASNAASSFHEENLIWCTLDLFFAGTETTSTTLRWGLLYMALYPDIQEKVHAE

IDRVIGQEQLPSVAARESLPYTNAVIHEVQRMGNIVPLNVPREVAADTTLAGYHLPKGTM

VLTNLTALHRDPAEWATPDTFNPEHFLENGQFKKREAFLPFSIGKRVCLGEQLARTELFI

FFTCLMQKFTFRPPDNEKLSPKFRMGVTLSPVKHRLCAVPRG

>296208098

MGWLPTAVSKSITSSTDFRLVYLECMELACQTSALAIKHAMVTIALPCVSATRIGNNHKC

QKRERMEKESCAKSSGKMIGLAAPLQGPRQLHLLLPLLGAGELMPLWPPLAPSVDPFPCI

IHPLLPSTLENVPCLCQNNICAPASLHVIDKPASSSPDPGGLWPLGFTAPRGTPKRTCGR

GLKRKNTIIMLTPHRFSAGPWTRPPSRERGNVGSSALSWDAREICGRGVQDQRGKSKKEA

GCLRGNQDEVRNSCHLDDEYLLPAFETAEEQKVRTVLAAMGSLAAALWAVLSPRTLLLGT

IAFLLFADFLKRRRPKNYPPGPWSLPFFGNFFHVDFEKSHLDTQLFVKKYGNLFSLELGN

ISAVLITGLPLIKEALIHMDQNFGSRPVTPIRERIFKKNGLIMSNGQVWKEQRRFTLTAL

RNFGLGKKSLEERMQEEAHHLTEAIKEENGQPFDPHFKINNAVSNIICSITFGKRFEYQD

GQFQELLKLLDEAIHLEASKTCQLYNIFPWIMKLLPGPHQTLFSHWQKLKLFVSHIIEKH

RRDWNPAETRDFIDAYLKEMSKHTDNPTSSFHEENLICSALDLFFAGTETTSTTLRWALL

YMALYPEIQEKVQAEIDRVIGQGQQPSTAARESMPYTNAVIHEVQRMGNIIPLNVPREVT

VDSTFAGYQLPKGTMILTNLTALHRDPTEWATPDTFNPEHFLENGQFKKREAFMPFSIGK

RACLGEQLARSELFIFFTSLVQKFTFRPPDNEKLSLKFRMGVTISPVSHRLCAVPRV

>AAV66395.1

MLEALGSLAAALWTALRPGTVLLGAVVFLLLSDLLKRQRPKNYPPGPPRLPFVGNFFQLD

FEQGHLSLQRFVKKYGNLFSLELGDLPSVVITGLPLIKEVLVHQDQNFVNRPITPIRERV

FKENGLIMSNGHIWKEQRRFSLTALRNFGLGRKSLEEHIQEEVAFLIQAIGEKNGQPFNP

HFKINNAVSNIICSIAFGERFDYQDDQFQELLRLLDEVTYLETTLWCQLYNVFPRIMNFL

PGPHQRLFSNWEKLKMFVARMIENHKKDWNPDEARDFIDAYLQETEKHKGNAASSFHEEN

LIYSTLDLFFAGTETTSTTLRWGLLYMALYPEIQEKVQAEIDKVLGKSRPPSTATRESMP

YTNAVIHEVQRMGNIIPLNVPREVTVDTILAGYHLPKGTMVLTNLTALHRDPAEWATPDT

FNPEHFLENGQFKKREAFLPFSIGKRMCLGEQLARTELFIFFTSLLQKFTFRPPDNEELS

LTFRMGLTLSPVSHRLCAVPRA

>347543811

MTPALGFLAEALWTALRPSTLLLGAVAFLFFADFLKRRSPKNYPPGPPRLPFLGNFFHLD

VEKGHLALQRFVKKYGNIISLDSSVFSSVVITGLPLIKEAFVHQDQHFANRPMIPIQERV

FKKNGLIMSNGQVWKEQRRFALTTLRNFGLGKKSLEERIQEEAQYLIQAIGEENGQPFNP

HFKINNAVSNIICSITFGKRFDYQDDQFQELLRLLDEVTCQHTSVQVQLYNMFPRIMKFL

PGPHQTLFSNWEKLQIFVACVIENHKRDWNPAEARDFIDAYLQEIEKHKGNATSSFQEEN

LIFTTLDLFFAGTETTSTTLRWSLLYMALYPEIQEKVQAEIDRVLGQSQQPSTAARESMP

YTNAVIHEVQRMGNIIPLNVPREVAEDTTLAGYHLPKGTMVLTNLTALHRDPAEWATPNI

FNPEHFLENGKFKKREAFLPFSIGKRACLGEQLARTELFVFFTSLLQKFSFRPPDNEKLS

LKFRVGLTLSPVTYCICAVPRA

>119890124

MLEALGSLAAALWPALRPGTVLLGAIVFLLLTDLLNRRRPKNYPPGPPRLPFVGNFFQLD

FEQGHLSLQRFVKKYGNLFSLEFGDLPSVVITGLPLIKEVLVYQDQNFVNRPISPIRERV

FKKNGLIMSNGHIWKEQRRFSLTALRNFGLGRKSLEERIQEEVAYLIQAIGEEKGQPFNP

HFKINNAVSNIICSITFGERFDYQDDQFQELLRLLDEVTYLETTVWCQLYNVFPRIMNFL

PGPHQMLFSNWRKLKMFVARVIENHKRDWNPAEARDFIDAYLQETEKHKGNAASSFHEEN

LIYNTLDLFFAGTETTSTTLRWGLLYMALYPEIQEKVQAEIDKVLGESQQPSTAARESMP

YTNAVIHEVQRMGNILPLNVPREVTVDTVLAGYHLPKGTMVLTNLTALHRDPAEWATPDT

FNPEHFLENGQFKKREAFLPFSIGKRMCLGEQLARTELFIFFTSLLQKFTFRPPDNEELS

LKFRMGLTLSPVSHCLCAVPRA

>DAA31249.1

MLEALGSLAAALWAALRPGTVLLGAIVFLLLTDLLNRRRPKNYPPGPPRLPFVGNFFQLD

FEQGHLSLQRFVKKYGNLFSLEFGDLPSVVITGLPLIKEVLVYQDQNFVNRPISPIRERV

FKKNGLIMSNGHIWKEQRRFSLTALRNFGLGRKSLEERIQEEVAYLIQAIGEEKGQPFNP

HFKINNAVSNIICSITFGERFDYQDDQFQELLRLLDEVTYLETTVWCQLYNVFPRIMNFL

PGPHQMLFSNWRKLKMFVARVIENHKRDWNPAEARDFIDAYLQETEKHKGNAASSFHEEN

LIYNTLDLFFAGTETTSTTLRWGLLYMALYPEIQEKVQAEIDKVLGESQQPSTAARESMP

YTNAVIHEVQRMGNILPLNVPREVTVDTVLAGYHLPKGTMVLTNLTALHRDPAEWATPDT

FNPEHFLENGQFKKREAFLPFSIGKRMCLGEQLARTELFIFFTSLLQKFTFRPPDNEELS

LKFRMGLTLSPVSHCLCAVPRA

>297664775

MLAAMGSLAAALWAVVHPRTLLLGTVAFLLAADFLKRRRPKNYPPGPWRLPFLGNFFLVN

FEQSHLEVQLFVKKYGNLFSLELGDISAVLITGLPLIKEALIHMDQNFGNRPVTPIREHI

FKKNGLIMSSGQVWKEQRRFTLTALRNFGLGKKSLEERIQEEAQHLTEAIKEENGQPFDP

HFKINNAVSNIICSITFGERFEYQDSRFQQLLKLLDEVTYLEASKTCQLYNVFPWIMKFL

PGPHQTLFSNWKKLKLFVSHMIDKHRKDWNPAETRDFIDTYLKEMSKHTGNPTSSFHEEN

LICSTLDLFFAGTETTSTTLRWALLYMALYPEIQEKVHAEIDRVIGQGQQTSTAARESMP

YTNAVIHEVQRMGNIVPLNVPREVTVDTTLAGYHLPKGTMILTNLTALHRDPTEWATPDT

FNPDHFLENGRFKKREAFMPFSIGKRACLGEQLARTELFIFFTSLMQKFTFRPPNNEKLS

LKFRMGITISPVSHRLCAVPRV

>BAG37780.1

MLAAMGSLAAALWAVVHPRTLLLGTVAFLLAADFLKRRRPKNYPPGPWRLPFLGNFFLVD

FEQSHLEVQLFVKKYGNLFSLELGDISAVLITGLPLIKEALIHMDQNFGNRPVTPMREHI

FKKNGLIMSSGQAWKEQRRFTLTALRNFGLGKKSLEERIQEEAQHLTEAIKEENGQPFDP

HFKINNAVSNIICSITFGERFEYQDSWFQQLLKLLDEVTYLEASKTCQLYNVFPWIMKFL

PGPHQTLFSNWKKLKLFVSHMIDEHRKDWNPAETRDFIDAYLKEMSKHTGNPTSSFHEEN

LICSTLDLFFAGTETTSATLRWALLYMALYPEIQEKVQAEIDRVIGQGQQPSTAARESMP

YTNAVIHEVQRMGNIIPLNVPREVTVDTTLAGYHLPKGTMILTNLTALHRDPTEWATPDT

FNPDHFLENGQFKKREAFMPFSIGKRACLGEQLARTELFIFFTSLMQKFTFRPPNNEKLS

LKFRMGITISPVSHRLCAVPQV

>332232030

MLAAMGSLAAALWAVVHPRTLLLGTVAFLLAVDFLKRRRPKNYPPGPWRLPFLGNFFLVD

FEQSHLEVQLFVKKYGNLFSLELGDISAVLITGLPLIKEALIGMDQNFGNRPVTPIREHI

FKKNGLIMSSGQVWKEQRRFTLTALRNFGLGKKSLEERIQEEAQHLTEAIKEENGQPFDP

HFKINNAVSNIICSITFGERFEYQDSQFQQLLKLLDEVTYLEASKTCQLYNVFPWIMKFL

PGPHQTLFSNWKKLKLFVSHMIDKHRKDWNPAETRDFIDAYLKEMSKHTGNPTSSFHEEN

LICSTLDLFFAGTETTSTTLRWALLYMALYPEIQEKVQAEIDRVIGQGQQPSTAARESMP

YTNAVIHEVQRMGNIVPLNVPREVTVDTTLAGYHLPKGTMILTNLTALHRDPTEWATPDT

FNPDHFLENGQFKKREAFMPFSIGKRACLGEQLARTELFIFFTSLMQKFTFRPPNNEKLS

LKFRMGITISPVSHRLCAVPRV

>BAB85489.1

MLAAMGSLAAALWAVVHPRTLLLGTVAFLLAADFLKRRRPKNYPPGPWRLPFLGNFFLVD

FEQSHLEVQLFVKKYGNLFSLELGDISAVLITGLPLIKEALIHMDQNFGNRPVTPMREHI

FKKNGLIMSSGQAWKEQRRFTLTALRNFGLGKKSLEERIQEEAQHLTEAIKEENGQPFDP

HFKINNAVSNIICSITFGERFEYQDSWFQQLLKLLDEVTYLEASKTCQLYNVFPWIMKFL

PGPHQTLFSNWKKLKLFVSHMIDKHRKDWNPAETRDFIDAYLKEMSKHTGNPTSSFHEEN

LICSTLDLFFAGTETTSTTLRWALLYMALYPEIQEKVQVEIDRVIGQGQQPSTAARESMP

YTNAVIHEVQRMGNIIPLNVPREVTVDTTLAGYHLPKGTMILTNLTALHRDPTEWATPDT

FNPDHFLENGQFKKREAFMPFSIGKRACLGEQLARTELFIFFTSLMQKFTFRPPNNEKLS

LKFRMGITISPVSHRLCAVPQV

>344278635

MLALLGSLASALWVEFHLRSLLLGVVVFLFFVDFRKRRRPKNYPPGPWSLPFLGNFFVLG

FEESHLVLQKFVKKYGNLLSLEFGDLHAVTVSGLPLIKEVLANQDQNFVNRPKTPIRERV

FKNHGLIMSNGQEWKEQRRFALTTLRNFGLGKKRLEERIQEEAHHLIEAIEEEKGQPFDP

HFKINNAVSNIICSITFGERFEYQDGQFQELLKLLDEVTCLETTKWCQLYDVFPWIMKFL

PGLHQKLFSDWEKLKIFVSRMIEKHKRDWNPDEPRDFIDSYLREMTKHTGNATSSFHEEN

LVCSTLDLFFAGTETTSTTLRWALLYLAIYPEIQEKVQAEIDRVVGQSRQPSLNDREHMP

YTNAVIHEVQRMGNIIPLNVPREVMFDTTVAGYQLPKGTMILTNLTALHRDPAEWATPET

FNPEHFLENGQFKKREAFLPFSIGKRVCLGEQLARSELFIFITSLVQKFTFRPPANEKLS

LKFRMGLTISPVSHRLCAVPRA

>397507595

MLAAMGSLAAALWAVVHPRTLLLGTVAFLLAADFLKRRRPKNYPPGPWRLPFLGNFFLVD

FEQSHLEVQLFVKKYGNLFSLELGDISAVLITGLPLIKEALIHMDQNFGNRPVTPIREHI

FKKNGLIMSSGQAWKEQRRFTLTALRNFGLGKKSLEERIQEEAQHLTEAIKEENGQPFDP

HFKINKAVSNIICSITFGERFEYQDSWFQQLLKLLDEVTYLEASKTCQLYNVFPWIMKFL

PGPHQTLFSNWKKLKLFVSHMIDKHRKDQNPAETRDFIDAYLKEMSKHTGNPTSSFHEEN

LICSTLDLFFAGTETTSTTLRWALLYMALYPETQEKVQAEIDRVIGQGQQPSTAARESMP

YTNAVIHEVQRMGNIIPLNVPREVTVDTTLAGYHLPKGTMILTNLTALHRDPTEWATPDT

FNPDHFLENGQFKKREAFMPFSIGKRACLGEQLARTELFIFFTSLMQKFTFRPPNNEKLS

LKFRMGITISPVSHRLCAVPRV

>114556842

MLAAMGSLAAALWAVVHPRTLLLGTVAFLLAADFLKRRRPKNYPPGPWRLPFLGNFFLVD

FEQSHLEVQLFVKKYGNLFSLELGDISAVLITGLPLIKEALIHMDQNFGNRPVTPIREHI

FKKNGLIMSSGQAWKEQRRFTLTALRNFGLGKKSLEERIQEEAQHLTEAIKKENGQPFDP

HFKINKAVSNIICSITFGERFEYQDSWFQQLLKLLDEVTYLEASKTCQLYNVFPWIMKFL

PGPHQTLFSNWKKLKLFVSHMIDKHRKDRNPAETRDFIDAYLKEMSKHTGNPTSSFHEEN

LICSTLDLFFAGTETTSTTLRWALLYMALYPEIQEKVQAEIDRVIGQGQQPSTAARESMP

YTNAVIHEVQRMGNIIPLNVPREVTVDTTLAGYHLPKGTMILTYLTALHRDPTXWATPDT

FNPDHFLENGQFKKREAFMPFSIGKRACLGEQLARTELFIFFTSLMQKFTFRPPNNEKLS

LKFRMGITISPVSHRLCAVPRV

>AAF21133.1

MLATAGSLIATIWAALHLRTLLVAALTFLLLADYFKTRRPKNYPPGPWGLPFVGNIFQLD

FGQPHLSIQPFVKKYGNIFSLNLGDITSVVITGLPLIKETFTHIEQNILNRPLSVMQERI

TNKNGLIFSSGQTWKEQRRFALMTLRNFGLGKKSLEQRMQEEAHYLVEAIREEKGKPFNP

HFSINNAVSNIICSVTFGERFEYHDSRFQEMLRLLDEVMYLETTMISQLYNIFPWIMKYI

PGSHQTVFRNWEKLKLFVSSMIDDHRKDWNPEEPRDFIDAFLKEMSKYPEKTTSFNEENL

ICSTLDLFFAGTETTSTTLRWALLYMALYPEVQEKVQAEIDRVIGQKRAASLADRESMPY

TNAVIHEVQRMGNIIPLNVPREVAMDTTLNGFHLPKGTMVLTNLTALHRDPKEWATPDVF

NPEHFLENGQFKKRESFLPFSMGKRACLGEQLARSELFIFFTSLMQKFTFKPPTNEKLSL

KFRNGLTLSPVTHRICAVPRE

>EHB10399.1

MLAAVGSLVAALWGALHLRTVLLGAVAFLFFADFLKRRRPKNYPPGPWRLPFLGNFFYLD

VEKSQMAVKRHVKKYGNLVSLEVADISAVLVTGLPLIKEVLTHTDQNFMNRPMTPTRDHV

FGKKGLIMSNGQIWKEQRRFTLTTLRNFGLGKKSLEERIQEEAYHLAEVIAEEKGQPFNP

HFKINNAVSNIICSIIFGERFDYQDAEFQELLRLLDEVTHLEGSPVGQLYNFFPRIMEFL

PGPHHTLFRDWDKLKSFVSRVIENHKRDWNPNEPRDFIDAYLKEMAQHPDKTISSFDEEN

LILSTLDLFFAGTETTSTTLRWGLLYMALYPEVQEKVHAEIDRVVGQGQQPSLANRESMP

YTNAVIHEVLRMGNIIPLNVPREVSVDTALGGYHLPKGTMIVTNLTALHRDPTEWATPDT

FNPEHFLENGQFKKRESFLPFSLGKRGCLGEQLARSELFIFFTSLLQKFTFKPPKNEKLS

LKIRVGLTLSPESYRICAVPRA

>395840734

MAGWKLGPSILETQLERSLRANQKGPGLRQSFNMWLKWGTQTRPVTSGHRRARVIPDLLL

AMGFLQAALWAVLHPQTLLLGAVTFLFLADFLKSRRRKNYPPGPWRLPFVGNFFFLFLHS

NYLTIFQIVKKYGNIVSLEFGSISSVVITGLPLIKEALIHMDQNFVNRPIFPMQERVLQG

NGLVLSKGQVWKEQRRFALTALRNFGLGRKSLEERIQEEACHLVEAMKEENGQPFDPHFK

INNAVSNIICSITFGKRFEYQDGEFQELLRLLDEIIYMETSFANQLYNFFPWIMKFLPGP

HQTVFRNWEKLKLFVSHIVEKHKRDWNPDETRDFIDAYLKEIAKNASNATSSFHEDNLIG

CTLDLFFAGTETTSTTLRWGLLYMALYPDIQETAQVPIDRVIGQEQLPSVAARESLPYTN

AVIHEVQRMGNIIPLNVPREVAADTTLAGYHLPKGTMVFTNLTALHRDPAEWARLDTFNP

EHFLENGQFKKREAFLPFSIGKRACPGEQLARTELFIFFTCLMQKFTFRPPNNEKLSPKF

RMGVTLSPVKHHLCAVPRHDIVKKGKEKGKWEDTYSEATDDCADMSGQNESDPEEK

>AAH91149.1

MLATAGSLVATIWAALHLRTLLVAALTFLLLADYFKTRRPKNYPPGPWGLPFVGNIFQLD

FGQPHLSIQPFVKKYGNIFSLNLGDITSVVITGLPLIKETFTHIEQNILNRPLSVMQERI

TNKNGLIFSSGQTWKEQRRFALMTLRNFGLGKKSLEQRMQEEAHYLVEAIREEKGKPFNP

HFSINNAVSNIICSVTFGERFEYHDSRFQEMLRLLDEVMYLETTMISQLYNIFPWIMKYI

PGSHQTVFRNWEKLKLFVSSMIDDHRKDWNPEEPRDFIDAFLKEMSKYPEKTTSFNEENL

ICSTLDLFFAGTETTSTTLRWALLYMALYAEVQEKVQAEIDRVIGQKRAASLADRESMPY

TNAVIHEVQRMGNIIPLNVPREVAMDTTLNGFHLPKGTMVLTNLTALHRDPKEWATPDVF

NPEHFLENGQFKKRESFLPFSMGKRACLGEQLARSELFIFFTSLMQKFTFKPPTNEKLSL

KFRNGLTLSPVTHRICAVPRE

>344278840

MLALLGSLASALWAGFHLRPLLLGVVVFLFFVGFLKRRRPKNYPPRPRSLPFLGNFFLLG

FEKPHLVLQKFVKKYGNLLSLEFGDIHAVIVSGLPLIKEVLANQDQNFVSRPKTPIRERV

FKNHGLIMSNGQEWKEQRRFALMTLRNFGLGKKRLEERIQEEAHHLVEAIEEEKGQPFDP

HFKINNAVSNIICSITFGERFEYQDGQFQELLKLLDEVTYLETTKCCQLYNIFPCVMKFL

PGLHQTLFSDWEKLKIFVSSMIEKHKRDWNPDKPRDFIDAYLKEMTKHTDNAISSFHEEN

LVCSTLDLFFAGTETTSTTLRWALLYLAIYPEIQEKVQTEIDRVIGQSRQPSLNDREHMP

YTNAVIHEVQRMGNILPVNVPREVTFDTTMAGYQLPKGTMILTNLTALHRDPAEWATPET

FNPEHFLENGQFKKREDFLPFSIGKRVCIGEQLARSELFIFITSLVQKFTFRPPANEKLS

LKFRMGVTISPVSHRLCAVPRA

>395530530

MQLSLGNMIPSLWEGTPLQSFLLLSAIILILIHYVQSRRRHLNYPPGPPSLPILGNFLQM

DFEKPQVTMQKFVQKYGNIVSMHVGSQIFVIVTGLPLIKEVLVNQGDVFIDRPQLPIHLH

IFKSLGLIMSNGQLWKQQRRFALMTLRNFGLGKKTLEDRIRVEAKYLIEAMREEKGQPFD

PHFVINNAVSNIICSVTFGHRFEYHDSKYRELLKTVDYVMKLLPRWECQLFNIIPSVMKF

LPGPHQKVFRESKKLESFVEDVIKQHKENLNPEEPQDFIDAYLIELSKDNIHSSFNEKNL

IYCTMDLFFAGTETTSTTLRWALLYMALYPEIQDKIQAEIDRVIGQTRQPTIADKENLPY

TNAAVHEVQRMGDIIPFNAPRMSVVDTTVAGYHVPKGAMMMTNLTALHRDPKEWATPETF

NPEHFLENGQFKKKESFVPFSIGKRACLGEQLARTELFLFFTSLLQKFTFQAPPNTQLSL

DFQISMAVSPLPYKICAIPREI

>O54750.2

MLAATGSLLATIWAALHPRTLLVAAVTFLLLADYFKNRRPKNYPPGPWGLPFVGNIFQLD

FGQPHLSIQPLVKKYGNIFSLNLGDITSVVITGLPLIKEALTQMEQNIMNRPLSVMQERI

SNKNGLIFSSGQIWKEQRRFALMTLRNFGLGKKSLEERMQEEASHLVEAIREEEGKPFNP

HFSINNAVSNIICSVTFGERFDYHDSRFQEMLRLLDEVMYLETTMISQLYNIFPWIMKYI

PGSHQKVFRNWEKLKLFVSCMIDDHRKDWNPDEPRDFIDAFLKEMTKYPEKTTSFNEENL

ICSTLDLFFAGTETTSTTLRWALLYMALYPEVQEKVQAEIDRVIGQKRAARLADRESMPY

TNAVIHEVQRMGNIIPLNVPREVAMDTNLNGFHLPKGTMVLTNLTALHRDPKEWATPDVF

NPEHFLENGQFKKRESFLPFSMGKRACLGEQLARSELFIFFTSLMQKFTFNPPINEKLSP

KFRNGLTLSPVSHRICAVPRQ

>334321654

MQFPSLWEGASLKSLLLFSAVFLMLASYLQRRRRHPNYPPGPFQLPFLGNFFHMDHKNPH

MAFYQLAKKYGNIFSLELVGCPVIVVTGLSLIKEVLVNQGQVFVDRPHTPLRSYVFKKLG

LIMSNGQEWKDQRRFTLMTLRNFGLGKRTLELRIQEEAMYLTEAIREEKGQPFDPHFQIN

NAVSNIICSVTFGNRFEYHDSQFRELLKILDEAMMLQTKWECQFFEMFPRIMKFLPGAHK

KLFREWKKLESFVLDIIRQHKENPNSEEAQDFIEAYLTELSKGNISPSFSEDNLVSCTLD

LFLAGTETTSTTLRWALLYMALCPEIQGKIQAEIDRVVGQSRLPTMADRENMPYTNAAVH

EIQRMGNIVPFNVPRMSTVDTTVAGYHVPKGTLLVTNLTALHRDPKEWATPDAFNPEHFL

EDGQFKKRESFLPFSAGKRVCLGEQLARTELFIFFTCLLQRFTFQAPPDTQLTLDFRIGL

TISPAPYKICAIPRETQSDLDLAIRVLASVSRRFSPPCVPHKHPPASLTRTDSKAFKA

>DAA31259.1

MLEALGSLVAALWTTLRPGIVLLGAFVFLLFADFLKRQHPKNYPPGPLRLPFIGNFFHLD

LGKGILVPQQVVKKYGNIIRLDFGVIHFIVITGLPYIKEALVNQEQNFVNRPMIPLQKHI

FNNKGLVRSNGQVWKEQRRFTLTTLRNFGLGRKSLEERIQEEVTYLIQAIGEENGQPFDP

HFIINNAVSNIICSITFGERFDYKDDQFQELLRLLDEILCIQASVCCQLYNAFPRIMNFL

PGSHHTLFRKWEKLKMFVANVIENHRKDWNPAEARDFIDAYLQEIEKHKGNATSSFDDEN

LICSTLDLFLAGTETTSTTLRWGLLFMALNPEIQEKVQAEIDRVLGQSQKVSTASRESMP

YTNAVIHEVQRMGNIVPMNVPREVTVDTVLAGYHLVKGTMVLTNLTALHRDPAEWATPDT

FNPEHFLENGQFKKRESFLPFSIGKRMCLGEQLARTELFIFFTSLLQKFTFRPPENEKLS

LKFRESLTSSPASYRLCAIPRA

>O54749.1

MIMFLSSLVTTFWEALHLKTLVLAVVTFLFLINILRSRHPKNYPPGPWRLPFVGNFFQID

TKQTHLVLQQFVKKYGNVFSLELGQSPVVVVSGLPLIKEMFTHLDQNFVNRFMTPVRERI

TGKNGLVVSNGQTWKEQRRLALMALRNFGLGKKSLEERIQEETHHLVEAIREEGGQPFNP

HLKLINAVSNIICSVTFGERFDYEDCQFQELLQLLDETMHLMGSSAGQLYNGFPCIMKYL

PGPHQKIFRNWGKLKLFVSHIVKKHEKDWNPDEPRDFIDAFLIEMQKDPDRTTSFNEENL

ISTTLDLFLGGTETTSSTLRWALLYMSSYPEIQENVQAEIDRVIGHKRQVSLSDRESMPY

TNAVIHEVQRMGNIVPLNSSREVTVDTKFNGFHLPKGTMILTNLTALHRDPKEWATPEVF

NPEHFLENGQFKKRESFLPFSMGKRACLGEQLAKSELFIFFSALMQKFTFKPPINEKLSL

KFRMGLILSPASYRICAIPRV

>AAI10021.1

MLEALGSLVAALWTTLRPGIVLLGAFVFLLFADFLKRQHPKNYPPGPLRLPFIGNFFHLD

LGKGILVPQQVVKKYGNIIRLNFGVIHFIVITGLPYIKEALVNQEQNFVNRPMIPLQKHI

FNNKGLVRSNGQVWKEQRRFTLTTLRNFGLGRKSLEERIQEEVTYLIQAIGEENGQPFDP

HFIINNAVSNIICSITFGERFDYKDDQFQELLRLLDEILCIQASVCCQLYNAFPRIMNFL

PGSHHTLFRKWEKLKMFVANVIENHRKDWNPAEARDFIDAYLQEIEKHKGNATSSFDDEN

LICSTLDLFLAGTETTSTTLRWGLLFMALNPEIQEKVQAEIDRVLGQSQKVSTASRESMP

YTNAVIHEVQRMGNIVPMNVPREVTVDTVLAGYHLVKGTMVLTNLTALHRDPAEWATPDT

FNPEHFLENGQFKKRETFLPFSIGKRMCLGEQLARTELFIFFTSLLQKFTFRPPENEKLS

LKFRESLTSSPASYRLCAIPRA

>BAC28391.1

MLAATGSLLATIWAALHPRTLLVAAVTFLLLADYFKNRRPKNYPPGPWGLPFVGNIFQLD

FGQPHLSIQPLVKKYGNIFSLNLGDITSVVITGLPLIKEALTQMEQNIMNRPLSVMQERI

SNKNGLIFSSGQIWKVQRRFALMTLRNFGLGKKSLEERMQEEASHLVEAIREEEGKPFNP

HFSINNAVSNIICSVTFGERFDYHDSRFQEMLRLLDEVMYLETTMISQLYNIFPWIMKYI

PGSHQKVFRNWEKLKLFVSCMIDDHRKDWNPDEPRDFIDAFLKEMTKYPEKTTSFNEENL

VCSTLDLFFAGTETTSTTLRWALLYMALYPEVQEKVQAEIDRVIGQKRAARLADRESMPY

TNAVIHEVQRMGNIIPLNVPREVAMDTNLNGFHLPKGTMVLTNLTALHRDPKEWATPDVF

NPEHFLENGQFKKRESFLPFSMGKRACLGEQLARSELFIFFTSLMQKFTFNPPINEKLSP

KFRNGLTLSPVSHRICAVPRQ

>AAB87636.1

MLAATGSLLATIWAALHPRTLLVAAVTFLLLADYFKNRRPKNYPPGPWGLPFVGNIFQLD

FGQPHLSIQPLVKKYGNIFSLNLGDITSVVITGLPLIKEALTQMEQNIMNRPLSVMQERI

SNKNGLIFSSGQIWKEQRRFALMTLRNFGLGKKSLEERMQEEASHLVEAIREEEGKPFNP

HFSINNAVSNIICSVTFGERFDYHDSRFQEMLRLLDEVMYLETTMISQLYNIFPWIMKYI

PGSHQKVFRNWEKLKLFVSCMIDDHRKDWNPDEPRDFIDAFLKEMTKYPEKTTSFNEENL

ICSTLDLFFAGTETTSTTLRWALLYMALYPEVQEKVQAEIDRVIGQKRAARLADRESMPY

TNAVIHEVRRMGNIIPLNVPREVAMDTNLNGFHLPKGTMVLTNLTALHRDPKEWATPDVF

NPEHFLENGQFKKRESFLPFSMGKRACLGEQLARSELFIFFTSLMQKFTFNPPINEKLSP

KFRNGLTLSPVSHRICAVPRQ

>354486752

MLAAAGSLVAAFWATFHLRTLILAVVTFLFLADYLKNRRPKNYPPGPRGLPFVGSIFQLD

FDKAHLSIQPFVKKYGNIFSLNLGDITSVVITGLPLIKEVFTHMEENILTRPVTLIRKRI

SNNNGLVFSSGQTWKEQRRFALMTLRNFGLGKKSLEQRMQEEARYLVEAIKVEGGQPFNP

HFSINNAVSNIICSVTFGERFEYHDSQFQEMLRLLDEATCLEASLMCQLYNIFPWVMKYL

PGSHQTVFRNWGKLKLFVSSVIDNHRKDWNPDEPRDFIDAFLKEMAKYPDKTTTSFNEEN

LICSTLDLFFAGTETTSTTLRWALLYMALYPEVQEKVQAEIDRVIGQKRQPSLADRESMP

YTNAVVHEVQRMGNIIPLNVPREVAKDTDLDGFHLTKGTMLLTNLTALHTDPKEWATPET

FNPEHFLENGQFKKRESFLPFSMGKRACLGEQLARSELFIFFTALMQKFTFKPPANEKLS

LKFRMGLTLSPVSHNLCAVPRL

>P52786.2

MVAALSSLAAALGAGLHPKTLLLGAVAFLFFAYFLKTRRPKNYPPGPWRLPFLGNLFTLD

MEKSHLQLQQFVKKYGNLFCLDLAGKSIVIVTGLPLIKEVLVHMDQNFINRPVPPIRERS

FKKNGLIMSSGQLWKEQRRFALMTLRNFGLGKKSLEERIQEEARHLTEAMEKEGGQPFDA

HFKINNAVSNIICSITFGERFEYHDGQFQELLKLFDEVMYLEASMLCQLYNIFPWIMKFL

PGAHQTLFSNWKKLELFVSRMLENHKKDWNPAETRDFIDAYLKEMSKYPGSATSSFNEEN

LICSTLDLFLAGTETTSDMRWGLLFMALYPEIQEKVHAEIDSVIGQWQQPSMASRESLPY

TNAVIHEVQRMGNILPLNVPREVTVDTTLAGYHLPKGTVVLTNLTALHKDPEEWATPDTF

NPEHFLENGQFKKKEAFIPFSIGKRACLGEQLAKSELFIFFTSLMQKFTFKPPSDEKLTL

NFRMGITLSPVKHRICAIPRA

>BAE28954.1

MIMFLSSLVTTFWEALHLKTLVLAVVTFLFLINILRSRHPKNYPPGPWRLPFVGNFFQID

TKQTHLVLQQFVKKYGNVFSLELGQSPVVVVSGLPLIKEMFTHLDQNFVNRFMTPVRERI

TGKNGLVVSNGQTWKERRRLALMALRNFGLGKKSLEERIQEETHHLVEAIREEGGQPFNP

HLKLINAVSNIICSVTFGERFDYEDCQFQELLQLLDETMHLMGSSAGQLYNGFPCIMKYL

PGPHQKIFRNWGKLKLFVSHIVKKHEKDWNPDEPRDFIDAFLIEMQKDPDRTTSFNEENL

ISTTLDLFLGGTETTSSTLRWALLYMSSYPEIQENVQAEIDRVIGHKRQVSLSDRESMPY

TNAVIHEVQRMGNIVPLNSSREVTVDTKFNGFHLPKGTMILTNLTALHRDPKEWATPEVF

NPEHFLENGQFKKRESFLPFSMGKRACLGEQLAKSELFIFFSALMQKFTFKPPINEKLSL

KFRMGLILSPASYRICAIPRV

>AAK59868.1

MLAAAGSLVAAIWAALHLRILLLSAVTFLFLADFLKNRRPKNYPPGPMRLPFVGCLFHLD

PKQPHLSLQQFVKKYGNVLSLDFANIPSVVITGMPLIKEIFTQMEHNFMNRPVTLLRKHL

FNKNGLIFSSDQTWKEQRRFALMTLRNFGLGKRSLEERIQEEAHYLVEAIRDEGGQPFDP

HFNISNAVSNIICSITFGERFDYHDSQFQEMLRLLDEAMCLESSMMCQLYNIFPRILQYL

PGSHQTLFRNWRKLQLFVSDIVNNHRRDWDPDEPRDFIDAFLTEMTKYPDKTTTSFNEEN

LICSTLDLFFAGTETTSTTLRWALLCMALYPEVQEKMQAEIDRVIGQARQPNLADRDSMP

YTNAVIHEVQRMGNIIPFNVPREVAVDTQLAGFNLPKGIMVLSNLTALHRDPSEWTTPDT

FNPEHFLENGQFKKRESFLPFSMGKRACLGEQLARSELFIFITTLFQKFTFKPPVNEKLS

LQFRMAATVSPVSHRLCAIPRL

>DAA31248.1

MLEALGSLAAALWAALRPGTVLLGAVVFLFLDDFLKRRRPKNYPPGPPPLPFVGNFFQLD

FDKAHLSLQRFVKKYGNVFSVDFGIFRSVLITGLPLIKEALVHQDQNFANRPLIPIEKRI

FNNKGLIMSNGHVWKEQRRFALTTLRNFGLGKKSLEERIQEEAAYLIQEIGEENGQPFDP

HFTINNAVSNIICSITFGERFDYQDDQFQELLRLFDEMMHLRTSTCCQLYNIFPRIMSFL

PGPQHALFSKWEKLKMFIAGVVENHKRDWNPAEARDFIDAYLQEIEKHKGNATSCFHEEN

LIYNTLDLFFAGTETTSTTLQWGLLYMALYPEIQEKVQAEIDRVLGQSQKPSMAARESMP

YTNAVIHEVLRMGNILPLNVPREVTVDTVLAGYRLPKGTMVTTNLTALHRDPAEWATPDT

FNPEHFLENGQFKKRESFLPFSIGKRMCLGEQLARTELFIFFTSLLQKFTFRPPENEKLS

LKFRMSMTLSPLSHRLCAIPRA

>395530526

MQLSLGNMLPSFWEGVPLQNLLLLSAIVLILAHYLQSRRQHPNYPPNPPRLPILGNFFQM

DLKNPQANILKLAKKYGNVFCLYLGNLNLMVVTGLPLIKEVLLNQDQVFIDRPQIPVHFH

VFKRFGLFMSNGPAWKHQRRFALMTLRNFGLGKKTLEDRIREEAMYLTEAIREEKGQPFD

PHFQLNNAVSNIICSITFGHRFEYHDSQFRELLKSLDDVTVLEGRWECQLFNIFPWIMKF

LPGGHQKFFGEWKKLESFVEQVIKQHKENLNPEEPQDYIDAYLKELSKDNIHSSFNEKNL

VFCTLDLFFAGTETTSTTLRWALLYMALYPEIQGKIQEEIDRVIGQSRQPTMADKENMPY

TNAAVHEVQRMGDIIPFNGPKVATVDTTVAGYYVPKGTVLTTNLTALHRDPKEWATPETF

NPEHFLENGQFKKRESFLPFSMGKRVCLGEQLARTELFLFFTSLLQKFTFQAPPNTQLNL

DSRTGLTISPAPYKICAIPREM

>338721666

MCIFSCDNFTVFQLVKKYGNIFSLDFGDVPSVVITGLPLIKEALVHQDPNFVNRPVIPLR

ERVFKQNGLIMSNGQVWKEQRRFALTTLRNFGLGKKNLEERIQEEAHHLIQAIEEENGQP

FNPHFKINNAVSNIICSITFGERFEYEDGQFRELLRMLDEVTHLEASLWCQLYNAFPRIM

NFLPGPHQTLFSNWDKLKLFVARVIENHKRDWNPAETRDFIDAYLKEMEKYKGDVTSSFH

EENLIYSTLDLFFAGTETTSTTLRWGLLYLALYPEVQEKVHAEINSVIGPGQQPSTAART

SMPYTNAVIHEVQRMGNIIPLNVPREVVVDTSLAGYHLPKGTMILTNLTALHRDPAEWAT

PNTFNPEHFLENGQFKKRETFLPFSAGKRMCLGEQLAKSELFIFFTSLLQRFTFRPPSDE

QLSLRFRMGLTIAPAGHRICAVPRQEGVAARGESEQEESSEGSGPFSEVREQDEGGSEER

S

>348519226

MLALRLAFQTRFRWTRVSVQPKSSIAWCVPPWHLTFLDWGKVLGGPEKKKRLAGQVYTYP

TSINLEVFRSPGPPTLLPRHLCEPWGVRAKGKQLEEDRLSYPFIIRSASYSSQCSPTHIT

MFVSIILLWICIFFIILQLKSKRPKNFPPGPPVFPILGTILQLSLENPLKDFERLRKSYG

DVYSIYIGQKPAVVINGLKAIKEAMVTKAADFAGRPQDLFINDVTKNKGVIMVDYGSSWR

EHRRFALMTLRNFGLGKNSMEDRIHEEIKYLVSTLEESVGKTMSPRVMFHNAASNIICQV

LFARRYEYDDVLIKQIVQCFTENTKMANGPWAMLYDSFPIIRNLPLPFQKAFQNMETCRN

ITIDLINEHKTTRVPGEPRDFIDCYLDELEKRGNDRSSFSDDQLIMYSLDLYVAGTDTTS

NTLLTGFLYLMNYPHIQEKCQQEIDQVLQGKDRASFEDRHKMPYTQAVIHEIQRIANTVP

LSVFHCTNRDTELMGYSIPKGTAIIENLTSVLNEEGQWKFPHEFNPENFLDVKGEFVKPE

AFMPFSAGPRTCLGEGLARMELFLMMVTLLHKFKFIWPEDAGQPDFTPVFGITMNPKPYD

MKVQLRMPQ

>327270884

MPLPFVGNLLHLDAEKPHFSTQKLADIYGNVFSLQLGNRHFVFVNGLEIVKEVLIHHGEN

FLDRPKFPIISDHAKTLGLVMSNGLPWKQQRRFALSTLRNFGLGKRSLEERIQEESRFLA

GAIENEKGQPFDPHYQINNAVSNVICSITFGNRFDYHDSQFQKLLHLLNETGIIQRSIWA

QLYNIFPALMKQLPGPHQTIFKNWEQLKYFVRTIIKKHQENRNPLETRDFIDAYLNEMTK

ENVSSSFHMENLLQSALDLFIAGTETTSTTLRWALLYMAIYPEIQERVQSEIDSVIGQSR

PPAMTDRDNLPYTNAVIHEIQRISNILPLNVPRLTTNNTEIAGFHLPKGTILICNLTSVL

FDKDEWDTPKKFNPNHFLSNGQFRIREAFVPFSAGKRACLGERLARMELFLFFTALIQKF

SFQAPKGVELSLDFKMSLTLSPNQYHICAVSR

>392340559

MLATVGSLLAKIWSAINFWTLLLTLLTFLLLADYLKNRRPNNYPPGPWRLPFVGNLFQFD

LNISHLHLRIQQFVKKYGNLISLDFGNISVVVITGLPLIKEALINNEQNFLKRPIVPSRY

RVFKDNGIFFANVHKWKEQRRFALTMLKNFGLGKKSLEQCIQEEAHHLVEVIGEEKGQPF

DPHFRINNAVSNIICSITFGERFEYDDSQFQELLKLADEVILLETSPWCQLYNVFPLIFK

YLPGPHQTVFKNWEKLKSIVANMIDRHRKDWNPDEPRDFVDAFLTEMTKYPDKTTTSFNE

ENLIATTLDLFFAGTETTSTTLRWALLYITLNPEVQEKVHSEIDRVIGHGRLPSTDDQDA

MPYTNAVIHEVLRMGNIIPLNVPREVTADSTLAGFHLPKGKMILTNLTALHRDPKEWATP

DTFNPEHFLENGQFKKRDSFLPFSVGKRACPGEKLAKSELFIFFTALMQNFTFKAPTNEK

LSLKLRKGLSLYPVSYRICAVPR

>154147742

MLATEDTFETAIWALLHFKTLVLAAVTFMLLAHYLKTQRPKNYPPGPWRLPFVGNLFQLD

LEQPHVVIQQFVKKYGNLTSLDFGNIPSVIITGLPLIKEVFTNMEQNFLNRPITPIRKRV

FNNNGLIISNGQTWKEQRRFALMTLKNFGLGKKSLEQRIQKEADCLVEAIGEEKGQPFDP

HFKINSAVSNIICTFAFGERFEYEDSHFHELLRLLDEATCLEASMMCQLYNVFPSIIQYV

PGSHQTVLRNWEKLKLFIYQMMESHRKDWNPDEPRDFIDAFLTEMSKYGDKTTTSFNEEN

LINSTLDLFFAGSETTTNTLRWALLYITTNPEVQEKVHSEIDRVIGHGRQPSTDDRDSMP

YTNAVIHEVLRMGNILPMNVPREVTADSTLAGFHLPKGTMVLTNLTALHRDPKEWATPNT

FNPEHFLENGQFKKRESFLPFSLGKRACPGEQLARSELFIFFTALMQKFTFKPPINEKLS

LKFRNGLTLSPVSHCICAVPRH

>157278596

MFATIGCLVSNICSEIHLWTLLLAALTLLLLVDYIKNRHPKNYPPGPWRLPFVGNLFQFD

LDVSHLHLGIQPFVKKYGNLISLDFGTIPSVIISGEPLIKEALTCMGQNFLKRPILAARQ

HIFKNNGIISSSGQTWKEQRRFTLMILKNFGLGKKSLEQRIQDEAHHLVEAIAEEKGRPF

DPHFMINNAVSNIICSITIGERFEYEDNQFQELLKLADETLCLEASKVLMLYNVFPSIFK

YLPGPHQKLFSNWEKLKLFFSHVMDSHRKDWNPSAPRDFIDAFLTEMAKYSDKTTTSFNE

ENLICTTLDLFFAGTETTSTALRWALLYITVNPEVQEKVHSEIDRVIGQGRHPTIDDRDS

MPYTNAVIHEVLRMGNIIPLNVPREVEADITLAGFHLPKGTMVLTNLTALHRDPKEWATP

DVFNPEHFLENGQFKKRESFLPFSVGKRGCPGEQLARSELFTFFTALMQKFTFKPPINEK

LSLNFKMGVALSPVSYCICAVPR

>62649347

MLATVGSLLAKIWSAINFWTLLLTLLTFLLLADYLKNRRPNNYPPGPWRLPFVGNLFQFD

LNISHLHLRIQQFVKKYGNLISLDFGNISVVVITGLPLIKEALINNEQNFLKRPIVPSRY

RVFKDNGIFFANVHKWKEQRRFALTMLKNFGLGKKSLEQCIQEEAHHLVEVIGEEKGQPF

DPHFRINNAVSNIICSITFGERFEYDDSQFQELLKLADEVICSEASMTSVLYNVFPLIFK

YLPGPHQTVFKNWEKLKSIVANMIDRHRKDWNPDEPRDFVDAFLTEMTKYPDKTTTSFNE

ENLIATTLDLFFAGTETTSTTLRWALLYITLNPEVQEKVHSEIDRVIGHGRLPSTDDQDA

MPYTNAVIHEVLRMGNIIPLNVPREVTADSTLAGFHLPKGKMILTNLTALHRDPKEWATP

DTFNPEHFLENGQFKKRDSFLPFSVGKRACPGEKLAKSELFIFFTALMQNFTFKAPTNEK

LSLKLRKGLSLYPVSYRICAVPR

>ABO21082.1

MWLYDFLLSFDLKAILLFIFIFVLIVDFLKYKNPPDYPPGPLALPFVGNIFSLDRKHPYM

YFTKLADIYGNVFSVRLGHDKMVFVSGYKMVKEAIVAQAENFVDRPYSSIVDRLFSGTAG

GLFASNGEIWKRQRRFALSTLRSFGLGKNSLEQSICEEIRHLQEEIEKEKGEPFNPAGLF

NNAVSNIICRLVMGKRFEYNDHKFQMMLKYQSEVFFLQGSIWGLLYESFPAVMKHLPGPH

NKIFSHFIALQDFIIEEVKIHKKDLDNNNPRDYIDTFIIEMEKHKETDLGFTETNLALCS

LDLFLAGTETTSTTLLWALVYLIKHPEVQDKVHAEIDRVIGQNRLPSMTDRANLPYTDAV

IHEIQRVGNIVPLGALRMTTKDTTLGGYFIPKGTSLMSNLTTVLFDKTEWETPDSFNPGH

FLDAEGKFVKKEAFLPFSAGKRVCLGEGLAKMELFLFLVGLLQKFSFSVPDGVELSTEGV

TGITRVPHPYKVYAKAR

>EGW10540.1

MLAAAGSLVAAIWAALHLRTLLLAAVTFLFLADYLKSRRPKNYPPGPRRLPFVGNILHMD

SGRPHLTIQQLVKKYGNVISLDFGIMSSVIISSMPLIKEAFTHLDDNFMSRPKFPIQKYV

FNENGLIFQTWKEQRRFTLMTLRNFGLGKKSLEQRIQEEAGHLVEAIAEEGGQPFDPHFK

INNAVSNIICSITFGERFEYHDRQFQELLRLLDKAMYSGSSVMVHFYNIFPWIMKYLPGQ

HQTVIATWRKLKSYVSDVIDNHRKDWDPNEPRDFIDAFLKEMTKYPDNTTSFKEENLICS

TLDLFLAGTETTSTTLRWALLYMALYPEVQEKVQAEIDRVIGQEKQPSLADRDSMPYTNA

VVHEVQRMGNIIPLNVPREVTVDTTLAGFHLPKGSTLHINLTSLHMDPKEWATPDIFNPE

HFLENGQFKKRESFLPFSMGKRACLGEQLARSELFIFFTALVQKFTFKPPVNEKLSLKSR

MGITISPVSHHLCAVPRL

>AAI68751.1

MLSTEDTLEAAIRALLHFRTLLLAAVTFLFLANYLKTRRPKNYPPGPWRLPFVGNLFQLD

VKQPHVVIQKFVKKYGNLTSLDFGTIPSVVITGLPLIKEAFTNTEQNFLNRPVTPLRKRV

FNNNGLIMSNGQTWKEQRRFTMTTLKNFGLGKRSLEQRIQEEANYLVEAIGVDKGQPFDP

HFKINSAVSNIICSITFGERFEYEDSLFQELLRLLDEASCLESSMMCQLYNVFPTIIKYL

PGSHQTVLRNWEKLKLFISCMMDSHQKDWNPDEPRDFIDAFLTEMAKYRDKTTTSFNKEN

LIYSTLDLFFAGSETTSNILRWSLLYITTNPEVQEKVHSEIDRVIGHRRQPSTGDRDAMP

YTNAVIHEVLRMGNIIPLNVPREMTADSTLAGFHLPKGTTILTNLTGLHRDPKEWATPDT

FNPEHFLENGQFKKRDSFLPFSMGKRACPGEQLARTELFIFFTALMQNFTFKPPVNETLS

LKFRNGLTLAPVSHRICAVPRQ

>363736833

MLRFLWDSISLQMLFVFLLVFLLVSDYMKKRKPKDFPPGPFALPFLGNVQLMVAKDPVST

VQKLTEKHGDIFSMQVGSMSFVIVNGLQMIKEALVTQGENFMDRPEFPMNAEVFNKFGLL

SSNGHLWKQQRRFTLTTLRNFGLGKRSLEERIQEECRFLTDAFRDEQGNPFNPHLKINNA

VSNVICSITFGNRFEYHDEDFQNLLRLMDETVTLHGKIMSQLYTFFPSIVKYLPGSHQTV

IKNGKLMKDFVCNVISKHKEDLNPSESRDFIDSYLQEMAKPDSSDFCEDNLVSCTLDLFF

AGTETTSTTIRWALLFMAMYPEIQARVQAEIDAVIGQARQPSLEDRNNMPYTNAVIHEVQ

RKGNIIPFNALRLTVKDTVLAGFRVSKGTILIPNLSSVMYDKKEWETPHSFNPGHFLKDG

QFWKREAFMPFSIGKRACLGELLARAELFLFFTSLLQKFTFQAPPDTILDFKFTMGITLA

PRPYKICAVPR

>BAJ83935.1

MLRFLWESISLQMLLIFLIVFLLVADHMKHRKPKHFPPSPFSLPFVGHIHLLNFSNPQIT

VQKLTEKYGDIFSMQMGSTSFVFVNGLRMVKEVLVNQGENFIDRPEIPLDRDVFSKMGLI

SSSGNLWKQQRRFTLTTLRNFGLGKRTLEERIQEECRCLVDVFGDEQGKPFNPHFKINNA

VSNIICSITFGNRFDYHDEEFQRLLQLMDETVSLHGTIMSQLYNSFPSIIKFLPGSHQTI

FKNWRLLKSFVKEKISKHKEDWNPSESRDFIDSYLQEIAKDNGDGIFQEENLVACTLDLL

FAGTETTSTTIRWALLYMALYPEIQARVQAEIDTVIGQARQPALEDRGNMPYTNAVIHEV

QRKANIIPFNVPRLTVKDTVLDGFHIPKGTVLLTNLTSVLFDKNEFETPDTFNPGHFLKD

GQFWKRESFVPFSIGKRACLGEVLARSELFLFFTALLQKFTFQVPPDTTLSLQFKLGITI

APQPYKICAMPR

>358420877

METGRHISNSSRYTDRQTQRLPSIQASCQLHTPSIPGFRDICILNCGSSIFQFVKKYGNV

FSVDFGIFRSVLITGLPLIKEALVHQDQNFANRPLIPIEKRIFNNKGLIMSNGHVWKEQR

RFALTTLRNFGLGKKSLEERIQEEAAYLIQEIGEENGQPFDPHFTINNAVSNIICSITFG

ERFDYQDDQFQELLRLFDEMMHLRTSTCCQLYNIFPRIMSFLPGPQHALFSKWEKLKMFI

AGVVENHKRDWNPAEARDFIDAYLQEIEKHKGNATSCFHEENLIYNTLDLFFAGTETTST

TLRWGLLYMALYPEIQEKVQAEIDRVLGQSQKPSMAARESMPYTNAVIHEVLRMGNILPL

NVPREVTVDTVLAGYRLPKGTMVTTNLTALHRDPAEWATPDTFNPEHFLENGQFKKRESF

LPFSIGKRMCLGEQLARTELFIFFTSLLQKFTFRPPENEKLSLKFRMSMTLSPLSHRLCA

IPRA

>P51590.1

MLVTAGSLLGAIWTVLHLRILLLAAVTFLFLADFLKHRRPKNYPPGPWRLPLVGCLFHLD

PKQPHLSLQQFVKKYGNVLSLDFANIPSVVVTGMPLIKEIFTQMEHNFLNRPVTLLRKHL

FNKNGLIFSSGQTWKEQRRFALMTLRNFGLGKKSLEQRIQEEAYHLVEAIKDEGGLPFDP

HFNINKAVSNIICSVTFGERFEYHDSQFQEMLRLLDEAMCLESSMMCQLYNIFPRILQYL

PGSHQTLFSNWRKLKLFISDIIKNHRRDWDPDEPRDFIDAFLKEMAKYPDKTTTSFNEEN

LICSTLDLFFAGTETTSTTLRWALLCMALYPEVQEKMQAEIDRVIGQGRQPNLADRDSMP

YTNAVIHEVQRIGNIIPFNVPREVAVDTYLAGFNLPKGTMILTNLTALHRDPKEWATPDT

FNPEHFLENGQFKKRESFLPFSMGKRACLGEQLARSELFIFITSLIQKFTFKPPVNEKLS

LQFRMSVTISPVSHRLCAIPRL

>354486750

MLVTKGSLVAAIWAMLHPKTLLLAAFTFLLLINYLKTRRPKNCPPGPWRLPFVGNLFQLD

LEQLHLVIQQFVKKYGNLISLDYGNIPSVVITGLPLIKEAFTHMEQNFLKRPITPIRERV

FSNNGLIMSYGQTWKEQRRFALITLRNFGLGRKSLEECIQEEAFHLTNAIGEEEGQPFDP

HFKITNAVSNIICSITFGERFGYEDGQFQELLRLLDEATCLETSMMCLLYNVFPSIFKYL

PGSHQTVFRNWEKLKLFVSRMMDSHRKDWNPVEPRDFIDAYLTEMTKHPDKTTTSFNEEN

LICSTLDLFFAGTETTSTTLRWALLCMALYPEVQEKVQAEIDRVIGLRRQPSTADRESMP

YTNAVIHEVQRIGNIIPLNVPREVTTDSTLAGFHLPKGTMVLTNLTALHRDPKEWATPDV

FNPEHFLENGQFKKRESFLPFSIGKRACLGEQLARSELFIFFTALMQKFTFKPPVNEKLS

LKFRMGLTLNPVSHHICAVPRL

>395530528

MQLSLGNMIPSLWEGASLQNLLLLSAIVLMAYYLQSRRKHLNYPPGPPGLPILGNFFQMD

FKTPQITIQELCKKYGNIFCIYLGNLSFMMVSGLPLIKEILLNQGQVFIDRPQVPVHFHV

FKSFGLIVSNGQAWKQQRRFALMTLRNFGLGKKTLEDRIREEARYLIEAIREEKGQPFDP

HYEINNAVSNIICSITFGHRFEYHDSQFRELLKTLEDIMMLETKWQCQFFNLFPGIMKHL

PGTHQKLFRKWKKVESFVEYVIKQHKEDLNPEEPRDFIDAYLKELSKDNIHSSFTEKNLV

FCTLDLFIAGTETTSTTLRWGMLFMALYPEIQGKIQAEIDRVIGQSRQPTMADKENMPYT

NAAVHEVQRMGNILPFNVPRVAVVDTTVAGYHVPKGTIVMTNLTALHRDPKEWATPETFN

PEHFLENGQFQKRESFLPFSMGKRVCLGEQLARAELFLFFTSLLQKFTFQPPPNTQLSLD

FQYSTTISPLPYQICAIPREMSTKPGLKHEL

>334321652

MLCSLWEGSFLQTFLLLFLIFLILASYMQRRQRRPNYPPGPPQLPIIGNFFQMDFKRPQI

TMQELVRKYGNVFYMEMGSISFILVTGLPLIKEVLVNQGQVFIDRPQVPLNFYVLKSFGL

VMSNGQAWKDQRRFALMTLRNFGLGKRTLEHRIQEEVRYLTEAIREEKGQPFDPHFQINN

AVSNIICSITFGHRFEYHDPQFRELLKSVDEVIILETTWQCQLFNVLPRIMRFLPGAHQK

YFQEWKKLKSFVVRVIQQHKEDQNTGEARDFIDAYLKEMSQDGNISPSFNEHNLVWCTLD

LFLAGTETTSTTLRWALLYMALYPEIQGKIQSEIDRVIGQSRQPTMADRENMPYTNAAIH

EVQRMGNIVPFNVPRVAVVDTIVAGYYVPKGTLLVTNLNALHRDPKEWATPDTFNPEHFL

ENGQFKKKESFLPFSMGKRVCLGEQLARAELFIFFTCLLQRFTFQAPPDTQLSLDFRAGL

TISPAPYKICALPRETQPKVGL

>ABO21086.1

MWLYNFLLSFDLKAIFLFIFIFILIADFLKYKNPPDYPPGPLALPFAGNIFSVDRKHPYM

YFTKLAEIYGNVFSIRLGRDKLVFVSGYKMVKEAIVAQGENFVDRPYNPMTERLYSGTKD

GLFTTNGETWKRQRRFALSTLRSFGLGKNSLEQSICEEIRHLQEEIEKEKGEPFDPAGPF

NNAVSNIICRLVMGKRFEYNDHKFQMMLKYLSEVIYLQGSIWGLLYLAFPAVMKHLPGPH

NKTFSHFIALQDFLAEEVKIHKKDLDYNNPRDYIDTFIIEMEKHKETDLGFTETNLALCS

LDLFLAGTETTSTTLLWAWVYLIKHPEVQYKVHAEIDRVIGQNRLPSMADRANMPYTDAV

IHEIQRVGNIVPLNGLRMATKDTTLGGYFIPKGTTLMPNLTTVLFDKTEWETPDSFNPGH

FLDAEGKFVKREALLPFSAGKRVCLGKGLAKMELFLFLVGLLQKFSFSIPNGVELSTEGV

TGVTRVPHPYKVYAKAR

>395537420

MIPSLCEGVPLHNLLLLSAIVLILAHYLKRRRIRKLNYPPGPPGLPILGNFLQMDFQKPQ

VTMQKFVRKYGNILCMHVGSQTFITVTGLPMIKEVLVNQGDVFIDRPQIPLYLYILKSSG

LIMSNGQVWKQQRRFALMTLRNFGLGKKTLEDRIQVEAKYLFEAIREEKGQPFDPHYVIN

NAVSNIICSVTFGNRFEYHDSQFRELLKSMDAVMKLLVRWECQLFNIFPSVMKFLPGPRQ

KLLREYKKLESFVEDVIKQHKENLNPEEPQDFIDAYLKELSKDNIHSSFNEKNLVYCTLD

LFFAGTETTSTTLRWGLLYMALYPEIQGKIQAEIDRVIGQSRQPTMADKENMPYTNAAVH

EVQRMGDIVPFNVPRMAVVDTTVAGYHVPKGTMLSTNLTALHRDPKEWATPETFNPEHFL

ENGQFKKKESFLPFSIGKRACLGEQLARAELFLFFTSLLQKFTFQPPPNTQLSLDVQCGT

TMSPLPYKICAIPREM

>AAF69028.1

METILNVLGLGWIDSRSILIFLFVFLLLADVLKNRVPRNFPPGPWSFPLVGDLPRIEASK

IHLQFKEFAGKYGNVFSLRLFGGRIAIINSYKFMTEALVQRGEDFTDRPSIPLFEDVFGN

RGLVGSSGYPWKQQRRFALHTLRNFGLGKKTLERSIQQECQYLTEAFADQQGQPFNAQKL

INNAVSNIICCLVFGNRFEYSDKQFQTILQLLNETLYLEGTVWAQMYNTMPWLMRWLPGP

HQRIFSITNELRSFVKVRINEHRENLDPSSPRDYIDSFLIEMGEKEDKDSGFDLDNLCFC

VLDLFVAGTETTTTTLHWGLLYMICNPQIQERVQAEIDAVIGPSRPPSMSDRDNMPYTDA

VIHEIQRMGNIIPLNVARMANKDTTVDQYTIPKGTMNLATLDSVLHDESMWETPNTFNPE

HFLEKDGTFRKREAFLPFSAGKRVCLGEQLARMELFLFFTSLLQRFKFSPPPGEQPSLEY

KLGVTHCPKPYRLCAVSR

>395530524

MLPSLWEEASLQTLLLLSAIILILAHYLKSRRRHLNYPPSPPGLPILGNFFHINYNDPQA

NIQKLAKQYGNVFITRAGTLIFMMVTGLPMIKEVLLNQDEAFIDRPQFALLVHVFKSFGL

TLSNGQAWKQQRRFALMTLRNFGLGKKTLEDRIREEAMFLIEAIREEKGQPFDPHFQINN

AVSNIICSVTFGHRFEYHDSQFRELLKSLDDITVLETRWECRLFNIFPWIMKFLPGPHQT

FIKEWKKIESFVEQVIKQHKEDLNPGEPQDFIDAYLKELSKFGDNIHYSFSEKNLVFCTL

DLFFAGTETTSTTLRWALLYMALYPEIQGKIQEEIDRVIGQSRQPTMADKENMPYTNAAV

HEVQRMGDILPFNVPRMAAFDTTLAGYHVPKGTIVLINLTALHRDPKEWATPETFNPEHF

LENGQFKKRESFLPFSMGKRVCLGEQLARAELFIFFTSLLQKFTVQPPPNTQLSLDFRFG

LTISPVPYKICAIPREM

>AAI34878.1

MILQFIYENFDFKSWILLGFGLLLLVDLIKYWNPSSFPPGPRPLPFLGNVFTEIQDFRNI

NKLAQVYGTIFSLRLGSEKLIIVSGYKMAKEALVTQNDSFIDRPNVPLFHKVFKGLGTIL

SNGYLWRMHRKFAVSHLRNFGVGNESLEVKIQQECIFLCDAFKAEKGPVDPMVTLNSAIS

NIISSLIFGQRFDYHDEYYQRILRLDTECIQLAGSPRAQLYNACPWLFEYLPGPHQTIFS

NYKKIKDFLRGEIIKHREDWDPANPRDFIDNYLTEMERKKSDPEAGFNIEGLVVTCLDLI

EAGTETATTTLRWGLLFMIKFPEIQEKVQAEIDRVIGQSRQPCLNDRVNMPYTEAVIHEI

QRFGDVVPLGFPKRAVKDTQLGKYFIPKGTAITVNLSSVLHDPNEWETPDTFNPGHFLDE

IGQFRKRDAFMPFSAGKRACLGENLARQELFLYFTSLLQQFKISKCPGEEPSLEGEIWFT

YAPAPFHICVSSR

>AAK26114.1

MILHLIYDSFDFKSWIIFFVVFLIIAEMIKNRTPSNYPPGPWPLPFLGTVFTKMDFKNIN

KLAKVYGKVFSLRVGSEKMIIVSGYKMVKEALVTQNDSFVLRPPVPLFHKVYKGIGLTMS

NGYIWRSHRRFAASHLRTFGEGKKNLELGIQQECVYLCDAFKAEKEPFNPIFILHGAVSN

TVACLTFGQRFDYNDEWYQEILRLDNQCVQLAGSPRVQLYNAFPKLLDYLPGPHQKVFSN

YKKITQSLKDEIIKHREDWDPANPRDFIDNYLTEMEKKKSDPQAGFNIESLIISCLDIVE

AGTETGATTLRWGLLFMIKFPEIQKKVQAEIDRVIGQSRQPCLDDRVNMPYTEAVLHEIQ

RFGDVVPLGFPKQAAVDTKIGNYFIPKGTSITTNLSSVLHDPNEWETPDTFNPGHFLDKN

GQFRKRDAFLPFSAGKRACVGELLARNVLFLFFTSLLQQFTLSKCPGEEPSLEGEIWFTY

APAPFRISVSVR

>AAH45948.1

MILHLIYDSFDFKSWIIFFVVFLIIAEMIKNRTPSNYPPGPWPLPFLGTVFTKMDFKNIN

KLAKVYGKVFSLRVGSEKMIIVSGYKMVKEALVTQNDSFVLRPPVPLFHKVYKGIGLTMS

NGYIWRSHRRFAASHLRTFGEGKKNLELGIQQECVCLCDAFKAEKEPFNPIFILHGAVSN

TVACLTFGQRFDYNDEWYQEILRLDNQCVQLAGSPRVQLYNAFPKLLDYLPGPHQKVFSN

YKKITQSLKDEIIKHREDWDPANPRDFIDNYLTEMEKKKSDPQAGFNIESLIISCLDIVE

AGTETGATTLRWGLLFMIKFPEIQKKVQAEIDRVIGQSRQPCLDDRVNMPYTEAVLHEIQ

RFGDVVPLGFPKQAAVDTKIGNYFIPKGTSITTNLSSVLHDPNEWETPDTFNPGHFLDKN

GQFRKRDAFLPFSAGKRACVGELLARNVLFLFFTSLLQQFTLSKCPGEEPSLEGEIWFTY

APAPFRISVSVR

>348504770

MVFQALFDCLNFTSWLLLAFVLLILFDLIRNRRPRNFPPGPRALPLLGNVFTGVDFKSMM

KLAEKYGPVFSVKRGSERMVYVAGYKMVKEALVNQLDSFIDRPVIPLFHIVYKGLGISLS

NGYLWRKQRKFANTHLRYFGEGQKALEKYIEVECSFLCEALKEEQGKPFNPHYTITNAVS

NVICCVVFGHRFEYTDPNYRKILELDNEAIVLSGSAQAQLLDAFPDLMKHLPGPHQTVQN

NYKELLAFVKKEIEKHQEDWNPEDPRDFIDTYLSEMEKKKMDPQAGFNLETLSLSSLDLL

EAGTETTATTLRWALVFMMTYPEIQEKVQAEIDSVIGQSRLPTLADRPNLPYTDAVVHEV

QRFANIAPLGFPRCANKDTTLGGYFIPKGTATTMILASVLLDKNEWETPDVFNPEHFLDS

QGQFRKRDAFLPFAAGKRMCLGEPLAKMELFLFFASLLQRFTFTPIPGEMPSMDGVLGFT

HTPQEFRMQAVPR

>348504422

MIFQAFFDCMNFTSWFLLGFILLILNDVIRNRRPRNFPPGPWAVPLLGNVFTGVDYKTMH

ELAQKYGPVFSLRRGSERMVFVAGYKMVKEALVSQLDSFVDRPIXXXXXXXKQRKFANTH

LRYFGEGQKSLEKYIEVECNFLCEAFKEEQGKPFNPHYIITNAVGNIISSVVFGHRFEYT

DPSFRKILELDNDAVVLAGSAQTQLYDAFPGLMKYLPGPHQTVLANYREIVAFLNREIEK

HQEEWNPDDPRDFTDAYLSEMEKKKEDPRAGFNIETLLVSTLDLIEAGTETASTTLRWAL

VFMMNYPEIQEKVQAEIDRVVGQSRLPTLADRPNLPYTDAVIHETQRVGNIVPLGFPKMA

SKDSTLGGYFIPKGTAITTILSSVLFDKNEWETPDVFNPEHFLDSEGKFRRRDAFLPFSA

GKRVCIGEPLAKMQLFLFFASLLQRFTFTPVPGEMPSLEGVMGFTYSPEEFRMLAVTR

>348504426

MKVSATLSSANVPPSNLTIQEKKAVASLSEDHDITILLADKGSFILLILSDVIRNRRPHN

FPPGPWALPLLGNIFTGVDYKTMHELAQKYGPVFSLRRGSERKVFVAGYKMVKEALVSQL

DSFFDRPIVPLIHVVFKGIGISMSNGYLWKKQRKFANTHLRYFGEGQKSLXXXXXXXXXX

XXGHRFEYTDPSFRKILELDNDAVVLAGSAQTQLYDAFPGLMKYLPGPHQTVLANYREIM

TFLNREIEKHQEEWNPDDPRDFLDTYLSEMEKKKEDPRAGFNIETLLVSTLDLIEAGTET

ASTTLRWALVFMMNYPEIQEKVQAEIDRVVGQSRLPTLADRPNLPYTDAVIHETQRVGNI

IPLGLPKMASKDSTLGGYFIPKGTAVTTVLSSVLFDKNEWETPDVFNPEHFLDSEGKFRR

RDAFLPFSAGKRVCIGEPLAKMELFLFLVSLLQRFTLTPVPGEMPSLEGVMGFIYSPEEF

RMLAVPR

>AAI62547.1

MDLWYLYEWIDIKSILIFLCVFLLLGDYIKNKAPKNFPPGPWSLPIIGDLHHIDNSKIHL

QFTKFAERYGNIFSFRLFGPRIVVLNGYNLVKEVYIKQGDNLADRPVLPLFYEIVGDKGL

ILSSGYKWKHQRRFALSTLRNFGLGKKSLEPSINVECGFLNEAISNEQGRPFDPRLLLNN

AVSNVICVLVFGNRFDYSDHHFQTLLKNISEAVYLEGSICNQLYNMFPWLMERLPGPHKT

IITLWRKVTDFVREKVNEHRVDYDPSNPRDYIDCFLTEMEKLKDDTAAGFDVENLCICSL

DLFVAGTETTSTTLYWGLLYMIKYPEIQAKVQEEIDAVVGGSRQPSVSDRDNMPYTNAVI

HEIQRMGNIAPINLARSTSEDTQIGNYSIPKGTMVTSNLTSVLFDESEWETPHSFNPGHF

LDAEGKFRRRDAFLPFSLGKRVCLGEQLARMELFLFFSSLLQRFTFSPPAGVEPSLDYKM

GGTHCPKPFKLCAVPR

>AAI62823.1

MILYSLYDCLDFKGWLFLTCFLLLLIDLIRNKNPANFPPGPWPLPILGNVFTDVNYKTVD

QLIEKYGNIFSVRIGSDKIVYVSGFKMVKDVLITQGENFTDRPVSPLFDTLYKGRGLSFN

NGYSWRKHQQFSMSHLKNFGEGRKTLEENNQRECYFLCGAFKEEQGRPFDPLVKINNAVA

NVIGFLVFGHRYEYSDVNFQKRLQMSAESVFLTGSVWNQLYDAFPGIMKWVPGPHQTIIS

NYQKLANFLEEKIEQHKPDWDISNPRDYIDAYLTEAEKKKNDTEAGFTVGSLVWSMVDLF

EGGTETTTNTLRWALLFLIKYPDIQKKVQAEIDEVIGSRLPSMSDKANMHYLNAFIHEVL

RRANLVPLNMARVAKKDTTLGGYFITKGTVMITNLTSVLYDKQEWETPDAFNPKHFLDSE

GHFCRRNAFFAFSAGKRQCPGEYLAHLELFIFLTILLQTFSFSPPDGEEPSLDSQVGFTQ

APLPYKICAHPR

>CAH69015.1

MVGLYIGRSCKHPRLVIRVITLIQTLNLPNRPNITVMDLLHIYEWIDIKAVLFFACVFLL

LSNYIQNKTPKNFPPGPWPLPIIGNLYHIDFNKIHLEVEKLSEKYGSVVSVHLFGQRTVI

LNGYKQVKEVYIQQGDNVADRPELPMIHDIAGDNGLVAPSGYKWKQQRRFALSTLRNFGL

GKKSLEPSINLECHYLNEAISNENGRPFDPHLLLNNAISNVICVLVFGNRFDYSDHHFQT

LLNNINEAMYLDGTIWAQLYNSHPRIMRLLPGPHKKNITLWNKVIDFARERVKEHRVDYD

PSNPRDYVDCFLAEMEKLKDDTAAGFDVENLCICTLDLFVAGTETTSTTLSWSLLYMIKY

PEIQAKVQEEIDRVIGSSRQPSVSDRDNMPYTNAVIHEIQRFGNIAALNLPRAAVKDIQV

GKYLIPKGTIVIGNLTSVLFDESEWETPHSFNPGHFLDAEGKFRRRDAFLPFSLGKRVCL

GEQLARMELFLFFTSLLQHFTFSSPAGVEPSFNYKLGTTRAPKPFKLCAVSR

>AAH44463.1

MDLLHIYEWIDIKAVLFFACVFLLLSNYIRNKTPKNFPPGPWPLPIIGNLYHIDFNKIHL

EVEKLSEKYGSVVSVHLFGQRTVILNGYKQVKEVYIQQGDNVADRPELPMIHDIAGDNGL

VAPSGYKWKQQRRFALSTLRNFGLGKKSLEPSINLECHYLNEAISNENGRPFDPHLLLNN

AISNVICVLVFGNRFDYSDHHFQTLLNDINEAMYLDGTIWAQLYNSYPRIMRLLPGPHKK

NITLWNKVIDFARERVKEHRVDYDPSNPRDYVDCFLAEMEKLKDDTAAGFDVENLCICTL

DLFVAGTETTSTTLSWSLLYMIKYPEIQAKVQEEIDRVIGSSRHPSVSDRDNMPYTNAVI

HEIQRFGNIAALNLPRAAVKDIQVGKYLIPKGTIVIGNLTSVLFDESEWETPHSFNPGHF

LDAEGKFRRRDAFLPFSLGKRVCLGEQLARMELFLFFTSLLQHFTFSSPAGVEPSFNYKL

GTTRAPKPFKLCAVSR

>AAH56816.1

MDLWDLYEWIDIKSILIFLCVFLLLGDYIKNKAPKNFPPGPWSLPIIGDLHHIDNSKIHL

QFTKFAERYGNIFSLRLFGPRIVVLNGYNLVKEVYIKQGDNLADRPVLPLFYEIIGDKGI

VLSSGYKWKHQRRFALSTLRNFGLGKKSLEPSINLECGFLNEAISNEQGQPFDPRLLLNN

AVSNVICVLVFGNRFDYSDHHFQTLLKHINEAIYLEGGICAQLYNMFPWLMQRLPGSHKK

VITLWKKVIDFIRQKVNEHRVDHDPLNPRDYIDCFLAEMDKLKDDTAAGFDVENLCICTL

DLFVAGTETTSTTLYWGLLYMMKYPGIQAKVQEEIDRVVGGSRQPSVSDRDNMPYTNAVI

HEIQRMGNIIPINVTRTTSEDIRIGKYSVPKGTMVTSNLTSVLFDESEWETPHSFNPGHF

LDAEGKFRRRDAFLPFSLGKRVCLGEQLARMELFLFFSSLLQRFTFSPPAGVEPSLDYKL

GATHCPQPYQLCAVPR

>CAH69018.1

MDLWYLYEWIDIKSILIFLCVFLLLGDYIKNKAPKNFPPGPWSLPIIGDLHHIDNSKIHL

QFTKFAERYGNIFSLRLFGPRIVVLNGYNLVKEVYIKQGDNLADRPVLPLFYEIIGDKGI

VLSSGYKWKHQRRFALSTLRNFGLGKKSLEPSINLECGFLNEAISNEQGRPFDPRLLLNN

AVSNVICVLVFGNRFDYSDHHFQTLLKHINEAIYLEGGICAQLYNMFPWLMQRLPGSHKK

VITLWKKVIDFIRQKVNEHKVDHDPLNPRDYIDCFLAEMEKLKDDTAAGFDVENLCICTL

DLFVAGTETTSTTLYWGLLYMMKYPVIQAKVQEEIDRVVGGSRHPSVSDRDNMPYTNAVI

HEIQRMGNIIPINVTRTTSEDIRIGKYSVPKGTMVTSNLTSVLFDESEWETPHSFNPGHF

LNAEGKFRRRDAFLPFSLGKRVCLGEQLARMELFLFFSSLLQRFTFSPPAGVEPSLDYKL

GATHCPQPYKLCAVPR

>348504416

METVYSVLGLEWIDFSSISLFLCVFLVLTDFLKNRVPRNFPPGPWALPFIGDLHRIRPSR

LHLQFAEFAEKYGKIFSLRLFGGRVVVLNGYKLLREALVQQGEDFTDRPAVPIFEDLFGN

NGLVMSNGYRWKQQRRFTLHTLRNFGLGKKTLELSIQQECQYLTEAFADHQGKPVNAQPL

LNNAVSNIICCLVFGDRFEYTDEQYQSILNNFTEIVRLQGTTVAQMYNTMPWLVKWLPGP

HQKILTLKRHITDFIKTKIREHIENFNPASPRDYIDAFLIEMEEKEGSDSVFDHSSLSAC

TMDLFFAGSETTTTTLNWGLLYMICYPDIQEKVQAEIDAVIGSSRQPSMTDRENMPYTDA

VIHEIQRMGNIIPFNVARSASKDTTIDKYTIPKGTIILASLDSVLHDESMWETPHSFNPE

HFLDQGGKFCKRDAFLPFSAGKRVCLGEQLARMELFLFFTSLLQRFSFSAPAGEQPSLEF

QLGGTRCPKPYRLCAGTMVIPVLNSVLNDETMWETPNSFTPQHFLNQDGKFRRREAFLPF

SAGKRVCLGEQLARMELFLFFTSLLQRFSFSAPAGEQPSFQSRLLSS

>CAH69009.1

MAYTAILETLDVKGILLFMVAFLLVADYLKNRNPPKYPPSPFSVPLLGNIFNVDSKEPHL

YLTKLGHAYNNIFSLRLGSDKTVFITGYKMVKEALVTQAENFVDRPNSPVLARVYSGNAG

LFFSNGEMWKKQRRFALSTLRNFGLGKKTMELAICEESRFLLEEIDEQKGAAFDPTILLY

NAVSNIICQMVFGQRFDYADHQFKTMLKYISKSIQLEGSVWGQIYEAFPAIMKHLPGPHN

DIFSNYDLLKSFVHEVIVKHKAKLDPSEPRDYIDTFLIEMKEKPHERVNGFEEPNLVACV

LDLFLAGTESTSNTLCWGLIYLIMYPDVQEKVQEEIDRVIGNSREPSIADKPNMPYTEAV

IHEIMRFGDVIPLNGLRVAARDTTLGECFIPKGTTVLPILHSVLFDENEWETPYKFNPGH

FLDKEGKFVRRDAFMPFSAGKRVCLGEQIARIELFLFFVSLFRKFRFSATEGEKLNMDGV

IGITRTPHPFKICATAR

>AAI54482.1

MDVLQFYKWLDIKTVLVFLVVFLFLSDYIRNKSPKNFPPGPWSLPFIGHIHHIEHKKVHL

QFLKFAEKYGKIFSIRLFGPRIVVLDGYKLVKEVYLQQGDNLADRPILPMFYDITEDKGL

IGSNGYKWKHQRRFALSTFRTFGLGKKSLEPSILLECSCLNDAFSNEQGRPFDPRLLLNN

AVSNVICALVFSNRFDYSDHHFQTLLKHINEVLYLEGTVWAQLYNFFPWLMRRLPGPHQK

IFVLLNKVIDFVREKVNEHRVDYDPSNPRDYIDCFLAEMEKLKDDTAAGFDVENLCICTL

DLFVAGTETTSTTLYWGLLYIIKYPEIQAKVQQEIDAVVGGSRQPSVSDRDNMPYTNAVI

HEIQRMGNIVPLNVFRITVEDTQIGEYSIPKGTLVIGSLTSVLFDESEWETPHSFNPGHF

LDAEGKFRRRDAFLPFSLGKRVCLGEQLARMELFLFFSSLLQRFTFSPPAGVEPSLDYKL

GGTHSPQPYKLCAVPR

>AAH85592.1

MAYTAMLETLDVKGILLFMVAFLLVADYLKNKNPPKYPPSPFSVPLLGNIFNVDSKEPHL

YLTKLGHAYNNIFSLRLGSDKTVFITGYKMVKEALVTQAENFVDRPNSPVLARVYSGNAG

LFFSNGEMWKKQRRFALSTLRNFGLGKKTMELAICEESRFLLEEIDEQKGAAFDPTILLY

NAVSNIICQMVFGQRFDYADHQFKTMLKYISKSIQLEGSVWGQIYEAFPAIMKHLPGPHN

DIFSNYDLLKSFVHEVIVKHKAKLDPSEPRDYIDTFLIEMKEKPLERVNGFEEPNLVACV

LDLFLAGTESTSNTLCWGLIYLIMYPDVQEKVQEEIDRVIGNSREPSIADKPNMPYTEAV

IHEIMRFGDVIPLNGLRVAARDTTLGECFIPKGTTVLPILHSVLFDENEWETPYKFNPGH

FLDKEGKFVRRDAFMPFSAGKRVCLGEQIARIELFLFFVSLFRKFRFSATEGEKLNMDGV

IGITRTPHPFKICATAR

>CAI20788.1

MDMLHFYEWIDIKSILIFVCVFLLLSDFIKNKTPKNFPPGPWSLPIIGDIHHIDPSKLHL

QLSEFAEKYGKIFSLRLFGPRIVVLNGYNLVKEVYTQEGDNLADRAILPLFHDIIGDKGL

VASSGYKWKQQRRFALSTLRNFGLGKKNLEPSINLESGFLNEAISNEQGQPFDPRLLLNN

AVSNVICALVFGNRFDYSDHKFQTLLKNINETVHLEGGICAQLYNMLPWLMRLLPGPHKR

IIALWGKLIEFVGEKVNEHKVDHDPLNPRDYIDCFLDEMEKHKDDTAAGFDVENLCICTL

DLFVAGTETTSTTLYWGLLYMMKYPEIQAKVQQEIDVIVGGSRQPSVSDRDNMPYTNAVI

HEIQRMGNIIPINLARTTSEDTQIEKYSIPKGTMVTSNLTSVLFDESEWETPHSFNPGHF

LDAEGKFRRRDAFLPFSLGKRVCLGEQLARMELFLFFSSLLQRFTFSPPAGVEPSLDYNL

GATHCPQPYKLCAVPH

>348504768

MESIYNLLGLEWIDGRSILIFLTVFLLVTDFLKNRVPKNFPPGPWALPFIGDLHRIQSTR

LHLQLTEFAEKYGNIFSLRLFGGRIVVVHGYKLVKEALVHQGEDFMDRPTIPLFESIIGN

KGLVMSNGYPWKQQRRFALHTFRNFGLGKKTLELSIQQECQYLTEAFAEHQGKPFNAKNL

INNAVSNIICCLVFGNRFEYTDKQYQYILQTINDIIRLQGSLTVQMYNTLPWLMRWLPGS

HQKTFTLIKKIAEFVETRIKEHLENFDPSSPRDYIDSFLIEMGEKEDKDSGFYLENLKAC

TMDLFGAGTETTTTTLHWGLLYMIHYPDIQKKVQAEIDAVVGSCRQPSTSDRENMPFTDA

VIHEIQRMGNIIPLNVVHMANKNTTLDKYSIPKGTKILAPLHSVLHDESMWETPHSFNPQ

HFLDQDGKFRKREAFLPFSAGKRVCLGEQLARMELFLFFTSLLQRFSLSAPAGEKPSLDF

ILGATLSPKPYRLCAVPR

>348504758

METLSSVLGFEWIDGRSILIFLCVFLLLSDIWKNRVPKNFPPGPWALPFLGDIHRIRPDR

IHLQFREFAKKYGDIFTIRIFGGRTVVINSYKFVKEALVQQGDDFMDRPTVPLFEELFGN

EGLAIANGYPWKEQRRFALHTLRNFGLGKKTLELYIQQECQYLAEAFTEHQGKPFNVQLL

INNAVSNIICCLVFGNRFEYTDRQYQSILHNFKEVVRLQGRISIQMYNIIPWLMKRLPGP

HKTIFTLTQEIKDFIEIRIREHRETLNPASPRDYIDAFLIEMAKNEEKDSSFSLSNLHAC

TMDLFFAGTETTATTLRWGLLYMIYYPDIQERVQAEIDAVVGSSRQPSVSDRENMPYTDA

VIHEIQRMANIIPLNVLRMTSNETTVDKYTIPKGTMILATLDSVLHDESMWETPHSFNPQ

HFLDKDGKFRKKDAFLPFSTGKRVCLGEQLARMELFLLFTSLLQRFSLSPPAGEQPSLEF

QLGGIRCPKPYRLCAVPR

>348504760

MKTLYSVLGLEWIDFSSVLLFLCVFLLLTDFLKNRVPRNFPPGPWGLPFIGNLHRIRPDR

FHLQFAEFAEKYGKIFSLRLFGGRLVVLNGYKLLRDVLVQQGEDFIDRPTLPSFENLLGN

NGLLMSNGYQWKQQRRFTLHTLRNFGLGKKTLELSIQQECQYLTEAFADHQGKPFNAQPL

LNNAVSNIICCLVFGSRFEYTDEQYQSILSDFIEMERLQGTIGTQMYNTMPWLVKWLPGP

HQKILTLTKRVTDFIKTKIREHIENFNPASPRDYIDAFLIEMGEKEGSDSVFDHSSLSAC

TMDLFIAGSETTTTTLNWGLLYMICYSDIQERVQAEIDAVIGSSRQPSMTDRENMPYTDA

VIHEIQRVGNIIPLNLAHSAIKDTTIDKYTIPKGTIILASLDSVLHDESMWETPHSFNPE

HFLDQDGKFRKRDAFLPFSAGKRVCIGEQLARMELFLFFTSLLQRFSFSAPAGEQPSLEF

QLGAIRCPKPYRLCAVPR

>AAF22000.1

MEAIRSVLGLEWIDARGVLLFFFVFLLLSDVLRNRKPKNFPPGPLALPFIRDLHRIRPAR

LHLQLTEFAETYGDIYSLHLFGGRAVIINGYKHVKEALVQKGEDFMDRPNIPLFADFFNN

KGLVMSNGYQWKVQRRFALHTLRNFGLGKKAMERYIQQECQYLNEAFSEQQGKPFNGQAL

INNAVSNIICCLVFGNRYEYNDKQYQTILQYFNEAVRLQGDLSVQIYNSIPGLMRWLPGS

HKKIFMILQKLVDFVEIRIKEHRENLDPSSPRDYIDSFLIEMGEKEDKDSGFELSNLCAC

TLDLFGAGTETTTTTLHWGLLYMIYYPQIQERVQAEIDAVIGPSRQPSVADRENMPYTDA

VIHEIQRMGNIIPLNLPRMANKDTTLDKYSIPKGTIIIPTLHSVLQDKSIWETPQTFNPQ

HFLDQDGQFRKRDAFMPFSTGKRVCLGEQLARMELFLFFTSLLQRFTFSAPAGEEPSLEF

KLGATRSPKPYRLCATPR

>AAH49521.1

MDMFYFYEWVDIKSILIFLCVFLLLSDYIKNKAPKNFPPGPWSLPFIGDLHHIDPNKIHL

QFTEFAEKYGKIFSFRLFGSRIVVLNGYNLVKEVYTQQGDNLADRPTLPITSAIIGDNRG

LVASSGYKWKHQRRFALTTLRNFGLGKKNLELSINFECGFLNEAISNEQGRPFNPRLLLN

NAVSNVICVLVFGNRFEYSDHHFQNLLNKINESVYLEGSIFVHLYNMFPWLMQLLPGPHK

KLITLWQRVTDFVREKVNEHRVDYDPSSLRDYIDCFLAEMEKHKDDTAAGFDVENLCMCT

LDLFVAGTETTSTTLYWGLLYMIKYPEIQAKVQQEIDAVVGSSRQPSGSDRDNMPYTNAV

IHEIQRMGNIIPLNVVRTTSEDTRIEKYSIPKGTLVIGSLTSVLFDESEWETPHSFNPGH

FLDAEGKFRRRDAFLPFSLGKRVCLGEQLARMELFLFFSSVLQRFTFSPPAGVEPSLDFK

MGFTRCPKPYKLCAVPR

>ABH05128.1

METIHKVLGLEWMNGTSLLIFLCVFLLLTDVLKNRVPTNFPPGPWALPLIGDLHRIQPSR

LHPQFTEFAEKYGNVFSLRLFGGRIVVINGYKLVREALVHKGEDYIDRPIIPLIEALNGN

RGLVISSGNPWKQQRRFALQTLRNSGVGKKSLEPSIQQECQYLTEAFALQQGKPFDAQSL

ITSAASNIICCLVFGDRFEYTDKQYQGILQKFNEIVYLQGSFWTQVYNTYPWLMKWVPGP

HQRIFTLTQNLDDFVEIKIKEHRESLDPSSPRDYIDCFLTEMGEKEDTDSGFDLKNLAVC

TLDLLAAGTETTTTTLHWGLLFMMYYPDIQERVQAEIESVVGSSRLPSVTDRENMPYTDA

VIHEIQRMGNIVPLNVVHMTNKDTVLDKYTIPKGTTIMPTLNSVLHDESMWETPHTFNPQ

HFLDQDGKFRKREAFLPFSAGKRVCLGEQLARMELFLFFTSLLQRFSFSACAGEQPSLEF

KLGATHCPKPYRVCAVPR

>CAH69019.1

MDMFYFYEWVDIKSILIFLCVFLLLSDYIKNKAPKNFPPGPWSLPFIGDLHHIDPNKIHL

QFTEFAEKYGKIFSFRLFGSRIVVLNGYNLVKEVYTQQGDNLADRPTLPITSAIIGDNRG

LVASSGYKWKHQRRFALTTLRNFGLGKKNLELSINFECGFLNEAISNEQGRPFNPRLLLN

NAVSNVICVLVFGNRFEYSDHHFQNLLNKINESVYLEGSIFVHLYNMFPWLMQLLPGPHK

KLITLWQRVTDFVREKVNEHRADYDQSSLRDYIDCFLAEMEKHKDDTAAGFDVENLCMCT

LDLFVAGTETTSTTLYWGLLYMIKYPEIQAKVQQEIDAVVGSSRQPSGSDRDNMPYTNAV

IHEIQRMGNIIPLNVVRTTSEDTRIEKYSIPKGTLVIGSLTSVLFDESEWETPHSFNPGH

FLDAEGKFRRRDAFLPFSLGKRVCLGEQLARMELFLFFTSVLQRFTFSPPAGVEPSLDFK

MGFTRCPKPYKLCAVPR

>348504772

MESIYNLLGLEWIDGSSILIFLTVFLLLTDFLKNRVPKNFPPGPWALPFIGDLHRIQSSR

LHLQLTEFAEKYGNIFSLRLFGGRIVVVHGYKLVKEALVHQGEDFMDRPIMPLFESIIGN

KGLVMSNGYPWKQQRRFALHTFRNFGLGKKTLESSIQQECQYLTEAFADQQGKPFNAKNL

INNAVSNIICCLTFGNRFEYTDKQYQYILQTFNDIIRLQGNLTVRMYNTIPWLMKWLPGS

HQKIFILTKKIAEFVETRIKEHQENLDPSSPRDYIDSFLIEMGEKEDKDSGFYVENLKAC

AMDLFGAGTETTTTTLHWGLLYMIHYPDIQEKVQAEIDAVVGSSRQPSTSDRENMPFTDA

VIHEIQRSGNIAPLNVLHVANKNTTLDKYSIPKGTKILAPLHSVLHDESMWETPHSFNPQ

HFLDQDGKFRKREAFLPFSAGKRVCLGEQLARMELFLFFTSLLQRFSLSAPAGEKPSLDF

ILGATLSPKPYRLCAVPR

>348504764

METIYSLLGSEWVDGRSILIFLFVLLLTTEVLKNRVPKNFPPGPWALPFIGDLHRIRPDR

IHLQFAEFAEKYGNIFSLRFFGQRTVIINGYKLVKEALIQRGEDFVDRPSLPMFEELVGN

KGLVVSNGYQWKQQRRFALHTLRNFGLGKKTLELYIQQECQYLTEAFADHQGKPFNTQSL

LNNAVSNIICCLVFGNRFEYTDKQFQSILQLFNEIVYLEGSVWAQIYNTLPWLMRWLPGP

HQNIFTLFQKIIDFVEVKIKEHRENLNPSSPRDYIDAFLIEMGEKEDEDSGFSLSNLCFC

TLDLFGAGTETTTTTLHWGLLYMIYYPDIQEKVQAEIDAVIGSSRQPSMTDRENMPYTDA

VIHEIQRMGNIIPLNVAHVANRETTVDKYTIPKGTMVIPVLNSVLNDETMWETPNSFNPQ

HFLNQDGKFRRREAFLPFSAGKRVCLGEQLARMELFLFLTSLLQRFSFSAPAGEQPSLEF

KLGVTRSPKPYRLCAVPR

>348504766

METIYSLLGSEWIDGRSILIFLFVLLLTTEVLKNRVPKNFPPGPWALPFIGDLHRIRPDR

IHLQFAEFAEKYGNIFSLRFFGQRTVIINGYKLVKEALIQRGEDFMDRPSLPMFEELVGN

KGLVISNGYQWKQQRRFALHTLRNFGLGKKTLELYIQQECQYLTEGFADHQGKPFNTQSL

LNNAVSNIICCLVFGNRFEYTDKQFQSILQLFSEIVYLEGGVWAQIYNTLPWLMRWLPGP

HQKIFTLTQKIIDFIEVKIKEHRENLNPSSPRDYIDAFLIEMGEKEDEESGFSLSNLCFC

TLDLFGAGTETTTTTLHWGLLYMIYYPDIQEKVQAEIDAVIGSSRQPSMTDRENMPYTDA

VIHEIQRMGNIIPLNVAHVANKDTTLDKYTIPKGTMIIPALHSVLNDETMWETPNSFSPQ

HFLDQDGKFRRREAFLPFSTGKRSCLGEQLARMELFLFFTSLLQRFSFSAPAGEQPSLEF

KLGATRSPKPYCLCAVPR

>ABH05127.1

MWLYNLLPGFDIKEVLLFSFIFLLIADFLKNRNPPNYPPGPLALPLVGSIFSADFKQPQI

YFAKLADIYGNVFSIRFGGEKMVLVSGYKMVKEAIVTQAENFVDRPYNAIIDRLYSGNSG

GLFMSNGEIWKRQRRFALSTLRNFGLGKSTLEQSICEEVRHLQEEIEKERGKPFKPAGLF

NNAVGNIICQLVMGKRFEYSDHNFQIMLKYLSELLWLEGSVWSMMYQSFPRVMKHLPGPH

NKIFSHFNTLLDFISQEVKWHKKDLDHNNPRDYIDAFLTEMENHKGSELGFTENNLAMCS

LDLFQAGTETTSTTLLWALVFLIKNPDIQDKVQAEIDRVIGQTRQPTMADRPNLPYTDAV

IHEIQRIGNIVPLNGFRTAAKDTTLGGYFIPKGTSLVPILTSVLFDKTEWETPDTFNPGH

FLDAEGRFVKREALLAFSAGKRVCLGQGLAKMELFLFLVGLLQKFSFFVPDGVELSTEGI

TGGTRVPHPFKVCAKAR

>224058330

MLNFLRDSISLQTFLIFLFIFLLIADYMKNRNPNNFPPTPFRLPFLGHVYLLDFKDPAVT

ARKLSKRYGDIFGIHMGSMKFVMVNGMRLVKEVLVNQGDKFLDRPDIPIDEEIFSKIGLI

SSIGHLWKAQRRFTLSTLRNFGLGKRSLEERIQEECRYLVDVFGDEQGNPFNPQMKVTNA

VANVICSLIFGNRFEYHDEDFQRLLKLMYEMTVLHGAVTSQLYNSFPSIMKYLPGAHHTI

FKNWRLLKKFMQEQINKHKEDWNPSESRDYIDSYLLEISKDHDSDTFQEEHLIACSLDLM

FAGTETTSSTLRWALLFMATHPEIQARVQAEIDTFIGQARPPALEDRNNLHYTNAVIHEV

QRKGNVIPFNVPRMASEDTYVDGYYIPKGTGIMANLSSLLLDENEWKTPNTFNPEHFLKD

GKFWKNDHFLPFSLGKRACLGELLARSELFLFFTCLLQKFTFQAPPDTTLTLQPLIGITV

APQPYKICAVPR

>348504762

METLYSVLGFEWIDGRSILIFLCVFLLLNNIWKNRVPKNFPPGPWALPFLGDIHRIRPDR

IHLQFREFVEKYGDIFTIRIFGGRTVVINSYKFVKEALVQQGDDLIDRPTVPLFEDLFGN

EGLVIANGYPWKQQRRFALHTLRNFGLGKKTLELYIQQECQYLSEAFTEHQGKPFNVQLL

INNAVSNIICCLVFGNRFEYTDRQYQSILHNFNEVMRLQGSMSIQMYNIIPWLMKRLPGS

HKTMFTLTQVIKDFIEIRIREHRETLNPASPRDYIDAFLIEMAKNEDKDSSFSLSNLHAC

TMDLFFAGTETTTTTLRWGLLYMIYYSDIQERVQAEIDAVVGSSRQPSVSDRENMPYTDA

VIHEIQRMANIIPLNVLRMTNKETTVDKYTIPKGTMIVATLDSVLHDESMWETPHSFNPQ

HFLDKDGKFRKRDAFLPFSAGKRVCLGEQLARMELFLFFTSLLQRFSLSPPAGEQPSLEF

ELGGTRCPKPYRLCAVPR

>118094633

MLRFLWDSISLQMLFVFLLVFLLVSDYMKRRKPKDFPPSPFSFPFLGNVQFMFAKDPVVA

TQKLTEKLGDIFSMQAGSQSFVIVNGLPLIKEALVTQGENFMDRPEIPLDTDIFSKLGLI

SSSGHLWKQQRRFTLTTLRNFGLGKRSLEERIQEECRFLTEAFRDEQGNPFNPHLKINNA

VSNIICSVTFGNRFEYHDENFQTLLRLMDETVTLHEKIMSQLYNAFPSIVKYLPGSHQTI

FKNWRLMKDFVNEKISKHKEDLNPSESRDFIDSYLQEMAKPSGSEFHEENLVACALDLLF

AGTETTSTTIRWALLFMAVYPEIQAHVQAEIDAVIGQARQPALEDRNNMPYTNAVIHEVQ

RKGNIIPFNVPRQAVKDTVLAGFRVPKGTILIPNLSSVMYDKKEWETPHSFNPGHFLKDG

QFWKREAFMPFSIGKRACLGELLARAELFLFFTSLLQKFTFQAPPDTILDFKFTMGITLA

PRPYKICAVPR

>ABQ44505.1

MEAITAVLGFEWIDSRSLLIFLFVFLLLSDYLANRRPKNFPPGPHSLPFIGDLHRINPSR

LHLQLTEFAEKYGNVFSLHLFGERAVILNGHKHVKEALVQRGDDFVDRPSIPLFEQFYSN

KGIVVSNGYPWKQQRRFALHTLRNFGLGKKTMEKYMQEECRYLTEAFGEYKVKPFNAQAL

INNAVSNIICCLVFGERYEYSDKQYQQILQDINEIMILQGGFAAQLFNSFPWLMKKLPGP

HQKILTLLAKLIDFAKVKISEHKENLDPSSPKDYIDSFLIEMAQNENQESSFDISNLCMC

TLDLFIAGTETTTTTLHWGLLYMIYYADIQEKVQAEIDAVIGSSRQPSMADKENMPYTDA

VIHEIQRMGNILPLGVLRMASKDTTLDKYTIPKGTMIIPTLNSVLHDESMWETPHSFNPK

HFLDKDGKFRKREAFNPFGAGKRVCLGEQLARMELFLFFTSLLQRFSFSAPAGEQPSLEN

RMGATRCPKPYRLCAVPR

>326925457

MLRFLWDSVSLQMLFVFLFVFLLVADYMKRRKPKDFPPSPFALPFLGNVQFMIAKDPVAV

AQKIIEEHGDIFSMQVGTQSFVILNGLQMIKEALLIQGENFMDRPEIPINTEVFNKLGLI

SSNGHLWKQQRRFTLITLRNFGLGKRSLEERIQEECRFLTDAFRDEQGNPFNPHLKINSA

VSNVICSITFGNRFEYHNGNFQNLLRLMDETVILHGKIMSQVHLYNVFPSIIKYFPGSHQ

TIFENWRLMKGFVKEKISKHKEDLNPSESRDFIDSYLQEMAKSNGSDFCEDNLVACTLDL

FFAGTETTSTTIRWALLFMALYPEIQARVQAEIDAVIGQCRQPALEDRNNMPYTNAVIHE

VQRKGNITPFSLPREAKKDTVLAGFHLPKGSIVVPNLSSVMLDKKEWETPHSFNPGHFLK

DGQFWKREAFMPFSIGKRACLGELLARAELFLFFTALLQKFTFQAPRDTILDLKFILGMT

LAPRPYKICAVPR

>ABO21094.1

MWLYNFLLSFDLKAIFLFIFIFVLIVDFLKYKNPPNYPPGPLALPFVGNMFSLDRKHPYM

YFTKLAEIYGNVFSIRLGRDKLVFVSGYKMVKEALVAQAENFVDRPYNPLTERFYSGTKD

GLFMSNGEIWKRQRRFTLSTLRSFGLGKNSLEQSICEEIRHLQEETEKEKGELFNPAGLF

NKAVSNIICQLVMGKRFEYNDHKFQMMLKYLSEVLYLQGSIWGLLYQAFPAAMKHLPGPH

NKMFSHFIALQDFLAEEVKSHKKDLDYNNPQDYIDTFIIEMEKHNETDLGFTETNLALCS

LDLLLAGTETTSTTLLWALVYLIKHPEVQYKVHAEIDRVIGQNRLPSMADRANLPYTDAV

IHEIHRVGNVIPLNGYRMAAKDTTLGGYFIPKGTTLMPNLTTVLFDKTEWETPDSFNPGH

FLDAEGKFVKREALLPFSAGKRVCLGEGLAKMELFLFLVGLLQKFSFSVPDGIELSTEGI

SGFSRVPHPYKVYAKAR

>ABO21079.1

MWLYDFLLSFDLKAIFLFIFIFILIADYLKYKNPPNFPPGPLALPFVGNFFSVDRKHPHI

YFTKLADIYGNVFSVRLGRDKMVFVSGYKMVKEAIVAQGENFVDRPYSPMMERLYSGTKD

GLFMSNGEVWTRQRRFALSTLRSFGLGKNSLEQSICEEIRHLQEEIEKEKGEPFSPAGLF

NNAVSNIICRLVMGKRFEYNDHKFQKMLKYLSEVAFLQGSIWGTLYESFPGVMKHLPGPH

NKMFSHFNALQDFITEEVKSHKKDLDYNNPRDYIDTFIIEMEKHKETDLGFTGTNLALCS

LDLLLAGTETTSTTLLWASVFLIKHPDIQDKVQAEIDRVIGQNRLPSMADRANLPYTDAV

IHEIQRVGNIVPLNALRMATKDTTLGGYFIPKGTSILPNLTSVLFDKTEWETPDSFNPGH

FLDAEGKFVKREALLPFSAGKRVCLGEGLAKMELFLFLVGLLQKFSFSVPEGVELSTEGV

TGGTRVPHPYKVYAKAR

>326925459

MLRFLWDSVSLQMLFVFLFVFLLVADYMKRRKPKDFPPSPFALPFLGNVQFMIAKDPVAV

AQKFIEKHGDIFSMQVGSMSFVVVNGLPLIKEALVTQGENFMDRPEFPANTELFNKYGLV

SSNGHLWKQQRRFTLTTLRNFGLGKRSLEERIQEECRFLTDAFRDEQGNPFNPHLKINSA

VSNVICSITFGNRFEYHNGNFQNLLRLMDETVILHGKIMSQVHLYNVFPSIIKYFPGSHQ

TIFENWRLMKGFVKEKISKHKEDLNPSESRDFIDSYLQEMAKSNGSDFCEDNLVACTLDL

FFAGTETTSTTIRWALLFMALYPEIQARVQAEIDAVIGQCRQPALEDRNNMPYTNAVIHE

VQRKGNITPFSLPREAKKDTVLAGFHLPKGSIVVPNLSSVMLDKKEWETPHSFNPGHFLK

DGQFWKREAFMPFSIGKRACLGELLARAELFLFFTALLQKFTFQAPRDTILDLKFILGMT

LAPRPYKICAVPR

>348504436

MQLRDLFSCLDIKEQLLIIFAGLLMVYFHFRKDKDPPGFPPGPPALPILGNIFNIDAKQP

HIYLTKLADFYGNVFCIRLGRHKTVFVSGWKMVKEALVTQADNFVDRPYSPMVTRIYSGN

SAGLFFSNGKVWRRQRRFAMATLRTFGLAKESTEQSICDESQHLKDAMEKEKGEPFDPVP

FLNNAVSNIICKIVFGRRFDYSDHNFQVMLSNLTEMAYLEGSIWALLYDAFPGLMKHLPG

PHNRIFSSSRSLVASIREEIEKHKLDLDPNNPRDYVDSFLIKEKHNRNTHLGFEEENLVL

CCLDLFLAGSETTSKTLQWGLIYLIMNPQIQCKVQEEIDRVIGQTRQPTMTDKPNLPYTD

AVIHEIQRMGNIVPLNGLRMAAKDTMLGGYFIPKGTSVMPNLTSVLFDKNKWETPDTFNP

EHFLDTEGKFVRREAFLPFSAGRRACLGEGLARMELFLFFVSLFQKFKFSTLDGVKLSTE

GVTGATRTPYPFKVYAKPRRTIPNKPHS

>ABO21085.1

MWLYDFLLSFDLKAIFLFIFIFILIADFLKYKNPPDYPPGPFPLPFVGNVFSVDNKHPHI

YFTKLADIYGNVFSVRFGRDKLVFVSGYKMVKEAIVAQAENFVDRPYSPMMDRLYSGTKD

GLFMSNGEVWKTQRRFALSTLRSFGLGKNSLEQSICEEIRHLQEEIEKEKGEPFSPAGLF

NNAVSNIICRLVMGKRFEYNDHKFQMMLKYLCEITFLEGSIWATLYDSFPGVMKHLPGPH

SKLFSHFNTLQDFITEEVKSHKKDLDYNNPRDYIDTFIIEMEKHKETDLGFTETNLALCS

LDLFLAGTETTSTTLLWALVFLIKHPDVQDKVHAEIDRVIGQSRLPSMADRANLPYTDAV

IHEIQRVGNIVPLSAPRMAVKDTTLGGYLIPKGTSILPNLTSVLFDKTEWETPDSFNPGH

FLDAEGKFVKREALLPFSAGKRVCLREGLAKMELFLFLVGLLQKFSFSVPDGVELSTEGI

TGSTRVPHPYKVYAKAR

>348504756

METIYSVLGLEWIDGRSILLFLCVFLLLTDFLKNRVPKNFPPGPWALAFIGDLWRIQPAR

LHLQFVEFAGKYGNVFSLRLFGGRIVVVNGYKSVKEALVQKGEDLIDRPVIPIFELTENK

GLVMSNGYLWKQQRRFALHTLRNFGLGKKTLDLSIQQECQYLIEAFADKQGKPFNTQSLI

NNAVSNIICCLVFGNRFDYTDKEFQSILQKFNKIMHLQGTLSVQMYNTIPWLMKWIPGSH

KEILFLVQQLIEFVKVKINEHKENIDPSSPRDYIDAFLIEMGEKENKESGFDLENLCFCT

LDLFGAGTETTTTTLRWGLLYMIYYPDIQERVQAEIDAVVGSSRQPSMSDREKMPYTDAV

IHEIQRMANIIPLNLAHMTSKDTTLNNYAVPKGTIIITLLHSVLCDESMWETPHTFNPQH

FLDQDGKFKKREAFMPFAAGKRVCLGEQLARMELFLFFTSLLQRFSFTAPAGEQPSLEFK

LGIARAPKPYRLCAVPR

>327270830

MWFHAFAIFWETISLQVILGFLATFLLLTDYVKRRRPRGFPPGPIPLPFLGNLLSYDAKK

PHLYNQKLVAIYGNVFSLQLGNIHIVFLNGLQAVKEALINQGESFLDRPKVPITYDVSKT

FGVITSNGQTWKQQRRFVMSTLRNFGLGKTYLEERIQEESRFLVAAIEDEKGQPFDPYHQ

INNAVSNVICSVTFGNRFDYHDSDFQKLLHLLDETGVFLRNIWSHLYNAFPSLMRRLPGP

HQTYFKNWEQLKSFVRKIIEKHKEDWNPLKTKDFIDAYLNEMAKENASSTFHMENLLQST

LDLFVAGTETTSATLHWAVLYMAVYPEIQAKVQAEIDSVIGQSHLPAMADRDNMPYTNAV

IHEIQRRSSIVVVNAPRLTANDTQVAGFHLPKVSLQNLTSILFDKNEWETPNVFNPNHFL

KNGQFMKREAFVPFSTGKRACPGEQMAKMELFLVFTTLLQKFTFQAPKGVKLSLDSKTGH

VLKPKPYQICAISR

>AAI09451.1

MALENILLHLTSKVWTDAGTILLLFILFLLVSVKLRNRNKPHKNLPPGPTPLPFIGNVFN

LDTSQPHIDLTKMSDHYGNIFSLRLGSLNTVVVNTYSMVKKVLNDQGNSFMYRPSNDTTE

RISKCQGLTFNNGYSWKQHRRFTLSTLKFFGVGKRSLEFIIMEEYKFLHQSIMDTNGLPF

NPHYIINNGVSNIICSMVFGRRFEYTDQRFLNMLSLISKALKLQTSVFIQLYAAFPRLMD

LLPGPHKELFSCFHQVRAFIKEEVDKHRADWDPSSPRDFIDCYLTEIEKKKDDLEAGFHD

EGLQYAVLDLFVAGTETTSTTLLWAFVYMMKYPEIQEKVQAEIDKVVGRYRRPSMDDRPC

MPYTDAVIHEIQRMGNVVPLSVPRMTNEDTILEGYFIPKGTQIIPNLTSVLFDQTKWKTQ

HSFDPQNFLNAQGKFEKPEAFIPFSLGKRSCPGESLARMELFLFFTSFLQSFSLSAPDET

QTSLDFKCGMTLSPKPFKICFTPR

>AAH81576.1

MALENILLHLNSKVWTDAGTILLLFILFLLVSVKLRNRNKPHKNLPPGPTPLPFIGNVFN

LDTSQPHIDLTKMSDHYGNIFSLRLGSLNTVVVNTYSMVKKVLNDQGNSFMYRPVNDITE

RISKCQGLTFNNGYSWKQHRRFTLSTLKFFGVGKRSLEFIIMEEYKFLHQSIMDTNGLPF

NPHYIINNGVSNIICSMVFGRRFEYTDQRFLNMLSLISKALKLQTSVFIQLYAAFPRLMD

LLPGPHKELFSCFHQVRAFIKEEVDKHRADWDPSSPRDFIDCYLTEIEKKKDDLEAGFHD

EGLQYAVLDLFVAGTETTSTTLLWAFVYMMKYPEIQEKVQAEIDKVVGRYRRPSMDDRPC

MPYTDAVIHEIQRMGNVVPLSVPRMTNEDTILEGYFIPKGTQIIPNLTSVLFDQTKWKTQ

HSFDPQNFLNAQGKFEKPEAFIPFSLGKRSCPGESLARMELFLFFTSFLQSFSLSAPDET

QTSLDFKFGMTLSPKPFKICFTPR

>AAS83983.1

MALENILLHLTSKVWTDAVTILLLFILFLLVSIKLRNRNKPHKNLPPGPTPLPFIGNVFN

LDTSQPHIDLTKMSDHYGNIFSLRLGSLNTVVVNTYSMVKKVLNDQGNSFMYRPVNDITE

RISKCQGLTFNNGYSWKQHRRFTLSTLKFFGVGKRSLEFIIMEEYKFLHQSIMDTNGLPF

NPHYIINNGVSNIICSMVFGRRFEYTDQRFLNMLSLISKALKLQTSVFIQLYAAFPRLMD

LLPGPHKELFSCFHQVRAFIKEEVDKHRADWDPSSPRDFIDCYLTEIEKKKDDLEAGFHD

EGLQYAVLDLFVAGTETTSTTLLWAFVYMMKYPEIQEKVQAEIDKVVGRYRRPSMDDRPC

MPYTDAVIHEIQRMGNVVPLSVPRMTNEDTILEGYFIPKGTQIIPNLTSVLFDQTKWKTQ

HSFDPQNFLNAQGKFEKPEAFIPFSLGKRSCPGESLARMELFLFFTSFLQSFSLSAPDET

QTSLDFKCGMTLSPKPFKICFTPR

>AAD54016.1

MWFYNLLLSLDVKGLFLFIFLFLLIADFYKSRKPANFPPGPKALPFVGNFFSLDSKHPHV

YFQKLAEIYGNVFSFRLGRDSIVFLNGYKAVREALVTQAENFVDRPFNAITDRFYTEPSA

GIFMSNGEKWKKQRRFALSTLRNFGLGKNSLEQSVSEEIQHLQEEMEIEKGKPFNPSGLF

TNAVSNIICQLVMGKRYDYTDHRFQMMLRCMSEAVLLEGNVWGQLYMAFPSVMRYMPGPH

NKIFSHFSSVEQFLYEEVEQHKKDLDRDNPRDYIDTFLIEMENHKESDLGFTEANLVYCA

IDLFLAGTETTATTLLWALVFLVKYPEVQEKVQAEIDSVIEQARLPSMADRSSMPYTDAV

IHEIQRIGNILPLNGMRVAAKDTTLGGYFIPKGTSLMPVLTSVLFDKAEWACPDTFNPGH

FLDDNGKFVKRDAFLPFSAGKRACIGESLAKMELFLFLVALLQKFTFSVPEGVELSTEGI

TGTTRVPHPYKVSAKIR

>CAG32696.1

MLRFLWDSISLQMLFVFLLVFLLVSDYMKKRKPKDFPPGPFSFPFLGNMEFIIAKDPVAV

TEKFIKKHGDIFSTQVGSMSFVIVNGLPLIKEALVTQGENFMDRPEFPINTEFLNKFGLV

FSNGHLWKQQRRFTLTTLRNFGLGKRSLEERIQEECRFLTDAFRDEQGNPFNPHLKVNNA

VSNIICSVTFGNRFEYHDEDFQNLLRLMDETVTLQGEPMSQLYAFFPSIIKYFPGSHQTV

LKNEKLMKRFVCKKISKHKEDLSPSESRDFIDSYLQEMAKPSGSDFCEDNMVSCTLDLFF

AGTETTSTTIRWALLYMAIYPEIQARVQAEIDAVIGQARLPALEDRSNMPYTNAVIHEVQ

RKGNIIPFNVPRQAVKDTVLAGFRVPKGTILIPNLSSVMYDKKEWETPHSFNPGHFLKDG

QFWKREAFMPFSIGKRACLGELLARAELFLFFTALLQKFTFQAPPDTILDLKFTVGITLA

PQPYKICAVPR

>354486754

MLAATGSLVAAIWAALHLQTLLLAAVVFLFLADYFKNRRPKNYPPGPRRLPFFGCLFYLD

PKQPHIALQKFVKKYGNVLSLDFANIPSVVVSGMPLIKEVFTQLEPNFLNRPVTLLRQHL

FSKNGLIFSSGQTWKEQRRFTLMTLRNFGLGKKSLEQRIQEEAQHLVDAIGEEGGQPFDP

HFNVNNAVSNIICTVTFGERFEYHDSQFQEMLRLLDEVMCLESSLMCQLYNIFPRIIHYL

PGSHQTLFRNWRKLKLFVSDIIDNHRKDWDPEEPRDFIDAFLKEMTKYPDKTTTSFNEEN

LICSTLDLFFAGTETTSTTLRWALLCMALYPEVQEKVQAEIDRVIGQGRQPILADRESMP

YTNAVIHEVQRIGNIIPFNVPREGTGDTKLAGFDLPKGIMILTNLTALHRDPKEWATPDI

FNPEHFLENGQFKKRESFLPFSMGKRACLGEQLARSELFIFFTALVQKFTFKPPADEKLS

LQFRMSVTVSPVSHRICAIPRL

>354486756

MLAATGSLVAAIWAALHLQTLLLAAVVFLFLADYFKNRRPKNYPPGPRRLPFFGCLFYLD

PKQPHIALQKFVKKYGNVLSLDFANIPSVVVSGMPLIKEVFTQLEPNFLNRPVTLLRQHL

FSKNGLIFSSGQTWKEQRRFTLMTLRNFGLGKKSLEQRIQEEAQHLVDAIGEEGGQPFDP

HFNVNNAVSNIICTVTFGERFEYHDSQFQEMLRLLDEVMCLESSLMSILYNIFPRIIHYL

PGSHQTLFRNWRKLKLFVSDIIDNHRKDWDPEEPRDFIDAFLKEMTKYPDKTTTSFNEEN

LICSTLDLFFAGTETTSTTLRWALLCMALYPEVQEKVQAEIDRVIGQGRQPILADRESMP

YTNAVIHEVQRIGNIIPFNVPREGTGDTKLAGFDLPKGIMILTNLTALHRDPKEWATPDI

FNPEHFLENGQFKKRESFLPFSMGKRACLGEQLARSELFIFFTALVQKFTFKPPADEKLS

LQFRMSVTVSPVSHRICAIPRL

>363736831

MPAQRSAPPAPCQHRPVAADPRPVSQRRCGSSVQPYLASLLWPGAVCGSARRGGGGSERG

ASRCTRTMLRFLWDSISLQMLFIFLLVFLLVSDYMKRRKPKDFPPGPFSFPFLGNVQFMF

AKDPVVAIQKFIEKHGDIFRTQVGSMSFVIVNGLPLIKEALVTQGENFMDRPEFPTNTEF

FNKFGLVFSNGHLWKQQRRFTLTTLRNFGLGKRSLEERIQEECRFLTDAFRDEQGNPFNP

HLKVNNAVSNIICSVTFGNRFEYHDEDFQNLLRLMNETAILQGKIMSQLYNFFPSVIKYF

PGSHQTVIKNGRLMKRFVCKKISKHKEDLSPSESRDFIDSYLQEMAKPNGRDFCEDNLVA

CTLDLFFAGTETTSTTIRWALLYMAIYPEIQARVQAEIDAVIGQARQPSLEDRSNMPYTN

AVIHEVQRKGNIIPFNVPRQAVKDTVLAGFRVPKGTILIPNLSSVMFDMKEWETPHSFNP

GHFLKDGQFWKREAFMPFSIGKRACLGELLARAELFLFFTALLQKFTFQAPPDTILDLKF

THGITLAPQPYKICAVPR

>395530532

MIPSIWERASLQNFLLLSAIILILTHYLKRRRRNLNYPPGPPGLPILGNFLQMDFQKPQV

TMQKFVKKYGNILYMNMISQNFITVTGLPLIKEVLANQGDVFIDRPQIPIQLQIFKSSGL

IMSNGQLWKHQRRFALMTLRNFGLGKKTLEDRIRMEAKYLIEAIREEKGQPFDPHYVINN

AVSNIICSVTFGNRFEYHDSQFRELLKTVDEVMKLLVRWECQLFNIFPSVMKFLPGTHQK

LFRNFKKLESFVEDVIKQHKENLNPEEPQDFIDAYLKELSKDNIHYSFNEKNLVYCTLDL

FFAGTETTSTTLRWGLLYMALYPEIQGKIQAEIDRVIGQSQQPTMADKENMPYTNAAVHE

VQRMGDIVPFNVPRMAVVDTTVAGYYVPKGTMLTTNLTALHRDPKEWATPETFNPEHFLK

NGQFKKRESFLPFSIGKRACLGEQLARAELFLFFTSLLQKFTFQPPPNTQLSLDVQYSTT

MSPLPYKICAIPREM

>EDL30914.1

MLAIATCLVANICSAIHLWTLLLTLLTLLLLADYLKNRRPKNYPPGPRRLPFVGNLFQFD

LDVSRLHLGIQPFVKKYGNVISVNFGYISSVIISGLPLIKEAITGMEQNFLKRPSLAARQ

HVFKNNGIVFSSGQTWKEQRKFALTILKNFGLGKKSLEQCIQEEAYHLVKAIGEEKGQPF

DPHFRINNAVGNIICSIIFGERFEYDDNQFQELLKLADEIICSEASMMSVLYNVFPSIFK

YLPGPQQKLFSNWEKLKLFVSRMMDSHREDWNPSAPRDFIDAFLTEMTKYPDKTTTSFNE

ENLICTALDLFFAGTETTSNTLRWALLYITVNPEVQEKVHSEIDRVIGHGRHPTLDDRDS

MPYTNAVIHEVLRMGNIIPLNVPREVTADSTLAGFYLPKGTMVLINLTDLHRDPKEWDTP

NVFNPEHFLENGQFKKKESFLPFSMGKRACPGEQLASCELFIFFTALMQKFTFKSPINEK

PSLKFRMGLTLAPVSYRICAVPRL

>344278842

MLLALGSLANALWAGFHLRPLLLGVVVFLFFVNFFKRRRPKNYPPGPWSLPFLGNFFVLG

FEKPPHVVLQKLVKKYGNLLSLEFGTLHTVTVSGLPLIKEVLANQDQNFLGRPKSAIRER

VFKKHGLFMSNGQEWKEQRRFALRTLRDLGLGKTRLEERIQEEAHHLIEAIEEEKGQPFD

PHFKINKAASNIICSITFGERFEYQDGEFQELLKLLDEVPSLETSKWSLLCVTFPWIMKF

LPGLHQTLFSDWEKLKIFVSRMIENHKRDWNPDKPRDFIDAYLKEMTKHTGNATSSFQEE

NLIYSTLDLLFAGTDTTSTTLRWALLYLTIYPEIQEKVQAEIDRVVGQSRQPSLNDREHM

PYTNAVIHEVQRMGNILPLNVPREVTFDTTLAGYHLPKGTMILTNVTGLHRDPAEWATPE

TFNPEHFLENGQFKKREAFLPFSMGKRACIGEPLARSELFIFLTSLVQKFTFRPPANEKL

SLKFRMGVTISPVSHRLCAVPRA

>BAE25645.1

MLAIATCLVANICSAIHLWTLLLTLLTLLLLADYLKNRRPKNYPPGPRRLPFVGNLFQFD

LDVSRLHLGIQPFVKKYGNVISVNFGYISSVIISGLPLIKEAITGMEQNFLKRPSLAARQ

HVFKNNGIVFSSGQTWKEQRKFALTILKNFGLGKKSLEQCIQEEAYHLVKAIGEEKGQPF

DPHFRINNAVGNIICSIIFGERFEYDDNQFQELLKLADEIICSEASMMSVLYNVFPSIFK

YLPGPQQKLFSNWEKLKLFVSRMMDSHREDWNPSAPRDFIDAFLTEMTKYPDKTTTSFNE

ENLICTALDLFFAGTETTSNTLRWALLYITVNPEVQEKVHSEIDRVIGHGRHPTLDDQDS

MPYTNAVIHEVLRMGNIIPLNVPREVTADSTLAGFYLPKGTMVLINLTDLHRDPKEWDTP

NVFNPEHFLENGQFKKKESFLPFSMGKRACPGEQLASCELFIFFTALMQKFTFKSPINEK

PSLKFRMGLTLAPVSYRICAVPRL

>AAH80831.1

MLAIATCLVANICSAIHLWTLLLTLLTLLLLADYLKNRRPKNYPPGPRRLPFVGNLFQFD

LDVSRLHLGIQPFVKKYGNVISVNFGYISSVIISGLPLIKEAITGMEQNFLKRPSLAARQ

HVFKNNGIVFSSGQTWKEQRKFALTILKNFGLGKKSLEQCIQEEAYHLVKAIGEEKGQPF

DPHFRINNAVGNIICSIIFGERFEYDDNQFQELLKLADEIICSEASMMSVLYNVFPSIFK

YLPGPQQKLFSNWEKLKLFVSRMMDSHREDWNPSAPRDFIDAFLTEMTKYPDKTTTSFNE

ENLICTALDLFFAGTETTSNTLRWALLYITVNPEVQEKVHSEIDRVIGHGRHPTLDDQDS

MPYTNAVIHEVLRMGNIIPLNVPREVTADSTLAGFYLPKGTMVLINLTDLHRDPKEWDTP

NVFNPEHFLENGQFKKKESFLPFSMGKRACPGEQLASCELFIFFTALTQKFTFKSPINEK

PSLKFRMGLTLAPVSYRICAVPRL

>118094727

MDFRFWPISQLGKLNVSMLLVVLVMFLLIIDFVRKRRPRNFPPGPQLFPLVGTIVDLRQP

LHLEMQKLTARYGNIFSVQFGGLTFVVVSGYQMVREALVHQAEIFADRPHIPLLQEIFRG

FGLISSNGHIWRQQRKFVSATLKSIAVSFESKVQEESRYLVEAMEEEKGQPFDPHYKINS

AVSNIICSITFGNRFNYHDSNFQELLHLLAETLLLIGSFWGQLYNAFPLIMRWLPGPFRK

IFRHWEKLQRFVRGVIAKHKEDLDQSDLGDYIDCYLKEIEKCKGDTNSYFHEENLLCSTL

DLFLTGTETTATAIRWALLYMAAYPHIQEKVQLEIDAVIGQCRQPTMEDKEHMPYTSAVL

SEVLRMGNIVPLGVPRMSTNDTTLAGFHVPKGTTLMTSLTSIMFDKNVWETPDTFNPEHF

LENGQYRRREAFLPFSAGKRACPGEQLARTELFIFFTALLQKFTFQAPSATVLSFAFTLS

LTRCPKPFQLCALPRC

>334321642

MLLSLWEGASLLTLLLVSTVLLILAFYVQRGRQHPNFPPGPPLLPIFGNIFQMDPKKPQD

TFQEFAKKYGNIFCLKLFGAPMICVTGLPLIKEVLLKQGQVFIDRPQTPWSSYAFKHHGI

SLSNGQIWKDQRRFTLMTLRNFGLGKKTLEFRIQEEATYLTEAIKEEKGQPFDPHFQINN

AVSNIICSVTFGYRFEYHDSQFRELLKILDEVMVLHGRWECQLFEMFPWIMRFLPGPHHK

LFREWKKLQSFVTQIIKQHKEDQNSEEAQDYIDAYLKELSKNKGDVSSSFSEGNLASCTL

DIFFAGTETTSTTLRWALLYMALYPEIQGKIQAEIDRVIGQSRQPTMADKENMPYTNAAI

HEVQRMGNIIPINVPRVATVDTTVAGYHVPKGTVLMTNLTALHRDPKEWATPETFNPEHF

LENGQFKKRESFLPFSAGKRVCLGEQLARAELFIFFTCLLQRFTFQAPPDTQLSLDFRTG

VTISPVPYKICALPRETQPKLDL

>224058583

MVLVVLVILFLIIDLVRKRRPRNFPPGPQLFPLVGTVVDFKQPLHLALQKLTGQYGNIFS

VQFGSLTFVVVSGYQMVREALVHQAETFADRPNIPLLQEIFRGFGLISSNGHIWRQQRKF

ASATLKSLAVNFEEKVQEESRYLVETIEEEKGQPFDPHYKINSAVSNIICSITFGNRFDY

HDNRFQELLHSLAETLLLIGSFWGQLYNAFPLIMRWLPGPFRKIFRHWEKLQYFVKEVIA

KHKEDLDQSKAGDYIDCYLKEIEKFKGDTSSYFHEENLLCCTLDLFLTGTETTATAIRWA

LLYMAAYPHIQEKVQQEIDAVVGQCRQPSMADKEKMPYTSAVLSEVLRVGNMVPLGVPRM

ATSDTTLAGFHLPKGTTLMTSLTSVMFDKNVWETPDTFNPEHFLENGLYRRREAFLPFSA

GKRACPGEQLARTELFIFFVALLQKFTFQAPAALSFAFTLSLTRCPKPFQLCAVPRH

>334321650

MQLPLGNVLPSLQTLLLFSAILLILASYVQRRRRRQQLKYPPGPLRLPIFGNVFQIDTTK

AHVSVQEFVRKFGNIFSMELFGVPMMTVTGLPLIKEVLINQGQVFVDRPQFPLQTYTFKS

VGLLMSNGQEWKDQRRFTLMTLRNFGLGKKTLELRIQEEATYLIEAIREEKGQPFDPHFQ

INNAVSNIICSVTFGNRFEYHDSQFRELLKSLDQVSVLQASWECQFFNIIPWIMKFLPGA

HKKLFREFKKLESFVVHVIKQHKEDQNSEEARDFIDAYLKELSKGNNSSSFNEDNLVSCT

LDLFFAGTETTSTTLRWALLYMALYPEIQGKIQAEIDRVIGHSRQPTMADRENMPYTYAA

IHEVQRMGNIIPLNAARVATVDTTLAGYHVPKGTWLLTNLTALHRDPKEWATPETFNPEH

FLENGEFKKRESFLPFSAGKRVCLGEQLARAELFIFFTCLLQRFTFQAPPDTQLSLDFRT

GMTVSPAPYKICAIPRETEPKLSL

>EDL30917.1

MLEAADSLAAAFWAALHLRTLLLAAVTFLFLADYLKSRRPKNYPPRPPRLPFVGNSFQMD

LTQSHLAVQKFVKKYGNVISLDFGIKSAVIISSLPLIKEAFSHLDENFINRRIFPLQRQI

FNGNGLIFSSGQTWKEQRRFALMTLRNFGLGKKSLEHRIQEEASHLVEAIGEEEGQPFDP

HFKIYSAVSNIICSITFGERFEYHDSQFQELLQLLDKAMYLGTTSLMVHLYNIFPWITKH

LPGQHQTLLATWRKLKSYIADIIDNHRKDWNPDEPRDFIDAFLNEMAKYPGKTTTSFNEE

NLICSTLDLFLAGTETTSSTLRWALLYMALYPEVQENVQAEIDRVIGQSKHPSLADRDSM

PYTNAVVHEILRMGNSVPLNIPREVAVDTTLAGFHLPKRSVVMTNLTALHMDPKEWATPD

IFNPEHFLENGQFKKRESFLPFSMGKRACLGEQLARSELFIFFTALVQKFTFKPPANENL

SLKFRLGITISPVSYRIGAVPRQ

>334321648

MDQNTNVEQEPYEFFKHFDWDVWDTNEQSEYLNLEQTKSYVDNDDDDDDDDDDIMEDRKS

YEKVEKLFAISRVNSYEELLDKLFFENNNADEVSIYTDSDFEFMKPHVALTGPPAPSTLV

LKPVFRGSLSWGAEGCLETLLLFSAILLILASYVQRRRRRQQSKYPPGPLRLPIFGNVFE

IDIKKPHISIQECVRKYGNIFSMDLLSAPMIVVTGLPLIKEVLVNQGQVFVDRPRSPLQS

YIFKINGLFMSNGQEWKDQRRFTLMTLRNFGLGKKTLELRIQEEAIYLTEAIREEKGKPF

NPHFQINNAVSNIICSVTFGNRFEYHDSQFRELLKSLNQVSVLQASWECQFFDIFPRIMK

LLPGAHKKLFREFQKLESFVVHVIKKHKEDQNSEEAQDFIDAYLKELSKYSGNISSSFNE

NNLVACTLDLFFAGTETTSTTLRWALLYMAFYPEIQGKIQAEIDRVIGHSRWPTMADREN

MPYTYAAIHEVQRMGNIVPLNAPRVATVDTTLAGYHVPKGTMLLTNLTALHRDPKEWATP

DTFNPEHFLEDGQFKKKEAFLPFSAGKRVCLGEQLARAELFIFFTCLLQRFTFQAPPDTQ

LSLDFQAGMTLSPVFYQICALPRETEPKLIL

>EAX06612.1

MDQNFGNRPVTPMREHIFKKNGLIMSSGQAWKEQRRFTLTALRNFGLGKKSLEERIQEEA

QHLTEAIKEENGQPFDPHFKINNAVSNIICSITFGERFEYQDSWFQQLLKLLDEVTYLEA

SKTCQLYNVFPWIMKFLPGPHQTLFSNWKKLKLFVSHMIDKHRKDWNPAETRDFIDAYLK

EMSKHTGNPTSSFHEENLICSTLDLFFAGTETTSTTLRWALLYMALYPEIQEKVQAEIDR

VIGQGQQPSTAARESMPYTNAVIHEVQRMGNIIPLNVPREVTVDTTLAGYHLPKGTMILT

NLTALHRDPTEWATPDTFNPDHFLENGQFKKREAFMPFSIGKRACLGEQLARTELFIFFT

SLMQKFTFRPPNNEKLSLKFRMGITISPVSHRLCAVPQV

>326679351

MSSVFSQLIGQWLDVQGFLIFLCVLLLVKHFRDVYSKNMPPGPFPLPFVGNLTNIGFSDP

LGSFQRIAEKYGDVCTLYLGTKPCILMTGYDTLKEAFVEQADIFTDRPYFPIVDKLGNGK

GLIMSSGHMWRQQRRFALATLKYFGVGKKTLENAILQECRFLCDSLQAERGLPFDPQHLV

TNAVSNIICGLVFGHRFEYDDHQFHLMQTYINNILQLPISNWGRRVLVCLVLCVSLQLYN

EFPTLMSLLPGKHQTAFASMSKLQPFLKEEITKHQQDREPSSPRDYIDCYLEEIEKCKDS

DAEFTEENLMFCVVDLFGAGTETTSNTLRWALAFMVKYPDVQEKVQSEIDQVIGQTRQPL

MDDRTNLPYTYAVIHEIQRFANIVTFTPPRVANKDTTVGGQLIPKGVIVLPMLKPILLDK

KEYSTPYDFNPDHFLDQNGKFLKKENFIPFSIGKRMCPGEQLARMELFLFFISLMQHFTF

LPVEGQKLSLKGTTSVSSAPQPFQIRAVPR

>350586160

MLGAVGFLAEVFGTALGPSALLLSAVAFLFVADILKRWRPKNYPPGPLRLPFVGNFLHLD

FEQWHLSLQRFVKKYGNVLSLDLGAFSSVVITGLPLIKEALVHQDQNFVNRPINLNQVFQ

KNGLIMSNGQVWKEQRRFALTTLRNFGLGKKSLEERIQEEAQYLIQAVREENGDLFDPHF

KINNAVSNIICSITFGERFDYQDDQFQELLRLLDEVTCLPKSVRVQLFNVFPRIMKLLPG

PHQIIFSNREKLRMFIARVIENHRRDWNPAEARDFIDAYLREIEKGSSPSVFNEENLICS

TLDLFFAGTETTSTTLRWGLLYMALYPEVQEVQAEIDRVLGQSQQPSTAARESMPYTNAV

IHEVQRMGNIIPLNVPREVAVDTTLAGYHLPKGTTVLINLTALHRDPAEWATPDTFNPEH

FLENGKFKKREAFLPFSIGKRASLGEQLARTELDTDIPTLQCQCHIRLEAEDLQAAEEQG

TQETLSFMERVQVGQGWTTGPTKRLQQRDHLPEPCLEVTK

>301620102

MEILGATAVLLVICAFFLLLNTIQVIRRQGKGKLPPGPTPLPFLGNFLQLRGEEVFKSLL

EFGKKYGPVYTIHLGMEPVVVLCSFDIVKEALNDNGDEFGARGHMPLLEKISHGGHGVVA

SNGERWKQLRRFSLMTLRNFGMGKRSIEERIQEEAHFLTNEFKYTKGQPVDPTFYFSKAV

SNVICSVVFGDRFEYEDTEFLRLLGLLNQVFRGFSSVWGQLYNIFPKVMGKLPGPHNMIF

KSVNSLQEFIMQRINMHQETLDPSSPRDFIDCFLIKMQQEKDVPQTEFHMQGALNTTFDM

FGAGTETVSTTLRYGLLILLKHPDIEERIQKEIDSVIGRNRAPCIEDRSRMPYTDAVIHE

IQRFVDIIPMGIPHKVTRDIQFQGYFIPKGTTVYPMLSSVLHDPKQFKYPDIFNPGHFID

ENGKFCKNDGFMPFSSGKRICVGEGLARMELFLFITTILQNYTLRSPVDTEDLDLTPELS

GFGNIPRPYKLCFIPR

>126272340

MEPWGLTTTVLLTCVLFLIFLSLWNYGTKKGKLPPGPTPLPIFGNLLQFDFKNMVSTMSK

LAKKYGSIYTLYFGTQRVVVLYGYNIVKEALIDKGDIFMERGNVPIFEDIVKGQGVVFSR

GEKWKQIRKFSLMTLRNFGMGKRSIEERVQEEAQCLVEELKKTKGQPNDPTFILGCAPCN

VICSILFRERFNYKDEKFLYLIGILNENFQLFAKPWIQLYNFLPAFRVHLPGKHKQLFKN

VEELKRFILERVKEHQEILDPNNPQDYIDCYLSKMQQEKNNPQSEFDVENLIMTALDLFA

AGTETTSSTLRYGLLLILKHPEVQAKIHEEINRVIGHNRIPSIKDRQDMPYMDAVVHEVQ

RFIDLLPLNLPHAVNQDIQLQQYTIPKGTNVFPLLSPVLRDSKEFSNPDKFDPQHFLDKN

GSFKKSDCFMPFSAGKRACLGEGLARMELFLFLTTILQNFTLKPVGDPNEISVKNNHVGF

TNVPPYYQLCFLPHKA

>AAH71042.1

MEILGATAGLLVICVLFLLLNTIQVIQRQGKGKLPPGPTPLPFLGNFLQLKGKEVFKSLL

ELSKKYGPVYTIHLGMEPVVVLCNCDIVKEALNDNGEEFGARGYMPLLDKMSHGGHGVIA

SNGERWKQLRRFSLITLRNFGMGKRSIEERIQEEARFLAKEFKNTKGQPVDPTFYFSKAV

SNVICSIVFGDRFEYEDKEYLRLLDFLNQTFRGVSSVWGQLYNIFPKVMGKLPGPHNTIF

QSVDVIHEFIKKRINMHKETLDPSSPRDFLDCFLIKMQQEKDVPQTEFHMLGAVNTTFDL

FGAGTETVSTTLRYGLLILLKHPDIEERIHKEIDSIIGRNRAPCIEDRSRMPYTDAVIHE

IQRFTDIIPMGLPHKLTRDIHFQGYSIPKGTTVYPMLSSVLHDPKQFKYPYSFNPGHFVD

ENGKFRKNDGFMPFSSGKRICVGEGLARMELFLFISTVLQNFTLSSPVDTDDLDLTPHLS

GFGNVPCPYKLCFIPR

>AAH45126.1

MVMDSAGTILLSFCVILLLYVLVKWGGSSKHKHLPPGPTAFPLLGNFLQVGFTEVPASLV

RLSKTYGPVYTVHLGGHRSIILTGYDAVKEALIDHSDVFSDRGDGGVSQMLFKNYGVILS

HGERWKTMRRFTLTTLRNFGMGKRSIEERIQEEARSLEEAFLKKKDEPFDPMYLLGLAVS

NIICSIIFGERFDYEDGKFMTLLMYLREFFQLLNSFFGMLFNFFPNMFCYIPGPHQKLFM

YFNKLKEFVRDEAKSHKATLDANCPRDFIDCFLIKMEEEKINPNSEFHNENLSGTIIDLF

LAGTETTSLTLRYALLILLKYPEIQEKVYKEIDQVIGQGRCPSVEDRSKMPYTEAVIHEV

QRFADIIPLGLEHAASKDTIFRGYYIPKGTVIFPVLTSVLKDPKYFKNPDQFDPGHFLDE

NGLFKKNDAFLPFSSGKRMCAGEGLARMELFIFLTTILQKCILKSTVDTRDITITPEPNT

NASRPWPYKMYVVPRS

>327280426

MELLGTCTALLVIWISFLLLSATWKSKMYRKGKMPPGPTPLPIIGNVLQLMGKYWDQEFS

KISEKYGPVFTLYLGMEPVVMLNDYESIKEALIDQGNDFSARPKIPLTYKVSKDGGIVFS

NGKTWKQLRQFSLTTLRNFGMGKRSIEERIQKEAQYLLEQFHDTKGQPFDPHHLITCATS

NVIGSIIFGKHYGYDNKKFQTFIKLIVESLDIFTSFYAQLFNAFPAFMEWVPGPHHHMIA

NYVKCTEFILEEAKEHRATLDPNSPRDFIDCFLIRMDQEKHDEASEFTTENMVTCCTDLF

GAGTETTSTTLKYGLLILQKYPEIEEKAQKEIDQVLGRSRMPSMADRRQMPYTDAVIHEI

QRFISLVSLSVPHAMVKDTPFRGYVIPKGTTVFPILTSVLHDGKEFPNPTEFDPGHFLNE

DGTFRKSDYFMPFSAGKRVCVGESMAHMELFLFFTSIIQNFKLKPITDPKDIDITPLEKP

LGRFPRPYEFCVIPR

>345785701

MVKAPGAWSLLLPLPVSLPLSLSLSLWVAPTEASLQLAESPEPWATGKAYPELQGTDLEQ

ITPQLPPKIQQIPAAAFTMDGVSTAILLGLLALAFLFLILNSRGKSQLPPGPRPLPFLGN

LLQLRSQDMLTSLTKLSKEYGSVYTVHLGPRRVVVLSGYQAVKEALVDQGEDFSGRGDYP

VFFNFTKGNGIAFSNGDRWKVLRRFSVQILRNFGMGKRSIEERILEEGSFLLAELRKTEG

KPFDPTFVLSRSVSNIICSVIFGSRFDYDDERLLTIIRLINDNFQIMSGPWGELYNIFPS

LLDWIPGPHRRLFQNFGCMKDLIARSVRDHQDSLDPRCPRDFIDCFLNKMAQEKQDPHSH

FHMDTLLMTTHNLIFGGTETVGTTLRHAFLVLMKYPKVQARVQEEIDRVVGRARLPALED

RAAMPYTDAVIHEVQRFADVIPMNLPHRVIRDTPFRGFLLPKGTDIITLLNTVHYDPNQF

LTPQEFNPEHFLDANQSFKKSPAFMPFSAGRRLCLGESLARMELFLYLTAILQSFSLQPL

GAPEDIDLTPLSSGLGNLPRPFQLRLRTR

>126272342

MEPWGLTTTVLLTCVLFLIFLSLWNHGTKKGKLPPGPTPLPIFGNLLQFDFKNMAATMSK

LAKKYGSIYTLYFGMERVVVLHGYNIVKEALIDKGDIFMERGNVPIFEDAIKGQGVIFSR

GERWKQLRRFSLMTLRNFGMGKRSIEERVQEEAQCLVEELRKTKGQPNDPTFILGCAPCN

VICSILFRDRFKYKDEKFLYLMSLLNENFQLFTKPWIQLYNFLPAFRVHLPGKHKQLFKN

VEELKCFILERVKEHQEILDPNNPQDYIDCYLSKMQQEKNNPQSEFDVENLIMTGVDLFS

AGTETTSSTLRYGLLLILKHPEVQAKIHEEINRVIGHNRIPSIKDRQDMPYMDAVVHEVQ

RFIDLVPLNVPHAVNQDIQLQQYTIPKGTNVFPLLSPVLCDSKEFSNPDKFDPQHFLDKN

GSFKKSDCFMPFSAGKRACLGEGLARMELFLFLTTILQNFTLKPVGDPNEISVKNNHVGF

TNVPPYYQLCFLPHKA

>327280406

MEPLGTTSVLLLVCISCLLLSAFWKSQANKRTKMPPGPTPLPIIGNALQLKTNHLDLTLC

KLSEKYGSVFTLHFGTKPVVVLHGYSAVKEALIDQAEDFAPRGRMPLVEKYFRGQGIIFS

NGERWKQLRRFALTTLRNFGMGKKSIEERIREEAQYLLERLQGTKEQPFDPTFLLNCATS

NIICSIVFGKHYDYDDKKFLAIMALMNDNFEILSSPWGQLANTFPSFMDWIPGPHHRVGT

NLEKSKAFVMEEMEAHRQTLDPSSPRDFIDCFFIKMDQEKNNEPSEFTTESLLMSTIDLF

GAGTETTSTTLRYGLLVLQKYPEIEEKVQEEIDRVVGRSRLPCMADRGQMPYTDAVIHEI

QRFISLVPLSLPHSVAKDTLFRGYIIPKDTSVFPLLTSVLHDGKEFPNPTEFDPQHFLNK

DGTFRKSDFFMPFSAGKRICAGEGLARMELFMFLTSILQNFKLKPLMDPQDIDIKPHLSG

IGNIPQPYRLCVVPR

>O35293.1

MDGVSTAILLLLLAVISLSLTFTSWGKGQLPPGPKPLPILGNLLQLRSQDLLTSLTKLSK

DYGSVFTVYLGPRRVIVLSGYQTVKEALVDKGEEFSGRGSYPIFFNFTKGNGIAFSDGER

WKILRRFSVQILRNFGMGKRSIEERILEEGSFLLDVLRKTEGKPFDPVFILSRSVSNIIC

SVIFGSRFDYDDERLLTIIHFINDNFQIMSSPWGEMYNIFPSLLDWVPGPHRRVFRNFGG

MKDLIARSVREHQDSLDPNSPRDFIDCFLTKMVQEKQDPLSHFNMDTLLMTTHNLLFGGT

ETVGTTLRHAFLILMKYPKVQARVQEEIDCVVGRSRMPTLEDRASMPYTDAVIHEVQRFA

DVIPMNLPHRVIRDTPFRGFLIPKGTDVITLLNTVHYDSDQFKTPQEFNPEHFLDANQSF

KKSPAFMPFSAGRRLCLGEPLARMELFIYLTSILQNFTLHPLVEPEDIDLTPLSSGLGNL

PRPFQLCMRIR

>327276369

MEMVSGFLLLPLCVCCLLIALTWKRQRGKGHLPPGPTPLPILGNFFQLDRKDMMKSLVKM

SEVYGPVFTIYLGMHPIVILCGYKAVKEALVDQAEEFSGRGQVPAFSKDFNKHGVVFSNG

ERWRKLRRFSLSTLRNFGMGKRSIEERIQEEAQCLVQEFHKMHGMPFDPISILSHAVSNV

ICSIVFGNRFEYHDKKFIRLNKLITKRFRVANSSQAMLYNMFPEFLEKLPGPHHTGSKCS

QEIIGFIMERIKMQQVSLDPSAPQNFIDCFLAKMEQEKHDPNTEFTMENLVMNTFNLFFA

GTETISTTLRYGFLLLLKHPQIQEKVHEEIDRVIGQDRNPNAQDRNKMPYTEAVLHEIQR

FGDVLPMSLPHAVTQDTQFRGYVIPKGTYVYALLNTVHYDQQHHANPEEFDPGRFLDSHG

CFKKLEAFMPFSVGKRACLGEGLARMELFLFFTTILQSFTLISPVPHSEISLEPTVSGLS

RLPMKYQVKMVPRRC

>126273206

MGPSVVTALGLLFCISCLLLILSWRKGFGKGKLPPGPVPLPIIGNMLQLNLKNIPESLCM

LAKEYGPVFTLQLGVQRIVVLHGYKAVKEALIEHGEQFAARGPMPIFELVSNGFGIGVSN

GERWKQLRRFSLMTLRNFGMGKRSIEERVQGEAKFLVEELKKTKGLPCDPTFILGCAPCN

VICSLIFQKHFEYNDQKFLYLMKLLHEQVRIGSSAWIQFYNCFPSLVQHLPGPHRKLLKL

FHFLHTFILEEIKEHQGTLDPSNPRDLIDCFLMKMEQEKQQPLSEFNIDNLVNTVADLFG

AGTETTSTTLRYGLLMLLKHPEITEKIHEEIDRVIGHNRSPCMEDRNKMPYTNAVVHEIQ

RYIDLIPTSLPHLVTEDTQFRQYIIPKGTTIIPFLSSVLYDEKEFPNPNQFDPGHFLDEN

GNFKKSDYFMPFSTGKRICLGEGLARMELFLFFTTILQNFTLKSLIDPKDIDTTPIDSGF

GKIPPSYKLCFLPSQNM

>334313994

MEHCRVKLRKAPGFGSIKGKISPIGRNSRMSIKKTAMDPSVVTALGLIFCVSCLLLISAW

RKGFGKGKLPPGPTPLPIIGNLLQLDTKNINKSFCELAKTYGSVFTLYLGSERAVVLHGQ

KAVKEALIGNGDAFAGRGSFPISETINKGLGLLFSNGERWKQIRRFSLMTLRNFGMGKRS

IEERVQEEAKRLVEALKNTNGLPCDPTFIFGCAPCNVICSVVFQKHFEYQDKKFLTLMEY

LNENLQILSSPWIQVYNLFPSLIHHLPGIHHKVIKNFRALNDFVLERVKEHQETVDPNDP

RDFIDCFLMKMEQEKQNPKSEFIIENLVSTTIDLFGAGTETTSTTLRYGFLLLLKHPQIV

DKIREEMDQVIGQNRSPCMKDRSSMPYTDAVIHEIQRYIDLVPTSLPHAVTQDVKFRQYL

IPKGTTIIPLLTSVLYDNEEFPNPEQFDPGHFLDESGNFKKSDYFVPFSIGKRACVGESL

AQMELFLFFTTILQNFTLKPLVDPKDIDVTPISNGFNHVPPCYELCFLPSQNM

>327280428

MELLGTCTALLVIWISFLLLSATWKSKMYRKGKMPPGPTPLPIIGNVLQLKGKYWDQQFS

KIGKKYGPVFTVYLGMEPAVVLNGYEAIKEVLIDQGNDFSARAKIPLADKVIKGGGIVFS

NGKTWKQLRQFALTTLRNFGMGKRSIEERIQTEAQFLLEKFHDTKGQPFDPHYLMINAVS

SVIGSVIFGKHYGYNDKMFQTFTTLIVESIDGLTSVWGQLFNTFPAFMEWVPGPHHHVIV

KYVKCREFILEEAKEHRATLDPNSPRDFIDCFLIKMDQEKHDEASEFTMENMVTCCIDLF

GAGTETTSSTLKYGLLLLQKYPEIEEKVHEEIDRVVGHSRMPSMADRRQMPYTDAVIHEI

QRFISLLSLSLPHAVVKDTPFRGYIIPKGTTVIPVLTSVLYDSKEFPNPTEFDPGHFLNE

DGTFRKSDYFMPFSAGKRVCAGESMARMELFLFFTSILQNFKLKPITDPKDIDLTPLEKP

VNRFPRRYEFCVIPR

>126329366

MEFGGAITLFLALCVPCLVILIAWKRMHKGGKLPPGPTPLPFLGNLLQVRTDATFQSFLE

LSKKYGPVFTVYMGPRRVVVLCGHEAVKEALVDQAEEFSGRGELASIDRNFQGHGVALAN

GDRWRILRRFSLTVLRNFGMGKRSIEERIQEEAGFLMEEFRKTKGTPIDPTFFLSRTVSN

VISSVVFGSRFDYEDKQFLYLLHLINESFIEMSTPWAQLYDMYSGIMQYLPGRHNKIYNL

IEELKDFIASRVKINEASLDPNNPRDFIDCFLVKMHQEKNNPKTEFNLKNLVLTTLNLFF

AGTETVSSTLRYGFLLLMKYPEVGAKVQKEIDHVIGQNRIPKAEDRMQMPYTDAVIHEIQ

RLTDIVPMGVPHTVTQDTNFRGYILPKGTDIFPLIGSALRDPKYFKKPEAFEPQHFLDEE

GRFKKNEAFVPFASGKRVCLGEAMARMELFLYFTTILQNFSLQPLVPPSEIDITPQISGF

GNIPPTYKLCIVAR

>297704850

MEMGGAVTIFLALCLSCLLILIAWKRMNKAGKLPPGPTPIPFLGNLLQVRTDATFQSFMK

LREKYGPVFTVYVGTRPVVVLCGHEAVKEALVDQADEFSGRGELASTERNFQGHGVAVAN

GERWRILRRFSLTILRDFGMGKRSIEERIQEEASYLLEELRKTKGAPIDPTFLLSRTVSN

VISSVVFGSRFDYEDKQFLNLLRLINESFIEMSTPWAQLYDMYSGIMQYLPGRHNRIYYL

IEELKDFIASRVKINEASFDPQNPRDFIDCFLIKMHQDKNNPRTEFNLRNLVLTTLNLFF

AGTETVSSTLRYGFLLLMKHPEVEAKIHEEINQVIGQHRLPRVDDRVKMPYTDAVIHEIQ

RLVDIVPMGVPHNVIRDTQFRGYLLPKGTDVFPLLGSVLKDPKYFRYPDAFYPQHFLDEQ

GRFKKNEAFVPFSSGKRICLGEAMARMELFLYFTSILQNFSLRSLVPPADIDITPKLSGF

GNIPPTYELCLVAR

>AAH59990.1

MFLGDPVTVLLTVVLCLILANLLYGRKRNNFKNFPPGPKPLPVIGNINIINLKRPYLTYL

ELWKKYGPVFSIQIGGQKMVVLCGYETVKDALVNYAEEFSERPKIPIFRDISKEYGVLFS

HGENWKVMRRFTLSTLRDFGMGRSSIEDRINEECDFLVEKFKSYKGKPFENTMIINAAVA

NIIVSIVLGHRFDYQDPIFLRLMSLINENIRLSGSPTVMLYNVFPSVMRWLPGSHKTIAK

NAAENQRFIRKTFTKHRDKLDVNDQRTLVDAFLVKQQEKNVNVQYFHDENLTMIVSNLFA

AGMETTSSTIRWGLLLMMKYPEIQKNVQNEIEKVIGQSQPQTEHRKSMPYTDAVLHEIQR

FGNIVPMNLPHATAQDVTFRGYFLPKGTFVIPLLTSVLYDQTHFEKPHEFYPQHFLDSEG

NFVKNEAFLPFSAGKRSCAGENLAKTELFLFFTSLLQNFTFQASPGEELDLTPAVGITTP

PLPYNICALSRT

>AAI27368.1

MFLGDPVTVLLAVALCLIVANTLYRQKRDSYKNFPPGPKPLPIIGNIHNINLKRPYLTYL

ELWKKYGPIFSVQIGSQKMVVLCGYETVKDALVNYAEEFSERPVIPIFLDAVKEYGVIFS

HGENWKVMRRFTLSTLRDFGMGRRTIEDRINEECDFLVEQFKSFKGEPFDNTMIMNAAVA

NIIVSIVLGHRFDYQDPIFLRLMSLINENIRLMGSPTVMLYNVFPSVMRWLPGNHQTVGK

NAAENRRFIRETFTKHRDKLDVNDQRNLIDAYLVRQQEKNGNAVYFHDDNLTVLVSNLFA

AGMETTSTSVRWGLLLMMKYPEIQKNVQNEIEKVIGQSRPQTEHRKSMPYTDAVIHEIQR

FGNIIPMNLPHATAQDVTFRGYFLPKGTYVIPLLTSVLYDQTRFEKPKEFYPQHFLDSEG

NFVKNEAFLPFSAGKRSCAGENLAKMELFLFFTSLLQNFTFQAPPGEELDLTPAIGITTP

PLPHNICALPRT

>126272338

MEPWGLTTTVLLTCVLFLIFLSLWNHGTKKGKLPPGPTPLPIFGNLLQFDFKNMASTFSE

LAKKYGSIYTLYFGTQRVVVLYGYNIVKEALIDKGDIFMERGNVPIFEDTVKGQGVVFSR

GERWKQIRRFSLMTLRNFGMGKRTIEERVQEEAQCLVEELRKTKGQPNDPTFILGCAPCN

VICSILFRDRFNYKDEKFLYLMGLLNENFQLFTKPWIQLYNFLPAFRVHLPGKHKQLFKN

VEELKCFILERVKEHQEILDPNNPQDYIDCYLSKMQQEKNNPQSEFDVENLLMTGADLFS

AGTETTSSTLRYGLLLILKHPEVQAKIHEEINRVIGHNRIPSIKDRQDMPYMDAVVHEVQ

RFIDLIPLNLPHAVNQDIQLQQYTIPKGTNIFPLLSPVLRDSKEFSNPDKFDPQHFLDKN

GSFKKSDYFMPFSAGKRACLGEGLARMELFLFLTTILQNFTLKPVGDPNEISVKNNHVGF

TNVPPYYQLCFLPHKA

>149722306

MELGRAFTIFLALCLSCLLLLIAWKRINKGGKLPPGPTPIPFLGNLLQVRTDSTFQSFMK

LREKYGPVFTVYMGPRPVVVLCGHEAVKEALVDQADNFSGRGELATIERNFQGHGLAMAN

GERWRILRRFSLTILRDFGMGKRNIEERIQEEAGFLLKELLKTKGAPIDPTFFLSRTVSN

VISAIVFGSRFDYEDKQFQKLMQMINESFTEMSTPWAQLYDMYSGIMQYLPGRHNHIYYL

IEELKDFIASRVKINEASLDPQNPRDFIDCFLIKMHQDKSNPHTEFNLKNLVLTTLNLFF

AGTETVSSTLRYGLLLMMKYPEVEAKIHEEIEQVIGSHRIPSVDDRVKMPYTDAVIHEIQ

RLTDIVPMGVPHNVIRDTHFRGYFLPKGTDVFPLLGSVLKDPKYFRYPDAFYPQHFLDDQ

GHFKKNEAFVPFSSGKRICLGEALARMELFLYFTSILQNFSLRSLVPPADIDITPKVSGF

GNIPPTYELCFMVR

>301625318

MSYITHFVSTDILVYLATCITLLLYLLKGFKNSALKMPPGPAPFPFIGNLNILNLKKPFQ

SLMKLSEKYGEVFTVHFGPKKMVVLAGYKAVKDALVTQADDFGERAEMPIFSKLTHGNGI

VFSNGESWKVIRRFTLSTLRDFGMGKRTIETRIQDELRPLIKLFKSHAGKPFDPKIILNS

AVSNVICSIIFGKRFEYNDTTFLTLLQLLNENARLFGTPPLLLFNFYPLLGSILGAHKRV

LRNTERLKHFFQNYINEHRAEFNANNITGFIDAYLLKQEQESSNAKTHFHDENLVYSVLD

LFAAGTETSSTTLRWGFLLMMKYPEIQKKVQKEIKEVVRPGQLPKVEDRRNMPYTDAVIH

EIQRFANIVPMNVSRATVRDVNFRGFLIPRGTEVIPMLTSVLYDNNHWKTPHEFNPQHFL

DANGKFVQRDAFLPFSTGRRACVGESLAKMELFLFFTGLLQSFTLCPPAGVSGADLDLTP

DIGFVLTPLPFMMCAIPNKQTPHFP

>AAI68071.1

MAMDSAGTILLSVCVIILLYLVKWRGKSESKNLPPGPTAYPLLGNFPQIGLREIPSSFVQ

LSKTYGPVYTLYLGGHRLIVLIGHDAVKEALIDQSDVFSDRGRLGISQVLFDEHGVIMSN

GERWKTMRRFTLTTLRNFGMGKRSVEERIQEEARSLEEAFRKKKDEPFDPVNLLGPAVSN

IICSIIFGDRFDYEDEKFTTLLKCMRELINLLNSLFGQLVNVFPNLSQHIPGPHQNIFTY

FNKIKQFVKDEAKSHKDTLDANCPRDFIDCFLIRMEEEKMNPNTEFHNDNLFAVIFDLFF

AGTETSSLTLRYAFLIFLKYPEVQEKVYKEIDQVIGQNRYPSFEDKIKMPYTEAVIHEVQ

RFADIVPTGLEHKTSKDTTFRGYDIPKGTSVFPVLTSVLKDPKYFKNPDQFDPGHFLDEN

GCFKKNDAFMPFSAGKRMCAGEGLARMELFIFLTSILQKFTLKPTVPAETIKITPQPKTN

ASQPWPYKMYAVPRC

>AAI61466.1

MAMDSAGTILLSVCVIILLYLVKWRGKSKSKNLPPGPTAYPLLGNFPQIGLREIPSSFVQ

LSKTYGPVYTLYLGGHRLIVLIGHDAVKEALIDQSDVFSDRGRLGISQVLFDEHGVIMSN

GERWKTMRRFTLTTLRNFGMGKRSVEERIQEEARSLEEAFRKKKDEPFDPVNLLGPAVSN

IICSIIFGDRFDYEDEKFTTLLKCMRELINLLNSLFGQLVNVFPNLSQHIPGPHQNIFTY

FNKIKQFVKDEAKSHKDTLDANCPRDFIDCFLIRMEEEKMNPNTEFHNDNLFAVIFDLFF

AGTETSSLTLRYAFLIFLKYPEVQEKVYKEIDQVIGQNRYPSFEDKIKMPYTEAVIHEVQ

RFADIVPTGLEHKTSKDTTFRGYDIPKGTSVFPVLTSVLKDPKYFKNPDQFDPGHFLDEN

GCFKKNDAFMPFSAGKRMCAGEGLARMELFIFLTSILQKFTLKPTVPAETIKITPQPKTN

ASQPWPYKMYAVPRC

>126272334

MEPWGLTTTVLLTCVLFLIFLSLWNHGTKKGKLPPGPTPLPIFGNLLQFDFKNMASTFSE

LAKKYGSIYTLYFGMQRVVVLHGYNIVKEALIDKGDIFMERGNVPIFEDTVKGQGVVFSR

GEKWKQIRRFSLMTLRNFGMGKRTIEERVQEEAQCLVEELRKTKGQPNDPTFILGCAPCN

VICSILFRERFNYKDEKFLYLMGLLNENFQLFNKPWIQLYNFLPAFRVHLPGKHKQLFKN

VEELKCFILERVKEHQEILDPNNPQDYIDCYLSKMQQEKNNPQSEFDVENLIMTALDLFA

AGTGTTSSTLRYGLLLILKHPEVQAKIHEEINRVIGHNRIPSIKDRQDMPYMDAVVHEVQ

RFIDLLPLNLPHAVNQDIQLQQYTIPKGTNIFPLLSPVLRDSKEFSNPDKFDPQHFLDKN

GSFKKSDYFMPFSAGKRACLGEGLARMELFLFLTTILQNFTLKPVGDPNEISVKNNHVGF

TNVPPYYQLCFLPHKA

>P33267.1

MDGVSTAILLLLLAVISLSLTFSSRGKGQLPPGPKPLPILGNLLQLRSQDLLTSLTKLSK

EYGSVFTVYLGSRPVIVLSGYQTVKEALVDKGEEFSGRGAYPVFFNFTRGNGIAFSDGER

WKILRRFSVQILRNFGMGKRSIEERILEEGSFLLEVLRKMEGKPFDPVFILSRSVSNIIC

SVVFGSRFDYDDERLLTIIHFINDNFKIMSSPWGEMYNIFPSVLDWIPGPHKRLFRNFGG

MKDLIARSVREHQDSLDPNSPRDFIDCFLTKMAQEKQDPLSHFNMDTLLMTTHNLLFGGT

ETVGTTLRHAFLILMKYPKVQARVQEEIDRVVGRSRMPTLEDRTSMPYTDAVIHEVQRFA

DVIPMNLPHRVTRDTPFRGFLIPKGTDVITLLNTVHYDSDQFKTPQEFNPEHFLDDNHSF

KKSPAFMPFSAGRRLCLGEPLARMELFIYFTSILQNFTLQPLVDPEDIDLTPLSSGLGNL

PRPFQLCMHIR

>AAD45178.1

MMLGGAFSIFMALCLSCLLILIAWKRTSKGGKLPPGPTPIPFLGNFLQVRTDATFQSFQK

LQKKYGSVFTVYFGPRPVVVLCGHEAVKEALVDQADDFSGRGEMPTLEKNFQGYGLALSN

GERWKILRRFSLTVLRNFGMGKRSIEERIQEEAGYLLEELHKVKGAPIDPTLYLSRTVSN

VICSVVFGKRFDYQDQRFQSLMRMINESFVEMSKPWAQLYDMYWKVMQYFPGRHNYLYNL

IEDLKDFIASRVKINEASFDPSNPRDFIDCFLIKMHQDKSDPHTEFNLKNLVLTTLNLFF

AGTETVSSTLRYGFLLLLKYPEVEAKIHEEINQVIGTHRTPRVDDRAKMPYTDAVIHEIQ

RLTDIVPLGVPHNVTRDTHFRGYLLPKGTDVYPLFGSVLKDPKYFRYPDAFYPQHFLDEQ

GRFKKNDAFVVFSSGKRICVGEALARMELFLYFTSILQRFSLRSLVPPADIDIAHKISGF

GNIPPVYELCFMAR

>EHH30060.1

MLASGLLLVALLACLTVMVLMSVWQQRNSKGKLPPGPTPLPFIGNYLQLNTEQMYNSIMK

ISERYGPVFTIHLGPRRIVVLCGYDAVKEALVDQAEEFSGRGEQATFNWVFKGYGVVFSN

GERAKQLRRFAIATLRDFGVGKRGIEERIQEEAGFLIEALRDTHGANIDPTFFLSRTVSN

VISSIVFGDRFDYEDKEFLSLLRMMLGSFQFTATSTGQLYEMFSSVMKHLPGPQQQAFKE

LQGLEDFIAKKVEHNRRTLDPNSPRDFIDSFLIRMQEEEKNPNTEFHLKNLVLTSLNLFF

GGTETVSTTLRYGFLLLMKHPEVEAKVHEEIDRVIGKNRQPKFEDQARMPYTEAVIHEIQ

RFGDMLPLGVAHRVIKDTKFRDFFLPKGTEVFPMLGSVLKDPKFFSNPQDFNPQHFLDEK

GQFKKSDAFVPFSIGKRNCFGEGLARMELFLFFTTIMQNFRFRFESPQLPKDIDVSPKHV

GFATIPPNYTMSFLPR

>114677397

MEMGGAVTIFLALCLSCLLILIAWKRMNKAGKLPPGPTPIPFLGNLLQVRTDATFQSFMK

LREKYGTVFTVYMGPRPVVVLCGHEAVKEALVDQADEFSGRGELASIEQNFQGHGVALAN

GERWRILRRFSLTILRDFGMGKRSIEERIQEEASYLLEEFRKTKGAPIDPIFLLSRTVSN

VISSVVFGSRFDYEDKQFLNLLRLINESFIEMSTPWAQLYDMYSGIMQHLPGRHNRIYYL

VEELKDFIASRVKINEASFDPQNPRDFIDCFLIKMHQDKNKPYTEFNLKNLVLTTLNLFF

AGTETVSSTLRYGFLLLMKHPEVEAKIHEEINQVIGPHRLPRVDDRVKMPYTDAVIHEIQ

RLVDIVPMGVPHNVIRDTQFRGYLLPKGTDVFPLLGSVLKDPKYFCYPDAFYPQHFLDEQ

GRFKKNEAFVPFSSGKRICLGEAMARMELFLYFTSTLQNFSLHSLVPPADIDITPKLSGF

GNIPPTYELCLVAR

>AAA37517.1

MDGVSTAILLLLLAVISLSLTFSSRGKGQLPPGPKPLPILGNLLQLRSQDLLTSLTKLSK

EYGSVFTVYLGSRPVIVLSGYQTVKEALVDKGEEFSGRGAYPVFFNFTRGNGIAFSDGER

WKILRRFSVQILRNFGMGKRSIEERILEEGSFLLEVLRKMEGKPFDPVFILSRSVSNIIC

SVVFGSRFDYDDERLLTIXHFINDNFKIMSSPWGEMYNIFPSVLDWIPGPHKRLFRNFGG

MKDLIARSVREHQDSLDPNSPRDFIDCFLTKMAQEKQDPLSHFNMDTLLMTTHNLLFGGT

ETVGTTLRHAFLILMKYPKVQARVQEEIDRVVGRSRMPTLEDRTSMPYTDAVIHEVQRFA

DVIPMNLPHRVTRDTPFRGFLIPKGTDVITLLNTVHYDSDQFKTPQEFNPEHFLDDNHSF

KKSPAFMPFSAGRRLCLGEPLARMELFIYFTSILQNFTLQPLVDPEDIDLTPLSSGLGNL

PRPFQLCMHIR

>397482613

MEMGGAITIFLALCLSCLFILIAWKRMNKAGKLPPGPTPIPFLGNLLQVRTDATFQSFMK

LREKYGPVFTVYMGPRPVVVLCGHEAVKEALVDQADEFSGRGELASIEQNFQGHGVALAN

GERWRILRRFSLTILRDFGMGKRSIEERIQEEASYLLEEFRKTKGAPIDPIFLLSRTVSN

VISSVVFGSRFDYEDKQFLNLLRLINESFIEMSTPWAQLYDMYSGIMQHLPGRHNRIYYL

VEELKDFIASRVKINEASFDPQNPRDFIDCFLIKMHQDKNKPYTEFNLKNLVLTTLNLFF

AGTETVSSTLRYGFLLLMKHPEVEAKIHEEINQVIGPHRLPRVDDRVKMPYTDAVIHEIQ

RLVDIVPMGVPHNVIRDTQFRGYLLPKGTDVFPLLGSVLKDPKYFRYPDAFYPQHFLDEQ

GRFKKNEAFVPFSSGKRICLGEAMARMELFLYFTSTLQNFSLHSLVPPADIDITPKLSGF

GNIPPTYELCLVAR

>292627241

MASVFSQLIGQWLDVQGFLIFLSVLLLVKHFRDVFTNNMPPGPFPLPFVGNVLSIGFGDP

MEVFPKVAEKYGDVCTFYIGTKPCILMTGYEVFKEAFVEQADIFTDRPFFPVNDRICKGK

GLIFSGGHMCRQQRRFALATLKYFGVGKKTLENSILQECRSVCESVQSERGLPFNPHYIL

TYAVGNVICSLVFGYKFDYGNDDFNKLLDYSDDTFKLPINIWGRLYNAFPSVMPLLPGKH

QTAFANLTKLKLFFQEEINKHKENRNPYSPRDYIDCYLEEIEKCKDKEAEFTEENLIHCV

LDLFGAGTESTAKTLVWALLYMAKYPDVQEKVHTEIDQVIGQSRQPLMEDRAHLPYTYAV

MHEVQRFANVLAFIPPRVASKDTTVAGFLIPKGVMVLPMLKPILEDKNEYCTPNEFNPAH

FLDENGKFVKRENFIPFSIGKRMCPGEQLAGMELFLFFISLMQHFTFLPPEGETLSLKRT

VAIASAPAPFRIKAVPRQCDNTAS

>AAI16042.1

MDLAVVLVLCLSCLLLLSLWKQSSGKGKLPPGPTPLPILGNILQLDVKNIGKSLSNLSNT

YGPVFTVYFGLRPTVVLHGYEAVKEALIDQGEEFSGRGNIPMSQRVNKGYGIIFSNGKRW

KEIRRFSLMTLRNFGMGKRSIEDRVQEEAHCLVEELRKTNGSPCDPTFILGCAPCNVICS

IIFQNRFDYTDQNFLNLLDKFNENLQVASSPWMQVCNTFPILIDYFPGSHNKLFKNFAYI

RSYVLEKVKEHQATLDINNPRDFIDCFLIKMEQEKHNQEMEFTFENLIASVSDLFGAGTE

TTSTTLRYGLLMLLKHPEVTAKVQEEIDRVIGRHRSPCMQDRSHMPYMDAVVHEIQRYID

LVPTNLPHAVTRDIKFRNYLIPKGTTVVTSLSSVLHDEKEFPNPKVFDPAHFLDESGNFK

KSDYFMAFSAGKRSCVGEGLARMELFLFLTTILQKFTLKSVVDPKDLDTTPVSSGFGHVP

PPYQLCFTPL

>327280430

MELLGTCTALLVIWISFLLLSATWKSKMYRKGKMPPGPTPLPIFGNVLQLKGKYWDQEFS

KISETYGPVFTFYLGMEPVVVLNGYEAIKEALIDQGNDFSFRAKIPLIDKLNKGGGIMFS

NGKTWKQLRQFTLTTFRNFGMGKRSIEERIQKEIQYLLEQFHDTKGQPFDPHYLMINAVS

SVIGSVIFGKHYGYDDKKFQTFITQIIESIEIFASICGQLFYAFPAFMEWFPGPHQHVIA

NYVKLKEFILEEAKEHRATLDPNSPRDFIDCFLIRMDQEKHDEASEFTMENMVTCCIDLF

GAGTETTSSTLKYGLLLLQKYPEIEEKVHEEIDRVVGRSRMPGMADRRQMPYIDAVIHEI

QRFMSLASVSIPHSVVKDTPFRGYVIPKGTTVFPVLISVLHDGKEFPNPTEFDPGHFLNE

DGTFRKSDYFMPFSAGKRLCVGEGMARMELFLFFTSILQNFKLKPITDPKDIDVTPLEKP

GGRFSPHYEFCVIPR

>DAA19879.1

MLASGLLLVALLACLTIMVLMSVWRQRNLKGKLPPGPTPLPFIGNYLQLNTEQMCNSLMK

ISEHYGPVFTVHLGTRQIVVLCGYDAVKEALVDQAEEFSGRGKQATFDWLFKGYGVAFSN

GERAKQLRRFSITTLRDFGVGKRGIEERIQEEAGFLIEAFRGTRGAFIDPTFFLSRTVSN

VISSIVFGDRFDYEDKEFLSLLRMMLGSFQFTATSTGQLYEMFYSVMKYLPGPQQQAFKE

LQGLEDFIAKKVEQNQRTLDPNSPRDFIDSFLIRMQEEKENPNTEFYRKNLVMTTLNLFF

AGTETVSTTMRYGFLLLMKHPDVEAKIHEEIDRVIGKNRQPKFEDRAKMPYTEAVIHEIQ

RFGDMIPMGLARRVTKDTKFRDFLLPKGTEVFPMLGSVLRDPKFFSNPRDFNPQHFLDEK

GQFKKSDAFVPFSIGRRLCLGEALARMELFLYLTAILQSFSLQPLGAPEDIDLTPLSSGL

GNLPRPYQLCVLAR

>P10610.1

MALGGAFSIFMTLCLSCLLILIAWKRTSRGGKLPPGPTPIPFLGNLLQVRIDATFQSFLK

LQKKYGSVFTVYFGPRPVVILCGHEAVKEALVDQADDFSGRGEMPTLEKNFQGYGLALSN

GERWKILRRFSLTVLRNFGMGKRSIEERIQEEAGYLLEELHKVKGAPIDPTFYLSRTVSN

VICSVVFGKRFDYEDQRFRSLMKMINESFVEMSMPWAQLYDMYWGVIQYFPGRHNRLYNL

IEELKDFIASRVKINEASFDPSNPRDFIDCFLIKMYQDKSDPHSEFNLKNLVLTTLNLFF

AGTETVSSTLRYGFLLLMKYPEVEAKIHEEINQVIGTHRTPRVDDRAKMPYTDAVIHEIQ

RLTDIVPLGVPHNVIRDTHFRGYFLPKGTDVYPLIGSVLKDPKYFRYPEAFYPQHFLDEQ

GRFKKNDAFVAFSSGKRICVGEALARMELFLYFTSILQRFSLRSLVPPADIDIAHKISGF

GNIPPTYELCFMAR

>327280404

MEPLGTSTVLLVICISCLLLSAFWKSQANKRKMPPGPTPLPIIGNALQLKTNHLDLTLCK

LSKSYGPIFTLYFGPRPVVVLHGYGAVKEALIERADEFAARGRMPSMEKYFQGKGIIFSN

GETWKQLRRFALTTLRNFGMGKKSIEERIQEEAQYLLEQLQGTKEEPFDPTFLLNCATSN

IICSVVFAKHYDYDDKKFLAIMALMHENFEILNSPWGQLANLFPSFMDLIPGPHHRVGTN

FKKAKVFVMEEMEAHRETLDPSSPRDFIDCFYIKMDQEKNTEASEFTIENLLMSAINLFG

AGTETTSTTLRYGLLILQKYPEIEEKVQEEIDRVVGRSRAPCMSDRGQMPYTDAVIHEIQ

RFISLIPLSLPHSVNKDTVFRGYTIPKDTTVFPLLTSVLHDDKEFPNPREFDPQHFLNKD

GTFRKSDYFMPFSAGKRICAGEGLARMELFLFLASILQNFKLKPLTDPQDIDIKPHLSSI

GNVPPPYRLCVVPR

>AAH82928.1

MDPISILLSIAVCVFLLNLIYGGKGDSKTFPPGPTPLPVIGNLLIMNMKKPHLTFMELAK

KYGSVFSVQLGTEKVVVLCGYDTVKDALINHADEFSERPKIPIFEDVSKGYGLIFAHGEN

WRVMRRFTLTTLRDFGMGKKTIEDRIYEESDCLVETFKSYKGEPFENTLVMNAAVSNIIV

SILLGHRFDYQDTALLKLIKIINENVKLMGSPMVMLYNTYPSVVQWLPGNHKTVAENTLK

LLNFLQETFTKHRDQLDVNDQRDLIDAFLVKQQEEKSSSTMFFHNQNLTLLVANLFGAGM

ETTSTTLRWGLLLMMKYPEIQKKIQDEIDRVIGSAQPQAEHRKQMPYTDAVIHEIQRFAN

IAPSNLPHATTKDVTFRGYFIPKGTQVIPLLTSVLQDEAYFKKPEEFYPEHFLDSEGHFV

KNEAFLPFSAGRRSCAGETLAKMELFLFFTKLLQNFTFQAPPGAEVQLTSGEGFTSSPLP

HKICALPRT

>402905628

MELGGAVTIFLALCLSCLLVLIAWKRMNKAGKLPPGPTPIPFLGNLLQVRTDAAFQSFMK

LREKYGPVFTVYMGPRPVVVLCGHEAVKEALVDQADEFSGRGELASIERNFQGHGVALAN

GERWRILRRFSLTILRDFGMGKRSIEERIQEEASYLLEEFLKTKGAPIDPTFLLSCTVSN

VISSVVFGSRFDYEDKQFLNLLRLINESFIEMSTPWAQLYDMYSGIMQYLPGRHNRVYYL

IEELKDFIASRVKINEASFDSQNPRDFIDCFLIKMHQDKNNPRTEFNLKNLVLTTLNLFF

AGTETVSSTLRYGFLLLMKHPEVEARIHEEINQVIGPHRLPSVDDRVKMPYTDAVIHEIQ

RLVDIVPMGVPHNVIRDTQFRGYLLPKGTDVFPLLGSVLKDPKYFRYPEAFYPQHFLDEQ

GRFKKNEAFVPFSSGKRICLGEAMARMELFLYFTSILQNFSLRSLVPPADIDVTPKLSGF

GNIPPTYELCLVAR

>301608092

MFLGDPVTLLLAVVLSLIVANTLYRKERVNVQNFPPGPKPLPIIGNIHNINAKRPYLTYL

ELWKKYGSVFSVQIGSQRMVLLCGYETVKDALVNHAEEFSDRPIIPLFHEITKGNGVVFA

NGENWKVMRRFTILALRDFGMGRRTIEYRINEECDFLVEKIKSYRGEPFENTMIMNAAVA

NIIVSILLGHRFDYQDPTILRLLSLINQSVKITGSPMVMLYNMFPSVMRWLPGSHKTLAI

NVAEIQSFIRETFTKYRDKLEINDQRNLIDAFLVKQQENKENGLYFHDDNLTMLVSNLFT

AGMETTSSTLRWGLLLMMKYPEIQKNVQNEIEKVIGQSRPQTEHRKSMPYTDAVIHEIQR

FGNIIPMNLPHATAQDVTFRGYFLPKGTFVIPLLMSVLYDQSHFENPNEFYPQHFLDSEG

NFVKNEAFLPFSAGKRSCAGENLARMELFLFFTSLLQNFTFQAPPGEELDLTPGTGLSAP

PSPYKICALPCS

>109124880

MELGGAVTIFLALCLSCLLVLIAWKRMNKAGKLPPGPTPIPFLGNLLQVRTDATFQSFMK

LREKYGPVFTVYMGPRPVVVLCGHEAVKEALVDQADEFSGRGELASIERNFQGHGVALAN

GERWRILRRFSLTILRDFGMGKRSIEERILEEASYLLEEFRKTKGAPIDPTFLLSRTVSN

VISSVVFGSRFDYEDKQFLNLLRLINESFIEMSTPWAQLYDMYSGIMQYLPGRHNRVYYL

IEQLKDFIASRVKINEASFDSQNPRDFIDCFLIKMHQDKNNPRTEFNLKNLVLTALNLFF

AGTETVSSTLRYGFLLLMKHPEVEARIHEEINQVIGPHRLPSVDDRVKMPYTDAVIHEIQ

RLVDIVPMGVPHNVIRDTQFRGYLLPKGTDVFPLLGSVLKDPKYFRYPEAFYPQHFLDEQ

GRFKKNEAFVPFSSGKRVCLGEAMARMELFLYFTSILQNFSPRSLVPPADIDITPKLSGF

GNIPPTYELCLVAR

>AAH60328.1

MAIDTAVTILLTVCVIILLYLVKWSGNSKQKNFPPGPTAFPLLGNFPQIGTTEIPASLVE

LSKTYGPMYTLYLGGHPLVMLIGYDAVKEALIDYGDVFSDRGRTGISQAIFSEYGVIMSN

GERWKTMRRFTLMTLRNFGMGKRSLEERIQEEARNLEEAFRKKRDEPFDPIYLLGLAVSN

IICSIIFGERFDYEDEKFKSLLMYMRETLKLLNSFLGQVLNLFPNLSLYIPGPHQKVFAN

FNKLKEFVKDEAKSHRDTLDANCPRDFIDCFLIRMEEEKINPDTEFHNENLFAVIFDLFF

AGTETSSLTLRYAFLILQKYPEIQEKVYKEIDEVIGQHRYPSVEDRSKMPYTEAVIHEIQ

RFADIIPSGLERSASKDTTFRGYYIPKGTSVFPVLTSVLKDPKYFKNPDQFDPRHFLDEN

GCFKKNEAFMPFSTGKRMCAGEGLARMEIFIFLTTILQKFILKPTVDTEAIKITPQPNTN

ASRPWPYKMFAVPRC

>73948302

MELGGAFTIFLALSLSCLLILIAWKRNSKGGKLPPGPTPIPFLGNVLQVRTDATFQSFMK

LREKYGPIFTVYMGPRPVVVLCGHEAVKEALVDRADEFSGRGELASIERNFQGHGVALAN

GERWRILRRFSLTILRDFGMGKRSIEERIQEEAGFLLEELRKTKGSPIEPTFFLSRTVSN

VISSVVFGSRFDYEDKQFLKLLQMINESFIEMSTPWAQLYDMYSGIMQYLPGRHNRIYYL

IEELKDFIASRVKINEASLDPQNPRDFIDCFLIKMHQDTNNPHTEFNLKNLVLTTLNLFF

AGTETVSSTLRYGLLLMMKHPEVEAKIHEEIDQVIGPHRIPSVDDRAKMPYTDAVIHEIQ

RLTDIVPMGVPHNVIRDTHFRGYLLPKGTDVFPLLGSVLKDPKYFRYPDAFYPQHFLDEQ

GRFKKNEAFVPFSSGKRICLGEAMARMELFLYFTSILQNFSLHSLVPPADIDITPRVSGF

GNIPPTYELCLKAR

>344274939

MDLAVVLVLCLSSLLLFSLWKQSYGKGKLPPGPTPLPIIGNSLQLNFKNISKSLHDLSKT

YGPVFTLYLGMKPTVVLHGYEAVKEALIDQGEEFSGRGSFPVSEKVHKGLGVVFSNGKMW

KETRRFSLMTLRNFGMGKRSIEDRVQEEACCLVEELRKTKASPYDPTFILGCAPCNVICS

IIFQNRFDYTDQDFLNLIGKLNENIQILSSPWVQVCNSFPLFIDYLPGSHNKLFNNIAYS

KSYILEKMKEHQETLDINNPRDFIDCFLIKMEQEKDNQESVFTIENLIITISDVFSAGTE

TTSTTLRYGLLLLLKHPEVTAKVREEIDRVVGRHRSPCMQDRSSMPYTDAVVHEIQRYID

LIPTNLPHAATQDIKFRNYLIPKGMTILTSLTSVLHDNKEFPNPETFDPAHFLDESGNFK

KSDYFMAFSAGKRICLGEGLARMELFLFFTAILQNFTLKPLVDPKDIDTNPVEKGFASVP

PKYQLCFIPV

>EDL30915.1

MLSTVGSLVARIWSAIHLWTLLLTLLVFRLLADYLKNRRPKNYPPGPWRLPFVGNLFQFD

LDVSQLHLRIQLFVKKYGNLISIDFGNISSVVITGLPLIKEAFTDMEQNLLKRPIIPSRK

HVFKDNGIIFSSGQKWKEQRRFALMILKSFGLGKKSLEQRIQEEAYHLVKAMEEEKGQPF

DPHFRINSAVSNIICSINFGERFEYGDNKFQELLKITNETLSAETSMMLVLYNALPSIMK

YLPGPHQTVFRNWERLKMIVYHMMESHRKDWNPDEPRDFIDAFLTEMTKYPDKTTTSFNE

ENLICSCLDLFIAGTETSSTALRWALLYMTLNPEVQERVHSEIDGVIGQWRQPSTGDRDS

MPYTNAVIHEVLRMGNIVPLNVPREGKMILTNLTALHRDPKEWATPDIFNPEHFLENGQF

KKRESFLPFSMGKRACPGEQLARTELFIFFTALMQKFTFKAPVNEKLSLKFRMSVPISPV

SYCICAVPRQ

>301620116

MALGFVGTILLTACVTILLFLFKWRGKIKIKNLPPGPTPIPLLGNIPQINTTELPNSLLE

LSKTYGPVYTLHLGSYRSVMLIGYDAVKEALIDRSEVFSDRGIMDLTELIFKNYGVIMTN

GERWKTMRRFTLMTLRNFGMGKRSIEERIQEESQSLAEAFEKNKGQPFDPMYLLVLAVSN

IICSIIFGERFDYEDQKFMTLLMYLREIIRLSNTFIGQLLNFFPKVLQYIPGPHQNIFTY

FDKLKEFVREEANAHKDTLDKNCPRDFIDCFLMRMEEEKMNPNSEFHNENLNEVIFDLFF

AGTETTSVTLRYAFSILLKYPEIQEKIHKEIDQVIGQDRCPSVEDRSKMPYTEAVIHEVQ

RFADIVPAGLAHAASKDTTFRGYNIPKGTLIFPVLTSVLKDPKFFKNPYQFDPGHFLDND

GNFKKNDAFMPFSAGKRVCAGEGLARMELFIFLTTILQKFILKPTVDKKDIEITPEPKTN

GSRPRSYKMFVVPRC

>301620114

MAMDSAGTVLLAACVIVLFYLVKWRGNNKRKNLPPGPTAFPLLGNFLQVSTTEIPSSCVE

LSKTYGPVFTLYLGGHRSIILIGYDAVKEALIDNSDVFSDRGEGGVSEMIFKNYGVILSN

GERWKTMRRFTLTTLRNFGMGKRSVEERIQEEARSLEEAFRKKKDEPFDPIYLLGLAVSN

IICSIIFGERFDYEDEKFMTLLMYIREFVKLLNSFFGMLFNFFPNLFCYIPGPHQNIFTY

FNKLKQFVKDEAKSHKDTLDANCPRDFIDCFLIRMEQEKNNPNSEFHYENLFGTILDLFL

AGTETTSSTLRYAFLILLKYPEIQENVYKEIVQVIGQHRYPSVEDRSKMPYTEAVIHEVQ

RIGDILPLGLEHAASKDTTFRGYDIPKGTLIFPLLTSVLKDPKYFKNPDQFDPEHFLDEN

GCFKKNDAFMPFSTGKRVCAGEGLARMELFIFLTTILQKFILKSTVATEEIKITPEPNTN

GSRPWPYKMFVVPRC

>395501826

MDLSMVIELGLLLCISCLLLILSWKKNFGKGKLPPGPISLPIIGNILQVNLKNIPESLSV

LAKKYGPVFTLQLGMEHVIVLHGYEAVKEALIDHGDKFADRGHIPILEYISNGLGIGVSN

GERWKQLRRFCLMTLRNFGMGKRSIEERVQEEAKNLVKELEKTKGLPCDPTFILGCAPCN

VICSIIFQKHFDYKDQKFLHLLKLFEEDMKIVSSPWIQVYNCFPSLLHHLPGSHHKVYKI

IHLIYEFIFEEVKEHQKTLDPSNPRDFIDCYLMKMEQEKQQPQSIFTIENLIYIAIDLFS

AGTETTSTTLRYGLLLLLKHPEVTGKIHEEIDRVIGRNRSPCMEDRNKMPYTYAVIHEIQ

RYIDLIPNSLPHAVTQDIHFRQYFIPKGTTIIPSLTSVLYDDKEFPNPNQFDPGHFLDES

GKFKKSDYFMPFSAGKRICLGESLARMELFLFFTTILQNFTLKSPMDPKDIDTTPTASGF

GKVPPFYELCFLSL

>395501851

MDPSVVTALSVILCLLLISVWRKGYGKGKLPPGPTPFPIIGNILQLDTKNINKSFCLLAK

AYGPVFTLYLGSKRAIVLHGYEAIKEALIGHGDAFAGRGSFPISDTINKGFGLLFSNGER

WKQIRRFSLMTLRNFGMGKRSIEERVQEEAKCLVEELKKTNGLPCDPTTILESAPCNVIC

SVVFQKRIEYQDEKFQRLMENLNENIRILSSPWIQVYNLFPSLVHYLPGIHHKLIENFRY

MNDFVLERVKEHQETLNANDPRDFIDCFLMKVEEEKQNPQSEFTIENLVYTTLDLFGAGT

ETTSTTLRYGLLLLLKHPHITKKIHEEIDRVIGPNQSPCMKDRNNMPYTNAVIHEIQRYI

DLVPASLPHAVTQDIQFRQYLIPKGTTIIPLLTSVLYDNEEFPNPDQFDPHHFLDESGNF

KKSNYFLPFSIGKRACVGEGLAQMELFLFFTTILQNFTLKPVTDLKDIDVTPVDNGFNHV

PPCYEICFLPSKNM

>348552508

MELSLLLFLALLLGLLLLLFKGHPKAHGNLPPGPHPLPFLGNILQMNRKGLFKSFLQFRE

KYGDVFTVYLGPRPVVMLCGAETIREALVDQADSFSGRGMIAVAEPTFQGYGVVFANGDR

WKALRRFSLATMRDFGMGKRTVEERIQEEAQCLVEEIKKSKGDLLDPLFFFQSATANIIC

SIVFGERFDYKDQQFLRLLDLFYQSFALGSSLSSQIFELFSTILKYFPGPHREIYKNLQE

VNRFIGHRVEKHRETLDPSNPRDFIDTFLLRMDKEKSNSHTEFHHKNLILTSLSLFFAGT

ETTSTTLRYGFLFLLKYPHVTERVQKEIEQVIGSHRQPALDDRSKMPYTEAVICEIQRFA

DLLPIGLPHMVTKDTHFRGFFIPKGTEVYPLLSTALHDPHHFEKPEFFNPDHFLDAKGAL

KKNEAFIPFSMGKRICLGEGVARAELFLFFTTILQNFSIASPVAPEDIDLTPKELGIGRV

PPTYQISFLPRTIDSREK

>348588102

MMLTGLLFVILVLCLSYLFFISLWRQSSGRKKLPPGPTPLPIIGNVLQVDFKNISKSLTN

LSKVYGPVFTLYLGGKPTVVLHGYEAVKEALVDLGEEFSGRGNLPIFEKVGETLGIIFSN

GKLWKETRRFSLMTLRNFGLGKRSIEDRVQEEAHCLVEELRKTNGSPCDPTFILGCAPCN

VICSIVFQNRYDYKDQKFLTLMKTVNENTKILSSKWMQVYNNFPKLIDYLPGSHNRFAKN

FFYLRNYILDKVKEHQESLDISNPRDFIDCFLIKMNQEKHNQCLEFTFENLVSTVTNLFV

AGTETTSTTLRYALLLLMKHPHVTDKVQKEIERVIGRNRTPCMQDRSRMPYTDAVIHEIQ

RYIDLVPNSLPHAVTRDIKFRNYLIPKGTTILTSLTSVLHDDKEFSNPETFDPNHFLDDS

GKFKKSDYFMPFSTGKRICLGEGLARMELFIFLTSILQHFNLKSVVDLKYIDTTPTVNGF

ASLPPLYKLCFIPV

>AFR33963.1

MDAVVILVLCLSYLFFISLWRQSSGRKKLPPGPTPLPIIGNVLQVDFKNISKSLTNLSKV

YGPVFTLYLGGKPTVVLHGYEAVKEALVDLGEEFSGRGNLPIFEKVGETLGIIFSNGKLW

KETRRFSLMTLRNFGLGKRSIEDRVQEEAHCLVEELRKTNGSPCDPTFILGCAPCNVICS

IVFQNRYDYKDQKFLTLMKTVNENTKILSSKWMQVYNNFPKLIDYLPGSHNRFAKNFFYL

RNYILDKVKEHQESLDISNPRDFIDCFLIKMNQEKHNQCLEFTFENLVSTVTNLFVAGTE

TTSTTLRYALLLLMKHPHVTDKVQKEIERVIGRNRTPCMQDRSRMPYTDAVIHEIQRYID

LVPNSLPHAVTRDIKFRNYLIPKGTTILTSLTSVLHDDKEFSNPETFDPNHFLDDSGKFK

KSDYFMPFSTGKRICLGEGLARMELFIFLTSILQHFNLKSVVDLKYIDTTPTVNGFASLP

PLYKLCFIPV

>327288002

MDMGVSLLSPFLALAVSCLAVLALWKRLSPQKGRLPPGPTPLPFLGNLLHVKTTNAFQSF

LALRDKYGPVFTVYLGPRRVVVLCGHDAVKEALVDQAEEFSGRGELASIDRNFNGFGVAL

ANGERWRQLRRFSLTALRNFGMGKRSIEERIQEEAQFLVEAFRETQGLPFDPTFFLSRTV

SNVISSVVFGHRFDYEDQTFLSLMHKMNESFLEMSTPWAQLYDMFSCVMRYLPGRHNRIY

YLLEDLKAFVADKAQANLETLDPNNPRDFIDCFLIQMEKKKGNPSSEFNMKNLVLTTLNL

FFAGTETVSSTLRYGLLFLMKHPEVEEKVHQEIDRVIGRHRLPGIEDRMWMPFTDAVIHE

IQRMTDIVPFGVPHTVIRDTHFRGFLLPKGTNVFPLLGSVLRDPKYFRNPDQFDPGHFLD

ADGRFKKNEAFVPFSSGKRACLGEALARMELFLYLAFILQNFSLKGLGPPEGIDLAPRVS

GFGNIPPAYKMRLVPRC

>ADM53746.1

MEMGGAVTIFLALCLSCLLVLIAWKRMNKAGKLPPGPTPIPFLGNLLQVRTDATFQSFMK

LREKYGPVFTVYMGPRPVVVLCGHEAVKEALVDQADEFSGRGELASIERNFQGHGVALAN

GERWRILRRFSLTILRDFGMGKRSIEERILEEASYLLEEFRKTKGAPIDPTFLLSRTVSN

VISSVVFGSRFDYEDKQFLNLLRLINESFIEMSTPWAQLYDMYSGIMQYLPGRHNRVYYL

IEQLKDFIASRVKINEASFDSQNPRDFIDCFLIKMHQDENNPRTEFNLKNLVLTTLNLFF

AGTETVSSTLRYGFLLLMKHPEVEARIHEEINQVIGPHRLPSVDDRVKMPYTDAVIHEIQ

RLVDIVPMGVPHNVIRDTQFRGYLLPKGTDVFPPLGSVLKDPKYFRYPEAFYPQHFLDEQ

GRFKKNEAFVPFSSGKRVCLGEAMARMELFLYFTSILQNFSPRSLVPPADINITPKLSGF

GNIPPTYELCLVAR

>Q05555.1

MLASGLLLAALLACLTVMILLSVWRQRKLWGKLPPGPTPLPFIGNYLQLNTEQMYDSLMK

ISERYGPVFTIHLGPRRIVVLCGQEAVKEALVDQAEDFSGRGELATFDWLFKGYGVVFSS

WERARPLRRFAISTLRDFGVGKRGIEERIQEEAGFLIEAFRDTRGAFIDPTFFLSRTVSN

VISSIVFGDRFDYEDKEFLSLLRMMLGSFQFTATPTGQLYEMFYSVMKHLPGPQQQAFKE

LEGLRDFIAKKVERNQRTLDPNSPRDFIDSFLIRMQEEKKDPKSEFHMKNLVMTTLNLFF

AGTETVSTTMRYGFLLLMKHPDVEAKVHEEIDRVIGRNRQPKFEDRAKMPYTEAVIHEIQ

RFTDMIPMGLAHRVTRDTKFRDFLLPKGAEVFPMLGSVLKDPKFFSKPREFYPQHFLDEK

GQFKKSDAFMPFSVGKRYCLGEGLARMELFLFFTTIMQNFRFRSQQAPQDIDVSPKHVGF

ATIPRTYTMSFVPR

>301776637

MELGGAFTIFLALSLSCLLILIAWKRNNKGGKLPPGPTPIPFLGNVLQVRTDATFQSFMK

LKEKYGPVFTVYMGPRPVVVLCGHEAVKEALVDRADEFSGRGELASIERNFQGHGVALAN

GERWRILRRFSLTILRDFGMGKRSIEERIQEEAGFLLEELRKTKGSPIEPTFFLSRTVSN

VISSVVFGSRFDYEDKQFLKLLQMINESFIEMSTPWAQLYDMYSGIMQYLPGRHNRIYYL

VEELKDFIASRVKINEASFDPQNPRDFIDCFLIKMYQDTNNPHTEFNLKNLVLTTLNLFF

AGTETVSSTLRYGFLLLMKYPEVEAKIHKEIDQVIGPHRIPSVDDRVKMPYTDAVIHEIQ

RLTDIVPMGVPHNVIRDTHFRGYLLPKGTDVFPLLGSVLKDPKYFRYPDAFYPQHFLDEQ

GRFKKNEAFVPFSSGKRICLGEAMARMELFLYFTSILQNFSLHSLVPPADIDITPRVSGF

GNIPPTYELRLKAR

>126272434

MEPWALTTFFLVVCVSFLVFLSLWRKDYKGRNLPPGPFPLPIIGNLLQLGHNLSMSLCKL

SEKYGPVYTVYFGPQPVVVLHGYKALKEALTDQGDIFGERGHLPIIDDIYRGQGIVFSHG

EKWKQIRRFSLMTLRNFGMGKRSIEERVQEEAQFLLEELRKTNSQPFDPTFILGCAPCNV

ISSILFHQRFNYDDEEFLSMLRILNENVTLLNTPMAQLYNNFPWFLHYFPGPHKNFFSNV

KKLREFILKNAKKYQQTLDPNNLKNYIDCFLHKMQQDQKNPDSVFDLENLATAGMDLFDA

GTETTSTTLRYGLLLILKYPEVQNKIHEEIDQVIGRHRIPSIKDKLEMPYTEAVLHEILR

FVDLVPFSLPHEVTHDTQLQQHFIPKGTTVYPLLSSVLYDAKEFPNPKEFDPRHFLNKDG

SFKKSDYFVPFSIGKRACLGEGLAKMELFIFLATILQNFTLKSVIDSKEIDIKPGSTGLL

NVPPKYQLCLLPR

>344298309

MLASGMLLVALLACLSVMVLMSVWRQRKLWGKLPPGPTPLPFIGNYLQLNTQQMYNSLMK

LSERYGLVFTVHLGSRRVVVLCGYDAVKEALVDQAEEFSGRGEQATFDWLFKGYGVAFSN

GERAKQLRRFSITTLRDFGVGKRGIEERIQEEAGFLIETFRGTRGTFIDPTYFLSRTVSN

VISSIVFGDRFDYEDKEFLSLLRMMLGSFQFTATATGQLYDMFYSVMKYLPGPQQQAFKE

LQGLEDFITKKVEQNQRTLDPNSPRDFIDSFLIRMREEKKNPNTEFYMKNLVLTTLNLFF

AGTETVSTTLRYGFLLLMKHPDVQAKLHEEIDQVIGKNRQPKFEDRAKMPYTEAVIHEIQ

RFADMIPMGVARRVTKDTKFRNFFIPKGTEVFPMLGSVLRDTKFFSNPQDFNPRHFLDEK

GQFKKNDAFVPFSIGKRYCFGEGLARMELFVFLTTILQNFCFKSPQSPKDIDVSPKHVGF

ATIPRTYTMSFLPR

>2Q6NA

MAKKTSSKGKLPPGPSPLPVLGNLLQMDRKGLLRSFLRLREKYGDVFTVYLGSRPVVVLC

GTDAIREALVDQAEAFSGRGKIAVVDPIFQGYGVIFANGERWRALRRFSLATMRDFGMGK

RSVEERIQEEARCLVEELRKSKGALLDNTLLFHSITSNIICSIVFGKRFDYKDPVFLRLL

DLFFQSFSLISSFSSQVFELFSGFLKYFPGTHRQIYRNLQEINTFIGQSVEKHRATLDPS

NPRDFIDVYLLRMEKDKSDPSSEFHHQNLILTVLSLFFAGTETTSTTLRYGFLLMLKYPH

VTERVQKEIEQVIGSHRPPALDDRAKMPYTDAVIHEIQRLGDLIPFGVPHTVTKDTQFRG

YVIPKNTEVFPVLSSALHDPRYFETPNTFNPGHFLDANGALKRNEGFMPFSLGKRICLGE

GIARTELFLFFTTILQNFSIASPVPPEDIDLTPRESGVGNVPPSYQIRFLARHHHHHH

>392345030

MLPNTIVYFLVQGQCEAMHMLLADQGQSWKEEVIPIDIRMQGLLKPTCLYGQLPKFEDGD

LTLYQSNAILRHLGLSLGLYGKDQREATSMAMNQGGKAFIMGDQIAFADYNLLDLLLIHR

VLALGCLDNLPLLSAYVEEEEEEEDEEGGGGGEGEGEGEGEGEGEGEGEGEGEGEGEEML

SSEVWLTIKMVHAEAFSRPLSRNEVVGLIFRLTIFGAVTYFTIKWMVDAIDPTRKQKVEA

QKQNLINFHHFSFFFSFLATGFLSLSLCLSVSLSLSTRNMELGVFLLLTFTVGFLLLLAS

QNRPKTHGHLPPGPRPLPFLGNLLQMNRRGLLRSFMQLQEKYGDVFTVHLGPRPVVILCG

TDTMREALVDQAEAFSGRGTVAVLHPVVQGYGVIFANGERWKILRRFSLVTMRNFGMGKR

SVEERIKEEAQCLVEELKKYKGALLNPTSIFQSIAANIICSIVFGERFDYKDHQFLRLLD

LIYQTFSLMGSLSSQVFELFSGFLKYFPGVHKQISKNLQEILNYIDHSVEKHRATLDPNT

PRDFIDTYLLHMEKEKSNHHTEFHHQNLVISVLSLFFAGTETTSTTLRYSFLIMLKYPHV

AEKVQKEIDQVISSHRLPTLDDRIKMPYTDAVIHEIQRFADLAPIGLPHRVTKDTMFRGY

LLPKNTEVYPILSSALHDPRYFDHPDTFNPEHFLDANGTLKKSEAFLPFSTGKRTCLGEG

IARNELFIFFTALLQNFSLASPVAPEDIDLTPINSGAGKIPSPYQINFLSRCVG

>BAA85463.1

MLASGLLLVTVLAFLVVLILMSVWKQRKLSGKLPPGPTPLPFIGNYLQLNTEQMYNSLMK

ISERYGPVFTIHLGPRPIVVLCGQEAVKEALVDQAEEFSGRGEQATFDWLFKGYGVAFSS

GERAKQLRRFSIATLRDFGVGKRGIEERIQEEAGFLLEAFRKTNGALIDPTFYLSRTVSN

VISSIVFGDRFDYEDKEFLSLLRMMLGSFQFTGTSTGQLYEMFSSVMKHLPGPQQQAFKE

LQGLEDFITKKVEQNQRTLDPNSPRDFIDSFLIRMKEEKKNPNTEFYMKNLVLTTLNLFF

AGTETVSTTLRYGFLLLMKHPDVEAKVQEEIDRVIGKNRQPKYEDRLKMPYTEAVIHEIQ

RFGDMIPMGLARRVTKDTKFRGFFIPKGTEVFPMLGSVLRDPKFFSNPKDFNPQHFLDDK

GQFKKNDAFVPFSIGKRYCFGEGLARMELFLFLTNILQNFHLRSPQAPQDIDVSPRLVGF

ATIPPNYTMSFLSR

>P00178.1

MEFSLLLLLAFLAGLLLLLFRGHPKAHGRLPPGPSPLPVLGNLLQMDRKGLLRSFLRLRE

KYGDVFTVYLGSRPVVVLCGTDAIREALVDQAEAFSGRGKIAVVDPIFQGYGVIFANGER

WRALRRFSLATMRDFGMGKRSVEERIQEEARCLVEELRKSKGALLDNTLLFHSITSNIIC

SIVFGKRFDYKDPVFLRLLDLFFQSFSLISSFSSQVFELFPGFLKHFPGTHRQIYRNLQE

INTFIGQSVEKHRATLDPSNPRDFIDVYLLRMEKDKSDPSSEFHHQNLILTVLSLFFAGT

ETTSTTLRYGFLLMLKYPHVTERVQKEIEQVIGSHRPPALDDRAKMPYTDAVIHEIQRLG

DLIPFGVPHTVTKDTQFRGYVIPKNTEVFPVLSSALHDPRYFETPNTFNPGHFLDANGAL

KRNEGFMPFSLGKRICLGEGIARTELFLFFTTILQNFSIASPVPPEDIDLTPRESGVGNV

PPSYQIRFLAR

>126329364

MLVSGLLLGALFFCLSALVVLSVWRQRKVRGSLPPGPTPLPFIGNYLQLNTKQMYDSIMK

IGEKFGPVFTVHLGPRPIVVLSGYEAVKEALVDQAEEFSGRGEQATFDWLFKGFGVAFSN

GERARQLRRFSITTLRDFGMGKRGIEERIQEEAGFLVEALRGTKGAPIDPTFFLSRTVSN

VISSIVFGDRFEYEDKQFLSLLRMMLGSFQFTATSMGQLYEMFSGVMKHLPGPQQQAFKE

LQGLEDFITEKVRENQATLDPNHPRNFIDSFLIKMQEEKKNPNTEFFMRNLTMTTLNLFF

AGTETVSTTLRYGFLLLMKHPEVEAKIHEEIDRVIGRNRSPKFEDKAKMPYTEAVIHEIQ

RFGDMIPMGLARRVTKDTKFRGYLIPKGTEVYPMLGSVLRDQSFFACPQDFNPQHFLDEK

GQFKKSDAFVPFSIGKRYCFGEGLARMELFLFLTTILQNFRFQSPLPKEAIDISPKMVGF

ATIPRSYTISFLPRG

>1SUOA

MAKKTSSKGKLPPGPSPLPVLGNLLQMDRKGLLRSFLRLREKYGDVFTVYLGSRPVVVLC

GTDAIREALVDQAEAFSGRGKIAVVDPIFQGYGVIFANGERWRALRRFSLATMRDFGMGK

RSVEERIQEEARCLVEELRKSKGALLDNTLLFHSITSNIICSIVFGKRFDYKDPVFLRLL

DLFFQSFSLISSFSSQVFELFSGFLKYFPGTHRQIYRNLQEINTFIGQSVEKHRATLDPS

NPRDFIDVYLLRMEKDKSDPSSEFHHQNLILTVLSLFFAGTETTSTTLRYGFLLMLKYPH

VTERVQKEIEQVIGSHRPPALDDRAKMPYTDAVIHEIQRLGDLIPFGVPHTVTKDTQFRG

YVIPKNTEVFPVLSSALHDPRYFETPNTFNPGHFLDANGALKRNEGFMPFSLGKRICLGE

GIARTELFLFFTTILQNFSIASPVPPEDIDLTPRESGVGNVPPSYQIRFLARHHHH

>354491949

MALLVILGICLSCLFVFSLWNQYHGKGKLPPGPTPLPIVGNILQVDIKNISKSLNMLAKE

YGPVFTVYLGMKPTVVLHGYETLKEALVDRGDEFSGKTQSSLLSRVKEGLGIIFSNGEIW

KQTRRFSLMVLRSMGMGKKTIEDRIQEEVLYLLDALRKTNGAPCDPSFLLACVPCNVISA

VIFQHRFDYNDEIFQKVTESFHRIIKALESPWAQLCSIYPVLYCLPGSHKSFLKHEAEQH

EFILKEIKRHQESLNPSNPQDFIDHFLIKMEKEKQNKKSEFTMENLVATISDLFSAGTET

TSSTIKYGLLLLLKHPEVTAKIQEEIARVVGRHRSPCMQDRNHMPYTDAVLHEIQRYIDL

VPIPLPRKTTQDVVFRGYHIPKGTSVMTCLNSVLHDDKEFPSPEKFDPGHFLDERGNFKK

SDHFMAFSAGRRACIGEGLARMEMFLILTSILQHFTLKPLVNPEDIDTTPAHTGLLYLPP

PYELCFIPI

>AAA31224.1

MEFSLLLLLAFLAGLLLLLFRGHPKAHGRLPPGPSPLPILGNLLQMDRKGLLRSFLQLRE

KYGDVFTVYLGSRPVVVLCGTDAIREALVDQAEAFSGRGKIAVVDPIFQGYGVIFANGER

WRALRRFSLATMRDFGMGKRSVEERIQEEARCLVEELRKSKGALLDNTLLFHSVTSNIIC

SIVFGKRFDYKDPVFLRLLDLFFQSFSLISSFSSQVFELFPGFLKHFPGTHRQIYRNLQE

INTFIGQSVEKHRATLDPSNPRDFIDVYLLRMEKDKSDPSSEFHHQNLIITVLSLFFAGT

ETTSTTLRYGFLLLLKYPHVTERVQKEIEQVIGSHRPPALDDRAKMPYTDAVIHEIQRLG

DLIPFGVPHTVTKDTQFRGYVIPKNTEVFPVLSSALHDPRYFETPNTFNPGHFLDANGAM

KRNEGFMPFSLGKRICLGEGIARTELFLFFTTILQNFSIASPVPPEDIDLTPRESGVGNV

PPSYQIRFLAR

>AEZ51809.1

MTLLLWLSSWSNISVLGVFLTVFTILVDFMKRRKKWSRYPPGPMPLPFVGTMPYVNYYNP

HLSFEKFRKKFGNIFSLQNCWTNVVVLNGYKTVKEALVNKSEDFADRPYMPVYEHLGYGH

KSEGLVLARYGHLWKELRKFTLTTLRNFGMGKKSLEERVTEEAGFLCSAISSEGGHPFDP

RFLVNNAVCNVICTITYGERFDYGDKTFKKLLTLFENSLNEEAGFLPQLLNVAPVLLRIP

GLPQKIFPCQKAYVDFTQMLIDKHKETWNPAYIRDFTDAFLKEMAKGKEAEENGFNKSNL

TLVTADLLVAGSETTATTLRWAFLFMLLYPEIQSKVHKEIDKVIGRNRPPTMADQVNMPY

TNAVIHEVQRFGDVVPMGLPHMTYRDTELQGFFIPKGTTIITNLTSVLKDETAWKKPNEF

YPEHFLNENGQFVRPEAFLPFSAGRRACLGEQLTRMELFIFFTTLMQKFTFVFPEDQPRP

REDSHFAFTNSPHPYQLRAVPR

>126273200

MEPSVVIFLILVLCISCLFLISSRKKSHGKGQLPPGPFPLPIVGNLLQLDTKHIDKSLGS

LTKVYGPVYTLHFGSERVVVLHGYEAVKEALIDHGEEFAARGSLPIIDAVSKGFGLVFSK

GERWKELRRFSLMTLRNLGMGKRSIEERVQEEAKYLVEEFKKSNGQPCDPKFILECVPCN

VICSVIFQKRFEYSDRKLQTLMELLDENIKILTSPWIQVYNFIPSLVHYLPGPHRTFLNN

CKIMHNFIEEKVKEHQETLDSNNPQDFIDYFLIQMGQKKQNQQSEFTMENLILTVSDLFI

AGSETTSTTLRYGLLLLLKYPEITDKIHEEIDRVIGRDRSPCMKDRNSMPYTDAVIHEIQ

RHLDLIPFNLPHAVKQDTRFREYVIPKDTTIFTSLSSVLYDEKEFPNADQFDPGHFLDES

GNFKKSDYFMPFSIGKRACVGEGLARMELFLFFTNILQNFTLKPLIDPKDIDTTPISNGF

GCVPPSYKLHFLPVVK

>402905624

MLASGLLLVALLACLTVMVLMSVWQQRNSKGKLPPGPTPLPFIGNYLQLNTEQMYTSLMK

ISERYGPVFTIHLGPRRIVVLCGYDAVKEALVDQAEEFSGRGEQATFDWLFKGYGVVFSN

GERAKQLRRFAIATLRDFGVGKRGIEERIQEEAGFLIEALRDTHGANIDPTFFLSRTVSN

VISSIVFGDRFDYEDKEFLSLLRMMLGSFQFTATSAGQLYEMFSSVMKHLPGPQQQAFKE

LQGLEDFIAKKVEHNRRTLDPNSPRDFIDSFLIRMQEEEKNPNTEFHLKNLVLTSLNLFF

GGTETVSTTLRYGFLLLMKYPEVEAKVHEEIDRVIGKNRHPKFEDWAKMPYTEAVIHEIQ

RFGDMLPLGVAHRVIKDTKFRDFFLPKGTEVFPMLGSVLKDPKFFSNPQDFNPQHFLDEK

GQFKKSDAFVPFSIGKRNCFGEGLARMELFLFLTTIMQNFRFKSPQSPKDIDVSPKHVGF

ATIPPNYTMSFLPR

>224048889

MFIFQPPLSSLLLFKFQIYVENATFSVPFYPLWKGEIESGKLEHSWTLALDFSQDIFQTL

RHLSWPVPQPTEDILSQCCVQFWDLQYQRDLDRLERGRKDAVVSVPPGNLMSCVAVATEK

TEVVGGAGSWNNNRRQNFPPGPRPLPIIGNLLLFDLKRPYRTYLQLSKIYGPVFSVQMGH

RKVVVISGYETVKEALINQADAFAERPKIPVFEDLTKGNGVIFAHGENWKVMRRFTLTAL

RDFGMGKKAIEDRIVEEYGHLADSIASHDGTPVDASKTINAAVANIIVSILLGKRFDYKD

SKFVRLINLTNESMRLAGKPLVTMYNIFPYLGFLIRANKALLRNRDEFHDFVRVTFVEHL

KNLDKNDQRSLIDAFLVKQQEEKSTTNGYFHNGNLLSLVSNLFTAGVETISTTLNWGFLL

MLKYPEIQKKVQEEIEQVIGSNPPRIEHRAQMPYTDAVIHEIQRFANILPLDLPHETAAD

VTLQGYFIPKGTYIIPLLTSVLKDQSQWEKPDMFYPEHFLDANGKFVKKDAFMPFSAGQR

MCAGETLAKMELFLFFTSLLQRFNFHPPPGVSSSDLDLSPAISFNVIPKPYKMCAVARS

>Q05556.1

MLASGLLLAALLACLTVMILLSVWRQRKLWGKLPPGPTPLPFIGNYLQLNTEQMYDSLMK

IRDRYGPVFTIHLGPRRIVVLCGQEAVKEALVDQAEDFSGRGEQATFDWLFKGYGVAFST

WERARPLRRFAISTLRDFGVGKRGIEERIQEEAGFLIEAFRDTRGAFIDPTFFLSRTVSN

VISSIVFGDRFDYEDKEFLSLLRMMLGSFQFTATPTGQLYEMFYSVMKHLPGPQQQAFKE

LEGLRDFIAKKVERNQRTLDPNSPRDFIDSFLIRMQEEKKDPKSEFHMKNLVLTTLNLFF

AGTETVSTTMRYGFLLLMKHPDVEAKVHEEIDRVIGRNRQPKFEDRAKMPYTEAVIHEIQ

RFTDMIPMGLARRVTRDTKFRDFLLPKGTEVFPMLGSVLKDPKFFSKPREFYPQHFLDEK

GQFKKSDAFMPFSVGKRYCLGEGLARMELFLFFTTIMQNFRFRSQQAPQDIDVSPKHVGF

ATIPRTYTMSFVPR

>301620118

MALGFVGTILLTACVTILLFLFKWRGKIKIKNLPPGPTPVPLLGNIPQINTTELPTSLLE

LSKTYGPVYTLHLGSYRSVMLIGYDAVKEALIDRSEVFSDRGIMDFTELIFKNNGVLMTN

GERWKTMRRFTLMTLRNFGMGKRSIEERIQEESQSLAEAFEKNKGQPFDPMYLLVLAVSN

IICSIIFGERFDYEDQKFLTLLKYLREIIRLSNTFIGQLLNFFPNVLQYIPGPHQNIFTY

FDKLKEFVREEANAHKDTLDKNCPRDFIDCFLMRMEEESMNPNSEFHNENLNQVIFDLFI

AGTETTSVTLRYAFLILLKYPEIQEKIHKEIDQVIGQDRCPSVVDRSKMPYTEAVIHEVQ

RFADIVPAGLAHAASKDTTFRGYNIPKGTLIFPVLTSVLKDPKFFKNPYQFDPGHFLDNE

GNFRKNDAFMPFSGGKRVCAGEGLARMELFIFLTTILQKFILKPTVDIKDIEITPEPKTN

GSQPRSYKMFVVPRC

>BAJ83934.1

MELLGAGTVVLLVCIACLLSVAAWRRRSGKGKMPPGPAPLPILGNVLQVKPKHLAKTLQK

LSEEYGPVFTVHLGSDPVVVLHGHDVVKEALVDRADEFAARGHMPIGDRANNGLGIIFSN

NKEWLEVRRFALSTLRNFGMGKRSIEERIQEETEYLLEEINKTKGTPFDPTFTLGCAVSN

VICSIVFGKRYDYKDKKFLALMTNMNNIFEMMNSHWGQLYQMFSRILDYLPGPHNKIFDE

FDALKAFVSEEVKIHQASLDPSSPQDFIDCFLSKMQEEKEHPNSSFHMKNLITSTFDLFI

AGTESTSTTIRYGFLLLLKYPKIQEKVQEEIDWVVGRSRRPCVADRTQMPYTDAVVHEIQ

RFISLVPVGLPHTVTKDTYFREYVIPKGTTIFPILSSVLHDSKEFPNANEFNPGHFLNKN

GTFRKSEFFMPFSAGKRICPGEGLARMEIFLITATILQNFTLKPVVNPQELNITPTLSGT

GNVPPAYQLCALPR

>BAB85662.1

MMMFSAILLLIVLIGVFLLWGHPSSQGHLPPGPRPLPFLGNILQMDRKGFLKSFQALRER

YGDVFTVYLGPRPVVMICGTEAIREALVDQAEAFSGRGKIAVVEPIFQGYGVIFSNGERW

KTLRRFSLATMRDFGLGKRSVEERIQEEAQCLVEELRKSQGALQDPTYYFHSSTANIICS

IVFGKRFAYRDPEFLQLLDLLFQTFLLISSFPSQLFELYSAFLKYFPGSHRQIYKNLQQV

NAFIGRSVEKHRETLDPSDPRDLIDSYLLRMDKEKSNPNSEFHEQNLILTVLSLFFAGTE

TTSTTLRYGFLLMLKYPHITERIHKEIDRVIGSHRPPALDDRAKMPYMDAVIHEMQRFGD

LIPMGVPHTVTKDTHFRGYLLPKGTEVYPILTSALHDPRYFENPEAFNPDHFLDATGALK

KNDAFMPFSIGKRICLGEGLARTELFLFFTTILQNFSVASPVAPEDIDLTPQAVGVGTVP

PPYQIQFLPRQRG

>395501147

MDSSGLTTTVLLAFVFFLILSVWIQGTKRGRLPPGPTPFPFIGNILQINLKNMLASFLQL

AEKYGPIYTIYIGTKRIVVLYGYTMVKEALIDQGDVFMERGDMPIFEDLFKGLGVIFSHG

ERWRELRRFSLMTLRNFGMGKRSIEERIQEEAQCLVEELRKTKGQPTDPTFILGCAPCNV

ICSILFHDRFKYDDEKFLYLMKLLNENFHRFNKPWAQLYNFLPAFRIYLPGEHKIIFKGG

EEVRKFILERVKEHQKTLDPNNPRDYIDCYLSKMHQEKDNPKSEFHLENLTVTGANLFSA

GTETTSNTLRYGLLLILKHPEVQAKIYEEIDRVIGNRVPSIKDRLDMPYMDAVVHEIQRF

IDLVPLNVPHALTQDTEFRQYFLPKGTTIFPLLSPILWDSKEFPNPGQFDPQHFLDENGK

FKKSDYFVPFSIGKRSCLGEGLARMELFLFFTTILQNFTLKLVGDPNEINIKTNFSGFNN

ITPHYQLCFLPR

>403259863

MDLFIILAICLSCLILLFLWNGSNAKGKLPPGPTPLPIIGNIQQLNSKNISKSISMLAKD

YGPVFTVYFGMKPTVVLHGYKAIKEALIDHGEEFSGRGSLPVVDRITQGLGVVFSNGERW

KQIRRFSLMVLRNMGMGKKTIEDRIQEEALCLVAALKKTNASPCDPTFLLGCVPCNVIST

IIFQNRFDYSDPKFQTLIKYFNENFENVSTPWIQLYNAFPFLRVLPGGHNVIFKNHALQV

NFILEKVKEHQESLDISNPRDFIDYFLIKMEKEKHNKQSEFTMDNLVATIWDMFSAGTET

TSTTMRYGLLLLLKHPEISAKVQEEIDQVVGKNRSPCMQDRSRMPYTDAVVHEIQRYTDL

VPTSLPHAVTQDTHFREYFIPKGTTILTDLSSVLNDDKEFPNPEKFDPGHFLDESGNFKK

SDYFMVFSAGKRACAGEGLARMELFLILTTILQNFTLKPLVDPKDIDTTPVHKGLGSVPP

SYELCFIPV

>1PO5A

MAKKTSSKGKLPPGPSPLPVLGNLLQMDRKGLLRSFLRLREKYGDVFTVYLGSRPVVVLC

GTDAIREALVDQAEAFSGRGKIAVVDPIFQGYGVIFANGERWRALRRFSLATMRDFGMGK

RSVEERIQEEARCLVEELRKSKGALLDNTLLFHSITSNIICSIVFGKRFDYKDPVFLRLL

DLFFQSFSLISSFSSQVFELFSGFLKHFPGTHRQIYRNLQEINTFIGQSVEKHRATLDPS

NPRDFIDVYLLRMEKDKSDPSSEFHHQNLILTVLSLFFAGTETTSTTLRYGFLLMLKYPH

VTERVQKEIEQVIGSHRPPALDDRAKMPYTDAVIHEIQRLGDLIPFGVPHTVTKDTQFRG

YVIPKNTEVFPVLSSALHDPRYFETPNTFNPGHFLDANGALKRNEGFMPFSLGKRICLGE

GIARTELFLFFTTILQNFSIASPVPPEDIDLTPRESGVGNVPPSYQIRFLARHHHH

>3TK3A

MAKKTSSKGKLPPGPSPLPVLGNLLQMDRKGLLRSFLRLREKYGDVFTVYLGSRPVVVLC

GTDAIREALVDQAEAFSGRGKIAVVDPIFQGYGVIFANGERWRALRRFSLATMRDFGMGK

RSVEERIQEEARCLVEELRKSKGALLDNTLLFHSITSNIICSIVFGKRFDYKDPVFLRLL

DLFFQSFSLISSFSSQVFELFSGFLKYFPGTHRQIYRNLQEINTFIGQSVEKHRATLDPS

NPRDFIDVYLLRMEKDKSDPSSEFHHQNLILTVLSLFFAGTETTSTTLRYGFLLMLKYPH

VTERVQKEIEQVIGSHRPPALDDRAKMPYTDAVIHEIQRLGDLIPFGVPHTVTKDTQFRG

YVIPKNTEVFPVLSSALHDPRYFETPNTFNPGHFLDANGALKRNEGFMPFSLGKRICAGE

GIARTELFLFFTTILQNFSIASPVPPEDIDLTPRESGVGNVPPSYQIRFLARHHHH

>AAZ29436.1

MLASGLLLVALLACLTVMVLMSVWQQRNSKGKLPPGPTPLPFIGNYLQLNTEQMYNSLMK

ISERYGPVFTIHLGPRRVVVLCGYDAVKEALVDQAEEFSGRGEQATFDWLFKGYGVVFSN

GERAKQLRRFSIATLRDFGVGKRGIEERIQEEAGFLIEALRDTQGANIDPTFFLSRTVSN

VISSIVFGDRFDYEDKEFLSLLRMMLGSFQFTATSAGQLYEMFSSVMKHLPGPQQQAFKE

LQGLEDFIAKKVEHNRRTLDPNSPRDFIDSFLIRMQEEEKNPNTEFHLKNLVLTSLNLFF

GGTETVSTTLRYGFLLLMKHPEVEAKVHEEIDRVIGKNRQPKFEDWAKMPYTEAVIHEIQ

RFGDMLPFGVAHRVIKDTKFRDFFLPKGTEVFPMLGSVLKDPKFFSNPQDFNPQHFLDEK

GQFKKSDAFVPFSIGKRNCFGEGLARMELFLFLTTIMQNFRFKSPQSPKDIDVSPKHMGF

ATIPPNYTMSFLPR

>EDL97782.1

MLVTAGSLLGAIWTVLHLRILLLAAVTFLFLADFLKHRRPKNYPPGPWRLPLVGCLFHLD

PKQPHLSLQQFVKKYGNVLSLDFANIPSVVVTGMPLIKEIFTQMEHNFLNRPVTLLRKHL

FNKNGLIFSSGQTWKEQRRFALMTLRNFGLGKKSLEQRIQEEAYHLVEAIKDEGGLPFDP

HFNINKAVSNIICSVTFGERFEYHDSQFQEMLRLLDEAMCLESSMMCQLYNIFPRILQYL

PGSHQTLFSNWRKLKLFISDIIKNHRRDWDPDEPRDFIDAFLKEMAKYPDKTTTSFNEEN

LICSTLDLFFAGTETTSTTLRWALLCMALYPEVQEKMQAEIDRVIGQGRQPNLADRDSMP

YTNAVIHEVQRIGNIIPFNVPREVAVDTYLAGFNLPKGTMILTNLTALHRDPKEWATPDT

FNPEHFLENGQFKKRESFLPFSMGILKMKTKRTKMN

>301608096

MFLGDPVTVLLAVALCLIVANTLYRQKRSKYQHFPPGPKPLPIIGNIHILNRKRPYLTFL

ELWKKYGSIFSVQIGSQKMVVLCGYETVKDALVNHGEEFSERPEIPIFHVIAKGYGVIFS

HGENWKVMRRFTLSTLRDFGMGKKSIEDKINEECDSLVEKLRSYGGKAFENSVTINAAVA

NIIVSLLLGRRFDYEDPTFLRLMSLMNANFRLMGSPMVMLYNLYPSIIRWLPGSHKTVGK

NAAETQRFIRETFTKRREKLDVNDQRNLIDAFLVRQQETKEDGCSFHDDNLTVLVSNLFA

AGMETTSSTLRWGLLLMMKYPEIQKNVQNEIEKVIGQSRPQTEHRKSMPYTDAVIHEIQR

FGNIIPMNLPHATAQDVTFRGYFLPKGTYVIPLLTSVLYDKDHFEKPNEFYPQHFLDSEG

NFVRNEAFLPFSAGKRSCAGENLAKMQLFLFFTSLLQNFTFQAPPGEELDLTPTTGFTTP

PLLHNICALPRT

>P15392.3

MLTSGLLLVAAVAFLSVLVLMSVWKQRKLSGKLPPGPTPLPFVGNFLQLNTEQMYNSLMK

ISQRYGPVFTIYLGSRRIVVLCGQETVKEALVDQAEEFSGRGEQATFDWLFKGYGIAFSS

GERAKQLRRFSITTLRDFGVGKRGIEERIQEEAGFLIDSFRKTNGAFIDPTFYLSRTVSN

VISSIVFGDRFDYEDKEFLSLLRMMLGSLQFTATSMGQVYEMFSSVMKHLPGPQQQAFKE

LQGLEDFITKKVEHNQRTLDPNSPRDFIDSFLIRMLEEKKNPNTEFYMKNLVLTTLNLFF

AGTETVSTTLRYGFLLLMKYPDIEAKVHEEIDRVIGRNRQPKYEDRMKMPYTEAVIHEIQ

RFADLIPMGLARRVTKDTKFRDFLLPKGTEVFPMLGSVLKDPKFFSNPKDFNPKHFLDDK

GQFKKSDAFVPFSIGKRYCFGEGLARMELFLFLTNIMQNFHFKSTQEPQDIDVSPRLVGF

VTIPPTYTMSFLSR

>AAB27705.1

MEFSLLLLLAFLAGLLLLLFRGHPKAHGRLPPGPSPLPVLGNLLQMDRKGLLRSFLRLRE

KYGDVFTVYLGSRPVVVLCGTDAIREALVDQAEAFSGRGKIAVVDPIFQGYGVIFANGER

WRALRRFSLATMRDFGMGKRSVEERIQEEARCLVEELRKSKGALLDNTLLFHSITSNIIC

SIVFGKRFDYKDPVFLRLLDLFFQSFSLISSFSSQVFELFSGFLKHFPGTHRQIYRNLQE

INTFITQSVEKHRATLDPSNPRDFIDVYLLRMEKDKSDPSSEFHHQNLILTVLSLFFAGT

ETTSTTLRYGFLLMLKYPHVTERVQKEIEQVIGSHRPPALDDRAKMPYTDAVIHEIQRLG

DLIPFGVPHTVTKDTQFRGYVIPKNTEVFPVLSSALHDPRYFKTPNTFNPGHFLDANGAL

KRNEGFMPFSLGKRVCLGEGIARTELFLFFTTILQNFSIASPVPPEDIDLTPRESGVGNV

PPSYQIRFLAR

>327261109

MDWIHPITIFFLITLIILLVFKMGYFWNYSSQNLPPGPKPLPILGNLHIIDQKRPHRTML

KLSKIYGPVFSIQMGFQKMVVLTGYEMVKEALVDQADAFAERPVIPLFEDFAQGFGILFA

HGENWKVMRRFTLSTLRDYGMGKRSIEDKIVEECSILTKKLESYKGKPFETTAIMNAAVA

NIIVSILLGRRYEYEDPTFQRLLKLFSDNIRLFGTPSVLFYNMFPALGFLSGGRKTVLDN

REELFAFIKATFMKHLKELDENDQRSFIDTFLIRQQEEKNNNVNEYFHNENLQSLVGNLF

GAGMETTSTTLRWALLLMMKHPEIQRKVQEEIAVTIGSAQPRAEHRKKMPYTDAVIHEVQ

RYANILPTSVPRATTVDVTLKGYFIPKGTHIIPLLSSVLHDDSQWKKPLRFYPEHFIDPE

GNFIKRDAFMPFAAGRRQCVGETLAKMELFLFFTTLMQRFTFQPAPGTSREDLDLTPAVG

FTTPPMPFDVCALPR

>345316047

MGQAFIPVSVFLIVFLLLVGLRKRRRPEGYPPGPFSLPFVGNALNMDFSHPLVHTEKFVR

KYGNLFSLDLGTMPFVLVTGMKQIKEVLARQDENLDNRPVFPIRTEIFTQETGLLMSNGP

VWKRHRRFTLMTLRNLGLGKRSLQERIQEEAAYLVQTMEEEGGEPFDPHFKINSAVSNII

CTVTFGERFEYSDPRFQELLHLLDAVILLEANTWGQLFNMFPSVMRKLPGPHWEIFRKWA

KLKAFTSDAIRRHREDRNPDEPRDFIDAYLDEMDKEKDVAGSYLNEDNLISSTMDLFLAG

TETSSTTLRWALLYMALNPEIQETAQAEIDGVVGRSRPPTMDDRESMPYTCAVINEVLRM

GNIIPLNVPRETGAHVTVAGYHLPKGTVVMTNLMALHRDPEEWETPDRFNPRHFLDNGKL

KKKESFLPFSTEKRMCPEEQLARTELFIFFTCLLRNFTFRAPEGVTLSLRARNGITLSPY

PYRIRAVPR

>P17666.1

MDPVVVLVLCLSCLLLLSLWKQSHGGGKLPPGPTPLPILGNILQIDFKDISKSLQNLSKV

YGNVFTVYMGMKPTVVMYGYEAVKEALVDLGEEFSGRNLSPINKKVNKGLGVIFSNGKRW

KEIRRFSLMTLRNFGMGKRSIEDRVQEEARCLVEELRKTNGSPCDPTFILGAAPCNVICS

VIFQNRFDYKDETFLNLMGKFNENFRILNSPWLQVCNIFPILMDYLPGTHKTVFENFDYV

RNFVLEKTKEHQESLDINNPRDFIDCFLIKMKQEKHNQQSEFTIENLMATVTDVFAAGTE

TTSTTLRYGLLLLMKHPEVTAKVQEEIERVIGRHRSPCMQDRSRMPYTDATVHEIQRYIN

LVPNNVPHATTCNVKFRNYFIPKGTAVLTSLTSVLHDNQEFLKPDKFDPGHFLDASGNFK

KSDYFMPFSTGKRVCMGEALARMELFLFLTAILQNFTLKPLVDPKDIDTTPLVSGARSCA

TLYQLSFIPA

>395529480

MEFGGIITLFLALCAPCLAILIAWKHMHKAGKLPPGPTPLPFLGNLLQVRTDATFQSFLE

LSKKYGPVFTVYMGPRRVVVLCGHEAVKEALVDQAEEFSGRGELASIDRNFQGHGVALAN

GNRWKILRRFSLAVLRDFGMGKRSIEERIQEEAGFLIEGLRKTNGAPIDPTFFLSRTVSN

VISSVVFGSRFDYEDKQFLYLLHLINESFIEMXXPNLQLYDMYSGIMQYLPGRHNKIYNL

IEELKDFIASRVKINEASLDPNSPRDFIDCFLIKMHQEKNNPQTEFNLKNLVLTTLNLFF

AGTETVSSTLRYGFLLLMKYPEVGAKVQEEIDHIIGRHRIPKAEDRIQMPYTDAVIHEIQ

RVTDIVPMGVPHTVIRDTHFRGYILPKGTDVFPLIGSALRDPKYFKKPDAFEPKHFLDEQ

GRFKKNEAFVPFASGKRVCLGEAMARMELFLYFTFILQNFSLQPLVPPSEIDITPKISGF

GNIPPTYQLCIVAR

>301615199

MISLILIGVLSALLLVVYSTWRRDSRLPPGPTPWPVIGNIHQIDKLAPYETLMQFGEKYG

PVYTIYFGWNPVVVLYGYDALKEALIGQAEDFSGRAIVPVFERVANRKGLVFSNGAHWQQ

QRRFSLATLRSFGMGKRSIEERVREESTNLLEFFQEKKGNPFNPGPHITAAVSNVICSIV

FGDRFDTEDGTFQTLLRMVNENITFLGKRGFQMYNTFPGILKHLPGEHNKIFQNVSKLKT

FLRGLIDNHTLSRDPNCPRDFVDSFLNKMDEEAGNPDSHFTMESLTYTTFNLFIAGTETT

SSTIRWALRFMLAYPHIQKRVQDEIDSVLGPDKCPSLEDRVNLPYTDAVIHEVLRYSSVV

PNGLPHEALYDIKFKGYTIPKGTQIITFLFSALNDKGYWDDPEQFNPERFLDKEGKFVKN

EAHLPFGAGKRACIGEALARTEIFIFFVNILQKFSLKSPPGEGGPIELAGGGTRAPRPFN

VCAEWRL

>301618939

MFGVNSYTLVLVSICLIYMYLLHKQKGNLPPGPQGLPIWGNLHQLDTKDLVKSLGDMSKI

YGPVFTIHLGPKPNVVLYGYKAVKEALVEQADIFSGRGEFKVVHRFTKGNGLVFSNGEKW

KTLRRFALTTLRNFGMGKRGVEERIKEEAQFLMEAFKHTNQNPFDPTFFLSRAVSNVICS

IVFGDRFDYGDKRFLMLLSLINESFQLMSSQWGMFYNIFPGVMKYLPGPHNQIFQNFEKI

KLFLLEMLKRHEETFQQDSPRDLMDCFLLEIKKENGKPLSHFNIETLVMTSFTLFFGGTE

TVSTTLRYGILILMKYTNITEKIQEEIDTVIGKDRFPTMDDRSRMPYTDAVIHEIQRFAS

IIPLSLPHSVTQDTYFRGYKLPKGTNVIPVLSSVHTDPTKYKEPQGFNPHNFLDENNQFK

NNEAFMPFSSGKRICIGESLARMELFIFFSTLLQNFTLQPTQNPERIDLKPRISGVGNVP

TPYQLCAFPR

>AFJ20720.1

MDLFIVLVICLSFLIFLFLWNQSYAKGKLPPGPTPLPIIGNILQINIKDVTKSLSKLAEV

YGPVFTVYFGMKPTVVLYGYEAVKEALIDQSEEFSGRGSLPIVDRINQGLGIVFSSGEKW

KQTRRFSLMVLRNMGMGKKTIEDRIQEEALCLVESLKKTNASPCDPTFLLSCAPCNVICS

IIFRNRFEYSDEEFLTLIKYINENARLASTSWIELYNAFPFLIHYLPGSHNTLFKNIIEQ

RKFIFENIKKHQEFLDLSNPQDFIDYFLIKMEKEKHNKQSEFTMDNLITTVWDVFTAGTE

TTSMTLRYGLLLLLKHPEVTAKVQEEIDRVIGRHRSPCMQDRSRMPYTDAVVHEIQRYID

LVPGSMPHATTQDVKFREYIIPKGTAIVTSLTSVLYDGKEFPNPEQFDPGHFLDESGNFK

KSDYFMTFSAGKRVCVGEGLARMELFLFLVSILQHFTLKSLVDPKDIDTTPALKGLGSVA

PFYEVRFIPV

>EDL24215.1

MEFSVLLLLALTTGFLIFLVSQSQPKTHGHFPPGPRPLPFLGNLLQMDRRGLLSSFIQLQ

EKYGDVFTVHLGPRPVVMLCGTDTIREALVNQAEAFSGRGTVAVLDPIVQGYGVIFSSGE

RWKTLRRFSLATMRDFGMGKRSVEERIKEEAQCLVEELKKYKGAPLNPTFYFQCIVANII

CSIVFGERFDYKDHQFLHLLNLIYQTFSLMSSLSSQVFELFSAILKYFPGAHRQISKNLQ

EILDYIGHSVEKHRATLDPSAPRDFIDTYLLRMEKEKSNHHTEFHHQNLVISVLSLFFAG

TETSSTTLRYSFLIMLKYPHVAEKVQKEIDQVIGSHRLPTLDDRTKMPYTDAVIHEIQRF

TDLAPIGLPHKVTKDTLFRGYLIPKNTEVYPILSSALHDPRYFEQPDSFNPEHFLDANGA

LKTNEAFMPFSTGKRICLGEGIARNELFLFFTTILQNFSLASPVAPENIDLIPNNSGATK

TPPQYQIHFLSR

>395501145

MDLWGLNTTALLTCVLFLIFFFLWNQGFKREKLPPGPIPLPFLGNIMQLNLKNITESFSQ

LAEKYGPIYTVYIGTLRVVVLHGYSITKEALLDQGEIFMERGNVPIFEEISRGDGVVFSH

GEKWKQIRRFSLMTLRNFGMGKRSIEERVQEEAHCLVEELRKTKGQPTDPTFILGCAPCN

VICSILFRDRFKYNDEKFLYLMNLLNENFHLFNKPWAQLYNFLPAFRRYLPGEHKKILKN

IDEIKRFILERIKEHQKTLDLNNPQDYIDCYLSKMHQEKDNPQSQFDLENLTMTAADLFA

AGTETTSSTLRYGLLLILKHPEVKAKIHEEIDRVIGHNRVPSIKDRQDMPYMDAVVHEVQ

RFIDLIPLNLPHEVTRDIQFQQYVLPKGTTIFPILSPILYDNKAFPNSDQFDPQHFLDKN

GKFKKSDYFLPFSIGKRSCLGEGLARMELFLFFTTILQNFTLKSIGDPNEINIKTNHVGF

TNVPPHYQLCFLPH

>O18809.1

MDSISTAILLLILALICLLLTTSSKGKGRLPPGPRALPFLGNLLQLRSQDMLTSLTKLSK

EFGAVYTVYLGPRRVVVLSGYQAVKEALVDQAEEFSGRGDYPAFFNFTKGNGIAFSNGDR

WKALRKYSLQILRNFGMGKRTIEERILEEGHFLLEELRKTQGKPFDPTFVVSRSVSNIIC

SVIFGSRFDYDDDRLLTIIHLINENFQIMSSPWGEMYNIFPNLLDWVPGPHRRLFKNYGR

MKNLIARSVREHQASLDPNSPRDFIDCFLTKMAQEKQDPLSHFFMDTLLMTTHNLLFGGT

ETVGTTLRHAFRLLMKYPEVQVRVQEEIDRVVGRERLPTVEDRAEMPYTDAVIHEVQRFA

DIIPMSLPHRVTRDTNFRGFTIPRGTDVITLLNTVHYDPSQFLKPKEFNPEHFLDANMSF

KKSPAFMPFSAGRRLCLGEALARMELFLYLTAILQSFSLQPLGAPEDIDLTPLSSGLGNV

PRPYQLCVRAR

>126329309

MLASGLLLVTLGFCLSTLVVLSVWRQRKLRGSLPPGPTPLPFIGNYLQLNTEQMYDSLMK

IGEKFGPVFTVHLGSRPIVVLSGYEAVKEALVDQAEEFSGRGKQATFDWLFKEYGVIFSN

GERSRQLRRFSITTLRDFGMGKRGIEERIQEEAGFLMEVLQDTKGAPIDPTFFLSRTVSN

VISSIVFGDRFEYEDKQFLSLLNMMLGSFQFTATSMGQLYEMFSGVMKYLPGPQQQAFKD

LKGLEDFITEKVRENQATLDPNHPRNFIDSFLIKMQEEKKNPSTEFFMRNLTMTSINLFF

AGTETVSTTLRYGFLLLMKHPEVEAKIHEEIDRVIGRNRSPKFEDKAKMPYTEAVIHEIQ

RFGDMIPMGVSRRVTKDTKFRSFLLPKGTEVYPMLGSVLRDRRFFACPEDFNPQHFLDEK

GQFKKSDAFVPFSIGKRYCFGEGLARMELFLFLTTILQNFHFQSPLPKEALDISPKMVGF

ATIPRSYTISFLPRG

>BAB85661.1

MLASGLLLVALLTCLTIMVLMSVWRQRKLQGKLPPGPTPLPFIGNYLQLNTEQMYNSLMK

ISQRYGPVFTVHLGPRRIVVLCGYDAVKEALVDQAEEFSGRGEQATFDWLFKGYGVAFSN

GERAKQLRRFSITTLRDFGVGKRGIEERIQEEAGYLIEAFRGTRGAFIDPTYFLSRTVSN

VISSIVFGDRFDYEDKEFLALLRMMLGSFQFTATSTGQLYEMFYSVMKHLPGPQQQAFKD

LQGLEDFIARKVEHNQRTLDPNSPRDFIDSFLIRMQEEKKNPDTEFYWKNLVLTTLNLFF

AGTETVSTTMRYGFLLLMKHPDVEAKVHEEIDRVIGRNRQAKFEDRAKMPYTEAVIHEIQ

RFGDMIPMGLARRVTKDTKFRDFLLPKGTEVFPMLGSVLRDPKFFSNPRGFNPQHFLDEN

GQFKKNDAFVPFSIGKRYCFGEGLARMELFLFLTNILQNFHLKSPQLPQDIDVSPKHVGF

ATIPPTYTMSFQPR

>327276367

MDLSGATPLLLMAALFCLALYWNGGKRKKGKLPPGPAPLPIFGNLFQLDPREMHRSLEKL

SKQYGPVYTIHLGSLPCVVLCGYLAVKEALVDQADDFSDRGDFPVFYRFTQGNGIAFSNG

EKWKILRRFALHTLKNFGMGKSSIEARIQEEAQCLVQEFQKTDGAPFNPINLVCRAVSNV

ICAVVFGNRFDYEDADFLRLLDHFNENFQIMSSFWGELYNIFPHVMDLLPGPHHKIFQNF

EKLRHFITERIRLHQKDLDPEAPRDFIDCFLIRMEEEKQDPLSYFNMETLVMTTHNLFFG

GTETTSTTLRFSFLILMKYPDVAVRVHQEIDGVIGTNRTPCLADRKSMPYTDAVIYEIQR

FISILPMGLPRAVTRDTPFKEFLLPKGTNVIPLLSSVHYDPTQFKDPMSFNPDHFLDEKS

TFKNTDAFMPFAPGKRICLGAGLARMEIFLFLTTILQHFSLQPLVAPAEIDLTPQCTGLG

NVPRPFQFRVLPR

>327280410

MEPLGMSTVLLLTCLSCLLLSAIWKSGARQKGKMPPGPTPLPIIGNALQLKTHHLDQVLQ

KMSEKYGPVFTLYFGMAPAVVLHGYEAIKEALLDRGNEFASRGKMHLIEKTNRGKGIIFS

NGETWKQLRRFALTTLRNFGMGKKSIEERIHEEAQYLLEQFRNTKQQPFDPHYLFSCATS

NVICSIVFGKRYDYKDKKFQAMLNLMNENFEMFNSAWAQLANMFPTLMEWIPGPHHRIVS

GSLRSEEFVLEEAKEHRATLDPSSPQDFIDCFYIKMDQEKHNEASEFTMENLIMSSLDLF

AAGTETTSTTLRYGLLILQKYPEIEEKVQEEIDRVVGRSRMPGMADRGQMPYTDTVLHEI

QRFVSLVPLGVPHTVDKDTPFRGYVIPKGTTIFPVLTSVLHDSKEFPNPTEFDPGHFLNE

DGTFRKSDYFVPFSTGKRICAGEGLARMELFLFLTSILQNFKLKPLKDPKDIDIMPRLSS

LNNFPQPYKFCLVPR

>O55071.1

MEFSVLLLLALTTGFLIFLVSQSQPKTHGHFPPGPRPLPFLGNLLQMDRRGLLSSFIQLQ

EKYGDVFTVHLGPRPVVMLCGTDTIREALVNQAEAFSGRGTVAVLDPIVQGYGVIFSSGE

RWKTLRRFSLATMRDFGMGKRSVEERIKEEAQCLVEELKKYKGAPLNPTFYFQCIVANII

CSIVFGERFDYKDHQFLHLLNLIYQTFSLMSSLSSQVFELFSAILKYFPGAHRQISKNLQ

EILDYIGHSVEKHRATLDPSAPRDFIDTYLLRMEKEKSNHHTEFHHQNLVISVLSLFFAG

TETTSTTLRYSFLIMLKYPHVAEKVQKEIDQVIGSHRLPTLDDRTKMPYTDAVIHEIQRF

TDLAPIGLPHKVTKDTLFRGYLIPKNTEVYPILSSALHDPRYFEQPDSFNPEHFLDANGA

LKTNEAFMPFSTGKRICLGEGIARNELFLFFTTILQNFSLASPVAPENIDLIPNNSGATK

TPPQYQIHFLSR

>AAH11233.1

MLTSGLLLVAAVAFLSVLVLMSVWKQRKLSGKLPPGPTPLPFIGNFLQLNTEQMYNSLMK

ISQRYGPVFTIYLGPRRIVVLCGQEAVKEALVDQAEEFSGRGEQATFDWLFKGYGVAFSS

GERAKQLRRFSIATLRDFGVGKRGIEERIQEEAGFLIDSFRKTNGAFIDPTFYLSRTVSN

VISSIVFGDRFDYEDKEFLSLLRMMLGSFQFTATSMGQLYEMFSSVMKHLPGPQQQAFKE

LQGLEDFITKKVEHNQRTLDPNSPRDFIDSFLIRMLEEKKNPNTEFYMKNLVLTTLNLFF

AGTETVSTTLRYGFLLLMKHPDIEAKVHEEIDRVIGRNRQPKYEDRMKMPYTEAVIHEIQ

RFADMIPMGLARRVTKDTKFRDFLLPKGTEVFPMLGSVLKDPKFFSNPKDFNPKHFLDDK

GQFKKNDAFVPFSIGKRYCFGEGLARMELFLFLTNIMQNFHFKSTQAPQDIDVSPRLVGF

ATIPPTYTMSFLSR

>AAA40426.1

MLTSGLLLVAAVAFLSVLVLMSVWKQRKLSGKLPPGPTPLPFVGNFLQLNTEQMYNSLMK

ISQRYGPVFTIYLGSRRIVVLCGQEAVKEALVDQAEEFSGRGEQATFDWLFKGYGIAFSS

GERAKQLRSFSIATLRDFGVGKRGIEERIQEEAGFLIDSFRKTNGAFIDPTFYLSRTVSN

VISSIVFGDRFDYEDKEFLSLLRMMLGSLQFTATSMGQVYEMFSSVMKHLPGPQQQAFKE

LQGLEDFITKKVEHNQRTLDPNSPRDFIDSFLIRMLEEKKNPNTEFYMKNLVLTTQNLFF

AGTETGSTTLRYGFLLLMKYPDIEAKVHEEIDRVIGRNRQPKYEDRMKMPYTEAVIHEIQ

RFADLIPMGLARRVTKDTKFRDFLLPKGTEVFPMLGSVLKDPKFFSNPKDFNPKHFLDDK

GQFKKSDAFVPFSIGKRYCFGEGLARMELFLFLTNIMQNFHFKSTQAPQDIDVSPRLVGF

VTIPPTYTMSFLSR

>344298313

MELSILLLLALFTGLLLLLARSYPKAEGHLPPGPSPLPFLGNLLQMDRGGLLKSFLKLQE

KYGDVFTVYLGPRPVVMLCGTEAIREALVDQAEVFSGRAHVAYVEQVFQGYGVIFANGDR

WKALRRFSLATMRDFGMGKRSVEERIQEEAQCLMEELRKSKGALLDPTFFFHAITANIIC

SIVFGERFAYKDPQFLRLLDTFYQTFALVSSFSSQVFELFQDFLRHFPGPHRQVYKNLQE

VNAFISCSVAKHRETLDPSAPRDFIDTYLLRMDKEKSNPDTEFHQRNLILTTLSLFFAGT

ETTSTTLRYGFLLMLKYPHIAEKVRKEIEQVIGLHRPPALDDRAKMPYTDAVIHEMQRFG

DLLPIGVPHMVTKDTYFRGYIIPKGTKVFPILSSALHDPRYFEKPDEFNPDHFLDDKGAL

KKNDAFMPFSVGKRICLGEGIAKAELFLFFTTILQNFSVASPVAPKDISLMPLESGFGKM

PPVYQIRFLAR

>327278202

MELEWVLSISLGIFLVLISVWTWRHKEGRFPPGPMPLPFFGNLLQLNPKDIPKSFLALSH

KYGPVFTLYLGPRRVVVLCGHEALKEALVDHGEQFCGRGEVPSVERMFKGFGIALANGER

WKKLRHFSLLTLKNFGMGKCSIEERIQEEAQFLLEKFRKTEGLPFDPTFLLNCTTSNIVC

SIVFGKRFEYEDKTFLSMLDLTNKMFVELSTPWAKLYDMYSGIMQYLPGGHKRVYNFLQD

LKAFIDDRIRINQETLDPKNPRDFIDCFLIEMEKEKGNPSTEFTMNNLVFTAINLFTAGT

DTVSFTLKYAFLLLMKYPEVAEKVKQEIDSVVGHNRVPAVKDRINMPYTNAVIHETQRLI

DIFPVGVPHKVTADTEFRGYLLPKDTNIIAVLGSALHDPKYFRDPKIFNPAHFLDEEGHF

KKNDAFVPFSTGKRSCVGESMARMELFLYLTTILQSFSLKSSLAPNDIDISPQLNGFLNI

PPIFQLCLIPH

>327280434

MEPLGMSTVLLLTCLSCLLLSAIWKSGARQKGKMPPGPTPLPIIGNALQLKTHHLDQVLQ

KMSEKYGPVFTLYFGMAPAVVLHGYEAIKEALLDRGNEFAFRGKIHLMEKTNKGKGIIFS

NGERWKQLRRFALTTLRNFGMGKKSIEERIHEEAQYLLEQFRNTKQQPFDPHYLFSCATS

NVICSIVFGKRYDYKDKKFQAMMNLMNENFEIFNSAWAQFANMFPTLMEWIPGPHHQIVS

GSLRSEEFVLEEAKEHRATLDPNSPRDFIDCFFIKMDQEKHNEASEFTMENLITCSLDLF

GAGTETTSTTLRYGLLILQKYPEIEEKVQEEIDRVVGRSRMPGMADRGQMPYTDAVLHEI

QRFVSLVPLGVPHTVDKDTPFRGYVIPKGTTIVPVLSSVLHDSKEFPNPTEFDPGHFLNK

DGTFRKSDYFVPFSAGKRICAGEGLARMELFLFLTSILQNFKLKPLTDPKDIDIMPRLSS

LSNVPQPYKFCLVPC

>395820854

MDPAVALVLCLSCLLLLSLWRQSSGRGKLPPGPTPLPIIGNILQLDIKDISECLSNLSKA

YGPVFTVYLGMERAVVLHGYEAVKEALIDHGEEFSGRGSFPVFERVTRGHGIIFSNGNRW

KEIRRFSLMTLRNFGMGKRSIEECVQEEARCLVEELRKTKGSPCDPTFILSCAPCNVICS

IVFRDHFDYTDQDFINLMKKFDENHRIMSSPWIQVYNNMPTLIDYLPGSHNKLFKNFAYI

ENYVLEKVKEHQESLDINNPQDFIDCFLVKMEQEKQNPQSEFTTENLLATIIDVFGAGTE

TTSTTLRYGLLLLLKHPEVTAKVREEIDRVVGRHRSPCMQDKSRMPYTDAVVHEIQRYID

LVPTNLPHAVTCDTKFRNYLIPKGTTIITLLTSALHDDKEFPNPEMFDPGHFLDECGNFR

KSDYFMPFSAGKRICVGEGLARMELFLFLTTILQNFTLKSLVDPEDIDTIPIVSGLARVP

PSYKLCFIPV

>AAI22662.1

MERLEITTLALVICVTCLVFLFVWKKSHKGLGKLPPGPTPLPIIGNLMQLNLKDIPASLS

KLAKQYGPVYTLHLGSQTTVVLHGYEVVKEALIDQGDEFLGRAHFPIIDDTQRGYGLIFS

NGDTWKQMRRFSLMTLRNFGMGKRSLEERIQEEAQFLVEEFRKSEAQPFNPAVTLSCATC

NIICSILFNERFHYQDKTLHSLLDLLNENFNRISSLWNQIYNLWPKLIKPLPGEHRAFSK

RLKDVHYFVLEKVKEHQKSLNHNNPRDYIDCFLSRMEQEKQNPESQFHLENLATCGSNLF

SAGVETTTATLSYGLLLLMKYPEVQAKVHEEIDRVIGRTRSPCMKDKMKLPYTEAVLHEI

QRYVTLVPSNLPHAVVQDTKFRQYVIPKGTTVLPLLSSILYDCKEFPNPEKFDPGHFLDK

NGSFRKTKYFVAFSIGKRACVGEGLAQMELFLFFTTILQNFVLKPLGETKDIETKPIVIG

LINMPPPFKLCLIPR

>327280402

MEPLGMSTVLLVVCISCLLLSAVWKRGAQGKGKMPPGPTPLPLIGNVLQLKGKSLDQALC

KISEEYGPVFTLYLGMNPAVVLYGYEAIKEALIDHGNDFADRAKAPLIEKMGDGKGIVFS

NGETWKQIRRFTLTTLRNFGMGKKSIEERIQEETQYLLEQFHEKKGQPFDPQNLFGCATA

NVICSVVFGKRYEYNDKRFQTLITVTVENNELFNSGWGQLYNTFPVLMEWIPGPYQRMIQ

RSDKCNKIVLEEAKEHRATLDPNSPRDFIDCFFIRMDQEKHNEASEFTMESMVNCCLELF

GAGTETTSTTLRYGFLILQKYQEIEEKVQEEIDRVVGRSRMPSMADRGQMPYTDAVIHEI

QRFISLSPISVPRSVVSDTPLRGYVIPKGTTILPVLTSVLHDGKEFPNPTKFDPGHFLNP

DGTFRKSNYFMPFSAGKRMCAGEGLARMELFLFFTSILQNFKLKPLTDPKDIDLSPMKGN

MNNVPHPYKFCVIPR

>AAP04405.1

MEPSTLLLLTLLLSFLVLLVRGYPKTRGHLPPGPRPLPFLGNLLQIDRGGLLNSFRRFQE

KYGDVFTLHLGSKPVVMLYGTEAIREALVDQAEAFSGRTTIAVLKPTFMDYGVIAANGER

WKTLRRFSLVTMRDFGMGKRSVEERIQEEAQCLVEELRKSQGAPLDPTFLFQCITANIIC

SIVFGQRFDYKDGQFLRLLGLFYQTFSIISSLYTQMFECFPGFLKYFPGTHVQVSKNLQE

LLDYIEHSVEKHRVNMDASNPRDFIDTYLLRMEKEKSNPHTEFHHRNLVTTVLSLFFAGT

ETSSTTLRYGFLLMLKYPHVAEKVQREIDHVIGSDRPPSLDDRPKMPYTEAVIYEIQRFS

DLVPTGVPHKVNKDTYFRGYLLPKDTEVYPILSSALHDPKYFEQPYTFNPDHFLDASGAL

KKNEAFMPFSIGKRICLGEGIARNELFLFFTTILQNFSVSSPMAPKDIDLNPKENGFGRL

PPEYQICFLAR

>EHB10398.1

MLAAVGSLAASLWDVLHLRTLLLAAVAFLFFADFLKRRRPKNYPPGPWRLPFLGNFFHLD

LEKTHLALKQNVEKYGNLVSIDFADIPSVLVTGLPLIKEVLTHTDQNYMNRPLTSIRKRV

FGKKGLIMSNGQIWKEQRRFTLTTLRNFGLGKKSLEERIQEEAYHLAEVIAEEKGQPFNP

HFKINNAVSNIICSIIFGERFDYQNAEFQELLRLLDEVTHLEASPMCQLYIFFPRIVEYL

PGSHNTLFRDWDKLKSFVSHVIENHKRDWNPDEPRDFIDAYLKEMAQHPDKTTSSFDEEN

LILSTLDLFFAGTETTSTTLRWGLLYMALYPEIQEKVHAEIDTVVGQGRQPSLANRESMP

YANAVIHEVLRMGNIIPLNVPRELSVDTALGGYHLPKGTMILTNLTALHRDPTEWATPDT

FNPEHFLENGQFKKRESFLPFSLALVFE

>P08683.1

MDPVLVLVLTLSSLLLLSLWRQSFGRGKLPPGPTPLPIIGNTLQIYMKDIGQSIKKFSKV

YGPIFTLYLGMKPFVVLHGYEAVKEALVDLGEEFSGRGSFPVSERVNKGLGVIFSNGMQW

KEIRRFSIMTLRTFGMGKRTIEDRIQEEAQCLVEELRKSKGAPFDPTFILGCAPCNVICS

IIFQNRFDYKDPTFLNLMHRFNENFRLFSSPWLQVCNTFPAIIDYFPGSHNQVLKNFFYI

KNYVLEKVKEHQESLDKDNPRDFIDCFLNKMEQEKHNPQSEFTLESLVATVTDMFGAGTE

TTSTTLRYGLLLLLKHVDVTAKVQEEIERVIGRNRSPCMKDRSQMPYTDAVVHEIQRYID

LVPTNLPHLVTRDIKFRNYFIPKGTNVIVSLSSILHDDKEFPNPEKFDPGHFLDERGNFK

KSDYFMPFSAGKRICAGEALARTELFLFFTTILQNFNLKSLVDVKDIDTTPAISGFGHLP

PFYEACFIPVQRADSLSSHL

>BAA83589.1

MLGSELLLVAILAYLSVMVLVSVWKQKVRGKLPPGPTPLPYIGNYLQLNTKDMYSSITEL

SERYGPVFTIHLGPRPVVVLYGYDAVKEALVDQAEEFSGRGEQATYNTLFKGYGVTFSSG

ERAKQLRRFSIATLRDFGVGKRSVEERIQEEAACLVKMLQSTSGAPIDPTVYLSQTVSNV

INSIVFGDRFDYEDKEFLELLRMMGQINRFAASPTGQLYDMFHSVMKYLPGPQQQIIKNT

KELEDFMVRKVKQNQSTLDLNSPRNFIDSFLIHMQEEKKNPDSEFNIQNLVMTSLNLFFA

GSETVSSTMRYGFLLLMKYPEVEAKVHEEIDRVIGRNRQPQFEDRMKMPYTEAVINEIQR

FANLAPLGIPRKTIQNTTFRGFFLPKDTEVFPILGSLLTDPKFFPSPKDFNPQNFLDDKG

QLKKNAAFVPFSVGKRFCLGDGLARMELFLFFTTILQNFRFKFPKKPEDIDESPKPLGFA

RIIPHYTMSFLPI

>ABX82805.1

MLASGLLLVALLSCLTLMVLMSVWRQRKLWGRLPPGPTPLPFIGNYLQLDTERICDSLMK

IGKRYGPVFTVHLGPRRVVVLCGYDAVKEALLDQAEEFSGRGEQATFNWIFKGYGVAFSN

GERAKQLRRFSITTLRDFGVGKRGIEERIQEEAGFLIEAFRSTCGNFIDPTFFLSQAVSN

VISSIVFGNRFDYEDKEFLSLLRMMLGNFQFTATSTGQLYEMFYSVMKHLPGPQQQAFKE

LQGLEDFIAKKVEQNQRTLDPNSPRNFIDSFLIRMQQEQTNPNTEFYLKNLVITTLNLFI

AGTETISTTLRYGFLLLMKHPDIEAKVHEEIDRVIGKNRQPKFEDRAKMPYTEAVIHEIQ

RFGDVIPMSLARRVTKDTKFRGFLIPKGTEVFPMLGSVLRDPKFFSNPHDFNPQHFLDEK

GQFKKSDAFVPFSIGKRYCFGEGLARMELFLFLTTIMQNFCFRYPQSPQDIDVSPKHVGF

ASIPRNYTMSLQPR

>DAA19880.1

MELGGAFTIFLALCLSCLLILIAWKRMSKGGKLPPGPTPIPFLGNVLQVRTDATFQSFMK

LKEKYGPVFTVYMGPRPVVVLCGHEAVKEALVDRADEFSGRGELASVERNFQGHGVALAN

GERWRILRRFSLTILRDFGMGKRSIEERIQEEAGFLLVELRKTRGARIEPTFFLSRTVSN

VISSVVFGSRFDYEDQQFLKLLQMINQSFIEMSTSWAQLYDMYSGIMQYLPGRHNRIYYL

IEELKDFIASKVKINEASLDPQNPRDFIDCFLIKMHQDKNNPHTEFNLKNLVLTTLNLFF

AGTETVSSTLRYGLLLMMKHPEVEAKIHEEIDQVIGPHRIPSVDDRAKMPYTDAVIHEIQ

RLTDIVPMGVPHNVIRDTHFRGYLLPKGTDVFPLLGSVLKDPKYFRYPDAFYPQHFLDEQ

GHFKKNEAFVPFSSGKRICLGEAMARMELFLYFTSILQNFSLRSLVPPADIDITPKVSGF

GNIPPTYELCFMVR

>348552446

MLASGLLVVALLACLSVMVLMSVWRRRKLLGKLPPGPTPLPFIGNYLQLNTEQMYNSLMK

ISKRYGPVFTIHLGPRRIVVLCGQDAVKEALVDQAEEFSGRGEQATFDWLFKGYGVAFSN

GERAKQLRRFSITTLRDFGVGKRGIEERIQEEAGFLIQAFRDTRGAFIDPTFYLSRTVSN

VISSIVFGDRFEYEDKEFLSLLRMMLGSFQFTATSTGQLYEMFSSVMKHLPGPQQQAFKE

LQGLEDFITKKVEQNQRTLDPNSPRDFIDSFLIRMLEEKQNPNTEFYMKNLVLTTLNLFF

AGTETVSTTLRYGFLLLMKHPEVEAKIHEEIDRVIGKNRQPKFEDRAKMPYTEAVIHEIQ

RFGDMIPMGLARRVTKDTKFRDFLIPKGTEVFPMLGSVLRDPKFFSNPRDFYPQHFLDEN

GQFKKSDAFVPFSIGKRYCFGEGLARMELFLFLTTIMQNFRFKSPQVPKEIDVSPKHVGF

ATIPPNYTMSFLLR

>EGW14144.1

MEASVLLFLTLLLGFVVLLLRGHPKAHGHLPPGPRPLPFLGNLLQMDRRGLLNSFMRLRE

KYGDVFTVHLGPRPVVILCGTEAIREALVDQAEAFCGRGTVEVVKPTFKGYGLIFANGER

WKALRRFSLATMRDFGMGKRSVEERIQEEAQCLVEELRKSQGAPLDPTFLFQCVXXXXTA

NIICSIVFGERFDYKDRQFLRLLDLFYKCFSHIGSFYTQVFELFSGFLKYFPGTHKQISR

ILQEVLDYIDHNVQKHRATLDPSNPRDFIDTYLLRMEKEKSNPYTEFQQKNLVLTVLSLF

FAGTETSSTTLRYGFLLMLKYPHVAEKLQKEVDQVIGSHRLPTLDDRTKMPYTDAVIHEI

QRFSDLIPIGVPHKVTKDTLFRGYLLPKDTEVYPILSSALHDPQHFEQPDTFNPDRFLDA

NGALKKNEAFMPFSIGKRICLGEGIARNELFLFFTTILQNFSLSSPVDPKDIDLSPKESG

FAKLPPIYKICFLSR

>327261115

MDWIHPITIFFLIILIIMLVLKMSNFRNYSTQNLPPGPKPLPIVGNLHIIDQERPHRTML

KLSKIYGPVFSIQMGFQKMVVLTGYEMVKEALVNQADAFAERPVIPLFEEFAQGFGIIFS

HGENWKVMRRFTLSTLRDYGMGKRSIEDKIVEECSILTKKLESYKGRPFETTTIMNAAVA

NIIVSILLGRRYEYEDLTFRKLLKLINENARLFGSPSVLFYNMFPALGFLSGGRKTCLDN

RKEFFAFINATFMKHLKELDENDQRSFIDTFLIRQQEEKSNNGNGYFHNENLRSVVGNLF

AAGMETTSTTLRWALLLMMKHPEVQRKVQEEIAVTIGSAQPRAEHRQKMPYTDAVIHEVQ

RYANIVPTSVPRATTMDVTLKGYFIPKGTHIIPLLSSVLHDDSQWKKPLRFYPEHFIDPE

GKFIKREAFMPFAAGRRQCAGENLAKMELFLFFTTLMQRFTFQPAPGTSREDLDLTPAVG

FTTPPMPFEVCALPR

>AAM10792.1

MLASGLLLVALLTCLTIMVLMSVWRQRKLQGKLPPGPTPLPFIGNYLQLNTEQMYNSLMK

ISQRYGPVFTVHLGPRRIVVLCGYDAVKEALVDQAEEFSGRGEQATFDWLFKGYGVAFSN

GERAKQLRRFSITTLRDFGVGKRGIEERIQEEAGHLIEAFRGTRGAFIDPTYFLSRTVSN

VISSIVFGDRFDYEDKEFLALLRMMLGSFQFTATSTGQLYEMFYSVMKHLPGPQQQAFKD

LQGLEDFIARKVEHNQRTLDPNSPRDFIDSFLIRMQEEKKNPDTEFYWKNLVLTTLNLFF

AGTETVSTTMRYGFLLLMKKPDVEAKVHEEIDRVIGRNRQAKFEDRAKMPYTEAVIHEIQ

RFGDMIPMGLARRVTKDTKFRDFLLPKGTEVFPMLGSVLRDPKFFSNPRGFNPQHFLDEN

GQFKKNDAFVPFSIGKRYCFGEGLARMELFLFLTNILQNFHLKSPQLPQDIDVSPKHVGF

ATIPPTYTMSFQPR

>ABB43281.1

MVASGILLVALLTCLTVMVLMSVRRQWKLLEKLPPGPTPLPFIGNYLQLNIQQMSDSFMK

ISKRYGPVFTIHLGPRRVVVLCGYEAVKEALVDQAEEFSGRGAQATFDTLFKGYGVTFSN

GERAKQLRRFSITTLRDFGVGKRGIEERIQEEAGFLIEALQGTRGAFIDPTFFLSRTVSN

VISSIVFGDRFDYEDKEFLSLLRMMLGSFQFTATSMGQLCEMFHSVIKYLPGPQQQAIKE

LQGLEDFITKKVEQNQRTLDPNSPRDFIDSFLIRMQEEQNNPNTEFHLKNLVLTTLNLFF

AGTETVSTTLRYGFLLLMKHPDVEAKVHEEIDRVIGKNRQPKFEDRAKMPYTEAVIHEIQ

RFGDIIPLSLARRVIKDTKFREFLLPKGTEVFPMLGSVLRDAKFFSNPQDFHPQHFLDEK

GQFKKSDAFVPFSIGKRYCFGEGLARMELFLFLTTILQNFHFKSPQLPQDIDVSPKLVGL

ATIPRNYTMSFQPR

>AAI35913.1

MDPISILLSIAVCVFLLNLFYGGKGDSKMFPPGPKPLPLIGNLLIMNMKKPHLTFMELAE

KYGSVFSVQLGTEKVVVLCGTDAVKDALINHADEFSERPKIPIFEDVSKGYGLIFSHGEN

WKVMRRFTLTTLRDFGMGKKTIEERICEESDCLVEAFKSYKGKPFENTLIMNAAVANIIV

SILLGHRFDYQDTALLKLIKIINENVRLMGSPMVMLYNTYPSVMQWLPGKHKTVAENTLK

LFKFLEETFTKHRDQLDVNDQRDLVDTFLVKQQEEKPSSSKFFHDQNLTLLVSNLFGAGM

ETTSTTLRWGLLLMMKYPDIQKKVQDEIDKVIGSAEPQTEHRKQMPYTDAVIHEIQRFAN

IAPSNLPHATTRDVTFRGYFIPKGTQVIPLLTSVLQDKNYFKKPEEFYPEHFLDSEGHFM

KNEAFLPFSAGRRSCAGETLAKMELFLFFTKLLQNFTFQPPPGVEVQLTSGEGFTSSPLQ

HNICALPRT

>224052440

MELLGGVTVVLLVCIACLLSFAAWKGRSGKGKMPPGPAPLPILGNLLQVKPSNMTKTLQK

LSEEYGPVFTVHLGSDPVVVLYGHDVVKEALVDRADEFAARGHMPIGDRTNKGLGIIFSN

NELWLQGRRFSLTTLRNFGMGKRSIEERIQEESDYLLEEINKTKRTPFDPTFMLSCAVSN

VICSIVFGKRYDYKDKKFLALMNNMNNIFEMMNSRWGQLYQMFSNILDYLPGPHNNIFAE

FDALKAFVAEEVKLHQASLDPSSPQDFIDCFLCKMQEEKDRPNSSFYMKNLITSTFDLFL

AGTETTSTTLRYGLLLLLKYPKIQEKIQEEIDQVVGQSRKPCVADRTQMPYTDAVVHEIQ

RFITLIPLALPHTVTKDTTFRDYIIPKGTTVFPVLASVLHDSKEFPNPHEFNPEHFLNKN

GSFRKSNFFMPFSAGKRICPGEGLARMEIFLLIATILQKFTLKSVVNPQELNITPTLSGT

GNVPPAYQLCAVPR

>DAA14791.1

MERLEITTLALVICVTCLVFLFVWKKSHKGLGKLPPGPTPLPIIGNLMQLNLKDIPASLS

KLAKQYGPVYTLHLGSQTTVVLHGYEVVKEALIDQGDEFLGRAHFPIIDDTQRGYGLIFS

NGDTWKQMRRFSLMTLRNFGMGKRSLEERIQEEAQFLVEEFRKSEAQPFNPAVTLSCATC

NIICSILFNERFHYQDKTLHSLLDLLNENFNRISSLWNQIYNLWPKLIKPLPGEHRAFSK

RLKDVHYFVLEKVKEHQKSLNHNNPRDYIDCFLSRMEQEKQNPESQFHLENLATCGSNLF

SAGVETTTATLSYGFLLLMKYPEVQAKVHEEIDRVIGRTRSPCMKDKMKLPYTEAVLHEI

QRYVTLVPSNLPHAVVQDTKFRQYVIPKGTTVLPLLSSILYDCKEFPNPEKFDPGHFLDK

NGSFRKTKYFVAFSIGKRACVGEGLAQMELFLFFTTILQNFVLKPLGETKDIETKPIVIG

LINMPPPFKLCLIPR

>AAP33890.1

MLASGLLLVALLTCLTIMVLMSVWRQRKLQGKLPPGPTPLPFIGNYLQLNTEQMYNSLMK

ISQRYGPVFTVHLGPRRIVVLCGYDAVKEALVDPAEEFSGRGEQATFDWLFKGYGVAFSN

GERAKQLRRFSITTLRDFGVGKRGIEERIQEEAGYLIEAFRGTRGAFIDPTYFLSRTVSN

VISSIVFGDRFDYEDKEFLALLRMMLGSFQFTATSTGQLYEMFYSVMKHLPGPQQQAFKD

LQGLEDFIARKVEHNQRTLDPNSPRDFIDSFLIRMQEEKKNPDTEFYWKNLVLTTLNLFF

AGTETVSTTMRYGFLLLMKHPDVEAKVHEEIDRVIGRNRQAKFEDRAKMPYTEAVIHEIQ

RFGDMIPMGLARRVTKDTKFRDFLLPKGTEVFPMLGSVLRDPKFFSNPRGFNPQHFLDEN

GQFKKNDAFVPFSIGKRYCFGEGLARMELFLFLTNILQNFHLKSPQLTQGEGWRGEEEGT

ERRSCSLY

>ABB43280.1

MLASGLLLVALLACLTIIVLMSVWKQRKLGGKLPPGPTPLPFIGNYLQLNTEQMYNSLMK

ISERYGPVFTIHLGPRPVVVLCGHEAVKEALVDQAEEFSGRGEQATFDWLFKGYGVAFSN

GERAKQLRRFSITTLRDFGVGKRGIEERIQEEAGFLIEALRGTRGAFIDPTFFLSRTVSN

VISSIVFGDRFDYEDKEFLSLLRMMLGSFQFTATSMGQLYEMFYSVMKHLPGPQQQAFKE

LQGLEDFITKKVEQNQRTLDPNSPRDFIDSFLIRMQEEQNNPNTEFYLKNLVLTTLNLFF

AGTETVSTTLRYGFLLLMKHPDVEAKVHEEIDRVIGKNRQPKFEDRAKMPYTEAVIHEIQ

RFGDMIPMGVARRVIKDTKFREFLLPKGTEVFPMLGSVLRDAKFFSNPQDFHPQHFLDEK

GQFKKSDAFVPFSIGKRYCFGEGLARMELFLFLTTILQNFHFKSPQLPQDIDVSPKHVGF

ATIPRNYTMSFQPR

>149722308

MALMAILLLIILIGLLLLWSQPTSRGRLPPGPWPLPFLGNILHLDHKDFLKSFQALREKY

GDVFTIYLGTQPAVILCGYEAVREALVDQAEAFSGRGHVAILDPILQGAGVAFANGKSWK

TLRRFSVATMKDFGMGKRSMEERIKEESQCLVEELRKSQGSYLDPTVLFNSVTANILCSI

IFGERFNYQDTQFLRLLNLMNEVLIILSSFYSQVFEFLSSILKHFPGPHTRLYRIIQEME

NFTSENIKRHWKTLDPSNPRDLIDSFLLRMDKECSDPETDFHHKNLIHTVLSLLFAGTET

SSSTLRYALLLLFKYPDVLEKVQAEIDRVIGPHRLPALEDQAKMPYTDAVIHEIQRFIDL

IPFGVPHLVIKDTDFRGYHLPKDTTVYVMLTSVLHDPRHFEKPDVFYPGHFLDAEGNFRK

HEAFIPFSMGKRLCLGEGLARSQLFLILTSMLQNFSLGSPQAPEDIDFTPRINGLTKLPS

VFQLSFLPRQGGGQGMQTPSESLHPAPTSAHNEFVEVS

>149689819

MDPFIVLVICLSCLILLFLWKQSYVKGKLPPGPTPLPIFGNVLQINTKDISKSLSKLAKD

YGPVFTVYFGMKPTVVLHGYEAVKEALTGQNEEFSGRGSMPVFDKVAQGLGIVFSNGEMW

KQTRRFSLTVLRNMGMGKNTIEDRIQEEALCLVEALRKTNASPCDPTFLLGCAPCNVICS

IIFQSRFEYSDKKLITLLHYSHENLRISSTPWITLYNAFPSLVHYLPGSHNELFKNIAGQ

QKFILEKIKEHQESLDLNNPRDFIDYFLIKMEKEKHNKQSEFTMDNLITTIWDVFSAGIE

TTSTTLRYGLLLLMKHPEVAGKVREEIDCVVGRHRSPSMQDRNRMPYTDAVVHEIQRYID

LVPNSLPHATTQDIKFREYLIPKGTEILTSLTSVLHDDKEFPNPEQFDPGHFLDESGNFK

KSDYFMAFSAGKRVCVGEGLARMELFLLLTNILQHFTLKPLVDPKDIDTTPIASGITSVP

PFYELCFIPV

>358411531

MVFPVVMYGYESWTVKKXXXXXXXXXFNTKGLIMSSGHIWKEQRRFALTTLRNFGLGKKS

LEERIQEEASYLIQMIREENGKPFDPHFIINNAVSNIICSITFGERFDYQDSQFRELLRL

LDEVLNLHTSLCCQLYSVFPRIMNFVPGPHQTLFSNLEKLKMFVAEMIENHKRDWNPAEA

RDFIDAYLQEIEKHKGGDASSFREENLIYSTLDLFLAGTETTSTSLRWGLLYMALNPEIQ

EKVQAEIDRVLGQSQQPSTAARESMPYTNAVIHEVLRMGNIIPLNVPREVAVDTTLAGYH

LPKGTVVVTNLTALHRDPAEWATPDTFNPEHFLENGQFKKRESFLPFSIGKWMCLGEQLA

RAELFIFFTSLLQKFTFRPPENEKLSLKFRVSLTLAPVSHRLCAVPRG

>AAA41007.1

MDPVLVLVLTLSSLLLLSLWRQSFGRGKLPPGPTPLPIIGNTLQIYMKDIGQSIKKFSKV

YGPIFTLYLGMKPFVVLHGYEAVKEALVDLGEEFSGRGSFPVSERVNKGLGVIFSNGMQW

KEIRRFSIMTLRTFGMGKRTIEDRIQEEAQCLVEELRKSKGAPFDPTFILGCAPCNVICS

IIFQNRFDYKDPTFLNLMHRFNENFRLFSSPWLQVCNTFPAIIDYFPGSHNQVLKNFFYI

KNYVLEKVKEHQESLDKDNPRDFIDCFLNKMEQEKHNPQSEFTLESLVATVTDMFGAGTE

TTSTTLRYGLLLLLKHVDVTAKVQEEIEHVIGRNRSPCMKDRSQMPYTDAVVHEIQRYID

LVPTNLPHLVTRDIKFRNYFIPKGTNVIVSLSSILHDDKEFPNPEKFDPGHFLDERGNFK

KSDYFMPFSAGKRICAGEALARTELFLFFTTILQNFNLKSLVDVKDIDTTPAISGFGHLP

PFYEACFIPVQRADSLSSHL

>327276373

MELGGVLTMLLGICLVIVIAWKWRHKEGSLFTMELEWVLSISLGIFLVLISAWKWRHKEG

RFPPGPMPLPFFGNLLQLNPKDLPKSFLALSHKYGTVYTLYLGPRRVVVLCGHEALKEAL

VDHAEQFCGRGEMPYVEQTFKGSGIVLANGERWKKLRHFTLITLKNFGMGKCSIEERIQK

EAQYLLEKFRKLKGLPFDPTFLLSCTTANIICSIVFGKRFEYEDKIFLSMLDLTNKIFFE

LSTPWAKLYDMYFGIMQYLPGGDSHIYNLLQELKALIGERIKLNQETLDPKNPRDFIDCF

LIEMNKEKRNPSTEFTVTNLVLTVLNLFTAGTETVSSTLKYALLLLMKYPKVEEKVHQEI

DSVVGRNRTPAVKDRMNMPYTNAVIHEIQRLVDILPAGLPHKVMEDTEFRGYLLPKDTNI

ITLLGSALHDPKYFCDPETFNPEHFLDQEGGFKKNDAFVPFSSGKRACVGESMARMELFL

YFTNILQSFSLKSSLAPTDIDISPQLNGFLNIPPVYQLCLIPR

>348552506

MELRGVFTIFWALCLSCLIILVAWKKTNKGGKLPPGPTPIPFLGNLLQVRIHGTFQSLMK

LHKKYGSVFTVYLGPRPVVVLCGFETLKEALVDQGDDFSGRGEIASIERNFQGYGVAMAN

GERWKILRRFSLTILRDFGMGKRSVEERIQEEAGFLVEELRKTKGAPVELTFILSRSVSN

VISSIVFGNRFDYEDKEFQHLLWMINQSFIEMSNPWAQAYDMYSGIMQYFPGRHNYLYNL

IEDLKDFVAHRVKINEASFDPQNPRDFIDYFLIKMHQEKNNPHTEFNLKNLVLTTLNLFF

AGTETVSSTLRYGILLLTKYPEVQAKVYEEINHVIGPHRIPGANDRPKMPYTDAVIHETQ

RMTNIVPMGVPHIVTRDTHFRGYFLPKGTDVYPLLGSVLQEPKYFRYPDTFYPQHFLDEQ

GRFKKNEAFVPFSYGKRLCLGESLARMELFIYFTSIIQNFSLHSLVPPDDVDISPQVSGF

GNIPPTYQLRVVAR

>327280408

MEPLGTTSVLLLVCISCLLLSAFWKSQANKRRKMPPGPTPLPIIGNALQLKTNHLDLTLC

KLSEKYGSVFTLHFGTKPVVVLHGYGAVKEALIDQAEDFAPRGSLPLIEKHFQGKGIIFS

NGERWKQLRRFALTTLRNFGMGKKSIEERIREEAQYLLERLQGTKEQPFDPTFLLNCTTS

NIICSIVFGKHYDYDNKKFLAMMAIMNDNFEIVSSPWGQLANTFPSFMDWIPGPHHRVGT

NLAKAKAFVMEEMEAHRQTLDPSSPRDFIDCFFIKMDQEKTNEASEFTTENLLLSTIDLF

VAGSETTSSTLRYGLLILQKYPEIEESVQEEIDRVVGRSRLPCMADRGQMPYTDAVIHEI

QRFISLVPLSVPHSVAKDTPFRGYTIPKDTSVFPLLTSVLHDGKEFPNLTEFDPQHFLNK

DGTFRKSDFFMPFSAGKRICAGEGLARMELFLFLASILQNFKLKPLTDPQDIDIKPHLSS

MGNVSPPYQLCVVPR

>334321640

MAKLTVFRALTGWLMVLPISSSEKRRSLAYLCQSSGKMQLPLGTVLPSLWEGAPLQTLLL

LSAILLILASYLQRKRRQANYPPGPSPLPLLGNFFQIDFKKPQVAFQEFVRKYGNVFCME

LGSFTFVFVTGMPLIKEVLVLFAIQIKLNISSSQLDKTIHKLGLIMSEGQAWKHQRRFTL

MTLKNFGLGKRTLEHRIQEEAMYLTEAIREEKGQPFDPHLQINNAVSNIICSITFGHRFE

YHDSQFRELLMSLDEVTVLAARWECLLFDTFPWIMRFLPGPHQKFFREWKKLESFVVNVI

KQHKEDQNSEEAQDFIDAYLKELSKDNISSSFNEDNLVCCTLDLFFAGTETTSTILRWAL

LYMALYPEIQDKIQAEIDRVIGQSRQPTMADRENMPYTNAAIHEVLRMGNILPCHVPRVA

RVDTTVAGYYVPKGTMLLTNLTALHRDPKVWATPETFNPEHFLENGQFTKREAFLPFSAG

KRVCLGEQLARAELFIFFTCLLQRFTFQAPPDTQLSLDFRAGLTISPVPYKICALPRKTH

LNFGLNLKLEFSSVSSMRLARQQEDESQESRLAERKHGESLSLLRAGGNALLLFVCSAPT

VGALLVTNLTALHRDPSEWDTPETFNPEHFLENGKRVCLGEQLARTELFIFFTCLLQRFT

FQAPPDTQLSLDFRVGLTISPVPYKICALPQESQPKLGL

>395529483

MLASGLLLGALLFCLSTLVVLSVWRQRKVRSSLPPGPTPLPFIGNYLQLNMEKMYDSLMK

IGQKYGPVFTVHLGHRTVVVLTGYEAVKEALVDQAEEFSGRGKQATLEWLFKGFGVAFGN

GERARQLRRFSITTLRDFGLGKRGIEERIQEEAGFLIEALRGTKGEAIDPTFFLSRTVSN

VISSIVFGDRFEYEDKQFLSLLRMMLGSFQFTATSMGQLYEMFSPVMKHLPGPQQQAFKD

LKGLEVFITEKVWQNQATLDPNSPRNFIDSFLIKMQEEKKNPNTEFFMRNLTMTTLNLFF

AGTETISTTLRYGFLLLMKHPEVEAKIHEEIDRVIGRNRSPKFEDKAKMPYTEAVIHEIQ

RFGDLIPMGLARRVIKDTKFRGYLLPKGTEVFPMLGSVLRDRNFFACPEAFNPQHFLDEK

GQFKKSDAFVPFSIGKRRCFGKGLARMELFLFLTTILQNFCFQSPVPNEAIDISPKAVGF

ATMPRSYTISFLPWE

>327280400

MEPLGMSTVLLVVCISCLLLSAVWKRGAQGKGKMPPGPTPLPLIGNALQLKGKSLDQALC

KIGEEYGPVFTLYLGMNPAVVLHGYEAIKEALIDHGNDFASRAIIPLVEKTSEGKGIIFS

NGERWKQIRRFTLTTLRNFGMGKKSIEERIQEETQYLLEQFHDTKGKPFDPRKLFGCATS

NVICSIVFGKRYEYNDKRFQTLVAITDENTELFNSGWGQLYNTFPALMEWIPGPFQHLMQ

SCVTCREFILEEAKEHRATLDPSSPRDFIDCFFIRMDQEKDNEASEFTMENLVMSSLDLF

GAGTETTSTTLRYGFLILQKFPQIEEKVQEEIDQVVGRSRIPSTADRGQMPYTDAVIHEI

QRFISLTPVALPHSVVNDTPFRGYVIPKGTTIFPVLTSVLHDSKEFPNPTEFNPGHFLNP

DGTFRKSNYFMPFSAGKRICAGEGLARMELFLFFTSILQNFKLKPLMDPKDIDLSPMKGS

MNNLPWPYKFCIIPR

>P20812.1

MLASGLLLVASVAFLSVLVLMSVWKQRKLSGKLPPGPTPLPFIGNYLQLNTEKMYSSLMK

ISQRYGPVFTIHLGPRRVVVLCGQEAVKEALVDQAEEFSGRGEQATFDWLFKGYGVAFSS

GERAKQLRRFSIATLRDFGVGKRGIEERIQEEAGFLIESFRKTNGALIDPTFYLSRTVSN

VISSIVFGDRFDYEDKEFLSLLRMMLGSFQFTATSTGQLYEMFSSVMKHLPGPQQQAFKE

LQGLEDFITKKVEQNQRTLDPNSPRDFIDSFLIRMLEEKKNPNTEFYMKNLVLTTLNLFF

AGTETVSTTLRYGFLLLMKHPDIEAKVHEEIDRVIGRNRQAKYEDRMKMPYTEAVIHEIQ

RFADMIPMGLARRVTKDTKFREFLLPKGTEVFPMLGSVLKDPKFFSNPNDFNPKHFLDDK

GQFKKSDAFVPFSIGKRYCFGEGLARMELFLFLTNIMQNFCFKSPQAPQDIDVSPRLVGF

ATIPPNYTMSFLSR

>BAF85442.1

MEPFVVLVLCLSFMLLFSLWRQSCRRRKLPPGPTPLPIIGNMLQIDVKDICKSFTNFSKV

YGPVFTVYFGMNPIVVFHGYEAVKEALIDNGEEFSGRGNSPISQRITKGLGIISSNGKRW

KEIRRFSLTTLRNFGMGKRSIEDRVQEEAHCLVEELRKTKASPCDPTFILGCAPCNVICS

VVFQKRFDYKDQNFLTLMKRFNENFRILNSPWIQVCNNFPLLIDCFPGTHNKVLKNVALT

RSYIREKVKEHQASLDVNNPRDFIDCFLIKMEQEKDNQKSEFNIENLVGTVADLFVAGTE

TTSTTLRYGLLLLLKHPEVTAKVQEEIDHVIGRHRSPCMQDRSHMPYTDAVVHEIQRYSD

LVPTGVPHAVTTDTKFRNYLIPKGTAIMALLTSVLHDDKEFPNPNIFDPGHFLDKNGNFK

KSDYFMPFSAGKRICAGEGLARMELFLFLTTILQNFNLKSVDDLKNLNTTAVTKGIVSLP

PSYQICFIPV

>AAB02144.1

MDPALVLVLTLSSLLLLSLWRQSFGRGKLPPGPTPLPIIGNTLQIYMKDIGQSIKKFSKV

YGPIFTLYLGMKPFVVLHGYEAVKEALVDLGEEFSGRGSFPVSERVNKGLGVIFSSGMQW

KEIRRFSIMTLRTFGMGKRTIEDRIQEEAQCLVEELRKSKGAPFDPTFILGCAPCNVICS

IIFQNRLDYKDPTFLNLMHRFNENFRLFSSPWLQVCNTFPAIIDYFPGSHNQVLKNFFYI

KNYVLEKVKEHQESLDKDNPRDFIDCFLNKMEQEKHNPQSEFTLESLVATVTDMFGAGTE

TTSTTLRYGLLLLLKHVDVTAKVQEEIERVIGRNRSPCMKDRSQMPYTDAVVHEIQRYID

LVPTNLPHLVTRDIKFRNYFIPKGTNVIVSLSSILHDDKEFPNPEKFDPGHFLDERGNFK

KSDYFMPFSAGKRICAGEALARTELFLFFTTILQNFNLKSLVDVKDIDTTPAISGFGHLP

PFYEACFIPVQRADSLSSHL

>348552448

MELSILLFLALLMGLLLLLFKGHSKTHGNLPPGPRPLPFLGNILQMNRKGLLKSFLKFRE

KYGDVFTVYLGPRPVVMLCGAETIREALVDQADSFSGRGMIATIESIFQGYGVVFANGDR

WKALRRFSLATMRDFGMGKRTVEERIQEEAQCLVQEMKKSKGGFLDPWFFFQCATANIIC

SIVFGERFDYKDQQFLRLLDLFYQSFSLLSSLSSQMFELFHSVLKYFPGTHSKIYKNVQE

INRFIGRNVEKHRETLDPSNPRDFIDTFLLRMDKEKSNSHTEFHHKNLILTSLSLFFAGT

ETTSTTLRYGFLFLLKYPHVTERVQKEIEQVIGSHRQPALDDRSKMPYTEAVICEIQRFA

DLIPIGVPHMVTKDTHFRGFFIPKDTEVYPLLSTALHDPRHFEKPDSFNPDHFLDAKGTL

KKNEAFIPFSIGKRICLGEGIAHAELFLFFTTILQNFSISSPVAPEDIDFTPQEFGFGRV

PPTYQISFLPRTIDSRET

>1408144A

MDPVLVLVLTLSSLLLLSLWRQSFGRGKLPPGPTPLPIIGNTLQIYMKDIGQSIKKFSKV

YGPIFTLYLGMKPFVVLHGYEAVKEALVDLGEEFSGRGSFPVSERVNKGLGVIFSNGMQW

KEIRRFSIMTLRTFGMGKRTIEDRIQEEAQCLVEELRKSKGAPFDPTFILGCAPCNVICS

IIFQNRFDYKDPTFLNLMHRFNENFRLFSSPWLQVCNTFPAIIDYFPGSHNQVLKNFFYI

KNYVLEKVKEHQESLDKDNPRDFIDCFLNKMEQEKHNPQSEFTLESLVATVTDMFGAGTE

TTSTTLRYGLLLLLKHVDVTAKVQEEIERVIGRNRSPCMKDRSQMPYTDAVVHEIQRYID

LVPTNLFHLVTRDIKFRNYFIPKGTNVIVSLSSILHDDKEFPNPEKFDPGHFLDERGNFK

KSDYFMPFSAGKRICAGEALARTELFLFFTTILQNFNLKSLVDVKDIDTTPAISGFGHLP

PFYEACFIPVQRADSLSSHL

>AAH92552.1

MDPVSVLLSVVVCIFLFKVFYDGEKESQNFPPGPKPLPLIGNLHIINMEKPYLTFMELAE

KYGSVFSFHLGTEKVVVLCGTDAVRDALINHAEEFSGRPKVAIFDQIFKGHGIIFADGEN

WKVMRRFSLSTLRDFGMGKKTIEEKISEESDCLVETFKSHGGKPFDNTMIMNAAVANIIV

ALLLSQRFDYQDPTLLKLVKSINKIVRITGSSMVMLYNTFPSIMQWIPGSHQNVVKNAEK

IYTFLIETFTKHRHQLDVNDQRDLIDTFLIKQQEEKSSSTKFFHDENLKVLLLNLFGAGM

ETTSTTLRWGILLMMKYPEVQKKVQDEIDRVIGSAEPRLEHQKQMPYTDAVIHEIQRFAD

LVPNNVPHATTKDVTFRGYFIPKGTHVIPLLTSVLKDKDYFKKPNEFYPEHFLDSEGHFV

KNEAFLPFSAGRRICAGETLAKMELFLFFTNLLQNFTFQPPPGVEVQLTRGVAITSIPTE

HKICALPRS

>395514710

MTAYKVLLQDLTISSLVYGALGLVAIFYLISLLERYKSKLPPGPFPMPFIGNLNLVDLNK

LYLSLTELSKTYGQVFTVYLGPKKKVVLVGYDVIKDAFLNHNEEFGNRPEIPIFKKIGEG

HGVVFSNGETWKMMRRFALHNLRDFGMGKKLIEYKIQDELHSLVTYFGSHKGKPFDTSII

LKHATSNIICSILLGKRYEYDDYEFQYLLKLLDENVKLCGSSMIELYNYYPFLGFMIGAH

RTVLRNVQEMNNFFHKLFEKHTLHLNINHLTGFVDSFLLKQQQETQNPKTEFHEKNLLFT

TMDLFGAGSETTSTTLCWGLLLMMKYPEVQRRVQAEINKVISLRRDPQVQDRKKMPYTEA

VIHEIQRFADIVPMGLSHSTTTDVKFRGYVIPKGTEVIPLFTSIFKDKTLWETPHKFNPA

HFLDAAGNFVKKDCFIPFGLGRRVCIGESLARMELFLLFVGLLQKFTFKPAPGVDPADLD

LTAEVGLTLTPVPHLVCAIPLEEVHDS

>EHA98995.1

MLVAVGSLAATLWGALHLHTLLLGAVAFLFFAGFLKSRRPKNYPPGPWRLNFLGSFFHLD

LEKIHLGLKQYVKKYGNIVSMDLAYTSAVLVTGLPLIKEVLTHPDQDYMNRPLSPIQERV

FSKRGLIMSSGQIWKEQRRFTLTTLRNFGLGKKSLEERIQEEAYNLTEVIAEEKGQPFNP

HFKINNAVSNIICSIIFGERFDYQDAEFQELLRLLDEVTYLEMSPICQLYNFFPRIMEFL

PGPHHTLFRDWDKLKSFVSRVIENHKRRDWNPNEPRDFIDAYLKEMAQHPDKTTSSFDEE

NLIRSTLDLFLAGTETTSTTLRWGLLYMALYPEIQEKVHAEIDRVVGQGQQSSLANRESM

PYTNAVIHEVLRMGNIIPLNVPREVSVDTALGGYHLPKGTMILTNLTALHRDPTEWATPD

TFNPEHFLENGQFKKRESFLPFSLAQQDSMLLLAAAQQSLDSTLVPILPETQSFLFL

>149689833

MDLVVVLGLCLSCWLLLSLWKQSSRKGKLPPGPTPLPIIGNILQLDVKNISKSLTNLSKV

YGPVFTLYIGMKPAVVLHGYEAVKESLIDLGEEFSGRGNSPGSERANKGLGIVFSNGKIW

REMRRFSLMTLRNFGMGKRSIEDRVQEEARCLVEELRKTNASPCDPTFILGCAPCNVICS

IIFQNRFDYTDQNFLNIMKKFDENVRIGSSPWMQLCNHFPVLLDYFPGSHNKLLKNIAYI

NNYVLEKTKEHQESLDINNPRDFIDCFLIKMEQEKENKQSEFTFENLTITVSDLFGAGTE

TTSTTLRYGLLLLLKHPEVTAKVQEEIDRVIGRHRSPCMQDRSHMPYMDAVIHEVQRYID

LIPTNLPHAVTRDVKFRNYLIPKGTNILISLTSVLHDDKEFPNPEVFDPGHFLDKSGNFK

KSDYFMAFSAGKRICVGEGLARMELFLFLTTILQKFNLKSLVDPKDIDVTPVVNGFASVP

PFYKICFIPV

>BAF37097.1

MDPFVVLLLCLSFLLLFSLWRQSSGRGKLPPGPTPLPIIGNILQISVKDIGKSFSNLSKV

YGPLFTVYFGTKPVVVLHGYEAVKEALIDNGEEFSGRSIFPVSQRTSKDLGIISSNGKRW

KEIRRFSLTTLRNFGMGKRSIEDRVQQEARCLVEELRKTKASPCDPTFILGCAPCNVICS

VVFQNRFDYKDENFLTLMKRFNENFKILSSPWIQFCNNFPLLMDYFPGPHNKLFKNVALT

KSYIWEKIKEHQASLDVNNPRDFIDCFLIKMQQEKDNQESEFTIESLVGTVADLFVAGTE

TTSTTLRYGLLLLLKHPEVTAKVQEEIDHVIGRHRSPCMQDRSHMPYTDAVMHEIQRYID

LVPTSVPHAVTTDIKFRNYLIPKGTAIMTSLTSVLHSDKEFPNPKTFDPGHFLDKNGNFK

KSDHFMPFSAGKRICAGEGLARMEIFLFLTTILQNFNLKSVGDIKNLNTTSASKSIVSLP

PPYQICFIPV

>BAA04555.1

MDLVVFLALTLSCLILLSLWRQSSGRGKLPPGPTPLPIIGNFLQIDVKNISQSFTNFSKA

YGPVFTLYLGSKPTVILHGYEAVKEALIDRGEEFAGRGSFPMAEKIIKGFGVVFSNGNRW

KEMRRFTLMTLRNLGMGKRNIEDRVQEEAQCLVEELRKTKGSPCDPTFILSCAPCNVICS

IIFQNRFDYKDKEFLILMDKINENVKILSSPWLQVCNSFPSLIDYCPGSHHKIVKNFNYL

KSYLLEKIKEHKESLDVTNPRDFIDYYLIKQKQVNHIEQSEFSLENLASTINDLFGAGTE

TTSTTLRYALLLLLKYPDVTAKVQEEIDRVVGRHRSPCMQDRSHMPYTDAMIHEVQRFID

LLPTSLPHAVTCDIKFRKYLIPKGTTVITSLSSVLHDSKEFPNPEMFDPGHFLNANGNFK

KSDYFMPFSTGKRICAGEGLARMELFLILTTILQNFKLKSLVHPKEIDITPVMNGFASLP

PPYQLCFIPL

>BAF98714.1

MEPFVVLVLCLSFMLLFSLWRQSCRRRKLPPGPTPLPIIGNMLQIDVKDICKSFTNFSKV

YGPVFTVYFGMNPIVVFHGYEAVKEALIDNGEEFSGRGNSPISQRITKGLGIISSNGKRW

KEIRRFSLTTLRNFGMGKKSIEDRVQEEAHCLVEELRKTKASPCDPTFILGCAPCNVICS

VVFQKRFDYKDQNFLTLMKRFNENFRILNSPWIQVCNNFPLLIDCFPGTHNKVLKNVALT

RSYIREKVKEHQASLDVNNPRDFIDCFLIKMEQEKDNQKSEFNIENLVGTVADLFVAGTE

TTSTTLRYGLLLLLKHPEVTAKVQEEIDHVIGRHRSPCMQDRSHMPYTDAVVHEIQRYSD

LVPTGVPHAVTTDTKFRNYLIPKGTTIMALLTSVLHDDREFPNPNIFDPGHFLDKNGNFK

KSDYFMPFSAGKRICAGEGLARMELFLFLTTILQNFNLKSVDDLKNLNTTAVTKGIVSLP

PSYQICFIPV

>327259162

MGEGLFCGCSQIAELPPKRDQDGSIHAGLLQASQDFPAGNEMKKEKLPPGPTPLPIIGNI

LQLKTNYLDQAIHKLSQKYGPVFTMYVGTERVVVLNGYDAVKEALIDRADEFSARGKLPL

ADKINKGKGIIFSNGERWKQLRRFALTTLRNFGMGKKSIEERIQDETQYVVEYLQNTKEK

PFDPTFMLSCSTSNVICSIVFGKRYEYNDKRFLSIMASMNENFEVFSSPWGQLYNIFPSL

MDFIPGPHHKVASNSNKNAEFVLEEAKEHRATLDPSSPRDYIDCFYIKMDQEEQNDASEF

TIENLIFCVLDLFTAGTETTSTTLRYGLLILQKYPEIEAKVQEEIDQVIGGARKPCMADR

GKMPYTDAVIHEIQRFISLVPLSVPHAVLKDTVFREYVIPKGTTIYPVLTSVLCDTKEFR

NPTKFDPQHFLHEDGSFRKSDYFMPFSAGKRICAGEGLARMELFLFLTTILQNFKLKPLT

DPKDIDISPQMSSIGSLPRSYQLCVVPR

>P56656.2

MDLVTFLVLTLSSLILLSLWRQSCGRGSLPPGPTPFPIIGNFLQIDIKNVSQSLTNFSKA

YGPVFTLYLGSRPTVVLHGYEAVKEALIDHGEEFSDRGSIPMVEKINNGLGIVFSNGNRW

KEIRRFTLTTLRNLGMGKRNIEDRVQEEAQCLVEELRKTKGSPCDPTFILSCAPCNVICS

IIFQDRFDYKDKDFLMLMEKLNENVKILSSPWLQVCNNFPLLIDYCPGSHHKVLKNVKYI

RSYLLEKIKEHQESLDVTNPRDFIDYYLIKQKQANHIQQAEFSLENLACTINNLFAAGTE

TTSTTLRYALLLLMKYPDVTAKVQEEIDHVIGRHRSPCMQDRNHMPYTDAMIHEVQRFIN

LVPNNIPRAVTCDIKFRNYLIPKGTTVVTSLTSVLHDSKEFPNPELFDPGHFLDANGNFK

KSDHFMPFSAGKRVCAGEGLARMELFLFLTTILQNFKLKSLVHPKDIDMIPFVNGLIALP

PHYQVCIIPR

>AAD13721.1

MDLVTFLVLTLSSLILLSLWRQSCGRGSLPPGPTPFPIIGNFLQIDMKNFSQSLTNFSKA

YGPVFTLYLGSRPTVVLHGYEAVKEALIDHGEEFSDRGSIPMVEKINNGLGIVFSNGNRW

KEIRRFTLTTLRNLGMGKRNIEDRVQEEAQCLVEELRKTKGSPCDPTFILSCAPCNVICS

IIFQDRFDYKDKDFLMLMEKLNENVKILSSPWLQVCNNFPLLIDYCPGSHHKVLKNVKYI

RSYLLEKIKEHQESLDVTNPRDFIDYYLIKQKQANHIQQAEFSLENLACTINNLFAAGTE

TTSTTLRYALLLLMKYPDVTAKVQEEIDHVIGRHRSPCMQDRNHMPYTDAMIHEVQRFIN

LVPNNIPRAVTCDIKFRNYLIPKGTTVVTSLTSVLHDSKEFPNPELFDPGHFLDANGNFK

KSDHFMPFSAGKRVCAGEGLARMELFLFLTTILQNFKLKPLVHPKDIDMIPFVNGLIALP

PHYQVCIIPR

>397510090

MEPFVVLVLCLSFMLLFSLWRQSSGRRKLPPGPTPLPIIGNMLQIDVKDICKSFSNFSKV

YGPVFTVYFGMNPIVVLHGYEAVKEALIDNGEEFSGRGSSPISQRITKGLGIISSNGKRW

KEIRRFSLTTLRNFGMGKRSIEDRVQEEAHCLVEELRKTKASPCDPTFILGCAPCNVICS

VVFQKRFDYKDQNFLTLMKRFNENFRILNSPWIQVCNNFPLLIDCFPGTHNKVLKNVALT

QSYIREKVKEHQASLDVNNPRDFIDCFLIKMEQEKDNQKSEFNIENLVGTVADLFVAGTE

TTSTTLRYGLLLLLKHPEVTAKVQEEIDHVIGRHRTPCMQDRSHMPYTDAVVHEIQRYSD

LVPTGVPHAVTTDTKFRNYLIPKGTTIMTLLTSVLHDDKEFPNPNIFDPGHFLDKNGNFK

KSDYFMPFSAGKRICAGEGLARMELFLFLTTILQNFNLKSVDDLKNLNTTAVTKGIVSLP

PSYQICFIPV

>301759261

MTKASKQHFPPGPRPLPIIGNLHILNLKRPYQTMLELSQKYGSIYSIQMGPKKVVVLSGY

ETVKDALVSYGDQFGERSQVPIFERLFEGKGIVFSHGETWRTMRRFSLTTLRNFGMGKRL

IEDTIIEECQHLVRSFESHRGKPFEVTTVMNASVANVIVSVLLGKRFDYQDTQFLRLLTL

IGENVKLVGSPRIALFNMFPVLGFLLKSHKTVLKNRDELFAFIRMTFLDHQHKFDKNDPR

SLIDAFLVRQQEEKRTSTAYFSDENLVALVSNLFAAGTETTASTLRWALLLMMRYPEVQK

KVCDEITKVVGSAQPRIAHRTQMPYTDAVIHEVQRFANILPTSLPHATTTDVMFKNYYIP

KGTEVITLLTSVLRDQTQWEKPDTFNPEHFLSSTGKFIKKEAFMPFSLGPRMCAGESLAK

MELFLFFTSLMQKFTFQPPPRVSHLDLDLTPDIGFTTRPMPHKLRAVLRA

>P10632.2

MEPFVVLVLCLSFMLLFSLWRQSCRRRKLPPGPTPLPIIGNMLQIDVKDICKSFTNFSKV

YGPVFTVYFGMNPIVVFHGYEAVKEALIDNGEEFSGRGNSPISQRITKGLGIISSNGKRW

KEIRRFSLTTLRNFGMGKRSIEDRVQEEAHCLVEELRKTKASPCDPTFILGCAPCNVICS

VVFQKRFDYKDQNFLTLMKRFNENFRILNSPWIQVCNNFPLLIDCFPGTHNKVLKNVALT

RSYIREKVKEHQASLDVNNPRDFIDCFLIKMEQEKDNQKSEFNIENLVGTVADLFVAGTE

TTSTTLRYGLLLLLKHPEVTAKVQEEIDHVIGRHRSPCMQDRSHMPYTDAVVHEIQRYSD

LVPTGVPHAVTTDTKFRNYLIPKGTTIMALLTSVLHDDKEFPNPNIFDPGHFLDKNGNFK

KSDYFMPFSAGKRICAGEGLARMELFLFLTTILQNFNLKSVDDLKNLNTTAVTKGIVSLP

PSYQICFIPV

>301608066

MDLVSVLLSVVICIFLYKVFYGGEKESQNFPPGPKPLPIIGNFHMINMKKPHLTFMELAK

KYGSVFSIQLGPEKLVVVCGADAVKDALVNHADEFSARPTIPVFDKTSKGHGVFFANGEN

WKVMRRFTLSTLRDFGMGKKTIEDRICEESDFLMETFKSYKGKPFDNTMIMNAAVANIIV

HILLNHRFDYQDPTLLKLINIVSENISIAAKPIVLLYNAYPSIMEWVPGTHKSVAENMLK

LYNFLRETFTQHRDQLDVNDQRDLIDVFLVKQQEEKPSSTKFFNDQNLTVLLADLFGAGM

ETTSTTLRWGLLFIMKYPDIQKKVQDEIDKVIGSAQPRLEHRKKMPYTDAVIHEIQRLGN

LAPNVGHETTTDVTFRGYFIPKGTQVIILLTSVLQDKDYFKKPEEFYPEHFLDSEGNFVK

NEAFLPFSAGRRICVGETLAKMELFLFFTKLLQNFTFQPPPGVEVDLTCADAITSKPLEH

QICALPRS

>AAA35739.1

MEPFVVLVLCLSFMLLFSLWRQSCRRRKLPPGPTPLPIIGNMLQIDVKDICKSFTNFSKV

YGPVFTVYFGMNPIVVFHGYEAVKEALIDNGEEFSGRGNSPISQRITKGLGIISSNGKRW

KEIRRFSLTNLRNFGMGKRSIEDRVQEEAHCLVEELRKTKASPCDPTFILGCAPCNVICS

VVFQKRFDYKDQNFLTLMKRFNENFRILNSPWIQVCNNFPLLIDCFPGTHNKVLKNVALT

RSYIREKVKEHQASLDVNNPRDFMDCFLIKMEQEKDNQKSEFNIENLVGTVADLFVAGTE

TTSTTLRYGLLLLLKHPEVTAKVQEEIDHVIGRHRSPCMQDRSHMPYTDAVVHEIQRYSD

LVPTGVPHAVTTDTKFRNYLIPKGTTIMALLTSVLHDDKEFPNPNIFDPGHFLDKNGNFK

KSDYFMPFSAGKRICAGEGLARMELFLFLTTILQNFNLKSVDDLKNLNTTAVTKGIVSLP

PSYQICFIPV

>AAH20596.1

MEPFVVLVLCLSFMLLFSLWRQSCRRRKLPPGPTPLPIIGNMLQIDVKDICKSFTNFSKV

YGPVFTVYFGMNPIVVFHGYESVKEALIDNGEEFSGRGNSPISQRITKGLGIISSNGKRW

KEIRRFSLTTLRNFGMGKRSIEDRVQEEAHCLVEELRKTKASPCDPTFILGCAPCNVICS

VVFQKRFDYKDQNFLTLMKRFNENFRILNSPWIQVCNNFPLLIDCFPGTHNKVLKNVALT

RSYIREKVKEHQASLDVNNPRDFIDCFLIKMEQEKDNQKSEFNIENLVGTVADLFVAGTE

TTSTTLRYGLLLLLKHPEVTAKVQEEIDHVIGRHRSPCMQDRSHMPYTDAVVHEIQRYSD

LVPTGVPHAVTTDTKFRNYLIPKGTTIMALLTSVLHDDKEFPNPNIFDPGHFLDKNGNFK

KSDYFMPFSAGKRICAGEGLARMELFLFLTTILQNFNLKSVDDLKNLNTTAVTKGIVSLP

PSYQICFIPV

>DAA14852.1

MDLPVVLVLCLCCLLLISLWKQSSGKGKLPPGPTPLPILGNILQLDVKDISKSVSNLSKV

YGPVFTLYFGMNPLVVLHGYEAVKEALIGLGEEFSGRGSCPVIQRASKGYGVIFSNGKIW

KETRRFSLMTLRDFGMGKRSMEDRVQQEACCLVEELRKTDGLPCDPTFILGCAPCNVICS

IIFQNHFDYKDQIFLDLMERLNENARILGSPWIQLCSSFPALIDYVPGKHKKFFENYACM

KSYVLEKTREHQASLDMNNPRDFIDCFLTKMEKEKHNQELEYTVENLAHTVLDLFVAGTE

TTSTTLRYGLLLLLKHPEVTAKVQEEIDHVIGRHRSPCMQDKSHMPYTDAVVHEIQRYID

LVPTNLPHAVTCDIKFRNYLIPKGTGILTSLTSVLYDDKEFPNPEVFDPGHFLDESGNFR

KSDHFMAFSTGKRICVGEGLARMELFLFLTTILQNFTLKSVVDPKDLDTTPVVNGLLSVP

PFYQLCFIPV

>Q64458.2

MDLVVFLALTLSCLILLSLWRQSSGRGKLPPGPTPLPIIGNFLQIDVKNISQSFTNFSKA

YGPVFTLYLGSKPTVILHGYEAVKEALIDRGEEFAGRGSFPMAEKIIKGFGVVFSNGNRW

KEMRRFTLMTLRNLGMGKRNIEDRVQEEAQCLVEELRKTKGSPCDPTFILSCAPCNVICS

IIFQNRFDYKDKEFLILMDKINENVKILSSPWLQVCNSFPSLIDYCPGSHHKIVKNFNYL

KSYLLEKIKEHKESLDVTNPRDFIDYYLIKQKQVNHIEQSEFSLENLASTINDLFGAGTE

TTSTTLRYALLLLLKYPDVTAKVQEEIDRVVGRHRSPCMQDRSHMPYTDAMIHEVQRFID

LLPTSLPHAVTCDIKFRKYLIPKGTTVITSLSSVLHDSKEFPNPEMFDPGHFLNGNGNFK

KSDYFMPFSTGKRICAGEGLARMELFLILTTILQNFKLKSLVHPKEIDITPVMNGFASLP

PPYQLCFIPL

>268370269

MDLVVVLVLSLSCLLLLSLWKESSGKGELPPGPTPLPILGNILQLDVKNISKSLSNLAEV

YGPVFTVYFGIKPTVVLYGYEAVKEALVDLGEEFSGRGHFPVAEKTHKGLGIIFSNGKKW

KEIRRFSLMTLRNLGMGKRSIEDRIQEEACCLVEELRKTNASPCDPTFILDCAPCNVICS

IIFQNRFDYTDQDFLTLLEKFHENLNILSSPWIQVCNNFPALIDYSPGIHNKLLKNIAYL

KSYVLKKVKEHQESLDINNPRDFIDCFLIKMEQEKHNQQSEYTIENLIATVSDMFSAGTE

TTSTTMRYGLLLLLKHPEVTAKVQEEIDRVIGRHRSACMQDRSHMPYTDAVVHEIQRYID

LVPTNLPHAVTCDIKFRNYLIPKGTTILTSLTSVLYDCKAFPNPEVFDPGHFLDESGNFK

KSDYFMPFSTGKRICVGEGLARMELFLFLTTILQKFNLKSVVDPKDIDTTPVANGFASVP

PFYQICFIPL

>1PQ2A

MAKKTSSKGKLPPGPTPLPIIGNMLQIDVKDICKSFTNFSKVYGPVFTVYFGMNPIVVFH

GYEAVKEALIDNGEEFSGRGNSPISQRITKGLGIISSNGKRWKEIRRFSLTTLRNFGMGK

RSIEDRVQEEAHCLVEELRKTKASPCDPTFILGCAPCNVICSVVFQKRFDYKDQNFLTLM

KRFNENFRILNSPWIQVCNNFPLLIDCFPGTHNKVLKNVALTRSYIREKVKEHQASLDVN

NPRDFIDCFLIKMEQEKDNQKSEFNIENLVGTVADLFVAGTETTSTTLRYGLLLLLKHPE

VTAKVQEEIDHVIGRHRSPCMQDRSHMPYTDAVVHEIQRYSDLVPTGVPHAVTTDTKFRN

YLIPKGTTIMALLTSVLHDDKEFPNPNIFDPGHFLDKNGNFKKSDYFMPFSAGKRICAGE

GLARMELFLFLTTILQNFNLKSVDDLKNLNTTAVTKGIVSLPPSYQICFIPVHHHH

>AAY51973.1

MEPFVVLVLCLSFVLLFSLWRQSSGRRNLPPGPTPFPIIGNMLQIDVKDICKSFSNFSKV

YGPVFTVYLGMNPVVVLHGYEAVKEALIDNAEEFSGRGILPISERITKGLGIISSNGKRW

KETRRFSLTTLRNFGMGKRSIEDRVQEEARCLVEELRKTKASPCDPTFILGCAPCNVICS

VVFQKRFDYKDENFLTLMKRFTENFRILTSPWIQVCNNFPLLIDCFPGTHNKLLKNVALT

KSYIREKVKEHQATLDINNPRDFIDCFLIKMEKEKDNQQSEFTIENLVGTVADLFVAGTE

TTSTTLRYGLLLLLKHPEVTAKVQEEIDHVIGRHRSPCMQDRSHMPYTDAVVHEIQRYID

LVPTGVPHAVTTDIKFRNYLIPKGTIIMTLLTSVLHDDKEFPNPKIFDPGHFLDENGNFK

KSDYFMPFSAGKRICAGEGLARMELFLFLTTILQNFNLKSVADLKNLNTTSATRGIISLP

PSYQICFIPV

>DAA14851.1

MDLPVVLVLCLCCLLLISLWKQSSGKGKLPPGPTPLPILGNILQLDVKDISKSVSNLSKV

YGPVFTLYFGMNPLVVLHGYEAVKEALIGLGEEFSGRGSCPVIQRASKGYGVIFSNGKIW

KETRRFSLMTLRDFGMGKRSMEDRVQQEACCLVEELRKTDGLPCDPTFILGCAPCNVICS

IIFQNHFDYKDQIFLDLMERLNENARILGSPWIQLCSSFPALIDYVPGKHKKFFENYACM

KSYVLEKTREHQASLDMNNPRDFIDCFLTKMEQEKHNQELEYTVENLAHTVLDLFVAGTE

TTSTTLRYGLLLLLKHPEVTAKVQEEIDHVIGRHRSPCMQDKSHMPYTDAVVHEIQRYID

LVPTNLPHAVTCDIKFRNYLIPKGTGILTSLTSVLYDDKEFPNPEVFDPGHFLDESGNFR

KSDHFMAFSTGKRICVGEGLARMELFLFLTTILQNFTLKSVVDPKDLDTTPVVNGLLSVP

PFYQLCFIPV

>ABO41976.1

MDSISTAILLLLLALVCLLLTLSSRDKGKLPPGPRPLSILGNLLLLCSQDMLTSLTKLSK

EYGSMYTVHLGPRRVVVLSGYQAVKEALVDQGEEFSGRGDYPAFFNFTKGNGIAFSSGDR

WKVLRQFSIQILRNFGMGKRSIEERILEEGSFLLAELRKTEGEPFDPTFVLSRSVSNIIC

SVLFGSRFDYDDERLLTIIRLINDNFQIMSSPWGELYNIFPSLLDWVPGPHQRIFQNFKC

LRDLIAHSVHDHQASLDPRSPRDFIHCFLTKMAEEKEDPLSHFHMDTLLMTTHNLLFGGT

KTVSTTLHHAFLALMKYPKVQARVQEEIDLVVGRARLPALKDRAAMPYTDAVIHEVQRFA

DIIPMNLPHRVTRDTAFRGFLIPKGTDVITLLNTVHYDPSQFLTPQEFNPEHFLDANQSF

KKSPAFMPFSAGRRLCLGESLARMELFLYLTAILQSFSLQPLGAPEDIDLTPLSSGLGNL

PRPFQLCLRLR

>P15123.1

MDPVVVLVLGLCCLLLLSHWKQNSGRGKLPPGPTPFPIIGNILQIDAKDISKSLTKFSER

YGPVFTVYLGMKPAVVLHGYQAVKEALVDLGEEFAGRGSFPMLDKVSKGLGIVFTNGKRW

KEIRRFSLMTLRNFGMGKRSIEDRVQEEARCLVEELRKTNASPCDPTFILGCAPCNVICS

IIFHNRFDYKDEEFLKLLEKFNENVRILSSPWLQVCNNFPALIDYLPGSHKTLLKNSDYV

KNFIMEKVKEHQKFLDVNNPRDFIDCFLIKMEQENHLEFTLESLVTTVFDLFGAGTETTS

TTLRYSLLLLLKHPEVADKVQEEIERVIGRHRSPCMQDRSRMPYTDAVIHEIQRFIDLVP

NNLPHTVTRDIKFRNYFIPKGTDIMTSLTSVLHDEKAFPNPKVFDPGHFLDESGNFKKSD

YFMPFSAGKRICVGEALARMELFLFLTSILQNFKLQSLVEPKDLDITAVLNGFVSVPPSF

QLCFIPV

>291404482

MDPVVVLGLCLSCLLLLSVWKQRHRGGKLPPGPTPFPILGNILQLDFKDMSKSITRLSAV

YGPVFTVYLGMKPAVVICGYDAVKEALVDLGEEFSERDVFPLTAKVNKGFGIIFSNGKRW

KEMRRFSLMTLRNFGMGKRGIEDRVQEEARCLVEELRKTNGSPCDPTFILGAAPCNVICS

VIFQNRFDYRDQTFLNLMGKFNENFKILNSPWVQVCNTFPILIDCLPGSPRKVINNLSYV

KNYVLEKIKEHQESLDINNPRDFIDCFLIKMEQEKHNQQSEFTIENLMTTVTDVFAAGTE

TTSTTLRYGLLLLMKHPEVTAKVQEEIERVIGRHRSPCMQDRSRMPYTDATVHEIQRYTD

LIPNNVPRATTCNVKFRNYFIPKGTAVITSLTSVLHDDKEFLNPDRFDPDHFLDASGNFK

KSDYFMPFSAGKRVCVGEALARMELFLFLTTILQNFTLKPLVDPKDIDITPLSTGFSRVP

PLYKLCLIPV

>402913352

MDLFIILVICLSCLILLSLWNRSYAKGKLPPGPTPLPIIGNILQLNNKNISKSIRMLAKD

YGPVFTVYFGMKPTVVLHGYKAIKEALIDQGEVFSGRGSFPVIEKITEGFGVIFSNGERW

KQIRRFSLMVLRNMGMGKKTIEDRIQEEALCLVEALKKTNASPCDPTFLLGCVPCNVISS

IIFQNRFDYRDQKFLTLMKYFNENFETVSTPWIQLYNAFPFLRVLPGSHNVLFKNYALQR

SFILEKVKEHQESLDINNPRDFIDYFLIRMEKEKHNKESEFTMDNLVATIWDMFSAGTET

TSTTMRYGLLLLLKHPEISAKVREEIDHVVGKNRSVCMQDRSRMPYTDAVVHEIQRYIDL

VPTSVPHAVTQDIRFREYLIPKGTTILTDLTSVLYDDKEFPNPEKFDPGHFLDESGNFKK

SDYFMAFSAGKRICAGEGLARMELFLILTTILQNFTLKPLVDPKDIDTTPVHKGFGTIPP

FYELCFIPV

>P00179.2

MDPVVVLVLGLCCLLLLSIWKQNSGRGKLPPGPTPFPIIGNILQIDAKDISKSLTKFSEC

YGPVFTVYLGMKPTVVLHGYEAVKEALVDLGEEFAGRGSVPILEKVSKGLGIAFSNAKTW

KEMRRFSLMTLRNFGMGKRSIEDRIQEEARCLVEELRKTNASPCDPTFILGCAPCNVICS

VIFHNRFDYKDEEFLKLMESLNENVRILSSPWLQVYNNFPALLDYFPGIHKTLLKNADYI

KNFIMEKVKEHQKLLDVNNPRDFIDCFLIKMEQENNLEFTLESLVIAVSDLFGAGTETTS

TTLRYSLLLLLKHPEVAARVQEEIERVIGRHRSPCMQDRSRMPYTDAVIHEIQRFIDLLP

TNLPHAVTRDVRFRNYFIPKGTDIITSLTSVLHDEKAFPNPKVFDPGHFLDESGNFKKSD

YFMPFSAGKRMCVGEGLARMELFLFLTSILQNFKLQSLVEPKDLDITAVVNGFVSVPPSY

QLCFIPI

>AAH19908.1

MDLVVFLALTLSCLILLSLWRQSSGRGKLPPGPTPLPIIGNFLQIDVKNISQSFTNFSKA

YGPVFTLYLGSKPTVILHGYEAVKEALIDRGEEFAGRGSFPMAEKMIKGFGVVFSNGNRW

KEMRRFTLMTLRNLGMGKRNIEDRVQEEAQCLVEELRKTKGSPCDPTFILSCAPCNVICS

IIFQNRFDYKDKEFLILMDKINENVKILSSPWLQVCNSFPSLIDYCPGSHHKIVKNFNYL

KSYLLEKIKEHKESLDVTNPRDFIDYYLIKQKQVNHIEQSEFSLENLASTINDLFGAGTE

TTSTTLRYALLLLLKYPDVTAKVQEEIDRVVGRHRSPCMQDRSHMPYTDAMIHEVQRFID

LLPTSLPHAVTCDIKFRKYLIPKGTTVITSLSSVLHDSKEFPNPEMFDPGHFLNGNGNFK

KSDYFMPFSTGKRICAGEGLARMELFLILTTILQNFKLKSLVHPKEIDITPVMNGFASLP

PPYQLCFIPL

>Q9D816.1

MDPVLVLVLTLSCLLLLSLWRQNSGRGKLPPGPTPFPIIGNILQIDIKNISKSFNYFSKV

YGPVFTLYFGSKPTVVVHGYEAVKEALDDLGEEFSGRGSFQIFERINNDLGVIFSNGTKW

KELRRFSIMTLRSFGMGKRSIEDRIQEEASCLVEELRKANGSLCDPTFILSCAPSNVICS

VIFHNRFDYKDEKFLNLMERLNENFKILNSPWMQVYNALPTLINYLPGSHNKVIKNFTEI

KSYILGRVKEHQETLDMDNPRDFIDCFLIKMEQEKHNPHSEFTIESLMATVTDIFVAGTE

TTNITLRYGLLLLLKHTEVTAKVQAEIDHVIGRHRSPCMQDRTRMPYTDAMVHEIQRYID

LIPNNVPHAATCNVRFRSYFIPKGTELVTSLTSVLHDDKEFPNPEVFDPGHFLDENGNFK

KSDYFMPFSIGKRMCVGEALARTELFLILTTILQNFNLKSLVDTKDIDTTPVANTFGRVP

PSYQLYFIPR

>114631924

MEPFVVLVLCLSFMLLFSLWRQSSGRRKLPPGPTPLPIIGNMLQIDVKDICKSFSNFSKV

YGPVFTVYFGMNPIVVLHGYEAVKEALIDNGEEFSGRGSSPISQRITKGLGIISSNGKRW

KEIRRFSLTTLRNFGMGKRSIEDRVQEEAHCLVEELRKTKASPCDPTFILGCAPCNVICS

VVFQKRFDYKDQNFLTLMKRFNENFRILNSPWIQVCNNFPLLIDCFPGTHNKVLTNVALT

QSYIREKVKEHQASLDVNNPRDFIDCFLIKMEQEKDNQKSEFNIENLVGTVADLFVAGTE

TTSTTLRYGLLLLLMHPEVTAKVQEEIDHVIGRHRTPCMQDRSHMPYTDAVVHEIQRYSD

LVPTGVPHAVTTDTKFRNYLIPKGTTIMTLLTSVLHDDKEFPNPNIFDPGHFLDKNGNFK

KSDYFMPFSAGKRICAGEGLARMELFLFLTTILQNFNLKSVDDLKNLNTTAVTKGIVSLP

PSYQICFIPV

>1DT6A

MAKKTSSKGKLPPGPTPFPIIGNILQIDAKDISKSLTKFSECYGPVFTVYLGMKPTVVLH

GYEAVKEALVDLGEEFAGRGSVPILEKVSKGLGIAFSNAKTWKEMRRFSLMTLRNFGMGK

RSIEDRIQEEARCLVEELRKTNASPCDPTFILGCAPCNVICSVIFHNRFDYKDEEFLKLM

ESLHENVELLGTPWLQVYNNFPALLDYFPGIHKTLLKNADYIKNFIMEKVKEHQKLLDVN

NPRDFIDCFLIKMEQENNLEFTLESLVIAVSDLFGAGTETTSTTLRYSLLLLLKHPEVAA

RVQEEIERVIGRHRSPCMQDRSRMPYTDAVIHEIQRFIDLLPTNLPHAVTRDVRFRNYFI

PKGTDIITSLTSVLHDEKAFPNPKVFDPGHFLDESGNFKKSDYFMPFSAGKRMCVGEGLA

RMELFLFLTSILQNFKLQSLVEPKDLDITAVVNGFVSVPPSYQLCFIPIHHHH

>194205831

MDLVVFLGLCLSCWFLLSLWKQSSRKGKLPPGPTPLPIIGNILQLDVKDISKSLSNLSKL

YGPVFTLYLGLKPTVVLHGYEAVKEALIDLGEEFSGRGSFPVAERVNKGHGILFSNGKRW

KEIRRFSLMTLRNFGMGKRSIEDRVQEEARCLVEELRKTNASPCDPTFILGCAPCNVICS

IIFQNRFDYTDQTFLNLLEKFNENLRIMSSPWIQVCNNLPALIDYLPGSHNKMLKNFDYL

KSYVLEKTKEHQESLDIDNPRDFIDCFLIKMEQEKHNQQSEFTFENLIAAVSDLFGAGTE

TTSTTLRYALLLLLKHPEVTAKVQEEIDRVIGRHRSPSMQDRSHMPYMDAVIHEIQRYTD

IVPTNLPHAVTCDVKFRNYIIPKGTTILTSLTSVLYDAKEFHNPEVFDPGHFLDESGNFK

KSDYFMAFSAGKRMCLGEGLARMELFLFLTTILQKFTLKSVVDPKDIDTTPAASGFGHVP

ASYQLCFIPV

>326923635

MGKGVLRAPRGCARTQRSERQPPPKTGWEPGNDATSAAFAAREPAGSPMPAPGRAETDSP

AASYASPRDPNRSRTPPRFPYSQNRSPFLSHIPLALTKVSKKYGPIFTIHLGPKKVVVLY

GYDVVKEALIDNGEAFSGRGNLPFFEKVFKGIGIVTSNGETWRQLRRFALTTLRDFGMGK

KGIEERIQEEARFLVERIRNTHEKPFNPGKFLIHAVSNIICSIVFGDRFDYEDTKFLDLI

EMLEENNKYQSKIQTQLYNFFPTILDSLPGPHKKLIKNIEKVDDFISEIVRAHQESFDPS

CPRDFIDAFINKMQQEKGNPIFTVESLTRTTLDLFLAGTGTTSTTLRYGLLILQKYPEIE

EKIHKEIDRVVGRDRSPCMADRSQLPYTDAVIHEIQRFIDFLPVNLPHAVTKDTKLRDYF

IPKDTMIFPLLSPILQDSKEFPDPEKFDPGHFLNANGTFRKSDYFMPFSAGKRICAGEGL

ARMEIFLFLTSILQNFTLRPVVDHKDVDISPIVTSAAKIPRPYEISFIPR

>BAA34652.1

MLASGLLLVSVLAFLVILVLMSVWKQRKLSGKMPPGPTPLPFIGNYLQLNTAQMYNSLMK

ISKRYGPVFTVHLGPRRRIVVLWGQEAVKEALVDQAEEFSGRGEQATFDSFFEGYGLVFS

SGERAKQLRRFSIATLRDFGMGKRGVEERILEEAGYLLEALQKTNGALIDPTFYLSRAVS

NVMSSIVFGDRFDYEDQEFLSLLRMMLETFQFTASSTGQLYEMFSSVMKRLPGPQQQAYK

GLKALEDFIAKKVEQHQSTLDPNSPRDFIDSFLIRMQEEKDPNTEFFMKNLVLTTLNLFA

AGTETISTTLRYGFLLLMKHPDVEAKVHEEIDRVIGRNRQPKYEDRTKMPYTEAVIHEIQ

RFGDVIPMGVPRRVTKDTKFRDFLIPKGTDVFPVLGSVLKDSKFFSNPKDFNPKHFLDGN

MQFKKNDAFVPFSLGKRYCFGEGLARMELFLFLTIILQNFRFRSSQAPQDIDVSPQLVGF

GTLPPNYTMSFLSR

>194205829

MDLIVVLVLSLSCLLLLSLWKQSSRKGKLPPGPSPLPVIGNILQIDVKDISKTLINLSKV

YGPVFTLYFGMKPTVVLHGYEAVKEALIDLGEEFSGRGTFPVAERANRKYGIILSNGKRW

KEMRRFSLMTLRNFGLGKRSIEDCVQEEARCLVEELRKTNASPCNPTFILGCAPCNVICS

IIFQNRFDYTDQNFLNLLELLNENVKILSSPWVQVCNTFPIFMDYFPGSHNDLLKNFAYI

KNYLLEKVKEHQESLDINNPRDFIDHFLIKMEQEKDNQQSEFTIENLIISAFDLFSAGSE

TTSTTLRYAILLLIKYPEVTVKVQEEIDRVIGRHRSPCMQDRSHMPYTDAVVHEIQRYID

IIPTNVPHAVTQDIKFRNYLIPKGTTILTSLTSVLHDDKEFPNPRVFDPGHFLDESGNFK

KSDYFMAFSAGKRICVGEGLARMELFLFLTNILQKFTLKSVVDPKDIDITPVANGFASVP

PSYQLHFIPV

>Q6GUQ4.1

MSALGVSVALLVWVAVLLLVSIWRQVHSSWNLPPGPFPLPIIGNLFQLELKNIPKSFTRL

AQRFGPVFTLYVGSRRVVVVHGYKAVREVLLDHKDEFSGRGDIPAFHAHRDRGIIFNNGP

TWKDIRRFSLTTLRNYGMGKQGNESRIQREAHFLLEALRKTQGQPFDPTFLIGCAPCNVI

ADILFRKHFDYNDEKFLRLMYLFNENFQLLSTPWLQLYNNFPSLLHYLPGSHRKVMKNVA

EIKEYVSERVKEHLQSLDPNCPRDLTDCLLVEMEKEKHSAERLYTMDGITVTVADLFFAG

TETTSTTLRYGLLILMKYPEIEEKLHEEIDRVIGPSRIPAIKDRQEMPYMDAVVHEIQRF

ITLVPSNLPHEATRDTIFRGYIIPKGTVIVPTLDSVLYDNQEFPDPEKFKPEHFLDESGK

FKYSDYFKPFSAGKRVCAGEGLARMELFLLLSAILQHFNLKPLVDPKDIDISPVNIGFGC

IPPRFKLCVIPRS

>149690200

MHLVVVLGLCLSCLLLLSLWKQRSRKGKLPPGPTPLPIIGNILQLDVKDISKSFINLSKV

YGPVFTLYLGLEPTVVLHGYKAVKEALIDLGEEFSGRGSFPLAERANKGHGILFSNGKRW

KEIRRFSLMTLRNFGMGKRSIEDRVQQEACCLVEELRKTSASPCNPTFILGCAPCNVICS

VIFHNRFDYTDQNVLNLLELFNENVNILSSPWAQVCNNFPAVIDYIPGRHKKLLKNVADI

KSYLLEKIKEHQESLDINNPRDFIDCFLIKMEQEKYNQYSEFTVENLVTTAFDLFAAGTE

TTSTTLRYGLLLLLKHPEVTAKVHEEIDRVIGRDRSPCMQDKSHMPYTDAVVHEIQRYIN

LVPNNLPHAVTCDIKFRNYLIPKGTTILTSLTSVLHDDKEFPNPEVFDPGHFLDKSGNFK

KSDYFMAFSAGKRMCAGEGLARMELFLFLTTILQKFTLKSVVDPKDIDTTPVANGFTSVP

PSYQLHFIPL

>395820858

MDPAVALVLCLSCLLLLSLWRQSSGRGKLPPGPTPLPIIGNILQLDIKDISKSLTNLSKA

YGPVFTVYLGMMPAVVLHGYEAVKEALIDHGEEFSGRGSFPLAERANRGYGIVFSNGNKW

KEIRRFSLMTLRNFGMGKRSIEERVQEEARCLVEELRKTKSSPCDPTFTLSCAPCNVICS

IIFKNRFDYRDQNFLNLMEKLNENIRILSSPWVQVCNNFPALIDYFPGSHNKLLKNIAFV

KSYILEKVKEHQESLDVNNPRDFIDCFLIKMEQEKHNQQSEFTVENLIITASDFFGAGTE

TTSTTLRYALLLLLKHPEVTAKVQEEINHVIGRHRSPCMQDRSHMPYTDAVVHEVQRYID

LIPTNLPHAVTRDIKFRNYLIPKGTTVLTSLTSVLHDCKEFPNPETFDPSHFLDESGNFR

KSDYFMPFSAGKRICAGEGLARMELFLFLTSILQNFNLKSLVDPKAIDTTPIFNGFASLP

PFYQLCFVPV

>EHB18323.1

MLTSGLLVVALLTCLTVMILMSIWRQRKLWGKLPPGPTPLPFIGNFLQLNTEQMYNSLMK

ISKRYGPVFTIHLGPRRVVVLCGQDAVKEALVDQAEEFSGRGEQATFDWIFKGYGVAFSN

GERAKQLRRFSITTLRDFGMGKRGIEERIQEEAGFLIQAFRDTSGAFIDPTFYLSRTVSN

VISSIVFGDRFEYQDKEFLSLLRMMLGSFQFTATSTGQLYEMFSSVMKHLPGPQQQAFKE

LQGLEDFITKKVKQNQRTLDPNSPRDFIDSFLIRMQEVHPTARVGRSKNPNTEFYMKNLV

MTTLNLFFAGTETVSTTLRYGFLLLMKHPDVEAKIHEEIDRVIGKNRQPKFEDRAKMPYT

EAVIHEIQRFGDMIPMGLARRVTKDTKFRDFLLPKGTEVFPMLGSVLKDPQFFSNPQDFH

PQHFLDEKGQFKKSDAFVPFSIGKRYCFGEGLARMELFLFLTTILQNFRFKSPQAPQDID

VSPKLVGFATIPPNYTMSFQLR

>Q8WNE1.2

MDSVSTAILLLLLALICLLLTLSSRDKGKLPPGPRPLPLLGNLLLLRSQDMLTSLTKLSK

EYGSMYTVHLGPRRVVVLSGYQAVKEALVDQGEEFSGRRDFPAFFNFTKGNGIAFSNGDR

WKVLRRFSIQILRNFGMGKRSIEERILEEGSFLLAELRKTEGEPFDPTFVLSRSVSNIIC

SVLFGSRFDYDDERLLTIIRHINDNFQIMSSPWGELYDIFPSLLDWVPGPHQRIFQNFKC

LRDLIAHSVHDHQASLDPRSPRDFIDCFLTKMAEENEDPLSHFHMDTLLMTTHNLLFGGT

ETVGTTLRYAFLALMKYPKVQARVQEEIDLVVGRARLPALKDRAAMPYTDAVIHEVQRFA

DIIPMSLPHRVTRDTAFRGFLIPKGTDIITLLNTVHYDPSQFLTPQEFNPEHFLDANQSF

KKSPAFMPFSAGRRLCLGESLARMELFLYLTAILQSFSLQPLGAPKDIDLTPLSSGLGNL

PRPFQLCLRPR

>395862705

MSGIDFSALFPILVLILIFILSIKISMAKGPKQHSPPGPRPLPVVGNLHILNLKRPYQTM

LELSQKYGSIYSIHMGPKKVVVLSGHETVKDALINYGDQFGERSQVPIFERLFEGKGKGI

AFSHGETWKTMRRFSLTTLRNFGMGKRVIEDRIIEECQHLILNLECQRGKPIEIRTIMNA

SVANVIVSVLLGKRFDYHDAQFLRLLTLIGENMKLLGGPRIALFNMFPVLGFLLKSHETV

LRNRDELFSFVRMTFLRHRRTLDKNDPRSFIDAFLIRQQEEKDTSTDYFSDENLVALVTN

LLAAGTETTASTLRWGILLMMRYPEIQKKVCDEIAKVVGAAQPRIAHRTQMPYTDAVIHE

IQRFANILPTGLPRATTTDIIFKNYYIPKGTEVIILLTSVLRDQTQWEKPDAFNPEHFLS

SEGKFIKKEAFMPFSVGRRMCAGESLAKMELFLFFTSLLQKFTFQPPPGVCHLNLDLTPD

MGFTTRPVPHEICALLRA

>ABS52843.1

MDLVVVLGLCLSCLLLLLLWKESSRKGKLPPGPTPLPIIGNILQLDVKNISKSLSNLSKV

YGPVFTLYFGMKPTVVLHGYEAVKEALIDLGEEFSGRGRFPVTERVNKGHGIISSNGKRW

KEIRRFSLMTLRNFGMGKRSIEDRVQEEARCLVEELRKTNASPCDPTFILGCAPCNVICS

IIFQNRFDYKDQNFLNIMKVFDENFKILSSPWMQICNAFPALLEYFPGSTDKLFKNVAYV

RSYILEKVKEHQASLDINNPRDFIDCFLIKMEQEKQNQQSEFTFENLKITVSDLFGAGTE

TTSTTLRYGLLLLLKHPEVIAKVQEEIDRVIGRHRSPCMQDKSHMPYTDAVVHEIQRYID

LLPTNVPHAVTRDVKFRNYFIPKGTTILISLTSVLHDDREFPNPEVFDPGHFLDESGNFK

KSDYFMAFSAGKRVCAGEGLARMELFLFLTTILQKFNLKSVVDPKDIDTTPVANGFAFVP

PSYQLYFIPV

>301784779

MDLFIVLVICLSCLIPFFLWNHNHAKGKLPPGPTPLPIIGNILQINTKNVSKSLSKLAES

YGPVFTVYFGMKPTVVLYGHEAVKEALTDCSEEFSGRGSFPAFDWIARGLGIVFSNGEKW

KQTRRFSLTILRNMGMGKKTIEDRIQEEALCLVEALKKTNASPCDPTFLLGCAPCNVICS

IIFQNRFEYDDKTFLSLLEYVHQDFLISSTSWIQLYNAFPLLIHYLPGSHHVLFKNIANQ

FKFIMEKIKEHQESLDFNNPRDFIDYFLIKIEKEKHNKQSVFTTENLVITLWDVLSAGTE

TTSTTLRYGLLMLLKYPEVTAKVQEEIHRVVGRHRSPCMQDRSRMPYTDAVLHEIQRYID

LVPNSLLHAVTQDIKFREYLIPKDTNILVSLTSVLHDDKEFPNPDRFDPGHFLDESGNFK

KSDYFMPFSAGKRACIGEGLARMELFLLLTNILQHFTLKPLVDPKDIDTTPTANALSSTP

PYFKLCFIPV

>AAP55508.1

MVLGVFLGLLLTCLLLLSLWKQNSQRRNLPPGPTPLPIIGNILQLDLKDISKSLRNFSKV

YGPVFTLYLGKKPAVVLHGYKAVKEALIDHGEEFAGRGTFPVADKFIRRCGVVFSSGRTW

KEMRRFSLMTLRNFGMGKRSIEDRVQEEARCLVDELRKTKGVPCDPTFILGCAPCNVICS

IVFQNRFDYKDQEFLTFIDILNENVEILSSPWIQVCNNFPAIIDYLPGRHRKLLKNFDFA

KHYFLAKVIQHKESLDINNPRDFIDCFLIKMEQEKHNPKTEFTCENLIFTASDLFAAGTE

TTSTTLRYSLLLLLKYPEVTAKVQAEIDCVIGRHRSPCMQDRHSMPYTDAVLHEIQRYID

LLPTSLPHAVTRDVKFREYLIPKGTTVIASLTSVLYDDKEFLNPERFDPSHFLDESGKFK

KSDYFFPFSTGKRICVGEGLARTELFLFLTTILQNFNLKSPVDLKDLDTTPVANGFVSVP

PKFQICFISI

>291404529

MRLPASDLPTADAMTVAKLSQEEEKTLVDPAMFLLLSLSCLFLLLLWRWNSTKGKLPPGP

TPLPIIGNILQLDLKDVSKSFSSFSKIYGPVFTVYLGMKPTVVLHGYEAVKEALIDLGEE

FSGRGSFPVAERINKGLGIILSNGKTWKEMRRFSLMTLRNFGMGRRSIEDRVQEEARCLV

EELRKTNASPCDPTFILGCAPCNVICSIIFQNRFEYTDQTFLKFLKMLNEGIDILNSPWI

QVCNNFPILIDYLPGSHKKFIEDMNYIPNFISEKVKEHQQSLDMNNPRDFIDCFLIKMEQ

EKHNQQSEFTLENLKYTASDLYSAGTETTSTTLRYGLLLLLKHPEVTAKVQEEIERVIGR

HRSPCMQDRSRMPYTDAVMHEIQRFIDLVPTSLPRAVTRDVKFRNYLIPKGTNIITSLTS

VLHDDKEFPNPESFDPGHFLDKSGNFKKSDYFMPFSTGKRICVGEGLVRMELFLFLTTIL

QNFKLKPLVDPKDINITPVVNGFASVPPPYQLCFIPV

>224058581

MVLVVLVILVLIIDLVRKRRPRNFPPGPQLFPLVGTVVDFKQPLHLALQKLTGQYGNIFS

VQFGSLTFVVVSGYQMVREALVHQAETFADRPNIPLLQEIFRGFGLISSNGHIWRQQRKF

ASATLKSLAVNFEEKVQEESRYLVETIEEEKGQPFDPHYKINSAVSNIICSITFGNRFDY

HDNRFQELLHSLAETLLLIGSFWGQLYNAFPLIMRWFPGPFRKIFRHWEKLQYFVKEVIA

KHKEDLDQSEAGDYIDCYLKEIEKFKGDTSSYFHEENLLCCTLDLFLTGTETTATAIRWA

LLYMAAYPHIQEKVQQEIDAVVGQCRQPSMADKEKMPYTSAVLSEVLRVGNVVPLGVPRM

ATSDTTLAGFHLPKGTTLMTSLTSVMFDKNVWETPDTFNPEHFLENGLYRRREAFLPFSA

EPLYSNQGVFQNSPQGLKSISETKGQINFQVQFKGDTSSYFHEENLLCCTLDLFLTGTET

TATAIRWALLYMAAESAAGDRRRVGQCRQPSMADKEKMPYTSAVLSEVLRVGNVVPLGVP

RMATSDTTLAGFHLPKAEGCPGEQLARTELFIFFVACCRSSLQAPAALSSPFTLSLTRCP

KPFQLCAVPRH

>CAD23244.1

MLLLGAASVVLLVCVACLLSIVQWRKRTGKGKMPEGPTPLPIVGNILEVKPKNLAKTLEK

LAEKYGPVFSVQLGSTPVVVLSGYEAVKEALIDRADEFAARGHMPIGDRANKGLGIIFSN

NEGWLHVRRFALSTLRNFGMGKRSIEERIQEEAEHLLEEITKTKRLPFDPTFKLSCAVSN

VICSIVFGKRYDYKDKKFLSLMNNMNNTFEMMNSRWGQLYQMFSYVLDYLPGPHNNIFKE

IDAVKAFVAEEVKLHQASLDPSAPQDFIDCFLSKMQEEKDNPKSHFHMTNLITSTFDLFI

AGTETTSTTTRYGLLLLLKYPKIQEKVQEEIDRVVGRSRRPCVADRTQMPYTDAVVHEIQ

RFITLIPTSLPHAVTKDIHFRDYIIPKGTTVMPLLSTALYDSKEFPNPTEFNPGHFLNQN

GTFRKSDFFIPFSAGKRICPGEGLARMEIFLLLTAILQNFTLKPVISPEELSITPTLSGT

GNVPPYYQLCAFPR

>AAZ29451.1

MDSLVVLVLCLSCLLLLSLWRQRSGRGKFPPGPTPLPVIGNILQIDIKDVSKSLTNLSKV

YGPVFTLYFGLERMVVLHGYEAVKEALIDLGEEFSGRGHFPLADRANRGFGIVFSNGKRW

KEIRRFSLMTLRNFGMGKRSIEDRVQEEARCLVEELRKTKGSPCDPTFILGCAPCNVICS

IIFHKRFDYKDQQFLKVMEKLNENVKILSSPWIQICNNFPPFIDYFPGAHNKLLKNIAFL

KSYILEKVKEHQESMDMSNPRDFIDCFLMKMEKEKHNQQSEFTIENLENTAVDLFGAGTE

TTSTTLRYALLLLLKHPEVTARVQEEIERVIGRNRSPCMQDRSHMPYTDAVVHEIQRYID

LLPTNLPHAVTCDVKFRNYLIPKGTTILISLTSVLHDNKEFPNPEMFDPRHFLDEGGNFK

KSNYFMPFSAGKRICVGEALARMELFLFLTSVLQNFNLKSLVDPKDLDTTPVVNGFASVP

PFYQLCFIPA

>AAI53846.1

MDLFVVLVICLSILIFLFLWNQRHAKGKLPPGPTPLPIVGNILQINIKNVSKSISKLAED

YGPVFTLYFGMKPTVVLHGYEAVKQVLIDQSEEFSGRGSLPVADNINQGLGIVFSNGEIW

KQTRRFSLMVLRNMGMGKRTIEHRIQEEALCLVEALKKTNGSPCDPTLLLSCAPCNVICS

IIFRNRFEYNDERLLTLIKYFNENSRLVSTPWVELYNTFPSLLHYFPGSHNTIFKNMTEQ

RKFILEEIKKHQESLDLNNPQDFIDYFLIKMEKEKHNKHSEFTMDNLITTVWDVFSAGTE

TTSLTLRYGLLLLLKHPEVTAKVQEEIDRVVGRNRSPCMQDKSCMPYTDAVLHEIQRYID

LVPSSMPHAATQDVKFREYLIPKGTVILTSLTSVLHDDNEFSNPGQFDPGHFLDESGNFK

KTDHFMAFSAGKRVCVGEGLARMELFLLLVSILQHFTLKSVVDPKHIDTAPSFKGLISIP

PFCEMCFIPV

>P24903.2

MDSISTAILLLLLALVCLLLTLSSRDKGKLPPGPRPLSILGNLLLLCSQDMLTSLTKLSK

EYGSMYTVHLGPRRVVVLSGYQAVKEALVDQGEEFSGRGDYPAFFNFTKGNGIAFSSGDR

WKVLRQFSIQILRNFGMGKRSIEERILEEGSFLLAELRKTEGEPFDPTFVLSRSVSNIIC

SVLFGSRFDYDDERLLTIIRLINDNFQIMSSPWGELYDIFPSLLDWVPGPHQRIFQNFKC

LRDLIAHSVHDHQASLDPRSPRDFIQCFLTKMAEEKEDPLSHFHMDTLLMTTHNLLFGGT

KTVSTTLHHAFLALMKYPKVQARVQEEIDLVVGRARLPALKDRAAMPYTDAVIHEVQRFA

DIIPMNLPHRVTRDTAFRGFLIPKGTDVITLLNTVHYDPSQFLTPQEFNPEHFLDANQSF

KKSPAFMPFSAGRRLCLGESLARMELFLYLTAILQSFSLQPLGAPEDIDLTPLSSGLGNL

PRPFQLCLRPR

>EHH25402.1

MSALGVSVALLVWVAVLLLVSIWRQVHSSWNLPPGPFPLPIIGNLFQLELKNIPKSFTRL

AQRFGPVFTLYVGSRRVVVVHGYKAVREVLLDHKDEFSGRGDIPAFHAHRDRGIIFNNGP

TWKDIRRFSLTTLRNYGMGKQGNESRIQREAHFLLEALRKTQGQPFDPTFLIGCAPCNVI

ADILFRKHFDYNDEKFVRLLYLFNENLNLLSTPWLQLYNNFPSLLHYLPGSHRKVMKNVA

EIKEYVSERVKEHLQSLDPNCPRDLTDCLLVEMEKEKHSAERLYTMDGITVTVADLFFAG

TETTSTTLRYGLLILMKYPEIEEKLHEEIDRVIGPSRIPAIKDRQEMPYMDAVVHEIQRF

ITLVPSNLPHEATRDTIFRGYIIPKGTVIVPTLDSVLYDNQEFPDPEKFKPEHFLDESGK

FKYSDYFKPFSAGKRVCAGEGLARMELFLLLSAILQHFNLKPLVDPKDIDISPVNIGFGC

IPPRFKLCVIPRS

>194205827

MDPVVVLGLCLSCLLFLSLWKQSSRKGKLPPGPTPLPIIGNILRLDVKDISKSLLNLSKV

YGPVFTLYLGLEPTVVLHGYEAVKEALIDQGEEFSGRGSFPIIERTNNKRHGIVLSNGKR

WKEMRRFSLMTLRNFGMGKRSIEDHVQQEASCLVEELRKTGASPCNPTFILGCAPCNVIC

SIIFQNRFDYTDQNLLNLLKLLNEAVSIMSSLWVQIGNAFPALMEYFPGSHNKLSKNFTY

TKSYLSEKIKEHQESLDINNPRDFIDCFLIKMEQDKHNLQSEFTTENLINAVFDLFSAGT

ETTSTTLRYGLLLLIKHPEVTAKIQEEIDRVIGRHRSPCMQDRSHMPYMDAVVHEIQRYI

DLIPTNLPHAVTHDIKFRNYLIPKGTTILTSLTSVLHDEKEFPNPKVFDPGHFLDESGNF

KKSDYFMAFSAGKRICVGESLARMELFLFLTTILQKFTLKSVVDPKDIDTTPVVNGFASV

PPSYQLCFVPV

>126273202

MDSSMVTAFGLVCCTLCLLLISALRKRCGKGKLPPGPGPLPIIGNILQLDTKNIPKSLCM

LAKVYGPVFTLYLGSKSVVVLHGYKAMKEALIDHGEEFAGRGSFPIIDAINKGLGLAFSN

GERWKQIRRFSLMTLKNLGMGKRSIEERVQEEAKCLVEALKKTNGMPCDPTFILGCAPCN

VICSIIFQKRFEYHDQKFLHLMKLLDEKVKILSSPWIQIYNLLPLLAQYLPGSHHKLFKI

SQMMHNFFLEKVKEHQDALDPNNPQDLIDSFLIKMEQEKEKPQSEFTMENLVCTVSDIFG

AGTQTTSTTLRYGLLLLLKHPEITGKIHEEIDRVIGHNRSPCLKDRNSMPYTDAVIHEMQ

RYIDLVPANLTHSVIQDVKFRQYIIPKGTTIIPLLTSVLYDNEEFPNPDQFDPGHFLDES

GNFKKSDYFMPFSAGKRICIGEGLARMELFLFFTTILQNFTLKSLIDPKDIDTTPIASGF

GNIPPSFKLCFLPSQNV

>296220855

MDLFIILATCLSCLTLLFLWNGSNAKGKLPPGPILSPLLENIRQLNSKNISKSISMLAKD

YGPVFTVYFGMKPTVVLHEYKAIKEALIDHGEEFSGRGSFPVMDRITQGLGVIFSNGERW

KQTRRFSLMVLRNMGMGKKTIEDRIQEEALCLVAALKKTNASPSDPTFLLGCVPCNVISA

IIFQNRFDYSDPKFQTLINYFNENFESVNAPWIQLYNAFPFLRFLPGSHNVIFKNYALQI

NFILEKVKEHQESLDISNPRDFIDYFLIKMEKEKHNKQSEFTMDNLVATIWDMFNAGTES

TSTTMRYGLLLLLKHPEISAKVQEEIDQVVGKNRSPCMQDRSCMPYTDAVVHEIQRYIDL

VPTSLPHEVTQDTHFREYFIPKGTTILADLTSVLYDDKEFPSPEKFDPGHFLDENGNFKK

SDYFMPFSTGKRACAGEGLARMELFLNLTTILQNFTLKPLVDPKDIDTTPVHKGLGSVPP

FYELCFIPV

>AAT49267.1

MDSLVVLVLCLSCLLLLSLWRQRSGRGKFPPGPTPLPVIGNILQIDIKDVSKSLTNLSKV

YGPVFTLYFGLERMVVLHGYEAVKEALIDLGEEFSGRGHFPLADRANRGFGIVFSNGKRW

KEIRRFSLMTLRNFGMGKRSIEDRVQEEARCLVEELRKTKGSPCDPTFILGCAPCNVICS

IIFHKRFDYKDQQFLKVMEKLNENVKILSSPWIQICNNFPPFIDYFPGAHNKLLKNIAFL

KSYILEKVKEHQESMDMNNPRDFIDCFLMKMEKEKHNQQSEFTIENLENTAVDLFGAGTE

TTSTTLRYALLLLLKHPEVTAKVQEEIERVIGRNRSPCMQDRSHMPYTDAVVHEIQRYID

LLPTNLPHAVTCDVKFRNYLIPKGTTILISLTSVLHDNKEFPNPEMFDPRHFLDEGGNFK

KSNYFMPFSAGKRICVGEALARMELFLFLTSVLQNFNLKSLVDPKDLDTTPVVNGFASVP

PFYQLCFIPV

>395828401

MDPAVALVLCLSCFLLLFFWRQSSGRGKLPPGPTPLPIIGNILQMDIKDIRKSIKNLSKV

YGPVFMVYFGMKPVVVLHGYEVVKEALTDHGEEFSGRGLLPISEKTSKGFGVIFSNGNRW

KEIRRFSLMTLRNFGMGKRSIEERVQEEAHCLVEELRKTKASPCDPTFFLGCAPCNVICS

IVFQKRFDYKDQNFLTLMGRFNENFRILSSPWIQLCNSFPALIDYFPGIHNKLLKNVALT

KSYILEKVKEHQESLDVNNPRDFIDCFLIKMEQEKNNSQSEFTMENLVGSVADLFVAGTE

TTSTSLRFALLLLLKHPEVTAKVQEEIDRVIGRHRSPCMQDRSHMPYTDAMVHEVQRYID

LVPLNLPHTVTHDIKFRNYLVPKGTMIMTSLTSVLHYDNEFPDPETFNPAHFLDESGNFK

KSDFFMPFSTGKRICLGEGLARMELFLFLTSILQNFNLKSSIDTRKIDITSVPSGTISLP

PLYKICFIPV

>EAW57017.1

MDSISTAILLLLLALVCLLLTLSSRDKGKLPPGPRPLSILGNLLLLCSQDMLTSLTKLSK

EYGSMYTVHLGPRRVVVLSGYQAVKEALVDQGEEFSGRGDYPAFFNFTKGNGIAFSSGDR

WKVLRQFSIQILRNFGMGKRSIEERILEEGSFLLAELRKTEGEPFDPTFVLSRSVSNIIC

SVLFGSRFDYDDERLLTIIRLINDNFQIMSSPWGELYDIFPSLLDWVPGPHQRIFQNFKC

LRDLIAHSVHDHQASLDPRSPRDFIHCFLTKMAEEKEDPLSHFHMDTLLMTTHNLLFGGT

KTVSTTLHHAFLALMKYPKVQARVQEEIDLVVGRARLPALKDRAAMPYTDAVIHEVQRFA

DIIPMNLPHRVTRDTAFRGFLIPKGTDVITLLNTVHYDPSQFLTPQEFNPEHFLDANQSF

KKSPAFMPFSAGRRLCLGESLARMELFLYLTAILQSFSLQPLGAPEDIDLTPLSSGLGNL

PRPFQLCLRPR

>126273208

MDPSVVNAFGLLFCISCLLLILAWKKDFRRGKLPPGPVPFPIIGNILQLDLKNIPESLCK

LAKEYGPVFTLQLGFTRTVVLHGYKAVKEALIDHGDQFAARGHMPVFEFISQGLGIVSSN

GERWKQLRRFSLMTLRNFGMGKKSIEENVQEEAKLLVEAIKQTKGLPCDPTFILGCAPCN

VICSLIFQKHFEYKDPKFLYLMKLLDDDLKLLSSPWIQVYNYFSPLIHYLPGLHHKLFKI

TDLQKKFILEEVKEHQQTLDPNNIRDFIDCFLMKMEQEKQKPLSEFTIGNLVNTAIDLFA

AGTETTSTTLKYGFLMLLKHPEITEKIHEEIDRVIGYNRSPCMEDRNKVPYTNAVVHEIQ

RYIDHIPTSLPHAVTEDVQFRQYLIPKGTTIIPLLTSVLYDDEEFPNPHQFDPGHFLDAS

GNFKKSDYFMPFSTGKRICLGEGLARMELFLFFTTVLQNFTLKSLIDPKDIDTTPVDSGF

GKIPPSYKLCFLPSQNM

>P19225.1

MALFIFLGIWLSCLVFLFLWNQHHVRRKLPPGPTPLPIFGNILQVGVKNISKSMCMLAKE

YGPVFTMYLGMKPTVVLYGYEVLKEALIDRGEEFSDKMHSSMLSKVSQGLGIVFSNGEIW

KQTRRFSLMVLRSMGMGKRTIENRIQEEVVYLLEALRKTNGSPCDPSFLLACVPCNVISS

VIFQHRFDYSDEKFQKFIENFHTKIEILASPWAQLCSAYPVLYYLPGIHNKFLKDVTEQK

KFILMEINRHRASLNLSNPQDFIDYFLIKMEKEKHNEKSEFTMDNLIVTIGDLFGAGTET

TSSTIKYGLLLLLKYPEVTAKIQEEITRVIGRHRRPCMQDRNHMPYTDAVLHEIQRYIDF

VPIPLPRKTTQDVEFRGYHIPKGTSVMACLTSALHDDKEFPNPEKFDPGHFLDEKGNFKK

SDYFMAFSAGRRACIGEGLARMEMFLILTSILQHFTLKPLVNPEDIDTTPVQPGLLSVPP

PFELCFIPV

>ADM73512.1

MSALGVSVALLVWVAVLLLVSIWRQVHSSWNLPPGPFPLPIIGNLFQLEWKNIPKSFTRL

AQRFGPVFTLYVGSRRVVVVHGYKAVREVLLDHKDEFSGRGDIPAFHAHRDRGIIFNNGP

TWKDTRRFSLTTLRNYGMGKQGNESRIQREAHFLLEALRKTQGQPFDPTFLIGCAPCNVI

ADILFRKHFDYNDEKFLRLMYLFNENFQLLSTPWLQLYNNFPSLLHYLPGSHRKVMKNVA

EIKEYVSERVKEHLQSLDPNCPRDLTDCLLVEMEKEKHSAERLYTMDGITVTVADLFFAG

TETTSTTLRYGLLILMKYPEIEEKLHEEIDRVIGPSRIPAVKDRQEMPYMDAVVHEIQRF

ITLVPSNLPHEATRDTIFRGYIIPKGTVIVPTLDSVLYDNQEFPDPEKFKPEHFLDESGK

FKYSDYFKPFSAGKRVCAGEGLARMELFLLLSAILQHFNLKPLVDPKDIDISPVNIGFGC

IPPRFKLCVIPRS

>ACB98739.1

MDPWGLTSTALLTCVLLLIFLSLWRQGFKRRKLPPGPIPLPIIGNILQLDLKNMPESLSK

LAEKYGPIYTLHIGTRRVVVLHGYDIMKEALIDQGDIFMDRGNLPMFEDVAEGHGVIFSS

GERWKQHRRFTLTTLRNFGMGKRSVEERVQEEAQCLVEELRKRKGQPTDPTFILSCAPCN

VICSILFRDRFKYNDEKFLHLMNLLNENFRLFNKPWTQLYNFLPAFRAYLPGEHKRILKI

NEEVKDFILERVKEHQKVLDPNNPQDFIDCYLSKMQQEKDNPQSEFDLENLKMTGVDLFS

AGTETTNSTIRYGLLLILKHPEVQAKIHEEIGRVIGHNRLPSIKDRQDMPYMDAVVHEVQ

RFIDLVPLNVPHAVNRDVHFQQYILPKGTTIFPLLTPVLHDKKEFPKADQFDPQHFLDEN

GKFKKSDHFMPFSIGKRSCAGEGLAKMEVFLFLTTILQNFTLKAVGDPNEIRIKPNYVGF

SKLPPRYQLCFLPQ

>AAX55817.1

MDSISTAILLLLLALVCLLLTLSSRDKXKLPPGPRPLPLLGNLLLLRSQNMLTSLTQLSK

EYGSVYTVHLGPRRVVVLSGYQAVKEALVDQGEEFSGRGDYPVFFNFTKGNGIAFSNGDR

WKVLRRFSIQILRNFGMGKRSIEERILEEGSFLLAELRKTEGEPFDPTFVLSRSVSNIIC

SVLFGSRFDYDDERLLTVIRLINDNFQIMSSPWGELYNIFPSLLDWVPGPHQRIFQNFKR

LRDLIAHXVHDQQASLDPRSPRDFIDCFLTKMAEEKEDPLSHFHMDTLLMTTHNLLFGGT

ETVGTTLRHAFLALMKYPKVQARVQEEIDLVVGRTRLPTLEDRAAMPYTDAVIHEVQRFA

DIIPMNLPHRVIRDTAFRXFLIPKGTDIITLLNTVHYDPSQFLXPQEFNPEHFLDANQSF

KKSPAFMPFSAGRRLCLGESLARMELFLYLTAILQSFSLQPLGAPEDIXLTPLSSGLGNL

PRXFQLCLCPR

>327276259

MDLSGAVILFLVIYLSFLAIVSFKRKLSNKGKLPPGPTALPLIGNFLQIKSSETLKSLLR

LSEIYGPVFTVYFGTRPIVVLCGHDAVKEALIDKAEEFSGRATNPTLERTFQGHGVVFAN

GERWKQLRRFSLSVLRDFGMGKKSIHERIQEEAHFLLDEFRKTKEKPFDPTYFLSRAVSN

VICSIVFGDRFDYENKEFQALMEMMNNSFREISTAWAQFYDMYESFLKYFPGPHTKIYNI

LEDMICFIAKKVKKNKETFDPNYPRDFIDCFLTQMEKEKDKASSEFNERNLELTTLNLFF

AGTETVSSTLRYGFLFLMKHPEVQAKVHEEIDRVIGHNRVPNIEDRSQMPYMDAVIHEIQ

RCSDLIPMDVAHRVICDTEFRGYIIPKGTEIYPILSSVLHDPTMFKRPFAFDPENFLDEN

GRFKKNDAFVPFSSGKRICLGEALARMELFLFFTTILQSFQLKSLVPPEDINIIPQESGF

ATIPPFYQLSVIPR

>363732465

MALVFILTFLFIMKIGGLWSNHWRKNFPPGPRALPIIGNLHLFDLKRPYRTYLQLSKEYG

PVFSVQMGQRKIVVISGYETVKEALINQADAFAERPKIPIFEDLTRGNGIVFAHGENWKV

MRRFTLTTLRDFGMGKRAIEDRIVEEYGYLIDNVGSQEGKPFDASKIINAAVANIIVSIL

LGKRFDYKDSRFIRLQHLTNESMRLAGKPLVTMYNIFPYLGFLLRANKTLLKNRDEFHAY

VKATFLENLKTLDKNDQRSFIDAFLVKQQEEKSTTNGYFHNGNLLSLVSNLFTAGVETIS

TTLNWSFLLMLKYPEIQRKVQEEIEQVIGSNPPRIEHRTQMPYTDAVIHEVQRFANILPL

DLPHETAEDVTLKDYFIPKGTYIIPLLTSVLRDKSQWEKPDMFYPEHFLDSKGKFVKKDA

FMPFSAGRRICAGETLAKMELFLFFTSLLQRFTFQPPPGVSSSDLDLSPAISFNVVPKPY

KICAVARS

>194205771

MELLGISTTLPLVICVTCLVFLFVWKKSYGWGRLPPGPTPLPIIGNLMQLNLKNLPASLS

KMAKEYGPVYTLYFGPQLTVVLHGYEVVKEALIDQADEFLGRGRFPIIEDNQKGHGILFS

NGETWKQMRRFSLMTLRNFGMGKRSIEERVQEEARCLVEELRKMEAQPFDPVFVFACAPC

NVICSILFNERFHYKNETFLSLMYLLTENFKQISSPWNQIYNLWPTLIRHLPGEHKAFSK

RLDGVKNFILEKVKAHQESLDPNNPRDYIDCFLSKMEQEKHKPESEFNLENLAVCGSNLF

TAGTETTSSTLRWGLLLLMKHPEVEAKVHEEIDRVIGRNQSPCMKDKMRLPYTEAVLHEI

QRYITLLPSDLPHAVTRDTKFKNYVIPKGTTVLPLLSSVLFDCKEFPNPEKFDPGHFLDK

NGSFRKTEYFVPFSLGKRACAGEGLARMELFLFFTTILQNFCLKPLVEPKEIDTTPVVTG

LLNIPPPYKLCLIPR

>297687075

MEPFVVLVLCLSFMLLFSLWRQSSGRRKLPPGPTPLPIIGNMLQIDIKDICKSFSNFSKV

YGPVFTVYFGMNPMVVLHGYEAVKEALIDNGEEFSGRGSSPISQRITKGLGIISSNGNRW

KEIRRFSLTTLRNFGMGKRSIEDRVQEEARCLVEELRKTKASPCDPTFILGCAPCNVICS

VVFQKRFDYKDQNFLTLMKRFNENFRILNSPWIQVCNNFPLLIDCFPGTHNKLLKNVALT

RSYIREKVKEHQTSLDVTSPRDFIDCFLIKMEQEKDNQKSEFNIENLVGTVADLFVAGTE

TTSTTLRYGLLLLLKHPEVTAKVQEEIDHVIGRHRSPCMQDRSHMPYTDAVVHEIQRYID

LVPTGVPHAVTTDIQFRNYLIPKGTTIMTLLTSVLHDDKEFPNPKIFDPGHFLDKNGNFK

KSDYFMPFSAGKRICAGEGLARMELFLFLTTILQNFNLKSVDDLKNLNTTAVTKGIVSLP

PSYQICFIPV

>327260049

MLAEAGQAMLVKAGQATEAGQAMPTEGRGAREGKGGRLHPHGAFRNHASPAAYQDEVARR

GSSSAVRVSQSAPGGEGGGMGSEGWAAAGGSVTATAALLLLLSALSLLLLVVRQLLKQRR

PAGFPPGPAGLPLVGNIHSLAAEQPHVYMRRQSQLHGQIFSLDLGGISAIVLNGYDTIKE

CLVQQSEVFADRPSLPLFKKLTKMGGLLNSRYGRGWTEHRKLAVTSFRTFGYGQKAFESK

ISEESVIFLEAIDTYKGKPFDMKYLITNAVSNISNLIIFGERFTYEDTEFQHMIDIFSEN

IELAASASAFLYNAFPWIGVLPFGKHQQLFKNAAEVYTFLLHLIQRFSQNRTPQSPRHFI

DAYLDEVAKNKNDPESTFSMENLIFSVGELMIAGTETTTNVLRWAVLFMALYPNIQGQVH

KEIDTVIGPNRTPSLEEKCKMPYTEAVLHEILRFCNVAPLGIFHATSKDTVVRGYSIPQG

TTVITNLYSVHFDEKYWNNPEMFCPERFLDSSGQFIKKEAFVPFSLGRRHCLGEQLARME

MFLFFTSLLQRFHLHFPSGLIPDLKPKLGMTLQPHPYLICAERRL

>AAA31209.1

MDPVVVLVLGLCCLLLLSIWKQNSGRGKLPPGPTPFPIIGNILQIDAKDISKSLTKFSEC

YGPVFTVYLGMKPTVVLHGYEAVKEALVDLGEEFAGTGSVPILEKVSKGLGIAFSNAKTW

KEMRRFSLMTLRNFGMGKRSIEDRIQEEARCLVEELRKTNASPCDPTFILGCAPCNVICS

VIFHNRFDYKDEEFLKLMESLNENVRILSSPWLQVYNNFPALLDYFPGIHKTLLKNADYI

KNFIMEKVKEHEKLLDVNNPRDFIDCFLIKMEQENNLEFTLESLVIAVSDLFGAGTETTS

TTLRYSLLLLLKHPEVAARVQEEIERVIGRHRSPCMQDRSRMPYTDAVIHEIQRFIDLLP

TNLPHAVTRDVRFRNYFIPKGTDIITSLTSVLHDEKAFPNPKVFDPGHFLDESGNFKKSD

YFMPFSAGKRMCVGEGLARMELFLFLTSILQNFKLQSLVEPKDLDITAVVNGFVSVPPSY

QLCFIPI

>ABB87189.1

MDLFIILVICLSCLILLSLWNRSYAKGKLPPGPTPLPIIGNILQLNTKNISKSISMLAKY

YGPVFTVYFGMKPTVVLHGYEAIKEALIDQGEVFSGRGSFPVIEKITQGFGVIFSNGERW

KQIRRFSLMVLRNMGMGKKTIEDRIQEEALCLVEALKKTNASPCDPTFLLGCVPCNVISS

IIFQNRFDYRDQKFLTLMKYFNENFETVSTPWIQLYNAFPFLRVLPGSHNVIFKNFALQR

SFILEKVKEHQESLDINNPRDFIDYFLIRMEKEKHNKESEFTMDNLVATIWDMFSAGTET

TSTTMRYGLLLLLKHPEISAKVREEIDHVVGKNRSVCMQDRSRMPYTDAVVHEIQRYIDL

IPTNVPHAVTQDIRFREYLIPKGTTILTDLTSVLYDDKEFPNPEKFDPGHFLDKSGNFKK

SDYFMAFSAGKRICAGEGLARMELFLILTTILQNFTLKPLVDPKDIDTTPVHKGFGTILP

FYELCFIPV

>327282461

MDVLGTTTIFLVVFLVLLVSWRKVEARRRNWPPGPTPLPIIGNLLQLKGFNISKHLKKLG

AKYGPIFMVYFGSNQVVAVHGYDVVKKVLVDNADDFLDRGSFPSAQKLSKGLGVLMSNGE

RWVQIRRFSLTTLRNFGMGKKGIEEWIQEEAQHLVKALRDTKGQPLSPSSLFNSATGNVI

NHILLGERFDYQDKEYQQIIYFILHSSQIESSFPGQLYNMFPSIMDHLPGPHQTMFQETY

SVQDFINRKIEEHIETFDATDTPRDFIDAFLLKMEQEKSNPRTEFTKEQLMLTIIDLFFA

GTETTSTTMRFILMVLLEYTSVQAKVQEEIDRMIGRERMPSMKDRPGMPYTEAVLHEGQR

YLDLVPSGLARRARRDVELEGVVIPKGATVLPLLTSLLNDSKQFKDPHCFNPEHFLDEKG

NFKKNGADIPFSAGKRNCLGEGLARMELFLFVTTILQNFHLKFPPGVTKIDLTPDISGFL

NIPRQVPFCFSPR

>AAT49266.1

MDPFVVLVLCLSFVLLFSLWRQSSGRRKLPPGPTPLPIIGNILQIDVKDICKSFSNFSKV

YGPVFTVYFGMNPVVVLHGYETVKEALIDNAEEFSGRGILPISERITNGLGIISSNGKRW

KETRRFSLTTLRNFGMGKRSIEDRVQEEARCLVEELRKTKASPCDPTFILGCAPCNVICS

VVFQKRFDYKDENFLTLMKRFTVNFRILTSPWIQVCNNFPLLIDCFPGTHNKLLKNVALT

KSYIREKVKEHQATLDVNNPRDFIDCFLIKMEQEKDNQESEFTIENLVGTVADLFVAGTE

TTSTTLRYGLLLLLKHPEVTVKVQEEIDHVIGRHRSPCMQDRSHMPYTDAVIHEIQRYID

LVPTGVPHAVTTDIKFRNYLIPKGTIIITLLTSVLHDDKEFPNPKIFDPGHFLDENGNFK

KSDYFMPFSAGKRICAGEGLARMELFLFLTTILQNFNLKSVADLKNLNTTSATRGIISLP

PSYQICFIPV

>308081811

MDLFIVLVICLSCLISFFLWNQNRAKGKLPPGPTPLPIIGNILQINTKNVSKSLSKLAEN

YGPVFTVYFGMKPTVVLYGYEAVKEALIDRSEEFSGRGHFPLLDWTIQGLGIVFSNGEKW

KQTRRFSLTVLRNMGMGKKTVEDRIQEEALYLVEALKKTNASPCDPTFLLGCAPCNVICS

IIFQNRFEYDDKDFLTLLEYFHENLLISSTSWIQLYNAFPLLIHYLPGSHHVLFKNIANQ

FKFISEKIKEHEESLNFSNPRDFIDYFLIKIEKEKHNKQSEFTMDNLIITIWDVFSAGTE

TTSTTLRYGLLVLLKHPDVTAKVQEEIHRVVGRHRSPCMQDRSCMPYTDAVVHEIQRYID

LVPNNLPHSVTQDIKFREYLIPKGTTILTSLTSVLHDEKGFPNPDQFDPGHFLDENGSFK

KSDYFMAFSAGKRVCVGEGLARMELFLLLTNILQHFTLKPLVDPKDIDTTPIANGLGATP

PSYKLCFVPV

>ABB87190.1

MDPAVALVLCLSCLFLLSLWRQSSGRGRLPSGPTPLPIIGNILQLDVKDMSKSLTNFSKV

YGPVFTVYFGLKPIVVLHGYEAVKEALIDHGEKFSGRGSFPVAEKVNKGLGILFSNGKRW

KEIRRFSLMTLRNFGMGKRSIEDRVQEEALCLVEELRKTNASPCDPTFILGCAPCNVICS

VIFHNRFDYKDQRFLNLMEKFNENLRILSSPWIQVCNNFPALIDYLPGSHNKVVKNFAYV

KSYVLERIKEHQESLDMDNPRDFIDCFLIKMEQEKHNLQSEFTIESLIATVTDMFGAGTE

TTSTTLRFGLLLLLKYPEVTAKVQEEIECVVGRNRSPCMQDRSHMPYTDAVVHEIQRYID

LIPTNLPHAVTCDVKFRNYLIPKGTTIITSLTSVLHNDKEFPNPEMFDPGHFLDRSGNFK

KSDYFMPFSAGKRMCVGEGLARMELFLFLTTILQNFNLKSQVDPKDIDITPIANAFGRVP

PLYQLCFIPV

>348552510

MELSLLLFLALLLGLLLLLFKGHPKAHGNLPPGPRPLPFLGNILQMNRKGLFKSFLKFRE

KYGDVFTVYLGPRPVVMLCGAETIREALVDQADSFSGRGIIATVESTFQGYGVFFANGDR

WKALRRFSLATMRDFGMGKRTVEERIQEEAQCLVEEMKKSKGDLLDPLFFFQCATANIIC

SIVFGERFGYKDQQFLRLLDLFYQSFALGSSLSSQMFELFYSVLKYFPGTHSVIYKNLQE

INSFIGRSVEKHRETLDPSNPRDFIDTFLLRMDKEKSNSHTEFHHKNLILTSLSLFFAGT

ETTSTTLCYGFLFLLKYPHVTERVQKEIEQLIGSHCQPALDDRSKMPYTEAVICEIQRFA

DLLLIGVPHMVTKDTHFRGFFIPKDTEVYPLLSTALHDPCHFEKPDSFNPDHFLDAKGTL

KKNEAFIPFSMGKRICLGEGIARAELFLFFTTILQNFSISSPVAPEDIDFTPQEFGFGRV

PPTYQISFLPRTIDSREK

>P20814.1

MDPVVVLLLSLFFLLFLSLWRPSSGRGKLPPGPTPLPIIGNFFQVDMKDIRQSLTNFSKT

YGPVYTLYVGSQPTVVLHGYEALKEALVDHGEEFSGRGRLPICEKVAKGQGIAFSHGNVW

KATRHFTVKTLRNLGMGKGTIEDKVQEEAKWLVKELKKTNGSPCDPQFIMGCAPGNVICS

IILQNRFDYEDKDFLNLIEKVNEAVKIISSPGIQVFNIFPILLDYCPGNHNIYFKNHTWL

KSYLLEKIKEHEESLDVSNPRDFIDYFLIERNQENANQWMNYTLEHLAIMVTDLFFAGIE

TVSSTMRFALLLLMKYPHVTAKVQEEIDHVIGRHRSPCMQDRSHMPYTNAMVHEVQRYID

IGPNGLLHEVTCDTKFRNYFIPKGTAVLTSLTSVLHDSKEFPNPEMFDPGHFLDENGNFK

KSDYFIPFSAGKRMCLGESLARMELFLFLTTILQNFKLKSLVDPKDINTTPICSSLSSVP

PTFQMRFIPL

>ABB87194.1

MDPAVALVLCLSCLFLLSLWRQSSGRGRLPSGPTPLPIIGNILQLDVKDMSKSLTNFSKV

YGPVFTVYFGLKPIVVLHGYEAVKEALIDHGEKFSGRGSFPVAEKVNKGLGILFSNGKRW

KEIRRFSLMTLRNFGMGKRSIEDRVQEEALCLVEELRKTNASPCDPTFILGCAPCNVICS

VIFHNRFDYKDQRFLNLMEKFNENLRILSSPWIQVCNNFPALIDYLPGSHNKVVKNFAYV

KSYVLERIKEHQESLDMDNPRDFIDCFLIKMEQERHNLQSEFTIESLIATVTDMFGAGTE

TTSTTLRFGLLLLLKYPEVTAKVQEEIECVVGRNRSPCMQDRSHMPYTDAVVHEIQRYID

LIPTNLPHAVTCDVKFRNYLIPKGTTIITSLTSVLHNDKEFPNPEMFDPGHFLDRSGNFK

KSDYFMPFSAGKRMCVGEGLARMELFLFLTTILQNFNLKSQVDPKDIDITSIANAFGRVP

PLYHLCFIPV

>118089176

MDWASVVPVGLLMILILLLILKTQDFWRSQGKFPPGPQPLPIIGNLHIMDLKKIGQTMLQ

LSETYGPVFTVQMGMRKVVVLSGYDTVKEALVNHADAFVGRPKIPIVEKAGKGKGVVFSS

GENWKVMRRFTLTTLRDFGMGKKAIEDYVVEEYGYLADVIESQKGKPLEMTHLMNSAVAN

VIVSILLGKRFEYEDPTFKRLVSLINENMRLFGSPSVSLYNMFPILGPFLKDNKSFLENV

KEVNDFIKVTFTKYLQVLDKNDQRSFIDAFLVKQQEQNEKANKFFDDENLTEVVRNLFTA

GMDTTATTLRWGLLLMMKYPEIQKKVQEEIDRVIGSNPPRTEHRTKMPYTDAVIHEIQRF

ANILPLNLPHETTMDVTIKGYFIPKGTYIIPLLNSVLQDKTQWEKPCSFHPEHFLNSEGK

FVKKDAFIPFSAGRRICAGETLAKMELFLFFTSLLQRFTFQPPPGISSSDLDLSAPPRFV

IAPVTHEVCAVSRS

>403305350

MGLGWAVTMFLELLLSCLLIFIAWKRMNKGGKLPPGPTPIPFLGNLLQVRTHATFQSLMK

LREKYGPVFTVYMGPRPVVVLCGHEAVKEALVDQADDFSGRGQLASVERNFQGHGVALSN

GDRWRSLRRFSLTILRDFGMGKRSIEERIQEEAGYLLEEFRKTKGAPVDPTVFLSRSVSN

VISSVVFGSRFDYEDKQFLKLVQLVNESFIEMSTPWTQLYDMYSGIMRYLPGRHHRIYYL

IEELKDFVASKVKINEATFDHQNPRDFIDCFLIKMHQDKNNPHTEFNLKNLVLTTLNLFF

AGTETVSSTLRYGFLVLMKHPEVEAKIHEEINQVIGPHRLPSVEDRVKMPYTDAVIHEIQ

RLIDIVPMGVPHNVIRDTQFGGYLLPKGTNIFALLGSVLKDPKYFRYPDAFYPQHFLDEK

GRFKKNEAFVPFSSGKRICLGEAMARMELFLYFIFILQNFSLRSLVPPADIDITPKLSGF

GNIPPTYKLCLVAR

>AAH97939.1

MDLVTFLVLTLSSLILLSLWRQSSRRRKLPPGPTPLPIIGNFLQIDVKNISQSLTKFSKT

YGPVFTLYLGSQPTVILHGYEAIKEALIDNGEKFSGRGSYPMIENVTKGFGIVFSNGNRW

KEMRRFTIMTFRNLGIGKRNIEDRVQEEAQCLVEELRKTKGSPCDPSLILNCAPCNVICS

ITFQNHFDYKDKEMLTFMEKVNENLKIMSSPWMQVCNSFPSLIDYFPGTHHKIAKNINYM

KSYLLKKIEEHQESLDVTNPRDFVDYYLIKQKQANNIEQSEYSHENLTCSIMDLIGAGTE

TMSTTLRYALLLLMKYPHVTAKVQEEIDRVIGRHRSPCMQDRKHMPYTDAMIHEVQRFIN

FVPTNLPHAVTCDIKFRNYLIPKGTKVLTSLTSVLHDSKEFPNPEMFDPGHFLDENGNFK

KSDYFLPFSAGKRACVGEGLARMQLFLFLTTILQNFNLKSLVHPKDIDTMPVLNGFASLP

PTYQLCFIPS

>P33262.1

MDPFVVLVLCLSFVLLFSLWRQSSGRRKLPPGPTPLPIIGNILQIDVKDICKSFSNFSKV

YGPVFTVYFGMNPVVVLHGYETVKEALIDNAEEFSGRGILPISERITNGLGIISSNGKRW

KETRRFSLTTLRNFGMGKRSIEDRVQEEARCLVEELRKTKASPCDPTFILGCAPCNVICS

VVFQKRFDYKDENFLTLIKRFTVNFRILTSPWIQVCNNFPLLIDCFPGTHNKLLKNVALT

KSYIREKVKEHQATLDVNNPRDFIDCFLIKMEQEKDNQQSEFTIENLVGTVADLFVAGTE

TTSTTLRYGLLLLLKHPEVTAKVQEEIDHVIGRHRSPCMQDRSHMPYTDAVIHEIQRYID

LVPTGVPHAVTTDIKFRNYLIPKGTIIITLLTSVLHDDKEFPNPKIFDPGHFLDENGNFK

KSDYFMPFSAGKRICAGEGLARMELFLFLTTILQNFNLKSVADLKNLNTTSATRGIISLP

PSYQICFIPV

>402881017

MDPFVVLVLCLSFVLLFSLWRQSSGRRKLPPGPTPLPVIGNILQIDVKDICKSFSNFSKV

YGPVFTVYFGMNPVVVLHGYETVKEALIDNAEEFSGRGILPISERITNGLGIISSNGKRW

KETRRFSLTTLRNFGMGKRSIEDRVQEEARCLVEELRKTKASPCDPTFILGCAPCNVICS

VVFQKRFDYKDENFLTLMKRFTVNFRILTSPWIQVCNNFPLLIDCFPGTHNKLLKNVALT

KSYIREKVKEHQATLDVNNPRDFIDCFLIKMEQEKDNQQSEFTIENLVGTVADLFVAGTE

TTSTTLRYGLLLLLKHPEVTAKVQEEIDHVIGRHRSPCMQDRSHMPYTDAVIHEIQRYID

LVPTGVPHAVTTDIKFRNYLIPKGTIIITLLTSVLHDDKEFPNPKIFDPGHFLDENGNFK

KSDYFMPFSAGKRICAGEGLARMELFLFLTTILQNFNLKSVADLKNLNTTSATRGIISLP

PSYQICFIPV

>BAB85663.1

MELLGITTLALMVCVTCLVFLSVWKKNHKRRRLPPGPTPLPIIGNLMQLNLKDIPASLSK

LAKQYGPVCTVYFGSQPAVVLHGYEAVKEALIDQGDEFLGRGIIPIIDDTQGGYGLVFSN

GERWKQIRRFSLMTLRNFGMGKRSLEERVQEEAQFLVEELRKTEAQPFDPTFILSCAPCN

VICSILFNDRFHYDNETFLSLMNLLNANFRQISSPWIQIYNLWPQIIKHLPGEHRAFSKR

LKDCRSFILEKVKEHQKSPNLNNPQDYIDCFLSKMEQEKQNPDSEFHLENLINSGSNLFA

AGTETTTSTLRYGLLLLMKHPEVQAKVHEEIDRVIGRSQRPCMQDKMKLPYTEAVLHEIQ

RYIALLPSNLPHATVRDTKFREYIIPKGTTVLPLLSSVLHDCKEFPNPEKFDPGHFLDKD

GSFRKTEYFVPFSIGKRACAGESLARMELFLFFTTILQHFVLKPLKEPKDLETKPISVGL

FNLPPPFKLCLIPR

>301608070

MDLVSVLLSVVVCIFLYKVFYGGEKESQNFPPGPKPLPLIGNLHIMNMKKPFLTFMELAE

KYGPVFSVQLGTKKVVVLCGTDAVKDALVNHADEFSGRPKIPMFDQTSKGHGVIFADGEN

WKVMRRFTLSTLRDFGMGKKTLEDRIGEESGCLVETFKSHEGKPFDNTLILNAAVANIIV

HILLNHRFDYQDPTLLKLIKSVSENVRIGGRPIVMLYNTYPSIMQWVPGSHKSIYENSQN

LLNFLKETFTEHRHQLDVNDQRDLIDTFLVKQQEEKSSSTKFFHDENLTILLSNLFGAGM

ETTSTTLRWGILLMMKYPDIQKKVQDEIDQVIGSAEPRLEHRKQMPYTDAVIHEIQRFAN

LAPNGLPHATTTDVTFRGYFIPKGTQVIPVLTSVLRDKAYFKKPEEFYPEHFLDSEGKFL

KNEAFLPFSAGKRICAGETLAKMELFLFFTKLLQNFTFQPPPGVEVQLTCGDAITSIPLD

HKICALSRS

>AAA41059.1

MDPVVVLLLSLFFLLFLSLWRPSSGRGKLPPGPTPLPIIGNFFQVDMKDIRQSLTNFSKT

YGPVYTLYVGSQPTVVLHGYEALKEALVDHGEEFSGRGRLPICEKVAKGQGIAFSHGNVW

KATRHFTVKTLRNLGMGKGTIEDKVQEEAKWLVKELKKTNGSPCDPQFIMGCAPGNVICS

IILQNRFDYEDKDFLNLIEKVNEAVKIISSPGIQVFNIFPILLDYCPGNHNIYFKNYTWV

KSYLLEKIKEHEESLDVSNPRDFIDYFLIERNQENANQWMNYTLEHLAIMVTDLFFAGIE

TVSSTMRFALLLLMKYPHVTAKVQEEIDHVIGRHRSPCMQDRSHMPYTNAMVQEVQRYID

IGPNGLLHEVTCDTKFRNYFIPKGTAVLTSLTSVLHDSKEFPNPEMFDPGHFLDENGNFK

KSDYFIPFSAGKRMCLGESLARMELFLFLTTILQNFKLKSLVDPKDINTTPICSSLSSVP

PTFQMRFIPL

>P05179.2

MDLVTFLVLTLSSLILLSLWRQSSRRRKLPPGPTPLPIIGNFLQIDVKNISQSLTKFSKT

YGPVFTLYLGSQPTVILHGYEAIKEALIDNGEKFSGRGSYPMNENVTKGFGIVFSNGNRW

KEMRRFTIMNFRNLGIGKRNIEDRVQEEAQCLVEELRKTKGSPCDPSLILNCAPCNVICS

ITFQNHFDYKDKEMLTFMEKVNENLKIMSSPWMQVCNSFPSLIDYFPGTHHKIAKNINYM

KSYLLKKIEEHQESLDVTNPRDFVDYYLIKQKQANNIEQSEYSHENLTCSIMDLIGAGTE

TMSTTLRYALLLLMKYPHVTAKVQEEIDRVIGRHRSPCMQDRKHMPYTDAMIHEVQRFIN

FVPTNLPHAVTCDIKFRNYLIPKGTKVLTSLTSVLHDSKEFPNPEMFDPGHFLDENGNFK

KSDYFLPFSAGKRACVGEGLARMQLFLFLTTILQNFNLKSLVHPKDIDTMPVLNGFASLP

PTYQLCFIPS

>334323982

MNGLDLLSILSGLSLILILILNMKLTLTKNFKKQSPPGPKPLPVIGNLHILNLKRPYQTM

LELSKKYGPIFSLRMGPKTVVVLSGYETVKDALVNYSEQFGERARIPIFERIFEGKGIVF

SHGENWKITRRFSLTTLRNFGMGKRVIEERILEECHHLIQVFESHQGKPFEISTIMSASV

ANIIVSILFGKRFDYKDPQFLRLLHLIGENIRLAGGPSITIFNMFPVLGFLLQDLKRVLR

NRDELFSFIRTTFLKHLRKLDKNDQRSFIDAFLIKQQEEKDKSDDYFNNDNLVALVSNLF

AAGTETTSSTLRWGILLMMKYPEIQKKVHNEITEVIGSAQPRIEHRTQMPYTDAVIHEIQ

RFSNILPMNLSRETTTDVIFKNYYIPKGTEVITLLTSVLQDQTQWEKPCTFHPQHFLTKE

GKFIKRDAFLPFSAGQRMCAGESLAKMELFLFFTSLLQKFTFCPSPGVSNSDLDLTPDIG

FTTRPQPYKICALPYF

>126273043

MDFWGLITLALVCFLFFQLIWNKGYKARRFPPGPIPLPILGNMLQMDSKDLFTFFDKMAE

KYGPVFTVYMGLQRIVVLHGYEAVKEALIDQGDNFLNRAQLPIFEDNFKGQGIFLSNGEK

WRQNRRFSLMTLRNFGMGKRSIEERVQEEAQYLVEELRKTKGQFFDPTFILGCAPCNVIC

SIVFNNRFNYDDEKFLRLMSLLNENFRLLNTPWIQLYNMYPSLIKHLPGSHRTINKNVLE

VRNFIMDEVKKHQETLDPNNPRDYIDGFLIKIQQEKLNPQSAFNYQELVANAINFLAAGT

ETTSSTLRYGLLLLMKYPKIQAKVHEEIDRVLGSSRKSSMQDRVKMPYVDAVVHEIQRYI

HLVSLIIPRLAAQDTHFQKYVIPKGTKVFPLLYSVLYDRKAFPNPYEFDPENFLDKSGNF

QKNDCFVPFSLGKRLCLGESLARMEVFLFLTTILQNFTLKPMVEPKELVTTPLRSGIVNI

PSIYKLSLIPR

>224052485

MEALGVTTVFLLVCISCLLFATWRSRSQKGKEPPGPTPFPIVGNLLQINPWNLPESMKEL

SEKYGPVFTVHLGPQKVVVLYGYDVVKEALIDQGDDFSGRGILPLIKKLFQGTGIVTSNG

ETWKQLRRFTLTTLRDFGMGKKGIEERIQEEAHFLVERLRNTHEQPLNPGSFLIHAVSNI

ICSIVFGDRFDYEDKSFLTLIDWLEENNKLQSSIQTQLYNFFPNVMDYLPGPHQQLIKNI

EKVDKFTTDIVMEHQKTLDPTCPRDFIDSFLNKMEQEKGNDDSKFTVETLSRTALDLFLA

GTGTTSITLRFAVLILHKYPEIVEKMQKEIDSVIGRDRSPRMSDRSQMPFTDAVIHEIQR

YIDFLPTNVPHAVIRDIKFRDYFIPKDTLIFPMLSSVLHDRKEFPNPEKFDPGHFLNANG

TFKKSDYFMPFSTGKRICAGEGLARMEIFIFLTSILQNFTLKPVVDHKDIDISPVITSLA

NMPRHYEVSFVPR

>397467781

MSALGVTVALLVWAAFLLLVSMWRQVHSSWNLPPGPFPLPIIGNLFQLELKNIPKSFTRL

AQRFGPVFTLYVGSQRMVVMHGYKAVKEALLDYKDEFSGRGDLPAFHAHRDRGIIFNNGP

TWKDIRRFSLTTLRNYGMGKEGNESRIQREAHFLLEALRKTQGQPFDPTFLIGCAPCNVI

ADILFRKHFDYNDEKFLRLMYLFNENFHLLSTPWLQLYNNFPSVLHYLPGSHRKVIKNVA

EIKEYVSERVKEHHQSLDPNCPRDLTDCLLVEMEKEKHSAERLYTMDGITVTVADLFFAG

TETTSTTLRYGLLILMKYPEIEEKLHEEIDRVIGPSRIPAIKDRQEMPYMDAVVHEIQRF

ITLVPSNLPHEATRDTIFRGYLIPKGTVVVPTLDSVLYDNQEFPDPEKFKPEHFLNENGK

FKYSDYFKPFSTGKRVCAGEGLARMELFLLLCAILQHFNLKPLVDPKDIDLSPIHIGFGC

IPPRYKLCVIPRS

>AAZ29453.1

MDLFIILVICLSCLILLSLWNRSYAKGKLPPGPTPLPVIGNILQLNTKNISKSISMLAKD

YGPVLTVYFGMKPTVVLHGYEAIKEALIDQGEVFSGRGSFPVAEKITQGFGVIFSNGERW

KQIRRFSLMVLRNMGMGKKTIEDRIQEEALCLVEALKKTNASPCDPTFLLGCVPCNVISS

IIFQNRFDYRDQKFLTLMKYFNENFETVSTPWIQLYNAFPFLRVLPGSHNVLFKNYALQR

SFILEKVKEHQESLDINNPRDFIDYFLIRMEKEKHNKESEFTMDNLVATIWDMFSAGTET

TSTTMRYGLMLLLKHPEISAKVREEIDHVVGKNRSVCMQDRSRMPYTDAVVHEIQRYIDL

IPTNVPHAVTQDIRFREYLIPKGTTILTDLTSVLYDDKEFPNPEKFDPGHFLDESGNFKK

SDYFMAFSAGKRICAGEGLARMELFLILTTILQNFTLKPLVDPKDIDTTPVHKGFGTIPP

FYELCFIPV

>AAH72157.1

MDPVSVLLSIVICIFIFKVFYGGNKESQNFPPGPKPLPLIGNLHMINMKKPHLTFMELAE

KFGSVFSFHFGTEKFVVLCNADTVKDALINYADEFSGRPAIPVFDKTTKGHGIFFANGEN

WKVMRRFTISTLRDFGMGKKTMEDRICEESEFLKQVFESYKGKPFDNTIIMNAAVANIIV

HILLNHRFDYDDATLKNLISIVSENISFAAKPIVLLYNAYPSILQWIPGSHKSVTKNMIK

LYNFLRETFTKHRDQLDVNDQRDLIDVFLVKQQEESSSTKFFHDQNLTVLLADLFGAGME

TTSTTLRWGLLFMMKYPEVQKKVQDEIDRVIGSAQPRLAHRKQMPYTEAVIHEIQRLGNL

APNVGHETTKDVTFRGYFIPKGTQVIILLTSVLQDKAYFKKPEEFYPEHFLDSEGKFVKN

DAFLPFSAGRRSCAGETLAKMELFLFFTKLLQNFTFQSPPGVEVDLTSADALTSKPVDHK

ICALPRN

>301608064

MDLVSVLLAVVICFFIFKVFYGGKNAFQNFPPGPKPLPIIGNFHMINMKKPYLTFMELAE

KYGPVFSIQLGTEKVVVLYGADAVKDALINHGDEFSGRPTIPVFDRISKGHGLFFANGEN

WKVMRRFTLSTLRDFGMGKKTIEDRICEESDFLMETFKSYKGKPFDNTMIMNAAVANIIV

HILLNHRFDYQDPTLLKLINTISENVRIAGKPMVVLYNAYPSIMQWFPGIHKSVAESILQ

FYDFLRETFTQHRDQLDVNDQRDLIDVFLVKQQEEKSSSTKFFNDHNLTALVADLFGAGM

ETTSTTLRWGLLFMMKYPDIQKKVQDEIDRVIGSAQPRLEHRKTMPYTDAVIHEIQRLGN

LAPFIGHETTTDVTFRGYFIPKGTQAIVLLASVLQDKDYFKKPEEFYPEHFLDSEGNFVK

NEAFLPFSAGRRMCVGETLAKMELFLFFTKLLQNFTFQPPPGVEVDLTCGDAVTSKPLDH

QICALPRS

>EHB18320.1

MELSILLLLTLLMGLLVLLFRGHPKAHGHLPPGPRPLPFLGNLLQMNRKGLLKSVLQVRK

KYGDVFTLYLGPRPVVMLCGTDTIREALVDQAEAFSGRGRIASIDAVFQGQSFNSASAEH

WKVLRRFTLATMRDFGMGKRSVEERIQEEAQCLVEELRKSQGGLVDPLFFFRPATANIIC

SIVFGKRFDYKDQQFLRLMDLIYQTFALMSSFSSLVFELYSSILKHFPGTHREIYKNFQE

ILTFIGHSVEKHQETLDPSAPRDFIDAYLLRMEKDKSDPHTEFHHRNLVLTSLSLFIAGT

ETTSTTLHYGFLFLLKYPHVTERVHKEIEQVIGSHRQPSLDDRNKMPYTEAVIYEIQRFA

DLSPMGVPRTVTKDTHFRGFIIPKNTDIYAVLTTALHDPCHFEKPDTFNPDHFLDAKGAL

KRSEAFMPFSIGKRICLGEGIARVELFLFFTTILQNFSISGTVAPQGIDLTPQEVGLGKV

PPTYEVRFLPRNVK

>AAH89651.1

MSLLSQLCPFALGCNVFTLGIIFTLLLLLLDFMKRRKPCTDFPPSPPSWPFVGNLLQMDF

RDLHNSFKQLSKQYGDVMSLRVFWKPTVVLNGFEVIKEALIQKSEDTADRPPFNLYEILG

FVGNNKAVVLANYGQSWKDLRRFTLSTLRDFGMGKKSLEERVRDEAGYLCDAFQSEQGGP

FDPHVLINTAVSNVICSIIFGERFEYDDHKFLKLLCLIEESIKAESGPVPQIISSLPWSS

KVPGLARLFFQPRIHMLQYLQEIINEHKQTWDSGHTRDFIDAFMLEMKKAKGVKDSNFND

QNLLLTTADLFSAGSETTTTTLRWGLLFMLLYPDVQRKVQEEIDQVIGRTRKPTMGDVLQ

MPYTNAVIHEIQRYADIIPLSVPHMAYRDTHIKGFFIPKGTVIMTNLSSVLKDEKVWEKP

FQFYPEHFLDRDGKFVKREAFMAFSAGRRVCLGEQLARMELFLFFTSLLQRFSFQIPDGE

PCLREDPVFVFLQVPHDYKICAKVR

>AAA40950.1

MALFIFLGIWLSCLVFLFLWNQHHVRRKLPPGPTPLPIFGNILQVGVKNMSKSMCMLAKE

YGPVFTMYLGMKPTVVLYGYEVLKEALIDRGEEFSDKMHSSMLSKVSQGLGIVFSNGEIW

KQTRRFSLMVLRSMGMGKRTIENRIQEEVVYLLEALRKTNGSPCDPSFLLACVPCNLISS

VIFQHRFDYSDEKFQKFIENFHTKIEILASPWAQLCSAYPVLYYLPGIHNKFLKDVTEQK

KFILMEINRHRASLNLSNPQDFIDYFLIKMEKEKHNEKSEFTMDNLIVTIGDLFGAGTET

TSSTIKYGLLLLLKYPEVTAKIQEEITRVIGRHRRPCMQDRNHMPYTDAVLHEIQRYIDF

VPIPLPRKTTQDVEFRGYHIPKGTSVMACLTSALHDDKEFPNPEKFDPGHFLDEKGNFKK

SDYFMAFSAGRRACIGEGLARMEMFLILTSILQHFILKPLVNPEDIDTTPVQPGLLSLPP

PFQLCFIPV

>296220836

MDPAVVLVFCLSCLLLLSLWSQSSARGRLPSGPTPLPIIGNILQLDVKDIGKSLTNFSKV

YGPVFTVYFGLKPIVVLHGYEAVKEALIDHGEEFSGRGSFPVAEKVNKGLGILFSNGKRW

KEIRRFSLMTLRNFGMGKRSIEDRVQEEARCLVEELRKTNASPCDPTFILGCAPCNVICS

IVFHKRFDYKDQRFLNLMEKFNENLRLLSSPWIQVCNNFPALIDYLPGSHNTVLKNFAYV

KNYVLERIKEHQESLDVNNPQDFIDCFLIKMEQEKHNQQSEFTIENLIATVTDVFGAGTE

TTSTTLRFGLLLLLKYPEVTAKVQEEIERVAGRNRSPCMQDRSHMPYTDAVVHEIQRYID

LIPTNLPHAVTCDVKFRNYLIPKGTTIIASLTSVLHNDKEFPNPEMFDPSHFLDKSGNFK

KSDYFMPFSTGKRMCVGEGLARMELFLFLTTILQNFNLKSQVDPKDIDITPTANAFGRVP

PLYQLCFIPV

>332212376

MEPFVVLVLCLSFILLFSLWRQSSGRRKLPPGPTPFPIIGNMLQIDIKNICKSFSNFSKV

YGPVFTVYFGMNPIVVLHGYEAVKEALIDNGEEFSGRGNSPISQRITKGLGIISSNGKRW

KEIRRFSLTTLRNFGMGKRSIEDRVQEEAHCLVEELRKTKASPCDPTFILGCAPCNVICS

VVFQKRFDYKDQNFLTLMKRFNENFRILNSPWIQVCNNFPLLIDCFPGTHNELLKNVGLT

KSYIMEKVKEHQASLDVNNPRDFIDCFLIKMEQEKDNQQSEFNIENLVGTVADLFVAGTE

TTSTTLRYGLLLLLKHPEVTAKVQEEIDHVIGRHRRPCMQDRSHMPYTDAVVHEIQRYID

LVPTGVPHAVTTDIKFRNYLIPKGTTIMTLLTSVLHDDKEFPNPKIFDPGHFLDKNGNFK

KSDYFMPFSAGKRICAGEGLARMELFLFLTTILQNFNLKSVDDLKNLNTTAVTKGIVSLP

PSYQICFIPV

>EGW10545.1

MLAATGSLVAAIWAALHLQTLLLAAVVFLFLADYFKNRRPKNYPPGPRRLPFFGCLFYLD

PKQPHIALQKFVKKYGNVLSLDFANIPSVVVSGMPLIKEVFTQLEPNFLNRPVTLLRQHL

FSKNGLIFSSGQTWKEQRRFTLMTLRNFGLGKKSLEQRIQEEAQHLVDAIGEEGGQPFDP

HFNVNNAVSNIICTVTFGERFEYHDSQFQEMLRLLDELYNIFPRIIHYLPGSHQTLFRNW

RKLKLFVSDIIDNHRKDWDPEEPRDFIDAFLKEMTKYPDKTTTSFNEENLICSTLDLFFA

GTETTSTTLRWALLCMALYPEVQEKVQAEIDRVIGQGRQPILADRESMPYTNAVIHEVQR

IGNIIPFNVPREGTGDTKLAGFDLPKGIMILTNLTALHRDPKEWATPDIFNPEHFLENGQ

FKKRESFLPFSMGCDVEEGKYEEMAYAWKTLVLART

>P33272.1

MEFGVLLLLTLTVGFLLFLVSQSQPKTHGHLPPGPRPLPFLGNLLQMNRRGFLNSFMQLQ

EKYGDVFTVHLGPRPVVILCGTDTIREALVDQAEAFSGRGTVAVLHPVVQGYGVIFATGE

RWKTLRRFSLVTMKEFGMGKRSVDERIKEEAQCLVEELKKYKGAPLNPTFLFQSIAANTI

CSIVFGERFDYKDHQFLHLLDLVYKTSVLMGSLSSQVFELYSGFLKYFPGAHKQIFKNLQ

EMLNYIGHIVEKHRATLDPSAPRDFIDTYLLRMEKEKSNHHTEFNHQNLVISVLSLFFAG

TETTSTTLRCTFLIMLKYPHVAEKVQKEIDQVIGSHRLPTPDDRTKMPYTDAVIHEIQRF

ADLTPIGLPHRVTKDTVFRGYLLPKNTEVYPILSSALHDPRYFEQPDTFNPEHFLDANGA

LKKSEAFLPFSTGKRICLGEGIARNELFIFFTAILQNFTLASPVAPEDIDLTPINIGVGK

IPSPYQINFLSR

>CAH93055.1

MSALGVTVALLVWAAFLLLVSMWRQVHSSWNLPPGPFPLPIIGNLFQLELKNVPKSFTRL

AQRFGPVFTLYVGSQRVVVMHGYKAVKEALLDYKDEFSGRGDLPAFHAHKDRGIIFNNGP

TWKEIRRFSLTTLRNYGMGKQGNESRIQREAHFLLEALRKTQGQPFDPTFLIGCTPCNVI

ADILFRKRFDYNDEKFLRLMYLFNENFYLLSTPWLQLYNNFPSFLHYLPGSHRKVIKNVA

EVKEYVSERVKEHHQSLDPNCPRDLTDCLLVEMEKEKHSAEPLYTMDGITVTVADLFFAG

TETTSTTLRYGLLILMKYPEIEEKLHEEIDRVIGPSRIPAIKDRQEMPYMDAVVHEIQRF

ITLVPSNLPHKATRDTIFRGYLIPKGTVIVPTLDSVLYDNQEFPDPEKFKPEHFLNENGK

FKYSDYFKPFSTGKRVCAGEGLARMELFLLLCAILQHFNLKPLVDPKDIDLSPIHIGFGC

IPPRYKLCVIPRHECVEDTLNPPLSNKFSNCLRSGFLKLIPFVA

>291412172

MVLSAFSLLLGLTGLLLLWAWGRPASPGHLPPGPWPLPLLGNILQIHHKGFLQSFQRLRE

KHGDVFTVYLGSRPVVVLCGYQAVREALVEQAEAFSGRGPIAITDAIFQGTGVVFANGKP

WKVLRRFCLATMKDLGMGRRSMEQRIKEEARCLVEELRKSQEAYLDPTSLFNAVTANVIC

SMVFGERFDYQDPAFLRLLRLLNETFILLSSFYSQVFELLSGILKHLPGPHARVDRIIQE

LKDFITKNIEKHRETLDPSCPQDFIDSFLLRMDKERFLPQSEFHHKNLVHTVLSLFFAGT

ESSSTTLRYGLLLFLKHPDVTEKVQAEIDRVIGPKRLPALEDRAKMPYTDAVIHEIQRYS

DLTPIGVPHSVIKDTHFRGYHLVKGTAVYPIMSSVLHDPHHFEKPDVFYPGHFLDAEGNF

RKREAFVPFSMGKRICLGESLARSELFLFFTSMLQNFSLGCPKAPEDIDLTPQVNGIGKL

PPVFQLRFLPRQREDEGTQSPSPLQASELPLPTGSCEKSCL

>Q08078.1

MDAVLVLVFILSSLVFLSLWRQSSERRKLPPGPTPLPIIGNFLQIDVKNISGSLTNFSKV

YGPVFTLYLGMKPTVVLHGYETVKEALIDHGEEFAGRGDFPMAERINKGLGIVFSNGNRW

KEIRRFSLMTLRNLGMGKRNIEDRVQEEAQCLVEELRKTNGSPCDPTFILSCAPCNVICS

IIFQNRFDYKDQDFLTFMKKVNENVRILSSPWLQVCNNFPSLIDYCPGSHHKITKNVNYL

KKYILEKIEEHQESLDIENPRDFIDYYLIKLKQANHNQQSEFSLENLTTTVSDLFGAGTE

TTSTTLRYALLLLLKHPHVTAKVQEEIDQVVGRHRKPCMQDRSHMPYTDAMIHEVQRFID

LIPISLPHAVTCDIKFRDYFIPKGTTVITSLSSVLHDNKEFPNPEVFDPGHFLDKNGNFK

KSDYFMPFSAGKRICAGEGLARMELFLFLTTILQNFKLKSMIHPKDIDTTPVVNGFASLP

PSYQLCFIPV

>P12789.1

MEFSLLLLLAFLAGLLLLLFRGHPKAHGRLPPGPPPLPVLGNLLQMDRKGLLRSFLRLRE

KYGDVFTVYLGSRPVVVLCGTDAIREALVDQAEAFSGRGKIAVVDPIFQGYGVFFANGEH

WRALRRFSLATMRDFGMGKRSVEERIQEEARCLVEELRKSKGALLDNTLLFHSVTSNIIC

SIVFGKRFDYKDPVFLRLLDLFFQSFSLISSFSSQVFELFPGFLKHFPGTHRQIYRNLQE

INTFIGQTVEKHRATLDPSNPRDFIDVYLLRMEKDKSDPSSEFHHRNLILTVLTLFFAGT

ETTSTTLRYGFLLMLKYPHVTERVQKEIEQVIGSHRPPALDDRAKMPYTDAVIHEIQRLG

DLVPFGAPHMVTKDTQFRGYVIPKNTEVFPVLSSALHDPRYFETPNTFNPGHFLDADGAL

KRNEGFMPFSLGKRICLGEGIARTELFLFFTTILQNFSIASPVPPEDIDLTPRESGVGNV

PPSYQIRFLAR

>DAA14854.1

MDLAVVLVLCLSCLLLLSLWKQSSGKGKLPPGPTPLPILGNILQLDVKNISKSLTNLSKV

YGPVFTVYFGMKPIVVLHGYEAVKEALIDLGEEFSGRGMFPLAERANIVNGILFSNGKTW

KEIRRFSLMTLRNFGMGKRSIEDRVQEEACCLVEELRKTNGLPCDPTFILGCAPCNVICS

IIFQNRFDYKDPVFLDLMERLNEILRILSSPWVQVCNNFPALFDYLPGSHNKVLKNVANL

KSFVLEKAMEHKASLDINNPRDYIDCFLIRMEQEKQNQQLEFTLENLTTTVFDLFGAGTE

TMSTTLRYGLLLLLKHPEVTAKVQEEIDRVIGRHRSPCMQDRSHMPYTDAVVHEIQRYID

LVPSSLPHMVTHDIELRNYIIPKGTGVLVSLTSVLYDDKVFPNPEMFDPGHFLDDSGNFK

KSDHFMPFSAGKRICAGESLARMEVFLFLTVILQKFTLKSVVDPKDIDTTPIANGFASVP

PPYKLCFIPL

>327280424

MEKAEVLSEGMQPEGGASGVEDAAECEVAQGKGKMPPGPTPLPLIGNVLQLKGKYLDQEL

CKISEEYGPVFTLYLGMNPAVVLHGYEAIKEALIDRGNDFASRAKIPLVEKMSEGKGIVF

SNGESWKQIRRFTLTTLRNFGMGKKSIEERIQEETQYLLEQFHDTKGQPFDPHNLFSYAT

ANVICSIVFGKRYKYNDKRFQTLIAITKENTELFNSAWGQLYNTFPVLMEWIPGPYQRMI

QCDIKCRXFILEEAKEHRATLDPNSPRGFIDCFFIRMDQEKHNEAFEFTMENMVICSLEL

FAAGTETINATLRYGLLILQKYQEIEEKVQEEIDRVVGRSRMPTMADRGQMPYTDAVIHE

IQRFTSPSPVALPHSVVNDTPFRGYLIPRGTTILPVLTSVLHDGKEFPNPTKFDPGHFLN

PDGTFRKSNYFMPFSAGKRICAGEGLALMELFLFFTSILQNFKLKPLMDPKDIDLSPMKG

NMDNIPQPYKFCVIPR

>327272618

MSICLIIFALALLLDYMKRRKKCGRCPPGPAPLPFIGNILWFNRKNPSESFRQVEKIFGP

IFLVQAGWQNFVIINGFKLTKEALGSKAEDFIERPALPLIFLLGRTKKYEGIILATSHNG

WREQKRFCVSTLKTFGMGKKTLEKKVCEEAWYLCSELKSKEGSPFDPKISIFNATGNIIS

TLAFGDRFEYHDETFLKLIHSTEEILKDLTRMVPEIVFARSWFSYLPGPHQKIKKHYDNF

TAVLKIMVDEHKKTRDPTFPRDLIDAFLEEIEKAKGNPETSFGEENLIHLMIDLFAAGTD

TTSVTLLWGLLKMILYPEVQKRVQEEIDMVIGRIKSPTMEDQSKLPYTNAVIHEIQRYAD

IAPTTIPYMTYRDTEVANFVIPKATVVICHLSSVLKDETMWEKPHDFYPEHFLDANGKFI

KREAFLPFSAGRRACTGEQLAKTELFIFFTTLLQHFTFCIPENCPKPTEERIYAVTVTPA

PFQLCAIPR

>332855851

MQGSQTRTMELSVLLFLALLTGLLLLLVQRHPNTHGRLPPGPRPLPLLGNLLQMDRRGLL

KSFLRFREKYGDVFTVHLGPRPVVMLCGVEAIREALVDKAEAFSGRGKIAMVDPFFRGYG

VIFANGNRWKVLRRFSVTTMRDFGMGKRSVEERIQEEAQCLIEELRKSKGEPFDPTFVLS

RSVSNIICSVLFGSRFDYDDERLLTIIRLINDNFQIMSSPWGELYNIFPSLLNWVPGPHQ

RIFQNFKCLRDLIAHSVHDHQASLDPRSPRDFIDCFLTKMAEKKEDPLSHFHMDTLLMTT

HNLLFGGTETVGTTLRHAFLALMKYPKVQARVQEEIDLVVGRARLPALKDRAAMPYTDAV

IHEVQRFADIIPMNLPHRVTRDTAFRGFLIPKGTDVITLLNTVHYDPSQFLTPQEFNPEH

FLDANQSFKKSPAFMPFSAGRRLCLGESLARMELFLYLTAILQSFSLQPLGAPEDIDLTP

LSSGLGNLPGPFQLCLRPR

>395828391

MDLFIVLVICLSCLILFSLWNASYGKGKLPPGPIPLPIVGNILQLNTKNISKSLSMLGKD

YGPVFTVYFGMKPTVVLYGYKAVKEALIDRGEEFSDRGSFPVLEKITQGLGIVFSNGKKW

KHTRCFSLMVLRNMGMGKKTIEDRIQEEALYLVELLRKTNASPCDPTSILSCVPCNVICF

VIFQTRFDHSDQKFQNFIKYFHENCEILSTPWIQLYNAFPLLHYLPGCHKDFFKNFAHQK

EFILGKIKEHQETLDINNPRDFIDYFLIKMEKEKHNKESEFTMENLVITILDLFGAGTDT

MSTTMRYGLLLLLKHPEIAAKVQKEIHHVIGRHRSPCMQDRSCMPYTDAVVHEIQRYIDL

IPTILPHAVTQDMQFREFLIPKGTNILIHLSSVLHDEKEFPNPERFDPGHFLDESGNFKK

SDYFMPFSAGKRICAGESLARMELFLILTSILQNFTLKPLVDPKDIDVTPISNGLSSIPP

FYELCFIPV

>AAI60556.1

MDPVSVLLSVVVCIFLFKFFYGGEKGSQNFPPGPKPLPLIGNLHMINMKKPYLTFMELAE

KYGPVFSVHLGANKAVVLCGTDAVKDALINYADEFSGRPKTPLFEQTFKGNGIVFADGEN

WKVMRRFTISTLRDFGMGKKTIEDRIIEESCCLVETFKSHKGKPFDNTMILNAAVANIIV

HILLNHRFEYQDPTLLKLIKGVNENVRNGARPIVMLYNAFPSIIQWIPGTHKRIFANTQH

FFNILKEIFIEHRDQLDVNDQRDLIDTFLVKQQEEKSSSTKFFHDENLKVLIGNLFAAGM

ETTSTTLRWGILLMMKYPEIQKKVQDDIDRVIGSAEPRLEHRKLMPYTDAVVHEIQRFAN

LVPNGLPHATTTDVTFRGYFIPKGTQVIPLLTSVLRDKAYFKKPEEFYPEHFLDSKGNFL

KNEAFLPFSAGKRTCAGETLAKMELFLFFTKLLQNFTFQPPPGVEVQLTRGVSLTSIPLD

HKICALSRS

>149722303

MDSINTPVLLLLLALVCLFLTLSSRSKGRLPPGPRPLPLLGNLLQLRSQNMLTSLTKLSK

KYGSVFTVHLGPRRVVVLSGYQTVKEALVDQAEEFSGRGDYPVFLNFTKGNGIAFSNGDR

WKVLRRFSIQILRNFGMGKRSIEERILEEGSFLLAELRNTDGEPFDPTFFLSRSVSNIIC

SVLFGSRFDYEDKRLLTIISLINENFQIMSSPWGELYNLFPSLLDWVPGPHRRLFQNFGC

VKDLIAHSVRDHQASLDPNSPRDFIDCFLTKMAQEKQDPLSHFNMDTLLMTTHNLLFGGT

ETVGTTLRFTFLVLMKYQEVQARIQEEIDRVVGRARLPALEDRTAMPYTDAVIHEVQRFA

DIIPMNLPHRVTQDTVFRGFLLPKGTDVITLLNTVHHDPTQFLTPREFNPEHFLDANRCF

TKSPAFMPFSAGRRQCLGESLARMELFLFLTAILQSFSLQPLGAPEDIDLTPLSSGLGNL

PRPFQLRLRAR

>EHH64900.1

MDPFVVLVLCLSFVLLFSLWRQSSGRRKLPPGPTPLPIIGNMLQIDVKDICKSFSNFSKV

YGPVFTVYFGMNPVVVLHGYEAVKEALIDNGEEFSGRGILPISQRTTKGLGRFSSNGKRW

KEIRRFSLTTLRNFGMGKRSIEDRVQEEARCLVEELRKTKASPCDPTFILGCAPCNVICS

VVFQKRFDYKDENFLTLMKRFTVNFRILTSPWIQVCNNFPLLIDCFPGTHNKLLKNVALT

KSYIREKVKEHQASLDINNPRDFIDCFLIKMEQEKDNQQSEFTIENLVGTVADLFIAGTE

TTSTTLRYGLLLLLKHPEVTAKVQEEIDHVIGRHRSPCMQDRSHMPYTDAVIHEIQRYID

LVPTGVPHAVTTDIKFRNYLIPKGTIIITLLTSVLHDDKEFPNPKIFDPGHFLDENGNFK

KSDYFMPFSAGKRICAGEGLARMELFLFLTTILQNFNLKSVADLKNLNTTSATRGIISLP

PSYQICFIPV

>P33263.1

MDAFVVLVFILSCLFLLSLWRQSSERGKLPPGPTPLPIIGNFLQIDVKDISGSLTNFSKV

YGPVFTLYLGMKPTVVLHGYEAVKEALIDHGEEFAGRGSFPVAERVNKGLGIVFSNGSRW

KETRRFSLMTLRNLGMGKRSIEDRVQEEAQCLVEELRKTNGSPCDPTFILGCAPCNVICS

IIFQNRFDYKDKDFLTFMKKLNENARILSSPWFQVCNNFPLLIDYCPGSHHRITKNINYI

RSYLSEKMKEHQESLDVANPRDFIDYYLIKLKQGNYNQQSEFSPENLATTVSDLFAAGTE

TTSTTLRYALLLLLKHPHVTAKVQEEIDQVVGRHRNPCMQDRSHMPYTDAMIHEVQRFID

LIPTNLPHAVTCDIKFRDYFIPKGTTIITSLSSVLHDSKEFPNPEVFDPGHFLDKNGNFK

KSDYFMPFSTGKRMCAGEGLARMELFLFLTTILQNFKLKSLVHPKDIDTTPVLNGFASLP

PSYQLCFIPV

>326916799

MALVFILTFLIVMKIGGVWSNHQRKNFPPGPRALPIIGNLHLFDLKRPYRTYLQLSKEYG

PVFSVQMGQRKIVVISGYETVKEALINQADAFAERPKIPIFEDLTRGNGIVFAHGENWKV

MRRFTLTTLRDFGMGKRAIEDRIVEEYGYLIDNIGSQEGKPFDASKIINAAVANIIVSIL

LGKRFDYKDSRFIRLQHLTNESMRLAGKPLVTMYNIFPFLGFLLRANKTLLKNRDEFHAY

VKVTFLENLKTLDKNDQRSFIDAFLVKQQEEKSTTNGYFHNGNLLSLVSNLFTAGVETIS

TTLNWSFLLMLKYPEIQRKVQEEIEQVIGSNPPRIEHRTQMPYTDAVIHEVQRFANILPL

DLPHETAEDVTLKDYFIPKGTYIIPLLTSVLRDKSQWEKPDMFYPEHFLDSKGKLVKKDA

FMPFSAGRRICAGETLAKMELFLFFTSLLQRFTFQPPPGVSSSDLDLSPAISFNVVPKPY

KICAVARS

>P56654.2

MDPILVLVLTLSCLFLLSLWRQSSERGKLPPGPTPLPIIGNILQIDVKDICQSFTNLSKV

YGPVYTLYLGRKPTVVLHGYEAVKEALVDHGEEFAGRGRFPVFDKATNGMGLAFSKGNVW

KNTRRFSLMTLRNLGMGKRSIEDRVQEEARCLVEELRKTNGSPCDPTFILGCAPCNVICS

IIFQDRFDYKDRDFLNLMEKLNEITKILSSPWLQICNTYPALLDYCPGSHKQFFKNYASI

KNFLLEKIKEHEESLDVTIPRDFIDYFLINGGQEDGNQPLQNRLEHLAITVTDLFSAGTE

TTSTTLRYAILLLLKYPHVTAKVQEEIEHVIGKHRSPCMQDRSRMPYTDAMIHEVQRFID

LIPNSLPHEVTSDIKFRNYFIPKGTTVITSLSSVLHDSTEFPNPEKFDPGHFLDENGKFK

KSDYFIPFSTGKRICAGEGLARMELFLFLTSILQNFNLKPLVHPKDIDVTPMLIGLASVP

PAFQLCFIPS

>AAH75418.1

MDTSWLWTLLLCLLISAMLIYSTWNKMYRKRNLPPGPTPIPLFGNVMQIKRGEMVKSLIE

LGKKYGDIYTLYFGPSPVVILCSYRAIKEALIDQAEEFSGRGAIPSFDQYFQGYGVVFTN

GEEWKNLRRFSLSTLRNFGMGKRGIEERIKEEAQFLVAEIKSYKEKPFDPTNILVQCVSN

VICSVVFGNRFEYANKDFQNLLSLFQSVFQETSSSWGQLLNMLPAVMNHVPGPHKNIIRD

MNKLEDFVLQRVKENEKTVDPNSPRDLIDSFLIKMQQENKNPTSPFHMKNLIATILSIFF

AGTETVSTTLRHGFLILLIHPEIEAKLQEEIDRVVGQNRSPTIEDRNKMPYTDAVIHEIQ

RLSDVIPMNVPHLVTKDTKFRGYTIPKGTNIYPLLCAVLRDPEQFDTPSKFNPNHFLDDK

GCFKSNDGFMPFSTGKRICLGEGLARMELFLFLTNILQNFKLHSESGLTEDNIAPKMKGF

ANYPTSYQLSFIPR

>326916797

MDWASLVPVGLLLILILLLILKTQHFWRSQVKFPPGPQPLPVIGNLHMMDLKKLGQTMLQ

LSETYGPVFTVQLGLRKVVVLSGYDTVKEALVNHADAFAGRPKIPIIEKTGKGKGIVFAR

GENWKVMRRFTLTTLRDFGMGKKAIEDHVVEEYGYLADIIESQKGKPLEMTQLMNSAIAN

VIVSILLGKRFDYEDPTFKRLVSLMNENMQLFGSPSVSLYNMFPILGLFLKDKNHFLENL

KEVNNFIKDTFTKYLKVLDKNDQRSFIDAFLVRQQEENGKANKFFDDVNLTEVVRNLFTA

GMDTTATTLRWGLLLMMKYPEIQKKVQEEIDQVIGSNPPRTEHRTKMPYTDAVVHEIQRF

ANILPLNLPHETTMDVTIKGYFIPKGTYVIPLLNSVLQDKTQWEKPCSFYPEHFLNSEGQ

FVKKDAFMPFSAGRRICAGETLAKMELFLFFTSLLQRFTFQPPPGVSPSDLDLSATTRFV

VAPVTHEVCAVSRS

>BAF64508.1

MALLEITTLALVICVTCLVFLFVWKKSHKAGRLPPGPTPLPIIGNLMQLNLKDVPASLSK

LAKEYGPVYTLYLGSQITVVLHGYEAVKEALIDQGDEFLCRGRIPIIDDTQRGYGIFFSN

GNRWKQMRRFSLMTLRNFGMGKRSLEERVQEEAQFLVEELRKTEAQPLDPVFTLSCASCN

VICSILFNERFHYNNKTLLSLLSLLNKNFNRINSPWNQIYNLWPKLIKHLPGEHKAFSKR

LNDIKYFILEKVKEHQKSLDHNNPRDYIDCFLSKMEQEKQNPESEFHLENLATCGSNLFS

AGIETTSITLSYGLLLLMKYPEVQAKVHEEIDRVIGCNQSPCMKDKIKLPYTEAVLHEIQ

RYITLLPSNMPRTVVRDTKFRQYFIPKGATVLPLLSSVLYDCKEFPNPEKFDPGHFLDKN

GSVRKTEYFVPFSMGKRACVGEGLARVELFLFLTTILQNFVLKPLGEPKNIETKPIVTGL

INIPQPYKLCFIPRQKKNFSLLTI

>BAB23748.1

MDPILVLVLTLSCLFLLSLWRQSSERGKLPPGPTPLPIIGNILQIDVKDICQSFTNLSKV

YGPVYTLYLGRKPTVVLHGYEAVKEALVDHGEEFAGRGRFPVFDKATNGMGLAFSKGNVW

KNTRRFSLMTLRNLGMGKRSIEDRVQEEARCLVEELRKTNGSPCDPTFILGCAPCNVICS

IIFQDRFDYKDRDFLNLMEKLNEITKILSSPWLQICNTYPALLDYCPGSHKQFFKNYASI

KNFLLEKIKEHEESLDVTIPRDFIDYFLXXGGQEDGNQPLQNRLEHLAITVTDLFSAGTE

TTSTTLRYAILLLLKYPHVTAKVQEEIEHVIGKHRSPCMQDRSRMPYTDAMIHEVQRFID

LIPNSLPHEVTSDIKFRNYFIPKGTTVITSLSSVLHDSTEFPNPEKFDPGHFLDENGKFK

KSDYFIPFSTGKRICAGEGLARMELFLFLTSILQNFNLKPLVHPKDIDVTPMLIGLASVP

PAFQLCFIPS

>395859750

MELSVLLSLAILTGLLLLLVKGRPKAHGHLPPGPYPLPFLGNLLQMDRGGLLKSFLKLQE

KYGDVFTVYLGPRPVVMLCGTEAIREALVDQAEYFSGRGKIAIVEPIFEGHGVVFANGDR

WKALRRFSLATMRDFGMGKRSVEERIQEEAQCLVEELRKSQGALLDPTFSFQAITANIIC

SIVFGKRFDYKDPEFLRMLDLFYQSFSLISSLSSQVFELFSSFLKYFPGIHRQIYKNLQE

INAFIGRSVEKHRETLDPSSPRDLIDVYLLRMEKEKSNPNSEFNHKNLILNTLSLFFAGT

ETTSTTLRYGFLLMLKYPHVTEKVYKEIVQVIGTHRPPALDDRAKMPYTDAVIHEIQRFA

DLIPIGLPHIVTKDTHFRGYVIPKNTEVFPILSSALHDPRHFEKPDAFNPDHFLDANGAL

KKNEAFIPFSLGKRICLGEGIARTELFLFFTTILQNFSLASSMAPEDIDLTPQQSGVGKV

APAYQIRFVPRGQFTVPLTVGGPDCRPQDELAAKQDRQRRQKHLAGRINVLGRIRHPDTT

LWPVTTMELGGALTIFLELCLTCLTIILVWKRINKGGKLPPGPTPIPFLGNLLQVRPDAT

FQSFMKLREKYGPVFTVYMGLRPVVVLCGHEVVKEALVDQGDDFSGRGGLAITERNFQGY

GVVMANGDRWKILRRFSLTILRDFGMGKRSIEERIQEEAGYLLEELRKTKGAPIEPTFFL

SRTVSNVISSVVFGSRFDYEDKQFLHLLQLMNETFTEMSTPWTQLYNTYPEIMQYLPGRH

NHMYHLIEELKDFVASKVKINEASLDPQNPRDFIDCFLIKMHQDKNNPHTEFNLRNLVLT

TLNLFSAGTETVSSTLRYGFLLLMKHPEVEGLGHVLVSVNDRVKMPYTDAVIHEIQRLLD

FVPMGVPHKVTRDTHFRGYLLPKGTDVFPMLGSVLKDPKYFRYPDAFYPQHFLDEQGRFK

KNEAFVAFSSGKRTCLGEALARMELFLYLTSVLQNFSLRPLVPPADIDVTPKLSGFVNVP

SPYELCLVAR

>395537397

MSEFDLLSILSGLSLILILILNIKLTLTKSFKRYSPPGPRPLPIVGNLHILNLKKPYQTM

QQLSQKYGNIFSLRMGPKKVVVLSGYETVKDALVNHSEEFGERAHVPIFEKIFEGKEQGI

VFSHGENWKIIRRFSLTTLRNFGMGKRVIEERIMEECQHLIQVIESYQRKPFETKTIMNA

SVANIIVSVLLGKRFDYQDPQFLRLLYLIGENVRLIGGPSIMLFNMFPALGFVLQDHKTV

LKNRDELFSFVRMTFLDHLQKLDKNDQRSFIDAFLIRRQEEKNKSDDCFSNDNLVALVTN

LFVAGTETTSTTLRWGILLMMKYPEIQKKVQNEIIKVIGSAQPRMEHRAQMPYTDAVIHE

IQRFSSILPMSVSRETTTDVIFKNYYIPKGTETIILLTSVLQDQTQWEKPHTFNPQHFLN

VEGKFVKRGAFLPFSAGQRMCAGESLVKMELFLFFTSLLQKFTFHPSPGVSTMDLDITAD

SGFTSRPKPHKICAVPYS

>BAF83511.1

MSALGVTVALLVWAAFLLLVSMWRQVHSSWNLPPGPFPLPIIGNLFQLELKNIPKSFTRL

AQRFGPVFTLYVGSQRMVVMHGYKAVKEALLDYKDEFSGRGDLPAFHAHRDRGIIFNNGP

TWKDIRRFSLTTLRNYGMGKQGNESRIQREAHFLLEALRKTQGLPFDPTFLIGCAPCNVI

ADILFRKHFDYNDEKFLRLMYLFNENFHLLSTPWLQLYNNFPSFLHYLPGSHRKVIKNVA

EVKEYVSERVKEHHQSLDPNCPRDLTDCLLVEMEKEKHSAERLYTMDGITVTVADLFFAG

TETTSTTLRYGLLILMKYPEIEEKLHEEIDRVIGPSRIPAIKDRQEMPYMDAVVHEIQRF

ITLVPSNLPHEATRDTIFRGYLIPKGTVVVPTLDSVLYDNQEFPDPEKFKPEHFLNENGK

FKYSDYFKPFSTGKRVCAGEGLARMELFLLLCAILQHFNLKPLVDPKDIDLSPIHIGFGC

IPPRYKLCVIPRS

>EDL24230.1

MEPSVLLLLALLVGFLLLLARGHPKSRGNFPPGPRPLPLLGNLLQMDRGGLLKSFIQLRE

KYGDVFTVHLGPRPVVMLCGTDTIREALVGQAEAFSGRGTVAVVEPTFKEYGVIFANGER

WKTLRRFSLATMRDFGMGKRSVEERIQEEAQCLVEELRKSQGAPLDPTFLFQCITANIIC

SIVFGERFEYTDRQFLRLLELFYQTFSLISSFSSQMFELFSGFLKYFPGAHRQISKNLQE

LLDYIGHSVEKHRATLDPSVPRDFIDIYLLRMEKEKSNQHTEFHHQNLMMSVLSLFFAGT

ETSSTTLRYGFLLMLKYPHVAEKVQKEIDQVIGSHRLPTLDDRTKMPYTDAVIHEIQRFS

DLIPIGVPHRVTKDTMFRGYLLPKNTEVYPILSSALHDPQYFEQPDSFNPDHFLDANGAL

KKSEAFLPFSTGQIFDQKSVGKRICLGESIARNELFLFFTSILQNFSVASHVAPKDIDLT

PKESGIGKIPPTYQICFLAR

>P12791.1

MEPSVLLLLALLVGFLLLLARGHPKSRGNFPPGPRPLPLLGNLLQMDRGGLLKSLIQLRE

KYGDVFTVHLGPRPVVMLCGTDTIREALVGQAEAFSGRGTVAVVEPTFKEYGVIFANGER

WKTLRRFSLATMRDFGMGKRSVEERIQEEAQCLVEELRKSQGAPLDPTFLFQCITANVIC

SIVFGERFEYTDRQFLRLLELFYQTFSLISSFSSQMFELFSGFLKYFPGAHRQISKNLQE

LLDYIGHSVERHKATLDPSVPRDFIDIYLLRMEKEKSNQNAEFHHQNLMMSVLSLFFVGT

ETSSTTLHYGFLLMLKYPHVTEKVQKEIDQVIGSHRLPTLDDRTKMPYSDAVIHEIQRFS

DLIPIGVPHRVTKDTLFRGYLLPKNTEVYPILSSALHDPQYFEQPDSFNPDQFLDANGAL

KKSEAFLPFSTGQIFDQKSVGKRICLGESIARSELFLFFTSILQNFSVASHVAPKDIDLT

PKESGIGKIPPTYQICFLAR

>P00176.1

MEPTILLLLALLVGFLLLLVRGHPKSRGNFPPGPRPLPLLGNLLQLDRGGLLNSFMQLRE

KYGDVFTVHLGPRPVVMLCGTDTIKEALVGQAEDFSGRGTIAVIEPIFKEYGVIFANGER

WKALRRFSLATMRDFGMGKRSVEERIQEEAQCLVEELRKSQGAPLDPTFLFQCITANIIC

SIVFGERFDYTDRQFLRLLELFYRTFSLLSSFSSQVFEFFSGFLKYFPGAHRQISKNLQE

ILDYIGHIVEKHRATLDPSAPRDFIDTYLLRMEKEKSNHHTEFHHENLMISLLSLFFAGT

ETSSTTLRYGFLLMLKYPHVAEKVQKEIDQVIGSHRLPTLDDRSKMPYTDAVIHEIQRFS

DLVPIGVPHRVTKDTMFRGYLLPKNTEVYPILSSALHDPQYFDHPDSFNPEHFLDANGAL

KKSEAFMPFSTGKRICLGEGIARNELFLFFTTILQNFSVSSHLAPKDIDLTPKESGIGKI

PPTYQICFSAR

>EDM07990.1

MEPSILLLLALLVGFLLLLVRGHPKSRGNFPPGPRPLPLLGNLLQLDRGGLLNSFMQLRE

KYGDVFTVHLGPRPVVMLCGTDTIKEALVGQAEDFSGRGTIAVIEPIFKEYGVIFANGER

WKALRRFSLATMRDFGMGKRSVEERIQEEAQCLVEELRKSQGAPLDPTFLFQCITANIIC

SIVFGERFDYTDRQFLRLLELFYRTFSLLSSFSSQVFEFFSGFLKYFPGAHRQISKNLQE

ILDYIGHIVEKHRATLDPSAPRDFIDTYLLRMEKEKSNHHTVFHHENLMISLLSLFFAGT

ETSSTTLRYGFLLMLKYPHVAEKVQKEIDQVIGSHRLPTLDDRSKMPYTDAVIHEIQRFS

DLVPIGVPHRVTKDTMFRGYLLPKNTEVYPILSSALHDPQYFDHPDSFNPEHFLDANGAL

KKSEAFMPFSTGKRICLGEGIARNELFLFFTTILQNFSVSSHLAPKDIDLTPKESGIGKI

PPTYQICFSAR

>0904267A

EPTILLLLALLVGFLLLLVRGHPKSRGNFPPGPRPLPLLGNLLQLDRGGLLNSFMQLREK

YGDVFTVHLGPRPVVMLCGTDTIKEALVGQAEDFSGRGTIAVIEPIFKEYGVIFANGERW

KALRRFSLATMRDFGMGKRSVEERIQEEAQCLVEELRKSQGAPLDPTELFQCITANIICS

IVFGERFDYTDRQFLRLLELFYRTFSLLSSFSSQVFEFFSGFLKYFPGAHRQISKNLQEI

LDYIGHIVEKHRATLDPSAPRDFIDTYLLRMEKEKSNHHTEFHHENLMISLLSLFFAGTE

TSSTTLRYGFLLMLKYPHVAEKVQKEIDQVIGSHRLPTLDDRSKMPYTDAVIHEIQRFSD

LVPIGVPHRVTKDTMFRGYLLPKNTEVYPILSSALHDPQYFDHPDSFNPEHFLDANGALK

KSEAFMPFSTGKRICLGEGIARNEIFLFFTTILQNFSVSSHLAPKDIDLTPKESGIGKIP

PTYQICFSAR

>AAA41046.1

MEPSILLLLALLVGFLLLLVRGHPKSRGNFPPGPRPLPLLGNLLQLDRGGLLNSFMQLRE

KYGDVFTVHLGPRPVVMLCGTDTIKEALVGQPEDFSGRGTIAVIEPIFKEYGVIFANGER

WKALRRFSLATMRDFGMGKRSVEERIQEEAQCLVEELRKSQGAPLDPTFLFQCITANIIC

SIVFGERFDYTDRQFLRLLELFYRRFSLLSSFSSQVFEFFSGFLKYFPGAHRQISKNLQE

ILDYIGHIVEKHRATLDPSAPRDFIDTYLLRMEKEKSNHHTEFHHENLMISLLSLFFAGT

ETSSTTLRYGFLLMLKYPHVAEKVQKEVDQVIGSHRLPTLDDRSKMPYTDAVIHEIHRFS

DLVPIGVPHRVTKDTMFRGYLLPKNTEVYPIRSSALHDPQYFDHPDSFNPEHFLDVNGAL

KKSEAFMPFSTGKHICLGEGIARNELFLFFTTILQNFSVSSHLAPKDIDLTPKESGIGKI

PPTYQICFSAR

>P04167.2

MEPSILLLLALLVGFLLLLVRGHPKSRGNFPPGPRPLPLLGNLLQLDRGGLLNSFMQLRE

KYGDVFTVHLGPRPVVMLCGTDTIKEALVGQAEDFSGRGTIAVIEPIFKEYGVIFANGER

WKALRRFSLATMRDFGMGKRSVEERIQEEAQCLVEELRKSQGAPLDPTFLFQCITANIIC

SIVFGERFDYTDRQFLRLLELFYRTFSLLSSFSSQVFEFFSGFLKYFPGAHRQISKNLQE

ILDYIGHIVEKHRATLDPSAPRDFIDTYLLRMEKEKSNHHTEFHHENLMISLLSLFFAGT

ETGSTTLRYGFLLMLKYPHVTEKVQKEIDQVIGSHRPPSLDDRTKMPYTDAVIHEIQRFA

DLAPIGLPHRVTKDTMFRGYLLPKNTEVYPILSSALHDPQYFDHPDTFNPEHFLDADGTL

KKSEAFMPFSTGKRICLGEGIARNELFLFFTTILQNFSVSSHLAPKDIDLTPKESGIAKI

PPTYQICFSAR

>AEO16875.1

MEPSVLLLLALLVGFVLLLVRGYPKARGHLPPGPRPLPLLGNLLQMDRGGFLNSFMRIRE

KYGDVFTVHLGPRPVVMLYGTEAIREALVDQAEAFSGRGTIAVIKPVIGDYGMIFSNGER

WKVLRRFSLATMRDFGMGKRSVEDRIQEEAQCLVEELQKSQGAPLDPTFLFQCITANIIC

SIVFGERYDYKDRQFLRLLDLFYRTFSLMSSFSSQVFELFSGFMKYFPGAHRQITRNLQE

ILDYVGQSVEKHRATLDPSNPRDFIDTYLLRMEKEKSNQHTEFHHQNLLISVLSLFFAGT

ETTSTTLRYGFLLMLKYPHVAEKVQKEIDQVIGSHRLPTLEDRTKMPYTDAVIHEIQRFS

DLAPIGAPHKVTKDTLFRGYLLPKNTEVYPILSSALHDPQYFEQPDTFNPDHFLDANGAL

KKSEAFMPFSLGKRICLGEGIARNELFLFFTAILQNFSLSSPVAPKDIDLSPKESGFGKV

PQTYQICFLAR

>AEO16878.1

MEPSVLLLLALLVGFVLLLVRGYPKARGHLPPGPRPLPLLGNLLQMDRGGFLNSFMRIRE

KYGDVFTVHLGPRPVVMLYGTEAIREALVDQAEAFSGRGTIAVIKPVIGDYGMIFSNGER

WKVLRRFSLATMRDFGMGKRSVEDRIQEEAQCLVEELQKSQGAPLDPTFLFQCITANIIC

SIVFGERYDYKDRQFLRLLDLFYRTFSLMSSFSSQVFELFSGFMKYFPGAHRQITRNLQE

ILDYVGQSVEKHRATLDPSNPRDFIDTYLLRMEKEKSNQHTEFHHQNLLISVLSLFFAGT

ETTSTTLRYGFLLMLKYPHVAEKVQKEIDQVIGSHRLPTLEDRTKMPYTDAVIHEIQRFS

DLAPIGAPHKVTKDTLFRGYLLPKNTEVYPILSSALHDPQYFEQPGTFNPDHFLDANGAL

KKSEAFMPFSIGKRICLGEGIARNELFLFFTTILQNFSVSSSVAPKDIDLSPKESGIGKV

PQTYQISFLAR

>AAA41056.1

MEPSILLLLALLVGFLLLLVRGHPKSRGNFPPGPRPLPLLGNLLQLDRGGLLNSFMQLRE

KYGDVFTVHLGPRPVVMLCGTDTIKEALVGQAEDFSGRGTIAVIEPIFKEYGVIFANGER

WKALRRFSLATMRDFGMGKRSVEERIQEEAQCLVEELRKSQGAPLDPTFLFQCITANIIC

SIVFGERFDYTDRQFLRLLELFYRTFSLLSSFSSQVFEFFSGFLKYFPGAHRQISKNLQE

ILDYIGHIVEKHRATLDPSAPRDFIDTYLLRMEKEKSNHHTEFHHENLMISLLSLFFAGT

ETGSTTLRYGFLLMLKYPHVTVKVQKEIDQVIGSHRPPSLDDRTKMPYTDAVIHEIQRFA

DLAPIGLPHRVTKDTMFRGYLLPKNTEVYPILSSALHDPQYFDHPDTFNPEHFLDADGTL

KKSEAFMPFSTGKRICLGEGIARNELFLFFTTILQNFSVSSHLAPKDIDLTPMESGIAKI

PPTYQICFSAR

>AEO16882.1

MEPSVLLLLALLVGFVLLLVRGYPKARGHLPPGPRPLPLLGNLLQMDRGGFLNSFMRIRE

KYGDVFTVHLGPRPVVMLYGTEAIREALVDQAEAFSGRGTIAVIKPIIGDYGMIFSNGER

WKVLRRFSLATMRDFGMGKRSVEDRIQEEAQCLVEELQKSQGAPLDPTFLFQCITANIIC

SIVFGERYDYKDRQFLRLLDLFYRTFSLMSSFSSQVFELFSGFMKYFPGAHRQITRNLQE

ILDYVGQSVEKHRATLDPSNPRDFIDTYLLRMEKEKSNQHTEFHHQNLLISVLSLFFAGT

ETTSTTLRYGFLLMLKYPHVAEKVQKEIDQVIGSHRLPTLEDRTKMPYTDAVIHEIQRFS

DLAPIGAPHKVTKDTLFRGYLLPKNTEVYPILSSALHDPQYFEQPGTFNPDHFLDANGAL

KKSEAFMPFSIGKRICLGEGIARNELFLFFTTILQNFSVSSSVAPKDIDLSPKESGIGKV

PQTYQISFLAR

>AEO16896.1

MEPSVLLLLALLVGFVLLLVRGYPKARGHLPPGPRPLPLLGNLLQMDRGGFLNSFMRIRE

KYGDVFTVHLGPRPVVMLYGTEAIREALVDQAEAFSGRGTIAVIKPVIGDYGMIFSNGER

WKVLRRFSLATMRDFGMGKRSVEDRIQEEAQCLVEELQKSQGAPLDPTFLFQCITANIIC

SIVFGERYDYKDRQFLRLLDLFYRTFSLMSSFSSQVFELFSGFMKYFPGAHRQITRNLQE

ILDYVGQSVEKHRATLDPSNPRDFIDTYLLRMEKEKSNQHTEFHHQNLLISVLSLFFAGT

ETTSTTLRYGFLLMLKYPHVAEKVQKEIDQVIGSHRLPTLEDRTKMPYTDAVIHEIQRFS

DLAPIGAPHKVTKDTLFRGYLLPKNTEVYPILSSALHDPQYFEQPGTFNPDHFLDANGAL

KKSEAFMPFSIGKRICLGEGIARSELFLFFTTILQNFSVSSSVAPKDIDLSPKESGIGKV

PQTYQISFLAR

>AEO16903.1

MEPSVLLLLALLVGFVLLLVRGYPKARGHLPPGPRPLPLLGNLLQMDRGGFLNSFMRIRE

KYGDVFTVHLGPRPVVMLYGTEAIREALVDQAEAFSGRGTIAVIKPVIGDYGMIFSNGER

WKVLRRFSLATMRDFGMGKRSVEDRIQEEAQCLVEELQKSQGAPLDPTFLFQCITANIIC

SIVFGERYDYKDRQFLRLLDLFYRTFSLMSSFSSQVFELFSGFMKYFPGAHRQITRNLQE

ILDYVGQSVEKHRATLDPSNPRDFIDTYLLRMEKEKSNQHTEFHHQNLLISVLSLFLAGT

ETTSTTLRYGFLLMLKYPHVAEKVQKEIDQVIGSHRLPTLEDRTKMPYTDAVIHEIQRFS

DLAPIGAPHKVTKDTLFRGYLLPKNTEVYPILSSALHDPQYFEQPGTFNPDHFLDANGAL

KKSEAFMPFSIGKRICLGEGIARNELFLFFTTILQNFSVSSSVAPKDIDLSPKESGIGKV

PQTYQISFLAR

>AEO16902.1

MEPSVLLLLALLVGFVLLLVRGYPKARGHLPPGPRPLPLLGNLLQMDRGGFLNSFMRIRE

KYGDVFTVHLGPRPVVMLYGTEAIREALVDQAEAFSGRGTIAVIKPVIGDYGMIFSNGER

WKVLRRFSLATMRDFGMGKRSVEDRIQEEAQCLVEELQKSQGAPLDPTFLFQCITANIIC

SIVFGERYDYKDRQFLRLLDLLYRTFSLMSSFSSQVFELFSGFMKYFPGAHRQITRNLQE

ILDYVGQSVEKHRATLDPSNPRDFIDTYLLRMEKEKSNQHTEFHHQNLLISVLSLFFAGT

ETTSTTLRYGFLLMLKYPHVAEKVQKEIDQVIGSHRLPTLEDRTKMPYTDAVIHEIQRFS

DLAPIGAPHKVTKDTLFRGYLLPKNTEVYPILSSALHDPQYFEQPGTFNPDHFLDANGAL

KKSEAFMPFSIGKRICLGEGIARNELFLFFTTILQNFSVSSSVAPKDIDLSPKESGIGKV

PQTYQISFLAR

>AEO16901.1

MEPSVLLLLALLVGFVLLLVRGYPKARGHLPPGPRPLPLLGNLLQMDRGGFLNSFMRIRE

KYGDVFTVHLGPRPVVMLYGTEAIREALVDQAEAFSGRGTIAVIKPVIGDYGMIFSNGER

WKVLRRFSLATMRDFGMGKRSVEDRIQEEAQCLVEELQKSQGAPLDPTFLFQCITANIIC

SIVFGERYDYKDRQFLRLLDLFYRTFSLMSSFSSQVFELFSGFMKYFPGAHRQITRNLQE

ILDYVGQSVEKHRATLDPSNPRDFIDTYLLRMEKEKSNQHTEFHHQNLLISVLSLFFAGT

ETTSTTLRYGFLLMLKYPHVAEKVQKEIDQVIGSHRLPTLEDRTKMPYTDAVIHEIQRFS

DLAPIGAPHKVTKDTLFRGYLLPKNTEVYPILSSALHDPQYFEQPGTLNPDHFLDANGAL

KKSEAFMPFSIGKRICLGEGIARNELFLFFTTILQNFSVSSSVAPKDIDLSPKESGIGKV

PQTYQISFLAR

>AEO16905.1

MEPSVLLLLALLVGFVLLLVRGYPKARGHLPPGPRPLPLLGNLLQMDRGGFLNSFMRIRE

KYGDVFTVHLEPRPVVMLYGTEAIREALVDQAEAFSGRGTIAVIKPVIGDYGMIFSNGER

WKVLRRFSLATMRDFGMGKRSVEDRIQEEAQCLVEELQKSQGAPLDPTFLFQCITANIIC

SIVFGERYDYKDRQFLRLLDLFYRTFSLMSSFSSQVFELFSGFMKYFPGAHRQITRNLQE

ILDYVGQSVEKHRATLDPSNPRDFIDTYLLRMEKEKSNQHTEFHHQNLLISVLSLFFAGT

ETTSTTLRYGFLLMLKYPHVAEKVQKEIDQVIGSHRLPTLEDRTKMPYTDAVIHEIQRFS

DLAPIGAPHKVTKDTLFRGYLLPKNTEVYPILSSALHDPQYFEQPGTFNPDHFLDANGAL

KKSEAFMPFSIGKRICLGEGIARNELFLFFTTILQNFSVSSSVAPKDIDLSPKESGIGKV

PQTYQISFLAR

>AEO16884.1

MGPSVLLLLALLVGFVLLLVRGYPKARGHLPPGPRPLPLLGNLLQMDRGGFLNSFMRIRE

KYGDVFTVHLGPRPVVMLYGTEAIREALVDQAEAFSGRGTIAVIKPVIGDYGMIFSNGER

WKVLRRFSLATMRDFGMGKRSVEDRIQEEAQCLVEELQKSQGAPLDPTFLFQCITANIIC

SIVFGERYDYKDRQFLRLLDLFYRTFSLMSSFSSQVFELFSGFMKYFPGAHRQITRNLQE

ILDYVGQSVEKHRATLDPSNPRDFIDTYLLRMEKEKSNQHTEFHHQNLLISVLSLFFAGT

ETTSTTLRYGFLLMLKYPHVAEKVQKEIDQVIGSHRLPTLEDRTKMPYTDAVIHEIQRFS

DLAPIGAPHKVTKDTLFRGYLLPKNTEVYPILSSALHDPQYFEQPGTFNPDHFLDANGAL

KKSEAFMPFSIGKRICLGEGIARNELFLFFTTILQNFSVSSSVAPKDIDLSPKESGIGKV

PQTYQISFLAR

>AEO16877.1

MEPSVLLLLALLVGFVLLLVRGYPKARGHLPPGPRPLPLLGNLLQMDRGGFLNSFMRIRE

KYGDVFTVHLGPRPVVMLYGTEAIREALVDQAEAFSGRGTIAVIKPVIGDYGMIFSNGER

WKVLRRFSLATMRDFGMGKRSVEDRIQEEAQCLVEELQKSQGAPLDPTFLFQCITANIIC

SIVFGERYDYKDRQFLRLLDLFYRTFSLMSSFSSQVFELFSGFMKYFPGAHRQITRNLQE

ILDYVGQSVEKHRATLDPSNPRDFIDTYLLRMEKEKSNQHTEFHHQNLLISVLSLFFAGT

ETTSTTLRYGFLLMLKYPHVAEKVQKEIDQVIGSHRLPTLEDRTKMPYTDAVIREIQRFS

DLAPIGAPHKVTKDTLFRGYLLPKNTEVYPILSSALHDPQYFEQPGTFNPDHFLDANGAL

KKSEAFMPFSIGKRICLGEGIARNELFLFFTTILQNFSVSSSVAPKDIDLSPKESGIGKV

PQTYQISFLAR

>AEO16898.1

MEPSVLLLLALLVGFVLLLVRGYPKARGHLPPGPRPLPLLGNLLQMDRGGFLNSFMRIRE

KYGDVFTVHLGPRPVVMLYGTEAIREALVDQAEAFSGRGTIAVIKPAIGDYGMIFSNGER

WKVLRRFSLATMRDFGMGKRSVEDRIQEEAQCLVEELQKSQGAPLDPTFLFQCITANIIC

SIVFGERYDYKDRQFLRLLDLFYRTFSLMSSFSSQVFELFSGFMKYFPGAHRQITRNLQE

ILDYVGQSVEKHRATLDPSNPRDFIDTYLLRMEKEKSNQHTEFHHQNLLISVLSLFFAGT

ETTSTTLRYGFLLMLKYPHVAEKVQKEIDQAIGSHRLPTLEDRTKMPYTDAVIHEIQRFS

DLAPIGAPHKVTKDTLFRGYLLPKNTEVYPILGSALHDPQYFEQPGTFNPDHFLDANGAL

KKSEAFMPFSIGKRICLGEGIARNELFLFFTTILQNFSVSSSVAPKDIDLSPKESGIGKV

PQTYQISFLAR

>AEO16892.1

MEPSVLLLLALLVGFVLLLVRGYPKARGHLPPGPRPLPLLGNLLQMDRGGFLNSFMRIRE

XYGDVFTVHLGPRPVVMLYGTEAIREALVDQAEAFSGRGTIAVIKPVIGDYGMIFSNGER

WKVLRRFSLATMRDFGMGKRSVEDRIQEEAQCLVEELQKSQGAPLDPTFLFQCITANIIC

SIVFGERYDYKDRQFLRLLDLFYRTFSLMSSFSSQVFELFSGFMKYFPGAHRQITRNLQE

ILDYVGQSVEKHRATLDPSNPRDFIDTYLLRMEKEKSNQHTEFHHQNLLISVLSLFFAGT

ETTSTTLRYGFLLMLKYPHVAEKVQKEIDQVIGSHRLPTLEDRTKMPYTDAVIHEIQRFS

DLAPIGAPHKVTKDTLFRGYLLPKNTEVYPILSSALHDPQYFEQPGTFNPDHFLDANGAL

KKSEAFMPFSIGKRICLGEGIARNELFLFFTTILQNFSVSSSVAPKDIDLSPKESGIGKV

PQTYQISFLAR

>AEO16904.1

MEPSVLLLLALLVGFVLLLVRGYPKARGHLPPGPRPLPLLGNLLQMDRGGFLNSFMRIRE

KYGDVFTVHLGPRPVVMLYGTEAIREALVDQAEAFSGRGTIAVIKPVIGDYGMIFSNGER

WKVPRRFSLATMRDFGMGKRSVEDRIQEEAQCLVEELQKSQGAPLDPTFLFQCITANIIC

SIVFGERYDYKDRQFLRLLDLFYRTFSLMSSFSSQVFELFSGFMKYFPGAHRQITRNLQE

ILDYVGQSVEKHRATLDPSNPRDFIDTYLLRMEKEKSNQHTEFHHQNLLISVLSLFFAGT

ETTSTTLRYGFLLMLKYPHVAEKVQKEIDQVIGSHRLPTLEDRTKMPYTDAVIHEIQRFS

DLAPIGAPHKVTKDTLFRGYLLPKNTEVYPILSSALHDPQYFEQPGTFNPDHFLDANGAL

KKSEAFMPFSIGKRICLGEGIARNELFLFFTTILQNFSVSSSVAPKDIDLSPKESGIGKV

PQTYQISFLAR

>AEO16918.1

MGPSVLLLLALLVGFVLLLVRGYPKARGHLPPGPRPLPLLGNLLQMDRGGFLNSFRRIRE

KYGDVFTVHLGPRPVVMLYGTEAIREALVDQAEAFSGRGTIAVVEPIFRGYGVVFANGER

WKALRRFSLVTMRDFGMGKRSVEERIQEEARCLVEEFRKSQGAPLDPTFLFQCITANIIC

SIVFGERYDYKDRQFLRLLDLFYRTFSLMSSFSSQVFELYSGFMKYFPGAHRQITRNLQE

ILDYVGQSVEKHRATLDPSNPRDFIDTYLLRMEKEKSNQHTEFHHQNLLISVLSLFFAGT

ETTSTTLRYGFLLMLKYPHVAEKVQKEIDQVIGPHRLPTLEDRTKMPYTDAVIYEIQRFS

DLIPIGVPHKVTKDTLFRGYLLPKNTEVYPILSSALNDPQYFEQPDTFNPDHFLDANGAL

KKSEAFMPFSIGKRICLGEGIARNELFLFFTTILQNFSLSSPVAPKDIDLCPKESGFGKL

PPTYQICFLAR

>AEO16913.1

MEPSVLLLLALLVGFVLLLVRGYPKARGHLPPGPRPLPLLGNLLQMDRGGFLNSFMRIRE

KYGDVFTVHLGPRPVVMLYGTEAIREALVDQAEAFSGRGTIAVIKPVIGDYGMIFSNGER

WKVLRRFSLATMRDFGMGKRSVEDRIQEEAQCLVEELQKSQGAPLDPTFLFQCITANIIC

SIVFGERYDYKDRQFLRLLDLFYRTFSLMSSFSSQVFELFSGFMKYFPGAHRQITRNLQE

ILDYVGQSVEKHRATLDPSNPRDFIDTYLLRMEKEKSNQHTEFHHQNLLISVLSLFFAGT

ETTSTTLRYGFLLMLKYPHVAEKVQKEIDQVIGSHRLPTLEDRTKMPYTDAVIHEIQRFS

DLAPIGAPHKVTKDTLFRGYLLPKNTEVYPILSSALHDPQYFEQPGTFNPDHFLDANGAL

KKSEAFMPFSIGKRICLGEGIARNELFLFFTTILQNFSVSSSVAPKDIDLSLKESGIGKV

PQTYQISFLAR

>AEO16888.1

MGPSVLLLLALLVGFVLLLVRGYPKARGHLPPGPRPLPLLGNLLQMDRGGFLNSFMRIRE

KYGDVFTVHLGPRPVVMLYGTEAIREALVDQAEAFSGRGTIAVIKPVIGDYGMIFSNGER

WKVLRRFSLATMRDFGMGKRSVEDRIQEEAQCLVEELQKSQGAPLDPTFLFQCITANIIC

SIVFGERYDYKDRQFLRLLDLFYRTFSLMSSFSSQVFELFSGFMKYFPGAHRQITRNLQE

ILDYVGQSVEKHRATLDPSNPRDFIDTYLLRMEKEKSNQHTEFHHQNVLISVLSLFFAGT

ETTSTTLRYGFLLMLKYPHVAEKVQKEIDQVIGSHRLPTLEDRTKMPYTDAVIHEIQRFS

DLAPIGAPHKVTKDTLFRGYLLPKNTEVYPILSSALHDPQYFEQPGTFNPDHFLDANGAL

KKSEAFMPFSIGKRICLGEGIARNELFLFFTTILQNFSVSSSVAPKDIDLSPKESGIGKV

PQTYQISFLAR

>AEO16915.1

MEPSVLLLLALLVGFVLLLVRGYPKARGHLPPGPHPLPLLGNLLQMDRGGFLNSFMRIRE

KYGDVFTVHLGPRPVVMLYGTEAIREALVDQAEAFSGRGTIAVIKPVIGDYGMIFSNGER

WKVLRRFSLATMRDFGMGKRSVEDRIQEEAQCLVEELQKSQGAPLDPTFLFQCITANIIC

SIVFGERYDYKDRQFLRLLDLFYQTFSLMSSFSSQVFELFSGFMKYFPGAHRQITRNLQE

ILDYVGQSVEKHRATLDPSNPRDFIDTYLLRMEKEKSNQHTEFHHQNLLISVLSLFFAGT

ETTSTTLRYGFLLMLKYPHVAEKVQKEIDQVIGSHRLPTLEDRTKMPYTDAVIHEFQRFS

DLAPIGAPHKVTKDTLFRGYLLPKNTEVYPILSSALHDPQYFEQPGTFNPDHFLDANGAL

KKSEAFMPFSIGKRICLGEGIARNELFLFFTTILQNFSVSSSVAPKDIDLSPKESGVGKV

PQTYQISFLAR

>AEO16890.1

MGPSVLLLLALLVGFVLLLVRGYPKARGHLPPGPRPLPLLGNLLQMDRGGFLNSFMRIRE

KYGDVFTVHLGPRPVVMLYGTEAIREALVDQAEAFSGRGTIAVIKPVIGDYGMIFSNGER

WKVLRRFSLATMRDFGMGKRSVEDRIQEEAQCLVEELQKSQGAPLDPTFLFQCITANIIC

SIVFGERYDYKDRQFLRLLDLFYRTFSLMSSFSSQVFELFSGFMKYFPGAHRQITRNXQE

ILDYVGQSVEKHRATLDPSNPRDFIDTYLLRMEKEKSNQHTEFHHQNLLISVLSLFFAGT

ETTSTTLRYGFLLMLKYPHVAEKVQKEIDQVIGSHRLPTLEDRTKMPYTDAVIHEIQRFS

DLAPIGAPHKVTKDTLFRGYLLPKNTEVYPILSSALHDPQYFEQPGTFNPDHFLDANGAL

KKSEAFMPFSIGKRICLGEGIARNELFLFFTTILQNFSVSSSVAPKDIDLSPKESGIGKV

PQTYQISFLAR

>AEO16883.1

MGPSVLLLLALLVGFVLLLVRGYPKARGHLPPGPRPLPLLGNLLQMDRGGFLNSFMRIRE

KYGDVFTVHLGPRPVVMLYGTEAIREALVDQAEAFSGRGTIAVIKPVIGDYGMIFSNGER

WKVLRRFSLATMRDFGMGKRSVEDRIQEEAQCLVEELQKSQGAPLDPTFLFQCITANIIC

SIVFGERYDYKDRQFLRLLDLFYRTFSLMSSFSSQVFELFSGFMKYFPGAHRQITRNLQE

ILDYVGQSVEKHRATLDPSNPRDFIDTYLLRMEKEKSNQHTEFHHQNLLISVLSLFFAGT

ETTSTTLRYGFLLMLKYPHVAEKVQKEIDQVIGSHRLPTLEDRTKMPYTDAVIHKIQRFS

DLAPIGAPHKVTKDTLFRGYLLPKNTEVYPILSSALHDPQYFEQPGTFNPDHFLDANGAL

KKSEAFMPFSIGKRICLGEGIARNELFLFFTTILQNFSVSSSVAPKDIDLSPKESGIGKV

PQTYQISFLAR

>EDM07984.1

MDPSVLLLFALFTGFLLLLIRGQGNGYGHLPPGPCPLPLLGNVLQMDRRGLLKSFIQLRD

KYGDVVTVHLGPRPIVMLYGTETIREALVDHAEAFSGRGTVAVVQPIIQDYGMIFANGER

WKILRRFSLATMRDFGMGKRSVEERIKEEAQCLVEELKKYKGAPLDPTFHLQCITANIIC

SIVFGERFDYTDHQFLHLLDLFYEILSLVSSFSSQVFELFPGFLKYFPGTHRHISKNIEE

ILNFIGHCVEKHRATLDPSTPRDFIDTYLLRMEKEKLNHHTEFHHQNLMMSVLSLFFAGT

ETSSTTLRYGFLLMLKYPHVAEKVQKEIDQVIGSHRVPTLDDRIKMPYTDAVIHEIQRFS

DLVPIGLPHRVTKDTLFRGYLLPKNIEVYPILSSALHDPQYFEHPDTFNPEHFLDANGAL

KKNEAFLPFSTGKRVCLGEGIARNELFLFFTTILQNFSVSSPVSPKDIDLTPKESGFAKI

PPTYQICFLSRQLG

>310923129

MEPSILLLLALLVGFLLLLVRGHPKSRGNFPPGPRPLPLLGNLLQLDRGGLLNSFMQFRE

KYGDVFTVHLGPRPVVMLCGTDTIKEALVGQAEDFSGRGTIAVIEPIFKEYGVFFANGER

WKALRRFSLATMRDFGMGKRSVEERIQEEAQCLVEELRKSQGAPLDPTFLFQCITANIIC

SIVFGERFDYTDRQFLRLLELFYRTFSLLSSFSSQVFEFFSGFLKYFPGAHRQISKNLQE

ILDYIGHIVEKHRATLDPSAPRDFIDTYLLRMEKVSPAWMREEKSNHHTEFHHENLMISL

LSLFFAGTETGSTTLRYGFLLMLKYPHVTEKVQKEIDQVIGSHRPPSLDDRTKMPYTDAV

IHEIQRFADLAPIGLPHRVTKDTMFRGYLLPKNTEVYPILSSALHDPQYFDHPDTFNPEH

FLDADGTLKKSEAFMPFSTGKRICLGEGIARNELFLFFTTILQNFSVSSHLAPKDIDLTP

KESGIAKIPPTYQICFSAR

>AEO16917.1

MEPSVLLLLALLVDFVLLLVRGYPKARGHLPPGPRPLPLLGNLLQMDRGGFLNSFMRIRE

KYGDVFTVHLGPRPVVMLYGTEAIREALVDQAEAFSGRGTIAVIKPVIGDYGMIFSNGER

WKVLRRFSLATMRDFGMGKRSVEDRIQEEAQCLVEELQKSQGAPLDPTFLFQCITANIIC

SIVFGERYDYKDRQFLRLLDLFYRTFSLMSSFSSQVFELFSGFMKYFPGAHRQITRNLQE

ILDYIGQSVEKHRATLDPSHPRDFIDTYLLRMEKEKSNQHTEFDHQNLVISVLSLFFAGT

ETSSTTLRYGFLLMLKYPHVAEKVQKEIDQVIGSHRLPTLEDRTKMPYTEAVIYEIQRFS

DLIPIGVPHKVTKDTLFRGYLPPKNTEVYPILSSALHDPQYFEQPDAFNPDHFLDANGAL

KKSEAFMPFSLGKRICLGEGIARNELFLFFTTILQNFSLSSPVAPKDIDLSPKESGFGKV

PQTYQICFLAR

>AEO16894.1

MEPSVLLLLALLVGFVLLLVRGYPKARGRLPPGPRPLPLLGNLLQMDRGGFLNSFMRIRE

KYGDVFTVHLGPRPVVMLYGTEAIREALVDQAEAFSGRGTIAVIKPVIGDYGMIFSNGER

WKVLRRFSLATMRDFGMGKRSVEDRIQEEAQCLVEELQKSQGAPLDPTFLFQCITANIIC

SIVFGERYDYKDRQFLRLLDLFYRTFSLMSSFSSQVFELFSGFMKYFPGAHRQITRNLQE

ILDYVGQSVEKHRATLDPSNPRDFIDTYLLRMEKEKSNQHTEFHHQNLLISVLSLFFAGT

ETTSTTLRYGFLLMLKYPHVAEKVQKEIDQVIGSHRLPTLEDRTKMPYTDAVIHEIQRFS

DLAPIGAPHKVTKDTLFRGYLLPKNTEVYPILSSALHDPQYFEQPGTFNPDHFLDANGAL

KKSEAFMPFSIGKRICLGEGIARNELFLFFTTILQNFSVSSSVAPKDIDLSPKESGIGKV

PQTYQISFLAR

>AEO16876.1

MEPSVLLLLALLVGFVLLLVRGYPKARGHLPPGPRPLPLLGNLLQMDRGGFLNSFMRIRE

KYGDVFTVHLGPRPVVMLYGTEAIREALVDQAEAFSGRGTIAVIKPVIGDYGMIFSNGER

WKVLRRFSLATMRDFGMGKRSVEDRIQEEAQCLVEELQKSQGAPLDPTFLFQCITANIIC

SIVFGERYDYKDRQFLRLLDLFYRTFSLMSSFSSQVFELFSGFMKYFPGAHRQITRNLQE

ILDYVGQSVEKHRATLDPSNPRDFIGTYLLRMEKEKSNQHTEFHHQNLLISVLSLFFAGT

ETTSTTLRYGFLLMLKYPHVAEKVQKEIDQVIGSHRLPTLEDRTKMPYTDAVIHEIQRFS

DLVPIGVPHKVTKDTLFRGYLLPKNAEAYPILSSALHDPQYFGQPDTFNPDHFLDANGAL

KKSEAFMPFSLGKRICLGEGIARNELFLFFTAILQNFSLSSPMAAKDIDLTPKESGFGKV

PQTYQICFLAR

>AEO16886.1

MGPSVLLLLALLVGFVLLLVRGYPKARGHLPPGPRPLPLLGNLLQMDRGGFLNSFMRIRE

KYGDVFTVHLGPRPVVMLYGTEAIREALVDQAEAFSGRGTIAVIKPVIGDYGMIFSNGER

WKVLRRFSLATMRDFGMGKRGVEDRIQEEAQCLVEELQKSQGAPLDPTFLFQCITANIIC

SIVFGERYDYKDRQFLRLLDLFYRTFSLMSSFSSQVFELFSGFMKYLPGAHRQITRNLQE

ILDYVGQSVEKHRATLDPSNPRDFIDTYLLRMEKEKSNQHTEFHHQNLLISVLSLFFAGT

ETTSTTLRYGFLLMLKYPHVAEKVQKEIDQVIGSHRLPTLEDRTKMPYTDAVIHEIQRFS

DLAPIGAPHKVTKDTLFRGYLLPKNTEVYPILSSALHDPQYFEQPGTFNPDHFLDANGAL

KKSEAFMPFSIGKRICLGEGIARNELFLFFTTILQNFSVSSSVAPKDIDLSPKESGIGKV

PQTYQISFLAR

>AEO16880.1

MEPSVLLLLALLVGFVLLLVGGYPKARGHLPPGPRPLPLLGNLLQMDRGGFLNSFMRIRE

KYGDVFTVHLGPRPVVMLYGTEAIREALVDQAEAFSGRGTIAVIKPVIGDYDMIFSNGER

WKVLRRFSLATMRDFGMGKRSVEDRIQEEAQCLVEELQKSQGAPLDPTFLFQCITANIIC

SIVFGERYDYKDRQFLRLLDLFYRTFSLMSSFSSQVFELFSGFMKYFPGAHRQITRNLQE

ILDYVGQSVGKHRATLDPSNPRDFIDTYLLRMEKEKSNQHTEFHHQNLLISVLSLFFAGT

ETTSTTLRYGFLLMLKYPHVAEKVQKEIDQVIGSHRLPTLEDRTKMPYTDAVIHEIQRFS

DLAPIGAPHKVTKDTLFRGYLLPKNTEVYPILSSALHDPQYFEQPGTFNPDHFLDANGAL

KKSEAFMPFSIGKRICLGEGIARNELFLFFTTILQNFSVSSSVAPKDIDLSPKESGIGKV

PQTYQISFLAR

>AEO16954.1

MEPSVLLPLALLVGFVLLLVRGYPKARGHLPPGPRPLPLLGNLLRMDRGGFLNSFKRIRE

KYGDVFTVHLGPRPVVMLYGTEAIREALVDQAEAFSGRGTIAVVRPVFRDYGVIFSNGER

WKALRRFSLATMRDFGMGKRSVEERIQEEAQCLVEEFRKSQGAPLDPTFLFQCITANIIC

SIVFGERFDYKDRQFLRLLDMFYQSFSLISSFSSQVFELFSGFLKYFPGAHRQISRNMQE

VLKYIGQSVEKHRATLDPNNPRDFIDTYLLRMEKEKSNQHTEFHHENLLISVLSLFFAGT

ETSSTTLRYGFLLMLKYPHVAEKVQKEIDQVIGSHRLPTLEDRTKMPYTDAVIYEIQRFS

DLIPIGVPHKVTKDTLFRGYLLPKNTEVYPILSSALNDPQYFEQPDTFNPDHFLDANGAL

KKSEAFMPFSIGKRICLGEGIARNELFLFFTTILQNFSLSSPVAPKDIDLCPKESGFGKL

PPTYQICFLAR

>AEO16881.1

MEPSVLLLLALLVGFVLLLVGGYPKARGHLPPGPRPLPLLGNLLQMDRGGFLNSFMRIRE

KYGDVFTVYLGPRPVVMLYGTEAIREALVDQAEAFSGRGTIAVIKPVIGDYGMIFSNGER

WKVLRRFSLATMRDFGMGKRSVEDRIQEEAQCLVEELQKSQGAPLDPTFLFQCITANIIC

SIVFGERYDYKDRQFLRLLGLFYRTFSLMSSFSSQVFELFSGFMKYFPGAHRQITRNLQE

VLDYVGQSVEKHRATLDPSNPRDFIDTYLLRMEKEKSNQHTEFHHQNLLISVLSLFFAGT

ETTSTTLRYDFLLMLKYPHVAEKVQKEIDQVIGSHRLPTLEDRTKMPYTDAVIHEIQRFS

DLAPIGAPHKVTKDTLFRGYLLPKNTEVYPILSSALHDPQYFEQPGTFNPDHFLDANGAL

KKSEAFMPFSIGKRICLGEGIARNELFLFFTTILQNFSVSSSVAPKDIDLSPKESGIGKV

PQTYQISFLAR

>AEO16916.1

MEPSVLLLLALLVGFVLLLVRGYPKARGHLPPGPRPLPLLGNLLQMDRGGFLNSFKRIRE

KYGDVFTVHLGPRPVVMLYGTEAIREALVDQAEAFSGRGTIAVVRPVFRDYGVIFSNGER

WKALRRFSLATMRDFGMGKRSVEERIQEEAQCLVEEFRKSQGAPLDPTFLFQCITANIIC

SIVFGERFDYKDRQFLRLLDMFYQSFSLISSFSSQVFELFSGFLKYFPGAHRQITRNLQE

ILDYVGQSVEKHRATLDPSNPRDFIDTYLLRMEKEKSNQHTEFHHQNLLISVLSLFFAGT

ETTSTTLRYGFLLMLKYPHVAEKVQKEIDQVIGSHRLPTLEDRTKMPYTDAVIHEIQRFS

DLAPIGAPHKVTKDTLFRGYLLPKNTEVYPILSSALHDPQYFEQPGTFNPDHFLDANGAL

KKSEAFMPFSIGKRICLGEGIARNELFLFFTTILQNFSVSSSVAPKDIDLSPKESGIGKV

PQTYQISFLAR

>AEO16952.1

MEPSVLLLLALLVGFVLLLVRGYPKARGHLPPGPRPLPLLGNLLQMDRGGFLNSFKRIRE

KYGDVFTVHLGPRPVVMLYGTEAIREALVDQAEAFSGRGTIAVVRPVFRDYGVIFSNGER

WKALRRFSLATMRDFGMGKRSVEERIQEEAQCLVEEFRKSQGAPLDPTFLFQCITANIIC

SIVFGERFDYKDRQFLRLLDMFYQSFSLISSFSSQVFELFSGFLKYFPGAHRQISRNMQE

VLKYIGQSVEKHRATLDPNNPRDFIDTYLLRMEKEKSNQHTEFHHENLLISVLSLFFAGT

ETSSTTLRYGFLLMLKYPHVAEKVQKEIDQVIGSHRLPTLEDRTKMPYTDAVIYEIQRFS

DLIPIGVPHKVTKDTLFRGYLLPKNTEVYPILSSALNDPQYFEQPDTFNPDHFLDANGAL

KKSEAFMPFSIGKRICLGEGIARNELFLFFTTILQNFSLSSPVAPKDIDLCPKESGFGKL

PPTYQICFLAR

>AEO16955.1

MEPSVLLPLALLVGFVLLLVRGYPKARGHLPPGPRPLPLLGNLLRMDRGGFLNSFKRIRE

KYGDVFTVHLGPRPVVMLYGTEAIREALVDQAEAFSGRGTIAVVRPVFRDYCVIFSNGER

WKALRRFSLATMRDFGMGKRSVEERIQEEAQCLVEEFRKSQGAPLDPTFLFQCITANIIC

SIVFGERFDYKDRQFLRLLDMFYQSFSLISSFSSQVFELFSGFLKYFPGAHRQISRNMQE

VLKYIGQSVEKHRATLDPNNPRDFIDTYLLRMEKEKSNQHTEFHHENLLISVLSLFFAGT

ETSSTTLRYGFLLMLKYPHVAEKVQKEIDQVIGSHRLPTLEDRTKMPYTDAVIYEIQRFS

DLIPIGVPHKVTKDTLFRGYLLPKNTEVYPILSSALNDPQYFEQPDTFNPDHFLDANGAL

KKSEAFMPFSIGKRICLGEGIARNELFLFFTTILQNFSLSSPVAPKDIDLCPKESGFGKL

PPTYQICFLAR

>AEO16959.1

MEPSVLLLLALLVGFVLLLVRGYPKARGHLPPGPRPLPLLGNLLQMDRGGFLNSFKRIRE

KYGDVFTVHLGPRPVVMLYGTEAIREALVDQAEAFSGRGTIAVVRPVFRDYGAIFSNGER

WKALRRFSLATMRDFGMGKRSVEERIQEEAQCLVEEFRKSQGAPLDPTFLFQCITANIIC

SIVFGERFDYKDRQFLRLLDMFYQSFSLISSFSSQVFELFSGFLKYFPGAHRQISRNMQE

VLKYIGQSVEKHRATLDPNNPRDFIDTYLLRMEKEKSNQHTEFHHENLLISVLSLFFAGT

ETSSTTLRYGFLLMLKYPHVAEKVQKEIDQVIGSHRLPTLEDRTKMPYTDAVIYEIQRFS

DLIPIGVPHKVTKDTLFRGYLLPKNTEVYPILSSALNDPQYFEQPDTFNPDHFLDANGAL

KKSEAFMPFSIGKRVCLGEGIARNELFLFFTTILQNFSLSSPVAPKDIDLCPKESGFGKL

PPTYQICFLAR

>AEO16951.1

MEPSVLLLLALLVGFVLLLVRGYPKARGHLPPGPRPLPLLGNLLQMDRGGFLNSFRRIRE

KYGDVFTVHLGPRPVVMLYGTEAIREALVDQAEAFSGRGTIAVVEPIFRGYGVVFANGER

WRALRRFSLVTMRDFGMGKRSVEERIQEEARCLVEEFRKSQGAPLDPTFLFQCITANIIC

SIVFGERFDYKDRQFLRLLYLFYQSFSLISSFSSQVFELFSGFLKYFPGTHRQISRNLQE

ILDYIGQSVEKHRATLDPSNPRDFIDTYLLRMEKEKSNQHTEFDHQNLVISVLSLFFAGT

ETSSTTLRYGFLLMLKYPHVAEKVQKEIDQVIGSHRLPTLEDRTKMPYTDAVIYEIQRFS

DLIPIGVPHKVTKDTFFRGYLLPKNTEVYPILSSALHDPQYFEQPDTFNPDHFLDANGAL

KKNEAFMPFSLGKRICLGEGIARNELFLFFTTILQNFSLSSPMAPKDIDLSPKESGFGKV

PPTYQICFLAR

>AEO16948.1

MEPSVLLLLALLVGFVLLLVRGYPKARGHLPPGPRPLPLLGNLLQMDRGGFLNSFRRIRE

KYGDVFTVHLGPRPVVMLYGTEAIREALVDQAEAFSGRGTIAVVEPIFRGYGVVFANGER

WKALRRFSLVTMRDFGMGKRSVEERIQEEARCLVEEFRKSQGAPLDPTFLFQCITANIIC

SIVFGERFDYKDHQFLRLLYLFYQSFSLISSFSSQVFELFSGFLKYFPGTHRQISRNLQE

ILDYIGQSVEKHRATLDPSNPRDFIDTYLLRMEKEKSNQHTEFDHQNLVISVLSLFFAGT

ETSSTTLRYGFLLMLKYPHVAEKVQKEIDQVIGSHRLPTLDDRTKMPYTDAVIYEIQRFS

DLVPIGVPHKVTKDTLFRGYLLPKNTEVYPTLSSALHDPQYFEQPDTFNPDHFLDANGAL

KKSEAFMPFSLGKRICLGEGIARNELFLFFTAILQNFSLSSPVAPKDIDLSPKESGFGKV

PQTYQICFLAR

>EDM07988.1

MEPSILLLLALLVGFLLLLVRGHPKSRGNFPPGPRPLPLLGNLLQLDRGGLLNSFMQFRE

KYGDVFTVHLGPRPVVMLCGTDTIKEALVGQAEDFSGRGTIAVIEPIFKEYGKTLKGWDD

VGWRMGTERVDSERGENKGGGRTQVSLNFLWDWTIHASPTLSGVFFANGERWKALRRFSL

ATMRDFGMGKRSVEERIQEEAQCLVEELRKSQGAPLDPTFLFQCITANIICSIVFGERFD

YTDRQFLRLLELFYRTFSLLSSFSSQVFEFFSGFLKYFPGAHRQISKNLQEILDYIGHIV

EKHRATLDPSAPRDFIDTYLLRMEKEKSNHHTEFHHENLMISLLSLFFAGTETGSTTLRY

GFLLMLKYPHVTEKVQKEIDQVIGSHRPPSLDDRTKMPYTDAVIHEIQRFADLAPIGLPH

RVTKDTMFRGYLLPKNTEVYPILSSALHDPQYFDHPDTFNPEHFLDADGTLKKSEAFMPF

STGKRICLGEGIARNELFLFFTTILQNFSVSSHLAPKDIDLTPKESGIAKIPPTYQICFS

AR

>AEO16936.1

MEPSVLLLLALLVGFVLLLVRGYPKARGHLPPGPRPLPLLGNLLQMDRGGFLNSFRRIRE

KYGDVFTVHLGPRPVVMLYGTEAIREALVDQAEAFSGRGTIAVVEPIFRGYGVVFANGER

WKALRRFSLVTMRDFGMGKRSVEERIQEEARCLVEEFRKSQGAPLDPTFLFQCITANIIC

SIVFGERFDYKDHQFLRLLYLFYQSFSLISSFSSQVFELFSGFLKYFPGTHRQISRNLQE

ILDYIGQSVEKHRATLDPSHPRDFIDTYLLRMEKEKSNQHTEFDHQNLVISVLSLFFAGT

ETSSTTLRYGFLLMLKYPHVAEKVQKEIDQVIGSHRLPTLEDRTKMPYTEAVIYEIQRFS

DLIPIGVPHKVTKDTLFRGYLLPKNTEVYPILSSALHDPQYFEQPDTFNPDHFLDANGAL

KKSEAFMPFSIGKRICLGEGIARNELFLFFTTILQNFSLSSPVAPKDIDLSPKESGFGKV

PQTYQICFLAR

>AEO16949.1

MEPSVLLLLALLVGFVLLLVRGYPKARGHLPPGPRPLPLLGNLLQMDRGGFLNSFRRIRE

KYGDVFTVHLGPRPVVMLYGTEAIREALVDQAEAFSGRGTIAVVEPIFRGYGVVFANGER

WKALRRFSLVTMRDFGMGKRSVEERIQEEARCLVEEFRKSQGAPLDPTFLFQCITANIIC

SIVFGERFDYKDHQFLRLLYLFYQSFSLISSFSSQVFELFSGFLKYFPGTHRQISRNLQE

ILDYIGQSVEKHRATLDPSNPRDFIDTYLLRMEKEKSNQHTEFDHQNLVISVLSLFFAGT

ETSSTTLRYGFLLMLKYPHVAEKVQKEIDQVIGSHRLPTLEDRTKMPYTDAVIYEIQRFS

DLIPIGVPHKVTKDTLFRGYLLPKNTEVYPILSSALNDPQYFEQPDTFNPDHFLDANGAL

KKSEAFMPFSIGKRICLGEGIARNELFLFFTTILQNFSLSSPVAPKDIDLCPKESGFGKL

PPTYQICFLAR

>AEO16944.1

MEPSVLLLLALLVGFVLLLVRGYPKARGHLPPGPRPLPLLGNLLQMDRGGFLNSFRRIRE

KYGDVFTVHLGPRPVVMLYGTEAIREALVDQAEAFSGRGTIAVVEPIFRGYGVVFANGER

WKALRRFSLVTMRDFGMGKRSVEERIQEEARCLVEEFRKSQGAPLDPTFLFQCITANIIC

SIVFGERFDYKDHQFLRLLYLFYQSFSLISSFSSQVFELFSGFLKYFPGTHRQISRNLQE

ILDYIGQSVEKHRATLDPSHPRDFIDTYLLRMEKEKSNQHTEFDHQNLVISVLSLFFAGT

ETSSTTLRYGFLLMLKYPHVAEKVQKEIDQVIGSHRLPTLEDRTKMPYTEAVIYEIQRFS

DLIPIGVPHKVTKDTLFRGYLLPKNTEVYPILSSALHDPQYFEQPDTFNPDHFLDANGAL

KKSEAFMPFSLGKRICLGEGIARNELFLFFTTILQNFSLSSPVAPKDIDLSPKESGFGKV

PQTYQICFLAR

>354486247

MEPSVLLLLTLLVGFLLLLVKGHTNAHGHLPPGPRPLPLLGNLLQMDRRGLLNSFMRFRE

KYGDVFTVHLGPRPVVMLYGTQAIREALVDQAEAFSGRGTVAVVEPVVQGYGVIFANGER

WKALRRFSLATMRDFGMGKQNVEEWIKEEARCLVEELRKSQGAPLDPTFLFQCITANIIC

SIVFGERFDYKDRQFLRLLDLFYRTFSLISSFSSQVFELFSGFLKYFPGAHKQIFINVQE

VLEYIGHSVEKHRATLDPSNPRDFIDTYLLRMEKEKSNPHTEFHHQNLMITVLSLFFAGT

ETSSTTLRYGFLLLLKYPHVAEKVQKEIDQVIGSHRLPTLDDRTKMPYTEAVIHEIQRFA

DLIPIGVPHKVTKDTLIRGYLLPKNTEVYPILSSALHDPQYFEQPDTFNPDRFLDANGAL

KKNEAFMPFSVGKRICLGEGIARNELFLFFTTILQNFSLSSPVDPKDIDLSPKENGFGRV

PPTYKICFLAR

>AEO16941.1

MEPSVLLLLALLVGFVLLLVRGYPKARGHLPPGPRPLPLLGNLLQMDRGGFLNSFRRIRE

KYGDVFTVHLGPRPVVMLYGTEAIREALVDQAEAFSGRGTIAVVEPIFRGYGVVFANGER

WKALRRFSLVTMRDFGMGKRSVEERIQEEARCLVEEFRKSQGAPLDPTFLFQCITANIIC

SIVFGERFDYKDHQFLRLLYLFYQSFSLISSFSSQVFELFSGFLRYFPGTHRQISRNLQE

ILDYIGQSVEKHRATLDPSHPRDFIDTYLLRMEKEKSNQHTEFDHQNLVISVLSLFFAGT

ETSSTTLRYGFLLMLKYPHVAEKVQKEIDQVIGSHRLPTLEDRTKMPYTEAVIYEIQRFS

DLIPIGVPHKVTKDTLFRGYLLPKNTEVYPILSSALHDPQYFEQPDTFNPDHFLDANGAL

KKSEAFMPFSIGKRICLGEGIARNELFLFFTTILQNFSLSSPVAPKDIDLSPKESGFGKV

PQTYQICFLAR

>AEO16937.1

MEPSVLLLLALLVGFVLLLVRGYPKARGHLPPGPRPLPLLGNLLQMDRGGFLNSFRRIRE

KYGDVFTVHLGPRPVVMLYGTEAIREALADQAEAFSGRGTIAVVEPIFRGYGVVFANGER

WKALRRFSLVTMRDFGMGKRSVEERIQEEARCLVEEFRKSQGAPLDPTFLFQCITANIIC

SIVFGERFDYKDHQFLRLLYLFYQSFSLISSFSSQVFELFSGFLKYFPGTHRQISRNLQE

ILDYIGQSVEKHRATLDPSHPRDFIDTYLLRMEKEKSNQHTEFDHQNLVISVLSLFFAGT

ETSSTTLRYGFLLMLKYPHVAEKVQKEIDQVIGSHRLPTLEDRTKMPYTEAVIYEIQRFS

DLIPIGVPHKVTKDTLFRGYLLPKNTEVYPILSSALHDPQYFEQPDTFNPDHFLDANGAL

KKSEAFMPFSIGKRICLGEGIARNELFLFFTTILQNFSLSSPVAPKDIDLSPKESGFGKV

PQTYQICFLAR

>AAI45896.1

MDPSVLLLLAVLLSLFLLLVRGHAKIHGHLPPGPRPLPLLGNLLQMDRGGLLKCFIHLQE

KHGDVFTVHLGPRPVVVLCGTQTIREALVNNAEAFSGRGTIAAAQLVMQDYGIFFSSGER

WKTLRRFSLATMKEFGMGKRSVEERIKEEAQCLVEELKKYQGAPLDPTFFFQCVTANIIC

SIVFGERFDYTDEQFLRLLNLMYQIYSLLRSFSCQMFELFSGLLKYFPGVHRQIAKNQQE

ILNFITHRVEKHRATLDPSEPRDFIDTYLLRMEKEKSNHNTEFHHQNLMMSVLSLFFAGT

ETTSTTLCCGFLLMLMYPHVAEKVQKEIDQVIGSHRLPTLDDRTKMPYTDAVIHEIQRFS

DIVPTGAPHRVTKDTMFRGYLLPKNTEVYPILSSALHDPQYFEQPDSFNPDHFLDANGAL

KKSEAFLPFSTGKRICLGESIARNELFLFFTSILQNFSVASPVASKDIDLTPKESGIGKI

PPTYQICFLAR

>AEO16945.1

MGPSVLLLLALLVGFVLLLVRGYPKARGHLPPGPRPLPLLGNLLQMDRGGFLNSFRRIRE

KYGDVFTVHLGPRPVVMLYGTEAIREALVDQAEAFSGRGTIAVVEPIFRGYGVVFANGER

WKALRRFSLVTMRDFGMGKRSVEERIQEEARCLVEEFRKSQGAPLDPTFLFQCITANIIC

SIVFGERFDYKDHQFLRLLYLFYQSFSLISSFSSQVFELFSGFLKYFPGTHRQISRNLQE

ILDYIGQSVEKHRATLDPSHPRDFIDTYLLRMEKEKSNQHTEFDHQNLVISVLSLFFAGT

ETSSTTLRYGFLLMLKYPHVAEKVQKEIDQVIGSHRLPTLEDRTKMPYTEAVIYEIQRFS

DLIPIGVPHKVTKDTLFRGYLLPKNTEVYPILSSALHDPQYFEQPDTFNPDHFLDANGAL

KKSEAFMPFSLGKRICLGEGIARNELFLFFTTILQNFSLSSPVAPKDIDLSPKESGFGKV

PQTYQICFLAR

>AEO16950.1

MGPSVLLLLALLVGFVLLLVRGYPKARGHLPPGPRPLPLLGNLLQMDRGGFLNSFRRIRE

KYGDVFTVHLGPRPVVMLYGTEAIREALVDQAEAFSGRGTIAVVEPIFRGYGVVFANGER

WKALRRFSLVTMRDFGMGKRSVEERIQEEARCLVEEFRKSQGAPLDPTFLFQCITANIIC

SIVFGERFDYKDRQFLRLLYLFYQSFSLISSFSSQVFELFSGFLKYFPGTHRQISRNLQE

ILDYIGQSVEKHRATLDPSNPRDFIDTYLLRMEKEKSNQHTEFDHQNLVISVLSLFFAGT

ETSSTTLRYGFLLMLKYPHVAEKVQKEIDQVIGSHRLPTLEDRTKMPYTDAVIYEIQRFS

DLIPIGVPHKVTKDTFFRGYLLPKNTEVYPILSSALHDPQYFEQPDTFNPDHFLDANGAL

KKNEAFMPFSLGKRICLGEGIARNELFLFFTTILQNFSLSSPMAPKDIDLSPKESGFGKV

PPTCQICFLAR

>AEO16927.1

MEPSVLLLLALLVGFVLLLVRGYPKARGHLPPGPRPLPLLGNLLQMDRGGFLNSFKRIRE

KYGAVFTVHLGTRPVVMLYGTEAIREALVDQAEAFSGRGTIAVVEPIFRDYGVIFSNGER

WKVLRRFSLATMRDFGMGKRSVEERIQEEARCLVEEFRKSQGAPLDPTFLFQCITANIIC

SIVFGERFDYKDRQFLRLLYLFYQSFSLISSFSSQVFELFSGFLKYFPGTHRQISRNLQE

ILDYIGQSVEKHRATLDPSNPRDFIDTYLLRMEKEKSNQHMEFDHQNLLISVLSLFFAGT

ETSSTTLRYGFLLMLKYPHVAEKVQKEIDQVIGSHRLPTLEDRTKMPYTDAVIYEIQRFS

DLVPIGVPHKVTKDTLFRGYLLPKNTEVYPILSSALHDPQYFEQPDTFNPDHFLDANGAL

KKSEAFMPFSLGKRICLGEGIARNELFLFFTAILQNFSLSSPMAAKDIDLSPKESGFGKV

PQTYQICFLAR

>AEO16933.1

MEPSVLLLLALLVGFVLLLVRGYPKARGHLPPGPRPLPLLGNLLQMDRGGFLNSFKRIRE

KYGAVFTVHLGTRPVVMLHGTEAIREALVDQAEAFSGRGTIAVVEPIFRDYGVIFSNGER

WKVLRRFSLATMRDFGMGKRSVEERIQEEARCLVEEFRKSQGAPLDPTFLFQCITANIIC

SIVFGERFDYKDRQFLRLLYLFYQSFSLISSFSSQVFELFSGFLKYFPGTHRQISRNLQE

ILDYIGQSVEKHRATLDPSNPRDFIDTYLLRMEKEKSNQHMEFDHQNLLISVLSLFFAGT

ETSSTTLRYGFLLMLKYPHVAEKVQKEIDQVIGSHRLPTLEDRTKMPYTDAVIYEIQRFS

DLVPIGVPHKVTKDTLFRGYLLPKNTEVYPILSSALHDPQYFEQPDTFNPDHFLDANGAL

KKSEAFMPFSLGKRICLGEGIARNELFLFFTAILQNFSLSSPMAAKDIDLSPKESGFGKV

PQTYQICFLAR

>AEO16930.1

MEPSVLLLLALLVGFVLLLVRGYPKARGHLPPGPRPLPLLGNLLQMDRGGFLNSFKRIRE

KYGAVFTVHLGTRPVVMLYGTEAIREALVDQAEAFSGRGTIAVVEPIFRDYGVIFSNGER

WKVLRRFSLATMRDFGMGKRSVEERIQEEARCLVEEFRKSQGAPLDPTFLFQCITANIIC

SIVFGERFDYKDRQFLRLLYLFYQSFSLISSFSSQVFELFSGFLKYFPGTHRQISRNLQE

ILDYIGQSVEKHRATLDPSNPRDFIDTYLLRMEKEKSNQHMEFDHQNLLISVLSLFFAGT

ETSSTTLRYGFLLMLKYPHVAEKVQKEIDQVIGSHRLPTLEDRTKMPYTDAVIYEIQRFS

DLVPIGVPHKVTKDTLFRGYLLPKNTEVYPILSSALHDPQYFEQPDTFNPDHFLDANGAL

KKSEAFMPFSLGKRICLGEGIARNELFLFFTAILQNYSLSSPMAAKDIDLSPKESGFGKV

PQTYQICFLAR

>AAF80366.1

MDPSVLLLFALLTGFLLLLIRGQGNGYGHLPPGPCPLPLLGNVLQMDRRGLLKSFIQLRD

KYGDVVTVHLGPRPIVMLYGTETIREALVDHAEAFSGRGTVAVVQPIIQDYGMIFANGER

WKILRRFSLATMRDFGMGKRSVEERIKEEAQCLVEELKKYKGAPLDPTFHLQCITANIIC

SIVFGERFDYTDHQFLHLLDLFYEILSLVSSFSSQVFELFTGFLKYFPGTHRHISKNIEE

ILNFIGHCVEKHRATLDPSTPRDFIDTYLLRMEKEKLNHHTEFHHQNLMMSVLSLFFAGT

ETSSTTLRYGFLLMLKYPHVAEKVQKEIDQVIGSHRVPTLDDRIKMPYTDAVIHEIQRFS

DLVPIGLPHRVTKDTLFRGYLLPKNIEVYPILSSALHDPQYFEHPDTFNPEHFLDANGAL

KKNEAFLPFSTGKRVCLGEGIARNELFLFFTTILQNFSVSSPVSPKDIDLTPKESGFAKI

PPTYQICFLSRQLG

>AEO16920.1

MEPSVLLLLALLVGFVLLLVRGYPKARGHLPPGPRPLPLLGNLLQMDRGGFLNSFKRIRE

KYGAVFTVHLGTRPVVMLYGTEAIREALVDQAEAFSGRGTIAVVEPIFRDYGVIFSNGER

WKVLRRFSLATMRDFGMGKRSVEERIQEEARCLVEEFRKSQGAPLDPTFLFQCITANIIC

SIVFGERFDYKDRQFLRLLYLFYQSFSLISSFSSQVFELFSGFLKYFPGTHRQISRNLQE

ILDYIGQSVEKHRATLDPSNPRDFIDTYLLRMEKEKSNQHMEFDHQNLLISVLSLFFAGT

ETSSTTLRYGFLLMLKYPHVAEKVQKEIDQVIGSHRLPTLEDRTKMPYTDAVIYEIQRFS

DLVPIGVPHKVTKDTLFRGYLLPKNTEVYPILSSALHDSQYFEQPDTFNPDHFLDANGAL

KKSEAFMPFSLGKRICLGEGIARNELFLFFTAILQNFSLSSPMAAKDIDLSPKESGFGKV

PQTYQICFLAR

>AEO16924.1

MEPSVLLLLALLVGFVLLLVRGYPKARGHLPPGPRPLPLLGNLLQMDRGGFLNSFKRIRE

KYGAVFTVHLGTRPVVMLYGTEAIREALVDQAEAFSGRGTIAVVEPIFRDYGVIFSNGER

WKVLRRFSLATMRDFGMGKRSVEERIQEEARCLVEEFRKSQGAPLDPTFLFQCITANIIC

SIVFGERFDYKDRQFLRLLYLFYQSFSLISSFSSQVFELFSGFLKYFPGTHRQISRNLQE

ILDYIGQSVEKHQATLDPSNPRDFIDTYLLRMEKEKSNQHMEFDHQNLLISVLSLFFAGT

ETSSTTLRYGFLLMLKYPHVAEKVQKEIDQVIGSHRLPTLEDRTKMPYTDAVIYEIQRFS

DLVPIGVPHKVTKDTLFRGYLLPKNTEAYPILSSALHDPQYFEQPDTFNPDHFLDANGAL

KKSEAFMPFSLGKRICLGEGIARNELFLFFTAILQNFSLSSPMAAKDIDLTPKESGFGKV

PQTYQICFLAR

>124487255

MEPSVLLLLALLTGFLLLLIRGQPKAYSRLPPGPRPLPFLGNLLQMDRGGLLKSFIKLRD

KHGDVFTVHLGPRPVVMLYGTETIKEALVDHSDAFSGRGAIAVIQPIVQDYGVIFSSGER

WKTLRRFSLATMRDFGMGKRSVEERIKEEAQCLVEELKKYEGAPLDPTFLFQCITANIIC

SIVFGERFDYTDHQFLHLLDLFYQTLSLISSFSSQLFELFSAVLKYFPGTHRQISKNIQE

ILNYIGHSVEQHKATLDPSAPRDFIDTYLLRMEKEKSNHHTEFHHQNLLISVLSLFFAGT

ETTSTTLRYGFLLMLKYPHVAEKVQKEIDQVISAHHVPTLEDRIKMPYTEAVIHEIQRFS

DLAPIGLPHTVTKDTVFRGYLLPKNTEVYPILSSALHDPQYFEQPDKFNPEHFLDANGAL

KKSEAFLPFSTGKRICLGEGIARNELFLFFTALLQNFSLSSPVAPEDIDLTPKESGFVKI

PPVYRICFLPR

>AEO16925.1

MGPSVLLLLALLVGFVLLLVRGYPKARGHLPPGPRPLPLLGNLLQMDRGGFLNSFKRIRE

KYGAVFTVHLGTRPVVMLYGTEAIREALVDQAEAFSGRGTIAVVEPIFRDYGVIFSNGER

WKVLRRFSLATMRDFGMGKRSVEERIQEEARCLVEEFRKSQGAPLDPTFLFQCITANIIC

SIVFGERFDYKDRQFLRLLYLFYQSFSLISSFSSQVFELFSGFLKYFPGTHRQISRNLQE

ILDYIGQSVEKHQATLDPSNPRDFIDTYLLRMEKEKSNQHMEFDHQNLLISVLSLFFAGT

ETSSTTLRYGFLLMLKYPHVAEKVQKEIDQVIGSHRLPTLEDRTKMPYTDAVIYEIQRFS

DLVPIGVPHKVTKDTLFRGYLLPKNTEVYPILSSALHDPQYFEQPDTFNPDHFLDANGAL

KKSEAFMPFSLGKRICLGEGIARNELFLFFTAILQNFSLSSPMAAKDIDLTPKESGFGKV

PQTYQICFLAR

>AAA03647.1

MDPSVLLLLAVLLSLFLLLVRGHAKIHGHLPPGPRPLPLLGNLLQMDRGGLLKCFIHLQE

KHGDVFTVHLGPRPVVVLCGTQTIREALVNNAEAFSGRGTIAAAQLVMQDYGIFFSSGER

WKTLRRFSLATMKEFGMGKRSVEERIKEEAQCLVEELKKYQGAPLDPTFFFQCVTANIIC

SIVFGERFDYTDEQFLRLLNLMYQIYSLLRSFSCQMFELFSGLLKYFPGVHRQIAKNQQE

ILNFITHRVEKHRASLDPSEPRDFIDTYLLRMEKEKSNHNTEFHHQNLMMSVLSLFFAGT

ETTSTTLCCGFLLMLMYPHVAGKVQKEIDQVIGSHRLPTLDDRTKMPYTDAVIHEIQRFS

DIVPTGAPHRVTKDTMFRGYLLPKNTEVYPILSSALHDPQYFEQPDSFNPDHFLDANGAL

KKSEAFLPFSTGKRICLGESIARNELFLFFTSILQNFSVASPVASKDIDLTPKESGIGKI

PPTYQICFLAR

>AEO16926.1

MEPSVLLLLALLVGFVLLLVRGYPKARGHLPPGPRPLPLLGNLLQMDRGGFLNPFKRIRE

KYGAVFTVHLGTRPVVMLYGTEAIREALVDQAEAFSGRGTIAVVEPIFRDYGVIFSNGER

WKVLRRFSLATMRDFGMGKRSVEERIQEEARCLVEEFRKSQGAPLDPTFLFQCITANIIC

SIVFGERFDYKDRQFLRLLYLFYQSFSLISSFSSQVFELFSGFLKYFPGTHRQISRNLQE

ILDYIGQSVEKHRATLDPSNPRDFIDTYLLRMEKEKSNQHMEFDHQNLLISVLSLFFAGT

ETSSTTLRYGFLLMLKYPHVAEKVQKEIDQVIGSHRLPTLEDRTKMPYTDAVIYEIQRFS

DLVPIGVPHKVTKDTLFRGYLLPKNTEVYPILSSALHDPQYFEQPDTFNPDHFLDANGAL

KKSEAFMPFSLGKRICLGEGIARNELFLFFTAILQNFSLSSPMAAKDIDLSPKESGFGKV

PQTYQICFLAR

>AEO16931.1

MEPSVLLLLALLVGFVLLLVRGYPKARGHLPPGPRPLPLLGNLLQMDRGGFLNSFKRIRE

KYGAVFTVHLGTRPVVMLYGTEAIREALVDQAEAFSGRGTIADVEPIFRDYGVIFSNGER

WKVLRRFSLATMRDFGMGKRSVEERIQEEARCLVEEFRKSQGAPLDPTFLFQCITANIIC

SIVFGERFDYKDRQFLRLLYLFYQSFSLISSFSSQVFELFSGFLKYFPGTHRQISRNLQE

ILDYIGQSVEKHRATLDPSNPRDFIDTYLLRMEKEKSNQHMEFDHQNLLISVLSLFFAGT

ETSSTTLRYGFLLMLKYPHVAEKVQKEIDQVIGSHRLPTLEDRTKMPYTDAVIYEIQRFS

DLVPIGVPHKVTKDTLFRGYLLPKNTEVYPILSSALHDPQYFEQPDTFNPDHFLDANGAL

KKSEAFMPFSLGKRICLGEGIARNELFLFFTAILQNFSLSSPMAAKDIDLSPKESGFGKV

PQTYQICFLAR

>EDM07989.1

MEPSILLLLALLVGFLLLLVRGHPKSRGNFPPGPRPLPLLGNLLQLDRGGLLNSFMQFRE

KYGDVFTVHLGPRPVVMLCGTDTIKEALVGQAEDFSGRGTIAVIEPIFKEYGKTLKGWDD

VGWRMGTERVDSERGENKGGGRTQVSLNFLWDWTIHASPTLSGVFFANGERWKALRRFSL

ATMRDFGMGKRSVEERIQEEAQCLVEELRKSQGAPLDPTFLFQCITANIICSIVFGERFD

YTDRQFLRLLELFYRTFSLLSSFSSQVFEFFSGFLKYFPGAHRQISKNLQEILDYIGHIV

EKHRATLDPSAPRDFIDTYLLRMEKVSPAWMREEKSNHHTEFHHENLMISLLSLFFAGTE

TGSTTLRYGFLLMLKYPHVTEKVQKEIDQVIGSHRPPSLDDRTKMPYTDAVIHEIQRFAD

LAPIGLPHRVTKDTMFRGYLLPKNTEVYPILSSALHDPQYFDHPDTFNPEHFLDADGTLK

KSEAFMPFSTGKRICLGEGIARNELFLFFTTILQNFSVSSHLAPKDIDLTPKESGIAKIP

PTYQICFSAR

>AEO16922.1

MEPSVLLLLALLVGFVLLLVRGYPKARGHLPPGPRPLPLLGNLLQMDRGGFLNSFKRIRE

KYGAVFTVHLGTRPVVMLYGTEAIREALVDQAEAFSGRGTIAVVEPIFRDYGVIFSNGER

WKVLRRFSLATMRDFGMGKRSVEERIQEEARCLVEEFRKSQGAPLDPTFLFQCITANIIC

SIVFGERFDYKDRQFLRLLYLFYQSFSLISSFSSQVFELFSGFLKYFPGTHRQISRNLQE

ILDYIGQSVEKHRATLDPSNPRDFIDTYLLRMEKEKSNQHMEFDHQNLLISVLSLFFAGT

ETSSTTLRYGFLLMLKYPHVAEKVQKEIDQVIGPHRLPTLEDRTKMPYTDAVIYEIQRYS

DLVPIGVPHKVTKDTLFRGYLLPKNTEVYPILSSALHDPQYFEQPDTFNPDHFLDANGAL

KKSEAFMPFSLGKRICLGEGIARNELFLFFTAILQNFSLSSPMAAKDIDLSPKESGFGKV

PQTYQICFLAR

>AEO16919.1

MEPSVLLLLALLVGFVLLLVRGYPKARGHLPPGPRPLPLLGNLLQMDRGGFLNSFKRIRE

KYGAVFTVHLGTRPVVMLYGTEAIREALVDQAEAFSGRGTIAVVEPIFRDYGVIFSNGER

WKVLRRFSLATMRDFGMGKRSVEERIQEEARCLVEEFRKSQGAPLDPTFLFQCITANIIC

SIVFGERFGYKDRQFLRLLYLFYQSFSLISSFSSQVFELFSGFLKYFPGTHRQISRNLQE

ILDYIGQSVEKHRATLDPSNPRDFIDTYLLRMEKEKSNQHMEFDHQNLLISVLSLFFAGT

ETSSTTLRYGFLLMLKYPHVAEKVQKEIDQVIGSHRLPTLEDRTKMPYTDAVIYEIQRFS

DLVPIGVPHKVTKDTLFRGYLLPKNTEVYPILSSALHDPQYFEQPDTSNPDHFLDANGAL

KKSEAFMPFSLGKRICLGEGIARNELFLFFTAILQNFSLSSPMAAKDIDLSPKESGFGKV

PQTYQICFLAR

>AEO16946.1

MGPSVLLLLALLVGFVLLLVRGYPKARGHLPPGPRPLPLLGNLLQMDRGGFLNSFRRIRE

KYGDVFTVHLGPRPVVMLYGTEAIREALVDQAEAFSGRGTIAVVEPIFRGYGVVFANGER

WKALRRFSLVTMRDFGMGKRSVEERIQEEARCLVEEFRKSQGAPLDPTFLFQCITANIIC

SIVFGERFDYKDHQFLRLLYLFYQSFSLISSFSSQVFELFSGFLKYFPGTHRQISRNLQE

ILDYIGQSVEKHRATLDPSHPKDFIDTYLLRMEKEKSNQHTEFDHQNLVISVLSLFFAGT

ETSSTTLRYGFLLMLKYPHVAEKVQKEIDQVIGSHRLPTLEDRTKMPYTEAVIYEIQRFS

DLIPIGVPHKVTKDTLFRGYLLPKNTEVYPILSSALHDPQYFEQPDTFNPDHFLDANGAL

KKSEAFMPFSLGKRICLGEGIARNELFLFFTAILQNFSLSSPMAVKDIDLSPKESGFGKV

PQTYQICFLAR

>EDL24217.1

MEPSVLLLLALLTGFLLLLIRGQPKAYSRLPPGPRPLPFLGNLLQMDRGGLLKSFIKLRD

KHGDVFTVHLGPRPVVMLYGTETIKEALVDHSDAFSGRGAIAVIQPIVQDYGVIFSSGER

WKTLRRFSLATMRDFGMGKRSVEERIKEEAQCLVEELKKYKGAPLDPTFLFQCITANIIC

SIVFGERFDYTDHQFLHLLDLFYQTLSLISSFSSQLFELFSAVLKYFPGTHRQISKNIQE

ILNYIGHSVEQHKATLDPSAPRDFIDTYLLRMEKEKSNHHTEFHHQNLLISVLSLFFAGT

ETTSTTLRYGFLLMLKYPHVAEKVQKEIDQVISAHHVPTLEDRIKMPYTEAVIHEIQRFS

DLAPIGLPHTVTKDTVFRGYLLPKNTEVYPILSSALHDPRYFEQPDKFNPEHFLDANGAL

KKSEAFLPFSTGKRICLGEGIARNELFLFFTALLQNFSLSSPVAPEDIDLTPKESGFVKI

PPVYRICFLPR

>AEO16935.1

MEPSVLLLLALLVGFVLLLVRGYPKARGHLPPGPRPLPLLGNLLQMDRGGFLNSFKRIRE

KYGAVFTVHLGARPVVMLHGTEAIREALVDQAEAFSGRGTIAVVEPIFRDYGVIFSNGER

WKVLRRFSLATMRDFGMGKRSVEERIQEEARCLVEEFRKSQGAPLDPTFLFQCITANIIC

SIVFGERFDYKDRQFLRLLYLFYQSFSLISSFSSQVFELFSGFLKYFPGTHRQISRNLQE

ILDYIGQSVEKHRATLDPSNPRDFIDTYLLRMEKEKSNQHMEFDHQNLLISVLSLFFAGT

ETSSTTLRYGFLLMLKYPHVAEKVQKEIDQVIGSHRLPTLEDRTKMPYTDAVIYGIQRFS

DLVPIGVPHKVTKDTLFRGYLLPKNTEVYPILSSALHDPQYFEQPDTFNPDHFLDANGAL

KKGEAFMPFSLGKRICLGEGIARNELFLFFTAILQNFSLSSPMAAKDIDLSPKESGFGKV

PQTYQICFLAR

>AEO16943.1

MEPSVLLLLALLVGFVLLLVRGYPKARGHLPPGPRPLPLLGNLLQMDRGGFLNSFRRIRE

KYGDVFTVHLGPRPVVMLYGTEAIREALVDQAEAFSGRGTIAAVEPIFRGYGVVFANGEH

WKALRRFPLVTMRDFGMGKRSVEERIQEEARCLVEEFRKSQGAPLDPTFLFQCITANIIC

SIVFGERFDYKDHQFLRLLYLFYQSFSLISSFSSQVFELFSGFLKYFPGTHRQISRNLQE

ILDYIGQSVEKHRATLDPSHPRDFIDTYLLRMEKEKSNQHTEFDHQNLVISVLSLFFAGT

ETSSTTLRYGFLLMLKYPHVAEKVQKEIDQVIGSHRLPTLEDRTKMPYTEAVIYEIRRFS

DLIPIGVPHKVTKDTLFRGYLLPKNTEVYPILSSALHDPQYFEQPDTFNPDHFLDANGAL

KKSEAFMPFSIGKRICLGEGIARNELFLFFTTILQNFSLSSPVAPKDIDLSPKESGFGKV

PQTYQICFLAR

>2004298A

MELGVLLLLTFTVGFLLLLASQNRPKTHGHLPPGPRPLPFLGNLLQMNRRGLLRSFMQLQ

EKYGDVFTVHLGPRPVVILCGTDTIREALVDQAEAFSGRGTVAVLHPVVQGYGVIFANGE

RWKILRRFSLVTMRNFGMGKRSVEERIKEEAQCLVEELKKYKGALLNPTSIFQSLAANII

CSIVFGERFDYKDHQFLRLLDLIYQTFSLMGSLSSQVFELFSGFLKYFPGVHKQISKNLQ

EILNYIDHSVEKHRATLDPNTPRDFINTYLLRMEKEKSNHHTEFHHQNLVISVLSLFFTG

TETTSTTLRYSFLIMLKYPHVAEKVQKEIDQVIGSHRLPTLDDRTKMPYTDAVIHEIQRF

ADLIPIGLPHRVTNDTMFLGYLLPKNTEVYPILSSALHDPRYFDHPDTFNPEHFLDVNGT

LKKSEAFLPFSTGKRICLGEGIAQNELFIFFTAILQNFSLASPVAPEDIDLSPINSGISK

IPSPYQIHFLSRCVG

>AEO16932.1

MEPSVLLLLALLVGFVLLLVRGYPKARGHLPPGPRPLPLLGNLLQMDRGGFLNSFKRIRE

KYGAVFTVHLGTRPVVMLYGTEAIREALVDQAEAFSGRGTIAVVEPIFRDYGVIFSNGVR

WKVLRRFSLATMRDFGMGKRSVEERIQEEARCLVEEFRKSQGAPLDPTFLFQCITANIIC

SIVFGERFDYKDRQFLRLLYLFYQSFSLISSFSSQVFELFSGFLKYFPGTHRQISRNLQE

ILDYIGQSVEKHRATLDPSNPRDFIDTYLLRMEKEKSNQHMEFDHQNLLISVLSLFFAGT

ETSSTTLRYGFLLMLKYPHVAEKVQKEIDQVIGSHRLPTLEDRTKMPYTDAVIYEIQRFS

DLVPIGVPHKVTKDTLFRGYLLPKNTEVYPILSSALHDSQYFEQPDTFNPDHFLDANGAL

KKSEAFMPFSLGKRICLGEGIARNELFLFFTAILQNFSLSSPMAAKDIDLSPKESGFGKV

PQTHQICFLAR

>AEO16921.1

MEPSVLLLLALLVGFVLLLVRGYPKARGHLPPGPRPLPLLGNLLQMDRGGFLNSFKRIRE

KYGAVFTVHLGTRPVVMLYGTEAIREALVDQAEAFSGRGTIAVVEPIFRDYGAIFSNGER

WKVLRRFSLATMRDFGMGKRSVEERIQEEARCLVEEFRKSQGAPLDPTFLFQCITANIIC

SXVFGERFDYKDRQFLRLLYLFYQSFSLISSFSSQVFELFSGFLKYFPGTHRQISRNLQE

ILDYIGQSVEKHRATLDPGNPRDFIDTYLLRMEKEKSNQHMEFDHQNLLISVLSLFFAGT

ETSSTTLRYGFLLMLKYPHVAEKVQKEIDQVIGSHRLPTLEDRTKMPYTDAVIYEIQRFS

DLVPIGVPHKVTKDTLFRGYLLPKNTEVYPILSSALHDPQYFEQPDTFNPDHFLDANGAL

KKSEAFMPFSLGKRICLGEGIARNELFLFFTAILQNFSLSSPMAAKDIDLSPKESGFGKV

PQTSQICFLAR

>AAA03648.1

MDPSVLLLLAVLLSLFLLLVRGHAKIHGHLPPGPHPLPLLGNLLQMDRGGLLKCFIQLQE

KHGDVFTVHLGPRPVVVLCGTQTIREALVDHAEAFSGRGTIAAAQLVMQDYGIFFASGQR

WKTLRRFSLATMKEFGMGKRSVEERIKEEAQCLVEELKKYQGVPLDPTFLFQCITANIIC

SIVFGERFDYTDDQFLHLLNLMYKIFSLLSSFSGQMFELFSGFLKYFPGVHRQIVKKQQE

LLDYIAHSVEKHKATLDPSAPRDYIDTYLLRMEKEKSNHNTEFHHQNLMMSVLSLFFAGT

ETTSATLHYGVLLMLKYPHVTEKVQKEIDQVIGSHRLPTLDDRTKMPYTDAVIHEIQRFS

DLVPIGLPHKVIKDTLFRGYLLPKNTEVYPVLSSALHDPQYFEQPDKFNPEHFLDANGAL

KKCEAFLPFSTGKRICLGESIARNELFIFFTTILQNFSVASPVAPKDIDLTPKESGIGKI

PPAHQIYFLAR

>AEO16923.1

MEPSVLLLLALLVGFVLLLVRGYPKARGHLPPGPRPLPLLRNLLQMDRGGFLNSFKRIRE

KYGAVFTVHLGTRPVVMLYGTEAIREALVDQAEAFSGRGTIAVVEPIFRDYGVIFSNGER

WKVLRRFSLATMRDFGMGKRSVEERIQEEARCLVEEFRKSQGAPLDPTFLFQCITANIIC

SIVFGERFDYKDRQFLRLLYLFYQSFSLISSFSSQVFELFSGFLKYFPGTHRQISRNLQE

ILDYIGQSVEKHRATLDPSNPRDFIDTYLLRMEKEKSNQHMEFDHQSLLISVLSLFFAGT

ETSSTTLRYGFLLMLKYPHVAEKVQKEIDQVIGSHRLPTLEDRTKMPYTDAVIYEIQRFS

DLVPIGVPHKVIKDTLFRGYLLPKNTEVYPILSSALHDPQYFEQPDTFNPDHFLDANGAL

KKSEAFMPFSLGKRICLGEGIARNELFLFFTAILQNFSLSSPMAAKDIDLSPKESGFGKV

PQTYQICFLAR

>P12790.1

MDPSVLLLLAVLLSLFLLLVRGHAKIHGHLPPGPHPLPLLGNLLQMDRGGLLKCFIQLQE

KHGDVFTVHLGPRPVVVLCGTQTIREALVDHAEAFSGRGTIAAAQLVMQDYGIFFASGQR

WKTLRRFSLATMKEFGMGKRSVEERIKEEAQCLVEELKKYQGVPLDPTFLFQCITANIIC

SIVFGERFDYTDDQFLHLLNLMYKIFSLLSSFSGQMFELFSGFLKYFPGVHRQIVKKQQE

LLDYIAHSVEKHKATLDPSAPRDYIDTYLLRMEKEKSNHNTEFHHQNLMMSVLSLFFVGT

ETTSATLHYGVLLMLKYPHVTEKVQKEIDQVIGSHRLPTLDDRTKMPYTDAVIHEIQRFS

DLVPIGLPHKVIKDTLFRGYLLPKNTEVYPVLSSALHDPQYFEQPDKFNPEQFLDANGAL

KKCEAFLPFSTGKRICLGESIARNELFIFFTTILQNFSVASPVAPKDIDLTPKESGIGKI

PPAHQIYFLAR

>354505853

MEASVLLFLTLLLGFVVLLLRGHPKAHGHLPPGPRPLPFLGNLLQMDRRGLLNSFMRFRE

KYGDVFTVHLGPRPVVMLYGTQAIREALVDQAEAFSGRGTVAVVEPVVQGYGVIFANGDH

WKALRRFSLATMRNFGMGKRTGEERRKSERQMKTDKLKEFKGAPLDPKFLFKCITANIIC

SIVFGERFDYKDRQFLRLLDLIFQIYSLISSFSSQVFELFSGFLKYFPGAQVRISKITQE

ILDYIERSVEQHQKTLDPSNPRDFIDTYLLRMEKEKSNPHTEFQQKNLVLTVLALFFAGT

ETSSTTLHYGFLLMLKYPHVAEKVQKEIEEVIGSHRLPTLDDRTKMPYTDAVIHEIQRLS

DVVPIGVPHKVTKDTLFRGYLLPKDTEVYPILSSALHDPQYFEQPDTFNPDRFLDANGAL

KKNEAFMPFSIGKRICPGEGIARNELFLFFTTILQNFSLSSPVDPKDIDLSPKESGFGRV

PPEYQICFLAR

>392337639

MEPSVLLLLAVLLSFLLLLVRGHAKIHGRLPPGPCPVPLLGNLLQMDRRGLLNSSLKLQE

KYGDVFTVHLGLRPVVVLCGTQTIREALVDHAEAFSGRGTIAGLEPVFQDYGIFFSSGEQ

WKTLRRFSMATMRDFGMRKKSVEERIKEESQCLVEELKKYQGAPLDPTFLFQCITSNIIC

SIVFGECFDYTDHQFLHLLDLMYQTFSLLSSIFSQVFELFPGVLKYFPGAHRQISRNLHE

ILDFIGQSVEKHRATLDPNAPRDFIYTYLLHMEKKSNHYTEFHHWNLLSSVLSLFFAGTE

TSSTTLRYGFLIMLKYPHITEKVQKEIDCVIGSHRLPTLDDRSKMPYTEAVIHEIQRFSD

LAPIGTPHRVIKDTIFRGYLLPKNTEVFPILSSVLHDPQYFEQPDIFNLQHFLDANGALK

IIEAFLPFSTGKRICLGESIARNELFLFFTTILQNFSVSSPVAPKDIDLTPKESGIGRIP

QVYQICFLAH

>354505851

MEASVLLFLTLLLGFVVLLLRGHPKAHGHLPPGPRPLPFLGNLLQMDRRGLLNSFMRFRE

KYGDVFTVHLGPRPVVMLYGTQAIREALVDQAEAFSGRGTVAVVEPVVQGYGVIFANGDH

WKALRRFSLATMRNFGMGKRTGEERVPFDALSPVLPLHPPGAPLDPKFLFKCITANIICS

IVFGERFDYKDRQFLRLLDLIFQIYSLISSFSSQVFELFSGFLKYFPGAQVRISKITQEI

LDYIERSVEQHQKTLDPSNPRDFIDTYLLRMEKEKSNPHTEFQQKNLVLTVLALFFAGTE

TSSTTLHYGFLLMLKYPHVAEKVQKEIEEVIGSHRLPTLDDRTKMPYTDAVIHEIQRLSD

VVPIGVPHKVTKDTLFRGYLLPKDTEVYPILSSALHDPQYFEQPDTFNPDRFLDANGALK

KNEAFMPFSIGKRICPGEGIARNELFLFFTTILQNFSLSSPVDPKDIDLSPKESGFGRVP

PEYQICFLAR

>354486245

MEPSVLLLLTLLVGFLLLLVKGHTNAHGHLPPGPRPLPLLGNLLQMDRRGLLNSFMRLRE

KYGDVFTVHLGPRPVVMLCGTQAIREALVDQAEAFSGRGTIAVFEPIVQGYGVVFANGER

WKALRRFSLATMKDFGMGKQSVEERIKEEARCLVEELQKSQGAPLDPMFLFQCITANIIC

SIVFGERFDYKDRQFLRLLDLFYQTFSLISSFSSQVFELFSGVLKYFPGVHRQVSKNLQE

VLDHIDHSVEQHQETLDPSNPRDLIDTYLLRMEKEKSNPHTEFHHQNLIISVLSLFFAGT

ETSSTTLRYGFLLMLKYPHVAEKVQKEIDHVIGSHRLPTLDDRTKMPYTDAVIHEIQRFA

DLVPIGVPHKSRGSLPPKGSPNTEVYPILSSALHDPQPSEQTDTFNPDHFLDANGALKKN

EAFMPFSIGKRICLGEGIARKELFLFFTTILQNFSLSSPVDPKDIDLSPKESGVSKIPPR

YRILFLPR

>P24460.1

MELSVLLLLALLTGLLLLMARGHPKAYGHLPPGPRPLPILGNFLQMDRKGLLKSFLRLQE

KYGDVFTVYLGPRRTVMLCGIDAIREALVDNAEAFSGRGKIAVVEPVFQGYGVVFANGER

WKTLRRFSLATMRDFGMGKRSVEERIQEEAQCLVEELRKTEGVLQDPTFFFHSMTANIIC

SIVFGKRFGYKDPEFLRLMNLFYVSFALISSFSSQMFELFHSFLKYFPGTHRQVYNNLQE

IKAFIARMVEKHRETLDPSAPRDFIDAYLIRMDKEKAEPSSEFHHRNLIDTALSLFFAGT

ETTSTTLRYGFLLMLKYPHIAERIYKEIDQVIGPHRLPSLDDRAKMPYTDAVIHEIQRFG

DLLPIGVPHMVTKDICFRGYIIPKGTEVFPILHSALNDPHYFEKPDVFNPDHFLDANGAL

KKNEAFIPFSIGKRICLGEGIARMELFLFFTTILQNFSVASPMAPEDIDLTPQEIGVGKL

PPVYQISFLSRGGC

>ACE79165.1

MELSVLLLLALLTGLLLLMARGHPKAYGHLPPGPRPLPILGNFLQMDRKGLLKSFLRLQE

KYGDVFTVYLGPRRTVMLCGIDAIREALVDNAEAFSGRGKIAVVEPVFQGYGVVFANGER

WKTLRRFSLATMRDFGMGKRSVEERIQEEAQCLVEELRKTEGVLQDPTFFFHSMTANIIC

SIVFGKRFGYKDPEFLRLMNLFYVSFALISSFSSQMFELFHSFLKYFPGTHRQVYNNLQE

IKAFIARMVEKHRETLDPSAPRDFIDAYLIRMDKEKAEPSSEFHHRNLIDTALSLFFAGT

ETTSTTLRYGFLLMLKYPHIAERIYKEIDQVIGPHRLPSLDDRAKMPYTDAVIHEIQRFG

DLLPIGVPHMVTKDICFRGYIIPKGTEVFPILHSALNDPHYFEKPDVFNPDHFLDANGAL

KKNEAFIPFSIGKRICLGEGIARMELFLFFTTILQNFSVASPMAPEDIDLTPQEIGVGKL

PPVYQISFLSRGGC

>301776633

MELSVLLLLALLMGLLLLLVRGHPKAYGRLPPGPRPLPFLGNVLQMDRRGLLKSFLRLRE

KYGDVFTVYLGPRPAVMLCGIEAIREALVDQAESFSGRGKIAIVEPVFQGYGVVFANGER

WKALRRFSLATMRDFGMGRRSVEERIQEEAQCLVEELRKTQGAFQDPTFFFHSMTANIIC

SIVFGKRFGYRDPEFLRLLDLFYQSFALISSFSSQVFELFHNFLKYFPGTHRQVYKNLQE

INAFIARAVEKHRETLDPNSPQDFIDVYLIRMDKEKADPSSEFHQRNLIYTTLSLFFAGT

ETTSTTLRYGFLLLLKYPHVAERIHKEIDQVIGPHRFPSLDDRAKMPYTDAVIHEMQRFG

DLLPIGVPHMVTKDTCFRGYIIPKGTEVFPILHSALYDPHYFEKPYAFNPDHFLDANGAL

KKNEAFIPFSIGKRICLGEGIARMELFLFFTTILQNFSVASPVAPEDIDLTPRESGVGKV

PPVYQIRFLARGGC

>P13107.1

MDTSVLLLLAVLLSFLLFLVRGHAKVHGHLPPGPRPLPLLGNLLQMDRGGFRKSFIQLQE

KHGDVFTVYFGPRPVVMLCGTQTIREALVDHAEAFSGRGIIAVLQPIMQEYGVSFVNEER

WKILRRLFVATMRDFGIGKQSVEDQIKEEAKCLVEELKNHQGVSLDPTFLFQCVTGNIIC

SIVFGERFDYRDRQFLRLLDLLYRTFSLISSFSSQMFEVYSDFLKYFPGVHREIYKNLKE

VLDYIDHSVENHRATLDPNAPRDFIDTFLLHMEKEKLNHYTEFHHWNLMISVLFLFLAGT

ESTSNTLCYGFLLMLKYPHVAEKVQKEIDQVIGSQRVPTLDDRSKMPYTEAVIHEIQRFS

DVSPMGLPCRITKDTLFRGYLLPKNTEVYFILSSALHDPQYFEQPDTFNPEHFLDANGAL

KKCEAFMPFSIGKRMCLGEGIARSELFLFFTTILQNYSVSSPVDPNTIDMTPKESGLAKV

APVYKICFVAR

>AAT49265.1

MELSVLLFLALLTGLLLLLVQRHPNAHGRLPPGPCPLPLLGNLLQMDRRGLLRSFLRFRE

KYGDVFTVYLGPRPVVMLCGVEAIREALVDNAEAFSGRGKIAITDPVFQGYGVVFANGNR

WKVLRRFSLTTMRDFGMGKRSVEERIQEEAQCLIEELRKSKGALVDPTFLFHSITANIIC

SIVFGKRFHYQDQEFLKILNLFYHTFSLASSMFGQLFELLSGFLKYFPGAHRQVYKNLQE

INAYIGHSVEKHRETLDPSAPQDLIDSYLLQMEKEKSNPHSEFSHRNLIINTLSLFFAGT

ETTSTTLRYGFLLMLKYPHVAERIYKEIEQVIGPHRPPALDDRAKMPYTEAVIHEIQRFA

DLLPMGVPHIVTQQTSFRGYIIPKDTEVFPLLSTALHDPHYFEKPDTFNPDHFLDANGAL

KKNEAFIPFSLGRRMCLGEGIARNELFLFFTTILQNFSVASPVAPEDIDLTPQESGVGKI

PPTYQIRFLPR

>3IBDA

MAKKTSSKGKLPPGPRPLPLLGNLLQMDRRGLLKSFLRFREKYGDVFTVHLGPRPVVMLC

GVEAIREALVDKAEAFSGRGKIAMVDPFFRGYGVIFANGNRWKVLRRFSVTTMRDFGMGK

RSVEERIQEEAQCLIEELRKSKGALMDPTFLFQSITANIICSIVFGKRFHYQDQEFLKML

NLFYQTFSLISSVFGQLFELFSGFLKHFPGAHRQVYKNLQEINAYIGHSVEKHRETLDPS

APRDLIDTYLLHMEKEKSNAHSEFSHQNLNLNTLSLFFAGTETTSTTLRYGFLLMLKYPH

VAERVYREIEQVIGPHRPPELHDRAKMPYTEAVIYEIQRFSDLLPMGVPHIVTQHTSFRG

YIIPKDTEVFLILSTALHDPHYFEKPDAFNPDHFLDANGALKKTEAFIPFSLGKRICLGE

GIARAELFLFFTTILQNFSMASPVAPEDIDLTPQECGVGKIPPTYQIRFLPRHHHH

>P20813.1

MELSVLLFLALLTGLLLLLVQRHPNTHDRLPPGPRPLPLLGNLLQMDRRGLLKSFLRFRE

KYGDVFTVHLGPRPVVMLCGVEAIREALVDKAEAFSGRGKIAMVDPFFRGYGVIFANGNR

WKVLRRFSVTTMRDFGMGKRSVEERIQEEAQCLIEELRKSKGALMDPTFLFQSITANIIC

SIVFGKRFHYQDQEFLKMLNLFYQTFSLISSVFGQLFELFSGFLKYFPGAHRQVYKNLQE

INAYIGHSVEKHRETLDPSAPKDLIDTYLLHMEKEKSNAHSEFSHQNLNLNTLSLFFAGT

ETTSTTLRYGFLLMLKYPHVAERVYREIEQVIGPHRPPELHDRAKMPYTEAVIYEIQRFS

DLLPMGVPHIVTQHTSFRGYIIPKDTEVFLILSTALHDPHYFEKPDAFNPDHFLDANGAL

KKTEAFIPFSLGKRICLGEGIARAELFLFFTTILQNFSMASPVAPEDIDLTPQECGVGKI

PPTYQIRFLPR

>402905626

MELSVLLFLVLLTGLLLLLVQRHPNAHGRLPPGPRPLPLLGNLLQMDRRGLLRSFLRFRE

KYGDVFTVYLGPRPVVMLCGVEAIREALVDNAEAFSGRGKIAITDPVFQGYGVVFANGNR

WKVLRRFSLTTMRDFGMGKRSVEERIQEEAQCLIEELRKSKGALVDPTFLFHSITANIIC

SIVFGKRFHYQDQEFLKILNLFYHTFSLASSMFGQLFELLSGFLKYFPGAHRQVYKNLQE

INAYIGHSVEKHRETLDPSAPQDLIDSYLLQMEKEKSNPHSEFSHRNLIINTLSLFFAGT

ETTSTTLRYGFLLMLKYPHIAERIYKEIEQVIGPHRPPALDDRAKMPYTEAVIHEIQRFA

DLLPMGVPHIVTQQTSFRGYIIPKDTEVFPLLSTALHDPHYFEKPDTFNPDHFLDANGAL

KKNEAFIPFSLGRRICLGEGIARNELFLFFTTILQNFSVASPVAPEDIDLTPQESGVGKI

PPTYQIRFLPR

>297704848

MELSVLLFLALLTGLLLLLVQGHPNTHGRLPPGPRPLPLLGNLLQMDRRGLLKSFLRFRE

KYGDVFTVHLGPRPVVMLCGVEAIREALVDKAEAFSGRGKIAMVDPVFRGYGVIFANGNR

WKVLRRFSVTTMRDFGMGKRSVEERIQEEAQCLIQELRKSKGALMDPTFLFQSITANIIC

SIVFGKRFHYQDQEFLKILNLFYQTFSLVSSVFGQLFELFSGFLKYFPGAHRQVYKNLQE

INAYIGHSVEKHRETLDPSTPKDLIDTYLLHMEKEKSNAHSEFSHQNLTLNTLSLFFAGT

ETTSTTLRYGFLLMLKYPHVAERVYREIEQVIGPHRPPELHDRAKMPYTEAVIHEIQRFA

DLLPMGVPHIVTQHTSFRGYIIPKDTEVFLILSTALRDPHYFEKPDAFNPDHFLDASGAL

KKNEAFIPFSLGKRICLGEGIARAELFLFFTTILQNFSVASPVAPEDIDLTPQECGVGKI

PPTYQIRFLPR

>AAH67430.1

MELSVLLFLALLTGLLLLLVQRHPNTHDRLPPGPRPLPLLGNLLQMDRRGLLKSFLRFRE

KYGDVFTVHLGPRPVVMLCGVEAIREALVDKAEAFSGRGKIAMVDPFFRGYGVIFANGNR

WKVLRRFSVTTMRDFGMGKRSVEERIQEEAQCLIEELRKSKGALMDPTFLFHSITANIIC

SIVFGKRFHYQDQEFLKMLNLFYQTFSLISSVFGQLFELFSGFLKYFPGAHRQVYKNLQE

INAYIGHSVEKHRETLDPSAPRDLIDTYLLHMEKEKSNAHSEFSHQNLNLNTLSLFFAGT

ETTSTTLRYGFLLMLKYPHVAERVYREIEQVIGPHRPPELHDRAKMPYTEAVIYEIQRFS

DLLPMGVPHIVTQHTSFRGYIIPKDTEVFLILSTALHDPHYFEKPDAFNPDHFLDANGAL

KKTEAFIPFSLGKRICLGEGIARAELFLFFTTILQNFSMASPVAPEDIDLTPQECGVGKI

PPTYQIRFLPR

>AAH67431.1

MELSVLLFLALLTGLLLLLVQRHPNTHDRLPPGPRPLPLLGNLLQMDRRGLLKSFLRFRE

KYGDVFTVHLGPRPVVMLCGVEAIREALVDKAEAFSGRGKIAMVDPFFRGYGVIFANGNR

WKVLRRFSVTTMRDFGMGKRSVEERIQEEAQCLIEELRKSKGALMDPTFLFQSITANIIC

SIVFGKRFHYQDQEFLKMLNLFYQTFSLISSVFGQLFELFSGFLTYFPGAHRQVYKNLQE

INAYIGHSVEKHRETLDPSAPKDLIDTYLLHMEEEKSNAHSEFSHQNLNLNTLSLFFAGT

ETTSTTLRYGFLLMLKYPHVAERVYREIEQVIGPHRPPELHDRAKMPYTEAVIYEIQRFS

DLLPMGVPHIVTQHTSFRGYIIPKDTEVFLILSTALHDPHYFEKPDAFNPDHFLDANGAL

KKTEAFIPFSLGKRICLGEGIARAELFLFFTTILQNFSMASPVAPEDIDLTPQECGVGKI

PPTYQIRFLPR

>AAZ29439.1

MELSVLLFLALLTGLLLLLVQRHPNAHGRLPPGPCPLPLLGNLLQMDRRGLLRSFLRFRE

KYGDVFTVYLGPRPVVMLCGVEAIREALVDNAEAFSGRGKIAITDPVFQGYGVVFANGNR

WKVLRRFSLTTMRDFGMGKRSVEERIQEEAQCLIEELRKSKGALVDPTFLFHSITANIIC

SIVFGKRFHYQDQEFLKILNLFYHTFSLASSMFGQLFELLSGFLKYFPGAHRQVYKNLQE

INAYIGHSVEKHRETLDPSAPQDLIDSYLLQMEKEKSNPHSEFSHRNLIINTLSLFFAGT

ETTSTTLRYGFLLMLKYPHVAERIYKEIEQVIGPHRPPALDDRAKMPYTEAVIHEIQRFA

DLLPMGVPHIVTQQTSFRGYIIPKDTEVFPLLSTALHDPHYFEKPDTFNPDHFLDANGAL

KKNEAFIPFSLGRRMCLGEGIARNELFLFFTTILQNFSVASPVALEDIDLTPQECGVGKI

PPTYQIRFLPR

>397482615

MELSVLLFLALLTGLLLLLVQRHPNTHGRLPPGPRPLPLLGNLLQMDRRGLLKSFLRFRE

KYGDVFTVHLGPRPVVMLCGVEAIREALVDKAEAFSGRGKIAMVDPFFRGYGVIFANGNR

WKVLRRFSVTTMRDFGMGKRSVEERIQEEAQCLIEELRKSKGALMDPTFLFQSITANIIC

SIVFGKRFHYQDQEFLKMLNLFYQTFSLVSSVFGQLFELFSGFLKYFPGAHRQVYKNLQE

INAYIGHSVEKHRETLDPSAPKDLIDTYLLHMTQEKSDAHSEFSHQNLTLNTLSLFFAGT

ETTSTTLRYGFLLMLKYPHVAERVYREIEQVIGPHRPPELHDRAKMPYTEAVIYEIQRFS

DLLPMGVPHIVTQHTSFRGYIIPKDTEVFLILSTALHDPHYFEKPDAFNPDHFLDANGAL

KKNEAFIPFSLGKRICLGEGIARAELFLFFTTILQNFSVASPEAPEDIDLTPQECGVGKI

PPTYQIRFLPR

>AAF13602.1

MELSVLLFLALLTGLLLLLVQRHPNTHDRLPPGPRPLPLLGNLLQMDRRGLLKSFLRFRE

KYGDVFTVHLGPRPVVMLCGVEAIREALVDKAEAFSGRGKIAMVDPFFRGYGVIFANGNR

WKVLRRFSVTTMRDFGMGKRSVEERTQEEAQCLIEELRKSKGALMDATFLFHSITANIIC

SIVFGKRFHYQDQEFLKMLNLFYQTFSLISSVFGQLFELFSGFLKYFPGAHRQVYKNPQE

INAYIGHSVEKHRETLDPSAPRDLIDTYLLHMEKEKSNAHSEFSHQNLNLNTLSLFFAGT

ETTSTTLRYGFLLMLKYPHVAERVYREIEQVIGPHRPPELHDRAKMPYTEAVIYEIQRFS

DLLPMGVPHIVTQHTSFRGYIIPKDTEVFLILSTALHDPHYFEKPDAFNPDHFLDANGAL

KKTEAFIPFSLGKRICLGEGIARAELFLFFTTILQNFSMASPVAPEDIDLTPQECGVGKI

PPTYQIRFLPR

>EHB18321.1

MELSVLLLLALLMGLLVLLFRGHPKAHGHLPPGPHPLPYLGNLLQMNRKGLVKSILQLRE

KYGDVFTVYLGPRPVVMLCGADTIQEALVDQAEAFSGRGPIASMDAVFQGYGLSFANGER

WKVLRRFTLATMRDFGMGKRSLEERIQEEAQCLVEELRKSRGGLVDPLFLFQSATANIIC

STVFGKRFDYKDHQFLRLLDLIYQTFEHMSSFSSQVFELYSSILKHFPGTHREIYKNLQE

IITFIGHSVEKHHETLDPSAPRDFIDAYLLRMEKDKSDPHTEFHLQNLILTSLSLFFAGT

ETTSTTLRYGFLFLLKHPHVTERVHKEIEQVIGTHHQPALDHRTKMPYTEAVMCEIQRFS

DLAPIGVPHVVTKDTHFRGFIIPKNTEIYPILSTALHDPHHFENPDTFNPDHFLDAMGAL

KRSEAFMPFSIGKRICLGEGIARAELFLFFTTILQNFSISSPVAPEDIDLTPQKMGLSRI

PPTYQVCFLPRTVE

>ABB05542.1

MELSVLLFLALLTGLLLLLVQRHPNSHGTLPPGPRPLPLLGNLLQMDRRGLLKSFLRFRE

KYGDVFTVHLGPRPVVMLCGVEAIREALVDNAEAFSGRGKIVIMDPVYQGYGMLFANGNR

WKVLRRFSVTTMRDFGMGKRSVEERIQDEAQCLIEELRKSKGALVDPTFLFHSITANIIC

SIIFGKRFHYQDQEFLKTLNLFCQSFLLISSISSQLFELFSGFLKYFPGAHRQVYKNLQE

INAYIGHSVEKHRETLDPSAPRDLIDTYLLHMEKEKSNPHSEFSHQNLIINTLSLFFAGT

ETTSTTLRYGFLLMLKYPHVAERVYKEIEQVVGPHRPPALDDRAKMPYTEAVIREIQRFA

DLLPMGVPHIVTQHTSFRGYTIPKDTEVFLILSTALRDPHYFEKPDAFNPDHFLDANGAL

KKNEAFIPFSLGKRICLGEGIARAELFLFFTTILQNFSVASPVAPEDIDLTPQECGVGKI

PPTYQICFLPR

>AAA31434.1

MEFSLLLLLAFLAGLLLLLLRGHPKAHGRLPPGPPPLPVLGNLLQMDRKGLLHSFLQLRE

KYGDVFTVYLGSRPVVVLCGTDAIREALVDQAEAFSGRGKIAVLDPIFQGYGVTFANGER

WRALRRFSLATMRDFGMGKRSVEERIQEEARCLVEELRKSKGALLDNTLLFHSVTSNIIC

SIVFGKRFDYKDPVFLRLLDLFFQSFSLTSSFSSQVFELFSGFLKHFPGTHRQIYRNLQE

INTFIGQSVERHRETLDPSNPRDFIDVYLLRMEKDKSDPSSEFHHQNLILTVLTLFFAGT

ETTSTTLRYGFLLLLKYPHVTERVQKEIEQVIGSHRPPALDDRAKMPYTDAVIHEIQRLG

DLIPLGVPHMVTKDTQFRGYVIPKNTEVFPVLSSALHDPRYFETPNTFNPGHFLDADGAL

KRIEGFMPFSLGKRICLGEGIARTELFLFFTTILQNFSIASPVPPEDIDLTPRESGVGNV

PPSYQIRFLAR

>332855744

MELSVLLFLALLTGLLLLLVQRHPNSHGTLPPGPRPLPLLGNLLQMDRRGLLKSFLRFRE

KYGDVFTVHLGPRPVVMLCGVEAIREALVDKAEAFSGRGKIAIMDPVYQGYGVLFANGNR

WKVLRRFSVTIMRDFGMGKRSVEERIQDEAQCLIEELRKSKGALVDPTFLFHSITANIIC

SIVFGKRFHYQDQEFLKMLNLFCQSFSLISSISSQLFELFSGFLKYFPGAHRQLYKNLQE

INAYIGHSVEKHRETLDPSAPRDLIDTYLLHMEKEKSNPHSEFSHQNLIINTLSLFFAGT

ETTSTTLRYGFLLMLKYPHVAERVYKEIEQVVGPHRPPALDDRAKMPYTEAVIHEIQRFA

DLLPMGVPHIVTQHTSFXGYTIPKDTEVFLILSTALRDPHYFEKPDAFNPDHFLDANGAL

KKNEAFIPFSLGKRICLGEGITRAELFLFFTTILQNFSVASPVAPEDIDLTPQECGVGKI

PPTYQICFLPR

>297704846

MELSVLLFLALLTGLLLLLVQRHPNTHGRLPPGPRPLPLLGNLLQMDRRGLLKSFLRFRE

KYGDVFTVHLGPRPVVVLCGVQAIREALVDKAEAFSGRGKIAIMDPVYQGYGVIFANGNR

WKVLRRFSVTTMRDFGMGKQSVEERIQEEAQCLIEELQKSKGALMDPTFLFHSITANIIC

SIVFGKRFHYQDQEFLKMLNLFCQSFSLISSISSQLFELFSGFLKYFPGAHRQLYKNLQE

INAYIGHSVEKHRETLDPSAPQDLIDTYLLHMEKEKSNPHSEFSHQNLIINTLSLFFAGT

ETTSTTLCYGFLLMLKYPHVAERVYKEIEQVVGPHCPPVLDDRAKMPYTEAVIHEIQRFA

DLLPMGVPHIVTQHTRFRGYIIPKDTEVFLILSTALRDPHYFEKPDAFNPDHFLDANGAL

KKNEAFIPFSLGKRICLGEGIARAELFLFFTTILQNFSVASPVAPEDIDLTPQECGVGKI

PPTYQIRFLPH

>EHB11773.1

MELSVLLLLTLLMGLLVLLFRGHPKTHGHLPPGPRPLPFLGNLLQMNRKGLVKSILQKSV

VMLCGPETIREALVDQAEAFSGRGTIASTEAIFQGYGLNFANGERWNVLRRFTLATMRDF

GMGKWSMEERIQEEAQCLVEELRKSQGGLVDPLFLFQSATANIICSTVFGKRFDYKDRQF

LRLLDLIYQSFALISSFSSQMFELFPSILKHFPGTHREISKVSQEIITFIGHSVEKHQET

LDPSAPRDFIDAYLLRMEKDKSDPHTEFHHKNLVLTSLSLFFAGTETTSTTLRYGFLFLL

KYPHVAERVHKEIEQVIGSHRQRALDDRTKMPYTEAVICEIQRFADLVPIGLPHVVTKDI

HFRGFIIPKMSLAGLPHLCNTEIFPILTTALHDPRHFEKPDTFNPDHFLDAKEALKKSEA

FMPFSIGKCICLGEGIARAELFLFFTTILQNFSISSPVAPEDIDLTPQKIGVGRIPPSYQ

IRFLPHVVE

>395526098

MMSRVAIPSLKYKVQGSCEEFTMGFSLLKALGKDGSSDLLYPQXTALLGLLVLLARGPPA

SRRGRLPPGPRPLPLLGNVLQMDRRGLLKSFLALRDKYGDVFTIYLGSRPVVVLCGPEAV

KEALVEKAEEFSGRGPIAMVHSVFQGQGVIFANGHTWKTLRRFSLATLRDFGMGKRSIEE

RIQEEAQCLVEELRKSQGALLDPTFLFHSITANIICSIVFGERFSYHDDQFQDLLNRFNE

TFALTSSTWGQLFELFSGFLKFFPGPHRRAYSNLAIINDYIADSVEKHKESLDPSAPRDF

IDVYLLRMEKEKGDPSTEFHYKNLILTVLSLFFAGTETTSTTLRYGCLMLLKYPWVAEKV

QDEIDQVIGRDRPPELKDRLQMPYTDAVIHEIQRFSDLIPLGVPHCVTQDTSFRGYLIPK

GTEVYPLLSTVLHDPKYFEKPYDFDPNHFLDTQGSLKKSEAFIPFSSGKRVCLGEGIARM

ELFLFLTTILQNFSLSSSMVPASIDLTPRESGVGTVPPIYKLGFLPRCKADRAPAMDSVW

LLLLTALLGLLVLLARGPPASRRGRLPPGPRPLLLLGNVLQMDRRGLLKSFLALRDKYGD

VFTVYLGCRPVVVLCGPEAVKEALVKKAEEFSGRGPIAMVDSVFQGQGITFANDHTWKTL

RQFSLATLWDFGMGKRSIEERTQEEAQCLVEELQKSQGALLDPTFLFHSIAANIICFIMF

GERFSYHDDLFHELLSKFNDMFALASSAWGQLFELFSGFLKFFPGPHRCAHNNLTFISAF

IADSVEKHKESLDPSTPRDFIDVYLLHMEKEKGDPKSEFHYKNLIITVLSLFFGGTETTS

TTLRYGCLLLLKYPWVAKKVQEEIDQVIGKNRSPELKDRAQMPYTDAVIHEIQRFSDLLP

MSIPHCVTQDTSFRGYLIPKGTEVYPLLSTVLHDPKYFEKPYDFDPNHFLDAQGSLKKSE

AFIPFSSGKRVCLGEGIARMELFLFLTTILQNFSLRSSMVPASIDLTPRESGVGTVPPIY

QLQFLPR

>EGW12122.1

MEASVLLFLTLLLGFVVLLLRGHSKARGHLPPGPRPLPFLGNLLQMDRRGLLNSFMRVRL

PWGLGLGFPCLCLWRSLISKSKGLLQALGSGVIFANGDHWKALRRFSLATMRDFGMGKRS

VEERIQEEAQCLAEELRKSQAAWSPPGPQVPLPMHLSQHHLPIVFGERFDYKDRQFLRLL

DLLHQSFSLLSSFSSQVFELFSGFLKYFPGTHVHISRITQEILDYIERSVEQHQETLDPS

NPRDLIDTYLLRMEKEKCNSHKGFHHQNLVFTVLSLFFAGTETSSTTLRYGFLLMLKYPH

VAEKVQKEIDEVIGSHRLPTLDDRTKMPYTDAVIHEIQRFSDLVPIGVPHKVTKDTLFRG

YLLPKNTEVYPILSSALHDPQYFEQPDTFNPDRFLDANGALKKNEAFMPFSVGKRICLGE

GIARHELFLFFTTLLQNFSLSSPVDPKDIDLSPKESGFAKLPPIYKICFLSR

>390479053

MELTVFLFFALLTGLLLLLLRRHPKAHGRLPPGPRPLPLLGNLLQLERRGLLKSFLKFRE

KYGDVFTVHLGPRPVVMLCGVDAIREALVDQAEVFSGRGKIAIVDPVFQGYGVVFANGDR

WKALRRFSLTTMRDFGMGKRSVEERIKEEAQCLVQELWKYKDRLGKRFEYQDKEVPEAAA

LFYQSFSLVSSLFSQLFELFSDFLKYFPGAHRQIRKNLQEIGAFIGHSVEKHREALDPSS

PQDLIDTYLLHMEKEKSNPHSEFNHQNLIFNTLSLFFAGTETTSTTLRYGFLLMLKYPHV

AERVYKEIEQVIGPHRPPALDDRAKMPYTDAVIHEIQRIADLLPMGLPHIVTQHTSFRGY

TIPKGTEVFPILSTALNDPHYFEKPDTFNPDHFLDTNGALKKNEAFIPFSLGKRICLGEG

IARTELFLFFTTILQNFSVASPVAPEDIDLTPQENGVGKLPPAYQIRFLPR

>403305472

MELSVFLFLALLTGLLLLLVQRHPKAHGRLPPGPRPLPLFGNLLQMDRRGLLKSFLKFRE

KYGDVFTVHLGPRPVVMLCGVDAIREALVDQAEVFSGRGKIAIVDPVFQGYGVVFASGDR

WKVLRRFSLTTMRDFGMGKRSVEERIQEEAQCLVQELQKSKGALVDPTFFFHSITANIIC

SIVFGKRFDYQDREFLKLLHLFYQSFSLVSSLFSQLFELFSDFLKYFPGAHRQIYKNLQE

ISAFIGRSVEKHREALDPSAPQDLIDTYLLHMEKEKSNPHSEFNHKNLILNTLSLFFAGT

ETTSTTLRYGFLLMLKYPHVAERVYKEIEQVIGPHRPPALDDRAKMPYTDAVIHEIQRIA

DLLPIGLPHMVTRNTSFRGYTIPKGTEVFPILSAALHDPHYFEKPDTFNPDHFLDASGAL

KKNEAFIPFSLGKWDPQFLSQTPEGRSYPQLEKNNDCFLFIEHLIYSNCFTCLIPFHLHY

NPGRGL

>DAA19905.1

MELSVLLLFALLTGLLVLLARGRPKAHGRLPPGPRPLPFLGNLLQMDRKGLLKSFLRGYG

VIFANGERWKALRRFSLATMRDFGMGKRSVEERIQDEAQCLVEELRKSQGALQDPVFYFH

SITANIICSIVFGKRFDYRDPEFLRLLELLFQSFVLISSLSSQLFELYSSFLKYFPGSHR

QIYKNLQEINVFIGRSVEQHRETLDPNAPRDFIDCYLLRMEKDKSNPQSQFDHQNLIMSV

LSLFFAGTETTSTTLRYGFLLMLKYPHITERIQKEIDQVIGSYRPPALDDRAQMPYTDAV

IHEIQRFADLIPIGVPHMVTKDTHFRGYILPKGTEVYPVLSSALHESCYFEKPDDFNPDH

FLDANGVVKKNDAFMPFSIGKRICLGEGIARIELFLFFTTILQNFSVASPVAPEDIDLTP

QESGVGNVPPNYRIQFLPRQRG

>AAI12660.1

MELSMLLLFALLTGLLVLLARGRPKAHGRLPPGPRPLPFLGNLLQMDRKGLLKSFLRGYG

VIFANGERWKALRRFSLATMRDFGMGKRSVEERIQDEAQCLVEELRKSQGALQDPVFYFH

SITANIICSIVFGKRFDYRDPEFLRLLELLFQSFVLISSLSSQLFELYSSFLKYFPGSHR

QIYKNLQEINVFIGRSVEQHRETLDPNAPRDFIDCYLLRMEKDKSNPQSQFDHQNLIMSV

LSLFFAGTETTSTTLRYGFLLMLKYPHITERIQKEIDQVIGSYRPPALDDRAQMPYTDAV

IHEIQRFADLIPIGVPHMVTKDTHFRGYILPKGTEVYPVLSSALHESCYFEKPDDFNPDH

FLDANGVVKKNDAFMPFSIGKRICLGEGIARIELFLFFTTILQNFSVASPVAPEDIDLTP

QESGVGNVPPNYRIQFLPRQRG

>119910553

MALFTILFLILFIGLLLLWGRPASLGCLPPGPWPLPFLGNVLQMDQKGLLKSFQALREKY

GDVFTIYLGSRPAVILWWYEAMKEALVDQAEAFSGRGHIAIIDPVFQGTGVVFANGKSWK

VLRRFSVTTMKDFGIGKRSIEERIKEEAQCLVEELQKSQGAYLDPHFLFNAITANIICSI

VFRERFNYQDPRLLQLLHLLNEIFIILCSFYTQMFEIFPGILKHFPGTHTRLYSMIEEVK

DFITENMERHQKMLDPSGPKDFIDSFLLRMDKERLDPESEFHQKNLVHTVLSLFFAGTET

SSSTLCFGFLLLLKNPGVLEKVQAEIDRVIGAHRLPTLEDRAKMPYTDAVIHEIQRFSDL

VPVGVPHSVIKDTHFRGYYLPKGITVYPVLSSALQDPRHFEKPNTFYPGHFLDAQGNFRK

PEVFIPFSMGKRLCLGESLARSELFLFLTTLLQNFSLGSPKAPEDIDLTPRENGLGRPPP

LFQVRFLPRR

>301776635

MALSALLLLTVLVGLLLLWGYPASRGRLPPGPWPLPFLGNILQIDNQGFLKTFQMLRKKY

GDVFTVYLGPRPVVILCGYKAVKEALVGQAEAFSGRGHIAIIDPILRGKGVVFANGKSWK

ALRQFSQLTMRDFGMGRQSIEKRIQEEAQCLVEELRKSRGAYLDPTFFFHSAMANIICTI

ILGQRFNYQDPRFLHLLHLMNDLSDISSSFYGQMFELLSGILKHFPGIHTRLYSKIQEME

DFVVESIERHREMLDPSTPKDFIDSFLVRMDKESSDPESEFHYKNLVYTVLSLFFAGTES

SSNTLLHGLLLLLKNPDVLEKVQMEIDRVIGSHHLPALEDRAKMPYTDAVIHEIQRLGDV

IPLGIPRSVIKDTHFRGYHLPKDTTVYPILSSVLHDPCHFEKPEAFHPGHFLDAEGNFRT

QGAFIPFSLGRRLCLGESLARSELFLILTSVLQNFTLGSPKALEDIDITSQRNGLLKMPP

EFKLCFLPRQGVGPGTQIP

>0901269A

MEFSLLLLLAFLAGLLLLLFRGHPKAHGRLPPGPSPLPVLGNLLQMDRKGLLRSFLRLRE

KYGDVFTVYLGSRPVVVLCGTDAIREALVDEAEASFGRIAVVDPIFQGYGVIFANGERWR

ALRRFSLATMRDGYMGKRGVEERIQEEARCLVEELRKSKGASIVFGKRFDYKDELFSGFL

KHFPGNLQEINTFIGQSVEKHRATLDPSNPRDFIDVYLLRMEKDKSDPEFQFLRQNLIML

KYPHVTERVQKEIEQVIGSHRPPALDDRAKMPYTDAVIHEIQRLGDLIPFGVPHTVTKDT

QFRGYVIPKNTEVFPVLSSALHDPRYFETPNTFNPGHFLDANGALKRNEGFMPFSLGKRI

CLGEGIARTELFLFFTTILQNFSIARIDLTPRESGVGNVPPSYQIRFLAR

>327276371

MELEWVLSISLGIFLVLISAWKWRHMEGRFPPGPMPLPFFGNLLQLNPKDIPKSFLELRD

KYGPVYTLYLGPRRVVVLCGHDVLKEALVDCGEQFSGRGEMATLDQTCRGFGFAFSNGER

WKKLRHFSLITLKNFGMGKCSIEERIQEESQYLLEKFRKTHGLPFDPTFLLSCTTSNIIC

SIVFGSRFEYEDKTFLYLLDVINKAFYEMSTPWAQMYEMYSGIMQHLPGGHKRLYNLLEE

LNTFIAERIKINQETLDPKNPRDFIDCFLIEMEKEKGDPSTEFTMTNLIITVLNLFFAGT

ETISSTLRYLFLLMLKYPEVEEKVHQEIDCVVGRNRIPAVKDRVNMPYTNAVIHEIQRLV

DILPVGLPHKVTEDIEFRGYLLPKDTNIITLLGSALHDPQYFSDPEIFNPEHFLDEEGHF

KKIDAFVPFSAGKRACIGEPMAHMELFLYFTTILQNFSMKFSLDPKDIDISPQLSGFAKI

PPVYQLCPMPH

>EHB18322.1

MELGGAFTIFLALYLSCLLILIAWKRTNKGGKLPPGPTPIPFLGNLLQVRTEGTFQSLMK

LHEKYGSVFTVYMGPRPVVVLCGHEAVKEALVDQADDFSGRGEIASIERNFQGYGVALAN

GERWRILRRFSLSILRDFGMGKRSIEERIQEEASYLMKEFWKTKGAPIEPTFFLSRTVSN

VISSVVFGSRFDYEDKQFLNLLQLINESFIEMSIPWAQLYDMYSGIMQYFPGRHNRIYYL

IEKLKDFIASRVKINEESLDPQNPRDFIDCFLIKMHQEKNNPHTEFNLKNLVLTTLNLFF

AGTETVSSTLHRDNLSIFSAMFSDLAKIYEEINQVIGPHRIPSVDDRSKMPYTDAVIHEI

QRLTDIVPMGVPHIVIRDTQFRGYFLPKGTDVYPLLGSVLRDPKYFCYPDAFYPQHFLDE

QGCFKKNEAFVPFSSGKRVCLGESMARMELFLYFTSILQNFSLHSLVPPADIDITPRISG

FGNIPPIYKLCLKAR

>EGW14145.1

MEASVLLFLTLLLGFVVLLLRGHPKAHGHLPPGPRPLPFLGNLLQMDRRGLLNSFMRLRD

KYGDVFTVHLGPRPVVMLYGTEAIREALVDQAEAFSGRGTIAVVKPTFKDYGVIFANGER

WKALRRFSLATMRDFGMGKRSVEERIQEEAQCLVEELQKSQGAPLDPKFLFQCITANIIC

SIVFGERFDYKDRQFLRLLDLIFQIYSLISSFSSQVFELFSGFLKYFPGTHKQIHRNMKE

ILDYIDHSVEKHRATLDASNPRDFIDTYLLRMEKEKSNPHTEFHHQNLIISVLSLFFAGT

ETSSTTLRYGFLLMLKYPHVAGAPLDPKFLFKCITANIICSIVFGERFDYKDRQFLRLLD

LIFQIYSLISSFSSQVFELFSGFLKYFPGAQVRISKITQEILDYIERSVEQHQKTLDPSN

PRDFIDTYLLRMEKEKSNPHTEFQQKNLVLTVLALFFAGTETSSTTLHYGFLLMLKYPHV

AEKVQKEIEEVIGSHRLPTLDDRTKMPYTDAVIHEIQRLSDVVPIGVPHKVTKDTLFRGY

LLPKDTEVYPILSSALHDPQYFEQPDTFNPDRFLDANGALKKNEAFMPFSIGKRICPGEG

IARNELFLFFTTILQNFSLSSPVDPKDIDLSPKESGFGRVPPEYQICFLAR

>126329368

MNAGDLLLLLAVFLGFLLLLAKRPQKVNLPPGPTPLPLLGNLLQLGRHGLIKSFLECRDK

YGDVFTVYLGTRRYFVICGPESVKKALVDNAEVFSGRGTLAISEMVFQRYGFFVTYDGWK

TLRRVSIATLRDFGMGKRSIEEQIKAEAQCLVEELQKSQGALIDPTFIFHSVTANVICSI

VFGERFSYQDTQFREILNMFVEVFTILSSLWMQFFEQFPSVLKLLPGPHYRVLKIAQHIK

DFISSKIEQHQESLDPNSPRDFIDSFLLRIEKEKETPDSKYHVKNLVLTVLSLFFAGTET

TSTTLRYGFLLLLKYPQVAEKVQEEIDQVVGRDRAPEIKDRAKMPYTDAVIHEIQRFSDL

LPMGIPHMVTEDTSFQGYFLPKGTDVFILLSASLKDPCYFEKPHIFDPNHFLDAQGALKK

NEAFIPFSMGKRACLGEGIARTELFLFFTTILQNFSLVSSKPLEDLDIRPQCSGLGTLPQ

IYKLGFLPR

>350585223

MELGGAFTIFLALCLSCLLILIAWKRISKGGKLPPGPTPIPFLGNLLQVRTDATFQSFMK

LREKYGPVFTVYMGPRPVVVLCGHEAVKEALIDRADEFSGRGELASVERNFQGHGVALAN

GDRWRILRRFSLTILRDFGMGKRSIEERIQEEAGFLLQELRKTKGAPIEPTFFLSRTVSN

VISSVVFGSRFDYEDKQFLKLLQMINQSFIEMSTPWAQLYDMYSGIMQYLPGRHNRIYYL

IEELKDFIASRVKINEASLDPQNPRDFIDCFLIKMHQDKDNPHTEFNLKNLVLTTLNLPI

SKSLMISATITSCFIACDVFWSFPAKIHEEIDQVIGPHRIPRVDDRVKMPYTDAVIHEIQ

RLTDIVPMGVPHNVIRDTHFRGYLLPKGTDVFPLLGSVLKDPKYFRYPDAFYPQHFLDEQ

GRFKKNEAFVPFSSGKRICLGEAMARMELFLYFTSILQNFTLRSLVPPAHIDITPQVSGF

GNIPPTYELCLIAR

>350585225

MDPYIFLALCLSCLLILIAWKRISKGGKLPPGPTPIPFLGNLLQVRTDATFQSFMKLREK

YGPVFTVYMGPRPVVVLCGHEAVKEALIDRADEFSGRGELASVERNFQGHGVALANGDRW

RILRRFSLTILRDFGMGKRSIEERIQEEAGFLLQELRKTKGAPIEPTFFLSRTVSNVISS

VVFGSRFDYEDKQFLKLLQMINQSFIEMSTPWAQLYDMYSGIMQYLPGRHNRIYYLIEEL

KDFIASRVKINEASLDPQNPRDFIDCFLIKMHQDKDNPHTEFNLKNLVLTTLNLPISKSL

MISATITSCFIACDVFWSFPAKIHEEIDQVIGPHRIPRVDDRVKMPYTDAVIHEIQRLTD

IVPMGVPHNVIRDTHFRGYLLPKGTDVFPLLGSVLKDPKYFRYPDAFYPQHFLDEQGRFK

KNEAFVPFSSGKRICLGEAMARMELFLYFTSILQNFTLRSLVPPAHIDITPQVSGFGNIP

PTYELCLIAR

>BAA93554.1

MLASGLLLVTVLAFLVILVLMSVWKQRELSGKMPPGPTPLPFIGNYLQLNTEEIYNSLMK

IRERYGPVFTVHLGPRRRIVILCGQEAVKEALVDHALEFSGRGENATFDWLFKGYGVAFS

PWERSKPLRRFSIATLRDFGMGKRGIEERIQEEADYLLEALQKTNGALIDPTFYLSRTVS

NVMSSIVFGNRFDYEDKEFLSLLKMMTESFQFTASSTGQLYEMFSSVMKHLPGPQQKAFK

ELQGLEDFITKKVEQHQSTLDSNSPRDFIDSFLIHMQAEKKNPNTEFYMKNLVLTTLNLF

FAGTETVSTTLRYGFLLLMKHPDVEAKIHEEINQVIGRNRQPKYEDRMKMPYTEAVIHEI

QRFADLIPMGLPRRVTKDTKFRDFLIPKGTEVFPMLGSVLNDPKFFSNPKDFNPKHFLDD

NGQFKKNDAFIPFSLGKRNCFGEGLARMELFLYLTNILQNFHLRSSQAPRDIDVSPKMVG

FGTIPPNYTMSFLSH

>390479051

MGLGWALTMFLELFFSCLLIFVAWKRMNKGGKLPPGPTPIPFLGNLLQVQTDAVFQSFMK

LSEKYGPVFTVYMGPRPVVVLCGHEAVKEALVDQADDFSGRGQLASVERNFQGYGVALSN

GERWRSPRRFSLTILQNFGMGKQSIEEQIQGRNSYLLEEFQKTKGAPIDPTFFLGRSVSN

VISSVVFGSRFDYEDKQFLKLVQLVNEXFIEMSTPWTQLYDMYSGIMQYLPGRHSRVYCL

IEELKDFVAPRVKINEATFDHQNPQDFIDCFLVKMHQDKNNPHTEFSLKNLVLTTLNLFF

AGTETVSSTLRYGFLLLMKHPEVEAKILEEINQVIGPHQLTSVDDRVKMPYTDAVIHEIQ

RLIDIVPVGVPHNVTRDTQFRGYLLPKGTNVLALLGSVLKDPKYFRYPDAFYPQHFLDEN

RRFKKNDAFVPFSSGKRICLGEAMARMELFLYFILILQNFSLRSLVPPADIDITPKLSGF

GNIPPTYKLCLVARSAPPPVGQGGSSEERSSPLLSSLLRLSNPGSIP

>327276271

MEWSGAVTALLVVYVSCLLLISAKRKLSQKGRLPPGPTPLPLIGNFLQIKSTKTLQSLLK

LRDEYGSVFTVYFGTRPILVLCGHQAVKEALIDKAEEFSGRSTLPTLERNFQGHGVVFAN

GERWKQMRRFSLTVLRNFGMGKKSIEERIKEEAQFLLEEFQKMKEKPFEPTYFLSRAVSN

IICSIVFGDRFDYEDKEFQALMEMMNNSFREMSTGWAQFYDIYVDFLKYFPGPHTKIYNI

LEDMRVFIAKRVKKNQETFDPNFPRDFIDCFLIQMEKEKGNPTTEFNVKNLELNTLNLFF

AGTETVSSTLRYGFLLLMKYPEVQAKMHEEIDRVIGHNRVPNIEDRSQMPYTDAVIHEVQ

RFSDLLPMDLAHRVIRDTEFRGYLLPKGMEVYPLLTTVLHDPTMFKSPNTFNPENFLDED

GRFKKNDAFVPFSSGKRMCLGEALARMELFLFFTTTLQSFQLKSLVLPEDIDLTPQESGF

ANIPPFYQLSIIPR

>327276263

MEWVGTIVLLLVYFSWRLLISSKRKLWHKGRLPPGPTPLPLIGNFLQIKASQTLKSLLKL

SEKYGPVFTVYFGSHPVLVLCGHQAMKEALIDKAEEFSGRTTLPVLEQTFQGYGVIFSNG

ECWKQMRRFSLSILRGFGMGKKSIEERIQEEAQFLLEEFRKMKEKPFDPTYRFSCALSNI

ICSIVFGDRFDYEDKEFQALMEMLCNTFREISTARSQFYNIYVSFLKYFPGPQTKVYDLM

LGMRVFICKRIKENQETLDPNFPRDFIDCFLIQMEKEKDHPSSEFHIKNLEMTTLNLFFA

GTESTSSTLRYGCLLLMKYPEVQVKVHEEIDRVIGRNRVPNSEDRKQMPYTDAVIHEVQR

WSDLIPMGVARMVIRDTEFRGYLLPKGMEVYPVLSSALHDPTMFKSPNAFNPENFLDENG

CFKKNEAFVPFSLGKRICLGEALAFMELFLFFTTILQNFQLKPLVPPQDLDINPLESGFA

NIPPFYQLSAIPR

>354505855

MEASVLLFLTLLLGFVVLLLRGHPKAHGHLPPGPRPLPFLGNLLQMDRRGLLNSFMRLRD

KYGDVFTVHLGPRPVVMLYGTEAIREALVDQAEAFSGRGTIAVVKPTFKDYGVIFANGER

WKALRRFSLATMRDFGMGKRSVEERIQEEAQCLVEELQKSQGAPLDPKFLFQCITANIIC

SIVFGERFDYKDRQFLRLLDLIFQIYSLISSFSSQVFELFSGFLKYFPGTHKQIHRNMKE

ILDYIDHSVEKHRATLDASNPRDFIDTYLLRMEKEKSNPHTEFHHQNLIISVLSLFFAGT

ETSSTTLRYGFLLMLKYPHVAGASQEKKNEAFMPFSIGKRICLGEGIARNELFLFFTTML

QNFSLSSPVDPKDIDLSPKESGFGRVPQEYQICFLSR

>AAA37084.1

MLVSGMLLVVVLTCLSVMIIMSVWRQRRLLRKMPPGPTPLPFIGNFLELDTEKFYDCLSK

MRERYGPVFTIHLGPRPAVMLWGYDAVKEALIDQAEELSDRGEQAFFDWFFKGYGVVFSS

GERAKQLRRFSIATLRDFGFGKRGIEERTIEETSFLIQALRDTNGATIDPTFYMSRTVSN

VISSIVFGNRFEYDDKEFLSLLGMIMRSFQFMSTSTGQLFEMFYSVMKHLPGCQHQAYKE

MQGLEDFIARKVEENQRTLDPNSPRDFIDSFLIRMQEEKKNPRTQFHMRNLLMTTLNLFF

AGTETVSTTTRYGFLLLMKYPHIAAKMHEEIDQVIGRNRQPKYEDHLKMPYTEAVIYEIQ

RFVDVVPLGLPRSTTKDIKFRDFLIPKGTDVFPVLSSVLKDPKFFSNPNDFNPQHFLDDK

GQFKKSNAFMPFSVGKRYCFGESLAKMELFIFFTTIMQNFCFKSPQAPQDIDVTPQYFSF

AAIPPKFTMSFLPR

>EHB18325.1

MLASGLPLVALLVFLSIVILMSVWQHRRLHGKLPPGPTPLPFIGNYLQLNTEQMYNSIMK

IKEQYGPVFTIHLGPRRVVVLCGYDAVKEALIDQAEEFSGRGKQATFDWIFKGYGVAFSS

WERARHLRRFSISTLRDFGMGKRGIEERIQEEIGFLIESFRETQGAFIDPTFYLSRAVSN

IISSIVFGDRFDYEDKEFLSLLRMMLGSFQFTATSTGQLYEMFYSVMKYLPGPQQQAFKE

LQGLENFIVKKVEQNQRTLDPSSPRNFIDSFLIRIQKVFCLCEKNSNAEFHMKNLVMTTL

SLFFAGTETVSTTLRYGFLLLMKHPDVEAKIHEEIDRVIGRNRHPMFEDKAKMPYTDAVI

HEIQRFGDMIPMNLAHSVTKDTKFRNFLLPKGTEVFPVLGSVLRDPKFFSNPQDFHPQHF

LDEKGQFKKNDAFVPFSLGKRYCLGEGLARMELFLFLTSILQNFRFKSPQARQDIDVSPK

LVGFATIPQNYTMSFLPR

>327276377

MEWTVSVTFFLVVFISFYLLLSSKRKRLHKKKLPPGPTPLPLIGNFLQIKSSETLKSLLK

IRDKYGPVFTVYFGSRPVLVLCGHDAVKEALIDKAEQFSGRITTPIFEQTFQQYGVVFAN

GERWKQLRRFSLTILKNFGMGRKSIEERIQEEAQFLLEEFQKTKEKPFEPSSFLSRAVSN

IICSIVFGDRFDYEDKEFQSLMEMIHNAFQEMSSTWSQLYDIYVSFLKYFPGPHSRVHHI

LEELRLFIAKRVKKNQETLDSNSPRDFIDSFLIQIEKEKGNPDSGFDIKNLELTTLCLFF

GGTETVSSTLRYGFLLLMKYPKVQAKVHDEIDRVIGHNRIPNIEDRSQMPYTDAVIHEIQ

RFSDLLPMSVAHMVTCDTEFRGYFIPKGMIVYPMLTSVLHDPTMFKSPNVFNPENFLDEN

GRFKTNDAFVPFSSGKRICLGESLARMELFIFFTTILQSFYLKSLVPPEDIDITPQECGF

ATIAPLYQLSIIPRRA

>P24454.1

MLVSGMLLVVVLTCLSVMIIMSVWRQRRLLRKMPPGPTPLPFIGNFLELDTEKFYDCLSK

MRERYGPVFTIHLGPRPAVMLWGYDAVKEALIDQAEELSDRGEQAFFDWFFKGYGVVFSS

GERAKQLRRFSIATLRDFGFGKRGIEERTIEETSFLIQALRDTNGATIDPTFYMSRTVSN

VISSIVFGNRFEYDDKEFLSLLGMIMRSFQFMSTSTGQLFEMFYSVMKHLPGCQHQAYKE

MQGLEDFIARKVEENQRTLDPNSPRDFIDSFLIRMQEEKKNPRTQFHMRNLLMTTLNLFF

AGTETVSTTTRYGFLLLMKYPHIAAKMHEEIDQVIGRNRQPKYEDHLKMPYTEAVIYEIQ

RFVDVVPLGLPRSTTKDIKFRDFLIPKGTDVFPVLSSVLKDPKFFSNPNDFNPQHFSDDK

GQFKKSNAFMPFSVGKRYCFGESLAKMELFIFFTTIMQNFCFKSPQAPQDIDVTPQYFSF

AAIPPKFTMSFLPR

>327276375

MELPGAVSLFLVIYTFVLVILTVMRDLSRRKTLPPGPTPLPFLGNIMQIKSTELLKSLLK

LHEKYGPVFTVHFGPRPVVVLCGHKAVKEALVDKAEEFSGRVTNATVDDTFQGYGVIFSN

GERWKQLRRFSLTIMRNFGMGKKTIEERIKEESQFLLQEFRKTNEKPFNPTFFLSRAVSN

VVSSILFGSRFDYEDKLFQNLMQSSNDIFRLMSTPSGQRYDVYYSFLKYFRGTQTKINEL

LKQVKDVIADLVKLNQETLDLNNPRNYIDCFLIQMEKEKDNPDSEFTIRNLELSVLILLF

AGTETVSSTLRYGFLFLMKYPEVQAKMHEEIDRVIGQDRPPNIGDRTLLPYVDAVVHEVQ

RFSDLIPLDLPHMVMRDTEFRGYMIPKGTEVYPLLNTVLFDPTMFKTPYAFNPEHFLDEN

GRFKKSDAFVPFSSGKRVCLGETLARMELFIFLATILQKFELKPLVAPKDIDLTPHEVGF

GRIPPYYQLCMISLMRDFSRRKTLPPGPTPLPFLGNILQVKSTELLTSLLKGRSSTPPSS

SAVLSNVVSSILFGSRFDYKDKVFLKLTQTSNDIFRLMSTPSGQRYDVYYSFLKYFRGTQ

TKINELLKQIQDVIVDLVKLNQETLDLNNPRNYIDSFLIQMEKEKDNPDSEFTIRNLELS

VLILLFAGTETVSSTLRYAFLFLMKHPEIQAKMHEEIDRVIGRDRPPNIGDRTLLPYVDA

VVHEVQRFSDLIPLDLPHMVMRDTEFRGYMLPKGTEVYPLLNTVLFDPTMFKTPYAFNPE

HFLDENGRFKKRKRVCLGETLARMELFIFLATILQKFELKPLVAPKDIDLTSHEDGTYDH

SAAWCGEFTYHFADKVAQIRCNLDANFDAIQQSDVSIAHSNELWCGRGIVTGILIDLGGN

GVRKLSSKGRLPPGPTPLPLIGNFLQIKSLEILKSLLKLREKYGPVFTVYFGTRPIVVLC

GHDAVKEALIDKAEEFSGRATNPTLERTFQGHGVVLSTGERWKQLRRFSLTVLRDFGMGK

KSIEERIQEEAQFLLEEFKKTKEKPFDPTYFLSRTVSNVICSIIFGDRFDYEDKEFQSLM

EMINITFREMSSGQAQFYDMYVSFLKYFPGPPVKVYDSLENMRLFIAKKVKKNKDTFDPN

FQRDFIDCFLNQMEKVFGTSEFHDRNLELTTLNLFVAGTETVSSTLRYGFLFLMKHPEVQ

AKVHEEIDKVIGRSRVPNIEDRSQMPYVDAVIHETQRCSDLVPMDVAHRVIRDTEFRGYL

IPKGTEIYPILSSVLHDPTMFKRPFAFDPENFLDENGRFKKNDAFIPFSSGKRICLGESL

ARMELFLFFTTILQSFHLKPTIPPEDIDLTPLESGLITVPPFYQLSVVPR

>395751224

MVPLLCLTVMVLMSVWRQRKSRGNLPPGPTPLPFIGNYLQLNTEQMYNSLMKISERYGPV

FTIHLGPRRVVVLCGHDAVKEALVDQAEEFSGRGEQATFNWLFKGYGVTCRTWERTKPLR

HFSIATLRDFGVGKRGIKXRIQEKAGFLIKALXGTRGDSSIDPTFFLSRTVSNVISSIVF

GDRFDYEDKEFLSLLRMMLESFQFTATSTGQLYEMFSSVMKHVPGPQQQAFKELQGLEDF

IAKKVEHNQRTLDPNSPRDFIDSFLIRMQEEEKNPNTEFYLKNLVMTTLNLFFAGTETVS

TTLRYGFLLLMKHPEVEAKVHEEINRVIGKNRQPKFEDRAKMPYTEAVIHEIQRCGDMLP

MGVARRVNKDTKFRDFFLPKGTEVFPMLGSVLRDPRFFSNPQDFYPQHFLDEKGQFKKSD

AFVPFSIGKRYCFGEGLARMELFLFFTTIMQNFRFKSPQSPKDIDVSPKHVGFATIPRNY

TMSFLPR

>301620108

MDITGLGTLVLILLISCIVIYSTWNSMYRKRNLPPGPTPLPLIGNLLQIKRGEMVKSLTE

FGKQYGPVYTLYLGPRPVIVLNGYQAVKEALIDQGEEFSGRGKLVVADLIFGGFGVVFSN

GDRWKQLRRFSLMTLRDFGMGKRSIEERIKEEAQCLQVELHKYKQTPTDPQNILVQAVSN

VICSVVFGNRFEYENSEFLKLLRLFNETFQMMSSTWGQLQQIIPFIMNYIPGPHQKIDKV

VARQLEFVSERVKKNQETIDFNSPRDFIDCFLIKMQQETQNPTSEFNLKNLLMTVLNLFV

AGTETVSSTLRNGILLLLKYPHIQEKLHKEIDVVIGQNRSPNIDDRSKMPYMDAVIHEIQ

RFTDILPMNLPHSVIKDTAFQGYTIPKDTDVYPMLCSVLRDPTQFTTPENFNPEHFLDDS

GCFKKSDAFMPFSTGKRICLGEGLARMELFLFLTTILQNFTLTSETQITESDITPRMAGF

ANVPISYKVSFVPR

>AAH76922.1

MDTTWLWSLQLFLLIATMLIYSTWNKMYRKRNLPPGPTPIPLFGNVLQIKRGEMVKSLLE

LGKKYGPVYTLYFGPSPVIILCDYQSIKEALNDQAEEFSGRGKIPSWDQFFQGYGESFSN

GDEWKQLRRFSLTTLRNFGMGKRGIEERIQEEAQFLVAEIKSYKGKPFDPTKILVQCVSN

VICSVVFGQRYEYSNKDFHKLLYMFQAVFEDTSSTLGQLMTLLPNIMNHIPGPHKTVVNK

LNKVNDFILQRVKENEKTLDPNSPRHFIDSFLIQMQKEKDNPVTKFHWKNLLCTIMNLFF

AGTETVSTTLRHGFLMLLIHPEIEEKLHEEIDRVVGQDRSPTIEDRSKMPYTDAVIHEIQ

RFSDVLPMSLPHLVMKDTQFRGYTIPKGTDVYPLICAALRDPKQFATPNKFNPQHFLDDN

GLFKSSNAFLPFSTGKRICLGEGLARMELFLFLTNILQNFKLHSENQFAEDDIAPKMNGF

ANYPLSYEFSLIPRVQSLLVL

>AAA52143.1

MELSVLLFLALLTGLLLLLVQRHPNSHGTLPPGPRPLPLLGNLLQMDRRGLLKSFLRFRE

KYGDVFTVHLGPRPVVMLCGVEAIREALVDNAEAFSGRGKIVIMDPVYQGYGMLFANGNR

WKVLRRFSVTTMRDFGMGKRSVEERIQDEAQCLIEELRKSKGALVDPTFLFHSITANIIC

SIIFGKRFHYQDQEFLKTLNLFCQSFLLISSISSQLFELFSGFLKYFPGAHRQVYKNLQE

INAYIGHSVEKHRETLDPSAPRDLIDTYLLHMEKEKSNPHSEFSHQNLIINTLSLFFAGT

ETTSTTLRYGFLLMLKYPHVAERVYKEIEQVVGPHRPPALDDRAKMPYTEAVIREIQRFA

DLLPMGVPHIVTQHTSF

>AAH74204.1

MDTSWLWTLLLSLLISCILIYSTWNKMYRKRNLPPGPTPIPLFGNVLQIKRGEMVKSLIE

YGKKYGDTYTLYFGPSPVIILCSYRATKEALIDQAEDFSGRGAMPSFDQYFQGYGVVFTN

GEEWKQLRRFSLTTLRNFGMGKRGIEERIQEEAQFVVEEIKSYKKKPFDPTDILVQCVSN

VICSVVFGNRFEYDNKDFQNLLSLFQSVFRESSSAWGQLLNMFPLIMNHIPGPHKKVIRD

MNKLEAFVLQRVKENEKTLDSNSPRDIIDSFLIKMQQENENPTSAFHMKNLLATVLSIFF

AGTETVSTTLRHGFLILLIYPEIEAKLREEIDRVIGQNRSPTIEDRSKMPYTDAVIHEIQ

RFSDVIPMNVPHLVTKDTQFRGYTIPKGTDVYPLLCAVLRDPEKFATPYEFNPNHFLDDN

GCFKSNDGFMPFSTGKRICLGEGLARMELFLFLTNILQHFKLHTESRLIEDDIAPKMNGF

ANYPTSYQLSFIPR

>327291296

LRDKYGPVFTVHLGPRPVVVLCGHEAVKEALVDQAEEFSGRGELASLDRNFNGTGVALAN

GERWRQLRRFSLTALRNFGMGKQSIEERIQEEAQFLVEAFRETQGLPFDPTFFLSRTVSN

VISSVVFGRRFDYEDQTFLSLLHKIHESLLEMSTPWAQVRNMFSCVMRYLPGRHNHIYSL

MEDLKAFIAEKAQANLETLDPDNPRDFIDCFLIQMEKEKGNPSSEFNMENLVPTALNLFF

GGTETVSSTLRYGFLLLMKHPDVEEKVHQEIDRVIGRERLPSIEDRKRMPFTDAVVHEIQ

RVTNIVPLGMPHSVVRDTHFRGFLLPKGTNVFPLLGSVLTDPKYFHNPEKFNPGHFLDAN

GCFKKNEAFVPFASGKRVCLGEAMARMELFLYVVIILQNFSLKALVPPEDIDLTPQVSGF

ANIPPEYRMCLVPRC

>327276265

MEWAGTLILLLAVFIFCHLLTSSKRKQLHKGRLPPGPTPLPLIGNLLQIKLSEPLKSLLK

LRENYGPVFTIHFGTRPVLILSGHQAVKEALIDKAEEFSGRTTMPSMEPSFQGYGMGFSN

GQRWKEMRRFSLTVLRNFGMGKKSIEERIQEEAQFLLEEFRNTKGKPCDPTFLLSRAFAN

IICSIAFGNRFDYEDKEFQALMVMANKFFWEMSTSWAQFYDMYANYLNYIPGIHNKLYDC

MDMKLFIAKRIKKNQETFDPNFPRDYIDCFLIQMEKEKDNPHSEFIMKNLEVNILNLILG

GTETGSSTMRYGFLLLMKYPEVQAKVQEEIDRVIGPNRVPNAEDRRQMPYTDAVIHEVQR

CSDVLPMDLVHMVIHDTEFRGYHIPKGMTVYPLLSTVLHDPTMFKSPNAFNPENFLDENG

CFKKNDAFVPFSSGKRICPGESLARMELFLFFTTILQNFQLKSLVPPEDIDLTPQMSGFY

NIPPFYQLCAIPR

>395859635

MELGGALTIFLELCLTCLTIILVWKRINKGGKLPPGPTPIPFLGNLLQVRPDATFQSFMK

LREKYGPVFTVYMGPRPVVVLCGHKAVKEALVDQGDEFSGRGELATIEQNFQGHGVVMAN

GDRWKILRRFSLTILRDFGMGKRSIEERIQEEAGYLLEEFRKTKGAPIEPTFFLSRTVSN

VISSVVFGSRFDYEDKQFLRLLQLMNETFIEMSTPWAQLYDMYSGIMQYLPGKHNHIYNL

IEELKDFVASKVKINEASLDPQNPRDFIDCFLIKMHQVPPPLPESFPLTSSLSTACTLTF

YWGQKCIIWSSFSNKNTSCPFFWMLPAKIHEEIDQVIGPQRIPSVNDRVKMPYTDAVIHE

IQRLLDFVPMGVPHSVIRDTHFRGYLLPKGTDVFPLLGSVLKDPKYFRYPDAFYPQHFLD

EQGCFKKNEAFVAFSSGKRVCLGEALARMELFLYLTSVLQNFSLRPLVPPADIDVTPKLS

GFGNIPPTYELCLVAR

>403259873

MDPAMALVFCLSCLLLLSLWSQRSGRGRLPSGPTPLPIIGNILQLDVKDISKSLTNFSKV

YGPVFTVYFGLKPIVVLHGYEAVKEALIDHGEEFSGRGSFPVAEKVNKGLGILFSNGKRW

KEIRRFSLMTLRNFGMGKRSIEDRVQEEARCLVEELRKTNASPCDPTFILGCAPCNVICS

VVFQKRFDYKDQRFLNLMEKFNENLRLLSSPWIQVCNNFPALIDYLPGSHNKVLKNFAYV

KSYVLERIKEHQESLDVNNPRDFIDCFLIKMEQEKHNQQSEFTTENLIATVTDMFGAGTE

TTSTTLRFGLLLLLKYPEVTAKVQEEIEHVVGRNRSPCMQDRSHMPYTDAVVHEIQRYID

LIPTNLPHAVTCDVKFRNYLIPKGTTIITSLNSVLHNDKEFPNPEMFDPGHFLDKSGNFK

KSDYFIPFSTGKRMCVGEGLARMELFLFLTTILQNFNLKSQVDPKDIDITPIANSFGRVP

PLYQLCFIPV

>407261476

MPKKLINKTASHLISQRYGPVFTIYLGPRRIVVLCGQEAVKEALVDQAEEFSGRGEQATF

DWLFKGYGVAFSSGERAKQLRRFSIATLRDFGVGKRGIEERIQEEAGFLIDSFRKTNGAF

IDPTFYLSRTVSNVISSIVFGDRFDYEDKEFLSLLRMMLGSFQFTATSMGQLYEMFSSVM

KHLPGPQQQAFKELQGLEDFITKKVEHNQRTLDPNSPRDFIDSFLIRMLEEKKNPNTEFY

MKNLVLTTLNLFFAGTETVSTTLRYGFLLLMKHPDIEAKVHEEIDRVIGRNRQPKYEDRM

KMPYTEAVIHEIQRFADMIPMGLARRVTKDTKFRDFLLPKGTEVFPMLGSVLKDPKFFSN

PKDFNPKHFLDDKGQFKKNDAFVPFSIGKRYCFGEGLARMELFLFLTNIMQNFHFKSTQA

PQDIDVSPRLVGFATIPPTYTMSFLSR

>327289003

MTDPHFVKVLALFMSDKRKRLHKGKLPPGPTPLPLIGNFLQMKSGETLKSLLKLHEKYGP

VFTVYLGTRPVLVLCGHQAVKEALIDKAEEFSGRTTKPTLERAVEGYGVCFCNGERWKQL

RRFSITVLRSFGMGKKSIEERIQEEAQFLLEELRKTKGKPLEPTDLLSRAVCNIISSIVF

GERFDYENEEFQALMTIIHNFFWEMSSTWSQLYDMFPTLLKYFPGPHTRVYNIVSDALRF

IGKRVKKNQETLDSNFPRDFIDCFLIQMEKEKDNPLSEFNIKNMELTIFDLFFAGTETVG

LTLRYGFLLLIKYPEVQAKVHEEIDRVIGHNRTPKSEDRRQMPYTDAVIHEIQRVSDIAP

MGVAHMVTCDTEFRGYFIPKGMEVFPLLSTVLHDPTMFKSPSVFNPENFLDENGCFKKND

ASVPFSSGKRICLGESLARMELFLFFTTILQSFQLKPLVPREDLDPTPLENGFLNVSPVY

HLSIIPR

>EDL24211.1

MLGSGLLLVAILAFLSVMVLVSVWQQKIRGKLPPGPIPLPFIGNYLQLNRKDVYSSITQL

QEHYGPVFTIHLGPRRVVVLYGYDAVKEALEDNAEEFSGRGEQATFNTLFKGYGVTFSNG

ERAKQLRRFSIATLKDFGLGKRGMEERIQEEAGCLIKMLQGTCGAPIDPTMYLSKTVSNV

ISSIVFGDRFNYEDKEFLSLLQMMSQMNQFAASPTGQLYDMFHSVMKYLPGPQQQIIKDS

HKLEDFMIQKVKHNHSTLDPNSPRGFIDSFLIHMQKEKNFNSEFHMKNLVMTSLNLFFAG

SETVSSLLRYGFLLLMKHPDVEAKVHEEIDRVIGRNRQPQYEDHMKMPYTQAVIHEIQRF

SNFAPLGIPRRITKDTSFRGFFLPKGTDVFPIMGSLMIDPKFFSSPKDFNPQHFLDDKGQ

LKKIPAFLPFSIGKRSCLGYSLGKMQLFLFFTTILQNFRFKFPRKLEDINESPKPEGFTR

IIPKYTMSFVPI

>403259867

MDPFVVLLLCLSFLLLFSLWRQSSGRGKLPPGPTPLPIIGNILQIDVKDTSKSFGNLSKV

YGPVFTVYFGMKPVVVLHGYEAVKEALIDNGEEFSGRGIFPVSQRTSKELGIIFSNGKRW

KEIRRFSLTTLRNFGMGKRSIEERVQQEARCLVEELRKNEASPFDPTFILGCAPCNVICS

VVFQNRFDYKDETFLTLMKRFNENFRILGSPWIQVCNIFPLFLDYFPGTHNKLFKNVALT

KSYIEEKIKEHQASLDINNPRDFIDCFLIKMQQEKDNQESEFTIENLVSTVADLFVAGTE

TTSTTLRYGLLLLLKHPEVTAKVQEEIDHVIGRHRSPCMQDRSHMPYTDAVVHEIQRYIN

LVPAGVPHAVTTDIKFRNYLIPKGTAIMSSLTSVLYSDKEFPNPTTFDPGHFLDKNGNFK

KSDYFMPFSAGKRICAGEGLARMEIFLFLTTILQNFNLKYVGDIKNLNTTAISNGMVSLP

PLYQICLIPV

>P24455.2

MLGSGLILVAILAYLSVMVLVFVWKQKFRGKLPPGPTPLPYIGNYLQLNTKDIYSSITEL

SERYGPVFTIYLGPRPVVVLYGYDAVKEALVDQAEEFSGRGEQATYNTLFKDYGVAFSSG

ERAKQLRRFSIATLRDFGVGKRGVEERIQEEAAYLIKMLRSTRGAPIDPNDYLSQTVSNV

ISSVVFGDAFDYEDKEFLELLHMMNEMNKFAASPVGQLYDMFHSVMKYLPGPQQQIIKNT

KELEDFMIRKVKQNQSTLDLNSARNFIDSFLIHMHEEKKNPTSEFNIKNLVMTSLNLFFA

GSETVSSTIRYGFLLLMKYPEVEAKVHEEIDRVIGRNRQPQFEDRMKMPYTEAVINEIQR

FANLAPLGIPRKTIKNTTFRGFFLPKDTDVYPILGSLLTDPKFFTSPKHFNPQNFLDDRG

QLKKIAAFVPFSVGKRFCLGDGLARMELFLFLTTILQNFRLKFPKKLEDIDASPKPLGFS

RIIPRYTMSFLPI

>405113047

MDPVLVLVLTLSCLLLLSLWRQSSGRGKLPPGPTPLPIIGNILQIDVKDISKSFTNFSKI

YGPVFTLYFGPKPTVVVHGYEAVKEALDDLGEEFSGRGSFPIVERMNNGLGVIFSNGTKW

KELRHFSLMTLRNFGMGKRSIEDRIQEEASCLVEELRKTNGSLCDPTFILSCAPSNVICS

VVFHNRFDYKDENFLNLMEKLNENFKILNSPWMQVCNALPAFIDYLPGSHNRVIKNFAEI

KSYILRRVKEHQETLDMDNPRDFIDCFLIKMEQEKHNPRTEFTIESLMATVSDVFVAGSE

TTSTTLRYGLLLLLKHTEVTAKVQEEIDHVIGRHRRPCMQDRTRMPYTDAMVHEIQRYIN

LIPNNVPHAATCNVRFRNYVIPKGTDLVTSLTSVLHDDKEFPNPEVFDPGHFLDENGNFK

KSDYFMPFSTGKRMCVGEALARMELFLLLTTIVQNFNLKSFVDTKDIDTTPMANTFGRVP

PSYQLCFIPR

>301620100

MEFNSTLVLFLLFLITFMLMLSKKKHRGRLPPGPVPLPYIGNMHQVKINDLIKSLVDLSK

KYGPMYTFYLGSRPTVVLCGYQILKEALIDHAEEFSGRGEFPAVQMYSKGNGIVYGTGER

WRQLRRFAITTLKNFGMGKRSLEERVKEEAQYLIAEFRNSEGEPFDPTFGFSSAGSNIIC

SLVFGDRFDYRNPEFLNLLYLINNSWHLMSSTWGQLLFVFPKIMQLIPGPHKKIYKNYLK

LGAFVAKRLKMNKETLDPNNPRDFIDCFLIKIQQEKKNPDSHFNYETMLKTTVNVFFAGT

ETVGSTLRYGFLILLKHPEVEEKVQGEIDKVIGRNRSPCMDDRSKMPYTEAVIYEILRFA

DIVPMSVPHTVTRDIEFRGYTLPKGLNIMPLICTSQYDVTKFKNPASFDPAHFLDENMDF

KKNEGFMAFSAGKRICVGEGLAQMELFIFFTTILQNFKLKRLMDPKNIDITPESSGLGNI

PRPYKLCLIPR

>P11711.2

MLDTGLLLVVILASLSVMLLVSLWQQKIRGRLPPGPTPLPFIGNYLQLNTKDVYSSITQL

SERYGPVFTIHLGPRRVVVLYGYDAVKEALVDQAEEFSGRGEQATYNTLFKGYGVAFSSG

ERAKQLRRLSIATLRDFGVGKRGVEERILEEAGYLIKMLQGTCGAPIDPTIYLSKTVSNV

ISSIVFGERFDYEDTEFLSLLQMMGQMNRFAASPTGQLYDMFHSVMKYLPGPQQQIIKVT

QKLEDFMIEKVRQNHSTLDPNSPRNFIDSFLIRMQEEKNGNSEFHMKNLVMTTLSLFFAG

SETVSSTLRYGFLLLMKHPDVEAKVHEEIEQVIGRNRQPQYEDHMKMPYTQAVINEIQRF

SNLAPLGIPRRIIKNTTFRGFFLPKGTDVFPILGSLMTDPKFFPSPKDFDPQNFLDDKGQ

LKKNAAFLPFSTGKRFCLGDGLAKMELFLLLTTILQNFRFKFPMKLEDINESPKPLGFTR

IIPKYTMSFMPI

>AAA41030.1

MLDTGLLLVVILASLSVMLLVSLWQQKIRGRLPPGPTPLPFIGNYLQLNTKDVYSSITQL

SERYGPVFTIHLGPRRVVVLYGYDAVKEALVDQAEEFSGRGEQATYNTLFKGYGVAFSSG

ERAKQLRRLSIATLRDFGVGKRGVEERILEEAGYLIKMLQGTCGAPIDPTIYLSKTVSNV

ISSIVFGERFDYEDTEFLSLLQMMGQMNRFAASPTGQLYDMFHSVMKYLPGPQQQIIKVT

QKLEDFMIEKVRQNHSTLDPNSPRNFIDSFLIRMQEEKNGNSEFHMKNLVMTTLSLFFAG

SETVSSTLRYGFLLLMKHPDVEAKVHEEIEQVIGRNRQPQYEDHMKMPYTQAVINEIQRF

SNLAPLGIPRRIIKNTTFRGFFLPKATDVFPILGSLMTDPKFFPSPKDFDPQNFLDDKGQ

LKKNAAFLPFSTGKRFCLGDGLAKMELFLLLTTILQNFRFKFPMKLEDINESPKPLGFTR

IIPKYTMSFMPI

>P33260.3

MDPAVALVLCLSCLFLLSLWRQSSGRGRLPSGPTPLPIIGNILQLDVKDMSKSLTNFSKV

YGPVFTVYFGLKPIVVLHGYEAVKEALIDHGEEFSGRGSFPVAEKVNKGLGILFSNGKRW

KEIRRFCLMTLRNFGMGKRSIEDRVQEEARCLVEELRKTNASPCDPTFILGCAPCNVICS

VIFHDRFDYKDQRFLNLMEKFNENLRILSSPWIQVCNNFPALIDYLPGSHNKIAENFAYI

KSYVLERIKEHQESLDMNSARDFIDCFLIKMEQEKHNQQSEFTVESLIATVTDMFGAGTE

TTSTTLRYGLLLLLKYPEVTAKVQEEIECVVGRNRSPCMQDRSHMPYTDAVVHEIQRYID

LLPTNLPHAVTCDVKFKNYLIPKGTTIITSLTSVLHNDKEFPNPEMFDPGHFLDKSGNFK

KSDYFMPFSAGKRMCMGEGLARMELFLFLTTILQNFNLKSQVDPKDIDITPIANAFGRVP

PLYQLCFIPV

>P33273.2

MDPVLVLVLTLSCLLLLSLWRQSSGRGKLPPGPTPLPIIGNILQIDVKDISKSFSYFSKI

YGPVFTLYFGPKPTVVVHGYEAVKEALDDLGEEFSGRGSFPIVERMNNGLGLIFSNGTKW

KELRHFSLMTLRNFGMGKRSIEDRIQEEASCLVEELRKTNGSLCDPTFILSCAPSNVICS

VVFHNRFDYKDENFLNLMEKLNENFKILNSPWMQVCNALPAFIDYLPGSHNRVIKNFAEI

KSYILRRVKEHQETLDMDNPRDFIDCFLIKMEQEKHNPRTEFTIESLMATVSDVFVAGSE

TTSTTLRYGLLLLLKHTEVTAKVQEEIDHVIGRHRRPCMQDRTRMPYTDAMVHEIQRYIN

LIPNNVPHAATCNVRFRNYVIPKGTDLVTSLTSVLHDDKEFPNPEVFDPGHFLDENGNFK

KSDYFMPFSTGKRMCVGEALARMELFLLLTTIVQNFNLKSFVDTKDIDTTPMANTFGRVP

PSYQLCFIPR

>297277116

MELSVLLFLALLTGLLLLLVQRHPNAHGRLPPGPCPLPLLGNLLQMDRRGLLRSFLRVLQ

SVSLTCCCFLGALVDPTFLFHSITANIICSIVFGKRFHYQDQEFLKILNLFYHTFSLASS

MFGQLFELLSGFLKYFPGAHRQVYKNLQEINAYIGHSVEKHRETLDPSAPQDLIDSYLLQ

MEKEKSNPHSEFSHRNLIINTLSLFFAGTETTSTTLRYGFLLMLKYPHVAERIYKEIEQV

IGPHRPPALDDRAKMPYTEAVIHEIQRFADLLPMGVPHIVTQQTSFRGYIIPKDTEVFPL

LSTALHDPHYFEKPDTFNPDHFLDANGALKKNEAFIPFSLGKEEKLCLTGIDREKLFLFF

TTILQNFSVASPVAPEDIDLTPQESGVGKIPPTYQIRFLPR

>AAD13719.1

MDPILVLVLTLSCLFLLSLWRQSSERGKLPPGPTPLPIIGNILQIDVKDICQSFTNLSKV

YGPVYTLYLGRKPTVVLHGYEAVKEALVDHGEEFAGRGRFPVFDKATNGMGLAFSKGNVW

KNTRRFSLMTLRNLGMGKRSIEDRVQEEARCLVEELRKTNGSPCDPTFILGCAPCNVICS

IIFQDRFDYKDRDFLNLMEKLNEITKILSSPWLQICNTYPALLDYCPGSHKQFFKNYACI

KNFLLEKIKEHEESLDVTIPRDFIDYFLINGGQEDGNQPLQNRLEHLAITVTDLFSAGTE

TTSTTLRYALLLLLKYPHVTAKVQKEIEHVIGKHRSPCMQDRSRMPYTDAMIHEVQRFID

LIPNSLPHEVTCDIKFRNYFIPKGTTVITSLSSVLHDSTEFPNPEVFDPGHFLDGNGKFK

KSDYFMPFSTGKRICAGEGLARMELFLFLTSILQSFNLKPLVHPKDIDVTPMLIGLGSVP

PAFQLCFIPS

>AAA02630.1

MDPAVALVLCLSCLFLLSLWRQSSGRGRLPSGPTPLPIIGNILQLDVKDMSKSLTNFSKV

YGPVFTVYFGLKPIVVLHGYEAVKEALIDHGEEFSGRGSFPVAEKVNKGLGILFSNGKRW

KEIRRFCLMTLRNFGMGKRSIEDRVQEEARCLVEELRKTNASPCDPTFILGCAPCNVICS

VIFHDRFDYKDQRFLNLMEKFNENLRILSSPWIQVCNNFPALIDYLPGSHNKIAENFAYI

KSYVLERIKEHQESLDMNSARDFIDCFLIKMEQEKHNQQSEFTVESLIATVTDMFGAGTE

TTSTTLRYGLLLLLKYPEVTAKVQEEIECVVGRNRSPCMQDRSHMPYTDAVVHEIQRYID

LLPTNLPHAVTCDVKFKNYLIPKGMTIITSLTSVLHNDKEFPNPEMFDPGHFLDKSGNFK

KSDYFMPFSAGKRMCMGEGLARMELFLFLTTILQNFNLKSQVDPKDIDITPIANAFGRVP

PLYQLCFIPV

>BAB85664.1

MDGAVVLVLCLSCLLLLSLWKQNSGKGRLPPGPTPLSILGNILQLDVKDISKSLSNLSKV

YGPVFTVYFGLKPAVVLHGYEAVKEALIDGGEEFSGRGHFPMAERVNKGHGIIFSSGKRW

KETRRFSLMTLRNFGMGKRSIEERVQEEARCLVEELRKTNASPCDPTFLLGCAPCNVICS

IIFHNRFDYTDPNFLTLLEKLNENFRILSSPWMQVCNNFPALIDYLPGSHNKVLKNSAYV

KSYILEKVKEHQASLDVKDPRDFIDCFLIKMEQEKHNHQVAFTFENLIATITDLFGAGTE

TTSTTLRYGLLLLLKHPDVTAKVQKEIDSVIERHRSPCMQDRSRMPYTDAVVHEIQRYID

LIPISVPHAVTRDIKFRNYLIPKGTTVLTSLTSVLRDDQEFPNPEVFDPGHFLDESGNFK

KSDCFMPFSTGKRICVGEGLARMELFLFLTSILQKFTLEPVVDLKDIDTTPILSGFSHMP

PSYQLRFIPV

>P00180.2

MDPVVVLGLCLSCLLLLSLWKQSYGGGKLPPGPTPFPILGNILQIGIQDISKSFTKLSEV

YGPVFTVYLGMKPTVVIHGYDAVKEALVDLGEEFSGRIVFPLTAKINKGYGIVFSNGKRW

KETRRFSLMTLRDFGMGKRSIEDRVQEEARCLVEELRKTNGSPCNPTFILGAAPCNVICS

VIFQNRFDYTDQDFLSLMGKLNENFKILNSPWVQMCNNFPILIDYLPGSHNKILRNNIYI

RNYVLEKIKEHQETLDINNPRDFIDCFLIKMEQEKDNQQSEFTIENLMTTLSDVFGAGTE

TTSTTLRYGLLLLMKHPEVIAKVQEEIERVIGRHRSPCMQDRSRMPYTDATVHEIQRYIN

LIPNNVPRATTCNVKFRSYLIPKGTAVITSLTSMLYNDKEFPNPDRFDPGHFLDASGKFR

KSDYFMPFSTGKRVCVGEVLARMELFLFLTAILQNFTPKPLVDPKDIDTTPLVSGLGRVP

PLYQLSFIPA

>EDL41824.1

MDPILVLVLTLSCLFLLSLWRQSSERGKLPPGPTPLPIIGNILQIDVKDICQSFTNLSKV

YGPVYTLYLGRKPTVVLHGYEAVKEALVDHGEEFAGRGRFPVFDKATNGMGLAFSKGNVW

KNTRRFSLMTLRNLGMGKRSIEDRVQEEARCLVEELRKTNGSPCDPTFILGCAPCNVICS

IIFQDRFDYKDRDFLNLMEKLNEITKILSSPWLQICNTYPALLDYCPGSHKQFFKNYASI

KNFLLEKIKEHEESLDVTIPRDFIDYFLINGGQEDGNQPLQNRLEHLAITVTDLFSAGTE

TTSTTLRYALLLLLKYPHVTAKVQEEIEHVIGKHRSPCMQDRSHMPYTDAMIHEVQRFID

LIPNSLPHEVTSDIKFRNYFIPKGTTVITSLSSVLHDSMEFPNPEKFDPGHFLDENGKFK

KSDYFIPFSTGKRICAGEGLARMELFLFLTSILQNFNLKPLVHPKDIDVTPMLIGLASVP

PAFQLCFIPS

>P24470.2

MELLGFTTLALVVSVTCLSLLSVWTKLRTRGRLPPGPTPLPIIGNLLQLNLKDIPASLSK

LAKEYGPVYTLYFGTSPTVVLHGYDVVKEALLQQGDEFLGRGPLPIIEDTHKGYGLIFSN

GERWKVMRRFSLMTLRNFGMGKRSLEERVQEEARCLVEELQKTKAQPFDPTFILACAPCN

VICSILFNDRFQYNDKTFLNLMDLLNKNFQQVNSVWCQMYNLWPTIIKYLPGKHIEFAKR

IDDVKNFILEKVKEHQKSLDPANPRDYIDCFLSKIEEEKDNLKSEFHLENLAVCGSNLFT

AGTETTSTTLRFGLLLLMKYPEVQAKVHEELDRVIGRHQPPSMKDKMKLPYTDAVLHEIQ

RYITLLPSSLPHAVVQDTKFRDYVIPKGTTVLPMLSSVMLDQKEFANPEKFDPGHFLDKN

GCFKKTDYFVPFSLGKRACVGESLARMELFLFFTTLLQKFSLKTLVEPKDLDIKPITTGI

INLPPPYKLCLVPR

>EHB18324.1

MLALGMLLVTLLACLSIRALMSAWRQRKLWGKLPPGPTPLPFIGNYLQLDTEKMYSCLLK

ISKHYGPVFTIYMGLRRVVVLCGYDIIKEALVDQAEEFNGRGEQATFNSFFNGYGVTFSN

GERSKQLRRFSIATLRDFGVGKRGIEERIKEEAGYFVQTIQATHGAFIDPTFYLSKSVSN

VMNSIVFGDRFDYEDKEFLSLLQMMTGIKKFVASPQGQLYEMFFSVMKYLPGPQQEIYKD

MQNLENYMAKKVEKNQRTLDPNAPRDFIDSFLIRMQQEKKNPNTEFNMKNLVMTTLSLFF

AGTETISSTLRFGLLLLLKHPDVEAKIHEEIDQVIGRNRQPQFEDRMKMPYTEAVIHEIQ

RFANFVPMGVARRITKNTTLRNFFLPKDTEVFPMLGTVLTDYKFFSNPEDFNPQHFLDDK

GQFKKSDAFVPFSLGKRNCFGEGLARMELFLFLTIIMQNFWFKSPQAAQDIYVPPQPLGF

TRILPNYTISFLPR

>348552442

MFISLPLRALEKCIPVPDIWIRKHYGPVFTIHLGPRPIVVLCGYDAVKEALMDQAEEFSG

RGTQATFDWLFQGYGIAFSNGERWKVLRRFSLQILRNFGMGNRSMEERILEEGSFLLEEL

RKTEGKPFNPLFMLSRSVSNIICSVVFGSRFHYDDERLLTIIRLINDNFQIMSTPWGELY

NIFPSLLDWLPGPHLRMFKNFRGMKELIAHSVREHQASLDPDSPRDFIDCFLTKMAQEKK

DPLSHFCMDTLLMTTHNLLFGGTETVGTTLRHAFLILMKYPKVQARVQEEIDRVVGRSRL

PSLNDRAAMPYTDAVIHEVQRFADIIPMNLPHRVTRDTIFRGFLLPKGTDVITLLNTVHS

DPSQFVKPQEFNPEHFLDANQSFKKSPAFMPFSAGRRLCMGESLARMELFLFFTAILQSF

TLQPLVAPEDIDLTPLSTGLGKVPRPFEMCMSLR

>AAH78707.1

MELLGFTTLALVVSVTCLSLLSVWTKLRTRGRLPPGPTPLPIIGNLLQLNLKDIPASLSK

LAKEYGPVYTLYFGTSPTVVLHGYDVVKEALLQQGDEFLGRGPLPIIEDTHKGYGLIFSN

GERWKVMRRFSLMTLRNFGMGKRSLEERVQEEARCLVEELQKTKAQPFDPTFILACAPCN

VICSILFNDRFQYNNKTFLNLMDLLNKNFQQVNSVWCQMYNLWPTIIKYLPGKHIEFAKR

IDDVKNFILEKVKEHQKSLDPANPRDYIDCFLSKIEEEKDNLKSEFHLENLAVCGSNLFT

AGTETTSTTLRFGLLLLMKYPEVQAKVHEELDRVIGRHQPPSMKDKMKLPYTDAVLHEIQ

RYITLLPSSLPHAVVQDTKFRDYVIPKGTTVLPMLSSVMLDQKEFANPEKFDPGHFLDKN

GCFKKTDYFVPFSLGKRACVGESLARMELFLFFTTLLQKFSLKTLVEPKDLDIKPITTGI

INLPPPYKLCLVPR

>344274935

MDLVVVLVLCLSSLLLFSLWKQSYGKGKLPPGPTPLPIIGNILQLDMKDISNSMSNFAKT

YGPVFTLYLGMKPTVVLHGYEAIKEALIDQGEEFSGRGHFAVAERTNKEFGVVFSNGKIW

KETRRFSLMTLRNFGMGKRSLENRVQEEARCLVEELRKTKALPCDPTFILACAPCNVICS

IIFQNRFEYTDQHFLNLIGILNENTEILSTPWMQMCNIFPMLLDYFPGIHNKLFNNAIFL

KSYILGKVKEHQESLDVNNPRDYIDCFLIKMEQEKHNQQSEFITENLLATIADLFGAGTE

TTSTTLRYGLLLLLKHPEVTAKVQEEIDRVIGRHRSPCVQDRGSMPYTNAVIHEILRYID

LIPLSLPHAVTRDIKFRDYIIPKGHDHMTSLSSVLYDKKEFSNPEMFDPGHFLDASGNFM

KSDYFMAFSAGKRICLGEGLARMEIFLFLTTILQKFTLKSLVDPKHIDTTPVVNGFASLP

PLYQLCFIPV

>AAH71029.1

MDTAWLWTLLLTLLISCMLIYSTWTKMYRNSNLPPGPTPIPLFGNVLQIKRGEMVKSLLE

LRKIYGPVYTLYFGPSPVIILCDYQSIKEALNDQAEEFSGRGKIPSWDQYFQGYGEAFTN

GEEWKQLRRFSLTTLRNFGMGKKGIEERIQEEAQFLVEEIKSYKEKSFDPAKLLVQCVSN

VICSVVFGKRYEYSNKDFHELLYMFQAVFEDTSSSWGQLMTMLPIIMKHIPGPHRRVLHE

LNRVNDFILQRVNENEKTLDPKSPRNFIDSFLIQMQQEKENPMTKFHRKNLICTIMNLFF

AGTETVSTTLRHGFLILLIHPEIEVKLHEEIDRVIGQGRCPTMEDKSKMPYTDAVIHEIQ

RVSDVIPMSLPHSVMKDTQLRGYTIPKGTDVYPMICTALRDPKQFATPNKFNPQHFLDDK

GNFKTSNAFMPFSTGKRICLGEGLARMELFLFLTNILQNFKLHSEKQFTEDDIAPKMQGF

ANYPLFYEFSLIPRI

>354492721

MELLGVTTLVLVVSVICLSFLSVWTKTRPGGRLPPGPTPLPVIGNILQLNFKDVPASLSK

LAKDYGPIYTLYFGSWPTVVLHGYDVVKEALLNQGDEFLGRGPFPIIEDSLKGSGLVFTN

GETWKVLRRFSLMTLKNFGMGKRSLEERVQEEAQCLVEELQKMEAGPFDPTFILACAPCN

VICSILFNERFPYRSKTILNLMDLLNKNFHQVNSVWCQMYNLWPTFMKYVPGKHKEVPQR

INDINNFILEKIKEHQESLDPANPRDYIDCFLSKIEEEKHNPKSEFDLKNLIASGASLFT

AGTETTSITLRFGILLLLKYPEVQAKIHEEIDHVIGRHQPPSMKDKMKLPYTNAVLHEIQ

RYTTLLPSSLPHAVVKDTKFRDYIIPKGTVVLPLLSSILLDHKEFPNPETFDPGHFLDKN

GCFKKTDYFVPFSLGKRSCMGESLAIMELFLFLTTILQKFSLKSPVEPRDLDIKPAATGL

INIPPPYKLCLIPR

>327289005

MEWVYVVTLLLVICVSCHLLISSKRKQLQKGKLPPGPTPLPLIGNFLQMKSGETLKSLLK

MYEKYGPVFTVYLGTHPVLVLCGHQAVKEALIDKAEEFCGRTIKPTLESAVEGYGVGFSS

GERWKQLRRFSITVLKSFGMGKKSIEERIQEEAQFLMEEFRKTKGKPVVLQENLLGCATS

NIISSIVFGERFDYENEEFQALMRMINNFFWEISTTWSQLYDMFPTLLKYFPGPHTRVYN

ILSDMFLFVAKRVEKNQETLDSNFPRDFIDCFLIQMEKEKDNPLSEFNIKNLEHTIFNLF

FGGTETVTSTLKYGFLLLMKYPEVQAKVHEEIDRVIGHNRIPNSEDRRQMPYTDAVIHEI

QRVSDLVPMSVAHMVTCDTEFRGYFIPKGMEVWPVLSTVLHDPTMFKSPSVFNPENFLDE

NGCFKKNDAFVPFSTGKRTCLGESLARMELFLFFTIILQSFQLKPLVPPEDLDPTPLENG

FLTVPPFYHLSIIPR

>327276267

MEWAGAVILLLVFFFSCPLLMSSKRKRLHKGKPPPGPTPLPLIGNFLQMKSSEILKSLLK

LNEKYGPVFTVYFGSRPVLVLCGHQAVKEALIDKAEKFSGRVCMPSMVPTFQGYGVGFAN

GERWKELRRFSLAVLRSFGMGKKSIEQRIQEEAQFLLEEFRKTKEKPFEPTTLLSHMFTN

IICSIVFGNRFDYEDKEFQALLVMVNEFFWEMSTSWAQFYDVYANYLNYIPGIYSKLYDS

KDLRLFVAKRIKKNQETFDPNFPRDYIDCFLIQMEKEKDNSDSEYNIKNLQLSILNLILG

GSETGSCTLKYGFLFLTKYPEVQAKVHEEIDRVIGHDRVPNTEDRRQMPYTDAVIHEVQR

CSDVLPMSLAHMVTCDTELRGYLIPKGMTVYPILSTVLHDPTMFKSPNAFSPENFLDENG

CFKKNDAFVPFSSGKRNCLGEVLARMELFLFFTTILQSFQLKSLVPPEDIDLTPQESGFA

NIPPFCHLSVIPR

>403259871

MDPFVVVVLSLSCLLLLSLWKQSSKRGKLPPGPTPLPIVGNLLQIDVKDITKSLTNFSKV

YGPVFTLHFGLERTVVLHGYEVVKEALIDRGDEFSGRGHFPVAERANRGYGVIFSNGNRW

KEIRRFSLMTLRNFGMGKRSIEDRVQEEARCLLEELRKTEASPCDPTFILGCAPCNVICS

IIFQKRFDYKDQQFLNLMERLNESTKILSTPWIQICNSFPPFIDYFPGTHNKLLKNIALT

KSFILEKVKEHQESVDINNPRDFIDCFLIKMKKEEHNQQSEFTIENLVHTAFDLFGAGTE

TTSTTLRYALLLLLKHPEVTAKVQEEIEHVVGRNRSPCTQDRSHMPYTDAVVHEVQRYID

LIPTNLPHAVTSDVQFRNYLIPKGTTILTSLTSVLHDNKEFPNPEKFDPHHFLDEGGNFK

KSNYFMPFSTGKRICAGEGLARMELFLFLTSILQNFNLKPLVDPKDLDTTPVVNGFASVP

PFYQLCFIPI

>P00181.2

MDLVVVLGLCLSCLLLPSLWKQSHGGGKLPPGPTPFPILGNVLQLDFKDLSKSLTNLSKV

YGPVFTVYLGMKPTVVVHGYEAVKEALVDLGHELSGRSRFLVTAKLNKGFGVIFSNGKRW

TETRRFSLMTLRNFGMGKRSIEERVQEEAHCLVEELRKTNASPCDPTFILGAAPCNVICS

VIFQNRFDYTDQDFLSLMGKFNENFKILNSPWVQFCNCFPILFDYFPGSHRKAVKNIFYV

KNYITEQIKEHQKSLDINNPRDFIDCFLIKMEQEKCNQQSEFTIENLLTTVSDVFMAGTE

TTSTTLRYGLLLLMKHPEVIAKVQEEIERVIGRHRSPCMQDRSRMPYTDATVHEIQRYIN

LIPNNVPHTTICNLKFRNYLIPKGTDVLTSLSSVLHDDKEFPNPDRFDPGHFLDASGNFR

KSDYFMPFSTGKRVCVGEALARMELFLFLTAILQNFTPKPLVNPNNVDENPFSSGIVRVP

PLYRVSFIPV

>EHB03591.1

MDPVVVLVLGLSCLLLFSLWRQSSGRRKLPPGPTPLPIIGNILQIDITDISKSFTNFSKA

YGPVFTLYFGMKPTVVVHGYEAVKEALDDLGEEFSGRGSFPIVEKTNSGQGIIFSNGTKW

KEIRRFTLMTLRNFGMGKRSIEDRIQEEACCLVEELRKTNGSSCDPTFILGCAPCNVICS

IIFQKRFDYKDENFLILMQKFNENFKILNSPWMQVCNIFPAFIDYLPGSHKKVLKNFAHI

KTYVLKKVKEHQESLDIHNPRDFIDCFLIKMEQEKHSQHSEFTIENLMATVTDLFIAGTE

TTSTTLRYGLLLLLKYPEVTAKVQEEIDRVIGRHRSPCMQDRSHMPYTDAMVHEIQRYIN

LIPNNVPRAATCNLRFRNYFIPKGTNLITSLTSVLHDDQEFSNPEIFDPGHFLDENSNFK

KSDYFMPFSIGKRMCVGEGLARMELFLFLTTILQNFNLKSLVDTKDIETIPIANTFGHVP

PLYEICFIPV

>AAH67993.1

MDWALEINGLPILLLIAALLLLLARKVGKKVKGCLPPGPKPLPILGNLLQLKSREIHKPL

LEFNKKYGPVYTLYMGSMPAVVLCGYEAVKEALVDNAEKFSGRAEVPIVNLTTQGYGIAF

SNGERWKELRRFSLTTLRNFGMGKRSIEERIQEEIHFLLEAFHETQGSFFSPAFIIRRSV

SNVICSVVFGKRFDYTDQKLQILLDLIAENLRRVDNIWVQVYNFIPKLLNILPGPHHKLT

ENYKAQLRYVEEIVQEHGKTLDPSAPQDYIDAFLLKMEQERKKAHTEYNVQNLLSCSLDI

FFAGQESTSSTLGYGLLILMKYPHIKEKVQAEIESVIGRSRRPCMDDRAKMPYTEAVIHE

IMRFIDFFPLGVPHSVTEDTLYRGYVIPKGTTIFPFLHSVLFDPSMFERPQEFYPGHFLN

QDGSFRKNEGFMAFSAGKRACPGKSLARVEIFLYLTSILQQFDPQPALSPKDIDLSPEYS

GFGKMAPSFQLKLVPH

>335302026

MDGAVVLVLCLSCLLLLSLWKQNSGKGRLPPGPTPLPILGNILQLNVKDISKSLSNLSKV

YGPVFTVYLGLKPAVVLHGYEAMKEALIDGGEEFSGRGHLPMAERVNKGHGIIFSSGKRW

KEIRRFSLMTLRNFGMGKRSIEERVQEEARCLVEELRKTNASPWDPTFLLGCAPCKVICS

IIFRNRFDYTDPNFLTLLEKLNENFRILSSPWIQVCNNFPALIDYLPGSHNKVFKNSAYV

KSYILEKVKEHQASLDVNDPRDFIDCFLIKMEQEKHNHQVAFTFENLIATISDLFGAGTE

TTSTTLRYGLLLLLKHPDVTAKVQKEIDSVIERHRSPCMQDRSRMPYTDAVVHEIQRYID

LIPISVPHAVTRDIKFRNYLIPKGMTVLTSLTSVLHDDQEFPNPEVFDPGHFLDESGNFK

ESDYFMPFSTGKRICVGEGLARMELFLFLTSILQKFTLEPVVDLKDINTTPVYSGFSHVP

RSYQLRFIPV

>301620120

MSPSIFTLLIFVLLVLLSIMWWKKNLKDRSLLPPGPTPLPFLGNLLQVKPKEFLKALDKL

KEKHGSVFTVYFGARPTVILCGYQTVKEALIDQADTFSSRGKMALAEHILKGYGITGSNG

ERWKQLRRFALTTLRNFGMGKRTIEKRIQEETTFLIEEFRNAEGMPFDPTFYLGCAVSNI

ICSIVFGERFDYNDKQFLFLLKNINKVLRFMNSTWGVVFFTFDKIMCHIPGPHQKAMKHL

VDLKAFVQQRVRESKEILDINSPQHFIDCFLIKMQEEQENPHSEFHMDNLIGSALNLFFA

GTETVSTTLRYGILILLKWPHIQGRIQEEIDDVIGRQQCPKIEDRSKMPYTDAVIHEIQR

FSDIVPTGLPHTATQDTTFRGHTIPKGTDVFALLTTVLKDPEVFQNPEEFNPERFLDENG

ILKKSQAFMPFSAGKRMCPGESLARMEIFLFLTTLLQKFTLIPTVPSVDLDVTPEISSSG

HLPREYKMCVLPRQ

>344298311

MLASGMLLVALLACLSVMVLMSVWRQRKLWGKLPPGPTPLPFIGNYLQLNTQQMYNSLMK

LSELYGLVFTVHLGSRRVVVLWAYDAVKEALVDQAEEFSGXGEESTFDWLFKGYGVAFSN

GERAKQLRRFSITTLLDLGVGKRGIEERIQEEAGFLLPPLLPSPGAFTDSTYFLSRTISN

VISSIVFGDRLDXEDKEFLALLRMMLGSFQFTATATXWLYDMFYSVMKYLPGPQQQAFKE

LQGLEDFITKKVEQNQRTLDPNSPWDFIDSFLIRMREEKKNPNTEFYMKNLVLTTLNLFF

AGTETISTTLRYGFLLLMKHPDVQAKLHEEIDQVIGKNRQPMFEDRAKMPYTEAVIHEIQ

RFADMIPMGVAHRVTKDTKFRDFFIPKGTELFPMLGSVLRDTKLFSNPQDFNPRHFLDEK

GQFKKNNIFVPFSIGKRYCFGEGLARMELFVFLTTILQNFCFKSPQSPKDIDVSPKHVGF

ATIPRTYTMSFLPR

>344274444

MELLEITTLALVICITCLIFLLVWRKSHKMRRLPPGPAPLPIIGNMLQLNPKDIPGSLCK

LAEEYGPVYTLHIGPQLTVVLHGYEAVKEALIDQGDEFLGRGPMPIIEDTLKGYGIVFSH

GERWKQMRRFSLMTLRNFGMGKRSIEERVQEEARYLVEELRQTEAQPFDPTFVLSCAPCN

TICSILFSERFQYTNEKFLYLMNLLNENFRQINSPWSQMYNLWPRLIKHLPGQHRAFSKR

SKDLKYFILEKVKEHQESLDHNSARDYIDCFLSKMEEEKNNPESEFDMENLVTCASNLFA

AGTETLSNTLRFGLLLLMKHPEVEAKVHEEIDSVIGRNQSPCMKDKMKLPYTEAVLHEIQ

RFINLLPSNLPHAVIRDTKFRQFVIPKGTTVLPLLSSIMFDCKEFPNPEKFDPGHFLDKN

GSFKKTEYFVPFSLGKRACVGESLARMELFLFFTTIMQNFSLKPLVEPKELTIKPVVTGL

LNIPPPYKLCCIPRXKKNFYLLIICIVNQSNCFPPLEIYYKSVSSL

>AAZ29452.1

MDSLVVLVLCLSCLLLLSLWRQRSGRGKLPPGPTPLPVIGNILKIGIKDVSKSLTNLSKV

YGPVFTLYFGLERMVVLHGYEAVKEALIDLGEEFSGRGHFPLFERANRRFGIVFSNGKRW

KEIRRFSLMTLRNFGMGKRSIEDRVQEEARCLVEELRKTKASPCDPTFILGCAPCNVICS

IIFHKRFDYKDQQFLNLMEKFNENAKILSSPWIQIYNNFSPIIDYFPGTHNKLLKNIAFV

KSYILEKVKEHQESMDMNNPRDFIDCFLIKMEKEKHNQQSEFNIENLENTAVDLFAAGTE

TTSTTLRYALLLLLKHPEVAAKVQEEIEHVIGRNRSPCMQDRSHMPYTDAVVHEIQRYID

LLPTSVPHAVTCDVKFRNYLIPKGTTILISLTSVLRDNKEFPNPEMFDPRHFLDEGGNFK

KSNYFMPFSAGKRICVGEALARMELFLFLTSILQNFNLKSLVDLKDLDTTPVFNGFVSVP

PVYQLCFIPV

>332834691

MDSLVVLVLCLSCLLLLSLWRQSSGRGKLPPGPTPLPVIGNILQIGIKDISKSLTNLSKV

YGPVFTLYFGLKPIVVLHGYEAVKEALIDLGEEFSGRGIFPLAERANRGFGIVFSNGKKW

KEIRRFSLMTLRNFGMGKRSIEDRVQEEARCLVEELRKTKASPCDPTFILGCAPCNVICS

IIFHKRFDYKDQQFLNLMEKLNENVKILSSPWIQICNNFSPIIDYFPGTHNKLLKNVAFM

KSYILEKVKEHQESMDMNNPQDFIDCFLMKMEKEKHNQPSEFTIESLENTAVDLFGAGTE

TTSTTLRYALLLLLKHPEVTAKVQEEIERVIGRNRSPCMQDRSHMPYTDAVVHEVQRYID

LLPTSLPHAVTCDIKFRNYLIPKGTTILISLTSVLHDNKEFPNPEMFDPHHFLDEGGNFK

KSNYFMPFSAGKRICVGEALARMELFLFLTSILQNFNLKSLVDPKNLDTTPVVNGFASVP

PFYQLCFIPV

>397510088

MDSLVVLVLCLSCLLLLSLWRQSSGRGKLPPGPTPLPVIGNILQIGIKDISKSLTNLSKV

YGPVFTLYFGLKPIVVLHGYEAVKEALIDLGEEFSGRGIFPLAERANRGFGIVFSNGKKW

KEIRRFSLMTLRNFGMGKRSIEDRVQEEARCLVEELRKTKASPCDPTFILGCAPCNVICS

IIFHKRFDYKDQQFLNLMEKLNENVKILSSPWIQICNNFSPIIDYFPGTHNKLLKNVAFM

KSYILEKVKEHQESMDMNNPQDFIDCFLMKMEKEKHNQPSEFTIESLENTAVDLFGAGTE

TTSTTLRYALLLLLKHPEVTAKVQEEIERVIGRNRSPCMQDRSHMPYTDAVVHEVQRYID

LLPTSLPHAVTCDIKFRNYLIPKGTTILISLTSVLHDNKEFPNPEMFDPHHFLDEGGNFK

KSNYFMPFSAGKRICVGEALARMELFLFLTSILQNFNLKSLVDPKNLDTTPVVNGFASVP

PFYQLCFIPV

>348552494

MLASGMLLVTLLTCLGLMALMSVWKQRKLQGKLPPGPTPVPLIGNYLQLDTKKMYSCLMK

MSKLYGPVFTIYLGLRRVVVLCGYDAIKEALVDQAEDFSGRGEHVTFNRLFQGYGVTFSN

GERSKQLRRFSISTLRDFGVGKRGIEERIKEEAEYLLQAIQGTRGAFIDPTYYLSKSVSN

VMNSIVFGDRFDYEDEEFLSLLQMTMGIKRFLASPVGQLYEIFYSVMKHLPGPQQEAFKN

MQGLENFMIKKVEQNQRTLDPNAPRNLIDSFLIRMQEEKKNPNTEFHMKNLVMTILSLFF

AGTETISSTLRFGFLLFMKYPEVEAKVHEEIDRVIGKNRQPQFEDRMKMPYMEAVIHEIQ

RFANFAPMGISRRITKNTTFQDFFLPKGTEVFPMLGSVLTDYNFFSNPEDFNPQHFLDEK

GQFKKNDGFIPFSLGKRNCFGEGLARMELFLFFTIIMQNFRLKSPQAPEDIYVPPKPVGF

TRVVPYFTMSFLPR

>AAH11222.1

MDPILVLVFTLSCLFLLSLWRQSSERGKLPPGPTPLPIIGNILQINVKDICQSFTNLSKV

YGPVYTLYLGNKPTVVLHGYEAVKEALVDHGEEFAGRGRLPVFDKATNGMGIIFSKGNVW

KNTRRFSLTTLRNLGMGKRSIEDRVQEEARCLVEELRKTNGSPCDPTFILGCAPCNVICS

IIFQDRFDYKDRDFLNLMEKLNEITKIMSTPWLQVCNTFPVLLDYCPGSHNKVFKNYACI

KNFLLEKIKEHEESLDVTIPRDFIDYFLINGGQEDGNYPLKNRLEHLAITVTDLFSAGTE

TTSTTLRYALLLLLKYPHVTAKVQEEIEHVIGKHRSPCMQDRSRMPYTDAMIHEVQRFID

LVPNSLPHEVTCDIKFRNYFIPKGTNVITSLSSVLRDSKEFPNPEKFDPGHFLDENGKFK

KSDYFMPFSTGKRICAGEGLARMELFLFLTSILQNFNLKPLVHPKDIDVTPMLIGLASVP

PAFQLCFIPS

>BAD97680.1

MDSLVVLVLCLSCLLLLSLWRQRSGRGKLPPGPTPLPVIGNILKIGIKDVSKSLTNLSKV

YGPVFTLYFGLERMVVLHGYEAVKEALIDLGEEFSGRGHFPLFERANRRFGLVFSNGKRW

KEIRRFSLMTLRNFGMGKRSIEDRVQEEARCLVEELRKTKASPCDPTFILGCAPCNVICS

IIFHKRFDYKDQQFLNLMEKFNENAKILSSPWIQIYNNFSPIIDYFPGTHNKLLKNIAFV

KSYILEKVKEHQESMDMNNPRDFIDCFLIKMEKEKHNQQSEFNIENLENTAVDLFAAGTE

TTSTTLRYALLLLLKHPEVAAKVQEEIEHVIGRNRSPCMQDRSRMPYTDAVVHEIQRYID

LLPTSVPHAVTCDVKFRNYLIPKGTTILISLTSVLRDNKEFPNPEMFDPRHFLDEGGNFK

NSNYFMPFSAGKRICVGEALARMELFLFLTSILQNFNLKSLVDLKDLDTTPVFNGFVSVP

PIYQLCFIPV

>1R9OA

MAKKTSSGRGKLPPGPTPLPVIGNILQIGIKDISKSLTNLSKVYGPVFTLYFGLKPIVVL

HGYEAVKEALIDLGEEFSGRGIFPLAERANRGFGIVFSNGKKWKEIRRFSLMTLRNFGMG

KRSIEDRVQEEARCLVEELRKTKASPCDPTFILGCAPCNVICSIIFHKRFDYKDQQFLNL

MEKLNENIKILSSPWIQICNNFSPIIDYFPGTHNKLLKNVAFMKSYILEKVKEHQESMDM

NNPQDFIDCFLMKMEKEKHNQPSEFTIESLENTAVDLFGAGTETTSTTLRYALLLLLKHP

EVTAKVQEEIERVIGRNRSPCMQDRSHMPYTDAVVHEVQRYIDLLPTSLPHAVTCDIKFR

NYLIPKGTTILISLTSVLHDNKEFPNPEMFDPHHFLDEGGNFKKSKYFMPFSAGKRICVG

EALAGMELFLFLTSILQNFNLKSLVDPKNLDTTPVVNGFASVPPFYQLCFIPIHHHH

>395828395

MDPAVALVLCLSCLLLLFLWRQSSGRGKLPPGPTPLPIIGNSLQMDIKDIRKSIKNLSKV

YGPVFTLYFGMKPIVVLHGYEAIKEALIDHGEEFSGRGLLPITEKTSKGLGIVFSNGNRW

KEIRRFSLMTLRNLGMGKRSIEERVQEEARCLVEELRKTKALSCDPTFILGCAPCNVICS

IIFQDRFDYKDQNFLTLMGRLNENFRILSSPWIQLCNNFPALIDYFPGIHNKLLKNVALT

KSYILEKVKKHQESLDINNPRDFIDCFLIKMEQEKNNSESAFTIENLVSSVSDLFGAGTE

TTSTSLRYALLLLLKHPEVTAKVQEEINHVIGRHRSPCMQDRSRMPYTDAMVHEVQRYID

LIPNNALRAVTHDIKFRNYLIPKGTMIMSSLTSVLHDDNEFPDPETFNPAHFLDESGNFK

KSDFFMPFSTGKRICLGEGLARMELFLFLTSILQNFNLKSSIDTRKIDITSVPSGTISLP

PLYKICFIPV

>AAB23864.2

MLLLSLWRQSSGRGKLPPGPTPLPVIGNILQIGIKDISKSLTNLSKVYGPVFTLYFGLKP

IVVLHGYEAVKEALIDLGEEFSGRGIFPLAERANRGFGIVFSNGKKWKEIRRFSLMTLRN

FGMGKRSIEDRVQEEARCLVEELRKTKASPCDPTFILGCAPCNVICSIIFHKRFDYKDQQ

FLNLMEKLNENIKILSSPWIQICNNFSPIIDYFPGTHNKLLKNVAFMKSYILEKVKEHQE

SMDMNNPQDFIDCFLMKMEKEKHNQPSEFTIESLENTAVDLFGAGTETTSTTLRYALLLL

LKHPEVTAKVQEEIERVIGRNRSPCMQDRSHMPYTDAVVHEVQRYIDLLPTSLPHAVTCD

IKFRNYLIPKGTTILISLTSVLHDNKEFPNPEMFDPHHFLDEGGNFKKSKYFMPFSAGKR

ICVGEALAGMELFLFLTSILQNFNLKSLVDPKNLDTTPVVNGFASVPPFYQLCFIPV

>291404525

MDPAVFLVLSLSCLLLLSLWRQHSARRKLPPGPTPLPIIGNILQMDLKDISKSFSNFSKV

YGPVFTLYIGTKPTVVLHGYEAVKEALIDLGEEFAGRGSFPVAERMNKDLGIFLSSGNRW

KEMRRFSLMTLRNFGMGKRSIEDRVQEEARCLVEELRKTNASPCDPTFILGAAPCNVICS

IIFQNRFEYTDQDFLTFLKLLNEGINILSSPWIQVCNNFPVLIDYFPGSHKTFMEDMTCI

QNFISEKVKEHQQSLDMNNPRNFIDCFLIKMEQHNQQSEFTLENLKYTVGDLFAAGTETT

STTLRYGLLLLLKYPEVTARVQEEIEHVIGRHRSPCMQDRSRMPYTDAVIHEIQRFIDLV

PTSLPRAVTRDVKFRNYLIPKGTTILTSLTSVLHDDKEFPNPESFDPGHFLDKSGNFKKS

DYFMPFSTGKRICVGEGLARMELFLFLTTILQNFRLKPLVDPKDINITPVVNGFASVPPP

YQLCLIPV

>296220840

MDPFVVFVLSLSCLLLLSLWKQGSKRGKFPPGPTPLPIVGNLLQIDIKDITKSLANFSKV

YGPVFTLYFGLKRTVVLHGYEVVKEALIDLGDEFSGRGLIPVVERANREYGIVFSNGNRW

KEIRRFSLMTLRNFGMGKRSIEDRVQEEAHYLLEELRKTKASPCDPTFILGCAPCNVICS

IIFQKRFDYKDQQFLSLMERLNENTKILSSPWLQICNSLPPIIDYFPGTHNKLLKNVALI

KSFILEKVKEHQESVDINNPRDFIDCFLIKMKKEEHNQQSEFIIENLVNTALDLFGAGTE

TTSTTLRYALLLLLKHPEVTAKVQEEIGHVVGRNRSPCMQDRGHMPYTDAVVHEVQRYID

LIPTNLPHAVTSDVQFRNYLIPKGTTILTSLTSVLHDNKEFPNPEKFDPHHFLDEGGNFK

KSNYFIPFSTGKRICVGEGLARMELFIFLTSILQNFNLKPLVDPKNLDITPVVNGFASVP

PFYQLCFIPI

>Q91X77.2

MDPILVLVFTLSCLFLLSLWRQSSERGKLPPGPTPLPIIGNILQINVKDICQSFTNLSKV

YGPVYTLYLGRKPTVVLHGYEAVKEALVDHGEEFAGRGRLPVFDKATNGMGIIFSKGNVW

KNTRRFSLTTLRNLGMGKRSIEDRVQEEARCLVEELRKTNGSPCDPTFILGCAPCNVICS

IIFQDRFDYKDRDFLNLMEKLNEITKIMSTPWLQVCNTFPVLLDYCPGSHNKVFKNYACI

KNFLLEKIKEHEESLDVTIPRDFIDYFLINGGQENGNYPLKNRLEHLAITVTDLFSAGTE

TTSTTLRYALLLLLKYPHVTAKVQEEIEHVIGKHRRPCMQDRSHMPYTDAMIHEVQRFID

LVPNSLPHEVTCDIKFRNYFIPKGTNVITSLSSVLRDSKEFPNPEKFDPGHFLDENGKFK

KSDYFMPFSTGKRICAGEGLARMELFLFLTSILQNFNLKPLVHPKDIDVTPMLIGLASVP

PAFQLCFIPS

>291404478

MDLVVVLVLSLSFLLLLSLWKQSHGRRRLPPGPTPFPILGNILQLDVKDITRSFIQLSKV

YGPVFTVYLGMEPTVVMHGYEAVKEALVDLGEEFSGRTNFPVTAKLNQASGVIFSNGKRW

KEMRRFSLMTLRNFGMGKRSIEERVQEEARCLVEELRKTSASPCDPTFILACAPSNVICS

VIFQNRFDYRDPKFLNLMGKFNENFKILNSPWLQFCNIFPILMDYLPGSHNKVIKNFAEI

RGYILEKIKEHQESLDINNPRDFIDCFLIKMEQENNNEQSEFTTESLIGTITDLFVAGTE

TTSTTLRYGLLLLLKHPEVTAKVQEEIERVIGRHRSPCMQDRSRMPYTDATVHEIQRYID

LIPNNVPRATTCNIKFRNYLIPKGTSIIASLTSVLYDDKEFSNPQTFDPGHFLDDRGNFK

KSDYFMPFSAGKRMCVGEGLARMELFLFLTAILQKFTLKSLVDPRDVDITPVFKGFGHVP

PLYQLSFIPV

>P11712.3

MDSLVVLVLCLSCLLLLSLWRQSSGRGKLPPGPTPLPVIGNILQIGIKDISKSLTNLSKV

YGPVFTLYFGLKPIVVLHGYEAVKEALIDLGEEFSGRGIFPLAERANRGFGIVFSNGKKW

KEIRRFSLMTLRNFGMGKRSIEDRVQEEARCLVEELRKTKASPCDPTFILGCAPCNVICS

IIFHKRFDYKDQQFLNLMEKLNENIKILSSPWIQICNNFSPIIDYFPGTHNKLLKNVAFM

KSYILEKVKEHQESMDMNNPQDFIDCFLMKMEKEKHNQPSEFTIESLENTAVDLFGAGTE

TTSTTLRYALLLLLKHPEVTAKVQEEIERVIGRNRSPCMQDRSHMPYTDAVVHEVQRYID

LLPTSLPHAVTCDIKFRNYLIPKGTTILISLTSVLHDNKEFPNPEMFDPHHFLDEGGNFK

KSKYFMPFSAGKRICVGEALAGMELFLFLTSILQNFNLKSLVDPKNLDTTPVVNGFASVP

PFYQLCFIPV

>EHH25617.1

MDSLVVLVLCLSCLLLLSLWRQRSGRGKLPPGPTPLPVIGNILKIGIKDVSKSLTNLSKV

YGPVFTLYFGLERMVVLHGYEAVKEALIDLGEEFSGRGHFPLADRANRGFGIVFSNGKRW

KEIRRFSLMTLRNCGMGKRSIEDRVQEEARCLVEELRKTKGSPCDPTFILGCAPCNVICS

IIFHKRFDYKDQQFLKVMEKLNENVKILSSPWIQICNNFPPFIDYFPGAHNKLLKNIAFV

KSYILEKVKEHQESLDINNPRDFIDCFLMKMEKEKHNQQSEFTIENLENTAVDLFGAGTE

TTSTTLRYALLLLLKHPEVTAKVQEEIERVIGRNRSPCMQDRSHMPYTDAVVHEIQRYID

LLPTNLPHAVTCDVKFRNYLIPKGTTILISLTSVLRDNKEFPNPEMFDPRHFLDEGGNFK

KSNYFMPFSAGKRICVGEALARMELFLFLTSVLQNFNLKSLVDPKDLDTTPVVNGFASVP

PFYQLCFIPV

>149689829

MDLVVFLGLCLSCLLLLSLWKRRSRKGKLPPGPTPLPIIGNILQLDVKNISKSLSKLSKL

YGPVFTVYLGLKPTVVLHGYEAVKEALIDLGEEFSGRGSVPLLERSNKGYGLVFSSGKRW

KEIRRFSLMTLRNFGMGKRSIEECVQQEAHCLVEELRKTNASPCDPIFILGCAPCNVICS

IIFQNHFDYTDQNLLSLLKLMNENVNILNSPWVQVCNIFPVFMDYFPGIQNKYLKNSDNI

KSYLLEKIKEHQESLDINNPRDFIDHFLIKVKQEKHNLHSEFTIENLLYTASDLFAAGIH

TTATTLRYGLLLLIKHPEVTAKVQEEIDRVIGRHRSPCMQDRSHMPYMDAVVHEIQRYID

LVPTNLPHAVTRDVKFRNYLIPKGTTILTSLTSVLHDDKEFPNPGVFDPGHFLDESGNFK

KSDYFMAFSAGKRMCVGEGLARMELFLFLTIILQKFTLKSVVEPKDINITPATSGLASLP

PSYQICFIPV

>EDL41828.1

MDPILVLVFTLSCLFLLSLWRQSSERGKLPPGPTPLPIIGNILQINVKDICQSFTNLSKV

YGPVYTLYLGRKPTVVLHGYEAVKEALVDHGEEFAGRGRLPVFDKATNGMGIIFSKGNVW

KNTRRFSLTTLRNLGMGKRSIEDRVQEEARCLVEELRKTNGSPCDPTFILGCAPCNVICS

IIFQDRFDYKDRDFLNLMEKLNEITKIMSTPWLQVCNTFPVLLDYCPGSHNKVFKNYACI

KNFLLEKIKEHEESLDVTIPRDFIDYFLINGGQEDGNYPLKNRLEHLAITVTDLFSAGTE

TTSTTLRYALLLLLKYPHVTAKVQEEIEHVIGKHRRPCMQDRSHMPYTDAMIHEVQRFID

LVPNSLPHEVTCDIKFRNYFIPKGTNVITSLSSVLRDSKEFPNPEKFDPGHFLDENGKFK

KSDYFMPFSTGKRICAGEGLARMELFLFLTSILQNFNLKPLVHPKDIDVTPMLIGLASVP

PAFQLCFIPS

>Q6XVG2.1

MDPILVLVLTLSCLFLLSLWRQSYERGKLPPGPTPLPIIGNILQIDVKDICQSFTNLSRV

YGPVYTLYLGRKPTVVLHGYEAVKEALVDHGDVFAGRGRLPVFDKATNGMGIGFSNGSVW

KNTRHFSLMTLRNLGMGKRSIEDRVQEEARCLVEELRKTNGSPCDPTFILGCAPCNVICS

IIFQDRFDYKDRDFLNLLEKLDEISKILSTPWLQVCNTFPALLDYCPGSHNQFFKNYAYI

KNFLLEKIREHKESLDVTIPRDFIDYFLIKGAQEDDNHPLKNNFEHLAITVTDLFIGGTE

SMSTTLRYALLLLLKYPHVTAKVQEEIEHVIGKHRRPCMQDRSHMPYTNAMIHEVQRFID

LVPNNLPHEVTCDIKFRNYFIPKGTTVITSLSSVLRDSKEFPNPEKFDPGHFLDENGKFK

KSDYFMPFSTGKRICAGEGLARMELFLFLTSILQNFNLKPLVHPKDIDITPMLIGLGSVP

PAFQLCFIPS

>AAI65950.1

MDPILVLVLTLSCLFLLSLWRQSYERGKLPPGPTPLPIIGNILQIDVKDICQSFTNLSRV

YGPVYTLYLGRKPTVVLHGYEAVKEALVDHGDVFAGRGRLPVFDKATNGMGIGFSNGSVW

KNTRHFSLMTLRNLGMGKRSIEDRVQEEARCLVEELRKTNGSPCDPTFILGCAPCNVICS

IIFQDRFDYKDRDFLNLLEKLDEISKILSTPWLQVCNTFPALLDYCPGSHNQFFKNYAYI

KNFLLEKIREHKESLDVTIPRDFIDYFLIKGAQEDDNHPLKNNFEHLAITVTDLFIGGTE

SMSTTLRYALLLLLKYPHVTAKVQEEIEHVIGKHRRPCMQDRSHMPYTNAMIHEVQRFID

LVPNNLPHEVTCDIKFRNYFIPKGTTVITSLSSVLRDSKEFPNPEKFDPGHFLDENGKFK

KSDYFMPFSTGKRICAGEGLARMELFLFLTSILQNFNLKPLVHPKDIDITPMLIGLGSVP

PAFQLCFIPS

>AAO52736.1

MDPILVLVFTLSCLFLLSLWRQSSERGKLPPGPTPLPIIGNILQINVKDICQSFTNLSKV

YGPVYTLYLGRKPTVVLHGYEAVKEALVDHGEEFAGRGRLPVFDKATNGMGIIFSKGNVW

KNTRRFSLTTLRNLGMGKRSIEDRVQEEARCLVEELRKTNGSPCDPTFILGCAPCNVICS

IIFQDRFDYKDRDFLNLMEKLNEITKIMSTPWLQVCNTFPVLLDYCPGSHNKVFKNYACI

KNFLLEKIKEHEESLDVTIPRDFIDYFLINGGQENGNYPLKNRLEHLAITVTDLFSAGTE

TTSTTLRYALLLLLKYPHVTAKVQEEIEHVIGKHRRPCMQDRSHMPYTDAMIHEVQRFID

LVPNSLPHEVTCDIKFRNYFIPKGTNVITSLSSVLRDSKEFPNPEKFDPGHFLDENGKFK

KSDYFMPFSTGKRICAGEGLARMELFLFLTSILQNFNQKPLVHPKDIDVTPMLIGLASVP

PAFQLCFIPS

>332212370

MDPFVVLVLCLSCLLLLSLCRQRSGRGKLPPGPTPLPVIGNILQIDIKDVSKSLTNLSKV

YGPVFTLYFGLERMVVLHGYEAVKEALIDLGEEFSGRGHFPLAERANRGFGIVFSNGKRW

KEIRRFSLMTLRNFGMGKRSIEDRVQEEARCLVEELRKTKASPCDPTFILGCAPCNVICS

IIFQKRFDYKDQQFLNLMEKLNENVRIVSTPWIQICNNFPPIIDYFPGTHNKLLKNIAFM

ESYILEKVKEHQESIDINNPRDFIDCFLIKMEKEKQNQQSEFTIENLVITAADLLGAGTE

TTSTTLRYALLLLLKHPEVTAKAQEEIERVIGRNRSPCMQDRGHMPYTDAVVHEVQRYID

LIPTNLPHAVTCDVKFRNYLIPKGTTILTSLTSVLHDNKEFPNPEMFDPRHFLDGGGNFK

KSNYFMPFSAGKRICVGEGLARMELFLFLTFILQNFNLKSLIDPKDLDTTPVVNGFASVP

PFYQLCFIPV

>327276257

MEFFSGTKMDFSGAITILLAMAVSCLLFLNFSSKKKYRTLPPGPTPLPFIGNIHQVDIKE

LIKSLRELSKTYGPMYTFYLGSRPCVVLSGYQVLKEALIDKAEEFSGRGDFPAVQMWSKG

NGIVYGTGECWRQLRRFAITTFKSFGMGKRSIEERIKEEAQFLIAEFHKTEGKPFDPTFC

LSCAGSNIICTLVFGDRFEYTDKKFLTLLDLINNNWKLMSSTWGQMLFTFPKIMRHIPGP

HRQIYKNYLKLAEFVGERLEMNKQTLDPNSPRDFIDCFLIKIQQEKNNPNTYFNEDTMSK

TTVNLFFAGTETVSSTLKYGLRILLRHPEVEEKLHEEIDRVIGPNRSPCMEDRIRMPYTD

AVIHEIQRYADIVPMGVPHTVTRDIDFRGYTLPKDLNIIPLLCTSQFDPTQFKNPNNFDP

THFLDKNGRFKRNDAFMAFSAGKRVCLGENLALMELFIFLTTILQNFKLKPLMDPKDIDI

TPESTGLGSIPRPYKFCLLPR

>354503136

MVLGVFLGLVLACLLLLSLWRQNSQRRKLPPGPTPLPIIGNILQVDLKNISKSLRDLSKV

YGPVFTLYLGSKPAVVLHGYEVVKEALIDHGEEFSGRGSLPAVDKIKKGLGIIFSNGKNW

KETRRFSLMTLRNFGMGKRSIEERVQEEARCLVEELRKTNGAPCDPTFILGCAPCNVICS

IIFQNRFNYKDQDFLNLINILNENANILSSPWMQVCNNFPVITDYLPGSHAKLLKNFTFL

KHYCLEKVKEHQESLDINNPRDFIDCFLIKMEQEKHNSNVECTYDNFAVTTSDLFAAGTE

TTSTTLRYSLLLLLKHPEVTAKVQEEIDHVIGRNRSPCMQDRTHMPYTDAVLHEIQRYID

LVPTSLPHATTSEIKFRNYLIPKGTTVITSLTSVLNDDKEFPNPERFDPGHFLDENGKFK

KSDYFFPFSAGKRICVGEGLARMELFLFLTTILQNFHLESPVDPKDLDTTPVVSGLIAVP

PKFQICFIPV

>P08682.2

MAVLGITVALLGWMVILLFISVWKQIHSSWNLPPGPFPLPIIGNLLQLDLKDIPKSFGRL

AERFGPVFTVYLGSRRVVVLHGYKAVREMLLNHKNEFSGRGEIPAFREFKDKGIIFNNGP

TWKDTRRFSLTTLRDYGMGKQGNEDRIQKEAHFLLEELRKTQGQPFDPTFVIGCTPFNVI

AKILFNDRFDYKDKQALRLMSLFNENFYLLSTPWLQVYNNFSNYLQYMPGSHRKVIKNVS

EIKEYTLARVKEHHKSLDPSCPRDFIDSLLIEMEKDKHSTEPLYTLENIAVTVADMFFAG

TETTSTTLRYGLLILLKHPEIEEKLHEEIDRVIGPSRMPSVRDRVQMPYMDAVVHEIQRF

IDLVPSNLPHEATRDTTFQGYVIPKGTVVIPTLDSLLYDKQEFPDPEKFKPEHFLNEEGK

FKYSDYFKPFSAGKRVCVGEGLARMELFLLLSAILQHFNLKPLVDPEDIDLRNITVGFGR

VPPRYKLCVIPRS

>AAS86751.1

MTALGITVALLVWLVTLLLISIWKHIHSSWKLPPGPFPLPIVGNIFQLDLKNIPKSFTML

AERYGPVFTVYLGSRRIVVLHGYKAVKEVLLHYKNEFSGRGEIPTFQVHKDKGVIFNNGP

TWRDTRRFSLTTLRDFGMGKQGNEQRIQREAHFLLEALRKTHGQPFDPTFLIGCAPCNVI

SDILFRQHFDYNDKTCLRLMSMFNENFYLLSTGWIQLYNNFSGYLRYLPGSHRKLMKNIS

EIKDYALERVKDHRDSLEPSCPRDFTDTLLMEMEKEKYSAEPIYTLDNIAVTVADMFFAG

TETTSTTLRYGLLILMKYPEVEEKLHEEIDRVIGPNRIPAIKDRLDMPYLDAVVHEIQRF

IDLIPSNLPHEATRDTVFRDYIIPKGTVVIPTLDSVLYDSQEFPEPEKFKPEHFLNENGK

FKYSDHFKAFSAGKRVCVGEGLARMELFLFMAAILQHFNLKSLVDPKDIDLSPIAIGFAK

IPPHYKLCVIPRSQV

>AAI53745.1

MELGVTWSLILAVIVSFLVYSFTWRRKLRKINMPPGPPLYPLLGNMLQISAKEFPQSLVK

LSEKYGTVFTVYLPSKPAVILSGYDCIKEALLDNNESFGARGESPLGYLLFKDYGVIFSN

GERWKQLRRFSLSCLRDFGMGKKSIEERIQEEARCLVEELGKNGDTPMDPTYMLTLAVSN

VICTVVFGERFDYKDEKFMTLISLLKIVSRDFSSAWGILLNLFPNTLSRLPGPPQRLFRN

FDKLKAFVAESLKSHQETLNSDCPRDFIDCFLIKMEKEKNNPQTEFHSDNLFGTVLDLFF

AGTETTSITLKYSFLMLLKYTEVTRKAMEEIDNIIGQERCPFYEDRIKMPYTNAVIHEIQ

RMADIVPLGVPHATTHDIIFRGYNIPKDTIIFPLMTSVLKDPKYFNDPKQFDPAHFLDEN

GSFKKNDAFQPFSIGKRSCLGEGLARMEIFLFITSILQAFNLKSDTAPQDIDITPEPDKN

GAIPRTYKMYFVPK

>EHH64899.1

MDSLVVLVLCLSCLLLLSLWRQRSGRGKFPPGPTPLPVIGNILKIGIKDVSKSLTNLSKV

YGPVFTLYFGLERMVVLHGYEAVKEALIDLGEEFSGRGHFPLFERANRRFGIVFSNGKRW

KEIRRFSLMTLRNFGMGKRSIEDRVQEEARCLVEELRKTKASPCDPTFILGCAPCNVIRS

IFFHKRFDYKDQQFLNLMEKFNENAKILSSPWIQIYNNFSPIIDYFPGTHNKLLKNIAFL

KSYILEKVKEHQESMDMNNPRDFIDCFLIKMEKEKHNQQSEFNIENLENTAVDLFAAGTE

TTSTTLRYALLLLLKHPEVTAKVQEEIEHVIGRNRSPCMQDRSHMPYTDAVVHEIQRYID

LLPTSVPHAVTCDVKFRNYLIPKGTTILISLTSVLRDNKEFPNPEMFDPRHFLDEGGNFK

KSNYFMPFSAGKRICVGEALARMELFLFLTSILQNFNLKSLVDLKDLDTTPVFNGFVSVP

PVYQLCFIPV

>AAB59426.1

MDPFVVLVLCLSCLLLLSIWRQSSGRGKLPPGPTPLPVIGNILQIDIKDVSKSLTNLSKI

YGPVFTLYFGLERMVVLHGYEVVKEALIDLGEEFSGRGHFPLAERANRGFGIVFSNGKRW

KEIRRFSLMTLRNFGMGKRSIEDRVQEEARCLVEELRKTKASPCDPTFILGCAPCNVICS

IIFQKRFDYKDQQFLNLMEKLNENIRIVSTPWIQICNNFPTIIDYFPGTHNKLLKNLAFM

ESDILEKVKEHQESMDINNPRDFIDCFLIKMEKEKQNQQSEFTIENLVITAADLLGAGTE

TTSTTLRYALLLLLKHPEVTAKVQEEIERVIGRNRSPCMQDRGHMPYTDAVVHEVQRYID

LIPTSLPHAVTCDVKFRNYLIPKGTTILTSLTSVLHDNKEFPNPEMFDPRHFLDEGGNFK

KSNYFMPFSAGKRICVGEGLARMELFLFLTFILQNFNLKSLIDPKDLDTTPVVNGFASVP

PFYQLCFIPV

>AAI02812.1

MDLAVVLVLCLSCLLLLSLWKQSSGKGKLPPGPTPLPILGNIFQLDVKNISKSLTSLSKV

YGPVFTVYFGMKPTVVLHGYEAVKEALIDLGEEFSRRGSFPVIERNVKGHGIVFSNGKTW

KETRRFSLMTLRNFGMGKRSIEDRVQEEARCLVEELRKTNGLPCDPTFILGCAPCNVICS

IIFQNRFDYKDQTFLNLMKTINENIKILGSPWIQVLNIFPVLLDFFPWSYSYKKLYTNTA

YVKNYVLEKTREHQASLDINNPRDFIDCFLIKMEQEKHNHQSEYTFENLTITVSDLFGAG

TETTSTTLRYGLLLLLKHPEVTAKIQEEIDRVIGRHRSPCMQDRTHMPYMDAVLHEIQRY

IDLAPTSVPHAVNCDVKFRNYLIPKGTDILTSLTSVLHDDKEFPNPEVFDPGHFLDENGN

FRKSDYFMAFSAGKRVCVGEGLARMELFLFLTTILQTFTLKSVVDPKDLDTTPAVTGIAN

VPPPYQLCFIPV

>P33264.1

MDAFVVLVFILSCLFLLSLWRQSSERGKLPPGPTPLPLIGNFFQIDVKDISGSLTNFSKV

YGPVFTLYLGRKPAVVLHGYEAVKEALIDHGEEFAGRGSFPMAERYNKGLGIVFSNGNRW

KDIRRFSLMALRSLGMGKRSIEDRVQEEAQCLVEELRKTNGSPCDPTFILSCAPCNVICS

IIFQNRFDYTDQDFLTFMEKVNENVRILSSPWLQVCNNFPSLIDYCPGSHHTITKNVNYI

RSYLSEKIKEHQETLDVANPRDFIDYYLIKLKQGNYNQQSEFTLENLATTVRDLFAAGTE

TTSTTLRYALLLLLKHPHVTAKVQEEIDQVVGRHRNPCMQDRSHMPYTDAMIHEVQRFID

LIPTNLPHAVTCDIKFRDYFIPKGTTIITSLSSVLHDSKEFPNPEVFDPGHFLDKNGKFK

KSDYFMPFSTGKRMCAGEGLARMELFLFLTTILQNFKLKSLVHPKDIDTTPVVNGLASLP

PSYQLCFIPV

>P33261.3

MDPFVVLVLCLSCLLLLSIWRQSSGRGKLPPGPTPLPVIGNILQIDIKDVSKSLTNLSKI

YGPVFTLYFGLERMVVLHGYEVVKEALIDLGEEFSGRGHFPLAERANRGFGIVFSNGKRW

KEIRRFSLMTLRNFGMGKRSIEDRVQEEARCLVEELRKTKASPCDPTFILGCAPCNVICS

IIFQKRFDYKDQQFLNLMEKLNENIRIVSTPWIQICNNFPTIIDYFPGTHNKLLKNLAFM

ESDILEKVKEHQESMDINNPRDFIDCFLIKMEKEKQNQQSEFTIENLVITAADLLGAGTE

TTSTTLRYALLLLLKHPEVTAKVQEEIERVVGRNRSPCMQDRGHMPYTDAVVHEVQRYID

LIPTSLPHAVTCDVKFRNYLIPKGTTILTSLTSVLHDNKEFPNPEMFDPRHFLDEGGNFK

KSNYFMPFSAGKRICVGEGLARMELFLFLTFILQNFNLKSLIDPKDLDTTPVVNGFASVP

PFYQLCFIPV

>403259877

MDPAMALVFCLSCLLLLSLWSQRSGRGRLPSGPTPLPIIGNILQLDVKDISKSLTNPIVV

LHGYEAVKEALIDHGEEFSGRGSFPVAEKVNKGLGILFSNGKRWKEIRRFSLMTLRNFGM

GKRSIEDRVQEEARCLVEELRKTNASPCDPTFILGCAPCNVICSVVFQKRFDYKDQRFLN

LMEKFNENLRLLSSPWIQVCNNFPALIDYLPGSHNKVLKNFAYVKSYVLERIKEHQESLD

VNNPRDFIDCFLIKMEQEKHNQQSEFTTENLIATVTDMFGAGTETTSTTLRFGLLLLLKY

PEVTAKVQEEIEHVVGRNRSPCMQDRSHMPYTDAVVHEIQRYIDLIPTNLPHAVTCDVKF

RNYLIPKGTTIITSLNSVLHNDKEFPNPEMFDPGHFLDKSGNFKKSDYFIPFSTGKRMCV

GEGLARMELFLFLTTILQNFNLKSQVDPKDIDITPIANSFGRVPPLYQLCFIPV

>1OG2A

MAKKTSSKGRPPGPTPLPVIGNILQIGIKDISKSLTNLSKVYGPVFTLYFGLKPIVVLHG

YEAVKEALIDLGEEFSGRGIFPLAERANRGFGIVFSNGKKWKEIRRFSLMTLRNFGMGKR

SIEDRVQEEARCLVEELRKTKASPCDPTFILGCAPCNVICSIIFHKRFDYKDQQFLNLME

KLNENIEILSSPWIQVYNNFPALLDYFPGTHNKLLKNVAFMKSYILEKVKEHQESMDMNN

PQDFIDCFLMKMEKEKHNQPSEFTIESLENTAVDLFGAGTETTSTTLRYALLLLLKHPEV

TAKVQEEIERVIGRNRSPCMQDRSHMPYTDAVVHEVQRYIDLLPTSLPHAVTCDIKFRNY

LIPKGTTILISLTSVLHDNKEFPNPEMFDPHHFLDEGGNFKKSKYFMPFSAGKRICVGEA

LAGMELFLFLTSILQNFNLKSLVDPKNLDTTPVVNGFASVPPFYQLCFIPVHHHH

>114631906

MDPFVVLVLCLSCLLLLSIWRQSSGRGKLPPGPTPLPVIGNILQIDIKDVSKSLTNLSKV

YGPVFTLYFGLERMVVLHGYEVVKEALIDLGEEFSGRGHFPLAERANRGFGIVFSNGKRW

KEIRRFSLMTLQNFGMGKRSIEDRVQEEARCLVEELRKTKASPCDPTFILGCAPCNVICS

IIFQKRFDYKDQQFLNLMEKLNENIRIVSTPWIQICNNFPTIIDYFPGTHNKLLKNLAFM

ERDILEKVKEHQESMDINNPRDFIDCFLIKMEKEKQNQQSEFTIENLVITAADLLGAGTE

TTSTTLRYALLLLLKHPEVTAKVQEEIERVIGRNRSPCLQDRGHMPYTDAVVHEVQRYID

LLPTSLPHAVTCDVKFRNYLIPKGTTILTSLTSVLHDKKEFPNPEMFDPRHFLDEGGNFK

KSNYFMPFSAGKRICVGEGLARMELFLFLTFILQNFNLKSLIDPKDLDTTPVVNGLASVP

PFYQLCFIPV

>P11371.1

MDPVAGLVLGLCCLLLLSLWKQNSGRGKLPPGPTPFPIIGNILQIDVKDISKSLTKFSER

YGPVFTVYLGMKPTVVLHGYKAVKEALVDLGEEFAGRGHFPIAEKVNKGLGIVFTNANTW

KEMRRFSLMTLRNFGMGKRSIEDRVQEEARCLVEELRKTNALPCDPTFILGCAPCNVICS

VILHNRFDYKDEEFLKLMERLNENIRILSSPWLQVYNNFPALLDYFPGIHKTLLKNADYT

KNFIMEKVKEHQKLLDVNNPRDFIDCFLIKMEKENNLEFTLGSLVIAVFDLFGAGTETTS

TTLRYSLLLLLKHPEVAARVQEEIERVIGRHRSPCMQDRSHMPYTDAVIHEIQRFIDLLP

TNLPHAVTRDVKFRNYFIPKGTDIITSLTSVLHDEKAFPNPKVFDPGHFLDESGNFKKSD

YFMPFSAGKRMCVGEGLARMELFLFLTSILQNFKLQSLVEPKDLDITAVVNGFVSVPPSY

QLCFIPI

>P79383.1

MTALGITVALLVWLVTLLLISIWKHIHSSWKLPPGPFPLPIVGNIFQLDLKNIPKSFTML

AERYGPVFTVYLGSRRIVVLHGYKAVKEVLLHYKNEFSGRGEIPTFQVHKDKGVIFNNGP

TWRDTRRFSLTTLRDFGMGKQGNEQRIQREAHFLLEALRKTHGQPFDPTFLIGCAPCNVI

SDILFRQHFDYNDKTCLRLMSMFNENFYLLSTGWIQLYNNFSGYLRYLPGSHRKLMKNIS

EIKDYALERVKDHRDSLEPSCPRDFTDTLLMEMEKEKYSAEPIYTLDNIAVTVADMFFAG

TETTSTTLRYGLLILMKYPEVEEKLHEEIDRVIGPNRIPAIKDRLVMPYLDAVVHEIQRF

IDLIPSNLPHEATRDTDFRDYIIPKGTVVIPTLDSVLYDSQEFPEPEKFKPEHFLNENGK

FKYSDHFKAFSAGKRVCVGEGLARMELFLFMAAILQHFNLKSLVDPKDIDLSPIAIGFAK

IPPHYKLCVIPRSQV

>AAI17532.1

MVLGVFLGLLLTCLLLLSLWRQNSQRRNLPPGPTPLPIIGNILQLDLKDISKSLRNFSKV

YGPVFTLYLGRNPAVVLHGYEAVKEAFTDHGEEFAGRGVFPVFDKFKKNCGVVFSSGRTW

KEMRRFSLMTLRNFGMGRRSIEDRIQEEARCLVDELRKTKGEPCDPTFILGCAPCNVICS

IVFQNRFDYKDQEFLTFLDILNENVEILSSPWIQICNNFPAVIDYLPGRHRKLHKNFAFA

EHYFLSKVKQHQESLDINNPRDFIDCFLIKMEQEKHNPKTEFTCENLVFTASDLFAAGTE

TTSTTLRYSLLLLLKYPEVTAKVQEEIDCVIGRHRSPCMQDRHSMPYTDAVLHEIQRYID

LLPTSLPHAVTRDVKFREYLIPKGTTVIASLTSVLYDDKEFPNPEKFDPSHFLDERGKFK

KSDYFFPFSTGKRICVGEGLARAELFLFLTTILQNFNLKSPVDLKDLDTTPVANGFASVP

PKFQICFIPI

>ADM53743.1

MDPFVVLVLCLSCLLLFSLWRQSSGRGKLPPGPTPLPIIGNILQLDIRNISKSFSNFSKL

YGPVFTVYFGLKPTVVLHGYEALKEALIDHGEEFSERGALPLIERSQKGHGIIFSTGKRW

KEMRRFSLLTLRNFGMGKRSIEYCVQKEARCIVEELRKTNASPCDPTFILGCAPCNVICS

IVFQNRFQYEDKNFLTLMERFNENFRIASTPWIQVCNIFPFLIDYFPGTHNKFLKNGAFT

KSYILEKVKEHQESLDINNPRDFIDCFLIKMEQEKDNQQSEFTVENLVSTVFDLFVAGTH

TTSTTLRYGLLLLLKHPEVTAKVQEEIDHVIGRHRSPCMQDRSHMPYMDAVLHEIQRYID

LIPNGLLHTVTSDIKFRNYLIPKGTNIVPSLTSVLYDDKEFPNPEVFDPGHFLDESGNFK

KSENFMPFSAGKRICVGEGLARMELFLFLTTILQNFNLKSLTDLKALNATPTAKGLVSML

PSYQICFTPV

>AAA31175.1

MDLLIILGICLSCVVLLSLWKKTHGKGKLPPGPTPLPVVGNLLQLETKNINKSLSMLAKE

YGSIFTLYFGMKPAVVLYGYETVKEALIDRGEEFSGRGIFPVFDRVTKGLGIVFSSGEKW

KETRRFSLTVLRNLGMGKKTIEERIQEEALCLIQALRKTNASPCDPTFLLFCVPCNVICS

VIFQNRFDYDDEKFKTLIKYFHENFELLGTPWIQLYNIFPILHYLPGSHRQLFKNIDGQI

KFILEKVQEHQESLDLNNPRDFVDHFLIKMEKEKHKKQSEFTMDNLITTIWDVFSAGTDT

TSNTLKFALLLLLKHPEITAKVQEEIEHVIGRHRSPCMQDRTRMPYTDAVMHEIQRYVDL

VPTSLPHAVTQDIEFNGYLIPKGTDIIPSLTSVLYDDKEFPNPEKFDPGHFLDESGNFKK

SDYFMPFSAGKRACVGEGLARMELFLLLTTILQHFTLKPLVDPKDIDPTPVENGFVSVPP

SYELCFVPV

>291404497

MDPVVVLVLGLCCLLLLSLWKQNSGRGKLPPGPTPFPIIGNILQIDVKDISRSLTKFSER

YGPVFTVYLGMKPTVVLHGYEAVKEALVDLGEEFAGRGSVPILDKVNKGLGIIFSNAKRW

KEMRRFSLMTLRNFGIGKRSIEDRVQEEPRCLVEELRKTNASPCDPTFILGCAPCNVICS

IIFHNRFDYKDEDFLKLMEKLNKNARLLSSPWLQVCNNFPALMDYFPGIHTTFLKNSDYT

KNFIMEKVKEHQKSLDVNSPRDFIDCFLIKMEQVSCSQQSEFTLESLVIAVSDLFGAGTE

TTSTTLRYSLLLLLKHPEVTAKVQEEIERVIGRHRSPCMQDRSCMPYMDAVVHEIQRYID

LLPYNLPHAVTRDIKFRNYFIPKSTNIITSLTSVLHDEKEFPNPKVFDPGHFLDESGNFK

KSDYFVPFSAGKRMCVGEALARMELFLFLTSILQNFKLQSLVEPKDLDITAVVNGFVSVP

PSFQLCFIPV

>344274931

MDLIIILVICLSCLILLSLSTQSYAKGKLPPGPTPLPIVGNILQLNFKNISKSLSALTKD

YGPVFTVHFGMKPTVVLHGYEAVKEALIDRAEEFSGRGVLPVADKVSQSYGIVFSNGEKW

KQTRRFSLVILRNMGMGKKTIEDRVQEEALCLVEALKKTNEFPCDPSFLLGCVPCNVICS

IIFQNRFDYSDEKLVTLINYFHENFSIVSTPWIQLYNAFPSVIHYLPGSHNKLFKNIDGQ

KKFILEKIQEHQESLDLNNPRDFIDYFLIKMEQEKHSKQSEFTMDNLVTTVWDLFTAGTE

TTSTTIRYGLLLLLKHPEVTAKVREEIDLVVGRHRSPCMQDRSSMPYTDAVVHEIQRYID

LIPTNLPHMVTQDIQFREYFIPKDTSVLVSLTSVLHDAKEFPNPEQFDPGHFLDKSGNFK

KSNYFMPFSAGKRVCAGEGLARMELFLILTNILQHFTLKPLVDPKDIDTTPVDNGLGTVP

PSYKLCFIPV

>397510086

MDPFVVLVLCLSCLLLLSIWRQSSGRGKLPPGPTPLPVIGNILQIDIKDVSKSLTNLSKV

YGPVFTLYFGLERMVVLHGYEVVKEALIDLGEEFSGRGHFPLAERANRGFGIVFSNGKRW

KEIRRFSLMTLRNFGMGKRSIEDCVQEEARCLVEELRKTKASPCDPTFILGCAPCNVICS

IIFQKRFDYKDQQFLNLMEKLNENIRIVSTPWIQICNNFPTIIDYFPGTHNKLLKNLAFM

ERDILEKVKEHQESMDINNPRDFIDCFLIKMEKEKQNQQSEFTIENLVITAADLLGAGTE

TTSTTLRYALLLLLKHPEVSAKVQEEIERVIGRNRSPCMQDRGHMPYTDAVVHEVQRYID

LLPTSLPHAVTCDVKFRNYLIPKGTTILTSLTSVLHDKKEFPNPEMFDPRHFLDEGGNFK

KSNYFMPFSAGKRICVGEGLARMELFLFLTFILQNFNLKSLIDPKDLDTTPVVNGLASVP

PFYQLCFIPV

>AAH77850.1

MALLGIETLLLVCGVTFLLYLITRRQRHLKLPPGPTPLPLIGNILQLVFPNQVKAFVKLG

SQYGPVSMVFLGQNPVLVLNGYDVVKEAFVENGEVFSNRGKNTFIEMLFKGRGVAFSNGE

TWRQMRRFSLSTLRDFGMGKRSIEERVQEEACSLVEEFNKTKGAPFDSTYLLTLAVSNVI

CSIVFGNRFEYKNETFLSVLALLKDTFRIVTSPWTQFFGFAPGFLQHFPGPHKMAAKNIG

RLKKFVTEIVTTHEETLDENSPRDYIDCFLIKMRQEKGNVNTEFDYENLFVTLLNLFFAG

TETTSITLRYGMLLLLKYPDIQKKIHDEIDCVVGLNRCPSMEDRPKLPYTDATIHEIQRF

ADIVPMGVPRSTNKDTTLRGYDIPKGTTVFPMLTSIMKDPRYFKDPESFNPCHFLDEKGS

LKKSDAFLPFSIGKRVCLGEGLARMEIFLFLTSILQRFELKCHMDPEDIDISPVPFKAAS

TPRPYELYITPR

>291404495

MDPVVVLVLGLCCLLLLSLWKQNSGRGKLPPGPTPFPIIGNILQIDVKDISRSLTKFSER

YGPVFTVYLGMKPTVVLHGYEAVKEALVDLGEEFAGRGSVPILDKVNKGLGIIFSNAKRW

KEMRRFSLMTLRNFGIGKRSIEDRVQEEPRCLVEELRKTNASPCDPTFILGCAPCNVICS

IIFHNRFDYKDEDFLKLMEKLNKNARLLSSPWLQVCNNFPALMDYFPGIHTTFLKNSDYT

KNFIMEKVKEHQKSLDVNSPRDFIDCFLIKMEQVRHETHLEFTLESLVIAVSDLFGAGTE

TTSTTLRYSLLLLLKHPEVTAKVQEEIERVIGRHRSPCMQDRSCMPYMDAVVHEIQRYID

LLPYNLPHAVTRDIKFRNYFIPKSTNIITSLTSVLHDEKEFPNPKVFDPGHFLDESGNFK

KSDYFVPFSAGKRMCVGEALARMELFLFLTSILQNFKLQSLVEPKDLDITAVVNGFVSVP

PSFQLCFIPV

>392338112

MDAVLVLVLILSSLLLLSFWRQNSERRKLPPGPTPLPIIGNILQIDVKDISKSIKKFSEV

YGPVFTLYFGLKPTVVVYGYEVVKEVLDGEEFSGRGVFPIVTKVNNDLGVIFSNGTKWKE

LRRFSLMTLRNFGMGKRSIEDRIQEEASCLVEELRKTNGSLCDPTFILSCAPSNVICSVI

FHNRFDYKDENFLNLMEKFNENFKILNSPWMQVCNAIPAFIDYLPGSHNKVIKNFAEIKS

YILRRVKEHQETLDMDNPRDFIDCFLIKIEQEKHNPCTEFTIQSLVATVTDVFVAGSETT

STTLRYGLLLLLKHTEVTAKVQEEIDHVIGRHRRPCMQDRTRMPYTDAMVHEIQRYINLI

PNNVPHAATCNVRFRNYVIPKGTDLITSLTSVLHDDKEFPNPEVFDPGHFLDEHGNFKRS

DYFMPFSSGKRMCVGEALARMELFLLLTTIVQNFNLKSFVATKDIDTTPLTNTFGCVPPS

YQLYFTPR

>BAA05139.1

MDLLIILGICLSCVVLLSLWKKTHGKGKLPPGPTPLPVVGNLLQLETKNINKSLSMLAKE

YGSIFTLYFGMKPAVVLYGYETVKEALIDRGEEFSGRGIFPVFDRVTKGLGIVFSSGEKW

KETRRFSLTVLRNLGMGKKTIEERIQEEALCLIQALRKTNASPCDPTFLLFCVPCNVMCS

VIFQNRFDYDDEKFKTLIKYFHENFELLGTPWIQLYNIFPILHYLPGSHRQLFKNIDGQI

KFILEKVQEHQESLDSNNPRDFVDHFLIKMEKEKHKKQSEFTMDNLITTIWDVFSAGTDT

TSNTLKFALLLLLKHPEITAKVQEEIEHVIGRHRSPCMQDRTRMPYTDAVMHEIQRYVDL

VPTTLPHAVTQDIEFNGYLIPKGTDIIPSLTSVLYDDKEFPNPEKFDPGHFLDESGNFKK

SDYFMPFSAGKRACVGEGLARMELFLLLTTILQHFTLKPLVDPKDIDPTPLENGFVSVPP

SYELCFVPV

>291404476

MDLVVVLVLSLSFLLLLSLWKQSHGRRRLPPGPTPFPILGNILQLDVKDISTSLTNLSKV

YGPVFTVYLGMEPTVVMHGYEAVKEALVDLGEEFSGRTNFPVTAKLNQASGVIFSNGKRW

KEMRRFSLMTLRNFGMGKRSIEERVQEEARCLVEELRKTSASPCDPTFILACAPSNVICS

VIFQNRFDYRDPKFLNLMGKFNENFKILNSPWLQFCNIFPILMDYLPGSHNKVIKNFAEI

RGYILEKIKEHQESLDINNPRDFIDCFLIKMEQENNNEQSEFTTESLIGTITDLFVAGTE

TTSTTLRYGLLLLLKHPEVTAKVQEEIERVIGRHRSPCMQDRSRMPYTDATVHEIQRYID

LIPNNVPRATTCNIKFRNYLIPKGTSIIASLTSVLYDDKEFSNPQTFDPGHFLDDRGNFK

KSDYFMPFSAGKRMCVGEGLARMELFLFLTAILQKFTLKSLVDPRDVDITPVFKGFGHVP

PLYQLSFIPV

>AAP55507.1

MVLGVFLGLLLTCLLLLSLWRQNSQRRNLPPGPTPLPIIGNILQLDLKDISKSLRNFSKV

YGPVFTLYLGRNPAVVLHGYEAVKEAFTDHGEEFAGRGVFPVFDKFKKNCGVVFSSGRTW

KEMRRFPLMTLRNFGMGRRSIEDRIQEEARCLVDELRKTKGEPCDPTFILGCAPCNVICS

IVFQNRFDYKDQEFLTFLDILNENVEILSSPWIQICNNFPAVIDYLPGRHRKLHKNFAFA

EHYFLSKVKQHQESLDINNPRDFIDCFLIKMEQEKHNPKTEFTCENLVFTASDLFAAGTE

TTSTTLRYSLLLLLKYPEVTAKVQEEIDCVIGRHRSPCMQDRHSMPYTDAVLHEIQRYID

LLPTSLPHAVTRDVKFREYLIPKGTTVIASLTSVLYDDKEFPNPEKFDPSHFLDERGKFK

KSDYFFPFSTGKRICVGEGLARAELFLFLTTILQNFNLKSPVDLKDLDTTPVANGFASVP

PKFQICFIPI

>AAA31221.1

MAVLGITVALLGWMAILLFISVWKQIHSSWNLPPGPFPLPIIGNLLQLDFKDIPKSFGRL

AERFGPVFTLYLGSRRVVVLHGYKAVREMLLNHKNEFSGRGEIPALREFKDKGIIFNNGP

TWKDTRRFSLTTLRDYGMGKQGNEDRIQQEAHFLLEELRKTQGQPFDPTFVIGCTPFNVI

AKILFNDRFDYKDKQALRLMSLFNENFYLLSTPWLQVYNNFSNYLQYMPGSHRKVIKNTC

EIKEYTLARVKEHQKSLDPSCPRDFIDCLLIEMEKDKHGTEPLYTLENIAVTVADLFFAG

TETTSTTLRYGLLILLKHPEIEEKLHEEIDRVIGPSRMPSVRDRVQMPYMDAVVHEIQRF

IDLVPSNLPHEATRDTTFQGYVIPKGTVVIPTLDSLLYDKQEFPDPEKFKPEHFLNEEGK

FKYSDYFKAFSAGKRVCVGEGLARMELFLLLSAILQHFNLKPLVDPEDIDLRSIAVGFGR

IPPRYKLCVVPRS

>301620104

MDFSGCGTIFLTIFITLLIFFMIWNKMYRRRKLPPGPTPLPLIGNLLQVRNGEMAKTLME

LGKQYGPVFTFYFGSHPVVVFCGYDAVKEALVDKGEDFVGRGKQPTVDRVFQGYGLITSE

GDRWRQLRRFSLKTLRNFGVGKRTIEERINEEASCLVEELRTYKELPVDPAIIISKAVTN

VISSVVFGTRFDYSDKRFHRMLDIFYETFELMSSVWGQIQDMVPMIMNHIPGPHQNIVTL

LEELNEFITERIKLNQDTLDPNSPRDYIDCFLIKMQEEKDNPASEFNYKNLMLTLNNLFF

AGGETVATTLKHGLLVLLKYPDIQAKLHEEIDRVIGQNRSPNIEDRNKMPYTEAVIHEIQ

RFANVIPMNAPHSATRDTNFRGYTIPQGTGVCALLCSVLGDPKYFVTPNKFNPNHFLDAD

GHFIKNEAFLPFSTGKRICLGEGLARTELFLFFTNILQNFQLTSDTHFTESDIAPRMTGF

ANVPIPYKLSFVPR

>291404493

MDPVVVLVLGLCCLLLLSLWKQNSGRGKLPPGPTPFPIIGNILQIDVKDISRSLTKFSER

YGPVFTVYLGMKPTVVLHGYEAVKEALVDLGEEFAGRGSVPILDKVNKGLGIIFSNAKRW

KEMRRFSLMTLRNFGIGKRSIEDRVQEEPRCLVEELRKTNASPCDPTFILGCAPCNVICS

IIFHNRFDYKDEDFLKLMEKLNKNARLLSSPWLQVCNNFPALMDYFPGIHTTFLKNSDYT

KNFIMEKVKEHQKSLDVNSPRDFIDCFLIKMEQETHLEFTLESLVIAVSDLFGAGTETTS

TTLRYSLLLLLKHPEVTAKVQEEIERVIGRHRSPCMQDRSCMPYMDAVVHEIQRYIDLLP

YNLPHAVTRDIKFRNYFIPKSTNIITSLTSVLHDEKEFPNPKVFDPGHFLDESGNFKKSD

YFVPFSAGKRMCVGEALARMELFLFLTSILQNFKLQSLVEPKDLDITAVVNGFVSVPPSF

QLCFIPV

>76641757

MLASGLLLVALLACLTIMVLMSVWRQRNLKGKLPPGPTPLPFIGNYLQLNTEQMCNSLMK

ISEHYGPVFTVHLGTRQIVVLCGYDAVKEALVDQAEEFSGRGKQATFDWLFKGYGVAFSN

GERAKQLRRFSITTLRDFGVGKRGIEERIQEEAGFLIEAFRGTRGAFIDPTFFLSRTVSN

VISSIVFGDRFDYEDKEFLSLLRMMLGSFQFTATSTGQLYEMFYSVMKYLPGPQQQAFKE

LQGLEDFIAKKVEQNQRTLDPNSPRDFIDSFLIRMQEEKENPNTEFYRKNLVMTTLNLFF

AGTETVSTTMRYGFLLLMKHPDVEAKIHEEIDRVIGKNRQPKFEDRAKMPYTEAVIHEIQ

RFGDMIPMGLARRVTKDTKFRDFLLPKGTEVFPMLGSVLRDPKFFSNPRDFNPQHFLDEK

GQFKKSDAFVPFSIGKRHCQLNTHRGLLYL

>1506290A

PDMDSSRXRNXIWRQSSGRGKLPPGPTPLPVIGNILQIGIKDISKSLTNLSKVYGPVFTL

YFGLKPIVVLHGYEAVKEALIDLGEEFSGRGIFPLAERANRGFGIVFSNGKKWKEIRRFS

LMTLRNFGMGKRSIEDRVQEEARCLVEELRKTKASPCDPTFILGCAPCNVICSIIFHKRF

DYKDQQFLNLMEKLNENIKILSSPWIQICNNFSPIIDYFPGTHNKLLKNVAFMKSYILEK

VKEHQESMDMNNPQDFIDCFLMKMEKHNQPSEFTIESLENTAVDLFGAGTETTSTTLRYA

LLLLLKHPEVTAKVQEEIERVIGRNRSPCMQDRSHMPYTDAVVHEVQRYIDLLPTSLPHA

VTCDIKFRNYLIPKGTTILISLTSVLHDNKEFPNPEMFDPHHFLDEGGNFKKSKYFMPFS

AGKRICVGEALAGMELFLFLTSILQNFNLKSLVDPKNLDTTPVVNGFASVPPFYQLCFIP

V

>P00182.2

MDLLIILGICLSCVVLLSLWKKTHGKGKLPPGPTPLPVVGNLLQLETKDINKSLSMLAKE

YGSIFTLYFGMKPAVVLYGYEGVIEALIDRGEEFSGRGIFPVFDRVTKGLGIVFSSGEKW

KETRRFSLTVLRNLGMGKKTIEERIQEEALCLIQALRKTNASPCDPTFLLFCVPCNVICS

VIFQNRFDYDDEKFKTLIKYFHENFELLGTPWIQLYNIFPILHYLPGSHRQLFKNIDGQI

KFILEKVQEHQESLDSNNPRDFVDHFLIKMEKEKHKKQSEFTMDNLITTIWDVFSAGTDT

TSNTLKFALLLLLKHPEITAKVQEEIEHVIGRHRSPCMQDRTRMPYTDAVMHEIQRYVDL

VPTSLPHAVTQDIEFNGYLIPKGTDIIPSLTSVLYDDKEFPNPEKFDPGHFLDESGNFKK

SDYFMPFSAGKRACVGEGLARMELFLLLTTILQHFTLKPLVDPKDIDPTPVENGFVSVPP

SYELCFVPV

>BAD07403.1

MSALGMTVALLIWAAILLLVSIWRQVHSSWNLPPGPFPLPIVGNLFNLELKNIPKSFTRM

AERFGPVFTLYLGARRVVVLYGYKAVREALLDYKSEFSGRGEIPAFREHKDRGIIFNNGP

TWKDIRRFSLTALRNYGMGKQGNENRIQREAHFLVEALRKTQGQPFEPTFLIGCAPCNVI

ADILFRKRFDYDDEKFLRLMHLFNENFYLLSTPWLQLYNNFSTYLHYLPGSHRKVIRNVA

EIKEYVSERVKEHYQSLDPNCPRDLTDCLLVEMEKEKPSAEPLYTMDGITVTVADLFFAG

TETTSTTLRYGLLILMKYPEIEEKLHEEIDRVIGPSRIPAVKDRLEMPYMDAVVHEIQRF

INLVPSNLPHEATRDAIFRGYVIPKGTVIIPSLDSVLYDKQEFPDPEKFKPEHFLNENGK

FKYSDYFKPFSTGKRVCAGEGLARMELFLLLSAVLQHFNLKSLVHPKDIDLSPVVTGFGR

IPPHYKLCVIPRSSV

>BAC34051.1

MDLVTFLVLTLSSLILLSLWRQRSRRGRLPPGPTPFPIIGNFLQIDVKNFNQSLTNFSKT

YGPVFTLYLGSRPIVVLHGYEAVKEALIDHGEEFSGRENIPMSEKINNGLGITFSNGNSW

KETRRFTLMTLRNLGMGKRNIEDRVREEAQCLVEELRKTKGSPCDPTFILSCAPCNVICS

IIFQDRFDYKDKDFLMLMKKLNENVKILSSPWLQVCNNFPLLIDYCPGSHHKVLKNFKYI

RSYLLEKVKEHQESLDATNPRDFIDYFLIKQKQANHIEQAEYSLENLVCTINNLFAAGTE

TTSTTLRYALLLLMKYPDVTAKVQEEIDHVVGRHRSPCMQDRSRMPYTDAMIHEVQRFIN

LVPNNLPHAVTCDIKFRNYIIPKGTTVVTSLTSVLHDSKEFPNPEMFDPGHFLDANGNFK

KSDYFMTFSAGKRVCAGEGLARMELFLILTTILQNFKLKSLVHPKDIDMIPFVNGLITLP

PHYQLCFIPL

>AAI50722.1

MDLVTFLVLTLSSLILLSLWRQRSRRGRLPPGPTPFPIIGNFLQIDVKNFNQSLTNFSKA

YGPVFTLYLGSRPIVVLHGYEAVKEALIDHGEEFSGRGNIPMSEKINNGLGITFSNGNSW

KETRHFTLMTLRNLGMGKRNIEDRVREEAQCLVEELRKTKGSPCDPTFILSCAPCNVICS

IIFQDRFHYKDKDFLMLMKKLNENVKILSSPWLQVCNNFPLLIDYCPGSHHKVLKNFKYI

RSYLLEKVKEHQASLDFTNPRDFIDYFLIKQKQANHIEQAEYSLENLVCTINNLFAAGTE

TTSTTLRYALLLLMKYPDVTAKVQEEIDHVVGRHRSPCMQDRSHMPYTDAMIHEVQRFIN

LVPNNLPHAVTCDIKFRNYIIPKGTTVVTSLTSVLHDSKEFPNPEMFDPGHFLDANGNFK

KSDYFMTFSAGKRVCAGEGLARMELFLILTTILQNFKLKSLVHPKDIDMMPFVNGLIALP

PHYQLCFIPL

>P56655.2

MDLVTFLVLTLSSLILLSLWRQRSRRGRLPPGPTPFPIIGNFLQIDVKNFNQSLTNFSKT

YGPVFTLYLGSRPIVVLHGYEAVKEALIDHGEEFSGRENIPMSEKINNGLGITFSNGNSW

KETRRFTLMTLRNLGMGKRNIEDRVREEAQCLVEELRKTKGSPCDPTFILSCAPCNVICS

IIFQDRFDYKDKDFLMLMKKLNENVKILSSPWLQVCNNFPLLIDYCPGSHHKVLKNFKYI

RSYLLEKVKEHQESLDVTNPRDFIDYFLIKQKQANHIEQAEYSLENLVCTINNLFAAGTE

TTSTTLRYALLLLMKYPDVTAKVQEEIDHVVGRHRSPCMQDRSRMPYTDAMIHEVQRFIN

LVPNNLPHAVTCDIKFRNYIIPKGTTVVTSLTSVLHDSKEFPNPEMFDPGHFLDANGNFK

KSDYFMTFSAGKRVCAGEGLARMELFLILTTILQNFKLKSLVHPKDIDMIPFVNGLITLP

PHYQLCFIPL

>AAI60285.1

MDLVTFLVLTLSSLILLSLWRQRSRRGRLPPGPTPFPIIGNFLQIDVKNFNQSLTNFSKT

YGPVFTLYLGSRPIVVLHGYEAVKEALIDHGEEFSGRENIPMSEKINNGLGITFSNGNSW

KETRRFTLMTLRNLGMGKRNIEDRVREEAQCLVEELRKTKGSPCDPTFILSCAPCNVICS

IIFQDRFDYKDKDFLMLMKKLNENVKILSSPWLQVCNNFPLLIDYCPGSHHKVLKNFKYI

RSYLLEKVKEHQESLDVTNPRDFIDYFLIKQKQANHIEQAEYSLENLVCTINNLFAAGTE

TTSTTLRYALLLLMKYPDVTAKVQEEIDHVVGRHRSPCMQDRSRMPYTDAMIHEVQRFIN

LVPNNLPHAVTCDIKFRNYIIPKGTTVVTSLTSVLHDSKEFPNPEMFDPGHFLDANGNFK

KSDYFMTFSAGKRVCAGEGLARMELFLILTTILQNFKLKSLVHPKDIDMIPFVNGLITLP

PHYQLCFIPL

>332252798

MSALGVTVALLVWVAFLLLVSMWRQVHSSWNLPPGPFPLPIIGNLFQLELKNIPKSFTRL

AQRFGPVFTLYVGSQRTVVLHGYKAVKEALLDHKDEFSGRGDLPAFHAHKDRGIIFNNGP

TWKDIRRFSLTTLRNYGMGKQGNESRIQREAHFLLEALRKTQGQPFDPTFLIGCAPCNVI

ADILFRKHFDYNDEKFLRLMYLFNENFYLLSTPWLQLFNNFPSFLHYLPGSHRKVIKNVA

EVKEYVSERVKEHHQSLDPNCPRDLTDCLLVEMEKEKHSAEPLYTMDGITVTVADLFFAG

TETTSTTLRYGLLILMKYPEIEEKLHEEIDRVIGPSRIPAAKDRQEMPYMDAVVHEIQRF

ITLVPSNLPHEATRDTIFRGYLIPKGTVIVPTLDSVLYDNQEFPDPEKFKPEHFLNENGK

FKYSDYFKPFSTGKRVCAGEGLARMELFLLLCAILQHFNLKPLIDPKDIDLSPTHIGFGC

IPPRYKLCVIPRS

>AAH79802.1

MDFSGCGTLFLAILISLLIFFMIWNRRSKLPPGPTPLPLIGNLLQVRNGEMAKTLMELGE

QYGPVFTFYFGPSPVIVFCGFDAVKEALVDYGEDFVGRGKQPTVDRVFQGYGLITSEGDR

WKQLRRFSLTTLRNFGVGKRTIEERIKDEASCLVEELQTYKQLPVNPAMIISKAVTNVIS

SVVFGTRFDYSDKRFHRMLDIFYETFELMSSIWGQIQDMVPWLMNHIPGPHQNIVTLLEE

LNEFIAERIKLNQDTLDPSSPRDYIDCFLLKMQEEKDNPASEFNYKNLILTLNNLFFAGG

ETVATTLKHGLLLLLKYPDIQAKLHEEIDSVIGQNRSPNIEDRNKMPYTEAVIHEIQRFA

NVIPMNAPHSATRDTYFRGYTIPQGTGVCALLCSVLGDPKYFATPNKFNPNHFLDSKGHF

IKNEAFLPFSTGKRICLGEGLARIELFLFLTNILQNFVLTSDTQFTEADITPRMTGFANV

PISYELSFVPR

>327259164

MEALGITTLFLVVFISCLVFSAVWKSRMKKEKLPPGPTPLPIIGNILQLKTNYLDQAIHK

LSQKYGPVFTMYVGTERVVVLNGYDAVKEALIDRADEFSARGKLPLADKINKGKGIIFSN

GERWKQLRRFALTTLRNFGMGKKSIEERIQDETQYVVEYLQNTKEKPFDPTFMLSCSTSN

VICSIVFGKRYEYNDKRFLSIMASMNENFEFQNIIQPLYNIFPSLMDFIPGPHHKVASNS

NKNAEFVLEEAKEHRATLDPSSPRDYIDCFYIKMDQEEQNDASEFTIENLIFCVLDLFTA

GTETTSTTLRYGLLILQKYPEIEAKVQEEIDQVIGGARKPCMADRGKMPYTDAVIHEIQR

FISLVPLSVPHAVLKDTVFREYVIPKGTTIYPVLTSVLCDTKEFRNPTKFDPQHFLHEDG

SFRKSDYFMPFSAGKRICAGEGLARMELFLFLTTILQNFKLKPLTDPKDIDISPQMSSIG

SLPRSYQLCVVPR

>354503132

MVLGVFLGLVLACLLLLSLWRQNSQRRKLPPGPTPLPIIGNILQVDLKNISKSLRDLSKV

YGPVFTLYLGRKPAVVLLGYEAVKEAMVDHGEEFAGRGSFPVADKIGQGFGIVFSSGKIW

KTMRRFSLVTLRNFGMGQRSIEERVQEEARYMVGELRKTNGSPCNPTFILGCGPCNVICS

IVFQNRFDYKDKEFLNFMHVLNENVEILSSPWMQICNNFPFMIDYLPGKHRKLLENFTFL

KHYFSAKATQHQESLDINNPRDFIDCFLIKMEQEKNNPKTEFIRENLIFSASDLFAAGTE

TTSTTLRYSLLLLLKYPEVTAKVQKEIDHVIGRNRSPCMQDRIHMPYTDAVLHEIQRYID

LLPTSLPHATTCDMKFRNYLIPKGTTVIASLSSVLYDDKEFPNPEKFDPGHFLDENGKFK

KSDYFFPFSTGKWICVGEGLARMELFLFLTTILQNFNLKSPVDLKDLDITPVANGFVSVP

PEFQICFTPI

>301620110

MAVLGIETLFLVCSFTFLVFLFSRRQRHARLPPGPTPLPLLGNVLQLDFSKQVKEFVKLG

SQYGPVSMVYLGPYPVLVLNGYDVVKEAFVDNGEVFSNRGKNAFIEMIFKGRGVAFSNGE

RWRQMRRFSLSTLRDFGMGKRRVEERVQEEACALVEEFKKTKGTPFNSTYLMTLAVSNVI

CSVVFGERFDYQNETFLSVLALLKDTFKIITSPWTQLFSFAPGLLKHLPGPHKKAAENLD

RLKTFVTEFVASHEETLEENFPRDYIDCFLIKMRQEKDNVNTEFDYENLFVTLMNLFFAG

TETTSITLQYGMLILLKYPDIQKKIHEEIDSVIGFNRCPSMEDRPKMPYTDATIHEIQRF

ADIVPMGVPRSTNKDTTLRGYDIPKGTTVFPMLTCILKDPRYFKDPESFNPCHFLDEKGC

LKKTDAFIPFSIGKRVCLGEGLARMEIFLFLTSILQRFELKCHMDPKDIDISPVPSKSAY

MPRPYELYITPR

>P51581.1

MAVFGVTIALLVWVATLLIVSIWKQIYSSWNLPPGPFPLPILGNIFQLDLKNIPKSLTKL

AERFGPVFTLHLGSKRIVVLHGYKAVKEVLLNHKNEFSGRGDIPVFQEYMNKGIIFNNGP

TWKDVRRFSLSILRDYGMGKQGNEARIQREAHFLMEELKKTNGQPFDPTFLVGCAPFNVI

SDILFHKRFDYNDKTCLRLMSLFNENFYLLSTPWIQAYNNFENYLRYLPGSHRKIMKNAS

EIRQYTLAKAKEHLQSLDSSCPRDVTDCLLIEMEKEKDSQEPMYTMENISVTLADLFFAG

TETTSTTLRYGLLILMKYPEVEEKLHEEIDRVIGPSRVPVFKDRLEMPYMDAVVHEIQRF

ISLIPSNLPHEATRDTMFRGYVIPKGTVVIPTLDSLLYDSQEFPDPEKFKPEHFLNENGK

FKYSDHFKAFSAGKRVCVGEGLARMELFLLLTAILQHFNLKSLVDPKDIDLNPVTIGFGC

VPPEFKLCVIPRS

>AAH67435.1

MSALGVTVALLVWAAFLLLVSMWRQVHSSWNLPPGPFPLPIIGNLFQLELKNIPKSFTRL

AQRFGPVFTLYVGSQRMVVMHGYKAVKEALLDYKDEFSGRGDLPAFHAHRDRGIIFNNGP

AWKDIRRFSLTTLRNYGMGKQGNESRIQREAHFLLEALRKTQGQPFDPTFLIGCAPCNVI

ADILFRKHFDYNDEKFLRLMYLFNENFHLLSTPWLQLYNNFPSFLHYLPGSHRKVIKNVA

EVKEYVSERVKEHHQSLDPNCPRDLTDCLLVEMEKEKHSAERLYTMDGITVTVADLFFAG

TETTSTTLRYGLLILMKYPEIEEKLHEEIDRVIGPSRIPAIKDRQEMPYMDAVVHEIQRF

ITLVPSNLPHEATRDTIFRGYLIPKGTVVVPTLDSVLYDNQEFPDPEKFKPEHFLNENGK

FKYSDYFKPFSTGKRVCAGEGLARTELFLLLCAILQHFNLKPLVDPKDIDLSPIHIGFGC

IPPRYKLCVIPRS

>291404491

MDPVVVLVLGLCCLLLLSLWKQNSGRGKLPPGPTPFPIIGNILQIDVKDISRSLTKFSER

YGPVFTVYLGMKPTVVLHGYEAVKEALVDLGEEFAGRGSVPILDKVNKGLGIIFSNAKRW

KEMRRFSLMTLRNFGIGKRSIEDRVQEEPRCLVEELRKTNASPCDPTFILGCAPCNVICS

IIFHNRFDYKDEDFLKLMEKLNKNARLLSSPWLQVCNNFPALMDYFPGIHTTFLKNSDYT

KNFIMEKVKEHQKSLDVNSPRDFIDCFLIKMEQVNGFYPLRFTLESLVIAVSDLFGAGTE

TTSTTLRYSLLLLLKHPEVTAKVQEEIERVIGRHRSPCMQDRSCMPYMDAVVHEIQRYID

LLPYNLPHAVTRDIKFRNYFIPKSTNIITSLTSVLHDEKEFPNPKVFDPGHFLDESGNFK

KSDYFVPFSAGKRMCVGEALARMELFLFLTSILQNFKLQSLVEPKDLDITAVVNGFVSVP

PSFQLCFIPV

>AAA41060.1

MAVLGITIALLVWVATLLVISIWKKIYNSWNLPPGPFPLPILGNIFQLDLKDIPKSFTKL

AKRFGPVFTLHLGSRRIVVLHGYKAVKEVLLNHKNEFSGRGDIPVFQEYKNKGIIFNNGP

TWKDVRRFSLSILRDWGMGKQGNEARIQREAQFLVEELKKTKGQPFDPTFLIGCAPCNVI

ADILFNKRFDYNDKKCLRLMSLFNENFYLLSTPWIQLYNNFADYLRYLPGSHRKIMKNVS

EIKQYTLEKAKEHLQSLDINCARDVTDCLLIEMEKEKHSQEPMYTMENVSVTLADLFFAG

TETTSTTLRYGLLILMKYPEIEEKLHEEIDRVIGPSRVPAVRDRLDMPYMDAVVHEIQRF

INLVPSNLPHEATRDTVFQGYVIPKGTVVIPTLDSLLYDSHEFPDPEKFKPEHFLNENGK

FKYSDYFKAFSAGKRVCVGEGLARMELFLLLSAILQHFNLKSLVDPKDIDLSPVTVGFGS

IPPQFKLCVIPRS

>P05182.4

MAVLGITIALLVWVATLLVISIWKQIYNSWNLPPGPFPLPILGNIFQLDLKDIPKSFTKL

AKRFGPVFTLHLGSRRIVVLHGYKAVKEVLLNHKNEFSGRGDIPVFQEYKNKGIIFNNGP

TWKDVRRFSLSILRDWGMGKQGNEARIQREAQFLVEELKKTKGQPFDPTFLIGCAPCNVI

ADILFNKRFDYNDKKCLRLMSLFNENFYLLSTPWIQLYNNFADYLRYLPGSHRKIMKNVS

EIKQYTLEKAKEHLQSLDINCARDVTDCLLIEMEKEKHSQEPMYTMENVSVTLADLFFAG

TETTSTTLRYGLLILMKYPEIEEKLHEEIDRVIGPSRVPAVRDRLDMPYMDAVVHEIQRF

INLVPSNLPHEATRDTVFQGYVIPKGTVVIPTLDSLLYDSHEFPDPEKFKPEHFLNENGK

FKYSDYFKAFSAGKRVCVGEGLARMELFLLLSAILQHFNLKSLVDPKDIDLSPVTVGFGS

IPPQFKLCVIPRS

>AAF13601.1

MSALGVTVALLVWAAFLLLVSMWRQVHSSWNLPPGPFPLPIIGNLFQLELKNIPKSFTRL

AQRFGPVFTLYVGSQRMVVMHGYKAVKEALLDYKDEFSGRGDLPAFHAHRDRGIIFNNGP

TWKDIRRFSLTTLRNYGMGKQGNESRIQREAHFLLEALRKTQGQPFDPTFLIGCAPCNVI

ADILFRKHFDYNDEKFLRLMYLFNENFHLLSTPWLQLYNNFPSFLHYLPGSHRKAIKNVA

EVKEYVSERVKEHHQSLDPNCPRDLTDCLLVEMEKEKHSAERLYTMDGITVTVADLFFAG

TETTSTTLRYGLLILMKYPEIEEKLHEEIDRVIGPSRIPAIKDRQEMPYMDAVVHEIQRF

ITLVPSNLPHEATRDTIFRGYLIPKGTVVVPTLDSVLYDNQEFPDPEKFKPEHFLNENGK

FKYSDYFKPFSTGKRVCAGEGLARMELFLLLCAILQHFNLKPLVDPKDIDLSPIHIGFGC

IPPRYKLCVIPRS

>AAY51974.1

MDSLVVLVLCLSCLLLLSLWRQRSGRGKLPPGPPPLPVIGNILKIGVKDVSKSLTNLSKV

YGPVFTLYFGLERMVVLHGYEAVKEALIDLGEEFSGRGHFPLFERANRRFGIVFSNGKRW

KEIRRFSLMTLRNFGMGKRSIEDRVQEEARCLVEELRKTKGSPCDPTFILGCAPCNVICS

IIFHKRFDYKDQQFLNLMEKFNENAKILSSPWIQIYNNFSPIIDYFPGTHNKLLKNVAFV

KSYILEKVKEHQESMDINNPRDFIDCFLIKMEKEKHNQQSEFNIENLENAAVDLFAAGTE

TTSTTLRYALLLLLKHPEVAAKVQEEIERVIGRNRSPCMQDRSHMPYTDAVVHEVQRYID

LLPTSVPHAVTCDVKFRNYLIPKGTTILISLTSVLRDNKEFPNPEMFDPRHFLDEGGNFK

KSNYFMPFSAGKRICVGEALARMELFLFLTSILQNFNLKSLVDLKDLDTTPVFNGFVSVP

PVYQLCFIPV

>268834986

MELLGLPTLALLVLVMSLSLLSVWTKMRTGGRLPPGPTPLPIIGNILQLDLKDIPASLSK

LAKEYGPVYTLYFGSWPTVVLHGYDVVKEALLNQGDEFLGRGPLPIIEDSQKGHGIVFSE

GERWKLLRRFSLMTLKNFGMGKRSLEERVQEEARCLVEELHKTEAQPFDPTFILACAPCN

VICSILFNERFPYNDKTFLNLMDLLNKNFYQLNSIWIQMYNLWPTIMKYIPGKHREFSKR

LGGVKNFILEKVKEHQESLDPANPRDYIDCFLSKIEEEKHNLKSDFNLENLAICGSNLFT

AGTETTSTTLRFGLLLLVKHPEVQAKVHEELDRVIGRHQPPSMKDKMKLPYTDAVLHEIQ

RYITLLPSSLPHAVVQDTKFRHYVIPKGTAVFPFLSSILLDQKEFPNPEKFDPGHFLDKN

GCFKKTDYFVPFSLGKRSCVGEGLARMELFLFFTTILQKFSLKALVEPKDLDIKPVTTGL

FNLPPPYKLRLVPR

>AAH25819.1

MELLGLPTLALLVLVMSLSLLSVWTKMRTGGRLPPGPTPLPIIGNILQLDFKDIPASLSK

LAKEYGPVYTLYFGSWPTVVLHGYDVVKEALLNQGDEFLGRGPLPIIEDSQKGHGIVFSE

GERWKLLRRFSLMTLKNFGMGKRSLEERVQEEARCLVEELHKTEAQPFDPTFILACAPCN

VICSILFNERFPYNDKTFLNLMDLLNKNFYQLNSIWIQMYNLWPTIMKYIPGKHREFSKR

LGGVKNFILEKVKEHQESLDPANPRDYIDCFLSKIEEEKHNLKSDFNLENLAICGSNLFT

AGTETTSTTLRFGLLLLVKHPEVQAKVHEELDRVIGRHQPPSMKDKMKLPYTDAVLHEIQ

RYITLLPSSLPHAVVQDTKFRHYVIPKGTAVFPFLSSILLDQKEFPNPEKFDPGHFLDKN

GCFKKTDYFVPFSLGKRSCVGEGLARMELFLFFTTILQKFSLKALVEPKDLDIKPVTTGL

FNLPPPYKLRLVPR

>3E4EA

MAKKTSSKGKLPPGPFPLPIIGNLFQLELKNIPKSFTRLAQRFGPVFTLYVGSQRMVVMH

GYKAVKEALLDYKDEFSGRGDLPAFHAHRDRGIIFNNGPTWKDIRRFSLTTLRNYGMGKQ

GNESRIQREAHFLLEALRKTQGQPFDPTFLIGCAPCNVIADILFRKHFDYNDEKFLRLMY

LFNENFHLLSTPWLQLYNNFPSFLHYLPGSHRKVIKNVAEVKEYVSERVKEHHQSLDPNC

PRDLTDCLLVEMEKEKHSAERLYTMDGITVTVADLFFAGTETTSTTLRYGLLILMKYPEI

EEKLHEEIDRVIGPSRIPAIKDRQEMPYMDAVVHEIQRFITLVPSNLPHEATRDTIFRGY

LIPKGTVVVPTLDSVLYDNQEFPDPEKFKPEHFLNENGKFKYSDYFKPFSTGKRVCAGEG

LARMELFLLLCAILQHFNLKPLVDPKDIDLSPIHIGFGCIPPRYKLCVIPRSHHHH

>CAA39087.1

MELLGFTTLALVVSVTCLSLLSVWTKLRTRGRLPPGPHPPSHYWESTATEPQGHPASLSK

LAKEYGPVYTLYFGTSPTVVLHGYDVVKEALLQQGDEFLGRGPLPIIEDTHKGYGLIFSN

GERWKVMRRFSLMTLRNFGMGKRSLEERVQEEAWCLVEELQKTKAQPFDPTFILACAPCN

VICSILFNDRFQYNDKTFLNLMDLLNKNFQQVNSVWCQMYNLWPTIIKYLPGKHIEFAKR

IDDVKNFILEKVKEHQKSLDPANPRDYIDCFLSKIEEEKDNLKSEFHLENLAVCGSNLFT

AGTETTSTTLRFGLLLLMKYPEVQAKVHEELDRVIGRHQPPSMKDKMKLPYTDAVLHEIQ

RYITLVGSSLPHAVVQDTKFRDYVIPKGTTVLPMLSSVMLDQKEFANPEKFDPGHFLDKN

GCFKKTDYFVPFSLGKRACVGESLARMELFLFFTTLLQKFSLKTLVEPKDLDIKPITTGI

INLPPPYKLCLVPR

>47550667

MELLGLPTLALLVLVMSLSLLSVWTKMRTGGRLPPGPTPLPIIGNILQLDLKDIPASLSK

LAKEYGPVYTLYFGSWPTVVLHGYDVVKEALLNQGDEFLGRGPLPIIEDSQKGHGIVFSE

GERWKLLRRFSLMTLKNFGMGKRSLEERVQEEARCLVEELHKTEAQPFDPTFILACAPCN

VICSILFNERFPYNDKTFLNLMDLLNKNFYQLNSIWIKMYNLWPTIMKYIPGKHREFSKR

LGGVKNFILEKVKEHQESLDPANPRDYIDCFLSKIEEEKHNLKSDFNLENLAICGSNLFT

AGTETTSTTLRFGLLLLVKHPEVQAKVHEELDRVIGRHQPPSMKDKMKLPYTDAVLHEIQ

RYITLLPSSLPHAVVQDTKFRHYVIPKGTAVFPFLSSILLDQKEFPNPEKFDPGHFLDKN

GCFKKTDYFVPFSLGKRSCVGEGLARMELFLFFTTILQKFSLKALVEPKDLDIKPVTTGL

FNLPPPYKLRLVPR

>AAH67433.1

MSALGVTVALLVWAAFLLLVSMWRQVHSSWNLPPGPFPLPIIGNLFQLELKNIPKSFTRL

AQRFGPVFTLCVGSQRMVVMHGYKAVKEALLDYKDEFSGRGDLPAFHAHRDRGIIFNNGP

TWKDIRRFSLTTLRNYGMGKQGNESRIQREAHFLLEALRKTQGQPFDPTFLIGCAPCNVI

ADILFRKHFDYNDEKFLRLMYLFNENFHLLSTPWLQLYNNFPSFLHYLPGSHRKVIKNVA

EVKEYVSERVKEHHQSLDPNCPRDLTDCLLVEMEKEKHSAERLYTMDGITVTVADLFFAG

TETTSTTLRYGLLILMKYPEIEEKLHEEIDRVIGPSRIPAIKDRQEMPYMDAVVREIQRF

ITLVPSNLPHEATRDTIFRGYLIPKGTVVVPTLDSVLYDNQEFPDPEKFKPEHFLNENGK

FKYSDYFKPFSTGKRVCAGEGLARMELFLLLCAILQHFNLKPLVDPKDIDLSPIHIGFGC

IPPRYKLCVIPRS

>354503134

MVLGVFLGLVLACLLLLSLWRQNSQRRKLPPGPTPLPIIGNILQVDLKNISKSLRDLSKV

YGPVFTLYLGRKPAVVLLGYEAVKEAMVDHGEEFAGRGSFPVADKIGQGFGIVFSSGKIW

KTMRRFSLVTLRNFGMGQRSIEERVQEEARYMVGELRKTNGSPCNPTFILGCGPCNVICS

IVFQNRFDYKDKEFLNFMHVLNENVEILSSPWMQICNNFPFMIDYLPGKHRKLLENFTFL

KHYFSAKATQHQESLDINNPRDFIDCFLIKMEQVSHISWTEFIRENLIFSASDLFAAGTE

TTSTTLRYSLLLLLKYPEVTAKVQKEIDHVIGRNRSPCMQDRIHMPYTDAVLHEIQRYID

LLPTSLPHATTCDMKFRNYLIPKGTTVIASLSSVLYDDKEFPNPEKFDPGHFLDENGKFK

KSDYFFPFSTGKWICVGEGLARMELFLFLTTILQNFNLKSPVDLKDLDITPVANGFVSVP

PEFQICFTPI

>354491953

MDPFTVLVLSLSFLLLLSLWRQKTERGKLPPGPTPLPLIGNFLQIDVKNIQKSFTKLSKV

YGPVFTLYFGQQPTVVLHGYNAVKEALIDHGEEFSGRGRIPFLDKNFNGLGVIFSNGKIW

KEMRRFSLITLRNLGMGKRSIEDRVQEEAQCLVEELRKTNGSPCDPSFILAYAPCNVICS

IVFQNRFDYKDKDFLNLLKFLEENTKILSSPWIQVCNSFPALLDYCPGSHKLFFKNYTYV

KSYFLEKIKEHVDSLDVTNPRDFIDYYLIKRQKENCNQELKYTLENLAVIVNDLFGAGTE

TTSSTLRYALLLLMKYPQVTAKVQEEIDRVVGRDRSACMQDKSRMPYTNATIHEVQRFID

LVPNSIPHAVTRDVKFRNYLIPKGTTVIVSLSSVLHDSKEFPNPETFDPGHFLDGNGNFK

KSDYFMPFSTGKRVCAGEALAQMELFLFLTTILQKFKLKSLLPPKDVNITPVVNGFSSVP

PPFQICFIPV

>354503138

MDPVLVLVLTVSCLLLLLVWRQNSGRGRLPPGPTPLPLIGNILQIDIKDISKSLANFSKV

YGPVFTLYFGMKPTVVVHGYEAVKEALDDLGEEFSGRGNFPIVERMNNGLGIVFSNGTKW

KELRRFSLMTLRNFGMGKRSIEDRIQEEARCLVEELRKTNGSLCDPTFILSCAPSNVICS

VVFHNRFDYKDENFLNLMEKLNENFEILNSPWMQVCNAIPAFLDYLPGSHNKALKNFAEI

KSYILKRVKEHQETLDMDNPRDFIDCFLIKMEQEKDNPHSEFTTESLMATVADVFVAGSE

TTSTTLRYGLLLLMKHTEVTAKVQKEIDHEIGRNRSPCMQDRTRMPYTDAMVHEVQRYVN

LIPNNVPHAATCNVKFRNYVIPKGTDLITSLTSVLHDDKEFPNPKIFDPAHFLDENGNFK

KSDYFMPFSTGKRMCVGEALARMELFLFLTTILQNFDLKSLVDTKDIDTTPVASTFGCVP

PSYQLYFIPR

>ADZ11091.1

MDLFVVLVICLSILIFLFLWNQKHGKGKLPPGPTPLPIVGNILQINIKNISKCISKLAED

YGPVFTLYFGMKPTVVLHGYEAVKQVLIDQSEEFSGRGSLPVADNINQGLGIVFSNGEKW

KQTRRFSLMVLRNMGMGKRTIEHRIQEEALCLVEALKKTNGSPCDPTLLLSCAPCNVICS

IVFRNRFEYSDEKLLTLIKYFNENAMLASTPWIELFNAFPSLLRHFPGSHNTIFKNMTEQ

RKFILEEIKKHQESLDLNNPQDFIDYFLIKMEKEKHNKHSEFTMDNLITTVWDVFSAGTE

TTSLTLRYGLLLLLKHPEVTAKVQEEIDHVVGRNRSPCMQDRSRMPYTDAVLHEIQRYID

LVPSNLPHVATQDVKFREYLIPKGTAILTSLTSVLHDGKEFPNPGQFDPAHFLDESGNFK

KTDHFMAFSAGKRVCVGEGLARMELFLLLVSILQHFTLKPVVDPKHIDIAPSFKGILSIP

PFCEMCFIPV

>O62671.1

MDPVVVLVLCLSCCLLLSLWKQSSRKGKLPPGPTPLPFIGNILQLDKDINKSLSNLSKAY

GPVFTLYFGMKPTVVLHGYDAVKETLIDLGEEFSARGRFPIAEKVSGGHGIIFTSGNRWK

EMRRFALTTLRNLGMGKSDLESRVQEEACYLVEELRKTNALPCDPTFVLGCASCNVICSI

IFQNRFDYTDQTLIGFLEKLNENFRILSSPWIQAYNSFPALLHYLPGSHNTIFKNFAFIK

SYILEKIKEHQESFDVNNPRDFIDYFLIKMEQEKHNQPLEFTFENLKTIATDLFGAGTET

TSTTLRYGLLLLLKHPEVTVKVQEEIDRVIGRHQSPHMQDRSRMPYTNAVLHEIQRYIDL

VPNSLPHAVTCDVKFRNYVIPKGTTILISLSSVLSDEKEFPRPEIFDPAHFLDDSGNFKK

SDYFMAFSAGKRICVGEGLARMELFLFLTTILQKFTLKPLVDPKDIDTTPLASGFGHVPP

TYQLCFIPV

>395529485

MLASGLLLGALFICLSVLMVHSVWKQRKVRSSLPPGPTPLPFIGNFLQLNTQQMYDSLMK

IGEKYGPVFTVHLGHRAVVVLTGYDAVKEALVDQAEEFSGRGEQATFDWLFKGFGVAFSN

GERARQLRRFSITTLRDFGMGKRGIEERIQEEAGFLIEAFQGTKGEAIDPTFFLSRTVSN

VISSIVFGDRFEYEDKQFLSLLRMMLGSFQFTATSMGQLYEMFYGVMKHLPGPQQQAFKE

LKGLDDFITEKVQQNQATLDPNSPRNFIDSFLIKMQEEKKNPNTEFFLKNLIMTTLNLFF

AGTETVSTTLRYGFLLLMKHPEVEAKIHEEIDRVIGRNRSPKFEDKAKMPYTEAVIHEIQ

RFGDMIPMGLARRVIKDTKFRGYLLPKGTEVYPMLGSVLRDRNFFACPEAFNPQHFLDEK

GQFKKSDAFVPFSIGEQEILFFSFPNPHSYP

>ADZ11094.1

MAALGITVALLVWMAALLLISIWKHIYSSWKLPPGPFPLPIIGNLLQLDIKNIPKSFTRL

AERFGPVFTLYLGSRRVVVVHGYKPVKEVLLDYKNEFSGRGENPGFQVHKNNGIIFNNGP

TWRDTRRFSLTILRDLGMGKQGNEQRIQREAHFLLDVLRKTQGQPFDPTFVIGFAPYNVI

SDILFHKRFDYKDKTGLRLMSLFNENFYLLSTPWIQLYNNFSDYLKYLPGSHRKLLKNVS

EVKDYALERTKDHQKSLEPSCPRGFLDTMLIEMAKEKHSADPMYTLENIAVTVADLLFAG

TETTSTTLRYGLLILMKYPEVEEKLHEEIDRVIGPSRIPAIKDRLDMPYLDAVVHEIQRF

IDLIPSNLLHEATQDTVFRGYVIPKGTLIIPTLDSVLYDKQEFPEPEEFKPEHFLNESGK

FKYSDHFKAFSAGKRVCVGEGLARMELFLFLAAILQHFNLKSLVDPKDIDLSPIAIGFGK

IPPHYKLCLIPRSEV

>350596291

MVLSAILFRIVLVGILLLWGHPSSQGHLPAGPWPLPFLGNILQMDHKGFLKSFQVLQEKY

GGIFTRHLGPQPVLKSLFLFLTVLQSFSLGSPKPLQDINPTPRTNGQGRLPPVVQENEEE

VEEEEGDQEEEAAFTPQHFSSPSSGLSLRILPIDDFQPPGAPIEPTFFLSRTVSNVISSV

VFGSRFDYEDKQFLKLLQMINQSFIEMSTPWAQLYDMYSGIMQYLPGRHNRIYYLIEELK

DFIASRVKINEASLDPQNPRDFIDCFLIKMHQDKDNPHTEFNLKNLVLTTLNLFFAGTET

VSSTLRYGLLLMMKHPEVEAKIHEEIDQVIGPHRIPRVDDRVKMPYTDAVIHEIQRLTDI

VPMGVPHNVIRDTHFRGYLLPKGTDVFPLLGSVLKDPKYFRYPDAFYPQHFLDEQGRFKK

NEAFVPFSSGKRICLGEAMARMELFLYFTSILQNFTLRSLVPPAHIDITPQVSGFGNIPP

TYELCLIARLKAQPPRVSPQSEMSGLEVSPSPTCTHPRLEVAPEQGCPTAVPVRCPPGAP

LRPT

>149689821

MDPFIVLVICLSCLILLFLWNQSYVKGKLPPGPTPLPIVGNILQINTKNISKSRSKLAED

YGPVFTVYFGMKPTVVLHGYEAVKEALVDQSEEFSGRGSMPVFDKVSQGLGIVFSNGERW

KQTQRFSLTVLRNMGMGKNTIEDRIQEEALCLVEALRKTNASPCDPTLLLGCAPCNVICS

VVFHCCFEYSDEKLLTLLNYLDENMKIASTAWIALYNALPFLIHYLPGSHNALFNNIAGQ

QKFILEKIKEHQESLDLNNPRDFIDYFLIKMEKEKHNKQSEFTMDNLITTIWDLFSTGTE

TTSTTLRYGLLLLLKHPEVTGKVQEEIDRVIGRHRSPCMQDRSRMPYTDAVVHEIQRYID

LVPNNVPHATTRDVKFREYLIPKGTAILLSLTSVLHDDKEFPNPEQFDPGHFLDASGNFK

KSDYFMAFSAGKRVCVGEGLARMELFLLLTNILQHFTLKPLVDPKDIDTTPPASGITSVP

PFYELCFIPV

>BAD86845.1

MAALGITVALLVWMATLMLISVWKQIYSSWKLPPGPFPLPIIGNLLQLDFKNVPKSLTKL

AERYGPVFTLYLGSQRTVVLHGYKAVKEVLLDYKNDFSGRGEIYAFEAHKEKGITFNNGP

SWKDTRRLSLSILRDYGMGKLANEERIQREVPFLMEAFRKTQGQPFDPTFVLGYAPCNVI

SDILFRKRFDYTDQTGLRIQKLFNENFHLLSTSWLQLYNIFPSYMRYLPGNHRKVIKNVY

EIKSYTAARVKEHEESLDPNCPRDFTDSLLLEMRKERYTKEPWFTFDNIAATIADLFFAG

TETTSTTLRYGLLILMKYPEIEEKLHEEIDRVIGPSRIPAIKDRLEMPYMDAVVHEIQRF

IDLLPSNLPHEAIQDTMFRGYVIPKGTVVVPTLDSLLFDNQEFPDPEKFKPEHFLNESGK

FKYSDYFKAFSAGKRVCVGEGLARMELFLFLSAILQHFNLKPLVDPKDIDLSPSTIGFAK

IPPRYKLCVIPRSGV

>397482729

MLASGLLLVALLACLTVMVLMSVWQQRKSRGKLPPGPTPLPFIGNYLQLNTEHICDSIMK

FSEHYGPVFTIHLGPRRVVVLCGHDAVKEALVDQAEEFSKRGIEERIQEEAGFLIEAIRS

THGANIDPTFFLSRTVSNVISSIVFGDRFDYEDKEFLSLLSMMLGIFQFTSTSTGQLYEM

FSSVMKHLPGPQQQAFKLLQGLEDFIAKKVEHNQRTLDPNSPRDFIDSFLIRMQEEEKNP

NTEFYLKNLMMSTLNLFIAGTETVSTTLRYGFLLLMKHPEVEAKVHEEIDRVIGKNRQPK

FEDRTKMPYMEAVIHEIQRFGDVIPMSLARRVKKDTKFRDFFLPKGTEVFPMLGSVLRDP

SFFSNPQDFNPQHFLDDKGQFKKSDAFVPFSIGKRNCFGEGLARMELFLFFTTVMQNFRF

KSSQSPKDIDVSPKHVGFATIPRKYTMSFLPREPFDPTFVLSRSGSNIICSVLFGSRFDY

DDERLLTIIHLINDNFQIMSSPQLYNIFPSLLNWVPGPHQRIFQNFKCLRDLIAHSVHDH

QASLDPRSPRDFIHCFLTKMAEKKEDPLSHFHMDTLLMTTHNLLFGGTETVGTTLRHAFL

ALMKYPKVQAHVQEEIDLVVGRARLPVLKDRAAMPYTDAVIHEVQRFADIIPMNLPHRIT

RDTAFHGFLIPKGTDVITLLNTVHYDPSQFLTPQEFNPEHFLDANQSFKKSPAFMPFLAG

RPSVPGRVAGVPKHIEPDSGRVELAPQPSVEVAWEALP

>AAI09597.1

MAALGITVALLVWMATLLFISIWKHIYSSWKLPPGPFPLPIIGNLLQLDIKNIPKSFTRL

AERYGPVFTLYLGSQRAVVVHGYKPVKEVLLDYKNEFSGRGENPGFQMHKNNGIIFNNGS

TWRDTRRFSLTTLRDLGMGKQGNEQRIQREAHFLLEVLRKTQGQPFDPTFVVGFAPYNVI

SDILFHKRFDYKDQTSLRLMSLFNENFYLLSSPWIQLYNNFSDYLQYLPGSHRKLLKNVS

EVKSYALERVKDHQKSLEPSCPRGFLDTMLIEMAKERHSVDPMYTLENIAVTVADLLFAG

TETTSTTLRYGLLILMKYPEVEEKLHEEIDRVIGPSRIPAVKDRLDMPYLDAVVHEIQRF

IDLLPSNLLHEATQDTVFRGYVIPKGTVVIPTLDSVLHDRQEFPEPEKFKPEHFLNENGK

FKYSDHFKAFSAGKRVCVGEGLARMELFLLLAAILQHFNLKSLVDPKDIDLSPIAIGFGK

IPPRYKLCLIPRSKV

>ABW89488.1

MAALGITVALLVWVATLLPISIWKQIYSSWNLPPGPFPLPIIGNLFHLDLKNIPKSFTRL

AERYGPVFTLYLGSQRVVVMHGYKAVKEVLLNYKNELSGRGEIAVFQAHKDNGVIFNNGP

SWKDTRRLSLTILRDYGMGKQRNEERIQRETHFLLEALRKTQGQPFDPTFVLGGGPFNVI

ADILFHKHFDYEDKTCQRLMHLFNENFYLLSTPWLQAYNYFSTYLRYLPGSHRKVMKNVS

EIKEFTSERVKEHHKSLDPNCPRDFTDNLLMEMEKEKHSAEPLFTLENITVTTADMFFAG

TETTSTTLRYGLLILLKHPEVEEKLHKEIDSVIGPSRIPAFKDRLEMPYMDAVVHEIQRF

INLVPSNLPHVATQDTAFRGYVIPKGTVVIPTLDSLLYDNQEFPDAEKFKPEHFLNEDGK

FKYSDHFKAFSAGKRVCVGEGLARMELFLFLTAILQHFNLKSLVDPKDIDLSPVTIGFGN

IPPNYKLCIIPRS

>297687085

MDLFITVVICLSCLILLSLWNGSYAKGKLPPGPTPLPIVGNILQLNIKNISKSISMLAKD

YGPVFTVYFGMKPTVVLHGYKAIKEALIDQGKEFSGRASFPVLDKIAQGYGVIYSNGETW

KQTRHFCLMVLRNMGMGKKTIEDRIQEEALCLVEALKKTNASPCDPTFLLGCVPCNVISS

IIFQNRFDYSDQKFQALMKCFNEIFENASTSWIQLYNAFPFLRVFPGSHNVIFKNYALQR

SFILEKVKEHQESLDINNPRDFIDYFLIKMEEEKHNKPSEFTMDNLVATIRDMFSAGTET

TSTTMRYGLLLLLKHPEISAKVQEEINHVVSKNRSPCMQDRSRMPYMDAVVHEIQRYIDL

FPTGLSHAATQDIRFREYLIPKGTAILTDLNSVLYNDKEFPNPEKFDPGHFLDESGNFKR

SDYFMAFSAGKRVCVGEGLACMELFLILTTILQNFTLKPLVDPKDIDTTPVHKGFGAVPP

FYELCFIPV

>O18963.1

MAALGITVALLVWMATLLFISIWKHIYSSWKLPPGPFPLPIIGNLLQLDIKNIPKSFTRL

AERYGPVFTLYLGSQRAVVVHGYKPVKEVLLDYKNEFSGRGENPGFQMHKNNGIIFNNGS

TWRDTRRFSLTTLRDLGMGKQGNEQRIQREAHFLLEVLRKTQGQPFDPTFVVGFAPYNVI

SDILFHKRFDYKDQTSLRLMSLFNENFYLLSSPWIQLYNNFPDYLQYLPGSHRKLLKNVS

EVKSYALERVKDHQKSLEPSCPRGFLDTMLIEMAKERHSVDPMYTLENIAVTVADLLFAG

TETTSTTLRYGLLILMKYPEVEEKLHEEIDRVIGPSRIPAVKDRLDMPYLDAVVHEIQRF

IDLLPSNLLHEATQDTVFRGYVIPKGTVVIPTLDSVLHDRQEFPEPEKFKPEHFLNENGK

FKYSDHFKAFSAGKRVCVGEGLARMELFLLLAAILQHFNLKSLVDPKDIDLSPIAIGFGK

IPPRYKLCLIPRSKV

>BAD86844.1

MAALGITVALLVWMATLMLISVWKQIYSSWKLPPGPFPLPIIGNLLQLDFKNVPKSLTKL

AERYGPVFTLYLGSQRTVVLHGYKAVKEVLLDYKNDFSGRGEIYAFEAHKEKGITFNNGP

SWKDTRRLSLSILRDYGMGKLANEERIQREVPFLMEAFRKTQGQPFDPTFVLGYAPCNVI

SDILFRKRFDYTDQTGLRIQKLFNENFHLLSTSWLQLYNIFPSYMRYLPGNHRKVIKNVY

EIKSYTAARVKEHEESLDPNCPRDFTDSLLLEMRKERYTKEPWFTFDNIAATIADLFFAG

TETTSTTLRYGLLILMKYPEIEEKLHEEIDRVIGPSRIPAIKDRLEMPYMDAVVHEIQRF

IDLLPSNLPHEAIQDTMFREYVIPKGTVVVPTLDSLLFDNQEFPDPEKFKPEHFLNESGK

FKYSDYFKAFSAGKRVCVGEGLARMELFLFLSAILQHFNLKPLVDPKDIDLSPSTIGFAK

IPPRYKLCVIPRSGV

>P33265.1

MDPVLVLVLTLSCLLLFSVWRQSSGRGRLPPGPTPLPLIGNILQIDIKDISKSLANFSKV

YGPVFTLYFGTKPTVVVHGYEAVKEALEDLGEEFSGRGNFPIVERMNSGLGIIFSNGTKW

KELRRFSLMTLRNFGMGKRSIEDCIQEEARCLVEELRKTNGSPCDPTFFLSCAPSNVICS

VVFHNRFDYNDKNFLNLMEKLNENFEILNSPWLQVCNVIPAFLDYLPGSHNKALKNFAEI

KSYILKRVKEHQETLDMNNPRDFIDCFLIKMEKEKDNPHSEFTTESLMATVADVFVAGSE

TTSTTLRYGLLLLLKHKEVTAKVQKEIDHVIGRDRSPCMQDRTRMPYTDAMVHEVQRYVN

LIPNNVPHAATCNVKFRNYVIPKGTDLITSLTSVLHDDKEFPNPKIFDPAHFLDENGNFK

KSDYFMPFSIGKRMCMGEALARMELFLLLTTILQNFDLKSLADTKDIDTTPVASTFGCVP

PSYQLYFIPR

>BAE24131.1

MEMETLRKVTTSCLSQPFSKAYGPVFTLYLGSKPTVILHGYEAVKEALIDRGEEFAGRGS

FPMAEKIIKGFGVVFSNGNRWKEMRRFTLMTLRNLGMGKRNIEDRVQEEAQCLVEELRKT

KGSPCDPTFILSCAPCNVICSIIFQNRFDYKDKEFLILMDKINENVKILSSPWLQVCNSF

PSLIDYCPGSHHKIVKNFNYLKSYLLEKIKEHKESLDVTNPRDFIDYYLIKQKQVNHIEQ

SEFSLENLASTINDLFGAGTETTSTTLRYALLLLLKYPDVTAKVQEEIDRVVGRHRSPCM

QDRSHMPYTDAMIHEVQRFIDLLPTSLPHAVTCDIKFRKYLIPKGTTVITSLSSVLHDSK

EFPNPEMFDPGHFLNGNGNFKKSDYFMPFSTGKRICAGEGLARMELFLILTTILQNFKLK

SLVHPKEIDITPVMNGFASLPPPYQLCFIPL

>397510094

MNPIVVLHGYEAVKEALIDNGEEFSGRGSSPISQRITKGLGIISSNGKRWKEIRRFSLTT

LRNFGMGKRSIEDRVQEEAHCLVEELRKTKASPCDPTFILGCAPCNVICSVVFQKRFDYK

DQNFLTLMKRFNENFRILNSPWIQVCNNFPLLIDCFPGTHNKVLKNVALTQSYIREKVKE

HQASLDVNNPRDFIDCFLIKMEQEKDNQKSEFNIENLVGTVADLFVAGTETTSTTLRYGL

LLLLKHPEVTAKVQEEIDHVIGRHRTPCMQDRSHMPYTDAVVHEIQRYSDLVPTGVPHAV

TTDTKFRNYLIPKGTTIMTLLTSVLHDDKEFPNPNIFDPGHFLDKNGNFKKSDYFMPFSA

GKRICAGEGLARMELFLFLTTILQNFNLKSVDDLKNLNTTAVTKGIVSLPPSYQICFIPV

>126273045

MDFWGLITLALVCFLFFQLIWNKGYKARRFPPGPIPLPILGNMLQMDSKDLFTFFDKMAE

KYGPVFTVYMGLQRIVVLHGYEAVKEALIDQGDNFLNRAQLPIFEDNFKGQGILSSSGEK

WRQNRRFSLMTLRNFGMGKRSIEERVQEEAQCLVEELRKTKGQFFDPTFILGCAPCNVIC

SIVFNNRFNYDDKKFLRLMSLLNENFRLLNTPWIQLYNMYPSLIKRLPGNHRTINKNVLE

VRNFIMDEVKKHQETLDPNNPQDYIDCFLIKMQQEKLNPQSAFNYQELMATGSNLFSAGT

ETTSSTLRYGLLLLMKHPKIQDKVHEEIDRVLGSSRKPSMQDRVKMPYVDAVVHEIQRYI

HLLPFSLPRLAAQDIHFQKYVIPKGTSVFPLLYSVLYDRKAFPNPYEFDPENFLDKSGNF

QKNDHFVPFSLGKRLCLGESLARMEVFLFLTTILQNFTLKPMVEPKELVTTPLRNGIVNI

PSIYKLSLIPR

>254281186

MPKRKILICECCIKCLECLIIPVSVRRVSMDPFVVLVLCLSFLLVLSLWRQRSARGNLPP

GPTPLPIIGNYHLIDMKDIGQCLTNFSKTYGPVFTLYFGSQPIVVLHGYEAIKEALIDHG

EEFSGRGRIPVFDKVSTGKGIGFSHGNVWKATRVFTVNTLRNLGMGKRTIENKVQEEAQW

LMKELKKTNGSPCDPQFIIGCAPCNVICSIVFQNRFDYKDKDFLSLIGKVNECTEILSSP

GCQIFNAVPILIDYCPGSHNKLFKNHTWIKSYLLGKIKEHEESLDVTNPRDFIDYFLIQR

RQKNGIEHMDYTIEHLATLVTDLVFGGTETLSSTMRFALLLLMKHTHITAKVQEEIDNVI

GRHRSPCMQDRNHMPYTNAMVHEVQRYIDLGPNGVVHEVTCDTKFRNYFIPKGTQVMTSL

TSVLHDSTEFPNPEVFDPGHFLDDNGNFKKSDYFVPFSAGKRICVGESLARMELFLFLTT

ILQNFKLKPLVDPKDIDMTPKHSGFSKIPPNFQMCFIPVE

>P56657.2

MDPFVVLVLCLSFLLVLSLWRQRSARGNLPPGPTPLPIIGNYHLIDMKDIGQCLTNFSKT

YGPVFTLYFGSQPIVVLHGYEAIKEALIDHGEEFSGRGRIPVFDKVSTGKGIGFSHGNVW

KATRVFTVNTLRNLGMGKRTIENKVQEEAQWLMKELKKTNGSPCDPQFIIGCAPCNVICS

IVFQNRFDYKDKDFLSLIGKVNECTEILSSPGCQIFNAVPILIDYCPGSHNKLFKNHTWI

KSYLLGKIKEHEESLDVTNPRDFIDYFLIQRRQKNGIEHMDYTIEHLATLVTDLVFGGTE

TLSSTMRFALLLLMKHTHITAKVQEEIDNVIGRHRSPCMQDRNHMPYTNAMVHEVQRYID

LGPNGVVHEVTCDTKFRNYFIPKGTQVMTSLTSVLHDSTEFPNPEVFDPGHFLDDNGNFK

KSDYFVPFSAGKRICVGESLARMELFLFLTTILQNFKLKPLVDPKDIDMTPKHSGFSKIP

PNFQMCFIPVE

>P05178.2

MDLVMLLVLTLTCLILLSIWRQSSGRGKLPPGPIPLPIIGNIFQLNVKNITQSLTSFSKV

YGPVFTLYFGTKPTVILHGYEAVKEALIDHGEEFAERGSFPVAEKINKDLGIVFSHGNRW

KEIRRFTLTTLRNLGMGKRNIEDRVQEEARCLVEELRKTNGSPCDPTFILGCAPCNVICS

IIFQNRFDYKDQDFLNLMEKLNENMKILSSPWTQFCSFFPVLIDYCPGSHTTLAKNVYHI

RNYLLKKIKEHQESLDVTNPRDFIDYYLIKWKQENHNPHSEFTLENLSITVTDLFGAGTE

TTSTTLRYALLLLLKCPEVTAKVQEEIDRVVGKHRSPCMQDRSRMPYTDAHDHEVQRFID

LIPTNLPHAVTCDIKFRNYLIPKGTTIITSLSSVLHDSKEFPDPEIFDPGHFLDGNGKFK

KSDYFMPFSAGKRMCAGEGLARMELFLFLTTILQNFKLKSVLHPKDIDTTPVFNGFASLP

PFYELCFIPL

>AAH89886.1

MDLVMLLVLTLTCLILLSIWRQSSGRGKLPPGPIPLPIIGNIFQLNVKNITQSLTSFSKV

YGPVFTLYFGMKPTVILHGYEAVKEALIDHGEEFAERGSFPVVEKINKDLGIVFSHGNRW

KEIRRFTLTTLRNLGMGKRNIEDHVQEEARCLVEELRKTNGSPCDPTFILGCAPCNVICS

IIFQNRFDYKDQDFLNLMEKLNENMKILSSPWTQFCSFFPVLIDYCPGSHTTLAKNMYYN

RNYLLKKIKEHQESLDVTNPRDFIDYYLIKWKQENHNPNSEFTLENLSITVTDLFAAGTE

TTSTTLRYALLLLLKCPEVTAKVQEEIDRVVGKHRSPCMQDRSRMPYTDAMIHEVQRFID

LIPTNLPHAVTCDIKFRNYLIPKGTTIITSLSSVLHDSKEFPDPEIFDPGHFLDGNGKFK

KSDYFMPFSAGKRMCAGEGLARMELFLFLTTILQNFKLKSVLHPKDIDTTPVFNGFASLP

PFYELCFIPL

>AAI58225.1

MDPVSVLLSVVICIFLYKVFYGGKETSKNFPPGPKPLPLIGNLHIMNMKKPHLTFMELAE

KYGSVFSFEFGLRKTVVLCGTDTVRDALINHAEEFSERARIPVFEDITKGHGIVFAHGEN

WKVMRRFTLSTLRDFGMGKKTIEDKICEESDCLVEIFKSYNGKPFDNTLIMNSAVANIIV

TILLGDRFDYKDPTMLKLVKVVNQNIRITGGLMARLYNIYPSIMRWIPGSHQTVSKNMAK

VFKFLNETFTEHRKQLDVNDQRDLIDAFLVKQREEKLSAKTFFHNDNLTVLVTNLFGAGM

ETTSTTLRWGLLLMMKYPVIQKKVQKEIDQVIGSAQPRLEHRKQMPYTDAVIHEIQRFAN

IAPINIPHETTQDVTFRGYFIPKGTQVIPVLTSVLQDKAYFKKPEEFYPEHFLDSEGKFV

KNEAFLPFSAGKRSCAGETLAKMELFLFFTKLLQNFTFQPPPGVEVQLTCGVALTSIPAD

HKICALPLS

>EGW13565.1

MAFLGVTIALLVWVATLLIISIWKQIYSSWNLPPGPFPLPILGNVFQLDLKNIPNSFTKL

AKRFGPVFTLHLGSRRIVVLHGYKAVKEVLLNHKNEFSGRGDIPVFHEYKNKGIIFNNGP

TWKDVRRFSLSILRDYGMGKQGNEARIQREAHFLVEELKKTNGQPFDPTFLIGCAPFNVI

SDILFHKRFDYNDKKCLRLMSLFNENFYLLSTPWIQAYNNFSDYLRYLPGSHRKIMKNVS

EIKQYTLEKAKEQLQSLDSNCPRDVTDCLLIEKEKEKHSKEPMYTMENISVTLADLFFAG

TETTSTTLRYGLLILLKYPEVEEKLHEEIDRVIGPSRVPAFKDRLEMPYMDAVVHEIQRF

INLIPSNLPHEATRDTMFRGYVIPKGTIVIPTLDSLLFDSQEFPDPEKFKPEHFLNENGK

FKYSDHFKAFSAGKRVCVGEGLARMELFLLLSAILQHFNLKSLVDPKDIDLNPVTVGFGC

VPPEFKLCVIPRS

>354498963

MDPAVVLVFILSCLVLLSLWRQRSERGKLPPGPTPLPLIGNFLQIDVKDISRSLTNLSKV

YGPVFTLYLGMKPTVVLHGYEAVKEALISSQNLFQCFTISSGIIFSNGSRWKETRRFTLM

TLRNLGMGKRSIEDCVQEEAQSLVEELRKTKGSPCDPTFILGCAPCNVICSIIFQNRFDY

KDHHFLTLMEKVNENSRILSSPWFQVCNNFSSLIDYCPGSHHTITKNFNYIRSYLLEKIK

EHQESLDDANPRDFIDCYLIKMKQANHNQQSEFSLENLAITLSDLFAAGTETTSTTLRYA

LLLLLKHPHVTAKVQEEIDQVVGRDRSPSMQDRSRMPYTDATVHEVQRFIDLIPTNLPHA

VTCDIKFRDFFIPKGTTIITSLSSVLHDSKEFPNPEVFDPGHFLDKNGNFKKSDYFMPFS

TGKRICAGEGLARMELFLFLTTILQNFKLKSLVHPKDIDTNPVVNGFASLPPPFQLCFIP

V

>348544071

MEFSGTLILIGAVLTLLWLFSFKSRRRLALPPGPSALPIIGNVLQMDKRAPFKTMLKLSD

KYGSVMTMYLGPQRTVILLGYDAVKEALVDQADDFTGRGPIPLLFKASKGYGLGVSNGEC

WQQLRRFTLTTLRDFGMGRKGMEEWIQEESSHLVGCITKMNAKPFDPTFFLSCTVSNVIC

CLVFGQRFSYDDKHFLYLLQIISDTLKFGSSSQGQLYNIFPRLMEWLPGSHHNVFAKLEE

MRAFIMNKIQEHQDRLDASSPRDYIDCFLLRLNQEKHIPISQFHYDNLVSTVLNLYLAGT

ETTSSTIRYALNVLIKYPKIQETIQQEIDTVVGQRCPCMEDKKSLPYTDAVLHEIQRFLD

IIPLSIPHYALHDISFRGYTIPKDTLIIPLLHSVLKDEKRWTTPWSFNPQHFLDNNGNFK

KNPAFLPFSAGKRSCVGESLARMEIFLFLVSLLQHFTFSCPGGPDSIDLSPEYSGFANVP

RRYTIIATPR

>354498967

MDPAVVLVFILSCLVLLSLWRQRSERGKLPPGPTPLPLIGNFLQIDVKDISRSLTNLSKV

YGPVFTLYLGMKPTVVLHGYEAVKEALXXXXXXXSLVSSQNLFQCFTISSGIIFSNGSRW

KETRRFTLMTLRNLGMGKRSIEDCVQEEAQSLVEELRKTKGSPCDPTFILGCAPCNVICS

IIFQNRFDYKDHHFLTLMEKVNENSRILSSPWFQVCNNFSSLIDYCPGSHHTITKNFNYI

RSYLLEKIKEHQESLDDANPRDFIDCYLIKMKQANHNQQSEFSLENLAITLSDLFAAGTE

TTSTTLRYALLLLLKHPHVTAKVQEEIDQVVGRDRSPSMQDRSRMPYTDATVHEVQRFID

LIPTNLPHAVTCDIKFRDFFIPKGTTIITSLSSVLHDSKEFPNPEVFDPGHFLDKNGNFK

KSDYFMPFSTGKRICAGEGLARMELFLFLTTILQNFKLKSLVHPKDIDTNPVVNGFASLP

PPFQLCFIPV

>BAH11491.1

MNPIVVFHGYEAVKEALIDNGEEFSGRGNSPISQRITKGLGIISSNGKRWKEIRRFSLTT

LRNFGMGKRSIEDRVQEEAHCLVEELRKTKASPCDPTFILGCAPCNVICSVVFQKRFDYK

DQNFLTLMKRFNENFRILNSPWIQVCNNFPLLIDCFPGTHNKVLKNVALTRSYIREKVKE

HQASLDVNNPRDFIDCFLIKMEQEKDNQKSEFNIENLVGTVADLFVAGTETTSTTLRYGL

LLLLKHPEVTAKVQEEIDHVIGRHRSPCMQDRSHMPYTDAVVHEIQRYSDLVPTGVPHAV

TTDTKFRNYLIPKGTTIMALLTSVLHDDKEFPNPNIFDPGHFLDKNGNFKKSDYFMPFSA

GKRICAGEGLARMELFLFLTTILQNFNLKSVDDLKNLNTTAVTKGIVSLPPSYQICFIPV

>P15149.1

MLDTGLLLVVILASLSVMFLVSLWQQKIRERLPPGPTPLPFIGNYLQLNMKDVYSSITQL

SERYGPVFTIHLGPRRIVVLYGYDAVKEALVDQAEEFSGRGELPTFNILFKGYGFSLSNV

EQAKRIRRFTIATLRDFGVGKRDVQECILEEAGYLIKTLQGTCGAPIDPSIYLSKTVSNV

INSIVFGNRFDYEDKEFLSLLEMIDEMNIFAASATGQLYDMFHSVMKYLPGPQQQIIKVT

QKLEDFMIEKVRQNHSTLDPNSPRNFIDSFLIRMQEEKYVNSEFHMNNLVMSSLGLLFAG

TGSVSSTLYHGFLLLMKHPDVEAKVHEEIERVIGRNRQPQYEDHMKMPYTQAVINEIQRF

SNLAPLGIPRRIIKNTTFRGFFLPKGTDVFPIIGSLMTEPKFFPNHKDFNPQHFLDDKGQ

LKKNAAFLPFSIGKRFCLGDSLAKMELFLLLTTILQNFRFKFPMNLEDINEYPSPIGFTR

IIPNYTMSFMPI

>354491957

MDPFTVLVLSLSFLLLLSLWRQKTERGKLPPGPTPLPLIGNFFQIDVKNIQKSFTKLSKV

YGPVFTLYFGQQPTVVLHGYNAVKEALIDHGEEFSGRGRFPVLDKIFNGLGVIFSNGNTW

KEMKRFSLTTLRNLGMGKRSIEDRVQEEAQCLVEELRKTNGLPCDPSFILTCAPCNVICS

IVFQNRFDYKDKHVLNLLKYLEENAKILSAPWIQVCNAFPAILDYCPGSHNIFHKNYTYL

KSYFLEKIKEHAESLDVTNPRDFIDYYLIKGRQVNHLKILKYTLENLTVMVNDLFGAGTE

TTGSTMKYALLLLMKYPHVTAKVQEEIDRVVGRERSPCMQDRNHMPYTNATIHEVQRFID

LVPNSVPHAVTCDVKFRNYLIPKGTTVIASLSSVLHDSKEFPNPETFDPGHFLDGNGNFK

KSDYFMPFSTGKRVCAGEGLAKMELFLFLTTILQKFKLKSLVPPKDVNITSVVNGISLVP

PHYQICFIPV

>BAD86843.1

MAALGITVALLVWMATLMLISVWKQIYSSWKLPPGPFPLPIIGNLLQLDFKNVPKSLTKL

AERYGPVFTLYLGSQRTVVLHGYKAVKEVLLDYKNEFSGRGEIYAFEAHKDKGITFNNGP

SWKDTRRLSLSILRDYGMGKRANEEQIQREVPFLLEAFRGTQGQPFDPTFVLGYAPCNVI

SDILFRKRFDYRDQTGLRIQKLFNENFHLLSTNWLQLYNIFPSYMRYLPGNHRKVIKNVY

EIKSYTAARVKEHEESLDPNCPRDFTDSLLLEMRKERYTKEPWFTFDNIAATIADLFFAG

TETTSTTLRYGLLILMKYPEIEEKLHEEIDRVIGPSRIPAIKDRLEMPYMDAVVHEIQRF

IDLLPSNLPHEAIQDTMFRGYVIPKGTVVVPTLDSLLFDNQEFPDPEKFMPEHFLNESGK

FKYSDYFKVFSAGKRVCVGEGLARMELFLFLSAILQHFNLKPLVDPKDIDLSPSTIGFAK

IPPRYKLCVIPRSGV

>AAA52156.1

MDSISTAILLLLLALVCLLLTLSSRDKGKLPPGPRPLSILGNLLLLCSQDMLTSLTKLSK

EYGSMYTVHLGPRRVVVLSGYQAVKEALVDQGEEFSGRGDYPAFFNFTKGNGIAFSSGDR

WKVLRQFSIQILRNFGMGKRSIEERILEEGSFLLADVRKTEGEPFDPTFVLSRSVSNIIC

SVLFGSRFDYDDERLLTIIRLINDNFQIMSSPWGELYDILDPRFPSLLDWVPGPHQRIFQ

NFKCLRDLIAHSVHDHQASSPRDFIQCFLTKMAEEKEDPLSHFHMDTLLMTTHNLLFGGT

KTVSTTLHHAFLALMKYPKVQARVQEEIDLVVGRARLPALKDRAAMPYTDAVIHEVQRFA

DIIPMNLPHRVTRDTAFRGFLIPKGTDVITLLNTVHYDPSQFLTPQEFNPEHFLDANQSF

KKSPAFMPFSAGRRLCLGELLARMELFLYLTAILQSFSLQPLGAPEDIDLTPLSSGLGNL

PRPFQLCLRPR

>AAD13722.1

MDPFVVLVLCLSFLLVLSLWRQRSARGNLPPGPTPLPIIGNYHLIDMKDIGQCLTNFSKT

YGPVFTLYFGSQPIVVLHGYEAIKEALIDHGEEFSGRGRIPVFDKVSTGKGIGFSHGNVW

KATRVFTVNTLRNLGMGKRTIENKVQEEAQWLMKELKKTNGSPCDPQFIIGCAPCNVICS

IVFQNRFDYKDKDFLSLIGKVNECTEILSSPGCQIFNAVPILIDYCPGSHNKLFKNHTWI

KSYLLGKIKEHEESLDVTNPRDFIDYFLIQRRQKNGIEHMDYTIEHLATLVTDLVFGGTE

TLSSTMRFALLLLMKHTHITAKVQEEIDNVIGRHRSPCMQDRNHMPYTNAMVHEVQRYID

LGPNGVVHEVTCDTKFRNYFILKGTQVMTSLTSVLHDSTEFPNPEVFDPGHFLDDNGNFK

KSDYFVPFSAGKRICVGESLARMELFLFLTTILQNFKLKPLVDPKDIDMTPKHSGFSKIP

PNFQMCFIPVE

>395863029

MAALGITMALLVWMAVLLLVSIWKQVYSSWNLPPGPFPLPFFGNLFQLEFKNIPKSLDKL

AERFGPVFTLYLGPRRTVVLHGYKAVKEALLDHKNELSGRGDIPAFREYRDKGIIFNNGP

TWKDVRRFSLTTLRDYGMGKKANEERIQKEMHFLLAELRKTQGQPFDPTFLIGCAPFNVI

ADILFHKHFDYNDKICQRLMHLFNENFYLLSTTWLEFYNNFSSYLHYLPGNHRKVIKNVS

EIKEYVTVKVKEHQQSLDPNCPRDFTDCLLVEMEKEKHSVEPLYTLDNIAVTVADILFAG

TETTSTTLRYGLLILMKYPEIEDKIHEEIDRVIGPRRTPATKDRLEMPYTDAVVHEIQRF

INLVPNNLPHEATRDTTFRGYVIPKGTIIIPTLSPILYDSQEFPDPEKFKPEHFLDEQGR

FKYSDYFKPFSTGKRVCVGEGLARMELFLLLTAILQNFNLRPLVDPKDIDLSPKTIGFGR

IPPHYKLCVIPRA

>354498965

MDPAVVLVFILSCLVLLSLWRQRSERGKLPPGPTPLPLIGNFLQIDVKDISRSLTNLSKV

YGPVFTLYLGMKPTVVLHGYEAVKEALTPIRSLVSSQNLFQCFTISSGIIFSNGSRWKET

RRFTLMTLRNLGMGKRSIEDCVQEEAQSLVEELRKTKGSPCDPTFILGCAPCNVICSIIF

QNRFDYKDHHFLTLMEKVNENSRILSSPWFQVCNNFSSLIDYCPGSHHTITKNFNYIRSY

LLEKIKEHQESLDDANPRDFIDCYLIKMKQANHNQQSEFSLENLAITLSDLFAAGTETTS

TTLRYALLLLLKHPHVTAKVQEEIDQVVGRDRSPSMQDRSRMPYTDATVHEVQRFIDLIP

TNLPHAVTCDIKFRDFFIPKGTTIITSLSSVLHDSKEFPNPEVFDPGHFLDKNGNFKKSD

YFMPFSTGKRICAGEGLARMELFLFLTTILQNFKLKSLVHPKDIDTNPVVNGFASLPPPF

QLCFIPV

>EHB02894.1

MAVLGITLALLVWVVTLLLISIWKQIYSSWNLPPGPFPLPIIGNLLQVDIKDIPKSFTKL

AKRYGPVFTLHLGSRRIVVLHGYKAVKENEFSGRGEIPVFQEYKDKGIIFNNGPTWKDVR

RFSLSILRDYGMGKQGNEARIQKEAGFLLQELRKTQGQPFNPTFLIGCAPCNVIADILFH

SRFDYKDKTCLRLMRLFNEDFYLLSTPWLQVKPFPSFPLIFNYYLCYMPGSHRKVMKNLS

EIKAYALERAKEHLQSLDPNCPRDVTDCLLAEMEKENQSGELSMYTMENISVTLADLFFA

GTETTSTTLRYGLLILMKYPEVEEKLHEEIDRVIGPTRIPAVKDRLEMPYMDAVVHEIQR

FINLVPSNLPHEATRDTMFQGYLIPKGTVVIPTLDSVLYDDQEFPDPEKFKPEHFLNESG

KFKYSDYFKAFSAGKRVCVGEGLARMEVFLLLTAILQHFNLKSLVEPKDIDLKPITVGFG

NIPPHYKLCLIPRS

>402881019

MNPVVVLHGYETVKEALIDNAEEFSGRGILPISERITNGLGIISSNGKRWKETRRFSLTT

LRNFGMGKRSIEDRVQEEARCLVEELRKTKASPCDPTFILGCAPCNVICSVVFQKRFDYK

DENFLTLMKRFTVNFRILTSPWIQVCNNFPLLIDCFPGTHNKLLKNVALTKSYIREKVKE

HQATLDVNNPRDFIDCFLIKMEQEKDNQQSEFTIENLVGTVADLFVAGTETTSTTLRYGL

LLLLKHPEVTAKVQEEIDHVIGRHRSPCMQDRSHMPYTDAVIHEIQRYIDLVPTGVPHAV

TTDIKFRNYLIPKGTIIITLLTSVLHDDKEFPNPKIFDPGHFLDENGNFKKSDYFMPFSA

GKRICAGEGLARMELFLFLTTILQNFNLKSVADLKNLNTTSATRGIISLPPSYQICFIPV

>114631928

MNPIVVLHGYEAVKEALIDNGEEFSGRGSSPISQRITKGLGIISSNGKRWKEIRRFSLTT

LRNFGMGKRSIEDRVQEEAHCLVEELRKTKASPCDPTFILGCAPCNVICSVVFQKRFDYK

DQNFLTLMKRFNENFRILNSPWIQVCNNFPLLIDCFPGTHNKVLTNVALTQSYIREKVKE

HQASLDVNNPRDFIDCFLIKMEQEKDNQKSEFNIENLVGTVADLFVAGTETTSTTLRYGL

LLLLMHPEVTAKVQEEIDHVIGRHRTPCMQDRSHMPYTDAVVHEIQRYSDLVPTGVPHAV

TTDTKFRNYLIPKGTTIMTLLTSVLHDDKEFPNPNIFDPGHFLDKNGNFKKSDYFMPFSA

GKRICAGEGLARMELFLFLTTILQNFNLKSVDDLKNLNTTAVTKGIVSLPPSYQICFIPV

>348543373

MEFSTTLILAGLILALLWIFNVKNKTNRRLPPGPTALPLIGNLLQIDKKAPFKSFVKFSE

TYGPVMTVYLGWQRTVVLVGYDAVKEALVDQADDFTGRAPTPFLHNPNKKYGIGSSNGER

WRQLRRFTLTTLRDFGMGRKGMEEWIQEESEHMRAHMLKLKGSPFDPTFLLSCTVSNVIC

CLVFSQRFSYEDKQFLRLLTILSHIGKLNSSPVGQMYNLFSKLMDHLPGPHHKVFAEYEE

VRKFIKMKIQEHKDTLDPSSPRDYIDSFLIRINQEKEISTTEFHYDNLVSTIMTLFLAGT

QTTSSTLRFALSVLIKYPKIQETMQQEIDSVTGKGRCPSMEDRKSLPFSLAVIHEMQRFL

DLVPLSLPHYTLNDISFRGYTIPKDTVIIPLLHSVLKEEKQWATPRSFNPQHFLDNNGSF

KKSPAFMPFSAGKRNCVGESLARMELFIFIVSLLQDFTFSCAGGPDSINLIPEYSSFLNV

PCRYHIMATPR

>AAH92260.1

MDPFVVLVLCLSFLLVLSLWRQRSARGNLPPGPTPLPIIGNYHLIDMKDIGQCLTNFSKT

YGPVFTLYFGSQPIVVLHGYEAMKEAFIDHGEEFSGRGRFPFFDKVTKGKGIGFSHGNVW

KATRVFTINTLRNLGMGKRTIENKVQEEAQWLMKELKKTNGLPCDPQFIIGCAPCNVICS

IVFQNRFDYKDKDFLSLIGKVNECTEILSSPGCQIFNAVPILIDYCPGRHNKFFKNHTWI

KSYLLEKIKEHEESLDVTNPRDFIDYFLIQRCQEKGIEHMEYTIEHLATLVTDLVFGGTE

SLSSTMRFALLLLMKHTHITAKVQEEIDNVIGRHRSPCMQDRNHMPYTNAMVHEVQRYVD

LGPISLVHEVTCDTKFRNYFIPKGTQVMTSLTSVLHDSTEFPNPEVFDPGHFLDDNGNFK

KSDYFVPFSAGKRICVGESLARMELFLFLTTILQNFKLKPLVDPKDIDMTPKHSGFSKIP

PNFQMCFIPVE

>AAA41031.1

MDPVVVLLLSLFFLLFLSLWRLSSGRGKLPPGPTPLPIIGNFFQVDMKDIRQSLTNFSKT

YGPVYTLYVGSQPTVVLHGYEALKEALVDHGEEFSGRGRLPICEKVAKGQGIAFSHGNVW

KATRHFTVKTLRNLGMGKGTIEDKVQEEAKWLVKELKKTNGSPCDPQFIMGCAPGNVICC

IILQNRFDYEDKDFLNLIEKVNEAVKIISSPGIQVFNIFPILLDYCPGNHNIYLKNYTWV

KSYLLEKIKEHEESLDVSNPRDFIDYFLIERNQENANQWMNYTLEHLAIMVTDLFFAGIE

TVSSTMRFALLLLMKYPHVTAKVQEEIDHVIGRHRSPSMQDRSHMPYTNAMVHEVQRYID

IGPNGLLHDVTCDTKFRNYFIPKGTAVLTSLTSVLHDSKEFPNPEMFDPGHFLDENGNFK

KSDYFIPFSAGKRMCLGESLARMELFLFLTTILQNFKLKSLVDPKDINTTPICSSLSSVP

PTFQMRFIPL

>Q9MZY0.1

MAALGITVALLVWMATLMLISIWKQIYSRWKLPPGPFPLPIIGNILQVDIKNVPKSLAKL

AEQYGPVFTLYLGSQRTVVLHGYKAVKEVLLDHKNDLSGRGEVFAFQSHKDRGITFNNGP

GWKDTRRLSLSTLRDYGMGKRGNEERIQREIPFLLEALRGTRGQPFDPTFLLGFAPFNVI

ADILFHKHFDYSDQTGLRIQKLFNENFHLLSTGWLQLYNIFPSYLHYLPGSHRKVLRNVA

ELKDYSLERVKEHQESLDPTCSRDFTDCLLQELQKERYGTEPWYTLDNIAVTVADLFFAG

TETTSTTLRYGLLILMKYPEVEEKLHEEIDRVIGPSRVPAIKDRLEMPYMDAVVHEIQRF

IDLLPSNLPHVANQDTMFRGYVIPKGTVVIPTLDSVLFDKQEFPDPEKFKPEHFLNENGK

FKYSDYFKAFSAGKRVCVGEGLARMELFLFLSAILQHFNLKSLVDPKDIDLSPCTIGFAK

IPPHYKLCVVPRSG

>AAA36660.1

MTKNSPTQRCLTLATFWIRVATLRKVTTSCLSQQLSKIYGPVFTLYFGLERMVVLHGYEV

VKEALIDLGEEFSGRGHFPLAERANRGFGIVFSNGKRWKEIRRFSLMTLRNFGMGKRSIE

DRVQEEARCLVEELRKTKASPCDPTFILGCAPCNVICSIIFQKRFDYKDQQFLNLMEKLN

ENIRIVSTPWIQICNNFPTIIDYFPGTHNKLLKNLAFMESDILEKVKEHQESMDINNPRD

FIDCFLIKMEKEKQNQQSEFTIENLVITAADLLGAGTETTSTTLRYALLLLLKHPEVTAK

VQEEIERVVGRNRSPCMQDRGHMPYTDAVVHEVQRYIDLIPTSLPHAVTCDVKFRNYLIP

KGTTILTSLTSVLHDNKEFPNPEMFDPRHFLDEGGNFKKSNYFMPFSAGKRICVGEGLAR

MELFLFLTFILQNFNLKSLIDPKDLDTTPVVNGFASVPPFYQLCFIPV

>Q29510.1

MDLFIVLVICLSCLLLVSLLKQSYARRKLPPGPTAFPLVGNVLQLDRKNISKSISMLAKE

YGPVFTVYFGTKPSVVLHGYEAVKEALVDHGEEFSGRVTVPVFDRYAQGAGLFASQGELW

KETRRFTLTVLRNMGMGKKTIEDRIQEEASCLVKALKETNASPCDPSSLLFCVPCNVICS

IIFQNRFEYNDQTFQTLVKHFLDSIRLFNTPWMQLYNTFPILHYLPGSHHELFKVFNDQM

KFFSEKIREHQESLDHNNPRDFIDYLLIKMEKEKHNKQSQFTMHHLLVTIWDVFDAGSQT

TSSTMQFGLLLLMKHPEIAAKVQEEIERVIGRDRSPCMQDRSCMPYTDAVIHEIQRYIDM

VPNNVPHAVTQDIQFRGYLIPKGTHILPSLTSVLYDEKEFPNPEKFDPGHFLDASGNFKK

SDYFLPFSIGKRACAGEGLARMELFLVLTTILQHFTLKPLVDPKDIDPTPVDNGFLSVPP

SYELCFVPV

>332212380

MNPIVVLHGYEAVKEALIDNGEEFSGRGNSPISQRITKGLGIISSNGKRWKEIRRFSLTT

LRNFGMGKRSIEDRVQEEAHCLVEELRKTKASPCDPTFILGCAPCNVICSVVFQKRFDYK

DQNFLTLMKRFNENFRILNSPWIQVCNNFPLLIDCFPGTHNELLKNVGLTKSYIMEKVKE

HQASLDVNNPRDFIDCFLIKMEQEKDNQQSEFNIENLVGTVADLFVAGTETTSTTLRYGL

LLLLKHPEVTAKVQEEIDHVIGRHRRPCMQDRSHMPYTDAVVHEIQRYIDLVPTGVPHAV

TTDIKFRNYLIPKGTTIMTLLTSVLHDDKEFPNPKIFDPGHFLDKNGNFKKSDYFMPFSA

GKRICAGEGLARMELFLFLTTILQNFNLKSVDDLKNLNTTAVTKGIVSLPPSYQICFIPV

>ACY69401.1

MAVLGITIALLVWVVTLLIISVWKQIYSSWNLPPGPFPLPFFGNVSQLDLKDIPKSFTKL

AKRFGPVFTLHLGSRRIVVLHGYKAVKEVLLNHKNEFSGRGDIPVFQEYKNKGIIFNNGP

TWKDVRRFSLSILRDYGMGKEGNEARIQRESRFLVEELKKTKGQPFDPTFLVGCAPFNVI

ADILFNKRFDYNDKKALRLMSLFNENFYLLSTPWIQLYNNFADYLRYLPGTHRKIMKNAS

EIKQYALEKAKEHLQSLDSHCPRDVTDCLLREMEKEKHSKEPMYTMENISVTLADMFFAG

TETTSTTLRYGLLILMKYPEIEEKLHEEIDRVIGPSRVPAVKDRLEMPYMDAVVHEIQRF

INLVPSNLPHEATQDTKFRGYVIPKGTIVIPTLDSLLYDSQEFPDPEKFNPEHFLNENGK

FKYSDYFKPFSAGKRVCVGEGLARMELFLLLSAILQHFNLKSLVDPKDIDLNPVTVGFGS

VPPEYKLCVIPRS

>403305348

MLASGLLLVALLTCLTVMLLMSVWRQRKSKGKLPPGPTPLPFIGNYLQLNTEQMYNSLMK

VSQGVAFSNGERAKQLRRFSITTLRDFGVGKRGIEERIQEEAGFLIEVLRGTHGASIDPT

FFLSRTVSNVISSIVFGDRFDYEDKEFLSLLRMMLGSFQFTATSMGQLYEMFSSVMKHLP

GPQQQAFKELQGLEDFIAKKVEHNQRTLDPNSPRDFIDSFLIRMREEEKNPNTEFYLKNL

VLTTLNLFFAGTETVSTTLRYGFLLLMKHPEVEAKVREEIDRVIGKNRQPKFEDRAKMPY

TEAVIHEMQRFGDMLPMGVARRVTKDTEFRDFMLPKGTEVFPLLGSVLRDSRFFSNPRDF

NPQHFLDEKGQFKKSDAFVPFSIGKRYCFGEGLARMELFLFLTTIMQNFRFKSPQSPKDI

DVSPKHVGFATIPPNYTMSFLPR

>397482611

MEATGTWALLLALALLLLLTLALSGTRARGHLPPGPTPLPLLGNLLQLRPGALYSGLMRL

SKKYGPVFTIYLGPWRPVVVLVGQEAVREALGGQAEEFSGRGTVAMLEGTFDGHGVFFSN

GERWRQLRKFTMLALRDLGMGKREGEELIQAEARCLVETFQGTEGRPFDPSLLLAQATSN

VVCSLLFGLRFSYEDKEFQAVVRAAGGTLLGVSSQGGQTYEMFSWFLRPLPGPHKQLLHH

VSTLAAFTVRQVQQHQGNLDASGPARDLVDAFLLKMAQEEQNPGTEFTNKNMLMTVIYLL

FAGTMTVSATVGYTLLLLMKYPHVQKRVREELNRELGAGQAPSLGDRTRLPYTDAVLHEA

QRLLALVPMGIPRTLMRTTRFRGYTLPQGTEVFPLLGSILHDPNIFKHPEEFNPDRFLDA

DGRFRKHEAFLPFSLGKRVCLGEGLAKAELFLFFTTILQAFSLESPCPPDTLSLKPTVSG

LFNIPPAFQLQVRPTDLHSTTQTR

>Q96SQ9.2

MEATGTWALLLALALLLLLTLALSGTRARGHLPPGPTPLPLLGNLLQLRPGALYSGLMRL

SKKYGPVFTIYLGPWRPVVVLVGQEAVREALGGQAEEFSGRGTVAMLEGTFDGHGVFFSN

GERWRQLRKFTMLALRDLGMGKREGEELIQAEARCLVETFQGTEGRPFDPSLLLAQATSN

VVCSLLFGLRFSYEDKEFQAVVRAAGGTLLGVSSQGGQTYEMFSWFLRPLPGPHKQLLHH

VSTLAAFTVRQVQQHQGNLDASGPARDLVDAFLLKMAQEEQNPGTEFTNKNMLMTVIYLL

FAGTMTVSTTVGYTLLLLMKYPHVQKWVREELNRELGAGQAPSLGDRTRLPYTDAVLHEA

QRLLALVPMGIPRTLMRTTRFRGYTLPQGTEVFPLLGSILHDPNIFKHPEEFNPDRFLDA

DGRFRKHEAFLPFSLGKRVCLGEGLAKAELFLFFTTILQAFSLESPCPPDTLSLKPTVSG

LFNIPPAFQLQVRPTDLHSTTQTR

>AAF88144.1

MAALGITVALLVWMATLMLISIWKQIYSRWKLPPGPFPLPIIGNILQVDIKNVPKSLAKL

AEQYGPVFTLYLGSQRTVVLHGYKAVKEVLLDHKNDLSGRGEVFAFQSHKDRGITFNNGP

GWKDTRRLSLSTLRDYGMGKRGNEERIQREIPFLLEALRGTRGQPFDPTFLLGFAPFNVI

ADILFHKHFDYSDQTGLRIQKLFNENFHLLSTGWLQLYNIFPSYLHYLPGSHRKVLRNVA

ELKDYSLERVKEHQESLDPTCSRDFTDCLLQELQKERYGTEPWYTLDNIAVTVADLFFAG

TETTSTTLRYGLLILMKYPEVEEKLHEEIDRVIGPSRVPAIKDRLEMPYMDAVVHEIQRF

IDLLPSNLPHVANQDTMFRGYVIPKGTVVIPTLDSVLFDKQEFPDPEKFKPEHFLNENGK

FKYSDYFKAFSAGKRVCVGEGLARMELFLFLSAILQHFNLKSLVDPKDIDLSPCTIGFAK

IPPHHKLCVVPRSG

>157074229

MDPFVVLVLCLSFMLLLSLWRQRSARRNLPPGPTPLPIIGNYHLIDMKDIGQCLTNFSKT

YGPVFTLYFGSQPIVVLHGYEAIKEALIDHGEVFSGRGSFPFFDKVSKGKGIGFSHGNVW

KATRVFTVNTLRNLAMGKRTIENKVQEEAQWLMKELKKTNGSPCDPQFIIGCAPCNVICS

IVFQNRFDYKDKDFLSLIGKVNECTEILSSPGCQIFNAVPILIDYCPGRHNKFFKNHTWI

KSYLLEKIKEHEESLDVTNPRDFIDYFLIQRRQDKGIEHMEYTIEHLATLVTDLVFGGTE

TLSSTMRFALLLLMKHTHITAKVQEEIDNVIGRHRSPCMQDRKHMPYTNAMVHEVQRYVD

LGPTSLVHEVTCDTKFRNYFIPKGTQVMTSLSSVLHDSTEFPNPEVFDPGHFLDDNGNFK

KSDYFVPFSAGKRICVGESLARMELFLFLTTILQNFKLKPLVDPKDIDTTPKYSGFSKIP

PKFQMCFIPVE

>297687079

MNPMVVLHGYEAVKEALIDNGEEFSGRGSSPISQRITKGLGIISSNGNRWKEIRRFSLTT

LRNFGMGKRSIEDRVQEEARCLVEELRKTKASPCDPTFILGCAPCNVICSVVFQKRFDYK

DQNFLTLMKRFNENFRILNSPWIQVCNNFPLLIDCFPGTHNKLLKNVALTRSYIREKVKE

HQTSLDVTSPRDFIDCFLIKMEQEKDNQKSEFNIENLVGTVADLFVAGTETTSTTLRYGL

LLLLKHPEVTAKVQEEIDHVIGRHRSPCMQDRSHMPYTDAVVHEIQRYIDLVPTGVPHAV

TTDIQFRNYLIPKGTTIMTLLTSVLHDDKEFPNPKIFDPGHFLDKNGNFKKSDYFMPFSA

GKRICAGEGLARMELFLFLTTILQNFNLKSVDDLKNLNTTAVTKGIVSLPPSYQICFIPV

>392344425

MESSAPEILSSISCILLVKLFSKTYGPVFTLYLGSQPTVILHGYEAIKKALIDHGEKFSG

RGSYPMIENVTKGFGIAFSNGNRWKEIRRFTIMTLRNLGMGKRNIEDRVQEEAQRLVEEL

RKTKGNTVYLWCSHLLLRCSFSVFVVAFSKTYGPVFTLYLGSQPTVILHGYEAIKKALID

HGEKFSGRGSYPMIENVTKGFGIAFSNGNRWKEIRRFTIMTLRNLGMGKRNIEDRVQEEA

QRLVEELRKTKGSPCDPSFILNCAPCNVICSITFQNHFDYKDKEILTFMEKVNENVKIMS

SPRMQVCNSFPSLIDYFPGTHHKIAKNINYMKSYLLKKIEEHQESLDVTNPRDFVEYYLI

KQKQANHIEQSEYSHENLACSIMDLIGAGTETMSSTLRYALLLLMKYPHVPAKVQEEIDH

VIGRYRSPCMQDRSHMPYTDAMIHEVQRFINFVPTNLLHAVTCDIKFRNYLIPKGTKVLT

SLTSVLHGSKEFPNPEMFDPGHFLDENGNFKKSDYFLPFSAGKRACVGEGLARMELFLFL

TTILQNFKLKSLVHPKDIDTRPVLNGFASLPPTYQFCFIPS

>EAW57021.1

MEATGTWALLLALALLLLLTLALSGTRARGHLPPGPTPLPLLGNLLQLRPGALYSGLMRL

SKKYGPVFTIYLGPWRPVVVLVGQEAVREALGGQAEEFSGRGTVAMLEGTFDGHGVFFSN

GERWRQLRKFTMLALRDLGMGKREGEELIQAEARCLVETFQGTEGRPFDPSLLLAQATSN

VVCSLLFGLRFSYEDKEFQAVVRAAGGTLLGVSSQGGQTYEMFSWFLRPLPGPHKQLLHH

VSTLAAFTVRQVQQHQGNLDASGPARDLVDAFLLKMAQEEQNPGTEFTNKNMLMTVIYLL

FAGTMTVSTTVGYTLLLLMKYPHVQKWVREELNRELGAGQAPSLGDRTRLPYTDAVLHEA

QRLLALVPMGIPRTLMRTTRFRGYTLPQGTEVFPLLGSILHDPNIFKHPEEFNPDRFLDA

DGRFRKHEAFLPFSLGKRVCLGEGLAKAELFLFFTTILQAFSLESPCPPDTLSLKPTVSG

LFNIPPPQQWAWTGLMSPECTLQAATFTRLQLFSGVCPTAHTLT

>P05180.1

MDFLGLPTILLLVCISCLLIAAWRSTSQRGKEPPGPTPIPIIGNVFQLNPWDLMGSFKEL

SKKYGPIFTIHLGPKKIVVLYGYDIVKEALIDNGEAFSGRGILPLIEKLFKGTGIVTSNG

ETWRQLRRFALTTLRDFGMGKKGIEERIQEEAHFLVERIRKTHEEPFNPGKFLIHAVANI

ICSIVFGDRFDYEDKKFLDLIEMLEENNKYQNRIQTLLYNFFPTILDSLPGPHKTLIKNT

ETVDDFIKEIVIAHQESFDASCPRDFIDAFINKMEQEKENSYFTVESLTRTTLDLFLAGT

GTTSTTLRYGLLILLKHPEIEEKMHKEIDRVVGRDRSPCMADRSQLPYTDAVIHEIQRFI

DFLPLNVPHAVIKDTKLRDYFIPKDTMIFPLLSPILQDCKEFPNPEKFDPGHFLNANGTF

RRSDYFMPFSAGKRICAGEGLARMEIFLFLTSILQNFSLKPVKDRKDIDISPIITSLANM

PRPYEVSFIPR

>AAI66952.1

MYVAGLGTILLVLISCVLIFSSWKTLYQKHNLPPGPTPLPLIGNLMNIKRGKLVSSLMKL

WEQYGAVYTLYFGIQPVIVLCGYDAVKEALVDQAEDFGARGKISSLDPVTQGYGLSFSNG

ERWRQLRHFTLKALRDFGMGKKSIEEKIQEEALCLVEEFRKSGEMPTDPEKPIMKAVSNI

FFTIVLGNRFEYNDETFSALLAKVEEMFRLMSNTWSQIENVLPKLMAYIPGPHKKRDALG

KQLILFLHERIKANQETFDPNAPRDFIDEFLIKMEQEKKNPNSEFTMRNTLLTFYSIFLG

GTETSTTTLKHGLLLLIKYPEIQAKLHMEIDNVIGRNRTANMIDRNSMPYMEAVINEIQR

FSDIIPLNVPRKVTKDVQFRGYCIPKDTEIYPLLCTVHHDAKYFSSPYEFNPSHFLDEQG

KFKKNNAMMAFSAGKRICPGESLTRMELFLFFTTILQNFTLTSPTHFTDNDVAPKMTGFI

NHPIQYKASFISR

>390479055

MEATGTWTLLLALALLLLLTLALSGTRARGHLPPGPTPLPLLGNLLQLRPGALYSGLMRL

SKKYGPVFTIYLGPWRPVVVLVGQDAVREALGGQAEEFSGRGTIAMLERTFDGHGVFFSN

GERWRQLRKFTTLALRDLGMGKREGEELIQAEAKCLVEAFQGTEGRPFDPSLLLAQATSN

VVCSLLFGLRFSYEDKEFQAMVQAARGTLLGVSSPGGQTYEMFSWFLWPLPGPHKQLLHH

VGALATFTVQQVQQHQANLDASSPASDLVDAFLLKMAQEEENSHTEFTEKNMLMTVIYLL

FAGTMTVSATVGYTLLLLMKYPHVQSKSLGAGQAPSLGDRTRLPYTDAVLHEAQRLLALV

PMGIPHTLMRTTRFRGYTLPQGTEVYPLLGSVLHDPNFFEQPEEFNPDRFLDADGRFRKH

EAFLPFSLGKRVCLGEGLANAELFLFFTSILQAFSLESPCPRGTLSLKPTISGLFNVPPA

FQLQVHPADLHPTTXTK

>402905632

MLASGLLLVALLACLTVMVLMSVWQQRNSKGKLPPGPTPLPFIGNYLQLNTEQMCTSLMK

VSQGVAFSNGERAKQLRRFSIATLRDFGVGKRGIEERIQEEAGFLIEALRGTRGANIDPT

FFLSRTVCNVISSIVFGDRFDYEDKEFLSLLRMMLGSFQFTATSTGQLYEMFSSVMKHLP

GPQQQAFKELQGLEDFIAKKVEHNQRTLDPNSPRDFIDSFLIRMQEEEKNPNTEFHLKNL

VMTSLNLFFAGTETVSTTLRYGFLLLMKHPEVEAKVHEEIDRVIGKNRQPKFEDRVKMPY

TEAVIHEIQRFGNVIPMSLAHRVIRDTKFRDFFLPKGTEVFPMLGSVLRDPRFFSNPQDF

NPQHFLDEKGQFKKSDAFVPFSIGKRNCFGEGLARMELFLFLTTIMQNFGFESPQLPKDI

DVSPKHVGFATIPRNYTMSFLPR

>BAB55227.1

MEATGTWALLLALALLLLLTLALSGTRARGHLPPGPTPLPLLGNLLQLRPGALYSGLMRL

SKKYGPVFTIYLGPWRPVVVLVGQEAVREALGGQAEEFSGRGTVAMLEGTFDGHGVFFSN

GERWRQLRKFTMLALRDLGMGKREGEELIQAEARCLVETFQGTEGRPFDPSLLLAQATSN

VVCSLLFGLRFSYEDKEFQAVVRAAGGTLLGVSSQGGQTYEMFSWFLRPLPGPHKQLLHH

VSTLAAFTVRQVQQHQGNLDASGPARDLVDAFLLKMAQEEQNPGTEFTNKNMLMTVIYLL

FAGTMTVSTTVGYTLLLLMKYPHVQKWVREELNRELGAGQAPSLGDRTRLPYTDAVLHEA

QRLLALVPMGIPRTLMRTTRFRGYTLPQGTEVFPLLGSILHDPNIFKHPEEFNPDRFLDA

DGRFRKHEAFLPFSLGKRVCLGEGLAKAELFLFFTTILQAFSLESPCPPDTLSLKPTVSG

LFNIPPASTVGMDRVNVSRVYTAGSHIYTPAVVFRSLSHGPHAHLTHAAKMHNRTPIHNY

KGHKATAGLAFHRHKYSPSAITST

>332242429

MEATGTWALLLALALLLLLTLALSGTRARGHLPPGPTPLPLLGNLLQLRPGALYSGLMRL

SKKYGPVFTIYLGPWRPVVVLVGQEAVREALGGQAEEFSGRGTVAMLERTFDGHGVFFSN

GERWRQLRKFTMLALRDLGMGKREGEELIQAEARCLLEAFQETEGRPFDPSLLLAQATSN

VVCSLLFGLRFSYEDKEFQAVVRAAGGTLLGVSSRGGQTYEMFSWFLRPLPGPHKQLLHH

ISTLAAFTVQQVQQHQGNLDASVPTRDLVDAFLLKMAQEEQNPDTEFTNKNMLMTVIYLL

FAGTMTVSATVGYTLLLLMKYPHVQKRVREELNRELGAGQAPSLGDRIRLPYTDAVLHEA

QRLLALVPMGIPRTLMRTTRFRGYTLPQGTEVFPLLGSILHDPNIFKHPEEFNPDRFLEA

DGRFRKHEAFXPFSLGKRVCLGEGLAKAELFLFFTTILQAFSLESPCPPGTLSLKPTISG

LFNIPPAFQLQVRPTDLHSITQTR

>AAH95374.1

MELSSSLLLVLVLTVLMLIRWRRKENGLSLPPGPLALPLIGNLLTLDKSAPFKSFMKWRK

TYGSVMTVHLGPQRMVVLVGYETVKEALVDQADDFAPRAPIPFMNRIVKGYGLAISNGER

WRQLRRFTLTTLRDFGMGRKRMEQWIQEESRYLLKSFEETKSKPVDPTFFFSRTVSNVIC

SLVFGQRFDYEDKNFLQLLQIISKLLRFLSSPWGQLYNIFPQVMERFSSRHHAILKDVEN

IRTFIRSKVKEHEQRLDFSDPSDFIDCFLIRLTQEKDKLDTEFHKDNLMATVLNLFVAGT

ETTSTTLRYALMLLIKHPQIQEQMQREIDRVIGQNRIPTMEDRKSLPFTDAVIHEVQRYM

DIVPLSLPHYAMKDITFRGYKIPKDTVIIPMLHSVLRDEGQWETPWTFNPEHFLDSNGNF

QKNPAFMPFSAGKRSCVGESLARMELFLFTVSLLQKFTFSSPNGPDGIDLSPELSSFANM

PRFYELIASPR

>327282465

MEVLGTTTLFLVVFLVLFVAWRKVEAKRRNWPPGPTPLPLIGNLLQLKPTNIAEQFKKMN

KKYGSVFMVYFGLDPVVVVYGYDVVKKVLLDSGEEFLNRGSFPLVDKTNKGLGIIMSNGE

RWVQLRRFSLMTLRNFGMGKKSIEERIQEEAEHLMKELRDKRGQAFNPQHLFNCITSNVI

SHVLLGERFDYHDEEYLQILKQLIDGVRLESSVSGQLYNFFPRIMDYLPGPHQTFFKNIY

GVQTFIARKVEEHEKTLDHTDVPQNFVDAFLLKMEQEKNNPTTEFTKENLMMTIYDLFIA

GTETTSTTIRYFFMTLVEHPDIQAKIQDEIDRVIGRERMPTMKDRQEMPFTEAAIHEGQR

FLDLVPLGLIRMVKRDIELEGFTIPKGATIYPILSSALHDPKQYANPYQFNPEHFLDKDG

RFKKNGADMPFSAGKRNCLGEGLARMELFIFITTVLQNFNLKHAPGVPKIDLTPDVSGIL

NVPRQVPFCFTPR

>402905637

MEATGTWALLLALALLLLLTLALSGTRARGQLPPGPTPLPLLGNLLQLRPGALYSGLMRL

SKKYGPVFTIYLGPWRPVVVLVGHEAVREALGGQAEEFSGRGTIAMLERTFDGHGVFFSN

GERWRQLRKFTMLALRDLGMGKREGEELIQAEARCLVEAFQGTEGRPFDPSLLLAQATSN

VVCSLLFGLRFSYEDKEFQAMVRAAGGTLLGVSSRGGQTYEMCSWFLWPLPGPHKQLLHH

ISTLAAFTVRQVQQHQGNLDASGPARDLVDAFLLKMAQEKQNPDTEFTNKNMLMTIIYLL

FAGTMTVSATVGYTLLLLMKYPHVQKRVHEELTRELGSGQAPSLGDRTRLPYTDAVLHEA

QRLLALVPMGIPRTLMRTTRFRGYTLPQGTEVFPLLGSILHDPSIFKHPEEFNPDHFLDA

DGRFRKHEAFLPFSLGKRVCLGEGLAKAELFLFFTTILQAFSLESSCPLDSLSLKPTISG

LFNIPPAFQLQVRPTDLHSTTQTT

>186287327

MDPFVVLVLCLSFLLLLSLWRQRSARGNLPPGPTPLPIIGNYHLIDMKDIGQCLTNFSKI

YGPVFTLYFGSQPIVILHGYEAMKEAFIDYGEEFSGRGRIPVFDKVSKGKGIGFSHGNVW

KATRVFTVNTLRNLGMGKRTIETKVQEEAQWLMKELKKTNGSPCDPQFIIGCAPCNVICS

IVFQNRFDYKDKDFLSLIGKVNECTEILSSPECQIFNAVPILIDYCPGSHNKFLKNHTWI

KSYLLEKIKEHEESLDVTNPRDFVDYFLIQRRQKNGIEHMDYTIEHLATLVTDLVFGGTE

TLSSTMRFALLLLMKHTHITAKVQEEIDNVIGRHRSPCMQDRNHMPYTNAMVHEVQRYID

LGPNGVVHEVTCDTKFRNYFIPKGTQVMTSLTSVLHDSTEFPNPEVFDPGHFLDDNGNFK

KSDYFMPFSAGKRICVGESLARMELFLFLTTILQNFKLKPLVDPKDIDMTPKHSGFSKIP

PNFQMCFIPVE

>301786655

MAALGITVALLVWAATLTLISVWKQIYSSWNLPPGPFPLPIIGNLLQLDLKNFPKSLTKL

AERYGPVFTLYLGSQRTVVLHGYKAVKEALLDHKNDLSGRGEIFAFQFHKDRGITFNNGP

GWKDTRRLSLTTLRDYGMGKRGNEERIRREIPFLLEALKGTQGQPFDPTFLLGFAPCNVI

ADILFRKHFDYTDETGLRIQRLFNENFHLLSTGWLQLYNIFPSCLQYLPGSHRKVLKNVS

EIKDYTSERVKEHQQSLDPNCLRDFTDCLLLELQKGRYSSEPWYTLDNIAVTVADLFFAG

TETTSTTLRYGLLILMKYPEIEEKLHEEIDRVIGPSRIPAIKDRLEMPYMDAVVHEIQRF

IDLLPSNLPHEANQDTVFRGYVIPKGTVVIPTLDSLLFDKQEFPDPEKFKPEHFLNENGK

FKYSDYFKAFSAGKRVCVGEGLARMELFLFLSAVLQHFNLKSLVDPKDIDLSPIVIGFAK

IPPHYKLRVIPRSG

>EHH59604.1

MEATGTWALLLALALLLLLTLALSGTRARGQLPPGPTPLPLLGNLLQLRPGALYSGLMRL

SKKYGPVFTIYLGPWRPVVVLVGQEAVREALGGQAEEFSGRGTIAMLERTFDGHGVFFSN

GERWRQLRKFTMLALRDLGMGKREGEELIQAEARCLVEAFQGTEGRPFDPSLLLAQATSN

VVCSLLFGLRFSYEDKEFQAMVRAAGGTLLGVSSRGGQTYEMCSWFLWPLPGPHKQLLHH

VSTLAAFTVRQVQQHQGNLDASGPARDLVDAFLLKMAQEEQNPDTEFTNKNMLMTVIYLL

FAGTMTVSATVGYTLLLLMKYPHVQKRVREELTQELGSGQAPSLGDRTRLPYTDAVLHEA

QRLLALVPMGIPRTLMRTTRFRGYTLPQGTEVFPLLGSILHDPSIFKHPEEFNPDRFLDA

DGRFRKHEAFLPFSLGKRVCLGEGLAKAELFLFFTTILQAFSLESPCPLDSLSLKPTISG

LFNIPPAFQLQVRPTDLHSTTQTT

>327276261

MELSEAVTLFLVICLSCIAFLSLKRKISNKGRLPPGPTPLPLIGNFLQIKSSETLKSLVK

VSEWEAFSNGERWKKLRRFTLCVLRDFGMGKRSIEERIQEEAQFLLDDFRKTKEKPFDVT

FFLSRAVSNVICSIVFGNRFDYEDKNFQTLVEMVNSSFREISTAQAQLYDLYESFLKYFP

GPQTKNYDILKVIRAFICKKVKQNQKTLDLNAPRDFIDSFLIQMEKEKENPSTEFYMKNL

ECTVLNLFLAGTETVSSTLRYGFLLLLKYPEVQEKVHEEIDRVIGQNCIPNMADRNRMPY

TDAVIHEVQRCADLIPMNLIHMVTRDTEFRGYLIPKGTEVHPLLSTVLNDPTKFKNAHIF

NPENFLDENGCFKKNDAFIPFSSGKRICLGETLARMELFLFFTTILQSFCLKSIIPPEDI

DITPQESGFSKIPPLYQLSIIPR

>348543371

MESCATVILAGLILALLWMFNIKHKRNWHLPPGPTAFPLIGNLLQIGKKAPFKSFVKFSE

TYGPVMTVYLGWQRTVVLVGYDVVKEALVDQADDFTGRAPIPFIHNPNKKYGIGSSNGER

WRQLRRFTLTTLKDFGMGRKGMDEWIQEESEHMRDYIHKLKGAPFDPTFLLSCTVSNVIC

CLVFGQRFNYEDKQFLRLLTILSHIEKVNSRPVGQMYNLFPKLMDHLPGPHQKIFAEYDE

VRGFIKMKIQEHKDTLEPSSPRDYIDSFLIQINQEKEISTTEFHYDNLVSTIMTLFLAGT

QTTSSTLRFALSVLIKYPKIQETMQQEIDSVTGKGRCPSMEDRKSLPFSLAVIHEMQRFL

DLVPLSLPHYTLNDISFRGYTIPKDTVIIPLLHSVLKEEKQWATPRSFNPQHFLDNNGSF

KKSPAFMPFSAGKRNCVGESLARMELFIFIVSLLQDFTFSCAGGPDSINLIPEYSSFINV

PCRYHIMATPR

>EHH30061.1

MEATGTWALLLALALLLLLTLALSGTRARGQLPPGPTPLPLLGNLLQLRPGALYSGLMRL

SKKYGPVFTIYLGPWRPVVVLVGQEAVRDALGGQAEEFSGRGTIAMLERTFDGHGVFFSN

GERWRQLRKFTMLALRDLGMGKREGEELIQAEARCLVEAFQGTEGRPFDPSLLLAQATSN

VVCSLLFGLRFSYEDKEFQAMVRAAGGTLLGVSSRGGQTYEMCSWFLWPLPGPHKQLLHH

VSTLAAFTVRQVQQHQGNLDASGPARDLVDAFLLKMAQEEQNPDTEFTNKNMLMTVIYLL

FAGTMTVSATVGYTLLLLMKYPHVQKRVREELTQELGSGRAPSLGDRTRLPYTDAVLHEA

QRLLALVPMGIPRTLMRTTRFRGYTLPQGTEVFPLLGSILHDPSIFKHPEEFNPDRFLDA

DGRFRKHEAFLPFSLGKRVCLGEGLAKAELFLFFTTILQAFSLESPCPLDSLSLKPTISG

LFNIPPAFQLQVRPTDLHSTTQTT

>327282447

MEVLGTTTLFLVVFLVLFVAWRKVEAKRRNWPPGPTPLPLIGNLLQLKPTNIAEQFKKMS

KKYGSVFMLYFGLDPVVIVYGYDVVKKVLVDSGDEFLNRGSFPTSDKTNKGLGIIMSNNE

RWVQLRRFSLMTLRNFGMGKKSIEERIQEEAEHLMKELRDKRGQAFNPQHLFNCVTSNVI

SYILLGKRFDYHDEEYLQILKQLIDGVRLESSVSGQLYNFFPWIMDYLPGPHQTFFKNIY

GVQAFIARKVEENEKILDHTDVPQNFVDAFLLKMEQEKNNPTTEFTKENLMMTIYDLFIA

GTETSSTTMRYFFMTLVEHPDVQAKIQDEIDRVIGRERMPTMKDRLEMPFTDAAIHEGQR

LLDFIPLGLIRMAKRDVEMEGFIIPKGATIYPILSSALHDPKQYANPYQFNPEHFLDKDG

RFKKNGADMPFSAGKRNCLGEGLARMELFIFITTVLQSFSLKHAPGVPKIDLTPDVSGFL

NIPRQVPFCFSPR

>AAH60472.1

MDPISVLLSVVVCIFLFNVFYGGKRESQNFPPGPKPLPLIGNLHMMNMKKPYLTFMELGK

KYGSVFSVQLGMKKAVVLCGTDAVKDALINHADEFSGRAKIPIFHQASKGFGIVFADGEN

WRVMRRFAISTLRDFGMGKKTIEDRISEESDCLVETFKSHEGKPFDNTLIMNAAVANIIV

HILLNQRFDYQDPTLLKLIKSISENVRISGRPIVMLYNTYPSIMQWLPGGHQTVFENTQK

LFNFLKETFTKRRDQLDVNDQRDLIDAFLVKQQEETSSSTKFFHDDNLKVLLGNLFGAGM

ETTSTTLRWGLLLMMKHPDIQKKVQDEINQVIGSAEPRLDHRKQMPYTDAVIHEIQRFAN

LVPNGLPHATTKDVTFRGYFIPKGTQVIPLLTSVLRDKAYFKKPEEFYPEHFLDSEGHFV

KNEAFLPFSAGRRICAGETLAKMELFLFFTKLLQNFTFQPPLGVEVQLTCAEAITSIPTD

HKICALPRN

>Q91W64.2

MALFIFLGIWLSCFLFLFLWNQHRGRGKLPPGPTPLPIVGNILQVDVKNISKSMGMLAKK

YGPVFTVYLGMKPTVVLHGYKAMKEALIDQGDEFSDKTDSSLLSRTSQGLGIVFSNGETW

KQTRRFSLMVLRSMGMGKKTIEDRIQEEILYMLDALRKTNGSPCDPSFLLACVPCNVIST

VIFQHRFDYNDQTFQDFMENFHRKIEILASPWSQLCSAYPILYYLPGIHNRFLKDVTQQK

KFILEEINRHQKSLDLSNPQDFIDYFLIKMEKEKHNQKSEFTMDNLVVSIGDLFGAGTET

TSSTVKYGLLLLLKYPEVTAKIQEEIAHVIGRHRRPTMQDRNHMPYTDAVLHEIQRYIDF

VPIPSPRKTTQDVEFRGYHIPKGTSVMACLTSVLNDDKEFPNPEKFDPGHFLDEKGNFKK

SDYFVAFSAGRRACIGEGLARMEMFLILTNILQHFTLKPLVKPEDIDTKPVQTGLLHVPP

PFELCFIPV

>CAG12753.1

MELSLTLVLVGLVLACLWFVLRQRNYNLPPGPTALPLIGNLPLIDRKQPFKSCVELSKTY

GPVMTLHMGWQRTVFLTGYDAVKEALVDQADDFTGRGPLPFLLKATKGYGLGISNGERWR

QLRRFTLSTLRDFGMGRKGMEEWIQEESKHLTDRIKTLKGKPFDPTFVISCAVSNVICCL

VFAERFSYDDQRFLHLLGVISKVLRFQSSFLGQMYNIFPSIMELLPGPHHTMFRNTDFLR

NFVMTKIQEHKDSLDPSSPRDFIDCFLIRMEQEKNLPTTEFQYENLVSTVLNLFLAGTET

TSTTIRYALQVLIKHPNIQEKMQQEIDTVVKQEHCPKMEDRKSLPFVDAAIHEVQRFLDI

VPFSLPHFALKDISFRGYTIPKGTMIIPFLHSVLKEDQWATPWSFNPKHFLEQNGSFKKN

PAFLPFSAGKRSCVGESLARMELFIVLVTLLKNFTFSCAEGPDSINLIPQYSGFANIPQD

YDIIATPR

>DAA14859.1

MELFVVLVTCLSFLIFLFLWNQRHAKGKLPPGPTPFPVVGNIFQINTKNVSKSISKLAED

YGPVFTLYFGMKPTVVLHGYEAVRQVLIDQSEEFSARGSLPAADNINQGLGIVFSNGEKW

KQTRRFSLMVLRNMGMGKRTIEHRIQEEALCLVEALKKPMVGSPCDPTFLLSYAPCNVIC

SIVFRNRFEYSDENFLTLAKYFDENAKILSTPWIELYNAFPSLLHYLPGSHNTLFKNMTE

QRKFILEKIKEHQESLDLNNPQDFIDYFLIKMEKEKHNKQSEFTMDNLITTIWDVLTAGI

ETTSLTLRYGLLLLLKHPEVTAKVQEEINHVVGRNRSPCMEDRSRMPYTDAVIHEIQRFI

DLVPNNLPHAAAQDIKFREYLIPKGTTILTSLTSVLHDGKEFPNPEQFDPGHFLDKSGNF

KKSDYFMAFSAGKRVCVGEGLARMELFLLLVSILQKFTLKPLVDPKNIDTAPLLKGVGSI

PHFYEVCFIPV

>402905606

MLASGLLLVALLACLTVMVLMSVWQQRNSKGKLPPGPTPLPFIGNYLQLNTEQMYNSIMK

VSQGVAFSNGERAKQLRRFSIATLRDFGVGKRGIEERIQEEAGFLIEALRGTRGANIDPT

FFLSRTVCNVISSIVFGDRFDYEDKEFLSLLRMMLGSFQFTSTSMGQLHEMFSSVMKHLP

GPQQQAFKELQGLEDFIAKKVEHNQRTLDPNSPRDFIDSFLIRMQEEEKNPNTEFYMQNL

LMTALNLFIAGTETVSTTLRYGFLLLMKHPEVEAKVHEEIDRVIGKNQQPKFEDRVKMPY

MEAVIHEIQRFGNVIPMSLARRVNKDTKFRDFFLPKGTEVFPMLGSVLKDPRFFSNPQDF

NPQHFLDEKGQFKKSDAFVPFSIGKRNCFGEGLARMELFLFFTTIMQNFRFKSPQSPKDI

DVSPKHVGFATIPRNYTMSFLPR

>301608082

MDPVSVLLSVVVCIFLFKVFYGGKRTLENFPPGPKPLPLIGNLHMMNMKKPHLTFMELAE

KYGSVFSVHLGTEKVVVLCGTDTVRDALINHAEEFSERAKMPIFEDFSKGLGVVFGHGEN

WKVMRRFTLSTLRDFGMGKKTIEERISEESDCLVETIKSYEGKPFDNTLIMNAAVANIIV

HILLNHRFDYQDPTLLKLINIVIDNIKIGGSPIVMLYNTYPSVVRWIPGSHKTLGENTAQ

LYKFLEETFTQHREQLDVNDQRDLIDAFLVKQQEEKPSSAKFFHNENLVALLANLFVAGM

ETSSTTLRWGLLLMMKYPDIQKKVQDEIDKVIGSAEPRLEHRKLMPYTDAVIHEIQRFAN

IAPISLPHATTTDVTFRGYFIPKDTQVMIVLTSVLQDKDYFKKPEEFYPEHFLNSKGNFV

KNEAFLPFSAGRRICAGETLAKMELFLFFTKLLQNFTFQPPPGVEVDLTCADAMTSKPQE

HQICALPRG

>327282467

MEVLGTTTLFLVVFLVLFVAWRKVEAKRRNWPPGPTPLPLIGNLLQLKPTNIAEQFKKMS

KKYGSVFMVYFGMDPVVVVYGYDVVKKVLVDSGDEFLNRGSFPSSDKTNKGLGIFMSNGE

RWVQLRRFSLMTLRNFGMGKKSIEERIQEEAEHLMKELRDKRGQAFNPQHLFNCVTSNVI

CHILLGKRFDYNDEEYLRALKQLIDGFRLESSVSGQLYNFFPRIMDYLPGPHQTFFKNMY

GVQAFIARKIEENEKTLDHTDVPQNFVDAFLLKMEQEKNNPTTEFTKENLMMAIYDLFIA

GTETSSTTIRYFFMTLLEHPEVQAKIQDEIDRVIGRERMPTMKDRLEMPFTEAAIHEGQR

FLDLVPLGFIRLAKRDVEMEGFTIPKGATIYPILSSALHDPKQYANPYQFKPEHFLDKDG

HFQKNGADMPFSAGKRSCLGEGLARMELFIFITTILQNFSLKHAPGVPKIDLTPDVSGVL

NIPHQVPFCFSPR

>AAH19468.1

MDPFVVLVLCLSFLLLLSLWRQRSARGNLPPGPTPLPIIGNYHLIDMKDIGQCLTNFSKI

YGPVFTLYFGSQPIVILHGYEAMKEAFIDYGEEFSGRGRIPVFDKVSKGKGIGFSHGNVW

KATRVFTVNTLRNLGMGKRTIETKVQEEAQWLMKELKKTNGSPCDPQFIIGCAPCNVICS

IVFQNRFDYKDKDFLSLIGKVNECTEILSSPECQIFNAVPILIDYCPGSHNKFLKNHTWI

KSYLLEKIKEHEESLDVTNPRDFVDYFLIQRCQKNGIEHMDYTIEHLATLVTDLVFGGTE

PLSSTMRFALLLLMKHTHITAKVQEEIDNVIGRHRSPCMQDRNHMPYTNAMVHEVQRYID

LGPNGVVHEVTCDTKFRNYFIPKGTQVMTSLTSVLHDSTEFPNPEVFDPGHFLDDNGNFK

KSDYFVPFSAGKRICVGESLARMELFLFLTTILQNFKLKPLVDPKDIDMTPKHSGFSKIP

PNFQMCFIPVE

>DAA14857.1

MDLSVVLVICLSILIFLFLWNQRHAKGTLPPGPTPLPIVGNILQINIKNVSKSISKLAED

YGPVFTLYFGMKPTVVLHGYEAVKQVLIDQSEEFSGRGSLPVADNINQGLGIIFSNGEIW

KQTRRFSLMVLRNMGMGKRTIEHRIQEEALCLVEALKKTNGCPCDPTFLLTYAPCNVICS

IIFRNRFEYSDESFLTLAKYIHENATIFSTPWIELYNAFPSLLHYLPGSHNTLFKNMTEQ

RKFILEKIKEHQESLDLNNPQDFIDYFLIKMEKEKHNKQSEFTMDNLITTVWDVFTAGIE

TTSISLKYGLLLLLKHPEVTAKVQEEINRVVGRNRSPCMQDRSRMPYTDAVIHEIQRYID

IVPNNLPHAAAQDIKFREYLIPKGTTILTSLTSVLHDGKEFPNPEQFDPGHFLDKSGNFK

KSDYFMVFSAGKRVCIGEGLARMELFLLLVSILQKFTLKPLVDPKNIDTTPLLKGVGSIP

HFYEVCFIPV

>AAI35863.1

MDPVSVLLSVFVCIFLFKVFYDGEKESQNFPPGPKPLPLIGNLHIMNMKKPYLTFMELAE

KYGPVFSVQLGTKKTVVLCGADTVKDALINYADEFAGRPKTPLTEQASKGNGIIFANGEN

WKVMRRFTLSTLRDFGMGKKTIEDRISEESDCLVETFKSHEGKPFDNTLILNAAVANIIV

HILLNHRFDYQDPTFLKLIKSVNENVRNGARPIIVLYNAFPSIIQWIPGTHKRIFASSQN

FFNFLKEIFMKRKDQLDVNDQRDLIDAFLVKQQEEKSSSTKFFHDENLKVLIGNLFGAGM

ETTSTTLRWGILLMMKYPDIQKKVQDEIDRVMGSTEPRPEHRKQMPYTDAVIHEIQRFAD

LVPNGVPHATTTDVTFRGYFIPKGTQVFPLLTSVLRDKAYFKKPDEFYPEHFLDSEGNFL

KNEAFLPFSAGKRICAGETLAKMELFLFFTKLLQNFTFQPPPGVEVQLTRGEAITSIPLD

HQICALSRS

>291412170

MEAAGTWALLLLLLLLLLLALALPRARGHLPPGPAPLPLLGNLLQLRPGALYPGLLRLSE

RYGPVFTVHLGPWRPVVVLVGHEAVKEALGGQAEDFAGRGALATLDQTFDGHGVFFANGE

RWQQLRKFTMLALRDRGMGKREGEELIQAEAQRLVEALQETQGHPFDPSLLLAQATSNIV

CSLIFGFRMPYEDEEFQAVVRAAGGTLLGTSSPWGQAYEMFSWFLRPLPSPHKQLLHHLG

TLAAFTVRQVRRHQESLDPSGPARDVVDAFLLKMAQEEKDPNTEFTEKNLLMTVTYLLFA

GTATVSATIRYALLLLLKHPHVHKRVQEELTRELGAGRAPGLGDRARLPYTDAVLHEAQR

LLALLPMGVPRALTRTTCFRGYTLPQGTEVFPLLGSVLRDPKVFEQPEEFNPGRFLDADG

RFRKHEAFLPFSLGKRVCLGEGLARAELFLLFTAILQGFSLDSPCPPQALSLQPAVSGLF

NIPSAFQLQVRPR

>Q05421.1

MAVLGITVALLVWIATLLLVSIWKQIYRSWNLPPGPFPIPFFGNIFQLDLKDIPKSLTKL

AKRFGPVFTLHLGQRRIVVLHGYKAVKEVLLNHKNEFSGRGDIPVFQEYKNKGIIFNNGP

TWKDVRRFSLSILRDWGMGKQGNEARIQREAHFLVEELKKTKGQPFDPTFLIGCAPCNVI

ADILFNKRFDYDDKKCLELMSLFNENFYLLSTPWIQAYNYFSDYLQYLPGSHRKVMKNVS

EIRQYTLGKAKEHLKSLDINCPRDVTDCLLIEMEKEKHSQEPMYTMENISVTLADLFFAG

TETTSTTLRYGLLILMKYPEIEEKLHEEIDRVIGPSRAPAVRDRMNMPYMDAVVHEIQRF

INLVPSNLPHEATRDTVFRGYVIPKGTVVIPTLDSLLFDNYEFPDPETFKPEHFLNENGK

FKYSDYFKAFSAGKRVCVGEGLARMELFLLLSAILQHFNLKSLVDPKDIDLSPVTIGFGS

IPREFKLCVIPRS

>AAP55509.1

MALFIFLGIWLSCFLFLFLWNQHRGRGKLPPGPTPLPIVGNILQVYVKNISKSMGMLAKK

YGPVFTVYLGMKPTVVLHGYKAMKEALIDQGDEFSDKTDSSLLSRTSQGLGIVFSNGETW

KQTRRFSLMALRSMGMGKKTIEDRIQEEILYMLDALRKTNGSPCDPSFLLACVPCNVIST

VIFQHRFDYNDQTFQDFMENFHRKIEILASPWSQLCSAYPILYYLPGIHNRFLKDVTQQK

KFILEEINRHQKSLDLSNPQDFIDYFLIKMEKEKHNQKSEFTMDNLVVSIGDLFGAGTET

TSSTVKYGLLLLLKYPEVTAKIQEEIAHVIGRHRRPTMQDRNHMPYTDAVLHEIQRYIDF

VPIPSPRKTTQDVEFRGYHIPKGTSVMACLTSVLNDDKEFPNPEKFDPGHFLDEKGNFKK

SDYFVAFSAGRRACIGEGLARMEMFLILTNILQHFTLKPLVKPEDIDTKPVQTGLLHVPP

PFELCFIPV

>334328668

MDWIGTGLLLLLLGITCLLLAKAAHKTKGRLPPGPRPLPLLGNLLQLRSRDMVTSLTKLS

QEYGSMFTVHLGTRPVVVLSGYDTVKEALVDKAEEFSGRGEYPVFYDFTKGNGIAFSNGE

KWKVLRRFSIQILRNFGMGKKSLEERILEEEAFLLEELKKTEGAPFDPTFVLSRSVSNII

CSVVFGSRFDYEDERLLKLVHLINENFKIMSSPWGEIYNIFPGLLQWIPGPHRSLFQNYG

SMKTLIDRIIHEHQINLDSTSPSDFIDCFLIKMAEEAKSPDSYFHMETLVKTTFILIFGG

TETVGTTLRHSFLLLMKYPQIQARVQAEIDSVVGRGRRPTLDDRSSMPYTDAVIHEVQRF

ADIIPMNLPHRLTRDTLFRGFLLPKGTDIITLLNTVHYDPSQFKTPNEFNPAHFLDSKEE

FKKSPAFMPFSAGRRLCLGEPLARMELFLYLTGILQNFTLQPLCSPEEIDLTPLSSGLGN

VPRPFQLRMVPR

>AAH16494.1

MALFIFLGIWLSCFLFLFLWNQHRGRGKLPPGPTPLPIVGNILQVYVKNISKSMGMLAKK

YGPVFTVYLGMKPTVVLHGYKAMKEALIDQGDEFSDKTDSSLLSRTSQGLGIVFSNGETW

KQTRRFSLMVLRSMGMGKKTIEDRIQEEILYMLDALRKTNGSPCDPSFLLACVPCNVIST

VIFQHRFDYNDQTFQDFMENFHRKIEILASPWSQLCSAYPILYYLPGIHNRFLKDVTQQK

KFILEEINRHQKSLDLSNPQDFIDYFLIKMEKEKHNQKSEFTMDNLVVSIGDLFGAGTET

TSSTVKYGLLLLLKYPEVTAKIQEEIAHVIGRHRRPTMQDRNHMPYTDAVLHEIQRYIDF

VPIPSPRKTTQDVEFRGYHIPKGTSVMACLTSVLNDDKEFPNPEKFDPGHFLDEKGNFKK

SDYFVAFSAGRRACIGEGLARMEMFLILTNILQHFTLKPLVKPEDIDTKPVQTGLLHVPP

PFELCFIPV

>ADT62516.1

MGITIALLVWMATLLLISIWKHIYSSRKLPPGPFPLPIIGNLFQLDIKDIPKSLTRLAER

FGPVFTLYLGSRRIVVLHGYKAVKEVLLDYKNEFSGRGDNPAFQVHKNKGIIFNNGPTWR

DTRRLSLTVLRDLGMGNWGNEERIRREVPFLLEALRKTQGQPFDPTFVIGFAPYNVISDI

LFHKHFDYSDKTGLRLMYLFNENFYLLSTPWIQLYNNFSNCLQYLPGSHRKLLKNVFRIK

DYASARVKEHQESLDPSCPQDFIDSLLVEMEKGKAQCTACAHLGGHRRDRRCLLFAGTET

TSTTLRYGLLILMKHPEVEEKLHEEIDRVIGPSRVPAIKDRLDMPYLDAVVHEIQRFIDL

LPSNLLHEATQDTVFRGYVIPKGTLVIPTLDSVLYDNQEFPEPEKFKPEHFLNEHGKFKY

SDYFKPFSAGKRVCVGEGLARMELFLFLAAILQHFNLKSLVDPKDIDLSPIATGFAKIPP

RYKFCVIPRLVELMIRSARLIISIGNLSAPGIVHRHSREKWFGFWALRSRPG

>334328691

MSMYLYDMDSTFIMIVLPWVELWRRVLRGHGTAEPAKGSKRLLVLVGKLRPGVPLRVSGR

AGAELGALGAGDGGGGGGGSVRGGVKLGGWPGRQVLEPAGCAESAWRPQGRRRRAPDFEP

STRFGPGSGRGLIPVASRGGAPQGRLYKRRGPSPRPPCCGLGMALATGVASPLLVLVLVL

VLLLALLVLRQGRPRSSRLPPGPPPLPLLGNVGQLRLSPLFNPSDCFSLQLSAKYGPVFT

VYLGSRPVVVLSGYRAVKEALVDQAEAFSGRGKIAGLEKTFHEHGLFFANGEQWRSLRKF

TTLSLRALGMGKKEGEEHIQEEARCLLEALRSTQGSPLDPSLLLSQAVSNITCLLLFGKR

FDYEDKKFQAMVLATAGILTEISSPWGQVCEMFMGPVCYLSDILKYLSAPHGRLTRHLST

LAAFVSDQIQQHQETLDPEAPVRDFIDDFLLKMRQEEKAQGVGPDHTDFLLTTVNLLFAG

TVTVSATLRFAFLLLLKYPEFQDRIHEELNQELGRERAPSLGDRGRLPYTDAFLHEVQRF

LALIPMGVPRTVTKPTIFQGYELPQGIEVFPLLGSVLHDPEFFERPKEFYPRHFLNADGR

FIKNEAFLPFSSGKRICLGEGLARTELFLFFTTILQNFSLESPSPLGALSLHPAISGFAN

IPPTFQLRFRPR

>358419094

MVCSGLVLFILVICLSILIFLFLWNQRHAKGTLPPGPTPLPIVGNILQINIKNVSKSISK

LAEDYGPVFTLYFGMKPTVVLHGYEAVKQVLIDQSEEFSGRGSLPVADNINQGLGIIFSN

GEIWKQTRRFSLMVLRNMGMGKRTIEHRIQEEALCLVEALKKTNGCPCDPTFLLTYAPCN

VICSIIFRNRFEYSDESFLTLAKYIHENATIFSTPWIELYNAFPSLLHYLPGSHNTLFKN

MTEQRKFILEKIKEHQESLDLNNPQDFIDYFLIKMEKEKHNKQSEFTMDNLITTVWDVFT

AGIETTSISLKYGLLLLLKHPEVTAKVQEEINRVVGRNRSPCMQDRSRMPYTDAVIHEIQ

RYIDIVPNNLPHAAAQDIKFREYLIPKGTTILTSLTSVLHDGKEFPNPEQFDPGHFLDKS

GNFKKSDYFMVFSAGKRVCIGEGLARMELFLLLVSILQKFTLKPLVDPKNIDTTPLLKGV

GSIPHFYEVCFIPV

>194215482

MLASGLLLVAFLACLTLMVLMSVWRQGKFLGKLPPGPTPLPFIGNYLQLNTEQMYNSLMK

VSLGVAFSNGERAKQLRRFSITTLRDFGVGKRGIEERIQEEAGFLIEAFRSTRGTLIDPT

FFLSRAVSNVISSIVFGDRFDYEDKEFLSLLRMMLGSFQFTATSTGQLYEMFYSVMKHLP

GPQQQAFKELQGLEDFIAKKVEQNQRTLDPNSPRNFIDSFLIRMQQEQKNPNTEFYLKNL

VLTTLNVFFAGTETVSTTLRYGFLLLMKHPDVEAKVHEEVDRVIGKNRQPKFEDRAKMPY

TEAVIHEIQRFGDMIPMGVARRVTKDTKFRDFLLPKGTEVFPMLGSVLRDPRFFSNPHDF

NPQHFLDEKGQFKKSDGFVPFSIGKRYCFGEGLARMELFLFLTTIMQNFHFKSPQSPQDI

DVSPKHVGFATIPRNYTMSFQPR

>15147328

MLASGLLLVALLACLTVMVLMSVWQQRKSRGKLPPGPTPLPFIGNYLQLNTEHICDSIMK

VSQGVAFSNGERAKQLLRFAIATLRDFGVGKRGIEERIQEESGFLIEAIRSTHGANIDPT

FFLSRTVSNVISSIVFGDRFDYEDKEFLSLLSMMLGIFQFTSTSTGQLYEMFSSVMKHLP

GPQQQAFKLLQGLEDFIAKKVEHNQRTLDPNSPQDFIDSFLIHMQEEEKNPNTEFYLKNL

MMSTLNLFIAGTETVSTTLRYGFLLLMKHPEVEAKVHEEIDRVIGKNRQPKFEDRTKMPY

MEAVIHEIQRFGDVIPMSLARRVKKDTKFRDFFLPKGTEVFPMLGSVLRDPSFFSNPQDF

NPQHFLDDKGQFKKSDAFVPFSIGKRNCFGEGLARMELFLFFTTVMQNFRLKSSQSPKDI

DVSPKHVVFATIPRNYTMSFLPR

>291412176

MLTCVTALLLLALVLALAWWGCGTRRARTPGPLPPGPTPLPLLGNLLQLESGRLDRALME

LSGRWGPVFTVWLGPRPAVVLCSYAALRDALVLQADTFSGRGAMAVFERFTRRNGILFSN

GQRWRTLRNFTLGALKEFGVGTRTIEERVLEEAVCLLDEFQATAGAPLNPRQLLDNAVAN

VICSVVFGHRYGYTDPEFRRLLDLFHDNFRIMSSRWGEIYNIFPSLLDWIPGPHHRIFGN

FTELRVFISEQIQRHRQTRQPEVARDFIDCFLAQMDKEQQDPESHFQRETLVMTTHNLFF

GGTETTSTTLRYGLLILVKYPEVAAKVQAELDAVVGRAHAPRLEDRARLPYTNAVLHEIQ

RFISVLPLGLPRALTRDAHLHGHFLPKSTFVIPLLASSHRDPTQFKDPDCFNPTNFLDDK

GEFQSNDAFMPFALGKRMCLGAGLARSEIFLFLTAILQRFHLLPVGSPANIDLTPQCTGL

GNVPPVFQLRLVARGGLSPATHTQ

>BAF64510.1

MVALLVWMATLLLISIWTHIYSNRRLPPGPFPLPFVGNIFQLEIKNIPKSFTRLAERFGP

VFTLYLGSRRFVVLHGYKAVKEVLLDYRNEFSGRGETPAFQVHQDKGIIFNNGPTWQDTR

RFSLTTLRDFGMGKQGNEQRIQSEAQLLLGALRKTHGQPFDPTFVIGFAPYNVISDILFH

KRADYNDKTALRMLSLFNENFYLLSSPWIQLYNNFPGYIRYLPGSHRKLIKNVSEIKEYA

LEGVKDHQKSLEPSCPRDFTDTMLMEMEKEKHSTDPVYTLDNIAVTVADLLFAGTETTNT

TLRYGLLILMKHPEVEEKLHEEIDRVIGPSRIPAVKDRLDMPYLDAVVHEIQRFIDIIPS

NLSHKATRDTVFRGYVIPKGTVIIPTLDSLLYDSQEFPEPEKFKPEHFLNENGKFKYSDH

FKPFSAGKRACVGEGLARMELFLFLASILQHFNLKSLGDPKDIDLSPIAIGFAKVPPHYK

LCVIPRSQV

>EAW57014.1

MLASGLLLVALLACLTVMVLMSVWQQRKSRGKLPPGPTPLPFIGNYLQLNTEHICDSIMK

VSQGVAFSNGERAKQLLRFAIATLRDFGVGKRGIEERIQEESGFLIEAIRSTHGANIEPT

FFLSRTVSNVISSIVFGDRFDYEDKEFLSLLSMMLGIFQFTSTSTGQLYEMFSSVMKHLP

GPQQQAFKLLQGLEDFIAKKVEHNQRTLDPNSPQDFIDSFLIHMQEEEKNPNTEFYLKNL

MMSTLNLFIAGTETVSTTLRYGFLLLMKHPEVEAKVHEEIDRVIGKNRQPKFEDRTKMPY

MEAVIHEIQRFGDVIPMSLARRVKKDTKFRDFFLPKGTEVFPMLGSVLRDPSFFSNPQDF

NPQHFLDDKGQFKKSDAFVPFSIGKRNCFGEGLARMELFLFFTTVMQNFRLKSSQSPKDI

DVSPKHVVFATIPRNYTMSFLPR

>AAH84884.1

MDVEGLGTFLLVIITCLYIFNTWNTMYKKANLPPGPTPLPLIGNLMNIKKGKMVHSLMKM

WEQYGAVYTLYFGTKPIIVLCGYDAVKEALVDQAENFGARGKIISLDKISQGYGISFSNG

ERWRQMRHFTLKTLKDYGMGKKSIEQKIQEEALCLVEQFRKSGETPVDPSKQIMDAVSRV

FCSIIFGSQFECDDKKFAILLAKVDEIFRLMSCTWGQIENFIPRLMAYIPGPHKKKDTLS

EEVISFLHERVKANQETFDPCSPRDFIDEFLMKLEKEKKNPNSEFTMKNILLTFFSIFLG

GTETSTTTLKHGLLLLIKYPEIQAKLHMEIDHVIGRNRTANITDRNAMPYMEAVLNEIQR

FCDIVPLNVPRKVTKDIQFRGYTIPKGTEIYPLLCTVHRDPKYFSTPYTFNPSHFLDEQG

RFKKSDAMMAFSAGKRICPGESLARMELFLFFTNILQNFTLTSPTHFTEDDIAPRLTGFI

NHPIKYKVSFIPR

>359323287

MALLGLPTFLVACVAFLLFIFVWRRGGTRGRLLPPGPPPLPIIGNILQVNLWDLPNSLSR

LAEQYGSVYSLRLGAHPVVVLHGYQALKEALCGQAVNFEGRGKFPIMDNALRGYGIVFSH

GERWKQMRRFTLMTLRNFGMGKRSIEDRIQEEAQHLMQALSHTQAQPVDPTFIFACAPCN

MIFSILFNERLDYQDKELQQLIMLLNENISIASSFWTQLYNLWPSFIHYLPGRHQKFFKN

IQNIKNFILEKVAQHQETLKPEQPRDYTDCFLDRMEEEKHNPYSEFNLENLVAVGFNLFS

AGTETVTNTLRLALLILLKHPEVEGKIHEEIDRVVGRDRVPCMNDRAQMPYTDAVVHEVQ

RYINLIPSNLPHAVTQDTKFRQFYIPKGTTVFPLLSSVLYDSKEFTNPQRFDPNHFLDEN

GSFQKSDFFVPFSIGKRACLGESLARMEVFLFLTTTLQNFTLKPAVDQRELNIDPMCNGL

LSIRQSFKLCFLPRLK

>327282463

MDVLGTTTIFLVVFLVLLVSWRKVEARRRNWPPGPTPLPIIGNLLQLKGFNISKHLKKLS

ATYGPIFTVFFGSDQMVVVFGYDLVKKVLVEKGDEFLNRGSLPSAEKASRGLGVLMSNGE

RWVQLRRFSLMTLRNFGMGKKSIEERIQEEAQHLVKALRETKGQPLTSSSIFNCATGNVI

SHILLGDRFDYQDKEYLRIINILTYGFRMESSLVGQLYNMFHWIIDYFPGPHLKILEEAF

SIQGFINQKIEEHVKTFDATDVPRDFIEAFLLKMEQEKNNPKTEFTTENLTMTINDLFVA

GMETTSTTLRFILMLLLEHPAVAAKVHEEIDQVIGGERMPTMADRSRMPYTEATLHEAQR

FLDLIPLGLARRARRDVELEGFVIPKGATVLPMLTSLLNDAKQFKNPHRFDPEHFLDEKG

NFKKNGADVPFSAGKRNCLGEGLARMELFLFVTTILQNFRLKFPPGVTKIDLTPDVSGFL

NIPRQVPFCFSPR

>301608090

MDPVSVLLSVVVCIFLYKVFYGGEKESQNFPPGPKPLPLIGNLHIMNMRKPHLTFMELAK

TYGSVFSVQLGLRKTVVLCGADTVRDALINHAEEFSERARIPVFEDITKGHGIVFAHGEN

WKVMRRFTLSTLRDFGMGKKTIEDKICEESDSLVEIFKSYNGKPFDNTLILNSAVANIIV

TILLGDRFDYKDPTLLKLVKVVNQNIRIGGGFMARLYNIYPSVMRWIPGDHKTVFKNIAK

VYKFLNKTFTEHRKVLDVNDQRDLIDAFLVKQQEEKLSSKKFFHNQNLTVLVANLFAAGM

ETTSTTLRWGLLLMMKYPEIQKKIQEEIDRVIGSAEPRLEHRKLMPYTDAVIHEIQRFAN

IAPNNVPHETTQDVTFRGYFIPKGTQVIPMLTSVLRDKAYFKKPEEFYPEHFLDSEGKFV

KNEAFLPFSAGRRSCAGETLAKMELFLFFTKLLQNFTFQPPPGVEVQLTCGVAMTSIPLY

HNICALSRS

>EDM07993.1

MEAASTWALLLALLLLLLALTLPRTPARGQLPPGPTPLPLLGNLLQLRPGALYSGFLRLS

KKYGPVFTVHLGPWRRVVVLVGHDAIREALGGQAEEFSGRGTLATLDKTFDGHGVFFANG

ERWKQLRKFTLLALRDLGMGKREGEELIQEEVQNLVKAFQRTEGRPFNPSMLLAQATSNV

VCSLIFGIRLPYEDKEFQAVIQAASGTLLGISSPWGQAYEMFSWLLQPLPGPHTQLQHHL

GTLAAFTIQQVQRHQGRSHTSGPARDVVDAFLQKMAQEKEDPGTEFTEKNLLMTVTYLLF

AGTMTIGATIRYALLLLLKYPQVQKRVREELIQELGPSRTPSLSDRVRLPYTDAVLHEAQ

RLLALVPMGMPHTVTKTTSFRGYTLPKGTEVFPLIGSVLHDPAVFRNPEEFHPSRFLDDD

GRIRKHEAFLPYSLGKRVCLGEGLARAELWLFFTSILQAFSLDTPCPPGDLSLKPAVRGL

FNIPPDFQLQVWPTGDQSR

>ACI33595.1

MDVLHILQTNFVSIVIGVVVIILLWMNRGKQSNSRLPPGPAQIPLLGNLLGMDVKAPYKL

YMELSKKYGSVFTVWLGSKPVVVISGYQAIKDAFVTQGEEFSGRANYPMIMTVSKGYGVL

VSSGKRSKDLRRFSLMTLKTLGMGRRSIEERVQEEAKMLVKAFSEYGDSVVNPKELLCNC

VGNVICSIVFGHRFENDDPMFQLIQKAVDAYFNVLSSPIGAMYNMFPRIIWCFPDKHHEM

FAVVNKAIAYIQEQAEIRLKTLDTSEPQDFIEAFLVKMLEEKDDPNTEFNNDNMVMTAWS

LFAAGTETTSSTLRQSFLMMIKYPHIQASVQKEIDEVIGSRVPTVDDRVKMPYTDAVIHE

VQRYMDLSPTSVPHKVIRDTEFYNYHIPEGTMVLPLLSSVLADPKLFKNPDEFDPENFLD

ENGVFKKNDGFFAFGVGKRACLGEALARVELFLFFTSLLQRFTFTGTKPPEEINIEPACS

SFGRMPRSYDCYIKLRTEE

>EDL41822.1

MDPFVVLVLCLSFMLLLSLWRQRSARRNLPPGPTPLPIIGNYHLIDMKDIGQCLTNFSKT

YGPVFTLYFGSQPIVVLHGYEAIKEALIDHGEVFSGRGRFPFFDKVSKGKGIGFSHGNVW

KATRVFTVNTLRNLGMGKRTIENKVQEEAQWLMKELKKTNGCAPCNVICSIVFQNRFDYK

DKDFLSLIGKVNECTEILSSPGCQIFNAVPILIDYCPGRHNKFFKNHTWIKSYLLEKIKE

HEESLDVTNPRDFIDYFLIQRRQDKGIEHMEYTIEHLATLVTDLVFGGTETLSSTMRFAL

LLLMKHTHITAKVQEEIDNVIGRHRSPCMQDRKHMPYTNAMVHEVQRYVDLGPTSLVHEV

TCDTKFRNYFIPKGTQVMTSLSSVLHDSTEFPNPEVFDPGHFLDDNGNFKKSDYFVPFSA

GKRICVGESLARMELFLFLTTILQNFKLKPLVDPKDIDTTPKYSGFSKIPPKFQMCFIPV

E

>395509811

MALIGATLALLFWFVFLLFASVWKQIYSSWKLPPGPFPLPIIGNIFNLDIKNIPKSFSKL

AKEYGPVFTIYIASRRIVVLHGYKVVKEALLDLKNEFAGRADVPAFEAHQDSGIIFNNSE

TWRDTRRFSLMVLRDYGMGKKSNEERIQRESQFLIEALRKTNSQPFDPTFILGGAPLNVI

FDILFHHRLDYSDKTCQRLLHLYNENFYLLSTPWLQIYNNVGSFLRYLPGSHWKVLKNIS

EAKQYAFERIKEHQELLDLNNPQDLTDCLLIQMEKDRKNQKIGFDLENVTVTVADLLFAG

TETTSTTLRYALLILLKHPEVEEKLHEEIDRVIGPERLPSMKDKVNMPYMDAVIHEILRF

INLVPSNLPHIMNQDIHFRGYLIPKGTTVYPTLSSIMLDSQEFANPEQFDPGHFLNENGK

FKYSDYFKAFSAGKRVCVGEGLARMELFLFLIIILQHFNLKPLISPEEIDIKPATIGFGS

IPPIYKLCLLPRSEM

>73948306

MMFTALLLLLLLLLLLALARRSWGAQGTRTQGALPPGPTPLPLLGNLLQLESRRLDRALM

ELSGRWGPVFTVRLGPRPAVVLCGYSALRDALVLQADAFSGRGAMAVFERFTHGNGIVFS

NGLRWRTLRNFALGALKEFGLGTRTIEERILEEAACLLGEFQATTGAPFDPRRLLGNAVS

NVICSVVFGNRYGYEDPEFQRLLDLFNDNFRIMSSRWGEMYNVFPTLLDWLPGPHHRIFQ

NFTELRVFISEQIQRHQQTRQPGKPRDFIDCFLDQMDKEQNDPESHFQEETLVMTTHNLF

FGGTETTSTTLRYGLLILLKYPEVAAKVQAELDAVVGQSRTPRLGDREHLPYTNAVLHEI

QRFISVLPLGLPRALTRDTHLHGYFLPKGTFVIPLLVSSHRDPTQFKDPDCFNPTNFLDD

KGEFQTNDAFMPFAPGKRMCLGAGLARSEIFLFFTAILQRFCLLPVGNPANIDLSPQCTG

LGNIPPAFQLRLVAR

>AAA41784.1

MDPFVVLVLSLSFLLLLYLWRPSPGRGKLPPGPTPLPIFGNFLQIDMKDIRQSISNFSKT

YGPVFTLYFGSQPTVVLHGYEAVKEALIDYGEEFSGRGRMPVFEKATKGLGISFSRGNVW

RATRHFTVNTLRSLGMGKRTIEIKVQEEAEWLVMELKKTKGSPCDPKFIIGCAPCNVICS

IIFQNRFDYKDKDFLSLIENVNEYIKIVSTPAFQVFNAFPILLDYCPGNHKTHSKHFAAI

KSYLLKKIKEHEESLDVSNPRDFIDYFLIQRCQENGNQQMNYTQEHLAILVTNLFIGGTE

TSSLTLRFALLLLMKYPHITDKVQEEIGQVIGRHRSPCMLDRIHMPYTNAMIHEVQRYID

LAPNGLLHEVTCDTKFRDYFIPKGTAVLTSLTSVLHDSKEFPNPEMFDPGHFLDENGNFK

KSDYFMPFSAGKRKCVGEGLASMELFLFLTTILQNFKLKSLSDPKDIDINSIRSEFSSIP

PTFQLCFIPV

>73948300

MEAAGTWTLLLALLLLLLLLALARPRTRGHLPPGPPPLPLLGNLLQLRPGALYSGLLRLS

KKYGPVFTVYLGPWRRVVVLVGHEAVQEALGGQAEEFSGRGMLATLDGTFGGHGVFFSNG

ERWRQLRRLTTLALRDLGMGKREGEELIQAEAQSLVEAFQGTVGRPFDPSLLLAQATSNI

ICSLTFGLRFPYEDKEFQAVVQAAGGTVLGVSSPWGQTYEMFSWLLQHLPGPHTQLLSHL

SVLATFAVQQVQRHKESLDTSGPPHDVVDAFLLKMAKEEQDPNTELTDKNLLMTVIYLLF

AGTVTVSTTVRYTLLLLLKYPQVQERVREELSRELGAGRAPGLGDRARLPYTDAVLHEAQ

RLLALVPMGVPRALARTTCFRGYTLPQGTEVFPLLGSVLHDPEIFDEPEEFNPDRFLDAD

GRFQKQEAFLPFSLGKRICLGEGLAHAELFLLLTTILQAFSLESPSPPGALSLQPAVSGL

FNIPPAFQLRVRPH

>344274486

MDPVVVLMLCLSSLLLFSLWKQSYGKGKLLPGPTPLPIIGNILQLDVKDINNSIHSFTKV

XGPVFTLYIGMKPTVVLHGYDAIKEALVDLEEEFSARSHFPIIERANKQFGVAFSNGKTW

KEMWRFCLMTLWNFGMGKRSIEERIQEEAHCLMEELRKTKASPCDPTFILTCAPCNVICS

IIFQKCFDYTDQNFLNLMEKLNENLSILSSPWMQVCNAFLMLIDYVPGSHEKFLNNDAYI

KSYILEKVKEYQESLDINDPWDIXCFLIKMEQGKHNQQSEFITENLLATIADLFGAGTET

TSTALRYGLLLLLKHPEVTAKVQEEVDHVVGQHRSPCMQDRGSMPYKNAIVHEIQRYIDL

IPQNTPRAMTCDIKFRNYLIPKGTTILTSLSSVLHDSKEFPNPEIFDPGRFLDESGNFKK

SDYFLAFSARKXICLGEGLARMELFLFLTTVLQKFTLKPLDDPKHIDITPVANGFVSMPP

SYXLCFIPA

>BAH14057.1

MERSFLEEAIPQYLKELLKDLVGAHISVSALVTGVRGMENRALKSFSAELSLSAWLPSVA

ALSSLAVNSPSFCPFFLLGIISSNGKRWKEIRRFSLTTLRNFGMGKRSIEDRVQEEAHCL

VEELRKTKASPCDPTFILGCAPCNVICSVVFQKRFDYKDQNFLTLMKRFNENFRILNSPW

IQVCNNFPLLIDCFPGTHNKVLKNVALTRSYIREKVKEHQASLDVNNPRDFIDCFLIKME

QEKDNQKSEFNIENLVGTVADLFVAGTETTSTTLRYGLLLLLKHPEVTAKVQEEIDHVIG

RHRSPCMQDRSHMPYTDAVVHEIQRYSDLVPTGVPHAVTTDTKFRNYLIPKGTTIMALLT

SVLHDDKEFPNPNIFDPGHFLDKNGNFKKSDYFMPFSAGKRICAGEGLAPMELFLFLTTI

LQNFNLKSVDDLKNLNTTAVTKGIVSLPPSYQICFIPV

>AAI47911.1

MEAAGTWALLLLLLLLVVTLVLPATWDRGHLPPGPTPLPLLGNLLQLRPGALYLGLLRLS

KKYGPVFTVYLGPWRRVVVLVGHEAVQEALGGQAEEFSGRGTVATLDGTFDSHGVFFSNG

ERWRQLRKFTTLALRDLGMGKREGEELIQAEARCLVEALQGTKGRPFDPSLLLAQATCNI

ICSLVFDLRLPYDNEEFQAVVRAAGGIAVGVSSPWGQTYEMFSRFLQRLPGPHTQLLRHL

GTVAAFAAQQVWQHKGSLGTSGPVRDLVDAFLLKMAKEKQDPNTEFTAKNLLMTVVYLLF

AGTVTVSTTIRYTLLLLLKYPQVQERVQEELMRELGAGQRPSLGDRARLPYTDAVLHEAQ

RLLALVPMGIPRALTKTTRFRGYTLPQGTEVFPLLGSILHDPAVFEEPKEFNPGRFLDAD

GKFKKHEAFLPFSLGKRVCLGEGLARTELFLLFTAILQAFSLEGPCPLGALSLQPAISGL

FNIPQAFQLQFRPR

>BAH11492.1

MFLQPIAKGIISSNGKRWKEIRRFSLTTLRNFGMGKRSIEDRVQEEAHCLVEELRKTKAS

PCDPTFILGCAPCNVICSVVFQKRFDYKDQNFLTLMKRFNENFRILNSPWIQVCNNFPLL

IDCFPGTHNKVLKNVALTRSYIREKVKEHQASLDVNNPRDFIDCFLIKMEQEKDNQKSEF

NIENLVGTVADLFVAGTETTSTTLRYGLLLLLKHPEVTAKVQEEIDHVIGRHRSPCMQDR

SHMPYTDAVVHEIQRYSDLVPTGVPHAVTTDTKFRNYLIPKGTTIMALLTSVLHDDKEFP

NPNIFDPGHFLDKNGNFKKSDYFMPFSAGKRICAGEGLARMELFLFLTTILQNFNLKSVD

DLKNLNTTAVTKGIVSLPPSYQICFIPV

>EAW50016.1

MAAECCSTFFLGCEFSQFLPLFLLGIISSNGKRWKEIRRFSLTTLRNFGMGKRSIEDRVQ

EEAHCLVEELRKTKASPCDPTFILGCAPCNVICSVVFQKRFDYKDQNFLTLMKRFNENFR

ILNSPWIQVCNNFPLLIDCFPGTHNKVLKNVALTRSYIREKVKEHQASLDVNNPRDFIDC

FLIKMEQEKDNQKSEFNIENLVGTVADLFVAGTETTSTTLRYGLLLLLKHPEVTAKVQEE

IDHVIGRHRSPCMQDRSHMPYTDAVVHEIQRYSDLVPTGVPHAVTTDTKFRNYLIPKGTT

IMALLTSVLHDDKEFPNPNIFDPGHFLDKNGNFKKSDYFMPFSAGKRICAGEGLARMELF

LFLTTILQNFNLKSVDDLKNLNTTAVTKGIVSLPPSYQICFIPV

>348552512

MEATGTWALLLVLLLLLLSLALPASRAPGHLPPGPAVLPLLGNLLQLRPGALYSGLLRLS

KKYGPVFTVHLGPWRRVVVLVGHEAVREALGDQAEEFSGRGTLATLDQIFNCHGVFFANG

ERWRQLRKFTMQALRDLGMGKPEGEELIQAEVQHLVEALQRTEGHPFDPSLLLAQATSNI

VCLLLFDLRFSYEDKEFQAVIQAAGGTLLGISSTYGQIYEMFSWILKLLPGPNTQLLRYL

DTLATFTSQQVQQHQGNLDTSGPARDLVDAFLLKMAQEEKNLSTEFTEKNLLMTVTYLLF

AGTMTTGATVRYALLLLLKYPEVQKQVREELMRELPAGQAPSLRDRPHLPFTDAVLHEAQ

RLLALVPMGMPHVITKTTSFRGYTLPKGTEVFPLLGSVLHDPTIFEQPEKFNPGRFLDAE

GRFKKPKAFLPYSLGKRICLGEGLAQAELFLLFTAILQAFYLDSPCPPDTLSLQPAVSGL

FNIPPAFQLQVHPADFHPTVQTR

>P11510.1

MDPFVVLVLSLSFLLLLYLWRPSPGRGKLPPGPTPLPIFGNFLQIDMKDIRQSISNFSKT

YGPVFTLYFGSQPTVVLHGYEAVKEALIDYGEEFSGRGRMPVFEKATKGLGISFSRGNVW

RATRHFTVNTLRSLGMGKRTIEIKVQEEAEWLVMELKKTKGSPCDPKFIIGCAPCNVICS

IIFQNRFDYKDKDFLSLIENVNEYIKIVSTPAFQVFNAFPILLDYCPGNHKTHSKHFAAI

KSYLLKKIKEHEESLDVSNPRDFIDYFLIQRCQENGNQQMNYTQEHLAILVTNLFIGGTE

TSSLTLRFALLLLMKYPHITDKVQEEIGQVIGRHRSPCMLDRIHMPYTNAMIHEVQRYID

LAPNGLLHEVTCDTKFRDYFIPKGTAVLTSLTSVLHARKEFPNPEMFDPGHFLDENGNFK

KSDYFMPFSAGKRKCVGEGLASMELFLFLTTILQNFKLKSLSDPKDIDINSIRSEFSSIP

PTFQLCFIPV

>334313780

METSGLPVSISNYGRDRIQMHGEQRSRLTSPAPYRLPETRTRSRQASLLSPVSYLMVKCR

APRSDALLVNAHSPNTDITFTLMTFTILTLLNIFFLSLWKKDYKGRNLPPGPVPLPIIGN

ILQIDHKNFFMSLNKLYEKYGPVYTIYLGSQPVVVLHGYKALKEALIDQGDIFGGRGKVP

VFENTFKGNGIVFSHGEKWKQIRQFSLMTLRKFGMGKRSIEERIQEEAQFLIKELKKTNS

QPFNPHVILQYAPFNVISSILFHQRFNYDNEDFLSMMHILNENAILLNSPMIQLYNYFPW

LFHYFMSHHKKFFSNTEATKKIFLDQVKKHQDTLDLNNPQNLVDCYFHKMQQEQKNPDSV

FDMENLAAIGVDLFGAGTTTTSTTLAFSLFLILKHPEVQDKIHEEIDQVIGPHRIPSIKD

KLEMPYTDAVLHEIQRYINIAPISMPHEVTHDTQFRQYFIPKGTMVFPFLSSVLFDPIEF

PNPYQFDPGHFLHQDGRFKNSDYFMPFSIGKRACLGEGLAKMELFLLLVTILQNFTIKSV

IDPKEIIIKPMSTRGVSCPPKYQLCFFPR

>395751226

MEATGTWALLLALLLLLTLALSGTRARGHLPPGPTPLPLLGNLLQLRPGALYSGLMRLSK

KYGPVFTIYLGPWRPVVVLVGQEAVREALGGQAEEFSGRGTVAMLERTFDGHGVFFSNGE

RWRQLRKFTMLALRDLGMGKREGEELIQAEARCLVEAFQGTEGRPFDPSLLLAQATSNVV

CSLLFGLRFSYEDKSSMAVVQAAGWYPAGQSAPGGGQTYEMFSWFLRPLPGPHKQLLHHV

STLGLPSQSGQVQQHQGNLDASGPARDLVDAFLLKMAQEEQNPDTEFTNKNMLMTVIYLL

FAGTMTVSATVGYTLLLLMKYPHVQKRVREELNRELGAGQAPSLGDRTRLPYTDAVLHEA

QRLLALVPMGIPRTLMRTTHFRGYTLPQGTEVFPLPALLGSILHDPNIFKHPEEFNPDRM

LDADGRFRKHEAFLPFSLGKRVCLGEGLAKAELFLFFTTILQAFSLESPCPPGTLSLKPT

ISGLFNIPPAFQLQVRPTDLHSTTQTR

>EGW03894.1

MELLGVTTLVLVVSVICLSFLSVWTKTRPGGRLPPGPTPLPVIGNILQLNFKDVPASLSK

LAKDYGPIYTLYFGSWPTVVLHGYDVVKEALLNQGDEFLGRGPFPIIEDSLKGSGLVFTN

GETWKVLRRFSLMTLKNFGMGKRSLEERVQEEAQCLVEELQKMEAGPFDPTFILACAPCN

VICSILFNERFPYRSKTILNLMDLLNKNFHQVNSVWCQMYNLWPTFMKYVPGKHKEVPQR

INDINNFILEKIKEHQESLDPANPRDYIDCFLSKIEEEKHNPKSEFDLKNLIASGASLFT

AGTETTSITLRFGILLLLKYPEVQAKIHEEIDHVIGRHQPPSMKDKMKLPYTNAVLHEIQ

RYTTLLPSSLPHAVVKDTKFRDYIIPKRNIYIYMMVTRGKRSCMGESLAIMELFLFLTTI

LQKFSLKSPVEPRDLDIKPAATGLINIPPPYKLCLIPR

>EDL24204.1

MLLLWWGGVVRRQAQMQKDLPPGPAPLPLLGNLLQLQSGDLDRVLMELSSHWGPVFTVWL

GPLPAVVLCGYEALRDALVLQADAFSGRGAMAVFDRFTCGNGIVFSNGPRWHSLRNFALG

VLRELGVGRSTIEDRILEEAACVLDEFQATMGAPFDPQQLLDSAVSNVICTVVFGKRYDY

GDPEFRRLLNLFSDNFCIMSSRWAEIYNMFPSFMDWIPGPHNRIFKNFQELRLFISEQIQ

WHWQSRQTGEPRDFIDCFLDQMDKEQQDLESHFQDETLVMTTHDLFFGGTETTSTTLRYG

LLIMLKYPEVAAKVQEELDATVGRTWAPRIEDRARLPYTNAVLHEIQRFISVLPLGLPRA

LTRDVNLKNHFLHKGTFVIPLLVSAHRDPTQFKDPDHFNPTNFLDDHGEFQNNDAFMPFA

LGKRMCLGAGLARSEIFLFLTAILQKFSLLPVGSPANINLNPQCTGLGNVPPAFQLRLVA

R

>395741846

MPMMTLAEEKPSVDPAVALVLCLSCLFLLSLWRQSSGRGRLLSGPTPLPIIGNILQLDVK

DMSKSLSNFSKVYGPVFTVYFGLKPIVVLHGYEAVKEALIDHGEEFSGRGSFPLAEKVNK

GLGILFSNGKRWKEIRHFSLMTLRNFGMGKRSIEDRVQEEALCLVEELRKTNGAARSFMI

VSGCWSSYGGWRFEAESQGRFCAPMLVVFVHNDYAYGVCNNFPALIDYLPGSHNKVAENF

AYIKSYVLERIKEHQESLDMNNARDFIDCFLIKMEQEKHNQQSEFTVENLIATVTDMFGA

GTETTSTTLRFGLLLLLKDPEVSAKVQEEIERVVGRNRSPCMQDRSHMPYTDAVVHEIQR

YIDLIPTNLPHAVTCDVKFRNYLIPKGMTIITSLTSVLHNDKEFPNPEMFDPGHFLDKSG

NFKKSDYFMPFSAGKWMCVGEGLARMELFLFLTTILQNFNLKSQVDPKDIDITPIANAFG

RVPPLYQLCFIPV

>CAJ83629.1

MDPVSVLLSVVVCIFLFKFFYGGEKGSQNFPPGPKPLPLIGNLHMINMKKPYLTFMELAE

KYGPVFSVHLGANKAVVLCGTDAVKDALINYADEFSGRPKTPLFEQTFKGNGIVFADGEN

WKVMRRFTISTLRDFGMGKKTIEDRIIEESCCLVETFKSHKGKPFDNTMILNAAVANIIV

HILLNHRFEYQDPTLLKLIKGVNENVRNGARPIVMLYNAFPSIIQWIPGTHKRIFANTQH

FFNILKEIFIEHRDQLDVNDQRDLIDTFLVKQQEEKSSSTKFFHDENLKVLIGNLFAAGM

ETTSTTLRWGILLMMKYPEIQKKAQDVDRVIGSAEPRLEHRKLMPYTDAVVHEIQRFANL

VPNGLPHATTTDVTFRGYFIPKGTQVIPLLTSVLRDKAYFKKPEEFYPEHFLDSKGNFLK

NEAFLPFSAGKRTCAGETLAKMELFLFFTKLLQNFTFQPPPGVEVQLTRGVSLTSIPLDH

KICALSRS

>AAI60414.1

MDPVSVLLSVVVCIFLYKVFYGGKGASQNFPPGPKPLPLIGNLHMMNMKKPHLTFMEFSK

KYGPVFSIQLGLNKAIVLCGADAVKDALINHGDEFSGRPKIPVFDQISKGYGVVFADGEN

WKVMRRFALSTLRDFGMGRKTIEDTIVEESGCLVETFKSHEAKPFDNTLILNAAVANIIV

HILLNHRFEYQDPTLIKLIKSVSENVKIAGSPIVMLYNTYPSIMGWIPGSHKTVFENFQK

LSNFLKETFTKRRDQLDVNDQRDLIDAFLVKQQEEKSSSKKFFHDENLKVLLGDLFAAGM

ETTSTTLRWGILMMMKYPDIQKKVQDEIDRVIGSAEPRLEHRKQIPYTDAVIHEIQRFAN

LVPIVLPHSITEDVTFRGYFLPKGTQVIPLLISVMQDKDYFQKPEEFYPEHFLDSKGNFV

KNEAFLPFSVGKRSCVGETLAKMELFLFFTKLLQNFTFQPPPGVEVQLTCGDALTSIPLD

HQICALPRS

>358419092

MDLAVVLVLCLSCLLLLSLWKQSSGKGKLPPGPTPLPILGNILQLDVKNISKSLTNFLFL

LLVGILFSNGKTWKEIRRFSLMTLRNFGMGKRSIEDRVQEEACCLVEELRKTNGLPCDPT

FILGCAPCNVICSIIFQNRFDYKDPVFLDLMERLNEILRILSSPWVQVCNNFPALFDYLP

GSHNKVLKNVANLKSFVLEKAMEHKASLDINNPRDYIDCFLIRMEQEKQNQQLEFTLENL

TTTVFDLFGAGTETMSTTLRYGLLLLLKHPEVTAKVQEEIDRVIGRHRSPCMQDRSHMPY

TDAVVHEIQRYIDLVPSSLPHMVTHDIELRNYIIPKGTGVLVSLTSVLYDDKVFPNPEMF

DPGHFLDDSGNFKKSDHFMPFSAGKRICAGESLARMEVFLFLTVILQKFTLKSVVDPKDI

DTTPIANGFASVPPPYKLCFIPL

>126272238

MAFLGATLALLVWFVFLLFVSVWKQIQSSWKLPPGPFPLPIIGNLFTLDIRNFPKSFAKL

AKEYGPVFTVYVGSRRIVVLHGYKVVKEALLDLKNEFAGRADIPAFEAHQNSGIIFNDSE

TWRDTRRFGLTVLRDYGMGKQSNEERIQRESHFLVEALRNTNSQPFDPTFVLGGGPLNVI

HDILFHSRLDYSDKMCQRLLHLYNEDFYLLSSPWIQVYNNIGSYLRYLPGSHRKLLKNIS

EAKQYVFEKVKEHQEVVDLNHPYDLTDTLLIQMEKDQKKKKLGFNLENVTVTVADLLFAG

TETTSTTLRYGLLLLLKHPKVEEKLHEEIDRVIGPHRLPSMKDKINMPYMEAVVHEIQRV

INLVPSNLPHVMHEDIHFRGYLLPKGTTVYPTLDSVMLDSEEFPNPEQFDPGHFLNENGK

FRYSDYFKAFSAGKRVCVGEGLARMEIFLFLTIILQHFNLKPLISPEKIDTSPLTVGFGT

IPPIYKLCVLPRS

>402881021

MFLQSLAKGIISSNGKRWKETRRFSLTTLRNFGMGKRSIEDRVQEEARCLVEELRKTKAS

PCDPTFILGCAPCNVICSVVFQKRFDYKDENFLTLMKRFTVNFRILTSPWIQVCNNFPLL

IDCFPGTHNKLLKNVALTKSYIREKVKEHQATLDVNNPRDFIDCFLIKMEQEKDNQQSEF

TIENLVGTVADLFVAGTETTSTTLRYGLLLLLKHPEVTAKVQEEIDHVIGRHRSPCMQDR

SHMPYTDAVIHEIQRYIDLVPTGVPHAVTTDIKFRNYLIPKGTIIITLLTSVLHDDKEFP

NPKIFDPGHFLDENGNFKKSDYFMPFSAGKRICAGEGLARMELFLFLTTILQNFNLKSVA

DLKNLNTTSATRGIISLPPSYQICFIPV

>297687083

MFFQPIAKGIISSNGNRWKEIRRFSLTTLRNFGMGKRSIEDRVQEEARCLVEELRKTKAS

PCDPTFILGCAPCNVICSVVFQKRFDYKDQNFLTLMKRFNENFRILNSPWIQVCNNFPLL

IDCFPGTHNKLLKNVALTRSYIREKVKEHQTSLDVTSPRDFIDCFLIKMEQEKDNQKSEF

NIENLVGTVADLFVAGTETTSTTLRYGLLLLLKHPEVTAKVQEEIDHVIGRHRSPCMQDR

SHMPYTDAVVHEIQRYIDLVPTGVPHAVTTDIQFRNYLIPKGTTIMTLLTSVLHDDKEFP

NPKIFDPGHFLDKNGNFKKSDYFMPFSAGKRICAGEGLARMELFLFLTTILQNFNLKSVD

DLKNLNTTAVTKGIVSLPPSYQICFIPV

>EAW61358.1

MVVMHGYKAVKEALLDYKDEFSGRGDLPAFHAHRDRGIIFNNGPTWKDIRRFSLTTLRNY

GMGKQGNESRIQREAHFLLEALRKTQGQPFDPTFLIGCAPCNVIADILFRKHFDYNDEKF

LRLMYLFNENFHLLSTPWLQLYNNFPSFLHYLPGSHRKVIKNVAEVKEYVSERVKEHHQS

LDPNCPRDLTDCLLVEMEKEKHSAERLYTMDGITVTVADLFFAGTETTSTTLRYGLLILM

KYPEIEEKLHEEIDRVIGPSRIPAIKDRQEMPYMDAVVHEIQRFITLVPSNLPHEATRDT

IFRGYLIPKGTVVVPTLDSVLYDNQEFPDPEKFKPEHFLNENGKFKYSDYFKPFSTGKRV

CAGEGLARMELFLLLCAILQHFNLKPLVDPKDIDLSPIHIGFGCIPPRYKLCVIPRS

>292621096

MELSSSLLLVLVLTVLMLIRWRRKENGLSLPPGPLALPLIGNLLTVETSAPFKSFMKWSK

SYGPVMTVHIGTQRMVVLVGYETVKEALVDQAEDFASRAPIPFTNRVLRGYGLIISNGER

WRQLRRFTLTTLRDFGMGRKRMEQWIQEESRYLLKSFEETKSKPVDPLFFMSRAVSNVIC

SLVFGQRFDYEDKNFLQLLQIISNLMRFASSPWGQLYNIFPKVMEILPGRHHTMFGEIDD

LKSSIMTIIKEHEENLDPSDPKDFIDCFLIRLNQEKHNPDTEFHKENMFATSLNLFTAGT

ETTSTTLRYALMLLIKHPHIQEQMQKEIDCVIGQNRIPTMEDRKSLPFTDAVIHEVQRCL

DIAPLNVPHYALKDITFRGYKIPKDTVIIPMLHSVLRDEGHWETPWTFNPEHFLDSNGNF

KKNPAFMPFSAGKRVCVGESLARMEIFLFIVSLLQKFSFSSPNGPDSIDPSPELSSFGNM

PRLYELIASPR

>332212382

MFLQPIAKRIISSNGKRWKEIRRFSLTTLRNFGMGKRSIEDRVQEEAHCLVEELRKTKAS

PCDPTFILGCAPCNVICSVVFQKRFDYKDQNFLTLMKRFNENFRILNSPWIQVCNNFPLL

IDCFPGTHNELLKNVGLTKSYIMEKVKEHQASLDVNNPRDFIDCFLIKMEQEKDNQQSEF

NIENLVGTVADLFVAGTETTSTTLRYGLLLLLKHPEVTAKVQEEIDHVIGRHRRPCMQDR

SHMPYTDAVVHEIQRYIDLVPTGVPHAVTTDIKFRNYLIPKGTTIMTLLTSVLHDDKEFP

NPKIFDPGHFLDKNGNFKKSDYFMPFSAGKRICAGEGLARMELFLFLTTILQNFNLKSVD

DLKNLNTTAVTKGIVSLPPSYQICFIPV

>301608062

MTAFLAMFPLEPTTLFVAIVLCLFLIYLLLHNGKGTPPNFPPGPKPLPFIGNLHIMNLNK

PHKTYMELGNKYGSVFSVQLGTEKVVVLCGYDAVKDALINHAEEFSERAVSTLSRKRLKG

YGIIFSHGENWKVMRRFTLATLRDFGMGKRTTEDTINEECNFLMETFKSYKGEPFETNLI

MNAAVANIIVSILLGHRFEYQDPTLLKLIGLVNEIVKLSGRPIIMIYDAFPSVVSWLPGS

HQKVLENTRGLRNFIKETFTEHKARLDINDQRDLIDVFLVKQREEKPNPGLFFHNENLIS

LVSNLFVAGMETTSTTLRWGLLLMMKYPEIQKKVQNEIDKVIGSAQPQMEHRKQMPYTDA

VIHEIQRFADIVPTNLPHATTMDVTFRGYLIPKGTRVIPLLTSVLRDKAYFEKPYEFYPE

HFLDSEGNFVKNEAFIPFSAGKRICAGETLAKMELFLFFTNLLQNFTFRSPPGQDLPLTT

AEGFTSIPMVHKICAVSRA

>395828397

MDPAVALVLCLSCLLLLFLWRQSSGRGKLPPGPTPLPIIGNSLQMDIKDIRKSIKNVSRG

IVFSNGNRWKEIRRFSLMTLRNLGMGKRSIEERVQEEARCLVEELRKTKALSCDPTFILG

CAPCNVICSIIFQDRFDYKDQNFLTLMGRLNENFRILSSPWIQLCNNFPALIDYFPGIHN

KLLKNVALTKSYILEKVKKHQESLDINNPRDFIDCFLIKMEQEKNNSESAFTIENLVSSV

SDLFGAGTETTSTSLRYALLLLLKHPEVTAKVQEEINHVIGRHRSPCMQDRSRMPYTDAM

VHEVQRYIDLIPNNALRAVTHDIKFRNYLIPKGTMIMSSLTSVLHDDNEFPDPETFNPAH

FLDESGNFKKSDFFMPFSTGKRICLGEGLARMELFLFLTSILQNFNLKSSIDTRKIDITS

VPSGTISLPPLYKICFIPV

>332835435

MSALGVTVALLVWAAFLLLVSMWRQVHSSWNLPPGPFPLPIIGNLFQLELKNIPKSFTRL

AQRFGPVFTLYVGSQRMVVMHGYKAVKEALLDYKDEFSGRGDLPAFHAHRDRGIIFNNGP

TWKDIRRFSLTTLRNYGMGKEGNESRIQREAHFLLEALRKTQGQPFDPTFLIGCAPCNVI

ADILFRKHFDYDDEKFLRLMYLFNENFHLLSTPWLQLYNNFPSVLHYLPGSHRKVIKNVA

EIKEYVSERVKEHHQSLDPNCPRDLTDCLLVEMEKEKHSAERLYTMDGITVTVADLFFAG

TETTSTTLRYGLLILMKYPEIEAYMDAVVHDVQRFITLVPSNLPLEATRDTIFRGYLIPK

GTVVVPTLDSVLYDNQEFPDPEKFKPEHFLNENGKFKYSDYFKPFSTGKRVCAGEGLARM

ELFLLLCAILQHFNLKPLVDPKDIDLSPIHIGFGCIPPRYKLCVIPRS

>332212374

MVLQWLAKGIVFSNGKRWKEIRRFSLMTLRNFGMGKRSIEDRVQEEARCLVEELRKTKAS

PCDPTFILGCAPCNVICSIIFQKRFDYKDQQFLNLMEKLNENVRIVSTPWIQICNNFPPI

IDYFPGTHNKLLKNIAFMESYILEKVKEHQESIDINNPRDFIDCFLIKMEKEKQNQQSEF

TIENLVITAADLLGAGTETTSTTLRYALLLLLKHPEVTAKAQEEIERVIGRNRSPCMQDR

GHMPYTDAVVHEVQRYIDLIPTNLPHAVTCDVKFRNYLIPKGTTILTSLTSVLHDNKEFP

NPEMFDPRHFLDGGGNFKKSNYFMPFSAGKRICVGEGLARMELFLFLTFILQNFNLKSLI

DPKDLDTTPVVNGFASVPPFYQLCFIPV

>EDL24205.1

MDGVSTAILLLLLAVISLSLTFSSRGAYPVFFNFTRGNGIAFSDGERWKILRRFSVQILR

NFGMGKRSIEERILEEGSFLLEVLRKMEGKPFDPVFILSRSVSNIICSVVFGSRFDYDDE

RLLTIIHFINDNFKIMSSPWGEMYNIFPSVLDWIPGPHKRLFRNFGGMKDLIARSVREHQ

DSLDPNSPRDFIDCFLTKMAQEKQDPLSHFNMDTLLMTTHNLLFGGTETVGTTLRHAFLI

LMKYPKVQARVQEEIDRVVGRSRMPTLEDRTSMPYTDAVIHEVQRFADVIPMNLPHRVTR

DTPFRGFLIPKGTDVITLLNTVHYDSDQFKTPQEFNPEHFLDDNHSFKKSPAFMPFSAGR

RLCLGEPLARMELFIYFTSILQNFTLQPLVDPEDIDLTPLSSGLGNLPRPFQLCMHIR

>BAE28959.1

MAEKIIKGFGVVFSNGNRWKEMRRFTLMTLRNLGMGKRNIEDRVQEEAQCLVEELRKTKG

SPCDPTFILSCAPCNVICSIIFQNRFDYKDKEFLILMDKINENVKILSSPWLQVCNSFPS

LIDYCPGSHHKIVKNFNYLKSYLLEKIKEHKESLDVTNPRDFIDYYLIKQKQVNHIEQSE

FSLENLASTINDLFGAGTETTSTTLRYALLLLLKYPDVTAKVQEEIDRVVGRHRSPCMQD

RSHMPYTDAMIHEVQRFIDLLPTSLPHAVTCDIKFRKYLIPKGTTVITSLSSVLHDSKEF

PNPEMFDPGHFLNGNGNFKKSDYFMPFSTGKRICAGEGLARMELFLILTTILQNFKLKSL

VHPKEIDITPVMNGFASLPPPYQLCFIPL

>332834695

MLLQWLAKGIVFSNGKKWKEIRRFSLMTLRNFGMGKRSIEDRVQEEARCLVEELRKTKAS

PCDPTFILGCAPCNVICSIIFHKRFDYKDQQFLNLMEKLNENVKILSSPWIQICNNFSPI

IDYFPGTHNKLLKNVAFMKSYILEKVKEHQESMDMNNPQDFIDCFLMKMEKEKHNQPSEF

TIESLENTAVDLFGAGTETTSTTLRYALLLLLKHPEVTAKVQEEIERVIGRNRSPCMQDR

SHMPYTDAVVHEVQRYIDLLPTSLPHAVTCDIKFRNYLIPKGTTILISLTSVLHDNKEFP

NPEMFDPHHFLDEGGNFKKSNYFMPFSAGKRICVGEALARMELFLFLTSILQNFNLKSLV

DPKNLDTTPVVNGFASVPPFYQLCFIPV

>327276269

MEWAGAVILLLVFFFSCPLLMSSKRKRLHKGKPPPGPTPLPLIGNFLQMKSSEILKSLLK

VSSGVGFANGERWKELRRFSLAVLRSFGMGKKSIEQRIQEEAQFLLEEFRKTKEKPFEPT

TLLSHMFTNIICSIVFGNRFDYEDKEFQALLVMVNEFFWEMSTSWAQFYDVYANYLNYIP

GIYSKLYDSKDLRLFVAKRIKKNQETFDPNFPRDYIDCFLIQMEKEKDNSDSEYNIKNLQ

LSILNLILGGSETGSCTLKYGFLFLTKYPEVQAKVHEEIDRVIGHDRVPNTEDRRQMPYT

DAVIHEVQRCSDVLPMSLAHMVTCDTELRGYLIPKGMTVYPILSTVLHDPTMFKSPNAFS

PENFLDENGCFKKNDAFVPFSSGKRNCLGEVLARMELFLFFTTILQSFQLKSLVPPEDID

LTPQESGFANIPPFCHLSVIPR

>301608080

MVSFEPITLFLAIVICLFLIYLVYGGKGTPPNFPPGPKPLPLIGNLHIINLKKPYMTFME

LGKKYGSVFRVQLGTEKVVVLCGYDAVKDALINHAEEFSDRPIIETFHRRSNGHGITFSH

GENWKVMRRFTIATLRDFGMGKRTIEDRINEECHSLVETFQSYKGEPFETNLIMNAAVAN

IIVSILLGHRFEYQDPTLLKLIGLSNEMVRILGSPIVLLYNAYPSVMKWLPGSHHNVIKN

TQKSHTFIKETFTEHKAQLDINDQRDFIDAFLAKQSEEKPNPGLFFHNENLVSLVDGLFV

AGMETTSTTLRWGLLLMMKYPEIQKKVQDEINKVIGSAQPQTEHRKQMPYTDAVIHEIQR

FADIIPANLPHATTKDVTFRGYFIPKGTQVIPMLTSVLRDKDYFERPYEFYPQHFLDSEG

NFVKNEAFLPFSAGKRSCAGETLAKMELFLFFTNLLQNFTFQPPPGQDLNLTTTGGFTSI

PMVHKICALSRN

>402912717

MDSIRLVVFLLVLCYKIKIPSALSGGRQDWEEKPSVDLTVALVLCLSCLLPRSLWSQISG

RARLPSSPTPLPIIGNILQLDVKDMSKSLTNFSKVYGPVFIVYFGLKPIVVLHGYEAVKE

ALIDHGEKFSGRGSFPVAEKVNKGLGILFSNGKRWKEIRRFSLMTLRNFRMGKRSIEDHV

KEEALCLVEELRKTNASPCDPTFILGCAPYNVICFIVFYNRFDYKDXRFLFLIKRFNENL

RILSFPWIQLMCHFSPFPSFLTCILSTKVQEEIECVVGRNWSPCMWDRSHMLYTDAVVHE

IQRYIDLIPTNLPHAVTCDVKFRNYLIPKLSKVYGPVFTLYFGLERMVVLHGYEAVKEAL

IDLGEEFSGRGHFPLFERANRRFGIVFSNGTRWKEIRRFSLMTLRNFGMGKRSIEDRVQE

EARCLVEELRKTKASPCDPTFILGCAPCNVICSIIFHKRFDYKDQQFLNLMEKFNENAKI

LSSPWIQICNNFSPIIDYFPGTHNKLLKNVAFVKSYILEKVKEHQESMDMNNPRDFIDCF

LIKMEKEKHNQQSEFTIENLENAAVDLFAAGTETTSTTLRYALLLLLKHPEVTAKVQEEI

ERVIGRNRSPCMQDRSHMPYTDAVVHEVQRYIDLLPTSLPHAVTCDVKFRNYLIPKGTTI

LISLTSVLRDNKEFXXXXXXXXRQCLSQSQLHTRSNCSLCFLFSGKRICVGEGLARMELF

LFLTSILQNFNLKSLVDLKDLDTTPVFNGFVSVPPVYQLCFVPV

>395859633

MEAVGTWALLLVLLLLLLLALVLPGTRARGHLPPGPAPLPLLGNLLQLRPGALYSGFVRL

SKKYGPVFTVYLGPWRRVVVLVGHKAVQEALGGQAEEFSGRGALATLDGTFDGHGVFFSN

GVRWKQLRKFTMLALRDLGMGKPEGEKLIQSEAQHLVEAFWETKGHPFDPSLLLAQATSN

VACSLIFGLRFPYEDEEFQAVVRAAGGTLLGVSSPWGQTYEMFFRLLRPLPGPHTQLFHH

LGTLAAFAIQQVQQHQGSLDTSGPARDLVDAFLLKMAQEEQDPNTEFTEKNLLMTVTYLL

FAGTMTVSATVGYALLLLVKYPQVQKCVHEELTRELGDGRAPGLEDRTRLPYTNAVLHEA

QRLLALVPMGIPRALTRTTCFRGYTLPQGTEVFPLLGSVLRDPEVFEQPEEFNPGRFLDV

DGRFKKHEAFLPFSLGKRVCLGEGLARAEIFLLFTTILQAFSLESPCLPGTLSLQPAVSG

LFNVPPAFQLQVQPADLHPTMQTR

>301786653

MALFSLPTLLVVYVAFLLFIFAWRRGNTRGLLLPPGPPPLPIIGNILQVKLWDLPTSLSR

LAEQHGPVYSLRLGPHPIVVLHGLQALKDALCGQAVNFGGRGRLPIMDNALRVYRLSFSH

GRRWEQMHQFTLTTLRNFGMGKRSMEERVQKEAQHLVRALAQTEAQPVDPAFILVCAPCN

IICSILFNEHFDYHDEEFLKLIRLLNENLSILSSFWVQLYSLWPSFIHYLPGRHQKVFKN

IQNIMDFILERVAQHQRTPKPVQPRDYTDCFIERMEEEKHDSYSEFNSENLAAVSLNLFS

AGTETVSRTLWLALLTLLKRPEVESRVHEEIDRVVGRDRVPCMKDRAQMPYTDAVVHEVQ

RCINLVPSNLPHVVIQETKFRQFFLPKGTTVFPLLSSVLYDSKEFANPQRFDPNHFLDKN

GNFRKSDFFIPFSIGKWACLGEGLARMEIFLFLTTTLQNFTLKPVVDQRELSTDPVYNGL

LRIIPASKLCFLPRPK

>154146204

MERARHDRMVTCLALLLLLLILMLLLWWGGVVRRQAQMQKDLPPGPAPLPLLGNLLQLQS

GDLDRVLMELSSHWGPVFTVWLGPLPAVVLCGYEALRDALVLQADAFSGRGAMAVFDRFT

CGNGIVFSNGPRWHSLRNFALGVLRELGVGRSTIEDRILEEAACVLDEFQATMGAPFDPQ

QLLDSAVSNVICTVVFGKRYDYGDPEFRRLLNLFSDNFCIMSSRWAEIYNMFPSFMDWIP

GPHNRIFKNFQELRLFISEQIQWHWQSRQTGEPRDFIDCFLDQMDKEQQDLESHFQDETL

VMTTHDLFFGGTETTSTTLRYGLLIMLKYPEVAAKVQEELDATVGRTWAPRIEDRARLPY

TNAVLHEIQRFISVLPLGLPRALTRDVNLKNHFLHKGTFVIPLLVSAHRDPTQFKDPDHF

NPTNFLDDHGEFQNNDAFMPFALGKRMCLGAGLARSEIFLFLTAILQKFSLLPVGSPANI

NLNPQCTGLGNVPPAFQLRLVAR

>BAJ83936.1

MDWVNIAPEGLLFILVLLLILKRQHVWNSHTRDNFPPGPRALPIIGNLHTVDLKRPYLTM

LELAKTYGPVFSIQMGPKKMVVLSGYDTVKEALVNQADAFAGRPKIPILEKAGKGREKGV

IFSYGENWKVMRRFTLTTLRDFGMGKKAIEDRVVEEYRCLADTIEAQKGKPLEMTLMLNA

AVANVIVSILLGKRYDYEDPTFKRLLLLVNENARILGAPSISIYNMFPFLGFLLKDYKIF

IQNVKEISAFIKVTFIEHLKALDRNDQRSFIDAFLVRQQEENGKANEYFSNENLIEVVRN

LFAAGMETTSTTLRWGLLLMMKYPEIQKKVQEEIEQVIGSTPPRIEHRTQMPYTDAVVHE

IQRFANILPLNLPHETTADVTLKGYFIPKGTYIIPLLASVLRDKSQWEKPDIFYPEHFLD

SEGKFVKKEAFIPFSAGRRICAGETLARMELFLFFTSLLQRFTFRPPPGVSISDLDLSPT

FGITIPPVTHEVCAVPRS

>268835091

MELLGLPTLALLVLVMSLSLLSVWTKMRTGGRLPPGPTPLPIIGNILQLDLKDIPASLSK

LAKEYGPVYTLYFGSWPTVVLHGYDVVKEALLNQGDEFLGRGPLPIIEDSQKGHGIVFSE

GERWKLLRRFSLMTLKNFGMGKRSLEERVQEEARCLVEELHKTEAQPFDPTFILACAPCN

VICSILFNERFPYNDKTFLNLMDLLNKNFYQLNSIWIQMYNLWPTIMKYIPGKHREFSKR

LGGVKNFILEKVKEHQESLDPANPRDYIDCFLSKIEEEKHNLKSDFNLENLAICGSNLFT

AGTETTSTTLRFGLLLLVKHPEVQAKVHEELDRVIGRHQPPSMKDKMKLPYTDAVLHEIQ

RYITLLPSSLPHAVVQDTKFRHYVIPKGTAVFPFLSSILLDQKEFPNPEKFDPGHFLDKN

GCFKKTDYFVPFSLGKYTRFGQLLASLNYVLRDPLM

>301608088

MDPVSVLLSVVVCIFLYKVFYGGKERPENFPPGPKPLPLIGNLHIMNMRKPHLTFMELST

TYGSVFSFQLGLEKIVVLCGTDTGKTLLSNLCCHLTNRLFFSGIVFAHGENWKVMRRFTL

SALRDFGMGKKTIEDKICEESDCLVETFKSYNGKPFDNTFILNSAAANIIVTILLGDRFD

YKDPKMLNLIKVVNQNMRIGGGFMVRLYNTYPTIMRWIPGSHQTVSKNVATIFKFLNETF

TEHRKVLDVNDQRDLIDAFLVKQQEEELSSKKFFYNQNLTVLVTNLFAAGMETTSTTLRW

GLLLMMKYPEIQKKIQKEIDQVIGSAQPRLEHRKQMPYTDAVIHEIQRFANIAPINIPHE

TTQDVTFRGYFIPKGTQVIPLLASVLRDKAYFKKPEEFYPEHFLDSEGNFVKNEAFLPFS

AGKRSCAGETLAKMELFLFFTKLLQNFTFQPPPGVEVQLTCGVALTSIPLDHKICALPRS

>348552496

MLASGMLLVTLLTCLGLMALMSVWKQRKLQGKLPPGPTPVPLIGNYLQLDTKKMYSCLMK

VSTGVTFSNGERSKQLRRFSISTLRDFGVGKRGIEERIKEEAEYLLQAIQGTRGAFIDPT

YYLSKSVSNVMNSIVFGDRFDYEDEEFLSLLQMTMGIKRFLASPVGQLYEIFYSVMKHLP

GPQQEAFKNMQGLENFMIKKVEQNQRTLDPNAPRNLIDSFLIRMQEEKKNPNTEFHMKNL

VMTILSLFFAGTETISSTLRFGFLLFMKYPEVEAKVHEEIDRVIGKNRQPQFEDRMKMPY

MEAVIHEIQRFANFAPMGISRRITKNTTFQDFFLPKGTEVFPMLGSVLTDYNFFSNPEDF

NPQHFLDEKGQFKKNDGFIPFSLGKRNCFGEGLARMELFLFFTIIMQNFRLKSPQAPEDI

YVPPKPVGFTRVVPYFTMSFLPR

>348587728

MELLGVTTLALVISVTCLIFIFLWTKGQKAVRLPPGPTPLPVIGNILQLNLKDVPASLSK

LAEQYGPVYTLYLGPRCTVVLHGYDAVKEALINQGDEFHGRGRFPIIDNAQKGYGLIFSH

GERWKLMRRFSLMTLRNFGMGKRSLEERVQEEARGLLQELRNTEGQPFDPTFALSCAPCN

VICSVLFNERFQYNDEKFLTLMDLLDENFKQVNSRWCQIYNLWPVLMKYLPGKHKEFSKK

IYDVKNFILEKVKEHQETLDPDNPRDYIDCFLTKMEQEKYKPESEFNLENLAACGSNLFT

AGTETTSITLRFGLLLLMKYPEVEAKVHEEIDRVIGRNRSPSMKDKMKLPYTEAFLHEIQ

RYITLFPSNLPHAVTQDTKFRQYVIPKGTTVLPLLSSVLLDCQEFPNPEKFDPGHFLDEN

GCFKKTDYFVPFSLVMIARGSSVEKTVQPEGEGREGPSAL

>BAC34255.1

MELLGLPTLALLVLVMSLSLLSVWTKMRTGGRLPPGPTPLPIIGNILQLDLKDIPASLSK

LAKEYGPVYTLYFGSWPTVVLHGYDVVKEALLNQGDEFLGRGPLPIIEDSQKGHGIVFSE

GERWKLLRRFSLMTLKNFGMGKRSLEERVQEEARCLVEELHKTEAQPFDPTFILACAPCN

VICSILFNERFPYNDKTFLNLMDLLNKNFYQLNSIWIQMYNLWPTIMKYIPGKHREFSKR

LGGVKNFILEKVKEHQESLDPANPRDYIDCFLSKIEEEKHNLKSDFNLENLAICGSNLFT

AGTETTSTTLRFGLLLLVKHPEVQAKVHEELDRVIGRHQPPSMKDKMKLPYTDAVLHEIQ

RYITLLPSSLPHAVVQDTKFRHYVIPKGTAVFPFLSSILLDQKEFPNPEKFDPGHFLDKS

GCFKKTDYFVPFSLGKYTRFGQLLASLNYVLRDPLM

>BAG60671.1

MDSLVVLVLCLSCLLLLSLWRQSSGRGKLPPGPTPLPVIGNILQIGIKDISKSLTNLSKV

YGPVFTLYFGLKPIVVLHGYEAVKEALIDLGEEFSGRGIFPLAERANRGFGIVFSNGKKW

KEIRRFSLMTLRNFGMGKRSIEDRVQEEARCLVEELRKTKASPCDPTFILGCAPCNVICS

IIFHKRFDYKDQQFLNLMEKLNENIKILSSPWIQICNNFSPIIDYFPGTHNKLLKNVAFM

KSYILEKVKEHQESMDMNNPQDFIDCFLMKMEKEKHNQPSEFTIESLENTAVDLFGAGTE

TTSTTLRYALLLLLKHPEVTAKVQEEIERVIGRNRSPCMQDRSHMPYTDAVVHEVQRYLD

LLPTSLPHAVTCDIKFRNYLIPKGTTILISLTSVLHDNKEFPNPEMFDPHHFLDEGGNFK

KSKYFMLVLKMIRTERTKRKRVGKLFWVSVRVT

>358419090

MDLPVVLVLCLCCLLLISLWKQSSGKGKLPPGPTPLPILGNILQLDVKDISKSVSNGYGV

IFSNGKIWKETRRFSLMTLRDFGMGKRSMEDRVQQEACCLVEELRKTDGLPCDPTFILGC

APCNVICSIIFQNHFDYKDQIFLDLMERLNENARILGSPWIQLCSSFPALIDYVPGKHKK

FFENYACMKSYVLEKTREHQASLDMNNPRDFIDCFLTKMEQEKHNQELEYTVENLAHTVL

DLFVAGTETTSTTLRYGLLLLLKHPEVTAKVQEEIDHVIGRHRSPCMQDKSHMPYTDAVV

HEIQRYIDLVPTNLPHAVTCDIKFRNYLIPKGTGILTSLTSVLYDDKEFPNPEVFDPGHF

LDESGNFRKSDHFMAFSTGKRICVGEGLARMELFLFLTTILQNFTLKSVVDPKDLDTTPV

VNGLLSVPPFYQLCFIPV

>345322441

MAVVKVLLHSLSASFLFYGVIVGLVVIYILTSSKRSRGKLPPGPPPLPIIGNLNLVDLNK

LYQSLMELSENYGSMFTVHLGPRKVVVLAGYDAIKDALFNNSEAFGDRPEIPIFQKVTGG

HGKGIGFSNGDTWKAMRRFALSTLRDFGMGKKSIEAKIQEELNSLVKYFQSHQGKPFDTK

IILNNAVSNIICSILFGDRFEYDDPEFLHLLQLLSDNTKLMGSSMVQLYNFYPFFGFLIG

AHKTVVRNIQEITSFIQKLFKEHILNFSPNHLTGFVDAFLLKQQQESKNPQTEFHNKNLL

FSTLDLFAAGTETTSTTLRWGLLLMMKYPEIQRRVHEEIDKVIVLGQVPSVEDRKKMPFT

DAVIHEMQRFADIVPMGLSRSPTKDIHFRGFVIPKGTEVIPLLSSVFCDPSHWATPYQFN

PSHFLDAAGNFVRKDAFIPFGLGRRACAGEGLARMELFLLFTGLLQKFTFQAAPEVDKAD

LDLTASVGFTLTPKPHLVCALPREEAKSQG

>EHB02542.1

MGISWPSLTLPASRARGHLPPGPAQLPLLGNLLQLRPGALYSGLLRLSKKYGPVFTVHLG

PWRRVVVLVGHKAVQEALGDQAEEFSGRGTLATLDQTFNGHGVFFANGERWRQLRKFTML

VLRDLGMGKREGEELIQAEVQHLVEVFQGTEGCPFDPSLLLVQATSNIVCLLLFGLRFSY

EDEEFQAVVRAAGGTLLGISSPWGQTYEMFSQLLQPLPGPHTQFLRHLDTLVAFTAQQVQ

RHQGNLDTSGPAPPLPAFSQEEQNLSTEFTEKNLLMTVTYLLFAGTMTVGATVRYALLLL

LKYPEVQKQVQEELMRELQAGQAPCLGDRVRLPYTDAVLHEAQRLLALVPMGMPHVVTRT

THFRGYTLPQGTEIFPLLGSVLHDPAVFEQPEEFNPGRFLDVEGRFKPAFLPYSLGKRVC

LGEGLARAELFLFFTAILQAFSLESPSSPDSLSLQPAVSGLFNIPPAFQLQVRPADHHPT

VQTR

>348552502

MLASGMLLLSLLTCLSVMALMSVWKQRKLQGKLPPGPIPLPLIGNYLQLNTKKMYSCLMK

VSSGVSFSNGERSKQLRRFSICTLRDFGVGKRGIEERIKEEAGYLIEAIQATRGAFIDPT

FYLSKSVSNVINSIVFGDRFEYEDKEFLSLLQLTQGIKRFSASPVGQLYEIFYSVMKHLP

GPQQEAFKNMQELENFIIKKVEQNQRTLDPNAPRNLIDSFLIRMQEEKEYPNTEFHMKNL

VMTTLSLFFAGSETVSTTLHFGFLLFMKYPEVEAKVHEEIDRVIGKNRQPRFEDRMKMPY

MEAVIHEIQRFANLAPMGISRKITKNTTFRDFFLPKGTEVFPMLGSVLTDYNFFSDPEDF

NPQHFLDEKGQFKKNDGFIPFSLGKRNCFGEGLTRMELFLFFTTIMQNFRFKSPQAPEDI

YVSPKPMGFTRIVPSFTMSFLPR

>348552504

MLPFLVLLTCLSVMALMSVWKQRKLQGKLPPGPIPLPLIGNYLQLNTKKMYSCLMKVSSG

VSFSNGERSKQLRRFSICTLRDFGVGKRGIEERIKEEAGYLIEAIQATRGAFIDPTFYLS

KSVSNVINSIVFGDRFEYEDKEFLSLLQLTQGIKRFSASPVGQLYEIFYSVMKHLPGPQQ

EAFKNMQELENFIIKKVEQNQRTLDPNAPRNLIDSFLIRMQEEKEYPNTEFHMKNLVMTT

LSLFFAGSETVSTTLHFGFLLFMKYPEVEAKVHEEIDRVIGKNRQPRFEDRMKMPYMEAV

IHEIQRFANLAPMGISRKITKNTTFRDFFLPKGTEVFPMLGSVLTDYNFFSDPEDFNPQH

FLDEKGQFKKNDGFIPFSLGKRNCFGEGLTRMELFLFFTTIMQNFRFKSPQAPEDIYVSP

KPMGFTRIVPSFTMSFLPR

>338710110

MLIAGIATLLLLFLLLALARPSWGGRATPMQGALPPGPTPLPLLGNLLQLGSGRLDHALM

ELSGRWGPVFTVRLGPRPAVILCGYAALRDALVLQADTFSGRGAMTVFERFTRGNGILFS

NGQRWRTLRNFAVGALKEFGLGTRTVEERVLEETACLLGEFQALAGAPFDPRRLLDNAVS

NVICFVVFGSRYGYQDPEFLKLLDLFSENFRIMSSRWGEMYNIFPSILHWLPGPHHRIFR

NFAELRVFISEQIQRHRQTRQPGEPRDFIDCFLDQMDQEQKDSERHFQEETLVMTTHNLF

FGGTETTSNTLRYGLLILLKYPEVAAKVQAELDAVVGRMRAPSLEDRGRLPYT

**CYP3**

>CAD91343.1

MDLIPNFAMETWVLVATSLVLLYIYGTHSHGLFKKLGIPGPTPLPFLGNILSYHKGFCMF

DMECHKKYGKVWGFYDGQQPVLAITDPDMIKTVLVKECYSVFTNRRPFGPVGFMKSAISI

AEDEEWKRLRSLLSPTFTSGKLKEMVPIIAQYGDVLVRNLRREAETGKPVTLKDVFGAYS

MDVITSTSFGVNIDSLNNPQDPFVENTKKLLRFDFLDPFFLSITVFPFLIPILEVLNICV

FPREVTNFLRKSVKRMKESRLEDTQKHRVDFLQLMIDSQNSKETESHKALSDLELVAQSI

IFIFAGYETTSSVLSFIMYELATHPDVQQKLQEEIDAVLPNKAPPTYDTVLQMEYLDMVV

NETLRLFPIAMRLERVCKKDVEINGMFIPKGVVVMIPSYALHRDPKYWTEPEKFLPERFS

KKNKDNIDPYIYTPFGSGPRNCIGMRFALMNMKLALIRVLQNFSFKPCKETQIPLKLSLG

GLLQPEKPVVLKVESRDGTVSGA

>AAS77822.1

MFDLSSLSVTWTLVVLVITLLLIYGVWPHGFFKKLGIPGPRPLPFVGTALSYSKGICNFD

IECSKKYGKVWGIYDGRLPLLLVTDLEMIKTILVKDCYSTFTNRRNMNPDLVGPFADGIT

LVKDERWRRIRSSLSPYFTSGRLKEIFPIAMTHADRFMENMEKKDPNLPLKIKDVVAPYS

LDVVASSSFSVDFDSINNPDDPFVTSIKSFFNINPLSPLFLLLAFCPSAANLLAKMGISV

FSRSTTDFYYKALRKIKDEHNESNGRVDFLKLMIQNQIPDDQVKDTASEQPVKGLTDHEI

LSQSFIFILGGYETTSTTLSYLLYNLATNPDCLEKLVEEIDKNFPLDTPITYDALMRMDY

LEMAIHESMRVFPAGPRLERVCKKTVEINGITIPKNTLVGIPLYVLSRDPDLWESPNEFK

PERFSPESKTEINQCAFMPFGLGPRNCIGMRFALMMMKLLVVKLLQKYTVETCKETQIPV

QLNFFFQPKVPITLKLIPRSHKEKQ

>P08684.4

MALIPDLAMETWLLLAVSLVLLYLYGTHSHGLFKKLGIPGPTPLPFLGNILSYHKGFCMF

DMECHKKYGKVWGFYDGQQPVLAITDPDMIKTVLVKECYSVFTNRRPFGPVGFMKSAISI

AEDEEWKRLRSLLSPTFTSGKLKEMVPIIAQYGDVLVRNLRREAETGKPVTLKDVFGAYS

MDVITSTSFGVNIDSLNNPQDPFVENTKKLLRFDFLDPFFLSITVFPFLIPILEVLNICV

FPREVTNFLRKSVKRMKESRLEDTQKHRVDFLQLMIDSQNSKETESHKALSDLELVAQSI

IFIFAGYETTSSVLSFIMYELATHPDVQQKLQEEIDAVLPNKAPPTYDTVLQMEYLDMVV

NETLRLFPIAMRLERVCKKDVEINGMFIPKGVVVMIPSYALHRDPKYWTEPEKFLPERFS

KKNKDNIDPYIYTPFGSGPRNCIGMRFALMNMKLALIRVLQNFSFKPCKETQIPLKLSLG

GLLQPEKPVVLKVESRDGTVSGA

>CAA30944.1

MALIPDLAMETWLLLAVSLVLLYLYGTHSHGLFKKLGIPGPTPLPFLGNILSYHKGFCMF

DMECHKKYGKVWGFYDGQQPVLAITDPDMIKTVLVKECYSVFTNRRPFGPVGFMKSAISI

AEDEEWKRLRSLLSPTFTSGKLKEMVPIIAQYGDVLVRNLRREAETGKPVTLKDVFGAYS

MDVITSTSFGVNIDSLNNPQDPFVENTKKLLRFDFLDPFFLSITVFPFLIPILEVLNICV

FPREVTNFLRKSVKRMKESRLEDTQKHRVDFLQLMIDSQNSKETESHKALSDLELVAQSI

IFIFAGYETTSSVLSFIMYELATHPDVQQKLQEEIDAVLPNKAPPTYDTVLQMEYLDMVV

NETLRLFPIAMRLERVCKKDVEINGMFIPKGWVVMIPSYALHRDPKYWTEPEKFLPERFS

KKNKDNIDPYIYTPFGSGPRNCIGMRFALMNMKLALIRVLQNFSFKPCKETQIPLKLSLG

GLLQPEKPVVLKVESRDGTVSGA

>AAA35744.1

MALIPDLAMETWLLLAVSLVLLYLYGTHSHGLFKKLGIPGPTPLPFLGNILSYHKGFCMF

DMECHKKYGKVWGFYDGQQPVLAITDPDMIKTVLVKECYSVFTNRRPFGPVGFMKSAISI

AEDEEWKRLRSLLSPTFTSGKLKEMVPIIAQYGDVLVRNLRREAETGKPVTLKDVFGAYS

MDVITSTSFGVNIDSLNNPQDPFVENTKKLLRFDFLDPFFLSITVFPFLIPILEVLNICV

FPREVTNFLRKSVKRMKESRLEDTQKHRVDFLQLMIDSQNSKETESHKALSDLELVAQSI

IFIFAGYETTSSVLSFIMYELATHPDVQQKLQEEIDAVLPNKAPPTYDTVLQMEYLDMVV

NETLRLFPIAMRLERVCKKDVEINGMFIPKGWVVMIPSYALHRDPKYWTEPEKFLPERFS

KKNKDNIDPYTYTPFGSGPRNCIGMRFALMNMKLALIRVLQNFSFKPCKETQIPLKLSLG

GLLQPEKPVVLKVESRDGTVSGA

>AAF13598.1

MALIPDLAMETRLLLAVSLVLLYLYGTHSHGLFKKLGIPGPTPLPFLGNILSYHKGFCMF

DMECHKKYGKVWGFYDGQQPVLAITDPDMIKTVLVKECYSVFTNRRPFGPVGFMKSAISI

AEDEEWKRLRSLLSPTFTSGKLKEMVPIIAQYGDVLVRNLRREAETGKPVTLKDVFGAYS

MDVITSTSFGVNIDSLNNPQDPFVENTKKLLRFDFLDPFFLSITVFPFLIPILEVLNICV

FPREVTNFLRKSVKRMKESRLEDTQKHRVDFLQLMIDSQNSKETESHKALSDLELVAQSI

IFIFAGCETTSSVLSFIMYELATHPDVQQKLQEEIDAVLPNKAPPTYDTVLQMEYLDMVV

NETLRLFPIAMRLERVCKKDVEINGMFIPKGVVVMIPSYALHRDPKYWTEPEKFLPERFS

KKNKDNIDPYIYTPFGSGPRNCIGMRFALMNMKLALIRVLQNFSFKPCKETQIPLKLSLG

GLLQPEKPVVLKVESRDGTVSGA

>EAW76636.1

MALIPDLAMETWLLLAVSLVLLYLYGTHSHGLFKKLGIPGPTPLPFLGNILSYHKGFCMF

DMECHKKYGKVWGFYDGQQPVLAITDPDMIKTVLVKECYSVFTNRRPFGPVGFMKSAISI

AEDEEWKRLRSLLSPTFTSGKLKEMVPIIAQYGDVLVRNLRREAETGKPVTLKDVFGAYS

MDVITSTSFGVNIDSLNNPQDPFVENTKKLLRFDFLDPFFLSIIFPFLIPILEVLNICVF

PREVTNFLRKSVKRMKESRLEDTQKHRVDFLQLMIDSQNSKETESHKALSDLELVAQSII

FIFAGYETTSSVLSFIMYELATHPDVQQKLQEEIDAVLPNKAPPTYDTVLQMEYLDMVVN

ETLRLFPIAMRLERVCKKDVEINGMFIPKGVVVMIPSYALHRDPKYWTEPEKFLPERFSK

KNKDNIDPYIYTPFGSGPRNCIGMRFALMNMKLALIRVLQNFSFKPCKETQIPLKLSLGG

LLQPEKPVVLKVESRDGTVSGA

>ABU85097.1

MALIPDLAMETWLLLAVSLVLLYLYGTHSHGLFKKLGIPGPTPLPFLGNILSYRKGFCMF

DMECHKKYGKVWGFYDGRQPVLAITDPDMIKTVLVKECYSVFTNRRPFGPVGFMKSAISI

AEDEEWKRIRSLLSPTFTSGKLKEMVPIIAQYGDVLVRNLRREADTGKPVTLKDVFGAYS

MDVITSTSFGVNIDSLNNPQDPFVENTKKLLRFDFLDPFFLSIIVFPFLIPILEVLNICV

FPREVTNFLRKSVKRMKESRLEDTQKHRVDFLQLMIDSQNSKETESHKALSDLELVAQSI

IFIFAGYETTSSVLSFIMYELATHPDVQQKLQEEIDAVLPNKAPPTYDTVLQMEYLDMVV

NETLRLFPVAMRLERVCKKDVEINGMFIPKGVVVMIPSYALHRDPKYWTEPEKFLPERFS

KKNKDNIDPYIYTPFGSGPRNCIGMRFALMNMKLALIRVLQNFSFKPCKETQIPLKLRLG

GLLQPEKPIVLKVESRDGTVSGA

>3UA1A

MAYLYGTHSHGLFKKLGIPGPTPLPFLGNILSYHKGFCMFDMECHKKYGKVWGFYDGQQP

VLAITDPDMIKTVLVKECYSVFTNRRPFGPVGFMKSAISIAEDEEWKRLRSLLSPTFTSG

KLKEMVPIIAQYGDVLVRNLRREAETGKPVTLKDVFGAYSMDVITSTSFGVNIDSLNNPQ

DPFVENTKKLLRFDFLDPFFLSITVFPFLIPILEVLNICVFPREVTNFLRKSVKRMKESR

LEDTQKHRVDFLQLMIDSQNSKETESHKALSDLELVAQSIIFIFAGYETTSSVLSFIMYE

LATHPDVQQKLQEEIDAVLPNKAPPTYDTVLQMEYLDMVVNETLRLFPIAMRLERVCKKD

VEINGMFIPKGVVVMIPSYALHRDPKYWTEPEKFLPERFSKKNKDNIDPYIYTPFGSGPR

NCIGMRFALMNMKLALIRVLQNFSFKPCKETQIPLKLSLGGLLQPEKPVVLKVESRDGTV

SGAHHHH

>1TQNA

MALYGTHSHGLFKKLGIPGPTPLPFLGNILSYHKGFCMFDMECHKKYGKVWGFYDGQQPV

LAITDPDMIKTVLVKECYSVFTNRRPFGPVGFMKSAISIAEDEEWKRLRSLLSPTFTSGK

LKEMVPIIAQYGDVLVRNLRREAETGKPVTLKDVFGAYSMDVITSTSFGVNIDSLNNPQD

PFVENTKKLLRFDFLDPFFLSITVFPFLIPILEVLNICVFPREVTNFLRKSVKRMKESRL

EDTQKHRVDFLQLMIDSQNSKETESHKALSDLELVAQSIIFIFAGYETTSSVLSFIMYEL

ATHPDVQQKLQEEIDAVLPNKAPPTYDTVLQMEYLDMVVNETLRLFPIAMRLERVCKKDV

EINGMFIPKGVVVMIPSYALHRDPKYWTEPEKFLPERFSKKNKDNIDPYIYTPFGSGPRN

CIGMRFALMNMKLALIRVLQNFSFKPCKETQIPLKLSLGGLLQPEKPVVLKVESRDGTVS

GAHHHH

>1W0EA

MAYGTHSHGLFKKLGIPGPTPLPFLGNILSYHKGFCMFDMECHKKYGKVWGFYDGQQPVL

AITDPDMIKTVLVKECYSVFTNRRPFGPVGFMKSAISIAEDEEWKRLRSLLSPTFTSGKL

KEMVPIIAQYGDVLVRNLRREAETGKPVTLKDVFGAYSMDVITSTSFGVNIDSLNNPQDP

FVENTKKLLRFDFLDPFFLSITVFPFLIPILEVLNICVFPREVTNFLRKSVKRMKESRLE

DTQKHRVDFLQLMIDSQNSKETESHKALSDLELVAQSIIFIFAGYETTSSVLSFIMYELA

THPDVQQKLQEEIDAVLPNKAPPTYDTVLQMEYLDMVVNETLRLFPIAMRLERVCKKDVE

INGMFIPKGVVVMIPSYALHRDPKYWTEPEKFLPERFSKKNKDNIDPYIYTPFGSGPRNC

IGMRFALMNMKLALIRVLQNFSFKPCKETQIPLKLSLGGLLQPEKPVVLKVESRDGTVSG

AHHHH

>AAA35747.1

MAVIPDLAMETWLLLAVSLVLLYLYGTHSHGLFKKLGIPGPTPLPFLGNILSYHKGFCMF

DMECHKKYGKVCGFYDGQQPVLAITDPDMIKTVLVKECYSVFTNRRPFGPVGFMKSAISI

AEDEEWKRLRSLLSPTFTSGKLKEMVPIIAQYGDVLVRNLRREAETGKPVTLKDVFGAYS

MDVITSTSFGVNIDSLNNPQDPFVENTKKLLRFDFLDPFFLSIIFPFLIPILEVLNICVF

PREVTNFLRKSVKRMKESRLEDTQKHRVDFLQLMIDSQNSKETESHKALSDLELVAQSII

FIFAGYETTSSVLSFIMYELATHPDVQQKLQEEIDAVLPNKAPPTYDTVLQMEYLDMVVN

ETLRLFPIAMRLERVCKKDVEINGMFIPKGWVVMIPSYALHRDPKYWTEPEKFLPERFSK

KNKDNIDPYTYTPFGSGPRNCIGMRFALMNMKLALIRVLQNFSFKPCKETQIPLKLSLGG

LLQPEKPVVLKVESRDGTVSGA

>397489490

MALIPDLAMETWLLLAVSLVLLYLYGTHSHGLFKKLGIPGPTPLPFLGNILFYRKGFCMF

DMECHKKYGKVWGFYDGRQPVLAITDPDMIKTVLVKECYSVFTNRRPFGPVGFMKSAISI

AEDEEWKRIRSLLSPTFTSGKLKEMVPIIAQYGDVLVRNLRREADTGKPVTLKHVFGAYS

MDVITSTSFGVNIDSLNNPQDPFVENTKKLLRFDFLDPFFLSIIVFPFLIPILEVLNICV

FPREVTNFLRKSVKRMKESRLEDTQKHRVDFLQLMIDSQNSKETESHKALSDLELVAQSI

IFIFAGYETTSSVLSFIMYELATHPDVQQKLQEEIDAVLPNKAPPTYDTVLQMEYLDMVV

NETLRLFPVAMRLERVCKKDVEINGMFIPKGVVVMIPSYALHRDPKYWTEPEKFLPERFS

KKNKDNIDPYIYTPFGSGPRNCIGMRFALMNMKLALIRVLQNFSFKPCKETQIPLKLRLG

GLLQPEKPIVLKVESRDGTVSGA

>AAA35742.1

MALIPDLAMETWLLLAVSLVLLYLYGTHSHGLFKKLGIPGPTPLPFLGNILSYHKGFCMF

DMECHKKYGKVWGFYDGQQPVLAITDPDMIKLVLVKECYSVFTNREPFGPVGFMKSAISI

AEDEEWKRLRSLLSPTFTSGKLKEMVPIIAQYGDVLVRNLRRERETGKPVTLKDVFGAYS

MDVITSSSFGVNVDSLNNPQDPLVENTKKLLRFDFLDPFFLSITVFPFLIPILEVLNICV

FPREVTNFLRKAVKRMKESRLEDTQKHRVDFLQLMIDSHKNSKETESHKALSDLELVAQS

IIFIFAGYETTSSVLSFIMYELATHPDVQQKLQEEIDAVLPNKAPPTYDTVLQMEYLDMV

VNETLRLFPIAMRLERVCKKDVEINGMFIPKGWVVMIPSYALHRDPKYWTEPEKFLPERF

SKKNKDNIDPYIYTPFGSGPRNCIGMRFALMNMKLALIRVLQNFSFKPCKETQIPLKLSL

GGLLQPEKPVVLKVESRDGTVSGA

>297679962

MDLIPDLAMETWILLAVSLVLLYLYGTHSHGLFKKLGIPGPTPLPLLGNVFSYRKGFWRF

DMECHKKYGKVWGFYDGRQPVLAITDPDMIKTVLVKECYSVFTNRRPFGPVGFMKSAISI

AEDEEWKRIRSLLSPTFTSGKLKEMVPIIARYGDVLVRNLRREAETGKPVTLKDVFGAYS

MDVITSTSFGVNIDSLNNPQDPFVENTKKLLRFDFLDPFFLSIIVFPFLTPILEILNISV

FPREVTSFLRKSVKRMKESRLKDTQKHRVDFLQLMIDSQNSKETESHKALSDLELVAQSI

IFIFAGYETTSSVLSFIMYELATHPDVQRKLQEEIDAVLPNKAPPTYDTVLQMEYLDMVV

NETLRLFPIAMRLERVCKKDVEINGMFIPKGVVVMIPSYALHHDPKYWTEPGKFLPERFS

KKNKDNIEPYVYTPFGTGPRNCIGMRFALMNMKLAVIRVLQNFSFKPCKETQIPLKLRLG

GLLQTEKPIVLKVESRDGTLSGA

>402862960

MDLIPDLAVETWLLLAVTLVLLYLYGTHSHGLFKKLGIPGPTPLPLLGNVLSYRKGFWTF

DMECYKKYGKVWGFYDGRQPVLAITDPNMIKTVLVKECYSVFTNRRPFGPVGFMKSAISI

AEDEEWKRIRSLLSPTFTSGKLKEMVPIIAKYGDVLVRNLRRETETGKPVTLKDVFGAYS

MDVITSTSFGVNIDSLNNPQDPFVENTKKLLRFDFLDPFFLSITIFPFLIPILEVLNISI

FPREVTSFLRKSVKRIKESRLKDTQKHRVDFLQLMIDSQNSKETESHKALSDLELVAQSI

IFIFAGYETTSSVLSFIMYELATHPDVQQKLQEEIDAVLPNKAPPTYDTVLQMEYLDMVV

NETLRLFPIAMRLERVCKKDVEINGIFIPKGVVVMIPTYALHHDSKYWTEPEKFLPERFS

KKNKDNIDPYIYTPFGSGPRNCIGMRFALMNMKLALIRVLQNFSFKPCKETQIPLKLRLG

GLLQTEKPIVLKIESRDGTVSGA

>332258046

MDLIPNLAVETWLLLAVSLVLLYLCGTHSHGLFKKLGIPGPTPLPFLGNILSYRKGFWTF

DMECHKKYGKVWGFYDGRQPVLAITDPDMIKTVLVKECYSVFTNRRPFGPVGFMKSAISI

AEDEEWKRIRSLLSPTFTSGKLKEMVPIIAQYGDVLVRNLRQEAETGKPVTLKDVFGAYS

MDVITSTSFGVNIDSLNNPQDPFVENTKKLLRFDFLDPFFLSIIVFPFLTPILEVLNISV

FPREVTSFLRKSVKRMKESRLKDTQKHRVDFLQLMIDSQNSKETESHKALSDLELVAQSI

IFIFAGYETTSSVLSFIMYELATHPDVQQKLQEEIDAVLPNKAPPTYDTVLQMEYLDMVV

NETLRLFPIAMRLERVCKKDVEINGMFIPKGVVVMIPSYVLHHDPKYWTEPGKFLPERFS

KKNKDSIDPYIYTPFGTGPRNCIGMRFALMNMKLALIRVLQNFSFKPCKETQIPLKLHSG

GLLQPEKPIVLKVESRDGTV

>AAY51970.1

MDLIPDLAVETWLLLAVTLVLLYLYGTHSHGLFKKLGIPGPTPLPLLGNVLSYRKGFWQF

DMECYKKYGKVWGFYDGRQPVLAITDPNMIKTVLVKECYSVFTNRRPFGPVGFMKSAISI

AEDEEWKRIRSLLSPTFTSGKLKEMVPIIAKYGDVLVRNLRRETETGKPVTMKDVFGAYS

MDVITSTSFGVNIDSLNNPQDPFVENTKKLLRFDFLDPFFLSITIFPFLIPILEVLNISI

FPRKVTSFLRKSVKRIKESRLKDTQKHRVDFLQLMIDSQNSKETESHKALSDLELVAQSI

IFIFAGYETTSSVLSFIMYELATHPDVQQKLQEEIDTVLPNKAPPTYDTVLQMEYLDMVV

NETLRIFPIAMRLERVCKKDVEINGIFIPKGVVVMIPSYALHHDPKYWAEPEKFLPERFS

KKNNDNIDPYIYTPFGSGPRNCIGMRFALMNMKLALIRVLQNFSFKPCKETQIPLKLRLG

GLLQTEKPIVLKIESRDGTVSGA

>AAY51971.1

MDLIPDLAVETWLLLAVTLVLLYLYGTHSHGLFKKLGIPGPTPLPLLGNVLSYRKGFWQF

DMECYKKYGKVWGFYDGRQPVLAITDPNMIKTVLVKECYSVFTNRRPFGPVGFMKSAISI

AEDEEWKRIRSLLSPTFTSGKLKEMVPIIAKYGDVLVRNLRRETETGKPVTMKDVFGAYS

MDVITSTSFGVNIDSLNNPQDPFVENTKKLLRFDFLDPFFLSITIFPFLIPILEVLNISI

FPRKVTSFLRKSVKRIKESRLKDTQKHRVDFLQLMIDSQNSKETESHKALSDLELVAQSI

IFIFAGYETTSSVLSFIMYELATHPDVQQKLQEEIDTVLPNKAPPTYDTVLQMEYLDMVV

NETLRIFPIAMRLERVCKKDVEINGIFIPKGVVVMIPSYALHHDPKYWAEPEKFLPERFS

KKNNDNIDPYIYTPFGTGPRNCIGMRFALMNMKLALIRVLQNFSFKPCKETQIPLKLRLG

GLLQTEKPIVLKIESRDGTVSGA

>332258077

MDLIPNLAMETWLLLAVSLILLYLYGTHSHGLFKKLGIPGPTPLPFLGNVWSFRKGFWTF

DMECYKKYRKVWGFYDGLQPVLAITDPDMIKTVLVKECYSVFTNRRPLGPVGFMKSAISR

AEDEEWKRIRSLLSPTFTSGKLKEMVPIIAQYGDVLVRNLRREADTGMPVTLKDIFGAYS

MDVITSTSFGVNIDSLNNPQDPFVENTKKLLRFNSLDPFILSIKIFPFLTPILEALNITV

FPRKVTSFLTKSVKRIKEGRLKETQKHRVDFLQLMIDSQNSKETESHKALSDLELVAQSI

IFIFAGYETTSSVLSFIMYELATHPDVQQKLQEEIDAVLPNKAPPTYDTVLQMEYLDMVV

NETLRLFPVAMRLERVCKKDVEINGMFIPKGVVVMIPSYVLHHDPKYWTEPEKFLPERFS

KKNKDNIDPYIYTPFGSGPRNCIGMRFALMNMKLALIRVLQNFSFKPCKETQIPLKLRFG

GLLQPEKPIVLTAESRDETVSGA

>307691239

MDLIPDLAVETWLPLAVSLILLYLYGTHSHGLFKKLGIPGPTPLPFFGNVLSFRKGFWTF

DMECYKKYRKVWGFYDCRQPVLAITDPNMIKTVLVKECYSVFTNRRPLGPVGFMKSAITV

AEDEEWKRIRSLLSPTFTSGKLKEMVPIIAQYGDVLVRNLRREAETGKPVTLKDVFGAYS

MDVITSTSFGVNVDSLNNPQDPFVENTKKLLRFNSLDPFFLSIKIFPFLTPIFEALNITV

FPRKVTSFLRKSVKRMKEGRLKETQKRRVDFLQLMIDSQNSKDTESHKVLSDLELMAQSI

MFIFAGYETTSSVLSFIIYELATHPDVQQKLQKEIDTVLPNKAPPTYDTMLQMEYLDMVV

NETLRLFPVAMRLERVCKKDVEINGMSIPKGVVVMIPSYVLHHDPKYWTEPEKFLPERFS

KKNKDNIDPYIYTPFGNGPRNCIGMRFALMNMKLALIRVLQNFSFKPCKETQIPLKLSFG

GLLKTEKPIVLTAESRDGTVSGA

>P33268.1

MDLIPDLAVETWLLLAVTLVLLYLYGTHSHGLFKKLGIPGPTPLPLLGNILSYRKGFWTF

DMECYKKYGKVWGFYDGRQPVLAITDPNMIKTVLVKECYSVFTNRRPFGPVGFMKNAISI

AEDEEWKRIRSLLSPTFTSGKLKEMVPIIAKYGDVLVRNLRREAETGKPVTLKDVFGAYS

MDVITSTSFGVNIDSLNNPQDPFVENTKKLLRFDFLDPFFLSITIFPFIIPILEVLNISI

FPREVTSFLRKSVKRIKESRLKDTQKHRVDFLQLMIDSQNSKETESHKALSDLELVAQSI

IFIFAGYETTSSVLSFIIYELATHPDVQQKLQEEIDTVLPNKAPPTYDTVLQMEYLDMVV

NETLRIFPIAMRLERVCKKDVEINGIFIPKGVVVMIPSYALHHDPKYWPEPEKFLPERFS

KKNNDNIDPYIYTPFGSGPRNCIGMRFALMNMKLAIIRVLQNFSFKPCKETQIPLKLRLG

GLLQTEKPIVLKIESRDGTVSGA

>AAP94642.1

MDLIPDLAVETWLLLAVTLVLLYLYGTHSHGLFKKLGIPGPTPLPLLGNILSYRKGFWTF

DMECYKKYGKVWGFYDGRQPVLAITDPNMIKTVLVKECYSVFTNRRPFGPVGFMKNAISI

AEDEEWKRIRSLLSPTFTSGKLKEMVPIIAKYGDVLVRNLRREAETGKPVTLKDVFGAYS

MDVITSTSFGVNIDSLNNPQDPFVENTKKLLRFDFLDPFFLSITIFPFIIPILEVLNISI

FPREVTSFLRKSVKRIKESRLKDTQKHRVDFLQLMIDSQNSKETESHKALSDLELVAQSI

IFIFAGYETTSSVLSFIIYELATHPDVQQKLQEEIDTVLPNKAPPTYDTVLQMEYLDMVV

NETLRIFPIAMRLERVCKKDVEINGIFIPKGVVVMIPSYALHHDPKYWPEPEKFLPERFS

KKNNDNIDPYIYTPFGSGPRNCIGMRFALMNMKLAIIRVLQNFSFKPCKETQIPLKLRLG

GLLQTEKPIVLKIESRDGTVSGA

>BAE45352.1

MDLIPDLAVETWLLLAVTLVLLYLYGTHSHGLFKKLGIPGPTPLPLLGNILSYRKGFWTF

DMECYKKYGKVWGFYDGRQPVLAITDPNMIKTVLVKECYSVFTNRRPFGPVGFMKNAISI

AEDEEWKRIRSLLSPTFTSGKLKEMVPIIAKYGDVLVRNLRREAETGKPVTLKDVFGAYS

MDVITSTSFGVNIDSLNNPQDPFVENTKKLLRFDFLDPFFLSITIFPFIIPILEVLNISI

FPREVTSFLRKSVKRIKESRLKDTQKHRVDFLQLMIDSQNSKETESHKALSDLELVAQSI

IFIFAGYETTSSVLSFIIYELATHPDVQQKLQEEIDTVLPNKAPPTYDTVLQMEYLDMVV

NETLRIFPIAMRLERVCKKDVEINGIFIPKGVVVMIPSYALHHDPKYWPEPEKFLPERFN

KKNNDNIDPYIYTPFGSGPRNCIGMRFALMNMKLAIIRVLQNFSFKPCKETQIPLKLRLG

GLLQTEKPIVLKIESRDGTVSGA

>AAS91645.1

MDLIPDLAVETWLLLAVTLVLLYLYGTHSHGLFKKLGIPGPTPLPLLGNILSYRKGFWTF

DMECYKKYGKVWGFYDGRQPVLAITDPNMIKTVLVKECYSVFTNRRPFGPVGFMKNAISI

AEDEEWKRIRSLLSPTFTSGKLKEMVPIIAKYGDVLVRNLRREAETGKPVTLKDVFGAYS

MDVITSTSFGVNIDSLNNPQDPFVENTKKLLRFDFLDPFFLSITIFPFIIPILEVLNISI

FPREVTSFLRKSVKRIKESRLKDTQKHRVDFLQLMIDSQNSKETESHKALSDQELVAQSI

IFIFAGYETTSSVLSFIIYELATHPDVQQKLQEEIDTVLPNKAPPTYDTVLQMEYLDMVV

NETLRIFPIAMRLERVCKKDVEINGIFIPKGVVVMIPSYALHHDPKYWPEPEKFLPERFS

KKNNDNIDPYIYTPFGSGPRNCIGMRFALMNMKLAIIRVLQNFSFKPCKETQIPLKLRLG

GLLQTEKPIVLKIESRDGTVSGA

>BAG16268.1

MDLIPDLAVETWLPLAVSLILLYLYGTHSHGLFKKLGIPGPTPLPFFGNVLSFRKGFWTF

DMECYKKYRKVWGFYDCRQPVLAITDPNMIKTVLVKECYSVFTNRRPLGPVGFMKSAITV

AEDEEWKRIRSLLSPTFTSGKLKEMVPIIAQYGDVLVRNLRREAETGKPVTLKDVFGAYS

MDVITSTSFGVNVDSLNNPQDPFVENTKKLLRFNSLDPFFLSIKIFPFLTPIFEALNITV

FPRKVTSFLRKSVKRIKEGRLKETQKRRVDFLQLMIDSQNSKDTASHKVLSDLELMAQSI

MFIFAGYETTSSVLSFIIYELATHPDVQQKLQKEIDTVLPNKAPPTYDTMLQMEYLDMVV

NETLRLFPVAMRLERVCKKDVEINGMSIPKGVVVMIPSYVLHHDPKYWTEPEKFLPERFS

KKNKDNIDPYIYTPFGNGPRNCIGMRFALMNMKLALIRVLQNFSFKPCKETQIPLKLSFG

GLLKTEKPIVLTAESRDGTVSGA

>EHH17167.1

MDLIPDLAVETWLLLAVTLVLLYLYGTHSHGLFKKLGIPGPTPLPLLGNILSYRKGFWTF

DMECYKKYGKVWGFYDGRQPVLAITDPNMIKTVLVKECYSVFTNRRPFGPVGFMKNAISI

AEDEEWKRIRSLLSPTFTSGKLKEMVPIIAKYGDVLVRNLRREAETGKPVTLKDVFGAYS

MDVITSTSFGVNVDSLNNPQDPFVENTKKLLRFDFLDPFFLSITIFPFIIPILEVLNISI

FPREVTSFLRKSVKRIKESRLKDTQKHRVDFLQLMIDSQNSKETESHKALSDLELVAQSI

IFIFAGHETTSSVLSFVIYELATNPDVPQKLQEEIDTVLPNKAPPTYDTVLQMEYLDMVV

NETLRIFPIAMRLERVCKKDVEINGIFIPKGVVVMIPSYALHHDPKYWPEPEKFLPERFS

KKNNDNIDPYIYTPFGSGPRNCIGMRFALMNMKLAIIRVLQNFSFKPCKETQIPLKLRLG

GLLQTEKPIVLKIESRDGTVSGA

>ABU85096.1

MDLIPNLAVETWLLLAVSLILLYLYGTHSHGLFKKLGIPGPTPLPFVGNALSFHKGFWTF

DMECYKKYRKVWGFYDGLQPVLAITDPDMIKTVLVKECYSVFTNRRPLGPVGFMKSAISR

AEDEEWKRIRSLLSPTFTSGKLKEMVPIIAQYADVLVRNLRREAETGKPVTLKDFFGAYS

MDVITSTSFGVNIDSLNNPQDPFVENTKKLLRFNSLDPLTLSIKIFPFLIPILEALNVTV

FPREVTSFLTKSVKRIKEGRLKETQKHRVDFLQLMIDSQNLKDTESHKALSDLELVAQSI

VFIFAGYETTSSVLSFIIYELATHPDVQQKLQEEIDAVLPNKAPPTYDTVLQMEYLDMVV

NETLRLFPVAMRLERVCKKDVEINGMFIPKGVVVMIPSYVLHHDPKYWIEPEKFLPERFS

KKNKDNIDPYIYTPFGNGPRNCIGMRFALMNMKFALVRVLQNFSFKPCKETQIPLKLRFG

GLLRTEKPIVLMAESRDETVSGA

>395738168

MDLIPNLAVETWLLLAVSLILLYLYGTHSHGLFKKLGIPGPTPLPFVGNALSFHKGFWTF

DMECYEKYRKVWGFYDGLQPVLAITDPDMIKTVLVKECYSVFTNRRPLGPVGFMKSAISR

AEDEEWKRIRSLLSPTFTSGKLKEMVPIIAQYGDVLVRNLRREAETGKPVTLKDFFGAYS

MDVITSTSFGVNVDSLNNPQDPFVENTKKLLRFNSLDPLTLSIKIFPFLIPILEALNVTV

FPREVTSFLTKSVKRIKEGRLKETQKHRVDFLQLMIDSQNLKDTESHKALSDLELVAQSI

VFIFAGYETTSSVLSFIIYELATHPDVQQKLQEEIDAVLPNKAPPTYDTVLQMEYLDMVV

NETLRLFPVAMRLERVCKKDVEINGMFIPKGVVVMIPSYVLHHDPKYWIEPEKFLPERFS

KKNKDNIDPYIYTPFGNGPRNCIGMRFALMNMKFALVRVLQNFSFKPCKETQIPLKLRFG

GLLRTEKPIVLMAESRDETVSGA

>O18993.1

MDFIPNLAVETWLLLAVSLVLLYLYGTHSHGLFKKLGIPGPTPLPFLGTVLYYRQGFWKF

DMECYKKYGKMWGIYDGRQPVLAITDPNIIKTVLVKECYSVFTNRRPFGPVGFMKSAISI

AQDDEWKRIRSLLSPTFTSGKLKEMVPIIAQYGEVLVRNLRREAEKGKPINMKDIFGAYS

MDVITGTSFGVNIDSLNNPQDPFVESTKKLLRFDFLDPFFLSITIFPFLTPILEALNISM

FPRDSTSFLRKSIKRIKESRLKDTHKHRVDFLQLMIDSQNSKETESDKALSDLELVAQSI

IFIFAGYETTSSTLSFIMYELATHPDVQQKLQEEIDAVLPNKAPATYDTVLQMEYLDMVV

NETLRLFPLAMRLERVCKKDVEINGVFIPKGVVVMIPSYALHYDPKYWTEPEKFLPERFS

KNNKDNIDPYIYTPFGTGPRNCIGMRFALMNMKLALIRVLQNFSFKPCKETQIPLKLRLG

GLLQTEKPIVLKVEPRDGTVSGA

>AAY51972.1

MALIPDLAMETWLLLAVSLILLYLYGTHSHGLFKKLGIPGPTPLPFFGNVLSFHKGLWTF

DMECYKKYRKVWGFYDCLQPVLAITDPNMIKTVLVKECYSVFTNRRPLGPVGFMKSALSR

AQDEEWKRIRSLLSPTFTSGKLKEMVPIIAQYGDVLVRNLRREAETGKPVTLKDIFGAYS

MDVITSTSFGVNVDSLNNPQDPFVENTKKLLRFNSLDPFFLSIKIFPFLTPIFEALNITM

FSRKVTNFLRKSVNKIKEDRLKETQKRRVDFLQLMIDSQNSKDTESHKALSDLELVAQSI

IFIFAGYETTSSVLSFIMYELATHPDVQQKLQKEIDAVLPNKAPPTYDTVLQMEYLHMVV

NETLRLFPVAMRLERVCKKDVEINGMFIPKGVVVMIPSYVLHHDPKHWTEPEKFLPERFS

KKNKDNIDPYIYTPFGSGPRNCIGMRFALMNMKLALIRVLQNFSFKPCKETQIPLKLRFG

GLLQPEKPIVLTAESRNGTVSGA

>ABP68411.1

MDLIPNLAVETWLLLAVSLILLYLYGTHSHGLFKKLGIPGPTPLPFVGNVWSFRKGFWTF

DMECYKKYRKVWGFYDGLQPVLAITDPDMIKTVLVKECYSVFTNRRPLGPVGFMKSAISR

AEDEEWKRIRSLLSPTFTSGKLKEMVPIIAQYGDVLVRNLRREADTGMPVTLKDVFGAYS

MDVITSTSFGVNIDSLNNPQDPFVENTKKLLRFNSLDPFILSIKIFPFLTPILEALNITV

FPRKVTSFLTKSVKRIKEGRLKETQKHRVDFLQLMIDSQNSKDMESHKALSDLELVAQSI

VFIFAGYETTSSVLSFIIYELATHPDVQQKLQEEIDAVLPNKAPPTYDTVLQLEYLDMVV

NETLRLFPVAMRLERVCKKDVEINGMFIPKGVVVMIPSYVLHHDPKYWTEPEKFLPERFS

KKNKDNIDPYIYTPFGNGPRNCIGMRFALMNMKFALVRVLQNFSFKPCKETQIPLKLRFG

GLLRTEKPIVLMAESRDETVSGA

>ABG79670.1

MDFIPNLAVETWLLLAVSLVLLYLYGTHSHGLFKKLGIPGPTPLPFFGTVLSYRQGFWKF

DMECYKKYGKVWGIYDGRQPVLAIADPNIIKTVLVKECYSVFTNRRPFGPVGFMKSAISI

AEDEEWKRIRSLLSPTFTSGKLKEMVPIIAQYGEVLVKNLRREAEKGKPINMKDIFGAYS

MDVITGTSFGVNIDSLNNPKDPFVENTRKLIRFDFSDPFFLSIILFPFLTPIFEALNISM

FPRDSTSFLRKSIKRIKESRLKDTHKHRVDFLQLMIDSQNSKETESHKALSDLELVAQSI

IFIFAGYETTSSTLSFIMYELATHPDVQQKLQEEIDAVLPNKAPATYDTVLQMEYLDMVV

NETLRLFPLAMRLERVCKKDVEINGMFIPKGVVVMIPSYALHYDPKYWTEPEKFLPERFS

KNNKDNIDPYIYTPFGTGPRNCIGMRFALMNMKLALIRILQNFSFKPCKETQIPLKLRSG

GLLQTEKPIVLKVEPRDGTVSGA

>402862962

MDLIPDLAVETWLLLAVSLILLYLYGMHSHGLFKKLGIPGPTPLPFFGNVLSFRKGLWTF

DMECYKKYRKVWGFYDCLQPVLAITDPNMIKTVLVKECYSVFTNRRPLGPVGFMKSAITV

AEDKEWKRIRSLLSPTFTSGKLKEMVPIIAQYGDVLVRNLRREAETGKPVTLKDVFGAYS

MDVITSTSFGVNVDSLNNPQDPFVENTKKLLRFNSLDPFFLSIKIFPFLTPIFEALNITV

FPRKVTSFLRKSVKRIKEGRLKETQKRRVDFLQLMIDSQNSKDTESHKALSDLELMAQSI

MFIFAGYETTSSVLSFIIYELATHPDVQQKLQKEIDTVLPNKAPPTYDTMLQMEYLDMVV

NETLRLFPVAMRLERVCKKDVEINGMFIPKGVVVMIPTYVLHHDPKYWTEPEKFLPERFS

KKNKDNIDPYIYTPFGNGPRNCIGMRFALMNMKLALIRVLQNFSFKPCKETQIPLKLSFG

GLLKTEKPIVLTAESRDETVSGA

>397489484

MDLIPNLAVETWLLLAVSLILLYLYGTHSHGLFKKLGIPGPTPLPFVGNVWSFRKGFWTF

DMECYKKYRKVWGFYDGLQPVLAITDPDMIKTVLVKECYSVFTNRRPLGPVGFMKSAISR

AEDEEWKRIRSLLSPTFTSGKLKEMVPIIAQYGDVLVRNLRREADTGMPVTLKDVFGAYS

MDVITSTSFGVNIDSLNNPQDPFVENTKKLLRFNSLDPFILSIKIFPFLTPILEALNITV

FPRKVTSFLTKSVKRIKEGRLKETQKHRVDFLQLMIDSQNSKDMESHKALSDLELVAQSI

VFIFAGYETTSSVLSFIIYELATHPDVQQKLQEEIDAVLPNKAPPTYDTVLQMEYLDMVV

NETLRLFPVAMRLERVCKKDVEINGMFIPKGVVVMIPSYVLHHDPKYWTEPEKFLPERFS

KKNKDNIDPYIYTPFGNGPRNCIGMRFALMNMKFALVRVLQNFSFKPCKETQIPLKLRFG

GLLRTEKPIVLMAESRDETFVDMEPIHMDFLRSLAFQGPHLCFFWELLCPTIRSR

>332867108

MDLIPNLAVETWLLLAVSLILLYLYGTRTHGLFKKLGIPGPTPLPFLGNALSFRKGYWTF

DMECYKKYRKVWGIYDCQQPMLAITDPDMIKTVLVKECYSVFTNRRPFGPVGFMKSAISI

AEDEEWKRIRSLLSPTFTSGKLKEMVPIIAQYGDVLVRNLRREAETGKPVTLKHVFGAYS

MDVITSTSFGVNIDSLNNPQDPFVENTKKLLRFNPLDPFVLSIKVFPFLTPILEALNITV

FPRKVISFLTKSVKQIKEGRLKETQKHRVDFLQLMIDSQNSEDSETHKALSDLELMAQSI

IFIFAGYETTSSVLSFIIYELATHPDVQQKVQKEIDTVLPNKAPPTYDTVLQLEYLDMVV

NETLRLFPVAMRLERVCKKDVEINGMFIPKGVVVMIPSYVLHHDPKYWTEPEKFLPERFS

KKNKDNIDPYIYTPFGSGPRNCIGMRFALVNMKLALVRVLQNFSFKPCKETQIPLKLRFG

GLLLTEKPIVLKAESRDETVSGA

>AAG32289.1

MDLIPNLAVETWLLLAVSLILLYLYGTRTHGLFKKLGIPGPTPLPFLGNALSFRKGYWTF

DMECYKKYRKVWGIYDCQQPMLAITDPDMIKTVLVKECYSVFTNRRPFGPVGFMKNAISI

AEDEEWKRIRSLLSPTFTSGKLKEMVPIIAQYGDVLVRNLRREAETGKPVTLKHVFGAYS

MDVITSTSFGVSIDSLNNPQDPFVENTKKLLRFNPLDPFVLSIKVFPFLTPILEALNITV

FPRKVISFLTKSVKQIKEGRLKETQKHRVDFLQLMIDSQNSKDSETHKALSDLELMAQSI

IFIFAGYETTSSVLSFIIYELATHPDVQQKVQKEIDTVLPNKAPPTYDTVLQLEYLDMVV

NETLRLFPVAMRLERVCKKDVEINGMFIPKGVVVMIPSYVLHHDPKYWTEPEKFLPERFS

KKNKDNIDPYIYTPFGSGPRNCIGMRFALVNMKLALVRVLQNFSFKPCKETQIPLKLRFG

GLLLTEKPIVLKAESRDETVSGA

>P24462.2

MDLIPNLAVETWLLLAVSLILLYLYGTRTHGLFKKLGIPGPTPLPFLGNALSFRKGYWTF

DMECYKKYRKVWGIYDCQQPMLAITDPDMIKTVLVKECYSVFTNRRPFGPVGFMKNAISI

AEDEEWKRIRSLLSPTFTSGKLKEMVPIIAQYGDVLVRNLRREAETGKPVTLKHVFGAYS

MDVITSTSFGVSIDSLNNPQDPFVENTKKLLRFNPLDPFVLSIKVFPFLTPILEALNITV

FPRKVISFLTKSVKQIKEGRLKETQKHRVDFLQLMIDSQNSKDSETHKALSDLELMAQSI

IFIFAGYETTSSVLSFIIYELATHPDVQQKVQKEIDTVLPNKAPPTYDTVLQLEYLDMVV

NETLRLFPVAMRLERVCKKDVEINGMFIPKGVVVMIPSYVLHHDPKYWREPEKFLPERFS

KKNKDNIDPYIYTPFGSGPRNCIGMRFALVNMKLALVRVLQNFSFKPCKETQIPLKLRFG

GLLLTEKPIVLKAESRDETVSGA

>332867110

MDLIPNLAVETWLLLAVSLILLYLYGTRTHGLFKKLGIPGPTPLPFLGNALSFRKGYWTF

DMECYKKYRKVWGIYDCQQPMLAITDPDMIKTVLVKECYSVFTNRRPFGPVGFMKSAISI

AEDEEWKRIRSLLSPTFTSGKLKEMVPIIAQYGDVLVRNLRREAETGKPVTLKHVFGAYS

MDVITSTSFGVNIDSLNNPQDPFVENTKKLLRFNPLDPFVLSIKVFPFLTPILEALNITV

FPRKVISFLTKSVKQIKEGRLKETQKHRVDFLQLMIDSQNSEDSETHKALSDLELMAQSI

IFIFAGYETTSSVLSFIIYELATHPDVQQKVQKEIDTVLPNKAPPTYDTVLQLEYLDMVV

NETLRLFPVAMRLERVCKKDVEINGMFIPKGVVVMIPSYVLHHDPKYWTEPEKFLPERFS

KKNKDNIDPYIYTPFGSGPRNCIGMRFALVNMKLALVRVLQNFSFKPCKETQIPLKLRFG

GLLLTEKPIVLKAESRDETFIDRKPIHMDLLKSLEFQGPDFCFFWELLCPTVR

>AAG48618.1

MDLIPNLAVETWLLLAVSLILLYLYGTRTHGLFKKLGIPGPTPLPFLGNALSFRKGYWTF

DMECYKKYRKVWGIYDCQQPMLAITDPDMIKTVLVKECYSVFTNRRPFGPVGFMKNAISI

AEDEEWKRIRSLLSPTFTSGKLKEMVPIIAQYGDVLVRNLRREAETGKPVTLKHVFGAYS

MDVITSTSFGVSIDSLNNPQDPFVENTKKLLRFNPLDPFVLSIKVFPFLTPILEALNITV

FPRKVISFLTKSVKQIKEGRLKETQKHRVDFLQLMIDSQNSKDSETHKALSDLELMAQSI

IFIFAGYETTSSVLSFIIYELATHPDVQQKVQKEIDTVLPNKAPPTYDTVLQLEYLDMVV

NETLRLFPVAMRLERVCKKDVEINGMFIPKGVVVMIPSYVLHHDPKYWTEPEKFLPERFS

KKNKDNIDPYIYTPFGSGPRNCIGMRFALVNMKLALVRVLQNFSFKPCKETQIPLKLRFG

GLLLTEKPIVLKAESRDETFVDMEPIHMDFLRSLAFQGPHLCFFWELLCPTIRSR

>EHH17166.1

MDLIPDLAVETWLLLAVSLILLYLYETHSHGLLKKLRIPGPTPLPFFGNVLSFRKGFWTF

DMECYKKYRKVWGFYDCRQPVLAITDPNMIKTVLVKEFYSVYTNRRPFGPVGFMKNAISI

AEDEEWKRIRSLVSPTFTSGKLKEMVPIIAQYGDVLVRNLRREAETGKPVTLKDVFGAYS

MDVITSTSFGVNVDSLNNPQDPFVENTKKLLRFNSLDPFFLSIKIFPFLTPIFEALNITV

FPRKVTSFLRKSVKRIKEGRLKETQKRRVDFLQLMIDSQNSKDTESHKVLSDLELMAQSI

MFIFAGYETTSSVLSFIIYELATHPDVQQKLQNEIDTVLPNKAPPTYDTMLQMEYLDMVV

NETLRLFPVAMRLERVCKKDVEINGMSIPKGVVVMIPSYVLHHDPKYWTEPEKFLPERFS

KKNKDNIDPYIYTPFGSGPRNCIGMRFALMNMKLAIIRVLQNFSFKPCKETQIPLKLRFG

GLLKTEKPIVLTAESRDGTFVDMEPIHMDFLRSLEFQGPHLCLFWELLCPTVR

>374532781

MDLIPNLAVETWLLLAVSLILLYLYGTRTHGLFKKLGIPGPTPLPFLGNALSFRKGYWTF

DMECYKKYRKVWGIYDCQQPMLAITDPDMIKTVLVKECYSVFTNRRPFGPVGFMKNAISI

AEDEEWKRIRSLLSPTFTSGKLKEMVPIIAQYGDVLVRNLRREAETGKPVTLKHVFGAYS

MDVITSTSFGVSIDSLNNPQDPFVENTKKLLRFNPLDPFVLSIKVFPFLTPILEALNITV

FPRKVISFLTKSVKQIKEGRLKETQKHRVDFLQLMIDSQNSKDSETHKALSDLELMAQSI

IFIFAGYETTSSVLSFIIYELATHPDVQQKVQKEIDTVLPNKAPPTYDTVLQLEYLDMVV

NETLRLFPVAMRLERVCKKDVEINGMFIPKGVVVMIPSYVLHHDPKYWREPEKFLPERFS

KKNKDNIDPYIYTPFGSGPRNCIGMRFALVNMKLALVRVLQNFSFKPCKETQIPLKLRFG

GLLLTEKPIVLKAESRDETFVDMEPIHMDFLRSLAFQGPHLCFFWELLCPTIRSR

>ABS52842.1

MDLIPSFSMETWVLLATSLVLLYLYGTYTHGLFKKLGIPGPTPLPFFGNVLSYHKGIWDF

DKKCFEKYGKMWGTYHGTKPVLAITDPDMIKTVLVKECYSVFTNRRPFGPFGFMKSAISL

SEDEQWKRIRTLLSPTFTSGKLKEMFPILGQYGDVLVRNLKKEAEKGKPITLKDIFGAYS

MDVITSTSFGVNIDSLNNPQDPFVENTKKLFSFDFLDPLLLSITLFPFLNAVFEVLNVFV

FPKSVTDFFIKSVKRMKESRLKDKEKHRVDFLQLMINSQNSKELDTHKALSDLELVAQSI

IFIFAGYETTSSSLSFLMYFLATHPDVQQKLQEEIDATFPNKAPPTYDALVQMEYLDMVL

NESLRLFPIAVRLERVCKKDVEINGVFIPKGTVVMVPTFALHKHPEFWPEPEEFRPERFS

KENKDSINPYIYLPFGAGPRNCIGMRFALMNMKLALVRMLQNFSFKPCKETQIPLKLGNQ

GLLQPQKPIVLKVESRDGTVSGA

>297679950

MDLIPNLAVETWLLLAVSLVLLYLYGTRTHGLFKRLGIPGPTPLPLLGNVLSYRQGLWKF

DTECYKKYGKMWGTYDGQLPVLAITDPDMIKTVLVKECYSVFTNRRSLGPVGFMKSAISL

AEDEEWKRIRSLLSPTFTSGKLKEMFPIIAQYGDVLVRNLRREAEKGKPVTLKDIFGAYS

MDVITGTSFGVNIDSLNNPQDPFVESTKKFLKFSFLDPLFLSIILFPFLTPVFEALNVSL

FPKDTINFLNKSVNRMKKSRLNDKQKHRLDFLQLMIDSQNSKETESHKALSDLELAAQSI

IFIFAGYETTSSVLSFTLYELATHPDVQQKLQKEIDAVLPNKAPPTYDAVVQMEYLDMVV

NETLRLFPVAIRLERTCKKDVEINGVFIPKGAMVVIPTYALHHDPKYWTEPEEFRPERFS

KKKDSIDPYIYTPFGTGPRNCIGMRFALMNMKLALIRVLQNFSFKPCKETQIPLKLDTQG

LLQPEKPIVLKVDSRDGTLSGE

>ABP68412.1

MDLIPNLAVETWLLLAVSLVLLYLYGTRTHGLFKRLGIPGPTPLPLLGNVLSYRQGLWKF

DTECYKKYGKMWGMYDGQLPVLAITDPDMIRTVLVKECYSVFTNRRSLGPVGFMKSAISL

AEDEEWKRIRSLLSPTFTSGKLKEMFPIIAQYGDVLVRNLRREAEKGKPVTLKDIFGAYS

MDVITGTSFGVNIDSLNNPQDPFVESTKKFLKFGFLDPLFLSIILFPFLTPVFEALNVSL

FPKDTINFLSKSVNRMKKSRLNDKQKHRLDFLQLMIDSQNSKETESHKALSDLELAAQSI

IFIFAGYETTSSVLSFTLYELATHPDVQQKLQKEIDAVLPNKAPPTYDAVVQMEYLDMVV

NETLRLFPVAIRLERTCKKDVEINGVFIPKGSMVVIPTYALHHDPKYWTEPEEFRPERFS

KKKDSIDPYIYTPFGTGPRNCIGMRFALMNMKLALIRVLQNFSFKPCKETQIPLKLDTQG

LLQPEKPIVLKVDSRDGTLSGE

>397489486

MDLIPNLAVETWLLLAVSLVLLYLYGTRTHGLFKRLGIPGPTPLPLLGNVLSYRQGLWKF

DTECYKKYGKMWGMYDGQLPVLAITDPDMIRTVLVKECYSVFTNRRSLGPVGFMKSAISL

AEDEEWKRIRSLLSPTFTSGKLKEMFPIIAQYGDVLVRNLRREAEKGKPVTLKDIFGAYS

MDVITGTSFGVNIDSLNNPQDPFVESTKKFLKFGFLDPLFLSIILFPFLTPVFEALNVSL

FPKDTINFLSKSVNRMKKSRLNDKQKHRLDFLQLMIDSQNSKETESHKALSDLELAAQSI

IFIFAGYETTSSVLSFTLYELATHPDVQQKLQKEIDAVLPNKAPPTYDAVVQMEYLDMVV

NETLRLFPVAIRLERTCKKDVEINGVFIPKGSMVVIPTYALHHDPKYWTEPEEFRPERFS

KKKDSIDPYIYTPFGTGPRNCIGMRFALMNMKLALIRVLQNFSFKPCKETQIPLKLDTQG

LLQPEKPIVLKVDSRDGTLSGE

>P20815.1

MDLIPNLAVETWLLLAVSLVLLYLYGTRTHGLFKRLGIPGPTPLPLLGNVLSYRQGLWKF

DTECYKKYGKMWGTYEGQLPVLAITDPDVIRTVLVKECYSVFTNRRSLGPVGFMKSAISL

AEDEEWKRIRSLLSPTFTSGKLKEMFPIIAQYGDVLVRNLRREAEKGKPVTLKDIFGAYS

MDVITGTSFGVNIDSLNNPQDPFVESTKKFLKFGFLDPLFLSIILFPFLTPVFEALNVSL

FPKDTINFLSKSVNRMKKSRLNDKQKHRLDFLQLMIDSQNSKETESHKALSDLELAAQSI

IFIFAGYETTSSVLSFTLYELATHPDVQQKLQKEIDAVLPNKAPPTYDAVVQMEYLDMVV

NETLRLFPVAIRLERTCKKDVEINGVFIPKGSMVVIPTYALHHDPKYWTEPEEFRPERFS

KKKDSIDPYIYTPFGTGPRNCIGMRFALMNMKLALIRVLQNFSFKPCKETQIPLKLDTQG

LLQPEKPIVLKVDSRDGTLSGE

>332258037

MDLIPNLALETWVLLAVSLVLLYLYGTRTHGLFKRLGIPGPTPLPLLGNVLSYRQGLWKF

DTKCYKKYGKMWGTYDGQLPVLAITDPDMIKTVLVKECHSVFTNRRSLGPVGFMKSAISL

AEDEEWKRIRSLLSPTFTSGKLKEMFPIIAQYGDVLVRNLRREAEKGKPVTLKDIFGAYS

MDVITGTSFGVNIDSLNNPQDPFVESIKKFLKFDFLDPLFLSIILFPFLTPVFEALNVSL

FPKDAINFLNKSVNRMKKSRLNDKQKHRPDFLQLMIDSQNLKETESHKALSDLELVAQSI

IFIFAGYETTSSVLSFTLYELATHPDVQQKLQKEIDAVLPNKAPPTYDAVVQMEYLDMVV

NETLRLFPIAIRLERTCKKDVEINGVFIPKGAMVVIPTYALHHDPKHWTEPEEFRPERFS

KKKDSIDPYIYTPFGTGPRNCIGMRFALMNMKLALIRVLQNFSFKPCKETQIPLKLGMQG

LLQPEKPIVLKVDSRDGTLSGE

>AAX42491.1

MDLIPNLAVETWLLLAVSLVLLYLYGTRTHGLFKRLGIPGPTPLPLLGNVLSYRQGLWKF

DTECYKKYGKMWGTYEGQLPVLAITDPDVIRTVLVKECYSVFTNRRSLGPVGFMKSAISL

AEDEEWKRIRSLLSPTFTSGKLKEMFPIIAQYGDVLVRNLRREAEKGKPVTLKDIFGAYS

MDVITGTSFGVNIDSLNNPQDPFVESTKKFLKFGFLDPLFLSIILFPFLTPVFEALNVSL

FPKDTINFLSKSVNRMKKSRLNDKQKHRLDFLQLMIDSQNSKETESHKALSDLELAAQSI

IFIFAGYETTSSVLSFTLYELATHPDVQQKLQKEIDAVLPNKAPPTYDAVVQMEYLDMVV

NETLRLFPVAIRLERTCKKDVEINGVFIPKGSMVVIPTYALHHDPKYWTEPEEFRPERFS

KKKDSIDPYIYTPFGTGPRNCIGMRFALMNMKLALIRVLQNFSYKPCKETQIPLKLDTQG

LLQPEKPIVLKVDSRDGTLSGE

>BAG36549.1

MDLIPNLAVETWLLLAVSLVLLYLYGTRTHGLFKRLGIPGPTPLPLLGNVLSYRQGLWKF

DTECYKKYGKMWGTYEGQLPVLAVTDPDVIRTVLVKECYSVFTNRRSLGPVGFMKSAISL

AEDEEWKRIRSLLSPTFTSGKLKEMFPIIAQYGDVLVGNLRREAEKGKPVTLKDIFGAYS

MDVITGTSFGVNIDSLNNPQDPFVESTKKFLKFGFLDPLFLSIILFPFLTPVFEALNVSL

FPKDTINFLSKSVNRMKKSRLNDKQKHRLDFLQLMIDSQNSKETESHKALSDLELAAQSI

IFIFAGYETTSSVLSFTLYELATHPDVQQKLQKEIDAVLPNKAPPTYDAVVQMEYLDMVV

NETLRLFPVAIRLERTCKKDVEINGVFIPKGSMVVIPTYALHHDPKYWTEPEEFRPERFS

KKKDSIDPYIYTPFGTGPRNCIGMRFALMNMKLALIRVLQNFSFKPCKETQIPLKLDTQG

LLQPEKPIVLKVDSRDGTLSGE

>402862964

MAMDLIPNLAMETWLLLAVSLVLLYLYGTRSYGLFKRQGIPGPTPLPFLGNVLSYRQGLW

KFDTECYKKYGKMWRTQDGQLPVLTITDPDMIKTVLVKECYSVFTNRRPFGPVGLMKSAI

SIAENEEWKRIRSLLSPTFTSGKLKEMFPIIAQYGDVLVRNLRREAEKGKPVTLKDIFGA

YSMDVITSTSFGVNIDSLNNPKDPFVESVKKFLKFDFLDPLFLLTILFPFLIPAFEALNV

SLFPKDAINFLNKSVNSMKKSRLNDKQKHRVDFLQLMIDSQNSKETESHKALSDQELVAQ

SIIFIFAGYETTSSVLSFIIYELATHPDVQQXLQEEIDAVLPNKAPATYDAMVQMEYLDM

VVNETLRLFPIAIRLERACKKDVEINGVFIPKGAMVVIPTYALHHDPKYWTEPEEFRPER

FSKKNKDSIDPYIYTPFGTGPRNCIGMRFALMNMKLAIIKVLQNFSFKPCKETQIPLKLG

NQGLLQSEKPIVLKVESRDGTLSGE

>AAZ29441.1

MDLIPNLAMETWLLLAVSLVLLYLYGTRSYGLFKRQGIPGPTPLPFLGNILSYRQGLWKF

DTECYKKYGKMWRTQDGQLPVLTITDPDMIKTVLVKECYSVFTNRRPLGPVGLMKSAISI

AEDEEWKRIRSLLSPTFTSGKLKEMFPIIAQYGDMLVRNLRREAEKGKPVTLKDIFGAYS

MDVITSTSFGVNIDSLNNPKDPFVESVKKFLKFDFLDPLFLLTILFPFLIPAFEALNVSL

FPKDAINFLNKSVNSMKKSRLNDKQKHRVDFLQLMIDSQNSKETESHKALSDQELVAQSI

IFIFAGYETTSSVLSFIIYELATHPDVQQKLQKEIDAVLPNKAPATYDAMVQMEYLDMVV

NETLRLFPIAIRLERACKKDVEINGVFIPKGAMVVIPTYALHHDPKYWTEPEEFRPERFS

KKNKDSIDPYIYTPFGSGPRNCIGMRFALMNMKLAIIKVLQNFSFKPCKETQIPLKLGNQ

GLLQSEKPIVLKVESRDGTLSGE

>ABU85101.1

MDLIPNLAVETWLLLALSLVLLYLYGTRSHGIFKKLGIPGPAPLPFVGNILSYRQGIWKF

DSECHKKYGKMWGSYDGQLPVLAITDPDIIKAVLVKECYSVFTNRRPFGPVGLMKSAISL

AQDDEWKRIRSLLSPTFTSGKLKEMFPIIAQYGDVLVRNLRREAGKGKPVTMKDIFGAYS

MDVITGTSFGVNIDSLNNPKDPFVESVKKFLKFDFLDPLFLSTIFFPFLTPVFEALNFSL

FPKDAINFLKQSVNRMKKSRLNDKQKHRVDFLQLMIDSQNSNETASHKALSDLELLAQSI

IFIFAGYETTSSVLSFTIYELATNPDVQQKLQEEIDVVLPNKAPATYDAVVQMEYLDMVV

NETLRLYPIAVRLERVCKKDVEINGVFIPKGALVVIPTYALHHDPKYWTEPKEFRPERFS

KKNKDSIDPYIYTPFGTGPRNCIGMRFALMNMKLALIRVLQNFSFKPCKETQIPLKLGVQ

GLLQAEKPIILKVESRDGTLSGE

>CBJ94506.1

MDLIPTFSMETWVLLATSLVLVYLYGTYTHGLFKKLGIPGPTPLPFFGNVLSYRKGISNF

DRKCFKKYGKMWGVYDGRRPVLAITDPDMIKTVLVKECYSVFTNRRPFRPVGFMKSAISL

SEDEEWKRIRTLLSPTFTSGKLKEMFPIIGQYGDVLVRNLRKEAEKGKPVTLKDIFGAYS

MDVITSTSFGVNIDSLNNPQDPFVENSNKLLRFDFLNPLILLMVLFPFLQPIFEVLNISL

FPKSAIDFFTKSVKRMKESRLKDKDKHRVDFLQLMINSQNSKELDTHKGLSDLELVAQSI

VFIFAGYETTSTSLSFLLYLLATHPDVQQKLQEEIDAIFPNKAPPTYDALVQMDYLDMVL

NESLRLFPVAVRLERVCKKDAEINGVFIPKGTVVMVPTFSLHRASEFWPEPEEFRPERFS

KKNKDNINPCIYMPFGNGPRNCIGMRFAMVNMKLALVRVLQNFSFKPCKETQIPLKLGNQ

GLLQPQKPIVLKVESRDGTVNGA

>CBJ94508.1

MDLIPNFSMETWVLLATSLVLLYLYGTYTHGLFKKLGIPGPTPLPFLGTVLGYLKGFWDF

DKKCFKKYGSMWGIYDGPQPVLAITDPDMIKTVLVKECYSVFTNRRSLGPVGFMKNAISL

SEDEQWKRIRTLLSPTFTSGKLKEMFPIIGQYGDVLVRNLRKETEKGKPVTLKNIFGAYS

MDVITSTSFGVNIDSLNNPQDPFVENTKKLLRFNFLDPFFLSITLFPFLKAVFEVIHIYM

FPKRVTDFFTKSVKRMKESRLKDKQKHRVDFLQLMINSQNSKEMDTHKALSDLELVAQSI

IFIFAGYETTSTSLSFLMYLLATHPDVQQKLQDEIDVTFPNKALPTYDTLLQMEYLDMVL

NESLRLFPIAGRLERVCKKDVEINGVLIPKGTVVMVPTFILHRASEFWPEPEEFCPERFS

KNNKDNINPYIYLPFGTGPRNCIGMRFAIMNMKLAIVRVLQNFSFKPCKETQIPLKIGNQ

GLIQPQKPIVLKVESRDGTWNGA

>395852793

MNLIPDFAMETWVLLAVSLVLLYLYGTHSHGLFKKLGIPGPTPLPFLGTVLGYRKGFWDF

DAECHRKYGKTWGLYDGRQPLLVITDPDMIKTVLVKECYSVFTNRRSFGPVGFMKNAISI

SENEEWKRIRALLSPTFTSGKLKEMFPIMSQYADVLVRNLRQEAEKDKPINLKDAFGAYS

MDVITGTSFGVNIDSLNNPQDPFVENTKKLLRFDFLDPFLLSIILFPFLTPVYEALSITA

FPKDVIDFFKKSVKRMKESRLKDKERHRVDFLQLMIDSQNTKETVSHKVLSDLELVAQSV

IFIFAGYETTSSALSFIMYELATHPDVQKKLQDEIDAALPNKAPATYDAMVQMEYLDMVV

NETLRLFPIAGRLERVCKKDVEVNGVLIPKGVTVMIPTYALHRDPKHWIEPEEFRPERFS

KKNKDSIDPYIYLPFGTGPRNCIGMRFALMNMKLALVGVLQNFSFKPCEETQIPLKLSRQ

GFLQPEKPIVLKVESRVGTVRGA

>AAT49270.1

MDLIPNLAMETWLLLAVSLVLLYLYGTRSYGLFKRQGIPGPTPLPFLGNILSYRQGLWKF

DTECYKKYGKMWRTQDGQLPVLTITDPEMIKTVLVKECYSVFTNRRPLGPVGLMKSAISI

AEDEEWKRIRSLLSPTFTSGKLKEMFPIIAQYGDMLVRNLRREAEKGKPVTLKDIFGAYS

MDVITSTSFGVNIDSLNNPKDPFVESVKKFLKFDFLDPLFLLTILFPFLTPAFEALNVSL

FPKDAINFLNKSVNSMKKSRLNDKQKHRVDFLQLMIDSQNSKETESHKALSDQELVAQSI

IFIFAGYETTSSVLSFIIYELATHPDVQQKLQKEIDAVLPNKAPATYDAMVQMEYLDMVV

NETLRLFPIAIRLERACKKDVEINGVFIPKGAMVVIPTYALHHDPKYWTEPEEFRPERFS

KKNKDSIDPYIYTPFGTGPRNCIGMRFALMNMKLAIIKVLQNFSFKPCKETQIPLKLGNQ

GLLQSEKPIVLKVESRDGTLSGE

>ABU85100.1

MDLIPNLAVETWLLLALSLVLLYLYGTRSHGIFKKLGIPGPAPLPFVGNVLSYRQGIWKF

DSECHKKYGKMWGSYDGQLPVLSITDPDMIKAVLVKECYSIFTNRRPLGPVGFMKSAITV

AQDDEWKRIRSLLSPTFTSGKLKEMFPIIAQYGDVLVRNLRREAEKGKLVTMKDIFGAYS

MDVITGTSFGVNIDSLNNPKDPFVENVKKFLKFDVLAPLFLSVVLFPFLIPVFEALNFSL

FPKDAINFLKQSVNRMKKSRLNDKQKHRVDFLQLMIDSQNSNETASHKALSDLELVAQSI

IFIFAGYETTSSVLSFTIYELATNPDVQQKLQEEIDVVLPNKAPATYDAVVQMEYLDMVV

NETLRLYPITVRHERVCKKDVEINGVFIPKGALVVIPTYALHHDPKYWTEPKEFRPERFS

KKNKDSIDPYIYTPFGTGPRNCIGMRFALMNMKLALIRVLQNFSFKPCKETQIPLKLGVQ

GILQAEKPIVLKVESRDGTLSGE

>297679952

MQLLMLELLLLLIVYGTRTHGLFKRLGIPGPTPLPLLGNVLSYRQGLWKFDTECYKKYGK

MWGTYDGQLPVLAITDPDMIKTVLVKECYSVFTNRRSLGPVGFMKSAISLAEDEEWKRIR

SLLSPTFTSGKLKEMFPIIAQYGDVLVRNLRREAEKGKPVTLKDIFGAYSMDVITGTSFG

VNIDSLNNPQDPFVESTKKFLKFSFLDPLFLSIILFPFLTPVFEALNVSLFPKDTINFLN

KSVNRMKKSRLNDKQKHRLDFLQLMIDSQNSKETESHKALSDLELAAQSIIFIFAGYETT

SSVLSFTLYELATHPDVQQKLQKEIDAVLPNKAPPTYDAVVQMEYLDMVVNETLRLFPVA

IRLERTCKKDVEINGVFIPKGAMVVIPTYALHHDPKYWTEPEEFRPERFSKKKDSIDPYI

YTPFGTGPRNCIGMRFALMNMKLALIRVLQNFSFKPCKETQIPLKLDTQGLLQPEKPIVL

KVDSRDGTLSGE

>EHH52026.1

MDLIPNLAMETWLLLAVSLVLLYLYGTRSYGLFKRQGIPGPTPLPFLGNILSYRQGLWKF

DTECYKKYGKMWRTQDGQLPVLTITDPDMIKTVLVKECYSVFTNRRPLGPVGFMKSAISI

AEDEEWKRIRSLLSPTFTSGKLKEMFPIIAQYGDMLVRNLRREAEKGKPVTLKDIFGAYS

MDVITSTSFGVNIDSLNNPKDPFVESVKKFLKFDFLDPLFLLTILFPFLIPAFEALNVSL

FPKDAINFLNKSVNSMKKSRLNDKQKHRVDFLQLMIDSQNSKETESHKALSDQELVAQSI

IFIFAGYETTSSVLSFIIYELATHPDVQQKLQKEIDAVLPNKAPATYDAMVQMEYLDMVV

NETLRLFPIAIRLERACKKDVEINGVFIPKGAMVVIPTYALHHDPKYWTEPEEFRPERYK

KNKDSIDPYRYIPFGSGPRNCIGMRFALMNMKLAIIRVLQNFSFKPCKETQIPLKLGNQG

LLQSEKPIVLKVESRDGTLSGE

>EHH17165.1

MDLIPNLAMETWLLLAVSLVLLYLYGTRSYGLFKRQGIPGPTPLPFLGNILSYRQGLWKF

DTECYKKYGKMWRTQDGQLPVLTITDPEMIKTVLVKECYSVFTNRRPLGPVGLMKSAISI

AEDEEWKRIRSLLSPTFTSGKLKEMFPIIAQYGDVLVRNLRREAEKGKPVTLKDIFGAYS

MDVITSTSFGVNIDSLNNPKDPFVESVKKFLKFDFLDPLFLLTILFPFLTPAFEALNVSL

FPKDAINFLNKSVNSMKKSRLNDKQKHRVDFLQLMIDSQNSKETESHKALSDQELVAQSI

IFIFAGYETTSSVLSFIIYELATHPDVQQKLQKEIDAVLPNKAPATYDAMVQMEYLDMVV

NETLRLFPIAIRLERACKKDVEINGVFIPKGAMVVIPTYALHHDPKYWTEPEEFRPERYK

KNKDSIDPYIYTPFGTGPRNCIGMRFALMNMKLAIIRVLQNFSFKPCKETQIPLKLGNQG

LLQSEKPIVLKVESRDGTLSGE

>397489488

MQLLMLELLLLFIVYGTRTHGLFKRLGIPGPTPLPLLGNVLSYRQGLWKFDTECYKKYGK

MWGMYDGQLPVLAITDPDMIRTVLVKECYSVFTNRRSLGPVGFMKSAISLAEDEEWKRIR

SLLSPTFTSGKLKEMFPIIAQYGDVLVRNLRREAEKGKPVTLKDIFGAYSMDVITGTSFG

VNIDSLNNPQDPFVESTKKFLKFGFLDPLFLSIILFPFLTPVFEALNVSLFPKDTINFLS

KSVNRMKKSRLNDKQKHRLDFLQLMIDSQNSKETESHKALSDLELAAQSIIFIFAGYETT

SSVLSFTLYELATHPDVQQKLQKEIDAVLPNKAPPTYDAVVQMEYLDMVVNETLRLFPVA

IRLERTCKKDVEINGVFIPKGSMVVIPTYALHHDPKYWTEPEEFRPERFSKKKDSIDPYI

YTPFGTGPRNCIGMRFALMNMKLALIRVLQNFSFKPCKETQIPLKLDTQGLLQPEKPIVL

KVDSRDGTLSGE

>73957820

MDLIPSFSMETWLLLATSLVLLYLYGTYTHGVFKKLGIPGPTPLPFVGTALGYRKGFSVF

DENCFRKYGRMWGFYDGRQPVLAITDPDMIKTVLVKECYSVFTNRRSFGPVGFMKSAISL

SEDEEWKRIRTLLSPTFTSGKLKEMFPIIGQYGDVLVRNLRKEAEKGKSINLKDIFGAYS

MDVITSTSFGVNIDSLNNPQDPFVENIKKLLKFDFLDPFFFSILLFPFLTPVFEVLNIWL

FPKSVTDFFTKSVKRMKENRLKDKQKHRVDFLQLMINSQNSKETDTHKALSDLELVAQSI

IFIFAGYETTSTSLSFLMYELATHPDVQQKLQEEIDATFPNKALPTYDALVQMEYLDMVL

NETLRLYPIAGRLERVCKKDVEISGVFIPKGTVVMVPTFTLHRDQSLWPEPEEFRPERFS

RKNKDSINPYTYLPFGTGPRNCIGMRFAIMNMKLALVRVLQNFSFKPCKETQIPLKLNAQ

GIIQPEKPIVLKVEPRDGSVNGA

>395852799

MDVILNFAMETWVLLAASLVLLYIYGTHSHGLFKKLGIPGPTPLPFFGTFLNYHRGVWDS

DLECYKKYGKMWGLYDGQQPVLAITDPEMIKTVLVKECHSVFTNRRSFGPLGLMKSAISL

SVDDEWKRIRTLLSPTFSSGKLKEMFPIMSQYGDVLVRNLRQEANKGKTVNLKDIFGAYS

MDVITSTSFGVNIDSLNNPQDPFVENIKKFLKFNFVTPFFLSILLFPFLIPVFEALNIFV

FPKDVMDFFEKSIKRIKESRLQDKQKHRVDLLQLMIDSQNSKETESHKVLSDVELVAQSI

IFIFAGYETTSSALSFLMYELATHPDVQQKLQDEIDAALPSKTPATYDAMVQMEYLDMVV

NETLRLFPIAGRLERVCKKDVEVNGVLIPKGVTVMIPTFALHRDPKHWTEPEEFRPERFS

KKNKDSIDPYMYMPFGSGPRNCIGMRFALMNMKIALIRILKNFSFKPCKETQIPLKLSKK

AILQPEKPIVLKIESREGTTNGA

>BAH12282.1

MQLLMLELLLLFIVYGTRTHGLFKRLGIPGPTPLPLLGNVLSYRQGLWKFDTECYKKYGK

MWGTYEGQLPVLAITDPDVIRTVLVKECYSVFTNRRSLGPVGFMKSAISLAEDEEWKRIR

SLLSPTFTSGKLKEMFPIIAQYGDVLVRNLRREAEKGKPVTLKDIFGAYSMDVITGTSFG

VNIDSLNNPQDPFVESTKKFLKFGFLDPLFLSIILFPFLTPVFEALNVSLFPKDTINFLS

KSVNRMKKSRLNDKQKHRLDFLQLMIDSQNSKETESHKALSDLELAAQSIIFIFAGYETT

SSVLSFTLYELATHPDVQQKLQKEIDAVLPNKAPPTYDAVVQMEYLDMVVNETLRLFPVA

IRLERTCKKDVEINGVFIPKGSMVVIPTYALHHDPKYWTEPEEFRPERFSKKKDSIDPYV

YTPFGTGPRNCIGMRFALMNMKLALIRVLQNFSFKPCKETQIPLKLDTQGLLQPEKPIVL

KVDSRDGTLSGE

>332258039

MQLLTLELLLLFIIYGTRTHGLFKRLGIPGPTPLPLLGNVLSYRQGLWKFDTKCYKKYGK

MWGTYDGQLPVLAITDPDMIKTVLVKECHSVFTNRRSLGPVGFMKSAISLAEDEEWKRIR

SLLSPTFTSGKLKEMFPIIAQYGDVLVRNLRREAEKGKPVTLKDIFGAYSMDVITGTSFG

VNIDSLNNPQDPFVESIKKFLKFDFLDPLFLSIILFPFLTPVFEALNVSLFPKDAINFLN

KSVNRMKKSRLNDKQKHRPDFLQLMIDSQNLKETESHKALSDLELVAQSIIFIFAGYETT

SSVLSFTLYELATHPDVQQKLQKEIDAVLPNKAPPTYDAVVQMEYLDMVVNETLRLFPIA

IRLERTCKKDVEINGVFIPKGAMVVIPTYALHHDPKHWTEPEEFRPERFSKKKDSIDPYI

YTPFGTGPRNCIGMRFALMNMKLALIRVLQNFSFKPCKETQIPLKLGMQGLLQPEKPIVL

KVDSRDGTLSGE

>P24463.1

MDLIPSFSTETWLLLAISLVLLYLYGTYTHGIFRKLGIPGPTPLPFVGTALGYRNGFYVF

DMKCFSKYGRMWGFYDGRQPVLAITDPDMIKTVLVKECYSVFTNRRTLGPVGFMKSAISL

SEDEEWKRMRTLLSPTFTTGKLKEMFPIIGQYGDVLVNNLRKEAEKGKAINLKDVFGAYS

MDVITSTSFGVNIDSLNHPQDPFVENTKKLLKFDFLDPFFFSILLFPFLTPVFEILNIWL

FPKKVTDFFRKSVERMKESRLKDKQKHRVDFLQLMINSQNSKEMDTHKALSDLELVAQSI

IFIFAGYETTSTSLSFLMYELATHPDVQQKLQEEIDATFPNKALPTYDALVQMEYLDMVL

NETLRLYPIAGRLERVCKKDVEISGVFIPKGTVVMVPTFTLHRDQSLWPEPEEFRPERFS

RKNKDSINPYTYLPFGTGPRNCIGMRFAIMNMKLALVRVLQNFSFKPCKETQIPLKLNAQ

GIIQPEKPIVLKVEPRDGSVNGA

>EHB00903.1

MDLIPNYSMETWVLLATCLVLLYLYGTSSHGLFKKLGIPGPKPLPFFGTILSYHKGFWVF

DTKCYKKYGNIWGFYDGQQPVLAITDPDMIKTVLVKECYSTFTNRRSFGPVGFMKKAISI

SEDEEWKRIRTLLSPAFTTGKLKEMFPIIEQYGDVLVKNLRQRAKKGEPVSMKDIFGAYS

MDVITGTSFGVNVDSLNNPQDPFVGKVKKILKFGIFDPLFMLIIFFPFLTPVLEALNISI

FPRDVTDFLKISIERMKESRLKEKEKHRVDFLQLMINSQNSKDTESHKALSDLELVAQSI

IFIFGGYETTSSALSFVMYELATHPDVQKKLQQEIDTVLPNKTPATYDALVQMEYLDMVV

NETLRLYPIAGRLERACKKDVEINRVVIPKGSVVLIPTYALHRDPKYWKEPEEFHPERFS

KKNKGNIDPYIYMPFGAGPRNCIGMRFALMNMKLAIIRVLQNFSFQTCKETQIPITFSKQ

GLLQPEKEVLLKVVSREEPLSGA

>73958043

MDLIPSFSTETWLLLATSLVLFYLYGTYTHGLFKKLGIPGPTPLPFLGTVLGYRNGFCDF

DEKCFRKYGRMWGFYDGRQPVLAIMDPDMIKTVLVKECYSVFTNRRSFGPVGFMKSAITV

SEDEEWKRIRTLLSPTFTSGKLKEMFPIIGQYGDMLVRNLRKEAEKGKAISLKDIFGAYS

MDVITSTSFGVNIDSLNNPQDPFVENAKKLLKFDFPDPFLLSIILFPFLTPLYEMLNIWL

FPKKITDFFTKSVKRMKESRLKDKQKHRVDFLQLMINSQNSKEMNTHKALSDLELVAQSI

IFVVAGYETTSTSLCLLMYELATHPDVQQKLQKEIDATFPNKAAPTYDTLVQMEYLDMVL

NESLRLYPITGRLVRVCKKDVEISGVFIPKGTVVMVPTFTLHQDPDIWPEPEKFQPERFS

KKNKDSINPYTYLPFGTGPRNCLGMRFAIMNMKLALIKVLQNFSFKPCKETQIPLKLSSQ

GLIRPEEPIILNVEPRDGSVRGA

>CBJ94509.1

MDLIPSLSMETWVLLATSLVLLYLYGTYSHGHFKKLGIPGPTPLPFFGTVLGYIKGVLAF

DKKCFKKYGKMWGFYDGRQPVLAITDPDMIKTVLVKECYSVFTNRRSFGPMGLMKNAITI

AEDEKWKRIRTLLSPTFTSGKLKEMFPIIGQYGDMLVRNLRKEAEKGKPITMKDIFGAYS

MDVITSTSFGVNIDSLNNPQDPFVENTKKLLRFDFLDPFFLSILFFPFLIPVYEALNIFL

FPKCVTDFFIKSVKKMKESRLKDKAKQRVDFLQLMINSQNSKEIDNLKVLSDLELVAQSI

TFIFAGYEPVSTSLSFLLYLLATHPDVQQKLQEEIDATFPNKAPPTYDALVQMEYLDMVL

NETLRLFPIADRLERVCKKDVEINGVFIPKGSTVMMPIFVLHQHPEFWSEPEEFRPERFS

KKNKDSINPYTYLPFGTGPRNCIGMRFAIVNIKLAVVRVLQNFSFKPCKETQIPLKLVSQ

GLIRPEKPIVLKVESRDGTMSGA

>395852901

MDLIPEFATETWVLLATSLVLLYLFGTHSHGLFKKLGIPGPTPLPILGNVLSYRKGFWKF

DEECYKKYGKTWGLYDGRQPLLAITDPEMIKTVLVKECYSAFTNRRPFGPVGFMKSAISI

SEDEEWKRIRALLSPTFTSGKLKEMFPIMSQYADVLVRNLRQEADKGKTITLKDVFGAYS

MDVITGTSFGVNVDSLNNPQDPFVEKTKKLLRFDFLDPLFLSIILFPFLTPVFEALNISM

FPKDVTNFLKNSVQKMKESRLHDKQKVKSGGVLSDLELVAQSIIFIFAGYETTSSVLSFI

MYELATHPDVQQKLQDEIDAALPNKTPATYDALAQMECLDMVVNETLRLFPIAGRLERVC

KKDVEIKGVLIPKGTVVMVPTYALHRDPKYWTEPEEFHPERFSKKNKDSIDPYTYMPFGS

GPRNCIGMRFALVNMKLALVRVLQNFSFKPCKETQIPMKLSPKGMLQPEKPIVLKIESRD

GTIHGA

>AFJ20721.1

MAVAGIDWNMDLIPSFSLETWVLLATSLVLLYLYGTYSHGFFKKLEIPGPRPLPYFGNVL

GYRKGFWAFDSKCFKKYGKIWGFYDGQQPVLAITDPDMIKTVLVKECYSVFTNRRFIGPR

GIMKNAMSVAQDEQWKRVRTLLSPTFTSGKLKEMFPIIGQYGDVLVRNMRKEAEKGNPVT

MKNIFGAYSMDVITSTAFGVNIDSLNNPQDPFVENSKKLFRFDFLDPFLLTIIFFPFLTL

VYEALNITLFPKSAVNFFTKSVKRMKENRLKDKQKHRVDFLQLMIDSQNSKETDTHKALT

DLEIVAQGIIFIFAGYETTSNSLSFIMYELATHPDVQQKLQKEIDATFPNKAPPTYDGLL

HMEYLDMVVNETLRIFPLPGRLERVCKKDVEINGVFIPKGTLVIVPVYALHKDPALWTEP

EEFRPERFSKKNQDSINPYTYLPFGTGPRNCIGTRFAMMSMKLALVKVLQNFSFKPCKET

QIPLELGTQGILQPKKPIILKVVPRDGTLNEA

>EHH52027.1

MDLIPDLAVETWLPLAVSLILLYLYGTHSHGLFKKLGIPGPTPLPFFGNVLSFRKGFWTF

DMECYKKYRKVWGFYDCRQPVLAITEPNMIKTVLVKECYSVFTNRRPLGPVGFMKSAITV

AEDEEWKRIRSLLSPTFTSGKLVFGAYSMDVITSTSFGVNVDSLNNPQDPFVENTKKLLR

FNTLDPFFLSIKIFPFLTPIFEALNITVFPRKVTSFLRKSVKRIKEGRLKETQKRRVDFL

QLMIDSQNSKDTESHKVLSDLELMAQSIMFIFAGYETTSSVLSFIIYELATHPDVQQKLQ

KEIDTVLPNKAPPTYDTMLQMEYLDMVVNETLRLFPVAMRLERVCKKDVEINGISIPKGV

VVMIPSYVLHHDPKYWTEPEKFLPERFSKKNKDNIDPYIYTPFGNGPRNCIGMRFALMNM

KLALIRVLQNFSFKPCKETQIPLKLSFGGLLKTEKPIVLTAESRDGTFLHMEPIHMDFLR

SLEFQGPHLCLFWELLCPTVR

>CBJ94510.1

MDLIPSFSVETWVLLATSLALLYLYGIYTHGLFKKLGIPGPKPLPFLGTLLGYRKGFGGF

DTECLKKYRKMWGFYDGRQPVLAITDPDLIKTVLVKECYSVFTNRRSFGPVGFMKNAISI

SEDEQWKRIRTLLSPTFTSGKLKEMFPIIGQYGDVLVRNLKKEAEKGKPIALKDIFGAYS

MDVITSTSFGVNIDSLNNPQDPFVENTKKLLRFDFLDPFILSITIFPFLNPVFELLNIFL

FPKSVTDFFTKSVKRIKESRLKDKEKQRVDFLQLMINSQNSKEMDTHKALSDLELVAQSI

IFIFAGYEPVSSSLSFLLYLLATHPDVQQKLQEEIDATFPNMAPPTYDALVQMEYLDMVL

NESLRLFPVAGRIERTCKKDVELGGVFIPKGTVVMVPSFALHRDTELWPQPEEFHPERFS

KENKDSINPYIYMPFGNGPRNCIGMRFALMNMKVAVVRVLQNFSFKPCKETQIPLKLVTY

GFLQPEKPIVLKVESRAGTVSGA

>ABQ12997.1

MELILSFSTETWVLLATGLVLLYLYGTYSYGLFKKLGVPGPRPLPYFGNILSYRKGVCEF

NEECFKKYGKIWGIFEGKQPLLVITDPDMIKTVLVKECYSVFTNRRVFGPSGVMKNAISV

AEDEQWKRIRTLLSPTFTSGKLKEMFPIIGKYGDVLVRNLRKEAEKGTSVDIKDIFGAYS

MDVITSTSFGVNIDSLSNPQDPFVENVKKLLRFSILDPFLLAVVLFPFLVPILDVLNITI

FPKSVVNFFTKSVKRIKESRLKDNQKPRVDFLQLMINSQNSKETDNHKALSDQELIAQSI

IFIFAGYETTSSTLSFLLYILATHPDVQQKLQEEIDATFPNKAPPTYDVLAQMEYLDMVV

NETLRMFPIAIRLERLCKKDVEIHGVSIPKGTTVMVPISVLHKDPQLWPEPEEFRPERFS

KKNKDSINPYVYLPFGTGPRNCIGMRFAIMNMKLAIVRVLQNFSFKPCKETQIPLKISSQ

GVLRPEKPVVLKVVLRDGTISGA

>AAI02567.1

MELIPNFSVETWVLLAISLVLLYLYGTYSHGLFKKLGVPGPRPLPLFGNVLSYRKGVCEF

DEECFKKYGKMWGIFEGKHPLLVITDPDMIKTVLVKECYSVFTNRRVFGPMGVMKNAVSV

AEDEQWKRIRTLLSPTFTSGKLKEMFPIIGKYGDVLVRNLRKEAEKGTSVDMKEVFGAYS

MDVITSTSFGVNIDSLGNPQDPFVENAKKLLRFDILDPFLLSVVLFPFLIPIFEVLNISI

FPKSAVNFLTTSVKKIKESRLKDTQKPRVDFLQLMINSQNSKETDNHKALSDQELMAQSI

IFIFGGYETTSTSLSFIIYELATHPDVQQKLQEEIDATFPNKAPPTYDVLAQMEYLDMVV

NETLRMFPIAVRLERFCKKDVEIHGVSIPKGTTVTVPISVLHRDPQLWPEPEEFRPERFS

KKNKDSINPYVYLPFGTGPRNCIGMRFAIMNMKLAVVRVLQNFSFKPCKETQIPLKIKSQ

GLLRPEKPIVLKVVLRDETISGA

>DAA15042.1

MELILSFSTETWVLLATGLVLLYLYGTYSYGLFKKLGVPGPRPLPYFGNILSYRKGVCEF

NEECFKKYGKIWGIFEGKQPLLVITDPDMIKTVLVKECYSVFTNRRVFGPSGVMKNAISV

AEDEQWKRIRTLLSPTFTSGKLKEMFPIIGKYGDVLVRNLRKEAEKGTSVDIKDIFGAYS

MDVITSTSFGVNIDSLGNPQDPFVENVKKLLRFSILDPFLLAVVLFPFLVPILDVLNITI

FPKSAVNFFTKSVKRIKESRLKDNQKPRVDFLQLMINSQNSKETDNHKALSDQELIAQSI

IFIFAGYETTSSTLSFLLYILATHPDVQQKLQEEIDATFPNKAPPTYDVLAQMEYLDMVV

NETLRMFPIAIRLERLCKKDVEIHGVSIPKGTTVMVPISVLHKDPQLWPEPEEFRPERFS

KKNKDSINPYVYLPFGTGPRNCIGMRFAIMNMKLAIVRVLQNFSFKPCKETQIPLKISSQ

GVLRPEKPVVLKVVLRDGTISGA

>403285938

MDLIPNLAVETWLLLALSLALLYLYGTRSHGIFKKLGIPGPTPLPFLGNVLSYRQGIWKF

DSECHKKYGKMWGSYDGQLPVLAITDPDMIKAVLVKECYSVFTNRRPIGPVGLMKSAITI

AQDEEWKRIRSLLSPTFTSGKLKEMFPIIAQYGDVLVRNLRRETEKGKPITVKDIFGAYS

MDVITGTSFGVNIDSLNNPKDPFVESVKKFLKFDVLDPLFLSIVLFPFLTPVFEALNFSL

FPKDAINFLRKSVNRMKKSRLNDKQKHRVDFLQLMIDSQNSKETASHKALSDLELVAQSI

IFIFAGYETTSSVLSFTIYELATHPDVQQKLQEEIDVVLPNKAPATYDAVVQMEYLDMVV

NETLRLYPITIRLERVCKKDVEINGLFIPKGALVVIPTYALHYDPKYWTEPKEFRPERYE

ASRKTELTPHTYTIYSTGPPGCLGARLSLAQIKLALIRVLQNFSFKPCKETQIPLKLGVQ

GLLQPEKPIVLKVESRDGTLSGE

>BAK52240.1

MDLIPSFSIEAWLLLATSLVLLYLYGTYTHGLFKKLGIPGPKPLPFVGTALGYRQGFCAF

DEKCFKTYGRMWGFYDGRQPVLAITDPDMIKTVLVKECYSVFTNRRSLGPMGFMKSAISL

SEDEQWKRIRTMLSPTFTSGKLKEMFPIVGQHGDVLVRNLRKEAEKGKPVNLKDIFGAYS

MDVITGTSFGVNIDSLNNPQDPFVENTKNLFKFSFLDPLFFSIILFPFLTPIFELLNIWI

FPKKVTDFFTKSVKSMKESRLKDKQKHRVDLLQLMINSQNSKETDTHKALSDLELVAQSI

MFIFGGYETTSTSLSFLVYDLATHPDVQQKLQEEIDATFPDKAPPTYDALVQMEYLDMVL

NETLRLYPIAGRLERVCKRDVEISGVFIPKGTVVMVPTFTLHRDLDLWPEPEEFCPERFS

KKNKDSINPYIYLPFGSGPRNCIGMRFAIMNMKLALVRVLQNFSFKPCKETQIPLKLNTL

SIIEPEKPIVLKVELRDGSVNGA

>AAN47145.1

MDLIPSFSTETWLLLAISLVLLYLYGTYTHGIFRKLGIPGPTPLPFVGTALGYRNGFYVF

DMKCFSKYGRMWGFYDGRQPVLAITDPDMIKTVLVKECYSVFTNRRTLGPVGFMKSAISL

SEDEEWKRIRTLLSPTFTTGKLKEMFPIIGQYGDVLVNNLRKEAEKGKAINLKDVFGAYS

MDVITSISFGVNIDSLNHPQDPFVENTKNLLKFDFLDPFLFSILLFPFLTPVFEILNIWL

FPKKVTDFFRKSVERMKESRLKDKQKHRVDFLQLMINSQNSKEMDTHKALSDLELVAQSI

VFIFAGYETTSSCLSFLMYELATHRDVQQKLQEEIDATFPNKAAPTYEALVQMEYLDMVL

NETLRLYSVAGRLERVCKKDVEISGVFIPKGTVVMVPTFILHRDQNLWPEPEEFRPERFS

RKNKDSINPYTYLPFGTGPRNCIGMRFAIMNMKLALVRVLQNFSFKSCKETQISLRINTR

GIIQPEKPVVLKVEPRDGSVSGA

>354488847

MDLVPNFSMETWMLLATGLVLLYLYGTHSHGLFKKLGIPGPKPLPFLGSILAYRKGFWEF

DVQCGKKYGKMWGFYEGRQPVLAITDPDMIKTVLVKECYSTFTNRRVFGPSGILKKAISI

AENEEWKRIRALLSPTFTSGKLKEMLPIINQYADVLVRNMRQGSENGNPISMKDIFGAYS

MDVITATSFGVNVDSLNNPQDPFVEKIKKLLKFDVFDPLFLSVTLFPFLTPVFDAVNICI

FPKDVINFFKTSVQRMKENRLQEKGKQRVDFLQLMINSQTSNVKDSHKALSDLEIVAQSV

IFIFAGYETTSSALSFALYSLATHPDVQKKLQDEIDATLPNKVPATYDTLVQMEYLDMVV

NETLRLYPIAGRLERVCKTDVEVNGVLIHKGTVVMVPIFVLHKDPKYWPEPEEFRPERFS

KKNQGSINPYTYLPFGDGPRNCIGMRFAVMNMKVALVSILQNFSFQPCEETQIPLKLGKQ

GLLQPEKPVLLKVVSRDGTISGA

>ACN69112.1

MDLIPNFSTETWVLLATSLVFLYLFGTYTHGLFKKLGIPGPTPLPFFGNILSYRKGIWDF

DKKCFKKYGKTWGFYEGRLPVLAITDPDMIKTVLVKECYSVFTNRRTFGPEGFMKNAITR

SEDEQWKRIRTLLTPTFTSGKLKEMFPIIGHYGDVLVRNLRNEAEKGKPVTLKNIFGAYS

MDVITSTSFGVNIDSLNNPQDPFVDNAKRLLRLDFLDPLILSITLFPFLRPVYEALNISV

FPKSVTDFFIKSVKRMKESRLKNKETNRVDFLQLMINSQNSKEMDTHKALSDLELVAQSI

VFIFAGYETTSTSLSFLMYLLATHPDVQQKLQKEIDVTFPNKVPPTYDALLQMDYLDMVL

NESLRLFPVAGRLQRICKKDVELNGVFIPKRTLVTVPTFVLHRASEFWPEPEEFRPERFS

KENKDSINPYIYLPFGTGPRNCIGMRFALMNMKLAVVRVLQNFSFKPCKETEIPIKLGTE

AIVKPQKPIVLKVEPRDGTVTGA

>AAD04628.1

MDLIPGFSTETWVLLATSLVLLYLYGTYSHGLFKKLGIPGPRPLPYFGNILGYRKGVDHF

DKKCFQQYGKMWGFFDGRQPVLAITDPDMIKTVLVKECYSVFTNRRSFGPRGAMRTALSL

AEDEEWKRIRTLLSPTFTSGKLKEMFPIISHYGDLLVSNLRKEAEKGKPVTMKDIFGAYS

MDVITSTAFGVNTDFLNNPQDPFVENSKKLLKFSFFSPLFLSIIFFPFLTPILEVLNVTL

FPKSVVNFFMRSIKRMKESRLKDKQTHRVDFLQLMINSQNSKETDTHKGLSDEELVAQGV

FFIFAGYETTSSSLSLLVYELATHPDVQQKLQEEIDATFPSKALPSYDALAQMEYLDMVV

NEILRLYPIAARLERVCKKDVEIHGVSVPKGTVMMVPVFSIHRDPELWPEPEEFRPERFS

KKNKDSINPYTYLPFGTGPRNCIGMRFALMNMKLALVRVLQNFSFKPCKETQTPLKLSSQ

GLIQPEKPILLKVVPRDGTVGGA

>Q29496.1

MELIPSFSLETWVLLAISLVLLYLYGTYSHGLFKKLGVSGPRPLPYFGNVLSYRKGVCEF

DEECFKKYGKMWGVFEGKQPLLVITDPDVIKTVLVKECYSVFTNRRVFGPMGIMKNAVSV

AEDEQWKRIRTLLSPTFTSGKLKDMFPIIGKYGDVLVRNLRKEAEKGKSVNMKDIFGAYS

MDVITSTSFGVNIDSLGNPQDPFVENAKKLLRFNILDPFLLSVVLFPFLVPIFEVLNITM

FPKSAVDFLTKSVKRIKESRLKDNQKPRVDFLQLMINSQNSKETDNHKALSDQELMAQSV

IFIFAGYETTSNTLSFLLYILATHPDVQQKLQEEIDATFPNKAPPTYDVLAQMEYLDMVV

NETLRMFPIAVRLDRLCKKDVEIHGVSIPKGTAVTVPIFVLHRDPQLWPEPEEFRPERFS

KKNKDSINPYVYLPFGTGPRNCIGMRFAIMNMKLAIVRVLQNFSFKPCKETQIPLKINSQ

GLIRPEKPIFLKVVLRDETISGA

>CBJ94511.1

MDLIPNFSTETWVLLATSLVFLYLFGTYTHGLFKKLGIPGPTPLPFFGNILSYRKGIWDF

DKKCFKKYGKTWGFYEGRLPVLAITDPDMIKTVLVKECYSVFTNRRTFGPEGFMKNAIIR

SEDEQWKRIRTLLTPTFTSGKLKEMFPIIGHYGDVLVRNLRNEAEKGKPVTLKNIFGAYS

MDVITSTSFGVNIDSLNNPQDPFVDNAKRLLRLDFLDPLILSITLFPFLRPVYEALNISV

FPKSVTDFFIKSVKRMKESRLKNKETNRVDFLQLMINSQNSKEMDTHKALSDLELVAQSI

VFIFAGYETTSTSLSFLMYLLATHPDVQQKLQKEIDVTFPNKVPPTYDALLQMDYLDMVL

NESLRLFPVAGRLQRICKKDVELNGVFIPKRTLVTVPTFVLHRASEFWPEPEEFRPERFS

KENKDSINPYIYLPFGTGPRNCIGMRFALMNMKLAVVRVLQNFSFKPCKETEIPIKLGTE

AIVKPQKPIVLKVEPRDGTMTGA

>Q64464.1

MDLIPNFSMETWMLLATSLVLLYLYGTHSHGIFKKLGIPGPKPLPFLGTILAYQKGFWEC

DIQCHKKYGKMWGLYDGRQPVLAITDPDIIKTVLVKECYSTFTNRRRFGPVGILKKAISI

SENEEWKRIRALLSPTFTSGRLKEMFPIINQFTDVLVRNMRQGLGEGKPTSMKDIFGAYS

MDVITATSFGVNIDSLNNPQDPFVEKIKKLLKFDIFDPLFLSVTLFPFLTPVFDALNVSL

FPRDVISFFTTSVERMKENRMKEKEKQRVDFLQLMINSQNYKTKESHKALSDVEIVAQSV

IFIFAGYETTSSALSFALYLLAIHPDVQKKLQDEIDAALPNKAPATYDTLLQMEYLDMVV

NETLRLYPIAGRLERVCKTDVEINGLFIPKGTVVMIPTFALHKDPKYWPEPEEFRPERFS

KKNQDSINPYMYLPFGSGPRNCIGMRFALINMKVALVRVLQNFTVQPCKETEIPLKLSKQ

GLLQPENPLLLKVVSRDETVSDE

>BAF64509.1

MDLIPSFSVETWVLLATSLVLLYLYGTYSHGLFKKLGIPGPRPLPYFGNILSYRKGLWEF

DNRCFKKYGKMWGCYEGQQPLLVITDPDIIKTVLVKECYSVFTNRRAFGPVGIMKNAISL

SEDEQWKRIRTLLSPAFTSGKLKEMFPIISQIGDVLVRNLKKEAEKGKPISMKDFFGAYS

MDVITGIAFGVNIDSLNNPQDPFVEYSKKILKFNLLSPLLLSIVLFPFLTPVFEALNITL

FPKSSLNFLTKSVKRIKESRLKDKQTHRVDFLQLMINSQNSKETDTHKVLSDTELMAQSI

IFIFAGYETTSNSLSFIIYELATHPDVQQKLQEEIDATFPNKAPPTYEALVQMEYLDMVL

NETLRIFPIAGRLERVCKKDVEVNGVFIPKGTTVVVPLFVLHNDPELWPEPQEFRPERFS

KKNKDSINPYTYLPFGTGPRNCIGMRFVIMNMKLALVRILQNFSFKPCKETQIPLKLLTQ

GLTQVEKPVVLKVVSRDATISGA

>ACG76385.1

MDLIPGFSTETWVLLATSLVLLYLYGTYSHGLFKKLGIPGPRPLPYFGNILGYRKGVDHF

DKKCFQQYGKMWGVYDGRQPLLAVTDPNMIKSVLVKECYSVFTNRRSFGPLGAMRNALSL

AEDEEWKRIRTLLSPTFTSGKLKEMFPIISHYGDLLVSNLRKEAEKGKPVTMKDIFGAYS

MDVITSTAFGVNIDSLNNPQDPFVENSKKLLKFSFFDPFLLSLIFFPFLTPIFEVLNITL

FPKSSVNFFTKSVKRMKESRLTDQQKRRVDLLQLMINSQNSKEMDPHKSLSNEELVAQGI

IFIFAGYETTSSALSLLAYELATHPDVQQKLQEEIEATFPNKAPPTYDALAQMEYLDMVV

NETLRLYPIAARLERACKKDVEIHGVFVPKGTVVVVPVFVLHRDPDLWPEPEEFRPERFS

KKNKDTINPYTYLPFGTGPRNCIGMRFALMNMKLALVKVLQNFSFKPCKETQIPLKLTTQ

GLTQPEKPVVLKILPRDGTVSGA

>344289642

MDLIPSFSAETWILLVTSLVLLYLYGTHSHNVLKKLRIPGPKPLPFVGSVLAHRKGLWDF

DMKCSKKYGKIWGFYHGLQPVIAITDPGMIKTIMVKECYSTFTNRRVFVPMGFMKSAISL

SKDEEWRRVRTLLSPTFTSGKLKEMLPIIGQYGEVLLKHLREEAEKGKPVTLKNIFGAYS

MDVITSTSFGVNIDSLNNPQDPFVQNIRKLMRFNIFDPLIFTITVFPFLTPILEALSISV

FPRAVTDFFTKSVKTIKESRLKDNKKHRVDFLQLMIDSQNSKETMSHKALTDMELVAQSI

IFIFAGYETTSTTLSFLMYLLATHPDIQQKLQKEIDAAFPNKASPTYDVMLQMEYLDMVV

NETLRLFPIVGRIERVCKKDVEISGVTIPKGAVAMVPAFALHRDPEHWPEPEKFIPERFS

KENKDSIDPYLYLPFGIGPRNCIGMRFALMNMKLAIIRVLQEFSVKPCKETQIPIKVGHG

AIIAPEVPVVIMFESRDGNASGA

>P79401.1

MDLIPGFSTETWVLLATSLVLLYLYGTYSHGLFKKLGIPGPRPLPYFGNILGYRKGVDHF

DKKCFQQYGKMWGVYDGRQPLLAVTDPNMIKSVLVKECYSVFTNRRSFGPLGAMRNALSL

AEDEEWKRIRTLLSPTFTSGKLKEMFPIISHYGDLLVSNLRKEAEKGKPVTMKDIFGAYS

MDVITSTAFGVNIDSLNNPQDPFVENSKKLLKFSFFDPFLLSLIFFPFLTPIFEVLNITL

FPKSSVNFFTKSVKRMKESRLTDQQKRRVDLLQLMINSQNSKEMDPHKSLSNEELVAQGI

IFIFAGYETTSSALSLLAYELATHPDVQQKLQEEIEATFPNKAPPTYDALAQMEYLDMVV

NETLRLYPIAARLERACKKDVEIHGVFVPKGTVVVVPVFVLHRDPDLWPEPEEFRPERFS

KKHKDTINPYTYLPFGTGPRNCIGMRFALMNMKLALVRVLQNFSFKPCKETQIPLKLTTQ

GLTQPEKPVVLKILPRDGTVSGA

>AAC52582.1

MDLIPNFSMETWLLLVISLVLLYLYGTHSHGIFKKLGIPGPKPLPFLGTILAYRKGFWEF

DKYCHKKYGKLWGLYDGRQPVLAITDPDIIKTVLVKECYSTFTNRRNFGPVGILKKAISI

SEDEEWKRIRALLSPTFTSGKLKEMFPIINQYTDMLVRNMRQGSEEGKPTSMKDIFGAYS

MDVITATSFGVNVDSLNNPQDPFVEKVKKLLKFDIFDPLFLSVTLFPFLTPLFEALNVSM

FPRDVIDFFKTSVERMKENRMKEKEKQRMDFLQLMINSQNSKVKDSHKALSDVEIVAQSV

IFIFAGYETTSSALSFVLYLLAIHPDIQKKLQDEIDAALPNKAHATYDTLLQMEYLDMVV

NETLRLYPIAGRLERVCKTDVEINGVFIPKGTVVMIPTFALHKDPHYWPEPEEFRPERFS

KKNQDNINPYMYLPFGNGPRNCIGMRFALMNMKVALVRVLQNFSFQPCKETQIPLKLSKQ

GLLQPEKPLLLKVVSRDETVNGA

>AAH86985.1

MDLIPNFSMETWLLLVISLVLLYLYGTHSHGIFKKLGIPGPKPLPFLGTILAYRKGFWEF

DKYCHKKYGKLWGLYDGRQPVLAITDPDIIKTVLVKECYSTFTNRRNFGPVGILKKAISI

SEDEEWKRIRALLSPTFTSGKLKEMFPIINQYTDMLVRNMRQGSEEGKPTSMKDIFGAYS

MDVITATSFGVNVDSLNNPQDPFVEKIKKLLKFDIFDPLFLSVTLFPFLTPLFEALNVSM

FPRDVIDFFKTSVERMKENRMKEKEKQRMDFLQLMINSQNSKVKDSHKALSDVEIVAQSV

IFIFAGYETTSSALSFVLYLLAIHPDIQKKLQDEIDAALPNKAHATYDTLLQMEYLDMVV

NETLRLYPIAGRLERVCKTDVEINGVFIPKGTVVMIPTFALHKDPHYWPEPEEFRPERFS

KKNQDNINPYMYLPFGNGPRNCIGMRFALMNMKVALVRVLQNFSFQPCKETQIPLKLSKQ

GLLQPEKPLLLKVVSRDETVNGA

>ABS58488.2

MDLIPGFSTETWVLLATSLVLLYLYGTYSHGLFKKLGIPGPRPLPYFGNILGYRKGVDHF

DKKCFQQYGKMWGFFDGRQPVLAITDPDMIKTVLVKECYSVFTNRRSFGPRGAMRSAVSL

AEDEEWKRIRTLLSPTFTSGKLKEMFPIISHYGDLLVSNLRKEAEKGKPVTMKDIFGAYS

MDVITSTAFGVNVDSLNNPQDPFVENSRKLLKFSFFSPFFLSIIFFPFLTPILEVLNITL

FPKSVVNFFTRSIKRMKESRLKDTQKHRVDLLQLMINSQNSKEMDAHKGLSNEELVAQGV

IFIFAGYETTSSSLSLLVYELATHPDVQQKLQEEIDATFPNKAPPTYDGLAQMEYLDMVV

NESLRIFPVTPRLERSCKKDVEIHGVFVPKGTVMMVPIFALHRAPELWPEPEEFRPERFS

KKNKDTINPYTYLPFGTGPRNCIGMRFALMNMKLALVRVLQNFSFKPCRETQIPLKISSQ

GLIQPEKPIVLMVVPRDGTTSGA

>P51538.2

MDLIPNFSMETWLLLVISLVLLYLYGTHSHGIFKKLGIPGPKPLPFLGTILAYRKGFWEF

DKYCHKKYGKLWGLYDGRQPVLAITDPDIIKTVLVKECYSTFTNRRNFGPVGILKKAISI

SEDEEWKRIRALLSPTFTSGKLKEMFPIINQYTDMLVRNMRQGSEEGKPTSMKDIFGAYS

MDVITATSFGVNVDSLNNPQDPFVEKVKKLLKFDIFDPLFLSVTLFPFLTPLFEALNVSM

FPRDVIDFFKTSVERMKENRMKEKEKQRMDFLQLMINSQNSKVKDSHKALSDVEIVAQSV

IFIFAGYETTSSALSFVLYLLAIHPDIQKKLQDEIDAALPNKAHATYDTLLQMEYLDMVV

NETLRLYPIAGRLERVCKTDVEINGVFIPKGTVVMIPTFALHKDPHYWPEPEEFRPERFS

KKNQDNINPYMYLPFGNGPRNCIGMRFALMNMKVALFRVLQNFSFQPCKETQIPLKLSKQ

GLLQPEKPLLLKVVSRDETVNGA

>348568280

MNLIPNYAMETWVLLVTILVLLYLYGTSSHGLFKKLGIPGPKPLPFFGTILGYRKGFWDF

DTNCYKKYGKIWGFYDGHQPVLAITDPDMIKTVLVKECYSVFTNRRTFGPVGFMKKAISI

SEDEDWKRIRTLLSPVFTTGKLKEMFPIIEQYGDVLVKNLRQKAKKGEPVSMKDVFGAYS

MDVITGTSFGVNIDSLNNPQDPFVGKVKKLLKFSFFDPLFLLGVLFPFLIPVMDALNLSV

FPRDVTDFLKKSVETMKESRLKEKQKHRVDFLQLMINSQNSKDTESHKALSDLELVAQSI

IFIFGGYETTSSTLSFAMYALATHPDVQKKLQQEIDTILPNKTPATYDVLMEMEYLDMVV

NEILRLYPIAGRLERTCKKDVEINGVVIPKGSLVLIPIYALHRDPKYWKEPEKFCPERFS

KKNKNNIDPYIYLPFGTGPRNCIGMRFALLNMKLAIIRVLQNFSFQTCKETQIPLTFKKQ

GLLQPEKEVLLKVVPRDQTIDGSLK

>AAZ29442.1

MDLIPNFAMETWVLVATSLVLLYIYGTHSHKLFKKVGIPGPTPLPFLGTILFYLRGLWKF

DRECNEKYGEMWGLYEGQQPMLVIMDPDMIKTVLVKECYSVFTNRMPLGPMGFMKSALSF

AEDEEWKRIRTLLSPAFTSVKFKEMVPIISQCGDMLVRSLRREAENSKPTNLKDFFGAYT

MDVITGTLFGVNLDSLNNPQDPFLKNMKKLLKLDFLDPFLLSISLFPFLTPVFEVLNIGL

FPKDVTRFLKNSIERMKESRLKDKQKHRVDFFQQMIDSQNSKETKSHKALSDLELVAQSI

IIIFAAYDTTSTTLPFIMYELATHPDVQQKLQEEIDAVLPNKATVTYDALVQMEYLDIVV

NETLRLFPVVSRVTRVCKKDIEINGVFIPKGLAVMVPIYALHHDPKYWTEPEKFCPERFS

KKNKDSIDPYRYIPFGAGPRNCIGMRFALTNIKLAVIKALQNFSFEPCEETQIPLKLNNL

PILQPEKPIVLKVHLRDGITSGP

>EHH52028.1

MDLIPNFAMETWVLVATSLVLLYIYGTHSHKLFKKLGIPGPTPLPFLGTILFYLRGLWKF

DRECNEKYGEMWGLYEGQQPMLVIMDPDMIKTVLVKECYSVFTNRMPLGPMGFMKSALSF

AEDEEWKRIRTLLSPAFTSVKFKEMVPIISQCGDMLVRSLRREAENSKPTNLKDFFGAYT

MDVITGTLFGVNLDSLNNPQDPFLKNMKKLLKLDFLDPFLLSISLFPFLTPVFEVLNIGL

FPKDVTRFLKNSIERMKESRLKDKQKHRVDFFQQMIDSQNSKETKSHKALSDLELVAQPI

IIIFAAYDTTSTTLPFIMYELATHPDVQQKLQKEIDAVLPNKAPVTYDALVQMEYLDIVV

NETLRLFPVVSRVTRVCKKDIEINGVFIPKGLAVMVPIYALHHDPKYWTEPEKFCPESRF

SKKNKDSIDPYRYIPFGSGPRNCIGMRFALTNIKLAVIKALQNFSFEPCEETQIPLKLNN

LPILQPEKPIVLKVHLRDGITSGP

>395738170

MDLIPNLAVETWLLLAVSLILLYLFYDGLQPVLAITDPDMIKTVLVKECYSVFTNRRPLG

PVGFMKSAISRAEDEEWKRIRSLLSPTFTSGKLKEMVPIIAQYGDVLVRNLRREAETGKP

VTLKDFFGAYSMDVITSTSFGVNVDSLNNPQDPFVENTKKLLRFNSLDPLTLSIKIFPFL

IPILEALNVTVFPREVTSFLTKSVKRIKEGRLKETQKHRVDFLQLMIDSQNLKDTESHKA

LSDLELVAQSIVFIFAGYETTSSVLSFIIYELATHPDVQQKLQEEIDAVLPNKAPPTYDT

VLQMEYLDMVVNETLRLFPVAMRLERVCKKDVEINGMFIPKGVVVMIPSYVLHHDPKYWI

EPEKFLPERFSKKNKDNIDPYIYTPFGNGPRNCIGMRFALMNMKFALVRVLQNFSFKPCK

ETQIPLKLRFGGLLRTEKPIVLMAESRDETVSGA

>AAL13316.1

MDLIPGFSTETWVLLATSLVLLYLYGTYSHGLFKKLGIPGPRPLPYFGNILGYRKGVDHF

DKKCFQQYGKMWGVYDGRQPLLAVTDPNMIKSVLVKECYSVFTNRRSFGPLGAMRNALSL

AEDEEWKRIRTLLSPTFTSGKLKEMFPIISHYGDLLVSNLRKEAEKGKPVTMKDIFGAYS

MDVITSTAFGVNIDSLNNPQDPFVENSKKLLKFSFFDPFLLSLIFFPFLTPIFEVLNITL

FPKSSVNFFTKSVKRMKESRLTDQQKRRVDLLQLMINSQNSKEMDPHKSLSNEELVGPGI

IFIFAGYETTSSALSLLAYELATHPDVQQKLQEEIEATFPNKAPPTYDALAQMEYLDMVV

NETLRLYPIAARLERACKKDVEIHGVFVPKGTVVVVPVFVLHRDPDLWPEPEEFRPERFS

KKNKDTINPYTYLPFGTGPRNCIGMRFALMNMKLALVKVLQNFSFKPCKETQIPLKLTTQ

GLTPTEKPVVLKILPRDGPLSGA

>EHH17168.1

MDLIPNFAMETWVLVATSLVLLYIYGTHSHKLFKKLGIPGPTPLPFLGTILFYLRGLWKF

DRECNEKYGEMWGLYEGQQPMLVIMDPDMIKTVLVKECYSVFTNRMPLGPMGFMKSALSF

AEDEEWKRIRTLLSPAFTSVKFKEMVPIISQCGDMLVRSLRREAENSKPTNLKDFFGAYT

MDVITGTLFGVNLDSLNNPQDPFLKNMKKLLKLDFLDPFLLSISLFPFLTPVFEVLNIGL

FPKDVTRFLKNSIERMKESRLKDKQKHRVDFFQQMIDSQNSKETSPIKALSDLELVAQSI

IIIFAAYDTTSTTLPFIMYELATHPDVQQKLQEEIDAVLPNKAPVTYDALVQMEYLDIVA

NETLRLFPVVSRVTRVCKKDIEINGVFIPKGLAVMVPIYALHHDPKYWTEPKKFCPESRF

SKKNKDSIDPYRYIPFGAGPRNCIGMRFALTNIKLAVIKALQNFSFEPCEETQIPLKLNN

LPILQPEKPIVLKVHLRDGITSGP

>P11707.2

MDLIFSLETWVLLAASLVLLYLYGTSTHGLFKKMGIPGPTPLPFIGTILEYRKGIWDFDI

ECRKKYGKMWGLFDGRQPLMVITDPDMIKTVLVKECYSVFTNRRSFGPVGFMKKAVSISE

DEDWKRVRTLLSPTFTSGKLKEMLPIIAQYGDVLVKNLRQEAEKGKPVDLKEIFGAYSMD

VITGTSFGVNIDSLRNPQDPFVKNVRRLLKFSFFDPLLLSITLFPFLTPIFEALHISMFP

KDVMDFLKTSVEKIKDDRLKDKQKRRVDFLQLMINSQNSKEIDSHKALDDIEVVAQSIII

LFAGYETTSSTLSFIMHLLATHPDVQQKLQEEIDTLLPNKELATYDTLVKMEYLDMVVNE

TLRLYPIAGRLERVCKKDVDINGTFIPKGTIVMMPTYALHRDPQHWTEPDEFRPERFSKK

NKDNINPYIYHPFGAGPRNCLGMRFALMNIKLALVRLMQNFSFKLCKETQVPLKLGKQGL

LQPEKPIVLKVVSRDGIIRGA

>BAK52239.1

MDLIPSFFTETWLLLATSLVLLYLYGTHTHGLFKKLGIPGPKPLPFLGTALGYRQGFCEF

DEKCFRTYGRMWGFYDRRQPVLAITDPDMIKTVLVKECYSVFTNRRSFGPVGFMKGAISL

SEDEQWKRIRTVLSPTFTSGKLKEMFPIVGQYGDVLVRNLRKEAEKGKPVNLKDILGAYS

MDVITSTSFGVNIDSLNNPRDPFVENTKKLLKFSFLDPFFFSLLLFPFLTPIFDLLNIWL

FPKSVINFFTKSVKRMKESRLKDKQKHRVDLLQLMINSQNSKETDTHKALSDLELVAQSV

IFIFAGYEPTSTSLSFLVYELATHPDVQQKLQEEIDATFPNKAPPTYDALVQMEYLDMVL

NETLRLYPIAGRLERVCKRDVEISGVFIPKGTVVMVPTFTLHRDLDLWPEPEEFHPERFS

KKNKDSINPYIYLPFGTGPRNCIGMRFAVMNMKLALVTLLQNFSFQPCEETQIPLKLNAQ

GIIQPEKPIVLKVELRDGSVSRA

>Q64459.1

MDLVSALSLETWVLLAISLVLLYRYGTRKHELFKKQGIPGPKPLPFLGTVLNYYKGLWKF

DMECYKKYGKTWGLFDGQTPLLAVTDPETIKNVLVKECFSVFTNRRDFGPVGIMSKAISI

SKDDEWKRYRALLSPTFTSGKLKEMFPVIEQYGDILVKYLRQKAKKGKPVTMKDVLGAYS

MDVITSTSFGVNVDSLNNPEDPFVEKAKKLLRFDFFDPLLFSVVLFPFLTPVYEMLNICM

FPKDSIEFFKKFVDRMKESRLDSKQKHRVDFLQLMMNSHNNSKDKVSHKALSDMEITAQS

IIFIFAGYETTSSTLSFTLHSLATHPDIQKKLQDEIDEALPNKAPPTYDTVMEMEYLDMV

LNETLRLYPIANRLERVCKKDVELNGVYIPKGSTVMIPSYALHHDPQHWSEPEEFQPERF

SKENKGSIDPYVYLPFGNGPRNCLGMRFALMNMKLALTKIMQNFSFQPCKETQIPLKLSR

QGLLQPEKPIVLKVVPRDAVITGA

>AAH94062.1

MDLVSALSLETWVLLAISLVLLYRYGTRKHELFKKQGIPGPKPLPFLGTVLNYYKGLWKF

DMECYKKYGKTWGLFDGQTPLLAVTDPETIKNVLVKECFSVFTNRRDFGPVGIMSKAISI

SKDDEWKRYRALLSPTFTSGKLKEMFPVIEQYGDILVKYLRQKAKKGKPVTMKDVLGAYS

MDVITSTSFGVNVDSLNNPEDPFVEKAKKLLRFDFFDPLLFSVVLFPFLTPVYEMLNICM

FPKDSIEFFKKFVDRMKESRLDSKQKHRVDFLQLMMNSHNNSKDKVSHKALSDMEITAQS

IIFIFAGYETTSSTLSFTLHSLATHPDIQKKLQDEIDEALPNKAPPTYDTVMEMEYLDMV

LNETLRVYPIANRLERVCKKDVELNGVYIPKGSTVMIPSYALHHDPQHWSEPEEFQPERF

SKENKGSIDPYVYLPFGNGPRNCLGMRFALMNMKLALTKIMQNFSFQPCKETQIPLKLSR

QGLLQPEKPIVLKVVPRDAVITGA

>ABU85093.1

MDLIPNFAMETWVLVATSLVLLYIYGTHSHKLFKKLGIPGPTPLPFLGTILFYLRGLWKF

DRECNEKYGEMWGLYEGQQPMLVIMDPDMIKTVLVKECYSVFTNRMPLGPMGFLKSALSF

AEDEEWKRIRTLLSPAFTSVKFKEMVPIISQCGDMLVRSLRQEAENSKSINLKDFFGAYA

MDVITGTLFGVNLDSLNNPQDPFLKNMKKLLKLDFLDPFLLLISLFPFLTPVFEALNIGL

FPKDVTRFLKNSIERMKESRLKDKQKHRVDFFQQMIDSQNSKETKSHKALSDLELVAQSI

IIIFAAYDTTSTTLPFIMYELATHPDVQQKLQEEIDAVLPNKAPVTYDALVQMEYLDMVV

NETLRLFPVVSRVTRVCKKDIEINGVLIPKGLAVMVPIYALHHDPKYWTEPEKFCPERFS

KKNKDSIDPYRYIPFGAGPRNCIGMRFALTNIKLAVIRALQNFSFKPCKETQIPLKLDNL

PILQPEKPIVLKVHLRDGITSGP

>BAD06180.1

MDLIPSFSVETWLLLATSLVLLYLYGTYSHGLFKKLGIPGPKPLPYLGSVLEYRKGMWHF

DNKCFKKYGKMWGFYDGRQPVLAMRDPEMIKAVLVKECYSAFTNRRNFGPAGVMKNAISV

AKDEQWKRVRTLLSPTFTSGKLKEMFPIIAQYGDMLVRNLRKEAEKGNSINTKEIFGAYS

MDVITSTAFGVDVDSLNNPRDPFVQYARKLLRFDFLDPFILSIILFPFLSPFFEVLDITL

FPRSSVKFFTQSVKRMKESRLKDQQMRRVDLLQLMINSQNSKETDAHKALSDQELVAQSI

IFICAGYETTSSSLSFLAYILATHPDVQQNLQEEIDATFPSKALPSYDALAQMEYLDMVV

NEILRLYPIAARLERVCKKDVEIHGVSVPKGTVMMVPVFSIHRDPELWPEPEEFRPERFS

KKNKDSINPYTYLPFGTGPRNCIGMRFALMNMKLALVRVLQNFSFKPCKETQTPLKLSSQ

GLIQPEKPILLKVVPRDGTVSGA

>Q9HB55.1

MDLIPNFAMETWVLVATSLVLLYIYGTHSHKLFKKLGIPGPTPLPFLGTILFYLRGLWNF

DRECNEKYGEMWGLYEGQQPMLVIMDPDMIKTVLVKECYSVFTNQMPLGPMGFLKSALSF

AEDEEWKRIRTLLSPAFTSVKFKEMVPIISQCGDMLVRSLRQEAENSKSINLKDFFGAYT

MDVITGTLFGVNLDSLNNPQDPFLKNMKKLLKLDFLDPFLLLISLFPFLTPVFEALNIGL

FPKDVTHFLKNSIERMKESRLKDKQKHRVDFFQQMIDSQNSKETKSHKALSDLELVAQSI

IIIFAAYDTTSTTLPFIMYELATHPDVQQKLQEEIDAVLPNKAPVTYDALVQMEYLDMVV

NETLRLFPVVSRVTRVCKKDIEINGVFIPKGLAVMVPIYALHHDPKYWTEPEKFCPERFS

KKNKDSIDLYRYIPFGAGPRNCIGMRFALTNIKLAVIRALQNFSFKPCKETQIPLKLDNL

PILQPEKPIVLKVHLRDGITSGP

>EDL89819.1

MDLIPNISLETWMLLATILVLLYLYGTSTHGNFKKLGISGPKPLPFVGNILAYRHGFWEF

DRHCHKKYGDIWGFYEGRQPILAITDPDIIKTVLVKECYSTFTNRRSFGPAGILKKAITL

SEDEEWKRLRTLLSPTFTSGKLKEMFPIINQYADLLVKNVKHEAEKGNPITMKDIFGAYS

MDVITGTSFGVNVDSLNNPQNPFVQKVKKLLKFNFLDPFFLSVILFPFLTPVFEAFDITV

FPKDVMKFFRTSVERMKENRMQEKVKQRLDFLQLMINSQSSGDKESHQGLTDVEIVAQSI

FFIFAGYETTSSALSFALYLLATHPDLQKKLQDEIDAALPNKAPVTYDVLVEMEYLDMVL

NETLRLFPVGGRLERVCKKDVEINGVFIPKGTVVMVPTFALHKDPKCWPEPEEFCPERFR

KKNQDSINPYIYLPFGNGPRNCIGMRFALMNMKIALVRVLQNFSFGPCKETQIPLKLRKK

GFFQPEKPIILRAVSRD

>344289644

MDLIPSFSMETWILLVTILVLLYLYGTHSHNVLKKLGIPGPKPLPFLGTSLAYLKGSWDF

DMKCSKKYGKFWGFYDGRQPVIAITDPVMIKTILVKECYSTFTNRRMIGPMGFMKSAISL

SEDEVWKRVRTLLSPTFTSGKLKEMFPIISQYGDVLVKHLREEEQKGKPVALKSIFGAYS

MDVITSTSFGVNIDSLHNPQDPFVQNARKLIKFDFLDPFVFLITLFPFLTPICEALNISL

FPRDATDFFTKSVQRMKESRLKDNEKHQVDLLQLMIDSQNSKEIMSHKALTDMELVAQSI

MFIFAGYETTSTTLSFVMYLLATHPDIQEKLQREIDAAFPDKAPPTYDGMLQMEYLDMVV

NETLRLFPIAGRLERVCKKDVEIKGLIIPKGTVMMVPIFVLQRDPEHWPEPDKFIPERFS

KENKDNIDPYLYMPFGIGPRNCIGMRFALMNMKLAIIRILQEFSIKPCNETQIPLKLGKR

ALIAPERPVVIMFDSRVGKVIGA

>P79102.1

MELIPSFSMETWVLLATSLVLLYIYGTYSYGLFKKLGIPGPRPVPYFGSTMAYHKGIPEF

DNQCFKKYGKMWGFYEGRQPMLAITDPDIIKTVLVKECYSVFTNRRIFGPMGIMKYAISL

AWDEQWKRIRTLLSPAFTSGKLKEMFPIIGQYGDMLVRNLRKEAEKGNPVNMKDMFGAYS

MDVITGTAFGVNIDSLNNPHDPFVEHSKNLLRFRPFDPFILSIILFPFLNPVFEILNITL

FPKSTVDFFTKSVKKIKESRLTDKQMNRVDLLQLMINSQNSKEIDNHKALSDIELVAQST

IFIFGGYETTSSTLSFIIYELTTHPHVQQKLQEEIDATFPNKAPPTYDALVQMEYLDMVV

NETLRMFPIAGRLERVCKKDVEIHGVTIPKGTTVLVPLFVLHNNPELWPEPEEFRPERFS

KNNKDSINPYVYLPFGTGPRNCLGMRFAIMNIKLALVRILQNFSFKPCKETQIPLKLYTQ

GLTQPEQPVILKVVPRGLGPQVEPDFL

>AAQ92352.1

MDLIPNFAMETWVLVATSLVLLYIYGTHSHKLFKKLGIPGPTPLPFLGTILFYLRGLWNF

DRECNEKYGEMWGLYEGQQPMLVIMDPDMIKTVLVKECYSVFTNQMPLGPMGFLKSALSF

AEDEEWKRIRTLLSPAFTSVKFKEMVPIISQCGDMLVRSLRQEAENSKSINLKDFFGAYT

MDVITGTLFGVNLDSLNNPQDPFLKNMKKLLKLDFLDPFLLLISLFPFLTPVFEALNIGL

FPKDVTHFLKNSIERMKESRLKDKQKHRVDFFQQMIDSQNSKETKSHKALSDLELVAQSI

IIIFAAYDTTSTTLPFIMYELATHPDVQQKLQEEIDAVLANKAPVTYDALVQMEYLDMVV

NETLRLFPVVSRVTRVCKKDIEINGVFIPKGLAVMVPIYALHHDPKYWTEPEKFCPERFS

KKNKDSIDLYRYIPFGAGPRNCIGMRFALTNIKLAVIRALQNFSFKPCKETQIPLKLDNL

PILQPEKPIVLKVHLRDGITSGP

>297679966

MDLIPNFAMETWVLVATSLVLLYIYGTHSHKLFKKLGIPGPTPLPFLGTILFYLRGLWKF

DRECNEKYGEMWGLYEGQQPMLVIMDPNMIKTVLVKECYSVFTNRMPLGPMGFLKSALSF

AEDEEWKRIRTLLSPAFTSVKFKEMVPIISQCGDMLVRSLRQEAENSKSINLKDFFGAYT

MDVITGTLFGVNLDSLNNPQDPFLKNMKKLLKLDFLDPFLLLISLFPFLTPVFEALNIGL

FPKDVTRFLKNSIERMKESRLKDKQKHRVDFFQQMIDSQNSKETKSHKALSDLELVAQSI

IITFAAYDTTSTTLPFIMYELATHPDVQQKLQEEIDAVLPNKAPVTYDALVQMEYLDMVV

NETLRLFPVVSRVTTVCKKDIEINGVLIPKGLAVMVPIYALHHDPKYWTEPEKFCPERFS

KKNKDSIDPYRYIPFGAGPRNCIGMRFALTNIKLAVIRALQNFSFKPCKETQIPLKLDNL

PILQPEKPIVLKVHLRDGITSGP

>AAI14004.1

MELIPSFSMETWVLLATSLVLLYIYGTYSYGLFKKLGIPGPRPVPYFGSTMAYHKGIPEF

DNQCFKKYGKMWGFYEGRQPMLAITDPDIIKTVLVKECYSVFTNRRIFGPMGIMKYAISL

AWDEQWKRIRTLLSPAFTSGKLKEMFPIIGQYGDMLVRNLRKEAEKGNPVNMKDMFGAYS

MDVITGTAFGVNIDSLNNPHDPFVEHSKNLLRFRPFDPFILSIILFPFLNPVFEILNITL

FPKSTVDFFTKSVKKIKESRLTDKQMNRVDLLQLMINSQNSKEIDNHKALSDIELVAQST

IFIFGGYETTSSTLSFIIYELTTHPHVQQKVQEEIDATFPNKAPPTYDALVQMEYLDMVV

NETLRMFPIAGRLERVCKKDVEIHGVTIPKGTTVLVPLFVLHNNPELWPEPEEFRPERFS

KNNKDSINPYVYLPFGTGPRNCLGMRFAIMNIKLALVRILQNFSFKPCKETQIPLKLYTQ

GLTQPEQPVILKVVPRGLGPQVEPDFL

>EHA98417.1

MDLITNYSMETWVLLATCLVLLYLYGTSSHGLFKKLGIPGPKPLPFFGNMLTSRKGIWDF

DTKCFKKYGKMWGFYDGRQPILAITDPDIVKTVLVKECYSVFTNRRSFGPLGFMKKAISL

SEDEDWKRIRTLLSPTFTTGKLKEMFPIIEQYGDVLVKNLRQRAKKGEPVSMKDIFGAYS

MDVTTGTSFGVNVDSLNNPKDPFVEKIKKILKFQFFDPLFLLILFFPFLTPVLEALNISV

FPRDVINFFKISIEGIKESRLKEKQKHRVDFLQLMMNSQKSKGTVSHKALSDLELVAQSI

IFIFAGYETTSSALSFIMYELATHLDVQKKLQQEIDTVLSNKTPATYDALVQMEYLDMVV

NETLRLYPIAGRLERACKKDVEINRVVIPKGSVVLIPTYALHRDPKYWKEPEEFHPERFS

KKNKDNINPYIYMPFGAGPRNCIGMRFAFMTMKLAIIRVLQNFSFQTCKETQIPMTFNNQ

ALLQPEKDILLKVVSREETLSGA

>BAC23085.1

MDLIPNISLETWMLLATILVLLYLYGTSTHGNFKKLGISGPKPLPFVGNILAYRHGFWEF

DRHCHKKYGDIWGFYEGRQPILAITDPDIIKTVLVKECYSTFTNRRSFGPAGILKKAITL

SEDEEWKRLRTLLSPTFTSGKLKEMFPIINQYADLLVKNVKHEAEKGNPITMKDIFGAYS

MDVITGTSFGVNVDSLNNPQNPFVQKVKKLLKFNFLDPFFLSVILFPFLTPVFEAFDITV

FPKDVMKFFRTSVERMKENRMQEKVKQRLDFLQLMINSQSSGDKESHQGLTDVEIVAQSI

FFIFAGYETTSSALSFALYLLATHPDLQKKLQDEIDAALPNKAPVTYDVLVEMEYLDMVL

NETLRLFPVGGRLERVCKKDVEINGVFIPKGTVVMVPTFALHKDPKCWPEPEEFCPERFR

KKNQDSINPYIYLPFGNGPRNCIGMRFALMNMKIALVRVLQNFSFGLCKETQIPLKLRKK

GFFQPEKPIILRAVSRD

>AAG33010.1

MDLIPNFAMETWVLVATSLVLLYIYGTHSHKLFKKLGIPGPTPLPFLGTILFYLRGLWNF

DRECNEKYGEMWGLYEGQQPMLVIMDPDMIKTVLVKECYSVFTNQMPLGPMGFLKSALSF

AEDEEWKRIRTLLSPAFTSVKFKEMVPIISQCGDMLVRSLRQEAENSKSINLKDFFGAYT

MDVITGTLFGVNLDSLNNPQDPFLKNMKKLLKLDFLDPFLLLISLFPFLTPVFEALNIGL

FPKDVTHFLKNSIERMKESRLKDKQKHRVDFFQQMIDSQNSKETKSHKALSDLELVAQSI

IIIFAAYDTTSTTLPFIMYELATHPDVQQKLQEEIDAVLPNKAPVTYDALVQMEYLDMVV

NETLRLFPVVSRVTRVCKKDIEINGVFIPKGLAVMVPIYALHHDPKYWTEPEKFCPESRF

SKKNKDSIDLYRYIPFGAGPRNCIGMRFALTNIKLAVIRALQNFSFKPCKETQIPLKLDN

LPILQPEKPIVLKVHLRDGITSGP

>114614907

MDLIPNFAMETWVLVATSLVLLYIYGTHSHKLFKKLGIPGPTPLPFLGTILFYLRGLWNF

DRECNEKYGEMWELYEGQQPMLVIMDPDMIKTVLVKECYSVFTNRLPLGPMGFLKSALSF

AEDEEWKRIRTLLSPAFTSVKFKEMVPIISQCGDMLVRSLRQEAENSKSINLKDFFGAYT

MDVITGTLFGVNLDSLNNPQDPFLKNMKKLLKLDFLDPFLLLISLFPFLTPVFEALNIGL

FPKDVTHFLKNSIERMKESRLKDKQKHRVDFFQQMIDSQNSKETKSHKALSDLELVAQSI

IIIFAAYDTTSTTLPFIMYELATHPDVQQKLQEEIDAVLPNKAPVTYDALVQMEYLDMVV

NETLRLFPVVSRVTRVCKKDIEINGVFIPKGLAVMVPIYALHHDPKYWTEPEKFCPERFS

KKNKDSIDLYRYIPFGAGPQNCIGMRFALTNIKLGVIRALQNFSFKPCKETQIPLKLDNL

PILQPEKPIVLKVHLRDGITSGP

>397489510

MDLIPNFAMETWVLVATSLVLLYIYGTHSHKLFKKLGIPGPTPLPFLGTILFYLRGLWNF

DRECNEKYGEMWELYEGQQPMLVIMDPDMIKTVLVKECYSVFTNRMPLGPMGFLKSALSF

AEDEEWKRIRTLLSPAFTSVKFKEMVPIISQCGDMLVRSLRQEAENSKSINLKDFFGAYT

MDVITGTLFGVNLDSLNNPQDPFLKNMKKLLKLDFLDPFLLLISLFPFLTPVFEALNIGL

FPKDVTHFLKNSIERMKESRLKDKQKHRVDFFQQMIDSQNSKETKSHKALSDLELVAQSI

IIIFAAYDTTSTTLPFIMYELATHPDVQQKLQEEIDAVLPNKAPVTYDALVQMEYLDMVV

NETLRLFPVVSRVMRVCKKDIEINGVFIPKGLAVMVPIYALHHDPKYWTEPEKFCPERFS

KKNKDSIDLYRYIPFGAGPQNCIGMRFALTNIKLGVIRALQNFSFKPCKETQIPLKLDNL

PILQPEKPIVLKVHLRDGITSGP

>354488851

MDLIPNFSMETWMLLATGLVLLYLYGTCTHGTFKNLGISGPKPLPFVGNILAYRNGFWEF

DRQCHQKYGEIWGFYEGRQPVLAIMNPDMIKTVLVKECYSTFTNRRIFGPVGILKKAITL

SENEEWKRLRTLLSPTFTSGKLKEMFPIINQYADLLVRNVRHGAENGDPITMKDIFGAYS

MDVITGTSFGVNVDSLNNPQNPFVQKVKKLLKFNFLDPFFLSVILFPFLIPLFDELKMTV

FPNDVINFFKTSVEQMKEKRMQDKKKQRLDFLQLMINSQNSKDKESHQGLSDLEIVAQSI

FFIFAGYETTSSALSFAMYLLATHPDVQKKLQDKIDVALPNKAPATYDALVQMEYLDMVV

NETLRLYPVGGRIERVCKTDVEVNGALIPKGTLVVVPTFALHKDPKCWPEPEEFCPERFN

KKNQGSINPYTFLPFGDGPRNCIGMRFALMNMKVALVRVLQNFSFQPCEETQIPLKLRKQ

GFFQPEKPIILRAILRDETISRA

>332258087

MIKTVLVKECYSVFTNRRPFGPVGFMKSAISIAEDEEWKRIRSLLTPSFTSGKLKEMVPI

IAQYGDVLVRNLRREAETGKPVTLKDVFGAYSMDVITSTSFGVNIDSLNNPQDPFVENTK

KLLRFNSLDPFTLSIKIFPFLTPILEALNITVFPRKVTSFLTKSVKRIKEVRLKETQKHR

VDFLQLLIDSQNSKDTESHKALSELELVAQSIIFIFAGYETTSSVLSFIVYELATHPDVQ

QKLQKEIDTVLPNKAPPTYDTVLQMEYLDMVVNETLRLFPVAMRLERVCKKDVEINGMFI

PKGVVVMIPSYVLHHDPKYWTEPEKFLPERFSKKNKDNIDPYIYTPFGTGPRNCIGMRFA

LVNMKLALVRVLQNFSFKPCKETQIPLKLHFGGLLRTEKPIVLMAESRDETVSGA

>P04800.1

MDLLSALTLETWVLLAVVLVLLYGFGTRTHGLFKKQGIPGPKPLPFFGTVLNYYMGLWKF

DVECHKKYGKIWGLFDGQMPLFAITDTEMIKNVLVKECFSVFTNRRDFGPVGIMGKAVSV

AKDEEWKRYRALLSPTFTSGRLKEMFPIIEQYGDILVKYLKQEAETGKPVTMKKVFGAYS

MDVITSTSFGVNVDSLNNPKDPFVEKTKKLLRFDFFDPLFLSVVLFPFLTPIYEMLNICM

FPKDSIEFFKKFVYRMKETRLDSVQKHRVDFLQLMMNAHNDSKDKESHTALSDMEITAQS

IIFIFAGYEPTSSTLSFVLHSLATHPDTQKKLQEEIDRALPNKAPPTYDTVMEMEYLDMV

LNETLRLYPIGNRLERVCKKDVEINGVFMPKGSVVMIPSYALHRDPQHWPEPEEFRPERF

SKENKGSIDPYVYLPFGNGPRNCIGMRFALMNMKLALTKVLQNFSFQPCKETQIPLKLSR

QGLLQPTKPIILKVVPRDEIITGS

>194218796

MNLIPSFSTDTWVLLATILGLLYLYGTYTHGLFKKLGIPGPTPLPFLGNVLSYRKGFWDF

DKTCFKKYGKIWGFYDGRQPVLAITDPEMIKTVLVKECYSLFTNRRPVGPVGILKSSITL

SEDEQWKRMRTLLSPTFTSGKLKEMFPTISQYGDVLVRNLRKEAEKGMPITLKEFFGAYS

MDVITGISFGVNIDSLNNPQHRFVENCKNILKFHFFDPLTILLSLLPFLSPVLEVLNILV

FPVSAIKFFTKSIKTIKESRLKDKQKQQVDFLQLMINSQNSKEMSRYKALSDLELVVQSI

SFIFAGYETTSSSLSFLTYALATHPEVQEKLQEEIDLAFPNKAPPTYDALLQMDYLDMVL

NENLRLFPIAVRLERVCKKNVKINGVFIPKGTVVMVPNFLLHRDPEFWPEPEEFHPERFS

KKNKDRINPYVYLPFGTGPRNCIAMRFAIMNMKLAVVKLLQNFSFKPCEETQIPLKLCNQ

GLMEPQKPIVLKVEFRDRTVSGP

>AAF15303.1

MELIPNLSIETWVLLVTSLVLFYIYGTYSHGLFKKLGIPGPKPLPLLGTIFNYYDGMWKF

DEDCYKKYGKIWGFYEGPQPILAIMDPEIIKIVLVKECYSVFTNRRFFGPVGFMKKAITI

SEDEEWKRLRTLLSPTFTSGKLKEMFPIMRQYGDILVRNLRREEEKGEPISMKDIFGAYS

MDVITGTSFGVNVDSLNNPQDPFVQKAKKILKFKVFDPFLLSIILFPFLTPIYEMLNFSI

FPRDSMNFFKKFVKRMKKERLASNQKNRVDFLQLMMNTQNSKGQESQKALSDLEMAAQAV

IFIFGGYDATSTSISLIMYELATHPDVQKKLQDEIDRTLPNKAPVTYDALMDMEYLDMVV

NESLRLYPIAIRLERVSKKDVEINGVFIPKGTVVMIPIYPLHRNPEYWPEPQEFCPERFS

KENKGNIDPYIYMPFGNGPRNCIGMRFALISIKLAVIGVLQNFTVQPCEETQIPLKISRE

PIFQPEKPIILKVVSRDKPRTGS

>O09158.1

MELIPNLSIETWVLLVTSLVLFYIYGTYSHGLFKKLGIPGPKPLPLLGTIFNYYDGMWKF

DEDCYKKYGKIWGFYEGPQPILAIMDPEIIKIVLVKECYSVFTNRRFFGPVGFMKKAITI

SEDEEWKRLRTLLSPTFTSGKLKEMFPIMRQYGDILVRNLRREEEKGEPISMKDIFGAYS

MDVITGTSFGVNVDSLNNPQDPFVQKAKKILKFKIFDPFLLSIILFPFLTPIYEMLNFSI

FPRDSMNFFKKFVKRMKKERLASNQKNRVDFLQLMMNTQNSKGQESQKALSDLEMAAQAV

IFIFGGYDATSTSISLIMYELATHPDVQKKLQDEIDRTLPNKAPVTYDALMDMEYLDMVV

NESLRLYPIAIRLERVSKKDVEINGVFIPKGTVVMIPIYPLHRNPEYWPEPQEFCPERFS

KENKGNIDPYIYMPFGNGPRNCIGMRFALISIKLAVIGVLQNFTVQPCEETQIPLKISRE

PIFQPEKPIILKVVSRDKPRTGS

>AAA31430.1

MDLIFSLETWVLLAASLVLLYLYGTSTHGLFKKMGIPGPTPLPFIGTILEYRKVINWDFD

IECRKKYGKMWGLFDGRQPLMVITDPDMIKTVLVKECYSVFTNRRSFGPVGFMKKAVSIS

EDEDWKRVRTLLSPTFTSGKLKEMLPIIAQYGDVLVKNLRQEAEKASPSTLKEIFGAYSM

DVITGTSFGVNIDSLRNPQDPFVKNVRRLLKFSFFDPLLLSITLFPFLTPIFEALHISMF

PKDVMDFLKTSVEKIKDDRLKDKQKRRVDFLQLMINSQNSKEIDSHKALDDIEVVAQSII

ILFAGYETTSSTLSFIMHLLATHPDVQQKLQEEIDTLLPNKELATYDTLVKMEYLDMVVN

ETLRLYPIAGRLERVCKKDVDINGTFIPKGTIVMPTYALHRDPQHWTEPDEFRPERFSKK

NKDNINPYIYHPFGAGPRNCLGMRFALMNIKLALVRLMQNFSFKLCKETQVPLKLGKQGL

LQPEKPIVLKVVSESGIIRGA

>CAA45743.1

MDLLSALTLETWVLLAVVLVLLYGFGTRTHGLFKKQGIPGPKPLPFFGTVLNYYMGLWKF

DVECHKKYGKIWGLFDGQMPLFAITDTEMIKNVLVKECFSVFTNRRDFGPVGIMGKAVSV

AKDEEWKRYRALLSPTFTSGRLKEMFPIIEQYGDILVKYLKQEAETGKPVTMKKVFGAYS

MDVITSTSFGVNVDSLNNPKDPFVEKAKKLLRIDFFDPLFLSVVLFPFLTPVYEMLNICM

FPKDSIEFFKKFVYRMKETRLDSVQKHRVDFLQLMMNAHNDSKDKESHTALSDMEITAQS

IIFIFAGYEPTSSTLSFVLHSLATHPDTQKKLQEEIDRALPNKAPPTYDTVMEMEYLDMV

LNETLRLYPIGNRLERVCKKDVEINGVFMPKGSVVMIPSYALHRDPQHWPEPEEFRPERF

SKENKGSIDPYVYLPFGNGPRNCIGMRFALMNMKLALTKVLQNFSFQPCKETQIPLKLSR

QGLLQPTKPIILKVVPRDEIITGS

>ACN69111.1

MDLIPSFSVETWVLLATSLALLYLYGIYTHGLFKKLGIPGPKPLPFLGTLLGYRKGFGGF

DTECLKKYRKMWGFYDGRQPVLAITDPDLIKTVLVKECYSVFTNRRSFGPVGFMKNAISI

SEDEQWKRIRTLLSPTFTSGKLKEMFPIIGQYGDVLVRNLKKEAEKGKPIALKDIFGAYS

MDVITSTSFGVNIDSLNNPQDPFVENTKKLLRFDFLDPFILSITIFPFLNPVFELLNIFL

FPKSVTDFFTKSVKGIKESRLKDKEKQRVDFLQLMINSQNSKEMDTHKALSDLELVAQSI

IFIFAGYEPSAVLFFLFCIFWPLTLMSSRSCRRRLMQLPPIWHLPTYDALVQMEYLDMVL

NESLRLFPVAGRIERTCKKDVELGGVFIPKGTVVMVPSFVLHRDTELWPQPEEFHPERFS

KENKDSINLYIYMPFGNGPRNCIGMRFVLMNMKVAVVRVLQNFSFNPCKETQIPLKLVTY

GFFHPEKPIVLKVESRAGTVSGA

>P05183.2

MDLLSALTLETWVLLAVILVLLYRLGTHRHGIFKKQGIPGPKPLPFLGTVLNYYKGLGRF

DMECYKKYGKIWGLFDGQTPVFAIMDTEMIKNVLVKECFSVFTNRRDFGPVGIMGKAVSV

AKDEEWKRYRALLSPTFTSGRLKEMFPIIEQYGDILVKYLKQEAETGKPVTMKKVFGAYS

MDVITSTSFGVNVDSLNNPKDPFVEKTKKLLRFDFFDPLFLSVVLFPFLTPIYEMLNICM

FPKDSIAFFQKFVHRIKETRLDSKHKHRVDFLQLMLNAHNNSKDEVSHKALSDVEIIAQS

VIFIFAGYETTSSTLSFVLYFLATHPDIQKKLQEEIDGALPSKAPPTYDIVMEMEYLDMV

LNETLRLYPIGNRLERVCKKDIELDGLFIPKGSVVTIPTYALHHDPQHWPKPEEFHPERF

SKENKGSIDPYVYLPFGNGPRNCIGMRFALMNMKLALTKVLQNFSFQPCKETQIPLKLSR

QAILEPEKPIVLKVLPRDAVINGA

>CAA55887.1

MDLLSALTLETWVLLAVILVLLYRLGTHRHGIFKKQGIPGPKPLPFLGTVLNYYKAVGRF

DMECYKKYGKIWGLFDGQTPVFAIMDTEMIKNVLVKECFSVFTNRRDFGPVGIMGKAVSV

AKDEEWKRYRALLSPTFTSGRLKEMFPIIEQYGDILVKYLKQEAETGKPVTMKKVFGAYS

MDVITSTSFGVNVDSLNNPKDPFVEKTKKLLRFDFFDPLFLSVVLFPFLTPIYEMLNICM

FPKDSIAFFQKFVHRIKETRLDSKHKHRVDFLQLMLNAHNNSKDEVSHKALSDVEIIAQS

VIFIFAGYETTSSTLSFVLYFLATHPDIQKKLQEEIDGALPSKAPPTYDIVMEMEYLDMV

LNETLRLYPIGNRLERVCKKDIELDGLFIPKGSVVTIPTYALHHDPQHWPKPEEFHPERF

SKENKGSIDPYVYLPFGNGPRNCIGMRFALMNMKLALTKVLQNFSFQPCKETQIPLKLSR

QAILEPEKPIVLKVLPRDAVINGA

>Q64417.2

MDLVPSFSLETWVLLALSLVLLYRYATYSHGFFKKLGIPGPKPLPLFGNVLSYRKGMWSF

DIECRKKYGNMWGLYDGPQPVLAITEPDMIKAVLVKECYSVFTNRRSLVPVGFMKKAVSL

SEDEEWKRIRTQLSPNFTSGKLKEMFPIIKQYGDVLVKNLRQEAEKGKPVQLKEIFGAYS

MDIIVATAFGVNVDSLNNPHDPFVSKARKLFRFDFLSPFLLSIVMFPFLTQLYEMLSISI

FPRDSLKFFTKFVKKTKENHLESNKKQRVDFLQMMLNSQNFKDTESHKALSDVEILAQSI

IFIFAGYETTSSTLSFIMYSLATHPDVQKKLQQEIDKTLPNKAFPTYDVMMEMEYLDMVV

NETLRLYPVTNRIERMSKKDFEINGMSFPKGTGVMIPSFALHRDSKYWPEPDEFRPERFS

KKNKENIDPYIYMPFGNGPRNCIGMRMALMNLKLALIRLLQNFSFYPCKETQIPLRLGSE

ALLQPAKPIILKVVSRDETIRGA

>EGV99122.1

MDLTSVLSLETWVLLAISLVLLYRLGTHKHDIFKKQGIPGPKPLPFLGTLLSYYKGVWKF

DIECRKKYGKIWGLFDGQTPLFSVTDTEMIKNVLVKECYSVFTNRRDFGPVGIMSKAVSI

SKDEEWKRIRALLSPTFTSGKLKEMFPIIEQYGDILVKYLRREAEKGKPVITKEVFGAYS

MDVITSTAFGVSVDSLNNPKDPFVEKARKLLRFDFFDPLFLSVVLFPFITPIYERLNVSL

FPKDSIEFFRKFVYKMKEHRLDYNQKHRVDFLQLMMNAHDNSKDKESHKALSDMEIIAQS

IIFIFAGYETTSSTLAFALYLLATHPDIQKKLQEEIDVALPNKTRPTYDKVMEMEYLDMV

MNETLRLYPIGNRLERVCKRDVQMDGVFVPKGSIVMVPVFALHYDPQYWPEPEEFRPERF

SKENKGSIDPYVFMPFGNGPRNCIGMRFALMNMKLALTKVLQNFSFQPCKETQMPMKLSR

KAMLQPEKPIVLKVVPRSAIITGA

>BAA03865.1

MDLVPSFSLETWVLLALSLVLLYRYATYSHGFFKKLGIPGPKPLPLFGNVLSYRKGMWSF

DIECRKKYGNMWGLYDGPQPVLAITEPDMIKAVLVKECYSVFTNRRSLVPVGFMKKAVSL

PEDEEWKRIRTQLSPNFTSGKLKEMFPIIKQYGDVLVKNLRQEAEKGKPVQLKEIFGAYS

MDIIVATAFGVNVDSLNNPHDPFVSKARKLFRFDFLSPFLLSIVMFPFLTQLYEMLSISI

FPRDSLKFFTKFVKKTKENHLESNKKQRVDFLQMMLNSQNFKDTESHKALSDVEILAQSI

IFIFAGYETTSSTLSFIMYSLATHPDVQKKLQQEIDKTLPNKAFPTYDVMMEMEYLDMVV

NETLRLYPVTNRIERMSKKDFEINGMSFPKGTGVMIPSFALHRDSKYWPEPDEFRPERFS

KKNKENIDPYIYMPFGNGPRNCIGMRMALMNIKLALIRLLQNFSFYPCKETQIPLRLGSE

ALLQPAKPIILKVVSRDETIRGA

>AAA41051.1

MDLLSALTLETWVLLAVILVLLYRLGTHRHGIFKKQGIPGPKPLPFLGTVLNYYKGLGRF

DMECYKKYGKIWGLFDGQTPVFAIMDTEMIKNVLVKECFSVFTNRRDFGPVGIMGKAVSV

AKDEEWKRYRALLSPTFTSGRLKEMFPIIEQYGDILVKYLKQEAETGKPVTMKKVFGAYS

MDVITSTSFGVNVDSLNNPKDPFVEKTKKLLRFDFFDPLFLSVVLFPFLTPIYEMLNICM

FPKDSIAFFQKFVHRIKETRLDSKHKHRVDFLQLMLNAHNNSKDEVSHKALSDVEIIAQS

VIFIFAGYETTSSTLSFVLYFLATHPDIQKKLQEEIDGALPSKAPPTYDIVMEMEYLDMV

LNETLRLYPIGNRLERVCKKDIELDGLFIPKGSVVTIPTYALHHDPQHWPKPEEFHPERF

SKENKGSIHPYVYLPFGNGPRNCIDMRFALMNMKLALTKVLQNFSFQPCKETQIPLKLSR

QAILEPEKPIVLKVLPRDAVINGA

>AAA41023.1

MDLLSALTLETWVLLAVVLVLLYGFGTRTHGLFKKQGIPGPKPLPFFGTVLNYYMGLWKF

DVECHKKYGKIWGLFDGQMPLFAITDTEMIKNVLVKECFSVFTNRRDFGPVGIMGKAISV

SKDEEWKRYRALLSPTFTSGRLKEMFPVIEQYGDILVKYLRQEKGKPVPVKEVFGAYSMD

VITSTSFGVNVDSLNNPKDPFVEKAKKLLRIDFFDPLFLSVVLFPFLTPVYEMLNICMFP

KDSIEFFKKFVYRMKETRLDSVQKHRVDFLQLMMNAHNDSKDKESHTALSDMEITAQSII

FIFAGYEPTSSTLSFVLHSLATHPDTQKKLQEEIDRALPNKAPPTYDTVMEMEYLDMVLN

ETLRLYPIGNRLERVCKKDVEINGVFMPKGSVVMIPSYALHRDPQHWPEPEEFRPERFSK

ENKGSIDPYVYLPFGNGPRNCIGMRFALMNMKLALTKVLQNFSFQPCKETQIPLKLSRQG

LLQPTKPIILKVVPRDEIITGS

>AAI38085.1

MNLFSALSLDTWVLLAIILVLLYRYGTRTHGLFKKQGIPGPTPLPFLGTVLNYYKGLWKF

DMECYEKYGKTWGLFDGQMPLFVITDPEMIKNVLVKECFSVFTNRREFGPVGIMSKAISI

SKDEEWKRYRALLSPTFTSGKLKEMFPVIEQYGDILVKYLMQEAEKGKPVTMKDVLGAYS

IDVITSTSFGVNVDSLNNPEDPFVEKAKGILRVDFFDPLVFSVVLFPFLTPVYEMLNICM

FPKDSIEFFKKFVNRMKESRLDSKQKHRVDFLQLMMNAHNNSKDKDSHKALSDMEITAQS

IVFIFAGYETTSSTLSFTLYCLATHPDIQKKLQEEIDETLPNKAPPTYDTVMEMEYLDMV

LNETLRLYPIGNRLERFCKKDVELNGVYIPKGSTVMIPSYALHHDPQHWPEPEEFQPERF

SKENKGSIDPYLYMPFGIGPRNCIGMRFAFMTMKLALTKVMQNFSFQPCQETQIPLKLSR

QGLLQPEKPIVLKVVPRDVVITGA

>BAA06233.1

MDLLSALTLETWVLLAVVLVLLYGFGTRTHGLFKKQGIPGPKPLPFFGTVLNYYMGLWKF

DVECHKKYGKIWGLFDGQMPLFAITDTEMIKNVLVKECFSVFTNRRYFGPVGIMGKAISV

SKDEEWKRYRALLSPTFTSGRLKEMFPVIEQYGDILVKYLRQEKGKPVPVKEVFGAYSMD

VITSTSFGVNVDSLNNPKDPFVEKAKKLLRIDFFDPLFLSVVLFPFLTPVYEMLNICMFP

KDSIEFFKKFVYRMKETRLDSVQKHRVDFLQLMMNAHNDSKDKESHTALSDMEITAQSII

FIFAGYEPTSSTLSFVLHSLATHPDTQKKLQEEIDRALPNKAPPTYDTVMEMEYLDMVLN

ETLRLYPIGNRLERVCKKDVEINGVFMPKGSVVMIPSYALHRDPQHWPEPEEFRPERFSK

ENKGSIDPYVYLPFGNGPRNCIGMRFALMNMKLALTKVLQNFSFQPCKETQIPLKLSRQG

LLQPTKPIILKVVPRDEIITGS

>Q9JMA7.2

MNLFSALSLDTWVLLAIILVLLYRYGTRTHGLFKKQGIPGPKPLPFLGTVLNYYKGLWKF

DMECYEKYGKTWGLFDGQMPLFVITDPEMIKNVLVKECFSVFTNRREFGPVGIMSKAISI

SKDEEWKRYRALLSPTFTSGKLKEMFPVIEQYGDILVKYLMQEAEKGKPVTMKDVLGAYS

IDVITSTSFGVNVDSLNNPEDPFVEKAKGILRVDFFDPLVFSVVLFPFLTPVYEMLNICM

FPKDSIEFFKKFVNRMKESRLDSKQKHRVDFLQLMMNAHNNSKDKDSHKALSDMEITAQS

IVFIFAGYETTSSTLSFTLYCLATHPDIQKKLQEEIDETLPNKAPPTYDTVMEMEYLDMV

LNETLRLYPIGNRLERFCKKDVELNGVYIPKGSTVMIPSYALHHDPQHWPEPEEFQPERF

SKENKGSIDPYLYMPFGIGPRNCIGMRFAFMTMKLALTKVMQNFSFQPCQETQIPLKLSR

QGLLQPEKPIVLKVVPRDVVITGA

>Q64406.1

MDLIPSFSLETWVLLALSLVLLYQYATYSHGFFKKLGIPGPKPLPLFGNVLSYRKGMWSF

DIECRKKYGNMWGLYDGPQPVLAITEPDMIKAVLVKECYSVFTNRRSLVPVGFMKKSVSL

SEDEEWKRIRTQLSPNFTSGKLKEMFPIIKQYGDVLVKNLRQEAEKGKPVQLKEIFGAYG

MDIIIATAFGVNVDSLNNPHDPFVSKANKLFRFDFLSPFLLSTVMFPFLTQLYEMLSISI

FPRDSLKFFTKFVKKTKENHLESNKKQRVNFLQMMLNSQNFKDTESHKALSDVEILAQSI

IFIFAGYETSSSTLSCIMYSLATHPDVQKKLHQEIDKTLPNKAFPTYDVMMEMEYLDMVV

NETLRLYPVANRIERMSKKDFEINGMSFPKGTLVMIPSFALHRDSKYWPEPDEFRPERFS

KKNKENIDPYIYMPFGNGPRNCIGRRMALMNLKLALIRLLQNFSFYPCKETQIPLRLSSE

ALLQPEKPLILKVVSRDETIRGA

>354495213

MELFLNLSIETWVLLATSLVLIYIYGTYSHGTFKKLGIPGPKPLPLFGTIFDYRDGTWKF

DEDCYKKYGKIWGFYDGRRPVLGIADPEVIKTVLVKECYSIFTNRRSFGPVGFMKKAITV

SEDEEWKRLRTLLSPTFTSGKLKEMFPIITQYGDTLVKNLRREEEKGKPINTKDILGAYS

MDVITGTSFGVNVDSLNNPEDPFVQKAKKILKFKFFDPFILSIVLFPFLTPIYELLNFSI

FPRQSTNFFKKFVTTMKKNRLDSNQKTRKDFFQLMMNTQNSKGNESQKALSDLEMAAQTI

IFIFGGYEATSTSISFMLYELATHPDVQKKLQDEIDRALPNKAPVTYDALMDMEYLDMIV

NETLRMYPIANRLERVSKKSVEINGVFIPKGTVVMVPIYPLHRDPEYWTEPEVFRPERFS

KENKGSIDPYVYLPFGSGPRNCIGRRFALISMKLAVISILQNFTLQTCEQTEIHPKFSRQ

PILQPENPIILKVVSRDKPITGA

>EDL05796.1

MELIPNLSIETWVLLVTSLVFFYIYGTYSHGLFKKLGIPGPKPLPLFGTIFNYFDGMWKF

DEDCYKKYGKIWGFYVGPKPILAIMDPEIIKIVLVKECYSVFTNRPTLGPVGFLKKSITI

SEDEEWKRLRTLLSPTFTSGKLKEMFPIMRQYGDILVRNLRREEEKGEPINMKDIFGAYS

MDVITGTSFGVNIDSLNNPQNPFVQKAKKILKFKIFDPFLLSTILFPFLTPIYEMLNLSI

FPRDSMNFFKKFVKRMKKERLASKQKNRVDFLQLMMNTQNSKGQESQKALSDLEMAAQAV

IFIFGGYDATSTSISLIMYELATHPDVQKKLQDEIDRTLPNKAPVTYDALMDMEYLDMVV

NESLRLYPTAIRLERVSKKDVEINGVFIPKGTVVMIPIYPLHRNPEYWPEPQEFCPERFS

KENEGNIDPYIYMPFGNGPRNCLDMRFALISMKLAVIGVLQNFTVQPCEETQIPLKISRE

PVFQPEKPIILKVVSRDKPRTAS

>O70537.1

MDLTSVLSLETWVLLAISLVLLYRLGTHKYDIFKKQGIPGPKPLPFLGNVLNYYKGIWTF

DIECHKKYGKIWGLFEGQRPLFTVTDTEMIKNVLVKECYSIFTNRRDFGPVGIMSKAVSI

SKDEEWKRIRALLSPTFTSGKLKEMFPIIEQYGDILVKFLRREAEKGNPVTTKEVFGAYS

MDVITSTAFGVSVDSLNNPKDLLWKGRKLLRFDFFDPLFLSVVLFPFLIPIYEKLNVSMF

PKDSISFFRKFVDKTKENRLDYNQKHRVDFLQLMMNSHDNSKDSHKALSDMEIIAQSIIF

IFAGYDTTSSTLSFALYLLATHPDVQKKLQEEIDIALPNKARPSYDKVMEMEYLDMVLNE

TLRLYPIGSRLERVCKQDVEMDGVFVPKGSIVMVPVFALHYDPQYWPEPEKFRPERFSKE

NKGSIDPYIFLPFGNGPRNCIGMRFALMNMKLALTKVLQNFSLQPCKETQIPMKLSRKAM

LQPEKPIILKVVPRDAIITGA

>157364980

MELIPNLSIETWVLLVTSLVFFYIYGTYSHGLFKKLGIPGPKPLPLFGTIFNYFDGMWKF

DEDCYKKYGKIWGFYVGPKPILAIMDPEIIKIVLVKECYSVFTNRPTLGPVGFLKKSITI

SEDEEWKRLRTLLSPTFTSGKLKEMFPIMRQYGDILVRNLRREEEKGEPINMKDIFGAYS

MDVITGTSFGVNIDSLNNPQNPFVQKTKKILKFKIFDPFLLSTILFPFLTPIYEMLNLSI

FPRDSMNFFKKFVKRMKKERLASKQKNRVDFLQLMMNTQNSKGQESQKALSDLEMAAQAV

IFIFGGYDATSTSISLIMYELATHPDVQKKLQDEIDRTLPNKAPVTYDALMDMEYLDMVV

NESLRLYPTAIRLERVSKKDVEINGVFIPKGTVVMIPIYPLHRNPEYWPEPQEFCPGRFS

KENEGNIDPYIYMPFGNGPRNCLDMRFALISMKLAVIGVLQNFTVQPCEETQIPLKISRE

PVFQPEKPIILKVVSRDKPRTAS

>Q64409.1

MDLIPSFSLETWVLLALSLVLLYQYATYSHGFFKKLGIPGPKPLPLFGNVLSYRKGIWNF

DIECHKKYGNMWGLYDGPRPVLSITEPDMIKAVLVKECYSVFTNRRSIFPAGFMKKALSI

SKDEEWKRIRTQLSQNFTSGKLKEMFPIIKQYGDVLVKNLRQEAEKGKPVQLKEIFGAYS

MDIIIATAFGVNVDSLNNPHDPFVSKASKLFRFDFLSPFLLSVVIFPFLTQLYEMLNISI

FPRDSLNFFTKFVKRTKENHLESNEKQRVNFLQMMLKSQNFKDTESHKALSDVEILAQSI

FFIVAGYETTSSTLCFIMYSLATHPDVQKKLQQEIDKTLPNKAFPTYDVMMEMEYLDMVV

NETLRLYPVTNRIERMSKKDFEINGMSFPKGTGVMIPSFALHRDSKYWPEPDEFRPERFS

KKNKENIDPYIYMPFGNGPRNCIGMRMALMNLKLALIRLLQNFSFYTCKETQIPLRLGSQ

VILQPAKPIILKVVSRDETIRGA

>395852795

MNLIPDFAMETWVLLAVSLVLLYLLYDGRQPLLVITDPDMIKTVLVKECYSVFTNRRSFG

PVGFMKNAISISENEEWKRIRALLSPTFTSGKLKEMFPIMSQYADVLVRNLRQEAEKDKP

INLKDAFGAYSMDVITGTSFGVNIDSLNNPQDPFVENTKKLLRFDFLDPFLLSIILFPFL

TPVYEALSITAFPKDVIDFFKKSVKRMKESRLKDKERHRVDFLQLMIDSQNTKETVSHKV

LSDLELVAQSVIFIFAGYETTSSALSFIMYELATHPDVQKKLQDEIDAALPNKAPATYDA

MVQMEYLDMVVNETLRLFPIAGRLERVCKKDVEVNGVLIPKGVTVMIPTYALHRDPKHWI

EPEEFRPERFSKKNKDSIDPYIYLPFGTGPRNCIGMRFALMNMKLALVGVLQNFSFKPCE

ETQIPLKLSRQGFLQPEKPIVLKVESRVGTVRGA

>Q64581.1

MEIIPNLSIETWVLLATSLMLFYIYGTYSHGLFKKLGIPGPKPVPLFGTIFNYGDGMWKF

DDDCYKKYGKIWGFYEGPQPFLAIMDPEIIKMVLVKECYSVFTNRRCFGPMGFMKKAITM

SEDEEWKRLRTILSPTFTSGKLKEMFPLMRQYGDTLLKNLRREEAKGEPINMKDIFGAYS

MDVITGTSFGVNVDSLNNPQDPFVQKAKKILKFQIFDPFLLSVVLFPFLTPIYEMLNFSI

FPRQSMNFFKKFVKTMKKNRLDSNQKNRVDFLQLMMNTQNSKGQESQKALSDLEMAAQAI

IFIFGGYDATSTSISFIMYELATRPNVQKKLQNEIDRALPNKAPVTYDALMEMEYLDMVV

NESLRLYPIATRLDRVSKKDVEINGVFIPKGTVVTIPIYPLHRNPEYWLEPEEFNPERFS

KENKGSIDPYVYLPFGNGPRNCIGMRFALISMKLAVIGVLQNFNIQPCEKTQIPLKISRQ

PIFQPEGPIILKLVSRD

>Q64148.2

MELIPNLSIETWVLLAISLVLIYIYGTYSHGTFKKLGIPGPKPLPFFGTILGYHDGTWKF

DETCYKKYGKIWGFYDGRVPVLAIADPEIIKTVLVKECYSNFTNRRSFGPVGFMKKSITI

SKDEEWKRLRTLLSPAFTSGKLKEMFPIIGQYGDTLVKNLRREEEKGKPVNMKEILGAYS

MDVITGTSFGVNVDSLNNPEDPFVQKARKILKFNFFDPFILSIILFPFLTTIYDLLRFSI

FPRQSTNFFKKFITTMKKNRLHSNQKTRMDFFQLMMNTQNSKGKESQKALSDLEMAAQAI

IFIFAGYESTSTSICLVLYELATHPDVQKKLHDEIDSALPNKAPVTYDVLMGMEYLDMVI

NEGLRLYPIANRLERISKKAVEINGLFIPKGITVMVPTYPLHRDPEYWPEPEEFRPERFS

KENKGSIDPYVYMPFGNGPRNCIGMRFALLSMKLAVVSVLQNFTLQTCEQTENHLKFARQ

IILQPENPIILKIISRDKPITGA

>EDL89594.1

MEIIPNLSIETWVLLATSLMLFYIYGTYSHGLFKKLGIPGPKPVPLFGTIFNYGDGMWKF

DDDCYKKYGKIWGFYEGPQPFLAIMDPEIIKMVLVKECYSVFTNRRCFGPMGFMKKAITM

SEDEEWKRLRTILSPTFTSGKLKEMFPLMRQYGDTLLKNLRREEAKGEPINMKDIFGAYS

MDVITGTSFGVNVDSLNNPQDPFVQKAKKILKFQIFDPFLLSVVLFPFLTPIYEMLNFSI

FPRQSMNFFKKFVKTMKKNRLDSNQKSRVDFLQLMMNTQNSKGQESQKALSDLEMAAQAI

IFIFGGYDATSTSISFIMYELATRPNVQKKLQNEIDRALPNKAPVTYDALMEMEYLDMVV

NESLRLYPIATRLDRVSKKDVEINGVFIPKGTVVTIPIYPLHRNPEYWLEPEEFNPERFS

KENKGSIDPYVYLPFGNGPRNCIGMRFALISMKLAVIGVLQNFNIQPCEKTQIPLKISRQ

PIFQPEGPIILKLVSRD

>344289839

MEWSITEKGHTRAPQHHPFNPCDIPEYPLVCGTYSHNVLKKLGIPGPKPLPFLGTLLAYR

KGMWDFDVKCSKKYGKLWGFYDGPQPVIAITDPGMIKTVLVKESYSTFTNRRMLGPTGFM

KSALSSSKDEQWKRLRTLLSPTFSSGKLKEMFPIISQYGDLVVKHLREKTQKGKPVTLKS

VFGAYSMDVITSTSFGVNIDSLSNPQDLFVKNARNLIRFDFLDPLIFLITLFPFLIPICE

ALKISVFPRAATDFFTKSVQRIKESRLKDNQKRRVDLLQLMMDSQETKEISPQKALTDTE

LVAQSIMFIFAGYETTSTALSFVMYLLATHPDIQEKLQREIDAAFPNKAPPTYDATLQMA

YLDMVVNETLRLFPIAGRLERVCKKDIEINGVTIPKGTIVMVPIFVLHRDAEHWPEPEKF

MPERFSKENKDNVDPYLYLPFGTGPRNCIGMRFALMNMKLAIIKILQEFSVKPCKETQIP

LKLGRERILAPEGPIVIKFESRDGHASGA

>BAA05133.1

MNLFSALSLDTLVLLAIILVLLYRYGTYTHGLFKKQGIPGPKPLPFLGTVLNYYKGLWKF

DMECYEKYGKTWGLFDGQIPLFVITDPETIKNVLVKECFSVFTNRQDFFPVGIMSKSISL

AKDEEWKRYRALLSPTFTSGNLKEMFPVIEQYGDILVKYLRQEAEKGKPVAVKDVLGAYS

MDVIISTTFGVNIDSLNNPEDPFVENAKKVLRFDYFDPLSLSVALFPFLTPIYEMLNICM

FPKDSIEFFKKFVDRMTENRLDSKQKHRVDFIYLMMEAYNKSKDKDSHKALSEIEITAQS

IIFIFAGYETTSSILSFTVYSLATHPDIQKKLQEEIDEALPNKAPPTYDTVMAMEYLDMV

LNETLRLYPITNRLQRVCKKDVEINGIYIPKGSTVIIPSYVLHHDPQHWPEPEEFQPERF

SKENKGSIDPYVYLPFGNGPRNCIGMRFALMNMKLALIKVLQNFSFQPCKETQIPLKLSR

ELLLQPVKPIVLKVVPRDAVITGA

>395852801

MDVILNFAMETWVLLAASLVLLYMLYDGQQPVLAITDPEMIKTVLVKECHSVFTNRRSFG

PLGLMKSAISLSVDDEWKRIRTLLSPTFSSGKLKEMFPIMSQYGDVLVRNLRQEANKGKT

VNLKDIFGAYSMDVITSTSFGVNIDSLNNPQDPFVENIKKFLKFNFVTPFFLSILLFPFL

IPVFEALNIFVFPKDVMDFFEKSIKRIKESRLQDKQKHRVDLLQLMIDSQNSKETESHKV

LSDVELVAQSIIFIFAGYETTSSALSFLMYELATHPDVQQKLQDEIDAALPSKTPATYDA

MVQMEYLDMVVNETLRLFPIAGRLERVCKKDVEVNGVLIPKGVTVMIPTFALHRDPKHWT

EPEEFRPERFSKKNKDSIDPYMYMPFGSGPRNCIGMRFALMNMKIALIRILKNFSFKPCK

ETQIPLKLSKKAILQPEKPIVLKIESREGTTNGA

>Q64481.2

MNLFSALSLDTLVLLAIILVLLYRYGTCTHGLFKKQGIPGPKPLPFLGTVLNYYKGLWKF

DMECYEKYGKTWGLFDGQIPLFVITDPETIKNVLVKECFSVFTNRQDFFPVGIMSKSISL

AKDEEWKRYRALLSPTFTSGNLKEMFPVIEQYGDILVKYLRQEAEKGKPVAVKDVLGAYS

MDVIISTTFGVNIDSLNNPEDPFVENAKKVLRFDYFDPLSLSVALFPFLTPIYEMLNICM

FPKDSIEFFKKFVDRMTENRLDSKQKHRVDFIYLMMEAYNKSKDKDSHKALSEIEITAQS

IIFIFAGYETTSSILSFTVYSLATHPDIQKKLQEEIDEALPNKAPPTYDTVMAMEYLDMV

LNETLRLYPITNRLQRVCKKDVEINGIYIPKGSTVIIPSYVLHHDPQHWPEPEEFQPERF

SKENKGSIDPYVYLPFGNGPRNCIGMRFALMNMKLALIKVLQNFSFQPCKETQIPLKLSR

ELLLQPVKPIVLKVVPRDAVITGA

>109496572

MDLVSALSLETWLLLAIILVLFYRFGTRTHGIFKKQGIPGPKPLPFLGTVLNYYRGLWKF

DMECYKKCGKIWGLFDGQTPVFAIMDTEMIKSVLVKECFSVFTNRRNIGPVGIMSKSISV

AKDEEWKRYRAFLSPTFTSGRLKEMFPIIEHYGDILVKYLKQKVEKGKPLAMKEVFGAYS

MDVITSTSFEVNINSINNPKDPFVEKVKKFQRFDFFDPLFLSVVLFPFLTPIYEMLNICL

FPKDSVAFFQKFVYRMKQTRLDSKHKHRVDFLQLMMNAHNNSKDKVSHKALSDIEIVAQA

IIFIFASYETTSSTLSFVLYSLATHPDSQKKLQEEIDRALPNKAPPTYDTVMEMEYLDMV

LNETPRLYPIGYRLERVCKKDIKLDGVFIPKGSVVMIPFYTLQHDPQHWPEPEEFLPERF

SKENKGSIDPYVYLPFGNGPRNCIGMRFALMNMKLALTKVLQNFSFQLCEETQIPLKLSR

QRLFGPEKPIVLKVVPRDAVITGA

>AAI13144.1

MNLFSALSLDTLVLLAIILVLLYRYGTCTHGLFKKQGIPGPKPLPFLGTVLNYYKGLWKF

DMECYEKYGKTWGLFDGQIPLFVITDPETIKNVLVKECFSVFTNRQDFFPVGIMSKSISL

AKDEEWKRYRALLSPTFTSGNLKEMFPVIEQYGDILVKYLRQEAEKGKPVAVKDVLGAYS

MDVIISTTFGVNIDSLNNPEDPFVENAKKVLRFDYFDPLSLSVALFPFLTPIYEMLNICM

FPKDSIEFFKKFVDRMTENRLDSKQKHRMDFIYLMMEAYNKSKDKDSHKALSEIEITAQS

IIFIFAGYETTSSILSFTVYSLATHPDIQKKLQEEIDEALPNKAPPTYDTVMAMEYLDMV

LNETLRLYPITNRLQRVCKKDVEINGIYIPKGSTVIIPSYVLHHDPQHWPEPEEFQPERF

SKENKGSIDPYVYLPFGNGPRNCIGMRFALMNMKLALIKVLQNFSFQPCKETQIPLKLSR

ELLLQPVKPIVLKVVPRDAVITGA

>AAI25523.1

MNLFSALSLDTLVLLAIILVLLYRYGTRTHGLFKKQGIPGPKPLPFLGTVLNYYTGIWKF

DMECYEKYGKTWGLFDGQTPLLVITDPETIKNVLVKDCLSVFTNRREFGPVGIMSKAISI

SKDEEWKRYRALLSPTFTSGRLKEMFPVIEQYGDILVKYLRQEAEKGMPVAMKDVLGAYS

MDVITSTSFGVNVDSLNNPEDPFVEEAKKFLRVDFFDPLLFSVVLFPLLTPVYEMLNICM

FPNDSIEFFKKFVDRMQESRLDSNQKHRVDFLQLMMNSHNNSKDKDSHKAFSNMEITVQS

IIFISAGYETTSSTLSFTLYCLATHPDIQKKLQAEIDKALPNKATPTCDTVMEMEYLDMV

LNETLRLYPIVTRLERVCKKDVELNGVYIPKGSMVMIPSYALHHDPQHWPDPEEFQPERF

SKENKGSIDPYVYLPFGIGPRNCIGMRFALMNMKLAVTKVLQNFSFQPCQETQIPLKLSR

QGILQPEKPIVLKVVPRDAVITGA

>126334466

MNIIPSLSAGTWTLIVLFLTLLYLYGTRTHKLFKNLGIPGPTPLPFFGTVLAYRKGFADF

DYGCFKKYGKTWGFFDGRQAVLAIMDPETIKTVLVKECYSVFTNRRMFGPSGFLEKAISI

AKDEQWKRIRTVLSPTFTSGKLKEMFPIINQYGDVLVKNMKKEAEKSKPVTMKEIFGAYS

MDVITSTSFGIHVDSLNNQNDPFVREIRKLIRFSFLDPLILSITIFPFLIPLFKKLDITV

FPKEATDFLAKSIIKIKEERTKSTEKHRVDFLQLMMDSQTSKNSESHSQKDLSDEEILAQ

SIIFIFAGYETTSSVLSFLFYHLATNPKIQEKLQKEIDAFLPNKEAVTYDALVQMEYLDM

VINENLRLYPIAGRIERVAKKTVELNGLTIPKGTVVMAPPYVLHRDPEYWPEPEEFRPER

FSKENKESINPYVYLPFGAGPRNCIGMRFALMSMKVAVSRLLQEFSFRPCKETQIPLKLS

NQPLLTPTVPIVLQAELRNKKGNQH

>402863012

MDLIPNFAMETWVLLATSLVLLYIYGTHSHKLFKKLGIPGPTPLPFLGTILFYLRGLWKF

DRECNEKYGEMWGLYEGQQPMLVIMDPDMIKTVLVKECYSVFTNRMPLGPMGFMKSALSF

AEDEEWKRIRTLLSPAFTSVKFKEMVPIISQCGDMLVRSLRREAENSKPTNLKEFFLNVT

LFPFLTPVFEVLNIGLFPKDVTRFLKNSIERMKESRLKDKQKHRVDFFQQMIDSQNSKET

KSHKALSDLELVAQSIIIIFAAYDTTSTTLPFIMYELATHPDVQQKLQEEIDAVLPNKAP

VTYDALVQMEYLDIVVNETLRLFPVVSRVTRVCKKDVEINGVFIPKGLAVMVPIYALHHD

PKYWTEPEKFCPERFSKKNKDSIDPYRYIPFGAGPRNCIGMRFALTNIKLAVIKALQNFS

FEPCEETQIPLKLNNLPILQPEKPIVLKVHLRDGITSGP

>126334470

MNIIPSLSAGTWTLIVLFLTLLYLYGTRTHKLFKNLGIPGPTPLPFFGTVLAYRKGFADF

DYGCFKKYGKTWGFFDGRQAVLAIMDPETIKTVLVKECYSVFTNRRMFGPSGFLEKAISI

AKDEQWKRIRTVLSPTFTSGKLKEMFPIINQYGDVLVKNMKKEAEKSKPVTMKEIFGAYS

MDVITSTSFGIHVDSLNNQNDPFVREIKKLIRFSFLDPLILSTTIFPFLIPLFNKLDITV

FPKEATDFLAKSIIKIKEERTKSTEKHRVDFLQLMMDSQTSKNSESHSQKDLSDEEILAQ

SIIFIFAGYETTSSVLSFLFYHLATNPKIQEKLQKEIDAFLPNKEAVTYDALVQMEYLDM

VINENLRLYPIAGRIERVAKKTVELNGLTIPKGTVVMAPPYVLHRDPEYWPEPEEFRPER

FSKENKESINPYVYLPFGAGPRNCIGMRFALMSMKVAVSRLLQEFSFRPCKETQIPLKLS

NQPLLTPTVPIVLQAELRNKKGNQH

>126334468

MNIIPSLSAGTWTLIVLFLTLLYLYGTRTHKLFKNLGIPGPTPLPFFGTVLAYRKGFADF

DYGCFKKYGKTWGFFDGRQAVLAIMDPETIKTVLVKECYSVFTNRRMFGPSGILEKAISI

AEDEQWKRIRTVLSPTFTSGKLKEMFPIINQYGDVLVKNMKKEAEKSKPVTMKEIFGAYS

MDVITSTSFGIHVDSLNNQNDPFVREIRKLIRFSFLDPLILSITIFPFLIPLFKKLDITV

FPKEATDFLAKSIIKIKEERKKSTGKHRVDFLQLMMDSQTSKNSESHSQKDLSDEEILAQ

SIIFIFAGYETTSSVLSFLFYHLATNPKIQEKLQKEIDAFLPNKEAVTYDALVQMEYLDM

VINENLRLYPIAGRIERVAKKTVELNGLTIPKGTVVMAPPYVLHRDPEYWPEPEEFRPER

FSKENKESINPYVYLPFGAGPRNCIGMRFALMSMKVAVSRLLQEFSFRPCKETQIPLKLS

NQPLLTPTVPIVLQAELRNKKGNQH

>154146256

MELIPNLSIQTWVLLVTSLVFFYIYGTYSHGLFKKLGIPGPKPLPLFGNIINYLDGMWKF

DDDCYKKYGKIWGFYEGPQPILAIMDPEIIKIVLVKECYSVFTNRRPFGPVGFLKKSITI

SEDEEWKRLRTLLSPTFTSSKLKEMFPIMRQYGDILVRNLRREEEKEEPINMKDIFGAYS

MDVITGTSCGVNIDSLNNPQDPFVQKIKKILKFKFFDPFLLSVVLFPFLTPIYEMLNLSI

FPRQSMNFFKKFVKKMKKDRLDYNQKNRVDFLQLMMNTQNSKGQESLKALSDLEMAAQTL

IFIFGGYDGTSTSISFIMYELATHPDVQKKLQDEIDRALPNKAPVTYDALMDMEYLDMVV

NESLRLYPVVSRIDRFSKKDVEIHGVFIPKGTIVMIPIYPLHRDPEYWPEPEDFCPERFS

KENKGNIDPYIYMPFGNGPRNCIGMRFALISVKLAVTGVLQNFTVQPCEETQIPVKISRQ

PIFRPEKPIILKFVSRDKPRTGP

>358421111

MELIPNFSVETWVLLAISLVLLYLYGTYSHGLFKKLGVPGPRPLPLFGNVLSYRKGVCEF

DEECFKKYGKMWGIFEGKHPLLVITDPDMIKTVLVKECYSVFTNRRVFGPMGVMKNAVSV

AEDEQWKRIRTLLSPTFTSGKLKEMFPIIGKYGDVLVRNLRKEAEKGTSVDMKDVLFCYL

FSIVLFPFLVPIFEVLNITMFPKSAVNFLAKSVKRIKESRLKDNQKPRVDFLQLMINSQN

SKETDNHKALSDQELMAQSVIFIFAGYETTSNTLSFLLYILATHPDVQQKLQEEIDVTFP

NKAPPTYDVLAQMEYLDMVVNETLRMFPITVRLDRLCKKDVKIHGVSIPKGTTVTVPISV

LHRDPQLWPEPEEFRPERFSKKNKDTISPYVYLPFGTGPRNCIGMRFAIMNMKLAVVRVL

QNFSFKSCKETQIPLKINSQGLIRPEKPIFLKVVLRDETISGA

>BAG60675.1

MALIPDLAMETWLLLAVSLVLLYLYGTHSHGLFKKLGIPGPTPLPFLGNILSYHKGFCMF

DMECHKKYGDVLVRNLRREAETGKPVTLKDVFGAYSMDVITSTSFGVNIDSLNNPQDPFV

ENTKKLLRFDFLDPFFLSITVFPFLIPILEVLNICVFPREVTNFLRKPVKRMKESRLEDT

QKHRVDFLQLMIDSQNSKETESHKALSDLELVAQSIIFIFAGYETTSSVLSFIMYELATH

PDVQQKLQEEIDAVLPNKAPPTYDTVLQMEYLDMVVNETLRLFPIAMRLERVCKKDVEIN

GMFIPKGVVVMIPSYALHRDPKYWTEPEKFLPERFSKKNKDNIDPYIYTPFGSGPRNCIG

MRFALMNMKLALIRVLQNFSFKPCKETQIPLKLSLGGLLQPEKPVVLKVESRDGTVSGA

>348568538

MDLIWNFSMETWVFLVLCLLLLYVYGTYTYGHFKKLGIPGPKPLPFLGNVLSYAQGSWKI

DVECYKKYGSIWGLFEGRQPVLLIMDPDIIKTVLVKECYSTFTNRREIRPAVLMKKSVLL

SQDEQWKRIRTLLSPTFTSGKLKEMFPIIEHYGDMLLKNLTRAAEKDVPVPIKDIFGAYS

MDVIIGTSFGVNVDSLSNPQDPFVKKTKKFLNFDNFNPLIFSGCIFSFLIPIYEMLGISL

FAKDSTEFFIDFVKKAIENRLESNQKHRVDFLQLMMNSQNSKDTESYKALSDVEITAQSA

NFILAGYETTSNSLSFIMYELATHPDIQKKLQQEIDAALPNKAFPTYDILVEMEYLDMVV

NETLRLYPIGNRLSRICKKDIEVNGVFIPKGTVVMVPIYVLHRDPKYWKNSKDFCPERFS

KKNKENIQPYVYMPFGAGPWNCIGMRFALMNMKLAVIIIMQHFSLQPCKETQIPIKLSRN

RFLQPEKPIVLKIVSRDGT

>348568540

MDLIWNFSMETWVFLVLCLLLLYVYGTYTYGHFKKLGIPGPKPLPFLGNVLSYAQGSWKI

DVECYKKYGSIWGLFEGRQPVLLIMDPDIIKTVLVKECYSTFTNRREIRPAVLMKKSVLL

SQDEQWKRIRTLLSPTFTSGKLKEMFPIIEHYGDMLLKNLTRAAEKDVPVPIKDIFGAYS

MDVIIGTSFGVNVDSLSNPQDPFVKKTKKFLNFDNFNPLIFSGLFFLSFSGIFSFLIPIY

EMLGISLFAKDSTEFFIDFVKKAIENRLESNQKHRVDFLQLMMNSQNSKDTESYKALSDV

EITAQSANFILAGYETTSNSLSFIMYELATHPDIQKKLQQEIDAALPNKAFPTYDILVEM

EYLDMVVNETLRLYPIGNRLSRICKKDIEVNGVFIPKGTVVMVPIYVLHRDPKYWKNSKD

FCPERFSKKNKENIQPYVYMPFGAGPWNCIGMRFALMNMKLAVIIIMQHFSLQPCKETQI

PIKLSRNRFLQPEKPIVLKIVSRDGT

>ABU85092.1

MDLIPNFAMETWVLVATSLVLLYMLYEGQQPMPVIMDPDMIKTVLVKECYSVFTNRMPLG

PMGFMKSALSFAEDEEWKRIRTLLSPAFTSVKFKEMVPIISQCGDMLVRSLRREAENSKP

TNLKDFFGAYTMDVITGTLFGVNLDSLNNPQDPFLKNMKKLLKLDFLDPFLLSISLFPFL

TPVFEVLNIGLFPKDVTRFLKNSIERMKESRLKDKQKHRVDFFQQMIDSQNSKETKSHKA

LSDLELVAQSIIIIFAAYDTTSTTLPFIMYELATHPDVQQKLQEEIDAVLPNKAPVTYDA

LVQMEYLDIVANETLRLFPVVSRVTRVCKKDIEINGVFIPKGLAVMVPIYALHHDPKYWT

EPEKFCPERFSKKNKDSIDPYRYIPFGAGPRNCIGMRFALTNIKLAVIKALQNFSFEPCE

ETQLPLKLNNLPILQPENPIVLKVHLRDGITSGP

>126334464

MNIIPNLSAGTWTLIILFLTILYLYGTRTHKLFKNIGIPGPTPFPFIGTILYYRKGIVGF

DYGCYKKYGKTWGFFDGTKPVLAIMDPETIKTVLVKECYSVFTNRRMLGLSGILEKAISI

AEDEEWKRIRTVLSPAFTSGKLKEMFPIINQYGDVLVKNMKKEAEKSKPVTMKEIFGAYS

MDIIISTSFGIHVDSLNNPNDPFVREIRKLIRFSFLDPLILSITIFPFLIPLFKKLDITV

FSKDATDFLGKSILRIKEERKKSTEKHRVDFLQLMMDSQTSKNSESHSQKDLSDEEILAQ

SIVFIFAGYESTSSVLSFLFYHLATNLEIQEKLQKEIDAVLPNKEAVTYDALVQMEYLDM

VINENLRLYPISGRIERIAKKTVQLNGLTIPQGTVVMTPPYVLHRDPEYWPEPEEFRPER

FSKENKESINPYVYLPFGAGPRNCIAMRFALMGMKVAVSRLLQEFSFIPCKETPIPLKLN

NQPLLTPSVPIVLQAVLRNKKGNQH

>395852903

MDLIPDFAMETWVLLAASLLLLYLLYDGRQPLLAITDPDVIKTVLVKECYSAFTNRRPLG

PVGFMKSAISVSKDEEWKRIRTLLSPTFTSGKLKEMFPIVSQYGDVLVRNMRQEAEKGKT

INLKDIFGAYSMDVIMGTSFGVNIDFLNSPQSPFVRKAKNLIKLDFSDPLFLSITFFPFL

TPIYEALNISVFPEDVTKFFINTVQKVKESRCQDKQKHRVDFLQLMIDSQNSKETESHKV

LSDLELVAQSIIFILAGYETTSSVLSFIMYELATHPDIQQKLQDEIDAALPNKTPTTYDA

LAQMEYLNMVVNETLRLFPVIGRLERVCKKDIEINGMFIPKGTIVMIPTYALHRDPKYWA

EPEKFHPERFGRKNKDHIDPHMYMPFGSGPRNCIGMRFAFMSMKLALVRVLQNFSFKPCE

ETQITMKLFTNGSLQLDKPIIVKVESRVGNLL

>EAW76641.1

MKSAISLAEDEEWKRIRSLLSPTFTSGKLKEMFPIIAQYGDVLVRNLRREAEKGKPVTLK

DIFGAYSMDVITGTSFGVNIDSLNNPQDPFVESTKKFLKFGFLDPLFLSIILFPFLTPVF

EALNVSLFPKDTINFLSKSVNRMKKSRLNDKQKHRLDFLQLMIDSQNSKETESHKALSDL

ELAAQSIIFIFAGYETTSSVLSFTLYELATHPDVQQKLQKEIDAVLPNKAPPTYDAVVQM

EYLDMVVNETLRLFPVAIRLERTCKKDVEINGVFIPKGSMVVIPTYALHHDPKYWTEPEE

FRPERFSKKKDSIDPYIYTPFGTGPRNCIGMRFALMNMKLALIRVLQNFSFKPCKETQIP

LKLDTQGLLQPEKPIVLKVDSRDGTLSGE

>395852797

MNLIPDFAMETWVLLAVSLVLLYLYGTHSHGLFKKLGIPGPTPLPFLGTVLGYRKGFWDF

DAECHRKYGKTWGLYDGRQPLLVITDPDMIKTVLVKECYSVFTNRRSFGPVGFMKNAISI

SENEEWKRIRALLSPTFTSGKLKEMFPIMSQYADVLVRNLRQEAEKDKPINLKDAFGAYS

MDVITGTSFGVNIDSLNNPQDPFVENTKKLLRFDFLDPFLLSIILSDLELVAQSVIFIFA

GYETTSSALSFIMYELATHPDVQKKLQDEIDAALPNKAPATYDAMVQMEYLDMVVNETLR

LFPIAGRLERVCKKDVEVNGVLIPKGVTVMIPTYALHRDPKHWIEPEEFRPERFSKKNKD

SIDPYIYLPFGTGPRNCIGMRFALMNMKLALVGVLQNFSFKPCEETQIPLKLSRQGFLQP

EKPIVLKVESRVGTVRGA

>ABI63602.1

MNIIPSLSAGTWALLVLFLTLLFLYGTWTHKLFKDLGIPGPAPLPFFGTVLSYRKGFTDF

DMTCFKKYGKTWGFFDGRKPVLSIMDPETIKLVLVKECFSVFTNRRSFGPAGIMESAISI

AEDDQWKRIRTILSPTFTSGKLKEMFPIIKQYGDVLVKNMGKEAEKSKPVSLKDIFGAYS

MDVITSTSFGVKTDSLNNQDDPFVREIRKLIKFNFLDPLLLTVSIFPFVIPLLKKMDVTI

FPKETLDFLTKSIKKIKEDRKKNSQKNRVDFLQLMMDSQTSKNSEMHSQKDLSDDEILAQ

SVIFIFAGYETTSSVLCFLFYHLATHPEIQQKLQGEIDAVLPNKEAVTYDALVQMEYLDM

VIHEILRLYPIAGRIERVAKKTVEINGVKIPKGTVVMVSPFVLHRDPDYWPEPEEFRPER

FSKENRESMNPYVYLPFGAGPRNCIGRRFALMSMKVATSLVLQEFSFRTCKETQIPVKLN

NQPLISPTIPIVLQAVPRSKKGNQH

>126334518

MAICPSQSSLLLPMELVLLYRYGTRTHKLFKNLGIPGPTPLPFIGTVLSYHKGIEGFDYG

CFKKYGKTWGFFDGTKPVLAIMDPETIKTVMVKECYSVFTNRRMLGLSGILEKAISIAED

EQWKRMRTVLSPAFTGGKLKEMFPIIKQYGDVLVKNMKKEAEKSKPVTLKDILGAYSMDV

ITSTSFGIHVDSLNNPNDPFVRKIRKLIRFSFRDPLILSIVIFPFLIPLFKKLDITIFSK

DVTDFLAKSILKIKEERKKTEKHRVDFLQLMMDSQTSKNSESHSQKDLSDEEILAQSIIF

IFAGYESTSSVLCFLFYQLATNPGIQEKLQKEIDAFLPNKEAVTYDALVQMEYLDMVINE

NLRLYPITGRIERIAKKPVELNGLMIPKGTVVMAPPYVLHRDPEYWPEPEEFRPERFSKE

NKESINPYVYLPFGVGPRNCLGMRFALMSMKVAVSRLLQEFSFRPCKETQIPLKLSYRPL

LAPSVPIVLQAVLRNKKGNQH

>DAA15144.1

MELIPSFSMETWVLLSISLVLLYLYGTYSHGLFKKLGVPGPRPLPYFGNVLSYRKVFGPR

GIMKNAVSVAEDEQWKRIRTLLSPAFTSGKLKEMFPIIGKYGDVLVRNLRKEAEKGKSVN

MKEVFGAYSMDVITSTSFGVNIDSLGNPQDPFVENAKKLLRFDILNPFLLSVVLFPFLVP

IFEVLNITMFPKSAVNFLAKSVKRIKESRLKDNQKPRVDFLQLMINSQNSKETDNHKALS

DQELMAQSVIFIFAGYETTSNTLSFLLYILATHPDVQQKLQEEIDVTFPNKAPPTYDVLA

QMEYLDMVVNETLRMFPITVRLDRLCKKDVKIHGVSIPKGTTVTVPISVLHRDPQLWPEP

EEFRPERFSKKNKDTISPYVYLPFGTGPRNCIGMRFAIMNMKLAVVRVLQNFSFKSCKET

QIPLKINSQGLIRPEKPIFLKVVLRDETISGA

>CAD91345.1

MDLIPNFAMETWVLVATSLVLLYIVFGAYSMDVITSTSFGVNIDSLNNPQDPFVENTKKL

LRFDFLDPFFLSITVFPFLIPILEVLNICVFPREVTNFLRKSVKRMKESRLEDTQKHRVD

FLQLMIDSQNSKETESHKALSDLELVAQSIIFIFAGYETTSSVLSFIMYELATHPDVQQK

LQEEIDAVLPNKAPPTYDTVLQMEYLDMVVNETLRLFPIAMRLERVCKKDVEINGMFIPK

GVVVMIPSYALHRDPKYWTEPEKFLPERFSKKNKDNIDPYIYTPFGSGPRNCIGMRFALM

NMKLALIRVLQNFSFKPCKETQIPLKLSLGGLLQPEKPVVLKVESRDGTVSGA

>BAG60667.1

MDVITSTSFGVNIDSLNNPQDPFVENTKKLLRFDFLDPFFLSITVFPFLIPILEVLNICV

FPREVTNFLRKSVKRMKESRLEDTQKHRVDFLQLMIDSQNSKETESHKALSDLELVAQSI

IFIFAGYETTSSVLSFIMYELATHPDVQQKLQEEIDAVLPNKAPPTYDTVLQMEYLDMVV

NETLRLFPIAMRLERVCKKDVEINGMFIPKGVVVMIPSYALHRDPKYWTEPEKFLPERFS

KKNKDNIDPYIYTPFGSGPRNCIGMRFALMNMKLALIRVLQNFSFKPCKETQIPLKLSLG

GLLQPEKPVVLKVESRDGTVSGA

>AAG33011.1

MDLIPNFAMETWVLVATSLVLLYIYGTHSHKLFKKLGIPGPTPLPFLGTILFYLRGLWNF

DRECNEKYGEMWGLYEGQQPMLVIMDPDMIKTVLVKECYSVFTNQMPLGPMGFLKSALSF

AEDEEWKRIRTLLSPAFTSVKFKEMVPIISQCGDMLVRSLRQEAENSKSINLKDFFGAYT

MDVITGTLFGVNLDSLNNPQDPFLKNMKKLLKLDFLDPFLLLISLFPFLTPVFEALNIGL

FPKDVTHFLKNSIERMKESRLKDKQKHRVDFFQQMIDSQNSKETKSHKALSDLELVAQSI

IIIFAAYDTTSTTLPFIMYELATHPDVQQKLQEEIDAVLPNKAPVTYDALVQMEYLDMVV

NETLRLFPVVSRVTRVCKKDIEINGVFIPKGLAVMVPIYALHHDPKYWTEPEKFCPERSH

>397489512

MDLIPNFAMETWVLVATSLVLLYIYGTHSHKLFKKLGIPGPTPLPFLGTILFYLRGLWNF

DRECNEKYGEMWELYEGQQPMLVIMDPDMIKTVLVKECYSVFTNRMPLGPMGFLKSALSF

AEDEEWKRIRTLLSPAFTSVKFKEMVPIISQCGDMLVRSLRQEAENSKSINLKDFFGAYT

MDVITGTLFGVNLDSLNNPQDPFLKNMKKLLKLDFLDPFLLLISLFPFLTPVFEALNIGL

FPKDVTHFLKNSIERMKESRLKDKQKHRVDFFQQMIDSQNSKETKSHKALSDLELVAQSI

IIIFAAYDTTSTTLPFIMYELATHPDVQQKLQEEIDAVLPNKAPVTYDALVQMEYLDMVV

NETLRLFPVVSRVMRVCKKDIEINGVFIPKGLAVMVPIYALHHDPKYWTEPEKFCPERSH

>114614909

MDLIPNFAMETWVLVATSLVLLYIYGTHSHKLFKKLGIPGPTPLPFLGTILFYLRGLWNF

DRECNEKYGEMWELYEGQQPMLVIMDPDMIKTVLVKECYSVFTNRLPLGPMGFLKSALSF

AEDEEWKRIRTLLSPAFTSVKFKEMVPIISQCGDMLVRSLRQEAENSKSINLKDFFGAYT

MDVITGTLFGVNLDSLNNPQDPFLKNMKKLLKLDFLDPFLLLISLFPFLTPVFEALNIGL

FPKDVTHFLKNSIERMKESRLKDKQKHRVDFFQQMIDSQNSKETKSHKALSDLELVAQSI

IIIFAAYDTTSTTLPFIMYELATHPDVQQKLQEEIDAVLPNKAPVTYDALVQMEYLDMVV

NETLRLFPVVSRVTRVCKKDIEINGVFIPKGLAVMVPIYALHHDPKYWTEPEKFCPERSH

>224070359

MKLLPFFSTETWALILLLVALLLVYGIWPFGVFKKLGIPGPRPLPFLGTSLEYRKGFLEF

DTECFKKYGKIWGIYDGRQPTLAVTDPQIIKSVLVKDCYTTFTNRRRTDLAGVLNNAVSL

AEDEHWKRLRTVLSPTFTSGKLKEMFPIMKHYGEALVENVQKQVKEGSSISVKDIFGSYS

MDVVTSTSFGVNIDSMNNPKDPFVREMKKLVKFDFFNPVFILSFAFPFLIPVMAKMNVNI

FPSDAVDFFMRSLAKIKQDREKEAHKGRVDFLQLMIESQRSASQGNNEANHSYKALTDTE

VLAQAFIFIFAGYEPTSNTLGFLAYELAMHPDVQEKVLQEIDTVLPNKAPLTYEAITKLE

YLDMTVNETLRIYPLGGRIERTCKKDVEINGVNIPKGVVVTIPPYVLHRDPEYWPNPDEF

RPERFSKENKESIDPYTYLPFGAGPRNCIGMRFALLILKVAIVSLLQHFTFQTCKETQTP

IKLSSVGLLTPEKPIILKLVPRTSTEPAKN

>297679968

MDLIPNFAMETWVLVATSLVLLYIYGTHSHKLFKKLGIPGPTPLPFLGTILFYLRGLWKF

DRECNEKYGEMWGLYEGQQPMLVIMDPNMIKTVLVKECYSVFTNRMPLGPMGFLKSALSF

AEDEEWKRIRTLLSPAFTSVKFKEMVPIISQCGDMLVRSLRQEAENSKSINLKDFFGAYT

MDVITGTLFGVNLDSLNNPQDPFLKNMKKLLKLDFLDPFLLLISLFPFLTPVFEALNIGL

FPKDVTRFLKNSIERMKESRLKDKQKHRVDFFQQMIDSQNSKETKSHKALSDLELVAQSI

IITFAAYDTTSTTLPFIMYELATHPDVQQKLQEEIDAVLPNKAPVTYDALVQMEYLDMVV

NETLRLFPVVSRVTTVCKKDIEINGVLIPKGLAVMVPIYALHHDPKYWTEPEKFCPERSH

>CAB62060.1

MNFLPFFSIETWIILLIFVVLLIIYGTWPFGLFKKLGIPGPRPLPFFGTCLEYRKGFLDF

DTECFQKYGKIWGIYDGRQPAVVVMDPQIIKTVLVKECYSTFTNRRRTDLAGVLRNAVSL

AEDDQWKRLRTVLSPTFTSGKLKEMFPTMKYFGEMLVKNIQKRVEKNSSVPVKDFFGSYS

MDVVTSTSFGVNIDSMNNPKSPFVREMQKLTKFDFFDPVFILSFVCPFLTPLMAKMNISF

FPSDAVDFFMRSIDKIKKDRERETHTGRVDFLQMMIESQKSDSNGSSDAKHSYKALSDIE

VLSQAFIFIFAGYEPTSNTLGYLAYQLALHPDVQQKVVNEIDTILPNKAPLTYEAIMQLE

YLDMAVNETLRLYPLGGRLERTCKRDVEINGVTIPKGTIVIIPPYTLHRSPEYWPNPEEF

RPERFSKENKDNIDPYTYLPFGAGPRNCIGMRFALLTLKVAITAVLQHFTFQVCKETQIP

LKLQSLGLTTPEKPIVLKLVPRTNTAKA

>327286988

MELLPSFSIETWSFLVVLLGLLLLYGIWPFGVFGKLGIPGPRPWPFLGTLLQYRKGFVKF

DSWCFQKYGRIWGIYDGRQPVMAILDPAIIKTVLVKECYTTFTNRRNFGATGVLKSAISL

AEDEHWKRVRTVLSPTFTSGKLKEMFPIIKHYGEALMRNVQKKVDMDEPLVVKDVFGAYA

MDVVTSTSFGVNIDSMNNPKDPFVKEIQKLTKFRSFDPRIILLFTFPILRTVFEALNIQI

FSAEAVDFFTRSFAKMKAGRAGETKEGRVDFLQLMIDSQTSNGHHQSNGIDHTYKGLTDD

EILAQAFIFVFAGYEPTSNSLGYMAYMLATHPDVQQKLQDEIDSVLPNKAPLTYDAIMQL

EYLDMVLNETQRLYPLGGRIERVCKKDVEINGVTIPKGVVVMIPPYNLHRDQEYWPEPEE

FRPERFSKENKESVDPYTYLPFGAGPRNCIGMRFALVTMKVAITILLQHFSFRVCKETPI

PLELSSQAFLVPEKPIVLKLVRRNNLQ

>EDL05793.1

MELIPNLSIETWVLLVTSLVLFYIYGTYSHGLFKKLGIPGPKPLPLLGTIFNYYDGMWKF

DEDCYKKYGKIWGFYEGPQPILAIMDPEIIKIVLVKECYSVFTNRRFFGPVGFMKKAITI

SEDEEWKRLRTLLSPTFTSGKLKEMFPIMRQYGDILVRNLRREEEKGEPISMKDIFGAYS

MDVITGTSFGVNVDSLNNPQDPFVQKAKKILKFKVFDPFLLSIILFPFLTPIYEMLNFSI

FPRDSMNFFKKFVKRMKKERLASNQKNRVDFLQLMMNTQNSKGQESQKALSDLEMAAQAV

IFIFGGYDATSTSISLIMYELATHPDVQKKLQDEIDRTLPNKVSGWNLLCLHLSDPNMTF

RVENITILDGIFVCLFVCLRHLLLFVSKTQCAYVLVSWGLYFPSPYFHLFCGSRFSKENK

GNIDPYIYMPFGNGPRNCIGMRFALISIKLAVIGVLQNFTVQPCEETQIPLKISREPIFQ

PEKPIILKVVSRDKPRTGS

>326928939

MNLLPFFSIETWILLLIFVALLIAYGTWPFGLFKKLGIPGPRPLPFFGTCLEYRKGFLEF

DTECFKKYGKIWGIYDGRQPAVVVMDPQIIKTVLVKECYSTFTNRRRVDLAGELRNAVSL

AEDDQWKRLRTVLSPTFTSGKLKEMFPTMKHFGEMLVKNVQKRVEKNSSVPVKDFFGSYS

MDVVTSTSFGVNIDSMNNPKSPFVREMQKLTKFDFFDPLFILAFVCPFLTPLMAKMNVSF

FPSDAVDFFLRSIDKIKKDREKETHKGRVDFLQMMIESQKSDSDGKNSHKALSDIEVLSQ

AFIFIFAGYEPTSNTLCYLAYLLALHPDVQQKVVNEIDTILPNKAPLTYEAIMQLDYLDM

AVNETLRLYPLGGRIERTCKKDVEINGVTIPKETIVVIPPYTLHRNSEYWPNPEEFRPER

FSKENKDNIDPYTYLPFGAGPRNCIGMRFALLTLKVAITSILQHFTFQVCKETQIPLKLL

SMGLMTPEKPIILKLVPRTNNAKA

>ABE28024.1

MNLLPFFSIETWILLLIFVALLIAYGTWPFGLFKKLGIPGPRPLPFFGTCLEYRKGFLEF

DTECFKKYGKIWGIYDGRQPAVVVMDPQIIKTVLVKECYSTFTNRRRVDLAGELRNAVSL

AEDDQWKRLRTVLSPTFTSGKLKEMFPTMKHFGEMLVKNVQKRVEKDSSVPVKDFFGSYS

MDVVTSTSFGVNIDSMNNPKSPFVREMQKLTKFDFFDPLFILAFVCPFLTPLMAKMNVSF

FPSDAVDFFLRSIDKIKKDREKETHKGRVDFLQMMIESQKSDSDGKNSHKALSDIEVLSQ

AFIFIFAGYEPTSNTLCYLAYLLALHPDVQQKVVNEIDTILPNKAPLTYEAIMQLDYLDM

AVNETLRLYPLGGRIERTCKKDVEINGVTIPKETIVVIPPYTLHRNSEYWPNPEEFRPER

FSKENKDNIDPYTYLPFGAGPRNCIGMRFALLTLKVAITSILQHFTFQVCKETQIPLKLL

SMGLMTPEKPIILKLVPRTNNAKA

>AAH60496.1

MNLIPHLSTGTWILLAALFLLILLYGIWPYGLFKKMGIPGPTPLPFIGTFLEFRKGLVQF

DTECFKKYGKTWGLYDGRQPVLAIMDPAIIKTILVKECYTNFTNRRNFGLSGPLESAITA

AEDEQWKRIRSVLSPTFTSGKLKEMFQIMKDYSDILVKNIQDNVGKDEPCTMKDVIGAYS

MDVITSTSFSVNIDSLKNPSHPFVVHIKNLLKGGLLNPLIILVVVFPFLRPIFEGLNINF

FPKNFTQFFTNAVTSFREKRKKGDHTGRVDFLQLMVDSSVTEGNDSGNQHKALTDVEIMA

QSIIFIFAGYETTSTALSYLFYCLATHPDVQQRLHEEIDSFLPDKASPTYDILMQMEYLD

MVIQETLRLYPPAGRLERVSKQNIEINGVSIPKGTVTMIPAYVLQRDPDYWPEPEEFRPE

RFSKENRETHTPFTFLPFGDGPRNCIGMRFALLSMKVAIVSMLQNFSVRPCAETLIPMEY

STIGVLQPKKPIVLKFLSRTSAQE

>Q8AXY5.1

MGYFYLTAETWTLLVAFVTLLLIYAYWPYGTFKRLGISGPKPVPFFGTMLHYRRGFFTFD

EECKKKYGKVWGIYDGRQPVLCVTDPEIIKAVLVKECLSFFTNRRNFHLNGPLYDALSVA

EDDQWKRIRSVLSPSFTSGRLKEMFEIMKNHSANLIRSMKKKADKDEPLDLKEFFGSYSM

DVVTSTAFSVDIDSLNNPSDPFVTNIKKMLKFDFLNPLFLAVAFFPFLGPILEKFELSFF

PKSVTDFFYASLEKIKSNREASQQKSRVDFLQLMIDSQKNSGAQQDKSLTDHEILSQSMI

FIFAGYETSSSSLTFLAYNLATNPEVMKKLQEEIDATFPNKAPVHYQPLMEMEYLDCVIN

ESLRLFPIAARLERVAKAAVEINGIVIPKDMVVMIPTWPLHRDPEIWPEPEAFKPERFSK

KNKDNIDPYIYMPFGSGPRNCIGMRFALVLIKLAVVEILQQYSFSVCKETEVPFEMDIQG

LLAPKRPIQLKLVPRS

>Q9PVE8.2

MGYFYLTAETWTLLVAFVTLLLVYAYWPYGTFKRLGISGPKPVPFFGTMLHYRRGFFTFD

EECKKKYGKVWGIYDGRQPVLCVTDPEIIKAVLVKECLSFFTNRRNFRLNGPLYDAVSIA

EDDQWKRIRSVLSPSFTSGRLKEMFEIMKNHSANLIRSMKKKADKDEPLDLKEFFGSYSM

DVVTSTAFSVDIDSLNNPSDPFVTNIKKMLKFDFLNPLFLAVAFFPFLGPILEKFELSFF

PKSVTDFFYASLEKIKSNREASQQKSRVDFLQLMIDSQKNSGAQQDKSLTDHEILSQSMI

FIFAGYETSSSSLTFLAYNLATNPEVMKKLQEEIDATFPNKAPVHYQPLMEMEYLDCVIN

ESLRLFPIAARLERVAKAAVEINGVVIPKDMVVMIPTWPLHRDPEIWPEPEAFKPERFSK

KNKDNIDPYIYMPFGSGPRNCIGMRFALVLIKLAVVEILQQYSFSVCKETEVPFEMDIQG

LLAPKRPIQLKLVPRS

>301608788

MNLIPHLSTGTWILLAVLLVLILLYGIWPYGLFKKMGIPGPTPLPFIGTFLEFRKGMIQF

DIECFKKYGRMWGMYDGRQPVLAIMDPAIIKTILVKECYTNFTNRRNFGLSGPLESAITS

AEDEQWKRIRSVLSPTFTSGKLKEMFQIMKDYSDILVKNVQVYVDKDEPCATKDVIGAYS

MDVITSTSFSVNIDSLQNPSNPFVIHIKNLLKTGLLSPVIILVVIFPFLRPIFEVLNISF

FPKNFTQFFMNAVTSFREKRKKGDHSGRVDLLQLMVDSGTTEGNDLSNQHKALTDAEIMA

QSVIFIFAGYETTSTALSYLFYNLATHPDVQQKLHEEIDSFLPDKASPTYDILMQMEYLD

MVIQETLRLFPPAGRLERVSKQNVEINGVSIPKGTVAMIPAYVLQRDPEYWPEPEEFRPE

RFSKENRATQTPFTFLPFGDGPRNCIGLRFALLSMKVAIATLLQNFSVRPCAETLIPMEF

STIGVLQPKKPIVLKFLSRAAAHE

>AAH89731.1

MNLIPHLSTGTWILLAALLVLILLYGIWPYGYFKKMGIPGPTPLPFIGTFLEFRKGMVQF

DTECFKKYGKMWGTYDGRQPVLAIMDPAIIKTILVKECYTNFTNRRNFGLNGPFESAITI

AEDEQWKRIRNVLSPTFTSGKLKEMFQIMKDYSDILVKNIQGYVEKDEPCATKDVIGAYS

MDVITSTSFSVNIDSLNKPSDPFVIHMKKLLKTGLLSPLLILVVIFPFLRPILEGLNLNF

VPKDFTEFFMNAVTSFREKRKKGDHSGRVDLLQLMMDSRTTGGNDLSNKHKALTDAEIMA

QSVIFIVAGYETTSTALSYLFYNLATHPDVQQRLHEEIDSFLPDKASPTYDILMQMEYLD

MVIQETLRLFPPAGRLERVSKQNVEINGVSIPKGIVTLIPAYVLQRDPEYWPEPEEFRPE

RFSKENRATHTPFTFLPFGDGPRNCIGLRFALLSMKVAIVTLLQNFSVRPCAETLIPMEF

STIGFLQPKKPIVLKFLSRAAAHE

>AFH08755.1

MGYFLYFSAETWTLLVAFVTLLLVYAYWPYGTFKKLGISGPKPVPFFGTMLNYRRGFFNF

DQECYRKYGKIWGIYDGRQPVLCVADPEIIKAILVKECYSFFTNRRNFHLDGPLYDSLFN

AEDDQWKRIRSVLSPSFTSGRLKEMFEVMKSHSANLIRSMKKKADKGEALELKEFFGSYS

MDVVTSTAFSVDIDSLNNPSDPFVTNIKKMLKFDFLNPLFLAVAFFPFLGPIFEKLEFSF

FPKSVTDFFYASLEKIKSNRETSQQKSRVDFLQLMIDSQKNNGLQKDKRLSDHEILSQSM

IFIFAGYETSSSSLSFLAYNLATNPEVMRKLQEEIDSTFPNKAPVQYQPLMEMDYLDSVI

NESLRLFPIAARLERVAKASVEINGLVIPKDMVILVPTWPLHRDPEIWPEPEKFKPERFS

KKNKEDIDPYTYMPFGLGPRNCIGMRFALVVMKLAVVEILQQYSFSVCRETEIPFEMDVQ

GLLAPKRPIQLKLIPRS

>327289798

MDFFSFFSIQTWSLLGIFLLLLMLYGIWPYNFFKKLGIPGPRPLPFIGTFLEYNKGVLDF

DERCRQKYGKIWGFFDGRQPVMAIMDPVIIKTILVKECYTHFTNRRDFGLNGDFDASVLV

AQDEHWKRIRTVLSPNFTSGRLKEMLPIINHYGEMLVKNVQKKGENGEYIDTKDTFGAYS

MDVITSTSFSIDIDSINNPKDPFVVSMKKIIKTNFVNPLLLLIVIFPFLRPVLEALNISL

APTSATGFLRGALRKIKSDRQKNVHQDRVDFLQLMVDSQISGDVFDGTNSYKALNDREIL

AQAFTFVFGGYETTSSTLGYMAYCLATHPDVQKKLQDEIDEVLPDQATPVYDALLQMEYL

DMVINETLRLYPPGGRIERVCKNTVEINGVTIPKGTVAMIPAFVLHRDPEFWPEPEEFRP

ERFSKENKEFLNPYVFLPFGIGPRNCIGMRFALLSLKVATVVLMQRFSFRTCKETPIPLE

LESKGFMRPKNPIRLKFVPRVMAESNE

>Q98T91.1

MGNFLYFSAETWTLLVAFITLLLVYAYWPYGTFKRLGIPGPKPVPFFGTMLAYRKGFFNF

DDECRKKYGKTWGIYDGRQPVLCITDPATIKAVLVKECYSLFTNRRNFRLNGPLYDAVSI

AEDDQWKRIRSVLSPSFTSGRLKEMFDIMKHHSANLIQSMKKKVDKDEPLEMKEYFGPYS

MDVVTSTAFSVDIDSLNNPSDPFVTNIKKMLKFDFLNPLFLAVAFFPFLGPILEKFEFSL

FPSSVMDFFFAALRKIKANHENNKQKSRIDFLQLMIDSQKNNQNGLQDKGLSDHEILSQA

MIFIFAGYETTSSSLTFLAYNLATNPEVMKKLQKEIDATFPNKAPVQYVPLMEMEYLDCV

VNESLRLYPIAARLERVAKATVEVNGLVIPKDMVVMVPTWPLHRDPELWPEPEKFKPERF

SKENKDTFDPYTYMPFGAGPRNCIGMRFALVMMKLAVVEILQTYDFSVCKETEVPFEMDV

QGFLAPKRPIQLKLTPRSASSS

>50755531

MGLLPDLPPITWLLLAAFLGLLVLYGIWPYQTFKKLGIPGPRPLPFLGTFLRYRQGLLNF

DQMCFEKYGKIWGIYDGRQPVMAILDPVLIKTILVKECYSTFTNRRVFGLSGRLESALSM

AMDDQWKRIRTVLSPTFTSGKLKEMFPIIESYGDKLVKNIEKKVANEEFLDIKSIFGAYS

MDVVASTSFSVDIDSMSKPSDPFVTNIRKFLKFSFLNPLLIFIVLFPFMIPVLEKMNVTL

LPTKVMDFFDAVFMKMKKEREEGYHVDRVDFLQLMIDSQSSQDSSKSAREKDSYKSLSDE

EILAQALIFVFAGYETTSSTLSYISYHLAIHPDVQKRLQDEIDANLPNKAAPTYNVVMQM

EYLDMVVNESLRLHPPGGRIERICKKTVEFNGVTIPKDMVVMIPAYVLHRDPAYWPKPEE

FRPERFSKENGENIDPYTFLPFGAGPRNCIGMRFALLIVKVAMVVLLQNFSFKPCKDTPI

PLVLDTKGFMQPKKPIILKMVPRAHSDPQN

>AAI29607.1

MNLIPHLSTGTWILLAALFLLILLYGIWPYGFFKKMGIPGPTPLPFIGTFLEFRKGMVQF

DTECFKKYGKMWGTYDGRQPVLAVMDPAIIKTILVKEFYTNFTNRRNFGLNGPFEFAITI

AEDEQWKRIRSVLSPTFTSGKLKEMFQIMKDYSDILVKNIQGYVDKDEPCATKDVIGAYS

MDVITSTSFSVNIDSLNKPSDPFVIHMKKLLTTGLMSPVLILVVIFPFLRPILEGLNLNF

VPNDFTQFFMNAVTSFREKRKKGDHSGRVDLLQLMVDSRTTEGNDLSNQHKALTDAEIMA

QAVIFIVAGYETTSTALSYLFYCLATHPDVQQRLHEEIDSFLPNKASPTYDILMQMEYLD

MVIQETLRLYPPAGRLERVSKQNVEINGVSIPKGTVTMIPAYVLQRDPEYWPEPEEFRPE

RFSKENRETHTPFTFLPFGDGPRNCIGMRFALLSMKVAIVTMLQNFSVRPCAETLIPMEF

STIGFLQPKKPIVLKFLSRTSAQE

>334333318

MSVLSNLQVASLRIVLAQGEMSLPNPWEEGVRAGSICCRSVKDTPGEGTVLLQEHASRWR

CQMEMHSRTPQPVKRNSTAAGNTRVSPRDQLQKTGTPRKTYFLKLSGLPPQQQHKCWSLA

HLTASMTSYSGLSTETWTLLVAFVTLLILLGCDISTWKILIDSLLSLESLNSLLLLSVLI

SFSSYYRYGIWPYGIFKKLGIPGPRPLPFFGTFLEYRKGILEFDKQCFQKYGKMWGFYDG

RLPILAILDPDIIKIVLVKEFYTLFTNRRNFGLNGILDSGITVAEGEKWKRIRSIISPTF

TTGKLKEMFPIIKHHIDVLVNNIEKKVAQDESVNMKDIFGAYSLDVITSTSFGVDIDSIN

KPNDPLLVHIKKLLSFSFMSPLLILILVFPSLVPILEKMNLSLLSKENIDFLVNASRRII

ADRKKSNSRDRVDFLQLMIDSQVMNGSRSEKRNNSPKALTEMEIVAQAVTFIFAGYETTS

TTLNFITYNLATHPEIQKKLLEEIDSTLPNKAVPTYDTIFQMEYLDMVVNETLRLFPLGG

RIERICQKTAEINGITIPKGTVMLIPVYVLHHDPEYWPEPEEFRPERFDQEGRKSIDPYV

FLPFGAGPRNCVGMRFALLTLKTALVTLLQNFTMEPCKETPIPLELETKGFMQPKKPIIL

KLVPRPRPDVED

>AAG35209.1

MGNFLYFSAETWTLLVAFITLLLVYAYWPYGTFKRLGIPGPKPVPFFGTMLAYRKGFPIF

DEECHKKYGKTWGIYDGRQPVLCITDPATIKAVLVKECYSLFTNRRNFHLNGPMYDSVFN

AEDDQWKRIRSVLSPSFTSGRLKEMFDIMKHHSANLVNSMKKKADKDEPLNLKDHFGPYS

MDVVTSTAFSVDIDSLNNPSDPFVTNIKKMLKFDFLNPLFLLVAFFPFLGPIMEKMELSF

FPASAFDFFYSALQKIKSDHKNNKQKSRIDFLQLMIDSQKNNQNGLQDKGLSDHEILSQA

MIFIFAGYETTSSSLTFLAYNLATNPEVMKKLQKEIDATFPNKAPIQYAPLLEMEYLDSV

VNESLRLYPIAARLERVAKATVEVNGLVIPKDMVVMVPTWPIQRDPELWPEPEKFKPERF

SKETKETFDPYTYMPFGAGPRNCIGMRFALVSMKLALVEILQQYSFSVCKETEIPFELDI

QGLIAPKRPIFLKLVPRSAC

>344289837

MAFLPSLGAETWVFLVTCGALLLLYGIWPYNFFRKLGIPGPRPLPFIGTYLEYRKGMLEF

DLECSKKYGKIWGLYEGRQPILAILDPDLIKTVLVKEFYTAFTNRRNLALSGNLKLAITE

VEDEMWKRIRAIISPTFSSGKLKEMFPLIKHHGDILMKNIEKKVAQDEVVNVSEIFGAYS

LDVITSTSFGIDTDSINNPDDIILRCVKKAVSVSFLSPLIFLTGLFPFLVPLLERMNVTL

LSRKELDFFVNVTQRLKEQRQASGRSDRVDLLQLMIDSQATVSLESSKSNHFPKALTDVE

ITAQSIIFIFAGFETSSLTLSFIAYNLATHPEVQEKLQEEIDSALPNKEDFTYDALFQME

YLDMVVNETLRLFPLGGRLERVCKKTIEINGVTVPKGTVVVIPTYVLHRDPAYWPEPEKF

CPERFSKDNKKGLDPYVFLPFGIGPRNCIGMRFSLLSLKAALVLLLQNFSLEICKETPIP

LELNTNSFMVPKKPIFLKLTPRTRVVSQE

>ADR00355.1

MGFLTSLSVETWTLLVVCGTLLVLYGIWPYNFFKKLGIPGPRPLPFVGTFLEYRNGILEF

DQKCFKKYGKMWGFYDGRLPILATLDPAFIKIVLVKEFYTLFTNRRNFGLNGSLESAITI

VKGEKWKWIRTIISPTFTSGKLKEMFPIIKHHGDVLMQNIEKRMAKDESLNMKDIFGAYS

LDTITSTSFGVDIDSINKPNDPLLVHIKGLFSFSFLNPLLILILVFPSLVPILEKLNVSL

FSKEVINLLVNATRHIVADRQKSNKRDRMDFLQLMIDSQATNDPGSKKRNNSPKALTEME

IVAQAVTFLFAGYETTSTTLTFISYNLATHPEIQKKLHEEIDSNLPNKASPTYDTIFQME

YLDMVVNETLRLFPLAGRLERICEKTAEINGIIIPKGTVVMVPVHILHHDPEYWPEPEEF

CPERFDREGRKSINPYVFLPFGAGPRNCIGMRFAMLTMKTALIMLLQNFTLETCKETPIP

LELDTKGFMKPKKPIILKLVPRIRFDAEEKKPRN

>ABB90404.1

MGYFFYFSAETWTLLVTLITLFFVYVYWPYGTFKKMGIPGPKPIPFFGTMLAYRKGFMNF

DSECFQKYGKTWGIYDGRQPVLCITDVSMIKTVLIKECYSLFTNRRNFRLNGPLYDAVSI

AEDDQWRRIRSVLSPSFTSGRLKEMFGIMKQHSANLISSMKKKADKDEPLELKEFFGPYS

MDVVTSTAFSVDLDSLNNPSDPFVTNIKKMLKFDLFSPLFLIVAFFPFMGPVFEKMEFSF

FPTYVTDFFYAALQKIKSNRETSKQKSRVDFLQLMIDSQKNNDLSGVEQDKGLNDHEILS

QAMIFIFAGYETSSSSLTFLAYNLATNPAVMKRLQQEIDSTFPNKAPIEYQALMQMEYLD

SVINESLRLYPIAPRLERVAKATVELNGIVIPKDMVVMIPTWPLHRDPDLWPEPEEFKPE

RFSKENKETIDPYTYMPFGAGPRNCIGMRFALVMMKLAIVEVLQRYSFSVCKETEVPFEM

DIQGLLMPKRPIKLKLEPRSEPSS

>ACA35026.1

MSYDLFFSAETWALLSLLVALLVLYGHWPHRQFKKLGIAGPKPIPFFGTMLRYREGFHNF

DLECFKKYGRIWGIYDARQPVLCITDQSIIKTILIKECYSLFTNRRNFRLNGPLYDAVSI

VEDDDWRRIRSVLSPSFTSGRLKEMFGIMKAHSQILVQNLGKSATRGENVDIKEVFGAYS

MDVVTSTAFSVDIDSLNNPKDPFVTNIKKMLKFDFLSPVFLISVLFPFTIPILEKMELGF

FPISVTDFFYAALQKIKSERVANDHKKKRVDFLQLMVDSQTAGKTQQGEEHTEKGLSDHE

ILSQAMIFIFAGYETSSSTLSFFFYNLATNPEAMKKLQEEVDETFPNKAPVDYEAVMNMD

YLDAALNESLRLFPVAARLERVCKKTVEINGVIIPKDMVVMIPTFALHRDPDYWSDPESF

KPERFTKGNKEAIDPYMYMPFGLGPRNCIGMRFAQVTMKLAIVEILQRFDVSVCAETQVP

LKLGKSGLLAPKDPINLKFEHRTAADVCNNES

>ABH05129.1

MGYFFFFSAETWTLLVALITLIFVYVCWPFGTFKKMGVPGPRPIPFFGTMLAYKKGFMHF

DSECFKKYGKTWGIFDGRQPVLCITDPAMIKTVLIKECYSLFTNRRNFRLNGELYDAVSI

AEDDQWRRIRSVLSPSFTSGRLKEMFDIMKHHSANLISSMKKTADKDEPLELKEFFGPYG

MDVVTSTAFSVDIDSLNNPTDPFVANIKKMLKFDLFNPIFLFIAFFPFMAPILEKLEFSF

FPKSVLDFFYAALKKIKANRETTKLKNRVDFLQLMINSQKNNDLSKVENDKGLNDHEILS

QAMIFLFAGYETTSSSLTFLAYNLARNPHVMKRLQEEVDATFPNKAPVEYQALMQMEYLD

SVVNESLRLYPIAPRLERVAKATVEINGLVIPKGMVVMVPTWPLHRDPDLWPEPEEFKPE

RFSKENKETIDPYTYMPFGAGPRNCIGMRFALVLMKLAVVEILQTYSFSVCKETEIPIEL

DIQGLISPKRPIKLKLVPRSEPSN

>ADO19749.1

MSYDLFFSAETWALLVLFGALLVIYGSWTHSIFKKLGIAGPKPIPFFGTMLRYREGFHNF

DLECFKKYGRVWGIYDARQPVLCIMDQSIIKTILIKECYSLFTNRRNFRLNGPLYDAVSI

VEDDDWRRIRSVLSPSFTSGRLKEMFGIMKAHSKILVENLGKSATRGENVDIKEFFGAYS

MDVVTSTAFSVDIDSLNNPKDPFVTNIKKMLKSDFLNPLFLISALFPFVIPVLEKMDFAL

FPTSVTDFFYAALQKIKSERVANDHKKKRVDFLQLMVDSQTAGKTQHDEEHTEKGLSDHE

ILSQSMIFIFAGYETSSSTLMFFFYNLATNPETMKKLQEEIDETFPDKAPVDYEAVMNMD

YLDAALNESLRLYPVAARLERVCKKTVDINGLIIPKDMVVMVPTFVLHRDPDYWSDPESF

KPERFTKGNKELIDPYMYMPFGLGPRNCIGMRFAQVTMKLAIVEILQRFDVSVCEETQVP

LELGLNGLLAPKDPIKLRLKPRTASDICNNNKS

>ADF87312.1

MSYGLFFSAETWALLILFVTLLFIYGSWPHGVFKKLGIPGPKPLPFFGTMLEYRKGFHNF

DMECFKKNGRVWGIYDARQPVLCIMDLSIIKTILVKECYSLFTNRRNFRLNGPLYDAVSI

VEDDDWRRIRSVLSPSFTSGRLKEMFGIMKTHSHTLVDNLGKTATRGEAVEIKEFFGAYS

MDVVTSTAFSVDIDSLNNPKDPFVTNIKKMLKFDFLNPVFLISAVFPFITPVLEKMGFAF

FPTSVTDFFYAALKKIKSERVSSEQKKKRVDFLQLMVDSQTAGGSEEHTEKGLSDHEILS

QSMIFIFGGYETSSSTLSFFFYNLATHPEAMEKLQGEIDQSFSREDPVDYEGIMNMEYLD

AALNESLRLFPIVARLERVCKKTVDINGLLVPKDVVVMIPTFALHRDPDYWSEPDSFRPQ

RFSKDNRESIDPYMFMPFGLGPRNCIGMRFAQVSIKLAIVEILQRFDVSVCEQTQVPLEL

DTSGLLAPKSPIKLQFKPRKPSLSEDICNNNNTS

>ACA35027.1

MSYDLFFSAETWALLSLLVALLVLYGHWPHRQFKKLGIAGPNPIPFFGTMLRYREGFHNF

DLECFKKYGRIWGIYDARQPVLCITDQSIIKTILIKECYSLFTNRRNFRLNGPLYDAVSI

VEDDDWRRIRSVLSPSFTSGRLKEMFGIMKAHSQILVQNLGKSATRGENVDIKEVFGAYS

MDVVTSTAFSVDIDSLNNPKDPFVTNIKKMLKFDFLNPVFLISVLFPFTIPILEKMELGF

FPISVTDFFYAALQKIKSERVANDHKKKRVDFLQLMVDSQTAGKTQQGEEHTEKGLSDHE

ILSQAMIFIFAGYETSSSTLTFFFYNLATNPEAMKKLQEEVDETFPNKAPVDYEAVMNMD

YLDAALNESLRLFPVAARLERVCKKTVEINGVIIPKDMVVMIPTFALHRDPDYWSDPESF

KPERFTKGNKEAIDPYMYMPFGLGPRNCIGMRFAQVTMKLAIVEILQRFDVSVCAETQVP

LKLGKSGLLAPKDPINLKFEHRTAADVCNNES

>345305286

MSSIFSFSIETWILLGTFLTLMILYGIWPYGVFTKRNIPGPWPVPFLGNIQEYSQGLMVF

DKKCSEKYGKIWGLYEGRQAIIAVLDLDIIKKVLVKEVFSSFTNRRNTGIEGILELAVSS

AEDEHWKELRTLMSRTFTSGKLKEMLPIMTHYGEVLVRNIWRKAEKNENVTVKDYFGAYS

LDVITSTSFGVDVDSLNKMNDTFVKEIKKISSIDFFSPLFALISTFPFMIPLLKLLNINL

FPNDSVEYFKRFLHKVKERRHQDNQQHRVDFLQLMLDSQNPKEGSKGKGGSSSWKGLSDA

ELLAQAFAFIFAGYETSSNTLSFISYNLATHPDVQEKLQAEIDTVLPNKSPLTYDALVRM

EYLDMVINESLRLYPISSRLERMCKKTTEINGLTIPKGTCVVIPLFILQKDPNYWLEPDE

FHPERFSKEMRDKRDPYTFMPFGAGPRNCIGMRFALLSIKVAMIGLLKNFSLRTCQETQI

PPEINSFGLLTAKKPIVLRYIPRADTKIKD

>AAI63925.1

MFFSAETWTLLAFLVALLVIYGSWPHRYFKKLGIPGPKAIPFFGTMLRYKDGFHVFDMDC

FEKYGRIWGIYDGRQPVLCIMDQTIIKTVLIKECYSLFTNRRNFRLNGALYDAVSIAEDD

DWRRIRSVLSPSFTSGRLKEMFGIMKKHSHILVDSMGKTAKRGESADIKEFFGAYSMDVV

TSTAFSVDIDSLNNPKDPFVTNIKKMLKFDLLNPLFLLIAFFPFMAPVLEKMDFALFPTS

VTDFFYAALQKIKSDRDTKTVAKDNTKKRVDFLQLMVDSQTGVKHKSDGEHTEKGLSDHE

ILSQSMIFIFAGYETSSSTLSFLFYNLATNPETMKKLQEEIDETFPNQAPVDYETLMSMD

YLDAALSESLRLFPVAARLERVCKKTVEINGLLIPKDLVVMVPTYALHRDPDYWSEPESF

KPERFTKGNKESIDPYMYMPFGLGPRNCIGMRFAQVTMKLAIVEILQRFDVSVCDETQVP

LELGFNGLLSPKDPIKLKLQPRKLSQSPDVCNNNQKS

>AAI09441.1

MFFSAETWTLLAFLAALLVIYGSWPHRYFKKLGIPGPKAIPFFGTMLRYKDGFHVFDMDC

FEKYGRIWGIYDGRQPVLCIMDQTIIKTVLIKECYSLFTNRRNFRLNGALYDAVSIAEDD

DWRRIRSVLSPSFTSGRLKEMFGIMKKHSHILVDSMGKTAKRGESADIKEFFGAYSMDVV

TSTAFSVDIDSLNNPKDPFVTNIKKMLKFDLLNPLFLLIAFFPFMAPVLEKMDFALFPTS

VTDFFYAALQKIKSDRDTKTVAKDNTKKRVDFLQLMVDSQTGVKHKSDGEHTEKGLSDHE

ILSQSMIFIFAGYETSSSTLSFLFYNLATNPETMKKLQEEIDETFPNQAPVDYETLMSMD

YLDAALSESLRLFPVAARLERVCKKTVEINGLLIPKDLVVMVPTYALHRDPDYWSEPESF

KPERFTKGNKESIDPYMYMPFGLGPRNCIGMRFAQVTMKLAIVEILQRFDVSVCDETQVP

LELGFNGLLSPKDPIKLKLQPRKLSQSPDVCNNNQKS

>ABE28023.1

MGFLPDLSPITWLLLVAFLSLLVLYGIWPYQTFKKLGIPGPQPVPFLGTFLGYQQGILNF

DQMCFEKYGKIWGIFDGRQPIMAILDPALIKTILVKECYSTFTNRRVFGLSGRLESAVSM

AVDDQWKRIRTVLSPTFTSGKLKEMFPIIESYGEKLVKNIEKKVANEELLDMKNIFGAYS

MDVVASTSFSVDVDSMSKPSDPFVTNIRKFLKFSFLNPLLMFIVLFPFMIPVLEKMNVTL

LSNEVMDFFDVFFMKMKKEREKGYHMNRVDFLQLMIDSQSSQDGSKTAEEKDSHKSLSDE

EILAQALIFVFAGYETTSSTLSYISYHLAIHPDVQKQLQDEIDANLPNKAAPTYNAVMQM

EYLDMVVNESLRLYPAGGRIDRVCKKTVEFNGVTIPKDMVVMIPIYVLHHDPEYWPKPEE

FRPERFSKENRENIDPYTYLPFGAGPRNCIGMRFALLVVKVAVVALLQNFSFRPCKDTPI

PLVLDTKGFMQPKKPIVLKMIPRAHGDLQN

>AAS09920.1

MFFSAETWTLLAFLVALLVIYGSWPHRYFKKLGIPGPKTIPFFGTMLRYKDGFHVFDMDC

FEKYGRIWGIYDGRQPVLCIMDQTIIKTVLIKECYSLFTNRRNFRLNGALYDAVSIAEDD

DWRRIRSVLSPSFTSGRLKEMFGIMKKHSHILVDSMGKTAKRGESADIKEFFGAYSMDVV

TSTAFSVDIDSLNNPKDPFVTNIKKMLKFDLLNPLFLLIAFFPFMAPVLEKMDFALFPTS

VTDFFYAALQKIKSDRDTKTVAKDNTNEERVDFLQLMVDSQTGVKHKSDGEHTEKGLSDH

EILSQSMIFIFAGYETSSSTLSFLFYNLATNPETMKKLQEEIDETFPNQAPVDYETLMSM

DYLDAALSESLRLYPVAARLERVCKKTVEINGLLIPKDLVVMVPTYVLHRDPDYWSEPES

FKPERFTKGNKESIDPYMYMPFGLGPRNCIGMRFAQVTMKLAIVEILQRFDVSVCDETQV

PLELGFNGLLSPKDPIKLKLQPRKLSQSPDVCNNNQKS

>AEV42266.1

MGYFPDFSIETWTLLAFVIALIAVYGYAPYGFFKKIGIPGPTPLPFIGTFLEYRRGIHNF

DTECFQKYGRLWGVYDGRQPLLGVMDTDMIKTILVKECYSVFTNRRDLGLNGPLRDAVSI

TEDEQWKRIRSVLSPSFTSGRLKEMYTIMMKHSSNLIQSLHKKVESDEVIEVKEVFGPYS

MDVVTSTAFSVDIDSINHPSDPFVTNIKRMVKFNFLNPLLVLVVLFPFLKPLFDKTDVSL

FPADVMDFFYKFLQMIKSDRNKNDHKNRVDFMQLMVDSQVSENNKVDNSSQKGLSDHEIL

SQAMFFIFAGYETSSSSLCFLAYNLATHPDTQKTLQREIDETFPDKGRPTYEALMQMEYL

DMVVNESQRLYPIANRLERMTKASVEVNGVIIPKGTVVMIPVYTLHRDPALWPEPEAFKP

ERFSKENKDNMDPYAYLPFGAGPRNCIGMRFALLSMKLALVEILQNFSFVTCKETQIPLE

LANDGLTSPKDPIILNLEPRGSAANSPSI

>O42563.1

MMSFLPYFSAETWTLLALLITLIVVYGYWPYGVFTKMGIPGPKPLPYFGTMLEYKKGFTN

FDTECFQKYGRIWGIYDGRQPVLCIMDKSMIKTVLIKECYNIFTNRRNFHLNGELFDALS

VAEDDTWRRIRSVLSPSFTSGRLKEMFGIMKQHSSTLLSGMKKQADKDQTIEVKEFFGPY

SMDVVTSTAFSVDIDSLNNPSDPFVSNVKKMLKFDLFNPLFLLVALFPFTGPILEKMKFS

FFPTAVTDFFYASLAKIKSGRDTGNSTNRVDFLQLMIDSQKGSDTKTGEEQTKGLTDHEI

LSQAMIFIFAGYETSSSTMSFLAYNLATNHHVMTKLQEEIDTVFPNKAPIQYEALMQMDY

LDCVLNESLRLYPIAPRLERVAKKTVEINGIVIPKDCIVLVPTWTLHRDPEIWSDPEEFK

PERFSKENKESIDPYTYMPFGAGPRNCIGMRFALIMIKLAMVEILQSFTFSVCDETEIPL

EMDNQGLLMPKRPIKLRLEARRNTPSNTTATTLKSPTT

>ACI33861.1

MMSFLPYFSAETWTLLALLITLIVVYGYWPYGVFTKMGVPGPKPLPYFGTMMEYKKGFTN

FDTECFQKYGRIWGIYDGRQPVLCTMDKSMIKTILIKECYNIFTNRRNFHLNGELFDAVS

VAEDDTWRRIRSVLSPSFTSGRLKEMFGIMKQHSANLLSGMKKQADKDQAIELKEFFGPY

SMDVVTSTAFSVDIDSLNNPSDPFVSNVKKMLKFDLFNPLFLLVALFPFTGPILEKMKFS

FFPTAVTDFFYASLAKIKSGRDTGNSTSRVDFLQLMIDSQKGNDTKTGEEQTKGLTDHEI

LSQAMIFIFAGYETSSSTMSFLAYNLATNPHVMAKLQEEIDTVFPNKAPIQYEALMQMDY

LDCVLNESLRLYPIAPRLERVAKKTVEINGIVIPKDCIVLVPTWTLHRDPEIWSDPEEFK

PERFSKENKESIDPYTYMPFGAGPRNCIGMRFALIMIKLAMVEILQSFTFSVCDETEIPL

EMDNQGLLMPKRPIKLRLEPRSNTPSNTTVTSL

>149409184

MSSILNFSIETWILLGTFLALMILYGIWPFRVFTKSGIPGPRPVPFFGNILEYSQGIIEF

DKKCFQKYGKIWRLFEGRQAMVAVLDLEIIKTVLVKEVFSSFTNRRRIGIEGIMEESIIG

AEDEQWKELRILISPTFSSGKLKEMLRIMIHYGEILVKTIWKKAEKNQNVIVKDYYGAYS

LDVITSTSFGVDVNSLNKMNDTYFKEVKKLSSVDFFSPLLALTSIFPFITPLLNLLDISL

FPSDSVEYIKRFVHKIRESRHQDNQQHRADLLKLLLDSQNPKEGLKGKKGLSDTGILAQA

VAFVFAGYETTSSALSFISYNLATHPDVQQKLQEEIDKVLPNKSPLTYDAIVQMEYLDMV

INETLRLYPIGPRLERMCKKTLEINGLTIHKGTCVVIPIAVLQHDPNYWTEPDEFRPERF

SKEMREKRDPYTFIPFGVGPRNCIGMRFALLNMKVAIIGLLQNFSLRTCQETQIPLELNS

MGFLTPKKPIVLQFIPRADTKSQD

>AAH84827.1

MTFLPDFSMETWTLLVLLLTLLAYYAIWPYRLFKRHGIPGPTPIPFIGTFLGNRNGIMEF

DMECFKKYGNVWGFYDGPKPLLAIVDPVIIKNIMIKECYTNFTNRRDFGLSGPLKSSVLI

SKDEQWKRIRTVLSPTFTSGKLKQMFPVMKHYGELLVKNIQKKIDNKEPLNMKYIFGSYS

MDTILSTSFSVNVDSMNNPNDPFVTNARNLFTFSFFNPLFLLTILCPFLVPLLDKMNFCF

LSLKILNFFKDAVASIKKKRQKDIHEDRVDFLQLMVDAQNNEGDSVPEGEKQRYKELSDD

EILAQSLIFIMAGYETTSTTLMFLAYNIAMHPDVQSRLEEEIDTLLPNKAPPTYEALMKM

EYMDMVINETMRLFPSAIRIDRVCKKTMEINGVTIPAGVVIVVPLFVLHLNPEVWPEPEK

FQPERFSKENQKNQDPYSFLPFGTGPRNCIGMRFALVNMKLALTLLLQNFRFEKCEDTPD

PLNICTKGYLKPTKPIILKLVPKTAQTMKE

>AAK58569.1

MMSFLPYFSAETWTLLALLITLIVVYGYWPYGVFTKMGIPGPKPLPYFGTMLEYKKGITN

FDTECFQKYGKIWGIYDGRQPVLCIMDKSIIKTVLIKECYNIFTNRRDFHLNGEMFDALS

VVEDDAWRRIRSVLSPSFTSGRLKEMFGIMKQHSANLLNGMKKQADKDQTIEVKEFFGPY

SMDVVTSTAFSVDIDSLNNPSDPFVSNVKKMVKFDLLNPLFLLVALFPFTGPILEKMKFS

FFPTAVLDFFYASLIKIKSGRDTGNSTNRVDFLQLMIDSQKGSDTKTGEEQTKGLTDHEI

LSQAMIFIFAGYETSSSTMSFLAYNLATNHHVMTKLQEEIDTVFPNKAPIQYEALMQMDY

LDCVLNGSLRLYPVAPRLERVAKKTVEINGIVIPKDCVVLVPTWALHRDPEIWSDPEEFK

PERFSKENKEPIDPYTYMPFGAGPRNCIGMRFAMIMMKLAMVEILQSFTFSVCDETEIPL

EIDNQGLLMPKRPIKLRLEPRNNTLSNTTATSLKSPTT

>CAF91666.1

MDLLPDFSIETWTLIVLVFTLITVYGYAPYGFFKNLGIHGPKPWPFIGTFLNYRKGVHHF

DQECYKKYGKVWSLFDGRQPLMCIMDTLMIKTILIKECYSNFTNRRDLGLNGPLRDAVSI

TEDEQWKRIRSILCPSFTSGRLKEMYTIMLQHPQNLINLLQKKVKADDVIDVKDVFGPYS

MDVVTSTAFSVDIDSINHPSDPFVANIKKMVKFNFMNPLLILVVLFPFTQTIFDKMDVSL

FPGHVLDFFYNFLRQIKSDRSKEKKKSRVDFMQLMVNAHMTEDNEKDSSLKGLTDKEILA

QAMIFIFAGYETTSSSLGFLAYSLATNPDIQKKLQEEIDKTFPGKVRPNYDDLMQLEYLD

MVVNESMRLYPIANRLERMAKTSVEINGVTIPKGTVVAIPVYALHRDPALWPEPEAFKPE

RFSKENSANIDPYTYLPFGAGPRNCIGMRFALVVMKLAIAEILQHYSFVPCKETEIPMVL

GSEGFVTPKTPIKLKLKPRTSS

>301616914

MTFLPDFSMATWTLLVLLLTLLAYYAIWPYRLFKRYGIPGPTPIPFIGTFLGNRKGLMEF

DMECFKKYGKVWGIYEGQKPLLAIVDPVIIKSIMIKECYSNFTNRRDLGLTGPLKSSVLV

SKDEQWKRIRTVLSPTFTSGKLKQMFPLMKHYGELLVKNIHKKIDNKEPLDMKHIFGSYS

MDIVLSTSFSVNVDSMNNPNDPFVTNARNLFKFSFFNPLFLIAILCPFLVPLLDKMNFCF

LSLKILNFFKDAVASIKKKRQKGTHEDRVDLLQLMVDAQSNEGESVPEEEKHGYKELSDT

EILAQSLIFIMAGYETTSTTLMFLAYNIATHPDVQRKLEEEIDALLPNKAPPTYDALMKM

EYMDMVINETLRMFPPAIRIDRVCKKTMEINGVTIPAGVVIVVPLFVLHLNPEIWPEPEE

FQPERFSKENQKNQDPYNFLPFGVGPRNCIGMRFALVNMKLVLTILLQNFRFETCKDTPV

SSNQLCTDGYLKPTKPIILNLIPKAGQTVEE

>ABG25922.1

MDLIPNLAVETWLLLAVSLVLLYLYGTRTHGLFKRLGIPGPTPLPLLGNVLSYRQGLWKF

DTECYKKYGKMWGTYEGQLPVLAITDPDVIRTVLVKECYSVFTNRRSLGPVGFMKSAISL

AEDEEWKRIRSLLSPTFTSGKLKEMFPIIAQYGDVLVRNLRREAEKGKPVTLKDIFGAYS

MDVITGTSFGVNIDSLNNPQDPFVESTKKFLKFGFLDPLFLSIILFPFLTPVFEALNVSL

FPKDTINFLSKSVNRMKKSRLNDKQKHRLDFLQLMIDSQNSKETESHKALSDLELAAQSI

IFIFAGYETTSSVLSFTLYELATHPDVQQKLQKEIDAVLPNKAPPYL

>345305284

MQLPVGFKVILLVIFHHRVEAALFTFCYCILVMPSAHIVSTFLALLILYGIWPYGVFTKL

RIPGPRPIPFLGNILDYSQGMDEFDKKCFQKYGKIWGLYEGRKAKIAVLDLDIIKSVLVK

EVFTNFTNRQMAGIEGIMSESIFSAKDERWKELRTLVSPTFTSGKLKEMVPMMIQYGEVL

VKNLQNKVEKNQNVTIKDYFGAYNLDVITSTSFGVDIDNLNKMNNTFVKEIKKLTSINFF

SPLFALISVLPFMIPLLKLLNINTFPNDSVEYFKRFMDKIKERTQQGNPQQRMNFLQLML

DTQNPKEGLKNKGGIISQKGLSDAEILAQAFSFIFAGYETSSTTLSFISYNLATHLDVQK

KLQEEIDEVLPNKAPLTYDALVQMEYLDMIINETLRLYTVGSRLERICKKTMKINGLTIP

KGMTVVIPVSCLHSDPNYWVNPDEFHPERFSKEMRDKRDPYTFMPFGAGPRNCIGMRFAL

LNIKVAIIGLLQNFSLRTCQETQIPLEVNSFGLLTTKKPIVLRFIPRAEIKRFLRFKSHH

PRPNLDQCRIPKPRLYEGWQAIIAILNLDIIKMILLKEMFSNFTNWLVCLELHKWQVYTR

ICWKTPHYFGVYSLDVITSTSLGMDVDWNKMNDTFVKEIKKISSVNLFSPLFAWKMILWN

TLKDLCLKYRRHRDDQQHRVDLLQLLLDSQDPKEDSKGKGGSSSRKDWEHPVDQRLNLIL

A

>392352295

MGDLGPLGIMSKAITFSKVEEWKRYRAFLSPTFTSGKLKEMFPIVEQHGDILVKYLRRED

EKGKVFGAYSMDEITSTSFGVSVDFLTNPKDTFVEKTKKLLRFDFFDPLFLVFGAYSMDE

ITSTSFGVSVDFLTNPKDTFVEKTKKLLRFDFFDPLFLSVALFPFLTPIYEMLNICMFPK

DSIAFLQKFVYRMKETRLDSKHKHRVDFLQLMLNAHNNSKDEVSHKALSDVEIIAQSVTF

IFAGYEITSSTLSFVLYSLATYPDIQKKLQEEIDGALPNKAPPTYDIVMEMEYLDMVLNE

TLRLYPVGNRLERVCKKDIELGGVFIPKGSVVMIPTYPLQRDPQHWTEPEEFHPERFSKE

NKGSIDPYVFLPFGHGPRNCIGMRFALMNMKLALTKVLQNFSFQPCKETQIPLKLSIQAI

LEPEKAIVLKVVPWDAVITGA

>327289800

MLGFFDGREPVMAILDPVIIKTILVKECYTHFTNRRDFGLNGDFDSSILTVQDEQWKRIR

TVLSPNFTSGRLKEMFPIINHYGEMLVKNIQKKVENGEYIDTKDIFGAYSMDVITSTSFS

IDIDSINNPNDPFVTNIKKIVKFNFVNPLLVLIVIFPFLTPVLEALNFSLTPTSVTNFIK

ESIKKLKKDRQKNLHKDRVDFLQLMVDSQISGEILDDSKSYKALNDKEILTQAIIFVFAG

YETTSSTLSYMAYCLATHPDVQQKLQEEIDEALPNQATPVYEVLLQMEYLDMVINETLRL

YPPGGRIERVCKTTVEINGVTIPKGTVTMIPAFVLHRDPEFWPEPEEFRPERFSKENKET

LDPYTFLPFGAGPRNCIGMRFALLSLKVAMIVLLQRFSFRTCEETPIPLELQSQGFMTPK

KPIRLKFVPRVMAESKE

>392332536

MGDLGPLGIMSKAITFSKVEEWKRYRAFLSPTFTSGKLKEMFPIVEQHGDILVKYLRRED

EKGKPAPVKQVFGAYSMDEITSTSFGVSVDFLTNPKDTFVEKTKKLLRFDFFDPLFLSVA

LFPFLTPIYEMLNICMFPKDSIAFLQKFVYRMKETRLDSKHKHRVDFLQLMLNAHNNSKD

EVSHKALSDVEIIAQSVTFIFAGYEITSSTLSFVLYSLATYPDIQKKLQEEIDGALPNKA

PPTYDIVMEMEYLDMVLNETLRLYPVGNRLERVCKKDIELGGVFIPKGSVVMIPTYPLQR

DPQHWTEPEEFHPERFSKENKGSIDPYVFLPFGHGPRNCIGMRFALMNMKLALTKVLQNF

SFQPCKETQVREKLFLQIVLTKEPFVVRMYRLELSITKSQ

>AFJ49833.1

MGDPVLGWALGTWALLLLLLALGLLYGTWPFNLFKKLGIPGQRPLPFIGTFHEYRHGVLK

FDQKCFEKYGKIWGLFDGRQPVMAILDPALIKTILVKEFYTYFTNRRDFGLNGNLDTALS

IVVDEQWKRIRSALSPTFTSGRLKEMMPIINHYNEILEKNLQKKVESDEAIDMKLIFSAY

SLDVVTSTSFGVDIDSLNHPNDPIVVHMKKFLRFTLLNPVLMLAVLFPFLIPVLNKLNLT

IFPKSSLDYFHGVVKKIKEDRQKENHTNRVDFLRLMMDSQAAANTSGEANSEKALTDTEI

LAQSVIFIFGGYETTGTALSFMSYCLAMYPDVQEKLSQEINETFPNQAPPTYEGIQQMEY

LDMVVNETFRLYPPGVRIDRVCKKTVEIHGVTIPEGTVVMIPVYVLHRIPEYWPEPEEFR

PERFTKENKESRDPYVFLPFGAGPRNCIAMRFALLVLKMGLVVALQRFRFQTCKETPIPL

ELDDRGFLKPKKPIKLKLVPRTAVEHEE

>344289835

MVWSWVGEEKKEGVGAGGGGKEKQEGVGSWEGEEKQKGVGLDGYGIWPYNFFRKLGIPGP

RPLPFVGTFLEYRKGMLEFDLECSKKYGKIWGIYHGRQPVLAITDPVLIKTVLVKEFYTV

FTNRRNLGLNGDLISSISIADDEKWKWIRALLSPAFSSGKLKKMFPLIKHHADMLVQNLE

KKARGEAVNMTEIFGAYSLDVIASTSFGVDIDSINNPDDILHHHVKKLISFPFNNPLIFL

IELFPFLVPLLERMDVSLLSWKERDFFVNVTQRLKEQRQASGCRDCVDLLQLMIDSQATG

SPESSEANQSPTALTDMEIAAQVITFIFAGYETSSLTLSFISYNLATHPEVQERLQEEID

SALPNKADPTYEVLFQMEYLDMVVNETLRLFPLGGRLERVCKKTVEINGVTIPKGTMVVI

PTYVLNHNSEYWPEPEEFCPERFSKENKKRLDPYVFLPFGIGPRNCIGMRFALLALKAAL

VLLLQNFSLETCKDTPIPLELDTNSFMVPKKPIFLKLTPRMKAVSQE

>301616916

MTFLPDFSKETWTLLVLLLTLLAYYAIWPYRLFKRYGIPGPTPIPFIGTFLGNRHVSIFF

YDGPQPVLAILDPVIIKSIMVKECYTNFTNRRDFGLSGPLKSSVLMSKDEQWKRIRTVLS

PTFTSGKLKQMFPLMKHYGELLVKNIHKKIDNKEPLDMKSIFGSYSMDTILSTSFSVNVD

SMNNPNDPFVTNARNLFTFSFFKPLFLITILCPFLVPLLDKMNFCFLSSKFLKFFKDAVA

SIKKKRQKGAHMDRVDFLQLMVDAQSNEGESVPEEEKHRHKELSDTEILAQSLIFIMAGY

ETTSTTLMFLTYNIARYPDVQRKLEEEINTLLPNKAPPTYDALMKMEYMDMVINETLRLL

PPAIRIDRVCKKTMEINGVTIPAGVVIVVPLFALHLNPEIWPEPEEFQPERFSKENQKNQ

DPYNFLPFGVGPRNCIGMRFALVNIKLALTILLQNFKLETCKDTPVPLKVCTRGNLKPTK

PIILNLVPKGAQTTEEQI

>EDL05795.1

MELIPNLSIETWVLLVTSLVLFYIYGTYSHGLFKKLGIPGPKPLPLLGTIFNYYDGMWKF

DEDCYKKYGKIWGFYEGPQPILAIMDPEIIKIVLVKECYSVFTNRRFFGPVGFMKKAITI

SEDEEWKRLRTLLSPTFTSGKLKEMFPIMRQYGDILVRNLRREEEKGEPISMKDIFGAYS

MDVITGTSFGVNVDSLNNPQDPFVQKAKKILKFKVFDPFLLSIILFPFLTPIYEMLNFSI

FPRDSMNFFKKFVKRMKKERLASNQKNRVDFLQLMMNTQNSKGQESQKALSDLEMAAQAV

IFIFGGYDATSTSISLIMYELATHPDVQKKLQDEIDRTLPNKVSG

>327289804

IKRTFLLYRYGIWPYGVFKKLGVPGPTPLPFIGTALAYRNGILQFDKACFKKYGKVWGFY

DGRTPVLAVTDPAVIKAVLVKECYTVFTNRRKFGPTGKLEKAVSLAEDDTWKRIRTVLSP

TFTSGKLKEMLPIIQHYAEILIKNFSEKADKGETVDIKDVLGPYSMDVVASSSFGVNINS

MNNPQDPFVKQAKKLVKFNFFSPLFVLMYMVPWLTPVLNKMNITLFSAEAVDFFAKAIGK

IKEQRKQEMDKGRVDFLALMIESQKAEAKTQINGMNQTYKALTDEEILANAVIFIFAGYE

ATSNILCYMLYELAIHPDVQQKLQDEIDAALPNKAPLVYETVMQMEYLDMTVSELLRMYP

LGGRIDRTCKKDVEINGITIPKGVSVMIPAHVLHFDPDLWEQPEEFRPERYGAGVLRLSA

LVSTLLSSHLQSFLLGRQWE

>348537068

MGFILFFSLETWILLITFICIFVMYGHWTFGIFEKMGIPGPKPVMYWGTIARHNRVHFLD

DYECAQKYERIWGVYEFRKPMLAVMDPDMLKTILVKECFTYFTNRRNFRLNGDLYDAVSL

AEDDQWRRIRNILSPSFTSGHLKEMFSIMKHHSRKLTDSLQSKAHNDEVIIVKDFFGPYS

IDVMASCIFSVDFDSIKNPSNPFITHANQMFKFPLLLYIFQACFPIFLPLLERLGVSLFP

KSSTAFLKSVAEKVKAERNSSSQTSDDMLEHLIKCQTASENDKEKKHKGLTEHEIISQVT

MFVFAGYETSAIALVFLAYSLARNPEIMKRLQREIDSTFPNKGPVEYEALMQMEYLDSVV

SECLRLYPSIPRLERVAKETVKISEITIPKGMLVMVPVYALHRDPELWPEPEEFKPDRFS

KENKQNINPYTYLPFGAGPRSCMGTRFALMMVKLAMVEVLQNFSFSVCKETEIPLEMDVA

GFVSPVRPIKLKLKMRSISSANVDPCN

>68358146

MIDLSSLSVTWILVVLVLTLLYMYGVWPHGFFKKLGIPGPRPWPFFGTFLSYTKGFFNFD

MECAKKYGKVWGIYDGRLPILMVTDLEMIKTIMVKECYSTFTNRREINVDLAGPLADGIT

AVKDERWKRIRGTLSPYFTSGRLKEIFPIAMTHADRFIKNMQKGDHEQPVKIKEVMASYS

LDVVTSSAFSVDIDSINNPDDPFVTNIKNFLKFNLFGLFVLLLTVFPSIANLLGKMGLSM

FSKPTVDFFYKALRKIKEEHKDSNGRVDFFRLMIQNEIPDDQVKDTTSEQPEKGLTDHEI

LSQSFIFIMGGYETTSTTLTFLLYNLAINPDCLEKLVEKIDKNFPLDTPISYDALMKIDY

LEMSINESMRLLPTAPRLERVCKKTVEMNGITIPKDTMITIPTYVLNQDPQLWDSPHEFR

PERFSPENKSEFLQYAFMPFGLGPRNCIGMRYALMIVKLLVVKLLQNFSVETCKETQIPL

ELNPVFQPKVPITLKLTPRKRNN

>ABH05130.1

MGYLPFLSSETWILLFTFICIFVTYGCWTFGTFEKLGIPGPKPLMYFGTIGRHNPVYYLA

DNECAQKYGRVWGMYELRRPMLAVMDPDMLKTILVKECFTYFTNRRNIRLNGDLYDAVST

VEDDHWKRIRNILSPFFTSGHIKEMFSIMKHHSHKLTASLQSKVHDDEVITIKDIFGPYS

IDVMASCAFSVDMDTINNPSSPLITHASKLFKFSKSLFLFQGCFPFFLPLLELLGVSFFP

KTSTGFFKTVVEKIRAERNKSSQQNSGDVLQHMMNSQQANEPNKEKQNKGLTDHEITSQA

TMFVFAGYETSATTLVFLAYNLARNPEVMKHLQEEIESTFPNKGPVQYEALMQMEYLDSV

VHESLRLYPPSPRLERIAKETVKISGITIPKDMLVMTPVYALHRDPELWPDPEEFKPERF

SKQNRQSINPYTYLPFGIGPRNCLGMRFALVMIKLALVEVLQNYSFSVCEETEFPLKMDP

LSLVGPLQPIKLKLVTR

>AAH52130.1

MFDLSSLSVTWTLVVLVITLLLIYGVWPHGFFKKLGIPGPRPLPFVGTALSYSKGICNFD

IECSKKYGKVWGIYDGRLPLLLVTDLEMIKTILVKDCYSTFTNRRNMNPDLVGPFADGIT

LVKDERWRRIRSSLSPYFTSGRLKEIFPIAMTHADRFIKNMEKKDPNLPLKIKDVVAPYS

LDVVASSSFSVDFDSINNPDDPFVTSIKSFFNINPLSPLFLLLAFCPSAANLLAKMGISV

FSRSTTDFYYKALRKIKDEHNESNGRVDFLKLMIQNQIPDDQVKDTASEQPVKGLTDHEI

LSQSFIFILGGYETTSTTLSYLLYNLATNPDCLEKLVEEIDKNFPLDIPITYDALMRMDY

LEMAIHESMRVFPAGPRLERVCKKTVEINGITIPKNTLVGIPLYVLSRDPDLWESPNEFK

PERFSPESKTEINQCAFMPFGLGPRNCIGMRFALMMMKLLVVKLLQKYTVETCKETQIPV

QLNFFFQPKVPITLKLIPRSHKEKQ

>AAI24296.1

MIDLSFLSVTWTLVVLVITLLFIYGVWPHRFFIKLGIPGPRPWPFVGTLLSYAKGLCNFD

MECAKKYGKVWGIYDGRLPILMVADLEVIKVVLVKECYSNFINRRKRTTGLAGPFSDGII

MVKDERWKRMRSTLSPYFTSGRLKEIFPIAMTHADRFIKNMQKKDPEQPVKIKEVVAPYS

LDVITSSSFSVDIDSINNPEDPFVINVKKFIQFNMFNPLLLLILLFPSLAIPLKKMGVTL

FSKSAMDFFYSVLKKIKDDHNKESEGRVDFLKLMLQNQIPDDQIQDSDTSEQPAKGLTDH

EILSQSLVFILGGYETTSTTLTFLLYNLATNPDCLEKLVEEIDKNFPLDTPITYDALMRM

DYLEMAINESMRLLPTAPRLERSAKKTVVINGLTIPEGTLVGIPTYVLSHDPDIWESPGE

FRPERFSPENKSEFLQYAFIPFGLGPRNCIGMRYAVMIMKLFVVKLLQNFSVETCKETQI

PLEMNVTFQPKVPITLKLIPRSCNKKQ

>AAH85438.1

MDPGGSGYNPPVYVWCLATWIFQKNWEFQDQDPGLFFGTFLSYTKGFFNFDMECAKKYGK

VWGIYDGRLPILMVTDLEMIKTIMVKECYSTFTNRREVNVDLAGPLADGISAVKDERWKR

IRGTLSPYFTSGRLKEIFPIAVTHADRFIKNMQKRDHEQPVKIKEVVAPYSLDVVTSSSF

SVDIDSINNPDDPFVTNIKNFLKFSLFSPLILFLTLFPSIANLLGKMGISVFSRSIMNFF

YTALRKIKDEHKDSNGRVDFLRLMIQNQIPDDQAKDTTSEQPVKGLTDHEILSQSFIFIL

GGYETTSTTLSFLLHNLATNPDCLEKLVEEIDKNFPLDTPISYDALMKMDYLEMSINESM

RLLPTAPRLERVCKKTVELNGITIPKDTLVAIPTYVLNRDPQLWDSPQEFRPERFSPENK

SEFLQYAFMPFGLGPRNCIGMRFALMIVKLLVVKLLQNFSLETCKETQIPLELTPVFQPK

VPITLKLTPRKRNN

>345305280

MVGPSKVMVFVIIIIIYGIWPFGVFTKLGIPGPRPIPFFGNILEYAQGTMHFDKKCFQKY

GKIWGFYEGWKAKIAVLDLDIIKRVLVKEVFTNFTNRQNSGIEGILKHSVFAAKDKHWKK

LRTLISSTFTSGKLKEMVPTIIHYGEILVKNIWYKAEKNQNVTVKDYFGAYSLDVITSTS

FGVDVDSLNKMNDRFVKEIRKLSSVDFFPPIFVLISVLPFITPLLKLLNINIFPNDSVEY

FKRFMDNIKGTHHQGNQQQQVNFLQMMLDTQNPKEGSKAKGGVSFGKVMLSDAEILTQAF

AFIFAGYESSSSVLSFIAYNLAIHPDVQQKLQEEIDTVLPNKLPLTYEALVQMEYLDMVI

NETLRLYSVGPRLQRVSKKTTVINGLTIPKGMTVMIPLTCLHREPKYWLEPDEFHPERWQ

AKIFEDAFLELFCNSASEWIPLELTARGFLIPQKPIVLQFIPRAEIKKG

>AAI00982.1

MDLIPNFAMETWVLVATSLVLLYIYGTHSHKLFKKLGIPGPTPLPFLGTILFYLRRSLNK

IPSWAWWLTPVIPALWEAEAGGSPKVRSSRPALPTWVFGILTENVMKNTEKCGALFPFLT

PVFEALNIGLFPKDVTHFLKNSIERMKESRLKDKQKHRVDFFQQMIDSQNSKETKSHKAL

SDLELVAQSIIIIFAAYDTTSTTLPFIMYELATHPDVQQKLQEEIDAVLPNKAPVTYDAL

VQMEYLDMVVNETLRLFPVVSRVTRVCKKDIEINGVFIPKGLAVMVPIYALHHDPKYWTE

PEKFCPERFSKKNKDSIDLYRYIPFGAGPRNCIGMRFALTNIKLAVIRALQNFSFKPCKE

TQIPLKLDNLPILQPEKPIVLKVHLRDGITSGP

>CAF97054.1

MLDLWGLSGTTWTLLALFFALFFLYGIWPYNHFKKLGIPGPGSLPFIGSLFYYRKGLVPF

ERWCQEQYGDIWGIFDGRLPILMVSDPEMVKTVLVKECYSAFTNRRDSLFSGPLDDGVTV

VKDERWKRIRSTMSPCFTSGRLKQAFPIVARYADRVTKKLEQSDLDKPIDVKEFLAPYSL

DTVTSASFSVEADAINNPNDPIIVNIKKVLKFNFVVLFLIAFFPFCSRLLSFLGIDPLPR

ASVNYFYNLIKKLKDEHHADESVRGDFLQVLIQSEIPQSEIKSEEDQPPKGLTEHEILSQ

AFIFIFGGYETTTTTLTNLLYCLAVNPDALETLHREIDANTSGDAPISYEDLMGLQYLDQ

VLNEAQRLYPTAPRLERMCKKTVQVQGVTIPEGTVVGIPSHLLHKDPRYWSSPEEFRPER

FSKENAEEVNPYAFVPFGLGPRNCVGMRYALLVMKMVIVRLLQSYTVKTCKDTMIPLEMD

WKYQPLKPIKLSFVPRQK

>348501405

MFFTLFSATTWTLLALLFTLILLYGIWPYRFFKKLGIPGPRPLPYIGTMLGIRKGFLGFD

LECHAKYGDVWGLYDGRTPLLMVADPEIVKTVMVKECYSVFTNRRDTGFFTGPMEDAVVV

VKDERWKRIRNSVSPCFTSGRLKQVFPLVARYAERLVAKLEEMNLDEPISVKQFVAPYSL

DTVTSASFSVEIDSINNPNDPVNVQAQKLLKLKMWLLLLIMAVPFGRRLAKFLNMEIIPK

DSVAFFYNLLKKFKNQHHEDKSAQADFLQVLIQSEIPEKTYKNDEEQPSKGLTEHEILSQ

ALVFIFGGYETTSTALTNILYNLATNPDALQTLHKEIDANLKKDAPISYEELTGLQYLDQ

VILESMRLIPPAPRIDRVCKKTVQVNGLTIPEGTTIVIPAWVLHKDPRYWDSPELFRPER

FSKDNMEEVNPYAYMPFGLGPRNCVGMRYAILVMKMVIVCLLKKYTLETCKETMIPLRYD

WKFQPVKPVKLKFVPRNK

>EEN48955.1

MLDLLPFSPTWVLVGLCVVLIYLYMVWPYSTFKKLGIPGPKPVLLFGNYLSYRKGGSKFD

KECYKKFNKVYGIFEGRHPLLMVGDLELVKDITVKEFNTFTNSYRRTFEGQEGSVLTVLK

DADWKRVRSAISPAFSPGKLKQMEMVVEKCANNFVVNLVEKAKQGVVFDLKELSGAYTMD

VISGTAFGVHLDSLHDRNHPFVANAKKMFDVNLFHPIIIILFLFPPLVKVLQKLGASMFP

REVLDFFSAAVDNVMDMRGSNEDEAKRVDLLQLMLKAHNEELDKEVPKDVVKHGQEIKDN

AMLFWLAGYETTADTLSLTAYNLALHQEAQNKVIEEVDAIVEKRGKLDHDAVNEMPFLEM

CVNETLRIFPVNQRFDRVCKEDIEVKGLHIPAGTVVAIPAYAIHHDPEIWPEPEKFKPER

FTKEEKESRDPYAYLPFGSGPRNCVGMRLALMELKFALAKALQKVRFVTCDKTVVSME

>392352293

MDLVSALSLETWLLLAIILVLFYRFGTRTHGIFKKQGIPGPKPLPFLGTVLNYYRGLWKF

DMECYKKCGKIWGXVFWKALVTPQSGGSSTVLTQCEGRSWRDGFAATLVTYSWDCFMIYL

NETCISNKTYLFSVPVLFPFLTPIYEMLNICLFPKDSVAFFQKFVYRMKQTRLDSKHKHR

VDFLQLMMNAHNNSKDKVSHKALSDIEIVAQAIIFIFASYETTSSTLSFVLYSLATHPDS

QKKLQEEIDRALPNKAPPTYDTVMEMEYLDMVLNETPRLYPIGYRLERVCKKDIKLDGVF

IPKGSVVMIPFYTLQHDPQHWPEPEEFLPERFSKENKGSIDPYVYLPFGNGPRNCIGMRF

ALMNMKLALTKVLQNFSFQLCEETQIPLKLSRQRLFGPEKPIVLKVVPRDAVITGA

>291242642

MGIPGPPPTPLFGNMLQIRKGMHTKEQEWIQKYGKVFGTYEGRRPVINFADPEICKQITV

KHFSSFVNRRPVPLRGKPMDSNLTVLRGDQWKEVRNTLTPSFTAAKMKLMAPLINQCAND

LVENMETHCKEEKTVKCKDLYGAFGMDSIASAGFGLDVSSQSQPGHPFVENAKEALKFTL

LSPVMIIVFFLPFLVPIMNYFNMSLYPSKVVKYFSCVIEDAIAMRKTTKDINNRIDFLQL

MVNAHEVYDQHMANKDKEIDEDEGENKVDFVKDGPSSQIKLHKGFTEDEILAQSLMFFFA

GYETTSSLMSFVSYNLATNPEAQERLQREIDDVISNSDDLGYNTIASMKYLDMVVMETLR

MYPSSIRFNRQCNEDVNINGISIPKGMLVAVSIYSIHHDSDIYPDPEKFIPERFSKEEKE

KRHPYAWIPFGAGPRNCIGMRFALMEAKIGLVRVLQKFTFEPCAETQIPPELGKMGLLTP

LDGIKLGVKRRK

>291223243

MEMSWFHSFSPTTFVLVVIVIVLLYLYSTWTFSTFKNMGVRGPTPWPFIGSMLEYRKGMV

IKDLEWSKKYGKVFGIYEGRSPMLLVTDPEMCKQICVKHFSSFRNRKAIPLRSKPMSSGL

FQLADSRWKVTRNTLIPSFSGSKMRLMSPLISRCADELIKNMEAHCKEGKTVQCKELFGG

YVVDSIASVGFGMDVSSQTQPEHPFVEHAKVAFTFGVFSPAFAIVFFFPFLVPLLNYFEI

PLLPKKTVDYFAQVVEEAIKLRKSDECASQRVDFLQLMINAHDVYDEYVKNKEDEEDRDE

GVNRVEFIKDGAHSPVNLSKGLSKDEMLAQSIVFFIAGYETTNTTMSFVCYNLATNPETQ

VKLQREIDEVMRNYDDVGYEAVSKMKYLDMVVSETLRIFPPPSRFNRECNQDVNINGINI

PKGMTVSVSPYVIHHDPDNYPDPEKFIPERFTKEEKEKRHPYAWIPFGAGPRNCIGMRFA

LIEAKIGLVRVLQKFTFEPCAETEIPPVFGKQLILSPPNGIKLSVKLRA

>CAF97055.1

MPLPFIGTMLYSINKNLIFDRECQAKYGDVWGTFEARMPVLMVTNPEMIKTIMVKECYSA

FTNHTDFFSGPLENSVFVAKDEKWKRMRASISPCFSSGRIKQAFPIIARYADRFIAKLSQ

NLEEATDIKQLFGPYSLDVVTSSSFSVDADSINNPDDPFILNVKKILQFKFWVLLIKSMF

PFSSYLFKLLGMELTPRPHCGLFYERHQEIQGDFLHVMIQNEIPESQIKSRQDQPSKGLT

EHEILSQSILFIFGGYDTTSVSITNALYNLAINPDALQTLHKEIDTCLPKDAPISYEDLM

GLQYLDQVLNESQRLMPTAIRLERVCKKTVQVEGVTIPKGATVVIPLLLLHKDPRFWSSP

EEFRPERFSKEEVNPYAFMPFGLGPRNCVGMRYALLVMKMVIARVLQIYTVETCKDTMIP

MELDWKFQPVKPIKLSLVPRQK

>EEN48959.1

MLELLPLSPTWVLVGVCLVLYYLYLTWPFYTFKRLGIPGPKPVVNFGNILSYTEGSHVFD

CKCYKQYGKVFGFFEGRTPMLMVGDLELIKEITVKQFNNFSNRRDLGSLLEAFAGGLFVI

EDTDWKRVRSAISPTFSSSKLKQMSALVEKCADGLVSSLMEKQEKRVMFDMKELSSAFSM

DVISSTAFGVEVDSFKHPDHPFATHAKAIFKINMLKGFFYVMLPPPFNHIFKLRGFSLFP

KFATDFFKQTVHNVIKMRRGNPDDDGLSVVVPCRMTKPSQCLSMTEIVGNSLAFWLAGYD

TTANTIALTAYNLAFNQEAQDKVREEIDRVVQARGKLDYEALNEMRFLEMCVNETLRIYP

GAKRFDRVCKEDIEVKGLHIPAGTIINIPAYAIHHDPEIWPEPEKFRPERFSKEQQESRD

PYAFLPFGSGPRACVGMRLAMLEIKHALAKVLEKFRFVTCEKTEVPLRLENNNGNQVTGG

VWLKVEARTHTPRVRMSSA

>ACQ90303.1

METLVKVPICWIYLEMFTQFSDLRSYKYSVYKQSYFWSLGIPGPTPIPFLGIIPSIAKMG

LKETDLRLMQKYGRHVGTFFGNLPQLLITDPEMIKEILVRRFSEFTNRAQSFRNVEFFDS

AVNVAPGEHWKFLRSTLSPTFSSGKIRNMTPLISRCLEKLTRNARNMSEGGKSVEMKSLF

GAFTLDVICSTGFGIEVDSQSNPEDPFVVNARKAMSASIIGIRILLSMLFPDFQIFFRNI

PLMDQKALDFFIEATKSVMQERRKSTSDNYKDLIQLMINAHKEQSDKTTENEEGMNYESY

KKRGLTDREILANALIFLLAAFDTTSSLLTFVSYCLAVNQDVQDKLISEIDKELCKKQPT

YDNVFKLQYLDMVVNETLRIYPPATRFNREASADTEVCGVKIPKGLDVTVVVSAVHYDPE

FWPNPRKFDPERFSPENKGNIKPFSFLPFGAGPRNCIGMRLALLEAKMAIVEMLQNFRFV

TAPETEIPPKLGKGALTKPANGVYLKLEAL

>348501409

MAISVLQKAGDTWTEAPALYWNDAGHKKGSFLLFRLYDGRTPVLMIADPEIVKTVMVKEC

YSAFTNRRDTSFVSGPMDDAITVVKDERWKRIRNSLSPCFTSGRLKQVFPLVARYAERLV

AKLEETNLDEPITVKQFVAPYSLDTVTSASFSVETDSINNPNDPVNVQVQKLLKMKIWLL

FLLMAFPFGGRLVELLKIDIVPKDSIEFFYNIIKRFKDQHHEEKSDRADFLQVLIQSEIP

EKTYKDDEEQPSKGLTEHEMLSQALIFIFGGYETTSTTLTNILYNLATNPDALQTLHKEI

DANLKKDAPISYEELTGLQYLDQVILESMRLIPTAPRIDRTCKKTVQVHGLTIPEGTIVG

VPVWMLHKDPRYWDSPELFRPERFSKDSGEEVNPYAFMPFGLGPRNCVGMRYAILVMKMV

IVCLLQKYTLETCKETMIPLQFDWKFQPVKPVTLKFVSRNQ

>EEN52982.1

MLELLPFSPTWVLVGLCALFLYMYLVWPFSTFKKLGIPGPKPVPLFGHYLEYGKGINIFD

QECYQKYNKIYGIFEGRQPILMIGDLDIVKAITVKGINTFTNHRDMPGQGEGKHALFFSK

DETWKRLRIAMSPAFSSGKLKQLTVHIERCAEGFVSNLAEDAEHGKVFDVKELCGAFSMD

VISSTAFGVEIDSLHNPDHPFIKFHAYHCSLFPKVAFPQLGKFLERFGMTVLPKKPMKFF

SEAVDSVMDMRKSNSGDEKPVDFLQLMLKAHNEELDNKASGEVAKQAGVSIKEIKGNAVL

FWVAGYDTTANTLTLTAYNLAVDQEAQDRAVQEVDAIIAKRGKLDYESVNELTYLEMCIN

ETLRMFPASQRFDRVCKEDTEVNGLHIPEGTIVTFPVWAIHYDADIWPEPEKFKPERFSK

KEKESRDPYAFLPFGAGPRNCLGMRIAMLELKFALAKALQKFRFVTCEKTEKFPVLSKFV

QNCG

>291223241

MEMSWFHSFPSTTFVLVVIVIVLLYLYSTWTFSTFKNMGVRGPTPWPLIGSMLEYRKGIV

VKDLEWTKKYGKVCGIYEGRRPMLLVTDPEMCKQICVKHFSSFKNRNTLPLKSKPMSSRL

FQLADDRWKVTRNTLTPSFSGSKMRLMSPLISRCADELIKNMEAHCKEGKTVQCKDLFGS

YVVDSIASVGFGMDVSSQSQPEHPFVKHANLTAILSLFNPVFAIVFFFPFLLPLLKYFEI

QLLPMKTIDYFSQVVGETIKLRNSDKGASQRVDFLQLMINAHEIYDEYVKSKEDDEDHDE

GVNRVEFIKDSTHSSVNLSKGLSEDEMLAQSIVFFIAGYETTNATMSFVCYNLATNPETQ

EKLQKEIDEVMCNYDDVGYEAVSKMKYLDMVVSETLRMFPPPSRFNRECNQDININGINI

PKGMTVSVSPYVIHHDPDNYPDPEKFIPERFSKEQKEKRHPYAWIPFGAGPRNCIGMRFA

LMELKMGLVRVLQKFTFEPCAETEIPPVLGKGIFLTPPNGIKLSVKLRE

>BAH24057.1

MDVLGLVDIPSWIFTILLFIFTLYLYTLWKHTLWRRLGVPGPIPFPILGSIKDLAVKGVS

KTDQDLVPKYGSVVGVYNGTIPVLLVTDTDLIKEIFIKEYQTFTNRVLILKTDDLLDSMI

TAAEDDHWKFIRSTMTPTFTSGKLKNMLPMILRCCDDLVNNINGKLEQNDTVEMTEVFGG

FTLDVIASTAFGLQVNSQKEPDNEFVTNTKKTLTDSVVKPYFVIVMIFPFLKNLMGTLFK

APVFGEGSEDFFRNIVQQLVAERKSNSNSHFRDFIQLMLNSHKDKTKEKENGRIDELGGL

DFSEYKNRGMNENEILTNSMVFFGAGFDTTKTTPSFAAYVLALHPDMQERLFTEIDNELG

KSPPTYDSLPNMKYLEMFLAEVLRLYAAVPRINRRAKSDITVNGMFIPKDTDVTIPISAL

HRNPKYWPEPEKFDPERFTDENKATRPMYSYLPFGLGPRVCIGMRLALIETKYALISTVQ

NFKFVVGSKTEIPMPLEKGFITRPLNGIHLKIEKR

>348501411

MFFTWTLLALLFTLILLYGISPYRFFKKLGIPGPRPLPFIGTMLGMRKGFLAFDRECHAK

YGDIWGLYDGQMPVLMVTDPEIVKTVMVKECYSLFTNRRVFPLVARYAERLVAKLEETNL

DEPINVKQFVAPYSLDTVTSASFSVEIDSINNPNDPINVQIMKVLKVNIGLFFLLVAFPL

GRRLLKQLKIEIIPKENMNFFLNIIKRFKDEHREDKSTRPDFLHVLIQSEIPEKNYKNNE

EQPSKGLTEHEILSQAFVFILGGYDTTSNTVTNILYNLATNPDVLQTLHKEIDDNLKEDA

PISYEELTGLQYLDQVILESMRLIPVVPRLDRVCKKTVKVNGLTIPEGTVVGIPMWLLHK

DPRYWNSPELFRPERFSKDSGEEVNPYVYMPFGLGPRNCVGRRYGLLTTKMVIVCLLQKY

TLETCKETMIPLQIDWKFEPVQPVKLKFVPRKE

**CYP4**

>BAA02864.1

MSVSVLSPSRLLGDVSGILQAASLLILLLLLIKAVQLYLHRQWLLKALQQFPCPPSHWLF

GHIQELQQDQELQRIQKWVETFPSACPHWLWGGKVRVQLYDPDYMKVILGRSDPKSHGSY

RFLAPWIGYGLLLLNGQTWFQHRRMLTPAFHYDILKPYVGLMADSVRVMLDKWEELLGQD

SPLEVFQHVSLMTLDTIMKCAFSHQGSIQVDRNSQSYIQAISDLNNLVFSRVRNAFHQND

TIYSLTSAGRWTHRACQLSHQHTRPKVIQLRKAQLQKEGELEKIKRKRHLDFLDILLLAK

MENGSILSDKDLRAEVDTFMFEGHDTTASGISWILYALATHPKHQERCREEIHSLLGDGA

SITWNHLDQMPYTTMCIKEALRLYPPVPGIGRELSTPVTFPDGRSLPKGIMVLLSIYGLH

HNPKVWPNPEVFDPFRFAPGSAQHSHAFLPFSGGSRNCIGKQFAMNELKVGQQALTLVRF

ELLPDPTRIPIPIARLVLKSKNGIHLRLRRLPNPCEDKDQL

>24653964

MHLRLLSPPQLERTTNLELCSILILLVISLSIYTFYATLNTYLRSVLLSLRLTGPPSLPF

LGNCMLVTDKDLMRRCAGKAFDLYGSLVRIWVLLFPFFAVLEPEDLQVILSSKKHTNKVF

FYRLMHNFLGDGLITSSGSKWSNHRRLIQPAFHHNLLEKFIDTFVDASQSLYENLDAEAV

GTEINIAKYVNNCVLDILNEAVLGVPIKKRGQDVAMMEDSPFRQGKIMMPARFTQPWLLL

DGIYHWTKMANDELNQKKRLNDFTRKMIQRRRQIQNNNNGNSERKCLLDHMIEISESNRD

FTEEDIVNEACTFMLAGQDSVGAAVAFTLFLLTQNPECQDRCVLELATIFEDSNRAPTMT

DLHEMRYMEMCIKEALRLYPSVPLIARKLGEEVRLAKHTLPAGSNVFICPYATHRLAHIY

PDPEKFQPERFSPENSENRHPYAFLPFSAGPRYCIGNRFAIMEIKTIVSRLLRSYQLLPV

TGKTTIAATFRITLRASGGLWVRLKERDHPLIAH

>156553274

MHSGCILSAISLPIQCGIVFLFAIAEYKSRSKELLDKIPGPPTVPILGNLLMFNVPIGKK

TWNVIRDLNKNYYPIAKFWLGPYPYVSIHHPDDLEILLSSTKHLEKGSAYTVLHPWLRTG

LLTSSGSKWQQRRKILTPAFHFKILRKYMDITNEHGKKFIEALRTENSETVQSLMPFCSN

YTLHIICETAMGIALDKIDTKISKEYKNAVADIANLIMYRVMRPYLKEWMMLPVWTFGRL

QTQTLRTLHKFTKTVLTERREYHKRSDNKYLKGFHDDTYDVNKDDVAIGGKKKLAMLDLL

LSAAQDGLTDDEGIKEEVDTFMFAGHDTTGIALVYAIMLLAEHKEIQEKARAEVIEVLTE

SNGEIGTLEIQKLHYLERCIKESLRIFPPVSALSRTVKEDIQLKNYVVPAGTEIGCHIFD

LHRDPNFWPEPEKYDPDRFLPENIQGRHPYAYIPFSAGSRNCIGQKFAMMELKSLTARIL

YNFELEPVSQTKDMKLTLDLVTRPLEPVYTKFIRIDNNN

>O46054.1

MLVVLLVALLVTRLVASLFRLALKELRHPLQGVVPSVSRVPLLGAAWQMRSFQPDNLHDK

FAEYVKRFGRSFMGTVLGHVVMVTAEPRHIDALLQGQHQLKKGTMYFALRGWLGDGLLLS

RGKEWHTMRKIITPTFHFSILEQFVEVFDRQSSILVERLRTLSYGNEVVNIYPLVGLAAL

DIITETAMGVNVDAQGADSEVVHAVKDLTNILATRFMRPHLLFPHLFRLCWPSGFRKQQA

GVICLHEFTNGIIEQRRRLLAREANQDKPTKPHALLDTLLRATVDGQPLTDKQIRDEVNT

FIFEGHDTTTSAVSFCLYLLSRHEAVQQKLFEELRMHYGQDLFRGVILSDFATLPYLSCV

VKESLRLYPPIPAVARCLEKDLVIDEGYIPVGTNVVVLLWQLLRDEAIFTDPLVFQPERH

LGEEAPRLSPYSYIPFSAGPRNCIGQKFALLEMKTMVTKVIRHYQLLPMGADVEPSIKIV

LRSKSGVNVGLRPRLY

>AAS87604.1

MWATQIRSNAAKASISCISVNTQNNRYLSACARLADDHEYLPVTFDERHLKKQARWSELF

DGLFSYCIGLYVCMELSHPSPLSKLLSTAEPKDRISYRTIKPWIGDGLLVSSGEKWARNR

RLLTPAFHFDVLKPYVGIFSSTANIMADKWRKILSKNDEPLEMFQHVSLMTLDSLLKCIF

GQTGSVQNEGRDGSYIRSVYQLTELVMKRFQFFPYLIDWIYYFTPSALQVSPMLQHCPRI

LAWSDSRQEGREEKKRSRRNQVDSQNLDFLDILLDAKDSDGRGLTDEEIQHEVDTFMFEG

HDTTASGLSWCLYNLARHPEYQDRCRKEVMDVMGDRSDVEWDDMSKLTFLTMCIKESLRL

HPAVPNIGRSLTKPMTFPDGRTVPAETDLGIAIYGCHHNSALWENPEQYDPERFNAENSK

DRPPHSFIPFSAGPRNCIGQHFAMHEMRSVLAVCLKNFQLRIDDTRILELLPQLVLKAKG

GLWLKVTPIE

>ABF06545.1

MLFWLFQVVFALAAAFCIAFCLKKYNFKKKISWVPFVSGYPVIGAALELRDRKKILENIE

NHLNKHNGLCYMEVGTIPMLMASNPDFLKWFLSSNLPLAKSYSYGFLHNWLGGGLLISAG

ERWRRSRKLLNPAFHFSILEQFIEVFEDATKDLVRILEQEVGKDEVDIHYILTRYTLDVI

CQTSMGVQLKIQDRENTDYINAVASMGELIMERTFNPFKTFDLIYKLTADYRQEMAYVKL

LHEVSEGVINKRKQEIEAENKEDGKKKRKMAFLDLLLMYKDENGQPLSQDFIRHEVDTFM

FAGHDTTAAALGFALYLLAKHSNVQAEAFREVEPLGSTSKITIWDLQNLKYLDLVIKETL

RLYPSVPMIARHITEDLTYDGDKVLPKGLTVTVVISAVNKDDSIYNKPNEFNPSRFLEEE

PKPFSYLPFSAGPRNCIGQKFAILEMKMALAMILLNFELFPANPTFEPILESATVLKSQN

GIKVRLEKRS

>AAH17758.1

MVPSFLSLSFSSLGLWASGLILVLGFLKLIHLLLRRQTLAKAMDKFPGPPTHWLFGHALE

IQETGSLDKVVSWAHQFPYAHPLWFGQFIGFLNIYEPDYAKAVYSRGDPKAPDVYDFFLQ

WIGRGLLVLEGPKWLQHRKLLTPGFHYDVLKPYVAVFTESTRIMLDKWEEKAREGKSFDI

FCDVGHMALNTLMKCTFGRGDTGLGHSRDSSYYLAVSDLTLLMQQRLVSFQYHNDFIYWL

TPHGRRFLRACQVAHDHTDQVIRERKAALQDEKVRKKIQNRRHLDFLDILLGARDEDDIK

LSDADLRAEVDTFMFEGHDTTTSGISWFLYCMALYPEHQHRCREEVREILGDQDFFQWDD

LGKMTYLTMCIKESFRLYPPVPQVYRQLSKPVTFVDGRSLPAGSLISMHIYALHRNSAVW

PDPEVFDSLRFSTENASKRHPFAFMPFSAGPRNCIGQQFAMSEMKVVTAMCLLRFEFSLD

PSRLPIKMPQLVLRSKNGFHLHLKPLGPGSGK

>AAR88241.2

MNSVTTSIAVVILLPVLYKVYQYLHWIYRTWFVIGTQVNKFPGEPLKWPYGNLHQNPGFN

EKLNGYQKSMAAKYNRLWRMWFGPFVPFISLCHPDTIKILVKTEEPKFTDGNAGYLLAEP

WLGNRLLLKCWTKVEEKQETPDTSFSFWHPQELHFCLQCGNRNSDEKSGQQKDRKERALT

FRRIFRLCTLSIILRCAFSYDEKIQETGERHPYAVAVLDLSRLMVERAFNPFVRMSKFLY

GLTANGRTFFKHCDYVHKVAETVISQRRETLQENPDALEDRKYLDFLDILLQARDEDGTG

LSDLEIRNEVDTFMFEGHDTTASGISWALYSLAKHPEFQKKAQQEIDELLADRKNKWIMW

DDLNQLPYLTMCLKESMRLWCPVPVISRQLLNPITIDGVTLPPHTLFDINIIALHHNPTV

WGEDHDEYKPERFLPENINKMDNFAFLPFSAGPRNCIGQNFAFNEMKTTIARIIQRFDLS

VDESHPVYPRPEVVTRAIHGIKLFMNPR

>ABF06546.1

MLFVLLALVTCILLGLFTLYIVAKIKKDTSKYLTNVPCSEQTFPFGNTLPFLKGSVVYLD

LIMQGVKELGRTSLFHDGPLSWVVITADPEFIELIYSSSTHITKGSQYDYFKRWLGQGLL

ISDGDKWRFHRKIITPTFHFSILQQFLTVFDTVGDNFVRKLQQHVGSTSVEISNLISLCT

LDIICETAMGVKMNALDMKDSENMEYIKGIRIMCKIIVDRMFSFLHPIFYPLTLNYYREK

RALKMVNGYVDNVISQKIQQRKELNQKEDKSQIDGIRTRLAFLDLLLEAKIDGTPLTKAE

LRDEVNTFMFEGHDTTSSAITFCLLMLATHPRVQDKVMAEQKEILEGDLKLAHPTSKELS

QMKYLENVIKETLRLYPSVPLFSRKLGEDVEFKGNLYPKGITLVLTPYATHRDPDIFPEP

EKFLPERFEESEMLKINPFAYTPFSAGSRNCIGQKFAMMEIKSTVSKVVRHFKLEPAHPE

HQIQLVSETTLNSKNGVKISLQAR

>ABF06547.1

MIKAWLGPVPILFVVDPDSAKVILESNTLITKSSFYDKVADWIGTGLLISTNEKWQSRRK

LLTPAFHFNILKGYTEVMVKEGEVFVDQLDKLADTGREFNLYPYVKRCALDIICETAMGT

HINAQIGMNSEYVDAVTRVSDLTWTYIRFPFFWIKPIWYASSYGFEFDRLVKLTNDFTRK

VINERREALREEGILESGPKISTKQRMAFLDLLLMHQAASNLNDEDIREEVDTFMFEGHD

TTAAGMAFAIWLIGQNAEAQAKVHAEVDSIFGDSSRPPEEADVTKLVYLERCIKESLRLF

PSVPLFARRLTHDITIKDTVLPEGLNLILAPLATHRDPEQYERPWEFYPDHFTQEAIAKR

HPYAYFPFSAGPRNCIGQKFALSEEKIVLSWFFRRFRVESSEPMPGNRPLPELILKPSDG

VLCKIYRRRH

>ABF06549.1

MGFWVPVVVLLLFVYATLRIWPWILKRKRLIQMVDRIPGPTAIPILGCAYQFRPKIEDFS

YELLEYARLHKDSEVVRFWLGPIPIVCAFGPESVKTVLESNKVITKGDEYDILERWLGTG

LLISTGNKWRSRRKMLTMAFHFNVLNGFMDTYDKEARIFLDQIREFADTNEPFDVCPFIK

RCALDIICETSMAHKIDAQVDHNHPYVNAVAQMNTLSFLYARSPWFWIKPIWRFFGHEEN

YERNLKLVTDFTQNVIAERRKELHTAKKTVQENKSGTEEIGGKTRRAFLDLLLSIQDEGK

LTDEDIREEVDTFMFEGHDTTSSGMSWTIWCLAHHLDYQNKVIQEIDAVFGNSDRNCTNE

DLKELKYLEQCIKEAMRLYPPVPLISRKVEEDFHCAGYDVPRDATILISPLVLHRDPALY

ENVESYNPENFSPSAIARRHAYSFIPFSAGPRNCIGQKFALMEERTVLSWFFRRYCVRSS

EEFLSNIPCAKIILKPSKGVPIKIYRRNAAH

>ABF06550.1

MKMFIGVLCGLVAVIFLFLLRKYNKLRKKRCRIPNPPGHWLLGNLTLNNLNSGELFDQLR

NFARDYGPIYRISIPFLDIVNFFHPADLEIILSQKKHMKKSLLYQFLEGWLGKGLLTSTG

SKWQFRRKLLTQAFHFNILQKFVRVFNEETTHLVKRIEEINLTGQSQAGISVLPLITHLT

LQSVTETSLGVSNIEKETLKAYRENIYKMGDFLIDRLRKPWRLFNFIYHFTEASKQEQLT

INKLHQFTYQVIKEREEVLQSDKTQSVMTSTYSGRKIYKMLDLLLHEKLQFGSIDYEGIR

EEVDTFMFEGHDTTSAALVFLLHNLASNLAIQEKVRQEIKTVERIPTFQTLQNLPYTDRV

IKESLRLYPSVPFISRIASEDFITHTGYSISKGTVLYMHIFDLHRNPEIYPDPLTFDPDR

FLPEKVKERHPFAYLPFSAGPRNCIGQKFAMLELKAVLWGLLHKFRLTLDPSTTQINFQV

DLILRTQGEIKINFQPLE

>ABF06551.1

MFFLTVFTALATVLAYYVGRWWMKLHNARNKLASWMPTAPGHPLIGSVFEFGDNTVFTTN

LHRLTDNPCKSCYVEVMGEWMILTRDHDLLDLVLSSNKILTKGGDYRTTAPWLGQGLLTS

NGLQWKSHRKMITPAFHFSILEQFVDIFNSQGDTLVNKLQRDALEKTSIDIYQYVTACAL

DIICETAMGVNLNSQENHDLEYVQAVKDMCRIIMDRTFSLWKSFDCLYNLSKLGKKQEQV

LEILHNFTNSVINKRRKETTEQLKNVKQDDVGRKRKMAFLDLLLSTNIDGRPPTQEEIRC

EVDTFMFAGHDTTSSAMSSAFYVLAKKTQIQKMVRDELQGVFGKDGSTQITHKSLQELKY

LELFIKEVLRLYPPATVISRVTEEDVQYKNHTIPKNTLIEMLIYSANRDPDYHENAEEFK

PERFLDTSGKKRPFAYIPFSAGPRNCIGQKFAMLEMKTIISKVLMNFEVLPSIPEREMVM

TTEVVLKAKNGVHVQLKPL

>ABF06552.1

MNPTVWLLVVFLTFLFLYWKRLWLAFRERRRFTHMINQLPGPMGLPLVGSALNFSPDTEK

CTYQMELYFRTYTEDIDSSGIMRVWIGPKPLVFIYKPETAKVILESQTLITKPGEYGILE

RWLGTGLLTSTGEKWHTRRKMLTPTFHFSILNNFMKVFNRQANTLIENLDRHAADGYPFD

FYKVIKALALDVICEAAMGMNIGAQRGQNTTTYRRCGKCASSASCGCAHRGYGRSWRGGC

PDDAQTTTAVTWRYKRFIRDVIADRKAKYQAAKQRGSEKKAATEEKKRLAFLDLLLDLQY

ETGRLNDEDIREEVDTFMFEGHDTVSSSMGFFVFSMSQRLHLQEKVYEEMLEIFGNDEER

DVTSEDISQMKYLEQCIREVMRLLPTVPIIGRIISEDTNIGGYMVPKNTTAMIAPFAVQR

DKRFYHNPDAFDPDHFTPERVKQRNPFAYIPFSAGPRNCIGQKI

>EFA04693.1

MEILFTLFILLLCLLLYFYNKYVYIFDKNLKDYPAPPQVPILGHTLDFVSGKGFLDVLLS

YTHKYGDIVRIRPGPIRQLLLTSNYKLFEAVLSNAKITKKSADYKFFHRWLGTGLLTSDG

AKWKKHRQIITPTFHFQILENFVDVFEKNGKILTKQLEKHLNEDSVNVYGFVNLCALDII

CEAAMGTSVKAQENMNSEYVRSVKDLLDTLMGRIFSPYKMIDFIYFFTEDYKKEMKALQV

IHSYTRNVIKSRQAAINSGEFEQKTKKNFLDLLLAANEQQMTLEEIREEVDTFMFAGHDT

TASTISFALFCLANHPDEQARVYREQKDIFGDDFKRAVTFQDLKKMKYLEYVIKETLRLY

PVGPFFSRELDKDVPFAGKVLPKGLTITLFIYAMHRNPEYFPDPEKFNPSRFETFDGKMP

FAFVPFGAGPRNCLGQKFAMLEMLSVVSRVVRTYKILPSIPRHEINVAAQVVLISTNGMR

MRFEKRSEFSRK

>Q9VA27.1

MSSKVITSLMAESILLSKVGQVISGYSPITVFLLGSILIFLVVYNKRRSRLVKYIEKIPG

PAAMPFLGNAIEMNVDHDELFNRVIGMQKLWGTRIGINRVWQGTAPRVLLFEPETVEPIL

NSQKFVNKSHDYDYLHPWLGEGLLTSTDRKWHSRRKILTPAFHFKILDDFIDVFNEQSAV

LARKLAVEVGSEAFNLFPYVTLCTLDIVCETAMGRRIYAQSNSESEYVKAVYGIGSIVQS

RQAKIWLQSDFIFSLTAEYKLHQSYINTLHGFSNMVIRERKAELAILQENNNNNNNNAPD

AYDDVGKKKRLAFLDLLIDASKEGTVLSNEDIREEVDTFMFEGHDTTSAAISWTLFLLGC

HPEYQERVVEELDSIFGDDKETPATMKNLMDMRYLECCIKDSLRLFPSVPMMARMVGEDV

NIGGKIVPAGTQAIIMTYALHRNPRVFPKPEQFNPDNFLPENCAGRHPFAYIPFSAGPRN

CIGQKFAILEEKAVISTVLRKYKIEAVDRREDLTLLGELILRPKDGLRVKITPRD

>AAF45736.1

MWLLLSLVLLLAIIALEMRRFLRNMRTIPGPLPLPLLGNAHIFLGLTPAEACLKIGELAE

RHGDTFGLFLGPSYSVMLFNPRDVERVLGSSQLLTKSQEYSFLGRWLNEGLLVSNGRKWH

RRRKIITPAFHFRILEPYVEIFDRQSLRLVEELALRISRGQERINLGEAIHLCALDAICE

TAMGVSINAQSNADSEYVQAVKTISMVLHKRMFNILYRFDLTYMLTPLARAEKKALNVLH

QFTEKIIVQRREELIREGSSQESSNDDADVGAKRKMAFLDILLQSTVDERPLSNLDIREE

VDTFMFEGHDTTSSALMFFFYNIATHPEAQKKCFEEIRSVVGNDKSTPVSYELLNQLHYV

DLCVKETLRMYPSVPLLGRKVLEDCEINGKLIPAGTNIGISPLYLGRREELFSEPNIFKP

ERFDVVTTAEKLNPYAYIPFSAGPRNCIGQKFAMLEIKAIVANVLRHYEVDFVGDSSEPP

VLIAELILRTKEPLMFKVRERVY

>Q27606.2

MWFVLYIFLALPLLLVAYLELSTFRRRRVLNKFNGPRGLPLMGNAHQMGKNPSEILDTVF

SWWHQYGKDNFVFWIGTYSNVLVTSSKYLEFILSSQTLITKSDIYQLTHPWLGLGLLTST

GSKWHKHRKMITPAFHFNILQDFHEVMNENSTKFIKHLKTVAAGDNIFDFQEQAHYLTLD

VICDTAMGVSINAMENRSSSIVQAFKDMCYNINMRAFHPLKRNELLYRLAPDYPAYSRTL

KTLQDFTNEIIAKRIEAHKSGAVSTNAGDEFTRKKMAFLDTLLSSTIDGRPLNSKELYEE

VSTFMFEGHDTTTSGVSFAVYLLSRHQDEQRKLFKEQREVMGNSELGRDATFQEISQMKY

LDLFIKEAQRVYPSVPFIGRFTEKDYVIDGDLVPKGTTLNLGLVMLGYNEKVFKDPHKFR

PERFELEKPGPFEYVPFSAGPRNCIGQKFALLEIKTVVSKIIRNFEVLPALDELVSKDGY

ISTTIGLPDAERKKRDPYRHKYDPILSAVLTLKSENGLYIRLKERH

>Q08477.2

MPQLSLSSLGLWPMAASPWLLLLLVGASWLLARILAWTYTFYDNCCRLRCFPQPPKRNWF

LGHLGLIHSSEEGLLYTQSLACTFGDMCCWWVGPWHAIVRIFHPTYIKPVLFAPAAIVPK

DKVFYSFLKPWLGDGLLLSAGEKWSRHRRMLTPAFHFNILKPYMKIFNESVNIMHAKWQL

LASEGSARLDMFEHISLMTLDSLQKCVFSFDSHCQEKPSEYIAAILELSALVTKRHQQIL

LYIDFLYYLTPDGQRFRRACRLVHDFTDAVIQERRRTLPSQGVDDFLQAKAKSKTLDFID

VLLLSKDEDGKKLSDEDIRAEADTFMFEGHDTTASGLSWVLYHLAKHPEYQERCRQEVQE

LLKDREPKEIEWDDLAQLPFLTMCIKESLRLHPPVPAVSRCCTQDIVLPDGRVIPKGIIC

LISVFGTHHNPAVWPDPEVYDPFRFDPKNIKERSPLAFIPFSAGPRNCIGQAFAMAEMKV

VLGLTLLRFRVLPDHTEPRRKPELVLRAEGGLWLRVEPLS

>Q9V3S0.1

MAVEVVQETLQQAASSSSTTVLGFSPMLTTLVGTLVAMALYEYWRRNSREYRMVANIPSP

PELPILGQAHVAAGLSNAEILAVGLGYLNKYGETMKAWLGNVLLVFLTNPSDIELILSGH

QHLTKAEEYRYFKPWFGDGLLISNGHHWRHHRKMIAPTFHQSILKSFVPTFVDHSKAVVA

RMGLEAGKSFDVHDYMSQTTVDILLSTAMGVKKLPEGNKSFEYAQAVVDMCDIIHKRQVK

LLYRLDSIYKFTKLREKGDRMMNIILGMTSKVVKDRKENFQEESRAIVEEISTPVASTPA

SKKEGLRDDLDDIDENDVGAKRRLALLDAMVEMAKNPDIEWNEKDIMDEVNTIMFEGHDT

TSAGSSFALCMMGIHKDIQAKVFAEQKAIFGDNMLRDCTFADTMEMKYLERVILETLRLY

PPVPLIARRLDYDLKLASGPYTVPKGTTVIVLQYCVHRRPDIYPNPTKFDPDNFLPERMA

NRHYYSFIPFSAGPRSCVGRKYAMLKLKVLLSTIVRNYIVHSTDTEADFKLQADIILKLE

NGFNVSLEKRQYATVA

>167234443

MILAIVIALISAPFIWWMLILSKYRSLSNVPGPKPSPVIGNSLHIGYRPQDLFNNIVKWA

KEYGTVYKIFVANDVRVLITNPELTELVLSSNVHITKSNAYDLLKPWLGIGLLISTGKKW

KSRRKLITPTFHFKILDQFLDVFNSCGNVLVQRLSSQVGKDSFDVYPFINLCSLDIICET

SMGVKIKAQQGNNTEYVEAVRRFLDIFIIRTFSVWKGIDALFKFSSAYSVYKRLLKILHQ

FTINIIVQRRGEKAQQKTQNVTSDDGIKRKVALLEMLLESEDNNMLSNEDIREEIDTFMF

EGHDTTTSGIAFAILCLAENPKVQEKLYEEVVAVIDNIENITMQQLQEMKYLEMVLKEAQ

RLYPSVPVIERRLEVDCNIGGYDFPKDTFLSLFIYGMHHNEKYFPEPEKFDPNRYLPENQ

AKRHNYAYVPFSAGPRNCIGQKFAMLEMKTTIAKIVKHFKILPVPDYKPDLGIAAILKSY

NGVCVRLQHRHGN

>ABZ81071.1

MYLILLICLLVLVLTVSWWSMLNRICKSNVPGPFPLPIIGNAHQFVVRSTEFLGLLKSFT

DKYGDVFRVHFFSYPYVLISHPKYAEALVSSADLITKGRSYSFLKAWLGEGLLTASGPRW

RLHRKFLTPAFHFNILQNFLPVFCKKSEILRDKIRRLADGQPIDLFPITALAALDNVAES

IMGVSVNAQQNSESEYVRAIETLSQITTLRMQIPLLGEDFIFNLTSYKKKQNIALEVVHG

QTKKVIEARRCELEKNNKTNISGTNEYGIKNKHAFLDLLLLAEIDGKLIDEQSVREEVDT

FMFEGHDTTTSGIVYTLFCLSKHPDIQEKLYEEQLTIFGEEMDRTPAYNELAQMKVLELV

IKESLRMYPSVPLIERLITKDAEVGGLKLSKGTSVVLNIFQMHRNPEVFEKPLEFIPERF

DSLEHKNPFSWLAFSAGPRNCIGQKFAMMEMKVTLSTLVRNFKLVPVDIEPILCADLILR

SQNGVKVGFLPRTQSNSKT

>AAM54722.1

MFWFLLFFVGFLCLLHLLLNYNERARLIRKLPGPEDSFILGNGPAVMLSSVEVMKLARKL

AQENSGIYRLWMYPVAAVSIYNPEDIETIVSSMKYNEKSQVYRFLKPWLGDGLLLSKGQK

WQQRRKILTPTFHFNILKQFCEVISENTQRFVENLKEVSGRPIDVVPVISEFTLNSICET

AMGTNLTEYDKTAASAYKEAIHNLGYIFYQRFIKVYYFFDFIFNLSSLSKKQDGYLKTVH

SFTKKVIDERSAYIEKHGIKIPDENDDDDTYVYKSKKKTAMLDVLISARKEGHISDTGVQ

EEVDTFMFEGHDTTAGGLTYCFMLLANHKEAQDKILEELKEILGDDKRPITMEDLPKMKY

LERCIKESLRLFPPVHFISRSLNETVTLSNYKIPAGTLCHIQIYDLHRRADLFKNPTSFD

PDRFLPENSVGRHPYAYIPFSAGPRNCIGQKFAMMEMKIAVAEVLREFELQPVTRPSDIR

MIADAVFRNDGPVEVTFVKRQ

>Q9V558.1

MIILWLILALSALLYWLHRANKDYHILSFFTKRIRLKDGTPVEIIAPIAKGKTIFGNTLD

LYGRDHAGVFNYSRERAKEMGTSYIEYVFGKAIYNIIDADSAENVLNHPNLITKGLVYNF

LHPFLRTGLLTSTGKKWHARRKMLTPTFHFNILNQFQEIFKTESQKFLLQFEGQDEVTIT

LHDVIPRFTLNSICETAMGVKLDEMAEKGDRYRENFSQIEECFIRRLSNPLLWGDKLFEM

FAAKDFASALDVVHRFSSEIIAKRRDLLKDELDKSSSTADDDGFVSKKRFAMLDTLIYAE

KDGLIDHIGICEEVDTLMFEGYDTTSIGLIFGLMNMSLNPDKQELCYQEIQEHIDDDLSN

LDVGQLNKLKYLEYFMKETTRLFPSVPIMGREAVQETELANGLILPKGAQITIHVFDIHR

NAKYWDSPEEFRPERFLPENVQDRHTYAYVPFSAGQRNCIGKKYAMQEMKTLMVVLLKQF

KVLKAIDPQKIVFHTGITLRTQDKIRVKLVRRT

>AAP94193.1

MFLAPIILCLVLLALLVSSLRKKGRFWECLAPIPQPPAYPIIGNLFDIMRTPEQMFLADR

ERGLKYYPIYKLDVCGTGGVNLLNPEDVELVLTDTKQNTKSFIYHFLHSWLGTGLLTSRG

PKWQNRRKILTPAFHFNILQEFIQIFNEETKRLVEDLEAESHKPYIDVVVPITQFTLLSI

GETAMGIKLNASDNDKDGYKRAVYKIGQLLTYRAPRPWIHNETIYSLTPQGRKEQKVLKS

LHSFSNNVIAERKKHFSSSSYSSRKRLAMLDLLLKYKSEGANIDDEGIREEVDTFMFEGH

DTTSVSICYTLMLLANHREVQEEILKEMEAVLDEEPPTYAKLQELKFMDRVIKESLRLYP

SVPFISRVSGSEIQTKTGYTIPKDCMVNLQIYDMHHNPNVFPDPEKFDPDRFLPENIQKR

HPFAYIPFSAGSRNCIGQKFAMLEIKTVLCGILKKFILEAVDTRKDMAFVSDLVLRPKGS

IKVKFVPRRAN

>Q9VXY0.1

MSTLALVAFVLWAAFLRYLPKILNFLRLQRFAKTLPGPTIGELIANVKKGEILNWLKELR

EKHGPVFRIWFGKDLMVMFTDPEDIKQLLGNNQLLTKSRNYELLEPWLGKGLLTNGGESW

HRRRKLLTPGFHFRILSEFKEPMEENCRILVRRLRTKANGESFDIYPYITLFALDAICET

AMGIKKHAQLQSDSEYVQAVQSICRVMHKQSFSFWQRLNVFFKHTKPGKEREAALKVLHD

ETNRVIRLRREQLIQERNEWKPEAEQDDVGAKRRLAFLDMLLLTQMEGGAELSDTDIREE

VDTFMFEGHDTTSSAIAFALSLLSKNPDVQQRAFEEASELEGREKESMPYLEAVIKETLR

IYPSVPFFSRKVLEDLEVGKLTVPKGASISCLIYMLHRDPKNFPDPERFDPDRFLVNEKQ

MHPFAFAAFSAGPRNCIGQKFAMLELKTSLAMLLRSYRFLPDKDHQPKPLAELVTKSGNG

IRLRILPRDENGTTA

>JE0361

MELTEAFLTLHWGLPRLHHLLALLCLVAVVYKLATLLAKRRDVFRSYEDFPGPPTHWLFG

HVLEFKQDGTDFDTLMAWTKQYPYAFPLWFGPFFCVLNIHHPDYVKTILASTEPKDDVSY

RFILSWIGEGLLVSSGQKWFRHRRFLTPGFHYDVLKPYVKLMSDSTKTMLDKWGSYANSN

ESFELFQHVSLMTLDSILKCAFSYNSNCQTESGTNVYIKAVYELSDLINLRLRTFPYHSD

LIFYLSPHGYRYRKAIKVAQSHTEEVIKKRKEALKEEKELDRIQAKKNLDFLNILLLARD

EKQQGLSDEDIRAEVDTFMFEGHDTTASGISFILYSLACHPEHQKICRDEIMQVLDGKDT

MDWEDLSKIPYTTMCIKESLRIYPPVPGMSRKITKPITFCDGRTLPEGSYIGTSVFGIHR

NGIVWENPDVFDHWRFLPENVSKRSPHAFVPFSAGPRNCIGQNFAMNEMKVVIALTLKKY

HLIEDPNWKPKIIPRLVLRSLNGIHIKIKPVDPQV

>ABB86762.2

MLTVALSCILLGALALYMHFRARIRPMMMLSERMPGPRLHPVVGNALQFGFKTEDFLENL

TRAVREHGTITRIWLGNELVILLWDPRYVETLLTNTRMLDKAFMYQFMHTWLGEGLLTAT

GTKWKEHRKLFTPAFHFKILEEFVGVFNSNDRIMIQKLDKHVNGPGFDIRPYISLCTLDM

ICETAMGVQVHAQEDSNSEYVRAVRGMADIIMQRTFKPWIQLDSLFRLSPLARQQTKYLA

ILHDMTNSVIKMSKEEYLQKRANSLTTEERNDIGAKRRLAFLDLLIQAQQDGATISDKEI

REEVDTFMFEGQDTTTSGICFTLWALAKHQDVQAKALQEQRAIFGGSDRDATYTDLQEMK

YLEQVIKEAHRLYPPVPLYGRRISENLTVGDYVLPAGSNVMVHAFMLHRNPDHFPDPERF

DPDRFLTENCKDRHPYCYIPFSAGSRNCIGQKFAMLEMKATISAVLRHYKLSLEDPSETP

WFVLEVVLTSLNGTRLKLEPRLWSAPGF

>Q9DBW0.1

MLWLWLGLSGQKLLLWGAASAVSLAGATILISIFPMLVSYARKWQQMRSIPSVARAYPLV

GHALYMKPNNAEFFQQLIYYTEEFRHLPIIKLWIGPVPLVALYKAENVEVILTSSKQIDK

SFLYKFLQPWLGLGLLTSTGSKWRTRRKMLTPTFHFTILENFLDVMNEQANILVNKLEKH

VNQEAFNCFFYITLCALDIICETAMGKNIGAQSNNDSEYVRTVYRMSDMIYRRMKMPWLW

FDLWYLVFKEGRDHKRGLKCLHTFTNNVIAERVKERKAEEDWTGAGRGPIPSKNKRKAFL

DLLLSVTDEEGNRLSQEDIREEVDTFMFEGHDTTAAAINWSLYLLGTNPEVQRKVDQELD

EVFGRSHRPVTLEDLKKLKYLDCVIKETLRVFPSVPLFARSLSEDCEVGGYKVTKGTEAI

IIPYALHRDPRYFPDPEEFRPERFFPENSQGRHPYAYVPFSAGPRNCIGQKFAVMEEKTI

LACILRQFWVESNQKREELGLAGDLILRPNNGIWIKLKRRHEDDP

>AAD40966.1

MNAVAFHVLHDVLPALLASFLLYSVAAAAAAFACLAGLLYYIKYKQRETLVYMGKFPGRT

EPIPLVTTWSIHRALSNEAHRLDISTHLFQVAFGFSRIYKNHGMFRFYDATHPVLVLFKA

EAVEELLTSNTVLKKGHEYELIKPWLGTGLLTSSGSKWRSRRKLLTPAFHFRILEDFLPA

VNDQSNILVRKLGQLGKDRSCNIVPLVTLCALDIICETIMGYTINAQSNQDSEYVQAIKV

LGHSFTRRLETPLYWIDAIFQLSKPGREFARKVKELHQFTLKVIRERKREILSCPELQES

YEETASESDVYGLKGTARKPFLDILLREHIKDPENFTEEHVREEVDTFMFEGHDTTAMGM

SWAIYLIALHTEHQDLIHQELDTIFGSDKTRPVTSEDLKQMKYMECCLKESQRLYPSVPF

ISRKCEEDAVICGRKVPKGTDIQVAIYNLHHDEQVFPKPDEFRPDRFFPENVRGRHAFAF

VPFSAGPRNCIGQRFAMMEEKVVIANILRNYKLVALHYRDKIHVMAELVLRPTSGLHVIF

IPRVSAKQQ

>Q8N118.1

MEFSWLETRWARPFYLAFVFCLALGLLQAIKLYLRRQRLLRDLRPFPAPPTHWFLGHQKF

IQDDNMEKLEEIIEKYPRAFPFWIGPFQAFFCIYDPDYAKTLLSRTDPKSQYLQKFSPPL

LGKGLAALDGPKWFQHRRLLTPGFHFNILKAYIEVMAHSVKMMLDKWEKICSTQDTSVEV

YEHINSMSLDIIMKCAFSKETNCQTNSTHDPYAKAIFELSKIIFHRLYSLLYHSDIIFKL

SPQGYRFQKLSRVLNQYTDTIIQERKKSLQAGVKQDNTPKRKYQDFLDIVLSAKDESGSS

FSDIDVHSEVSTFLLAGHDTLAASISWILYCLALNPEHQERCREEVRGILGDGSSITWDQ

LGEMSYTTMCIKETCRLIPAVPSISRDLSKPLTFPDGCTLPAGITVVLSIWGLHHNPAVW

KNPKVFDPLRFSQENSDQRHPYAYLPFSAGSRNCIGQEFAMIELKVTIALILLHFRVTPD

PTRPLTFPNHFILKPKNGMYLHLKKLSEC

>154146258

MSISALSPIRFLECVSGFLQVIFLIGLLLLLFKAAQHYLRRQWLLRALYQFPSPPSHWLF

GHCLQFKVDEELRWILRYLEKFPGAFLHWIWGSHVFIKVCDPDYMKVILGRADPKASLYR

FLTPWLGHGLFFLNGDEWFQHRRLLTPAFHYDILKPYVGIMADSVQVMLEKWEQIACQDI

TLEIFHPISLMMLDTIMKYAFSYQGSVQLDRSSQAYLQAVSDLNNLVTSRMKNVFLHNDI

IYNLTSHSRRTNSACQIAHEHTDRVVKLRKAQLQDNKSMKKLRGKRCLDFLDILLLSRMD

DGSSLSDKALRAEVDSLMFGGHDTPASGISWVFYALATHPDHQQRCREEVQSLLGDGSPI

TWDHLHQMPYTTMCIKEALRLYPPIPSVGRKLSTPVTFPDGRSLPKGITVLLHFYALHHN

PKVWPNPEVFDPSRFAMNSVQHSHAFLPFSGGSRNCIGKHLAMNVLKVAVALTLLRFELL

PDPSRVPIPTQQLVLKSKNGIYLHLRKLQ

>Q9VMS9.1

MWIALLGIPILLAVLTLLLKHINKTYFILSLTKRVRTEDGSPLESKVAIMPGKTRFGNNL

DILNFTPASVFNFVRESTAKAKGQNYLWYFLYAPMYNVVRPEEAEEVFQSTKLITKNVVY

ELIRPFLGDGLLISTDHKWHSRRKALTPAFHFNVLQSFLGIFKEECKKFLNVLEKNLDAE

LELNQVIPPFTLNNICETALGVKLDDMSEGNEYRKAIHAIEEVLIQRVCNPLMYYNWYFF

VYGDYRKHLQNLRIVHDFSSRIIERKRQQFQQKQLGEVDEFGRKQRYAMLDTLLAAEADG

QIDHQGICDEVNTFMFEGYDTTSTCLIFTLLMLALHEDVQKKCYEEVENLPEDSDDISMF

QFNKLVYLECVIKESLRMFPSVPFIGRQCVEETVVNGMVMPKDTQISIHIYDIMRDPRHF

PKPDLFQPDRFLPENTVNRHPFAYVPFSAGQRNCIGQKFAILEMKVLLAAVIRNFKLLPA

TQLEDLTFENGIVLRTQENIKVKLSKRVK

>Q9V4T3.1

MFLIAIAIILATILVFKGVRIFNYIDHMAGIMEMIPGPTPYPFVGNLFQFGLKPAEYPKK

VLQYCRKYDFQGFRSLVFLQYHMMLSDPAEIQNILSSSSLLYKEHLYSFLRPWLGDGLLT

SSGARWLKHQKLYAPAFERSAIEGYLRVVHRTGGQFVQKLDVLSDTQEVFDAQELVAKCT

LDIVCENATGQDSSSLNGETSDLHGAIKDLCDVVQERTFSIVKRFDALFRLTSYYMKQRR

ALSLLRSELNRIISQRRHQLAAENTCQQGQPINKPFLDVLLTAKLDGKVLKEREIIEEVS

TFIFTGHDPIAAAISFTLYTLSRHSEIQQKAAEEQRRIFGENFAGEADLARLDQMHYLEL

IIRETLRLYPSVPLIARTNRNPIDINGTKVAKCTTVIMCLIAMGYNEKYFDDPCTFRPER

FENPTGNVGIEAFKSVPFSAGPRRCIAEKFAMYQMKALLSQLLRRFEILPAVDGLPPGIN

DHSREDCVPQSEYDPVLNIRVTLKSENGIQIRLRKR

>ADC44459.2

MLFCAILLLFFILALLNYVTKQRIRRGVLRNMPGPYCYPFIGAVQLYLQLSMNNMLDHMA

MFHEQHGSIIAAWVANRVGISSMDLELNEQILASPQHIAKHFNYTILRQWLGTGLLLSDG

RKWFVRRKIITPAFHFKILEQFVEVFDQQSTILLSCLAKKADGRNVFDVYPFVCLAALDI

IAETAMGTKMGAQTDARSEYALAVNKTTKIIAWRFIKFHLNNEILFSILHPYLKWQQMRH

IKTLHEFTTNMIKQRREALERNRNVAPLTGDLTEDSHCVGNKKRMALLDMLLQSTIDDQP

LSDDDIREEVDTCMFEGHDTITSGICFTLYLLSRHPEAQQKVLQEVAAVLGNDRKQAISL

RELSELKYVECVIKETLRLYPSVPLVGRQLTEDFKYTHSQIGGGIIPAGAAILISLYGMF

RQARYFERPTDFVPDRHLNNGSASAFLFIPFSSGPRNCIGQKFAMLEMKMITAKTVREYE

LLPFGEPVQIESNIVMRSTTGFQLGMRKRE

>EEB10123.1

MINPWVLGIIIIAIYAVNYFKNWDYYKALRKFPGPAALPILGNSLDFATNSHGIFLKLVD

FGQKYGRFYRVWNNGKAYVICNDEKDAEIILSSNEHLHKNEAYKFFIPWIGEGLISATDI

NKWKMRRKIITPTFHFKILNEFFEVFVRNAEILCHQLRLKSGKGSLNICKFIKLMALDNI

CETAMGVDIKAQENSENTYVKAVQDITSVISLRMFKVWLMIDSLFNLSSESKVQAQALKI

LHGTTDEVIKNAREKFKEKNKNDNNNINNLDETFDEKRKKAFLHLLLASEEGSQLSDKAI

REEVDTFMFAGHDTISSGLSFAIYSLAENPDVQELAYKEQMDIFDGSTRKPTYNDVQEMK

YLERVLKEVQRVYPSIPIIGRNIKKDLQLQGNYIVPKGTQLCINIYSLHHNPNIWPNPEK

FNPDNFLPEAIQSRSPYAFIPFSAGPRNCIGQKYAMLVMKVTLSTLLRQFKILPDPHSRE

KPMLAGEIVLLSTNGLNVCVEPRF

>195581531

MFLIAIAIILATILVFKGVRIFNYIDHMAGIMEMIPGPTPYPFVGNLFQFGLKPAEYPKK

VLQYCRKYDFQGFRSLVFLQYHMMLSDPAEIQNILSSSSLLYKEHLYSFLRPWLGDGLLT

SSGARWLKHQKLYVPAFERSAIEGYLRVVHRTGGQFVQKLDGLSDTQEVFDAQELVAKCT

LDIVCENATGQDSSSLNGEPSDLHGAIKDLCDVVQERTFSIVKRFDALFRLTSYYMKQRR

ALSLLRSELNRIISQRRHQLAAENTCQQGQPINKPFLDVXXXXXXXXXXXXXXXXXXXXX

XXXXXXXXXXXXXXXXXXXXXXXXXXXXXXXXXXXXXXXXXXXXXXXXXXXXXXXXXLEL

VIRETLRLYPSVPLIARTNRKPIDINGTKVAKRTTVIMCLIAMGYNEKYFDDPCTFRPER

FENPTGNVGIEAFKSVPFSAGPRRCIG

>ADD19990.1

MLLFFVVLLIILILLLRKVNENYFVLALCKRIKCVDGKPLEEKVCVIPGKTIFGNNFDVL

NLTPGKLFELTRGWAKFSRERSYVLHTLIGSIYSIINAEDAEQVFLSQTLTTKGITYDLL

QPFVGEGLLRSSEEKWHLRRKMLTPTFHFNILQTFNEIFKEESLKLLEKLKHLQHDSVNI

SDVIEEFALNNICETALGVKLNDIDGASEYRTALHNVGTIIAKRACNPLMFYRIIFYLFG

PYSENKHSIEITHNFSGKIISKRREEFLKDKGCNIDKENSYVKTRYAMLDTLLHQEAQGL

IDHQGICEEVDTFMFGAYETTSKCLEFVLLNLAEYPDIQEKCYQEIENLTTFEILTVFDF

NNLEYLGCVIKETLRMYPSAPLITRRCTKETVLNGLILPTNTQINIYISDIMRDSKHFPE

PSVFKPERFLAENTRNMHPFAFVPFSAGSRNCIGQKFAMLEIKAALVAILKTYRVLPVMQ

AKDLTLEYGITLKTKQKLFVKLEPRVI

>ACI25368.2

MIFYVIVTTILLLLYIKYRLTIRHLVVVDTMFPGPTCLPIIGNALMLRGTSEDFYKRFTE

TCFYYRRSFRVWIAHRLFIACLYPEDAEVILGNNFEITKADVYEFLLPWIGDGLITSTGK

KWRTHRKMLTPAFHFRILEEFTKVFNKNGKIFCEVLSKIPENKIFDVYEYVKMYAMDNIC

ETAMSVSINAQKNPNSAYIRAVKDMCTVAFKRMRTLWLRSDFLFRMSPWAKVHDDALSLI

HSQTRDVIKKRKQELERNPDLRLTMENDNFSIKGKLAFLDILLHAAQEYRLTDEDIREEV

DTFMFEGHDTITSAISFAMYYISRDQEVQKKILQETESIFRGEDREPTVKDFGAMKYLET

VIKETLRLHPSVPFIARKISQDFQIDMKAYAPRGAEVLVVIAALHRNPYQWEKWDQFYPE

HFLPEATQKRHPYSFVPFSAGPRNCIGQKFAMIEMKSVLSKVVKEFELIPSPHPEHAIRE

VPDLILTSGTGMHVGLRKRISGAA

>ACI25370.2

MVLHGLFVTFVCATLIYVLERIIHRRVYKAEKQFDGPPGYWFFGNSFDFINKSSKEIFDQ

VVENLKTYGNGRFYKIRIMHRLGIACSDHRDMEVILGSNTLIDKGVHYHFILPWIGFGPL

TSNGSRWRKHRKIITPTFHFKILEQFIDVFNKNGAIMCEKMEKEVNGEPFNVYNYVNLAA

LDNIFESAMGINMNVQKNSETTYVRAIKEMCNVVNKRVFWWILRIYPIFRLSKYYKMQTD

ALNVLHSTTVNIIEQRKKELAVRKKLEKDDEKDEEIEFYQKKRMAFLDLLLESAEASNIS

DEDIRQEVDTFMFEGHDTISSGLSFALWALANNPDVQQKVYEEQMEIFGDSNRPPTFNDL

QNMKYLERTLKESQRLFPSVPMITRKLNEDVDLPGGYHLPKGTNVGMIIYSLHRDPKVWP

NPEKFDPDNFTPDAIQGRNPYSYVPFSAGPRNCIGQKFAMLEMKSTVSKVVRQYKLLPSP

YEKHKLQLTSELVLMSLSGVHVKIQRRSNSFS

>CAX94852.1

MFVFVTEPQTIVLPASIVSEPASAAEDDKCFVRFVTKMLLELLGVCLLLLIVLIVHFHEW

PSLRTMRVMKRLPGPPEAFFFGHSPTLAKIKFEDLLVFFGQLIREYPPVFKIWTLGIPIV

VLTEPEDVEVLLSSVQYIKKGIDYDAFLDWLNEGLLVSTGSKWQYRRKLLTPAFHFKILE

HCIPIFNTNGALLCNELLETKNEEIEIEPFISKCTLDIICEAAMGCSLRNLPDKGEQYVR

ATKKMNNLIIERILTPWLAKDWLFHLSPTGREQKKIVDILHSFTQMIIEERKTQWRKSSK

EDLEDDEYGRHRPKTFLDCLIELSEKDSESLTLTDIREEVDTFMFEGHDTTSAAITATIF

MIGHHPEVQERIHEELDDVFGGSEREITMDDLHYLTYLERVIKESLRLFPSVPMMTRRLQ

TDLQLNSSQHMVPSTANVIVFSYWLHRNPKHFPEPDLFNPDRFLPDEVPRRHPFAYIPFS

GGPRNCIGQKFAMMEMKIVLATVMRKVRMESITKMEAIKLIPAVILRPQKPFKIKVSPRT

K

>ADY45110.1

MATLVQLLFLLVFTFFCYVRFWPYIVELRRRRKIAANIAGPTTSPLLGNALQFSRNPFEF

LEHLLRLANEAVQSGEHLLRLWVADKLVVFPLDGEALKPIIESNTELVKGRDYDFFRPWL

GDGLLTSSGNKWKSRRKMLTPAFHFKMLQEFVSVFDTESKILVEQLDHFANTDCEVDILP

FIKRCALDIICSTAMGVKVNAQIKHDSPYVIAVEKVTLLGFEYSITPFYWLQPVWYASGK

AQETKNAVEVLKSFTEKVIKERRANYSRVKKVDLHDKKKAAFLDMLLEMQYDNKLSDEDI

REEVDTFMFAGHDTTSTGIGWTLWCLATHPDVQEKAIEEVDSIFGDGEMRITIDSLQQLK

YVERCIKEAMRLFAPVPHVQRQLKNDILMGGKIVPRGTNCIISPILVHRNLKVFPNANDF

DVDNFLPERIAQRHPYSYIPFSAGPRNCIGQKFALLEEKTVIVSILRAFTMKCNLKLAEN

RCGAQVVLRPEQGFPIIFERRQTYP

>391332629

MCGLAEIYDSARIFRFWLGARPVVAFFKPETVEVLLNSNKHIDKSFDYTLLHPWLGTGLL

TSCGRKWRPRRKMLTPAFHFRILEDFVPIFNEQAQIFASLLEKHVNRNPVDIVPFITNCT

LDIICETAMGVKIGAQSNSESHYVRALYEVGKTFIYRVVRPWLWIPLVFLMTTKGRRFED

NLRDLHNFTRKVIQERKEEIQSETWLEGKEGLKETADIPDASHIGAKKRKAFLDLLLTEH

LQKNSLSLEDIREEVDTFMFEGHDTTAMGIAWSLYLIALHQDVQDKIHDELDFIFGEDRE

RDLTTDDLKNMKYLECAIKEAQRLFPSVPFIGRELKEDVVVNGFTVPRGTTCFVFTYMLH

RDKRTFPNPEAFIPERFLPENSIGRHPFSYVPFSAGPRNCIGQKFALMEEKLVCATILRR

YQLQATHHRDHIRLKPELVIRPEEGLRVRVRLRPPFTGRSICSSQLHRPYREPFYCDDAY

YGGAQHSI

>CBL51707.1

MVLVQVLCAIAVAIFAYAVHWYMKIKSRMHHWKKLPSKSPSFLFGNLRQVIKVYTRKNKY

HWGVYILERMMGQGLLYQKEGLYAFWVGPTPVIPVSCPVLMEKVLGSSVSIEKSFEYDFF

HKWLGLGLLTSTGTKWRNRRKLLTPCFHFRILEDFLPAFDDQSRILVPKIEQRRGDDHVH

VMPLVTLCTLDIVCETVMGYRIGAQTGQNLDYTTAIQNLGDFFNQRTIRPWLFSDFLFDL

SQIGRGFNKDLRTIHSFTRNVIKQKKDAFKRQESEQSFPESVKEANEDGRRRRQALMDLL

LNMFFNGGQLTEEDIQEEVETFMFAGHETTAAGVTYALYCIGLYQDIQERLHEELEAIFQ

GDVARAVTMDDVREMKYMECVLKESQRIYPSVPMVGRKTAEDIEHNGFTIPSGSEVHLNF

MCLHRHPDSFPNPEVFDPDRFLPENVLKRHPYAYVPFSAGPRNCIGQKFALLEMKVIVAN

ILRKFCVVSLDPRDKVFVKVEFTLKPAEPMRLKFLPRTPVCC

>EDW39228.1

MSSKVISSLMAESILLTKMAQVISGYSPITIFLLASIALFLVVYNKRRSRLVKHIEKIPG

PAAMPFLGNAIEMNVDHDELFNRVIGMQKLWGTRLGINRVWQGTSPRVLLFEPETVEPIL

NSQKFVNKSHDYDYLHPWLGTGLLTSTDRKWHSRRKILTPAFHFKILDDFVDVFNEQSAV

LARKLAVEVGSEAFNLFPYVTLCTLDIVCETAMGRRIYAQSDSESEYVKAVYGIGSIVQS

RQSKIWLQSDLIFRLTDDYKLHQSYINTLHGFSNMVIRERKSELALLQDNNNNNNNTPDA

YDDVGKKKRLAFLDLLIDASKDGTVLTNEDIREEVDTFMFEGHDTTSAAISWTLFLLGCH

PEYQEQVAEELDSIFGNDKETPATMKNLMDMRYLECCIKDSLRLFPSVPMMARMVGEDVN

IGGKLVPAGTQAIIMTYALHRNPRVFPKPEQFNPDNFLPENCAGRHPFAYIPFSAGPRNC

IGQKFAILEEKAVISTVLRKYKIEAVDRREDLTLLGELILRPKDGLRVKITPRD

>EDW67623.1

MSSKVFSSLMAESILLSKAAQIISGYSPITIFLLATISIFLVVYNKRRARWVKYIEKIPG

PAALPFLGNAIEMNVDHDELFNRVIGMQKLWGTRIGINRVWQGTSPRVLLFEPETVEPIL

NSQKFVNKSHDYDYLHPWLGTGLLTSTDRKWHSRRKILTPAFHFKILDDFIDVFNEQSAV

LARKLAVELGTEAFNIFPYVTLCTLDIVCETAMGRKIYAQSNSESEYVKAVYGIGSIVQS

RQAKIWLQSDLLFRLTEDYKTHQRYINTLHGFSNMVIRERKSELANNNNNNNNNNNETPD

AYDDLGKKKRLAFLDLLIDASKEGTVLSNEDIREEVDTFMFEGHDTTSAAISWTLFLLGA

NPEYQERVVEELESIFGDDTETPATMKNLLDMRYLECCIKDALRLFPSVPMMARMVGEDV

TIGGKLVPAGTQAIIMTYALHRNPRVFPKPEQFNPDNFLPENCAGRHPFAYIPFSAGPRN

CIGQKFAILEEKAVISTVLRKYKIEAVDRREDLTLLGELILRPKDGLRVKITPRH

>EAL26721.1

MSSKVISSLMAESILLTKMAQVISGYSPITIFLLASIALFLVVYNKRRSRLVKHIEKIPG

PAAMPFLGNAIEMNVDHDELFNRVIGMQKLWGTRLGINRVWQGTSPRVLLFEPETVEPIL

NSQKFVNKSHDYDYLHPWLGTGLLTSTDRKWHSRRKILTPAFHFKILDDFVDVFNEQSAV

LARKLAVEVGSEAFNLFPYVTLCTLDIVCETAMGRRIYAQSDSESEYVKAVYGIGSIVQS

RQSKIWLQSDLIFRLTADYKLHQSYINTLHGFSNMVIRERKSELALLQDNNNNNNNTPDA

YDDVGKKKRLAFLDLLIDASKDGTVLTNEDIREEVDTFMFEGHDTTSAAISWTLFLLGCH

PEYQEQVAEELDSIFGNDKETPATMKNLMDMRYLECCIKDSLRLFPSVPMMARMVGEDVN

IGGKLVPAGTQAIIMTYALHRNPRVFPKPEQFNPDNFLPENCAGRHPFAYIPFSAGPRNC

IGQKFAILEEKAVISTVLRKYKIEAVDRREDLTLLGELILRPKDGLRVKITPRD

>391327448

MGVKVGAQRGTESQYVRDVYAVGKFFLERLVRPWLYLDSLYLLTEAGRVFDQHVRGIHAF

TKGVIRERKKQKIRENTHGTSGGKKRLAFLDLLLEEHFANPSGLPEHDIREEVDTFMFEG

HDTTAMALSWTIFLLGHHPEIQRRCQDELDQIFGSEKRQPDMEDLKNMKYLECCIKEALR

LFPSVPIVGREVHTTFNLNKYQVPEGSVVLVFAYQLHRNKESFPKPEEFIPDRFFPENCN

GRHPFAYVPFSAGPRNCIGQRFALMEEKVVLSSLLRHYTVKSLVGFDSLELELSLVEKIL

VIYCGLFVRWASAVVYRHFRMWKALRPMPGPFDRGLSGFIPPAYNIFCLILQKADSEYWD

TSTGIFRLLTGLSAWYHKEKVYKVFLGFQPTVIVFSPEGVEAVLNHTTNLKKPILYSLLN

TWLGTGLLTSCGEKWRARRKMLTPAFHFRILEDFLPVMNEQADVFVANLQTKIDADFDIV

PEITKCTLDIICETAMGVKVGAQSGTESQYVTDVYAVGKFFLERLVRPWLYLDYLYLLTE

AGRTFRRHVKGIHAFTEGVIKERKQQAVAERKLDPQYRGKRLAFLDLLLEQHFANPKSLP

ESDIREEVDTFMFEGHDTTAMALSWTIFMMGLHPDVQRRCQDEQDRIFGSDERQPSMADL

RSMKYLDCCIKEALRLFPSVPVVGREVHAAFRLNGYEIPAGTVVLVFSYQLHRDKQSFPK

PEEFIPERFLPENSNGRHPFAYVPFSAGPRNCIGQRFALMEEKVVLSRFLRNFSVKSMVG

LESIELSAEMVLRSKTGLPVKISRRTPSWV

>AAI25941.1

MGILFGLYILGILFTAVLLLLLASTAYNPLKNYIGKWNEMRPIPGMAGAYPIIGNALQFK

TNAGDFFNQIIEGTNENRHLPLAKVWVGPVPFLILYHAENIEVVLSNSRHLDKSYSYRFL

HPWLGTGLLTSTGEKWRNRRKMLTPTFHFSILSDFLEVMNEQTDILIQKMQKLEDGEPFN

CFNFITLCALDIICETAMGKKIYAQSNADSEYVQSVYKMSDIITKRQRAPWLWPDWIYNK

LKEGKEHAKRLKILHSFTANVIRERAEFMSSEPDSDSDQGERKRQAFLDMLLKTTYENGQ

KLSHEDIQEEVDTFMFEGHDTTAASMNWALHLIGSHPEVQKAVQAELQEVFGSSERHVGV

EDLKKLRYLECVIKESLRIFPSVPLFARSICEACHINGFKVPKGVNAVIIPYALHRDPRY

FPEPEEFQPERFMPENSKGRHPYAYIPFSAGPRNCIGQRFAMMEEKVVLATILRHFDVEA

CQSREELRPLGELILRPEKGIWIKLQRRSK

>348524528

MAVLIGSYSTTLLGVSIFVAFLTYLTYKLLSDYLHKWFEMKPIPELGTTYPFIGNALLFK

NNAGDFFRQIQDFTWEFRDKPLFKLWVGPVPFVVLYHAETVEPVLANSIHMEKAFAYKFL

HPWLGTGLLTSTGTKWRQRRKMLTPTFHFSILEDFLEVMNEQAEILVEKLEQHAGKGPFN

CFSLITLCALDIICETAMGKKIYAQSNSDSEYVRSVYRMSDIVSRRQRSPWLWPDFIYTY

FSEGRDHDKTLKVLHSFTYKVIHERAENMSCNESDSENDQGKRKRRAFLDMLLSTTYEDG

SKMSHEDIQEEVDTFMFRGHDTTAAAMNWALHLLGSHPEVHKKVQQELQEVFGTSSRPTS

TDDLKKLKYLECVIKEALRLFPSVPFFARSLGEDCYINGFKVPKGANAIIITYSLHRDPR

YFPEPEEFRPERFLPENSVGRPAYAYVPFSAGLRNCIGQRFALMEEKVVLAAILRNFTVE

ACQKREDLRPVGELILRPEKGIVIKLEKKTPQTSSN

>AAI25969.1

MVAFVGVYTYGFVAASVCVICFAYITYQLLKSYLHKWKELKPIPGIGNTFPFIGNALQFK

SNGDFFLQLVGYTTEFQNSPLLKIWIGPIPFLILFHAETVETVLNNPVHIDKAYAYQFLH

PWLGTGLLTSTGDKWRRRRKMLTPTFHFSILTEFLEVMNEQAEVLIEKLEKQAGKGPFNC

FSHITLCALDIICETAMGKRIYAQSNYDSEYVRTVYRMSDIITRRQRMPWYWPDFVYNYV

GEGREHNRSLKILHSFTESVINERAEYLSYVESDSESDQGMRKRRAFLDMLLKTKDEDGK

MLTHKDIQEEVDTFMFEGHDTTAAAMNWAIHLLGSHPEVQRKAQQELFEVFGESERPVNT

EDLKKLRYLECVIKESLRLFPSVPFFARTICDDTQINGFKVPKGTNIVVITYALHRDPRF

FPDPEEFRPERFLPENCVGRHPYAYIPFSAGLRNCIGQRFAIMEEKVILAYILRYFNIVA

CQKREELRPLGELVLRPEQGIWITLERRKLTMS

>301776066

MLALWLGHLGQKLLLWGALSAVSVAGATLILNLLQMLASYAHKWQQMRALPTLPGAYPLV

GHSLVMKPDGREFFQQLICYSEENRHQPLLKLWLGPIPIVAIYNAENVEVILTSSRQIDK

SYVYKFLEPWLGLGLLTSTGNKWRSRRKMLIPTFHFTILEDFLDVMNEHANILVNKLEKH

VNQEAFNCFFYITLCALDIICETAMGKNIGAQSNDDSEYVRAVYRMSDMIHRRMKMPWLW

LDFLFLMFKEGREHKKSLEILHNFTNNVITERTSEMKKDKERRSADKDSSPSKNTRRAFL

DLLLNVTDDEGNKLSHEDIREEVDTFMFEGHDTTAAAINWSLYLLGSYPEVQEQVHSELE

EVFGKSDRAATLEDLKKLKYLECVIKESLRIFPSVPLFARNLNEDCEVGGYKIVKGSQAI

IIPYALHRDPRYFPDPEEFQPERFFPENLQGRHPYAYVPFSAGPRNCIGQKFAIMEEKTI

LSCILRHFWVESNQKREELGLAGELILRPANGIWIKLKRRNADES

>395839911

MAWLWLWLGGQKLLLWGAAIAVSMAGATLALSVLQMVASYARKWHQMRPIPTVARAYPLV

GHALMMKRDGREFFQQLSQYTEEYRHMPLLKLWVGPVPMLALYNAENVEVILTSSKQIDK

SSMYKFLEPWLGLGLLTSTGNKWRYRRKMLTPTFHFTILEDFLDVMNEQANILVSKLDSH

VNQEAFNCFFYITLCALDIICETAMGKNIGAQSNEDSEYVRAVYRMSDLIFQRMKMPWLW

LESWYLMFKEGREHKKGLKILHTFTNNVIAERASEMRTNEEYNEEPSKNKRRAFLDLLLS

VTDEEGNKLSHEAIREEVDTFMFEGHDTTAAAITWALYLLGCNPEVQKKVDNELDEVFGK

SDRPATSEDLKKLKYLECVIKETLRLFPSVPLFARTLNEDCTVAGYKVLKGTEVIIIPYA

LHRDPRYFPNPEEFQPERFFPENAQGRHPYAYVPFSAGPRNCIGQKFAIMEEKTILSCVL

RHFWVESNQKREELGLAGELILRPCNGIWIKLKRRNADDS

>EFV50316.1

MGAVAVAVVATGIITALICWMLKLRKLTALVDRFPGPRYFPILGNIPYFKKDPTEFFEQT

IGTAYAYNNPRIFRVWMFWYPFFVVSGAEEAEVLLSSTKHINKGMEYRMLHPWMGLGLLT

SDAFKWRPRRKLLTPSFHYDILKDFIEVFNQQSKIMLKKLSDNLDISNEGFNMYQYITLC

ALDIICETAMGRHVNAQKNSDSDYVRAIYKLNRIIHERQKKPHLWPNIIFRLFGEGKEQE

ECLKILHSFTWKVIQERREEVKQLGGWAKVLDRQMTDFEMTSQKRRLAFLDLLLQIAEQG

KLTDQDIREEVDTFMFEVNQKKLQIQFDDDRDATFEDVKNMTYLECCLKEALRLFPSVPL

FARFINEDFDIGGLTIPSGSEVIVSPYGVHRDPRHWPDPEIFDPDRFLPKNANGRHPFAY

LPFSAGSRNCIGQRFALMEEKVVVSWILRYFEVTSVQRRDQIFPKAELIIRPTETILIKL

KRRQPLSFA

>327273760

MLSPPPIEPASIGPLQPLGPAPSREAPPPPPQGFHFLQRRVRPERSRGFGLSSGGGMSSS

PSWPEGGRILAWGAGAGALLAGLLALAFSRFLLDAFRRWKVLKPVPGINPCYPLLGNALL

FEKQGEAFFQQLMKYSEQFRNIPLYKLWVGPIPFLILYHADTVEVILSSSKYIEKPYLYK

FLHPWLGTGLLTSTGDKWRSRRKMLTPTFHFAILLDFLDVMNEQASILVQKLEKHVDKEP

FDCFLYITLCALDIICETAMGKNIGAQNNKDSEYVRAVYRMSDLIHHRQKSPWLWSDFIY

SICQEGRDHSKNLKILHTFTDSVIAEKAQQLKNQEHKSDVIINGEQSKTKGRRAFLDMLL

STTDENGKKMSYLDIREEVDTFMFEGHDTTAAAMNWVIYLLGSYPEVQRNVHKELDEVFG

DSDRPITMDDLKQLRYLESVIKESLRLFPSVPLFARTFTEEVHIKGFKIPQGTDCIIVPY

ALHRDPDVFPEPEEFRPERFFPENSNGRNPYAYVPFSAGPRNCIGQRFAQIEEKTILATI

LRRFQIETKQKRDELYPVGELILRPNRGIWIQLKRRSSSNF

>402871001

MAGIWLGLVWQKLLLWGAASAVSLAGASLVLSLLQRVASYVRKWQQMRPIPTVARAYPLV

GHALLMKRDGREFFQQIIEYTEEYRHMPLLKLWVGPVPMVALYNAEKVEVILTSSKQIDK

SSMYKFLEPWLGLGLLTSTGNKWRSRRKMLTPTFHFTILEDFLDIMNEQANILVKKLEKH

VNQEAFNCFVYITLCALDIICETAMGKNIGAQSNDDSEYVRAVYRMSEMIFRRIKMPWLW

LDLWYLMFKEGWEHKKSLKILHAFTNNVIAERANEMNADEDRKGDGRDSAPSKNKRRAFL

DLLLNVTDDEGNRLSHEDIREEVDTFMFEGHDTTAAAMNWSLYLLGSNPEVQKKVDHELD

DVFGRSDRPATVEDLKKLRYLECVIKETLRLFPSVPLFARSVSEDCEVAGYRVLKGTEAV

IIPYALHRDPRYFPNPEEFQPERFFPENAQGRHPYAYVPFSAGPRNCIGQKFAVMEEKTI

LSCILRHFWIESNQKREELGLEGQLILRPTNGIWIKLKRRNADEP

>EAX04624.1

MAGLWLGLVWQKLLLWGAASAVSLAGASLVLSLLQRVASYARKWQQMRPIPTVARAYPLV

GHALLMKPDGREFFQQIIEYTEEYRHMPLLKLWVGPVPMVALYNAENVEVILTSSKQIDK

SSMYKFLEPWLGLGLLTSTGNKWRSRRKMLTPTFHFTILEDFLDIMNEQANILVKKLEKH

INQEAFNCFFYITLCALDIICETAMGKNIGAQSNDDSEYVRAVYRMSEMIFRRIKMPWLW

LDLWYLMFKEGWEHKKSLKILHTFTNSVIAERANEMNANEDCRGDGRGSAPSKNKRRAFL

DLLLSVTDDEGNRLSHEDIREEVDTFMFEGHDTTAAAINWSLYLLGSNPEVQKKVDHELD

DVFGKSDRPATVEDLKKLRYLECVIKETLRLFPSVPLFARSVSEDCEVAGYRVLKGTEAV

IIPYALHRDPRYFPNPEEFQPERFFPENAQGRHPYAYVPFSAGPRNCIGQKFAVMEEKTI

LSCILRHFWIESNQKREELGLEGQLILRPSNGIWIKLKRRNADER

>Q6ZWL3.2

MAGLWLGLVWQKLLLWGAASALSLAGASLVLSLLQRVASYARKWQQMRPIPTVARAYPLV

GHALLMKPDGREFFQQIIEYTEEYRHMPLLKLWVGPVPMVALYNAENVEVILTSSKQIDK

SSMYKFLEPWLGLGLLTSTGNKWRSRRKMLTPTFHFTILEDFLDIMNEQANILVKKLEKH

INQEAFNCFFYITLCALDIICETAMGKNIGAQSNDDSEYVRAVYRMSEMIFRRIKMPWLW

LDLWYLMFKEGWEHKKSLQILHTFTNSVIAERANEMNANEDCRGDGRGSAPSKNKRRAFL

DLLLSVTDDEGNRLSHEDIREEVDTFMFEGHDTTAAAINWSLYLLGSNPEVQKKVDHELD

DVFGKSDRPATVEDLKKLRYLECVIKETLRLFPSVPLFARSVSEDCEVAGYRVLKGTEAV

IIPYALHRDPRYFPNPEEFQPERFFPENAQGRHPYAYVPFSAGPRNCIGQKFAVMEEKTI

LSCILRHFWIESNQKREELGLEGQLILRPSNGIWIKLKRRNADER

>AAH60857.1

MAGLWLGLVWQKLLLWGAASALSLAGASLVLSLLQRVASYARKWQQMRPIPTVARAYPLV

GHALLMKPDGREFFQQIIEYTEEYRHMPLLKLWVGPVPMVALYNAENVEVILTSSKQIDK

SSMYKFLEPWLGLGLLTSTGNKWRSRRKMLTPTFHFTILEDFLDIMNEQANILVKKLEKH

INQEAFNCFFYITLCALDIICETAMGKNIGAQSNDDSEYVRAVYRMSEMIFRRIKMPWLW

LDLWYLMFKEGWEHKKSLKILHTFTNSVIAERANEMNANEDCRGDGRGSAPSKNKRRAFL

DLLLSVTDDEGNRLSHEDIREEVDTFMFEGHDTTAAAINWSLYLLGSNPEVQKKVDHELD

DVFGKSDRPATVEDLKKLRYLECVIKETLRLFPSVPLFARSVSEDCEVAGYRVLKGTEAV

IIPYALHRDPRYFPNPEEFQPERFFPENAQGRHPYAYVPFSAGPRNCIGQKFAVMEEKTI

LSCILRHFWIESNQKREELGLEGQLILRPSNGIWIKLKRRNADER

>397506028

MAGLWLGLVWQKLLLWGAASAVSLAGASLVLSLLQRVATYARKWQQMRPIPTVARAYPLV

GHALLMKPDGREFFQQIIEYTEEYRHMPLLKLWVGPVPMVALYNAENVEVILTSSKQIDK

SSMYKFLEPWLGLGLLTSTGNKWRSRRKMLTPTFHFTILEDFLDIMNEQANILVKKLEKH

INQEAFNCFFYITLCALDIICETAMGKNIGAQSNDDSEYVRAVYRMSEMIFRRIKMPWLW

LDLWYLMFKEGWEHKKSLKILHTFTNSVIAERANEMNANEDCRGDGRGSAPSKNKRRAFL

DLLLSVTDDEGNRLSHEDIREEVDTFMFEGHDTTAAAINWSLYLLGSNPEVQKKVDHELD

DVFGKSDRPATVEDLKKLRYLECVIKETLRLFPSVPLFARSVSEDCEVAGYRVLKGTEAV

IIPYALHRDPRYFPNPEEFQPERFFPKNAQGRHPYAYVPFSAGPRNCIGQKFAVMEEKTI

LSCILRHFWIESNQKREELGLEGQLILRPSNGIWIKLKRRNADER

>AFE66738.1

MAGIWLGLVWQKLLLWGAASAVSLAGASLVLSLLQRVASYVRKWQQMRPIPTVARAYPLV

GHALLMKRDGREFFQQIIEYTEEYRHMPLLKLWVGPVPMVALYNAENVEVILTSSKQIDK

SSMYKFLEPWLGLGLLTSTGNKWRSRRKMLTPTFHFTILEDFLDIMNEQANILVKKLEKH

VNQEAFNCFVYITLCALDIICETAMGKNIGAQSNDDSEYVRAVYRMSEMIFRRIKMPWLW

LDLWYLMFKEGWEHKKSLKILHAFTNNVIAERANEMNVDEDCRGDGRDSAPSKNKRRAFL

DLLLSVTDDEGNRLSHEDIREEVDTFMFEGHDTTAAAMNWSLYLLGSNPEVQKKVDHELD

DVFGRSDRPATVEDLKKLRYLECVIKETLRLFPSVPLFARSVSEDCEVAGYRVLKGTEAV

IIPYALHRDPRYFPNPEEFRPERFFPENAQGRHPYAYVPFSAGPRNCIGQKFAVMEEKTI

LSCILRHFWIESNQKREELGLEGQLILRPTNGIWIKLKRRNADEP

>AAH74131.1

MEFKGDVQPLVWMAAAVVLLTLLAFSALPPLLDYFRKWKLMRPIPGPGPNYPLVGDALFL

KPNGGDFFLQMCEFTELFRVEPLIKLWIGPIPFVIVYHADTVEPILSTSKHMDKSYAYKF

LHPWLGQGLLTSTGEKWRSRRKMITPTFHFAILSEFLEVMNEQSKVCVEKLQKHVDGESF

NCFMDVTLCALDIISETAMGRKIQAQNNRDSEYVQAIYKMSDIIQRRQKMPWLWLDFIYA

HLKDGKEHNKNLKNLHAFTDKAILERAEELKKTEAKKGHFDSEPESEKPKKRSAFLDMLL

MATDDAGNKLSYKDIREEVDTFMFEGHDTTAAALNWSLFLLGSHPEAQRQVHKELDEVFG

KSDRPVTMDDLKKLRYLEAVIKEALRIYPSVPFFARTITEDCIIRGFHVPKGVNVVIIPY

ALHRDPEYFPEPEVFRPERFFSENASGRNPYAYIPFSAGLRNCIGQRFALMEEKVVLSSI

LRNYWVEATQKREELCLLGELILRPQDGMWIKLKNRKTASSA

>291386017

MWLWLGLVGQKLLFWGAASAVSLAGASLFLNLLQMVASYARKWQQMRPIPTIGRPYPLVG

HALYMKPSGKEFFQQLIQYTEEYRHLPLLKLWLGPLPIVALYNAENVEVILNSSKQINKS

SMYQFLEPWLGLGLLTSTGYKWRSRRKMLTPTFHFTILEDFLDIMNEQANILVHKLEKHV

DQEAFNCFFYITLCALDIICETAMGKNIGAQSNEDSEYVRAVYRMSDVIFRRMKMPWLWL

DLWYLMFKEGWEHKRCLKILHRFTNNVIAERVSEMKTDEEHRDADSNCAPSTMKRKAFLD

LLLTVTDEEGNKLSHEDIREEVDTFMFEGHDTTAAAINWSLYLLGSHPEVQRKVDDELDE

VFGKSDRPATSEDLKKLKYLECVIKETLRLFPSVPLFARSLSDDCEVAGFRVVKGTQAVI

VPYALHRDPKYFPNPEEFRPERFFPENAQGRHPYAYVPFSAGPRNCIGQKFAIMEEKTIL

SCILRKLWVESNQKMEELGLAGELILRPTNGIWIKLKRRNADKA

>BAE29740.1

MLWLWLGLSGQKLLLWGAASAVSLAGATILISIFPMLVSYARKWQQMRSIPSVARAYPLV

GHALYMKPNNAEFFQQLIYYTEEFRHLPIIKLWIGPVPLVALYKAENVEVILTSSKQIDK

SFLYKFLQPWLGLGLLTSTGSKWRTRRKMLTPTFHFTILENFLDVMNEQANILVNKLEKH

VNQEAFNCFFYITLCALDIICETAMGKNIGAQSNNDSEYVRTVYRMSDMIYRRMKMPWLW

FDLWYLVFKEGRDHKRGLKCLHTFTNNVIAERVKERKAEEDWTGAGRGPIPSKNKRKAFL

DLLLSVTDEEGNRLSQEDIREEVDTFMFEGHDTTAAAINWSLYLLGTNPEVQRKVDQELD

EVFGRSHRPVTLEDLKKLKYLDCVIKETLRVFPSVPLFARSLNEDCEVGGYKVTKGTEAI

IIPYALHRDPRYFPDPEEFRPERFFPENSQGRHPYAYVPFSAGPRNCIGQKFAVMEEKTI

LACILRQFWVESNQKREELGLAGDLILRPNNGIWIKLKRRHEDDP

>114597206

MAGLWLGLVWQKLLLWGAASAVSLAGASLVLSLLQRVATYARKWQQMRPIPTVARAYPLV

GHALLMKPDGREFFQQIIEYTEEYRHMPLLKLWVGPVPMVALYNAENVEVILTSSKQIDK

SSMYKFLEPWLGLGLLTSTGNKWRSRRKMLTPTFHFTILEDFLDIMNEQANTLVKKLEKH

INQEAFNCFFYITLCALDIICETAMGKNIGAQSNDDSEYVRAVYRMSEMIFRRIKMPWLW

LDLWYLMFKEGWEHKKSLKILHTFTNSVIAERANEMNANEDCRGDGRGSAPSKNKRRAFL

DLLLSVTDDEGNRLSHEDIREEVDTFMFEGHDTTAAAINWSLYLLGSNPEVQKKVDHELD

DVFGKSDRPATVEDLKKLRYLECVIKETLRLFPSVPLFARSVSEDCEVAGYRVLKGTEAV

IIPYALHRDPRYFPNPEEFQPERFFPKNAQGRHPYAYVPFSAGPRNCIGQKFAVMEEKTI

LSCILRHFWIESNQKREELGLEGQLILRPSNGIWIKLKRRNADER

>395542284

MGGDMWVVPVGQKLLYWGATGAVTLVGTILALTMLQMLVAYVRKWQQMKAIPTVATPYPL

LGHSLLMKSDSQEFFEQVNYYSEEYRHEPLLKLWLGPVPMVALYHADNVEVLLSSSKQID

KSYMYKFLEPWLGLGLLTSTGNKWRSRRKMLTPTFHFMILEEFLDIMNEQANVLISKLEK

HVDQEAFNCFLDITLCALDIICETAMGKNIGAQANGDSEYVRSVFRMTDIILRRIKMPWL

WLDVWYLLFKEGWEHRRSLKILHDFTDKVIEERAREKKENEAQKNGDVKAKNIKRQAFLD

LLLNATDEDGSTLSHQDIREEVDTFMFEGHDTTATAMNWIIYLLGSYPEAQRKVHNELDE

VFGKSDRPATMDDLKKLKYLDCVIKESLRLFPSVPFFARTLSSDCIMAGYKVPKGTEALV

LPYALHRDPKHFPDPEDFQPERFFPENSHGRHPYAYVPFSAGPRNCIGQKFAIMEEKTLI

SSVLRRFQVESIQKREELGLMGEMILRPKNGIWIKLKTRT

>403285089

MAGLWLGLGGQELLLWGAASVASLAGASLVLNLLQWAASYAWKWRQMRPIPTVARAVPLL

GHALLMKRDAREFFQQIIQYTEEYRHMPLLKLWVGPVPMVALYNAENVEVILTSSKQIDK

SSMYKFLEPWLGLGLLTSTGNKWRSRRKMLTPTFHFTILEDFLDIMNEQANILVKKLEKH

VNQEAFNCFFYITLCALDIICETAMGKNIGAQSNDDSEYVRAVYRMSEMIFQRIKMPWLW

LDLWYLMFKEGWEHKRSLKILHTFTNDVIAERANEITAGGECRGDGRDSAPSRNKRRAFL

DLLLSVTDDEGNRLSHEDIREEVDTFMFEGHDTTAAAINWSLHLLGSNPEVQKKVDNELD

DVFGKSVRPATVEDLKKLRYLECVIKETLRLFPSVPLFARSLSEDCEVAGYRVLKGTEAV

IIPYALHRDPRYFPNPEEFQPERFFPENAQGRHPYAYVPFSAGPRNCIGQKFAMMEEKTI

LSCILRHFWIESNQKREELGLEGQLILRPSNGIWIKLKRRNADEP

>BAF64512.1

MLALWLLSVGQKLLLWGGLCAVSLAGAILTLNLLRMAASYAWTWQRMRAVPTLEGAYPFL

GHALLLKPDARDFFQQMIQYTEEHRHLPLLKLWLGPIPVVFLYNAENVEVILTSSKHIDK

SYMYKFLEPWLGLGLLTSTGNKWRSRRKMLTPTFHFTILEDFLDVMNEQANILVNKLEKY

VNQEAFNCFSYITLCALDIICETAMGKNIGAQSNNDSEYVQAVYRMSDSIHQRMKMPWLW

LDLLFFIFKDGWEHKRSLKILHNFTKNVITERANEMKRHEEGRSNDKDFPPHNNKRRGFL

DLLLNVTDDQGNKLSYEEIREEVDTFMFEGHDTTAAAINLSLYLLGSYPEVQQKVDNELE

EVFGRSDRPATLDDLKKLKYLECVVKESLRLFPSVPFFARNLNEDCEVAGYKIVKGSQVI

IMPYALHRDQRYFPNPEEFKPERFFPENSKGRHSYAYVPFSAGPRNCIGQKFAMMEEKTI

LSCILRHFWVESNQKREELGLAGELILRPSNGIWIKLKRRNTNES

>AAI23987.1

MELGGEVHLLVWVAAAVVLLTLLALSILPALQDYVRKRRILKPIPGPGPNYPLIGDALFL

KNNGGDFFLQICEYTESYRLQPLLKVWIGTIPFIVVYHADTVEPVLSSSKHMDKAFLYKF

LHPWLGKGLLTSTGEKWRSRRKMITPTFHFAILSEFLEVMNEQSKILVEKLQTHVDGESF

DCFMDVTLCALDIISETAMGRKIQAQSNRDSEYVQAIYKMSDIIQRRQKMPWLWLDFLYA

HLRDGKEHDKNLKILHSFTDKAILERAEELKKMGEQKKEHCDSDPESDKPKKRSAFLDML

LMATDDAGNKMSYMDIREEVDTFMFEGHDTTAAALNWSLFLLGSHPEAQRQVHKELDEVF

GKSDRPVTMDDLKKLRYLEAVIKESLRIYPSVPLFGRTVTEDCSIRGFHVPKGVNVVIIP

YALHRDPEYFPEPEEFRPERFFPENASGRNPYAYIPFSAGLRNCIGQRFALMEEKVVLSS

ILRNYWVEASQKREELCLLGELILRPQDGMWIKLKNRETAPTA

>DAA14531.1

MLAPWLLSVGPKLLLWSGLCAVSLAGATLTLNLLKMVASYARKWRQMRPVPTIGDPYPLV

GHALMMKPDARDFFQQIIDFTEECRHLPLLKLWLGPVPLVALYNAETVEVILSSSKHIEK

SYMYKFLEPWLGLGLLTSTGNKWRSRRKMLTPTFHFTILEDFLDVMNEQANILVTKLEKH

VNQEAFNCFFYVTLCTLDIICETAMGKNIGAQRNDDSEYVRAVYRMSDSIHQRMKMPWLW

LDLIFYMFKNGREHRRSLKIVHDFTNNVITERANEMKRHEEGTSNDKEKDFPPRKTKCRA

FLDLLLNVTDDQGNKLSHEDIREEVDTFMFEGHDTTAAAINWSLYLLGWYPEVQQKVDTE

LEEVFGKSDRPVTLEDLKKLKYLDCVIKESLRLFPSVPFFARNLTEDCEVAGHKIVQGCQ

VIIVPYALHRDPKYFPDPEEFKPERFFPENLKGRHTYAYVPFSAGPRNCIGQKFAIMEEK

TILSCILRHFWVESNQKREELGLAGELILRPSNGIWIKLKRRNTDES

>390348048

MEYGVVLPIILLVISFVVTFLLAKAIRIIVLISRFQGPLALPLVGNAYAFKSDQREFFVD

IQSIFNEYRAKTGGIIRMWFGPAPVLVLYGAKYMEVILSSSQHVRKGFLYRLLEPWLGLG

LLTSTGQKWFHRRKMLTPTFHFSILQCFMDVFNEQSTILVKKLEKFADKSETINIFPLVT

NCVLDIICDTAMGRCTNAQEDGDNEYVQAVGRMGELVIARAKNPLTWPDYLFGKLNAGKE

HDKTLKILHDVTDNMIQERLKEPPSVTHEDEDETVARRRKRIAFLDLLLAMHREDASFTL

KDIREEVDTFMFEGHDTTAAAISWAILEIGQHPDIQERLHAELDEVFGDSIRPVTSDDLS

RLSYLTRIVKESLRIIPAVPMVARSLDEDIVLDGKVVPKEAMIMLHIYALHQDPQQFPDP

DQFDPDRFLPENAEKRHPYAFVPFSAGPRNCIGQKFAMMETKLTLANIFRRFSIESVQTI

EGAKPAGQLILRPVEGNILVKLSWRK

>AAI05151.1

MLAPWLLSVGPKLLLWSGLCAVSLAGATLTLNLLKMVASYARKWRQMRPVPTIGDPYPLV

GHALMMKPDARDFFQQIIDFTEECRHLPLLKLWLGPVPLVALYNAETVEVILSSSKHIEK

SYMYKFLEPWLGLGLLTSTGNKWRSRRKMLTPTFHFTILEDFLDVMNEQANILVTKLEKH

VNQEAFNCFFYVTLCTLDIICETAMGKNIGAQRNDDSEYVRAVYRMSDSIHQRMKMPWLW

LDLIFYMFKNGREHRRSLKIVHDFTNNVITERANEMKRHEEGTSNDKEKDFPPRKTKCRA

FLDLLLNVTDDQGNKLSHEDIREEVDTFMFEGHDTTAAAINWSLYLLGWYPEVQQRVDTE

LEEVFGKSDRPVTLEDLKKLKYLDCVIKESLRLFPSVPFFARNLTEDCEVAGHKIVQGCQ

VIIVPYALHRDPKYFPDPEEFKPERFFPENLKGRHTYAYVPFSAGPRNCIGQKFAIMEEK

TILSCILRHFWVESNQKREELGLAGELILRPSNGIWIKLKRRNTDES

>301609782

MELRGEVHLLVWVAAAVVLLTLLALSILPALQDYVRKWKLMKPIPGPGPNYPIVGDALFL

KPNGGDFFLQMCEFTESFRSEPLIKIWIGPIPFIIVYHADTVEPILSTSKHMDKAYSYKF

LHPWLGKGLLTSTGEKWRSRRKMITPTFHFAILSEFLEVMNEQSKILVEKLQKHVDGESF

NCFMDVTLCALDIISETAMGRKIQAQSNRDSEYVHAIYKMSDIIQRRQKMPWLWLDFFYA

YLKDGKEHDKNLKILHAFTDKAILERAEELKKMGEQKKEHCDSDPESDKPKKRSAFLDML

LMATDDAGNKMSYMDIREEVDTFMFEGHDTTAAALNWSLFLLGSHPEAQRQVHKELDEVF

GKSDRPVTMDDLKKLRYLEAVIKEALRIYPSVPFFARTVTEDCSIRGFHVPKGVNVVIIP

YALHRDPEYFPEPEEFRPERFFPENASGRNPYAYIPFSAGLRNCIGQRFALMEEKVVLSS

ILRNYWVEASQKREDLCLLGDLILRPQDGMWIKLKNRETAPTA

>BAD02915.1

MELKGDVNVLLWTAVIVVLLTLLVFSALPVLLDYVRKCKVMRLIPGPGPNYPLVGDALLL

KSDAREFFLQMCEFAEDFRSEPLLKLWIGPIPFLIVYHADTLEPFLSTSKHVDKAYLYKF

LHPWLGKGLLTSTGEKWRIRRKMITPTFHFAILSEFLEVMNEQSNVLVEKLQKHADGESF

DCFIDVTLCVLDIISETAMGRKIEAQSNKDSEYVQAIYKMADFIQNRQTKPWLWSDSLYA

YLKEGKEHNKTLNILHTFTDKAILERAEELKKMEVKKGDSDPESEKPKKRSAFLDMLLMA

TDDAGNKMSYKDIREEVDTFMFEGHDTTASALNWTLFLLGSHPEAQRQVHKELDEVFGKS

DRPVTMDDLKKLRYLEAVIKESLRIFPPVPMFGRTVTEDCTVRGFKVPKGVNIIVITYSL

HRDPEYFPEPEEFRPERFFPENASGRNPYAYIPFSAGLRNCIGQRFALMEEKVVLSSILR

KYWVEATQKRDECLLVGELILRPQDGMWIKLKNRETASSA

>126331227

MGGDMWLVPVGQKVLYWGVTGAITLLGTVLTLILLQMLVAYVRKWQQMKTIPAFPYALPL

VGHSLLMKSNSRDFFEQLIRYSEEFRKEPLLKLWIGPVPIVTLYHADNVEVLLSSSRQID

KSYFYKFLEPWLGLGLLTSTGNKWRSRRKMLTPTFHFTILEEFLDVMNEQANVLVNKLEK

HVDQEAFNCFMDITLCALDIICETAMGKNIGAQVNGDSEYVRSVFRMTNIIIRRMKMPWL

WLDLWFLMFKEGWEHRKRLKILHDFTDKVISERVKKMKENEEQKEDDLKPKTIKRRAFLD

LLLNTTDEDGNALSHQDIREEVDTFMFEGHDTTASAMNWVIYLLGSHPEAQRKVDNELDE

VFGNSDRPVTVDDLKKLKYLDCVIKESLRLFPSVPFFARTLNSDCFIAGYKVPKDTEAIV

FSYALHRDPKHFPDPEDFQPERFFPENSHGRHPYAYVPFSAGPRNCIGQKFAVMEEKTVI

SWVLRRYWVESIQKREELGLMGELILRPHDGIWIKLKKRK

>Q5RCN6.1

MAGLWLGLVWQKLLLWGAASAVSLAGASLVLSLLQRVASYARKWQQMRPIPTVARAYPLV

GHALLMKRDGREFFQQIIEYTEEYRHMPLLKLWVGPVPMVALYNAENVEVILTSSRQIDK

SSMYKFLEPWLGLGLLTSTGNKWRSRRKMLTPTFHFTILEDFLDIMNEQANILVKKLEKH

VNQEAFNCFFYITLCALDIICETAMGKNIGAQSNDDSEYVRAVYRMSQMIFQRIKMPWLW

LDLWYLMFKEGWEHEKGLKILHTFTNNVIAERANEMNADEDCRGVGRGSAPSKNKRRAFL

DLLLSVTDDEGNRLSHEDIREEVDTFMFEGHDTTAAAINWSLYLLGCNPEVQQKVDHELD

DVFGKSDRPATVEDLKKLRYLECVIKETLRLFPSVPLFARSVSEDCEVAGYRVLKGTEAV

IIPYALHRDPRYFPNPEEFQPERFFPENAQGRHPYAYVPFSAGPRNCIGQKFAVMEEKTI

LSCILRHFWIESNQKREELGLEGQLILRPSNGIWIKLKRRDADEP

>EHB10964.1

MLASYARKWLQMKPIPTVACAYPLVGHALLMKPNSTEFFQQLIQYTEEFRHMPLLKLWIG

PVPMVVLYHAENVEVILASSKQIDKSFVYKFIQPWLGLGLLTSTGIKWRSRRKMLTPTFH

FTILEDFLDVMNEQAKILVNKLEKHVNQEAFNCFFYITLCALDIICETAMGKNIGAQSNE

DSKYVRAVYRMSDKIFRRMKMPWLWLDLWYLMFKEGREHKRDLKILHTFTKNVIAERVNE

MKTNEESKGADSGPVFSKKKGKAFLDLLLSVTDDEGNKLSPEDIREEVDTFMFEGHDTTA

SAINWSLYLLGSYPEIQKKVDNELDEVFGKSDRPVDLEDLKQLKYLDLVIKESLRIFPPV

PLFARRLNEDCEVAGYKVVKGTEAVIVPYALHRDPRYFPNPEEFQPERFFPENAQGRHPY

SYVPFSAGPRNCIGQKFAVMEEKTILSYILRQFWVESNQKREELGLSGELILRPNNGIWI

TLKRRNPDESQLCTPAVPLS

>A2RRT9.1

MLWLWLGLSGQKLLLWGAASAVSVAGATVLLNILQMLVSYARKWQQMRPIPSVARAYPLV

GHALFMKPNNTEFFQQIIQYTEEFRHLPIIKLWIGPVPLVALYKAENVEVILTSSKQIDK

SFMYKFLQPWLGLGLLTSTGSKWRARRKMLTPSFHFTILEDFLDVMNEQANILVNKLEKH

VNQEAFNCFFPITLCALDIICETAMGKNIGAQSNGDSEYVRTVYRMSDMIYRRMKMPWFW

FDLWYLMFKEGRDHKKGLKSLHTFTNNVIAERVNARKAEQDCIGAGRGPLPSKTKRKAFL

DLLLSVTDEEGNKLSHEDIREEVDTFMFEGHDTTAAAINWSLYLLGSNPEVQRKVDKELD

DVFGRSHRPVTLEDLKKLKYLDCVIKETLRVFPSVPLFARSLSEDCEVAGYKISKGTEAV

IIPYALHRDPRYFPDPEEFQPERFFPENSQGRHPYAYVPFSAGPRNCIGQKFAVMEEKTI

LACILREFWIESNQKREELGLAGDLILRPNNGIWIKLKRRHEDDP

>AFK24489.1

MAVGLLITVCSFLFAYFLWKTLNSRDRKWLSKVSGPPTLPVFGNAHMLKTGGSDFFDQVL

EYSTLYESQTMFKILLGSHAIVVLIKAESVEKLLNSSSHITKSVEYRFLEPWLGTGLLTS

TGAKWHSRRKLLTPTFHFRILQDFLEVFNEQSQIMVENLKKKVGGNKFDIFPYITHCALD

IICDTAMGVNVDAQNDSDTQYVKDVYKISELVHRRQKAPWLWPDFLYSLLPAGRQTKECL

RNLHSFTQSVIKERQRDLQASFENEETLSSTSSIEDFLRITKRKRVAFLDMLLLYQRNSN

LSDEDIREEVDTFMFEGHDTTAAAANWAMHLIGSHPNVQEKIHKELDEVFDGSNRAITDE

DLKKMKYLECVIKETLRLFPSVPMYGRELKEDFNIDGVDIPKTTTLLVLTSALHRDPRYF

PDPEKFDPDRFTLENTVGRHPYAFVPFSAGQRNCIGQKFAMNEEKVLLASILRNFTVKAH

QKYCDLRPMGELILRPENGIWVSLQNRDV

>296195032

MAGLWLGLEGQKLLLWGAASVVSLAGASLVLNLLHWAASYARKWRQMRHIPTVARAYPLL

GHALLMKRDGREFFQQIIQYTEEYRHMPLLKLWVGPVPMVALYRAENVEVILTSSKQIDK

SSMYKFLEPWLGLGLLTSTGNKWRSRRKMLTPAFHFTILEDFLDIMNEQANILVKKLEKH

VNQEAFNCFFYVTLCALDVICETAMGKNIGAQSNDDSKYVRAVYRMSEMIFRRIKMPWLW

LDLWYLMFKEGWEHRKSLKILHTFTNDVIAERANEMTADGECGGDGTGSVHPKNKRRAFL

DLLLSVTDDEGNRLSHEDIREEVDTFMFEGHDTTAAAINWSLYLLGSNPEVQKKVDHELD

DVFGNSLRPATIEDLKKLRYLECVIKETLRLFPSVPLFARSLSEDCEVAGYRVLKGTEAV

IIPYALHRDPRYFPNPEEFQPERFFPENAKGRHPYAYVPFSAGPRNCIGQKFAMMEEKTI

LSCILRRFWVESNQKREELGLEGQLILRPSNGIWIMLKKRNTDRP

>344281440

MPSFVFTTARGAAEAAFAFAWSELRGPGAGPSNSPAGCVLRQPAWPEPVPRAAAAPAARV

AGPEAAPTPAPGWRRERAWRPWVPEGQDCWPPVTRHRIILCRMINSILQMELDSLTDLLE

VTELGSSIDPGEHARKWHQMRPVPTVARAYPLVGHALLLKADGKEYFQQIIQYSEEYRYM

PLLKLWMGPVPMVVLYKAENVEVILTSSKQIDKSSMYKFLEPWLGQGLLTSTGNKWRTRR

KMLTPAFHFTILEDFLDVMNEQADILVNKLEKHVNQEAFNCCFYITLCALDIICETAMGK

NIGAQNNGDSDYVRAVYRMSDMILRRIKMPWLWLDLWYLMLKEGREHKRSLKILHTFTDN

VIAERVRQMKSPEECKDRHERDSRPTKNKRRAFLDLLLNAAGDDGNKLSRENIREEVDTF

MFEGHDTTAAAINWSLYLLGSYPEVQKKVDMELDEVFGKSEGPATLEDLKKLRYLECVIK

ESLRLFPPVPLFARTLNEDCEVAGYKISKGTEAVILAYALHRDPRYFPDPEEFQPERFLP

ENMQGRHPYAYVPFSAGPRNCIGQKFAIIEEKTILSCILRRFWVECNQKREELGLTGELI

LRPYNGIWIKLKKRNFSES

>BAC86562.1

MAGLWLGLVWQKLLLWGAASALSLAGASLVLSLLQRVASYALLMKPDGREFFQQIIEYTE

EYRHMPLLKLWVGPVPMVALYNAENVEVILTSSKQIDKSSMYKFLEPWLGLGLLTSTGNK

WRSRRKMLTPTFHFTILEDFLDIMNEQANILVKKLEKHINQEAFNCFFYITLCALDIICE

TAMGKNIGAQSNDDSEYVRAVYRMSEMIFRRIKMPWLWLDLWYLMFKEGWEHKKSLKILH

TFTNSVIAERANEMNANEDCRGDGRGSAPSKNKRRAFLDLLLSVTDDEGNRLSHEDIREE

VDTFMFEGHDTTAAAINWSLYLLGSNPEVQKKVDHELDDVFGKSDRPATVEDLKKLRYLE

CVIKETLRLFPSVPLFARSVSEDCEVAGYRVLKGTEAVIIPYALHRDPRYFPNPEEFQPE

RFFPENAQGRHPYAYVPFSAGPRNCIGQKFAVMEEKTILSCILRHFWIESNQKREELGLE

GQLILRPSNGIWIKLKRRNADER

>50657412

MAMEITLGSMEGTQLLPWVAGAITLLLTVVTVHFLPSLLNYWWWWWVMKPIPGIRPCYPF

VGNALLLERNGEGFFKQLQQYADEFRKMPMFKLWLGPLPVTVLFHPDSVEVILSSSKHIK

KSFLYTFLHPWLGTGLLTSTGDKWRSRRKMITPTFHFAILNDFLEVMNEQGGVLLEKLEK

HVDKEPFNIFTDITLCALDIICETAMGKNLGAQDNKDSEYVRAVYRMSDLIQQRQKSPWL

WHDLMYLLFKEGREHERNLKILHGFTDTVIAEKVAELENTKLTKHDTDVNTEEESGSKKR

EAFLDMLLNATDDEGKKLSYKDIREEVDTFMFEGHDTTAAAMNWVLYLLGHHPEAQKKVH

QELDEVFGNTERPVTVDDLKKLRYLECVVKEALRLFPSVPMFARSLQEDCYISGYKLPKG

TNVLVLTYVLHRDPEIFPEPDEFRPERFFPENSKGRHPYAYVPFSAGPRNCIGQRFAQME

EKTLLALILRRFWVDCSQKPEELGLSGELILRPNNGIWVQLKRRPKTVTE

>390331656

MAAVLSAGIGVTVCIVALSAFLFYYIARLLRIFLLISKFDGPPAVPIFGNTLQFKSDPRD

IFLYYQRNFERYRQETGGITRIWYGPLPAVIVYSAKHGEALMNSSHHTSKGIQYKCLEPW

LGKGLLTSSGKKWTHRRKLLTPTFHFSILQNFMEVFNEQSIILVKKLEKFAEKSETFNIF

PFVTHCVLDIICDTAMGKRTNAQEDSENEYVRAVDSMSRLVVRRMRNPLCWSDWIYYNMG

VGKEHEKSLNVLHKVTNEMIQERLSDIPSNENGTNQDDAVTRKRKRIAFLDLLLQMHRED

ATFTLEDIREEVDTFMFEGHDTTSAAVSWALLLIGQHPEVQVRLHDEIDQVFGESERPVT

SDDLSKLPYLSRVIKETLRITPPVPGLSRELDEDIVVDGKVLPKEAMVIINIYGIHHDPE

QFPDPDRFDPDRFLPENSTKRHPFAFIPFSAGPRNCIGQKFAMMEDKVILINLLRRFSVK

SLQTLDEAKPAGLLILRPAEGTILVKLSLRK

>326919104

MAMEIMLGTTEGMQLLPWVAGAITLLLTVVTVRSLPSLLNYWWWWRVMKPIPGISPCYPF

VGNALLLERKGEGFFKQLQQYADEFRKMPMFKIWLGPLPVTVLFHSDTVEVILTSSKHIQ

KSFLYRFLHPWLGTGLLTSTGDKWRSRRKMITPTFHFAILNYFLEVMNEQGGILLEKLEK

HVDKEPFNIFIDITLCALDIICETAMGRNVGAQDNKDSEYVRAVYRMSDLIQQRQKSPWL

WHDLLYVLFKEGREHERNLKILHNFTDTVIAEKVAELENTKLTKHDTDVSSEEKSGSKKR

EAFLDMLLNATDDEGKKLSYKDIREEVDTFMFEGHDTTAAAMNWVLYLLGHHPEAQKKVH

QELDEVFGNTERPVTVDDLKKLRYLECVVKEALRLFPSVPMFARSLQEDCYIRGYKLPKG

TNVLILTYVLHRDPKIFPEPEEFRPERFFPENSKGRHPYAYVPFSAGPRNCIGQRFAQME

EKTLLALILRRFWVDCSQKPEELGLSGELILRPNNGIWVQLKRRPKTITD

>348566843

MASVLHCCCFLIIFSFPCITIRLHPKPSPLTCLPSGLLLGLASLSLEAVLESACFTGSCS

SPGSQPTQPDRRFPEGHVPKTPLNQSQIAAISHPDAEVRGRGVEPRLQSKRGLLQPPGWK

PGPGRRSAPSRVPLPSGHRRKRAAAPPSGPGAPPRPEADLASPGLHRSPRRAAAAERMEL

WLEPVAQKLFLWGAASAVSLTGATLLLSVLQMLASYARKWQQMKPIPEVARAYXLVGHAL

QMKPSSAEFFQQLIKYTEEFRHMPLLKLWIGPVPMVALYHAENVEVILTSSKQIDKSFMY

KFIEPWLGLGLLTSTGIKWRSRRKMLTPTFHFTILEDFLDIMNEQANILANKLEKHVNQE

AFNCFFYITLCVLDIICETAMGKRIGAQSNKDSKYVRAVYRMSDKIFRRMKMPWLWPDLW

YLMFKEGREHKRDLEILHTFTKNVIAEKVNEMKAKEENKSASSAPGISKRKGKAFLDLLL

SVTDEQGNKLSHEDIREEVDTFMFEGHDTTAAAINWSLYLLGSYPEVQKKVDNELDEVFG

KSDHPVTLEDLKKLKYLDCVIKETLRIFPPVPLFARRLNEDCEVAGYKVVKDTEAVIIPY

ALHRDPRYFPNPEEFQPERFFPENAKGRHPYAYVPFSAGPRNCIGQKFAVMEEKTILSCI

LRRFWVESNQKSEELGLSGELILRPHNGIWITLKRRNVDKS

>390331696

MAVVLPAGIGVTVCIVALSAFLFYYIARFLRIFLLISKFDGPPAVPIFGNTLQFKSDPRE

HFLYDVGVREKYRHQSGGVIRMWFGPLPIVLIYNAVHCEALMNSSRHINKGTEYTFMEPW

LGKGLLTSGGQKWFHRRKLLTPTFHFSILQSFMEVFNEQSMILVKKLGKFADESETVNIF

PLVTHCVLDIICDTAMGKRTNAQSDNENEYVKAVGRMSHLIVSRIRNFMLWPDWIYNRTD

AGKDHEKALQILHGVTNKMIQERLHDPLHSPNGGTYEDDAVTRKRKRIAFLDLLLQMHRE

DATFTLEAIREEVDTFMFEGHDTTAAAVNWALLLIGQHPTVQARLHDEIDQVFGDSERPI

TSDDLSELSYLSCVVKESLRLLPSVPGIGRDLDEDIIVNGKVVPKGASVFLSIYGIHHDP

EQFPDPERFDPDRFLPENSTKRHPFAFIPFSAGPXXCILFTGQKFAMMEDKVLLINILRR

FSIKSLQTLDETKPAGQIVLRPADGNILVKLSHRK

>AFJ49834.1

MESRMALASGAFFFEWAVGVASTLTVLAAAVVIVSFLVLRNYWKWKEMQAIPEVKPWYPI

LGNSLIMDRDAKEFWKQVIFYSEEFRYEPLLKLWIGPFPHIILYHQDTVEVVLRSSTFIE

KSNLYQFLQPWLGTGLLTSTGKKWHSRRKMLTPTFHFTILVDFLEVMKEQANILFQNLEQ

HLDKDPFNCTLYIALCTLDIICETAMGKNIGAQKNKKSDYVKAVQSMSDLIHHRQKSPWL

WPNLLYNMSGKGKQHYMNLKILHSFTDKIIEEKVCKIKQQEQYQNSAASSNHLRKNKERK

TFLDMLLNARDEDGNKLSYIDIREEVDTFMFEGHDTTAAALSWAIYLLASHSEIQRKVHN

ELDEVFGDSDHHITMEDLKKLRYLECVIKEALRLFPSVPFFARILNEECHIRGYKIPKGT

DVIILPYALHRDPHNFPDPEEFRPERFFPENSTGRHPYSYIPFSAGPRNCIGQRFALLEE

KTILATILRHFWIETKQTCDEVGMAAELILRPSKGIWIQLKKRPTPSS

>194226510

MQSTSREAAGPSSLEHCRLRKETRLDAQLRKYFFQQLIYYTEENRHLPLLKLWLGPVPVV

IFYNAENVEVILTSSRQIDKSYMYKFLKPWLGLGLLTSTGNKWRSRRKMLTPTFHFTNLE

DFLDVMNEQANILVNKLEKHVNQEAFNCFLYITLCALDIICETAMGKNIGAQRNNDSEYV

RAVYRMSDMIHRRMKMPWLWLDIFFLMFKEGREHRRLLKILHNFTNNVIVERASEMKKDE

ERSRSDDGGSAPSKNKRRAFLDLLLNVTDDEGNKLSHEDIRQEVDTFMFEGHDTTAAAIN

WSLYLLGCYPEVQKKVDSELEEVFGKSDRPATLEDLKKLKYLECVMKETLRLFPSVPLFA

RNLNEDCEVAGYKIVKGSQAIIVSYALHRDSRYFPNPEEFKPERFFPENSQGRHPYAYVP

FSAGPRNCIGQKFAVMEEKIILSCILRHFWVESNQKREELGLAGELILRPSNGIWIKLKR

RNTEES

>EDS28996.1

MLEMEFVKSSFVLSRAAKVFTYFSPLTVILVSAFLGAMFVYNRRRSRLVKMIEKIPGPAS

MPIIGNSLHINVDHDEIFNRIISIRKLYGRQQGFSRAWNGPMPYVMISKAAAVEPILSSP

KHIEKSHDYEFLKPWLGTGLLTSQGRKWHPRRKILTPAFHFKILDDFVDIFQEQSAVLVQ

RLERELGNREGFNCFPYVTLCALDIVCETAMGRLVNAQMNSDSEYVKAVYQIGGIVQNRQ

QKIWLQPDFIFKRTKDYRDHQRCLSILHEFSNRVIHERKEEIRKQKLSNNNSVEQAPNAD

GNNNAEEFGRKKRLAFLDLLIEASQDGTVLSHEDIREEVDTFMFEGHDTTSAAISWILLL

LGAEPAIQDRIVEEIDEIMGGDRDRFPTMKELNDMKYLECCIKEGLRLYPSVPLIARKLV

EDCVVQDYTIPAGTTAMIVVYQLHRDPAVFPNPDKFNPDHFAPENCRGRHPYAYIPFSAG

PRNCIGQKFAVLEEKSIISAVLRKYRIEAVDRRENLTLLGELILRPKDGLRIKISRRV

>EFX87732.1

MKLFNTWSKKSVTRLWIGPFPMITIGSAEGAEVVLSSNKIIDKGPEYDFIQPWLATGLLT

STGQKWHQRRKLLTPTFHFKILEDFVQVFNEQSAVLVQRLNEKVGQDFDVFPYITLCTLD

VICETAMGRNIDAQSNTDSEYVKAVYNMGHIIQARQTRPWLQPDWLFRLFPLGSEQKRCL

SILHGFTDQVIRERKAEHRKIGQQRQDVSPEKDDDEFVSKKPRLAFLDLLIEASQDGKVL

SDLDIREEVDTFMFEGHDTTAAAINWSLFLIGNHPEVQEKVSEELTRVFGESDRPITMAD

LSELKYLECCIKEALRLYPSVPIYARELMEDANICGYTLPVGASVLIVPYIIHRDPIYFP

DPEGFKPERFFPENIQGRHPYAYVPFSAGPRNCIGQQFALMEEKIVLASVLRRFQIKSLD

KPEDLPLLVEVILRPRDGIRLQLTHKTLLNN

>EDS45242.1

MLEMEFVKSSFVLSRAAKVFTYFSPLTVILVSAILGAMFVYNRRRSRLVKMIEKIPGPAS

MPIIGNSLHINVDHDEIFNRIISIRKLYGRQQGFSRAWNGPMPYVMISKAAAVEPILSSP

KHIEKSHDYEFLKPWLGTGLLTSQGRKWHPRRKILTPAFHFKILDDFVDIFQEQSAVLVQ

RLERELGNREGFNCFPYVTLCALDIVCETAMGRLVNAQMNSDSEYVKAVYQIGGIVQNRQ

QKIWLQPDFIFKRTKDYRDHQRCLSILHEFSNRVIHERKEEIRKQKLSNNNSVEQAPAAD

GNNNAEEFGRKKRLAFLDLLIEASQDGTVLSHEDIREEVDTFMFEGHDTTSAAISWILLL

LGAEPAIQDRIVEEIDQIMGGDRDRFPTMKELNDMKYLECCIKEGLRLYPSVPLIARKLV

EDCVVQDYTIPAGTTAMIVVYQLHRDPAVFPNPDKFNPDHFAPENCRGRHPYAYIPFSAG

PRNCIGQKFAVLEEKSIISAVLRKYRIEAVDRRENLTLLGELILRPKDGLRIKISRRV

>156550311

MFMYLANKCLQWILDVKRFNILLMVLFTIFGWLILRWWQMKRRCWKYLAKIPGPFSLPLI

GNLVQLKVDHDELFSLIVGMRLMWGTQEGINKAWLGRKPYVFLSKASTVEQILGSNRHID

KSCDYKFLQPWLGTGLLTGSGHKWYSRRKILTPTFHFNILEDFVQVFGEQTEVLVRKMSS

ELGRPSFNIFPYVTLCTLDIICETAMGRRISAQSHSDSDYVRAVYDIGSIIQTRQASVFY

QSNIFFRLSPLYKKHQECIRILHQFSNEVIKERRKEISESIREINGNEVPKKRLAFLDLL

IKASENGAILSNSDIREEVDTFMFEGHDTTSSAVCWTLYLLGCHPEVQEEVVNELEMIFS

EGDYDRRPSLKDLKRMKYLDKCIKEALRLYPSVPILGREISEDVEIGGYTVPKGTTALVV

LPVLHRDPEIFSNPEKYDPERFAAENMIGRHPYSYIPFSAGPRNCIGQKFALLEEKAIIS

GILRKFVVEATEQRDDISVTAELVLRTKSGLHLRIQPRMN

>198432600

MAVALLLIIVVTCVIALHYRKGIARHFKNRLMLSRLPSLPDPAYPLIGHAYLLRGDPSRF

FEFVTETTNAMVTEVNEKIGILWLGPVPLMVVCHPEAAEVVLRSSKHMEKSYLYKFLHPW

LGTGLLTSGGEKWKQRRRLITPSFHFNILQDFLEVMNEESGKLINNLKKKLKSGVKVDVG

KAVTMCALDIICETAMGQTVNAQENDDSSYVRALYRISELIQLRQKTPTLWWDPAFSRMK

LGKEHENLLSILHGFTRDVITKRAKTRDQKIVENPRRMAFLDVLLHAETEDGKTLSLNDI

QEEVDTFMFEGHDTTAAAMTWAIYVIGRHPDIQKKIHEELDAVFGEDRGGTITNNQLQKL

SYLERVIKECLRLYPSVPFYARVLSEDCKVGDYMVPKGTQTVIFAHTIHHHPYVWEDPEK

FDPDRFLAENCVKRHPYAYIPFSAGPRNCIGQKFALMEEKVILSKLLHNYFVVSHDKKED

LVINGDLILRSSTPLNITLEARK

>291238005

MKIGGRAQHEGQRELSVAARLLILVAEMAIVGGISLLLVVAAFLVYPLYRYFKLWQLVNR

IPGPVALPFIGNALQFERDGQAFFVQILKWVDDYRDTRVFRIWIGPKPVIFVYRADAAEC

IFNSSKHITKAFVYDFLHPWLGTGLLTSTGDKWKSRRRLLTPTFHFKILNDFVGVFNEHS

SVLVTKLNNYAGKEEFNVFPLITHCVLDIICETAMGIKTNAQVDCDSDYVQAVSGMSTVL

QDRMKSPWFWPDTFYNLTEDGKKHGKFLQILHKMTNKVIKDRSAELSKHLASQGQTVTED

TEMTTIGGRKHLAFLDMLLYMHEADPEFTFTDIREEVDTFLFEGHDTTAAALTWATYLIA

SHPEVQERIFEELDGIFGDSNRPVTMDDLKEMKYLDNTIKESLRMYPSVPIFARQLDEDV

TLAGFKIPSEANILVAPYALHRDEKYFPNPEVFDPDRFSSSRSKHRHPYAYVPFSAGLRN

CIGQKFALYEEKVVLSSIFRKFKIETAMRREDLKPTGEIILRPLNGINIKLSLRN

>BAF35771.1

MDVTSGGESSVWISSFSVYTVTTILVTLVVLAVVKRYNFMQRCNKVLGSPTDIPLFGGGS

LIFVPPEEIMNLLLLLHVVFGRLSPSGIIRAWIGPLPMFFATTAEAVEAVLSSNKIITKS

REYDFLHPWLNTGLLTSTGSKWQTRRKLLTPAFHFKILEDFVHVFNEQSLILVNKLNQAV

AKDKDLNIFPFVTLCTLDIICETAMGRNVEAQSKTDSAYVQAVYNMSQLIQHRQVRFYLW

LDWMFKLSSHWPEQRKTLGILHGFTNKVIQERKAEHQQRSSDIAEPSKDVTEDAVFSKRR

LAFLDLLIEFSQGGTVLSASDIREEVDTFMFEGHDTTSAAITWSIFLIGSHPEVQEMVNE

ELDRVFGDSDRPATMADLSELKYLECCVKEALRLYPSVPIISRTCVEDTVIGGDEIPAGT

SVSICSYYLHRDPKYFPDPELYQPKRFLAEHAERRHPYSYVPFSAGPRNCIGQRFALMEE

KAVLSAILRNFHVQSLDKREEIILLAELILRPRDGIRVRLEPKKKQ

>EKC23987.1

MESFLKLYADRGIFRLWLGTTLLVLIFKAEYAEVILQSSKHLDKAEEYKFLHPWLGTGLL

TSTGSKWKSRRRMLTPTFHFKILNDFVGVFHDQASIMVKKLSQVADGKEFDIFNYITLCA

LDIICETAMGRSIEAQSNSNSDYVKSVYKMSELVLQRQRTPWYWPDFLYNTIGYGKEHDR

CLKILHGFTLKVIKEKMDAVSDASGNSMEEMLNQHSDEDVYRGKGQRLAFLDMLLCTTDD

GTHLSMEGIREEVDTFMFEGHDTTAAAVNWAVHLIGANPDVQAKVHEEMDQLFGGSDKPA

SMNDLKEMRYLECCIKEALRLFPSVPGFGRKLKEDCKFGEYSIPKDTTVIIVPPDLHRDE

RYFPDPEKFDPNRFLPENSLKRHPYCYVPFSAGPRNCIGQKFAILEEKVMLSNIFRNFTV

TSKQSREELFPIGDLIMRPEHGIIVELRPRTT

>EDO31776.1

MAAIEAALVFGSIIILLFLLVNHRARRVREMHKFQSPPPYRMLFGNTLEFKDEPSEFLEQ

IMGWCSQFKGIFCLWLSFAKPFLLIYEPKTVEVILSSSKHITKAQFYDFLLPWLGTGLLT

STGEKWKKRRRLITPTFHFKILNDFVQVFDQQSQLMVSIIKSQPSDKVFNIFPLIACCAL

DIIGKTAMGVNINAQKNSSSEYVKGVIRMSELIQSRQKRPWVHPAFLYCLTNEGREHNKI

IKFLHDFTNQVITERIQERLYSPKEQDETEESDEFTGRRKRRLVAFLDMLLNAYDAGEID

VEGVREEVDIFMFEGHDTTAAAMAWAVQLLGEHPDVQRKAQAEVDEFFATNSGKLTADSL

KGLKYLECVIKETLRIFPSVPFFGRSLVEDLELEGRLIPKGTDVGVITIGLHRNPEVWPS

PMKFNPDRFLPENSEGRHPYAFVPFSAGSRNCIGQRFALLEEKVVLAYILHNFDIVSTEK

STKIKTCAELITRPRDGIFVSLTTREQGL

>EDV30459.1

MSSKVISSLMAESILLSKTAQVISGYSPITIFLAASVIIFLVVYNKRRSRLVKHIEKIPG

PAAMPFLGNAIEMNVDHDELFNRVIGMQKLWGTRIGINRVWQGTAPRVLLFEPETVEPIL

NSQKYVNKSHDYDYLHPWLGEGLLTSTDRKWHSRRKILTPAFHFKILDDFIDVFNEQSAV

LARKLAVEVGSEAFNLFPYVTLCTLDIVCETAMGRRIYAQSNSESEYVKAVYGIGSIVQS

RQAKIWLQSDLIFRLTSDYKLHQSYINTLHGFSNMVIRERKAELAILQENNNNNNNDAPD

AYDDLGKKKRLAFLDLLIDASKEGTVLSNEDIREEVDTFMFEGHDTTSAAISWTLFLLGC

HPEYQERVVEELDSIFGDDKETPASMKNLLDMRYLECCIKDSLRLFPSVPMMARMVGEDV

NIGGKLVPAGTQAIIMTYALHRNPRVFPKPEQFNPDNFLPENCAGRHPFAYIPFSAGPRN

CIGQKFAILEEKAVISTVLRKYKIEAVDRREDLTLLGELILRPKDGLRVKITPRD

>EDW99163.1

MSSKVISSLMAESILLSKVGQVISGYSPITVFLLGSILIFLVVYNKRRSRLVKYIEKIPG

PAAMPFLGNAIEMNVDHDELFNRVIGMQKLWGTRIGINRVWQGTAPRVLLFEPETVEPIL

NSQKFVNKSHDYDYLHPWLGEGLLTSTDRKWHSRRKILTPAFHFKILDDFIDVFNEQSAV

LARKLAVEVGSEAFNLFPYVTLCTLDIVCETAMGRRIYAQSNSESEYVKAVYGIGSIVQS

RQAKIWLQSDFIFSLTADYKLHQSYINTLHGFSNMVIRERKAELAILQENNNNNNNNAPD

AYDDLGKKKRLAFLDLLIDASKEGTVLSNEDIREEVDTFMFEGHDTTSAAISWTLFLLGC

HPEYQERVAEELDSIFGDDKETPATMKNLLDMRYLECCIKDSLRLFPSVPMMARMVGEDV

NIGGKIVPAGTQAIIMTYALHRNPRVFPKPEQFNPDNFLPENCAGRHPFAYIPFSAGPRN

CIGQKFAILEEKAVISTVLRKYKIEAVDRREDLTLLGELILRPKDGLRVKITPRD

>198436000

MALTLFILIGALAVTIAVYWKKIKEQFRKRQLINKLPTIMETAYPLVGHSYLFPKSAEEL

FVFITGRLNWVMTEAVEKIAVLWLGPIPLIGVVHPEAAEVILRSSKHIEKSFVYTFVHPW

LGTGLLTSGGEKWKQRRRLITPSFHFNILQEFLEVMNEQSKKMVDKLDASIASGSKIYVG

KAITMCALDIICETAMGQTVNAQDHQDSEYVKALYRISDLVQFRQRTPALWWDAVFSRSK

LGIEHDTILCTLHGFTRNVITERAQGKGKKEIENPRRLAFLDVLLNAETEDGKSLSLNDI

QEEVDTFMFEGHDTTAAAMTWTVYLIGRYPDIQEKLHEEIDSVFHDDKEGVISNSQLQKL

SYLERVIKESLRLYPSVPFIGRVTTEECIIADHVIPVGTQVALFIESMHRNPAVWPDAEK

FDPDRFTAENCVGRHPYAYIPFSAGPRNCVGQKFAMMEEKVILAQILRRFSLVSHDKEED

LKKQADLILRSSKPLNITLTPRV

>AFA26603.1

MTWLKEDVFSLVPHAVPYVFLTAIIGLVITWFIRRMLSKSMLNPIPGPRGLPILGREFFQ

VVKGLSYMWGLITPMWRVWLGPIPAVQIQRASEAEVILSSNKHIDKSRDYTFLHPWLGTG

LLTATGPKWRSRRKLLTPAFHFKILEDFVEIFSKQSDVMVQKLEKKADGKQFDIFPYVTL

CALDIICETAMGRCVHAQDNSDSDYVEALYRIGALVQQRQARPWLQPDILFKLLGYAKEH

DACLKVLHDMSYSCIKERRVQFQERKLRKKKQDGGNEDDAFVGKKERLAFLDLLLEAAET

DPSITDTDIREEVDTFMFEGHDTTAAAINWSLYLLGTHPEIQARVHEELDTVFGDEDRPV

TMSDLRELKTTENCIKEALRLFPSVPFIGRELTEEVVIDNYRIPKGTTIMIVPFRIHRDP

EQFPRPEVFDPDRFLAENCKDRHPYAYVPFSAGPRNCIGQKFALLEEKLLLCSILRKFKV

ESDIRREDLKLLGELILRPEDGNFVKLTPRRK

>EDW16611.1

MSSKVFSSLMAESILLSKAAQIISGYSPITIFLLATIIIFLVVYNKRRARWVKYIEKIPG

PAALPFLGNAIEMNVDHDELFNRVIGMQKLWGTRIGINRVWQGTSPRVLLFEPETVEPIL

NSQKFVNKSHDYDYLHPWLGTGLLTSTDRKWHSRRKILTPAFHFKILDDFIDVFNEQSAV

LARKLAVELGSEAFNIFPYVTLCTLDIVCETAMGRKIYAQSNSESEYVKAVYGIGSIVQS

RQAKIWLQSDLLFRLTEDYKTHQSYINTLHGFSNMVIRERKAELANNNNNNNNNEMPDAY

DDLGKKKRLAFLDLLIDASKEGTVLSNEDIREEVDTFMFEGHDTTSAAISWTLFLLGANP

EYQERVVEELESIFGNDTETPATMKNLLDMRYLECCIKDALRLFPSVPMMARMVGEDVTI

GGKLVPAGTQAIIMTYALHRNPRVFPKPEQFNPDNFLPENCAGRHPFAYIPFSAGPRNCI

GQKFAILEEKAVISTVLRKYKVESVDRREDLTLLGELILRPKDGLRVKITPRH

>ADD63783.1

MTWLRTELLRWAPTDLHYVFLTSILALTLFWLFRRQRKISMIEKIPGPKGLPILGSALDV

NVAPSELFVKLCDFCEYGRVAKVWIGPQPYLLISGARAAEVILSSQKHLDKSRDYSFLHP

WLGTGLLTSTGTKWHSRRKLLTPAFHFKILEDFVEVFNQQSSVMVQRLQKKADGNTFDIF

PYITLCALDVICETAMGRVVGAQADSDSEYVKALYRIGSLVQQRQARPWLQPDLFFKLSG

YQKEHDACLKVLHDFSNDTIRSRRFEYLEGKKNEDQNTTEDEAIGKKKRLAFLDLLLEYS

ETVSRLSNEDIREEVDTFMFEGHDTTAAAINWSLYLIGSNPEIQARVHEELDSIFGGSDR

PITMADLREMKLTENCIKEALRLFPSVPSLARELKEDAVIDDYRIPTGTTVTVVTYCLHR

DPEQFPNPEVFDPDRFLPENCKSRHPYAYVPFSAGPRNCIGQKFALMEEKILLSHILRSF

RVESTVKREDLRLIGELVLRPENGNPVKLLPREAH

>EFW45517.1

MLLALLEGLLQLGGGSPGAGACSTTTIVLLVLTALLGGLVAYTQLSRTRRLVSLLPAMPA

LPLIGSVPWFELDDARFFVQFVGLVQQFGSVFTIWLGPIPIAVLTAPESVEAVLRGSHHI

EKSFIYWFLTPWLGRGLLTSGGDKWRTRRRMLTPAFHFDILRGMVEVFREQAEVLIGVLD

ASADTKKPIDVFPLVSLCALDSICETAMGKKLHALKQSESTYVQAVFRISELIHHRQKFP

WNWPDFIYSWLPDGRDHTRVLAELHNFTDSVIAERRVFLNDNPDVTDEQADSSKKRRLAF

LDLLISARDEDGNHLSNMDIREEVDTFMFEGHDTTAAALAWSLHLIGSHPDVQARIHEEL

DRVLGSEPSPSFEQLKAHELPYLEMTLKEALRLFPSVPAISRVLDQDIDVCGYKIPAGLT

VGLIPYAVHRDPKHWPDPEAFNPDRFLPENSANRHPYAYIPFSAGPRNCIGQRFAEFEER

VVMASILKRFRIVSTQTRDQLAPLGEIILRPRDGVWVTLERR

>P29981.1

MEFITILLSTALFIVTFLFLFRQGAKRARFVYLVNKLPGPTAYPVVGNAIEAIVPRNKLF

QVFDRRAKLYGPLYRIWAGPIAQVGLTRPEHVELILRDTKHIDKSLVYSFIRPWLGEGLL

TGTGAKWHSHRKMITPTFHFKILDIFVDVFVEKSEILVKKLQSKVGGKDFDIYPFITHCA

LDIICETAMGIQMNAQEESESEYVKAVYEISELTMQRSVRPWLHPKVIFDLTTMGKRYAE

CLRILHGFTNKVIQERKSLRQMTGMKPTISNEEDELLGKKKRLAFLDLLLEASENGTKMS

DTDIREEVDTFMFEGHDTTSAGICWALFLLGSHPEIQDKVYEELDHIFQGSDRSTTMRDL

ADMKYLERVIKESLRLFPSVPFIGRVLKEDTKIGDYLVPAGCMMNLQIYHVHRNQDQYPN

PEAFNPDNFLPERVAKRHPYAYVPFSAGPRNCIGQKFATLEEKTVLSSILRNFKVRSIEK

REDLTLMNELILRPESGIKVELIPRLPADAC

>EDX15067.1

MSSKVITSLMAESILLSKVGQVISGYSPITVFLLGSILIFLVVYNKRRSRLVKYIEKIPG

PAAMPFLGNAIEMNVDHDELFNRVIGMQKLWGTRIGINRVWQGTAPRVLLFEPETVEPIL

NSQKFVNKSHDYDYLHPWLGEGLLTSTDRKWHSRRKILTPAFHFKILDDFIDVFNEQSAV

LARKLAVEVGSEAFNLFPYVTLCTLDIVCETAMGRRIYAQSNSESEYVKAVYGIGSIVQS

RQAKIWLQSDFIFSLTADYKLHQSYINTLHGFSNMVIRERKAELAILQENNNNNNNSEPD

AYDDLGKKKRLAFLDLLIDASKEGTVLSNEDIREEVDTFMFEGHDTTSAAISWTLFLLGC

HPEYQERVVEELDSIFGNDKETPATMKNLLDMRYLECCIKDSLRLFPSVPMMARMVGEDV

NIGGKIVPAGTQAIIMTYALHRNPRVFPKPEQFNPDNFLPENCAGRHPFAYIPFSAGPRN

CIGQKFAILEEKAVISTVLRKYKIEAVDRREDLTLLGELILRPKDGLRVKITPRD

>EDW84505.1

MSSKVFGSLMAESVLLTKTAKVISGYSPVTVFLVAAITLFLVIYNKRRARLVKHIEKIPG

PAAMPFLGNAIEMNVDHDELFNRVIGMQKLWGTRIGINRVWQGTSPRVLLFEPETVEPIL

SSQKFVNKSHDYDYLHPWLGTGLLTSTDRKWHSRRKILTPAFHFKILDDFIDVFNEQAAV

LVRKLSVELGSEAFNIFPYVTLCTLDIVCETAMGRRIYAQSNSESEYVKAVYGIGSIVQS

RQAKIWLQSDFLFRLTDDYKLHQSYINTLHGFSNMVIRERKTELALANDNNNNNNMPDAY

DDLGKKKRLAFLDLLIDASKEGTVLSNEDIREEVDTFMFEGHDTTSAAISWTLFLLASHP

EYQERVVEEMESIFGDDKETPATMKNLLDMRYLECCIKDSLRLFPSVPMMARMVGEDVTI

GGKLVPAGTQAIIMTYALHRNPRVFPKPEQFNPDNFLPENCAGRHPYAYIPFSAGPRNCI

GQKFAILEEKAVISSVIRKYKIEAVDRREDLTLLGELILRPKDGLRVKITPRV

>EDW53575.1

MSSKVITSLMAESILLSKVGQVISGYSPITVFLLGSILIFLVVYNKRRSRLVKYIEKIPG

PAAMPFLGNAIEMNVDHDELFNRVIGMQKLWGTRIGINRVWQGTAPRVLLFEPETVEPIL

NSQKFVNKSHDYDYLHPWLGEGLLTSTDRKWHSRRKILTPAFHFKILDDFIDVFNEQSAV

LARKLAVEVGSEAFNLFPYVTLCTLDIVCETAMGRRIYAQSNSESEYVKAVYGIGSIVQS

RQSKIWLQSDFIFSLTADYKLHQSYINTLHGFSNMVIRERKAELAILQENNNNNNNSEPD

AYDDLGKKKRLAFLDLLIDASKEGTVLSNEDIREEVDTFMFEGHDTTSAAISWTLFLLGC

HPEYQERVVEELDSIFGADKETPATMKNLLDMRYLECCIKDSLRLFPSVPMMARMVGEDV

NIGGKIVPAGTQAIIMTYALHRNPRVFPKPEQFNPDNFLPENCAGRHPFAYIPFSAGPRN

CIGQKFAILEEKAVISTVLRKYKIEAVDRREDLTLLGELILRPKDGLRVKITPRD

>CAQ57674.1

MKMITLILTSFALAIIVTYLVKLSYRRYQFLRKLKGLPGPKAYPIVGDSLEMLYLKRNEL

MKMNSEKRELYKSIYLEWSGPFAEIHLLRPEYVEVALKSTVNITKSMAYDFLHDWLGTGL

LTSSGRKWQERRKMITPAFHFGILEDFVEIFGEKSRTLVEILKKQKFGEEFDIYPMITNC

ALDIICESAMGTTVNAQEKKDSDYVRAVYEVSELILYRALRPWLYAEFIWKMSSHGKAFY

RNLKTLHDFTNKVIVERREATSKKCSLNESDDGVGKKKAFLDLLLEATENGHELSQADIR

EEVDTFMFEGHDTTAASIGWAIFLLGNNPEVQDRVVEELNDIFGDSDRLATIHDLNDMKY

LEMVIKETLRLYPSVPFIGRLVTEDMVVGEHLIPAGVWVNIELFSVHRCRDHYSDPEKFN

PDNFLPENTKSRHPFAYVPFSAGPRNCIGQKFALLEEKTILSSILRKFRVESTEKQEDIC

LMMDLVLRPESGVKIKMYPREQ

>EDV53009.1

MSSKVITSLMAESILLSKVSQVISGYSPITVFLLGSILIFLVVYNKRRSRLVKYIEKIPG

PAAMPFLGNAIEMNVDHDELFNRVIGMQKLWGTRIGINRVWQGTAPRVLLFEPETVEPIL

NSQKFVNKSHDYDYLHPWLGEGLLTSTDRKWHSRRKILTPAFHFKILDDFIDVFNEQSAV

LARKLAVEVGSEAFNLFPYVTLCTLDIVCETAMGRRIYAQSNSESEYVKAVYGIGSIVQS

RQAKIWLQSDLIFSLTADYKLHQSYINTLHGFSNMVIRERKAELAILQESNNNNNNNAPD

AYDDLGKKKRLAFLDLLIDASKEGTVLSNEDIREEVDTFMFEGHDTTSAAISWTLFLLGC

HPEYQERVVEELDAIFGKDDKETPATMKNLLDMRYLECCIKDSLRLFPSVPMMARMVGED

VNIGGKIVPAGTQAIIMTYALHRNPRVFPKPEQFNPDNFLPENCAGRHPFAYIPFSAGPR

NCIGQKFAILEEKAVISTVLRKYKIEAVDRREDLTLLGELILRPKDGLRVKITPRD

>EFX87731.1

MGVGGARLVVEDLLVVVGASVVVVVVVGFVVVEEGNILPDVVLLDKAVLTTFSSSSESDS

GESTDSSNFGLGVEEVAGYLPHGLHLFQTWNKRSVVRAWIGPLAIFAVGSAEGVEVVLGS

NKIIDKSREYNFLQPWLATGLLTSTGSKWHKRRKLLTPTFHFKILEDFIHVFNEQSAVLV

KKLGEKVGQDFDIFPFITRCTLDVICETAMGRHVNAQSNTDSEYVEAVYKMTRIVQTRQS

RPWLQPDWLFRLFPMASDQSRYLSILHGFTDQVIRERKIEFKQRNKEQQSGSLKEEDEEY

LLKKPRLAFLDLLIEASQDGKVLSDLDIREEVDTFMFEGHDTTAAAITWSLFLIGNHPEV

QEKVSEELTRVFGDSNRPVTMADLSELKYLECCIKEALRLYPSVPLMARELMEDTTICGY

DLPVGSTLMIVPFIVHRDPTYFPDPESFKPERFFPENIQGRHPYAYVPFSAGPRNCIGQK

FAQMEEKVILASLLRRFHVNSLDKPEDISLLVELILRPRDGIRLHLTPKMQHC

>EKC31870.1

MEPKMVYLWAWGSEVILQSSKHLDKAEEYKFLHPWLGTGLLTSTGSKWKSRRRMLTPTFH

FKILNDFVGVFHDQASIMVKKLSQVADGKEFDIFNYITLCALDIICETAMGRSIEAQSNS

NSDYVKSVYKMTELVLQRQRTPWYWPDFLYNTIGYGKEHDRCLKILHDFTLKVIKEKMDA

VSDASSNSMEEMLNQHSDEDVYRGKGQRLAFLDMLLCTADDGSHLSMEGIREEVDTFMFE

GHDTTAAAMNWAVHLIGANPDVQAKVHEEMDQIFGGSDRPASMNDLKEMRYLECCIKEAL

RLFPSVPFFGRKLTEDCKFGEYSIPKDTTVIITPPGLHRDERYFPDPEKFDPNRFLPENS

LKRHPYCYIPFSAGPRNCIGQKFAILEEKVMLSNIFRNFTVTSKQSREELFPIGDLIMRP

EHGIIVELRPRTT

>JC8026

MALLLGREFVWWSSVASYSLGTACLALLLTWFIRRQQTVWLIEKLPGPRSLPILGNALDV

NVAPRELFLKIMEFCEYGNTVKIWLGMYPYCLVSEAKSAEVLLSSNKHLDKSRDYNFLHP

WLGTGLLTSTGKKWHSRRKILTPAFHFKILEDFVEVFNSQSNKMLDKLTPKADGKAFDIF

PYITLCTLDIICETAMGININAQGNSNSEYVNAVYRIGALVQHRQTRPWIQPDFLFRLFG

YAKLHDEYLRVLHHFSNSAIENRRKEYQLEKLNAKENIDDDVIGKKRRLAFLDLLLNYSE

TQMPLSNEDIREEVDTFMFEGHDTTAAALNWSVYLLGCHPEIQAKVHEELDALFGDSDRP

VTMADLREMKYTENCIKEALRLFPSVPFLARELREEAVINNYRIPVGTTVMVITYRLHRD

PEQFPNPETFDPDRFLPENVAKRHPYSYVPFSAGPRNCIGQKFAIMEEKIVLSSIMRRFR

VESTTRREELKLLGELILRPENGNTVKLIPRTPKV

>390342677

MAIGIGIIAFIMALVAIVTVPLLKFIRLWIVFNKFHGPPAIPLLGNALQFKQDPNELVEQ

VSSWVEEYRTKSKGLMRLWLGPFPVLFIYNPHEVEPGKEPTRHIYEESEGKRLGRQITGK

KWFHRRKMLTPTFHFAILHNFIEVFDEQSNTLCQKFGEHAQKQDLVNICPLVTLCVLDII

SETAMGKQLHAQDESNNDYVNAVMRMSDIIQQRQKKPWFWPDPLFDNTKSGKEHAQCLQI

LHKMTNTLIDERSKEENQKVDSNDNDGSKRRIAFLDLLLKMQRDDPSFTKSDIREEVDTF

MFEGHDTTAALASWAMFLIGHHTRVQKKLHQELDSVFGDSDRPVTADDLQKLPYLTCVLK

ETLRIFPSVPIVGRDLQEDCIIDGKLAPRGTLLIIAIGSLHRDPTQFPEPLMFDPDRFLP

EFSSKRHPFSYVPFSAGPRNCIGQRFALMEDKVLLANVLRCFSLESTQSLKDTMPIAELI

LRPSEGIHMKITRRKGISNLDSKLLQRKQNNQRSRLVTV

>EDV90601.1

MSSKVFGSLMAESILLSKATQIISGYSPLTIFLLATIAIFLVVYNKRRARWVKYIEKIPG

PAALPFLGNAIEMNVDHDELFNRVIGMQKLWGTRIGINRVWQGTAPRVLLFEPETVEPIL

NSQKFVNKSHDYDYLHPWLGTGLLTSTDRKWHSRRKILTPAFHFKILDDFIDVFNEQSVV

LARKLNVELGNEAFNIFPYVTLCTLDIVCETAMGRKIYAQSNSESEYVKAVYGIGSIVQS

RQAKIWLQSDLLFRLTEDYKTHQSYINTLHGFSNMVIRERKSELADNNNNNNNNNNAEMP

DAYDDLGKKKRLAFLDLLIDASKDGTVLSNXXXXXXXXXREEVDTFMFEGHDTTSAAISW

TLFLLGANPEYQERVYEELESIFGDDTETPATMKNLLDMRYLECCIKDALRLFPSVPMMA

RMVGEDVTISGKLVPAGTQAIIMTYALHRNPRIFPKPEQFNPDNFLPENCAGRHPFAYIP

FSAGPRNCIGQKFAILEEKAVISTVLRKFKIESVDRREDLTLLGELILRPKDGLRVKITP

RH

>AAF09264.1

MSWLLDGALDLETGSVITYLIVTTLITLTLVWYFKRQQKVWLLEQIPGPRGLPILGNVLY

LNVDPPELFERFLAVAEYGEVSRLWLCNMCTCLLSSATTAEVILSSTKHLDKSEDYTLLH

PWLGESLLNSAGSRWHARRKLLTPAFHFKILEQFMEVFNSQTNKLVHKLLKKADGSPFDI

SDDITHCVLDIICETAMGRSINAQDNSESEYVQAVRKISGLIQYRQFRPWMYYEFLFKLM

GPIKEYNACFKTLHDMSNSTIKERKESRKDKANTEVLEEEEVFGKKKRQAFLDLMLEYAE

DNPELTDEEIRKEVDTFMFAGHDTTASAINWVLYTLGLHPDIQTRVQEELDDIFGSSDRP

ATMDDLRQMKYAEMCIKETMRLFTPVPVISRDIKEEVVINNYRIPANTIVAVVIYKIHRD

PEQFPDPEVFDPDRFLPENALKRHPYAYVPFSAGPRNCIGQKFAMLELKTVVSSIFRKLR

VESVIPRKDLKMTAEIILRPANGNILKLSPRTK

>AAL56662.1

MEWLRSQVVVMEEVGVVTYLVITALLALTLAAFFRRQYEVWIINRIPGPISLPLVGNALS

VTTDSEVLFKLGVWLVREYGQMVRVWIGMSPPVIISGARQAEVVLNNTKHLDKSHQYDFF

HPWLGTTGLFISKTSDWHTRKKLLTPAFHLKVLEQFVDVFNIQSNKLVSKLKKEADGCVF

DIFPYITNCTLDIICETAMGCSVNAQDNPESDYIMAIHRIQHLIQQRMIVLWMQPDFIFR

LLGYAREQEELLKTLHSFTRNIVKARRKLYEQQKQQGGAGSDDEQHLGKKQRLAFLDLLL

EYSEGGTVLTDEDIREEVDLFVFAGHDTTTVAINWCLYILGRHPEIQARVHEELDSIFEG

TDRPATMDDIRQMKYTENCIKEALRLFPSVPYVGRQLSGDINIGKYRIPAGASVMVFTYA

LHRDPEQFPDPEVFDPDRFLPENASKRHPFAYNAFSAGPRNCIGQKFGMIEEKVMVSSVL

RKFRIESITPMKKLKLLSEIVLRPKDGNHVRLFPRTAESPTSARGSNSVQDNNTRCQGEG

S

>EAT40234.1

MNHNIAAKIASLFSVLSPITTVILVVMVCAIITYKKKRARLVHHINRIPGPFMLPIIGNG

LHVTLGCKDEFLDRVISAQKMYGRRIGMSRAWNGPIPYVMISKASAVEPILSNPKLVEKS

VDYDFMKPWLGNGLLTSRASVWHPRRKTLTPAFHFKILSEFVNIFHKQALVMNEKLAEQL

DNTAGFDIVPFTTLCALDIFCETAMGCPVNAQRNSDSEYVRAHKLIGKIIRNRLQKVWLR

PDFIFKHTEDYRKHHECLQVLHSFSDRVVQERKAEIVAKRRQAEDLIDLNNNNESEELTS

CCRKKQLAFLDLLIEGSLDGNGLTDLDVREEVDTFVIGGHDTTAAAMAWILLLLGSDQKI

QDRVIDEIDGIMNGDRDRKPTMQELNDMKYLECCIKEGLRLYPSIPLIARRLTEDVQVDD

YIIPSGTTTLIVVYQLHRDPSVFPNPDKYNPDNFLPENCSGRHPYAYIPFSAGPRNCIGQ

KFAILEEKMVLSTVLRKFRIEAVERREDVKLLGDLVLRPRDGLKIRVSRRL

>EAT40232.1

MIEATVKSSFVLSKVAKMLSYFSPITIILATMIAGAIYVYNKRRARLVKLIEKIPGPASM

PLIGNSLHINVDHDEIFNRIISIRKLYGRQQGFSRAWNGPIPYVMISKASAVEPILGSPR

HIEKSHDYEFLKPWLGTGLLTSQGKKWHPRRKILTPAFHFKILDDFVDIFQEQSAVLVQR

LQRELGNEEGFNCFPYVTLCALDIVCETAMGRLIHAQKNSDSDYVKAVYQIGSIVQNRQQ

KIWLQPDFIFKRTEDYRNHQRCLSILHEFSNRVIRERKEEIRKQKQSNNNTINGNANNAV

EANILDGNNNAEEFGRKKRLAFLDLLIEASQDGTVLSNEDIREEVDTFMFEGHDTTSAAI

SWILLLLGAEPAIQDRIVEEIDHIMGGDRDRFPTMKELNDMKYLECCIKEGLRLYPSVPL

IARKLVEDVQIEDYTIPAGTTAMIVVYQLHRDPAVFPNPDKFNPDNFLPENCRGRHPYAY

IPFSAGPRNCIGQKFAVLEEKSVISAVLRKYRIEAVDRRENLTLLGELILRPKDGLRIKI

SRRE

>EFX87734.1

MFFTFVAICVFSKKRLAFIEHCNSIPGPPAPIPLLGNALELMRDPDELFQFLIDYLHEWR

QHFPVLRFWLGPFPIFLLYTPEGTEALLGSNKLIDKSREYQYLHPWLSTGLLTSTGTKWH

GRRKMLTPTFHFKILEDFIDIFNKQSVVLVEKLKEAHQDMVANNHDRINLFPYVARCTLD

IICETAMGRHVDAQLNNDSEYVKAVCTLGRVVQTRQAQPWLQPELLFQCHPMAKTQQKCL

DILHGFTDKVLQERKAEHRIRKAQELAQKENKTSPASADVDDNVDLIKKPRLAFLDLLIE

ASQDGTLLSDLDIREEVDTFMFEGQDTTAAAVSWCLYLIGSHPEVQERISEELNRIFGTS

DRPITMTDILQLKYLECCIKEALRLFPSVAMYGRTLSEDATIHGYVIPSGSTVAVIPYSL

HRDPVQFPDPERFDPERFMGDNKRSRHPYAYVPFSAGPRNCIGQKYAVMEEKVVLATVLR

NFHLESLEKREDLVLIGELVLRPRDGVQVRLTPKQITPSSPSC

>Q02928.1

MSVSVLSPSRLLGDVSGILQAASLLILLLLLIKAVQLYLHRQWLLKALQQFPCPPSHWLF

GHIQELQQDQELQRIQKWVETFPSACPHWLWGGKVRVQLYDPDYMKVILGRSDPKSHGSY

RFLAPWIGYGLLLLNGQTWFQHRRMLTPAFHYDILKPYVGLMADSVRVMLDKWEELLGQD

SPLEVFQHVSLMTLDTIMKCAFSHQGSIQVDRNSQSYIQAISDLNNLVFSRVRNAFHQND

TIYSLTSAGRWTHRACQLAHQHTDQVIQLRKAQLQKEGELEKIKRKRHLDFLDILLLAKM

ENGSILSDKDLRAEVDTFMFEGHDTTASGISWILYALATHPKHQERCREEIHSLLGDGAS

ITWNHLDQMPYTTMCIKEALRLYPPVPGIGRELSTPVTFPDGRSLPKGIMVLLSIYGLHH

NPKVWPNPEVFDPFRFAPGSAQHSHAFLPFSGGSRNCIGKQFAMNELKVATALTLLRFEL

LPDPTRIPIPIARLVLKSKNGIHLRLRRLPNPCEDKDQL

>AAO16078.1

MSVSVLSPSRLLGDVSGILQAASLLILLLLLIKAVQLYLHRQWLLKALQQFPCPPSHWLF

GHIQELQQDQELQRIQKWVETFPSACPHWLWGGKVRVQLYDPDYMKVILGRSDPKSHGSY

RFLAPWIGYGLLLLNGQTWFQHRRMLTPAFHYDILKPYVGLMADSVRVMLDKWEELLGQD

SPLEVFQHVSLMTLDTIMKCAFSHQGSIQVDRNSQSYIQAISDLNNLVFSRVRNAFHQND

TIYSLTSAGRWTHRACQLAHQHTDQVIQLRKAQLQKEGELEKIKRKRHLDFLDILLLAKM

ENGSILSDKDLRAEVDTFMFEGHDTTASGISWILYALATHPKHQERCREEIHSLLGDGAS

ITWNHLDQMPYTTMCIKEALRLFPPVPGIGRELSTPVTFPDGRSLPKGIMVLLSIYGLHH

NPKVWPNPEVFDPFRFAPGSAQHSHAFLPFSGGSRNCIGKQFAMNELKVATALTLLRFEL

LPDPTRIPIPIARLVLKSKNGIHLRLRRLPNPCEDKDQL

>AAB29502.1

MSVSVLSPSRLLGDVSGILQAASLLILLLLLIKAVQLYLHRQWLLKALQQFPCPPSHWLF

GHIQELQQDQELQRIQKWVETFPSACPHWLWGGKVRVQLYDPDYMKVILGRSDPKSHGSY

RFLAPWIGYGLLLLNGQTWFQHRRMLTPAFHYDILKPYVGLMADSVRVMLDKWEELLGQD

SPLEVFQHVSLMTLDTIMKCAFSHQGSIQVDRNSQSYIQAISDLNNLVFSRVRNAFHQND

TIYSLTSAGRWTHRACQLAHQHTDQVIQLRKAQLQKEGELEKIKRKRHLDFLDILLLAKM

ENGSILSDKDLRAEVDTFMFEGHDTTASGISWILYALATHPKHQERCREEIHSLLGDGAS

ITWNHLDQMPYTTMCIKEALRLYPPVPGIGRELSTPVTFPDGRSLPKGIMVLLSIYGLHH

NPKVWPNPEVFDPSRFAPGSAQHSHAFLPFSGGSRNCIGKQFAMNELKVATALTLLRFEL

LPDPTRIPIPIARLVLKSKNGIHLRLRRLPNPCEDKDQL

>297665030

MSVSVLSPSRLLGGVSGILPAASLLILLLLLIKAAQIYLHRQWLLKALQQFPCPPSHWLF

GHIQEFQHDQELQRIQKWVETFPSACPHWLWGGKVRVQLYDPDYMKVILGRSDPKSHGSY

RFLAPWIGYGLLLLNGQTWFQHRRMLTPAFHYDILKPYVGLMADSVRVMLDKWEKLLGQD

SPLEVFQHVSLMTLDTIMKCAFSHQGSIQVDRNSQSYIQAISDLNNLVFSRVRNAFHQND

TIYSLTSAGRWTHRACQLAHQHTDQVIQLRKAQLQKEGELEKIKRKRHLDFLDILLLARM

ENGSILSDKDLRAEVDTFMFEGHDTTASGISWILYALATHPKHQERCREEIHGLLGDGAP

ITWNHLDQMPYTTMCIKEALRLYPPVPGIGRELSTPVTFPDGRSLPKGIMVLLSIYGLHH

NPKVWPNPEVFDPSRFAPGSAQHSHAFLPFSGGSRNCIGKQFAMNELKVATALTLLRFEL

LPDPTRIPIPMARLVLKSKNGIHLHLRRLPNPCEDKDQL

>114556407

MSVSVLSPSRLLGGVSGILQVTSLLILLLLLIKAAQLYLHRQWLLKALQQFPCPPSHWLF

GHIQEFQHDQELQRIQERVKTFPSACPYWIWGGKVRVQLYDPDYMKVILGRSDPKSHGSY

RFLAPWIGYGLLLLNGQTWFQHRRMLTPAFHYDILKPYVGLMADSVRVMLDKWEELLGQD

SPLEVFQHVSLMTLDTIMKCAFSHQGSIQVDRNSQSYIQAISDLNSLVFCRMRNAFHEND

TIYSLTSAGRWTHRACQLAHQHTDQVIQLRKAQLQKEGELEKIKRKRHLDFLDILLLAKM

ENGSILSDKDLRAEVDTFMFEGHDTTASGISWILYALATHPKHQERCREEIHGLLGDGAS

ITWNHLDQMPYTTMCIKEALRLYPPVPGIGRELSTPVTFPDGRSLPKGIMVFLSIYGLHH

NPKVWPNPEVFDPSRFAPGSAQHSHAFLPFSGGSRNCIGKQFAMNELKVATALTLLRFEL

LPDPTRIPIPMARLVLKSKNGIHLRLRRLPNPCEDKDQL

>AAQ21367.1

MSVSVLSPSRRLGGVSGILQVTSLLILLLLLIKAAQLYLHRQWLLKALQQFPCPPSHWLF

GHIQEFQHDQELQRIQERVKTFPSACPYWIWGGKVRVQLYDPDYMKVILGRSDPKSHGSY

KFLAPWIGYSLLLLNGQTWFQHRRMLTPAFHYDILKPYVGLMADSVRVMLDKWEELLGQD

SPLEFFQHVSLMTLDTIMKSAFSHQGSIQVDRNSQSYIQAISDLNNLVFSRVRNAFHQND

TIYSLTSAGRWTHRACQLAHQHTDQVIQLRKAQLQKEGELEKIKRKRHLDFLDILLLAKM

ENGSILSDKDLRAEVDTFMFEGHDTTASGISWILYALATHPKHQERCREEIHSLLGDGAS

ITWNHLDQMPYTTMCIKEALRLYPPVPGIGRELSTPVTFPDGRSLPKGIMVLLSIYGLHH

NPKVWPNPEVFDPFRFAPGSAQHSHAFLPFSGGSRNCIGKQFAMNQLKVARALTLLRFEL

LPDPTRTPIPMARLVLKSKNGIHLRLRRLPNPCEDKDQL

>397518937

MSVSVLSPSRLLGGVSGILQVTSLLILLLLLIKAAQLYLHRQWLLKALQQFPCPPSHWLF

GHIQEFQHDQELQRIQERVKTFPSACPYWIWGGKVRVQLYDPDYMKVILGRSDPKSHGSY

RFLAPWIGYGLLLLNGQTWFQHRRMLTPAFHYDILKPYVGLMADSVRVMLDKWEELLGQD

SPLEVFQHVSLMTLDTIMKCAFSHQGSIQVDRNSQSYIQAISDLNSLVFCRMRNAFHEND

TIYSLTSAGSWTHRTCQLAHQHTDQVIQLRKAQLQKEGELEKIKRKRHLDFLDILLLAKM

ENGSILSDKDLRAEVDTFMFEGHDTTASGISWILYALATHPKHQERCREEIHGLLGDGAS

ITWNHLDQMPYTTMCIKEALRLYPPVPGIGRELSTPVTFPDGRSLPKGIMVFLSIYGLHH

NPKVWPNPEVFDPSRFAPGSAQHSHAFLPFSGGSRNCIGKQFAMNELKVATALTLLRFEL

LPDPTRIPIPMARLVLKSKNGIHLHLRRLPNPCEDRDQL

>EAX06886.1

MSVSVLSPSRLLGDVSGILQAASLLILLLLLIKAVQLYLHRQWLLKALQQFPCPPSHWLF

GHIQELQQDQELQRIQKWVETFPSACPHWLWGGKVRVQLYDPDYMKVILGRSDPKSHGSY

RFLAPWIGYGLLLLNGQTWFQHRRMLTPAFHYDILKPYVGLMADSVRVMLDKWEELLGQD

SPLEVFQHVSLMTLDTIMKSAFSHQGSIQVDRNSQSYIQAISDLNSLVFCRMRNAFHEND

TIYSLTSAGRWTHRACQLAHQHTDQVIQLRKAQLQKEGELEKIKRKRHLDFLDILLLAKM

ENGSILSDKDLRAEVDTFMFEGHDTTASGISWILYALATHPKHQERCREEIHGLLGDGAS

ITWNHLDQMPYTTMCIKEALRLYPPVPGIGRELSTPVTFPDGRSLPKGIMVLLSIYGLHH

NPKVWPNLEVFDPSRFAPGSAQHSHAFLPFSGGSRNCIGKQFAMNQLKVARALTLLRFEL

LPDPTRIPIPMARLVLKSKNGIHLRLRRLPNPCEDKDQL

>AAZ29443.1

MSVSVLSPSRLLGSVSGILQVTSLLILLLLLIKAAQLYLHRQWLLKALQQFPCPPSHWFF

GHFQKFQQDQELQRIRKWVEMFPSACPLWLWGGKVRVQLHDPDYMKVILGRSDPKSHGSY

RFLAPWIGYGLLLLNGQTWFQHRRMLTPAFHYDILKAYVALMADSVRVMLDKWEKLLGQD

SPLEVFQHVSLMTLDTIMKCAFSHQGSIQVDRDSQSYIQAISDLNNLVFSRVRNVFHQND

TIYSLTSTGRWTHRACQLAHQHTDQVIQLRKAQLQKEGELEKVKRKKHLDFLDILLLAKM

ENGSILSDKDLRAEVDTFMFEGHDTTASGISWILYALATHPKHQERCREEIHGLLGDGAS

ITWNHLDQMPYTTMCIKEALRLYPPVPGISRELSTPVTFPDGRSLPKGIAVMLSIYGLHH

NPKVWPNPEVFDPSRFAPGSAQHSHAFLPFSGGSRNCIGKQFAMNELKVATALTLLRFEL

LPDPTRIPIPMARLVLKSKNGIHLRLRRLPNPCEDKDQL

>397483490

MSVSVLSPSRLLGDVSGILQAASLLILLLLLIKAAQLYLHRQWLLKALQQFPCPPSHWLF

GHIQEFQQDQELQRIQKWVETFPSACPHWLWGGKVRVQLHDPDYMKVILGRSDPKSHGSY

RFLAPWIGYGLLLLNGQTWFQHRRMLTPAFHYDILKPYVGLMADSVRVMLDKWEELLGQD

SPLEVFQHVSLMTLDTIMKCAFSHQGSIQVDRNSQSYIQAISDLNNLVFSRMRNAFHQND

TIYSLTSAGRWTHRACQLAHQHTDQVIQLRKAQLQKEGELEKIKRKRHLDFLDILLLAKM

ENGSILSDKDLRAEVDTFMFEGHDTTASGISWILYALATHPXHQERCREEIHGLLGDGAS

ITWNHLDQMPYTTMCIKEALRLYPPVPGIGRELSTPVTFPDGRSLPKGIMVFLSIYGLHH

NPKVWPNPEVFDPSRFAPGSAQHSHAFLPFSGGSRNCIGKQFAMNELKVATALTLLRFEL

LPDPTRIPIPIARLVLKSKNGIHLRLRRLPNPFLLESGTAGQHLSSFHPADLISFLFLMY

PLLKWYFESPVLPRGHLLWVYERPAFSLAQFSQAFQSSGPLRRREPSWLRELMAQPFLLL

ILLCMSLLLFQVIRLYQRRRWTIRAMHLFPAPPVHWFYGHKEFYPVKEFEVYPELMEKYP

CAVPLWVGPFTMFFNIHDPDYVKILLKRQEKVIQDRKESLKDKLKQDTTQKRRRDFLDIL

LSAKSENTKDFSEADLQAEVKTFMFAGHDTTSSAIFWIFYCLAKYPEHQQRCRDEIRELL

GDGSSITWEHLSQMPYTTMCIKECLRLYAPVVNISRLLDKPITFPDGRSLPAGITVFINI

WALHHNPYFWEDPQVFNPLRFSRESSEKMHPYAFIPFSAGLRNCIGQHFAIIECKVAVAL

TLLRFELAPDYSRPSQPVRQMLLKSKNGIHVFAKKVC

>AAF76722.1

MSVSVLSPSRRLGGVSGILQVTSLLILLLLLIKAAQLYLHRQWLLKALQQFPCPPSHWLF

GHIQEFQHDQELQRIQERVKTFPSACPYWIWGGKVRVQLYDPDYMKVILGRSDPKSHGSY

RFLAPRIGYGLLLLNGQTWFQHRRMLTPAFHNDILKPYVGLMADSVRVMLDKWEELLGQD

SPLEVFQHVSLMTLDTIMKSAFSHQGSIQVDRNSQSYIQAISDLNSLVFCCMRNAFHEND

TIYSLTSAGRWTHRACQLAHQHTDQVIQLRKAQLQKEGELEKIKRKRHLDFLDILLLAKM

ENGSILSDKDLRAEVDTFMFEGHDTTASGISWILYALATHPKHQERCREEIHGLLGDGAS

ITWNHLDQMPYTTMCIKEALRLYPPVPGIGRELSTPVTFPDGRSLPKGIMVLLSIYGLHH

NPKVWPNLEVFDPSRFAPGSAQHSHAFLPFSGGSRNCIGKQFAMNQLKVARALTLLRFEL

LPDPTRIPIPIARLVLKSKNGIHLRLRRLPNPCEDKDQL

>AAQ21368.1

MAVSVLSPSRRLGGVSGILQVTSLLILLLLLIKAAQLYLHRQWLLKALQQFPCPPSHWLF

GHIQEFQHDQELQRIQERVKTFPSACPYWIWGGKVRVQLYDPDYMKVILGRSDSKSHGSY

KFLAPWIGYSLLLLNGQTWFQHRRMLTPAFHYDILKPYVGLMADSVRVMLDKWEELLGQD

SPLEFFQHVSLMTLDTIMKSAFSHQGSIQVDRNSQSYIQAISDLSSLVFCRMRNAFHEND

AIYSLTSAGRWTHRACQLAHQHTDQVIQLRKAQLQTEGELEKIKRKRHLDFLDILLLAKM

ENGSILSDKDLRAEVDTFMFEGHDTTASGISWILYALATHPKHQERCREEIHGLLGDGAS

ITWNHLDQMPYTTMCIKEALRLYPPVPGIGRELSTPVTFPDGRSLPKGIMVLLSIYGLHH

NPKVWPNPEVFDPSRFAPGSAQHSHAFLPFSGGSRNCIGKQFAMNQLKVARALTLLRFEL

LPDPTRIPIPMARLVLKSKNGIHLRLRRFPNPCEDKDQL

>Q5TCH4.1

MSVSVLSPSRRLGGVSGILQVTSLLILLLLLIKAAQLYLHRQWLLKALQQFPCPPSHWLF

GHIQEFQHDQELQRIQERVKTFPSACPYWIWGGKVRVQLYDPDYMKVILGRSDPKSHGSY

KFLAPRIGYGLLLLNGQTWFQHRRMLTPAFHNDILKPYVGLMADSVRVMLDKWEELLGQD

SPLEVFQHVSLMTLDTIMKSAFSHQGSIQVDRNSQSYIQAISDLNSLVFCCMRNAFHEND

TIYSLTSAGRWTHRACQLAHQHTDQVIQLRKAQLQKEGELEKIKRKRHLDFLDILLLAKM

ENGSILSDKDLRAEVDTFMFEGHDTTASGISWILYALATHPKHQERCREEIHGLLGDGAS

ITWNHLDQMPYTTMCIKEALRLYPPVPGIGRELSTPVTFPDGRSLPKGIMVLLSIYGLHH

NPKVWPNLEVFDPSRFAPGSAQHSHAFLPFSGGSRNCIGKQFAMNQLKVARALTLLRFEL

LPDPTRIPIPMARLVLKSKNGIHLRLRRLPNPCEDKDQL

>109004076

MSVSVLSPSRLLGGVSGILQVASLLILLLLLIKAAQLYLHRQWLLKAFQQFPCSPSHWLF

GHKQEFQQDQELQRIRKWVEMFPSACPLWLWGGKARVQLHDPDYMKVILGRSDPKSQDPY

RFLAPWIGYGLLLLNGQTWFQHRRMLTPAFHYDILKAYVALMADSVRVMLDKWEKLLGQD

SPLEVFQHVSLMTLDTIMKCAFSHQGSIQVDRDSQSYIQAISDLNNLTFSRARNAFHQND

TIYSLTSTGRWTHRACQLAHQHTDQVIQLRKAQLQKEGELEKVKRKKHLDFLDILLLAKM

ENGSILSDKDLRAEVDTFMFEGHDTTASGISWILYALATHPKHQERCREEIHGLLGDGAS

ITWNHLDQMPYTTMCIKEALRLYPPVPGISRELSTPVTFPDGRSLPKGITVMLSIYGLHH

NPKVWPNPEVFDPSRFAPGSAQHSHAFLPFSGGSRNCIGKQFAMNELKVATALTLLRFEL

LPDPTRIPIPMARLVLKSKNGIHLRLRRLPNPCEDKDQL

>AAB29503.1

MSVSVLSPSRLLGDVSGILQAASLLILLLLLIKAVQLYLHRQWLLKALQQFPCPPSHWLF

GHIQELQQDQELQRIQKWVETFPSACPHWLWGGKVRVQLYDPDYMKVILGRSDPKSHGSY

RFLAPWIGYGLLLLNGQTWFQHRRMLTPAFHYDILKPYVGLMADSVRVMLDKWEELLGQD

SPLEVFQHVSLMTLDTIMKCAFSHQGSIQVDRNSQSYIQAISDLNNLVFSRVRNAFHQND

TIYSLTSAGRWTHRACQLAHQHTDQVIQLRKAQLQKEGELEKIKRKRHLDFLDILLLAKM

ENGSILSDKDLRAEVDTFMFEGHDTTASGISWILYALATHPKHQERCREEIHSLLGDGAS

ITWNHLDQMPYTTMCIKEALRLYPPVPGIGRELSTPVTFPDGRSLPKGIMVLLSIYGLHH

NPKVWPNPEVFDPSRFAPGSAQHSHAFLPFSGGSRNCIGKQFAMNELKVATALTLLRFEL

LPDPTRIPIPIARLVLKSKMESTCVSGGSLTLVKTRTSFEGLHLPSCLPDPRFCPLPVCP

YPVFCLPTFPSSHLPAVPQSACPSLSHLSPGLPTCLSTCLLPTCISCWEKS

>CAH93009.1

MSVSVLSPSRLLGGVSGILPAASLLILLLLLIKAAQLYLHRQWLLKALQQFPCPPSHWLF

GHTQEFQHDQELQRIQEWVKTFPSACPYWIWGGKVRVQLYDPDYMKVILGRSDPKSHGSY

RFLAPWIGYGLLLLNGQTWFQHRRMLTPAFHYDILKPYVGLMADSVRVMLDKWEKLLGQD

SPLEVFQHVSLMTLDTIMKCAFSHQGSIQVDRNSQSYIQAISDLNSLVFCRMRNAFHEND

TIYSLTSAGRWTHRACQLAHQHTDQVIQLRKAQLQKEGELEKVKRKRHLDFLDLLLLAKM

ENGSILSDKDLRAEVDTFMFEGHDTTASGISWILYALATHPKHQERCREEIHGLLGDEAS

ITWNHLDQMPYTTMCIKEALRLYPPVPGIGRELSTPVTFPDGRSLPKGIMVLLSIYGLHH

NPKVWPNPEVFDPSRFAPGSAQHSHAFLPFSGGSRNCIGKQFAMNELKVATALTLLRFEL

LPDPTRIPIPMARLVLKSKNGIHLRLRRLPNPCEDKDQL

>EAX06885.1

MSVSVLSPSRLLGDVSGILQAASLLILLLLLIKAVQLYLHRQWLLKALQQFPCPPSHWLF

GHIQELQQDQELQRIQKWVETFPSACPHWLWGGKVRVQLYDPDYMKVILGRSDPKSHGSY

RFLAPWIGYGLLLLNGQTWFQHRRMLTPAFHYDILKPYVGLMADSVRVMLDKWEELLGQD

SPLEVFQHVSLMTLDTIMKSAFSHQGSIQVDSLTSAGRWTHRACQLAHQHTDQVIQLRKA

QLQKEGELEKIKRKRHLDFLDILLLAKMENGSILSDKDLRAEVDTFMFEGHDTTASGISW

ILYALATHPKHQERCREEIHGLLGDGASITWNHLDQMPYTTMCIKEALRLYPPVPGIGRE

LSTPVTFPDGRSLPKGIMVLLSIYGLHHNPKVWPNLEVFDPSRFAPGSAQHSHAFLPFSG

GSRNCIGKQFAMNQLKVARALTLLRFELLPDPTRIPIPMARLVLKSKNGIHLRLRRLPNP

CEDKDQL

>403291733

MSVSVLSPSRLLGDISRILQLASLLILLLLLIKVAQFYLHRQWLLKVLQEFPCPPSHWLF

GHNQEYPQDQELQRIRKWVETFPSACPHWLWGSKARVQLYDPDYIKVILGRSDPKSHSTY

RFLPPFIGYGLLLLNGQTWFQHRRMLTPAFHYDVLKPYVEFMADSVRVMLDKWEELIGQD

SPLEVFQHVSLMTLDTIMKCAFSHQGSIQLDRDSQSYIQAIMDMNDLVFFRVRNAFYQND

IIYSLTTAGRWSHRACQLAHQHTDRVIQQRKAHLQEQGELEKVRKKRHLDFLDIVLLAKM

ENGNSLSDKDLRAEVDTFMFAGHDTTASGISWILYALATHPKHQQRCREEIQGLLGDGDS

ITWHHLDQMPYTTMCIKEALRLYPPVPGISRDLVTPITFPDGRSLPKGISVVLSIYGLHH

NPKVWPNPEVFDPSRFAPGSAQHSHAFLPFSGGARNCIGKQFAMNELKVAVALTLLRFEL

LPDPTRIPIPTPRIVLKSENGIHLHLRRLPNPCKDKDQL

>390465895

MSVSVLSPSRLLGDVSRILQLTSLLILLLLLIKVAQFYLHRQWLLKVLQEFPCPPSHWLF

GHNKEYSQDQELQRIRKWVEIFPSACPYWLWGSKARVQLYDPDYIKVVLGRSDPKSYGSY

RFLAPWIGHGLLLLNGQTWFQHRRMLTPAFHYDILKPYVEFMADSVRVMLDKWEELIGQD

SPLEIFQHVSLMTLDTIMKCAFSHQGSIQLDRDSQSYIQAVTDLNNLFYFRVRNAFYQND

TIYSLTTAGRWSHRACQLAHQHTDRVIQQRKALLQEQGELEKVRKKRHLDFLDILLLAKM

ENGNSLSDKDLRAEVDTFMFEGHDTTASGISWVLYALATHSKHQQRCREEIQGLLGDGAS

ITWHHLDKMPYTTMCIKEALRLYPPVPGIGRELSTPITFPDGRSLPKGISIILSIYGLHH

NPKVWPNPEVFDPSRFAPGSAQHSHAFLPFSGGARNCIGKQFAMNELKVAVALTLLRFEL

LPDPTRIPIPTPRIVLKSENGIHLHLRRLPNPC

>332220075

MSVSVLSPSRLLGGVSGILQAASLLILLLLLIKAAQLYLHRQWLLKALQQFPCPPSHWLF

GHIQEFQEDQELQQTRKWVETFPSACPRWLWGGKVRVQLYDPDYMKVILGRSDPKSHGSY

RFLAPWIGYGLLLLNGQTWFQHRRMLTPAFHYDILKPYVGLMADSVRVMLDKWEKLLGQE

DSPLEVFQHISLMTLDTIMKCAFSHQGSIQVDRNSQSYIQAIRDLNDLLFSRVRNAFYQN

DTIYSLTSAGRWTHRACQLAHQYTDQVVQLRKAQLQKEGELEKIKRKRHLDFLDILLLAK

MENGSILSDKDLRAEVDTFMFEGHDTTASGISWIFYALATHPKHQERCREEIHGLLGDGA

SITWNHLDQMPYTTMCIRRHCIMVVLSIYGLHYNPKVWPNPEVFDPFRFAPGSAQHSHAF

LPFSGGSRNCIGKQFAMNELKVATALTLLRFELLPDPTRTPIPMARLVLKSKNGIHLRLR

RLPNPCEDKDQL

>AAH41158.1

MSVSVLSPSRLLGDVSGILQAASLLILLLLLIKAVQLYLHRQWLLKALQQFPCPPSHWLF

GHIQELQQDQELQRIQKWVETFPSACPHWLWGGKVRVQLYDPDYMKVILGRSDPKSHGSY

RFLAPWIGYGLLLLNGQTWFQHRRMLTPAFHYDILKPYVGLMADSVRVMLDKWEELLGQD

SPLEVFQHVSLMTLDTIMKCAFSHQGSIQVDRNSQSYIQAISDLNNLVFSRVRNAFHQND

TIYSLTSAGRWTHRACQLAHQHTDQVIQLRKAQLQKEGELEKIKRKRHLDFLDILLLAKM

ENGSILSDKDLRAEVDTFMFEGHDTTASGISWILYALATHPKHQERCREEIHSLLGDGAS

ITWNHLDQMPYTTMCIKEALRLYPPVPGIGRELSTPVTFPDGRSLPKGIMVLLSIYGLHH

NPKVWPNPEVFDPFRFAPGSAQHSHAFLPFSGGSR

>332808912

MSVSVLSPSRLLGDVSGILQAASLLILLLLLIKAAQLYLHRQWLLKALQQFPCPPSHWLF

GHIQEFQQDQELQRIQKWVETFPSACPHWLWGGKVRVQLHDPDYMKVILGRSDPKSHGSY

RFLAPWIGYGLLLLNGQTWFQHRRMLTPAFHYDILKPYVGLMADSVRVMLDKWEELLGQD

SPLEVFQHVSLMTLDTIMKCAFSHQGSIQVDRNSQSYIQAISDLNNLVFSRVRNAFHQND

TIYSLTSAGRWTHRACQLAHQHTDQVIQLRKAQLQKEGELEKIKRKRHLDFLDILLLAKM

ENGSILSDKDLRAEVDTFMFEGHDTTASGISWILYALATHPKHQERCREEIHGLLGDGAS

ITWNHLDQMPYTTMCIKEALRLYPPVPGIGRELSTPVTFPDGRSLPKGIMVFLSIYGLHH

NPKVWPNPEVFDPSRFAPGSAQHSHAFLPFSGGSR

>AAI42608.1

MSVSVLSPSRRLGGVSGILQVTSLLILLLLLIKAAQLYLHRQWLLKALQQFPCPPSHWLF

GHIQEFQHDQELQRIQERVKTFPSACPYWIWGGKVRVQLYDPDYMKVILGRSDPKSHGSY

KFLAPWIGYSLLLLNGQTWFQHRRMLTPAFHYDILKPYVGLMADSVRVMLDKWEELLGQD

SPLEFFQHVSLMTLDTIMKSAFSHQGSIQVDRNSQSYIQAISDLNSLVFCRMRNAFHEND

TIYSLTSAGRWTHRACQLAHQHTDQVIQLRKAQLQTEGELEKIKRKRHLDFLDILLLAKM

ENGSILSDKDLRAEVDTFMFEGHDTTASGISWILYALATHPKHQERCREEIHGLLGDGAS

ITWNHLDQMPYTTMCIKEALRLYPPVPGIGRELSTPVTFPDGRSLPKGIMVLLSIYGLHH

NPKVWPNPEVFDPSRFAPGSAQHSHAFLPFSGGSR

>CAI19738.1

MSVSVLSPSRRLGGVSGILQVTSLLILLLLLIKAAQLYLHRQWLLKALQQFPCPPSHWLF

GHIQEFQHDQELQRIQERVKTFPSACPYWIWGGKVRVQLYDPDYMKVILGRSDPKSHGSY

KFLAPRIGYGLLLLNGQTWFQHRRMLTPAFHNDILKPYVGLMADSVRVMLDKWEELLGQD

SPLEVFQHVSLMTLDTIMKSAFSHQGSIQVDRNSQSYIQAISDLNSLVFCCMRNAFHEND

TIYSLTSAGRWTHRACQLAHQHTDQVIQLRKAQLQKEGELEKIKRKRHLDFLDILLLAKM

ENGSILSDKDLRAEVDTFMFEGHDTTASGISWILYALATHPKHQERCREEIHGLLGDGAS

ITWNHLDQMPYTTMCIKEALRLYPPVPGIGRELSTPVTFPDGRSLPKGIMVLLSIYGLHH

NPKVWPNLEVFDPSRFAPGSAQHSHAFLPFSGGSR

>395858256

MSVSVLSPSRLPGGIGLLQVASLLVLFLLLLKSAQLFLQRQWLLRALQQFPGSPSHWFFG

HSRQIQKDKELQQILKWVEKFPSACPYWVWGSHARVQIYDPDYLKLILGRSDPKDHGFYK

FLAPWIGYGLLLLNGQTWFQHRRMLTPAFHYNILKPYVELMVDSVQVMLDKWEELSSQDS

PLEIFQHISLMTLDTIMKCAFSHQGSIQLDRNSRSYIQAVGDLNSLYFHRVRNAFYHYNI

IYSLTTDGRSAQRAYELAHQHTDRVIQLRKAHLQEEGELEKVQRKRHLDFLDILLLSKME

NGSSLSDKDIRAEVDTFMFEGHDTTASGISWILYALAAHPEHQQRCREEIQSLLGDGASI

TWAHLDQMPYTTMCIKEALRLYPPVPSIARELSKPITFPDGRSLPKGIVVSLSIYGLHHN

PKVWPNPEVFDPSRFTPGSAQHSHAFLPFSGGARNCIGKQFAMNELKVAVALTLLRFELL

PDPTRIPIPILRIVLKSKNGINLYLRKLPKPYGNRL

>P14579.1

MSVSALSPTRLPGSLSGLLQVAALLGLLLLLLKAAQLYLRRQWLLRALQQFPCPPFHWLL

GHSREFQMNQELQQILKWVEKFPRACPHWIGGNKVRVQLYDPDYMKVILGRSDPKSRGSY

TFVAPWIGYGLLLLNGQPWFQHRRMLTPAFHYDILKPYVGLMVDSVQIMLDKWEQLVSQD

SSLEVFQDISLMTLDTIMKCAFSYQGSVQLDSRNSQSYIQAVGDLNNLVFARVRNIFHQS

DTIYRLSPEGRLSHRACQLAHEHTDRVIQQRKAQLQQEGELEKVRRKRRLDFLDVLLFAK

MENGSSLSDQDLRAEVDTFMFEGHDTTASGVSWIFYALATHPEHQHRCREEIQGLLGDGA

SITWEHLDQMPYTTMCIKEAMRLYPPVPAISRDLSSPVTFPDGRSLPKGFTVTLSIYGLH

HNPNVWPNPEVFDPGRFTPGSARHSHAFLPFSGGARNCIGKQFAMNELKVAVALTLVRFE

LLPDPTRIPKPTARLVLKSNNGIHLRLRKLQ

>CAA40493.1

MSVSALSPTRLPGSLSGLLQVAALLGLLLLLLKAAQLYLRRQWLLRALQQFPCPPFHWLL

GHSREFQMNQELQQILKWVEKFPRACPHWIGGNKVRVQLYDPDYMKVILGRSDPKSRGSY

TFVAPWIGYGLLLLNGQPWFQHRRMLTPAFHYDILKPYVGLMVDSVQIMLDKWEQLVSQD

SSLEVFQDISLMTLDTIMKCAFSYQGSVQLDSRNSQSYIQAVGDLNNLVFARVRNIFHQS

DTIYRLSPEGRLSHRACQLAHEHTDRVIQQRKAQLQQEGELEKVRRKRRLDFLDVLLFAK

MENGSSLSDQDLRAEVDTFMFEGHDTTASGVSWIFYALATHPEHQHRCREEIQGLLGDGA

SITWEHLDQMPYTTMCIKEAMRLYPPVPAISRDLSSPVTFPDGRSLPKGFTVTLSIYGLH

HNPNVWPNPEVFDPSRFTPGSARHSHAFLPFSGGARNCIGKQFAMNELKVAVALTLLRFE

LLPDPTRIPKPTARLVLKSNNGIHLRLRKLQ

>P14580.1

MSVSALNPTRLPGSLSGLLQVAGLLGLLLLLLKAAQLYLHRQWLLRALQQFPCPPFHWLL

GHSREFQNGHELQVMLKWVEKFPSACPRWLWGSRAHLLIYDPDYMKVILGRSDPKAQGSY

RFLAPWIGYGLLLLNGQTWFQHRRMLTPAFHYDILKPYVGLMADSVQIMLDKWEQLVSQD

SSLEVFQDISLMTLDTIMKCAFSHQGSVQLDRNSQSYIQAVGDLNNLFFSRVRNVFHQSD

TIYRLSPEGRLSHRACQLAHEHTDRVIQQRKAQLQQEGELEKVRRKRRLDFLDVLLFAKM

ENGSSLSDQDLRAEVDTFMFEGHDTTASGISWIFYALATHPEHQHRCREEIQGLLGDGAS

ITWEHLDQMPYTTMCIKEALRLYPPVPGVGRQLSSPVTFPDGRSLPKGVIVTLSIYALHH

NPKVWPNPEVFDPFPFAPGSARHSHAFLPFSGGPRNCIGKQFAMNELKVAVALTLVRFEL

LPDPKRVPDQKPRLVLKSSNGIHLRLRKLR

>194207489

MSVSGLSPPRPLGSVSGLLQVASLLGLVLLLLKAAQLYLRRQWLLKAFQEFPCPPSHWLY

GHSQELRNDQGLQDLLKRVEKFPCASPRWLGGTSVQLLVYDPDYMKVILGRSDPKSLGTY

KFLAPWIGYGLLLLNGQTWFQHRRMLTPAFHYDILKPYVGLMADSVRVMLDKWEELINQD

SHLDIFGHISLMTLDTIMKCAFSHQGSIQTDRNSQSYTHAVEDLNNRFYVRLRNVFYHSD

IIYRLTREGSWNHRACKLAHQHTEQVIKLRKAQLRKEGELEKVRKKRHLDFLDILLFARM

ENGSSLSDKDLRAEVDTFMFEGHDTTASGISWILYALAMHPEHQQKCREEIQGLLGDGTS

ISWDHLDQMPYTTMCIKEALRLYPPVPGIGRELSKPITFPDGRSLPRGIMVMLSFYALHH

NPKVWPNPEVFDPFRFAPGSSQHSHAFLPFSGGSRNCIGKQFAMNELKVAVALTLLRFEL

APDPSRVAVPVPRIVLKSKNGIHLHLRKLL

>EAX06887.1

MKVILGRSDPKSHGSYRFLAPWIGYGLLLLNGQTWFQHRRMLTPAFHYDILKPYVGLMAD

SVRVMLDKWEELLGQDSPLEVFQHVSLMTLDTIMKSAFSHQGSIQVDRNSQSYIQAISDL

NSLVFCRMRNAFHENDTIYSLTSAGRWTHRACQLAHQHTDQVIQLRKAQLQKEGELEKIK

RKRHLDFLDILLLAKMENGSILSDKDLRAEVDTFMFEGHDTTASGISWILYALATHPKHQ

ERCREEIHGLLGDGASITWNHLDQMPYTTMCIKEALRLYPPVPGIGRELSTPVTFPDGRS

LPKGIMVLLSIYGLHHNPKVWPNLEVFDPSRFAPGSAQHSHAFLPFSGGSRNCIGKQFAM

NQLKVARALTLLRFELLPDPTRIPIPMARLVLKSKNGIHLRLRRLPNPCEDKDQL

>P10611.3

MSVSALSPTRLPGSLSGLLQVAALLGLLLLLLKAAQLYLHRQWLLRALQQFPCPPFHWLL

GHSREFQNDQELERIQKWVEKFPGACPWWLSGNKARLLVYDPDYLKVILGRSDPKAPRNY

KLMTPWIGYGLLLLDGQTWFQHRRMLTPAFHYDILKPYVGLMVDSVQIMLDRWEQLISQD

SSLEIFQHVSLMTLDTIMKCAFSYQGSVQLDRNSHSYIQAINDLNNLVFYRARNVFHQSD

FLYRLSPEGRLFHRACQLAHEHTDRVIQQRKAQLQQEGELEKVRRKRRLDFLDVLLFAKM

ENGSSLSDQDLRAEVDTFMFEGHDTTASGVSWIFYALATHPEHQHRCREEIQGLLGDGAS

ITWEHLDQMPYTTMCIKEALRLYPPVPSVTRQLSKPVTFPDGRSLPKGVILFLSIYGLHY

NPKVWQNPEVFDPFRFAPDSAYHSHAFLPFSGGARNCIGKQFAMRELKVAVALTLVRFEL

LPDPTRIPIPIARVVLKSKNGIHLRLRKLH

>AAA31234.1

MSVSALNPTRLPGSLSGLLQVAGLLGLLLLLLKAAQLYLHRQWLLRALQQFPCPPFHWLL

GHSREFQNGHELQVMLKWVEKFPSACPRWLWGSRAHLLIYDPDYMKVILGRSDPKAQGSY

RFLAPWIGYGLLLLNGQTWFQHRRMLTPAFHYDILKPYVGLMADSVQIMLDKWEQLVSQD

SSLEVFQDISLMTLDTIMKCAFSHQGSVQLDRNSQSYIQAVGDLNNLFFSRVRNVFHQSD

TIYRLSPEGRLSHRACQLAHEHTDRVIQQRKAQLQQEGELEKVRRKRRLDFLDVLLFAKM

ENGSSLSDQDLRAEVDTFMFEGHDTTASGISWIFYALATHPEHQHRCREEIQGLLGDGAS

ITWEHLDQMPYTTMCIKEALRLYPPVPGVGRQLSSPVTFPDGRSLPKGVIVTLSIYALHH

NPKCGPNPEVFDPSPFAPGSARHSHAFLPFSGGPRNCIGKQFAMNELKVAVALTLLRFEL

LPDPKRVPDQKPRLVLKSSNGIHLRLRKLR

>BAF64511.1

MSVSVLSPTRALGGVSGLLQVASLLGLVLLLLKAAQLYLHRQWLLKALQQFPSPPSHWLY

GHMQEFQEETELRPLLKRVEKYPSACARWLWGTKALVLVYDPDYMKVVLGRSDPKSHDSY

RLLIPWIGNGLLLLERQTWFQHRRMLTPAFHYDILKPYVGLMADSVRVMLDKWEELVSLD

SHLEVLGHVSLMTLETIMKCAFSHQGSIQTDRNIQSYIQAIRDLGNLTFSRIRNAFHQND

FIYRLTPEGRWSHRACQLAHQHTDAVIKLRKAHLQKEGELEKVRSKRHLDFLDILLFARM

ENGSSLSDVDLRAEVDTFMFEGHDTTASGISWILYALASHPEHQQRCREEIQSLLRDGAS

ITWDHLDKMPYTTMCIKEALRLYPPVPVISRDMSKPITFPDGRSLPAGIPLSLSFYGLHH

NPKVWPNPEVFDPSRFAPGSDRHSHAFLPFSGGSRNCIGKQFAMNEMKVAVALTLLRFEL

APDPSRVPVPIPRVVLKSKNGIHLQLRKLL

>EDL30645.1

MSVSALSPTRFADSLSGFLQVASVLGLLLLLVKAVQFYLHRQWLLKAFQQFPSPPFHWFF

GHEQFKGDQELQEIVSCIENFPSAFPRWFWGSKAYLTVYDPDYMKVILGRSDPKANGAYR

LLAPWIGYGLLLLNGQPWFQHRRMLTPAFHYDILKPYVKNMADSIRLMLDKWERLAGQDS

SIEIFQHISLMTLDTVMKCAFSHKGSVQVDGNYRTYLQAIGDLNNLFHSRVRNIFHQNDT

IYKLSSNGRLAKQACQLAHDHTDGVIKLRKDQLQDEGELEKIKKKRRLDFLDILLFARME

NGDSMSDKDLRAEVDTFMFEGHDTTASGVSWIFYALATHPDHQQRCREEVQSLLGDGSSI

TWDHLDQIPYTTMCIKEALRLYPPVPGIVRELSTSVTFPDGRSLPKGVQVTLSIYGLHHN

PKVWPNPEVFDPSRFAPDSPRHSHSFLPFSGGARNCIGKQFAMSELKVIVALTLLRFELL

PDPTRVPMPLARLVLKSKNGIYLHLKKLH

>O88833.2

MSVSALSPTRFADSLSGFLQVASVLGLLLLLVKAVQFYLHRQWLLKAFQQFPSPPFHWFF

GHEKFKGDQELQEIVSCIENFPSAFPRWFWGSKAYLTVYDPDYMKVILGRSDPKANGAYR

LLAPWIGYGLLLLNGQPWFQHRRMLTPAFHYDILKPYVKNMADSIRLMLDKWERLADQDS

SIEIFQHISLMTLDTVMKCAFSHKGSVQVDGNYRTYLQAIGDLNNLFHSRVRNIFHQNDT

IYKLSSNGRLAKQACQLAHDHTDGVIKLRKDQLQDEGELEKIKKKRRLDFLDILLFARME

NGDSMSDKDLRAEVDTFMFEGHDTTASGVSWIFYALATHPDHQQRCREEVQSLLGDGSSI

TWDHLDQIPYTTMCIKEALRLYPPVPGIVRELSTSVTFPDGRSLPKGVQVTLSIYGLHHN

PKVWPNPEVFDPSRFAPDSPRHSHSFLPFSGGARNCIGKQFAMSELKVIVALTLLRFELL

PDPTRVPMPLARLVLKSKNGIYLHLKKLH

>157384975

MSVSALSLTRFAGSLSGFLQVASVFCLLLLLVKAVQVYLHRKWLLKALQQFPSPPFHWFF

GHEQFKGDQELQEIVSCIENFPSAFPRWFWGSKAYLTVYDPDYMKVILGRSDPKANGAYR

LLAPWIGYGLLLLNGQSWFQHRKMLTPAFHYDILKTYVKNMADSIRLMLDKWERLAGQDS

SIEIFQHISLMTLDTVMKCAFSHKGSVQVDGNYKTYLQAIGDLNNLVHSRVRNMFHQNDT

IYKLSSNGRLSNQACQLAHDHTDGVIKMRKDQLQDEGELENIKKKRRLDFLDILLFARME

NEDSMSDKDLRAEVDTFMIEGHDTTASGVSWIFYALATHPEHQQRCREEVQSLLGDGSSI

TWDHLDQIPYTTMCIKEALRLYPPVPSIGRELSTSVTFPDGCSLPKGVQVTLSIYGLHHN

PKVWPNPEVFDPSRFAPDSPRHSHSFLPFSGGARNCIGKQFAMSELKVIVALTLLRFELL

PDPTRVPMSLARFVLKSKNGIYLHLKKVH

>ABA03106.1

MSVSLLSLTRPLDTVSGFLQVASLLGLALLLLKAAQFYWHRQWLLKAIQQFPSPPSHWFF

GHRQEFQKGRELQELKKRTEKYPCASPRWLWGSRVSLVVYDPDYMRMILGRSDPKSDGSY

RLMAPWIGYGLLLLNGEIWFQHRRMLTPAFHYDILKPYVRLMADSVQVMLDKWEELLTQN

SHLEIFEHVSLMTLDTIMKCAFSYQGNHQADRNSQAYIQAIRDLNNLVFARVRNVFYQKD

IFYGLTSEGRRNHKACQLAHEHTDRVIKLRKAQLQDEGELEKIRNKRHLDFLDILLFAKM

ENGRGLSDKDLRAEVDTFMFEGHDTTASGISWILYALATHPEHQQRCREEIQSLLGDGAS

ITWEHLDQMPYTTMCIKEALRLYPPVPGVGRELSKPITFPDGRSLPRGFLVLLSFYALHH

NPNVWPNPEVFDPSRFAPDASRHSHAFLPFSGGSRNCIGQHFAMNELKVAVALTLLRFEL

APDPFRIPVPTPRIVLMSKNGIHLHLRKLL

>AAH10747.1

MSVSALSPTRFADSLSGFLQVASVLGLLLLLVKAVQFYLHRQWLLKAFQQFPSPPFHWFF

GHEQFKGDHELQEIVSCIENFPSAFPRWFWGSKAYLTVYDPDYMKVILGRSDPKANGAYR

LLAPWIGYGLLLLNGQPWFQHRRMLTPAFHYDILKPYVKNMADSIRLMLDKWERLADQDS

SIEIFQHISLMTLDTVMKCAFSHKGSVQVDGNYRTYLQAIGDLNNLFHSRVRNIFHQNDT

IYKLSSNGRLAKQACQLAHDHTDGVIKLRKDQLQDEGELEKIKKKRRLDFLDILLFARME

NGDSMSDKDLRAEVDTFMFEGHDTTASGVSWIFYALATHPDHQQRCREEVQSLLGDGSSI

TWDHLDQIPYTTMCIKEALRLYPPVPGIVRELSTSVTFPDGRSLPKGVQVTLSIYGLHHN

PKVWPNPEVFDPSRFAPDSPRHSHSFLPFSGGARNCIGKQFAMSELKVIVALTLLRFELL

PDPTRVPMPLARLVLKSKNGIYLHLKKLH

>354469986

MSVSDLSATRFPGSISGFLQVASVLSLLLVLFKVVQFYLHRQWLLRNTQQFPCPPSHWFF

GHKLDFLVDQELQDILTRVKNFPSACPHWLWGTKLRFQVYDPDYMKVILGRSDPKAHGSY

RFLAPWIGYGLLLLNGETWFQHRRMLTPAFHYDILKPYVKTMADSVHVMLDKWEQIVGQD

STLEIFQHISLMTLDTIMKCAFSHEGSVQLDKKYQSYIQAVGDLNNLFFFRVRNIFHQND

TIYSLSSNGRQAISAYQLAHDHTDKVIKSRKAQLKDEGELEKARKKRRLDFLDLLLFARM

ENGSILSDKDLRAEVDTFMFEGHDTTASGISWIFYALATHPEYQKRCREEVQSLLGDGSS

ITWDHLDQMSYTTMCIKEALRLYPPVPGVSRDLSTPVTFPDGRSLPKGISVLLSFYGLHH

NPTVWPNPEVFDPSRFAPESSRHSHSFLPFSGGARNCIGKQFAMNELKVAVALTLLRFEL

LPDPTRIPVPIPRLVLKSKNGIHLRLKKLQ

>348551406

MSISALSPTRFLGGISGLLQVAFLLSLLLLLFKAAQFYLHRQWLFKALQQFPCPPSHWLF

GHKYQDQELQQVLKWAKEFPSACPRWLWGSRLTILLYDPDYIKVILGRSDPKAHGTYRLI

SPWIGYGLLLLNGPTWFQHRRMLTPAFHYDILKPYVAVMADSVQGMLDKWEQLISEDTPL

EIFQHVSLMTLDSLMKCAFNDQSCAQFNRKPHPYIQAVGDLNYLTFSRIRNVFHQNDTIY

SLSSNGRLFKQACKIAHEHTDQVIKQRKSQLQDERELEKIKRKRRLDFLDILLFARMEDG

SSLSNKDLRAEVDTFMFEGHDTTASGISWILYALAAHPKHQQKCREEVQSLLGNEASITW

EHLDQMPYTTMCIKEALRLYPPVPSVSRELSEPVTFPDGRSLPKGITVSLAIYALHHNPK

VWPNPEVYDPSRFALDSTRHSHSFLPFSGGSRNCIGKQFAMNELKVAVALTLLRFELLPD

PSRIPLPVSRIVMKSKNGIHLHLRKLP

>CAA30245.1

MSVSALSSTRFTGSISGFLQVASVLGLLLLLVKAVQFYLQRQWLLKAFQQFPSPPFHWFF

GHKQFQGDKELQQIMTCVENFPSAFPRWFWGSKAYLIVYDPDYMKVILGRSDPKANGVYR

LLAPWIGYGLLLLNGQPWFQHRRMLTPAFHYDILKPYVKNMADSIRLMLDKWEQLAGQDS

SIEIFQHISLMTLDTVMKCAFSHNGSVQVDGNYKSYIQAIGNLNDLFHSRVRNIFHQNDT

IYNFSSNGHLFNRACQLAHDHTDGVIKLRKDQLQNAGELEKVKKKRRLDFLDILLLARME

NGDSLSDKDLRAEVDTFMFEGHDTTASGVSWIFYALATHPKHQQRCREEVQSVLGDGSSI

TWDHLDQIPYTTMCIKEALRLYPPVPGIVRELSTSVTFPDGRSLPKGIQVTLSIYGLHHN

PKVWPNPEVFDPSRFAPDSPRHSHSFLPFSGGARNCIGKQFAMSEMKVIVALTLLRFELL

PDPTKVPIPLPRLVLKSKNGIYLYLKKLH

>AAA41061.1

MSVSALSSTRFTGSISGFLQVASVLGLLLLLVKAVQFYLQRQWLLKAFQQFPSPPFHWFF

GHKQFQGDKELQQIMTCVENFPSAFPRWFWGSKAYLIVYDPDYMKVILGRSDPKANGVYR

LLAPWIGYGLLLLNGQPWFQHRRMLTPAFHYDILKPYVKNMADSIRLMLDKWEQLAGQDS

SIEIFQHISLMTLDTVMKCAFSHNGSVQVDGNYKSYIQAIGNLNDLFHSRVRNIFHQNDT

IYNFSSNGHLFNRACQLAHDHTDGVIKLRKDQLQNAGELEKVKKKRRLDFLDILLLARME

NGDSLSDKDLRAEVDTFMFEGHDTTASGVSWIFYALATHPKHQQRCREEVQSVLGDGSSI

TWDHLDQIPYTTMCIKEALRLYPPVPGIVRELSTSVTFPDGRSLPKGIQVTLSIYGLHHN

PKVWPNPEVFDPSRFAPDSPRHSHSFLPFSGGARNCIGKQFAMSEMKVIVALTLLRFELL

PDPTKVPIPLPRLVLKSKNGIYLYLKKLH

>354469982

MSVSDLSATRFPGSISGFLQVASVLSLLLVLFKVVQFYLHRQWLLRNTQQFPCPPSHWFF

GHKVPKDQELQDILTRVKNFPSACPHWLWGTKLRFQVYDPDYMKVILGRSDPKAHGSYRF

LAPWIGYGLLLLNGETWFQHRRMLTPAFHYDILKPYVKTMADSVHVMLDKWEQIVGQDST

LEIFQHISLMTLDTIMKCAFSHEGSVQLDKKYQSYIQAVGDLNNLFFFRVRNIFHQNDTI

YSLSSNGRQAISAYQLAHDHTDKVIKSRKAQLKDEGELEKARKKRRLDFLDLLLFARMEN

GSILSDKDLRAEVDTFMFEGHDTTASGISWIFYALATHPEYQKRCREEVQSLLGDGSSIT

WDHLDQMSYTTMCIKEALRLYPPVPGVSRDLSTPVTFPDGRSLPKGISVLLSFYGLHHNP

TVWPNPEVFDPSRFAPESSRHSHSFLPFSGGARNCIGKQFAMNELKVAVALTLLRFELLP

DPTRIPVPIPRLVLKSKNGIHLRLKKLQ

>P08516.2

MSVSALSSTRFTGSISGFLQVASVLGLLLLLVKAVQFYLQRQWLLKAFQQFPSPPFHWFF

GHKQFQGDKELQQIMTCVENFPSAFPRWFWGSKAYLIVYDPDYMKVILGRSDPKANGVYR

LLAPWIGYGLLLLNGQPWFQHRRMLTPAFHYDILKPYVKNMADSIRLMLDKWEQLAGQDS

SIEIFQHISLMTLDTVMKCAFSHNGSVQVDGNYKSYIQAIGNLNDLFHSRVRNIFHQNDT

IYNFSSNGHLFNRACQLAHDHTDGVIKLRKDQLQNAGELEKVKKKRRLDFLDILLLARME

NGDSLSDKDLRAEVDTFMFEGHDTTASGVSWIFYALATHPEHQQRCREEVQSVLGDGSSI

TWDHLDQIPYTTMCIKEALRLYPPVPGIVRELSTSVTFPDGRSLPKGIQVTLSIYGLHHN

PKVWPNPEVFDPSRFAPDSPRHSHSFLPFSGGARNCIGKQFAMSEMKVIVALTLLRFELL

PDPTKVPIPLPRLVLKSKNGIYLYLKKLH

>AAH60945.1

MSASALSSIRFPGSISEYLQVASVLSLLLLLFKTAQLYLHRQWLLSSTQQFPSPPSHWLF

GHKILKDQDLQDILTRIKNFPSACPQWLWGSKVRIQVYDPDYMKLILGRSDPKAHGSYRF

LAPWIGRGLLLLDGQTWFQHRRMLTPAFHYDILKPYTEIMADSVHVMLDKWEQIVGQDST

LEIFQHITLMTLDTIMKCAFSHEGSVQLDRKYKSYIQAVEDLNNLFFLRVRNIFHQNDII

YRVSSNGCLANSACQLAHDHTDQVIKSRRSQLQDEEELEKLKMKRRLDFLDILLFARMEN

GKSLSDKDLRAEVDTFMFEGHDTTASSISWIFYALATNPEHQQRCRKEIQSLLGDGASIT

WNDLDKMPYTTMCIKEALRIYPPVPSVSRELSSPVTFPDGRSLPKGIHVMLSFYGLHHNP

TVWPNPEVFDPSRFAPGSSRHSHSFLPFSGGARNCIGKQFAMNELKVAVALTLLRFELLP

DPTRVPIPIPRIVLKSKNGIHLHLKKLQ

>P14581.1

MSVSALSSTRLPGSFSGFLQAAALLGLLLLLLKAAQLYLRRQWLLRALQQFPCPPSHWLL

GHSREFPIDSELQQVLKRVEKFPSACPRWLWGSELFLICYDPDYMKTILGRSDPKARVSY

SFLAPWIGYGLLLLEGQTWFQHRRMLTPAFHYDILKPYVGLMVDSVQVMLDKLEKLARKD

APLEIYEHVSLMTLETIMKCAFSHQGSVQLESRTSKSYIQAVRELSDLALQRVRNVFHQS

DFLYRLSPEGRLSHRACQLAHEHTDRVIQQRKAQLQQEGELEKVRRKRRLDFLDVLLFAK

MENGSSLSDQDLRAEVDTFMFEGHDTTASGISWIFYALATHPEHQHRCREEIQGLLGDGA

SITWEHLDKMPYTTMCIKEALRLYPPVPGVGSKLSSPVTFPDGRSLPKGIIITLSIYGLH

HNPKVWPNPEVFDPSRFAPGSARHSHAFLPFSGGSRNCIGKQFAMNELKVAVALTLVRFE

LLPDPTRVPIPITRLVLKSKNGIHLRLRKLH

>DAA30988.1

MSVSALSPSRALGGVSGLLQVVSLLGLVLLLIKAAQLYLRRQWLLKALHHFPSPPSHWFY

GHKREFQEEGELPHLLKRVEKYPRACVRWMWGTRALLLVYDPDYMKMVLGRSDPKAQIIH

RFVKPWIGTGLLLLEGQTWFQHRRMLTPAFHYDILKPYVGIMADSVRVMLDKWEELVSQD

SHLEIFGHVSLMTLDTIMKCAFSQQGSVQTDRNSQSYIQAIKDVSHLIISRLRNAFHQND

LIYRLTPEGHWNHRACQLAHQHTDAVIKERKVRLQKEGELEKVRSRRHLDFLDILLFARM

ENGSSLSDEDLRAEVDTFMFEGHDTTASGISWILYALASHPEHQQRCREEIQSLLADGAS

ITWDHLDQMPYTTMCIKEAMRLYPPVPVISRELSKPITFPDGRSLPAGILVSLSIYGLHH

NPKVWPNPEVFDPTRFAPGSTRHSHAFLPFSGGSRNCIGKQFAMNELKVAVALTLLRFEL

SPDSSRVPVPMPVIVLRSKNGIYLQLRKLSDPGT

>AAA31233.1

MSVSALSSTRLPGSFSGFLQAAALLGLLLLLLKAAQLYLRRQWLLRALQQFPCPPSHWLL

GHSREFPIDSELQQVLKRVEKFPSACPRWLWGSELFLIVYDPDYMKTILGRSDPKARVSY

SFLAPWIGYGLLLLEGQTWFQHRRMLTPASHYDILKPYVGLMVDSVQVMLDKLEKLARKD

APLEIYEHVSLMTLETIMKCAFSHQGSVQLESRTSKSYIQAVRELSDLALQRVRNVFHQS

DFLYRLSPEGRLSHRACQLAHEHTDRVIQQRKAQLQQEGELEKVRRKRRLDFLDVLLFAK

MENGSSLSDQDLRAEVDTFMFEGHDTTASGISWIFYALATHPEHQHRCREEIQGLLGDGA

SITWEHLDKMPYTTMCIKEALRLYPPVPGVGRQLSSPVTFPDGRSLPKGIIITLSIYGLH

HNPKVWPNPEVFDPSRFAPGSARHSHAFLPFSGGSRNCIGKQFAMNELKVAVALTLLRFE

LLPDPTRVPIPITRLVLKSKNGIHLRLRKLH

>AAI18406.1

MSVSALSPSRALGGVSGLLQVVSLLGLVLLLIKAAQLYLRRQWLLKALHHFPSPPSHWFY

GHKREFQEEGELPHLLKRVEKYPRACVRWMWGTRALLLVYDPDYMKMVLGRSDPKAQIIH

RFVKPWIGTGLLLLEGQTWFQHRRMLTPAFHYDILKPYVGIMADSVRVMLDKWEELVSQD

SHLEIFGHVSLMTLDTIMKCAFSQQGSVQTDRNSQSYIQAIKDVSHLIISRLRNAFHQND

LIYRLTPEGHWNHRACQLAHQHTDAVIKERKVRLQKEGELEKVRSRRHLDFLDILLFARM

ENGSSLSDEDLRAEVDTFMFEGHDTTASGISWILYALASHPEHQQRCREEIQSLLADGAS

ITWDHLDQMPYTTMCIKEAMRLYPPVPVISRELSKPITFPDGRSLPAGILVSLSIYGLHH

NPKVWPNPEVFDPTRFAPSSTRHSHAFLPFSGGSRNCIGKQFAMNELKVAVALTLLRFEL

SPDSSRVPVPMPVIVLRSKNGIYLQLRKLSDPGT

>311259135

MSVSALSPIGALGSVSGLLQVASVFGLLLLLLKATQLYLHRQWLLKALQQFPSPPSHWLY

GHSREHLKDNDLQEALRRVKNFPRACPYWLWGNKAHLVIYDPDYMKVVLGRSDPKLTDVH

KFLSPWIGYGLLLSDGQTWFQHRRMLTPAFHYDILKPYVNLMADSVKVMLDKWEQLVTQD

SHLEIFGHVSLMTLDTVMKCAFSYQGSVQTDRNSHSYIQAVGNLNSLFVARLRSALYQND

IIYRLSPEGRLSRQACQLAHQHTDQVIRLRKAHLQKEGEMENLRKKRHLDFLDILLFARM

ENGSSLSDTDLRAEVDTFMFEGHDTTASGISWIFYALASHPEYQQRCREEIRSLLGDGTS

ITWDHLGQMPYTTMCIKEAMRLYPPVPAIIRELSKPITFPDGRSLPAGIIISLSIYALHH

NPKVWPNPEVFDPSRFAPGSAQHSHAFLPFSGGSRNCIGKQFAMNEMKVAVALTLLRFEL

ALDASRVPIPLRRVVLKSKNGIHLYLRKLP

>EDL30641.1

MSVSALSPTRFADSLSGFLQVASVLGLLLLLVKAVQFYLHRKWLLKALQQFPSPPFHWFF

GHEQFKGEQELKEVVSCIEHFPSAFPCWFWGSNAYLTVYDPDYMKVILGRSDPKANGIYR

LLAPWIGYGLLLLNGQPWFQHRRMLTPAFHYDILKPYVKNMADSIRLMLDKWERLAGQDS

SIEIFQHISLMTLDTVMKCAFSHKGSVQVDGNYKTYLQAIGDLNNLFHSRVRNIFHQNDT

IYRLSSNGRLAKQACQLAHDHTDGVIKMRKDQLQDEGELENIKKKRRLDFLDILLFARME

NGDSMSDKDLRAEVDTFMFEGHDTTASGVSWIFYALATHPEHQQRCREEVQSLLGDGSSI

TWDHLDQIPYTTMCIKEALRLYPPVPGIVRELSTSVTFPDGRSLPKGVQVTLSIYGLHHN

PKVWPNPEVFDPSRFAPDSPRHSHSFLPFSGGARNCIGKQFAMSELKVIVALTLLHFELL

PDPTRVPEPLARIVLKSKNGIYLHLKKLH

>P24464.2

MSGSALSFTIFPGSILGFLQIATVLTVLLLLFKTAQFYLHRRWLLRATQQFPSPPSHWFF

GHKIPKDQEFQDILTRVKNFPSACPQWLWGSNVRIQVYDPDYMKLILGRSDPKSHHSYRF

LAPWIGYGLLLLNGQTWFQHRRMLTPAFHYDTLKPYVGIMADSVRIMLDKWEQIVGQDST

LEIFQHITLMTLDTIMKCAFSQEGSVQLDRKYKSYIKAVEDLNNLSFFRIRNIFHQNDII

YSLSSNGRKARSAWQLAHEHTDQVIKSRKAQLQDEEELQKVKQKRRLDFLDILLFARIEN

GSSLSDKDLRAEVDTFMFEGHDTTASGISWIFYALATNPEHQQGCRKEIQSLLGDGASIT

WDDLDKMPYTTMCIKEALRIYPPVTAVSRMLSTPVTFPDGRSLPKGITVMLSFYGLHHNP

TVWPNPEVFDPYRFAPESSRHSHSFLPFSGGARNCIGKQFAMNELKVAVALTLLRFELLP

DPTRIPIPIPRLVLKSKNGIYLRLKKLQ

>EDL30643.1

MSVSALSLTRFAGSLSGFLQVASVFCLLLLLVKAVQVYLHRKWLLKALQQFPSPPFHWFF

GHEFKGDQELQEIVSCIENFPSAFPRWFWGSKAYLTVYDPDYMKVILGRSDPKANGAYRL

LAPWIGYGLLLLNGQSWFQHRKMLTPAFHYDILKTYVKNMADSIRLMLDKWERLAGQDSS

IEIFQHISLMTLDTVMKCAFSHKGSVQVDGNYKTYLQAIGDLNNLVHSRVRNMFHQNDTI

YKLSSNGRLSNQACQLAHDHTDGVIKMRKDQLQDEGELENIKKKRRLDFLDILLFARMEN

EDSMSDKDLRAEVDTFMIEGHDTTASGVSWIFYALATHPEHQQRCREEVQSLLGDGSSIT

CPPDLKDGLYFRDHLDQIPYTTMCIKEALRLYPPVPSIGRELSTSVTFPDGCSLPKGVQV

TLSIYGLHHNPKVWPNPEVFDPSRFAPDSPRHSHSFLPFSGGARNCIGKQFAMSELKVIV

ALTLLRFELLPDPTRVPMSLARFVLKSKNGIYLHLKKVH

>AAA63485.1

MSGSALSFTIFPGSILGFLQIATVLTVLLLLLKTAQFYLHRRWLLRATQQFPSPPSHWFF

GHKIPKDQDFQDILTRVKNFPSACPQWLWGSNVRIQVYDPEYMKLILGRSDPKAHGSYRF

LAPWIGYGLLLLNGQTWFQHRRMLTPAFHYDTLKPYVGIMADSVRIMLDKWEQIVGQDST

LEIFQHITLMTLDTIMKCAFSQEGSVQLDRKYKSYIKAVEDLNNLFFFRVQNMFHQNDFI

YSLSSNGRKAHNAWQLAHDYTDQVIKSRKAQLQDEEELQKVKQKRRLDFLDILLFARIEN

GSSLSDKDLRAEVDTFMFEGHDTTASGISWIFYALATNPEHQQGCRKEIQSLLGDGASIT

WDDLDKMPYTTMCIKEALRIYPPVTAVSRMLSTPVTFPDGRSLPKGITVMLSFYGLHHNP

TVWPNPEVFDPYRFAPESSRHSHSFLPFSGGARNCIGKQFAMNELKVAVALTLLRFELLP

DPTRIPIPIPRLVLKSKNGIYLRLKKLQ

>AAI42396.1

MSVSVLSPTRALGGVSGLLQVVSLLGLVLLLLKAAQLYLRRQWLLKALHQFPSPPSHWFY

GHKKEFQKESELPPLLERVEKYPKACVRWLWGTKALVLVYDPDYMKVFLGRSDPKPHRTY

KYLAPWIGTGLLLLEGQKWFQHRRMLTPAFRYDILKAYVGIMADSVRVMLDKWEELVSQD

SHLEIFGHVSLMTLDTIMKCAFSHQGSVQMDRSSQSYIQAIRDLSHLIVSRLRNAFHQND

LIYRLTPEGRWNHRACQLTHQHTDAVIKERKAHLQKEGELEKVRSRRHLDFLDILLLARM

ENGSSLSDEDLRAEVDTFMFEGHDTTASGISWILYALASHPEHQQRCREEIQSLLGDGAS

ITWDHLDQMRYTTMCIKEAMRLYPPVPFIGRELRKPITFPDGRSLPAGILVSLSFYGLHH

NPNVWPNPEVFDPTRFSPGSTQHSYAFLPFSGGSRNCIGKQFAMNELKVAVALTLLRFEL

SPDPSRVPVPTPIMVLRSKNGIHLQLRKLSDPGLL

>ABA03107.1

MSVSVLSLTRPLDTVSGLLQLASLLGLALLLLKAAQLYQRRQWLLKAVQQFPSPPSHWLF

GHKRELQKGDELQLLLKWIEDFPCAFSRWIWGTEAEIMIYDPDYMKLILGRSDPKPDGSY

KLMAPWIGYGLLLLNGETWSQHRRMLTPAFHYDILKPYVRLMADSVQVMLDKWEELLSQN

SHLEIFDHVSLMTLDTIMKCAFSYQGNHQADRNSQAYIQAIRDLNNLVFARMRNVFYQKD

IFYGLTSEGRRNHKACQLAHQHTDRVIKLRKAQLQDKGELEKVRNKRHLDFLDILLFAKV

ENGRGLSDKDLRAEVDTFMFEGHDTTASGISWILYALATHPEHQQRCREEIQSLLGDGAS

ITWEHLDQMPYTTMCIKEALRLYPPVPGVGRELSKPITFPDGRSLPKGFLVLLSFYALHH

NPNVWPNPEVFDPSRFAPGVSRHSHAFLPFSGGSRNCIGQHFAMNEMKVAVALTLLRFEL

APDPFRIPVPTPKIVLMSKNGIHLHLRKLL

>ABA03105.1

MSVSVLSLTRPLDTVSGLLQLASLLGLALLLLKAAQLYQRRQWLLKAVQQFPSPPSHWFF

GHKQEFQTGQELQVFQKWTEKYPCASPRWLWGSRVSLVVYDPDYMKMILGRSDPKAGGSY

TFITPWIGYGLLLLHGEAWFQHRRMLTPAFHYDILKPYVRLMADSVQVMLDKWEELLTQN

SHLEIFEHVSLMTLDTIMKCAFSYQGNHQADRNSQAYIQAIRDMNNLVFARVRNVFYQKD

IFYGLTSEGRRNHKARQIAHQHTDRVIKLRKAQLQDEGELEKVRNKRHLDFLDILLFAKM

ENGKGLSDKDLRAEVDTFMFEGHDTTASGISWILYALATHPEHQQRCREEIQSLLGDGAS

ITWEHLDQMPYTTMCIKEALRLYPPVPGVGRELSKPITFTDGRSLPKGFLVSLSFYALHH

NPNVWPNPEVFDPSRFAPDASRHSHAFLPFSGGSRNCIGQHFAMNELKVAVALTLLRFEL

APDPFKIPVPTPRIVLMSKNGIHLHLRKLL

>BAC05239.1

MSASALSSIRFPGSISEYLQVASVLSLLLLLFKTAQLYLHRQWLLSSTQQFPSPPSHWLF

GHKILKDQDLQDILTRIKNFPSACPQWLWGSKVRIQVYDPDYMKLILGRSDPKAHGSYRF

LAPWIGRGLLLLDGQTWFQHRRMLTPAFHYDILKPYTEIMADSVRVMLDKWEQIVGQDST

LEIFQHITLMTLDTIMKCAFSHEGSVQLDRKYKSYIQAVEDLNNLFFLRVRNIFHQNDII

YRVSSNGCLANSACQLAHDHTDQVIKSRRSQLQDEEELEKLKKKRRLDFLDILLFARMEN

GKSLSDKDLRAEVDTFMFEGHDTTASGISWIFYALATNPEHQQRCRKEIQSLLGDGASIT

WNDLDKMPYTTMCIKEALRIYPPVPSVSRELSSPVTFPDGRSLPKGIHVMLSFYGLHHNP

TVWPNPEVFDPSRFAPGSSRHSHSFLPFSGGARNCIGKQFAMNELKVAVALTLLRFELLP

DPTRVPIPIPRIVLKSKNGIHLHLKKLQ

>EDL30650.1

MSASALSSIRFPGSISEYLQVASVLSLLLLLFKTAQLYLHRQWLLSSTQQFPSPPSHWLF

GHKILKDQDLQDILTRIKNFPSACPQWLWGSKVRIQVYDPDYMKLILGRSDPKAHGSYRF

LAPWIGRGLLLLDGQTWFQHRRMLTPAFHYDILKPYTEIMADSVHVMLDKWEQIVGQDST

LEIFQHITLMTLDTIMKCAFSHEGSVQLDRKYKSYIQAVEDLNNLFFLRVRNIFHQNDII

YRVSSNGCLANSACQLAHDHTDQVIKSRRSQLQDEEELEKLKKKRRLDFLDILLFARMEN

GKSLSDKDLRAEVDTFMFEGHDTTASGISWIFYALATNPEHQQRCRKEIQSLLGDGASIT

WNDLDKMPYTTMCIKEALRIYPPVPSVSRELSSPVTFPDGRSLPKGIHVMLSFYGLHHNP

TVWPNPEVFDPSRFAPGSSQHSHSFLPFSGGARNCIGKQFAMNELKVAVALTLLRFELLP

DPTRVPIPIPRIVLKSKNGIHLHLKKLQ

>CAM21084.1

MSASALSSIRFPGSISEYLQVASVLSLLLLLFKTAQLYLHRQWLLSSTQQFPSPPSHWLF

GHKILKDQDLQDILTRIKNFPSACPQWLWGSKVRIQVYDPDYMKLILGRSDPKAHGSYRF

LAPWIGRGLLLLDGQTWFQHRRMLTPAFHYDILKPYTEIMADSVHVMLDKWEQIVGQDST

LEIFQHITLMTLDTIMKCAFSHEGSVQLDRKYKSYIQAVEDLNNLFFLRVRNIFHQNDII

YRVSSNGCLANSACQLAHDHTDQVIKSRRSQLQDEEELEKLKKKRRLDFLDILLFARMEN

GKSLSDKDLRAEVDTFMFEGHDTTASGISWIFYALATNPEHQQRCRKEIQSLLGDGASIT

WNDLDKMPYTTMCIKEALRIYPPVPSVSRELSSPVTFPDGRSLPKGIHVMLSFYGLHHNP

TVWPNPEVFDPSRFAPGSSRHSHSFLPFSGGARNCIGKQFAMNELKVAVALTLLRFELLP

DPTRVPIPIPRIVLKSKNGIHLHLKKLQ

>CAM21091.1

MSVSALSPTRFADSLSGFLQVASVLGLLLLLVKAVQFYLHRQWLLKAFQQFPSPPFHWFF

GHEKFKGDQELQEIVSCIENFPSAFPRWFWGSKAYLTVYDPDYMKVILGRSDPKANGYGL

LLLNGQPWFQHRRMLTPAFHYDILKPYVKNMADSIRLMLDKWERLADQDSSIEIFQHISL

MTLDTVMKCAFSHKGSVQVDGNYRTYLQAIGDLNNLFHSRVRNIFHQNDTIYKLSSNGRL

AKQACQLAHDHTDGVIKLRKDQLQDEGELEKIKKKRRLDFLDILLFARMENGDSMSDKDL

RAEVDTFMFEGHDTTASGVSWIFYALATHPDHQQRCREEVQSLLGDGSSITWDHLDQIPY

TTMCIKEALRLYPPVPGIVRELSTSVTFPDGRSLPKGVQVTLSIYGLHHNPKVWPNPEVF

DPSRFAPDSPRHSHSFLPFSGGARNCIGKQFAMSELKVIVALTLLRFELLPDPTRVPMPL

ARLVLKSKNGIYLHLKKLH

>EGV94558.1

MSVSALSPSRFVGSISGFLQVVSVLGLLLLLLKVVQFYLHRQWLLKAFQQFPSPPFHWFL

GHKQFQGDQEIEQMMKCVENFPSAFPRWFWGSKAYFTVYDPDYMKVILGRSDPKANGAYR

LLAPWIGYGLLLLNGQPWFQHRRMLTPAFHYDILKPYVKTMADSVRLMLDKWEQLAGQDS

SIEIFQHISLMTLDTVMKCAFSHKGSVQVDGNYKTYIQAIGNLNNLFYSRVRNIFHQNDT

IYSLSSNGRMANQACQLAHDHTDGVIKLRKDQLQDEEELEKVKKKRRLDFLDILLFARME

NGDSLSDKDIRAEVDTFMFEGHDTTASGVSWIFYALATHPEHQQRCREEVQSLLGDGSSI

TWDHLDQMPYTTMCIKEALRLYPPVPAIVRELITPVTFPDGRSLPKGVSVTLSIYALHHN

PKVWPNPEVFDPSRFASDSPRHSHSFLPFSGGARNCIGKQFAMNELKVVVALTLLRFELL

PDPTRVPIPLPRLVLKSKNGIHLHLKKIH

>AAH14721.1

MSASALSSIRFPGSISEYLQVASVLSLLLLLFKTAQLYLHRQWLLSSTQQFPSPPSHWLF

GHKILKDQDLQDILTRIKNFPSACPQWLWGSKVRIQVYDPDYMKLILGRSDPKANGSYRF

LAPWIGRGLLLLDGQTWFQHRRMLTPAFHYDILKPYTEIMADSVRVMLDKWEQIVGQDST

LEIFRHITLMTLDTIMKCAFSHEGSVQLDRKYKSYIQAVEDLNDLVFSRVRNIFHQNDII

YRVSSNGCKANSACKLAHDHTDQVIKSRRIQLQDEEELEKLKKKRRLDFLDILLFARMEN

GKSLSDKDLRAEVDTFMFEGHDTTASGISWIFYALATNPEHQQRCRKEIQSLLGDGTSIT

WNDLDKMPYTTMCIKEALRIYPPVPSVSRELSSPVTFPDGRSLPKGIHVMLSFYGLHHNP

TVWPNPEVFDPSRFAPGSSRHSHSFLPFSGGARNCIGKQFAMNELKVAVALTLLRFELLP

DPTRVPIPIPRIVLKSKNGIHLHLKKLQ

>Q91WL5.2

MSASALSSIRFPGSISEYLQVASVLSLLLLLFKTAQLYLHRQWLLSSTQQFPSPPSHWLF

GHKILKDQDLQDILTRIKNFPSACPQWLWGSKVRIQVYDPDYMKLILGRSDPKANGSYRF

LAPWIGRGLLMLDGQTWFQHRRMLTPAFHYDILKPYTEIMADSVRVMLDKWEQIVGQDST

LEIFRHITLMTLDTIMKCAFSHEGSVQLDRKYKSYIQAVEDLNDLVFSRVRNIFHQNDII

YRVSSNGCKANSACKLAHDHTDQVIKSRRIQLQDEEELEKLKKKRRLDFLDILLFARMEN

GKSLSDKDLRAEVDTFMFEGHDTTASGISWIFYALATNPEHQQRCRKEIQSLLGDGTSIT

WNDLDKMPYTTMCIKEALRIYPPVPSVSRELSSPVTFPDGRSLPKGIHVMLSFYGLHHNP

TVWPNPEVFDPSRFAPGSSRHSHSFLPFSGGARNCIGKQFAMNELKVAVALTLLRFELLP

DPTRVPIPIPRIVLKSKNGIHLHLKKLQ

>EDL30646.1

MSVSALSPTRFADSLSGFLQVASVLGLLLLLVKAVQFYLHRQWLLKAFQQFPSPPFHWFF

GHEQFKGDQELQEIVSCIENFPSAFPRWFWGSKAYLTVYDPDYMKVILGRSGYGLLLLNG

QPWFQHRRMLTPAFHYDILKPYVKNMADSIRLMLDKWERLAGQDSSIEIFQHISLMTLDT

VMKCAFSHKGSVQVDGNYRTYLQAIGDLNNLFHSRVRNIFHQNDTIYKLSSNGRLAKQAC

QLAHDHTDGVIKLRKDQLQDEGELEKIKKKRRLDFLDILLFARMENGDSMSDKDLRAEVD

TFMFEGHDTTASGVSWIFYALATHPDHQQRCREEVQSLLGDGSSITWDHLDQIPYTTMCI

KEALRLYPPVPGIVRELSTSVTFPDGRSLPKGVQVTLSIYGLHHNPKVWPNPEVFDPSRF

APDSPRHSHSFLPFSGGARNCIGKQFAMSELKVIVALTLLRFELLPDPTRVPMPLARLVL

KSKNGIYLHLKKLH

>P20817.2

MGFSVFTPTRSLDGVSGFFQGAFLLSLFLVLFKAVQFYLRRQWLLKALEKFPSTPSHWLW

GHDLKDREFQQVLTWVEKFPGACLQWLSGSKTRVLLYDPDYVKVVLGRSDPKASGIYQFL

APWIGYGLLLLNGKKWFQHRRMLTPAFHYGILKPYVKIMADSVNIMLDKWEKLDDQDHPL

EIFHYVSLMTLDTVMKCAFSHQGSVQLDVNSRSYTKAVEDLNNLTFFRVRSAFYGNSIIY

NMSSDGRLSRRACQIAHEHTDGVIKMRKAQLQNEEELQKARKKRHLDFLDILLFAKMEDG

KSLSDEDLRAEVDTFMFEGHDTTASGISWVFYALATHPEHQERCREEVQSILGDGTSVTW

DHLDQIPYTTMCIKEALRLYPPVPSVSRELSSPVTFPDGRSIPKGITTTILIYGLHHNPS

YWPNPKVFDPSRFSPDSPRHSHAYLPFSGGARNCIGKQFAMNELKVAVALTLLRFELLPD

PTRIPVPMARLVLKSKNGIHLRLKKLR

>EGV94556.1

MGFSVFSFTRNLGGVSGFFQVVFVVSLFLVLFKAVRFYLRRQWLLKTLQKFPSTPSHWLW

GHQLKDKELEQILIWVEEFPSACLHWLSGSKARVVLYDADYVKMILGRSDPKAPSIYRLA

APWIGYGLLLLNGKKWFQHRRMLTPAFHYDILKPYVRIMADSVNTMLDRWEKLDGENCPL

EIFHYISSMTLDTVMKCAFSHQGSVQLDENSRSYTKAVEDLNSLVFFRMRSAFYENNTIY

KMSSDGRCFYRACQLAHEHTDGVIKMRKAQLQNEEELERVKKKRRLDFLDILLFAKMEDG

SSLSDEDLRAEVDTFMFEGHDTTASGISWIFYALATHPEYQQRCREEVQSILGDGTSVTW

DHLDQMPYTTMCIKEALRLYPPVPNVSRELNTPITFPDGRSLPKGITVAISIYGLHHNPS

LWPNPQVFDPSRFAPDSSRHSHAFLPFSGGARNCIGKQFAMNELKVAVALTLLRFELLPD

PTRVPVPIPRVVLKSKDGIYLRLKKLR

>EHH14757.1

MSVSVLSPSRLLGSVSGILQVTSLLILLLLLIKAAQLYLHRQWLLKALQQFPCPPSHWFF

GHFQKFQQDQELQRIRKWVEMFPSACPLWLWGGKARVQLHDPDYMKVILGRSDPKSHGSY

RFLAPWIGIYTHTRLWQTWFQHRQMLTPAFHYDTLKPYVGLMAESMQVMLDKREELVGQD

SPLEVSEHISLMTLDITMKCAFSHQGSVQLDRNSQSYIQVIGDLNNLIFSHLRNPFHHND

TFYSLTSDGCWNHNACQLTHQHTGYRVIQLRKAHLQEEGELEKVRRKRHLDFLDILLLAK

MENGSSLSNKDLCAEVDTFMFEDHNTTASGISWILYALATHPKHQQRCQEEIQGLLGDGA

SITWDHLDQMPYTTMCIKEALKLYQPVPGTGRELSTPITFPDGCSCPEGITVVLSIYGLH

HNPKAWPNPEMFDPSHFASGSAQHSHAFLPFSGGSR

>348550117

MSVSAFSPARFPGSISGFLQVVSLLSLLLLLFKAAHFYLCRQWLLKAFQQFPCAPSHWLL

GHKILPEDELQQTLKWVKEFPSAYPRWFWGSEAHLIIYDPDYMKVILGRSDPKAISFYRF

LAPWIGYGLLLLNGPTWFQHRRMLTPAFHYDILKPYVEIMANSVRVMLDKWEKLISQDAS

MEIFQPVSLMTLDSIMKCAFSYQGSVQLDWKSRPYLQAIEEMNNMFFSRVRNMFHQNDTI

YSLSSNGQRCKRATQIAHHHTDQIIKQRKAQLKDDKELENIRKKRHLDFLDILLFARTEN

GSSLSDEDLRAEVDTFMFEGHDTTASGICWTLYALATHPSHQQRCREEVQNLLGDKGSIT

WEHLDQMSYTTMCIKEALRLYPPVPSVGRELSKPVTFPDGKSLPKGINVILSFHGLHHNP

KVWPNPEIFDPSRFAPDSSRHSHSFLPFSGGPRNCIGKQFAMNELKVAVAQTLLRFELLP

DPTRVPIPIPQIVLKSKNGIHVYLRKLH

>EDL90312.1

MGFSVFTPTRSLDGVSGFFQGAFLLSLFLVLFKAVQFYLRRQWLLKALEKFPSTPSHWLW

GHDLKDREFQQVLTWVEKFPGACLQWLSGSKTRVLLYDPDYVKVVLGRSDPKASGIYQFL

APWIGYGLLLLNGKKWFQHWRMLTPAFHYGILKPYVKIMADSVSIMLDKWEKLDDQDHPL

EIFHYVSLMTLDTVMKCAFSHQGSVQLDVNSRSYTKAVEDLNNLTFFRVRSAFYGNSIIY

NMSSDGRLSRRACQIAHEHTDGVIKMRKAQLQNEEELQKARKKRHLDFLDILLFAKMEDG

KSLSDEDLRAEVDTFMFEGHDTTASGISWVFYALATHPEHQERCREEVQSILGDGTSVTW

DHLDQISYTTMCIKEALRLYPPVPSVSRELSSPVTFPDGRSIPKGITTTILIYGLHHNPS

YWPNPKVFDPSRFSPDSPRHSHAYLPFSGGARNCIGKQFAMNELKVAVALTLLRFELLPD

PTRIPVPMARLVLKSKNGIHLRLKKLR

>EHB14339.1

MSISAHSPTRFPGGISGLLQVASLLGLILLLFNSPALYLHRQWLLKAVQQFPCPPSHWLF

GHKKEFQKDQELQQVLKWAEKFPSACPRWLAGSRLSVLVYDPDYMKVILGRSGYGLLLLN

GPTWFQHRRMLTPAFHFDILKPYVAVIANSVQVMLDRWEQLISQDSPLEIFQHVSLMTLD

SIMKCAFSHKSCAQLDRNSRSYIQAIEDLKDLSFSRIRSVFQQSDIIYSLSSNGQLFKQA

CKLAHEHTDRVIKQRKSQLQNERELEKIKRKRRLDFLDILLFAKMDDGNGFSNKDLRAEV

DTFMFEGHDTTASGISWILYALAAHPEHQQKCREEVQTILGDEASITWEHLDQMPYMTMC

IKEALRLYPPVPSVSRELSKPVTFPDGRSLPKGIIVSLSIYTLHHSPKVWPNPEVFDPSR

FAVDSTQHSHSFLPFSGGSRNCIGKQFAMNEMKVAVALTLLRFELLPDPTRIPLPMARIV

LKSKNGIYLHLRKLK

>EGV94557.1

MGFSVFSLTRNLGGVSGFFQVVFVVSLFLVLFKAVRFYLRRQWLLKTLQKFPSTPSHWLW

GHQLKVGIKDKELEQILIWVEEFPSACLHWLSGSKARVVLYDADYVKMILGRSDPKASGI

YGLFAPWIGYGLLLLNGKKWFQHRRMLTPAFHYDILKPYVRIMADSVNTMLDRWEKLDGE

NCPLEIFHYISSMTLDTVMKCAFSHQGSVQLDENSRSYTKAVEDLNSLAFFRMRSAFYEN

NTIYKMSSDGRWFYRACQLAHEHTDGVIKMRKAQLQNEEELERVKKKRRLDFLDILLFAK

MEDGSSLSDEDLRAEVDTFMFEGHDTTASGISWIFYALATHPEYQQRCREEVQSILGDGT

SVTWDHLDQMPYTTMCIKEALRLYPPVPIVSRELNTPITFPDGRSLPKGITVAISIYGLH

HNPSLWPNPKEFDPSRFAPDSSRHSYAFLPFSGGARNCIGKQFAMNELKVAVALTLLRFE

LLPDPTRVPVLIQRLVLKSKDGIYLRLKKLR

>354469998

MGFSVFSLTRNLGGVSGFFQVVFVVSLFLVLFKAVRFYLRRQWLLKTLQKFPSTPSHWLW

GHQLKDKELEQILIWVEEFPSACLHWLSGSKARVVLYDADYVKMILGRSDPKASGIYGLF

APWIGYGLLLLNGKKWFQHRRMLTPAFHYDILKPYVRIMADSVNTMLDRWEKLDGENCPL

EIFHYISSMTLDTVMKCAFSHQGSVQLDENSRSYTKAVEDLNSLAFFRMRSAFYENNTIY

KMSSDGRWFYRACQLAHEHTDGVIKMRKAQLQNEEELERVKKKRRLDFLDILLFAKMEDG

SSLSDEDLRAEVDTFMFEGHDTTASGISWIFYALATHPEYQQRCREEVQSILGDGTSVTW

DHLDQMPYTTMCIKEALRLYPPVPIVSRELNTPITFPDGRSLPKGITVAISIYGLHHNPS

LWPNPKEFDPSRFAPDSSRHSYAFLPFSGGARNCIGKQFAMNELKVAVALTLLRFELLPD

PTRVPVLIQRLVLKSKDGIYLRLKKLR

>348553417

MSVSAFSPTRFPGSISGLLQVVSLLSLLLLLFKAAHFYLCRQWLLKAFQQFPCAPSHWLL

GHKILPEDELQQTLKWVKEFPSAYPRWFWGSEAHLIIYDPDYMKVILGRSDPKVISFYRF

LAPWIGYGLLLLNGPTWFQHRRMLTPTFHYDILKPYVKIMANSVRVMLDKWEKLISQDAS

MEIFQPVSLMTLDSIMKCAFSYQGSVQLDRKSRPYLQAIEEMNNMFFSRVRNMFHQNDTI

YSLSSNGQRCKRATQIAHHHTDQIIKQRKAQLKDDKELENIRKKRHLDFLDILLFAKTEN

GSSLSDEDLRAEVDTFMFEGHDTTASGICWTLYALATHPSHQQRCREEVQNLLGDKGSIT

WEHLDQMSYITMCIKEALRLYPPVPFVGRELSKPVTFPDGKSLPKGINVILSFHGLHHNP

KVWPNPEIFDPSRFAPDSSRHSHSFLPFSGGPRNCIGKQFAMNELKVAVALTLLRFELLP

DPTRVPIPIPQLVLKSKNGIQLYLRKLH

>P20816.2

MGFSVFSPTRSLDGVSGFFQGAFLLSLFLVLFKAVQFYLRRQWLLKALEKFPSTPSHWLW

GHNLKDREFQQVLTWVEKFPGACLQWLSGSTARVLLYDPDYVKVVLGRSDPKPYQSLAPW

IGYGLLLLNGKKWFQHRRMLTPAFHYDILKPYVKIMADSVSIMLDKWEKLDDQDHPLEIF

HYVSLMTLDTVMKCAFSHQGSVQLDVNSRSYTKAVEDLNNLIFFRVRSAFYGNSIIYNMS

SDGRLSRRACQIAHEHTDGVIKTRKAQLQNEEELQKARKKRHLDFLDILLFAKMEDGKSL

SDEDLRAEVDTFMFEGHDTTASGISWVFYALATHPEHQERCREEVQSILGDGTSVTWDHL

DQMPYTTMCIKEALRLYSPVPSVSRELSSPVTFPDGRSIPKGIRVTILIYGLHHNPSYWP

NPKVFDPSRFSPDSPRHSHAYLPFSGGARNCIGKQFAMNELKVAVALTLLRFELLPDPTR

IPVPMPRLVLKSKNGIHLRLKKLR

>O35728.1

MGFFLFSPTRYLDGISGFFQWAFLLSLFLVLFKAVQFYLRRQWLLKTLQHFPCMPSHWLW

GHHLKDKELQQILIWVEKFPSACLQCLSGSNIRVLLYDPDYVKVVLGRSDPKASGIYQFF

APWIGYGLLLLNGKKWFQHRRMLTPAFHYDILKPYVKIMADSVNIMLDKWEKLDGQDHPL

EIFHCVSLMTLDTVMKCAFSYQGSVQLDENSKLYTKAVEDLNNLTFFRLRNAFYKYNIIY

NMSSDGRLSHHACQIAHEHTDGVIKMRKSQLQNEEELQKARKKRHLDFLDILLFARMEDR

NSLSDEDLRAEVDTFMFEGHDTTASGISWIFYALATHPEHQQRCREEVQSILGDGTSVTW

DHLGQMPYTTMCIKEALRLYPPVISVSRELSSPVTFPDGRSIPKGITATISIYGLHHNPR

FWPNPKVFDPSRFAPDSSHHSHAYLPFSGGSRNCIGKQFAMNELKVAVALTLLRFELLPD

PTRIPVPIARLVLKSKNGIHLCLKKLR

>BAE28849.1

MGFFLFSPTRYLDGISGFFQWAFLLSLFLVLFKAVQFYLRRQWLLKTLQHFPCMPSHWLW

GHHLKDKELQQILIWVEKFPSACLQCLSGSNIRVLLYDPDYVKVVLGRSDPKASGIYQFF

APWIGYGLLLLNGKKWFQHRRMLTPAFHYDILKPYVKIMADSVNIMLDKWEKLDGQDHPL

EIFHCVSLMTLDTVMKCAFSYQGSVQLDENSKLYTKAVEDLNNLTFFRLRDAFYKYNIIY

NMSSDGRLSHHACQIAHEHTDGVIKMRKSQLQNEEELQKARKKRHLDFLDILLFARMEDR

NSLSDEDLRAEVDTFMFEGHDTTASGISWIFYALATHPEHQQRCREEVQSILGDGTSVTW

DHLGQMPYTTMCIKEALRLYPPVISVSRELSSPVTFPDGRSIPKGITATISIYGLHHNPR

FWPNPKVFDPSRFAPDSSHHSHAYLPFSGGSRNCIGKQFAMNELKVAVALTLLRFELLPD

PTRIPVPIARLVLKSKNGIHLCLKKLR

>403292098

MEYAAQLDTDKVNNVPNLLLINGDGIGKRINIVIRWMILHNQSTLNSEVQALPSPFNPPQ

RWVGQPSQDLSTGGEEQSGTDRYTQRSSRCCIVGVPVPILTGFPDSISGLLQVILLCLLL

LLLKAAQFYLHRQWLLKAIQQFPSHPSHWLFGHSQELQQNQELQQIQKWVETFPSACPHW

LWGDKVLVQLYDPDYMKVILGRSDPKVTEISRLLAPXIGYGLPLLNRQKWRQHRRMLIPA

FHFNILKPYVGLMVDSVQAMLDKWEELIGQDSPLDVSQHVSLMTLDIIMKCAFSYQGSVQ

LDWDIQSYIQAIGDLKNQFISCMGNVFHQNDTIYNLTPTGCSNHHTCQLVHQHTDQVIQL

RKARLQEERELEKVRRKRHLDFPDILLLAKMENGSSLSDKDLRAEVDTFMFGGHDTTASS

ISWIFCALATHPKVQQMCPEEIQSLLGNEASVTWNHLDQMPYTTICIKEALRLYPPVPSI

SRELITPVTFPDGRSLPKGLKVSLSIYGLHHNPKVWPNPEVCDPSRFAPSSAQHSHAFLP

FSGESRNCIGKQFAMNQLKVAMALTLLRCELLPDLTRIPIPMSXIVLESSNGIHLHLRRL

PNPCGNKDQL

>354470010

MSVSALSPSRFVGSISGFLQVVSVLGLLLLLLKVVQFYLHRQWLLKAFQQFPSPPFHWFL

GHKQFQGDQEIEQMMKCVENFPSAFPRWFWGSKAYFTVYDPDYMKVILGRSDPKANGYGL

LLLNGQPWFQHRRMLTPAFHYDILKPYVKTMADSVRLMLDKWEQLAGQDSSIEIFQHISL

MTLDTVMKCAFSHKGSVQVDGNYKTYIQAIGNLNNLFYSRVRNIFHQNDTIYSLSSNGRM

ANQACQLAHDHTDGVIKLRKDQLQDEEELEKVKKKRRLDFLDILLFARMENGDSLSDKDI

RAEVDTFMFEGHDTTASGVSWIFYALATHPEHQQRCREEVQSLLGDGSSITWDHLDQMPY

TTMCIKEALRLYPPVPAIVRELITPVTFPDGRSLPKGVSVTLSIYALHHNPKVWPNPEVF

DPSRFASDSPRHSHSFLPFSGGARNCIGKQFAMNELKVVVALTLLRFELLPDPTRVPIPL

PRLVLKSKNGIHLHLKKIH

>BAE25803.1

MSASALSSIRFPGSISEYLQVASVLSLLLLLFKTAQLYLHRQWLLSSTQQFPSPPSHCLF

GHKILKDQDLQDILTRIKNFPSACPQWLWGSKVRIQVYDPDYMKLILGRSDPKANGSYRF

LAPWIGRGLLMLDGQTWFQHRRMLTPAFHYDILKPYTEIMADSVRVMLDKWEQIVGQDST

LEIFRHITLMTLDTIMKCAFSHEGSVQLDRKYKSYIQAVEDLNDLVFSRVRNIFHQNDII

YRVSSNGCKANSACKLAHDHTDQVIKSRRIQLQDEEELEKLKKKRRLGFLDILLFARMEN

GKSLSDKDLRAEVDTFMFEGHDTTASGISWIFYALATNPEHQQRCRKEIQSLLGDGTSIT

WNDLDKMPYTTMCIKEALRIYPPVPSVSRELSSPVTFPDGRSLPKGIHVMLSFYGLHHNP

TVWPNPEVFDPSRFAPGSSRHSHSFLPFSGGARNCIGKQFAMNELKVAVALTLLRFELLP

DPTRVPIPIPRSCVEVQEWDPLASQKAPIIFTGQDSSNGMLPAILSFCHLLFSPILLLTS

HSFFSPCSPPPTFCWASSLLACQSFSTSSEIPTCFSLYLSLT

>Q9GJX5.1

MTVPALASVSGLLQVASLLGLLLLLLKAAQLYLRRQWLLKALQQFPSPPSHWLYGHSREF

QEESELQPLLKRVEKYPSACARWLWGTRAMVLVYDPDYMKVVLARSEPKAPVLYRLLIPW

IGCGLLLLNGQMWFQRRRMLTPAFHYDILKPYVGLMAKSVQVMLDKWEQLVAQDPRLEIV

GPVSLMTLDTIMKCAFSHQGSAQTDGDSQSYIQAIWDLKNLIFSRLRSAFLQNDIIYRLS

PEGRQCQRACQKVHQHTDRVIQLRKTHLQKEGEMENVKKKRHLDFLDILLFARMENGNSL

SDTDVRAEVDTFMAAGHDSTASGISWVLYALASNPEHQQRCREEIQGLLGDGTSITWDHL

DQMPYTTMCIKEALRLYPPVPSVGRELSKPITFPDGRSLPAGIILSLSIYGLHHNPQVWP

NPEEFDPSRFAPGSARHSHAFMPFSGGSRNCIGKQFAMNEMKVVVALTLLRFELAPDPSR

IPVPIQGIVLKSKNGIHLNLRKIP

>AAK64456.1

MTVPALASVSGPLQVASLLGLLLLLLKAAQLYLHRQWLLKALQQFPSPPSHWLYGHSREF

QEESELPPLLKRVEKYPSACARWLWGTRAMVLVYDPDYMKVVLARSDPKTSVVYRLLIPW

IGCGLLLLNGQTWFQRRRMLTPAFHYDILKPYVGLMAKSVQVMLDKWEQLVAQDPRLEIV

GPVSLMTLDTIMKCAFSHQGSVQTDGDSHSYIQAIWDLKNLFSTRTKSAFLQNDIIYRLS

PEGHKNHRAARIAHQHTDRVIQLRKAQLQKQGEMENVRKKRHLDFLDILLLAQMEKGNSL

SDTDLRAEVDTFMFEGHDTTASGISWILYALASHPEHQQRCREEIQGLLGDGTSITWDHL

DQMPYTTMCIKEALRLYPPVPGVSRELSKPITFPDGRSLPAGIILSLFIYGLHHNPQVWP

NPEEFDPSRFAPGSARHSHAFMPFSGGSRNCIGKQFAMNEMKVAVALTLLRFELAPDPSR

KPIATPEVVLNSKNGIHLKLRKLP

>Q8SPK1.1

MTVPALASASGLLQVASLLGLLLLLLKAAQLYLHRQWLLKALQQFPSPPSHWLYGHSREF

QEESELPPLLKRVEKYPSACALWRWGTRAMVLVYDPDYMKVVLARSDPKNSVVYRLLIPW

IGCGLLLLNGQTWFQRRRMLTPAFHYDILKPYVGLMAKSVQVMLDKWEQLVAQDPRLEIV

GPVSLMTLDTIMKCAFSHQGSAQTDGDSHSYIQAIWDLKNLFSIRTKSAFLQNDIIYRLS

PEGRKNHRAARIAHQHTDRVIQLRKAQLQKQGEMENVRKKRHLDFLDILLLARMEKGNSL

SDTDLRAEVDTFMFEGHDTTASGISWILYALASHPEHQQRCREEIQGLLGDGTSITWDHL

DQMPYTTMCIKEALRLYPPVPGVSRELSKPITFPDGRSLPAGITLSLSIYGLHHNPQVWP

NPEEFDPSRFAPGSARHSHAFMPFSGGSRNCIGKQFAMNEMKVAVALTLLRFELAPDPSR

KPIATPEVVLNSKNGIHLKLRKLP

>Q8SPK0.1

MTVPALASASGLLQVASLLGLLLLLLKAAQLYLRRQWLLKALQQFPSPPSHWLYGHSREF

QEESELQPLLKRVEKYPSACARWLWGTRAMVLVYDPDYMKVVLARSEPKAPVLYRLLIPW

IGCGLLLLNGQTWFQRRRMLTPAFHYDILKPYVGLMAKSVQVMLDKWEQLVAQDPRLEIV

GPVSLMTLDTIMKCAFSHQGSAQTDGDSHSYIQAIWDLKNLFSIRTKSAFLQNDIIYRLS

PEGRKNHRAARIAHQHTDRVIQLRKAQLQKQGEMENVRKKRHLDFLDILLLARMEKGNSL

SDTDLRAEVDTFMFEGHDTTASGISWILYALASHPEHQQRCREEIQGLLGDGTSITWDHL

DQMPYTTMCIKEALRLYPPVPGVSRELSKPITFPDGRSLPAGIILSLSVYSLHHNPQVWP

NPEEFDPSRFAPGSARHSHAFMPFSGGSRNCIGKQFAMNEMKVAVALTLLRFELAPDPSR

KPTVIPEVVLHSKNGIHLKLRKLP

>390465907

MSVSVLSPSRLLGGISGLLQAASLLILLLLLLKAAQLYLHRQWLLKALQQFPSPPSHWLY

GYNQEFQKDQELQQILKRVERFPRACPQLLCGNRVRLLFYDPDYMKVILGRSDPKVHDFC

RYAAPWIGYGLLLLNGQTWFQHQWMLTPAFHYDILKPYVVLMVNSMRVMLDKWEELVSRN

SSLEVSEHISLMTLDTIMKCAFSHEGSVQLDRNSHSYNXAIGDLNNLISSCLRNPFYQND

TIYSLTPDGRWNHNSCQLTHQYTDRVIQLRKACLQEQGELDNVRGKRHLDFLDIFLVGQT

QQGQSLESKDLRAEVDTFMFEGHNTTASSIFWILYALATHPKHQQRCPEEIQGLLGHGTS

ITWDHLDQIPYTTMCVKETLRCYPPVPSIGKEFGTPITLPDGHSLSKGLLVSLSIYGLHH

NLKVXPNPEVKRLNDASHLVPGSAXHNHTFLPFSGGPRNCIGKQLAMNELKVALTLIRFE

LLPDPTRIPNPRPQIVLKSENGIHLHLRRLFNHCGNKDQL

>BAG60530.1

MKVILGRSDPKSHGSYRFLAPWIGYGLLLLNGQTWFQHRRMLTPAFHYDILKPYVGLMAD

SVRVMLDKWEELLGQDSPLEVFQHVSLMTLDTIMKCAFSHQGSIQVDRNSQSYIQAISDL

NNLVFSRVRNAFHQNDTIYSLTSAGRWTHRACQLAHQHTDQVIQLRKAQLQKEGELEKIK

RKGHLDFLDILLLAKMENGSILSDKDLRAEVDTFMFEGHDTTASGISWILYALATHPKHQ

ERCREEIHSLLGDGASITWNHLDQMPYTTMCIKEALRLYPPVPGIGRELSTPVTFPDGRS

LPKGIMVLLSIYGLHHNPKVWPNPEVFDPFRFAPGSAQHSHAFLPFSGGSR

>354469990

MLASALAAFPGTFISGFLQVISLLGLLLLLYKAVQYYLHRQWLLKCLQEFPSTPSHWLFG

NHLKDQDLQQTLLWVEKFPSACSRWFWGNKAHAVIYDPDYMKVILGRSDPKPVHSYSFFA

PWIGYGLLILEGKKWSQHRKMLTPAFHYDSLKPYVGIMVDSVHVMLDKWEQFIVHDCPLE

IYQHISLMTLEIIMKCAFSYQGSVQLDENVRSYSEAVQELTERIYSRMRNAFHQSNIIYN

LSSNGRSFYRACQVAHEYTDRMISMRKAQLQNEEELENVQKKRLLDFLDILLFAQTEDGK

SLSDEDLRAEVDTFMFEGHDTTASGISWILYALATNPEHQQRCREEVQNVLGDGTSVTWN

HLSQMPYTTMCIKEALRLYPPVPAIGRELSTPVTFPDGRSLPKGFLITLSLYGLHHNPRV

WPNPEVFDPSRFAPDAARHKYAFLPFSGGRRNCIGKQFAMSELKVAVALTLLRFELLSDP

TRVPVLKTRTVLKSKNGIYLCLKKLK

>154146229

MSIFELSHITNVFGIFGLLQVVSLLGLLLLLLKSAQYYLHRQWLIKSLQQFPPAPPPQWL

FGNTLKDQDLQQILLCVEKFPSTYVRWLWGNHARVVIYDPDYMKVILGRSDPKVVRSYSF

FAPWIGYGLLLLNGKKWFQHRRMLTPAFHYDILKPYVGIMVDSVHVMLDKWEQLVDQDCP

LEIYQDISLMTMETMMKCAFSYQGSVQLEENSKSYIKAVEDLTHLIYLRVRNGFHQNNTI

YSLSSNGRSFYHACQIAHKHTERVIRMRKAQLQNEAELEKFRKKKRLDFLDILLFAQTED

GKSLSDEDVRAEMDTFMFEGHNTTASGISWIFYALATHPEHQQRCREEVKSILGNGTSVT

LNHLDQMPYTTMCIKEALRLYPPIPGASRELSSPVTFPDGCSLPKGFLVTLSFYGLHHNP

RLRPNAEVFDPSRFAPGAPRQTHAFLPFSAGTRNCIGKQFTMNELKVAVALTLLRFELLP

DPTRVPILVSKIVLKSKNGIYLHTKKLK

>EDL30652.1

MSISALSPIRFLECVSGFLQVIFLIGLLLLLFKAAQHYLRRQWLLRALYQFPSPPSHWLF

GHCLQFKVDEELRWILRYLEKFPGAFLHWIWGSHVFIKVCDPDYMKVILGRAGHGLFFLN

GDEWFQHRRLLTPAFHYDILKPYVGIMADSVQVMLEKWEQIACQDITLEIFHPISLMMLD

TIMKYAFSYQGSVQLDRSSQAYLQAVSDLNNLVTSRMKNVFLHNDIIYNLTSHSRRTNSA

CQIAHEHTDRVVKLRKAQLQDNKSMKKLRGKRCLDFLDILLLSRMDDGSSLSDKALRAEV

DSLMFGGHDTPASGISWVFYALATHPDHQQRCREEVQSLLGDGSPITWDHLHQMPYTTMC

IKEALRLYPPIPSVGRKLSTPVTFPDGRSLPKGITVLLHFYALHHNPKVWPNPEVFDPSR

FAMNSVQHSHAFLPFSGGSRNCIGKHLAMNVLKVAVALTLLRFELLPDPSRVPIPTQQLV

LKSKNGIYLHLRKLQ

>350586250

MRVFTISLLNSVSPKLQRSVSLFVAVPRVLDSGSSRFRXEGEQQSLLKRIGKYPSACARW

RWGTRALVLVYDPDYMKVVLARSEPKPHITYKYLIPWLGNGLLLLDGQAWFQHRRMLTPA

FHHDILKPYVRLMADSVQVMLDKWEQLVAQDPRLEIVGPVSLMTLDTIMKCAFSHQGSVQ

TDGNSHSYIQAIRDLNDLLFSRVRNVFQQNDVIYRLSPEGRRSHRACQLAHQHTDRVIQL

RKAQLQKQGEMENVRKKRHLDFLDILLLAQMEKGNSLSDTDLRAEVDTFMFEGHDTTASG

ISWILYALASHPEHQQRCREEIQGLLGDGTSITWDHLDQMPYTTMCIKEALRLYPPVPVI

GRELSKPITFPDGRSLPAGIILSLSVYSLHHNPKVWPNPEEFDPSRFAPGSARHSHAFMP

FSGGSRNCIGKQFAMNEMKVAVALTLLHFELAPNPSRIPVPIQRVVLKSNNGIHLNLRKL

P

>350586236

MTVPALASVSGLLQVASLLGLLLLLLKAAQLYLRRQWLLKALQQFPSPPSHWLYGHSREF

QEESELPPLLKRVEKYPSACALWRWGTRAMVLVYDPDYMKVVLARSDPKTSVVYRLLIPW

IGCGLLLLNGQTWFQRRRMLTPAFHYDILKPYVGLMAKSVQVMLDKWEQLVAQDPRLEIV

GPVSLMTLDTIMKCAFSHQGSVQTDGDSHSYIQAIWDLKNLFSTRTKSAFLQNDIIYRLS

PEGRKSHRAARIAHQHTDRVIQLRKAQLQKQGEMENVRKKRHLDFLDILLLAQMEKGNSL

SDTDLRAEVDTFMFEGHDTTASGISWILYALASHPEHQQRCREEIQGLLGDGTSITWDHL

DQMPYTTMCIKEALRLYPPVPGVSRELSKPITFPDGRSLPAGITLSLSIYGLHHNPQVWP

NPEEFDPSRFAPGSARHSHAFMPFSGGSR

>EDL30649.1

MSIFELSHITNVFGIFGLLQVVSLLGLLLLLLKSAQYYLHRQWLIKSLQQFPPAPPPQWL

FGNTLKDLQQILLCVEKFPSTYVRWLWGNHARVVIYDPDYMKVILGRSGYGLLLLNGKKW

FQHRRMLTPAFHYDILKPYVGIMVDSVHVMLDKWEQLVDQDCPLEIYQDISLMTMETMMK

CAFSYQGSVQLEENSKSYIKAVEDLTHLIYLRVRNGFHQNNTIYSLSSNGRSFYHACQIA

HKHTERVIRMRKAQLQNEAELEKFRKKKRLDFLDILLFAQTEDGKSLSDEDVRAEMDTFM

FEGHNTTASGISWIFYALATHPEHQQRCREEVKSILGNGTSVTLNHLDQMPYTTMCIKEA

LRLYPPIPGASRELSSPVTFPDGCSLPKGFLVTLSFYGLHHNPRLRPNAEVFDPSRFAPG

APRQTHAFLPFSAGTRNCIGKQFTMNELKVAVALTLLRFELLPDPTRVPILVSKIVLKSK

NGIYLHTKKLK

>395537005

MSFLLSCAWLQGDISGILQTSFLLILILLILKGAQLYLCRQRLVRDFQSFPGPPAHWLYG

HFWEFPVEEELHKMNSLAEKFPCAFPRWFSGFMTFLQVYDPEYIKVILSKRDPKAHAIYR

FLTPWIGKGLLVLNGSEWHQHRRLLTPAFHSDILKPYVALMADSVRVMLDKWEKLSDQGS

SLEIFEHVSLMTLDSLMKCAFSHQSNCQMDRNSNSYIQAVHDLTYLLTYRIENGFLHSDI

IYWLSPVGHRTRRAIQRAHQYTDQVIQRRKWQLQQGELETISKKRHLDFLDVLLCAKTES

GDSLSDEELRAEVDTFMFEGHDTTASGISWVLYALATHPEHQQKCREEIRRVLGNGASIT

WDHLGQLPYTTMCIKEALRLYPPVPSIGRDLKETIIFPDGRILTAGNSVSLNIYTLHHNS

RVWPDPEVFDPLRFSPENVMLQHSHAYLPFSAGSRNCIGQKFAMNEIKVVLALTLLLFEL

KPDTMKPPLPISELVLRSKNGIHLWMKRLH

>126305758

MGTGLGLAWLPRDFSSFLQTSVLLSLVLLLLKGVQLYRRRQWLLRTFQNFPGPPAHWFNG

HFWEYQKADEITVTLFWAKQFPSAFPRWFSGFTVALQVYDPEYMRILLGRPDPKADKFYR

LLAPWIGKGLLILNGSTWFQHRRLLTPAFHYDILKPYVALMVDSVLVMLKKWEKLITQDS

SLEIFEHVSLMTLDTIMKCAFSYHSNCQMERNADDYIQAVKEQAILVFSRVRNDLYHNDF

VYWFSPQGYQARRWAHLAHNHTDQVIKKRKQHLHQEGGLEAILKKRHLDFLDILLCSKTE

NGDSLSDKELRAEVDTFMFEGHDTTASGISWLLYSLAMNPEHQQKCREEIRDLLGDEMAI

GWEHLNRMPYTTMCIKESLRLYPPVTSISRDLSKPLTLSDGRYLPAGTIVTLHIHALHHN

PSVWPEPEVFNPLRFSPENLTSRHTHSFLPFSAGTRNCIGQQFAMNEMKVAVALTLLHFH

LEPDATQPPQLFPRVVLRSKNGIHLKLTRI

>EHB09009.1

MSISALSPTRFPGGISGLLQVASLLGLILLLFKAAQLYLHRQWLLKAVQQFPCPPSHWLF

GHKKEFQKDQELQQVLKWAEKFPSACPRWLAGSRLSVLVYDPDYMKVILGRSGYGLLLSN

GPTWFQHRWMLTSAFHFDILKPYVAVIANSVQVMLDRWEQVISQDSPLEIFQHVSLMTLD

SIMKCAFSHKSCAQLDRFRPIELAMTPQAEPGPLVDLEDLGHTYVSTSLMQGSCDISVSD

PVIKQRKSQLQNERELEKIKRKRRLDFLDILLFAKMDDGNGFSNKDLRAEVDTFMFAGHD

TTACDELHSREHLDQMPYMTMCIKEALRLYPPVPSVSRELSKPVTFPDGRSLPKGIIVSL

SIYTLHHSPKVFDPSRFAVDSTQHSHSFLPFSGGSRNCIGKQFAMNEMKVAVALTLLRFE

LLPDPTRIPLPMARIVLKSKNGIYLHLRS

>224058125

MKLLWLGVDVSRVLHLAAVFCLTCVLLKVIQLFHRRRELLRALADFPGHPTHWLFGHVHE

FLKEEEMLDKVEVWAQKYPHAHAMWFGSFSAFLVITDPDYAKVLLNRGDPKDNISYKHLI

PWIGNGLLILHGPKWHQHRKLLTPGFHYDVLKPYVALMAESTNVMLEKWEKLAADGKPVE

LFEHISLMTLDSIMKCAFSCHSNCQTNRKNTYIQAVYDLCHRVHQRLRIFPYHNDIIYWL

SPQGFQFRKACRIAHDHTDKVIRERKESLKDEREFEKIQKKRHLDFLDILLCAKDENGAA

LSDEDLRAEVDTFMFEGHDTTASGISWLFYCLAVHPEHQQRCREEIQGILGDRDTIQWED

LGKMTYSTMCIKESLRLYPPVPGVSRQLSKPVTFPDGRTLPEGLVTAISIYLIHRNPEVW

KDPLVFDPLRFSPENVSGRHSHAFLPFSAGMRNCIGQQFAMNEMKVALALTLRLFELSPD

PATPPLKITRVILRSKNGIHLYLKKIC

>327279462

MSSILERIRAGPSPSLLYLLIGFILICALLKALQLYWRKKKLLRNFKSFPGPPSHWFFGN

NKQVRKMTRVVSKGNEFNKSLEWAEKYPYAFTRWFGGFSAVLIVSHPDYAKAIFGRGDPK

SMVTYKFLLPWIGKGLLILHGPKWFQHRRLLTPGFHYDILKPYIALMADSVKSMLDKWEK

LIVKDDMKSLEMFEHVSLMTLDSIMKCAFSSQSSSQTSRELNIYIKAIYDLTYLVAERVR

NVLYRSELIYPFTSAGRQFQKACKLAHKHTEKVIEERKILFKEEGELKKIQKKRHLDFLD

ILLSAKGENGVELSKEDLRAEVDTFMFEGHDTTASGIAWMLHAMAQNPEHQQRCREEIKE

MMGDRDTVQWDDLGKMPYTTMCIKECLRLYPPVPVVSRQLSKPITFCDGRILPEDAVISI

SIYNIHRNPSIWEDPEVFDPTRFSPERSSHRHSHAFVPFAAGPRNCIGQQFAMNEMKVAL

AQILLRFEILPDPANIPIPIPQIVLKSANGIHLFLKKLN

>BAA89459.1

MSVSALSPTRFADSLSGFLQVASVLGLLLLLVKAVQFYLHRQWLLKAFQQFPSPPFHWFF

GHEQFKGDHELQEIVSCIENFPSAFPRWFWGSKAYLTVYDPDYMKVILGRSDPKANGYGL

LLLNGQPWFQHRRMLTPAFHYDILKPYVKNMADSIRLMLDKWERLADQDSSIEIFQHISL

MTLDTVMKCAFSHKGSVQVDGNYRTYLQAIGDLNNLFHSRVRNIFHQNDTIYKLSSNGRL

AKQACQLAHDHTDGVIKLRKDQLQDEGELEKIKKKRRLDFLDILLFARMENGDSMSDKDL

RAEVDTFMFEGHDTTASGVSWIFYALATHPDHQQRCREEVQSLLGDGSSITWDHLDQIPY

TTMCIKEALRLYPPVPGIVRAQYICHLP

>AAI29578.1

MASNVLFGLFPTQLYSWAASLCLLLVLYKATQLYLRRRFLLTAFSSFPGPKCHWLYGNAL

QFLQVGKELDLILKWAQVYPYGVSMWLGNFHAALFLTHPDYAKAILARQDPKDETAYKYI

VPWIGEGLLVLSGPKWFQHRRLLTPGFHYDVLKPYVTLMSNCTRVMLDKWDKLMPNEKTV

ELFHHVSLMTLDTIMKCAFSYNSSCQNNRDNSYIKAVYELSYLLDQRSRFFLYHNDVIFY

LSPLGFRFRRALTVAHQHTDKVIKHRKQSLMNETELDKIKQKRHLDFLDILLCAKDENGK

GLSDKDLRAEVDTFMFEGHDTTASGISWILYCMAKYPEHQHKCREEIRELLGDRETMEWG

DLGQMPYTTLCIKESIRLYPPVPTIGRRLSKPITFCDGRSLPEGTEVLTSIYAINRSPSV

WNDPEVFDPLRFSPENSDNRHSHAFVPFSAGARNCIGQNFAMNEMKVAVALTLQRYELFP

DPDNEPQVIPQVVLRSLNGIHIKIRRVEKEKNKKGM

>345317133

MVSRMDGEMDKGPSAWPALLFLALALALIKGTQLFLRRQKLLKTTAQFPGPPPHWLFGHL

FQNELSQSNSWIKYCPFGTPLWIGSFAVIFNIYHPDYLKVLLKRGDPKNKAAYRFAVPWI

GHGLLILNGEKWFQHRKMLTPGFHYSILRPYVKIMADSTNVMLDKWEKLTAQGETLELFQ

HVSLMTLDTLMKCSFSNNSSGQKDSDSKAYIQAVNNLTSMISQRIFKFYQHNNFIYWLSP

LGRCFRQACKTAHQHSEQVIAERKKTLQNVFELETIQAKRHLDFLDILLCAKTKEGKGLS

DEELRAEVDTFMFEGHDTTASAISWILYALALYPEHQQRCREEAQEILGERDTVQWEDLA

QLTFTTMFIKESLRLYPPVPAISRELSSPITFFDGRSLPKGSFIRIHIYSLHRNPLVWKD

PEVFDPQRFTPENISQRHPYAFLPFSAGPRNCIGQQFAMNEIKVSVVQTLLRFQLEPDHS

RPPEPISQLILKSKTGIHLHLRKLP

>363736770

MKATWFGVDVARVLHLAAVFCLTCVLLKAIQLYHRRRELLRALSAFPGHPTHWLLGHVQE

FLKEEDVLEKAEAWALKYQHAHPIWFGGFSAVLVINDPEYAKALFARGDPKDNLSYKHLI

PWIGNGLLILHGPKWHQHRKLLTPGFHYDVLKPYVALMAESTNVMLDKWEKLITNGKSVE

LFEHVSLMTLDSIMKCAFSYHSNCQTDRQNTYIQAVYDLCRMVHERLRIFPYHNDFIYWL

SPHGYQFRKVCQLAHDHTDKVIQERKESLKDEREFEKIKKKRHLDFLDILLCAKDETGAG

LSDEDLRAEVDTFMFEGHDTTASGLSWVLYCLASHPEHQARCREEIKDILGSRDTIQWED

LGKMTYSTMCIKESLRLYPPVPGVSRQLSKPITFHDGRTLPEGTITAISIYLIHRNPLVW

KDPLVFDPLRFSPENVSGRHSHAFLPFAAGMRNCIGQQFAMIEMKVALALILLRFELSPD

LTNPPHKIPRLILRSKNGIHLYLKKIH

>327279466

MEGNGETPGSTGKSGNTVSVSVSNSWLPQDSLQIFHLVLLLTLTYAILKAIQLYWHRQHL

INALQGFSLPPTHWLYGHIHKMTPEEEFLTAAAWTEKYPQCHQLWYGQFLAAIVINHPEY

AKVVLSRSDPKSFLIYGFLIPWIGKGLLILEGPKWQQHRKLLTPGFHYDILKPYTSVMVE

SVKQMLDILGKLVSKNNTASVEIFEHVSLMTLDTIMKCAFSSKSNCQTERDNAYIKAVYD

LTCLFSQRLKSPWLHNDLMYWFSSQGRRFRKACRLAHHHTDSVIRERKESLRNEIELEKI

LKKRHLDFLDILICAKDENGNSLSDEDLRAEVDTFMFEGHDTTASGISWLFYCMAQNPEH

QQRCREEITELLGKEGHVQWDDLGKMTYTTMCIKESLRLYPPVPQVARELNSPVTFPDGR

TLPEGLLTIMSIYSLHQNPEVWENPEVFDPLRFSSENSSRRHPYAFLPFAAGPRNCIGQQ

FAMNELKVALALTLLRFELLPDHSKTHIPVAQIVTRSRNGIHLKMKPIH

>363736772

MKATWFGVDVARVLHLAAVFCLTCVLLKAIQLYHRRRELLRALSAFPGHPTHWLLGHVQE

FLKEEDVLEKAEAWALKYQHAHPIWFGGFSAVLVINDPEYAKALFARGDPKDNLSYKHLI

PWIGNGLLILHGPKWHQHRKLLTPGFHYDVLKPYVALMAESTNVMLDKWEKLITNGKSVE

LFEHVSLMTLDSIMKCAFSYHSNCQTDRSVSNPWLKAVYDLCRMVHERLRIFPYHNDFIY

WLSPHGYQFRKVCQLAHDHTDKVIQERKESLKDEREFEKIKKKRHLDFLDILLCAKDETG

AGLSDEDLRAEVDTFMFEGHDTTASGLSWVLYCLASHPEHQARCREEIKDILGSRDTIQW

EDLGKMTYSTMCIKESLRLYPPVPGVSRQLSKPITFHDGRTLPEGTITAISIYLIHRNPL

VWKDPLVFDPLRFSPENVSGRHSHAFLPFAAGMRNCIGQQFAMIEMKVALALILLRFELS

PDLTNPPHKIPRLILRSKNGIHLYLKKIH

>326925340

MKASWFGVDVARVLHLAAVFCLTCVLLKAIQLYHRRRELLRALSPFPGHPTHWLLGHVQE

VRRGEEDLLEKAETWAQKYQYAHPVWFGSFSAFLVVSNPEYAKAIFARGDPKDNLSYKHL

IPWIGNGLLILHGPKWHQHRKLLTPGFHYDVLKPYVALMAESTNVMLDKWEKVITNGKSV

ELFEHISLMTLDSIMKCAFSYYSNCQTDRQNTYIQAVYDLCLMVHQRLSIFPYHNDFIYW

LSPHGYRFRKVCQLAHDHTDKVIQERKESLKDEREFEKIKKKRHLDFLDILLCAKDETGA

GLSDEDLRAEVDTFMFEGHDTTASGLSWVLYCLASHPEHQARCREEIQDILGSRDTIQWE

DLGKMTYSTMCIKESLRLYPPVPGVSRQLSKPITFHDGRTLPEGTITAISIYLIHRNPSV

WKDPLVFDPLRFSPENVSGRHSHAFLPFAAGTRNCIGQQFAMIEMKVALALILLRFELSP

DLTNPPQKIPRIILRSKNGIHLHLKKIH

>224058127

MASLLDNITRPSAAVLQLSVLFGIIFVLLKVLQLYWERKKLIKALEAFPGPPKHWLYGHN

HLIKSESFLHQIVAWGQEYPYAFPRWFGPTLPSIIIHHPEYAKTILGRADPKASAVYKLL

VPWIGKGLLILDGSKWFQHRKMLTPAFHYDVLKSYVTLMSDSVKVMLDKWDKKITERRSV

ELFQDVSLMTLDTIMKSAFSFNSNCQTQRSDYIRAIFDVTYLLSNRIRNLSFTDVFYNFT

CNGRAFQNACKLARTHTDQVIKERKMLLSNEKELEKIQKKRHLDFLDILLCSKDENGVGL

SDEDLRAEVDTFMFEGHDTVASALSWLFYCMSLHPEHQQRCREEVQGILGHRDTIEWEDL

GKMTYTTMCIKESLRLFPPVTSVSRELSKPITFPDGRSLPAGCQVGVNIFAIHRNRDVWE

NPEIYDPLRFSPENSAQRHSHAFLPFSAGSRNCIGLQFAMNEIKVALALTLQRFELCPDP

SKLPIMISQIILRSSNGIHLHLKKIF

>301603599

MASNILFGLFPAQLYSWAASLCLLLVLYKATQLYLKRRFLVAALSPFPGPKSHWLYGNAH

EFFQVGKELDLILRWAQVYPYGVSMWLGNFHAALFVTHPDYAKAILGRQEPKDDTAYKYL

VPWIGKGLLVLSGPKWFQHRRLLTPGFHYDVLKQYVSLMSDCTRDMLDKWDKLMPNEKSI

ELFHHVSLMTLDTIMKCAFSYNSSCQNNRDNEYIKAVYELSYLVYERSSFFPYHNDVIFS

LSPLGFRFRKALSIAHQHTDKVIKHRKESLINETELDKISQKRHLDFLDILLFAKDENGK

GLSDEDLRAEVDTFMFAGHDTTASGISWILYCIAKYPEHQQKCREEITELLGDRETMEWG

DLGQIPYTTSCIKESLRLYPPVPIIGRRLSKSITFSDGRSLPEGSVIFINIFCIHRNPTV

WKDPEVFDPLRFSSENSSKRHSHAFVPFAAGPRNCIGQNFAMNELKVAVALTLNRYELSP

DLSKAPLKSPQLVLRSKNGIHVYLKKASY

>AAH98079.1

MASTLWKALSSPWLSVNIYQIGQFVALLCVLLLLLKAYALHSRGRRFAAALAPFPGPPAH

WLYGHVNQFRRDGKDLDRLMVWVNKYPNAFPLWIGKFFGTLIITDPDYAKVVFGRSDPKT

STGYNFLVPWIGKGLLILSGNTWFQHRRLITPGFHYDVLKPYVSLISDSTKIMLDELDVY

SNKDESVELFQHVSLMTLDSIMKCAFSYHSNCQTDKDNDYIQAVYDLSWLTQQRIRTFPY

HSNLIYFLSPHGFRFRKACRIAHLHTDKVIGQRKKLLESKEELEKVQKKRHLDFLDILLC

SKDENGQGLSHEDLRAEVDTFMFEGHDTTSSGISWILYCMATHPEHQQKCREEISEALGE

RQTMEWDDLNRMPYTTMCIKESLRLYPPVPSVSRELAKPITFHDGRSLPAGMLVSLQIYA

IHRNPNVWKDPEIFDPLRFSPENSSKRHSHAFVPFAAGPRNCIGQNFAMNEMKVAVALTL

KRFELSPDLSKPPLKQPQLVLRSKNGIHVYLKKAS

>118094503

MVSLLDNIARPSAVILQLSVVLGVIFVLLKVVRFYQGRKKILKALEAFPGPPAHWLYGHN

HLITGDKVLNLLVSLGEEYPYAFPRWFGPVLPSLVVHHPEYAKTILGRGDPKPPVPYQFL

VPWIGKGLLVLSGTKWFQHRKLLTPAFHYDVLKSYVSLMSDSVKVMLDKWEKKKSVELFE

DVSLMALDSIMKCAFSYNANCQTQSNSDTYIKAVFDLGYLINKRIQNFSYQDAFYDLTHS

SREFQDACRRAHAHTDKVIQERKILLSNDKELDKIRKKRHLDFLDILLCTKDENGVGLSD

EDLRAEVDTFMFEGHDTTASGISWLLYCMALHPKHQALCREEIQGIMGNRDTIEWEDLGK

MTYSTMCIKESLRLFPPVPAVSRRLSKPVTFSDGRSLPEGSQVSLNIFGIHRNRDVWEDP

EVFDPLRFSPENSAHRHSHAFLPFSAGSRNCIGQQFAMNEMKVALALTLLRFELFPNASK

PPMLTQQLILKSRSGIHLHLKKIC

>327279444

MNTVLESVNNSWLPRDSSQIFHLVLLLSFTYVILKVIKVFWRRQQDINALRCFPLPPTHW

LYGHVHKMSPEEEFLTTAAWAEKYPQCHQLWFGQFLYALYINHPEYAKVVFSKNDPKVLL

VYGFLIPWIGKGLLILNGPKWQQHRKLLTPGFHYDILKPYINVMVESVKQMLDILEKQVS

KDSTASVEIFKHVSLMTLDTIMKCAFSNKSNCQTERDNAYIKAVYDLTYLFDQRLKSPLL

HNDFTFQFSSQGRRFYNACKIAHHHTDNVIRERKESLKNEKELEKILKKRRLDFLDILIC

AKDENGNSLSDEDLRAEVDTFMFEGHDTTASGISWLFYCMAQNPEHQQRCREEITELLGK

EGHIQWDDLGKMPYVTMCVKESLRLYPPVYQIGRELNVPVTFPDGRTIPKGIFVILGINT

LHRNPEVWENPEVFDPLRFSPENSSRRHPFAYLPFAAGPRNCIGQQFAMNELKIALALTL

LRFELLPDNSQPPIPIPQTVTRSKNGIHLKLKSIH

>301603595

MASNLWEVLSSPGLPGNIYQFGQFAALLCLILLLLKACGLFIRWKSLKDALQNFPGPPKH

WLYGNANQFRNDGTDMDRILLWANKYPKAFPLWVGQFFASLVITNPEYAKAAFARSDPKT

PTGYDFLIPWIGKGLLVLSGDTWFQHRRLLTPGFHYDVLKPYAKLISDSTKVMLDKWVPF

SNKGEPVELFHHVSLMTLDSIMKCAFSYHSNCQTDNDNSYTKAVYDLSYLTHHRARTFPY

HNNLIYYLSPHGFLFRKACRIAHQHTDKVIKQRKTLLQNKEEFEKVKQKRHPDFLDILLC

ARDENGKGLSDEDLRAEVDTFMFEGHDTTASGISWILYCMAKYPEHQQKCREEIRDVLGE

KESFEWEDLNKIPYTTMCIKESLRLYPPVPAVSRELNKPITFSDGRSLPAGSVIFINIFC

IHRNPTVWKDPEVFDPLRFSSENSSKRHSHAFVPFAAGPRNCIGQNFAMNELKVAVALTL

NRYELSPDLSKAPLKSPQLVLRSKNGIHVYLKKASY

>AAI29579.1

MASNLWEVLSSPGLFGNIYQFGQFAAQLCLVLLLLKAWGLFIRWKSLKDALQNFPGPPKH

WLYGHANQFRGDGTDMDRVLAWADKYPKAFPMWVGQFFANLIITNPEYAKAVFARSDPKT

PTGYNFLIPWIGKGLLVLSGDKWFQHRKLLTPGFHYDVLKPYVRLISDSTNVMLDKWVSF

SNKGETVELFHHVSLMTLDSIMKCAFSYHSNCQTDKDNSYTKAVYDLSFLAHHRARTFPY

HNNLIYYLSPHGFLFRKACRIAHQHTGKVIKQRKTLLQNKGEFEKVKQKRHPDFLDILLC

ARDENGKGLSDEDLRAEVDTFMFEGHDTTASGISWILYCMAKYPEHQQKCREEIREVLGE

KDSFEWEHLSKIPYTTMCIKESLRLYPPVPGVSRELNKPITFYDGRSLPAGSVIFINIFC

IHRNPSVWKDPEVFDPLRFSSENSSKRHSHAFVPFAAGPRNCIGQNFAMNELKVAVALTL

NRYELSPHLSKPPLKSPQLVLRSKNGIHVYLKKAS

>CAJ83608.1

MASTLWKALSSPWLSVNIYQIGQFVALLCVVLLLLKAYALYSRGRRFAAALVPFPGPPAH

WLYGHVNQFRRDGKDLDRLMVWVNKYPNAFPLWIGKFFGTLIITDPDYAKVVFGRSDPKT

STGYNFLVPWIGKGLLILSGNTWFQHRRLITPGFHYDVLKPYVSLISDSTKIMLDELDVY

SNKDESVELFQHVSLMTLDSIMKCAFSYHSNCQTDKDNDYIQAVYDLSWLTQQRIRTFPY

HSNLIYFLSPHGFRFRKACRIVHLHTDKVIGQRKKLLESKEELEKVQKKRHLDFLDILLC

SKDENGQGLSHEDLRAEVDTFMFEGHDTTSSGISWILYCMATHPEHQQKCREEISEALGE

RQTMEWDDLNRMPYTTMCIKESLRLYPPVPSVSRELAKPITFHDGRSLPAGMLVSLQIYA

IHRNPNVWKDPEIFDPLRFSPENSSKRHSHAFVPFAAGPRNCIGQNFAMNEMKVAVALTL

KRFELSPDLSKPPLKQPQLVLRSKNGIHVYLKKAS

>338721711

MSVSGLSTPRPLGSISGFLQVASLLGLVLLLLKAAQLYLRRQWLLKAFQEFPCPPSHWLY

GHSQELQEDQDLPELLKRVEKFPCACPHWLWGSKVLFTVYDPDYMKVILGRSDPKPLGTY

RFLAPWIGYGLLLLNGQTWFQHRRMLTPAFHYDILKPYVGLMADSVRVMLSKGHSRGQQD

KLTMVNLSSGSFSGGMDKPGCHSVINLRKAHLQKEGELEKVRKKRHLDFLDILLFSKMEN

GSSLSDEDLRAEVDTFMFEGHDTTASGISWILYALAMHPEHQQRCREEIQGLLGDGASIS

WDHLDQMPYTTMCIKEALRLYPPVPAIGRELTKPITFPDGRSLPKGIMIALSFYALHHNP

EVWPNPEVFDPSRFAPGSTQHSHAFLPFSGGSRNCIGKQFAMKELKVAVALTLLRFELSP

DPSRVPVPIPRIVLMSKSGIHLHLRKLL

>EHB14338.1

MLTPAFHYDILKPYVGIMANSVQVMLDRWEQLISQDSPLEIFQHISLMTLDSIMKCAFSY

QGSIQLDRTSQFYIQAIEYLNNLIYSRIRNIFHQNDTIYSLSSNGRWSKYTPSCPHEHTD

QVIKQRKAELQNEGELEKVRKKRHLDFLDILLFAKMENGSSLSDEDLRAEVDTFMFEGHD

TTASGISWAFYALAMHPNHQQKCREEVQNLLGDGSSIASEHLDQMPYTTMCLKEALRLYP

PVPTVSRELSKPITFPDGHFLPKGILISLSIYALHHNPKVFDPSRFAPDSPRHSHSFLPF

SGGSRNCIGKQFAMNELKLAVALTLLHFELLPDPTRVPFPIQRSTCISGSSTNPEVCVPT

AIMQLLQELPVKDHWVIM

>326925342

MVSLLDNIARPSAIILQLSVVLGVIFVLLKVVKFYQQRKKILKALEAFPGPPAHWLYGHN

HLITGDKMLSLVVSLGEQYPYAFPRWFGPVLPSLVIHHPEYAKTILGRGDPKPPVPYHFL

VPWIGKGLLVLGGTKWFQHRKLLTPAFHYDVLKSYVSLMSESVKVMLDKWEKKKSVELFE

DVSLMALDSIMKCAFSYNTNCQTQSNSDTYIKAVFDLGSLINKRIQNFSYHDAFYDLSRS

SREFQDACRRAHAHTDKVIQERKILLSNDKELDKILKKRHLDFLDILLCTKDENGVGLSD

EDLRAEVDTFMFEGHDTTASGISWLLYSMALHPEHQARCREEIRGIVGKRDTIEWEDLGK

MTYSTMCIKESLRLFPPVPAVSRRLSKPVTFSDGRSLPEGSQVSLNIFGIHRNRDVWEDP

EVFDPLRFSPENSAHRHSHAFLPFSAGPRNCIGQQFAMNEIKVALALTLLRFELFPNASK

PPTLTQQLILKSSSGIHLHLKKIH

>Q64462.1

MALSFLSPSLSRLGLWASVVILMVTVLKLLSLLFRRQKLARALDSFPGPPKHWLFGHALE

IQKTGGLDKVVTWTEQFPYAHPLWLGQFIVFLNIYEPDYAKAVYSRGDPKAAYVYDFFLQ

WIGKGLLVLEGPKWFQHRKLLTPGFHYDVLKPYVAIFAESTRVMLDKWEKKASENKSFDI

FCDVGHMALDTLMKCTFGKGDSGLSHSDNSYYLAVSDLTLLMQQRIDSFQYHNDFIYWLT

PHGRRFLRACQIAHDHTDHVIRQRKAALQDEKEQKKLQERRHLDFLDILLGARDESGIKL

SDADLRAEVDTFMFEGHDTTTSGISWFLYCMALYPMHQQRCREEVREILGDRDSFQWDDL

AQMTYLTMCMKECFRLYPPVPQVYRQLSKPVTFVDGRSLPAGSLISLHIYALHRNSAVWP

DPEVFDPLRFSPENMTGRHPFAFMPFSAGPRNCIGQQFAMNEMKVVTALCLLRFEFSPDP

SKIPIKVPQLILRSKNGIHLYLKPLGPGSGK

>301603601

MVPDVFFGLFSFGVPSQLCSWAAWLCLLLVLYKAAQLYVWRRKLVAAFSPFPGPKCHWLY

GNTYEFLQIGKDLDLVLGWAQVFPYGMPLWLGNFYATLIITHPDYAKAVLARQDPKDDMA

YKFIVPWIGEGLLVLSGPKWFQHRRLLTPGFHYDVLKPYVTLMSDCTRVMLDKWDKLMPN

EKSVELFHYVSLMTLDTIMKCAFSYNTSCQNNRDNAYINAVYELSYLVDQRFRFFPYHNK

LIFHLSPLGFRFRKALSTAHQHTDKVIKQRKESLMHETELDKIRQKRHLDFLDILLCAKD

ENGKGLSDEDLRAEVDTFMFEGHDTTASGISWILYCIAKYPEHQQKCREEITELLGERET

MGWDDLGKIPYTTLCIKESLRLYPPVPGIGRRLSKPITFCDGRSLPEGASIILSIYSINR

SPSLWKDPEVFDPLRFLPENSDNRHPHAFLPFSAGPRNCIGQNFAMNEMKVAVALTLQRY

ELFPDPDNEPQKVPQIVL

>395530268

MGSDAAPSWLETHRARPLHLALTFALALLLLQVVKLYLRRQGLLRALRLFPGPPTHWLLG

NQREFYLEKELQQFDMLVEKYPCAFPRWVGAFQVLLNIYDPEYVKILLNRRDPKIQLGYK

FIVPWVGKGLLSLEGKKWYQHRHLLTPAFHFSILKPYIHVINDSVCRMLDAWGKLSTQDS

SVEICEPIRLMTLDSIMKCAFNVQTSSQTESFSINYLSTVTKLSELIFHRLHSYLHHNDL

IYRWSSQGQEFQALCQIAHQYAAKIIQERREALKNDSEQDKIRKKKYLDFLDVLLCAKSE

IGEGLSDEELEAELNTFVFGGHDTTASSLSWLFYCMAMNPDHQHQCREEIRDILKSGDTI

TWDHLDQMPYSTMCIKEALRLYPPEITIARELSKPITFPDGRSLPTGMIVVLNIWALHHN

PAVWENPQVFDPQRFSQENSMKRHSYAFLPFSAGPRNCIGQQFAMLELKVGLALTLLRFE

LVSDPEKPPIPMPHLVLRSKNGIHLYLKPFH

>AAI61776.1

MVYQTAIGSMWNTLVWQQVAALLCLLAVLLKATQLYLSQKRQENIFKQFPGPPRHWLLGN

VDQIRRDGKDLDLLVNWTHKNGGAFPVWFGNFSSFLFLTHPDYAKVIFGREEPKSSISYD

FLVPWIGKGLLVLTGPKWFQHRRLLTPGFHYDVLKPYVNLISKCTTDMLDNWEKLITKQK

TVELFQHLSLMTLDSIMKCAFSYDSNCQKDSNNAYIKAVFDLSYVANLRLRCFPYHNDTV

FYLSPHGYRFRKACRITHEHTDKVIQQRKESMKLEKELEKIQQKRHLDFLDILLFARDEK

GHGLSDEDLRAEVDTFMFEGHDTTASGISWILYCMAKYPEHQQKCREEIKEVLGDRQIME

WEDLGKIPYTNMCIKESLRMYPPVPGVARQLRNPVTFFDGRSVPAGTLIGLSIYAIHKNP

AVWEDPEVFNPLRFSPENSANRHSHAFLPFAAGPRNCIGQNFAMNEMKVAVALTLNRFHL

APDLENPPIKIPQLVLKSKNGIHVHLTKVQ

>AAH84618.1

MANEAVLGSMWNALVWQQVAALLCLLAVLLKATQIYLSKKRQERILEQFPGPPRHWLLGN

VDQIRRDGKDLDLLVNWTQSHGGAYPVWFGNFSSFLFLTHPDYAKVIFGREEPKSSLSYN

FLVPWIGNGLLVLTGPKWFQHRRLLTPGFHYDVLKPYVKLISKCTTDMLDNWEKRITKQK

TVELFQHVSLMTLDSIMKCAFSYESNCQKDSDNAYIKAVFDLSYLANLRLRCFPYHNDTI

FYLSPHGYRFRQACKITHEHTDKVIQQRKESMKHEKELEKIQQKRHLDFLDILLFARDEK

GHGLSDEDLRAEVDTFMFEGHDTTASGISWILYCMAKYPEHQQKCREEIKEVLGDRQTME

WEDLGKIPYTNMCIKESLRIYPPVPGVARMLRNPVTFFDGRSIPAGTLVGLSIYAIHKNP

AVWEDPEVFNPLRFTPENSANRHSHAFVPFAAGPRNCIGQNFAMNEMKIAVALTLNRFHL

AADLENPPILIPQLVLKSKNGIHVHLNKVQ

>AAI58511.1

MVYQTAIGSMWNTLVWQQVAALLCLLAVLLKATQLYLSQKRQENIFKQFPGPPRHWLLGN

VDQIRRDGKDLDLLVNWTHKNGGAFPVWFGNFSSFLFLTHPDYAKVIFGREEPKSSISYD

FLVPWIGKGLLVLTGPKWFQHRRLLTPGFHYDVLKPYVNLISKCTTDMLDNWEKLITKQK

TVELFQHLSLMTLDSIMKCAFSYDSNCQKDSNNAYIKAVFDLSYVANLRLRCFPYHNDTV

FYLSPHGYRFRKACRITHEHTDKVIQQRKESMKLEKELEKIQQKRHLDFLDILLFARDEK

GHGLSDDDLRAEVDTFMFEGHDTTASGISWILYCMAKYPEHQQKCREEIKEVLGDRQIME

WEDLGKIPYTNMCIKESLRMYPPVPGVARQLRNPVTFFDGRSVPAGTLIGLSIYAIHKNP

AVWEDPEVFNPLRFSPENSANRHSHAFLPFAAGPRNCIGQNFAMNEMKVAVALTLNRFHL

APDLENPPIKIPQLVLKSKNGIHVHLTKVQ

>327279442

MCKNKTATESLMSASWNFAQLFQLTVALILTCVALKAIQLFQTRKKLLKGLRDFPGPPTH

WLYGHIHMMGLQKELISIQKWTEEFPRCFPVWYGGFLAGLAINHPEYVKAVYSKGDPKCP

IFYHGLIPWIGKGLLLLEGPKWQQHRKLLTPGFHYEILKSYVIPVAESVKVMLAKWETLS

SQDNEVSVEIYNHVNLMTLDSILKCAFSFQSNCQMDRDNFHVKIIHDLAFLINERTLVPL

YQNDFIYSLSSQGRWFHKTCKLAHLHTDKIIQKRQESLKAENKTLFKGKHFDFLDILLLT

KDERGNPLPQEDLRAEVATFMFAGHDTTASGISWLFYCMAQNPEHQERCREEIKEVLGDQ

ETIQWDNLGKLTYTTMCIKESLRLYPPVPLIARTLDSPLTFDDGRTLPKGFLVGVCIFAL

HRNSEVWDNPKVYDPMRFSPENSCLRHPYAYLPFSAGSRNCIGQQFAMMEMKVALALTLL

RFELKPDPANPSIPVAQIVIRSKNGVHLKLKKLQ

>126305752

MGADKIPSWLETHWTRPLHLALTFVLALLLLQVVKLYLRRQGLLRALRLFPGPPPHWLFG

NQREFYLEKELQQFNVLPEQYPCALPLWVGAFQVLLNIYDPEYAKILLNRRDPKIQHGYK

FIIPWIGGGLLSLEGKKWYQHRHLLTPAFHLSILKPYIHVMNDSVCRMLDTWEKLSTQDN

SVEICEPIRLMTLDSIMKCAFSVQTSSQTESFSTNYLSTVTKLSELIFCRLNNYLHHNDL

IYRWSSQGQEFQALCQIAHQLPAKIIQERREALKNNSEQDKIRKKKFLDFLDVLLCAKSE

NGEGLSNEELEAEVNTFVFGGHDTTASSLSWIFYCMAMNPEHQHQCREEIRNIIKYGDTI

TWDHLDQMPYSTMCIKEALRLYPPSITIARELSKPITFPDGRFLPTGMTVVLNIWALHHN

PTVWENPQVFNPERFSQENSMKRHSYAFLPFSAGPRNCIGQQLAMLELKVGLALTLLRFE

LLPDLEKPPIPMPHLVLRSKNGIHLYLRPLH

>AAH77479.1

MASGLWKVLSSSWLPVNVAQIGQFAILLCVVLLVLKACALYSRGRKFTAALTPFPGPPSH

WLYGHVNQFRRDGKDLDRLMVWAKKYPNAFPLWIGKFFGTLIITHPEYAKLVFGRPDPKT

STGYSFLIPWIGKGLLVLSGDTWFQHRRLITPGFHYDVLKPYVGLISDSTKIMLDKLDAF

SNKDEPVELFQHVSLMTLDSIMKCAFSYQSNCQTDIDNDYIQAVYDLSWLTQQRIRTFPY

HSNLIYYLSPHGFRFRKACRIAHLHTDKVIGQRKKLLESKEELEKVQKKRHLDFLDILLC

AKDENGQRLSDEDIRAEVDTFMFEGHDTTSSGISWILHCMATHPEHQQKCQEEINEVLGE

RQTMEWDDLNRIPYTTMCIKESLRLYPPVPSVSRELAKPITFHDGKSLPAGILIFDPLRF

SPENSSKRHSHAFVPFAAGPRNCIGQNFAMNEMKVAVALTLKRFELLPDLSKPPLKLPQL

VLRSKNGIHVYLKKAS

>P15129.3

MVLNFLSPSLSRLGLWASVVILMVIVLKLFSLLLRRQKLARAMDSFPGPPTHWLFGHALE

IQKLGSLDKVVSWAQQFPHAHPLWFGQFVGFLNIYEPDYAKAVYSRGDPKAADVYDFFLQ

WIGKGLLVLDGPKWFQHRKLLTPGFHYDVLKPYVAIFAESTRMMLDKWEKKASENKSFDI

FCDVGHMALDTLMKCTFGKGDSGLGHRDNSYYLAVSDLTLLMQQRIDSFQYHNDFIYWLT

PHGRRFLRACKIAHDHTDEVIRQRKAALQDEKERKKIQQRRHLDFLDILLGVRDESGIKL

SDAELRAEVDTFMFEGHDTTTSGISWFLYCMALYPEHQQLCREEVRGILGDQDSFQWDDL

AKMTYLTMCMKECFRLYPPVPQVYRQLNKPVTFVDGRSLPAGSLISLHIYALHRNSTVWP

DPEVFDPLRFSPENAAGRHPFAFMPFSAGPRNCIGQQFAMNEMKVVTALCLLRFEFSLDP

SKMPIKVPQLILRSKNGIHLYLKPLASRSGK

>327279468

MDLARLWHLAAAFCLTWLLLKALQLHRRRKELLKSLSSFPGPPAHWLYGNMFELLPYSVV

FKKTEQWMLLYPYAFPLWFGRYFASVTITHPDYAKTLLARADPKDRLTYPSMIPWIGKGL

LVLDGPKWAQHRKLLTPGFHYNILKPYVTLMAESTKVMLDKWEQLIIQQNSVELFEHVSL

MTLEIIMKCAFSYNTNCQLDRENSYVKAVFELCSLVFEKLFSIFGHHDVTYWFTPQAYRF

RKACKVAHRHTEKIIKERKESLGDEEELQKIQQKRYLDFLDIILCAKDENGIGLSDEDIR

AEVDTFMFAGHDTTASGISWMLYCLAMNPEHQGRCREEIKSILGDRGTIEWDDLSKMTYC

TLCIKESLRLYPPVPGVSREIAKPITFFDGKILPEGTMIGVAIQLIHRNPNIWIDPEVFD

PQRFTPENVSSRNSHAFLPFSAGPRNCIGQQFAMNEMKIALAMTLLRFEISPDALKPPPV

PIPQIVLRSQNGIHLCLKKIM

>EGV94559.1

MVLSHLSPNLSRLGLWASVVILIVSALKLFSLLLRRQKLARAMDSFPGPPTHWLFGHALE

IQKLGSLDTVVSWSQQFPYAHPLWVGQFVGYLNIYEPDYAKAVYSRGDPKAPDVYDFLLQ

WLGKGLLVLDGPKWFQHRKLLTPGFHYDVLKPYVAIFAESTHIMLDKWEKKASENKSFDI

FCDVGHMALDTLMKCTFGKGDSGLGHRDNSYYRAVSELTLLTQQRIESFQYHNDFIYWCT

PHGRRFLRACQTAHDHTDQVIRQRKAALQDEKEQKKIQQRRHLDFLDILLGARDESGIKL

SDADLRAEVDTFMFEGHDTTTSGISWFLYCMALYPEHQQRCREEVREILGDQDSFQWDDL

AKMTYLTMCMKECFRLYPPVPQVFRQLNKPVTFVDGRSLPAGSLISMHIYALHRNSAVWP

DPEVFDPLRFSPENVTGRHPFAFMPFSAGPRNCIGQQFAMNEMKVVTALCLLRFEFSLDP

SKMPIKIPQLVLRSKNGIHLYLKSLGSRK

>BAA85387.1

MWNALVWQQVAALLCLLAVLLKATQIYLSKKRQERILEQFPGPPRHWLLGNVDQIRRDGK

DLDLLVNWTQSHGGAYPVWFGNFSSFLFLTHPDYAKVIFGREEPKRQLSYNFLVPWIGNG

LLVLTGPKWFQHRRLLTPGFHYDVLKPYVKLISKCTTDMLDNWEKLITKQKTVELFQHVS

LMTLDSIMKCAFSYESNCQKDSDNAYIKAVFDLSYLANLRLRCFPYHNDTIFYLSPPWVS

ISPSLQNNSEHTDKVIQQRKESMKHEKELEKIQQKRHLDFLDILLFARDEKGHGLSDEDL

RAEVDTFMFEGHDTTASGISWILYCMAKYPEHQQKCREEIKEVLGDRQTMEWKDLGKIPY

TNMCIKESLRIYPPVPGVARMLRNPVTFFDGRSIPAGTLVGLSIYAIHKNPAVWEDPEVF

NPLRFTPENSANRHSHAFVPFAAGPRNCIGQNFAMNEMKIAVALTLNRFHLAADLENPPI

LIPQLVLKSKNGIHVHLNKVQ

>348549852

MAPPFLSPSLSYLSLWASGVILAIVFLKLFHLLLRRHKLAKAMDNFPGPPTHWLFGHALE

IQKTGSLDKMVSWAHQFPYAFPLWVGPFIGFLNIYEPDYAKAVYSRGDPKAPDVYSFLLQ

WTGKGLLVLDGSKWFQHRKLLTPGFHYDVLKPYVAVFTSSANAMLDKWEEKARENKSFDI

FCDVGHMALDSLMKCTFGKGDSGLSYRDSSYYQTVADLTLLVQQRVDSFQYHNDFIYWLT

PHGRRFLRACQAAHDHTDHVIRNRKAVLQDKKEQEKIQHRRHLDFLDILLGARDESGIKL

SDADLRAEVDTFMFEGHDTTTSGISWFLYCMALYPEHQQRCREEAREILGDQDSFKWDDL

GKMTYLTMCIKECFRLYPPVPQVYRQLSKPVDFVDGRSLPAGSLISLHIYALHRNSAVWP

DPEVFDPLRFSPENASGRHPFAFMPFSAGPRNCIGQQFAMNEMKVVTALCLLRFEFSLDP

SQLPIKLPQLVLRSKNGIHLHLKSLRPESGK

>344278925

MVPVLLSLSLFHLGLWASGLILVLGFFKLACLLLRRQKLARAMDCFPGPTTHWLFGHALE

IQQTGSLDKAVSWAHQFPYAHPLWLGPFLGFLNIYEPDYVKAVYSRGDPKASDVYDFFLQ

WIGKGLLLLEGPKWFQHRKLLIPAFHHDVLKPYVAVFVDSTRIMLDKWEKKSREDKCFDI

FCDVGHMALDTLMKCTFGRGDSGLSHRDNSYYLAVSDLTLLMQQRVDSFQYHNDFIYWLT

PHGRRFLRACQVAHDHTDQVIRERKAALQDEKEQEKIQKRRHLDFLDILLGAWDENGSKL

SDEDLRAEVDTFMFAGHDTTTSGICWFLYCMARYPEHQDRCREEIREILGDRDTVQWDDL

GKMTYLTMCIKESFRLYPPVPQVYRQLSKPVNFVDGRSLPAGSLISLHIYALHRNGNVWS

DPEVFDPLRFTPENTAGRHPFAFIPFSAGPRNCIGQQFAMNEVKVVTALCLLRFELSLDP

LQPPIKMPQLILRSKNGFHLHLKPLGPGSGMQLHRE

>395858227

MVSSFVTRLGLWASGLILILSFLKLISLLLRRQRLARAMDNFPGPPTHWLFGHALEIQQT

GSLDKVVSWAHQFPYAHQLWLGQFVGFLNIYEPEYAKAVYSRGDPKASDVYDFFLQWIGK

GLLVLEGPKWFQHRKLLTPGFHYDVLKPYVAVFAESANAMLDKWEEKARKEKSFDIFCDV

GHMALDTLMKCTFGKGDKGLGQRDSNYYLAVSDLTLLMQQRLESFQYHNDFIYWLTPHGR

RFLRACQVAHEHTDQVIRERKAALQDEKEQKKIQNRRHLDFLDILLGAQDGDGIKLSDTD

LRAEVDTFMFEGHDTTTSGISWFLYCMAKYPEHQRLCREEIREILGDQNSIQWDDLSKMT

YLTMCIKESFRLYPPVPQVYRQLSKPVAFVDGRSLPAGSLISLHIYALHRNSAVWPDPEV

FDPLRFSPENASGRHPYAFMPFSAGPRNCIGQQFAMNEMKVVTALCLLRFEYSLDPSRLP

IKIPQLILRSKNGIHLHLKSLGTGSK

>344278923

MESSWLEARWARPFYLAFVFCLALVLLQAIKLYLRRQRLLRDLRCFPAPPTHWLYGHQKF

LQEDEMEKLEELVEKYPCAFPRWVGPFQAFFYIYDPEYAKTFLSRTDPKSQFPNRFLSPC

IGQGLLNLEGPKWFQHRHLLTPGFHLNILKPYAEVMVHSVNTMLDKWEKMLSTQHATVEV

FEHINLMTLDILLKCAFTQETNCQINGTNDPYVKAVFELGKIIFYRMYSFLHHHDRIFNF

SPEGHRFQKLNQVLHQYTEKIVQDRKKSLQDEKKQDNTQKRKYQDFLDIVLSAQAENEDS

FSDTDLLSEVNTFILSGHDTTAGGISWLLYCLALNPEHQQRCREEIRDILGDGSSIIWDH

LGEMSYTTMCIKEALRLIPPIPSISRELSKPITFPDGHSLPAGMNVVLSIWGLHHNPAIW

ENPKVFDPLRFSPENSDQRHPHAFLPFSAGPRDCIGQQIAMIQLKVTVALILLHFEVTPD

PTRPPILSPQIVLKSKNGIHLCLKKIP

>395537288

MHVKQDTTGHLDPWGSHHASSLTLCLYFLGSGRGLLILNGPTWFQHRRLLTPAFHYDILK

SYVVLMAESVRVMLNKWEKLISQGSNLEVFEHVSLMTLDTIMKCAFSQKSNCQMERNADY

YIQAVWEQSALIFARLRFALYHNDFIYWLTPQGYQARQAARRAHEHTDKVIRKRKEHLQQ

EGTLEAVSKKRHLDFLDILLYARKENGDSLSDEELRAEVDTFMFGGHDTVASGISWLFYS

LAMNPEHQEKCREEIRSILGDGKSITWEHLSQMPYTTMCIKESFRLYPPAPNIFRELSKP

LTFPDGCSLPAGTSVSLNIYALHHNPAVWPEPEVFDPLRFSPENSAFRHSHAFLPFSAGG

RNCIGQQFAMAEVKVAVALTLLHFHLAPDDTQPPELTFKLILRSTNGIHLKLKKL

>296207875

MEPSLLQELMAHPFLLLILFCMSLLLFQVIRLYQRRRRLIRALHLFPAPPAHWFYGHKEF

YPVKEFEVYHELMEKYPCAIPLWVGPFTMFFIVHDADYAKMLLKRNDPKTTISHKILESW

IGRGLVTLHGSKWKKHRQIVKPGFNISILKIFVTMMSESVRVMLNKWEEHIAQNSCLELF

QHVSLMTLDSLMKCAFSHEGSIQLDSTLDSYLNAVFNLAKLSFQRMNNFLHHNDLIFKFS

SQGQIFSKFNKVLQQFTEKVIQDRKESLTDELKQDTIQKRYQDFLDILLSAKIENSKDFS

EADLQAEVKTFMFAGHDTTSSAISWIFYCLANHPEHQQKCRDEIRELLGDGSSITWEHLS

QMPYTTMCIKECLRLYPPAPNISRLLDKPITFPDGRSLPAGITLFINIWALHHNPYFWED

PEVFNPLRFSRENSEKIHPYAFIPFSAGSRNCIGQHFAIIECKVAVALTLLRFELASDHS

RPPQLVRRVVLKSNNGVHVFVKKVC

>345319929

MNLSGLFPWLAGLFLTLLLLKLSKLCVRRKLFVQAFGSFPGPPAHWLFGHALEIQRGGLD

KVMVWAQDYPHAFPLWLGKFIGYLNIHEPDYAKAVFSRGDPKAPDVYDFFLQWIGKGLLV

LEGSKWFQHRKLLTPGFHYDILKPYIRVFVDSSKVMLDKWEKKITAGSSLEIVHDVGLMA

LDSLMKCTFGKGNSSQLESENAYYLAVHDLTFLMQERIDRFQYHNDFIYWLTPHGRRFLR

ACKATHDHTDQVIRERKEALQNERELAVLQKKRHLDFLDILLCAKDESGEGLSDADLRAE

VDTFMFEGHDTTTSGISWFLYCLALHPEHQRRCREEVQEILGNRDAFQWEDLGKMTYLTQ

CIKESFRLYPPVPQVYRQLNKPVQFVDGRSLPEGALVSLHIYALHRNPKVWSDPEVFDPL

RFSPENSVGRHPYAFLPFSAGPRNCIGQQFAMSEMKVVSAQCLLRFEFLPDPSRPPEPIP

QLVLKSKTGIHLRLRKLQ

>P15128.1

MLGFLSRLGLWASGLILILGFLKLLRLLLRRQRLARAMDSFPGPPTHWLFGHALEIQKTG

SLDKVVTWTQQFPYAHPLWVGQFIGFLNIYEPDYAKAVYSRGDPKAPDVYDFFLQWIGKG

LLVLDGPKWFQHRKLLTPGFHYDVLKPYVAIFADSTRIMLEKWEKKACEGKSFDIFSDVG

HMALDTLMKCTFGKGDSGLNHRDSSYYVAVSELTLLMQQRIDSFQYHNDFIYWLTPHGRR

FLRACRAAHDHTDRVIRQRKAALQDEKEREKIQNRRHLDFLDILLDVRGESGVQLSDTDL

RAEVDTFMFEGHDTTTSGISWFLYCMALYPEHQQRCREEVREILGDQDSFQWEDLAKMTY

LTMCMKECFRLYPPVPQVYRQLSKPVSFVDGRSLPAGSLISLHIYALHRNSDVWPDPEVF

DPLRFSPENSSGRHPYAFIPFSAGPRNCIGQQFAMNEMKVVTALCLLRFEFSVDPLRLPI

KLPQLVLRSKNGIHLYLKPLGPKAEK

>327279464

MESLMDILCFWLPGNLSQLFQLTFVLILTCVMLKAIQLFQRRQKLLKDFRDFPGPPSHWL

YGNSHMLKGGAELRNLVKWTEEYPYYFPLWFGGFLAFLGINHPESAKVYSRGDPKCLILY

NFAVPWIGRGLLVLNGPKWQQHRKMLTPGFHYEILKSYVTPITESVKVMLGNWEKLILED

PEVSLEMYEHVSLMTLDSIMKCAFSCQSNCQMDRENPYIKAIFELTFLVYQRAKTPLYHS

DLIYWFTSPGRRFRKACRLAHLHTDKVIRERVKVLQDEQKSGKSLKKRRLDFLDILLQAK

DEKGNPLPHEDLRAEVDTFMFTGHDTTACGIAWLFYCMAQNPEHQERCREEIKEVLGDQE

IIRWDDLGKMPYVTMCIKESLRLYSVVPLIARVLESPLTFDDGKTLSEGCVIGLNIFGLH

RNRDVWGNPEVFDPMRFSPENSCLRHPYAYLPFSAGPRNCIGQQFALIEMKVVLALTLLH

FELEPDPANPPIPVAQVVTRSENGIHLKLKKLL

>301792653

MSVSVLSITRLLGGVSGLLQVASLLGLALLLLKAAQLYLHRQRLLKAVQEFPSPPSHWLF

GHKQELQKGQEIQELQKRAEKYPRASPRWLWGSKVSLVVYDPDYMKVILGRSDPKSEGSY

RFMAPWIGYGLLLLNGQTWFQHRRMLTPAFHYDILKPYVGLMADSVQVMLKPQNIFYQKD

IIYRLTPEGRLNHRACQLAHQHTDRVIKMRKAQLQEEGELEKVRSKRHLDFLDILLFAQR

ENGNSFSDKDLRAEVDTFMFEGHDTTASGISWILYALATHPEHQQRCREEVQSLLGDGAS

ITWDHLDQMPYTTMCIKEALRLYPPVPGVGRELSKPITFPDGRSLPKGFLVLLSFYALHH

NPKVWPNPEVFDPSRFAPDSSRHSHAFLPFSGGSR

>194207491

MVPVLLSLSVSCLGLWASGLILVLGFLKLLSLLLRRQKLARAMDSFPGPPTHWLFGHSLE

IQQTGSLDKVVSWAHQFPYAHPLWYGQFVGFLNIYEPDYAKAVYSRGDPKAPDVYDFLLQ

WIGKGLLVLHGPKWFQHRKLLTPGFHYDVLKPYVALFADSTRAMLDKWEEKARENKSFDI

FCDVGHMALDTLMKCTFGKADTGLGHRDSSYYQAVGELTLLTQQRIESFQYHNDFIYWLT

LHGRRFLRACRVAHDHTDQVIRERKADLQDEKEQEKIQNRRHLDFLDILLGARDEDGIKL

SDAELRAEVDTFMFEGHDTTTSGISWFLYCMALYPEHQQCCREEVREVLGDRDSFQWDDL

GKMTYLTMCIKESFRIYPPVPQVFRQLSKPVSFVDGRSLPAGSLVSLHIYALHRNSAVWP

DPEVFDPLRFSSENVARRHPFAFIPFSAGPRNCIGQQFAMNEMKVVTALCLLRFEFALDP

LRLPVPLPQLVLRSRNGIHLHLKPLGPGSGKQLC

>P13584.2

MVPSFLSLSFSSLGLWASGLILVLGFLKLIHLLLRRQTLAKAMDKFPGPPTHWLFGHALE

IQETGSLDKVVSWAHQFPYAHPLWFGQFIGFLNIYEPDYAKAVYSRGDPKAPDVYDFFLQ

WIGRGLLVLEGPKWLQHRKLLTPGFHYDVLKPYVAVFTESTRIMLDKWEEKAREGKSFDI

FCDVGHMALNTLMKCTFGRGDTGLGHRDSSYYLAVSDLTLLMQQRLVSFQYHNDFIYWLT

PHGRRFLRACQVAHDHTDQVIRERKAALQDEKVRKKIQNRRHLDFLDILLGARDEDDIKL

SDADLRAEVDTFMFEGHDTTTSGISWFLYCMALYPEHQHRCREEVREILGDQDFFQWDDL

GKMTYLTMCIKESFRLYPPVPQVYRQLSKPVTFVDGRSLPAGSLISMHIYALHRNSAVWP

DPEVFDSLRFSTENASKRHPFAFMPFSAGPRNCIGQQFAMSEMKVVTAMCLLRFEFSLDP

SRLPIKMPQLVLRSKNGFHLHLKPLGPGSGK

>AAM09532.1

MVPSFLSLSFSSLGLWASGLILVLGFLKLIHLLLRRQTLAKAMDKFPGPPTHWLFGHALE

IQETGSLDKVVSWAHQFPYAHPLWFGQFIGFLNIYEPDYAKAVYSRGDPKAPDVYDFFLQ

WIGRGLLVLEGPKWLQHRKLLTPGFHYDVLKPYVAVFTESTRIMLDKWEEKAREGKSFDI

FCDVGHMALNTLMKCTFGRGDTGLGHRDSSYYLAVSDLTLLMQQRLVSFQYHNDFIYWLT

PHGRRFLRACQVAHDHTDQVIRERKAALQDEKVRKKIQNRRHLDFLDILLGARDEDDIKL

SDADLRAEVDTFMFEGHDTTTSGISWFLYCIALYPEHQHRCREEVREILGDQDFFQWDDL

GKMTYLTMCIKESFRLYPPVPQVYRQLSKPVTFVDGRSLPAGSLISMHIYALHRNSAVWP

DPEVFDSLRFSTENASKRHPFAFMPFSAGPRNCIGQQFAMSEMKVVTAMCLLRFEFSLDP

SRLPIKMPQLVLRSKNGFHLHLKPLGPGSGK

>AAL57720.1

MVPSFLSLSFSSLGLWASGLILVLGFLKLIHLLLRRQTLAKAMDKFPGPPTHWLFGHALE

IQETGSLDKVVSWAHQFPYAHPLWFGQFIGFLNIYEPDYAKAVYSRGDPKAPDVYDFFLQ

WIGRGLLVLEGPKWLQHRKLLTPGFHYDVLKPYVAVFTESTRIMLDKWEEKAWEGKSFDI

FCDVGHMALNTLMKCTFGRGDTGLGHRDSSYYLAVSDLTLLMQQRLVSFQYHNDFIYWLT

PHGRRFLRACQVAHDHTDQVIRERKAALQDEKVRKKIQNRRHLDFLDILLGARDEDDIKL

SDADLRAEVDTFMFEGHDTTTSGISWFLYCMALYPEHQHRCREEVREILGDQDFFQWDDL

GKMTYLTMCIKESFRLYPPVPQVYRQLSKPVTFVDGRSLPAGSLISMHIYALHRNSAVWP

DPEVFDSLRFSTENASKRHPFAFMPFSAGPRNCIGQQFAMSEMKVVTAMCLLRFEFSLDP

SRLPIKMPQLVLRSKNGFHLHLKPLGPGSGK

>AAI18399.1

MVPVLLSLSLSHLSLWAFALVSVLVFLKLTRLLLRRQMLARAMDRFSGPPTHWLFGHALE

IQQTGSLDKVVSWTHEFPYAHQLWVGQFLGFLNIYDPDYAKAVYSRGDPKAPDFYDFFLQ

WIGKGLLVLQGPKWFQHRKLLTPGFHYDVLKPYVALFAESTRVMLDKWEKKAREQKSFDI

YSDVGHMALDSLMKCTFGKGTSGLNDRDNNYYLSVKELTLLMQQRIDSFQYHNDFIYFLT

PHGRRFLRACQVAHDHTDQVIRERKAALQDEKERERIQSKRHLDFLDILLGAWDEEGIKL

SDEDLRAEVDTFMFEGHDTTTSAISWVLYCMSLYPEHQRRCREEIQEILGDRDTLKWDDL

AEMTYLTMCIKESFRLYPPVPQVYRQLSQPVNFVDGRSLPEGSLISLHIYALHRNSTVWP

DPEVFDPLRFSPENVAGRHSFAFIPFSAGPRNCIGQQFAMAEVKVVTALCLLRFEFSPDP

SRLPIKMPQLVLRSKNGIHLHLKPLGPGSEK

>109004067

MVPSFLSLRLSCLGLWASGLILVLGFLKLIRLLLRRQRLAKAMGNFPGPPTHWLFGHALE

IQQTGSLDKVVSWAHQFPYAHPLWFGQFIGFLNIYEPDYAKAVYSRGDPKAPDVYDFFLQ

WIGRGLLVLEGPKWFQHRKLLTPGFHYDVLKPYVALFAESTRVMLDKWEEKAQEGKSFDI

FCDVGHMALNTLMKCTFGRGDTGLGHRDSSYYLAVSDLTLLMQQRLVSFHYHNDFIYWLT

PHGRRFLRACQVAHDHTDQVIRERKAALQDEKVRKKIQNRRHLDFLDILLGARDEDDSKL

SDADLRAEVDTFMFEGHDTTTSGISWFLYCMALYPEHQHRCREEVREILGDQDSFQWDDL

GKMTYLTMCIKESFRLYPPVPQVYRQLSKPVTFVDGRSLPAGSLISMHIYALHRNSAVWP

DPEVFDPLRFSTENASKRHPFAFMPFSAGPRNCIGQQFAMSEMKVVTAMCLLHFEFSLDP

SRLPIKMLQLVLRSKNGIHLHLKPLGPGSGK

>AAA35712.1

MVPSFLSLSFSSLGLWASGLILVLGFLKLIHLLLRRRTLAKAMDKFPGPPTHWLFGHALE

IQETGSLDKVVSWAHQFPYAHPLWFGQFIGFLNIYEPDYAKAVYSRGDPKAPDVYDFFLQ

WIGRGLLVLEGPKWLQHRKLLTPGFHYDVLKPYVAVFTESTRIMLDKWEEKAREGKSFDI

FCDVGHMALNTLMKCTFGRGDTGLGHRDSSYYLAVSDLTLLMQQRLVSFQYHNDFIYWLT

PHGRRFLRACQVAHDHTDQVIRERKAALQDEKVRKKIQNRRHLDFLDILLGARDEDDIKL

SDADLRAEVDTFMFEGHDTTTSGISWFLYCMALYPEHQHRCREEVREILGDQDFFQWDDL

GKMTYLTMCIKESFRLYPPVPQVYRQLSKPVTFVDGRSLPAGSLISMHIYALHRNSAVWP

DPEVFDSLRFSTENASKRHPFAFMPFSAGPRNCIGQQFAMSEMKVVTAMCLLRFEFSLDP

SRLPIKMPQLVLRSKNGFHLHLKPLGPGSGK

>AAL57721.1

MVPSFLSLSFSSLGLWASGLILVLGFLKLIHLLLRRQTLAKAMDKFPGPPTHWLFGHALE

IQETGSLDKVVSWAHQFPYAHPLWFGQFIGFLNIYEPDYAKAVYSRGDPKAPDVYDFFLQ

WIGRGLLVLEGPKWLQHRKLLTPGFHYDVLKPYVAVFTESTRIMLDKWEEKAREGKSFDI

FCDVGHMALNTLMKCTFGRGDTGLGHRDSSYYLAVSDLTLLMQQRLVSFQYHNDFIYWLT

PHGRRFLRACQVAHDHTDQVIRERKAALQDEKVRKKIQNRRHLDFLDILLGARDEDDIKL

SDADLRAEVDTFMFEGHDTTTGGISWFLYCMALYPEHQHRCREEVREILGDQDFFQWDDL

GKMTYLTMCIKESFRLYPPVPQVYRQLSKPVTFVDGRSLPAGSLISMHIYALHRNSAVWP

DPEVFDSLRFSTENASKRHPFAFMPFSAGPRNCIGQQFAMSEMKVVTAMCLLRFEFSLDP

SRLPIKMPQLVLRSKNGFHLHLKPLGPGSGK

>311259368

MVPALLSLSLSHLSLWALGLILALGVLKLTSLLLRRQMLARAMDKFPGPPTHWIFGHALE

IQQTGSLDKMVSWAHQFPHAHPLWLGPFLGFLNIYEPEYAKAVYSRGDPKASDLYDFFLQ

WIGKGLLVLHGPKWFQHRKLLTPGFHHDVLKPYVAVMANSARVMLDKWEEKAREDKSFDI

YHDVGHMALELLMKCTFGKGTSGLNYSDNAYHLAVRDLTLLMQQRLSSFQYHNDFIYWLT

PHGRRFLRACRVAHDHTDQVIRERKAALQDKEEQERIQSRRHLDFLDILLGAQDEDRVKL

SDADLRAEVDTFMFEGHDTTTSGISWFLYCMALNPEHQHRCREEIREILGDRDSIQGEDL

GKMTYLTMCIRESFRLYPPVPQVYRQLSEPVSFVDGRSLPAGSLISLHIYALHRNSAVWP

DPEVFDPLRFSLENMAGRHPFAFLPFSAGPRNCIGQRFAMKEMKVVAALSLLRFEFAPDP

SRPPIPMPQLTLCSKTGIHLRLTSLGQGSGK

>149694470

MVPVLLPLSLSYLGLWASGLILVLGFLKLLSLLLRRQKLARAMDSFPGAPTHWLFGHALE

LQQTGSLDKVVSWAHQFPYAHPLWYGQFLGFLNIYEPDYAKAVYSRGDPKAPDVYDFLLQ

WTGEGLLVLHGPKWFQHRKLLTPGFHYDVLKPYVAVFANSAHTMLDKWEEMAREDKSFDI

FCDVGHMALDTLMKCTFGKADTGLCHRDSSYYQAVCELTLLTQQRIDSFQYHNDFIYWLT

PHGRRFLRACQVAHDHTDRVIRERKAALQDEKEQEKIRKRRHLDFLDILLGARDEDGIKL

SDAELRAEVDTFMFEGHDTTTSGISWFLYCMALYPEHQQRCREEVREVLGDRDSFQWDDL

GKMTYLTMCIKESFRLYPPVPQVFRQLSKPVSFVDGRSLPAGSLVSLHIFALHRNSAVWP

DPEVFDPLRFSSENVARRHPFAFIPFSAGPRNCIGQQFAMNEMKVVTALCLLRFEFALDP

LRLPILLPQLVLRSRNGIHLHLKPLGPGSGKQLC

>EHH49890.1

MVPSFLSLRLSCLGLWASGLILVLGFLKLIRLLLRRQRLAKAMGNFPGPPTHWLFGHALE

IQQTGSLDKVVSWAHQFPYAHPLWFGQFIGFLNIYEPDYAKAVYSRGDPKAPDVYDFFLQ

WIGRGLLVLEGPKWFQHRKLLTPGFHYDVLKPYVALFAESTRVMLDKWEEKAQEGKSFDI

FCDVGHMALNTLMKCTFGRGDTGLGHSRDSSYYLAVSDLTLLMQQRLVSFHYHNDFIYWL

TPHGRRFLRACQVAHDHTDQVIRERKAALQDEKVRKKIQNRRHLDFLDILLGARDEDDSK

LSDADLRAEVDTFMFEGHDTTTSGISWFLYCMALYPEHQHRCREEVREILGDQDSFQWDD

LGKMTYLTMCIKESFRLYPPVPQVYRQLSKPVTFVDGRSLPAGSLISMHIYALHRNSAVW

PDPEVFDPLRFSTENASKRHPFAFMPFSAGPRNCIGQQFAMSEMKVVTAMCLLHFEFSLD

PSRLPIKMLQLVLRSKNGIHLHLKPLGPGSGK

>EHH14756.1

MVPSFLSLRLSCLGLWASGLILVLGFLKLIRLLLRRQRLAKAMGNFPGPPTHWLFGHALE

IQQTGSLDKVVSWAHQFPYAHPLWFGQFIGFLNIYEPDYAKAVYSRGDPKAPDVYDFFLQ

WIGRGLLVLEGPKWFQHRKLLTPGFHYDVLKPYVALFAESTRVMLDKWEEKAQEGKSFDI

FCDVGHMALNTLMKCTFGRGDTGLGHSRDSSYYLAVSDLTLLMQQRLVSFHYHNDFIYWL

TPHGRRFLRACQVAHDHTDQVIRERKAALQDEKVRKKIQNRRHLDFLDVLLGARDEDDSK

LSDADLRAEVDTFMFEGHDTTTSGISWFLYCMALYPEHQHRCREEVREILGDQDSFQWDD

LGKMTYLTMCIKESFRLYPPVPQVYRQLSKPVTFVDGRSLPAGSLISMHIYALHRNSAVW

PDPEVFDPLRFSTENASKRHPFAFMPFSAGPRNCIGQQFAMSEMKVVTAMCLLHFEFSLD

PSRLPIKMLQLVLRSKNGIHLHLKPLGPGSGK

>EAX06891.1

MVPSFLSLSFSSLGLWASGLILVLGFLKLIHLLLRRQTLAKAMDKFPGPPTHWLFGHALE

IQETGSLDKVVSWAHQFPYAHPLWFGQFIGFLNIYEPDYAKAVYSRGDPKAPDVYDFFLQ

WIGRGLLVLEGPKWLQHRKLLTPGFHYDVLKPYVAVFTESTRIMLDKWEEKAREGKSFDI

FCDVGHMALNTLMKCTFGRGDTGLGHRDSSYYLAVSDLTLLMQQRLVSFQYHNDFIYWLT

PHGRRFLRACQVAHDHTDQVIRERKAALQDEKVRKKIQNRRHLDFLDILLGARVNDIKLS

DADLRAEVDTFMFEGHDTTTSGISWFLYCIALYPEHQHRCREEVREILGDQDFFQWDDLG

KMTYLTMCIKESFRLYPPVPQVYRQLSKPVTFVDGRSLPAGSLISMHIYALHRNSAVWPD

PEVFDSLRFSTENASKRHPFAFMPFSAGPRNCIGQQFAMSEMKVVTAMCLLRFEFSLDPS

RLPIKMPQLVLRSKNGFHLHLKPLGPGSGK

>395536933

MVLSWLASGLPLSLPRLCLWSAALVLALGILQLTRLLARRQKLLRALDSFPGPPTHWLLG

HANEIKDGGGLDKIMTWANTYPSAHPLWFGKFIGFLNIYDPDYAKAVYSRGDPKAWDVYS

FFLPWIGNGLLVLDGPKWFQHRKLLTPAFHNEVLKPYVGLFAESTHAMLDKWKEKVSVNK

SVEIFSDVGFMALDTLMKCIFGKTSKGQTQSDMDYYLAIADLTLLMQERIFTFQYHNDFL

YWLTPHGRNFMRACKIAHDHTDKVIRERKAALNEEKLDKTPKKRHLDFLDILLGVKDENK

TNLSDMDLRAEVDTFMFEGHDTTTSSLSWFLYCMALYPEHQDRCRKEIQEILQGRDAVQW

EDLAKMTYLTLCIKESFRLYPAVPQVYRQLSKPVTFADGRSLPEGGLVSLHIYALHRNPA

VWPDPEVFDPQRFTPENSSGRHPFAFMPFSAGPRNCIGQHFAMMEMKVVTALCLLNFEFS

PDLTQPPIKQLQLILRSKNGIHLNLKRLT

>73977060

MVSTLLSLSVSHLVLWATGLILVLGFLKLIRLLLRRQMLARAMDSFPGPPTHWLFGHAQE

IQQTGSLDKVVTWAHQYDYAHPLWLGPFLGFLNIYEPDYAKAVYSRGDPKAADVYDFFLQ

WIGKGLLVLQGPKWYQHRKLLTPGFHYDVLKSYVAVFTNSTHAMLDKWEEKAREDKSFDI

FCDVGYMALDSLMKCTFGKGDSSLGHRDSSYYSAVRDLTLLMQQRIESFQYHNDFIYWLT

PHGRRFLRACQAAHDHTDQVIRERKAALQDEKEQEKIQNRRHLDFLDILLGARDENGIKL

SDADLRAEVDTFMFEGHDTTTSGISWFLYCMALYPQHQHRCREEVCEILGDRDSFQWDDL

VKMTYLTMCIKESFRLYPPVPQVYRQLSKPVNFVDGRSLPAGSLISLHIYALHRNSAVWP

DPEVFDPLRFSSENVARRHPFAFMPFSAGPRNCIGQQFAMNEMKVVTALCLLRFEFALDP

LQLPVKMPQLILRSKNGIHLHLKSLRLGSGK

>AAN72309.1

MVPVLLSLSLSHLSLWAFGLVSVLVFLKLTRLLLRRQMLARAMDRFSGPPTHWLFGHALE

IQQTGGLDKVVYWTHQFPYAHQLWLGRFLGFLNIYDPEYAKAVYSRGDPKAPDVYDFFLQ

WIGKGLLVLQGPKWFQHRKLLTPGFHYDVLKPYVAVFAESARVMLDKWEKKAREQKSFDI

FSDVGHMALDSLMKCTFGKGTSGLNDRDNNYYLAVSELTLLMQQRIDSFQYHNDFIYFLT

PHGRRFLRACQVAHDHTDQVIRERKAALQDKKEREKIQSKRHLDFLDILLGARDEEGIKL

SDEDLRAEVNTFMFAGHDTTTSAISWVLYCMSLYPEHQRRCREEIQEILGDRDSLKWEDL

AEMTYLTMCIKESFRLYPPVPQVYRQLSKPVNFVDGRSLPAGSLISLHIYALHRNSTVWT

DPEVFDPLRFSPENVAGRHSFAFIPFSAGPRNCIGQQFAMNEVKVVTALCLLRFEFSPDP

SKLPIQMPQLVLRSKNGIHLHLKPVGPGSEK

>EAX06888.1

MVPSFLSLSFSSLGLWASGLILVLGFLKLIHLLLRRQTLAKAMDKFPGPPTHWLFGHALE

IQETGSLDKVVSWAHQFPYAHPLWFGQFIGFLNIYEPDYAKAVYSRGDPKAPDVYDFFLQ

WIGRGLLVLEGPKWLQHRKLLTPGFHYDVLKPYVAVFTESTRIMLDKWEEKAREGKSFDI

FCDVGHMALNTLMKCTFGRGDTGLGHSRDSSYYLAVSDLTLLMQQRLVSFQYHNDFIYWL

TPHGRRFLRACQVAHDHTDQVIRERKAALQDEKVRKKIQNRRHLDFLDILLGARVNDIKL

SDADLRAEVDTFMFEGHDTTTSGISWFLYCIALYPEHQHRCREEVREILGDQDFFQWDDL

GKMTYLTMCIKESFRLYPPVPQVYRQLSKPVTFVDGRSLPAGSLISMHIYALHRNSAVWP

DPEVFDSLRFSTENASKRHPFAFMPFSAGPRNCIGQQFAMSEMKVVTAMCLLRFEFSLDP

SRLPIKMPQLVLRSKNGFHLHLKPLGPGSGK

>301768212

MLSVLLSLSVSRLGLWAAGLILVLGLLKLIRLLLRRQMLARAMDSFPGPPTHWLFGHAQE

IQQTGSLDKVVSWAHQFPYAHPLWLGQFLGFLNIYEPDYAKAVYSRGDPKAADLYDFFLQ

WIGKGLLVLQGPKWYEHRKLLTPGFHYDVLKPYVAVFTNSTHTMLDQWEEKAREDKSFDI

FCDVGHMALDSLMKCTFGKGDSGLDHRDSSYHSAVRDLTLLMQERIDSFQYHNDFVYWLT

PHGHRFLRACQAAHDHTDHVIRGRKAALKDEKEWEKIQSRRHLDFLDILLGARDENGIKL

SDEDLRAEVDTFMFEGHDTTTSGICWFLYCMALYPEHQHRCREEVCEILGDRDSFQWDDL

GKMTYLTMCIKESFRLYPPVPQVYRQLSKPVSFVDGRSLPAGSLISLHIYALHRNSAVWP

DPEVFDPLRFSPENVATRHPFAFMPFSAGPRNCIGQQFAMNEMKVVTALCLLRFEFALDP

LRPPIKMPQLVLRSKNGIHLHLKSLRPDSGKQLC

>402854451

MVPSFLSLRLSCLGLWASGLILVLGFLKLIRLLLRRQRLAKAMGNFPGPPTHWLFGHALE

IQQTGSLDKVVSWAHQFPYAHPLWFGQFIGFLNIYEPDYAKAVYSRGDPKAPDVYDFFLQ

WIGRGLLVLEGPKWFQHCKLLTPGFHYDVLKPYVALFAESTRVMLDKWEEKAQEGKSFDI

FCDVGHMALNTLMKCTFGRGDTGLGHRDSSYYLAVSDLTLLMQQRLVSFHYHNDFIYWLT

PHGRRFLRACQVAHDHTDQVIRERKAALQDEKVRKKIQNRRHLDFLDILLGAQDEDDSKL

SDADLRAEVDTFMFGGHDTTTSGISWFLYCMALYPEHQHRCREEVREILGDQDSFQWDDL

GKMTYLTMCIKESFRLYPPVPQVYCQLSKPVTFVDGRSLPAGSLISMHIYALHRNSAVWP

DPEVFDPLRFSTENASKRHPFSFMPFSAGPRNCIGQQFAMSEMKVVTAMCLLHFEFSLDP

SRLPIKMLQLVLRSKNGIHLHLKPLGPGSGK

>ADJ68241.1

MVFSWLSSWLPVSLPRLWLCPTALVLALGLLQLTQLLARRQKLLRALDSFPGPPIHWLFG

HAKEIKEGGGLDKMISWANTYPSAHPLWFGRFVGFLNIYDPDYAKAVYSRGDPKAWDVYG

FFIPWIGNGLLVLDGPKWFQHRKLLTPAFHNEVLKPYVSLFAESTHAMLDKWKEKVCVNM

CVEIFNDVGFMALDTLMKCIFGKTHKGQTRSDTDYYLAITNLTLLMQERIQTFQYHNDIL

YWLTPHGRNFLRACKIAHNHTDKVIRERKAALKGEELEKIQKKGHLDFLDILLGVKDENK

TSLSDTDLRAEVDTFMFEGHDTTTSSISWFLYRMALYPEHQHRCRKEIQEILQGRDTIQW

EDLAKMTYLTYCIKESFRLYPTVPQIYRQLSKPVTFTDGRSLPEGSLVSLHIYALHRNHT

VWTDPEVFDPQRFTPENSSGRHPFAFMPFSAGPRNCIGQQFAMTEMKVVAALCLLHFEFS

PDPTQPPIKQLQLTLRSKNGIHLNLKKLAK

>397483177

MVPSFLSLSFSSLGLWASGLILVLGFLKLIRLLLRRQMLAKAMDNFPGPPTHWLFGHALE

IQETGSLDKVVSWAHQFPYAHPLWFGQFIGFLNIYEPDYAKAVYSCGDPKAPDVYDFFLQ

WIGRGLLVLEGPKWLQHRKLLTPGFHYDVLKPYVAVFTESTRIMLDKWEEKAREGKSFDI

FCDVGHMALNTLMKCTFGRGDTGLGHRDSSYYLAVSDLTLLMQQRLVSFQYHNDFIYWLT

PHGHRFLRACQVAHDHTDQVIRERKAALQDEKVRKKIQNRRHLDFLDILLGARDEDDIKL

SDADLRAEVDTFMFEGHDTTTSGISWFLYCMALYPEHQHRCREEVREILGDQDSFQWDDL

GKMTYLTMCIKESFRLYPPVPQVYRQLSKPVTFVDGRSLPAGSLISMHIYALHRNSAVWP

DPEVFDPLRFSTENASKRHPFAFMPFSAGPRNCIGQQFAMSEMKVVTAMCLLHFEFSLDP

SRLPIKMPQLVLRSKNGFHLHLKPLGPGSGN

>332635021

MESSWLETRWARPLYLAFAFCLALGLLQAIKLYLRRLRLLRDLCPFPAPPTHWFYGHQKL

LQDGKIEKLEELVEKYPCAFPYWVGPFQAFFYIYDPDYAKTFLGRTDPKSKYLYKFLIPC

LGNGLVNLEGSKWFQHRCLLTPGFNVNILKSYIKVMAHSVNTMLGKWEKICGTQDTVVEV

FEHINLMTLDVLMKCAFSQETNCQISSNHDLYVKTTFEASKINFYRLYSFLHHHDIIFKF

SPQGHRLQELVKILHQYTEKVIQDRKKLLKDEKKHGNTQKRKDQDYLDIILSAQAENGNS

FSDTDLRSEVNTFILAGHDTMAGSISWLLYHLALNPEHQERCRQEIRGILRDRSSITWDQ

LGEMSYTTMCIKESLRLAPPIPSISRELSKPITFPDGRSLPAGITVVLSIWGLHHNSAIW

ENPKVFDPLRFSQENSDQRHTHSFLPFSAGPRNCIGQHFAMVKLKVAIALILLHFKVSPD

PTRPLVFLHQIVLKPKNGVHLHLKKLP

>126305756

MVLSWLSIWLPVSFPRLWLWSIALVLALGLLQLTRLLARRQKLLRALDSFPGPPIHWLFG

HAKEIKGEGALDKIISWANTYPSAHPLWFGRFIGFLNIYDPDYAKAVYSRGDPKAWDIYG

FFIPWIGKGLLVLEGPKWFQHRKLLTPAFHNAVLKSYVGLFAESTQDMLDKWEKKVRVNK

CVEIFNDVGFMALDTLMKCIFGKTSRGQTKSDTDYYLAIADLTLLMQERVHAFQYHNDFL

YWLTPHGRNFLRACKVAHDHTDKVIRERKAILKGEMLEKTQKKRHLDFLDILLGVKDENK

IHMSDMDLRAEVDTFMFEGHDTTTSSISWFLYCMALYPEHQNRCRKEIQEILQGRDTVQW

EDLGKMTYLTLCIKESFRLFPAVPQVYRQLSKPVTFADGRSLPEGSLVSLHIYALHRNRT

VWTDPEVFDPQRFTPENTSTRHPFAFLPFSAGPRNCIGQQFAMMEMKVVTALCLLHFEFS

PDPTQPPIKQTQLILRSKNGIHLNMNKLKMNTRAPSQGPRENMEQLVGEA

>403291735

MEFSWLETRWARPFYLAFVFCLALGLLQAIKLYLRRQRLLRDLRPFPTPPTHWFFGHQKF

IQDDNMEKLEEIIEKYPRAFPLWIGPFQAFFYICDPDYAKIFLNRTDPKSQYVQKFLTPL

TGKGLVTLDGPKWFQHRRLLTPAFHFNILKAYIEVMAHSVKTMLDKWEKICSAQDTTVEV

YKHINLMSLDIIMKCAFSKETNCQTNSTHDPYEKALLEVSRIVFYRLYSFLHHSDTIFKL

SPQGHRFQKLNQVLYQYTDTIIQDRKKSLQTGVKQNNTQKRKYQDFLDIVLSAKDENGSS

FSDTDVHSEVSTFLLAGHDSVAASISWVLYCLAQNPEHQERCREEVRDILGDGSSITWDQ

LGEMSYTTMCIKETLRLIPAVPSISRDLSKPLTFPDGCTLPAGITVVLSIWGLHHNPAVW

KNPKVFDPLRFSKENSEQRHPYAYLPFSAGSRNCIGQQFAMIELKVTIALILLHFRVTLD

PTRPLTFSNHFILKPKNGLYLHLKKLPKC

>194207487

MESSWLATRWARPFYLAFVFCLALGLLQAIKLYLRRQRLLRDLRPFPAPPTHWFYGHQKL

LQDGKMEKLEELVEKYPCAFPCWVGPFQAFFYIYDPDYAKTFLSRTDPKSKYPCKVLLPC

LGKGLVSLDGPKWFQHRCLLTPGYSVNILKSYVEVMAHSVNMMLGKWEKICGTQDTIVEV

FEHINLMALDILMKCAFSQETNCQINSHCDLYVKSTFEASKIIFYRLYSFLRHHNILFNF

SPEGHRLQETSQMLHQHTEKAIQDRKKALEEEKRQDNTQKRKHQDFLDVVLSVQAENRDS

FSDTDLRSEVNTFLLAGQDTMPGAISWLLYHLALNPEHQERCREEIRSILGDGSSITWDQ

LDEMSYTTMCIKESLRLAPPVPSISRELSKPITFPDGLSLPAGITVVLSIWGLHHNPAIW

KKPKVFDPLRFSQENSDQRHSHSFLPFSAGPRNCVGQHFAMAELKVAIALILLRFKVTPD

PTRPLVFLSQVIRKPKNGVHLHLKKLS

>109004070

MVPSFLSLRLSCLGLWASGLILVLGFLKLIRLLLRRQRLAKAMGNFPGPPTHWLFGHALE

IQQTGSLDKVVSWAHQFPYAHPLWFGQFIGFLNIYEPDYAKAVYSRGGRGLLVLEGPKWF

QHRKLLTPGFHYDVLKPYVALFAESTRVMLDKWEEKAQEGKSFDIFCDVGHMALNTLMKC

TFGRGDTGLGHRDSSYYLAVSDLTLLMQQRLVSFHYHNDFIYWLTPHGRRFLRACQVAHD

HTDQVIRERKAALQDEKVRKKIQNRRHLDFLDILLGARDEDDSKLSDADLRAEVDTFMFE

GHDTTTSGISWFLYCMALYPEHQHRCREEVREILGDQDSFQWDDLGKMTYLTMCIKESFR

LYPPVPQVYRQLSKPVTFVDGRSLPAGSLISMHIYALHRNSAVWPDPEVFDPLRFSTENA

SKRHPFAFMPFSAGPRNCIGQQFAMSEMKVVTAMCLLHFEFSLDPSRLPIKMLQLVLRSK

NGIHLHLKPLGPGSGK

>AAN72312.1

MVPSFLSLSFSSLGLWASGLILVLGFLKLIHLLLRRQTLAKAMDKFPGPPTHWLFGHALE

IQETGSLDKVVSWAHQFPYAHPLWFGQFIGFLNIYEPDYAKAVYSRGGRGLLVLEGPKWL

QHRKLLTPGFHYDVLKPYVAVFTESTRIMLDKWEEKAREGKSFDIFCDVGHMALNTLMKC

TFGRGDTGLGHSRDSSYYLAVSDLTLLMQQRLVSFQYHNDFIYWLTPHGRRFLRACQVAH

DHTDQVIRERKAALQDEKVRKKIQNRRHLDFLDILLGARDEDDIKLSDADLRAEVDTFMF

EGHDTTTSGISWFLYCMALYPEHQHRCREEVREILGDQDFFQWDDLGKMTYLTMCIKESF

RLYPPVPQVYRQLSKPVTFVDGRSLPAGSLISMHIYALHRNSAVWPDPEVFDSLRFSTEN

ASKRHPFAFMPFSAGPRNCIGQQFAMSEMKVVTAMCLLRFEFSLDPSRLPIKMPQLVLRS

KNGFHLHLKPLGPGSGK

>395536931

MSRTELFMFLWLSSWLPVSLPTLWLWAVSLILTLGLLQLTRLLTKRQKLFQALKSFPEPP

THWLFGHAMEIKKEGELDKVLSWANIYPCAHQLWMGGFIGFLNIYDPDYTKAVFSRGDPK

AFDVYGFFLPWIGKGLLVLDGPKWFQHRKLLTPEFHSEVLRPYVDLFAESTKAMLNKWEK

KIQTDKCVNIFPDVGHMTLDSLTKCIFGRSSKTQTQSDIGYFQAIGQLTLLMQQRLANFH

YHNDLFYYWLTLHGRKFLQACKVAHDYTDKVIRTRKAALKEEEEQKKIQRKKRLDFLDIL

LGVGDENKIKLSDADLRAEVDTFIFEGHDTTTSAISWFLYCISLHPEHQQRCREEVQEIL

GNRNTIQWEDLGKMTYLTLCIKESFRLYPPVPQIYRQLNKPITFMDGKSLPEGSLVSLHI

YALHRNPAVWDKPEVFDPQCFSPENSSTRHPYAFMPFSAGPRNCIGQQFAMMEVKAVTAL

CLLHFEFSPEPSRLPIKRLQLILGAKDGIYLNLKKLM

>EHH25590.1

MEFSWLETRWARPFYLAFVFCLALGLLQTIKLYLRRQRLLRDLRAFPTPPTHWFLGHQKF

IQDDNMEKLEEIIEKYPRAFPFWIGPFQAFFYIYDPDYAKTFLSRTDPKSQYLQKFLPPL

IGKGLLALDGPKWFQHRRLLTPGFHFNILKAYIEVMAHSVKTMLDKWEKICSTQNTSVEV

YEHINLMSLDIIMKCAFSKETNCQTNSTHDPYVKAIFELGKIIFHRLYSFLYHSDIIFKL

SPQGYRFQKLSRVLNQYTDAIIQERKKSLQAGEKQDNTQKRKYQDFLDIVLSAKDENGSS

FSDTDVHSEVSMFLLGGHDSLAASISWILYCLALNPEHQERCREEVRGILGDGCSITWDQ

LGEMSYTTMCIKETCRLIPAVPSISRDLSKPLTFPDGCTLPAGITVVLSIWGLHHNPAVW

KNPKVFDPLRFSQENCDQRHPYAYLPFSAGSRNCIGQQFAMIQLKVAIALILLHFRVTPD

PTRPPTFSNHFILKPKNGMYLHLKKLSEC

>AFJ70614.1

MEFSWLETRWARPFYLAFVFCLALGLLQTIKLYLRRQRLLRDLRAFPTPPTHWFLGHQKF

IQDDNMEKLEEIIEKYPRAFPFWIGPFQAFFYIYDPDYAKTFLSRTDPKSQYLQKFLPPL

IGKGLLALDGPKWFQHRRLLTPGFHFNILKAYIEVMAHSVKTMLDKWEKICSTQNTSVEV

YEHINLMSLDIIMKCAFSKETNCQTNSTHDPYVKAIFELGKIIFHRLYSFLYHSDIIFKL

SPQGYRFQKLSRVLNQYTDAIIQERKKSLQAGEKQDNTQKRKYQDFLDIVLSAKDENGSS

FSDTDVHSEVSMFLLGGHDSLAASISWILYCLALNPEHQERCREEVRGILGDGCSITWDQ

LGEMSYTTMCIKETCRLIPAVPSISRDLSKPLTFPDGCTLPAGITVVLSIWGLHHNPAVW

KNPKVFDPLRFSQENCDQIHPYAYLPFSAGSRNCIGQQFAMIQLKVAIALILLHFRVTPD

PTRPPTFSNHFILKPKNGMYLHLKKLSEC

>402854454

MEFSWLETRWARPFYLAFVFCLALGLLQTIKLYLRRQRLLRDLRAFPTPPTHWFLGHQKF

IQDDNMEKLEEIIEKYPRAFPFWIGPFQAFFYIYDPDYAKTFLSRTDPKSQYLQKFLPPL

VGKGLLALDGPKWFQHRRLLTPGFHFNILKAYIEVMAHSVKTMLDKWEKICSTQNTSVEV

YEHINLMSLDIIMKCAFSKETNCQTNSTHDPYVKAIFELGKIIFHRLYSFLYHSDIIFKL

SPQGYRFQKLSRVLNQYTDAIIQERKKSLQAGEKQDNTQKRKYQDFLDIVLSAKDENGSS

FSDTDVHSEVSMFLLGGHDSLAASISWILYCLALNPEHQERCREEVRGILGDGCSITWDQ

LGEMSYTTMCIKETCRLIPAVPSISRDLSKPLTFPDGCTLPAGITVVLSIWGLHHNPAVW

KNPKVFDPLRFSQENCDQRHPYAYLPFSAGSRNCIGQQFAMIQLKVAIALILLHFRVTPD

PTRPPTFSSHSILKPKNGMYLHLKKLSEC

>EAX06890.1

MVPSFLSLSFSSLGLWASGLILVLGFLKLIHLLLRRQTLAKAMDKFPGPPTHWLFGHALE

IQETGSLDKVVSWAHQFPYAHPLWFGQFIGFLNIYEPDYAKAVYSRGGRGLLVLEGPKWL

QHRKLLTPGFHYDVLKPYVAVFTESTRIMLDKWEEKAREGKSFDIFCDVGHMALNTLMKC

TFGRGDTGLGHSRDSSYYLAVSDLTLLMQQRLVSFQYHNDFIYWLTPHGRRFLRACQVAH

DHTDQVIRERKAALQDEKVRKKIQNRRHLDFLDILLGARVNDIKLSDADLRAEVDTFMFE

GHDTTTSGISWFLYCIALYPEHQHRCREEVREILGDQDFFQWDDLGKMTYLTMCIKESFR

LYPPVPQVYRQLSKPVTFVDGRSLPAGSLISMHIYALHRNSAVWPDPEVFDSLRFSTENA

SKRHPFAFMPFSAGPRNCIGQQFAMSEMKVVTAMCLLRFEFSLDPSRLPIKMPQLVLRSK

NGFHLHLKPLGPGSGK

>301603597

MSSPEFVDPLKMFNTDGKDMDIIMNYAKKYPYAYPMWLGNFFASLIICHPDYAKAILSRQ

DPKDDFGYYFLTPWIGKGLLVLSGQKWFQHRRLLTPAFHYDVLKPYVKLMADCSNVMLDK

WDKEISDKKPVELFHHVSLMTLDSIMKCAFSYHSNCQNNSENKYIKAVYELSYLVDHRFT

FLPYHSDFIFYLSPHGFRFRRALKVAHDHTDKVIKQRKKSLHEQNELEKIQQKRHLDFLD

ILLCAKDENGKGLSDEDLRAEVDTFMFEGHDTTASGISWTLYCMAKYPEHQQKCREEIRD

VLGGKQTVDWDDLGKMPYTTLCIKESLRLYPPVPGIARQLTQPITFCDGRSLPKGLLVFD

PMRFSAENSAKRNSHAFLPFSAGSRNCIGQNFAMNEMKVALALTLQRFELFPDTGIEPLT

APQLVLRSLNGIHLKLKRVGGD

>358411567

MESSWMESSWMESSWLETHWARPFYLTLVFCLALGLLQAIKLYLQRQRLLRDLRLFPSPP

THWLYGHSKLFKDGKMEILEKIVEKYPCAFPRWIGPFQAFLYIYDPDYAKTFLSRTDPKS

NFLYKFMTASVGKGLVNLSGPKWSQHRRLLTPGFHFNTLKSFVEVMAQSVNIMLNKWEKI

CGSQNTLLDIYEHINLMTLDVLMKCIFSWETNCQTDSSHDNYVKATSEGSNILMQRLFNY

FYHYDLIFKLSPLGHRLREVNKILHHHTDKIVKNRRNSLKDENKQTNTQKRKYRDFLDIV

LSAQAENDSFTDADLWSEVNTFMVAGHDSVSAGISWLLYHLALYPEHQEKCREEIRAILG

DGSSITWDQLSEMSYTTMCIKESLRLAPPAVSISRQLSKPITFPDGRSLPAGMTVVLSIW

GLHHNPAVWENPEVFDPLRFSQECNKRHSHAYLPFSSGPRNCIGQNFAIAEIKVIVALIL

LRFQMRVEPTKPVVFVPYIFIKPKKGIYLHLKKLP

>395858229

MESSWLETHWARPFYLAFSFCLALGLLQAAKLYLRRQRLLRDLRDFPTPPVHWYHGHQKV

FHNDNFDKIGEIIEKYPRAFPCWIGRFQAFFFIYDPDYAKTFLSRTDPKSQYLYKFIIPT

LGKGLINLEGPKWFQHRRLLTPGFHFNVLKTYVDIMICSVNIMLDKWEKLCSTQDTSIEV

ADHINLMALDIIMKCAFSQETNCQINSTHDVYVKATFELSRIIYQRVYNFLYHHDMIFKL

SPLSRRFEKLNQVTRRYTENIIQERKKSIRAGTNQDNIQKRKYQDFLDIVLSAQAKDGEI

FSDIDVQSEVKTFMFAGHDTTASSLTWLLYCLAQHPEHQDRCREEIRAILGDGSSISWDQ

LAEMSYTTMCIKEMFRFISPIPTISRELSQPLTFPDGCSLPAGMNVFLSIWGLHHNPTVW

KNPKVFDPLRFSPENSDQRHPHAFLPFSAGPRNCIGQQFAMVELKVAIALILLRFEVSLD

PTKPVAFMNCVVLKPKHGLYLHLKKLPEC

>358411569

MESSWMESSWMESSWLETHWARPFYLTLVFCLALGLLQAIKLYLQRQRLLRDLRLFPSPP

THWLYGHSKLFKDGKMEILEKIVEKYPCAFPRWIGPFQAFLYIYDPDYAKTFLSRTDPKS

NFLYKFMTASVGKGLVNLSGPKWSQHRRLLTPGFHFNTLKSFVEVMAQSVNIMLNKWEKI

CGSQNTLLDIYEHINLMTLDVLMKCIFSWETNCQTDSSHDNYVKATSEGSNILMQRLFNY

FYHYDLIFKLSPLGHRLREVNKILHHHTDKIVKNRRNSLKDENKQTNTQKRKYRDFLDIV

LSAQAENDSFTDADLWSEVNTFMVAGHDSVSAGISWLLYHLALYPEHQEKCREEIRAILG

DGSSITWDQLSEMSYTTMCIKESLRLAPPAVSISRQLSKPITFPDGRSLPAGMTVVLSIW

GLHHNPAVWENPEVFDPLRFSQECNKRHSHAYLPFSSGPRNCIRQHFAMAKCKMTLALTL

LHFELTLDCYKPPILMCQIILKSKDGIHVFLKKVC

>301785808

LLQDGKMEKFEEVVEKYHCAFPCWVGPFQAFFYIYDPDYAKTFLGRTDPKSKYLYKFLIP

CLGKGLLSLDGPKWFQHRRLLTPGFNVNILKSYVKVMAHSVNTMLGEWEKICSPQDTAVE

VYEHVNLMTLDILMKCAFSQETNCQISSTRDLYVKAMFEASKIIFYRSYSFLHHHDIIFK

FSPQGHRLQEMVKILHQYTEKVIQDRKKFLKDDKKHGNTQKQKYQDFLDIILSAQAENDN

SFSDTDLRSEVNTFMLAGHDTTAGSISWLLYHLALNPEHQERCREEIRGILGDGSSITWD

QLGEMAYTTMCIKESLRLAPPIPSISRELSKPITFPDGRSLPAGITVVLSIWGLHHNPAI

WENPKVFDPLRFSQENSEQRHPHSFLPFSAGPRNCIGQHFAMIELKVAIALILLRFKVAP

DPTRPLVFLHQIVLKAKDGVHLHLKKLS

>350586248

MEEGMEGFHSYIQAIWDLKNLFSTRTKSAFLQNDIIYRLSPEGRKNHRAARIAHQHTDRV

IQLRKAQLQKQGEMENVRKKRHLDFLDILLLARMEKGNSLSDTDLRAEVDTFMFEGHDTT

ASGISWILYALASHPEHQQRCREEIQGLLGDGTSITWDHLDQMPYTTMCIKEALRLYPPV

PGVSRELSKPITFPDGRSLPAGIILSLSIYGLHHNPQVWPNPEEFDPSRFAPGSARHSHA

FMPFSGGSRNCIGKQFAMNEMKVAVALTLLRFELAPDPSRKPIATPEVVLNSKNGIHLKL

RKLP

>397483173

MEFSWLETRWARPFYLAFVFCLALGLLQAIKLYLRRQRLLRDLRPFPAPPTHWFLGHQKF

IQDDNMEKLEEIIEKYPRAFPFWIGPFQAFFYIYDPDYAKTLLSRTDPKSQYLQKFSPPL

LGKGLAALDGPKWFQHRRLLTPGFHFNILKAYVEVMAHSVKMMLDKWEKICSTQDTSVEV

YEHINLMSLDIIMKCAFSKETNCQTNSTHDPYAKAIFELSKIIFHRLYSLLYHSDIIFKL

SPQGYRFQKLSRVLNQYTDTIIQERKKSLQAGVKQDNTPKRKYQDFLDIVLSAKDESGSS

FSDIDVHSEVSTFLLAGHDTLAASISWILYCLALNPEHQERCREEVRGILGDGSSITWDQ

LGEMSYTTMCIKETCRLIPAVPSISRDLSKPLTFPDGCTLPAGITVVLSIWGLHHNPAVW

KNPKVFDPLRFSQENSDQRHPYAYLPFSAGSRNCIGQEFAMIELKVTIALILLHFRVTPD

PTRPLTFPNHFILKPKNGMYLHLKKLSEC

>332266359

MEFSWLETRWARPFYLAFVFCLALGLLQAIKLFLRRQRLLRDLRPFPTPPTHWFLGHQKF

IQDDNMEKLEEIIEKYPRAFPFWIGPFQAFFYIYDPDYAKTLLSRTDPKSQYLQKFSPPL

LGKGLAALDGPKWFQHRRLLTPGFHFNILKAYIEVMAHSVKTMLDKWEKICSTQDTSVEV

YAHINLMALDIILKCAFSKETNCQTNSTHDPYAKAIFELSKIIFHRLYSLLYHSDIIFKL

SPQGYRLQKLSRVLNQYTDTIIQERKKSLQAGVKQDNTQKRKYQDFLDIVLSAKDESGST

FSDIDVHSEVSTFLLAGHDTLAASISWILYCLALNPEHQERCREEVRGILGDGSSITWDQ

LGEMSYTTMCIKETCRLIPAVPSISRDLSKPLTFPDGCTLPAGITVVLSIWGLHHNPAVW

KNPKVFDPLRFSQENSDQRHPYAYLPFSAGSRNCIGQEFAMIELKVTVALILLHFRVTPD

PTRPLTFSNHFILKPKNGMYLHLKKLSEC

>297665028

MEFSWLETRWARPFYLAFVFCLALGLLQAIKLYLRRQRLLRDLRPFPTPPTHWFLGHQKF

IQDDNMDKLEEIIEKYPRAFPFWIGPFQAFFYIYDPDYAKTLLSRTDPKSQYLQKFSPPL

LGKGLAALDGPKWFQHRRLLTPGFHFNILKAYIEVMAHSVKMMLDKWEKICSTQDTSVEV

YEHINLMSLDIIMKCAFSKETNCQTNSTHDPYAKAIFELSKIIFHRLYSLLYHSDIIFKL

SPQGYRFQKLSRVLNQYTDTIIQERKKSLQAGVKQDNTQKRNYQDFLDIVLSAKDESGSS

FSDIDVHSEVSTFLLAGHDTLAASISWILYCLALNPEHQERCREEVRGILGDGSSITWDQ

LGEMSYTTMCIKETCRLIPAVPSISRDLSKPLTFPDGCTLPAGITVVLSIWGLHHNPAVW

KNPKVFDPLRFSQENSDQRHPYAYLPFSAGSRNCIGQEFAMIELKVTIALILLHFRVTPD

PNRPLTFPSHFILKPKNGMYLHLKKLSEC

>332808908

MEFSWLETRWARPFYLAFVFCLALGLLQAIKLYLRRQRLLRDLRPFPAPPTHWFLGHQKF

IQDDNMEKLEEIIEKYPRAFPFWIGPFQAFFYIYDPDYAKTLLSRTDPKSQYLQKFSPPL

LGKGLAALDGPKWFQHRRLLTPGFHFNILKAYVEVMAHSVKMMLDKWEKICSTQDTSVEV

YEHINSMSLDIIMKCAFSKETNCQTNSTHDPYAKAIFELSKIIFHRLYSLLYHSDIIFKL

SPQGYRFQKLSRVLNQYTDTIIQERKKSLQAGVKQDNTPKRKYQDFLDIVLSAKDESGSS

FSDIDVHSEVSTFLLAGHDTLAASISWILYCLALNPEHQERCREEVRGILGDGSSITWDQ

LGEMSYTTMCIKETCRLIPAVPSISRDLSKPLTFPDGCTLPAGITVVLSIWGLHHNPAVW

KNPKVFDPLRFSQENSDQRHPYAYLPFSAGSRNCIGQEFAMIELKVTIALILLHFRVTPD

PTRPLTFPNHFILKPKNGMYLHLKKLSEC

>291398956

MEASWLETRWVRSLYLAFVFCLALGLLQAIKLHLRRQRLQRDLRSFPAPPAHWFHGHQQF

LQDENMEQLEEFMDKYPCAFPCWVGPFQACLFIYDPEYAETVLSRTDPKSQYLSKFMTPC

LGRGLGNLDGPTWFQHRRLLTPGFHSNILKTYVAVMARSVNTMLGKWEKIGGAQDTAVEV

HEHINLMALDIIMKCAFSKETNCQTNSTHDPYVKAMFELDKILSHRFYNCLHHHDIIFKF

SPQGYHFQKLSQVLRQYTEQVLQDRRKSLQGGVKQASAGKTEYQDFLDIVLSAQRENEDS

FSDADLQSEMNTFILAGHSTTAASLSWFLYCLALNPEHQERCREEIRDVLGNGSSTTWEQ

LDELCFTSMCISETFRLFPPVPAVSRELSKPLTFPDGRALPAGMIVVLSIWGLHHNPAVW

KNPKVFDPSRFSKENSAQRHPHAFLPFSAGSRNCIGQQFAMLELKVAIALILLRFKVAPA

SPTPLASSNHVILKPKNGIHLRLQKLPACPIPGYGD

>Q8K4D6.1

MEASWLENRWARPLHLALVFCLALVLMQAVKLYLRRQRLLRDLRPFPGPTAHWLLGHQKF

LQEDNMEKLDEIVKEYPCAFPCWVGPFQAFFYIYDPDYAKIFLSRTDPKTQYLHQLMTPF

LGRGLLNLDGPRWFQHRCLLTPAFHQDILKPCVDMMAHSVNMMLDKWEKTWTTQETTIEV

FEHINLMTLDIIMKCAFGQETNCQINGTYESYVKATFELGEIISSRLYNFWHHHDIIFKL

SPKGHCFQELGKVIHQCTEKIIQDRKKTLKDQVNQDDTQTSQNFLDIVLSAQAGDEKAFS

DADLRSEVNTFMWAGHDASAASISWLLYCLALNPEHQDRCRTEIRSILGDGSSITWEQLD

EIPYTTMCIKETLRLIPPIPSISRELSKPLTLPDGHSLPAGMTVVLSIWGLHHNPAVWKD

PKVFDPLRFTKENSEQRHPCAFLPFSSGPRNCIGQQFAMLELKVAIALTLLRFRVAADLT

RPPAFSSHTVLRPKHGIYLHLKKLPEC

>ABM82582.1

MEFSWLETRWARPFYLAFVFCLALGLLQSIKLYLRRQRLLRDLRPFPAPPTHWFLGHQKF

IQDDNMEKLEEIIEKYPRAFPFWIGPFQAFFCIYDPDYAKTLLSRTDPKSQYLQKFSPPL

LGKGLAALDGPKWFQHRRLLTPGFHFNILKAYIEVMAHSVKMMLDKWEKICSTQDTSVEV

YEHINSMSLDIIMKCAFSKETNCQTNSTHDPYAKAIFELSKIIFHRLYSLLYHSDIIFKL

SPQGYRFQKLSRVLNQYTDTIIQERKKSLQAGVKQDNTPKRKYQDFLDIVLSAKDESGSS

FSDIDVHSEVSTFLLAGHDTLAASISWILYCLALNPEHQERCREEVRGILGDGSSITWDQ

LGEMSYTTMCIKETCRLIPAVPSISRDLSKPLTFPDGCTLPAGITVVLSIWGLHHNPAVW

KNPKVFDPLRFSQENSDQRHPYAYLPFSAGSRNCIGQEFAMIELKVTIALILLHFRVTPD

PTRPLTFPNHFILKPKNGMYLHLKKLSEC

>CAH10751.1

MEASWLETRWARPLHLALVFCLALVLMQAMKLYLRRQRLLRDLSPFPGPPAHWLLGHQKF

LQEDNMETLDEIVKKHPCAFPCWVGPFQAFFYIYDPDYAKIFLSRTDPKMQYLHQLLTPC

IGRGLLNLDGPRWFQHRCLLTPAFHQDILKPCVDTMAHSVKVMLDKWEKMWTTQETTIEV

FEHINLMTLDIIMKCAFGQETNCQINGTYESYVKATFELGEIISSRLYNFWHHHDIIFKL

SPKGHCFQELGKVIHQYTEKIIQDRKKILKNQVKQDDTQTSQIFLDIVLSAQAEDERAFS

DADLRAEVNTFMWAGHDASAASISWLLYCLALNPEHQDRCRTEIRSILGDGSSITWEQLD

EMSYTTMCIKETLRLIPPVPSISRELSKPLTLPDGHSLPAGMTVVLSIWGLHHNPAVWND

PKVFDPLRFTKENSDQRHPCAFLPFSSGPRNCIGQQFAMLELKVAIALILLHFQVAPDLT

RPPAFSSHTVLRPKHGIYLHLKKLLEC

>AAI65947.1

MEASWLETRWARPLHLALVFCLALVLMQAMKLYLRRQRLLRDLSPFPGPPAHWLLGHQKF

LQEDNMETLDEIVKKHPCAFPCWVGPFQAFFYIYDPDYAKIFLSRTDPKMQYLHQLLTPC

IGRGLLNLDGPRWFQHRCLLTPAFHQDILKPCVDTMAHSVKVMLDKWEKMWTTQETTIEV

FEHINLMTLDIIMKCAFGQETNCQINGTYESYVKATFELGEIISSRLYNFWHHHDIIFKL

SPKGHCFQELGKVIHQYTEKIIQDRKKILKNQVKQDDTQTSQIFLDIVLSAQAEDERAFS

DADLRAEVNTFMWAGHDASAASISWLLYCLALNPEHQDRCRTEIRSILGDGSSITWEQLD

EMSYTTMCIKETLRLIPPVPSISRELSKPLTLPDGHSLPAGMTVVLSIWGLHHNPAVWND

PKVFDPLRFTKENSDQRHPCAFLPFSSGPRNCIGQQFAMLELKVAIALILLHFQVAPDLT

RPPAFSSHTVLRPKHGIYLHLKKLLEC

>ADJ68242.1

MLSWLSSWLPGSLSKLWLWAVSLGLTLSLLQLIRLLSRRQKLLQGLKSFPGPPTHWLFGH

VKEMKKEGELDKVLSWTNIYPCAHQLWMGNFIGFLNIYDPDYTKAVYSRGDPKAWDIYGF

FLPWMGKGLLVLDGPKWFQHRKLLTPAFHSEVLKPYVDVFVESTKAMLNKWDKKIQEDKC

VNIFPDVGHMALDSLMKCIFGRSSGSQPESDTKYYLAIGELTLLMQEPMAAFQYHNDIFY

YWFTLHGRKFLQACKVAHDHTDKVIRARKAALKEEEEQEKIQEKKRLDFLDILLGAGDEN

KTKLSDADLRSEVDTFMFEGHDTTTSAISWFLYCIALNPEHQKRCREEIQNILGNRDTIQ

WEDLGKMTYLTMCIKVSFRLYPPVPQVYRQLNKPVTFPDGRSLPEGSLVSLHIYALHRNP

AIWDKPEMFDPQRFSPENSSTRHPYAFIPFSAGPRNCIGQQFAMMETKVVTALCLLHFEF

SPEPSRPPIKRLKLVLGSENGIHLNLKKLI

>CAM18519.1

MQAMKLYLRRQRLLRDLSPFPGPPAHWLLGHQKFLQEDNMETLDEIVKKHPCAFPCWVGP

FQAFFYIYDPDYAKIFLSRTDPKMQYLHQLLTPCIGRGLLNLDGPRWFQHRCLLTPAFHQ

DILKPCVDTMAHSVKVMLDKWEKMWTTQETTIEVFEHINLMTLDIIMKCAFGQETNCQIN

GTYESYVKATFELGEIISSRLYNFWHHHDIIFKLSPKGHCFQELGKVIHQYTEKIIQDRK

KILKNQVKQDDTQTSQIFLDIVLSAQAEDERAFSDADLRAEVNTFMWAGHDASAASISWL

LYCLALNPEHQDRCRTEIRSILGDGSSITWEQLDEMSYTTMCIKETLRLIPPVPSISREL

SKPLTLPDGHSLPAGMTVVLSIWGLHHNPAVWNDPKVFDPLRFTKENSDQRHPCAFLPFS

SGPRNCIGQQFAMLELKVAIALILLHFQVAPDLTRPPAFSSHTVLRPKHGIYLHLKKLLE

C

>397483179

MVPSFLSLSFSSLGLWASGLILVLGFLKLIRLLLRRQMLAKAMDNFPGPPTHWLFGHALE

IQETGSLDKVVSWAHQFPYAHPLWFGQFIGFLNIYEPDYAKAVYSCGGRGLLVLEGPKWL

QHRKLLTPGFHYDVLKPYVAVFTESTRIMLDKWEEKAREGKSFDIFCDVGHMALNTLMKC

TFGRGDTGLGHSRDSSYYLAVSDLTLLMQQRLVSFQYHNDFIYWLTPHGHRFLRACQVAH

DHTDQVIRERKAALQDEKVRKKIQNRRHLDFLDILLGARDEDDIKLSDADLRAEVDTFMF

EGHDTTTSGISWFLYCMALYPEHQHRCREEVREILGDQDSFQWDDLGKMTYLTMCIKESF

RLYPPVPQVYRQLSKPVTFVDGRSLPAGSLISMHIYALHRNSAVWPDPEVFDPLRFSTEN

ASKRHPFAFMPFSAGPRNCIGQQFAMSEMKVVTAMCLLHFEFSLDPSRLPIKMPQLVLRS

KNGFHLHLKPLGPGSGN

>109004089

MMWGGGLDLRSMPGHLRFSLYLSRCLFWESPSLYLTQPTSSPAEPQAPMCMNSSSSGLFI

QDDNMEKLEEIIEKYPRAFPFWIGPFQAFFYIYDPDYAKTFLSRTDPKSQYLQKFLPPLI

GKGLLALDGPKWFQHRRLLTPGFHFNILKAYIEVMAHSVKTMLDKWEKICSTQNTSVEVY

EHINLMSLDIIMKCAFSKETNCQTNSTHDPYVKAIFELGKIIFHRLYSFLYHSDIIFKLS

PQGYRFQKLSRVLNQYTDAIIQERKKSLQAGEKQDNTQKRKYQDFLDIVLSAKDENGSSF

SDTDVHSEVSMFLLGGHDSLAASISWILYCLALNPEHQERCREEVRGILGDGCSITWDQL

GEMSYTTMCIKETCRLIPAVPSISRDLSKPLTFPDGCTLPAGITVVLSIWGLHHNPAVWK

NPKVFDPLRFSQENCDQIHPYAYLPFSAGSRNCIGQQFAMIQLKVAIALILLHFRVTPDP

TRPPTFSNHFILKPKNGMYLHLKKLSEC

>EHH62195.1

MMWGGGLDLRSMPGHLRFSLYLSRCLFWESPSLYLTQPTSSPAEPQAPMCMNSSSSGLFI

QDDNMEKLEEIIEKYPRAFPFWIGPFQAFFYIYDPDYAKTFLSRTDPKSQYLQKFLPPLI

GKGLLALDGPKWFQHRRLLTPGFHFNILKAYIEVMAHSVKTMLDKWEKICSTQNTSVEVY

EHINLMSLDIIMKCAFSKETNCQTNSTHDPYVKAIFELGKIIFHRLYSFLYHSDIIFKLS

PQGYRFQKLSRVLNQYTDAIIQERKKSLQAGEKQDNTQKRKYQDFLDIVLSAKDENGSSF

SDTDVHSEVSMFLLGGHDSLAASISWILYCLALNPEHQERCREEVRGILGDGCSITWDQL

GEMSYTTMCIKETCRLIPAVPSISRDLSKPLPFPDGCTLPAGITVVLSIWGLHHNPAVWK

NPKVFDPLRFSQENCDQRHPYAYLPFSAGSRNCIGQQFAMIQLKVAIALILLHFRVTPDP

TRPPTFSNHFILKPKNGLYLHLKKLSEC

>348551414

MEPSWLETRGARPLYLAFVFGLALGLLQVIKLYLRRQRLLRDLRPFPAPPTHWFHGHQKL

LQEDKMEKLEELVEKYPCAFPCWIGPFQAFFFIYDPDYAQTFLNRTDPKSQYPYKFMTPC

FGKGLLSLDGPKWFQHRRLLTPGLQCDILKTYVEVMAHSVNTMLAKWEKICGTQDTAVEV

YEDISMMALDIIMQCAFSQETNCQINGTYDPYLKATTELSKIIFYRLYNFWNHHDLIFKF

SPKGQRFQELSKVLHQYTEKVIQDKKKSLKSETKQGTTQKKYQDILDIILSAQAESEDSF

PDADLQAEVSTFMLAGHDASAASLSWLLYCLALHPEHQERCREEIRSTLGEGSSIHWDQL

DEMSYTTMCIKEMFRLIPPVPSISRELSKPLTFPDGRSLPTGMNVVLSIWGLHHNPAVWN

SPQVFDPLRFTKENSDQRHPYAFLAFSAGPRNCIGQQFAMVELKVAIALILLHFRVSADL

TRPLSFSNHTVFKPKDGMHLHLKKLSGLIPPVATPQTASLGSVLFDAQVYKLCHLVAVQY

TASNRGKWNKNGHAVPWPQQAKGLIIHLRSQVQICTSLSDAERMGIASSSTLKLQEMQEG

KWAQHIPYHQPHGLSACSSHPSSRDLGLFSINSCYRANVTSKGSRGPENLRLPEKGSQST

EKQTWNWNRHLCDSQA

>297278643

MEKLEEIIEKYPRAFPFWIGPFQAFFYIYDPDYAKTFLSRTDPKSQYLQKFLPPLIGKGL

LALDGPKWFQHRRLLTPGFHFNILKAYIEVMAHSVKTMLDKWEKICSTQNTSVEVYEHIN

LMSLDIIMKCAFSKETNCQTNSTHDPYVKAIFELGKIIFHRLYSFLYHSDIIFKLSPQGY

RFQKLSRVLNQYTDAIIQERKKSLQAGEKQDNTQKRKYQDFLDIVLSAKDENGSSFSDTD

VHSEVSMFLLGGHDSLAASISWILYCLALNPEHQERCREEVRGILGDGCSITWDQLGEMS

YTTMCIKETCRLIPAVPSISRDLSKPLTFPDGCTLPAGITVVLSIWGLHHNPAVWKNPKV

FDPLRFSQENCDQIHPYAYLPFSAGSRNCIGQQFAMIQLKVAIALILLHFRVTPDPTRPP

TFSNHFILKPKNGMYLHLKKLSEC

>354469978

MEASWLETRWARPLHLALVFCLALGLMQALKLYLRRQRLLRDLRPFPGPPAHRLLGHQKF

LQDDKMEKLDEIVKKYPCAFPCWIGPFQAFFYIYDPDYAKIFLSRTDPKAQYVHQLMTPC

IGRGLLNLDGPRWFQHRCLLTPAFHHDILKTCVDMMAHSVNTMLDKWEKSWSAQETTMEV

FEHINLMALDIIMKCAFGQETNCQINRTYEPYMKATFELGEIISSYLYNFWHHHDIIFKF

SPKGHCFQELGKVIHQCTEKIIQDRKKLLKDGIKQDDTQKSQDFLDIVLSAQAEDERAFS

DTDLRSEVNTFMWAGHDASAVSISWLLYCLALNPEHQDRCRTEIRSILGDGSSITWDQLD

EMSYTTMCIKETLRLIPPIPSISRELSKPLTLPDGHSLPGGMTVVLSIWGLHHNPAVWKD

PKVFDPLRFTKENSDQRHPCAFLPFSSGPRNCIGQQFAMLELKVAIALILLHFQVAPDLT

RPPAFSSHTVLRPKHGIYLHLKKLH

>397483175

MMWGGGLDLCPMPGQLKFPPCLSRCLLWEPPSLYLTQPTSSLAEPQALICMTSSSSGLFI

QDDNMEKLEEIIEKYPRAFPFWIGPFQAFFYIYDPDYAKTLLSRTDPKSQYLQKFSPPLL

GKGLAALDGPKWFQHRRLLTPGFHFNILKAYVEVMAHSVKMMLDKWEKICSTQDTSVEVY

EHINLMSLDIIMKCAFSKETNCQTNSTHDPYAKAIFELSKIIFHRLYSLLYHSDIIFKLS

PQGYRFQKLSRVLNQYTDTIIQERKKSLQAGVKQDNTPKRKYQDFLDIVLSAKDESGSSF

SDIDVHSEVSTFLLAGHDTLAASISWILYCLALNPEHQERCREEVRGILGDGSSITWDQL

GEMSYTTMCIKETCRLIPAVPSISRDLSKPLTFPDGCTLPAGITVVLSIWGLHHNPAVWK

NPKVFDPLRFSQENSDQRHPYAYLPFSAGSRNCIGQEFAMIELKVTIALILLHFRVTPDP

TRPLTFPNHFILKPKNGMYLHLKKLSEC

>EAX06882.1

MMWGGGLDLCPMPGQLKFPPCLSRCLLWEPPSLYLTQPTSSLAEPQALICMTSSSSGLFI

QDDNMEKLEEIIEKYPRAFPFWIGPFQAFFCIYDPDYAKTLLSRTDPKSQYLQKFSPPLL

GKGLAALDGPKWFQHRRLLTPGFHFNILKAYIEVMAHSVKMMLDKWEKICSTQDTSVEVY

EHINSMSLDIIMKCAFSKETNCQTNSTHDPYAKAIFELSKIIFHRLYSLLYHSDIIFKLS

PQGYRFQKLSRVLNQYTDTIIQERKKSLQAGVKQDNTPKRKYQDFLDIVLSAKDESGSSF

SDIDVHSEVSTFLLAGHDTLAASISWILYCLALNPEHQERCREEVRGILGDGSSITWDQL

GEMSYTTMCIKETCRLIPAVPSISRDLSKPLTFPDGCTLPAGITVVLSIWGLHHNPAVWK

NPKVFDPLRFSQENSDQRHPYAYLPFSAGSRNCIGQEFAMIELKVTIALILLHFRVTPDP

TRPLTFPNHFILKPKNGMYLHLKKLSEC

>BAD18508.1

MMWGGGLDLCPMPGQLKFPPCLSRCLLWEPPSLYLTQPTSSLAEPQALICMTSSSSGLFI

QDDNMEKLEEIIEKYPRAFPFWIGPFQAFFCIYDPDYAKTLLSRTDPKSQYLQKFSPPLL

GKGLAALDGPKWFQHRRLLTPGFHFNILKAYIEVMAHSVKMMLDKWEKICSTQDTSVEVY

EHINSMSLDIVMKCAFSKETNCQTNSTHDPYAKAIFELSKIIFHRLYSLLYHSDIIFKLS

PQGYRFQKLSRVLNQYTDTIIQERKKSLQAGVKQDNTPKRKYQDFLDIVLSAKDESGSSF

SDIDVHSEVSTFLLAGHDTLAASISWILYCLALNPEHQERCREEVRGILGDGSSITWDQL

GEMSYTTMCIKETCRLIPAVPSISRDLSKPLTFPDGCTLPAGITVVLSIWGLHHNPAVWK

NPKVFDPLRFSQENSDQRHPYAYLPFSAGSRNCIGQEFAMIELKVTIALILLHFRVTPDP

TRPLTFPNHFILKPKNGMYLHLKKLSEC

>AAH56734.1

MLLYGISPFVLSVNHVFALIFLACLLTVVKLLIVRRKGVKTMERFPGPPAHWLFGHVKEF

RQDGHDLEKIVKWMELYQFAFPLWFGPSLAVLNIHHPSYAKTILTTTEPKDDYAYKFFIP

WLGDGLLVSTGQKWFRHRRLLTPGFHYDVLKPYVKLISDSTKVMLDKWEVHSRSEESFEL

FKHVSLMTLDSIMKCAFSCNSNCQTDSGTNPYIQAVFDLCHLVNLRFRVFPYHSKAIFHL

SPHGYRFRKAASIAHNHTAEVIRKRKEVLKMEEEQGIVKNRRYLDFLDILLSARDEHQQG

LSDEDIRAEVDTFMFEGHDTTASGISWIFYNLACNPEHQEKCRQEIQQALDGKDTLEWED

LNKIPYTTMCIKESLRLHPPVPGISRKLTKPLTFFDGRTVPEGCTIGVSIYGIHMNSTVW

ENPYKFDPLRFLPENAANRSPHAFVPFSAGPRFVTRSV

>332808910

MEKLEEIIEKYPRAFPFWIGPFQAFFYIYDPDYAKTLLSRTDPKSQYLQKFSPPLLGKGL

AALDGPKWFQHRRLLTPGFHFNILKAYVEVMAHSVKMMLDKWEKICSTQDTSVEVYEHIN

SMSLDIIMKCAFSKETNCQTNSTHDPYAKAIFELSKIIFHRLYSLLYHSDIIFKLSPQGY

RFQKLSRVLNQYTDTIIQERKKSLQAGVKQDNTPKRKYQDFLDIVLSAKDESGSSFSDID

VHSEVSTFLLAGHDTLAASISWILYCLALNPEHQERCREEVRGILGDGSSITWDQLGEMS

YTTMCIKETCRLIPAVPSISRDLSKPLTFPDGCTLPAGITVVLSIWGLHHNPAVWKNPKV

FDPLRFSQENSDQRHPYAYLPFSAGSRNCIGQEFAMIELKVTIALILLHFRVTPDPTRPL

TFPNHFILKPKNGMYLHLKKLSEC

>ACH53593.1

MQLYEIFPLNLEALSINHAFPLALLVCLVIVDRLMTMKRRGMRTMEPFPGPPAHWLFGHV

KEFHQDGHDLEKIIMWCEQYPFAFPLWFGPSLSILNIHHPSYVKTILTTTEPKDDYAYKF

FIPWLGDGLLVSAGQKWFRHRRLLTPGFHYDVLKPYVKLISDSTKVMLDKWELYAKSEES

FELFKHVSLMTLDSIMKCAFSCNSNCQTDSGTNPYIQAVFDLCHLVNLRFRVFPYHSKAI

FHLSPHGYKFRKAAGMAHNHTAEVIRKRKEVLKMEKEQGIVKNKRYLDFLDILLSARDEN

QQGLSDEDIRAEVDTFMFEGHDTTASGISWIFYNLACNPEHQEKCREEILRALDGKDTLE

WEDLNKIPYTTMCIKESLRLCPPVPGISRKLTKPLTFFDGRTVPEGCTIGVSIYGIHLNA

TVWENPYVFDPLRFLPENTAKRSPHAFVPFSAGPRKGKFFKELFPVQ

>EAX06884.1

MEKLEEIIEKYPRAFPFWIGPFQAFFCIYDPDYAKTLLSRTDPKSQYLQKFSPPLLGKGL

AALDGPKWFQHRRLLTPGFHFNILKAYIEVMAHSVKMMLDKWEKICSTQDTSVEVYEHIN

SMSLDIIMKCAFSKETNCQTNSTHDPYAKAIFELSKIIFHRLYSLLYHSDIIFKLSPQGY

RFQKLSRVLNQYTDTIIQERKKSLQAGVKQDNTPKRKYQDFLDIVLSAKDESGSSFSDID

VHSEVSTFLLAGHDTLAASISWILYCLALNPEHQERCREEVRGILGDGSSITWDQLGEMS

YTTMCIKETCRLIPAVPSISRDLSKPLTFPDGCTLPAGITVVLSIWGLHHNPAVWKNPKV

FDPLRFSQENSDQRHPYAYLPFSAGSRNCIGQEFAMIELKVTIALILLHFRVTPDPTRPL

TFPNHFILKPKNGMYLHLKKLSEC

>EEN58594.1

MWKMLKWGQTDSITLYLCLIILVFRFLRALLWKRYIQKVLAPFPGQPAHWLFGHMREVRP

DETGFTIVPQWAAKFKFAFPLWIGPFRVVLSLVHPDYIKEIVNSPEPKDRVSYAWLKPWI

GDGLLVSEGQKWFRNRRLLTPGFHFDVLKPYVKVFSECTNIMLDRWADLAPGTPVEMFHY

ASAMTLDSLMRCALSVRSDCQRDSDGSPYIRAVYDLTKCVVERGRFPPFHIPLIFHLSPT

GFRFRKACKTAHDFSDEVIRKRRTELQQQGCHQNDTANSSEDGGKKRYLDFLDILLQARD

EDGKGLSEREIRDEVDTFMFEGHDTTASGVSWILYNLAKHPACQDRCRAEVDAVLQGRAE

VKWWDLSKLPYTTMCIKESLRMHSPVPGVTRLTTQPHTFPDGRSIPAGVSVSIGVHSLHH

NIHVWGDNVMEFDPERFSPENSKGRSSHAFIPFSAGSRNCIGQHFAMNELKVTVALTLQR

YRLELDETRPPYRVARLITRTRDGLWLKVYPRGADN

>AAN72310.1

MVPVLLSLSLSHLSLWAFGLVSVLVFLKLTRLLLRRQMLARAMDRFSGPPTHWLFGHALE

IQQTGGLDKVVYWTHQFPYAHQLWLGRFLGFLNIYDPEYAKAVYSRGDPKAPDVYDFFLQ

WIGKGLLVLQGPKWFQHRKLLTPGFHYDVLKPYVAVFAESARVMLDKWEKKAREQKSFDI

FSDVGHMALDSLMKCTFGKGTSGLNDRDNNYYLAVSELTLLMQQRIDSFQYHNDFIYFLT

PHGRRFLRACQVAHDHTDQVIRERKAALQDKKEREKIQSKRHLDFLDILLGARDEEGIKL

SDEDLRAEVNTFMFAGHDTTTSAISWVLYCMSLYPEHQRRCREEIQEILGDRDSLKWDDL

AEMTYLTMCIKESFRLYPPVPQVYRQLSKPVNFVDGRSLPAGSLISLHIYALHRNSTVWT

DPEVFDPLRFSPENVAGRHSFAFIPFSAGPR

>EEN67093.1

MATEITLFSGGAVTASLWVFLVLIAVKLVTLVRKQREDARVLEKFPGPPRHWLLGHVHKV

RMQLMSLQWADDMAAEYVYGYPVWFGPVGYLNVTHPEYTKTILATTEPKDEFVYRFIKPW

IGDGLLVSHGQKWFRNRRLLTPAFHFGVLQPYTRLFSDSTNIMMAKWRQLGAGASIDMFE

HVSLMTLDSMLKCALTVESNCQVDRKQNSYIAAVFSLTKLALQRFHLFPLHSDLIYYLTP

MGYRFWKLCKVVHQHSESVIRDRREALKKEQVQDSGQRRKYLDFLDILLKTKDEDGNGLS

DVEIRDEVDTFMFEGHDTTASGLSWTLYNLAKHPEHQERCRQEARSVLQGRSVVNSTDLS

HLPYITVCIKESLRIHTAVPSIARKLTKSITFPDGKSLPPGSLVGISVWNIHHNPHVWRD

PKVYDPSRFLPENCKGRHSHAFIPFSAGPRNCIGQHFAMNELKTAVALIVQSFRLSVDEN

HVAKGVMSLVLKAAEPGLFLKVTPLD

>332808939

MTSPLSPIPSPGRGLLVLEGPKWLQHRKLLTPGFHYDVLKPYVAVFTESTRIMLDKWEEK

AREGKSFDIFCDVGHMALNTLMKCTFGRGDTGLGHRDSSYYLAVSDLTLLMQQRLVSFQY

HNDFIYWLTPHGRRFLRACQVAHDHTDQVIRERKAALQDEKVQKKIQNRRHLDFLDILLG

AWDEDDIKLSDADLRAEVDTFMFEGHDTTTSGISWFLYCMALYPEHQHRCREEVREILGD

QDSFQWDDLGKMTYLTMCIKESFRLYPPVPQVYRQLSKPVTFVDGRSLPAGSLISMHIYA

LHRNSAVWPDPEVFDPLRFSTENASKRHPFAFMPFSAGPRNCIGQQFAMSEMKVVTAMCL

LRFEFSLDPSRLPIKMPQLVLCSKNGFHLHLKPLGPGSGK

>EGV94554.1

MEASWLETRWARPLHLALVFCLALGLMQALKLYLRRQRLLRDLRPFPGPPAHRLLGHQKF

LQDDKMEKLDEIVKKYPCAFPCWIGPFQAFFYIYDPDYAKIFLSRTDPKAQYVHQLMTPC

IGRGLLNLDGPRWFQHRCLLTPAFHHDILKTCVDMMAHSVNTMLDKWEKSWSAQETTMEV

FEHINLMALDIIMKCAFGQETNCQINRTYEPYMKATFELGEIISSYLYNFWHHHDIIFKF

SPKGHCFQELGKVIHQCTEKIIQDRKKLLKDGIKQDDTQKSQDFLDIVLSAQAEDERAFS

DTDLRSEVNTFMWAGHDASAVSISWLLYCLALNPEHQDRCRTEIRSILGDGSSITWDQLD

EMSYTTMCIKETLRLIPPIPSISRELSKPLTLPDGHSLPGGLHSFPKHEILKVFDPLRFT

KENSDQRHPCAFLPFSSGPRNCIGQQFAMLELKVAIALILLHFQVAPDLTRPPAFSSHTV

LRPKHGIYLHLKKLH

>AAI24947.1

MLQFLDHFLDSLNPSHTTFRVYIFAAIILIFSLIIFRTILKMVKFIYAYIINARRLRCFP

EPPRRSWLLGHMGLIKNTEEGLLVVDSLVKTYIYACSWWFSLCYPIVRLFHPSSIKPILQ

VSAAIAQKDELFYGFLRPWLGDGLLLSHGEKWGRHRRLLTPAFHFDILKNYVKIFNQSTD

IMLAKWRRLAVEGPVSLDMFEHVSLMTLDTLLKCTFSYDSDCQEKPSDYITAIYELSSLV

VKREHYLPHHFDFIYNLSSNGRKFRLACKKVHKFTAGVVQQRKKALQETGAEEWIKSKQG

KTKDFIDILLLSKDEDGNQLSDEDMRAEVDTFMFEGHDTTASGLSWILYNLARHPEYQEK

CRKEITELLEGKDTKHLEWDELSQLPFTTMCIKESLRLYPPVTAISRRCTEDIKLPDGKV

IPKGNSCLISIYGTHHNPEVWPNPQVYDPYRFDPEKLQERSAHAFVPFSAGPRNCIGQNF

AMAEMKIVLALTLYNFHVRLDETKPVRRKPELILRAENGLWLQVEELKS

>AAH90091.1

MLPSLDHFLDSLNMSRSSFRVYIFAAVILLFCLIMFRTILKMAIYIYAYIINARRLRCFP

EPPRRSWLLGHLGLFMPTEEGLTEVSNTISNFRKSFLTWMGPISLVSMVHPDTIKPMVAA

SAAIAPKDELFYGFLRPWLGDGLLLSRGEKWGRQRRLLTPAFHFDILKNYVKIFNQSTDI

MLAKWRRLAAVGPVSLDMFEHVSLMTLDTLLKCTFSYDSDCQEKPSDYIAAIYELSSLVV

KREHYLPHHFDFIYNLSSNGRKFHQACKTVHEFTAGVVQQRKKALQEKGIEEWIKSKQGK

TKDFIDILLLSKDEDGNQLSDEDMRAEVDTFMFEGHDTTASGLSWILYNLACHPEYQEKC

RKEITELLEGKDTKHLEWDELSQLPFTTMCIKESLRLHPPVTAVSRRCTEDIKLPDGKVI

PKGNSCLISIYGTHHNPDVWPNPQVYDPYRFDPEKLQERSSHAFVPFSAGPRNCIGQNFA

MAEMKIVLALTLYNFYMRLDETKTVRRKPELILRAENGLWLQVEELKQ

>344283159

MSLFNLSWLGLWPGAASPWLLLLLAMASWLLARFLAWSYTFNDICLRLRCFPQPPVRNWF

LGHLGLIQSSEEGLQYTQDLASTFGDVCCWWVGPLYPVVRIFHPTFIKPVLLAPAAIAPK

DVVFYGFLKPWLGEGLLLSSGDKWSRHRRMLTPAFHFNILKPYVKIFNESVNIMHAKWKC

LVSGGSTRLDMFEHISLMTLDSLQKCVFSFDTHCQEKPSEYIAAILELSALVAKRHQQIF

LHFDFLYHLTTDGRRFRKACRLVHDFTDAAIQERRRTLSEQGVDDFLEAKAKAKTLDFID

VLLLSKDEDGKELSDEDIRAEADTFMFEGHDTAASSISWVLYNLAKHPEYQERCREEVQA

LLEDRKPDEIEWDDLAHLPFLTMCIKESLRMYPPVTIISRCCTQDIVFPDGRVIPKGVIC

LISIFGTHHNPSVWPNPEVYDPFRFNSENTQKRSPLAFIPFSAGPRNCIGQIFAMTEMKV

VLALTLLRFRVLPDRTEPRRKPELVLRAEGGLWLQVEALNSGPHGPPAAAKTGECVPWAA

FLEEVPQTRWG

>P51869.1

MPQLDLSWLGLRLETSLPWLLLLLIGASWLLVRVLTQTYIFYRTYQHLCDFPQPPKWNWF

LGHLGMITPTEQGLKQVTKLVATYPQGFMTWLGPILPIITLCHPDVIRSVLSASASVALK

EVIFYSFLKPWLGDGLLLSDGDKWSCHRRMLTPAFHFNILKPYVKIFNDSTNIMHAKWQD

LASGGSARLDMFKNISLMTLDSLQKCVFSFDSNCQEKPSEYISAILELSALVAKRYQQLL

LHTDSLYQLTHNGRRFHKACKLVHNFTDAVIQGRRRALPSQHEDDILKAKARSKTLDFID

VLLLTKDEDGKELSDEDIRAEADTFMFEGHDTTASGLSWILYNLARHPEYQERCRQEVRE

LLRDRESTEIEWDDLAQLPFLTMCIKESLRLHPPVTVISRRCTQDIVLPDGRVIPKGVIC

IINIFATHHNPTVWPDPEVYDPFRFDPENIKDRSPLAFIPFSAGPRNCIGQTFAMNEMKV

ALALTLLRFRVLPDDKEPRRKPELILRAEGGLWLRVEPLSTQ

>AAH21377.1

MGFFRMPQLDLSWLGLRLEASSPWLLLLLIGASWLLARVLTQTYIFYRTYHHLCDFPQPP

KWNWFLGHLGMITPTEHGLKEVTNLVATYPQGFMTWLGPIIPIITLCHPDIIRSVLNASA

SVALKEVVFYSFLKPWLGDGLLLSDGDKWSSHRRMLTPAFHFNILKPYVKIFNDSTNIMH

AKWQHLASGGSARLDVFENISLMTLDSLQKCVFSFDSNCQENPSEYISAILELSALVTKR

YHQLLLHIDSLYQLTCSGRRFHKACHLVHSFTDAVIQDRRRTLPSKHEDDVLKAKAKSKT

LDFIDVLLLSKDEDGKELSDEDIRAEADTFMFEGHDTTASGLSWILYNLARHPEYQERCR

QEVQELLRDRESTEIEWDDLAQLPFLTMCIKESLRLHPPVTVISRRCTQDIVLPDGRVIP

KGVICIINIFATHHNPTVWPDPEVYDPFRFDPENIKDRSPLAFIPFSAGPRNCIGQTFAM

NEMKVALALTLLRFRVLPDDKEPRRKPELILRAEGGLWLRVEPLSTQ

>AAH81808.1

MSQLSLSWLGLGPEVAFPWQTLLLFGASWILAQILTQIYAAYRNFRRLRGFPQPPKRNWL

MGHVGMVTPTEQGLKELTRLVGTYPQGFLMWIGPMVPVITLCHSDIVRSILNASAAVALK

DVIFYTILKPWLGDGLLVSAGDKWSRHRRMLTPAFHFNILKPYVKIFNDSTNIMHAKWKR

LISEGSSRLDMFEHVSLMTLDSLQKCVFSFDSNCQEKSSEYIAAILELSALVAKRHQQPL

LFMDLLYNLTPDGMRFHKACNLVHEFTDAVIRERRRTLPDQGLDEFLKSKAKSKTLDFID

VLLLTKDEDGKELSDEDIRAEADTFMFEGHDTTASGLSWILYNLAKHPEYQERCRQEVQE

LLRDRDPEEIEWDDLAQLPFLTMCIKESLRLHPPVTVISRCCTQDILLPDGRTIPKGIIC

LISIFGIHHNPSVWPDPEVYNPFRFDPENIKDSSPLAFIPFSAGPRNCIGQTFAMSEMKV

ALALTLLRFRLLPDDKEPRRQPELILRAEGGLWLRVEPLSAGAQ

>334327008

MLPESKWLSHQLGLLGLEPKVSSALLLIFLAFSTWILGKGLICIHQFRTNCQWLRCFPEP

PEWHWLNGHAGVIKPSEEGIKRVLQFVAAFREVFVMWGGPFFPIVFICHPDYIKPLLSAS

ANIAPKDKLFYGFFEPWLGDGLLLSSGDKWSRHRRLLTPAFHFDILKPYVKFFNQSTDIM

HMKWRHLCVGDNVRLNIFEHISLMTLDSLQKCVFSHDSHCQEKPSSYISAILELSALVSR

RNNQPLLYWNSLYYLTSQGRHFSRACNAVHVFTDDVIQNRRRVLAEQGSEAFLRNKRKGK

TLDFIDVLLLAKDEDGKALSDEDIRAEADTFMFEGHDTTASGLSWALYNLSQHQEYQDRC

RQEIQELLKGRQLEEIEWDDLSQMPFLTMCIKESLRLHPPVVAIARRCTKDIKLPDGRII

PKGNTCLVSIFGTHHNPTVWPNPEVYDPNRFDPNNTQISPLAFLPFSAGPRNCIGQNFAM

AELKVALALTLLRFRILPDEHPPRRKPEVILRSEDGLWLRVEPLQHPSTLEIHTPETSQD

>EHH29754.1

MPQLSLSSLGLGPVAVSPWLLLLLIGVSWLLARVLAWTYTFYDNCRRLRCFPQPPKRNWF

WGHLGLIHSSEEGLLYIQSLVCTFGDVCCWWVGPWHAIVRIFHPTYIKPVLFAPAAIVPK

DKVFYRFLEPWLGDGLLLSAGDKWSRHRRMLTPAFHFNILKPYMKIFNESVNIMHAKWQL

LASEGSARLDMFEHISLMTLDSLQKCVFSFDSHCQEKPSEYIAAILELSALVAKRHQQFF

LYIDFLYYLTHDGQRFRRACRLVHDFTDAVIQERRRTLPSQGVEDFLQAKAKSKTLDFID

VLLLSKDEDGKKLSDEDIRAEADNFMFEGHDTTASGLSWVLYHLAKHPEYQERCRQEVQE

LLKDREPKEIEWDDLAQLPFLTMCIKESLRLHPPVPAISRCCTQDIVLPDGRVIPKGVIC

HISVLGTHHNPAVWPDPEVYDPFRFDPENIKERSPLAFIPFSAGPRNCIGQAFAMAEMKV

VLALTLLRFRVLPDHTEPRRKPELVLRAEGGLWLRVEPLS

>EEN69383.1

MEWSKIYKYAHVIRMGPLLQHCSVYHPDYVKAVMSRADRKDDYVYSFLRPWLGDGLLTSA

GPKWFRNRRLLTPGFHFEILKPYVRLFSESTNVMLKNWEELKSGSSIDVFHHTSLMTLDS

MLKCALSQHTDCQTRKTNDYIAAVYDLADLTMKRGRTLLLRSDLIYALSADGKKYRKACN

LVHEYAKRIITERREALKQRHTQDDEEATNKKKYLDFLDILLKARDEDGNGLTDAEIRDE

VDTFMFEGHDTTASGLAWTLYCLARHPGHQEKCRKEAQEMLQGRTEVTWDLLPSLKYITM

CVKEAIRLFPPVPMIFRHLESPLTFPDGRTLPEGSRVGISTNTLHHNPHVWENPMEFDPL

RFSTENSKGRHPFAFIPFAAGPRNCIGQHFAMNELKTAVALILQRFSLTPDNTLPEPIPV

PKLIMRAKKPGLFLKIKSLTA

>390465905

MVPSFLSPSLSRLVLWASGLILVLGFLKLIXLLLQKQKLAKAMEKFAGPPTHWLFGHALE

ISEMGILDAVVTWAHQFPYAHPLWFGQFIGFLTIYEPDYAKAVYSHGDPKAPDMYVFFLQ

WIRRGLLVLEGPKWFQHFKLLTPGFHYDVLKPYVALFAESTRNMLDKWEEKALEDKSIDI

FSDVGHIVLDSLMKCTFGRGDASLGHGDSSYYLAVSDLTLLXQQRLETFQYHNVFIYWLT

PHGRXFLRACQVAHDHTEQVIREWKAALKDKKVREKIQKQRHQDFLDILLDAQDDNDNKL

SDADLRAEVDTFMFEGHDATTSGISWFLYCMALYPEHQHRCREEVCEILGDRDSFQWDDL

SKMTYLTMCIKESFRLYPPVPQVYCLLSKPVIFVDGWSLPAGGMGGFGGGNGGLCMVFDP

LRFSTENVSNRHPLVFMPFSAGPRNCIGQQFVMNEMKVVTAMCLLSFEFSLDPSRLPIKM

AQLILRCLVT

>402904592

MPQLSLSSLGLGPVAVSPWLLLLLIGVSWLLARVLAWTYTFYDNCRRLRCFPQPPKRNWF

LGHLGLIHSSEEGLLYIQSLVCTFGDMCCWWGGPWHAIVRIFHPTYIKPVLFAPAAIVPK

DKVFYSFLKPWLGDGLLLSAGDKWSRHRRMLTPAFHFNILKPYMKIFNDSVNIMHAKWQL

LASEGSARLDMFEHISLMTLDSLQKCVFSFDSHCQEKPSEYIAAILELSALVAKRHQQIF

LYIDFLYYLTHDGQRFRRACRLVHDFTDAVIQERRRTLPSQGVDDFLQAKAKSKTLDFID

VLLLSKDEDGKKLSDEDIRAEADTFMFEGHDTTASGLSWVLYHLAKHPEYQERCRQEVQE

LLKDREPKEIEWDDLAQLPFLTMCIKESLRLHPPVPAISRCCTQDIVLPDGRVIPKGVIC

HISVLGTHHNPAVWPDPEVYDPFRFDPENIKERSPLAFIPFSAGPRNCIGQTFAMAEMKV

VLALTLLRFRVLPDHTEPRRKPELVLRAEGGLWLRVEPLS

>AAZ29444.1

MSQLSLSWLGLGPVAVSPWLLLLLVGTSWLLARVLAWTYTFYDNSRRLRGFPQPPKRNWF

WGHQGMVKSTEEGMRVLTQLVATYPQGFRVWMGPVFPLLSLCHPDIIRSVINASAAIVPK

DKLFYRFLEPWLGDGLLLSAGDKWSRHRRMLTPAFHFNILKPYMKIFNESVNIMHAKWQL

LASEGSARLDMFEHISLMTLDSLQKCVFSFDSHCQEKPSEYIAAILELSALVSKRHQQFL

LHIDFLYYLTPDGQRFRRACRLVHDFTDAVIQERRRTLPSQGVEDFLQAKAKSKTLDFID

VLLLSKDEDGKELSDEDIRAEADTFMFEGHDTTASGLSWVLYHLAKHPEYQERCRQEVQE

LLKDREPKEIEWDDLAQLPFLTMCIKESLRLHPPVPVISRRVTQDIVLPDGRVIPKGITC

LLSVFGTHHNPTVWPDPEVYDPFRFDPENIKERSPLAFIPFSAGPRNCIGQAFAMAEMKV

VLALTLLRFRVLPDHTEPRRKPELVLRAEGGLWLRVEPLS

>P33274.1

MSQLSLSWLGLGPEVAFPWQTLLLFGASWILAQILTQIYAAYRNFRRLRGFPQPPKRNWL

MGHVGMVTPTEQGLKELTRLVGTYPQGFLMWIGPMVPVITLCHSDIVRSILNASAAVALK

DVIFYTILKPWLGDGLLVSAGDKWSRHRRMLTPAFHFNILKPYVKIFNDSTNIMHAKWKR

LISEGSSRLDMFEHVSLMTLDSLQKCVFSFDSNCQEKSSEYIAAILELSALVAKRHQQPL

LFMDLLYNLTPDGMRFHKACNLVHEFTDAVIRERRRTLPDQGLDEFLKSKAKSKTLDFID

VLLLTKDEDGKELSDEDIRAEADTFMFEGHDTTASGLSWILYNLANDPEYQERCRQEVQE

LLRDRDPEEIEWDDLAQLPFLTMCIKESLRLHPPVTVISRCCTQDILLPDGRTIPKGIIC

LISIFGIHHNPSVWPDPEVYNPFRFDPENIKDSSPLAFIPFSAGPRNCIGQTFAMSEMKV

ALALTLLRFRLLPDDKEPRRQPELILRAEGGLWLRVEPLTAGAQ

>EHH59316.1

MSQLSLSWLGLGPVAVSPWLLLLLVGSSWLLARVLAWTYTFYDNSRRLRGFPQPPKRNWF

WGHQGMVKSTEEGMRVLTQLVATYPQGFRVWMGPVFPLLSLCHPDIIRSVINASAAIVPK

DKLFYRFLEPWLGDGLLLSAGDKWSRHRRMLTPAFHFNILKPYMKIFNESVNIMHVKWQR

LALEGSARLDMFEHISLMTLDSLQKCVFSFDSHCQEKPSEYIAAILELSALVSKRHQQFL

LHIDFLYYLTPDGQRFRRACRLVHDFTDAVIQERRRTLPSQGVEDFLQAKAKSKTLDFID

VLLLSKDEDGKKLSDEDIRAEADTFMFEGHDTTASGLSWVLYHLAKHPEYQERCRQEVQG

LLKDREPKEIEWDDLAQLPFLTMCIKESLRLHPPVPVISRRVTQDIVLPDGRVIPKGITC

LLSVFGTHHNPTVWPDPEVYDPFRFDPENIKERSPLAFIPFSAGPRNCIGQAFAMAEMKV

VLALTLLRFRVLPDHTEPRRKPELVLRAEGGLWLRVEPLS

>AAI71055.1

MLPFLDHFLDSLNMSRTSFRVYIFAAVILMFCLIMCRTIFKMATYIYAYIINARRLRCFP

EPPRRSWLLGHLGMFMPTEEGLTEISSAICNLRRTLLTWLGPIPEVSLVHPDTVKPVVAA

SAAIAPKDELFYGFLRPWLGDGLLLSRGEKWGQHRRLLTPAFHFDILKNYVKIFNQSTDI

MLAKWRRLTAEGPVSLDMFEHVSLMTLDTLLKCTFSYDSDCQEKPSDYISAIYELSSLVV

KREHYLPHHFDFIYNLSSNGRKFRQACKTVHEFTAGVVQQRKKALQEKGMEEWIKSKQGK

TKDFIDILLLSKNEDGSQLSDEDMRAEVDTFMFEGHDTTASGLSWILYNLACHPEYQEKC

RKEITELLEGKDTKHLEWDELSKLPFTTMCIKESLRLHPPVVAVIRRCTEDIKLPKGDIL

PKGNCCIINIFGIHHNPDVWPNPQVYDPYRFDPENLQERSSYAFVPFSAGPRNCIGQNFA

MAEMKIVLALILYNFQVRLDETKTVRRKPELILRAENGLWLQVEELKR

>354485209

MPQLDLSCLRWSLEASSLWLLVLLIGASWILARVLAQTYSFYGTYHHLCGFPQPPKRSWF

LGHLGMVTPTEQGLREVTKLVATYPQGFMTWMGPILPIITLCHPDIIRAVLGASAAVAIK

EVVFYTFLKPWLGDGLLLSAGDKWSRHRRMLTPAFHFNILKPYVKIFNDSTNIMHAKWQR

LTSGGSTRLDMFENISLMTLDSLQKCVFSFDSNCQEKPSEYISTILELSALVNKRYQQLL

LHIDSLYQLTPNGMRFHKACRLVHDFTDAVIQDRRRALPSKHGDDVLKAKAKSKTLDFID

VLLLAKDEDGKELSDGDIRAEADTFMFEGHDTTASGLSWTLYNLARHPEYQERCRQEVRE

LLRDRESQEIEWDDLAQLPFLTMCIKESLRLHPPVTVISRCCTQDIGLPDGRVIPKGVVC

IINIFATHHNPTVWPDPEVYDPFRFDPENVKNRSPLAFIPFSAGPRNCIGQTFAMNEMKV

ALALTLLRFRVLPDKEPRRKPELILRAEGGLWLRWPPLATQAPGTQDVLSSDTSSSTAVV

NTLSCK

>CAJ83432.1

MLPFLDHFLDSLNMSRTSFRVYIFAAVILMFCLIMCRTIFKMAIYIYAYIINARRLRCFP

EPPRRSWLLGHLGMFMPTEEGLTEISSAICNLRRTLLTWLGPIPEVSLVHPDTVKPVVAA

SAAIAPKDELFYGFLRPWLGDGLLLSRGEKWGQHRRLLTPAFHFDILKNYVKIFNQSTDI

MLAKWRRLTAEGPVSLDMFEHVSLMTLDTLLKCTFSYDSDCQEKPSDYISAIYELSSLVV

KREHYLPHHFDFIYNLSSNGRKFRQACKTVHEFTAGVVQQRKKALQEKGMEEWIKSKQGK

TKDFIDILLLSKNEDGSQLSDEDMRAEVDTFMFEGHDTTASGLSWILYNLACHPEYQEKC

RKEITELLEGKDIKHLEWDELSKLPFTTMCIKESLRLHPPVVAVIRRCTEDIKLPKGDIL

PKGNCCIINIFGIHHNPDVWPNPQVYDPYRFDPENLQERSSYAFVPFSAGPRNCIGQNFA

MAEMKIVLALILYNFQVRLDETKTVRRKPELILRAENGLWLQVEELKR

>402904614

MSQLSLSWLGLGPVAVSPWLLLLLVGTSWLLARVLAWTYTFYDNSRRLRGFPQPPKRNWF

WGHQGMVKSTEEGMRVLTQLVATYPWGFRVWMGPVFPLLSLCHPDIIRSVINASATLAPK

DKFFYSFLEPWLGDGLLLSAGDKWSRHRRMLTPAFHFNILKPYMKIFNDSVNIMHAKWQL

LASGGSARLDMFEHISLMTLDSLQKCVFSFDSHCQEKPSEYIAAILELSALVSKRHQQIL

LYIDFLYYLTPDGQRFRRACRLVHDFTDAVIQERRRTLPSQGVEDFLQAKAKSKTLDFID

VLLLSKDEDGKELSDEDIRAEADTFMFEGHDTTASGLSWVLYHLAKHPEYQERCRQEVQE

LLKDREPKEIEWDDLAQLPFLTMCIKESLRLHPPVPVISRHVTQDIVLPDGRVIPKGITC

LLSVFGTHHNPTVWPDPEVYDPFRFDPENIKERSPLAFIPFSAGPRNCIGQTFAMAEMKV

VLALTLLRFRVLPDHTEPRRKPELVLRAEGGLWLRVEPLS

>BAD02914.1

MLPFLDHFLDSLNLSHSTFRVYIFYAVILFFSLIMFRTILKMVTYIYAYIINARRLRCFP

EPPRRSWLLGHLGLFMPTEEGLTEVSDAISSFRKTFLTWMGPISLVSVFHPDTVKPIVAA

SAAIAPKDDLFYGFLRPWLGDGLLLSHGEKWGRHRRLLTPAFHFDILKSYVKIFNQSTDI

MLAKWRRMTVEGPVSLDMFEHISLMTLDTLLKCTFSYDSDCQEKPSDYIAAIYELSSLMV

KREHYLPHHLDFIYNLSSNGRNFRQACKKVHEFTAGVVQQRQKALKEKGMEEWIKSKQGK

TKDFIDILLLSKVEDGNQLSDEDMRAEVDTFMFEGHDTTASGLSWILYNLARHPEYQEKC

RKEIIELLEGKILKHLEWDELSQLPFTTMCIKESLRLHPPVTAVSRRCTEDIKLPDGKVI

PKGNTCLISIYGTHHNPEIWPNPQVYDPYRFDPENVQERSSHAFVPFSAGPRNCIGQNFA

MAEMKIVLALILYKFHVRLDETKAVRRKPELILRAENGLWLQVEELKR

>Q99N16.2

MSQLSMSWMGLGHTAASPWLLLLLAGASCLLAYILTPIYGVFENSLRLRCFPQPPKRNWI

LGHLGLIQSSEEGLLYIQSLVRTFRDACCWWVGPLHPVIRIFHPAFIKPVVLAPALVAPK

DTVFYRFLKPWLGDGLLMSTGDKWSRHRRMLTPAFHFNILKPYVKVFNDSTNIMHAKWQR

LASKGSAYLNMFEHISLMTLDSLQKCVFSFDSNCQEKPSEYITAILELSTLVARRHQRLL

LHVDLFYYLTHDGMRFRKACRLVHDFTDAVIRERRRTLLDQGGVDVLKAKAKAKTLDFID

VLLLSKDEHGKALSDEDIRAEADTFMFGGHDTTASGLSWILYNLARHPEYQERCRQEVRE

LLRDREPEEIEWDDLAQLPFLTMCIKESLRLHPPVTAISRCCTQDIVLPDGRVIPKGVIS

RISIFGTHHNPAVWPDPEVYDPFRFDADNVKGRSPLAFIPFSAGPRNCIGQTFAMSEMKV

ALALTLLRFRVLPDDKEPRRKPELILRAEGGLWLKVEPLSAGAQ

>291231925

MADYGLFGLFISNVNVTTLFGLLWPTFLLAICIKVLVSVVRLFQKRWQLEKELACFPSLD

VPRHWLLGNLSTIKNNEDTYAVFPSLVKDIGPVFILWCGPFLPRLICRHPSSIKTLTATT

EPKEELVYGFLRPWLGDGLLVSSGPKWFRNRRLLTPGFHFDILKPYVKIFNECVHTMANK

WESVCRSAPDGVVLEMFEDVSLMTLDTLLKCIFGQDSHCQTQRERNPYIKSVYTLSALVI

ERARFPPYFNDFIYYISPSGFRFRRAAKILHNYSSKVIQDRKMAMKMEEKSGIKRTKNYI

DFLDILLNARDENGQGLEDKEIRDEVDTFMFEGHDTTASGISWIFYNLASHPEHQEKCRR

EIDDILDKKDTDEIEWDDLRRIPYTTMCIKESLRIRPPVPGFSRQLTSPLNFDGRIAPAG

SFVSGSAFLVHHNADVWPNPTKYDPLRFLPENTRDRSPHAFIPFSAGPRNCIGQNFAMNE

MKVATCIVLRHFQLTVDKQRPPRRVNNLVSRSESGIHLNIKKRN

>AAH89709.1

MLPFLDHFLDSLNMSRTSFRVYIFAAVILMFCLIMCRTIFKMATYIYAYIINARRLRCFP

EPPRRSWLLGHLGMFMPTEEGLTEISSAICNLRRTLLTWLGPIPEVSLVHPDTVKPVVAA

SAAIAPKDELFYGFLRPWLGDGLLLSRGEKWGQHRRLLTPAFHFDILKNYVKIFNQSTDI

MLAKWRRLTAEGPVSLDMFEHVSLMTLDTLLKCTFSYDSDCQEKPSDYISAIYELSSLVV

KREHYLPHHFDFIYNLSSNGRKFRQACKTVHEFTAGVVQQRKKALQEKGMEEWIKSKQGK

TKDFIDILLLSKNEDGSQLSDEDMRAEVDTFMFEGHDTTASGLSWILYNLACHPEYQEKC

RKEITELLEGKDIKHLEWDELSKLPFTTMCIKESLRLHPPVVAVIRRCTEDIKLPKGDIL

PKGNCCIINIFGIHHNPDVWPNPQVYDPYRFDPENLQERSSYAFVPFSAGPRNCIGQNFA

MAEMKIVLALILYNFQVRLDETKTVRRKPELILRAENGLWLQVEELKR

>BAB25315.1

MSQLSMSWMGLGHTAASPWLLLLLAGASCLLAYILTPIYGVFENSLRLRCFPQPPKRNWI

LGHLGLIQSSEEGLLYIQSLVRTFRDACCWWVGPLHPVIRIFHPAFIKPVVLAPALVAPK

DTVFYRFLKPWLGDGLLMSTGGKWSRHRRMLTPAFHFNILKPYVKVFNDSTNIMHAKWQR

LASKGSAYLNMFEHISLMTLDSLQKCVFSFDSNCQEKPSEYITAILELSTLVARRHQRLL

LHVDLFYYLTHDGMRFRKACRLVHDFTDAVIRERRRTLLDQGGVDVLKAKAKAKTLDFID

VLLLSKDEHGKALSDEDIRAEADTFMFGGHDTTASGLSWILYNLARHPEYQERCRQEVRE

LLRDREPEEIEWDDLAQLPFLTMCIKESLRLHPPVTAISRCCTQDIVLPDGRVIPKGVIS

RISIFGTHHNPAVWPDPEVYDPFRFDADNVKGRSPLAFIPFSAGPRNCIGQTFAMSEMKV

ALALTLLRFRVLPDDKEPRRKPELILRAEGGLWLKVEPLSAGAQ

>AAK15013.1

MSQLSLSWMGLGHTAASPWLLLLLAGASCLLAYILTQIYGVFENSLRLRCFPQPPKRNWI

LGHLGLIQSSEEGLLYIQSLVRTFRDACCWWVGPLHPVIRIFHPAFIKPVVLAPALVAPK

DTVFYRFLKPWLGDGLLMSTGDKWSRHRRMLTPAFHFNILKPYVKVFNDSTNIMHAKWQR

LASKGSAYLNMFEHISLMTLDSLQKCVFSFDSNCQEKPSEYITAILELSALVARRHQRLL

LHVDLFYYLTHDGMRFRKACRLVHDFTDAVIRERRRTLLDQGGDDVLKAKAKAKTLDFID

VLLLSKDEHGKALSDEDIRAEADTFMFGGHDTTASGLSWILYNLARHPEYQERCRQEVRE

LLRDREPEEIEWDDLAQLPFLTMCIKESLRLHPPVTAISRCCTQDIVLPDGRVIPKGVIS

RISIFGTHHNPAVWPDPEVYDPFRFDADNVKGRSPLAFIPFSAGPRNCIGQTFAMSEMKV

ALALTLLRFRVLPDDKEPRRKPELILRAEGGLWLKVEPLSAGAQ

>354485205

MLQLSLSWLGLGPVAASPWHLLLLGGASLFLARILAWIYAFYDNCRRLRCFPQPPSCHWL

WGHLDIMQTDEEGMQLLTELSHYFRDVYLSWFGAFYPILRLSHPKFIAPVLQAPAAVAPK

EMTFYGFLKPWLGDGLLLSSGDKWSRHRRLLTPAFHFDILKSYVKVFNKSVNIMHAKWQR

LTAKGSTRLDMFEHISLMTLDSLQKCVFSFDSNCQESPSEYIAAILELSSLIMKRHRQLL

LYVDFLYYLTANGRRFRKACDLVHDFTDAVIRERRHTLNSQGVDEFLKAKTKSKTLDFID

VLLLAKDEHGKELSDEDIRAEADTFMFGGHDTAASALSWILYNLARHPEYQERCRQEVQE

LLRGREPQEIEWDDLAQLPFLTMCIKESLRLHPPVISISRCCTKDIVLPDGRVIPKGNDC

IISIFGVHHNPSVWPDPEVYDPFRFDPENSQKRSPLAFIPFSAGPRNCIGQTFAMNEMKV

ALALTLLRFRVLPDDKEPRRQPELILRAEGGLWLRVEPQSTGAP

>EHB14401.1

MTQLSMFWLGLGPVTASLWLLPMLFGIFWLLTRILVQTYAFYENVQHLQCFPQPPKRNWF

LGHLGQEPPTEEGLKFVEQMAHTYPQGFVMWFGPILPIITLCHPDSLRFVLSASVQVAVK

DVFFYGFLKPWLGDGLLLSAGDKWSRHRRMLTPAFHFNILKPYLKIFTDSTKVMHAKWQR

LISQGSTHLDMFEHISLMTLDSLQKCVFSSESNCQEKPSEYIAAILELSALVAKRVDQIL

LHKDFLYYLTHDGQKFCKACRLVHDFTDSVIQERRHTLLSQGIDDFLKGKAKSKALDLID

VLLLSKDEDGKELSDEDIRAEADTFMFEGHDTTASGLSWALYNLARHPEYQERCRQEVRE

LLRGREPEEIEWEDLAQLPFLTMCIKESLRLHPPVTGISRCCTQDVVLPDGRVIPKGVTC

LISIFGLHHNPAVWPDPEVYDPFRFDPENSKDRSPLAFIPFSAGPRNCIGQTFAMAEMKV

ALALTLLCFRVLPDNKDTEPRRKPELILRAEGGLWLRVEPLS

>EGV95496.1

MPQLDLSCLRWSLEASSLWLLVLLIGASWILARVLAQTYSFYGTYHHLCGFPQPPKRSWF

LGHLGMVTPTEQGLREVTKLVATYPQGFMTWMGPILPIITLCHPDIIRAVLGASAAVAIK

EVVFYTFLKPWLGDGLLLSAGDKWSRHRRMLTPAFHFNILKPYVKIFNDSTNIMHAKWQR

LTSGGSTRLDMFENISLMTLDSLQKCVFSFDSNCQEKPSEYISTILELSALVNKRYQQLL

LHIDSLYQLTPNGMRFHKACRLVHDFTDAVIQDRRRALPSKHGDDVLKAKAKSKTLDFID

VLLLAKDEDGKELSDGDIRAEADTFMFEGHDTTASGLSWTLYNLARHPEYQERCRQEVRE

LLRDRESQEIEWDDLAQLPFLTMCIKESLRLHPPVTVISRCCTQDIGLPDGRVIPKGVVC

IINIFATHHNPTVWPDPEVYDPFRFDPENVKNRSPLAFIPFSAGPRNCIGQTFAMNEMKV

ALALTLLRFRVLPDKEPRRKPELILRAEGGLWLAXXXXXXXXXXXXXXXXXXXXXXXXXX

XXXXXXXXXXXXXXXXXXXXXXXXXXXXXXXXXXXXXXXXXXXXXXXXXXXXXXXXXXXX

XXXXXXXXXXXXXXXXXXXXXXXXXXXXXXXXXXXXXXXXXXXXXXXXXXXXXXXXLEEQ

SVLLTSEPSLQRRQ

>403303354

MSQLSLSWLGLGPVAASPWLLLLLVGVSWLLARVLAWTYTFYDNRRRLQCFPQPPKRNWF

LGHLGLVNPTEEGMRVLTQLVATYPQGFKVWMGPITPIISLCHPNILRSVVNASAAIAPK

DKVFYSFLKPWLGDGLLLSSGDKWSRHRRMLTPAFHFNILKPYMKIFNESVNIMHGKWQR

LASEGSARLDMFEHISLMTLDSLQKCVFSFDSHCQEKPSEYIAAILELSALVVKRHQHLL

LHSDFLYYLTPDGRRFLRACRLVHDFTDAVIQERRRTLPSQGVDDFLQAKAKAKTLDFID

VLLLSKDEDGKELSDEDIRAEADTFMFEGHDTTASGLSWVLYHLAKHPEYQERCRQEVQE

LLKDREPKEIEWDDLAKLPFLTMCVKESLRLHPPVPAVSRCCTQDIALPDGRVIPKGVIC

LISVLGTHHNPTVWPDPEVYDPFRFDPENSKERSPLAFIPFSAGPRNCIGQAFAMAEMKV

VLALTLLRFRVLLDHTEPRRKPELVLRAEGGLWLRVEPLS

>BAA02144.1

MPQLSLSSLGLWPMAASPWLLLLLVGASWLLARILAWTYTFYDNCCRLRCFPQPPKRNWF

LGHLGLIHSSEEGLLYTQSLACTFGDMCCWWVGPWHAIVRIFHPTYIKPVLFAPAAIVPK

DKVFYSFLKPWLGDGLLLSAGEKWSRHRRMLTPAFHFNILKPYMKIFNESVNIMHAKWQL

LASEGSARLDMFEHISLMTLDSLQKCVFSFDSHCQEKPSEYIAAILELSALVTKRHQQIL

LYIDFLYYLTPDGQRFRRACRLVHDFTDDVIQERRRTLPSQGVDDFLQAKAKSKTLDFID

VLLLSKDEDGKKLSDEDIRAEADTFMFEGHDTTASGLSWVLYHLAKHPEYQERCRQEVQE

LLKDREPKEIEWDDLAQLPFLTMCIKESLRLHPPVPAVSRCCTQDIVLPDGRVIPKGIIC

LISVFGTHHNPAVWPDPEVYDPFRFDPKNIKERSPLAFIPFSAGPRNCIGQAFAMAEMKV

VLGLTLLAFRVLPDHTEPRRKPELVLRAEGGLWLRVEPLS

>P78329.1

MSQLSLSWLGLWPVAASPWLLLLLVGASWLLAHVLAWTYAFYDNCRRLRCFPQPPRRNWF

WGHQGMVNPTEEGMRVLTQLVATYPQGFKVWMGPISPLLSLCHPDIIRSVINASAAIAPK

DKFFYSFLEPWLGDGLLLSAGDKWSRHRRMLTPAFHFNILKPYMKIFNESVNIMHAKWQL

LASEGSACLDMFEHISLMTLDSLQKCVFSFDSHCQEKPSEYIAAILELSALVSKRHHEIL

LHIDFLYYLTPDGQRFRRACRLVHDFTDAVIQERRRTLPSQGVDDFLQAKAKSKTLDFID

VLLLSKDEDGKKLSDEDIRAEADTFMFEGHDTTASGLSWVLYHLAKHPEYQERCRQEVQE

LLKDREPKEIEWDDLAHLPFLTMCMKESLRLHPPVPVISRHVTQDIVLPDGRVIPKGIIC

LISVFGTHHNPAVWPDPEVYDPFRFDPENIKERSPLAFIPFSAGPRNCIGQTFAMAEMKV

VLALTLLRFRVLPDHTEPRRKPELVLRAEGGLWLRVEPLS

>AAK15011.1

MGFFRMPQLDLSWLGLRLEASSPWLLLLLIGASWLLARVLTQTYIFYRTYHHLCDFPQPP

KWNWFLGHLGMITPTEHGLKEVTNLVATYPQGFMTWLGPIIPIITLCHPDIIRSVLNASA

SVALKEVVFYSFLKPWLGDGLLLSDGDKWSSHRRMLTPAFHFNILKPYVKIFNDSTNIMH

AKWQHLASGGSARLDVFENISLMTLDSLQKCVFSFDSNCQENPSEYISAILELSALVTKR

YHQLLLHIDSLYQLTCSGRRFHKACHLVHSFTDAVIQDRRRTLPSKHEDDVLKAKAKSKT

LDFIDVLLLSKDEDGKELSDEDIRAEADTFMFEGHDTTASGLSWILYNLARHPEYQERCR

QEVQELLRDRESTEIECSCAVFLRDDLAQLPFLTMCIKESLRLHPPVTVISRRCTQDIVL

PDGRVIPKGVICIINIFATHHNPTVWPDPEVYDPFRFDPENIKDRSPLAFIPFSAGPRNC

IGQTFAMNEMKVALALTLLRFRVLPDDKEPRRKPELILRAEGGLWLRVEPLSTQ

>332253678

MPQLSLSSLGLGPVAASPWLLLLLVGASWLLARVLAWTYTFCDNCRRLRCFPQPPKRNWF

LGHLGLIHSSEEGLLYTQSLACTFGDMCCWWVGPWHAIVRIFHPTYIKPVLFAPAAIVPK

DKVFYSFLKPWLGDGLLLSAGDKWSRHRRMLTPAFHFNILKPYMKIFNESVNIMHAKWQL

LASEGSARLDMFEHISLMTLDSLQKCVFSFDSHCQEKPSEYIAAILELSALVAKRHQQIL

LYIDFLYYLTHDGQRFRRACRLVHDFTDAVIQERRRTLPSQGVDDFLQAKAKSKTLDFID

VLLLSKDEDGKKLSDEDIRAEADTFMFEGHDTTASGLSWVLYHLAKHPEYQECCRQEVQE

LLKDREPKEIEWDDLAQLPFLTMCIKESLRLHPPVPAVSRCCTQDVVLPDGRIIPKGVIC

LISIFGTHHNPAVWPDPEVYDPFRFDPKNIKERSPLAFIPFSAGPRNCIGQAFAMAEMKV

VLGLTLLRFRVLPDHTEPRRKPELVLRAEGGLWLRVEPLS

>291223969

MLGFLRPVLQELVSKGYDLFNYRFLGLFILVALFSNVLCKLYQQLRTRWRAERDLIEIPS

PHRHWLFGHLHLFKNNEDSLAEVPRRALEFSPAAVIWLGPFVPRVMVNHPTTIKAILTTS

EPKDEFLYGLLKPWLGDGLLISSRQKWFRNRRLLTPGFHFDILRPYVQIYNDCVKTMLDK

WSNLCASSSSKSVSVEMFGDLSLMTLDSLLKCIFSQESHCQTAKSQNPYISSVYALSHLI

SERGRFVPYHSDIIYNLSISGYKFRKALRAVHGYSVRVIQERKQALQQSGDDKPARKYID

FLDILLRAKDEDGNGLSDREIRDEVDTFMFEGHDTTASGLSWCLYNFAKYPEHQRRCQEE

IDALLVKTKKEDIEWGDLSHLPYTTLCIKESLRIRSPVPMISRELKSPLTLPDGRFIPAG

YNVLISINGVHNNSLVWDNPTEFDPSRFLPENTRYRSPYAYVPFSAGPRNCIGQNFAMNE

MKVVVARTLRRFDLSPELSRQPQRISNLVLRSSNGIYVHVKPRN

>BAF83479.1

MSQLSLSWLGLWPVAASPWLLLLLVGASWLLAHVLAWTYAFYDNCRRLRCFPQPPRRNWF

WGHQGMVNPTEEGMRVLTQLVATYPQGFKVWMGPISPLLSLCHPDIIRSVINASAAIAPK

DKFFYSFLEPWLGDGLLLSAGDKWSRHRRMLTPAFHFNILKPYMKIFNESVNIMHAKWQL

LASEVSACLDMFEHISLMTLDSLQKCVFSFDSHCQEKPSEYIAAILELSALVSKRHHEIL

LHIDFLYYLTPDGQRFRRACRLVHDFTDAVIQERRRTLPSQGVDDFLQAKAKSKTLDFID

VLLLSKDEDGKKLSDEDIRAEADTFMFEGHDTTASGLSWVLYHLAKHPEYQERCRQEVQE

LLKDREPKEIEWDDLAHLPFLTMCMKESLRLHPPVPVISRHVTQDIVLPDGRVIPKGIIC

LISVFGTHHNPAVWPDPEVYDPFRFDPENIKERSPLAFIPFSAGPRNCIGQTFAMAEMKV

VLALTLLRFRVLPDHTEPRRKPELVLRAEGGLWLRVEPLS

>AAH67440.1

MSQLSLSWLGLGPVAASPWLLLLLVGASWLLAHVLAWTYAFYDNCRRLRCFPQPPRRNWF

WGHQGMVNPTEEGMRVLTQLVATYPQGFKVWMGPISPLLSLCHPDIIRSVINASAAIAPK

DKFFYSFLEPWLGDGLLLSAGDKWSRHRRMLTPAFHFNILKPYMKIFNDSVNIMHAKWQL

LASEGSACLDMFEHISLMTLDSLQKCVFSFDSHCQEKPSEYIAAILELSALVSKRHHEIL

LHIDFLYYLTPDGQRFRRACRLVHDFTDAVIQERRRTLPSQGVDDFLQAKAKSKTLDFID

VLLLSKDEDGKKLSDEDIRAEADTFMFEGHDTTASGLSWVLYHLAKHPEYQERCRQEVQE

LLKDREPKEIEWDDLAHLPFLTMCMKESLRLHPPVPVISRHVTQDIVLPDGRVIPKGIIC

LISVFGTHHNPAMWPDPEVYDPFRFDPENIKERSPLAFIPFSAGPRNCIGQTFAMAEMKV

VLALTLLRFRVLPDHTEPRRKPELVLRAEGGLWLRVEPLS

>397484932

MSQLSLSWLGLGPVAASPWLLLLLVGASWLLAHVLAWTYAFYDNCRRLRCFPQPPRRNWF

WGHQGMVNPTEEGMRVLTQLVATYPQGFKVWMGPISPLLSLCHPDIIRSVINASAAIAPK

DKFFYSFLEPWLGDGLLLSAGDKWSRHRRMLTPAFHFNILKPYMKIFNESVNIMHAKWQL

LASEGSACLDMFEHISLMTLDSLQKCVFSFDSHCQEKPSEYIAAILELSALVSKRHHEIL

LHIDFLYYLTPDGQRFRRACRLVHDFTDAVIQERRRTLPSQGVDDFLQAKAKSKTLDFID

VLLLSKDEDGKKLSDEDIRAEADTFMFEGHDTTASGLSWVLYHLAKHPEYQERCRQEVQE

LLKDREPKEIEWDDLAHLPFLTMCMKESLRLHPPVPVISRHVTQDIVLPDGRVIPKGIIC

LISVFGTHHNPAVWPDPEVYDPFRFDPENIKERSPLAFIPFSAGPRNCIGQTFAMAEMKV

VLALTLLRFRVLPDHTEPRRKPELVLRAEGGLWLRVEPLS

>291410589

MPQQGLSWLSLGLGAASPWLLLLLLGTSWLLARILTWAYTFYENSQRLRCFPQPPKRNWF

MGHLGQITPTEQGLREVTRMVTTFPQAFLSWMGPIVPVITLWHPDVVRSVLNAPAVVVPK

DMDFYGFLKLWLGEGLLLSTGDKWNSHRRMLTPAFHFEILKPYMKIFNRSTNTMHAKWQR

LASEGNACLDMFEHISLMTLDSLQKCVFSFDSNCQESSSDYIAAILDLSALVLKRDQQIL

MHMDFLYYLTPDGRRFRKACDLVHSFTDAIIQERRRTIASQGVDDFLKAKAKAKTLDFID

VLLLTKSEDGKELSDDDIRAEADTFMFAGHDTTASGLSWVLYNLARHPEYQQRCRQEVQE

LLKNRDAEEIEWDDLAQLPFLTMCIKESLRLHPPVTVISRRCTQDVKLPDGRVIPRGNVC

VISIFGIHHNPAVWPDPEVYDPFRFDPENPQKRSPLAFIPFSAGPRNCIGQTFAMAEMKV

ALALTLLRFRVLPASAEPRRKPELILRAEGGLWLRVEPLSGHPH

>Q3MID2.1

MPLLSLSWLGLGHTAASPWLLLLLVGASCLLAYILPQVYAVFENSRRLRRFPQPPPRNWL

FGHLGLIQSSEEGLLYIQSLSRTFRDVCCWWVGPWHPVIRIFHPAFIKPVILAPASVAPK

DRVFYRFLRPWLGDGLLLSTGDKWSRHRRMLTPAFHFNILKPYVKIFNDSTNIMHAKWQR

LASQGSARLDMFEHISLMTLDSLQKCVFSFDSNCQEKPSEYITAILELSALVARRHQSLL

LHVDLFYHLTRDGMRFRKACRLVHDFTDAVIRERRCTLPDQGGDDALKAKAKAKTLDFID

VLLLSKDEHGEALSDEDIRAEADTFMFGGHDTTASGLSWILYNLAKHPEYQERCRQEVRE

LLRDREPEEIEWDDLAQLPFLTMCIKESLRLHPPATAISRCCTQDIMLPDGRVIPKGVIC

RISIFGTHHNPAVWPDPEVYNPFRFDADNGKGRSPLAFIPFSAGPRNCIGQTFAMSEMKV

ALALTLLRFRVLPDDKEPRRKPELILRAEGGLWLRVEPLSAGAH

>348552031

MTQLSLAWLGLGPVAASPWLIGLLVGVSWLLARVLSRIYTIYENSGRLQCFPQPPKRNWL

LGHLGMIQSSEEGLLYTQGLARTYGTVCCWWVGPWSPVVRLFHPSYIKPVLLAPAAVAPK

DSVFYRFLKPWLGDGLLLSAGDKWSRHRRMLTPAFHFNILKPYVKIFTDSMNVMHAKWQR

LISQGSTRLDMFEHISLMTLDSLQKCVFSFESNCQEKPSEYIAAILELSALVAKRHQQLL

LHLDFLYHLSPDGQRFRRACRVVHDFTDAVIQERRRTLKNQGTDDSLKAKAKSKTLDFID

VLLMNEDKEGKELSDEDIRAEANTVMFGGHDTTASGLSWVLYNLAKHPEYQERCRQEVQE

VLRGREPEDIEWDDLAQLPFLTMCIKESLRLHPPVLVASRCCTQDIVLPDGRVIPKGVIC

LVSIFGLHHNPAVWPDPEVYNPFRFDSENSKGRSPLAFIPFSAGPRNCIGQTFAMTEMKV

ALAMTLQRFRVLPDATEPLRKPELILRAEGGLWLRVEPLSARAP

>291413164

MLQLSQSWLGLGLGLGLEAVSPWLLLLLVGASWLLARILTWTHAFHKMSRTLRSFPEPPR

WNWFLGHLHLIQSSEEGLLYTQGLARTFGDVCCWWVGPWHAVVRIFHPTHIKPVLFAPAA

IAPKDRVIYRFLEPWLGDGLLLSAGDKWSRHRRMLTPAFHFNILKPYMKIFNKSVNVMHA

KWQRLASEGSARLDMFEHVSLMTLDSLQRCVFSFDSNCQEKPSDYIAAILELSALVVKRH

QQILLHTDLLYYLTLNGQRFRRACRLVHDFTDAVIQERRRTLPNLEIDDFLKAKAKTKTL

DFIDVLLLTKDEDGKHLSDDDIRAEADTFMFEGHDTTASGLSWVLYNLAQHPEYQERCRQ

EVRELLRDRELKEIEWDDLAQLPFLTMCIKESLRLHPAVTVISRCCTQDVALPDGRVIPK

GVICVISIFGTHHNPAVWPDPEVYDPSRFEPEKVKDRSPLAFIPFSAGPRNCIGQSFAMA

EMKVALALTLLRFRVLPDRAEPRRKPELILRAEGGLWLRVEPLSAGAQ

>EEN60332.1

MATWMILPLAVIASVLLVQFVVSQVRQRRVTNQFVSPPSHWLYGHLMQLLPDESSWPVLK

QMMRDYSSHVLQLWVGPFLPLFIFCHPETSRAILSTSGDGLLLSHGSKWHRNRRLLTPAF

HFEILKPYVTLFSQSTNVLIDKWTKLGSGCSVEMFEHVSLMTLDSILKCSLSYHSNCQTD

SQSSAYIRAVYDITRLFVERIRFPPYYSDFIYSLSPSGRRFYKACDLIHNFSENVIRERK

AQLAEVKKLGLPRRGKGKCLDFLDILLEARDEDGTGLTDAEIRDEVDTFLFEGHDTTASG

ISWALYHLAKHPEYQDRCRGEAEGLLQGRTEMSWEDMSKLPFTTMCIKESLRLRPPVPGI

GRTLTQTLALPDGKKIPAGFQVLTGIMTAHRNPLIWPDPEVYDPLRFSPENSKDRHSHAF

IPFSAGPRNCIGQHFAMNEMKVAVALILQSFQLEPHGPPPFYEERIVLRARNGIWLKLHK

RE

>311249179

MLQLSLSWLGLGPVEASPWLLLLLVGASWLLARVLVWTCTSLDNVRRLRGFPQPPKPNWL

LGHMGQIESSEEGLLYTQDLASTYGDVCCWWVGPWHAVIRIFHPTCIKPVLFAPAAVAPK

DVVFYDFLKPWLGDGLLLSAGDKWSSHRRMLTPAFHFNILKPYMKIFNDSVNVMHAKWQR

LVTEGHNRLDMFEHISLMTLDSLQKCVFSFDSNCQEKPSEYIAAILELSALVAKRHQQIF

LHLDFLYYLTPDGWRFHKACRLVHDFTDAVIQERRNTLPTEGIDDFLKAKAKAKTLDIID

VLLLTKDEDGKGLSDEDIRAEADTFMFEGHDTTASGLSWVLYNLAKHPEYQERCRQEVHE

LLRDREPKEIEWDDLAQLPFLTMCIKESLRLHPPVTVISHRCTQDIVLPDGRIIPKGVIC

LISIFGTHHNPLVWQDPEVYDPFRFDPENIKERSPLAFIPFSAGPRNCIGQTFAMTEMKV

VLALTLLRFRVLPVEEEPRRKPELILRAEGGLWLRVEPLSASPQ

>EGV97526.1

MPQLSLSCLGLGPAAASLWLFLPLAGASCLLAYILTQAYAMYENSHHLRCFPQPPKRNWL

LGHLGLIQASEEGLLYIQNLVRTFRDVCCWWVGPCQPVIRIFHPAFIKPVLLAPASVAPK

DTVFYSFLKPWLGDGLLMSAGDKWSRHRRMLTPAFHFNILKPYVKIFSDSANIMHAKWQR

LASKGSARLDMFEHISLMTLDSLQKCVFSFNSNCQEKPSEYIAAILELSALVARRHQKLH

LHVGLFYHLTRDGTRFRKACRLVHDFTDAIIQERRCTLPDQGDDDVLKAKAKAKTLDFID

VLLLSKDEHGKALSDEDIRAEADTFMFGGHDTTASGLSWVLYNLARYPEYQERCRQEVRE

LLRDRDPEEIGWDDLAQLPFLTMCIKESLRLHPPVTAISRCCTQDIVLPDGRVIPKGVIC

RISIFGTHHNPAVWPDPEVYNPFRFDADNAKGRSPLAFLPFSAGPRNCIGQTFALSEMKV

VLALTLLRFRVLPDDKEPRRQPELILRAEGGLWLRVEPLSSQ

>301792136

MSQMNLSWLGLGQLAASPWLLLLLAGATWLLARILDXSYSLYDICSRLRCFPQPPKRNWL

WGHVGLIQSSEEGLLYTQDLASTYGDVCCWWVGPWHAVIRIFHPTCIKPVLFAPAAIAPK

DKVFYSFLKPWLGDGLLLSAGDKWSSHRHMLTPAFHFNILKPYVKIFNGSVNVMHAKWKH

LVSEGRTHLDMFEHISLMTLDSLQKCVFSFDSNCQEKPSEYIAAILDLSALVAKRHQQIL

VHTDFLYYLTPDGQRFRRACRLVHNFTDAVIQERRRTLPSQGVDDFLQAKAKNKTLDFID

VLLLTKDKDGKQLSDEDIRAEADTFMFEGHDTTASGLSWVLYNLAKHPEYQERCRQEVQE

LLRDREPQEIEWDDLAQLPFLTMCIKESLRLHPPVTVISRCCTQDTVLPDGRVIPKGVIC

LISIFGTHHNPSVWPDPEVYNPFRFDPENIKERSPLAFIPFSAGPRNCIGQTFAMTEMKV

VLALTLLRFRVLPDEEEPRRKPELILRAEGGLWLRVEPLSAGPRMSQLRLSWLGLGQLAA

SPWLLLLLAGATWLLARILD

>Q9EP75.1

MSQLSLSWLGLGPEVAFPWKTLLLLGASWILARILIQIYAAYRNYRHLHGFPQPPKRNWL

MGHVGMVTPTEQGLKELTRLVGTYPQGFLMWIGPMVPVITLCHSDIVRSILNASAAVALK

DVIFYSILKPWLGDGLLVSAGDKWSRHRRMLTPAFHFNILKPYVKIFNDSTNIMHAKWQR

LISDGSARLDMFEHVSLMTLDSLQKCVFSFDSNCQEKSSEYIAAILELSALVAKRHQQPL

MFMDLLYNLTPDGMRFRKACNVVHEFTDAVIRERHRTLPDQGLDDFLKSKAKSKTLDFID

VLLLSKDEDGKELSDEDIRAEADTFMFEGHDTTASGLSWILYNLARHPEYQERCRQEVQE

LLRGREPEEIEWDDLAQLPFLTMCIKESLRLHPPVTVISRCCTQDILLPDGRTIPKGIIC

LISIFGIHHNPSVWPDPEVYDPFRFDPENIKDSSPLAFIPFSAGPRNCIGQTFAMSEMKV

ALALTLLRFRLLPDDKEPRRQPELILRAEGGLWLRVEPLSAGAH

>AAH67437.1

MSQLSLSWLGLWPVAASPWLLLLLAGASWLLAHVLAWTYAFYDNCRRLRCFPQPPRRNWF

WGHQGMVNPTEEGMRVLTQLVATYPQGFKVWMGPISPLLSLCHPDIIRSVINASAAIAPK

DKFFYSFLEPWLGDGLLLSAGDKWSRHRRMLTPAFHFNILKPYMKIFNESVNIMHAKWQL

LASEGSACLDMFEHISLMTLDSLQKCVFSFDSHCQEKPSEYIAAILELSALVSKRHHEIL

LHIDFLYYLTPDGQRFRRACRLVHDFTDAVIQERRRTLPSQGVDDFLQAKAKSKTLDFID

VLLLSKDEDGKKLSDEDIRAEADTFMFEGHDTTASGLSWVLYHLAKHPEYQERCRQEVQE

LLKDREPKEIEWDDLAHLPFLTMCMKESLRLHPPVPVISRHVTQDIVLPDGRVIPKGIIC

LISVFGTHHNPAVWPDPEVYDPFRFDPENIKERSPLAFIPFSAGPRNCIGQTFAMAEMKV

VLALTLLRFRVLPDHTEPRRKPELVLRAEGGLWLRVEPLS

>EDL40284.1

MPVPERSRMSQLSLSWLGLGPEVAFPWKTLLLLGASWILARILIQIYAAYRNYRHLHGFP

QPPKRNWLMGHVGMVTPTEQGLKELTRLVGTYPQGFLMWIGPMVPVITLCHSDIVRSILN

ASAAVALKDVIFYSILKPWLGDGLLVSAGDKWSRHRRMLTPAFHFNILKPYVKIFNDSTN

IMHAKWQRLISDGSARLDMFEHVSLMTLDSLQKCVFSFDSNCQEKSSEYIAAILELSALV

AKRHQQPLMFMDLLYNLTPDGMRFRKACNVVHEFTDAVIRERHRTLPDQGLDDFLKSKAK

SKTLDFIDVLLLSKDEDGKELSDEDIRAEADTFMFEGHDTTASGLSWILYNLARHPEYQE

RCRQEVQELLRGREPEEIEWDDLAQLPFLTMCIKESLRLHPPVTVISRCCTQDILLPDGR

TIPKGIICLISIFGIHHNPSVWPDPEVYDPFRFDPENIKDSSPLAFIPFSAGPRNCIGQT

FAMSEMKVALALTLLRFRLLPDDKEPRRQPELILRAEGGLWLRVEPLSAGAH

>354485173

MSQLSLSWLGLAPEVASPWQLMMLVGALWLLARILTQIYAAHGNYHRLRGFPQPPKQNWL

MGHVGMVTPTEQGMKKLTQLVVTYPQGFMMWMGPMFPIITLCHSDIVRSILNASAAVALK

DVIFYSFLKPWLGDGLLVSAGDKWSRHRRMLTPAFHFNFLKPYVKIFNDSTNIMHAKWQH

LISKGSTRLDMFEHVSLMTLDSLQKCVFSVNSNCQEKSSEYIAAILELSALVAKRHQQPL

LYMDLLYHLTPDGMRFRKACSLVHEFTDAVIQERRRTLPDQGLDDFLKTKAESKTLDFID

VLLLIKDEDGKELSDEDIRAEADTFMFEGHDTTASGLSWILYNLAKHLEYQERCRQEVQE

LLRDREPQEIEWDDLNQLPFLTMCIKESLRLHPPVPVISRCCTQDVGLPDGRVIPKGVIC

LISIFGIHHNPSVWPDPEVYDPFRFDPENIKDRSPLAFIPFSAGPRNCIGQTFAMNEMKV

ALALTLLRFRVLLDDKEPRRQPELILRAEGGLWLRVEPLSTDSK

>291413162

MLQLSQSWLGLGLGLGLEAVSPWLLLLLVGASWLLARILTWTHAFHKMSRTLRSFPEPPR

WNWFLGHLHLVNSTEEGMKEVTQLVTAFPQGFLIWMGPIMPLVMLCHPDVVRPVLNASAA

IAPKDRVIYRFLEPWLGDGLLLSAGDKWSRHRRMLTPAFHFNILKPYMKIFNKSVNVMHA

KWQRLASEGSARLDMFEHVSLMTLDSLQRCVFSFDSNCQEKPSDYIAAILELSALVVKRH

QQILLHTDLLYYLTLNGQRFRRACRLVHDFTDAVIQERRRTLPNLEIDDFLKAKAKTKTL

DFIDVLLLTKDEDGKHLSDDDIRAEADTFMFEGHDTTASGLSWVLYNLAQHPEYQERCRQ

EVRELLRDRELKEIEWDDLAQLPFLTMCIKESLRLHPAVTVISRCCTQDVALPDGRVIPK

GVICVISIFGTHHNPAVWPDPEVYDPSRFEPEKVKDRSPLAFIPFSAGPRNCIGQSFAMA

EMKVALALTLLRFRVLPDRAEPRRKPELILRAEGGLWLRVEPLSAGAQ

>AAI13219.1

MLELSLSWLGLGPVAASPWLLLLLVGASWILARILAWIYAFYDNCCRLRCFPQPPKRSWF

WGHLGLAQSNEESMRLVEELGHYFRDVHLWWMGPFFPILRLVHPNFVAPLLQASATIIPK

DMFFYSFLKPWLGDGLLLSAGDKWSSHRRLLTPAFHFEILKPYMKIFNKSADIMHAKWQR

LALEGSTRLDMFEHISLMTLDSLQKCVFSYDSNCQEKPSEYIAAILELSALVMKRIKHIF

LHVDFLYYLTRDGQRFYRACRLVHDFTDAIIQKRRRTLISQGSQEFLKTKTKAKTLDFID

VLLLAKDEDGKGLPDEDIRAEADTFMFEGHDTTASGLSWILYNLAKHPEYQERCRQEVQE

LLRDREPKEIEWEDLAQLPFLTMCIKESLRLHPPVAVISRLCTHDVVLPDGRVIPKGNIC

VISIFGIHHNPSVWPDPEVFNPFRFDPEAPKRSPLAFIPFSAGPRNCIGQTFAMNEMKVA

LALTLLRFRILPDEEEPRRKPELILRAEGGLWLQVEPLNTGQ

>BAA75823.1

MSQLSLSWLGLGPVAASPWLLLLLVGASWLLAHVLAWTYAFYDNCRRLRCFPQPPRRNWF

WGHQGMVNPTEEGMRVLTQLVATYPQGFKVWMGPISPLLSLCHPDIIRSVINASAAIAPK

DKFFYSFLEPWLGDGLLLSAGDKWSRHRRMLTPAFHFNILKPYMKIFNESVNIMHAKWQL

LASEGSACLDMFEHISLMTLDSLQKCVFSFDSHCQEKPSEYIAAILELSALVSKRHHEIL

LHIDFLYYLTPDGQRFRRACRLVHDFTDAVIQERRRTLPSQGVDDFLQAKAKSKTLDFID

VLLLSKDEDGKKLSDEDIRAEADTFMFEGHDTTASGLSWVLYHLAKHPEYQERCRQEVQE

LLKDREPKEIEWDDLAHLPFLTMCMKESLRLHPPVPVISRHVTQDIVLPDGRVIPKGIIC

LISVFGTHHNPAMWPDPEVYDPFRFDPENIKERSPLAFIPFSAGPRNCIGQTFAMAEMKV

VLALTLLRFRVLPDHTEPRRKPELVLRAEGGLWLRVEPLS

>402904590

MPQLSLSSLGLGPVAVSPWLLLLLIGVSWLLARVLAWTYTFYDNCRRLRCFPQPPKRNWF

LGHLGLVTPTEQGMRVLTQLVATYPQGFKVWMGPIIPVIRFCHPNLIRSVINASAAIVPK

DKVFYSFLKPWLGDGLLLSAGDKWSRHRRMLTPAFHFNILKPYMKIFNDSVNIMHAKWQL

LASEGSARLDMFEHISLMTLDSLQKCVFSFDSHCQEKPSEYIAAILELSALVAKRHQQIF

LYIDFLYYLTHDGQRFRRACRLVHDFTDAVIQERRRTLPSQGVDDFLQAKAKSKTLDFID

VLLLSKDEDGKKLSDEDIRAEADTFMFEGHDTTASGLSWVLYHLAKHPEYQERCRQEVQE

LLKDREPKEIEWDDLAQLPFLTMCIKESLRLHPPVPAISRCCTQDIVLPDGRVIPKGVIC

HISVLGTHHNPAVWPDPEVYDPFRFDPENIKERSPLAFIPFSAGPRNCIGQTFAMAEMKV

VLALTLLRFRVLPDHTEPRRKPELVLRAEGGLWLRVEPLS

>DAA28218.1

MLELSLSWLGLGPVAASPWLLLLLVGASWILAHVLAWTYTSYNNSLRLRCFPQPPKRNWF

LGHLGLVPPTEQGLSKVTQLVTNYPQGYLMWMGPIIPLVIFCHPDLIRTFASASAAIAPK

NVVFYNFLKPWLGDGLLLSAGDKWSSHRRMLTPAFHFNILKPYMKIFTKSADIMHAKWQR

LIIEGHTHLDMFEHISLMTLDSLQKCVFSYDSNCQEKPSDYIAAILELSALVAKRYQQIF

LHMDFLYYLTPDGWRFRRACRLVHDFTGAVIQERRRTLPKEDIDDFLKAKEKTKTLDFID

VLLLTKDEDGKGLSDEDIRAEADTFMFEGHDTTASGLSWVLYNLAKHPEYQERCRQEVQE

LLKDRESKEIEWDDLAQLPFLTMCIKESLRLHPPVTVISRSCTQDITLPDGRVIPKGVIC

LISIFGTHHNPYVWPDPEVYDPLRFKPENIKGRSPLAFIPFSAGPRNCIGQTFAMTEMKV

VLALTLLRFRILPDEEEPCRKPELILRAEGGLWLRMELLSTGQQ

>114675897

MSQLSLSWLGLGPVAASPWPLLLLVGASWLLAHVLAWTYAFYDNCRRLRCFPQPPRRNWF

WGHQGMVNPTEEGMRVLTQLVATYPQGFKVWMGPISPLLSLCHPDIIRSVINASAAIAPK

DKFFYSFLEPWLGDGLLLSAGDKWSRHRRMLTPAFHFNILKPYMKIFNESVNIMHAKWQL

LASEGSACLDMFEHISLMTLDSLQKCVFSFDSHCQEKPSEYIAAILELSALVSKRHHEIL

LHIDFLYYLTPDGQRFRRACRLVHDFTDAVIQERRRTLPSQGVDDFLQAKAKSKTLDFID

VLLLSKDEDGKKLSDEDIRAEADTFMFEGHDTTASGLSWVLYHLAKHPEYQERCRQEVQE

LLKDREPKEIEWDDLAHLPFLTMCMKESLRLHPPVPVISRHVTQDIVLPDGRVIPKGIIC

LISVFGTHHNPAVWPDPEVYDPFRFDPENIKERSPLAFIPFSAGPRNCIGQTFAMAEMKV

VLALTLLRFRVLPDHTEPRRKPELVLRAEGGLWLRVEPLS

>EAW84489.1

MPQLSLSSLGLWPMAASPWLLLLLVGASWLLARILAWTYTFYDNCCRLRCFPQPPKRNWF

LGHLGLVTPTEQGMRVLTQLVATYPQGFKVWMGPIFPVIRFCHPNIIRSVINASAAIVPK

DKVFYSFLKPWLGDGLLLSAGEKWSRHRRMLTPAFHFNILKPYMKIFNESVNIMHAKWQL

LASEGSARLDMFEHISLMTLDSLQKCVFSFDSHCQEKPSEYIAAILELSALVTKRHQQIL

LYIDFLYYLTPDGQRFRRACRLVHDFTDAVIQERRRTLPSQGVDDFLQAKAKSKTLDFID

VLLLSKDEDGKKLSDEDIRAEADTFMFEGHDTTASGLSWVLYHLAKHPEYQERCRQEVQE

LLKDREPKEIEWDDLAQLPFLTMCIKESLRLHPPVPAVSRCCTQDIVLPDGRVIPKGIIC

LISVFGTHHNPAVWPDPEVYDPFRFDPKNIKERSPLAFIPFSAGPRNCIGQAFAMAEMKV

VLGLTLLRFRVLPDHTEPRRKPELVLRAEGGLWLRVEPLS

>291413160

MLQLSLSSPWLLLPLIGACWLLAHILTWTHAFYENTRRLRCFPQPPKRNWFLGHLGMIQS

SEEGLLYTQGLARTFGDVCCWWVGPWHAVVRIFHPAHIKPVLFAPAAIAPKNVVFYSFLK

PWLGDGLLLSAGDKWSSHRRMLTPAFHFNILKPYMRIFNKSVNVMHAKWQRLASEGSARL

DMFEHISLMTLDSLQKCVFSFDSNCQEKPSEYIAAILELSALVAKRHQQILLHTDLLYYL

TSDGQRFRRACHLVHDFTDAVIQERRRTLPNQGIDDFLKAKAKTKTLDFIDVLLLTKDED

GKEMSDEDIRAEADTFMFEGHDTTASGLSWVLYNLAKHPEYQERCRQEVQELLRDRELKE

IEWDDLAQLPFLTMCIKESLRLHPPVTVISRCCTQDVVLPDGRVIPKGVICLISIFGTHH

NPAVWTDPEVYDPSRFEPEKVKDRSPLAFIPFSAGPRNCIGQSFAMAEMKVALALTLLRF

RVLPDSAKPRRKPELILRAEGGLWLQVEPLSTGAQ

>AAC50052.2

MSQLSLSWLGLCRVAASPWLLLLLVGASWLLAHVLAWTYAFYDNCRRLRCFPQPPRRNWF

WGHQGMVNPTEEGMRVLTQLVATYPQGFKVWMGPISPLLSLCHPDIIRSVINASAAIAPK

DKFFYSFLEPWLGDGLLLSAGDKWSRHRRMLTPAFHFNILKPYMKIFNESVNIMHAKWQL

LASEGSACLDMFEHISLMTLDSLQKCVFSFDSHCQEKPSEYIAAILELSALVSKRHHEIL

LHIDFLYYLTPDGQRFRRACRLVHDFTDAVIQERRRTLPSQGVDDFLQAKAKSKTLDFID

VLLLSKDEDGKKLSDEDIRAEADTFMFEGHDTTASVLSWVLYHLAKHPEYQERCRQEVQE

LLKDREPKEIEWDDLAHLPFLTMCMKESLRVHPPVPVISRHVTQDIVLPDGRVIPKGIIC

LISVFGTHHNPAVWPDPEVYDPFRFDPENIKERSPLAFIPFSAGPRNCIGQTFAMAEMKV

VLALTLLRFRVLPDHTEPRRKPELVLRAEGGLWLRVEPLS

>311249181

MLQLSLSWLGLGPVEASPWLLLLLVGASWLLARVLVWTCTSLDNVRRLRGFPQPPKPNWL

LGHMGQVPPTEEGMTKLTQMMNTYPNGFMIWMGPITPIIVLCHPDLIRTMANASAAVAPK

DVVFYDFLKPWLGDGLLLSAGDKWSSHRRMLTPAFHFNILKPYMKIFNDSVNVMHAKWQR

LVTEGHNRLDMFEHISLMTLDSLQKCVFSFDSNCQEKPSEYIAAILELSALVAKRHQQIF

LHLDFLYYLTPDGWRFHKACRLVHDFTDAVIQERRNTLPTEGIDDFLKAKAKAKTLDIID

VLLLTKDEDGKGLSDEDIRAEADTFMFEGHDTTASGLSWVLYNLAKHPEYQERCRQEVHE

LLRDREPKEIEWDDLAQLPFLTMCIKESLRLHPPVTVISHRCTQDIVLPDGRIIPKGVIC

LISIFGTHHNPLVWQDPEVYDPFRFDPENIKERSPLAFIPFSAGPRNCIGQTFAMTEMKV

VLALTLLRFRVLPVEEEPRRKPELILRAEGGLWLRVEPLSASPQ

>332853570

MPQLSLSSLGLWPMAASPWLLLLLVGASWLLARILAWTYTFYDNCRRLRCFPQPLKRNWF

LGHLGLIHSSEEGLLYTQSLACTFGDMCCWWVGPWHAIVRIFHPTYIKPVLFAPAAIVPK

DKVFYSFLKPWLGDGLLLSAGEKWSRHRRMLTPAFHFNILKPYMKIFNESVNIMHAKWQL

LASEGSARLDMFEHISLMTLDSLQKCVFSFDSHCQEKPSEYIAAILELSALVTKRHQQIL

LYIDFLYYLTHDGQRFRRACRLVHDFTDAVIQERRRTLPSQGVDDFLQAKAKSKTLDFID

VLLLSKDEDGKKLSDEDIRAEADTFMFEGHDTTASGLSWVLYHLAKHPEYQERCRQEVQE

LLKDREPKEIEWDDLAHLPFLTMCMKESLRLHPPVPAVSRCCTQDIVLPDGRVIPKGIIC

LISVFGTHHNPAVWPDPEVYDPFRFDPKNIKERSPLAFIPFSAGPRNCIGQAFAMAEMKV

VLGLTLLRFRVLPDHTEPRRKPELVLRAEGGLWLRVEPLS

>AAC08589.1

MPQLSLSSLGLWPMAASPWLLLLLVGASWLLARILAWTYTFYDNCCRLRCFPQPPKRNWF

LGHLGLVTPTEQGMRVLTQLVATYPQGFKVWMGPIFPVIRFCHPNIIRSVINASAAIVPK

DKVFYSFLKPWLGDGLLLSAGEKWSRHRRMLTPAFHFNILKPYMKIFNESVNIMHAKWQL

LASKGYARLDMFEHISLMTLDSLQKCVFSFDSHCQEKPSEYIAAILELSALVTKRHQQIL

LYIDFLYYLTPDGQRFRRACRLVHDFTDAVIQERRRTLPSQGVDDFLQAKAKSKTLDFID

VLLLSKDEDGKKLSDEDIRAEADTFMFEGHDTTASGLSWVLYHLAKHPEYQERCRQEVQE

LLKDREPKEIEWDDLAQLPFLTMCIKESLRLHPPVPAVSRCCTQDIVLPDGRVIPKGIIC

LISVFGTHHNPAVWPDPEVYDPFRFDPKNIKERSPLAFIPFSAGPRNCIGQAFAMAEMKV

VLGLTLLRFRVLPDHTEPRRKPELVLRAEGGIWLRVEPLS

>EDL89476.1

MRHLDLSWLGLGPMSASPWLLLSLVGVSWFLTRCLTQIYTLYAKCQRLCGFPQPPKRSWF

WGHLGMSPPTEEGMKQMTELVATYPQGFMTWLGPIVPLITLCHPDIIRSVLSASAAVAPK

DGIFYSFLKPWLGDGLLVSASDKWSRHRSMLTPAFHFNILKPYVKIFNDSTNIMHAKWLR

LASGGSAHLDMFENISLMTLDTLQKCVFSFNSNCQEKPSEYIAAILELSALVVKRNEQLL

LHMDLLYRLTPDGRRFYKACHLVHDFTYAVIQERRRTLPKHGGDDVIKAKAKSKTLDFID

VLLLSKDEDGKELSDEDIRAEADTFMFEGHDTTASGLSWILYNLAKHPEYQERCRQEVQE

LLRDRDSEEIEWDDLAQLPFLTMCIKESLRLHPPVTMVSRCCTQDISLPDGRVIPKGIIC

IINIFATHHNPTVWQDPEVYDPFRFDPENIQARSPLAFIPFSAGPRNCIGQTFAMNEMKV

AVALTLLRFRVLPDDKEPRRKPELILRAEDGLWLRVEPLSAQA

>291233332

MVQVESASQLAAAKGKRRAPQAQKAQRPRTSSAKQSQPGGKRWYPSAGGKGSDAPPGKRG

PGGGSSSSGGHHPRQQVNHKKSHPDPTQQLLFLGMEFDTEVGLVRPSQARCLALSNLAER

LVSVPSVSLRELMRLIGMMTSVRRVSSPGRTTQPPGPTLVIGIPSSPTGTKQTGIDPGPS

KGNPVLVVVTDQPQTGGPLGPTRHNHDSGNRRLPPRVGSASIGQTSPRNMAPAMGSGPPY

QLARAESSAPSTFTLSTRITLVLVVLLTFGPRIVEGFKRRKWLEEVLSGFPTEPRHWLFG

HLHLRSLDLSAEWGSKHVPGFVVWIGPFNASYVCSHPTTVKALLSTAEPKDDFGYGMLKS

WLGDGLLISNGSKWARDRKLLTPGFHFDVLKPYAKIFNDCSKVLLSKWHEQSQEGSVDVF

HHVSLLTLDCLMNCVFSQDGDCQRQENNPYIRSVYSASQLFAKRFFNMFHYNDVIYYLSS

NGRQWRKTLDILHSHSHSVITQRRRALQQEQRSGIKNTRKYIDFLDILLSAKDEDGNGMT

DREIQDQVDTFMFEGHDTTASGISWCLYNLAKHAHHQRKCQTEIDDCFAKKTNADLSWDD

LNNFPYLTMCIKESLRITPPVLNHSRELTIPLHFPDGTSLPAGMWTSINVYATHHNNSVW

ENPEVYDPSRFLPEKVKLRSPHAFIPFSAGPRNCIGQNFAMNEMKITLANILHNFELSAD

ESKPVVPIAEIIYKTRNGMFLNL

>332853602

MSQLSLSWLGLGPVAASPWPLLLLVGASWLLAHVLAWTYAFYDNCRRLRCFPQPPRRNWF

WGHQGMIHSSEEGLLYAQSLACTFGDMGCWWVGPWQAIVRIFHPTYIKPVLFAPAAIAPK

DKFFYSFLEPWLGDGLLLSAGDKWSRHRRMLTPAFHFNILKPYMKIFNESVNIMHAKWQL

LASEGSACLDMFEHISLMTLDSLQKCVFSFDSHCQEKPSEYIAAILELSALVSKRHHEIL

LHIDFLYYLTPDGQRFRRACRLVHDFTDAVIQERRRTLPSQGVDDFLQAKAKSKTLDFID

VLLLSKDEDGKKLSDEDIRAEADTFMFEGHDTTASGLSWVLYHLAKHPEYQERCRQEVQE

LLKDREPKEIEWDDLAHLPFLTMCMKESLRLHPPVPVISRHVTQDIVLPDGRVIPKGIIC

LISVFGTHHNPAVWPDPEVYDPFRFDPENIKERSPLAFIPFSAGPRNCIGQTFAMAEMKV

VLALTLLRFRVLPDHTEPRRKPELVLRAEGGLWLRVEPLS

>297276377

MSQLSLSWLGLGPVAVSPWLLLLLVGTSWLLAHVLAWTYTFYDNSRRLRGFPQPPKRNWF

WGHQGMVKSTEEGMRVLTQLVATYPQGFRVWMGPVFPLLSLCHPDIIRSVINASAAIVPK

DKLFYRFLEPWLGDGLLLSAGDKWSRHRRMLTPAFHFNILKPYMKIFNESVNIMHAKWQL

LALGSSARLDMFEHISLMTLDSLQKCVFSFDSHCQEKPSEYIAAILELSALVSKRHQQFL

LHIDFLYYLTPDGQRFRRACRLVHDFTDAVIQERRRTLPSQGVEDFLQAKAKSKTLDFID

VLLLSKDEDGKELSDEDIRAEADTFMFEGHDTTASGLSWVLYHLAKHPEYQERCRQEVQE

LLKDHEPKEIEWDDLAQLPFLTMCIKESLRLHPPVPVISRHVTQDIVLPDGRVIPKGITC

LLSVFGTHHNPTVWPDPEVYDPFRFDPENIKERSPLAFIPFSAGPRNCIGQTFAMAEMKX

VLALTLLRFRVLPD

>114675867

MSLLSLSWLGLRPVAASPWLLLLVVGASWLLAHILAWTYAFYHNCRRLRCFPQPRKQNWF

LGHLGLVTPTEEGLRVLTQLVATYPQGFVRWLGPITPIINLCHPDIVRSVINTSDAITDK

DIVFYKTLKPWLGDGLLLSVGDKWRHHRRLLTPAFHFNILKPYIKIFSKSANIMHAKWQH

LAMEGSTCLDVFEHISLMTLDSLQKCIFSFESNCQEKPSEYITAIMELSALVVKRNNQFF

RYKDFLYFLTPCGRRFHRACRLVHDFTDTVIQERRRTLTSQGVDDFLQAKAKSKTLDFID

VLLLSEDKNGKELSDEDIRAEADTFMFGGHDTTASGLSWVLYNLARHPEYQERCRQEVQE

LLKDREPKEIEWDDLAQLPFLTMCLKESLRLHPPIPIFARGCTQDVVLPDSRVIPKGNVC

NINIFAIHHNPSVWPDPEVYDPFRFDPENAQKRSPMAFIPFSAGPRNCIGQKFAMAEMKV

VLALTLLRFRILPDHREPRRTPEIVLRAEDGLWLRVEPLG

>397484945

MSLLSLSWLGLRPVAASPWLLLLVVGASWLLAHILAWTYAFYHNCRRLRCFPQPRKQNWF

LGHLGLVTPTEEGLRVLTQLVATYPQGFVRWLGPITPIINLCHPDIVRSVINTSDAITDK

DIVFYKTLKPWLGDGLLLSVGDKWRHHRRLLTPAFHFNILKPYIKIFSKSANIMHAKWQR

LAMEGSTCLDVFEHISLMTLDSLQKCIFSFDSNCQEKPSEYITAIMELSALVVKRNNQFF

RYKDFLYFLTPCGRRFHRACRLVHDFTDAVIQERRRTLTSQGVDDFLQAKAKSKTLDFID

VLLLSEDKNGKELSDEDIRAEADTFMFGGHDTTASGLSWVLYNLARHPEYQERCRQEVQE

LLKDREPKEIEWDDLAQLPFLTMCLKESLRLHPPIPIFARGCTQDVVLPDSRVIPKGNVC

NINIFAIHHNPSVWPDPEVYDPFRFDPENAQKRSPMAFIPFSAGPRNCIGQKFAMAEMKV

VLALTLLRFRILPDHREPRRTPEIVLRAEDGLWLRVEPLG

>P98187.1

MSLLSLSWLGLRPVAASPWLLLLVVGASWLLARILAWTYAFYHNGRRLRCFPQPRKQNWF

LGHLGLVTPTEEGLRVLTQLVATYPQGFVRWLGPITPIINLCHPDIVRSVINTSDAITDK

DIVFYKTLKPWLGDGLLLSVGDKWRHHRRLLTPAFHFNILKPYIKIFSKSANIMHAKWQR

LAMEGSTCLDVFEHISLMTLDSLQKCIFSFDSNCQEKPSEYITAIMELSALVVKRNNQFF

RYKDFLYFLTPCGRRFHRACRLVHDFTDAVIQERRRTLTSQGVDDFLQAKAKSKTLDFID

VLLLSEDKNGKELSDEDIRAEADTFMFGGHDTTASGLSWVLYNLARHPEYQERCRQEVQE

LLKDREPKEIEWDDLAQLPFLTMCLKESLRLHPPIPTFARGCTQDVVLPDSRVIPKGNVC

NINIFAIHHNPSVWPDPEVYDPFRFDPENAQKRSPMAFIPFSAGPRNCIGQKFAMAEMKV

VLALTLLRFRILPDHREPRRTPEIVLRAEDGLWLRVEPLG

>334327012

MSPKWSAPSLPSVTQRVRVPFLASLAQPTRIAEIFLCSFHIRLRMVVPARAWLAAQPPSP

VYLEEEECRMLPGSKWLSHHLCLSSLEAEVSSILILAGLTLSTWILVQGLAWAHRYYINC

QRLRCFPGPPCQNWFLGHLGMVSTSEEGIRFIQDVTRHYSDVSLFWFGPLIPVIRLHHPQ

VISSLVNSSAFILPKDDFFYSFLEPWLGNGLLLSKGDKWSQHRRLLTPAFHFGILKSYVK

IFSQGADIMHAKWRRLCAEGNTRLDMFEHVSLMTLDSLQKCIFSYDSNCQEKPSAYISAI

LELSSLVTKRFQKPLLFWDALYSLTSEGRCFSRACRLVHHFSEAVIQDRRQILTKQGTES

FLREKGKRKTMDFIDILLLAKDKDGKTLSNKDIQAEADTFMFGGHDTTASGISWALYNLA

QHPEHQNRCRQEIEELLKGRQPEEIEWDDLANMPFLTMCIKESLRIHPPVPMVSRYCTKD

IQLPDGRVIPKGNICLISIFGTHHNPAVWPNPEVYNPYRFDTNNAEKIPPLAFIPFSAGP

RNCIGQNFALYEMKVVLALTLLRFRVFPDDHPVRRKSELILRADNGLWLKLEPVLGPSGF

DSQEEFLKKLMVKHLENVEAYQQKTLRNLNIFKLVSNRKTQGPK

>109123748

MPQLSLSSLGLGPVAVSPWLLLLLIGVSWLLARVLAWTYTFYDNCRRLRCFPQPPKRNWF

WGHLGLVTPTEQGMRVLTQLVATYPQGFKVWMGPIIPVIRFCHPNLIRSVINASAAIVPK

DKLFYSFLKPWLGDGLLLSAGDKWSRHRRMLTPAFHFNILKPYMKIFNESVNIMHAKWQL

LASEGSARLDMFEHISLMTLDSLQKCVFSFDSHCQEKPSEYIAAILELSALVAKRHQQFF

LYIDFLYYLTHDGQRFRRACRLVHDFTDAVIQERRRTLPSQGVEDFLQAKAKSKTLDFID

VLLLSKDEDGKKLSDEDIRAEADTFMFEGHDTTASGLSWVLYHLAKHPEYQERCRQEVQE

LLKDREPKEIEWDDLAQLPFLTMCIKESLRLHPPVPAISRCCTQDIVLPDGRVIPKGVIC

HISVLGTHHNPAVWPDPEVYDPFRFDPENIKERSPLAFIPFSAGPRNCIGQAFAMAEMKV

VLALTLLRFRVLPDHTEPRRKPELVLRAEGGLWLRVEPLS

>397484939

MPQLSLSSLGLWPMAASPWLLLLLVGASWLLACILAWTYTFYDNFRRLRCFPQPPKRNWF

LGHLGLVTPTEQGMRVLTQLVATYPQGFKVWMGPIFPVIHFCHPNIIRSVINASAAIVPK

DKVFYSFLKPWLGDGLLLSAGEKWSRHRRMLTPAFHFNILKPYMKIFNESVNIMHAKWQL

LASEGSARLDMFEHISLMTLDSLQKCVFSFDSHCQEKPSEYIAAILELSALVTKRHQQIL

LYIDFLYYLTHDGQRFRRACRLVHDFTDAVIQERRRTLPSQGVDDFLQAKAKSKTLDFID

VLLLSKDEDGKKLSDEDIRAEADTFMFEGHDTTASGLSWVLYHLAKHPEYQERCRQEVQE

LLKDREPKEIEWDDLAHLPFLTMCMKESLRLHPPVPAVSRCCTQDIVLPDGRVIPKGIIC

LISVFGTHHNPAVWPDPEVYDPFRFDPKNIKERSPLAFIPFSAGPRNCIGQAFAMAEMKV

VLGLTLLRFRVLPDHTEPRRKPELVLRAEGGLWLRVEPLS

>348552087

MPQLSESWLGLGLAAASPWLLPVIFGVFWLLTRILAWTYTFYTNLQHLRRFPQPPKRNWF

LGQLGQMPSTEQGLKFVVQTVRTCPQSFVMWFGPVLPIVTLCHPXRVVLSAPAQVAAKDD

LFYGFLKPWVGDGLLLSAGDKWSRHRRMLTPAFHFNILKPYMKIFNDSVNVMHAKWQRLI

SQGSTRLDMFEHISLMTLDSLQKCVFSFESNCQEKPSEYIAAILELSGLTAKRVNQILLH

TDFLYNLSPDGRRFCKACRVVHDFTDAVVQERRRTLKNQGVDDFLKAKAKAKTLDFIDVL

LTSKDEDGKELSDEDIRAEADTFMFEGHDTTASGLSWVLYNLAKHPEYQERCRQEVQEVL

RGRESEEVEWDDLTQLPFLTMCLKESLRLHPPVTGISRRCTQDVVLPDGRVVPKGVSCFL

SIFGTHHNPAVWPDPEVYDPFRFDPENIKDRSPLAFIPFSAGPRNCIGQAFAMAEMKVVL

ARTLLRFRALPDDAEPIRKPELILRTEGGLWLRVEPLSARAQ

>AAI58090.1

MRYLDLSWLGLGPISASPWLLFFLVGISWFLARFLTQLYTLYAKCQRLCGFPQPPKKNWF

WGHLGMSPPTEEGMKQVTELVTTYPQGFMTWLGPIVPLITLCHPDIIRSVLSASAAVAPK

DDIFYSFLKPWLGDGLLVSAGDKWNRHRRMLTPAFHFNILKPYVKIFNDSTNIMHAKWLR

LASGGSTRLNMFENISLMTLDTLQKCVFSFNSNCQEKPSQYIAAILELSTLAVKRNEQLL

MHVDLLYRLTPDGMRFYKACRLVHDFTNAVIQERRRTLLKHGGDDIIKAKAKSKTLDFID

VLLLTKDEDGKELSDEDIRAEADTFMFRGHDTTASGLSWILYNLARHPEHQERCRQEVQE

LLRDRDPKEIEWDDLAQLPFLTMCIKESLRLHPPVTMVSRCCTQDISLPDGRIIPKGVIC

IINIFGTHHNPTVWRDPEVYDPFRFDPENIQARSPLSFIPFSAGPRNCIGQTFAMSEMKV

ALALTLLRFRILPDDKEPRRKPELILRAEGGLWLRVEPLSTQAQ

>EAW84508.1

MSQLSLSWLGLWPVAASPWLLLLLVGASWLLAHVLAWTYAFYDNCRRLRCFPQPPRRNWF

WGHQGMIHSSEEGLLYTQSLACTFGDMGCWWVGPWQAVIHIFLPTCIKPVLFAPAAIAPK

DKFFYSFLEPWLGDGLLLSAGDKWSRHRRMLTPAFHFNILKPYMKIFNESVNIMHAKWQL

LASEGSACLDMFEHISLMTLDSLQKCVFSFDSHCQEKPSEYIAAILELSALVSKRHHEIL

LHIDFLYYLTPDGQRFRRACRLVHDFTDAVIQERRRTLPSQGVDDFLQAKAKSKTLDFID

VLLLSKDEDGKKLSDEDIRAEADTFMFEGHDTTASGLSWVLYHLAKHPEYQERCRQEVQE

LLKDREPKEIEWDDLAHLPFLTMCMKESLRLHPPVPVISRHVTQDIVLPDGRVIPKGIIC

LISVFGTHHNPAVWPDPEVYDPFRFDPENIKERSPLAFIPFSAGPRNCIGQTFAMAEMKV

VLALTLLRFRVLPDHTEPRRKPELVLRAEGGLWLRVEPLS

>EDL40300.1

MRYLDLSWLGLGPMSASPWLLFFLVGISWFLARFLTQLYTLYAKCQRLCGFPQPPKKNWF

WGHLGMSPPTEEGMKQVTELVTTYPQGFMTWLGPIVPLITLCHPDIIRSVLSASAAVAPK

DDIFYSFLKPWLGDGLLVSAGDKWNRHRRMLTPAFHFNILKPYVKIFNDSTNIMHAKWLR

LASGGSTRLNMFENISLMTLDTLQKCVFSFNSNCQEKPSQYIAAILELSTLAVKRNEQLL

MHVDLLYRLTPDGMRFYKACRLVHDFTDAVIQERRRTLLKHGGDDIIKAKAKSKTLDFID

VLLLTKDEDGKELSDEDIRAEADTFMFRGHDTTASGLSWILYNLARHPEHQERCRQEVQE

LLRDRDPKEIEWDDLAQLPFLTMCIKESLRLHPPVTMVSRCCTQDISLPDGRIIPKGVIC

IINIFGTHHNPTVWRDPEVYDPFRFDPENIQARSPLSFIPFSAGPRNCIGQTFAMSEMKV

ALALTLLRFRILPDDKEPRRKPELILRAEGGLWLRVEPLSTQAQ

>EHB00068.1

MTELSLSWLGLGPVTASPWLIGLLVGASWLLGQFLTWIYAFYANSRRLRCFPEPPRRNWL

LGHLGMIQSSEDGLLYTQGPARTYGDVCCWWVGPWHVVVHIFHPSCIKPVLFAPAAIALK

DVLFYSFLRPWLGDGLLLSAGDKWSQRRHMLTPAFHFNILKPYVKIFTESTNTMLAKWQR

VASWGSTRLDMFEHINLMTLDSLQKCVFSFESNCQEKPSEYIAAILELSALVAKRQRQLW

LHPDSLYHLTCDGHRFRKACRLVHDFTDAVIRERRHTLPNHGTDDFLKAKARSKTLDFID

VLLLSKDEDGKELSDEDIRAEADTFMFEGHDTTASGLSWALYNLARHPEYQERCRQEVRE

LLRGREPEDIEWEDLAQLPFLTMCIKESLRLHPPVSVISRCCTQDVVLPDGRVIPKGVIC

LISIFGLHHNPAVWPDPEVYDPFRFDPENSKDRSPLAFIPFSAGPRNCIGQTFAMAEMKV

ALALTLLRFRVLPDDTEPLRKPELILRAEGGLWLRVEPLS

>126324069

MVPITDQILKVLRLEKTSFRTYAISALLLSLLFIFFRFLVKVFQICHKFHITCQRLRCFP

QPPGRNWLLGHLGMYLPNESGLRDEMKVLDTLRHVVLVWIGPFLPLVVLVHPDYIKPLLG

ASAAIAPKDKLFYGFLTPWLGDGLLLSKGEKWSRHRRLLTPAFHFDILKPYMKIYNQCTD

IMHAKWHRLTAGATASLDMFEHVSLLTLDSLQKCVFSYNSDCQEKNSDYITAIIELSALV

VKRQYRIHHHLDFIYYRTADGRRFKKACDIVHRFTTEVIQERRQALNQQGAEAWLKSKQG

KTLDFIDVLLLAQDEDGKQLSDEDIRAEADTFMFEGHDTTSSGLSWVLFNLARYPEYQDK

CREEIQEIMKGRETDEIEWDDLAQMPFITMCIKESLRLFPPVTLISRRCTEDIKMPDGRV

IPKGIICLVSIYGTHYNPSVWPDSKVFNPYRFDPENSQQRSPLAFIPFSAGPRNCIGQSF

AMSEMKVVVALTLLRFRLSVDRTKKVRRKPELILRTENGIWLNVEPLSGQTTATPMAQPQ

Q

>354485167

MQHLDLSWLGLGPMSAAPWLLFVLIGASWLLARCLIWIFTLYEKCRRLRGFPQPPKRNWF

WGHLGTSPPTEEGMKEMTMRVATYPQGFMTWLGPVVPLIHLCHPDIIRSVVSASAAVAPK

DDNLYSFLKPWLGDGLLLSAGDKWSRHRSMLTPAFHFNILKPYVKIFNDSTNIMHAKWQR

LTSGGSTHLDMFEHISLMTLDTLQKCVFSFNSNCQEKPSEYIAAILELSALVVKRNEQLL

LHLDMLYRLTPDGRRFYKACRLVHDFTDAVIQERRRTLPSHGGDDVIKAKSKSKTLDFID

VLLLSKDEDGKELSDEDIRAEADTFMFRGHDTTASGLSWILYNLAKHPEYQERCRQEVQE

LFKGRESMDIEWDDLAQMPFLTMCIKESLRLHPPVTLASRCCTEDISLPDGRVIPKGVIC

IINIFGTHHNPTVWRDPEVYDPFRFDPENMQGRSPLAFIPFSAGPRNCIGQTFAMNEMKV

ALALTLLRFRVLPDKEPRRKPELILRAEGGLWLRVEPLINGAQ

>AAZ29445.1

MPQLSLSSLGLGPVAVSPWLLLLLIGVSWLLARVLAWTYTFYDNCRRLRCFPQPPKRNWF

WGHLGLVTPTEQGMRVLTQLVATYPQGFKVWMGPIIPVIRFCHPNLIRSVINASAAIVPK

DKLFYSFLKPWLGDGLLLSAGDKWSRHRRMLTPAFHFNILKPYMKIFNESVNIMHAKWQL

RASEGSARLDMFEHISLMTLDSLQKCVFSFDSHCQEKPSEYIAAILELSALVAKRHQQFF

LYIDFLYYLTHDGQRFRRACRLVHDFTDAVIQERRRTLPSQGVEDFLQAKAKSKTLDFID

VLLLSKDEDGKKLSDEDIRAEADTFMFEGHDTTASGLSWVLYHLAKHPEYQERCRQEVQE

LLKDREPKEIEWDDLAQLPFVTMCIKESLRLHPPVPAISRCCTQDIVLPDGRVIPKGVIC

HISVLGTHHNPAVWPDPEVYDPFRFDPENIKERSPLAFIPFSAGPRNCIGQAFAMAEMKV

VLALTLLRFRVLPDHTEPRRKPELVLRAEGGLWLRVEPLS

>332253674

MPQLSLSSLGLGPVAASPWLLLLLVGASWLLARVLAWTYTFCDNCRRLRCFPQPPKRNWF

LGHLGLVTPTGQGMRVLTQLVATYPQGFKVWMGPIFPVIRFCHPNIIRSVINASAAIVPK

DKVFYSFLKPWLGDGLLLSAGDKWSRHRRMLTPAFHFNILKPYMKIFNESVNIMHAKWQL

LASEGSARLDMFEHISLMTLDSLQKCVFSFDSHCQEKPSEYIAAILELSALVAKRHQQIL

LYIDFLYYLTHDGQRFRRACRLVHDFTDAVIQERRRTLPSQGVDDFLQAKAKSKTLDFID

VLLLSKDEDGKKLSDEDIRAEADTFMFEGHDTTASGLSWVLYHLAKHPEYQECCRQEVQE

LLKDREPKEIEWDDLAQLPFLTMCIKESLRLHPPVPAVSRCCTQDVVLPDGRIIPKGVIC

LISIFGTHHNPAVWPDPEVYDPFRFDPKNIKERSPLAFIPFSAGPRNCIGQAFAMAEMKV

VLGLTLLRFRVLPDHTEPRRKPELVLRAEGGLWLRVEPLS

>354485169

MQHLDLSWLGLGPMSAAPWLLFVLIGASWLLARCLIWIFTLYEKCRRLRGFPQPPKRNWF

WGHLGTSPPTEEGMKEMTMRVATYPQGFMTWLGPVVPLIHLCHPDIIRSVVSASAAVAPK

DDNLYSFLKPWLGDGLLLSAGDKWSRHRSMLTPAFHFNILKPYVKIFNDSTNIMHAKWQR

LTSGGSTHLDMFEHISLMTLDTLQKCVFSFNSNCQEKPSEYIAAILELSALVVKRNEQLL

LHLDMLYRLTPDGRRFYKACRLVHDFTDAVIQERRRTLPSHGGDDVIKAKSKSKTLDFID

VLLLSKDEDGKELSDEDIRAEADTFMFRGHDTTASGLSWILYNLAKHPEYQERCRQEVQE

LFKGRESMDIEWDDLAQLPFLTMCIKESLRLHPPVTVISRCCTQDIGLPDGRVIPKGVVC

IINIFATHHNPTVWPDPEVYDPFRFDPENVKNRSPLAFIPFSAGPRNCIGQTFAMNEMKV

ALALTLLRFLVLPDDKEPHRKPELILRAEGGLWLRVEPLSSQ

>300795285

MLQLSLSWLGRGPVTVSPWQLLLVVGTSLLLARILAWISAFYDNYCRLRCFPQPPSRHWF

WGHLNLVKNNEEGLQLLAEMSHQFQDIHLCWIGIFYPILRLIHPKFIGPILQAPAAVAPK

EMIFYGFLKPWLGDGLLVSAGEKWSRQRRLLTPAFHFDILKPYVKNFNKSVNIMHAKWQR

LTAKGSARLDMFEHISLMTLDSLQKCVFSFDSNCQESPSEYIAAIQELSSLIVKRHHQPF

LYMDFLYYLTADGRRFRKACDLVHNFTDAVIRERRRTLSSQSVDEFLKSKTKSKTLDFID

VLLLAKDEHGKELSDEDIRAEADTFMFGGHDTTASALSWILYNLARHPEHQERCRQEVRE

LLRDREPEEIEWDDLTQLPFLTMCIKESLRLHPPVTVISRCCTQDVVLPDGRVIPKGNDC

IISIFGVHHNPSVWPDPEVYDPFRFDSENPQKRSPLAFIPFSAGPRNCIGQTFAMNEMKV

AVALTLLRFRLLPDDKEPRRKPELILRAEGGLWLRVEPLSTGAQ

>354485165

MLQSSVSALGPTSVLASPWHLLLFGAASWLLAKILAWTYSFCENCSRLRCFPQTPRRNWF

LGHLGMIQSNEEGMQMVTEMGKTFRDVQVLWLGPVIPVLRLVDPAFVAPLLHAPALVAPK

DMTFLGFLKPWLGDGLFLSSGDKWSQRRRLLTPAFHFDILKPYVKIFNKSVNIMHDKWKR

LSSEGSARLEMFENISLMTLDSLQRCLFGFDSNCQDSPSEYIAAILELSSLIVKRSQKLF

LFSDFLYYLTANGQRFHKACDLVHDFTDAVIRERRRILSSQSVDEFLKSKAKSKTLDFID

VLLLAKDEHGKELSDEDIRAEADTFMFGGHDTTASGLSWVLYNLARHPEYQERCRQEVRE

LLRDREPQEIEWDDLAQLPFLTMCIKESLRLHPPVIDLLRRCTQDIVLPDGRVIPKGNIC

IISVFGIHHNPSVWPDPEVYDPFRFDPENPQKRSPLAFIPFSAGPRNCIGQTFAMTEMKV

VLALTLLRFRILPDNKEPRRKPELILRAEGGLWLRVEPLSSQ

>395848036

MLQLSLSWLGLGPVAASPWLLLLLVGASWLLARILTWTYAFYDNCRRLHCFPQPPMRNWF

LGHMGLVSPTEEGLREMTQLVATYPQIFKRWLGPTIPVITLCHPDFVRSVLNASATITHK

NMFLSRFLKPWLGDGLLLSRGDKWRHHRQMLTPAFHFNILKPYVKIFNKSVNIMHAKWQR

LTSEGSTHLDMFEHISLMTLDSLQKCIFSFDSNCQESPSEYIAAILDLSTLVTKRYYQFF

LYMDFLYYLTPEGRRFCRACDLVHDFTDAIIQERRRTLATQGIDDFLKIKTKSKSLDFID

VLLLSKDENGKELSDEDIRAEADTFMFGGHDTTASGLSWVLYNLARHPEYQEHCRKEVQE

LLRDREPKEIEWEDLAQLPFLTMCIKESLRLHPPAPFLSRHCIQDIVLPDGRIIPKGNIC

SINVFAIHHNPSVWSDPEVYDPFRFDPKNTQKRSPLAFIPFSAGPRNCIGQAFAMAEMKV

VLALTLLRFCFLPDTEPRRQPDLILRAEHGLWLRVEPLETSPQ

>302563559

MSLLSLSWLGLGPVAASPWLLLLLVGASWLLACILMWTYTFYDNCRRLRCFPQPPKQNWF

LGHLGLVTPTEEGLRVLTQLVATYPQGFVKWMGPIFPVINLYHPDTIRSVINTSDAITDK

DVVFYKMLKPWLGDGLLLSAGDKWRHHRRMLTPAFRFNILKPYIKIFNKSTNIMHAKWQR

LALEGSTRLDMFEHISLMALDSLQKCIFSIDSHCQEKPSEYIAAIMEISALIVKRNHKFF

LYKDFLYFLTPCGRRFLRACRLVHDFTDAVIQERRRTLPSQGVDDFLQAKAKSKTLDFID

VLLLSKDKNGKELSDEDIRAEADTFMFGGHDTTASGLSWVLYNLARHPEYQERCRQEVQE

LLKDREPKEIEWDDLAQLPFLTMCLKESLRLYPPVPTITRGCTQDVVLPDSRVIPKGNIC

SINIFAMHHNPSVWPDPEVYDPFRFDPENIQKRSPMAFIPFSAGPRNCIGQSFAMAEIKV

VLALTLLRFRILPDHREPRRTPEIILRAEDGLWLRVEPLG

>332253666

MLLLSLSWLGLGPVAASPWLLLLLVGASWLLVRILAWTYAFYDNCRRLRCFPQPPKQNWF

LGHLGLVTPTEEGLRVLTQLVATYPQGFVRWLGPIFPVINLCHPDTVRSVINTSDAITDK

DIIFYKTLKPWLGDGLLSSVGDKWRHHRRMLMPAFHFNILKPYIKIFSKSANIMHDKWQR

LAMEGNTRLDVFEHISLMTLDSLQKCIFSFDSNCQEKPSEYIATIMELSALVVKRNSQFF

WYKDFLYFLTPYGRRFRRACRLVHDFTDAVIQERRRTLTSQGVDDFLQAKAKCKTLDFID

VLLLSEDKNGKGLSDEDIRAEADTFMFAGHDTTASGLSWVLYNLARYPEYQERCRQEVQE

LLKDSEPKEIEWDDLAQLPFLTMCLKESLRLHPPVPTFSRGCTQDVVLPDSRVIPKGNVC

SINIFAIHHNPSVWPDPEVYDPFRFDPENAQKRSPMAFIPFSAGPRNCIGQTFAMAEMKV

VLALTLLRFRILPDHREPRRTSEIVLRAEDGLWLRVEPLD

>334327010

MVVLLQLLGQAGLPGIEQVPKVVLKNMELPLSVYGHIRMLPDSEWLSRQLGLAVLWPGVS

SVLLLVFLAISTWVLSRVITSVHRYYINCQRLRCFPQPPLQNWFLGHLRLIDSNEEGLIR

TVQLVNTFREIFLMWMGPFHPLVILCHPDYIRPLTSASANIAPKDKIFYGFLEPWLGDGL

LLSKGDKWSHHRRLLTPAFHFDILKSYVKIFNQSTNIMHEKWKHLCAGSSTHLDMFEHIS

LMTLDSLQKCIFSYDSGCQKKSNPYISAILELSSLVACRSKQLLFFWDSLYYLTSQGRRF

SRACHLVHDFSDAVIQARRRILDKHGCDSYFSSKDKGKTMDFIDVLLLAKDENGNTLSDK

DIQAEADTFMFEGHDTTASGISWALYNLAQHQEHQDRCRQEIQELLRGRHPEEIEWDDLS

QMPFLSMCIKESLRLHPPVVTVTRFCTKDIQLPDGRIIPKGNRCLINIFGTHHNPSVWTN

PEVYDPYRFDSNKSQKMSPLAFIPFSAGPRNCIGQNFAMSEMKIVLALTLLQFRVFSDGF

LPQRKPELILRAEGGLWLRVEPILNSSSPGLHRLETPQE

>327264045

MLPLMDWILENLTLEKASFQMVAVSGLLLLVLAALLHLLLKGFGFCYHYYVLCQKLRCFS

EPPRVNWFLGHLGLVYPNEEGMVTVAKLVATFSPAFLTWMGPFLPIVSLVHPDYIKPVVT

ASAAVAPKDKLLYGFLKPWLGDGLLLSSGKKWARHRRMLTPAFHFDILKPYMKIFNQSTD

IMHAKWRRLTTAGPTSIDMFEHISLMTLDSLQKCVFSYNSNCQEKPSDYIASILLLSSLV

TRRPHKLYNHIDWIYYLTSDGRRFRHACEVVHHFTADVIQKRRMALRDLGQNASLKPKQE

KTLDFIDVLLMAKDEEGCHLSDEDIAAEADTFMFEGHDTTASGLSWVLYNLAQHPHYQDQ

CREEIQELLHDRDIEEIEWEDLSQMPFTTMCIKESLRLHPAVTAISRSCNEDVKLPSGQI

IPKGVICLISIYGTHHNPAVWPEPEIYNPHRFDPAASKQQSPLAFVPFSAGPRNCIGQNF

AMAELKVTLALTLLRFVVRLDKSQTVRRKPELILRAENGLWLQVEPLPAEP

>354485171

MQHLDLSWLGLGPMSAAPWLLFVLIGASWLLARCLIWIFTLYEKCRRLRGFPQPPKRNWF

WGHLGTSPPTEEGMKEMTMRVATYPQGFMTWLGPVVPLIHLCHPDIIRSVVSASAAVAPK

DDNLYSFLKPWLGDGLLLSAGDKWSRHRSMLTPAFHFNILKPYVKIFNDSTNIMHAKWQR

LTSGGSTHLDMFEHISLMTLDTLQKCVFSFNSNCQEKPSEYIAAILELSALVVKRNEQLL

LHLDMLYRLTPDGRRFYKACRLVHDFTDAVIQERRRTLPSHGGDDVIKAKSKSKTLDFID

VLLLSKDEDGKELSDEDIRAEADTFMFRGHDTTASGLSWILYNLAKHPEYQERCRQEVQE

LFKGRESMDIEWDDLAQMPFLTMCIKESLRLHPPVTLASRCCTEDISLPDGRVIPKGVIC

IINIFGTHHNPTVWRDPEVYDPFRFDPENMQGRSPLAFIPFSAGPRNCIGQTFAMNEMKV

ALALTLLRFLVLPDDKEPHRKPELILRAEGGLWLRVEPLSSQ

>332853568

MPQLSLSSLGLWPMAASPWLLLLLVGASWLLARILAWTYTFYDNCRRLRCFPQPLKRNWF

LGHLGLVTPTEQGMRVLTQLVATYPQGFKVWMGPIFPVIRFCHPNIIRSVINASAAIVPK

DKVFYSFLKPWLGDGLLLSAGEKWSRHRRMLTPAFHFNILKPYMKIFNESVNIMHAKWQL

LASEGSARLDMFEHISLMTLDSLQKCVFSFDSHCQEKPSEYIAAILELSALVTKRHQQIL

LYIDFLYYLTHDGQRFRRACRLVHDFTDAVIQERRRTLPSQGVDDFLQAKAKSKTLDFID

VLLLSKDEDGKKLSDEDIRAEADTFMFEGHDTTASGLSWVLYHLAKHPEYQERCRQEVQE

LLKDREPKEIEWDDLAHLPFLTMCMKESLRLHPPVPAVSRCCTQDIVLPDGRVIPKGIIC

LISVFGTHHNPAVWPDPEVYDPFRFDPKNIKERSPLAFIPFSAGPRNCIGQAFAMAEMKV

VLGLTLLRFRVLPDHTEPRRKPELVLRAEGGLWLRVEPLS

>402904604

MSLLSLHWLGLGPVATSPGLLLMLVAGSWLLARVLAWIYAFYDNCRRLQCFPQPPKRHWF

WGHLGLITPTEEGLKNLTQLSATYSHGFMIWMGPIIPFIVLCHPDTIRSITNASAAIAPK

DDLSIRFLKPWLGEGILLSGGDKWSHHRRMLTPAFHFNILKPYIKIFNKSATIMLDKWQH

LASEGSSRLDMFEHISLMTLDSLQKCIFSFDSRCQERPSEYIATILELSALVDKRSQHIL

QYMDFLYYLTQDGRRFRRACRLVHDFTDAVIQERRRTLPTQGIDDFLKDKAKSKTLDFID

VLLLSKDEDGKALSDEDIRAEADTFMFAGHDTTASGLSWVLYNLARHPEYQERCRQEVQE

LLKDRDPKEIEWDDLAQLPFLTMCLKESLRLHPPAPFISRRCAQDVVLPDGRVIPKGNIC

VINIIALHHNPTVWPDPEVYDPFRFDPENSKERSPLAFIPFSAGPRNCIGQAFAMAEMKT

VLALMLLHFRFLPDHTEPRRKPELILRAEGGLWLRVEPLNAGLQ

>296233173

MSQLSLSWLGLGPVAASPWLLLLLVGVSWLLAHVLTWTYTFYDNCRRLQCFPQPPKLNWF

WGHQGLATPTEEGVKKFIQLVATYPQSFKLWLGPTFPLLILCHPDTVRSITSASAAVAPK

DMLFYGFLKPWLGDGLLLSAGDKWSRHRRMLTPAFHFNILKPYVAIFNKSVSIMHDKWQR

LASEGSTRLDMFEHISLMTLDSLQKCVFSFDSNCQEKPSKYIATILELSAFVEKRNQQIL

LHSDFLYYLTPDGQRFRRACRLVHDFTDAVIQERRRTLPTQGTDDFFKDKAKSKTLDFID

VLLLSKNEDGRELSDEDIRAEADTFMFGGHDTTASGLSWVLYHLSKHPEYQECCRQEVQE

LLKDREPIEIEWDDLAKLPFLTMCIKESLRLHPPVPAITRRCTQDMVLPDGRVIPKGVAC

LISIIGIHYNPTVWPDPEVYDPFRFDPEKSKERSPLAFIPFSAGPRNCIGQVFAMAEMKV

VLALTLLRFLVLPDCTEPRRKPEVIMRAEGGLWLRLESLGANPQ

>402904594

MSLLSLSWLGLGPVAASPWLLLLLVGASWLLACILRWTYTFYDNCRRLRCFPQPPKQNWF

LGHLGLVTPTEEGLRVLTQLVAAYPQGFVKWMGPIFPVINLYHPDTVRSVINTSDAITDK

DVVFYKMLKPWLGDGLLLSAGDKWRHHRRMLTPAFRFNILKPYIKIFNKSTNIMHAKWQR

LALEGSTRLDMFEHISLMALDSLQKCIFSIDSHCQEKPSEYIAAIMEISALIVKRNHKFF

LYKDFLYFLTPCGRRFLRACRLVHDFTDAVIQKRRRTLTSQGVDDFLQAKAKSKTLDFID

VLLLSKDKNGKELSDEDIRAEADTFMFGGHDTTASGLSWVLYNLARHPEYQERCRQEVQE

LLKDREPKEIEWDDLAQLPFLTMCLKESLRLYPPVPTITRGCTQDVVLPDSRVIPKGNIC

SINIFAMHHNPSVWPDPEVYDPFRFDPENIQKRSPMAFIPFSAGPRNCIGQSFAMAEIKV

VLALTLLRFRILPDHREPRRTPEIILRAEDGLWLRVEPLG

>302563729

MSLLSLPWLGLGPVATSPGLLLMLVVGSWLLARVLAWTYAFYDNRRRLQCFPQPPKQNWF

WGHLGLITPTEEGLKNLTQISATYSQGFMIWMGPIIPFIVLCHPDTIRSITNASAAIAPK

DALSIRFLKPWLGEGILLSGGDKWSHHRRMLTPAFHFNILKPYIKIFNKSATIMLDKWQH

LASEGSSRLDMFEHISLMTLDSLQKCIFSFDSRCQERPSEYIATILELSALVEKRSQHIL

QYMDFLYYLTQDGRRFRRACRLVHDFTDAVIQERRRTLPTQGIDDFLKDKAKSKTLDFID

VLLLSKDEDGKALSDEDIRAEADTFMFAGHDTTASGLSWILYNLARHPEYQERCRQEVQE

LLKDRDPKEIEWDDLAQLPFLTMCLKESLRLHPPAPFISRHCTQDIVLPDGRVIPKGIIC

VINITGVHHNPTVWPDPEVYDPYRFDPENSKERSPLAFIPFSAGPRNCIGQAFAMAEMKT

VLALMLLHFRFLPDHTEPRRKPELILRAEGGLWLRVEPLSAGLQ

>EDL89477.1

MELVLGSPGHASVALKEVIFYSFLKPWLGDGLLLSDGDKWSCHRRMLTPAFHFNILKPYV

KIFNDSTNIMHAKWQDLASGGSARLDMFKNISLMTLDSLQKCVFSFDSNCQEKPSEYISA

ILELSALVAKRYQQLLLHTDSLYQLTHNGRRFHKACKLVHNFTDAVIQGRRRALPSQHED

DILKAKARSKTLDFIDVLLLTKDEDGKELSDEDIRAEADTFMFEGHDTTASGLSWILYNL

ARHPEYQERCRQEVRELLRDRESTEIEWDDLAQLPFLTMCIKESLRLHPPVTVISRRCTQ

DIVLPDGRVIPKGVICIINIFATHHNPTVWPDPEVYDPFRFDPENIKDRSPLAFIPFSAG

PRNCIGQTFAMNEMKVALALTLLRFRVLPDDKEPRRKPELILRAEGGLWLRVEPLSTQ

>CAH93059.1

MSLLSLPWLGLRPVATSPWLLLLLVVGSWLLARVLAWTYAFYNNCRRLQCFPQPPKRNWF

WGHLGLITPTEEGLKNSTQMSATYSQGFTIWLGPIIPFIVLCHPDTIRSITNASAAIAPK

DDLSIRFLKPWLGEGILLSGGDKWSRHRRMLTPAFHFNILKSYITIFNKSANIMLDKWQH

LASEGSSRLDMFEHISLMTLDSLQKCIFSFDSHCQERPSEYIATILELSALVEKRSQHIL

QHMDFLYYLSHDGRRFHRACRLVHDFTDAVIRERRRTLPTQGIGDFLKDKAKSKTLDFID

VLLLSKDEDGKALSDEDIRAEADTFMFAGHDTTASGLSWVLYNLARHPEYQERCRQEVQE

LLKDRDPKEIEWDDLVQLPFLTMCVKESLRLHPPAPFISRRCTQDIVLPDGRVIPKGIIC

VINIIGVHHNPTVWPDPEVYDPFRFDPENSKERSPLAFIPFSAGPRNCIGQAFAMVEMKV

VLALMLLHFLFLPDHTEPRRKPELIMRAEGGLWLRVEPLNVGLQ

>CAH91950.1

MSLLSLPWLGLRPVATSPWLLLLLVVGSWLLARVPAWTYAFYNNCRRLQCFPQPPKRNWF

WGHLGLITPTEEGLKNSTQMSATYSQGFTIWLGPIIPFIVLCHPDTIRSITNASAAIAPK

DDLSIRFLKPWLGEGILLSGGDKWSRHRRMLTPAFHFNILKSYITIFNKSANIMLDKWQH

LASEGSSRLDMFEHISLMTLDSLQKCIFSFDSHCQERPSEYIATILELSALVEKRSQHIL

QHMDFLYYLSHDGRRFHRACRLVHDFTDAVIRERRRTLPTQGIDDFLKDKAKSKTLDFID

VLLLSKDEDGKALSDEDIRAEADTFMFAGHDTTASGLSWVLYNLARHPEYQERCRQEVQE

LLKDRDPKEIEWDDLAQLPFLTMCVKESLRLHPPAPFISRRCTQDIVLPDGRVIPKGIIC

VINIIGVHHNPTVWPDPEVYDPFRFDPENSKERSPLAFIPFSAGPRNCIGQAFAMVEMKV

VLALMLLHFLFLPDHTEPRRKPELIMRAEGGLWLRVEPLNVGLQ

>344283161

MSLLNLSWLGLWPGAASLWLLLLLAVASWPLAHFLAQTYTFYDICRRLRCFPQPPMRNWF

LGHLGLVSPTEEGLMEVTRLVATYPQGFKTWLGPINPIITLCHPDIIQPVVYASAAIAPK

NAKFYGFLKPWLGDGLLLSYGDKWSLHRRMLTPAFHFNILKPYMKIFNQSTNIMHAKWKH

LISGGSTRLDMFEHISLMTLDSLQKCVFSFDSHCQEKPSEYIAAILELSALAVKRNQQIL

LHSDFLYYFTPSGWRYRRACRMVHGFTDAAIQERRHTLSEQGVDDFLKAKAKAKTLDFID

VLLLSKDEDGKELSDEDIRAEADTFMFEGHDTTASGISWVLYNLAKHREYQERCRQEVRE

LLRDRQLEEIEWDDLTQLPFLTMCIKESLRMYPSVTAISRCCTQDIVLPDGRVIPKGVIC

LISIFGTHHNPSVWPNPEVYDPFRFDVQNTEKRSPLAFIPFSAGPRNCIGQTFAMTEMKV

VLALTLLRFRVLPDDAEPRRKPELILRAEGGLWLRVEPLGAGLQ

>EHH59310.1

MWTYTFYDNCRRLRCFPQPPKQNWFLGHLGLVTPTEEGLRVLTQLVATYPQGFVKWMGPI

FPVINLYHPDTIRSVINTSDAITDKDVVFYKMLKPWLGDGLLLSAGDKWRHHRRMLTPAF

RFNILKPYIKIFNKSTNIMHAKWQRLALEGSTRLDMFEHISLMALDSLQKCIFSIDSHCQ

EKPSEYIAAIMEISALIVKRNHKFFLYKDFLYFLTPCGRRFLRACRLVHDFTDAVIQERR

RTLPSQGVDDFLQAKAKSKTLDFIDVLLLSKDKNGKELSDEDIRAEADTFMFGGHDTTAS

GLSWVLYNLARHPEYQERCRQEVQELLKDREPKEIEWDDLAQLPFLTMCLKESLRLYPPV

PTITRGCTQDVVLPDSRVIPKGNICSINIFAMHHNPSVWPDPEVYDPFRFDPENIQKRSP

MAFIPFSAGPRNCIGQSFAMAEIKVVLALTLLRFRILPDHREPRRTPEIILRAEDGLWLR

VEPLG

>EDL40309.1

MLPLTDYLLQLLGLEKTAFRVYVVSALLLLVLFFFFRLLVRAFKLFSDFRITCRKLSCFP

EPPGRHWLLGHMSMYLPNEKGLQNEKKVLDTMHHIILAWVGPFLPLLVLVHPDYIKPVLG

ASAAIAPKDEFFYSFLKPWLGDGLLISKGNKWSRHRRLLTPAFHFDILKPYMKIFNQCTN

IMHAKWRRHLAEGSVTSFDMFEHISLMTLDSLQKCVFSYNSDCQERMSDYISSIIELSAL

VVRRQYRLHHYLDFMYYLTADGRRFRQACDTVHNFTTEVIQERRQALRQQGAEAWLKAKQ

GKTLDFIDVLLLAKDEEGKELSDEDIRAEADTFMFEGHDTTSSGLSWALFNLAKYPEYQE

KCREEIQEVMKGRELEELDWDDLTQLPFTTMCIKESLRQFPPVTLISRRCTEDIKLPDGR

VIPKGIICLVSIYGTHHNPIVWPDSKVYNPYRFDPDTPQQRSPLAFVPFSAGPRNCIGQS

FAMAEMRVVVALTLLRFRLSVDRTHKVRRKPELILRTENGLWLNVEPLPSRA

>EHH29755.1

MSLLSLPWLGLGPVATSPGLLLMLVVGSWLLARVLAWTYAFYDNRRRLQCFPQPPKQNWF

WGHLGLITPTEEGLKNLTQISATYSQGFMIWMGPIIPFIVLCHPDTIRSITNASAAIAPK

DALSIRFLKPWLGEGILLSGGDKWSHHRRMLTPAFHFNILKPYIKIFNKSATIMLDKWQH

LASEGSSRLDMFEHISLMTLDSLQKCIFSFDSRCQERPSEYIATILELSALVEKRNQHIL

QYMDFLYYLTQDGRRFRRACRLVHDFTDAVIQERRRTLPTQGIDDFLKDKAKSKTLDFID

VLLLSKDEDGKALSDEDIRAEADTFMFAGHDTTASGLSWILYNLARHPEYQERCRQEVQE

LLKDRDPKEIEWDDLAQLPFLTMCLKESLRLHPPAPFISRHCTQDIVLPDGRVIPKGIIC

MINIIGVHHNPTVWPDPEVYDPYRFDPENSKERSPLAFIPFSAGPRNCIGQAFAMAEMKT

VLALMLLHFRFLPDHTEPRRKPELTMRAEGGLWLRVEPLSAGLQ

>354485175

MLQLSLSWLGLGPMAASLWHLLLLGGASWILARILAWSYVFYDNCCRLRCFPQPPKPNWF

WGHLAMVSSNEEGMQFAAHLGHYFRDIHLCWVGPVYPVLRLIHPDTIALLLQAPAAVAPK

EMTLYGLLKPWLGDGLLLSSGGKWSHHRRMLTPAFHFDILKPYVKIFNKSVNIMHAKWQR

LTAKGIARLDMFEHISLMTLDSLQKCIFSFDSNCQESPSKYIAAILELSSLIMKRHRQLF

LHMDFLYYLTPDGRRFRKACDLVHDFTDAVIRERRRTLNSQGVDEFVKAKAKSKTLDFID

VLLMAKDEHGKELSDEDVRAEADTFMFGGHDTTASGLSWILYNLARHPEYQERCRQEVRE

LLRDREPQEIEWDDLAQLPFLTMCIKESLRLHPPVTMILRCCTQDIVLPDGRVIPKGNVC

AISIFGVHHNPSVWPDPEVYDPFRFDPETPQKRSPLAFIPFSAGPRNCIGQTFAMNEMKV

ALALTLLRFRVLLDDKEPRRQPELILRAEGGLWLRVEPLSSGAQ

>AAI45756.1

MLPLTDYLLQLLGLEKTAFRVYVVSALLLLVLFFFFRLLVRAFKLFSDFRITCRKLSCFP

EPPGRHWLLGHMSMYLPNEKGLQNEKKVLDTMHHIILAWVGPFLPLLVLVHPDYIKPVLG

ASAAIAPKDEFFYSFLKPWLGDGLLISKGNKWSRHRRLLTPAFHFDILKPYMKIFNQCTN

IMHAKWRRHLAEGSVTSFDMFEHISLMTLDSLQKCVFSYNSDCQERMSDYISSIIELSAL

VVRRQYRLHHYLDFMYYLTADGRRFRQACDTVHNFTTEVIQERRRALRQQGAEAWLKAKQ

GKTLDFIDVLLLAKDEEGKELSDEDIRAEADTFMFEGHDTTSSGLSWALFNLAKYPEYQE

KCREEIQEVMKGRELEELDWDDLTQLPFTTMCIKESLRQFPPVTLISRRCTEDIKLPDGR

VIPKGIICLVSIYGTHHNPIVWPDSKVYNPYRFDPDTPQQRSPLAFVPFSAGPRNCIGQS

FAMAEMRVVVALTLLRFRLSVDRTHKVRRKPELILRTENGLWLNVEPLPSRA

>62651806

MLPITDYLLYLLGLEKTAFRVYVLSALLLFLLFLLFRLLLQAFKLFSDFRITCRRLSCFP

EPPGRHWLLGHMSMYLPNEKGLQNEKKVLDTMHHIILAWVGPFLPLLVLVHPDYIKPVLG

ASAAIAPKDEFFYSFLKPWLGDGLLISKGNKWSRHRRLLTPAFHFDILKPYMKIFNQSVN

IMHAKWRRHLAEGSVTSFDMFEHVSLMTLDSLQKCVFSYSSDCQEKLSDYISSIIELSAL

VVRRQYRLHHYLDFIYYLTADGRRFRQACDTVHNFTTEVIQQRRRALRELGAEAWLKAKQ

GKTLDFIDVLLLAKDEEGKELSDEDIRAEADTFMFEGHDTTSSGLSWALFNLAKYPEYQD

KCREEIQEVMKGRELEELDWDDLTQLPFTTMCIKESLRQFPPVTLISRRCTEDIKLPDGR

IIPKGIICLVSIYGTHYNPLVWPDSKVYNPYRFDPDIPQQRSPLAFVPFSAGPRNCIGQS

FAMAEMRVVVALTLLRFRLSVDRTRKVRRKPELILRTENGLWLNVEPLPSRAGVPRGPTE

PEVQAPPAQA

>BAH12844.1

MCCWWVGPWHAIVRIFHPTYIKPVLFAPAAIVPKDKVFYSFLKPWLGDGLLLSAGEKWSR

HRRMLTPAFHFNILKPYMKIFNESVNIMHAKWQLLASEGSARLDMFEHISLMTLDSLQKC

VFSFDSHCQEKPSEYIAAILELSALVTKRHQQILLYIDFLYYLTPDGQRFRRACRLVHDF

TDAVIQERRRTLPSQGVDDFLQAKAKSKTLDFIDVLLLSKDEDGKKLSDEDIRAEADTFM

FEGHDTTASGLSWVLYHLAKHPEYQERCRQEVQELLKDREPKEIEWDDLAQLPFLTMCIK

ESLRLHPPVPAVSRCCTQDIVLPDGRVIPKGIICLISVFGTHHNPAVWPDPEVYDPFRFD

PKNIKERSPLAFIPFSAGPRNCIGQAFAMAEMKVVLGLTLLRFRVLPDHTEPRRKPELVL

RAEGGLWLRVEPLS

>AAZ29447.1

MSLLSLPWLGLGPVATSPGLLLMLVVGSWLLARVLAWTYASYDNRRRLQCFPQPPKQNWF

WGHLGLITPTEEGLKNLTQISATYSQGFMIWMGPIIPFIVLCHPDTIRSITNASAAIAPK

DALSIRFLKPWLGEGILLSGGDKWSHHRRMLTPAFHFNILKPYIKIFNKSATIMLDKWQH

LASEGSSRLDMFEHISLMTLDSLQKCIFSFDSRCQERPSEYIATILELSALVEKRSQHIL

QYMDFLYYLTQDGRRFRRACRLVHDFTDAVIQERRRTLPTQGIDDFLKDKAKSKTLDFID

VLLLSKDEDGKALSDEDIRAEADTFMFAGHDTTASGLSWILYNLVRHPEYQERCRQEVQE

LLKDRDPKEIEWDDLAQLPFLTMCVKESLRLHPPAPFISRHCTQDIVLPDGRVIPKGIIC

VINITGVHHNPTVWPDPEVYDPYRFDPENSKERSPLAFIPFSAGPRNCIGQAFAMAEMET

VLALMLLHFRFLPDHTEPRRKPELILRAEGGLWLRVEPLSAGLQ

>397484951

MLPITDRLLHLLGLEKTAFRIYAVSTLLLFLLFFLFRLLLRFLRLCRSFYITCRRLRCFP

QPPRRNWLLGHLGMYLPNEAGLQDEKKVLDNMHNVLLVWMGPVLPLLVLVHPDYIKPLLG

ASAAIAPKDDLFYGFLKPWLGDGLLLSKGDKWSRHRRLLTPAFHFDILKPYMKIFNQCTD

IMHAKWRHLAEGSAVSLDMFEHISLMTLDSLQKCVFSHNSNCQEKMSDYISAIIELSALS

VRRQYRLHHYLDFIYYRSADGRRFRQACDMVHHFTTEVIQERRRALRQQGAEAWLKAKQG

KTLDFIDVLLLARDEDGKELSDEDIRAEADTFMFEGHDTTSSGISWLLFNLAKYPEYQEK

CREEIQEVMKGRELEELEWDDLTQLPFTTMCIKESLRQYPPVTLVSRQCTEDIKLPDGRI

IPKGIICLVSIYGTHHNPTVWPDSKVYNPYRFDPDNPQQRSPLAYVPFSAGPRNCIGQSF

AMAELRVVVALTLLRFRLSVDRTRKVRRKPELILRTENGLWLKVEPLPPRA

>AAK15012.1

MLRLSVSGLDLGSVVTSSWHLLLLGVASWILARILAWTYSFYENCSRLSCFPQPPKKNWF

SGHLGMIQSNEEGMQLVTEMGQTFQDVHLFWLGPVIPVLRIVDPAFVAPLLQAPALVAPK

DMTFLRFLKPWLGDGLFLSSGDKWSRHRRLLTPAFHFDILKPYVKIFNQSVNIMHAKWKH

LSSEGSARLEMFEHISLMTLDSLQKCLFGFDSNCQESPSEYISAILELSSLIIKRSLQLF

LFVDFLYYHTADGRRFRKACDLVHNFTDAVIRERRHTLSSQNHDEFLKSKTKSKTLDFID

VLLLAKDEHGKELSDEDIRAEADTFMFGGHDTTASALSWILYNLARHPEYQERCRQEVQE

LLRDREPEEIEWDDLAQLPFLTMCIKESLRLHSPVIDLLRRCTRDIVLPDGRVIPKGNIC

VISIFGIHHNPSVWPDPEVYDPFRFDPENPQKRSPLAFIPFSAGPRNCIGQTFAMSEMKV

ALALTLLRFRILPDDKEPRRKPEIILRAEGGLWLRVEPLSKGAQ

>395847816

MPQLSLSWLGLGPVAASPWTLLLLVGASWLLARILAWTYTFYDNYRHLRCFPQPPKRSWF

WGHQGMIQSSEEGILYTQGLASTFGAVCLWQVGPLHAVISIFHSTFIRPVLFAPANIALK

DLVFYSFLKPWLGDGLLLSAGDKWSRHRRMLTPAFHFNILKPYMKIFNESANIMHAKWQR

LVSQGSDNLDMFEHISLMTLDSLQKCVFSFDSNCQEKPSEYIAAILELSALVAERQQQVL

LHLDFLYHLTPDGWRFRKACKLVHDFTDAVIQERRCSLPSQGIDEFLKTKAKTKTLDFID

VLLLSKDEDGKGLSDEDIRAEADTFMFEGHDTTASGLSWVLYHLAKHPEYQERCRQEVQE

LLRDREPLEIEWDDLAQLPFLTMCIKESLRLHPPVPVISRYCTQNIELPDGRVIPKGVIC

LLSIFGTHHNPAVWPDPEVYDPSRFDPENIKERSPLAFVPFSAGPRNCIGQTFAMTEMKV

ILALTLLRFRILPDNDEPRRKPELVLRAEGGLWLRVEPLSGGQQ

>BAH12428.1

MRVLTQLVATYPQGFKVWMGPILPVIRFCHPNIIRSVINASAAIVPKDKVFYSFLKPWLG

DGLLLSAGEKWSRHRRMLTPAFHFNILKPYMKIFNESVNIMHAKWQLLASEGSARLDMFE

HISLMTLDSLQKCVFSFDSHCQEKPSEYIAAILELSALVTKRHQQILLYIDFLYYLTPDG

QRFRRACRLVHDFTDAVIQERRRTLPSQGVDDFLQAKAKSKTLDFIDVLLLSKDEDGKKL

SDEDIRAEADTFMFEGHDTTASGLSWVLYHLAKHPEYQERCRQEVQELLKDREPKEIEWD

DLAQLPFLTMCIKESLRLHPPVPAVSRCCTQDIVLPDGRVIPKGIICLISVFGTHHNPAV

WPDPEVYDPFRFDPKNIKERSPLAFIPFSAGPRNCIGQAFAMAEMKVVLGLTLLRFRVLP

DHTEPRRKPELVLRAEGGLWLRVEPLS

>114675863

MLPITDRLLHLLGLEKTAFRIYAVSTLLLFLLFFLFRLLLRFLRLCRSFYITCRRLRCFP

QPPRRNWLLGHLGMYLPNEAGLQDEKKVLDNMHNVLLVWMGPVLPLLVLVHPDYIKPLLG

ASAAIAPKDDLFYGFLKPWLGDGLLLSKGDKWSRHRRLLTPAFHFDILKPYMKIFNQCTD

IMHAKWRHLAEGSAVSLDMFEHISLMTLDSLQKCVFSHNSNCQEKMSDYISAIIELSALS

VRRQYRLHHYLDFIYYRSADGRRFRQACDMVHHFTTEVIQERRWALRQQGAEAWLKAKQG

KTLDFIDVLLLARDEDGKELSDEDIRAEADTFMFEGHDTTSSGISWMLFNLAKYPEYQEK

CREEIQEVMKGRELEELEWDDLTQLPFTTMCIKESLRQYPPVTLVSRQCTEDIKLPDGRI

IPKGIICLVSIYGTHHNPTVWPDSKVYNPYRFDPDNPQQRSPLAYVPFSAGPRNCIGQSF

AMAELRVVVALTLLRFRLSVDRTRKVRRKPELILRTENGLWLKVEPLPPRA

>Q9HCS2.2

MSLLSLPWLGLRPVATSPWLLLLLVVGSWLLARILAWTYAFYNNCRRLQCFPQPPKRNWF

WGHLGLITPTEEGLKNSTQMSATYSQGFTIWLGPIIPFIVLCHPDTIRSITNASAAIAPK

DNLFIRFLKPWLGEGILLSGGDKWSRHRRMLTPAFHFNILKSYITIFNKSANIMLDKWQH

LASEGSSCLDMFEHISLMTLDSLQKCIFSFDSHCQERPSEYIATILELSALVEKRSQHIL

QHMDFLYYLSHDGRRFHRACRLVHDFTDAVIRERRRTLPTQGIDDFFKDKAKSKTLDFID

VLLLSKDEDGKALSDEDIRAEADTFMFGGHDTTASGLSWVLYNLARHPEYQERCRQEVQE

LLKDRDPKEIEWDDLAQLPFLTMCVKESLRLHPPAPFISRCCTQDIVLPDGRVIPKGITC

LIDIIGVHHNPTVWPDPEVYDPFRFDPENSKGRSPLAFIPFSAGPRNCIGQAFAMAEMKV

VLALMLLHFRFLPDHTEPRRKLELIMRAEGGLWLRVEPLNVSLQ

>BAC04868.1

MLPITDRLLHLLGLEKTAFRIYAVSTLLLFLLFFLFRLLLRFLRLCRSFYITCRRLRCFP

QPPRRNWLLGHLGMYLPNEAGLQDEKKVLDNMHHVLLVWMGPVLPLLVLVHPDYIKPLLG

ASAATAPKDDLFYGFLKPWLGDGLLLSKGDKWSRHRRLLTPAFHFDILKPYMKIFNQSAD

IMHAKWRHLAEGSAVSLDMFEHISLMTLDSLQKCVFSYNSNCQEKMSDYISAIIELSALS

VRRQYRLHHYLDFIYYRSADGRRFRQACDMVHHFTTEVIQERRRALRRQGAEAWLKAKQG

KTLDFIDVLLLARDEDGKELSDEDIRAEADTFMFEGHDTTSSGISWMLFNLAKYPEYQEK

CREEIQEVMKGRELEELEWDDLTQLPFTTMCIKESLRQYPPVTLVSRQCTEDIKLPDGRI

IPKGIICLVSIYGTHHNPTVWPDSKVYNPYRFDPDNPQQRSPLAYVPFSAGPRNCIGQSF

AMAELRVVVALTLLRFRLSVDRTRKVRRKPELILRTENGLWLKVEPLPPRA

>BAB18270.1

MSLLSLPWLGLRLVAMSPWLLLLLVVGSWLLARILAWTYAFYNNCRRLQCFPQPPKRNWF

WGHLGLITPTEEGLKDSTQMSATYSQGFTVWLGPIIPFIVLCHPDTIRSITNASAAIAPK

DNLFIRFLKPWLGEGILLSGGDKWSRHRRMLTPAFHFNILKSYITIFNKSANIMLDKWQH

LASEGSSRLDMFEHISLMTLDSLQKCIFSFDSHCQERPSEYIATILELSALVEKRSQHIL

QHMDFLYYLSHDGRRFHRACRLVHDFTDAVIRERRRTLPTQGIDDFFKDKAKSKTLDFID

VLLLSKDEDGKALSDEDIRAEADTFMFGGHDTTASGLSWVLYNLARHPEYQERCRQEVQE

LLKDRDPKEIEWDDLAQLPFLTMCVKESLRLHPPAPFISRCCTQDIVLPDGRVIPKGITC

LIDIIGVHHNPTVWPDPEVYDPFRFDPENSKGRSPLAFIPFSAGPRNCIGQAFAMAEMKV

VLALMLLHFRFLPDHTEPRRKLELIMRAEGGLWLRVEPLNVGLQ

>333033782

MSLLSLPWLGLRPVATSPWLLLLLVVGSWLLARILAWTYAFYNNCRRLQCFPQPPKRNWF

WGHLGLITPTEEGLKNSTQMSATYSQGFTVWLGPIIPFIVLCHPDTIRSITNASAAIAPK

DNLFIRFLKPWLGEGILLSGGDKWSRHRRMLTPAFHFNILKSYITIFNKSANIMLDKWQH

LASEGSSRLDMFEHISLMTLDSLQKCIFSFDSHCQERPSEYIATILELSALVEKRSQHIL

QHMDFLYYLSHDGRRFHRACRLVHDFTDAVIRERRRTLPTQGIDDFFKDKAKSKTLDFID

VLLLSKDEDGKALSDEDIRAEADTFMFGGHDTTASGLSWVLYNLARHPEYQERCRQEVQE

LLKDRDPKEIEWDDLAQLPFLTMCVKESLRLHPPAPFISRCCTQDIVLPDGRVIPKGITC

LIDIIGVHHNPTVWPDPEVYDPFRFDPENSKGRSPLAFIPFSAGPRNCIGQAFAMAEMKV

VLALMLLHFRFLPDHTEPRRKLELIMRAEGGLWLRVEPLNVSLQ

>Q6NT55.1

MLPITDRLLHLLGLEKTAFRIYAVSTLLLFLLFFLFRLLLRFLRLCRSFYITCRRLRCFP

QPPRRNWLLGHLGMYLPNEAGLQDEKKVLDNMHHVLLVWMGPVLPLLVLVHPDYIKPLLG

ASAAIAPKDDLFYGFLKPWLGDGLLLSKGDKWSRHRRLLTPAFHFDILKPYMKIFNQSAD

IMHAKWRHLAEGSAVSLDMFEHISLMTLDSLQKCVFSYNSNCQEKMSDYISAIIELSALS

VRRQYRLHHYLDFIYYRSADGRRFRQACDMVHHFTTEVIQERRRALRQQGAEAWLKAKQG

KTLDFIDVLLLARDEDGKELSDEDIRAEADTFMFEGHDTTSSGISWMLFNLAKYPEYQEK

CREEIQEVMKGRELEELEWDDLTQLPFTTMCIKESLRQYPPVTLVSRQCTEDIKLPDGRI

IPKGIICLVSIYGTHHNPTVWPDSKVYNPYRFDPDNPQQRSPLAYVPFSAGPRNCIGQSF

AMAELRVVVALTLLRFRLSVDRTRKVRRKPELILRTENGLWLKVEPLPPRA

>DAA28167.1

MLELSLSWLGLGPVAASPWLLLLFTGASWLLARILAWTYTFYNNSRRLQCFPQPPKRNWF

LGHLGLVPPTEQGMSKLTELVAKYSQGFRIWMGPITPIIVFCHPDLIRIVANASAAVAPK

DVIFYEVLKPWLGDGLLLSAGDKWSRHRRMLTPAFHFNILKPYMKIFTKSTDIMHAKWQH

LIKEGHTHLDMFEHISLMTLDSLQKCIFSYDSNCQEKPSEYIAAILELSALVAKRHQEIF

LHMDFLYYLTPDGRRFRRACRLVHDFTDAVIQERHRTLPSESIDDFLKAKAKTKTLDFID

VLLLTKDEDGKGLSDEDIRAEADTFMFEGHDTTASGLSWILYNLAKHKEYQERCRQEVQE

LLKDREPKNIEWDDLAQLPFLTMCIKESLRLHPPVSVISRRCTQDTVLPDGRVIPKGVIC

LISIFGTHHNPSVWPDPEVFDPFRFDPENIKGRSPVAFVPFSVGPRNCIGQTFAMTEMKV

VLALTLLRFRVLPDKEEPRRKPELILRAEGGLWLRVEPLSTGQQ

>P51871.1

MLQLSLSRLGMGSLTASPWHLLLLGGASWILARILAWIYTFYDNCCRLRCFPQPPKPSWF

WGHLTLMKNNEEGMQFIAHLGRNFRDIHLSWVGPVYPILRLVHPNVIAPLLQASAAVAPK

EMTLYGFLKPWLGDGLLMSAGEKWNHHRRLLTPAFHFDILKSYVKIFNKSVNTMHAKWQR

LTAKGSARLDMFEHISLMTLDSLQKCIFSFDSNCQESNSEYIAAILELSSLIVKRQRQPF

LYLDFLYYLTADGRRFRKACDVVHNFTDAVIRERRSTLNTQGVDEFLKARAKTKTLDFID

VLLLAKDEHGKGLSDVDIRAEADTFMFGGHDTTASALSWILYNLARHPEYQERCRQEVRE

LLRDREPEEIEWDDLAQLPFLTMCIKESLRLHPPVLLISRCCSQDIVLPDGRVIPKGNIC

VISIFGVHHNPSVWPDPEVYNPFRFDPENPQKRSPLAFIPFSAGPRNCIGQTFAMSEIKV

ALALTLLRFCVLPDDKEPRRKPELILRAEGGLWLRVEPLSTVTSQLPWDLLAHPPTS

>BAG37237.1

MSLLSLSWLGLRPVAASPWLLLLVVGASWLLARILAWTYAFYHNGRRLRCFPQPRKQNWF

LGHLGLVTPTEEGLRVLTQLVATYPQGFVRWLGPITPIINLCHPDIARSVINTSDAITDK

DIVFYKTLKPWLGDGLLLSVGDKWRHHRRLLMPAFHFNILKPYIKIFSKSANIMHAKWQR

LAMEGSTCLDVFEHISLMTLDSLQKCIFSFDSNCQEKPSEYITAIMELSALVVKRNNQFF

RYKDFLYFLTPCGRRFHRACRLVHDFTDAVIQERRRTLTSQGVDDFLQAKAKSKTLDFID

VLLLSEDKNGKELSDEDIRAEADTFMFGGHDTTASGLSWVLCNLARHPEYQERCRQEVQE

LLKDREPKEIEWDDLAQLPFLTMCLKESLRSHPPIPTFARGCTQDVVLPDSRVIPKGNVC

NINIFAIHHNPSVWPDPEVYDPFRFDPENAQKRSPMAFIPFSAGPRNCIGQKFAMAEMKV

VLALTLLRFRILPDHREPRRTPEIVLRAEDGLWLRVEPLG

>291221931

MSDQLKYLMSWKDSTVMAVHQYASVVWVTICVVLVLNTLLRLAQLMRKRRRAERDLALFP

SPMRHWFFGHLLLMKNNEQSFLLLESFTKHHSTSRVSWTGPFEVTVILYHPSVIKPLLAS

TGAIEPKDDLFYGFLRPWLGDGLLLSRGKKWFRNRKLLTPGFHFDILKPYVQVFNECANK

MLDNWSGIYKSSGNSDGVTMEMFEHISLMTLDSLLRCIFSQESNCQMHRTHPYIQAVYAL

SYLVVERGRFPPFYSDFIYALTPSGYRHRQALKVLHGHTNAVIKERRETLKSEEAPGIGG

KEKKFIDFLDILLKAKDADGHGLTDKEIQDEVDTFMFEGHDTTASGISWCLYNLAKYKEH

QDRCREEVNDLLTIKDRTNIEWEDLGKLPYLTMTLKESLRLHPPVPMTGRMLTKPLVLPD

GRTIPPGYRVTVNADCLHRNQHVWKDPLVFDPLRFTPENKKTRSPHAYIPFSAGPRNCIG

QNFALNEMKVTIATILNRFELDVDESQPPNRTMRLVLRSINGIHLRVKPLTK

>395847822

MPQLSLSWLGLGPEGASPSLLLLLVGVSCILARILAWTYSFYDNFRRLRCFPQPPKRNWF

LGHLGLIQSSEEGLLYTQGLASTFGTLCLWRVGPLHTVIRIFHPTFIKPVLFAPANIALK

DMLYYSFLKPWLGDGLLLSAGDKWSRHRRMLTPAFHFNILKPYMKIFNESANIMHAKWQR

LVSEGSTHLDMFEHISLMTLDSLQKCVFSFDSNCQEKPSEYIAAILELSTLVAERHLQIL

LHLDFLYYLTSEGRRFRKACRLVHDFTDAVIQERRCTLSSQGIDDFLKAKAKTKTLDFID

VLLLSKDEDGKGLSDEDIRAEADTFMFEGHDTTASGLSWVLYHLARHPEYQERCRQEVQE

LLRDRKPKEIEWDDLAQLPFLTMCIKESLRLHPPVPLVSRCCTQDIELPDGRVIPKGVIC

LISIFGTHHNPAVWPDPEVYNPSRFNPENIKERSPLAFIPFSAGPRNCIGQTFAMTEMKV

VLALTLLRFCILCDHTEQRRKPELVLRAEGGLWLQVEPLSSGP

>EAW84492.1

MLAWETGGFLVNGLLANSQGFRMSLLSLPWLGLRPVAMSPWLLLLLVVGSWLLARILAWT

YAFYNNCRRLQCFPQPPKRNWFWGHLGLITPTEEGLKDSTQMSATYSQGFTVWLGPIIPF

IVLCHPDTIRSITNASAAIAPKDNLFIRFLKPWLGEGILLSGGDKWSRHRRMLTPAFHFN

ILKSYITIFNKSANIMLDKWQHLASEGSSRLDMFEHISLMTLDSLQKCIFSFDSHCQERP

SEYIATILELSALVEKRSQHILQHMDFLYYLSHDGRRFHRACRLVHDFTDAVIRERRRTL

PTQGIDDFFKDKAKSKTLDFIDVLLLSKDEDGKALSDEDIRAEADTFMFGGHDTTASGLS

WVLYNLARHPEYQERCRQEVQELLKDRDPKEIEWDDLAQLPFLTMCVKESLRLHPPAPFI

SRCCTQDIVLPDGRVIPKGITCLIDIIGVHHNPTVWPDPEVYDPFRFDPENSKGRSPLAF

IPFSAGPRNCIGQAFAMAEMKVVLALMLLHFRFLPDHTEPRRKLELIMRAEGGLWLRVEP

LNVGLQ

>AAH35350.1

MSLLSLPWLGLRPVAMSPWLLLLLVVGSWLLARILAWTYAFYNNCRRLQCFPQPPKRNWF

WGHLGLITPTEEGLKDSTQMSATYSQGFTVWLGPIIPFIVLCHPDTIRSITNASAAIAPK

DNLFIRFLKPWLGEGILLSGGDKWSRHRRMLTPAFHFNILKSYITIFNKSANIMLDKWQH

LASEGSSCLDMFEHISLMTLDSLQKCIFSFDSHCQERPSEYIATILELSALVEKRSQHIL

QHMDFLYYLSHDGRRFHRACRLVHDFTDAVIRERRRTLPTQGIDDFFKDKAKSKTLDFID

VLLLSKDEDGKALSDEDIRAEADTFMFGGHDTTASGLSWVLYNLARHPEYQERCRQEVQE

LLKDRDPKEIEWDDLAQLPFLTMCVKESLRLHPPAPFISRCCTQDIVLPDGRVIPKGITC

LIDIIGVHHNPTVWPDPEVYDPFRFDPENSKGRSPLAFIPFSAGPRNCIGQAFAMAEMKV

VLALMLLHFRFLPDHTEPRRKLELIMRAEGGLWLRVEPLNVSLQ

>ABM81736.1

MSLLSLPWLGLRPVAMSPWLLLLLVVGSWLLARILAWTYAFYNNCRRLQCFPQPPKRNWF

WGHLGLITPTEEGLKDSTQMSATYSQGFTVWLGPIIPFIVLCHPDTIRSITNASAAIAPK

DNLFIRFLKPWLGEGILLSGGDKWSRHRRMLTPAFHFNILKSYITIFNKSANIMLDKWQH

LASEGSSCLDMFEHISLMTLDSLQKCIFSFDSHCQERPSEYIATILELSALVEKRSQHIL

QHMDFLYYLSHDGRRFHRACRLVHDFTDAVIRERRRTLPTQGIDDFFKDKAKSKTLDFID

VLLLSKDEDGKALSDEDIRAEADTFMFGGHDTTASGLSWVLYNLARHPEYQERCRQEVQE

LLKDRDPKEIEWDDLAQLPFLTMCVKESLRLHPPAPFISRCCTQDIVLPDGRVIPKGITC

LIDIIGVHHNPTVWPDPEVYDPFRFDPENSKGRSPLAFIPFSAGPRNCIGQAFAMAEMKV

VLALMLLHFRFLPDHTEPRRKLELIMRAEGGLWLRVEPLNVSLQ

>BAE26998.1

MLRLSVSGLDLGSVVTSSWHLLLLGVASWILARILAWTYSFYENCSRLSCFPQPPKKNWF

SGHLGMIQSNEEGMQLVTEMGQTFEDVHLFWLGPVIPVLRIVDPAFVAPLLQAPALVAPK

DMTFLRFLKPWLGDGLFLSSGDKWSRHRRLLTPAFHFDILKPYVKIFNQSVNIMHAKWKH

LSSEGSARLEMFEHISLMTLDSLQKCLFGFDSNCQESPSEYISAILELSSLIIKRSLQLF

LFVDFLYYHTADGRRFRKACDLVHNFTDAVIRERRHTLSSQNHDEFLKSKTKSKTLDFID

VLLLAKDEHGKELSDEDIQAEADTFMFGGHDTTASALSWILYNLARHPEYQERCRQEVQE

LLRDREPEEIEWDDLAQLPFLTMCIKESLRLHSPVIDLLRRCTRDIVLPDGRVIPKGNIC

VISIFGIHHNPSVWPDPEVYDPFRFDPENPQKRSPLAFIPFSAGPRNCIGQTFAMSEMKV

ALALTLLRFRILPDDKEPRRKPEIILRAEGGLWLRVEPLSKGAQ

>AAG33247.1

MSLLSLPWLGLRPVAMSPWLLLLLVVGSWLLARILAWTYAFYNNCRRLQCFPQPPKRNWF

WGHLGLITPTEEGLKDSTQMSATYSQGFTVWLGPIIPFIVLCHPDTIRSITNASAAIAPK

DNLFIRFLKPWLGEGILLSGGDKWSRHRRMLTPAFHFNILKSYITIFNKSANIMLDKWQH

LASEGSSRLDMFEHISLMTLDSLQKCIFSFDSHCQERPSEYIATILELSALVEKRSQHIL

QHMDFLYYLSHDGRRFHRACRLVHDFTDAVIRERRRTLPTQGIDDFFKDKAKSKTLDFID

VLLLSKDEDGKALSDEDIRAEADTFMFGGHDTTASGLSWVLYNLARHPEYQERCRQEVQE

LLKDRDPKEIEWDDLAQLPFLTMCVKESLRLHPPAPFISRCCTQDIVLPDGRVIPKGITC

LIDIIGVHHNPTVWPDPEVYDPFRFDPENSKGRSPLAFIPFSAGPRNCIGQAFAMAEMKV

VLALMLLHFRFLPDHTEPRRKLELIMRAEGGLWLRVEPLNVGLQ

>348552029

MLPVTHHLLDLLGLEKTAFRACAVSALLLALLFSLFRGLLGFLQLCWAFRRTCRRLRCFR

QPQRCHWLLGHLGMYLPNEKGLQDEKKVLDEIHDVILVWAGPVLPVLVLVHPDYIKPVLS

ASAAVAPKDEFFYSFLKPWLGDGLLLSKGDKWSRHRRLLTPAFHFDILKPYMKIFNQCTH

TMHSKWRRQLGQKPVASFDMFEHVSLMTLDSLQKCVFSYNSDCQEKMSDYISAIIELSAL

VVRRQYRLHHYLEALYYLSADGRRFRRACAVVHRFTTEVIQERRRALSRQGAEGWLKAKQ

GRTLDFIDVLLLARDEDGNELSDEDIRAEADTFMFEGHDTTSSGLSWVLFNLAKYPEYQE

KCREEIQEIMKGRELEELEWDDLTQLPFTTMCIKESLRQFPPVTLVSRRCTQDIALPDGR

VIPKGIICMINIYGTHHNPTVWPDAKVYNPYRFDPVNPQQRSPLAYVPFSAGPRNCIGQS

FAMAEMRVAVALTLLRFRLSVDRTRKVRRKPELILRTENGIWLNVEPLPRQGAQAPPLRA

HAPPPRN

>EHH29753.1

MSLLSLSWLGLGSVAASPWLLLLLVGASWLLACILMWTYTFYDNCRRLRCFPQPPKQNWF

LGHLGLVTPTEEGLRVLTQLVATYPQGFVKWMGPIFPVINLYHPDTIRSVINTSDAITDK

DVVFYKMLKPWLGDGLLLSAGDKWRHHRRMLTPAFRFNILKPYIKIFNKSTNIMHAKWQR

LALEGSTRLDMFEHISLMALDSLQKCIFRIDSHCQEKPSEYIAAIMEISALIVKRNHKFF

LYKDFLYFLTPCGRRFLRACRLVHDFTDAVIQERRRTPPSQGVDDFLQAKAKSKTLDFID

VLLLSKDKNGKELSDEDIRAEADTFMFGGHDTTASGLSWVLYNLARHPEYQERCRQEVQE

LLKDREPKEIEWDDLVQLPFLTMCLKESLRLYPPVPTITRGCTQDVVLPDSRVIPKGNIC

SINIFAMHHNPSVWPDPEVYDPFRFDPENIQKRSPMAFIPFSAGPRNCIGQSFAMAEIKV

VLALTLLRFRILPDHREPRRTPEIILRAEDGLWLRVEPLG

>296233167

MRVLTQLVATYPQGFKVWMGPFTLIIRLCHPNIIRSVINASVIFLFLPCIHQFLSQHLLF

PLPSDPLSWCSGVPRDLNPRLSPGGTPKLKEMDLDRDIPNFMGSGKGQRIHSSEEGLLYT

QSLACTFGDVCCWWVGPWHAIVRIFHPTCIKPVLFAPAAIAPKDEVFYGFLKPWLGDGLL

LSAGDKWSRHRRMLTPAFHFNILKPYMKIFNESVNIMHGKWQRLASEGSARLDMFEHISL

MTLDSLQKCVFSSDSHCQEKPSEYIAAILELSALVVKRHQHLLLHTDFLYYLTPDGRRFR

RACRLVQDFTDAVIRERRRTLPSQGVDDLLQIKAKSKTLDFIDVLLLSKDEDGKELSDED

IRAEADTFMFEGHDTTASGLSWVLYHLAKHPEYQERCRQEVQELLKGREPKEIEWDDLAR

LPFLTMCMKESLRLHPPVPAVSRCCTQDIALPDGRVIPKGVICLISVFGTHHNPAVWPDP

EVYDPFRFDPENIKERSPLAFIPFSAGPRNCIGQAFAMAEMKVVLALTLLRFRVLPDHME

PRRKPELVLRAEGGLWLRVEPRS

>344283155

MSLLNLSWLGLWPGAASPWLLLLLAVASWLLARFLAWIYTFYDICRRLRNFPQPPVRNWF

LGHLGLGSPTEEGLMEVTHLVATYPQGFKTWMGPIRPIITLCHPEIIRPVINASAAIAPK

DVIFYSFLKPWLGDGLLLSGGDKWSRHRRMLTPAFHFNILKPYMKIFNESTNIMHAKWKH

LVSGGSTRLDMFEHISLMTLDSLQKCVFSFDSHCQEKPSEYIAAILELSALVSKRNQQIS

LHFDFLYHLTPDGRRFRRACRLVHDFTDAAIQERRRTLSEHGVDDFLKAKTKAKTLDFID

VLLLSKDEDGKELSDEDIRAEADTFMFEGHDTTASGISWVLYNLAKHPEYQERCRQEVRE

FLRDRQPEEIEWDDLTQLPFLTMCIKESLRMYPSVTVIARCCTQDIVFPDGRIIPKGVIC

LISIFGTHHNPSVWPNPEVYDPFRFNLDNSEKRSPLAFIPFSAGPRNCIGQTFAMTEMKV

VLALTLLRFRVLPDDAEPRRKPELILRAEDGLWLRVEPLGGGQK

>DAA28219.1

MLPITEQLLHLLGLEKTAFRLYAVSGLLLTLLFFLFRLLLQFLRLCWGFYITCRRLRCFP

QPPRRNWLLGHLGMYLPNERGLQDEKKVLDDMHHVILVWLGPVLPLVVLVHPDYIKPLVG

ASAAVAPKDDLFYGFLKPWLGDGLLLSRGDKWSRHRRLLTPAFHFDILKPYMKIFNQCAD

TMHAKWRGLAEGSEVSLDMFEHVSLMTLDCLQKCVFSYNSNCQEKMSDYISAIIELSALV

VRRQYRLHHYLDFIYYRTADGRRFRQACDTVHGFTTEVIQERRRALRQQGAEAWFKAKQG

KTLDFIDVLLLAKDEDGKELSDEDIRAEADTFMFEGHDTTSSGLSWVLFNLAKYPEYQEK

CREEIQEVMKGRELEELEWDDLTQLPFTTMCIKESLRQFPPVTLVSRRCTEDIKLPDGRI

IPKGIICLVSIYGTHYNPAVWPDSKVYNPYRFDPDNPQQRSPLAYVPFSAGPRNCIGQSF

AMAEMRVAVALTLLRFRLSVDRTRKVRRKPELILRTENGIWLKVEPLPPRA

>EGV95494.1

MKEMTMRVATYPQGFMTWLGPVVPLIHLCHPDIIRSVVSASAAVAPKDDNLYSFLKPWLG

DGLLLSAGDKWSRHRSMLTPAFHFNILKPYVKIFNDSTNIMHAKWQRLTSGGSTHLDMFE

HISLMTLDTLQKCVFSFNSNCQEKPSEYIAAILELSALVVKRNEQLLLHLDMLYRLTPDG

RRFYKACRLVHDFTDAVIQERRRTLPSHGGDDVIKAKSKSKTLDFIDVLLLSKDEDGKEL

SDEDIRAEADTFMFRGHDTTASGLSWILYNLAKHPEYQERCRQEVQELFKGRESMDIEWD

DLAQMPFLTMCIKESLRLHPPVTLASRCCTEDISLPDGRVIPKGVICIINIFGTHHNPTV

WRDPEVYDPFRFDPENMQGRSPLAFIPFSAGPRNCIGQTFAMNEMKVALALTLLRFRVLP

DKEPRRKPELILRAEGGLWLRVEPLINGAQ

>ACI33584.1

MAVIDAVLDRLLDGLLALGRLLSPLCSLFVLLQLSALVVLLLLSLRVVCLLWSHAQFTRR

LRCFSKPPTQNWLMGHLGEMRSTEEGLQAVDQMVRTYSHSCSWFLGPFYSLVRLFHPDYI

KPLLLAPASITVKDELFYGFLRPWLGQSLLLSNGEDWSRRRRLLTPAFHFDILKNYVKIF

NHSSNIMHSKWRHLVAEGESRQDMFSHISLMTLDSLLRCTFSYNSNCQESSSEYIAAIFE

LSTLVIERRGRILHHWDWLYWRSPEGQRFKQACNVVHRFTRTVVQERRAQLLHQGEPESH

TDTTGGEEKRKRVADFIDLLLLSKDEEGHGLTDEEIKAEADTFMFGGHDTTASGISWVLY

NLSQHQDYQDRCRAEVNDLLQDRETEDLDWEDLSSLPFTTMCIKESLRLHSPVSAVTRRY

TKDITVPGGRVIPQGSICLVSIYGTHHNPEIWPDPDVYNPMRFDPENSKDRSSHAFIPFS

SGPRNCIGQKFAMAELRVVVALTLRRFHLTPGGVEVRRLPQLVLRAEGGLWLTLETLDTP

QD

>335282854

MLPITEYILHLLGLEKTAFRVYALSGLLLSLLFFLFRLLLQFLRFCWCFYITCRRLRCFP

QPPRRNWLLGHLGMYLPNEMGLRAEKKVLDNMHHVILVWIGPVLPLLVLVHPDYIKPVVG

ASAAIAPKDDLFYGFLKPWLGDGLLLSKGDKWSRHRRMLTPAFHFDILKPYMKIFNQCTN

IMHAKWRRLAEGSVVSLDMFEHVSLMTLDSLQKCVFSYNSNCQEKMSDYISAIIQLSALV

VQRQYRLHLHIDFIYYCTAEGRRFRKACDTVHRFTTEVIQERRRALRQQGAEAWLKAKQG

KNLDFIDVLLLARDEDGKELSDEDIRAEADTFMFEGHDTTSSGLSWVLFNLAKYPEYQEK

CREEIQEVMKGRELEELEWDDLTQLPFTTMCIKESLRQFPPVTLVTRRCTEDIKLPDGRV

IPKGIICLISIYGIHYNPRVWPDSKVYNPYRFDPDNPQERSALAYLPFSAGPRNCIGQNF

AMAEMRVAVALTLLRFRLSVDRTHKVRRKPELILRTENGIWLKVEPLPPRA

>EDL40302.1

MLRLSVSGLDLGSVVTLSWHLLLLGAASWILARILAWTYSFCENCSRLRCFPQPPKRNWF

LGHLGMIEHNEEGMQLMTEAGQTYRDVHLCWLGPVIPVLRIVDPAFVAPLLLASALVAPK

DTTFHTFVKPWLGDGLFLNSGDKWSRHRRLLTPAFHFDILKPYVKIFNQSVNIMHAKWKH

LSSEGSARLEMFEHISLMTLDSLQKCLFGFDSNCQESPSEYISAILELSSLVIKRSHQLF

LFVDFLYYHTADGRRFRKACDLVHNFTDAVIRERRHTLSSQNHDEFLKSKTKSKTLDFID

VLLLAKDEHGKELSDEDIRAEADTFMFGGHDTTASALSWILYNLARHPEYQERCRQEVQE

LLRGREPQEIEWDDLAQLPFLTMCIKESLRLHPPVIDLLRRCTRDIVLPDGRVIPKGNIC

VISIFGIHHNPSVWPDPEVYDPFRFDPENAHKRPPLAFIPFSAGPRNCIGQTFAMNEMMV

ALALTLLRFRILPDDKEPRRKPEIILRAEGGLWLRVEPLSTVLKDSKPDLEASLPHPPIV

LSGSQN

>EDL40308.1

MLPLTDYLLQLLGLEKTAFRVYVVSALLLLVLFFFFRLLVRAFKLFSDFRITCRKLSCFP

EPPGRHWLLGHMSMYLPNEKGLQNEKKVLDTMHHIILAWVGPFLPLLVLVHPDYIKPVLG

ASAAIAPKDEFFYSFLKPWLGDGLLISKGNKWSRHRRLLTPAFHFDILKPYMKIFNQCTN

IMHAKWRRHLAEGSVTSFDMFEHISLMTLDSLQKCVFSYNSDCQEYVWPPGRMSDYISSI

IELSALVVRRQYRLHHYLDFMYYLTADGRRFRQACDTVHNFTTEVIQERRQALRQQGAEA

WLKAKQGKTLDFIDVLLLAKDEEGKELSDEDIRAEADTFMFEGHDTTSSGLSWALFNLAK

YPEYQEKCREEIQEVMKGRELEELDWDDLTQLPFTTMCIKESLRQFPPVTLISRRCTEDI

KLPDGRVIPKGIICLVSIYGTHHNPIVWPDSKVYNPYRFDPDTPQQRSPLAFVPFSAGPR

NCIGQSFAMAEMRVVVALTLLRFRLSVDRTHKVRRKPELILRTENGLWLNVEPLPSRA

>354485163

MQSLSLSWLGMGPVLASSWHLLLLFGASWLVARILAWTYSFYEKCARLRCFPQAPKRNWF

LGHLGTIQNNEEGMRMVTEMGKTFRDVQLCWLGPVIPVLKLVDPAFIAPLLQAPALVAPK

DMTFLGFLKPWLGDGLFLSSGDKWSQRRRLLTPAFHFDILKPYVKIFNKSVNIMHAKWKR

LASEGNACLEMFENISLMTLDSLQKCLFGLDSNCQESPSEYIAAILELSALVAKKYQQVF

LYVDFLYYLTADGRRFHKACDLVHDFTDAVIRERRHILSSQSVDEFLKSKAKSKTLDFID

VLLLAKDEHGKELSDEDIRAEADTFMFGGHDTTASGLSWTLYNLAKHPEYQERCRQEVQE

LLRDREPQEIEWDDLAQLPFLTMCIKESLRLHPPVTDLVRGCIQDIVLPDGRIIPKGSAC

VISIFGVHHNPSVWPDPEIYDPFRFDPENPQKRSPLSFIPFSAGPRNCIGQTFAVNEMKV

ALALTLLRFRVLPDKEPRRQPELILRAEGGLWLRMEPLSSQ

>DAA28217.1

MLELSLSWLGLRPVAASPWLLLVLVGASWILAHVLAWTYTFYNNSRHLQCFPQPPKRNWF

LGHMGQYPPTEKGLRDFTQLVANYPQGFRICLGPAIPIIIFCHPDMIRIVANASAAAAPK

DVIFYDFLKPWLGDGLLLSAGDKWSRHRRMLTPAFHFNILKPYMKIFTKSADIMHAKWQR

LITEGHTHLDMFEHISLMTLDSLQKCVFSYDSNCQEKPNEYIASILELSALVAKRNQQIF

LHMDFLYFLTPDWRRFRRACRLVHDFTDAVIQERRHTLPSEGTDDFLKAKVKTKTLDFID

VLLLTKDEDGKGLSDEDIRAEADTFMFEGHDTTASGLSWVLYNLAKHPEYQERCRQEVQD

LLRDRESKEIEWDDLAQLPFLTMCIKESLRLHPPVSVISRRYAQDTLLPDGRVIPKGVIC

LINIIGTHHNPSVWPDPEVYDPFRFEPENIKGRSPLAFIPFSVGPRNCIGQTFAMTEMKV

VLALTLLRFRVLPGEEPRRKPELILRAEGGLWLRVEPLSADQQ

>ABG81479.1

MLELSLSRLGLGPLAASPWLLPLLAGVSWILARVLAWTYTFYNNSRRLRCFLQPPKPNWF

LGHMNLVPSTEQGLIYFTQMAANYPRGYLIWFGPIIPMVIFCHPDMLRSITNASAAIAPK

DMQFYGTLKPWLGDGLLLSAGDKWSSHRRMLTPAFHFNILKPYIKIFTKSAEIMHAKWEH

LITEGHTHLDMFEHISLLTLDNLQKCVFSFDSNCQEKPSEYIAAILELSALVSKRNQQLF

LYMDFLYYLTSDGQRFRNACRLVHDFTDAVIQERRRTLPKENIDDFLKAKAKTKTLDFID

VLLLTKDEDGKGLSDEDIRAEADTFMFEGHDTTASGLSWILYNLAKHPEYQERCRQEVQD

LLRDRESKEIEWDDLAQLPFLTMCIKESLRLHPPVTSISRRCTQDIVLPDGRVIPKGVVC

LIDIFGTHHNPSVWQDPEVYDPFRFDPENIKGRSPLAFIPFSAGPRNCIGQTFAMTEMKV

ILALTLLRFRVLPDKEPCRKPELILRTEGGLWLRVEPLSAGQQ

>402904588

MLPITDHLLHLLGLEKTTFRMYAVSSLLLLLLFFLFRLLLRFLRLCRSFYITCRRLRCFP

QPPRRNWLLGHLGMYLPNEAGLQDEKKVLDNMHHILLVWMGPVLPLLVLVHPDYIKPILG

ASAAIAPKDDLFYGFLKPWLGDGLLLSKGDKWSRHRRLLTPAFHFDILKPYMKIFNQCTD

IMHAKWRRLAEGSVVSLDMFEHISLMTLDSLQKCVFSYNSNCQEKMSDYISAIIELSALA

VRRQYRLHHYLDFIYYRSADGRRFRQACDMVHHFTTEVIQERRRALRQQGSEAWLKSKQG

KTLDFIDVLLLARDEDGKELSDEDIRAEADTFMFEGHDTTSSGLSWVLFNLAKYPEYQEK

CREEIQEVMKGRELEELEWDDLTQLPFTTMCIKESLRQYPPVTLVSRQCTEDIKLPDGRI

IPKGIICLVSIYGTHHNPTVWPDSKVYNPYRFDPDNPQQRSPLAYVPFSAGPRNCIGQSF

AMAEMRVVVALTLLRFRLSVDRTRKVRRKPELILRTESGIWLKVEPLPPRA

>354485161

MLPITDYLLHLLGLEKTAFRVYAVSALLLSLLFFLFRLLLQLFKLCWDFRTTCRRLSCFP

EPPGRHWLLGHVSMYLPNEKGLQNEKKVLDAMHHIILAWVGPFLPLLVLVHPDYIKPVLG

ASAAIAPKDEFFYSFLKPWLGDGLLLSKGNKWSRHRRLLTPAFHFDILKPYMKIFNQCTD

VMHAKWRRHLSEGSVASFDMFEHVSLLTLDSLQKCVFSYNSDCQERMSDYISSIIELSAL

VVRRQYRLHHYLDFIYYLTADGRRFRQACDTVHKFTTEVIQERRRALREQGAGAWLKAKQ

GKTLDFIDVLLLAKDEEGKELSDEDIRAEADTFMFEGHDTTSSGLSWALFNLAKYPEYQE

KCRKEIQEVMKGRELEELDWDDLTQLPFTTMCIKESLRQFPPVTLISRRCTEDIKLPDGR

IIPKGIICLLSIYGTHYNPLVWPDSKVYNPYRFDPDIPQQRSPLAYVPFSAGPRNCIGQS

FAMAEMRVVVALTLLRFRLSVDRTRKVRRKPELILRTENGIWLNVEPLPPRA

>EGV95491.1

MSPRMLPITDYLLHLLGLEKTAFRVYAVSALLLSLLFFLFRLLLQLFKLCWDFRTTCRRL

SCFPEPPGRHWLLGHVSMYLPNEKGLQNEKKVLDAMHHIILAWVGPFLPLLVLVHPDYIK

PVLGASAAIAPKDEFFYSFLKPWLGDGLLLSKGNKWSRHRRLLTPAFHFDILKPYMKIFN

QCTDVMHAKWRRHLSEGSVASFDMFEHVSLLTLDSLQKCVFSYNSDCQERMSDYISSIIE

LSALVVRRQYRLHHYLDFIYYLTADGRRFRQACDTVHKFTTEVIQERRRALREQGAGAWL

KAKQGKTLDFIDVLLLAKDEEGKELSDEDIRAEADTFMFEGHDTTSSGLSWALFNLAKYP

EYQEKCRKEIQEVMKGRELEELDWDDLTQLPFTTMCIKESLRQFPPVTLISRRCTEDIKL

PDGRIIPKGIICLLSIYGTHYNPLVWPDSKVYNPYRFDPDIPQQRSPLAYVPFSAGPRNC

IGQSFAMAEMRVVVALTLLRFRLSVDRTRKVRRKPELILRTENGIWLNVEPLPPRA

>403308901

MLPLTDHLLHHLGLEKTAFRVYAVSALLLFLLFFLFRLLLRLLRLCQSFYITCRRLRCFP

QPPRRNWLLGHLGMYLPNEAGLQHEKKVLDSMHHVLLVWMGPVLPLLVLVHPDYIKPLLG

ASAAIAPKDDLFYGFLKPWLGDGLLLSRGDKWSRHRRLLTPAFHFDILKPYMKIFNQCTD

TMHAKWRRLAEGPVVSLDMFEHVSLMTLDSLQKCVFSYNSNCQEKMSDYISAIIELSALA

VRRQYRLHHYLDFIYYRTADGRRFRQACDMVHHFTTEVIQERRRALHQQGAEVWLKAKQG

KTLDFIDVLLLARDEDGKELSDEDIRAEADTFMFEGHDTTSSGLSWVLFNLAKYPEYQEK

CREEIQEVMKGRELEELEWDDLTQLPFTTMCIKESLRQYPPVTLVSRRCTEDIKLPDGRI

IPKGIICLVSIYGTHHNPTVWPDSKVYNPYRFDPDNPQQRSPLAYVPFSAGPRNCIGQSF

AMAEMRVVVALTLLRFRLSVDRTRKVRRKPELILRTENGIWLKVEPLPPRA

>347543818

MLQLSLSWLGLGPVSASPWLLLQLVGATWLLSRILSWIYDFYDNCCRLRCFPQPPKRNWF

WGHQDLAKSNEECLQLMEDVGHYFHDVYLWWLGPFYPILRLVHPNFVAPLLQAPATIIHK

DMFLYIFLKPWLGVGLLLSAGEKWSHQRRLLTPAFHFDILKSYVKIFNRSADIMHAKWQH

LASEGHTRLDMFEHISLMTLDSLQKCVFSFDSNCQEKPSEYIAAISELSALVTKRNEQIF

LHKDFLYYLTPEGRRFRKACRLVHDFTDAVIQERRRTLISQGSQDFLRAKAKAKTLDFID

VLLLAKDEDGKGLSDEDIQAEADTFMFAGHDTTASGLSWVLYNLAKHPEYQERCRQEVQE

LLRDREPKEIEWDELAQLPFLTMCIKESLRLHPPVTGISRCCTQDVVLPDGRIIPKGNIC

VISIFGIHHNPSVWPDPEVYNPFRFDPESPQKRSPLAFIPFSAGPRNCIGQTFAMTEMKV

VLALTLLRFRVLPDEEEPRRKPEIVLRAEGGLWLRVEPLRGGQQ

>73986392

MLPITEHLLHLLGMEKTAFRLYAVSMLLLSLLFFSFRLLMQFLRFCWRFYITCRRLSCFP

QPPRRNWLLGHLGMYLPNETGLQDEKKVLDNMHHVILVWIGPVLPLLVLVHPDYIKPLVG

ASAAIAPKDDLFYGFLKPWLGDGLLLSKGDKWSRHRRLLTPAFHFDILKPYMKIFNQCTD

IMHGKWRRLAQGSVISLDMFEHVSLMTLDSLQKCVFSYNSNCQEKMSDYISAIIELSALV

VRRQYRLHHHIDFIYYLTADGRRFRQACDTVHRFTTEVIQERRQALRQQGAETWLKGKQG

KTLDFIDVLLLARDEDGRELSDEDIRAEADTFMFEGHDTTSSGLSWVLFNLAKYPEYQEK

CREEIQEVMKGRELEELEWDDLTQLPFTTMCIKESLRQFPPVTLVSRRCTEDIKLPDGRI

IPKGIICLVSIYGTHHNPTVWPDSKVYNPYRFDPDNPQQRSPLAYVPFSAGPRNCIGQSF

AMAEMRVAVALTLLRFRLSVDRTRKVRRKPELILRTESGIWLNVEPLPPRA

>332253680

MRVLTQLVATYPQGFKVWMGPIFPVIRFCHPNIIRSVINASAAIVPKDKVFYSFLKPWLG

DGLLLSAGDKWSRHRRMLTPAFHFNILKPYMKIFNESVNIMHAKWQLLASEGSARLDMFE

HISLMTLDSLQKCVFSFDSHCQEKPSEYIAAILELSALVAKRHQQILLYIDFLYYLTHDG

QRFRRACRLVHDFTDAVIQERRRTLPSQGVDDFLQAKAKSKTLDFIDVLLLSKDEDGKKL

SDEDIRAEADTFMFEGHDTTASGLSWVLYHLAKHPEYQECCRQEVQELLKDREPKEIEWD

DLAQLPFLTMCIKESLRLHPPVPAVSRCCTQDVVLPDGRIIPKGVICLISIFGTHHNPAV

WPDPEVYDPFRFDPKNIKERSPLAFIPFSAGPRNCIGQAFAMAEMKVVLGLTLLRFRVLP

DHTEPRRKPELVLRAEGGLWLRVEPLS

>302563521

MLPITDHLLHLLGLEKTTFRIYAVSTLLLLLLFFLFRLLLRFLRLCRSFYITCRRLRCFP

QPPRRNWLLGHLGMYLPNEAGLQDEKKVLDNMHHILLVWMGPVLPLLVLVHPDYIKPILG

ASAAIAPKDDLFYGFLKPWLGDGLLLSKGDKWSRHRRLLTPAFHFDILKPYMKIFNQCTD

IMHAKWRRLAEGSVVSLDMFEHISLMTLDSLQKCVFSYNSNCQEKMSDYISAIIELSALA

VRRQYRLHHYLDFIYYRSADGRRFRQACDMVHHFTTEVIQERRRALRQQGAEAWLKSKQG

KTLDFIDVLLLARDEDGKELSDEDIRAEADTFMFEGHDTTSSGLSWVLFNLAKYPEYQEK

CREEIQEVMKGRELEELEWDDLTQLPFTTMCIKESLRQYPPVTLVSRQCTEDIKLPDGRI

IPKGIICLVSIYGTHHNPTVWPDSKVYNPYRFDPDNPQQRSPLAYVPFSAGPRNCIGQSF

AMAEMRVVVALTLLRFRLRVDRTRKVRRKPELILRTESGIWLKVEPLPPRA

>EHH59307.1

MLPITDHLLHLLGLEKTTFRIYAVSTLLLLLLFFLFRLLLRFLRLCRSFYITCRRLRCFP

QPPRRNWLLGHLGMYLPNEAGLQDEKKVLDNMHHILLVWMGPVLPLLVLVHPDYIKPILG

ASAAIAPKDDLFYGFLKPWLGDGLLLSKGDKWSRHRRLLTPAFHFDILKPYMKIFNQCTD

IMHAKWRRLAEGSVVSLDMFEHISLMTLDSLQKCVFSYNSNCQEKMSDYISAIIELSALA

VRRQYRLHHYLDFIYYRSADGRRFRQACDMVHHFTTEVIQERRRALRQQGAEAWLKSKQG

KTLDFIDVLLLARDEDGKELSDEDIRAEADTFMFEGHDTTSSGLSWVLFNLAKYPEYQEK

CREEIQEVMKGRELEELEWDDLTQLPFTTMCIKESLRQYPPVTLVSRQCTEDIKLPDGRI

IPKGIICLVSIYGTHHNPTVWPDSKVYNPYRFDPDNPQQRSPLAYVPFSAGPRNCIGQSF

AMAEMRVVVALTLLRFRLSVDRTRKVRRKPELILRTESGIWLKVEPLPPRA

>194238911

MPWLSLSWLGLGPLAASPWLLLLLVGASWLLARVLAWTYTFYDNCCRLRCFPQPPKRNWF

WSHLGLVTPTEQGMRVVTQLVANYPKGFVTWLGPVIPLVTLCHPDMVRTVLSASAAIAPK

DMVFYSFLKPWLGDGLLLSGGDKWSRHRRMLTPAFHFNILKSYVRIFNDSVNIMHAKWKR

LASEGSAHLDMFEHISLMTLDSLQKCVFSFDSDCQEKPSEYISGVLELSALVRKRDEQIF

LYMDSLYHLTSEGQRFRRVCRLVHDFTDSIIQERRRTLPDQGLDDLLKAKAKVKTLDFID

VLLLAKDEDGKELSDEDIRAEVDTFMFAGHDTTASGLSWVLYNLARHPEHQERCRQEVRE

LLRDREPKEIEWDDLAQLPFLTMCIKESLRLHPPAVGISRRCTQDIVLPDGRVIPKGVTC

VISIFGTHHNPSVWPDAEVYDPFRFDPENTKERSPLAFIPFSAGPRNCIGQTFAMTEMKV

VLALTLLRFRVLPAGEEPRRKPELILRAEGGLWLRVEPLSAGPQ

>301792134

MSQLSLSWLGLGQLAASPWLLLLLAGATWLLARVLAWSYSLYDNCCRLRCFPQPPKRNWL

WGHVGLVKSNEEGLRMIEDLGHCFRDVHLWWIGPFYPVLRLVHPTFVAPLLQAPATIIPK

DMFFYNFLKPWLGDGLLLSAGDKWSHHRRLLTPAFHFEILKPYVKVFNKSAGIMHAKWQC

LALEGSAHLDMFEHISLMTLDSLQKCVFSFDSNCQESPSEYIAAILELSSLVVKRQEQIF

LHMDFLYNLTPDGRHFRRACNLVHNFTDAVIRERRRALISGGSHDFLKAKAKAKTMDFID

VLLLAKDEDGKRLSDEDIRAEADTFMFEGHDTTASGLSWVLYNLAKHPEYQERCRQEVQE

LLRDREPQEIEWDDLAQLRFLTMCIKESLRLHPPVTVIARHCTQDVRLPDGRVIPKGNIC

VISIFGIHHNPSIWPDPEVYNPFRFDPETPQKRSPLAFIPFSVGPRNCIGQTFAMTEMKV

VLALTLLRFRVLPDEEEPRRKPELILRAEGGLWLRVEPLSAGPR

>P51870.1

MPWLTVSGLDLGSVVTSTWHLLLLGAASWILARILAWTYSFCENCSRLRCFPQSPKRNWF

LGHLGTIQSNEEGMRLVTEMGQTFRDIHLCWLGPVIPVLRLVDPAFVAPLLQAPALVAPK

DTTFLRFLKPWLGDGLFLSSGDKWSRHRRLLTPAFHFDILKPYVKIFNQSVNIMHAKWKH

LCLEGSARLEMFENISLMTLDSLQKCLFGFDSNCQESPSEYISAILELSSLIIKRSQQLF

LYLDFLYYRTADGRRFRKACDLVHNFTDAVIRERRRLLSSQGTDEFLESKTKSKSKTLDF

IDVLLLAKDEHGKELSDEDIRAEADTFMFGGHDTTASALSWILYNLARHPEYQERCRQEV

WELLRDREPEEIEWDDLAQLPFLTMCIKESLRLHPPAIDLLRRCTQDIVLPDGRVIPKGN

ICVISIFGIHHNPSVWPDPEVFDPFRFDSENRQKRSPLSFIPFSAGPRNCIGQTFAMNEM

KVVVALTLLRFRVLPDDKEPRRKPEIILRAEGGLWLRMEPLSTDTQ

>EHH29750.1

MLPITDHLLHLLGLEKTTFRIYAVSTLLLLLLFFLFRLLLRFLRLCRSFYITCRRLRCFP

QPPRRNWLLGHLGMYLPNEAGLQDEKKVLDNMHHILLVWMGPVLPLLVLVHPDYIKPILG

ASAAIAPKDDLFYGFLKPWLGDGLLLSKGDKWSRHRRLLTPAFHFDILKPYMKIFNQCTD

IMHAKWRRLAEGSVVSLDMFEHISLMTLDSLQKCVFSYNSNCQEKMSDYISAIIELSALA

VRRQYRLHHYLDFIYYRSADGRRFRQACDMVHHFTTEVIQERRRALRQQGAEAWLKSKQG

KTLDFIDVLLLARDEDGKELSDEDIRAEADTFMFEGHDTTSSGLSWVLFNLAKYPEYQEK

CREEIQEVMKGRKLEELEWDDLTQLPFTTMCIKESLRQYPPVTLVSRQCTEDIKLPDGRI

IPKGIICLVSIYGTHHNPTVWPDSKVYNPYRFDPDNPQQRSPLAYVPFSAGPRNCIGQSF

AMAEMRVVVALTLLRFRLSVDRTRKVRRKPELILRTESGIWLKVEPLPPRA

>149759130

MLQLSLSWLGLGPVAASPWLLLLLGGASWLLAHVLAWTYTFYDNCCRLRCFPQPPKRNWF

WGHLGLAKSNEEGLRLVEDLGHYFRDVHLWWMGPLYPILRLVHPKFVAPLLQAPAAIAPK

DMYFYSFLKPWLGDGLLLSAGDKWSHHRRLLTPAFHFEILKSYIKIFNKSADIMHAKWKR

LASEGSAHLDMFEHISLMTLDSLQKCVFSFDSNCQENPSEYIAAILELSALVTKRHHQIF

MRMDFLYYLTPDGRRFRRACDLVHDFTDAVIQERRRTLFSQDSHDLLKAKAKTKTLDFID

VLLLAKDEDGKELSDEDIRAEADTFMFGGHDTTASGLAWVLYNLARHPEHQERCRQEVRE

LLRDREPKEIEWDDLAQLPFLTMCIKESLRLHPPVAAISRCCTQDVVLPDGRVIPKGNMC

VISIFGIHHNPSVWQDPEVYDPFRFNPENPQKRSPLAFIPFSAGPRNCIGQTFAMTEMKV

VLALTLLRFRVLPAGEEPRRKPELILRAEGGLWLRVEPLSAGPQ

>291413170

MLPITDHLLHLLGLENTTFRVYAVSALLLSFLFFLFRLLLWFLRLCWRFHSLCRQLRCFP

QPPRRNWLLGHLGMYLPNESGLQDEKKVLDSMHHVVLVWIGPVLPLLVLVHPDYIKPVVG

ASAAVAPKDDFFYGFLKPWLGDGLLLSKGNKWSRHRRLLTPAFHFDILKPYMKIFNQCTD

ILHAKWRRLADGSVVSLDMFEHISLMTLDSLQKCVFSYNSNCQEKMSDYISAIIELSALV

VRRQYRLHHYLDFVYYHLTADGRRFRQACSTVHRFTTEVIQERRRALSEQGPKAWLKAKQ

GKALDFIDVLLLARDEDGKELSDEDIRAEADTFMFEGHDTTSSGLSWVLFNLAKYPEYQE

KCREEIREVMKGRELEELQWDDLTQLPFTTMCIKESLRQFPPVTLVSRRCTEDVKLPDGR

IIPKGIICLVSIYGTHHNPTVWPDSKVYNPYRFDPDTPQQRSPLAYVPFSAGPRNCIGQS

FAMAEMRVVVALTLLRFRLSVDRTRKVRRKPELILRTENGIWLNVEPLPPRA

>114675869

MSLLSLPWLGLRQVAISPWLLLLLVVGSWLLTRILAWTYAFYNNCRRLQCFPQPPKRNWF

WGHLGLITPTEEGLKDSTQMSATYSQGFTIWLGPIIPFIVLCHPDTIRSITNASAAIALK

DDLSIRFLKPWLGDGLLLSGVDKWSRHRRMLTPAFHFNILKSYITIFNKSANIMLDKWQH

LASEGSSRLDMFEHISLMTLDSLQKCIFSFDSHCQERPSEYIATILELSALVEKRNQHIL

QHMDFLYYLSHDGRRFHRACRLVHDFTDAVIRERRRTLPTQGIDDFLKDKAKSKTLDFID

VLLLSKDEDGKALSDEDIRAEADTFMFGGHDTTASGLSWVLYNLARHPEYQERCRQEVQE

LLKDRDPKEIEWDDLAQLPFLTMCVKESLRLHPPAPFISRCCTQDIVLPDGRVIPKGITC

VINIIGVHHNPTVWPDPEVYDPFRFDPENSKGRSPLAFIPFSAGPRNCIGQAFAMAEMKV

VLALMLLHFRFLPDHTEPRRKPELIMRAEGGLWLRVEHLNVGLQ

>AAI29086.1

MPWLTVSGLDLGSVVTSTWHLLLLGAASWILARILAWTYSFCENCSRLRCFPQSPKRNWF

LGHLGTIQSNEEGMRLVTEMGQTFRDIHLCWLGPVIPVLRLVDPAFVAPLLQAPALVAPK

DTTFLRFLKPWLGDGLFLSSGDKWSRHRRLLTPAFHFDILKPYVKIFNQSVNIMHAKWKH

LCLEGSVRLEMFENISLMTLDSLQKCLFGFDSNCQESPSEYISAILELSSLIIKRSQQLF

LYLDFLYYRTADGRRFRKACDLVHNFTDAVIRERRRLLSSQGVDEFLESKTKSKSKTLDF

IDVLLLAKDEHGKELSDEDIRAEADTFMFGGHDTTASALSWILYNLARHPEYQERCRQEV

WELLRDREPEEIEWDDLAQLPFLTMCIKESLRLHPPAIDLLRRCTQDIVLPDGRVIPKGN

ICVISIFGIHHNPSVWPDPEVFDPFRFDSENRQKRSPLSFIPFSAGPRNCIGQTFAMNEM

KVVVALTLLRFRVLPDDKEPRRKPEIILRAEGGLWLRMEPLSTDTQ

>344282664

MLPITDHLLCLLGLEKTMFRRYAVSVILLSLLFFLFRLLLQFLCVCRHFYVTCRRLRCFP

QPPRRNWLLGHLGMYLPNEAGLRDETKLLNDMRHALLIWMGPVLPLVVLVHPDYIKPLLG

ASAAIAPKDDLFYGFLKPWLGDGLLLSKGDKWSRHRRLLTPAFHFDILKPYMKIFNQCTD

IMHAKWRRRAEGSVVSLDMFEHISLMTLDGLQKCVFSYNSNCQEKMSDYISAIIELSALV

VRRQYRVHHHLDFIYYLTADGRRFRQACDTVHHFTTEVIQQRRQALRKQGAEAWLKAKQG

KTLDFIDVLLLAKDEDGKELSDEDIRAEADTFMFEGHDTTSSGLSWVLFNLAKYPEYQEK

CRKEIQDILKGRELDELDWDDLTQLPFITMCIKESLRQFPPVTLVSRRCTEDVKLPDGRI

IPKGVICLVSIYGTHHNPLVWPDYKVYNPYRFDPENPQQRSPLAYVPFSAGPRNCIGQSF

AMAEMRVVVALTLLRFRLSVDRTRKVRRKPELILRAENGIWLNVEPLPPRA

>AAI02057.1

MLELSLSWLGLGPVAASPWLLLLFTGASWLLARILAWTYTFYNNSRRLQCFPQPPKRNWF

LGHLGLVPPTEQGMSKLTELVAKYSQGFRIWMGPITPIIVFCHPDLIRIVANASAAVAPK

DVIFYEVLKPWLGDGLLLSAGDKWSRHRRMLTPAFHFNILKPYMKIFTKSTDIMHAKWQH

LIKEGHTHLDMFEHISLMTLDSLQKCIFSYDSNCQEKPSEYIAAILELSALVAKRHQEIF

LHMDFLYYLTPDGRRFRRACRLVHDFTDAVIQEWHRTLPSESIDDFLKAKAKTKTLDFID

VLLLTKDEDGKGLSDEDIRAEADTFMFEGHDTTASGLSWILYNLAKHQEYQECCRQEVQE

LLKDREPKNIEWDDLAQLPFLTMCIKESLRLHPPVSVISRRCTQDTVLPDGRVIPKGVIC

LISIFGTHHNPSVWPDPEVFDPFRFDPENIKGRSPVAFVPFSAGPRNCIGQTFAMTEMKV

VLALTLLRFRVLPDKEEPRRKPELILRAEGGLWLRVEPLSTGQQ

>403308903

MLQLSLSWLGLRPVAESPWLLLLLAGASWLLARVLAWTYAFYSNCRRLRCFPQPPKHSWF

WGHMGLVQSNEDGMRLMEHLSHCFRDVHLWWFGPFFPVVRLIHPKFIAPVLQAPAEVAPK

DMTFYGFLKPWLGTGLLLKDGDNWRRQRRLLAPAFQFNILKPYVKIFNKSANIMHAKWQR

LALEGSARLDMFEHISLMTLDSLQKCIFSFDSNCQEKPSEYIAAILELSALIAKRYRQII

LYSDFLYFLTPNGRRFRRTCDIVHNFTDAIIQERRRTLASQSVDDFLQAKAKSRTLDFID

VLLLAKDENGKELSNEDIRAEADTFMFAGHDSTASGLSWVLYNLAKHPEYQEHCRQEVQE

LLKGRDPKEIEWDDLAQLPFLTMCLKESLRLHPPVPLISRRCIQDVVLPDGRVIPKGNIC

TISITGVHHNPSVWPDPEVYDPFRFSPENPQKRSPLAFIPFSAGNRNCIGQAFGMAEMKV

VLALTLLRFRVLPDHVEPRRKLELVLRAENGLWLRVEPLSADLQ

>397484941

MSLLSLPWLGLRQVAISPWLLLLLVVGSWLLTRILAWTYAFYNNCCRLQCFPQPPKRNWF

WGHLGLITPTEEGLKDSTQMSATYSQGFTIWLGPIIPFIVLCHPDTIRSITNASAAIALK

DDLSIRFLKPWLGDGLLLSGVDKWSRHRRMLTPAFHFNILKSYITIFNKSANIMLDKWQH

LASEGSSRLDMFEHISLMTLDSLQKCIFSFDSHCQERPSEYIATILELSALVEKRNQHIL

QHMDFLYYLSHDGRRFHRACRLVHDFTDAVIRERRRTLPTQGIDDFLKDKAKSKTLDFID

VLLLSKDEDGKALSDEDIRAEADTFMFGGHDTTASGLSWVLYNLARHPEYQERCRQEVQE

LLKDRDPKEIEWDDLAQLPFLTMCVKESLRLHPPAPFISRCCTQDIVLPDGRVIPKGITC

VINIIGVHHNPTVWPDPEVYDPFRFDPENSKGRSPLAFIPFSAGPRNCIGQAFAMAEMKV

VLALMLLHFRFLPDHTEPRRKPELIMRAEGGLWLRVEPLNVGLQ

>395847813

MLPLTDHLLHLLRLEKTTFRIYTVSALLLFLLFFFFRLLLQFLRLCWSFYITCRRLRCFP

QPPRRNWLLGHLGMYLPNETGLQDEKKVLDNMHHVVLVWMGPILPLLVLVHPDYIKPVVG

ASAAIAPKDDLFYGFLKPWLGDGLLLSKGDKWSRHRRLLTPAFHFDILKPYMKIFNQCTD

VMHAKWRRLVEGSVVSLDMFEHVSLMTLDSLQKCVFSHNSNCQEKMSDYISAIIELSALA

VKRQYHLHHYLDFIYYRTADGWRFRQACDTVHRFTTDVIQERRWALHQQGAEAWLKAKQG

KTLDFIDVLLLAKDEDGKELSDEDIRAEADTFMFEGHDTTSSGLSWVLFNLAKHPEFQEK

CREEIQEVMKGRELEELEWDDLTQLPFTTMCIKESLRQYPPVTLISRRCTEDIKLPDGRI

IPKGIICLVSIYGTHHNPTVWPDSKVYNPYRFDPENPQQRSPLAYVPFSAGPRNCIGQSF

AMAEMRVVVALTLLRFRLSVDRTRKVRRKPELILRTESGIWLNVEPLSPRA

>EEN58725.1

MKVVQISNHKQYGDGLLTTSGAKWFRNRRLLTPGFHFEVLRPYVKLFSDSTNVMLENWEE

FGAGASIDVFQHVSLMTLDSMLKCALSQNTGCQKRKKFNSYISAIHEISELFMARALSIM

AIVSDFVYNNSSAGKRYKKACNEVHQFSEKIIQQRKQDLDNLSTTETTRRQKYLDFLDIL

LMAKDEDGNGLTNTEIRDEVDTFMFEGHDTTASGLAWTLYCLARHPGHQEKCRKEAQEVL

QGRTDVTWEDLPSMKYITMCIKESLRIYPPVPKILRELEEPLTFPDGKTVQKGTTVFIGL

QLMHLNPNVWERPEEYDPLRFSPKNSSSRHPYAFLPFSAGQRNCIGQHFAMNELKTAVAL

ILQRFRLTPDDSLQEPVPVHKIVLRADSPGLFLKINPL

>332253735

MLQLSLSWLGLGPVAASPWLLLLLVGGSWLLARVLAWTYTFYDNCRRLQCFPQPPKQNWF

WGHQGLVTPTEEGMKTLTQLVATYPQGFKLWLGPTFPLVILCHPDIIRPVTSASAAVAPK

DMLFYGFLKPWLGDGLLLSGGDKWSRHRRMLTPAFHFNILKPYMKIFNKSVNIMHDKWQR

LASEGSTRLDMFEHISLMTLDSLQKCVFSFESNCQEKPSEYIAAILELSAFVEKRNQQIL

LHTDFLYYLTPDGQRFRRACHLVHDFTDAVIQERRRTLPTQGIDDFLKNKAQSKTLDFID

VLLLSKDEDGKELSDEDIRAEADTFMFEGHDTTASGLSWVLYHLAKHPEYQERCRQEVQE

LLKDREPIEIEWDDLAQLPFLTMCIKESLRLHPPVPVISRCCTQDIVLPEGRVIPKGIVC

LISIIGIHYNPTVWPDPEVYDPFRFDQENIKERSPLAFIPFSAGPRNCIGQAFAMAEMKV

VLALTLLRFRVLPTHNEPRRKPELILRAEGGLWLRVEPLGANSQ

>AAK15009.1

MLQLCLSWLGMGSLTASPWHLLLLGGASWILARILAWIYAFYDNCSRLRCFPQPPKPSWF

WGHLALMKNNEESMQFITHLGHDFHDVHLSWVGPVYPILRLVHPNFIAPLLQASAAVAPK

EMTLYGFLKPWLGDGLLMSAGDKWSHHRRLLTPAFHFDILKSYVKIFNKSVNIMHAKWQC

LASKGTSRLDMFEHISLMTLDSLQKCIFSVDSNCQESDSKYIAAILELSSLVVKRHRQPF

LYLDLLYYLTADGRRFRKACDLVHNFTDAVIKERRSTLNTQGVEFLKAKAKTKTLDFIDV

LLMAEDEHGKGLSNEDIRAEADTFMFGGHDTTTSALSWILYNLARHPEYQERCRQEVQEL

LRDRDSEEIEWDDLAQLPFLTMCIKESLRLHPPVLLISRCCTQDVLLPDGRAIPKGNICV

ISIFGVHHNPSVWPDPEVYNPFRFDPENPQKRSPLAFIPFSAGTRNCIGQTFAMSEIKVA

LALTLLRFRILPDDKEPRRKPELILRAEGGLWLRVEPLSAGAQ

>EDL40280.1

MLQLCLSWLGMGSLTASPWHLLLLGGASWILARILAWIYAFYDNCSRLRCFPQPPKPSWF

WGHLALMKNNEESMQFITHLGHDFHDVHLSWVGPVYPILRLVHPNFIAPLLQASAAVAPK

EMTLYGFLKPWLGDGLLMSAGDKWSHHRRLLTPAFHFDILKSYVKIFNKSVNIMHAKWQC

LASKGTSRLDMFEHISLMTLDSLQKCIFSVDSNCQESDSKYIAAILELSSLVVKRHRQPF

LYLDLLYYLTADGRRFRKACDLVHNFTDAVIKERRSTLNTQGVEFLKAKAKTKTLDFIDV

LLMAEDEHGKGLSNEDIRAEADTFMFGGHDTTTSALSWILYNLARHPEYQERCRQEVQEL

LRDRDSEEIEWDDLAQLPFLTMCIKESLRLHPPVLLISRCCTQDILLPDGRAIPKGNICV

ISIFGVHHNPSVWPDPEVYNPFRFDPENPQKRSPLAFIPFSAGTRNCIGQTFAMSEIKVA

LALTLLRFRILPDDKEPRRKPELILRAEGGLWLRVEPLSAGAQ

>126324075

MDGVGRPHGDAQDGRNSTATTPFPPTSAPVQPPPAQLEEPRRAAGPRWHKSWWQRSYRRR

RLKGMVPGSEWLSHQLGLGGLKSGVHSVMILFFLALSTWILAQVLFFLNRYYTNIQQLRC

FPQPLIWNWFSGHLGLMDFTEKSLIRIIDYVSTFREVFLMWMGPFRPVIVLCHSDYIRPL

TSASAHIAPKDNFIYGFLKPWLGDGLLLSGGDKWSRHRRLLTPAFHFDILKPYVKIFNQC

ADIMHEKWKRLCAEESTQLDVFDHVSLMTLDTLQKCIFSHNSNCQEKPSKYISTILELSV

LSTKRSNQFFLYWDSVYYLTSQGRSFTQACHLVHDFTDAVIKARQKVLAEQGVEAFLKDK

GKGKTMDFIDILLLSKDEDGKPLSDKDIRAEADTFMFEGHDTTASGISWVLYNLAQHQEY

QDRCRQEIQELMKGRETEEIEWNDLSQMPFLTMCIKESLRLHPPVPIIFRQCTKDIQLPD

SRVIPKGSVCLISIFGTHHNPTVWPNPEVYDPYRFDTNNPQKMSPLAFMPFSAGPRNCIG

QNFAMSEMKVVLALTLLRFRVFPHGNPPRRKLELVLRTESGLWLKVEPVQHSSSSEADLQ

EAPQN

>291243277

MANTTWTANVGTLCLTILILIVLQLVFTLWKLFRQRQSHRIALAAFPCPPARHWFYGHHW

NTSAEQGIQDQLSLGKLYSHYYQRWVGPFRGTLSLIHPDSVKAILSTAEPKSPNSYGVLK

EWLGDGLLISSGAKWKRNRRLLTPAFHFDILKPYVKVYNDCVEVLMDKWCKGDLDKPVEL

FKDVSLLTLDSLLKCIFSVESNCQTDSGHNEYVRSVKQLALLFIFRYRTFLPSFLFRRLV

SHGRQWAETLDILHGYTKQVIDQKRNNRKKEQATEKTYVDFLDILLDAKDEDGDGLTDKE

IQDEVDTFMFRGHDTTASGISWCLYNLARYPEYQQKCREEIDELVEGKDNKEIEWEDLHC

LNYTTMFIKESLRFHPPVSNISRRTTTPLHFPDGKVLPAGFSVAIFILGVHHNPHVWDDP

EVFDPMRFSSENSKNRTPYAFIPFAAGPRNCIGQNFAMNELKVVISRILHRFELSIDNSC

PTPKRRFGFVLRAENGIYIKLKPRSSF

>AAH03954.1

MLQLCLSWLGMGSLTASPWHLLLLGGASWILARILAWIYAFYDNCSRLRCFPQPPKPSWF

WGHLALMKNNEESMQFITHLGHDFRDVHLSWVGPVYPILRLVHPNFIAPLLQASAAVAPK

EMTLYGFLKPWLGDGLLMSAGDKWSHHRRLLTPAFHFDILKSYVKIFNKSVNIMHAKWQC

LASKGTSRLDMFEHISLMTLDSLQKCIFSVDSNCQESDSKYIAAILELSSLVVKRHRQPF

LYLDLLYYLTADGRRFRKACDLVHNFTDAVIRERRSTLNTQGVEFLKAKAKTKTLDFIDV

LLMAEDEHGKGLSNEDIRAEADTFMFGGHDTTTSALSWILYNLARHPEYQERCRQEVQEL

LRDRDSEEIEWDDLAQLPFLTMCIKESLRLHPPVLLISRCCTQDILLPDGRAIPKGNICV

ISIFGVHHNPSVWPDPEVYNPFRFDPENPQKRPPLAFIPFSAGTRNCIGQTFAMSEIKVA

LALTLLRFRILPDDKEPRRKPELILRAEGGLWLRVEPLSAGAQ

>395517884

MESEKQVGSQDIKTETPRISRATFIGREFIRMLTGFIWLSHHLGLAGLKSWIPSILLLVF

LVLSTWILIYGLTCVQRYRINCKRLRCFPQPPLKNWLLGHLGLELSSLFHDVHIYWLGPF

IPIIRLFHPKVISPLLMASGTVIPKDKLFYNFLEPWLGEGLLLSEGEKWRRHRHMLTPAF

HFDILKPYVKIFNQSVDIMHAKWRRLCAEGTNYLEMFEHISLMTLDSLQKCIFSYNSNCQ

EKPSAYISAILELSSLVAKRHNNPLLYWDTLYNLTSQGRRFSRACHLVHDFSNAVIQERR

HILAQQGTEAFLGNKRKSKTIDFIDILLLAKDEDGKALSDKDIQAEADTFMFAGHDTTAS

GISWALYNLAQHQEYQDRCRQEIKELLGDRQPEEIECSLDLVSRDDLVQMPFLTMCIKES

LRLHSPVSMVSRYCTKDIQLPDGRIIPKGNICLISIFGIHHNPAVWPKPEVYDPHRFDTN

NPEKISPLAFIPFSVGSRNCIGQNFALSELKVVLALTLLRFRVLPDKHPVRRKPEIILRE

ENGLRLKVEPLLDLSSPDMHLSETSQE

>345787069

MLQLSLSWLGLGQLAASPWVLLLLVGATWLLTRVLAWSYSFYDIRRRLQCFPQPPKRNWF

WGHVGLIQSSEEGLLYTQELASTYGDVCCWWVGPWHAVIRIFHPTYIKPVLFSSAAIAPK

DMVFYSLLKPWLGDGLLLSAGDKWSSHRRMLTPAFHFNILKPYVKIFNYSTNVMHAKWKR

LVSEGSTHLDMFEHISLMTLDSLQKCVFSFDSNCQEKPSEYIAAILELSALVAKRHQQIL

HMDFLYYLTPDGQRFRRACRLVHNFTDAVIQERRRTLPVQGVDDFLMTKAKSKTLDFIDV

LLLSKDEDGKQLSDKDIRAEADTFMFEGHDTTASGLSWVLFNLAKHPEYQERCRQEVQEL

LRDREPQEIEWDDLAKLPFLTMCIKESLRLHPPVTVISRCCTQDIVLSDGRVIPKGVTCL

ISIFGTHHNPSVWPDPEVYNPLRFDPENIKERSPLAFIPFSAGPRXAKRVSFSMSEMKVV

LALTLLRFRVLPHEEEPRRKPELILRAEGGLWLRVEPLSARPQ

>395848027

MLQLSLSWLGLGPVAASPWLLLLLVGASWLVAYVLAWFYSFCDNCRRLRCFPQPPKRNWF

WGHLGLVQSNEEGMRLVENLGHYFRDVHLWWMGPFCPILRLVHPKFVAPLLQAPATVTPK

DMTFYGFLKPWLGDGLLLSAGNKWSHHRRLLTPAFHFEILKLYMKIFNKSADIMHGKWQR

LVSGGSACLDMFEHISLMTLDILQKCVFSFDSDCQENPSEYIAAILELSALIVKRYRQIF

LHLDFLYYLTPDGRRFRKACNMVHEFTDTIIQERRRTLQHQSVDDILKAKTKSKTLDFID

VLLLSKDENGKELSDESIRAEADTFMFAGHDTTASGLSWILYNLARHPEYQDRCRQEVRE

LLKDRELKEIEWEDLAQLPFLTMCIKESLRLHPPVTVMSRCCTQDVVLPDGRVIPKGNIC

MVSIFGIHHNPSVWLDPKVYDPFRFDPENPQKRSPLAFIPFSAGPRNCIGQTFAMTEMKV

VLALTLLRFRVLPDNTEARRKPELILRAEGGLWLRVEPLSSDPQ

>EDL40307.1

MLQLGLSWLGLGPGAAFPWQLLQLVGASLFLARILTWICAFYDNYCRLRCFPEPPSRHWF

WGHMSMVKNNEEGLQLLTERSHQFHDVHLCWIGPFYPILRLIHPKFIGPILQASAAVAPK

EMIFYGFLKPWLGDGLLVSAGEKWSRHRHLLTPAFHFDILKPYMKNFNKSVNIMHAKWQR

LTTKGTACLDMLEHISLMTLDSLQNCVFSFDSNCQESPSEYIAAIQELSSLIVKRHHQPF

LYLDFLYYCTADGRRFRKACDLVHNFTDAVIRERRRTLSSQNLDEFLKSKTKSKTLDFID

VLLLAKDEHGKELSDEDIRAEADTFMFGGHDTTASALSWILYNLARHPEYQERCRQEVQE

LLRGREPQEIEWDDLAQLPFLTMCIKESLRLHPPVTVISRCCTQDVVLPDGRVIPKGTDC

VISIFGVHHNPEVWPDPEVYDPFRFDPENPQKRSPLAFIPFSAGPRNCIGQTFAMREMKV

ALALTLLRFRVLPGDKEPRRKPELILRAEGGLWLRVEPLSKGTQ

>291413168

MLQLSLSWLGRSLGATSPWLLLLLIGASGLLVCILPQACAFYNKCRRLRSFPQPPKQNWL

FGHLGLITLTEEGLRILTQLVATFPQGFVYWMGPIIPSIVLCHPDTIRPILNASAAIAPK

DIVFYSFLKPWLGDGLVVSAGDKWSRHRRMLAPTFHFNILKPYVKIFNNATNIMLAKWQR

LTSEGSARLEMYEDISLMTLDSLQKCIFSFDSNCQEKPSDYITANVELTYLFARRQQNFL

LYPDILYYLTPDGQRFRRACRVVHAFTEAVIQERRRTLLSQGIDDILKAKAKTKTLDFID

VLLLTKDEDGKRLSDEDIRAEADSFMFAGHDSTASSLTWILYNLAKHPEYQERCRKEARE

LLRDRGDEEIEWDDLAQLPFLTMCIKESLRLHPPLLGISRSCTQDMTLPDGRVIPKGVIC

FINIFGTHHNPAVWPDPEVYDPFRFEPEKVKDRSPLAFIPFSAGPRNCIGQTFAMIEMKV

ALVLLLLHFRFLPDSTETRRKPDLGMRAEGGLWLWVEPLSSSRQ

>345787071

MLQVSLSWLGLGQLAASPWVLLLLAGATWLLARVLTWSYSFYDNCCRLRCFPQPPKRNWF

WGHLGLVKSNEEGLRMIEDLGHYFRDVHLWWIGPFYPVLRLVHPKFVAPLLQAPATIVPK

DMFFYNFLKPWLGDGLLLSAGDKWSHHRRLLTPAFHFEILKSYVKIFNRSADIMHAKWKR

LVSEGSTHLDMFEHISLMTLDSLQKCVFSFDSNCQESPSEYIAAILELSALVVKRNEQVL

LYLDFLYNLSPDGRRFRRACELVHNFTDAIIQERRHTLISRGSCDFLKSKTMDFIDVLLL

AKDEAGKQLSDEDIRAEADTFMFEGHDTTASGLSWVLFNLAKHPEYQERCRQEVQELLRD

REPQEIEWDDLAQLPFLTMCIKESLRLHPPVTVIARRCTQDIVLPDGRVIPKGNNCVLSI

FGIHHNPSVWPDPEVYNPLRFDPEIPQKRSPLAFIPFSAGPRNCIGQAFAMSEMKVVLAL

TLLRFRVLPHEEEPRRKPELILRAEGGLWLRVEPLSARPQ

>194223692

MLPVAEPLLRLLGLERTAFRLGAASALLLSLLFVFFRLLLRFLRLCWRFRVTCRRLRCFP

QPPRRSWLLGHLGMYLPNEMGLQDEKKVLDNMHHVLLVWIGPILPLLVLVHPDYIKPVVG

ASAAIAPKDDLFYGFLKPWLGDGLLLSKGDKWSRHRRLLTPAFHFDILKPYMKIFNQCTN

TMHAKWRCLAEGSVVSLDMFEHISLMTLDSLQKCVFSYNSNCQEKMSDYITAIIALSALV

VRRQYTLHHHLDFIYYLTADGRRFRQACDTVHRFTTEVIQERRRGLRQQGAEAWLKARQG

KTLDFIDVLLLARDEEGKELSDEDIRAEADTFMFEGHDTTSSGLSWLLFNLAKYPEYQEK

CREEIQEVMKGRELEELEWDDLTQLPFTTMCIKESLRQFPPVTLVSRRCTEDIKLPDGRI

IPKGIICLVSIYGTHHNPTVWPDSKVYNPYRFDPDNPQQRSPLAFVPFSAGPRNCIGQSF

AMAEMRVVLALTLLRFRLSVDRTHKVRRKPELILRSESGIWLNVEPLPRRA

>AAI42691.1

MSVSVLSPSRCLGGVSGILQVTSLLILLLLLIKAAQLYLHRQWLLKALQQFPCPPSHWLF

GHIQEFQHDQELQRIQERVKTFPSACPYWIWGGKVRVQLYDPDYMKVILGRSDPKSHGSY

KFLAPRIGYGLLLLNGQTWFQHRRMLTPAFHYDILKPYVGLMADSVRVMLDKWEELLGQD

SPLEVFQHVSLMTLDTIMKSAFSHQGSIQVDRPSDPTEEGSTTEGGGAGEDQEEEALGFS

GHPPLGQRAPGLPRPCWCSGWNCFRNHLDQMPYTTMCIKEALRLYPPVPGIGRELSTPVT

FPDGRSLPKGIMVLLSIYGLHHNPKVWPNLEVFDPSRFAPGSAQHSHAFLPFSGGSR

>395517882

MLPDSEWLSHQLGLKGLGSGGSLVLLLLFLALSTWMMFQILVFVHKYYINCQQLRCFPQP

PLQNWFFGHLGLIDPTEEGLIRTIQLVNTFKKIFLMWMGPFYPLVILCHPDYIQPLTSAS

VYIAPKDKLFYGFLKPWLGDGLLLSEGDKWKRHRHLLTPAFHFDILKSYVKIFSQSTNIM

HEKWKHLCARSSNHLDMFKHISLMTLDSLQKCIFSYNSDCQEKPNSYISAILELSALVAN

RNKHLLLYWNGLYYLTSQGRHFSRACRLVHDFSEAVIQARRHILDTYGPEAYFSDKGKGK

IMDFIDILLLAKDEDGKPLSDKDIQAEADTFMFEGYDTTASGISWVLYNLAQHQEHQDHC

RQEIQELLRGRQPNEIKWDDLSQMPFLSMCIKESLRLYPPVVTVVRCCTKDIQLPDGHII

PKGISCLVNIFGTHHNPTVWTNPEVFNPYRFDSNNSQKMSPLAFMPFSAGPRNCIGKNFA

MAEMKVVLALTLLHFRVFSDGYLSQRKPELILRAKDGLWLKVEPILDPSSPGPHLTKLAF

LSQVHASVMCPSRIKSYHKTLLHLQLCVTGDLIAGSKCDKCTTYILSVLLLLKKDTVPSS

RENSPPTGALSRDGKRGQRRNSVATQL

>AAG47670.1

MPWLTVSGLDLGSVVTSTWHLLLLGAASWILARILAWTYSFCENCSRLRCFPQSPKRNWF

LGHLGTIQSNEEGMRLVTEMGQTFRDIHLCWLGPVIPVLRLVDPAFVAPLLQAPALVAPK

DPTFLHFLKPWLGDGLFLSSGDKWSRHRRLLTPAFHFDILKPYVKIFNQSVNIMHAKWKH

LCLEGSVRLEMFENISLMTLDSLQKCLFGFDSNCQESPSEYISAILELSSLIIKRSQQLF

LYLDFLYYRTADGRRFRKACDLLHNFTDAVIRERRRLLSSQGVDEFLESKTKSKSKTLDF

IDVLLLAKDEHGKELSDEDIRAEADTFMFGGHDTTASALSWILYNLARHPEYQERCRQEV

WELLRDREPEEIEWDDLAQLPFLTMCIKESLRLHPPAIDLLRRCTRHIVLPDGRVIPKGN

ICVISIFGIHHNPSVWPDPEVFDPFRFDSENRQKRSPLSFIPFSAGPRNCIGQTFAMNEM

KVVVALTLLRFRVLPDDKEPRRKPEIILRAEGGLWLRMEPLSTDTQ

>332853605

MPQLCLSWLGLGPVAASPWLLLLLVGASWLLARVLAWTYTFYDNCRRLQCFPQPPKQNWF

WGHQGLVTPTEEGMKTLTQLVTTYPQGFKLWLGPTFPLLILCHPDIIRPITSASAAVAPK

DMIFYGFLKPWLGDGLLLSGGDKWSRHRRMLTPAFHFNILKPYMKIFNKSVNIMHDKWQR

LASEGSARLDMFEHISLMTLDSLQKCVFSFESNCQEKPSEYIAAILELSAFVEKRNQQIL

LHTDFLYYLTPDGQRFRRACHLVHDFTDAVIQERRRTLPTQGIDDFLKNKAKSKTLDFID

VLLLSKDEDGKELSDEDIRAEADTFMFEGHDTTASGLSWVLYHLAKHPEYQEQCRQEVQE

LLKDREPIEIEWDDLAQLPFLTMCIKESLRLHPPVPVISRCCTQDIVLPDGRVIPKGIVC

LINIIGLHYNPTVWPDPEVYDPFRFDQENIKERSPLAFIPFSAGPRNCIGQAFAMAEMKV

VLALTLLHFRILPTHTEPRRKPELILRAEGGLWLRVEPLGANSQ

>293348508

MLPITDYLLYLLGLEKTAFRVYVLSALLLFLLFLLFRLLLQAFKLFSDFRITCRRLSCFP

EPPGRHWLLGHMSMYLPNEKGLQNEKKVLDTMHHIILAWVGPFLPLLVLVHPDYIKPVLG

ASAAIAPKDEFFYSFLKPWLGDGLLISKGNKWSRHRRLLTPAFHFDILKPYMKIFNQSVN

IMHAKWRRHLAEGSVTSFDMFEHVSLMTLDSLQKCVFSYSSDCQEKLSDYISSIIELSAL

VVRRQYRLHHYLDFIYYLTADGRRFRQACDTVHNFTTEVIQQRRRALRELGAEAWLKAKQ

GKTLDFIDVLLLAKDEEGKELSDEDIRAEADTFMFEGHDTTSSGLSWALFNLAKYPEYQD

KCREEIQEVMKGRELEELDWDDLTQLPFTTMCIKESLRQFPPVTLISRRCTEDIKLPDGR

IIPKGACSAGIYGTHYNPLVWPDSKVYNPYRFDPDIPQQRSPLAFVPFSAGPRNCIGQSF

AMAEMRVVVALTLLRFRLSVDRTRKVRRKPELILRTENGLWLNVEPLPSRAGVPRGPTEP

EVQAPPAQA

>397484929

MLQLCLSWLGLGPVAASPWLLLLLVGASWLLARVLAWTYTFYDNCRRLQCFPQPPKQNWF

WGHQGLVTPTEEGMKTLTQLVTTYPQGFKLWLGPTFPLLILCHPDIIRPITSASAAVAPK

DMIFYGFLKPWLGDGLLLSGGDKWSRHRRMLTPAFHFNILKPYMKIFNKSVNIMHDKWQR

LASEGSARLDMFEHISLMTLDSLQKCVFSFESNCQEKPSEYIAAILELSAFVEKRNQQIL

LHTDFLYYLTPDGQRFRRACHLVHDFTDAVIQERRRTLPTQGIDDFLKNKAKSKTLDFID

VLLLSKDEDGKELSDEDIRAEADTFMFEGHDTTASGLSWVLYHLAKHPEYQEQCRQEVQE

LLKDREPIEIEWDDLAQLPFLTMCIKESLRLHPPVPVISRCCTQDIVLPDGRVIPKGIVC

LINIIGIHYNPTVWPDPEVYDPFRFDQENIKERSPLAFIPFSAGPRNCIGQAFAMAEMKV

VLALTLLHFRILPTHTEPRRKPELILRAEGGLWLRVEPLGANSQ

>332253596

MLPITDRLLHLLGLEKTAFRIYEVAGGRQWGKLGLGTSSLLLPYLLQYLPNEVGLQDEKK

VLDSMHHVLLVWMGPVLPLLVLVHPDYIKPLLGASAAIAPKDDLFYGFLKPWLGDGLLLS

KGDKWSRHRRLLTPAFHFDILKPYMKIFNQCTDIMHAKWRHLAEGSVVSLDMFEHISLMT

LDSLQKCVFSYNSNCQEKMSDYISAIIELSALSVRRQYRLHHYLDFIYYRSADGRRFRQA

CDMVHHFTTEVIQERRRALRQQGAEAWLKAKQGKTLDFIDVLLLARDEDGKELSDEDIRA

EADTFMFEGHDTTSSGISWMLFNLAKYPEYQEKCREEIQEVMKGRELEELEWDDLTQLPF

TTMCIKESLRQYPPVTLVSRQCTEDIKLPDGRIIPKGIICLVSIYGTHHNPTVWPDSKVY

NPYRFDPDNPQQRSPLAYVPFSAGPRNCIGQSFAMAELRVVVALTLLRFRLSVDRTRKVR

RKPELILRTENGLWLKVEPLPPRA

>345326880

MLPITDQVLKLLNLEKTSFRTYTISALLLSLLVVFFRLLLHLFQVCRQFYVTCQRLRCFP

QPPRRNWLLGHLGMGKGAGVGRAAVAPKDELFYGFLRPWLGDGLLLSRGDKWGRHRRMLT

PAFHFDILKPYMKIFNQCTDIMHAKWRRLSAGAVVSLDMFEHVSLMTLDSLQKCVFSYNS

DCQEKMSDYISAIIELSALVVKRQYRLHHHLDFIYYRTADGRRFQQACDIVHHFTTEVIQ

GRRHALNQQGAEAWLKSKQGKTLDFIDVLLLAKDEEGKYLSDEDIRAEADTFMFEGHDTT

SSGLSWILFNLARYPEYQEKCREEIQEIMKGREMDEIEWEDLTQMPFITMCIKESLRLYP

PVTLISRRCTEDIKLPDGRVIPKETICLVSIYGTHHNPTVWPEPKVFNPYRFDPENPQQR

SPLAYVPFSAGPRNCIGQSFAMSEMKVVVALTLLRFKLVVDRTKKVRRKPELILRTENGI

WLNVEPLPARQ

>395848033

MPQLSLSWLGLGPVATSPWLLLLLVGASWLLAHILAWTYSFYDNCRRLRCFPQPPKQNWF

WGHQGLISPTEESLRERIELVANYPQGFKLWLGPILPLINLCHPDILRPVLNASAAIIPK

DMSFYGFLKPWLGDGLLLSGGDKWSQHRRMLTPAFHFNILKPYVKIFNRSVNIMHAKWQR

LALEGRAHLDMFEHISLMTLDSLQKCIFSFDSNCQEKSSEYIATILELSSLIEKRNQQVL

LLMDFLYYLTPNGRRFLKACRLVHNFTDAVIQERRRTLPTQDIDDFLEAKAKTKTLDFID

VLLLAKDEDGKALSDEDIRAEADTFMFEGHDTTASGLSWVLYNLARHPEYQERCRQEVQE

LLRDREPEEIEWDDLAQLPFLTMCIKESLRLHPPVPLVSRCSTQDIELPDGRVIPKDVIV

LINILGTHHNPAVWPDPEVYDPFRFDPDKVKERSPLAFIPFSAGPRNCIGQAFAMAEMKV

VLALTLLRFRVLPGHSEPRRLPELILRAEGGLWLHVEPLKAGGHRLCPNLIHGQQGALGH

CGDPGGGLDGWRRGWSLRATPRQPRWLSSALLRLSLVLSESKYGLYSTGDKEPWEVFEHE

RATVKAKFKRQDEGSESERGLLEADPFTY

>Q9HBI6.3

MPQLSLSWLGLGPVAASPWLLLLLVGGSWLLARVLAWTYTFYDNCRRLQCFPQPPKQNWF

WGHQGLVTPTEEGMKTLTQLVTTYPQGFKLWLGPTFPLLILCHPDIIRPITSASAAVAPK

DMIFYGFLKPWLGDGLLLSGGDKWSRHRRMLTPAFHFNILKPYMKIFNKSVNIMHDKWQR

LASEGSARLDMFEHISLMTLDSLQKCVFSFESNCQEKPSEYIAAILELSAFVEKRNQQIL

LHTDFLYYLTPDGQRFRRACHLVHDFTDAVIQERRCTLPTQGIDDFLKNKAKSKTLDFID

VLLLSKDEDGKELSDEDIRAEADTFMFEGHDTTASGLSWVLYHLAKHPEYQEQCRQEVQE

LLKDREPIEIEWDDLAQLPFLTMCIKESLRLHPPVPVISRCCTQDFVLPDGRVIPKGIVC

LINIIGIHYNPTVWPDPEVYDPFRFDQENIKERSPLAFIPFSAGPRNCIGQAFAMAEMKV

VLALTLLHFRILPTHTEPRRKPELILRAEGGLWLRVEPLGANSQ

>CAI19736.1

MSVSVLSPSRRLGGVSGILQVTSLLILLLLLIKAAQLYLHRQWLLKALQQFPCPPSHWLF

GHIQEFQHDQELQRIQERVKTFPSACPYWIWGGKVRVQLYDPDYMKVILGRSDPKSHGSY

KFLAPRIGYGLLLLNGQTWFQHRRMLTPAFHNDILKPYVGLMADSVRVMLDKWEELLGQD

SPLEVFQHVSLMTLDTIMKSAFSHQGSIQVDRPSDPTEEGSTTEGGGAGEDQEEEALGFS

GHPPLGQRAPGLPRPCWCSGWNCFRNHLDQMPYTTMCIKEALRLYPPVPGIGRELSTPVT

FPDGRSLPKGIMVLLSIYGLHHNPKVWPNLEVFDPSRFAPGSAQHSHAFLPFSGGSRNCI

GKQFAMNQLKVARALTLLRFELLPDPTRIPIPMARLVLKSKNGIHLRLRRLPNPCEDKDQ

L

>291223965

MADKAKLRVPRLCKSGCIVGTCLQRGMFSWGANSYGQLGLGHQDDKSKPEKILKIPDGKI

VDVTGGGGHTLLLTDCGAIYSCGWNSKGQLGLGHTDNLSVFKRIDHLPPVIKVTCGWDFC

LAITEVGDLYAWGCNVFGQLGIPEIDSFTAIPTKIQIFSRNVTSIAAGLRHSLAVTNDGK

LWSWGSARKGQLGNDQSKTGYQLLQRVPKQVHKLENISSVSSGSYHSTALTACGNMYLWG

SNKHNQLTNSVQGVVQSGGSSKEDILPVPTKLNLELFGGRQILAVHSGWTHILAITHDSN

VFGWGRNDYSQLGISLTGNNCAKKDVTKPKELVILKDIKQIACGSEHNLALSDDNKVLAW

GWNEHGSCGNGKEENVLTPQVIDDLAKEKIRLIDKFTTHRYYLRKDPPKIVNEIGVSSTV

LLHTVVSNKAYGSYIKQDKWSDLKKSVGSRTCSIEVFEHAGLLTLDILLKCIFSQDSNCR

TTSSQHPYVSSVHALTYLIAERVSCIPYHNDMVYNLSISGFKFRKALRSVHGYSARVIYE

RNQSLLQDGDVTLKRKNIDFLDILLTAKDEDGNGLFDQEIQDEVDTFMFEGHDTTLKNTE

AALAKFYSRSKELSPASVLWLGPFAPVVLLNHPQTVKVILCATDPKDDLFYHMLKPWLGD

GLLMSKGQKWFRNRLLVTPGFHFGILRPYVQIYNECVKTMLDKWSDLWKSVGSRTCSIEV

FEHAGLLTLDILLKCIFSQDSNCQTTSSQHPYVSSVHALTYLIAERIRCIPYHNDMIYNL

SISGYKFRKALRSVHGYSARVIQERKQALRQDGGVTLKRKYIDFLDILLTAKDEDDNGLS

DKEIQDEVDTFMFEGHDTTASGLSWCMYYLATHPEHQRKCQEEVDNLLNERAMDEIEWDD

LSHLPYTTLCIKESMRIRSPVPVIGRELTTPLTLPNGRVIPAGYNVIIPICLLHHNELIW

ENPTRFDPTRFLPENVKDRSPFAFTPFSAGPRNCIGQHFAMNEMKIVIARTLRRFDLSPV

PDNPPQRVHTLVTRSSNGIHLHVQPRNMG

>EHB14402.1

MTQLSLSWLGPVPLAASLWQLPLLAGACWLLVRVLARIIAFYGYCCRLRCFPQPPRRSWF

WGHLGLAKSDEEGMKLISDLGRDFLDVHLWWMGPVYPVLRLIHPKFVAPLLQAPGDGLLV

SAGDKWSLHRRLLTPAFHFDILKPYVKTFNKSVNIMHAKWQRLALESSTRLDMFEHISLM

TLDSLQKCVFSFDSNCQEHPSEYIAAILELSSLIIKRHQQLFQYIDFLYHFTPDGRRFRR

ACDLVHNFTDAVIQERRCTLASQGVDDFLKAKAKLKTLDFIDVLLLAKDENGKELSDSDI

RAEADTFMFAGHDTTASGLSWALYNLARHPEYQERCRQEVQELLRAREPEEIEWEDLAQL

PFLTMCIKESLRLHPPVVIISRCCAQDVALPDGRVIPKGTTCGISIFGIHHNPSVWPDPE

VYDPFRFDPENPQKRSPLAFIPFSAGSRNCIGQTFAMAEMKVALALTLLRFRVLPDDTEP

LRKPELILRAEGGLWLRVEPLS

>395517878

MQLGDMIDFTEENLVRFLSIVATFRETFLLWVGPFLPVVTLCHPDYIRPLTSASAQVVPK

DQFIYGFLEPWLGDGLLLSGGEKWSRHRRLLTPAFHFDILKPYMKLFNQSANIMHKKWRR

LCAEGKNHLDMFEHISLMTLDSLQKCIFSYDSNCQEKPSSYISTILELSTLSTKRLNQVF

LHWDRLYFLTSEGRRYSQACRVVHDFTDTIIQARKKALFEQGPEAFLKNKGKGKTTDFID

VLLLAKDEDGKSLSDKDIRAEADTFMFEGHDTTASGISWLFYNLAQHQEYQDRCRQEIEE

LLRGRQLEEIEWDDLSQMPFLTMCIKESLRLHPPVPAVLRRCTKDIKLPDSRIIPKGIIC

FISIFGTHHNPTVWPNPEVYDPYRFDPQNTQKMSPLAFIPFSAGPRNCIGQNFAMSEMKI

VVALTLLRFRILPHQSLPRRKAELVLRAEDGLWLTVEPLQHSSSSETRLQKDPQD

>AAG15889.1

MPQLSLSWLGLGPVAASPWLLLLLVGGSWLLARVLAWTYTFYDNCRRLQCFPQPPKQNWF

WGHQGLVTPTEEGMKTLTQLVTTYPQGFKLWLGPTFPLLILCHPDIIRPITSASAAVAPK

DMIFYGFLKPWLGDGLLLSGGDKWSRHRRMLTPAFHFNILKPYMKIFNKSVNIMHDKWQR

LASEGSARLDMFEHISLMTLDSLQKCVFSFESNCQEKPSEYIAAILELSAFVEKRNQQIL

LHTDFLYYLTPDGQRFRRACHLVHDFTDAVIQERRRTLPTQGIDDFLKNKAKSKTLDFID

VLLLSKDEDGKELSDEDIRAEADTFMFEGHDTTASGLSWVLYHLAKHPEYQEQCRQEVQE

LLKDREPIEIEWDDLAQLPFLTMCIKESLRLHPPVPVISRCCTQDFVLPDGRVIPKGIVC

LINIIGIHYNPTVWPDPEVYDPFRFNQENIKERSPLAFIPFSAGPRNCIGQAFAMAEMKV

VLALTLLHFRILPTHIEPRRKPELILRAEGGLWLRVEPLGANSQ

>AAH16853.1

MPQLSLSWLGLGPVAASPWLLLLLVGGSWLLARVLAWTYTFYDNCRRLQCFPQPPKQNWF

WGHQGLVTPTEEGMKTLTQLVTTYPQGFKLWLGPTFPLLILCHPDIIRPITSASAAVAPK

DMIFYGFLKPWLGDGLLLSGGDKWSRHRRMLTPAFHFNILKPYMKIFNKSVNIMHDKWQR

LASEGSARLDMFEHISLMTLDSLQKCVFSFESNCQEKPSEYIAAILELSAFVEKRNQQIL

LHTDFLYYLTPDGQRFRRACHLVHDFTDAVIQERRRTLPTQGIDDFLKNKAKSKTLDFID

VLLLSKDEDGKELSDEDIRAEADTFMFEGHDTTASGLSWVLYHLAKHPEYQEQCRQEVQE

LLKDREPIEIEWDDLAQLPFLTMCIKESLRLHPPVPVISRCCTQDFVLPDGRVIPKGIVC

LINIIGIHYNPTVWPDPEVYDPFRFNQENIKERSPLAFIPFSAGPRNCIGQAFAMAEMKV

VLALTLLHFRILPTHTEPRRKPELILRAEGGLWLRVEPLGANSQ

>EAW84512.1

MPQLSLSWLGLGPVAASPWLLLLLVGGSWLLARVLAWTYTFYDNCRRLQCFPQPPKQNWF

WGHQGLVTPTEEGMKTLTQLVTTYPQGFKLWLGPTFPLLILCHPDIIRPITSASAAVAPK

DMIFYGFLKPWLGDGLLLSGGDKWSRHRRMLTPAFHFNILKPYMKIFNKSVNIMHDKWQR

LASEGSARLDMFEHISLMTLDSLQKCVFSFESNCQEKPSEYIAAILELSAFVEKRNQQIL

LHTDFLYYLTPDGQRFRRACHLVHDFTDAVIQERRRTLPTQGIDDFLKNKAKSKTLDFID

VLLLSKDEDGKELSDEDIRAEADTFMFEGHDTTASGLSWVLYHLAKHPEYQEQCRQEVQE

LLKDREPIEIEWDDLAQLPFLTMCIKESLRLHPPVPVISRCCTQDFVLPDGRVIPKGIVC

LINIIGIHYNPTVWPDPEVYDPFRFDQENIKERSPLAFIPFSAGPRNCIGQAFAMAEMKV

VLALTLLHFRILPTHTEPRRKPELILRAEGGLWLRVEPLGANSQ

>CAH90021.1

MLQLSLSWLGLGPVAASPWLLLLLVGGSWLLARVLAWTYTFYDNCRHIKCFPQPPKQNWF

WGHQGLITPTEEGMKTLTQLVATYPQGFKLWLGPTFPLLILCHPDIIRPITSASAAVAPK

DMIFYGFLKPWLGDGLLLSGGDKWSRHRHMLTPAFHFNILKPYMKIFNKSVNIMHDKWQR

LASEGSARLDMFEHISLMTLDSLQKCVFSFESNCQEKPSEYIAAILELSAFVEKRNQQIL

LHTDFLYYLTPDGQRFRRACHLVHDFTDAVIQERRRTLPTQGIDDFLKNKAKSKTLDFID

VLLLSKDEDGKELSDEDIRAEADTFMFEGHDTTASGLSWVLYHLAKHPEYQERCRQEVQE

LLKDREPIEIEWDDLAQLPFLTMCIKESLRLHPPVPVISRCCTQDIVLPDGRVIPKGIVC

LISIIGIHYNPTVWPDPEVYDPFRFDQENIKERSPLAFIPFSAGPRNCIGQAFAMAEMKV

VLALTLLHFRILPTHTEPRRKPELILRAEGGLWLRVEPLGVNSQ

>297276360

MLTLSLSWLGLGSVAGSPWLLLLLVGASWLLARVLAWTYAFYDNCRRLRCFQQPPKRNWF

WGHLSIIQGNEEDMRLIEDLGHHFHDVQLWWLGPFHPALHLVHPTFAAPVLQASVAVAPK

DTISYSPLKLWLGDGLLISAGDKWRWHRHLLTPAFHFKILKPYVKIFNDSANIMHVKWQR

LALEGSARLDMFEHISLMTLDILQKCAFSFDSHCQEKPSEYIDAILELSALIVKRNQHIF

LQADFLYFLTPNGRRFRRACDIVHNFTDAIIQERRRTLTSQGVNDLLQAKAKSKTLDFID

VLLLAKDENGKELSDEDIRAEADTFMFGGHDTTASGLSWVLYNLARHPKYQEHCRQEVQE

LLKNRDPKEIEWDDLVQLPFLTMCLKESLRLHSPVSRIQRCCTQDVVLPDGRVIPKGNTC

TVSIFGIHHNPSVWPDPEVYDPFRFDPENLQKRSPLAFIPFSAGPRNCIGQTFAMAEMKV

VLALTLLRFRILPDLAEPRRKSEVILRAEDGLWLRMEPLSANPQ

>EHH59311.1

MSLLSLPWLGLGPVATSPGLLLMLVVGSWLLARVLAWTYTFYDNCRRLRCFPQPPKQNWF

WGHLGLITPTEEGLKNLTQISATYSQGFMIWMGPIIPFIVLCHPDTIRSITNASAAIAPK

DALSIRFLKPWLGEGILLSGGDKWSHHRRMLTPAFHFNILKPYIKIFNKSATIMHVRRQH

LASEGSSRLDMFEHISLMTLDSLQKCIFSFDSHCQERPSEYIATILELSALVEKRSQHIL

QYMDFLYYLTQDGRRFRRACRLVHDFTDAVIQERRRTLPTQGIDDFLKDKAKSKTLDFID

VLLLSKDEDGKALSDEDIRAEADTFMFAGHDTTASGLSWVLYNLARHPEYQEHCRQEVQE

LLKDRDPKEIEWDDLAQLPFLTMCLKESLRLHPPAPFISRHCTQDIVLPDGRVIPKGIIC

VINITGVHHNPTVWPDPEVYDPYRFDPENSKERSPLAFIPFSAGPRNCIGQAFAMAEMKT

VLALMLLHFRFLPDHTEPRRKPELTLRAEGGLWLRVEPLSAGLQ

>403303356

MSLLSLSWLGLGPVVASPWLLLPLVGAYWLLARVLAWTYTFYDKCRRLQCFPEPPKRNWF

WGHLGLIGPTEEGLKNMTQISATYSPGFRIWLGPIIPFVVLCHPDIIRSITNASAAIVPK

DELFIRFLKPWLGEGILLTAADKWSHRRRMLTPAFHFNILKPYVKIFNESVNIMLDKWQR

LASEGSSRLDMFEHISLMTLDSLQKCIFSFDSHCQERSSEYIATILELSGLVEKRTQHLL

QHMDFLYYLTHDGRRFRRACRLVHDFTDAVIQERRRTLPTQDIDDFLKDKAKSKTLDFID

VLLLSKDEDGKPLSDEDIRAEADTFMFAGHDTTASGLSWVLYNLARHPEYQERCRQEVQE

LLKDREPKEIEWDDLAQLSFLTMCLKESLRLHPPAPFISRRCAQDIVLPDGRVIPKDIIC

VINIIGIHHNPSVWPDPEVYDPFRFDPENSKERSPLAFIPFSAGSRNCIGQAFAMAEMKA

VLALTLLHFRFRPDHREPRRKPELIMRAEGGLWLRVEPLNAGLQ

>BAF82113.1

MPQLSLSWLGLGPVAASPWLLLLLVGGSWLLARVLAWTYTFYDNCRRLQCFPQPPKQNWF

WGHQGLVTPTEEGMKTLTQLVTTYPQGFKLWLGPTFPLLILCHPDIIRPITSASAAVAPK

DMIFYGFLKPWLGDGLLLSGGDKWSRHRRMLTPAFHFNILKPYMKIFNKSVNIMHDKWQR

LASEGSARLDMFEHISLMTLDSLQKCVFSFESNCQEKPSEYIAAILELSAFVEKRNQQIL

LHTDFLYYLTPDGQRFRRACHLVHDFTDAVIQERRRTLPTQGIDDFLKNKAKSKTLDFID

VLLLSKDEDGKELSDEDIRAEADTFMFEGHDTTASGLSWVLYHLAKHPEYQEQCRQEVQE

LLKDREPIEIEWDDLAQLPFLTMCIKESLRLHPPVPVISRCCTQDFVLPDGRVIPKGIVC

LINIIGIHYNPTVWPDPEVYDPFRFNQENIKERSPLALIPFSAGPRNCIGQAFAMAEMKV

VLALTLLHFRILPTHTEPRRKPELILRAEGGLWLRVEPLGANSQ

>291237979

MAREGFDRFSWGATTLAVRTSTFAQRRQESLDRHAIWASKHKYGYPIWIGPFDVTYMVTH

PMTMKAILSTSEPKDKMAYDMLKPWLGDGLLLSKGSKWFRNRKLLTPGFHFDVLKPYAKI

FNECSKALVCKWHGENNSIEVFHDVSLLTLDCLMKCIFSHDSQNQDGDSNSYTQSVYQAG

TLFSKRFLNLLHHSDSIYHLSSNGRKWRKALKILHSHSSRVIKQRHNEILNQKENGVSNK

RKYIDFLDILLSARDEDGNGLTDKEIQDEVDTFMFEGHDTTSSGISWCMYNLAKHAEYQQ

KCQQEIDEYFSKKGSKDLEWDDLHNLPYLTLCIKESLRINPAVPFIGRSLTKALYLPDGR

FLPAGMSITINIYGLHHNNTVWDNPEVYDPSRFLPENVKDRSPHAYVPFSAGPRNCIGQN

FAMSELKIVMATILHNFDLSVDTTKQINSISEMVYKTRNGMFLFITKRRK

>395848029

MPQLSLFWLGLGPVAAASWLLLLLVGASWLLARILTWTYTFYDNCRRLHCFPQPPMRSWF

WGHLGLIPPTEEGMRDLTQLVVTYPWGFKRWMGPTIPIITLYHPDFIRSVLSASATIASK

DVIFSSFLKPWLGDGLLLSGGDKWSHHRRMLTPAFHFNILKPYVKVFNESANIMHAKWQR

LASEDSIYLDMFEHISLMTLDSLQKCVFGFNSNCQENPSEYIAAILELSALVVKRNRQIF

LYTDFLYYLTPDGKRFCRDCDVVHEFTDAVIQKRRRTLESQGIDDFLKAKAKSKTLDFID

VLLLAKDENGKELSDKDIRAEADTFMFAGHDTTASGLSWVLYHLARHSEYQEHCREEVRV

LLRDREPKEIEWEDLAQLPFLTMCIKESLRLHPPVTVMSRCCNQDIVLPDGLVIPKGNIC

VISIFGIHHNPSVWSDPEVYDPSRFDPENPQTRSPLAFIPFSAGPRNCIGQTFAMAEMKV

VLALTLLRFRIMCDHIEPRRKPELVLRTEDGLWLRVEPLSMPAGEARSQMTSEQDDLQLR

GGPSGHSSIGATCGRRAGAFST

>348537148

MTLLHTVLALVLSWTGLYSVLYIFSIGLITVVALWTARLLLRHAWYTHKLSCFSKPQTRS

WLLGHLGKMQSTEEGLQRVDDLVQTYKHSCCWFIGPFYHLVRLFHPDYVKPLLMAAASIT

VKDELIYHHLQPWLGNSVLISNGEVWSRKRRLLTPAFHFDILKSYIAVFNSSAKTMHDKW

CRLVAEGKTNLEMFDHVSLMTLDSLLKCAFSYDSNCQESSSEYVSAIVELSDLIIERREK

NILHHWDWIYWKTQQGKQFKQALNTVHRFTRDVVEKRHTLLNQQRETETQSEIAPTAQKR

KDFVDILLLTKDEDGQGLTDEEMQAEANTFMFAGHDTTASAICWTLYNLARHEHYQEKCR

QEVMDLMEGRDGQEIEWEDLSNLPFTTMCIKESLRLHSPVQAVTRSYTQDMVLPGNRTVP

AGTICLVSIYGTHHNPTVWTNPHEYDPQRFDPSNKKSQTSHAFIPFSSGPRNCIGQKFAM

AELRVVVALTLLRFRLTPGVNPELGSSRVRRLPQLVLRAEGGLWLQLEPLNTPT

>AAG09778.1

MLELSVSRLGFGLVAASPWLLLLVVGASWLLARVLAWTYTFYNNCRLLQCFPQPPKQNWF

FAHLYLVPPTEQGLRKSTQLAANYSHGYLIWFGPITPMIVFCHPDMLRSIANASAAVAPK

NMDFYKFLKPWLGDGLLLSAGDKWSRHRHLLTPTFHFNILKPYMKIFTKSTDIMHTKWER

LITQGHTRLDMFEHLSLLTLDSLQKCVFSFDSNCQELSSCRKPSKYITAILELSELVAKR

NRQIFLHADFLYFLTLDGWRFLRACRLVHDFTDAVIQERCRTLPENVDDFLKAKAKTKTL

DFIDVLLLTKDEDGKRLSDEDIRAEADTFMFEGHDTTASGLSWILYNLAKHPEYQERCRQ

EVQELLRDRESKEIEWDNLAQLPFLTMCIKESLRLHPPVTIISRCCTQDIVLPNGWVIPK

GVICIIDIFGTHHNQSVWPDPEVYDPFRFDQENIKGRSPLAFIPFSAGPRNCIGQTFAMT

EMKVVLALTLLRFRFLPDKEEPRRKRELILRAEGGLWLQVEPLSASPQ

>392349263

MPWLTVSGLDLGSVVTSTWHLLLLGAASWILARILAWTYSFCENCSRLRCFPQSPKRNWF

LGHLGTVSMVADRLGLSSSMAALVAPKDPTFLHFLKPWLGDGLFLSSGDKWSRHRRLLTP

AFHFDILKPYVKTFNQSVNIMHAKWKHLCLEGSARLEMFENISLMTLDSLQKCLFGFDSN

CQESPSEYISATLELSSLTRKRSYKLFLYLDFLYYRTADGQRFRKACDLVHSFTDAVIRE

RRRLLSSQGVDEFLESKTKSKSKTLDFIDVLLLAKDEHGKELSDEDIRAEADTFMFGDES

PCVGLTEEVDVFQSQVEDVVDHVVAMCKCIEESHALSGDLCSPWRCNLVWSCLPSFLFHG

SFVPVPFLPSSLVAPKDTTFLRFLKPWLGDGLFLSSGDKWSRHRRLLTPAFHFDILKPYV

KIFNQSVNIMHAKWKHLCLEGSVRLEMFENISLMTLDSLQKCLFGFDSNCQESPSEYISA

ILELSSLIIKRSQQLFLYLDFLYYRTADGRRFRKACDLVHNFTDAVIRERRRLLSSQGVD

EFLESKTKSKSKTLDFIDVLLLAKDEHGKELSDEDIRAEADTFMFGGHDTTASALSWILY

NLARHPEYQERCRQEVWELLRDREPEEIEWDDLAQLPFLTMCIKESLRLHPPAIDLLRRC

TQDIVLPDGRVIPKGNICVISIFGIHHNPSVWPDPEVFDPFRFDSENRQKRSPLSFIPFS

AGPRNCIGQTFAMNEMKVVVALTLLRFRVLPDDKEPRRKPEIILRAEGGLWLRMEPLSTD

TQDDLAQLPFLTMCIKESLRLHPPAVYDPFRFDPESRQKRSPLSFIPFSAGPRNCIGQTF

AMNEMKVAVALTLLRFRLLPDDKEPRRKPEIILRAEGGLRLLVEPLSGGA

>395848042

MTQLSLSSLGLGPVAASPWLLLLLVGTSWLLARVLAWTYTFYDNCLRLRCFLQPPMKNWF

LGHLGLIPPTEEGLRELTQLVATYPKGFKSWLGPIFPIIILCHPDIVRPVLNASAAIALK

EELAARFMKPWLGDGLLLSGGDKWSQHRRMLTPAFHFNILKPYVKIFNKSINIMHAKWQH

LASEGSARLDMFEHISLMTLDSLQKCIFSFDSNCQEKPSEYIATILELSSLIEKRSQNPL

LFPNFLYYLTPNGRRFLKACRLVHDFTDAVIQERRRTLPRQGIDDFLKAKAKTKTLDFID

VLLLSKDEDGKTLSDEDIRSQADTFMFGGHDTTASALSWVLYNLARHPEYQERCRQEVQE

LLRNREPEEIEWDDLAQLPFLTMCIKESLRLHPPVPFITRRCTQDIELPDGRVIPKGVIC

LISILGTHHNPTVWSDPEVYDPSRFDPDKVKERSPMAFIPFSAGPRNCIGQAFAMAEMKV

VLALTLLRFRVLPDHTEPRRLPELILRAEGGLWLHIEQLKAGGQ

>EDW48049.1

MHLRLLSSPQLERTTNLELCSILILLVISLSIYTFYATLNTYLRSVLLSLRLTGPPSLPF

LGNCMLVTDKDLMRRCAGKAFDLYGSLVRIWVLLFPFFAVLEPEDLQVILSSKKHTNKVF

FYRLMHNFLGDGLITSSGSKWSNHRRLIQPAFHHNLLEKFIDTFVDASQSLYENLDAEAV

GTEINIAKYVNNCVLDILNEAVLGVPIKKRGQDVAMMEDSPFRQGKIMMPARFTQPWLLL

DGIYHWTKMANDELNQKKRLNDFTRKMIQRRRQIQNNNNGNSERKCLLDHMIEISESNPD

FTEEDIVNEACTFMLAGQDSVGAAVAFTLFLLTQNPECQDRCVLELATIFEDSNRAPTMT

DLHEMRYMEMCIKEALRLYPSVPLIARKLGEEVRLAKHTLPAGSNVFICPYATHRLAHIY

PDPEKFQPERFSPENSENRHPYAFLPFSAGPRYCIGNRFAIMEIKTIVSRLLRSYQLLPV

TGKTTVAATFRITLRASGGLWVRLKERDHPLIEH

>EDX07270.1

MHLRLLSSPQLERTTNLELCSILILLVISLSIYTFYATLNSYLRSVLLSLRLTGPPSLPF

LGNCMLVTDKDLMRRCAGKAFDLYGSLVRIWVLLFPFFAVLEPEDLQVILSSKKHTNKVF

FYRLMHNFLGDGLITSSGSKWSNHRRLIQPAFHHNLLEKFIDTFVDASQSLYENLDAEAV

GTEINIAKYVNNCVLDILNEAVLGVPIKKRGQDVAMMEDSPFRQGKIMMPARFTQPWLLL

DGIYHWTKMANDELNQKKRLNDFTRKMIQRRRQIQNNNNGNSERKCLLDHMIEISESNPD

FTEEDIVNEACTFMLAGQDSVGAAVAFTLFLLTQNPECQDRCVLELATIFEDSNRAPTMT

DLHEMRYMEMCIKEALRLYPSVPLIARKLGEEVRLAKHTLPAGSNVFICPYATHRLAHIY

PDPEKFQPERFSPENTENRHPYAFLPFSAGPRYCIGNRFAIMEIKTIVSRLLRSYQLLPV

TGKTTVAATFRITLRASGGLWVRLKERDHPLIEH

>Q9V7G5.2

MFVEKVLERTTNLELCSILILLVISLSIYTFYATLNTYLRSVLLSLRLTGPPSLPFLGNC

MLVTDKDLMRRCAGKAFDLYGSLVRIWVLLFPFFAVLEPEDLQVILSSKKHTNKVFFYRL

MHNFLGDGLITSSGSKWSNHRRLIQPAFHHNLLEKFIDTFVDASQSLYENLDAEAVGTEI

NIAKYVNNCVLDILNEAVLGVPIKKRGQDVAMMEDSPFRQGKIMMPARFTQPWLLLDGIY

HWTKMANDELNQKKRLNDFTRKMIQRRRQIQNNNNGNSERKCLLDHMIEISESNRDFTEE

DIVNEACTFMLAGQDSVGAAVAFTLFLLTQNPECQDRCVLELATIFEDSNRAPTMTDLHE

MRYMEMCIKEALRLYPSVPLIARKLGEEVRLAKHTLPAGSNVFICPYATHRLAHIYPDPE

KFQPERFSPENSENRHPYAFLPFSAGPRYCIGNRFAIMEIKTIVSRLLRSYQLLPVTGKT

TIAATFRITLRASGGLWVRLKERDHPLIAH

>EDV55849.1

MTVGMMLERTTKLELCSILILLVISLSIYTFYATLNTYLRSVLLSLKLTGPPSLPFLGNC

MLVTDKDLMRRCAGKAFDLYGSLVRIWVLLFPFFAVLEPEDLQVILSSKKHTNKVFFYRL

MHNFLGDGLITSSGAKWSNHRRLIQPAFHHNLLEKFIDTFVDASQSLYENLDAEAVGTEI

NIAKYVNNCVLDILNEAVLGVPIKKRGQDVVMMEDSPFRQGKIMMPARFTHPWLLLDGIY

HWTKMANDELNQKKRLNDFTRKMIQRRRQIQNNNNGNSERKCLLDHMIEISESNPDFTEE

DIVNEACTFMLAGQDSVGAAVAFTLFLLTQNPECQDRCVQELAAIFEDSNRAPTMTDLHE

MRYMEMCIKEALRLYPSVPLIARKLGEEVRLAKHTLPAGSNVFICPYATHRLAHIYPDPE

KFQPERFSPENSETRHPYAFIPFSAGPRYCIGNRFAIMEIKTIVSRLLRSYQLLPVSGKT

TIAATFRITLRASGGLWVRLKPRDHALTKH

>EDW92017.1

MLVTDKDVMRRCAGKAFDLYGSLVRIWVLLFPFFAVLEPEDLQVILSSKKHTNKVFFYRL

MHNFLGDGLITSSGTKWSNHRRLIQPAFHHNLLEKFIDTFVDASQSLYENLDAEAVGTEI

NIAKYVNNCVLDILNEAVLGVPIKKRGQDVDMMEDSPFRQGKIMMPARFTHPWLLLDGIY

HWTKMANDELNQKKRLNDFTRKMIQRRRQIQNNNNGNSERKCLLDHMIEISESNPDFTEE

DIVNEACTFMLAGQDSVGAAVAFTLFLLTQNPECQDRCVQELESIFEDSSRAPTMTDLHE

MRYMEMCIKEALRLYPSVPLIARKLGEEVRLAKHTLPAGSNVFICPYATHRLAHIYPDPE

KFQPERFSPENSENRHPYAFIPFSAGPRYCIGNRFAIMEIKTIVSRLLRSYQLLPVSGKT

TIAATFRITLRASGGLWVRLKARDHPLTEH

>EDV37199.1

MYVFQFERATKTELCTVLVLLALSLAIYTFYGNLQTYLRSVLLSLRLTGPPSVPLLGNCL

LVTEKDLMRKWAAKAFDLYGSLVRVWVLLFPFFAVLQPEDLQVILSSKKHTNKVFFYRLM

HNFLGDGLITSSGSKWSDHRRLIQPAFHLSLLEKFIDTFVDASQSLYENLDASAAGTEIN

IAKYVNSCVLDILNEAVLGVPVRKKGDQLVNMEESPFRQGKLMMPRRFTHPWLLLDGIYH

WTKMASDELNQKKRLNDFTRQMIQRRRLIHKSSNNNNERKCLLDYMIEISDSNPHFGEED

IVNEACTFMLAGQDSVGAAVAFTLFLLAQNPDCQERCHLELEQIFDSSNRAPSMGDLREM

RYMEMCIKEALRLYPSVPLIARKLGEEVRLGNHTLPAGSNIFICPYATHRLAHIYPDPEK

FQPERFSPENSEHRHPYAFIPFSAGPRYCIGNRFAIMEIKTIVSRLLRSFQLLPVPGKTT

FEATFRITLRASGGLWVRLKPRDHPITAH

>EDW30231.1

MFVEKVLERATRTELCSILALLVISLSIYLFYAHLDTYLRSVILSLRLSGPPTLPILGNC

LLIKDKDLMRNQAAKAFDLYGSLVRIWVLFFPFFVVLQPEDLQVILSSQKHTNKVFIYQL

MHNFLGDGLITSSGLKWSTHRRLIQPAFHLSLLEKFIGTFVDASQSLYESVGASAVGTDI

NIVSIVNNCVVDILNEAVLGVPIKKKGLDLVKIDESPFRQGKLMIPTRFSHPWLLFDGIY

HWTKLANDELNQKKRLHDFTRQMIKIRREILANTGNNNAERKCLLDYMIEISESNPDFTE

QDIVNEACTFMLAGQDSVGGAVAFTIFLLAQNAECQEQCHEELERIFDYSDRAPTMSDLR

EMRYLEMCIKEALRLYPTVPLIARKLGEEVRLSDHTLPAGSNIFICPYATHRLNNIYPEP

EKFNPERFSPENSKDRHPYAFIPFSAGPRYCIGNRFAIMEMKTIVSRLLRSFQLLPVAGK

TTFDVSYRITLRASGGLWVRLKPRDHPISQTRDSDCFGL

>EAL26607.2

MFVEKVLERATRTELCSILALLVISLSIYLFYAHLDTYLRSVILSLRLSGPPTLPILGNC

LLIKDKDLMRNQAAKAFDLYGSLVRIWVLFFPFFVVLQPEDLQVILSSQKHTNKVFIYQL

MHNFLGDGLITSSGLKWSTHRRLIQPAFHLSLLEKFIGTFVDASQSLYESVGASAVGTDI

NIVSIVNNCVVDILNEAVLGVPIKKKGLDLVKIDESPFRQGKLMIPTRFSHPWLLFDGIY

HWTKLANDELNQKKRLHDFTRQMIKIRREILANTGNNNAERKCLLDYMIEISESNPDFTE

QDIVNEACTFMLAGQDSVGGAVAFTIFLLAQNAECQEQCHEELERIFDYSDRAPTMSDLR

EMRYLEMCIKEALRLYPTVPLIARKLGEEVRLSDHTLPAGSNIFICPYATHRLNNIYPEP

EKFNPERFSPENSKDRHPYAFIPFSAGPRYCIGNRFAIMEMKTIVSRLLRSFQLLPVAGK

TTFDVSYRITLRASGGLWVRLKPRDHPISQTRDSDCFGL

>EDW77109.1

MKCLQRATKSELCTVLALILVSLSVYRFYAHLQTYLRSVLLSLRLSGPPTLPFLGNCMLI

KEKDLMRKRAAKAFDLYGSLIRIWVLLFPFFVVLQPEDLQVILSSKKHTNKVFFYRLMHN

FLGDGLITSSGSKWSSHRKLIQPAFHLSLLGKFIDTFVDASQSLYEHLDDAAAVGSDLNI

AKYVNNCVLDILNEAVLGVPIRKKNQELVDMEESPFRQGKLMIPTRFANPWLLWDGLYHW

TKLANDELNQKKRLNDFTRQMIKRRREIMVNCGNNNTDERKCLLDYMIEISESNPDFTEE

DIVNEACTFMLAGQDSVGAAVAFTIFLLAQNPECQEKCCEELERIFDYTNRAPSMSDLRE

MRYMEMCIKESLRLYPSVPLIARKLGEEVSLNGGAYTLPAGSNVFICPYATHRLAHIYPE

PEKFMPERFSPENAEHRHPYAFLPFSAGPRYCIGNRFAILEIKTIVSRLLRSYQLLPVPG

RTTFEATFRITLRASGGLWVRLKPREHPITHTHTHTNSKNDMEIF

>EDW61836.1

MFIQNFYGHNAQLTLNLVLVLERATKSELCSALALLVLSLSIYAFYTQLLSYLKSVLLSL

RLSGPPSLPFLGNCLLINEKDLMRKRAARAFDLYGSLVRIWVLLFPFFVVLQPEDLQVIL

SSKKHTNKVFFYRLMHNFLGNGLITSSGVKWSTHRKLIQPAFHLSLLEKFIDTFVDASQS

LNEHLDASALDIEINIAKYVNNCVIDVLNEAVLGVPIKKKGLGLMEDSPFRKGKLMMPAR

FAQPWLLLDAIYQLTSLASDELNQKKRLNDFTRQMIKRRREIMADAANNNVERKCLLDYM

IEISESNPDFSEEDIVNEACTFMLAGQDSVGAAVAFTIFLLAQNADSQEQCHEELERIFD

YSNRAPTMSDLREMRYLEMCIKEALRLYPSVPLIARKLGEEVRLGAYTLPAGSNIFICPY

ATHRLAHIYPEPEKFKPERFSTDNVEQRHPYAFIPFSAGPRYCIGNRFAILEIKTIVSRL

LRSYQLLPVPGKTTFEATFRITLRASGGLWVRLKPRQQHPEAV

>EDW09262.1

MIDRERERERERGRLYLYLYLYLYLYLYLYLYLYLYLYLYLYLYLYLYLYLYLYLYLYLY

LYLYLYLYLYLYLYLYLYLYLYLYLYLYLYLYLYLYLYLYLYLYLYLYLYLYLYLYLYLY

LYLYLYLYLYLYLYLYLYLYLYLYLYLYLYLYLYLYLYLYLYLYLYLYLYLYLYLYLYLY

LYLYLYLYLYLYLYLYLYLYLYLYLYLYICICICICIFAFLERATRSELYSALALLVLSL

SIYTFYTQLQTYLRSVLLSLRLSGPPTLPFLGNCMLINEKDLMRKRAANAFDLYGSLVRI

WVLLFPFFVVLQPEDLQVILSSKKHTNKVFIYRLMHNFLGNGLITSSGDKWSTHRKLIQP

AFHLSLLEKFIDTFVDASQSLNEHLDASALDMEVNIAKYVNNCVVDVLNEAVLGVPIKKK

GLMEDSPFRKGKLMIRARFLHPWLLLDAIYQLTSLASDELKQKKRLNDFTRQMIKRRREI

MADAANNNVERKCFLDYMIEISESNPDFSEEDIVNEACTFMLAGQDSVGAAVAFTIFLLA

QHTDSQAQCYEELERIFDHTNRAPTMSDLREMRYLEMCIKEALRLYPSVPLIARKLGEEV

RLADYTLPAGSNIFICPYATHRLAHIYPEPEKFKPERFSTENMEQRHPYAFIPFSAGPRY

CIGNRFAIMEIKTIVSRLLRSYQILPVPGKTTFEATFRITLRASGGLWVRLRPRQQQQET

I

>EAA08827.5

MTFFQALEKSSAVELYTYLTLILVCVLLYIFSDYLKICALAFRLPGPKSYPIIGNCLDIA

RKDLITREMAQAYKNYGPLARVWIFVFPMFVVFDPNDLKMILSSKKHTNKSMFYKLLHNF

LGRGLITSSGHKWSSHRKLIQPSFNIAILEKFIDTFTDSASFLIDKLPKEETVLNVTEYV

NNCVLDILNEAVLGVPVKSVDKKEMEQSPFRQGKVVAPYRITHPWLLFNSIYKLTDAATA

ELNQKKQLDDFSRKMIRRRREILKTLSPSDRRCLLDFMIEISNENPEFTEDDIIDEACTF

MLAGQDSVGAAVAFTLFLLARHQDQQAKCYEEIERHIGTDCSKPPSAEGIRELRHLEACI

KESLRLYPSVPLMARKIGEDVRVGKYNLPTGTEIMILPYATHRLEHIYPDPERFDPERFG

DGAPHQNPYAFLPFSAGPRNCIGYKFAYIEMKTVIARVLQNFHLSPAPGKEEVQPIFRMT

LRARGGLWVKMTPRKVVS

>383853257

MAVVEKDFSRKVVKWDQTPGGVWTYLVILASIYILYILKSYIRTVIFVSRLDGPKTVPFL

GNARCVLKDNLLYRLAHENQMYGRIVRIWLTGLPYVILVEPEDIQIVLGSMKHTRKIFFY

KLLDNFLGKGLITRDVDTWRTHRKFLQPAFHLHILERFTSSFAECADCLMNEFLKQDRQE

INITSFINDSVYDILSETVLGINRRSGLASDDDLPFRKGQIMLLYRMVRPWLLIEWIYRL

TKHGRQEEKQRKDLFDTCFKMMKEKRDVLRNKDATTNVTNTKKISLLEYMVEMNEKNPCF

TDEDIVEECCTFMLAGQDSVGTATAMTIFLLANHPEWQEKCLEEVDNIFDGDSRPPTMKD

LREMRCLEMCIKEALRLYPSVPIIARILGEDVKIGKHVVPAGCGVFISPYSTHRLAHHFP

DPEAFKPERFSPENSEKRHPYAYIPFSAGPRNCIGYKFAMLEMKCMVSAILRKCRLESIP

GKQEVRPKFRMTIRAQGGLWVKVVARDKKLNTVVT

>EGI65786.1

MWTYLAFLAVIYVIYTLKNYVRILRFIFTLDGPKTVPIFGNANIILEGNLIQRMINEAQN

YGRIFRIWLTLLPYVILVEPEDIQVVLSSIKHTQKIFFYKLLDNFLGKGLITQNVDQWKV

HRRFLQPIFHRQVLEKFVGIFGKCADCLIDKLLENDGKDINITVFINNSVYDILIETILG

TPANQRKNDGENDPFRKGQVMMPYRFARPWLLIDWIYRLTAAGKSEEQQQRDLFNFCFKK

MKEKREFLKKNGSLIVDDNTIRKKSLLEYMVEISERNPEFTERDIIEECCTFMLAGQDSV

GTATAITLFLLANNPKWQERCVTELHEIFNDDTRSPTMQDLMKMTCLDMCIKESLRLHPS

VPLFARTLGEDVKLGKYIIPAGCGVFIAPYCTHRLSHHFPDPHDFKPERFSSENSEGRHP

YAYIPFSAGPRNCIGYKFAILEIKSIISAILRKCRLEPTLGKEKMIAKFRLTVRAQGGLW

VKVRACDIN

>345490271

MRPPDFKSLDTRDSSRASWIFRSARRLLLQISPPTSLFALTMGAVIRQIWDRDAQELWIY

VALIVGTLAIYLLKDQIRIYWTIFQLNGPPTVPLLGNVDCLLRKDILEVMSHTAYKDYGP

VARFWVTLIPYVILLEPDDIQAVLGSAKHTSKIFVYRLLDNFLGKGLITSDVDTWRTHRR

LLQPAFHLHVLQKFVGSFAERADRLADRLARYDNQQLDITKFVNDAVYEILNETVLGVNV

ASTANEDGVVDLDDLPFRKGQVLMTHRLIRPWLIFDWIYKLTSTGQQEKKQSQDLSAACH

RIIQEKRRLQRDSSSLTKKTSLLEYMMETSERNPEWFSDEDIINECCTFMLAGQDSVGTA

TAMTLFLLANHPDWQDKCREELDEIFAEGETNRPPTMQDLRAMRWLECCIKEALRLYPSV

PIFARKLGEDVKVGKHVIPSGCGVIILPYSTHRLPHHFPDPHSFRPERFSPENSEKRHPY

AYLPFSAGPRNCIGNKFAILEMKAVISAILRRYRLGGVEGKTEVRPKFRLTVRASGGLWL

KISQRVDAR

>EFN66590.1

MWTYLAFLVIVYVIYTLRNYIRILKFIFTLDGPKTVPILGNANAVLEGNLIQRMTNEVQN

YGRIFRIWITLLPYVVLVEPDDIQVVLSSMKHTRKIFFYKLLDNFLGKGLITRDVDKWKI

HRKILQPAFHLHILEEFAGTFAKCADHLIEKLLEKDGKDVNVTVFINNSVYDILTETILG

MKVDRRNSNAEDDTPFRKGQVMIPYRFARPWLLIDWIYRLTAAGKSEEQQQKDLLDFCFR

KMKEKRELLRQNNSFVIDDETMSAEGRKISLLEFMVEISERNSCFTDRDIIEECCTFMLA

GQDSVGTAAAMTLFLLANNPIWQEKCIAELDEIFGSDERSPTIQDLKNMKCLDMCIKESL

RLYPSVPIFARTLGEDVRIGKHVIPAGCGVFIAPYCTQRLAHHFPNPHDFKPERFSLENS

ETRHPYAYIPFSAGPRNCIGYKFATLEMKSIISAVLRKCRLESILGKEKVIAKFRMTIRA

HGGLWVKVRSRKS

>380023000

MVVTAQQIWEHTPGEAWVYLVILTSIYILYILRTYIRSVIHISRLNGPKTIPIIGNVNCI

LKENFLFRLAYESQIYGRIVKIWLTVFPYVMFLEPEDIQLILNNAKHTRKVFFYKLLDNF

LGKGLITRDIKSWRIHRRLLQPAFHLHVLEKFTNTFAKHADHLMNKILERNNQEINITTF

INDSVYNILSETVLGINRINRINEIDDLPFRKGQIMLLYRMIRPWLLIEWIYRLTKHGRQ

EEKQRKDLFDTCFKMVKEKRDLLQSKEHIPNNDIKKNRNISLLEYMVEINEKNPCFSDED

IVEECCTFMLAGQDSVGTATAMTIFLLANHPEWQNKCIEEIDEIFNGDTRFPTINDLKXM

KCLEMCIKESLRLYPSVPIIGRILGEDIKIGKHIIPAGCSVLISPYSTHHLPHHFPDPDA

FKPERFSPENSEKRHPYAYIPFSAGPRNCIGYKFAMLEMKSIISAILRRCRLQSIPGKKX

IRPKFRMTIRAQGGLWVKIVERDQILKSIAAQT

>350420628

MVVTAQQIWEHTPGEAWIYLVILTSIYILYILKNYIRCVIHVSRLNGPKAVPIIGNVNIA

LEENFLYRLANESKIYGRFVKVWVTILPYVVLLEPEDIQLVLGSTKHTRKVFFYKLLDNF

LGKGLITRDMETWRVHRKILQPAFHLHILEKFTNIFAEHADSLMKKFLEKKDQEINITTF

INDSVYNILSETVLGINQRTSKMDDLPFRKGQIMLLYRMMRPWLLMEWIYRLTKYGREEQ

KQRNELFDTCFKMMKEKRELLRNKKCEPDDQTEETRKMSLLEYMVEINEKHPCFSDKDIV

EECCTFMLAGQDSVGTATAMTLFLLANNPEWQEKCIEELDRIFDGNPKLPTINDLKDMKC

LEMCIKESLRLYPSVPIIARKLGEDVKIGKYVIPTGCSVLISPYATHRLPHHFPDPEAFK

PERFDTENSEKRHPYAHIPFSAGPRNCIGYKFAMLEMKSMICAILRRCRLQSVPGKEVIR

PKFRMTIRAQGGLWVKVIVRDETPKNVVI

>350420631

MVFLEWEHTPGEAWIYLVILTSIYILYILKNYIRCVIHVSRLNGPKAVPIIGNVNIALEE

NFLYRLANESKIYGRFVKVWVTILPYVVLLEPEDIQLVLGSTKHTRKVFFYKLLDNFLGK

GLITRDMETWRVHRKILQPAFHLHILEKFTNIFAEHADSLMKKFLEKKDQEINITTFIND

SVYNILSETVLGINQRTSKMDDLPFRKGQIMLLYRMMRPWLLMEWIYRLTKYGREEQKQR

NELFDTCFKMMKEKRELLRNKKCEPDDQTEETRKMSLLEYMVEINEKHPCFSDKDIVEEC

CTFMLAGQDSVGTATAMTLFLLANNPEWQEKCIEELDRIFDGNPKLPTINDLKDMKCLEM

CIKESLRLYPSVPIIARKLGEDVKIGKYVIPTGCSVLISPYATHRLPHHFPDPEAFKPER

FDTENSEKRHPYAHIPFSAGPRNCIGYKFAMLEMKSMICAILRRCRLQSVPGKEVIRPKF

RMTIRAQGGLWVKVIVRDETPKNVVI

>340716884

MVVTAQQIWEHTPGEAWVYLVILASIYILYILKNYIRCVIHVSRLNGPKTVPIIGNVNYA

LEENFLYRLANESKIYGRFVKVWVTILPYVVLLEPEDIQMVLGSTKHTRKVFFYKLLDNF

LGKGLITRDMETWRVHRKILQPAFHLHILEKFTNIFAEHADCLMKKFLEMKDQEINITTF

INDSVYNILSETVLGINQRTSEMDDLPFRKGQIMLLYRMMRPWLLMEWIYRLTKHGREEQ

KQRNELFDTCFKMMKEKRELLRNKKRGPDDQTEETGKISLLEYMVEINEKNPCFSDKDIV

EECCTFMLAGQDSVGTATAMTLFLLANNPEWQEKCIEELDRIFDGNPKLPTINDLKDMKC

LEMCIKESLRLYPSVPIIARKLGEDVKIGKYVIPTGCSVLISPYATHRLPHHFPDPETFK

PERFDAENSEKRHPYAHIPFSAGPRNCIGNKFAMLEMKSMICAVLRRCRLQSVPGKEEVR

PKFRMTIRAQGGLWVKVIVRDETPKSVVI

>340716886

MVFLKWEHTPGEAWVYLVILASIYILYILKNYIRCVIHVSRLNGPKTVPIIGNVNYALEE

NFLYRLANESKIYGRFVKVWVTILPYVVLLEPEDIQMVLGSTKHTRKVFFYKLLDNFLGK

GLITRDMETWRVHRKILQPAFHLHILEKFTNIFAEHADCLMKKFLEMKDQEINITTFIND

SVYNILSETVLGINQRTSEMDDLPFRKGQIMLLYRMMRPWLLMEWIYRLTKHGREEQKQR

NELFDTCFKMMKEKRELLRNKKRGPDDQTEETGKISLLEYMVEINEKNPCFSDKDIVEEC

CTFMLAGQDSVGTATAMTLFLLANNPEWQEKCIEELDRIFDGNPKLPTINDLKDMKCLEM

CIKESLRLYPSVPIIARKLGEDVKIGKYVIPTGCSVLISPYATHRLPHHFPDPETFKPER

FDAENSEKRHPYAHIPFSAGPRNCIGNKFAMLEMKSMICAVLRRCRLQSVPGKEEVRPKF

RMTIRAQGGLWVKVIVRDETPKSVVI

>189235279

MWTDICTYLVILAVMLMSIYFKNYIRSVYLAFQLSGPPAVPLIGNVLLMKNDRKLEEIAE

KMSEMYSPFMRCWLSIFPVFFIYEPKALQLIMGTNRYTSKNIFYNCLHNVVGDGLITSSG

EIWKQHRKLIQPYFHISVLKVYFDIFYKWSMAMVQDLENETEVNITKYANKFVFDVQEES

ILGISGKRPESSPYRGGQFLMLHRITRPWLLFNTIFKRTDFSKVEENQKNILQQYTKSIL

DGKKQNSHGTAMNNLMEMMMEISNMNPEFTDDDIVNETCTFMLAGQDSVGAALSFALYSL

AAHQDIQEKVVQELNGIFKDGNQAATFEDVAEMKYLEQCIKETLRLYPSVPMITRKITED

VPLGKYTLPTGTNIVISPFVTHRLPHVFPDPLKFDPDRFSPENKAKIHPYGFIPFSAGPR

NCIGYKFAIIELKTVLSQILRKYHVSLVPGREKLILSYRMTLKAKKGIWLRLKKRSV

>EFN76645.1

MWMYVTFLFVIYALYTLWDYVRIYRFVFTLNGPKTVPILGNANALLEKNLMQRFANEKSD

YGRIFRIWVIFLPYVVLIEPEDIQVVLSSMKHTRKVGFYRLLNNFIGKGLISLEVDKWKV

HRKILQPAFHLHVLKSFIATFSECADHLVNKLLERNGEEVNLTVFINNSVYDILLETILG

ISASRGDNNSKADMPFGKGQFMAIQRFVRPWLLINWIYRLTEAGKMEQQQQKNLINFCCR

KMKEKQEFLQKNSSLIVEDESDAPIKKITLLEYMIKINEKNPSFIEQDIIEECCTFMLAG

MESVSTSTALTLFLLALNRNWQERCIAELDEIFGDDRRSPTMQDLKEMKCLEMCIKESLR

LYPSVPLFARVLGEDVRIGKQIIPAGCGIFILPYSTHRLPNHFPDPHDFKPERFSPENSK

GRHPYAYLPFSAGPRDCIGQKFAILEMKSIISAILRRCRLESICGKEEVIPKFRMTIRVH

GGLWVKVKPRDECNY

>EFA01330.1

MWTDICTYLVILAVMLMSIYFKNYIRSVYLAFQLSGPPAVPLIGNVLLMKNDRKLEEIAE

KMSEMYSPFMRCWLSIFPVFFIYEPKALQLIMGTNRYTSKNIFYNCLHNVVGDGLITSSG

EIWKQHRKLIQPYFHISVLKVYFDIFYKWSMAMVQDLENETEVNITKYANKFVFDSQYWA

FLGKDQSRLLIGGNLVQHIHQCQFLMLHRITRPWLLFNTIFKRTDFSKVEENQKNILQQY

TKSILDGKKQNSHGTAMNNLMEMMMEISNMNPEFTDDDIVNETCTFMLAGQDSVGAALSF

ALYSLAAHQDIQEKVVQELNGIFKDGNQAATFEDVAEMKYLEQCIKETLRLYPSVPMITR

KITEDVPLGKYTLPTGTNIVISPFVTHRLPHVFPDPLKFDPDRFSPENKAKIHPYGFIPF

SAGPRNCIGYKFAIIELKTVLSQILRKYHVSLVPGREKLILSYRMTLKAKKGIWLRLKKR

SV

>345483339

MIIYFLLILLGLWVFKVYFQNKRSKELLDKIPGPPTVPILGNLLMFNVPIEKTWNVIRDL

NKNYYPIAKFWLGPYPYVSIHHPDDLEILLSSTKHLEKGSAYTVLHPWLRTGLLTSSGSK

WQQRRKILTPAFHFKILRKYMDITNEHGKKFIEALRTENSETVQSLMPFCSNYTLHIICE

TAMGIALDKIDTKISKEYKNAVADIANLIMYRVMRPYLKEWMMLPVWTFGRLQTQTLRTL

HKFTKTVLTERREYHKRSDNKYLKGFHDDTYDVNKDDVAIGGKKKLAMLDLLLSAAQDGL

TDDEGIKEEVDTFMFAGHDTTGIALVYAIMLLAEHKEIQEKARAEVIEVLTESNGEIGTL

EIQKLHYLERCIKESLRIFPPVSALSRTVKEDIQLKNYVVPAGTEIGCHIFDLHRDPNFW

PEPEKYDPDRFLPENIQGRHPYAYIPFSAGSRNCIGQKFAMMELKSLTARILYNFELEPV

SQTKDMKLTLDLVTRPLEPVYTKFIRIDNNN

>289177037

MTIYFLLIFLVLLFHRIYARNIKYRSLLKKIPGPPTLPLLGNLLIFNVPVEKQWDIVRSL

TKQYYPICKIWLGPRHAVVSLLHPDDIEVLLTSSKHINKGDSYTYLHTWLKTGLLTSSGD

KWRQRRKILTPAFHFNILKKYMDITNENSINFIEALRNEGEETIQNLTPLCSKYTLNIIL

ESAMGIVLDKVDKKTADDYKNAVYEIGRIIMYRLARPYIREWMLNPLMNLARVQKRVLKT

LHDFTEKVVKDRREYHERTENKYLDNFTNESHCDDVYAGTGGRKKRMAMLDLLLSAERDG

LIDDEGIKEEVDTFTFEGHDTTAMAMTFALLLLAENKKAQDKAREEVTEILDRSEGNMGM

AQIQEFNYLERCIKESLRLFPPVATMARTITEDLQLKNYLVPAGTEVMYHLWEIHRDPNF

WEEPLKFDPNRFLPERSQGRHPFSYVPFSAGPRNCIGQKFAMMELKSLIGRILYNFKLEP

IDKTADMPMLLDIVIRPAKPVYTRFVRIDK

>289177048

MLMYILLCVLGLIFCYIYNNRDDRKVKLINRIPGPPGLPVIGNLLIGLTPVDQLWDVVRS

YTETYYPTIRIWLGNYYSIVSISDPDDVETLLTSQKHIEKGYSYKNLQPWLKMGLLTSTG

EKWRRRRKILTPAFHFNILKKYVDITNENAERFVEKMRDDEGDDTVQDLAPLTSKYTLNI

ICESAMGVALDEIESKAAEKYKNAVYTMGNITIYRITRPYLTDWVMNICWRLKKLQEKTL

KTLHEFTDKVIMERKAQHKNTDYKYIKNLADDIKESEFDYVTSGRKKRLAMLDLLLSAEM

DGLIDDDGIREEVDTFMFEGHDTTGMAMTFTLMLLAENEEIQEKARAEVIKVLTESSGKI

GMRQLQEFNYLECCIKESLRLYPPVANISRYITEDLQLKKYLVPANTEVFVQLYPIHRDR

KFWREPNKFDPDRFLPENLQGRHPFSYIPFSAGPRNCIGQKFALMELKSLIARILYNFKL

EPIDRSADMKILLDIVIRPASPVRTRFVKINH

>289177033

MLMHLLLFIVLALTVFHFYIRYRRIGRLASAIPGPSPYPFIGNMLSFDISSPVSMWLVLR

QLSNQYYPITRVWMTTEALYSVRHPDDIEIALTSRKTIDKGWVYKYLHPWLRTGLLTSSG

EKWRHRRKILTPAFHFNILRKYMDITQEQGEKLIKFLQCDGKESVQSLIPFCSKFTLNII

CESAMGVALDSLDTKTIEKYKQAIYKIGEITVYRMPRPYIHDWMLKLVPFLPINRHRKQA

LSILHEFTAKVLKERKEYHDRTGNKYLQGLTEEDKDGDEDVYMGCKKRLAMLDLLLSAEK

DGLIDDEGIKEEVDTFTFEGHDTTGMAMTFILMLLAENKNAQDKARAEVNEMLKKNNGIL

GIAEVQELHYLERCIKESMRLYPPVPTIFRDVSEDLQFKHGLIPGGSCVVCHFYDLHRDP

NFWDEPDKFDPDRFLLENSSKRHPFAYIPFSAGSRNCIGQKFALLELKSILGRILQNFYL

EPVTRLADIKLIADLVLRPSEPVHLKFVKIEKNT

>289177125

MFYLLLVILGVLIFHISTRYSRMGKLVNKIPGPRPIPILGNMLLLNVSSDKLFGIMRELI

NSYYPILKVWFCTNAMVGLRHPDDLEVILSSQKAIDKSYVYGYLEPWLKTGLLTSTGDKW

RARRKILTPAFHFQILKKYMDITSEQGEKMIDALRQKKEMVENIVPMCSTFTLNIICESA

MGVALDKIDAKELKEYKMAIHDMGDVVVQRSVQPYVLDWMMSPLIKLGRMQKNALTVLHK

FTDKVLKERRNYHNRTENKYLHELDDNAHDDDSDKVALGRKKRLAMLDLLLLAEKKGLID

EEGIKEEMDTFTFEGHDTTGIALIFILLMLAENKEAQDKAREEVSEMLNSTGGKISQTEI

QDFNYLERCIKETLRLYPSVPNVLRHLTEDLQLKTHTLPAGVDVICFLYDVHRDPNFWPD

PEKFDPDRFLPESSAGRHPYAYVPFSAGPRNCIGQKFAMMELKSLVARILYNFQLEPIDR

SADVKFTTDLVLRPTNPIRVKFVRIDK

>345490638

MYLLFILFLGPLLFHIITRTNRRARILKKIPGLKPYPFVGNLLTFNVNCLNKFWELGRQF

SNEYYPITRFWLFKTAVISVRHPDDLEIIFTSQKFIDKGYIYDYLHPWLKTGLLTSTTNK

WRQRRKILTPAFHFSILKKYLENMNEEGKKIVNELKNEGQIIEKSLMPFCSSCTLNIICE

SALGVSLDSLDGKIVNKYKNAVHDIGNVVLYRMPRPFLTNWMMNFVWKMGSVQRNAVRVL

HRFTDEIIKERKTYHKRMNNKYLKEYADNIKQNRSYEKDINQKKKYAMLDLLLAAEYNGL

IDYNGIKEEVDTFTFEAHDTTGMAMAFALMLLAENHEAQNKARVEIIEVLDRHNGEMELT

NLQEMNYLDRCIKEALRLFPPVATVMRYTAEEVQLKNALVPADSHIMIHLYDTHRDPNFW

AEPEKFDPDRFLPERSINRHPFAYLPFSAGPRNCIGQKFAMIELKSLISLILYDFYLEPI

DRLDDMKLIADIILRPLNPIRIKLIRIKK

>345490636

MIFYLSSLLLVLCIFHIITRYNRRGRLLSKIPGPTAYPIVGNLLMFNMSSMHDFWQLGRL

TMKKYYPITRFWGLHSALISVGHPEDLEILLSSQKVIEKGYVYWYLQPWLRTGLLTSTDD

KWRQRRKILTPAFHFSILQKYFDITSEQGERFIEDLKAEGDETIKSLMPLCSNYTLNIIC

ESILGVVLNKMNDQEVKKYKQAILDVGSVVLYRTVRPFIANWMLPFVWKIGHMQRNALKI

LHDFTDKVLKERRKYHESTGYRYLNDEYTGEVKYDLGIGRKRRLAMLDLLLAAEKNGLIQ

EDGIKEEVDTFTFEGHDTSSMAMTFTLMLLAENKEAQDLARVEVIRVLDKSGGKIGVTEI

QELHYLERCLKESFRLYPPVATLMRYVSEELELKNATVPADSHVMIHFWDTHRDVNFWPE

PDKFDPDRFLPENAKNRHPYAYVPFSAGPRNCIGQKFAIMELKSLIARILYDFYLEPVDR

TTDMRLIADIILRPLDPVHLKFIKINKC

>289177039

MAYYYLFTVLLGLVGLLFHFYIQLFSRTGKLVNKIPGPTAIPFFGNLLELHVPLVEINNA

LRKWSKIYYPIYRLWFGPKALVFILHPDDLEIMLTSSKHINKQFAYEHFHLWLHSGLLTS

NGQKWHHRRKILTPAFHFNILKKYMEITNEEGERAVTNTRKTTKETKVDLLQFCSKYTLN

IICESAMGVPLDGDHKDKEASNKYKNAVYRMGNIIFFRIIRPYIWDWMVPFIPKHNREVK

EVVANLNNFTDMIVEERREYHASTNYEHLNFEKSEVNAYIGKSKRLAMLDLLLAAERDGK

IDDEGIKEEVSTFIFEGHDTTAMSMCFIIMLLAENKHCQDLAREEVEIILGPKNGQLETA

DLKHMNYLERCIKESLRLFPSVPSITRYLHEDVQLKNYKIPAGVNIIMHIIDVHRDPNFW

PEPEKFVPDRFLPEEIAKRHNFAYLPFSAGSRNCIGQKFAMMELKSLISRILYNFHLEPI

DYTRDVKLISDVVIRPSKPVYTKFVRIDRQSNI

>289177041

MIRIFVLLLLLTLIACHIFTRYTRKGRLLNIIPGPAPLPIIGNLLQFAVTSAHDLWKIAR

KYSTDYYPITRFWTTGLCVVSVRHPDDLEILLNSQKTITKGYIYNYLHSWLKSGLLTSTG

DKWRQRRKLLTPAFHFNILKEYLGPMNEEGLRCVDDLKNDGESTIKDLVEFLSESTLNVI

CESTMGVSLNRIETGLTTRYKKAIHEMGYIVSFRLCRPYIFDWIMNLPWKFGNLQRKSLK

ILHHFTNKFIQEKKIFNEESKGNYSDVTQHSEDNGEHSGSKKRLAFLDLLLTAKKNNMID

DEGIQEEVDTFTFEGHDTTAMALTFTLMLLAENKEAQEKARAEAKEVLDCSHGKLDVSDV

QKLNYLDRCIKESLRLYPPVSTIMRYSADELQLKNALVPADTHIVVNFFDTHRDPNFWPE

PNKFDPDRFLPERSVGRHPYAFVPFSAGSRNCIGQKFAMMEMKILIARILYDFRLEPTEH

LADGYADIPLLADIVIRPLNKIFTRFIKL

>156543774

MWLQILLTLLVVLFVIHYYIRGGRIGHYVNKMPGPPAYPLVGNLLSFMVPFDQLWQVVRK

MNDDYYPLYRIWSTNVAVINLYHPDDAEVLLSSTNHIEKSFIYELLHPWFGTGLLTSTGT

KWQHRRKILTPAFHFNVLQKYMDIIIENDERFIDTLKAKGESVQNLLPLITNYTLNVICE

AAMGTSLQGKEELQARYRRAIHDMGNILVYRLVRPWLRWDRLFNLTPKGHEQKKALQVLH

GFSTDIINERKEYHEKTESKFLNNTLASDESSKDNADTEVFGVRKRRLAMLDLLIAAYRE

KQIDDLGIREEVDTFMFEGHDTSAMGVCFVLLLLAEHKDIQARAREEVEEVLGKTGGRID

LSAINQMSHLERCIKEALRLYPSVPFISRNINEDLHLKDYTVPRGTIAHIHVYDLHRDAN

FWPEPLKYDPDRFLPERTRNRHPFSYIPFSAGSRNCIGQKFAMMELKSITAHLLHDFHLE

PIDLAHEVPIVCDLVLRPARPIYMKFVPIEK

>345481035

MLFYIVLSALVAALVYFHISIRYSRQGRMLSKIPGPFSFPIIGSLHLFTDRTQRGLWITI

RMITDTWYPICKGWAMGLPLLSVRHPDDLEVILSSQKACYKEFPYKYLRSWLNDGLLTNK

GDTYRARRRMLTPAFHFNMLRRYVDIMNEQSGRLMTELRANGKEFVLDVLPIYADVSLNI

IIESAMGIDLDKTDKKVIKDYKEAIEDMGIVLTYRMLRPYILDWMMTPFTEIGRKQIHAL

KVLREFTDKIIKERRKYHEMLGGIKGYKEFTDSLENQDESESIYAIGHKKKLPMLDILLS

AEKDGLLDEKGIKEEVDTFLLGGTDTTRMVMAYFTMLMAENKKCQDKARAEVCKVLERSG

GKRGMTELSQMPYLEQCIKESMRLYPIASTIFRHTVDDIQLKNYTIPAGVVVICQIIDAH

RDPNFWTEPEKFDPDRFLPENCRHRHPFAYLPFSAGPRNCIGQKFGWMEVKAVCSRLLYN

FYLEPIDSTRDMQLIGDFVLRPADPFHTKFIRIDRE

>289177143

MLIETIVVICMLLYAFHHYTRNDRIGRYVNKIPGPPTWPIVGNLPALSAPHEQLWPLGIK

WDKQYYPIYKIWTTHKAVIVILHPDDVETLLTSMEHIEKSIMYDMVHPWFGTGLLTSTGA

KWQKRRKILTPAFHFHVLQKYFDIIVENNEKVMHSLKSNGSVINNLVELLTYYTLDVICE

TAMGTTLLDKEHQQKYRNVLHDVSNAVIYRLMRPWFQVDWTFRWTSVAEKQKKALEVIHG

FSKKIIKERKEYHEKTKGHYLNQLESDQSDDKKEDEEYFGYRKKRLAMLDLLIAKQKDGN

QIDDAGIREEVDTFIFEGHDTSAMAMCFAILLIAEHNDVQDRIRAEVKEVLEKSEGKMGV

PEIQQLNYLELCIKESLRLYPSVPFISRGVTKDLHLKNYIVPKGTLIQINIFSLHRDPNF

WPDPEKFDPDRFLPDRFQGRHPYSYIPFSAGPRNCIGQKFAMMELKAFIAHLISEFYLEP

IDLAHEVLITPDLVLRPARPVKVKIVPVNK

>289177139

MIVEFLLLLLLAACIFHYYTRCGRVAKLTENIPGVRAWPIVGSLPGLMKSQDELWPIFRK

MSNDYYPTYKFWTTHEVVINIRHPDDIEILLSSVQHLEKSTAYKLLHPWLKTGLLTSAGN

KWQARRKILTPAFHFNVLKKYMDIIVENSERMVRSLKTKASDGPIIDLLQFSTNYTLNVI

CETAMGTSLHGMEEFQEKYRNAIHKMGDVLIYRIARPWFRKESTFKWTQKGQEQTAALKV

LHGFSEKIISERKEYHDQTKYNYLNQFMTDEVNDEEDNEIYGIRKKRLAMLDLLISLFRN

GQIDDEGIQEEVDTFIFEGHDTAAMGLSFALLLLAEHTDSQSRARDEVKEMFNKSGGKMG

YSEIQQLQYLEMCIKESLRLYPSVPFISRQLKKDLQLKHYLIPSGAIMHVHIYDLHRDAN

FWPEPEKYDPERFSPDSIRNRHPFSYIPFSAGPRNCIGQRFAMMELKASIAHLLHHFILE

PIDYAHEVPIRSDLVIRPARPIRIKFVPIKHDALL

>156552065

MLVLTTLLLGILILGAIHVHIRGRRGRILAKMPGPSGWPIIGVLPSVLVPIEKLWMVGRN

MDKQYYPLYKFWLANHVIVAILNPDDAEVMLTSMQNIDKSSVYNFLHAWFGTGLLTSTGE

KWQKRRKILTPAFHFNVLERYLTITVENSERAVKSLRGKGECVQDLVQFLTQHTLNIICE

AAMGASLSRKEESQKKYRKAVHDMGSALVYRLVRPWLYSSWTFGCTAAGRMQTDALKILH

RFTKNIIAERKEFHEKTDGRFLKNIVSKSSGADDDDEAVENEELHGYRKKRMAMLDLLIA

AQRDGQQIDELGIREEVDTFMFEGHDTSAMGLCFALLLIAEHKDVQERIRQEVNEVLKNA

DGKLEMSELNKFNYLERVIKESLRLYPSVPFISRNITEDMQLKDYLIPRGTLVDVRIYLI

HRDPKHWPDPLKFDPDRFLPERIQGRHPFSYIPFSAGPRNCIGQKFAMMELKVFVALIVK

NFILEPIDFAHEVPILPDIVLRPARSVHVKFVPIAEK

>EFN68668.1

MIFIILILCTFILLIYKYYMNYKRNWRLINLIPGPPGYPIIGNLLQFLVSAEEQWKIICT

LLDKYYPIFNIWGCFLFAVVIRHPDDLKTILNSTNHIEKGIIYNVLHPWFGTGLLTSTGV

KWQTRRKILIPTFHFNILNQFVDILIKEGDCMTKSLMDVGGTVVKDLLPFISEHTLNAIC

ETAMGVSLRTIGEFQQQYRNAVHDMIKLIFYRAFRPWLYSDMIFSLLPQGRKQKKILKIL

HGFTEKIIAERRLYHQRTNGQYLKNFESDKEAEINDVEIFGIKKKRIAMLDLLIATSLEN

SLTDLDIREEVDTFMFEGHDTTAMGIMFALLLLAEHKNIQERVRNEINNVMQENGEKLDM

KSLQNLSYLDRCLKESLRLYPSVFVISRYAADDVKLQSYTVPARTILYLHIYAVHRDPNF

WPNPEVFDPDRFLPERMQNRHPYCYLPFSAGPRNCIGQRFGLLEMKAMIAPLVHNFYLEP

VEHLKDVRLKTDMIIRPSHPIHMKFIPITCK

>283135238

MTSVYLALILFGLVIFHAWIRYRRMGRIIDKMPGMRPLPFLGNMLSLMGDKDQLWNIMRS

LAKEYAITRIWFFTRAWVAVRDPDDIEILLTNKANLNKRAVYELALKWLETGLLTSGGEK

WVHRRKIVNTGFHFNIMKKYVKISGEHAEKFIEKIKSHGDEYVLDLVPLFSDLTLQIICE

SAFGVDLNKLDQKLVQQYKGALHVMYDMVIYRSTRPYIRDWMMNFLPIGWKQNRAINILH

NFTNKVIQERREYYNKHASENRSYQSFVDDVDIESDYVADGRKKRLVMLDVLLSAEREGL

IDAKGVREEVDIFTVAGHDTTAVTMAFMMMLLAENPEIQDRARAEVINHFEENGGKLNIT

EIQKFEYLDRCIKETLRLYPPISQFVRYMDKDIQLKKYLIPAGVDVLFLTYDTQRDPRHW

TEPDKFDPDRFLPENAKKRHPFAYIPFSAGLRSCIGQKFAMLELKAMLAHILYNFYVKPV

DLTANMKLETYIVLQPSHPVHVKSIRRNVN

>345486403

MDLMIACLILFHLCIRYGRVGRIIDEIPGMYPLPIFGNILSLNNLLAAMALLYLLLVFIV

IGLIAFHVRVRYGRVGSMVAKIPGPYPLPFLGNLLSFAGNREELWNKLRALMKGCSIKRM

WFPTRAWIFIQDADDMEVLLTSKVNVNKGQAYNLALKWLHTGLLTSKREKWTERRKIVNT

GFHYNVMKKYVEISIEYTKQFVEEIKSHGDECVLNILPLLSDLTLQIICESALGISLDKL

DNKVLQEYKEAIREIDDIFVYRGARPYITDWMMYFLPLGRRQDHAIKILHEFTDRIIRER

KEYHANFQENRSYQHFVDDVEESAEDDSFKGTKKRLVMLDVLLSAEQDGLIDEKGIREEV

DLLVLAGHDTTSISMVIMLMLLAENPEAQDRARAEVIQVFSENGGKLDIKEIQKFEYLDR

CIKEAMRLYPAIGNFIRHLNEDVQLKKYLLPAGVDVAFFVYDLHRDPKHWQEPEKFDPDR

FLEENVKKRHPFAYMPFSAGPRNCIGKKFAMLEMKIMLAHILYNFYLEPVDFPANVKLET

NIILHLTHPVHVKFIR

>EFN72811.1

MFINISLLFIFILSIYYYYVHYGKKGRLLHLIPGPPSVPIFGHVFLVQVSTVEQWKLLCT

VSDKYYPTFKLWGFIEFTVSIRHPDDLEKILGSTKHIDKSRAYSVLHPWFGTGLLTSVKW

QTRRKILTPAFHFNILNQFVDILIKESDCMTKSLKDVGGTVVKDLLPFISEYTLNAICET

AMGVSLQKLGEFQQQYRNAIHEIIELIVYRALKPWFLSDFLFSLSPQGRKQKKILKILHG

FTEKVIAERKLYHERTGNRYLNLEDDKETKEEDVEVFGIKKKRLAMLDLLIAASRESSLT

DLDMREEVDTFMFEGHDTTAVGIMFALLLLAEHKDIQERVRVEVNTVMQENGGKLTMKAL

QNLSYLERFLKEALRLYPSVFLILRDAAEDVKLHSYVVPAGTIIHPNIYGVNRDPNFWPN

PEVFDPDRFLPENIRNRHPYSYLPFSAGPRNCIGQRFGLLELKAMIAPLVHNFYLEPVEH

LKDIQLKADIILRPSHPIHMRFIPISYK

>EFN71969.1

MFVTILVLSIFILLIYNYYVHYGRNGRLLNLIPGPLGLPIVGNLFQYYVSAEEQWKIFCT

LPNEYYPIFKIWGFFTFIVCIRHPDDLETILSNIKHIEKGRLYNVLHPWLNTGLLTSTGV

KWQTRRKILTPTLHFNILNQFVDILIKEGECMTKSLNGVEGTVVKDLLSFVSEHTLNAIC

ETIMGVSLQTLGEIQQQYRNAIHDIIELIVYRGLRPWLYNDLLFSLSPQARKQKKILKIL

HGSTETIITERKLYHERTNDRYLKNLEGNKEIEEMVGIKKKRLAMLDLLIAASRENSLTD

LDIREEVDTFMFGGHDTTAASIMFTLLLLAEHKDIQERVRIEVDNVMQENEGKLNMSLLQ

NLSYLERCIKEALRLYPSGFLISRSPGEDVKLQSYVVPAGTILYLNIYGVHRDPNFWPNP

EVFDPDRFLPERIKNRHPYSYIPFSAGPRNCIGQRFGLLKMKALIAPLVHNFYLEPIDYL

KDIRLKFDMLIRASHPVHLRFLPIKQPFKTNADTARSDVL

>AAQ90477.1

MFIALLLIIIVVYLMCHCYAQYGPNGRLINKIPGPPGYPIIGNLFNLLESREQFWETLSI

LNEQYYPIYKLRAFFNSLVFIRHPDDLEIILNNTKHISKSNLYDALRPWLGMGLLTSGGS

KWHLRRKILTPTFHFNILQQFVEILIEEGESMTRSLKNAGGTITKDLVPFISEYTLNAIC

ETAMGTSLQEMGSFQQQYRKAVHRMGELLVYRAMRPWLKYDWIFSLTSKGREQKKLLKIL

HGFTEKVIAKRKDYHKRTKGQYLKNLNKDVVPNETETIGIKKKRFAMLDLLIQASQEGLL

TDFDIREEVDTFMFEGFDTTGMAMCFILSLLAEHKDIQDCVRKEIDAVMQENQGKLNMKS

LQDLQYLERCIKEALRLYPSVYFISRITSEETELKTYLIPAGTLVILNFYITHRDSNFWP

NPEVFDPDRFLPDNIRNRHPYSYLPFSAGPRNCIGQRFALLELKAMLVPLIYNFYLEPVD

YLENQRFGIDMILRSLDPCRLKFVPIATEGI

>EFN68667.1

MFTILILCMFILLMYYLYNKYYINYERNWRLIKLIPGPPGLPIVGNVFQFHVSAEELWKL

FYTLHNEYYPIFKVWSFSIFVVSIRHPDDLETILSNTNIKRIEKTVTYDILHPWFGTGLL

TSTGVKWQTRRKILTSTFHFYILNQFVDILIKEGDCMTKSLMDIEGTVVKDLLPFISEYT

LNAICETAMGVSLQKLGEFQQQYRNAIHDMIELIIYRIFRPWLHNDMIFLLSSQGRKQKK

ILKILHGFTEKIIAERKLYHERTNGQYLKNLESDKEGATNDVESFGVKKKRLAMLDLLIA

ASQENSLTDLDIREEVDTFMFEGHDTTATGIMFTLLLLAEHKNIQERVRLEIDNVMQDNG

GKLNMRSLQNLSYLDRCLKEALRLYPSVHLIERKLTDDVKLQSYVVPAGTIIRLNIYAVH

RDPNFWPNAEVFDPDRFLPEKKENRHPYSYLPFSAGPWNCIGQRFGLLEMKAMIAPLVHN

FYLEPIEYLKDIRKNNGSSSIHYIMTLYYYPIFKTWGFFLLAVFIRHPDDLETILSNTNH

IEKGLIYDVLHPWFGTGLLTSTETAMGVSLQKLGEFQQQYRNAIHDLIELLFYRAFRPWL

YNDMLFSLLPEGRKQKKILKILHGFTEKIITERKLYHERTNDRYLKNLESNKETEDIEVF

GIKKKRLAMLDLLIAASRENSLTDLDIREEVDTFMFEGHDTTATAIMFTLLLLAEHKDIQ

EALRLYPSVFLISRYTGDDIKLQSYVVPAGMMIYLNIYAVHRDPNFWPNPEIFEPDRFLP

ERIENRHPYCYLTFSAGPRNCIGQRFGLLEMKAMIAPLVHNFYLEPVEYLKNIQLKADIF

IRPSHPVHVKFVPIICK

>289177134

MIVYFQLILLSLFVLHIWIRYRRMGRVVDKIPGLRPLPFLGNMLSFAVDKHQLWLTMRGL

AKIYTITRVWFFTRAWVVTRDPDDFQILLTSKVNLNKRALYEHCLKFAKTGLLTSNREKW

SQRRKIVSTGFHFNIMKKYVEITGEHAELFTKKIKSNGKESSLNVVPVCSDLMLRIICES

AFGVNLNKLDPQLVDQYSEALHVVYDTVVYRATRPYIREWMMDFLPIGWKLNRAIKILHS

FSDKIIQERRDYYNKQAESRTYQSFVDDVDTDNENTLGERKKRLVMLDVLLSAEREGLID

AEGVREEVDIFTVAGHDTTAVTMAFMLMLFAEHSEAQDRVRKEVTKVFNECGGKLTITET

QKLEYLDRCVKETLRLYPPFSIFARYIDEDVQLKKYLVPAGVDVLFLSYDAHRDHRHWPD

PDKFDPDRFLPENVKKRHPYAYVPFSAGPRNCIGQKFAMHEVKSVLAHVLYNFNLEPVDN

IADIKLETYIITQPCHPFHVKFIARNAN

>289177129

MLLYFLIVALCSLACFHVYVRHGKYGRAIGKMRGPPALPIFGNMLSLNCGPVEFWNYLAD

AIKIYYPCGRIWFFTCAVVITQDPDDLRILLTDKTNITKGFVYEYFHLMAKTGLITSTGE

KWQYRRKILTPAFHTHIIKKYMKITFEQGERFVKSIKAQGDETVQSLIPVCSDLTLSIIL

EAAMGIQFQTIGNKAEEYRKAIEHYFSSIAYRATRPYIKDWMLRYFSEGRRILATVKVLR

EFTDKIIDERREYHKQTKQESFQKYVDDDMEHECTDGEGRKKRLAMLDLLLMAEKEGLID

EEGIKEEVDTFTAAGYDTTGMEMVYLLMLMAENKDQQELARAEANRILDASGGKISMKEI

NQMEYIERCVKESLRLFPTAPHIVRAVTEDIQLKNYMVPAGTDIFVPIHILHRDPKYWSD

PLKFDPDRFLPGEAEKRYPFTYLPFSHGPRNCIGQKFAIAELKSLLACVLRNFYLEPVSY

TKDLQFTLHVVLLPTVRPHIKFISIKKND

>EGI58147.1

MIVIVLILFVIILLSYYYYVHYGRNGRLINLIPGPPGYPIIGNTLLLQGSRENLWKRLIT

ITDKYYPICKIWTFFIPIVSIRDPDDMEVILNNMKHINKSIIYDVLRPWFNTGLLTSGGT

KWQTRRKILTPAFHFNILQHFIESLIEEGENMTKSLKNTGGTVVKDLIPFISEHTLNAIC

ETAMGTSLRDQSAYQQRYRKAVHQIGEIIIYRLMRQWLRYNWIFSLMPKGKKQAETLRIL

HGFTERIIEKRKLCQEYTDDQYLKDFDSNTMTETSDVETIKIRKKRLALLDILIAASRKG

LLTDSDIREEVDTFMFEGHDTVAKGICFALLLLAEHKDIQNRVRDEIRTTIEKTGEKFTI

NVLQNLSYLDRCIKEALRLYPSVYFISRITSEDVKFKSYIIPAKTIVHLNIYGLHRDPNF

WPNPEIFDPDRFLSENIRNRHPYSYLPFSAGPRNCIGQRFALLEMKAMIASLIHNFYLEP

IDYLKDLQMEVDLVLHPAHPLQDLWKFQRRLCKNYYPIYKLWCGPIAFVSIHHPDDLEKV

LGSAKEHLSKGYLYDALIPWLGTGLLTSEVKKRRLAMLDLLIAAVRNDEMDDVDIREEVD

TFVFEGHDTVAVGLTYAILLLAEHEDVQKRARNEISAIMEANGGKLTMSALNNMPYLERC

LKESLRLYPSVPFISRVLSKDLQTQTYLVPSGTIVHLNIYDIHRDPNFWPNPDVFDPDRF

LLEKIQKRHPYSYLPFSAGPRNCIGQRFAMMELKAIIATLIYNFYLEPIDYLKDLQFKTD

LTSRVTQPIRTRFVPINRTSKVI

>AAP97090.1

MPRQHLINLLFSLCDKYYPIFRTWAFFEGFVSIRHPDDLEVILNSSTNIEKSRIYSVLHP

WFGLGLLTSGGSKWHSRRKILTPTFHFNILQQFVEILIEEGESMTNSLKNAGSTITKDLV

PLISEHTLNAICETAMGTSLQGMNSVQQEYRKAVHRMGELFIYRLVRHWLISDWIFALTS

QGREQVKILKILHGFTNKIIAERKDYHKRTNGQYLKNFNQSIITDNEEIVGSKKKRLAML

DFLIYASQEGHLTDVDIREEVDTFMFEGHDTTSTSLCYTLALLAENKDIQDRVRQEVDLV

MQENEGKLTIKSLQNLQYLERCIKESLRLYPSVYFISRITTEEAQLKSHLIPVGTIMHLH

IYGVHRDPNFWPNPDVFDPDRFLPENSRNRHPYSYIPFSAGPRNCIGQRFAMLEMKAMIA

PLIHNFCLEPVDLLKNLRVGPDLVLRPLGGHRIKFIPIATKCI

>289177132

MLLYLLIAVLCVVGFHAYVRYGRYGKLINKLPGPTPLPILGNLLLLNKGPVDLWNFLHVK

MKEYYPFGKIWFCTSAIILTQDPDDLKILLSDKVNIVKGFMYKPLHLWAETGLITGTGEK

WQHRRKIITPAFHANIIKKYIEITSEQGERFVEYLKTKGDETVESLIPICSNLTLSIIIE

AAMGTKFNTMGDKAEEYKKAIEDYFHGVAYRATRPYIQNWMLRYFGRGRRILDCIKSLRG

FADKIIEERREYHNQTKHQSFQNYVDDVDDTNLLDGGRKKRLAMLDLLLLAEKEGLIDEQ

GIKEEVDTFTAAGYDTTGMGMVYMLLLLAENKDVQKLARAESDRILSASGGRITMTEIQQ

MDYIERCVKESLRLFPTAPNISRTIIKDIQLKNCMVPAGTDVFVPIYDVHRDPKYWPDPL

KFDPDRFLPEEVHKRHPFSYLPFSHGPRNCIGQKFAIAEMKALVARIVYNFYLEPVTYTK

DLQFTAHIVLLSTVPPCTKFIKRLRSKD

>328784475

FQLILISIYLLHYFLLHHRKFGKMINLIPGPEPLPILGNIPTFHNISPSELWKFLTQLSK

QYYPIYRMWTFLEAYVHICHPDDIETILGNIKFTKKGFGYKYLKPWFNTGLLTSSGHKWH

VRRKILTSAFHFNVLRQFVDIFIEDAERLIKTLESEEGIFVENLLQLTSEHTLNVICETA

MGTSLKNKEKFQYEYRKAVYNMGCIFANRIVKPWFYYDFFFNLSPEGWQQSKLLKILHNF

TRKIIQERKEYHDKTNGRYLNDFHENINENDNNNDYNDFRRKRLAMLDLLIEAHRNNKID

DEGIREEVDTFMFRGHDTTAISFCFSIMLLAEHKEIQDRARAEIKAAIEENGGKLNITVL

QNLPYLERCIKESLRLFPSVPRISRKLETSVKLSNYEIPSNTIINVNIFDTHRDPKFWPN

PNKFDPDRFLPENSKKRHPYAYVPFSAGPRNCIGQRFAMLELKTYLGLLLYNYYFEPIDY

LKDVTFVSGIVLRLENPVRMKFIPVKKIC

>EFN74805.1

MFIIILLVFILTLLSYNYYVHHRRNGRLINLIPGPPSFLIVGNIFEFIVSPKKQWKVLHS

MLNEYYPITKTWTFFLPFVSIRHPDDLETIMSSTKHIEKSAMYSLLHPWFSTGLLTSTGA

KWQLRRKILTPTFHFNILNQFVDILSKESAHMIKSLKDEEGAIVKDLIPFISEHTLNIIC

ETAMGISLHDLGAFQQQYREAVYQMTELVVYRLVRPWLYNNLLFALSPQGRRQKKILKIL

HGFTEKIIAERKLYHERTNDRYLKNFEKEETDDAEVFGISKKRLAMLDLLIAASREGSLT

DLDIREEVDTFMFEGHDTTAMGITFALLLLAEHKDIQERVRIEANTVIQENEGKLTMKSL

QNLPYLDRCLKEALRLYPSVYFISRYAAEDVKLQSYVIPARTGIHLNIYGVHRDPNFWPN

PEVFNPDRFLPDRIQNRHPFCYLPFSAGPRNCIESYVISAGTSIHLNIFGIHRDPNFWPN

PEVFNPDRFLPDRIQARHPYSYLPFSAGPRNCIGRRYGMLEMKAIMALLVHNFYSKPVDC

LKDIQLKTDIILRPFHPVHIKFVPIKC

>350399463

MIITILFSLFLFVSLHYIILHYGKYGRILNSLPGPPILPIIGNVHHALVSSSNLWIFMNK

MSDQYYPIFKLWGFTAALVHTRHPDDFQILFGNTKFTQKGYMYKLLAPWFSTGLLTSSGR

KWQIRRKILTPAFHSDMLNKFVDVFVKEGEYLVKSLKSEEGVVVNDLFHTISKHTLNMIC

ETAMGISLNDEDEFQHKYLKAASDMEGVFAYKTARPWLFYDFFLKFDPKGRRQSELLKTL

HGFTRKIIQERKEYHYQSNGSTDFNKNSSDDNDNVESNDVGIRKRRLALLDILIEAHRNN

QIDDEGIREEVDTFMFTGHDTTAIAVCYTIMLLAEHKEAQDRVRAEVKAVLKENEGKLNM

STLQDLSYLERCIKESLRLYPSVPRIGRKTEKELKLGNCKLPSSTEVLVDIYNIHRDPRY

WPNPDIFDPDRFLPENSKSRHPYVYVPFGAGSRNCIGKRFAMLELKIIMSFLLNNYFFES

VDYLKDISFLTGIIMKPAHRIRTKFIPIKDTC

>340721101

MIITILFSLFLFVSLHYIVLHYGRYGRIINSLPGPPVLPIIGNIHHFLVSSSDLWILMNK

LSDQYYPIFKLWSFTAALVHIRHPNDFQTLLGNMKFTQKGYMYNLLGPWFSTGLLTSSGR

KWQIRRKILTPAFHSDMLNKFVNVFVKEGEYLVQSLKSEEGVVVNDLLHTISKHTLNMIC

ETAMGISLKDGDEFQHKYLKAASDMEGVFAYKTARPWLFYDFFLKFDPKGWKQCKLLKTL

HGFTRKIIQERKEYHYQSNGRCPTDFNKNSSDDNDNVENNDVGIRKRRLALLDILIEAHR

NNLIDDEGIREEVDTFMFAGHDTTAIAVCYIIMLLAEHKEIQDRVRAEVKVVWKENEGKL

NMSTLQDLSYLERCIKESLRLYPSVPRIGRKTEKELKLSNCRLPSNTEVLVDIYSIHRDP

RYWPNPDVFDPDRFLPENSENRHPYVYVPFGAGSRNCIGKRFAMLELKIIMSFLLNNYFF

EPVDYLKDISFLTGIIMKPTHRIRTKFIPIKDTC

>EGI58966.1

MFVIVLLSFVFILVCNYYVHYGRTGRLINLIPGPLCYPIIGSLYVLFGSRETLWKRMITL

SNRYYPIYKIWGFFVPVISIRHPDDLETILSNPKYIKKTFMYNIFKPWLGTSLVISEGAK

WHSQRKILTSTFHFNILQRFVEILDKESKNMIKSLKNADGTVVKNLSPFISEHTLNAICE

TAMGTSLKDFGEFQQQYLKAIRQISELFVYRVKRQWLYFNWIFSLTPKGREQAKVLKILH

GFTDRIIAERKLYHEQTNGQYLKSFDNDTSAEEDDPKMIGTQRKRLAMLDLLIAASREGL

MTDLEIRQEVDTIMFAGHDTTASSLCFILALLAEHKDIQDRVRNEVDIAMQDNEDKLTMK

FLNQLLYLERCIKEALRLHSVIFFISRICEEDVKLQSYLVPAGTNLVIDINGVHKDPNFW

PNPEVFDPDRFLPERIRNRHPYSYIPFSAGPRNCIGQRYAMLQMKMMVTSLIHHFYLEPV

DYIKDVRLQVDLILHPHPLRVRFVPILQN

>AAM54723.1

MWLYLCAIIIFLCVLDLCLNYNERARMIKKLPGPPDDFIVGNGLRIIKSSVDVMKVRREY

AKKWNGIYRIWAYPFGAVLVFNPEDVELIMSSMKYGEKSIIYTILKPWLQDGLLLSNGAK

WQERRKILTPAFHFNILRQFCDIIQENTQRFLEKLEQTAGQDVDVVPILSEYTLNSICET

AMGTQLSEDLTGTVQSYKQAIYDLGYVFCQRFIRIYLYPETVFNLTSLGRMQKRSVKTVK

NFTEDVIKQRRDYVEQYGFHTIGDNNDSEEVFVYKKKKKTAMLDLLLSAEKEGLIDKTGI

QEEVDTFMFEGHDTTASGLTFMFMLLAHHKDIQDKIVEELNEILGDSDRWLTMDDLAKMK

YMERCIKESLRIYPPVHFISRKLNETTVLSGYQVPADTLCHIPIYDMHHREDLFEDPERF

DPDRFLPENSVGRHPYAYIPFSAGPRNCIGQKFAMLEMKIAVAEVLRKFELKPVTKPSEI

EFIADLVLRNNGPVKVNFVKRK

>ABK27872.1

MWMYFILILLLLLTIHFLLNYNYRARLLRRIPGPRGYFIVGNALDVILSPAELFALTRKN

ATQWPNLNRFWSFGIGALNIYGPDEIEAIISSTQHITKSPVYNFLSDWLRDGLLLSTGTK

WQKRRKILTPAFHFNTLKQFCVILEENSQRFTENLKDTEGKSINVVPAISEYTLHSICET

AMRTQLGSETSEAGRSYKNAICELGNQFVHRLARIPPHNNFIYNLFTLGKQNEHLNIVHS

FTKRVIKDRRQYIRRNGGNNFDDEKDTQADEHSIYFNKKKTAMLDLLLKAERDGLIDEIG

VQEEIDTFMFEGHDTTATGLTYCIMLIANHKSIQDKIIEELDEIFGESTRAADIEDLSKM

RYLERCIKESLRLYPPVPSMGRILSEEINLNGYTVPAGTYCHIQIFDLHRREDLFKDPLV

FDPDRFLPHNTEGRHPYAYIPFSAGPRNCIGQKFAILEMKSLLSAVLRRYNLYPITKPED

LKFVLDLVLRTTEPVHVRFVKRNKV

>ABK27871.1

MWMYFILILLLLLTIHFLLNYNYRARLLRRIPGPRGYFIVGNALDVILSPAELFASTRKN

AAQWPNLNRFWSFGIGALNIYGPDEIEAIISSTQHITKSPVYNFLSDRLRDGLLLSTGTK

WQKRRKILTPAFHFNILKQFCVILEENSQRFTENLKGTEGKSINVVPAISEYTLHSICET

AMRTQLGSETSEAGRSYKNAICELGNQFVHRLARLPLHNNFIYNLYTLGKQNKHLNIVHS

FTKKVIKDRRQYIRGNGGNNFDDEKDTQADEHSIYFNKKKTAMLDLLLKAERDGLIDEIG

VQEEIDTFMFEGHDTTATGLTYCIMLIANHKSIQDKIIEELDEIFGESTRAADIEDLSKM

RYLERCIKESLRLYPPVPSMGRILSEEINLNGYTVPAGTYCHIQIFDLHRREDLFKDPLV

FDPDRFLPHNTEGRHPYAYIPFSAGPRNCIGQKFAILEMKSLLSAVLRRYNLYPITKPED

LKFVLDLVLRTTEPVHVRFVKRNKV

>EFA10753.1

MFASTLILTTIFAIVSVLAFKRIKYHVKNYILLNKLPGPPPDGLFVGNMPYLQTTPEDIF

NRLREANKKFYPIYKLFAVHRMGANILSPEDSEIVLSNPIHNEKGYIYKLLHHWLKEGLL

TSKGEKWQLRRKILTPAFHFNILQQFIMILNEEAEKLVEGLRKECHKPYINITPHISQFT

LKSITETAMGTKLDFTTKKEIRYKEAVYKIGKILTYRITHPWFIEPLLNIFSPCFIQERR

VTSTLHKFTKEVIEDREKNFKDFELPTEEHDVYKGKKRLAMLDLLLSAKKKDGIIDNKGI

QEEVDTFMFEGHDTTAVALNFALMLIACHKDVQETILQEMRDVLGDIHAKPTYSDLQNLK

YLERCIKESLRLYPSVHLISRALGEDVRTQKGYLIPKDTITIIHIYDLHHNPDIYPDPEK

FDPDRFLPENCQNRHPFAYLPFSAGPRNCIGQRFAMLELKAAICAILANFVLEPIDTPET

IVVVVDIVLRTKEGIKIRFVPREQ

>ABC72321.2

MFAYLVVAVIFLCVAHILLNYNRKARLIRKIPGPRDDFIIGNGFTVIRSPVETMQMRRQL

CTEFDGIFRIWICPMGAVCIYNPEDIEILMSGMKYGEKSFVYNFLKNWLRDGLLVSNGAK

WQTRRKILTPAFHFNILKQFCEIIDENSERFLEVLYQTAGKPIDVVPVLSEFTLNSICET

AMGTQLSDDNSAARSYKKATYDIGCLVFDRFVRIYLYPDFIFNLTSMGRKESKYLKVVHS

FTENVIEQRREYIEKNGININEQIEADDDDSYVYKKKKKTAMLDLLLSAQKEGHIDKIGV

QEEVDTFMFEGHDTTVSGLTYCFMLLANHRKIQDKIIQELDDIFGDEDRPIKMEDLAKMK

YLDCCIKESLRLYPPVHFISRNINEETVLSNYTIPAGTLCHILISDLHLRPDLFKNPTVF

DPDRFLPENSVGRHPYSYIPFSAGPRNCIGQKFAMIEMKIAVARVLRKFHLSPVTRPCDI

TFTADLVLRNNGPVLVNFIKRES

>AAC21661.1

MFVIIAIFALFLCLIHILFNNNKKARLLKQIPGSKYNFIIGNALDFLKSPEQLFYFMREY

YETWKPLNRFWAFQIAFVNVYEPHDIEVVISSTKHNAKSPPYYFLKNWLRDGLLLSKGPK

WQSRRKILTPTFHFNILRQFCGILEDNSERLVQNVGKSLGKPVNIIPTISEYTLYSICET

AMGSRLGEESKESQKSYKQSICALGRQFVYRITRIYLHSDFIYNLITFGKVKKDLDVVHN

FTTKVIKDRKEYVEKYGINMFGVSDIDDNNVYKKNKKIAMLDLLITAQKEGFIDDIGIQE

EVDTFMFEGHDTIALALTYTLMLLANHRSIQHTVIAEIDEIFGDSERQADLDDLSKMRYL

ERCIKESLRLYPPVPAIGRLLSEDVTLSGYRVPEGAYCHIQCFDLHRRGDLYKDPLVFDP

DRFLPENCSDRHPYAYIPFSAGPRNCIGQKFAILEMKSAISSLLRHYELLPVTKPEDLKF

TADLVLRTTNPVYVKFVKKEKNK

>ABP87671.1

MFVYLIFIASFFLLIHLAFNYNSKAVMMNKVPGPKLSFILGNAPEIMMLSSVELMKLARK

FASRWDGIYRIWAFPLSIINIYNPDDVEVIVSTTKHNEKSSVYKFLKPWLGDGLLISKGE

KWQQRRKILTPAFHFSILRQFSVIIEENSQRLVESLEKCIGKPIDIVPVVSEYTLNSICE

TSMGTQLSDKTEDAWKAYKDAIYELGPYFFQRFTRVYLYFDIIFYLTSLWRKMKKPLKSL

HGFTSTVIKERKIYVEQNGVKFGEDVNDDDLYIYKKRRKTAMLDLLIAAQKDGEIDDHGI

QEEVDTFMFEGHDTTASGLTFCFMLLANHRAVQDKIVEEINDIMGDSTRRANLEDLSKMK

YLECCIKESLRLYPPVHFISRNLNEPVVLSNYEIPAGSFCHIHIFDLHRRADIYEDPLVY

DPDRFSPENSKGRHPYAYIPFSAGPRNCIGQKFAMIEMKSAVAEVLRKYELVPVTRPSEI

ELIADIILRNSGPVEITFNKRTK

>ADE05576.1

MFVIIAIFALFLCLIHILFNNNKKARLLKQIPGSKYNFIIGNALDFLKSPEQLFYFMREY

YETWKPLNRFWAFQIAFVNVYEPHDIEVVISSTKHNAKSPPYYFLKNWLRDGLLLSKGPK

WQSRRKILTPTFHFNILRQFCGILEDNSERLVQNVGKSLGKPVNIIPTISEYTLYSICET

AMGSRLGEESKESQKSYKQSICALGRQFVYRITRIYLHSDFIYNLITFGKVKKDLDVVHN

FTTKVIKDRKEYVEKYGINMFGVSDIDDNNVYKKNKKIAMLDLLITAQKEGFIDDIGIQE

EVDTFMFEGHDTIALALTYTLMLLANHRSIQHTVIAEIDEIFGDSERQADLDDLSKMRYL

ERCIKESLRLYPPVPAIGRLLSEDVTLSGYRVPEGAYCHIQCFDLHRRGDLYKDPLVFDS

DRFLPENCSDRHPYAYIPFSAGPRNCIGQKFAILEMKSAISSLLRHYELLPVTKPEDLKF

TADLVLRTTNPVYVKFVKKEKINNISTIKIK

>ABP99018.1

MFVYLIFIASFFLLIHLAFNYNSKAVMMNKVPGPKLSFILGNAPEIMMLSSVELMKLARK

FASRWDGIYRIWAFPLSIINIYNPDDVEVIVSTTKHNEKSSVYKFLKPWLGDGLLISKGE

KWQQRRKILTPAFHFNILRQFSVIIEENSQRLVESLEKCVGKPTDIVPVVSEYTLNSICE

TSMGTQLSDKTEDAWKAYKDAIYELGPYFFQRFTRVYLYFDIIFYLTSLWRKMKKPLKSL

HGFTSTVIKERKIYVEQNGVKFGEDVNDDDLYIYKKRRKTAMLDLLIAAQKDGEIDDHGI

QEEVDTFMFEGHDTTASGLTFCFMLLANHRAVQDKIVEEINDTMGDSTRRANLEDLSKMK

YLECCIKESLRLYPPVHFISRNLNESVVLSNYEIPAGSFCHIHIFDLHRRADIYEDPLVY

DPDRFSQENSKGRHPYAYIPFSAGPRNCIGQKFAMIEMKSAVAEVLRKYELVPVTRPSEI

ELIADIILRHSGPVEITFNKRTK

>CAX94850.1

MIIIYLLIILVTVCILHFIFNHNEAARILKKIPGPKDVFLVGNAGDINVDPVALFKLRNK

YAKEFNGIYRIWIYPLGIVNIYNPEDIEIVTSTNKHSDKSIIYKFLKPWLNDGLLVSKGT

KWHERRKILTPTFHFNILRQFCTIMEENSSRLVNKLDTVLGQPVDVTPIITEYTLSTICE

TAMGTQLNKETTECGKSYKEAIYAVGDVLVNRFTNLRYYSDTYFNLSSLGRKQKKHLKTV

HYFTKKVIEERKKLVTETNITETEDESVYMFKKKRRTAMLDLLIAAQRDGLIDQAGVQEE

VDTFMFEGHDTTAVGLIFFLMVLANRKDIQDKIVAELKEIFGDSQKNITIEDLSKMRYLE

CCIKESLRLYPPVHFISRNISETVKLSNYTVPAGTICHIHIYDLHRQENLFENPLEFIPE

RFLPEKCIGRHPYAYIPFSAGPRNCIGQRFAIYEMKSFVAAILRNYKLMPVTRPEDIEFV

SDIVLRSNGPVYVQFEKRVDSV

>ADE05575.1

MFIYLFAIIVILCVIHIIFNYNASARLLRRIPGPPDRFIVGNSPELLLSSVELMKLPRKY

AERWAKSGICRFWTFPWAAVMIYDPKDIEIVLSGMKHHEKSVIYKFLSPWLGDGLLVSAG

EKWQHRRKILTPAFHFNILRQFSVILEENSQRLVERLEKTVGQPIDVTPILSEFTLNSIC

ETAMGTQLNKETTGAGKAYKDAIYELGQILVQRFISFHLYSDIIFFSTSLGRKLKKHLNA

LHRFTERVISDRKDYLDYFGLNFGDEIVDDDKFIYKKRKKTAMLDLLIAAERDGEIDSQG

IQEEVDTFMFEGHDTTASGLTFCFMLLANNKHIQDKIVAELDDIFGDSTRPANMEDFAKM

RYLECCIKESLRLYPPVHFISRNINEPVKLNNYEVPGGTSCFVFIYDLHRRSDLFKNPSV

FDPDRFLPENSVGRHPYSYIPFSAGPRNCIGQKFAIMEMKSAVSEVLRKYELRPVTRPSD

IEFIADIVLRNSGPVEVTFVKRQK

>BAI45222.1

MFVYLIFIASFFLLIHLAFNHNSKAVMMNKVPGPKLSFILGNAPEIMMLSSVELMKLARK

FASRWDGIYRIWAFPLSIINIYNPDDVEVIVSTTKHNEKSSVYKFLKPWLGDGLLISKGE

KWQQRRKILTPAFHFNILRQFSVIIEENSQRLVESLEKCIGKPIDIVPVVSEYTLNSICE

TSMGTQLSDKTEDAWKAYKDAIYELGPYFFQRFTRVYLYFDIIFYLTSLWRKMKKPLKSL

HGFTSTVIKERKIYVEQNGVKFGEDVNDDDLYIYKKRRKTAMLDLLIAAQKDGEIDDHGI

QGEVDTFMFEGHDTTASGLTFCFMLLANHRAVQDKIVEEINDIMGDSTQRANLEDLSKMK

YLECCIKESLRLYPPVHFISRNLNEPVVLSNYEIPAGSFCHIYIFDLHRRADIYEDPLVY

DPDRFSQENSKGRHPYAYIPFSAGPRNCIGQKFAMIEMKSAVAEVLRKYELVPVTRPSEI

ELIADIILRNSGPVEITFNKRTK

>ABX64439.1

MFVYLIFIASFFLLIHLAFNYNSKAVMMNKVPGPKLSFILGNAPEIMISSPVELMKLGRK

FASRWDGIYRIWAFPLSVINIYNPDDVEVIVSTAKHNEKSSVYKFLKPWLGDGLLISKGE

KWQPRRKILTPAFHFNILRQFSVIIEENSQRLVESLEKCIGKPIDIVPVVSEYTLNSICE

TSMGTQLSDKTEDAWKAYKDAIYELGPYFFQRFTRVYLYFDIIFYLTSLWRKMKKPLKSL

HGFTSTVIKERKIYVEQNGVKFGEDVNDDDLYIYKKRRKTALLDLLIAAQKDGEIDDHGI

QEEVDTFMFEGHDTTASGLTFCFMLLANHRAVQDKIVEEINDIMGDSTRRANLEDLSKMK

YLECCIKESLRLYPPVHFISRNLNEPVVLSNYEIPAGSFCHIHIFDLHRRADIYEDPLVY

DPDRFSQENSKGRHPYAYIPFSAGPRNCIGQKFAMIEMKSAVAEVLRKYELVPVTRPSEI

ELIADIILRNSGPVEITFNKRTK

>EFA10757.1

MIQSTLLLIFIVILSVIIYIWWYQNFSRFFSLLNKVPGPPGYPIIGNIIQFLATPEELFK

IDRELGRRFYPIYKEWTLTYGAVNLLHPDDIELVLSNSKYNDKSAIYDFLHCWLGTGLLT

SSGTKWQTRRKVLTPAFHFNILQQFLPIFNEETVKLIKNIKNDQPIDVIPPVTQFTLLSI

AETSMGVKLDASPKSCDDYKNAIHVFGYALTYRLGRPWLHNPFVFFNLTRLGHLTKKSIK

ILHDFSRNVIEQKKRTFEGEKRGKRLAMLDLLLLAKHQGADIDDEGIAEEVDTFMFEGHD

TTSIAICYTLLLLANHPDIQDELYSELKSVLSDPTQTPSYSDLKQLNLMERCIKESLRIF

PSVPFISRLLTEDLTTASGYVIPRGSMAHIHIYDLHNNPEIYPDPKKFDPDRFLPENCQK

RHPFAYLPFSAGPRNCIGQKFAMLELKVVLSGILGNFVLEAVDKPKDVTMITDLVLRCKG

PIRVKFVPRYKIN

>EFA10754.1

MLLSTTLVCAIGLLGVFHLLAWIKFHASNYMKMCVIPGPPQKGILIGNMTYLQTTPEKIF

LRLREATKNFYPIYKLNALHKCAANILNPEDCELIMSNSAHNQKGQIYDLLRNWLKDGLL

TSFGAKWQTRRKILTPAFHFSILQQFVQIFNEEAEILVEDLKKDCSKSYISISSHITKFT

LKTIAETAMGSKLKFETQKEIDYYQAVYDVGKILLYRLTHPWFIFHYVNFFSPWYLQEVK

VTKTLHNFTREVIKHREENFKDIELPTEEHEVYKGKKRLAMLDLLLSAKHKEGIVENDGI

QEEVDTFMFKGHDTTSAALCFALMLIASHSEVQESIVAEMREVLGDLSKKPSYNDLQNLK

YLERCIKETLRLYPSVHFISRTLGQDLITTGGYTLPKESNAIIHIYDVHHNADIYPDPEK

FDPDRFLPENVQKRHPYAYLPFSAGPRNCIGQRFAMLELKTAICAILANFTLQPIDTPET

IILVVDIILRTKEPIKIKFVPRS

>AAF70496.1

MLVLTTVLTAAAVFLLFYLFYCYKTIKQHIYSLISLSYLPGPPVNDIISGNILPLYTSAE

NIFKQLREWGRLYYPIYKLNAAHLAAANILSPEDCELVLSNPTHMEKSAIYNLLHDWLGT

GLLTSTGLKWQTRRKILTPAFHFSILQQFVAIFNEETDKLVEVLKEECYKPFINVNAHVA

QFTLKTIAETAMGTKLRFTTRKETIYKQSIVDMGEFLLYRLLRCWLISKCIYVFNPRYYL

EKKVTRRLHRFTKSVIAERQENFKEIVVPETDEVYTGKRRLAMLDLLLTAKNKEGLIDDE

GIREEVDTFMFEGHDTTAAALGFALMVLAGHKEVQDKIVEEMNEVLGDIKKKPTYQDLQE

MKYLERCVKEVLRLYPSVHFISRKLGEDLVTHSGHKLAKGSIVNLHIYDLHHNPAIYPDP

EKFDPDRFLPENCQKRHPFAYLPFSAGPRNCIGKKFAMLELKAAICGILANFTLEPIDIP

ETIVLVVDIVLRTKEGIKVRFIPRV

>AAP94192.1

MLVLTTVLTAAAVFLLFYLFYCYKTIKQHIYSLISLSYLPGPPVNDIISGNIPPLYTSAE

NIFKQLREWGRLYYPIYKLNAAHLAAANILSPEDCELVLSNPTHMEKSAIYNLLHDWLGT

GLLTSTGLKWQTRRKILTPAFHFSILQQFVAIFNEETDKLVEVLKEECYKPFVNVNAHVA

QFTLKTIAETAMGTKLRFTTRKETIYKQSIVDMGEFLLYRLLRCWLISKCIYVFNPRYYL

EKKVTRRLHRFTKSVIAERQENFKEIVVPETDEVYTGKRRLAMLDLLLTAKNKEGLIDDE

GIREEVDTFMFEGHDTTAAALGFALMVLAGHKEVQDKIVEEMNEVLGDIKKKPTYQDLQE

MKYLERCVKEVLRLYPSVHFISRKLGEDLVTHSGHKLAKASIVNLHIYDLHHNPAIYPDP

EKFDPDRFLPENCQKRHPFAYLPFSAGPRNCIGKKFAMLELKAAICGILANFTLEPIDIP

ETIVLVVDIVLRTKEGIKVRFIPRV

>AAF70178.1

MFLAPIILCLVLLALLVSSLRKKGRFWECLAPIPQPPAYPIIGNLFDIMRTPEQLFLAER

ERGLKYYPIYKLDVCGNGGVNLLNPEDVELVLTDTKQNTKSFIYHFLHSWLGTGLLTSAG

PKWQNRRKILTPAFHFNILQEFIQIFNEETKRLVEDLEAECHKPYIDVVVPITQFTLLSI

GETAMGIKLNASDNDKDGYKRAVYKIGQLLTYRAPRPWIYNETIYSLTPQGRKEQKVLKS

LHSFSNNVIAERKKHFSSSSYSSRKRLAMLDLLLKYKSEGANIDDEGIREEVDTFMFEGH

DTTSVSICYTLMLLANHREVQEEILKEMEAVLDEEPPTYAKLQELKFMDRVIKESLRLYP

SVPFISRVSGSEIQTKTGYTIPKDCMVNLQIYDMHHNPNVFPDPEKFDPDRFLPENIQKR

HPFAYIPFSAGSRNCIGQKFAMLEIKTVLCGILKKFILEAVDTRKDMAFVSDLVLRPKGS

IKVKFVPRCAN

>EFA10756.1

MFLAPIILCLVLLALLVSSLRKRGRFWECLAPIPQPPAYPIIGNLFDIMRTPEQLFLAER

ERGLKYYPIYKLDVCGNGGVNLLNPEDVELVLTDTKQNTKSFIYHFLHSWLGTGLLTSAG

PKWQNRRKILTPAFHFNILQEFIQIFNEETKRLVEDLEAECHKPYIDVVVPITQFTLLSI

GETAMGIKLNASDNDKDGYKRAVYKIGQLLTYRAPRPWIYNETIYSLTPQGRKEQKVLKS

LHSFSNNVIAERKKHFSSSSYSSRKRLAMLDLLLKYKSEGANIDDEGIREEVDTFMFEGH

DTTSVSICYTLMLLANHREVQEEILKEMEAVLDEEPPTYAKLQELKFMDRVIKESLRLYP

SVPFISRVSGSEIQTKTGYTIPKDCMVNLQIYDMHHNPNVFPDPEKFDPDRFLPENIQKR

HPFAYIPFSAGSRNCIGQKFAMLEIKTVLCGILKKFILEAVDTRKDMAFVSDLVLRPKGS

IKVKFVPRCAN

>EFA10781.1

MLSFYLLLTGVGLILLLQTWRFFRKYFKNKTILKKIRGPPETPIFGHMYIFTQDPDLLFS

RIRKYSREYYPIFRFSALYVDIVNFLEPNDIELILSTTKHLSKSKIYTFLNNWLGTGLLT

STGAKWHQRRKILTPAFHFNILQQFLNIFNDETEKLVLRLGEDCGRTIDVVPPVTNFTLQ

SIAETAMGLSNINELTQKEYKKAIYKIGHIFLKRLSKPWYRLDAIYNYSKLAEEENATVK

ILHDFSNGIISEREKERTTNLSQNLASYSKKKRMAMLDLLLAAKNEGADIDYEGIREEVD

TFMFEGHDTTSMAISFILLTLANLQDVQTKVREEILSVVGKEKIPTYNDLQELKYTERCI

KETLRLFPSVPFISRYASEDFVTKTGYTIPEGTVLHIHIFDLHRNAEIYPDPLKFDPDRF

LPEKVNERHPFAYIPFSAGPRNCIGQKFAFLELKTVLCGILRKFKLEKVDDMYEIEFRPD

LVLRPKNDVKVRIEKL

>EHJ71112.1

MLTIICVIVLSLCLIHLAFNYNYKARLLRKIPGPDESFIVGNAPKLFVSPGLELFAMSRQ

WSSTFKGIYRFYAFPVASVNIYNPEDVETITSNMKYHEKSLIYKFLEPWLKDGLLISNGT

KWQNRRKVLTSAFHFDVLKKYFDSIDGNSQRLVRVLQQTNGQSVDVVPILSEYTLNTICE

SAMGTALSDESSSEGKMYKNSIYQMGHTLFQRFINIYLHPDFIFNLSFLGRKQKQHLSVI

HNFTKNVIKNRRKLLDIDNISETKVAQEDGNISNVFKKKKAAMLDLLLLAEKEDLIDGDG

IQEEVDTFMFEGHDTTQAALTYCLMSLANEEFVQQKAYAEQECIFAGDNRPATLADLSEM

TYLECCIKESLRLYPPVPFISRKINEPTTLSNYTVPAGASCHIHIYDLHRQESIYKNALK

FDPDRFLKENSVGRHTYAYIPFSAGPRNCIGQKFAMMEMKSSLSAVLRNFKLVPVTSPDD

LCFMSDIILRNHAPVYLKFIKRNRIC

>AAF67724.2

MDVFKNLSAVLAAVFVIYIVYKFLKIRSVLRKVYKLPGPPKLPILGNFNDLFYSDSVQLF

KNFREWSRKYSPLYSVVVLDIPVVVVTGPDEFEKIASGSKHITKGMIYGLVEPWLGKGLL

TNSGSLWQQRRKILTPAFHFSILQEFVKVFNKETARLVETIKQENKKSATNIIPLISQTA

LNTIAETSFGTTLDLTKKDDKNYVSAIHEMGKILIYRMVRPWFYSLFVFYILSSVGAKLK

QVLSTLHSFTERIIPERSKDFKPFEVNTDGETKRKKLAFLDLLLNAKLSKGIIDDQGIKD

EVNTFMFEGHDTTATGISWILRQLATHSEYQDQIYEEIITVLGDAQKQPDLNDLNELKVM

ERFIKETLRLFPPVPYIARTLDEDIELNGYLIPKEASIDIWIYDIHRNPKHWPEPEKFDP

DRFLPENCVNRHPFAYVPFSAGPRNCIGQRFAMYEMKAIICGIMQNFSVKLADKNEKVEI

MTDLVLRSAHEINLNFIPRTN

>EDX01405.1

MLVVLLVVLLVTRLVASLFRLVLKELRHPLQGVVPSVSRVPLLGAAWQMRSFQPDNLHDK

FAEYVRRFGRCFMGTVLGHVVVVTAEPRHVDALLQSQHQLKKGTMYMALRGWLGDGLLLS

RGREWHTMRKIITPTFHFSILDQFVEVFDRQSSILVERLKVLSRGDQVVNIYPLVGLAAL

DIITETAMGVSVGAQGGDSEVVHAVRDLTNILATRFMRPHLLFRHLFRLCWPSGFRKQQA

GVLCLHQFTNGIIEQRRRSLAREAQQDQPTKRHALLDTLLRATVDGKPLTDKQIRDEVNT

FIFEGHDTTTSAVSFCLYLLSRHEAVQQKLFEELTMHYGQDLSRGVLLSDFAALPYLSCV

VKESLRLYPPIPAVARCLEKDLVIDEGYIPVGTNVVVLLWQLLRDEELFADPLVFQPERH

LGEEAPRLSPYSYIPFSAGPRNCIGQKFALLEMKTMVTQVIRHYQLLPMGADVEPSIKIV

LRSKNGVNFGLRPRLY

>EDV45581.1

MLVALLVALLVTRLVASLFRLALKELRHPLQGVVPSVSRVPLLGAAWQMRSFQPDNLHDK

FAEYVKRFGRCFMGTVLGQVVVVTAEPRHIDALLQSQHQLKKGTMYMALRGWLGDGLLLS

RGKEWHTMRKIITPSFHFSILEQFVEVFDKQSSILVERFKALSRGDQVVNIYPLVGLAAL

DIITETAMGVSVGAQEGDSEVVHAVKDLTNILSTRFMRPHLLFRHLFRLCWPSGFRNQEA

GVLCLHQFTNKIIEQRRRLLAMEANQDQPTKRHALLDTLLRASVDGKPLTDKQIRDEVNT

FIFEGHDTTTSAVSFCLYLLSRHEAVQQKLFEELRMLYDQDLSRRVILSDFAALPYLNCV

VKESLRLYPPIPAVARCLEKELVIDEGYIPVGTNVVVLLWQLLRDEEIFADPLVFQPERH

QADEAPRLSPYSYIPFSAGPRNCIGQKFALLEMKTMVTKVIRHYQLLPMGADVEPSIKIV

LRSKSGVNVGLRPRLY

>EDY72000.1

MLMALLLTMALTLIVAWLLQLALREMRHPLRGFVPSVSRVPLVGAAWQMRSFQPDNLHEK

FAEYVERFGRSFMASVCGQVIMVTAEPSHVDALLHSQKQLRKSSIYGALRGWLGNGLLLS

QGEQWHGMRKIITPTFHFSILEEFVEVFDKQSQLLVERLQPLSNGGMVAINIYPYVGLAA

LDIITETAMGVCVNAQLDADSAVVQAVKAVTNTLATRFMRPHLLPPLLFRLCWPSGFRKQ

QEGIKCLHLFTNSIIEQRRQLLRRENAPGEPVARDRRNALLDTLLRATVDGQPLTDKQIR

DEVNTFIFEGHDTTTSAISFCLYLLSRHAAVQERLFDEVKGHFGRELDRCVVYSDFAALP

FLNCVVREALRLYPPIPALGRCLETDLAVEGGRIPAGTNVILLLWQLLRDEMLFADPLIF

RPERHLSNKATSKKGAYSNIPFSAGPRNCIGQKFAMFEVKTMIIKMLRNFILLPIGPDVE

PSIKIVLRSRNGVHFGLRPRVY

>EDW30776.1

MLMALLLTMALTLIVAWLLQLALREMRHPLRGFVPSVSRVPLVGAAWQMRSFQPDNLHEK

FAEYVERFGRSFMASVCGQVIMVTAEPSHVDALLHSQKQLRKSSIYGALRGWLGNGLLLS

QGEQWHGMRKIITPTFHFSILEEFVEVFDKQSQLLVERLQPLSNGGMVAINIYSYVGLAA

LDIITETAMGVCVNAQLDAESAVVQAVKAVTNTLATRFMRPHLLPPLLFRLCWPSGFRKQ

REGIKCLHLFTNSIIEQRRQLLRRENAPGEPVARDRRNALLDTLLRATVDGQPLTDKQIR

DEVNTFIFEGHDTTTSAISFCLYLLSRHAAVQERLFDEVKGHFGRELDRCVVYSDFAALP

FLNCVVREALRLYPPIPALGRCLETDLAVEGGRIPAGTNVILLLWQLLRDEMLFADPLIF

RPERHLSNKATSKKGAYSNIPFSAGPRNCIGQKFAMFEVKTMIIKMLRNFILLPLGPDVE

PSIKIVLRSRNGVHFGLRPRVY

>EDV32761.1

MLVAWLLALLLARLVAWLIQLAVKEVRHPLRGIVPHVSNIPVVGSAWQMRSFQPDNLHAK

FAEYCQRFGRSFMGTVFGHCVVVTAEPQYVDALLHSQQHLRKGTLYGALRGWLGNGLLLS

RGEDWRTMRKIITPTFHFSILEQFVEVFDRQSRVLVQQLEPLADGQQVVNIYPYAGLAAL

DIIMETAMGVRVEAQKDADSKVVRAVKDLTNILAIRFMRPHLLPLFLFRWFWPSGYKNQI

EGVQCLHQFTDTVIEQRRKLLEDHPDDAETGKHHALLDTLLRATCDGKPLTDRQIRDEVN

TFIFEGHDTTTSAASFCLYLLSRHADVQQRLFEELREHYGQDMEQRVVYGDFAVLPYLNC

VVKEALRLYPPIPAVARFLEKDLMIDGGHIPARTNVVVLLWQLLRDDKLFPDPLSFRPER

HLDVETSNLAAYSYIPFSAGPRNCIGQKFALLEMKTMVIQLLRHYQLLNLGVDVEPSIKI

VLRSRTGVNFGLRRRDY

>EDW86241.1

MIALLLFGICAIWLTRFLLRLVRLEQRLQRDLIGKVPSVSRVPLIGSAWQLRNFQPYNLH

EKFTEYVKRFGPTFMATVFGHLIVITANRSHVEALLFSQHHLRKGSIYGALRKWLGNGLL

VSRGQNWRAMRKIITPTLHFAILEQFVDVFDRQALVMVNKLRPLANGREVINIYPYMGLA

ALDIITETAMGVHINAQLDEESAVVQAVKDVTNILATRFMRPHLLPPSLFRLCWPSGYRR

QESGIQCLHKFTNSIIEQRRQLLQEKTVNEPNDRRSVLLDTLLRATMSGESLTDAQIRDE

VNTFIFEGHDTTTSAASFCLYLISRHGDVQQLLFDEIKSYYGSDVQRPIVYGDFQNFPYL

NCVVKESLRLYPPIPAVSRCLEQDLPIDNGCRIPANTNVIVLLWQLLRDENIYPDALSFR

PERHLYQSENTSSASFISYIPFSAGPRNCIGQRFALFEVKTIVIRMLRHFQLLPLGEDVK

PTIKIVLRSRLGVNFALKPRTY

>EDV95416.1

MLVIIAVIGLLVSLLRLLLQLLWQERKQREQLSNLAPNVSQLPLIGAIWQMRDFQPDNLH

EKFAAYVRRYGRSFVATTCGRIVLVTSDPGHVDALLMSKHQLRKGVLYGALTSWLGNGLL

LSRGESWHAMRKIITPTFHFSILEQYIEIFDRQSNILVTKLKPFAAAAAAADPREAINIY

PHVCLAALDIISEAAMGVCVNAQLNVDAPVVQAVKDVTNILATRFMRPHLLPPLLFRLLW

PAGYRKQIAGLQCLHHFTNAIIERRRQLLEQQQQLKQEAQQLGKRFALLDTLLQARLAGA

SLTDDQIRDEVSTFIFAGHDTTTSAVSFCLYLLSRHATIQQRLFEELAGYYGKDLEQPVC

YGDFAALPYLHCVVKESLRLYPPIPAVSRCLDKDLLLGESRLPSGSNVIVLLWQLLRDEQ

LYADPLRFWPERHLDAAGRDYNIPFSAGPRNCIGQRFALLELKTIVIRMLRHFELLPLGL

DVKPSIKIVLRSATGVNLGLKLRSKN

>EDW62656.1

MLLILLVIALLVFLLRFLLQLFWTERRHRQQLEAVAPNVSQVPLIGTLWQMRDFQPDNLH

EKFAAYVKRYGRSFVATTCGRMVLVTAEPRHADALLLSKQQLRKSVVYGALGGWLGNGLL

LSRGDRWHAMRKIITPTFHFSILEQYIEVFDRQCNVLVDRLQPLAANSNRPQAFNIYPYM

CLAALDIISEAAMGVSVDAQRNVDAPVVQAVKDVTNILATRFMRPHLLPPLLFRLLWPSG

HRKQWDGVKCLHSFTDDIIRRRRQLLLHEQQLGKRSALLDTLLQARLEGAPLTDAQIRDE

VSTFIFAGHDTTTSAASFCLFLLSRHASVQRRLFEELHAHYGPAVDRPVIYGDFADLPYL

HCVIKESLRLYPPIPAVGRCLESDLMLDGAHLVPAGTNVIVLLWQLLHDEQLYEDPLRFW

PERHLDSHKTAEAGAARACSSYIPFSAGPRNCIGQRFALLELKTIVIKMLRHFELLPLGV

DVKPSIKIVLRSATGVNLGLKKRSYAN

>EDW06510.1

MLLGLLLFALLVVGLRFVLQLIKREWRHRQLLQHVAPNVSRIPVIGGVWQMRSFQPDNLH

EKFAEYVARYGRSFVATTFGRMVLVTADPKHVDALLLSKQHLRKSVVYGALGGWLGNGLL

LSRGEHWHAMRKVITPSFHFSILEQYVKIFDSQCNVLVGKLKPLAAMGSESAQAFNIYPY

MCLTALDIISEAAMGVSLDAQENVEAPVVQAVKDVTNILATRFMRPHLLPPLFFRLLWPS

GYRKQWMGIKCLHDFTDEIIKRRCQLLHTEQQGTAALLDTLLQARLDGAPLTDAQIRDEV

STFIFAGHDTTTSAASFCLYLLSRHAAVQQRLYEEIHSHYGTAMDRPVVHADFAELPYLH

CVIKESLRLYPPIPAVGRCLEKDLPLASGARVPAGTNVLVLLWQLQRDEELYAEPLRFWP

ERHLDNRMTDSGSGKCTTSYIPFSAGPRNCIGQRFALLELKTIVIKLLRHFELLPLGAEV

KPSIKIVLRSATGVNLGLKMRA

>EDV32760.1

MLGVIGLFLAAFAALLLWDYLWRKRRNDMLSYMPGPRPLPLLGNVLMYRGLDAEQIMDFV

AKNQAKYGKIYKVWVIHQLAVFSTDPRDIEFLLSSQQHITKNNLYKLLNCWLGEGLLMST

GRKWHGRRKIITPTFHFKILEQFVEIFDQQSAVMVEKLKSRADGKTAFNIFPVVCLTALD

IIAETAMGTKINAQMNPNLPYVKAVNDVTNIMTTRFINAWQRVDWIFRLIQPKEAKRQDT

AIKIMHDFTENIIRQRRQALVNSQDPNSPSELVDDLGQKRRMALLDVLLQSTIDGAPLSD

EDIREEVDTFMFEGHDTTTSAISFCLYEISRHPEVQKRLVQEIHEVLGDDTQRPVTLRDL

GELKYMENAIKESLRLHPPVPMIGRWFAEDVEIRGKRIPAGTNFTVGIYVLLRDPEYFDS

PDEFRPERFEADVPQTHPYAYIPFSAGPRNCIGQKFAMLEMKSTISKLLRHFELLPLGPD

PRLSMNIVLRSANGVHLGLKPRSAAKED

>Q27589.2

MLGVVGVLLLVAFATLLLWDFLWRRRGNGILPGPRPLPFLGNLLMYRGLDPEQIMDFVKK

NQRKYGRLYRVWILHQLAVFSTDPRDIEFVLSSQQHITKNNLYKLLNCWLGDGLLMSTGR

KWHGRRKIITPTFHFKILEQFVEIFDQQSAVMVEQLQSRADGKTPINIFPVICLTALDII

AETAMGTKINAQKNPNLPYVQAVNDVTNILIKRFIHAWQRVDWIFRLTQPTEAKRQDKAI

KVMHDFTENIIRERRETLVNNSKETTPEEEVNFLGQKRRMALLDVLLQSTIDGAPLSDED

IREEVDTFMFEGHDTTTSAISFCLYEISRHPEVQQRLQQEIRDVLGEDRKSPVTLRDLGE

LKFMENVIKESLRLHPPVPMIGRWFAEDVEIRGKHIPAGTNFTMGIFVLLRDPEYFESPD

EFRPERFDADVPQIHPYAYIPFSAGPRNCIGQKFAMLEMKSTVSKLLRHFELLPLGPEPR

HSMNIVLRSANGVHLGLKPRA

>CAA15698.1

MRGNGILPGPRPLPFLGNLLMYRGLDPEQIMDFVKKNQRKYGRLYRVWILHQLAVFSTDP

RDIEFVLSSQQHITKNNLYKLLNCWLGDGLLMSTGRKWHGRRKIITPTFHFKILEQFVEI

FDQQSAVMVEQLQSRADGMTPINIFPVICLTALDIIAETAMGTKINAQKNPNLPYVQAVN

DVTNILIKRFIHAWQRVDWIFRLTQPTEAKRQDKAIKVMHDFTENIIRERRETLVNNSKE

TTPEEEVNFLGQKRRMALLDVLLQSTIDGAPLSDEDIREEVDTFMFEGHDTTTSAISFCL

YEISRHPEVQQRLQQEIRDVLGEDRKSPVTLRDLGELKFMENVIKESLRLHPPVPMIGRW

FAEDVEIRGKHIPAGTNFTMGIFVLLRDPEYFESPDEFRPERFDADVPQIHPYAYIPFSA

GPRNCIGQKFAMLEMKSTVSKLLRHFELLPLGPEPRHSMNIVLRSANGVHLGLKPRA

>AAF45741.1

MLGVVGVLLLVAFATLLLWDFLWRRRGNGILPGPRPLPFLGNLLMYRGLDPEQIMDFVKK

NQRKYGRLYRVWILHQLAVFSTDPRDIEFVLSSQQHITKNNLYKLLNCWLGDGLLMSTGR

KWHGRRKIITPTFHFKILEQFVEIFDQQSAVMVEQLQSRADGMTPINIFPVICLTALDII

AETAMGTKINAQKNPNLPYVQAVNDVTNILIKRFIHAWQRVDWIFRLTQPTEAKRQDKAI

KVMHDFTENIIRERRETLVNNSKETTPEEEVNFLGQKRRMALLDVLLQSTIDGAPLSDED

IREEVDTFMFEGHDTTTSAISFCLYEISRHPEVQQRLQQEIRDVLGEDRKSPVTLRDLGE

LKFMENVIKESLRLHPPVPMIGRWFAEDVEIRGKHIPAGTNFTMGIFVLLRDPEYFESPD

EFRPERFDADVPQIHPYAYIPFSAGPRNCIGQKFAMLEMKSTVSKLLRHFELLPLGPEPR

HSMNIVLRSANGVHLGLKPRA

>EDX16905.1

MLGVIGVLLLVAFATLLLWDFLWRRRGNGILPGPRPLPFLGNLLMYRGLDPEQIMDFVKK

NQRKYGRLYRVWILHQLAVFSTDPRDIEFVLSSQQHITKNNLYKLLNCWLGDGLLMSTGR

KWHGRRKIITPTFHFKILEQFVEIFDQQSAVMVEQLQSRADGKTPINIFPVICLTALDII

AETAMGTKINAQKNPNLPYVQAVNDVTNILIKRFIHAWQRVDWIFRLTQPTEAKRQGKAI

KVMHDFTENIIRERRQTLVNNSKDTTPEEEVNCLGQKRRMALLDVLLQSTIDGAPLSDED

IREEVDTFMFEGHDTTTSAISFCLYEISRHPEVQQRLHQEIRDVLGEDRKSPVTLRDLGE

LKFMENVIKESLRLHPPVPMIGRWFAEDVEIRGKRIPAGTNFTMGIFVLLRDPEYFESPD

EFRPERFETDVPQIHPYAYIPFSAGPRNCIGQKFAMLEMKSTVSKLLRHFELLPLGPEPR

HSMNIVLRSANGVHLGLKPRA

>EDW43939.1

MLGVIGVLLLVAFATLLLWDFLWRRRGNGILPGPRPHPFLGNLLMYRGLDPEQIMDFVKK

NQRKYGRLYRVWILHQLAVFSTDPRDIEFVLSSQQHITKNNLYKLLNCWLGDGLLMSTGR

KWHGRRKIITPTFHFKILEQFVEIFDQQSAVMVEQLQSRADGKTPINIFPVICLTALDII

AETAMGTKINAQKNPNLPYVQAVNDVTNILIKRFIHAWQRVDWIFRLTQPTEAKRQGKAI

KVMHDFTENIIRERRQTLVNNSMETTPEEEVNCLGQKRRMALLDVLLQSTIDGSPLSDED

IREEVDTFMFEGHDTTTSAISFCLYEISRHPEVQQRLHQEIRDVLGEDRKSPVTLRDLGE

LKFMENVIKESLRLHPPVPMIGRWFAEDVEIRGKRIPAGTNFTMGIFVLLRDPEYFESPD

EFRPERFETDVPQIHPYAYIPFSAGPRNCIGQKFAMLEMKSTVSKLLRHFELLPLGPEPR

HSMNIVLRSANGVHLGLKPRA

>EDW60801.1

MFLEIVALLLTVLLVWDYLTMRRQNNMMAAAGVKGPPQFPLLGSAPICMMGESAETMLDL

ETRLIEKYGKNIQLTMFHEYGFITADARMIESILSSQQTITKNNLYNLLQCWLGTGLLLS

TGKKWFRRRKIITPTFHFKILEQFVEVFDQQSAIMAENLYDRADGKTVINMFPVACLAAL

DIIAETSMGVKINAQGEPNFPYVQSVKIVSNMMAERFMNPLQRFDSTMRIFFPLLFRKLN

QHIKAMHDFTDKVISERRDTLQKSLNEQSSTGTEENDVGSKRRMALLDVLLQSSVDGQPL

SNNDIREEVDTFMFEGHDTTTSAISYTLYLLARHPELQTRAFREIVDVIGADKSKPITMR

DLGELKYLECVIKESLRLYPPVPMIGRQLTEDVTLDGKRFPALTNIIMLTYHAQRDPEYF

PDPEKFNPERFSSESSSNIDVFAYAPFSAGPRNCIGQKFAMLEMKSTVSKMLRHFELLPL

GEPVQPIMNLILRSKTGINLGLKPRVY

>EDX01406.1

MLGVVGVLLLVAFATLLVCDFLWRRRGNGVLPGPRPLPLLGNLLMYRGLDPEQIMDFVKK

NQRKYGRLYRVWILHQLAVFSTDPRDVELVLSSQQHITKNNLYKLLNCWLGDGLLMSTGR

KWHGRRKIITPTFHFKILEQFVEIFDQQSAVMVEQLQSRADGKTPINIFPVVCLTALDII

AETAMGTKINAQENPNLPYVQAVNDVTNILIKRFIHAWQRVDWIFRLTQPTEAKRQDKAI

QVMHDFTENIIRERRQTLVNNSKDTTPEEQVDCLGQKRRMALLDVLLQSTIDGAPLSDED

IREEVDTFMFEGHDTTTSAISFCLYEISRHPEVQQRLVQEIRDVLGEDRKRPVTLRDLGE

LKYMENVIKESLRLHPPVPMIGRWFAEDVEIRGKRIPAGTNFTMGIYVLLRDPEYFESPD

EFRPERFEADVPQIHPYAYIPFSAGPRNCIGQKFAMLEMKSTVSKLLRHFELLPLGLEPR

HSMNIVLRSANGVHLGLKPRAS

>EDW00775.1

MIVEITVLLVAALFIWHHLKTRRAKAMFHAAGIKGPIALPLLGNAPLIMIGESAETMLDL

LWRLINKYGKFISFSLFNEIAFITADPRMIEAVLSSQKPITKNKLYNLLTDWLGTGLLLS

TGKKWFKRRKIITPTFHFKILEQFVEVFDQQSAVMVEKLYDRADGKTIINMFPVACLAAL

DIIAETSMGVKLNAQHKPDFPYVQSVKIVSNIMAERFMRPLQRFNFTMRIFYPLIYRELN

QHIKSMQDFTDSVISERRYTLQKSLKESNQNSKSAEDDDVGSKRRMALLDVLLQSTVDGQ

PLSNEDIREEVDTFMFEGHDTTTSAISYTSYLLARHPEVQERAFREVVEVIGNDKSKPIT

MRDLGELKYLECVIKESLRLYPPVPMIGRNLTEDITLDGKRFPADSNLILLIYHAQRDPE

YFPEPEKFNPDRFSPENINNIDVFAFAPFSAGPRNCIGQKFAMLEMKSTISKMLRHFELL

PLGEPVKPVMNLILRSTTGVNIGLKPRVY

>EDW10178.1

MFLEIVVLLLTALGFWNYLNTRRQKRMIDEAGIKGPVELPVLGNAPVLMMGESAKTMLDL

VSRLIAKYGKNVKLTLLHEYGFITADVRMMEAILSSQQTITKNSLYNLLQYWLGNGLLLS

TGKKWFRRRKIITPTFHFKILEQFVEVFDQQSAIMAEKLYDRADGKTVINMFPVACLAAL

DIIAETSMGVKINAQGEPDFPYVQAVKTVSSIMAERFVSPLQRFDETMRIFYPLLFRKLN

RNIKAMHDFTDKVIAERRATLQKTLGESTQSADDEDVGSKRRMALLDVLLQSTADGQPLS

NQDIREEVDTFMFEGHDTTTSAISYTLYLLARHPEVQARAFQEIVDVIGTDKAKPTTMRD

LGELKYLECVIKESLRLYPPVPMIGRHLTEDVTLNGKRFAAGTNIILIIYNAQRDPDFFP

EPEKFNPDRFSPENNGNIDVFAYAPFSAGPRNCIGQKFAMLEMKSTVSKMLRHFELLPLG

EPVQPIMNLILRSTTGINMGLKPRTY

>EDW86243.1

MLNKAGIVGPPTVPFLGNALTLLKESPATVFDLQQRLINQYGRNIRMTILFEKGFMTADP

RMIEAILSSQKVIQKNNLYDLLHSWLGTGLLLSTGKKWFRRRKIITPTFHFKILEQFVEV

FDQNSAIMAEQLYEKADGKTVINMFPVACLCALDIIAETAMGVQVHAQRKPDFPYVQSVK

TASNMLAERFLSPAQRFDTTMKIFYPKLYNTLTKTIQTMHDFTDSVIADRRAALQKSIAD

GSYDRSESKATDVGEDVGQKRRMALLDVLLQASVDGQPLSNADIREEVDTFMFEGHDTTT

SGISYTLYLLSRNPEAQARCFQEIREVIGDDKTRPVDMKDLGQLKYLECAIKESLRLFPP

VPLIGRYVAEDTELNGKLIPANTNVILLTYHAQRDPEFFEEPNKFNPDRFAIENKGDIDP

FAYTPFSAGPRNCIGQKFAMLEMKSTISKMLRHFELLPLGPEVQPLMNLILRSTTGINVG

IKPRVY

>EDV45580.1

MLGIVGVLLLAAFATLLVWDFLWRRRGNGILPGPRPLPLLGNLLMYRGLDPEQIMDFVKK

NQRKYGRLYRVWILHQLAVFSTDPRDVEFVLSSQQHITKNNLYKLLNCWLGDGLLMSTGR

KWHGRRKIITPTFHFKILEQFVEIFDQQSAVMVEQLQSRADGKTPINIFPVVCLTALDII

AETAMGTKINAQKNPNLPYVQAVNDVTNIMIKRFIHAWQRVDWIFRLTQPTEAKHQDKAI

KVMHDFTENIIRERRQTLVNNLKKTTTEELVDCLGQKRRMALLDVLLQSTIDGAPLSDED

IREEVDTFMFEGHDTTTSAISFCLYEISRHPEVQQRLVQEIRDVLGEDRKRPVSLRDLGE

LKYMENVIKESLRLHPPVPMIGRWFAEDVEIRGKRIPAGTNFTIGIFVLLRDPEYFESPD

EFRPERFEADVAQIHPYVYIPFSAGPRNCIGQKFAMLEMKSTVSKLLRHFELLPLGPEPR

HSMNIVLRSANGVHLGLKPRAS

>EDX16904.1

MYLELFVIFLATVFAWDYMRNRRHNKMYAAAGIRGPKSYPLVGNALMLINESPKTIFDLQ

SRLISEFGKNIRMQMLGERGFMTADSKMIEAIMSSQQTIQKNNLYGLLVNWLGDGLLLSK

GKKWFRRRKIITPAFHFKILEDFVEVFDQQSATMVQQLYDRADGKTVINMFPVACLCAMD

IIAETAMGVKINAQLQPQFSYVQSVTTASSMLAERFMNPLQRVDFSMKLFYPKLLAKLND

SVKNMHDFTNSVITERRELLQKSIADGGDADAALLNDVGQKRRMALLDVLLKSTIDGAPL

SNDDIREEVDTFMFEGHDTTTSSIAFTCYLLARHPEVQARVFQEVRDVLGEDKSAPVTMQ

LLGELKYLECVIKESLRLFPSVPLIGRYISQDIVLDGKLIPADSNVVILIYHAQRDPDYF

PDPEKFIPERFSMERKGEINPFAYTPFSAGPRNCIGQKFAMLEMKSTISKMVRHFELLPL

GEEVQPVLNLILRSSTGINCGLKPRVY

>EDW43938.1

MYLELFVIFLATVFAWDYMRNRRHNKMYAAAGIRGPRSYPLVGNAFMLINESPKTIFDLQ

SRLIAEFGKNIRMQMLGERGFMTADSKMIEAIMSSQQTIQKNNLYGLLVNWLGDGLLLSK

GKKWFRRRKIITPAFHFKILEDFVEVFDQQSATMIQQLYDRADGKTVINMFPVACLCAMD

IIAETAMGVKINAQLQPQFSYVQSVTTASSMLAERFMNPLQRVDFSMKLFYPKLLAKLND

SVKNMHDFTNSVITERRELLQKSIADGGDADVALLNDVGQKRRMALLDVLLKSTIDGAPL

SNGDIREEVDTFMFEGHDTTTSSIAFTCYLLARHPEVQARVFQEVRDVLGEDKSAPVTMQ

LLGELKYLECVIKESLRLFPSVPLIGRYISEDTVLDGKLIPADSNVVILIYHAQRDPDYF

PDPEKFIPERFSMERKGEINPFAYTPFSAGPRNCIGQKFAMLEMKSTISKMVRHFELLPL

GEEVQPVLNLILRSSTGINCGLKPRVY

>EDW30777.1

MLALVVFLVALTTVLVWDFFWRKRRNDILHYMPGPRGLPLVGNALMYRGLDAEQIMGFVS

KNRNKYGPLYKVWVLNQLAVFSTDPHDIEFILSSQQHITKNNLYELLHCWLGTGLLMSTG

KKWHSRRKIITPTFHFKILEQFVEIFDQQSAVMVGQLQQRADGKTAINIFSVVCLLALDI

IAETAMGTKIQAQMSPNLPYVQAVTDVTNIMTTRFIHAWQRIDWLFRLVHPGMAKRQDSA

IKVMHDFTENIIKQRRQALVSAGKQEEEEEVKEDDDLGQKRRMALLDVLLQSTIDGASLS

NDDIREEVDTFMFEGHDTTTSAISFCLYEISRHPQVQQLLVEEIHEVLGVDRQRPVTLRD

LGELKYLENVIKESLRLHPPVPMIGRWFNEDVEIRGKRIPAGTNYTVGIFVLLRDPEYFE

SPDEFKPDRFDSTSPQTHPYAYIPFSAGPRNCIGQKFALLEMKSTISKLLRNFELLPLGP

EPRPAMNIVLRSANGVHLGLQPRSVLAS

>EDY71999.1

MLALVVFLVALTTVLVWDYFWRKRRNDILHYMPGPRGLPLVGNALMYRGLDAEQIMGFVS

KNRNKYGPLYKVWVLNQLAVFSTDPHDIEFILSSQQHITKNNLYELLHCWLGTGLLMSTG

KKWHSRRKIITPTFHFKILEQFVEIFDQQSAVMVGQLQQRADGKTAINIFSVVCLLALDI

IAETAMGTKIQAQMSPNLPYVQAVTDVTNIMTTRFIHAWQRIDWLFRLVHPGMAKRQDSA

IKVMHDFTENIIKQRRQALVSAGKQEKEKEEIKEDDDLGQKRRMALLDVLLQSTIDGASL

SNDDIREEVDTFMFEGHDTTTSAISFCLYEISRHPQVQQLLVEEIHEVLGVDRHRPVTLR

DLGELKYLENVIKESLRLHPPVPMIGRWFNEDVEIRGKRIPAGTNYTVGIFVLLRDPEYF

ESPDEFKPDRFDSTSPQTHPYAYIPFSAGPRNCIGQKFALLEMKSTISKLLRNFELLPLG

PEPRPAMNIVLRSANGVHLGLQPRSVLAS

>CAA53568.1

MLGVVGVLLLVAFATLLLWDFLWRRRGNGILPGPRPLPFLGNLLMYRGLDPEQIMDFVKK

NQRKYGRLYRVWILHQLAVFSTDPRDIEFVLSSQQHITKNNLYKLLNCWLGDGLLMSTGR

KWHGRRKIITPTFHFKILEQFVEIFDQQSAVMVEQLQSRRDGMTPINIFPVICLTALDII

AETAMGTKINAQKNPNLPYVQAVNDVTNILIKRFIHAWQRVDWIFRLTQPTEAKRQDKAI

KVMHDFTENIIRERRETLVNNSKETTPEEEVNFLGQKRRMALLDVLLQSTIDGAPLSDED

IREEVDTFMFEGHDTTTSAISFCLYEISRHPEVQQRLQQEIRDVLGEDRKSPVTLRDLGE

LKFMENVIKESLRLHPPVPMIGRWFAEDVEIRGKHIPAGTNFTMGIFVLLRDPEYFESPD

EFRPERFDADVPQIHPYAYIPFSAGPRNCIGQKFAMLEMKSTVSKLLRHFELLPLGPEPR

HSMNIVCGRPTAFILA

>O46051.1

MYLELFAILLATALAWDYMRKRRHNKMYAEAGIRGPKSYPLVGNAPLLINESPKTIFDMQ

FRLIAEFGKNIKTQMLGESGFMTADSKMIEAIMSSQQTIQKNNLYSLLVNWLGDGLLISQ

GKKWFRRRKIITPAFHFKILEDFVEVFDQQSATMVQKLYDRADGKTVINMFPVACLCAMD

IIAETAMGVKINAQLQPQFTYVQSVTTASAMLAERFMNPLQRLDFTMKLFYPKLLDKLND

AVKNMHDFTNSVITERRELLQKAIADGGDADAALLNDVGQKRRMALLDVLLKSTIDGAPL

SNDDIREEVDTFMFEGHDTTTSSIAFTCYLLARHPEVQARVFQEVRDVIGDDKSAPVTMK

LLGELKYLECVIKESLRLFPSVPIIGRYISQDTVLDGKLIPADSNVIILIYHAQRDPDYF

PDPEKFIPDRFSMERKGEISPFAYTPFSAGPRNCIGQKFAMLEMKSTISKMVRHFELLPL

GEEVQPVLNVILRSTTGINCGLKPRVY

>EDV45579.1

MYLELLALFLATVIAWDYMRNRRHNKMYAAAGIRGPRSYPLVGNAPMMINESPKTVFDLQ

DRLIAQYGKNIRIRMLGERGFMTADSKMIEAIMSNQQTIQKNNLYGLLVNWLGDGLLLSK

GKKWFRRRKIITPAFHFKILEDFVEVFDQQSAIMVQQLYDRADGKTVINMFPVACLCAMD

IIAETAMGVKINAQLQPQFSYVQSVTTASSMLAERFMNPLQRVDASMQIFYPKLLAKLND

SVKNMHDFTNSVITDRRELLQKSIADGGDADAPLLNDVGQKRRMALLDVLLQSTIDGAPL

SNGDIREEVDTFMFEGHDTTTSSIAFTCYLLARNPEVQARVFQEVRDVLGDDKSAPVTMQ

LLGELKYLECVIKESLRLFPSVPLIGRYISQDTVLDGKLIPADSNVIILIYHAQRDPDYF

PDPKKFIPERFSMERKGEINPFAYTPFSAGPRNCIGQKFAMLEMKSTISKMVRHFELLPL

GEEVQPVLNLILRSSTGINCGLKPRVY

>EDX01407.1

MYAAAGIRGPRSYPLVGNAPMLINESPKTVFDLQNRLIAEFGKNIRIQMLGERGFITADP

KMIEAIMSSQQTIQKNNLYGLLVNWLGDGLLISKGKKWFRRRKIITPAFHFKILEDFVEV

FDQQSAIMVQQLYDRADGKTVINMFPVACLCAMDIIAETAMGVKINAQLQPQFSYVQSVT

TASSMLAERFMNPLQRSELSMKILYPKLTAKLNESVKNMHDFTNSVITERRELLQKSIAD

GGDADAALLNDVGQKRRLALLDVLLKSTIDGAPLSNSDIREEVDTFMFEGHDTTTSSIAY

TCYLLARHPEVQARVFQEVRDVLGDDKSAPVTMQLLGELKYLECVIKESLRLFPSVPIIG

RYITQDTLLDGKLIPADSNVMILIYHAQRDPDYFPDPEKFIPERFSMERKGEINPFAYTP

FSAGPRNCIGQKFAMLEMKSTISKMVRHFELLPLGEEVQPVMNLILRSSTGINCGLKPRV

Y

>EDV32759.1

MYLELIALLLTAVLVWDHLRNRRHNQMFAAAGFKGPKRYPLVGNAPMIINESPRTVFDLQ

ARLIRDHGKNIHIQMLGERGFMTACPKMIEAIMSSQQTIQKNNLYGLLVNWLGDGLLLST

GKKWFHRRKTITPAFHFKILEDFVEVFDQQSAIMAEKLYSRADGKTVINMFPVACLCALD

IIAETAMGVKVNAQLQPDFPYVQSVTTASGMVAERLMNPLQRMDATMKILYPKLFAKLNN

AVKSMHDFTNKVITERRDLLQKSISEGKEEELVNDVGQKRRMALLDVLLKSTINGEPLTN

EDIREEVDTFMFEGHDTTTSSIAFTCYLLARHPEAQARVFQEIRDVIGDDKSRPVDMKIL

GELKYLEMVIKESLRLFPSVPMIGRHINQDTMLDGKLIPGNSDILILIYHAQRDPDLFPE

PLKFKPERFSFENKGEINPFAYTPFSAGARNCIGQKFAMLEIKSTISKLLRHFEFLPLGE

EVQPVLNLILRSTTGINVGIKPRVY

>EDW86242.1

MLSLALFLVVSAIATLFLWDYLTRKRRNDMLHYMPGPPALPLLGNVLMYRGLKAEQLMDF

IQKNRDLYGTMYRVWVLKQLAVFSTDPRDIEIILSSPQHITKNNLYELLHCWLGTGLLMS

TGKKWHTRRKIITPAFHFKILEQFVEIFDQQSSILVEQLRKHADGETPINVFPFICLEAL

DIIAETSMGTRINAQMNPNLPYVQAVTDVTNIMITRFINAWQRIEWLYRLVKPSMAKKQD

LAIQTLHKFTDDIIQKRRQALINGPETKTQETNADDVGQKQRMALLDVLLQATVNGQPLS

NEDIREEVDTFMFEGHDTTTSAISFCLYEISRDARVQKLLLEEIQDKLSPGKPTTQRDLG

ELKYLECVIKESLRMHPPVPMIGRWFHEDVEIRGKRIPAGTNFTVGIYFLMRDPLYFDSP

NEFRPERFLNDQTTNPYAYIPFSAGPRNCIGQKFAVLEMKSTISKLLQHYELLPLGPDPL

PLMNIVLRSVNGVHLGLRPRKIAM

>EAL32280.1

MFVELLVLLLTVALVWDYLVGRKHKRLFDRAGITGPKAWPLVGNAPLMINESPKTVFDLQ

WRLIQTHGKNVRCQILQDRGFMTADPKMIEAIMSSQQTIQKNNLYGLLVNWLGDGLLLSK

GKKWFRRRKIITPAFHFKILEQFVEVFDQQSAIMAKNLYDRADGKTVINMFPVACLCAMD

IIAETAMGVKINAQLQPNFPYVQSVKTASGMLAERFVNPAQRLDISMRLFFPSAFAKLSA

NIKAMQDFTNNVISERRDLLQKSISDGTYETTNASPLDDVGQKRRMALLDVLLQSSIDGA

PLSNEDIREEVDTFMFEGHDTTTSSIAFTCYLLARHPEVQARVFQEVRDVLGDEKDAPVN

IQLLGELKYLECVIKESLRLFPSVPIIGRHIVEDTLLDGKLIPAKTDVMILIYHSQRDPD

YFPEPNKFVPERFSPERKGEINPFAYTPFSAGPRNCIGQKFAMLEMKSTISKMVRHFELL

PLGEDVQLVLNLILRSTTGINVGLKPRVY

>EDW30778.1

MFVELLVLLLTVALVWDYLVGRKHKRLFDRAGITGPKAWPLVGNAPLMINESPKTVFDLQ

WRLIQTHGKNVRCQILQDRGFMTADPKMIEAIMSSQQTIQKNNLYGLLVNWLGDGLLLSK

GKKWFRRRKIITPAFHFKILEQFVEVFDQQSAIMAKNLYDRADGKTVINMFPVACLCAMD

IIAETAMGVKINAQLQPNFPYVQSVKTASGMLAERFVNPAQRLDISMRLFFPSAFAKLSA

NIKAMQDFTNNVISERRDLLQKSISDGTYETTNASPLDDVGQKRRMALLDVLLQSSIDGA

PLSNEDIREEVDTFMFEGHDTTTSSIAFTCYLLARHPEVQARVFQEVRDVLGDEKDAPVN

IQLLGELKYLECVIKESLRLFPSVPIIGRHIVEDTLLDGKLIPAKTDVMILIYHSQRDPD

YFPEPNKFVPERFSPERKGEINPFAYTPFSAGPRNCIGQKFAMLEMKSTISKMVRHFELL

PLGEDVQLVLNLILRSTTGINVGLKPRVY

>ADP22303.1

MFLALLIGLLTLLIVGDYLRKRRHNEIFKKNGINSMPSLPIIGVAHHLIKKTPADMIEFT

GEVLKNMGKTCNLWFLNQSTILTADPVFLEPLLSSQKVLTKNNLYELLTPWLREGLLLST

GSKRHSRRRIITPTLHSKILEEFVGVFERQSNVMMDLLRDKADGKTVVNLYSFVCAMALD

VIAETAMGVKIHAQLQPDLPYVKAVRTVSAITSARFVSMLQRFDFWLWLTANSTYKQLHS

NIALMHDFTGKVIEERRKLLEQNIKNKLPEDQHDDDEVVGRKKHTAFLDMLLLAEINGQP

LTNNDIREEVDTFMFEGHDTTTSGISFTCYLLSRHPNVQQKVFEEIRAVIGDDKQRPITL

RDLQELKYLECVIKESMRLYPPVPTIGRHIEQDVYLNGKLYPANTNVTVMIYHALRDPAY

CKDPEKFIPERFYSDNAEKINTFAYVPFSAGPRNCVGQKFALLEMKSTISTILRHYELLP

LGEEVRPLLNLVLVSSNGVNMGLISRIYPN

>ABV48807.1

MLFEFLAGLLTLLLVVDTLYKRRRHVMIAKAGIRGPTPLPLFGNMLHFVGNTTANAYDLF

DKLTAKYGKVVRMWMGNHCVLFVRDAKFFEDIFKSQQLITKNNLYDLLSGWLGQGLLLST

GPKWFSRRKIITPTFHFKILEEFVEIFDQQSHVMVKSLMEKADGKTVVHMFPKVCLMALD

IITETAMGVKVHAQEHPEFPYAKAVTDTSNIMSDRFVKPFDRFDGYFRIFHNQKYHQLQN

NIKIMHDFTDKVINERREALQKSIDSGTYQAGSSVDEMGIKKRMAFLDVLLQSTVGGKPL

TNKDIREEVDTFMFEGHDTTTSGICFTLYLLSRHPDVQQKVLEEIHSVIGEDKEKPVTMK

DLQELKYLDCVIKESQRLYPSVPTIGRVTEQDVVINGVTIPANTNITLLMYAAMKDPDYF

PKPEEFLPERFMNTEDKINPFAYVPFSAGPRNCIGQKFAMAEMKSTVSKMVRHFELLPLG

EEVKPVMNMVLRSETDAQLGLRVRK

>EDW69010.1

MLLIAIVVFLLTAAFRFHRSQGARRAAVANLPGPYCPPVIGAVHRMIKLNPKSFIRESRK

QVEKHGNLQRVWILNRLLITSSDTEFNEQLLFSQEHLVKHQLYQLLGQWLGNGLLLSHGR

VWHQRRKIITPTFHFSILEQFVEVFDQQSSICIQRLQQRADGQTCFDVYPFICLAALDII

AETAMGTKVNAQLAESTPYANAVNECTALMAWRFMSIYLQNELLFTLTHPHLKWRQTQLI

RTMHEFTIGVIERRRQALEQAQQLGGGSECENAVGSKRRMALLDVLLQASVNGRPLKNEE

IREEVDTFMFEGHDTTTSAISFCLWCISRHANVQAKLLAEILQVLGTDRNRAINMRDLGE

LKYMECVMKESLRQFPPVPLVGRTLQADFKYSHSKVGDGVIPAGSEVIMGIIGMHNDPAY

FPEPLRFKPERHESGERAATFAFVPFSAGPRNCIGQKFAQLEMKMLLAKIVREFELLPLG

EPVQPVVNIVLRSDNGFQLGMRRRNDASDSKQ

>EDW73146.1

MLLFAIVALLLTAAWFIHYGQRSRRQAVANLPGPYCPPLVGALHIMFQMNPQNFFEVGRE

QVKKMGALQRVWVFNRLLVISADNEMNEQILSSQEHLVKHPVYRVLGQWLGNGLLLSDGK

VWHQRRKIITPTFHFAILEQFVEVFDSQSNTCINRLSQRANGEVFDVYSYICLAALDIIA

ETAMGTKVDAQKNEGTPYANAVNECTALMSWRFLSVYLQNELLFTLTHPHLKWRQMRMIR

TMHEYTINVIKQRRTTLEEHLKNKRPEQANEDDVGTKRRMALLDVLLQSTVDGRPLSNDE

IREEVDTFMFEGHDTTTSAISFCLTAISRHPAVQAKLLNEIHEVLGQDRNRPITIRDLNE

LKYMECVIKESLRMYPSVPLVGRKLQTDFKYSHSKYGDGILPAGTEVLLGIYGLQNSSEN

FPDPDRFMPERHESISTDRGSAFTYIPFSAGPRNCIGQKFAQLEMKTMIAKIVREFELLP

MGEPVKCILNIVLRSSTGFQLGMRKRNA

>EAL30748.2

MLLLVLVLLLVAVGWLVHRGQAERRRAVSNLPGPMCPPLLGALHIMSSMNPKTFIKVSGE

NMRKYGPLQRVWILNRLLIMSADSEINEQLLSSQEHLVKHPVYRVLGKWLGNGLLLSDGK

VWHQRRKIITPTFHFSILEQFVEVFDQQSNVCVERLAPRANGQTFDVYRPVCMAALDIIA

ETAMGTKIHAQAAESTPYAEAVNECTALLSWRFISVYLQVELLFTLTHPYMKWRQTQLIR

TMHDFTTKVIEERRRTLEEQQGSKLSPSPDDDVGRKRRMALLDVLLLATVDGRPLTNDEI

REEVDTFMFEGHDTTTSALSFCLHVLARHPQVQEKMLEEILRVIGTDRKRPVTIRDLNEM

KYLECVIKESLRLYPPVPLVGRKLQTDFKYSHSKYGDGVIPAGSEFLIGIYGMQRNKNEF

AQSEEFIPERHENGERFSPFTFIPFSAGPRNCIGQKFAQLEMKMMLAKIVREYELLPLGE

PVECVINIVLRSDTGFQLGMRRRNDTQ

>EDW93770.1

MLLFLLVVLLCGAGWIIHLGQADRRRKVANLPGPICPPLIGALHLILRLSPKTFLKVGRE

YVQKFGHLQRVWIFNRLLILSGDAELNEQLLSSQEHLVKHPVYKVLGQWLGNGLLLSDGK

VWHQRRKIITPTFHFSILEQFVEVFDQQSNICVQRLAQKANGDTFDVYRSICAAALDIIA

ETAMGTKVYAQANESTPYAEAVNECTALLSWRFMSAYLQVELLFTLTHPHLKWRQTQLIR

TMQEFTIKVIEQRRQALEDQQKQSRAAVATGDEDVGSKRRMALLDVLLMATVDGRPLTND

EIREEVDTFMFEGHDTTTSALSFCLHELSRHPEVQEKMLEEILRVLGTDRSRPVSIRDLG

ELKYMECVIKESLRMYPPVPIVGRKLQTDFKYTNSVYGDGVLPAGTEIIIGIFGIHRQPE

TFSNPDEFIPERHENGSRVAPFRMIPFSAGPRNCIGQKFAQLEMKMMLAKIVREYELLPM

GKRVECVVNIVLRSETGFQLGMRKRNSN

>EDV97038.1

MLLIAVVVFLLTAAILIHRGQRRSREAVANLPGPLCPPLVGALHRVFKLNPKSFLSEAKK

NLEKLGRLQRIWIFNRLQIVSSDVEFSEQVLANQEHLVKHTVYKLLGQWLGNGLLLSDGK

VWHQRRKIITPAFHFSILDQFVEVFDQQSTICIKRLEQKADGKTIFDVYPFICMAALDII

AETAMGTKVNAQLAESTPYANAVNECTALMAWRFMSAYLQFELLFTLTHPHLKWRQIKLI

RTMHEFTIKVIEQRRQALQEELEQKQQLDKDQDQDQVDNEVGSKRRMALLDVLLQAKVDG

RPLSNDEIREEVDTFMFEGHDTTTSAISFCLWCISRHPDVQDKMQAEILHVLGTDRSRTI

TTRDLSELKYTECVMKESLRQFPPVPLLGRKLHSDFKYTHSKVGDGIIPAGAEILVAIIG

MQNDALYFPEPELFKPERHENGERSSNFAYVPFSAGPRNCIGQKFAQLEMKMMLAKIVRE

FELLPMGDPVQPVVNIVLRSDNGFQMGMRRRNATGETK

>EDX09580.1

MLLFLLVVLLCVAGWIIHLGQADRRRKVANLPGPMCPPLIGAMQLMLRLNPKSFIKVGRE

YVLKFGHLQRVWIFNRLLIMSGDAELNEQLLSSQEHLVKHPVYKVLGQWLGNGLLLSDGK

VWHQRRKIITPTFHFSILEQFVEVFDQQSNICVQRLAQKANGETFDVYRNICAAALDIIA

ETAMGTKIYAQANESTPYAEAVNECTALLSWRFMSVYLQVELLFTLTHPHLKWRQTQLIR

TMQEFTIKVIEKRRQALEDQQKQSKLMDTVDEDVGSKRRMALLDVLLMATVDGRPLTNDE

IREEVDTFMFEGHDTTTSALSFCLHELSRHPEVQEKMLEEILQVLGNDRSRPVSIRDLGE

LKYMECVIKESLRMYPPVPIVGRKLQTDFKYTHSVHGDGVIPAGSEIIIGIFGVHRQPET

FPNPDEFIPERHENGSRVAPFKMIPFSAGPRNCIGQKFAQLEMKMMLAKIVREYELLPMG

RRVECIVNIVLRSETGFQLGMRKRNNK

>EDW50852.1

MLLFLLVVLLCGAGWIIHLGQADRRRKVANLPGPICPPLIGAMQLMFRLNPKSFIKVCRE

YVLKFGHLQRVWVFNRLLIMSGDAELNEQLLSSQEHLVKHPVYKVLGQWLGNGLLLSDGK

VWHQRRKIITPTFHFSILEQFVEVFDQQSNICVQRLAQKANGDTFDVYRNICAAALDIIA

ETAMGTKIYAQANESTPYAEAVNECTALLSWRFMSVYLQVELLFTLTHPHLKWRQTQLIR

TMQEFTIKVIEKRRQALEDQQKQSKLMGTVDEDVGSKRRMALLDVLLMATVDGRPLTNDE

IREEVDTFMFEGHDTTTSALSFCLHELSRHPEVQEKMLEEILQVLGTDRSRPVSIRDLGE

LKYMECVIKESLRMYPPVPIVGRKLQTDFKYTHSVHGDGVIPAGSEIIIGIFGVHRQPET

FPNPDEFIPERHENGSRVAPFKMIPFSAGPRNCIGQKFAQLEMKIMLAKIVREYELLPKG

QRVECIVNIVLRSETGFQLGMRKRNNK

>EDX04106.1

MWILLGIAVLIMTLVWDNSRKQWRVNTFEKSRILGPFTIPIVGNGLQALTLRPENFIQKF

GDYFNKYGKTFRLWILGECLIYTKDLKYFESILSSSTLLKKAHLYRFLRDFLGDGLLLST

GNKWSSRRKVLAPAFHFKCLENFVEIMDRNSGIMVEKLRNYADGKTCVDLFKFVSLEALD

VTTETAMGVQVNAQNEPNFPYTKALKSVVYIESKRLASVSMRYNWLFPLAAPLVYRRLQK

DIAIMQDFTDKVIRERRAILERERADGTYKPLIMGDDDIGGKAKMTLLDILLQATIDNKP

LSDVDIREEVDVFIFAGDDTTTSGVSHALHAISRHPKVQERIYEELVSVLGPDPDASVTQ

TKLLELKYLDCVIKETMRLHPPVPILGRYIPEDLQIGEKTIPGNTSILLMPYYVYRDPEY

FPDPLAFKPERWMDMKTTTHTPLAYIPFSSGPKNCIGQKFANLQMKALVSKVIRHYELLP

LGADLKATYTFILSSSTGNNVGLKPRTRVK

>EDW87740.1

MWLLLGIGVLIMILVWDTGRKRWRINVFAKSKISGPFSLPILGNGLQALTFRPENFIQRF

GDYFNKYGNTFRLWVLGECLIYTKDLKYFEGILSSSTLLKKAQLYRFLRDFLGDGLLLST

GNKWTSRRKVLAPAFHFKCLENFVEIMDRNSGIMVEKLRGVADGKTCVDLFKFVSLEALD

VTTETAMGVQVNAQNDPNFPYTKALKSVVYIESKRLASVSMRYDWLFPLAAPLVYRRLQK

DIAIMQDFTNKVIRERRAILERTKADGTYKPLIMGDEEIGGRAKMTLLDILLQATIDNKP

LSDVDIREEVDVFIFAGDDTTTSGVSHALHAISRHPKVQKCIHEELLSILGTDPDAPVTQ

TKLLELKYLDCVIKETMRLHPPVPILGRYIPEDLHIGDKTIPGNTSILLMPYYVYRDAEY

FPDPLVFKPERWLDMKASSYAPLAYIPFSSGPKNCIGQKFANLQMKALISKVIRHYELLP

LGEDLQPTYTFILSSSTGNHVGLRPRTRVQ

>EDW52008.1

MWILLGIAVLIMTLVWDNSRKQWRVNTFEKSRILGPFTIPIVGNGLQALTLRPENFIQKF

GDYFNKYGKTFRLWILGECLIYTKDLKYFESILSSSTLLKKAHLYRFLCDFLGDGLLLST

GNKWSSRRKVLAPAFHFKCLENFVEIMDRNSGIMVEKLRNYADGKTCVDLFKFVSLEALD

VTTETAMGVQVNAQNEPNFPYTKALKSVVYIESKRLASVSMRYNWLFPLAAPLVYRRLQK

DIAIMQDFTDKVIRERRAILERARADGTYKPLIMGDDDIGGKAKMTLLDILLQATIDNKP

LSDVDIREEVDVFIFAGDDTTTSGVSHALHAISRHPKVQERIYEELVLVLGPDPDASVTQ

TKLLELKYLDCVIKETMRLHPPVPILGRYIPEDLQICEKTIPGNTSILLMPYYVYRDPEY

FPDPLVFKPERWMDMKTTTHTPLAYIPFSSGPKNCIGQKFANLQMKALVSKVIRHYELLP

LGADLKATYTFILSSSTGNNVGLKPRTRVK

>EDV59231.1

MWLLLGIGVLILSLVWDTRRKQWRMNVFEKSKISGPFSIPIVGNGLQALTLRPENFIQRF

GDYFNKYGKTFRLWILGECLIYTKDLKYFEGILSSSTLLKKAQLYRFLRDFLGDGLLLST

GNKWSSRRKVLAPAFHFKCLENFVEIMDRNSGIMVEKLRRVADGKTCVDLFKFVSLEALD

VTTETAMGVRVNAQNDSNFPYTKALKSVVYIESKRLASVSMRYNWLFPLAAPLVYRRLQK

DIAIMQDFTDKVIRERREILERTRADGTYKPLIMGDEEIGGKAKMTLLDILLQASIDNKP

LSDVDIREEVDVFIFAGDDTTTSGVSHALHAISRHPEVQECIYEELVSVLGPDPDASVTQ

SKLLELQYLDCVIKETMRLYPPVPILGRYIPEDLNIGDKTIPGNTSILLMPYYVYRDPEY

FPDPLVFKPERWMDMKMTSQTPLAYIPFSAGPKNCIGQKFANLQMKALISKVIRHYELLP

LGEDLKATYTFILSSSSGNNVGLKPRTRVK

>EDV50459.1

MLLLLLVVLLCGAGWIIHLGQADRRRKVANLPGPMCPPLIGALQLMLRLNPKTFLKVGRE

YVQKFGHLQRVWIFNRLLIMSGDAELNEQLLSSQEHLVKHPVYKVLGQWLGNGLLLSDGK

VWHQRRKIITPTFHFSILEQFVEVFDQQSNICVQRLLQKANGDTFDVYRSICAAALDIIA

ETAMGTKVYAQANESTPYAEAVNECTALLSWRFMSAYLQVELLFTLTHPHLKWRQTQLIR

TMQEFTIKVIEKRRQALEDQQKQSKLVEHVDEEVGSKRRMALLDVLLMATVDGRPLTNDE

IREEVDTFMFEGHDTTTSALSFCLHEISRHPEVQEKMLAEILSVLGTDRSRPVSIRDLGE

LKYMECVIKESLRMYPPVPIVGRKLQTDFKYTNSVYGDGVIPAGSEIIIGIFGVHRQPET

YPNPDEFIPERHENGCRVAPFKMIPFSAGPRNCIGQKFAQLEMKMMLAKIVREYELLPMG

QRVECIVNIVLRSGTGFQLGMRKRNSN

>EDW33184.1

MLLLTLAFGALAVLLTWDFARKRQRAATLERSKISGPLSLPVLGNGLQALDLGAENIVDW

VEESFRLYGKTFRMWILGESLIYTKDLKYFEAILGSTTLLQKGQLYKFLRPFLNDGLLLS

VGKKWHARRKVFTNAFHFNVLGHYVDIMDRQAAVMVEKLRQVADGETTVDMLPYVSLAAL

DVITEAAMGVQVNAQSDSEFPYVQALKSVVYIQSDRMFRFLQRYDWLFRLTCPVLRRKML

KDVRVMHDFTDKVIRERRAAVEQAKADGSYRPLSLGDPEVGRKSQMALLDILLQATIEGQ

PLSDADIREEVDTFMFEGDDTTSSGVSFALYAIARHPQVQERIYRELLQVLGRDPSTPVT

QAKLQDLKYLDCVIRETLRLYPAVPAVGRYTSKEIQIGGQTIPANTSIYLVLYFAHREAE

FFPDPLAFKPERFLERADSEDRETFAYLPFSAGPKNCIGQKFAMLELKALVSKVLRYYEL

LPRGPEVKPMMNFILRSSSGMNVGLRPRRSP

>Q9W011.1

MWLTLITGALILLLTWDFGRKRQRVLAFEKSAIPGPISIPILGCGLQALHLGAENIIGWV

GEKFDKYGKTFRFWILGESLIYTKDLQYFETILSSTTLLEKGQLYEYLRPFLNDGLLVST

GRKWHARRKIFTHAFHFKVLEHYVEIMDRHSSVMVDNLRKVADGKTAVDMLKYVSLAALD

VITEAAMGVQVNAQNDPDFPYIKALKSVVYIQPDRMFRFSRRYNWLFPLAAPLLHRQLLS

DIRVMHDFTDKVISERRETVRRAKADGTYRPLSLGDAEIGSKSQMALLDILLQSSINNQP

LSDADIREEVDTFMFEGDDTTSSGVSHALYAIARHPEVQQRIFEELQRVLGPDASAPVTQ

AQLQDLKYLDCVIKETMRLYPPVPAIGRHAQKELEIGDKTIPANTSIYLVLYYAHRDANY

FPDPLSFRPERFLEDQEQGHNTFAYVPFSAGPKNCIGQKFAVLEMKVLISKVLRFYELLP

LGEELKPMLNFILRSASGINVGLRPRKALR

>EDV40713.1

MLLWLLVASLLAAAWLFHRGQERRRRAVSNLSGPFCPPVFGAIHLMMGMTPKTFLKKSKE

YYFRWGDLQRVWVFNRLLIMSANAEFNEQLLSSQEHLVKHPVYRVLGQWLGNGLLLSDGK

VWHQRRKIITPTFHFSILEQFVEVFDQQSNVCVQRLAQKANGEVFDVYKNVCLAALDIIA

ETAMGTKICAQSKESTPYAEAVNECTAMMSWRFMSVYLQSEILFTLTHPHLKWRQMQLIR

TMHDFTIKVIEQRRQALEEQQKRSNLTETSNDVGSKQRMALLDVLLMATVDGKPLTNDEI

REEVDTFMFEGHDTTTSALSFCLYEVSRHSEVQEKLLEEILRVIGTDSSRPVTIRDLGEL

KYMESVIKESLRMYPPVPIVGRKLVNDFKYTNSKHGDGIIPAGSEIILGIYGIHRQLDAF

ADPEKFDPERFETGDRVPPFHMIPFSAGPRNCIGQKFAQLEMKMMLAKIVREYELLPLGD

PLECVVNIVLRSDTGFQLGMRKRNNS

>AAL48745.1

MWLTLITGALILLLTWDFGRKRQRVLAFEKSAIPGPISIPILGCGLQALHLGAENIIGWV

GEKFDKYGKTFRFWILGESLIYTKDLQYFETILSSTTLLEKGQLYEYLRPFLNDGLLVST

GRKWHARRKIFTHAFHFKVLEHYVEIMDRHSSVMVDNLRKVADGKTAVDMLKYLSLAALD

VITEAAMGVQVNAQNDPDFPYIKALKSVVYIQPDRMFRFSRRYNWLFPLAAPLLHRQLLS

DIRVMHDFTDKVISERRETVRRAKADGTYRPLSLGDAEIGSKSQMALLDILLQSSINNQP

LSDADIREEVDTFMFEGDDTTSSGVSHALYAIARHPEVQQRIFEELQRVLGPDASAPVTQ

AQLQDLKYLDCVIKETMRLYPPVPAIGRHAQKELEIGDKTIPANTSIYLVLYYAHRDANY

FPDPLSFRPERFLEDQEQGHNTFAYVPFSAGPKNCIGQKFAVLEMKVLISKVLRFYELLP

LGEELKPMLNFILRSASGINVGLRPRKALR

>Q9VS79.2

MQLMLRLNPKTFIKVGREYVLKFGHLQRVWIFNRLLIMSGDAELNEQLLSSQEHLVKHPV

YKVLGQWLGNGLLLSDGKVWHQRRKIITPTFHFSILEQFVEVFDQQSNICVQRLAQKANG

NTFDVYRSICAAALDIIAETAMGTKIYAQANESTPYAEAVNECTALLSWRFMSVYLQVEL

LFTLTHPHLKWRQTQLIRTMQEFTIKVIEKRRQALEDQQSKLMDTADEDVGSKRRMALLD

VLLMSTVDGRPLTNDEIREEVDTFMFEGHDTTTSALSFCLHELSRHPEVQAKMLEEIVQV

LGTDRSRPVSIRDLGELKYMECVIKESLRMYPPVPIVGRKLQTDFKYTHSVHGDGVIPAG

SEIIIGIFGVHRQPETFPNPDEFIPERHENGSRVAPFKMIPFSAGPRNCIGQKFAQLEMK

MMLAKIVREYELLPMGQRVECIVNIVLRSETGFQLGMRKRKHN

>EDW57109.1

MWLTLLVGALTLLLVWDFVRKRHSCATLKRSGITGPLSLPLLGCGIQALQLGAENIIDYV

GACFNKYGATFRLWILDESLIYTKDLGHFEAILSSTTLLEKGQLYQFLRPFLNDGLLLSV

GRKWHSRRKVFSNVFHFKILEHYVDIMDSQSAVLVEKLQPVADGKHVVNMLKYVSLAALD

VITETAMGVQVNAQSDPEFPYIKALKSVVNIQPDRMFKFSQRYEWLFRLTAPLLHMKLVR

DIRIMHDFTDKVIRERREAVERAKADGSYRPLCLGDGDIGRRPQMALLDILLQSSIQGRP

LTDTDIREEVDTFMFEGDDTTSSGVSHAFYCLARHPEVQANLYDELLQVLGKNRMEPISQ

AQLQQLKYLECVIKETMRLYPPVPAIGRHTRKDLQIGEQTIPANTSIYLVLYFAHRDPKY

FPDPLSFKPERFLDDTWEAEGKRQTFAYLPFSAGPKNCIGQKFAMLEMKTLISKVIRHYE

LLPEGPEVSPMMNFILRSPTGMNVALKPRLE

>EDX08950.1

MWLTLITGALILLLTWDFGRKRQRVLAFEKSEIPGPISIPILGCGVQALHLGAENIIGWV

GEKFDKYGKTFRFWILGESLIYTKDLQYFETILSSTTLLEKGQLYEYLRPFLNDGLLVST

GRKWHARRKIFTHAFHFKVLEHYVEIMDRHSGVMADNLRKVADGKTAVDMLKYVSLAALD

VITEAAMGVQVNAQNDPDFPYIKALKSVVYIQPDRMFRFSRRYNWLFPLAAPLLHRQLLS

DIRVMHDFTDKVISERRETVRRAKADGTYRPLSLGDAEIGSKSQMALLDILLQSSINNQP

LSDADIREEVDTFMFEGDDTTSSGVSHALYAIARHPEVQQRIYEELQRVLGPDASASVTQ

AQLQDLKYLDCVIKETMRLYPPVPAIGRHAQKELKIGDKTIPANTSIYLVLYYAHRDPTY

FPDPLSFKPERFLEDQEQGHDTFAYVPFSAGPKNCIGQKFAVLEMKVLISKVLRFYELLP

LGEELKPMLNFILRSASGINVGLKPRKS

>EDW93926.1

MWLTLITAALILLLTWDFGRKRQRVLAFEKSGIPGPLSLPILGCGLQALHLGAENIIAWV

GEQFDQYGKTFRFWILGESLVYTKDLKYFETILSSTTLLAKGQLYQFLRPFLNDGLLVST

GRKWHARRKIFTHAFHFKVLEHYVEIMDRNSGVMVDHLRKLADGKTTVDMLKHVSLAALD

VITEAAMGVQVNAQNDPDFPYIKALKSVVYIQPDRMFRFSRRYNWLFPLAAPWLHRQLLS

DIRVMHDFTDKVIRERRETVRRAKADGTYRPLSLGDAEIGSKSQMALLDILLQSSIDNQP

LSDADIREEVDTFMFEGDDTTSSGVSHALYAIARHPEVQQRIYEELQRILGPDPSAPVTQ

AQLQELKYLECVIKETMRLYPPVPAVGRHTQKELKIGNKTIAADTSIYLVLYYAHRDPDY

FPDPLSFKPERFLDGEEQGHDTFAYVPFSAGPKNCIGQKFAVLEMKALISKVLRFYELLP

LGEELKPMLNFILRSASGINVGLRPRKS

>291232911

MGVNIIFDFLSSAQKNVSFLDDFAEKLPSVRTWQVIVVFICSVIVIQAIAIVFKLFKMKK

TSEMAFKPFATPSLDIFKSPILGHLGLLSVNKDEIMQMAGDITHEFKYALPLWLGPFQAA

LICHHPSTVQPILATTEPKDDFSYGMLKPWLGDGLLISSGNKWSRNRKLLTPGFHFDILR

PYVKVFNECAITMTDKWSTMCDTGPLEMFQHISLMTLDSLLKCIFSQESHCQTDSDVNPY

IKAVYTLTDLIMERINFPPYYSDTVYSLTYEGVKWRKALNDVHNHSRRVIKERKSALKDE

VERGTVNKRKYIDFLDILLAAKDEDGNGLTDKEIQDEVDTFMFEGHDTTASGISWCFYNL

ARHPKYQQMCRDEVDQLLDKKENDELDWDDYAKLPFLTMCIKESLRLHPTVPFVGRRNNK

PLTFPELGITIPAGQFLGISVIGLHHNVHLWEEPLVYNPYRFTTENTKVRQNYSFLPFSA

GPRNCIGQNFAMNEMKTAIALVLRKFILSVEDDYPVRRMYNVVLRAEEGLHIVVKPREVK

IQN

>EEN60380.1

MVFSIGDGLLTSKGSKWHRNRRLLTPAFHFEILKPYVTLFSQSTNVLIDKWSRVAAGSSV

ELFDHVSLLTLDSMLKCSLGYRSDCQTDGQSAPYIRAVFELSRLIVERVRFFPYHFDFIY

YLTSSGRRFRQQCDIVHGVSEHLIRQRKKALQDGDTKEEKSKKYLDFLDILLQAKDEDGT

GLTDAEIRDEVDTFLFEGHDTTASGISWALYHLAKHPEYQDRCRREAEGLLQGRTEMTWE

DMSKLPFITMCIKESLRIRPPVPSISRSLTKTLTFPDGKTMPEGSLVSARIMSTHHNHLI

WPDPEVYDPLRFSPERSKDRHHHAFVPFSAGPRNCIGQNFAMNEMKVAVALILQRFCLEL

DETKPSPFFLQQLILRAKDGIWVKLTPNN

>291238355

MGSLAFKPLIQASTFTNNPYVDFIFVMFVVLFIIKATTSFYGLLKMKWATEKAFVGFPIE

NRHPVFGHLHMLKLNTEETMHIAGERSDNFPYTGLLWFGPFHGILICHHPDTIQPIMSTA

EPKDEFTYGMVKPWLGDGLLISKGSKWFRNRRLLTPGFHFDILRPYVHIFNECTHTMMDK

WMKQSDNGMIEMFEHVSLMTLDSLLKCIFSIESHCQTDVSRNPYITAVYALSEMVLKRVQ

FPPYFSDLVYHLTYSGYKWRRALRQVHGHSKTVIHQRKQALQDQKKLGIKHNRKYIDFLD

ILLEARDESGKGLTDQEIRDEVDTFMFEGHDTTASGISWILYNMAKHTEHQLKCQEEIDE

LMDQKDKDEIEWDDLSKLPYLTMCIKESLRLHPPVPFIGRKLSKPIEMRSPTGKGKVVIP

AGERIGIAITGLHHNQHVWEDPEVYNPLRFTPENCKGRSPHAYLPFSAGPRNCIGQNFAM

NEMKIAVGLVLRKFNLAVDDFIRIRRYNSIVLRGERGIPLHVKPRKIKTT

>403303515

MSLLSLSWLGLGPVAPSLWLLLLLVGVSWLLAHVLAWTYTFYDNCRRLQCFPHPPKLNWF

WGHQGLESALSAFLYAGTQLGQKRSVGISRCLLFLRHLPPTLLFLTDKSQSASQIHVRRE

QSMLEADRVSWARIKAFSRALSYAQVLENTLEHAGVLSWCAGACWVIALLCRCSLVRSLL

PSSAAVAPKDMLFYGFLKPWMGDGLLLSAGDKWSRHRCMLTPAFHFNILKPYVAIFNKTV

NIMHDKWQCLASVGTTRLDMFEHISLMTLDTLQKCVFSFDSNCQEKPSDYIAATLELSSS

AEKRNQHFLLHSDFLYYLTPDGRRFRRACRLVHDFTDAVIQERRRTLHTQGTDDFLKDKA

KSKTLDFIDVLLLSKDEDGRELSDEDIRAEADTFTFGGSDTTASGLSWILYHLSRHPEYQ

ERCRQEVRELLKDREPIEIEWDDLAKLPFLTMCIKESLRLHPPVPAITRRCTQDMVLPDG

RVIPKGVSCHISIIGIHHNPTVWPDPEVYDPFRFDPENSKERSPLAFIPFSAGPRNCIGQ

TFATAEMKVVLALTLLRFRVLPDRIEPRRKPEMVMRAEGGLWLRLESLGANPQ

>291238492

MFFEGILWRSVVGEVLVIVIVLKVTLTLVNLNKQRRYNERALSFFPGFKKHFLLGDLDKF

RNDEASFQWTTNALKNVKYGFQLWAGPFIACLSCIHPDTIRPVLVSTEPKDEFTYGMVKP

WLGDGLLLSKGNKWFRNRRLLTPGFHFDILRPYVGIFNDCTNTLIEKWSQRCHTGSLEMF

EHISLLTLHSLIKCLFSQEIHCQVDGKRNSYVDSVKELALMVDSRIQNPLYYSNLIYHLT

YSGYKWRKALNVVHTYSRNVIQQRKETIKLKREKTDCENRRYVDFLDILLAARDENGEGL

TDREIRDEVDTFMFEGHDTTASGLSWCLYNLAKNPEHQKTCQQEIDALLEKKGEDKIEWN

DLNHLNYVTMCIKESLRITPAVPFVGRKLSKSMTFPELNVTIPEGIWIGIGIMGLHENRF

IWDNPEVYDPLRFTPENCQGRSPYAFLPFSAGPRNCIGQNFAMSEMKTVIALLLRKFEFV

IDESVPARRRIALVMRSEGGLRLFVKPRHTSGHSS

>291240224

MAIIWNNLAVLVVTVSVLLATNALIKIWSLIKQRRQNESDLSVFPCPPRHWLFGHEHLGL

RKGKGIQMMFEFTKNYSHYFQIWIGPFRALLFCVHPDTVKTILATPEPKDPLAYGRLSEW

LGDGLLISSGAKWKRNRRLLTPSFHFEVLKPYIKLYNECAQKMLTKWSHHSSDTPMEMFD

CVSLMTLDTLLNCIFGINSSCQERGDDNTVTYVKAVRNLSQLFVERMAAFYLPSFVFLHL

TPRGRKYTHNLKVLHNFTRSIIQQKKRLRAEGSSIKKEYVDFLEMLLDARDDDGVGLSDQ

EIQDEVDIFMFEGHDTTASAISWCLYNLARYPDYQYKCRDEIDEMMVVKDNDEIEWNDLQ

CMPETTMFIKESLRMNPPVPATSRQLTSPMTFPDGKVAPVGSIVVVAVNALHHNSHVWEN

PDIFDPMRFSSENSKKRSPHAFVPFAAGARNCIGQNFAMNEIKVVIGRVLHQFELYVDDS

CPFPQPKNTLVLKADPGIFIKFKQRKLKI

>EAW84511.1

MSAAVAPKDMIFYGFLKPWLGDGLLLSGGDKWSRHRRMLTPAFHFNILKPYMKIFNKSVN

IMHDKWQRLASEGSARLDMFEHISLMTLDSLQKCVFSFESNCQEKPSEYIAAILELSAFV

EKRNQQILLHTDFLYYLTPDGQRFRRACHLVHDFTDAVIQERRRTLPTQGIDDFLKNKAK

SKTLDFIDVLLLSKDEDGKELSDEDIRAEADTFMFEGHDTTASGLSWVLYHLAKHPEYQE

QCRQEVQELLKDREPIEIEWDDLAQLPFLTMCIKESLRLHPPVPVISRCCTQDFVLPDGR

VIPKGIVCLINIIGIHYNPTVWPDPEVYDPFRFDQENIKERSPLAFIPFSAGPRNCIGQA

FAMAEMKVVLALTLLHFRILPTHTEPRRKPELILRAEGGLWLRVEPLGANSQ

>291223967

MWLGPFVPRVAVNHPSTVKAILTTTGDGLLISSGQKWFRNRRLLTPGFHFDILRPYVQIY

NDCVKTMLDKWSNLCELSSSRSYSIEMFENLSLMTLDSLLKCIFSQESHCQTAKSQNPYI

SSVYALSHLISERSRFVPYHSDIIYNLSISGYKFRKALRAVHGYSARVIQERKQALRQRG

DDKPARKYIDFLDILLSAKDEDGHGLSDKELRDEVDTFMFEGHDTTASGLSWCLYNLAKY

PEHQQKCQDEIDTLLAKTRKKDIEWEDLSKLSYTNLCIKESLRIRNPVPMISRELKSSLT

LPDGRAIPAGYNVLIAINALHHNSLVWDNPLEFDPSRFLPENSKSRSPYAYVPFSAGPRN

CIGQNFAMNEMKVAVARTLHRFDLSPVLSRPPQRVNNIVLRSSNGIYVQVTPRNR

>EHH29759.1

MSQLSLSWLGLGPVAVSPWLLLLLVGTSWLLARVLAWTYTFYDNSRRLRGFPQPPKRNWF

WGHQGMVKSTEEGMRVLTQLVATYPQGFRVWMGPVFPLLSLCHPDIIRSVINASGTIWNT

WCSEEGLLYIQSLVCTFGDMCCWWVGPWQAVVHIFLPTCIKPVLFAPGDGLLLSAGDKWS

RHRRMLTPAFHFNILKPYMKIFNESVNIMHAKWQLLASEGSARLDMFEHIGLMPLDSLHK

FVFSFDSHCQEKPSEYIAAILELSALVSKRHQQFLLHIDFLYYLTPDGQRFRRACRLVHD

FTDAVIQERRRTLPSQGVEDFLQAKAKSKTLDFIDVLLLSKDEDGKELSDEDIRAEADTF

MFEGHGTTASGLSWVLYHLAKHPEYRERCRQEVQELLKDREPKEIEWDDLAQLPFLTMCI

KESLRLHPPVPVISRHVTQDIVLPDGRVIPKGITCLLSVFGTHHNPTVWPDPEVYDPFRF

DPEHIKDRSPLAFIPFSAGPRNCIGQTFAMAEMKVVLALTLLRFRVLPDHTEPRRKPELV

LRAEGGLWLRVEPLS

>ABY26093.1

MPLLQSVLSQVLSWTGLRQVLFVVCALVAVWTAKLLVRHAWYAHRLSCFSKPPADSWLLG

HLGQMKSTEEGLLEVDELVRTYKHSCSWFLGPFYHFVRLFHPDYVKPLLMAPASITVKDE

LIYGHLRPWLGQSLLISNGEEWSRKRRLLTPAFHFDILKNYVATFNASTNIMHDKWRRLV

SEGTTNLEMFDHVILMTLDSLLKCAFSYNSNCQRSTSEYVLAIVELSDLIIDRRQKILHH

WDWIYWRTEQGKRFKKALSIVHRFTREVVQKHRAQISQQREKETDFTTAPRRKKDFVDII

LLSKDEDGQGLTDEEIRAEANTFMFAGHDTTASAICWTLYNLARHDHYQGKCRQEVTDLM

QGRDGHAIEWEDLSNLPFTTMCIRESLRLHSPVQAVTRKYTQDMALPGDRTVPQGAICLV

SIYGTHHNPAVWQNPHEFNPLRFDPTNKEGLASHAFIPFSSGPRNCIGQKFALAELRVVV

ALTLLRFRLTPGVNPELGNKSGGVRRLPQLVLRAEGGLWLQVEPLDQHNLEGDPTR

>348537144

MALQHSVLSPVFTCLCSLMYIFSTGLVVVVAVLTVRHIWYSRRLSCFSKPQTRSWLLGHL

GKIRNTEEGLQQMDDLVKTYKHSCFWFLGPFYHLVRLFHPDYVKPLLMVPASMTVKKGLI

YNHLQPWLGHSVLISNSEVWSRKRRLLTPAFHFDVLKNYITIFNSSSKIMHDKWCRLVAE

GKTNLEMFDHVSLMTLDSLLKCAFSYDSNCQESPSEYVSAIVQLCDLVAQRQHNILHHWD

WIYWKTQQGKLFKQTLNIVHRFSRDVVQKRRALHKKQREIITQSVAPTSQRRKDFVDILL

LTRDEDGRGLTDEEIQAEANTFMFAGHDTTASAICWMLYNLARHEHYQEKCRQEVMDLME

GRDGQEIKWEDLSNLPFTTMCIKESLRLHTPVQAVSRSYTQDMVLPGNRTVPAGTVCLVS

IYGTHHNPIVWTNPNEFDPHRFDPGNKMSQASHAFIPFSSGPRNCIGQKFAMTELQVVVA

LTLLRFRLTPGVNPELGSSRVRRLPQIVLRAEGGLWLQLEPLNTLRLNKQSEE

>EHB14403.1

MGGTEDSGKAWKAGVPVAVLPRRMLPITDHLLHFLGLEKTAFRAYAVSALLLSLLFSFFR

LMLRFLRICWAFHNTCRRLHCFPQPPRNNWLMGHLGMYLPNEKGLQDEKKLLDKMHDVVL

VWAGPVLPLLVLVHPDYIKPVLSASASKNKGRISQYLANKCSVACRTDCFSEVPTSVFGE

KLREQVEEGLCFYETGKIPRKNLDVMKEAMVQAEEAAAEITRKLEKQEKKRLKKEKKGLA

ALAALVSSENGSAPKEHEETHEKPQKKKKPKLQEAPQEDRMDDPYVLVSRPRKNKSFSKE

ELVSSDLEGTAGGSLSPPKGNRSLPKEEMATAIAPKDDFFYSFLKPWLGDGLLLSKGDKW

SRHRRLLTPAFHFDILKPYMKIFNQCTNIMHAKWRRHLGENSVASFDMFEHISLMTLDSL

QKCVFSFNSNCQEKMSDYISAIIELSALVVRRQYRLHHYLNFIYYLSADGRRFRRACDIV

HHFTTEVIQERRKALCQQGAEMWLKTKQGKALDFIDVLLLARDEDGKELSDEDIRAEADT

FMFEGHDTTSSGLSWVLFNLAKYPEYQEKCREEIQEVMKGRELEELEWDDLTQLPFTTMC

IKESLRQFPPVTLVSRRCTEDIKLPDGRIIPKGIICLVSIYGTHHNPTVWPDSKVYNPYR

FDPDNPHQRSPLAYVPFSAGPRNCIGQSFAMAEMRVAVALTLLRFRLSVDRTRKVRRKPE

LILRTENGIWLNVEPLTPLGPQEWHLAHRGAAASSGAPSPAQGDPGPAPEGPGPAPQVLS

A

>291238357

MGSSVFKPLIHLSTITNYPYVDFIFVTFAVLFIINAVTTFYRLLKMRWASEKAFADFPIE

NRHPIFGHVLLVKLNSEETIQDIAERADIFPYTDQLWLGPFHALLICYHPDTMKPVLSSS

EPKDEFTYGMVKPWLGDVLLISKGSKWFRNRRLLTPGFHFDILRPYVHIFNECTHTMMDK

WMKQYDNGMIEMFEHVSLMTLDSLMKCIFSIETHCQTEVSTNPYITAVYELSEMVVNRIN

FPPYFSDLIFHLTYSGYKWRRALRQVHGHSKTVIHQRKQALEEQKELGIKHKRKYIDFLD

ILLEARDENGKGLTDQEIQDEVDTFMFEGHDTTASGISWILYNIAKHTEHQRKCQEEIDE

LMEQKDKDVIEWDDLSKLPYLTMCIKESLQLHPPVPFIGRKLSKPIEMPSPTGNGKVTIP

AGERIGLPIIGLHHNPHVWEDPEVYNPLRFTPENSKGRSPHAYLPFSAGPR

>198430206

MTLFEAASEVVFLNRGFVLSSLISEVVLLIAIVAVTKVYLVPAIRYYKRGLLYTKQGCSP

PRHWLWGHLTVFPLSHDGMLKRLRTTAQYAGMFVSWAGPFTYWIETQHPTTCAVLANSSA

PKAEFLYGCLRPWIGDGLLTSKGSKWKRNRRLLTPAFHFEILKPYLNIFNKSSYAMLEKW

KRHSGQSYDVYGDVSNLTLDTMMQCAMSTDTDSGGEDGGYGYINAVHELTLLVMERVYNP

LHMIDWIYHFSSNGRRFRKLVDFVHETSETIIKERKKELETLVQEDNDNASTATNNEISS

FKKRGTKHLDFLDILLQTKDENGNGLTLKEIRDEVDTFLFEGHDTTASGIAWCLYNLAKH

PEYQQRCREEVLEEVGEKEKIEWEDLSKLTYLAMCIKESLRLNPPVFCVGRELNEDLTFN

DKYTPCKKMKVESGNTVLMQIMSMHRNPHVWENPEVYDPERFSAENMKNRHSMSFLAFSA

GPRNCIGRNFAMNELRVVIGQTLRQYKLWCDDDTPQPKMQPNIILRSANGIHIKFKPV

>AAZ29446.1

MLQLSLSWLGLGPVAASPWLLLLLVGGSWLLARVLAWTYTFYDNCRHLQCFPQPPKQNWF

WGHQGLVTPTEEGMKKVTQLVATYPQGFKFWMGPTFPLLTLCHPDTIRPITSASAAVAPK

DMIFYGFLKPWLGDGLLLSGGDKWSRHRRMLTPAFHFNILKPYMKIFNKSVNIMHDKWQR

LASEGSARLDMFEHLSLMTLDSVQKCVFNFESNCQEKPSEYIAAILELSALIEKRNQHIL

VYPDFLYHLTPDGQRFRRACRLVHDFTDAVIQERRQTLRTQGIDDFLKNKAKSKTLDFID

VLLLSKDEDGKELSDEDIRAEADTFMFGGHDTTASGLSWVLYHLAQHPEYQERCRQEARE

LLKDCEPVEIEWDDLAQLPFLTMCIKESLRLHPPVPAVSRCCTQDIVLPDGRIIPKGIIC

IISIFGIHHNPTVWPDPEVYDPFRFYQENIKERSPLAFIPFSAGPRNCIGQVFAMAEMKV

VLALTLLRFRILPTHPEPCRKPELVLRTEGGLWLRVEPLGVNSQ

>348537146

MALLHSVLSPVLSWMCLCSLMYIFGTGLLAVVAVLTVRKVWYSHRLSCFSKPPANSWLLG

HLGQIQSTEEGLQQMDDLVKTYKHSCYWVLGPFYHLVRLFHPDYVKPLLMAPASMTVKNG

LIYHHLQPWLGHSVLISNGEVWSRKRRLLTPAFHFDILKSYITIFNSSSKIMHDKWCRLV

AEGKTNLEMFDHVSLMTLDSLLKCAFSYDSNCQESPSEYVSAIVQLCDLVTQRHHNILHH

WDWIYWKTQQGKLFKQTLNIVHRFSRDVVQKRRALLKAQREITQCEVAPTSQRRKDFVDI

LLLTRDEDGQGLTDEEIQAEANTFMFAGHDTTASAICWTLYNLARHEHYQEKCRQEVMDL

MEGRDGHEIKWEDLPKLPFTTMCIKESLRLHTPVQAVSRSYTQDMVLPGNRTVPAGEXXX

XXXXGTHHNPIVWTNPNEFDPHRFDPGNKMSQASHAFIPFSSGPRNCIGQKFAMTELQVV

VALTLLRFRLTPGVNPELGSSRVRRLPQIVLRAEGGLWLQLEPLNTLRLNKQSEE

>198428826

MAWTSVGGIVTTTAVGDFLLVACIVVVIKSFLIPAVRGYQDAKKRARSVGEVKKHWLYGS

VKMFPQNEEGLMKRVASSFEKPVWTVNWFGPYISEVVIHHADLAITLLSSSAPKNNLVYG

FFHPWIGDGLLTSSGRKWQRNRHLLTPAFHFSILKPYTNVSNACVRVMLDKWSKKVGTSM

EIYLDVNLMTLDTILQCAMSTKSDCQNRSKKNEYIEAVHDVSKYIMSRVHKPLLHIDWIY

WLTAEGRKFKQLVKVLHDQSEKVIRERRKTLENRKFEEESSGKKRLDFLDILLHTKDEDG

KGLNDSEIRDEVDTFLFAGHDTTASGIAWALYNLAVNVDCQDKCREELKSVVGDKENIEW

EDLSKLSYLTLCIKESLRLCPPVPFIGRELNEPLKFRSKLKEPNETTIDANSNIALHIFT

LHRNVHVWDSPEEFIPERFKPENMKGRSPHAYLPFSAGPRNCIGQNFAMNEMKIAIGQTL

RKFKVIPDESFPKPSITPQVVLRPKDGIFIKLMEI

>AFI45019.1

MFLSVSHTFLAVLVLLLSVCYRRSRNIKEKLWWVPLIPGYPILGNALEFADSTKILERMN

HYVLKYQGYCYFTLLFRPLMLISDAKFCEWLLSSTTILTKSKDYQSLYNWLEGGMLISSG

PEWRKARKIVTPAFHFAILEQFVEVFEKPTAILVDCLRKQLDKDAVNVHPLIANYTLDII

CQTAMGVDLKVQEESQNDYVAAVDAMCKTVVERAFNPLKSFDIIYRLSPDYYREMRNVKL

LHSVSNTVIQKRRKEMESEKNNVESVNEDGTKRKMAFLDLLLKSRDEHGQPLSQDFIRRE

VDTFMFAGHDTTASAISFVLFCLANHPDVQNQVLNEIKEVRGEGQKITYKELQEMKYLEM

VIKESMRLYPSVPFYSRQTTEEVLYEDGKVIPQGITLIISAYAIHRNPHVYEQPDKFIPS

RFFDLESKPFTYLPFSAGPRNCIGQKFAMLLMKFALINMLSNFEILPSNPPCEMVLSAES

VLKAHNGVNIRLKSRT

>AEL88541.1

MFLSISHIFLAVLVLLVSVCYRRSRNFKEKLWWVPVIPGYPILGNALEFRDSTKTLERMN

HYMLKYQGLCCFTLLFRPLILISDAKFCEWLLSSTTILTKSKDYQFLYNWLEGGMLISTG

SEWRKSRKILTPAFHFAILEQFVEVFEKPTAIFLDCLRKQMHNEAINVHPLIVNYTLDII

CQTAMGVDLKVQKESQNDYVAAVDAMCKTVVERAFNPLKSFDITYRLSPDYYREMRNVKL

LHSVSNAVIDKRRKEMESEKDNVEIVNDDGTKRKMAFLDLLLKSRDEHGQPLSQEFIRRE

VDTFMFAGHDTTASAISFIFFCLANHPDEQNQVLSEIKEVLGEGQKITYKELQEMRYLEM

VIKESLRLYPSVPFYSRQTTEDVLYEDGKVIPQGITLIVSSYAIHRNPHVYDQPGKFIPS

RFLNLESKPFTYLPFSAGPRNCIGQKFAMLLIKFALINVLSNFEILPSNPSCEVVLSAES

VLKARNGVNIRLKSRT

>ABF06544.1

MILFEVVVVILTIWYIRKCRFREKVKWVATVPGHPILGVALDLTDPTKTLDRMTEYLTKY

NGMCYTEFMLCPTLVVSDLSFLKWFLTSNIPIHKGDIYAVLKNWLGGGLLISGGEEWRNS

RKILTPAFHFTILEQFIEVFEDATQVLGDVLSAEVTSKGVVDIYPYLTRYTLDVICQTSM

GVKLNIQQQSHSDYINAVAEMGKIIVERAFNPLKVYDFTYIFTSDYWKEKQYVKLLHQVS

NSIIEQRRQALEDQKFPEREGGKKKMAFLDHLLQYRDEQGKPLSDAFIRHEVDTVMFAGH

DTTAVALAFALYLLAKHPEVQAKARAEAREIVEGREKLTIKDIQNLNYLDLVVKETLRLY

PSVPFYSRALKEDMVYQEGKILPKGLVVLIVAYTVNRNPAVYQNPDQFIPTRFLDKEPVP

FSLLSFSAGPRNCIGQKFALLELKVALSKLLLRFEFVIPDSDASEPVVEAVTVLKPKNGI

RIKLNKLGSSN

>194473697

MGALALVVFAVTTLLFLIWFIRLVRKNNDYYKDIPGPRPLFFFGNVFEVSTTRDILNVMH

KYALQFNGLYKIHLGPVRKLIIASDYKFLECVLSSMKILNKSEDYLYLRPWLGTGLLTSD

GPKWKKHRKIITPAFHFQILEQFVEVFESGGNRFVQQLQKEVGKKSVDIYPYVTLCTLDI

ICESAMGISINAQFDDNSDYVRSVKAMCRITMERLFHLLEMNDVTYPLTKNYYTQKKSLN

VLHNQTNSVINRRRKELENKTEAINVTNEDKADNNDTLFGRNKKAFLDLLLQATVDGRPL

TQEEIREEVDTFMFEGHDTTASAISFAIYCLANNADVQAKAYEEQIALFGGNKSPAVTYS

DLQSMKYLELVIKETLRLYPSVPMFARKTSEPVQYENIFIPEGVTVNIFAYGIHRDPKYF

KDPEKFDPSRFETVDGKLPYAYIPFSAGPRNCIGQKFAMLEMKSTISKVLRNFELQPATP

THTVQLAAESVLKSANGVKIALKNRN

>EFA04617.1

MILPILLCLIFSFLIWSLYQTCKKNSFYKNIPGPSGVPIFGIAFEFKSTQDVLINLTSYI

KTYGDIVTAKIGPFRRYLLVSDYNFLECVLSSTKLIKKSHHYTFFQGWLGTGLLTADGAK

WKTHRRILTPAFHFQILEQFIEVFEKCGDVLVKKFENEVGRKSFDIYPYVTLHTLDVICE

SIMGISVNAQNNSTSEYVNSVKNICKIFVERSVSPLQMCRFMYPFTKNYWAEQKALKILH

QHTNSVIDARRKELHKAENGHNTNPKKSKKPFLDLLLETKIDGIPLTQEEIREEVDTFMF

EGHDTTASAISFTLYSLANNLHVQEKAVDEQKKIFGERKDVTAAYADLQNMKYLENIIKE

SLRLYPSVPFYNREITDDIMFDNKLLPKGDTIMVFAFAIHRNAKYFDNPEQFNPDRFNDL

ENKLPYAYIPFSAGPRNCIGQKFAMLEMKSTISKILRKYKLLPADPQHELNLVSELILKS

SNGIKISIEPRC

>EFA04615.1

MTIITEVFFALLISGLVILWFRLKNKNKEFYDKIPGPPPIPFFGNILEFTSTTVVLDIMM

KYMKIYGGVVKFHGGPIDKTLLVSDYNFLENVLSSTKILNKTDDYKFLHSWLGTGLLTSA

GPKWKKHRRILTPAFHFKILEQFIDVFESAGNKLVKKLEKVVGKDSVDIYPFVTLCTLDI

ICETAMGIKINAQDNGESEYVRSVKQMCKIIIERSFSLLQMINLTYPLTKNFYIEKNALK

ILHQQTNSVINQRRQELKNQSKNIEQENDLGTKTRKAFLDLILEATVDGRPLTDTEIREE

VDTFMFEGHDTTASAISFALFCLATHPEVQARALEEQKALFGDTKNPTPTYTDLQNMKYL

EQVIKEALRLYPSVPFHGRKTNEAVEFNNGTVVPKDVTITVFTYGIHRNPEYFKDPEKFD

PSRFDTIDGKLPYSFIPFSAGPRNCIGQKFAMLELKSTLSKVVRKFELRPATPEHKLQLT

AETVLKSVNGIKISLKLRQ

>EFA04616.1

MALLTDVFLPLVIILLIFWYWYRSQKNKKYYQNVATPPTVPILGNALDFTTTTELLGTFM

RYRKDYGGLVKVHIGPLRHFLLVSDYKMLEYLLSSPKIVDKSEDYKFLSSWLGTGLLLAD

GGPKWKKHRKILTPAFHFQILEQFVDVFDSCSNVLIQKLDKEVGNTSVDVYPFVTLFTLD

VICESTMGTKINAQDDVTSDYVQSVKHMCRIIIERSISPVQMYDFLFVFTRNYFIQRRAL

RILHDKADGVISQRLRELQAQTKTDSEGDSEGIKKKKAFLDLILEAKVDGKPLSQDDIRQ

EVETFMFAGHDTTASAISFTLYCLANYPEVQKMAYEEQLSIFEDNNEPDVTYANLQSMKY

LELVIKETLRLYPSVPIIGRQSGEDFQFDNSWIPKGDTMLLFLYGIHRDPKYFKDPEVFD

PNRFENPDNKMPYSYIPFSAGPRNCIGQKFAMLEMKCVLSKILRKFELQPAVPQHNLLLT

AETVLKSANGIKIGIKLRK

>395730546

MVPSFLSLSFSSLGLWASGLILVLGFLKLIYLLLRRXKLAKAMDNFPGPPTHWLFGHALE

IQQTGSLDKVVSWAHQFPYAHPLWFGQYIGFLNIYEPDYAKAVYSCGDPKAPDVYDFLLQ

WIGEGLLVLEGPKWLQHRKLLTPGFHYDVLKPYVAMFTESTRIMLDKWEEKAREGKSLDI

FCDVGHMVLNTLMKCTFGRGDTGLGHRDSSXYLAVSDLTLSMQQCLVSFQYHNDFIYWLT

PHGCCFLRACQVAHDRTECSSCVAGRDDLGKMTYLTMCIKESFRLYPPVPQVYRQLSKLV

TFVDGRSLPAGSLISMHVYDLHXNSAVWPDPEVFDPLRFSTENASKHHPFAFMPFSAGPR

NRIGQQFAMSEMKVVTAMCLLHFEFFLDPSRLPIKMPQLVLCSKNGFHLHLKPLGPGSGK

>126305754

MGMRMRERTTTSRNKKMDFGRILEKARETEADVGRASISSFGIPKSKKGDGVPHTRRTAS

QDFQQPERQRGTSGLCHSTEAEDVLDSVLRLGLEVWPLPAVAVHRQALHLVLATNPSPAL

LPSTPNTMALWQREGGKGLLSLEGEKWHYHRRLLTPAFHFNILKDYIYIMRNTVSLMLDK

WEKLRTKDSSIDVFDFISYMSLDTSLKATFGLQDFNKEESLFSYFQNMNKLLYLVRKRME

TFLYYSDFIYKLTSDHYEFQTTCKELKEEPAKIIRHRRALYKNQSEKDKVPKKKYLNFLD

ILFQAKDEDGGGFTNEELENEVNTFRFAGQDTVAVGFSWTLYCLAMNPEYQKKCREEIQG

ILKDGESITWDHLTQMTYSTMCIKESFRMYSPIPKVARTLNQPITFPDGQTLPSDTTVTI

NIWALHYNPAIWENPKVFDPERFTPENTKKRHPYAFLPFSAGLRNCIGQQFAMNLIKVSL

ALTLLRFELLPDLEKPPIPISQIVLRSTSGFHFYLKPLR

>ADI52567.1

MYRTWFIIGKAVNKFPGEPVEWPYGNLHQSPGFNEKLILHMKEMATKFNRAWRQWFGPFV

PFITLCHPDTVKILAKTEEPKFTEGNSAYLLAQPWLGDGLLLTSGRKWKRNRRLLTPAFH

FDILKSYISVYNEQSEILMKKWTEKCSKGESFDIQTDVCLCTLSIILQCAFSYGENIQEA

GKRHPYAAAVLELSHLMVKRALNPLVRMSKFLYGLTSDGRLFFKHCDYVHEVAKNVISHR

RQALQENPEALKDKKYFDFLDILLQARDQDGTGLTDLEIRDEVDTFLFEGHDTTASGISW

TLYSLAKHPEFQKKAQQELDELLADRPNKWILWEDLNQLPYLTMCIKESMRLWCPVPLIS

RQLSQPITIEGVTLPPHTIIDINIVALHHNPTVWGEDHDEYKPDRFLPENINKMDNFAFL

PFSAGPRNCIGQNFAMNEQKVTIARIIQRFDLSVDESHPVQPRPELVTRAIQGIKLFMKP

R

>ACD75826.1

MDLGFSSMSLTLQAVVVLVVTTLVVGGIKRLLRYRSYLKAMSLLPGETDHSWIWGDLHRL

QNMTCEQRISVYGKFTEKFPKFYRFWMGPFHANIVLVHPDTVKDLFKTADPKPFNYEFGI

PWLGEGLLIAGGSKWARSRRLLSPAFHFDILKPYVKVGNEASDKLLEKVKKYADKGESIE

MYNNISLCTLDMIMRCAMSYSNDIQTKGESHPYVVAVSELNDLLIQRIRNPLVYNDFVYG

LTKNGRRFKEQCHYVHGISEQIIQERQKILEREGPSKKRYLDFLDILLTAKDDTGTGLTP

LEIRSEVDTFLFAGHDTTASAISWILYSLCQHPDIQEKVQQEIDTVLKGRDSDEIEWSDL

PKFKFMTMVIKEGMRLHCPVPFISRISQKPMTLEGFSIPAGSLCSIHMFNIHHNPVVWPD

PWAFKPERFHPDNTTDRDSFAFVPFSAGPRNCIGQHFAMDEEKVMLSRLLRRYTFRLDPK

HPVVRKMTIIMKTESGMRMFATPRTPTVYNNWEH

>ACD75825.1

MDLGFSSMSLTLQAVVVLVVTTLVVGGIKRLLRYRSYLKAMSLLPGETDHSWIWGDLHKL

RNMTSEQRISVFGKFTEKFPKFYRFWLGPFQANIVLLHPDTVKDLFKTADPKPFGYQFGI

PWLGEGLLIAGGSKWARSRRLLSPAFHFDILKPYVKVGNEASDKLLEKVKKYADKGESME

MYNNISLCTLDMIMRCAMSYSNDIQAKGESHPYVVAVSELADLLIQRIRNPLAYNDFVYG

LTKNGRRFKEQCHYVHGISEQIIQERQKILEREGPPKKRYLDFLDILLTAKDDTGTGLTP

LEIRSEVDTFLFEGHDTTASAISWILYSLCQHPDIQEKVQQEIDTVLKGRDSDEIEWSDL

PKFEFMTMVIKEGMRLHCPVPFISRVTQKPMILEGFSIPAGSVCTIHIFNIHHNPVVWPD

PWEFKPERFHPDNTKDRDSFAFVPFSAGPRNCIGQHFAMNEEKVMLSRLLRRYTFRLDPK

YPVVRKMTAIMKTESGMRMFATPRTPTV

>ACM16804.2

MFGLSNCNTYKRYYVWWTTFMKPSLSVCHPDTVKMIMKTSEPKPIGFMGIYRLGLPWLGE

GLLIAGGKKWARARRLLTPAFHFDILKPYMSVYNDAADLLVGNLERYMEKKERFEVFDYV

SRATLDVIMRCAFSYQTDCQKDKGTRHPYVQAVEEIADAWNYRARKPWLYPDWLYNMTRH

GKQFRKNCDYVHTVAEDVIKQRKEALDKSRNSDRKYLDFLDILLTAKDENGQGLTPLEIR

NEVDTFMFEGHDTTASAISWILYSLAKYPEYQKKCQQEIDELLQGRDTDDIEWSDIPKLE

YLTMCIKEGMRDHSPVPFIQREFTHDFEIDGKTFPAGTTVSLHIFGLHHNKNVWENPMEF

VPERFSKENIAKMDTFQFVPFSAGPRNCIGQHFAMNEEKVIISKLLRRYWFELDETHLVR

RKIGAVMRAENGIYLYVRPRDL

>EKC34228.1

MLITQKIYLYDTSIKICHDITASAISWILFSLAENSECQQKCPEVIDKVVSETKFGQLKW

LAVISVVVIIIFQLLIKLLRVKYAYHGLPEPLEDKHWLLGHLPIFSNKIAGQSVVDKFLE

WTAKYPRMYVLWFGPFDARVVLNHPDPIKKILKTSDPKPVNFGQVYRHGIPWLGEGLLIA

GGAKWKRSRRLLTPAFHFDILRPYVKIYKSCADILARNIEISVEKNESVEIFSLVSSCTL

DIILQCAFSYKTDCQNFCGTIHPYISAVNEISATWNRRNRAPWLHPDFIFYRTAEGKSFK

AKCDYVHQVAEDVIDKRRNTLETQGISSQKYLDFLDILLTAKDENGKGMSKEDIRSEVDT

FMFEGHDTTASAISWILYSLAENPECQRKCQEEIDKVISETKSGQLEWKDLGGLEYLTQC

IKEGMRLHSPVPGILRDIQSPLREGDHEIPARTSVLISINGLHHNPTVWGKDHAQFKPER

FSPENTEERDSFAFCPFSAGPRNCIGQNFAMSEERTVLATLLQRFTFSVDKTHKVEKQIS

AVMRARYGIKLFAAPR

>AEE62164.1

MWILLVLPIVAYILVHIASSRIKSQSKYLRNVPGKKPFLIFGNLLDFLPGSIVFLDNLMG

YLKTYGDTILFHDGLFSWMLVSTDYEFNEFMHSSSVHIEKSNLYHFFHGWLGQGLLTSFG

SKWRNHRKVITHSFHFSILQQFIGSFDGVGDNLLKKLESDAGKDSVEISQLISLYTLDVI

CEAAMGVKIHALDSGNSQYVKSIKDMCNIVADRIFSYLSPRLYMLTANYYKEKRALRIIH

KHVDAVISQRIEEHNQKAKKSESSQADDFGVKKRLAFLDMLLDARIDGKPLSRKELRDEV

NTFMFEGHDTTSSAISFALFAIATHPEVQGKLFDEQTQIFPSDWKSAHASHKQLMEMKYL

DMVIKETLRLYPPVPFYGRKLAQDVDFKGTLYPKGLTVFLFPYGCHRSAKYFPEPEKFIP

ERFENWTGKLPYAYTPFSAGPRNCIGQKFAMLEMLAIISKIIRKFKLAPARPEHEMQLAG

ETILISKNGVNISLEKRF

>AFI45016.1

MWILLVLPIVAYILVHIASSRIKSQSKYLRNVPGKKPFLIFGNLLDFLPGSIVFLDNLMG

YLKTYGDTILFHDGLFSWMLVSTDYEFNEFMHSSSVHIEKSNLYHFFHGWLGQGLLTSFG

SKWRNHRKVITHSFHFSILQQFIGSFDGVGDNLLKKLESEAGKDSVEISQLISLYTLDVI

CEAAMGVKIHALDSGNSQYVKSIKDMCNIVADRIFSYLSPRLYMLTANYYKEKRALRIIH

KHVDAVISQRIEEHNQKAKKSESSQADDFGVKKRLAFLDMLLDARIDGKPLSRKELRDEV

NTFMFEGHDTTSSAISFALFAIATHPEVQEKLFDEQTQIFPSDWKSAHASHKQLMEMKYL

DMVIKETLRLYPPVPFYGRKLVQDVDFKGTLYPKGLTVFLFPYGCHRSAKYFPEPEKFIP

ERFENWTGKLPYAYTPFSAGPRNCIGQKFAMLEMLAIISKIIRKFKLAPARPEHEMQLAG

ETILISKNGVNISLEKRF

>AFI45017.1

MWILLVLPIVAYILVHIASSRIKSQSKYLRNVPGKKPFLIFGNLLDFLPGSIVFLDNLMG

YLKTYGDTILFHDGLFSWMLVSTDYEFNEFMHSSSVHIEKSNLYHFFHGWLGQGLLTSFG

SKWRNHRKVITHSFHFSILQQFIGSFDGVGDNLLKKLESEAGKDSVEISQLISLYTLDFI

CEAAMGVKIHALDSGNSQYVKSIKDMCNIVADRIFSYLSPRLYMLTANYYKEKRALRIIH

KHVDAVISQRIEEHNQKAKKSESSQADDFGVKKRLAFLDMLLDARIDGKPLSRKELRDEV

NTFMFEGHDTTSSAISFALFAIATHPEVQEKLFDEQTQIFPSDWKSAHASHKQLMEMKYL

DMVIKETLRLYPPVPFYGRKLVQDVDFKGTLYPKGLTVFLFPYGCHRSAKYFPEPEKFIP

ERFENWTGKLPYAYTPFSAGPRNCIGFGVLRQKFAMLEMLAIISKIIRKFKLAPARPEHE

MQLAGETILISKNGVNISLEKRL

>AEL88542.1

MWILLLLPVVAYILIQIAISQVKSQKKYLRNVPGKKPFLIFGNMLDFMPGSTVLLDNLMG

YLKTYGDTILVHDGSFSWVLLTIDYEFNEFLHTSTVHIEKSNLYNFFKGWLGQGLLTSFG

PTWRSHRKVITPSFHFSILQQFISVFDSVGNKLMKKLESEAGKDSVEVSQLISLYALDVI

CEAAMGVKIHALESGNSEYVKSIKEMCNIVVDRMFSIISTRFYKLTSTYQKEKKALKIIH

EHVDAVISKRLEEYSQKPEKSSQSDDFGVKKRLAFLDMLLEARIDGKPLTKTELRDEVNT

FMFEGHDTTSSAMSFALYLIATHPEVQDKLFEEQTQIFPSDCKNARPSHNELLEMKYLDL

VIKETLRLFPPVTFYGRKLAHDVEFKGTLYPKGLSVLLFPYGSHRSPKYFTEPDKFIPER

FENWTGKLPFAYTPFSAGPRNCIGQKFAVLEMLATLSKIRPQVQAGASKARAQTRTGSRN

DFNFQKRSEHFVGKAIIYSVKCNE

>EFA04665.1

MHKYALQFNGLYKIHLGPVRKLIIASDYKFLECVLSSMKILNKSEDYLYLRPWLGTGLLT

SDGPKWKKHRKIITPAFHFQILEQFVEVFESGGNRFVQQLQKEVGKKSVDIYPYVTLCTL

DIICESAMGISINAQFDDNSDYVRSVKAMCRITMERLFHLLEMNDVTYPLTKNYYTQKKS

LNVLHNQTNSVINRRRKELENKTEAINVTNEDKADNNDTLFGRNKKAFLDLLLQATVDGR

PLTQEEIREEVDTFMFEGHDTTASAISFAIYCLANNADVQAKAYEEQIALFGGNKSPAVT

YSDLQSMKYLELVIKETLRLYPSVPMFARKTSEPVQYENIFIPEGVTVNIFAYGIHRDPK

YFKDPEKFDPSRFETVDGKLPYAYIPFSAGPRNCIGQKFAMLEMKSTISKVLRNFELQPA

TPTHTVQLAAESVLKSANGVKIALKNRN

>189237253

MCCCFPLALAALHWPRDARVDDCRLCYPMPMRDAGQQLLQHQKLSGTLRMDTQAAAKTPT

LALRHDAMQSFNRPTFYRPKWKKHRRILTPAFHFKILEQFIDVFESAGNKLVKKLEKVVG

KDSVDIYPFVTLCTLDIICETAMGIKINAQDNGESEYVRSVKQMCKIIIERSFSLLQMIN

LTYPLTKNFYIEKNALKILHQQTNSVINQRRQELKNQSKNIEQENDLGTKTRKAFLDLIL

EATVDGRPLTDTEIREEVDTFMFEGHDTTASAISFALFCLATHPEVQARALEEQKALFGD

TKNPTPTYTDLQNMKYLEQVIKEALRLYPSVPFHGRKTNEDVVDNGTVVPKDVTITVFTY

GIHRNPEYFKDPEKFDPSRFDTIDGKLPYSFIPFSAGPRNCIGQKFAMLELKSTLSKVVR

KFELRPATPEHKLQLTAETVLKSVNGIKISLKLRQ

>EFA04535.1

MNIILEFFSALAVIIVLSWWYLFLKNYKVGKFFKNFSGPPGVPLLGNALDFKNKTKGVLP

AFLNYNKNFGGLVKVQIGPFRKLLLVSDYKFLEFVLSSTKIINKSQNYRTMLPWLGTGLL

TSEGIKWKKHRRIITPTFHFKILEQFINSFDAAGDVMINKLRKKVGIESVDIYPFVTLCA

LDIICETAMGTTINAQNNEESEYVTSVKEMGRIIIERAIAPQKNNEFLFRFTKDYQLQKS

ALKVLHNYTNNVISKRREELLKDQASKVSENVIDMGIKKKMAFLDLLLQATVDGRPLTNE

EIREEVDTFMFEGHDTTASGISFALYCLANNPEAQEKAYEEQVALFGKEKKPIVSYSDLQ

EMKYLELVIKEALRLYPSVPFYARETNQEVEFGDIKIPKGVNITIFAYGIHRDPKYFPEP

DKFDPGRFETIDGKLPYAYIPFSAGPRNCIGQKFAMLEMKSTISKVLRNFKLCPATPHHT

LDLVAETVLKSDNGVRLSLMERQ

>EFA04628.1

MFIFAALFFLIIFYLYHQNVKLNRILCTFPEPPKKFLLGHVLDITSTTDVLNVFSKYINN

YGPTIKLRAATLFAGLGTTDYKLCELLLSNNRILSKSLNYQYARNWLGNGLLISDGDYWR

RHRKILTPAFHFEILKQFVETFESVGKVFVEKLDISQGPSVDLPPLVTLCTLDVICETAM

GTQINAQKGQNAKYVQSVREMCRILIDRGLSPLKILNNTYWMTKDYYIEKKSLKVLHSFT

SSVIEKRKAERNERNCTKLAFLDLLLKFSNEGELLTDQELREEVDTFMFEGHDTTASSIS

FVLFCLANHPEVQEKVLREQNELFGDDKDPSVTYHELQKMKYLEQVIKETLRLYPAVPII

GRCTSEDITFGEHFIPKDTNIAIYIYGIHRNPEHFPEPETFNPDRFKNSNSLPPYAYIPF

SAGPRNCIGQKFAMLEIKSIVSRVVRCFELRPAEPYHSLVLSAETVLKSANGIKIGIRKR

I

>91082229

MMKDRKIKFTENYSINYQLGLAYINNYGPTIKLRAATLFAGLGTTDYKLCELLLSNNRIL

SKSLNYQYARNWLGNGLLISDGDYWRRHRKILTPAFHFEILKQFVETFESVGKVFVEKLD

ISQGPSVDLPPLVTLCTLDVICETAMGTQINAQKGQNAKYVQSVREMCRILIDRGLSPLK

ILNNTYWMTKDYYIEKKSLKVLHSFTSSVIEKRKAERNERNCTKLAFLDLLLKFSNEGEL

LTDQELREEVDTFMFEGHDTTASSISFVLFCLANHPEVQEKVLREQNELFGDDKDPSVTY

HELQKMKYLEQVIKETLRLYPAVPIIGRCTSEDITFGEHFIPKDTNIAIYIYGIHRNPEH

FPEPETFNPDRFKNSNSLPPYAYIPFSAGPRNCIGQKFAMLEIKSIVSRVVRCFELRPAE

PYHSLVLSAETVLKSANGIKIGIRKRI

>EFA04629.1

MYLIAIVIVLISLYLIVKPVKKTGNIDDFPEPPSKVFFGHALDAKSTTEILNVTTNYVKK

YGKIVKLRLGPFFKALVVTDYKLVEFFLSSNSLLTKSQNYAFARSWLGNGLILANGTHWK

QHRKILTPAFHLEILREYLETFDSVGRIFIEKLKSLDNVQSVDLDALVPLCTLDVICETA

MGTKINAQKGENSEYVSSVKEMCRIIVARFLSPLKILKCTYWMTHDFYTERKVVNILHSF

TSKVIDGRKTNRNETEKSGKKKAFLDLLLKFSDIEKLSDRDIREEVDTFMFAGHDTTATG

VCFALYLLANNPEVQEKVLSEQKELFGDEKNPCVTYQELQNMKYLEYVIKETLRLYPSVP

VIGRYLKEDTTFGDRVISAKTNVAIFIYGIHRNPDYFPEPEKFIPERFENMTNLPPYAYI

PFSAGPRNCIGQKFAMLEMKSLISKVIRHFELTPANPHHELVLAAETVLKSANGIKIGLR

ARSENKM

>189237251

MALLTDVFLPLVIILLIFWYWYRSQKNKKYYQNVATPPTVPILGNALDFTTTTELLGTFM

RYRKDYGGLVKVHIGPLRHFLLVSDYKMLEYLLSSPKIVDKSEDYKFLSSWLGTGLLLAD

GGPKWKKHRKILTPAFHFQILEQFVDVFDSCSNVLIQKLDKEVGNTSVDVYPFVTLFTLD

VICESTMGTKINAQDDVTSDYVQSVKHMCRIIIERSISPVQMYDFLFVFTRNYFIQRRAL

RILHDKADGVISQRLRELQAQTKTDSEGDSEGIKKKKAFLDLILEAKVDGKPLSQDDIRQ

EVETFMFAGHDTTASAISFTLYCLANYPEVQKMAYEEQLSIFEDNNEPDVTYANLQSMKY

LELVIKETLRLYPSVPIIGRQSGEDFQFGKVFIKNGDTMLLFLYGIHRDPKYFKDPEVFD

PNRFENPDNKMPYSYIPFSAGPRNCIGQKFAMLEMKCVLSKILRKFELQPAVPQHNLLLT

AETVLKSANGIKIGIKLRK

>AAT38512.1

MFQTAILSLLCSLILWLVYWYYKINKYEKYLKTVPGPARTPIMGNIPDIGSPVSFLEDLL

KLSYMYKGNFKLYIGAQPRVFLVEPKDLEFLLNSTSLLTKSKSYKFLYRWLGAGLLTSSG

NKWKKHRKIITPAFHFQILEEFIDVFNSASDVLVEKLNAAPNKSSIDIYPFIARCTLDII

CETAMGTSVDAQNDINSEYVNSVKILLGILVQRSLSPILANDLLYPFTTTYQKEKAALKV

VHGYTKSVINKRKIEFYNNSKSEDRNVDSFGRKKKQAFLDLLLEYSANDPSFTEQHIQEE

VDTFMFEGHDTTATSITFALYALAMNPHIQEKAYAELKEIFSNNSKRHASYRDLQEMKYL

EMVIKETLRIYTTVPFYSRALEEDVNMNGQILPKGTMLNVFAYGVHHNPKIYKDPETFDP

ERFSIENSKERSPFAFIPFSAGPRNCIGQKFAMLEMKSSISDVLRNFKLLPSVPAHKVVL

KSEAVLKSDNGVFVRLQKRMDS

>EFA09279.1

MITIVIILIFILVSINFITKYLKKLKQNKDFYQNIRGPRGLPILGSALDFKTTKDVLQNL

INYTRDYGEVVTAQIGPFHRYILVSNYDFLECVLSSTKLSSKSHNYNFLRPWLGTGLLTS

DGAKWKTHRRILTPAFHFQILEQFIEVFEKCGNTLLEKLRNEVDKETCDIYPYVTMCTLD

IICESIMGISINAQEDSTSDYVQSVKNVCRIMVERSISPLQMCPFMYPLTKNYWTEKKSL

KILHKQTNDVINARRKELESSKEVSSYEENFTKKKKPFLDLLLETKIDNRLLTQEEIREE

VDTFMFEGHDTTASAVSFTLFCLANNLESQQKAFEEQQAIFGNIQNVTASYTDLQNMKYL

EQVIKEALRLYPSVPFYGREITENVEYDGKLLPKGDILLIFAFGIHRNEKYFPNPEKFDP

DRFHNMDNKTPYAYIPFSAGPRNCIGQKFAMLEMKSTVSKVLRQYKLLPTTPQHELELVG

ETILKSTNGIKIRVTLRN

>91093475

MGVISHYYVLQNLINYTRDYGEVVTAQIGPFHRYILVSNYDFLECVLSSTKLSSKSHNYN

FLRPWLGTGLLTSDGAKWKTHRRILTPAFHFQILEQFIEVFEKCGNTLLEKLRNEVDKET

CDIYPYVTMCTLDIICESIMGISINAQEDSTSDYVQSVKNVCRIMVERSISPLQMCPFMY

PLTKNYWTEKKSLKILHKQTNDVINARRKELESSKEVSSYEENFTKKKKPFLDLLLETKI

DNRLLTQEEIREEVDTFMFEGHDTTASAVSFTLFCLANNLESQQKAFEEQQAIFGNIQNV

TASYTDLQNMKYLEQVIKEALRLYPSVPFYGREITENVEYDGKLLPKGDILLIFAFGIHR

NEKYFPNPEKFDPDRFHNMDNKTPYAYIPFSAGPRNCIGQKFAMLEMKSTVSKVLRQYKL

LPTTPQHELELVGETILKSTNGIKIRVTLRN

>EGK96892.1

MFVATLTLLALIASVAFYVYVERFSKILKHSGKLGGPRAYPLIGNGLLFAGKTPAEFLQT

VGRLIQQYGKCFRIWLGTQMLIVITEPKDVEAILSSNKYIDKSIEYDFIRPWLGEGLLTS

TGRKWHTHRKVITPTFHFKILEQFVEIFDQQSSTFVQVLAAHAKSGDTFDIFRPVTLCAL

DVICETAMGTKVNAQLNTESEYVQAVKDITNLVQIRLFNFLIRYEIFYRFSANRRKQLEA

LKVLHGYTDSVIKIRREELNRAAGSGKNTVVDENENELGIKKKMAFLDMLLQSKIDGRPL

TDLEIREEVDTFMFEGHDTTTSAISFLLQNLAKHPAIQQKVFDEVRNVVGDDRTRPVTIA

MLNDMHYLDLVIKETLRLYPSVPMFGRKMMEDAEINGKVFPAGSNTIILPFFLGRNPEFF

PNPEKFDPERFNVETSAEKTNPYQYVPFSAGPRNCIGQKFAVAEIKSLVSKLLRNYEILP

PVGRYDETFIAELILRPEKGIYVRLQPRVY

>EAT35570.1

MSELTTFIYGILVFLIFAPFLQWWVKRARLVQIIDKIPGPKAYPFIGTTYTFFGKKHYEL

FYIIDERTRRYPDIHRIWTGMRPEIRISKPEYVETIIGASKHMEKSHGYDFLFDWLGEGL

LTSKGERWFQHRKLITPTFHFNILDGFCDVFAEQGAVLAERLEPFANTGKPVDVFPFITK

AALDIICETAMGVKVNAQTGGENNYVNAIYRMSEIFVDRSIKPWLHPEFIFKRTEYGRQH

KKALDIVHGYTKKVIRDRKEALQVKENSTGAGDTGEDLYFGTKKRLAFLDLLLEGNAKHK

QLTDDDVREEVDTFMFEGHDTTTAGMSWALFLLGLHPDWQDRVHQEIDSIFAGSDRPATM

KDLGEMKLLERCLKETLRLYPSVSFFGRKLSEDVTLGQYHIPAGTLMGIHAYHVHRDERF

YPDPEKFDPDRFLPENTEHRHPFAYIPFSAGPRNCIGQKFAILEEKSIVSSVLRKFRVRS

ANTRDEQKICQELITRPNEGIRLYLEKRQ

>ACD75823.1

MFVALLLVGIVTIVGTLVWMLQTAKFRQQISLIPGPPTIPLLGNVHQMTTGPGWYKQVLK

WGDEFRKQGIFRIMLISKPVIGVTCAETAEVMLNSSKHIDKAQEYGYLHAWLGTGLLTST

GDKWRMRRKMLTPTFHFRILHDFVEVFNQQTCVLIEKLKHHVDKGQFNVFQDIALCALDI

ICETAMGQHVNAQTKESAYVQSVYKMCGFVEKRMRTPWHYNKTMYDLFGPGEVHDHHLKI

LHEFTIKVIKDRMSNFNAERAQRLLDDANKKKDWVNCSESETNCRKTRLAFLDMLLFMSD

NGKVLSIDDIREEVDTFMFEGHDTTAAGMNWCTYLIGSDEKVQGKVCEELDRVFGNSDRM

PTMDDLKELKYLECCIKEAQRLFPSVPYFGRTTTEEAQISSFRVPKDVTVIVFTSAIHRD

TRWFPNPEHFDPDRFLPENSVGRHPFAYIPFSAGLRNCIGQKFAMMEEKVILSSIFRNFK

VKSCQSREELLPVGELILRPQKGIFIELSAR

>EFX88057.1

MSESVWTDWGSLAFKWLLLVTIPVLYYWIWSRSTFVQLVNAIPGPRPIPFLGNVLDLYVD

RDEFLKIVHIDWIRKYGSIYRAWGGTRPVVVISSPELMEPILVSQKLITKAAEYSFLSTW

LGECMFLTTGTRWKNRRRLLTPAFHFQILNSFVDVFNEKSFDCGREFERAIVQHGGKEFD

VFPIITQCALDIICETSMGKQTRGEDEKALYVQNLHRIGQIVMERGIRPWLRLDWIYQFS

ALGRENKRCVKALHNFTNKVINDRREALQKELSSPESNNNFNNNYTDHSSNDSLTSKKRL

AFLDLLIAASENGANLSDDDIREEVDTVMFAGHDTTASAMTWFLYCLAMHPHHQDLVTEE

LDQIFGDSDRPCTIQDVAELKYLECCIKETLRLYPSVPAVMRYITEDIHVGGYKIPAGVS

VSLMIYGMHHNPLVYPDPQTFNPERFLPENVLGRHPYAFVPFSAGPRNCIGQKYGLLEIK

IVLANLLRRFRFSVADPSKPMLIPSSEVVLKPKHGVPLIVSKRAAH

>CAQ57675.1

MAKTANGSIKMDYTTTILSLVLFIFSALYLLRQAFRRIKIINMVDQLPGPRAYPIIGNAL

DFMVPRSELMNVFDSRTKKYGPLFRTWAGPVPQIHITRPEHMEIVMSSLKHIDKSKAYTF

LQPGLGTGLLTGTGAKWHSHRKMITPTLHFKILDVFVEVFGEKCQTLIENLLKKADGQEF

DIYPFITHCALDIICETAMGTQINAQNESDSDYVRAIYDISELTTERTTKPWLHSDLIWK

SSKRGARYAHDLSILHGFTNRVISERKVARLADKERIKNHEDDDEFLGKKKRMAFLDLLL

EASELGQKLTDDEIREEVDTFMFEGHDTTTAGICWSLFMLGNHPEYQDQVAQELDQIFGD

SNLPPTMKDLNEMKYLERVIKESLRLFPSVPFIGRYLGEDTKFDNYIVPAGCVMNLQIFH

VHRCPDQFPDPEKFNPDNFLPERTQGRHPYAYIPFSAGPRNCIGQKFAVLEEKTVLSSIL

RNYRVESVEKLEDLNLMNELILRPESGIRMRIYPRKKTQS

>AEL88543.1

MLEAQVVLWVLVALVSYLLIQAFRFRKVLSHIPSPPGHWLIGNLEFLMKNPYPGKIFEYL

RECAQIYGPIYRFSTLHIVVINLLHPDNMELVLSQMKHMEKSRLYRFLGRWLGEGLLTSS

GQKWQRRRKLPTPAFHFSALQKFLDIFNEETSNMVKQIEEINAQNIGRKPLINLMPIVTN

LTLQSITETALGVSSVEEDTMKNYRSNIYKMGQFVFGRVTKPWSLIPLVYSLSNDYEKES

ETIHSLHQFTYRVMKEREKQLVDGRDEQVVSTTYSGRKIMKMLDLLLHAEINEGTIDYEG

IREEVDTFMFEGHDTTAAAMTFLLYALAEHPDIQENVSKEIQSNIIPTRAPTYHDLQNLP

YTEMVIKESLRLYPSVPLITREASEDFISHTGSRIPKGTVLYLHIFDLHRNPDIYPDPQR

FDPDRFLPENCANRHPFAYLPFSAGPRNCIGQKFAILEIKAVIVGLLRKFRLESSSSAPP

SFFVDLILRPEGAINVKFVPI

>EFA10752.1

MLLSSSVLFVISFFLVLYFFKKLKCVSAKLYNLGKLDGPSNHNIIIGNVLRLHAAPEVIF

KKLRQWARDYYPVYQLRAIHIPAACILAPEDFEAILSNPRHIEKSMVYLLLHDWLGTGLL

TSGGQKWATRRKILTPAFHFSILQQFITIFNEETDKLVDTFKQQCDQPIEITSHITQFTL

KTIGETAMGTKFEFTTRKQIEYKQAILEIGEILLYRLLNSWLISDYLNFFSPHRLREKRL

VKTLHTFTRDVITEREKTFEKFQLPTEEHDVYIGKKRLAMLDLLLTAKNEERTIDEDGIR

EEVDTFMFEGHDTTSAALSFALMLLANHKNVQDQIVDEMVTVLGDLHQKPTYNNLQEMKY

LERAIKESLRLYPSVHFISRKLGEDFVTCNGLKLPKSTITHLHIYDLHHNPDIYPDPEKF

DPERFRPENSQKRHPFAYLPFSAGPRNCIGQKFAMLELKAAICGILANFILEPVDTPESI

VLVVDLVLRTKNGIKIGFIPRVK

>EFA10751.1

MIILLIIVALVLALFLKPLGHYISIIKYVQQLPGPPCENFLHGNITHLYGPPEKMFKTVR

KWGLEYYPIYQNWIAHVAVANVMNPYDFETILSNMKHTSKSMIYDMLHCWLGTGLLTSDG

IKWQTRRKILTPAFHFNILQEFIKIFNEETETLVQQLKKHSNSSVDVTEYISAFTLNTIG

ETAMGTSFGTETSTGREYKKSIQEIGEILKYKLLRPWLLSKFVYVCDPNYWKEIKLVKIL

HNFTNNVIANRQSNFKPVEQKSDEFSYSRRKRMAMLDLLLTAKNEDNLIDDEGIREEVDT

FMFEGHDTTAVAICFALMCIACHPDIQERIFEEIEETFSDDTKPDYKSLQELKYMERCIK

EVLRLYPSVPFIARSLGEDIVTYSGHKLKAGSMVHLHIYDMHHNPQVYPDPEKFDPDRFL

PENCLKRHNFAYVPFSAGPRNCIGQKFAILEMKAVLVGILKEFTLEPVDCRYT

>EFA10749.1

MALEIILIALAAITAICYFLKQLIDMTLVIFYVKKMPGPPKFNLFFGNMRPLHSTPAQIF

NVLRKWGLEYYPIYKLWLFFVSVANILSPEDLALILPDMKHSKKSKAYDLIRRWLGNGLL

TSFGTHWQVRRKILTPAFHFSILQDFILTFNESTEELVELLKKEVEKPFVEITPLITQFT

LKVIGETAMGVKFDFSTKQAKEYKEAFHKLVSIVLHRIFRPYLAYDIFYVFSPKFYEERK

LLKLLHNFTNDIIARRRNEFHQGTVNTQKKKRLAMLDLLLTAQKEEGIIDDEGIREEVDT

FVFEGHDTVSAAINFTLMMLANHPEVQEEIVQEMKDVLGDIKKKPVYNDLQELKYMERVI

KEVLRLYPSVHYISRELGEDMITTTGYKLKKGTILQLHIYDLHYNPVIYPEPEKFDPDRF

LPENCDKRHPYAYIPFSAGPRNCIGQRFAMLELKAVLCGILSNFKLLWIPLRVLLWSKI

>189241026

MPGPPKFNLFFGNMRPLHSTPAQIFNVLRKWGLEYYPIYKLWLFFVSVANILSPEDLALI

LPDMKHSKKSKAYDLIRRWLGNGLLTSFGTHWQVRRKILTPAFHFSILQDFILTFNESTE

ELVELLKKEVEKPFVEITPLITQFTLKVIGETAMGVKFDFSTKQAKEYKEAFHKLVSIVL

HRIFRPYLAYDIFYVFSPKFYEERKLLKLLHNFTNDIIARRRNEFHQGTVNTQKKKRLAM

LDLLLTAQKEEGIIDDEGIREEVDTFVFEGHDTVSAAINFTLMMLANHPEVQEEIVQEMK

DVLGDIKKKPVYNDLQELKYMERVIKEVLRLYPSVHYISRELGEDMITTTGYKLKKGTIL

QLHIYDLHYNPVIYPEPEKFDPDRFLPENCDKRHPYAYIPFSAGPRNCIGQRFAMLELKA

VLCGILSNFKLLWIPLRVLLWSKI

>EFA01272.1

MSVLTEFFIALVALILLPFYFYYTKIRRIENFFKHFPSPAGVPILGVAPEFKTTSSVLDN

FHKYAKAHGTMVHLKFGPFKHALLCTDTKFLEFLLSSNKLLKKSVNLRFLEPWIGTGLVT

ADGGPKWKTHRKLITPAFHFKILEQFVDIFESGGDILVKKLSEVSGSKSCDIYPYITKCT

LDIICETAMGTKVDVQNTENSAYFESIKEMCRIFTVRTVGAFLSNDFIFRFSKDYKIQTK

AVKVLHNHSNEVIVNKRKELKEKNNENTSDIDELGRKKKKVFLDLILSATTDGQSLSPEE

IREEVDTFMFGGHDTTGTATSFILYCIANNKEAQEKILEEQKQLFGDERSPKVTYSTLQE

MKYLENAIKEGLRLYSPVPIYGRLIDQDTEYNGTMIPKGVGVIIFAHGIHMNPKYYPNPE

KFDPSRFENTTGKDPFTFIPFSAGPRNCIGQKYAMLEIKSLVSKVVRNFELFPASPTHEM

HLAPETVLKSLNGVKIGLKMR

>EAT40475.1

MFLLVTVFFAVVSLAVFVYQKFANQLYYGAKIGGPKCYPLVGNAFRFINKSPPDFFLTIE

RTVREAGKCFRLWLGPELLIIVTDAKVAEGVLSSPKFIEKSGEYNFIRPWLGDGLLTSSY

RKWHSHRKIITPTFHFKILEQFVEIFDSQSNILIDKLTPFMESGETFDVFPLVTLCALDV

ICESAMGTKVNAQIHSDSEYVQAVKEITTIIHIRTYDVLARYDFLFNLSSYRKRQDKVLE

VLHGYTNSVIRSRRRELSDAKEANPDNNATSELGIRRKVAFLDMLLQATVDGRPLTDVEI

REEVDTFMFEGHDTTTSAISFLLYRLAKHPEVQHKVYDEIKAVIGEGMTGPVTLSMLNEL

HYLELVIKETLRLYPSVPFYGRKVLENSEIEGTTFPAGSNLILMPMFMGRDPEYFDDPLE

FRPERFEKEISAEKVNPYRYIPFSAGPRNCIGQKFAMAELKSVASKVLRHFEVLPPEGGQ

EESFIGEMILRPTYGVLLRLKKRQ

>EDS33498.1

MAIAALVALALVGLALAVLLYRKFANALYYGAKIGGPRGYPLIGNALLFLYKSPPDFFLA

IEKTIREAGRCFRLWLGPELLVIVTDAKVAEGILSSPKYIEKSGEYDFMRPWLGEGLLTS

SGRKWHSHRKIITPTFHFKILEQFVEIFDHQSDVFVEQLRAQAASGQAFDVFPLVTLYAL

DVICESAMGTKINAQIHSDSEYVKAVKEITCIIHIRTYDVMARYNSLFNLSPYRRQQDKV

LKVLHGYTNSVIQSRRRDLVENNQKSQQDRDREAELGIRRKVAFLDMLLQTTVDGRPLTD

VEIREEVDTFMFEGHDTTTSAISFLMYRLARHPEVQRKVYEEIVAVIGADRDRPVTLAML

NELHYLELVVKETLRLYPSVPFYGRKILENCEIEGRTFPAGANVIIMPFFMGRDSEYFED

PQEFRPERFAVETSAEKTNPYRYIPFSAGPRNCIGQKFAMAEIKNVISKVLRHFELLAPD

HVPEESFIAEMILRPTYGISLRLKERT

>157117004

MLILLASVVVLSIGLAVYFYQQFANRLHYAAKIGGPKGYPLLGNSIQYGTKSPVEFLQEV

QKTNEQCGKFYRLWIGPDLIFPITDAKLVEAILSSQKLLDKSVQYDFIRPWLGNGLLTST

GRKWHSRRKIITPTFHFKILEQFVEIFDQQSNIFVGQLKSKAQSGEDFDVFPVVTLCALD

VICESAMGTKVNAQLNSDSKYVRAVKDMATVAMARSFKAFARFNFTFYFTPYRRMQDKAL

KVLHDYTDSVIRSRRLELAKGAFTKSDENENDVGIRKKVAFLDMLLQATVDGRPLDDLEV

REEVDTFMFEGHDTTTSAISFLIGILAKHPDVQQKVYDEVRNVIGDDLNVSVTLSMLNQL

NYLDLVIKETLRLYPSVPIYGRMLLENQEINGTVFPAGSNLAIFPYFMGRDPEYFENPLE

FRPERFAVETSAEKANPYRYVPFSAGPRNCIGQKFAVAEIKSLISKLVRHYEVLPPKQPN

SERMIAELVLRPEGGVPVRIRSRVR

>BAI77922.1

MAFLLVLILVAIAVTIVVYQKFANLVQYGSKIAGPKAYPVVGNAIRFLSLTPAGFLEELH

ALSKEWGKCFRIWLGVDLLIFLMDARKVETILSSQKFLDKSIEYDFIRPWLGDGLLTSSG

RKWHSRRKIITPTFHFKILEEFVEIFDQQSTVFVDQLKLMAASGEPFDVYPRVTLMALDV

ICESAMGTKVNAQLNADSEYVRAVKAMTNIVIVRAFKILARLDFTFKYTSYAKIQKQALA

VLHGYTDNVIHSRRKELAKTHSQNDFSENDVGQRKKVAFLDMLLQATTTDGQPLTDLEIR

EEVDTFMFEGHDTTTSAMSFLLGVLAKHPEVQKKVYDEVRNVFGDDPSKPATLAMLNELN

YLDMVIKETLRLYPSVPMFGRKMMENHDIDGTIFPAGSNVIVMPYIMGRDPDYFEDPLEF

RPERFAVETSAEKSNPYRYVPFSAGPRNCIGQKFAVAEIKSLVSKTLRHYEILPDTSEEP

EKLYSEATLKNEKGVILRLKKRSFGIFTSK

>282721123

MIVFTGYLIAIVLILFPLYYYYAKCYALEKFYHQFPSPPKTPFFGNALDLKTTKDLFQSF

HRYVKAYGTMVHLKIGPFDHMLLCSNPKFLEFLLTSNTLLKKSPNLRYLVPWIGTGVATA

YGPKWKLHRKLVTPAFHYSVLSQFVNTFESSGNILVEKISKLCDGQKVDNFDLYPHITRC

TFDIICETAMGTKSNVQTNETSVYFESIKEMCRIFMSRTMAVFQRYDFIFRFSKDFHLQK

KALDIIHNYSNEVIATKRKELDENVKQKESVDDLGRKEKMVFLDMILNKAIDGHIFSQEA

IRGEVDTFMFAGHDTTATAISFILLCIANHNEVQDKIIEEQERLFGCEKNPKVTFAHLQE

MQFLENVIKEGLRLYTPVPLYGRQIDKNVEFEGMLIPKGVNVVIFNHGVHMNPEFYPNPE

KFDPNRFESMEDKHPFAFIPFSAGPRVCIGKKFAILEIKSILSKIVRNFELFPASPSYEL

DLAAETVLKSLNGIRIGIKRRETF

>EDS33501.1

MFWSLVLVPLVVLLFNYLVRDAKKKYSVMLPGPAKYPVFGSVHIYRKRDPEDTWSFITNL

SRKYGKLFCVWIGSRLALFCSNHSDTETVLSSQKLIRKSELYKFLIPWLGDGLLLSTGQK

WFNKRKILTPAFHFKILEQFVEVFDKQGTVLVQRLREQATGKLVNIYPFVTLAALDIICE

TAMGTSINAQTDTDSKYVRAVTELSYLLAGRFMKVWQRSDFLFNLSTDKKRQDKVIKVLH

DFTTSIIRSRREELLAEKIETNDTADEEGVGTRKKMAFLDVLLQASIDGRPLTDKEIQEE

VDTFMFEGHDTTTIAITFTLMLLAKHPDIQEKVYQEVTEIIGKDLNAPTTYRNLQDMKYL

ELVIKESLRLYPPVPIIGRKFTEKTEIDGKVVPEDSNFNVGIILMHRDPTQFEEPERFDP

ERFSPERTTELSSPYAYIPFSAGPRNCIGQKFAMLELKSTLSKIIRNYRLTDTGPEPKLI

IQLTLKPKDGLKVAFVPRAAA

>EDS33497.1

MAIWLLSILAIVFAVGVHLYQKFANLVHYGSKITGPKAYAVVGNAARFFSMTPAEFLQEV

HRLSKEWGKCFRIWLGPDLLIVVTDAKKVETVLGSQKYLDKSTEYDFIRPWLGDGLLTSS

GRKWHSRRKIITPTFHFKILEQFVEIFDQQSTVFVDQLKPMAASGEPFDVYPRVTLCALD

VICESAMGTKVNAQLNADSEYVRAVKAMSNVAFQRTYKILARLELTFKYTSYAQIQEKAL

AVLHGYTDSVVKNRRKELTKSNGDHNQNNVNDNDVGQRKKVAFLDMLLQATTTDGQPLTD

LEIREEVDTFMFEGHDTTTSAMSFILGVLAKHPEVQKKVYDEVRNVFGDDPSKPATLAML

NELNYLDMVIKETLRLYPSVPIFGRKMLENHDIDGTIFPAGSNVIIMPYIMGRDPDYFED

PLEFRPERFAVETSAEKSNPYRYVPFSAGPRNCIGQKFAVAEIKSLVSKTLRHYEILPDT

LAPPEKLLVELILRAENGVTLRLKKRN

>BAI77921.1

MAIWLLSILAIVFAVGVHLYQKFANLVHYGSKITGPKAYAVVGNAARFFSMTPAEFLQEV

HRLSKEWGKCFRIWLGPDLLIVVTDAKKVETVLGSQKYLDKSTEYDFIRPWLGDGLLTSS

GRKWHSRRKIITPTFHFKILEQFVEIFDQQSTVFVDQLKPMAASGEPFDVYPRVTLCALD

VICESAMGTKVNAQLNADSEYVRAVKAMSNVAFQRTYKILARLELTFKYTSYAQIQEKAL

AVLHGYTDSVVKNRRKELTKSNGDHNQNNVNDNDVGQRKKVAFLDMLLQATTTGGQPLTD

LEIREEVDTFMFEGHDTTTSAMSFLLGVLAKHPEVQKKVYDEVRNVFGDDTSKPATLAML

NELNYLDMVIKETLRLYPSVPIFGRKMLENHDIDGTIFPAGSNVIVMPYIMGRDPDYFEN

PLEFRPERFAVETSAEKSNPYRYVPFSAGPRNCIGQKFAVAEIKSLVSKTLRHYEILPDT

LAPPEKLLVELILRAENGVTLRLKKRN

>AAT38513.1

MLTVILLVILSPLIGWVIYWYYKVHYFEKYLHKIPGPKRLPLTGNALDIPSTLTMLPVLM

DYYKQFRTNFKIYIGAQPYLFLTEPKDLEFVMIRRLYWRNPIYTSSSQRWLGFGLLTSGG

NQWRKHRKIITPAFHFQILEEFIEVFNSQADVLVKKLKEESKKGTIDIYPFIARCTLDII

CETAMGTSVNAQDDYDSEYVKCVNILLEIAMLRSFSPILRSVLYPFTSMCRRENSALKVV

HDYTKSVIASRKQQFLSDADRNVESSDSLGRKRRRAFLDVLLEYSKTDPSFTEDHIREEV

DTFMFEGHDTTATSITFALQAIARHPEIQKKVYEELQTVFADDPNRKATYRDLQEMKYLE

MVIKESLRIYTTVPLLGRRIEKDVEWNGMTLPKGLMITMFVYCAQNSDSTFKDPAVFDPG

RFNAENSKGRHPYAYVPFSAGARNCIGQKFAMFEMKATMSSILRNLELLPPVPDHKIALK

NDGVLKSDNGVLIRLKMR

>EAA06490.5

MESLLGGSLLLSKLSKVFTLFSPVTLLLLTTVSCAIYVYNRRRAHIVRHIDKIPGPAGLP

ILGNTLHINVDHDEIFNRIIAIRKLYGRIQGFSRAWNGPLPYVMISKASAVERILGSQKH

IEKSHDYEFLKPWLGTGLLTSAGKKWHPRRKILTPAFHFKILDDFVDIFQEQSAVLVKRL

EAELGNEQGFNCFPYVTLCALDVVCETAMGRQVNAQCNSDSDYVKAVYQIGSIVQNRQQK

IWLQPDFIFKLTQDYKNHQKCLAILHEFSNRVIHERKEEIRRQKQQLGDKNNNNSNNNNG

GGGAAGTDQDGNNNKDDYLSHEELGRKKRLAFLDLLIEASQDGAVLSHEDIREEVDTFMF

EGHDTTSAAISWILLLLGTEPTIQDRIVEEIDQIMGGDRERFPTMQELNEMKYLEACIKE

GLRLYPSVPLIARRLTEDVDIDGYVLPAGTTAMIVVYQLHRNPEVFPNPDKFNPDHFLPE

NCRGRHPYAYIPFSAGPRNCIGQKFAVLEEKSIISAVLRRYRVEAVDRRENLTLLGELIL

RPKNGLRIRIARRT

>EAA06725.5

MAYGDSVLLTSSIGAGSNSWTRQQFIESVPKTSVLLLLSFIFIVCCLAAYLERYRRLVRH

INCIPGPATLPIIGNALLINADREELFNRIIASRKLYGRRQGITRIWNGMTPYVLISQAQ

AVEKILSSTKNIEKGRDYEFLQPWLGTGLLTSPASKWQHRRKILTPTFHFRILADFVEVF

NKQATVLVEKLAKELDNEAGFDCVRYITLCSLDIICETAMGCPVYAQRQSDSEYVRAHEK

IGEIMLNRLQKLWLHPDIIFRCTRQYREQQKCLDILHRFSYRMITERRSIIQTGSVVKQA

NTEDGLDANNNNCEGTGRKQLAFLDLLIEASDGGRILSDTDIREEVDTFILGGHDTTATS

ISWTLFLLGTDPTVQERVVQEIECVMGDGGERWPTMRELNEMRYLEACIKEGLRLYPSIP

IIGRRLTEDVRLADHVLPAGTNAVIVVYQLHRDPAVFPNPDRFNPDHFMVDASSSQEPRH

PFAYIPFSAGPRNCIGQKFGALEAKAVLVAVLRQYRVEAVDRRENLTLYGELVLRSKDGL

RIRITKRK

>EDS28597.1

MDVHLVAAKIANLLSVLSPVTTIILVMIVGAYVTYRKRLAKLEYHINKLPGPYSLPLIGN

GLQVSLGSKDDFLDRVVSAQKMYGRRIGLSRAWNGPFPYVLISKASAAEPILSNPRQVEK

SADYDFLKPWLGNGLLTSPASIWHPRRKTLTPAFHFKILSDFIPIFHRNALILAEKLEEQ

LGNRTGFDVVPLTTLCALDVFCETAMGIPVNAQRNSDSEYVRAHQLIGKIIRNRVQKFWL

QPDAIFKLTDEYRQHQECLQVLHSFSDRVIRDRHESLLKRRQAADLNNNNDPADDQPFRR

KQLAFLDLLIEGALDGNGLTLLDVREEVDTFVIGGHDTTAAAMAWLLLLLGTDQTIQERC

FAEIDQIMGGDRDRMPTLHELNEMKYLELCLKETLRLYPSIPMIARKLAEDVKIHNQYTI

PAGTNVMIVVYQLHRDPAVFPNPEKFNPDNFLPENSAGRHPYAYIPFSAGARNCIGQKFA

VLEEKTVLSTILRKFRIEAIERREDVSLLGDLVLRPRDGLRIRVSRRV

>EDS28598.1

MDKLVLLTVLSQLLSLVREFPLVATAAFVGVLFFYVKTFGVGPPGKSLTQKSLKSARQYI

NELPGPACLPLLGNSLMMATDREDMFNRLIAARCLYGRQQGMTRVWNGPVPYVLVSRAAV

VERILSSSVNIEKGRDYDFLRPWLGDGLLTAPAAKWYHRRKALNPTFNYKMLSDFLGVFN

KQAAVMARMLGKELGNGEGFDCTNYATLCSLDILCGNGGKTAMGCSIHAQEQHESEYVKA

HEEIGQIMLYRLQNIWLHPDLVFRFTKYYKRQNECLRVLHSFSEQVIQQRRALRESAPAD

FEDSSEIGRKRQLAFLDLLIETTQNGKPLSDRDIREEVDTFILGGHDTTATAIGWLMYLL

GTDAAVQDRLFEEIDSVMGQDRDREPSMIELNEMRYLDCCIKEALRLFPSIPLIARRLTE

DVQVENYVIPKATNAVIVVYQLHRDAKVFPNPEAFNPDRFLPENCCGRHPYAYIPFSAGP

RNCIGQKFGALEEKAVMVAVLRKFRIESLDRREDLTLYGELVLRSKNGLRVRIAKRD

>EAT40233.1

MLKEPLLLVITIASQLLHAVKEFPLPATVLLGVVIVVYLFAHADRDQLKSLLRINGAKDG

SKKSVKFYLNQLPGPQCIPLLGNSLMMATDREDMFNRLTTARKLYGRKQGICRIWNGRTP

YVLISKAEPVERILSSSVNIEKGRDYGFLRPWLGNGLLTCPGSRWYKRRKALNPTFNYKM

LSDFLEVFNRQAQTMVRLMEKELNRENGFNCTPYATLCSLDILCETAMGYPIQAQEQFGS

DYVKAHEEIGRIMLERLQKIWLHPDFIYKRTNFYKRQSECLKILHGFSENVIKQRRLQRD

ASLANKHDEDPSIEIGRKRQLAFLDLLLEATQDGQPLSDRDIRDEVDTFILGGHDTTATA

IGWLLYLLGTDLQVQDRVFEEIDSIMGQDRDRPPTMIELNEMKYLECCIKEALRLFPSIP

LIARKLTESVNVGDYTIPAGTNAVIVVYQLHRDTQVFPNPDKFNPDRFLPENSQGRHQYA

YIPFSAGPRNCIGQKFGLLEEKAVAVAVLRKYRITSLDRREDLTLYGELVLKSKNGLRIS

ISQRQ

>ACZ48687.1

MEEGRSWLGVGSPGLPTYILLTTCLAVVMTWIYKRQQKVWLVEQMPGPKALPLVGNSLFF

WGSPEVLFQQLYKVTEFGAVARFWLGPKPYCLLSSAKAVEAILSSQKHLHKSWDYSLLHP

WLGEGLITSAGKKWHSRRKLLTPAFHFRILEDFLDVFTSQTDTLVRRLRAQADGRPFDVF

HYITLCALDIICETAMGRRVNAQEDSESDFVRAVHDLSSLIQFRQFRPWLHPDFVFHLTS

HGRKHDACLKVIHGLAKQTISMRRKVRRTKGFGAQKKAQEDDIGQKTRQAFLDLLLEYSE

KDPTIINEDILEEVNTFMFAGHDTTTAAMNWFLYAMGTHKEIQTRVQEELDEVFQGSDRP

PTMADLRELKYLELCMKESLRVFPSVPSIIREIKEEIQINNYRIPAGTSIAIHVYRIHRD

PEQFPNPEVFDPDRFLPESCNKRHPYAYIPFSAGPRNCIGQKFAQLEMKVVLSSILRNFR

VESDIPWKDMKVLGELILRPKEGNPLKLHPRK

>EFX87569.1

MNLLLVLHNIFGRKSASGMVRSWIGPLPMFFATTAEAAEVVLSSQKIITKSREYEFLQPW

LNTGLLTSTGSKWQGRRKLLTPAFHFKILEDFVHVFDEQSRILVNKLNQAVVKSNELNIF

PFVTLCTLDVICETAMGRNVDAQSKTDSDYVQAVYNMSQLIQHRQIRFYLWPDWMFKLSS

HWPEQCKALDILHGFTNKVSALYANDYFKYLEPVIRERKLEHQQNKRQTPDIVEDSQKTE

DAFTSPKRRRLAFLDLLIEASKDGTVLSDSDIREEVDTFMFEGHDTTSAAITWSILLIGS

HPEVQELVNEELDRIFGDSDRPVTMTDLNELKYLECCIKEALRLYPSVPIISRTCQEDVI

IGDDEIPAGTSVSICPYFLHRDPKYFPDPELFQPKRFQAENSEKRHPYSYVPFSAGPRNC

IGQRFALLEEKSIISAIFRNFHVRSLDKREEIILMAELILRPRDGIRVHLEPKKKQ

>EFX78587.1

MYHLQEAERDPKSYCIQEILSGYSNVTLFSLTAIFSIFVLVALIKRVIFVKRINRIPGLP

CGITIIGNAPTFLVPPEEILNRLTGYVAAMQSSGPVLRSWAGPFPFFILFTAESFEVILS

SRKLIDKSRDYNYLQPWLNTGLLTSTGRKWLKRRKMLTPTFHFKILEDFVQVFNEQSQNL

IQQIHDAIKLKNEIDVYPFITRCTLDIICDTAMGCNVDAQAKSDSEYVKAVYTMSEIVAA

RQSRPWIQPNILFQMSEYSSKQRKVLRVLHSFTDEVIRRRKMERIEQTTNKITAQYQEDD

SFVAKKKRLALLDLLLDASKNGEVLSDLDIREEVDTFMFEGHDTTTAAINWSLLLIGSYP

QVQERLNEELDRVFGGSDRPATMADLSELKYLECCIKEALRLYPSVPIIGRKLNEDTVIH

GYKLPANTTVGLMTYILHRDPKHFPDPELYQPERFFETNSRGRHPYAYVPFSAGPRNCIG

QKFALMEEKVILSSMFRNFHIKALDKREELILLIELILRPRDGIRLLLTPKQKD

>AFP49818.1

MELITLMLTTTLLAFIVMYFFTHANKRIQLVRTMNKLPGPKEYPLVGNSLELAVPRNQFF

KIFDDRTRKWGPIFRTWEGPLAVLHITRPEHAEIILASSKHIDKSLVYTFLHPWLGTGLL

TGTGAKWHSHRKMITPTFHFKILDIFQEVFVEKCQLLVEKLKSKANNEPFDIYPFITRCA

LDIICETAMGTEINAQEKTDSDYVRAIYDISELTLKRSFQPWFWPDLVFNMTDYGKRYSE

CLSVLHGFTTRVIKERKALRSSSNGKHIEQTVDEDAELLGKKKRLAFLDLLLEASENSNG

SALTDIEIREEVDTFMFEGHDTTTAGICWTLFLLGSHPEYQDKVAEELNNIFQGDNRLAT

MKDLNDMKYLERCIKDSLRLFPSVPFIGRTLKEDTSFDNYQVPKGTLVNLQIYHIHRCKD

QWPNPEKFDPDNFLPERISKRHPYAYVPFSAGPRNCIGQKFALLEEKTMLSAVLRNYRVE

SHEKFEDLTLMNELILRPESGIILKLTPRS

>EFX87776.1

MRSCGPVLRVWAGPFPNFLLFTPEAFETVLSSKKLIDKGTEYGFLHPWLGTGLLTSTGSK

WSTRRKMLTPAFHFKILEDFFHVFNEQSQVLIRKLGAISTTEKDGFDIYPFISRCTLDII

CETAMGCKIEAQTHIDSEYLQAVLSMCRILNVRTSQPLMRSDILFKLSSHGAQQRRNLKI

LHGFTDGVIQKKKLERKQLNLINKEPTSVVNNSEKDDFVPTKKRLAFLDLLIEASKDGEL

LSDSDIREEVDTFMFEGHDTSTAAISWSLHLIGSHPPVMELVNKELERVFGNSNRSVTMN

DLNELKYLECCIKEALRLFPSVPITARNLREDTVIHDYILPANTTVLLVTYFLHRDPKYY

PDPELFQPERFFEENSRGRHPYVYVPFSAGPRNCIGQKFAMMEQKVILANIFRNFHLQAK

DKRDEIVLLNEVVLRPRDGIRVHLTPRLKESL

>EDS30083.1

MYGRRIGLSRAWNGPFPYVLISKASAAEPILSNPRQVEKSADYDFLKPWLGNGLLTSPAS

IWHPRRKTLTPAFHFKILSDFIPIFHRNALILAEKLEEQLGNRTGFDVVPLTTLCALDVF

CETAMGIPVNAQRNSDSEYVRAHQLIGKIIRNRVQKFWLQPDAIFKLTDEYRKHQECLQV

LHSFSDRVIRDRHESLLKRRQTTDLNNNNADDDQPFRRKQLAFLDLLIEGSLDGNGLTLL

DVREEVDTFVIGGHDTTAAAMAWLLLLLGTDQTIQDRCFAEIDQIMGGDRDRMPTLHELN

EMKYLELCLKETLRLYPSIPMIARKLAEDVKIHNQYTIPAGTNVMIVVYQLHRDPAVFPN

PDKFNPDNFLPENSAGRHPYAYIPFSAGARNCIGQKFAVLEEKTVLSTILRKFRIEAIER

REDVSLLGDLVLRPRDGLRIRLLSLVREFPLVATAAFVGVLFFYVKTFGVGPPGKSLTQK

SLKSARQYINELPGPACLPLLGNSLMMATDREGS

>EFX87944.1

MSAVLMLASYSWIGWGPFTTWTVLAVLLVVYYLMWSQSRNVRLVNTLPGPDFLPFLGNFL

DLNVDLDEFLKLVHFDWVKKYGPIYRAWGGFRPVAILSSPELMEPILVSQKLITKATEYS

YLSPWLGNCMFLTTGARWKNRRRLLTPAFHFQILNSFVDVFNEKSVDCARQLNRAIDIHR

DAEFDVFPIMTQCALDIICETSMGRQTRSEEEKAIYVKNLHRIGQIVMERGIRPWLTFDW

IYQFSALGRENQRCVTALHAFTNQVIKDRREALKREAETTSQDNNNVQEDKCDAPKERLA

FLDLLIKASETNADFNDDDIREEVDTVMFAGHDTTASAMTWFLYCIAKHPEHQQMVMEEV

DQVFGGDAERPCSTQDAAQLKYLECCIKETLRLYPSVPAVMRSLTEDIDIGGYTLPAGVS

VALMIYGMHHSPLVYPDPDAFKPERFLPENSVGRHPYAFIPFSAGPRNCIGQKYGILEIK

IVLANLMRQFRFAVADASQPMLTPSSEVVLKPKHGVPLIVSKRTTTALMA

>EFR25255.1

MFSTITGARMLYGRRQGFNRVWAGLKPYVLISKASAAEVGLDLKPQVSEAVFFTQYLRFT

QHLTWATWQRILSSSKNIEKGRDYDLLKPWIGTGLLTSHAVKWHQRRKLLTPTFHFKILS

NFVEVFNKQSYELVRQLEKQLGNEDGFDCTIYATLTSLDIICETAMGFPINALQKSDSEY

VQAHEKIAEIILDRLQKFWLRPDWIFRFTKSYAEHERCLKVLHDFAYNMIRERRALYRQR

KQEAEQEKEKQAAGDTGSEATDQGTVGGRRQLAFLDLLLELSEDGQLLTDDDIREEVDTF

ILGGHDTTATALAWMLYLFGTAPAVQEQAYQEIVSIVGPDPARHPTMAELTEMRYLECCI

KESLRLFPSIPMLSRTLVQEVDIDGYHIPAGTNAVIMVYQLHRDPQYFPNPEKFNPDRFL

PEAVVGRHPYAFIPFSAGPRNCIGQKFGALEEKAVLSAVIRHYRIEAVHRREDLTLYGDL

VMRTKDGLKIRLTRRD

>EFX68624.1

MDLFYNIYLCYLLVFVSSLKLTAGLSLYEKVQLLETNYDEIKGNLECKILNLETKVSTLE

EKITELVEKVELQGSKITAQESKITAHESKITAQGSKITAQESKITTHESRITAQESQIT

AQESKITAQGSKITAQGSKITAQESKITAQDSLITVLKNQLENATKSVPNSVVIDRQQVG

NSNPIYRTCHEINEANPSLDSGIHWIDPDGKGIGDDAIAVYCNMTSGSTSIFHDLTDIKT

DVGHCAERGCYSRKINYNVTNRQIAALIGLSFECHQSITYDCIGAPLQFNGVDFSCAELI

KIVLNFTNAVNDKNNSLLRTSYGGTLGYLVLSRVLSFFLVKSQITWANYVCRADVYWFVD

RIRSRFVRLINALPGPDALPFLGNFMDLNVDHDEYLKIVHFDWIKKYGAIYRLWLGHPAV

LISSPEFMEPILTSQKLISKAVEYRYLNPWLGNCMFLTTGSRWRNRRRLLTPAFHFQILN

SFVDVFNEKSADCAREFTKAIEAHGDENEIDIFPMMTQCALDIICETSMGRQTRNETEKA

IYVKNLHRIGQIVMERGIRPWLAFDWIYKLSSLGRENQRCVKALHTFTNQVIRDRREILK

RKANCHTDMPKSSLYNNNNVIFQDTQEFHPKERLAFLDLLIKASETNADFSDDDIREEVD

TVMFAGHDTTASAMSFFLHCIAKYPEHQQLVLQEVDAVFGDSDRDCSVQDAAELKYLECC

IKETLRLYPSVPAIMRCLTEDIEIGGYKLPSGTSVALMIHGMHHSPEVFPDPETFDPKRF

LPENSIGRHPYAFVPFSAGPRNCIGQKYGMLEIKVVLANLMRRFRFSVSDPSAPLIIPSS

EVVLKPKHGIPLILSKRSFGE

>EAA44936.4

MVVVVQIGLALLVVILTINYLLVRQNIKYGRHIPGPVPLPIVGCFYLYINLKPEDIIDFV

SDLRKRYGNLFRVWIGNRLALFCTNVKYNETVLSSQKLIRKSELYKFLIPWLGDGLLLST

GQKWFGKRKILTPAFHFKILDQFIEVFHKQSSILADRLRPEANGQLVNIYPFVTLAALDI

ICETAMGTSINAQTDADSAYVKAITELSLVLTGRFVKVWQRVDFLFNLSPDKRRQDRIIK

VLHDFTTKIIQSRRRELMEQGGGGGGTVEDDDMADLGTKRRMAFLDVLLQATIDGRPLTD

REIQEEVDTFMFEGHDTTTIAISFTLLLLARHPEVQERVYREVVAIVGNDPATPATHRNL

QDMKYLELVIKESLRLYPPVPIIARRFTENVELGGKIVPEGSNFNIGIMHMHRDPTLFPD

PERFDPERFAPDRTMEQSSPYAYVPFSAGPRNCIGQKFAMLELKSTVSKVIRHFKLTAAG

PEPKLTMQLTLKPRDGLYIGFVPRR

>EEB15501.1

MFSVILSIIVIFLIWKLFKLIEKEYILSKIPGPFTYPLLGNVEVFEPIEVTLGNILKRKE

KYGPVYKMRAGFLVGVFLTRAQKIEDLIKGMKHLEKSHVYKFVHPWLGTGLLTAHGEKWA

SHRKLITPSFHFTVLQSFIEIFQENSNILINKLNDVADSDKAVDIFPFITLCLLDVICET

AMGTKINAQTDSENEYVKSVYSLSSLIIHRLNRAWLHPDFIYNLTSHGREHKKHLDIVHN

FTDSVIRARKLLFENESSTNFDPSEKRKLSFLDLLLKASINEASTPLTDVELREEVDTFM

FEGHDTTAAGVNWAILMLSHHPEIQEQAYEEVKTIFENKKGKDLTLGDLSEMALLERIIK

ETLRLYPSVPTIGRHIDEDTQIGDYLIPKGSNTVLVIYAVHRDPKVFPNPDVFDPDRFLP

ENSADRHPFAFIPFSAGPRNCIGQKFAMYEEKVVLSNLIYNYRFESVGKLEDVIKIPELV

LRPKNGIPVKVYKRN

>157116996

MFWSFILVSLLLLVINLCLKDAAKQRSVKIAGPASVPILGNAYLYWNRRPDDVMDFIFTL

SRKYGTMFRVWVGTRLALFCTNTPDTETVLSSQKLIRKSELYKFLVPWLGNGLLLSTDQK

WFNKRKIITPAFHFKILEQFIEVFDRQSGILVQKLKPEASGKLVNVYPYVTLCALDVICE

TAMGTPINAQTDVDSKYVRAVTELSYLLTTRFVKVWQRSDFLFNLSPDRKRQDKVIKVLH

DFTTNIIQKRRKELMDHGDSGISGDDSIGSKKKMAFLDVLLQASVDGKPLTDKEIQEEVD

TFMFEGHDTTTIAIAFTLLLLARHPEVQEKVYKEVTEIIGTDLSIPATYRNLQDMKYLEM

VIKESLRLYPPVPIIGRKFTEKTTIGGNVIPEDSNFNLGIIVMHRDPKLFDDPEKFDPER

FSPERTMEQSSPYAYIPFSAGPRNCIGQKFAMLELKSTLSKVIRNYRLTEAGPEPQLIIQ

LTLKPKDGLKIAFVPRA

>EAA14939.4

MSSYSTVDMLLVVLVLLLLTPVLQWLIRRVQMSQIYDKIPGPKAYPFVGTLYLLFGKKSH

GKIFSHTRARVTRQYDGIHRIWVGMTPEVRLSRAEYVEQIIGASKHTEKATLYRFLGDWL

GEGLLTSKGERWFQHRKLITPTFHFNILDGFCEVFAENGAVLVERLQRHANTGQPVNIYP

YVTKAALDVICETAMGVQVHAQTAGEDNAYVNAVYELSSLFLERLVRPWLHPEWTFRRSA

LGRRQAQLLAILHGYTRKVIQERRRTLDQQHAGERNQPEAEGDGLGRRKRLAFLDLLLQS

ATTGTGSPLLTDEDVREEVDTFMFEGHDTTTAGMSWALFLLALHPEVQERVHQEIDSIFG

GSDRPATMQDLTAMRLLERCLKETLRLYPSVAFFGRTTSKDVTLGGYHVPAGTIVGIHAY

NVHRDERFFPDAETFDPDRFLPERTAENRHPYAYIPFSAGPRNCIGQKFALLEEKCLVSS

ILRRFRIRSHRTRAEQLIVNELITRPKDGILLYLEEREW

>EKC32053.1

MAAAIVIILAVAVVLFTTWLLKTELARKKVNTIPGPVAVPILGNAHQVILQSSKHLDKAE

EYKFLHPWLGTGLLTSTGSKWKSRRRMLTPTFHFKILNDFVGVFHDQASIMVKKLSQVAD

GKEFNIFNYITLCALDIICETAMGRSIEAQSNSNSDYVKSVYKMSELVLQRQRTPWYWPD

FLYNTIGYGKEHDRCLKILHDFTLKVIKEKMDAVSDASGNSMEEMLNQHSDEDVYRGKGQ

RLAFLDMLLCTADDGSHLSMEGIREEVDTFMFEGHDTTAAAVNWAVHLIGANPDVQAKVH

QEMDQLFGGSDRPASMNDLKEMRYLECCIKEALRLFPSVPGFGRKLTEDCKFGEYSIPKD

TTVIITPPDLHRDKRYFPDPEKFDPNRFLPENSLKRHPYCYVPFSAGPRNCIGQKFAILE

EKVMLSNIFRNFTVTSKQSREELFPIGDLIMRPEHGIIVELRPRTT

>EAT40472.2

MSILDWILVITGAVLAINYLLVRRNLKYQSQWPGPAAVPLIGCYYLYFNKKPEDVMDFIF

TLSRKYGTMFRVWVGTRLALFCTNTPDTETVLSSQKLIRKSELYKFLVPWLGNGLLLSTD

QKWFNKRKIITPAFHFKILEQFIEVFDRQSGILVQKLKPEASGKLVNVYPYVTLCALDVI

CETAMGTPINAQTDVDSKYVRAVTELSYLLTTRFVKVWQRSDFLFNLSPDRKRQDKVIKV

LHDFTTNIIQKRRKELMDHGDSGISGDDSIGSKKKMAFLDVLLQASVDGKPLTDKEIQEE

VDTFMFEGHDTTTIAIAFTLLLLARHPEVQEKVYKEVTEIIGTDLSIPATYRNLQDMKYL

EMVIKESLRLYPPVPIIGRKFTEKTTIGGNVIPEDSNFNLGIIVMHRDPKLFDDPEKFDP

ERFSPERTMEQSSPYAYIPFSAGPRNCIGQKFAMLELKSTLSKVIRNYRLTEAGPEPQLI

IQLTLKPKDGLKIAFVPRA

>391339635

MQSASVVATLLSTICLGVLVLYVFHLLRRKYFEHKMKLPGLRPEWWNPFGIEYELWNMMR

DNGYNGGQAIFNFLLRVTREFKEYPTFMGWVFHVPILFVTTPESMEFVMSSTTLIEKSRF

YELLHDWLGTGLLTSEGAKWRTRRKMLTPAFHFKILEDFLDVFNEQGIILADKLKGRSRT

GEMFDVTKDVTSCTLDIICETAMGVRIGAQTNPESDYVKSLYTLGESFMDRSFAPYYWTK

PAYKLSRKGREFFKSIDTMQSFTMKVIRERRAEIQNTLENDKSTDNDIGRKTRKPFLNLL

LERHIKEGDLSLEDIQEEVDTFMFEGHDTTAMGISWTLFLLAQNPEAQRRVYEELEEIFR

GDQKRHATNEDLARMKYLECCIKESQRLYPSVPFIGRKFTTDTEFKKKTIPAGTQALLVI

FTLHRDEKTFPDPERFDPDRFLPENCEGRHPYAYVPFSAGPRNCIGQKFAMMEEKVVLSW

VFRKVALETNLRREDLRVAGELVTRSLNGLSLKSKALQDTVI

>EGK96893.1

MVYSTLAVLLALGSLAAVYLYRRFGEMLKHTGKLGGPPAYPILGNGLMFLNKSPADFLRI

IGELLQRYGDCFRVWLGTQLVIVMGDPKDIEVLLSSPKYIDKSTEYDFIRPWLGEGLLTS

RGRKWHTHRKVITPTFHFKILEQFVEIFDRQSSTFIKVLEPFAASGKSFDIFPQVTLCAL

DVICESAMGTKVNAQINSTSDYVLAVKEITNLIQLRFYDFLIRYDIFFRLSANARKQRKV

LQVLHSYTDSVIKGRRQQLASIQQSGEQEEASDQDLGIKKRMAFLDMLLQSTIDGRPLTD

LEIREEVDTFMFEGHDTTTSAISFLLHSLAQNPTIQEKVFDEVRNVVGDDRTRPVTMAML

NDMHYLDLVIKETLRLYPSVPMIGRKMQQTAEINGKIIPAGANLIIMPFFLGREARYFPE

PEKFDPERFNVERSAEKTNPYQYIPFSAGPRNCIGQKFAVAELKSLVSKVLRHYEILPPT

GKQDESFIAELILRPEHGVFVRLKPRAY

>ACD75824.1

MFVALLLVGIVTIVGTLVWMLQTAKFRQQISLIPGPPTIPLLGNVHQMTTGPGWYKQVLK

WGDEFRKQGIFRIMLISKPVIGVTCAETAEVMLNSSKHIDKAQEYGYLHAWLGTGLLTST

GDKWRMRRKMLTPTFHFRILHDFVEVFNQQTCVLIEKLKHHVDKGQFNVFQDIALCALDI

ICETAMGQHVNAQTKESAYVQSVYKMCGFVEKRMRTPWHYNKTMYDLFGPGEVHDHHLKI

LHEFTIKVIKDRMSNFNAERAQRLLDDANKKKDWVNCSESETNCQKTRLAFLDMLLFMSD

NGKVLSIDDIREEVDTFMFEGHDTTAAGMNWCTYLIGSDEKVQGKVCEELDRVFGNSDRM

PTMDDLKELKYLECCIKEAQRLFPSVPYFGRTTTEEAQISKSSNCSFRVPKDVTVIVFTS

AIHRDTRWFPNPEHFDPDRFLPENSVGRHPFAYIPFSAGLRNCIGQKFAMMEEKVILSSI

FRNFKVKSCQSREELLPVGELILRPQKGIFIELSAR

>EKC32054.1

MESFMNLYADRGIFRLWLGTTFLVVIFKAEYAEVILQSSKHLDKAEEYKFLHPWLGTGLL

TSTGSKWKSRRRMLTPTFHFKILNDFVGVFHDQASIMVKKLSQVADGKEFDIFNYITLCA

LDIICETAMGRSIEAQSNSNSDYVKSVYKMTELVLQRQRTPWYWPDFLYNTIGYGKEHDR

CLEILHDFTLKVIKEKMDAVSDASSNSMEEMLNQHSDEDVYRGKGQRLAFLDMLLCTADD

GSHLSMEGIREEVDTFMFEGHDTTAAAMNWAVHLIGANPDVQAKVHEEMDQIFGGSDRPA

SMNDLKEMRYLECCIKEALRLFPSVPFFGRKLTEDCKFGEYSIPKDTTVIITPPGLHRDE

RYFPDPEKFDPNRFLPENSLKRHPYCYIPFSAGPRNCIGRNFTVTSKQSREELFPIGDLI

MRPEHGIIVELRPRTT

>EDS33496.1

MALLLVLISVAIAVTIVVYQKFANLVQYGSKVAGPKAYPVVGNAIRFLSLTPAGFLEELH

ALSKEWGKCFRIWLGVDLLIFLMDARKVETILSSQKFLDKSIEYDFIRPWLGDGLLTSSG

RKWHSRRKIITPTFHFKILEEFVEIFDQQSTVFVDQLKPMAASGEPFDVYPRVTLMALDV

ICESAMGTKVNAQLNAYSEYVRAVKAMTNIVIVRAFKILARLDFTFKYTSYAKIQKQALA

VLHGYTDNVIHSRRKELAKTHSQNDFSENDVGQRKKVAFLDMLLQATTTDGQPLTDLEIR

EEVDTFMFEGHDTTTSAMSFLLGVLAKHPEVQKKVYDEVRNVFGDDPSKPATLAMLNELN

YLDMVIKETLRLYPSVPMFGRKMMENHDIDGTIFPAGSNVIVMPYIMGRDPDYFEDPLEF

RPERFAVETSAEKSNPYRYVPFSAGPRNCIGQKFAVAEIKSLVSKTLRHYEILPDTSEEP

EKLYSEATLKNEKGVILRLKKRSFGIFTSK

>383859846

MSAAGPEIVAGSVAAASGFSATTVFLSLLIPAVVLYLIYFRISRRHLIELGEKLPGPPAL

PVIGNALDLLGSSDTIFQNVYRRSFEYQDVIRLWVGPKLVVFLIDPRDVEVILSSHVYID

KSAEYRFFQPWLGDGLLISTGPKWRAHRKLIAPTFHLNVLKSFIDLFNANSRAVVEKMRK

EGNKEFDCHHYMSELTVEILLETAMGVSKSTQDRSGFEYAMAVMKMCDILHLRHTKIWLR

PDWLFNLTKYGKDQIKLLEIIHGLTKKVIQRKKEEYKSGKRNIIDTSNQKSDAKTTSVEG

VSFGQSAGLKDDLDVEDDVGEKKRQAFLDLLIEAGENGVVLTDREVKEQVDTIMFEGHDT

TASGSSFFLAMMGCHPDIQEKVIQELDEIFGDSDRPATFQDTLQMKYLERCLLETLRMYP

PVPIIAREIKTDVKLASGDYTVPAGSTVVVATFKLHRQPHIYPNPDTFDPDNFLPEKTAN

RHYYAFVPFSAGPRSCVGRKYAMLKLKIVLSTILRNFRVRSDIKESDFRLQADIILKRAE

GFKVRLEPRKVVAATA

>189238680

MDFTYLLLFTFFTIGFVNFCKWLKDRKRIWDAIEPIGGEKWYPLIGTCLELIKTKRKDFY

DVYCARNMKYGPFFRTWMGFIPAVHVMKPDHVERILSSTVNLTKGDNYRFVVPWLGEGLI

TGSNHKKWRVHRKLITPTFHFSILDNMMEVMAEKGQFLAEQLVPKANGQFFNIYPFLTLC

ELDIICETAMGVEVNAMKHSQSEYVRSVYGISDIMLYRMFHPYLHPDFVFNLSSKGRQHK

KYLSILHGFTRKVIQERKEKLTSGRDVVQELSEEDKLLGKKKRLAFLDLLLEANKNSEGG

LTDEDIREEVDTFMFAGHDTSTVTVGWTLFTLSNYPEYQEKVHQELDEIFQGEERPITPQ

DVLKMQYLDKVIKETQRLIPVVPVIARTLDQDLEIGGRTIPAGVMVVIHLARLHKDPDQF

PEPDRFDPERFLPENVSKRHPYSFVPFSAGPRNCLGQKFALRNTKVLLASILRKYKVRAE

KKIDEMKYNIEIVLRPQGGLSVALEPRRR

>340722435

MSTAGPEIVAGSMAAASTTGFSATTVFLSLFIPAVVLYLIYFRLSRQHMLKLAESLPGPP

ALPIIGNALDLLGSSDTIFQNLLAKAADYKDVVRLWIGPKLVVFLFDPRDIEIILSSNVY

IDKSAEYRFFYPWLGDGLLISTGQKWRSHRKLIAPTFHLNVLKSFINLFNANARDVVERM

RKEGSKEFDCHDYMSELTVEILMETAMGVSKTTQDRSGFEYAMAVMKMCDILHLRHTKVW

LRPDWLFNLTKYGKDQIKLLEIIHGLTKKVISRKKEDYKSGKRNIIDTSVQKNEKGDNAT

VVEGVSFGQSTGLKDDLDVDDDVGEKKRQAFLDLLIEAGQEGVILTDKEVREQVNTIMFE

GHDTTAAGASFFLALMGCHPDVQEKVIQELDEIFGDSDRPATFQDTLEMKYLERCLLETL

RLYPPVPIIAREIKTDLKCMSGNYTIPAGCTVVIATYKLHRHPSIYPNPDVFNPDNFLPE

NTAKRHYYAFVPFSAGPRSCVGRKYAMLKLKIILSTILRNFRIKSNSKESDFRLQADIIL

KRADGFNIKLEPRKVVAATA

>EEB15502.1

MLQLLGEKLNYASFPLIILLIFILLKLFGEDEKTKIINKLPGPFAYPLIGNIEILEPVEV

FMDNILIRKKKYGPIFKMRAGPRYGIFITRAREVEDLITGMKNTEKSFVYKFVQPWLGTG

LLTAHGKKWASHRKLITPSFHFTVLQSFIEIFQENSNILINKLNKMADTNEVIDIYPFIT

LCLLDIICETAMGTKINAQTDKTNEYVKSVYRLSTLIVKRMGRIWLHPDFIYNLTSHGKE

NRKHLDIVHNFTDSVIQTRKLLFENESSTNIDPSEKRKLSFLDLLLKASSNEASTPLTDV

ELREEVDTFMFEGHDTTAAAVNWAILMLSHHPEIQEKAYEEVKTVLENKQEEGLSLGDLS

EMKLLERVIKETLRLHPSVPMIGRRIEVDTRLGEYFIPEGVSAVISIYALHRDPEVFPNP

DVFDPDRFLPENSADRHPFAYIPFSAGPRNCIGQKFAMYEEKVILSNLIYNYRFESVGKL

NDVIKIPDLVLRPKNGIFVKIYNR

>EAU77127.3

MWPIIVSGTALLLSIAIWWTVANFGSALRYAGKLGGPVAYPLVGNGFLFINKTPAEFLQL

LGRLIAQYGKCLRVWLGTQLIVLITDPKDIEILLSSPKYIEKSSEYDFVRPWLGEGLLIS

SGRKWQTHRKIITPTFHFKILEEFMDIFDQQGNTFVDILHLFAKSGETFDVFPLVTLCAL

DVICESAMGTKVNAQMNSESEYVKAVKEITNLIQLRIYDFVIRYEFFFRLSANRRRQRKA

LAILHGYTDSVIRTRRQELNERGAGHNDVPEEDVLGRKKRMAFLDMLLQSTVDGRPLTDL

EIREEVDTFMFEGHDTTTSAISFMLHSLAQNPDVQEKAFNEVRNIVGDDRKQPVTMAMLN

DMHYLDLVIKETLRLYPSVPLFGRKMLQNTEINGKIFPAGSNVIVLPFFMGRDPDCFANP

EKFDPERFNVERSAEKTNPYQYIPFSAGPRNCIGQKFAVTELKSLVSKVLRNYELLPPEQ

VREETFIAELILRPEHGIPIRVKQRVY

>350416638

MSTAGPEIVAGSMAAASATGFSATTVFLSLFIPAVVLYLIYFRLSRQHMLKLAESLPGPP

ALPIIGNALDLLGSSDTIFQNLLAKAADYKDVVRLWIGPKLVVFLFDPRDIEIILSSNVY

IDKSAEYRFFYPWLGDGLLISTGQKWRSHRKLIAPTFHLNVLKSFINLFNANARDVVERM

RKEGSKEFDCHDYMSELTVEILMETAMGVSKTTQDRSGFEYAMAVMKMCDILHLRHTKVW

LRPDWLFNLTKYGKDQIKLLEIIHGLTKKVISRKKEDYKSGKRNIIDTSAQKNEKGDNAT

VVEGVSFGQSTGLKDDLDVDDDVGEKKRQAFLDLLIEAGQEGVILTDKEVREQVNTIMFE

GHDTTAAGASFFLALMGCHPDVQEKVIQELDEIFGDSDRPATFQDTLEMKYLERCLLETL

RLYPPVPIIAREIKTDLKCMSGNYTIPAGCTVVIATYKLHRHPSIYPNPDVFNPDNFLPE

NTAKRHYYAFVPFSAGPRSCVGRKYAMLKLKIILSTILRNFRIRSNSKESDFRLQADIIL

KRADGFNIKLEPRKVVAATA

>Q964T1.1

MDITVFLSLTVVVFLAVIIFYGRNEERKRMLQKIPGAPSLPLIGTALPILYRRKEDRMTW

IEEILEKYKPLILWYFGNRPFVNISSPELIEVVLRNTQLIDKAFLYDLFHSWLGTGLLTS

SGAKWHQHRKIITPTFHFSILEGFITIFAEKSEILVRKLQKEVGRGPFFIRQYVSNCALD

IICETAMGTSVNAQDEGFSEYVTAINKMTDVLSDRMANPLLYPEFIFKLTPYYWTHKKCL

KVLNGFVNKIIQERKEERKKSKVTQTSEDADIGKKKRVPFLDTLLDASEDDNKLTDTDIL

EEVHTFMFEGHDTVSAAMTWLLFELGHHPEIQEEAYKEVQDIFQGSDRVPTMADLNNMNY

LERVIKESLRLHPSVIYFVREAHQDFELGGYTIPAGTNIDFSVPFIHRNPEIFPNPRCFN

PDNFLPDRVVNRHPYAYIPFSAGPRNCIGQRFALLEEKVVLSYLLRHYRFRTVNKREDSK

FKLEMINTPVKPIQLIIEARN

>ACT34901.1

MLTSASLIYGKERIVSMWLGTRPFVFLFTPEAVEVVLSSNTLTDKAAEYDFLKPWLGLGL

VTSKGNKWKQRRKLLTPAFHFRILNDFLPIINENAEVLINKLNQLTDNKSGESKVIDILD

YITLTTLDVICETAMGIKVHCRTKESEYVSCLHQVSELFLIRISRPWLWPDWSFALSSHG

QRYKRALNVMKTFTMKVIKQRKAEWIESHSKCGQQSKSSSTNQLDHDDQISKKTTTEYGA

KRMAFLDLLMEHHLQNNVMTIEDVREEVDTFMFAGHDTTAMSISWTLYILGLYKDIQDKV

RDEIDSISESDYNNKDEQFTGLTINQLKQMKYLDCVLKEVQRVYPVAPFIGRELSEDTMI

NGYLVPKGTTCAIFTYLLHRNEETFPNPEHFDPDRFLPENCSGRHPYSYIPFSAGPRDCI

GQKFAVMEEKAILAMVLRSFSVRSVDERDKLVISGEMVLRSRNGLRIVLSRRKLGKDFDE

TSIGSKCNNNQLNCKHNSLSNQPVTRNI

>BAM17765.1

MGYTATENVVQSSTWAATNMFYVLLVPALILWYAYWRMSRRRLYELAEKLEGPPGLPLIG

NALDFTGGSDEIFRNIVSKSYPYDRESVIKIWIGPRLIVFLYDPRDVELILSSHVHIDKA

DDYRFFKPWLGDGLLISTGQKWRSHRKLIAPTFHLNVLKSFIDLFNANSRAVVDKLKKES

GVFDCHDYMSECTVEILLETAMGVSKSTQDQSGFEYAMAVMKMCDILHLRHTKIWLRPDL

LFNFTQYAKVQNKLLDVIHGLTKKVITRKKAEFQSGKKPTIVTEVEVPKNSSSKTTSVEG

LSFGQSAGLKDDLDVDDDVGQKKRLAFLDLLLESSQSGVVISDEEIKEQVDTIMFEGHDT

TAAGSSFFLSMMGIHQDIQDKVVEELDQIFGDSDRPATFQDTLEMKYLERCLMETLRMFP

PVPIIARHLKQDVTLPSNGKKVPAGATIVIATYKLHRRSDVYPNPDTFDPDNFLPERSAN

RHYYAFVPFSAGPRSCVGRKYAMLKLKIILSTILRNFRIHSDLKESDFRLQADIILKRAE

GFKVRLEPRKRMAKAF

>EFA07488.1

MDFTYLLLFTFFTIGFVNFCKWLKDRKRIWDAIEPIGGEKWYPLIGTCLELIKTKRKDFY

DVYCARNMKYGPFFRTWMGFIPAVHVMKPDHVERILSSTVNLTKGDNYRFVVPWLGEGLI

TGSNHKKWRVHRKLITPTFHFSILDNMMEVMAEKGQFLAEQLVPKANGQFFNIYPFLTLC

ELDIICETAMGVEVNAMKHSQSEYVRSVYGISDIMLYRMFHPYLHPDFVFNLSSKGRQHK

KYLSILHGFTRKVIQERKEKLTSGRDVVQELSEEDKLLGKKKRLAFLDLLLEANKNSEGG

LTDEDIREEVDTFMFAGHDTSTVTVGWTLFTLSNYPEYQEKVHQELDEIFQGEERPITPQ

DVLKMQYLDKVIKETQRLIPVVPVIARTLDQDLEIVASSRTIPAGVMVVIHLARLHKDPD

QFPEPDRFDPERFLPENVSKRHPYSFVPFSAGPRNCLGQKFALRNTKVLLASILRKYKVR

AEKKIDEMKYNIEIVLRPQGGLSVALEPRRR

>EGI61202.1

MSAAEPEMIAGSAAAASYGLSSSAIFLSLLIPALILYYIYFRISHRHMIELAEKLPGPEG

LPLIGNALMFLGSSDTMFRTLYRKSFEYDQIIKIWLGPKLLVFLMDPRDVEIILSSHVYI

DKSTEYRFFQPWLGNGLLISTGSKWRAHRKLIAPTFHLNILKSFIDLFNANSRAVVERMR

KEGNKEFDCHDYMSECTVEILLETAMGVSKSTQDRSGFEYAMAVMKMCDILHLRHTKVWL

RPDWLFNLTKYGKDQIRLLEIIHGLTKKVIARKKQEFKNGKRNVIDESKNSNGKTIKSTS

VEGLSFGQAAGLKDDLDIEDNDVGEKKRQAFLDLLMEAGQNGTVLTDEEVKEQVDTIMFE

GHDTTAAASSFFLSIMGCHPDIQEKVIQELDEIFGDSDRPATFQDTLEMKYLERCLMETL

RMYPPVPIIARTIKTDLKLVSGDYTIPAGSTVIVTTFKMHRQPHIYPNPEIFDPDNFLPE

KTANRHYYAFVPFSAGPRSCVGRKYAMLKLKIILSTIMRNYRVKSDIKESDFRLQADIIL

KRSEGFKIKLVPRKQAAATV

>380014369

MSTTGPEIVAGSIATASATGFSASTVFFSLLIPALVLYFIYFRISRRHMLELAEKIPGPP

TLPLIGNALELFGSSDAIFSKVMKKAENFKDVVKIWVGPKLMIFLIDPRDVEIILSSNVY

IDKSTEYRFFKPWLGDGLLISTGQKWRSHRKLIAPTFHLNVLKSFINLFNANAKSVVEKM

RKENGKEFDCHDYMSELTVDILLETAMGVSKPSRDHSAFEYAMAVMKMCDILHLRHTKIW

LRPDWLFNLTKYSKNQIKLLEIIHGLTKKVIQIKKEEYKSGKRNIIDNTQKTESKGNNIV

VEGVSFGQSVGLKDDLDVDDDVGEKKRQAFLDLLIEAGQNGVLLSDKEVKEQVDTIMFEG

HDTTASGSSFFLAMMGCHPDIQEKVIQELDEIFGDSDRPATFQDTLEMKYLERCLLXTLR

MYPPVPIIAREIKTDLKLASGDYTIPAGCTVIIGTFKLHRQPHIYPNPDVFDPDNFLPEK

TANRHYYAFVPFSAGPRSCVGRKYAMLKLKIVLSTILRNFRIKSDVKESDFRLQADIILK

RADGFKIRLEPRKPVATTA

>BAD81026.1

MSYTTAENVVPSSTFSAINLFYVLLVPAIILWYTYWRMSRRRLYELAEKLSGPEPLPIIG

NALEFVGGSNDIFNNIIAKSLPFDDEAVVRLWIGPRLLVFIYDPRDVEVILSSHVHIDKA

DEYRFFKPWLGNGLLISTGQKWRSHRKLIAPTFHLNVLKSFIDLFNANSRAVVDKLKKES

GTFDCHDYMSECTVEILLETAMGVSKSTQDQSGFEYAMAVMKMCDILHLRHTKIWLRPDL

LFKLTDYAKNQTRLLDVIHGLTKKVIKRKKEEFQSGKKATIMPEGNDVTNEVPSSKLTSV

EGLSFGQSSGLKDDLDVDDDVGQKKRLAFLDLLLESSQSGVVITDEEIKEQVDTIMFEGH

DTTAAGSSFFLSMMGIHQHIQDKVIEELDHIFGDSDRPATFQDTLEMKYLERCLMETLRL

YPPVPIIARHLKEEITLPSNGKKVPIGTTLIVGTYKLHRRPDVYPNPHKFDPDNFLPERS

ANRHYYAFVPFSAGPRSCVGRKYAMLKLKIILSTILRNFRVYSDLNESDFKLQADIILKR

AEGFKVRLQPRKKQAKV

>ACZ97413.1

MSYTTENVIQGGSWAFTSMFYVLLVPALILWYAYWRMSRRHMYELAEKLNGPKGLPLFGN

ALEFTGGSHDIFRNIVSKSTEFDDESVVKIWIGPRLIVFLYDPRDVELILSSHVHIDKAE

DYRFFKPWLGNGLLISTGQKWRSHRKLIAPTFHLNVLKSFIDLFNANSRAVVEKLKKESG

DFDCHDYMSECTVEILLETAMGVSKSTQGQSGFEYAMAVMKMCDILHLRHTKIWLRPEIL

FKFTDYAKKQTKLLDVIHSLTKRVIKKRKEEFKSGKKISLVSENSDGSSSVDAVTNTTTS

VEGLSFGQSSGLKDDLDVDDNDVGQKKRLAFLDLLLEASQSGVVISDEEIKEQVDTIMFE

GHDTTAAGSSFFLSMMGIHQDIQDKVIEELDLIFGDSDRPATFQDTLEMKYLERCLMETL

RMFPPVPIIARDIKRDIILPSNNKIVPAGATIVIGTYKLHRRPDVYPNPDKFNPDNFLPE

KSANRHYYAFVPFSAGPRSCVGRKYAMLKLKIILSTILRNFRVHSDLKEEDFKLQADIIL

KRAEGFRVSLTPRKKIAKAC

>EDV34238.1

MFLILGAVLASALFVGLLLYQLKFKRLINIISYMPGPPALPLVGHGHHFIGKPPHEMVKR

IFEFMELYGSKDQTLKVWLGPELNVLMGSPKDVEVVLGTLRFNDKAGEYKALEPWLKEGL

LVSRGRKWHKRRKIITPAFHFKILDQFVDVFEKGSRDLLRNMERDRLKQGETGFNLYDWL

NLCTMDTICETAMGVSIDAQTNADSEYVQAVKTISMVLHKRMFNIFYRFDLTYMFTPLAW

AEKKALNVLHQFTEKIIVQRREELIKSGGQAAAEADADVGAKRKMAFLDILLQSTIDDRP

LTNLDIREEVDTFMFEGHDTTSSALMFFFYNIATHPEAQKKCFEEIRSVVGDDKATPVTY

ELLNKLHYVDLCVKETLRLYPSVPLLGRKVLEDCEINGKLIPAGTNIGISPLYIGRREDL

FTEPNAFKPERFDVVTSAEKLNPYAYIPFSAGPRNCIGQKFAMLEIKAIVANVLRHYDVS

FVGDTSEPPVLIAELILRTKDPLMFKLKERVY

>EFX88383.1

MQSFYSQWISLIGFNWTTWFVLASCAVLAWICWRNSRYVRLINAVPGPKGIPILGNLLEL

NVGQVGNRIVPKFLRIVHREWVEKHGPIYRGWGGSRSIVCISSPELMEPILASQKLITKG

KEYSYLSRWLGACMFLTTGLRWRSRRRLLTPAFHFQILESFFDVFNDQSQQLIKELDAAA

AAARPAASVDGSVNVYKILTQCALDIICDSSMGRQVRKREETSGYLHSINRITQIVMERS

LRPWLDSDLLFNLSALGRENQRCVKILHDFTNQVIQDRKRMLNNNQSDSAADDADVKSQE

ESPKRRLAFLDLLIQASENGDKLSDDDIREEVDTFMFAGHDTTATAMSWFLYCIARNPEE

QKLLLDEVDEVFEDSDRPCTPQDAANLKYLDCCIKETLRMYPSIPGVMRTITEDTEIGGY

VLPAGLSVALLIYGMHRNPKVYPEPDAFKPERFLPEHSANRHPYAFIPFSAGPRNCIGQK

YAQFELKVVLSWVLRKFEFSLSDPSCPAVSASSEIVLKPVDGIHLSVKRRQPTIAQQSPL

KISTTTQQQQQQPN

>ADE05582.1

MSYAAAEHVVQSSTFSAVNLFYVLLVPALILWYTYWRMSRRRLYELAEKLNGPSGWPLLG

NALEFTGGSAEIFKNIVEKSIPYDQESVVKLWIGPRLLVFIYDPRDVEVILSSHVHIDKA

DEYRFFKPWLGNGLLISTGQKWRSHRKLIAPTFHLNVLKSFIDLFNANSRAVVDKLKKEA

KDFDCHDYMSECTVEILLETAMGVSKTTQDQSGFDYAMAVMKMCDILHLRHTKIWLRPDL

LFKFTEYAKNQTKLLDVIHGLTKKVIKRKKEEFQSGKKPSVLNNDVTSTEVPTSKTTSVE

GLSFGQSAGLKDDLDVEEDVGQKKRLAFLDLLLESSQSGVVITDEEIKEQVDTIMFEGHD

TTAAGSSFFLSMMGIHQDIQDKVIEELDQIFGDSDRPCTFQDTLEMKYLERCLMETLRMY

PPVPIIARHLKQEVTLPSNGKKVPAGTTLVVATFKLHRRPDVYPNPEKFDPDNFLPERSA

NRHYYAFVPFSAGPRSCVGRKYAMLKLKIILSTLLRNFRIHSDLKESDFKLQADIILKRA

EGFKVRLEPRKRLAKA

>289177152

MSAAGPEVVAGSVAAAAASGFSATSVFFTLLVPAILLYYVYFRISRRHMIELSDKIPGPK

GLPLLGNALELIGSSDTIFRNVYKRSFEFDQVIKLWVGPKLVIFLIDPRDVEVILSSHVY

IDKSPEYRFFQPWLGNGLLISTGQKWRAHRKLIAPTFHLNVLKSFIDLFNANSRAVVQKM

RKEDEREFDIHDYMSETTVEILLETAMGVSKSTQDKSGFEYAMAVMKMCDILHLRHTRVW

LRPDWLFNLTKYGKEQVHLLDIIHGLTKKVIARKKEDYKSGKRNFVDTSAAAKDNKSTTV

VEGLSFGQSAGLKDDLDVDDNDVGEKKRQAFLDLLVEASQNGVVLTDEEVKEQVDTIMFE

GHDTTAAGSSFFLSMMGCHPDIQEKVIQELDEIFGDSDRPATFQDTLEMKYLERCLMETL

RMYPPVPIIAREVKTDLKLASGDYTIPAGCTVVVATFKLHRQPHIYPNPDVFNPDNFLPE

KTANRHYYAFVPFSAGPRSCVGRKYAMLKLKILLSTILRNFRVRSTVKEEDFRLQADIIL

KRAEGFKVKLEPRKRAAGLKA

>EFX88387.1

MSFDFCACVFALGFILAFLSWCLYIRFSPFHVSVNRIPGPKFHLPLLGNSLELAGGLDNV

LHMFQEKWPEKYGEVYRIFLGNHCYVVISTPELLEAILSSQKIIDKGSSYANLVPWLGLG

LLVSSGDLWRSRRKMLTPAFHFSILNSFVEVFNEQSRVLCGIIGEICQSFADGKSEMDVY

PLITRCSLDIICEASMGTTINAQTKDSEYVRAVYRMGQLIVQRFQQPWLDIPWIFSVSAL

GREHNRLLKILHGFTEDVIQKRREALNKKGKNKTGEIETGMRNRLPLLDLLIKTSDDGKV

LSNQDIRNEIDTFMFEGHDTVSSLMGWFLYCMATNTDCQDKVRTELNDLFGDSERDCSEE

DIPNLKYLECCIKETLRLYPSVPLFDRTVLEDVQIGKYLIPAGCTVACPSFATHRNPKTF

PDPLVFSPERFFPDEAVGRHPYAYIPFSAGPRNCIGQRFAMLETKVVLSTLLRRFKFEVS

ANTKPPISSTQLILKSMNGINLVVSRR

>391339740

MAVGIMARADTAVTDIVAIGDTTVGIDTTAVMEDIIVMAAGTTETITIATVDTEIVTEVT

GKSGTTTIIAAIITTAMREDDIDTPTTTELGVGRGVITAMTTTRTEVTETMVIETPTIER

IRRITASTTTIITMAIIGEDTTTIIGITIDTAGEPGTANITASTMVDIDTETIRTDMEEV

TTGTITENTEDEDTSTTRSTASIDTTVEDTTAEDITITAATTIIKAITNTANIDTMVDTE

PLSYKLGATRGRLSFCAGITFLDMLSVKLITLGVQLRTQDFCQPVILTLSVSVIAIIVFK

MQQRWLFDRRLGIPHGWSPSRWMPLGFELTCLAMHRENENLGNVMMTSIQGFSTVYENVG

LYRFYLLFRPIVVLTAPETAEQILKDSSNIEKGCFYDLLRPWLRTGLLTSSGPKWRSRRK

LLTPAFHFRILEDFMVIFNEQSMLMADLLARDCGAKPIEISKRVTKCTLDIICETAMGVE

IKAQENSNSSYVRAVYSLGASFSQRCIRPWQWLSSIYPLTPEGRKYQKDLATLHAFTEEV

CGRAELPKVINERKKHRGEFLVTEGPRTADDCVIGIKRRRAFLDLLLARHSENGELSLLD

IQEEVDTFMFEGHDTTAMGISWCLYLIGQDLDVQRKIHEELDSVFGFDRHRFATSNDLSR

LKYLECCLKEAQRLFPSVPFIARELQRDIHIGQYTIPRGTTCLVNIFHIHRNKKHFPNPE

MFDPDRFHPENSVARHPYAFIPFSAGSRNCIGQKFAQLEEKVILANLLRRFEIRSMLPRD

KLLLVGEMVVRSHNGLMVRIRER

>ACZ97444.1

MSYTTAENVVPSSTFSAINLFYVLLVPALILWYTYWRISRRRLYELAEKLGGPKPLPIIG

NALEFVGGSADIFNNIIAKSLPFDHESVVRLWIGPRLLVFIYDPRDVEVILSSHVHIDKA

DEYRFFKPWLGNGLLISTGQKWRSHRKLIAPTFHLNVLKSFIDLFNANSRAVVDKLKKES

GTFDCHDYMSECTVEILLETAMGVSKTTQDQSGFEYAMAVMKMCDILHLRHTKIWLRPDL

LFKLTDYAKNQTKLLDVIHGLTKKVIKRKKEEFQSGKKATIMPEANDVTNEVPSSKSTSV

EGLSFGQSSGLKDDLDVDDDVGQKKRLAFLDLLLESSQSGVVITDEEIKEQVDTIMFEGH

DTTAAGSSFFLSMMGIHQHIQDKVIEELDHIFGDSDRPATFQDTLEMKYLERCLMETLRL

YPPVPIIARQLKEEITLPSNGKKVPIGTTLVVGTYKLHRRPDVYPNPHKFDPDNFLPERS

ANRHYYAFVPFSAGPRSCVGRKYAMLKLKIILSTILRNFRVYSDLTESDFKLQADIILKR

AEGFKVRLQPRKKQAKV

>EEB17245.1

MDVYQIYSNARLLCYYFAGLFLLFIIFRYIIKRMIFVQGLSPIPGPFGIPLLGNSLSLTG

GQDDFFRLLLNYRKIYGSIFKLWIGMRPFVFISDAQYVSPILNSSLHIDKSYEYTFLHPW

LGTGLLTATGSKWHSRRKLLTPTFHHSLLEGFIQPIIEKSKILISLLENEVGQPPFDVLK

YTKLCALDIICVTAMGKDVNAQLCHGTEYVQAVEGLNKILQRRFITPWLKPDFIFKRCQL

GRQQENYINTINNFVSQVIEDKKNELKKTETESEQKSTSKHPAFLDLILKTRKDGQALSD

NDIREEVNTFMFAGHDTTSVAISWCLFALGKHQSIQKNILEEYETVVKNKIPTFDEIQKL

EYLENCIKETLRLYPVVPLIARDIKHKIDIDGKTRLLPGVTALIFTPSLHRDCKVFQEPN

EFMPDRFKENKTRNPFSYIPFSAGPRNCIGAKFAMIEVKIVLYNILKNYEIISVDSEKDL

NLMSEIVLSNKEGIRIILEKRKRKICLE

>ABF51415.1

MSYTNAENVVPTSTFSAINLFYVLLVPAVILWYAYWRMSRRRLYELADKLNGPPGLPLLG

NALEFVGGSADIFRNIVQKSADYDHESVVKIWIGPRLLVFLYDPRDVEVILSSHVYIDKA

EEYRFFKPWLGNGLLISTGQKWRSHRKLIAPTFHLNVLKSFIDLFNANSRAVVDKLKKEA

SNFDCHDYMSECTVEILLETAMGVSKSTQDQSGFEYAMAVMKMCDILHLRHTKIWLRPDL

LFKFTDYAKNQTKLLDIIHGLTKKVIKRKKEEFASGKKPSNLNETATTSEPSTGKLTSVE

GLSFGQSSGLKDDLDVDDDVGQKKRLAFLDLLLESSQSGVAISDEEIKEQVDTIMFEGHD

TTAAGSSFFLSMMGIHQDIQDKVIEELDQIFGDSDRPVTFQDTLEMKYLERCLMETLRLY

PPVPIIARQVNQEITLPSNGKKIPAGTTLVIATYKLHRRPDVYPNPNKFDPDNFLPERSA

NRHYYAFVPFSAGPRSCVGRKYAMLKLKVILSTILRNFRVISVLKESDFKLQADIILKRA

EGFQVRLQPRKRMAKA

>EFX88390.1

MPVYSYFIAFCVAVLLFILAYIGWWIYIHTSPFHVAVNRIPGPKYLPLIGNALELAGGLD

RLQHTLQTKWVEEYGDIYRIFVGTHCYINISSPELMESVLSSQKIIDKGVSYNELIPWLG

QGLLLSSGDLWRSRRKLLTPAFHFSILNSFVEVFNEQSRILCGIIGDICQSFADGKSEMD

VYPLITRCSLDIICEAAMGTNINAQTETSDYIRAVYRIGQVVTEQFRLPWLRNPTILSLT

ALGKERDQLLKTLHGFTEEVINNRREVIKKKENNVAQETGTGIKNRLPLLDLLLKASEDG

KVLSNQDIRQEIDTFMFEGHDTTTSLLSWFLYAMASNPDVQERVWIELQNEFGDSERDCT

QEDIPNLKYLECCIKETLRLYPSVPGFERAVKEDVQIGKYFLPAGCTIGILSFAAHRNPE

IFPDPLVFNPERFFPDESVGRHPYAYIPFSAGPRNCIGQRFAMLESKIVLSTLLRRFKFE

VSANTKPPIIATQLVLKSLNGINLIVSRR

>ADP22308.1

MFLTLSLLAIATVLTVAALIYKLRFKYLIDVTRDIPSAPTLPIIGHAHYFVNKPPRVQIQ

KLNELLEIYGNTMKVWLGTELNVIMGDVQDVEAVLGSMTFIEKAGEYKRLEPWLKEGLLV

SGGRKWHKRRKAITPAFHFKILDQFIEVFERESRALVANLERECRLQSNSGFNLYDWINL

CTLDTICETAMGVSVHAQTNTDSEYVQAVKTISTVIHKRMFDILYRFDLTYRFTKLARAE

KRALSVLHGFTERIIMQRRAELLRAQENTLNAAADKNSTNGYNATDNDADVGAKRKQAFL

DILLHAEIDGKPLSNLDIREEVDTLMFEGHDTTSSAITFFFYNIATYPECQRKCYAEIVD

IFGKDTSKPVTYEALNGLTYVELCIKETLRLFPSVPLLGRKVTQECEINGKVLPAGTNIG

ISPLYLGRQESIFPDANIFKPERFDISNDAKKMNPYAYIPFSAGPRNCIGQRFAMLEVKS

IVTNVLRHFEVEFVSDGTEGPTLIAELILRTKDPLMFRLKPRI

>EFX88056.1

MIVGILVAVLLFLLALLFLVHHRWECSSFVKTIDLIPGPKRKPIVGNAMDLPKESHQIMQ

TIQGKWVKQFGRVYRSWLGFRTFVHISTPMLMEKMLTSQTFIDKGKSYSILRPWLGEGLL

LASGNKWRRSRRLLTPAFHFQILDNFFDVFNKNADILCEQLTIANTPVKSDSVEEVDVFP

YLKKCTLDIICEAAMGIKINAQLEDSEYIRNVHKISEIVVERFFSFGHFLPDWMYHLTPK

GREHMKLLKQIHGFTSKVIRERKEEIAREEIQKENDDGAPDEVKKRRRAFLDLMLLSVKD

GVELSDLDIRNEVDTFMFEGHDTTASALVWFLYCMATNSEQQALVQEELNEVFGDSDRPC

TMEDTTKLKYLECCIKESLRLYPAVPNITRYMSEDSELGGYKIPSGASVSLQIYALHRNE

EYFPDPDVFNPERFQTNESIGRHAFAFVPFSAGPRNCIGQRFAMFEEKVLASSLLRRFKF

SYDIAKHGPPRANAELVLRPRDGMPLKLVSCPRCLNFI

>O16805.1

MFLVIGAILAGALFVGLLLYQLKFKRLIDLISYMPGPPVLPLVGHGHHFIGKPPHEMVKK

IFEFMETYSKDQVLKVWLGPELNVLMGNPKDVEVVLGTLRFNDKAGEYKALEPWLKEGLL

VSRGRKWHKRRKIITPAFHFKILDQFVDVFEKGSRDLLRNMEQDRLKHGDSGFSLYDWIN

LCTMDTICETAMGVSINAQSNADSEYVQAVKTISMVLHKRMFNILYRFDLTYMLTPLARA

EKKALNVLHQFTEKIIVQRREELIREGSSQESSKDDADVGAKRKMAFLDILLQSTVDERP

LSNLDIREEVDTFMFEGHDTTSSALMFFFYNIATHPEAQKKCFEEIRSVVGNDKSTPVSY

ELLNQLHYVDLCVKETLRMYPSVPLLGRKVLEDCEINGKLIPAGTNIGISPLYLGRREEL

FSEPNSFKPERFDVVTTAEKLNPYAYIPFSAGPRNCIGQKFAMLEIKAIVANVLRHYEVD

FVGDSSEPPVLIAELILRTKDPLMFKVRERVY

>ABB36785.1

MAAASATGFSASSVFLSLLIPALILYFIYFRISRRHLLELAEKIPGPPALPLIGNALDLF

GSPDAMFSQVLKKAENFKDVVKIWVGPKLVICLIDPRDVEIILSSNVYIDKSTEYRFFKP

WLGDGLLISTGQKWRNHRKLIAPTFHLNVLKSFIDLFNANARSVVEKMRKENGKEFDCHN

YMSELTVDILLETAMGVSKPTRDHNAFEYAMAVMKMCDILHLRHTKIWLRPDWLFNLTKY

GKNQIKLLEIIHGLTKKVIQLKKEEYKSGKRNIIDNSAQKTESKTNNIVVEGVSFGQSVG

LKDDLDIDDDVGEKKRQAFLDLLIEAGQNGVLLTDKEVKEQVDTIMFEGHDTTASGSSFF

LAVMGCHPDIQEKVIQELDEIFGDSDRPATFQDTLEMKYLERCLLETLRMYPPVPLIARE

IKTDLKLASGDYTIPAGCTVVIGTFKLHRQPHIYPNPDVFDPDNFLPEKTANRHYYAFVP

FSAGPRSCVGRKYAMLKLKIVLSTILRNFRVRSDVKESEFRLQADIILKRADGFKIRLEP

RKQVASTA

>EDW82406.1

MLSLVIVVACCIVILTIVTLNCWGPLCRRLRVYQLAARLPGPRSLPLLGNANLFLGLTPA

EACLLMGKLAEEHGDTFKLWLGPWFSVMLFDPRDVEHVLSSTQLLDKAFEYDFLGQWLNQ

GLLVSGGRKWHQRRKIITPAFHFRILEQYVEIFDRQTRQFVKNLDTALRKEAANSWNGVD

LGRFIHLCTLDVICVVGLVLASAIFAGLLLYQLKYKHIIDVISYMPGPPTLPLVGHAHHF

IGKAPHEMIVVITDFMKTYSKGQAMKVWLGPELNILMGDPKDVEVVLGTLRFNDKAHEYK

ALEPWLKEGLLVSRGRKWHRRRKIITPAFHFKILDQFVEIFERESRVLIKQLEKERQRNG

SDGFNLYDWINLCTMDTICETAMGVSIHAQTNADSDYVRAVKTISMVLHKRMFNIFYRFD

LTYMLTPLARAERRALNVLHEFTEKIIVQRREELLRGTPSETETSSADADVGAKRKMAFL

DILLQSTVDEKPLTNLDIREEVDTFMFEGHDTTSSALMFFFYNLATHPESQQKCFEEIKS

VIGTDVQTPVTYELLNKLHYVELCIKETLRLYPSVPLLGRRVVEECEINGKVIPAGTNIG

ISPLFMGRREDLFVEPNSFKPERFDVVTTAEKLNPYAYIPFSAGPRNCIGQKFAMLEIKA

IVANTLRHFEIDFVGDTSEPPVLIAELILRTRDPLMFKLKERVY

>EDS33837.1

MSFKFGPPFKNFEHPWYTWSFITNLSRKYGKLFCVWIGSRLALFCSNHSDTETVLSSQKL

IRKSELYKFLIPWLGDGLLLSTGQKWFNKRKILTPAFHFKILEQFVEVFDKQGTVLVQRL

REQATGKLVNIYPFVTLAALDIICETAMGTSINAQTDTDSKYVRAVTELSYLLAGRFMKV

WQRSDFLFNLSTDKKRQDKVIKVLHDFTTSIIRSRREELLAEKIETNDTADEEGVGTRKK

MAFLDVLLQASIDGRPLTDKEIQEEVDTFMFEGHDTTTIAITFTLMLLAKHPDIQEKVYQ

EVTEIIGKDLNAPTTYRNLQDMKYLELVIKESLRLYPPVPIIGRKFTEKTEIDGKVVPED

SNFNVGIILMHRDPTQFEEPERFDPERFSPERTTELSSPYAYIPFSAGPRNCIGQKFAML

ELKSTLSKIIRNYRLTDTGPEPKLIIQLTLKPKDGLKVAFVPRATA

>EDX01410.1

MWLLLSLVLLLVILALEMRRFLRNMRTIPGPLPVPLLGNAHLFLGLTPAEACLKIGELAE

RHGDTFGLFLGPSYSVMLFNPRDVERVLGNSQLLTKSQEYSFLGRWLNEGLLVSNGRKWH

RRRKIITPAFHFRILEPYVEIFDRQSLRLVEELRLRMSRGQERINLGEAIHLCTLDAICA

IEENQRNMFLVIGAILASALFVGLLLYQLKFKRLIDLISYMPGPPALPLVGHGHHFIGKP

PHEMVKKIFDFMETYSKDQVLKVWLGPELNVLMANPKDVEVVLGTLRFNDKAGEYKALEP

WLKEGLLVSRGRKWHKRRKIITPAFHFKILDEFVEVFEKGSRDLLRNMEQDRLKHGDSGF

SLYDWINLCTMDTICETAMGVSINAQSNADSEYVQAVKTISMVLHKRMFNILYRFDLTYM

LTPLARAEKKALNVLHQFTEKIIVQRREELIRGGSGQESSNEDADVGAKRKMAFLDILLQ

STVDERPLSNLDIREEVDTFMFEGHDTTSSALMFFFYNIATHPEAQKKCFEEIRSVVGND

KSTPVSYELLNKLHYVDLCVKETLRMYPSVPLLGRKVLEDCEINGKLIPAGTNIGISPLY

LGRREELFSEPNSFKPERFDVVTTAEKLNPYAYIPFSAGPRNCIGQKFAMLEIKAIVANV

LRHYEVDFVGDSSEPPVLIAELILRTKDPLMFKVRERVY

>AAB71161.1

MFLVIGAILASALFVGLLLYHLKFKRLIDLISYMPGPPVLPLVGHGHHFIGKPPHEMVKK

IFEFMETYSKDQVLKVWLGPELNVLMGNPKDVEVVLGTLRFNDKAGEYKALEPWLKEGLL

VSRGRKWHKRRKIITPAFHFKILDQFVEVFEKGSRDLLRNMEQDRLKHGDSGFSLYDWIN

LCTMDTICETAMGVSINAQSNADSEYVQAVKTISMVLHKRMFNILYRFDLTYMLTPLARA

EKKALNVLHQFTEKIIVQRREELIREGSSQESSNDDADVGAKRKMAFLDILLQSTVDERP

LSNLDIREEVDTFMFEGHDTTSSALMFFFYNIATHPEAQKKCFEEIRSVVGNDKSTPVSY

ELLNQLHYVDLCVKETLRMYPSVPLLGRKVLEDCEINGKLIPAGTNIGISPLYLGRREEL

FSEPNSFKPERFDVVTTAEKLNPYAYIPFSAGPRNCIGQKFAMLEIKAIVANVLRHYEVD

FVGDSSEPPVLIAELILRTKEPLMFKVRERVY

>P33269.2

MFLVIGAILASALFVGLLLYHLKFKRLIDLISYMPGPPVLPLVGHGHHFIGKPPHEMVKK

IFEFMETYSKDQVLKVWLGPELNVLMGNPKDVEVVLGTLRFNDKAGEYKALEPWLKEGLL

VSRGRKWHKRRKIITPAFHFKILDQFVEVFEKGSRDLLRNMEQDRLKHGESGFSLYDWIN

LCTMDTICETAMGVSINAQSNADSEYVQAVKTISMVLHKRMFNILYRFDLTYMLTPLARA

EKKALNVLHQFTEKIIVQRREELIREGSSQESSNDDADVGAKRKMAFLDILLQSTVDERP

LSNLDIREEVDTFMFEGHDTTSSALMFFFYNIATHPEAQKKCFEEIRSVVGNDKSTPVSY

ELLNQLHYVDLCVKETLRMYPSVPLLGRKVLEDCEINGKLIPAGTNIGISPLYLGRREEL

FSEPNSFKPERFDVVTTAEKLNPYAYIPFSAGPRNCIGQKFAMLEIKAIVANVLRHYEVD

FVGDSSEPPVLIAELILRTKEPLMFKVRERVY

>EEB15503.1

MLRHFLDLDIFSLTITILLFLIFWKFFQNDKKTLHINKLPGPLALPIFGNIIEVLQPKDK

FLENSMKRRKKFGPIFKMWFGRNPSVFLSRAKEIEVVIKGTKHMEKSFVYKFVQPWLGTG

LLTAHGKKWASHRKLITPSFHFTVLQSFIEIFQENSNILINKLNKMADTNEVIDIYQYIT

LCLLDIICETAMGTKVNAQSDSQSEYVKSVYRLSALVVERTGRVWLHPDFIYNLTSHGKE

NRKHLDIVHSFTDRVIQKRKLSFENESSKNFDSPDRKKLSFLDLLLKASMNEASTPLTDM

ELREEVDTFMFEGHDTTAAGVNWAILMLSHHPEIQEKVYEEVKTIFENKQEENLTLGDLS

EMKLLDRVIKETLRLCPSVTSIGRIAEEDIHLGEYTIPKGANTVINIYALHRDPTVFPDP

DVFDPDRFLPENMSGRHPFAYIPFSAGPRNCIEKFK

>AAB71165.1

MFLVIGAILASALFVGLLLYHLKFKRLIDLISYMPGPPVLPLVGHGHHFIGKPPHEMVKK

IFEFMETYSKDQVLKVWLGPELNVLMGNPKDVEVVLGTLRFNDKAGEYKALEPWLKEGLL

VSRGRKWHKRRKIITPAFHFKILDQFVEVFEKGSRDLLRNMEQDRLKHGDSGFSLYDWIN

LCTMDTICETAMGVSINAQSNADSEYVQAVKTISMVLHKRMFNILYRFDLTYMLTPLARA

EKKALNVLHQFTEKIIVQRREELIREGSSQESSNDDADVGAKRKMAFLDILLQSTVDERP

LSNLDIREEVDTFMFEGHDTTSSALMFFFYNIATHPEAQKKCFEEIRSVVGNDKSTPVSY

ELLNQLHYVDLCVKETLRMYPSVPLLGRKVLEDCEINGKLIPAGTNIGISPLYLGRREEL

FSEPNIFKPERFDVVTTAEKLNPYAYIPFSAGPRNCIGQKFAMLEIKAIVANVLRHYEVD

FVGDSSEPPVLIAELILRTKEPLMFKVRERVY

>AAC03111.2

MLLVALGLLLACLLAVLFLNDFKTRSRMQLADKIPGPKALPVLGNLLDFGLRPDRYRELV

EGLIYKHGTIVRLWSGAYLIVILTEAKYVEALLSSTSQIDKAYTYRFVWPWLGSGLLTST

GQALGNPPQAADSSFPLQGTREFRGCVQQKWKILVEKFSRHVNGPEFDVTPYMTLCALDN

MSETSMGVTLNAQKDSDSEYVRAIHSLGEIVFTRSGKPWYHSDTTFRLSTLGREQQKNLA

ILHSFTRSVIRSRKQELLVHLNNQSGEGVQNELGLKRRHAFLDLMLQASQDGASLTDEEI

REEVDTFMFEGHDTTTSALSFTMWCLAKYQDVQEKAVVELKQIFGDSTRDATFRDLQEMK

YLEQVIKETLRLYPSVNCFGRQLTENFTVGDYVNPAGANVWIYPYHLHRRPEYFPDPERF

DPDRFLPENCVGRHPYCYVPFSAGPRNCIGQKFAILELKSTISQVLRSFKVIESDCNGNI

RYKLDFVLRSASGLKVKLQPR

>EFX81321.1

MILALFCLAVAVIFFFYLKFEYSSYVRTIDLIPGPPKVPFFGNALSIPLDPYGGLQKISV

EWPKTYGHFRRGWFGFKGYIDISCPLVTEEILSSQKFIDKGKDYDMLMPWLGEGLLTSAG

NKWRKRRRLLTPAFHFQILDNFFDTFNKSADILCQQLQCSLSKKAELNQTEEIEVFPYLK

RCTLDIICEAAMGVQINAQIEDSEYIYAVQRYSMVLFEYFTSIWSFLPQRIYFMTKHGKE

YQKCLKIIHDFTSKVIQERRKEIDQELETKEVKEKQDGPEESQFKSKKRRAFLDLMLIAA

KEGADLTDMDIRNEVDTFMFEGHDTTACAAVWFLYCMGIHPDCQELAREELNDVFGDSDR

PCTLEDASKLKYLECCIKETLRLYPSVPHIKRYNTEDFVLSNGFKIPAGASYSIHIYTLH

RNEEFFPDPLSFKPERFYSDQCSGRHPFAFVPFSAGPRNCIGQRFALYEEKVIFSTLLRR

FRFTYNTTNHGPAKACADMLLKPHHDMPLGITPLKTKEIVREL

>391335060

MRDFGVDTLYEDFWQVFVELQSRMKHKVYLTLLAQRLYNEFKYPMMTLPGPYNLFRLAFP

FFDDLRFLFGKGDLDLQKVFHTIIQGFSQVAREQGLCRIYVALKPFVFIFDAVAVESLIT

SNEHIDKADVYRILHPWLGTGLLTSSSAKWRSRRKLLTPAFHFSILDEFVPSVNQQSLIL

VGRLRGHAEEDFDLVNDVTLCTLDVICETAMGISVQAQQGFNSDYVQAIYDLGNSMYVRL

ARPWLKADFVFKMSKEGRKFQKSLVILHGFTRKIIRERKQKRLAQMNGTTTDDLGIKKRV

AFLDLLMDIHVEEGSLSLEDIAEEVDTFMFEGHDTTAAGISFCLYLLGLSPDKQERAQRE

LDEVVGDASHITTEHLPRLKYLEAVIKESQRLYPSVPNVGRNLRKDLTMPNGVTIPAGST

CIVAISELHRNPKYFEYPLEFIPERFLPEGKHLMAKNPFCFIPFSAGPRNCIGQRFAILE

EKIIIGHVLRNFSIRSLQERDELFLSVELVTRSLCGLRVRLNERQRRKTFRSDDLR

>ABV48806.1

MAAAALINVLLCAGGLFTILSTIGLVLYLVKFKHMIDITNNLPSPPYLPIVGHGHHFAGK

PPHENMYTIYEFASKYGPTFKVWLGTELNIWTSDLKDVEVILGTTRFNDKAHEYDMLEPW

LKEGLLTSRGRKWNSRRKVLTPAFHFKILESFIDVFEKESRTLVANLDKEYRMQTDKGFD

LNDWVNLCTLDTICETAMGVSVNAQTNADSEYVRAVKTIAMVLHKRMFDILCRFELTYRF

TRLAREEKKALAVLHGFTEKVIVQRREELLKVQEAGKLDAADKDTGSKRKMAFLDILLQS

QVDGKPLTNMDIREEVDTFMFEGHDTTSSAVTFCFYNLANYPECQQKCFEEIVQVLGKDK

SKPVTFEDLNNLHYLDLCIKETLRMFPSVPLLGRKVTEECEINGKIIPAGTNIGISPLQL

GRLEELFPDPDTFKPERFEAGYSTEHLNPYAYIPFSAGPRNCIGQKFAVLEIKTILSNVL

RHYSVEYVGPGEEEIHLIAELILRTKDPLMFKIKPRVYY

>EFX88388.1

MTLYSVFVAFCVVALLLVLAYIGWWIYIHTSPFHVTVNRIPGPKYLPIVGNVLEIAGGLD

HLLHTFQIKWAQQYGGIYRIFVGTHCYIPISSPELMEVVLSSQKTIDKGMSYSELVPWLG

QGLLISSGDLWRSRRKLLTPAFHFSILNSFVEVFNEQSRILCGIIGDICQSFADGKGEMD

VYPLITRCSLDIICEAAMGTKINAQTETSDYIKAVYRIGQVITEQFQQPWLRNSTILSLS

ALGKERNQLLKTLHGFTEEVINHRREILKKKEDIEVDDKETGIRNRLPLLDLLLKASEGG

KVLSNQDIRNEIDTFMFEGHDTTTSLLSWFLYVMAMNPDIQERAWIELQNEFGDSERDCT

QEDIPNLKYLECCIKETLRMYPSVPAFERTVQEDLQIGKYLIPAGCTIGFLILAAHRNPE

IFPDPLVFNPERFFQDEVVGRHPYAYVPFSAGPRNCIGQRFAMLESKIVLSTLLRRFKFE

TLSNTKPPIIANQLVLKSMNGINLVVSRR

>EGI70283.1

MVTLSVVALMIRRAKFLYALKNVPYPTALPIVGNAYQLNCTQEGFFQKLTKWANKFGDIY

LIWVGMRPFIFLYRIESVQPLLSSSVHIDKSLEYQYLKPWLGTGLVTSSGEKWHIRRKLL

TPTFHSGLLETYLKTIKEETNVLISCLEKEVDKWFNIVPFAKRAALDIICDTAMGYKLNS

QKNFKVDYVEAVDKIASIVQMRFTNVWLSFDSIFKLTKSGKEHDHSIQIIQEFVSKVIVK

RKAEWKSDTNFNNSKKNRRALLDLLLQISQNGTILSDEDICEEVNTFMYAGHDTLATSIS

WTLYALGRNPEYQQKILEEYNDMAVDTNEITLENIHKLTWLEACIKEQWRLYPVTPLIAR

QIYKPIEIMSSQIPPGSTVLINSYLLHRDDRFFPEPHVYRPERFLPDGPKLPPYAFIPFS

AGSRNCIGWKFATLVVKIAILSVLKAFRVEALDKEDELRFISELVLVNANGLRLKITPR

>EFX70986.1

MFLVVCLAAAVIFLLYLRWEYSTYVKTIDLIPGPPKVPVMGNALLIPRNPHGSLEILQEK

WPKIYGRIYRVWVAFQAFIDISSPSLMEEIMSSQKFINKGEVYDPLLPWLGEGLLTAKGN

KWRKRRRLLTPAFHFQILDNFFDTFNKNADILCQQLHRSLSKEGELNEREIEVFPFLKRC

TLDIICEAAMGIQVNAQLEDSEYLNAVQKFSLLLFENFFSVWRLVPRWIFFLTTKGKEYK

KSLKVIHDFTSKVLSNSNNFIVEHFSMISFADRRAFLDLMLIAAKEGADLTDTDIRNEVD

TFMFEGHDTTASAAVWFLYCMGTHPEHQERVREELSHVFGDSNRPCTLEDTTKLKYLECC

IKESLRLYPSVPNIKRYISEDIVLNGYKVPAGSTISMHIYSLHRNEEVFPDPLVFKPERF

ENQQLVGRHPFSFVPFSAGPRNCIGQRFALFEEKVIMSTLLRRFRFTYDTDKHGPAKPSA

DLVLKPHHGMPMILSPLVDNFLGSE

>EAT40476.2

MLILLASVVVLSIGLAVYFYQQFANRLHYAAKIGGPKGYPLLGNSIQYGTKSPVEFLQEV

QKTNEQCGKFYRLWIGPDLIFPITDAKLAILSSQKLLDKSVQYDFIRPWLGNGLLTSTGR

KWHSRRKIITPTFHFKILEQFVEIFDQQSNIFVGQLKSKAQSGEDFDVFPVVTLCALDVI

CESAMGTKVNAQLNSDSKYVRAVKDMATVAMARSFKAFARFNFTFYFTPYRRMQDKALKV

LHDYTDSVIRSRRLELAKGAFTKSDENENDVGIRKKVAFLDMLLQATVDGRPLDDLEVRE

EVDTFMFEGHDTTTSAISFLIGILAKHPDVQQKVYDEVRNVIGDDLNVSVTLSMLNQLNY

LDLVIKETLRLYPSVPIYGRMLLENQEINGTVFPAGSNLAIFPYFMGRDPEYFENPLEFR

PERFAVETSAEKANPYRYVPFSAGPRNCIGQKFAVAEIKSLISKLVRHYEVLPPKQPNSE

RMIAELVLRPEGGVPVRIRSRVR

>EFA01322.1

MLILSKYRSLSNVPGPKPSPVIGNSLHIGYRPQDLFNNIVKWAKEYGTVYKIFVANDVRV

LITNPELTELVLSSNVHITKSNAYDLLKPWLGIGLLISTGKKWKSRRKLITPTFHFKILD

QFLDVFNSCGNVLVQRLSSQVGKDSFDVYPFINLCSLDIICETSMGVKIKAQQGNNTEYV

EAVRRFLDIFIIRTFSVWKGIDALFKFSSAYSVYKRLLKILHQFTINIIVQRRGEKAQQK

TQNVTSDDGIKRKVALLEMLLESEDNNMLSNEDIREEIDTFMFEGHDTTTSGIAFAILCL

AENPKVQEKLYEEVVAVIDNIENITMQQLQEMKYLEMVLKEAQRLYPSVPVIERRLEVDC

NIGGYDFPKDTFLSLFIYGMHHNEKYFPEPEKFDPNRYLPENQAKRHNYAYVPFSAGPRN

CIGQKFAMLEMKTTIAKIVKHFKILPVPDYKPDLGIAAILKSYNGVCVRLQHRHGN

>EDW62835.1

MLYCLPLIIAAVCIALALATLSWLPLRPWLRRRRRAHELAALLPGPRTLPLIGNFNMFLG

LEPSQLPQLIGELADKYDGTFKLKMGTNFSVMMFKPRDVETVLGSTQLLDKAVEYNFLRG

WLNEGLLVSRGRKWHKRRKIITPAFHFRILDQYVEIFDRQTRLLVNHLAQARGDGSNLVE

LGHAAHLCALDVICALGLFLASALFLGLLIYQLKFKHLIEVTRDLASPPAWPLLGHGHHF

IGKEPHEMMQTLDDFMRLYARDETLKVWLGPELNMLMANPKDVELVLGTLRFNDKAGEYK

ALEPWLKEGLLVSRGRKWHKRRKIITPAFHFKILEQFVDIFERESRVLLHNLDKERQRQG

QTGFNLYDWINLCTMDTICETAMGVSINAQTNVDSEYVRAVKTISMVLHKRMFNIFYRFE

LTYMLTPLARAERRALNVLHNFTEKIIVQRREELLRASNAGEPSAQSADDADVGAKRKMA

FLDILLQSSIDDKPLTNLDIREEVDTFMFEGHDTTSSAIMFFFYNIATYPECQRKCVDEI

ISVLGRDKATPVTYDLLNKLHYVDLCIKETLRMYPSVPLLGRKVLEECEINGKIIPAGTN

IGISPLYLGRREELFSEPDTFIPERFDVVTSAEKLNPYAYIPFSAGPRNCIGQKFATLEI

KAIVANVLRHFEIEFVGNTAEPPVLIAELILRTKDPLMFKLKERII

>66562674

MSSSTIIMGVSWLTMILSICLMTIVVLLLVRRGKFLYALRKVPCPPAFPIIGNAYELCCS

PEEAFKKMIKWGKELGDMYLIWVGMRPFIFLYKAEAIQPLLSSSVHIDKSLEYQYLQPWL

GSGLVTSTGEKWHFHRKLLTPTFHSGLLELYLKTTIREAQILISCLRKEIGKPEFDIVPY

AKRAALDIICDSSMGCNINAQKNFENEYVQAVNTLASISQRRFLNVWMSFDPIFKLTSWG

KRHDHALSVTHGFVNKIIAERKAEWKDRKDTNFNEKSHKRQALLDLLLELSKDGKVLTDD

DIRDEVNTFMFAGHDTTATSVSWILYALGRHPQYQELIIEEYDETVGTKELTLDILSKLT

WLEACIKESWRLYPVTPLIARQIYHPITILGHEIPIGSTVLVNSFLLHRDSRYFPEPDIY

RPERFLPDGPKYPSYAFVPFSAGSRNCIGWKYGTMIVKVLILYILKNFHVESLDTEDQLR

FISELVLHNADGLRLKITPRK

>EDW27551.1

MFLLLGALLASGLFVALLVYQLKFKRVIEVISYMPGPPVLPLVGHGHHFIGKPPHELVKQ

ITEFMSKYAKDETLKVWLGPELNVLMANPKDVEVVLGTMRFNDKAGEYKALEPWLKEGLL

VSRGRKWHKRRKIITPAFHFKILDQFVDVFESGSRDLLRNMEMDRLRQGQTGFNLYDWIN

LCTMDTICETAMGVSINAQTNADSEYVQAVKTISMVLHKRMFNIFYRFDLTYMLTPLARA

EKRALNVLHEFTEKIIVQRREELIRGGSKDAAAASNDADADVGAKRKMAFLDILLQSTID

EKPLTNLDIREEVDTFMFEGHDTTSSALMFFFYNVATHPEAQKKCFEEISSVIGQDKSTP

VTYELLNKLHYVDLCIKETLRMYPSVPLLGRRVVEECEINGKRIPAGTNIGISPLYLGRR

EELFSEPNSFKPERFDVVTSAEKLNPYAYIPFSAGPRNCIGQKFAMLEIKAIVANVLRHY

HIDFVGDTTEPPVLIAELILRTKDPLLFKLQERVY

>EFN66742.1

MILSVCLLSLLLALLIKRGRFLYALRKIPYPIALPIIGNAYQLNCSPEEFFQNLIKWAEE

FGNIYLIWVGLRPFIFLYKVEAIQPLLSSSTHIDKSLEYEYLKPWLGTGLVTSTGETWQF

RRKLLTPTFHSGLLATYFKIAKEEMNVLISCFEKESNKWFDVVPYLKRATLDIICESAMG

YKLNAQVNSENEYVEAVDKIASIVQMRFTNVWVSNDKIFKLTKAGKEHDHALRIIQDFVD

KVIAQKKIEWQQKHDGNLNEPPNKKQALLDLLLDISKNGTVHLSDADIRDEVNTFMYAGH

DTMATSISWTLYALGRHPEYQEKILDEYYNVLGTTEVTLQNIHKLTWLDACIKEQWRIYP

VAPLIARQIYKPINLMGNKIPPGSTVLINSYLLHRDSRFFPDPHIYRPERFLPNIPKLPP

YSFIPFSAGSRNCIGSRFATSVIKVAVLSVLKAFRVEAFDTEDQLRFISELVLVNANGLR

LKITPH

>EFR27937.1

MGRQVNAQYNSDSEYVKAVYQLPGHQKCLAILHEFSNRVIHERKEEIRQQRLSKGDSNNN

HSAGRTSTADGNNNGDFLSHEELGRKKRLAFLDLLIEASQDGTVLSHEDIREEVDTFMFE

GHDTTSAAISWILLLLGGDPAIQDRIVEEIDLIMGGDRERFPTMQELNEMKYLEACIKEG

LRLYPSVPLIARHLTEDVDIDGYTLPAGTTAMIVVYQLHRNPDVFPNPDKYNPDHFLPEN

CRGRHPYAYIPFSAGPRNCIGQKFALLEEKSIISAVLRKYKIEAVDRRENLTLLGELILR

PKNGLRIRISRRT

>EDY72689.1

MFLLLGALLASGLFVALLVYQLKFKRVIEVISYMPGPPVLPLVGHGHHFIGKPPHELVKQ

ITEFMSKYAKDETLKVWLGPELNVLMANPKDVEVVLGTMRFNDKAGEYKALEPWLKEGLL

VSRGRKWHKRRKIITPAFHFKILDQFVDVFENGSRDLLRNMEMDRLRQGQTGFNLYDWIN

LCTMDTICETAMGVSINAQTNADSEYVQAVKTISMVLHKRMFNIFYRFDLTYMLTPLARA

EKRALHVLHEFTEKIIVQRREELIRGGSKDAAAASDDADADVGAKRKMAFLDILLQSTID

EKPLTNLDIREEVDTFMFEGHDTTSSALMFFFYNVATHPEAQKKCFEEISSVIGQDKSTP

VTYELLNKLHYVDLCIKETLRMYPSVPLLGRRVVEECEINGKRIPAGTNIGISPLYLGRR

EELFSEPNSFKPERFDVVTSAEKLNPYAYIPFSAGPRNCIGQKFAMLEIKAIVANVLRHY

HIDFVGDTTEPPVLIAELILRTKDPLLFKLQDRVY

>EFX88053.1

MIAAYLISVLLIVLTLLFFVHRKWENSSYVKTIDLIPGPPKQFFVGNALALPKETNEILQ

TIHSKWVKQYGKIFRVWLGLRPFVMTSSAVLIEKMMTSNTFIDKKDAYSILTPWLGEGLL

LASGNKWKKNRRLLTPAFHFQILDNFFEVFNKNADILCEQLIKANTSIQGDSVEEIDVFP

YLKRCALDIICEAAMGIQVNAQLEDSEYIRNVQRISEIVVERFFSFGHFMPDWMYECTTS

GREHKKILKQIHDFTSKVIRERKVEIALEDEILPEEDTSEVSNRSKKRRAFLDLMLLANI

NGVELSDLEIRNEVDTFMFEGHDTTASALVWFLYCMAINPKHQALVQEELNEVFGGSDRP

CTIEDTTKLKYLECCIKESLRLYPAVPIISRYISEDFELGGYKIPVGASVVIEIYALHRN

DEYFPEPDVFNPERFQTNESIGRHAFAFLPFSAGSRNCIGQRFAMFEEKVLASSLLRRFK

FSYDVAKHGPPKANADLVLKPRDGMPLQIVSYLQ

>AAO20251.1

MSVTVETATVESPASSISTFMVLLVSAVALFFAYWKISRRRFLQLAEKIPGPKGYPIIGN

ALDFLGSSSQVTDRMIQIGFQFTTIAKVWILHKLVVFIADPRDIELILGNSTHLEKSEEY

RFFKPWLGDGLLISSGQKWKSHRKLIAPTFHLNVLKSFVDLFNANSRAVCDKMAKENGRT

FDCHDYMSECTVEVLLETAMGVSKKTQNKSGFEYAMAVMKMCNILHLRHSKVWLRPDWLF

NLTKYGKEQVDLLDVIHGLTKKVIKNKKEIISSGTKKYIEESVTQEEKAIASTPVKGLRD

DLDEQDENDVGQKKRLAFLDLMIESAQNGVVLTDEEIKEEVGTIMFEGHDTTAAGSSFFL

CLMGIHQKYQDMCVQELNQIFGDSDRPATFADTLEMKFLERCLLEALRMYPPVPVIARKL

AEDLTLASTGVVIPQGTTIVVSTVKTHRLEEHWPNPDVYDPDNHLPEKAAERHYYSFVPF

SAGPRSCVGRKYALLKLKIILSTILRNFKVHSDISEDEFKLQGDIILKRADGFMIRLEPR

KKTVAA

>EGI65081.1

MDFIFLGLCILCIVLIFSILWNYHQYIIRQKFRNIPEVKGYPFIGVAFEFMKLSDYDRIK

RYSSFIEEFKEGIFMYQIGTKPFINIFKPEYLELIFPSTVNITKGNSYDLLKPWLGNGLL

TSTGKQWFHDRKLIGPTFHFSILDQFAVILSEKAEILTKCLEKKIKDNPGKAVDIFPFMI

NVTLDIICETAMGVDVHAQEIVNEYTSTVHQISKLISNRMIQPWYWIDWLYYLLPTGKQF

KSMLDILHGFTKKVISKKKIDRQSQNAKLENEDDEFNIGKRKRKAFLDLLLDQNEKDYCT

PLTDDELRAQVDTFMFEGHDTTAVAITWALFLLGNNLEHQEKVHKELEEIFGDSEVPASV

KELSQLKYLERVIKETLRIFPSVPLIVRELVEDVKIDNYTLMKGTSVILTILLAHRNPAV

WPDPLKFDPDRFLPENSQNRNPYAYIPFSAGPRNCIGQRFALLEEKTVLTAILRKWRVKS

VKTIDTIEYGGSLITRPVEEVFIHFTPKK

>EFX88391.1

MIVTHRPELLAQWKCAVLHVCKCSLRDEDFRIQIPASTWEIGCLNNSLLDTLPGKQLEDL

IDLMYSLLGLLLILAYTGWWIYIHTSPFHVAINRIPGPKYLPLIGNSLEMFGGLDHLISL

FHVTFVEKYGEIYRAFAGRNCYVCISSPELLKDVFTSQKIIDKGVSYDQLAPWLGQGLLL

SSGDLWRSRRKLLTPAFHFSILNTFVEVFNEQSRVLCGIIGDICQSFADGKSEMDVYPFL

AKCSLDVICEVAMGTKINAQTDDSDYYKAVCRMGQVMITKFQKPWLENKRIFSLSALGKE

HDQLLKTLHGFTEDIIRKRRKSFSYVSNNNEKNESGIKNRLPLLDLLLQASDDGKFLSDL

DIRNEIDTFMFEGHDTVSSLMGWFLYCMAINPDCQENAWIELSGVFDNSERDCTQEDIPN

LKYLECCIKETLRMYPSVPAFERTVQEDVQIGKYLIPAGCTLGCLTLATHRNPEVFPDPL

VFNPERFFADEANGRHPYAYFPFSAGPRNCIGQRFALLELKIILSSLVRRFKFEFSSDAE

LLIPSHQITLKSLTGINLIVSRR

>EFX87943.1

MWLLLVTTLVFYYWTWSQSRFVRLINAIPGPKSLPLLGNLLDLDVYNDNFLKMTTIDWVQ

KYGPMYRVWLCTRPFIALSSPELVQKILASNKLITKSPDYSNFSSWLGNCMFTSTGVHWK

NRRRLVAPGFHFQNHTSFIDIFNEKSAECAREFERTIDTHGDVEIDVNPFMAKCALNIIC

ETAMGQQTRIEVEKAIYVNNVHRICQIFVERVSRPWLSIDWIYKLSSLGRESQKCISSLH

AFTNKVTRDRREMLKEEEQNRGNVQNKVMDVEKESKRRFAFVDGLIKASNEGADLNDNGI

REEIDLITFAGYDTTSAAMVWFLYLMAKHPEHQKLILDELDVVFSGDVERPCTTQDIAEL

KYLECCIKEAMRIYPSIPFVMRNLTEDVEIDGHTLPAGVTVAMVFYAIHHNPLIYPDPEV

FRPERFFPENSVGRHPYAFIPFSAGPRNCIGQKYAMLELKVVFANLLRKVKFSVPDPTKP

LSDAPDLGFVLKPKHEVRLNLSKRLNKLG

>EDW06072.1

MFLVLGLILASALFVGLLIYQLKFKHLIEVTRDFGSAPTLPVVGHGYHFVGKEPHELIIK

VRGFMETYGKDYTLKVWLGPELNLLMGNLKDVEVVLGTLRFNDKAGEYKALEPWLKEGLL

ISRGRKWHKRRKIITPAFHFKILYQFVDIFERESRVLLKNLEREREQQDQGGFNLYDWIN

LCTMDTICETAMGVSINAQTNKDSDYIRAVKTISMVLHKRMFNIFYRFDLTYMLTPLARA

ERRALKVLHSFTEKIIVQRRQELLRPNSSTQSSDDTDVGAKRKMAFLDILLQSKIDDKPL

TNADIREEVDTFMFEGHDTTSSAIMFFFYNIALYPECQRKCVEEIISVLGNDRETPVTYD

LLNNLHYMDLCIKETLRMYPSVPLLGRKVVQECEINGKIIPAGTNIGISPLLLGRREDLF

SEPNTFKPERFDVVTSAEKLNPYAYIPFSAGPRNCIGQKFAVLEIKAIAANVLRHYEVEF

VGNAEEPPVLIAELILRTKDPLMFKLKQRML

>156548380

MVPLLLLFTLLAIVGFVISRWIYEYVGLRQKLSTIPGPTLLPFVGIVLERMRIPKEDVWQ

WFNDLCFKFESGMVLAWVGTQPIVILRRPQQMEVILPSNTLITKAATYDYMKPWLGDGLV

TSTGELWFSHRRLITPAFHFGVLEEYGAVMREKVEIFKECIESELKTDPKAPINIFGLVV

KYTLDTICETAMGVNIDTQRNPESAYVKAVHTYARLTVERFYKPWLKWNAIYYRTNKGKE

ALNAVKIMHSFTEKVIRQKQAERKEKSFSNKELSDEVDEFGKRKRKAFLDLLLSASENAS

NPLTFEELREEVDTFMFAGHDTTSSAISWGLFALANAPEIQAKVHKELQEIFGDSGETAN

SKQLSELKYLDRVIKEVLRLYPSAPMVSRRLTHDTVIDNHHVPKGTFVNIHIYQMHHDPK

VWKDPETFDPDRFLPENIRSRHPYSYVPFSAGPRNCIGQKFALLEVKTALTAILRKWQIS

SVLKPTEIKMIHTFILRPQNESLELYFTPIVK

>EDV91972.1

MSFSLPLIIAAVCIVLALATLSWLPLRSWLRRRRRAYELAALLPGPKTLPLLGNVNLFLG

LEPSQVLLVIVELAEKYGDTFKLKMGFNFSFMMFKPRDVEVVLGSTFLLDKAMEYDFLSD

WLNEGLLVSGGRKWHQRRKIITPAFHFRILESYVEIFERQTRQMLERLHQARGSDGTNLI

ELGHAAHLLTLDVICELDSPSLEGLLLVISSLLKFKHLIEVTRDLGSPPSLPLVGHAHHF

IGKAPHELMQTIFNFLEKYGKDMTLKIWLGPELNLLMSNPEDLKIILGTLLFNDKAGEYK

ALEPWLKEGLLVSGGRKWHNRRKIITPAFHFKILEQYINIFQQQSVLLLSNLEKERRQQG

QTGFNLYDWINLCTMDTICETAMGVSVNAQTNVDSEYVQAVKTISMVLHKRMFNIFYRFD

LTYMLTPLARAEKRALNVLHNFTEKIIVQRREELLRAGQQDTEPQSAGDADVGAKRKMAF

LDILLQSTIDDKPLTNMDIREEVDTFMFEGHDTTSSAIMFFFYNIATYPDCQRKCLDEII

SVMGKDKESPVTYNMLNKLSYVDLCIKETLRMYPSVPLLGRKVLKECEINGKIIPAGSNI

GISPLYLGRREDLFSDPNTFKPERFDVVTSAEKQNPYAYIPFSAGPRNCIGQKFAILEIK

AVVANVLRHYEIEFVGNAAEPPVLIAELILRTKDPLMFKLKERVY

>EFX88241.1

MLNLLLFFFVALIVWYWSRERSYFFQQIDNIPGPLKLPFLGNILALPRDGPGLLQIFNVK

WTRAYGHIYRFWRGMFPIVNISSPSDVEAILASQVHINKSVTGAFLLPWLGDGLLLSGGN

KWRKDRKLLTPAFHFQILDGFFDVFNRNSQIFVEQISKRILDENEVDIYPMTSRCTLDII

CEAAMGIQINAQVNYDSEYINAIDRVESMIQGRVNSLLGLLPDWIYFQFTPHGRECQTYL

DIVHSFTRKVVVARKTIVNQQVVDEVECDSGPKKRRAFLDLLLESARNGESDMSEADIIN

QVDTFMFEGHDTTSAAVTWFLYCMATHPAEQQDRVYEELYECFGDSDRPCSLEDLSKLKY

LECCIKESLRRHPPVPLIRRRVNEDVRLSGFNVPADTSLGIQIYALHRNEEFFPDPEAFK

PERFQPDQVIGRNPFAYVPFSAGPRNCIGQKFAMYEDKVIVSTLLRQFRFGIDVHRLPIK

ESLNMILKPEGGMPLLIAPRR

>EAT44585.1

MSATVAPADPVMANANIASPMNVFYFLLAPALLLWFIYWRISRQHMLKLAEKIPGPPGLP

LLGNALELIGTSHSVFRNVIEKGKDFNQVIKIWIGPKLIVFLVDPRDVELLLSSHVYIDK

SPEYRFFKPWLGNGLLISTGHKWRQHRKLIAPTFHLNVLKSFIDLFNENSRLVVEKMHKE

AGKTFDCHDYMSECTVEILLETAMGVSKKTQDQSGFDYAMAVMKMCDILHLRHRKMWLYP

DLFFNMSQYAKRQVKLLDTIHSLTRKVIRNKKAAFATGTRGSLATTSIKTAEFEKPKSNI

NTNSVEGLSFGQSANLKDDLDVDENDVGEKKRLAFLDLLLESAENGALISDEEIKNQVDT

IMFEGHDTTAAGSSFFLSMMGIHQHIQDKVIQELDDIFGDSDRPATFQDTLEMKYLERCL

METLRMYPPVPIIARSLKQDLKLASSDLVVPSGATIVVATYKLHRLETIYPNPNVFDPDN

FLPERQANRHYYAFVPFSAGPRSCVGRKYAMLKLKVILSTILRNFRVISDLKEEDFKLQA

DIILKREEGFQIRLEPRQRKPKAAKA

>ACZ97414.1

MSNIVDDDVAYHYSTSRLVFYPLLLFASGLWLLYRWQQQSRLFKMGNRLPGPMALPLFGN

ALLAVGKRPDDLVNVALEYADKYGTVIRGWLGSKLIIFLTDPDDIEVILNSQIHIDKASE

YRFFKPWLGEGLLISSGEKWRSHRKMIAPTFHINVLKSFIPIFNQNSKNVVEKLKPEIGR

VFDVHDYMSEATVDILLETAMGITKKTQTESGFDYAMAVMKMCDIIHQRHYKLWLRFDAL

FKLTSFYKQQIKLLNIIHSLTNKVIKSKQKVYLENKAKGITPPTLEELTRSSDSHEPGAE

GDTKTLADQVFKGYRDDLDFNDENDVGEKKRLAFLDLMIESAQNGTNKISDHEIKEEVDT

IMFEGHDTTAAGSSFILSLLGVHQDIQEKVYQELYEIFGTSDRPVTFGDTLRMKYLERVI

FESLRMYPPVPIIARKLKRDVKIATNNYVLPAGSTIVIGTLKLHRDPKYYKNPNVFNPDN

FLPENTQERHYYSYIPFSAGPRSCVGRKYALLKLKVLLATILRNYKTISVVPEEEFKLQA

DIILKRSDGFRVRIEPRNRVPMSTA

>EFX88054.1

MIGVFLVSVLLLVLALLFLAHHRWENSAYVRTIDLIPGVRRKFIVGNVTALPKEIDENLE

IVQGKWVKQYGRIYRIWLAFRPFVQISSPDFIEKILTSHTNIDKGKSYSILRPWLGDGLL

LASGDKWRRSRRLLTPAFHFQILDNFFDVFNKNAEILCEQLAKVTKTFTPGEFAEDVDVF

PFLKKCTLDIICEAAMGITVNAQLEDSEYIRNVHRISEIVIDRFLSGKGMFPDWLYHLTP

SGREHKKILKQMHDFTSKVIRERKVEIALEEDLPEVKKEKRRAFLDLMLLANKNGVELSD

LDIRNEVDTFMFEGHDTTASAIVWFLYCMAINPKHQALVQEELNEVFGDSDRSCTMEDAT

KLKYLECCIKESLRLYPPVPIFARYMTEEIELGGYSIPKGTFISLQTFALHRNEEYFPDP

DVFKPERFQTNEAIGRHSFAYVPFSAGSRNCIGQRFAMFEEKVLSSTLLRRFRFYYDLDK

LGPRKAIPDLVLKPKNGMPLQIAPYLQ

>383847823

MEGSWVTLVLSSFLMTIVLLLLVRRGKFLYALRKVPYPPALPIIGNAYQLCCSPEEAFKK

MIKWGRELGDIHLIWVGMRPFIFLYNAEAIQPLLSSSIHIDKSLEYKYLRPWLGTGLVTS

SGNKWHFRRKLLTPTFHSGLLEVYLKTTIREAEILISCLKKEIDKPAFDIVPYIKRATLD

IICDSSMGCNVNAQTNFKNEYVQAVNTLASISQRRFLNVWMSLDPIFKLTSWGQKHDHAL

RVTHEFVNKIIAERKEEWKAKKYENSNVQFNKHRALLDLLLELSQDGKVLTDDDIRDEVN

TFMFAGHDTTANSVSWILYVLGRHPQYQEKILNEYYEVTKEKKLTLDLLSKLTWLEACIK

ECWRIYPVVPLIARQIYHPITILGHNIPIGSTVVVNSFLLHRDPRYFPDPDAYKPERFLP

NGLKYPSYAFLPFSAGSRNCIGSKYAIMMVKVLSLFVLKNFHVISLDAEDQLRFISELVL

HNANGLRLKITPRKQETDVIILQCMHSNKKC

>EDV45575.1

MWLLLSLVLLLAILALEMRRFLRKMSTIPGPLPLPLLGNAHLFLGLSAAEACLKMGELAE

RHGDTFGLFLGPSYSVLLLNPRDVERVLGNSQLLTKSHEYSFLGRWLNEGLLVSNGSKWH

RRRKIITPAFHFRILEPYVEIFDRQSLRLVEQLGLRMRRGQERINLGEAIHLCTLDAICE

TAMGVSINAQSNADSEYVQAVKTISMVLHKRMFNILYRFDLTYMLTPLARAEKKALDVLH

QFTEKIIVQRREELIRGGSSQESSNEDADVGAKRKMAFLDILLQSTVDERPLSNLDIREE

VDTFMFEGHDTTSSALMFFFYNIATHPEAQKKCFEEIRSVVGNDKSTPVSYELLNKLHYV

DLCVKETLRMYPSVPLLGRKVLEDCEINGKLIPAGTNIGISPLYLGRREELFSEPNSFKP

ERFDVVTTAEKLNPYAYIPFSAGPRNCIGQKFAMLEIKAIVANVLRHYEVDFVGDSSEPP

VLIAELILRTKDPLMFKVRERVY

>340727270

MDSSYSFRMSLPMIAIGGSWLTLILSTCLMLIVLLLVVRRGRFLYALRKVPYPPALPIIG

NAYQLCCSPEEAFKKMLKWGKELGDMYLVWVGMRPFIFLYKAEAIQPLLSSSVHIDKSLE

YGYLRPWLGSGLVTSTGEKWHFRRKLLTPTFHSGLLEVYLKTAIKEAEILISCLNQEVDK

PEFDIVPYAKRATLDIICDSSMGCNINAQKNFENEYVLAVNTLASISQRRFLNVWMSFDP

IFKLTSWGKEYDQALRVTHGFINKIIAERKAEWKARKDGNCHERSHKRQALLDLLLELSK

DGEVLSDIDIRDEVNTFMFAGHDTTATSISWFLYALGRHPQYQEKILEEYYEIAKTEELS

LDILSKLTWLEACIKESWRIYPVAPLIARQIYHPITILGHEIPVGSTVLVNSFLLHRDTR

YFPEPDTYRPERFLPNGPKYPSYAFIPFSAGSRNCIGWKYATMIVKVLILHILKNFKVES

LDTEDQLRFTSELVLHNANGLRLKITPRK

>EGI65082.1

MQIETVSVIADRYHQHPMHTDCIQYTDRNSLLGDPESWSLMNSLYDKALVEASRIFTLEI

TDGDFSAELFGDAAPLDSLNNKWFLNLSNIDIPIEVQYLLQLGEERLKLFLTIMENCKEG

IFIQYIGATPNINIFKPEYLEHIFPSTVNITKGDFYDMLKPWLGNGLLTSTDEQWFHDRK

LIGPTFHFNILDQFAGIMFEKAEILTKCLQKEIEKEPGKAVNIFPFIINAALDIICETAM

GVDVHAQEVVTKYTSTVHLASSLIMKRLFRPWYWIEWLFYSVPTGKQFKSSLDILHGFTK

EVINKRKIERQSQNGHMEFENEDNEFNIGKRKRQAFLDLLLDQNAKSDTPLTDDELRAQV

DTFMFEGHDTTAVAITWTLFLLGDNLEHQAKVHEELEEVFGASETSASIKELSKLKYLDR

VIKETLRIFPSVPMISRTLTEDVKIDNYILPKGVMITLAILLTHRNSMVWPDPLKFDPDR

FLPENSKNRNPYAYVPFSAGPRNCIGQKFAQLEEKIVLTTILRKWRVKSVKSVDTIKFGG

SLILRPSEDVLLHFTPKK

>EFX88055.1

MLGVFLVSVFLLLLALLFLAYHRWENSSYVKTIDLIPGVRRKFLVGNITALPKESDEVLE

TAQIKWVKQYGRILRTWIAFRPFVQISSPVLIEKILTSHTLIDKGKSYSILRPWLGDGLL

LASGDKWRRSRRLLTPAFHFQILDNFFDVFNKNAEILCEQLAKVTKTFTPGEFAEDVDVF

PFLKKCTLDIICEAAMGIQINAQLQDSEYIRNVHRISEIIIERFLSGKGMLPDWLYNLTQ

NGREQNKILKQMHDFTSKVIRERKVEIALEEDLPEEAKDEEITGTKKRRAFLDLMLLANQ

NGVELSDLDIRNEVDTFMFEGHDTTASAIVWFLYCMAINPKHQALVQEELNEVFGDSNRP

CTMEDTTKLKYLECCIKESLRLYPPVPVFSRYMTEEIELGGYKIPTEAFVNLQIYALHRY

EEYFPEPEVFNPERFQTNESIGRHAFAFVPFSAGSRNCIGQRFAMFEEKVLSSTLLRRFR

FSYDLGKRGPRKAIPELVLKPKDGMPLQIVAI

>EFN62598.1

MDFISLILCTLFVILIIPVIILTYHQYILRRKLKNIPQYGRFPFGVVFEVMKLTNYELVQ

WYLQRAVQFKEGIFIHWVTIFPFINLYKPELLEIIMTSTVNIEKGHPYQLLESWLGKGLL

TSTGKQWFHDRKLIGPTFHFSILDQFAVVMYEKAKILIKCLEKEIAKNPGMAINIYPFIN

NAALDVICETAMGVNIHAQEDETKYTAVIHRVSKLVAKRLIEPWLWPDWLYNLISEGIEY

KSMVNKLHKFTREIIHKKKIIRQSQNDSTKAENENDELDIGKLEKKAFLDLLLDQNEKDE

IPLTDDELRAQVDTFMFEGHDTTAVAITWALFLLGNNLEHQNKVHEELKEVFKDSETPAN

IKELSQLKYLDRVIKEALRLFPSVPAITRKLSEDVKIGDYTFPKGITVVLAIATVHRNPE

VWSDPLKFDPDRFLPENSKHRNPYAYIPFSAGPRNCIGQKFALLEEKMMLTAILRKWRVE

SVKELIEFEATLILRPTEKIFIHFTPKK

>O18596.1

MSLSLPPLIAVACLVVALARISWLPLRSWLRRRRRTHQLAAQLPGPRNLPLLGNFHMFFG

LEPWQVPHLINQLAKKYDGTFKLKMGSNFSLMMFQPRDIEVVLGSSQLLDKAVEYSFLRG

WLNDGLLLSGGRKWHRRRKIITPAFHFRILESYDEIFDRQTRLLIHKWQQTLGHSFDLGH

DVHLFTLDVICETAMGVSTNAQTNADSDYVRAVKTISTVLHKRMFNIFYRFDLTYMLTPL

AWAERRALNVLHKFTEKIIVQRREELLRGGVTQTTDGADVGAKSKMVFLDILLQSNIDDK

PLTNLDIREEVDTFMFEGHDTTSSGITFFFYNIALYPECQRKCVEEIVSVLGKDTETPVT

YDLLNNLNYMDLCIKETLRMYPSVPLLGRKVLQECEINGKIIPAGTNIGISPLFLGRSED

ISSEPNTFKPERFDVVTSAEKLNPHAYIPFSAGPRNCIGQKFAMLEIKAIAANVLRHYEI

EFVGNAEESPVLIAELILRTKDPLMFKLKKRVI

>CAA47887.1

MFLVIGAILASALFVGLLLYHLKFKRLIDLISYMPGPPVLPLVGHGHHFIGKPPHEMVKK

IFEFMETISKDQVLKVWLGPELNVLMGNPKDVEVVLGTLRFNDKAGEYKALEPWLKEGLL

VSRGRKWHKRRKIITPAFHFKILDQFVEVFEKGSRDLLRNMEQDRLKHGDSGFSLYDWIN

LCTMDTICETAMGVSINAQSNADSEYVQAVKTISMVLHKRMFNILYRFDLTYMLTPLARA

EKKALNVLHQFTEKIIVQRREELIREGSSQESSNDDADVGAKRKMAFLDILLQSTVDERP

LSNLDIREEVDTFMFKGHDTTSSALMFFFYNIATHPEAQKKCFEEIRSVVGNDKSTPVSY

ELLNQLHYVDLCVKETLRMYPSVPLLGRKVLEDCEINGKLIPAGTNIGISPLYLGRREEL

FSEPNIFKPERFDVVTTAEKLNPYAYIPFSAGPRNCIGQKFAMLEIKPSWPMCSGTTRLT

LWATSFGTTRADRRTYSAYQGPLSSRCGRVY

>EDW06073.1

MTLSLSLLIAAVCIVVALAGISWLPLVAWLRRRRRTYELAAQLPGPRNLPLLGHFHMFFG

LEPWQVPQLIAQLAEQYDGTFKLKMGSNFSLMMFQPRDMEVVLGSSQLLDKAIEYSFLHG

WLNDGLLLSSGQKWHRRRKIITPAFHFRILEPYVEIFDRQTRVLIRKWQQTLGHSFDLGH

DVHLFTLDVICDVYAKDHTLNVWLSPELNVLMGDAKDLEIVLGTALWHKRRKIITPAIHF

RILEEFVDICEHESRVLLKNLKKERQQQGQGGFDLSEWIDLFTMDTICETAMGVSTNAQT

NADSDYVRAVKTISMVLHKRMFNIFYRFNLTYMLTPLARAERRALKVLHSFTEKIIVQRR

QELLRANSSTQSSDDTDVGAKRKMTFLDILLQSKIDDKPLTNADIREEVDTFMFEGHDTT

SSAIMFFFYNIALYPECQRKCVEEIISVLGNDRETPVTYDLLNNLHYMDLCIKETLRMYP

SVPLIGRKVLQECEINGKMIPAGTNIGISPLFLGRREDLFSEPNTFKPERFDVVTSAEKL

NPYAYIPFSAGPRNCIGQKFAVLEIKAIAANVLRHYEVEFVGNAEEPPVLIAELVLRTRD

PLMFKLKERVL

>EAT40473.2

MWIYLSLLTVGFVAVVIYRKFARTLEVAQQYAGPPALPILGNGLWFLNKQPDEFLPIIHK

LTSTYGDVVRFWQGPQFTLYVGNPSMIETILTNKHLTDKSGEYDYLSNWLGDGLLLSKRH

KWHARRKAITPAFHFKILEQFVDVFDRNAAELVDVLEKHADDGKTFDMFPYVLLYALDVI

CESAMGTSVNALRNADSEYVRAVKEAAHVSIKRMFDIIRRTSLFYLTPSYQKLRKALKVL

HGYTDNVIVSRRNQLMSKTDSGGVSDEFGAKKKDAFLDMLLRTSINGKPLTNLEIREEVD

TFMFEGHDTTTSAVVFTLFNLAKHPEIQQKVYDEIVSVIGKDPKEKIELSHLHDLSYTEM

AIKETLRLFPSVPLIGRRCVEEITIEGKTIPAGANIIVGIYFMGRDTKYFENPSQFIPER

FEGEFSVEKFNPYKYIPFSAGPRNCIGQKFALNEMKSVISKLLRHYEFILPPDSVEEPPL

ASELILKPHQGVPLQIRHRALN

>EAT40474.1

MWFLLSLVAAACLAWAIYRKFARTLEISGQHTGPPALPILGNGLWFLNKQPDEFLPIIQR

LTDEYGDVFRFWQGPEFTLYVGRPSMIETLLTDKNLTDKSGEYGYLSNWLGDGLLLSKRN

KWHARRKAITPAFHFKILEQFVDVFDRNASELVDVLGKHADSGEVFDIFPHVLLYALDVI

CESAMGTSVNALRNADSEYVRAVKEAANVSIKRMFDFIRRTPLFYLTPSYQQLRKSLKVL

HGYTDNVITSRRKQLSNSSNKNHKDSDDFGFRRKEAFLDMLLKTNINGKPLTDLEIREEV

DTFMFEGHDTTTSAVVFTLLNLAKHPAIQQKVYDEIESVIGNDLQKPIELSDLHDLSYLE

MVIKETLRLYPSVPLIGRRCVEETTIEGKTIPAGANIIVGVFFMGRDPNYFEKPLDFIPE

RFSGEKSVEKFNPYKYIPFSAGPRNCIGQKFALNEMKSVISKLLRHYEFILPAGSPAEPL

LASELILKPHHGVPLQIRRRGH

>EDS33500.1

MQYDFVNLIQKLTDDYGDVVRFWMGPQFTIYTGNPKWIEAILTNRNLTNKSDEYGFLSNW

LGDGLLLSKRHKWHARRKIITPAFHFKILDQFVEVFDRNAAELVDVLGKFAANGKTFDVF

PYILLYTLDVICETAMGTSVDAMRNSDSEYVKAVKEAASISITRMYDVYMRTPLFYLTPG

YQKLRKAIKMLHAYTDNVIVSRRKQLEASSNQSAEVASEEHEYGGKKKEAFLDLLLKTSI

EGRPLTNLEIREEVDTFMFEGHDTTTSGVSFTLYNLAKYPAIQQKVHDEIIAVLGTDPHK

PITMANLNELTYLEMVIKETLRLFPSVPIIGRKCVEEVTIEGKTVPAGTNIIIGIYYMGR

DPNYYDNPLEFIPERFEGEKSVEKFNPYKYVPFSAGQRNCIGQKFALNEVKSVISKLLRH

YEFVLPADQNHEPPVLSSELILKPHHGIPLQIKPRAPLTH

>EDS33499.1

MGRMVDPDIVYMHLNFVNLIQKLTDDYGDVVRFWMGPQFTIYTGNPKWIEAILTNRNLTN

KSDEYGFLSNWLGDGLLLSKRHKWHARRKIITPAFHFKILDQFVEVFDRNAAELVDVLGK

FAANGKTFDVFPYILLYTLDVICETAMGTSVDAMRNSDSEYVKAVKEAASISITRMYDVY

MRTPLFYLTPGYQKLRKAIKMLHAYTDNVIVSRRMQLEASSNQSAEVASEEHEYGGKKKE

AFLDLLLKTSIEGRPLTNLEIREEVDTFMFEGHDTTTSGVSFTLYNLAKYPAIQQKVHDE

IIAVFGTDPHKPITMANLNELTYLEMVIKETLRLFPSVPIIGRKCVEEVTIEGKTVPAGT

NIIIGIYYMGRDPNYYDNPLEFIPERFEGEKSVEKFNPYKYVPFSAGQRNCIGQKFALNE

VKSVISKLLRHYEFVLPADQNHEPPVLSSELILKPHHGIPLQIKPRAPLTH

>EAT37902.1

MIAIIAFTAIFVLFVYVWQWRRRLSRPFRTVPGPPGLPLIGNCHQFIGKSSTNIFHMLIE

LERLYGSVFKVDVATGIWLFYMSPGDIERIMTGPEFNCKSDDYDMLLEWLGTGLLISNGN

KWFTHRKALTPAFHFKILDNFVQVFDEKSTILARKFLSYSGKVVGIFPLVKLCTLDVIVE

TAMGTESNAQTEESGYTMAVEDISEIVFWRMFNNVYNTEFMFKLSNKYGTYKKCLETIRE

FTLSIIEKRRSTLNVFDKNGGTSEVCNDSTGLKKKMALLDILLQTEIDGRPLTNEEVREE

VDTFMFAGHDTTASAITFLLYAMAKYPDVQQKVYEEAVSVLGDSIDTPITLSALNDLKYL

DLVIKESLRMFPPVPYISRSTIKEVELSGCTIPTGTNITVGIFNMHHNPKYFPDPEEFIP

ERFEVERGVEKQHPYAYVPFSAGGRNCIGQKFAQYEIKSTISKVIRLCRIELIRPNYEPP

LKAEMILKPQDEMPLRFFPR

>EAL29795.2

MLLLTLAFGALAVLLTWDFVRKRQRAATLERSKISGPLSLPVLGNGLQALDLGAENIVDW

VEESFRLYGKTFRMWILGESLIYTKDLKYFEAILGSTTLLQKGQLYKFLRPFLNDGLLLS

VGKKWHARRKVFTNAFHFNVLGHYVDIMDRQAAVMVEKLRQVADGETTVDMLPYVSLAAL

DVITEAAMGVQVNAQSDSEFPYVQALKSVVYIQSDRMFRFLQRYDWLFRLTCPVLRSKLL

KDVRVMHDFTDKVIRERRAAVEQAKADGSYRPLSLGDPEVGRKSQMALLDILLQATIEGQ

PLSDADIREEVDTFMFEGDDTTSSGVSFALYAIARHPQVQARIYRELLQVLGRDPSTPVT

QAKLQELKYLDCVIRETLRLYPAVPAVGRYTSKEIQIGGQTIPANTSIYLVLYFAHREAE

FFPDPLAFKPERFLDRADSEDRETFAYLPFSAGPKNCIGQKFAMLEMKALVSKVLRCYEL

LPRGPEVKPMMNFILRSSTGMNVGLRPRRSP

>EDV50308.1

MWLTLITGTLILLLTWDFRRKRQRVLAFKKSGIPGPISIPILGCGLQALHLGAENIIGWV

GEKFDQYGKTFRFWILGESLIYTKDLKYFETILSSTTLLEKGQLYQYLRPFLNDGLLVST

GRKWHARRKIFTQAFHFKVLEHYVEIMDRNSAVMVDNLRKVADGKSVVDMLKYVSLAALD

VITEAAMGVQVNAQKDPDFPYVKALKSVVYIQPDRMFRFSRRYNWLFPLAAPLLHRQLLS

DIRVMHDFTDKVISERRETVRRAIADGTYRPLSLGDDEIGSKSQMALLDILLQSSINNQP

LSDADIREEVDTFMFEGDDTTSSGVSHALYAIARHPEVQQRIYEELQRVLGPDPSAPVTQ

AQLQDLKYLDCVIKETMRLYPPVPAIGRHAQQELKIGNKTIPANTSIYLVLYFAHRDPDY

FPDPLSFKPERFLEGSEQGHDTFAYVPFSAGPKNCIGQKFAVLEMKALISKVLRFYELLP

LGEELKPMLNFILRSASGINVGLRPRKS

>EDW46936.1

MWFVLYIFLALPLLLVAYLELSTFRRRRVLNKFNGPSGLPLMGNAHQMGKNPSEILDTVF

SWWHQYGKDNFVFWIGTYSNVLVTSSKYLEFILSSQTLITKSDIYQLTHPWLGLGLLTST

GSKWHKHRKMITPAFHFNILQDFHEVMNENSTKFIKHLKTVAAGDNIFDFQDQAHYLTLD

VICDTAMGVSINAMENRSSSIVQAFKDMCYNINMRAFHPLKRNELLYRLAPDYPAYSRTL

KTLQDFTNEIIAKRIEAHKSGAVSTSTGDEFTRKKMAFLDTLLSSTIDGRPLNSKELYEE

VSTFMFEGHDTTTSGVSFAVYLLSRHQDEQSKLIKEQREVMGNSELGRDATFQEISQMKY

LDLFIKEAQRVYPSVPFIGRYTEKDYVIDGDLVPKGTTLNLGLVMLGYNEKVFKDPHMFR

PERFELEKPGPFEYVPFSAGPRNCIGQKFALLEIKTVVSKIIRNFEVLPALDELVSKDGY

INTTIGLPDAERKKRDPYRHKYDPILSAVLTLKSENGLYIRLKERH

>EDW89347.1

MWFVLYIFLALPLLVVAYLELSTFRRKKMLNKFKGPSGLPLVGNAHQMGKNPTEILDTVF

SWWHRYGKDNFVFWIGTYSNVLVTSTKYLEFILSSQTLITKSDIYQLTHPWLGLGLLTST

GSKWHKHRKMITPAFHFNILQDFHEVMNENSTKFIKHLKTVAAGDNIFDFQDQAHYLTLD

VICDTAMGVSINAMENRNSSIVQAFKDMCYNINMRAFHPLKRNELLYRLAPDYPAYSRTL

KTLQDFTNEIIAKRIEAHKSGAISTSAGDEFTRKKMAFLDTLLSSTIDGRPLNSKELYEE

VSTFMFEGHDTTTSGVSFAVFLLSRHQDEQRKLFEEQREVMGNSELGRDATFQDISQMKY

LDLFIKEAQRVYPSVPFIGRFTEKDYVIDGDIVPKGTTLNLGLVMLGYNEKVFKDPHKFR

PERFNLEKPGPFEYVPFSAGPRNCIGQKFALLEIKTVVSKIIRNFEVLPSLDELVSKDGY

ISTTIGLPDAERKKRDPYRHKYDPVLSAVLTLKSENGLYIRLKERQ

>AAC47424.1

MGNAHQMGKNPSEILDTVFSWWHQYGKDNFVFWIGTYSNVLVTSSKYLEFILSSQTLITK

SDIYQLTHPWLGLGLLTSTGSKWHKHRKMITPAFHFNILQDFHEVMNENSTKFIKHLKTV

AAGDNIFDFQEQAHYLTLDVICDTAMGVSINAMENRSSSIVQAFKDMCYNINMRAFHPLK

RNELLYRLAPDYPAYSRTLKTLQDFTNEIIAKRIEAHKSGAVSTNAGDEFTRKKMAFLDT

LLSSTIDGRPLNSKELYEEVSTFMFEGHDTTTSGVSFAVYLLSRHQDEQRKLFKEQREVM

GNSELGRDATFQEISQMKYLDLFIKEAQRVYPSVPFIGRFTEKDYVIDGDLVPKGTTLNL

GLVMLGYNEKVFKDPHKFRPERFELEKPGPFEYVPFSAGPRNCIGQKFALLEIKTVVSKI

IRNFEVLPALDELVSKDGYISTTIGLPDAERKKRDPYRHKYDPILSAVLTLKSENGLYIR

LKERH

>EDV59522.1

MWIVLYILLALPLLVVAYLELSTYRRKKVLNKFNGPSGLPLVGNAHQMGKNPAEILDTLF

SWWHQYGKGNFAFWIGTYSNVLVTSSTYLEFILSSQTLITKSDIYELTHPWLGLGLLTST

GSKWHKHRKMITPAFHFNILQDFHEVMNENSAKFIKHLKSVAAGDNIFDFQEEAHYLTLD

VICDTAMGVSINAMENRSSSIVQAFNDMCYNINMRAFHPLKRNEMLYRLAPDYPAYRRTL

KTLQDFTNEIIAKRIEAHKSGAVSTSEGDEFTRRKMAFLDTLLSSTIDGRPLNSRELYEE

VSTFMFEGHDTTTSGVSFAVYLLSRHQDEQRKLFDEQRQIMGDSKLGRDATFQDISQMKY

LDLFIKEAQRVYPSVPFVGRFTEKDYVIDGDLVPKGTTLNLGIVMLGYNEKVFKDPHKFR

PERFELEKPGPFEYVPFSAGPRNCVGQKFALLEIKTVVSKIIRNFEVLPALDELVSKDGY

ISTTIGLTDAERKKRDPNRHKYDPILSAVLTLKSENGLHIRLKERH

>EDV35666.1

MWVVLILILALPLLAAAYLQLTTYRRRKVLKKFNGPKGLPLIGNAHQMGKNPSEILDQVF

AWWHQFGKDNYVFWIGSYSNLLVTNPKFLEYILSSQTLISKSDIYDLTHPWLGLGLLTST

GSKWHKHRKMITPAFHFNILQDFHEVMNENSSKFIKHLQKVAAGDTIFDFQEQAHYLTLD

VICDTAMGVSINAMENRNSSIVQAFKDICYNINMRAFNPLKRNEWLYRLAPDYPAYSRTL

KTLRDFTNEIIDKRIEAHKSGSVTTSQGDEFSRKKMAFLDTLLSSTIDGRPLNSTELYEE

VSTFMFEGHDTTTSGVSFAVYLLSRHPDEQKKLFAEQCEVMGTSELNRDATYQEIAQMKY

LDLYIKEAQRVYPSVPFIGRYTDKDYEIDGKLVPKGSTLNLGIVMLGYNDKVFKDPLKFS

PERFEQQKPGPFEYVPFSAGPRNCIGQKFALLEIKTVVSKIIRNFKVLPALDELVSKDGY

ISTSVGLPPAERAKREEHRHKYDPILSAVLTLKSENGLHIRLKERN

>EDW31620.1

MWFVLYLFLALPLLLVAYLELSTFRRRRALNKFKGPSRLPIVGNAHQAGKNPTEILDQVF

SWWHQYGKDNFRFWIGSYSNLIITNHKYLEFILSSQTLITKSDIYNLTHPWLGLGLLTST

GSKWHKHRKMITPSFHFNILQDFHEVMNENSTKFIKQLKEVAAGDSIFDFQNETHYLTLD

VICDTAMGVSVNAMENRSSSIVQAFKDMCYCINMRAFHPFKRNDLFFRLAPDYPAYCKTL

KTLQDFTNNIIEKRINAHKDGTVSASQGDEFSRKKMAFLDTLLSSTIDGRPLTTQELYEE

VSTFMFEGHDTTTSGVSFAVFLLSRHQDEQRKLFDEQREVMGDSGMDRDATFQEISEMKY

LDLFIKEAQRVYPSVPFIGRYTEKDYIIEGDLVPKGTTLNLALVMLGYNDRVFKDPHKFR

PERFELEKPGPFEYVPFSAGPRNCIGQKFALLEIKTVVSKIIRHFEVLPALDELASKDGY

ISTSLGYPPEERKKRDLLRHKYDPLLSAVLTLKAENGLFIRLKERH

>EAL24874.1

MWFVLYLFLALPLLLVAYLELSTFRRRRALNKFKGPSRLPIVGNAHQAGKNPTEILDQVF

SWWHQYGKDNFRFWIGSYSNLIITNHKYLEFILSSQTLITKSDIYNLTHPWLGLGLLTST

GSKWHKHRKMITPSFHFNILQDFHEVMNENSTKFIKQLKQVAAGDSIFDFQNETHYLTLD

VICDTAMGVSVNAMENRSSSIVQAFKDMCYCINMRAFHPFKRNDLFFRLAPDYPAYCKTL

KTLQDFTNNIIEKRIKAHKDGTVSASQGDEFSRKKMAFLDTLLSSTIDGRPLTTQELYEE

VSTFMFEGHDTTTSGVSFAVFLLSRHKDEQRKLFDEQREVMGDSGMDRDATFQEISEMKY

LDLFIKEAQRVYPSVPFIGRYTEKDYIIEGDLVPKGTTLNLALVMLGYNDRVFKDPHKFR

PERFELEKPGPFEYVPFSAGPRNCIGQKFALLEIKTVVSKIIRHFEVLPALDELASKDGY

ISTSLGYPPEERKKRDLLRHKYDPLLSAVLTLKAENGLFIRLKERH

>EDX15482.1

MWIVLCAFLALPLFLVTYFEFGLLRRKRMLNKFQGPSMLPLVGNAHQMGKTPTEILNRFF

GWWHEYGKDNFRYWIGYHSNIMVTSPKYMEFILSSQTLISKSDVYDLTHPWLGLGLLTST

GSKWHKHRKMITPAFHFNILRDFHEVMNENSSKFIDQLKKVADGGNIFDFQEEVHYLTLD

VICDTAMGVSINAMENRSSSVVQAFKDITYTIKMRAFSPWKRNEYLFRFAPEYPEYSKTL

KTLQDFTNEIIAKRIEVRKSGLEDDIKADEFSRKKMAFLDTLLSSTVDGRPLTSQELYEE

VSTFMFEGHDTTTSGVGFAVYLLSRHPDEQEKLFKEQCDVMGSSELGRDATFQEISTMKH

LDLFIKEAQRLYPSVPFIGRFTEKDYVIDGDIVPKGTTLNLGLLMLGYNDRVFKDPHKFQ

PERFDREKPGPFEYVPFSAGPRNCIGQKFALLEIKTVVSKIIRNFEVLPALDELVSKDGY

ISTTLGLPPEEKKSRDAHIHKYDPILSASMTLKSENGLHLRMKPRLV

>EDW89348.1

MWFVLCAFLALPLFLVTYFEFGLLRRKRMLNKFQGPSMLPVVGNAHQMGKTPTEILNRFF

GWWHEYNKDNFRYWIGFYSNIMVTSPKYMEFILSSNTLISKSDVYDLTHPWLGLGLLTST

GSKWHKHRKMITPAFHFNILQDFHEVMNENSTKFIDQLKKVADGGNIFDFQEEAHYFTLD

VICDTAMGVSINAMENRSSSVVQAFKDITYTIKMRAFSPWKRNEYLFRFAPEYPEYTRTL

KTLQDFTNEIIAKRIEVRKSGLDSAIKPDEFSRKKMAFLDTLLSSTVDGRPLTSQELYEE

VSTFMFEGHDTTTSAVGFAVYLLSRHPDEQKKLFNEQCEVMGASGLGRDATFQEISTMKL

LDLFIKEAQRLYPSVPFIGRFTEKDYVIDGDTVPKGTTLNLGLLMLGYNDRVFKDPHKFQ

PDRFDRVKPGPFDYVPFSAGPRNCIGQKFALLEIKTVVSKIIRNFEVLPALDELVSKDGY

ISTTLGLPPAEKKKRDALIHKYDPILSASMTLKSENGLHLRMKQRLVRDST

>Q9V4T5.1

MWIVLCAFLALPLFLVTYFELGLLRRKRMLNKFQGPSMLPLVGNAHQMGNTPTEILNRFF

GWWHEYGKDNFRYWIGYYSNIMVTNPKYMEFILSSQTLISKSDVYDLTHPWLGLGLLTST

GSKWHKHRKMITPAFHFNILQDFHEVMNENSTKFIDQLKKVADGGNIFDFQEEAHYLTLD

VICDTAMGVSINAMENRSSSVVQAFKDITYTIKMRAFSPWKRNKYLFHFAPEYPEYSKTL

KTLQDFTNEIIAKRIEVRKSGLEVGIKADEFSRKKMAFLDTLLSSKVDGRPLTSQELYEE

VSTFMFEGHDTTTSGVGFAVYLLSRHPDEQEKLFNEQCDVMGASGLGRDATFQEISTMKH

LDLFIKEAQRLYPSVPFIGRFTEKDYVIDGDIVPKGTTLNLGLLMLGYNDRVFKDPHKFQ

PERFDREKPGPFEYVPFSAGPRNCIGQKFALLEIKTVVSKIIRNFEVLPALDELVSKDGY

ISTTLGLQPAEKKSRDAHNHKYDPILSASMTLKSENGLHLRMKQRLVCDST

>EDV59521.1

MWIVLCAFLALPLFLVTYFELGILRRKRMLDKFQGPRMLPVVGNAHQMGKTPTEILNRFF

GWWHEYNKDNFRYWIGHYSNIMVSSPKYMEFILSSHTLISKSDVYDLTHPWLGLGLLTST

GSKWHKHRKMITPAFHFNILQDFHEVMNENSTKFIDQLRKVADGGNIFDFQEEAHYLTLD

VICDTAMGVSINAMENRSSSVVQAFKDITYTIKMRAFSPWKRKEYLFRFAPEYSEYCRTL

KTLQDFTNEIIARRIEVRKSGIDVGIKADEFSRKKMAFLDTLLSSTVDGRPLNSQELYEE

VSTFMFEGHDTTTSGIGFAVYLLSRHQDEQKKLFDEQCEVMGASGLGRDATFQEISTMKH

LDLFIKEAQRVYPSVPFIGRFTEKDYVIDGDIVPKGTTLNLGLLMLGYNDRVFKDPHKFQ

PDRFDREKPGPFDYVPFSAGPRNCIGQKFALLEIKTVVSKIIRNFEVLPALDELGSKDGY

ISTTVGLPPPEKKRRDAHIHKYDPILSASMTLKSENGLHLRMKQRLVCDST

>EDV35665.1

MWVVLCILVGLPLLLVAYFELGLMRRKRMLNKFQGPRMLPVVGNAHQMGKTPTEILNRFF

GWWHEYGKGNFRYWIGSYSNILITCPKYMEFILSSQTLISKADSYDLMHAWLGLGLLTST

GSKWHKRRKMITPAFHFNILQDFHEVMNENSSKFIDQLRKVADGDNIFDFQEEAHYLTLD

VICDTAMGVSINAMENRSSSVVQAFKDMCYIIKMRAFNPWKRNEKLFRWASDYPAYKRTL

KTLQDFTNEIIEKRIEARNLNNTPESPGTEFVRKKMAFLDTLLSSKIDGRPLTSQELYEE

VSTFMFEGHDTTTSGVGFAVYLLSRHQDEQAKLFKEQCDIMGESSLCRDATFQEISQMKY

LDLFIKEAQRLYPSVPFIGRFTEKDYVIDGEVVPKGTTLNLGILFLGYNDRVFKDPHKFR

PERFEEEKPGPFEYVPFSAGPRNCIGQKFALLEIKTVVSKIIRNFEVLPALDELASKDGY

VATTVGLPAAEKAKKDALIHKYDPILSAAMTLKSENGLFLRMKSRL

>EDX06174.1

MWIVLCAFLALPLFLVTYFEFGLLRRKRMLNKFQGPSMLPLVGNAHQMGKTPTEILNRFF

GWWHEYGKDNFRYWIGYHSNIMVTSPKYMEFILSSQTLISKSDVYDLTHPWLGLGLLTST

GSKWHKHRKMITPVFHFNILRDFHEVMNENSTKFIDQLKKVADGGNIFDFQEEAHYLTLD

VICDTAMGVSINAMENRSSSVVITYTIKMRAFSPWKRNEYLFRFAPEYPEYSKTLKTLQD

FTNKIIAKRIEVRKSGLEDDIKADEFSRKKMAFLDTLLSSTVDGRPLTSQELYEEVSTFM

FEGHDTTKSGVGFAVYLLSRHPDEQEKLFKEQCDVMGSSGLGRDATFQEISTMKHLDLFI

KEAQRLYPSVPFIGRFTEKDYVIDGDIVPKGTTLNLGLLMLGYNDRVFKDPHKFQPERFD

REKPGPFEYVPFSAGPRNCIGQKFALLEIKTVVSKIIRNFEVLPALDELVSKDGYISTTL

GLPPAEKKSRDAHIHRYDPILSASMTLKSENGLHLRMKARLV

>EDW31622.1

MWLVLYAFLALPLLVWLYQELTTFRRRRLLKKFHGPKRLAIVGNAHQMGKNPTEILDQLF

AWWYQYDKDNFSFWIGPKPNIMVTKPKYIEYILTSQTLIKKAYNYALTQPWLGLGLVNSM

GSRWHKHRKMITPSFHFNILQDFHKVMDEYSTKFVQKLKNVSAGDSIFDIQDQVAYLTLD

VICNTAMGVSINAMENRTSDIAQAFRDICENVSVRSFSVWKRNQMLYRLAPDYPAYCKTL

KTLRDFTHDVIEKRVKAHKDGTASTSQGDEFSGKKMAFLDTLLSATVDGRPLTSQELYEE

VSTFIFAGHDTTTSGISFAMYLLSRHQDEQRKLFDEQREVMGDSEMGRDATYQEIAQMKY

LDLFIKEAQRVYPSVPFIGRYTEKDHLIDGDLVPKGTTLNLALIMLGYDDRVFKDPHKFR

PERFELEKPGPFEYVPFSAGPRNCIGQKFAILQIKTVVSKIVRNFEVLPAVDGLESKDGY

LNTTMGLPTAEKIKKEAYRHKYDPILSSAITLKSINGLHIRLRERN

>EDY68785.1

MWLVLYAFLALPLLVWLYQELTTFRRRRLLKKFHGPKRLAIVGNAHQMGKNPTEILDQLF

AWWYQYDKDNFSFWIGPKPNIVVTKPKYIEYILTSQTLIKKAYNYALTQPWLGLGLVNSM

GSRWHKHRKIITPSFHFNILQDFHKVMDEYSTKFVQKLKNVSAGDSIFDIQDQVAYLTLD

VICNTAMGVSINAMENRTSDIAQAFRDICENVSVRSFSVWKRNQMLYRLAPDYPAYCKTL

KTLRDFTHDVIEKRVKAHKDGTASTSQGDEFSGKKMAFLDTLLSATVDGRPLTSQELYEE

VSTFIFAGHDTTTSGISFAMYLLSRHQDEQRKLFDEQREVMGDSEMGRDATYQEIAQMKY

LDLFIKEAQRVYPSVPFIGRYTEKDHLIDGDLVPKGTTLNLALIMLGYDDRVFKDPHKFR

PERFELEKPGPFEYVPFSAGPRNCIGQKFAILQIKTVVSKIVRNFEVLPAVDGLESKDGY

LNTTMGLPTAEKIKKEAYRHKYDPILSSAITLKSINGLHIRLRERN

>EDW60848.1

MWFILYAFLALPLLIFAYFELSVAKKRRALNKIKGPPTVPLLGNAHQVGKNPAEILNTFF

EWWHKYDKDNFRIWIGYYANILVANPKHLEVIMNSNTLIKKSDIYDMLHPWLGEGLLTST

GSKWHKHRKMITPAFHFNILQDFHQVMNENSTKFIKRLKKVSAGDSIIDFQDETHYLTLD

VICDTAMGVPINAIEQRDTVEVVKAFKDMCYNLNMRAFHPFKRSELLYRLAPDYPANAKT

LKILQDFTNEIIEKRIEAHRTGIAKKTEDNEFSRKKMAFLDTLLSSTIDGRPLNQQEIYE

EVSTFMFEGHDTTTSGVAFAGYLLSRFPEEQRKLFEEQRSVMGNEMHRDATFQEIAEMKY

LDLFIKEAQRVYPSVPFIGRYTEKDYVIHGTTMPKGTTLNLALIILGYDDRTFKEPHRFY

PERFETEKPGPFEYLPFSAGPRNCIGQKFALLEIKTVVSKLVRTFEILPAVDELVSKDGY

LNTYVGLPKEEKEKKESQGHKYDPILSAVLTLKSENGLHLRLRERK

>EDW31621.1

MWFALYLVLALPLLWLAYLELKSLRRRRVLNKFKGPKVLPVVGNAHQLGRTPPEILGQMF

EWWYQHDKDNFRYWIGYYPNLMVTRTKYFEFILSSQTLISKAEVYSLMQPWLGLGLLTST

GSKWHKHRKMITPSFHFNVLQDFHEVMNEKSTKFIKHLKNVSGGGSIFDFQEQAHFLTLD

IICETAMGVSINAMENQDSELVQAFKDMCYNIKVRTFSSWKRNPTLYQFASDYPAYCKTL

KTLQDFTHDVIEKRLNAHKEGTVLASHGDEFSRKKMVFLDTLLSATVDGRPLTSQELYEE

VSTFIFAGHDTTTSAISFALYLVSRHQDEQRKLFEEQREVMGDSGLDREATFQEISEMKY

LDLFIKEAQRVYPSVPFVGRYTEKDYVIDGDLVPKGTTLHMGIFMLGYDDRVFKEPHKFR

PQRFEEEKPGPFEYVPFSAGPRNCIGQKFALLEIKTVVSKIIRHFEVLPALDELASKDGY

IRTTLGLPPTEKRKRDLQQHKYDPILLVAMSLKSENGIYLRLKERF

>EDY68784.1

MWFALYLVLALPLLWLAYLELKSLRRRRVLNKFKGPKVLPVVGNAHQLGRTPPEILGQMF

EWWYQHDKDNFRYWIGYYPNLMVTRTKYFEFILSSQTLISKAEVYSLMQPWLGLGLLTST

GSKWHKHRKMITPSFHFNVLQDFHEVMNEKSTKFIKHLKNVSGGGSIVDFQEQAHFLTLD

IICETAMGVSINAMENQDSELVQAFKDMCYNIKVRTFSSWKRNPTLYQFASDYPAYCKTL

KTLQDFTHDVIEKRLNAHKEGTVLASHGDEFSRKKMVFLDTLLSATVDGRPLTSQELYEE

VSTFIFAGHDTTTSAISFTLYLVSRHQDEQRKLFEEQRDVMGDSGLDREATFQEISEMKY

LDLFIKEAQRVYPSVPFVGRYTEKDYVIDGDLVPKGTTLHMGIFMLGYDDRVFKEPHKFR

PQRFEEEKPGPFEYVPFSAGPRNCIGQKFALLEIKTVVSKIIRHFEVLPALDELASKDGY

IRTTLGLPPTEKRKRDLQQHKYDPILLVAMSLKSENGIYLRLKERF

>EDW60847.1

MWFILYAFLALPLLVFAYFELSVAKKKRVLNKIKGPPTLPILGNAHQVGKSPAAILNTLF

DWWYEYDKDNYRMWIGYNGNICVTNPKHLEVILNSITLIKKSGIYDLLNPWLGEGLLTST

GSKWHKHRKMITPAFHFNILQDFHQVMNENSTKFIKRLKKVSAGDSIIDFQDETHYLTLD

VICDTAMGVTINAIEQRDTVEVVKAFKDMCYNINMRAFHPFKRSELLYRLAPDYPANVKT

LKILQDFTNEIIEKRIEAHRTGIAKKTEDNEFSRKKMAFLDTLLSSTIDGRPLNQQEIYE

EVSTFMFEGHDTTTSGVAYAAYLLSRFPEEQRKLFEEQRAVMGNEMHRDATFQEIAEMKY

LDLFIKEAQRVYPSVPFIGRYTEKDYVIHGTTIPKGTALTLVLIILGYDDRIFKEPHRFY

PERFETEKPGPFEYLPFSAGPRNCIGQKFAMLEIKTVVSKLVRTFEILPAVDELVSKDGY

LNTYLGLPKAEKEKKESQGHKYDPILSAVLTLKSENGLHLRLHERK

>EDW10127.1

MWLIVYAFVALPIILYVLFELRVAKKKRLLSKLNGPPTLPFLGNAHQMGKSPAEILNTIF

EFWHKYDRDNFRFWIGYYSNILVANPKHLEVIMSSNTLIEKSDIYDMLHPWLGEGLLTSK

GSKWHKHRKMITPAFHFNILQDFHQVMNENSAKFIKRLKEVSAGDNIIDFQDETHYLTLD

VICDTAMGVPINAIENRDTVEVVKAFKDMCYNINMRAFNPLKRTHMIYRLAPDYAAHART

LKILQDFTNDIIEKRIKAHRTGAAKTDEGSEFSRKKMAFLDTLLSATIDGRPLNQQEIYE

EVSTFMFEGHDTTTSGVAFAGYILSRFPDEQRKLYEEQKAVMGNELHRDATFQEISEMKY

LDLFIKEAQRVYPSVPFIGRYTDKDYDILGTIMPKGTTLNLALIILGYDDRVFKDPHRFQ

PERFEQQKPGPFQYVPFSAGPRNCIGQKFALLELKTVISKLVRTFEVLPAVDELLSKDGT

LNTYLGLPKLEKERRERQGHKYDPILSAVLTLKSENGLHLRLRERK

>EDW74217.1

MWLILLGLLALPVIIFAYIELRVAHRRRLVNKFNGPATVPIVGNGFQMGKNPSDILNQVF

NWWHQYGKDNFRFWIGYYSNILITNPKYLEYILSSQTLIQKSDIYDLLHPWLGFGLLTST

GKKWHKHRKMITPSFHFNILQDFHEVMNENSTKFIKKLKRVAANDNIIDFQDQTHYLTLD

VICDTAMGVPINAMENSDSKVVHAFKDMCYNINMRAFHPFKRTEFLYRFFPEYSKYCETL

KTLQDFTNDIIEKRVEAYKLETNKKTDSNEFSRKKMAFLDTLLSSTIDGRPLTNVEIYEE

VSTFMFEGHDTTTSGVAFAVYLLSRHPEEQRKLFEEQCAVMGSDLSRDATFQEIADMKYL

DLFIKEAQRVYPSVPFIGRYTDKDYEIDGTTIPKGSTLNLALIILGYDERTFKDPHVFRP

ERFEQERPAPFEYLPFSAGPRNCIGQKFALLEIKTVVSKIIRTFQVLPALDELISKDNRL

STFLGPEEERLKFEQKRHKYDPILSAVLTLKSENGLHLRLKERVICEIFLFCSLSTLFAY

LLRSFAEFPLLSLALCLY

>EDW74216.1

MWLILIGVLTFPVIIFAYIELRVAHRRRLVNKFNGPSPVPIVGNALQLGNNLPETINTYF

NWWHQFGKDNFRFWIGYYSNILITNAKYLEYILSSQTLIQKSDIYDLLHPWLGFGLLTSK

GEKWHKHRKMITPSFHFNILQDFHEVMNENSTKFIKKLRIVAANDNIIDFQDQTHYFTLD

VICDTAMGVPINAMENSDSKVVHAFNDMCYNINMRAFHPFKRSKFCYRFFPEYSKYCETL

KTLQDFTNDIIEKRVEAYKLETTEKTESNEFSRKKMSFLDTLLSSTIDGRPLTNVEIYEE

VSTFMFEGHDTTTSGVAFAVYLLSRHPDEQRKLFEEQCAVMGSDLSRDATFQEIADMKYL

DLFIKEAQRVYPSIPFIGRYTDKDYEIDGTIIPKDTTLVLAILIINYDKRTFKDPHVFRP

ERFEQERPGPFEYLPFSAGPRNCIGQKFALLEIKTVISKIIRTFEVLPALDELISKDNRL

STFLGPEEERLKFEQKRHKYDPILSAVLTLKSENGLHLRLKERV

>EDW00809.1

MWFILYAILALPLLVWMYFEVKFAHKKRMLNKLRGPSTLPLLGNAHQIGKNPTEMLNKLF

EWWHKYDKDNFRIWASYYGNIIVCNPKHLEVILSSNTLIQKSDIYDMLHPWLGEGLLTSK

GSKWHKHRKMITPAFHFNILQDFHQVMNETSTTFINQLKKISAGDNIIDFQNETHYLTLD

VICNTAMGVNINAIEKRDTVNVVKAFKDICYNINMRAYDPLMRTNWIHRLTGSYVEFSKT

LKILQDFTNEIIEKRIEAHRTGTAKKIEGDEFSRKKMAFLDTLLSSTIDGRPLNQQEIYE

EVSTFMFEGHDTTTSGVGFAAYILSRFPEEQRKLYEEQRTVMGNQMHRDATFQEIAEMKY

LDLFIKEAQRVYPSVPFIGRYTDKDYDIHGTIMPKGVTLNLPLIMLGYDDRTFKEPHRFY

PERFELQKPGPFDYIPFSAGPRNCIGQKFALLEIKTVISKLVRTFEVLPAVDELVSKDGY

LNTYLGLPKAEQEMKESQGHKYDPILSAVLTLKSDNGLHLRLRERK

>EDW46937.1

MWTVLCAFLALPLFLVTYFEFGLLRRKRMLNKFQGPSMLPLVGNAHQMGKTPTEILNRFF

GWWHEYGKDNFRYWIGYHSNIMVTSPKYMEFILSSQTLISKSDVYDLTHPWLGLGLLTST

GSKWHKHRKMITPAFHFNILRDFHEVMNENSTKFIDQLKKEEAHYLTLDVICDTAMGVSI

NAMENRSSSVVQAFKDITYTIKMRAFSPWKRNEYLFRFAPEYPEYSKTLKTLQDFTNQII

AKRIEVRKSGLEDDIKADEFSRKKMAFLDTLLSSTVDGRLLTSQELYEEVSTFMFEGHDT

TTSGEKLFKEQCDVMGASGLGRDATFQEISTMKHLDLFIKEAQRLYPSVPFIGRFTEKDY

VIDGDIVPKGTTLNLGLLMLGYNDRVFKDPHKFQPERFDREKPGPFEYVPFSAGPRNCIG

QKFALLEIKTVVSKIIRNFEVLPALDELVSKDGYISTTLGLPPEEKKSLDAHIHK

>EDW78102.1

MWFVLFFLLALPSLIILYFELSVVRKRRIMKTINGPPTVPVLGNAHKLGKTPVEILEKFF

EWWIDYGKDNYAYWIGYNSGIIVTNPKHLEYILNSQELIQKSSVYQLLKPWLGFGLLTSH

GIKWHKHRKMITPSFHFNILQDFHQVMNENSTKFIEQLKKAAANDSIFDFQDQAHYLTLD

VICDTAMGVSINAMEERDSNIVQAFKDMCYMTNMRAFHPFKRSHTIYSFLPEYAVYQKTL

KTLKNFTYDIIEKRIQSLRNSVSQTGHQTDDLSMSRKKMAFLDTLLSSTIDGRPLNQQEI

YEEVSTFMFEGHDTTTSGVAFAGYLLSRHQDIQQKLYEEQCQIMGDNMQRDATYKEINQM

KYLDLFIKEAQRVYPSVPIIGRFTDKEYMINGTLVPKHTTLNVAIVLLGYNDRVFKEPHL

FRPERFEEEKPGPFEYVPFSAGPRNCIGQKFALLEIKTAISKVIRTFKVLPAVAELESKD

GYLNTYLGLPHSERQKREANRHKYDPILSAVLTLKSENGLHLRLKERI

>EDW88676.1

MWLAVLALLFLPLCALVYFERKASHRRQLLKEFNGPTPVPILGNANRIGKNPAEILSTFF

DWWYDYGKDNFLFWIGYSSHIVMTNPKQLEYILNSQQLIQKSTIYDLLHPWLGHGLLTSY

GSKWHKHRKMITPSFHFNILQDFHEVMNENSSKFMSQLKKASAGETIIDFQEHANYLTLD

VICDTAMGVPINAMEQRDSSIVQAFRDMCYNINMRAFHPFKRSNRVFSLTPEFAAYQKTL

KTLQDFTFDIIEKRVDALQNGGSKKEHDPSLPRKKMAFLDTLLSSTIDGRPLTRQEIYEE

VSTFMFEGHDTTTSGVSFSVYLLSRHPDVQRKLFEEQCEVMGSDMNRNVSFQEIAQMKYL

DLFIKEAQRVYPSVPFIGRYCDKDYDINGSLVPKGTTLNLALILLGYNDRIFKDPHHFRP

ERFEEEKPAPFEYLPFSAGPRNCIGQKFALLELKTVISKVVRSFEVLPAVDELVSKDGRL

NTYLGLSPGEKLKCEAGRHKYDPILSAVLTLKSDNGLHLRLRERRSLQISNINKEYAYC

>EAL33424.2

MWLAVLALLALPLLVLVYFELRASRRRRMLKVFNGPAPVPVLGNANRVGNTPAEILATIF

DWWQEYGKDNFVFWIGYTPHLAVTNPKYLEYILNSQSLIQKSTIYELLHPWLGYGLLTSH

GSKWHKHRKMITPSFHFNILQDFHEVMNENSTKFIAQLRRDAAGDSIIDFQDHAHYLTLD

VICDTAMGVPINAMEHRNSAIVQAFKDMCLSVNMRAFHPFKRSNRIYSLSPEYAAYQRTL

KTLQDFTYDIIEKRVETLQAGQNSSSADSSSPRKKMAFLDTLLSSTVDGRPLTRQEIYEE

VSTFMFEGHDTTTSGVAFAGYLLSRHPEAQQRMYQEQCNVMGSELDRDATFQEIAQMKYL

DLFIKEAQRVYPSVPMIGRYADKDYMINGTLVPKGTTINLALVLLGYNDRIFKDPHHFRP

ERFEEEKPGPFEYVPFSAGPRNCIGQKFALLELKTVISKLVRSFEVLPAVDELLSTDGQL

NTYLGLSPAEKQKREAGRHKYDPILSAVLTLKSDNGLHLRLRERRH

>EDW52292.1

MWLAVFALLVLPLITLVYFERKASQRRQLLKEFNGPTPVPILGNANRIGKNPAEILSTFF

DWWYDYGKDNFLFWIGYSSHIVMTNPKQLEYILNSQQLIQKSTIYDLLHPWLGHGLLTSY

GSKWHKHRKMITPSFHFNILQDFHEVMNENSAKFMTQLKKASAGDTIIDFQEHANYLTLD

VICDTAMGVPINAMEQHDSSIVQAFRDMCYNINMRAFHPFKRSNRVFSLTPEFSAHQKTL

KTLQDFTYDIIEKRVDALQNGGSKEEHDPSLPRKKMAFLDTLLSSTIDGRPLTRQEIYEE

VSTFMFEGHDTTTSGVSFSVYLLSRHPDVQMKLYREQCEVMGNDMNRSVSFQEIAKMKYL

DLFIKEAQRVYPSVPFIGRYCDKDYAINGSIVPKGTTLNLALILLGYNDRIFKDPHHFRP

ERFEEEKPAPFEYLPFSAGPRNCIGQKFALLELKTVISKVVRSFEVLPAVDELVSTDGRL

NTYLGLAPGEKLKREAGRHKYDPILSAVLTLKSDNGLHLRLRERRS

>EDW28480.1

MWLAVLALLALPLLVLVYFELRASRRRRMLKVFNGPAPVPVLGNANRVGNTPAEILATIF

DWWQEYGKDNFVFWIGYTPHLAVTNPKYLEYILNSQSLIQKSTIYELLHPWLGYGLLTSH

GSKWHKHRKMITPSFHFNILQDFHEVMNENSTKFIAQLRRDAAGDSIIDFQDHAHYLTLD

VICDTAMGVPINAMEHRNSAIVQAFKDMCLSVNMRAFHPFKRSNRIYSLSPEYAGYQRTL

KTLQDFTYDIIEKRVETLQAGQNSSSSDPSSPRKKMAFLDTLLSSTVDGRPLTRQEIYEE

VSTFMFEGHDTTTSGVAFAGYLLSRHPEAQQKMYQEQCNVMGSELDRDATFQEIAQMKYL

DLFIKEAQRVYPSVPMIGRYADKDYMINGTLVPKGTTINLALVLLGYNDRIFKDPHHFRP

ERFEEEKPGPFEYVPFSAGPRNCIGQKFALLELKTVISKLVRSFEVLPAVDELLSTDGQL

NTYLGLSPAEKQRREAGRHKYDPILSAVLTLKSDNGLHLRLRERRH

>Q9VL92.1

MWLAVLALLVLPLITLVYFERKASQRRQLLKEFNGPTPVPILGNANRIGKNPAEILSTFF

DWWYDYGKDNFLFWIGYSSHIVMTNPKQLEYILNSQQLIQKSTIYDLLHPWLGHGLLTSF

GSKWHKHRKMITPSFHFNILQDFHEVMNENSAKFMTQLKKASAGDTIIDFQEHANYLTLD

VICDTAMGVPINAMEQRDSSIVQAFRDMCYNINMRAFHPFKRSNRVFSLTPEFSAYQKTL

KTLQDFTYDIIEKRVYALQNGGSKEDHDPSLPRKKMAFLDTLLSSTIDGRPLTRQEIYEE

VSTFMFEGHDTTTSGVSFSVYLLSRHPDVQRKLYREQCEVMGHDMNRSVSFQEIAKMKYL

DLFIKEAQRVYPSVPFIGRYCDKDYDINGSIVPKGTTLNLALILLGYNDRIFKDPHHFRP

ERFEEEKPAPFEYLPFSAGPRNCIGQKFALLELKTVISKVVRSFEVLPAVDELVSTDGRL

NTYLGLAPDEKLKREAGRHKYDPILSAVLTLKSDNGLHLRLRERRS

>O44221.1

MWFIVYILLALPIMLFVFLSCEWPKRNDAEQIEWSSGVPFLGNAHQMGKTPAEILNTFFE

FWHKYNKDNFRIWIGYYANILVSNPKHLEVIMNSTTLIEKLDIYDMLHPWLGEGLLTSKG

SKWHKHRKMITPTFHFNILQDFHQVMNENSAKFIKRLKEVSAGDNIIDFQDETHYLTLDA

ICDTAMGVTINAIEKRDTVDVVKAFKDMCHIINMRAFRPLQRSDFLYRFSPEYATYAKTL

KTLKDFTNDIIAKRIKVHRTAAAKTNQEGSEFSRKKMLPDTLLSATIDGRPLNQQEIYEE

VSTFMFEGHDTTTSGVAFAGYILSRFPEEQRKLYEEQQAVMGNELNRDATFQEISAMKYL

DLFIKEAQRVYPSVPFIGRYTDKDYNIHGTIMPKGTTLNLGIIVLGYDDRVFEEPHRFYP

ERFEKQKPGPFEYVPFSAGPRNCIGQKFALLELKTVISKLVRTFEVLPAVDELVSKDGNL

NTYVGLPKEEKERKERMGYKYDPILSAVLTLKSENGLHLRLR

>EDX04391.1

MWLVVLALLVLPLITLVYFERKASQRRQLLKEFNGPTPVPILGNANRIGKNPAEILSTFF

DWWYDYGKDNFLFWIGYSSHIVMTNPKQLEYILNSQQLIQKSTIYDLLHTWLGHGLLTSY

GSKWHKHRKMITPSFHFNILQDFHEVMNENSAKFMTQLKKASAGDTIIDFQEHANYLTLD

VICDTAMGVPINAMEQRDSSIVKAFRDMCYNINMRAFHPFKRSNRVFSLTPEFSAYQKTL

KTLQDFTYDIIEKRVDALQNGGSKEEHDPSLPRKKMAFLDTLLSSTIDGRPLTRQEIYEE

VSTFMFEGHDTTTSGVSFSVYLLSRHPDVQRKLYQEQCEVMGDDMNRSVSFQEIAKMKYL

DLFIKEAQRVYPSVPFIGRYCDKDYDINGSIVPKGTTLNLALILLGYNDRIFKDPHHFRP

ERFEEEKPAPFEYLPFSAGPRNCIGQKFALLELKTVISKVVRSFEVLPAVDELVSTDGRL

NTYLGLAPGEKLKREAGRHKYDPILSAVLTLKSDNGLHLRLRERRS

>EDV33048.1

MWFFVLALLASPLLVFLYFELKTSNHRRRLKKFPGPTPVPVLGNANRIGKTPAEILATFF

NWWYDYGKENFLFWIGYSAHLVVTEPTDLEYILNSQELIQKSTIYNLLHPWLGSGLLTSY

GSKWHKHRKMITPSFHFNILQDFHQVMNENSTKFISQLKKAAAGSSILDFQEHANYLTLD

VICDTAMGVPINAMEERDSTIVQAFRQMCYILNMRAFHPFKRSHRIYSMLPEFADFQRTL

KTLQDFTYDIIEKRVIALQNGISTTEKDPSMPKKKLAFLDALLSSTVDGRPLTRKEIYEE

VSTFMFEGHDTTTSGVSFGVYLLSRHPDIQQKLYQEQIEVMGNDMNRDATFQEIAQMKHL

DLFIKEAQRVYPSVPFIGRYCDKDYTINGTVIPKGTTINIPLALMGYNDRVFKDPHHFRP

ERFLDEKPGPFECVPFSAGPRNCIGQKFALLELKTVISKMIRSFEVLPAIEGLYSRDGRL

NTYLGLSPGERLKREAGRHKYDPILSAVLTLKSDNGLHLRLKERVY

>ADP22302.1

MWLLLLYSVLLAPILAILYLEVKNYSKVKQLNKLPGPPALPVLGNAHQVGKTPSELLNKL

FQWWVESDHGNYRVLIGPYRNMVVSDAKDLEYILTSKSLIDKSDIYDMLHPWLGTGLLTS

TGRKWHTHRKIISPSFHFKILQNFHVDMNKNSNKFIEKLRKVSQHDSIFDFQDMTHYLTM

DVICDTAMGVHINAMDHHDHKLVQAFKDMCYNINMRAFHPLKRSNLLYRFAPDYPKYCKV

LKTLQDFINEVITKRRETLKLETMEGEENEFSRKKQAFLDTLLSSTIDGKPLTQEEIYEE

VSTFMFEGHDTTTSAVAFTVYLLAMHPEIQRKVYAEQKQLLGDNFKDEATFQQIADMQYL

DMVIKESLRLYPSVPIVARRTDKEYDINGFVVPVDTTINLFLMALGYNEKYFPDPYRVDP

ERWCATKRSQNPFEYLPFSAGPRNCIGQKFALLEVKTVVSKVVRTFEILPPLDELASTDG

YSRHFIGLSQAEQRERLPNPSKYDPILSAVLTLKAENGIFLRLRERP

>EGV95492.1

MLQLSLSWLGLGPVAASPWHLLLLGGASLFLARILAWIYAFYDNCRRLRCFPQPPSCHWL

WGHLDIMQTDEEGMQLLTELSHYFRDVYLSWFGAFYPILRLSHPKFIAPVLQAPAAVAPK

EMTFYGFLKPWLGDGLLLSSGDKWSRHRRLLTPAFHFDILKSYVKVFNKSVNIMHAKWQR

LTAKGSTRLDMFEHISLMTLDSLQKCVFSFDSNCQESPSEYIAAILELSSLIMKRHRQLL

LYVDFLYYLTANGRRFRKACDLVHDFTDAVIRERRHTLNSQGVDEFLKAKTKSKTLDFID

VLLLAKDEHGKELSDEDIRAEADTFMFGGHDTAASALSWILYNLARHPEYQERCRQEVQE

LLRGREPQEIEWDDLAQLPFLTMCIKESLRLHPPVISISRCCTKDIVLPDGRVIPKGNDC

IISIFGVHHNPSVWPDPEVRPSPPNSGTSGGVWLLSGKPELSFFCLIAIVSGAGWVCRKT

WPISLVLFGPQVYDPFRFDPENSQKRSPLAFIPFSAGPRNCIGQTFAMNEMKVALALTLL

RFRVLPDDKEPRRQPELILRAEGGLWLRVEPQSTGAPDDLAQLPFLTMCIKESLRLHPPV

TDLVRGCIQDIVLPDGRIIPKGVSPSFLSFMLTRRAGDPVRLCPRTTVWVPAQSRVPTLL

PGRNCIGQTFAVNEMKVALALTLLRFRVLPDKEPRRQPELILRAEGGLWLRMEPLSSQ

>402904616

MLQLSLSWLGLGPVAASPWLLLLLVGGSWLLARVLAWTYTFYDNCRHLQCFPQPPKQSWF

WGHLGLVTPTEEGMKKVTQLVATYPQGFKFWMGPTFPLLILCHPDTIRPITSASAAVAPK

DMIFYGSLKPWLGDGLLLSGGDKWSRHRRMLTPAFHFNILKPYMKIFNKSVNIMHDKWQR

LASEGSARLDMFEHLSLMTLDSLQKCVFNFESNCQEKPSEYIAAILELSALIEKRNQHVL

VYPDFLYHLTPDGQRFLRACRLVHDFTDAVIQERRRTLPTQGIDDFLKNKAKSKTLDFID

VLLLSKPRNPVLLGCPSGHDTTASGLSWVLYHLAQHPEYQERCRQEVRELLKDREPVEIE

WDDLAQLPFLTMCIKESLRLHPPVPAVSRCCTQDIVLPDGRIIPKGIICIISIFGIHHNP

TVWPDPEVYDPFRFYQENIKERSPLAFIPFSAGPRNCIGQAFAMAEMKVVLALTLLRFRI

LPTHPEPSRKPELILRAEGGLWLRVEPLGVVNSQ

>BAH12435.1

MLTPAFHFNILKPYMKIFNESVNIMHAKWQLLASEGSARLDMFEHISLMTLDSLQKCVFS

FDSHCQEKPSEYIAAILELSALVTKRHQQILLYIDFLYYLTPDGQRFRRACRLVHDFTDA

VIQERRRTLPSQGVDDFLQAKAKSKTLDFIDVLLLSKDEDGKKLSDEDIRAEADTFMFEG

HDTTASGLSWVLYHLAKHPEYQERCRQEVQELLKDREPKEIEWDDLAQLPFLTMCIKESL

RQHPPVPAVSRCCTQDIVLPDGRVIPKGIICLISVFGTHHNPAVWPDPEVYDPFRFDPKN

IKERSPLAFIPFSAGPRNCIGQAFAMAEMKVVLGLTLLRFRVLPDHTEPRRKPELVLRAE

GGLWLRVEPLS

>297276366

MSLLSLPWLGLGHVAASPWLLLLLAGGSWLLAHVLAWTYTLYDNYRRLQCFPQPPKRNWF

WGHLGLIAPTEEGLKNLTQLSATHSQGFTIWMGPIIPFIVLCHPDTIRSITNASAATAPK

DDLFTRFLKPWLGEGIILSNGDKWSRHRRMLTPAFHFNILKPYIKIFNESVNIMLDKWQH

LASEGSSRLNMSEHISLMTLDSLQRCIFSFDSHCQXXTQDGRRFRRACRLVHNFTDAVIQ

ERRRTLPTQGIDDFLKDKAKSKTLDFIDVLLLSKDEDGKALSDEDVRAEADTFMFADPTI

PGCSSGHDTTASGLSWVLYNLARHPEYQERCRQEVKELLKDREAKEIEWDDLAQLPFLTM

CVKESLRLHPPAPFISRRCTQDTVLPDGRVIPKGIICMINIIGVHHNPTVWPDPEVYNPF

RFDPENSQKRSPLAFIPFSAGPRNCIGQTFAMAEMKTVLALLLLHFRFLPDHTEPRRKPE

LTMRPEGGLWLRVEPLNTGLQ

>332253602

MLQLSLSWLGLGLVAASPWLLLLLVGASWLLGRALAWTSAFYDNCRRLQCFQQPPKRNWF

WGHLSMFLPEMQGNEEDLRLMEYLGHYFRDVQFWWLGPFYPVLHLVHPTFAAPVLQASAA

VAPKDMSFYGFLKPWLGDGLMISAGDKWRRHRHLLTPAFHFKILKPYVKIFNKSTNIMHA

KWQRLALEGSARLEMFEHISLMTLDSLQKCIFSFDSNCQEKPREYTDAILELSALILKRQ

QHIFLRVDFLYFLPPNGRCFHRASDIVHNFTDAVIQERRRTLTSQGVDDFLQAKAKSKTL

NLIDVLLLAKHPRDPSLPGCPSGHDTTASGLSWVPYNHARYPEYQEHCRQEVXEFLKNRD

PKEIGWDDLAQLPFLTMCLKESLRLHSPVSRIHRCCPQDVVLPDGRVIPKGNTCTVSIFG

IHHNPSVWPDPEVYDPFRFDPENLQKTSLLAFVPFSAGPRNCIGQAFAMAEMKVVLALTL

LPFRILPDHAEPRRKLELIVHAEDGLWLRVETLSADLQ

>BAG58928.1

MLTLAFHFNILKPYMKIFNESVNIMHAKWQLLASEGSACLDMFEHISLMTLDSLQKCVFS

FDSHCQEKPSEYIAAILELSALVSKRHHEILLHIDFLYYLTPDGQRFRRACRLVHDFTDA

VIQERRRTLPSQGVDDFLQAKAKSKTLDFIDVLLLSKDEDGKKLSDEDIRAEADTFMFEG

HDTTASGLSWVLYHLAKHPEYQERCRQEVQELLKDREPKEIEWDDLAHLPFLTMCMKESL

RLHPPVPVISRHVTQDIVLPDGRVIPKGIICLISVFGTHHNPAMWPDPEVYDPFRFDPEN

IKERSPLAFIPFSAGPRNCIGQTFAMAEMKVVLALTLLRFRVLPDHTEPRRKPELVLRAE

GGLWLRVEPLS

>390478678

MSLLSLSWLGFGPVAASPSLWLLPLLVGVSWLLARVLAWTYAFYDNCHRLQCFPQPPKRN

WFLGHLGLIGPTEEGLKNLTQISATYSPGFRIWLGPIIPFVVLCHPDIIRSITNASAAIV

PKDELFIRFLKPWLGEGILLAAADKWSHRRRMLTPAFHFNILKPYVKTFNESVNIMLDKW

QRLASEGSSRLDMFEHISLMTLDSLQKCIFSFDSHCQERSSEYIATILELSALVEKRTQH

LLQHMDFLYYLTHDGQRFRKACRLVHDFTDAVIQERRRTLPTQDIDDFLKDKAKSKTLDF

IDVLLLSKXXXXXXXXHPEYQERCRQEVQELLKDRGPKEIEWDDLAQLPFLTMCLKESLR

LHPPAPFISRRCAKDIVLPDGRVIPKGIICVINIIGIHHNPSVWPDPEVYDPFRFDPENS

KERSPLAFIPFSAGPRNCIGQAFAMAEMKVVLALTLLHFRFLPDHREPRRKPELIMRAEG

GLWLHVEPINAGSQ

>405113045

MPWLTVSGLDLGSVVTSTWHLLLLGAASWILARILAWTYSFCENCSRLRCFPQSPKRNWF

LGHLGVVSMAADRLGPETQICLSAYPSGHDTTASTLSWILYNLARHPEYQESCLQEVWEL

LRDREPEEIEWMPWLTVSGLDLGSVVTSTWHLLLLGAASWILARILAWTYSFCENCSRLR

CFPQSPKRNWFLGHLGTIQSNEEGMRLVTEMGQTFRDIHLCWLGPVIPVLRLVDPAFVAP

LLQAPALVAPKDTTFLRFLKPWLGDGLFLSSGDKWSRHRRLLTPAFHFDILKPYVKIFNQ

SVNIMHIQSNEEGMRLVTEMGQTFRDIHLCWLGPVIPVLRLVDPAFVAPLLQAPGMECRE

LKGKMHQSPEGSAVADGPWVCLVAGEGRIKSSWSPSEYISATLELSSLTRKRSYKLFLYL

DFLYYRTADGQRFRKACDLVHSFTDAVIRERRRLLSSQGVDEFLESKTKSKSKTLDFIDV

LLLAKDEHGKELSDEDIRAEADTFMFGGHDTTASALSWILYNLASHPEYQERCRQEVWEL

LRDREPEEIEWDDLAQLPFLTMCIKESLRLHPPAVDLLRRCTQDIVLPDGRVIPKGNICV

ISIFGIHHNPSVWPDPEVYDPFRFDPENRQKRSPLSFIPFSAGPRNCIGQTFAMNEVKVA

VGLTLLRFRFLPDDKEPRRKPELILRAEGGLWLRVELLSRDTQ

>402913698

MSLLSLPWLGLGPVATSPGLLLMLVVGSWLLARILAWTYTFYDKCRRLQCFPQPPKRHWF

WGHLGLITPTEEGLKNLTQLSATYSHGFMIWMGPIIPFIVLCHPDTIRSITNASAAIAPK

DDLFIRFLKPWLGEGILLSGGDRWSHHRRMLTPAFHFNILKPYIKIFNKSATIMLDKWQH

LASEGSSRLDMFEHISLMTLDSLQKCIFSFDSHCQERPSEYIATILELSALVDKRSQHIF

QHMDFVYYLTQDGRRFRRACRLVHDFTDAVIQERRRTLPTQGIDDFLKDKAKSKTLDFID

VLLLSKNIYIIHPFSLHSANSSIHVSNASSVPSTFLCSGDLERTLPMDVSSRDDLAQLPF

LTMCLKESLRLHPPAPFISRHCTQDIVLPDGRVIPKGIICVINIIGVHHNPTVWPDPEVY

DPYRFDPENSKERSPLAFIPFSAGPRNCIGQAFAMAEMKTVLALMLLHFRFLPDHTEPRR

KPELILRAEGGLWLRVEPLNTGLQ

>CAD38795.2

MPQLSLSWLGLGPVAASPWLLLLLVGGSWLLARVLAWTYTFYDNCRRLQCFPQPPKQNWF

WGHQGLVTPTEEGMKTLTQLVTTYPQGFKLWLGPTFPLLILCHPDIIRPITSASAAVAPK

DMIFYGFLKPWLGDGLLLSGGDKWSRHRRMLTPAFHFNILKPYMKIFNKSVNIMHDKWQR

LASEGSARLDMFEHISLMTLDSLQKCVFSFESNCQEKPSEYIAAILELSAFVEKRNQQIL

LHTDFLYYLTPDGQRFRRACHLVHDFTDAVIQERRRTLPTQGIDDFLKNKAKSKTLDFID

VLLLSKDEDGKELSDEDIRAEADTFMFEGHDTTASGLSWVLYHLAKHPEYQEQCRQEVQE

LLKDREPIEIEWDDLAQLPFLTMCIKESLRLHPPVPVISRCCTQDFVLPDGRVIPKGLRP

LPFQPREHQGEVTSGFYSLLGRAQKLHRAGVRHG

>332253604

MSLLSLPWPGLRPVATSPWLLLLLVVGSWLLAHVLAWTYAFYNNCRRLQCFPQPPKRHWF

WGHLGLMTPTEEGLKNSTQMVATYSQGFTIWLGPIIPFIVLCHPDTIRSITNASAAIAPK

DDLSIRFLKPWLGEGVLLSGGDKWSRHRRMLTPAFHFNILKPYITIFNKSANIMLDKWQH

LASEGSSRLDMFEHISLMTLDSLQKCIFSFDSHCQERPSEYIATILELSALVEKRSQHIL

QHMDFLYYLTHDGRRFHRACRMVHDFTDAVIRERRRTLPTHGIDDFLKDKAKSKTLDFID

VLLLSKKYQFFRTSSLLECLLRDDLAQLPFLTMCVKESLRLHPPAPFISRRCTQDIVLPD

GRVIPKGIICVITIIGVHHNPTVWPDPEVYDPFRFDPENSKQRSPLAFIPFSAGPRNCIG

QAFAMAEMKVVLALMLLHFRFLPDHTEPRRKPELIMRAEGGLWLRVEPLNVGLQ

>EAW84498.1

MLTPAFHFNILKSYITIFNKSANIMLDKWQHLASEGSSRLDMFEHISLMTLDSLQKCIFS

FDSHCQERPSEYIATILELSALVEKRSQHILQHMDFLYYLSHDGRRFHRACRLVHDFTDA

VIRERRRTLPTQGIDDFFKDKAKSKTLDFIDVLLLSKDEDGKALSDEDIRAEADTFMFGG

HDTTASGLSWVLYNLARHPEYQERCRQEVQELLKDRDPKEIEWDDLAQLPFLTMCVKESL

RLHPPAPFISRCCTQDIVLPDGRVIPKGITCLIDIIGVHHNPTVWPDPEVYDPFRFDPEN

SKGRSPLAFIPFSAGPRNCIGQAFAMAEMKVVLALMLLHFRFLPDHTEPRRKLELIMRAE

GGLWLRVEPLNVGLQ

>EDL90827.1

MLTPAFHFNILKPYVKIFNDSTNIMHAKWQRLASQGSARLDMFEHISLMTLDSLQKCVFS

FDSNCQEKPSEYITAILELSALVARRHQSLLLYVDLFYHLTRDGMRFRKACRLVHDFTDA

VIRERRRTLPDQGGDDALKAKAKAKTLDFIDVLLLSKDEHGEALSDEDIRAEADTFMFGG

HDTTASGLSWILYNLAKHPEYQERCRQEVRELLRDREPEEIEWDDLAQLPFLTMCIKESL

RLHPPATAISRCCTQDIMLPDGRVIPKGVICRISIFGTHHNPAVWPDPEVYNPFRFDADN

GEGRSPLAFIPFSAGPRNCIGQTFAMSEMKVALALTLLRFRVLPDDKEPRRKPELILRAE

GGLWLRVEPLSAGAH

>EHB14400.1

MSMLCELSFRAGDREKEVPEKEKVEGKAAPDRRMTQMSVFWRELSLEEAYPLLLLLLAGA

LIQILKWIYCFYEYYSHLCCFPQIPKQNWLLGHLGMVPLTEQNLKDVARLVATYPQGFMM

WMGPMLPIITLCHPDNIQCVLSASAEVAVKDVFAYNFLKPWLGDGLLLISGHKWSHRRCV

LTPSFHFNVLKPYVKIFCACTNVMLAKWQCLALEGSARLNMFEHISLMTLDSLQKSIFCF

IDNGQNNWKRYILTISELSALVTERINQPHLHSDSLYNLTHDGKRFQEICRLLLSFTDTV

ILERCLTLHNLKIKTLNFIDMFLLSEDEDGKEMSLKAIRGEIATFMFKGYETTASGLSWA

LYNLARHPEYQERCRQEVRELLRGREPEEIEWEDLAQLPFLTMCIKESLRLHPPVTGISR

CCTQDVVLPDGRVIPKGVTCLISIFGLHHNPAVWPDPEVYDPFRFDPENSKDRSPLAFIP

FSAGPRNCIGQTFAMAEMKVALALTLLRFRVLPDNKDTEPLRKPELILRAEGGLWLRVEP

LSASERKAQWSGTPLTPLTFGVAQE

>390478676

MLPITDHLLHHLGLEKTAFRVYTVSTFFLFLLFFLFRLLLQFLRLCQSFYITCRRLRCFP

QPPRRNWLLGHLGMYLPNEAGLQDEKKVLDSMHHVVLVWMGPILPLLVLVHPDYIKPLLG

ASAAIAPKDDLFYGFLKPWLGDGLLLSRGDKWSRHRRLLTPAFHFDILKPYMKIFNQCTD

TMHAKWWRLAEGSVVSLDMFEHVSLMTLDSLQKCVFSYNSNCQEKMSDYISAIIELSALA

VRRQYHLHHYLDFIYYRTVDGRRFRQACDMVHHFTTEVIQERRRALHQQGAKAWLKAKQG

KTLDFIDVLLLARDEDGKELSDEDIRAEADTFMFEGHDTTSSGLSWVLWPFTTMCIKESL

RQYPLVTLVSRRCTEDIKXPDGRIIPKGIICLVSIYGTHHNPTVWPDSKVYNPYRFDPDN

PQQRSPLAYVPFSAGPRNCIGQSFAMAEMRVVVALTLLRFRLSVDRTRKVRRKPELILRT

ENGIWLKVEPLPQ

>224154700

MAVATALGSLGAVALGTFAVALLLQWLWNALLAVTRFRATCRQLNKFPIPPWRSWVLGHT

GMVRSTEEGLQQVDSLVARYRHGCLWWGLPWLPVLRLFHPSTLRPLLSASAFVAPKDKIF

YGFLKPWLGEGLLLSGGERWAQHRRLLTPAFHGDVLRNYLGIFNQSTRVLLAKWGAAAAA

AAAAAGGGPVELEVLQPLSLLTLDTLQKCIFSHESHCQERPSEYIQAILELSSLVVRRQF

RPLLHPWWLYRLSSDGRRFARACATVHNFTADVVQRRRRALTCLGHQARLDGHQGHRMDF

IDLLLLSKDENGHTLSDEDIAAEAETFMFEGHDTTASGLAWLFYNLAGHPEHQERCRQEV

QELLAGRDTADIEWEDLSQLPFTTMCIKESLRLHPPVTAVSRRCTEDIPLRDGRVIPKGV

ICLMSIYGTHHNPDLWPEPEVFNPLRFSPENSKGRSPSSFIPFSAGPRNCIGQSFAMAEM

KVVVALTLSRFVLRRDTARPPPRRKPELILRAEDGLWLLLEPLLAVP

>AAH78713.1

MFEHISLMTLDSLQKCIFSFDSNCQESNSEYIAAILELSSLIVKRQRQPFLYLDFLYYLT

ADGRRFRKACDVVHNFTDAVIRERRSTLNTQGVDEFLKARAKTKTLDFIDVLLLAKDEHG

KGLSDVDIRAEADTFMFGGHDTTASALSWILYNLARHPEYQERCRQEVRELLRDREPEEI

EWDDLAQLPFLTMCIKESLRLHPPVLLISRCCSQDIVLPDGRVIPKGNICVISIFGVHHN

PSVWPDPEVYNPFRFDPENPQKRSPLAFIPFSAGPRNCIGQTFAMSEIKVALALTLLRFC

VLPDDKEPRRKPELILRAEGGLWLRVEPLSTVTSQLPWDLLAHPPTS

>AAI34145.1

MLFGLSVTGILALSVAALLCALVTRIIINRRALRCFNQPPLRNWIMGHMGLMGHNEEGLQ

RVDDLVCKYGHSCSWFLGPFYNMVRLFHPDYIRSLLTASASITLKDRIFYGFMKPWLGNC

LLLQSGQEWSRHRRLLTPAFHFDILKKYVHIFNQSTNIMHDEWRRLLAKGEHSVDMFEQI

SSLTLDSLLKCTFSCDTHSQEKPRQYISAILDLSRLLVQRQHYLPYHWDWLYWRSAQGRR

FQQACAVVHQFTADIVQERRTQLDQQSDPESHPENTGRYRKRKNTDLIDLLLLAKDDKGE

GLTNEEIKAHADMFMFAGHDTTASALSWIFYNLAMNQDYQERCRAEVRDLLADRDTHTIG

WEDLSQLTFTTMCIKESLRLHSPVLALTRYYSQNMKTPGDCVIPHGCLCLISIYGVHRNP

QVWPDPLVFDPTRFDPHNSDSRSPHAFIPFSAGPRNCIGQNFAMAEMKVVVALTLARFKI

LPGPKPVRRLYQLVLRAEGGMILHFQELNEE

>CAX15403.1

MLQLCLSWLGMGSLTASPWHLLLLGGASWILARILAWIYAFYDNCSRLRCFPQPPKPSWF

WGHLALAKWQCLASKGTSRLDMFEHISLMTLDSLQKCIFSVDSNCQESDSKYIAAILELS

SLVVKRHRQPFLYLDLLYYLTADGRRFRKACDLVHNFTDAVIKERRSTLNTQGVEFLKAK

AKTKTLDFIDVLLMAEDEHGKGLSNEDIRAEADTFMFGGHDTTTSALSWILYNLARHPEY

QERCRQEVQELLRDRDSEEIEWDDLAQLPFLTMCIKESLRLHPPVLLISRCCTQDVLLPD

GRAIPKGNICVISIFGVHHNPSVWPDPEVYNPFRFDPENPQKRSPLAFIPFSAGTRNCIG

QTFAMSEIKVALALTLLRFRILPDDKEPRRKPELILRAEGGLWLRVEPLSAGAQ

>EAW84486.1

MKPSEYITAIMELSALVVKRNNQFFRYKDFLYFLTPCGRRFHRACRLVHDFTDAVIQERR

RTLTSQGVDDFLQAKAKSKTLDFIDVLLLSEDKNGKELSDEDIRAEADTFMFGGHDTTAS

GLSWVLYNLARHPEYQERCRQEVQELLKDREPKEIEWDDLAQLPFLTMCLKESLRLHPPI

PTFARGCTQDVVLPDSRVIPKGNVCNINIFAIHHNPSVWPDPEVYDPFRFDPENAQKRSP

MAFIPFSAGPRNCIGQKFAMAEMKVVLALTLLRFRILPDHREPRRTPEIVLRAEDGLWLR

VEPLG

>BAG62247.1

MPSITTAAASGVSRSPGNRTGSWVTWAWKPSEYITAIMELSALVVKRNNQFFRYKDFLYF

LTPCGRRFHRACRLVHDFTDAVIQERRRTLTSQGVDDFLQAKAKSKTLDFIDVLLLSEDK

NGKELSDEDIRAEADTFMFGGHDTTASGLSWVLYNLARHPEYQERCRQEVQELLKDREPK

EIEWDDLAQLPFLTMCLKESLRLHPPIPTFARGCTQDVVLPDSRVIPKGNVCNINIFAIH

HNPSVWPDPEVYDPFRFDPENAQKRSPMAFIPFSAGPRNCIGQKFAMAEMKVVLALTLLR

FRILPDHREPRRTPEIVLRAEDGLWLRVEPLG

>EAW84499.1

MLTPAFHFNILKSYITIFNKSANIMLDKWQHLASEGSSRLDMFEHISLMTLDSLQKCIFS

FDSHCQERPSEYIATILELSALVEKRSQHILQHMDFLYYLSHDGRRFHRACRLVHDFTDA

VIRERRRTLPTQGIDDFFKDKAKSKTLDFIDVLLLSKDEDGKALSDEDIRAEADTFMFGG

HDTTASGLSWVLYNLARHPEYQERCRQEVQELLKDRDPKEIEWDDLAQLPFLTMCVKESL

RLHPPAPFISRCCTQDIVLPDGRVIPKGITCLIDIIGVHHNPTVWPDPEVLPSPFTTTTP

ILYFCVCVCEFQGS

>EEN42804.1

MLLSLPQDRLLSLEEKDRCSVWRLGPFLPVLSCSHPDTVRPILGGKTQKTSWVYGFFRPW

LGDGLLISEGQKWQRNRRLLTPAFHFDILKHYVTLFSESTQVLLEKWLSRGPGASVELFD

QIGLMTLDNILKCSLGYHSNCQTDGQSAPYIQAVHDLTSLIDERVDRPLQHIDFIYYLTA

KGRRFRQACKVVHSFSEQVIMTRKEERKKEELKEEDFTSPGYKSGKCLDFVDILLQAKDE

DGTGLSVSEIRDEVNTFLFAGHHTTASGVSWTLFYLAQHQDYQDECRREAEGVLEGRTEI

TWHDVGKLPFLTMCIKESLRCVPPVARSARTLQLDLTFADGKWLPKGTSAVADFPGLHRN

SEIWENPTVYDPYRFSPENSERRPPHAFLPFSAGPRNCIGQNFAMNEMKVTLALTLQRFR

LEPDVTKPPVIPCDGITLQARGGIWVKVHPLKTNT

>EEN64406.1

MDHNDRYRVFWVTPFLPITYGIHPSTARSILSSKSDIIPSTPCFCTLYVGDGLLLSDGYK

WQRNRRLLTPAFHFDILKHYVQLFSESTAVLLDKWMSRGPDASVELLDDIGLMTLDNILK

CSLGYNSRCQTDGKSAPYILAVNDLTRLFAERGDQPLHYFDFIYYLSSDGRRFRKACKVV

HSFAEQVIKARKEELKKGGGTLPKPRGGKCMDLLDVLLQAKDKDGTGLSVSEIQDEVNTF

LFAGHHTTASGISWTLHHLAKHKDYQDKCRREVEEVLRGRAEVTWHDIGKLPFITMCIKE

SMRCVPPVRRLARTLKQDLIFSDGKSLPKGSTTFVDFGDLHRNRDVWPNPKVYDPYRFSP

ENSENRHPHAFLPFSAGPRNCIGQNFAMNEMKVSVALILQRFQLELDGTKPPAVPCTRVT

NQAEDGIWVKFHPAKTNT

>198435282

MLLFYLQYAVYATVCAFAVFLLPSVFTSLKRFYTVRKNLNSQWPGPKGNWFWGSSAKVIA

EVQKDSAYYFTYIKEMTKQFPQGFRGQLGPIFLSLNAYHPNMIKAVLCAPASDAPKLNRA

YKMLFEWLGHGLLVLEDKVWFRHRRLLTPSFHFEVLKPYVKTMNESAHVMVENWLGKTSE

NKVAKIEIFHSASLMTLDTILKCLMSYQSNCQEEKSTNTYVAKIYQLSNTVVKRMRDLNL

FKRIDPIYYLTKNGKLYKKLCDDVHKFSESVIDRRNCDIGQPAHNGTRKHFDFLDTLLKA

RDSDGKGLSDSEIRAEVDTFMFEGHDTTASGISWTFYCLAMHPEHQEKCFQEIKKVMADR

TDIEWNDLSNLPHLTLCIKESLRQYPPVPIIFRKLNKDIEVDGKTIVKDTDVILHIYALH

HHEEFWKDPHIFDPSRFTQENMKSMSSYAYVPFSAGPRNCIGQRFAMNEIKIAVAQVLSK

FQLKPDLSKKIQHSVDVIYRATTGLYLKAERRV

>344283153

MSLLNLSWLGLWPGAASPWLLLLLAVASWLLACFLAWIYTFYDICRRLRSFPLPPMRNGF

LGHLGLGSPTEEGLMEVTHLVATYPRGFKTWLGPICPIITLCHPEIIRPIINASAAIAPK

DVIFYSFLKPWLGDGLLLSGGDKWSHHRRMLAPAFHFNILKPYMKIFNESTNIMHAKWKH

LVSGGSTRLDMFEHISLMTLDSLQKCVFSFDSHCQEIMVVLPSFQDEDGKELSDEDIRAE

ADTFMFEGHDTTASGISWVLYNLAKHPEYQERCRQEVRELLRDRQPEEIEWDDLTQLPFL

TMCIKESLRMYPSVTAIARCCTQDIVFPDGRIIPKGIICLISIFGTHHNPSVWPNPEVYD

PFRFDLDNSEKRSPLAFIPFSAGPRNCIGQTFAMTEMKVVLALTLLRFRVLPDDAEPRRK

PELILRAEDGLWLRVEPLGGGQK

>198419762

MQSSGLSSIWDQLQINDWLPSSFLVETTLLILLIYLITHYGLLFRETYTKSRKISERVGV

DEKNRHWFFGHLKHITNNEEGLLKFVELAKAFPLMVSLWLGPALTTVQTYHPDSTTALLN

SGAPKNDLGYRFIRTWIGDGLLTSKGKKWFRNRRLLTPAFHYKVLKPYTEVFNKNSLVMV

NKLNKLCDQPVNIYTHVGLMTLDTMLQCAMTSKTNCQHVTDHPYLLAVKEISDLIIARVR

QPLHFFDWMYNLSAAGKRNKEVTKILHEHTEKIISERKAVLAKTANIDPEPSDVEGFRKT

EGKTLDFLDILLQCKDENGEGLSDIEIRDEVDTFLFAGHDTTSSGIAWAMFHLAKYPQFQ

TKCREELKEVLGNKDEIEWSDLPQLVYLTMFLKESMRLRPPVYAVGRYLEHPITIKDKNT

KRDTVIPAGTNVSMHIFTLHRHPDFWDEPSKFDPERFTKENIAKRPAFAYVPFSAGSRNC

IGQNFAMNEMKISLAHVIRNLRLYIDDETPVPKMQPRLILQSSTGIFVKIEKLERQ

>198424634

MLLPDYLEFSLSLYSIGAVCVVVFLYKSRKLIGRFLKERRIRKELTKEIDGPQPHWLFGH

LNLVQPNEETILKTCEQTQKYPKLGMVWFGPALAYLMVFHPHVVRPVLNVEHPKDNLAYR

FIKPWIGDGLLVSHGSKWRRNRHLLTKAFHFDILKQYTKVFDACCKTMLEKWSRKCDGST

VEVFRPVSLMTLDSMLQCAISCETECQQEGNSNAYTDAVFTLTTTAMKRIFNPFCHVDWI

FAMTKSGRQFKAAAKLIHEFDEKVIRERRQENKKILGRSDDEDLTQEDLEKITSWRKSDG

RYLDFLDMLILTRDDDGKGLTEREIRDEVDTFLFEGHDTTASGIAWSLYCLAANEEYQEK

CREEIEQVVGSKDALEWQDLSKLPFVTMFIKEVLRLYPPVFAVARRTEQPVKFPRGFGGD

QFNVGDEQLPKSDCSRVVDAPLNIGIPIMTLHRNEQVWEDAKVFDPYRFSTENCAKRSPY

AYLPFSAGPRNCIGQNFAMAELKTVLVRTLLKYKLYVDNQCPDPKMSPMIVLKSLNGIHI

KLKKI

>340372923

MSFLVTLLSSHWLELLLVICSVVFLASIPYIVSVCKRQWRLQKCLKALPSSDETDSHWLF

GQGPMSIKMIKSGEFLDYYAKYWKFGKITVVPLGPLFNVIFCNHPDALKISLAKDPKADF

VYFPLMPWLGDSLLTGNGHKWARTRRLLTPAFHFDILKPYVRVYQSCTKELVTNWKKLAL

SKEPFDVFSSISLLTLDIMLRSTCSFKSNCQTEKTHSPYVAAVYELSHLATERMLFSPAY

FDWVYALMPSKWRYDRACRLVHKFSMDVIKERRKTDVLKGDKKRKYIDFIDILLEARDDD

GSGLTDEEIRAEVDTFMFEGHDTTASGITWTLYNLARYPEHQQKCREEVDAAFEDGDELS

WETVKGFTYLKYCIKESLRLFPPVPIIVRTLAEDTKFEDYTLPKGAWISSNIYGVHHSPE

IWEDPEAFDPLRFAPENAKDRHTHAFVPFSAGPRNCIGQEFALNEEKVVLAYILRNFEIS

LPDDERKNVTKLFALILRPKGGLYLQLKPRNI

>198435280

MLFMFIQYAVYALFSLLALFILHSLFRSIQEFYTVTKYLKSNWPEPESHWLWGSFGAVML

KMSADPSYYFTYMNDCMKKYPYGFRTQLGPFIKSLNTYHPTIAKAILSGSAADVPKLKRA

YKLLFEWLGHGLLVLEDKVWLRHRRLLTPSFHFEVLKPYVKTMNESAHVMVENWLSKTSD

NKVAKVEIFHYASLMTLDTTLRCLMSYQSNCQDENSTNDYVAKIYELSETIVRRQRNLNI

LNRIDPIYNVTKEGRKYLKLCDDVHKFSESVINRRKCDSEQPAHNETRKYFDFLDTLLKA

RDSDGKGLSDSEIRAEVDTFMFEGHDTTASGISWTFYCLAMHPEHQEKCFKEIEKVMADR

TDIEWNDLSNLPHLTLCIKESLRQYPPVPIIFRKLNKDIEVDGKTIVKDTNVVLHIYALH

HHEEFWKDPHIFDPSRFTQDNMKSMNSYAYVPFSAGPRNCIGQKFAMNKMKIAVAQVLRQ

FQIKPDLTRTIKRSADMIYKTTSGLYLNIERRY

>291243941

MLTLPGVSLFNGVVYIVVITAVVKYVIALVTFIGDIRKKTRCVQQWPGLQTELLTGSLRY

CPKNKEDTLIWRRQMSEKWTNGYQFWYGPIYPSIICNSPTLLRAVCKNIETKPGNSAGYG

VLGPWLGSGLLVSNGQRWFRSRKLLTPAFHFEILKPYAKIYNQSVDILLNKMEKYSQTGE

SFEVIQNISLLTLDILLRCAFTWKDNNCQIGGDNNPYVAAVRELADIAQERIITPLWYND

IIFYLTSAGKRFKKSCDFVHGTSDDIIKRRRQELEDRTVERTEKTYLDFLDILLTARDSD

GEGLTDLEIRSEADTFLFEGHDTTASSLSWVLYALASHPEFQYKCQQEIDDLLQDRNTDE

IRWDDLAKLTYLTQCIKESNRMYPPVCGVLRTADKEIKVDGKTIVPGVRVEINIYGLHHN

PDVWPEHMEYKPERFDPDRVATMDPYAYLPFAAGPRNCIGQNFALNEEKVVLARILRKYT

LEVDPTHTVSPALYLVLKATNGIKLKIKARR

>EDX16353.1

MAVEVVQETLQQAAASSSTTVLGFSPMLTTLVGTLVAMALYEYWRRNSREYRMVANIPSP

PELPILGQAHVAAGLSNAEILAVGLGYLNKYGETMKAWLGNVLLVFLTNPSDIELILSGH

QHLTKAEEYRYFKPWFGDGLLISNGHHWRHHRKMIAPTFHQSILKSFVPTFVDHSKAVVA

RMGLEAGKSFDVHDYMSQTTVDILLSTAMGVKKLPEGNKSFEYAQAVVDMCDIIHKRQVK

LLYRLDSIYKFTKLREKGDRMMNIILGMTSKVVKDRKENFQEESRAIVEEISTPVASTPA

SKKEGLRDDLDDIDENDVGAKRRLALLDAMVEMAKNPDIEWNEKDIMDEVNTIMFEGHDT

TSAGSSFALCMMGIHKDIQAKVFAEQKAIFGDNMLRDCTFADTMEMKYLERVILETLRLY

PPVPLIARRLDYDLKLASGPYTVPKGTTVIVLQYCVHRRPDIYPNPTKFDPDNFLPERMA

NRHYYSFIPFSAGPRSCVGRKYAMLKLKVLLSTIVRNYIVHSTDTEADFKLQADIILKLE

NGFNVSLEKRQYATVA

>EDV45360.1

MAVEVVQETLQQAAASSSTTVLGFSPMFTTLVGTLVAMALYEYWRRNSREYRMVANIPSP

PELPILGQAHVAAGLSNAEILAVGLGYLNKYGETMKAWLGNVLLVFLTNPSDIELILSGH

QHLTKAEEYRYFKPWFGDGLLISNGHHWRHHRKMIAPTFHQSILKSFVPTFVDHSKAVVA

RMGLEAGKSFDVHDYMSQTTVDILLSTAMGVKKLPEGNKSFEYAQAVVDMCDIIHKRQVK

LLYRLDSIYKFTKLREKGDRMMNIILGMTSKVVKDRKENFQEESRAIVEEISTPAASTPA

SKKEGLRDDLDDIDENDVGAKRRLALLDAMVEMAKNPDIEWNEKDIMDEVNTIMFEGHDT

TSAGSSFALCMMGIHKDIQAKVFAEQKAIFGDNMLRDCTFADTMEMKYLERVILETLRLY

PPVPLIARRLDYDLKLASGPYTVPKGTTVIVLQYCVHRRPDIYPNPTKFDPDNFLPERMA

NRHYYSFIPFSAGPRSCVGRKYAMLKLKVLLSTIVRNYIVHSTDTEADFKLQADIILKLE

NGFNVSLEKRQYATVA

>EDX00782.1

MAVEVVQETLQQAAASSSTTVLGFSPMFTTLVGTLVAMALYEYWRRNSREYRMVANIPSP

PELPILGQAHVAAGLSNAEILAVGLGYLNKYGETMKAWLGNVLLVFLTNPSDIELILSGH

QHLTKAEEYRYFKPWFGDGLLISNGHHWRHHRKMIAPTFHQSILKSFVPTFVDHSKAVVA

RMGLESGKSFDVHDYMSQTTVDILLSTAMGVKKLPEGNKSFEYAQAVVDMCDIIHKRQVK

LLYRLDSIYKFTKLREKGDRMMNIILGMTSKVVKDRKENFQEESRAIVEEIATPVASTPA

SKKEGLRDDLDDIDENDVGAKRRLALLDAMVEMAKNPDIEWNEKDIMDEVNTIMFEGHDT

TSAGSSFALCMMGIHKDIQAKVFAEQKAIFGDNMLRDCTFADTMEMKYLERVILETLRLY

PPVPLIARRLDYDLKLASGPYTVPKGTTVIVLQYCVHRRPDIYPNPTKFDPDNFLPERMA

NRHYYSFIPFSAGPRSCVGRKYAMLKLKVLLSTIVRNYIVHSTDTEADFKLQADIILKLE

NGFNVSLEKRQYATVA

>EAL31842.1

MTVDTVQETLQHAATSTSGLGFSPMLTTLVGTIVALGLYEYWRRNTREYRMVANIPSPPG

LPLLGQAHMVAGLSNAEILNVGLGYLNKYGETMKAWLGNVLLVFLTNPNDIELILSGHQH

LTKAEEYRYFKPWFGDGLLISNGHHWRHHRKMIAPTFHQSILKSFVPTFVDHSKSVVGRM

GLETGKSFDVHDYMSTTTVDILLSTAMGVKKLPEGNKSFEYAQAVVDMCDIIHKRQVKLL

YRLDSIYKFTKLREKGDRMMNIILGMTSKVVKDRKQNFQEESRAIVDEVQAVSTPATKKE

GLRDDLDDIDENDVGAKRRLALLDAMVEMAKNPDIEWNEKDIIDEVNTIMFEGHDTTSAG

SSFALCMMGIHKDIQEKVFAEQKAIFGDNMLRDCTFADTNEMKYLERVILETLRLYPPVP

LIARRLDYDLKLASGPYTVPKGTTVIVLQYCVHRRADIYPNPTKFDPDNFLPERMANRHY

YSFIPFSAGPRSCVGRKYAMLKLKVLLSTIVRNYIVHSTDTEADFKLQADIILKLENGFN

ISLEKRKYATVA

>EDW66352.1

MSVETVQETLQQATGSTGSFMLSPLLTGLVGTMLIMALYEYWHRNTREYRMVANIPSPPS

LPIIGMAHLAAGLSNAEILAVGLGYLNKYGETMKAWLGNVLIVFLTNPSDIELILSGHQH

LTKAEEYRYFKPWFGDGLLISNGHHWRHHRKMIAPTFHQSILKSFVPTFVDHSKAVSARM

AKEAGKSFDVHDYMSQTTVDILLSTAMGVKKLPEGNKSFEYAQAVVDMCDIIHKRQVKLL

YRLDSIYKFTKLREKGDRMMNIILGMTRKVVKDRKDNFQNETHAIIEEVEETPVKQSRVT

SATKKEGLRDDLDDIDENDVGAKRRLALLDAMVEMAKNPDIEWNEKDIMDEVNTIMFEGH

DTTSAGSSFALCMLGIHKHIQERVFAEQKSIFGDNMQRDCTFADTMEMKYLERVILETLR

MYPPVPLIARRLDHDVKLVSGPYTVPKGTTVVLLQYCVHRRPDIYPNPTEFDPDNFLPER

AANRHYYSFIPFSAGPRSCVGRKYAMLKLKVLLSTIVRNFIVHSTDTEADFKLQADIILK

LENGFNISLEPRKYQTVA

>EDW05602.1

MSVETVQETLQQAAKSSGGFSPILTGLLGTIIVMALYEYWHRNTREYRMVANIPSPPSLP

LIGMAHLAAGLSNAEILSVGLGYLNKYGETMKGWLGNVLLVFLTNPNDIELILSGHQHLT

KAEEYRYFKPWFGDGLLISNGHHWRHHRKMIAPTFHQSILKSFVPTFVAHSKAVSARMAK

EAGKSFDVHDYMSQTTVDILLTTAMGVKKLPEGNKSFEYAQAVVDMCDIIHTRQVKLLYR

LDSIYKFTKLREKGDRMMNIILGMTRKVVKDRNENYSPESRAIIEDVAEPTPAKQATKTE

GLRDDLDDIDENDVGAKRRLALLDAMVEMAKNPDIEWNEKDIMDEVNTIMFEGHDTTSAG

SSFALCMMGIHKDVQERVFAEQKAIFGDNMLRDCTFADTMEMKYLERVILETLRMYPPVP

LIARRLDHDVKLASGPYTVPKGTTCVVLQYCVHRRPDIYENPTKFDPDNFLPERAAKRHY

YSFIPFSAGPRSCVGRKYAMLKLKVLLSTIVRNFIIHSTDTEADFKLQADIILKLENGFN

ISLEPRQYPTAA

>EDV91896.1

MSVETVQETLQQAASGGGGYILSPLLTGVLGTILIMALYEYWHRNSREYRMVENIPSPPT

LPLVGMAHLVVGLSNAEILSVGLGYLNKYGETMKAWLGNVLVVFLTNPSDIELILSGHQH

LTKAEEYRYFKPWFGDGLLISNGHHWRHHRKMIAPTFHQSILKSFVPTFVDHSKAVSARM

GKESGKPFDVHDYMSQTTVDILLSTAMGVKKLPEGNTSFEYAQAVVDMCDIIHKRQVKLL

YRLDSIYKFTKLREKGDRMMNIILGMTRKVVKDRKDNFITESRPIIDEVEETPNPKLSRA

TPPAAAAKKEGLRDDLDDIDENDVGAKRRLALLDAMVEMAKNPEIEWNEKDIMDEVNTIM

FEGHDTTSAGSSFALCMLGIHKDIQERVFAEQKAIFGDKMQRDCTFADTMEMKYLERVIL

ETLRMYPPVPLIARRLDHDVKLTSGPYTVPKGTTVVVLQYCVHRRADIYPNPTKFDPDNF

LPERAANRHYYSFIPFSAGPRSCVGRKYAMLKLKVLLSTIVRNYIVHSTDTEADFKLQAD

IILKLENGFNISLEPRKYQTVA

>EDW82168.1

MVSNIPSPPGLPLLGQAHLAAGLSNAEIMSVGMGYLNKYGETVKAWLGHVLLVFLTNPND

IELILSGHQHLTKAEEYRYFKPWFGDGLLISNGHHWRHHRKMIAPTFHQSILKSFVPTFV

DHSKNVVARMDTEAGKSFDVHDYMSQTTVDILLSTAMGVKKLPEGNKSFEYAQAVVDMCD

IIHKRQIKLLYRLDSIYKFTKLREKGDKMMNIILGMTSKVVKDRKENFQADTRAIIEEEL

TKPAATSPSAKKEGLRDDLDDIDENDVGAKRRLALLDAMVEMAKNPDIEWNEKDIIDEVN

TIMFEGHDTTSAGSSFALCMMGIHKHIQERVFAEQKAIFGDNMQRDCTFADAMEMKYLER

VILETLRLYPPVPLIARRLDHDLKLASGPYTVPKGTTVIVLQYCVHRRPDIYPNPTTFDP

DNFLPERMANRHYYAFIPFSAGPRSCVGRKYAMLKLKVLLSTIVRNYIIHSTDTEADFKL

QADIILKLENGFNISLEKRKYPTVA

>ABV48808.1

MDSANNSTAGPATVLNPIWTALLGIAVVVSLYEIWLRNTRKYKLTANMPNPPMLPLIGNG

HLVAHLTNAEILARGIGYMQTYGGAMRGFLGPMLVVFLWNAPDIELILSTHTHLEKSIEY

RFFKPWFGDGLLISNGHHWQHHRKMIAPTFHQSILKSFVPAFVQHSKKVVERMAKELGKE

FDVHDYMSQTTVEILLSTAMGVKKVPEDNKSLEYAKAVVDMCDIIHKRQLKFFYRMDALY

NLSSMSEKGKKMMDIILGMTRKVVTERQQNFNAESRAIVEEDDEISKQKQQAKKKEGLRD

DLDDIDENDVGAKKRLALLDAMMAMSKNPDVEWTDKDVMDEVNTIMFEGHDTTSAGSSFV

LCMLGIYKDIQEKVLAEQKAIFGDNFLRDCTFADTMEMKYLERVIMETLRLYPPVPLIAR

RAEFDVKLASGPYTIPKGTTVVIAQFAVHRNPQYFPNPEKFDPDNFLPERMANRHYYSFI

PFSAGPRSCVGRKYAMLKLKVLLSTIIRNYSVQSNQQEKDFKLQADIILKIENGFNIMLN

RRPEAMKAM

>ADD20180.1

MSMDILNDTVQENVANQSFASSSIFNSLLTAAFGLVLTLALYDYWRRNTRAYRLLKSMPT

VKEYPVVGHGHIVLLRNNPEVLDIFVDLMREGGDTLRLSLAHINVVAISNPVDVEIFLSG

QKHLEKSVEYRFFHPWFGDGLLVSKGHHWRHHRKMIAPTFHQSILKSFVPTFVQHSKAVC

DRLFAKVGKDFDCHKYMSETTVNILLTTAMGVKTQPEMKTSAEYAQAVMDMCDIIHNRQV

KMMYRMDALYRWTTMRQKDNKLMNIILGMTQKVVEERKSNFNADERAVIDKVAAVSGKKT

IKEKKEGLRDDLDEIDENDVGEKKRLALLDAMMEMEKNPNITWTDKDIRDEVNTIMFEGH

DTTAAGSSFALCMLGIHQDIQQRVVEEQEAIFGHDMQRDCTFADTLQMNYLERVICETLR

LFPPVPLIARKAEEDVKLASAPYIVPKGTTVLISQFIIHRRASVYPDPDKFDPDRFLPER

TAQRHYYSFIPFSAGPRSCVGRKFAMLQLKVLLSTIIRKYKVFSSRTDKDFRLQGDIILK

LANGFQISLEPRAYA

>ADD20183.1

MSPSTANAGFTESSTLFNPLSILLVAVPLILALYEYWRRNTRSYKLMSNIPTIPEMPVIG

HGHVVIGKSSHDITEYIIKQSEIYGTTLRVSLGHVNVVLLGDPRDIEILLSGSKHLEKAY

EYRYFQPWFGDGLLISKGHHWRHHRKMIAPTFHQSILKSFVPTFVQHSKAVCDRFNNKIG

HEFDVHEYMSETTVNILLTTAMGIKRAPEIEKSSEYAKAVMDMCDIIHNRQIRLLYRLDA

LYKWTNMYEKDNKLMNIILGMTQKVVEERKSNFNANERAVIDKVAAVSGKKTIKEKKEGL

RDDLDEIDENDVGEKKRLALLDAMMEMEKNPNITWTDKDIRDEVNTIMFEGHDTTAAGSS

FALCMLGIHQDIQQRVVEEQEAIFGLDMQRDCTFADTLQMNYLERVINETLRLYPPVPII

ARKVEEDVKLASGPYTIAKDTTVIVSQFSVHRQADLFPDPEKFDPDRFLPERTAQRHYYS

FIPFSAGPRSCVGRKFAMLQLKVLLSTIVRRYKIFSTRTQSDFQLQGDIILKLANGFKIS

IVPRLNV

>AAK40120.1

METLQRESSQLPLGAATSSAGTWLSLVFGVGVIAILYEYYRRNTRAYKLMDTIPTPKQMP

LIGNGHMAFGQNNHQILKLMSQVFKDCGETVRILLGHMNVVFLTNPADLEIFLNGYKHLE

KSVEYRYFQPWFGNGLLISKGHHWRHHRKMIAPTFHQSILKSFVPTFVKHSKAVSGRFAS

KVGQEFDCHKYMSETTVDILLTTAMGCKVQPETEKSAEYAQAVMDMCDIIHIRQLKLFYR

LDSTIRFTKLREHDNKLMNVILSMTRKVVEDRKKNFNAEERGIVDRTAEDAKLKAKAAKK

EGLRDDLDDIDENDIGEKRRLALLDAMMEMEKNPDIKWTDKDVMDEVNTIMFEGHDTTAA

GSSFALCMLGIHQDVQQRVFEEQQAIFGDDLNRDCTFADTLQMSYLERVICETQRLFPPV

PLIARKAEEDVNLKSGPYTIAKGTTVVLLQYFVHRRADIYPDPEKFNPDNFLPERTANRH

YYAYIPFSAGPRSCVGRKFAMLQLKVLLSTIIRNYRVSSTRTQKDFQLQGDIILKMANGF

NITLEKRPTAKV

>ADD19771.1

MLISSATEKTVSAIVPNSWFTLFVGLVTIGALYEYWRRNTREYKLMKNIPTFREMPIVGH

GHLGIGLNTVEVGLNLVAALAELGETARGNLFHIIAVVITNPKDIELFLNSHKHLEKSEE

YRYFQPWFGDGLLISKGQHWRHHRKMIAPTFHQSILKSFVPTFVKHSKAVCNRLNATIGR

EFDAHKYMSETTVDVLLTTVMGIKTEPETEKSAEYAQAVMDMCQIIHNRQYRPLYRLNAT

YKWTEMRQKGEKLLNIILNMTREVVKERKLNFNADERAIKEKLDDSLLLSRKISPAKKTG

LRDDLDDIDDNDVGEKKRLALLDSMLEMENDPNIEWTDKDINDEVNTIMFEGHDTVAAGS

SFVLCLLGIHQDVQKKVYDEQKQIFGNDMLRDCTFGDTLHMNYLERVICETFRLYPPVPA

IARKVEEDTKLISGPYTIAKGTTVIIPQYFTHRRPDIYPEPDKFDPDRFLPECAIKRHYY

SFIPFSAGPRSCVGRKYAMLQLKVLLSTMTRRFRIISGRTEEDFLLQADIILKIANGFNI

SLEPRVII

>AAL13523.1

MCDIIHKRQVKLLYRLDSIYKFTKLREKGDRMMNIILGMTSKVVKDRKENFQEESRAIVE

EISTPVASTPASKKEGLRDDLDDIDENDVGAKRRLALLDAMVEMAKNPDIEWNEKDIMDE

VNTIMFEGHDTTSAGSSFALCMMGIHKDIQAKVFAEQKAIFGDNMLRDCTFADTMEMKYL

ERVILETLRLYPPVPLIARRLDYDLKLASGPYTVPKGTTVIVLQYCVHRRPDIYPNPTKF

DPDNFLPERMANRHYYSFIPFSAGPRSCVGRKYAMLKLKVLLSTIVRNYIVHSTDTEADF

KLQADIILKLENGFNVSLEKRQYATVA

>EAT39885.1

MSAEIVAERGSSLVSLAVPMVIFMTLVLVASALFHFWMISRRYVQLGNKIPGPRAYPFIG

NANMLLGMNHNEIMERAMQLSYIYGSVARGWLGYHLVVFLTEPADIEIILNSYVHLTKSS

EYRFFKPWLGDGLLISSGEKWRSHRKLIAPAFHMNVLKTFVDVFNDNSLAVVERMRKEVG

KEFDVHDYMSEVTVDILLETAMGSQRTSESKEGFDYAMAVMKMCDILHSRQLKFHLRMDS

VFNFTKIKQEQERLLGIIHGLTRKVVKQKKELFEKNFADGKLPSPSLSEIIAKEESESKE

PLPVISQGSLLRDDLDFNDENDIGEKRRLAFLDLMIETAKSGADLTDEEIKEEVDTIMFE

GHDTTAAGSSFVLCLLGIHQDVQDRVYKEIYQIFGNSKRKATFNDTLEMKYLERVIFETL

RMYPPVPVIARKVTQDVRLASHDYVVPAGTTVVIGTYKVHRRADIYPNPDVFNPDNFLPE

RTQNRHYYSYIPFSAGPRSCVGRKYAMLKLKVLLSTILRNYRVVSNLKESDFKLQGDIIL

KRTDGFRIQLEPRV

>EDS28283.1

MNVEFVHERSSLAALAMPTVIVMTLVLVVSVLFHMWMLSRRYVKLGNMIPGPRAYPLIGN

ANMLLGKSHDEIMKRAIELSFVYGSVARGWLGYHLVVFLTEPADIELILNSYVHLTKSNE

YRFFKPWLGDGLLISSGDKWKSHRKLIAPAFHQNVLKTFIDVFNDNSLAVVERMRKEVGK

VFDVHDYMSEVTVDILLETAMGSNRTGENKEGFDYAMAVMKMCDILHSRQIKIHLRMDPI

FNMTKTKKEQERLLGIIHGLTRKVVKQKKELFEKNLAEGKLPSPSLSEIIGKEEESSGTT

KVEEPAVISQGSMLRDDLDAIDENDIGEKRRLAFLDLMIETAKTGADLSDEEIKEEVDTI

MFEGHDTTAAGSSFVLCLLGIHQDIQDRVYKEIKQIFGDSKRKATFNDTMEMKYLERVIF

ETLRMYPPVPAIARKLTQEVRLASHDYVVPSGTTVVIGTYKLHRREDIYPNPDVFNPDNF

LPERTSNRHYYSYIPFSAGPRSCVGRKYAMLKLKVLLTTILRNYRVVSNLKESDFKLQAD

IILKRTDGFRIQLEPRV

>AAW78325.1

MAVEQIIQSSVFSSPLLMPLLAIVFVLAAVHFWQMSRRERKIGDLLPGPPTVPIIGNAYL

FMNGTNHEMFKKAVDLVNCYGSVVRGWVGHKLLVGLSDPRDVEIILGSQVHIDKSDEYRF

FRPWLGNGLLISSGDKWRTHRKLIAPAFHMNVLKSFMATFNDNSRFVIKKLMKEAGKEFD

CHDYMSEATVDILLETAMGSKRTSESEEGFKYAMAVMKMCDILHRRQFKIFSRFEPFFTL

TGMKEQQKKLLGIIHGMTQRVLNEKKAIFDKNLSEGNLPSPSLQEIIKTDASVDQAIKKA

KAKAQNIDAGLHDDLDDIDENDVGEKRRLAFLDLMIETSHYNPQQLSQEEIKQQVDTIMF

EGHDTTAAGSSFTLCMLGCHPDIQEKVYQEQKAIFGDSDRDCTFADTLEMKYLERVIFET

LRMYPPVPLIARKINKDIRLASCDQVVPAGTTIIIATVKIHRRPDIYPNPDKFDPDNFLP

ERTSNRHYYGFIPFSAGPRSCVGRKYAMLKLKVLLSTIVRNFYVKSTVPEKDFKLQADII

LKRTDGFRIKLEPRKTKAN

>AEK25132.1

MAVEQIIQSSVFSSPLLMPLLAIVFVLTAVHFWQMTRRERKIGDLLPGPPTVPIIGNAYY

FLNSTNHEIFNKAIDLVNCYGSVVRGWVGHKLFVGLSDPRDVEIILGSHDHIDKSDEYKL

FQPWFGNGLLISTVEKWRTHRKLIAPAFHMNVLKSFMSTFNDNSRFVIKKLMKEAGKEFD

CHDYMSEATVDILLETAMGSKRTSESEEGFKYAMAVMKMCDILHRRQFKIFSRFEPFFTL

TGMKEQQKKFLGIIHGMTQRVLNEKKAVFDKNLSEGNLPSPSLQEIIKSDASVDQAIKKA

KAKAQNLDAGLRDDLDDIDENDVGEKRRLAFLDLMIETSHYNPHQLSKDEIKQQVDTIMF

EGHDTTAAGSSFALCILGCHPDIQEKVYQEQKAIFGDSDRDCTFADTLEMKYLERVIFET

LRMYPPVPIIARKINKDIRLASCDQIVPAGSTMIIATVKIHRRPDIYPNPDKFDPDNFLP

ERTSNRHYYGFIPFSAGPRSCVGRKYAMLKLKVLLSTIIRNFHIKSTVAEKDFKLQADII

LKRTDGFRIKLEPRKTKAY

>EAL39767.4

MGIETIPERMAMDETVPGSWVLSVTVATVLLLVAGTLFHLWMQTRRYVQLGNLIPGPVAY

PLIGNANMLLGKTHNQIMEKAMELSYIYGTVARGWLGYHLVVFLTEPADVEIILNSYVHL

EKSSEYRFFKPWLGDGLLISSGDKWKSHRKLIAPAFHQNVLKTFIDVFNDNSLAVVKRMS

KEVGHVFDCHDYMSEVTVDILLETAMGSTRTGENKEGFEYAMAVMKMCDILHKRQLKIHL

RLDPLFNLTGVKKEQERLLQIIHGLTRKVVREKKQLYERQMAEGKMPSPSLTEIIGKEEK

PGEGQLGGSPAFISQGSMLRDDLDDNDENDIGEKRRLAFLDLMIETANNGANISDEEIKE

EVDTIMFEGHDTTAAGSSFVLCLLGIHQHVQEQVYAELRQIFGDSKRKATFGDTLEMKYL

ERVIFETLRMFPPVPMIARKINEDVQLASKNYTIPAGTTVVIGTYKIHRREDLYPHPETF

NPDNFLPERTQNRHYYSYIPFSAGPRSCVGRKYAMLKLKVLLSTVLRHYRVVSNLTEKDF

KLQADIILKRTDGFQIQLEPRA

>289177154

MDAVPMSTSYLPATLFWPLVLIAAALAAVHYYIETSRIVRLGNKLPGPKTVPFFGNALMA

LGVQPKDVLTEVMKYDIYGNVARAFLGPKLVVFLVDPRDVEIILGSHVHIDKSPEYRYFA

PWLGEGLLISTGEKWRSHRKIIAPTFHLNVLKSFVPLFYENSIDLVKRLKSEVGKEFDCH

DYMSGITVDILLETAMGVRGTQKEKSSYDYAMAVMKMCNIIHQRQYNFMLRLDTFFQFTS

FAKQQTKFLDIIHGLTKRVIKKRNVEFKDKMDSPMMNSIMKELKKDSTEIVDEKQPEEQK

MRYVRDDLDEIDENDVGEKRRLAFLDLMLEMRKNGEQLTDEEIKEEVDTIMFEGHDTTAA

GSSFVLCVLGIHQDVQDRVIEELNEIFKGSDRPCTFQDTLEMKYLERVILETLRLFPPVP

AIARQLNQDVKLASGDYILPSGCTVVIPQFKIHRLKEYYPNPDVFDPDNFLPDKTQDRHY

YAYIPFSAGPRSCVGRKYAMLKLKVLLSTILRNYKINSDLTEEDFKLQVDIILKRSDGFR

IQIEPRNQAVIV

>ADE05583.1

MTSFVDESTGYHVNSRLIFYPLLLLASGFWILYRWQQKSHIYKLGEKIPGPAAVPLFGNA

LIVLKKKPHELVDLGLNYAKQFGNVVRVWLGSKLIVFLTDADDVEVILNSHVHIDKSTEY

RFFKPWLGEGLLISSGEKWRSHRKMIAPTFHINILKSFVSVFNQNSKNVVEKMRSEVGKT

FDVHDYMSGVTVDILLETAMGITRKTQDESGFDYAMAVMKMCDIIHQRHYKFWMRFDAVF

KLTPFFKKQINLLNIIHGLTNKVIKSKKQTYLENKAKGIIPLTIEELTRNVESDENVLAN

SAKTLSDAVFKGYRDDLDFNDENDIGEKKRLAFLDLMIESAQNGTNQITDHEIKEEVDTI

MFEGHDTTAAGSSFTLCLLGIHQHIQDKVYDELYEIFGDSDRPATFADTLKMKYLERVIL

ESLRLYPPVPIIARQLKRDVKIPTKNYVLPAGSTVVVGTYKIHRHEKYYNNPDKFDPDNF

LPENTQNRHYYSYIPFSAGPRSCVGRKYALLKLKILLSTILRNYKSVSDIPEEKFSLQAD

IILKRADGFRMRIEPRKRVQSTAA

>EDW65231.1

MEVLKKDAALGSPSSVFYLLLLPTLVLWYTYWRLSRAHLYRLAARLPGPRGLPIVGHLFD

VIGPASSVFKTVIRKSTPFEHIAKMWIGPKLVVFIYDPRDVELLLSSHVYIDKASEYKFF

KPWLGDGLLISTGQKWRAHRKLIAPTFHLNVLKSFIELFNENSRNVVRKLRAEAGGTFDC

HDYMSEATVEILLETAMGVSKKTQDKSGFEYAMAVMRMCDILHARHRSIFLRNEFIFTLT

RYYKEQGRLLNIIHGLTTKVIRSKKAAFEQGTRGSLAQSELKAAALEQEERELREQQQRS

SAGGAKPVELASPVAGLSYGQSAGLKDDLDVEDNDIGEKKRLAFLDLMLESAQNGALITD

TEIKEQVDTIMFEGHDTTAAGSSFFLSLMGIHQDIQDRVIAELDGIFGDSQRPATFQDTL

EMKYLERCLMETLRMFPPVPLIARELQEDLKLNSGPYVIPRGATVTVATILLHRNPKVYD

NPNVFDPDNFLPERQANRHYYAFVPFSAGPRSCVGRKYAMLKLKILLSTIMRNYRVYSDL

SESDFRLQADIILKREEGFRVRLQPRQPGQATTTGSTAAS

>EDV46045.1

MEVLKKDAALGSPSNVFYFLLLPTLVLWYIYWRLSRAHLYRLAGRLPGPRGLPIVGHLFD

VIGPASSVFRTVIRKSAPFEHIAKMWIGPKLVVFIYDPRDVELLLSSHVYIDKASEYKFF

KPWLGDGLLISTGQKWRSHRKLIAPTFHLNVLKSFIELFNENSRNVVRKLRAEDGRTFDC

HDYMSEATVEILLETAMGVSKKTQDKSGFEYAMAVMRMCDILHARHRSIFLRNEFVFTLT

RYYKEQGRLLNIIHGLTTKVIRSKKAAFEQGTRGSLAQCELKAAALEREQGSEDKDKDRK

KDTDKDKEKEKATPVAGLSYGQSAGLKDDLDVEDNDIGEKKRLAFLDLMLESAQNGALIT

DTEIKEQVDTIMFEGHDTTAAGSSFFLSLMGIHQDIQDRVLAELDSIFGDSQRPATFQDT

LEMKYLERCLMETLRMYPPVPLIARELQEDLKLNSGNYVIPRGATVTVATVLLHRNPKVY

ANPNVFDPDNFLPERQANRHYYAFVPFSAGPRSCVGRKYAMLKLKILLSTILRNYRVYSD

LTESDFKLQADIILKREEGFRVRLQPRTS

>ABF51451.1

MTSLVDETEGYHVNSRVIFYPLLGLTTAIWILYRWQQNSHMHKLAELIPGPAPIPIFGNA

LTLMRKDPHELVNLALGYAQTFGNVVRVWLGSKLIVFLADADDIEIILNSHVHIDKATEY

KFFKPWLGEGLLISSGPKWRSHRKMIAPTFHINILKSFVGIFNQNSNNVVEKLKSEVGKT

FDVHDYMSGTTVDILLETAMGISRKTQDESGFDYAMAVMKMCDIIHQRHYKFWMRSEIVF

KLTSFFKQQTKLWGIIHGLTNKVIKNKKETYLENKAKGIIPPTLEEWTHHSGEILANNAK

TLSDTVFKGYRDDLDFNDENDVGEKKRRAFWDLMIESSQNGTNKISDHEIKEEVDTIMFE

GHDTTAAGSSFVLCLLGIHQDVQARVYDELYQILGDSDRPATFADTLEMKYLERVILESL

RLYPPVPVIARKLNRDVTISTKNYVIPAGTTVVIGTFMLHRQPKYHKDPEVFNPDNFLPE

NTQNRHYYSYIPFSAGPRSCVGRKYALLKLKILLSTILRNFRTISEIPEKEFKLQGDIIL

KRAEGFQMKVEPRKRVPTNVAR

>EDV99545.1

MEVLKKDAAMGSPSSVFYLLLLPTLVLWYIYWRLSRAHLNKLAARLPGPRGLPIVGHLFD

VIGPASSVFKTVIRKSTPFEHIAKMWIGPKLVVFIYDPRDVELLLSSHVYIDKASEYKFF

KPWLGDGLLISTGQKWRAHRKLIAPTFHLNVLKSFIELFNENSRNVVRKLRAEDGRTFDC

HDYMSEATVEILLETAMGVSKKTQDKSGFEYAMAVMRMCDILHARHRSIFLRNEFIFTLT

RYYKEQGRLLNIIHGLTTKVIRSKKAAFEQGTRGSLAQNELKAAALEQDQREQQQQAPVV

KPEPAIPFAGLSYGQSAGLKDDLDVEDNDIGEKKRLAFLDLMLESAQSGALITDTEIKEQ

VDTIMFEGHDTTAAGSSFFLSLMGIHQDIQDRVIAELDGIFGDSQRPASFQDTLEMKYLE

RCLMETLRMFPPVPLIARELQEDLKLNSGPYVIPRGATVTVATILLHRNPKVYANPNVFD

PDNFLPERQANRHYYAFVPFSAGPRSCVGRKYAMLKLKILLSTIMRNYRVYSDLSESDFR

LQADIILKREEGFRVRLQPRQAGQATTAAS

>AAZ94273.1

MSAATASVDLENPTTLLTPKNIFYFLLIPALVLWYAYWKISRRHMVELASKIPGPEGLPL

LGSALEFVGTSADIFKRMYAKSFEYGNTVKVWIGPKLLIFLVDPRDVEIILSSHVHIDKA

SEYRFFQPWLGDGLLISTGQKWRAHRKLIAPTFHLNVLKSFIDLFNANSREVVQKLKKEV

GKEFDCHDYMSEATVEILLETAMGVSKKTQDQSGYDYAMAVMKMCDILHLRHTKVWLRPD

FIFNLTNYAKKQEGLIGIIHSLTRKVIKRKRADFEKGIRGSTAEVPEELKTKNFDKNVSS

KTVVEGLSYGQAAGLKDDLDVDDDVGEKKRMAFLDLMIEASQNGVVINDEEIKEQVDTIM

FEGHDTTAAGSSFFLSMMGVHQDIQDKVVQEIDEIFGDSDRPATFADTLEMKYLERCLME

TLRMYPPVPIIARQLRQDVKLASGDYTLPAGATIVIGTFKIHRQEDVYPNPDKFDPDNFL

PERSANRHYYSFIPFSAGPRSCVGRKYAMLKLKILLSTILRNYRIYSTVEEKDFQLQGDI

ILKRADGFRIKLEPRKRVLKA

>AFI45022.1

MTVLGAESFNNASFMPNMWATIFIGILLMLTIYHFWLQSLRYIKLGNKIPGPAPWPIVGN

MLMAIGRNPIDMMKMVLDIYHNSKSDVLKVYLGPKLYVALSNPQDIELILGSHVHLEKSW

EYELFEPWLGEGLLISKGEKWRTHRKMIAPTFHSSILKSFMPVFNKNARVLLQQLDKVKG

QEFDVHDYMSGTTVDILLETAMGVKKTEEDNTGFEYAKAVMDMCNILHLRHYKVWLRPSF

IFKWTKMASEQTKLLQLIHSLTRKVIKRKKDDYFVRASKQESSLYKEALKEEMLDLNAPA

KPNERTENYKYIRDDLEDIEDNDVGEKKRLAFLDFMIEASQTSGTKLTDEEIKEEVDTIM

FEGHDTTAAGSSFVLSMLGVHQDVQEKCVAELKEIFQDDWTRPITFFDTLQMKYLERVIM

ETLRLYPPVPIISRKINEDVQLVTCDLTIPAGSTILISQFATHKHPKYWENPDVFNPDNF

LPEKCQDRHYYAYFPFSAGPRSCVGRKYAMLKLKVILASVLRQFVVTSLKQEKDFMLQAD

IILKRADGFGITIKDRVVN

>383850343

MAAVTTVTTFWQTAVYYSLIAITTTLLAVYLYIENLRVVRFGNKIPGPRKVPILGNALST

VGLHPNTVLETLLTYDVYGPVIRAFLGSKLVIFMYHPRDIEIILSSHVHIDKADEYRYFK

PWLGDGLLISTGEKWRTHRKIIAPTFHLNVLKTFVPLFYENSRDLVIRLRDQVGKEFDCH

DYLSAVTVDILIETAMGLRETEKHKTGYDYAMAVMKMCDIIHRRQYTVRLRPDCLFNLSN

LKKQQEKLLSVIHGLTSKVIQKKKQEAFQSIGSNVEGRQKKSSEAESIKQISQNSETKTN

NEGVTKMHYARDDLDDIDDNDVGEKRRLAFLDLMLELSRNGAKLTDEEIKEEVDTIMFEG

HDTTAAGSSFVLCLLGIHQDIQDRVYEELNEIFKDSNRPCTFQDTLEMKYLERVILETLR

LYPPVPAIARLLKEDVQLVTGNYVLPKDCTILISPYKVHRLEEYYPNPEEFNPDNFLPER

TQNRHYYAFIPFSAGPRSCVGRKYAMLKLKVLLSTILRNYKILSDHSEKDFRLKVDIILK

RVDGFRIKIEPRNKTSQEILV

>EFA02800.1

MVVVEESLNHSLNLGNSVLISLGVVAVILAVYHFWLQSLRYTKLGNKIPGYDPLPIIGNA

HMVMNKNPTQVMELALRVASEKGSVVRFWFGSKLGVALLDPRDIELILGSNVHLEKSSEY

RFFEPWLGDGLLISKGDKWRSHRKMIAPTFHQSILKTFVPVFNKNAMDLVEQLRNEALDQ

ICDVHDYLSGATVDVLLETVMGVKKTKEARTSYKYAKAVMDMCTILHFRHVKLWLRSDWI

FSFTKLFKEQTSLLRIIHNLTDRVIKQKKKAYFERVKDGDVSLYNNAVKETEEENLKIKN

EQTFNFGSGLRDDLDENDENLGEKKRLAFLDFMVEASQTEGNKLNDEEIREEVNTIMFEG

HDTTAAASSFFICILGVYPEIQEKVYQELRDIFQDSDRPITFNDTLQMKYLERVLLETLR

MYPPVPIITRVINEEVKLASGDYTLPVGTTVGIGQFLVHRNPKYFPNPDKFDPDNFLPER

CQQRHYYSFIPFSAGPRSCVGRKYAMLKLKILLASIVRNFKIKSVVKEKDFQLQADIILK

RADGFRVILTSRT

>EDS33030.1

MSATVAPADQLTANANIASPMNVFYFLLVPALLLWFIYWRLSRRHMLELAEKIPGPPGLP

LFGNALELVGTSHSVFRNVIEKGKDFDQVIKLWIGPKLIVFLVDPRDVELLLSSHVYIDK

SPEYRFFKPWLGNGLLISTGHKWRQHRKLIAPTFHLNVLKSFIDLFNENSRLVVEKMRKE

AGKTFDCHDYMSECTVEILLETAMGVSKKTQDQSGFDYAMAVMKMCDILHLRHRKMWLYP

DLFFNMTQYAKRQVQLLDTIHSLTRKVIRNKKAAFETGTRGTLATTSIKTAEFEKPKSTA

NTNTVEGLSFGQSANLKDDLDVEENDVGEKKRLAFLDLLLESAENGALISDEEIKNQVDT

IMFEGHDTTAAGSSFFLSMMGIHQHIQDKVIQELDTIFGDSDRPATFQDTLEMKYLERCL

METLRMYPPVPIIARSLKQDLKLASSDLVVPSGATIVVATYKLHRLETIYPNPNVFDPDN

FLPERQANRHYYAFVPFSAGPRSCVGRKYAMLKLKIILSTILRNFRVISDLKEDEFKLQA

DIILKREEGFQVRLQPRQRKPKAAQA

>AFP20598.1

MSYTTAESVVPTSTWAATNLFYVLVVPALILWYAYWRMSRRHFYELAAKLHGPPGLPLLG

NALEFTGGSHDIFRNVIEKSIPFDGESVVKIWIGPRLLVFLYDPRDVELILSSHTHIDKA

DEYRFFKPWLGDGLLISTGQKWRSHRKLIAPTFHLNVLKSFIDLFNANSRAVVNKLKKES

GEFDCHDYMSECTVEILLETAMGVSKTTQDQSGFEYAMAVMKMCDILHLRHTKIWLRPDL

LFKFTDYAKTQTKLLDVIHGLTKKVIRRKKEEFNSGKRPTILNDYSSTAEETTKTTSVEG

LSFGQAAGLKDDLDVDDSDVGQKKRLAFLDLLLESSQSGVVISDEEIKEQVDTIMFEGHD

TTAAGSSFFLSMMGIHQDIQDKVIEELDKIFGDSDRPATFQDTLEMKYLERCLMETLRMF

PPVPIIARHLKQDITLPSCGKQVPAGTTVVVATYKLHRRPDVYENPTKFDPDNFLPEKSA

NRHYYAFVPFSAGPRSCVGRKYAMLKLKIILSTILRNFRVYSDLKESDFKLQADIILKRA

EGFKVRLQPRKMAKAC

>EFA02880.1

MSTTVPTPDISTPSGILSASNVFYFLLIPALLLWYAYWRISKRHMLELAAKIPGPPGLPI

LGNALDLVGKPHQVFSHVYQKSFEYKKVVKMWAGPKLLVFLTDPSDIELILSSYVHIDKS

SEYRFFKPWLGDGLLISTGQKWKAHRKLIAPTFHLNVLKSFIDLFNANSRDVIRKLQKEI

GKEFDCHDYMSEATVEMLLETAMGVSKKTQDQSGYDYAMAVMKMCDILHLRHTKFWLRPD

IIFNQTKYAEYQKSLINTIHSLTRKVIKRKRADFDKGIRGSTAEVPPELQTKNYDKTESK

TVVEGLSYGQSAGLKDDLDVDDNDIGEKKRMAFLDLMIEASQNGVVINDEEIKEQVDTIM

FEGHDTTAAGSSFFLSMMGVHQDIQDKVVQELYDIFGDSDRPATFADTLEMKYLERCLME

TLRMFPPVPIIARQLNQDLKLASGDYTVPAGCTVVIGTFKVHRLEEYYPNPDKFDPDNFL

PERTANRHYYSFIPFSAGPRSCVGRKYAMLKLKILLSTILRNYRIYSDLKEKDFQLQGDI

ILKRAEGFKVRLEPRKMAKA

>EAL40625.3

MSATIAHTDGLNSSANIISPINMFYFLLTPALLLWFFYWRLSRRHMLELAERIPGPKGLP

LIGNALDLVGSSHSVFRTIIEKGKEYNEVIKIWIGPKLIVFLVDPRDIELLLSSHVYIDK

SPEYRFFKPWLGNGLLISTGHKWRQHRKLIAPTFHLNVLKSFIDLFNENSRLVVKKMQKE

NGKVFDCHDYMSECTVEILLETAMGVSKKTQDQSGYDYAMAVMKMCDILHLRHRKMWLYP

DLFFNLTQYAKKQVKLLNTIHSLTKKVIRNKKAAFDTGTRGSLATTSINTVNIEKSKSDS

TKTNTVEGLSFGQSSNLKDDLDVEENDVGEKKRLAFLDLLLESAENGALISDEEIKNQVD

TIMFEGHDTTAAGSSFFLSMMGVHQQIQDKVIQELDEIFGESDRPATFQDTLEMKYLERC

LMETLRMYPPVPIIARSLKQDLKLASSDIVVPAGATITVATFKLHRLESIYPNPDVFNPD

NFLPEKQANRHYYAFVPFSAGPRSCVGRKYAMLKLKIILSTILRNFRVYSDLKEEEFKLQ

ADIILKREEGFQIRLEPRQRKSKTL

>AEL88545.1

MTVLGAESYNNVSFMPNMWATVFVGILLMLTIYHFWLQSLRYIKLGNKIPGPAPWPIVGN

MLMAIGRNPIDMMKMVLDIYHNSKSDVLKIYLGPKLYVALSNPQDIELILGSHVHLEKSW

EYELFEPWLGEGLLISKGEKWRTHRKMIAPTFHSSILKSFMPIFNKNARVLLQQLDKVKG

QEFDVHDYMSGTTVDILLETAMGVKKTEEDNTGFEYAKAVMDMCNILHLRHYKVWLRPSF

IFKWTKMASEQTKLLQLIHSLTRKVIKRKKDDYFVRASKEESSLYKEALKEEMLDLNAPA

KPNERTENYKYIRDDLEDIEDNDVGEKKRLAFLDFMIEASQTSGTKLTDEEIKEEVDTIM

FEGHDTTAAGSSFVLSMLGVHQDVQEKCVAELKEIFFFDTLQMKYLERVIMETLRLYPPV

PIISRKINEDVQLVTCDLTIPAGSTILISQFATHKHAKYWENPDVFNPDNFLPEKCQDRH

YYAYFPFSAGPRSCVGRKYAMLKLKVILASVLRQYVVTSLKQEKDFMLQADIILKRADGF

GITIKDRVIN

>EGI64338.1

MDADPVTTSYLSIIIYSLLGTIITALIALYYYVETFRAVRLIKKLPGPSSIPIMGHSLLI

VKESPEFLFQLAVKYYNLYGNVIGAYLGTKTVVFLSDPQDIEIILSSSEHIEKAEDYKLF

KPWLGDGLLITSGDKWRRHRKVIAPTFHMNILKTFVPLFYENSVDLVNRLRDKIGKEFDC

HDYLSAVTVDILTETAMGVKKERRSKTGYEYAMAVMKMSDILHRRHFDVSLRFDIFFKFS

KFANLQKKFLKTIHTLTEHVIKEKSKDFEEKYEKCQQLKDAQNDKSIEDTKVTENVENAK

INAANYMHYVRDDLDDIDENDVGEKKRLAFLEMMLELKKNGQMTDEEIWEEVNTIMFEGH

DTTAAGSSFALCVLGNHPDIQARVHEELDTIFGDSNRQCTFQDTIEMKYLERVIMETLRL

FPPVPMIARRLNQDVKIVTGDYILPKSTTVVILQYQIHRLEKYYSNPTVFNPDNFLPENI

QKRHYYAYIPFSAGPRSCVGRKFAMLKLKVMLSTILRNYRIISEIPEKDFLLRGDIILKR

HDGFKIKIEPRTPTSCKK

>EEB10761.1

MTSTVISPETPVGYSASFIFYSLVVSTLVLWYAYFKISRRRMYELAEKLPGPPGIPLFGN

AFEFIGEAPAIFKHVYSHSNTFTDVCKFWIGSKLVVFLINPKDIELILGSNVHIDKASEY

KFFKPWLGNGLLISTGSHWRSHRKLIAPTFHLNVLKSFITLFNQNSRAVCDKMNSLNGKE

FDCHDYMSEATVEILLETAMGVNKKTQDKSGYEYAMAVMKMCNILHLRHRKFWLKPDVIF

NMTKYKKEQESLLDIIHSLTKKVIKKKKEEWNSGIKKTLASVSDMEQTTEQFKGKSSEVA

ASAHSYGQSAGLKDDLDVDDNDVGEKKRMAFLDLMIESAQSGVVMNDNDIKEEVDTIMFE

GHDTTAAGSSFFLSLMGLHQDIQEKIVEELNEIFGDSDRPATFQDTLEMKYLERCIMETL

RMYPPVPLIARQINEDLKLESGDYTIPAGTTVVVATYRLHRDANIYPNPEKFDPDNFLPE

KSANRHYYAFIPFSAGPRSCVGRKYAMLKLKILLSTILRKFRVHSNIAEKDYQLQADIIL

KRAEGFKIRLEPRNKPFAAAAAAAVA

>EHJ74357.1

MSYAAAETVAASSTWAATNLFYVLLVPALLLWYTYWRMSRRHMYELAEKIAGPPGYPLIG

NALEFTGGSDEIFKNIMKRSFEFKNETAVRIWIGPRLLVFLYDPRDVELILSSHVHIDKA

EEYRFFKPWLGDGLLISTGQKWRSHRKLIAPTFHLNVLKSFIDLFNANSRAVVNKLKKES

GEFDCHDYMKTAMGVNKNTQDSGFEYAMAVMKMCDILHLRHTKIWLRPDLLFNFTQYAKV

QNKLLSIIHGLTTKVIKRKKEEFKSGKKPSILETEVTTKDTKTTSVEGLSFGQSAGLKDD

LDVDEDVGQKKRLAFLDLLLESAQGGIVISDTEIKEQVDTIMFEGHDTTAAGSSFFLSLM

GIHQDIQAKVVEELDQIFGDSDRPATFQDTLEMKYLERCLMETLRMFPPVPIIARHLNQD

ITLPSSGKKVPAGTTVVIGTYKLHRSESIYPNPDKFDPDNFLPERSANRHYYAFVPFSAG

PRSCVGRKYAMLKLKIILSTILRNFRVISDLKEEDFKLQADIILKRAEGFKVRLEPRKRL

VKA

>ABF06553.1

MSTATLSSAAAPGLLTSTNLFLFLLAPALALLYVYWKVSRKHMVELAERIPGPSGLPILG

NALEFIGTPNQIFNTIYQKSFEFGRTIKVWVGPRLLIFLTDPRDVEIILSSHVHIDKSPE

YRFFKPWLGDGLLISTGQKWRAHRKLIAPTFHLNVLKSFIDLFNKNSIETVNKLEKELGK

EFDCHDYMSEATVEILLETAMGVSKKTQDQSGYDYAMAVMKLCDILHLRHTKLWFRPDII

FNLTSTAKYQEKLINVIHSLTRKVIQKKKADFEKGIRGSTAEVPEELKTQKYETAVPTKT

TTLEGTSYGQSVGLKDDLDVDDDIGEKKRMAFLDLMIEASQNGVVINDEEIKEQVDTIMF

EGHDTTAAGSSFFLCQMAAHPEIQEKVLQEIDEIFQGSDRPATFADTLEMKYLERCLLET

LRLFPPVPIIARQLQQDVKLASNPSYVLPSGATIIIGTFKVHRLEEIYGPNADKFDPDNF

LPERAANRHYYSFIPFSAGPRSCVGRKYAMLKLKILLSTILRNYKIKSNLKESDYKLQGD

IILKRADGFKIMLEKRKPIVSVKA

>AAR26517.1

MSYAAAESVVPTSTWAATSLFYVLLVPALILWYAYWRMSRRHMYELAAKLHGPPGLPLLG

NALEFTGGSHDIFRNVIEKSIPYDGESVVKIWIGPRFWCSCTILVTWSLILSSHTHIDKA

DEYRFFKPWLGDGLLISTGQKWRSHRKLIAPTFHLNVLKSFIDLFNANSRAVVSKLKKEA

GEFDCHDYMSECTVEILLETAMGVSKSTQDQSGFEYAMAVMKMCDILHLRHTKIWLRPNL

LFKLTDYAKKQTKLLDVIHGLTKKVIRRKKEEFNSGKRPTILQDCTTTTTEEANKTTSVE

GLSFGQSAGLKDDLDVDDADVGQKKRLAFLDLLLESSQSGVVISDEEIKEQVDTIMFEGH

DTTAAGSSFFLSMMGIHQDIQDKVIDELDKIFGDSDRPATFQDTLEMKYLERCLMETLRM

FPPVPIIARHLKQDITLPSCGKQVPAGTTVVVATYKLHRRPDVYPNPTEFDPDNFLPEKS

ANRHYYAFVPFSAGPRSCVGRKYAMLKLKIILSTILRSFRVHSDLKESDFKLQADIILKR

AEGFKVRLEPRKTTKAY

>AFI45021.1

MSVTMENPEFVRNTGLFTASNTFPLLLVSVISLYYVYWRISRRRMLELAENLPGPKGWPI

LGNALEFTGSPHEIFMRLYQKVDEFGRVIKLWIGPRLLIFLIDPRDVEIVLSSHVHIDKS

PEYRFFKPWLGDGLLISTGQKWRAHRKLIAPTFHLNVLKSFIDLFNKNSRETVDKLKKEV

GKEFDCHDYMSEATVEILLETAMGVSKKTQDQSGYDYAMAVMKLCDILHIRQTKIWLYSD

YIFNLTKLSKYQASLISVIHNLTRKVIQKKKAYFQEGIRGSMAEVPEELKMQKFDSNVPT

KTTTVEGTSYGQSVGLKDDLDVDDDVGEKKRMAFLDLLIEASQNGVVINDEEIKEQVDTI

MFEGHDTTAAGSSFFLCQMAAHPEIQERVVQELNEIFKGSDRPATFSDTLEMKYLERCLL

ETLRLFPPVPIIARQLQQDVQLASNPKFKLPAGATIIVAQFGVHRLEEYYPEPEKFNPDN

FLPERAAARHYYSFIPFSAGPRSCVGRKYAMLKLKILLSTILRNYKIYSNLRENDYKLQG

DIILKREDGFRIKLEPRKTCPC

>AEL88544.1

MSVAMENPEFVRNTGLLTASNTFTLLLVSVISLYYVYWRVSRRRMVELAENLPGPKGWPI

LGNALEFTGTPHEIFMRLYQKVNDFGRVIKLWIGPRQLIFLLDPRDVEIVLSSHVHIDKS

PEYRFFKPWLGDGLLISSGQKWRAHRKLIAPTFHLNVLKSFIDLFNKNSRETVDKLRKEV

GKEFDCHDYMSEATVEILLETAMGVGKKTQDQSGYDYAMAVMKLCDILHIRQTKIWLYSD

YIFNLTNLSKHQASLISVIHNLTRKVIQKKKAYFQEGIRGSMAEVPEELKTQKFDLNVPT

KTTTVEGTSYGQSVGLKDDLDVDDDVGEKKRMAFLDLLVEASQNGVVINDEEIKEQVGTI

MFEGHDTTAAGSSFFLCQMAAHPEIQERVVQELNEIFKGSDRPATFSDTLEMKYLERCLL

ETLRLFPPVPIIARQLQQDVQLASDPKLRLPAGATIIVAQFGVHRLEEYYPEPEKFNPDN

FLPERAAARHYYSFIPFSAGPRSCVGRKYAMLKLKILLSTILRNYKIYSNLRGDDYKLQG

DIILKREDGFRIKLEPRKTSPC

>EDW27627.1

MSTVEVLLHEFYQPTTSNARKREIETNLLAFKSQPEAWQLCLRVATAGNSFTDNQFLWFF

STSTLEHTITRRWAQLTPSDRAQLRETLWNTYAQLGMLNGARRHRDTLAQLIALMGKREF

PEQDPNYMQHCMELTKTRFALGINLLRVTSEEVVSNRGDLTTEWKQYFYSCISMCIPDVM

DLVTKYLLIAVCHINGKDIQSTIPNTLMDFSLTSALPNDNQLSSSILELLGCVQHLVSWI

RTELISEYFLMSILDLSQWRPANEPISLAALSVLNELLYLQKPLPYAGTLMGGVSSLLEQ

HNVNKQQSEMYSDKLRELLRLYTTKYAGKLMQEPEVLETFLNQLYGCTTELHGALDFTEK

LDIWSPIIKAIAQQPAKITRFNQVFTQLVDEIMRRTQFEANKPELEVLDNELMEDDTPTT

EWQQFLDQCFECLALLASTRGAHIVFAQVFAHWSRPQMYLMSLEHALDHGSSRSYEAARK

LKHANVGEILRDFATVCQAVVRLAPLMDTSTAAPGVADEMEAQLQMLSDSLCRRCSFWPA

IASEEQIWTRPPFRLIWITSATKKEGLRDDLDDIDENDVGAKRRLALLDAMVEMAKNPDI

EWNEKDIIDEVNTIMFEGHDTTSAGSSFALCMMGIHKDIQEKVFAEQKAIFGDNMLRDCT

FADTNEMKYLERVILETLRLYPPVPLIARRLDYDLKLASGPYTVPKGTTVIVLQYCVHRR

ADIYPNPTKFDPDNFLPERMANRHYYSFIPFSAGPRSCVGRKYAMLKLKVLLSTIVRNYI

VHSTDTEADFKLQADIILKLENGFNISLEKRKYATVA

>EFN85367.1

MDAVSITSYLSSIGYTILAMFITTLLALYYYIETNRAVRMSTKMPNPPRIPILGHILITL

KIKPEDFVCKGIKYTETYGSVVSAHLGPRVFTFLSDPHDIEIILSSNVHLEKSMEYRLFK

PWLGDGLLITKGDKWRKHRKAIEPAFHMSILKTFVPLFYESGLDLVRRLREKVGQQFDCH

DYLSAVTVDILTETAMGVRREKRQNPGYDYAMAVMKLSDIVHRRHYDMSLRPDTLFKFSK

LAKLQKRLLHTVHSLTENVIQEKWKDIEEKQQNETQGEHIETNKSNAGDKNNIANYTKMH

YVRDDLDDIDENDLGEKKRLAFLEMLIDMKKNGGQMTDEEIWEEVNTIMFEGHDTTAAGS

SFALCTLGCLAEIQARVHEELDKIFGDSDRQCTFQDTLEMKYLERVILETLRLFPPVPFI

ARKLNEDVRIGNYVIPKDTTTVLVQFLVHRNEKYYPNPLVFNPDNFLPEKMQQRHYYAFI

PFSAGPRSCVGRKYAMLKLKVLLSTLLRNYRITSNVSYQDFVLRSDIILKRGDGFPIKIE

PRKPASHTGVTIRA

>EFN85148.1

MSVTEPKIIAGSVAAAASAGLSATTVFLSLLIPALVLYYIYFRISRRHMIELAAKLPGPD

GLPFIGNALMFLGNSDTIFRNIYRKSFEFDQIIKLWLGPKLIVFLMDPRDVEIILSSYIY

LDKSTEYRFFKPWLGNGLLISTGQKWRAHRKLIAPTFHLNVLKSFIDLFNANSRAVVERM

RKEGGKEFDCHDYMSECTVEILLETAMGVSKNTQDRSGFEYAMAVMKQVSEKICGAQRDK

YRDNVSQFSINTMCDILHLRHTKIWLRPDWLFNLSKYGKDQIRLLEIIHGLTKKVIARKK

QDYKSGKRNLIDVTMQNGTVQAAKNTTVEGLSFGQSAGLKDDLDVDDNDVGEKKRQAFLD

LLMEAGQKGSVLTDEEVKEQVDTIMFEGHDTTAAASSFFLSVMGCHLDIQEKVIQELDEI

FGDSDRPATFQDTLEMKYLERCLMETLRIYPPVPVIARNIDKDLKLASGDYTIPAGSTVV

VTTFKMHRQPHLYPNPEVFNPDNFLPEKTANRHYYAFVPFSAGPRSCVGRKYAMLKLKII

LSTIMRNFRVKSDILESDFRLQADIILKRAEGFKIRLQPRKLAATATA

>EFN60325.1

MSTAEPEIITAAAAAAAAGLSATTVFLSLLIPALVLYYIYFKLSHRHLFELGDKLPGPDG

LPLIGNALMFTGSADTVFRTIYRKSFEFDQIIRLWLGPKLMVFLMDPRDVEIILSSQVYI

DKSSEYRLFEPWLGNGLLISTGPKWRAHRKLIAPTFHLNVLKSFIDLFNANSRAVVEKLR

KEDGKEFDVHDYMSECTVEILLETAMGVSKSTQDRSGLEYAMAVMKMSNILHLRHMKVWL

RPDFLFNLTKYGKDQIHLLEIIHGLTKKIIARKKQEYKSGKRNVIDVSAQNGSATQNGKA

IQNGNAKNKDVPVEGLSFGQSANLKDDLDVDDNDIGEKKRQAFLDLLMEAGQNGSVLTDT

EVKEQVDTIMFEGHDTTAAASSFFLSVMGCHPEIQEKVIQELDDIFGDSDRPVTFQDTME

MKYLERCLMETLRMYPPVPLIARTINTDLKLASGDYTIPAGCTVVVTTFKMHRQPHIYPN

PEVFDPDNFLPEKTANRHYYAFVPFSAGPRSCVGRKYAMLKLKIILATVMRNFRVKSDIK

ESDFRLQADIILKRAEGFKIRMEPRKLVAATA

>328699379

MVTNVQGVNPLFALSAFNLFFYLLTPAIVLWYIYFRMSRKQLYDLASKIPGSEGLPLLGN

ALDFMQDPHTIFEKIYERSFEFEKNSPIKMWIGPRLLVFLTDPRDVEVILSSNVYIDKSP

EYRLFEPWLGNGLLISTGDKWRAHRKLIAPTFHLNVLKSFVTLFNVNSRDTVSKLRKMGS

STFDIHDFMSECTVEILLETAMGVSKKTQKKSGFEYAAAVMKMCDILHMRHTNLWLKPDF

IFNFTKYAKEQVGLLDLIHGLTNNVLAKKKEEFLKKKSLMKEVSDIPAASEEIVETSSTL

EVEEVPYGNSFGQSAGLKDDLDVEDDGIGEKKRVAFLDLLIECSENGVVLSDEEVREQVD

TIMFEGHDTTAAGSSFFLCLMGAHQDVQQKVVDELYSIFGDSDRPVTFQDTLQMKYMERC

IMETLRMYPPVPIISRQIKEKVKLASRDITLPVGATIVIATFKIHRNEDVFPNPEVFNPD

NFLPEKSASRHYYAYVPFSAGPRSCVGRKYAMLKLKIILSTILRNFKINSNLTEKDWKLQ

ADIILKRTDGFKLSLEPRKSLAKTAA

>EGK97046.1

MSATIAHTDGLNSSANIISPINMFYFLLTPALLLWFFYWRLSRRHMLELAERIPGPKGLP

LIGNALDLVGSSHSVFRTIIEKGKEYNEVIKIWIGPKLIVFLVDPRDIELLLSSHVYIDK

SPEYRFFKPWLGNGLLISTGHKWRQHRKLIAPTFHLNVLKSFIDLFNENSRLVVKKMQKE

NGKVFDCHDYMSECTVEILLETAMGVSKKTQDQSGYDYAMAVMKMCDILHLRHRKMWLYP

DLFFNLTQYAKKQVKLLNTIHSLTKKVIRNKKAAFDTGTRGSLATTSINTVNIEKSKSDS

TKTNTVEGLSFGQSSNLKDDLDVEENDVGEKKRLAFLDLLLESAENGALISDEEIKNQVD

TIMFEGHDTTAAGSSFFLSMMGVHQQIQDKVIQELDEIFGESDRPATFQDTLEMKYLERC

LMETLRMYPPVPIIARSLKQDLKLASSDIVVPAGATITVATFKLHRLESIYPNPDVFNPD

NFLPEKQANRHYYAFVPFSAGPRSCVVKDTNIYITSFFQDVNMLC

>EHJ74962.1

MGITKETQDQSGFDYAMAVMKMCDIIHQRHYKIWLRFDAIFKFTSFFEKQKKLLAIIHGL

TNKVIKNKKERYLQNKAKGIIPPTIDEISKSAPKTENYNVLANEKTLADTVFKGYRDDLD

FNDENDVGEKKRLAFLDLMIESAQNGSNKITDFEIKEEVDTIMFEGHDTTAAGSSFVLCL

LGIHQDIQARVYDELYSIFGDSDRPATFEDTLQMKYLERVIFESLRMYPPVPIIARKINR

DVKIATNDYVLPAGCTVVIGTYGIHRNPKYYENPDVFNPDNFLPEKTQNRHYYSYIPFSA

GPRSCVGRKYAILKLKILLSTILRNYKMVSDITEDKFVLQADIILKRHDGFRVQIEPRKR

VPSTA

>EFA01323.1

MFVVAFFVTLTVILCTPLIWWYLTLAKYSSLKKVPGPPPLPVIGNSNVIGKTTVDLLHSF

MNLQDKYGTVVKVWLGPRLHLLITKPEMVEFFLNSTVHLNKSDGYDLFKPWLGDGLLVST

GSKWKTRRKLITPTFHFKILENFLETSFNKQINILLDVLLKEASQTDKSIEIHSLINLCS

LDIICETAFGTELNAQAKCNPKYVEAVTGFLEIFTLRFFSAWLRHPLIFRLSDKYAKYME

YLKILHDFTNTIIKRRKEEFKLLQDNAKISEEGIKRRAALLDMLLEVSDNGKNLTDEDIR

EEVDTFMFEGHDTTTTSICFVLYAIAQNPDVQKKIYDELVSVLGPDCKKEITFSDIQELK

YLDVVIKEAHRLYPPVPLIERSLEEDCTIDGLTIPKNTNISIFLYGMNYNKDVYPEPHVF

DPDRFLPEKQGERHTFAYVPFSAGPRNCIGQKFALLELKTTIAKLLRCFEISPDPKNPPQ

VGMCSVLKSRNGIHMFLKKKN

>EAT40430.1

MVPLLMLISLLASALIWVLSALVKNLLVYRELQRKLPNFVSTPTVLLLGNTHLFKKDPTP

PGIFATFNQFHRTYGNDLIVQGLLNRPALQITSAPVVEQVLQARTIKKSIIYEFMRPWLN

EGLITSLGKKWAQRRKIITPAFHFKILEEFLAIFNERTEVFVDKIKDQVGKGDFNIYEHV

TLCTLDIISESAMGVKLNAQDDPNSSYVQAVKEMSEIIFQRLFGLLRMHKFFFQMSEAAQ

RQRAALKVLHKFTDSVIFQRKDQLDDEQARQESKQKLEETDIYGKRKMTLLELLLNVSVE

GHHLSNSDIREEVDTFMFAGHDTTTSCISFSAYHIARHPEVQQKLYDEMVQVIGKDFKNA

ELSYSTLQELKYLEMTIKEVLRIHPSVPIIGRKTTGDMRIDGETVPAGVDIAVLIYAMHN

NPEVFPEPEKFDPERFNEENSAKRHPYSYIPFSAGPRNCVGQKYALLEIKVTLVKLLGHY

RLLPCEPENEVKVKSDITLRPVNGTFVKIVPR

>EDS32953.1

MFTALLLALLLLVVTWLVTTLVRNYATTRNLRRNLPSFNVLPSVPLLGSSHLFKDGTEPP

EIFATSSGFHRRFGNDLIAQSFLNQPSLQVTSPEVVEQIINTRTINKSQIYDFMKPWINE

GLLTAVGKKWAQRRKIITPAFHFKILEEFLGTFNQQAEVLVDKLREQVGQGDFNIYKYIT

LCTLDVISETAMGVKINAQDNPDSSYVKAVKEMADLILRRLFSVLREFRWLFPFLKMAKQ

QKRLLNVLHSFTDSVIVTRKNQLENESAQLITQKKLEESDIYGKRKLTLLDLLLNVSIDG

HPLSNPDIREEVDTFMAAGHDTTTSALSFGAYHIARNPPVQQKLYDEMVQVLGPDFKNTT

LTNSMLQDLKYLDMTIKEILRIHPSVPIIGRMSTSDMTINGTKLPTGIEVIIFIYAMHNN

PEVFPEPDRFDPDRFNEENSAKRHPYAYIPFSAGARNCIGQKYALLEAKTILVKLLGSYR

LLPCDPGNTVRIKSDITLRPVNGAFVKIVER

>AAL48300.1

MIVLLVFVVVVLALLVSWISLVRVSRRFNVPGPSPLPLVGNAHLFVVKSSEFLNLVQRLS

EKYGHAFRVHFFSTPYVVICHSKYAEPLVSSTEHITKGRSYSFLTCWLGQGLLTATGQRW

KSHRKFLTPAFHFNILQNFLPVFCKNQRILTEKIRGMADGRPIDMFPIIALAALDNVNES

IMGVCMDAQKHSQSEYVKSIEELSAIVTMRMQIPFFGEDAIFNLLPYKTKQDKALKVLHG

QTNKVINARRAELKKANITTLNDSSDIGTKNKHTFLDLLLLAEIDGKKIDDESVREEVDT

FMFEGHDTTTSGIVYTLHCLSKRRDVQEKIYEELKTIFGSEIHRDPTYHELQQMKYLELV

IKESMRLFPPVPLIERRIMKDCEVGGLKLVKGTSVVMNIFQIQRQPDLFDDPLEFRPERF

EAPLKNPFSWLAFSAGPRNCIGQKFAMMELKITISEIVKNFFILPAAEEPELSADLVLRS

KNGVNVKFLPRK

>EHJ76614.1

MIIIFSLLALVVTGLISWTILLRDSKKYNVPGPTPYPIIGNGYLFISKSSDFLPQLRRLM

LEYGNAYRVFLLHEPYIVLSHPKYVEPLLSHSELIVKGKSYSYLLSWLGNGLLTSTGQRW

RNTRKFLTPAFHFNILQNFLPVFLKNEKILIKKLRDHADGRAFNVLPIMALTALDNVTES

IMGVAVNAQTNRESKYVKSIETLAKITSSRMRNPLLGEDIIFNMLSAKKQQDEAVKYVHS

ETNKVIKARRQELKLSKITRLEKNNDMGIKNKHAFLDLLLLAEVDGKPINDEHVREEVDT

FMFEGHDTTASGLAFSLYCMSLYPNVQEKILEEQKTILGDDLTRDPTYSEVQQMKYLDCV

IRESLRIFPSVPLIERMITEDSQVGELRIPKNTSVIINILELQRHPDLYEDPMEFRPERF

ETMNAKNAFSWIAFSAGPRNCIGQKFAMLELKATLASIVQKFRILPADSAEPILCAELVL

RSENGVRIKLMPRKSE

>CAX94851.1

MIVYVILAVLVIVAVLSWLSFMSDRKKTNLRGPVPYPFIGNGHLLAVDPCEFIKVLHRLQ

EEYEDTVLIYLASRRYVLSSDPRVAEAVLTSNELITKGNSYTFLQPWLGNGLLTSTGQKW

KAHRKFLTPAFHFNILQNFLPIFCKNGRVLRNKLSGLADGSSVDIFPILALLALDNVTES

IMGVSVNAQNDQHPEYVQAVEAVSQISSMRMRNPFVAPDAVFNLLPYKTAQDKALKVLHG

QTMKVISSRKEELRKSNITDLGPNADTGIKNKHAFLDLLLLAEVDGRKISDENIREEVDT

FMFEGHDTTTSGITYCLYCLSNREDIQEKVFMEQKSIYGDDFGRDPTYAEIQKMRYLESV

IKESLRLYPSVPIIERAIAEDSDLAGYHVPKGTNLVLDFLNMQRNAALYEDPLEFRPERF

LEVSRNAFHWLPFNAGARNCIGQKFAMMEIKVTVAMVVNNFKILPSSEEPELASTLILRS

RNGVNIKLIPRK

>ACZ97411.2

MYLVLAVIVLFLYVTFSSWLRLINEAKRMNVPGPTPLPFIGNGYLFFGGITDFMDALLRL

ENKYRGGAALIHILSERHLVLSHPKYIKGIMTNPETITKGDGYAVSKPWLGLGLLTSTGD

LWRSHRKFLTPAFHFSILQTFLPVMIKNEKILLTKLRSLADGTSFDIFPIIALTAMDNIT

ESTMGVHVGAQSNSESKYVTAIQMLTKILALRFFNPIFKSDMLFNLIPHKIKHDKYVDII

QSEIKSIIQSRRQKIIKEQVTGRLQKDSTENEIKSRNAFLDLMLLSGEKMDDESIKDEVN

TFMFAGHDTISSTSSFCLFCLSKYQDAQKKVLEEQISIFGKGLQRETTFSDLQQMVYLDC

FIKEALRLYSPVPFIIRKITRDIDIDGLLITKDTNVLIDIFNMHRNPEVYEDPLTFKPER

FEKEVSLFSWLVFSAGPRNCIGKKYAMMELKLILSTIVRNFHILPSGIEPKLTVALVLTS

ANGVNIKLKPRGNLMI

>ADE05577.1

MFSFVLIVLFISVIIFGLYIRYSHAGRLMAKIPGPKGFFIIGNTLAIVRERDDLFNYGRE

LYAKYGKLSKITGFHIRGVNVYSPEDAETILSSPRYNEKQIPYIFLEPWLGDGLLISKGE

KWHDRRKMLTPAFHFNILKKFTKVFCEETEEFLNLVKEETKKDKTEIMPLIMKSTIRIMC

ETSMGTSMDEDIHTVLKKYLKAIHVLGECVVYRFSRSWFYTNFTFFLSKVAGIQRRAVKD

LHIFTKQIIQERRRYRKQGKIVDINDDDEVYGKKSRMAMLDLLLEQEKLGNIDEDGIREE

VDTFMFEGHDTTSMLLTFMIMRIANEQHVQDLIYEEMQRIFGDSRRSPTMEDFSEMKYLE

CCIKEALRLYPSVPFMSRILNEEVTLSGYKVPEGTQCNIHVFDIHRLEEYYPEPEKFVPE

RFLAENKSTRHPFAYIPFSAGPRNCIGQRFAMLEIKTMMSGLIRRFHLQPVTKHEDVAFL

SDLVLRPKYPIYVRFRERKM

>EHJ68707.1

MSEKNLFHYLRTLHNTYGELNQVHALSVQAVNVFSPEDMEVILSSTKHNDKQLPYTFLKP

WLGEGLLTSNGLKWHQRRKLLTKAFHFNILKKYSATFTEQTQEFIKKVHEETKKSKTDVL

PLICSATLHIMCETAIPATRNEGIQTITQKYFKSIHTVGEAVVERMCRVWLYFDPFFKLT

KTAKEQETALKELHTFTNKIIADRKEFVKNFDVSKYIDSDEYDNSKGKLTMLDLLLENEK

TGNIDLESIREEVDTFMFEGHDTTAMALSYFIMAIANEPAIQRKIYEEMEQIFGDSKRLA

TMADLHEMRYLECCIKESLRLYPSVPFIARNLTQETVLSGYTVPANTFVHLFIYDLHRRP

DLFPDPERFIPERFLPQNSLNRHPYAYIPFSAGSRNCIGQKFAMLEMKTVLSSLIRQFHI

EPVTKPSELRFRTDLVLRTTHPIYVKFKNRE

>EHJ71113.1

MEQTMILLSGKKHSSKGFLYNFLRPWLKDGLLLSDGIKWHSRRKILTPTFHFNILRHFNS

VLLEKVQAFVERLNSEVGNSATEIFPLITEFSLNSICETAMGTALEDEKCDMGQKYKDSI

HTLGTYVFSRSTKFWIYPDFIFNLSSLGMNQKKVLEVISQFRNNVIAKRKLDTNVRQIED

DDCDDEYFISKKQRLAMLDLLLQAEKEGHIDSDGISEEVDTFMFEGHDTVATGLQYIFML

LANHEDIQDKVFLECEEILSRNNGRPSINDMAQMKFLEACIKEALRLYPPVYFISRIGDE

PIELSNGHTCPAGTDFNILIYELHRQADQFVEPYKFLPERFLKEPTWHPFSYLPFSAGPR

NCIGQKFAMMEMKLLLSKVLTVYRLLPITKPQDIIFYADILLRSNQVKVKIEKRKK

>AFP20600.1

MIFELLLAALVAAVVWLIFFRDDDNPVDKLPGPPRKPWIGNAWELLQMPSDKLLDILVDY

TNTYGDRYVFKVFSLRVLHISGPPDVETVLSHSKNIKKSAPYDFLKGWLGTGLLLSTGLH

WHKRRKILTPTFHFNILKNFAHVIEEKTQDLVKMLKDKNGADVSLMPTISDFTLFTICET

AMGTKLDDDKTSATVDYKNAILNIGMQMLARITRSWLHNDFIFNNSSRGKEFTKTLEVAR

AFADNVIMDRKAQRIQNKGEDISIADASDGIGTKKRLAMLDLLLEAEEKGEIDMDGIRDE

VNTFMFEGHDTTALALTFGLMLLADHEDVQERIYEECQTILGDSEHVTMSNLSDMKYLEA

VIKEILRLYPSVPFIGREITEDFKLGDITVKKGTTVDVHIYELHRRADMFPEPEKFMPER

FLGTEMKHPYAYVPFSAGPRNCIGQRFAMQEMKTTLSELVRHFKIVPKVKGARPRIMADL

VLRPVDPIYVKFIPR

>AAL48299.1

MLLTLALLGFITFVIWRLLADEENTLDQLPGPPRRPIIGCALEFLKISPRELFLKMRQYK

KEYGDRYVIKMMKRRILHLSNEKDVEVVLSHSRNIKKSKPYTFLEAWLGTGLLLSSGAKW

HRRRKILTPTFHFNILKSFTVVMKEKSRSLVETLNNLEGTEVNLLPVISDYTLYTICETA

MGTQLDADKSSKTLEYKTAILEIGNLLLGRLTRVWLHNEYLFKKFPIGRRFENCLEKVHS

FADDVIMERKKNWKPGQSEFTEDDVSIGGKKRYAMLDLLLEAENKGEIDLEGIREEVNTL

MFEGHDTTAMAIVLGLMLIADHEEVQDRIFEECQKILGDSDRTPTMSDLADMKYLEAVIK

ETLRLYPSVPFIAREITEDFMLDDLPVKKGSEVSIHIYDLHRRPDLFPEPDAFIPDRFLS

GAPMHPYAFVPFSAGPRNCIGQRFAMLEMKCVFSAICRNFRLAPKVQGYRPALLADMLIR

PEGPVYVKFSRR

>ABC84370.2

MIATLLLVGFIGLVIYRLLAEEENPLDKLPGPPRKPIVGCVFEFLNLTPRVMFKKMRLYS

KVYGGRYLVKILRRRILHLHNVNDVEIVLSHPRNIKKSKPYSFLEGWLGTGLLLSSGAKW

HRRRKILTPTFHFNILKNFTNVMEERSRGLVDKLKEYDGKEVNLMPVISDCTLYTICETA

MGTQLDSDSSTKTQEYKTAILQIGGLLMGRLTRVWLHNEYIFRKFPMGKQFEKCLEKVHP

FADNVIMERKKNWKPGQSNAGEDDVGGKKRLAMLDVLLEAERKGEIDLEGIREEVNTFMF

EGHDTTAMALVFGLMLLADHPEVQERIYEECQTILGDSDTSPTMSDLAEMKYLEAVIKEI

LRLYPSVPFIAREVTEDFMLGDVLVKKGTEVSIHIYDLHRLPELYPDPEAFKPERFLNQQ

PTHPYAFVPFSAGPRNCIGQRFAMLEMKCMLSGVCRKFKLSPIVPGERPKLLADMVLRPV

GPVCVKMHQR

>EDW47078.1

MIVLWLVLALSALLHWLHRANKDYHILSFFTKRIRSKDGTPVEIIAPMPKGKTIFGNTFD

LYGRDHAGVFDHSRERAKELGTSYIEYTFGSAIYNIIDADSAENVMNHPNLITKGLVYNF

LHPFLKTGLLTSTGKKWHARRKMLTPTFHFNILNQFQEIFKTESQKFLQQFEGQDEVTIT

LHDVIPRFTLNSICETAMGVKLDEMAEKGDRYRENFSQIEECFIRRLSNPLFWGDKLFEM

FAAKDYASALDVVHGFSSEIIAKRRDLLKDELDKSSSTQTADDDGFVSKKRFAMLDTLIY

AEKDGLIDHSGICEEVDTLMFEGYDTTSIGLIFGLMNMSLNPDKQELCFQEIQEHIDDDL

SNLDVSQLNKLKYLEYFMKETMRLFPSVPIMGREAVQETELANGLILPKGAQITIHVFDI

HRNAKYWDSPEEFRPERFLPENVQDRHTYAYVPFSAGQRNCIGQKYAMQEMKTLMVVLLK

QFKVLKAIDPQKIVFHTGITLRTQDKIRVKLVRRQ

>EDW89481.1

MIALLLVLALGALLHWLYRANKDYHVLSFFTKRIRSKDGTPVEIIAPTPKGKTIFGNTFD

LYGRDHAGVFNHSRERAKEMGTSYIEYAFGTAIYNIIDADSAENVMNHPNLITKGLVYNF

LHPFLRTGLLTSTGKKWHARRKMLTPTFHFNILNQFQEIFKTESQKFLQQFEGQDEVTIS

LHEVIPRFTLNSICETAMGVKLDEMAEKGDRYRENFSQIEECFIRRLSNPLFWGDKLFNI

FAAKDYASALDVVHGFSSEIIAKRRVLLKDELNKNPSTQTGDDDGFVSKKRFAMLDTLIH

AEKDGLIDHIGICEEVDTLMFEGYDTTSIGLIFGLMNMSLNPDKQEICFQEIQEHIDDDL

SNLDVSQLNKLKYLEYFMKETMRLFPSVPIMGREAVQETELANGLILPKGAQITLHVFDI

HRNAKYWDSPEEFRPERFLPENIQDRHTYAYVPFSAGQRNCIGQKYAMQEMKTLMVVLLK

QFKVLKAIDPQKIVFHTGITLRTQNKIRVKLVRRK

>EDV59394.1

MIVVWLVLALSALLHWLYRVNKDYHVLSLFAKRIRSKDGTPMELIAPTPKGKSIFGNTFD

LYGRDHAGVFNHSRERAAQMGTSYIEYSFGTAIYNIIDAETAENVMNHPNLITKGLVYNF

LHPFLRTGLLTSTGKKWHARRKMLTPTFHFNILNQFQEIFKTESQKFLQQFEGQDDITVC

LHDVIPRFTLNSICETAMGVKLDEMAEKGDRYRENFGQIEECFIRRLSNPLFWGDKLFNI

FAAKDYAAALDVVHGFSSEIIAKRRVLLKDELDKSPSTQTADDDGFVSKKRFAMLDTLIH

AEKDGLIDHIGICEEVDTLMFEGYDTTSIGLIFGLMNMSLYPDKQELCFQEIQEHIDDDL

GNLDVSQLNKLKYLEYFMKETMRLFPSVPIMGREAVQETELANGLILPKGAQITLHVFDI

HRNTKYWDSPEEFRPERFLPENVLDRHTYAYVPFSAGQRNCIGQKYAMQEMKTLMVVLLK

QFRVVKAINPQKIVFHMGITLRTRNKIRVKLVRRK

>EDV36701.1

MLVLWLVIAVSALVHWLYRLNKDYYVMSFFAKRVRTKDGQPLSSLVALPKGVTIFGNSFD

LYGKDHAGVFNMSRQRSKLGQSYVEYGMGTPIYNLIDADSADIVLNNQNLITKGLVYNFL

HPFLRTGLLTSTGKKWHARRKMLTPTFHFNILGQFQEIFKTESQKFLQQFKGQDETIISL

NDVIPRFTLNSICETAMGVKLDEMAEKGDRYRENFASIEECFIRRLSNPLYWGDTLFNLF

AAKDYASALDVVHGFSSEIIAKRRVLLEDELESRKETQTADEDLYAGKKKRFAMLDTLIC

AEKDGLIDHTGICEEVDTLMFEGYDTTSIGLIFGLMNMGLYADKQELCFQEIQEHIEDDL

SNLDISQLNKLKYLDYFVKETMRLYPSVPIMGRATVQETELSNGLILPKATQITIHVFDI

HRNPKFWDSPEEFKPERFSPENSQNRHTYAYIPFSAGQRNCIGQKYAMQEMKTLLVAVLK

QFKILPVTDPESIVFTTGITLRTKNKIQVKLQRRK

>EDV36700.1

MFVVWLIVAVSLLAHWLYRVNKDYYILAFFAKRIRTKDGTPVENIVAIAKGNTIFANSFD

LYGQDHAGVFTHTREGAKLGRNYLEYVMGTPIYNVIDAEGSDAILNNPNLITKSVVYNFL

HPFLRTGLLTSTSKKWHARRKMLTPTFHFNILGQFQEIFKTESQKFLLQFKGQDDITISL

NEVIPRFTLNSICETAMGVKLDEMAEKGDRYRENFTSIEECFIRRMSNPLYWGDTLFNIF

AAKDYASALDVVHSFSSEIIAKRRILLEDELESRRETQTADDDLYAGKKKRFAMLDTLIC

AEKDGLIDHIGICEEVDTLMFEGYDTTSIGLTFGLLNMSLYADKQELCYQEIQEHIQDDL

SNLDISHLNKLKYLEYFVKETMRLYPSVPGMGRETVEETELENGLILPKGSQIVIHNFDV

HRNPKYWDSPEEFRPERFTPENSQNRHTYAYIPFSAGQRNCIGQKYAVQEMKTLLVVLLK

EFKVLPVTDPKSIVFTTGITLRTQNKIQVKLVRRK

>EDW47079.1

MLFLWLVGAFIMLFQWIYRLNRDYCILGFFAKRIRAKNGQPTESIAPFVKGSTIFANSFD

LYGKDHSGVFEHSRDCAKKLGKSYVEYAMGTAIYNVIDADSAEHVLNDPNLINKGTVYYF

LHPFLRTGLLTSTGKKWHARRKMLTPTFHFNILNQFQEILITESLKFLEQFKGKDEAVIS

LNEVIPRFTLNSICETAMGVKLDEMAEKGDRYRENFRQIEECFIRRMSNPLLWSDTLFKM

FAEKDYTSALDVVHGFSSEIIAKRRDQLNDDMDSRGKTQTAEDELFTSKKRFAMLDTLIL

AEKDGLIDHIGICEEVDTLMFEGYDTTSIGLIFGLMNMSLYPKEQEKCYQEIQANINDEL

NNLDIGQVNKLKNMEYFIKETMRLFPSVPAMGRETTRETELGNGLILPKGSQIAVHVFDI

HRNPEYWDSPEEFRPERFLPENSQNRHTYAYIPFSAGQRNCIGQKFAMQEMKTLMVALLK

QFQILPEIDPKTIVFQTGLTLRTKNQIHVKLVRRK

>Q9V559.3

MLILWLVGAFIVLIQWIYRLNRDYCILGFFAKRIRTKNGQNPESIAPLVKGSTIFANSFD

LYGKDHSGVFEHSRDCAKKLGKSYAEYAMGTAIYNVIDADSAERVLNDPNLINKGTIYDF

LHPFLRTGLLTSTGKKWHARRKMLSPTFHFNILNQFQEIFITESLKFLEQFKGNDEAIIS

LNEVIPRFTLNSICETAMGVKLDEMAEKGDRYRENFRQIEECFIRRMSNPLLWSDTLFKM

FAEKDYASALDVVHGFSSEIIAKRRDQLNDEIDSRGNTQTAEDELFTSKKRFAMLDTLIL

AEKDGLIDHIGICEEVDTLMFEGYDTTSIGLMFGLMNMSLYPEEQEKCYQEIQANIDDEL

NILNIGQLNKLKNLEYFIKETMRLFPSVPAMGRETTRETELSNGLILPKGSQIFVHVFDI

HRNPEYWDSPEEFRPERFLPENSQNRHTYAYIPFSAGQRNCIGQKFAMQEMKTLMVALLK

QFQILPEIDPKTIVFQTGLTLRTKNQIHVKLVRRK

>EDW89482.1

MVFLWLVGASIVLLQLIYRLNRDYCILGFFAKRIRTKKGQPPESIAPLVKGSTIFANSFD

LYGKDHAGIFKHSRDCAQALGKSYVEYAIGAAIYNVIDADNAEHVLNDPNLINKGTVYYF

LHPFLRTGLLTSTGKKWHARRKMLTPTFHFNILNQFQEILITESLKFLQQFKDKDEVIIS

LNEVIPRFTLNSICETSMGVKLDEMAEKGDRYRENFSQIEESFIRRMSNPLLWSDTLFKM

FAEKDYASALDVVHGFSSDIIAKRREQLNDEMDNIRNTQTADDELFTTKKRFAMLDTLIH

AEKDGLIDHIGICEEVDTLMFEGYDTTSIGLIFGLMNMSLYPDEQEKCFQEIQAHIDDEL

TNLDSGQLNKLKNLEYFIKETMRLYPSVPAMGRETARETELANGLILPKGSQIAVHVFDI

HRNPEYWDSPEEFRPERFLPENSQNRHTYAYIPFSAGQRNCIGQKFAMQEMKTLMVAVLK

QFQILPEIDPKSIVFQTGLTLRTQNQIHVKLLRRK

>EDV59393.1

MLFLWLVGASIVLLRWIYRLNRNYCILGFFAKRIRTRNGQPPESIAPFVKGSTIFANSFD

LYGKDHSGIFEHSRDCAKILGRSYVEYAMGTAIYNVIDADNAEHVLNDPNLINKGMVYYF

LHPFLRTGLLTSTGKKWHARRKMLTPTFHFNILNQFQDILITESLKFLQQFKDKDEAIIS

LNEVIPRFTLNSICETSMGVKLDEMAEKGDRYRANFSQIEECFIRRMSNPLLWSDTLFKM

FAEKDYAAALDVVHGFSSEIIAKRRDQLNDDMDDRRNTQAADDELFTTKKRFAMLDTLIR

AEKDGLIDHIGICEEVDTLMFEGYDTTSIGLIFGLMNMSLYPDEQEKCFKEIQAHIDDEL

NNLDIGQLNKLKNLEYFIKETMRLYPSVPAIGREATRETELANGLIFPKGSQLAVHIFDI

HRNPEYWDAPEEFRPERFLPENSQSRHTYAYIPFSAGQRNCIGQKFAMQEMKTLMVAVLK

QFQILPQTDPKSIVFQTGLTLRTQKQIHVKLLRRK

>EDY69101.1

MIVVLWLTVALCVIVHWLYRVNKDYYIMAFFSRRVRTKDGRPVESIVPVPKGRTIFANCL

DLYGKDHAGVFSYSRELSQRLGESYLQYGMGLPIFNIIDAENAENILNHPKLITKGLVYD

FLQPALRTGLLTSTEKKWHARRKMLTRTFHFNILEQFQEIFQAESLKFVQQFQSQDESIV

SLSDLIPRFTLNSICETAMGVKLDEMAEKGDRYREGIHMIEMSFIRRLSNPLLWNKTIYN

WFAAKTYDAFLKTVHEFSSEIIAKRRILLEEELEHRRLTQTADDDIFVSRKKRFAMLDTL

ICAEKDGLIDHAGICEEVDTLMFEGFDTTSIGLIFSLMNMSLYPEKQELCYKELEEHIDD

NFDNLDHNQLSKLKYLEYFIKETMRLFPSVPIMARQTIQETELANGLILPERSQITIHIF

DVHRNPKYWDSPNEFRPERFLPENCQNRHTYAYIPFSAGQRNCIGQKFAMQEMKTLIVVL

LKKFRILPLIDPKTIVFNVGITLRTQNNIQVKLVRRN

>EDY69102.1

MIVFLLTVALCALVLWLYRINKDYYILAFFSRRVRTKDGRPVESILPSPKGRTIFANCFD

MYGKDLAGVFSYSRELSQRLGESYLEYGMGVPLLNIIDAENAENILNHPKLITKSLVYDF

LQPALRTGLLTSTEKKWHARRKMLTPTFHFNILGQFQEIFRAESLKFVQQFQSQDESIVS

LSDLIPRFTLNSICETAMGVKLDEMAEKGDRYREGIHMIEMSFIRRLSNPLLWNKTIYNW

FAAKTYDAFLKTVHEFSSEIIAKRRILLEEELEHRRSTQTADDDIFGSRKKRFAMLDTLI

CAEKDGLIDHAGICEEVDTLMFEGYDTTSIGLIFSLMNMSLYPEKQELCYKEIEEHIDDS

FDNLDNNQLSKLKYLEYFIKETMRLFPSVPIMARQTIQETELANGLILPERSQITIHVFD

VHRNPKYWDSPNEFRPERFLPENCQNRHTYAYIPFSAGQRNCIGQKFAMQEMKTLIVVLL

KKFRILPLIDPKTIVFNVGITLRTQNNIQVKLVRRK

>EDW61516.1

MDLGLLILIGLGTLIFWLYRINKDYYVLAFFAKRVRTKDGRPIESIAPVPEGRTIFGNSF

DLYGRNDAEMFNDMRVRVKQMGRSFIEYVMGTPVYNIIDADNFDLILNDQNLITKGLVYG

FLHPALRTGLLTSTDKKWHTRRKMLTPTFHFNILGQFEETFKAESLKFVKQFEGKAETCI

SLSELIPRFTLNSICETAMGIKLDDMAAKGDRYRESFSMIEKSFIRRMSNPLIWNNTIYN

LFGSSEDARFLKEVHDFSSEIIAKRRILLEQVLNEKLSTQAADDDIYINKKQRFAMLDTL

ILAEKDGLIDHEGICEEVDTLMFEGFDTTSIGLIFGLMNMSLNQDKQELCYQEISEHIAD

DFNNLDTNQLSKLKYLECFVKETMRLFPSVPIMARQTVRETELANGLILPAGAQITLHVF

DLHRNPKYWSDPDEFQPERFLPENSKDRHTYAYMPFSAGQRNCIGQKYAMLEMKTLLIVV

LKQFKILPLVDPKEFVFQTGITLRCKNNIKVKLVRRS

>EDW61515.1

MELGLLILIGLGTLIFWLYRINKDYYVLAFFAKRVRTKDGRPVESIAPVPEGRTIFGNSF

DLYGRNDAELFNDMRVRVKQMGRSFIEYVMGTPVYNIIDAENFDLILNDQNLITKGLAYG

FLHPALRTGLLTSTDKKWHTRRKMLTPTFHFNILSQFEETFKAESLKFVKQFEGKAETCI

SLSELIPRFTLNSICETAMGIKLDDMAAKGDRYRESFSMIEKSFIRRMSNPLIWNNTIYN

LFGSSEDARFLKEVHDFSSEIIAKRRILLEQVLKEKLSTQAADDDIYINKKQRFAMLDTL

ILAEKDGLIDHEGICEEVDTLMFEGFDTTSIGLIFGLMNMSLNQDKQELCYQEISEHIDD

DFNNLDINQLSKLKYLECFVKETMRLFPSVPAMGRKAVRETELANGLILPAGAEIALHVF

ELHRNPKYWSDPDEFQPERFLPENSKDRHTYAYMPFSAGQRNCIGQKYAMLEMKTLLIVV

LKQFKILPLVDPKEFVFHTGITLRCKNNIKVKLIRRI

>EDW36031.1

MITLWLTVALCVVIHWLYQVNKDYYILAFFSRRVYTRDDRPVESLVPSPKGRTIFAHCFD

LYGKDNAGVFKFTRDLSKTLGKSYVRYVVGMPIYNVIDAETAEMILTDQKSLIGKGCVYN

FLHPALKTGLLTSQGDKWHARRRMLTPTFHFNILGQFQEIFQAESLKFVQQFQSQDENIV

SLSELIPRFTLNTICETAMGVKLDEMAEKGDRYRESFHMIEKSFVRRLSNPFLRSTTIYN

LFVAKEYAPFLKVVHDFSSEIIAKRRILLEEELEHRRSTQTADDDIFVSRHKRFAMLDTL

ICAEKDGMIDHAGICEEVDTLMFEGYDVSSITLIFTLMNMSLYPDKQELCFKEIQEHIED

DFSNLDINQMSKLKYLEYFIKETMRLFPPIPLMGRQAVQETELANGLILPSGTQIHIHTF

DIHRNPKHWDSPNEFQPERFLPENSQNRPVYAYIPFSAGQRNCIAQKFAMQEMKTLMTVI

LKKFEIQPLVDPKSIVFHVGITLRIKNHIRVKLVRRK

>EDY69103.1

MITLWLTVALCVVIHWLYQVNKDYYILAFFSRRVYTRDDRPVESLVPSPKGRTIFAHCFD

LYGKDNAGVFKFTRDLSKTLGKSYVRYVVGMPIYNVIDAETAEMILTDQKSLIGKGCVYN

FLHPALKTGLLTSQGDKWHARRRMLTPTFHFNILGQFQEIFQAESLKFVQQFQSQDENIV

SLSELIPRFTLNTICETAMGVKLDEMADKGDRYRESFHMIEKSFVRRLSNPFLRSTTIYN

LFVAKEYAPFLKVVHDFSSEIIAKRRILLEEELEHRRSTQTADDDIFVSRHKRFAMLDTL

ICAEKDGMIDHAGICEEVDTLMFEGYDVSSITLIFTLMNMSLYPDKQELCFKEIQEHIED

DFSNLDINQMSKLKYLEYFIKETMRLFPPIPLMGRQAVQETELANGLILPSGTQIHIHTF

DIHRNPKHWDSPNEFRPERFLPENSQNRSVYAYIPFSAGQRNCIAQKFAMQEMKTLMTVI

LKKFEIQPLVDPNSIVFHVGITLRIKNHIRVKLVRRM

>EDW47077.1

MVWILWLSVAISVVVHWIYKVNKDYNILAFFARRVRTKDGKPLDSVVPMTKGRTVFANCF

DLFGKDSDQVFTYWRKLAKKNGDSYLQYAMGFTTFNVIDAHNAGNILNHPNLITKGVIYN

FLHPFLRTGVLTATEKKWHTRRGMLTRTFHMDILNQFQEIFIAESLKFVSEFEGQNECIV

SLRDRIAKFTLNSICETAMGIKLDEMAEKGDRYRENFHIIDEGFTRRIVNPLYWDDCVYN

IFAGHKYNAALKVVHEFSSELIAKRRVLLEEELENRRATQTADDDICVIRKKRFAMLDTL

ICAQKDGLIDDIGICEEVDTLMAEGYDTTSIGLVFGLMNMSLYAAEQELCYQEIQEHILD

DLSNLNLSQLSKLNHLGYFIKETMRLYPSIPIMGRQTLQETELENGLILPKRSQINIHVF

DIHRNAKYWESPEEFRPERFLPENCLKRHPYAYIPFSAGQRNCIGQKYAMQEMKTLMVVI

LKQFKILPVIDPKSIVFQVGITLRFKNKIKVKLVRRNCV

>EDV59395.1

MIWLLWLSVAMSVVVHWIYKVNKDYYILAFFARRVRTKDGKPLDSLVPMPKGRTLFANCF

DLYGKDSDQVFTHLRQLARKSGNSYLQYSMGFSNFNVIDAHNAAHILNHPNLITKGVIYN

FLHPFLRTGVLTATEKKWHTRRSMLTRTFHLDILNQFQEIFIAESLKFVNHFEGQNECVV

SLRDHIARFTLNSICETAMGIKLDEMAEKGDRYRENFHIIDEGFTRRIVNPLYWDDCVYN

IFAGHKYNAALRVVHEFSSEIIAKRRILLEEELESRRATQTADDDICVVRKKHFAMLDTL

ICAQKDGLIDHIGICEEVDTLMAEGYDTTSIGLVFGLMNMSLYADAQELCYQEIQEHIRD

DLSNLNLSQLSKLNQLSYFIKETMRLYPSIPIMGRQTLEETELENGLILPKRSQINIHVF

DIHRNPKYWESPEEFRPERFLPENSRKRHPYAYIPFSAGQRNCIGQKYAMQEMKTLMVVI

LKQFKILPVIDPKSIVFQVGITLRFKNKIKVKLVRRNCAQSLSI

>EDW79782.1

MIIACLWFTALAIVIHWLYKLNNEYFILSFCSRRVRAKPGKTVESIAALPPGRTIFGNCL

DLYGKDDAGVFNYSRNLAKTLGKSYIQYGMGVPIYNIIDAENAELILNDQNLITKGLVYE

FIHPALKTGLLTSTGKKWHMRRKMLTPTFHFNILGQFQEIFKSESIKFVQPFIGQEETEI

SLSDLIPRFTLNSVCETAMGIKLDDMAEKGDRYRESFKMIEKCFIRRLSNPLYWNNHLYN

LFAASDYESHLKEVHDFSSEIIAKRRILFEDELAERKKTQTADDDIFINKKQRFAMLDTL

LLAEKDGFIDHAGICEEVDTLMFEGFDTTSMGLIFGLMNMAQYPEKQELCYKEIQEYIED

DFSNLDITQLSKMKYLECFIKENLRLYPSVPIMGRQTTRETELANGLILPAYTQITLHVF

DIHRNPKYWSQPDEFQPERFLNENSNDRHTFAYLPFSAGQRNCIGQKYAILEMKTLLVVV

LKHFKVLPLVEPKSFEFHMGITLRTKNNIIVKLVKRK

>EDW89480.1

MVWLLWLSVAISVVVHWIYKVNKDYNILAFFARRVQTKDGKPLDSLVPMPKGRTLFANCF

DLYGKDSDQVFNHLRQLAKKSGDSYLLYYMGYSNFNVIDAHNAANILNHPNLITKGVIYN

FLHPFLRTGVLTATEKKWHTRRSMLTRTFHLDILNQFQEIFIAESLKFVNHFQGQNECVV

SLKDHIARFTLNSICETAMGIKLDEMAEKGDRYRENFLIIDEGFTRRILNPLYWDDCVYN

IFAAHKYNAALKVVHEFSREIIAKRRVLLEEELENRRVTQTADDDICVIRKKRFAMLDTL

ICAQKDGLIDNIGICEEVDTLMAEGYDTTSIGLVFGLMNMSLYADAQERCYQEIQEHIQD

DLGNLNLSQLSKLNHLSYFIKETMRLYPSIPIMGRQTLQETELENGLILPKSSQIDIHVF

DIHRNPKYWESPEEFCPERFLPENCKKRHPYAYIPFSAGQRNCIGQKYAMQEMKTLMVVI

LKQFKILPVIDPKSIVFQVGITLRFKNNIKVKLVRRNCV

>Q9V557.1

MMICLLWISVAILVVIHWIYKVNKDYNILAFFARRVQTKDGKPLDSLVPMIKGRTVFANC

FDLLGKDTDQVFTHLRQLAKNSGDSYLQYSMGFSNFNVIDAHNAANILNHPNLITKGVIY

NFLHPFLRTGVLTATEKKWHTRRSMLTRTFHLDILNQFQEIFIAESLKFVSQFQGQNEVV

VSLKDRISRFTLNSICETAMGIKLDEMAEKGDRYRANFHIIDEGLTRRIVNPLYWDDCVY

NMFTGHKYNAALKVVHEFSREIIAKRRVLLEEELENRRATQTADDDICVIRKKRFAMLDT

LICAEKDGLIDDIGISEEVDTLMAEGYDTTSIGLVFGLMNMSLYAAEQELCYQEIQEHIL

DDLSNLNLSQLSKLNYLGYFIKETMRLYPSIPIMGRQTLQETELENGLILPKRSQINIHV

FDIHRNPKYWESPEEFRPERFLPQNCLKRHPYAYIPFSAGQRNCIGQKYAMQEMKTLMVV

ILKHFKILPVIDPKSIVFQVGITLRFKNKIKVKLVRRNCV

>EDW09153.1

MSLGLLLLIGLGTLIFWLYRLNKEYYVLAFFAKRVRTKDARPLESIAPIAEGRTIFGNSF

DLYGRNDAEVFTDLRAKIKKIGRSYIEYAMGRAKFCIIDAENFDNILNDQNMISKGLGYQ

FLHPFLRTGLLTSTGQKWHSRRKMLTPTFHFNILSQFVEIFKAESFKFVKQFEEKDEVSI

SLSELIPRFTLNSICETAMGIKLDDMAGKGDRYRENFTMIEKRFISRISNPIYWNDTIYR

LFGASKDKQFLEEVHGFSSEIIAKRRKLLAEEIKEKKNNQEGVDDIYINKKQRFAMLDTL

IFAEKDGLIDHVGICEEVDTLMFEGFDTTSIGLIFGLMNMSLYQDKQELCYQEISEHIAD

DFNNLDLNQLSKLKYLECFIKETMRLFPSVPVMSRKAVKETELPNGLILPPGAVTMLHIY

ELHRNPKYWSSPEEFQPERFLPENSKDRHTYAFMPFSAGQRNCIGQKYAMLEMKTLLIVV

LKKFKVLPLIDPRDLVFQTGITLRCKNNIKVKLVKRA

>EDW36030.1

MIVVLWLTVALCVIVHWLYRVNKDYYIMAFFSRRVRTKDGRPVESIVPVPKGRTIFANCL

DLYGKDHAGVFSYSRELSQRLGESYLQYGMGLPIFNIIDAENAENILNHPKLITKGLVYD

FLQPALRTGLLTSTEKKWHARRKMLTPTFHFNILGQFQEIFQAESLKFVQQFQSQDESIV

SLSDLIPRFTLNSICAGVFSYSRELSQRLGESYLEYGMGVPLLNIIDAENAENILNHPKL

ITKSLVYDFLQPALRTGLLTSTEKKWHARRKMLTPTFHFNILGQFQEIFRAESLKFVQQF

QSQDESIVSLSDLIPRFTLNSICETAMGVKLDEMAEKGDRYREGIHMIEMSFIRRLSNPL

LWNKTIYNWFAAKTYDAFLKTVHEFSSEIIAKRRILLEEELEHRRSTQTADDDIFGSRKK

RFAMLDTLICAEKDGLIDHAGICEEVDTLMFEGFDTTSIGLIFSLMNMSLYPEKQELCYK

EIEEHIDDNFDNLDNNQLSKLKYLEYFIKETMRLFPSVPIMARQTIQETELANGLILPER

SQITIHIFDVHRNPKYWDSPNEFRPERFLPENCQNRHTYAYIPFSAGQRNCIGQKFAMQE

MKTLIVVLLKKFRILPLIDPKTIVFNVGITLRTQNNIQVKLVRRK

>EDW00739.1

MVCGWCIATVLLSILIYWLIKINENYNILAFFARRVHTKDGRPVESIAPTPKGKTIFANC

FDLYGKDAAGVFQHSRHLAKEMGTNYLSYSLGTAMYMIIEPEIVEFVLTNPQLITKGMVY

NFMKSALRTGLLTSTGKKWHTRRKMITHTFHFNILEQFEEIFKAESQKFVDKFKDQSESV

ISLTDLIPRFTLNSICETAMGVNLDEMTEEGDRYRESFKLVEQCFVKRIKNPLQYSDTMY

KLFGAKYDAHFLKVVHDFSSNIIAKRRILLEKELQERRDDQLPDDDIYINKKRRFAMLDT

LICAEKDGLIDHEGICEEVDTLMFAGYDTTSMGLTFNLMNLSLHEDMQEMCYQEISENID

DDLSKLDINQLSKLKYMDRFIKETIRMYPSAPVMGRQTTSETELPNGLILPPGTQCVIHV

YDLHRNPKYWNAPEQFDPDRFLPENSMDRHNFAYVPFSAGQRNCMGQKYAMLEIKTLLIY

ILKQFKILPITKAEDLILHSGITLCVKNDVKVKFVVRK

>EDW60768.1

MVCASIVIIAACVLLYWLYKINEEYYILAFFARRVRAKHGQSLESIVSVPKGRTIFANCF

DLFGKDDAGVFQYSRRLAREMGSSYLIYSLGTPIYSIIDPVNAEFVLNNASLITKGAIYD

FMQPALRTGLLTSTGKKWHTRRKMITPTFHFNILGQFMEIFKAESQKFVRRFENQEESVI

SISEHIPRFTLNSICETAMGINLDELTEKGDRYRESIKMIEKCFVKRISNPLYYNNTMYK

LLAASEDAPFLKMVHEFSSDIIAKRRVLLKAELDERRKNQSPDDDIYINKKRRFAMLDTL

ICAEKDGLIDHDGICEEVDTLMFGGFDTTSMSLIFTLLNLSLYEDMQELCYQEIAEYIDD

PSDLDITQLSNLKYLDRFVKETIRMFPPVPIMSRQTLNETELPNGLILPAGTQISMHIFD

IHRNPKYWDSPEEFDPDRFLPENSMKRHTYAYIPFSAGQRNCIGQKYAMMEIKTLVIYIL

KRFKILPITDPDEFILHSGITLCVKNNIKVKLVLRK

>EDV36702.1

MLILWLLAVLSVLIHWLYKVNKDYYILSFFARRARTKDGRSVESLVPTPKSRTIFGNCFD

CIGKDNAEVFQFFRDQAVRYGECYLHYTPGKASFNVIDAPTVENILNHPKLINKGFMYDF

LHPFLKTGVLTAKEKKWHTRRGMLQRTFHYNILNQFQEIFIAESLKFVEQFKGKESCIVS

LRDPIYRFTLNSICETAMGVKLDEMGDSGSHYRDNLHHIDEGFVRRIRNPFYWGDFWYNL

FAARDYAAALKQVNGFSSDIIAKRRILLEEELENSSGFTSKKPFAMLDTLICAEKDGLID

HKGICEEVDTLIYEGYDSPFHVIKFGLMNMSLYPDHQENCYREVQELIRDLSNLDTRQLN

NLKNLDCFLKETMRLFPSGPILARQAVEETELANGLILPKGSHINIHIFDIHRNPKHWDS

PEEFRPDRFLTENCQWRHPYAYIPFSAGSRNCLGKKFAMQEIKTLLVVILKHFRIVPITN

PEPIVFQMGIQLRTTNTLKVKFVRRT

>EDW60767.1

MVCAFIVIITLCVLLQWLYKINEEYYILSFCARRVRAKDDRSVESIAPIPKGRTIFANCF

DLYGKDGVGIFQYHRRLAREMGSSYLIYSLGAPIYSIIDPVNAEFVLNNASLITKGALYD

FIQPALRYGMLTSTGKKWHTRRKMITPTFHFNILGQFVEIFKAESQKFLKLVESQPESII

SLSELIPRFTLNNICETAMGINLDELTEKGDRYRESIKMIERCFVKRLCNPLLHNNTMYK

LLAASEDAPFLKVVHEFSSDIIAKRRVLLKAELDERRKHQSPDDDIYINKKRRFAMLDTL

ICAEKDGLIDHDGICEEVDTLMFGGFDTTSMSLIFTLLNLSLYEDMQELCCQEISEYIDD

PSDLDITQLSNLKYLDRFIKETIRMFPPVPFIGRQTLSETELPNGLILPARTQIIMHIFD

IHRNPKYWDSPEEFDPDRFLPENSMKRQTYAYIPFSAGQRNCIGQKYAMLETKTLLIFIL

KRFKILPITDPKELVLFNGITLCVKNNIKVKLVLRK

>EDW80605.1

MFTIWLTFAFALFYWLYRVNKNYHVLGFFAKRVYTQNGKPVESLVGVPKGLSGLRNLFAN

FGKDNASVFTYLREVSKRMGHRSYIQYGMGRTMLSVVDAEYAEIVLNDQNLINKGLVYTF

LEPALKNGLLIASGQKWHSRRKMLTPTFHFNIMGQFQEVFKTESLKFVQPFLKEEEIDIT

LNDIIPRFTLNTICETAMGIKLDDMAEEGDRYRNSFSKIDQCFARRLTNPFLWNRKIYDL

IAAKDYEPHLKEVHRFSSEIIAKRKVLFEKELEQRRNTKTTEDDLFINKKQRYAMLDTLL

CAESDGLIDHAGICEEVDTLMFAGFDTTSMGLIFGLMNMSVYPELQDLCFKEIEEYVDDD

FSNLDSNLLGKLKHLECFVKETMRMYPSVPIISRKTIQDTTLSNGLVLPKNTDVTLLIFD

ILRNPKHWDSPDEFKPERFLPENSQNRPTYAFVPFSAGQRNCIGQKYAMQEMKTLMVVIL

KSFKILPLVDPKALL

>EDX06322.1

MGFSTFNVIDAHNAAYILNHPNLITKGVIYNFLHPFLRTGVLTATEKKWHTRRGMLTRTF

HMDILNQFQEIFIAESLKFVSQFEGQNECIVSLRDRIAKFTLNSICETAMGIKLDEMAEK

GDRYRENFHIIDEGFTRRIVNPLYWDDCVYNIFAGHKYNAALKVVHKFSSEIIAKRRVLL

EEELENRRATQTADDDICVIRKKRFAMLDTLICAQKDGLIDDIGICEEVDTLMAEGYDTT

SIGLVFGLMNMSLYAAEQELCYQEIQEHILDDLSNLNLSQLSKLNQLGYFIKETMRLYPS

IPIMGRQTLQETELENGLILPKRSQINIHVFDIHRNAKYWESPEEFRPERFLPENCLKRH

PYAYIPFSAGQRNCIGQKYAMQEMKTLMVVILKQFKILPVIDPRSIVFQVGITLRFKNKI

KVKLVRRNCV

>EDW10215.1

MVYPFIVIIAVFVLLYWLLKINKEYYVLAFFARRARATDGRFVDNIAPILKGNTIFGNVF

DLCGKDDAGTFRYYRDAALEMGKSYLIYACGSPMFNIIDPVNAEFVLSNPTLITKSIDYD

FMQPALHKGLLTSSGKKWHTRRKMITPTFKHNVLVQFEEIFRAESQKFVKQFDHQEESIV

SLPELIPRFSLNSICETAMGINLDDMTERGDRYRESMKMFEKCFVKTFTNPLHYYGAIYK

LFPINENIPYLKVVHDFTNDIIAKRQILLEEELNERRCHQLPDDDIYVNKKRRFAMLDTL

ICAEKDGLIDHAGICEEVNTLMFGGFDPSSIALMFTIFNLSLYEDMQELCYQEILEHIHD

HNNLDINQLSNLKYLERFIKETLRLFPPVPIIGRQTSEETVLPNGLILPTGSQIIMHVFD

LHRNPKYWDQPDVFDPDRFLPQNSVKRHAYAYIPFSMGLRNCLAQQYFMILIKTLLCFIL

KKFKILPVTHSEDLVFHMGLTLRVENNIKVKFVLRNK

>EDW02205.1

MGLCLLLLIGLGTLIFWLYRINKDYYVLAFFARRVRTNDGRPLESIVPVAKGRTIFGNSF

DLYGKSDAEVFEVMSNRAKQIGRSYLAYAMGVTVYNIIDAENVELILNDPSLISKSLVYE

FLHPAVRTGLLTSTAEVFEVMSNRAKQIGRSYLAYAMGVTVYNIIDAENVELILNDPSLI

SKSLVYEFLKPALRTGLLTSTDKKWHTRRKMLTPTFHFNILGQFEEIFKVESLKFVKQFE

GQSEISISLSELIPRFTLNSICETAMGVKLDDMAAKGDTYRVNFSMIEKTFVRRLSNPIL

WNNTLYNLFGARKDAPFLDGVHEFSSEIIAKRRIMLEELLKDNKNVQNADDDIYIKKKQR

FAMLDTLILAEKDGLIDHEGICEEVDTLMFEGFDTTSIGLIFGLMNMSLYADKQELCYQE

INEHIADDFSNLDINQISKLKYLECFVKENLRMFPSVPIMGRKAVRETELANGLILPGGC

NISVHIFALHRNPKYWTSPEEFQPERFLPENSKDRHTFAYVPFSAGQRNCIDDFSNLDIN

QLSKLKYLECFVKETLRMFPSVPIISRKAVRETELANGLILPAGCNITVHIFALHRNPKY

WTSPEEFQPERFLPENSKDRHPFAYVPFSAGQRNCIGQKYAMLEMKTLLIVILKQFKVLP

LVDPKDLGFNVGITLRSRNDIKVKLVKRN

>ADC44461.2

MWLYVFIVVLAFLAAWLYKLNRDYCVMAFFAKRLKTVDGTPLEHIVPQAKAETIFANVFD

CFGADNVVLFDSCRISAATLRKSYVEYNLFTTFYNVITAEDAELLLNDKNLITKGIAYYF

LEPFLHTGLLTSTDKKWHTRRKMLTPAFHFNILGQFEEVFKEESQKFVDQLEAIDCDSVT

VNEIIPKFTLNSVCEAALGVKLDEQLDDQEYRASFKMIEEAFILRISNPFYMIDTIYDIF

MAPKMKKHLETVHGFSSNIIHKRRQLFNAKLEQSPVKEAKDEDFGFTKKKRYAMLDTLLH

AERDGLIDHAGICEEVDTFMFEGFDTTSMALIFALMNLSLYPEMQELCYQELVEHIEDDL

HKLDVNDLGKLKYLDCFIKETLRLYPSVPGIMRKVMRDTSLANNVFLPADTQISIHIFDI

QRDPKYFPEPNKFDPTRFTPENSEGWHPYAYIPFSAGQRNCIGQKFAILEIKTLLVYMLK

KFKILPLMDPKDLRFETGIILRTPNAIKVKLQKRSSS

>EDW72088.1

MNSDFDPDYTVSGTRRNLRQRQRKAENEDVPTEMEKSKRRGKKKGNYYGKTSAYDEYGNI

RSNGRDVCDCMNDDCDGLFVAIFIALISLCLWHLHKDYELLSLCRRFKTTDGSEFVKGKG

NSIFGNTFDTIGMTSEELFYYSRDYAEELKRSYVQYIVGFPIYNIIDAADAELVLNDNNL

ITKGDMYRFLHPFLQTGLLTSTGQYWNTRRKLLTPAFHFKILEQFLEVFKKESVKFIESL

RKSTNKTDEINLNELVPRFTLNSICETALGVKLDDCADGDDYRKNIAIAEHSFIKRVMNP

LMASDTLYNLGEGRKDVSTLKKLHAFSSNIIQKRRLNFDEASVRKAQEMDSSDLNTKQRY

AMLDTMLLAETEGLIDHAGICEEVNTFMFGGYDTTAMTLILCLAFLGSYPEKQQRCFEEL

KEFVDDDLANLDNRTLSQLKYLECVIKETQRLCPSVPGIMRECHTETKLNNLILPKGAQI

IVHIFDIHRNPKYYDRPNEFIPERFLNDSTVQRHPYAFIPFSAGRRNCIGQKYAMTEMKT

LLTYILKNFEILPVDDPLTYRFLAGILIRTKSDVYVKIRKRL

>EDW01979.1

MNSEQLFYYNRDYATELKRSYIQYIIGMPVYNIIDAADAELVLNDNNLITKGDLYRFLQP

FLKSGLLTSTGKHWHSRRKLLTPAFHFKILEQFLEVFKQESVKFIDLLQNMSTNTVGPCE

VNLNNVIPRFTLNSICETALGVRLDDCADGDEYRNNITIAEHSFINRVINPLLGSDTLYN

NFGNGRKDIPTLKNLHAFSSNIIKKRRQNFNREAYETESSDLNTKQRYAMLDTMLLAEMD

GLIDHAGICEEVDTFMFEGFDTTSMNIILALAYLASHPNKQQLCYEEITQFISNNGELND

LDSRTLGQLKYLECVIKETQRLCPSVPSIMRECHEDTKLANNLIMPKGTQIIVHIFDIHR

NPLYFEEPNEFIPERFLPENSTQRHPFAFIPFSAGRRNCIGQKFAMLEVKTLLVYILSHF

MVLPVSNPKDYRYLAGILIRTKEDVFIKITKRKN

>EDW88947.1

MWIALLGTPILLAALALLLKHINKTYFILSLTKRVRTEDGSPLESKVALMPGKTRFGNNL

DILNFTPASVFNFVRESTAKAKGQNYLWYFLYAPMYNVVRAEEAEEIFQSTKLITKNVVY

ELIRPFLGDGLLISTDHKWHSRRKALTPAFHFNVLQSFLGIFKEECKKFLNVLEKNLDAE

LELNQVIPPFTLNNICETALGVKLDDMSEGNEYRKAIHAIEEVLIQRVCNPLMYYNWYFF

LYGDYRKHVQKLRIVHDFSSRIIERKRQQFRQKQLGEVDEFGRKQRYAMLDTLLAAEAEG

QIDHQGICDEVNTFMFEGYDTTSTCLIFTLLMLALHEDVQKKCYEEVESLPEDSDDVSVF

QFNELVYLECVIKESLRMFPSVPFIGRQCVEETVVNGMVMPKDTQISIHIYDIMRDPRHF

PKPNQFQPERFLPENTVNRHPFAFVPFSAGQRNCIGQKFAILEMKVLLAAVVRNFRLLPA

TQLEDLTFENGIVLRTQQNIKVKLTKRVT

>EDW54353.1

MWIALLGTPILLAVLTLLLKQINKTYFILSLTKRVRTEDGSPLESKVAIMPGKTRFGNNL

DILNFTPASVFNFVRESTAKAKGKNYLWYFLHAPMYNVVRPEEAEEVFQSTKLITKNVVY

ELIRPFLGDGLLISTDHKWHSRRKALTPAFHFNVLQSFLGIFKEECKKFINVLEKNLDAE

LELNRVIPPFTLNNICETALGVKLDDMSEGNEYRKAIHAIEEILIQRVCNPLMYYNWYFF

LYGDYRKHSQNLRIVHDFSSRIIERKRQQFQQKQLGEVDEFGRKQRYAMLDTLLAAEADG

QIDHQGICDEVNTFMFEGYDTTSTCLIFTLLMLALHEDVHKKCYEEVESLPEDSDDISMF

QFNKLVYLECVIKESLRMFPSVPFIGRQCVEETVVNGMVMPKDTQISIHIYDIMRDPRHF

PKPDLFQPDRFLPENTVNRHPFAYVPFSAGQRNCIGQKFAILEMKVLLAAVIRNFKLLPA

TQLEDLTFENGIVLRTQENIRVKLLKRVK

>EDV31772.1

MWIALLGTPALLVVLWLLLKKLNESYFILSLSRRVRTEDGSPLESKVAVMPGKTIFGNNL

DILNFTPASVFNFVRESTAKAKGRNYLWYFLFAPMYNVVRAEEAEEIFQSTKLITKNVVY

ELIRPFLGDGLLISTDQKWHSRRKALTPAFHFNVLQSFLAIFKEECNKFIKVLNENLDGE

LDLNQVIPQFTLNNICETALGVKLDDMSEGNGYRKAIHAIEEILIQRVCNPLMYYNWYFF

LNGDYRKHEHNLRIVHDFASGIIQRKRELFKQKQLGHVDEFGKKQRYAMLDTLLAAEAEG

QIDHEGICDEVNTFMFEGYDTTSTCLIFTLLMLALHEDVQTRCYEEVQNLPEDSDETTVF

QYNELVYLECAIKESLRMFPSVPFIGRQCVEECVVNGMVMPKDTQISLHIYDIMRDPRHF

PKPNQYQPERFLPENTVNRHPFAFVPFSAGQRNCIGQKFAILEMKVLLAAVLRNFRLLPA

TQLEDLTFENGIVLRTQENIKVKLVKREASS

>EDW29166.1

MLIALLGTPLLLLGVWLLLKRLNRTYFILSLTRRVRTEDGSPLESKVAVMPGTTRFGNNL

DVLNCTPSSLFNFLRDSAAKAQGRNFLWYFLFAPMYNVIRAEEAEEIFQSTKLITKNVIY

ELLRPFLGEGLLISTDQKWHSRRKALTPAFHFNVLQSFLAIFKEECNKLIKVLDKKVDGE

LDLNQVIPQFTLNNICETALGVKLDDMSEGNQYRRAIHAIEEVLIQRVCNPLMFYNWYFF

LIGDYRKQVQNLRIVHDFSSRIIERKRQQFKRKQLGVVDDFGKKQRYAMLDTLLAAEADG

QIDHQGICDEVNTFMFEGYDTTSTCLIFTLLMLSLHKDVQERCLEEVSALPEDTDSISVF

QFNELVFLECVIKESLRMFPSVPFIGRTCVEECVVNGLILPKDTQISIHIYDIMRDPRHF

PNPNAFQPERFLPENTVDRHPFAFVPFSAGQRNCIGQKFAILEIKVLLAAVLRNFRILPV

TRLEDLTFENGIVMRTQQNVKVKLVRR

>EDY70183.1

MFELLLGVPLIVLLCCVLLRRLNRKYFVLSLCKRLQAEDGSPLESKIAVMPGRTRFGNNF

DLLNFTPSTVFNFMRDSCANARGRNYLWYFFGSPMYNVIRAEEAEEIFQSTKLLTKNVVY

DLLRPFLGEGLLTSTDQKWHARRKALTPAFHFNVLQSFLAIFKEECHKLVKVLHESVESE

ALVLNQVIPQFTLNNICETALGVKLDDMAEGFQYRESIHAIEAVILQRICNPFLYNSLYF

YLYGDYRKHIDSLKIAHDFSSSIIERRRRDIKRKELGVVDDFGKKQRYAMLDTLLAAEAD

GQIDHQGICDEVNTFMFEGYDTTSTCLIFTLLMLSLHKDVQERCLEEVSALPEDTDSISV

FQFNELVFLECVIKESLRMFPSVPFIGRQCTEECVVNGLVMPKDTHINIHIYDIMRDPRH

FPNPNAFQPERFLPENTVDRHPFAFVPFSAGQRNCIGQKFAILEIKVLLAAVLRNFKILP

DTRFEDLTIENGIVLRTQQKVKVKLVQRVG

>Q9VMS8.4

MFLEVLFAAPLVIFIFRKLWAHLNRTYFILSLCKRIRTEDGSLLESKIYVAPSKTRFGNN

FDLVNFTSESIFNFMRDASAKAKGRNYLWYFFHAPMYNIVRAEEAEEILQSSKLITKNMI

YELLKPFLGEGLLISTDQKWHSRRKALTPAFHFKVLQSFLIIFKEECNKLVKVLHQSVNM

ELELNQVIPQFTLNNVCETALGVKLDDLSEGIRYRQSIHAIEEVMQQRLCNPFFYNIVYF

FLFGDYRKQVNNLKIAHEFSSNIIEKRRSLFKSNQLGQEDEFGKKQRYAMLDTLLAAEAD

GQIDHQGICDEVNTFMFEGYDTTSTCLIFTLLMLALHEDVQKKCYEEIKYLPDDSDDISV

FQFNELVYMECVIKESLRLFPSVPFIGRRCVEEGVVNGLIMPKNTQINIHLYEIMRDARH

FSNPKMFQPDRFFPENTVNRHPFAFVPFSAGQRNCIGQKFAILEIKVLLAAVIRNFKILP

VTLLDDLTFENGIVLRTKQNIKVKLVHRENK

>EDW29167.1

MFELLLGVPLIVLLCCVLLRRLNRKYFVLSLCKRLQAEDGSPLESKIAVMPGRTRFGNNF

DLLNFTPSTVFNFMRDSCANARGRNYLWYFFGSPMYNVIRAEEAEEIFQSTKLLTKNVVY

DLLRPFLGEGLLTSTDQKWHARRKALTPAFHFNVLQSFLAIFKEECHKLVKVLHESVESE

ALVLNQVIPQFTLNNICETALGVKLDDMAEGFQYRESIHAIEAVILQRICNPFLYNSLYF

YLYGDYRKHIDSLKIAHDFSSSIIERRRRDFKRKELGGVDDFGKKQRYAMLDTLLAAEAD

GQIDHQGICDEVNTFMFEGYDTTSTCLIFTLLMLALHEDVQERCLEEVSALPEDTDSISV

FQFNELVFLECVIKESLRMFPSVPFIGRQCTEECVVNGLVMPRDTHINIHIYDIMRDPRH

FPNPNAFQPERFLPENTVDRHPFAFVPFSAGQRNCIGQKFAILEIKVLLAAVLRNFKILP

VTRFEDLTIENGIVLRTQQKVKVKLVQRVG

>EDV57843.1

MWIALLGTPILLAVLALLLKQINKTYFILSLSKRVRTEDGSPLESKVAIIPGKTRFGNNL

DILNFTPSSVFNFVRESTAKAKGQNYLWHFLYAPMYNVVRPEEAEEIFQSTKLITKNVVY

ELIRPFLGDGLLISTDHKWHSRRKALTPAFHFNVLQTFLGVFKEECKKFLDVLETHLDAE

LELNHVIPPFTLNNICETALGVKLDDMSEGNEYRKAIHAIEEVLIQRVCNPLMYYNWYFF

LYGDYRKHVHNLRIVHDFSSRIIERKRQQFRQKQLGEVDEFGRKQRYAMLDTLLAAEAEG

QIDHQGICDEVNTFMFEGYDTTSTCLIFTLLMLALHEDVQKKCYEEVEGLPEDSDDISVF

QFNELVYLECVIKESLRMFPSVPFIGRQCVEECVVNGMVMPKDTQISIHIYDIMRDPRHF

PKPDLFQPERFLAENTVNRHPFAFVPFSAGQRNCIGQKFAILEMKVLLAAVVRNFRLLPA

TQLEDLTFENGIVLRTQQNIKVKLSKRVK

>EDX03805.1

MYLEVLFAVPLVIFIFRKLWTHLNRTYFILSLCKRIRTEDGSLLESKIYVAPSKTRFGNN

FDLVNFTSESIFNFMRDASAKAKGRNYLWYFFHAPMYNIVRAEEAEEILQSTKLITKNMI

YDLLKPFLGEGLLISTDQKWHSRRKALTPAFHFKVLQSFLIIFKEECSKLVKVLHQSVDT

ELELNQVIPQFTLNNVCETALGVKLDDLSEGIRYRQSIHAIEEVMQQRLCNPFFYNIVYF

FLFGDYRKQVNNLKIAHEFSSNIIEKRRSLFKNNQLGQEDEFGKKQRYAMLDTLLAAEAE

GQIDHQGICDEVNTFMFEGYDTTSTCLIFTLLMLALHEDVQKKCYDEIKYLPEDSDDISV

FQFNELVYMECVIKESLRLFPSVPFIGRRCVEEGVVNGLIMPKNTQINIHLYEIMRDARH

FSNPKQFQPDRFFPENTLNRHPFAFVPFSAGQRNCIGQKFAILEIKVLLAAVIRNFKILP

VTLLDDLTFENGIVLRTKQNIKVKLVHRANK

>EDW54354.1

MYLEVLFAIPLVVFLFRKLWAHLNRTYFILSLCKRIRTEDGSLLENKIYVAPSKTRFGNN

FDLVNFTSESIFNFMRDASAKAKGRNYLWYFFHAPMYNIVRAEEAEEILQSTKLITKNMI

YDLLKPFLGDGLLISTDQKWHSRRKALTPAFHFKVLQSFLIIFKEECSKLVKVLHQSVDT

ELKLNQVIPQFTLNNVCETALGVKLDDLSEGIRYRQSIHAIEEVMQQRLCNPFFYNIVYF

FLFGDYQKQVNNLKIAHEFSSNIIEKRRSLFKNNQLGQEDEFGKKQRYAMLDTLLAAEAD

GQIDHQGICDEVNTFMFEGYDTTSTCLIFTLLMLALHEDVQKKCYAEIKYLPEDSDDISV

FQFNELVYMECVIKESLRLFPSVPFIGRRCVEEGVVNGLIMPKNTQINIHLYEIMRDARH

FSNPKMFQPDRFFPENTVNRHPFAFVPFSAGQRNCIGQKFAILEIKVLLAAVIRNFKILP

VTLLDDLTFENGIVLRTKQNIKVKLVHRESK

>EDV98799.1

MFMTLLTYGLLIVALWTLLKCLNRKYFILSLVKRVQTQDGSPLDSKVFVIPGKTRFGSNF

DLLKMTPSMVFQFVRDAHKQAKGRNYLWYFLFAPLYNVVRAEEADEILQSTKLLTKNIVY

DLLKPFMGEGLLISTDRKWHSRRKMLTPAFHFNVLQMFLDIFKEECHKLLNILHQNVGDE

LELNHVIPQFTLNNVCETALGVKLDDMEEGNRYRNAIHALEDVMIERVCNPLLYYKPYFY

LYGSYRKHIENLKIAHDFSSRIIEQKRQQSKQKRTGSIDGIDEFGRKSRYAMLDTLLEAE

NEGLIDHQGICDEVNTFMFEGYDTTATCLIFTLLMLALHEDVQQRCFEEVQPLSDKDNEE

LSVFDFNGLVYLECVIKESLRLFPSVPFVGRFCTEECVVNGLILPKNSQICLHIYDIMRD

PVHFPNPSQFQPERFLPENSVNRHPFAFIPFSAGQRNCIGQKFAILEMKVLLVAILQNFQ

LLPVTRLEDIIFEYGIVLRSQKNIAIKLKRRQQS

>EDW84760.1

MFIICLATVVLLIAVWRLLEILNRKYYILCLCKRVRPVDGSPLESKTVVIPGTTPFGNNF

DILQNTPSGVFNYVRNASARAQLKGMNYFFYFLMAPMYNVIRAEEAEEIFQSPKLITKNV

IYELLRPFLGEGLLISTDQKWHSRRKALTPAFHFNVLQSFLIIFKEECMKLVDILNEKID

TELELNQIIPSFTLNNICETALGVNLDDLAEGKQYRDAIHALESVMVQRLCNPLFYFWLY

FYLFGEYGKHVKNLKIADDFSQNIIKRRRQEFKQQQRHGSDDYGRKKRYAMLDTLLAAEE

DGQIDHQGICDEVNTFMFEGYDTTSTCLIFTLLMLSLHEDVQERCFEELQQLAGDDIDDH

SVFDFNELIYLECVIKESLRMFPSVPFIGRLCTEETVVNGFIMPKDTQINIHIYDIMRDP

RHFPQPNEYRPERFLPENTVNRHPFAFVPFSAGQRNCIGQKFAILEIKVLLASILKNFRI

LPVTRFEDIIFEYGIVLRTQQNTSDMNSLMDTHPNCNGYQYLTGYQNLVYNLIKKG

>EDX03806.1

MWIALLGFSLLIGAIGFLLRQLNKTYFILSLCKRVRTADGSPLESKVFVIPGKTRFGNNL

DLLNLTPATIFNYIRESTAKANGQNYIWNFIFAPEYNIVRAEDAEEIFQSTKITTKNMAY

ELIRPFLGDGLLISIDQKWHSRRKTLTPAFHFNILQTFLSIFKEESKKFIKILDKDVGCE

LELNQIIPQFTLNNICETALGVKLDDMSEGNEYRKAIHDFEIVFNQRMCNPLMYYNWYFY

LFGDYKKYSRILRTIHGFSSGIIQRKRQQFKRKQLGQVDEFGKKQRYAMLDTLLAAEAEG

KIDHQGICDEVNTFMFGGYDTTSTSLIFTLLLLALHDDVQERCYEELQNLPEDIDEISMF

QFNELIHLECVIKESLRLFPSAPIIGRTCMEESVVNGLVLPKNAQISIHIYDIMRDPRHF

PKPNQFLPERFLPENAVNRHPFAFVPFSAGPRNCIGQKFGILEMKVLLAAVIRNYKLLPA

TQLEDLTFENGIVLRTQQNIKVKFEARVK

>EDV57845.1

MWIALLGSSLLIGALWLMLRQLNKTYFILSLCKRVRTADGSPLGRKVFVIPGKTRFGNNL

DLLNLTPATIFNYIRECTAKANGQNYLWYFLFAPEYNIVRAEDAEEIFQSTKITTKNMAY

ELIRPFLGDGLLISTDRKWHSRRKTLTPAFHFNILQSFLSIFKEESKKFIKIVDKNVGCE

LELNQIIPQFTLNNICETALGVKLDDMSEGNEYRKAIHDFEIVYNERMCNPLMYYNWYFF

LFGDYQKLSKILRTIHGFSSGIIQRKRQQFKQKQLGEVDEVGKKQRYAMLDTLLAAEAEG

KIDHQGICDEVNTFMFGGYDTTSTSLIFTLLLLALHEDVQQRCYEEIQNLPEDIDEISMF

QFNELIHLESVIKESLRMFPSAPIIGRKCTEESVMNGLVLPKNSQISIHIYDIMRDPRHF

PKPNQFLPERFLPENAANRHPFAFVPFSAGPRNCIGQKFGILEMKVLLAAVIRNFKLLPA

TRLEELTLENGIVLRTQQNIKVKFEARAK

>Q9VMS7.2

MWIALLGSSLLIGALWLLLRQLNKTYFILSLCKRVRTADGSPLESKVFVVPGKTRFGNNL

DLLNLTPANIFSYIRESTAKANGQNYIWNFLFAPEYNIVRAEDAEEIFQSTKITTKNMSY

ELIRPFLGDGLLISIDQKWHTRRKTLTPAFHFNILQSFLSIFKEESKKFIKILDKNVGFE

LELNQIIPQFTLNNICETALGVKLDDMSEGNEYRKAIHDFEIVFNQRMCNPLMFFNWYFF

LFGDYKKYSRILRTIHGFSSGIIQRKRQQFKQKQLGQVDEFGKKQRYAMLDTLLAAEAEG

KIDHQGICDEVNTFMFGGYDTTSTSLIFTLLLLALHADVQERCYEELQDLPEDIDEVSMF

QFNELIHLECVIKESLRLFPSAPIIGRTCIEESVMNGLVLPKNAQISIHIYDIMRDARHF

PKPNQFLPERFLPENSVNRHPFAFVPFSAGPRNCIGQKFGVLEIKVLLAAVIRNFKLLPA

TQLEDLTFENGIVLRTQQNIKVKFEARVK

>EDW88945.1

MWIALLGSTLLIGVLWLLLRQLNKTYFIFSLCKRVRTADGSPLESKVFLIPGKTRFGNNF

DLLNLTPATIFNFIRESTAKANGQNYLWYFLFAPEYNIVRAEEAEEIFQNTKITTKNMAY

DLIRPFLGDGLLISTDQKWHSRRKTLTPAFHFNILQSFLFIFKEESKKFIQILDKNVGCE

LELNQIIPQFTLNNICETALGVKLDDMSEGNEYRKAIHDFEIVFNQRMCNPLMFYNWYFF

LFGDYRKYSKILRTIHGFSSGIIQRKRQQFKQKQLGEVDEFGKKQRYAMLDTLLAAEAEG

KIDHQGICDEVNTFMFGGYDTTSTSLIFTLLLLALHEDVQQRCYEEIQNLPEDVDEISMF

QFNELVHLECVIKESLRLFPSAPIIGRKCVEESVMNGLILPKNAQISIHLYDIMRDPRHF

PNPNQFLPERFLPENAVNRHPFAFVPFSAGPRNCIGQKFGILEMKVLLATVIRNFKLLPA

TRLEDLTLESGIVLRTAQSIKVKFEARV

>EDW17009.1

MLIALLVNAVLLAAVWALLRYINRRYYILSLAKRIKAVDGSLLSGKVVVLPGKTRFGNNF

DVLKMTPAMLFRFVRDAYTKANGQSYIWYFLFAPMYNVVCAEDAEEIFQSTKLITKNVVY

ELLRPFMGDGLLISTDRKWHIRRKMLTPAFHFNILQEFLDIFKEECNKLIDVLYRNVDNE

VDLSKLIPQFTLNNVCETALGVKLDDMIEGAQYREAIHALETVMVERTCNPLMYYLPIFY

LCGSYKKHTTNLKLAHNFASRIIKRKRSQSKQQTEFKSDIGKKQRYAMLDTLLEAENEGL

IDHQGICDEVNTFMFEGYDTTSTCLIFTLLMLALHEDVQNRCVEELQHISRDHNELQVCN

FNELDYLECVIKESLRLFPPVPFLGRVCTEDTVINGLIMPKGTQINIHVFDVMRDPRHFS

NPCQFQPERFLKENSLSLHPFSFVPFSAGQRNCIGQKFAILEIKVLLVSILRNFKLIPVT

QLEDISLEYGIVLRSQQNVRIKLKKREV

>EDV31771.1

MIMKILISLPLVLIILYKLWSYLNKKYFILCLCKRVRALDGSPLESRIYIPPSKTPFGNN

FDLLDFTPASVFKFMRDAALQAKGRSYLWYFFGTPMYNIVRAEDAEEVFQSSKLITKNVI

YDLLKPLLGEGLLTSTDQKWHSRRKTLTPAFHLSVLQSFLNIFKEECKKLVKILHQNPGE

ELVLNQLIPQFTLNNMCETVLGIKLDDIEEGFQYRQSIHALEEVMLQRIANPLMYNIVYF

YLFGDYRKHVDNLKTALDFSSRIIEERRKLFQHKDDVGKRQRYAMLDTLLAAEAEGQIDH

QGICDEVNTFMFAAYDTTSTCLIFSLLMLALREDVQERCFEEVLHLPQDHDEASMLQFNE

LFYLECVIKESLRMLPSIPVIGRKVTEECVVNGLIMPRNTQINLHIYDIMRDPRHFPDPE

SFQPDRFLTENTTNRHRFGFVPFSAGKRSCIGQKFAILEMKVLLAAILRSFRILPVTTLQ

SLTFETGIGLRTQQDVKVKLQLRE

>EDX03804.1

MWIALLGTPILLAVLTLLLKQINKTYFILSLIKRVRTEDGSPLENKVAIVPGKTPFGNNL

DILNFTPASVFNFARESTAKAEGKNYLLYFLYAPMYNVVRPEEAEEIFQSTKLRTKNVVY

ELIRPFLGDGLLISTETALGVKLDDMSEGNEYRKAIHAIEEILIQRVCNPLMHYNWYFFL

YGDYRKHSQNLRIIHDFSSRIIERKRQQFQQKQLGEVDEFGRKQRYAMLDTLLAAEADAS

VFNFVRESTAKAKGKNYLWYFLYAPMYNVVRPEEAEEVFQSTKLITKNVVYELIRPFLGD

GLLISTDHKWHSRRKALTPAFHFNVLQSFLGIFKEECKKFLNVLEKNLDAELELNQVIPP

FTLNNICETALGVKLDDMSEGSEYRKAIHAIEEILIQRVCNPLMYYNWYFFLYGDYRKHS

QNLRIVHDFSSRIIERKRQQFQQKQLGEVDEFGRKQRYAMLDTLLAAEAEGQIDHQGICD

EVNTFMFEGYDTTSTCLIFTLLMLALHEDVQKKCYEEVEGLPEDSDDISMFQFNKLVYLE

CVIKESLRMFPSVPFIGRQCVEETVVNGMVMPKDTQISIHIYDIMRDPRHFPKPDLFQPD

RFLPENTVNRHPFAFVPFSAGQRNCIGQKFAILEMKVLLAAVIRNFKLLPATQLEDLTFE

NGIVLRTQENIKVKLSKRVK

>ADC44464.2

MHPTRWELSHMVDLQAAANSLVIMALFTFLLGFSIILLGLTLYLKRLSKEYYLLAFCKLL

KTVDGSKVTDKVYVVPGRTIFGNNFDILNSNPEDIYYWSRDLYQRANGKPYALYYYQGVI

YSITSPEAVQEVFQSQTLNTKGLLYDLVRPFLGEGLLVSSDQQWHDRRKLLTPAFHFNIL

QSFIEVFKNETLKLVDLLSAKQDQNLNLNEIIPEFTLNSVCETALGICLDDITGSSEYRK

VIHAIELVMVDRTCNPFMYFPWIFKLFGDHKGFYKNLKVAHDISSKIITKKHEEFQKRQF

ANETVQGNDDETGKRKRYAMWDTLFHAESQGAIDHGGICDEVNTFMFEGYDTTATCLIFT

LFNLAVYPDIQEKCYREVKNCDLLSMSISDFNRLAYLECVVKETLRLQPSVPFISRYCRE

DTELNGVILSAGSQINIHILDIMHDERHFPDPLKYDPDRFLPENSLNRHPFAFVPFSAGS

RNCIGQRFAMLEIKAMLVGILQNFRLLPVTTPEDIRYEYGLVLRTKENILVKLVKRAN

>EDV57844.1

MIFEVLFSAPLVIFLFYKLWAHLNKTYFILSLCKRIRPEDGSLLESKIYVTPTKTRFGNN

FDLVNFTSESIFNFVRDASVQAKGRNYLWYFFRAPMYNVVRAEEAEEIFQSPKLITKNMI

YDLLKPFLGEGLLTSTAQSPFREECNKLVKVLHQGVDKELELNQVIPQFTLNNVCETALG

VKLDDMTEGIRYRKSIHAIEEVMQQRLCNPFFYNIVYFYLFGDYRKQVNNLKIAHEFSSN

IIEKRRSLFKSKDLGQKDEFGKRQRYAMLDTLLAAEAEGQIDHQGICDEVNTFMFEGYDT

TSTCLIFTLLMLALHEDVQHRCYEEIKYLPEDSDDISVFQFNELVYMECVIKESLRLFPS

VPTIGRRCVEEGVVNGLIMPKDTQINIHIYEIMRDPRHFANPKMFQPDRFLSENTVNRHP

FAFVPFSAGQRNCIGQKFAILEIKVLLAAVIRNFKILPVTLLDDLTFENGIVLRTKQNVK

VKLVHRENK

>EDW88946.1

MILEALFSVPLLIFIFCKLWTHLNKTYFILSLCKRIRPEDGSLLESKIYVTPTKTRFGNN

FDLLNFTSESIFNFMRDASAEAKGRNYLWYFFRAPMYNVVRAEEAEEIFQSPKLITKNMI

YDLLKPFLGEGLLTSTARSLFREECSKLVKVLHRSVDKDLELNQVIPQFTLNNVCGKSNN

IIAETALGVKLDDMTEGIRYRKSIHAIEEVMQQRLCNPFFYNVVYFYLFGDYRKQVNNLK

IAHEFSSNIIEKRRSLFKSKQLGQEDEFGKKQRYAMLDTLLAAEAEGQIDHQGICDEVNT

FMFEGYDTTSTCLIFTMLMLALHEDVQRRCYEEIKYLPEDSDDISVFQFNELIYMECVIK

ESLRLFPSVPFIGRRCVEEGVVNGLIMPKDTQINIHIYEIMRDPRHFANPKMFQPDRFLP

ENTVNRHPFAFVPFSAGQRNCIGQKFAILEIKVLLTAVIRNFRILPVTLLEDLTFENGIV

LRTRQNVKVKLVFRDNK

>EDY70182.1

MLIALLGTPLLLLGVWLLLQRLNRTYFILSLTRRVRTEDGSPLESKVAVMPGTTRFGNNL

DVLNCTPSSLFNFLRDSAAKAQGRNFLWYFLFAPMYNVIRAEEAEEIFQSTKLITKNVIY

ELLRPFLGEGLLISTDQKWHSRRKALTPAFHFNVLQSFLAIFKEECNKLIKVLDKKVDGE

LDLNQVIPQFTLNNICETALGVKLDDMSEGNQYRRAIHAIEEVLIQRVCNPLMFYNWYFF

LIGDYRKQVQNLRIVHDFSSRIIERKRQQFKRKQLGVVDDFGKKQRYAMLDTLLAAEAEG

QIDHQGICDEVNTFMFEGYDTTSTCLIFTLLMLALHEEVQERCLEEVSTLPEDTDSISVF

QFNELVFLECVIKESLRMFPSVPFIGRTCVEECVVNGLILPKDTQISIHIYDIMRDPRHF

PNPHEFQPERFLPENTVDRHPFAFVPLRSKYSWLLF

>EAA11717.4

MLLAVLVCVLGSCIAILLVRDYRSKQTDGYRAALRYPGGTIVPVFGSLFELLFKNPVQTF

AYARANATRYGASYRQWIDGSVILNVTRVQEAEKILSSTQHTRKSILYRFLHPLMGDGLL

CSKGAKWQQRRRILTPAFHFNILPKFLTIFQEESEQLVRRLDRLADGVQDVVLQPIVTSF

ALHTICETAMGVKLDAYREADEYKQKVYEVGEMLVHRTMSPWLYSDRVYRLLGYDGPLAK

SLKPIHHFTRSIIRQRKETFQAAQLTADSTTEENMYFGSKQRYAMLDTLLAAEAKQQIDE

EGIREEVDTFMFEGHDTTAAAIMFTLILLAIEQDVQERCYGELQEVLDRSSSDPRSVQDY

QNLPYLDRVIKESLRLYPPVAFISRTTSGELVVDGTTFPHNTMSHIHIYDLHRDPVQFPD

PERFDPDRFLPEVAEKRNPYAYVPFSAGPRNCIGQKFAQLEMKTVLVAVLERFRLKPVTR

REEIVFMADLVLRAKTPLKVRLERR

>EAT32932.1

MFSSVVSLVIITLIVLLAVYEWYLRQRDGYRAALQYPGGPMLPVLGNILEVLIKDTVQTF

NYARSNALKYGRSYRQWIFGNVILNVIRIREAEPILSSTKHTRKSILYRFLEPLMGDGLL

CSKGSKWQARRKILTPAFHFSILNDFLQVFQEEAEKLVGLLDSCADAEEEVVLQSIVTRF

TLNTICETAMGVKLDTYNGADKYRSQVYVVGERIVHRTMTPWLYDDGVYNLFGYQKPLED

AIEPIHDFTRSIIRQKREQLKQDSTMHIVDSDGIYGSKQRYAMLNTLLMAEENDAIDEEG

IREEVDTFMFEGHDTTAAGLIFSILLLATEQEAQQRVYDELLKARSTKSESEAFTIADYN

NLKYLDRFVKEALRLYPPVSFISRNLSGPLEVDSTTFPHGTIAHIHIYDLHRDPEQFPDP

ERFDPDRFLPEVAAKRNPYAYVPFSAGPRNCIGQKYALLEMKTVLCALLINYRILPVTTR

QEVIFIADLVLRAKTPIKVQFAKRKANATRS

>EAT34185.1

MFSSVLSLVIITLIVLLAVYEWYLRQRDGYRAALQYPGGPMLPVLGNILEVLIKDTVQTF

NYARSNALKYGRSYRQWIFGNVILNVIRIREAEPILSSTKHTRKSILYRFLEPLMGDGLL

CSKGSKWQARRKILTPAFHFSILNDFLQVFQEEAEKLVGLLDSCADAEEEVVLQSIVTRF

TLNTICETAMGVKLDTFIGADKYRSQVYDVGERIVHRTMTPWLYDDGVYNLFGYQKPLED

AIEPIHDFTRSIIRQKREELKQDSTMHIEDSDGIYESKQRYAMLNTLLMAEENDVIDEEG

IREEVDTFMFEGHDTTAAGLIFSILLLATEQEAQQRVYDELLKARSTKSESEAFTIADYN

NLKYLDRFVKEALRLYPPVSFISRNLSGPLEVDSTTFPHGTIAHIHIYDLHRDPEQFPDP

ERFDPDRFLPEVAAKRNPYAYVPFSAGPRNCIGQKYALLEMKTVLCALLINYRILPVTTR

QEVIFIADLVLRAKTPIKVQFAKRKANATRS

>EDS31720.1

MLSPWLAAIFAALAALFLYDWRARQSESYRAALQYPGNRMLPVVGNLLEVLVKDPAQAFA

YARSSAAKFGRSYRQWIFNDVIVNVTRVREAEPILSSVKHTRKSVIYRFLAPLMGDGLLC

SRGDKWQGRRKILTPAFHFNILSKFLLVFQEEADKLVEGLEESAASGDDVVLQSIVTRFT

LNTICETAMGVKLDTYKSADIYRSKVYEVGEMLVHRTMTPWLYDDGVYNFFGYLKPLEDA

IVPIHEFTRDIIQQKRKQFLQDSTFVDKLDDHGGSKQRYAMLNTLLMAEADNAIDEEGIQ

EEVDTFLFEGHDTTAAGIIFTVLLLANEQDAQRRVYEELSKARRLKPEHEAFTIADYTNL

KYLDRFVKEALRLYPPVSFISRSLTGRLDVDSTTTLPHGTIANIHIFDLHRDPEQFPDPE

RFDPDRFLPEVSAKRNPYAYVPFSAGPRNCIGQKFALLELKVVVCALLSSFRVLPVTTRD

EVVFVADLVLRAKIPIKVRFARR

>ABF18193.1

MYVFTLXAGLLVFILILYEIYLRSLPSYRAAKFFPGYPVYPIVXNLFTALFKTQTGSFQL

ARQWARTFNNRTYRLLIQGVLFVQVIHHKDVEMLLSSSRLITKSPLYKLTVPFIGEGLLN

STGEKWHQRRKILTPAFHFNILQGFLQIFHDECRKLVNQLDKDAAQGIATTLQPLSTQVT

LNTICETAMGLKLDTSESAXLYKSNIREVGKIIQQRIMNPLLFEDWIYXITGYQAEFDKI

LRPIHAFTNSIIRQRRETFHETMKNVDSPSDENIYTNIKQRYAMLDSLLLAEAKQQIDGE

GIREEVDTFTFEGHDTTGSAFVFTFLLIAHEQLVQQRLFEEIERMFNLQPNPTQQDYNDL

KYMDRVIQESLRIYPPVPFISRLITEDVQYDGKLVPRGTIMNIEIYDLHRDPEQFPDPER

FDPDRFLPEEVQRRSPYAYVPFSAGPRNCIGQRFAMLELKAILIGVLREFRVLPVTKRED

VVFVGDMVLRSRDPIVVKFERR

>EFR27681.1

MLLAAVLVLLVLGCFIALAIVRDFQWRRTDAYRAANLYPGAPILPLIGNLCELLFKNAVQ

TFAYARENANRYGGSYRQCFNGNIILNITRVREAEKILSSTQHIRKSILYGFLHSLMGDG

LLCSKGTKWQHRRRILTPAFHFNILPKFLAIFQEESVKLVHQLDSLADGVQEIVLQPIVT

SFALHTICETAMGVKLDAYEEADEYKKRVYEVGEMLVHRTMSPWLYSDWVYRILGYEGPL

SRALKPIHHFTRSIIRQRRTEFHAQRQLVQDSEENMYFGRKDRYAMLDTLLTAEAKQQID

EEGIREEVDTFMFEGHDTTAAAIMFTLLVLANEPDVQDRCYAELRNCDSTLAPLTVHDYQ

QLPYLDRVLKESLRLYPPVAFISRTTTGDLTIGKRNHNENLSCMIQSMNIPRIDEATFGH

NTIAHIHIYDLHRDPVQFPDPERFDPDRFLPEHVAKRNPYAYVPFSAGPRNCIGQKFALL

EIKTVLVTLLNHFQLLPVTRREDVVFAADLVLRAKTPLIVKLVRRAA

>EFR25561.1

MLLAAVLVLLVFGCFIALAIVRDFQWRRTDAYRAANLYPGAPILPLIGNLCELLFKNAVQ

TFAYARENANRYGGSYRQCFNGNIILNVTRVREAEKILSSTQHIRKSILYGFLHSLMGDG

LLCSKGSKWQHRRRILTPAFHFNILPKFLAIFQEESVKLVHQLDSLADGVQEIVLQPIVT

SFALHTICETAMGVKLDAYEEADEYKKRVYEVGEMLVHRTMSPWLYSDWVYRILGYEGPL

SRALKPIHHFTRSIIRQRRTEFHAQRQLVQDSEENMYFGRKDRYAMLDTLLTAEAKQQID

EEGIREEVDTFMFEGHDTTAAAIMFTLLVLANEPDVQDRCYAELRNCDSTLAPLTVHDYQ

QLPYLDRVLKESLRLYPPVAFISRTTTGDLTIGKRNHNENFSCMIQSMNILLIDEATFGH

NTIAHIHIYDLHRDPVQFPDPERFDPDRFLPEHVAKRNPYAYVPFSAGPRNCIGQKFALL

EIKTVLVTLLNHFQLLPVTRREDVVFAADLVLRAKTPLIVKLVRRAA

>EJY58076.1

MYVFTTVAGLLVFIFILYEIYLRSLPSYRAAKYFPGYPVYPIVQNLFTALFKSQTGSFQQ

ARQWARIFNHRTYRLLIQGVLFVQIIHHKDVEMLLSSSRLITKSPLYKLIVPFIGKGLLN

STGEKWHQRRKILTPTFHFNILQGFLQIFHEECRKLVYQLDKDAAQGITTTLQPLSTQVT

LNTICETAMGLKLDTSETAEVYKSNIREVGKVIQQRIMNPLLFEDWIYKITGYQAKFDKI

LRPIHAFINSIIRQRRETFHETMKNVDTPSEENIYTNIKQRYAMLDSLLLAESKQQIDAE

GIREEVDTFTFEGHDTTGSAFVFTFLLIAHEQLVQQRLFEEIERMFNLQPNPALQDYNDL

KYMDRVIKESLRIYPPVPFISRLITEDVQYDGKFVPRGTIMNVEIYDLHRDPEQFPDPER

FDPDRFLPEDVQRRSPYAYVPFSAGPRNCIGQRFAMLELKAILTAVLREFRVLPVTKRED

VVFVADMVLRSRDPIVVKFERR

>EAT33703.2

MDWLTIVLLLILALLALYEVHLRLLLSNRAAKQFPGPRRLPVLGNALALLFNDQVSTFKL

PRRWAQRYKESYRLVIRGGFVINAIRARETEALLSSTKLIDKSILYTFLYPFMGKGLLTS

TGPKWFHRRKILTAAFHFNILPKFLVTFQEECDKLLRKLDADVKAGNTTTLQSVAARFTL

NTICETAMGVKLDSMSMADEYRAKIQEVIKLLLLRVMNPWLVEEFPYRLLGFRRRLMKVL

KPIHAFTRSIIKQRRDLFHANVKNVDDFSEENIYVNTNQRYALLDTLLASEAKNQIDEEG

IREEVDTFMFEGHDTTASAFTFIFLVIANHQEAQRQLVEEIETMIAGRSNPTEPLSMHDY

GELKFMDRVIKECLRLYPPVPFISRAVLEDAQLGDRFIPKDSMANVHIFDLHRDPEQFPD

PERFDPDRFLPENVEKRNPYAYVPFSAGPRNCIGQRFAMLELKAILTAVLREFRVLPVTK

REDVVFVADMVLRSRDPIVVKFERR

>AAL49205.1

MSTLALVAFVLWAAFLRYLPKILNFLRLQRFAKTLPGPTIGELIANVKKGEILNWLKELR

EKHGPVFRIWFGKDLMVMFTDPEDIKQLLGNNQLLTKSRNYELLEPWLGKGLLTNGGESW

HRRRKLLTPGFHFRILSEFKEPMEENCRILVRRLRTKANGESFDIYPYITLFALDAICET

AMGIKKHAQLQSDSEYVQAVQSICRVMHKQSFSFWQRLNVFFKHTKPGKEREAALKVLHD

ETNRVIRLRREQLIQERNEWKPEAEQDDVGAKRRLAFLDMLLLTQMEGGAELSDTDIREE

VDTFMFEGHDTTSSAIAFALSLLSKNPDVQQRAFEEASELEGREKESMPYLEAVIKETLR

IYPSVPFFSRKVLEDLEVGKLTVPKGASISCLIYMLHHDPKNFPDPERFDPDRFLVNEKQ

MHPFAFAAFSAGPRNCIGQKFAMLELKTSLAMLLRSYRFLPDKDHQPKPLAELVTKSGNG

IRLRILPRDENGTTA

>EDV47047.1

MSTLALVAFVLWAAFLFYLPKILNFLRLQRFAKTLPGPTIGELIANVKKGEILNWLKELR

EKHGPVFRIWFGKDLTVMFTDPEDIKQLLGNNQLLTKSRNYELLEPWLGKGLLTNGGESW

HRRRKLLTPGFHFRILSEFKEPMEENCRILVRRLTTRANGESFDIYPYITLFALDAICET

AMGIKKHAQLQSDSEYVQAVQSICRVMHKQSFSFWQRLNVFFKHTKPGKEREAALKVLHD

ETNRVIRLRREQLIQERSESRPEAEQDDVGAKRRLAFLDMLLLTQMEGTAELSDTDIREE

VDTFMFEGHDTTSSAIAFALSLLSKNPAVQQRAFEEASELEGREKESMPYLEAVIKETLR

IYPSVPFFSRKVLQDLEVGKLTVPKGASISCLIYMLHRDPKNFPDPERFDPERFLLNEKQ

MHPFAFAAFSAGPRNCIGQKFAMLELKTSLSMLLRNYRFLADEDHQPKPLAELVTKSGNG

IKLRILPRDQNGSTA

>EDX01739.1

MSTLALVAFVLWAAFLRYLPKILNFLRLQRFAKTLPGPTIGELIENVKKGEILNWLKELR

EKHGPVFRIWFGKDLMVMFTDPEDIKQLLGNNQLLTKSRNYELLEPWLGKGLLTNGGESW

HRRRKLLTPGFHFRILGEFKEPMEENCRILVRRLSTRANGESFDIYPYITLFALDAICET

AMGIKKHAQLQSDSEYVQAVQSICRVMHKQSFSFWQRLNVFFKHTKPGKERAAALKVLHE

ETNRVIRLRREQLIQERNESRPEADQDDVGAKRRLAFLDMLLLTQMEGGAELSDTDIREE

VDTFMFEGHDTTSSAIAFALSLLSKNPAVQQRAFEEATELEGREKESMPYLEAVIKETLR

IYPSVPFFSRKVLEDLEVGKLTVPKGASISCLIYMLHRDPKNFPDPERFDPDRFLLNEKQ

MHPFAFAAFSAGPRNCIGQKFAMLELKTSLSMLLRSYRLLPDEDHQPQPLAELVTKSGNG

IRLRLLPRDQNATIRP

>EDV34270.1

MNTLALLAFAFWVLFLRYLPRILNFLRMHRFANTLPGPSIGELIATVKKGEILNWLKEMR

EKHGSVFRIWFGKDLTVLFSDPEDIKQLLSNNTLLTKSRNYELLEPWLGKGLLTNGGESW

HRRRKLLTPGFHFRILGEFKEPMEENCRILVRRLQEKANGESFDIYPYITLFALDAICET

AMGIKKHAQLQSDSEYVKAVQTICRVLHKQSFSFWQRLNVFFKYTQAGRERDNCLKVLHD

ETTRVIRLRREQLLQERAESRPEAEQDDVGGKRRLAFLDMLLLTQMDGGAELSDTDIREE

VDTFMFEGHDTTSSAIAFTLSLLSKNAAVQQRAYEEACEMEGREKEPMPYLEAVIKESLR

IYPAVPFFSRKLLEDLQMGKMTLPKGTAISCLLYMVHRDPKNFPEPEKFDPDRFLLNEKE

IHPFAFAAFSAGPRNCIGQKFAMLELKTSLSMLLRSYRFLPDEEHKPRPLAEIVMKSGNG

IHLRILPRDEKESPSSA

>EAL32368.1

MNTWALMAFALWALFLRYLPQILTFLRLQRFAKTLPGPSVSELIANVKKGEILNWLKELR

ERHGPVFRIWFGKDLMVMFSDPEDIRQLLGNNSLLYKSRNYALLEPWLGKGLLTNGGESW

HRRRKLLTPGFHFRILSEFKEPMEENCRILVSQLKERANGEAFDIYPYITLFALDAICET

AMGIKKHAQLQSESEYVKAVQTICRVMHKQSFSFWQRLNIFFKYTQAGQERDGALRVLHD

ETNRVIRLRREQLVKERQLQQRTEAAQDDVGSKRRLAFLDMLLLTQLEGGDELSDRDIRE

EVDTFMFEGHDTTSSAIAFALSLLSKHSEVQQRAYEEARDLEGREKESMPYLEAIIKETL

RLYPSVPFFSRKVFEDIQVGKHTIPKGASVNCLIYMLHRDCNNFPDPERFDPDRFYLNEK

QMHPFAFAAFSAGPRNCIGQKFAMLELKCSLSSLLRSFQFMPDEDHQPIPLAELVMKSGN

GVRLRIQPRSQH

>EDW78415.1

MNTLALLAFVLWALFLRFIPQILTFLRLQRFAKTLPGPSVSELIANVKKGEILNWLKELR

SRHGSTFRIWFGKDLMVMFTDPEDIKQLLGNNSLVYKSRNYELLEAWLGKGLLTNGGESW

HRRRKLLTPAFHFRILSEFKEPMEQNCRILVSRLKERANGEVFDIYPYITLFALDAICET

AMGIKKHAQMQSDSEYVKAVQTICRVLHKQSFSFWQRLNVFFKYTQMGRDRDNALKILHD

ETNRVIRQRRQQLQKEQQESRPEAEQDDIGVKRRLAFLDMLLLAQMEGGTEELSDRDIRE

EVDTFMFEGHDTTSSAIAFALSLLSKHPEVQQRVYEEAVELEGREKDSMPYLEAVIKETL

RLYPSVPFFSRKVNEDLNVGKLLVPKGASISCLIYMLHRDPNSFPEPERFDPDRFYLNET

KMHPFAFAAFSAGPRNCIGQKFAMLELKLSLSMLLRHYQLMPADNHQPNPLAELVMKSGN

GIQLRMKPRLPLS

>EDW66164.1

MNTLALFAFALWALFLRYLPHILKFVQLQRFSRTLPGPSVGELIANVKKGEVLNWLKELR

ARHGPVFRIWFGKDLMVFFTTPEDIKQLLGNNTLLSKSRNYQLLEPWLGKGLLTNGGESW

HRRRKLLTPGFHFRILSEFKEPMEENCRILVSRLREHADGEPFDIYPYITLFALDAICET

AMGIKKHAQMQSDSEYVRAVQTICRILHRQSFSFWQRFNLLFNLSAAGRQRNAALRVLHE

ETHRVINMRRKLLQQQQLDKQNGKEEKEEEHNDIGGKRRLAFLDMLLLSQMEGGGDLSDV

DIREEVDTFMFEGHDTTSSAIAFAISLLSKHADVQQRVYEEALELEGREKESMPYLEAVI

KETLRLYPSVPFFSRNVHEDLQVGQVTVPKGASISCLVYMLHRDPDSFPEPERFDPDRFY

LNEHQLHPFAFAAFSAGPRNCIGQKFAMLELKCSLAMLLRHYQFLPVDEHQPQPLAELVM

KSGNGIRVRIRPRPSNPL

>EDW06367.1

MNTWVLFAFAVWALFMRFLPQIFNFMKLQRFSRTLPGPTVGELIANVKKGQILNWLKELR

LKHGPVFRIWLGKDLVVFFSAPDDIKQLLSNNTLLTKSRNYQLLEPWLGKGLLTNSGESW

HRRRKLLTPGFHFRILGEFKEPMEENCRILVNRLRAHANGEPFNIYPYITLFALDAICET

AMGIKKNAQMQSDSEYVKAVQTICRILHHQSFSFWQRFNILFNLSAAGQERNAALRVLHG

ETNRVIQQRRKQLQLAKVEQGKAQQQEDNDVGGRRRLAFLDMLLLAQMEGNGSELSDVDI

REEVDTFMFEGHDTTSSALAFAISLLSKHADVQQRVYEEAVELEGREKESMPYLEAVIKE

TLRLYPSVPFFSRGVLEDLQVGDVTVPKGASVSCLVYMLHRDPESFPDPERFDPDRFYLN

ENKLHPFAFAGFSAGPRNCIGQKFAMLELKCTLAMLLRHYRFLPVADHQPMPLAELVMKS

GNGIQVRIQPRPQSN

>EDV91948.1

MNTVALLAFAVWAIFLRYLPQILRFVHLIRFSKTLPGPTVSELIANVKRGEVLKWLKELR

ARHGPVFRIWFGKDLMVFFTTPEDMKQLLSSNALLYKSNSYQQLECWLGKGLLTNGGETW

HRRRKLLTPGFHFRILSEFKEPMEENCRILVSKLREKANGEPFNIYPYITLFALDSILET

AMGIKKHAQMQSDSEYVQAVQAICRILHQQSFSFWQRFPIIFKFSSAGRARNAALRVLHA

ETHRVIQMRRKQLQQESQQKVEANNDNDVGAKRRLAFLDMLLLSQMEGGGVELTDLDIRE

EVDTFMFEGHDTTSSAIAFALYLLSKHADVQQRAYEEAVEREGHEKESMPYLEAVIKETL

RLYPAVPFYSRQVRDDLQVGNVTVPKGASVSCLVYMLHRDPDSFPNPERFDPDRFYLNEQ

NLHPFAFAAFSAGPRNCIGQKFAMLELKCSLSMILRHYQFLPVAGYEPQPLAELIMKSGN

GIQVRMQPRP

>348526159

MKLTKAFAEFQLGWPHMHYVFAVLCLFVVVYKLTILLAKRRETLRNFQAFWGPPPHWLFG

HVLECAFSCYSNCQTEGGTNAYIKAVYDLSFLINLRLRMFPYHSDLIFHLSPHGFRYRKA

RQVALSHTEEVIRKRREALREEKELGQIQAKRYLDFLDILLFARDENQQGLSDEDMRAEV

DTFMFEGHDTTASGLSFILYCLACNPEHQKICRKEIMEALHDKETMEWEDLSKIPYTTMC

IKEALRLYPPVPGIARKTTKTVTFCDGRTVPAGSVVGISVYGIHRNASVWENPNVFDPLR

FLPENIAKRSPHAFVPFSAGPRNCIGQNFAMNEMKVVTALTLKRYQLILIAEPTMKPKII

PRLVLRSLNGIHIKIKPLDAES

>EEN61809.1

MDLKKILADQGYLLEFLLEQTQRFPKCFQQWVGPFRGILVVVHPELAKDVLKTIEPKSRV

YEYLRPWLGDGLLLSKGEKWRRNRRLLTPAFHFEILRPYITVYNQATDVFMEKMSSFAMK

NEAVEITEHLSLLTLDIILQCAFSHNIDCQRIGAKHPYVAAVHAMSQLVILRAMNPWMHV

WPMYRLTPQGRKFVEMYNLVHQEANSIIKARREVLNSEIREKMGRESRYLDFLDILLTAR

DPDGEGLTDEEIRAEVDTFLFEGHDTTATGISWSLYCLAKHPEHQDRVREEVDAVMADKD

ELVWEDICKLKYLAMCLKEAMRLYPPVPIVSRRITRDFDFQGHRLPAGTNVDLNVWCMHR

NPAVWGKDVMDYKPFRFSPENMTKMDPYAFIPFSAGPRNCIGQNFALNEEKVVISRILHR

FKVELVPDHPVVPSLQIVSRAANGIKVKFIPRS

>EKC42662.1

MPVLGLSSLSSWGSLVSVALLIVFSYVVIKLTLAYLDYLRWTPFYTGLPMDPYASKLLGH

LLMYPGPNEEGLRFQMERCDKFPKFHLLWISFRPLLIVHHPETVKVLLKSTEPKPRQLGT

VYILGLDWLGEGLLIANGQKWLRNRRLLTPAFHFDILQSYIALKNKAASVLVDKIDQQVK

AGNSFELFSFIGLASLDIILQCAFSYESNCQQLGETHPYVHAVNVLSDIWVARSLKPWFY

PDFLFYLSPSGREFKKNCDYVHKVAEDIIAKRKKSLEEEGSKPKSRHLDFLDILLTAKDE

NSQGLTSLEIRNEVDTFLFEGHDTTTSGMCWTLYLLAKHPEHQRECQREIDELMEGRENR

DLQWSDLPKLSYLTQCIKESMRLYPPVTFIQRVTTKDIVLDDHQIPAGTTIGVQIYNLHH

NKAVWEDPYEFKPERFSPDKERKYDNFAFVPFSAGPRNCIGQHFAMNEMKIILVHVLQRF

NLSLDPTGEVNIKIGVVLRTKNGIKVIAERR

>EKC34227.1

MEAVRGDWLHLLLLLTLACLVIVNVRLVVGLYHRRILFRGFPEPIEERHWLFGHLLVFAG

KTSRLDKVLEWTAKFPKMFIIRPGPFRVRVILHHPECVKQVLVTSDPKPVGFGEPYRHGL

PWLGEGLLMSSGQKWKRSRRLLTPAFHFDILKPYVKIYKTCADILSENLNEHADTGKSVE

IYNLVSSCTLDIILRCAFSYTTDCQSKRETTHPYVSAVQDIAMTWLLRNKSPWLYPDFIF

YRTKQGQKFKADCDYVHKIAEDIIDKRRRELESEDLSHKRYLDFLDILLTAKDESGNGLT

KEDIRNEVDTFLFEGHDTTASAISWILYSLAENHECQRKCQEEIDKVISETKSGRLEWKD

LGKLDYLTECIKEGMRVHSPVPGVSRVNTQPIKVDDVTIPPGTIILINFYALHHNPTVWG

QDHMEFKPERFNRDNSEKRDSFAFCPFSAGPRNCIGQNFAMSEEKVVLASLLRRFSFSLD

EDHKVDKVIAAVMRPVDGIKLYIRSRDN

>EDS33572.1

MWLVLSLIIATFGYLLFNYHKTRQKLLAIQSHFNGPPTHYFLGNFPIFIGKSIPDIFNII

TKMHKVYGEDIAIIAAFNELVIDLSSSRNVEKVLLAKSIKKSFVYNFLEPWLGTGLLTVP

TGEKWFQRRKIITPTFHFKMLDNFLEVFNREADVLVEKLKVRAGKGEFDIYDYVTLYALD

SICETSMGVQIHAQDDPGNEYAIAVKQMSTFILRRIFNPLRSFPRLFFLFPFAKEQKNVI

RKLHNFTNSVIDSRRKVLEREQASSTVAFDLQEENMYSKRKITFLDLLLSVTVEGKPLSR

EDIREEVDTFMFEGHDTTTSGISFTIWHLAKHQDVQQKLCDEIDQILGTEKKTAELTNVK

IQEFEYLDMVVKESLRLIPPVPIIGRQLIEDLEMNGTTIPAGTQINIKIYNIHRNPKIWP

DPERFDPDRFSKTNEDKRGPYDYIPFSAGSRNCIGQRYAMMELKVTLIKLLASYRILPGE

SMEKMRFKTDLVIRPNETIPIKLVERG

>224049819

MEVLEGLSGRPQLLPWGAGIIALLLTIVTVCSLPSLMEYWRQWRMMKSIPGVSPCYPVVG

NALLFKRKGEEFFKQIQFYSEEFRSWPLFKLWIGPVPVMFLYHPDSVESWKGKVRIGLDD

ADIQESEQETAMGRNVDAQKNKDSDYVSAIYRMSDLIQRRQISPWLWPDFLYVLFKEGRE

HKRNLNILHNFTDMVIAEKAEELKNIQQKKQDNDGNSEETGSKKRKAFLDMLLNATDDEG

KKLSYRDIREEVDTFMFEGHDTTAAAINWVLYLLGRNPEAQKKVHRELDEVFDDAERPVT

VDDLKNLRYLECVVKEALRLYPSVPIFARTLREDCCIKGYQIPRGANVLILTYALHRDPE

VFPDPEEFRPERFFPENSKGRHPYAYVPFSAGPRNCIGQRFAQMEEKALLALILRRFWVE

SCQKPEELGLCGELILRPNKGIWIKLKRRPNAGSE

>72098778

MDVFNEQSFIMAKKMEKFADQSEPFNIFPQITYCVLDIICDTAMGKSINAQGEGDNEYVT

AVISMTNLVQERMKKPWFWPDLLYDNIQSGKKHANNLRVLHDMTTKIIKQRLQEPPRALD

GSEEDAVAGKRRRIAFLDLLLQMHREDPSFTLEDIREEVDTFMFEGHDTTAAAASWTILM

IGRHPEVQTRLHEELDEVFGDSDRPITADDLQKLQYLNCVFKETLRLCPSVPMIGRDLEE

DCVIDGKVVPKGTLVVLGIYALHRDPEQFPDPEKFDPDRFLLENSTKRHPYAYVPFSAGP

RNCIGQKFAMMEDKVILANLMRKFSVQAIQTMEETNPLGELIMRPRDGIYVKLSRRK

>221127769

MVSVFYILFSGLVFYVVSKILWKLWRNSYGLSSIVTPPNVPFFGTSLYLHSDARKFFFQL

YDYTRRYGDVFCIWLGPKPVICSSSVKFSEAVLSSQKVITKGFSYDFLHDWLKTGLLTST

GSKWKTRRRLLTPSFHFSILNNFIKIFEEQASILVDKLAVAADNKEVVDVQVPIGLATLD

IICETSMGVKVNAQSHPDSEYVKAITVLNEEIQMRQKFPWLWFDAIYKLLPCGKRFYKAL

DVAHKLSFDVINERMQMKIQESYCETASDEKKFFLDLLLDIYRKGKIDTEGIQEEVDTFM

FEGHDTTSAALGWTLWLLGKNPDVQKKLHKEIDEIELNGGSLYDKVRQSKYLEIILKESL

RMHPPVPMYGRTVEEDMTIDGQFVPKGAQIVLLVLILHSNPDYWENPNDFIPERFEADSY

EKRNPYSYVPFSAGPRNCIGQKFAMIEEKILLYSIMKNFHLKSMQNENEVFGTLDIIHKS

INGINIKFTRR

>221103870

MIYASYLVLVGLFVFFVSKILWKLWKSSYGLETIATPPNIPVFGTSLYLHSDARKFFFQL

SEFTKKYGTVFCIWLGPKPMIISSSVKFSEAVLSSQKVITKGFSYDFLHDWLKTGLLTST

GSKWKTRRRLLTPSFHFSILNNFIKIFEEQASILVDKLAVAADNKEVVDVQVPIGLATLD

IICETSMGVKVNAQSHPDSEYVKAITVLNEEFVMRIKYPWLWFDVIYKLLPCGKRFYKAL

DVAHKLSFDVINERMQMKIRESYCETASDEKKFFLDLLLDIYRKGEIDTEGIQEEVDTFM

FEGHDTTSAALGWTLWLLGKNPDVQRKLHKEIDEIELNGGSLYDKVRQSKYLENILKESL

RMHPPVPMYGRTVEEDMTIDNQFIPKGAQIILLVLMLHSNPEYWENPNDFMPERFEADSY

EKRNPYSYVPFSAGPRNCIGQKFAMIEEKILLYSIMKNFHLKSMQDENEVFGTVDVIHKS

INGINIMFTRR

>391329482

MMVVYLGSEPNFVLFRADHAEKALNNSINISKGHAYNLLLPWLGEGLLTSSGKKWKTRRR

LLTPAFHFQILDEFSLVINRQAQIFVEQMSKKSRDDDILPYVAAATLDIVCETIMGVNLD

SQTTGAGKIYLKSIQALGELFFKRVQSPAKWIDFIYGLTADGRQNVGYIRQVHEFTLKVI

NDRKRELTENPAELEKLASIDGNLLGSKKPFLDLLLVEHLKNKTLSVSDIREEVDTFMFE

GHDTTSMGITWATYLIGLHPEVQEKIFEEMESVFGGDHTCTVTNEHLRQLKYLDMVLKET

QRIYPPVPMIARRVTTEFELLGKTVPTSSELNINIIAMHRDPKTFPRPELFIPERFSPES

SARRSPYAFIPFSAGPRNCIGQRFALMEEKIVLVWLLRRFRLKSLVLRDEVLMVAEMVLR

PKSAIKVECIPRNFETA

>391327918

MIWMCLPLLRFLAFSLFGFIVAFILLLVVVYLRQLPFRRSALARSCVRDPFPLLRYIGVH

LSLLEISKHCGSYDTHLTNFLLFEASSRPAEGWVMLFIGVVPELLLYRADHVEKVFGNSK

NIRKGPSYRLLDPWLGEGLLTSYGSKWKSRRKFLTPAFHFKVLDTFADAMNRQAKIFADQ

IGRKKPDEDMLPYVEAFTLDVVCETIMGIDMKCQLEGSGRDYLNKVKRISHQLVKRFADP

LSWIDFIYFNFTGDGREFKKIVEEVHDFTTQVINERKRELSATPEILESISNAEEAMTKS

KKPFLDLMLVEHLKTQNLSIEDIREEVDTFMFEGHDTTSMGLTWTLHFIGLHQDVQAKLH

EEIDRVFQGNSTCDVTAEHIKELKYLEMVIKESQRLCPSVPFAARLVTEEFRIDDKPVPI

GTEVIVFIRKLHEDPKVFPKPHEFDPQRFSAENSRNRNPYAFVPFSAGPRNCIGQKFALL

EEKILLVWVLRKFQIKSLDYRDQILVKIDIVLRPQSPIRIAVKNR

>EDL30655.1

MEASWLETRWARPLHLALVFCLALVLMQAMKLYLRRQRLLRDLSPFPGPPAHWLLGHQKF

LQEDNMETLDEIVKKHPCAFPCWVGPFQAFFYIYDPDYAKIFLSRTGRGLLNLDGPRWFQ

HRCLLTPAFHQDILKPCVDTMAHSVKVMLDKWEKMWTTQETTIEVFEHINLMTLDIIMKC

AFGQETNCQINGTYESYVKATFELGEIISSRLYNFWHHHDIIFKLSPKGHCFQELGKVIH

QYTGKNTDISWAEDERAFSDADLRAEVNTFMWAGHDASAASISWLLYCLALNPEHQDRCR

TEIRSILGDGSSITWEQLDEMSYTTMCIKETLRLIPPVPSISRELSKPLTLPDGHSLPAG

MTVVLSIWGLHHNPAVWNDPKVFDPLRFTKENSDQRHPCAFLPFSSGPRNCIGQQFAMLE

LKVAIALILLHFQVAPDLTRPPAFSSHTVLRPKHGIYLHLKKLLEC

>EDL90318.1

MEASWLENRWARPLHLALVFCLALVLMQAVKLYLRRQRLLRDLRPFPGPTAHWLLGHQKF

LQEDNMEKLDEIVKEYPCAFPCWVGPFQAFFYIYDPDYAKIFLSRTGRGLLNLDGPRWFQ

HRCLLTPAFHQDILKPCVDMMAHSVNMMLDKWEKTWTTQETTIEVFEHINLMTLDIIMKC

AFGQETNCQINGTYESYVKATFELGEIISSRLYNFWHHHDIIFKLSPKGHCFQELGKVIH

QCTGKNTGMSWAGDEKAFSDADLRSEVNTFMWAGHDASAASISWLLYCLALNPEHQDRCR

TEIRSILGDGSSITWEQLDEIPYTTMCIKETLRLIPPIPSISRELSKPLTLPDGHSLPAG

MTVVLSIWGLHHNPAVWKDPKVFDPLRFTKENSEQRHPCAFLPFSSGPRNCIGQQFAMLE

LKVAIALTLLRFRVAADLTRPPAFSSHTVLRPKHGIYLHLKKLPEC

>EAA44145.4

MDILTVVLLLVAFLLILYELRVQLSTHYRAGKRFPGPRALPLLGNAHNLLFNDQRRTFQL

PRGWAATYGDTYRILVRGILNLNAFQAKDVEPLLSSPKLIEKSMIYSLLHRFLGIGLLNS

TGSKWQHRRRILTPAFHFNILPSFLLTFQEECRRLVAQIGQYADKGAPVALQPLATKFTL

NTICETAMGIKLDSMTMANEYRSKIEAIGTMLLQRLMNPWLFEDFTYKLSGLQGRFEKLL

QPVHAFTRSIIQQRRELFHQNVRNIGDFSEENIYTNLKQRYAMLDTLLAAEAKEQIDEDG

IREEVDTFTFEGHDTTSAAVIFTLLLLAHSPDVQQRLYEELQEVAQSRTDADDEFTQRDY

TELRYMDMVLKESLRLYPPVPFISRNISEDTMFGDRLVPKDTLFNVHIFDLHRDPAVFPD

PERFDPDRFLPECVAERSPYAYVPFSAGPRNCIGQRFAILELKTVLAAILMHFRILPVTK

REELVFVADLILRTKDPIMVRFERR

>EDW46935.1

MFLIAIAIILATILVFKGVRIFNYIDHMAGIMEMIPGPTPYPFVGNLFQFGLKPAEYPKK

VLQYCRKYDFQGFRSLVFLQYHMMLSDPAEIQNILSSSSLLYKEHLYSFLRPWLGDGLLT

SSGARWLKHQKLYVPAFERSAIEGYLRVVHRTGGQFVQKLDGLSDTQEVFDAQELVAKCT

LDIVCENATGQDSSSLNGEPSDLHGAIKDLCDVVQERTFSIVKRFDALFRLTSYYMKQRR

ALSLLRSELNRIISQRRHQLAAENTCQQGQPINKPFLDVLLTAKLDGRALKEREIIEEVS

TFIFTGHDPIAAAISFTLYTLSRHSEIQQKAAEEQRRIFGENFAGEADLARLDQMHYLEL

VIRETLRLYPSVPLIARTNRKPIDINGTKVAKRTTVIMCVIAMGYNEKYFDDPCTFRPER

FENPTGNVGIEAFKSVPFSAGPRRCIAEKFAMYQMKALLSQLLRRFEILPAVEGLPPGIN

DHSREDCVPQSEYDPVLNIRVTLKSENGIQIRLRKR

>EDV59523.1

MFLIAIGIVLATILVFKGVRIFNYIDHMAGIMEMIPGPTPYPFVGNLFQFGLKPAEYPKK

VLQYCRKYDFQGFRSLVFLQYHMMLSDPAEIQNILSSSSLLYKEHLYSFLRPWLGDGLLT

SSGARWLKHQKLYVPAFERSAIEGYLRVVHRTGGQFVLKLDVLSDTQEIFDAQELVAKCT

LDIVCENATGLDSSSLNGEPSDLHGAIKDLCDVVQERTFSIVKRFDALFRLTSYYMKQRR

ALSLLRSELNRIISQRRHQLAAGDACQQGEPINKPFLDVLLTAKLDGRALKEREIIEEMS

TFIFTGHDPIAAAISFTLYTLSRHPEIQQKAAEEQRRIFGENCSAEADLGRLDQMHYLEL

VIRETLRLYPSVPLIARTNRQPIDINGTKVAKRTTVIMCLIAMGYNEKYFDDPCTFRPTR

FENPTGDVGIEAFKSVPFSAGPRRCIAEKFAMYQMKALLSQLLRRFEILPAVDGLPPGIN

DHSREDCVPQSEYDPVLNIRVTLKSENGIQIRLRKR

>EDW89346.1

MFLIAIAIILGTILVFKGVRIFNYIDHMAGIMEMIPGPTPYPFVGNLFQFGLKPAEYPKK

VLQYCRKYDFQGFRSLVFLQYHMMLSDPAEIQNILSSSSLLYKEHLYSFLRPWLGDGLLT

SSGARWLKHQKLYVPAFERSAIEGYLRVVHRTGGQFVQKLDVLSDTQEIFDAQELVAKCT

LDIVCENATGLDSSSLNGEPSDLHGAIKDLCDVVQERTFSIVKRFDALFRLTSYYMKQRR

ALSLLRSELNRIISQRRQHLAAENPGHQGQPINKPFLDVLLTAKLDGRALKEREIIEEMS

TFIFTGHDPLAAAISFTLYTLSRHPEIQEKAVEEQRRIFGDNFSGEADLARLDQMHFLEL

IVRETLRLYPSVPLIARTNRKPIDINGTKVAKRTTVIMCLIAMGYNEKYFDDPCTFRPER

FENPTGNVGIEAFKSVPFSAGPRRCIAEKFAMYQIKALISQLLRRFTILPAVDGLPPGIN

DHSREDCVPQSEYDPVLNIRVTLKSENGIQIRLRKR

>EDV35667.1

MFLIAIAIILATILVFKGVKIFNYIDHMASIMEMIPGPTPYPFVGNLFQFGLKPAEYPKK

VLQYCRKYDFQGFRSMVFLQYHMMLSDPAEIQNILSSSSLLYKEHLYSFLRPWLGDGLLT

SSGARWLKHQKIYLPAFERSAIEGYLRVIQRTGGQFIQKLSSLSETQEIFDAQELVAQCT

LDIVCENATGLDSSSLNGEPSDLHGSVKDLCDVVQERTFSIVKRFDALFRLTSYYMKQRR

ALSLLRSELGRIIAQRRHQLAKENASGTNTQNINKPFIDVLLAAKLDGRALKEREIIEEI

STFIFTGHDPVAAAISFTLYTLSRHSEIQQKAFEEQQRIFGKDLAGEADLARLDQMHYLE

LIIRETLRLYPSVPLIARTNRQAIDINGTQVAKRTTVIMCLIAMGYNEKYFEEPCAFRPE

RFENSSGNVGIEAFKSVPFSAGPRRCIAEKFAMYQLKSILSQLLRNYEILPAVDGLPPGI

NDHSRVDCVPQSEYDPILNIRVTLKSENGIQIRLKKR

>EAL24873.2

MFLIAIAIILATILVFKGVKIFNYIDHMAGIMEMIPGPTPYPFVGNIFQFGLKPAEYPKK

VLQYCRKYDFQGFRSLVFLQYHMMLSDPAEIQNILSSSSLLYKEHLYSFLRPWLGDGLLT

SSGARWLKHQKLYMPAFERSAIEGYLRVVHRTGGKFVQKLNELTETLEVFDAQELVAKCT

LDIVCENATGLVSNSLNDEPSDLHGAIKDLCDVVQERTFSIVKRFDALFRLTSYYMKQRR

ALSLLRREIYRIITQRRQQLADENTCGERTKKINKPFLDVLLAAKLDGRALKEREIIEEI

STFIFAGHDPVAAAISFTLYTLSRHTEIQQRVFEEQQRIFGQDLTAEADIGRLDQMRYLE

LVIRETLRLYPSVPLIARTNRKPIDINGTKVAKRTTVIMCLIAMGYNEKYFEEPCVFRPE

RFETAGADVGIEAFKSVPFSAGPRRCIAERFAMYQLKALLSQLLRNFEILPPVNGLSSGI

NDHSQVECVPQSEYDPVLNIRVTLKSENGIQIRLGKRAFVE

>EDW31619.1

MFLIAIAIILATILVFKGVKIFNYIDHMAGIMEMIPGPTPYPFVGNIFQFGLKPAEYPKK

VLQYCRKYDFQGFRSLVFLQYHMMLSDPAEIQNILSSSSLLYKEHLYSFLRPWLGDGLLT

SSGARWLKHQKLYMPAFERSAIEGYLRVVHRTGGKFVQKLNELTETLEIFDAQELVAKCT

LDIVCENATGLVSNSLNDEPSDLHGAIKDLCDVVQERTFSIVKRFDALFRLTSYYMKQRR

ALSLLRREIYRIITQRRQQLADENTCGERTKQINKPFLDVLLAAKLDGRALKEREIIEEI

STFIFAGHDPVAAAISFTLYTLSRHTEIQQRVFEEQQRIFGQDLTAEADIGRLDQMRYLE

LVIRETLRLYPSVPLIARTNRKPIDINGTKVAKRTTVIMCLIAMGYNEKYFEEPCVFRPE

RFETAGADVGIEAFKSVPFSAGPRRCIAEKFAMYQLKALLSQLVRNFEILPPVNGLSSGI

NDHSQVECVPQSEYDPVLNIRVTLKSENGIQIRLGKRAFVE

>EDW00808.1

MFLLAAAIILATILVFKGVRIFNYLDHMAGIMEMIPGPTPYPFVGNLFQFGLKPAEYPKK

VLQYCRKYDFRGFRSLVFLQYHIMLSDPAEIQNMLSSSSLLYKEHLYSFLQPWLGDGLLT

SSGARWLKHQKIYVPAFERSAIEGYLRVVHRTGNKFMQKLRQQAKASRPFDAQELVGKCT

LDIVCENATGNVSNSLNDDPSDFHSAIKDLCDVVQERTFSIVKRFDALFRLTPYYMKQRR

ALKLLRTEIRRIITQRQEHLAKAKENKNAVELSNKPFIDVLMAAKLDGCALKEREIIEEL

STFIFTGHDPVASAISFTIYTLSRHPEIQQKVFDEQQRIFGKDLTGESDIARLQQMHYLE

LVIRETLRLYPSVPLIARTNRKSIDINGTKVAKRTTVIMCLIAMGYNERYFDEPCVFRPE

RFEKTNSNSDIEAFKSVPFSAGPRRCIAEKFAMYQMQALLSLLVRHFEILPAVDNLPPGI

NDHSREDCVPQSEYDPVLNIRITLKSENGIQIRLKQRTGMQ

>EDW60846.1

MFLLAAAIVLATILVFKGVKIFNYIDHMAGIMEMIPGPTPYPFVGNIFQFGLKPAEYPKK

VLQYCRKYDFQGFRSLVFLQYHIMLSDPAEIQNMLSSSSLLYKEHLYSFLRPWLGDGLLT

SSGARWLKHQKLYGPAFERSAIEGYLRVVHRTGDRFVQKLRQQTEASKPFDAQELVAKCT

LDIVCENATGMASNSLNDEPSDLHSAIKDLCEIVQERTFSIVKRFDALFRLTPYYLKQRR

ALHLLRNEIKRIISDRQRQQQLTKSNDHKNKSFLDVLLCSKLDGDALKEREIVEELSTFI

FTGHDPVASAISFTIYTLSRHPEIQQKVYDEQLRIFGKNMSEEADMARLEQMHYLELVIR

ETLRLYPAVPLIARTNRKSIDINGTKVAKRTTVIMCLIAMGYNERYFDEPCVFRPERFER

ANRNEGIEAFKSVPFSAGPRRCIAEKYAMYQLQALLSLLVRHFEILPAVDGLPPGINDHS

QVDCVPQSEYDPVLNIRVTLKSENGIQIRLKQRTAMQ

>EDW10128.1

MFLLVFGIILATILVVKGVRIFNYIDHMAGIMEMIPGPTPYPFVGNIFQFGLKPAEYPKK

ILQYCRKYDFHGYRSLVFLQYHIMLSDPAEIENMLSSSSLLYKEHLYSFLRPWLGDGLLT

SSGARWLKHQKIYAPAFERSAIEGYLRVVHRTGERFVEKLRQESESSKPFDAQELVAKCT

LDIVCENATGSVSNSLNDEPSDLHSAIKDLCDVVLQRTFSIVKRCDALFRLSPYYLKQYR

ALQLLRREVKEIISRRKHELATKKPIENKPFIDVLLSAKLDGDALKERDIVEELSTFIFT

GHDPVASAISFTIYTLSRHPEVQQKVFEEQQRIFGKNITGEADIARLQQMHYLELVIRET

LRLYPSVPLIARTNRKSIDINGTKVAKRTTVIMCLLAMGYNERYFDEPCVFRPERFEGAN

TNSGLDAFKSVPFSAGPRRCIAEKFAMYQLQALLSLLVRHFEILPAVDGLPPGINDHTRV

DCVPQSEYDPVLNIRVTLKSENGIQIRLKQRVEPQQSAL

>EDW74218.1

MFLLAAAILLATILVFKGVKIFNYLDHMAGIMEMIPGPTPYPFVGNLFQFGLKPDEYPKK

VLQYCRKYDFRGFRSLVFLQYHIMLSDPAEIQNMLSSSSLLYKEHLYSFLRPWLGDGLLT

SSGARWLKHQKLYIPAFERSAIEGYLRVVHRTGSKFVQKLTEEQAETRETFDAQQLVAKC

TLDIVCENATGLPSNSLNDEPSDLHSAIKDLCDVTQKRTFSIVKRFDALFRLTPYFMKQR

RALQLLRNEINRIIAQRRHQLDSDKSKAINKPFLDVLLAAKLDGRALKEREIIEELSTFI

FTGHDPVAAAISFTLYTLSRHAEIQQKVFEEQKAIFGKDLSTEAGIPHLDQMRYLELVIR

ETLRLYPSVPLIARTNRNSIDINGTKVAKRTTVIMCLIAMGYNERYFDEPCVFRPERFEK

AHLDRGMEAFKSVPFSAGPRACIAEKFAMYQLKSLISLLVRHFEILPAIDGLPPGINDHS

RVDCVPQI

>EDX06172.1

MFLIAIAIILATILVFKGVRIFNYIDHMAGIMEMIPGPTPYPFVGNLFQFGLKPAEYPKK

VLQYCRKYDFQGFRSLVFLQYHMMLSDPAEIQNILSSSSLLYKEHLYSFLRPWLGDGLLT

SSGARWLKHQKLYVPAFERSAIEGYLRVVHRTGGQFVQKLDGLSDTQEVFDAQELVAKCT

LDIVCENATGQDSSSLNGEPSDLHGAIKDLCDVVQERTFSIVKRFDALFRLTSYYMKQRR

ALSLLRSELNRIISQRRHQLAAENTCQQGQPINKPFLDVXXXXXXXXXXXXXXXXXXXXX

XXXXXXXXXXXXXXXXXXXXXXXXXXXXXXXXXXXXXXXXXXXXXXXXXXXXXXXXXLEL

VIRETLRLYPSVPLIARTNRKPIDINGTKVAKRTTVIMCLIAMGYNEKYFDDPCTFRPER

FENPTGNVGIEAFKSVPFSAGPRRCIG

>EDW17782.1

MLLIAIVVLLLTSWVHWRQRDRRAAVANLPGPFCLPLVGAWHLAINMTPKTFIERSQKYF

RQLGKLQRAWVLNRLVIVSGDVEFNEQLMSSQEHLVKHPVYKLLGQWLGNGLLLSDGKLW

RQRRKIITPTFHFSILEQFVEVFDQQSNVCVERLEQRATGECFDVYPFVCLAALDIISET

AMGTKVHAQQAESTPYANAVTECTALMAWRFMSVIQQNELLFTLTHPHLKLRQMQLIRIM

HEFTAQVIKQRRQALEAMQVAGTEQQEDEQVGGKRRMALLDVLLQARVGGRALRDEEICE

EVDTFMFEGHDTTTSGISFCLWCISRHPEVQAKMLAEILEVLGSDRSRAVSMRDLGQLKY

LECVIKESLRRYPPLALVGRKLQADFRYTHSKFGAGVIPSGSEIFMIIYGMHNDADYFPE

PDLFKPERHESSDRSATNFSYAPFSAGPRNCVGQKFALLEMKMLLAKIVREYELLPLGEP

VQPVVNIVLRSDTGFQMGMRKRGQQ

>ABG34549.1

MYFAVVLLVLFILICVWAFHIHISTKYRRKLTDHIPGPYCYPIVGCVGEATTLTPKRLLA

KSYEVNELYGNIIKGWILNSLFIFTSNVEFMEQILSHTTQTRKSRLYSILKPWLGESLLL

SKEQKWHTRRKIITPTFHFSILEQFLKVFDRQTLVLIDCLAERADGRSAFDVMPYICSAA

LDIITETAMGVNVNAQTDKTMPYTMAVREMTNLVMWRFLRAYLNDERLFSILCPLKKLRQ

TTLIKTMHKFTGNVIEKRRRELENYMESESRHDDHDPDDIGIRKHRAFLDVLLQATIDGE

PLADEDIREEVETFMFEGHDTTTTALSFTPEVQQKLLAEIYAIFGEKSVEPFTLAKLSDL

KYMECVIKESLRLYPPVPLIGREITEDFPYTHSVIGDGIVPASTQFVISIFHALREPSVY

DRPLEFIPDRHKEASVNSPFIFVPFSAGPRNCIGQRFAMFEMKVVLCKILREYELLPLGD

DVEPIFGIVLRSDNGIQLSMRRRSAS

>ADD19966.1

MRYFREQYGKVCQVWFFNFLFVIVQEVEYLEPILSSPRLIKKNFVYDVLRGWLNDGLLLS

SGSKWHARRKIITRSYHFAILEQFIEVFDRQSETFVERLRGKADGKTVIEMFSEVCPTAL

DIITETAMGVKIDAQKNPNLPYVKALAHTTHLIAARITQPAYYLDIIYHITHMLDNIRLK

RNVRIMHEFTDSIIRERRKRLHKSITENHQKNAEAEAHLEFGIKKGVAFLDLLLQSTIDG

KPLSNEDIREEVDTFMFEGHDTTTSGVAHTLYRLARHPNAQQKAYEEVQQVLNKSKTEPI

TMKDLQNLKYLECVIKEALRLHPSVPLISRVSEEEFKIGNLTLPPNTQIVIPLHAVARDP

KYFSNPDDFLPERFLLENTTKMHPFAFVPFSAGPRNCVGQKFAMLEMKMIVGKVLRDYEI

LPLGPDIAPIVTLILRSTTGCNVGLRQRA

>EDW17638.1

MLLTLLVGALTLLLTWDFVCKRHSYAVLSKSGITGPLSLPLVGCGIQALQLGADNIIDYV

GSCFSSYGKTFRLWILNESQVYTTQLEHFEAILSSTTLLEKGQLYEFLRPFLNDGLLVSV

GKKWHSRRKVFSTVFHFKILEHYVGIMDKQADILVDSLQQVADGEQVVDTLKYVSLAALD

IITETAMGVQVNAQSDPDFPYIKALKSVVYIQPDRMLKFSQRYEWLFRVTAPLLHRKLVS

DIRIMHDFTDKVIRERREAVERAKADGTYKPLSLGDEEIGRKPQMAFLDILLQASIQGSP

LTDADIREEVDTFMFEGDDTTSSGVAHALYSIARHPAVQAKIYEELVRVLGNDPRAAISQ

SQLQQLKYLECVIKETMRLYPPVPAVGRYTRKDLKIGDQIIPAHTSIYMVLYFAHRDPKY

FPDPFSFKPERFLDDTAEVDGKPQAANFSYLPFSAGPKNCIGQKFAMLEMKMLLGKVVRY

YELLPKGPEIRPMMNFILRSATGMHIALRPRLK

>EDW62657.1

MLWLALLLVGIPCLLLWDYMRRKRRNDMLHYMPGPATLPFLGNVLLYRGIDGERECLTNP

FVLRLEQSIACTVYRLQAYGRMYRIWALYQLAIFSTDPRDVQFVLSSPQLITKNNLYNLL

RPWLGTGLLMSTGSKWRARRKIITPTFHFKILERFVDIFDQQSTILVLQLQQHADGRTPL

NIFPFIFLAALDIIAETAMGTKVNAQRDPHLPYVQDVYELTSIMTDRFIKPWQRNEWIFR

LLKPTLAKKQDALIRIMHEFTECIIKQRREALVQKQQQQQQLKSGDEGDVPGQRRRLALL

DVLLQAIALNGAPLSDEEIREEVDTFMFEGHDTTTSGISFCLYEISRHAAVQQRLIDEID

KVFGGDRQRALTLRDLNDLKYLECVIKEALRLYPSVPIIARTFSEDVQIRGKRIPAGTDF

FVGIFALLRDAREFQAPNEFRPERFASDFAMLEMKCTICRLLQRFQLLPLGPQPRPMLSL

VLRSVNGVHLGLRPCLDK

>EDW78816.1

MLLTLIIGTLILLLTWDFGRKRQRVAIFRKSHITGPRTLPLLGCGLEALHLGAENIIDYV

GNAFEKYGKTFRFWILNESLIYTKDLKYFETILSSTTLLEKAQLYQFLRPFLNDGLLLSV

DRKWHARRKVFTNAFHFKVLERYVEIMDKQSEIMVEKLKQDADGKTVVDMLKYVSLAALD

VITETAMGVQVNAQSNEDFPYIKALKSVVNIQPDRMFKFSQRYGWLFRLTCPLLHRKLVR

DIGIMHDFTDKVIRERRAAVEQAKANGSYQPLSLADADIGRKSQMALLDILLQANINGEP

LTDADIREEVDTFMFEGDDTTSSGVSHALYSISRHPKVQAKLYDELQQVLGKDPTAPITQ

SQLQELKYLDCIIKETMRLYPPVPAIGRHTTKELRIGDQIIPPNTSIYLVFYFAQRDAEY

FPDPLSFKPERWLTDDEDTKPNAKHQTFAYVPFSAGPKNCIGQKFAILEMKALISKVIRY

YHLLPLGEDVKPMMNFILRSSVGMNVGLKPRSL

>EDW50205.1

MWLTLITGALILLLTWDFGRKRQRVLAFEKSKIPGPISIPILGCGVQALHLGAENIIGWV

GEKFDKYGKTFRFWILGESLIYTKDLQYFETILSSTTLLEKGQLYEYLRPFLNDGLLVST

GRKWHARRKIFTHAFHFKVLEHYVEIMDRHSGVMADNLRKVADGKTAVDMLKYVSLAALD

VITEAAMGVQVNAQNDPDFPYIKALKSVVYIQPDRMFRFSRRYNWLFPLAAPLLHRQLLS

DIRVMHDFTDKVISERRETVRRAKADGTYRPLSLGDAEIGSKSQMALLDILLQSSINNQP

LSDADIREEVDTFMFEGDDTTSSGVSHALYAIARHPEVQQRIYEELLRVLGPDASAPVTQ

AQIQDLKYLDCVIKETMRLYPSVPAIGRHAQKELKIGDKTIPANTSIYLVLYYAHRDPAY

FPDPLSFKPERFLEDQEQGHDTFAYVPFSAGPKNCIGQKFAVLGMKILISKVLRFYELLP

LGEELKPMLNFILRSASGINVGLRPRKF

>ACI25369.2

MLGLLILLVIIFALVIGLDYYNHYKDPRRIIDRIFPGPFRIPIVGNALIAACSREAFYRV

YLNLSKSYDYKPFRLWFLKQPYVFISDYENTKKILSSNDLLDKAEDYRYFEPWLGQGLLT

SKGDKWRRDRKIITPAFHFQILHRFFQIFYDNGEIFCEKLTAHDGGDVFDVFEYIEMLTL

DNLCNAALKLDIQAQLNPFSALVCAIQNMGVVIYNRIFTLWLRYDAMFRLSSTARLQKKC

LEIIHGNDVKIIRERKEEMKTKQPKLKTMEFDEKEKVAFLDLLLEMQDDVGYTDEDIREQ

LDTFMFAGHDTVTATLGFLIYSLAEHPDVQEKVYRELLDIYGESERCPNFSDLQDMKYTE

QVIKETLRLYTVVTAVARRVEEDFELSEHQVVPKGVEIVLLLSALHRNPEIFPNPDIFDP

DRFSPEVNQERDSFAFVPFSAGSRNCIGQKFAMHEMKITVYKIVKKFKLSISDKPEDKVR

TRTGVVLSSTNGIRIKVQSRKKKTPSKLADQ

>EAL31680.1

MEVLKKDAAMGSPSSVFYFLLLPTLVLWYIYWRLSRAHLYRLAGRLPGPRGLPIVGHLFD

VIGPASSVFKTVIRKSAPFEHIAKMWIGPKLVVFIYDPRDVELLLSSHVYIDKASEYKFF

KPWLGDGLLISTGQKWRSHRKLIAPTFHLNVLKSFIELFNENSRNVVRKLRAEDGRTFDC

HDYMSEATVEILLETAMGVSKKTQDKSGFEYAMAVMRMCDILHARHRSIFLRNEFVFTLT

RYYKEQGRLLSIIHGLTTKVIRSKKAAFEQGTRGSLAQCELKAAALERERGQEQKQEQKQ

EQAEMGSAGGGKAAGEGTPVAGLSYGQSAGLKDDLDVEDNDIGEKKRLAFLDLMLESAQN

GALITDTEIKEQVDTIMFEGHDTTAAGSSFFLSLMGIHQEIQDRVIAELDGIFGDSQRPA

TFQDTLEMKYLERCLMETLRMYPPVPLIARELQEDLKLNSGDYVIPRGATVTVATVLLHR

NPKVYTNPNVFDPDNFLSERQANRHYYAFVPFSAGPRSCVGRKYAMLKLKILLSTILRNY

RVYSDLSESDFKLQADIILKREEGFRVRLQAR

>Q9VYY4.1

MEVLKKDAALGSPSSVFYFLLLPTLVLWYIYWRLSRAHLYRLAGRLPGPRGLPIVGHLFD

VIGPASSVFRTVIRKSAPFEHIAKMWIGPKLVVFIYDPRDVELLLSSHVYIDKASEYKFF

KPWLGDGLLISTGQKWRSHRKLIAPTFHLNVLKSFIELFNENSRNVVRKLRAEDGRTFDC

HDYMSEATVEILLETAMGVSKKTQDKSGFEYAMAVMRMCDILHARHRSIFLRNEFVFTLT

RYYKEQGRLLNIIHGLTTKVIRSKKAAFEQGTRGSLAQCELKAAALEREREQNGGVDQTP

STAGSDEKDREKDKEKASPVAGLSYGQSAGLKDDLDVEDNDIGEKKRLAFLDLMLESAQN

GALITDTEIKEQVDTIMFEGHDTTAAGSSFFLSLMGIHQDIQDRVLAELDSIFGDSQRPA

TFQDTLEMKYLERCLMETLRMYPPVPLIARELQEDLKLNSGNYVIPRGATVTVATVLLHR

NPKVYANPNVFDPDNFLPERQANRHYYAFVPFSAGPRSCVGRKYAMLKLKILLSTILRNY

RVYSDLTESDFKLQADIILKREEGFRVRLQPRTS

>AAF76522.1

MEVLKKDAALGSPSSVFYFLLLPTLVLWYIYWRLSRAHLYRLAGRLPGPRGLPIVGHLFD

VIGPASSVFRTVIRKSAPFEHIAKMWIGPKLVVFIYDPRDVELLLSSHVYIDKASEYKFF

KPWLGDGLLISTGQKWRSHRKLIAPTFHLNVLKSFIELFNENSRNVVRKLRAEDGRTFDC

HDYMSEATVEILLETAMGVSKKTQDKSGFEYAMAVMRMCDILHARHRSIFLRNEFVFTLT

RYYKEQGRLLNIIHGLTTKVIRSKKAAFEQGTRGSLAQCELKAAALEREREQNGGVDQTP

STAGSDEKDREKDKEKASPWRDLSYGQSAGLKDDLNVEDNDIGKKKRLAFLDLMLESAQN

GALITDTEIKEQVDTIMFEGHDTTAAGSSFFLSLMGIHQDIQDRVLAELDSIFGDSQRPA

TFQDTLEMKYLERCLMETLRMYPPVPLIARELQEDLKLNSGNYVIPRGATVTVATVLLHR

NPKVYANPNVFDPDNFLPERQANRHYYAFVPFSAGPRSCVGRKYAMLKLKILLSTILRNY

RVYSDLTESDFKLQADIILKREEGFRVRLQPRTS

>EDW51392.1

MEVLKKDAALGSPSNVFYFLLLPTLVLWYIYWRLSRAHLYRLAGRLPGPRGLPIVGHLFD

VIGPASSVFRTVIRKSAPFEHIAKMWIGPKLVVFIYDPRDVELLLSSHVYIDKASEYKFF

KPWLGDGLLISTGQKWRSHRKLIAPTFHLNVLKSFIELFNENSRNVVRKLRAEDGRTFDC

HDYMSEATVEILLETAMGVSKKTQDKSGFEYAMAVMRMCDILHARHRSIFLRNEFVFTLT

RYYKEQGRLLNIIHGLTTKVIRSKKAAFEQGTRGSLAQCELKAAALEREREQDGGVGGGD

QTASTAGSEEKDREKDKEKASPVAGLSYGQSAGLKDDLDVEDNDIGEKKRLAFLDLMLES

AQNGALITDTEIKEQVDTIMFEGHDTTAAGSSFFLSLMGIHQDIQDRVLAELDSIFGDSQ

RPATFQDTLEMKYLERCLMETLRMYPPVPLIARELQEDLKLNSGNYVIPRGATVTVATVL

LHRNPKVYANPNVFDPDNFLPERQANRHYYAFVPFSAGPRSCVGRKYAMLKLKILLSTIL

RNYRVYSDLTESDFKLQADIILKREEGFRVRLQPRTR

>EDX17646.1

MEVLKKDAALGSPSNVFYFLLLPTLVLWYIYWRLSRAHLYRLAGRLPGPRGLPIVGHLFD

VIGPASSVFRTVIRKSAPFEHIAKMWIGPKLVVFIYDPRDVELLLSSHVYIDKASEYKFF

KPWLGDGLLISTGQKWRSHRKLIAPTFHLNVLKSFIELFNENSRNVVRKLRAEDGRTFDC

HDYMSEATVEILLETAMGVSKKTQDKSGFEYAMAVMRMCDILHARHRSIFLRNEFVFTLT

RYYKEQGRLLNIIHGLTTKVIRSKKAAFEQGTRGSLAQCELKAAALEREREQDGGVGGGD

QTASTAGSEEKDREKDKEKASPVAGLSYGQSAGLKDDLDVEDNDIGEKKRLAFLDLMLES

AQNGALITDTEIKEQVDTIMFEGHDTTAAGSSFFLSLMGIHQDIQDRVLAELDSIFGDSQ

RPATFQDTLEMKYLERCLMETLRMYPPVPLIARELQEDLKLNSGNYVIPRGATVTVATVL

LHRNPKVYANPNVFDPDNFLPERQANRHYYAFVPFSAGPRSCVGRKYAMLKLKILLSTIL

RNYRVYSDLTESDFKLQADIILKREEGFRVRLQPRTR

>EDX01967.1

MEVLKKDAALGSPSSVFYLLLLPTLVLWYIYWRLSRAHLYRLAGRLPGPRGLPIVGHLFD

VIGPASSVFRTVIRKSAPFEHIAKMWIGPKLVVFIYDPRDVELLLSSHVYIDKASEYKFF

KPWLGDGLLISTGQKWRSHRKLIAPTFHLNVLKSFIELFNENSRNVVRKLRAEDGRTFDC

HDYMSEATVEILLETAMGVSKKTQDKSGFEYAMAVMRMCDILHARHRSIFLRNEFVFTLT

RYYKEQGRLLNIIHGLTTKVIRSKKAAFEQGTRGSLAQCELKAAALEREREQLGGGGGGG

RDELMAGSDDKDKDKDQEREKDKDREKEKATPVAGLSYGQSAGLKDDLDVEDNDIGEKKR

LAFLDLMLESAQNGALITDTEIKEQVDTIMFEGHDTTAAGSSFFLSLMGIHQDIQDRVLA

ELDSIFGDSQRPATFQDTLEMKYLERCLMETLRMYPPVPLIARELQEDLKLNSGNYVIPR

GATVTVATVLLHRNPKVYANPNVFDPDNFLPERQANRHYYAFVPFSAGPRSCVGRKYAML

KLKILLSTILRNYRVYSDLTESDFKLQADIILKREEGFRVRLQPRTR

>EDW07639.1

MEVLKKDAAVGSPSSVFYLLLLPTLVLWYIYWRLSRAHLYKLAARLPGPRGLPIVGHLFD

VIGPASSVFKTVIRKSTPFEHIAKMWIGPKLVVFIYDPRDVELLLSSHVYIDKASEYKFF

KPWLGDGLLISTGQKWRAHRKLIAPTFHLNVLKSFIELFNENSRNVVRKLRAEDGRTFDC

HDYMSEATVEILLETAMGVSKKTQDKSGFEYAMAVMRMCDILHARHRSIFLRNEFIFTLT

RYYKEQGRLLNIIHGLTTKVIRSKKAAFEQGTRGSLAQSELKAAALEQEERELREQREKQ

MATGTQPKQEQSAPVAGLSYGQSAGLKDDLDVEDNDIGEKKRLAFLDLMLESAQNGALIT

DTEIKEQVDTIMFEGHDTTAAGSSFFLSLMGIHQNIQDRVIAELDGIFGDSQRPATFQDT

LEMKYLERCLMETLRMFPPVPLIARELQEDLQLNSGPYVIPRGATVTVATILLHRNPKVY

ANPNVFDPDNFLPERQANRHYYAFVPFSAGPRSCVGRKYAMLKLKILLSTIMRNYRVYSD

LSESDFRLQADIILKREEGFRVRLQPRQPGQATTAGSTAAS

>EDW75014.1

MEMLRKDAPAALGTPSSVFYLLLLPTLVLWYIYWRLSRAHLYRLADRLPGPRGLPIVGHL

FDVIGPASSVFKTVIRKSAPFEHIAKMWIGPKLVVFIYDPRDVELLLSSHVYIDKASEYK

FFKPWLGDGLLISTGQKWRSHRKLIAPTFHLNVLKSFIELFNENSLNVVRKLRAEDGRTF

DCHDYMSEATVEILLETAMGVSKKTQDKSGFEYAMAVMRMCDILHARHRSIFLRNEFIFT

LTRYYKEQGRLLNIIHGLTTKVIRSKKAAFEQGTRGSLAQTELQQKLQEQQIQQAKEKEK

EHGELITSPPSTTATNETKANQTQTQTQTQSQAPVAGLSYGQSAGLKDDLDVDDNDIGEK

KRLAFLDLMLESAQNGALITDTEIKEQVDTIMFEGHDTTAAGSSFFLSLMGIHQDIQDRV

IAELDGIFGDSKRPATFQDTLEMKYLERCLMETLRMFPPVPLIARELQEDLKLNSGNYII

PRGATVTVATILLHRNPKVYANPNVFDPDNFLPERQVNRHYYAFVPFSAGPRSCVGRKYA

MLKLKILLSTILRNYRVYSDLSESDFKLQADIILKREEGFRVRLQPRGAS

>AAQ20834.1

MIVEFLLLILLMAILLMAAALPLHLIDLRPKTIWKMSKLPGPRGLPFFIGLAIRLAKLRD

IDILAFFQNIHQEYARLVAFWLAGIPTVWLFDPDDLEVILASSQAIQKGNEYHCLFPWMQ

DGLVLSKGEKWHQRRKLLTPAFHFKILENNMQSLNKNARCLLRNMLKKEGRPFVTQELVV

RCTLDVISETAMGHALNLQEREENNDYLNAVERACSLTFDRSKNVFYSNDGVYFLTLDGR

KFSKNLKYLHNFSENIIQNRKLKYLLEKKYSENNIEEDNNFGKGKKAFLNILIDLEDKSK

GTLTDKDIREEVDNFMFAGHDTTASCIMFTLYLLGRHPHVQEKAFEELYEIFGESDREVN

NKDLHGMHYLEMIIKESIRIYPPAPYFSRNLIQDLVLKDKTVLPEGANVGIFAFIMHRDP

KYFPNPEVFDPERFSAENCKKRHPYAYLPFSAGPRNCIGQKFAMMELKVVLSTILRFAKI

ESVNDEISARSLTPLILLKPCDPIRIKVFSRF

>EEB17116.1

MFESLIILFILIFIFHLFIYHEYHLPKYVNLINKIPGPVSHSYFGSSLKFYRLKHEEILY

DLIEGFKKWGPTYRLWYSGVAVVFVSDLEDVQTILSSREANAKGMLYRNILPWLGCGLLT

SCGEKWQKMRKIITPAFHFTVLCDYIEIFNKNTKIMLDVLKKKKDDDEGKKCDLSKYLTR

CTLDNICETAMGTSINALRTGSESKYVKAIFRMGQIVVERMFSPLHFTNMSFMFSLKGLE

HSRLLKILHGFTYEIINEKMMNLIKEKSGGRVEKKTGNVYKTKSSFLDILLENQKIHCLS

FEDIRQEVDTFMFEGHDTTAASLTWTLFELGHHPEVQERCYSELIEIFGDSNELPSYNDL

MKMNYLKRVIQETLRLYPSVPVISRKFKVDMQLNDYLVPANTEIILILYAIQRNEKIFKN

PDKFDPDRFLQEEIIKRHAFAYVPFSAGQRNCIGQKFAMLEELVVLSSIIRNFKIESLND

RNSIRVVPEMILRPHQNLKFKLIKRQ

>328708149

MIFSNVIGALTSDSNTQWMALLSLVVLGVYFLFSDRFSENRGRQISLLPSITRSQWTSLI

LSLKLASFGPRDILPYFDNVIKKYGSLIHLKIIARHYIIINDPDDIKVLLSSVQHITKGP

DYEMLEPWLNKGLLTSTDQKWHSRRKLLTNTFHFKILETYVPSLNKHSRSLVKNLINASD

NGKSIADIDSHVTLCALDIVCETIMGVNLRTQEGKSMNYVKAIKNVSQILIKRIFTFWYW

NEIVFNLSSIGREFRKSLKLLHDFTENVIRERRKILENVEQKKVDENGKKRIYSFLDLLV

GVSKENPGAMTDKDIREEVDTFLFEGHDTSSIAITMAIIHLGLDQNIQNLVRDELYEIFG

DSDRDATMEDLKAMTNLERVIKETMRLYPSVTGITRTLKQPLHLDKYTIPSKSVMVVVPH

LLHRDKNIYPNPEKFDPDRFLPEQCNGRHPYAYIPFSAGPRNCIGQKFAMYQMKTVLSTI

LRYTNVETLGTQKSIVISTQLILRADYLPSVKITPITNTTRHIIL

>30023836

MEPSWLQELMAHPFLLLILLCMSLLLFQVIRLYQRRRWMIRALHLFPAPPAHWFYGHKEF

YPVKEFEVYHKLMEKYPCAVPLWVGPFTMFFSVHDPDYAKILLKRQDPKSAVSHKILESW

VGRGLVTLDGSKWKKHRQIVKPGFNISILKIFITMMSESVRMMLNKWEEHIAQNSRLELF

QHVSLMTLDSIMKCAFSHQGSIQLDSTLDSYLKAVFNLSKISNQRMNNFLHHNDLVFKFS

SQGQIFSKFNQELHQFTEKVIQDRKESLKDKLKQDTTQKRRWDFLDILLSAKSENTKDFS

EADLQAEVKTFMFAGHDTTSSAISWILYCLAKYPEHQQRCRDEIRELLGDGSSITWEHLS

QMPYTTMCIKECLRLYAPVVNISRLLDKPITFPDGRSLPAGITVFINIWALHHNPYFWED

PQVFNPLRFSRENSEKIHPYAFIPFSAGLRNCIGQHFAIIECKVAVALTLLRFKLAPDHS

RPPQPVRQVVLKSKNGIHVFAKKVC

>EDV34702.1

MTVEAAATSTSLLGYSPTLTTLVATMVALGLYEYWRRNTREYRMVANIPSPPELPLLGQA

HLAAGLSNAEIMNVGLGYLSKYGETLKAWLGSVLLVFITNPNDIELILSGHQHLTKAEEY

RYFKPWFGDGLLISNGHHWRHHRKMIAPTFHQSILKSFVPTFVNHSKAVVDRMGLEAGKS

FDVHDYMSQTTVDILLSTAMGVKKLPEGNKSFEYAQAVVDMCDIIHKRQIKLLYRLDSIY

KFTKLREKGDRMMNIILGMTSKVVKDRKENFQEESRAIVEEITTPATPAAKKEGLRDDLD

DIDENDVGAKRRLALLDAMVEMAKNPDIEWNEKDIIDEVNTIMFEGHDTTSAGSSFALCM

MGIHKDIQEKVFAEQKAIFGDNMLRDCTFADTMEMKYLERVILETLRLYPPVPLIARRVD

YDLKLASGPYTVPKGTTVIVLQYCVHRRPDIYPNPTKFDPDNFLPERMANRHYYAFIPFS

AGPRSCVGRKYAMLKLKVLLSTIVRNYIVHSTDTEADFKLQADIILKLENGFNISLEKHM

>Q86W10.1

MEPSWLQELMAHPFLLLILLCMSLLLFQVIRLYQRRRWMIRALHLFPAPPAHWFYGHKEF

YPVKEFEVYHKLMEKYPCAVPLWVGPFTMFFSVHDPDYAKILLKRQDPKSAVSHKILESW

VGRGLVTLDGSKWKKHRQIVKPGFNISILKIFITMMSESVRMMLNKWEEHIAQNSRLELF

QHVSLMTLDSIMKCAFSHQGSIQLDSTLDSYLKAVFNLSKISNQRMNNFLHHNDLVFKFS

SQGQIFSKFNQELHQFTEKVIQDRKESLKDKLKQDTTQKRRWDFLDILLSAKSENTKDFS

EADLQAEVKTFMFAGHDTTSSAISWILYCLAKYPEHQQRCRDEIRELLGDGSSITWEHLS

QMPYTTMCIKECLRLYAPVVNISRLLDKPITFPDGRSLPAGITVFINIWALHHNPYFWED

PQVFNPLRFSRENSEKIHPYAFIPFSAGLRNCIGQHFAIIECKVAVALTLLRFKLAPDHS

RPPQPVRQVVLKSKNGIHVFAKKVC

>EDW43699.1

MAVEVVQETLQQAAASSSTTVLGFSPMLTTLVGTLVAMALKGLRDDLDDIDENDVGAKRR

LALLDAMVEMAKNPDIEWNEKDIMDEVNTIMFEGHDTTSAGSSFALCMMGIHKDIQAKVF

AEQKAIFGDNMLRDCTFADTMEMKYLERVILETLRLYPPVPLIARRLDYDLKLASGPYTV

PKGTTVIVLQYCVHRRPDIYPNPTKFDPDNFLPERMANRHYYSFIPFSAGPRSCVGRKYA

MLKLKVLLSTIVRNYIVHSTDTEADFKLQADIILKLENGFNVSLEKRQYATVA

**CYP5**

>BAG58828.1

MMEALGFLKLEVNGPMVTVALSVALLALLKWYSTSAFSRLEKLGLRHPKPSPFIGNLTFF

RQGFWESQMELRKLYGPLCGYYLGRRMFIVISEPDMIKQVLVENFSNFTNRMASGLEFKS

VADSVLFLRDKRWEEVRGALMSAFSPEKLNELGLLIMQERIKGHMGGQQAPQRIPPTRLS

KPSGIYVNLHYATLPFCMVPLISQACDLLLAHLKRYAESGDAFDIQRCYCNYTTDVVASV

AFGTPVDSWQAPEDPFVKHCKRFFEFCIPRPILVLLLSFPSIMVPLARILPNKNRDELNG

FFNKLIRNVIALRDQQAAEERRRDFLQMVLDARHSASPMGVQDFDIVRDVFSSTGCKPNP

SRQHQPSPMARPLTVDEIVGQAFIFLIAGYEIITNTLSFATYLLATNPDCQEKLLREVDV

FKEKHMAPEFCSLEEGLPYLDMVIAETLRMYPPAFRFTREAAQDCEVLGQRIPAGAVLEM

AVGALHHDPEHWPSPETFNPERFTAEARQQHRPFTYLPFGAGPRSCLGVRLGLLEVKLTL

LHVLHKFRFQACPETQVPLQLESKSALGPKNGVYIKIVSR

>397484567

MEALGFLKLEVNGPMVTVALSVALLALLKWYSTSAFSRLEKLGLRHPKPSPFIGNLTFFR

QGFWESQMELRKLYGPLCGYYLGRRMFIVISEPDMIKQVLVENFSNFTNRMASGLEFKSV

ADSVLFLRDKRWEEVRGALMSAFSPEKLNELGLLIMQERIKGHMDGQQAPQRIPPTRLLK

PSGIYVNLHYATLPFCMVPLISQACDLLLAHLKRYAESGDAFDIQRCYCNYTTDVVASVA

FGTPVDSWQAPEDPFVKHCKRFFEFCIPRPILVLLLSFPSIMVPLARILPNKNRDELNGF

FNKLIRNVIALRDQQAAEERRRDFLQMVLDARHSASPMGVQDFDIVREVFSSTGCKPNPS

RQHQPSSMARPLTVDEIVGQAFIFLIAGYEIVTNTLSFATYLLATNPDCQEKLLREVDVF

KEKHMAPEFCSLEEGLPYLDMVIAETLRMYPPAFRFTREAAQDCEVLGQRIPAGAVLEMA

VGALHHDPEHWPSPETFNPERFTAEARQQHRPFTYLPFGAGPRSCLGVRLGLLEVKLTLL

HVLHKFRFQACPETQVPLQLESKSALGPKNGVYVKIVSR

>332869446

MMEALGFLKLEVNGPMVTVALSVALLALLKWYSTSAFSRLEKLGLRHPKPSPFIGNLTFF

RQGFWESQMELRKLYGPLCGYYLGRRMFIVISEPDMIKQVLVENFSNFTNRMASGLEFKS

VADSVLFLRDKRWEEVRGALMSAFSPEKLNELGLLIMQERIKGHMDGQQAPQRIPPTRLL

KPSGIYVNLHYATLPFCMVPLISQACNLLLAHLKRYAESGDAFDIQRCYCNYTTDVVASV

AFGTPVDSWQAPEDPFVKHCKRFFEFCIPRPILVLLLSFPSIMVPLARILPNKNRDELNG

FFNKLIRNVIALRDQQAAEERRRDFLQMVLDARHSASPMGVQDFDIVREVFSSTGCKPNP

SRQHQPSSMARPLTVDEIVGQAFIFLIAGYEIVTNTLSFATYLLATNPDCQEKLLREVDV

FKEKHMAPEFCSLKEGLPYLDMVIAETLRMYPPAFRFTREAAQDCEVLGQRIPAGAVLEM

AVGALHHDPEHWPSPETFNPERFTAEARQQHRPFTYLPFGAGPRSCLGVRLGLLEVKLTL

LHVLHKFRFQACPETQVPLQLESKSALGPKNGVYIKIVSR

>332243371

MMEALGFLKLEVNGPMVTVALSVALLALLKWYSTSAFSRLEKLGLRHPKPSPFIGNLTFF

RQGFWESQMELRRLYGPLCGYYLGRRMFIVISEPDMIKQVLVENFSNFTNRMASGLEFKS

VANSVLFLRDKRWEEVRGALMSAFSPEKLNELGLLIMQERIKDHMGGQQAPQRIPPTHLL

KLSGIYVNLHYAILPFCMVPLISQACDLLLAHLKRYAESGDAFDIQRCYCNYTTDVVASV

AFGTPVNSWQAPEDPFVKHCKRFFEFCIPRPILVLLLSFPSIMVPLARILPNKNRDELNG

FFNKLIRNVIALRDQQAAEERRRDFLQMVLDARHSVSPMGVQDFDIVRDVFSSTGCKPNP

SRQHQPRPMARPLTVDEIVGQAFIFLIAGYEIVTNTLSFATYLLATNPDCQEKLLREVDL

FKEKHMAPEFCSLEEGLPYLDMVIAETLRMYPPAFRFTREAAQDCEVLGQRIPAGAVLEM

AVGALHHDPEHWPSPETFNPERFKAEARQQHRPFTYLPFGAGPRSCLGVQLGLLEVKLTL

LHVLHKFRFQACPETQVPLQLESKSALGPKNGVYIKIVSR

>EHH17731.1

MMEALGFLKLEVNGPMVTVALSVALLALLKWYSTSAFSRLEKLGLRHPKPSPFIGNLMFF

RQGFWESQMELRKLYGPLCGYYLGRRMFIVISEPDMIKQVLVENFSNFTNRMASGLEFKS

VADSVLFLRDKRWEEVRGALMSAFSPEKLNELGLLIMQERIKGHMGGRQAPQRIPPTRLL

KLRGIYVNLHYAIPPFCMTPLISQACDLLLAHLKRYAESGDAFDIQRCYCNYTTDVVASV

AFGTPVDSQQAPEDPFVKHCKRFFEFCIPRPILVLLLSFPSIMVPLARILPNKNRDELNG

FFNKLIRNVIALRDQQAAEERRRDFLQMVLDARHSASPVGVQDFDMVGDVFSSTRCKPNP

SRQHQAGPMARPLTVDEIVGQAFIFLIAGYEIVTNTLSFATYLLATNPDCQEKLLREVDL

FKEKHMVPEFCSLEEGLPYLDMVIAETLRMYPPAFRFTREAAQDCEVLGQRIPAGAVLEM

AVGALHHDPEHWPSPETFDPERFTAEAQQQHRPFTYLPFGAGPRSCLGVRLGLLEVKLTL

LHVLHKFQFQACPETQVPLQLESKSALGPKNGVYIKIVSR

>390467071

MMEALGFLKFEVNGPMVMVALSVVLLALLKWYSMSAFSRLETLGIRHPKPSPFIGNLTFF

RQGFWESQMELRKQYGPLCGYYLGRRMFIVISEPDMIKQVLVENFSNFTNRMASGLEFKS

VANSVLFLRDKRWEEVRGALMSVFSPEKLNELGLLIMQKRIKGHMGAQQAPQRIPPTHLL

KLSAIYVNLHYTILPFCMAPLISQACDLLLAHLKRYGDSGDAFDIQRCYCRYTTDVVASV

AFGTPVDSQQAPEDPFVKHCKRFFEFCIPRPILVLLLSFPSIMVPLARILPNKNRDELNG

FFNKLIRNVIALRDQQAAEERRRDFLQMVLDARHSASPVDVQDFDIVRDVFSFIECKPNP

SRQHQPRALARPLTVDEIVGQAFIFLIAGYEIVTNTLSFATYLLATHPDCQEKLLREVDL

FKEKHVAPEFCSLEEDLPYLDMVIAETLRMYPPAFRFTREAAQDCEVLGQHIPAGAVLEM

AVGALHHDPEHWPSPETFDPERFTAEARQQHRPFTYLPFGAGPRSCLGVRLGLLEVKLTL

LHVLHKFRFQACPETQVPLQLESKSALGPKNGVYIKIVPR

>P24557.3

MEALGFLKLEVNGPMVTVALSVALLALLKWYSTSAFSRLEKLGLRHPKPSPFIGNLTFFR

QGFWESQMELRKLYGPLCGYYLGRRMFIVISEPDMIKQVLVENFSNFTNRMASGLEFKSV

ADSVLFLRDKRWEEVRGALMSAFSPEKLNEMVPLISQACDLLLAHLKRYAESGDAFDIQR

CYCNYTTDVVASVAFGTPVDSWQAPEDPFVKHCKRFFEFCIPRPILVLLLSFPSIMVPLA

RILPNKNRDELNGFFNKLIRNVIALRDQQAAEERRRDFLQMVLDARHSASPMGVQDFDIV

RDVFSSTGCKPNPSRQHQPSPMARPLTVDEIVGQAFIFLIAGYEIITNTLSFATYLLATN

PDCQEKLLREVDVFKEKHMAPEFCSLEEGLPYLDMVIAETLRMYPPAFRFTREAAQDCEV

LGQRIPAGAVLEMAVGALHHDPEHWPSPETFNPERFTAEARQQHRPFTYLPFGAGPRSCL

GVRLGLLEVKLTLLHVLHKFRFQACPETQVPLQLESKSALGPKNGVYIKIVSR

>AAA60618.1

MMEALGFLKLEVNGPMVTVALSVALLALLKWYSTSAFSRLEKLGLRHPKPSPFIGNLTFF

RQGFWESQMELRKLYGPLCGYYLGRRMFIVISEPDMIKQVLVENFSNFTNRMASGLEFKS

VADSVLFLRDKRWEEVRGALMSAFSPEKLNEMVPLISQACDLLLAHLKRYAESGDAFDIQ

RCYCNYTTDVVASVPFGTPVDSWQAPEDPFVKHCKRFFEFCIPRPILVLLLSFPSIMVPL

ARILPNKNRDELNGFFNKLIRNVIALRDQQAAEERRRDFLQMVLDARHSASPMGVQDFDI

VRDVFSSTGCKPNPSRQHQPSPMARPLTVDEIVGQAFIFLIAGYEIITNTLSFATYLLAT

NPDCQEKLLREVDVFKEKHMAPEFCSLEEGLPYLDMVIAETLRMYPPAFRFTREAAQDCE

VLGQRIPAGAVLEMAVGALHHDPEHWPSPETFNPERFTAEARQQHRPFTYLPFGAGPRSC

LGVRLGLLEVKLTLLHVLHKFRFQACPETQVPLQLESKSALGPKNGVYIKIVSR

>AAF99275.1

MMEALGFLKLEVNGPMVTVALSVALLALLKWYSTSAFSRLEKLGLRHPKPSPFIGNLTFF

RQGFWESQMELRKLYGPLCGYYLGRRMFIVISEPDMIKQVLVENFSNFTNRMASGLEFKS

VADSVLFLRDKRWEEVRGALMSAFSPEKLNEMVPLISQACDLLLAHLKRYAESGDAFDIQ

RCYCNYTTDVVASVPFGTPVDSWQAPEDPFVKHCKRFFEFCIPRPILVLLLSFPSIMVPL

ARILPNKNRDELNGFFNKLIRNVIALRDQQAAEERRRDFLQMVLDARHSASPMGVQDFDI

VRDVFSSTGCKPNPSRQHQPSPMARPLTVDEIVGQAFIFLIAGYEIITNTLSFATYVLAT

NPDCQEKLLREVDVFKEKHMAPEFCSLEEGLPYLDMVIAETLRMYPPAFRFTREAAQDCE

VLGQRIPAGAVLEMAVGALHHDPEHWPSPETFNPERFTAEARQQHRPFTYLPFGAGPRSC

LGVRLGLLEVKLTLLHVLHKFRFQACPETQVPLQLESKSALGPKNGVYIKIVSR

>AAF99276.1

MMEALGFLKLEVNGPMVTVALSVALLALLKWYSTSAFSRLEKLGLRHPKPSPFIGNLTFF

RQGFWESQMELRKLYGPLCGYYLGRRMFIVISEPDMIKQVLVENFSNFTNRMASGLEFKS

VADSVLFLRDKRWEEVRGALMSAFSPEKLNEMVPLISQACDLLLAHLKRYAESGDAFDIQ

RCYCNYTTDVVASVPFGTPVDSWQAPEDPFVKHCKRFFEFCIPRPILVLLLSFPSIMVPL

ARILPNKNRDELNGFFNKLIRNVIALRDQQAAEERRRDFLQMVLDARHSASPMGVQDFDI

VRDVFSSTGCKPNPSRQHQPSPMARPLTVDEIVGQAFIFLIAGYEIITNTLSFATYLLAT

NPDCQEKLLREVDVFKEKHMAPEFCSLEEGLPYLDMVIAETLRMYPPAFRFTREAAEDCE

VLGQRIPAGAVLEMAVGALHHDPEHWPSPETFNPERFTAEARQQHRPFTYLPFGAGPRSC

LGVRLGLLEVKLTLLHVLHKFRFQACPETQVPLQLESKSALGPKNGVYIKIVSR

>AAF99277.1

MMEALGFLKLEVNGPMVTVALSVALLALLKWYSTSAFSRLEKLGLRHPKPSPFIGNLTFF

RQGFWESQMELRKLYGPLCGYYLGRRMFIVISEPDMIKQVLVENFSNFTNRMASGLEFKS

VADSVLFLRDKRWEEVRGALMSAFSPEKLNEMVPLISQACDLLLAHLKRYAESGDAFDIQ

RCYCNYTTDVVASVPFGTPVDSWQAPEDPFVKHCKRFFEFCIPRPILVLLLSFPSIMVPL

ARILPNKNRDELNGFFNKLIRNVIALRDQQAAEERRRDFLQMVLDARHSASPMGVQDFDI

VRDVFSSTGCKPNPSRQHQPSPMARPLTVDEIVGQAFIFLIAGYEIITNTLSFATYLLAT

NPDCQEKLLREVDVFKEKHMAPEFCSLEEGLPYLDMVIAETLRMYPPAFRFTREAAQDCE

VLGQRIPAGAVLEMAVGALHHDPEHWPSPKTFNPERFTAEARQQHRPFTYLPFGAGPRSC

LGVRLGLLEVKLTLLHVLHKFRFQACPETQVPLQLESKSALGPKNGVYIKIVSR

>AAF99273.1

MMEALGFLKLEVNGPMVTVALSVALLALLKWYSTSAFSRLEKLGLRHPKPSPFIGNLTFF

RQGFWESQMELRKLYGPLCGYYLGRRMFIVISEPDMIKQVLVENFSNFTNRMASGLEFKS

VADSVLFLRDKRWEEVRGALMSAFSPEKLNEMVPLISQACELLLAHLKRYAESGDAFDIQ

RCYCNYTTDVVASVPFGTPVDSWQAPEDPFVKHCKRFFEFCIPRPILVLLLSFPSIMVPL

ARILPNKNRDELNGFFNKLIRNVIALRDQQAAEERRRDFLQMVLDARHSASPMGVQDFDI

VRDVFSSTGCKPNPSRQHQPSPMARPLTVDEIVGQAFIFLIAGYEIITNTLSFATYLLAT

NPDCQEKLLREVDVFKEKHMAPEFCSLEEGLPYLDMVIAETLRMYPPAFRFTREAAQDCE

VLGQRIPAGAVLEMAVGALHHDPEHWPSPETFNPERFTAEARQQHRPFTYLPFGAGPRSC

LGVRLGLLEVKLTLLHVLHKFRFQACPETQVPLQLESKSALGPKNGVYIKIVSR

>AAF99279.1

MMEALGFLKLEVNGPMVTVALSVALLALLKWYSTSAFSRLEKLGLRHPKPSPFIGNLTFF

RQGFWESQMELRKLYGPLCGYYLGRRMFIVISEPDMIKQVLVENFSNFTNRMASGLEFKS

VADSVLFLRDKRWEEVRGALMSAFSPEKLNEMVPLISQACDLLLAHLKRYAESGDAFDIQ

RCYCNYTTDVVASVPFGTPVDSWQAPEDPFVKHCKRFFEFCIPRPILVLLLSFPSIMVPL

ARILPNKNRDELNGFFNKLIRNVIALRDQQAAEERRRDFLQMVLDARHSASPMGVQDFDI

VRDVFSSTGCKPNPSRQHQPSPMARPLTVDEIVGQAFIFLIAGYEIITNTLSFATYLLAT

NPDCQEKLLREVDVFKEKHMAPEFCSLEEGLPYLDMVIAETLRMYPPAFRFTREAAQDCE

VLGQRIPAGAVLEMAVGALHHDPEHWPSPETFNPERFTAEARQQHQPFTYLPFGAGPRSC

LGVRLGLLEVKLTLLHVLHKFRFQACPETQVPLQLESKSALGPKNGVYIKIVSR

>397484569

MFKQKAEQQQSTVPIRAARSASCPIWKTTTLGSQRKMEALGFLKLEVNGPMVTVALSVAL

LALLKWYSTSAFSRLEKLGLRHPKPSPFIGNLTFFRQGFWESQMELRKLYGPLCGYYLGR

RMFIVISEPDMIKQVLVENFSNFTNRMASGLEFKSVADSVLFLRDKRWEEVRGALMSAFS

PEKLNEMVPLISQACDLLLAHLKRYAESGDAFDIQRCYCNYTTDVVASVAFGTPVDSWQA

PEDPFVKHCKRFFEFCIPRPILVLLLSFPSIMVPLARILPNKNRDELNGFFNKLIRNVIA

LRDQQAAEERRRDFLQMVLDARHSASPMGVQDFDIVREVFSSTGCKPNPSRQHQPSSMAR

PLTVDEIVGQAFIFLIAGYEIVTNTLSFATYLLATNPDCQEKLLREVDVFKEKHMAPEFC

SLEEGLPYLDMVIAETLRMYPPAFRFTREAAQDCEVLGQRIPAGAVLEMAVGALHHDPEH

WPSPETFNPERFTAEARQQHRPFTYLPFGAGPRSCLGVRLGLLEVKLTLLHVLHKFRFQA

CPETQVPLQLESKSALGPKNGVYVKIVSR

>AAF99274.1

MMEALGFLKLEVNGPMVTVALSVALLALLKWYSTSAFSRLEKLGLRHPKPSPFIGNLTFF

RQGFWESQMELRKLYGPLCGYYLGRRMFIVISEPDMIKQVLVENFSNFTNRMASGLEFKS

VADSVLFLRDKRWEEVRGALMSAFSPEKLNEMVPLISQACDLLLAHLKRYAESGDAFDIQ

RCYCNYTTDVVASVPFGTPVDSWQAPEDPFVKHCKRFFEFCIPRPILVLLLSFPSIMVPL

ARILPSKNRDELNGFFNKLIRNVIALRDQQAAEERRRDFLQMVLDARHSASPMGVQDFDI

VRDVFSSTGCKPNPSRQHQPSPMARPLTVDEIVGQAFIFLIAGYEIITNTLSFATYLLAT

NPDCQEKLLREVDVFKEKHMAPEFCSLEEGLPYLDMVIAETLRMYPPAFRFTREAAQDCE

VLGQRIPAGAVLEMAVGALHHDPEHWPSPETFNPERFTAEARQQHRPFTYLPFGAGPRSC

LGVRLGLLEVKLTLLHVLHKFRFQACPETQVPLQLESKSALGPKNGVYIKIVSR

>AAF99278.1

MMEALGFLKLEVNGPMVTVALSVALLALLKWYSTSAFSRLEKLGLRHPKPSPFIGNLTFF

RQGFWESQMELRKLYGPLCGYYLGRRMFIVISEPDMIKQVLVENFSNFTNRMASGLEFKS

VADSVLFLRDKRWEEVRGALMSAFSPEKLNEMVPLISQACDLLLAHLKRYAESGDAFDIQ

RCYCNYTTDVVASVPFGTPVDSWQAPEDPFVKHCKRFFEFCIPRPILVLLLSFPSIMVPL

ARILPNKNRDELNGFFNKLIRNVIALRDQQAAEERRRDFLQMVLDARHSASPMGVQDFDI

VRDVFSSTGCKPNPSRQHQPSPMARPLTVDEIVGQAFIFLIAGYEIITNTLSFATYLLAT

NPDCQEKLLREVDVFKEKHMAPEFCSLEEGLPYLDMVIAETLRMYPPAFRFTREAAQDCE

VLGQRIPAGAVLEMAVGALHHDPEHWPSPENFNPERFTAEARQQHRPFTYLPFGAGPRSC

LGVRLGLLEVKLTLLHVLHKFRFQACPETQVPLQLESKSALGPKNGVYIKIVSR

>BAA07011.1

MEALGFLKLEVNGPMVTVALSVALLALLKWYSTSAFSRLEKLGLRHPKPSPFIGNLTFFR

QGFWESQMELRKLYGPLCGYYLGRRMFIVISEPDMIKQVLVENFSNFTNRMASGLEFKSV

ADSVLFLRDKRWEEVRGALMSAFSPEKLNEMVPLISQACDLLLAHLKRYAESGDAFDIQR

CYCNYTTDVVASVAFGTPVDSWQAPEDPFVKHCKRFFEFCIPRPILVLLLSFPSIMVPLA

RILPNKNRDELNGFFNKLIRNVIALRDQQAAEERRRDFLQMVLDARHSASPMGVQDFDIV

RDVFSSTGCKPNPSRQHQPSPMARPLTVDEIVGQAFIFLIAGYEIITNTLSFATYLLATN

PDCQEKLLREVDVFKEKHMAPEFCSLEEGLPYLDMVIAETLRMYPPAFRFTREAAQDCEV

LGQRIPAGAVLEMAVGALHHDPEHWPSPETFNPERFTAEARQQHRPFTYLPFGAGPRSCL

GVHLGLLEVKLTLLHVLHKFRFQACPETQVPLQLESKSALGPKNGVYIKIVSR

>AAF99272.1

MMEALGFLKLEVNGPMVTVALSVALLALLKWYSTSAFSRLEKLGLRHPKPSPFIGNLTFF

HQGFWESQMELRKLYGPLCGYYLGRRMFIVISEPDMIKQVLVENFSNFTNRMASGLEFKS

VADSVLFLRDKRWEEVRGALMSAFSPEKLNEMVPLISQACDLLLAHLKRYAESGDAFDIQ

RCYCNYTTDVVASVPFGTPVDSWQAPEDPFVKHCKRFFEFCIPRPILVLLLSFPSIMVPL

ARILPNKNRDELNGFFNKLIRNVIALRDQQAAEERRRDFLQMVLDARHSASPMGVQDFDI

VRDVFSSTGCKPNPSRQHQPSPMARPLTVDEIVGQAFIFLIAGYEIITNTLSFATYLLAT

NPDCQEKLLREVDVFKEKHMAPEFCSLEEGLPYLDMVIAETLRMYPPAFRFTREAAQDCE

VLGQRIPAGAVLEMAVGALHHDPEHWPSPETFNPERFTAEARQQHRPFTYLPFGAGPRSC

LGVRLGLLEVKLTLLHVLHKFRFQACPETQVPLQLESKSALGPKNGVYIKIVSR

>114616291

MMEALGFLKLEVNGPMVTVALSVALLALLKWYSTSAFSRLEKLGLRHPKPSPFIGNLTFF

RQGFWESQMELRKLYGPLCGYYLGRRMFIVISEPDMIKQVLVENFSNFTNRMASGLEFKS

VADSVLFLRDKRWEEVRGALMSAFSPEKLNEMVPLISQACNLLLAHLKRYAESGDAFDIQ

RCYCNYTTDVVASVAFGTPVDSWQAPEDPFVKHCKRFFEFCIPRPILVLLLSFPSIMVPL

ARILPNKNRDELNGFFNKLIRNVIALRDQQAAEERRRDFLQMVLDARHSASPMGVQDFDI

VREVFSSTGCKPNPSRQHQPSSMARPLTVDEIVGQAFIFLIAGYEIVTNTLSFATYLLAT

NPDCQEKLLREVDVFKEKHMAPEFCSLKEGLPYLDMVIAETLRMYPPAFRFTREAAQDCE

VLGQRIPAGAVLEMAVGALHHDPEHWPSPETFNPERFTAEARQQHRPFTYLPFGAGPRSC

LGVRLGLLEVKLTLLHVLHKFRFQACPETQVPLQLESKSALGPKNGVYIKIVSR

>332243369

MMEALGFLKLEVNGPMVTVALSVALLALLKWYSTSAFSRLEKLGLRHPKPSPFIGNLTFF

RQGFWESQMELRRLYGPLCGYYLGRRMFIVISEPDMIKQVLVENFSNFTNRMASGLEFKS

VANSVLFLRDKRWEEVRGALMSAFSPEKLNEMVPLISQACDLLLAHLKRYAESGDAFDIQ

RCYCNYTTDVVASVAFGTPVNSWQAPEDPFVKHCKRFFEFCIPRPILVLLLSFPSIMVPL

ARILPNKNRDELNGFFNKLIRNVIALRDQQAAEERRRDFLQMVLDARHSVSPMGVQDFDI

VRDVFSSTGCKPNPSRQHQPRPMARPLTVDEIVGQAFIFLIAGYEIVTNTLSFATYLLAT

NPDCQEKLLREVDLFKEKHMAPEFCSLEEGLPYLDMVIAETLRMYPPAFRFTREAAQDCE

VLGQRIPAGAVLEMAVGALHHDPEHWPSPETFNPERFKAEARQQHRPFTYLPFGAGPRSC

LGVQLGLLEVKLTLLHVLHKFRFQACPETQVPLQLESKSALGPKNGVYIKIVSR

>109068420

MMEALGFLKLEVNGPMVTVALSVALLALLKWYSTSAFSRLEKLGLRHPKPSPFIGNLMFF

RQGFWESQMELRKLYGPLCGYYLGRRMFIVISEPDMIKQVLVENFSNFTNRMASGLEFKS

VADSVLFLRDKRWEEVRGALMSAFSPEKLNEMTPLISQACDLLLAHLKRYAESGDAFDIQ

RCYCNYTTDVVASVAFGTPVDSQQAPEDPFVKHCKRFFEFCIPRPILVLLLSFPSIMVPL

ARILPNKNRDELNGFFNKLIRNVIALRDQQAAEERRRDFLQMVLDARHSASPVGVQDFDM

VGDVFSSTRCKPNPSRQHQAGPMARPLTVDEIVGQAFIFLIAGYEIVTNTLSFATYLLAT

NPDCQEKLLREVDLFKEKHMVPEFCSLEEGLPYLDMVIAETLRMYPPAFRFTREAAQDCE

VLGQRIPAGAVLEMAVGALHHDPEHWPSPETFDPERFTAEAQQQHRPFTYLPFGAGPRSC

LGVRLGLLEVKLTLLHVLHKFQFQACPETQVPLQLESKSALGPKNGVYIKIVSR

>BAE72925.1

MMEALGFLKLEVNGPMVTVALSVALLALLKWYSTSAFSRLEKLGLRHPKPSPFIGNLMFF

RQGFWESQMELRKLYGPLCGYYLGRRMFIVISEPDMIKQVLVENFSNFTNRMASGLEFKS

VADSVLFLRDKRWEEVRGALMSAFSPEKLNEMTPLISQACDLLLAHLKRYAESGDAFDIQ

RCYRNYTTDVVASVAFGTPVDSQQAPEDPFVKHCKRFFEFCIPRPILVLLLSFPSIMVPL

ARILPNKNRDELNGFFNKLIRNVIALRDQQAAEERRRDFLQMVLDARHSASPVGVQDFDM

VGDVFSSTRCKPNPSRQHQAGPMARPLTVDEIVGQAFIFLIAGYEIVTNTLSFATYLLAT

NPDCQEKLLREVDLFKEKHMVPEFCSLEEGLPYLDMVIAETLRMYPPAFRFTREAAQDCE

VLGQRIPAGAVLEMAVGALHHDPEHWPSPETFNPERFTAEAQQQHRPFTYLPFGAGPRSC

LGVRLGLLEVKLTLLHVLHKFQFQACPETQVPLQLESKSALGPKNGVYIKIVSR

>Q2PG45.2

MEALGFLKLEVNGPMVTVALSVALLALLKWYSTSAFSRLEKLGLRHPKPSPFIGNLMFFR

QGFWESQMELRKLYGPLCGYYLGRRMFIVISEPDMIKQVLVENFSNFTNRMASGLEFKSV

ADSVLFLRDKRWEEVRGALMSAFSPEKLNEMTPLISQACDLLLAHLKRYAESGDAFDIQR

CYRNYTTDVVASVAFGTPVDSQQAPEDPFVKHCKRFFEFCIPRPILVLLLSFPSIMVPLA

RILPNKNRDELNGFFNKLIRNVIALRDQQAAEERRRDFLQMVLDARHSASPVGVQDFDMV

GDVFSSTRCKPNPSRQHQAGPMARPLTVDEIVGQAFIFLIAGYEIVTNTLSFATYLLATN

PDCQEKLLREVDLFKEKHMVPEFCSLEEGLPYLDMVIAETLRMYPPAFRFTREAAQDCEV

LGQRIPAGAVLEMAVGALHHDPEHWPSPETFNPERFTAEAQQQHRPFTYLPFGAGPRSCL

GVRLGLLEVKLTLLHVLHKFQFQACPETQVPLQLESKSALGPKNGVYIKIVSR

>403276225

MFSWLPVPFSTHRTRFPQGQQDQPPLSSGRPPLWGLRGMMEALGFLKLEVNGPMVTVALS

VVLLALLKWYSTSAFSRLEKLGIRHPKPSPFIGNLTFFRQGFWESQMELRKLYGPLCGYY

LGRRMFIVISEPDMIKQVLVENFSNFTNRMASGLEFKSVADSVLFLRDKRWEEVRGALMS

VFSPEKLNKMVPLISQACDLLLAHLKRYGDSGDAFDIQRCYCCYTTDVVASVAFGTPVDS

QQAPEDPFVKHCKRFFEFCIPRPILVLLLSFPFIMVPLARILPNKKRDELNGFFNKLIRN

VIALRDQQAAEERRRDFLQMVLDARHSASPVDVQDFDIVRDVFSFTKCKPNPSRQHQPRA

MARPLTVDEIVGQAFIFLIAGYEIVTNTLSFATYLLATHPDCQEKLLREVDLFKEKHAAP

EFCSLEEGLPYLDMVIAETLRMYPPAFRFTREAAQDCEVLGQQIPAGAVLEVAVGALHHD

PEHWPSPETFDPERFTAEARRQHRPFTYLPFGAGPRSCLGVRLGLLEVKLTLLHVLHKFR

FQACPETQVPLQLESTSALGPKNGVYIKIVPR

>390467069

MMEALGFLKFEVNGPMVMVALSVVLLALLKWYSMSAFSRLETLGIRHPKPSPFIGNLTFF

RQGFWESQMELRKQYGPLCGYYLGRRMFIVISEPDMIKQVLVENFSNFTNRMASGLEFKS

VANSVLFLRDKRWEEVRGALMSVFSPEKLNEMAPLISQACDLLLAHLKRYGDSGDAFDIQ

RCYCRYTTDVVASVAFGTPVDSQQAPEDPFVKHCKRFFEFCIPRPILVLLLSFPSIMVPL

ARILPNKNRDELNGFFNKLIRNVIALRDQQAAEERRRDFLQMVLDARHSASPVDVQDFDI

VRDVFSFIECKPNPSRQHQPRALARPLTVDEIVGQAFIFLIAGYEIVTNTLSFATYLLAT

HPDCQEKLLREVDLFKEKHVAPEFCSLEEDLPYLDMVIAETLRMYPPAFRFTREAAQDCE

VLGQHIPAGAVLEMAVGALHHDPEHWPSPETFDPERFTAEARQQHRPFTYLPFGAGPRSC

LGVRLGLLEVKLTLLHVLHKFRFQACPETQVPLQLESKSALGPKNGVYIKIVPR

>AAH14117.1

MELRKLYGPLCGYYLGRRMFIVISEPDMIKQVLVENFSNFTNRMASGLEFKSVADSVLFL

RDKRWEEVRGALMSAFSPEKLNEMVPLISQACDLLLAHLKRYAESGDAFDIQRCYCNYTT

DVVASVAFGTPVDSWQAPEDPFVKHCKRFFEFCIPRPILVLLLSFPSIMVPLARILPNKN

RDELNGFFNKLIRNVIALRDQQAAEERRRDFLQMVLDARHSASPMGVQDFDIVRDVFSST

GCKPNPSRQHQPSPMARPLTVDEIVGQAFIFLIAGYEIITNTLSFATYLLATNPDCQEKL

LREVDVFKEKHMAPEFCSLEEGLPYLDMVIAETLRMYPPAFRFTREAAQDCEVLGQRIPA

GAVLEMAVGALHHDPEHWPSPETFNPERFTAEARQQHRPFTYLPFGAGPRSCLGVRLGLL

EVKLTLLHVLHKFRFQACPETQVPLQLESKSALGPKNGVYIKIVSR

>397484573

MELRKLYGPLCGYYLGRRMFIVISEPDMIKQVLVENFSNFTNRMASGLEFKSVADSVLFL

RDKRWEEVRGALMSAFSPEKLNEMVPLISQACDLLLAHLKRYAESGDAFDIQRCYCNYTT

DVVASVAFGTPVDSWQAPEDPFVKHCKRFFEFCIPRPILVLLLSFPSIMVPLARILPNKN

RDELNGFFNKLIRNVIALRDQQAAEERRRDFLQMVLDARHSASPMGVQDFDIVREVFSST

GCKPNPSRQHQPSSMARPLTVDEIVGQAFIFLIAGYEIVTNTLSFATYLLATNPDCQEKL

LREVDVFKEKHMAPEFCSLEEGLPYLDMVIAETLRMYPPAFRFTREAAQDCEVLGQRIPA

GAVLEMAVGALHHDPEHWPSPETFNPERFTAEARQQHRPFTYLPFGAGPRSCLGVRLGLL

EVKLTLLHVLHKFRFQACPETQVPLQLESKSALGPKNGVYVKIVSR

>109068426

MFFRQGFWESQMELRKLYGPLCGYYLGRRMFIVISEPDMIKQVLVENFSNFTNRMASGLE

FKSVADSVLFLRDKRWEEVRGALMSAFSPEKLNEMTPLISQACDLLLAHLKRYAESGDAF

DIQRCYCNYTTDVVASVAFGTPVDSQQAPEDPFVKHCKRFFEFCIPRPILVLLLSFPSIM

VPLARILPNKNRDELNGFFNKLIRNVIALRDQQAAEERRRDFLQMVLDARHSASPVGVQD

FDMVGDVFSSTRCKPNPSRQHQAGPMARPLTVDEIVGQAFIFLIAGYEIVTNTLSFATYL

LATNPDCQEKLLREVDLFKEKHMVPEFCSLEEGLPYLDMVIAETLRMYPPAFRFTREAAQ

DCEVLGQRIPAGAVLEMAVGALHHDPEHWPSPETFDPERFTAEAQQQHRPFTYLPFGAGP

RSCLGVRLGLLEVKLTLLHVLHKFQFQACPETQVPLQLESKSALGPKNGVYIKIVSR

>114616301

MELRKLYGPLCGYYLGRRMFIVISEPDMIKQVLVENFSNFTNRMASGLEFKSVADSVLFL

RDKRWEEVRGALMSAFSPEKLNEMVPLISQACNLLLAHLKRYAESGDAFDIQRCYCNYTT

DVVASVAFGTPVDSWQAPEDPFVKHCKRFFEFCIPRPILVLLLSFPSIMVPLARILPNKN

RDELNGFFNKLIRNVIALRDQQAAEERRRDFLQMVLDARHSASPMGVQDFDIVREVFSST

GCKPNPSRQHQPSSMARPLTVDEIVGQAFIFLIAGYEIVTNTLSFATYLLATNPDCQEKL

LREVDVFKEKHMAPEFCSLKEGLPYLDMVIAETLRMYPPAFRFTREAAQDCEVLGQRIPA

GAVLEMAVGALHHDPEHWPSPETFNPERFTAEARQQHRPFTYLPFGAGPRSCLGVRLGLL

EVKLTLLHVLHKFRFQACPETQVPLQLESKSALGPKNGVYIKIVSR

>332243377

MELRRLYGPLCGYYLGRRMFIVISEPDMIKQVLVENFSNFTNRMASGLEFKSVANSVLFL

RDKRWEEVRGALMSAFSPEKLNEMVPLISQACDLLLAHLKRYAESGDAFDIQRCYCNYTT

DVVASVAFGTPVNSWQAPEDPFVKHCKRFFEFCIPRPILVLLLSFPSIMVPLARILPNKN

RDELNGFFNKLIRNVIALRDQQAAEERRRDFLQMVLDARHSVSPMGVQDFDIVRDVFSST

GCKPNPSRQHQPRPMARPLTVDEIVGQAFIFLIAGYEIVTNTLSFATYLLATNPDCQEKL

LREVDLFKEKHMAPEFCSLEEGLPYLDMVIAETLRMYPPAFRFTREAAQDCEVLGQRIPA

GAVLEMAVGALHHDPEHWPSPETFNPERFKAEARQQHRPFTYLPFGAGPRSCLGVQLGLL

EVKLTLLHVLHKFRFQACPETQVPLQLESKSALGPKNGVYIKIVSR

>395837562

MEALGFLKLEVSVPMVMVALLVMLLALLKWYSMSAFSRLEKLGIRHPKPSPFIGNLTFFR

QGFWESQLELRRLYGPLCGYYLGRRIFIVISEPKMIKQVLVENFSNFTNRTALGLEFKPV

ANSVLFLRDKRWEEVRGVLTPAFSPEKLNELTPLISQACDLLLAHLKGYAESGEAFDIQR

CYCCYTTDVVASVAFGTQVDSWRAPEDPFVEHCRRFFQFSIPKPLLVLILSFPSIMVPLA

RILPNKNRDELNGFFNKLIRNVIALRDQQAAEERQRDFLQMVLDARNSSSPVGVEDFDIV

REVFSSTKCQVDPSRQHPPRALSQPLSVDEVVGQAFIFLIAGYEIVTNTLSFATYLLATN

PDCQEKLLREVDLFQEKHAAPEYCSLQEGLPYLDMVIAETLRMYPPAFRFTREAAQDLEV

LGRRIPAGTVLEMAVGALHHDPEHWPSPEIFNPERFTDEAQQQRQPFTYLPFGAGPRSCL

GVRLGLLEVKLTLLHILSKFRFQVCPKTQVPLQLESKSALGPKNGVYVKIVPR

>EAW83936.1

MMEALGFLKLEVNGPMVTVALSVALLALLKWYSTSAFSRLEKLGLRHPKPSPFIGNLTFF

RQGFWESQMELRKLYGPLCGYYLGRRMFIVISEPDMIKQVLVENFSNFTNRMASGLEFKS

VADSVLFLRDKRWEEVRGALMSAFSPEKLNEMVPLISQACDLLLAHLKRYAESGDAFDIQ

RCYCNYTTDVVASVAFGTPVDSWQAPEDPFVKHCKRFFEFCIPRPILVLLLSFPSIMVPL

ARILPNKNRDELNGFFNKLIRNVIALRDQQAAEERRRDFLQMVLDARHSASPMGVQDFDI

VRDVFSSTGCKPNPSRQHQPSPMARPLTVDEIVGQAFIFLIAGYEIITNTLSFATYLLAT

NPDCQEKLLREVDVFKEKHMAPEFCSLEEGLPYLDMVIAETLRMYPPAFRFTREAAQDCE

VLGQRIPAGAVLEMAVGALHHDPEHWPSPETFNPERYRCS

>AAA60617.1

MMEALGFLKLEVNGPMVTVALSVALLALLKWYSTSAFSRLEKLGLRHPKPSPFIGNLTFF

RQGFWESQMELRKLYGPLCGYYLGRRMFIVISEPDMIKQVLVENFSNFTNRMASGLEFKS

VADSVLFLRDKRWEEVRGALMSAFSPEKLNEMVPLISQACDLLLAHLKRYAESGDAFDIQ

RCYCNYTTDVVASVPFGTPVDSWQAPEDPFVKHCKRFFEFCIPRPILVLLLSFPSIMVPL

ARILPNKNRDELNGFFNKLIRNVIALRDQQAAEERRRDFLQMVLDARHSASPMGVQDFDI

VRDVFSSTGCKPNPSRQHQPSPMARPLTVDEIVGQAFIFLIAGYEIITNTLSFATYLLAT

NPDCQEKLLREVDVFKEKHMAPEFCSLEEGLPYLDMVIAETLRMYPPAFRFTREAAQDCE

VLGQRIPAGAVLEMAVGALHHDPEHWPSPETFNPERYRCS

>397484571

MVLHISILKTGEVRPQTSQAFSFYWKLDIFPPGFLGKPNGAQKAVWTSVWVRRKLNVSIM

YYLGRRMFIVISEPDMIKQVLVENFSNFTNRMASGLEFKSVADSVLFLRDKRWEEVRGAL

MSAFSPEKLNEMVPLISQACDLLLAHLKRYAESGDAFDIQRCYCNYTTDVVASVAFGTPV

DSWQAPEDPFVKHCKRFFEFCIPRPILVLLLSFPSIMVPLARILPNKNRDELNGFFNKLI

RNVIALRDQQAAEERRRDFLQMVLDARHSASPMGVQDFDIVREVFSSTGCKPNPSRQHQP

SSMARPLTVDEIVGQAFIFLIAGYEIVTNTLSFATYLLATNPDCQEKLLREVDVFKEKHM

APEFCSLEEGLPYLDMVIAETLRMYPPAFRFTREAAQDCEVLGQRIPAGAVLEMAVGALH

HDPEHWPSPETFNPERFTAEARQQHRPFTYLPFGAGPRSCLGVRLGLLEVKLTLLHVLHK

FRFQACPETQVPLQLESKSALGPKNGVYVKIVSR

>BAG62753.1

MVLHISILKTGEVRPQTSQAFSFHWKLDIFPPGFLGKPNGAQKAVWTSVWVRRKLNVSIM

YYLGRRMFIVISEPDMIKQVLVENFSNFTNRMASGLEFKSVADSVLFLRDKRWEEVRGAL

MSAFSPEKLNEMVPLISQACDLLLAHLKRYAESGDAFDIQRCYCNYTTDVVASVAFGTPV

DSWQAPEDPFVKHCKRFFEFCIPRPILVLLLSFPPIMVPLARILPNKNRDELNGFFNKLI

RNVIALRDQQAAEERRRDFLQMVLGARHSASPMGVQDFDIVRDVFSSTGCKPNPSRQHQP

SPMARPLTVDEIVGQAFIFLIAGYEIITNTLSFATYLLATNPDCQEKLLREVDVFKEKHM

APEFCSLEEGLPYLDMVIAETLRMYPPAFRFTREAAQDCEVLGQRIPAGAVLEMAVGALH

HDPEHWPSPETFNPERFTAEARQQHRPFTYLPFGAGPRSCLGVRLGLLEVKLTLLHVLHK

FRFQACPETQVPLQLESKSALGPKNGVYIKIVSR

>114616299

MVLHISILKTGEVRPQTSQAFSFYWKLDIFPPGFLGKPNGAQKAVWTSVWVRRKLNVSIM

YYLGRRMFIVISEPDMIKQVLVENFSNFTNRMASGLEFKSVADSVLFLRDKRWEEVRGAL

MSAFSPEKLNEMVPLISQACNLLLAHLKRYAESGDAFDIQRCYCNYTTDVVASVAFGTPV

DSWQAPEDPFVKHCKRFFEFCIPRPILVLLLSFPSIMVPLARILPNKNRDELNGFFNKLI

RNVIALRDQQAAEERRRDFLQMVLDARHSASPMGVQDFDIVREVFSSTGCKPNPSRQHQP

SSMARPLTVDEIVGQAFIFLIAGYEIVTNTLSFATYLLATNPDCQEKLLREVDVFKEKHM

APEFCSLKEGLPYLDMVIAETLRMYPPAFRFTREAAQDCEVLGQRIPAGAVLEMAVGALH

HDPEHWPSPETFNPERFTAEARQQHRPFTYLPFGAGPRSCLGVRLGLLEVKLTLLHVLHK

FRFQACPETQVPLQLESKSALGPKNGVYIKIVSR

>332243375

MYYLGRRMFIVISEPDMIKQVLVENFSNFTNRMASGLEFKSVANSVLFLRDKRWEEVRGA

LMSAFSPEKLNEMVPLISQACDLLLAHLKRYAESGDAFDIQRCYCNYTTDVVASVAFGTP

VNSWQAPEDPFVKHCKRFFEFCIPRPILVLLLSFPSIMVPLARILPNKNRDELNGFFNKL

IRNVIALRDQQAAEERRRDFLQMVLDARHSVSPMGVQDFDIVRDVFSSTGCKPNPSRQHQ

PRPMARPLTVDEIVGQAFIFLIAGYEIVTNTLSFATYLLATNPDCQEKLLREVDLFKEKH

MAPEFCSLEEGLPYLDMVIAETLRMYPPAFRFTREAAQDCEVLGQRIPAGAVLEMAVGAL

HHDPEHWPSPETFNPERFKAEARQQHRPFTYLPFGAGPRSCLGVQLGLLEVKLTLLHVLH

KFRFQACPETQVPLQLESKSALGPKNGVYIKIVSR

>73978856

MEVLSFLKLEVNGPMVTVALSVVLLALLKWYSTAAFSRLEKLGIRHPKPSPFIGNLPFFC

QGFWESQMELRKLYGPLCGYYLGRRMFIVISEPDMIKQVLVENFHNFTNRMVSGLESKPV

MDSVLFLRDKRWEEVRSVLTSAFSPEKLNEMTPLISQACDLLLAHLKHYAESGDAFDIQR

CYSCYTTDVVASVAFGTQVDSRRAPGDPFVKHCRRFFAYSIPRPILVLILSFPSIMVPLA

RILPNKNRDELNGFFNKLIRNVIALRDQQAAEERRRDFLQLILDARHLATSLGVDSFDMV

RQVFSSTDCTVGPSRPHQPRHLSQPLTLDEIVGQAFIFLIAGYEIITNTLSFATYLLATN

PDCQEKLLAEVDSFKEKYTALDYCSLQEGLPYLDMVIAETLRIYPPAFRFTREAARDCEV

RGQRIPAGAVVEVAVGALHRDPEYWPQPETFNPERFKAEAQRRQQPFTYLPFGAGPRSCL

GVRLGLLEVKLTLLQVLHQFRFEACPETQVPLQLDSKSALGPKNGIYIKIVSR

>EHB04422.1

MEVLGFLKLEVNGLTVTVALSVVLVALLKWYSTSAFSTLEKLGIRYPKPSPFIGNLTFFR

QGFWDGQLELRRLYGPLSGYYLGRRMHIVISDPDMIKQVLVENFSNFTNRMASGLEPKPV

ANSILCLRDKRWEEVRGVLTSVFSPEKLNELGHTQKRIKSHMGVTHCSKVTDTLPAWRSN

YSFVIHYQMMPLINQACDLLLAHLRRCAESGDPCNIQRCYCAYTTDVVASVAFGTQVDSQ

ETPEDPFVLHCRRFFAFSIPSSLLVLILSFPSIMVPLARILPNKNREELNGFFTKLIRNG

IALRDQQAAEERPRDFLQMVLDARCSASSVGVDHFDLVGEALSSAARSSDPSAQRQPRPL

SQPLTVDEIVGQAFLFLIAGHKIVTNTLSFATYLLATNLDCQERLLREVDLFHERQVAPD

YCSLQEGLPYLDMVIAETLRMYPPAFRFTREAARDCEVLGQRIPAGAVLEVAVGALHHDP

ELWPCPETFDPERFTADAQRQRQPFTYLPFGAGPRSCLGVRLGLLEVKLTLLRILSKFRF

DVCPETQVPLQLESTSALGPKNGVYIKLVPR

>301756787

MEVLGFLKLEVNGPMVTVALSVVLLALLKWYSTSAFSRLEKLGIRHPKPSPFIGNLTFFF

QGFWEGQMELRKLYGPLCGYYLGRRMFIVISEPDMIEQVLVENFHNFTNRMVSGLESKPV

MDSILFLRDRRWEEVRSVLTSAFSPEKLNEMTPLISQACDLLLAHLKRCAESGDAFDIQR

CYSCYTTDVVASVAFGTQVDSRRAPEDPFVTHCRRFFTYSVPRPVLVLILSFPSIMVPLA

RILPNKNRDELNGFFNKLIRNVIALRDQQASEERRRDFLQLILDARHSAPSVGVDSFDMV

RQVFSSTDYATGPSQPHQPGRLSKPLTVDEVVGQAFIFLIAGYEIVTNTLSFATYLLATN

PDCQEKLLTEVDGFHEKYPAPDYCSLQEGLPYLDMVIAETLRIYPPAFRFTRETARDCEV

RGQRIPAGTVVEVAVGALHHDPEYWPQPETFNPERFKAEARRRQRPFTYLPFGAGPRSCL

GVRLGLLEVKLTLLHVLRRFRFEACAETQVPLPLESKSALGPKSGIYIKIVSR

>Q2KIG5.1

MEVLGLFRLEVSGPMVTVALSVVFLALLKWYSTSAFSRLEKLGIRHPKPSPFIGNLAFFR

QGFWESHMELRKQYGPLSGYYLGRLMFIVISEPDMIEQVLVEKFSNFTNRMATGLEPKPV

ADSVLFLRDKRWEEVRSVLTVAFSPEKLSEMTPLISRACDVLLAHLERHAQSGEAFDIQR

TYCCYTTDVVASVAFGTEVNSQEAPEHPFVEHCRRFFASSIPKPLLVLLLSFPSIMVPLA

RILPNKNRDELNGFFNKLIRNVIALRDQQAAEERRRDFLQMVQDVRHSAATVGVEKFDIV

RQVFSATKCPANPPRRHSPRPLSKPLSVDEVVGQAFIFLIAGYEIVTNTLSFATYLLATN

PECQEKLLEEVDCFSKEHLAPEYCSLQEGLPYLDMVIKETLRMYPPAFRFTRVAAQDCEV

LGQRIPAGAVLETAVGALHYDPEHWPNPENFNPERFTAEAQQRRRPYTYLPFGAGPRSCL

GVRLGLLELKLTLLHILRKFRFEACPETQVPLQLESKSALGPKNGVYIRIVSR

>CAH92354.1

MMEALGFLKLEVNGPMVTVALSVALLALLKWYSTSAFSRLEKLGLRHPKPFPFIGNLTFF

RQGFWESQMELRKLYGPLCGYYLGRRMFIVVSEPDMIKQVLVENFSNFTNRMASGLEFKS

VADSVLFLRDKRWEEVRGALMSAFSPEKLNEMVPLISQACDLLLAHLKRYAESGDAFDIQ

RCYCNYTTDVVASLAFGTPVDSWQAPEDPFVKHCKRFFEFCIPRPILVLLLSFPSIMVPL

ARILPNKNRDELNGFFNELIRNVIALRDQQAAEERRRDFLQMVLDARHSASPMGVQDFDI

IRDVFSSTGCKPNPPRQHQPRPMARPLTVDEIVGQAFIFLIAGYEIVTNTLSFATYLLAT

NPDCQEKLLREVDLFKEKHMAPEFCSLEEGLPYLDMVIAETLRMYPPAFRFTREAAQDCE

VLGQHIPAGAVLEMAVGALHHDPEHWPSPETFNPERYRCS

>DAA30276.1

MEVLGLFRLEVSGPMVTVALSVVFLALLKWYSTSAFSRLEKLGIRHPKPSPFIGNLAFFR

QGFWESHMELRKQYGPLSGYYLGRLMFIVISEPDMIEQVLVEKFSNFTNRMATGLEPKPV

ADSVLFLRDKRWEEVRSVLTVAFSPEKLSEMTPLISRACDVLLAHLERHAQSGEAFDIQR

TYCCYTTDVVASVAFGTEVNSQEAPEHPFVEHCRRFFASSIPKPLLVLLLSFPSIMVPLA

RILPNKNRDELNGFFNKLIRNVIALRDQQAAEERRRDFLQMVQDVRHSAATVGVENFDIV

RQVFSATKCPANPPRRHLPRPLSKPLSVDEVVGQAFIFLIAGYEIVTNTLSFATYLLATN

PECQEKLLEEVDCFSKEHLAPEYCSLQEGLPYLDMVIKETLRMYPPAFRFTRVAAQDCEV

LGQRIPAGAVLETAVGALHYDPEHWPNPENFNPERFTAEAQQRRRPYTYLPFGAGPRSCL

GVRLGLLELKLTLLHILRKFRFEACPETQVPLQLESKSALGPKNGVYIRIVSR

>338724406

MSAFSRLEKLGIRHPKPSPFIGNLMFFRQGFWESQMELRKLYGPRCGYYFGRRMVIVISE

PDMIKQVLVENFSNFTNRMASGLESQPVANSILFLRDKRWEEVRSVLTSALSPEKLNEMM

PLISQACDLLLAHLQRYAESGDACNIQRCYCCYTTDVVASIAFGTQVDSQTAPEDPFVEH

CRRFFAFSIPRPILVLILSFPSIMVPLARILPNKNRDELNGFFNKLIRNVIALRDQQAAE

ERRRDFLQMVLDARHSAASVGVENFDIVRQVFSSTKSTANPSQRHQPRPLSRPLTVDEVV

GQAFIFLIAGYEIVTNTLSFATYLLATNPDCQEKLLREVDHFNEKHTAPEYCSLQEGLPY

LDMVIRETLRMYPPAFRFTREAVQDCELLGQRIPAGTVIETAVGALHHDPEHWPRPEAFD

PERFTAEAWRRQRPFTYLPFGAGPRSCLGVRLGLLEVKLTLLHLLRKFRFEACAETQVPL

QLESKSALGPKNGVYIKIVPR

>P47787.1

MEVLGFLSPELNGPMVTMALAVVLLALLKWYSTSAFSRLEKLGIRHPKPSPFIGNLTFFR

QGFWESHMELRKQYGPLSGYYLGRRMIVVISDPDMIKQVLAEKFSNFTNRMATGLESKPV

ADSILFLRDKRWEEVRSVLTSAFSPKKLNKLTPLISQACDLLLAHLERYAESGDAFDIQR

CYCCYTTDVVASVAFGTQVNSSEEPEHPFVKHCRRFFAFSVPRLILVLILSFPSIMVPLA

RILPNKKRDEVNGFFNKLIRNVIALRDQQAAEERRQDFLQMVLDLRHSAPSVGVENFDIV

RQAFSSAKGCPADPSQPHLPRPLSKPLTVDEVVGQAFLFLIAGYEIITNTLSFVTYLLAT

NPDCQEKLLREVDDFSKKHPSPEHCSLQQGLPYLDMVLSETLRMYPPAFRFTREAARDCE

VLGQRIPAGTVLEVAVGALHHDPKHWPHPETFDPERFTAEAQRLQQPFTYLPFGAGPRSC

LGVQLGLLEIKLTLLHILRKFRFEACPETQVPLQLESKSALSPKNGVYIRIVPR

>109068422

MMEALGFLKLEVNGPMVTVALSVALLALLKWYSTSAFSRLEKLGLRHPKPSPFIGNLMFF

RQGFWESQMELRKLYGPLCGYYLGRRMFIVISEPDMIKQVLVENFSNFTNRMASGLEFKS

VADSVLFLRDKRWEEVRGALMSAFSPEKLNEMTPLISQACDLLLAHLKRYAESGDAFDIQ

RCYCNYTTDVVASVAFGTPVDSQQAPEDPFVKHCKRFFEFCIPRPILVLLLSFPSIMVPL

ARILPNKNRDELNGFFNKLIRNVIALRDQQAAEERRRDFLQMVLDARHSASPVGVQDFDM

VGDVFSSTRCKPNPSRQHQAGPMARPLTVDEIVGQAFIFLIAGYEIVTNTLSFATYLLAT

NPDCQEKLLREVDLFKEKHMVPEFCSLEEGLPYLDMVIAETLRMYPPAFRFTREAAQDCE

VLGQRIPAGAVLEMAVGALHHDPEHWPSPETFDPERYRCS

>AAC01761.1

MMEALGFLKLEVNGPMVTVALSVALLALLKWYSTSAFSRLEKLGLRHPKPSPFIGNLTFF

RQGFWESQMELRKLYGPLCGYYLGRRMFIVISEPDMIKQVLVENFSNFTNRMASGLEFKS

VADSVLFLRDKRWEEVRGALMSAFSPEKVNEMVPLISQACDLLLAHLKRYAESGDAFDIQ

RCYCNYTTDVVASVRFGTPVDSWQAPEDPFVKHCKRFFEFCIPRPILVLLLSFPSIMVPL

ARILPNKNRDELNGFFNKLIRNVIALRDQQAAEERRRDFLQMVLDARHSASPMGVQDFDI

VRDVFSSTGCKPNPSRQHQPSPMARPLTVDEIVGQAFIFLIAGYEIITNTLSFATYLLAT

NPDCQEKLLREVDVFKEKHMAPEFCSLEEGLPYLDMVIAETLRMYPPAFRFTREAAQDCE

VLGQRIPAGAVLEMAGGCPAP

>AAH29014.1

MEVLGLLKFEVSGTIVTVTLLVALLALLKWYSMSAFSRLEKLGIRHPKPSPFVGNLMFFR

QGFWESQLELRERYGPLCGYYLGRRMHVVISEPDMIKQVLVENFSNFSNRMASGLEPKMV

ADSVLLLRDRRWEEVRGALMSSFSPEKLDEMTPLISQACELLMAHLKRYAASRDAFNIQR

CYCCYTIDVVASVAFGTQVDSQNSPEDPFVQHCRRASTFCIPRPLLVLILSFPSIMVPLA

RILPNKNRDELNGFFNTLIRNVIALRDQQAAEERRRDFLQMVLDAQHSMNSVGVEGFDMV

PESLSSSECTKEPPQRCHPTSTSKPFTVDEIVGQAFLFLIAGHEVITNTLSFITYLLATH

PDCQERLLKEVDLFMGKHPAPEYHSLQEGLPYLDMVISETLRMYPPAFRFTREAAQDCEV

LGQRIPAGAVLEIAVGALHHDPEHWPNPETFDPERFTAEARLQRRPFTYLPFGAGPRSCL

GVRLGLLVVKLTILQVLHKFRFEASPETQVPLQLESKSALGPKNGVYIKIVSR

>P36423.2

MEVLGLLKFEVSGTIVTVTLLVALLALLKWYSMSAFSRLEKLGIRHPKPSPFVGNLMFFR

QGFWESQLELRERYGPLCGYYLGRRMHVVISEPDMIKQVLVENFSNFSNRMASGLEPKMV

ADSVLLLRDRRWEEVRGALMSSFSPEKLDEMTPLISQACELLVAHLKRYAASRDAFNIQR

CYCCYTIDVVASVAFGTQVDSQNSPEDPFVQHCRRASTFCIPRPLLVLILSFPSIMVPLA

RILPNKNRDELNGFFNTLIRNVIALRDQQAAEERRRDFLQMVLDAQHSMNSVGVEGFDMV

PESLSSSECTKEPPQRCHPTSTSKPFTVDEIVGQAFLFLIAGHEVITNTLSFITYLLATH

PDCQERLLKEVDLFMGKHPAPEYHSLQEGLPYLDMVISETLRMYPPAFRFTREAAQDCEV

LGQRIPAGTVLEIAVGALHHDPEHWPNPETFDPERFTAEARLQRRPFTYLPFGAGPRSCL

GVRLGLLVVKLTILQVLHKFRFEASPETQVPLQLESKSALGPKNGVYIKIVSR

>P49430.1

MEVLGLLKFEVSGTVVTVTLSVVLLALLKWYSTSAFSRLRKLGIRHPEPSPFVGNLMFFR

QGFWESHLELRERYGPLCGYYLGRRMYIVISDPDMIKEVLVENFSNFSNRMASGLEPKLI

ADSVLMLRDRRWEEVRGALMSAFSPEKLNEMTPLISQACELLLSHLKHSAASGDAFDIQR

CYCCFTTNVVASVAFGIEVNSQDAPEDPFVQHCQRVFAFSTPRPLLALILSFPSIMVPLA

RILPNKNRDELNGFFNTLIRNVIALRDKQTAEERRGDFLQMVLDAQRSMSSVGVEAFDMV

TEALSSAECMGDPPQRCHPTSTAKPLTVDEIAGQAFLFLIAGHEITTNTLSFITYLLATH

PECQERLLKEVDLFMEKHPAPEYCNLQEGLPYLDMVVAETLRMYPPAFRFTREAAQDCEV

LGQHIPAGSVLEIAVGALHHDPEHWPNPETFDPERFTAEARLQQKPFTYLPFGAGPRSCL

GVRLGLLVVKLTLLQVLHKFRFEACPETQVPLQLESKSALCPKNGVYVKIVSR

>403276227

MFSWLPVPFSTHRTRFPQGQQDQPPLSSGRPPLWGLRGMMEALGFLKLEVNGPMVTVALS

VVLLALLKWYSTSAFSRLEKLGIRHPKPSPFIGNLTFFRQGFWESQMELRKLYGPLCGYY

LGRRMFIVISEPDMIKQVLVENFSNFTNRMASGLEFKSVADSVLFLRDKRWEEVRGALMS

VFSPEKLNKMVPLISQACDLLLAHLKRYGDSGDAFDIQRCYCCYTTDVVASVAFGTPVDS

QQAPEDPFVKHCKRFFEFCIPRPILVLLLSFPFIMVPLARILPNKKRDELNGFFNKLIRN

VIALRDQQAAEERRRDFLQMVLDARHSASPVDVQDFDIVRDVFSFTKCKPNPSRQHQPRA

MARPLTVDEIVGQAFIFLIAGYEIVTNTLSFATYLLATHPDCQEKLLREVDLFKEKHAAP

EFCSLEEGLPYLDMVIAETLRMYPPAFRFTREAAQDCEVLGQQIPAGAVLEVAVGALHHD

PEHWPSPETFDPERCHCS

>AAB87704.1

MEVLGLLKFEVSGTIVTVTLLVALLALLKWYSMSAFSRLEKLGIRHPKPSPFVGNLMFFR

QGFWESQLELRERYGPSVWVLSWPSIDVVISEPDMIKQVLVENFSNFSNRMASGLEPKMV

ADSVLLLRDRRWEEVRGALMSSFSPEKLDEMTPLISQACELLVAHLKRYAASRDAFNIQR

CYCCYTIDVVASVAFGTQVDSQNSPEDPFVQHCRRASTFCIPRPLLVLILSFPSIMVPLA

RILPNKNRDELNGFFNTLIRNVIALRDQQAAEERRRDFLQMVLDAQHSMNSVGVEGFDMV

PESLSSSECTKEPPQRCHPTSTSKPFTVDEIVGQAFLFLIAGHEVITNTLSFITYLLATH

PDCQERLLKEVDLFMGKHPAPEYHSLQEGLPYLDMVISETLRMYPPAFRFTREAAQDCEV

LGQRIPAGTVLEIAVGALHHDPEHWPNPETFDPERFTAEARLQRRPFTYLPFGAGPRSCL

GVRLGLLVVKLTILQVLHKFRFEASPETQVPLQLESKSALGPKNGVYIKIVSR

>291411688

MEAPGLPKLELNVPMVTVALLVALLALLKWYSTSAFSRLEKLGISHPKPSPFIGNLPFFC

QGFWEGHLELRKLYGPLCGYYLGRQMFIVISDPDMIKQVLVENFSNFTNRMASGLEPKLV

ADSALFLRDKRWEEVRGVLTAALHPERLNEMLPLVSEACDLLLAHFQRCAESGAAFDVQR

CYCYYTTDVVASVAFGTQVDSREVPEHPLVEHCRRFFQVSLPRPLLALILSFPSVMVPLA

RILPNKNRDELNGFFNQLIRSVIALRDRQAADERRRDFLQMILEAQHCARPASLAAFDIV

SQVLPAERPGAASLQHPPAALSQPLTVDEIVGQACIFLIAGYEIITNTLSFATYLLATNP

DCQDRLLREVDRFKEKHPALEYRGLQEGLPYLDMVIAETLRMYPPAFRFTREAAQDCEVL

GQRIPAGAVLETAVGALHYDPEHWPNPETFDPERFTAEAREQRRPFTYLPFGAGPRSCLG

VRLGLLEVKLTLLQVLHKFRLEACAEIQVPLQLDSKSALSPKNGVYIRIVPR

>344297248

MAVTVGLGYGVMTCTQSSEAGPRARCRGSDGNWSGLGCTQELVAQSEMELLKYSSASNVL

TATLHFSDGRFLLESGTEMCRTTFPDFGYSTSAFSRLEKLGIRHPKPSPFIGNLTFFRQG

FWESQLELRRRYGPLCGYYLGRRMFIVISEPDMIRQVLAENFSNFSNRAASGLESKAVAD

SVLFLRNTRWEEVRGVLTPAFSPEKLNEVTPLINQACDLLLTHLQPYADSGAAFNIQRCY

CCYTTDLVASVAFGTQLDSQETPEHPFVTHCRRFFEFSIPRPILALILSFPSIMIPLARI

LPNKNRDELNGFFNKLIRNVATSRDQQAANKRRKDFFQMILDARHSVSSVGVDSFDIVRQ

VFSSAGCPEPPPRPHAPRVLSKPLTMDEIVGQAFIFLIAGYEIVTNTLSFATYLLAIHPD

CQEKLLREVDRFYEKHPALQYCSLQEGLPYLDMVIAETLRLYPPAFRFTRESAKDCKLLG

QHIPAGAVVEMAVGALHHDPEHWPQPEAFDPERFTAEAQRRRRPFTYLPFGAGPRSCLGV

RLGLLEVKLTLLQVLHKFRFQACAETQVPLQLESKSALAPKNGVYIKIVPR

>348579245

MEVLDFLKLQVNGLMVAVALSVALVALLKWYSTSVFSTLEKLGIRHPKPSPFIGNLTFFR

QGFWDGQLELRKLYGPLCGYYLGRRMYIVISEPDMIKQVLVENFSNFTNRMASGLEPKPV

ANSILLLRDKRWEEVRGVLTSVFSPEKLNEMIPLISQACDLLLAHLRRCAESGNACDMQR

YYRAFATDVVASVAFGTRVDSQKDPEDPFVLHCRRFFAFSMARPLLVLILSFPSIMVPLA

RILPNKNRDELNHFFTELIRNKIALRDQQAAEERPRDFLQMVLDTRCSPSSVGVDSFDLV

QEALSSEACTADTSAPRQPRALSQPLTVDEVVGQAFLFLIAGHEFISNTLSFATYLLATN

PHCQEKLLREVDLFQQRKVAPDYGSLQEGLPYLDMVILETLRMYPPAFRFTREAARDCKV

LGQHILAGTVLEVAVGALHHDPEFWPHPETFNPERFTAEARQQRRSFTYLPFGAGPRSCL

GVRLGLLEVKLLLLQILSRFRFDACSQTQVPLQLESTSALSPKNGVYIKLVLR

>BAG64487.1

MMEALGFLKLEVNGPMVTVALSVALLALLKWYYLGRRMFIVISEPDMIKQVLVENFSNFT

NRMASGLEFKSVADSVLFLRDKRWEEVRGALMSAFSPEKLNEMVPLISQACDLLLAHLKR

YAESGDAFDIQRCYCNYTTDVVASVAFGTPVDSWQAPEDPFVKHCKRFFEFCIPRPILVL

LLSFPSIMVPLARILPNKNRDELNGFFNKLIRNVIALRDQQAAEERRRDFLQMVLDARHS

ASPMGVQDFDIVRDVFSSTGCKPNPSRQHQPSPMARPLTVDEIVGQAFIFLIAGYEIITN

TLSFATYLLATNPDCQEKLLREVDVFKEKHMAPEFCSLEEGLPYLDMVIAETLRMYPPAF

RFTREAAQDCEVLGQRIPAGAVLEMAVGALHHDPEHWPSPETFNPERSKLNPEI

>350596053

MVHEPGSWLVPEAHKRAISMLKIHSEGILEGRYSTSAFSRLEKLGIRHPKPSPFIGNLTF

FRQGFWESHMELRKQYGPLSGYYLGRRMIVVISDPDMIKQVLAEKFSNFTNRMATGLESK

PVADSILFLRDKRWEEVRSVLTSAFSPKKLNKLTPLISQACDLLLAHLERYAESGDAFDI

QXXXXXXXXXCRRFFAFSVPRLILVLILSFPSIMVPLARILPNKKRDEVNGFFNKLIRNV

IALRDQQAAEERRQDFLQMVLDLRHSAPSVGVENFDIVRQAFSSAKGCPADPSQPHLPRP

LSKPLTVDEVVGQAFLFLIAGYEIITNTLSFVTYLLATNPDCQEKLLREVDDFSKKHPSP

EHCSLQQGLPYLDMVLSETLRMYPPAFRFTREAARDCEVLGQRIPAGTVLEVAVGALHHD

PKHWPHPETFDPERFTAEAQRLQQPFTYLPFGAGPRSCLGVQLGLLEIKLTLLHILRKFR

FEACPETQVPLQLESKSALSPKNGVYIRIVPR

>297289461

MMEALGFLKLEVNGPMVTVALSVALLALLKWYYLGRRMFIVISEPDMIKQVLVENFSNFT

NRMASGLEFKSVADSVLFLRDKRWEEVRGALMSAFSPEKLNEMTPLISQACDLLLAHLKR

YAESGDAFDIQRCYCNYTTDVVASVAFGTPVDSQQAPEDPFVKHCKRFFEFCIPRPILVL

LLSFPSIMVPLARILPNKNRDELNGFFNKLIRNVIALRDQQAAEERRRDFLQMVLDARHS

ASPVGVQDFDMVGDVFSSTRCKPNPSRQHQAGPMARPLTVDEIVGQAFIFLIAGYEIVTN

TLSFATYLLATNPDCQEKLLREVDLFKEKHMVPEFCSLEEGLPYLDMVIAETLRMYPPAF

RFTREAAQDCEVLGQRIPAGAVLEMAVGALHHDPEHWPSPETFDPERSKLNPDI

>334348483

MRQAFLDQSNEEICLTVPVCCKSFAYLVFTEYTMGVRSGVVTASSTHTMIYDLGYSSLPL

THNQEHXVVLFSLFRYSTSAFSSLERVGIRHPKPLPFIGNLTFFRQGFWESHMELIKTYG

PLCGYYLGRRMYIVVYEPQMIKEVLVKNFGNFTNRMMSGLETKPIADSILFLRNKRWEEV

RSTLTPAFSPKKLDEVIPLISQACDLLLTHLKPYAESGEAFDIQRSYGCFTLDVVASVAF

GFQVDSQKRPEHPFVKHCRRFFQFSIPKCVLILIFAFPSIMIPLSRVLPNKNRDELSAFF

NNVIRNMITLRDKQAEEERRRDFLQILLDARGAPSSAEVSQSAQNGDSSIPQATSRVLQK

TLTTDEIVGQAFIFLIAGYETITNTLSFVTYLLATNSECQEKLLREVDEFSERNAVPKFC

TLEEELPYLDMVIAETLRMYPPAFRFTRETEKDCDVMGLHIPAGAIVEMAVGYLHHNPEY

WPNPETFIPERFSLEARQQHLPFTYLPFGAGPRSCIGVKMAMLEMKLTLLRLLQKFKFEI

CPETQVPLQLKSRSALGPQNGVYIKIVSR

>395539528

MSAKHNQAKVLEMPELQEQSREFLRADTVSILSLYPWYSVSAFSSLEKLGIRHPKPLPFI

GNMTFFRQGFWESHMKLIKTYGPLCGYYLGRQMYIVVYDPQMIKEVLVKNFSNFTNRMMS

GMETKPVADSILFLRNKRWEEVRSVLTPAFSPKRLDEVIPLISQACDLLLTHLKPYAESG

EAFDIQRCYGCYTLDVVASMAFGFQVDSQKTPDDPFVTHCRRFFKVSFPRFVLILILSFP

SIMIPLARVLPNKNRDEVNAFLSKVIGNVIALRDKQAEEQRRRDFLQILLDARSSPSSVG

TELFDNVRQNAQDGELPTHQTTSNVQQKALTTDEIMGQAFIFLIAGYETITNTLSFVTYL

LATNSECQEKLLREVDQFSERNTELKFSTLEEELPYLEMVIAETLRMYPPAFRFTREAAK

DCEMMGLCIPAGTIVEIAVGYLHHNPEYWPNPESFIPERFSQEARQRQLPFTYLPFGAGP

RSCIGAKLAILEIKLTLLGILQKFKFETCSETQIPLQLKSRSALGPQNGVYIKIVPR

>BAE24030.1

MEVLGLLKFEVSGTIVTVTLLVALLALLKWYSMSAFSRLEKLGIRHPKPSPFVGNLMFFR

QGFWESQLELRERYGPLCGYYLGRRMHVVISEPDMIKQVLVENFSNFSNRMASGLEPKMV

ADSVLLLRDRRWEEVRGALMSSFSPEKLDEMTPLISQACELLVAHLKRYAASRDAFNIQR

CYCCYTIDVVASVAFGTQVDSQNSPEDPFVQHCRRASTFCIPRPLLVLILSFPSIMVPLA

RILPNKNRDELNGFFNTLIRNVIALRDQQAAEERRRDFLQMVLDAQHSMNSVGVEGFDMV

PESLSSSECTKEPPQRCHPTSTSKPFTVDEIVGQAFLFLIAGHEVITNTLSFITYLLATH

PDCQERLLKEVDLFMGKHPAPEYHSLQEGLPYLDMVISETLRMYPPAFRFTREAAQDCEV

LGQRIPAGTVLEIAVGALHHDPEHWPNPETFDPER

>363728085

MPFHQPRLAEGTMGTTVSGAGWCPAVSTGPLSHHPTASPACCRYSTSAFSKLSKVGIRHP

PPLPFIGNLLFFREGFWENHTKLITEYGPVCGYYIGRQMFVVISTPEVIKQILVTDFGNF

TNRTKPNLISKPMLDSILCLRDERWKYVRSLLTPAFSDAKLKEMIPLINQACDVLLSNLK

VYADSGRAFDIQRCYNCFTLDVVGSVAFGTEVDSQKNPDDPFVKNCRTFFEMSLFKPLLF

LILSFPFIMIPLLRILPNKKQKELNGFFIQTIKNAIAFRDQQDAAERRRDFLQWMLDSRN

SADALAGGYFDQICPATTSSHNEVPLVDKTPSEKAQKMLTEDEIAGQAFLFLIAGYETTT

STLSFATYLLATNPECQEKLLQEIDEFSAKHMAQDLCALEENLPYLDMVIAETLRMFPPA

FRFTREAAKDCVVLGQRIPAGAVIETAVGHLHHNPEFWPEPEKFIPERFTEEAKKERHPF

AYLPFGAGPRGCIGRKMGLLATKMTLLRILQMFRFKTCPETEIPLQLKSKATLGPKNGVY

IILESR

>224095495

MVRVDWSLFRYSTSAFSKLSRVGIRHPPPLPFIGNLLFFREGFWENHTKLIKEYGPVCGY

YIGCQMFVVVSSPEMIKQILVADFSNFTNRAKPNLISKPMLDSILCLRDDRWKYVRSLLT

PAFSDTKLKEMTPLINQACDILLCNLKVYADSSKAFDIQRCYNCFTLDVVGSVAFGIEVD

SQKNPDDPFVKNCRTFFEMSLFKPLLILILSFPSIIIPLLRIFPNKKQKELNGFFIQTIK

NAIVYRHQQDAAQRRRDFLQWMLDACDSADSLAAGCSDVLSPSAAPRQNEAPLVGTAPSE

KAQKTLTEDEIAGQAFLFLIAGYETTTSTLSFATYLLATNPECQEKVLQEVDEFSAKHMI

PDHQNIQELPYLDMVIAETLRMYPPAFRFTREAAKDCIVLGQHIPAGAVIETAVGHLHHD

PEFWPQPERFIPERFTEEAKKERHPFAYLPFGAGPRGCLGMKMGLLETKMTLLRILQKFR

FKTCPETEIPLQLKSKATLGPKNGVYIMLESR

>327272302

MGLMDILTACSLEINATAVTITCLVTFLVLLYWSLKPEVLCAYPFMQTIKKRTTKTYQKL

LAVMRVGLPAVSERNNISFALTWLSFTFPFRYSTFSYSRLQNVGIRHPPPLPFIGNLLYF

RKGFWEGHNKLINKYGPACGYYIGRRMFIVISEPDMIKFILTEDFQNFTNRMTVDLASKP

LADSILILRDERWKGVRGVLTPAFSTARIKEMAPLINQACDILLSNLKVYADSGRAFDIQ

RNYSCFTLDIVVSMAFGIQLDSFKNSNDTFVKNSKIFFEPAFSRPLLILTIAFPFIMIPL

LRILPNKKREEVNRFFIDTVKNTIALRDQQDPNERRRDFLQLMLDARSSDNDVTVEHFDF

INQADFDVKTSETAANQSLPKKQQKRLSEDEIAGQASLFLIAGYETTNSILSFATYVLAT

NPSCQEKLLQETDEFFFKHDFPDYKNIHELPYLDMVIAETLRMYPPAFRFTREAAKDCLV

LKQHIPAGAVVEVAVGHLHYNPKIWPEPHKFIPERFTTEAKQQRNPFSYLPFGAGPRSCI

GLKLALMEVKITLLRILQKFKFQTCPETQIPLQLKSQGTLGPLNGIYIKMVSR

>326912215

MHDYLFLGRYSTAPFSKLSKVGIRHPPPLPFIGNLLFFREVASYSYNTEVKYYIGRQMFV

VISAPEVIKQILVTDFSNFTNRTKPNLISKPMLDSILCLRDERWKYVRSLLTPAFSDAKL

KEMIPLINQACDVLLSNLKAYADSGRAFDIQRCYNCFTLDVVGSVAFGTEVDSQKNPDDP

FVKNCRTFFEMSLFKPLLFLILSFPFIMIPLLRILPNKKQKELNGFFIQTIKNAIVFRDQ

QDAAERRRDFLQWMLDSRNSADALAGGYFDKICPATTSSQNELPLVDKTPSEKAQKMLTE

DEIAGQAFLFLIAGYETTTSTLSFATYLLATNPECQEKLLQEIDEFSAKHPYPNSCSLQE

ELPYLDMVIAETLRMFPPAFRFTREAAKDCVVLGQRIPAGAVIETAVGHLHHNPEFWPEP

EKFIPERFTEEAKKERHPFAYLPFGAGPRGCVGRKMGLLETKMTLLRILQMFRFKTCPET

EIPLQLKSKATLGPKNGVYIILESR

>AAH60001.1

MEEPTLWGLDGCTVTFALVAGFLGLLYWYSVSAFSQLDKVGIKHPKPLPFIGNVMLFKKG

FWEGDRHLIKTYGPICGYYMGRRPMIVIAEPDAIKQVLQKDFVNFTNRMKLNLVTKPMSD

SLLCLRDDKWKRVRSVMTPSFSAIRMKEMCPLINQCCDVLVDNLLEYASSGEACNVQRCY

ACYTMDVVASVAFGTQVDSQRDPDHPLVQNCKRFLELFTPFKPLILLCLAFPSIMIPIAR

RLPNKQRDRINSFFLKVIRDIIAFRENQPPNERRRDFLQLMLDAQDSVSHVTVDHFDIVN

QADLSVPQNSPSEKQDRGQDPPRKSSKKLNKEEILGQAFIFLIAGYETTCSLLSFTSYLL

ATHPDCQEKLLKEVDEFSQEHKEADYNTVHDLPYMDMVINETLRMYPPAYRFAREAARDC

TVMGQNIPAGAVVEIPIGCLQNDPRFWHEPEKFNPERFTAEEKQKRHPFLFLPFGAGPRS

CIGMRLALLEAKITLYRVLQKFRFQTCDLTQIPLQLSAMSTLRPKDGVYVTVVAR

>AAH59313.1

MEMLRLWGLDGCTVTFALVAGFLGLVYWYSVSAFSQLEKVGIKHPKPLPFIGNVMLFQKG

FWEGDRHLLKTYGPICGYYMGRRPMIVIAEPDAIKQVLQKDFVNFTNRMKFNLATKPMSD

SLLCLRDDKWKRVRSVLTPSFSAIRMKEMCPLINQCCDVLVDNLQEYASSGEACNVQRCY

ACFTMDVVASVAFGTQVDSQKDPEHPLVQNCKRFLEQFTPFKPLVLLCLAFPSIMIPIAR

RLPNKQRDRINSFFLKVIRDIIAFRENQPPNERRRDFLQLMLDARDSAGHVTVDHFDIVN

QADLSVPRNSPSEKQDRGQDSPRKSTKKLNEEEILGQAFIFLIAGYETTCSLLSFASYLL

ATHPECQKKLLREVDEFGREHKEADYNTVHELPYMEMVINETLRMYPPAYRFAREAARDC

TVMGLNIPAGAVVEIPIGCLQNDPRFWHEPEKFNPERFTAEEKQKRHPFLFLPFGAGPRS

CIGMRLALLEAKITLYRILKNFRFQTCDLTQIPLQLSAMSTLRPKDGVYVRVVAR

>AAI59144.1

MGESWLWGLDGCTVTLTLVAGFLGLLYWYSVSAFWQLEKAGIKHPKPLPFIGNIMLFQKG

FWEGDRHLLKTYGPICGYYMGRRPMIVIAEPDAIKQVLQKDFVNFTNRMRLNLVTKPMSD

SLLCLRDDKWKRVRSVLTPSFSAARMKEMCPLINQCCDVLVENLMEYASSGEACNVQRCY

ACFTMDVVASVAFGTQVDSQRDSDHPLVQNCKRFLELFTPFKPVVLLCLAFPSIMIPIAR

RLPNKHRDRINSFFLKVIRDIIAFRENQPPNERRRDFLQLMLDARDSAGHVSVDHFDIVN

QADLSVPQNQDRGQDPPRKSTQKTLNEEEILGQAFIFLIAGYETTCSLLSFASYLLATHP

DCQEKLLKEVDEFSQEHEEADYNTVHDLPYMEMVINETLRMYPPAYRFAREAARDCTVMG

LGIPAGAVVEIPIGCLQNDPRFWHEPEKFNPERFTAEEKQKRHPFLFLPFGAGPRSCIGM

RLALLEAKITLYRVLRKFRFQTCDLTQIPLQLSAMTTLRPKDGVYVRVVAR

>ACN10451.1

MEIVMNILKMPVPSGVSVSVGLFMIFLALLYWYATFPYSALARCGIRHPKPSPFFGNMFL

FRQGFFGVHTDLIHKYGRVCGYYLGRRPVVVVADPDMLRQIMVKDFSTFPNRMTIRSATK

PMSDCLLMLRNEHWKRVRSILTPSFSAAKMKEMGPLINMATDTLLTNLLGHVESAESFDI

HRCFGCFTMDVIASVAFGTQVDSQKDPDDPFVHHAQKFFSFSFFRPIMFVFIAFPFLAPL

ARVIPNKRRDEMNNFFINCIQKIIRQRDEQPVEERRRDFLQLMLDTRSTKECVPLEHFDV

VNHADELAHTHDSGEQENGGAGSHESPNRRSVQTQKRMMSEDEIVGQAFVFFLAGYETSS

NTLAFTCYLLALHPECQSKLQAEVDDFFTRYDSPDYTNVQDLKYLDMVISEALRLYPPGF

RFARDVDEDCMVNGQFLPKGATLEIPAGYLHYDPEYWPEPEKFIPDRFTAEAKASRHPFV

YLPFGAGPRSCVGMRLAQLEIKMALVHVFRKFNIVASTDTKVPLELKSHTTLGPKNGIYV

KITKRETEGEPVTPPAEEE

>AAQ97855.1

MQLLVDGLKWFGVEASGLSLTVCLFLLFLSLLYWYSISPFSNLERCGIKHPKPLPFIGNL

MMFRNGFFKSQADLINKYGRICGYYIGRRSTVIIADPDMLRQVMVKEFNKFPNRMTARGI

TKPMSDSLIMLKGEQWKRVRSILTPTFSAAKMKEMVPLINTATETLLRNLKSHAESENSF

NIHKCFGCFTMDVIASVAFGTQVDSQNNPDDPFVHQASKFFAFSFFRPIMIFFMAFPFLL

RPLAGLLPNKSRDEMNSFFTQCIQKMIKQRDDLSPEQRRKDFLQLMLDVRTNNKFLSVEH

FDVVNDADEEAYDGHENSPANESTKRSQQKRMMTEDEIVGQSFIFLVAGYETSSNTLAFT

CYLLAVHPECQKKLQEEVDEFFSRHEMVDYANVQELKYLDMVICESLRLYPPAFRVARDV

EEDTVLNGQFLPKGASLEIPTGFLHYDPEHWTEPTKFIPERFTPEAKARRHPFVYLPFGA

GPRSCVGMRLAQLEIKVALVHIFRRFNVLACEDTEIPLELKSHTTLGPKNGVMVKITERE

NLEDAS

>CAK04594.1

MQLLVDGLKWFGVEASGLSLTVCLFLLSLSLLYWYSISPFSNLERCGIKHPKPLPFIGNL

MMFRNGFFKSQADLINKYGRICGYYIGRRSTVIIADPDMLRQVMVKEFNKFPNRMTARGI

TKPMSDSLIMLKGEQWKRVRSILTPTFSAAKMKEMVPLINTATETLLRNLKSHAESENSF

NIHKCFGCFTMDVIASVAFGTQVDSQNNPDDPFVHQASKFFAFSFFRPIMIFFMAFPFLL

RLLAGLLPNKSRDEMNSFFTQCIQKMIKQRDDLSPEQRRKDFLQLMLDVRTNKKFLSVEH

FDVVNDADEEAYDGHENSPANESTKRSQQKRMMTEDEIVGQSFIFLVAGYETSSNTLAFT

CYLLAVHPECQKKLQEEVDEFFSRHETVDYANVQELKYLDMVICESLRLYPPAFRVARDV

EEDTVLNGQFLPKGASLEIPTGFLHYDPEHWTEPTKFIPERFTPEAKARRHPFVYLPFGA

GPRSCVGMRLAQLEIKVALVHIFRRFNVLACEDTEIPLELKSHTTLGPKNGVMVKITERE

NLEDAS

**CYP6**

>ACV91647.1

KIIRLSMALTYILFQVAVALLAILTYYIHRKLTYFKRRGIPFVAPHLIRGNMEELQKTKN

IHEIFQDHYNKFRESKAPFVGFFFFQSPAAFVIDLELAKQILIKDFSNFSNKGIFYNEKD

DPISAHLFNLDGAQWRLLRNKLSSTFTSGKMKLMYPTVVSVANEFMTVMHEKVPKNSVLE

IRDLVARFTVDVIGTCAFGIQCNSLRDEKAEFLYFGKRSLVDKRHGTLLNGFMRSYPKLA

RKLGMVRTAPHIQEFYSRIVTETVAVREKEHIKRNDFMDMLIELKNQKEMTLENGDVVRG

LTMEEVLAQAFVFFIAGFETSSSTMGFALYELAKNPDIQDKVRAEVEEVIEQHDQNFTYE

CTKDLKYLNQVLDETLRLYTIVPNLDRMAAKRYVVPGHPNFVIEAGQSVIIPSSAIHHDP

SIYPEPFEFRPERFSPEESACRPSVAWLPFGDGPRNCIGLRFGQMQARIGLALLIRNFKF

STCSKTPNPLVYDPKSFVLGVKDGIYLKVETV

>AAM34435.1

MGYVSVVLFLVVPALTLLYLYLKQHYRHWANRNLPQLEASFPLGNMKGVGSEIHFNDVLN

EAYAKGKAQSAPLVGLYFMLKPILIVTELDMVKRILVKDFSSFHDRGLYVNERDDPLSGH

LFALDGERWRYLRNKLSPTFTSGKIKLMFSTICEIGDEFLATVNRFVERDEPLDVKLLSQ

CFTCDVVGSCAFGLQCNSLKNGGSKLLEIGDKVFKPPAWRNMLAFALISFKKLAKRLRLP

VLPGEVTSFFMPLVTETVADRERNSIERPDFLNLLIQLKNKGTVEDETTEGLQKLTLDEV

AAQAFVFFFAGFETSSTTLSFALFELANNPDIQERVRAEMLEKLQLHDGKITYDALKEMT

YLDQVINETLRMYPPVPQLIRVTTQPYKVEGANVSLEPDTMLMIPIYAIHHDASIYPDPE

RFDPDRFALAATHARHTHAFLPFGDGPRNCIGMRFGLLEVKFGIVQMVSKLRFTVSERMQ

MPLRMSKTASILEAEGGIWLNATKL

>AAP83688.1

MHFLIALGILLIILYLYGIKNHKYWEKKGVPYVKPIPFFGTNFKVFMQRICISDQLCKYY

EQFPNEKFVGAFIGDRIGLVLREPELIKRVMVTDFQYFHPRGVNPHKTVYEPLLKNLFTG

DGDMWKLLRQRITPAFTSGKLKAMFPLIVERAERLQIIAATAAESHGEVDVRELMARFTT

DFIGACGFGIDADTLNDEESTFRRLGKRIFTLTKRDGFVFMLKTIAPEIFKNLHMFAPEI

EKTTVDLVTSIMQQRKYKHSGRNDFIDFLLELKGKGKIVGESVEKRNPDGTPKIVEMELD

DMIMAAQVFIFFAAGFETSSSTTSYTLHQLAFHPEEQKKCQDQIDEVLSRHGGKLSYEAI

KEMTYLDMIFKESMRMYPSLGILTRRCVQKYTFPGTNLTIDEDVLVCIPVHALHNDEKYF

DEPEKFKPERFSPENIKNIPKYVYLPFGDGPRACIGERLGHMQSLAGLAALLSKFSVAPS

KNTLRQPITDPTCTVVKSIKGGLPLSLVARKV

>ABX64440.1

MFLLINICVILFVIYYLLTKKYSYWRNRNVSHEKPVLLLGNYGDLILQKKNFGEMAQAIC

QKFPGEPVVGAFFGTEPVLIPQDPEVIKTILTKDFYYFNGREISEHVHKELLSYNLFATY

GDEWKILRQNLTPIFSTAKLKSMFTLIEKCSKSFQNLLEDETKISKELEVRTLMQRFTIE

CIGSCIFGVDTDTLGNDKMNPFKTVGSQLSDFSRFVFVKGIVRAIWPTLFYALGFKTLTT

ELDIFKKLVNAVFTQRKHKPTTRNDFIDIILTWKNNNAITGDSIGSFKNSDKTKFSIDVN

DDLLLAQCLVFFAAGFETSAMTSSYTLHELAKNQRALKKACDEVDAYLLRHGNKINYDCV

TELPYLEACIEETLRLYPVLGIITREVMEDYVLLDKIHLKKGDRIHVPVFHLHHNPEHFP

NPEEYRPERFYGEEKRKVKPYTYLPFGEGPRICIGMRFAKMQSIAGLITILKKFRLELPE

GAPTKIEFKPEAFVTTPKDLIKIKFLEREGWQQRVFV

>AAV41248.1

MLFLAFAIFVLFAIIQIVYHFRYWMRRGVPQLRPSFPFGDFGEFFRQKHGIPMTYANIYA

RTRHLPYVGIYLSMRPVLFVNDPQMVKDILSRDFEHFHDRGLHVNEETDPLSGNLFSLGG

VKWKNMRAKLTPTFTSGCLKGMLAILIDKATVLQKQFAKEIATHNTIEVKDLFARYTTDV

IASVAYGIDNDSINNDHDLFRQMGIKVFQQDFKTSLRLALTFFIPKIKALLGFSLVAKDV

EDFMINLVSKTIEHRERNGIQRKDMMQLMLQLRNSGSVSINDQQWNLDSSATVKNLTINQ

VAAQVFVFFVAGYETSSTLMSFCVWELARNPEIQVKVHQEIDSVLSNYGGALTYEALADM

EYLECCMEETLRKHPPVSFLNRECTKTYRIPETDVIIDKGTAVVVSLLGMHRDPQHFTQP

TEFKPERFSSDEQSNESNKAYFPFGGGPRLCIGMRLGMLQAKVALVTLLAKFEFSLGKEH

VKDMELPLKANTLLLVPQDGIQLVVKKR

>ADE05585.1

MILIITFVLFLYALYVYSTKNHNYWKKRNIKHDPPIPLFGNHFVNQFGYKSITQVATELY

NRYPEEKVVGYYQGTTPLLLVRDLEIVRRILTVDFSHFYLRGLGRNPDVEPIFTNLFHTE

GDSWKLLRQRLTPTFTTAKLKAMFPLVVECAKKLQGVAEGVAKRNESCDVRELVARFTTE

FIGACGFGINMDTINNEKSMFRNLGKLITSSRSLGLIAKYMLWENFPYVRKFLYVGDRRI

EESIVEILQQIRKKRNYKPSGRNDFIDLLLELEAHGKITGESIEKRNSDGTPKLIEMDMD

MNCMVGQVFVFFAAGFETSSSASSYTLHQLAYNPDIQRQVQENIDEVLNKYNGELCYDAI

AEMKLLSMAFKEAMRMFPSLGVLSRVCARRYTIPELNITINPGVKILIPLEGIQNDEKYF

TNPKEFRPQRFSPDAMKEQQKFSYMPFGEGPRACIGARLGEMQSLAGLAAILHKFTVEPA

PSSRLEVQIDTTMNVIQKLEGGLYLTLKSRKSKEE

>289177169

MEFSAFELLTALALLFLAIYYYFTSTYDFWSSRHVEHPKPELFFGNFKKMMLGKSSLGDY

LYEQYQYYKNEPMFGLYARRTPVLVVTDPEYIKDVLIKDFSSFSDRGMTIHEKVEPLSQH

LFSLEPARWRPLRIKLSPTFTSGKLKEMFYLLAECGNSFETLIDNMVKKNPVIECRELTA

KFTTEVIGICVFGLKAEAMADGDSEFRKIGRKVFENSWMKFIKFRIRDAMPWLFNLLGPF

FYDHELNGFFINLMKQTMEYRKKNNVRRNDFVDLLMDIKDDPSKVGDIEMTDALITAQAF

VFFIAGFETSSTTISNALYELALNPSVQEKLREEIIEELANDNGSLKYETIKGMKYLHKV

FCETLRKYPPVTVMMRKSMQPYTFSGTKVTIPKGMRVWIPAYAIQRDPAIYPDPDTFDPE

RFSEESIKQRHPSFYLPFGDGPRNCIGARFANYQSKIGIIQVLRNYKVQVCDKTCIPYVN

DPRAFLLAPIGGINLKFTRL

>ABX64441.1

MLTAYLLYVFASIVVLIYFYLNNKYKYWKNKNIAGPEPEFLFGNLKESFHRRKHIATVFK

EIYDQYPDEKVVGMFRMTSPTLIVRDLDIVKQIMIKDYAKFNERGIKFSKEGLGDNLFHA

DADAWKAVRSHLTPMFSSGKLKNMVRVLSQTGDRFVDHVTNEVRLRPEQELVSLFLKYNI

ATIMACSFSMETDINDMQVYEMLNVAVFENSYISELDMVFPGILRKTNRSIFGPTVNNFC

YEIVEFIKNERNGKPANRNDMMDMLLGVNCDKLKMKNGSEDFVEITEHVMAGQVFVFFAA

GYVNNTITLTFSLYHLAKDQSIQERLMREIETVLENHNNVLTLEAINEMSYLEMIFLETL

RMHPTTNTLQRSALEDYTIPGTDIEIEKGTLVLIPPLAFHHDEKIYPEPEKFDPGRFSVE

NHKSRHACAFLSFGIGPRTCIGIRLAKLQYKVCMVKFLTKYRVAPSTRTKDAYKIDPLRT

ALEPEGGVYVDILPRS

>193657145

MFTANWWINVITPCTIIVTIAYYFCVSTFKRWEKLNVPYIKPIPLFGNFLNIALGKDHPL

EFYNKIYYEFAGRKYGGLFQMRTPYLMVRDPEIINDVMIKDFSSFPDRGIYSDFTANPLS

NNLFFMENPQWKTIRNKLSPAFTSGKLKTMYDQIKKCGDELMKNIDIDLNKNGNEIEVRD

ILGKYSTDVIGTCAFGLKLNAISDDESPFRKYGKSIFTPSLRMLFRELCLMITPALLKVI

RVKDFPTAATDFFHAAFKETMTYRIENKIVRNDFVHCLMQARNDLVLNTDLPKHEKFTET

QIVANAFVMFAAGFETVSTTVSYSLYELALDKSIQDRAREEIQLKLSKNDGQINHEFLMD

LNYLDMVIAETLRKYPPLVALFRKASQTYRIPNDSLIIEKGQKIIIPIYAIHYDTKYYPE

PEKFIPERFSVEEKAKRPSGIYLPFGDGPRMCIGKRFAEMEMKLAFVEILTKFEVFPCEK

TEVPLKYSNKVLTLMPKHGIWLRFNRIN

>CAH65682.2

MIEIGLLIIVCLLIYHYCTATFRYWKQRSVPLVEPTALFGNYYDVVSLKTSPAACHESIY

RNFPNEKYVGMIQLRTPALLIRCPEMVKQILVKDFNHFMDRGFHADEEREPITAHLVNLQ

GEKWRMLRQKISPVFTSGKLKAMFPLLETCSSQLSNYIESALGEQDSSLLEMRDVMARFT

TDVIGSVAFGLHFNSFTEKESDFQLMGRRVLDSSQTSVITKAIRVFFPQLFHFLRLRTFP

EEIATFFQTVIHDTIENRERNDVQRNDFIPLLMQLRKKSPDYDGTEANDLEITESVIAAQ

AFVFFMAGYETSSTTLSFCLYELAKNLDVQEKACNEIKKVLNKHGKLSHEALMDLDYLEM

ILLETMRKYPPVSVLARVCTKPYTIPGTKISIDPGTSVAIPVYSFHHDHKYFPDPETFDP

ERFSPENQEKSINYTYLPFGAGPHVCEGTKFRTGRSGIGLRHLLRLFRFRYVDSTLTADC

TQHNDSRLSGFTGDDLDVEGL

>EDS44711.1

MLFYLALVAVSLTFFWVGRRFSYWKQLGVPYVEASFPFGNLKGMNKRHLGYIAQDLYVKL

KSTGKKFGGAFFFVSPTALILDLDFAKDVFVKDFQYFHDRGVYCNEKADPITAHLVTMSG

TKWKNLRTKLTPTFTSGKMKMMFPTIVGVAEEFRKGLLVEAEAGGEVEMKEFLARFTTDV

IGTCAFGLECNSLKDPDAEFRRMGKKALTLSTMDFLRRILTVTFKDVAMKFNVRLSNPDV

ANFFMNAVRETVDYREKNKIQRNDFMDLLIRMKNAEPIEGGDPNQVGQLTIEEIAAQSFV

FFLAGFETSSTAMTFCLYELAQNQELQDKARKNVLDVLKEHGSISYEAVHDMKYIEMCIN

ESLRKYPPIANILREVTKDYHVPDMNVTLPKGHRIMLPIYAIHHDPEYYPAPDQYDPERF

TPAAVAARHQMAFVPFGEGPRVCIGQRFGMMQARVGLAYLLKNFRFRLSSKTAVPLKILA

SSTVLATEGGLWLNIEKL

>AAW69913.1

MGLLTVLIATLLAAIVALLGYLKWIQNYWERRAIPAAKNTHILTGNMAGMGTKISLAIAL

QKIYREFRERGLPFGGFNNFFAPTILAVDPEFVKLVLVKEFNVFHDHGFYTDAESDPLNS

HLFNLDGAKWRVMRQKLSPTFTSGKMKMMFNTVQSVADELKKFVEANYHREDLEIRDLLQ

RFTTDVIGSVAFGIECNSFKDPDSKLREMAGKVFDLSPKTLVKTFVGIQFKGLARWMKMK

LTDDEVEKFFMELVRTTVESREKSDVQRNDFMKLLLEIKNSGKLSDAPDSGGDGLTMNEL

AAQCFVFFAAGFETSSTAMNFCLYELAINEDIQDRLREEINEVLGENGGQLTYDALMKMD

YLDRVFNETLRKYPPLDNTFRTNSVDYTVPGTNYTIPAGTFVQIPMYALQRDPEHFPAPE

RFDPDRFLPEVVKTRHPYAYVPFGEGPRICIGMRFGVMQAKIGLVTLLQNFKFRVNSRTP

IPMEFEPASVTLSPKNGMYLQIERVK

>289177053

MLDPSFLVNLVTVLAMLLTTFYLWARYKQSYWSRRGIPTAPNSHWLFGHFRDAIFMRSAP

PTLLGELHKSCPSDEPILGVYIMQKPFLLLRNAELIKQLLVKDSQYFPNRYFASKRKSDV

IGSQNLFSVDNPMWKYLRVKLSPAFTTGKQKRLFELMLESSQNMSKYLEANLPKDGTEID

MEMRRVSSKYTTDVISSLAFGIKTDSFTEPEAEFYLRSRKLFEQSIINTLKLFYNFFFPK

LNDIVGSSFLGAEEDYFRKIFWESMNQREKSGVSRGDAMDLLLELKNLKQSKEFRFEGDR

LVAQSVILFAAGLETSATTMSFTLLEIAKQPEIQRKVREEIRDKLNGKPLTYERISEMNY

LHQVVSETLRLYPPAPLLDRVAIDDYKIPGTDIVLEKGSVVYVALNGVHRDPDYHSDPLT

YDPDRFSEMRKKDMKPCTYMPFGDGPRICIGMRTGMLQTMVGLITLLSQYEVKLNPMYKN

EISKRAIFLAPANGIHLYIKRSPIM

>EFA12636.1

MLFSDSLTKDLFGCVLTFAILIIAYFKWSYQYWKRKNLPYLKPTFPFGNITGLLTRKETF

GVIIQKVYQEMKNCNWNHGGIYLTLNPAYCAIDLEYVKNIMTKDFQHFVDRGSYYNEKDD

PLSAHLFAIGGQKWRNLRTKLTPTFTSGKMKQMFQTLVDCESNLAKKIDMEYEKKHPIDI

KEVLGCFTTDIIGSCAFGLDCKTFEHENSPFRVYGKKLFKGSTLRNIKRSLLTQNFPDLA

QKLGVLALPEDISNFFMKVVKDTVRYREKNGFTRSDFLQLLINLKNSKVAEEEGYKHDGN

TLTLEEIAAQSFVFFIAGFETSSTTMTFALYELAKNQEIQSQVREEIDAVLKKHDGKITY

DSIQDMKFMNQVIDETLRKYPPIPFVTRKCVNNYQIPGENIVIEKGTTVIIPILGIHYDK

DYYPNPEKFDPERFTEKNKNARHHYAHIPFGEGPRICIGARFGLMQTKVGLISLLRNYKF

TVNNMTREPLRMQVDSFVLAAEGEIWLDAHKL

>EFA05684.1

MLTSCLATLFITLLSIFAYFYKRFYFSYWERKKIPNLQQHVPRTKPHLGIQLQQLYKIMK

EKKWKHGGFYQQLSPVYVICDLNYVKNIMTRDFNYFPYRGLYYNETSDPLSAHIFNTSGA

KWKRMRRKLDQIFTSSKLKTIFPLLPNCGEKLAGKCEPVDIKKVLDDFISDVIELCVFGS

ECKVFKQLGRKILNRSRLRRLKGIIINNFPKLAKILDIVLIPRDASTFFKKVVAEIVEER

KKGQTGRNDFLQVLVEMEKKFEITIDEIAAQCFIFLIAGMSTSPTAMTFALYELARHQDI

QEKVRQDIAKFGKVTYDSLQEMKYLGQVFDETLRMYPPAAYLNRKCERNYEIPDGSGIIV

EKGTIVAIPVLGIHYDRDYYPDPEKFDPEHFSEENKRLRPNFAFLPFGEGPRICIGMRFG

LMQSKLGLVSLLKRYKFTVSEKTREPLKMKPNSLVLSAEGEIWLDVTEIKI

>EFA12638.1

MILLVLSVVLLVALRIFFFWRFQYWEKLQIPYLRPYCPFGCLPNPLINKEKGGIILKRLY

RNVRDKGWKHAGIFVFVTPVYLPTNLDYIKNILTKDFEHFDDRGLYYNEKDDPLSGHLIN

LGGPKGVTMRKKLNSLFTPSSLKMMFQTLNTSIEDLLQQIHSRNTIDIHKLFTCFTIHVI

ASCTLGLECNIFKEEHLLFESYANKFLVWSRGKWCLQLLAAMYIDTARKLKVRTVPKETE

DFFVKIVTKNIKYREENDYFRQDMMQFFIEQKNANTMSINEIIAQCFIFFIAAYETSTTT

LTFALYELAQNLDIQDRVRREITSVLLKHQKYSYEAIAEMKYLNQIVDVRRCTKDYTIPG

EDVTIKKGIFALISILGIHHDEEIYPNPQKFDPDRFSKENKKMRHPYAYLPFGEGPRICI

GKRFALMQIKFGLAALLEKYKFTLNEKTKVPLEIRTDTLVVAPKGDVLLDIQKIN

>EFA02818.1

MLLVNGSLTYDLVAILATVLAGLVVYYKWAFTYWKRRGLEYLEPSIPFGNCASLLKLKYS

FGEQFAEFYKQFREKNLRHGGVFMFARPFYIPVDPAIMKDIMQKDFQHFVDRGFYVNEEH

DPLSGHLFSLEGNKWKNLRAKLTATFTSGKMKMMFETMSKCAEELDNYVNEAAKREETID

IKETLARFTTDIISSCAFGLESNCLKNPDTQFRYYGKRTFQNTTWENFKDVIQFITPTYL

LDLVKFKITKPDVEDYMLNLVKKTVDYREKNNVYRKDFLHLLLQLKNKGKITDDETFLDH

NNKSKEVALTINELAAQVFVFFAAGYETSSTAMTFTLYELCLNQKIQDKLREEIKTVLAK

HNNKISYEAIMEMTYMDQALNETLRKYPPVPVLNRKCTKAYDVAGTNLHLDEGTMVVLPI

LGLQHDPEYYPDPSKYDPDRFSEENKNSRPPFTWMPFGEGPRICIGLRFGMLQAKVGLAT

LLGNYKVTLSQKTQTPLEMDCKAFITTTKEGIWLDFTRLN

>EFA12627.1

MMSVFTEFWPQILVFLALFLLVLVNYFNYCYRYWSKKGVATIKPRFPLGNTNIILPQGLS

IGILSKQFYDEFKAKGHKFGGVYLVTEPNLLIVDPEYVKDIMSKDFQHFVDRGFYYNKEK

DVLSAHLFAIDGSDWRNLRIKLTPTFTSGKMKMMFEAVVQCSKHMIENLDKNLGSDIEIK

EVLGRFTTDVIGTCAFGIECNSFKYPDAEFRMMGKKIFEYDLWKSVKAFFAVNVPKVALQ

LGLTSTPDDVIDFFTRIVADAVKMRESNNIRRNDFLQILIDLKNTTSLTLDEMSAQVFLF

FVAGFETSSTTMTFALYELARNEKMQEKLRFEICQVLDKTGGQITYESLIEMKYLQQVID

ETLRMYPPLPTLNRRCTKDYVLRDTNIIIEKDTPILISALGLHMDPEFFPKPEKFDPERF

TEEKKKERHPFVHLPFGDGPRNCIGLRFGVMQSKIGIITIIKNFKLTVSSNMKPVQFNPY

VFLLNINDKVLLQVGKL

>EFA05733.1

MLLLAESLITLVLLLIAYYKFSFQYWKRQNVPFIQPSIPWGNYPSALKRQDNFGELVVRM

YHQMKKQGHKYFGIYLFFQPCFVPTDPKFIKLILAKDFQHFNFRNFYYNEKDDPISAHLL

SLNGEKWKRLRSKLTPTFTAGKIKMVFKIFVECGKIIEHTLEHHAQNRTSFDVKEMFSSY

STDVIVNCAFGVEVDRLKKTEFKNYSQKALDFNTRRCIVMYLGAVFPKLCRKLGFRQIAK

SAGDFFINIVRNTVEFREKTNTIRNDFLQLLINLKNDESDDWGQFSIEEVAAQCFIFFLA

GFETSATTMSFALYEIAANPHVYEQIMDEMRRVLDKYNGEMCHDAIKEMTYLGQVVDETL

RIHSPAYAVSRTCTQDYHVPETNIVLKKGFQVLIPIRGIHSDPEFYPEPEKFKPERFHPD

NRQNIEPYSYLPFGEGPRICIG

>Q9W223.1

MWTILLTILIAGLLYRYVKRHYTHWQRLGVDEEPAKIPFGVMDTVMKQERSLGMALADIY

ARHEGKIVGIYMLNKRSILIRDAQLARQIMTSDFASFHDRGVYVDEDKDPLSANLFNLRG

ASWRNLRQKLTPSFSSGKIKGMFGTIDDVGDKLVQHLEGALDQSDEVEIKDVMTTYAVDI

IGSVIFGLEIDSFRNPKNEFREISSSTSRDESLLLKIHNMSMFICPPIAKLMNRLGYESR

ILTSLRDMMKRTIEFREEHNVVRKDMLQLLIRLRNTGKIGEDDDQVWDMETAQEQLKSMS

IEKIAAQAFLFYVAGSESTAAASAFTLYELSMYPELLKEAQEEVDAVLMKHNLKPKDRFT

YEAVQDLKFLDICIMETIRKYPGLPFLNRECTEDYPVPGTNHIIAKGTPILISLFGMQRD

PVYFPNPNGYDPHRFDSNNMNYDQAAYMPFGEGPRHCIALRMGKVNSKVAVAKILANFDL

VQSPRKEVEFRFDAAPVLVTKEPLKLRLTKRK

>ADJ57460.1

MVLTEVLFVVVAALVALYTWFRRNHSYWQRKGIPYIPPTPIIGNTKVVFKMENSFGMHLS

EIYNDPRLKDEAVVGIYSMNKPGLIIRDIELIKSILIKDFNRFHNRYARCDPHGDPLGYN

NLFFVRDAHWKGIRTKLTPVFTSGKVKQMYTLMQEIGKDLELALQRRGEKNTGSFITEIK

EICAQFSTDSIATIAFGIRANSLENPNAEFRNYGRKMFTFTVARAKDFFVAFFLPKLVSL

MRIQFFTADFSHFMRSTIGHVMEERERSGLLRNDLIDVLVSLRKEAAAEPSKPHYAKNQD

FLVAQAGVFFTAGFETSSSTMSFALYEMAKHPEMQKRLRDEINEALVEGGGSLSYEKIQS

LEYLAMVVDEVLRMYPVLPFLDREYESVEGQPDLSLKPFYDYTLENGTPVFIPIYALHHD

PKYWTNPSQFDPERFSPANRKNIVAMAYQPFGSGPHNCIGSRIGLLQSKLGLVSLLKNHS

VRNCEATMKDMKFDPKGFVLQADGGIHLEIVNDRLYDQSAPSLQ

>EDS36418.1

MLLTITLVVSAVVALYVYLTWNFSYWKKRNVPGPSPHPLFGNFPSFILRNHPIAVEMDNV

YREYKSKYNFVGVFSTRSPRIFVTSAELAKSILIKDFKNFHDNEFAGMTDKELDPILGRN

PFMLNGDEWKAKRAEVTPAFTTSRMKSLFSIVEDVGSRMTKYIRQNKNTPLESKELAAKF

TTDVVSSCIFDTDAQSFTNEKSEISEQGRKMFDSSFLFVIVMIFMSLFPKLAKLLKIGMV

SKSVEKFFTKLMAEAIQYREKNGVQRVDYLEHLISLRNKKEISDLDMAAHGVTFFIDGFE

TSSVAMSFILYELAKNPDVQERLRKELLNASNDQGTITYDLLLELPYLDQVINESLRLWP

PAAFLSKKCTEPIELNLTLTEKKLIETDVCAIIPVWSIHRDPDHFEDPATFNPDRFSPER

GGASPYREKGCFMPFGDGPRQCLGIRFARMQVKRGVYEILTHFELTVDSKTVEPLQLDPK

QFLTMALGGIWLNFKPIAV

>EDS32857.1

MLVTISLLVSAVAAAYIYLVWNFNYWKKRHVPGPDPKPLVGNFPAFILRTRTVMEEMDEI

YNDYKAKYNFVGVFSNRSPRILITCPALAKDILTKHFRSFHDNEFGEFTESAGDAESVLS

ERGQVEGETSGDHASVHDVEGRMKALFPIVEFVSTSMSAYIVKNRNSELDARELAAKFTT

DVVSSCIFDTDAQSFTNEKAQIREMGRRLTNSSFTAFVTMMLLSTCPKLAKMLNIGSGVK

KVEQFFIDLMSEAAMYREKNGTKRVDYLEHLISLRNEKQITEVDMAAHGVTFFFDGFETS

SVVMSFILYELARNPTVQTRLREDCSKASNDQGTISYEDLLELPYLEQVINETLRLWPVG

PFLSKTCTEPVDLELCPGQSVRIEPGVCAVIPFGGIHRDPQFYQDPLTFNPDRFSPETGG

VNPYREMGCFMPFGEGPRQCLGMRFARMQMKRAIYEVVKNFELSVSDKTVQPLRMDPKQL

FTAAVGGIWLNFEPILEQS

>Q9GQM9.1

MTLVDWFLDTIALLLGLLHIWIARHFQHWEEKGVHHVAPQPLVGSMLRLLTFSVSPAVFI

QGVHEAAGSHPYIGFYIFGRPALLVRDPSLLQHILVKDFSNFTDRLTSSNKHKDPVGAAN

LFCIKGNRWRQIRACITHTFSTARLKIMFSRVLNSASVTRDYILERGNQPINVKDLFVRT

SLDSMCSTLFGIESSTLYNSEAQFLHYGHKMMRWTPYRALEALAHFFSPELLNVFDTRLF

ECESTEFLINAFSEAITEREKTGLYCSDLVDSLIQLKKQLIDVSEHEVLGQAMLFFAAGF

ETTSSAMAFAMYELALHPEIQDRLRTEIQEVTACHGGQVTYEGVKKMSYLDMVVSEVLRK

YPPMSFIDRVCLHDYHIPGTDVVIEKGTPIFVSLLGLHRDSIFYPDPEDFNPERFNKEKK

SGVHPFSYIPFGDGPRNCIGLRLGLMSVKLVIIMLLQSLRVETHESTPVPLQLSPYVLFL

KNLGRLPLMFVPIGEDNVETSLA

>AAP76391.1

QLYNELKGKHPFGGIYMFTKPVALVTDLELVKNVFVKDFQYFHDRGTYYNEKHDPLSAHL

FNLEGYKWKSLRNKITPTFTSGKMKMMFPTVVAAGKQFKDYLEDAIDEQEEFELKELLAR

YTTDVIGTCAFGIECNSMRNPDAEFRVMGKKIFGRSRSNLQLLLMNAFPSVAKLVGIKLI

LPEVSDFFMNAVRDTIKYRVENNVQRNDFMDILIRMRSDKETKSDDGTLTFHEIAAQAFV

FFVAGFETSSSLMAFTLYELALDQDMQDKARKCVTDVLERHNGELTYEAAMEMDYLECVL

KECLRKHPPISVHFRITAKDYIVPGTTSVLEAGTSVMIPVLGIHHDPEHFPEPERFDPER

FTAEQESKRHPYAWTPFGEGPRICVGPRFGLLQARIGLIYLLTSFRFVRCSKTPVPLRYA

LKNQ

>AAK32956.1

MLLYLLLLAVTAAIWYVRRKYTFWADRGVPYVKPRFPFGNIQGIGRRMHSSQLMTKFYNE

MKTSGRPFGGIYFFTNPVALALELDFVKNVLVRDFAHFHDRGVYYNEKDDPISGHLFNIE

GTKWTNLRKKLIPTFSSGKMKMMCPTIVSVGGQFRECLERCIAHSDGEVEMKELLARFTT

DVIGTCAFGIECNSLNDPNAEFLRMGRKVFEVPRGRILKFFFMATFKDFSRRIHIKGTAE

DVSQFFFKVVRETIEYREQNNIVRNDFMNLLMQLKNSGQLDGSGEEVGKLSLNEIVAQAF

VFFLGGFETSSTTMSYCLYELALNEAIQQRARECVVEAVKKHGGLTYEALMDMPYIDQCI

NESLRKYPPGANLIRQVSQDYRVPGTDVTFPKGMNVMIPVYAIHHDADNYPDPERYDPDR

FAPEACESRKPYSFIPFGEGPRICIAARFGMLEARVGLAVLLMHFSFARCSKTNVPLVIS

SRHAVLTPEGGLWLKVEKLKE

>AAK32959.1

MSLLGVLLLGVVLLLSLAYLFLRSKHNYWRSRGFPCKPSPSLLYGQMQGNGTTRHAAYVT

QEIYRYAQDRGERYMGYSFFFMPIVMVCDIELVKTVLVKDFAVFHDRGMYSNPRADPLSG

NLFALEGHEWRALRQKLTPTFTSGRMKQMFGTMLQVATELHRHLLEQIDRELEMKAVLAR

FTTDVIGTCAFGIECNTLRNPDSDFLKYGRRVFEHRMLAMVKMTFAMLFRAQAVKLGVKV

TDDDLEAFFMNLVHDTVEHRERNGVQRNDFLNLLLQIKNKGCLEEQEQEGSASADRTGMT

MNELAAQVFIFFVAGFETSSTVMNFCLYELAKNPHIQERLRDELNRSIDANGGELTYDMV

MGHEYLGQVVNETLRKYPPLETTLRVAGQDYTIPGTRHVIPRHVGVQIPVYAIHHDPAHY

PEPECFDPDRFSAEACRNRTPYTFLPFGEGPRVCIGMRFGMMQVKVGLVSMVRAFRFLPT

AQTPDRIVFDPKSFILSPAGGNYLRIVQV

>AAL58568.1

MLIVAVFVAFFGIFWIYFHFRQRYLFWLNRDVPFLEPTFPFGNMIDIFNPNIHFAHLIQK

LYRQLKDRGDYVGLFFFRDPVLLVLSPEFARTVMVKDFNYFVDRGVYSNEEFDPLSANLF

FMEGAKWRKLRSKLTPTFTSGKLKAMFHTIVDVANRLDQHLGERCVRMQRIDIKELLARF

MTDVIGSCAFGIECNTLENPNSQFRLMGKRMINLPKLKSLKVFFAMMFRKQAQALGIRYN

DKDVADFFMNVVRDTIRYRAENGVRRDDFMQLLIDMMQENGAGAGESLTFEEIAAQAFVF

FFGGFETSTTTLTFALHLLAQHPKAQRKARKCVRSTLAKHGNEMTYEAIKDMYYLECVIH

ETLRLYPPVASIHRMTSQPYQLPNGEVIPEGVGVIISNLAFQHDPTLFPDPLAFKPERFE

DKTFAKTNPSYLAFGDGPRMCIAMRFGKLQTCLGLAMLLKSYTFSLEDCDVDRPLLIDPI

NLMYGPAGPVWLNVERYVEGSKSS

>AAM94461.1

MIYFILALAIIWAFVKLYTHVFNYWEQRGFPYVEGKFPLGSDPCLSRPSKFLGLEVQEHY

RKLSGHPLGGIYVGRRPDLIVRDPKIIKNIMVKDFAHFRNRGVEIPSKDSPLTQHLFALE

GTKWRALRVKLTPTFTSGKLKLMYSLFVECAQRLERKLNEDSMKNEGVVDIKDTIARFTT

DIIGSCAFGLEIDSLNNPDEPFRKIGMRLFQRNLKGRLIELIYSLAPNLRNYLKLSRTSK

ETEKMFMSGIGQTIEYREKNNVRRNDFLDLLIELKNQGHLYVDRQKDSNIENDHSQDGEA

QEKIEMDLGMITAQCFVFFIAGFETSSSVQSYALYELAYHQVIQDKVRREIEEVTSKFGG

LNYQALHEMPYLAQVINETIRKYPTLHTLTRVCTKPYAVPGTKYVIEEGIRVLIPVYSLH

HDRQYFPDPERFDPDRFSDTNKSSITPFTFLPFGEGPRNCIGMRFGLLQTKIGLVTLLKS

KRVLPTAQTKSPLVFSSSLSVISQNEGSIVLKLENI

>AAL93297.1

MFLQLVGVVLAIVLSCLAWIHRRYHFWKDRSVAYIKPRFPFGNFATLGKVEHIAPITQRH

YDHFKRQNVPYGGVFMLTSPLLYIFDTKLIKQLLVKDFHHFPNRGVYFNERDDPLSAHMF

AIEGQKWRTLRAKLSPTFTSGRIKMTLPLITQVCERFCEHLNESLQSSDEIEVHDLLSRY

TIDVIGACAFGIECNSFREPDNEFRRYGKIAFDKLRHSPLVVYLMKAFRAHANALGMKQL

HDDVSGFFMRVVKDTVEYREREQIVRNDFMDLLLKLKNTGRLEADGEEIGRLTFEEIAAQ

AFIFFTAGYDTSSTAMSYTLYELALNPEVQERARECVKQTLQKYDGKLSYEAVSEMSYLE

QCISETLRKHPPVAILERNADKDYRLPDSGLLLRRGQKIMIPIYAMHHDPAHFPEPEQYR

PERFSPDEVARRDPYCYLPFGEGPRVCIGMRFGSIQAKLGLASLLDRFRFSACDRTQIPV

QYSRTNFILGPANGVWLRVEVLDA

>AAO24698.1

MFVYTLALVAAVIFLVLRYIYSHWERHGLPHLKPEIPYGNIRTVAEKKESFGIAINNLYH

KSSDRLLGIYLFFRPAILIRDPHLAKRIMVNDFQNFHDRGVYCNEEHDPFSANLFALPGQ

RWKNLRAKLTPTFTSGQLRNMLPTLLDVGNKLIDRMNKVADEKAIVDMRDIASRFVLDTI

ASVFFGFEANCIHNSEDPFLSTLQRLTKSRKFMDNFRTSGVFICPGLLKLTRITSLPPEL

ISFVMEIITHQIDHREKNQITRKDFVQLLIDLRREAENGSEKALSIEQCAANVFLFYIAG

AETSTATISFTLHELSHNPEAMAKLQQEIDEMMERYNGEITYENIKEMKYLDLCVKETLR

KYPGLPILNRECTIDYKVPDSDVVIRKGTQVIIPLWSISMNEKYFPDPELHSPERFDEAT

KNYDADAYYPFGAGPRNCIGLRQGVFVSKIGLVLLLSKYNFQATTPAKVKFAVATVVVTP

EDGFPMRVEHRC

>ADD19432.1

MSVLQIFSYVLVTLITIAYYFIRRHHTYFAVRNIPHIKPVLLYGNMKKVGNSQHLKEPFL

QLYKKFKAEAPFAGFYLSIRKAVVLLDLDLIKRVLITDFSEFSHRGNYYNEKDDPLSGHL

FNLDGPKWKLMRAKLTPTFTSGKMKFMFSTVVEVGQRFVEVLKEILEKEPQENGVEIREL

LARFTTDVIGCCAFGLDCNSLKDPETKFRTMGKKAFTTRRHGRFVVAFMQAFPNISRRLG

LCMAPKDVSDFFMKVVKETVEYREKEDVQRNDFMNILLELRKRNGMEGLSLEEIAAQSFV

FFLAGFETSSSSMAFALYELAVNQQMQEQSRQEVRSVLEKYNNVLTYEGLKELHYLQKVL

NETMRKYSIASVIIRKALTNYQVPGSKLIIEKDEIVIIPVDAIHHDPDIYPNPEEFQPER

FNADQINQRHAMSWLPFGEGPRNCIGLRFGKLQAMIGLTLLLNNFYFTLSPQTPVPMIFD

PKSFVLAPKGGIFLNIQRVE

>ADD19579.1

MVDATTLLLVVITILTGMFIWSRRTNVYWRRRRVKFVPPSYFLGNLKDVLKMQNSFALQL

RDYYFNERFTREPLVGIYLFHQPALLLRDLRLIHTVLVEDYVNFSHRFAQCDRAQDKMGA

LNLFFARNPEWRDIRTKLAPVFSAGKMRQMFSLMEEIGYDLETYLAKLTKDIKNKNARST

IVQIKDICDLYNTDMIASIAFGLKTYSLRNTVRSQLGGHSHDMFVISYRHALDLCLIFLC

PKLVKLIKPKLFTPEHTQFLRNLVLSMLEERERSGIMRNDLIETLLTLKKEAALHQDTSH

FGHQPDYLVAQAATFELAGIQTCSSTLAFALYELARHPAIQERLHAEIFRILGNDEATLT

YEKVEHMTYLRMVIEETLRKYPIVPFLERQCTPLHKKRFVTFRPLAECMARRGMPVFISN

LAIHYDPQFWPNPEEFDPERFSLEQKKTHLPMTYLPFGAGPHQCIGMPVAHMQIKLGIIH

FLRRHRLEICDKTVENITFDKRYALLSQEGGIYLKLVKI

>Q9VRI9.1

MIAVFSLIAAALAVGSLVLLPVVLRGGCLLVVTIVWLWQILHFWHWRRLGVPFVPAAPFV

GNVWNLLRGACCFGDQFRELYESKEAAGRAFVGIDVLHNHALLLRDPALIKRIMVEDFAQ

FSSRFETTDPTCDTMGSQNLFFSKYETWRETHKIFAPFFAAGKVRNMYGLLENIGQKLEE

HMEQKLSGRDSMELEVKQLCALFTTDIIASLAFGIEAHSLQNPEAEFRRMCIEVNDPRPK

RLLHLFTMFFFPRLSHRVGTHLYSEEYERFMRKSMDYVLSQRAESGENRHDLIDIFLQLK

RTEPAESIIHRPDFFAAQAAFLLLAGFDTSSSTITFALYELAKNTTIQDRLRTELRAALQ

SSQDRQLSCDTVTGLVYLRQVVDEVLRLYPPTAFLDRCCNSRTGYDLSPWNGGSPFKLRA

GTPVYISVLGIHRDAQYWPNPEVFDPERFSAEQRQQHHPMTYLPFGAGPRGCIGTLLGQL

EIKVGLLHILNHFRVEVCERTLPEMRFDPKAFVLTAHNGTYLRFVKNSL

>Q9VRB3.1

MVYSTNILLAIVTILTGVFIWSRRTYVYWQRRRVKFVQPTHLLGNLSRVLRLEESFALQL

RRFYFDERFRNEPVVGIYLFHQPALLIRDLQLVRTVLVEDFVSFSNRFAKCDGRSDKMGA

LSLFLAKQPEWREIRTRLAPAFAGAKLKQMFSLMEEIGCDLEWYLKRLTRDLRRGDAERG

AIVSIKDVCDLYNTDMIASIAFGLRSYSLRNTQSEIGSHCQDLFRPNVRRIIDLFVIFYL

PKLVPLLRPKLFTEPHAEFLRRVIQLVIEERERGGDLRNDLIEMLLTLKKEADLQQDKSH

FTHHRDFLAAQAASFEVAGIETCSASMSFALYELAKQPLMQSRLRREIREAFASNPNGRL

TYEAVARMEFLDMVVEETLRKYPIVPLLERECTPINKKRFYSLRPHAECYTRRGMPVFIS

NLAIHHDPKYWPDPDRFDPERFSAANKALQAPMSYMPFGAGPRNCIGMQIGLLQIKLGLV

YFLHQHRVEICDRTVERIQFDAKFALLASEQRIYLKVDCL

>Q9V9L1.1

MLLLLLLGSLTIVFYIWQRRTLSFWERHGVKYIRPFPVVGCTREFLTAKVPFFEQIQKFH

EAPGFENEPFVGVYMTHRPALVIRDLELIKTVMIKKFQYFNNRVLQTDPHNDALGYKNLF

FARSPGWRELRTKISPVFTSGKIKQMYPLMVKIGKNLQDSAERLGSGTEVQVKDLCSRFT

TDLIATIAFGVEANALQDAKSEFFYHNRAIFSLTLSRGIDFAIIFMIPALASLARVKLFS

RETTKFIRSSVNYVLKERERTGEKRNDLIDILLALKREAAANPGKMSKEVDLDYLVAQAA

VFQTAGFETSASTMTMTLYELAKNEALQDRLRQEIVDFFGDEDHISYERIQEMPYLSQVV

NETLRKYPIVGYIERECSQPAEGERFTLEPFHNMELPHGMSIYMSTVAVHRDPQYWPDPE

KYDPERFNSSNRDNLNMDAYMPFGVGPRNCIGMRLGLLQSKLGLVHILRNHRFHTCDKTI

KKIEWAPTSPVMASKRDIILRVEKVSGKKDFGQK

>289177186

MLEFTYSQLFWITAVFAAVYAYLKYYLYNYWNRYNVPNETPSIPLGNVPLDFLRGKIHYG

GFIDSIYKKFKHYRFCGIYVFHKPILCINDPELIKLILVKDFDLFPDRGWFYDGKIDPMS

SHLFLLPGEKWKRLRVKFAPIFTSGKLRQMYPFLVEISKHMIKTCDAELKTTDTVDVNDI

LARFTTDVISSIVYGLDSKSLDDPKSEFRTKGVKALSFGSFNLLVALFAPEMMSWATVPL

YQENVSEFFLNIFKEAVNYRRKEKIQKKDFLDLIMQLIDSGKVDDENKILQTNGKNSEHF

DKLTIEEAAANAFVFFLGGYETSSSTTSFCLYELAQNPEVQEKLQAEIDEVVRSLTGLTY

ESIAEMEYLDMVLSETLRKYPSGATLNRIAKEDYPLPNTDFVIKKGMRIIIPLSGIQNNP

EYYPDPEKFDPLRFTKEKVAARNKYVNIPFGDGGRICIGKRFAVMQVKIVLTAMLYNYQF

SISQKTQVPPKYETRNFTQIARNGVHLRVERR

>ACA51846.1

MELLEIVKSAMDTHSVLLIFLSVMVYLLYVYRDKFHYWSKRGVPCQSPAQSIVRTFRLVL

RMDSFTDNFYGVYKAFDGHPYVGSLELTKPILVVRDPELARIVLVKSFSSFSGRLKSPDT

TLDPLSNHLFTLNGEKWRQVRHKTATAFSTAKLKNMFHSLKDCAREMDAYMERAIGDKGD

VEFDALKVMSNYTLEVIGACAMGIKCDSIHDEETEFKRFSRDFFRFDARRMIFTLLDLLH

PKLPVLLKWKAVRPEVENFFREAIKEAASLKESEAAARTDFLQILIDFQKSEKASKTDAG

NDTELVFTDNIIGGVIGSFFSAGYEPTAAALTFCLYELARNPQVQAKLHEEILAVKEKLG

DDIEYETLKEFKYANQVIDETLRLYPASGILVRTCTEPFKLPDSDVVIEKGTKVFVSSYG

LQTDPRYFPEPEKFDPERFSEENKEKILPGTYLPFGDGPRLCIAMRLALMDVKMMMVRLV

SKYEIHTTPKTPKKITFDTNSFTVQPAEKVWLRFRRRASTP

>EDS36419.1

MILTITLVVTAVAALYVYLTWNYGYWKKRNVPGPDPLPFLGSFPSLILRHRTIMEEMDKI

YSDYKNKFNFVGIFSNRSPSIFVASPALAKDVLIKDFKNFHDNEFGGFTDKDTDPILSRN

PFLLNGEEWKEKRSEVTPAFTTSRMKALFPIIEDVGTRMTKYVKQKGKTALDTRELTAKF

TTDVVSSCIFDTDAESFTNEKAVIREMGRKLLDNSFSFFLIAMFIGVMPTLAKLLNVGLI

PKSVEKFFTNLMSEALKHREQKGIKRVDYLEHLISLRNKKNISELDMAAHGVTFFIDGFE

TSSIAMAFVLYEVARNPDVQKQLREELLKASNDQGSISYDTLLELPYLEQVINESLRLWP

PAAFLSKKCTEPIELDLTSSQKVHIEKGVYAMIPVWSIHRDPECYENPTMFNPDRFSPEQ

GGVSPYREKGCFIPFGDGPRQCLGMRFARMQMKRGVYEILKNFELSVDPKTIQPLQMDPK

QFLTMPLGGIWLTYKAITK

>EAT41341.1

MFLTITLIVTAVAAIYLYLTWNYNYWKKLNVPGPSPLPGLGSFPSFITQRRPVADEMDEI

YREYKPKYNFVGVFSNRSPRIMITSSELAKDILSKNFKNFHDNEFGEMTNKEIDPLFGRN

PFMLTGDEWKAKRAEITPAFTTSRMKALFPLVEDVCSRMTKYVTQNRGSVLDSKELSAKF

TTDVVSSCIFACDAQSFTSGKPEIREQGRKLMEQSFSSFLILLFIINFPTLAKIFKIGLV

PKSLEKFFTDLMKEAISHRDASGTNRVDYLDYLISLRNKKEISELDMAAHGVTFFIDGFE

TSSVAISFMLYEIAKNPEVQKRLRKELQKVTTDQGTVSYDSLLELSYLDQVVNESLRLWP

PAAFISKKCTEPMDLPLTANQNVTIGKEICAIINIWSLHRDPEYYDDPLTFNPDRFSPET

GGTAPYREKGCFIPFGDGPRQCLGMRFARMQVKRCLYELVSNFKITVNEKTKQPMKLDPK

QFLTMPLGGIWLDFEPISK

>EAL40433.4

MFVTVTLLVTAITFVYVYLAWYSNYWTKRGVPGPAPRLLFGNFPSFILRNRPFVDDFQDI

YDKYRSKGNVIGVFNGRQPAVLVMKPELIKDVLIKNFKQFQDNEFADSVSKDSDPIFGRN

PFMLKGEEWKEKRAEVTPAFTTSRMKALFPFVEDVSNRMKQYIAQHTTKGPIETRELCAK

YTTDVVSSCIFAADAQSFTKEKAEIREMGRKLLEPNFQIVMMFLLIGLIPALKKLIKISF

VSKEIEQFFTDLMEKAVAYREQNNVKRVDYLDHLMALRAKKQLSSLDMAAHGVTFFIDGF

ETSSLAMCFTLYELARNPDCQETLRSELMAARNEDGSIDYDALLELPYLDQVLYEALRIW

PPAAFLSKVCTVPTELEVTDGHKVLIERGLPVMLPVWAVHRDKEYYEEPERFNPDRFSPA

TGGTKVYREKGCFLPFGDGPRQCLGMRFALMQVKRGLFEVITKYEVSVNSKSKQPIQLDP

KAFIVCPLGGIWLDYKPIEA

>EAT41342.1

MFITITLIVSAVTAIYLYLTWHFNYWKKLNVPGPSPLPGLGNFPSFITQKRPVAEEMDEI

YREYKPKYNFAGVFSNRSPRIMITSAELAKDILVKNFKNFHDNEFGELTNKEIDPLLGRN

PFLLDGSEWKAKRAEVTPAFTTSRMKALFPLVEDVCSRMTKYLIKNRGSVIDAKELSAKF

TTDVVSSCIFACDAQSFTSEKPEIREQGRKLIEQTFSSFMLLLFIVNFPTLAKIFHVGFI

PKSMEKFFTNLMKDAVRYRDASETNRADYLDYLITLKKKKELSELDMAAHGVTFFIDGFE

TSSVAISFMLYEIAKNPTVQKRLRQELKKVTTDNGTVSYDSLLELSYLDQVVNESLRLWP

PAAFMSKKCTEPMELPLTANRSVTIGKEVCAIINIWSLHRDPEYFDDPLTFNPDRFSPET

GGTSPYREKGCFVPFGEGPRQCLGMRFARMQVKRCLYEAVTNFAITVNPKTMEPMRLDPK

QVLTMPLGGIWLNFEPISK

>157114918

MWLIVISILVTIVSLVYHYLTWNFNYWKYRGVPGPLPKPFLGTFSSTFTQKEHPIEENNR

IYREYRKDVPFIGGFSFRSPQLFALSPTLVKDILVKYHKHFRANEVGGTFDSKADPLLAR

NPFFLDGEEWRSKRAQITPAFTNSRLKALLPIMDNICNNMVSYIDRHIPNGPIESKELSA

KYTTDVVSSCIFGAEGGSLTSDRSEIREMGNALFQQTFMFIVLAVISSIAPILKRFVKLS

LIPKSIENYFVGLMTEAVRKRKASGTKQVDYLDHLINLQEQKEISILDMAAHGVTFFIDG

FETTSEVLGFSLLELSIDKEIQNRLRQEIHSAEDGQLTFETIMELPYLDQIVNETLRKWP

PAYALSKRCTEEITFRLKDNHEVLIEKGITAILPIWAIHLDKEFYPDPNRFNPDRFSEED

GGHSVRYYQEKGVFLPFGDGPRACIGRRIGLLQVKRALVEIVKNYDFTVNSKTVLPIKID

PKNIAVTPLGGIWIDYRKL

>EDS36420.1

MLCTCLTVLGGLAAVVYLILVWNFNYWKKAGINGPKPKILAGNLPNMWTQKQHIFYDLKK

IYDDFKNEPVVGYFSVRTPQLMIRDPELIKEVLTKSFRYFASNQFSDLVDEKSDPLFSRN

PFSLSGEKWKTRRAEITPAFTNNRIKALSTLVDEVCVRLSDYVKQNMEKNGSTFDVKELM

SKYTTDVVSNCVFAIDAQSFTKENPEIREMGRRIMDFNFKAQVIMFLTTFFPSITKFYKF

TFIAHDVEQFFIRIMKDAIRHRKQNNIVRNDYLDHLLSLEEKKQVTEIDMAGHGVSFFAD

GFETSSSVITYCLFDLATNPAIQTQLRQEIRDVQANKGGLTYDNVGEMTFLEQVLNESLR

MHVVIPTLAKRCTETVELISSKGKKIRVEAGTAVHIPYMVHFDSQYYDEPDKFKPERFSP

ENGGTKPFREKGVFFPFGEGPRMCLGMRFALMQAKRGIVEIIDKFEISVDPKMKMPIVYD

PKSFMLYPDGGTWLKFKVL

>Q27593.2

MALTYILFQVAVALLAILTYYIHRKLTYFKRRGIPFVAPHLIRGNMEELQKTKNIHEIFQ

DHYNKFRESKAPFVGFFFFQSPAAFVIDLELAKQILIKDFSNFSNKGIFYNEKDDPISAH

LFNLDGAQWRLLRNKLSSTFTSGKMKLMYPTVVSVANEFMTVMHEKVPKNSVLEIRDLVA

RFTVDVIGTCAFGIQCNSLRDEKAEFLYFGKRSLVDKRHGTLLNGFMRSYPKLARKLGMV

RTAPHIQEFYSRIVTETVAVREKEHIKRNDFMDMLIELKNQKEMTLENGDVVRGLTMEEV

LAQAFVFFIAGFETSSSTMGFALYELAKNPDIQDKVRAEVEEVIEQHDQNFTYECTKDLK

YLNQVLDETLRLYTIVPNLDRMAAKRYVVPGHPNFVIEAGQSVIIPSSAIHHDPSIYPEP

FEFRPERFSPEESAGRPSVAWLPFGDGPRNCIGLRFGQMQARIGLALLIRNFKFSTCSKT

PNPLVYDPKSFVLGVKDGIYLKVETV

>EDX07155.1

MALTYILFQVAVALLAIFTYYLHRKLTFFKRRGIPFVAPHPIRGNMEELQKTKNIHEIFQ

DHYNKFRDSKAPFVGLFFFQNPAAFVIDLELAKQILIKDFSNFSNKGIFYNEKDDPISAH

LFNLDGAQWRLLRNKLSSTFTSGKMKLMYPTVVSVANEFMTVMHEKVPKNSVLEIRDLVA

RFTVDVIGTCAFGIQCNSLRDEKAEFLHFGKRSLIDKRHGTLLNGFMRSYPKLARKLGMV

RTAPHIQEFYRRIVTETVAVREKEHIKRNDFMDMLIEMKNQKEMTLENGDVVRGLTMEEV

LAQAFVFFIAGFETSSSTMGFALYELAKNPHIQDKVRAEVEEVMEQHDQNFTYECTKDLK

YLNQVINETLRLYTIVPNLDRMAAKRYVVPGQPKFVIEAGQSVIIPSSAIHHDPSIYPEP

FEFRPERFSPEESAGRPSVAWLPFGDGPRNCIGLRFGQMQARIGLALLIRNFRFSTCSKT

PNPLVYDPNSFVLGVKDGIYLKVEVV

>AAK71286.1

MALTYILFQVAVALLAILTYYIHRKLTYFKRRGIPFVAPHLIRGNMEELQKTKNIHEIFQ

DHYNKFRESKAPFVGFFFFQSPAAFVIDLELAKQILIKDFSNFSNKGIFYNEKDDPISAH

LFNLDGAQWRLLRNKLSSTFTSGKMKLMYPTVVSVANEFMTVMHEKVPKNSVLEIRDLVA

RFTVDVIGTCAFGIQCNSLRDEKAEFLYFGKRSLVDKRHGTLLNGFMRSYPKLARKLGMV

RTAPHIQEFYSRIVTETVAVREKEHIKRNDFMDMLIELKNQKEMTLENGDVVRGLTMEEV

LAQAFVFFIAGFETSSSTMGFALYELAKNPDIEQHDQNFTYECTKDLKYLNQVLDETLRL

YTIVPNLDRMAAKRYVVPGHPNFVIEAGQSVIIPSSAIHHDPSIYPEPFEFRPERFSPEE

SAGRPSVAWLPFGDGPRNCIGLRFGQMQARIGPALLIRNFKFSTCSKTPNPLVYDPKSFV

LGVKDGIYLKVETV

>EDW47933.1

MPNQSYSKITRLSMALTYMLFQVAVALLAIFTYYLHRKLTFFKRRGIPFVAPHPIRGNME

ELQKTKNIHEIFQNHYNKFRDSKAPFVGLFFFQNPAAFVIDLELVKQILIKDFSNFSNKG

IFYNEKDDPISAHLFNLDGAQWRLLRNKLSSTFTSGKMKIMYPTVVSVANEFMTVMHEKV

PKNSVLEIRDLVARFTVDVIGTCAFGIQCNSLRDEKAEFLHFGKRSLIDRRHGTLLNGFM

RSYPKLARKLGMVRTAPHIQEFYHRIVTETVAVREKEHIKRNDFMDMLIEMKNQKEMTLE

NGDVVKGLTMEEVLAQAFVFFIAGFETSSSTMGFALYELAKNPHIQDKVRAEVEEVMEQH

DQNFTYECTKDLKYLNQVISETLRLYTIVPNLDRMAAKRYVVPGQPKFVIEAGQSVIIPS

SAIHHDPSIYPEPFEFRPERFSPEESTGRPSVAWLPFGDGPRNCIGLRFGQMQARIGLAL

LIRNFRFSTCSKTPNPLVYDPNSFVLGVKDGIYLKVEVV

>AAB05550.1

MALAYILFQVAVALLAILTYYIHRKLTYFKRRGIPFVAPHLIRGNMEELQKTKNIHEIFQ

DHYNKFRESKAPFVGFFFFQSPAAFVIDLELAKQILIKDFSNFSNKGIFYNEKDDPISAH

LFNLDGAQWRLLRNKLSSTFTSGKMKLMYPTVVSVANEFMTVMHEKVPKNSVLEIRDLVA

RFTVDVIGTCAFAIQCNSLRDEKAEFLYFGKRSLVDKRHGTLLNGFMRSYPKLARKLGMV

RTAPHIQEFYSRIVTETVAVREKEHIKRNDFMDMLIELKNQKEMTLENGDVVRGLTMEEV

LAQAFVFFIAGFETSSSTMGFALYELAKNPDIEQHDQNFTYECTKDLKYLNQVLDETLRL

YTIVPNLDRMAAKRYVVPGHPNFVIEAGQSVIIPSSAIHHDPSIYPEPFEFRPERFSPEE

SAGRPSVAWLPFGDGPRNCIGLRFGQMQARIGPALLIRNFKFSTCSKTPNPLVYDPKSFV

LGVKDGIYLKVETV

>EDV55965.1

MALTYLLFQVAVALLAIFTYYLHRKLTYFKRRGIPFVAPHPIRGNTEELQKTKNIHEILQ

DHYNKFRESKAPFVGFYFFQNPTAFVIDLELAKQILIKDFSNFSNKGIFYNEKDDPISAH

LFNLDGAQWRLLRNKLSSTFTSGKMKLMYPTVVSVANEFMAVMHEQVPNNSVLEIRDLVA

RFTVDVIGTCAFGIKCNSLRDEKAEFLYFGKRSLVDKRHGTLLNGFMRSYPKLARKLGMV

RTAPHIQEFYRRIVTETVAVREKEHIKRNDFMDMLIEMKNQKEMTLENGDVVRGLTMEEV

LAQAFVFFIAGFETSSSTMGFALYELARNPHIQDKVRAEVEEVMEQHGQNFTYECTKDLK

YLNQVINETLRLYTIVPNLDRMAAKRYVVPGQPEFVIEAGQSVIIPSSAIHHDPSIYPEP

FEFRPERFSPEESSSRPSVAWLPFGDGPRNCIGLRFGQMQARIGLALLIKNFRFSTCSKT

PDPLVYDPNSFVLGVRDGIYLKVELVQQID

>EDW91091.1

MALTYILFQVAIALLATFTYYLHRKLTYFKRRGIPFVAPHLVRGNMQELQRTKNFHEILQ

DHYNKFRESKAPFVGFFFFQNPTAFVIDLDLAKQILIKDFSNFSNKGIFYNEKDDPISAH

LFNLDGAQWRLLRNKLSSTFTSGKMKLMYPTVVSVANEFMAVMHEKVPKSPVLEIRDLLA

RFTVDVIGTCAFGIKCNSLRDEKAEFLYFGKRALVDKRHGTLLNGFMRSYPNLARKLGMV

RTAPHIQQFYRRIVTETVAVREKEHIKRNDFMDMLIEMKNQKELTLENGDVVKGLTMEEV

LAQAFVFFIAGFETSSSTMGFALYEMARNPHIQDKVRAEVEEVMEQHGQNFTYECTKDLK

YLNQVINETLRLYTIVPHLDRMAAKRYVVPGHPEFVIEAGQSVIIPSSAIHHDPSIYPEP

FEFRPERFSPEESSSRPSVAWLPFGDGPRNCIGLRFGQMQARIGLALLIKNFRFSTCSKT

PDPLVYDPNSFVLGVKDGIYLKVEVV

>EDX14653.1

MPLTYFLFQVAVALLAIVTYILHRKLTYFKRRGIPYDKPHPLRGNMEGYKKTRTVHEIHQ

EYYNKYRNSKAPFVGFYLFQKPAAFVIDLELAKQILIKNFSNFTDKGIYYNEKDDPMSAH

LFNLDGPQWRLLRSKLSPTFTSGKMKFMYPTIVSVAEEFMTVMHEKVAENSILDVRDLVA

RFTVDVIGTCAFGIKCNSLRDEKAEFLHFGRRALVDSRHGNLVSGLMRSYPNLARRLGLC

RNTAQIQEFYQRIVKETVTLREKENIKRNDFMDMLIGLKNQKDMTLENGEVVKGLTMDEI

IAQAFVFFIAGFDTSSSTMAFALYELAKNPSIQDKVRAELEQVLEQHDQKFTYECIKDLK

YLDQVINETLRHYTIVPNVDRVAAKRFVVPGKPKFVIEAGQSVIIPSSAIHHDPSIYPEP

NEFRPERFSPEESAKRPSVAWLPFGEGPRNCIGLRFGQMQARIGLAMLIKNFMFSPCSET

PDPLIFDPHSAILLGIKGGIQLKVEAI

>Q9VB31.1

MQLTYFLFQVAVALLAIVTYILHRKLTYFKRRGIPYDKPHPLRGNMEGYKKTRTVHEIHQ

EYYNKYRNSKAPFVGFYLFQKPAAFVIDLELAKQILIKNFSNFTDKGIYYNEKDDPMSAH

LFNLDGPQWRLLRSKLSSTFTSGKMKFMYPTVVSVAEEFMAVMHEKVSENSILDVRDLVA

RFTVDVIGTCAFGIKCNSLRDEKAEFLHFGRRALLDSRHGNLVSGLMRSYPNLARRLGLC

RNTAQIQEFYQRIVKETVTLREKENIKRNDFMDMLIGLKNQKNMTLENGEVVKGLTMDEI

VAQAFVFFIAGFDTSSSTMGFALYELAKNPSIQDKVRAELGQVLEQHDQKFTYECIKDLK

YLDQVINETLRHYTIVPNVDRVAAKRFVVPGNPKFVIEAGQSVIIPSSAIHHDPSIYPEP

NEFRPERFSPEESAKRPSVAWLPFGEGPRNCIGLRFGQMQARIGLAMLIKNFTFSPCSAT

PDPLTFDPHSAILLGIKGGIQLKVEAI

>ACL91251.1

MQLTYFLFQVAVALLAIVTYILHRKLTYFKRRGIPYDKPHPLRGNMEGYKKTRTVHEIHQ

EYYNKYRNSKAPFVGFYLFQKPAAFVIDLELAKQILIKNFSNFTDKGIYYNEKDDPMSAH

LFNLDGPQWRLLRSKLSSTFTSGKMKFMYPTVVSVAEEFMAVMHEKVSENSILDVRDLVA

RFTVDVIGTCAFGIKCNSLRDEKAEFLHFGRRALLDSRHGNLVSGLMRSYPNLARRLGLC

RNTAQIQEFYQRIVKETVTLREKENIKRNDFMDMLIGLKNQKNMTLENGEVVKGLTMDEI

VAQAFVFFIAGFDTSSSTMGFALYELAKNPSIQDKVRAELGQVLEQHDQKFTYECIKDLK

YLDQVINETLRHYTIVPNVDRVAAKRFVVPGNPKFVIEAGQSVIIPSSAIHHDPSIYPEP

NEFRPERFSPEESAKRPSVAWLPFGEGPRNCIGLRFGQMQARIGLAMLIKNFTFSPCSAT

PDPLTFDPHSAILLGIKGGIQLKVEAI

>EDW98503.1

MSLTYFLFQVAVALLAILTYILHRKLTYFKRRGIPYDKPHPIKGNMEGYKKTRTVHEIHQ

EYYNKYRNSKAPFVGFYLFQKPAAFVIDLELAKQILIKNFSNFTDKGIYYNEKDDPMSAH

LFNLDGPQWRLLRSKLSSTFTSGKMKFMYPTVVSVAEEFMAVMHEKVSENSILDVRDLVA

RFTVDVIGTCAFGIQCHSLRDEKAEFLHFGRRALVDSRHGNLVSGLMRSYPNLSRRLGLC

RNAAHIQEFYQRIVKETVTLREKENIKRNDFMDMLIALKNQKNVTLENGEVVKGLTLDEV

VAQAFVFFIAGFDTSSSTMGFALYELARNPSIQDKVRAELEQVLEQHDQKFTYECIKELK

YLDQVINETLRHYTIVPNVDRVATKRFVVPGNPKFVIEAGQPVIIPSSAIHHDPSIYPEP

NEFRPERFSPEECAKRPSVAWLPFGEGPRNCIGLRFGQMQARIGLAMLIKNFSFSPCSAT

PDPLIFDPHSAILLGIKGGIHLKVKAL

>EDV35769.1

MALTYILFQVVVALLAVATYILHRRLTYFKRRGIPYDPPHPLKGNLQGLMKTKGFHEVLR

DHYNKFKDVKAPFVGFYIFQKPAVLVKDQELAKNILIKDFSNFSEKGLYYNEKDDPISAH

LFNLDGPKWRSLRSKLSSTFTSGKMKYMYPTVVSVANEFIAVMNEKVEQNSVLDIRDLVA

RFTVDVIGTCAFGIQCNSLRDEKAEFLQFGKRSLVEKRHGVLILGFMRTYPKLAKKLGFV

RTAKPIQDFYRRIVTETVTIREKENIRRNDFMDMLIDMKNKKELTLENGEVVKGLTMDEV

LAQAFVFFIAGFETSSSTMGYALYELAKNPQIQDKLRAEINDVLVEHNKELTYECIKDLH

YLHQVINETLRRYTIVPQLDRMAKNRYVIPGHPNFVIEAGQSVVIPSSAMHLDPDIYPEP

YEFRPERFSPQESANRSSVAWLPFGDGPRNCIGLRFGQMQARIGLALLIKNFKFSTCPDT

PDPLVYDPKSFVLSVKDGIHLKLEKVPQ

>EAL26035.2

MGVTYVLLQVAVALLAIVTYYVYRRLSYFKSRGIPHDTPHLILGNMQGVFTKRNVHEILK

KYYEKYRESKVPFVGFYFFQKPSAFILDLDLAKHILIKDFSNFSDKGLFYNEKDDPISAH

LFNLDGPQWKVLRNKLTSTFTAGKMKFMYPTVVSVAEEFIDVMHEKMKQNSVLEIRELMA

RFTVDVIGTCAFGIKCNSLRDENAEFLRFGKRTFVDKRHGSFLTGFMTSYPKLARRLRLV

RTPTHIRNFYERIVHETVAMREKENIKRNDFMDLLIDMKNQKVKLENGDLVDGLTIDEIL

AQAFVFLIAGFETSSSTMGYALYELAKQPHIQDKVRAEVEEVLEQHEQKFTYESTKDLKY

LEQVIFETLRLHTIVPHLDRMAHKRYVVPGHPNFVIEAGQSVIIPASAIHVDPNIYPDPY

KFRPERFSPEESAKRNSLAWLPFGDGPRNCIGLRFGKMQTTIGLALLIKNFKFSTCPKTP

DTLSYSPKSFVLAVDGIHLKVEAV

>EDW74196.1

MGLTFLLFQVAVALFAIASYYIYRNLSYFKRRNIPHETPHWLHGNLKGLRSTHNFHDIFG

AYYNKFKTLQVPFVGLFVHQKPAVFVTDLELVKHVLIKDFSNFSDKGLYYNEKDDPISAH

LFTLDGPQWRLLRNKMSSTFTSGKMKLMFPTVVSVANEFIAVMHEKSQKNSVLEIRDLMG

RFTVDVIGTCAFGIECNSLRDENAEFLQYGKLTLVEKRHGNLIIAFMESFPKLARRLRLA

RTAKRVEEFYTRIVKQTVAIREKQNIKRNDFMDLLIELKNKKNSNPDNNEGLTMEEVIAQ

AFVFFIAGFETSSSTMGFALYELAKNPHIQDKLRAEVEEVFEKHNQQFTYECMKDLKYLT

QVMFETLRLYTIVPHLNRQALKRYEVPGHPNFIIEAGQSVIIPAAAIHRDPKLYPDPEEF

RPERFSPEESANRESVAWLPFGDGPRNCIGLRFGQMQARIGLALLIKNFKFSTCPETPDP

LVFNPKSFALGMTSGINLKVEAI

>EDW45247.1

MPLTYFLFQVAVALLAIVTYILHRKLTYFKRRGIPYDKPHPLRGNMEGYKKTRTVHEIHQ

EYYNKYRNSKAPFVGFYLFQKPAAFVIDLKLAKQILIKNFSNFTDKGIYYNEKDDPMSAH

LFNLDGPQWRLLRSKLSPTFTSGKMKFMYPTIVSVAEELMAVMHEKVAENCILDVRDLVA

RFTVDVIGTCAFGIKCNSLRDEKAEFLHFGRRALVDSRHGNLVSGLMRSYPNLARRLGLC

RNTAQIQEFYQRIVKQTVTLREKENIKRNDFMDMLIGLKNQKNMTLENGEVVKGLTMDEI

VAQAFVFFIAGFDTSSSTMTFALYELAKNPSIQDKVRAELEQVLEQHDQKFTYECIKDLK

YLDQVINETLRHYTIVPNVDRVAAKRFVVPGNPKFVIEAGQSVIIPSSAIHHDPSIYPEP

NEFRPERFSPEESAKRPSVAWLLFGEGPRNCIGLRFGQMQARIWTSHAH

>EDV53413.1

MEGFRKTRTVHEIHQEYYNKYRNSKAPFVGFYLFQRPAAFVIDLELAKQILIKNFSNFTD

KGIYYNEKDDPMSAHLFNLDGPQWRLLRSKLSPTFTSGKMKFMYPTVVSVAEEFMAVMHE

KVSENCILDVRDLVARFTVDVIGTCAFGIQCNSLRDEKAEFLHFGRRALVDSRHGNLVSS

LMRSYPNLARRLGLCRNAAHIQKFYQRIVRETVTLREKENIKRNDFMDVLIGLKNQKNVT

LENGEVVKGLTMDEIVAQSFVFFIAGFDTSSSTMGFALYELAKHPSIQDKVRAELEQVLE

QHDQKLTYECIKDLKYLDQVISETLRHYTIVPNVDRVAAKRFVVPGHPEFVIEAGQSVII

PSSAIHHDPSIYPEPNEFRPERFSPEESAKRPSVAWLPFGEGPRNCIGLRFGQMQARIGL

ALLIRNFLFSPCSETPDPLIFDPHSSILLGLKGGIRLKVEAI

>EDW32566.1

MGVTYILLQVAVALLAIVTYYVYRRLSYFKSRGIPHDTPHLILGNMQGPSAFILDLDLAK

HILIKDFSNFSDKGLFYNEKDDPISAHLFNLDGPQWKVLRNKLTSTFTAGKMKFMYPTVV

SVAEEFIAVMHEKMKQNSVLEIRELMARFTVDVIGTCAFGIKCNSLRDENAEFLRFGKRT

FVDKRHGSFLTGFMTSYPKLARRLRLVRTPTHIRNFYERIVHETVAMREKENIKRNDFMD

LLIDMKNQKVKLENGDLVDGLTIDEILAQAFVFLIAGFETSSSTMGYALYELAKQPHIQD

KVRAEVEEVLEQHEQKFTYESTKDLKYLEQVIFETLRLHTIVPHLDRMAHKRYVVPGHPN

FVIEAGQSVIIPASAIHVDPNIYPDPYKFRPERFSPEESAKRNSLAWLPFGDGPRNCIGL

RFGKMQTTIGLALLIKNFKFSTCPKTPDTLSYSPKSFVLAVDGIHLKVEAV

>EDW74195.1

MAVTFLLFQVVVALLAVVLYYVHRNLSYFKRRNIPHESPHWLKGNLRELRSTRNFHDIYE

AYYNKFKKLRVPFVGLFIHQRPAVFVMDLELVKHILIKDFSNFSDKGIYYNEKDDPISAH

LFNLDGPQWRVLRNKMSSTFTSGKMKFMFPTVVAVANEFMAVMNEKSQENSVLEIRDLMA

RFTIDVIGTCAFGLKCNSLREEDAEFLQMGRATFQEKNHCNLVLAFMESYPYLGRKLGLV

RTTKRVQDFYSRIVKETVAIREKENIKRSDFMDLLIELKNKKNSDQDHAEESKGITIEEV

IAQAFIFFVAGFETSSSTMGYALYELAKNPHIQDKVRAEVEQVFEKHNQQFTYECMKDLK

YLKQVISETLRLYTIVPHLNRQALKRYVVPGNPNYVIEAGQAVIIPAAAIHRDPEIYPDP

EEFQPERFSPEECAKRDSVTWLSFGDGPRNCIGLRFGEMQSRIGLALLIKNFKFSTCAET

PKTLEYDPNSFVLGVKGGIQLKVEAISRS

>EDW08929.1

MLFIIYLLSAISALLVYLLHRNMNYWKSRGIPQEPPHLIYGNVVGFRKTRVLNDLVIEYY

NKFRKSGHPFVGFAFIQKQSAFIMDTKLAKNILIKDFTNFADRGQFHNERDDPLTGHLFN

LDGKRWREMRQKLSPTFTSGKMKFMFPTVIKVSEEFVEVMMENVPAKTRGAIIEIKDLMA

RFTTDVIGTCAFGIECNTLRTPVTDFRTMGQKSFTEMRHGVLLTAFEFSFPSLARKLGMR

MIPEDVHQFFMGLVKETIAIREKENIKRNDFMQMLIELKQKGSFTMDNGEVVTGLDIGEL

AAQVFVFYLAGFETSSSTMTYALYELAQHTDIQDRLREDIQEVLQQHDGKLTYECVKAMR

YLDQVISETLRLYTLVPFLERKALNDYVVPGHPKYVIEKGTQIILPAAAYHRDEDLYPEP

EKFDPERFSPEQVAARDSVEWLPFGDGPRNCVGMRFGQMQTRVGLAQLIRNFKFTVCDKT

DIPLTYDPKSLIIGTIGGIYLRVERV

>EDW08931.1

MLFFIYLLTAVSALVAYFLYYNMNYWKNRGIPQDPPHPVYGNMIGFRRNRIVHEIMGDYY

MKFRKSGHPFVGFSFLQRRSAFIMDIKLAKNVLIKDFTNFTDRGQFHNERDDPLTGHLFN

LDGKRWKIMRQKLSPTFTSGKMKFMFPTVIKVSEEFIKIMMEKVPAKTGGAIIEIKDLMA

RFTTDVIGTCAFGIECNTLRNPDTDFRKMASKSLVEMRHGVLLTAFQFSFPNLARKLGMR

AIPEDVHQFFMGLVKATIALRERENIKRNDFMQMLIELKQKGSFTMDNGEVVSGLDVGEL

AAQVFVFYLAGFETSSSTMTYALYELAQHTDIQDRLREDIQEVLQQHDGKLTYECVKAMR

YLDQVISETLRLYTLVPFLERKALNDYVVPGHPKYVIEKGTQIILPAAAYHRDEDLYPEP

EKFDPERFSPEQVAARDSVEWLPFGDGPRNCVGMRFGQMQTRVGLAQLIRNFKFTVCDKT

DIPLTYDPKSFVLGTVGGIHLRVERV

>EDW77031.1

MWIIVYLVIAIASLIAYLLHRNLNYWKYRGIPHETPHPFYGNLIGFRKNRIVHDLIIDYY

NKFRKTGNPFVGFYLLNKPTAFIVDTKLAKNILIKDFSNFADRGQFHNEKDDPLTQHLFN

LDGKRWKELRQKLSATFTSGKMKFMFPTVIKVSEELVKVLNEKVPAGNKNGTVLEIKELV

ARFTTDVIGTCAFGIECNTLRTPETEFRKMGIKAFTEVRHGPLLTTFIVSFPSLAKKLRM

RMVPEDVHKFFLHLVKDTISYREKENIKRNDFMEMLIELKKKGSMTLDNGEVVKGMDLGE

LTAQVFVFYLAGFETSSSTMGYCLFELAQHQDVQDKLRAEVTEVLDKHDGKLSYECIKDM

HYLDQVLSETLRLYTIVPHLERKALSDYNVPGSPNLVIEKDTQIVIPACAYHRDEDLYPN

PEKFDPDRFSAEEVASRDSVEWLPFGDGPRNCIGMRFGQMQARIGMAQIIKNFKFSICDQ

TEMPLKYDPKSFVLGTSHGINLRVDRV

>EDV38107.1

MFVLIYLVIAIVSLLAYVLHRNFTYWTRRGVPHDAPHLIYGNMVGFRKDRVMHNIFIDYY

NKYRKTGNPFVGFYFLHKPAAFIVDPKLAKNILIKDFSNFTDRGQFHNERDDPLTQHLFN

LDGKKWKDMRQRLSPTFTSGKMKFMFPTVIKVSEEFVKVMTSQVPASQGGAVLEIKDLMA

RFTTDVIGNCAFGIECSTLSNPVSDFRTMGQKVFTELRHGRLLTMFQFSFPKLARRLRMR

MMPEDVHQFFMRLVNDTIALREKENFKRSDFMNLLIELKQKGKVTLENGDVIEGMNIGEL

AAQVFVFYVAGFETSSSTMSYCLYELAQNQDIQDRLRSEVQSVLEQHEGKLTYEAIKDMR

YLDQVISETLRLYTLVPHLERKALDDYTVPGHPNLVIQSGTQILIPACAYHRDEDLYPDP

MTFDPERFSAEKVAARDSVEWLPFGDGPRNCIGMRFGQMQVRIGLAQLINKFRFSLCDET

EIPLKYTPMSIVLGTIGGIHLRVERV

>EAL24730.1

MLILIYLVIAVGSLVAYLLHRNFQYWKRKGVPHDPPHPFYGNLVGFRKNRVIHDIFYDYY

NKFRTSGSPFVGFFFLQKPAAFIVDTKLAKNILIKDFSNFADRGQFHNERDDPLTQHLFN

LDGKRWKELRQRLSPTFTSGKMKLMFPTVIKVSEEFASVMEEQVPPTQGGAIIEIKEMMA

RFTTDVIGTCAFGIECNTLRTPVTDFRTMGQKAFTELRHGQLLTFFIFSFPKLARRLRMR

IMPEDVHQFFMRLVNDTVAVREKENFKRHDFMDMLIEIKQKGSVTLENGEVMKAMDIGEL

AAQVFVFYLAGFETSSSTMSYCFYELAQHQDIQDKLRSEVLTVLAEHDGKLTYECVKEMR

YLDQVFSETLRLYTLVPHLERRALSDYVVPGHPDLVIEKDTQIIIPACAYHRDENLYPDP

LRFDPDRFSAEQVAARDSVEWLPFGDGPRNCIGMRFGQMQARIGMAQLLSRFKLSLCDKT

EIPLKYEPKSFVLGSIGGIYLRLERI

>EDW31412.1

MLILIYLVIAVGSLVAYLLHRNFQYWKRKGVPHDPPHPFYGNLVGFRKNRVIHDIFYDYY

NKFRTSGSPFVGFFFLQKPAAFIVDTKLAKNILIKDFSNFADRGQFHNERDDPLTQHLFN

LDGKRWKELRQRLSPTFTSGKMKLMFPTVIKVSEEFASVMEEQVPPTQGGAIIEIKEMMA

RFTTDVIGTCAFGIECNTLRTPVTDFRTMGQKAFTELRHGQLLTFFIFSFPKLARRLRMR

IMPEDVHQFFMRLVNDTVAVREKENFKRHDFMDMLIEIKQKGSVTLENGEVMKAMDIGEL

AAQVFVFYLAGFETSSSTMSYCFYELAQHQDIQDKLRSEVLTVLAEHDGKLTYECVKEMR

YLDQVFSETLRLYTLVPHLERRALSDYVVPGHPDLVIEKDTQIIIPACAYHRDENLYPDP

LRFDPDRFSAEQVAARDSVEWLPFGDGPRNCIGMRFGQMQARIGMAQLLSRFKLSLCDKT

EIPLKYEPKSFVLGSIGGIYLRLERI

>EDW08930.1

MFFLIYLLSAISALLVYLVHQQMTYWKRRGIPQDPPHPIYGNAVGFRKTHTLSEIMIEYY

KKFRKSGHPFVGFAFIQKQCAFIMDTKLAKNILIKDFTNFADRGQFHNERDDPLTGHLFN

LDGKRWREMRQKLSPTFTSGKMKFMFPTVIKVSEEFVEVMMENVPAKTGGAIIEIKDLMA

RFTTDVIGTVAFGIECNTLRNPDTDFRKMGQKSFTELRHGIILTAFQFSFPNFARKLGMR

MIPEDVHQFFMRLVKETIALREKENIKRNDFMQMLIELKQKGSFTMDNGEVVTGLDIGEL

AAQVFVFYLAGFETSSSTMTYALYELAQHTDIQGRLRENIQEVLQKHDGKLTYECIKEMQ

YAHQVISETLRLYTIGPFLERKALDDYVVPGHPKYVIEKGTQIILPAAAYHRDEDLYPEP

EKFDPERFSPEQVAARDSVEWLPFGDGPRNCVGMRFGQMQTRIGLVQLIRNFKFTVCDKT

DIPLTYDPKSLVLGTIGGIYLRAERV

>EDW61677.1

MLFLIYLLIAISALVVYMLHRNMNFWKSRGIPQEPPHPFYGNLVGFRKNRIIHNALNEYY

NKFRKTGHPFVGFSFIQRQSAFIMDIKLAKNILIKDFTNFADRGQFHNERDDPLTGHLFN

LDGKRWREMRQKLSPTFTSGKMKFMFPSVIKVSEEFVKVLQEEVPAKSGGAIIEIKDLMA

RFTTDVIGTCAFGIECNTLRTPVTDFRKMGQKSFTEMRHGIFLTAFSFSFPNLARKLRMR

MIPEDVHQFFMGLVQETIALREKENIKRNDFMEMLIELKQKGSFTMDNGEIVTGLDVGEL

AAQVFVFYLAGFETSSSTMTYSLYELAQHTEIQDKLREDIKDVLQQHDGKLTYESIKAMR

YLDQIISETLRLYTIVPFLERKALNDYVVPGHPKYAIEKGTQVIIPAAAYHRDEDLYPDP

EKFDPERFSAEQVAARDSVEWLPFGDGPRNCVGMRFGQMQTRIGLAQLIRNFKFSVCDKT

DIPLIYDPKSFVLGTIGGIYLRVERVQN

>EDW61676.1

MLLFLIYLLTGLSALVAYFLHHNMNYWKNRGIPQDAPHPLYGNLVGFRKNRVINDIVVEY

YNKFRKTGHPFVGFNFLQRRSAFIMNIKLAKNVLIKDFTNFADRGQFHNERDDPLTGHLF

NLDGKRWREMRQKLSPTFTSGKMKFMFPSVIKVSEEFVKVLQEEVPAKSGGAIIEIKDLM

ARFTTDVIGTCAFGIECNTLRTPETDFRKMARKALVELRHGPLLTAFQFSFPNLARKLRM

RMIPDDVHEFFMGLVQETIALREKENIKRNDFMEMLIELKQKGSFTMDNGEVVTGLDVGE

LAAQVFVFYMAGFETSSSTMSYCLYELAQHTDIQDRLREDIHIILQQHDGKLTYESILAM

RYLDQVISETLRLYTIVPFLERKALNDYVVPGHPKYVIEKGTQVILPAAAYHRDEDLYPD

PEKFDPERFSAEQVAARDSVEWLPFGDGPRNCVGMRFGQMQTRIGLAQLIKNFKFSVCDK

TDIPLIYDPKSFVLGTIGGIYMRVEPA

>EDW02011.1

MLFLIYLFVAVSALVAYFLHYNLNYWKNRGIPQDPPHPFYGNMVGFRKNRIVHDIFKEYY

NKFRKTNHPFVGFSFLQKRSAFVMDIKLAKNILIKDFTNFTDRGQFHNERDDPLTGHLFN

LDGKRWKELRQKLSPTFTSGKMKFMFPTIIKVSEEFVRVMFDEVPVKSGGSIIEIKDLMA

RFTTDVIGTCAFGIECNTLRTPESDFRKMGQKFFTETRHGTLLSGLIFSFPNLARKLRMR

VISEDVHQFFMGLVNETVAYREKENIKRNDFMEMLIELKQKGSFTMDNGEVVTGLDVGEL

AAQVFVFYLAGFETSSSTMSYCLYELAQHTDIQQKLREDIKNVLQQHDGKLTYESIKAMR

YLDQVISETLRLYTLVPFLMRKALSDYVVPGNSKYIIEKGTQVMVPAAAYHRDEDFYPDP

EKFDPDRFSAEKVAARDSVEWLPFGDGPRNCIGMRFGQMQSRVGLAQLIQNFKFSVCEKT

DIPLKHDPKSLVLGTVGGIYLRVERV

>EDX05917.1

MFVLIYLLIAISSLLAYLYHRNFNYWNRRGVPHDAPHPLYGNMVGFRKYRVMHDFFYDYY

NKYRKSGYPFVGFYFLHKPAALIVDTQLAKNILIKDFSNFADRGQFHNGRDDPLTQHLFN

LDGKKWKDMRQRLTPTFTSGKMKFMFPTVIKVSEEFVKVITEQVPATQNGAVLEIKELMA

RFTTDVIGTCAFGIECNTLRTPVSDFRTMGQKVFTEMRHGKLLTMFMFSFPKLARRLRMR

MMPEDVHQFFMRLVNDTIALRERENFKRNDFMNLLIELKQKGRVTLDNGEVIEGMDIGEL

AAQVFVFYVAGFETSSSTMSYCLYELAQNQDIQDRLRNEIQTVLEEHEGQLTYESIKAMT

YLNQVISETLRLYTLVPHLERKALNDYVVPGHEKLVIEKGTQILIPACAYHRDEDLYPNP

ETFDPDRFSPEKVAARESVEWLPFGDGPRNCIGMRFGQMQARVGLAQIISRFRVSVCDKT

EIPLQYSPLSIILGTVGGIYLRVERV

>P33270.2

MFVLIYLLIAISSLLAYLYHRNFNYWNRRGVPHDAPHPLYGNMVGFRKNRVMHDFFYDYY

NKYRKSGFPFVGFYFLHKPAAFIVDTQLAKNILIKDFSNFADRGQFHNGRDDPLTQHLFN

LDGKKWKDMRQRLTPTFTSGKMKFMFPTVIKVSEEFVKVITEQVPAAQNGAVLEIKELMA

RFTTDVIGTCAFGIECNTLRTPVSDFRTMGQKVFTDMRHGKLLTMFVFSFPKLASRLRMR

MMPEDVHQFFMRLVNDTIALRERENFKRNDFMNLLIELKQKGRVTLDNGEVIEGMDIGEL

AAQVFVFYVAGFETSSSTMSYCLYELAQNQDIQDRLRNEIQTVLEEQEGQLTYESIKAMT

YLNQVISETLRLYTLVPHLERKALNDYVVPGHEKLVIEKGTQVIIPACAYHRDEDLYPNP

ETFDPERFSPEKVAARESVEWLPFGDGPRNCIGMRFGQMQARIGLAQIISRFRVSVCDTT

EIPLKYSPMSIVLGTVGGIYLRVERI

>AAR36858.1

MFVLIYLLIAISSLLAYLYHRNFNYWNRRGVPHDAPHPLYGNMVGFRKYRVMHDFFYDYY

NKYRKSGYPFVGFYFLHKPAALIVDTQLAKNILIKDFSNFADRGQFHNGRDDPLTQHLFN

LDGKKWKDMRQRLTPTFTSGKMKFMFPTVIKVSEEFVKVITEQVPATQNGAVLEIKELMA

RFTTDVIGTCAFGIECNTLRTPVSDFRTMGQKVFTEMRHGKLLTMFMFSFPKLARRLRMR

MMPEDVHQFFMRLVNDTIALRERENFKRNDFMNLLIELKQKGRVTLDNGEVIEGMDIGEL

AAQVFVFYVAGFETSSSTMSYCLYELAQNQDIQDRLRNEIQTVLEEHEGQLTYESIKAMT

YLNQVISETLRLYTLVPHLERKALNDYVVPGHEKLVIEKGTQILIPACAYHRDEDLYPNP

ETFDPDRFSPEKVAARESVEWLPFGDGPRNCIGMRFGQMQARVGLAQIISRFRVSVCDKT

EIPLKYSPLSIILGTVGGIYLRVERI

>AAR23114.1

MFVLIYLLIAISSLLAYLYHRNFNYWNRRGVPHDAPHPLYGNMVGFRKNRVMHDFFYDYY

NKYRKSGFPFVGFYFLHKPAAFIVDTQLAKNILIKDFSNFADRGQFHNGRDDPLTQHLFN

LDGKKWKDMRQRLTPTFTSGKMKFMFPTVIKVSEEFVKVITEQVPAAQNGAVLEIKELMA

RFTTDVIGTCRFGIECNTLRTPVSDFRTMGQKVFTDMRHGKLLTMFVFSFPKLASRLRMR

MMPEDVHQFFMRLVNDTIALRERENFKRNDFMNLLIELKQKGRVTLDNGEVIEGMDIGEL

AAQVFVFYVAGFETSSSTMSYCLYELAQNQDIQDSVRNEIQTVLEEQEGQLTYESIKAMT

YLNQVISETLRLYTLVPHLERKALNDYVVPGHEKLVIEKGTQVIIPACAYHRDEDLYPNP

ETFDPERFSPEKVAARESVEWLPFGDGPRNCIGMRFGQMQARIGLAQIISRFRVSLCDTT

EIPLKYSPMSIVLGTVGGIYLRVERI

>AAB36782.1

MFVLIYLLIAISSLLAYLYHRNFNYWNRRGVPHDAPHPLYGNMVGFRKNRVMHDFFYDYY

NKYRKSGFPFVGFYFLHKPAAFIVDTQLAKNILIKDFSNFADRGQFHNGRDDPLTQHLFN

LDGKKWKDMRQRLTPTFTSGKMKFMFPTVIKVSEEFVKVITEQVPAAQNGAVLEIKELMA

RFTTDVIGTCRFGIECNTLRTPVSDFRTMGQKVFTDMRHGKLLTMFVFSFPKLASRLRMR

MMPEDVHQFFMRLVNDTIALRERENFKRNDFMNLLIELKQKGRVTLDNGEVIEGMDIGEL

AAQVFVFYVAGFETSSSTMSYCLYELAQNQDIQDRLRNEIQTVLEEQEGQLTYESIKAMT

YLNQVISETLRLYTLVPHLERKALNDYVVPGHEKLVIEKGTQVIIPACAYHRDEDLYPNP

ETFDPERFSPEKVAARESVEWLPFGDGPRNCIGMRFGQMQARIGLAQIISRFRVSVCDTT

EIPLKYSPMSIVLGTVGGIYLRVERI

>EDW46660.1

MFVLIYLLIAISSLLAYLYHRNFNYWNRRGVPHDAPHPLYGNMVGFRKYRVMHDFFYDYY

NKYRKSGYPFVGFYFLHKPAALIVDTQLAKNILIKDFSNFADRGQFHNGRDDPLTQHLFN

LDGKKWKDMRQRLTPTFTSGKMKFMFPTVIKVSEEFVKVITEQVPATQNGAVLEIKELMA

RFTTDVIGTCAFGIECNTLRTPVSDFRTMGQKVFTEMRHGKLLTMFMFSFPKLARRLRMR

MMPEDVHQFFMRLVNDTIALRERENFKRNDFMNLLIELKQKGSVTLDNGEVIEGMDIGEL

AAQVFVFYVAGFETSSSTMSYCLYELAQNQDIQHRLRNEIQTVLEEHEGQLAYESIKAMT

YLNQVLSETLRLYTLVPHLERKALNDYVVPGHEKLVIEKGTQVLIPACAYHRDENLYPNP

ETFDPDRFSPDKVAARESVEWLPFGDGPRNCIGMRFGQMQARVGLAQIISRFRVSVCDKT

EIPLQYSPLSIILGTVGGIYLRVERI

>EDW89090.1

MFVLIYLLIAISSLLAYLYYRNFNYWSRRGVPHDAPHPLYGNMVGFRKDRVMHDFFYDYY

NKYRKSGNPFVGFYFLHKPAAFIMDPQLAKHILIKDFSNFADRGQFHNGRDDPLTQHLFN

LDGKKWKDMRQRLTPTFTSGKMKFMFPTVVKVSEEFVKVITEQVPAAQNGAVLEIKELMA

RFTTDVIGTCAFGIECNTLRTPVSDFRTMGQKVFTELRHGKLLTMFMFSFPKLASRLRMR

MMPEDVHQFFMRLVNDTIALRERENFKRNDFMNLLIELKQKGRATLDNGEVIEGMDIGEL

AAQVFVFYVAGFETSSSTMSYCLYELAQNQDIQDRLRDEIQTVLEEHEGQLTYDSIKAMT

YLDQVISETLRLYTLVPHLERKALNDYVVPGHEKLVIEKGTQIVIPACAYHRDEDLYPNP

EVFDPERFSPEKVAARESVEWLPFGDGPRNCIGMRFGQMQARIGLAQIISRFRLSVCDKT

EIPLKYSPFSIVLGSVGGIYLRVERI

>AAA28438.1

MFVLIYLLIAISSLLAYLYHRNFNYWNRRGLPHDAPHPLYGNMVGFRKNRVMHDFFYDYY

NKYRKSGFPFVGFYFLHKPAAFIVDTQLAKNILIKDFSNFADRGQFHNGRDDPLTQHLFN

LDGKKWKDMRQRLTPTFTSGKMKFMFPTVIKVSEEFVKVITEQVPAAQNGAVLEIKELMA

RFTTDVIGTCRFGIECNTLRTPVSDFRTMGQKVFTDMRHGKLLTMFVFSFPKLASRLRMR

MMPEDVHQFFMRLVNDTIALRERENFKRNDFMNLLIELKQKGSSFTLDNGEVIEGMDIGE

LAAQVFVFYVAGFETSSSTMSYCLYELAQNQDIQDRLRNEIQTVLEEQEGQLTYESIKAM

TYLNQVISETLRLYTLVPHLERKALNDYVVPGHEKLVIEKGTQVIIPACAYHRDEDLYPN

PETFDPERFSPEKVAARESVEWLPFGDGPRNCIGMRFGQMQARIGLAQIISRFRVSVCDT

TEIPLKYSPMSIVLGTVGGIYLRVERI

>AAB24525.1

MFVLIYLLIAISSLLAYLYHRNFNYWNRRGLPHDAPHPLYGNMVGFRKNRVMHDFFYDYY

NKYRKSGFPFVGFYFLHKPAAFIVDTQLAKNILIKDFSNFADRGQFHNGRDDPLTQHLFN

LDGKKWKDMRQRLTPTFTSGKMKFMFPTVIKVSEEFVKVITEQVPAAQNGAVLEIKELMA

RFTTDVIGTCRFGIECNTLRTPVSDFRTMGQKVFTDMRHGKLLTMFVFSFPKLASRLRMR

MMPEDVHQFFMRLVNDTIALRERENFKRNDFMNLLIELKQKGSSFTLDNGEVIEGMDIGE

LAAQVFVFYVAGFETSSSTMSYCLYELAQNQDIQDRLRNEIQTVLEEQEGQLTYESIKAM

TYLNQVISETLRLYTLVPHLERKALNDYVVPGHEKLVIEKGTQVIIPACAYHRDEDLYPN

PETFDPERFSPEKVAARESVEWLPFGDGPRNCIGMRFGQMQARIGLAQIISRFRVSVCDT

TEIPLKYSPMSIVLGTVGGIYLRVERI

>EDV59784.1

MFVLIYLLIAISSLLAYLYHRNFNYWRRRGVAHDAPHPLYGNMVGFRKNRVMHDCFYEYY

NKYRRSGNPFVGFYFLHKPAAFIVDTQLAKNILVKDFANFADRGQFHNGRDDPLTQHLFN

LDGKKWKEMRQRLTPTFTSGKMKFMFPTVIAVSEEFVKVITEQVPAAQNGAVLEIKELMA

RFTTDVIGTCAFGIECNTLRKPVSDFRTMGQKVFTEMRHGKLLTMFIFSFPELASRLRMR

MMPEDVHQFFMRLVNDTIALRERENFKRNDFMNLLIELKQKGRATLDNGEVIEGMDIGEL

AAQVFVFYVAGFETSSSTMSYCLYELAQNQGIQDRLRDEIQTVLDEHEGQLTYDSVKAMT

YLNQVISETLRLYTLVPHLERKALDDYVVPGNEKFLIEKGTQVIIPACAYHRDEDLYPNP

EAFDPERFSPEKVAARESVEWLPFGDGPRNCIGMRFGQMQVRIGLAQIISRFRVSVCDQT

DIPLKYTPMSIVLGTVGGIYLRMERI

>EDW02010.1

MVGFRKTRTVHDILEEYYNKFRKTNHPFVGFNFLQKRSAFVMDIKLAKNILIKDFTNFTD

RGQFYNERDDPLTGHLFNLDGKRWKELRQKLSPTFTSGKMKFMFPTIIKVSEEFVRVMFD

EVPVKSGGSIIEVKNLMARFTTDVIGTCAFGIECNTLRTPESDFRKMGQKAFSETRQGSL

ITAFILSFPNLARKLRMRVVPEDVHQFFMGLVNETVAYREKENIKRNDFMEMLIELKQKG

SFTMDNGEVVTGLDVGELAAQVFVFYLAGFETSSSTMSYCLYELAQHTDIQQKLREDIKN

VLQQHDGKLTYESIKAMRYLDQVISETLRLYTIVPFLVRKALSDYVVPGNPKYVIEKGTQ

VIMPAAAYHRDEDFYPDPEKFDPDRFSAENVAARDSVEWLPFGDGPRNCIGMRFGQMQSR

VGLAQLIRNFKFSVCEKTDIPLKHDPKSFVLGTVGGIYVRAERV

>EDW00827.1

MGVGIWLLTVVIALIGYFFLRLRRNMQHWQNLGIPCEEPSLMLGTMTGLRTKRGFSEVWM

EYYNKFRGTGPFAGFYWFQRPAAFILEPFLVKLILMKDFNNFTDRGFYSNAEDDPLSGQL

FLLDGHKWRSMRGKLSSTFTSGKMKYMFPTVVNICNEFIQVFGDMVSKSSIVEVKELLAR

FTTDVIGSCAFGIECSSLKDPEAEFRIMGRKSLTDQRHGNLGIALLNSFPNFSRRIHMKM

TAEHIEKFFMRIVKETVDYREKNNVRRNDFMDQLIDLKNKPLMKSETGESMNLTIEEISA

QAFVFFAAGFETSSTTMGFALYELARAEDVQNRLRKECNEVLARHNGDLSYECIKDMKYL

DQVISETLRLYTVLPILNRQCLEDYVVPGYPNYVIKKGMPVLIPAGAMHRDERYYPEPNR

FNPDNFDEERVKNRDSVEWLPFGEGPRNCIGMRFGQMQARIGLSMLIKNFKFSVCDQTPI

PIKYNKESFLIASESGIFLKVERV

>EDW10109.1

MGVVTLLLTALIAVIGYLFIRLHQRMHYWQRKGIPCEEPSLLMGSMKGVQTERAFIDIWT

HHYNKFKGTGPFAGFYWFQRPAAFVLEPFLVKHILIKDFNKFSDRGFFSNPEDDPLSGQL

FVLDGHKWRSMRNKLSSTFTSGKMKYMFPTVVKISHEFIKVFGDMADKSSVIEVRELLAR

FTTDVIGNVAFGIECNSLKDPEAEFRVMGRKSLTDKRHGLVGIAVLTSFPKFAKRLHLKG

TAEHIEKFFMKIVRETVDFREKNNIRRNDFMDQLIDLKNNRLLKTETGEDTSLTIEEIAA

QTFLFFAAGFETSSTTMGFALYELAQNVEIQNRLRQECKEVLAKYNGDLTYECIKDMQYL

NQVISETLRLYTVLPILNREALEDFVVPGYPKYVIKQGTMVIIPAGPMHRDEKLYPEPNR

FNPDNFEPEKVKNRDSVEWLPFGDGPRNCIGKRFGEMQARIGLAMLIKDFKFSVCDKTVI

PIKYDKTNFVVSSEGGIHLKVERV

>EDW74197.1

MAVCTLLVTALIGLIGYVFVLIRRRFNYWQDLGIACEEPHWLLGSLRGLRTTRSFPQVWT

EYYNKFKGTGPFAGFYWFQRPAAFILEPSLIKHVLIKDFNKFTDRGFFHNEEDDPLSGQI

FLLDGHKWKSMRNKLSSTFTSGKMKYMFPTVLKVAHEFIDVFGEMVNKSSEIEVKELLAR

FTTDVIGTCAFGIECSSLKDPEAEFRVMGRKSITDLRHGAIGIAFLNSFPQLSRRLHMKM

TPEHIEKFFMRIVRETVAYREQNNIKRNDFMDQLIELKNNRLMKSATGESMNLTIEEVAA

QAFVFFNAGFETSSTTMGFALYELAQHPDIQQRLRDECQKVLADNEFTYECMKDLVYTDQ

VISETLRLYTVLPLLNRQCIEDFVVPENPKFVIKKGMPVLIPAGALHRDEKYYPEPNVFN

PDNFSEERVKERDSVEWLPFGDGPRNCIGMRFGQMQARIGLALLIKNFKFSVCKDTPIPI

EQQMDSFLIASKSGIYLKVERV

>EDW10110.1

MAFGIILLTAVVALLGYLFLRMNQRMHHWENAGIPCEKPNIIVGCLRGVQTERTFLDIWM

QYYKKFKGTGPFAGFYWFQRPTAFILEPFLVKHILIKDFNKFTDRGFFSNPEDDPLSGQL

FLLDGHKWRSMRNKLSSTFTSGKMKYMFPTVVKISHEFVEVFGDMADKSSVVEVKELLAR

FTTDVIGSVAFGIECSSLKDPEAEFRVMGRSALTDQRHGALGIAILNSFPNFSRRMHLKM

TPEHIEKFFMRIVRETVDFREKNNIRRNDFMDQLIDLKNNRLLKAETGEDTSLTIEEIAA

QVFVFFAAGFETSSTTMGFVLYELAQNVDIQDRLREECKEVLAKHNGDLTYECIKDMQYL

NQVISETLRLYTVLPVLNRECLEDFVVPGYPNYVIKKGMTVIIPSAAMHRDEKLYPEPNR

FNPDNFEPEKVKNRDSVEWLPFGDGPRNCIGMRFGEMQARIGLAMLIQHFKFSVCDKTMI

PIKYNKKTFLVGSESGIYLKVERV

>ABG34552.1

MSLSFVLFTIVCGLLAYLVSVLNKKRSYWQSLGIPCEEPHFLFGSLWGIQTSRGFWEIWE

QYYNRFKGMGPFAGFYWFLRPAVFVLDPELVKNILIKDFTKFTDRGFYHNEKDDPLTGQL

FLLDGSKWKNMRNKLSPTFTSGKMKFMFPTVTKVGEEFIEVLNHMVAEEGSVVEVKDLLA

RFTTDVIGTCAFGIECSSLKDPNAEFRVMGKKFLVEQRHNRLVIAFMASFVDLARKMGLK

QTPDDIEAFFMRIVRETVEYREKNNIRRNDFMDMLIDLKNKKLMKSDHGDELTNLSLEEI

AAQAFVFFNAGFETSSTTLGFTLYELAQNQEIQDKARKEVLEKLEKYQGELSYECMKEMH

YLEQILSETLRMYTVLPILNRMALEDYVVPGNPKYVIKKNMQILIPAGAIHRDERYYPNP

NTFNPDNFSHDKVTERDSVLFLPFGEGPRNCIGLRFGKMQALVGLALLLKNFRFSVCQET

QIPLTYSKESFLISTDKGIYLRVEKN

>EDW00828.1

MGIATLLFTAVLAVLGYLFLRLRRNMQHWQNLGIPCEEPSLMLGTMTGLRAKRGFSEIWM

EYYNKFRGTGPFAGFYWFQRPAAFILEPFLVKHILIKDFNKFTDRGLFNNPEDDPLSGQL

FLLDGHKWKSMRGKLTSTFTSGKMKYMFPTVVNICNEFIQVFGDMVSKSSIVEVTELLAR

FTTDVIGSCAFGIECSSLKDPEAEFRIMGRKSLTDQRHGNLGNALLNGFPNFSRRIHMKL

TPEHIEKFFMRIVKETVDYREKNNVRRNDFMDQLIDLKNKPLMKSETGESMNLTIEEISA

QALVFFAAGFETSSTTMGFALYELARAEDVQNRLRKECNEVLARHNGDLTYESIKDMKYL

DQVISETLRLYTVLPILNRQCLEDYVVPGYPNYVIKKGMPVMIPAGAMHRDERYYPEPNR

FNPDNFDEERVKNRDSVEWLPFGEGPRNCIGIRFGQMQARIGLSMLIKNFKFSVCDQTPI

PIKYNKESFLIASESGIFLKAERV

>EDW05266.1

MAFGIILLTAVVALLGYLFLRMNQRMHHWENAGIPCEKPNIIVGCLRGVQTERTFLDIWM

QYYKKFKGTGPFAGFYWFQRPTAFILEPFLVKHILIKDFNKFTDRGFFSNPEDDPLSGQL

FLLDGHKWRSMRNKLSSTFTSGKMKYMFPTVVKISHEFVEVFGDMADKSSVVEVKELLAR

FTTDVIGSVAFGIECSSLKDPEAEFRVMGRSALTDQRHGNLGLAILNSFPNFSRRMHLKM

TPEHIEKFFMRIVRETVDFREKNNIRRNDFMDQLIDLKNNRLLKAETGEDTSLTIEEIAA

QVFVFFAAGFETSSTTMGFVLYELAQNVDIQDRLREECKEVLAKHNGDLTYECIKDMQYL

NQVISETLRLYTVLPVLNRECLEDFVVPGYPNYVIKKGMTVIIPSAAMHRDEKLYPEPNR

FNPDNFEPEKVKNRDSVEWLPFGDGPRNCIGKRFGEMQARIGLAMLIQQFKFSVCDKTTI

PIKYDKKSFLIASEGGIYLKVERV

>EDW04923.1

MVGFRKNRVVHDILEKYYNKFRKTNHPFVGFHFLQKRSAFVMDIKLAKNVLIRDFNNFAD

RGQFHNERDDPLTGHLFNLDGKRWKEMRQKLSPTFTSGKMKFMFPTVIKVSEEFVRVMLD

EVPVKSGGSIIEIKDLMARYTTDVIGTCAFGIECNTLRTPESDFRTMARKAIVELRHGPV

LTFFHFSFPNLARKLRMRMIPDDIHEFFMRLVKDTLAYREKENIKRNDFMEMLIELKQKG

SFTMDNGEVVTGLDVGELAAQVFVFYLAGFETSSSTMSYCLYELAQHTDIQQKLREDIKN

VLQQHDGKLTYESIKAMRYLDQVISETLRLYTIVPFLERRALNDYVVPGNPKYVIEKGTQ

VIVPAAAYHRDEDFYPNPEKFDPDRFSAENVAARDSVEWLPFGDGPRNCVGMRFGQMQTR

IGLAQLIQKFKFTVCEKTDIPLKHDPKSFVLGTVGGIYLRVERV

>EDW02009.1

MVGFRKNRVVHDILEEYYNKFRKTNHPFVGFHFLQKRSAFVMDIKLAKNVLIRDFNNFAD

RGQFHNERDDPLTGHLFNLDGKRWKEMRQKLSPTFTSGKMKFMFPTIIKVSEEFVRVMFD

EVPVKSGGSIIEIKDLMARFTTDVIGTCAFGIECNTLRTPESDFRTMARKAIVELRHGPV

LTFFHFSFPNLARKLRMRMIPDDIHEFFMRLVKDTLAYRENENIKRNDFMEMLIELKQKG

SFTMDNGEVVTGLDVGELAAQVFVFYLAGFETSSSTMSYCLYELAQHTDIQQKLREDIKN

VLQQHDGKLTYESIKAMRYLDQVISETLRLYTIVPFLERRALNDYVVPGNPKYVIEKGTQ

VIVPAAAYHRDEDFYPNPEKFDPDRFSAEKVAARDSVEWLPFGDGPRNCVGMRFGQMQTR

IGLAQLIQKFKFTVCEKTDIPLKHDPKSFVLGTVGGIYLRVERV

>EDV55968.1

MGVYMVLLTIAVVLVGYLLLKWRRALHYWQSLGIPCEEPHILMGSLTGVQTSRSFSDIWM

DYYNKFRGTGPFAGFYWFQRPGILVLDIALAKLILIKEFNKFTDRGFYHNTEDDPLSGQL

FLLDGQKWRSMRSKLSSTFTSGKMKYMFPTVVKVGHEFIEVFGQAVEKSSIVEVKDILAR

FTTDVIGTCAFGIECSSLKDPEAEFRVMGRRAVFEQRHGPIGIAFINSFQNLARRLHMKI

TLEDAEHFFLRIVRETVAFREEHNIRRNDFMDQLIDLKNSPLTKSESGESVNLTIEEMAA

QAFVFFGAGFETSSTTMGFALYELAQHQDIQDRVREECQEVFKKCNGELNYESMKDLVYL

DQVISETLRLYTVLPVLNRECLEDYVVPGHPKYVIKKGMPILIPCGAMHRDEELYANPNT

FDPDNFSPDRVKERDSVEWLPFGDGPRNCIGMRFGQMQARIGLALLINNFKFSVCDQTTI

PIVYSKKTFLISSETGIFLNVERV

>EDW91088.1

MGVYIVLLTIVAVLVGYLALKWRRTLHYWQNLGIPCEEPHILMGSLTGVQTSRSFSDIWM

DYYNKFRGTGPFAGFYWFQRPGLLVLDITLAKLILIKEFNKFTDRGFYHNTEDDPLSGQL

FLLDGQKWKSMRSKLSSTFTSGKMKYMFPTVVKVGHEFIEVFGQAVEKSPVVEVKDILAR

FTTDVIGTCAFGIECSSLKDPEAEFRVMGRRAVFEQRHGPIGIAFINSFQNLARKLHMKI

TLEEAEHFFLRIVRETVAFREANNIRRNDFMDQLIDLKNSPLTKSESGESVNLTIEEMAA

QAFVFFGAGFETSSTTMGFALYELAQHQDIQERVRVECQEVIKKCNGELSYESMKDLVYL

DQVISETLRLYTVLPVLNRECLEDYVVPGHPKYVIKKGMPVLIPCGAMHRDEKLYANPNT

FDPDNFSPERVKERDSVEWLPFGDGPRNCIGMRFGQMQARIGLALLINNFRFSVCDQTTI

PMVYSKKTFLISSDTGIFLKVQRV

>EDW05265.1

MAIGILLLTAVIALLGYLYLRMHRNMQHWQNAGIPCEEPHILMGNLKGVQTERSFLDIWM

QYYKKFKGTGPFAGFYWFQRPAAFILEPFLVKHILIKDFNKFTDRGFFSNPEDDPLSGQL

FLLDGHKWRSMRNKLSSTFTSGKMKYMFPTVVKISHEFVEVFGDMADKSSVIEVRELLAR

FTTDVIGSVAFGIECSSLKDPEAEFRVMGRSALSDQRHGALGIAILNSFPNFSRRMHLKM

TPEHIEKFFMRIVRETVDFREKNNIRRNDFMDQLIDLKNNRLLKTETGEDTSLTIEEIAA

QAFVFFAAGFETSSTTMGFVLYELAQNVDIQDRLREECKEVLAKHNGDLTYECIKDMQYL

NQVISETLRLYTVLPVLNRECLEDFVVPGYPNYVIKKGMTIVIPSAAMHRDEKLYPEPNR

FNPDNFEPEKVKNRDSVEWLPFGDGPRNCIGMRFGEMQARIGLAMLIQHFKFSVCDKTMI

PIKYNKKTFLVGSESGIYLKVERV

>EDV35770.1

MDIGTVLFTAVLALVVYLLAKFRQSMKYWQNEGIPCEEPDQLMGSLKGLLTKRSFNDVWT

GYYKKFRGTGPFAGFYWFRRPAVFVLEPSLAKHILIKEFNKFTDRGFYHNTKDDPLTGQL

FLLDGQKWKSMRNKLSPTFTSGKMKFMFPIVTKVGHEFIDVFSEMVDQSSTVEVKELLAR

FTTDVIGTCAFGIECSSLKDPEAEFRVMGRRSLTEQRLGSLAIGLVNSFPNVARRLGVKT

TAEPIEKFFMRIVRETVSFREQNNIRRNDFMDQLIDLKNNTLMKSDTGENVNLTIEEVAA

QAFVFFTAGFETSSTTMGFALYELAQHQDIQDRVRKECQEVFEKYNGELTYESTKELVYL

DQVISETLRLYTVLPVLNRECLEDFVVPENPKYVIKKGMPILIPAGAMHRDEKYYPNPDT

FNPDNFTAERVRDRDSVLWLPFGDGPRNCIGMRFGQMQSRIGLALLIKNFKFSVCSQTTI

PMKFDKKLFLIGSESGIYLKVERV

>EDV35772.1

MLLLLAAGFFYRYSDVMGVQAFLISAVVLLLSYLLLRWRRTLNYWKNAGIPCEKPHILMG

SLAGVQSKRSFCDIWEDYYKKFRGTGPFAGFYWFQKPAAFVLEPSLAKLILIRDFNKFTD

RGFYHNTEDDPLSGHLFMLDGQKWKSMRNKLSSTFTSGKMKYMFPTVVKVGHEFAEVFGQ

TLEKDNIVEVKDLLARFTTDVIGTCAFGIECSSLKDPNAEFRVMGRKSVTDQRHGALGIA

FINSFPNLARRLHMKITKEEVEHFFLRIVRETVSFREQNNIRRNDFMDQLIDLKNSPLTK

SESGESVNLTIEQMAAQAFVFFGAGFETSSTTMGFALYELAQHQDIQDRVRKECNEVFGK

SGEFSYENMKDLIYLDQVLSETLRLYTVLPILNRECLEDYVVPDNPKYVIKKGMPVLIPC

GAMHRDEKLYPNPDVFNPDNFLPERVKERDSVEWLPFGDGPRNCIGMRFGQMQARIGLAL

LIKDFRFSICKETTIPMKYSKETFLISSESGIYLKVERV

>AAM51056.1

MGVYSVLLAIVVVLVGYLLLKWRRALHYWQNLDIPCEEPHILMGSLTGVQTSRSFSAIWM

DYYNKFRGTGPFAGFYWFQRPGILVLDISLAKLILIKEFNKFTDRGFYHNTEDDPLSGQL

FLLDGQKWKSMRSKLSSTFTSGKMKYMFPTVVKVGHEFIEVFGQAMEKSPIVEVRDILAR

FTTDVIGTCAFGIECSSLKDPEAEFRVMGRRAIFEQRHGPIGIAFINSFQNLARRLHMKI

TLEEAEHFFLRIVRETVAFREKNNIRRNDFMDQLIDLKNSPLTKSESGESVNLTIEEMAA

QAFVFFGAGFETSSTTMGFALYELAQHQDIQDRVRKECQEVIGKYNGEITYESMKDMVYL

DQVISETLRLYTVLPVLNRECLEDYEVPGHPKYVIKKGMPVLIPCGAMHRDEKLYANPNT

FNPDNFSPERVKERDSVEWLPFGDGPRNCIGMRFGQMQARIGLALLINRFKFSVCEQTTI

PIVYSKKTFLISSETGIFLKVERV

>EDX07152.1

MGVYSVLLAIVVVLVGYLLLKWRRALHYWQNLGIPCEEPHILMGSLTGVQTSRSFSDIWM

DYYNKFRGTGPFAGFYWFQRPGILVLDISLAKLILIKEFNKFTDRGFYHNTEDDPLSGQL

FLLDGQKWKSMRSKLSSTFTSGKMKYMFPTVVKVGHEFIEVFGQAMEKSPIVEVKDILAR

FTTDVIGTCAFGIECSSLKDPEAEFRVMGRRAIFEQRHGPIGIAFINSFQNLARRLHMKI

TLEEAEHFFLRIVRETVAFREENNIRRNDFMDQLIDLKNSPLTKSESGESVNLTIEEMAA

QAFVFFGAGFETSSTTMGFALYELAQHQDIQDRVREECQEVIGKCNGEITYESMKDMVYL

DQVISETLRLYTVLPVLNRECLEDYEVPGHPKYVIKKGMPVLIPCGAMHRDEKLYANPNT

FNPDNFSPERVKERDSVEWLPFGDGPRNCIGMRFGQMQARIGLAFLINKFKFSVCDQTTI

PIVYSKKTFLISSETGIFLKVERV

>AAL48149.1

MSVGTVLLTALLALVGYLLMKWRSTMRHWQDLGIPCEEPHILMGSMEGVRTARSFNEIWT

SYYNKFRGSGPFAGFYWFRRPAVFVLETSLAKQILIKEFNKFTDRGFFHNPEDDPLSGQL

FLLDGQKWRTMRNKLSSTFTSGKMKYMFPTVVKVANEFTDVFGQNVAKSPVVEVRELLAR

FTTDVIGTCAFGIECSSLKDPDAEFREMGRRSLTEQRLGPVGIGFVNSFPNLARRLHMKM

TAEPIERFFMRIVRETVAFREQNNIRRNDFMDQLIDLKNKPLMVSQSGESVNLTIEEIAA

QAFVFFAAGFETSSTTMGFALYELAQNQDIQNRVRKECQEVIEKCNGELNYESMKDLVYL

DQVVSETLRLYTVLPVLNRECLEDYEVPGHPKYVIKKGMPVLIPCGAMHRDEKLYANPNT

FNPDNFSPERVKERDSVEWLPFGDGPRNCIGMRFGQMQARIGLALLIKDFKFSVCEKTTI

PMTYNKEMFLIASNSGIYLKAERV

>Q9V774.1

MSVGTVLLTALLALVGYLLMKWRSTMRHWQDLGIPCEEPHILMGSMKGVRTARSFNEIWT

SYYNKFRGSGPFAGFYWFRRPAVFVLETSLAKQILIKEFNKFTDRGFFHNPEDDPLSGQL

FLLDGQKWRTMRNKLSSTFTSGKMKYMFPTVVKVANEFTDVFGQNVAKSPVVEVRELLAR

FTTDVIGTCAFGIECSSLKDPDAEFREMGRRSLTEQRLGPVGIGFVNSFPNLARRLHMKM

TAEPIERFFMRIVRETVAFREQNNIRRNDFMDQLIDLKNKPLMVSQSGESVNLTIEEIAA

QAFVFFAAGFETSSTTMGFALYELAQNQDIQNRVRKECQEVIEKCNGELNYESMKDLVYL

DQVVSETLRLYTVLPVLNRECLEDYEVPGHPKYVIKKGMPVLIPCGAMHRDEKLYANPNT

FNPDNFSPERVKERDSVEWLPFGDGPRNCIGMRFGQMQARIGLALLIKDFKFSVCEKTTI

PMTYNKEMFLIASNSGIYLKAERV

>Q27594.3

MGVYSVLLAIVVVLVGYLLLKWRRALHYWQNLDIPCEEPHILMGSLTGVQTSRSFSAIWM

DYYNKFRGTGPFAGFYWFQRPGILVLDISLAKLILIKEFNKFTDRGFYHNTEDDPLSGQL

FLLDGQKWKSMRSKLSYTFTSGKMKYMFPTVVKVGHEFIEVFGQAMEKSPIVEVRDILAR

FTTDVIGTCAFGIECSSLKDPEAEFRVMGRRAIFEQRHGPIGIAFINSFQNLARRLHMKI

TLEEAEHFFLRIVRETVAFREKNNIRRNDFMDQLIDLKNSPLTKSESGESVNLTIEEMAA

QAFVFFGAGFETSSTTMGFALYELAQHQDIQDRVRKECQEVIGKYNGEITYESMKDMVYL

DQVISETLRLYTVLPVLNRECLEDYEVPGHPKYVIKKGMPVLIPCGAMHRDEKLYANPNT

FNPDNFSPERVKERDSVEWLPFGDGPRNCIGMRFGQMQARSGLALLINRFKFSVCEQTTI

PIVYSKKTFLISSETGIFLKVERV

>EDV35774.1

MSLFLSLICVLISLVVFAVRRRLGYWQQRGIPHDAPHPIFGNLKDWPKKRHFSVIFKDYY

NKYKSSNNPFAGFYFFLTPTAIVTDLELVKRVLIKDFNHFENRGVFHNEKDDPLSGTLFS

IEGQKWRHLRRKLSPTFTSGKMKYMFPIVAKIGEELEKVFNQKTASSQVVEVVDLVARFT

SDVIGSCAFGLNCNSLHDPKAEFVAMGKRAIVERRYGGWLDLFFFGFPKLSRRLHLKINV

QDVEDFYSRIVRETIDYRLKTKEKRNDFMDSLIELYQKEQEGNSEDGLSFNEVLAQAFIF

FLAGFETSSTTMGFALYELALNQDVQDKLRKEVTEGLAKTNNEFTYEGVREMKYLNQVIM

ETLRKYPVLAHLTRDTETDFSPEDPKYFIAKGTSVLIPSLGIHYDPDIYPEPNEFKPERF

TEEAIAARPSCTWLPFGEGPRNCIGLRFGLMQSQVGIAYLIRSFRFSVAPETQIPMKFVT

PNILLSAENGIHLKVEKISN

>EDW60866.1

MGIGTLLLTAVIALIGYLFLQIRRNMQHWQNLGIPCEEPNFLMGNLTGVRIKRAFPDIWI

HYYNKFRGTGPFAGFYWFQRPAAFILEPFLVKHILIKDFNKFTDRGFFHNPEDDPLSGQL

FLLDGHKWRSMRNKLSSTFTSGKMKYMFPTIVKIGHEFVDVFGEMTDKSSIIEVKELMAR

FTTDVIGTCAFGIECSSLKDPEAEFRVMGRKALTDQRHGDLGMAFINSFPKFSRRIHLKM

TPEHIEKFFMRIVRETVAYREENNVRRNDFMDQLIDLKNNQLMKSETGESMSLTIEEIAA

QAFVFFAAGFETSSTTMGFALFELAQNEDIQKRLREDCNEVLARYNGELTYECIKDMEYL

NQVISETLRLYTVLPVLNRECLEDFVVPGYPNYVIKKGMPVLIPCGAIHRDEKFYPEPNR

FNPDNFKAERVKNRDSGEWLPFGDGPRNCIGMRFGEMQARIGLAMLIKNFKFSVCDKTII

PIKNHKESFLIASESGIYLKVERV

>ABK91491.1

MEVTAALLYLIIAIVSVALYFVKRNLNYWKDQGIPHEEPHLLMGNFRGVRTKYQIGEIIG

NYYKKFKGTGPFAGIHIVQRPGVVLLDKGLIKNVLIKDFNNFVNRGMYYNEKNDPLTGNL

FFMDGQLWRVLRNKLSPTFTSGKMKYMYPTVLNVAEKFMQVLKEKVNEDPIVDVRDMLGR

FTTDVISSVAFGIEVNSLRNPNSDFPRLGRKLTEQPRHNALIMAFIDGFPNLSRKLGMRI

VPEDVHQFFMKTIKDTIAYREKNQIERNDFMKLLLDIKNNKELSGLTIEEMCAQVFVFFL

GGFETSSSTMSFALFELALNTHLQDRLREEVNEAYDKQQGFNYECLKNMTYLDQVLSETL

RKYPIVPQLNRQAAQDYAVPGHPEYVIKKGMPVLIPVLGIHHDPEFYPNPQEFDPERFDP

EVAKQRDPMEYLPFGDGPRNCIGERFGKMQSHLGLANLIRNFRFSTCSKTEVPIKLDPKA

VTYSPKNTIYLKVEAI

>EDW91090.1

MSVAAVLLTALLALVGYLLMKWRSTMRHWQDLGIPCEEPHMLMGSMLGVRTKRSFNEIWT

SHYKKFRGTGPFAGFYWFRRPAVLVLETSLAKQILIKEFNKFTDRGFFHNPEDDPLSGQL

FLLDGQKWKSMRNKLSSTFTSGKMKYMFPTVVKVANEFIDVFGQSVAKSPVVEVRELLAR

FTTDVIGTCAFGIECSSLKDPDAEFREMGRRSLTEQRLGPLGVGFVNSFPNLARRLHMKM

TAEPIEKFFMRIVRETVAFREQNNIRRNDFMDQLIDLKNKPLMMSQSGENVNLTIEEIAA

QAFVFFAAGFETSSTTMGFALYELAQHQDIQERVREECQEVIRKCNGELNYESMKDLVYV

DQVISETLRLYTVLPVLNRECLEDYVVPGHPKYVIKKGMPVLIPCGAMHRDEKLYANPNT

FDPDNFSPERVKERDSVEWLPFGDGPRNCIGMRFGQMQTRIGLALLIKDFKFSVCEKTTI

PMKYNKEMFLIASESGIYLKAERV

>AAB37340.1

MSVLQFLPGRVLRLFGYGFYLLRKRFMYWEVRGIAYEKPNYLLGNLNGVRSTRSCLEIWM

STYKKFKGTGPFCGFFWFQRPAAFILDPKLAKSILIKDFNNFVDRGFYNNPEDDPLTGQL

FLLDPHRWRVMRNKLSPTFTSGKMKYMSPAVCRVSEEFVRVVSEAIKEEPILEMRDYLAR

FTTDVIAPCAFGIDVNSLKNPNEEFRQMGKRVFSDLRNGRIGLAMINSFPELCRRLHVKQ

TPDHITEFYMRVVRENIEYRERNKIRRNDFFDMLLDLKNNKLMKSEDGHDMSITVEELAA

QAFVFLVAGFETSSTTMGFALYELAQDEEIQQRVRTEVNEALEKHNGEFTYDCMKDMVYL

DQVISETLRLYTVLPILNRECMEEYPVPGHPGFVIGKGMQVIIPAAPFHRDADIYPNPNT

FDPDNFSPDRVAQRDGVYWLPFGEGPRNCIGMRFGQMQARVGLALLLKNFKFSVCGKTPI

PMKYNKVNFLLQSEHGISLYIEKV

>EAL26034.2

MAVGVGALLLWTLVALVVYLLLKWLLGRQYWQNLGIPCEEPHWLLGSMWGLRTTRTFNDV

WTDYYKKYRLSGPFAGFYWFQRPAVFVLEPSLAKLILIKEFNKFTDRGFFHNPEDDPLSG

QLFLLDGHKWKSMRHKLSSTFTSGKMKYMFPTVVKVSHEFTEVFGQMADKSPIVEVRELL

ARFTTDVIGTCAFGIDCSSLKDPDAEFRKMGRRALTENRHGSLGIAFLNSFPKLSRKLHL

KMTAEPIEQFFMRIVKETVKFREQNQIRRNDFMDQLIDLKNNQIIKTESGDNINLTIEEI

AAQAFVFFAAGFETSSTTMGFALYELAQHPDIQQRVREECQEVIQKYGGELTYESMKELV

YLDQVISETLRLYTVLPVLNRECLEDFVVPDNPKYVIKKGMPILIPAGAMHRDERFYPNP

DAFNPDNFSPERVKNRDSVEWLPFGDGPRNCIGMRFGQMQARIGMAMLLKDFKFSVCDET

AFPMKYDLESFLIASKTGISLRVERV

>EDV55966.1

MSVGTVLLTALLALVGYLVMKWRSTMRYWQDLGIPCEEPHILMGSLKGVRTAHSFNEIWT

RNYRKFRGTGPFAGFYWFTRPSVLVLETSLAKQILIKEFNKFTDRGFFHNAEDDPLSGHL

FLLDGQKWKSMRNKLTSTFTSGKMKYMFPTVVKVANEFTDVFGQYVAKSPVVEVKELLAR

FTTDVIGTCAFGIECSSLKEPDSEFREMGRRSLTERRLGAVGIVFVNSFPKLARRLHMKM

TAEPIESFFMRIVRETVTYREQHNIRRNDFMDQLIDLKNKPLMMSQSGETVNLTIEEIAA

QAFVFFAAGFETSSTTMGFALYELAQHQDIQDRVREECQEVFKKCNGELNYESMKDLVYL

DQVISETLRLYTVLPVLNRECLEDYVVPGHPKYVIKKGMPILIPCGAMHRDEKLYANPNT

FDPDNFSPDRVKERDSVEWLPFGDGPRNCIGMRFGQMQARIGLALLINNFKFSVCEKTTI

PMAYDKEMFLIASESGIYLKAERV

>EDW32567.1

MAVGVGALLLWTLVALVVYLLLKWLLGRQYWQNLGIPCEEPHWLLGSMWGLRTTRTFNDV

WTDYYKKYRLSGPFAGFYWFQRPAVFVLEPSLAKLILIKEFNKFTDRGFFHNPEDDPLSG

QLFLLDGHKWKSMRHKLSSTFTSGKMKYMFPTVVKVSHEFTEVFGQMADKSPIVEVRELL

ARFTTDVIGTCAFGINCSSLKDPEAEFRKMGRRALTENRHGSLGIAFLNSFPKLSRKLHL

KMTAEPIEQFFMRIVKETVKFREQNQIRRNDFMDQLIDLKNNQIIKTESGDNINLTIEEI

AAQAFVFFAAGFETSSTTMGFALYELAQHPDIQQRVREECQEVIQKYGGELTYESMKELV

YLDQVISETLRLYTVLPVLNRECLEDFVVPDNPKYVIKKGMPILIPAGAMHRDERFYPNP

DAFNPDNFSPERVKNRDSVEWLPFGDGPRNCIGMRFGQMQARIGMAMLLKDFKFTVCDET

AFPMKYDLESFLIASKTGISLGVERV

>EDW10113.1

MSTILVLIGIIVTLLYYAARHRHNYWNRRGIPHDEPRFPKGNIADWQTKRQLGVIFRDYY

LKYKNRNAPFAGFYFFFTKTVIVTDMELVKRVLIKDFSNFENRGIFHNELDDPLSATLFS

IEGQKWRQLRHKLTPTFTSGKMKNMQPIVLKVAEEMAKIFKEKASTAQVLEVTDLVGRFT

ADVIGNCAFGLDCNSQRNPDADFVRMGKRAVTERRYRGILDFFIFGFPKLARRMHLKITV

EEVEEFYLRIIRDTIKYRLSMEEKRNDFMDMLIEMYQKQKAGNTEEGLTFEELAAQAFIF

FVAGFETSSTTMGFALYELALHQDIQNKLRAEINEVLSKYNNEYTYDNVKEMKYLEQVVM

ETLRKYPVLPHLTRKAISDYSPGDPKHYIEKNTSVVIPALGIHYDPDIYPEPDKFKPERF

TEAEIAARPACSWLPFGDGPRNCIGSRFGMMQTSVGLAHLIRDFKFSVSPQTQIPMKWVT

KNILLSAENGIYLKVERVSI

>EDW60865.1

MGVGTLLLTAVLALIGYLFLRLRRNMQHWQNLGIPCEEPNFLMGNLAGVRIKRAFPDIWI

HYYNKFKGTGPFAGFYWFQRPAAFILEPFLVKHILIKDFNKFTDRGFFHNPEDDPLSGQL

FLLDGHKWRSMRNKLSSTFTSGKMKYMFPTIVKIGHEFVDVFGEMIAKSSIIEVKELMAR

FTTDVIGTCAFGIECSSLKDPEAEFRVMGRKALTDHRHGALGIAFLNSFPKFSRRIHLKM

TPEDIEKFFMRIVRETVAYREENNVRRNDFMDQLIDLKNNQLMKSETGESMSLTIEEIAA

QAFVFFAAGFETSSTTMGFALFELAQNEDIQKRLRDDCNEVLARYNGELTYECIKDMEYL

NQVISETLRLYTVLPVLNRECLEDFVVPGYPNYVIKKGMPVLIPCGAIHRDEKFYPEPNR

FNPDNFKAERVKNRDSGEWLPFGDGPRNCIGMRFGEMQARIGLAMLIKNFKFSVCDKTII

PIKNHKESFLIASESGIYLKVERVQV

>EDW47927.1

MSLLLTLIAILVSLLLFVARRRHGYWQRRGIPHDVPHPIYGNMKDWPKKRHIAMIFRDYY

LKYKSSDYPFAGFYFFFTRSAVITDLELVKRVLIKDFNNFENRGIFYNEIDDPLSATLFS

IEGQKWRHLRHKLTPTFTSGKMKNMFPIVVKVGEEMEKVFSAKTATGQGQVLEIVDLVAR

YTADVIGNCAFGLNCNSLQNPKAEFVAIGKRAIIERRYGGLLDFLIFGFPKLSRRLRLKL

NVQDVEDFYTGIVRNTIDYRLRTNEKRNDFMDSLIEMYEKEQAGNTEDGLSFNEILAQAF

IFFLAGFETSSTTMGFALYELARNQDIQDQLREEINSVLGKHNNEFTYEGIKEMKYLEQV

VMETLRKYPVLAHLTRMTQTDFSPEDPKYFIAKGTIVVIPALGIHYDPEIYPEPEVFKPE

RFTDEAIAARPSCTWLPFGEGPRNCIGLRFGLMQACVGLAYLIRGYKFSVSAETQIPMKI

VVKSILLSAENGIHLKVEKLSK

>EDX07150.1

MSLLLTLIAILVSLLLFVARRRHGYWQRRGIPHDVPHPIYGNMKDWPKKRHIAMIFRDYY

LKYKSSDYPFAGFYFFFTRSAVITDLELVKRVLIKDFNHFENRGVFYNEIDDPLSATLFS

IEGQKWRHLRHKLTPTFTSGKMKNMFPIVVKVGEEMEKVFSAKTTTGQGQVLEIVDLVAR

YTADVIGNCAFGLNCNSLQNPKAEFVAIGKRAIIERRYGGLLDFLIFGFPKLSRRLRLKL

NVQDVEDFYTGIVRNTIDYRLRTNEKRNDFMDSLIEMYEKEQAGNTEDGLSFNEILAQAF

IFFVAGFETSSTTMGFALYELARNQDIQDQLREEINSVLGKHNNEFTYEGIKEMKYLEQV

VMETLRKYPVLAHLTRMTQTDFSPEDPKYFIAKGTIVVIPALGIHYDPEIYPEPEKFKPE

RFTDEAIAARPSCTWLPFGEGPRNCIGLRFGLMQACVGLAYLIRGYKFSVSAETQIPMKI

VVKSILLSAENGIHLKVEKLSK

>EDW32570.1

MSLVLALIAIVVSLVLFAARRRHGYWQRRGIPHDVPHPLFGNMGDWPKKRHIAIIFRDYY

RKYKSANYPFAGFYFFFTRTAVVTDLDLVKRVLIKDFNHFENRGIFYNEIDDPLSATVFS

IEGQKWRHLRHKLTPTFTSGKMKHMFPIVVKVGEEMDKVFSNKVGDGKVLEIVDLVARYT

ADVIGNCAFGLNCNSQQNPNADFVTFGKRALSERRYKGLLDFFIFGFPKLSRSLRLKLNV

QEVEDFYTKIVRDTIDYRLKTKEKRNDFMDSLIEMYQKEQEGNSDEGLTFNEILAQAFIF

FVAGFETSSTTMGFALYELAQNPDTQDKLRNEINDVLAKHNNQFTYEGIKEMKYLEQVVM

ETLRKYPALAHLTRKTRLDFSPEDPKNFIAKGTTVVIPALGIHYDPDIYPEPEKFKPERF

TEEAIASRPACAWLPFGEGPRNCIGLRFGIMQTCVGLAYLIKGYRFSVAPETQIPIKFVL

KSFLLSAENGIYLKVETVSK

>AAA57305.1

MDFGSFLLYAIGVLASLALYFVRRNFGYWKRRGIPHEEPHLVMGNVKGLRSKYHIGEIIA

DYYRKFKGSGPFAGIFLGHKPAAVVLDKELRKRVLIKDFSNFANRGLYYNEKDDPLTGHL

VMVEGEKWRSLRTKLSPTFTAGKMKYMYNTVLEVGQRLLEVMYEKLEVSSELDMRDILAR

FNTDVIGSVAFGIECNSLRNPHDRFLAMGRKSIEVPRHNALIMAFIDSFPELSRKLGMRV

LPEDVHQFFMSSIKETVDYREKNNIRRNDFLDLVLDLKNNPESISKLGGLTFNELAAQVF

VFFLGGFETSSSTMGFALYELAQNQQLQDRLREEVNEVFDQFKEDNISYDALMNIPYLDQ

VLNETLRKYPVGSALTRQTLNDYVVPHNPKYVLPKGTLVFIPVLGIHYDPELYPNPEEFD

PERFSPEMVKQRDSVDWLGFGDGPRNCIGMRFGKMQSRLGLALVIRHFRFTVCSRTDIPM

KINPESLAWTPKNNLYLNVQAIRRKMK

>EDW60863.1

MSFILALVGIIISLLFYAVRHRHNYWNRRGIPHDVPKFPKGNIGDWPSKRQIGVVFRDYY

LKYKNANSPFAGFYFFFTKTVVVTDMELVKRVLIKDFNNFENRGVFYNEIDDPLSATLFS

IEGQKWRQLRHKLTPTFTSGKMKHMLPIVLQVAEEMVKVFNDKVSAPQVLEITDLVGRYT

ADVIGTCAFGLDCNSQRNPDADFVSMGKRAVTERRYHGLLDFILFGFPKLSRRLHLKITT

EEVEEFYMRIVRDTINYRLKSNENRGDFMDMLIEMYQKQQKGNTDEGLTFEELAAQAFIF

FVAGFETSSTTMGFALYELAQHQDIQDKLRAEINDVLGKHNNKYSYDNVKQMEYLEQVVM

ETLRKYPVLAHLTRMAISDYSPGDPKYYIEKGSIVVIPALGIHYDPDIYPEPQKFKPERF

TEAEISARPACSWLPFGDGPRNCIGSRFGLMQTCVGLAHLIKDFKFSVSPETQIPMKLVT

KNILLSSENGIYLNVERVSK

>EDW74201.1

MALVLALFALLVSLLLFSARRRHSYWQRRGIPHDVPHPIWGNIGDWPKKKHIAYVFKDYY

NKYKSSKSPFAGFYFFFTKTAVLTDLELVKRVLVKDFNHFENRGVFYNEIDDPLSATLFS

IEGQKWRHLRHKLTPTFTSGKMKHMFPIVTKVAEEMEKVFKDKVGNASGGKTLEVVDLVA

RYTADVIGTCAFGLKCNSLYEPKAEFVQIGRRAVTDRRYYGLLDFFIFGFPKLARRLHLK

LNVQDVSDFYTRIVRDTINYRLKTKEKRGDFMDSLIEMYQKELEGDSEEGLTFDELLAQA

FIFFVAGFETSSTTMGFALYELAQHQDIQNKIRKEVNEVLAKHNNEFTYESIKQMKYLEK

SVMETLRKYPVLAHLTRLTNTDYTPDDSKYHIDKGTVVVIPALGIHYDPSIYPQPEKFQP

ERFTEEEIQARPPCTWLPFGDGPRNCIGLRFGLMQTCVGLAYLIKNYNFSVAPETPIPMK

LVTKNILISAENGIPLKVEKL

>EDW74203.1

MALVLALFALLVSLLLFSARRRHSYWQRRGIPHDVPHPIWGNMGEWQKTKHFSFIFKDYY

IKYKNSKSPFAGFYFFFGKTAVVTDLDLVKRVLIKDFNHFENRGIFYNEIDDPLSASLFS

IEGQKWRHLRHKLTPTFTSGKMKHMFPIVVKVAEEMEKVFRENIGSAKNGKVLEVVDLVA

RYTSDVIGTCAFGLNCNSLHDPDAEFVQIGRKAITERRYYGLLDFFIFGFPTLARRLHLK

FNVQEVSDFYTRIVRDTINYRLKTKEKRGDFMDSLIEMYQKELEGDSEEGLTFDELLAQA

FIFFVAGFETSSTTMGFALYELAQHQDIQSKIRKEVNEVLAKHSNEFTYDSIKQMKYLEQ

SVMETLRKYPVLAHLTRLTNTDYTPDDSKYHIDKGTVVVIPALGIHYDPSIYPQPEKFQP

ERFTEEEIQARPPCTWLPFGDGPRNCIGLRFGLMQTCVGLAYLIKNYNFSVAPETPIPMK

FVTKNILISAENGIPLKVEKL

>EDY69281.1

MSLVLALIAIVVSLVLFAARRRHGYWQRRGIPHDVPHPLFGNIGDWPKKRQIAVIFRDYY

RKYKNANYPFAGFYFFFTRSAVVTDLDLVKRVLIKDFNHFENRGIFYNEIDDPLSATVFS

IEGQKWRHLRHKLTPTFTSGKMKHMFPIVVKVGEEMDKVFSNKVGDGKVLEIVDLVARYT

ADVIGNCAFGLNCNSQQNPNADFVTFGKRALRERRYKGLLDFFIFGFPKLSRSLRLKLNV

QEVEDFYTKIVRDTIDYRLKTKEKRNDFMDSLIEMYQKEQEGNSDEGLTFNEILAQAFIF

FVAGFETSSTTMGFALYELAQNQDTQDKLRNEINDVLAKHNNQFTYEGIKEMKYLEQVVM

ETLRKYPALGHLTRKTRLDFSPEDPKNFIAKGTTVVIPALGIHYDPDIYPEPEKFKPERF

TEEAIASRPACAWLPFGEGPRNCIGLRFGLMQTCVGLAYLIKGYRFSVAPETQIPIKFVL

KSFLLSAENGIYLKVETVSK

>AAA29293.1

MDFGSFLLYALGVLASLALYFVRWNFGYWKRRGIPHEEPHLVMGNVKGLRSKYHIGEIIA

DYYRKFKGSGPFAGIFLGHKPAAVVLDKELRKRVLIKDFSNFANRGLYYNEKDDPLTGHL

VMVEGEKWRSLRTKLSPTFTAGKMKYMYNTVLEVGQRLLEVMYEKLEVSSELDMRDILAR

FNTDVIGSVAFGIECNSLRNPHDRFLAMGRKSIEVPRHNALIMAFIDSFPELSRKLGMRV

LPEDVHQFFMSSIKETVDYREKNNIRRNDFLDLVLDLKNNPESISKLGGLTFNELAAQVF

VFFLGGFETSSSTMGFALYELAQNQQLQDRLREEVNEVFDQFKEDNISYDALMNIPYLDQ

VLNETLRKYPVGSALTRQTLNDYVVPHNPKYVLPKGTLVFIPVLGIHYDPELYPNPEEFD

PERFSPEMVKQRDSVDWLGFGDGPRNCIGMRFGKMQSRLGLALVIRHFRFTVCSRTDIPM

QINPESLAWTPKNNLYLNVQAIRKKIK

>EDW74202.1

MALVLALFALLVSLLLFFARRRHSYWQRRGIPHDVPHPIWGNIGDWPKKKHIAYVFKDYY

NKYKSSKSPFAGFYFFFTKTAVVTDLELVKRVLIKDFNHFENRGVFYNEIDDPLTASLFS

IEGQKWRHLRHKLTPTFTSGKMKHMFPIVVKVGEEMEKVFQDKVGNAKGGKVLEVVDLVA

RYTSDVIGTCAFGLNCNSLHDPQAEFVQVGKKAITERRYYGLLDFFIFGFPKLARRLRLK

LNVQEVSDFYTRIVRDTINYRLKTKEKRGDFMDSLIEMYQKDLEGDSEEGLTFDELLAQA

FIFFVAGFETSSTTMGFALYELAQHQDIQSKIRKEVNEVLAKHSNEFTYDSIKQMKYLEQ

SVMETLRKYPVLAHLTRLTNTDYTPDDSKYHIDKGTVVVIPALGIHYDPSIYPQPEKFQP

ERFTEEEIQARPPCTWLPFGDGPRNCIGLRFGLMQTCVGLAYLIKNYNFSVAPETPIPMK

LVTKNILISAENGIPLKVEKL

>EDV35773.1

MSLVLTLIAVVFSLILFAARRRLGYWQRRGIPHDVPHPVFGNIKDWPKKRHIAHIFRDYY

LKYKGSEYPFAGFYFFFTRTAVITDLELVKRVMIKDFNHFENRGIFHNEIDDPLSGTLFA

IEGQKWRHLRHKLTPTFTSGKMKHMFPIVVKVGEQMEKVFREKTVAGQSQVLEVVDLVAR

FTADVIGTCAFGLDCNSLHNPKAEFVTMGRRAIAERRYGGWLDFFIFGFPKLSRRLHLKL

NVQEVEDFYTGIVRETIDYRLKTKEKRNDFMDSLIEMYQKEQEGNSEDGLTFNELLAQAF

IFFVAGFETSSTTMGFALYELAQNLDIQDKLRQEINQVLAKHNNEFTYEGIKEMKYLEQV

VMETLRKYPVLAHLTRKTETDFSPEDPKYFIAQGTVVVIPALGIHYDPEIYPEPEKFKPE

RFTEDAIAARPSCTWLPFGDGPRNCIGLRFGLMQTCIGLAYLIRGYKFSVAPETQIPMKV

VIKNILISAENGIHLKVERISK

>AAL49218.1

MSLLLTLIALLVSLLLFMARRRHGYWQRRGIPHDVPHPIYGNMKDWPKKRHIAMIFRDYY

TKYKRSVYPFAGFYFFFTRSAVITDLELVKRVLIKDFNHFENRGIFYNEIDDPLSATLFS

IEGQKWRHLRHKLTPTFTSGKMKNMFPIIVKVGEEMEKIFSAKTTTGEGQVLEIVDLVAR

YTADVIGNCAFGLNCNSLQNPNAEFVTIGKRAIIERRYGGLLDFLIFGFPKLSRRLRLKL

NVQDVEDFYTSIVRNTIDYRLRTNEKRHDFMDSLIEMYEKEQDGNTEDGLSFNEILAQAF

IFFVAGFETSSTTMGFALYELALDQDIQDQLRAEINNVLSKHNNEFTYEGIKEMKYLEQV

VMETLRKYPVLAHLTRMTQTDFSPEDPKYFIAKGTTVVIPALGIHYDPEIYPEPEKFKPE

RFTDEAIAARPSCTWLPFGEGPRNCIGLRFGLMQACVGLAYLIRGYKFSVSTETQIPMKF

VVKSILLSAENGIHLKVEKLSK

>Q9V771.2

MSLLLTLIALLVSLLLFMARRRHGYWQRRGIPHDVPHPIYGNMKDWPKKRHIAMIFRDYY

TKYKRSVYPFAGFYFFFTRSAVITDLELVKRVLIKDFNHFENRGIFYNEIDDPLSATLFS

IEGQKWRHLRHKLTPTFTSGKMKNMFPIIVKVGEEMEKIFSAKTTTGEGQVLEIVDLVAR

YTADVIGNCAFGLNCNSLQNPNAEFVTIGKRAIIERRYGGLLDFLIFGFPKLSRRLRLKL

NVQDVEDFYTSIVRNTIDYRLRTNEKRHDFMDSLIEMYEKEQAGNTEDGLSFNEILAQAF

IFFVAGFETSSTTMGFALYELALDQDIQDQLRAEINNVLSKHNNEFTYEGIKEMKYLEQV

VMETLRKYPVLAHLTRMTQTDFSPEDPKYFIAKGTTVVIPALGIHYDPEIYPEPEKFKPE

RFTDEAIAARPSCTWLPFGEGPRNCIGLRFGLMQACVGLAYLIRGYKFSVSTETQIPMKF

VVKSILLSAENGIHLKVEKLSK

>EDW91086.1

MSLLLTLIAVLVSLLLFVARRRHGYWQRRGIPHDVPHPVYGNIKDWPRKRHVAKIFRDYY

LKYKNSDYPFAGFYFFFTRTAVITDLELVKRVMIKDFNHFENRGIFYNEIDDPLSASLFS

IEGQKWRHLRHKLTPTFTSGKMKTMFPIVVKVGEEMEKTFSAKTSAGQGQVLEVVDLVAR

YTADVIGNCAFGLNCNSLHNPKAEFVAIGKRAIRERRYGGLLDFFIFGFPKLSRRLRLKA

NVQEVEDFYTGIVRNTIDYRLKTKEKRHDFMDSLIEMYQKEQAGNTEDGLSFNEILAQAF

IFFVAGFETSSTTMGFALYELARNQDIQDQLRAEINKVLGKHNNEFTYEGIKEMKYLERV

VMETLRMYPVLAHLTRMTETDFSPEDPKLFIAKGTVVVIPALGIHYDPEIYPEPGKFNPE

RFTDEAIAARPSCTWLPFGEGPRNCIGLRFGLMQTCVGLAYLIRGYKFSVSPDTQIPMKI

VLKNILLSAENGIHLKVEKLSQ

>EDX07149.1

MLLVALIVVILSLLVFAARRRHGYWQRRGIPHDEAHPLFGNIKDWPKKRHIAKIFRDYYF

KYKNSDYPFAGFFFFFTRTAVVTDLELVKSVLIKDFNHFENRGVFYNEIDDPLSATLFSI

EGQKWRHLRHKLTPTFTSGKMKNMFPIVVKVGEEMDKVFSSKTTADRGQVLEVVDLVARY

TADVIGNCAFGLNCNSLYDPKAEFVEIGKRAITEHRYGNMLDIFLFGFPKLSRRLRLKLN

IQEAEDFYTKIVRETIDYRLRTKEKRNDFMDSLIEMYKNEQSGNSEDGLTFNELLAQAFI

FFVAGFETSSTTMGFALYELARNQDVQDKLREEINNVFGKHNKEFTYEGIKEMKYLEQVV

METLRKYPVLAHLTRMTDTDFSPGDPKYFIAKGTIVVIPALGIHYDPDIYPEPETFKPER

FTDEEIAARPSCTWLPFGEGPRNCIGLRFGMMQTCVGLAYLIRGYKFSVSTETQIPMKIV

VKNILISAENGIHLKVEKLAK

>ABK91490.1

MIFEILILTLLAILAYFYKVLRQRYSYWQTLGIACDEPTFLLGNITGIGSKRAFVEIWQQ

FYDKYKNTGPFAGFYWFFKPAVFVFDPALLKLILIKDFSKFVDRGFYVNEEDDPLSGMLF

NLEGNKWRHMRNKLSPTFTSGKMKTMFPIIINISKEFVQVFGNAAKENDIIEVGDLMARF

TTDVIGSCAFGLEMSSLRDPNNKFRMMGRKSIVEQRYGRIGIAFRNSFPQLAKKLHMKDT

LQEVEDFFMGIVKETVRYREENNVRRNDFMDMLIDLKNNKLMKSETGEELTNLTLEEIAA

QAFVFLVAGFETSSTTMGFALYELAQHGDIQQKAREEVRQVLEKHNQEFTYEAMKEMVYL

EQIIAETLRLYTVLPLITRYASEDYPVPGHSKFVIKKDMLVLIPAGAIHRDERYYPNPNV

FNPDNFTADKVAQRDSVLHMPFGDGPRNCIGMRFGKMQALVGLAVLLKNFKFSVCDKTQI

PMKYDKNNFLCSSEGGIHLRVNRL

>ADP22304.1

MAITQILLLLVLGLVSYFAYWFRKRMNTWQDLGIACDKPNYLLGNLVGMRETRSFSDIGR

STYSKFKGTGPFCGFYWFHRPAVFVLETSLAKAVLIKEFNKFTDRGFYTNPDDDPLSGHL

FALDGHKWRAMRNKLSPTFTSGKMKQMFPTVTRVAAELVNVYNEAVNGERRSTLEVRDSL

GRYTTDVIGSCAFGIDCNSLKNPNDPFRQMGRRVFTAQRHGPLALALIILFPKLSRRLHI

KITPDHITDFYMRIVRENIAYREANNIKRHDFFDMLMDLKNNKMMKSEDGQAMMNITVEE

LAAQAFVFMVAGFETSSTTMGFALYELAQREDLQQRARQEVVEVLQKHNGEFTYECMNEM

VYLNQVISETLRKHTIMPLLVRECLEDYQVPDQPKYEIKKGTLVIIPAVGIHYDEDYYPN

PEEFNPDHFTPEKVALRDSIEWLPFGDGPRNCVGLRFGLMQTRVGLAYLLKNFKFSVSEK

TQIPIKYSKESFVSMAEGDIYLSVEKLAA

>EDY69282.1

MSLVLALIAIVVSLVLFAARRRHGYWQRRGIPHDVPHPLSGNMGDWPKKRHIAVIFRDYY

RKYKSANYPFAGFYFFFTRTAVVTDLDLVKRVLIKDFNHFENRGIFYNEIDDPLSATVFS

IEGQKWRHLRHKLTPTFTSGKMKHMFPIVVKVGEEMDKVFSNKVGDGKVLEIVDLVARYT

ADVIGNCAFGLNCNSQQNPNADFVTFGKRAIGERRYWGLLDFFIFGFPKLSRSLRLKLNV

QEVEDFYTKIVRDTIDYRLKTKEKRNDFMDSLIEMYQKEQEGNSDEGLTFNEILAQAFIF

FVAGFETSSTTMGFALYELAQNQDTQDKLRNEINDVLAKHNNQFTYEGIKEMKYLEQVVM

ETLRKYPVLAHLTRKTQADFSPEDPKTFIAKGTVVVIPALGIHYDPDIYPEPEKFKPERF

TEEAIASRPACTWLPFGEGPRNCIGLRFGIMQTCVGLAYLIKGYRFSVAPETQIPMKLIL

KSILLSAENGIYLKVKKVSK

>EDW32569.1

MSLVLALIAIVVSLVLFAARRRHGYWQRRGIPHDVPHPLFGNMGDWPKKRHIAIIFRDYY

RKYKSANYPFAGFYFFFTRTAVVTDLDLVKRVLIKDFNHFENRGIFYNEIDDPLSATVFS

IEGQKWRHLRHKLTPTFTSGKMKHMFPIVVKVGEEMDKVFSNKVGDGKVLEIVDLVARYT

ADVIGNCAFGLNCNSQQNPNADFVTFGKRAIGERRYWGLLDFFIFGFPKLSRALRLKLNV

QEVEDFYTKIVRDTIDYRLKTKEKRNDFMDSLIEMYQKEQEGNSDEGLTFNEILAQAFIF

FVAGFETSSTTMGFALYELAQNQDTQDKLRNEINDVLAKHNNQFTYEGIKEMKYLEQVVM

ETLRKYPVLAHLTRKTQADFSPEDPKTFIAKGTVVVIPALGIHYDPDIYPEPEKFKPERF

TEEAIASRPACAWLPFGEGPRNCIGLRFGIMQTCVGLAYLIKGYRFSVAPETQIPMKLIL

KSILLSAENGIYLKVKKVSK

>EDW00825.1

MSLIVTFIGIIVALLIYAVRRRYNYWNRRGIPHDVPKFPKGNIGDWPSKRQIGVIFGDYY

KKYKNSKSPFAGFLFFFTKSVVITDMELLKRVLIKDFQNFENRGIFYNEIDDPLSATLFS

IEGQKWRHLRHKVTPTFTSGKMKHMLPIVLKVAEELANVFKEKVTSPKVLEITDLMGRYT

ADVIGTCAFGIDCNSQRNPDADFVSMGKRAVTERAYHGLLDFFIFGFPKLARFLHLRLTV

PEVEDFYMRIIKDTIDYRVKNNERRNDFMDMLIQMYKKQQMGNTDEGLTFNELAAQAFIF

FVAGFETSSTTMGFALYELAQNQDVQDKLRKEIHEVLAKHNNVYSYDNIKEMAYLEQVVM

ETLRKYPVLAHLTRKTIADYSPEDPKYYIEKDTAVIVPAIGIHYDPDIYPEPHKFKPERF

TEAEIAARPSVSWLPFGDGPRNCIGSRFGMMQSCVGLAHLISDYKFSVSPQTTIPMKFVP

QNILLCSENGIYLNVERVGK

>P13527.1

MDFGSFLLYALGVLASLALYFVRWNFGYWKRRGIPHEEPHLVMGNVKGLRSKYHIGEIIA

DYYRKFKGSDPLPGIFLGHKPAAVVLDKELRKRVLIKDFSNFANRGLYYNEKDDPLTGHL

VMVEGEKWRSLRTKLSPTFTAGKMKYMYNTVLEVGQRLLEVMYEKLEVSSELDMRDILAR

FNTDVIGSVAFGIECNSLRNPHDRFLAMGRKSIEVPRHNALIMAFIDSFPELSRKLGMRV

LPEDVHQFFMSSIKETVDYREKNNIRRNDFLDLVLDLKNNPESISKLGGLTFNELAAQVF

VFFLGGFETSSSTMGFALYELAQNQQLQDRLREEVNEVFDQFKEDNISYDALMNIPYLDQ

VLNETLRKYPVGVGSALTRQTLNDYVVPHNPKYVLPKGTLVFIPVLGIHYDPELYPNPEE

FDPERFSPEMVKQRDSVDWLGFGDGPRNCIGMRFGKMQSRLGLALVIRHFRFTVCSRTDI

PMQINPESLAWTPKNNLYLNVQAIRKKIK

>EDW10114.1

MLDVIALLLVTLAVSFWFVRTRLSYWARRRISHVRPSFPMGNLEGFRSTKHFKDILTPLY

DSFKSTGAPFAGFYFMLRPVVLVLDLELAKQVLIQDFANFEDRGMYHNERDDPLTGHLFR

IDGPKWRPLRQKMSPTFSSGKMKFMFPTVSAVGEQLAQVCGELASNSICGILEINELMAR

YTSDVIGNCAFGLECNGLRNPEAEFAVMGRRAFTDRRHNKLIDGFIESFPNLARRLRLCQ

IHKDITDFYMRIVSDTVKERESKGIVRNDFMNLLIEMKQRGELTLPEIAAQSFIFFAAGF

DTSASTLAFALYELAKQPQIQVKLREEIELALQAHGGQFTYECMQELRYMELVIAETLRK

YPTLPHLSRVSKKYYAAKGNRHFYIEPGQMLYIPVYGIHHDPALYPEPHRFIPERFLADQ

MAQRPTASWLPFGDGPRNCIGMRFGKMQTSIALFHLLRRFQFSVCPRTEPKIKFQNSNLL

LCPATGIYLKVEELKK

>ABV48809.1

METSGVLIYLILVIASAVVYLLRRNLSYWKRLGIPHEEPTALMGNMNGLRTQRCMGDILA

EYYRKFKGCGPFAGIFNGQRPGVVLLDKQLIKQVLIKDFTQFTDRGLYYNEKSDPLTGHL

FFVDGDKWRSLRNKLSPTFTSGKMKYMYPTVLEVAERFMEVLAQKVRENSIVDVRDMLGR

FTVDVIASVAFGIECNSLRNAEDKFLQLGRKSIEVQRHNALIMTLIDGFPRLSRMLGMRT

LPQDVHEFFMQTIRETVEYRERNNIQRSDFLNILIELKNTVDDKSGLGGMELEELAAQVF

VFFLAGFETSSSTMAYALYELAQNQQIQERLREEINEAFEGDTKPSYETIMNLSYLDQVI

SETLRKYPILPFLNRQALNDYVVPGHPKFRIPKGTPIFIPVMGIQHDPEFYPQPDEFDPE

RFSPDMVKQRDSIEWMPFGDGPRNCIGARFAKMQTRLGLACVLKYFRVVVCPKTPLHLTF

QAKPLVLTPRHNVYLKLEAI

>EDW74915.1

MALLLGYVTLVLIAALAALFYYFLVSTYSYWQRRGVVHDKPVWLKGNLPTIGKTTTVREP

FREFYAKYKGKAPFGGFYFALREAAVLLDLELVKLVMIKDFNNFAYRGNFYNERDDPISA

HLFNLDGPGWREMRMKLTPTFTSLKMHQMLPTVVEIGQRFIKVVEKQLPTQGRAYPVEMK

DLLARFTTDVIGSCAFGLDCNSLDDPQVEFRRMGSKVFTDRRHGRLAFAFIQGFPKLAKM

MRMKVARDDVSDFYMRVVKDTLDYRDKHHVQRNDFFNLLMELRKEENGGLTFNQLAAQAF

VFFLAGFETSSSTMGFALYLLALHPEIQDKAREEVNEVFAKHKEFNYEAMKELKYLQQIL

YETMRKFSIAPILVRKAINDYPVPNSSYVIEAGTAVVIPVDAIHHDPEIYPEPEKFDPER

FSPEAIEQRSSVAWLPFGAGPRNCIGLRFGEMQSIVGLGMLLKNFKFSAAKETDIPIKIN

KTSFVLSSEGGIILNVEKV

>BAB43954.1

METSGVLIYLILAIASAVVYLLRRNLSYWKRLGIPHEEPTALMGNMHGLRTQRCMGDILA

DYYRKFKGCGPFAGIFNGQRPGVVLLDKQLIKQVLIKDFTQFTDRGLYYNEKSDPLTGHL

FFVDGDKWRSLRNKLSPTFTSGKMKYMYPTVLEVAERFMEVLAQKVRENPIVDVRDMLGR

FTVDVIASVAFGIECNSLRNAEDKFLQLGRKSIEVQRHNALIMTLIDGFPRLSRMLGMRT

LPQDVHEFFMQTIRETVEYRERNKIQRSDFLNILIELKNTVDDKLGLGGMELEELAAQVF

VFFLAGFETSSSTMAYALYELAQNQQIQERLREEINEAFEGDTKPSYETIMNLSYLDQVI

SETLRKYPILPFLNRQALNDYVVPGHPKFRIPKGTPIFIPVMGIQHDPEFYPQPDEFDPE

RFSPDMVKQRDSIEWMPFGDGPRNCIGARFAKMQTRLGLACVLKYFRVVVCPKTPLHLTF

QAKPLVLTPRHDVYLKLEAI

>EDW74919.1

MAFCCLSLYLIGAVVALILLYLHRRYTYWSRRGVVQLNPKWIYGNMDKVGKTQSLRDPFR

ELYNTFKGKEKFGGFYVGMRQTAVLLDLDLIKLVMIKDFQNFANRGNYYNEQDDPLMAHL

FNLDGQKWRNMRTKLTPTFTSLKMHHMYPTIVEIGDRFINLMNEEMLKARAVSNNGEAAV

EVKDLLARFTTDVIGSCAFGLDCNSLKDPNAEFRSVSRRMLAQRRHGPIAMSFMGTYPNL

ARKLHMKFLPDYAAEFYLRVIKESIDHRDKNNIERGDFLNLVMEMREAVDGMTFNQIAAQ

CFVFLLAGFETSSSTMGFALYELALNQDVQEKARQEVKEILAKHGEFTYEAMKELKYLQQ

VLYETMRKWPILPILTRLTEKDYPVPGTNSQVFEAGTQLIIPADAIHHDPEIYPDPEKFD

PERFSHDAVEKRHAMTWLPFGSGPRNCIGSRFGEMQSIVGLALLLKKFKFLPAKETPIPL

QLNKKSFLLTSDSGIVLDVQSI

>EDV35771.1

MLILIGLIVGVLTVAIWWVHQNFTYWKRRGIPHDPPQLILGNTRELMKTLQLAELIKQTY

VKYKNKTNGPFVGFYVYIKKFVIITDIDFVKTVLIREFDKFHDRGVFHNEKDDPLTANLT

TIEGQKWKVLRQKLTPTFTSGKMKNMFPLVQNVGDELVQVFSEKIEATPTPLEITDLLAR

FTADVIGVCAFGLDCNSLRDPRAEFVRMGNSALTERRHGKLVHMLLFGAPKLSAKLGIKS

LLQPVEDFYMNIIKDTVDYRVKNNVKRNDFMDMLIEMKLKYDNGNKQDGLTFNEIAAQAF

IFFLAGFETSSTTMGFALYELALNQDVQDKLRLEVNSVLEKHDGRLSYDSMRELVYMDKV

IDETMRKRPVVGHLIRIATQRYEHSNPKYYIEPGTGVMVPSYAIHHDPEFYPEPEKFIPE

RFDEEQIMQRPACTFLPFGDGPRNCIGLRFGRMQVIVGMALLIKNFKFEVHPTKTQVPLK

YRCDDILISAEGGIHLKVSKV

>EDW60862.1

MLDVIALLLITLAVGFWFVRTRLSYWARRSISHARPTFPMGNLEGFRKDKHFKDILTPLY

ESFKPTGAPFAGFYFMLRPVVLVLDLELAKEVLIKDFANFEDRGLYHNERDDPLTGHLFR

IDGPKWRPLRQKMTSTFSSGKMKFMFPTVCAVGEELAQVCAEQAQNSICGILEINDLMAR

YTSDVIGSCVFGLDCNGLRNPEAEFAVMGRRAFIERRHNKLIDGFIESFPNLARRLRLRQ

IHQDITDFYMRIVRDTVNERESKGIVRNDFMNLLIEMKHRGELTLPEMAAQSFIFFAAGF

DTSASTLGFALYELAKQPHLQLKLRQDIEQALQAHGGQFTYECMQELRYMELVIAETLRK

YPVLPHLSRVSRNFYAAKGNKHFYIEPGQMVFVPVYGIHHDPELYPEPHKFIPERFLADQ

MAQRHTASWLPFGDGPRNCIGMRFGKMQTSVALFYLLRRFQFSVCPRTASKIEFVKSNIL

LCPANGIYLKVEELRK

>ABG34551.1

MIALTILLSVLLILIAYIGRLLSEHFNYWRHQGVACEPPNWLLGNLRGLSTSRSFNQIIR

EYYEKYRNSAGPFAGFYWLHKKAVFVLDPELIKQILIKDFNKFTDRGFFSNEEDDPLSGQ

LINLSGNKWRNMRNKLSPAFTSGRMKAMFPLVIKLGYDLVEVVGERLKKHDDVVEVRDLA

AFFTSDVIGTCAFGLNMNCMKNSDAEFLKMGRKALNEQRYGTLGFVLRFSFPDLCRRLHM

KETLDDVEKYFMQIVQETVDYRERENVKRNDFMDMLIDLKNNKLIKDESGEEFINLTFGQ

IAAQAFVFLLAGFETSSTTMSFALYELAQHLEVQQRSREEVENVLKAHNGTFDYECLKEM

VYLEQVIQETLRFYTTIPTINRLASEDYVVGDNPKYVIKKGMAVVIPAAALHRDERYYPQ

PDVFNPEHFAASQVAERDSVLNLSFGDGPRNCIGMRFGKMQAMIGLALLLKNFRFTVCEK

TQIPLKLDKKSIIVAPESGIYLRVERI

>EDW47926.1

MLLLALIVVILSLLVFAARRRHGYWQRRGIPHDEAHPLFGNIKDWPNKRHIAKIFRDYYF

KYKNSDYPFAGFFFFFTRTAVVTDLELVKSVLIKDFNHFENRGVFYNEIDDPLSATLFSI

EGQKWRHLRHKLTPTFTSGKMKNMFPIVVKVGEEMHKVFSSKTTADRGQVLEVVDLVARY

TADVIGNCAFGLNCNSLYDPKAEFVEIGKRAITEHRYGNMLDIFLFGFPKLSRRLRLKLN

IQEAEDFYTKIVRETIDYRLRTKEKRNDFMDSLIEMYKNEQSGNSEDGLTFNELLAQAFI

FFVAGFETSSTTMGFALYELARNQDVQDKLREEINNVFGKHNKEFTYEGIKEMKYLEQVV

METLRKYPVLAHLTRMTDTDFSPGDPKYFIAKGTIVVIPALGIHYDPDIYPEPEIFKPER

FTDEEIAARPSCTWLPFGEGPRNCIGLRFGMMQTCVGLAYLIRGYKFSVSPETQIPMKIV

VKNILISAENGIHLNVEKLAK

>EDX07154.1

MSVGTVLLTALLALVGYLLMKWRSTMRHWQDLGIPCEEPHMMMGSMKGVRTARSFNEIWT

SYYKKFRGTGPFAGFYWFRRPAVLVLETSLAKQILIKEFNKFTDRGFFHNPEDDPLSGQL

FLLDGQKWKAMRNKLSSTFTSGKMNVTKSPVVEVRELLARFTTDVIGTCAFGIECSSLKD

PDAEFREMGRRSLTEQRLGPIGIGFVNSFPNLARRLHMKMTAEPIERFFMRIVRETVAFR

EQNNIRRNDFMDQLIDLKNKPLVVSQSGESVNLTIEEIAAQAFVFFAAGFETSSTTMGFA

LYELAQNQDIQERVREECQEVIAKCNGELNYESMKDLVYLDQVISETLRLYTVLPVLNRQ

CLEDYEVPGHPKYVIKKGMPVLIPCGAMHRDEKLYANPNTFNPDNFSPERVKDRDSVEWL

PFGDGPRNCIGMRFGQMQARIGLALLIKDFKFSVCEKTTIPMTYNKEMFLISSDSGIYLK

AERV

>Q9V770.1

MLLLALIVVILSLLVFAARRRHGYWQRRGIPHDEVHPLFGNIKDWPNKRHIAEIFRDYYF

KYKNSDYPFAGFFFFFTRTAVVTDMELLKRVLIKDFNHFENRGVFYNEIDDPLSATLFSI

EGQKWRHLRHKLTPTFTSGKMKNMFPIVVKVGEEMDKVFRSKTAADRGQVLEVVDLVARY

TADVIGNCAFGLNCNSLYDPKAEFVSIGKRAITEHRYGNMLDIFLFGFPKLSRRLRLKLN

IQEAEDFYTKIVRETIDYRLRTKEKRNDFMDSLIEMYKNEQSGNSEDGLTFNELLAQAFI

FFVAGFETSSTTMGFALYELARNQDVQDKLREEIGNVFGKHNKEFTYEGIKEMKYLEQVV

METLRKYPVLAHLTRMTDTDFSPEDPKYFIAKGTIVVIPALGIHYDPDIYPEPEIFKPER

FTDEEIAARPSCTWLPFGEGPRNCIGLRFGMMQTCVGLAYLIRGYKFSVSPETQIPMKIV

VKNILISAENGIHLKVEKLAK

>EDW91089.1

MAILIVLLIGVITFAAWYVHQHFNYWKRRGIPHDKPKIPFGNTSELMKTVQVSQIFVNTY

NKYKSKIDGPFVGFYMYFKQMVVVTDIDFVKTVLIREFEKFHDRGVFHNERDDPLSANLV

NVDGQKWKTLRQKLTPTFTSGKMKSMFPTILTVGDELIRVFDQTVSASSDSTEITHLLAR

FTADVIGSCAFGLDCHSLSDPKAEFVQMGTAAITERRYGKSMDLLLFGAPQLAAKLRMKA

TVQKVEDFYMNIIRDTVDYRIKNNVKRNDFMDMLIEMKLKYDNGDKANGLTFNEIAAQAF

IFFLAGFETSSTSMGFALYELASNQDVQDKLRIEIDAVMKKHNGKLDYDSMRELTYLEKV

IDETMRKRPVVAHLIRIATQRYEHTNPKYYIEKGTGVIIPTLAIHHDPEFYPEPEKFIPE

RFDEDQVQQRPACTFLPFGDGPRNCIGLRFGRMQVIIGMALLIHNFKFELHPTKTVVPMV

YRIDDILMSSKDGIHLKVSRVGKF

>EDW91085.1

MLLLALIVILLSLLVLAARRRHGYWQRRGIPHDEAHPLFGNIKDWPNRRHIAEIFRDYYF

KYKNSDYPFAGFFFFFTRTAVVTDLELVKRVMIKDFNHFENRGVFYNEIDDPLSATLFSI

EGQKWRHLRHKLTPTFTSGKMKNMFPIVVKVGEEMDKVFSSKTAEGQSQVLEVVDLVARY

TADVIGNCAFGLNCNSLYDPKAEFVAIGKRAITEHRYGNMLDIFLFGFPKLSRRLRLKLN

IQEAEDFYTKIVRETIDYRLRTKEKRNDFMDSLIEMYKNEQSGNSEDGLTFNELLAQAFI

FFVAGFETSSTTMGFALYELARNQDVQDKLREEIDTVLGKHKNEFTYEGIKEMKYLEQVV

METLRKYPVLAHLTRMTETDFSPEDPKYFIAKGTIVVIPALGIHYDPEIYPEPEMFTPER

FTDEEIAARASCTWLPFGEGPRNCIGLRFGMMQTCVGLAYLIRSYKFSVSPETQIPMKIV

VKNILISAENGIHLKVEKLAK

>EDX07148.1

MLDVVALLLIALAVGFWFVRTRYSYWTRRGIGCEPARFPVGNMEGFRKNKHFIDIVTPIY

EKFKGNGAPFAGFFMMLRPVVLVTDLELAKQILIQDFANFEDRGMYHNERDDPLTGHLFR

IDGPKWRPLRQKMSPTFTSAKMKYMFPTVCEVGEELTQVCGELADNAMCGILEIGDLMAR

YTSDVIGRCAFGVECNGLRNPEAEFAIMGRRAFSERRHCKLVDGFIESFPEVARLLRMRQ

IHQDITDFYVGIVRETVKQREEQGIVRSDFMNLLIEMKQRGELTIEEMAAQAFIFFVAGF

DTSASTLGFALYELAKQPALQAKLREEIDQALKLHQGEFTYDSMQELRYMELVIAETLRK

YPILPQLTRISRHLYAAKGDRHFYIEPGQMVLIPVYGIHHDPALYPEPHKFIPERFLADQ

LAQRPTAAWLPFGDGPRNCIGMRFGKMQTTIGLVSLLRNFHFSVCPRTDPKIEFVKSNIL

LCPANGIYLKVQQLSQLSR

>EDW74925.1

MSTIIVLFAGVLTLIAWWFRQRFSYWQRRGISHDTPRLLIGNMHEWKKTKQISYIMKATY

DKFKGSGPFAGFYFFMAPAVVALELDFIKEVLIKQFDKFHDRGVFHNERDDPLSASLITI

EGQKWRQLRQKLTPTFTSGKMKFMYPTVEKVAKELLQAFDQQLENSSLLEVHDLAARYTA

DVIGSCAFGLECNSLSNPQAEFVIMGGKAIKERHHGNFINSMIFSFPNLAKALRMRINLY

SVEDFYTNIVRETVDYRIKNGVKRNDFMDSLIDLIRKHEEGSKTEGLTFNEVVAQAAIFF

LAGFETSSTTMGFALYELALNQDIQDKLRKEISSVLAKYNNEFTYECMMDQRYLSQVVDE

TLRKYPVAAHLFRRTNQRYVNNDSKYYIEPGTLVFIPTLALHYDPEYYEEPEKFKPERFS

DDAIQQRPSCAYLPFGEGPRNCIGMRFGKMQTVIGLAKLISIYKFEVHPEKTQLPLELDT

TNFLLSAKGGIHLNVSKVV

>EDW91084.1

MLDVVALLLIALAVGFWFVRTRYSYWSRRGIGNVLARFPVGNMEGFRTTKHFIDIVTPIY

EKFKGNGAPFAGFYMMLRPVVLVTDLELAKQILIQDFANFEDRGMYHNARDDPLTGHLFR

IDGPKWRPLRQKMSPTFTSAKMKYMFPTVCEVGMELTQVCGELADNAMCGILEIGDLMAR

YTSDVIGRCAFGVECNGLRNPQAEFAIMGRRAFAERRHCKLIDGFIESFPDLARFLRMRQ

IHQDITDFYVGIVRETVKQREEQGIVRSDFMNLLIEMKQRGELTIEEMAAQAFIFFVAGF

DTSASTLGFALYELAKQPALQEKLREEIDQALKLHQGEFTYDSMQELRYMELVIAETLRK

YPILPQLTRISRHLYAAKGDRHFYIEPGQMLLIPVYGIHHDPALYPEPHKFIPERFLADQ

LSQRPTAAWLPFGDGPRNCIGMRFGKMQTTIGLVSLLRNFHFSVCPRTDPKIEFLKSNIL

LCPANGIYLKVQQLSQLSR

>EDW47925.1

MLDVVALLLIALAVGFWFVRTRYSYWTRRGIGCEPARFPVGNMEGFRQNKHFIDIVTPIY

EKFKGNGAPFAGFFMMLRPVVLVTDLELAKQILIQDFANFEDRGMYHNERDDPLTGHLFR

IDGPKWRPLRQKMSPTFTSAKMKYMFPTVCEVGEELTQVCGELADNAMCGILAIGDLMAR

YTSDVIGRCAFGVECNGLRNPEAEFAIMGRRAFSERRHCKLVDGFIESFPEVARLLRMRQ

IHQDITDFYVGIVRETVKQREEQGIVRSDFMNLLIEMKQRGELTIEEMAAQAFIFFVAGF

DTSASTLGFALYELAKQPALQAKLREEIDQALKLHQGEFTYDSMQELRYMELVIAETLRK

YPILPQLTRISRHLYAAKGDRHFYIEPGQMVLIPVYGIHHDPALYPEPHKFIPERFLSDQ

LAQRPTAAWLPFGDGPRNCIGMRFGKMQTTIGLVSLLRNFHFSVCPRTEPKIEFLKSNIL

LCPANGIYLKVQQLSQVSR

>EDW00824.1

MLDVIALILITLAIGFWYVRTRLSYWVRRQISHEQPTFPMGNFRGFRNTVHFKDILTPLY

ERFKSTGSPFAGIYFMLRPVVLVLDLELAKEVLIRDFANFEDRGMYHNERDDPLTGHLFR

IDGPKWRPLRQKMTPTFSSGKMKFMFPIVCAVGKELAQICAEQSQNSICGILEVSDLMSR

YTSDVIGSCAFGLDCNGLRNPEAEFRTMGRRALTERHHNKLIDGFIESFPHWARRLRLRQ

VHPDITKFYQRIVRDTVKERESQRIVRNDFMNLLIEMKQRGELTLPEMAAQSFIFFVAGF

DTSASTLTFALYELAKQPQIQHKLRQEIQESLEKHEGQFTYECMQELRYMELVIAETLRK

YPILPHLSRICRNYYAAKGNRHFYIEPGQMLLIPVYGFHHDPELYPEPHKFIPERFMADQ

MAERPTASWLPFGDGPRNCIGMRFGKMQTSIALFHLLQRFQFSVCPRTVSKIDFVKSNIL

LSPSSSIYLKVEELQNI

>EDV55970.1

MLDVVALLLIALAVGFWFVRTRYSYWSRRGIGNDPARFPVGNMEGFRRTKHFIDIVTPIY

EKFKGNGAPFAGFYMMLRPVVLVTDLELAKQVLIQDFANFEDRGMYHNERDDPLTGHLFR

IDGPKWRPLRHKMSPTFSSAKMKYMFPTVCEVGVELSQVCGELADNAMCGILEIGDLMAR

YTSDVIGRCAFGVECNGLRNPEAEFATMGRRAFLERRHCKLIDGFIESFPEVARFLRMRQ

IHQDITDFYVGIVRETVKQREEQGIVRSDFMNLLIEMKQRGELTIEEMAAQAFIFFVAGF

DTSASTLGFALYELAKQPALQEKLREEIDQALKLHHGEFTYDSMQELRYMELVIAETLRK

YPILPQLTRISRHLYAAKGDRHFYIEPGQMLLIPVYGIHHDPALYPEPHKFIPERFLADQ

LAQRPTAAWLPFGDGPRNCIGMRFGKMQTTIGLVSLLRNFHFSVCPRTDPKIEFLKSNIL

LCPANGIYLKVQQLSQLSR

>EDW47930.1

MGSLTGVQTSRSFSDIWMDYYNKFRGTGPFAGFYWFQRPGILVLDISLAKLILIKEFNKF

TDRGFYHNTEDDPLSGQLFLLDGQKWKSMRSKLSSTFTSAMEKSPIVEVKDILARFTTDV

IGTCAFGIECSSLKDPEAEFRVMGRRAIFEQRHGPIGIAFINSFQNLARRLHMKITLEEA

EHFFLRIVRETVAFREENNIRRNDFMDQLIDLKNSPLTKSESGESVNLTIEEMAAQAFVF

FGAGFETSSTTMGFALYELAQHQDIQDRVREECQEVIGKCNGEITYESMKDMVYLDQVIS

ETLRLYTVLPVLNRECLEDYEVPGHPKYVIKKGMPVLIPCGAMHRDEKLYANPNTFNPDN

FSPERVKERDSVEWLPFGDGPRNCIGMRFGQMQARIGLAFLINKFKFSVCDQTTIPIVYS

KKTFLISSETGIFLKVERV

>EDV55969.1

MLLLALIVVLLSLIVFAARRRHGYWQRRGIPHDEVHPLYGNIKDWPNRRHIAEIFRDYYL

KYKNSDYPFAGFFFFFTRTAVVTDLELVKRVMIKDFNHFENRGVFYNEIDDPLSATLFSI

EGQKWRHLRHKLTPTFTSGKMKNMFPLVVKVGEEMDKVFSSKTAAGRSQVLEVVDLVARY

TADVIGNCAFGLNCNSLYDPKAEFVAIGKRAITEHRYGNMLDIFLFGFPKLSRRLRLKLH

IQEAEDFYTKIVRETIDYRLRTKEKRNDFMDSLIEMYKNEQSGNSEDGLTFNELLAQAFI

FFVAGFETSSTTMGFALYELARNQDVQDKLRKEIDGVFGKYKNEFTYEGIKEMKYLEQVV

METLRKYPVLAHLTRMTQTDFLPEDPKQFIAKGTIVVIPALGIHYDPEIYPEPEKFKPER

FTDEAITARPSCTWLPFGEGPRNCIGLRFGLMQTCVGLAYLIRGYKFSVSPETQIPMKIV

LKNILISAENGIHLNVEKLSK

>EDX07153.1

MAVLIVLLIGVITFVAWYVHQRFNYWKRRGIPHDEPKIPFGNTSELMKTVQLSDIFKRTY

NKYKNKTDGPFVGFYMYFKQMVVVTDIDFVKTILIREFDKFHDRGVFHNERDDPLSAHLV

NIEGQKWKTLRQKLTPTFTSGKMKSMFPTILTVADDLIRVFGQTASAEGDSMEITNVVAR

FTADVIGTCAFGLDCHSLSDPKAEFVQMGTAAITERRYGKSMDLLLFGAPKLAAKLRMKA

TVQKVEDFYMNIIRDTVDYRVKNNVKRNDFVDMLIEMKLKYDNGDKANGLTFNEIAAQAF

IFFLAGFETSSTTMGFALYELACHQDIQDKLRTEIDAVLKKHNGKLDYDSMREMTYLEKV

IDETMRKRPVVGHLLRVATQDYQHTNPKYNIEKGTGVVIPTLAIQHDPEFYPEPEKFIPE

RFDEDQVQQRPPCTFLPFGDGPRNCIGLRFGRMQVIIGMALLIHNFKFEFHPTKTVVPLE

YRTDDILLSAKGGIHLKVSRV

>EDW74198.1

MAVLILLLLGVVSLAVWWVHQQFNYWQRRGIPHDPPKIPAGNMVELMKTRHIADIFQRTY

FKYKNKVDGPFVGFYFYFKRVALTVDINFVKTVLMKDFDKFHDRGIFHNERDDPLTCNLV

GVEGQKWKSLRHKLTPTFTSGKMKHMFPTLLKVGDEMVQVFNSKITSDTQPLEITNLVAR

FTADVIGSCAFGLECNSLHNPEAEFVKMGNAAINERRHGRALDLFLFGAPKLAAKLRMKQ

LVQKVEDFYMNIIRDTVNYRVKNNVKRNDFMDMLIELKKKYDEGNKEEGLSFNELAAQAF

VFFLAGYETSSTTMGFALHELALNQDIQNRLRNEIDEVLAKNNGEFSYDSMREMKYLEKI

IDETLRKHPVVGHLIRLATQRYVHPDNPKYYIEPGTGVIIPVKAIHHDPEFYPEPEKFIP

ERFDEDQVKQRPACTFLPFGEGPRNCIGLRFGRMQTTIGLAKLIHNFKFEVNPTLTSVPM

KFKVENILLSAEGGITLNVSKVIRK

>EDW60931.1

MSLWWFSLGLLVAIFGLLYACLERTYSYWARRGVLHEKPVWFYGNLRSVGKTTTVLEPFR

RFYEKYKGQAPFGGFYFALRGAAVLLDLELVKLVMVKEFHNFAHRGNYYNEQDDPISAHL

FNLDGRKWREMRNKLTPTFTSLKMHQMLPTVVEIAERFVQVVRQKVEQHQGGEESPVEVK

DLLARFTVDIIGSCAFGLDCNSLNDPNVEFRRLGQKTFTERRHGRLAFALIQAFPRLAKR

LHIKMVQDDVSAFYMRVVRETLDYRERNGVQRNDFLNLLMELRNEPNGGLTFNQIAAQAF

VFFLGGFETSSSTMGFALHLLALHPEVQQRGREEVQQVLARHGQLNYAALRELKYIKQII

YETLRKYSIASILLRKTIEDFQIPGSTFVLEAGTPVVIPVDAIHHDPDIYPEPDKFDPDR

FTPEAMEARPSTAWLGFGAGPRNCIGLRFGEMQALVGLAVLLKNFKFSPAKETEIPLQIH

KTSFFLQSQGGIVLNVEKV

>ADP22309.1

MTFLSLWIPVYLFVAAATLLFLWYRKGLNYWAERGVVHDKPSLIYGNMRGVGRTQTLRDP

FQNFYQKYKHVAPFGGFYLSLRAAVVLFDLDLIRNVLVKDFANFVNRGNYYNVKDDPISG

HVFNLDGASWRHMRAKLTPTFTSAKMKYMFPTVVAVAKEFVKVLEREATTLASDADASKQ

GIEIRDLMARFTTDVIGGCAFGLECNSLNEPNTEFRVMGKKVFSQRRHGRFVFAFAQAYP

NLARKLGIKMTPNDVSTFFRKVVHDTVSYRERTGIQRNDFLNLLLELKKQENGLSLDEIA

AQSFVFFLAGLETSSSTMSFALYELALHPEIQDKARAEIESVLARYDGEITYEGVREMQY

LYQIFCETLHKYSIASITMRRTMNDYHVPNTKHVIEKGVIVIIPIDAIHRDPEIYPDPER

FDPTRFEPEAAAKRHPMTGLPFGEGPRNCIGLRFGKMQALVGMALLLKNFKFTLAPQTKV

PLEIKMDSFVLSSKDGIHLNVERIASS

>EDX07151.1

MAILLGLVVGVLTLAAWWVLQNYTYWKRRGIPHDPPNIPLGNTSELWRTMPLASILKRTY

LKFSKQTDGPFVGFYLYAMKYIVITDVDFLKTVLIRDFDKFHDRGVYHNEKDDPLTNNLA

TIEGQKWKNLRQKLTPTFTSVKMKSMFSTVLNVGDEMIRVVDERLSSSSQTLEVTDLVSR

FTSDVIGICAFGLECNSLRDPKAEFVQMGYSALRERRHGWLVDLLIFGMPKLAVKLGFQF

LLPSVQKFYMKIVQDTIDYRVKSKVTRNDFMDTLIEMKLQYDKGDKENGLAFNEVAAQAF

VFLLAGFEAGSTTMGFTLYELACNPDVQDKLRAEIDSVLEKHNGKLEYDSMQELTYMEKV

INESLRKHPVVAHLARIATKPYQHSNPKYYIEAGTGVLVSTLGIHHDPEFYPEPEKFIPE

RFDEDQVKKRPTCAFLPFGAGPRNCIGLRFGRMQVVIGLALLIHNFRFELHPKTPVPMKY

TIKNLLLGSEGGIHLNVTKVVRD

>EDV35775.1

MSLLLSLVVIVLSLIIFAARRRHGYWRRRGIPHDVPNPLFGNIKDWPSQRHIATIFKDYY

EKYKGSGYPFAGFFFFFTRTAVLTDLDLVKRVMIKDFNHFENRGVFYNEIDDPLTGNLFS

IEGQKWRHLRHKLTPTFTSGKMKHMFPIVVKVGEEMDAVFSEKVAPGQSEVLEVVDLVAR

YTADVIGNCAFGLNCNSLHNPQADFVTIGKRAVTEHRYGGLLDIFIFGFPKLSRRLHLKL

NVQDVEDFYTGIVRETIDYRLKTKEKRNDFMDGLIEMYQKELEGNSEDGLTFNELLAQAF

IFFVAGFETSSTTMGFALYELALNQDVQDKLRKEINDVLAKNNNEYTYEGIKEMKYLEQV

VMETLRKYPVLAHLTRMAETDYSPEDPKYFIAKGTIVVIPALGIHYDPDIYPEPEKFKPD

RFTDEAIAARPSCTWLPFGEGPRNCIGLRFGLMQTCVGLAYLIRSYRFSVAPETQIPMKV

VVKSILISAENGIHLKVEKLVK

>P82711.1

MAILLGLVVGVLTLVAWWVLQNYTYWKRRGIPHDPPNIPLGNTGELWRTMPLAGILKRTY

LKFRKQTDGPFAGFYLYAMKYIVITDVDFVKTVLIRDFDKFHDRGVYHNEKDDPLTNNLA

TIEGQKWKNLRQKLTHTFTSAKMKSMFSTVLNVGDEMIRVVDEKISSSSQTLEVTDIVSR

FTSDVIGICAFGLKCNSLRDPKAEFVQMGYSALRERRHGWLVDLLIFGMPKLAVKLGFQF

LLPSVQKFYMKIVQDTIDYRMKRKVTRNDFMDTLIDMKQQYDKGDKENGLAFNEVAAQAF

VFFLAGFEAGSTTMGFTLYELACNQDVQDKLRAEIDSVLERYNGKLEYDSMQDLFYMEKV

INESLRKHPVVAHLARIATKPYQHSNPKYFIEAGTGVLVSTLGIHHDPEFYPEPEKFIPE

RFDEEQVKKRPTCAFLPFGAGPRNCIGLRFGRMQVIIGLALLIHNFRFELHPKTPVPMKY

TINNLLLGSEGGIHLNITKVVRD

>EAL26032.1

MAIIIVLLVGVISLAVWWVHQNFSYWKRRGIPHDPPNIPKGNTSELMKTRQIADLFRETY

FKYKGKTDGPFVGFYLYLKKIAIVTDIDFVKTVLIREFDKFHDRGLFHNEKDDPLTNHLV

AIEGQKWRQLRQKMTPTFTSGKMKIMFPTVLEIGNEMIRVFAEKIGPQPLNVADLVSRFS

ADVIGSCAFGLECNSLRNPNAEFVTMGKAAITERRYGKTADLFFFGAPKLAAKLRMKETV

QKVEDFYMKIVRETVDYRLKNNVKRNDFMDMLIDMKINYDKGNKQDGISFNELAAQAFIF

FLAGFDTSSTSLGFALHELAVNQDIQDKLRTEIDTVMAKHNGQLNYDSMRELTYLDKVID

ETFRKNPVVGHLIRICTQRYEHPNGKYFIEPGTGVVIPTLGIHHDPEFYPEPEKFKPERF

DEDQVLQRPPCTFLPFGDGPRTCIGLRFGRMQIVVGLALLIHNFKFEVHPTKTTVPLKFK

VTDVLLCSEGGVHLNVSKVPR

>EDW91087.1

MAILLGLVVGVLTLAAWWVLQNYTYWKRRGIPHDPPNIPLGNISDLWKTMPLAGILKRTY

LKYRKQTDGPFVGFYLYSMKYIVITDVDFLKTVLIRDFDKFHDRGVYHNEKDDPLTNNLA

TIEGQKWKSLRQKLTPTFTALKMKNMFSTVLSVGDEMIRVVDEKISSSSQTLEVTDLVSR

FTADVIGICAFGLECNSLRDPKAEFVQMGYSALRERRHGWLVDLLIFGMPKLAVKLGLQF

LLPSVQEFYMKIVQDTIDYRVKSKVTRNDFMDTLIDMKLQYDKGDKENGLTFNEVAAQAF

IFFLAGFEAGSTTMGFTLYELACNQDVQDKLRSEIDSVLQKHNGKLEYDAMQELTYTEKV

INESLRKHPVVAHLARIATKPYQHSNPKYFIEEGTGVLVSTMGIHYDPEFYPEPEKFIPE

RFDEEQVKKRPTCAFLPFGAGPRNCIGLRFGRMQVVVGLALLIHNFRFELHPETPVPMKY

TAKNLLLGSEAGIHLNVSKVVRD

>EDW32568.1

MAIIIVLLVGVISLAVWWVHQNFSYWKRRGIPHDPPNIPKGNTSELMKTRQIADLFRETY

SKYKGKTDGPFVGFYLYLKKIAIVTDIDFVKTVLIREFDKFHDRGLFHNEKDDPLTNHLV

AIEGQKWRQLRQKMTPTFTSGKMKIMFPTVLEIGNEMIRVFTEKITGGPQPLNVADLVSR

FSADVIGSCAFGLECNSLRNPNAEFVTMGKAAITERRYGKTADLFFFGAPKLAAKLRMKE

TVQKVEDFYMKIVRETVDYRLKNNVKRNDFMDMLIDMKINYDKGNKQDGISFNELAAQAF

IFFLAGFDTSSTSLGFALHELAVNQDIQDKLRTEIDTVMAKYNGQLNYDSMRELTYLDKV

IDETFRKNPVVGHLIRICTQRYEHPNGKYFIEPGTGVVIPTLGIHHDPEFYPEPEKFKPE

RFDEDQVLQRPPCTFLPFGDGPRTCIGLRFGRMQIVVGLALLIHNFKFEVHPTKTTVPLK

FKVTDVLLCSEGGVHLNVSKVPR

>EDV55967.1

MAVLIVLLIGAITFAAWYVRQHFNYWKRRGIPHDEPKIPFGNTSELMKTIQLSEIMKRTY

NKYKNKTDGPFVGFYMYFKRMVVVTDIDFVKTVLIREFEKFHDRGVFHNERDDPLSANLV

NIDGQKWKSLRQKLTPTFTSGKMKSMFPTILTVGDDLIRVFDQTASASGDSMEITHLLAR

FTADVIGSCAFGLDCHSLSDPKAEFVQMGTAAITERRYGKSMDLLLFGAPKLAAKLRMKA

TVQKVEDFYMNIIQDTVDYRVKNNVKRNDFMDMLIEMKLKYDNGDKENGLTFNEIAAQAF

IFFLAGFETSSTTMGFALYELASNQDIQDKLRIEIDTVLKNHNGKLDYDSMRDMTYLEKV

IDETMRKRPVVGHLIRVATQAYEHTNPKYNIEKGTGIIIPTLAIQHDPEFYPEPEKFIPE

RFDEDQVQQRPACTFLPFGDGPRNCIGLRFGRMQVIVGMALLVHNFKFELHPTKTVVPLE

YRIDDILMSSKGGIHLKVSRVGKL

>EAT39044.2

MEPLDVAITVVMVALAVYMYLDKKHSYWADRKVPFVKPKFFYGNAKEISQTMQVGQVFQQ

FYHELKGRSPFGGIYMFTAPVAVVTDLELLKCIFVKDFQYFHDRGTFYSEKGDPLSAHMF

NLEGNKWKMLRNKLSPTFTSGKMKMMFPTIVAAGKQFHDFMDEKVKQESEFELKDLLARF

TTDVIGMCAFGIECNSIKDPDAQFRVMGRKLFTTGRSKPKSFLMNTMPKVAKLLRLRIFP

ADVSDFFMKVVRETIDYRMANNVHRNDFMDLLIQMRNPDENKSSEGLLSFNEIAAQAFVF

YLAGFETSSTLLTWTLYELAVNQDIQEKGRQHVKEVLKKHDGEMTYESITSMKYLDQILN

EALRKYPPVPVHFRETSKDYTVPDSNIVIEGGTRLFVPVYAIHHDPEIFPNPEQFNPDRF

TPEEEQKRHPYAWTPFGEGPRICIGLRFGMMQARIGLAYLLNSFKFSIGEKCKVPLEFDV

KSFILAPKGGLWLKVEKI

>157120820

MMEPLDVAITVVMVALAVYMYLDKKHSYWADRKVPFVKPKFFYGNAKEISQTMQVGQVFQ

QFYHELKGRSPFGGIYMFTAPVAVVTDLELLKCIFVKDFQYFHDRGTFYSEKGDPLSAHM

FNLEGNKWKMLRNKLSPTFTSGKMKMMFPTIVAAGKQFHDFMDEKVKQESEFELKDLLAR

FTTDVIGMCAFGIECNSIKDPDAQFRVMGRKLFTTGRSKPKSFLMNTMPKVAKLLRLRIF

PADVSDFFMKVVRETIDYRMANNVHRNDFMDLLIQMRNPDENKSSEGLLSFNEIAAQAFV

FYLAGFETSSTLLTWTLYELAVNQDIQEKGRQHVKEVLKKHDGEMTYESITSMKYLDQIL

NEALRKYPPVPVHFRETSKDYTVPDSNIVIEGGTRLFVPVYAIHHDPEIFPNPEQFNPDR

FTPEEEQKRHPYAWTPFGEGPRICIGLRFGMMQARIGLAYLLNSFKFSIGEKCKVPLEFD

VKSFILAPKGGLWLKVEKI

>EAL26028.2

MLDVIALLLITLAIGFWYVRTRLSYWARRGIGHVRPTFPMGNMDGLRRTKHFKDIVTPLY

ERFKDSGAPFAGIYMMLRPVVLVLDLDLAKEVLIRDFANFEDRGMYHNERDDPLTGHLFR

IDGPKWRPLRHKMSPTFTSGKMKFMFPTICEVGVDLAEVCGELAANAICGILEISDIMGR

YTSDVIGRCVFGLECNGLRNPEAQFAVMGRRAFTERRHNQLIDGFIESFPDLARRLRMCQ

IHQDITDFYLGIVKETVKLREEQGIVRNDFMNLLIEMKHRGELTLEEMAAQAFIFFAAGF

DTSASTLGFALFELARQPQLQDKLRQEIDAALQLHQGQFTYDSMQELRYMELVIAETLRK

YPPLPHLTRISKNLYTAKGNRHFYIEPGQMLLIPVYGIHHDPAIYPEPHKFIPERFMGDQ

LAQRPTAAWLPFGDGPRNCIGMRFGKMQTSIALVNLLRRFHFSVCPRTESKIEFVKSNLL

LCPANGIYLKVQEISKLK

>EDW32571.1

MLDVIALLLITLAIGFWYVRTRLSYWARRGIGHVRPTFPMGNMDGLRRTKHFKDIVTPLY

ERFKDSGAPFAGIYMMLRPVVLVLDLDLAKEVLIRDFANFEDRGMYHNERDDPLTGHLFR

IDGPKWRPLRHKMSPTFASGKMKFMFPTICEVGVDLAEVCGELAANAICGILEISDLMGR

YTSDVIGRCVFGLECNGLRNPEAQFAVMGRRAFTERRHNQLIDGFIESFPDLARRLRMCQ

IHQDITDFYLGIVKETVKLREEQGIVRNDFMNLLIEMKHRGELTLEEMAAQAFIFFAAGF

DTSASTLGFALFELARQPQLQDKLRQEIDAALQLHQGQFTYDSMQELRYMELVIAETLRK

YPPLPHLTRISKNLYTAKGNRHFYIEPGQMLLIPVYGIHHDPAIYPEPHKFIPERFMGDQ

LAQRPTAAWLPFGDGPRNCIGMRFGKMQTSIALVNLLRRFHFSVCPRTESKIEFVKSNLL

LCPANGIYLKVQEISKLK

>EDW00887.1

MSLWWLSLALLSALLSLLCIYLERTYSYWARRGVLYDKPVWFYGNLRSVGRTTTVLEPFR

RFYEKYKGRAPFGGFYFALRGAAVLLDLELVKLVMVKDFHNFAHRGNYYNEADDPISAHL

FNLDGRDWREMRNRLTPTFTSLKMHQMLPTVVEIANRFVKVVQQKIEPLQHAEESPVMVK

DLLARFTMDVIGSCAFGLECNSLNDPNDQFCRLGQRTFQERRHGRLAFAFMQAFPQLARR

LHFKMVQDDVSAFFMSVVRETLDYRDKHQVQRNDFLNLLMELRNDPNGGLTFNQIAAQAF

VFFLGGFETSSSTMGFALHLLALHPEVQQRGREEVQQVFAKHKELNYEALRDLKYIKQIV

YETLRKYSIASILIRMTIEDYEVPGTDYVLEAGTPLVIPVDAIHHDPDIYPEPEKFDPDR

FTPEAIEERHSAAWLGFGAGPRNCIGLRFGEMQTLVGLAVLLKNFKFSAAKDTEIPLRIN

KTSFFLQSEGGIVLNVQKV

>EDW74199.1

MVVLIALLLGVATLAIWLVLRNFSYWERRGIPHDPPQIPSGSLSGMMTTIHISDIFKRTY

FKYKNKVSSPFVGFYVFFKRAVIVVDIDFVKTVLIKEFDKFHDRGVFHNERDDPLSGNLV

NIEGQKWKSLRHKLTPTFTSGKMKHMFPTVLDVGEELVKIFNAKTSSEPQPLEITDLVSR

YTADVIGSCAFGLECNSLHNPEAEFVKMGTVALTKRRHGRAMDLFLFGAPRLAAKFGIKA

TLQKVEDFYMKIVKDTVEYRTKNNVKRNDFMDMLIDLKKKYDEGNGEDGLSFNELAAQAF

VFFLAGFETSSTTMGFALHELAINQDIQSRLKKEIDEVLAKNNGEFNYESMHEMKYLEKV

IDETLRKHPVVGHLIREATQRYVHRDNPDYYIEPGTGVIIPVRAIHHDPEFYPEPEKFIP

ERFDEEQVNQRPACTFLPFGDGPRNCIGLRFGRMQTTIGLAKLIHNFKFEVNPTLTSVPM

KFKVENLLWSAEGGITLNVSKFART

>ACR19216.1

MWLSGILVGLVVTLISYLVLLMKRRLNYWHSRNVPCERPSLLLGNFKGMRTKYSFPEIWM

NYYKKFKGSGPFAGFFWFSHPAVFVLDLELIKNILTRDFNKFMDRGFFHNEQDDPLTGHL

FFLDGLKWKSLRQKLTPTFSSGKIAKMFPMVKNLTGRLMETMEEKLEDSEKQEDENAKHV

LEMKDLLARFGTDVIGCCAFGIDCNSLSDPEAKFHIMGQRLFSEPRNGQLGNALAFNFPE

LVQKLHMKVIPDEISEFFMDLVKKTIQSREENPTERDDFLALLMELRESKQIKTEDGEET

KSLTLEEIAAQIVLFFLAGYETSSTTVGFALYELARHQEIQNRLRQEVNEIWVKYGKDFT

YESVKDMTYLQQVIQETLRLYIPVPVLNRKCLEDYPVPGHDEKYLIKKGMNVIIPVLAIQ

RDEEFFPQPEEFNPDNFEASRCKDRESVVYMPFGEGPRNCIGKRFGEMQTGLVLATLIKK

FKFSTCPQTQIPVIFNKETYFLGAGHGIHLRVEKI

>ABV48810.1

MLSLVQTILLAFVAVLIGYLYNLYTYWKRRGVVYERPWPLFGNFWGIGTKYHIREINQRL

YRKFKGQAPFVGTYMFVRRASLIIDLDLIKNILIKDFANFHDRGVFNNVEDDPLTGHLVA

LEGEQWRAMRTKLSPVFTSARMKYMFSTVVRVGENLHRVMGDMLSEKSDQILEIKDVCAR

FTTDVIGTCAFGIECNSLRDPDAEFRQKGKDIFGKPRHSPLVQIFTITNSNLAKKLHMKL

FPDDVADFFMSVIRQTVEYREKNNVKCNDFMDLLIEMKAKNEEEAKAGKGIDLSLGLTLE

QMAAQTFVFFLAGFETSSTTMSFALYELAKHPEVQEQLRKEIRESLEKTKGELTYESLHE

MQYLEQVIAETLRIYPVLPNLIRLTKSDYQVPNTNHVLEKGIMTVIPVHAIHHDPEYYEN

PDEFRPSRFTPEECLKRHPSAYLPFGDGPRNCIGMRFGKMQTKIGLVSLLRHYRFECSPL

TEIPLEMDKRNFLAATKNGIFLKVIPL

>AAA82161.1

MLSLVQTILLAFVAVLIGYLYNIYTYWKRRGVVYERPWPLFGNFWGIGTKYHIREINQRL

YRKFKGQAPFVGTYMFVRRASLIIDLDLIKNILIKDFANFHDRGVFNNVEDDPLTGHLVA

LEGEQWRAMRTKLSPVFTSARMKYMFSTVVRVGENLHRVMGEMLSERSDQILEIKDVCAR

FTTDVIGTCAFGIECNSLRDPDAEFRQKGKDIFGKPRHSPLVQIFTITNSNLAKKLHMKL

FPDDVADFFMSVIRQTVEYRQKNNVKCNDFMDLLIEMKAKNEEEAKAGKGIDLSLGLTLE

QMAAQTFVFFLAGFETSSTTMSFALYELAKHPEVQEQLRKEIRESLEKTKGELTYESLHE

MQYLEQVIAETLRIYPVLPNLIRLTKSDYQVPNTNHVLEKGIMTVIPVHAIHHDPEYYDN

PEEFRPSRFTPEECLKRHPSAYLPFGDGPRNCIGMRFGKMQTKIGLVSLLRHYRFECSPL

TEIPLEMDKRNFLAATKNGIFLKVIPL

>EFR28296.1

MEPLDVVISIVMFCFVVYLMLDKRIQYWKDRGVPQSKPELIFGNVREVSRKMHIAVKFQE

LYNELKGKHPFGGIYMFLKPVALITDLELLKCVFVKDFQYFHDRGLYYNEKHDPLSAHLF

NIEGQKWKSLRNKLSPTFTSGKMKMMFSTIEAAGKQFRDFMEETVEKQNEFELKDLLARF

TTDVIGMCAFGIECNSMRDPEAEFREMGRKIFQQPRGKVRGLLTTSMPRVAKALGITNIL

PEVSSFFLGVVRDTIKYRVENNVQRNDFMDILIRMRSDKDVKSEDGTLTFHEIAAQVFVF

FLAGFETSSTLLTFTLYELALQPEVQEKGRQCVKEVLKRHNGQLTYESVLEMQYLDQILK

ESLRKYPPVPVHFRVTSKEYQVPGEKTVLEAGTSVMIPVYAIHRDPENFPDPERYDPDRF

SPEEEAKRHPYAWTPFGEGPRICVGLRFGMMQARIGLAYLLNSFQFSRGVKTVVPLKLDV

KSFILSPEGGMWLKVEKLK

>EDS44707.1

MLDFWDVFTAVLVICLGVLYYIKKQHSYWADRNVPFSKPVFLYGNAKPMGRTEQAGTLFK

RLYNELKGKHPFGGIYMFTKPVALITDLELLKCVFAKDFQYFHDRGTYYNEKDDPLSAHM

FNLPGSKWKNLRHKLSPTFTSGKMKMMFPTIVAAGKQFNDFLHEKVSHECEFELKDLLAR

YTTDVIGMCAFGIECNSLKDPDAQFRVMGRKIFETPRGRIKSMLTNATPNFARFIGVKLS

VPEVEQFFLRVVRETIDYRVKNNVKRNDFMDLLIQMRSPDDTKSDDGLLSFNEIAAQAFV

FYLAGFETSSTLLTWTLYELALNQDIQEKGRQHVKEVLPKYNGEMSYESITAMKYLDQIL

NESLRKYPPVPIHFREVAKDYQVPNTKSVLEAGTQVFIPVYGIHHDPEVFPDPEKFDPER

FSPEQEANRNPYAWTPFGEGPRICIGLRFGMMQARIGLAYLLTNFRFSIGEKCKVPLELN

KKSFILAPEGGLWLKVEKL

>EDW10042.1

MSLWWLTLGLLFALVALVCVLLHRSYSYWAREGVLHEKPVWFYGNLRSVGQTTTVLEPFR

RFYEKYKGQAPFGGFYFALRGAAVLLDLELVKLVMVKEFQNFSHRGNYYNAQDDPISAHL

FNLDGQEWREMRNKLTPTFTSLKMHQMLPTVVDISERFVRVVREKVEVFEHAEESPLEVK

DLLARFTMDIIGSCAFGLDCNSLNDPNVEFCRLGQKVFTQRRHGRLAFALLQTFPNLAKR

LHIKMVPDDVSNFYMRVVREAVDYRDKNQVQRKDFLNLLMELRNDPNGGLTFNQLAAQAF

VFFLGGFETSSSTMGFSLHLLATHPEVQERAREEVQQVFEKHKELNYEALRELKYIKQIV

QETLRKYSIASILIRRTLEDFPVPGSNYVLKAGTPVVIPVDAIHHDPDIYPEPEKFDPDR

FAPEAIEARHSAAWLGFGAGPRNCIGLRFGEMQTLVGLAVLLKNFKFSPAKETEIPLQLN

KTSFFLQSQGGIVLNVQKV

>AFM08398.1

MLSLFDFTFLVAALVAGLYYYLDRKRSYWKDRDVPGPKGELLLGNFGKVGTKEHITVPMK

KIYDENKGKHPFAGIYQFVKPVALITDLELLKCVFVKDFQYFHDRGTFYNERDDPLSAHL

FNLEGQKWRSLRNKLSPTFTSGKMKMMFPTIVTAGKQFKDFMEETVKRENVFELKDLLAR

FTTDVIGMCAFGIECNSMRNPDAEFRAMGRKIFEISPGTFKTMLMNGMPSLAKMLRMKQT

DQDVSDFFMNAVRDTINYRVANKVKRNDFVDLLITMMSKDETKSDDESLTFNEIAAQAFV

FFLAGFETSSTLLTWTLYELALNPEVQEQGRKCVREVLKKHNGEMSYDAVVDMKYLDQIL

NESLRKYPPVPVHFRVASKDYQVPGTKSVLEAGTAVMVPVHAIHHDPAVFPDPERYDPER

FSPEQEAKRHPYAWTPFGEGPRICVGLRFGMMQARIGLAYLLDGFQFAPSSKTVIPMELS

TESFIMAPKGGLWLKVDKI

>EDS32053.1

MLDFWDVFTAVLVICLGVVYYIKKQHSYWADRNVPFSKPVFLYGNAKPMGRTEQAGTLFK

RLYNELKGKHPFGGIYMFTKPVALITDLELLKCVFAKDFQYFHDRGTYYNEKDDPLSAHM

FNLPGSKWKNLRHKLSPTFTSGKMKMMFPTIVAAGKQFNDFLHEKVSHESEFELKDLLAR

YTTDVIGMCAFGIECNSMKDPDAQFRVMGRKIFETPRGRIKSMLTNATPNFARFIGVKLS

IPEVEQFFLRVVRETIDYRVKNNVKRNDFMDLLIQMRSPDDTKSDDGLLSFNEIAAQAFV

FYLAGFETSSTLLTWTLYELALNQDIQEKGRQHVKEVLPKYNGEMSYESVTAMKYLDQIL

NESLRKYPPVPIHFREVAKDYQVPNTKSVLEAGTQVFIPVYGIHHDPEVFPDPEKFDPDR

FSPEQEANRNPYAWTPFGEGPRICIGLRFGMMQARIGLAYLLTNFRFSIGEKCKVPLELN

KKSFILAPEGGLWLKVEKL

>AAO62002.1

MFSLLDFTFLVAALAAGLYYYLDRKRSYWQDRGVPGPKGELLFGNFGSIGTKEHITVPFK

RIYDDNKGKHPFAGMYQFVKPVALITDLELLKCVFVKDFQYFHDRGTFYNERDDPLSAHL

FNLEGQKWRSLRNKLSPTFTSGKMKMMFPTIVTAGKQFKDFMEETVLRENEFELKDLLAR

FTTDVIGMCAFGIECNSMRNSDAEFRAMGRKIFEISPGTFKTMMMNGMPELAKMLRMTQT

DKDVSDFFMNAVRDTINYRVKNNVQRNDFVDLLIKMMSKDGEKSDEDSLTFNEIAAQAFV

FFLAGFETSSTLLTWTLYELALNPEVQEKGRECVREILQKHNGEMSYDAVVEMKYLDQIL

NESLRKYPPVPVHLRVASKDYHVPGTKSVLEAGTAVMIPVHAIHHDPEVFPNPEQFDPER

FSPEQEAKRHPYAWTPFGEGPRICVGLRFGMMQARIGLAYLLDGFRFAPSSKTVIPMELS

KESFIMAPKGGLWLKVDKI

>Q9V769.1

MLDVVALLLIALAVGFWFVRTRYSYWTRRGIGSEPARFPVGNMEGFRKNKHFIDIVTPIY

EKFKGNGAPFAGFFMMLRPVVLVTDLELAKQILIQDFANFEDRGMYHNERDDPLTGHLFR

IDGPKWRPLRQKMSPTFTSAKMKYMFPTVCEVGEELTQVCGELADNAMCGILEIGDLMAR

YTSDVIGRCAFGVECNGLRNPEAEFAIMGRRAFSERRHCKLVDGFIESFPEVARFLRMRQ

IHQDITDFYVGIVRETVKQREEQGIVRSDFMNLLIEMKQRGELTIEEMAAQAFIFFAAGF

DTSASTLGFALYELAKQPALQAKLREEIDQALRLHNGEFTYDSMQELRYMELVIAETLRK

YPILPQLTRISRHLYAAKGDRHFYIEPGQMLLIPVYGIHHDPALYPEPHKFIPERFLADQ

LAQRPTAAWLPFGDGPRNCIGMRFGKMQTTIGLVSLLRNFHFSVCPRTDPKIEFLKSNIL

LCPANGIYLKVQQLSQMSS

>EAA12401.2

MFSLLDFTFLVAALAAGLYYYLDRKRSYWQDRGVPGPKGELLFGNFGSIGTKEHITVPFK

RIYDDNKGKHPFAGMYQFVKPVALITDLELLKCVFVKDFQYFHDRGTFYNERDDPLSAHL

FNLEGQKWRSLRNKLSPTFTSGKMKMMFPTIVTAGKQFKDFMEETVLRENEFELKDLLAR

FTTDVIGMCAFGIECNSMRNPDAEFRAMGRKIFEISPGTFKTMMMNGMPELAKMLRMTQT

DKDVSDFFMNAVRDTINYRVKNNVQRNDFVDLLIKMMSKDGEKSDDDSLTFNEIAAQAFV

FFLAGFETSSTLLTWTLYELALNPEVQEKGRECVREILQKHNGEMSYDAVVEMKYLDQIL

NESLRKYPPVPVHFRVASKDYHVPGTKSVLEAGTAVMIPVHAIHHDPEVFPNPEQFDPER

FSPEQEAKRHPYAWTPFGEGPRICVGLRFGMMQARIGLAYLLDGFRFAPSSKTVIPMELS

KESFIMAPKGGLWLKVDKI

>BAI77925.1

MVLPALLFIVAGLLLYVYYRYRKQRSFWADRGVPHVPANFRRNLDPAPMHMARRFQGYYQ

QFKEQYPFCGIYFFTKPVALAIDLELLKCIFVKDFQYFHDRGTYYNERDDPLSAHLFNLE

GTKWKNLRTKISPTFTSGKMKMMYPTMIAAGKQFSEYMDEKVSQENEFELKDLLARFTTD

VIGMCAFGIECNSMKDPDAQFRVMGRKHFDTPRVRWRDSLCAVAPKLARFLGMRQIVPEL

SDFFIGIVRETIDYRVKNNVRRNDFMDLLIAMMQGENSELGPLTFNEIAAQAFVFFIAGF

ETSSTTMTWALYELSVNQEVQERARKCVQDVLQKYNGEMSYDAVMEMAYIDQILQETLRK

YPPVPVHFRVVSKDYQVPDTNTILPAGTSLMIPVYAVHHDPEIFPEPERFDPDRFTPEEI

AKRHPYAWTPFGEGPRICIGMRFGMMQARIGLALLLKSFRFSTGQKSTVPLDFTAKSFIL

SPEEGLWLKVDKV

>EDV35776.1

MRDLIAVVLIALAVGFWFVRTRYSYWTRRRIGNVPATFPTGNMDGFRKTKHFIDIVTPIY

EKFKDCGAPFAGFFIMLRPVVLILDLEVAKQVLIRDFANFEDRGMYHNERDDPLTGHLFR

IDGPKWRPLRTKMTPTFSSGKMKFMFPTVCEVGVELTGVCGELAENSMCGILEVGGLMAR

YTSDVIGRCAFGVDCNGLRNPEAEFAVMGRRAFSERRHSKLVDLFIESFPNLARRLRFCQ

IHQDITDFYVGIVRETVRMREEQGIVRNDFMNLLIEMKQRGELTLEEIAAQAFIFFAAGF

DTSASTLGFALYELAKQPELQEKLRTEIDEAQKKHQGEFTYECMQELRYMELVIAETLRK

YPVLPQLTRVSKQYYAAKGDRNFYIEPGQMLLIPVYGIHHDPRIYHEPNRFIPERFLADQ

LALRPTASWLPFGDGPRNCIGMRFGKMQTTVALVHLLRRFHFSVCPRTEPKIEFVKSNIL

LCPANGIYLKVQELTCEA

>EDW74200.1

MVVLIVLLVGVVSLAIWWVREQFSYWEKRGIPHDPPKIPVGNVAGMMRTIQVSDIFKRSY

FKYKNKVDGPFVGFYMFFKRIALVVDIDFVKTVLIKDFEKFHDRGIFHNETDDPFSNNLV

TIEGEKWKNLRNKLTPTFTSGKMKHMFPIFLKVGEEMVQVLNEKTSNVPQPLEITDLVSR

YTVDVIGNCAFGLECNSLHNPEAEFVKMGNAVFTLKRHGRAMDLFLFGAPKLAAKLRIKV

TSQKVEDFYMNIIKDTVDYRMKNNIQRNDFMDMLIDFKRKYDEGNKEDGLSFNELAAQAF

VFFLGGHETSATTMGFALHELAINQDIQNRLRNEIDEVLAKNNGEFNYESMNEMKYLEKV

IDETLRKHPVVGHLIRKATQRYVHPDNPKYYIEAGTGVTIPVRAIHHDPEFYPEPEKFIP

ERFDEEQVKQRPPCSYLPFGDGPRNCIGLRFGRMQTTIGLAKLIHNFKFEVNPTLTSVPM

KFKFETILLSAEGGITLNVSKVTKK

>AFM08397.1

MEFTDVAITLGMLCIILYLILVRRFQYWTERNVPQLKPELLFGDAREVGKVCHIGEKIHQ

LYNELKGKHPFGGIYLFTKPVALITDLELLKCVFVKDFQYFHDRGTYYNEKHDPMSAHLF

NLEGQKWKRLRNKISPTFTSGKMKMMFPTIVAAGKQFKNYMEDTIQQQGEQELKDVLARF

TTDIIGTCAFGIECNSMRDPDAKFRVMGRKIFTRTRGTLQQLLMNAFPSVAKMVGIKLIV

PEVSDFFMKVVRDTIKYRVENNVQRNDFMDILIRMRSDKETKSDDGTLTTNEIAAQSFVF

FLAGFETSSSLMTFTLYELALNPEIQEQARNCVKEVLDRYGGELTYEAALEMDYLDRVLK

ECLRKYPPVSVHFRITAKDYLVPGTNSILERGTSVMIPVLGIHRDAEHFPNPERFDPDRF

TPEQEAKRHPYAWTPFGEGPRICVGLRFGMMQARIGLIHLLTSFRFACCSKTPIPMKFDI

TNTILSPKGGMWLRMEKLTKDCE

>EDW00820.1

MELLTVILLGVVLALVYHFYQNTYKYWERRGVPFEPPVPILGNMRGVGYKYHFRDINQRL

YDRFKGTTPFGGMFTFLKRVAFIVDLDLVKQVLIKDFTLFQDRGTFSNVRDDPLTGHLFS

LEGEEWRSMRHKLTPVFTSGKMKHMFGIVVDVGKRLAETMDKAVVGSSSEMEIKELCARF

TTDVIGTCAFGLECNSLADPHAKFRAKGRQLFEKPRHYRIVQSFMFTNPKLARKLRMKTF

PDDVSEFFLQAVRDTVDHRKKHNIKRNDFLDQLIELRAENEEAARLGKGIDLSHGLTIEQ

MAAQAFVFFIAGFETSSSTMAFFLYELALHQDVQERVRQEIITVLKETSNGEQLTYDAML

NMPYLDQVLAETLRKYPIVPHLTRVANKDYKVDGTQLVIEKDTMVLIPVHSIHHDPQIYP

SPETFDPSRFEPEAIKARHQYAYLPFGDGPRNCIGERFGKMQAKIGLVALLRSFKFGVSK

KTEVPLTFSRRAFTLSTQAGIHLMVERI

>EDW89361.1

MLFTIALLGVVLALAYSLHVKIFSYWKKKGVPHEKPLPLVGNMKGISKKYHFRDINQRIY

KKFKGKAPIAGMYMFFKRTAMITDLDFIKQVMIKDFSYFQDRGAFNNPRDDPLTGHLFSL

EGDEWRAMRHKLSPVFTSGKMKQMSKVIVDVGLRLGDAMDKAVKEAKVEGGNVEIKDLCA

RFTTDVIGSCAFGLECNSLQDPNAEFRQKGREIFTKRRHSTLVQTFMFTNARLARKLRMK

VLPDDVTEFFLSTVKSTVDYRLKNGIKRNDFIEQMIELRAEDQEAAKKGRGIDLSHGLTL

EQMAAQAFVFFVAGFETSSSTMSFCLYELALQPEIQQRLREEIESVLANVEGGELSYDVL

AEMSYLDQVLSETLRKHPLLPHLVREANRDYKIPNTDIVLDKGVLALIPVHNIHHDPEIY

PEPEKFDPSRFDPEEVKNRHPMAYLPFGDGPRNCIGLRFGKIQAKIGLVSLLRRFKFSVS

NRTEVPLILSNKNFLLGTDNGMYLKVESI

>EDS32051.1

MLEPLDAIITVAMICLGLYFYMDKKYSYWKDRDVPYLKPEFFYGNSRTMTRTEQTGQLFA

RFYEELKGEDHPFGGAYVFTRPVAIVTDLELAKCVFAKDFQYFHDRGTYYNERYDPLSAH

MFNLSGPKWKNLRHKLSPTFTSGKMKMMFSTIIAAGKQFRDFLDEKVSHESEFELKDLLA

RYTTDVIGTCAFGLECNSMRDPDAQFRVIGRKIFGNPRGMVKGFLIATMPRFAQFIGVKE

ILPEVSEFFFKVVRETVDYRVKNNVKRNDFMDLLIQMRNPDENKSDDGLLSFNEIAAQAF

VFYLAGFETSSTLLTWTLYELALNQDIQEKGRQHVREVLSKYNREITYESVTAMKYLDQI

LQESLRKYPPVPIHFREVAKDYQVPETKTVLTAGTRVFIPVLGIHHDPDIFPDPERFDPD

RFLPEQEAKRNPYAWTPFGEGPRICIGMRFGMMQARIALGYLLHSFKFSIGEKCQVPLQM

DEKSFVLSPKGGLWLKVEKI

>EDS44705.1

MLEPLDAIITVAMICLGLYFYMDKKYSYWKDRDVPYLKPEFFYGNSRTMTRTEQTGQMFA

RFYEELKGEDHPFGGAFVFTRPIAIVTDLELAKCVFAKDFQYFHDRGTYYNEKYDPLSAH

MFNLSGPKWKNLRHKLSPTFTSGKMKMMFSTIIAAGKQFRDFLDEKVSHESEFELKSLLA

RYTTDVIGICAFGLECNSMRDPDAQFRVIGRKIFGNPRGMVKGFLIATMPRFAQFIGVKE

ILPEVSEFFFKVVRETVDYRVKNNVKRNDFMDLLIQMRNPDESKSDDGLLSFNEIAAQAF

VFYLAGFETSSTLLTWTLYELALNQDIQEKGRQHVREVLSKYNREITYESVTAMKYLDQI

LQESLRKYPPVPIHFREVAKDYQVPETKTVLTAGTRVFIPVLGIHHDPDIFPDPERFDPD

RFSPEQEAKRNPYAWTPFGEGPRICIGMRFGMMQARIALGYLLHSFKFSIGKKCQVPLQM

DEKSFVLSPKGGLWLKVEKI

>EDW74204.1

MLDVIALLLITLAIGFWFVRTRLNYWARRGIGYVKPSFPMGNMQGFRTTKHFKDIITPLY

ELFKDSGAPFAGVFIMLRPVAVILDLELAKHILIRDFANFEDRGMYHNERDDPLTGHLFR

IDGPKWRPLRQKMSPTFSSGKMKYMFPSVCAVGEDLAVVCGELADNAICGILEITDLMAR

YTTDVIGRCVFGLECNGLRNPEAEFAVMGRRAFTDRRHSKLIDGFIESFPNLARTLRMCQ

IHQDITDFYVGIVEETVKMREEQNIVRNDFMNLLIEMKQRGELTLKEMAAQAFIFFAAGF

DTSASTLGFALFELAKQPELQEKLRQEIEAALQLHNGTFTYEAMQELRFMELVIAETLRK

YPVLPHLSRISRQYYACKGNKHFYIEPGQMVIIPVYGIHHDPAVYPEPHKFIPERFLADQ

LAQRPTAAWLPFGDGPRNCIGMRFGKMQTSIALYLSTD

>EDV59508.1

MLFTIALLGVVLALAYSLHVKIFSYWKRKGVPHERPLPLVGNMKGIVKKYHFRDINQRIY

KKFKGKAPIAGMYMFFRRTAMITDLDFIKQVMIKDFSYFQDRSAFNNPRDDPLTGHLFAL

EGDEWRAMRHKLSPVFTSGKIKQMSKVIVDVGLRLGDAMDKAVQEAQVEDGNVEIKDLCA

RFTTDVIGSCAFGLECNSLQDPSAEFRQKGREIFTRRRHSTLVQAFIFTNARLAKKLRMK

ILPDDVTEFFMAAVKNTVDYRLRNGIKRNDFIEQMIELRAEDQEAARKGQGIDLSHGLTL

EQMAAQAFVFFVAGFETSSSTMSFCLYELALQPEIQRRLREEIESVLASVEGGELSYDVL

AEMSYLDQVLSETLRKHPLLPHLIREANRDYKIPHSDIVLDKGVLALIPVHNIHHDPEIY

PDPERFDPSRFDPEEVKNRHPMAYLPFGDGPRNCIGLRFGKIQAKIGLVSLLRRFKFSVS

SRTEVPLIFSKKSFLLGTENGIYLKVESI

>AFM08396.1

MEPLDILISVLMVCFGVYLLIDRRFQYWTNRNVPQSKPELVFGNVREVSQTIHLADKFRE

IYNELKGKHPLVGVYLFFKPVALITDLELLKCVFVKDFQYFHDRGTYYNEKHDPLTAHLF

NIEGQKWKNLRNKLSPTFTSGKMKMMFPTIIAAGKQFKDFMEETVHEQVDFELKDVMARF

TTDVIGMCAFGIECNSMSNPDAEFRVMGRKIFARPRGKVKSLVINSMPRLAKLIGLRTLD

PEVSDFFMKAVRDTIKYRVENNVQRNDFMDILIRMRSDKETKSDDGTLTTNEIAAQAFVF

FLAGFETSSTLLTFTLYELALNQDAQDKGRRCVKEVLERHNGELTYDAVMEMHYLDQILK

ESLRKYPPVPVHFRTTSKEYQVPGTKTVLEAGTSVMVPVYAIHRDPEHFPNPDLFDPDRF

TPEEEAKRHPYAWTPFGEGPRICVGLRFGMMQARIGLAYLLTGFRFVPGAKTMVPMKLDV

KSFILSPQGGLWLKVEKLST

>ADD19316.1

MFGLLLFCVAISLLLYLVNNYTYWKARGVLHERPLPLLGNFKGVGRKYSIRDVSERVYKK

FKNLAPLAGMYAFLTPTLLIFDLELVKRILIKDFNNFHDRSIFNNVKDDPLTGHLFTLEG

EHWKAMRTKLSPVFTGARMKFMLPTMVKVGQQFIEALSDAFESSSDQIFEIKDFCARFTT

DVIGNCVFGVECNSCKDPQAEFRVMGRSIFNEPRHHPIVQTFIATNPKLARRLRMKIIPD

KISAFILNLIHQTIDYRIKNKIKRNDFVQLLMELRNETELEQKNEKLEKIDLSLGLSIEQ

MAAQAFVFFIAGFETSSTTMSFCLYELAKHPDIQAKLRLEIETVLKQHNNELTYEALNSM

SYMEQVVAETLRLYPILPILVRKCCQFYQVPNTTLSIEKGTVIWLPVSAIHRDADFYEEP

ENFKPERFEPSQIEKRHPCAYLPFGDGPRNCIGMRFGKMQTKIGLISFLREYSVGLCDKT

EIPVELDPKNFLISAKNGIYLKMSKV

>EFR28295.1

MVNFVDLAFLVAVVAAGVYYYLDRKRSYWKDRGVPGPQGDLLFGNFRTVGTEEHITVPMK

KIYDDYKGKHPFAGMYQFVKPVALITDLELLKCVFVKDFQYFHDRGTFYNERDDPLSAHL

FNLEGQKWRALRNKLSPTFTSGKMKMMFPTIVTAGKQFKDFMEETVQRQSEFELKDLLAR

FTTDVIGMCAFGIECNSMRDPDAKFRAMGRKIFEVPPGKFKTILINGMPKLARMLRMKLT

DTEVSDFFLGAVRETIDYRLKNNVQRHDFVDILIKMLSKDDSKSDDESLTFNEIAAQAFV

FFLAGFETSSTLLTWTLYELALNQEVQEKGRQCVREVLERHDGKMSYDAVVEMKYLDQIL

NETLRKYPPVPIHFRIASKDYQVPGTKSVLEAGTAVMVPVHAIHHDPEIFPEPARYDPER

FSPEEESKRHPYAWTPFGEGPRICVGLRFGMMQARIGLAYLLDGFRFAPSSKTSIPMELS

ISSFILAPKGANVRVISQFCLVMMNDPKRWPAHITVVCAHYVRRNPNSVVVIREITNTTT

GSSRSQRASR

>AAL29138.1

MKTVHFADIFKRTYNKYKNKTDGPFVGFYMYFKRMVVVTDIDFAKTVLIREFDKFHDRGV

FHNERDDPLSANLVNIDGQKWKTLRQKLTPTFTSGKMKNMFPTILTVGDELIRVFGETAS

ADSDSMEITNVVARFTADVIGSCAFGLDCHSLSDPKAKFVQMGTTAITERRHGKSMDLLL

FGAPELAAKLRMKATVQEVEDFYMNIIRDTVDYRVKNNVKRHDFVDMLIEMKLKFDNGDK

ENGLTFNEIAAQAFIFFLAGFETSSTTMGFALYELACHQDIQDKLRTEINTVLKQHNGKL

DYDSMREMTYLEKVIDETMRKRPVVGHLIRVATQHYQHTNPKYNIEKGTGVIVPTLAIHH

DPEFYPEPEKFIPERFDEDQVQQRPACTFLPFGDGPRNCIGLRFGRMQVIVGMALLIHNF

KFEFHPTKTVVPLEYRTDDFLLSSKGGIHLKVTRV

>EDW10112.1

MSTILVLIGIIVTLLYYAARHRYNYWNRRGIPHDAPRFPKGNLAGWPTKRQMGVIYRDYY

LKYKIRNAPFAGFYFFFRKTAVVTDMELVKRVIEGQKWRRLRHKLTPTFTSNKMKNMLPI

ILSVAEEMANVFKEKTSTAQVLEVTDLVGRFTADVIGNCAFGLDCDSLRDPNAHFVRVGK

RALTERRYHGLFDFFIFGFPKLARRMHLKLTVEEVEEFYMRIIRDTIDYRLSTKEKHNDF

MDMLIEMYYKQKAGNTEEGLTFEELAAQAFIFFVAGFETSSTTMGFALYELALHQDIQNK

LRAEINEVLSRYNNEYTYDNVKEMKYLEQVVMETLRKYPVLPHLTRKAISDYSPGDPKHY

IEKNTTVVIPGLGIHYDPDIYPEPDKFKPERFTEAEIAARPACSWLPFGDGPRNCIGSRF

GMMQTSVGLAHLIRAFKFSLSPQTQIPMKLITRNIFLTPENGIYLKVESVSK

>EAT39031.1

MLIYLTVLALTLAVLWIRKRYSYWMDRGIPYVEPSFPAGNLRGMGRKEHLSSQMQRCYKE

LKGKGPVGGMFFFINPVALAMDLDLIKSVLVKDFQYFHDRSVYYNEKDDPLSAHLFTMEG

AKWKNLRAKLTPTFTSGKMKMMYPTIIGVADEFQKLMKSEVSSNAEIEMKEILARFTTDV

IGTCAFGLECNSLHDPDAKFRAMGRKIFSFANGRFLKAVIAQQFRSLARSLHIALVDKEV

SDFFLGAVRDTIKYREENKIERNDFMSLLMKLKDDGNTGNTETLTVEEIAAQAFVFFLAG

FETSSTAMSYCLYELAQNSDLQNKARKSVMDSIKKHGSLTYEAMQDMQYIDQCINESLRK

YPPASTLTRSVSKDYKLPNSNVVLQQGSTLIVPVYALHHDAEYYPDPEKYNPDRFTPEEV

AKRNPYCFLPFGEGPRICIGMRFGMMQARVGLAYLLRDFSFTLSSKTPVPLKISPRSPVL

TSEGGLWLKVQKL

>AFM08404.1

MYLQVVGILVAVVVTCLAWIKRRYQYWKARSVAFIAPEFPYGNFGTLGKLEHIAPITQRH

YNYFQQLKVPYGGVFMLTTPLLYIFDTKLIKTLLVKDFHYFPNRGVYFNERDDPLSAHMF

AIEGQKWRTLRAKLSPTFTTGRIKMTLPLVKEVCQRFCDHLHQSLQTSAEVEMHDLLSRY

TIDVIGACAFGIECNSFRDPDNEFRRYGKIAFDKLRHSPLVVYLMKAFRKHANALGMKQL

HDDVSSFFMRVVKDTIEYREREQIVRNDFMDLLLKLKNTGRLESGGEDLGRLTFEEIAAQ

AFIFFTAGYDTSSTAMTYTLYELALKQDVQDKARQCVKETLQKYDGKLSYEAVSDMSYLD

QCINETLRKHPPVAILERNADKDYRLPDSGLLLRRGQKIMIPIYAMHHDSTHFPDPEQYR

PERFSPAEVAKRDPYCYLPFGEGPRICIGMRFGSLQAKMGLASMLDRFQFSTCERTQIPV

QYSRTNFILGPANGVWLRAEEL

>Q9V4U7.2

MLFTIALVGVVLGLAYSLHIKIFSYWKRKGVPHETPLPIVGNMRGIVKKYHFRDINQRIY

KKFKGQGPIAGMYTFFKRTALITDLDFIKQVMIKDFSYFQDRGAFTNPRDDPLTGHLFAL

EGEEWRAMRHKLTPVFTSGKIKQMSKVIVDVGLRLGDAMDKAVKEAKVEEGNVEIKDLCA

RFTTDVIGSCAFGLECNSLQDPSAEFRQKGREIFTRRRHSTLVQSFIFTNARLARKLRIK

VLPDDLTQFFMSTVKNTVDYRLKNGIKRNDFIEQMIELRAEDQEAAKKGQGIDLSHGLTL

EQMAAQAFVFFVAGFETSSSTMSLCLYELALQPDIQQRLREEIESVLANVDGGELNYDVL

AQMTYLDQVLSETLRKHPLLPHLIRETTKDYQIPNSDIVLDKGILALIPVHNIHHDPEIY

PEPEKFDPSRFDPEEVKNRHPMAYLPFGDGPRNCIGLRFGKIQAKIGLVSLLRRFKFSVS

NRTDVPLIFSKKSFLLTTNDGIYLKVERV

>EFR25764.1

MEYALALTGYLWLLAGTLGLIASYLFVWRRYTFWKRHNVPYAEPELPYGNFKELGKTLHP

AHLSQRFYERYKDLPGSPGFVGLYIFLNPVLMVTNLRLAKRILIEDFHYFPDRGVYFNEK

DDPLSAHLFSLEGAKWRDLRAKITPTFTSGRMKAAFPMVLAIAQQFCDHLSEGVRSDGAV

MEIRDLMARFTTDVIGSYAFGLDCNSFREPENEFRRIGRKHFDTPRNHPLKVFIMKTFRG

LANALGMKQIHDDVAEFFSGIIRETIAHRERSGESRQDFLELLIRLKNTGMLDGASEIVG

RLTFEEIAAQAFIFFTAGFETSSGAMTYTLYELALNEEVQQRARDCVLTELDRHDGELTY

DSFKNMLYLDQCIYETLRKYPPVAILERIVSKPYRMPDTGIVLPVGMKIMIPAYAIHHDP

ALYPEPASYRPERFTPEQMAKRDSCAFLPFGEGPRICIGLRFGMMQARIGLALLLRHFRV

MVCDKTEVPLSFSTTAFVLMPEKGVWLRLERYE

>157120808

MWLVYLVWLVAAVLLAVYLWIKKRFNFWKDRGVEYIEPEFPFGNFKTLGKVEHIAPITQR

HYDYFKQKGVPYGGVFMLTSPLLYILDTKLIKTLLVKDFNHFPNRGVYFNEKDDPLSAHM

FAIEGNKWKTLRNKLSPTFTSGRIKMTFPLVVGVCQQFCDHLGEVVQQSNEVEMHDLLSR

YTIDVIGTCAFGIDCNSFREPDNEFRKYGKIAFDKLPHSPLVVYLMKAFRSYANAFGMKQ

LHEDVSSFFSKVVKDTIEYRESNNVVRNDFMDLLLKLKNTGRLEESGEEIGKISFEEIAA

QAFIFFTAGYDTSSTAMTYTLYELALNQKAQEKARKCVLDIFAANNGTLTYESVGNMGYL

DQCINETLRKHPPVAILERNADRDYKLPDSDIVIKKGRKIMIPTFAMHHDAEHFPDPERY

DPDRFSPEQVACRDPYCYLPFGEGPRICIGMRFGTIQARVGLASLLKRFRFRVCDKTQIP

VRYSKTNFILGPANGVWLRVEKL

>EDW74206.1

MWFTIFLVGAVVALVYHYFHNTYSYWERRHVPNERPLPLIGNMKGIGRTKHFRDVNQRIY

DTFKGKTPIAGMFLFFKRAAFIIDLDFIKQVLIKDFTVFHDRGVFSNVEDDPLTGHLLTL

EGDEWRSMRNKLSPVFSSGKIKHMSKVVVDVGRHLISVMETAVKAAAVDDGDVEIKEYCA

RFTTDVIGSCAFGLECNSLNDPDAEFRKRGRMLFEQPRYSQLVTLFIFTNPKLAKKLHLK

ALPDELSSFFLKAVRDTVDYRIKNGIKRNDFLDQLIELKAANEELAKQSKGIDLTLGLTI

EQMAAQSLVFFVAGFETSSSTMAFCLYELALHQDIQQRLRDEIETVLSGVENQELTYDAI

TQMTYLDKVLAETLRKYPILSQLIREARQDYKFPESEFVIEKGTSVLIPVHNIHHDPELY

PQPERFDPSRFDPELVKNRHPCAYLPFGDGPRNCIGLRFGKMQAKIGLISLLRNFKFSAS

KKTEIPLIFSKKTPTLSTENGIYLKVERI

>AAX48014.1

MLTILLAGFILLLTAVVLYIHRRNRFWSDRGIPCAPNPRFFFGHVKGQVTDRHASYVNRE

LYQHFRSRGEPFGGFSFFAVPAVMIVDPELVKTVLVRDFNVFHDRGIFNNPKDDPLSGQL

FLLEGLQWKVLRQMLTPTFTSGRMKAMFGTILNVAEELREYLVQNHEREPVVEMKEVLAR

FTTDVIGTCAFGIECNTLKNPDSDFLKYGKRVFEQKPWNVFKFIFASLFKDLSKALGVKL

TDSGVEKFFMGLIRETVEFREKNNVQRNDFMNLLLQLKNKGRLEDMDEKEELSKRGLTMD

ELAAQCFVFFIAGYETSSTTMNFCLYELAKNQDVQERLREDIQEAVAANGGQVTYEMVMG

LPYLDNVINETLRKYPPIESLNRVPTADYTVPGTNHVLPKGTMTAIPVYALHHDPEYYPE

PERFDPDRFAPEAVQSRPAYVFLPFGEGPRNCIGLRFGLMQTKIGLITLLRNFRFSPSAK

TPDALVFDVKSFVLSPEGGNYLRYDKI

>EJY57790.1

MEVITITLLTILVLLIAYASHLLRRQIRFFKDRNVPHIPASFELLDKTIHPAKHFLRWYK

QFKGQYPLTGVIMFIKPIAIPLDLDLIKRILVKDFQYFQNRGIYYNERDDPLSAHLFSLE

GAKWRSLRAKISPTFTSGKMKMMYPTMVAAGKQFSEYLEEKVEDGNELEMRDLLARFTTD

MIGTCAFGIECNSMKEPNSKFREMGRKHFEAPRNALKDAFKMTAPGLARFLRVTEILPDV

SEFFMDVVKSTVEYRMKNNVRRNDFMDLLIAMLDDKTEGSESLTINEIAAQAYVFFIAGF

ETSSTTMTWALHELSRNPDIQEEGRKCVQEVLEKYNGVMSYEAIMEMTYIDQIINETLRL

YPPVPMHFRVVSKDYHVPETDTILPAGTFTMIPVYAIHHDEDIFPEPEKFDPTRFTPEEV

NKRHAFAWTPFGEGPRVCIGLRFGMMQARIGLALMLKNLRFSPGPKTCTEMEFQPQNFIL

SPKEGLWLNVEKI

>EDX06195.1

MLFTIALVGAVLALAYSLHIKIFSYWKRKGVPHEKPLPIVGNMKGVVKKCHFRDINQRIY

EKFKGQGPIAGMYMFFKRTALITDLDFIKQVMIKDFSYFQDRGAFNNPRDDPLTGHLFAL

EGEEWRAMRHKLSPVFTSGKIKQMSKVIVDVGLRLGDAMDKAVKEAKVEDGNVEVKDLCA

RFTTDVIGSCAFGLECNSLQDPSAEFRQKGREIFTRRRYSTVVQSFILTNAKLARKLRMK

VLPDDLTQFFMSTVKNTVEYRLKNGIKRNDFIEQMIELRAEDQEAAKKGQGIDLSHGLTL

EQMAAQAFVFFVAGFETSSSTMSLCLYELAVQPEIQQRLREEIESVLANVDGGELNYDVL

AQMTYLDQVLSETLRKHPLLPHLIRETNKDYQIPNTDIVLDKGVLALIPVHNIHHDPEIY

PEPEKFDPSRFDPEEVKNRHPMAYLPFGDGPRNCIGLRFGKIQAKIGLVSLLRRFKFSVS

NRTEVPLIFSKKSFLLATDNGIYLKVESV

>AAX48015.1

MLTILLAGFILLLTAAVLYIHRRNRFWSDRGIPCAPNPRFFFGHVKGQVTDRHASYVNRE

LYQHFRSRGEPFGGFSFFAVPAVMIVDPELVKTVLVRDFNVFHDRGIFNNPKDDPLSGQL

FLLEGLQWKVLRQMLTPTFTSGRMKAMFGTILDVAEELREYLVQNHEREPVVEMKEVLAR

FTTDVIGTCAFGIECNTLKNPDSDFLKYGKRVFEQKPWNVFKFIFASLFKDLSKALGVKL

TDSGVEKFFMGLIRETVEFREKNNVQRNDFMNLLLQLKNKGRLEDMDEKEELSKRGLTMD

ELAAQCFVFFIAGYETSSTTMNFCLYELAKNQDVQERLREDIQEAVAANGGRVTYEMVMG

LQYLDNVINETLRKYPPIESLSRVPTADYTVPGTNHVLPKGTMTAIPVYALHHDPEYYPE

PERFDPDRFAPEAVQSRPAYVFLPFGEGPRNCIGLRFGLMQTKIGLITLLRNFRFSPSAK

TPDALVFDVKSFVLSPEGGNYLRYDKI

>AEB77683.1

MLLFLLLSVVTLTYFWIGRRYSYWKQRSIPYVEPSFPLGNLQGLNKRHFGLLAQDVYLKL

KATGSKFGGMFFFVNPVVLILDLDFAKDVFVKDFQYFHDRGVYSNEKVDPITAHLVTMEG

TKWKNLRAKLTPTFTSGKMKMMFPTITAVAEEFRKCMVNEVDKGQEIEMKEFLARFTTDV

IGNCAFGLECNSLVDPEAEFRRMGRKALTPSPMGFLRRILSVTFRDAAKALGVRISDPDV

AEFFMNVVKSTIEYRERDKIQRNDFMDLLIKLKNAESVDGRPSQLEALTFNEIAAQAFVF

FIAGFETSSTAMTFCLYELAKNQELQDKARSNINEVLAKHGSMTYEAVHEMRYIENCINE

SLRKYPPLPNILRNVTKPYQVPGMNVTLEKDCRVLLPVYAIHHDPQIYPDPHQYDPDRFN

PDQCAARHPMAFVPFGEGPRICIGQRFGMMQARVGLTYLLKNFRVALSDRTPSPLNILAN

STVLASEGGLWLKLQKL

>EAA07790.1

MDLLSYVLTAFVFVVSIAYLYLRSRHNYWRDRCFPYTRQKPHLLYGHMEQFQSKHASYIN

EELYWEFKNRGEPIGGMSMFFLPGLIVADPELVKSILVKDFSVFHDRGVFNDAKADPLSA

HLFALEGHEWRVLRQKLTPTFTSGRMKQMFGTIQQVAGEFLKYMNEHCHREIEMKDVLAR

FTTDVIGTCAFGIECNTLKNPDSDFRKYGNKVFEQDTLLMLKFMFATTFKGLAKRIGMKL

TDEGVERFFLQVVRETVEYREMNNVQRNDFMNLLLQIKNTGSLDGGDVPIKGAAGLTMNE

LAAQVFVFFLAGFETSSTTMNFCLYELAKNPDIQDRLREEIERAVEENGGEVTYDMVMNV

QYLDNVINETLRKYPPIESLSRVPMRDYTVPGTKHVIPKDTFIQIPVYALHRDPEFYPEP

DQFNPDRFLPEEVKKRHPYVFLPFGEGPRICIGLRFGVMQAKIGLITLLRNFRFTPSSQT

PAKIVFDPKSFILSPSTGNYLKVDKI

>EDW46952.1

MLFTIALVGAVLALAYSLHIKIFSYWKRKGVPHEKPLPIVGNMKGVVKKYHFRDINQRIY

EKFKGQGPIAGMYMFFKRTALITDLDFIKQVMIKDFSYFQDRGAFNNPRDDPLTGHLFAL

EGEEWRAMRHKLSPVFTSGKIKQMSKVIVEVGVRLGDAMDKAVKEAKVEDGNVEIKDLCA

RFTTDVIGSCAFGLECNSLQDPSAEFRQKGREIFTRRRYSTVVRSFIFTNAKLARKLRMK

VLPDDLTQFFMSTVKNTVEYRLKNGIKRNDFIEQMIELRAEDQEAAKKGKGIDLSHGLTL

EQMAAQAFVFFVAGFETSSSTMSLCLYELAVQPEIQQRLREEIESVLANVDGGELNYDVL

AQMTYLDQVLSETLRKHPLLPHLIRETNKDYQIPNTDIVLDKGVLALIPVHNIHHDPEIY

PEPEKFDPSRFDPEEVKNRHPMAYLPFGDGPRNCIGLRFGKIQAKIGLVSLLRRFKFSVS

NRTEVPLIFSKKSFLLATDNGIYLKVESF

>EDS44715.1

MWIYLLITVITLSIWFVRRKYSFWKRNNVPFIEPRFPFGNIQAVGRRVHSSQLMTRFYTQ

TKGKHPFVGIFFFTNPVALALDLEFVKSVLVRDFAYFHDRGVYFNEKDDPISGHLFNIEG

TRWTNLRKKLIPTFSSGKMKMMCPTIVTVADRFRECIQKHISEDSEVEMKDLLARFTTDV

IGTCAFGIDCNSLNDPDVEFRKMGKKVFELPPRRMLKFFFMATFKELARKFHIKGTAEDV

SEFFFKVVRETIEYRERNNVQRNDFMNLLIQLKDNGELDGSDESVGSLSFNEVVAQAFVF

FLGGFETSSTTMSYCLYELSLNEEIQERARQCVRDAVKKHGGLNYEAAMDMDYIEQCINE

SLRKYPPGANLIRSVTKDYAVPDSDLIFHKGMNVMIPVYAIHHDPEYWPDPEQFNPDRFT

PEETAKRRPFTFMPFGEGPRICIAARFGILETKIGLATLLQNFRFSRCSKSVVPLVISPR

HAVLTPEGGLWLKVEKLEE

>EAT39030.1

MWIYLTVLALTLAVLWVRKRYAYWKERGIPYVEPSFPAGNIRGMGRKEHFSTQMQRCYKE

LKGKGPVGGVFFFINPVPLALDLDFIKTVLVKDFQYFHDRSIYYNEKDDPLSAHLVALEG

AKWKNLRTKLTPTFTSGKMKTMFPTIIGVADEFQKMMKNEVVGNTEIEMKDILARFTTDV

IGTCAFGIECNSLQDPNAQFRRMGRKIFSVAKGRLLKLITAQQFRSLARMLGITLIDKDV

SDFFIGAVRDTIKYREENKIERNDFMSLLMKLKNDESSQDTNSGDVETLTVEQIAAQAFV

FFLAGFETSSTAMSNSLYELAQNSDLQNKARKSVMDAIKKYGSLTYEAMQDMQYIDQCIN

ESLRKYPPASNLTRTVSTDYKLPDSNVVLQQGSTLIVPVYALHHDAEYYPDPEKYDPDRF

TPEEVAKRNPYCFLPFGEGPRNCIGMRFGMLQARVGLAYLLRDFSFTLSNKTPVPLKISP

HSPILTSEGGLWLNVRKL

>EDV35648.1

MLFALLVVGVVLALAYNFYYNKYTYWTRKGVPNEPPLPLIGNMKGLAKKYHLRDIIQRFY

EQFKGKSPIAGLFMFFKPTAMIMDLDLIKQVLIKDFHHFQDRGLFNNPEDDPLTGHLLNL

QGEEWKAMRQKLTPVFTSGKIKQMSEVVVEVAHHLVDTMDKEVTHAAVDDGDIEIKEICA

RYTTDVIGTCAFGLECYSLKDPQAEFRKKGRMILETPRHSMLVQLFILTNGKLAKQLRMK

VLPDELTEFFLSAVKSTVEYRLKNNIKRNDFMDQLIQLRAEDQEAAKKGKGIDLSHGLTI

EQMAAQAFVFFVAGFETSSSTMGFCLYELALQPDIQDRVREEIQSVLNGGEITYDALAQM

TYLEQVIAETLRKHPIISSLLRETNLDYKVPNTNVIIERGSTVLIPIHNIHHDPELYPNP

ELFDPSRFDPEEVKSRHPFSYLPFGDGPRNCIGLRFGKMQAKIGLVSLLQRFKFGVSKRT

EIPLILDTRSPFLSAKNGIHLKLERI

>AAX48013.1

MLTILLAGFILLLTAAVLYIHRRNRFWSDRGIPCAPNPRFFFGHVKGQVTDRHASYVNRE

LYQHFRSRGEPFGGFNFFAVPAVMIVDPELVKTVLVRDFNVFHDRGIFNNPKDDPLSGQL

FLLEGLQWKVLRQMLTPTFTSGRMKAMFGTILDVAEELREYLVHNHEREPVVEMKEVLAR

FTTDVIGTCAFGIECNTLKNPDSDFLKYGKRVFEQKPWNVFKFIFASLFKDLSKALGFKL

TDSGVEKFFMGLIRETVEFREKNNVQRNDFMNLLLQLKNKGRLEDMDEKEELSKRGLTMD

ELAAQCFVFFIAGYETSSTTMNFCLYELAKNQDVQERLREDIQEAVAANGGRVTYEMVMG

LPYLDNVINETLRKYPPIESLSRVPTADYTVPGTNHVLPKGTMTAIPVYALHHDPEYYPE

PERFDPDRFAPEAVQSRPAYVFLPFGEGPRNCIGLRFGLMQTKIGLITLLRNFRFSPSAK

TPDALVFDVKSFVLSLEGGNYLRYDKI

>AAX48016.1

MLTILLAGFILLLTAAVLYIHRRNRFWSDRGIPCAPNPRFFFGHVKGQVTDRHASYVNRE

LYQHFRSRGEPFGGFNFFAVPAVMIVDPELVKTVLVRDFNVFHDRGIFNNPKDDPLSGQL

FLLEGLQWKVLRQMLTPTFTSGRMKAMFGTILDVAEELREYLVHNHEREPVVEMKEVLAR

FTTDVIGTCAFGIECNTLKNPDSDFLKYGKRVFEQKPWNVFKFIFASLFKDLSKALGFKL

TDSGVEKFFMGLIRETVEFREKNNVQRNDFMNLLLQLKNKGRLEDMDEKEEPSKRGLTMD

ELAAQCFVFFIAGYETSSTTMNFCLYELAKNQDVQERLREDIQEAVAANGGRVTYEMVMG

LPYLDNVINETLRKYPPIESLSRVPTADYTVPGTNHVLPKGTMTAIPVYALHHDPEYYPE

PERFDPDRFAPEAVQSRPAYVFLPFGEGPRNCIGLRFGLMQTKIGLITLLRNFRFSPSAK

TPDALVFDVKSFVLSPEGGNYLRYDKI

>EFR25762.1

MFWHLLLVAVLLGVLWIRKRFAYWKDRGVPYVEPTFPHGNLSGMGSTVHMSDITRKVYQQ

LKPKATGTGFVGMYFFTNPVTLVIDLELIKAVLVKDFQYFHDRAVYHNEKDDPLSGHLFA

MEGNKWRNLRSKLTPTFTSGKMKMMYPTILTVAEEFNKCLANEIRVQDELELKELLARFT

TDVIGNCAFGLECNSLKDAEAEFRRMGRKAFEGSRIKIVKRFLSSQYPNLARKLGVTSTA

PDVSKFFLGAVRDTVQYRLKHNVQRNDFMDLLMELMKEQDREGLSIEEAAAQAFVFFLAG

FETSSTAMAFCLYELAGSEELQSKARANVTEVLARHEGKLTYEALQDMKYIEMCINGAYR

RSEEEYYPVPGTKHIIPKGQTVLIPVHGLHHDPEYYPNPDRYQPERFDEATKSQRNPYTF

LPFGEGPRNCIGLRFGLMQARIGLAVLLRNFRFTVSPKMATPLPISKASTVYSIEGGLYL

KVEKI

>EAT39040.1

MEVITITLLTILILLLIYVLHLLRRQIHFFKDRNVPYKPASFERLDKTIHPAMHFLRWYK

QFKGQYPLSGVFMFIKPIVIPLDLELIKRILVKDFQYFQNRGIYYNERDDPLSAHLFSLE

GAKWRNLRAKISPTFTSGKMKMMYPTMVAAGKQFSEYLEEKVGDGNELEMRDLLARFTTD

MIGTCAFGIECNSMKEPNSKFREMGRKHFEAPRNVLKDAFKMTAPGLARFLRVTEILPDV

SEFFMDVVKSTVEYRMKNNVRRNDFMDLLIAMLDDKTEGSESLTISEIAAQAYVFFIAGF

ETSSTTMTWALHELSRNPDIQEEGRKCVQEVLEKYNGVMSYEAIMEMTYIDQIINETLRL

YPPVPMHFRVVSKDYHVPETDTILPAGTFTMIPVYAIHHDEDIFPEPEKFDPTRFTPEEV

NKRHAFAWTPFGEGPRVCIGLRFGMMQARIGLALMLKNLRFSPGPKTCTEMEFQPQNFIL

SPKEGLWLNVEKI

>EAT39038.2

MWLVYLVWLVAAVLLAVYLWIKKRFNFWKDRGVEYIEPEFPFGNFKTLGKVEHIAPITQR

HYDYFKQKGVPYGGVFMLTSPLLYILDTKLIKTLLVKDFNHFPNRGVYFNEKDDPLSAHM

FAIEGNKWKTLRNKLSPTFTSGRIKMTFPLVVGVCQQFCDHLGEVVQQSNEVEMHDLLSR

YTIDVIGTCAFGIDCNSFREPDNEFRKYGKIAFDKLPHSPLVVYLMKAFRSYANAFGMKQ

LHEDVSSFFSKVVKDTIEYRESNNVVRNDFMDLLLKLKNTGRLEESGEEIGKISFEEIAA

QAFIFFTAGYDTSSTAMTYTLYELALNQKAQEKARKCVLDIFAANNGTLTYESVGNMGYL

DQCLFAETLRKHPPVAILERNADRDYKLPDSDIVIKKGRKIMIPTFAMHHDAEHFPDPER

YDPDRFSPEQVACRDPYCYLPFGEGPRICIGMRFGTIQARVGLASLLKRFRFRVCDKTQI

PVRYSKTNFILGPANGVWLRVEKL

>AAX48012.1

MLTILLAGFILLLTAAVLYIHRRNRFWSDRGIPCAPNPRFFFGHVKGQVTDRHASYVNRE

LYQHFRSRGEPFGGFSFFAVPAVMIVDPELVKTVLVRDFNVFHDRGIFNNPKDDPLSGQL

FLLEGLQWKVLRQMLTPTFTSGRMKAMFGTILDVAEELREYLVQNHEREPVVEMKEVLAR

FTTDVIGTCAFGIECNTLKNPDSDFLKYGKRVFEQKPWNVFKFIFASLFKDLSKALGVKL

TDSGVEKFFMGLIRETVEFREKNNVQRNDFMNLLLQLKNKGRLEDMDEKEELSKRGLTMD

ELAAQCFVFFIAGYETSSTTMNFCLYELAKNQDVQERLREDIQEAVAANGGRVTYEMVMG

LPYLDNVVNETLRKYPPIESLSRVPTADYTVPGTKHVLPKGTMTAIPVYALHHDPEYYPE

PERFDPDRFAPEAVQSRPAYVFLPFGEGPRNCIGLRFGLMQTKIGLITLLRNFRFSPSSK

TPDALVFDVKSFVLSPEGGNYLRYDKI

>ADY68482.1

MLAYLLAVVVALLTAAALYIRSRHRFWSDRGIPCAPNPEFLLGHVRGVVTNKHAAHVNRE

LYQDFKKRGESFGGFSFFAVPAVLVVDPELVKTMLVRDFAVFHDRGIYNNPKDDPLSGQL

FLLEGLQWRVLRQMLTPTFTSGRMKAMFGTIMDVAEEFRRFMVECRERESVIEMKEVLAS

FTTDVIGTCAFGIECNTLKNPDSDFLKYGKKVFEQKLSTLVKFIFASLFKDLARTMGIKI

TDAGVEKFFMGLVRETVEFREKHNVQRNDFMNLLLQLKNKGRLVDQLDEAEEAAARGLTM

DELAAQCFVFFIAGYETSSTTMNFCLYELAKNPDIQERLREDIEEAVAVNDGQVTYDLVM

GLRYLDNVVNETLRKYPPIESLNRVPNSDYTVPGTNHVLPAQTMITIPVYALHHDPEYYP

DPDRFDPDRFLPEVVQGRHPYAYIPFGEGPRNCIGMRFGVMQTKIGLITLLRNFRFSPSP

RTPDKIVFDVKSFVLSPEGGNNLRYDKI

>EDW60860.1

MVLLTIALLGVALALFYNFYLNVYKYWDRRGVPYERPLPILGNMRGIGTRLHFRDINQRL

YDRFKGKTPIIGMFMFFKRVAMIVDLDLIKQMLIKDFNTFQDRGVFSNVRDEPLTGHLFT

LEGDEWRSMRHKLTPVFTSGKMKHMFGVVVEVGHHLADTMDKAVTAARADGGDVEIKEFC

ARFTTDVIGTCAFGLECNSLADPHAEFRAKGRMLFEKPRHHQLVQAFIFTNSKLSKKLHM

KVFPDDLSNFFMEAVRTTVDHRLKHGIKRNDFLDQLIELRAENEEAARRGNGIDLSQGLT

IEQMAAQAFVFFIAGFETSSSTMAFCLYELALQPDVQHRLREEIETVIKATADGELTYDA

IGQMSYLEQVLAETLRKHPILPHLMRQTNQDYKVPGTDLVIEQETSIIIPVHSIHHDPDI

YPDPERFDPSRFEPDAIKARHQFAYLPFGDGPRNCIGERFGKMQAKIGLICLLRRFKFGV

SNRTEIPLILNNRNFPLATKYGIHLKVERV

>EDW10115.1

MLFLTIALLGVVLALLYSLYQNTYSYWERMGVPYEQPLPIIGNMKGIGSRLHFRDINQRI

YDQFKGKTPFAGLYMFFKRVAFIIDLDLIKQLLIKDFNVFQDRGIFNNVRDEPLTGHLFT

LEGDEWRSMRHKLTPVFTSGKMKHMFGIIVDVGRHLADTMDKEVAAASAEKGDVEIKELC

ARYTTDVIGSCAFGLECNSLADPFAEFRSKGRLIFENPRHPQIIQSFIFTNPNLARKMHL

KVLRDDVANFFMTAVRSTVDHRIKNGIKRNDFLDQLIELRAENEEAARQGKGIDLSHGLT

IEQMAAQAFVFFIAGFETSSSTMAFCLYELALQQDIQRRLREEIETVLKQTADGELTYEA

VSQMSYLEKVLAETLRKHPILPHLMRLAKTDYPDPGTNLVIEKGTSIVIPVHNIHHDPEI

YPQPERFDPERFEPAAIKARHQFAYLPFGDGPRNCIGERFGKMQAKVGLISLLRRFKFSV

SEHTEIPLKFNKRNFSLSTQDGIILKVERI

>ADW54425.1

MWLVYLVWIVVFILLAGFRWVRRRFDFWKDRGVEYIEPEFPYGNFKTLGKVEHIAPITQR

HYDYFKQKGVPYGGVFMLTSPLLYILDTKLIKTLLVKDFNYFPNRGVYFNEKDDPLSAHM

FAIEGNKWKTLRNKLSPTFTSGRIKMTFPLVVGVCQQFCDHLGEVVLQSNEVEMHDLLSR

YTIDVIGTCAFGIDCNSFREPDNEFRKYGKIAFDKLPHSPLVVYLMKAFRSYANALGMKQ

LHEDVSSFFSKVVKDTIDYRESNNVVRNDFMDLLLKLKNTGKLEEAGEEIGKLSFEEIAA

QAFIFFTAGYDTSSTAMTYTLYELARNQEAQEKARKCVLDIFAANNGTLTYESVGNMGYL

DQCINETLRKHPPVAILERNADRDYKLPDSDIVIKKGRKIMIPTFAMHHDADHFPDPDRY

DPDRFSQEQVAKRDPYCYLPFGEGPRICIGMRFGTIQARVGLASLLKRFRFSVCDKTQIP

VKYSKTNFILGPANGVWLRVEKL

>EFR28294.1

MWIYLLLLVLLAAIYWVRQRLAYWKRRGVAYVEPSFPQGNLQGVGKQHHIAQVLQRCYRQ

LKATSGGPYGGMYFFINPVALLLDLDLIKAVLVKDFQYFHDRNLYHNDRDDPLSRHLVAM

EGTKWKNLRAKLTPTFTSGKMKLMFSTVTAVADEFTKCLSHELERSTELEMKDLLARFTT

DVIGTVAFGLDCNSLADPQAKFRMMGRKIFEPSPSRVMKNFLASQFPGLARMLRVVLTPT

DITDFFMTVVKDTVQFREKNNVQRNDFMTLLMKVMKDQEQSGTAGTVGERLTMDDMAAQA

FVFFLAGFETSSTTMSFCLYELALNQAIQDRARQEVRDVLQQHDSISYEAVHEMKYIEMC

INESLRKYPPATTLTRKVTKDYRIGNGNQVLPKGLMVAVPVYALHHDPDYFPEPDRFDPE

RFSVQQTEKRHPFAFLPFGEGPRVCIGLRFGMMQARIGLVYLLKHFRFTLASKMSVPLKV

TPSSPILSIDGGLWLNVEKL

>EAT39043.1

MEPITIILVTILVLLLTYGFHLIRRQLRFFKDHNVPHIAGNFVLIDKTQHPANHFLRWYK

QSKGQYPLTGVFMFIKPIAIPLDLELIKRILVKDFQYFQNRGMYYNERDDPLSAHLFSLE

GAKWRSLRAKISPTFTSGKMKMMYPTMMAAGKQFSEHLEEKMSEENELEMRDLLARFTTD

MIGTCAFGIECNSMKEPNSKFREMGRKHFESPRSGLKDLLKITAPGLARFFGVTEILPDV

AEFFMDVVKSTVEYRMKNNVRRNDFMDLLIAMLDDETEGSESLTISEIAAQAYVFFIAGF

ETSSTTMTWALHELSRNPEIQEEGRKCVQEVLEKYNGVMSYEAIMEMTYIDYIINETLRL

YPPVPLHFRVVTKDYPVPGTDTVLPAGTFTMIPVYAIHHDEDIFPEPEKFDPTRFTPEEV

SKRHAYAWTPFGEGPRICIGLRFGMMQARIGLALLLNNLRFSPGPKSCTKMEFQPENLIL

TPKQGLWLKVEKV

>EAT39039.1

MDVFLLIAAFVLLVAYGLHLLRKQVNFWADRNVPHNPVNFRQTVDQTVHMARRFQGYYHQ

FKGQYPFAGMYLFTKPVALAIDLELLKCIFVKDFQYFHDRGTYYNEKDDPLSAHLFNLEG

NKWRNLRSKISPTFTSGKMKMMYPTMIAAGKQFSEYMDEKVGVEQELELKDLLARFTTDV

IGMCAFGIECNSMKDPNAEFREKGRMHFETPRNRKKDMMCSIAPKLARMMGLKQIIPDLS

DFFLGVVRETIDYRVKNGVRRNDFMDLLIGMLTGENVELGPLTFNEVAAQAFVFFVAGFE

TSSTTMTWALYELSVNQDIQEKGRKCVRDVLEKYNGELSYETIMEMSYIDHILHETLRKY

PPVPVHFRIVTKDYKVPNTETVLPAGTSVMIPVYAVHHDPEIFPDPKRFDPDRFTTEEIN

KRHPYAWTPFGEGPRICIGMRFGMMQARIGLALLLNNFRFSSGKKSTVPLDFTAKSFILS

PDEGLWLKVEKL

>EDW89362.1

MLLTLALLGLVLLLAYRFYHNTYTYWARMGVPHERPLPLIGNMKGIGTKYHLRDINQRVY

DKFKGKAPIAGMFLFIKRAAMVTDLDLIKQVLIKDFHHFQDRGLFNNTRDDPLTGHLLTL

EGDEWKSMRHKLTPVFTSGKMKKMSEVVVEVGHHLVDATEKAVKAGEVENGDVEIKDLCA

RFTTDVIGSCAFGLECNSLQDPNAEFRKKGRMVFDEPRHSTLIQNFIFTNAKWAKKLRMK

ALRDDLTDFFMSAVKNTVDYRLKNGIKRNDFMDQLIELRAEDQEAAKKGQGIDLSHGLTL

EQMAAQAFVFFVAGFETSSSTMAFCLYELALQPEIQNQVRDEIERVLDGGTITYDALAEM

SFLEQVLSETLRKHPILPQLLRETNESYKVPNTEFIIEKGTTLLIPVHSIHHDQDLYPQP

ELFDPSRFETDNSKSRHPFAYLPFGDGPRNCIGLRFGKMQAKIGIVSLLQRFKFGVSELT

EIPLILDTRSPTLSAKNGIHLKVERI

>AAF97936.1

MLIYLTVLALTLAVLWVRKRYSYWKERGIPYVEPSFPSGNIRGMGRKEHFSTQMQRCYKE

LKGKGPVGGVFFFINPVALAMDLDFIKTVLVKDFQYFHDRSIYYNEKDDPLSAHLVALEG

TKWKNLRAKLTPTFTSGKMKSMYPTIIGVAEEFQKMMESEVGGNTEIEMKDVLARFTTDV

IGTCAFGLECNSLYDPDAQFRCMGRKIFAIAKGRFVKLIAAQQFRSLARSLHVTLIDKEV

SDFFIGAVKDTIKYREENNIERNDFMSLLMKLKKVDNSDNTGEDSEALTDEQIAAQAFVF

FLAGFETSSTAMSNSLYELAQNSELQDKARKSVMDSIKKHGSLTYEAIQDMQYIDQCINE

SLRKYPPASNLTRIVSKDYKLPNCDVVLQQGSTIIVPVYALHHDAEYYPNPEKYDPDRFT

PEEVAKRNPYCFLPFGEGPRNCIGMRFGIMQARVGLAYLLKDFSFTLSSKTPVPLKISPH

NPILTSEGGLWLNVEKL

>AEF79984.1

MDIFLLITAIILLVAYGLHRIRKQVNFWADLNVPHNPVNLRQYVDQSIHMARRFQGYYHR

FKGQYPFAGMYLFTKPVALAIDLELLKCIFVKDFQYFHDRGTYYNEKDDPLSAHLFNLEG

TKWRNLRSKISPTFTSGKMKMMYPTMIAAGKQFSEYMEEKVGKECELELKDLLARFTTDV

IGMCAFGIECNSMKDPNAEFRQKGRMHFEAPRNRKKDMLCSIAPRLARLMGLKQIIPDLS

EFFLGVVRETIDYRVKNGVRRNDFMDLLIGMLTGSNTELGPLTFNEVAAQAFVFFVAGFE

TSSTTMTWALYELSVNQDIQEKGRKCVRDVLVKYNGDLNYDAIMEMTYIDQILQETLRKY

PPVPVHFRVVTKDYKVPNTETTLPTGTSVMIPVYAIHHDPEVFPDPEKFDPNRFSTEEIN

KRHPYAWTPFGEGPRICIGMRFGMMQARIGLALLLNNFRFSSGEKSTVPLDFTAKSFILS

PEEGLWLKVEKL

>AFM08395.1

MWFLFVEILVALVALLGGLVYLVKRKQSYWKDRNVPHPSPAFFFGSFKDTGTKIHFTEYL

EQYYTTYKGKHPFMGLYMLVSPIVLPTDLELVKSIFVRDFQYFHDRGTYYNEKDDPLTAH

LFNLEGQKWKNLRNKLTPTFTSGKMKMMFPTVVAAGKQLKEFMDENVQKNSELEMKDIMG

RFTTDVIGTCAFGIECNSMRDPNAEFRAMGKQFIDRQPSQFVNLMVQFSTKLSRMMGIRL

IDKEVSTFFLKVVKDTIDYRVKNGIQRNDFMDLMIRMLQNTENPEESLTFNEVAAQAFVF

FFAGFETSSTLLTWTLYELALNPEIQEKGRQCVKEILEKHNGEMTYEAILDMKYLDQILK

SLRKYPPVPMHFRTAAQDYHVPNTDSIIEAGTMILTPTFAIQRDPDIFPEPEKFDPERFS

PEEEAKRHPFAWIPFGEGPRVCIGLRFGMMQARIGLAYLLQGFSFAPYEKTSVPMKFVTN

SFMLAPRDGLWLKVNKL

>AAF97937.1

MLIYLTVLALTLAVLWVRKRYSYWKERGIPYVEPSFPSGNIRGMGRKEHFSTQMQRCYKE

LKGKGPVGGVFFFINPVALAMDLDFIKTVLVKDFQYFHDRSIYYNEKDDPLSAHLVALEG

TKWKNLRAKLTPTFTSGKMKSMYPTIIGVAEEFQKMMESEVGGNTEIEMKDVLARFTTDV

IGTCAFGLECNSLHDPDAQFRRMGRKIFAIAKGRFVKLIAAQQFRSLARSLHVTLIDKEV

SDFFIGAVKDTIKYREENNIERNDFMSLLMKLKKVDNSDNTGEDSEALTDEQIAAQAFVF

FLAGFETSSTAMSNSLYELAQNSELQDKARKSVMDSIKKHGSLTYEAIQDMQYIDQCINE

SLRKYPPASNLTRIVSKDYKLPNCDVVLQQGSTIIVPVYALHHDAEYYPNPEKYDPDRFT

PEEVAKRNPYCFLPFGEGPRNCIGMRFGIMQARVGLAYLLKDFSFTLSSKTPVPLKISPH

NPILTSEGGLWLNVKKL

>EDW04922.1

MKFMFPTIIKVSEEFVRVMFDEVPVKSGGSIIEVKNLMARFTTDVIGTCAFGIECNTLRT

PESDFRKMGQKAFSETRQGSLITAFILSFPNLARKLRMRVVPEDVHQFFMGLVNETVAYR

EKENIKRNDFMEMLIELKQKGSFTMDNGEVVTGLDVGELAAQVFVFYLAGFETSSSTMSY

CLYELAQHTDIQQKLREDIKNVLQQHDGKLTYESIKAMRYLDQVISETLRLYTIVPFLVR

KALNDYVVPGNPKYVIEKGTQVIMPAAAYHRDEDFYPDPEKFDPDRFSAENVAARDSVEW

LPFGDGPRNCIGMRFGQMQSRVGLAQLIQNFKFSVCEKTDIPLKHDPKSFVLGTVGGIYV

RAERV

>AAF97938.1

MLIYLTVLALTLAVLWVRKRYSYWKERGIPYVEPSFPSGNIRGMGRKEHFSTQMQRCYKE

LKGKGPVGGVFFFINPVALAMDLDFIKTVLVKDFQYFHDRSIYYNEKDDPLSAHLVALEG

TKWKNLRAKLTPTFTSGKMKSMYPTIIGVAEEFQKMMESEVGGNTEIEMKDVLARFTTDV

IGTCAFGLECNSLHDPDAQFRRMGRKIFAIAKGRFVKLIAAQQFRSLARSLHVTLIDKEV

SDFFIGAVKDTIKYREENNIERNDFMSLLMKLKKVDNSDNTGEDSEALTDEQIAAQAFVF

FLAGFETSSTAMSNSLYELAQNSELQDKARKSVMDSIKKHGSLTYEAIQDMQYIDQCINE

SLRKYPPASNLTRIVSKDYKLPNCDVVLQQGSTIIVPVYALHHDAEYYPNPEKYDPDRFT

SEEVAKRNPYCFLPFGEGPRNCIGMRFGMMQARVGLAYLLKDFSFTLSSKTPVPLKISPH

NPILTSEGGLWLNVEKL

>EDS44709.1

MSPIQLIVNGLLVILFIALGALIFIKHRYGYWRRRGVSYLEPEFPFGNFKEMGKSIHPAH

LTQQIYTQFKGRSSFVGLYIFLNPVLLVTDLSLIKRILIKDFNHFPNRGVYFNERDDPLS

AHLFSLEGRKWHDLRAKISPTFSSGRIRMTFPAVLEVAQRFCTYLEQSCIGKEAAEIRDA

MARFTTDVIGSCAFGIECNSFKEPDNEFRRVGRKHFDTPRNHPLKVFVMKTFRGLANGLG

IKLIHDDVAEFFLDVVRRTISHREENNVSRNDFLDLLIKLKNTGRLEADGGDIGQLSFSE

IAAQAFIFFTAGFETSSSALTYTLYELALNPVIQEAARVSVFETFDKFNGELSYECCQAM

TYLDQCINETLRKYPPVVNLERAVDKDYLIEESNLILPKGLKIMIPAFAIQHDPDFYPNP

ERYNPTRFSPDQQAARDQCTFLPFGEGPRICIGLRFGMLQARVGLAMILKNFRILRCEKT

EDPVVYSTRAFVLTPINGMWLKLEKLSK

>EAT39028.1

MIALLLIGAVTLVFLFVKQRFNYWKVRGVPYVRPTFPLGNLWGIGTKKHLSEGLEDLYVQ

LKGKAQLGGIYFFINPVVLVTDLDLIKTILIKDFNFFHDRSIYYNEKDDPLTAHLFTMEG

IKWKNMRVKLTPTFTSGKMKLMFPIVRDCANELEKCISKEIVDGKEIEVKDILARYTTDV

IGNCAFGLECNSLHNPNAEFREMGRKVFQLQGLGFLKLLLTQQFSTLSRALGATVLQPDV

AKFFLKTVSDNVDYREKNKIERNDFIDLMIKLKNGQTLEHDKSDQRVEKLSIEQVAAQSF

VFFFAGFETSSTLMSFCLYELAQNQDLQDKARKDILDTLNKHGSLSYEAVHEMKYLENCV

SETLRKHPPASNIFRTATQDYTVPGTSLTIEKGTSVMIPTLAIHRDPEYYPDPMKFDPDR

FTADQVAARHPFAFLPFGEGPRVCIGMRFGLMQARVGLATLLKNFRFTVGERLETPAQLD

PSSAILLIKGGLWLKVDKI

>EJY57332.1

MSALLIILALTPLFLFIIYVKQKYAYWARRNVPFLKPHFPYGNFEALDRKSIADVAREAY

EEMKNRGPFYGAYFFLQPLITITDPDLIKMVLIKDFNTFPDRGLYFNERDDPLSAHMFAI

EGNKWRSLRQRLSPTFTSGKMKMMFPTLAAVGDQFSAFLDEEIGSGKVVEVKDFMAKFTT

DIIGSCAFGIECNSFKDPHGRFRQFGKMVFETPVHGSLVRFALKSFPEISRRLRIKALHE

EASKFFYGVVEDTVKYREKNGVERKDFLSLLIDMKKDGVDFTMDEIAANSFIFFGAGFET

SSSNQTFCLYELARNPECQDKARQSVLDALRNHGGMTYDAACDMQYLDQCINETLRLYPS

VPVLERRAFQDYKIPGHDVVIPKGMKINIPAYAIQRDERFYPDPDVFNPDRFHQKEVAKR

HICTFIPFGEGPRICIGLRFGMMQSRVGLATILSKFRISICSETANPLEYSSKTSVLIPK

EGLWLRVDPL

>EAL26027.2

MMLTFALLGMALALAYNFYRNTYSYWERKGVPNERPLPLIGNVKGIGTKYHFRDINQRIY

DKFKGVAPFAGMFTFFKRSALIIDLDLIKQVLIKDFQYFQDRGVFNNVRDDPLTGHLLTL

EGDEWRSMRQKLTPVFTSGKIKHMSGVVVEVGHRLAAAMDKAVSAAKVDDGDVEIKDICA

RFTTDVIGTCAFGLECHSLSDPKAEFRSKGRMIFEKPRHSPIVQFFVFTNAKLAKKLRMK

LLPDDVSEFFMNAVKNTVDYRMKNGIKRNDFLDELIELRAENQEAAKHGKVIDLSHGLTI

EQMAAQAFVFFLAGFETSSSTMAYCLYELAQHPDIQRRVREEIDAVLKNVEGGEITYDAL

AEMNYLEKVLAETLRKHPIVPQLIREANQNYKVPNTDITIDKGTTVLIPVHSIHHDPEIY

PQPESFAPSRFEADQVKSRHPMAYLPFGDGPRNCIGLRFGKIQAKIGLVSLLRRFKFGIS

KRTEIPLILDTRSFPLTTKNGIHLRVERI

>EDW74917.1

MAVWLVPLLLVGAILALAALFLQHRYGYFARRGILHDKPVWIYGNTAEVGKTKTFRDPFR

ELYTKYKGKAKFAGFYVGLRPNPVLLDLDLIKQVMIKDFQNFANRGNYYNEEDDPVTAHL

FNLDGQKWRNMRTKLTPTFTALKMHQMLPIIVEIGDRFVDLLNKEVAKVKKNGEAPVEIK

DWLSRFTIDVIGSCAFGLDCNSLKDPNAEFRVITRTLFTVRRHGPLAMGLINTFPKIAEK

LHVKIAPDNVSEFYTRVVRDTVDYRDKNNVKRNDFLNLLMGLREGKDGGLTFNQILAEAF

VFLVAGFETSSSTMGFTLYELAINQDIQDKAREEVKQVLAKHGEFNYEAIKELKYIQQIF

NETLRKWAILPILIRVAEKDYPVPGTNDVFEAGTQLIIPADAIHHDPEIYPDPEKFDPDR

FTPEAVEQRHSMAWQPFGQGPRNCIGARFETLGVAFKQLLKDARWMHPGQAMSGKVSSAS

KCASAYASALGGCVATFPSCC

>EDV59507.1

MLTLLVLVLIAGLLLYAKLRWHYSYWSRRGVAGEAPVYFRGNMSGLGKDLHWTDINMRIY

RKFRGEERYCGYFTFMTKSLFIMDLELIREIMIRDFASFADRGLFHNVRDDPLTGNLLFL

DGPEWRWLRQNLTQVFTSGKMKFMFPNMVEVGEKLTQACRLQVGEIEAKDLCARFTTDVI

GSCAFGLECNSLQDPESQFRSMGRSVTTEPLHSVLVQAFMFAQPDLARMLRFRLFRPEVS

EFFLDTVRQTLDYRRRENIQRNDLIQLLMELGEQGVKDALSFEQIAAQALVFFLAGFDTS

STTMSFCLYELALNPDVQEKLRVEVLDVLKRNNQKLTYDSVQEMPYLDQVVAETLRKYPI

LPHLLRRSTKEYQVPDSSLVLEPGTKIIIPVHSIHHDPELYPDPEKFDPSRFEPEEIKAR

HPFAYLPFGEGPRNCIGERFGKLQVKVGLVYLLRDFRFSRSKKTQIPLKFSSRSFLISTQ

EGVHLRMEGLAAP

>ADY68483.1

MIALLLIAAVTLVFLFIKQRFNYWKVRGVPYVQPTFPLGNLGGVGRKKHLSEALEDLYIQ

LKGKASIGGIYFFINPVVLVTELDLAKTILVKDFNFFHDRSFYYNEKDDPLTAHLFTMEG

VKWKNMRIKLTPTFTSGKMKLMLPIIRDCANELEKCIEKETANGEEVEIKDILARYTTDV

IGNCAFGLECNSLRDPNAEFREMGRKIFQLEGFGFLKLLFTQQFRTISRALGATILQPDV

AKFFLKTVKDNVEYREKNKVERNDFIDLMVKLKNGQALEHENSEHRMEKLTIEQVAAQSF

VFFFAGFETSSTLMSFCLYELAENQDLQEKARKDVLDTLKKHGSLSYETIHEMKYLENCI

NETLRKHPPASNIFRTATQDYIVPGTSITIEKGTSVMIPTLAIHMDPEYYPDPERFDPDR

FNADQVAARHPFAFLPFGEGPRVCIGMRFGLMQTRVGLATLLKNFRFTVGKRLETPVKLE

PSSGILLIKGGLWMKVDKI

>EDS26136.1

MALLSLMLTAFVLVASLIYFYLKRRHRFWSNRGIPCAPDPHLFYGHVKGAISVAHGSYIN

QKLYNSLKQRNLSFGGYSFFTIPSIMVVDPEMVKTVMVRDFNVFHDRGIYNDPKSDPLSG

HLFLLEGAPWRALRQKLTPTFTSGRMKSMFGTILEVAEEFRKFMVENHDRGTRELEMKDV

LARFTTDVIGTCAFGLECNTLENPDSDFLKYGSKVFEQTPFQLAKFILASMFKEQAKKLG

VKLTDVGVESFFMGITRETVEFREKNNVQRNDFMNLLLQIKNKGRLTDQPDEPGTMEKVE

PGLTIEELAAQCFVFFVAGFETSSTTMNFCLYELAMNPDIQERLRNDIEKAIAANDGKLT

YEIVMGMQYLDNVVNETLRKYPPVESLNRIPLTDYTIPGTDHVIPSQTMFLIPVYALHHD

PEIYPDPERFDPDRFLPEVVATRHAYSYLPFGEGPRICIGLRFGVMQTKIGLITLLRSFR

FKPTAATPNPLVFDPKSFVLSPIGGNHLRVDKI

>EDW88440.1

MRQRLTPTFTSGKMKYMFPTMVKVSEELVKVITELVPAAHNGAVLEIKDLMARYTIDVIG

NCAFGIECNTLRNPVSDFRTMGLKVFTNFRHGKLLTMFMFSFPKLASRLRMRMLPEDVHQ

FFMRLVNDTIALRERENFKRNDFMNLLIELKQKGRATLDNGEVIEGMNIGELAAQVFVFY

VAGFETSSATMSYCLYELAQNQGIQDRLRNEIQTVLEEHEGQLTYDAVKAMTYLNQVISE

TLRLYTLVPHLERQALNDYVVPGHEKLLIEKGTQIIIPACAYHRDEDLYPNPEVFDPERF

SPEKVAARESVEWLPFGDGPRNCIGMRFGQMQARIGLAQIIRRFRISVCDKTEIPLKFNP

ASVAMVPIGGVYLRMERI

>EDV35651.1

MLFALLLLAVVLALAISFYLNIYTYWSRRGVPRESPLPLVGNMKGIGSKYHFRDVNQRIY

DQFKGKVPFAGMHVFFKKTAMILDLDLVKQVLIKDFHYFQDRGVFNNTKDDPLTGHLFAL

EGDEWKSMRKMLSPVFTSGKIKHMSEIIVAVSHHLVDAVEKELKTASLEDGEVEIKDICA

RYTTDVIGSCAFGLECFSLKDPNSEFRNKGRMIFEKPRHHQLIQAFIFTNAKLARKLRMK

VLPDEVTEFFIATVKNTVDYRLKNNVKRNDFVDQLIELRAEDQEAAKKGKGIELSHGLTL

EQMAAQAFVFFIAGFETSSSTMSFCLYELALQQDIQDRVREEINSVLGKVEGGEITYDAL

GEMTYLEQVIAETLRKHPILPHLVREINRNYQVPNTEFVIEKGNNILIPVHNIHHDPEIY

PQPEKFDPSRFNRDEVSSCHPMAYLPFGDGPRNCIGLRFGKIQSKIGLVALLRNFKFSPS

KQTEIPLILSNKTFTLSTKNGIHLKVERI

>AFM08394.1

MWFLSLEAFVALLVLLGALVYFIVRKQSYWKDRKVPHPSPTFLIGSFKDAFSKVHFSEYL

EQYYTTYKGKLPFMGVYMLVSPVVIVTDLELVKSILVRDFQYFHDRGTYHNEKHDPLTAH

LSNLEGQRWKHLRNKLSPTFTSGKMKMMFPTVVAAGKQLKEFMDENVQKNSELEMKDIMA

RFTTDVIGTCAFGIECNSMRDPNAEFRAMGKQFVDRPPSQFVNLMIQVSPKLSRMMGIRL

IDKEVSTFFLKVVKDTIDYRVKNSIQPNDFMDLMIRMLQNTENPEESLTFNEVAAQAFVF

FFAGFETSSTLLTWTLYELALNPEIQEKGRKCVKEILEKHNGEMTYEAILDMKYLDQILN

EALRKYPPVPMHFRMTTQDYHVPNTDSIIEAGTRVLIPTFAIQRDPDIFPEPEKFDPERF

SPEEEAKRHPFAWIPFGEGPRVCIGLRFGMMQARIGLAYLLQGFSFTPYEKTSVPKKFIK

NNIFLSPKDGLWLKVNKL

>ADY68484.1

MIALLLIAAVTLVFLFIKQRFNYWKVRGVPYVQPTFPLGNLGGVGRKKHLSEALEDLYIQ

LKGKASIGGIYFFINPVVLVTELDLAKTILVKDFNFFHDRSFYYNEKDDPLTAHLFTMEG

VKWKNMRIKLTPTFTSGKMKLMLPIIRDCANELEKCIEKETANGEEVEIKDILARYTTDV

IGNCAFGLECNSLRDPNAEFREMGRKIFQLEGFGFLKLLFTQQFRTISRALGATILQPDV

AKFFLKTVKDNVEYREKNKVERNDFIDLMVKLKNGQALEHENSEHRMEKLTIEQVAAQSF

VFFFAGFETSSTLMSFCLYELAENQDLQEKARKDVLDTLKKHGSLSYETIHETKYLENCI

NETLRKHPPASNIFRTATQDYIVPGTSITIEKGTSVMIPTLAIHMDPEYYPDPERFDPDR

FNADQVAARHPFAFLPFGEGPRVCIGMRFGLMQTRVGLATLLKNFRFTVGKRLETPVKLE

PSSGILLIKGGLWMKVDKI

>AFM08400.1

MLIYLLLIAVSAAVWYVHRKYSFWVDRGVPYVQPRFPFGNIQGIGRRMHSSQLMTKFYNE

LKPSGRSFGGIYFFTNPVALALELDFVKNVLVRDFAHFHDRGVYYNEKDDPISGHLFNIE

GTKWTNLRKKLIPTFSSGKMKMMCPTIVSVGGRFRECLERCIANDGEVEMKELLARFTTD

VIGTCAFGIDCNSLNDPNAEFLRMGRKVFEVPRGRILKFFFMATFKDFSRRIHIKGTSED

VSQFFFKVVRETIEYREQHNIQRNDFMNLLMQLKNSGQLDDSGETVGKLSLNEIVAQAFV

FFLGGFETSSTTMSYCLHELALNEQIQQRARQCVLDAVKKHGGLSYEALMDMPYIDQCIN

ESLRKYPPGANLIRQVSQDYRVPGTDVTFPKGMNVMIPVYAIHHDPEHYPDPERYDPDRF

AADASEARKPYSFIPFGEGPRICIAARFGMLEARVGLSVLLMHFSFTRCSKTNVPLVISS

RHAVLTPEGGLWLKVEKLQA

>EFR25763.1

MWLTIALLFASLVVLCYGWVRRRSRFWHDRSVAYIEPQFPYGNFKTLGKVEHIAPITQRH

YDHFKRLGVPYGGVFMLISPVLYIFDPALIKTLLVKDFHYFPNRGVYFNERDDPLSAHMF

AIEGQKWRTLRAKLSPTFTSGRIRLTLPLVLEVSHRLTDHLRDTIRTSSNVVEMHDLLAR

YTIDVIGACAFGIECNSFREPDNEFRRYGKIAFDKLPHSPLVVYLMKAFRAYANALGMKQ

LNDDVSSFFSKVVKDTVEFREREQFVRNDFMDLLLKLKNTGRLESDGEEIGRLTPEEVAA

QAFIFFTAGYDTSSTAMTYTLYELALNQQAQEKARECVQRTLEKYGGELTYEAVSDMPYL

EQCINETLRKHPPVALLERNADKDYRVPDSGLVLRRGQKIIIPIYAMHRDERYFPEPDAY

RPERFTPEEVAKRPSHTYLPFGEGPRICIGMRFGSLQAKVGLASLLSRFRFTICEETQIP

VQYSRTNFILGPANGVWLRVEQL

>EAT39029.1

MLLFLLLSVVTAAYLWVIWRYSYWKRRSVPYVEPSFPFGNLQGLNKRHFGLLTQDVYSKL

KGTGCKFGGMFFFVNPMVVILNLDFAKDVFVKDFQYFHDRGEYSNEKADPIMAHLVTMEG

TKWKNLRTKLTPVFTSGKMKMMFPIITAVAEEFRKCMAKEADKGEDIEMKELLARFTTDV

IGNCAFGLECNSLMDPEAEFRKMGRKAMAMSSADFLRRKLCNSFRGLAKLLGVRLSDPDV

SDFFMNAVRSTIEYRERNKVQRNDLMDLLIKLKNAELIDEKSDRLGPLTFNEIAAQAFVF

FLAGFESSSTAMSFCLYELAKNQELQDKARRNINEVLVKHGTLTYEALYEMTYIENCINE

SLRKYPPVTNIVRNVSKPYRVPGMNVTLEEDCRVLLPVYAIHHDPSLYPNPDQFDPERFN

PENSAARHPMAFVPFGEGPRICIGLRFGSMQARIGLTYLLKNFRFTLSEKMHDPLKMMSN

TIILASEGGLWMRIEKL

>EDW10111.1

MSATISLIACVLVAFYLWMRHRFSYLKRRGIAHEDPRPVFGNVFGWRITQHTSQMLQKLY

VKYKDSGPLFGIYFFMKPVYVVTDMDLLKQIFIKDFSNFDSRGLFYNEQDDPLSAHLFAI

DGPRWHNLRPRLTPIFSGAKMKLMFPSVLRIARELSRAVHERCAGHVAALEVTKLLACYT

MDTIGSAIFGLECNSLRDAKAPFVQMGLNAMLKKRIDYLFCLFFPELSLKLHIKRTPADV

EAFYMRLVKDTISHREAHHIKRNDLVDIMVEMKQKYERGDTAEGLTINEIAAQMYVFIVA

GFETTATALVLALYELARHEDIQCQLREEIEEVIANYGENGELTYEAMQKMKYLEQVMLE

TLRMYPVASEHLRRVNEHFEVPNYPKHYLPAGSQLIIPVYSIHHDATYYPEPEKFQPERF

TQEAIQQRPTCAYLPFGQGPRICIGMRFGRMKLAVGLITLIRNFRFSLAPDTPQPMQFKN

HSFLLHPKEFRLQLEPLS

>157125281

MWIYLLIAAITLSVLLVRRKYSYWKRHGVPYIKPTFPFGNIRPAGNRVHSSQLMTRYYNE

LKGKHQFGGIFFFTNPVALALDLEFIKDVLVRDFQYFHDRGMYYNERDDPISGHLFNIEG

TQWTNLRKKLLPTFSSGKLKMMSPTIISVAERFQECLEKCITVDTEIEMKDLLARFTTDV

IGTCAFGIDCNSLNDPEVEFRKMGNKMFELPTGRILKFFFISTFKNLARKARLKSVPEDV

SEFFFRVVRETIDYREKSHIQRNDFMNLLMQLREKGALEGSDEKVGTLSMNEVVAQAFVF

FLGGFETSSTTMSYCLYELSLHEDIQERARECVQSAIAKHGGFNYDAVMDMNYLEQCINE

SLRKYPPGANLVRCATKDYQVRNSSVVFKKGMSVMVPIYAIHHDAEYYPDPERYDPERFG

VEELAKRPPFTFMPFGEGPRICIAARFGMMESKIGLAALLMNFKFSKCSKSIVPLVISNK

HVVLTPAGGLWLKVEKLEQ

>EAT35332.2

MLPLLLAVVTILLTAAALYIRSRHRFWSDRGIPCAPNPEFLFGHVRGQVTNKHAAYVNRE

LYQQFKARGEGFGGYSFFAVPAVIIVDPELVKTILVRDFAVFHDRGIYNNPKDDPLSGQL

FLLEGLQWKILRQMLTPTFTSGRMKAMFGTIMDVAEEFRQFLVDSRKRESVIEMKEILAS

FTTDVIGTCAFGIECNTLKNPDSDFLKYGKKVFEQRVSTLMKFIFASLFKDLARKLRIKI

TDAGVEKFFLGLVRETVEFREKNNVLRNDFMNLLLQLKNKGRLVDQLDEADEVAARGLTM

EELAAQCFVFFIAGYETSSTTMNFCLYELAKNPDIQEKLREDIEEAVASNSGRVTYDLVM

GLRYLDNVVNETLRKYPPIESLNRVPTSDYTVPGTKHVLPKQTMITIPIYALHHDPDFYL

DPDNFDPDRFLPEAAQARHPYAFIPFGEGPRNCIGMRFGLMHTKIGLITLLRNFRFSPSP

KTPDKIAFDVKSFVLSPDGGNYLRYDKI

>EDW00826.1

MFVSYSLCVFFLICAYIWMQQRLSYFKRRGIAHETPWPLVGNMWGWRITKHTSEILQKLY

FRYKHLGPYVGAYFLMTPIWIVTDMELLKRIFIKDFANFDSRGLFYNERDDPLSAHLFAI

DGPKWHSLRHRMTPIFSGAKMKLMFPSVLRTASELVNVLQERCVGQAANLEVTKILACYT

SDTIGTAIFGLECNSLRDPKAPFVQMGLNAMLKKRLDYIFCLLFPKLSLKLRLKRTPRDV

EAFYMGLVKDTISHREKYNVKRNDFIDIMLQMKHKYDAGNLEEGLSLNEIAAQMYVFLVA

GFETTATALSLALYELARNEHIQSQLRGEIEEVIANYGVNGELSYEALQKMKYLEQVISE

TLRMYPVASEHLRRANAHYEFPNHPNYYIPAGAQLIIPVYSIHHDPEYYPEPEKFLPERF

TEEAIQQRPTCAFLPFGMGPRICIGMRFGKIKLAVGLISLLRHFRFSLSPDTPQPMEFTK

HSFLLHTKCFKLLVEPLK

>EDW32572.1

MWTLLALVLVAGALIYSRLLEHYDYWRRKGVAGEKPVLFLGNMSGLGKGLHWTDINRRIY

EKFSKTERFCGYFTFFSKAFFVMDLELIKKITISDFSSFADRGLFHNVKDDPLTGNLLFL

DGPEWRWLRQNLTQVFTSGKMKFMFPTMLSVGEKLTKACELQVGEIEAKDLCARFSTDVI

GSCAFGLECNSLNDPNSEFRRMGRSVTTNPLHTGMVQGFMFAQPKLARMLHMRLFRPEVS

EFFLKTVRQTLAYRKRENVQRSDLIQLLMDLGEEGTKDGLSFEQIAAQAMVFFLAGFDTS

STTMSFCLYELALNPDVQDRLRSEILEVLAKNNHQLTYESIQEMPYLDQVVAETLRKYPI

LPHIVRQTTKKYDVPDSRLTLEPGMRVMIPVHSIQHDPELYPEPENFDPSRFEPDQVKAR

HPMAYLPFGEGPRNCIGERFGKMQVKIGIAYILRDFQFRQSERTQIPLKFSSRTFLIGTQ

EGIHLIMEKLTAP

>EFR21004.1

MELIGYVLTALLFVVSIAYLYVRSRHNFWRDRGIAYPREQPHLLYGHMKGALDARHECLI

NQDLYNDYKQRGEAIGGKSFFIIPSLIVIEPELVKTILVKDFNVFHDRGVYNDAKADPLS

AHLFALEGQPWRLLRQKLTPTFTSGRMKQMFGTIQEVAEEFRKYMEENCDQEEIEMKDVL

ARFTTDVIGTCAFGIECNTLKNPDSDFRKYGNKVFEQDLLLMVKFIFASLFKGISRRIGV

KLTDEGVERFFLQVVQQTVQYREKNNVQRNDFMNLLLQIKNQGKLAEQDEVQLGKGEAGL

TMNELTAQAFVFFIAGFETSSTTMNFCLYELAKNPDIQERLREEINRAIEENGDKVTYDV

VMNIQYLDNVINETLRKYPPVESLTRVPVRDYTIPGTKHVIPKETLVQIPVYALHHDEDH

YPEPEQFNPDRFLPEEIQRRHPYVFLPFGEGPRICIGLRFGLMQAKLGLITLLRNFRFAP

TSRTPANIEFDPKSFILSPVTGNFLKVDKIV

>Q9V4U9.1

MLTLLVLVFTVGLLLYVKLRWHYSYWSRRGVAGERPVYFRGNMSGLGRDLHWTDINLRIY

RKFRGVERYCGYFTFMTKSLFIMDLELIRDIMIRDFSSFADRGLFHNVRDDPLTGNLLFL

DGPEWRWLRQNLTQVFTSGKMKFMFPNMVEVGEKLTQACRLQVGEIEAKDLCARFTTDVI

GSCAFGLECNSLQDPESQFRRMGRSVTQEPLHSVLVQAFMFAQPELARKLRFRLFRPEVS

EFFLDTVRQTLDYRRRENIHRNDLIQLLMELGEEGVKDALSFEQIAAQALVFFLAGFDTS

STTMSFCLYELALNPDVQERLRVEVLAVLKRNNQKLTYDSVQEMPYLDQVVAETLRKYPI

LPHLLRRSTKEYQIPNSNLILEPGSKIIIPVHSIHHDPELYPDPEKFDPSRFEPEEIKAR

HPFAYLPFGEGPRNCIGERFGKLQVKVGLVYLLRDFKFSRSEKTQIPLKFSSRNFLISTQ

EGVHLRMEGLERP

>AFM08393.1

MWFLTIEALVAVLALLGGLVYLVKRKQSYWKDRNVPHPSTTFFLGSFKDAGTKMHFSDYL

EQYYTTYKGKHPFTGAYMLISPIVLPTDLELVKSILVKDFQYFHDRGTYYNEKHDPLTTH

LFNLEGQKWKNLRNKLTPTFTSGKMKMMFPTVVAAGKQLKEFMDENVQKNSELEMKDIMA

RFTTDVIGTCAFGIECNSMRDPNAEFRAMGKLFVERQPSQFVNIMVQFSHKLSRMLGIRL

IDKEVSTFFLKVVKDTIDYRVKNSIQRNDFMDLMIRMLQNTENPEESLTFNEVAAQAFVF

FFAGFETSSTLLTWTLYELALNPEIQEKGRQCVKEILEKHNGEMTYEAILDMKYLDQILK

EALRKYPPLPIHIRVAKQDYRVPNTDSVIEAGTLVLVPIYAIQRDPDIFPEPEKFDPERF

SPEEEAKRHPFAWIPFGEGPRVCIGLRFGMMQARIGLAYLLQGFSFAPYEKTSVPMKFVT

NNILLSPKDGLWLKVNKL

>EFR21006.1

MELVSIIIAVIALCATVVYLFLRRQQNYWKSRGFPTLPDEPHLLYGHTKGVNTVRHASAV

SAENYQRLKRSGAAFGGINILILPVVTLVDPEVIKTILVKDFNVFHDRGMYSDADADPLS

ATLFALAGKSWRSLRHKLTPTFTSGKMKFMFGTILEVADELQRYVDEHAVQGELEMKDIL

VRFTTDIIGTCAFGIECNTLRNPDSEFLKYGHRVFEQTSYKMLKVMFIMTLPKLAKKVGV

KVTDTELETFFLGLVRETVEHREKHNVQRNDFLNLLIEIKNKGSVQDGGDAIAPEEPGMT

MNELAAQVFIFFLAGFETSSTTMNFCLYELVKHPDIQERLRNEINAAIEANGGQLTYDVV

MNIQYLDHVINETLRKYPPLETITRAPERDYTVPGTTHVIPKGAMIQIPIYGLHHDPDYY

PNPERFDPERFTPEEVKKRPAYVFLPFGEGPRNCIGLRFGLMQTKVGLITLLRNFRFSAT

ERTPESITFLPSVFVLTPDKGNYLQVDKL

>EDW00822.1

MELLTVILLGVVLALVYHFYQNTYKYWERRGVPFEPPVPILGNMRGVGYKYHFRDINQRL

YDRFKGTTPFGGIFTFLRRVAFIVDLDLVKQVLIKDFTLFQDRVTFSNVRDDPLTGHLFS

LEGEEWRSMRHKLTPVFKSGKMKHMVGIVVDVGKRLAETMDKAVVGSSSEVEIKELCACF

TTDVIGTCAFGLECNSLADPNAKFRANGRQLFEKPRHNQFVQAFIFSNVKLARMLRMKTF

PDDVSEFFMQAVRDTVDHRKQHNIKRNDLLDQLIELRAENEEAARLGKGIDLSHGLTIEQ

MAAQAFIFFIASFENSANAMAFFLYELALHQDMQERVRQEIETVLKETSNGEQLTFDAMQ

NMPYLDQVLAETLRKYPIVPHLTRVANKDYKVDGTQLVIEKDTMVLIPVHSIHHDPQIYP

SPETFDPSRFEPEAIKARHQYAYLPFGDGPRNCIGERFGKMQAKIGLVTLLRSFKFGVSK

KTDVPLIFSTHNFTLRPQAGIHLKVERI

>EAL26030.1

MWTLLALVLVAGALIYSRLLEHYDYWRRKGVAGEKPVLFLGNMSGLGKGLHWTDINRRIY

EKFSKTERFCGYFTFFSKAFFVMDLELIKKITISDFSSFADRGLFHNVKDDPLTGNLLFL

DGPEWRWLRQNLTQVFTSGKMKFMFPTMLSVGEKLTQACELQVGEIEAKDLCARFSTDVI

GSCAFGLECNSLNDPNSEFRRMGRSVTTNPLHTGMVQGFMFAQPKLARMLHMRLFRPEVS

EFFLETVRQTLAYRKRENVQRSDLIQLLMDLGEEGTKDGLSFEQIAAQAMVFFLAGFDTS

STTMSFCLYELALNPDVQDRLRSEILEVLAKNNHQLTYESIQEMPYLDQVVAETLRKYPI

LPHIVRQTTKKYDVPDSRLTLEPGMRVMIPVHSIQHDPELYPEPENFDPSRFEPDQVKAR

HPMAYLPFGEGPRNCIGERFGKMQVKIGIAYILRDFQFRQSERTQIPLKFSSRTFLIGTQ

EGIHLIMEKLAAP

>ADY68485.1

MIALLLIAAVTLVFLFIKQRFNYWKVRGVPYVQPTFPLGNLGGVGRKKHLSEALEDLYIQ

LKGKASIGGIYFFINPVVLVTELDLAKTILVKDFNFFHDRSFYYNEKDDPLTAHLFTMEG

VKWKNMRIKLTPTFTSGKMKLMLPIIRDCANELEKCIEKETANGEEVEIKDILARYTTDV

IGNCAFGLECNSLRDPNAEFREMGRKIFQLEGFGFLKLLFTQQFRTISRALGATILQPDV

AKSFLKTVKDNVEYREKNKVERNDFIDLMVKLKNGQALEHENSEHRMEKLTIEQVAAQSF

VFFFAGFETSSTLMSFCLYELAENQDLQEKVRKDVLDTLKKHGSLSYEPIHEMKYLENCI

NETLRKHPPASNIFRTATQDYIAPGTSITIEKGTSVMIPTLAIHMDPEYYPDPERFDPDR

FNADQVAARHPFAFLPFGEGPRVCIGMRFGLMQTRVGLATLLKNFRFTVGKRLETPVKLE

PSSGILLIKGGLWMKVDKI

>EDW60864.1

MFASTLLLACVLIGFYIWIRQRLSYFERRGIAHEAPLPLVGNLWGWRVTRHTSEIVQNLY

VRYKHLGPLIGFYFLMTPAYIISDMELLKRVFIKDFANFDGRGLFYNERDDPLSAHLFAI

DGPKWHSLRPRMSPIFSSAKMKLMFPAVLRTARELSKVLHERCSGKAASLEVTKLLACYT

ADTIGSAIFGLDCNSLRNPKAPFVQMGLNAMLKKRIDYIFCLLFPELSLRLHIKRTPADV

EAFYMGLVKDTISHREQHKIKRNDFVDIMLEMKQKYDSGNTAEGLTVNEIAAQMYVFLVA

GFETTSTALTLALYELARHEHIQSQLREEIEEVIANYGENGELNYEAMQKMKYLEQVISE

TLRMYPVATEHMRRANGHFEVPDHPKHYIPAGSHLIIPVYAIHHDPEYYPEPEKFLPERF

TEEAIQKRPSCAFLPFGLGPRICIGMRFGRIKLAVGLIALIRNFRFAFAPTTPQTMQFEK

HSFLLHTKGFQLQVEPLG

>EDW05267.1

MKYMFPTVVKISHEFIKVFGDMADKSSVIEVRELLARFTTDVIGNVAFGIECNSLKDPEA

EFRVMGRKSLTDKRHGLVGIAVLTSFPKFAKRLHLKGTAEHIEKFFMKIVRETVDFREKN

NIRRNDFMDQLIDLKNNRLLKTETGEDTSLTIEEIAAQTFLFFAAGFETSSTTMGFALYE

LAQNVEIQNRLRQECKEVLAKYNGDLTYECIKDMQYLNQVISETLRLYTVLPILNREALE

DFVVPGYPKYVIKQGTMVIIPAGPMHRDEKLYPEPNRFNPDNFEPEKVKNRDSVEWLPFG

DGPRNCIGKRFGEMQARIGLAMLIKDFKFSVCDKTVIPIKYDKTNFVVSSEGGIHLKVER

V

>ADY68481.1

MLAYLLAVVVALLTAAALYIRSRHRFWSDRGIPCAPNPEFLLGHVRGVVTNKHAAHVNRE

LYQDFKKRGESFGGFSFFAVPAVLVVNPELVKTMLVRDFAVFHDRGIYNNPKDDPLSGQL

FLLEGLQWRVLRQMLTPTFTSGRMKAMFGTIMDVAEEFRRFMVECRERESVIEMKEVLAS

FTTDVIGTCAFGIECNTLKNPDSDFLKYGKKVFEQKLSTLVKFIFASLFKDLARTMGIKI

TGRRGGESSSWGWCERRWEFREKNNVLRNDFMNLLLQLKNKGRLVDQLDEAEEAAARGLT

MDELAAQCFVFFIAGYETSSTTMNFCLYELAKNPDIQERLREDIEEAVAANDGQVTYDLV

MGLRYLDNVVNETLRKYPPIESLNRVPNSDYTVPGTNHVLPAQTMITIPVYALHHDPDYY

PDPDRFDPDRFLPEVVQGRHPYAYIPFGEGPRNCIGMRFGVMQTKIGLLTLLRNFRFSPS

PKTPDKIVFDVKSFVLSPEGGNYLRYDKI

>ACG68822.1

MDLLGYVLTAFVFVVSIAYLYLRSRHNYWRDRGIPYARGKPHLFMGHMEQSNTKHTSYIN

QEIYQDLKSRGETFGGMNILFLLGLVVVDPELVKTILVRDFNVFHDRGVFNDAKSDPLSA

HLFALEGPEWRVLRQKLTPTFTSGRMKQMFGTIQQVAQEFLKYMNENSHREMEMKDVLAR

FTTDVIGTCAFGIECNTLKNPDSEFRKYGNKVFEQDAVIMVKFVFVMMFKGLAKRIGVKL

TDEGVERFFLQVVRDTVQYREMNNVQRNDFMNLLLQIKNKGYLDERDIGANDNLKGETAM

TLNELAAQVFVFFLAGFETSSTTMNFCLYELAKNPDIQERLREEIERGVEDHGGQVTYEM

VMNNQYLDNVINETLRKYPPIESLSRVPIRDYTIPGTKHVIPKNTLVQLPVYAIQRDPEF

YPEPDQFNPDRFLPEEVKQRHPYAFLPFGEGPRICIGLRFGMMQAKLGLITLLRNFRFSP

SSQTPSELVFDPKSFILSPSTGNYLKIDKL

>EDW60861.1

MLTFLLLLVTAGGVIYVWLNQHYTYWRRRGVPGERPVFLLGNMRGFAQRFHWMDINWRIY

DKFRGRERFCGFYTFLSKALFVLDLELIKRIMISDFNSFADRGLFHNVRDDPLTGNLLFL

DGEQWRWLRQHLTPVFSSGKMKYMFPTMVQVGEQLALSAEQQLGEFEAKDLCARFTTDVI

GSCAFGLECNSLANPGSEFREMGRSITERPLHQGIVQAFMFAQPQLARKLRLRLFRPEVS

QFFMDTVRQTLEYRKREHVKRNDLIQLLIELGEHTDETKAALSFEQIAAQAMVFFLAGFD

TSSTTMSFCLYELALNPAVQQQLRQEIQSTLKRHKQQLGYECLQEMTYLNQVVAETLRKY

PVLPHLVRRTTNAYNVPESSLILEPGTRVMIPVHSIHHDPDIYPEPESFDPARFEPEAIQ

SRHPFAYLPFGNGPRACIGERFGLMQVKIGIVTLLRHYQFRSTQRTQIPLKFSKKNFLIA

TESGVHLQMERLDIDLP

>ABC87786.1

MELINAVLAAFIFVVSAVYLFIRNKHNYWKDNGFPYAPNPHFLFGHAKGQAQTRHAADIH

LELYKKFKQRRERYVGMSQFMIPSLLVIDPELVKTILVKDFNVFHDHGVFNNARDDPLSA

HLFALEGNPWRLLRQKLTPTFTSGRMKQMFGTLWDVALELDKYMEENYRQPDIEMKDVLG

RFTTDVIGTCAFGIECNTLKTPDSEFRKYGNKAFEFNLMIFLKTFLASAYPSLVRKLRMK

ITFDDVEQFFLKIVKETVEYRESNNIKRNDFMNLLLQIKNKGKLDDSDDGSVGKGEVGMT

QRELAAQAFIFFLAGFETSSTTQSFCLYELAKNPDIQERLRQEINQAIEENDGQVTYDVA

MNIQYLDDVINETLRKYPPVESLSRVPSVDYVIPGTKHVIPKRTLVQIPVHAIQHDPEHY

PDPERFDPDRFSPEEVKKRHPFTFLPFGEGPRVCIGLRFGVMQTKVGLITLLRKFRFSPS

ARTPDCVKFDPKMIILSPIAGNYLKVEKL

>AFM08405.1

MELVVASLVSLGAILATFCLGVYLFVTRKYSFWRAHNVPYVEPELPYGNFKEMGKSIHPA

HLSQRFYEQYKSDEGPGFVGLYIFVNPVLLVTNLRLAKRILIEDFHHFPDRGVYYNEKDD

PLSAHLFAIEGQRWKDLRAKITPTFTSGRMKAAFPLVLDIAEQFCDFLRERYTSSDMVEV

RDLMARFTTDVIGSYAFGLECNSFRDPQNEFRRIGRKHFDTPRNHPLKVFIMKTFRGLAN

RLGLKLLHDDVATFFQSVIRQTIEHRERHGIGRSDFLDLLIRLKNTGSLEGSNEIVGRLS

GDEIAAQAFIFFTAGFETSSSAMTYTLYELALNQELQQRARDCVMDALEKHAGVLSYESS

KNMLYLDQCIYETLRKYPPVAILERTVAKPYRLSDTNIVLHRGMKIMIPAYAIHHDPDIY

PNPATYDPDRFTPERMARRDPCAYLPFGEGPRICIGLRFGMMQARIGLALLLKHFQVLPC

KETDEPLTYSTQAFVLTPVNGVRLRLAKYELNEP

>EDX03842.1

MDFTLLLLTSLLSFLLGYLRYRFTYWELRGIPQLRPHFLFGHFFRLQSVHLSELLQETYD

AFRGSAQVAGTYVFLRPLAVVLDLDLVKAVLIRDFNKFVDRRSFHGDTLTANLFNLQGEE

WRALRTKLSPTFTSGKMKYMFGTVSSVAHQLGGTFDELVGSQGAVLELHDLMARYTTDVI

GSCAFGTECNSLRDPQAEFRQVGRRLFQNSNRSIRWRIFRMTYLSSLVKLGLPVRIFHPD

ITKFFNRIVRETVELREREHIRRNDFMDLLLDLRRQENGKGLTMEQMAAQVFVFFVAGFE

TSSSNMSYALFELAKNQDVQQKLRMEINDSLAKHGKLTYEAMMEMPYLDQTITETLRKYP

ALSSLTRLASEDYEIPSPDGGDPVVLEKGTSVHIPVLAIHYDPEVYPEPHEFRPERFAPD

ACRERHPTAFLGFGDGPRNCIGLRFGRMQVKVGLITLLRRFRFRLPPGSPTQLKVTKRNV

ILLPSEGVRLQVDPIESRLM

>EAT37909.2

MWIYLLIAAITLSVLLVRRKYSYWKRHGVPYIKPTFPFGNIRPAGNRVHSSQLMTRYYNE

LKGKHQFGGIFFFTNPVALALDLEFIKDVLVRDFQYFHDRGMYYNERDDPISGHLFNIEG

TQWTNLRKKLLPTFSSGKLKMMSPTIISVAERFQECLEKCITVDTEIEMKDLLARFTTDV

IGTCAFGIDCNSLNDPEVEFRKMGNKMFELPTGRILKFFFISTFKNLARKARLKSVPEDV

SEFFFRVVRETIDYREKSHIQRNDFMNLLMQLREKGALEGSDEKVGTLSMNEVVAQAFVF

FLGGFETSSTTMSYCLYELSLHEDIQERARECVQSAIAKHGGFNYDAVMDMNYLEQCIES

LRKYPPGANLVRCATKDYQVRNSSVVFKKGMSVMVPIYAIHHDAEYYPDPERYDPERFGV

EELAKRPPFTFMPFGEGPRICIAARFGMMESKIGLAALLMNFKFSKCSKSIVPLVISNKH

VVLTPAGGLWLKVEKLEQ

>AAL91655.1

MDLLSYVLTAFVFVVSIAYLYLRSRHNYWRDRCFPYTRQKPHLLYGHMEQFQSKHASYIN

EELYWEFKNRGEPIGGMSMFFLPGLIVADPELVKSILVKDFSVFHDRGVFNDAKADPLSA

HLFALEGHEWRVLRQKLTPTFTSGRMKQMFGTIQQVAGEFLKYMNEHCHREIEMKDVLAR

FTTDVIGTCAFGIECNTLKNPDSDFRKYGNKVFEQDTLLMLKFMFATTFKGLAKRIGMKL

TDEGVERFFLQVVRGTVEYREMNNVQRSDFMNLLLQIKNTGSLDGGDVPIKGAAGLTMNE

LAAQVFVSFLAGSETSSTTMNFCLYELAKNPDIQGRLREEIERAVEENGGEVTYDMVMNV

QYLDSVINETLRKYPPIESLSRVPMRDYTVPGTKHVIPKDTFIQIPVYALHRDPEFYPEP

DQFNPDRFLPEEVKKRHPYVFLPFGEGPRICIGLRFGVMQAKIGLITLLRNFRFTPSSQT

PAKIVFDPKSFILSPVPPVNYLKVDKI

>EDW54394.1

MDFTLLLLTSLLSFLLGYLRYRFTYWELRGIPQLRPHFLFGHFFRLQSVHLSELLQETYD

AFRGSAKVAGTYVFLRPLAVVLDLDLVKAVLIRDFNKFVDRRSFHGDTLTANLFNLQGEE

WRALRTKLSPTFTSGKMKYMFGTVSSVAKQLGGTFDELVGSQGAVLELHDLMARYTTDVI

GSCAFGTGCNSLRDPQAEFRQVGRRLFQNSNRSIRWRIFRMTYLSSLVKLGLPVRIFHPD

ITKFFNRIVSETVELREREQIRRNDFMDLLLDLRRQENGKGLTMEQMAAQVFVFFVAGFE

TSSSNMSYALFELAKNQDVQQKLRMEINDSLAKHGKITYEAMMEMPYLDQTITETLRKYP

ALSSLTRLASEDYEIPSPDGGDPVVLEKGTSVHIPVLAIHYDPEMYPEPHEFRPERFAPD

ACRERHPTAFLGFGDGPRNCIGLRFGRMQVKVGLITLLRRFRFSLPPGSPTQLKVTKRNV

ILLPSEGVRLQVDPVESRLM

>AAO62001.1

MELVVTSVISLGVLLATLCLCVYLLVVRKYSFWRSYHVPYVEPELPYGNFKEMGKSIHPA

HLSQRFYEQYKAVPGSPGFVGLYIFLNPVLLVTDLKLAKRILIEDFHHFPDRGVYFNEKD

DPLSAHLFAIEGQRWKDLRAKITPTFTSGRMKAAFPLVLGIAEQFCGFLREQYTSDDVVE

VRDLMARFTTDVIGSYAFGLELNSFRDPQNEFRRIGRKHFDTPRNHPLKVFIMKTFRGLA

NRLGLKLLHDDVAAFFQSVIRETIHHREGHGVRRNDFLDLLIRLKNTGSLEGSHEIVGRL

SGDEIAAQAFIFFTAGFETSSSAMTYTLYELALNQEAQRKARECVLEALAKHDGVVSYES

SKNMLYLDQCIYETLRKYPPVAILERIVTKPYRIPDTSVTLHPGMKIMIPAYAIHHDPDI

YPEPATYDPDRFTPERMARRDPCAYLPFGEGPRICIGLRFGMMQARIGLALLLKHFQVLP

CKETDVPLTYSPRAFVLTPVNGVRLRLVKYDAHGAAEGN

>EAA12475.1

MELVVTSVISLGVLLATLCLCVYLLVVRKYSFWRSYHVPYVEPELPYGNFKEMGKSIHPA

HLSQRFYEQYKAVPGSPGFVGLYIFLNPVLLVTDLKLAKRILIEDFHHFPDRGVYFNEKD

DPLSAHLFAIEGQRWKDLRAKITPTFTSGRMKAAFPLVLGIAEQFCGFLREQYTSDDVVE

VRDLMARFTTDVIGSYAFGLELNSFRDPQNEFRRIGRKHFDTPRNHPLKVFIMKTFRGLA

NRLGLKLLHDDVAAFFQSVIRETIHHREGHGVRRNDFLDLLIRLKNTGSLEGSHEIVGRL

SGDEIAAQAFIFFTAGFETSSSAMTYTLYELALNQEAQRKARECVLETLAKHDGVVSYES

SKNMLYLDQCIYETLRKYPPVAILERIVTKPYRIPDTSVTLHPGMKIMIPAYAIHHDPDI

YPEPATYDPDRFTPERMARRDPCAYLPFGEGPRICIGLRFGMMQARIGLALLLKHFQVLP

CKETDVPLTYSPRAFVLTPVNGVRLRLVKYDAHGAAEGN

>AAA69817.1

MFALLLLFCICLLVGYLLNLYTYWERQGVPHETPIPLLGNFKGIGSKYHVRDINQRLYRR

FKGKAPFAGVYMFVRRAALVIDLNLIKNIMVKDFANFHDRGGLSNVEDDPLTGHLVSLEG

EQWRAMRTKLTPVFTSARMKYMFPTVVKVGENFARTVGEMLEEGSGTQILEIRDLCARFT

TDVIGNCAFGIECNSLKDPNSEFRLKGKEIFSRPRNGPLLHLFAITNDKLASKFHMKILP

DDITEFFMSLVRQTVEYRVKNEVKCNDFMDLLIEMRAKDEELARASKGIDLSHGLTLEQM

GAQAFVFFFAGFETSSITMTFALYELARHQEVQDRLRKEILESLRENKGELTYEAINNME

YLDRVVAETLRFYPPLATVVRVTKNDYQIPNTRYVIKKDIMTIIPIHAIHHDPQYYAEPE

RFNPDRFTPEECLKRHPSAYLPFGDGPRNCIGLRFGKMQVKVGLVSLLSHYRFEFCPLTE

QPLQFNNHHMMVAPKNGVYLKVTPV

>EDW89363.1

MLTLLVLVLTAGLLLYAKLRWHYSYWSRRGVAGERPVYFRGNMSGLGRDLHWTDINLRIY

RKFRGVERYCGYFTFMTKSLFIMDLELIREIMIRDFASFADRGLFHNVRDDPLTGNLLFL

DGPEWRWLRQNLTQVFTSGKMKFMFPNMVEVGEKLTQACRLQVGEIEAKDLCARFTTDVI

GSCAFGLECNSLQDPESQFRRMGRSVTTEPLHSVLVQAFMFAQPDLARKLRFRLFRPEVS

EFFLDTVRQTLDYRRRENIQRNDLIQLLMELGEEGVKDALSFEQIAAQALVFFLAGFDTS

STTMSFCLYELALNPDVQERLRVEVLDVLNSNNQKLTYDSVQEMPYLDQVVAETLRKYPI

LPHLLRRSTKEYQVPGSNLILEPGTKIIIPVHSIHHDPELYPDPEKFDPSRFEAEKIRAR

HPFAYLPFGEGPRNCIGERFGKLQVKVGLVYLLRDFKFSRSQKTQIPLKFSSRNFLISTQ

EGVHLRMDALAAQ

>ABO77953.1

MELINAVLAAFIFVVSAVYLFIRNKHNYWKDNGFPYAPNPHFLFGHAKGQAQTRHAADIH

LELYKKFKQRGDRYVGMSQFIIPSVFVIDPELVKTILVKDFNVFHDRGIFTNARDDPLSG

HLFALEGNPWRLLRQKLTPTFTSGRMKQMFGTLWDVALELDKYMEENYHQQEIEMKDVLG

RFTTDVIGTCAFGIECNTLKTPDSEFRKYGNKAFELDLLIMMKFFFASAYPSLVRKLRMK

ITFDDVEEFFVKIVRETVNYREMNNVKRNDFMNLLLQIKNKGKLDDSDDGSVGKGEVGMT

QLELAAQAFVFFLAGFETSSTTQSFCLYELAKNPEIQERLRQEINQAIEENDGQVTYDVA

MNIQYLDNVINETLRKYPPVESLSRVPSVDYVIPGTKHVIAKRTLVQIPVHAIQHDPEHY

PDPERFDPDRFSPEEVKKRHPFTFLPFGEGPRVCIGLRFGVMQTKVGLITLLRKFRFSPS

ARTPDCVTFHPKMITLSPIAGNYLKVEKL

>EDW46954.1

MLTLLVLLFTVGLLLYAKLRWHYSYWSRRGAAGERPVYFRGNMKGLGRDLHWTDINLRIY

RKFRGVERYCGYFTFMTKSLFIMDLELIRDIMIRDFSSFADRGLFHNVRDDPLTGNLLFL

DGPEWRWLRQNLTQVFTSGKMKFMFPNMVEVGEKLTQACRLQVGEIEAKDLCARFTTDVI

GSCAFGLECNSLQDPESEFRRMGRSVTKEPLHSLLVQAFMFAQPELARKLRFRLFRPEVS

EFFLDTVRQTLDYRRRENIHRNDLIQLLMELGDEGVKDALSFEQIAAQALVFFLAGFDTS

STTMSFCLYELALNPDVQERLRVEVLAVLKRNNQKLTYDSVQEMHYLDQVVAETLRKYPI

LPHLLRRSTKQYQIPDSNLILEPGSKIIIPVHSIHHDPELYPDPEKFDPSRFEPEEIKAR

HLFAYLPFGEGPRNCIGERFGKLQVKVGLVHLLRDFRFTKSEKTQIPLKFSSKTFLISTQ

EGVHLRMDGLARP

>EDW12192.1

MGFVLIVFSLVLTWLFGYLRHCYTYWEQRGVPQVRTHFLFGNFFKLKSIHHSELLQEAYD

AFKGKSKVAGTYVYTSPMAVVTDLDLVKTILIKDFNKFVDRMNTPFNLEKNPLSGHLFNL

HGEQWRALRTKLSPTFTSGKIKYMFSTITSVAQQLDETCRQEVAESAGNGNILELHDLLG

RYTVDVIGNCAFGIECNSLKNPKAEFREVARQLFTGHNRSVRWSLFKRTYAKFLEKMPIR

PRHPHEDFVSFFLRLVRDTVEVRERENIKRNDFMDLLLGLRKTEEKDGLTVDQLAAQVFV

FFVAGFETSSSNMSYALYELAKNPDIQSQLRAEIQSVLQKHGKLTYEAMMEMSYLDQVVN

ETLRKYPALASLTRVPSEDYKVPGDEANIVLERGLSVHIPVCAIHYDPEYYPEPHEFRPE

RFEPAALQQRHPMAFLGFGDGPRNCIGLRFGRMQVKVGLITLLSSYRFLLPADGSGKLTI

GSKNFLLIPDQGIRLRVEPLAV

>AEF79990.1

MSALLLILALTPLLLAIIYIKQKYIYWARRNVPFLKPRFPYGNFHALDRKSIADVSREAY

EQMKNFGPFYGAYFFLQPLITITDPDLIKLVFIKDFNFFPDRGMYFNERDDPLSAHMFAI

EGNKWRSLRQRLSPTFTSGKMKMMFPTLAAVRENFSEFLIEEIGGGKVVEVKDFMAKLTT

DIIGSCAFGIECNSFKDPQGRFREFGKKGFETPPHGSLVRLALKSFPDIARRLRIKALHE

DASQFFYDVVRDTVNYREKSGVERKDFLSLLIDMKKDGVDFTMDEIAANSFIFFGAGFVT

SSSNQTFCLYELARHPDYQEKARQNVLDALRKHGGLTYEAACDMQYLDQCINETLRLYPS

VPVLERRAFKDYKIPGHGVVIPKGMKINVPAFAIHRDERFYPDPDVFNPDRFHPAEVAKR

HICTFIPFGEGPRICIGLRFGMMQSRVGLATILSKFRISTCSETPNPLEYSSKTSVLIPK

EGLWLRVDPL

>EFR27732.1

MELINLVLAGFIFVVSAAYLYLRNKHNYWKDHGFPYAPNPHILFGHAKGQTQTKHGLEIH

QEWYDYFKKLGVPYGGMSQFIAPALVVMDPDLVKTILVKDFNVFHDHGVFVNEKDDPLSA

HLFALEGTPWRLLRQKLTPTFTTGRMKQMFGTIWEVGQHLEKFMLQNYRQSEVEMKDILG

RFTTDVIGTCAFGIECNTLDNPDSDFRKYGNKSFELNPMIMFKFFLASGYPKLIRALKMK

ITFDDVERFFLGIVRETVEYREQNNVKRNDFMNLLLQIKNKGKLDENDNEVVGKGEVGMT

NEELAAQVFVFFIAGFETSSTTQSFCLYELAKNPDIQERLREEINRAIDENGGQVNYDVV

MGIQYLDQVINETLRMYPPVEGLNRVADVDYTIPGTKHVIPKRTLVQIPVYALQHDPDYY

PDPERFDPNRFSPEEVKKRHPYVFLPFGEGPRICIGLRFGLMQTKVGLITLLRKFRFSPS

AKTPAKVTFDPKIITLAPLNGNHLKARRWPIGGAVRYLTPCVIPIDREVIEYLLSEEFIK

IPTIRWNRELIRSWQEFEDVHYEALLGISAEESETIATSVSSSDEGQCVKAIVEQFTMRM

LLQSVLGSNCAQQLEAIKMLLFENRTSLFEKIIVTAFPDSTNVQQLLDALIPNQKSKWMQ

QLKDLLMHADHDRGTNFLTFLKQKRTDGELIGIVASDDTQHSLLELMRTIFYTTSGTIVA

CLYELARHHDIQDRLHEALKNSDNTLTDYLDNVITETLRKYPPQDEISFTSLTSCRLVEC

DLVIPRNTRIIIPTYALHRDAENYPDPMSFNPGRTVASSSHFYSPLGGDGVNLGSTFAML

MVRVALTKLLLHWQIDCIEESCCYEGYVLYFQDRPIVSTTAGQVQGTTEQCGLFCTYYSF

KGIPYAEPPLGALRFSDTVPKAAWSGVRDASNHGATCPSQDMAPTESEDCLFLNVYTPSL

IGTRSIMVFIPGGGFSAGSGDDALYGGQYFMGEDVVLVTLNYRLGALGFLSTGDRAAAGN

WALKDCIEALRWVQRNALAFGGDPSSVTIFGQSAGGVLVHILTLSPLSNGLFQKAIAHSG

LAFNSWAFQPNPRFYANRLAQALGVPSGDTVAMVTALRSIPYRQIVALQDNIVTLTVDAL

LSPIDFGPVVEPE

>EAU76336.1

MWFPTIEVLVALLALLGGAVYFIVRKQSYWKERGVPHPKPTFFFGSFKDAGTKIHFTEEV

ERHYAIYKGKHPFIGVYMLTTPVVLPLDLELIKAIFVKDFQYFHDRGTYYNEKHDPLTAH

LFNLEGQKWRNLRNKMTPTFTSGKMKMMFPTVVAAGQQLRDFMEENVQKHGEMELKDVMA

RYTTDVIGTCAFGIECNSMRDPDAEFRAMGKLFMERQPSQFVNMMVQFSPKLSRLLGIRF

IDKEVSTFFLKVVRDTIDYRVKNGIQRNDFMDLMIRMLQNTENPEEALTFNEVAAQAFVF

FFAGFETSSTLLTWTLYELALNPEVQEKGRQCVQEVLAKHNGEMTYDAIHDMKYLDQILK

ESLRKYPPVPLHFRMTAQNYRVPDTDSVIEAGTMLFIPIFSIQRDASLFPEPEKFDPERF

SAEEEAKRHPFAWTPFGEGPRVCIGLRFGMMQARIGLAYLLQGFSFAPYEKTSIPMKFIT

NSFILGPREGLWLKVNKLESKQG

>ACA42438.1

MELINAVLAAFIFEVSAVYLFIRNKHNYWKDDGFPYAPNPHFLFGHAKGQAQTRHAADIH

LELYKKFKQRRERYVGVSQFMIPSLLVIDPELVKTILVKDFNVFHDHGVFNNARDDPLSA

HLFALEGNPWRLLRQKLTPTFTSGRMKQMFGTLWDVALELDKYMEENYRQPDIEMKDVLG

RFTTDVIGTCAFGIECNTLKTPDSEFRKYGNKAFEFNLMIILKTFLASAYPSLVRKPRMK

ITFDDVEQFFLKIVKETVEYRESNNIKRNDFMNLLLQIKNKGKLDDSDDGSVGKGEVGMT

QRELAAQAFIFFLAGFETSSTTQSFCLYELAKNPDIQERLRQEINQAIEENDGQVTYDVA

INIQYLDNVINETLRKYPPVESLSRVPSVDYVIPGTKHVIPKRTLVQIPVHAIQHDPEHC

PDPERFDPDRFSPEEVKKRHPFTFLPFGEGPRVCIGLRFGVMQTKVGLITLLRKFRFSPS

ARTPDCVKFDPKMIILSPIAGNYLKVEKL

>EDW32574.1

MILTFALLGMALALAYNFYRNTYSYWERKGVPNERPLPLIGNVKGIGTKYHFRDINQRIY

DKFKGVAPFAGMFTFFKRSALIIDLDLIKQVLIKDFQYFQDRGVYNNVRDEPLTGHLLSL

EGDEWRSMRQKLTPVFTSGKIKHMSGVVVEVGHRLAAAMDKAVSAAKVDDGDVEIKDICA

RFTTDVIGTCVFGLECHSLSDPNAEFRSKGRMIFEKPRHYAIVEFFIFTNAKLAKKLRMK

ILPDDLSDFFLDAVKNTVDYRMKNGIKRNDFLDELIELRAENQEAAKQGKVINLSNGLTI

EQMAAQAFVFFLAGFETSSSTMAYCLYELAQHPDIQRRVREEIDAVLKNVEGGEITYDAL

AEMNYLEKVLAETLRKHPIVPQLIREANQNYKVPNTDITIDKGTTMLIPVHNIHHDPEIY

PEPERFDPSRFEENQVKSRHPMAYLPFGDGPRNCIGLRFGKIQTKIGLVSLLRRFKFGIS

KRTEIPLILDTRSFLLATKNGIHLRVERI

>AFM08399.1

MWINLLVLVLLVAVFWVWQRFSYWKRRNIPYITPSFPQGNLTGVGKKRHMSAILQNCYKQ

LKSSGQSYGGIYFFINPVALLIDPELIKVVLVKDFPYFHNRNLYYNDRDDPLSHHLVAME

GTKWRNLRAKLTPTFTSGKMKLMFSTVTAVAEQFHKCMAEEIRHSSVVEMKDLLARFTTD

VIGSVAFGLECNSLQDPDAEFRVMGRKVFQSSPYRALKLFLAAQFPHIARAFHVTLTLPE

VANFFTGVVNDTIDFRKTNNVQRNDFMTLLMKILKEQEESGSIDDGQKDHLTLDDIAAQA

FVFFLAGFETSSTAMSFCLYELALHQDLQDKARQNITEVLKKYDSISYEALHEMKYIDMC

INESLRKYPPATTLTRRVEKDYRVPGTDQVLQKGIMVAIPVYALQHDPDHFPNPERFDPE

RFTPEQSEKRHPFTFLPFGEGPRVCIGLRFGMVQARIGLVYLLKHFRFTLATDKTSVPLK

ITPSSTILTIDGGLWLNVEKL

>AAL58569.1

MWFPTIEVLVALLALLGGAVYFIVRKQSYWKERGVPHPKPTFFFGSFKDAGTKIHFTEEV

ERYYAIYKGKHPFMGVYMLTTPVVLPLDLELIKAIFVKDFQYFHDRGTYYNEKHDPLTAH

LFNLEGQKWRNLRNKMTPTFTSGKMKMMFPTVVAAGQQLRDFMEENVQKHGEMELKDVMA

RFTTDVIGTCAFGIECNSMRDPDAEFRAMGKLFVERQPSQFVNMMVQFSPKLSRLLGIRF

IDKEVSAFFLKVVRDTIDYRVKNGIQRNDFMDLMIRMLRDTENPEEALTFNEVAAQAFVF

FFAGFETSSTLLTWTLYELALNPEVQEKGRQCVQEVLAKHNGEMTYDAIHDMKYLDQILK

ESLRKYPPVPMHFRMTAQDYRVPDTDSVIEAGTMLFIPIFSIQRDASLFPEPEKFDPERF

SAEEEAKRHPFAWTPFGEGPRVCIGLRFGMMQARIGLAYLLQGFSFAPYEKTSIPMKFIT

NSFILGPREGLWLKVNKLESKQG

>AAK32960.1

MWISLLVLTLLAAVYWIRQRLAYWEKRGVPYVPPSFPHGNLGGFGKKLHMSAILQNCYKQ

LKSSGQPFGGIHFFINPVALLIDPDLIKTVLVKDFSYFHDRNLYYNERDDPLSHHLVAME

GTRWKNLRAKLTPTFTSGKMKLMFSTVTAVAEEFDRCMMNEIKTSPVVEMKDLLARFTTD

IIGSVAFGLECNSLKDPEAKFRAMGRKVFQLSPYRALKIFLSAQFPKIARTLRVKLTQPD

VAEFFMGAVKDTVEFRQINNVQRNDFMMLLMKMLKEQMEQDGTAGDLKDRITIEDVAAQA

FVFFLAGFETSSTAMSFCLYELALNQELQDKARQNITDVMKQHSSITYEAVHEMKYIEMC

INESMRKYPPITTLTRRVEKDYRVPDTDKVLQEGIMAAIPVYALHHDPEHFPNPEQFDPD

RFTAEQEAKRHPFVYLPFGEGPRICIGLRFGMMQARIGLVYLLKHFRFTLAKDRMSVPLK

ITPSSTILTIDGGLWLNVEQL

>EFR21003.1

MESISVWLTGFIFLVSIAYLYIRSRHNYWKDNGVPYAPDPHFFFGHVKGQAQTRHGADIN

QELYNQFKKRNLPYGGISLFVMPSLIVVDPELVKTILVKDFNVFHDRGVFTNPKHDPFTG

NLFGLEGNPWRLLRQKLTPTFTSGRMKQMFGTIWQVALELEQFMEDNHRQPDLEMKNILG

RFTTDVIGTCAFGIECNTLKNPDSDFRKYGNKAFEVDAVRLMKIFLAASYPRIGRKLGVA

ITDYGVTKFFMNIVDETINFREQNDVKRNDFMNLLLQIKNKGKLDDENGASVGKGGVGLT

REELAAQVFIFFLAGFETSSTTMNFCLYELVKNPDIQERLREEINRAIDENGGEVTYDVV

MNMPYLDNVINGKLRSSPCWLYPRLISNSNFSILETLRKYPPVETLTRKTSKDYLIPGTK

HVIPEGTIVQIPAYAIQRDPDHYPDPERFDPDRFTPEEVKKRHPYVFIPFGEGPRICIGL

RFGVMQTKVGLITLLRKFRFTPSHRTPDKIVFDPKVFTLSPTAGSYLKVEKVATK

>EDS44714.1

MIYLALIAVATLLVLFVRQRYNYWKVRGVPYLDPVFPAGNVWGLGTRLHLSEVLQKCYEK

LKGKGPFGGVYFFLGPTVLATDLDFIKTILVKDFQYFHDRSFYYNEKDDPLTGHLFTIEG

QRWKNLRAKLTPTFTSGKMKLMFPIVADVAVELKKCLVTEKDDGGEVELKDVLARYTTDV

IGKCAFGLDCNSLENPNAEFREMGRKIFTTTPFSIIKLFFVQQIKPLARKLGVTVLNQEV

TKYFLKAVKDTVEYRESNNVERNDFMNLLIKLKNAEPVDEGSSRSMEKLSLNEISAQAFV

FFFAGFETSSTLMSFCLYELAMNQDLQDRARQNVRDVLSQHGSLTYEAIHDMKYLENCIF

ETLRIYPPASILFRTATQDYRVPNTDFTIEKGTATNIPVLAIHRDPEIYPDPMKFDPERF

NADQVAKRHPFAYLPFGEGPRVCIGMRFALMQTRVGLATLLQNFRFTVSPRTRIPAKINP

ASGIIMAEGGLYLQVDRIGDCEVEIK

>EDS26787.1

MLLYLAVLTLSLAVLWIRKRYSYWSDRGVKYLKPTFPFGNIRGLGRKEQLALAFQRLYLQ

LKGSGPFGGAFFFVNPVAMALDLDFVKNVLVKDFQHFHDRSIYYNERDDPLSAHLVTIEG

TKWRNLRTKLTPTFSSGKMKMMFPTIVGVAEQFRELLLEEVESGGEVEMKEILARFTTDV

IGTCAFGLECNSLHDSDAAFRRMGRKVMEPPPGRLFVVILAQQFRKIARALRVKTIDTDV

SQFFMNAVRETVEYREKNHVERNDFMNLLIKMKNGQQVEDGTNVDTEGLTLEEIAAQAFV

FFLAGFETSSTAMTYCLYELARNPELQQKARDDILKTIEKHGSLSYEAVHDMHYIEMCIN

GQLTRTVSKHYKVPSTEHTLEKGTTVAIPVYGIHHDPELYADPERYNPDRFLPEELAKRN

PYCFLPFGEGPRNCIGLRFGMMQARIGLATLLKDFRIRMSSKTQEPLRIDAKKVVLTFEG

GLWLHIEKIQGK

>EFA05693.1

MFSCFLTNIIGIFVAFFAVIIAFYKWSFGYWKQKNVPYLPPKIPFGNLANPLRPAENMGV

QVQHFYAQMRAKKWKHGGLFFLANPTYMVVDLEYVKNILTKDFQHFVDRGVYYNEDVDPI

GAHLFALGGTKWRNLRTKLTPTFTSGKMKMMFQTLIDCESNLHKKIQAQKNKPIDIKDVL

GCFTTDIIGSCAFGLDCNTFEDEDSPFRKYGKKVFVATPLERLKIAIFMNFPKVGRFFKM

RQVSKDISDFYSKVVKDTVEYREKHNYSRKDFMQLLIDLKNDGNALTLDEITAQSFIFFL

AGFETSSTTMTFALYELAKNSEVQEKVREEVLAVLGKHGGKITYEAIQDMKYMNQVLNET

LRKYPPVPFITRQCIKEYKIPDQEIIIETGTRVIIPILGIHYDPEYYPDPQKFDPERFSE

ENVNKRHHYAHLPFGEGPRICIGLRFGLMQSKVGLASLLSKYRFKLNERTKQPMKFQVKS

FVLAAEGEVWLDAESL

>EAA07776.4

MEPVTIVLTAFIFVVSIVYLFVRSKHRFWKDQGVPHAPKPHLFYGHVKGQSRTRHGADIN

QELYRYFKQRGVPYGGISLFIMPSLIVVDPELVKTILVKDFNVFHDRGVYSNPRDDPFTG

NLFGLEGTPWRLLRQKLTPTFTSGRMKQMFGTIWAVALELEKYMEENYNQPEVEMKDVLG

RFTTDVIGTCAFGIECNTLKTPDSDFRKYGNKAFELDPITLTKFFFATSYPQLARKLHVR

TTQQDVEDFFMKIVRETVDYRETNNVQRNDFMNLLLQIKNKGKLDDHGTVVGKGEVGLTH

NELAAQVLIFFLAGFETSSTTQSFCLYELAKNPDIQDRLREEINRAIEENGGEVTYDVAM

NIQYLDNVINETLRKYPPVETLTRKPSSDYVIPGTRHIVPKDTVVQIPIYAIQRDPDHYP

DPERFDPDRFLPEEVKKRHPYVFLPFGEGPRICIGLRFGMMQTKVGLINLLRRFRFSPSA

RTPERVVYDPKMFTLSPIGGNYLKVDKIA

>AAA69816.1

MMCCPLVVLLLGSLTLFVVYLYRLYTYWQRRGILQETPLPLLGNFKGLGTSHHLRDIYQR

LYGEFKGKAQMVGSYMFMQRSALLLDLDLIKNVLIKDFAYFQNRGGFHNVEDDPLTGHLS

NLEGEQWRALRAKLTPVFTSARMKYMFPTVVRVGEILSDTIRGEISRESPKDQVLEIKDF

CSRYTTDVIANCAFGIECSSLTDPRAEFRQRGIDIFTKPRHHPLLLLLIITNPKVAQRLR

LKLFPDELTEFFMSVIRQTVEYRERNQVKCNDFMDLLMEIRAKEGEEGLSFEQMAALTFD

FFLAGFETSSTTMAFTLYELARNPEIQEKLRTEILDILKDSNDELSYEALQKMSFLEQSI

SETLRLYPVLATLVRVANRDYPVPDSQHIIEKDIQVVIPVHAIHHDPEYYENPEEFNPNR

FAAEECERRHSSTYLPFGDGPRSCIGMRFGKMQTKIGLVSLLRHYRFECCPLTEIPLELD

KKSFFVGSKNGIFVRVVDLKCEK

>EEZ99338.1

MAILTYSWLLDIFGILTALIAVFIAWAQWSHTYWSRRNLIAPKTTFFLGNSKGIVTQKQS

FGDFTVEMYEYIRKHNKPHGGFYLTLMPNYMPVDPEIIKHIMLNDFDHFVDRGIYYNEEV

DPLSAHLFSLDGAKWRNLRIKLTPTFTSGKMKMMFDTLVKCSDQLLVEMNQTAGKAPVDA

KNILARFTTDIIGSCAFGIECNSLKNPNDEFTQYLKLFFVEGFWQNLNGIITFVAPELAK

KLNMKVVNPVLSNFFMKVVEDTVNMRERNNIYRKDFMHLLLQLKNRGELVDDDSILQNNT

DAENKEVTLTLNELAAQAFVFFLAGYETSSTTMTFCLYELASNPEIQDKLRAEINEVLQK

HGKLTYDAIMEMRYMDKVVNETLRKYPPLPGLNRVCNKEYKVPGTDFVIEKGTKIWIPVL

GLHRDPEYFPNPEKFDPERFTEENKRQRHPYTYLPFGEGPRICIGLRFGMMQTKVGLSVL

LKNYKFSINSKTKLPLQLDPKSFIMSTEGGIWLDYTRV

>EAT39034.1

MWLNLLVMVFALSVILVRRRYSYWKRIGVPFIQPRFPLGSIGSIGTRIHSSQLLAQFYQQ

LKGSHPFAGIFYFLQPVALALDLEFVKNVMVRDFQYFHDRGLYYNEKDDPLSSHLFNIEG

TKWTTLRRKLVPTFSSGKLKMMCPTVVSVADRFKMCIEKSIAKEEAIEMRELLARFTTDV

IGSCAFGIECNSLENPDDKFRKMGEKVFDVSPFAILAFFFLSTFKDLARKCRISITDSEV

AAFFSTIVQKTITYREKNNVQRNDFMNLLMQMMKKNKEDESEENSVTLTLDEVVAQSYVF

FLGGFETSRTTMSYCLYELSLNQEVQNRARKCIQSAVAKHDGLNYEALMDMPYLEQCINE

SLRKYPPISNALRSTTKDYAVPGTEVILKKGTDVIVPIYAIHHDPEYYPDPELFDPDRFS

ADQCAKRKPFTFMPFGEGPRMCVASRFGMMETKIGLAAMLMSFRFSKCEKSIVPLKISPN

HLMLTPAGGLWLKVEQLESDETEMGFSKLISDERVNRLGYSM

>EDV35647.1

MLVFLFLVFATGFLVYLKLRWHYSYWSRRGVAGEKPEYFLGNMKGFGKNLHWTDINLRIY

KKFQNTERYCGFFTFVTKALFVMDLELIRRIMVSDFGSFADRGLFHNVRDDPLTGNLLFL

DGPEWRWLRQNLTQVFTSGKLRYMFSNMLKVGEELAESCQKQEGEIEAKDLCARFTTDVI

GSCAFGLECNSLRDPESKFRQMGKSVTTNPHHSIIVQAFMFGQPKLARKFRLRLFRPEVS

EFFLDTVRQTLDYRRRENIKRNDLIQLLMELGEGEGAEGKKEEALSFEQIAAQAMVFFLA

GFDTSSTTMSFCLYELALNPEIQGKLREEIVEVFERYNQELTYESFKEMPYLDKVISETL

RKYPILSHLLRRSTKDYAVPDTDLVLEAETKIIIPVHSIHHDPKIYPQPEVFDPSRFDPE

EVRKRHPFSYLPFGEGPRNCIGERFGKMQVKVGIAYLIRDFKFNRLDKTQVPLKFSNRTF

LITTQKGIHLQMERL

>EFR25765.1

MFIYLLLAVITVAVWYVHRKYSFWADRGVPFVKPRFPFGNIQGIGRRMHSSQLMTKFYNE

LKPSGRSFGGIFFFTNPVALALDLDFVKSVLVRDFAYFHDRGVYYNEKDDPISGHLFNIE

GSKWTNLRKKLVPTFSSGKIKQMCPTIVSVGDRFRECLEGICNSTGGEEVEMKELLARFT

TDVIGTCAFGIDCNSLNDPNAEFLRMGRKVFEVPRGRILKFFFMASFKEFSRRIHIKGTA

EDVSAFFFKVVRETIEYREKHNVQRNDFMNLLMQLKNSGQLLDDSGDEVVGKLSLNEVVA

QAFVFFLGGFETSSTTMSYCLHELSLNHEIQQRARQCVLEAVQKHGGLTYEALMDMPYID

QCINGKPSNEADVRSFFRIITSLSASLLESLRKYPPGANLIRQVNQDYRVPGTDVIFPKG

MNVMIPVYAIHHDPEHYPDPERYDPDRFATERTPFTFLPFGEGPRICIAQRFGMLEARIG

LSVLLMNFAFSRCSKTSVPLVISSRHAVLTPEGGLWLKVEKLKA

>EFR27796.1

MDYISVLLYLVLPILGLGYYYTRQLYSYWSARGIPQLEASFPVGNMKDVGRKVHFNDVMK

VAYDMGKSLGKPFVGLYFMLKPVLILTDLDMVKRVLVKDFNSFHDRGLYVNERDDPLSGH

LFAIDGERWRYLRNKLSPTFTSGKIKQMFVTIREIGDEFLGSVTKYVERDEPIDIKLLCQ

CFTCDVVGSCAFGLQCNSLKNEGSKLLTIGDEVFKPPAWRNMYTFLLISFQELARKLRLR

ALPSSVSDYFVGLVSDTVAHREKNLIERPDFLNMLIQLKNKGTVEGDTPASGTDSAHDKL

TLDEVSAQAFVFFFAGFETSSTTLSFALYELANNPDIQEKVREEIIGKLQLHDNQITYEA

LKEMTYLDQIINETLRMYPPVPQLIRVATKPYPVETVNMTLEKDCMLMIPIYSIHHDPNI

YPNPQQFDPDRFTPEAVHARHTNAFIPFGDGPRNCIGMRFGLLEVKFGIVQMLSKLRFTV

SSRMQMPLKLSKSASILEAEGGIWLNGTKL

>EDS38462.1

MAVALIVLLVCVLLLCIGYVKYQYSYWSRRNVPFIKPRFPYGNFADANRKSIADVAIEQY

NQMKNKGRFFGLYFFLQPLVMITDLDLIKTIFITDFTYFPDRGVYHNFKDDPLSAHLFSL

EGNKWRSLRARLTPTFTLGKMKMMFPTLKAVGDNLSEYLSTAVGSGTELELKDYMVRFTM

DVVGNCAFGIECNSFLEPNSEFRMCGKEFFDSPRHSTMMRLFLRLFPELGQKLRIKWLND

HAAGFFYKLVRDTIDYRQKNSVERNDFMSLLIAMKNKGELSMDETAAQSFIFFLAGFETS

SSNQTYCLYELAKNPEYQDKARECVLKALQKHGGLTYEAVSNMQYLDQCINETFRLYPSV

PVLERKAFRDYKIPGTDVIIPKGMKVHVPSFAIHRDEQHYPDPLRFNPDRFHPEEVAKRH

LCTFLSFGEGPRICIGKRFGMLQSRVGLANVLSKFRIFPCSKTAIPLEYSTRSSVLQPKG

GLWLRLEPL

>AAN05456.1

MELINLVLAAFIFVWSVVYLFIRNKHNYWKDNGFPYAPNPHFLFGHAKGQTLTKHAADIH

LELYKQFKQRGDRYVGMSQFIIPSVFVIDPELVKTIMVKDFNVFHDRGVFTNAKDDPLSG

HLFALEGNPWRLLRQNVTPTFTSGRMKQMFGTLWDVALELDKYMEENYRQPDMEMKDVLG

RFTTDVIGTCAFGIECNTLKTPDSEFRKYGNKAFEFNLSIMIKIFLASSYPELVRALKMK

ITFDDVERFFLKIVRETVDYREQNNVKRNDFMNLLLQIKNKGKLDDSEDIVGKGEVGMTQ

LELAAQAFVFFLAGFETSSTTQSFCLYELAKNPEIQERLRQEINQAVEENDGQVTYDVAM

NIQYLDNVINETLRKYPPVESLNRVPSVDYLIPGTKHVIPKRTLVQIPVHAIQNDPDHYP

DPERFDPDRFNPEEVKKRHPFTFIPFGEGPRICIGLRFGVMQTKVGLITLLRKFRFSPSA

RTPDRVTFEPKMITLSPNAGNYLKVEKV

>EDV57889.1

MDFTLLLLTSLLSFLLGYLRYRFSYWELRGIPQLRAHFLLGHCSRLQSVHLSELLQDTYD

AFRGSAKVAGSYVFLRPLAVVLDLDLVKAVLIRDFHKFVDRRSFHGDALTANLFNLQGEE

WRALRTKLSPTFTSGKMKYMFGTVATVAQQLGATCEELVGAQGAVLELHDLMARYTTDVI

GSCAFGTECNSLREPQAEFRQVGRRIFQRHGKGIRWRIFKMTYLTSLAKLGLPVRIFPPD

ISEFFNRIVRQTVELREREHIRRNDFMDLLLALRRQENGAGLTVEQLAAQAFVFFVAGFE

TSSSNMSYALFELAKNQAVQQKLRLEICDALARHGELTYEAMMEMPYLDQTVTETLRKYP

ALSSLTRLATEDYEILQPDGGEPVVLEKGTSVHIPVLAIHYDPEVYPEPQEFRPERFAPA

ACRQRHPTAFLGFGDGPRNCIGLRFGRMQVKVGLVELLCRFRFSLPPGAPTQLRLPKEHV

ILLPSEGIRLQVEPVDSVESRLM

>EJY58114.1

MFVREVLTRLTNPQLREFCSARNLPTSGKKEKLIRRLLSFSENSQEETVLHTAKGIVTST

PKADEKESESEMSSEDESVGSRGSVRAPQFTFNDIGDSLERFSGERTDRDVHDWLDEFEE

TSENFGWSDAHKFVYSRRLLKGTAKLFVNSSSGLNSWKKLKAALQEEFGGKVSSAEIHEL

LRNRKKKSDESFLQYIYHMQNIAKRGRIEEEAVCEYVANGVNDISVNKVCLYGAKTVAEL

KERVKQYEKMKIQMCELSSQAVKNTNTGGRQKEKMESKSSVKDSTGTANVSDSVRCYNCG

RRGHYANDCDTKSQGPKCFGCGEHGHRAKDCEREKKKAEVNALSDGMPVCEIQVGGSALR

TLFDTGSRHNLLCESVYVKVGKPNLHPTMMSFSGFGAAKTPARGTVDVDVCVGVDKFPSM

TFYVVPPGSKETIVSQESSRNLRASVLLQQRTSLVHTMAFLFTTLCLLLPLLGLLYYYVR

RKFAYWADRGVPYVPGSLPMGSFNDMGSTKHIVELLDAIYKQYRNTHKAVGMFLSINPIL

LAVDLELVKQILVKDFNSFHDRGMYFNERDDPLASHMFSVEGERWRFLRNKLSPTFSSGK

IKYMFLTVREIGLEFLASFEPFMERKEPVEIGIQAQKFTCDVIGSCAFGLSCNALKDEST

ELLDIADRVFNPKPLEMMYMLLLICFRKWAVKLRLKQTPADIERFFVGMVHKTVEHREKN

NITRSDFLQLLMQLKNKGTLEEGEEDSKETISMNDVIAQAFLFFFGGFETSSKALSFALF

ELALNPELQEKARDEVQRTLDKHDGLLTYEALKDMTYVEQIVHESLRKYAPIGNVIRKAN

EPYQIHSPDIILEKGTMVMIPVHSIHHDPEIYPDPSRFDPDRFTPEAISARHSHSFLPFG

DGPRNCIGMRFALLEVKFGIAQLLSRLRFTVNEKTQLPLRYDPKANVASALGGLWLDVER

I

>EDW88907.1

MDFTLLLLTSLLSFLLGYLRYRFSYWELRGIPQLRPHFLLGHFSRLQSVHLSELLQETYD

AFGGSAKVAGTYVFLRPLAVVLDLDLVKAVLIRDFNKFVDRRSFHGDSLTANLFNLQGEE

WRVLRTKLSPTFTSGKMKYMFGTVSTVAQQLGATFEELVGSQGAVLELHDLMARYTTDVI

GSCAFGTECNSLREPQAEFRQVGRRLFQNNGRSIRWRIFKMTYLSTLVKLGLPVRIVHPE

ITKFFNRIVRETVELREREDIRRNDFMDLLLDLRRQEQGKGLSVEQMAAQAFVFFVAGFE

TSSSNMSYALFELAKNQAVQQKLRLEISDAMARHGELTYEAMMEMPYLDQTITETLRKYP

ALSSLTRLASEDYEIASFDGGDPVVLEKGTSVHIPVLAIHYDPELYPEPHEFRPERFAPD

ACRARHPTAFLGFGDGPRNCIGLRFGRMQVKVGLIALLRRFHFSLPPGSPTQLRVTKRHV

ILLPSEGVRLQVDPVESRLM

>EDS26140.1

MSLLNTLLYLVLPVAALIYLFVRRKLSYWADRNVPHAPGSFPMGSLQGMGTKYHMSHILE

RIYQQFKNESPAVGFYLTMKPTLMVTDLELVKQVLVKDFNSFRDRGVYYNEKDDPVSAHL

FAIDGEKWRFLRNKLSPTFTSGKIKYMFNTMQEIGDEFVECFGRYEEREEAVDVKTLCGW

FTCDVIGSCAFGLNCNSLKNEESELITIANRLFKPSPIDAMWQFFFMSFRDLSAKLRLLI

LPRDIAKYFMTLIPETVTYREQNNVTRPDFLQLLIQLKNKGTVDGAEQESSGETLTMNQV

VAQSFVFMLGGFETTSTALTFALFELANNPEIQEKVRVEVSSVLARHDGKITYDALKEMT

YLEQVVNETLRMYPPVPFVARNSNHSYHITHPDVTLDADTMVLVPVYAIHYDPDIYQNPT

RFDPDRFTPEATQARHTHAFLPFGDGPRNCIGLRFALLEVKFGIAVLLSKFRFSTSAKMI

LPLELDISAPMMAAKGGVWLDVNKINSKE

>EAA07732.1

MELINAVLAAFIFAVSIVYLFIRNKHNYWKDNGFPYAPNPHFLFGHAKGQAQTRHGADIH

QELYRYFKQRGERYGGISQFIVPSVLVIDPELAKTILVKDFNVFHDHGVFTNAKDDPLTG

HLFALEGQPWRLMRQKLTPTFTSGRMKQMFGTIWDVGIELEKCMEQSYNQPEVEMKDILG

RFTTDVIGTCAFGIECNTLKTTDSEFRKYGNKAFELNTMIMMKTFLASSYPTLVRNLHMK

ITYNDVERFFLDIVKETVDYREANNVKRNDFMNLMLQIKNKGKLDDSDDGSLGKGEVGMT

QNELAAQAFVFFLAGFETSSTTQSFCLYELAKNPDIQERLREEINRAIAENGGEVTYDVV

MNIKYLDNVIDETLRKYPPVESLTRVPSVDYLIPGTKHVIPKRTLVQIPAYAIQRDPDHY

PDPERFNPDRFLPEEVKKRHPFTFIPFGEGPRICIGLRFGLMQTKVGLITLLRKFRFSPS

ARTPERVEYDPKMITIAPKAGNYLKVEKL

>AAL93295.1

MELINAVLAAFIFAVSIVYLFIRNKHNYWKDNGFPYAPNPHFLFGHAKGQAQTRHGADIH

QELYRYFKQRGERYGGISQFIVPSVLVIDPELAKTILVKDFNVFHDHGVFTNAKDDPLTG

HLFALEGQPWRLMRQKLTPTFTSGRMKQMFGTIRDVGLELEKCMEQSYNQPEVEMKDILG

RFTTDVIGTCAFGIECNTLKTTDSEFRKYGNKAFELNTMIMMKTFLASSYPTLVRNLHMK

ITYNDVERFFLDIVKETVDYREANNVKRNDFMNLMLQIKNKGKLDDSDDGSLGKGEVGMT

QNELAAQAFVFFLAGFETSSTTQSFCLYELAKNPDIQERLREEINRAIAENGGEVTYDVV

MNIKYLDNVIDETLRKYPPVESLTRVPSVDYLIPGTKHVIPKRTLVQIPAYAIQRDPDHY

PDPERFNPDRFLPEEVKKRHPFTFIPFGEGPRICIGLRFGLMQTKVGLITLLRKFRFSPS

ARTPERVEYDPKMITIAPKAGNYLKVEKL

>EFR28291.1

MWFSLEALVAIAAVVGGFVYYVVRKQSYWKDCGVPHPKPAFFFGSFKDAGTKEHFTTQLE

QYYVDYKGKYPFIGAYMFLSPTVLLTDLDLIKTVLVKDFQYFHDRGLYYNEKHDPLSAHL

FNLDGQKWRNLRNKISPTFTSGKMKMMFPTIVGAGKQLNDFMLATVERQDEFELKDILAR

FTTDVIGTCAFGIECNSMKDPDAKFRAIGKRLIEEQPSNWKNILVQLVPKLCKIFGLKII

DTEVSDFLLKVVRETIDYRVQNGIQRNDFMDLLIKMLRNTENPEESLTFNEVAAQAFVFF

FAGFETSSTLLTWTLYELAQNQDIQQRGRQCVKDVLGRHNGEMTYESVVEMKYLDQILQE

SLRKYPPVPMLFRLTAKDYRVPDTDSTILAGTPVFVPIYAIQRDPDLFPEPLKFDPERFS

PEEEAKRHPFAFSPFGEGSRNCIGLRFGMMQARVGLAYLLKGFQFCTYEKTSIPMRFVEN

SFILAPKDGLWLKVNKL

>AAZ94272.1

MVFSTDNLLLDLFGVLVALSAVLFVYIKRSFQYWDRKGVPYLPPNIPWGNLQPPHSRDIP

EGDDVANIYYKAKAKGWKYIGIYVMTGSVFLPVDLELIKHITTKDFQHFVDRGTYVNEKD

EPITAHLFSIGGKKWRNLRTKFTPTFTSGKMRQMFETIANCGHILEKYIEHEVDHHEPLD

IKNVLACYTTDIIGSCAFGLDCNSFKEPNSPFTQFGQRVFRTDGIRNLKITFMGAFPNLS

KMLRMRLTEKEVEDFYTKVVEDTVRYREKEGVTRPDFLQMLIDIKNKTNEHTGDGTSLTM

DEIVAQSFVFFIAGFETSSTTMTFALYQLATHPEIQEKVRSEINSVLEKHNNQITYDALN

ELKYMGKVIDETLRMYPALPVVTRRCVEDYRIPDSDVIIEKGIEVFIPIKAIHYDPEYYE

NPEVFDPERFNEENIQGRHPYAHIPFGEGPRICIGLRFGVMQSKVGLVSILKNFRVTLSS

KTKLPLKIDVNSFIPTTEGGMWLNLERIGK

>EFA12631.1

MFTFVGVAIALLATLFAYFKWTYKYWERRNLQYLEPKIPYGNGTGPFKRTENAGFRFKRQ

YEELKSRGWKHGGLYSILNPIYLVADLDYIKNIMTKDFHYFTDRGIYYNEKNDPLSAHLF

ALGGPKWRHLRTKLSPTFTSGKMKQMFQTLVDCVPQLLQQIDKNVPVDIKEVLGCFTTDI

IGSCAFGLECKTFEDENSPFREYGRKFFQVSNLKIAKFLFSGNFPNVARTLGVTITSKDV

SDFFISVVEDTVKYREKHNLVRKDFMQLLIDLKNTEKEQMLTIEELAAQCFVFFIAGFET

SSTTMTFALFELAKRPDLQQQVRDEIETVLAKHGNITYDAIQDLKFMDQVIDETLRMYPP

VPVLTRKCVKDYKIPDQDVIIQKGTRVFIPVLGIHYDSDLYPNPSQFDPDRFSEEKKKSR

HGYAHLPFGEGPRICIGMRFGLMQTKVGLTALLKNYKFSVNSKTQTPLKMKPNSFILAAE

GDVWLNAQKV

>EDS38448.1

MSLVLVVLLVTVLLLCIGYVKHKYSYWARQNAPFIEPQFPYGNFKDSNKTSIADLSIQQY

NAMKDCGSFFGLYFFLQPLLMITDLDLIKTIFIKDFNYFPDRGIYHNERDEPLSGHLFSI

EGNKWRSLRTRLTPTFTSGKMKMMFPTLKAVGDNFAEYLTNMVGSGAEIEVKDGVARFTT

DVIGSCAFGIECNTFLEPKSQFREFGIQVFDDPLHSGMVRMFLRLFPELGRKLRIKVFRE

EAAKFFHKLVADTIAYRETNSVERNDFMSLLIAMKNKGDLTLDETAAQSFIFFLAGFETS

SSNQTYCLYELAFKPEYQEKARACVLKAMEKHGGLTYEAVNDMQYLDQCINETLRLYPSV

PVLERKTFQDYRIPNSDVIIPKGMKVQIPVFAIQRDEQYYPNPTVFNPDRFHPDEMAKRH

MCTFLSFGEGPRICIGLRFGMLQSRVGLATVLSKYRISPCSRTAIPLEFSVKSGVLQPKE

GLWLKVEPL

>AAR88142.1

MTLLGVILLGVVLLLSVAYLFLRERHSFWRKRGFPCMPNPSLLFGQMEGNGSTKHAAYVT

QEIYNNAKDRGERFLGYSFFFMPLVMVYDIELVKTILVKDFTVFHDRGIYSNARIDPLSG

NLFALEGHEWRELRQKLTPTFTSGRMKQMFGTMLEVAEKLHKYILTNVDREMEMKDVLGR

FTTDVIGTCAFGIECNSLDNPNSEFLQYGKRVFEHRLFALVKMTFAMLFRNTATKLGVKV

TDADLETFFLNLVHETVEYRERNDVQRNDFLNLLLQIKNKGCLEEQEDGQTVPNGSTGMT

MNELAAQVFIFFVAGFETSSTVMNFCLYELAKNADIQERLREEINRTIENNGGELTYEVV

MGQEYLGQVVNETLRKYPPLETTLRVTSQDYTIPGTEHVIPRKVGVQIPVFAIHRDPELY

PDPECFDPDRFTKEECKKRPAYTFLPFGEGPRMCIGMRFGMMQVKVGLATLLRSFRFFPS

EKTPERIVFDPKSFILSPTGGNYLRVEQV

>AEN19672.1

MSLLNTLLYLVLPVAALIYLFVRRKLSYWADRNVPHAPGSFPMGSLQGMGTKYHMSHILE

RIYQQFKNESPAVGFYLTMKPTLMVTDLELVKQVLVKDFNSFRDRGVYYNEKDDPVSAHL

FAIDGEKWRFLRNKLSPTFTSGKIKYMFNTMQEIGDEFVECFGRYEEREEAVDVKTLCGW

FTCDVIGSCAFGLNCNSLKNEESELITIANRLFKPSPIDAMWQFFFMSFRDLSAKLRLLI

LPRDIAKYFMTLIPETVTYREQNNVTRPDFLQLLIQLKNKGTVDGAEQESSGETLTMNQV

VAQSFVFMLGGFETTSTALTFALFELANNPEIQEKVRVEVSSVLARHDGKITYDALKEMT

YLEQVVNETLRMYPPVPFVARNSNHSYHITHPDVTLDADTMVLVPVYAIHYDPDIYQNPT

RFDPDRFTPEATQARHTHAFLPFGDGPRNCIGLRFALLEVKFGIAVLLSKFRFSPSAKMI

LPLELDISAPMMAAKGGVWLDVNKINSKE

>EDS35555.1

MILYLLLSILVLSFLWIQRQYSYWERRGVPYVKASFPRGNLQGVGKKFHMSTAIQACYNK

LKGKGPFGGIFFFLNPVVLVMDLELVKHVLVKDFQFFHDRSLYYNEKDDPLTGNLLNIEG

TRWRNLRAKITPTFTSGKMKMMFPTIVAIADQFHALLVRESAEGVEMEMKEALARFTTDV

IGRCAFGLECDSLSNPESIFRQMGSRLFSKRSFRLVVAQQFRELARFLGYVTFDKEVSTF

FLNIVRETIEYREESGVSGKDFMDLMIKLKNSESIDDSEGSSLGALTFNEIAAQAVVFFA

AGFETSATTMSYCLYELALNPALQGKARDEVTHVIRKHGILTYEAAQEMQYVGACIDEAL

RKYPPGPSLSRAVTKNYKVPNTNTTLEKGTSVLIPVYAIHHDPEYFPEPERYVPDRFLPE

QQAARNPYSYLPFGEGPRNCIGLRFGLTVARIGVAYVLKGFRISLSRRTPVPLELSRQKM

ILTIEGGLWLHLEKL

>ACI25098.1

MAILTYSWLLDIFGILTALIAVFIAWVQWSHTYWSRRNLIAPKTTFFLGNSKGIVTQKQS

FGDFTVEMYEYIRKHNKPHGGFYLTLMPNYMPVDPEIIKHIMLNDFDHFVDRGIYYNEEV

DPLSAHLFSLDGAKWRNLRIKLTPTFTSGKTKMMFDTLVKCSDQSLVEMNQTAGKAPVDA

KNVLARFTTDIIGSCAFGIECNSLKNPNDEFTQYLKLFFVEGFWLNLNGIITFVAPELAK

KLNMKVVNPVLSNFFMKVVEDTVNMRESNNIYRKDFMHLLLQLKNRGELVDDDSILQNNT

DAENKEVTLTLNELAAQAFVFFLAGYETSSTTMTFCLYELAANPEIQDKLRAEINEVLQK

HGKLTYDAIMEMRYTDKVVNETLRKYPPLPGLNRVCNKEYKVPGTDFVIEKGTKIWIPVL

GLRRDPEYFPNPEKFDPERFTEENKRQRHPYTYLPFGEGPRICIGLRFGMMQTKVGLSVL

LKNYKFSINSKTKLPLQLDPKSFIMSTEGGIWLDYTRV

>EAT32868.1

MDPVTVILTIFVGLTGLVYFFLRREQQKWPRLGVPFAKNPHLLFGNVRGIFQKEHSCEIL

QRLYWEFKGRGLKLGGIMNFFQPAVLVIDPEISKSILVKDFNKFHDRGIFVDPAGDPLSA

NLFSLEGAQWKAMRTKMSPTFTSGKMKYMFESVLNVAERLKDYLAENCLKEDIELKNILQ

RFTMDVIGNVAFGVECNSIKNPSSEFRLMGLKANRFDGVRFLKFFIGGAYKNFAKKIKLK

VVEDDVHKFFMSLVHSTVHYREGNNVKRNDFLNLLMEIKNKGKFSDEPNSGGEGITMNEI

AAQCFIFFTAGFETSSTTINFCLYELANNPDIQDRLRNEIEDVVAKDGGELKYDTLLGMN

YLDRVVSETLRKYSAVDNLFRISNSPYTPDGCNFTIPAGTLFQIPIHSMHHDPEYFPDPG

RFDPDRFLPEVAKSRHPYCYLPFGEGPRVCIGMRFGLMQTKIGLVTLLRNFRFGPRSETP

DRLQFEAKTFVLTPQTGIYLKIEPIGI

>EAT39033.1

MWIFLLLSIAVLLILQVRRKYSYWKRHGVPFIQPRFPFGSITPVGDRVHSSQLMARFYNQ

LKGTYPFAGMYFFTNPVVLALDLDFIKNVLVRDFQYFHDRGLYHNEKDDPLTCHLFNIEG

TKWTNLRRKLLPTFSSGKMKMMCPTILAIADRFRTAIENSISDQNEIEMRDFLARFTTDV

IGTCAFGIDCNSLENPDAEFLKMGNKIFEVPTSRIIAYFFVSTFQELSKKLHIKAVPEDV

SRFFYKVVRETMAYRQSSGVQRNDFMNLLMQLKEKGELEGSDEKLGTLTLDEVVAQAYVF

FLGGYETSSTNMCFCLYELALNGEIQEKARECVQKAVAKHGGLNYEALMDMPYLEQCIYE

ALRKYPPIANLFRSVTQDYNVPNSNVMLPKGMNVWIPIYAIHHDPEFFPEPELFDPERFT

QEECEKRKPFTYMPFGEGPRTCIATRFGMMETKTGLATLLMNFKFTKSARLEVPPKFSTK

HVMLTPVGGLWVKVEKIEQ

>EFA12637.1

MFSVLVFLIIVVVFLYYKWAFTYWHRRNIPYLEPSFPFGNLPNLFQRKENIGLTVEKFYN

EMKRRKWKHGGVYFLVSPVYCVIDLEYVKNIMNRDFEYFTNRSIYYNEKDDPLSAHLFAI

GGQKWRNLRTKLTATFTSGKMKAMFQTLLECETNLLKQIEDCNNPINIKEILSCFTTDVI

GSVGFGLECKTFEEKNSPFRIYGKKVFKDSVLRSLKRAFLTSNFPELARKLGILAFPSEI

TDFFLKVVADTVKYREENNRSRNDFLQLLIDLKNSGTLSLEEIAAQCFLFFLAGFETSST

TMTFALYELAQHQDIQDKVREEIDAVLKKYGGKITYEAIHDMKYMNQVIDETLRKYPAAS

IITRTCVKDYKIPDQDIVIEKGTSVIIPVLGIHHDEKFYPNPEKFDPERFTEENKAARHH

YAHLPFGEGPRICIGMRFGLVQSKVGLTSLLKNYIFKVNKKTIEPLKMQARSLILAAEGE

IWLDVYKL

>EDW00823.1

MLCLAFLLAIAVGAIYALLDQHYSYWRRRHVPGERPRFLLGNMGGLGRHWHWTDIDWRIY

QRFAGRERFCSFYTFLTKALFLLDLELIKRIMISDFNSFADRGLFHNVRDDPLTGNLLFL

DGEQWRWLRQHLTPVFSSGKMKFMFPTMVQVAKQLALSAEQQLGEFEAKDLCARFTTDVI

GSCAFGLDCNSLANPQSEFREMGRSITERPLHQGVVQAFMFSEPQLARRLRLRLFRPEVS

RFFMETVRQTLDYRKREHIKRNDLIQLLIELGAETEEDKLSFEQIAAQAMVFFLAGFDTS

STTMSFCLYELALNPEIQQRLRLEIKETLKRHGEQLSYECLQEMTYLNQVVAETLRKYPI

LPHLLRRTTKEYQVPGCSLILEKGIRVMIPVHSIHHDPEIYPDPERFDPDRFEVEAIKSR

HPFAYLPFGQGPRACIGERFGLIQVKIGIATLLHHCQIQSTPRTQIPLRFQKKNFLIASE

SGIHLKIERFAAD

>189238292

MFFTQSLLTDVTTLVVTLTVILVTYYKWSYTYWRRRGVPSLTPKIPWGNLSNPLNRKDTI

GVDVKKMYDSMRRQNWKHGGVFFMASPVYLVTDLEYVKDILTKNFQNFVDRGLYYNEKDD

PISAHLFAIGGPKWRNLRTKLTPTFTSGKMKAMFQTLLDCESNLQEKVAAELGKNQAIDI

KDVFGCFTTDVIGSCAFGLDCNTFKEADSPFRKYGKKVFTNTPIRQLKSSFSNSFPKLAQ

QFGISVMPKDVSDFFTKVVKDTVEYRQKNNYTRKDFMQLLIDLKGEGKQDGKSLTMDEIT

AQSFLFFAAGFETSSTTVTFALYELAKHQDIQQKVREEIETVLAKFDGKVTYEAIQEMHY

MNQVLNETLRKYPPLSFLTRECVQDYKIPDQDVIIEKGTKVVISILGMHRDQEFYPDPET

FDPGRFSEGNVVSRHQYAYIPFGEGPRICMGLRFGMMQTKVGLVSLLRKFKFTVNGRTKE

PIKFDVRGFILAAEGDIWLNAQKVC

>EDW74205.1

MSVILLLLVISIIGLIFVWLHQHYGYWRRKGVAQERPLYFLGHMKGFGRSLHWTDINQRI

YEKFSGTQRYCGFFTFLSKALFVMDLDLIRHIMVSDFGSFADRGLFHNVRDDPLTGNLLF

LDGAEWRWLRQHLTPVFSSGKMKFMFPTMVEVSEKLIKVCQSQLGDMEAKDLCARFTTDV

IGSCAFGLECNSLNDPDALFRHMGRSVTEKPLHSGIVIALMFAQPNWARALRLRLFRPEV

SEFFLNTVSQTLEYRRREHIQRPDLMQLLMELKAEDKTSDGLSFEQITAQALMFFLAGFD

TSSTTMSFCLYELALNPTVQERLRQEIIKKLGSNQQLNYEIIQQMPYLDQVISETLRKYP

ILPHLLRRSTKEYSIPDSGLKIKPGMRVMIPVHSIHHDPQLYPEPEVFDPSRFEPEEIRK

RHPFAYLPFGEGPRACIGERFGKMQVKIGIISLIRDFQFSKSERTQIPLQFSKVNFLIGS

ESGIHLKMERLSNI

>BAI77926.1

MGLLSVVLLALAGAAAVLFAFLKRQQHFWRSCGIPAARNPQLLAGNVAGMASTEHMAITL

QRLYREFKQRGLTFGGFNSFFAPTVLLVDPELVKHVMVKDFGVFHDRGLYVDPEGDPLSG

NLFNLEGHQWKVMRQKLSPTFTSGKMKLMFDTVASVAKELNQFVDDNCHREDLELREVLQ

RFTVDVIGNVAFGIDCNTMKNPDSEFRKMGNKAFALTTTSVIKSFIGIQFRSLAKFMKMK

ITEDDVEEFFLELTRSTVQMREQNGSQRNDFMKLLLEIRDQGTFSDAPNSAGEGLTMREL

AAQCFVFFLAGFETSSSTMTFCLYELALNPDVQYRLRKEIKEALKEGGGNLTYEALMKMD

YLDRVVSETLRKHPPLDNTFRTNEVDYVIPGTNYTIPAGTFVQIPIYAIQQDPDNFPEPD

KFDPDRFLPEAVKSRHPYAYIPFGEGPRICIGMRFGLMQAKLGLATLLANFHFSKSSQTS

VPLVYDPPSITLGPKGGMYLRIERV

>AAW69910.1

MGLLTVLIATLLAAIIALLGYLKWIQNYWERRAIPAAKNTHILTGNMAGMGTKISLAIAL

QKIYREFRERGLPFGGFNNFFAPTILAVDPEFVKLVLVKEFNVFHDHGFYTDAESDPLNS

HLFNLDGAKWRVMRQKLSPTFTSGKMKMMFNTVQSVADELKKFVEANYHREDLEIRDLLQ

RFTTDVIGSVAFGIECNSFKDPDSKLREMAGKVFELSPKTLVKTFVGIQFKGLARWMKMK

LTDDEVEKFFMELVRTTVESREKSDVQRNDFMKLLLEIKNSGKLSDAPDSGGDGLTMNEL

AAQCFVFFAAGFETSSTAMNFCLYELAINEDIQDRLREEINEVLGENGGQLTYDALMKMD

YLDRVFNETLRKYPPLDNTFRTNSVDYTVPGTNYTIPAGTFVQIPMYALQWDPEHFPAPE

RFDPDRFLPEVVKSRHPYAYVPFGEGPRICIGMRFGVMQAKIGLVTLLQNFKFRVNSRTP

IPMEFEPASVTLSPKNGMYLQIERVK

>EFA05683.1

MLLTNSICGDLVTIFLTLVTVLTLYFKWCHQYWEKLNLPSLPPSIPFGTSLGPKGHFGLR

MKHIYDKMRQKGWKHGGIYLMQRPFYVVVDLDYVKNIMTKDFHHFSDRGIFHNEKADPLS

AHLFNISGPKWRNLRSKLTPTFTSGKMKLMFQTLLDCEVNLRKQVEQHYEAKKPIDIKEV

LGCFTTDIIGSCAFGLDCNSFKEENSPFRVYGRRVFDRSGFIRTLKRTFASNFPNLAKLV

NIALVPRDISDFFINIVKDTVEYREKNKIVRNDFLQLLLDLKTEESGNDGTALTFEEIAA

QSFVFFIAGFETSSTTMAFALYELARHQDIQDQVRDEIESVLKKYDGKITYEAIQEMSFL

GQVIDEALRMYPPGPLVPRRCVKDYKVPNTDVVIEKGVMVLIPILGIHYDEKYYPDPEKF

DPERFSEENKALRHSYAHIPFGEGPRICIGLRFGLMQSKVGLVSLLRNFKFTVNEKTQEP

LKMKTNSLVLAVEGEIWLNVHRL

>EDS26134.1

MGLLSVVLLALAGAAAILFAFLKRQQHFWRSCGIPAARNPQLLAGNVAGMASTEHMAITL

QRLYREFKQRGLTFGGFNSFFAPTVLLVDPELVKHVMVKDFGVFHDRGLYVDPEGDPLSG

NLFNLEGHQWKVMRQKLSPTFTSGKMKLMFDTVASVAKELNQFVDDNCHREDLELREVLQ

RFTVDVIGNVAFGIDCNTMKNPDSEFRKMGNKAFALTTTSVIKSFIGIQFRSLAKFMKMK

ITEDDVEEFFLELTRSTVQMREQNGSQRNDFMKLLLEIRDQGTFSDAPNSAGEGLTMREL

AAQCFVFFLAGFETSSSTMTFCLYELALNPDVQYRLRKEIKEALNEGGGNLTYEALMKMD

YLDRVVSETLRKHPPLDNTFRTNEVDYVIPGTNYTIPASTFVQIPIYAIQRDPDNFPEPD

KFDPDRFLPEAVKSRHPYAYIPFGEGPRICIGMRFGLMQAKLGLATLLANFQFSKSSRTS

VPLVYDPPSITLGPKGGMYLRIERV

>ABX64450.1

MSLLISFIVKLISVLVSLIAVTLTYIKWKHQYWSKRNIPFFPPTIPFGNLENPRRKKYSI

GYHIKNFYDEAKRKGWKHCGLYFFASPVYLIVDVEYLKNVMSNDFQYFVDRGMYYNEKAD

PISAHLFALGGDRWKNLRSKLTPTFTPAKMKMMFPTLVECVSLLLDAMEKESVNIDIKEL

LGRFTTDIIGSCAFGLDCNTIKEENSPFRVYGKKVFNSTRMRTFKLSFASSFPNLGRLLR

IKQVSSDVSDFFSKIVKDSIEYREKNNFVRPDFLQMLIDLRKKGAGLTLEEVTAQCFIFF

LGGFETSSTTMTFTLYELAKNEEIQEKLREEILRILDKHEGKITYEGISEMKYLDQVIDE

SLRKYPPFPFVTRTCVMDYKVPNSDVIIQKGRRVVVPILALHLDKEYWPDPQKFDPERFS

DDNKPLIQQYSYIPFGEGPRYCIGMRFGLTQTKVGLVALLRDYKFSVNPRTLDPLKMAVN

SFILNAEGGIWLDSQKLLKNNHRKQ

>EFR21005.1

MGVFSSVFIAIIALVALVYFYLRHKHAFWRKRGFPCKPNPKLLTGHLDPSVHGSYVNQEL

YHYAKGRGDRFIGISFFFMPLVVLCDLELIKTILVKDFTVFHDRGMYNNPEADPLSGHLF

SLDGPEWRTLRQKLTPTFTSGRMKQMFGTMLLVADRLREVLGAADQKTSFSAGREMKDVL

ARFTTDVIGTCAFGIECNSLADPDSEFLRYGNKVFQTKLSTMIKFIFIMMYKETAKRLGI

KGTEDDAEAFFMNLVRETVEYREKHNVQRNDFLNLLLEIKNKGTLSEPGHDDSTGGDASV

TGTEHGMTMNELTAQVFIFFLAGFETSSTTMNFCLYELAKSPDIQQRLREEINSAIEANG

GQLTYDVAMNIAYLDQVINETLRKYPPLESVNRSPVRDYTIPDTTHVIPRGVLVEVPIYA

IHHDPEYYPEPERFDPERFTEEAVKKRVPYTFLPFGEGPRICIGMRFGMMQVKVGLITML

RDFRFHPSSQTPDRITFDKNIAILSPSKGNYLHIEKL

>AAW69911.1

MGLLTVLIATLLAAIIALLGYLKWIQNYWERRAIPAAKNTHILTGNMAGMGTKISLAIAL

QKIYREFRERGLAFGGFNNFFAPTILAVDPEFVKLVLVKEFNVFHDHGFYTDAESDPLNS

HLFNLDGAKWRVMRQKLSPTFTSGKMKMMFNTVQSVADELKKFVEENYHREDLEIRDLLQ

RFTTDVIGSVAFGIECNSFKDPDSKLREMAGKVFELSPKTLVKTFVGIQFKGLARWMKMK

LTDDEVEKFFMELVRTTVESREKSDVQRNDFMKLLLEIKNSGKLSDAPDSGGDGLTMNEL

AAQCFVFFAAGFETSSTAMNFCLYELAINEDIQDRLREEINEVLGENGGQLTYDALMKMD

YLDRVFNETLRKYPPLDNTFRTNSVDYTVPGTNCTIPAGTFVQIPMYALQWDPEHFPAPE

RFDPDRFLPEVVKTRHPYAYVPFGEGPRICIGMRFGVMQAKIGLVTLLQNFKFRVNSRTP

IPMEFEPASVTLSPKNGMYLQIERVK

>EAT35330.1

MVFLFTTLCLLLPHLGLLYYYVRRKFAYWADRGVPYVPGSLPMGSFNGMGSTKHFVELLD

PVYKQYRNTHKAVGMFLSINPVLLAVDPDLVKQILVKDFNSFHDRGMYFNERDDPLASHM

FSVEGERWRFLRNKLSPTFSSGKIKYMFLTVREIGLEFLASFEPFMERKEPVEIGIQAQK

FTCDVIGSCAFGLSCNALKDESTELLDIADRVFNPKPLEMMYMLLLICFRKWAVKLRLKQ

TPADIERFFVNMVRKTVEHREKNNISRPDFLQLLMQLKNKGTLEESKEDSKETISMNDVI

AQAFLFFFGGFETSSKALSFALFELALNPELQEKARDEVLRTLDKHDGLLTYEALKDMTY

VEQIVHESLRKYAPIGNVIRKANEPYQIHSPDIILEKGTMVMIPVHSIHHDPEIYPDPSR

FDPDRFTPEAISARHSHSFLPFGDGPRNCIGMRFALLEVKFGIAQLLSRLRFTVNEKTQL

PLRYDPKTNVASALGGLWLDVERI

>ABC86306.1

MIKDFSYFQDRGAFTNPRDDPLTGHLFALEGEEWRAMRHKLTPVFTSGKIKQMSKVIVDV

GLRLGDAMDKAVKEAKVEEGNVEIKDLCARFTTDVIGSCAFGLECNSLQDPSAEFRQKGR

EIFTRRRHSTLVQSFIFTNARLARKLRIKVLPDDLTQFFMSTVKNTVDYRLKNGIKRNDF

IEQMIELRAEDQEAAKKGQGIDLSHGLTLEQMAAQAFVFFVAGFETSSSTMSLCLYELAL

QPDIQQRLREEIESVLANVDGGELNYDVLAQMTYLDQVLSETLRKHPLLPHLIRETTKDY

QIPNSDIVLDKGILALIPVHNIHHDPEIYPEPEKFDPSRFDPEEVKNRHPMAYLPFGDGP

RNCIGLRFGKIQAKIGLVSLLRRFKFSVSNRTDVPLIFSKKSFLLTTNDGIYLKVERV

>AAW69914.1

MGLLTVLIATLLAAIIALLGYLKWIQNYRERRAIPAAKNTHILTGNMAGMGTKISLAIAL

QKIYREFRERGLPFGGFNNFFAPTILAVDPEFVKLVLVKEFNVFHDHGFYTDAESDPLNS

HLFNLDGAKWRVMRQKLSPTFTSGKMKMMFNTVQSVADELKKFVEANYHREDLEIRDLLQ

RFTTDVIGSVAFGIECNSFKDPDSKLREMAGKVFELSPKTLVKTFVGIQFKGLARWMKMK

LTDDEVEKFFMELVRTTVECREKSDVQRNDFMKLLLEIKNSGKLSDAPDSGGDGLTMNEL

AAQCFVFFAAGFETSSTAMNFCLYELAINEDIQDRLREEINDVLGENGDQLTYDALMKMD

YLDRVFNETLRKYPPLDNTFRTNSVDYTVPGTNYTIPAGTFVQIPMYALQWDPEHFPAPE

RFDPDRFLPEVVKTRHPYAYVPFGEGPRICIGMRFGVMQAKVGLVTLLQNFKFRVNSRTP

IPMEFGPASVTLSPKNGMYLQIERVK

>EFA02817.1

MLLVNGSLTYDLVAILVTVLAGLVVYYKWAFTYWKKRGLEYLEPSIPFGNCASLFKAKYN

FGEQFAEFYKQFREKNLRHGGVFMFARPFYIPVDPAIMKDIMQKDFQHFVDRGFYVNEEI

DPLSGHLFSLEGNKWKNLRAKLTATFTSGKMKMMFETMSRCAEELDNYVNEASKRKETID

IKETLARFTTDIISSCAFGLESNCLKNPDTKFRYYGKRTFQNTTWENLKNLIQFMTPMYL

LNLVKFKTTKQDVEDYMLNLVKKTVDYREKNNVYRKDFLHLLLQLKNKGKITDDETFLDN

NNKSKEVALTINELAAQVFVFFAAGYETSSTAMTFTLYELCLNQKIQDKMREEIKTVLAK

HNNKISYEALMEMTYMEQALNETLRKYPPVPFLNRKCTKAYDVAGTNLHLDEGTMVVLPI

LGLQHDPEYYPDPSKYDPDRFSEENKNSRPPFTWMPFGEGPRICIGLRFGMLQAKVGLAT

LLENYKVTLSQKTKTPLEMDCKTFITTTKEGIWLDFTRLN

>EFA12630.1

MSLLLSFLLRLGSVIISLIVVVLSVIKWKHQYWANRNIPFFPPSVPFGNLQNPRRKKHSI

GYHIKNFYDQAKRKGWKHCGLYFFASPVYLVIDVNYVKHIMNTDFHHFVDRGMYSNEKID

PISAHLFALGGTRWKNLRNKLSPTFTSSKMKMMFPTIVECVSLLLDAMTEKSEQIDIKEL

LGRFTTDIIGSCAFGLDCNTIKEENSPFRLYGKKVFFSTKMRTFKLTFASSFPTLGRLLR

IRQVSSDVSDFFRKIVKDTIEYRAQNQFSRPDFLQMLIDLRRDGAEITLDEIIAQCFIFF

LAGFETSSTTMTFTLYELAKNHQIQDKLRQEIMTILTKYHGEITYDAISEMKYLDQVIEE

SLRKYPPLPFVTRTCVMDYKVPNTDLVIEKGRRVILPILALHHDPEFWPEPQNFDPERFN

DQNRNLRHQFSYIPFGEGPRFCIGKKFGLTQTKVGLVALIQNYKFSVNSRTLDPLKMAPN

TFILSAAGGIWLDSEKL

>EFA12634.1

MLITTSLTGDLLAVFLTLVLIIVTYCKWSYQYWERKHLPYLEPQIPFGNVGDSIKGKTNF

GITIKKIYEEMKHNGWKHGGLYVFTEPNYMVLDLEYVKNIMTKDFQYFVDRSTYYNKKED

PLNAHLLNLGGTKWRNLRTKLTPTFTSGKMKMMFQTLADCQTGLLKRMEKERLTRQPIDI

KEVLACFTTEIIGSCAFGLNCKAFEDENSPFRVYGRKVFATSKLQRLKRTIATIFPTLGY

LFNISITPKDITNFYLNVVKDTVEYREKNNYNRNDFMQLLIDLKNNKLEGGTSNGGFTLK

EVAAQSFVFFLAGFETSSTLMTFALYELARHQEIQDIVREEINEVLRKHNGNVTYDSIND

MKYLSQVIDETLRLYPPASLVNRKCIKDYQVPDCDLVIEKGTTVLIPIMGIHYDKDYYPD

PEKFDPERFTEENKNARHNYAHIPFGEGPRICIGMRFGLMQTKVGLSCLLKHYKFTVNKR

TQEPLKMQPSPLILSAEGEIWLDAEKL

>EDW03066.1

MGFLILVLTSLLTFVLGYLRHCYNYWELQGVPQLRPHFIYGHFFKLKSLHLSELLQETYD

AFKGKARLAGTYLFTRPIAVVTDLDLAKVILIKDFNKFVDRSSSASLNLQDNPLAGHLFN

LHGEEWRALRTKLSPTFTSGKIKYMFSTITEVADQLQQTFDQEVGDDGAVLELHDLLGRY

TIDVIGSCAFGVKCNSLKNPQAEFRVVGRKLFNNPKFNVRWALFRMTYANLFRKLPIKFK

SFGQEHTKFFLRLVQDTVDLRERENIKRNDFMDLLLNLRKTGESSGLSVEQLAAQVFVFF

VAGFETSSSNMSWALFELTKNQSIQAKLRDEILSVLQKHGKLTYEAMMEMTYLDQVVNET

LRKYPALASLTRIPAEDYKLPSDDESNSDGHIVLERGIKVHIPVRAIHYDPEIYPEPHEF

RPERFEPAATQQRHPLAFLGFGDGPRNCIGLRFGRMQVKVGLITLLAAFQFSQKGSEIPI

VTNSHFVLVPKNGVKIRVDRLPADWWSDYKSRQV

>157125279

MWIYLLIGIITSLVLFVRRKYLFWERQGVPFIKPKFPFGNLLVNGKRVHTSQLTTYYYNA

LKGKKHPIGGVFFFTTPFAVVLDRELMRNVLIQDFQHFHDRGLYYNEKDDPISCHLFNIE

GSYWTNLRKKLSPIFSSGKLKLMCPMVITIAERFQKCLSKSITQNQQEAEMKEWLNRFTI

DVIGTCAFGIECNSLTNPEEKFRKMGVKMFHVANSRIIKFFFISLFKNLAKKVHIKSVPE

DVSEFFFKVIRKTIAFREMNHVLRNDFINLSMQLMADGKLEGSDEDVGKITLNEVVAQSF

VFFLAGYETSSTVMMFCLYELSLQEDIQRRARENVITAVSRHGGLNYDALMDMGYLDQCV

NETMRKYPPAGNLGRCVTKDYNIPNTNITLRKGLNVVIPVHGIHHDAEYYPDPERFDPER

FSAEESTKRLPFTFMPFGEGPRNCIAARFGMLMAKVGVASMLMRFQFSKCSKTAVPLVIS

PKHASMSPEGGMWLKVKEIK

>EDS38451.1

MYVELIILIVSLLILSSIAVWRKITFWSRQNVPFIKPQFPAGNFHEADQLSVADISVKQY

NAMKDRGRFFGLYFFLQPLLMITDLDLIKTMLIKDFNSFPSRGVYYNARDDPLSAHIFSI

EGEKWRSLRTRLSPTFTSGKLKIMFPTLKAVGDSFGEYLTKIAGVGSEIGVKDAFARFTT

DVIGSCAFGIECNSFVEPNNEFREFGHKIVNEQLHAGTMRIFWKLFPVLGNLLRVKTLDS

NATQFFHKLVADTIDYRQKHSINRKDFMSSLIELKNKGELTLDEVAAQSLVFFVAGFETS

SANQTYCLYELARNVECQEKARESVLKAIETHGGLTYEAVNDMQYLDQCINGCFILETLR

LYPAVPVLERKSSQNYKIPDTDVTIPKGTKIHIPVFAIHRDPQLYPEPLKFNPDRFLPEE

AAKRHPNSFLTFGDGPRACIGFRFAILQSKVGLATMLSKFRISTSAKTAVPLEYTHKSPF

LQPKKELVLKIESL

>EFA12632.1

MFRFLDLVGVFIALFAILFAYFKWTYQYWSQKNVPFIQPTIPFGNSNLFRQKESGGIRMK

NFYDEMKAQGWKHGGLYSILKPSYMVIDLDYLKNIMTKDFDYFSDRGFYYNEKDDPLSAH

LFAIGGEKWRNLRIKLTPTFTSGKMKMMFQTLVDCAPGLVKQIDRKMESIDIKEVLGCFT

TDIIGSCAFGLDFNTSNDNNSPFREYGRKAFVPTKFDIVKIIFAMSFPRLAQLFRLTLTR

KDVSEFFLKVVRETVEYREKNNYRRKDFIQLLIDLKGEDGKTLTINEIAAQSFVFFIAGF

ETSSTTMAFVLYELSRRPDLQQKLRDEINTVLSRYEGSITYEATQEMKYMDQVINEALRM

YPPVPMLGRKCVKDYKIPDQDVIIEKGTSILIPVLGIHYDQEYYPDPKTFDPERFNEENR

KARHHYAHLPFGEGPRICIGMRFGLMQTKVGLATLLKNYKFKVGERTQEPLKFKVASFVL

AAEGEIWLDAEKL

>AAA69818.1

MIPIPLAIFTLIIFLAIIWLHYQLTYWRRRKAPTVPTTGLYKLRNREHIAYSYQRAYHQM

KRENKGYAGIYTLITPTLLIADLEILKLILVTEFEVFPDRGFFVNYKSDPLSRNMARLHG

EMWRKIRTKITPTFTAAPMRQMFGNVEAIGRHFMQVMEKQAEPKRNVVEVRDLCARFTTD

VIGNVAFGLDCNSLEDPNTEFRLKGDRTFSDVNPFWDMLASHFPKLFTWLDYKVATPELI

SFYSNIVCRTVEYREKNGVRRNDFLDLLIELKNRPQQEGEYQLEMDDLIAQSFAFFSAGF

ETSSNTMAFALYELAKNPRVQERARENIAEALRKHQGVFSYDSLNEMTYIRQVVQETLRK

YPPVPSTKRVCRRSYKFPDRQGLTVEPYVHIIIPIYAIHHDPEYYPQPEVFRPERFSAEE

RQRRHPMAYLPFGAGPRICIAERFGMMETMMGVALLLMHFKFSPCKETAENLIFDPKNVF

VFSIRGGIHLRVEKVQ

>EFA12635.1

MFVSNSLLGDLIALFVTLGVLLTFYFKWTYQYWTRRNVPQIDPHIPFGNISESIKGREIV

GVTFKKIYQQLKSQGCRHGGIYIFTQPSYFPIDLDYIRNILTKDFQYFVDRPFYVNEKDP

LQAHLLNLSGTRWRNMRSKLTPTFTSGKMKLMFHIMAESQSDLLKRMDKEAESLEPINIK

EVLACFTTNIIGSCAFGLDCKALEDENSPFRQFGKKLFKISVLHRLKRTFAATFPDFAKF

LRMSNSRKEIVKFIMDMVEGTIEYREKNNYSRNDFMQLLIDMKNNKPKGDHDGKPLTLDE

VASQAIVFFAAGFETSSTLMTFAFYELAKNPHIQDKLREEINLILAEHNNEISYDAIQEM

KYLSQVIDETLRLHPPAAQTSRKCIKDYKIPDQDITIEKGTSVTVSILGIHHDPDYYPDP

EKFDPERFTEENKSLRHNYAFLPFGEGPRNCIGMRFGLLQSKMGLVSLIKNYKFTVNEKT

KEPITYQPAHVVLAAQGEIWLNAQKV

>EDS38450.1

MYIELLILVVTLLVLGVFFLRQKYTYWKRQNVPFIEPKFPYGNFQEANQISTADISSKQY

RSMKTSGRFFGMYFFFEPLVMLTDLDLIKTMLVKDFNFFPDRGMYFNEKDDPLSAHMFAI

EGKKWRSLRTRLTPTFSSGKMKMMFPILKAVGDTYSDYVAKIIGSGAELEVKDAVARFTT

DVIGNCAFGIDCNSFAEPNNEFREFGVKIINEPISAGIMRIFWKLFPELGNLLGIKALNV

SATTFFRRLVADTIDYRQKSMVDRKDFMSMLVELKNKGDLTLDEAAAQSLVFFVAGFETS

SSNQAYCLYELALNPDCQEKARESVLKAIEKHGGLNYEAVNDMLYLDQCINESLRLYPSV

PVLERKTFQSYKIPDSDVTIPKGMKIHIPVFAIQRDEQYYPEPLKFNPDRFHPSEVAKRH

SSTFLPFGDGQRACIGLRFGMLQSRMGLAAMLSKFRFSISERTVVPLEYSVQAPILQPKS

DLVLKVEPL

>AAW69912.1

MGLLTVLIATLLAALIALLGYLKWIQNYWERRAIPAAKNTHILTGNMAGMGTKISLAIAL

QKIYREFRERRLAFGGFNNFFAPTILAVDPEFVKLVLVKEFNVFHDHGFYTDAESDPLNS

HLFNLDGAKWRVMRQKLSPTFTSGKMKMMFNTVQSVADELKKFVEENYHREDLEIRDLLQ

RFTTDVIGSVAFGIECNSFKDPDSKLREMAGKVFELSPKTLVKTFVGIQFKGLARWMKMK

LTDDEVEKFFMELVRTTVESREKSDVQRNDFMKLLLEIKNSGKLSDAPDSGGDGLTMNEL

AAQCFVFFAAGFETSSTAMNFCLYELAINEEIQDRLREEINDVLGENGGQLTYDALMKMD

YLDRVFNETLRKYPPLDNTFRTNSVDYTVPGTNYTIPAGTFVQIPMYALQRDPEHFPAPE

RFDPDRFLPEVVKTRHPYAYVPFGEGPRICIGMRFGVMQAKIGLVTLLQNFKFRVNSRTP

IPMEFEPASVTLSPKNGMYLQIERVK

>AAN05727.1

MGYVNVVFYLVLPALGLLYYYVKQHYRYWANRNIPQLEASFPVGNMKGVGSKLHFNDVLG

EAYDKGKSKTAPLVGLYFMLKPVLIVTDLDMVKRILVKDFNSFHDRGLYVNERDDPLSGH

LFALDGERWRYLRNKLSPTFTSGKIKLMFTTICEIGDEFLASVTRYVDREAPIDVKLLSQ

CFTCDVVGSVAFGLKCNSLKNEGSKLLEIGDKVFKPPAWRNMLTFMLISCKKMAKRLHLP

ALPSEVGSFFMPLVSETVHDRERNAIVRPDFLNLLIQLKNKGTVEDESSEGLEKLTLDEV

AAQAFVFFFAGFETSSTTLSFALFELANNPAIQERVRAEVLEKLKLHDGQITYDALKEMT

YLDQVINETLRMYPPVPQLIRVSTQPYTVEATNVTLDRDTMLMVPIYAIHHDANIYPEPE

RFDPDRFAPDAVHSRHTHAFLPFGDGPRNCIGMRFGLLEVKFGIVQMLSKLRFTVNSRMQ

LPIKLSKAAAMLEVEGGIWLNATKL

>EDW74918.1

MIKDFQNFANRGNYYNEADDPITAHLFNLDGQKWRNMRTKLTPTFTALKMHQMLPIVVQI

SDRLVQLLGKEVDQVKASSKNGEASIEIKDWLARFTTDGIGSCAFGLDCNSLEDPNAEFR

AVSRRLFTERRLNPIIMGLLNAYPSIGRKLHIKVAPKFASDFYTRVVKESIDYRDKNNVK

RNDFLNILMEMREGKDGGLTFNQILAQAFVFLLAGFETSSSTMGFALYELATNQDVQDKA

REEVKQVLAKHGELNYEAIKELKYMQQVFYGESIKCLLIIVILIHKFLLFPETMRLWPIL

PILFRQAEKEYPVPGTNDVFEAGTHLVIPVEAIQHDPEIYPDPEKFDPERFTPEAVEQRH

AMAWLPFGSGPRNCIGARFGEMQSIVGLATLVHNFKFVPSIDTQIPLKLSKTVFLTSPEA

GIKLDVQKL

>EAT37908.2

MWIYLLIGIITSLVLFVRRKYLFWERQGVPFIKPKFPFGNLLVNGKRVHTSQLTTYYYNA

LKGKKHPIGGVFFFTTPFAVVLDRELMRNVLIQDFQHFHDRGLYYNEKDDPISCHLFNIE

GSYWTNLRKKLSPIFSSGKLKLMCPMVITIAERFQKCLSKSITQNQQEAEMKEWLNRFTI

DVIGTCAFGIECNSLTNPEEKFRKMGVKMFHVANSRIIKFFFISLFKNLAKKVHIKSVPE

DVSEFFFKVIRKTIAFREMNHVLRNDFINLSMQLMADGKLEGSDEDVGKITLNEVVAQSF

VFFLAGYETSSTVMMFCLYELSLQEDIQRRARENVITAVSRHGGLNYDALMDMGYLDQCV

ETMRKYPPAGNLGRCVTKDYNIPNTNITLRKGLNVVIPVHGIHHDAEYYPDPERFDPERF

SAEESTKRLPFTFMPFGEGPRNCIAARFGMLMAKVGVASMLMRFQFSKCSKTAVPLVISP

KHASMSPEGGMWLKVKEIK

>EFA12628.1

MGLFLEDFWTEVFFLLTTLAILVLSYFHNAYKYWEKRGVPTIKPTFPLGNGDTLLPEGFS

VGMLSRKFYEELKKRGEKFGGVYLVTQPNLVLVDPEYVKDILATDFQYFVDRGFYFNEKT

DPLSAHLFALDGTSWRNLRIKLSPTFSSGKMKMMFKTVLECCGYMRDYIEDILDKDLDIK

EIFSRYTTDVIGSCAFGIECNSFKYPDAEFRKMGKKVFSFGLWRSICSFCIIYFPNLSYH

LGVPTNEKDVQNFFRGIVAESVNTRKEQNIKRNDFLQLLIDLKDHSALSLNEMAAQVFLF

FAAGFESSSTTMTFVLYELAKHPDIQQELRNEIRKTLDKHQGQLTYEAVMEMKYLQQVID

ETLRLYPPLATLNRRCIKDYTLRNTGIKIEKGTSVIIPSLGLHMDPELYPDPEIFNPDNF

SEENKKKRPSFVHLPFGDGPRNCIGLRFALMQSKCGIATVVNNFRFSLSPATKPLRLNPN

SFVLNTYDKIYLRAERLAM

>EDS38434.1

MAALVVALLITVFTFAIWFVKRKYSYWSRQNVPHVEPSIPYGNFKELGQVSIFELSSRFY

NQMKTCGRYFGLYFFLEPILVVTDLDLIKTIMIKDFAHFPDRGQYYNARDDPLSAHLFNI

EGNAWRSLRARLTPTFTSGKMKMMFPTLKVVADRFAEFLTRSVESGDADMEVKDCFQRFT

IDVIGSCAFGIECNSFSDPDSRFRKVGNQIFSNPRNSTAVRLFLKTFPDLGRMLRIKSLN

SEATEFFYKTVQDTVGYRKANGVERNDFLNLLLELEKKGVDLSMDEIAAQAFLFFIGGFE

TSSSTQTFCMYELSLHPDIQEKARQCVLEGIQKHGGLTYEAVSDMPYLDQCINETLRKYP

TLPILERKTFKDYHIPDSNVVIQKGTRIQIPVYALQRDERYHPEPDRFDPGRFTADEMAT

RHVSTFLPFGEGPRVCIGQRLGMMQSRVGLATVLSNFKVSTCAKSQVPLQISSKSAVLQP

QQGVWVRVEPLK

>EDS38449.1

MFLITLLITLFFLSITFIKYKFSYWSRQNVPYIEPNFPYGNFKDSGRISIADISAQQYNE

MKSRGQFFGMFFFLQPMVMITNLDLIKTILIKDFNYFPDRGVYHNLRDDPLSGHLFSIEG

NKWRSLRTRLTPTFTSGKMRMMFPTLKAVGDNFGEFLESAVGGGIEMELKDVVQRFTTDV

IGSCAFGIECNTFVDGNSQIRSVSRLIFDSPKHSGKKRLFLKLFPEIGNKLRIKQLNENA

TEFFQKLVGDTIAYRVKNSTDRNDFISLLIGLMNNGDLTLDEAAAQSLIFFLAGLETSSS

NQTFCLYELALNQGYQQKARECIHEAMEKHGGLTYEAVSDMQYLDQCINETFRLYPAVPV

LERKTFQNYQIPGTKVIIPKGMKVHIPVYGIQRDEQYYPNPTVFNPDRFYPDAVAKRHMC

AFLPFGEGPRICIGLRFGMLQSRVGLATVLSRFRIMPCSQTTIPLEYSTKSTVLQAKGGL

WLKVEPL

>189241880

MPTLFDILGVSVALLVITYAYFKWRFQYWRQKNVPFLPPTVPFGNFNMLRQRDNGGILLK

KFYDEMKSHGWKHGGFYSLFQPGYLVIDLDYLKNIMTKDFDHFTDRGFYYNEKDDPLSAH

LFALGGEKWRNLRTKITPTFTSGKMKQMFQILVDCTPDLIERIDSFCREKKPIDIKELLG

CFTTDIIGSCAFGLQFNTSKDKNSPFREYGRKAFTPTYFDIVKMTFAMAFPRLALALKLR

MTRKDVSDFFLQLVRDTVKYRVENNYSRKDFIQLLIDLKSLTIEEIAAQCFVFFIAGFET

SSTTMTFVLYELSRRHDLQQKLRNEINTVLAKYDGHMTYEAIQDMKYMDQVINEALRMYP

PVPLLSRKCVKDYKIPDQEIIIDKGTTVCIPIWGIHYDKDYYPEPEIFDPERFNEDNKKS

RHHYAHLPFGEGPRICIGLRFGLMQTKVGLATLLQNYNFRVAGRTQEPLKYKVASFVLAA

EGEIWLDTEVTPSFTLGKIKQMFKILVDCTPDLIERIDSFCREKKPIDIKEVLGCFTTDI

IGSCAIGLQFNTSKDKNSLFREYGRKAFTPTYFDLIKMSFAMAFPRLALAFKLRMTRKDV

SDFFLQLVRDTVKYRVENNYSRKDFIQLLIDLQNLTNEEIAAQCFVFFIAGFETSSTTMT

FVLYELSRRQDLQQKLRNEINTVLAKYDGHMTYEAIQDMKYMDQVINEALRMYPPVPLLS

RKCVKDYKIPDQEIIIDKGTTVCIPIWGIHYDKDYYPEPEIFDPERFNEDNKKSRHHYAH

LPFGEGPRICIGLRFGLMQTKVGLATLLQNYNFWVAGRTQEPLKYKVASFILAAEGEIWL

DAEKL

>AAR88141.1

MELVTTILSLLFVLASGLYWFLRNRYNYWTNRGFPTVPNQKLLYGHVKGTNTERHASYIT

AEIYQQLKKRGDSFGGLNVFIIPAVMVVDPELIKTILVKDFHIFHDRGLFSDPEVDPLSG

TLFALQGKPWKILRQKLTPTFTSGKMKQMFTTIRDVAEQLGTYVDEHLHQEELEMKEILV

RYTTDVIGTCAFGIECNTLKDPDSEFLKYGNKVLDQKVSTMIKIIFIMVMRKFKSNFVLK

ITDADVESFFLNLVRETVEYRENNNVKRNDFLNLLMQIKNTGKLWEAEEEHVGKGEVGMT

QNELAAQVFIFFLAGFETSSTTMNFCLYELAKNSELQERLRKEINRALEANGGELTYDVV

MGIEYLNKVVDETLRKYPPLESITRAPEQDYTIPGTKHVIPKGTMVQIPIYALHHDPEYY

PEPERFDPERFQPEVANARPPYVYMPFGEGPRICIGMRFGMMQTKVGLITLLRQFRFSPT

AKTPETIRFMPNVFILSPDRGNYLHVEKI

>EDS44716.1

MNVLLLLLASLLFALLKWIYSHFQSRYQFWKDRNVPYLEPRFPVGNVADTLKPTIHFAHI

MDTLYKKLTKLGDDYAGIYFFRDPVLVVLSPEFAKTVLVKDFNYFVDRGVYSNERDDPLS

ANLFFMEGHQWRKLRAKLTPTFTSGRLKAMFHTILAVGEQFDRFLGGYVDGVNKVEVKDL

LARFTTDVIGSCAFGIDCNSLENPKSEFREMGKRMINFPKLKALKVFFAMMFRDTARKLG

IRFNDEDVSEFFFTVVRDTIKYREENNVQRKDFMQLLIELKNKGYMDGEVDGAAEEMSGQ

RLEKLTFEEIAAQAFVFFFAGFETSATTMTFALHLLASHPEIQEKGRSCVLDVLSRHDNK

FSYEAIMEMTYLDWIINETLRIYPPVATLHRMTTKPYKLPNGSIIPKGTGLCVPNLAIQR

DPQHFPDPLKFRPERFSEEEKSTRHPFSYLPFGEGPRICIGMRFGLLQTRMGLALLLKSY

RFSLCSRSPVPLVIDPVNLIHGPAGDVWLGIEKVQ

>EFA12529.1

MPTLFDILGVSVALLVITYAYFKWRFQYWRQKNVPFLPPTVPFGNFNMLRQRDNGGILLK

KFYDEMKSHGWKHGGFYSLFQPGYLVIDLDYLKNIMTKDFDHFTDRGFYYNEKDDPLSAH

LFALGGEKWRNLRTKITPTFTSGKMKQMFQILVDCTPDLIERIDSFCREKKPIDIKELLG

CFTTDIIGSCAFGLQFNTSKDKNSPFREYGRKAFTPTYFDIVKMTFAMAFPRLALALKLR

MTRKDVSDFFLQLVRDTVKYRVENNYSRKDFIQLLIDLKSLTIEEIAAQCFVFFIAGFET

SSTTMTFVLYELSRRHDLQQKLRNEINTVLAKYDGHMTYEAIQDMKYMDQVINEALRMYP

PVPLLSRKCVKDYKIPDQEIIIDKGTTVCIPIWGIHYDKDYYPEPEIFDPERFNEDNKKS

RHHYAHLPFGEGPRICIGLRFGLMQTKVGLATLLQNYNFRVAGRTQEPLKYKVASFVLAA

EGEIWLDAEKL

>EAT35331.1

MAILELYLAIGVTLVLATAGCVFLFLDKKRSFWKDRNFPCTGRAKMIYGDYKNMNQTEHM

QYINQRIYNEFKARKLPIGGTVLFLVPSTVVVDPDLIKAMLVKDFNFFHDRGVYNNPEVD

PLTGHLFSLEGQAWRQLRAKLSPTFTSGKMKMMFSTILSVADDLKEFLLEKTESGPTELE

MKNVLAGFTTDVIGSCAFGIECNSLRATHCRFREVSRKIFEQSVGQMLWMIVLMLFKGVA

TKLKLKATPAEVENFFTNMVQETIDHRERNNVQRSDFMNILIQMKNSTNLEEKLTLNEIT

AQSFIFFVAGFETSSTTMVNCLFELAMNPDIQEKLRAEIFKVCGEGDLTYESVSSVEYLN

MVIDETLRKHPVVDSLLRTSTQPYNIPNTDLKIPKGTFVFIPVHALHHDPEYYPDPDRFD

PERFNAENRASRHPFVYLPFGEGPRNCIGMRFGLMQTRVGLITVLRNFRVRPSSNTPERL

VVNPKSGIPAPLGGIPLLIERI

>EFA02819.1

MNTLLHNFVYDFVALFATVLIGVLLYYKWKFTYWAKVGLPTLAPTFPFGDAKEVMLGRLT

FGEQFLEFYKKFRANGFKHGGVYFCLQQFYVPVDPEIIKHIMQKDFHHFMNHGNYFDSEN

DPLSGHLFNLEDNKWKNMRVKLTPTFTSGKIKMMFQTLADCASGLNTIMDEAAKNQTSLD

IKDILARFTTDVIGSVAFGLECNSLKDPDAEFRKFGKRIFQIDVLDRIKILLQLIFPRNV

LQTMKMKTTKSEVEEFFMKAIRDTVNYREKNNVYRKDFMHLLIQLKNRGAVTDDGNITSD

QSKTDKALSLNELAAQAFVFFIAGFETSSTTMTFALYEIASNPQVQEKLREEIVTTLSKY

NGELNYNAMMEMTYMEKVIFETLRKYPPLPLLTRQCTKDYLIPNTTIRLKKGDMVGIAAQ

ALHYDPEYHPDPEKFDPERFSEENKSKRPDFTWIPFGEGPRLCIGLRFGMLQSKVGLTTL

LRNYKIALNKKTKVPLEMDKKAFVSSPEGGVWLDVEKLE

>EFA02824.1

MSLITDNLTFDLVAILATILVGIIVYFKWQLSYWDRLGVPSLNPVLFFGDIKDFILAKCT

LGEQFKEFYGKFKSKGYKHGGIFLGPKPFYVPIDPELVKHIMQKDFQHFVNHGNYFDEDA

DPLSGHLFNLEDAKWKNMRIKLTPTFTSGKIKMMFQTLADCTRGLKEIMDHSALNHAPAD

IKDILGRFTTDIIGSVAFGIECNSLKDPDAEFRKYGRKVFEVGFIDRLKTICILSLPHPV

LRFFKLKFTKSDVEKFFMSAIRDTVNYREKNNIYRKDFMHLLLQLKNRGFVADDGKVTDE

KENVKEKALTLNELSAQAFVFFLAGFETSSTTMTWALYELATNQDVQEKLRNEINNVLSR

HDNKLTYEAMMEMTYMEKVIHETLRKYPPLPILTRKCNKDYTIPNTSIKLSRGTAVGIPV

LALHTDPEYYSNPEKFDPEHFSEENVKTRPGFTWLPFGDGPRVCIGLRFGMLQSKVGLTA

LLKNYKIKLSKKTELPIKLDPKSFITTAKGGIWLDVEKLD

>EFA02822.1

MLLNNFTLNILAVFITVLVGVIVYFKWHLSYWDRLGVPSLSPVLFFGDTKNLILSKCTIG

EQFRVFYNKFKSKGYKHGGIFFGPIPFYIAIDPEIIKHILQKDFQHFMNHGYYINEEDDP

LTGHLLNLENVKWKNMRAKLTPTFTSGKMKIMFQTLADCTTGLKKVMDDSALNHTPVDIK

DIFGRFTTDIIGSVAFGIECNSLENPDAEFRKYGKKVFEIDFFGRIKTLCTFAIPHPILR

LFRFKFYNSDVATFFMDAIRETVNYREKNNIYRKDFMHLLLQLKNRGLVTDDEKITGDKD

IVTEALTMNELAAQAFVFFLAGFETSSTAMTWALYELAINPDVQQKLRAEINDVLRKHNK

LTYEAMMDMTYMEKVICETLRKYPPIPVLTRKCTKDYTIPNTSIQLQRGVSVSVPVLALH

TDPEYYPNPEKFDPDRFNDENVKARPGFTWLPFGEGPRICIGLRFGLLQSKVGLTAVLKH

YRIKLNHKTQLPVTLNPRSFITSAKGGVWLDVEKID

>EDS26138.1

MPQTCSFMNQLFALFYLFLRRKYSYWADRNVPHEPGQFPMGSLKGMGSKYHLIQITDRIY

SKFKGTGSMAGFYVTLSPTVLVTDLELVKQILVKDFNSFRDRGMYYNEKEDPLSAHLFSI

EGEKWRYMRNKLSPTFTSGKIKYMFLTMQEIGDEFVHSFDKYAEAQEPVDMKALAQRFTC

DVIGSCAFGLNCNSLKNEGSELLAMGDKVFKLNQWQMLKAFFVMSYRDLSLKLGLRQLPV

EVTDYFMNVIQGTVDHREKNNVVRPDFLQLLMQLKNKGTIEDHAEDSADKITMDQAAAQA

FLFFFAGFETSSTALSFAIFELANNPDIQEKARAEVQRVLAVHGGHLTYEAIKDMTYLEQ

VVNESLRMYPPVGNIIRIANQPYQLTSPNVVIEKGTMVMIPAYSIQRDPDIYPNPSQFDP

DRFTPEAIQARHTHTFLPFGDGPRNCIGMRFALLEVKFGIAQLLSRLSFTLNSRVRLPLE

LETKSSMMEIKGGLWLDVKKI

>91081159

MNTLLHNFVYDFVALFATVLIGVLLYYKWKFTYWAKVGLPTLAPTFPFGDAKEVMLGRLT

FGEQFLEFYKKFRANGFKHGGVYFCLQQFYVPVDPEIIKHIMQKDFHHFMNHGNYFDSEN

DPLSGHLFNLEDNKWKNMRVKLTPTFTSGKIKMMFQTLADCASGLNTIMDEAAKNQTSLD

IKDILARFTTDVIGSVAFGLECNSLKDPDAEFRKFGKRIFQIDVLDRIKILLQLIFPRNV

LQTMKMKTTKSEVEEFFMKAIRDTVNYREKNNVYRKDFMHLLIQLKNRGAVTDDALSLNE

LAAQAFVFFIAGFETSSTTMTFALYEIASNPQVQEKLREEIVTTLSKYNGELNYNAMMEM

TYMEKVIFETLRKYPPLPLLTRQCTKDYLIPNTTIRLKKGDMVGIAAQALHYDPEYHPDP

EKFDPERFSEENKSKRPDFTWIPFGEGPRLCIGLRFGMLQSKVGLTTLLRNYKIALNKKT

KVPLEMDKKAFVSSPEGGVWLDVEKLE

>EDW63574.1

MDFTLVLLSSLLVIVLGCLRHCYNYWELQGVRQLRPHFLFGHIFKLKSVHLSELLQETYD

AFKGKSRVAGTYLFLRPIAVVTDLDLVKAILIKDFNKFVDRSNTPLNLPENPLAGHLFNL

HGEEWRALRTKLSPTFTSGKIKYMFSTITGVAQQLEETFEQEVSEAGSTLELHDLLGRYT

VDVIGSCAFGIECNSLKDPQAQFRVLARKLFANQKRNVRWNLFKVNYMKLLLRLHIKMRT

FDQEYTDFFLRLVRDTVELREREKIRRNDFMDLLLDLRRTDQKMGLSVEQLAAQVFVFFI

AGFETSSSNMSYALFELAKNRSIQQKLREEIRRVLQVHGKLTYEAMMEMPYLDQVINETL

RKYPALASLTRVPSEDYKLPSDEGYESDGHIVLERGIKVHIPVRAIHYDPEIYPEPHEFR

PERFEAAACQQRHPLAFLGFGDGPRNCIGLRFGRMQVKVGLITLLSRYRFSLPPDGPKEL

AISNKNLLLVPGEGVRLRVDRLQTNSRDECK

>EDS32054.1

MLSSPAAVHLDVKLIKTLLVKDFGYFPNRGVYFNERDDPLSAHMFAIEGQKWKTLRNKLS

PTFTSGRIKMTFPLVVEVCKQFCDHLEEVVQGVNEVEMHDLLSRYTIDVIGTCAFGIECN

SFRDPDNEFRRYGKIAFDKLPHSPLVVYLMKAFRKYANALGMKMLHEDVSSFFSNVVRDT

IDYREKNNVVRNDFMDLLLKLKNTGRLEEAGEEIGKLSFDEIAAQAFIFFNAGFDTSSTA

MTYTLYELAMNREAQEKARKCVLDIFAANGGQLTYESVANMGYLDQCISETLRKHPPVAI

LERNADKDYRIPDTDLVIAKGRKIMVPIFAMHHDAEHFPDPEAYKPERRFAKRDPYCYLP

FGEGPRICIGMRFGQIQARVGLANLLRRFRFSVCDRTQIPVKYSRTNFILGPANGVWLKV

EKLATVSLKQSTSMSPIQLIVNGLLVILFIALGALIFIKRRYGYWRRRGVSYLEPEFPFG

NFKEMGKSIHPAHLTQQIYTQFKGRSPFVGLYIFLNPVLLSANEPESGSSGPCVPNRLLV

YLV

>EFA02821.1

MLFSDSLIVDLVTIISTIVVVSIAYCKWKLSYWNNTGLPTLSPVFPFGDTKELLLGQKSF

GEQFQDLYKEIRAKGWKHAGVYLAIQPFYLPVDPKIIKHIWQVDFQHFVNHGNYINEEVD

PLSGHLFNLEDAKWKEMRVKLTPTFTSGKMKMMFQTLADCTHGLKTIMNECATSGTPIEI

KDVLGRFTTDIIGSAAFGLECNSLKDPDSTFRKYGKKVFELQGWERIKVLAQFALPHSFL

RAIKFKQTQSDVEKFFMKVVRDTADYREKNKIYRKDFMHLLLQLKNRGKVSDDEKVTQEN

GKTGEKALTMNEVAAQAFVFFLAGFETSSTLMTFTLYELATNPDIQGKLREEINTVLAKH

DGQMTYEAMMEMTYMEKVLNETLRKHPPIPFLFRRCTKDYTIPETSVKLRKGDDVGISIV

GIHNDPEYYPNPEKFIPERFNEENKNARPPFTWIPFGEGPRICIGLRFGMLQSKVGLTAL

LKNYKISLSSKTKMPLEMEKSGLVTTVEGGMWLNVEKIN

>189236550

MLFSDSLIVDLVTIISTIVVVSIAYCKWKLSYWNNTGLPTLSPVFPFGDTKELLLGQKSF

GEQFQDLYKEIRAKGWKHAGVYLAIQPFYLPVDPKIIKHIWQVDFQHFVNHGNYINEEVD

PLSGHLFNLEDAKWKEMRVKLTPTFTSGKMKMMFQTLADCTHGLKTIMNECATSGTPIEI

KDVLGRFTTDIIGSAAFGLECNSLKDPDSTFRKYGKKVFELQGWERIKVLAQFALPHSFL

RAIKFKQTQSDVEKFFMKVVRDTADYREKNKIYRKDFMHLLLQLKNRENGKTGEKALTMN

EVAAQAFVFFLAGFETSSTLMTFTLYELATNPDIQGKLREEINTVLAKHDGQMTYEAMME

MTYMEKVLNETLRKHPPIPFLFRRCTKDYTIPETSVKLRKGDDVGISIVGIHNDPEYYPN

PEKFIPERFNEENKNARPPFTWIPFGEGPRICIGLRFGMLQSKVGLTALLKNYKISLSSK

TKMPLEMEKSGLVTTVEGGMWLNVEKIN

>EDX15485.1

MLLTLALLSLVLLLAYRFYYNTYTYWARMGVPHERPLPVIGNTKGIGTKYHLRDISKRIY

DKFKGQAPIAGMFLFFKRTAVVTDLDLIKQVLIKDFHYFQDRGLFNNTRDDPLTGHLLTL

EGDEWKSMRHKLTPVFTSGKMKKMSEVVIEVGHRLADAMEKAVKVGEVDNGDVEIKDLCA

RFTTDVIGTCAFGLECHSLADPKAEFRRKGRMIFEEPRHSALIQNFILTNSKLAKKLRMK

ILRDDLTDFFLSAVKNTVDYRLKNGIKRNDFMDQLIELRAEDQEAAKKGQGIDLSHGLTL

EQMAAQAFVFFLAGFETSSSTMAFCLYELALQPEIQNQVRDEIERVLDGEPITYDALAEM

TYLEQVLSETLRKHPIIPQLLRETNENYKVPNTEIIIEKGTSVLIPVHSIHYDPDLYPQP

ELFDPSRFEADKSSSRHPFAYLPFGDGPRNCIGSAFRQNAGQDWHCVLASALQVRCFRPN

TDPLSFGHSQPHLGGQAWNSSKSRTHIDSEFY

>EEZ99243.1

MESALMEAGIIFLTIVSFVYLYFKHAYTYWKARNVPQLSPTFPFGDMASVIFRRHNMGDK

IKEIYDETKARGHKYVGLYFFSRKAFLPIDPVLIKKILAPDFQHFHDRGIYYDEENDPIS

AHLFSLAGPKWKNLRARLTPAYSPGKLKFMFDTVLACGEEMADILKEIAKREGKVEIKEI

LARYSTDVIGCCAFGIDCNSMRNPTAEFRLMGRRAFTQTIGDVLKMIVIRSFPRLAKLLG

FGVFSPTVTGFFQRVVNETIRYREENNVTRNDFMQLLIQLKNKGKLEGDGEEKDGAPAVG

TSLTLDEAAAQAFIFFLAGFETTSTTISFALFEMARNESIRTKARTEINKVLGKHGGKLT

YEATMEMEYLDTVISETLRKYPPAPVFLRKCTKTYRVPDTDVIIEEGLSVLIPAYGLHRD

PEYFPDPETFDPDRFNEDNRSKIWDYTYIPFGDGPRICIGMRFAMIQIKIALALILKSFE

FPLSEKTILPMKMENSGIILAPIGGIWLNLIPLE

>EFA12629.1

MNIPSPYFEILGTVLALLVAIIFYYHHAFQFWARKNIPFIPPRFPLGNTNVVLPRGLTIG

LLSKTFYDYFKQRGHKIGGVFLVTEPNLMILDPDYIKNILLKDFHHFVDRGFYFNEKTDP

ISANLFTIDGDQWRNLRTKLTPTFTSSKMKIMFQTVKNCCNNMLEAIASNTDLDKDLKEF

LGRYTVDVVGSCAFGIECNSFKHPDAEFRKMGLRAFHFSFLRSIRAFFIAYLPKLSRQMG

LPSNNKDIREFFYKVVMETIQKREETQIKRNDFLQILMDLRKTEILSLDEVAAQVFLFFT

AGFETSSSTMAMCLYEIAKEPEYQEKLRQEICEITNGEITYENLFEMKYLDQVFSETLRK

YPPGQTLNRRCVKDYTLPGTSTIIEKGTPILISAIGVHRDPEYYPDPEKFDPERFSEENK

KLRHPFVYLPFGDGPRNCIGMRFGTMQSKLGIASVVKNFKVSVSPITKRVELDPNTFLLN

TIDKIYFRVEKIVK

>AAL93298.1

MELLTLTLSLLVALATGLYLFVRNRYNYWSNRQFPTLPNQKLLFGHVKGVNTERHASYIS

SEIYREFKKRGDAFGGFNMFVIPAVMAIDPEFIKTILVKDFHIFHDRGLFSDPEIDPLSG

TLFALQGKAWKILRQKLTPTFTSGKMKQMFATVLEVAERLGQHVATHTGQMEMKDVLARY

TTDVIGTCAFGIECNTLRNPDSDFLKYGNKVFEQKVSTMIKIILVLMGRKISSKFTLKIT

DADVETFFMNLVRETVEYRERNNVKRNDFLNLLLQIKNTGKLWEGEEDHIGKGEVGMTMN

ELAAQVFIFFLAGFETSSTTMNFCLYELAKHPDIQERLRREIERAVEENGGELTYDVVMG

TEYLNWVVDETLRKYPPLETVTRAPEHDYTVPGTAHVIPKGTMIQIPIYALHHDAQYYPD

PERFDPERFRPEVANARPAYVYMPFGEGPRICIGLRFGMMQTKVGLITLLRQFRFSPTEQ

TPDRIRFMPNVFVLSPDSGNYLEVEKL

>EFA02823.1

MTLITNNITLDLVAIIATFLVGIIVYFKWQLSYWDRLGVPSLNPTLVFGDFKNYIFAKCS

LGEQFKELYDQFKSKGYKHGGVFVGPKPFYVPIDPEIVQHIMQKDFHHFMNHGNYFDENA

DPLSGHLFNLEDSKWKNMRVKLTPTFTSGKIKMMFQTLADCTRGLDEIMYNSALNHAPAD

IKDILGRFTTDIIGSIAFGIECNSLKNPDAEFRKYGRRVFEVGIIDRIKIICILALPDSV

LRLLKLKFTKSDVENFFMNAIRDTVNYREKNNIYRKDFMHLLLQLKNRGSVTDDEKVTDD

KDNVKEKALTLNELSAQAFVFFLAGFETSSTTMTWALYELATNQDVQEKLRNEINNVLSR

HDNKLTYEAMMEMTYMEKVIHETLRKYPPLPILTRKCNKDYTIPNTCIKLRRGTTVAIPV

LGLHTDPEYYSNPEKFDPEHFSEENVKSRPGFTWLPFGDGPRICIGLRFGMLQSKVGLTA

ILKNYKVTLSNKTKFPVTLDPKSFITTAKDGIWLDVKKLD

>345493076

MLSLIKDCLWEPLPIVLIMLGYALYAFLTSTFDYWLVRGVPYRKPTPLLGNFGDLLLFRK

SQPEGISEMYNWFGNERFFGVFRVRSPILIVRDPELIKCVCVKDFHVFCNRGIPVNSTKD

PLSGHLFNLEGKHWKSLRSKLTPAFSSGKLKNMFYLLVECSDDLTRLVERRLEMLENSSS

SSSSSFPDASIVEVRELAANFTIDVIGSCAFGIQINALSDEDSEFRKAARRLSKPSYKAT

LWRMLRTSMPKLYKLLGVQVIDPSVTKFFMDVVSQMVKERENKALKRHDFMDLLIELKNR

GTLELDNGNGLRAHNDEEVPVAEEIVLDENTIAAQAFVFFVAGYETSSNTIAFCLYELAV

NPEIQEKARRDIIDALDKRDSKLTYDAVQDMKYLDMVILETLRKYPPAPLLSRRCEYPYK

LPGSDVELSKGMRVVIPIYAIHHDPKHYPEPDKFRPERFGDEEKRARHPYTFLPFGEGPR

NCIGTRFALLQTKVGVITFLRKYQVEVCEKTDIPIKFSRRSLVTASETGVSLNISRLSE

>ABN80241.2

MALLTSCWCTDFALLFIGAFIGVYYFYQKKFKHWDKYKIPHTTPKFPFGDIGLTTINENI

VISLDRIYKSFPKEKVVGLWSFFQPILIARDAGVIKDILVKDFMNFHDRGIPVDEENDPL

SAHLFSLGGSKWRNLRAKLSPTFTSGKMKMMFPILVQTGNEMPKILEPKAANKETVEMKD

ILSRFTTDIIASCAFGIDSNSLKDPNVEFRTMGKKITEPTLGNVLRNLVFLVPKMAKALK

IRIFPADMSDFFLNLVRETIDYREKNNVKRNDFMQLLIQLKNKGTLDDVDEIKAKLDNVE

SNEEEKKTVQTFSVEEAAAQAFLFFIAGFETSSTTMIFALYELALHPEIQEKLRNEIKKD

LAKNNGEITYEAVFGNEYLGNVVDETLRKYPVAGLLLRECNKDYKVPNSDMVIPKGTSIQ

VPIYSLHYDPKYFPDPQRFDPDRFKEEVKSQRHRYAYLPFGEGPRNCIGMRFGLMQTRVG

LVQLILNYKFEICEKSPVPLKFSNVSLTPKEGMHLRVSKLN

>EFA02816.1

MNYTFLALFVTFLLGVISYLKWKLRYWDKKGLPTIPPKLPFGNLTDVIFGRKSFGEQFVE

FYQILKAKGYRHGGIYFGASPLYVPTDPEIVKHIMLVDFAHFSSHGGYVDEESDPLSGHL

FNLDDIKWRNLRVKLTPTFTSGKMKMMFQTLAEVSYNLKNVLDESVINKTPVDARDVLSR

FGVDVISSVAFGLECDSLKDPNVSFRKYGKKAVDFDIWVRIKLFMQVLLPHCVLRAIGHK

FTKTEVEEFFMKSIRDVVEYREKNNIFRKDFMHLLIQLKNLGTVTDDGQILDETSGSKEV

GLTMNQVAAQAFVFFMAGYETSSSTITFALYELAMNPPLQDKLRDEINTILAKHDNKLTY

AAMMEMTYMEKVIQETLRKYPPLPIIMRLCTKDYVVPGTDIEIKKGVGVMIPVLGLQTDP

EYFPDPDVFDPDRFSEEKKKERPGFTWLPFGDGPRICIGMRFGMLQSKVALTTFIRNYRV

KLNEKTKIPLKIDKGSFTTRAEGGVWLNLEKLN

>189236553

MRQSPVDDATAAVATIEPLFEIRFTFSCLHCFILTLRRRLLLRANYLIGFTLNMRLDCDN

ELGKKCKNTIRGSLSAKDLFNLENSLATVQKNPDEKRRDKTSLRFPVRKFGPKTMNYTFL

ALFVTFLLGVISYLKWKLRYWDKKGLPTIPPKLPFGNLTDVIFGRKSFGEQFVEFYQILK

AKGYRHGGIYFGASPLYVPTDPEIVKHIMLVDFAHFSSHGGYVDEESDPLSGHLFNLDDI

KWRNLRVKLTPTFTSGKMKMMFQTLAEVSYNLKNVLDESVINKTPVDARDVLSRFGVDVI

SSVAFGLECDSLKDPNVSFRKYGKKAVDFDIWVRIKLFMQVLLPHCVLRAIGHKFTKTEV

EEFFMKSIRDVVEYREKNNIFRKDFMHLLIQLKNLGTVTDDGQILDETSGSKEVGLTMNQ

VAAQAFVFFMAGYETSSSTITFALYELAMNPPLQDKLRDEINTILAKHDNKLTYAAMMEM

TYMEKVIQETLRKYPPLPIIMRLCTKDYVVPGTDIEIKKGVGVMIPVLGLQTDPEYFPDP

DVFDPDRFSEEKKKERPGFTWLPFGDGPRICIGMRFGMLQSKVALTTFIRNYRVKLNEKT

KIPLKIDKGSFTTRAEGGVWLNLEKLN

>189242381

MSVFTEFWPQILVFLALFLLVLVNYFNYCYRYWSKKGVATIKPRFPLGNTNIILPQGLSI

GILSKQFYDEFKAKGHKFGGVYLVTEPNLLIVDPEYVKDIMSKDFQHFVDRGFYYNKEKD

VLSAHLFAIDGSDWRNLRIKLTPTFTSGKMKMMFEAVVQCSKHMIENLDKNLGSDIEIKE

VLGRFTTDVIGTCAFGIECNSFKYPDAEFRMMGKKIFEYDLWKSVKAFFAVNVPKVALQL

GLTSTPDDVIDFFTRIVADAVKMRESNNIRRNDFLQILIDLKNTTSLTLDEMSAQVFLFF

VAGFETSSTTMTFALYELARNEKMQEKLRFEICQVLDKTGGQITYESLIEMKYLQQVIDE

TLRMYPPLPTLNRRCTKDYVLRDTNIIIEKDTPILISALGLHMDPEFFPKPEKFDPERFT

EEKKKERHPFVHLPFGDGPRNCIGLRFGVMQSKIGIITIIKNFKLTVSSNMKPVQFNPYV

FLLNINDKVLLQVGKL

>EGK96742.1

MTVWLLLVVVVATLLYYYVWRRYRYWRDRGIAELDPSFPLGDMKGVGTTRGFNDMLDEAY

GRVRGKSAAVGVYFLISPILIVADLDLAKQILVKDFHLFHDRGMYVNERDDPLSGHLFSI

GGERWRYLRNKLSPTFTSGKIKSMFFTIREIGDELLACFDKYIDCGDPIDIKLLCQCFTC

DVVGSCAFGLQCNSLKNGGSKLLEIGDKVFRASPMRMVYSMAIGIFPRLSRALRLPALPG

DVSAFFRPLVQSTVEHRERNAVERPDFLNLLIQLKNKGTVEDEATEELQKLTLDEVAAQA

FVFFFAGFETSSTTLSFALFELANNPNIQERVRAEVLEKLQLHDGRITYDALKEMTYLDQ

VINETLRLYTPVPQLFRVTNESYHLPSLNVTLEKGTMVLIPVHSYHHDPALFPEPYRFDP

DRFTADAMKARHSHAFLPFGDGPRNCIGMRFGLLEVKFGLVQVLNKLRLTVNERTALPLR

MSKRSGFPEAEGGIWLNASRL

>EFR27797.1

MIGVFLVLLVTAAGLLYYFSLRRFQYWRDRGIPQLDGSLPIGSMKGIGRELSMNDLLDRV

YYRFQKQSPVAGLYFLINPVLLILDLDVVKQVLVKDFNSFHDRGMYVNERDDPMSGHLFS

IGGERWRYLRNKLSPTFTSGKIKQMFGTIQEIGDEIQATVEGCMDRGEPLDMKQLCQWFT

CDVVGSCAFGIQCGSLKNGGSELLEIGSRVFRQTPPRMLYNIAVSTFPSLSRALRLPLFP

LQFRTYFREMVRSTVEHREQHQIERNDFLNLLMQLKNRGRLEPTNEEPTDQEPWTSEQPT

DSKRLTLDEVSAQSFVFFFAGFETSSTTLTFALFLLASHPEEQERCRREILDKLATGGVD

GHPITYEALKQMTYLDQVIHETLRIYPAVGMLMRVVSKRVHLEAANLTLEKGTKVMIPIN

AFHHDPELYPEPYSFRPERFTPEAIKERHTHAYLPFGDGPRNCIGMRFGLLEVKFGIVQL

LSKLRFTVHPKTTLPLRMAKNSGFLEAEGGIWLSATRL

>EDS26139.1

MSLLSTVLYLVLPAIGLLYAYLRRKYAYWNDRNVPNDPGQFPMGTFAGTGTKYHFTQTMD

SVYRQFKRVSPVAGMYMTVSPVLLLIDLELVKQVLVKDFNFFRDPHLFSIEGDKWKFLRN

KLSPTFTSGKIKYMFLTVREIGDQFLQGFDKYVDSKSPVDLKALAKRFTCDVIGSCAFGL

NCNSLKNEGSELLTIGDKVFNAGPLQFFKIIFMTSCTAISRKLGLKVVPKDVTEYFMNVV

RGTVDHREKNNVMRSDFLQLLMQLKNKGTVEDHEEESQDKISMDEVAAQASLFFFAGFET

SSTALSFALFELANNQGIQEKTRAEVKRVLAAHGGQITYEALKDMSYLEQVVNETLRLHP

PVGNLFRVANQPYRLSKTDLTIEKGTLLMIPVTSIHQDPEIYPNPWQFDPDRFTPEAIQA

RHSHAFLPFGDGPRNCIGMRFALLEVKFGIAALLSKLKFTVNSRTQVPLELNVKSTLIEA

KGGLWLDVARI

>EFR28297.1

MTTFHILVIVAILLYFLIKKRYRYWTDRKVPQAQPELFFGNVRNVRRTVHLAERFRQLYD

ELKGKHPFGGIYMFFKPIVLITDLELLKCVFVRDFQYFHDRGNYYNEKDDPISAHLFNLE

GQKWRNLRNKISPTFTSGKMKMMYPTIVAAGEQLREFMEESVRDKVEFELKDLLTRFTTD

VIGMCAFGIECNSMRNPEAEFRLMGRKIFQRPTTTLRNLVVNTSPRLARAIGIRVIERDV

SNFFMKVVRETINYRVENNVERHDFMNILIGMRSDNETKSEDDKLTFNEIAAQAFLFFLA

GFDTSSALLAFTLYELAMNQELQEKARSSIEEVLQRHDGQLTYEAIMEMHYMEKVMKGLS

LESFATISMIRSFIHFLISFHSRSPETLRKYPPIAVHFRVAAKDYLVPGTDTVISAGTSV

MVPVYGIHHDPQYFPDPERYDPERFSAEEEAKRHPYAWTPFGEGPRICVGLRFGLMQARV

GLVLLLRSFRFGASEKTVYPMQLDPKSVILTLDGGMWLKVEKLK

>EDS45780.1

MLLYLVTIVTVMIYVWIKRRYSYWKDRGVPSLEVSFPAGNLQGIGHRHLGLIMQDLYGKL

KGSGAKFGGIYSFLKPMVMVLDLDFAKDVLVREFQYFHDRGMYYNEKDDPLSAHLVSLEG

DKWKSLRTKLTPTFTSGKMKMMFGTIEEVVDRLEGCIRVRVESGECIEIRDIISRFAMDV

IGSCAFGLDCNSLVLSDPPFWKMSLKASTSTKLQSLISLFATTYRKFSNQIGICVLPNDV

SDFYLGAVRDTIKFRMDNQVSRKDFMDLLIKLEDNFTFNEIAAQAFVFFQAGYETSSITM

TFCLYELALNQELQERARKSVEDVLKRHGSFSYDAIQDMEFLNCCVKETLRKYPPVANLF

REITKNYKVPETDITLEKGYRVVIPVYGIHHDPDIYPNPEVFNPERFIPEQSTNRHPMAY

LPFGEGPRTCIGERFALMETKICLSRLLQKFRFKLAPQTSTRIELNKTGVFLSIQGDLWM

KSKKTCHNLTVVTEPAAEN

>BAA28946.1

MLLYLVTIVTWLVYVWIKRRYSYWKDRGVPSLRVSFPAGNLQGIGHRHLGLIMQDLYGKL

KGSGAKFGGIYSFLKPMVMVLDLDFAKDVLVREFQYFHDRGMYYNERDDPLSAHLVSLEG

DKWKSLRTKLTPTFTSGKMKMMFGTIEEVVDRLEGCIRVRVESGECIEIRDIISRFAMDV

IGSCAFGLDCNSLVLSDPPFWKMSLKASTSTKLQFLISLFATTYRKFSNQIGICVLPNDV

SDFYLGAVRDTIKFRMDNQASRKDFMDLLIKLEDNFTFNEIAAQAFVFFQAGYETSSITM

TFCLYELALNQELQERARKSVEDVLKRHGSFSYETIQDMEFLNCCVKETLRKYPPVANLF

REITKNYKVPETDITLEKGYRVVIPVYGIHHDPDIYPNPEVFNPERFIPELSTNRHPMAY

LPFGEGPRTCIGERFALMETKIGLSRLLQKFRFKLAPQTSTRIELNKTGVFLSIQGNLWM

KVKKTCHNLTVVTEPAAEN

>EFA05731.1

MLFSAIILILALNYFYYKWKFTYWQCKKIPTLAPKIPFGDLPNPLKQAEATGVHIKRLYD

QIKGKGWKHAGLYFLARPVYMVVDLEYVKNILTKDFHHFVDRGFYFNEKDDPISAHVFAV

GGQKWRNLRTKLTPTFTSGKMKMMFQTLLECEANLLKRFEKEHKSAIDIKDILGCFTTDI

IGSCGFGLDFNTFEDENSPFRKYGKKVFASNLTRRLQIFFFTHFPSMARALKVRHFSKDV

ADFFTKVVKDTVEFRERNNYKRKDFMQLLIDLKNCNSGLTLNEITAQSLIFFLAGFETSS

TTMTFALYELAKNEKIQERVRDEIFSILGEEKITYEALQKMKYLALVINETLRKYPPLSF

LTRECVQDYKIPDQDVIIEKGTKVVISILGMHRDQEFYPDPETFDPGRFSEGNVVSRHQY

AYIPFGEGPRICMGLRFGMMQTKVGLVSLLRKFKFTVNGRTKEPIKFDVRGFILAAEGDI

WLNAQKVC

>EAT39035.1

MWIYLLLPILTLSFLLIRRRFSYWQRQGVPFIPARFPVGSFDGVGVRNHPSQLLAKFYRQ

MKGLHPFVGVYYFLQPVVVVLDLDFAKTILIRDFQYFHDRGLYYNEKDDPISGNLLHLEG

SRWTNQRKKLIPTFSSGKLRMMCPTILKVADNLKVSFERYVAERDEIEIKDILARFSTDV

IASCAFGLDCSSLLEADDEFRRMGTKVFDISGWKLLKLFFVFAFGNVARRCHMKLIDEDI

SQFFFKVVRETIDFRKKNHVHRKDFLNLLIQLKDNGELEGSNEKLGTLTLNEVVAHSFVF

FLGGFETASTTMSYCLYELSLNEEVQERARQCVKAAIHKYGDLNYDALLDMPYLEQCINE

TLRKYPPSTIYRIVTQNYHVPDSSIVFPKGMSVMIPVYAIHHDPEFWPSPELYDPDRFAP

EECVSRNPLTFIPFGEGPRMCVAARLGVLQTKIGLATLLMNFRFSRCKNSTEPLQYSPKH

FILTPVGGLKMRVEKIQ

>BAD84176.1

MLETLDRWLLQFAVLSTIILAALYVYFKISYSYWEKRGIPTLKFTAPFGNFTYSFLFGKN

PEYEVTEMYKAFEGHRVGGLYRFDSPSLLIRDTDIMKDILVKDFGHFVTRGLKFDEVSEP

LDGHLFSLSGAKWRNLRVKMTPIFTSGKLKTMFGTVVECGKDLQVALQKAANNEETVEIK

DFLARYSTDVISSCAFGIQCNCLKKPNAEFRNWGRRIFEPHFRQRLTSILNMLYPSLVYT

LKLSLIPKDVGNYFRKMVNETVEYRENNSVKKNDFMQMMIQLKNKTLGTAEENDMNFVEK

ETEELKSNASFDVTMNVIAAQAFVFYVAGFETSSTTMTFCLYELALNPDIQERLHAEIDT

MLEKHGGNITYESIFEMEYLDKVVNETLRKYPPVVILTRECTKPIKLRRTDVTLEKGLQV

LLPVLGLHHDPKYYPDPERFDPERFSEEEKKKRPHFSFLPFGEGPRMCIGMRFALMQTKV

GLISLLSNYEVRVSEKTPVPLVFEKSALVLTAAGGMWLMIENRQERSN

>EDS44713.1

MYIILLLVITLAILFIKQRYNYWKVRGVPYLDPAFPTGNVRGLGTRLNLAEVLHSCYERL

KDRGGPFGGIFFFLGPQVVATDLDFVKTILVRDFQYFHDRSLFYNERDDPLSAHLFTIEG

SRWRALRAKLTPTFTSGKMKLMFPIVRDVAGELEKCLGQELAMDSNVEMKDILARFTTDV

IGNCAFGLECNSLKNPGAEFREMGRRVSTLPPFTMVKFFLAQQIKPEARALGVTIMERDV

TDFFMSTVKDVVEYREKSKVQRDDFMNLLIELKNSAKDSERLTLNEIAAQVFLFFFAGFD

TSSTLMLFCLYELALNQDVQDKARQNVLEVLKHHGALSYDAVFDMKYLDKCISETLRMYP

PAAVIFRTATKDYPGFTVTLELYPDPMKFDPERFNPDQVAKRHPFAYLPFGEGPRLCIGM

RFAQMQTRVGLATLLQNFRFTVSPRTPIPPKIVPSSGLFVPQDNFWLNIERIE

>340720441

MLLATWLISDILAISSVVIAGWYIYYKLHIFKFWQKRGVFYVEPTFPTGNIMPIVIGKRS

QAEFFGDIYHRYKQHPLVGIYMLHKPFLIINDLDLTRDVLAKQFTNFHDRGFFCNEKTDP

LSGHLFQLPGKKWRNLRVKLTPTFTAGKLKQMFPIMKDIGNTLATYLEEKAQTRSTIDVK

DVFSRYAVDIIMSAAFGITCDSLENPDNEFRYWGKKIFDPKPLWNALIVWAPQVFDFFSI

PYTDKDVTNFFINVFKETVEYRESNNIERKDFLNLLMQLMRNGYVEADDNSEAATLAKNK

LTMTEAAAQAFVFYLAGFETSSTTVTFCLYELAMHQDIQNKVREEIQTHLEKHGGDLTYD

AVNEMTYLHKVISETLRKYPPVTVLNRICTNETTMEGTKFVIPKGIAVTIPVFGIHRDAN

IYPNPDKFDPERFSEENIKTRHPYAYLPFGEGPRICIGLRFGLIQTKVAVINALLKNKMT

LAPETPTTLDYEPGSLILIPKGGVHLTIEPLK

>350410091

MLLATWLISDILAISSVIIAGWYIYYKLHIFKFWQKRGVFYVEPTFPTGNIMPIVIGKRS

QAEFFGDIYHRYKQHPLVGIYMLHKPFLIINDLDLIRDVLAKQFTNFHDRGFFCNEKTDP

LSGHLFQLPGKKWRNLRVKLTPTFTAGKLKQMFPIMKDIGNTLATYLEEKAQTRSTIDVK

DVFSRYAVDIIMSAAFGITCDSLENPDNEFRYWGKKIFDPKPLWNALIVWAPQVFDFFSI

PYTDKDVTNFFINVFKETVEYRESNNIERKDFLNLLMQLMRNGYVEADDNSEAATLAKNK

LTMTEAAAQAFVFYLAGFETSSTTVTFCLYELAMHQDIQNKVREEIQTQLEKHGGDLTYD

AVNEMTYLHKVISETLRKYPPVTVLNRICTNETTMEGTKFVIPKGIAVTIPVFGIHRDAN

IYPNPDKFDPERFSEENIKTRHPYAYLPFGEGPRICIGLRFGLIQTKVAVINALLKNKMT

FAPDTPTTLDYEPGSLILIPKGGVHLTIEPLK

>AFI45023.1

MLLVVMLAVMLVLAYIYFKDSYKYWRRLGIPQLNPQFPFGDMADVIFRKQNMGDKINQIY

AHQRRQGHDYVGLYFFSRKAFLPLDPALIKDILGKDFQHFYDRGIYYDEKNDPLSAHLFS

IAGPKWKNLRAKLTPAYSPVKLKGMFNTILNCGKQMSEVIGEISEKQGEVEIKEILARYS

TDVIGCCAFGLDCNTMRNPEAEFRRMGRRAFTQTIGDVLKMIVIRSFPPIAKALRLGVFS

DSVTSFFRKVVDETITFRETNGIQRKDFMQLLVELKNNRSIVDDELEPAKQTEPGTALTV

DEAAAQAFIFFLAGFETTSTTTSFALFEMARCPHIQQRAREEALGILAQHGGEITYDALM

EMKYLDMIFNETLRKYPPAPVFLRKCTKSYRIPNTNAFIEEGQSVLIPCIGLHRDPVYFP

NPDLFDPERFSDQNKSKVKDGTYIPFGSGPRNCIGMRFAMIQSKVALVLSLVHYELRLSE

RTQLPLKMETKGIILAPIGGLWIEFKHR

>ABX64399.1

MAILTSCWCIDLGIILIGAFIGIYSFYQKKFKHWKKYNIPHSKPKFPFGDLISITTMGEN

FVLTLDRIYKSFEKEKVGGIWAFFHPILIVRDVEVIKDILVKEFMSFHDRGVPVDEKNDP

LSAHLFSISGSKWRNLRAKLTPTFTSGKMKMMFPILVESGREMISILEPNAINKDIVEMK

DILARFTTDVIASCAFGIDSNSLKNPDTEFRKMGRKIVEPSFANILRNFSFMAMPTLVKI

FKIKLIPEYLANFFMGSVRETMDYREQNNVKRNDFMQLLIQLKNKGKLDDVDEIKEKTDD

GNEIKELEVEENRSDQKFSFEEAAAQAFIFFVAGFETSSTTMVLALYELALQPEIQEKVR

NEIKTVLGKHNGKITYEAAFGMDYLGNVIDETLRKYPVAGVIIRQCNKDYKIPDSDMVIP

KGTSTHIPIYSLHYDSKYFPNPEKFDPDRFTEEVKSQRPRYAYLPFGEGPRHCIGMRFGL

MQTRVGLIELLLNYKFEICEKSPVPLKFSSMSFTPKEGMHLRLSKVN

>ABN80240.1

MAILTSCWCIDLGIILIGAFIGIYNFYQKKFKHWKKYNIPHSKPKFPFGDLISITTMGEN

FVLTLDRIYKSFEKEKVGGIWAFFHPILIVRDVEVIKDILVKEFMSFHDRGVPVDEKNDP

LSAHLFSISGSKWRNLRAKLTPTFTSGKMKMMFPILVESGREMISILEPNAINKDIVEMK

DILARFTTDVIASCAFGIDSNSLKNPDTEFRKMGRKIVEPSFANILRNFSFMAMPTLVKI

FKIKLIPEYLANFFMGSVRETMDYREQNNVKRNDFMQLLIQLKNKGKLDDVDEIKEKTDD

GNEIKELEVEENRSDQKFSFEEAAAQAFIFFVAGFETSSTTMVLALYELALQPEIQEKVR

NEIKTVLGKHNGKITYEAAFGMDYLGNVIDETLRKYPVAGVIIRQCNKDYKIPDSDMVIP

KGTSTHIPIYSLHYDSKYFPNPEKFDPDRFTEEVKSQRPRYAYLPFGEGPRHCIGMRFGL

MQTRVGLIELLLNYKFEICEKSPVPLKFSSMSFTPKEGMHLRLSKVN

>ABX64400.1

MAILTSCWCIDLGIILIGAFIGTYRFYQKKFKHWDKYNIPHSKPNSPFGDLISITTMGEN

FVLTLDRIYKSFEKEKVGGIWAFFHPILIVRDVEVIKDILVKEFMSFHDRGVPVDEKNDP

LSAHLFSIEGSKWRNLRAKLTPTFTSGKMKMMFPILVESGREMISILEPNAINKDIVEMK

DILARFTTDVIASCAFGIDSNSLKNPDTEFRKMGRKIVEPSFANILRNFSFMAMPTLVKI

FKIKLIPEYLANFFMGSVRETMDYREQNNVKRNDFMQLLIQLKNKGKLDDVDEIKEKTDD

GNEIKELEVEENRSDQKFSFEEAAAQAFIFFVAGFETSSTTMVLALYELALQPEIQEKVR

NEIKTVLGKHNGKITYEAAFGMDYLGNVIDETLRKYPVAGVIIRQCNKDYKIPDSDMVIP

KGTSTHIPIYSLHYDSKYFPNPEKFDPDRFTEEVKSQRPRYAYLPFGEGPRHCIGMRFGL

MQTRVGLIELLLNYKFEICEKSPVPLKFSSMSFTPKEGMHLRLSKVN

>EAT39032.1

MILILLLLAATALFFRWINAYRARYQFWKEHNVPHLEPRFPVGNAGDILKSTIHFAHIMD

NLYRELKHFGDYAGIYFFRDPVLVVLSPEFAKTVLVKDFNYFLDRGVYSNEKDDPLSANL

FFMEGHRWRKLRAKLTPTFTTGKLKAMFHTILAVGEQFDRYLQDYTKQKDEVEVKDLLAR

FTTDIIGSCAFGIDCNSLENPESKFRQMGKRMINFPKLKALKIFFAMMYRKQARWLRIRF

NDEDVSDFFFAVVRDTIRYREENNFERKDFMQLLIELKNKGYMEDDGEYVEELQGGRLEK

LTFEEIAAQAFVFFFAGFETSATTMTFALHLLASNQEVQDRGRKCVYEVLERHDGKLSYE

ALMEMTYIDCIIQETLRIYPPVATIHRITTKPYKLPNGSVLPEGVGVVIPNLAFQRDPEF

FPEPMQFRPERFFEDEKDKRHNFCHLPFGEGPRICIGMRFGLLQTRMGIAMLLKNYRFRL

CPKSVFPLKTDPINLIYGPAGDVWLGIEKIQ

>383849310

MSVTTWLIPEILSAVIIGTIGLYVYFKLFIFNFWRKRGVFYVEPTFPTGNILPLVLGTRS

QAEFTKDIYDDTKKHRVTGIFMFNKPFLVVNDLDLIRNVLTKDFMSFHDRGIFCNEKTDP

LSGHLFQLPGKKWRNLRVKLTPTFTSGKLKSIFPVLQESGDKLSKYLDDKASVRGVIDVK

DVFAMYSVDIIMSAAFGITCDSFKNPNNEFNYWGKKVFEPKPFWNAVVIWAPQVLDLLSL

PFNERGVTNFFLKMFEDTVTYRENNNIVRKDFLNLLIQLKTTETKLTMTEAAAQAFVFYL

AGFETSSTTVTFCLYELAMHQDIQDKVREEIRTVLKKHGELSYNAVNDMPYLHKVVSETL

RKYPPVVFLNRICTKDIQIETTDFHIPTGTSILIPVFGIHRDPDIYPDPDKFDPERFSEE

NVKARHPYAYLPFGEGPRICIGLRFGLIQTKVGIIRSLFKNRFKLAPNTPTSLEFKEGSL

ILMAKGGVNLTIESVE

>EFA02820.1

MTFMTESLIFDIVTIISTIVVLTIAFWKWKLSYWDRVGLPTLSPILFFGDTKDVLLGRLT

FGEQFQELYKKLKAKGLKHGGVYLGTRPFYLPTDPEIIKHIWQKDFQHFVNHGNYFDEEA

DPLSGHLFNLEDAKWKNMRVKLTPTFTSRKMKMMFQTLADCTHGLKDIMDEGASSGSPVD

IKEILGRFTTDIIGSVAFGLECNSLKDPNTLFRMYGKKIFELSTGRRLHLFLQFILPERV

LHAMKFKVTPSDVETFFMKVIRDTVGYREKNNVYRKDFLHMLIQLKNRGTVTDDEKITED

NGKTGEKALTMNELAAQSFVFFAAGFETSSTTVTFALLELALNQDIQQKLREEINTCLAK

SNGELTYQAIMEMTYMDKVLQETLRKYPPVPFLTRRCTKDYTIPETSIKLRKGDHVGISV

VGIQNDPEYYPDPEKFVPERFNEENKNSRHPFTWMPFGEGPRICIGLRFGMLQSKVGLAA

LLKNYKITLNSKTKMPIEMEKSSFITSVNGGVWLNVEKLS

>ADH29767.1

MTLITNNLTLDLVAIIATFLVGIIVYFKWQLSYWDRLGVPSLNPTLVFGDFKNYIFAKCS

LGEQFKELYDQFKSKGYKHGGIFVGPKPFYIPVDPDLGKHIMQKDFQHFMNHGFYINEEV

DPLTGNLFSLEDAKWRNMRVKLTPTFTSGKMKMMFQTLADCTRGLKEIMDNLALNHAPAD

IKEILGRFTTDIIGSVAFGIECNSLKNPDAEFRKYGRKIVETGFIDRIKVFLVLSIPHAL

LRFWRFKFTNTEVETFFMGAIQDTVNYREKNNVYRKDFMHLLLQLKNRGLVADDQKITDD

KGNIKENDLITINELAAQAFVFFLGGFETSSTTVSWALYELATNQDIQEKLRKEINDVLS

RHNNKLSYDAMMEMTYMDKVINETLRKYPPLPIIPRVCNKDYTIPNTSTKLSRGTSVAIP

VLAIHTDPEYYPNPEKFDPEHFSEENVKARPGFTWLPFGDGPRICIGMRFGMMQSKVGLA

TILKNYKIKLNNKTEFPIKVDPKNFITTAKGGVWLDVEKLD

>EFA02825.1

MAIFTSSIFIDIITIITTVLALTIAFFKWKLTFWEKLGVPTLNPVLFFGDAKNILLGKRQ

VGVEFIDYYKQFKPRGLKHGGVYLGPRPFWVITDPGLIKHILQIDFPHFINHGTYADEEN

DPLSAHLFSLEGTKWKNMRTKMTPTFTSGKMKMMFQTLVDCTPGLKDIMDEHASKKKAVN

IKNILERFTTDIIGSVGFGIECNSLKDPDSLFPKYGKKVFDLTPAEQLKQSLLFFLPNPV

LKAMKFRITNKDVEDFFMKTVRETVLYREKNNIYRKDFMHLLLQLKNRGTVTDDEKIIDH

SNQKQEPALTMNELTAQAFVFFLAGFETSSTTMTFALYELATNPDIQEKLRNEICTVLEK

HNNELTYNAIAEMTYMDQVLHETLRKYPPLPILQRQCNKDYIVPETNIRIPKGVDVAITI

LALHRDPEFYPNPEVFDPERFNEENKNARTPFTWLPFGDGPRVCIGLRFGMMQSKVGLTA

LLKDYRVTLNKKTKVPLEMSKNSFIIAAEGGIWLDIEKINT

>EDS26137.1

MDGLQSIFLAIFVTLFLGLATSAWLFLDRRRNYWKTRNFPCSSNPVLFFGDYKDTNKTEH

MSKTNQRLYQDFKARQLPVGGSILFVIQSAIVIDPELIKAILVKDFSTFHDRGVYNNPEV

DPLSGHLFALEGQPWRQLRMKLSPTFTSGKMKMMFETILGVANELRSYLGEKVGSEGSAE

VMMKDLLVGFTTDVIGSCAFGLECNSLRSEKCRVREIALQVFQQSKLQVLKMMFMMIFKG

IATTFKMRSLPAAVEKFFIELVHDTVRQREKNNVQRNDFMNLLIQLKNSEDPDARITMDE

MAAQSFVFFLAGSETSSTAMVNCMYELAMNQDIQDKLRNEITRVCGKGKLTYEAVNSVEY

LNMVIDETLRKHPSVDFLMRTSNSDFPVPNSDLTIPKGTFLIVPTYALQHDPDHYPDPDR

FDPERFNETNCASRHPFVYLPFGEGPRNCIGMRFGLMQIRVGLITMLREFRVLPGVNTPV

PLELDVTSNMPMPKGGVPLLVERI

>EAT33065.1

MTLLSIGVALLCVAAFAFLNYVFGYWKRRGIRQLTPHFPFGNFTDLFFGKASFPKVCENL

YERSKQWRLLGGYVLLRPILLVNDPQLAKDIMVKDFQHFHDRGPHVDEENDPLSGHLFSL

AGEKWKHLRAKLTPTFTSGRLKGMFQTLVDTGEVLQEYIQKYAEGEDVVEIREILARYNT

DNIASVAFGIKIDSINNPNEPFRHIGRKVFEPNFRNNMRGLITFMVPKLNKYLKIKSVDD

DVEKFILKVVQETLEYREKNGIVRRDMMQLLLQLRNTGTVSVDERWDVETSDKFKKLTLK

EVAAQAHVFFLAGFETSSTTMSFCLYELAKHPEIQRRVQAEIDSVTALHDGKLTYDSIND

MRYLECCIDETLRKYPPVPVLNRECTQDYKVPGMDFTIEKGTAIVLQIAGMQHDPQYYPD

PMQFKPERFQDPEVKSKPYAPFGDGPRVCIGMRMGKIQTKVGLCLLLSKFDFELFGHDEP

ELVMDPNNFVLTPVDGINLKVSCRE

>EAL39623.1

MFVQFLLVVSLGWLWIYLHFKQRYRFWAERQVPFLEPSFPAGNVPDTLKPTIHFAYIIEK

LYKRLKSKGDYVGIYFFRDPVLLVLSPEFARTVLVKDFNYFVDRGVYSNEEVDPLSANLF

FLEGNRWRKLRSKLAPTFTSGKLKAMFHTIVDVGNRLDQHLAEKCQQVKRIDVRQLMACF

LTDVIGSCAFGIDCNSLENPNSQFRVMGKRMINLPKLKALKVFFAMMFRKQARALGIRFN

DEDVSDFFMNVVRDTIRYREEHGERRDDFMQLLIDMMKQDGGAEEDPLKFEEVAAQAFVF

FFAGFETSATTLTFVLHLLAKHPEVQLEGRECVRDVLAKHDNKLSYDAVMEMEYLGWIVN

ETLRLYPPVATLHRITTQPYQLPNGTILPEGVGVILPNLAFHYDPDYFPDPYDFKPERFA

VKNDFKNNFSYLPFGEGPRMCIGMRFGLLQTRLGLAMLLRNYHFTIDPSDAARPLRIDPI

NLTHGPEGQVWLNVERIA

>ABL60878.1

MYFLIALGILLIILYLYGIKNHKYWEKKGVPYVKPIPFFGTNFKVFMQRICISDQLCKYY

EQFPNEKFVGAFIGDRIGLVLREPELIKRVMATDFQYFHPRGVNPHKTVYEPLLKNLFTG

DGDMWKLLRQRITPAFTSGKLKAMFPLIVERAERLQIIAATAAQSHGEVDVRELMARFTT

DFIGACGFGIDADTLNDEESTFRRLGKRIFTLTRRDGFVFMLKTIAPEIFKNLHMFAPEI

EKTTVDLVTSIMQQRKYKHSGRNDFIDFLLELKGKGKIVGESVEKRNPDGTPKIVEMELD

DMLMAAQVFIFFAAGFETSSSTTSYTLHQLAFHPEEQKKCQDQIDEVLSRHGGKLSYEAI

KEMTYLDMIFKESMRMYPSLGILTRRCVQKYTFPGTNLTIDEDVLVCIPVHALHNDEKYF

DEPEKFKPERFSPENIKNIPKYVYLPFGDGPRACIGERLGHMQSLAGLAALLSKFSVAPS

KNTLRQPITDPTCTVVKSIKGGLPLSLVARKV

>ABL60877.1

MYLLIALGVLLIILYLYGIKNHKYWEKKGVTYVKPIPFFGTNFRVFMQKISFSDQLYKYY

QQFPNEKFVGIFLGDRIGLLLRDPELIKSVMSTDFHHFHRRGINSHKTVHEPLHKNLFTG

DGDMWKLLRQRITPAFTSGKLKAMFPLIVERAERLQIIAATTAESQSEVDVRELMARFTT

DFIGACGFGIDADTLNDEESTFRRLGKRIFTVTKRDGLVLMLKSIAPETFKYLHMLPPEV

EKTTVGLVKSIMQQRNYKHSGRNDFIDLLLELKGKGKIVGESVEKRNSDGTPTIVEMELD

DLLMAAQVFIFYAAGFETSSSTTSYTLHQLAFHPEEQKKCQDQIDEVLSRHGGKLSYDAI

KEMTYLDMIFKESMRMYPSLGFLMRRCTQKYTLPGTNVTVDEDVVVLIPVHALHNDEKYF

DEPEKFKPERFSPENIKNIPKYVYLPFGDGPRACIGERLGQMQSLAGLAAVLSKFSVAPS

KNTLRHPITDPSSLIVQSIKGGLPLSLVARKF

>AFP20592.1

MITLAIALVALVALYLYGTRNFSYWQKKGVKHDKPIAFFGNNVASYLMKKSITQMAVDAY

WKYPKEKVVGFYRSTSPELVIRDPEIVKRILITDFQYFYPRGLNVHKTAIEPLMRNLFFA

DGDLWRMLRQRITPAFTSGKLKAMFPLIVERAEKLQARALSAATANREMDARDLMARYTT

DFIGACGFGLDADSLNEEDSAFRKLGVSIFDFKPKDVFVAMLKEMFPEACKNLKYLERLE

KPMFELVTAILKQRNYEPVGRNDFIDLLLECKKKGKMVVESVEKVNPDGSAETVSMELTD

ELIAAQVFVFFAAGFETSSSATSYTLHQLAYHPEVQKKVQDEIDRVLAKHNNKLSYDAIK

EMTYLECAFKEGMRMFPSLGFLMRQSAHKYTIPELDLTIDPGVNVVIPLQALHNDPQYFE

EPHVFRPERFLPDQVDEKTKFVYLPFGDGPRACIGLRLGLMQSLAGLAALLSRFTVAPGP

STLRYPVVEPKSGIVQSIKHGLPLSFTVRNPESVATTL

>AEL87782.1

MITLAIALVALVALYLYGTRSFSYWQKKGVKHDKPIAFFGNNAAAYLMKKSITQMAVDAY

WKYPKEKVVGFYRSTSPELVIRDPEIVKRILITDFQYFYPRGLNVHKTAIEPLMRNLFFA

DGDLWRMLRQRITPAFTSGKLKAMFPLIVERAEKLQARALSAATANREMDARDLMARYTT

DFIGACGFGLDADSLNEEDSAFRKLGVSIFDFKPKDVFVAMLKEMFPEACKNLKYLERLE

KPMFELVTAILRQRNYEPVGRNDFIDLLLECKKKGKMVVESVEKVNPDGSPETVSMELTD

ELIAAQVFVFFAAGFETSSSATSYTLHQLAYHPEVQKKVQDEIDRVLAKHNNKLSYDAIK

EMTYLECAFKEGMRMFPSLGFLMRQSARKYTIPELDLTIDPDVNVVIPLQALHNDPQYFE

EPHVFRPERFLPDQVDEKTKFVYLPFGDGPRACIGLRLGLMQSLAGLAALLSRFTVAPGP

STLRYPVVEPKSGIVQSINTACRFPSQ

>ABJ97708.1

MLTAAIFVIIVALVYLYSTRTFRYWEKRGIKHDKPVPFFGTDSEGYLLRKSMTQTAVDAY

LKYPNEKVIGFFRSSRPELIIRDPDIIKRILTTDFAYFYPRGLNPHKKVIEPLMRNLFFA

DGDLWRLLRQRMTPAFTSGKLRAMFPLVVERAERLQSRTLEIASQPLDARELMARYTTDF

IGACGFGLDADSLNDEDSAFRKLGAAIFNITVQQAIVAALKEIFPGIFKHFKYSSKYETD

FVSLVSSILKQRNYKPSGRNDFIDLLLECRMKGEIVVESIEKMKPDGTPEIIRMELTEQL

LAAQVFIFFAAGFETSSSATSFTLHQLAFHPEIQEKVQKELDQVLAKYNNKLCYDAIKEM

RYLESAFKEAMRMFPSLGFLIRECARQYTFPELNLTIDEGVGIIIPLQALHNDPKYFDSP

NEFRPERFMPSEYNHNKTKFVYLPFGDGPRGCIGARLGLMQSLAGLAAVLSKFTVKPAPN

TKRHPVVEPKSSIVQSFKDGLPLLFIERTKP

>ABK27873.1

MLTAAIFVIIVALVYLYSTRTFRYWEKRGIKHDKPVPFFGTDSEGYLLRKSMTQTAVDAY

LKYPNEKVIGFFRSSRPELIIRDPDIIKRILTTDFAYFYPRGLNPHKKVIEPLMRNLFFA

DGDLWRLLRQRMTPAFTSGKLRAMFPLVVERAERLQSRTLEIASQPLDARELMARYTTDF

IGACGFGLDADSLNDEDSAFRKLGAAIFNITVRQAIVAALKEIFPGIFKHFKYSSKYETD

FMSLVSSILKQRNYKPSGRNDFIDLLLECRMKGEIVVESIEKMKPDGTSEVVRMELTEQL

LAAQVFIFFAAGFETSSSATSFTLHQLAFHPEIQEKVQKELDQVLAKYNNKLCYDAIKEM

RYLESAFKEAMRMFPSLGFLIRECARQYTFPELNLTIDEGVGIIIPLQALHNDPEYFDSP

NEFRPERFMPSEYNHNKTKFVYLPFGDGPRGCIGARLGLMQSLAGLAAVLSKFTVKPAPN

TKRHPVVEPKSSIVQSFKDDLPLLFIERTKP

>BAI47531.1

MLTAAIFVIIVALVYLYSTRTFRYWEKRGIKHDKPVPFFGTDSEGYLLRKSMTQTAVDAY

LKYPNEKVIGFFRSSRPELIIRDPDIIKRILTTDFAYFYPRGLNPHKKVIEPLMRNLFFA

DGDLWRLLRQRMTPAFTSGKLRAMFPLVVERAERLQSRTLEIASQPLDARELMARYTTDF

IGACGFGLDADSLNDEDSAFRKLGAAIFNITVQQAIVAALKEIFPGIFKHFKYSSKYETD

FMSLVSSILKQRNYKPSGRNDFIDLLLECRMKGEIVVESIEKMKPDGTSEVVRMELTEQL

LAAQVFIFFAAGFETSSSATSFTPHQLAFHPEIQEKVQKELDQVLAKYNNKLCYDAIKEM

RYLESAFKEAMRMFPSLGFLIRECARQYTFPELNLTIDEGVGIIIPLQALHNDPEYFDSP

NEFRPERFMPSEYNHNKTKFVYLPFGDGPRGCIGARLGLMQSLAGLAAVLSKFTVKPAPN

TKRHPVVEPKSSIVQSFKDDLPLLFIERTKP

>ABN71370.1

MLTAAIFVIIVALVYLYSTRTFRYWEKRGIKHDKPVPFFGTDSEGYLLRKSMTQTAVDAC

LKYPNEKVIGFFRSSRPELIIRDPDIIKRILTTDFAYFYPRGLNPHKKVIEPLMRNLFFA

DGDLWRLLRQRMTPAFTSGKLRAMFPLVVERAERLQSRTLEIASQPLDARELMARYTTDF

IGACGFGLDADSLNDEDSAFRKLGAAIFNITVQQAIVAALKEIFPGIFKHFKYSSKYETD

FMSLVSSILKQRNYKPSGRNDFIDLLLECRMKGEIVVESIEKMKPDGTSEVVRMELTEQL

LAAQVFIFFAAGFETSSSATSFTLHQLAFHPEIQEKVQKELDQVLAKYNNKLCYDAIKEM

RYLESAFKEAMRMFPSLGFLIRECARQYTFPELNLTIDEGVGIIIPLQALHNDPEYFDSP

NEFRPERFMPSEYNHNKTKFVYLPFGDGPRGCIGARLGLMQSLAGLAAVLSKFTVKPAPN

TKRHPVVEPKSSIVQSFKDDLPLLFIERTKP

>ADE05584.1

MLFVLLVIVFTCLYYYGVRTFDYWKKKGVKHDPPLPYFGNNLKQFMQQASMCMMATEVYK

KYPEEKVVGFYRGTSPELVVRDPEIIKRVLVTDFNCFYARGFNPHKKVIEPLLKNVFFAD

GDLWKLIRQRFTPAFSTGKLKAMFPLITERAEKLQVLAEDIVDEEYYDVRELMARYTTDF

IGVCGFGINMDTMSDDNSQFRRLGKRIFERNFRDGVCGALKLMFPELCKNLTLLSPELEI

SMVKLVQSVLRDRNYKPSGRNDFIDLMLELKEKGKIIGESIESRNPDGSKKIVELEMDDL

LMTAQAFVFFGAGFETSSTASSYTLHQLAFNPDCQKKVQQEIDTVLAKYNNKLTYEAVKE

MHYLEMAFYEAMRMYPSVGYLIRECTVPEYTIPEINLTINEGVKLMIPMQAIHKDEKYFR

DPENFDPERFQDGAKEDIKNFVFLPFGEGPRACVGARLGQMQSMAGIAALLHKFSVEPAA

CSLKNLLPEPTGIVSEGFIGGLPLKLRRR

>ABQ45551.1

MFFYLLIVILVALYYYGIRTFDYWKKKGVNHDPPLPFFGNNIRQFMQKASMAMMATETYK

KYPEEKVVGFYRGTSPELVVRDPELIKRILVTDFSSFYARGFNPHKKVIEPLLKNLFFAD

GDLWRLIRQRFTPAFSTAKLKAMFHIITERAEKLQMIAENEAYENFCDVRELMARYTTDF

IGACGFGLNIDSLSDENSQFRKLGKRIFKRDLSDAVRAALKLMFPELCKHLTFLTPELEK

SMTYLVQNVIREKNYKPSGRNDFIDLMLELKQKGKLLGESIEAKNANGTPKQVELEFDDL

LMTAQVFVFFGAGFETSSTASSYTLHQLAFNPKCQEKTQKEIDKVLSKHNNKITYDAIKE

MTYLEMAFNEAMRLYPSVGYLVRMCTVPEYTFPEINLTINEDVKLMIPIQAIHKDEKYFK

DPERFHPERFSSGAKANLKPYTFLPFGEGPRACVGERLGQMLSMAGLVAVLQKYTVEPVE

ISLRDPIPDPTTTVSEGFVHGLPLKLRRRERRI

>ACN88551.1

MFFYLLIVILVALYYYGIRTFDYWKKKGVNHDPPLPFFGNNIRQFMQKASMAMMATEMYK

KYPEEKVVGFYRGTSPELVVRDPELIKRILVTDFNSFYARGFNPHKKVIEPLLKNLFFAD

GDLWRLIRQRFTPAFSTAKLKAMFHIITERAEKLQIIAENEADGNFCDVRELMARYTTDF

IGACGFGLNIDSLSDENSQFRKLGKRIFERDLSDAVRGALKLLFPELCKHLTFLTPELEK

SMTYLVQNVIREKNYKPSGRNDFIDLMLELKQKGKLLGESIEAKNANGTPKKIELEFDDL

LMTAQVFVFFGAGFETSSTASSYTLHQLAFNPECQEKTQKEIDEVLSKHNNNITYDAIKE

MTYLEMAFNEAMRLYPSVGYLVRMCTVPEYTFPEINLTINEDVKLMIPIQAIHKDEKYFK

DPERFHPERFSSGAKANLKPYTFLPFGEGPRACVGERLGQMLSMAGLVAVLHKYTVEPVG

ISLRDPIPDPTTIVSEGFVHGLPLKLRRRERRI

>AET11927.1

MITLAIIVVLLISLYLYGTKNFKYWEKRGIKHDKPLPLFGNNIKGYFFYSSMTQVADELY

WKYPNERVVGFFRASEPELPIRDPEIAKRLLSNEFSHFFQRGFTPNKTVFEPMMQNLFFA

EGDLWKLLRQRMTPAFTSGKLKAMFPLIVERAERLKTRALAAAADGKSLDARDLMARYTT

DFIGACGFGLDADTLNDENSPFRQLGINIFKIKPFELVKLVLKEMFPDLCQNVKIWTRVE

KDINELVGEIMAKRNNKPSGRGDFIDLMLECKMKGTMVGESIESRKPDGSPETASLEFND

GIIAAQVFVFFAAGFETSSSATSFTLHQLAYHPEVQKKAQEEVDRILAKHDGMLSYDSIK

EMNYLEMAFKEGLRMFPSLGFLLRQCTRPYTFPEFNMTIDETCKILIPLQSLHNDPKYFP

NPEVFRPERFSPEEFDSNNKFVYLPFGLGPRACIGERLGLMQSLAGLAAVLSEFTVEPAP

ETLRYPVVDPQSSIVQSVQGGLPLMFRPRKKLA

>AFP20591.1

MIVIAVVVVLLITLYFYGTRNFKYWEDRGVKHDKPLPLFGNNLQGYLFRSSMTQVADEIY

WRYPNEKVVGFYRSSRPELLIRDPEIAKRILITDFAHFFERGFTPHKTVFEPLMQNLFFA

EGDLWKLLRQRMTPAFTTGKLKAMFPLIVERAEKLKTRALTAAAEGKSLDARDLMARYTT

DFIGACGFGLDADSLNDEDSPFRKLGIKIFKMRPQEIITAIAKELFPELGRNLKIWTRVE

NDINELVVEILKKRDYKPCGRGDFLDLMLECKQKGVMVGESIQNKKPDGTAETVTLEFTD

SLIPAQVFVFFAAGFETSSSATSYTLHQLAYHQDVQKKAQEEIDRVLAKHDGKLSYDAVR

EMHYLENVFKEGLRMFPSLGFLLRQCTKSFTFPDLNLTIDEGVRALIPLQSLHNDPKYFP

EPHVFRPERFEPDEFDANNKYVYLPFGGGPRACIGERLGLMQSLAGLAAVLSCFTVEPAK

ETVRHPVVDPLSSVVQSIKAVCHSCSGRERNMLELT

>EHJ69768.1

MVKHDKPVPFLGNNARVYTMQMSVTQLSDEVYWRYPTERVVGFYRGLLPELVVRDPELVK

RVLVTDFASFYPRGLNRHREVELLLRNLFFVDGDLWRLLRQRMTPAFTSGKLKAMFPLIE

ERAERLRSRALSASAKGRPVDARDLMARYTTDFIGACGFGLDSDSLQDENSAFRKLGAKI

FDIPIKDVILSVLKEMFPSTFKNIRVMARVEKDLMALVNEVLRQRNHEPSGRNDFIDLML

ECKKKGIITGDSIEKVNSNGSPEKASVEMTNELIAAQVFIFFAAGFETSSSATSTTLHEL

AYHPEVQKKVQNEIDTVLAKYNNKLCYEAIKEMTYLEWAFKEGMRIFPSLGFLIRQCAKP

YTFEDLGLTIDEGVKIIIPLQALHNDPQYFDNPSEFRPERFDPQNFDSRNRFVYLPFGEG

PRACIGERMGMMQSLAGLAAILSRFSVQPAPGAPRKPTIDTRSNIVQVIRGGLPLIFTER

RPD

>EHJ70413.1

MFLIILACLVALYFYTTRNHRYWAKKNIKHERPIPLFGTHFRNIVALKSASELIHEFYVK

FGDEKVIGAYRGNDKELIVKDPDIIRDILMVDFNSFHNRGLGRDPEKEPLLKNLFHAEDD

LWKLLRHKLTPAFTTAKLKSMFPLIVNCAEKLVGLGENIIAKGGDCDIREVMAKFTTEFI

GACGLGIEMDTINNESSIFRQIGKKMFSRSDMEVFLFAVWDVFPEIRNLMNLSNKKVEQV

FFDMVTKIFEQRNYKPIGRNDFVDLLLDYLSQGKIQSESIKYKNPDGTAKQVEMEMDIEV

LVAQVFVFFAAGFETSSSTTSYTLHELAYHPETQKKIQDEIDRVLSKYDNKLCYDAVNEM

TLLDMAMKEALRIMPAVGILNRQCVKPYTIKQVGLTIDPDVNIVIPVQSLHLDEKYFDEP

YEFKPERFADEDFNQRTKNVYLPFGAGPRACIGARLGQMQSLAGLAVMLHNFSVEPSENT

KRTLDINPRLNVVQGVLHGVPIKLVKRK

>AFP20585.1

MSLLLLPLVLVLVYHYVTRNHDYWEKRNVKYEKPVPIFGTLYANLVAKKSITEISVEMYN

KYPNEPAVGIYRGTTPELIIRDLDVVRRILNVDFAYFYPRGVGRNPKEEPVFLNLFHVDG

DMWKLLRQRLTPTFTTAKLKNMFPLVVQCAEKLQTVGENIVNRGGDCDVRELMARFTTEF

IGACGFGIQMDSINDEHSLFRALGKKMFQRSWKNYLVVALYDLFPEFRTIIQKSLRETEI

MDAISEIVENIRKQRNYKPIGRNDFIDLLLELESKGKIVGESVEKRDANGKPEQVEMEMD

LTCMVAQVFVFFAAGFETSSSATSFMLHQLAFHPEEQRKIQENIDQVLAKYDNKLCYDSI

SEMTALSNGFKEAMRLFPSLGTLHRVCAQKYTIPEMGITLDPGVKIIVPVQAIQVDGKYF

ENPTEFNPDRFNDTSADRHKFAYLPFGEGPRQCIGARLGEMQSLAGLAAVLHKFSVEPAA

NTKRHLEVNHGSNTVQSIKGGLPLKLRLRSKQSQVAA

>BAM18399.1

MRGINTHKTVIEPMAKNLFLAEGDLWKLLRQRMTPAFTSGKLKAMFPLIVERAERMQTHA

INAPPTGRVLDSREIMARYTTDFIGACGFGLDMDSLTTENSSFRKLGMDIFKFEVSQIIK

QMLKEMFPEACKHFRIFSKIEKDANALVKTILEKRNYEPSGRHDFVDLLLEYRKKGKIVG

QSIEIMKSNGMPEEVSLELTEELITAQIMVFFGAGFETSSSSTSYTLHELAYNPEEQQKV

QEEIDIVLAKYNNKLCYEAVSEMTYLHCAFKEGIRMFPSLGHLVRKCARKYTFPDLDLTI

DEGVSILIPVQALHMDPLYFEEPEKFKPSRFLTDFINPLTKHIYLPFGEGPRACIGERLG

LMQSLAGLAAVLSRYSVEPAPETLRHPIVDPTSNIVQMCCRWITFDV

>EHJ75236.1

MFPLIVERTEKLAKLAEGMAETCDELDIRDLMARYTTDFIGACGFGIDSAALNDENSDFR

KLGKRIVTVTKRDHFVSLLKRMMPVLCKDLHFLAPEIENNTVSIVQQIMAQRNYKPSGRN

DFVDMMLELREKGNLVELDDQLIAAQVFIFFVAGFETSSSASSFLLHMLAYHPEVQEKCR

KEVDEVLKNHEGKLSFDAVKDMKYLEMSLKESVRFLTSPGFLIRRTVNKCTLPGTNFTLD

ENMVMIVSSQAMNMDGELFENPEEFRPERFNPDNIGDIKKCTFMPFGDGPRSCIGERLGI

MQSLAGVATILSKFTLSPSRSSLRKPRIDPMSTIVQTVIGGLPLSLKRRKDIL

>AEY75583.1

MITSLLLTAVFVIIFTIYLVSKKKFQYWEKRKVPHLPPVPLLGNFGKFILQRQFLGYTLQ

DICEKFPKAPYVGAYFGTEPALLIQDPELIKLVITKDFYFFSGREVSEYVDKERFTQNLF

STSGNKWKVLRQNLTPVFTSAKMKNMFHLIEKCSHVFEDFLDKEAKSDDVEMRALIARYT

MDCIGTCAFGVETKTMNETENNPFTAVGNSIFTMSWFQGFKFVLRGIYPALFYALGFRSL

PPEVDAFFSNLMTGVFKGRNYTPTSRNDFVDFVIKFKQNKTMTGDSLTNMKSDSQKKVSL

EVDDDLLVAQCFIFFAAGYETSATTLSFTLYELAKNPEAQKRAIAEVDDYLRRHNNELKY

ECLSEMPFIEACIDETLRIYPVLSMLTREVVEDYTFPTGLKVEKGLRIFLPLYHLHHNPE

FFPNPEEYRPERFLPENKDNIKPYTYMPFGDGPRICIGMRFAKMQMAAGIITLLKKYRLE

LAPGMSSKIEFEPNSFVSQVVGGIKLKMIEREGWEGRLLKNLEKAY

>ABI84381.1

MITSLLLTAVFVIIFTIYLVSKKKYQYWEKRKVPHLPPVPLLGNFGNFILQRQFLGYTLQ

QICGKFPNVPYVGAYFGTEPALIVQDPEHIKLVMTKDFYFFSSREISEYADRERFTQNLF

STSGNKWKVLRQNLTPVFTSAKMKNMFHLIEKCSHVFEDFLDKEAKSNEVEMRALVARYT

MDCIGTCAFGVETKTMNVTENNPFTAVGNSIFMLSRVQGFKFVLRGIYPSLFYLLGFRTL

PPEVNAFFSNLMTGVFKGRNYTPTSRNDFVDFVLKWKQNKTMTGDSLTNMKYDSQKKVTL

EVDDDLLVAQCFIFFAAGYETSATTLSFTLYELAKHPEAQKRAIAEVDDYLRRHNNELKY

ECLSEMPFVEACFDETLRKYPVLSLLTREVVEDYTFPSGLKVEKGLRIFLPLYHLHHNPE

FFPDPEEYRPERFLPENKDKIKPYTYMPFGEGPRLCIGMRFAKMQMTAGIITLLKKYRLE

LAPGMPQNIEFEPNSFVSQVAGGINLKMIKRESWEGRLLKNLEKAY

>ADW23116.1

MITSLLLTAVFVIIFTIYLVSKKKFQYWEKRKVPHLPPVPLLGNFGNFILQRQFLGYTLQ

QICGKFPNVPYVGAYFGTEPALIVQDPEHIKLVMTKDFYFFSSREISEYADRERFTQNLF

STSGNKWKVLRQNLTPVFTSAKMKNMFHLIEKCSHVFEDFLDKEAKSNEVEMRALVARYT

MDCIGTCAFGVETKTMNVTENNPFTAVGNSIFMLSRVQGFKFVLRGIYPSLFYLLGFRTL

PPEVNAFFSNLMTGVFKGRNYTPTSRNDFVDFVLKWKQNKTMTGDSLTNMKYDSQKKVTL

EVDDDLLVAQCFIFFAAGYETSATTLSFTLYELAKHPEAQKRAIAEVDDYLRRHNNELKY

ECLSEMPFVEACFDETLRKYPVLSLLTREVVEDYTFPSGLKVEKGLRIFLPLYHLHHNPE

FFPDPEEYRPERFLPENKDKIKPYTYMPFGEGPRLCIGMRFAKMQMTAGIITLLKKYRLE

LAPGMPQNIEFEPNSFVSQVAGGINLKMIKRESWEGRLLKNLEKAY

>AEY75585.1

MITSLLLTAVFVIIFTIYLVSKKKFQYWEKRKVPHLPPVPLLGNFGKFILQRQFLGYTLQ

DICEKFPKAPYVGAYFGTEPALLIQDPELIKLVITKDFYFFSGREVSEYVDKERFTQNLF

STSGNKWKVLRQNLTPVFTSAKMKNMFHLIEKCSHVFEDFLDKEAKSDDVEMRTLIARYT

MDCIGTCAFGVETKTMNETENNPFTAVGNSIFTMSWFQGFKFVLRGIYPALFYALGFRSL

PPEVDAFFSNLMTGVFKGRNYTPTSRNDFVDFVIKSKKNKTMTGDSLTNMKSDSQKKVSL

EVDDDLLVAQCFIFFAAGYETSATTLSFTLYELAKNPEAQKRAIAEVDDYLRRHNNELKY

ECLSEMPFIEACIDETLRIYPVLSMLTREVVEDYTFPTGLKVEKGLRIFLPLYHLHHNPE

FFPDPEEYRPERFLAENKDNIKPYTYMPFGDGPRICIGMRFAKMQMAAGIITLLKKYRLE

LAPGMSSKIEFEPNSFVSQVVGGIKLKMIEREGWEGRLLKTL

>ADE05581.1

MLPLFLILFLSSLLLIIYYVSKRQFGYWERKNVPYMKPAPVLGNYSGYILLKEYIGHSVD

KIYRKFPNAPYVGAFYGTEPTLIVRDPELIKIVMTKDFYYFSSREIAKYCHKEVITQNLF

FTFGDRWKVMRQNLTPLFSSGKMKNMFHLIEKCSRVFEKVLDNETAMSNVLESRSLLIRF

TMDCIGSCAFGVDTNTLVNAEGNPFTTMGNEIFASTTSRGIKNITRAVWPFVFYGLGFKS

FPPTIEQFFSKLLTGVFEARGYKPSQRNDFIDLILNLKKNNFIEGDAITNLKTGDADKVQ

LEVNNDLLVAQCIVFFAAGFETSATTSCLTLYELAKCPEAQKRVHEEVDSYMKRHGNKLD

YSCVSELPYLLACIDETLRLYPVIGVLTREVVEDYTFPTDLTVEKGMRVHVPVFSLHRDP

EYFPDPEEYRPERFYGEAKHNVKPYTYMPFGDGPRTCIGMRFAKMQMLAGLITLLKKFSV

DLADDMPSAVGFEPKAIVTQPMGGIYLKFNKREGWEQRMLV

>ABQ08711.1

MFLLINICVILFVIYYLVTKKYSYWRNRNVSHEKPVLLLGNYGDLILQKKNFGEMARAIC

QKFPGEPVVGAFFGTEPVLIPQDPEVIKTILTKDFYYFNGREISEHVHKELLSYNLFATY

RDEWKILRQNLTPIFSTAKLKSMFTLIEKCSKSFQNLLEDETKISKELEVRTLMQRFTIE

CIGSCIFGVDTDTLGNDKMNPFKAAGSQLSDFSRLVFVKGIVRAIWPTLFYALGFKTFTT

ELDIFKKLVNAVFAQRKHKPTTRNDFVDLILTWKNNNTITGDSIGSFKNSDKTKFSIDVN

DDLLLAQCLVFFAAGFETSAMTSSYTLHELAKNQRALKKACDEVDAYLLRHGNKINYDCV

TELPYLEACIEETLRLYPVLGIITREVMEDYVLLDKIHLKKGDRIHVPVFHLHHNPEHFP

NPEEYRPERFYGEEKRKVKPYTYLPFGEGPRICIGMRFAKMQSIAGLITILKKFRLELPE

GAPTKIEFKPEAFVTTPKDLIKIKFLEREGWQQRVFV

>AFP20588.1

MFLYLFVTLLILFYYVITRKYDYWKVRNVAHSKPTFLLGNYGDMILQKKSFGENIQDCCN

KFPEAKYIGVFFGTEPVLIPKDPEIIKLIVTKDFYYFNGRELSKYNHLEPAALNVFATYG

DNWRVLRQNMTPLFSSAKMKNMFHLIEKCSYVFEEMLDREIKISNELEVRSMTSRYTMEC

IGACAFGVDTNTMAEGVEENPFKKIAAFVEPPTYVVYVRIIRSIWPSIFYLFGGRLLKKQ

LEFFKNLITSVMSKREYKPSGRHDFVDLVMSWKQSHQIVGDSIKNMKTGEHKKVTLEAND

DLLVAQCFVFFAAGFETSSTTLSYTLYELAKHEEIQTKVLDEVDAYLARHNNKLNYDCVT

ELPYLEAVIDEALRFYPVLGMIPRELMEDYTMPDGTKLTKGLRVHLPVFYLHHNPENYPE

PEVFRPERFLGEEKRNINPYVYLPFGEGPRTCIGMRFAKMQMLAGLITMLKKYRPEFLAD

GMPTKLKFKAQSFITHPVGGIKIKFIEREGWESRVFAKSS

>ADE05580.1

MFNILLIVFGVLFVVYYLTTRKFNYWKKINVPHLPPVPLFGNYASMILQRKTFGEVVSDI

CEKFPKAPVVGAYFGTEPVLIPRDPEVIKTILTKDFYYFNGREVSEYAHRDGDLFTLFAT

YGDHWKVLRQNLTPLFSSAKMKNMFKMITQNAHAFEKLLEQDTKQEVLDVKNMMERFTIE

CIGSCVFGVATNTIEKGEANPFKKAGNDIIKPNKMLAFKGVMRAIWPSIFYGLRIPLFTN

ELNLFRQVLTAVFKERETNPTKTQDFINLIMKWKQQSHIEGDKLRPAHSAENGKTSVDVT

DELVRAQCVVFFATGFETSAVTLTHTLYELSKNERVMQKACEEVDAFMKRYNNHMTYESL

SELQYLDACLDEALRKYPVLGCITREVMHDYILSPGIHIEKGMRVHIPVQYLQNNANLFP

EPEEYRPERFIGEEKRNIIPYSYMPFGEGPRICIGMRFAKMQMLAGLVTVLKSYKVTLGD

NVPKKLRFLPESFVTVSADRIKLKFTRRNGWEKALYAQHS

>ABB69054.1

MITLLLSLVIPLVFFAIYITSKRKYEHWEKKKIPHLKPVPILGNFSEYILQKTYLGQVAQ

KICKKFPNEPFIGAYYGTEPALIVQDPELIKIVMTKDFYFFSGREVSNYSHKEMFTQNLF

FTYGDRWKVLRQNLTPLFSSAKMKNMFHLIEKCSRVFEKVLDKETQKCDEIEVRALMAGF

TMDCIGSCAFGVDTNVMEKTENNIFRKIADLIFDIQGFRAFKNVARAIWPSVFYGLGFRA

FPREIDNFFNNLMSGIFKGREYKPTNRNDFVDLVLKLSNNKTMTGDSLLNMKSDADKKVV

LEVDEEMLVAQCVLFFAAGFETSSTTLSYALYELAKNPEAQEKALAEVDEYLRRHDNTLK

YECVAGTPYLEACVDEALRLYPVLGLLTREVAEDYTFPSGLEVEKGLRVHLPVYHLHHHP

EHFPDPEQFRPERFLAENKQNIKPYTYMPFGEGPRICIGMRFARMQMAAGLLTLLKKYRV

ELAPGMKRELVFEPGAIVTQPIGGIKLKFVERENWKERLLKTP

>AFP20594.1

MIFLYLILVFIFLFYYVISRKYKYWKIRNVPYKEPTFLLGNFTDMFLDKKFNGEIMDDIC

KQFPDDKYVGAFNGIEPVLIPKDPEIIKLIMTKDFYYFHGRALSKYNDKEPGGLNLFGTF

GDNWRVIRQNMTPLFTSAKMRNMFHLIEKCSHEYEAMIDREIKISKEHDVMPMTTRFTMD

CIGACAFGIDTKTMTEGEDDNPFRRIIRSSKPRKSMIYQGMLRSLYPEIYYALGFKMPST

QLDFLPKLVTSVMNQRDNKPSERNDFVDLIMSWKQKHQIVGDSIRNMITGEHEKVVLKAS

DDLLIAQCYVFFAAGFKTSTTALSYTLYEIAKHEDVQNKVFNEIDSYLARHNNKINYDCV

SELPYLDAVIDETFRVYPVLSVIPRELMEDYTMPDGTKLDKGVTVQLPVYHLHHNPKFYP

EPEVFRPERFMGEEKKKINPYVYLPFGEGPRVCIGMRFAKMQMLAGLITMLKKYRLELAD

GMPTKLKFKAQSFITHPVGGIKIKFIEREGWESRVFAKSS

>AAP83689.1

MSVILILSSILTLLITSIYLISTRKFNYWKKKNVPHLKPKLILGNYGDFLLMKKPNGLVV

QEICQAFPNEPYIGAYYGTEPALIVQDAEIIKLITIQDFSYFNSREVGDHTHKEDMSKNI

FSTNGDRWKVVRQHMTPLFTSSKMKNMFHLIEKCSHEFERMLDREFKMSNVQEVRALTAR

FTMDCIVSCGFGLDANTMGDNSKENPFTKLSNSIFEVSNLRFLAMQARNLWPSIFYGLGL

RTFPKSIDDFFHELITNVFKQRQYTPTSRNDFVDLIMNWTKKDYLTADSFKHDKEKGSTK

ISLKVDDKLLVAQCSIFFGAGHETSATTLSYTLFELAKNPEAQKRAIEEVDAYLLKNNNK

LGYDCVTELPFLEACLDETLRLYPVLSVLTREVVEDYTLPTGLHLDKGVRIHLPLYHLHH

NPDNFPEPEKYRPERFVGDERKNIKPYTYMPFGEGGRTCIGMRFSKMQTLSGLVTFLKKY

RVELAKGTSPTVEFEPRALVFISKNGINLQMMEREGWEKRLFAK

>AFP20589.1

MITLYLLLAIPLALYGVYLVSKRKFRYWEQKKVPHLPPKPILGNFSEYILQQKYYGHVEQ

DICDKFPEEPYVGSYFGTEPALIVQDPEFIKTVMTKDYYFFSGREASAYSKNEALTQNLF

FTYGDRWKVLRQNLTPLFSSAKMKNMFHLIEKCARIFENMIDQDVQKCKDIEVRTLTAKF

TMDAIGNCAFGVETWTMVKTENNPFTPFTKIGDIIFDTARLRALKVVMRSIWPAIFYGCG

GKSLPADVDNFFYNLMTGIFKGRNYKPTPRNDFVDLLLKFYNNKTVTTGDSMKNMKGDSE

EKVTLKVDEEFLIGQCFLFFAAGYETSATTLSYTLYELAKNPKAQELAIQDVDNYLRRND

NELKYECATELPYVEACVDEALRLYPVLGVLTREVIEDYTFSTGLKLEKGLRVHLPVYHM

QHNPKYFPEPEQYRPERFLGEEKNNITPYTYFPFGEGPRLCIGMRFAKMQITAGIITILK

KYRVELAPGMSEKLQFEPRSIITAPIGGIRLKFIEREGWQQRVFKAPSEA

>CAX94849.1

MLPIYLILCVAVIYCLHRLATLKYNYWKKRNVPHPPPTPVLGNYGPYLLMQKYYGQVAQE

IRQKYPNAPYIGAYYGTEPTLIVQDPELIKIITTKDFYYFNSREISEHTHKEVTTQNLFF

TYGDQWKAVRQNMTPLFSSAKMRNMFPLIAKCSVSFQEHVDQETSSSDVIDVKSLMARYT

MACIVSCAFGAEVDTLTPEGKDSQFVKIGKLIFLNSYLRGGLTVFRAIWPTLFYKLGLKN

FPDEMTNFFKRFVTNVFEARKYTPTRRNDFVDLLLNLRQQKSIVADSLSNGKTGREDKVE

LPVTDDLLVSQCVLFFAAGFETSSTATSFLLYELSKKPEIQERVLQEVDAFLAKHDNKLT

YDCVTELPYTQACIDESLRLYPALGVITREVMEEYTLPDGLVLEKGVRIHIPVYGLHYNP

DHFPDPEEFRPERFEGSNRNNIKPHTYIPFGDGSRICIGLRFAKMQMLAGLISLLKKYRV

EMTKDTPTKLDYDPRALVTIPLQSVNLKLVPREGWEKRIFVQ

>ACF17813.2

MIVFISTFIVIALSLLYYVTTKNHNYWKKKNVVSCPNPKPIFGNYSDYILFKKFVGEVSQ

DICRALPNEPYIGTFYGSDPALLVQDPDILKIILTKDFYYFSSREVSQYTHKEVVTHQNL

FFTYGDRWKVLRQNLTPIFTTAKMKLMFGLIVDCCRILEKSLDLDTKASDAIEVRALAAK

YTIETIGSCGFGIETNSMLQSEQNPFIIIGNKIIDNSTIRGLVWNARVMWPSVFYGLGLK

SFPAEVYKFFDTVSTKIFKARDYKPTARNDFIDCILNLKNNKYITGDSIRSVKDGDNKKI

VMDVDNDMLVGLCMMFFTGGFETSSSTMSYTLYELAKNKEAQRRAQQEIDEYLKAHDNKL

AYDCVTSLTYTEACVDEALRLYPVLAVLTREVVEDYTFPSGLTLEKNSRVHIPLYHLHHN

PKYFPDPEAFKPERFLPENKQNIKPYTYMPFGDGPRICIGLRFAKMQTLAGLLTILKKYS

VELADGTPEKLQFDPRAVVTTPIGGIPLKFTPRDGWEQRTFNK

>CBB07053.1

MLAIILSTFAAIFAVLYYLLQRRYNYWKKRNVPYIKPTPLLGNYGDFILQKKDISSVMED

LCKKMPNEPYIGVFYGTGPTLIVRDLELLKLITTKDFYYFHGREVSDHVHKEELTNNLFN

SAGDEWKVMRQNLTPVFTSAKMKSMFPLIRNCSYEFEKLLDSESKMDGFEARIQVGRFTM

ACILSCGFGVDSGTMNGEYDKNNPFVNMGDAFFSVSPLSSLAFTLRSLWPTIFYALGLEI

APARMSQIMKNFMNSIFEARNYKSTSRNDFIDFILKFKQSTYLVGDSISNSKTGDNKKEQ

VLVTDTLLTAQCIVFFLAGYETSATTSNFTLYELAKHPEVQRKVQEEIDAYLQKTGNQLN

YDVVTELPYTYACIEETMRLFPVLGMITREVMDAYTMPNGVLLEKGTRIYIPIREIQRDP

KHFPEPEVFRPERFLGEERQKIAPYSYMPFGEGSRICLGMRFAKMQMLAGLVTLLKKFNV

ELADKNQTIVFDPLSGVTSPKIPLKFKVTPREGWESRVFTKN

>ACZ97416.2

MVWMMLLLIVLVVILYYIATFKHDYWKKRGVPYLQPSLVFGNYRDFILMRKNMGEVAESI

CKEFPDAPVVGTFYGTDPALIVKDPELIKLITTKDFYYFSGRENSDHINKEMFIKNLFFS

YGDYWKVVRQNLTSLFSTSKMKTMFYLIEKCTTQMHNLLEIETKTDDILEVKSFITRFTM

DCIGSCVFGVETDAMGKDNNRNPFRAISDKIFDFGISRTLKTIGRAMWPGIFYGLGYKLL

PDDVESFFDNLLNGVFEQRNHEPTARNDFIDLILKFSKNKCITGDSIKTTKVKDEQTKVS

VEVEKDFLVSQCFLFFAAGFETTASTLTFTLYELAKNEKAQKLVIQEVDEYLRQRDGKLE

YDCVSQLPYLDACVNEALRLYPVLTILTREVTDDYTLPTGLRLEKKTRIHFPMLYIHKNP

EYFPNPEEYRPERHLPGERNNIKACTFFPFGEGPRMCIGMRFAKMMMASCLVTFFRRYRV

ELAEGMPSTVQLHPRYLGSQSINGVKLKFLKRF

>ABO21682.1

MILTIIFILSLCVYILYRISTRKFDYWQKKNVSYVEPAPFLGNYSGYILLKENLLDVVHN

LSKLFPNDPYVGAFFGTKPTLIVKDPEFIKLVLIKDFYYFHGREGSKYTHNEVITQGIFF

TYGDRWKVIRQNLTPLFSSSKIRNMFRTIEKCSGVFENLLDEESLAPEVEMRSVMSRFTM

DCIGGCAFGVDTNAMQEPKDNIFKTMGYLFFESTTRRGIKNVFKAIWPEIFYGLGFKVFP

TDLNEFFSKLLVGIFEARDYKPSSQNVFINLLLNLKKNRHIVGDRLLKIKTGNVRAESKI

KLEVDDELLVSQCVAFFIAGFETSSTISRFTLYELAKNPDVQKRAQKEVDEYIKKHNNKL

DYDCVKELPFVEACIDEALRLYPVLGVLTREVMEQYTFPTGLTLDKGDRVHIPVYHLHRD

PEYFPEPELFKPERFCGEEKRNIRPFTYLPFGAGPRTCIGQRFAKMQMLAGLVTILKRYT

VCLAEGMPETINFEQRAIVTQPNIGIRLNLLPRNN

>ADD70250.1

MSVSALVFFAFVLLVTYIYYWSTRKFDYWKRKNVPYAKPVPFFGNYMRYITLQSFLGDVM

QKLCQQFPDRPYFGSFYGTEPALVLQNPEIIKQVFTKDFYYFNSRENRDYNHKEVFTQNL

FFANGDRWKVIRQNLTPLFSSSKMKNMFYLVEKCNHSLEDMLDKETKDLQSIEIRSAMIR

YTLDSICSSAFGIETNTLSEGAENSPFPSMGSTIFSSSITRGLKLIGRSMWPGIFYKLGL

RCFPTEIDDFFERLLTEVFENRGYKPTNRNDFVDLILSLKQNDYLTGDGLVPKNVDAKKV

TVKVDDALLIAQCVVFFGAGFETSATTLSAALYELAKNPEAQRRAQEEVDELLLKHNNKL

NYDCLAELPYLEACMNEAMRLYPVFPNITRETVADYTFPDGLRIDKGMRVHVPVYAIHRN

PDNFPDPEEFRPERHLGDAKNDIKQFTFFPFGEGPRICIGMRFGKMQTIAGLITCLKKFN

FELADGMPRTLAFRSTTLLTQPSTGLFLKATPRDGWEQRIFAR

>ACJ10074.1

MSVSALVFSAFVLLVTYIYYWSTRKFDYWKRKNVPYAKPVPFFGNYMRYITLQSFLGDVM

QKLCQQFPDRPYFGSFYGTEPALVLQNPEIIKQVFTKDFYYFNSRENRDYNHKEVFTQNL

FFANGDRWKVIRQNLTPLFSSSKMKNMFYLVEKCNHSLEDMLDKETKDLQSIETRSAMIR

YTLDSICSSTFGIETNTLSEGAENSPFPSMGSTIFSSSITRGLKLIGRSMWPGIFYKLGL

RCFPTEIDDFFERLLTEVFENRGYKPTNRNDFVDLILSLKQNDYLTGDGLVPKNVDAKKV

TVKVDDALLIAQCVVFFGAGFETSATTLSAALYELAKNPEAQRRAQEEVDELLLKHNNKL

NYDCLAELPYLEACMNEAMRLYPVFPNITRETVADYTFPDGLRIDKGMRVHVPVYAIHRN

PDNFPDPEEFRPERHLGDAKNDIKQFTFFPFGEGPRICIGMRFGKMQTIAGLITCLKKFN

FELADGMPRTLAFRSTTLLTQPSTGLFRKATPRDGWEQRIFAR

>ABY40426.1

MSVSALVFAAFVLLVTYIYYWSTRKFEYWKRKNVPYAKPVPFFGNYMRYITLQSYIGDVM

QKLCQQFPDRPYFGSFYGTEPALVLQNPEIIKQVFTKDFYYFNSRDNSDYNHNEVVTQNL

FFANGDKWKVVRQNLTPLFSSSKMKNMFYLVEKCNHSLEDMLDKETKDLQIIEIRSAMIR

YTLDSICSSAFGIETNTLSEGGEYSPFANMGSIIFSPSITRGLKWISRSMWPGIFYKLGL

QCFPAEIDDFFERLLTEVLENRGYKPTNRNDFVDLILSLKQNDYLTGDGLVPKNVDAKKV

TVKVDDALLIAQCVVFFGAGFETSATTLSAALYELAKNPEAQRRAQEEVDELLLKHNNKL

NYDCLAELPYLEACMNEAMRLYPVFPNITRETVADYTFPDGLRIDKGMRVHVPVYAIHRN

PDNFPDPEEFRPERHLGDAKNDIKQFTFFPFGEGPRICIGMRFGKMQTIAGLITCLKKFN

FELADGMPRTLAFRSTTLLTQPSTGLFLKATPRDGWEQRIFAR

>AFP20593.1

MIQKLCQKFKSEPYFGAYYGTEPVLIVQDPEIIKHVLTKDFYYANGRESSNYSNGELITQ

NLFFSAGDNWKVVRQNLTPLFSSLKMKNMFHLIEKCTIGFEDMLDDEIKMSNVIEVRRIA

ARYTMDCICSCAFGVESKTMSQKENNPFQLMAEEIFESSRYRGFKLIFRAVWPSIFYRIG

LKIFPSTINRFFFKLLTGVFESRGYKPSPRNDFVDLLLNLKKEDYITGDSISNVKTGDDK

KVHLKVDDELLVSQCILFFSAGFETSATTMGFTLYELAKHPEVQKKAAEEVDAFMCRHKN

KLVYDCVTELPYLEACMYETLRMYPVLGNLTREVMDDYVLPTGLKLDVGVRLHIPVYHMH

YNSQYFPEPYKYKPERFLPEHKNEVNPNTFFPFGSGPRLCIGMRFAKMQVLSGLITFLKK

YKVELADGMATELDFDPKTILTQPLDNCIGLKLTEREGWEQRRFVRT

>ABQ42339.1

MSVSAVVFAAFVLLVTYIYYWSTRKFDYWKRKNVPYAKPVPFFGNYMRYITLQSYIGDVS

QKLCQQFPDRPYFGSFYGTEPALVLQNPEIIKQVFTKDFYYFNSRENSDYNHKEIVTQNL

FFANGDKWKVVRQNLTPLFSSLKMKNMFYLVEKCNHSLEDMLDKEIKDLQIIEIRSAMIR

YTLDSICSSAFGIESNTLSEGGENSPFANMGSIVFSSSITRGLKWISRSMWPGIFYKLGL

QCFPAEIDGFFERVLTEVFENRGYKPTNRNDFVDLILSLKQNDYLTGDGLVPKNMDAKKV

TVKVDDALLIAQCVVFFGAGFETSASTLSAALYELAKNPEAQRRAQEEVDELLLKHNDKL

NYDCLAELPYLEACMNEAMRLYPVLPNITREAVADYTFPDGLRIEKGMRVHVPVYAIHRN

PDNFPDPEEFRPERHLGDAKNDIKQFTFFPFGEGPRICIGMRFGKMQTIAGMVTCLKKYN

FELADGMSKTVPFRSTTVLTQPSTGLFLKATPRDGWKQRIFAR

>ABO21683.1

MSVSALVFAAFVLLVTYIYYWSTRKFEYWKRKNVPYAKPVPFFGNYMRYITLQSYIGDVM

QKLCQQFPDRPYFGSFYGTEPALVLQNPEIIKQVFTKDFYYFNSRDNSDYNHKEVVTQNL

FFANGDKWKVVRQNLTPLFSSSKMKNMFYLVEKCNHSLEDMLDRETKDLQIIEIRAAMIR

YTLDSICSSAFGIETNTLSEGGEYSPFANMASIIFSPSIARGLKWISRSMWPGIFYKLGL

QCFPAEIDDFFERLLTEVFENRGCKPTNRNDFVDLILSLKQNDYLTGDGLVPKNVDAKKV

TVKVDDALLIAQCVVFFGAGFETSATTLSAALYELAKNPEAQRRAQEEVDELLLKHNNKL

NYDCLAELPYLEACMNEAMRLYPVFPNITRETVADYTFPDGLRIDKGMRVHVPVYAIHRN

PDNFPDPEEFRPERYLGDAKNDIKQFTFFPFGEGPRICIGMRFGKMQTIAGLITCLKKFN

FELADGMPRTLAFRSTTLLTQLSTGLFLKATPRDGWEQRIFAR

>ABX64438.1

MSVSAVVFAAFVLLVTYIYYWSTRKFDYWKRKNVPYAKPVPFFGNYMRYITLQSYIGDVT

QKLCQQFPDRPYFGSFYGTEPALVLQNPEIIKQVFTKDFYYFNSRENSDYNHKEIVTQNL

FFANGDKWKVVRQNLTPLFSSLKMKNMFYLVEKCNHSLEDMLDKEIKDLQIIEIRSAMIR

YTLDSICSSAFGIESNTLREGGENSPFANMGSIVFSSSITRGLKWISRSMWPGIFYKLGL

QCFPAEIDGFFERVLTEVFENRGYKPTNRNDFVDLILSLKQNDYLTGDGLVPKNVDAKKV

TVKVDDALLIAQCVVFFGAGFETSASTLSAALYELAKNPEAQRRAQEEVDELLLKHNNKL

NYDCLAELPYLEACMNEAMRLYPVLPNITREAVADYTFPDGLRIEKGMRVHVPVYAIHRN

PDNFPDPEEFRPERHLGDAKNDIKQFTFFPFGEGPRICIGMRFGKMQTIAGMVTCLKKYN

FELADGMSKTVPFRSTTVLTQPSTGLFLKATPRDGWKQRIFAR

>ABP99020.1

MSVSAVVFAAFVLLVTYIYYWSTRKFDYWKRKNVPYAKPVPFFGNYMRYITLQSYIGDVT

QKLCQQFPDRPYFGSFYGTEPALVLQNPEIIKQVFTKDFYYFNSRENSDYNHKEIVTQNL

FFANGDKWKVVRQNLTPLFSSLKMKNMFYLVEKCNHSLEDMLDKEIKDLQIIEIRSAMIR

YTLDSICSSAFGIESNTLSEGGENSPFANMGSIVFSSSITRGLKWISRSMWPGIFYKLGL

QCFPAEIDGFFERVLTEVFENRGYKPTNRNDFVDLILSLKQNDYLTGDGLVPKNMDAKKV

TVKVDDALLIAQCVVFFGAGFETSASTLSAALYEPAKNPEAQRRAQEEVDELLLKHNNKL

NYDCLAELPYLEACMNEAMRLYPVLPNITREAVADYTFPDGLRIEKGMRVHVPVYAIHRN

PDNFPDPEEFRPERHLGDAKNDIKQFTFFPFGEGPRICIGMRFGKMQTIAGMVTCLKKYN

FELADGMSKTVPFRSTTVLTQPSTGLFLKATPRDGWKQRIFAR

>ABN71367.1

MSVSALVFAAFVLLVTYIYYWSTRKFDYWKRKNVPYAKPVPFFGNYMRYITLQSYIGDVT

QKLCQQFPDRPYFGSFYGTEPALVLQNPEIIKQVFTKDFYYFNSRDNSDYNHKEVVTQNL

FFANGDKWKVVRQNLTPLFSSLKMKNVFYLVEKCNHSLEDMLDKEIKDLQIIEIRSAMIR

YTLDSICSSAFGIESNTLREGGENSPFANMGSIVFSSSITRGLKWISRSMWPGIFYKLGL

QCFPAEIDGFFERVLIEVFENRGYKPTNRNDFVDLILSLKQNDYLTGDGLVPKNVDAKKV

TVKVDDALLIAQCVVFFGAGFETSASTLSAALYELAKNPEAQRRAQEEVDELLLKHNNKL

NYDCLAELPYLEACMNEAMRLYPVLPNITREAVTDYTFPDGLRIDKGMRVHVPVYAIHRN

PDNFPDPEEFRPERHLGDAKNDIKQFTYFPFGEGPRICIGMRFGKMQTIAGMVTCLKKYN

FELADGMSKTVPFRSTTVLTQPSTGLFLKATPRDGWKQRIFAR

>ABP99019.1

MYVLTFLLHFILFLVLVVYYITKRNHDYWKNKKVPFEKPYPILGNYGDYILLKIFFGNVT

KRLCEKFPESPYVGTFYGTEPALVVQDPDLIRLILVKDFFYFHGREVCIYVDREITTQSI

FFTYGDDWRVLRHSLTPLFTTSKMKNMFHLIKNCCRVFENVLEEKATVKSFEINSIIKYF

IMDCVGACLFGVEINAMEKHSQNPIVKISKEIFPKSTTRGIINVLRGIWPSLFYALRLKL

FPEVITMFFTDLLECAFKDRQRNLSQRQDFIDLFMKLRQNKYLVGDSIPDIKNGRVQKVN

LEVTDELLIAQCVSFFGAAFETSSTTLSLTFYELAKNPKYQKRAIEEVDDYFAKHNNEIE

FDIVSETPYLNACIDETLRLYPSLANLTREVMEDYTFPTGLKVEKGTRIHIPVYHLQRNP

KYFPEPNKFDPRRFLPEAKQTIYPFTYMPFGEGHRICIAMRFAKMQMLAGFATLLKKYEV

AVDDTTPQELTIDPRIIVTTPIENIQLKLIARQHAL

>BAE45261.2

MPYLDVAVALLAAFIAFTLWTNRRWNYWKKQNVKYLTPIPFLGNVADVIFQRDTFGAVTQ

RICQQFPDEAVVGMFYCSNPAALVQCPDMLKTVMVKDYAYCSSKEVSVHSHKEPMTKNMF

FTFGDKWKLIRQNLTPVYTSAKMKNMFPLVQDCCRIFQKVLDDEIGKGRGVEVKSLIARY

TMDCITSCAFGVDSGTMSKGEEGNPFTETGHLLFDERPIAGVKNVLRYGYPSFFYSVGLE

LYSSKIYRFFRSVILDVINSRNGAKSSRNDMVDLISDWKKNKYITGDSIDNGIDGGNKKV

RIEVDDELLVSQCVLFFQAGFQPSALTSAYLLYELAKNQDIQERVLAEVDEYWSTRDEVQ

TDCVTALPFLAQCMEESLRMYPPVSVLMREIYKDYALPNGVHLKKGMMIHIPVYHLHHNP

KYFPEPEVFRPERFSEEGRKNIVPYTYLPFGEGPRMCIGYRFARLEIFSSLAVLLKKYRV

ELAPHMPRKLQFLTTSRVLTSVHGIHLRLVDRVN

>AAY89329.1

MPYLDVAVALLAAFIAFTLWTNRRWNYWKKQNVKYLTPIPFLGNVADVIFQRDTFGAVTQ

RICQQFPDEAVVGMFYCSNPAALVQCPDMLKTVMVKDYAYCSSKEVSVHSHKEPMTKNMF

FTFGDKWKLIRQNLTPVYTSAKMKNMFPLVQDCCRIFQKVLDDEIGKGRVVEVKSLIARY

TMDCITSCAFGVDSGTMSKGEEGNPFTETGHLLFDERPIAGVKNVLRYGYPSFFYSVGLE

LYSSKIYRFFRSVILDVINSRNGAKSSRNDMVDLISDWKKNKYITGDSIDNGIDGGNKKV

RIEVDDELLVSQCVLFFQAGFQPSALTSAYLLYELAKNQDIQERVLAEVDEYWSTRDEVQ

TDCVTALPFLAQCMEESLRMYPPVSVLMREIYKDYTLPNGVHLKKGMMIHIPVYHLHHNP

KYFPEPEVFRPERFSEEGRKSIVPYTYLPFGDGPRMCIGYRFARLEIFSSLAVLLKKYRV

ELAPHMPRKLQFLTTSWVLTSIHGIHLRLVDRVN

>AAX84943.1

MPYLDVAVALLAAFIAFTLWTNRRWNYWKKQNVKYLTPIPFLGNVADVIFQRDTFGAVTQ

RICQQFPDEAVVGMFYCSNPAALVQCPDMLKTVMVKDYAYCSSKEVSVHSHKEPMTKNMF

FTFGDKWKLIRQNLTPVYTSAKMKNMFPLVQDCCRIFQKVLDDEIGKGRVVEVKSLIARY

TMDCITSCAFGVDSGTMSKGEEGNPFTETGHLLFDERPIAGVKNVLRYGYPSFFYSVGLE

LYSSKIYRFFRSVILDVINSRNGAKSSRNDMVDLISDWKKNKYITGDSIDNGIDGGNKKV

RIEVDDELLVSQCVLFFQAGFQPSALTSAYLLYELAKNQDIQERVLAEVDEYWSTRDEVQ

TDCVTALPFLAQCMEESLRMYPPVSVLMREIYKDYTLPNGVHLKKGMMIHIPVYHLHHNP

KYFPEPEVFRPERFSEEGRKSIVPYTYLPFGDGPRMCIGYRFARLEIFSSLAVLLKKYRV

ELAPHMPRKLQFLTTSRVLTSIHGIHLRLVDRVN

>EHJ77671.1

MVKDPELIKLILSKDFYYFNMREIADFIHNEPVTQNMFFGGGDRWRVIRQNLSPLFSSIK

LKSMFYIIENCAKTMEATIREEMNESGTFEVKSLLTRYTIDCVGSCVFGVNTGTLGKNYD

KNPFTLIAGQLFDVTSYGGFKLVTRAMWPSIFYGLGLKMFSDNVNNFFSNLLIGVFESRD

HKESGRHDFVDLLLNWKQKDFLCGDSITNMKTGEKKTIQLNIDEGLIIGQCALLFAAGYE

TTATTTSLLLYELAKNKDVQARVIDEVDEYYKRHHGKIEFEAINEMPYLQACIDETLRFY

PVLGLLTREAVEGYTLPTGLHLPKGTRIHIPVYHIHHNPKYFSEPERWLPERFYGEGKHF

ITPFTYMPFGEGPRVCLGKRFSKMPMYAGLLTIFKNYELELDPKTPSSYKFQPRTFVTQA

LGGIFLKFIPRK

>ACO55746.1

MVAMDRHETPRYNHQLYVYYITKRNHDYWKNKKVPFEKPYPILGNYGDYILLKNFFGNVT

KRLCEKFPESPYVGTFYGTEPALVVQDPDLIRLILVKDFFYFHGREVCKYVDREITTQSI

FFTYGDDWRVLRHSLTPLFTTSKMKNMFHLIKNCCRVFENVLEEKATVKSFEINSIIKYF

IMDCVGACLFGVEINAMEKHSQNPIVKISKEIFPKSTTRGIINVLRGIWPSLFYALRLKL

FPEVITMFFTDLLECAFKDRQRNLSQRQDFIDLFMKLRQIKYLVGDSIRIIKNGRVQKVN

LGVTDELLIAQCVSFLGAAFETPSTTLSTTFYDLAKNPKYQKRAIESDDNYFASNMNEIE

FDSRIDDAACRLLSDETLRLLPSLANLTREVMEDYTFPTGLKVEKGTRIHIPVYHLQRNP

KYFPEPNKFDPRRFLPEAKQTIYPFTYMPFGEGHRMCIAMRFAKMQMLAGFATLLKKYEV

AVDDTTPQELTIDPRIIVTTPIENIQLKLIARQHAL

>EHJ77668.1

MFFTGGDQWKVLRQNLSPMFSSKKLKNMFYLIEVCSKTITEYMRSEIQHSNVIEVRSLLA

KYTMDCIGNCVFGVNTGTLGKNTDNNPFTIMGNKIFDISTYRGFKMVARAMWPSIFYSLQ

LKMFSEDINKFFHNLLTDVFKTRKYKESGRHDFVDLLLCWKQKDFIVGDSVSNNIKGEKS

QVQIKVDDECLISQCVLLFAAGYETTATTTSFILYELAKHKDAQIRVVKEIDEYYERHEG

KIEFESINEMPYLQACICETLRLYPVLGVITREVFEDYTLPTGLLLEKGTRIHIPIYHNQ

MNTKYFPNPEEFRPERFLGEEKKNIKPYTYLPFGEGPRMCIGMRFAKMPMVTALLAIFKD

YRIETTERTPKSFDFEPRAISTQPLGGIYLKFLSRN

>EHJ77667.1

MTKDFYYFNGREVTQYNTREELTNNMFFHSGDKWKVVRQNLTPLFTSMKMKKMFHLVKDI

GDSFEKILEDELKSSSDVEIKSFLVKYTLECILSCGFGISGKLLEKGNKKDDNVFFMIGS

SIFDSSPSRGMKQVFRSIWPKLFYGIGLNVFDPKIFAMFKNLIAEVFQSRLSEQSNRNDF

VDLILSWKKEHYIYGDSLNPNSEKYKKISIEVTNELLIPQCVLFFAAGFETTSTTLSFLM

YELSKHRDVQDRLIEEVDDYYKKHNGVVEYECIGEMPYLDACIDEVLRLYPVLGVVAREV

MEDYTLPTGLKLEKGNRIHIPLYHIHRDPKFFPNPEEFRPDRFFGEERKNIVPYSFMPFG

EGPRICIGQRFAKMVMYNLILKIFKKYRIESSSLTPKSLEFNNISLVTQCNTSIYVKLVE

R

>EAT39305.1

MLFLAFAIFVLFAIIQIVYHFRYWMRRGVPQLRPSFPFGDFGEFFRQKHGIPMTYANIYA

RTRHLPYVGIYLSMRPVLFVNDPQMVKDILSRDFEHFHDRGLHVNEETDPLSGNLFSLGG

VKWKNMRAKLTPTFTSGCLKGMLAILIDKATVLQKQFAKEIATHNTIEVKDLFARYTTDV

IASVAYGIDNDSINNDHDLFRQMGIKVFQQDFKTSLRLALTFFIPKIKALLGFSLVAKDV

EDFMINLVSKTIEHRERNGIQRKDMMQLMLQLRNSGSVSINDQQWNLDSSATVKNLTINQ

VAAQVFVFFVAGYETSSTLMSFCVWELARNPEIQVKVHQEIDSVLSNYGGALTYEAFADM

KYLECCMEETLRKHPPVSFLNRECTKTYRIPETDVIIDKGTAVVVSLLGMHRDPQHFTQP

TEFKPERFSSDEQSNESNKAYFPFGGGPRLCIGMRLGMLQAKVALVTLLAKFEFSLGKEH

VKDMELPLKANTLLLVPQDGIQLVVKKR

>EAT38452.1

MLLAFLALSAPLVTVLIWLQFRYWTRCGVPQLDPSFPFGNFSEFFCQKNGIPSTYANLYH

RTKHLPFVGIYLSLRPALLINDPELVKNILTRDFEHFHDRGIHVDEETDPMSGHLFALGG

VKWKNLRAKLTPTFSSGSLKEMFPLLVEKATVLQKRFLKEIATSEVVEVKELAACYTSDV

IASVAYGIDMDSINNRDDLFRRMGEKVLAHDLITSLRLALAFWFPKLKVMLGSKSIAPVI

QEFMTELVRKTIEHREKEGVHRKDMMQLLLQLRNGVSLKRNGVQWTEDSAPKNAIKSLSI

DEVTAQVMVFFVAGYETSSSTVSFCLFELARHQDIQAKVHQEIDTVLAEHEGNLTYASLA

SMKYLEQCLEETVRKYPPVAILNRECTKTYRIPETDVIVEKGTPIVVPLMGMHRDPQYFP

QPNDFQPDRFEGGAQSKAYFGFGAGPRLCIGMRLGILQSKVAVVTLLRKFKFSLANPEDQ

HTELRMKPRSFILTTEGGIQLVVQQRHVCET

>EDS36035.1

MFAWIICAAAAVPLVYFLIVYQFSYWKRRGITQLTPSFPFGDLGPFFRQRSSLGVVYADV

YRLCKRLPFVGIYLSLRPMLVVNDPELIKNVLVRDFDHFHDRGLYVNEEKDPLSGHLFAL

GGEQWRHHRSKLTPTFTSGRLKEMFTNLVQIGRVLQDHVAKRAGEDIEIRDVMARYTTDI

IASVGFGIENDSINEKGNIFREMGTKVFSPDLKTILRLTSTFFTPKLNALFGFKFIAQEI

EDFIMNVVRETLEYRESNKVVRKDMMQLLMQLRNSGTVSIDDRWDIEVSTNKKKLSLEQV

TAHAFVFFIAAYETSSTTISFCLFELARNPEIQKKVQQEIDQVLASHNGEITYDNINEMK

YLENCIDETLRKYPAVPFLNRECSKDYKIPGTDTTIEKGTSLVIPVLGLHRDPDHYPEPD

RFIPERFSNFEDISTKPYLPFGAGPRNCIGLRLGKLQTKAGLVMMLSKFNVRLADETYAS

KELALDARSVVLMPVGGIKVSISERRAS

>AAT72405.1

MFAWIICAAAAVPLVYFLIVYQFSYWKRRGITQLTPSFPFGDLGPFFRQRSSLGVVYADV

YRLCKRLPFVGIYFSLRPMLVVNDPELIKNVLVRDFDHFHDRGLYVNEEKDPLSGHLFAL

GGEQWRHHRSKLTPTFTSGRLKEMFTNLVQIGRVLQDHVAKRAGEDIEIRDVMARYTTDI

IASVGFGIENDSINEKGNIFREMGTKVFSPDLKTILRLTSTFFTPKLNALFGFKFIAQEI

EDFIMNVVRETLEYRESNKVVRKDMMQLLMQLRNSGTVSIDDRWDIEVSTNKKKLSLEQV

TAHAFVFFIAAYETSSTTISFCLFELARNPEIQKKVQQEIDQVLASHNGEITYDNINEMK

YLENCIDETLRKYPAVPFLNRECSKDYKIPGTDTTIEKGTSLVIPVLGLHRDPDHYPEPD

RFIPERFSNFEDISTKPYLPFGAGPRNCIGLRLGKLQTKAGLVMMLSKFNVRLADETYAS

KELALDARSVVLMPVGGIKVSISERRAS

>AAX47271.1

MFAWIICAAAAVPLVYFLIVYQFSYWKRRGITQLTPAFPFGDLGPFFRQRSSLGVVYADA

YNLCKRLPFVGIYFSLRPMLVVNDPEVIKNVLVRDFDHFHDRGLYVNEEKDPLSGHLFAL

GGEQWRHHRSKLTPTFTSGRLKEMFTNLVQIGRVLQDHVAKRVGEDIEIRDVMARYTTDI

IASVGFGIENDSINEKGNIFREMGTKVFSTDLKTILRLTSTFFTPKLNALFGFKFIAQEI

EDFIMNVVRETLEYRESNNVVRKDMMQLLMQLRNSGTVSIDDRWDIEVSTNKNKLSLEQV

TAHAFVFFIAAYETSSTTISFCLFELARNPEIQKKVQQEIDQVLASHNGEITYDNINEMK

YLENCIDETLRKYPAVPFLNRECSKDYKIPGTDTTIEKGTSLVVPVLGLHRDPDYYPEPD

RFIPERFSNFEDISTKPYLPFGAGPRNCIGLRLGKLQTKTGLVMMLSKFSVRLADETYVR

KELALDARSVVLMPVGGIKASISERRAS

>EDS42304.1

MSSWLNAALAVPLLYLLVARQFSYWKRRGVAQLVPVFPFGNLGPFFRQRSCLGVTYAELY

HRWKALPFVGIYFSLRPLLLINDPELIKNVLTRDFEYFHDRGLHVNEGKDPLSGHLFALG

GDKWRHHRSKLTPTFTSGRLKEMFQNLVEIGLVLQEYVGKRAAAGEEVDVRELMARYTTD

VIASVGFGIENDSINDSDNMFRKMGTKVFAQDLKTIVRLASTFFLPKLNALFNFKFIAQD

IEDFMMNVVRETLEYRESNNVVRKDIMQLLMQLRNTGTVSMDKIWKVEAATPSAKQLSLE

QVTAHAFVFFAAGYETSSSTISFCLFELARNVNIQRKLQADIDQVLAKHNGAITYDSINE

MKYLENCIDETLRKYPAVPFLNRECTKDYVIPGTSSTVEKGTTVIIPVLGLQHDPEFYSE

PQKFIPERFSEEATTDKPYLPFGTDPRVCIGLRLGKLQTKMGLVMMLSKFNIRLVDEAYH

SKELELDSKGLFLMPIGGIKVAVSTRSD

>EAT33064.1

MTPLSIGVALLCVAAFAFLNYVFSYWNRRGVQQLTPYFPFGNFSDLFLGKASFPRVCETL

YERTKKWRLLGVYILLRPVLLVNDPQLAKDIMVKDFQHFHDRGTHVDEENDPLSGHLFSL

AGEKWKHLRAKLTPTFTSGRLKGMFQTLVDTGEVLQDYIHTCAKNEEVVEIREILARYNT

DNIASVAFGIKIDSINNPNEPFRQIGRKFFESNFRNNMRLMITFMVPKLNKYFKIKSVDA

EVEQFILGMAKETLEYREKNGVVRKDMMQLLIQLRNTGTVSVDERWDVETSTNSKKLTIG

EVAAQAHVFFLAGFETSSSTMSFCLYELAKNPEVQRKVQSEIDSVTALHDGKLTYDSINE

MRYLECCIDETLRKYPPVPVLNRECTKDYKVPDSDITIEKGTAVILQISAMHHDPQYYPD

PLRFVPERFLDPDMKGKPYAPFGDGPRICIGLRMGKIQTKVGLCLLLSKFNFELYGHKES

ELVMSPNNFLNTPVNGINLKVSCRE

>EAT46777.1

MLLPILLVVLVVYLFQKWTYSHWKRRGVPQLNPAFPFGNVADTFKQRTSYSNRLAELHHQ

AVRDGHRFVGIYTLLQPILLVTDVELVKRMLTVDFEHFVDRGAHVNEKRDPLSGHLFSLT

GAKWRRMRLKLTPAFTTAKLKAMFPTMMACGRTLSAVIDDHVGRALAIRDLMTRFTMDVI

ASVGFGLECNSMRNPDELFRQMGGRFFSKSWKTSVRMLLAFVAPKVNRYLQVKLNDDDVE

EYMLNLVRDTIAKREGGGEVRKDFIQLLVQLRNQVEVKDGGSWEMNKVDQNKTLTVEEMA

AQSFVFLNAGYETTSSTVTFCLFELCRNKDLIGKVQEEIDRVMDGGREISYEALAEMTYL

ESCIDETLRKYPISPVLFRVCTKPYKIPETDVVIEKDTLVQISLVGLQRDTRYYEDPMKF

DPDRYGERKSETMPHYSFGDGPRVCIGLRMGKVMAKMALVELLSRYDFELESPAADSGEI

ELDPSLLMLQAKHDVKLIPRLRTK

>EAT39154.1

MLLPILLVVLVVYLFQKWTYSHWKRRGVPQLNPAFPFGNVADTFKQRTSYSNRLAELHHQ

AVRDGHRFVGIYTLLQPILLVTDVELVKRMLTVDFEHFVDRGAHVNEKRDPLSGHLFSLT

GAKWRRMRLKLTPAFTTAKLKAMFPTMMACGRTLSAVIDDHVGRALAIRDLMTRFTMDVI

ASVGFGLECNSMRNPDELFRQMGGRFFSKSWKTSVRMLLAFVAPKVNRYLQVKLNDDDVE

EYMLNLVRDTIAKREGGGEVRKDFIQLLVQLRNQVEVKDGGSWEMNKVDQNKTLTVEEMA

AQSFVFLNAGYETTSSTVTFCLFELCRNKDLIRKVQEEIDRVMDGGREISYEALAEMTYL

ESCIDETLRKYPISPVLFRVCTKPYKIPETDVVIEKDTLVQISLVGLQRDTRYYEDPVKF

DPDRYGERKSETMPHYSFGDGPRVCIGLRMGKVMAKMALVELLFRYDFELESPAADSGEI

ELDPSLLMLQAKHDVKLIPRFRAK

>328793308

MFLTTWLIPDIIAVASLITVGLYFYYKLYLFKFWHKKGIFYVKPSFPTGNIMPIINGKLS

LAEFFRDIYEHNKHHRLVGIYMLYKPYLIVNDPNLIRDILTKEFTNFHDRGIFYNEEVDP

LSGHLFQLPGKKWRNLRVKLTPTFTSGKIKQFFPILNEAGNILAKYLEEEARKGSTIDVK

DIFARYSTDIIMSVAFGISCDSFKEPNNEFRYWGKKIFDPKPLWNALILFAPQILNFFSI

SYTEKSVTKFFTNMFKQTVKYRESNNIERKDFLNLLIQLMKNGYVDADDESLSNNVNAAK

NKLTMMEAAAQAYVFFLAGFETSSTTVTFCLYELAKNQDIQNKVREEIQTMIKKNGDLTY

NALNDMNYLHKVISETLRKYPPVVILNRICTNDVKLSTTDFCIPKGTCIAIPVFGLHRDS

NIFPNPEKFDPERFSEENIKTRHPYVYLPFGEGPRICIGLRFGLIQTKIAIINALLKNKF

KFGPNTPSTLEFEKGSLILIGKGGIHLNIEPI

>AAX35340.1

MVFSVSSVLSIIALLIVLAISSLYIYLKYVYSYWERHGVKSFAGKIPYGNFEKTIKQKRS

VGEVLTDLYRSTTEPFIGFYGPLKPILLIRDPALVRQILIKDFQYFANRGIYSDEKNDPL

SAHLFSLDGDKWKKLRYKVSPTFTSGKLKAMFSTLVRCGESLQKVVKSTVEKGEEIDMRE

LVACFSTNIIASIAFGWDVDAITDSNTPFRKYGRQYFQKSLKNGVRNLAKFVSPKLMHVL

RLKAIDADIEEFFFDMIRQTMALRENNNVVRKDFFQLLFQLRNMGSIQSDDDWETKTISD

EEKDKNLTINELAATAFIFYAAGFESSSSTVSFCLYELAKNSEMQRIAQRKINAVLERHN

GQYTFDAINEMKYLGCCVDETLRKHSPIAMLNREVSQSYKVPGMGLTLETGTAIMVPVYG

IHHDEKYYPNPEVFEPNRFSVENAKNKTFVEMPYLPFGDGARNCIGMRLGLMVFKVCLAM

LLKEFNYSLGEQHIGKELEYSPTSFVMAPIGTLKLKATYRH

>EDV92646.1

MIVILLLLASAALLYIYLKRTFSFWERKGFPYIEGKIPFGAMDSVRQKKRSFGLAIYDMY

LASTKPFVGIYLTLRPALLVRDAQMAHDILTTDFGHFHDRGVYVDEKHDPLSADLFSMEG

SSWRTMRTKLSPSFTSGKLKGMFGTAEDVADKMMGHLNGQMQRSGTVEVDMKNLMSSYAI

DIIASTIFGLDVDSFSNPDNEMRRISGEVMKPTAKNLLRGTTSFLYPKVEKIFNRLGWHI

DGRRGMRDLVYHTISLREKNNIERRDLMQLLLQLRNTGQISNDDAVWSSQAAAHGIKSLS

KDNIAAQMFLFFIAGYETTASTAAFTLYELSQNPDVLAKLMDDINQTLAKHNGELNYESI

QDMKYLELCVMETTRKYPALPLLNRMCNKDYPLPNSKLVLEKGTEIIISLLGLHRDPEYF

PDPLKYQPERFTEEHKNYTPAAYMPFGVGPRQCIAARMGKMNVKIALTKILTNYTIETRS

EVTEIEFTVNGIGLLAKGGVPVKLSRKLKFTNVQ

>380026862

MLLTTWLIPDIIAVARIFYVKPSFPTGNIMPIITGKTSFAEFFKDIYEHNKHHPLVGIYM

FYKPFLIVNDPNLIRDVLTKEFTSFHDRGIFCNEKIDPLSGHLFQLPGKKWRNLRVKLTP

TFTSGKIKQIFPILKETGNILEKYLEEEALKGSIIDVKDIFARYSTDIIMSVAFGISCDS

FKEPNNEFRYWGKKIFDPKPFWNALILFAPQVLDFFSISYTEKSVINFFTNMFKQTVEYR

ESNNIERKDFLNLLIQLMKNGYVDADDESFVSNNINAATAAQAYVFFLAGFETSSTTVTF

CLYELAKNQDIQNKLREEIRTMIEKNGDLTYSVLNDMNYLHKVISETLRKYPPIVILNRI

CTNDIKLNTTDFCIPKGTSVAIPVFGLHRDSNIYPNPEKFDPERFSEENIKTRHPYVYLP

FGEGPRICIGLRFGLIQTKIAIISALLKNKFKLGPNTPSTLEFEEGSLILVGKGGIHLSI

EPI

>EDW58667.1

MIVIVYLLGAVALLYIYLKWTFSFWQRKGFPSTVGTIPFGALDSVRRNKRSFGLAIYDMY

LSTTKPFLGIYLTLRPALLVRDAQLVHDMMVKDFASFHDRGVYVDEKHDPLSANLFSMEG

KSWKTMRTKLTPSFTSGKLKGMFGTAEDVADKMLAHLNAKIQQSGTVEIDVKNLMSCFVI

DIIASTIFGLDVDSFSQPDNDLRRISKAVTQNTAKNILRGTTSFLYPGVEKIFNRLGWKL

DGVEDMRQLVHHTISMREKSKVERRDLMQLLLQLRNTGEVSKDDSIWSAKATVDTFKALS

KDNVAAQLFLFFVAGYETTASTAAFTLYELAQNPDVLAKLLDDINQSLAKHNGVLNYDAI

QDMKYLELCIMETTRKYPALPILNRMCNQDYPLPNSNLVLKKGTEIIISLLGMHRDGEYF

PDPLSYQPERFTEEHKNYNPIAYMPFGVGPRQCIAARMGKLNVKIALTKILTNYNLETRQ

QKREIEFTVYGVPLLSKGGVPIKLSKKK

>ADH29768.1

MTLITNNLTFDLIAILATLLVCVIAYLKWHLTYWNRLGVPSLNPALFFGDATDYLFGKCG

VGEQFAKFYGKFKSKGYKHGGVFLGPKCYYVPVDPDLVKHIMQKDFQHFMNHGFYINEEV

DPLTGNLFSLEDAKWRNMRVKLTPTFTSGKMKMMFQTLADCTRGLKEIMDNLALNHAPAD

IKEILGRFTTDIIGSVAFGIECNSLKNPDAEFRKYGRKIVESGFIDRIKVFLVLSIPHAL

LRFWRFKFTNTEVETFFMGAIQDTVNYREKNNVYRKDFMHLLLQLKNRGLVADDQKITDD

KGNIKENDLITINELAAQAFVFFLGGFETSSTTMSWALYELATNQDIQEKLRKEISDVLS

RHNNKLSYDAMMEMTYMDKVINETLRKYPPLPIIPRVCNKDYTIPNTSTKLSRGTSVAIP

VLAIHTDPEYYPNPEKFNPEHFNEENIKSRPGFTWLPFGDGPRICIGMRFGMMQSKVGLA

TILKNYKIKLNNKTEFPIKVDPKNFITTAKGGVWLDVEKLD

>328706125

MVFFDSSLLNLVAYGIVISTTFYFYLRYRYTFWQRQGCPVPLKPHIIYGHTKEVTKMKTW

VGKHYANIYYNTDGYKFVGFYQFQKPKLMLRDLNIIKDVFTKEFSTFPNRGIVFDDKLEP

LTGNLLTLEGHRWKVLRNKLTPAFTIGKIKNMIDLIDGRAQEMVRVLEKSAVIGEQVEFK

ELLARFSTDVISIVAFGFETNSLTNPDAEFRRVGRMLFSTSLETIIRNALNALAPSLIGL

LKVRSIKKEYADFFYNVVNDTVKYREENGIQRNDFLDLLMKIKRGQNLASDEDSKSIFDE

NDGKEDFKFTMDVLAAQCFVWFIGGYETSSVTLTFTFFELAQNLDVQMRAQDEIDSVLSK

YDGKLTYEILQEMPYLDMIVSEALRKYPPVPNLTRKAVKPYKLPNSDFTLDKGLQVVIPV

YGIHNDPEYWPEPEKFIPERFTEEEKRNRPQYAYLPFGAGPRLCIGMRFGMMQVKVALFR

ILSTYNISLSKSMKLPMKMNPKTIPANPDGGMFLHITKRKN

>EDX13005.1

MIGIYLLIAAVTLLYVYLKWTFSYWDRKGFPSTGASIPFGALESVTKGKRSFGMAIYDMY

KSTKEPVIGLYLTLRPALLVRDAQLAHDVLVKDFASFHDRGVYVDEKNDPMSASLFQMEG

ASWRSLRTKLTPSFTSGKLKAMFETSDSVGDKLVDSIRKQLPETGAKELELKKLMATYAI

DIIATTIFGLDVDSFADPNNEFQVISRKVNRKNFEDIVRGASSFLYPGLEKFFVQIGWKQ

EATERMRELSNRTVDLREQNNIVRKDLLQLLLQLRNQGSVNADDNIWSAESTKNGVKSMS

KDLIAGQLFLFYVAGYETTASTASFTLYELTQNPEVMEKAKEDVRSAIEKHGGKLTYDAI

SDMKYLEACVLETARKYPALPLLNRICTKDYPVPDSQLVIKKGTPIIISLIGMHRDEEYF

PDPLAYKPERYLENGKDYTQAAYLPFGEGPRMCIGARMGKVNVKIAIAKVLSNFDLEIRK

EKCEIEFGVQGIPLIPNSGVPVRMSLKK

>EDW42241.1

MIGIYLLIAAVTLLYVYLKWTFSYWDRKGFPSTGASIPFGALESVTKGKRSFGMAIYDMY

KSTKEPVIGLYLTLRPALLVRDAQLAHDVLVKDFASFHDRGVYVDEKNDPMSASLFQMEG

ASWRALRTKLTPSFTSGKLKAMFETSDSVGDKLVDSIRKQLPETGAKELELKKLMATYAI

DIIATTIFGLDVDSFADPNNEFQVISRKVNRKNFEDIVRGASSFLYPGLEKFFVQIGWKQ

EANERMRELSNRTVDLREQNNIVRKDLLQLLLQLRNQGKVNTDDNIWSAESTKNGVKSMS

KDLIAGQLFLFYVAGYETTASTASFTLYELTQNPEVMEKAKQDVRSAIEKHGGKLTYDAI

SDMKYLEACVLETARKYPALPLLNRICTKDYPVPDSKLVIKKGTPIIISLIGMHRDEEYF

PDPLAYKPERYLENGKDYTQAAYLPFGEGPRMCIGARMGKVNVKIAIAKVLSNFDLEIRK

EKCEIEFGVHGVPLMPKSGVPVRMSLKK

>EDV48829.1

MIGIYLLIGAVTLLYVYLKWTFSYWDRKGFPSTGVSIPFGALESVTKGKRSFGMAIYDMY

KSTKEPVIGLYLTLRPALLVRDAQLAHDVLVKDFASFHDRGVYVDEKNDPMSASLFQMEG

TSWRALRNKLTPTFTTGKLKAMFETSDSVGDKLVDSIKKQLPETGAKELELKKLMATYAI

DIIATTIFGLDIDSFADPNNEFQVISRKVNRNNFEDIVRGASSFLYPGLEKFFVRIGWKQ

EATERMRELSNRTVDLREQNNIVRKDLLQLLLQLRNQGQVNTDDNVWSAESSKNGVKSMS

KDLIAGQLFLFYVAGYETTASTASFTLYELTQNPEVMEKAKEDVRSAIEKHGGMLTYEAI

SDMKYLEACVLETARKYPALPLLNRICTQDYPVPDSKLVITKGTPIIISLIGMHRDEEYF

PDPLAYKPERYLEDGRDYNQAAYLPFGEGPRMCIGARMGKVNVKIAIAKVLSNFDLEIRK

EKREIEFGVHGIPLMPKSGVPVRLSLKK

>156546729

MTLGFFEVAAGLVVLGLAVYYYFTSNMDFWSSRGVKGPKPVLLFGTTRDLVFGRMSIAQH

TTLTYEAFQGQPLVGLFTNQKPILMVRDPELIKDVLIKDFSVFSYRGLPIASKAEPLSQH

LFLLEPKRWRPLRQKLSPTFTSGKLKDMFYLLRECAEHLGSFLEDFVEKNPVIECRELAG

KFTTDVIGVCAFGLNMNAIAEDDSKFRKIARKPFDSSTLSLMKNYLRFAAPWLMDLLSPI

FYDRELNDFFIGTITQTMEYRKKSNVKRNDFIDLLMAIKDDPSKLNDIELTDTLLAAQAF

VFFIAGFETSSSTISHTLYELAQNQPIQDKLREEIVEGLKSHNGELTYESVKSMKYLHKI

FCETLRKYPPLSVLQRCSLEPYTFAGTKVTIPENTLLWIPAHAIQHDPENYPEPEKFDPD

RFEEENVKHRHPSLYLPFGDGPRNCIGARFGIYQTKVGLIQILKSYKVEVCAETCIPYVV

DPKKFLLTPLDGIKLKFRKVVDK

>156553634

MSGLFEVAAGLIALGLAVYYYLSSNLDFWSKLDVKGPKPVPLFGTTSDVLLGRLSLFQYA

AREYGRFRDEPLVGIFSYRKPILLLRDPDLIRNVLVKDFSVFSCRGMKAATKAEPLAQHL

VFLEPERWRPLRYKLSPAFTSGKLKEMFYLLKECAQHLDRLLEDCVARDPVIECRELAGK

FTTDAIGVCAFGLDMHAIDGEDNEFRKTSRKMFSTSILNRIRIHLKITAPWLLDLLSPIF

YDREMNDFFIDTVAQTMDYRKKNGVKRHDFIDLLMDIRDNPSKVNDIEITETLIASQAFV

FFLAGFETSSTTISNALYEMALNPSIQDKLREEILEELKKHNGEMTYESIKGMKYLHKIF

CETLRLYPPAGLLSRRSLEPYTFAGTKVTIPKNTSVVVPIFGIHHDPEIYPQPDKFDPER

FDEEAVNHRHPSFYLPFGDGPRNCIGSRFGTYQAKVALVQILKSYKVEVCDQTVIPYVID

PKTVILLAPRGGIKLKFKKIK

>EGI66978.1

MESFEILCGIATVILFLYYFLTSTFDFWKSRGVPGPRPIPGFGNFKDVMFNKISSGDYLT

KIYNTYKDEPLVGIFTRKTPILIVKDPNLIKDVLIKDFTKFADRGFPIFDKAEPLSQHLF

SLEPKRWRPLRTNLSPVFTSGKLKEMFSLMSECADHLIQYIEKVSSKNEPIECRELSAKY

TTDVIGSCAFGIDMNALSNEESEFRRMGRKIFALTWARMLRIRIRQISPWLYNMLGYVLP

HTDVTRFFIRVITENMDYRETNNVIRNDFIDMLRELKRHPDQFGDFDLTDNLITSQAFVF

FIAGFETSSTTISNALYELALNQKMQNSLREEIDEVYAKYDGDFTYDNIKKMSYLDKVFK

ETLRKYPVVTFLMRQSITSYTFDGTKVNVPEGQKVWIPVYAIHRDPSIYPKPEVFDPERF

SDDAVQSRHPMSYLPFGDGPRNCIGSRFAIYQTKVGLVKILRNYKIETCEKTPIPYVNDP

KAFLLAPKDGLHLKIIKINRT

>380021411

MASSFEILCGIAILILTLYYYFTSTFDFWKSRGVVGPKPIPFFGSTKDLILIKKSMAHFV

KDIYENYKNEPMIGLYANRSPFLLLNDPELIKDILIRDFSKFANRGLGVFEKTEPLSPHI

LNLEVERWRPLRSRLSPIFTSGKLKEMFYLIIECSLNLETYLDNLIEKNEPIECRELTAR

FTTDVIGSCAFGIDMSSMTNENSEFRKMGRKVFDVNLINIIRLKLKQFMPRLYDLLGYVM

PDRTFAPFFTRVVKDTIKYRNDNNIVRPDFINMLMELQKNPHKLENIKLTDSLIAAQAFV

FFLAGFETSSTTMSNALYELALNQDVQKKLREEINTFCAKNNRELKYDDIKEMEYLDKVF

KETLRMYPPATILMRKATSDYTFNDTKVTIPKDTTIWVPAFAIHRDSAIYPNPDSFDPER

FDEDAMASRHPMHYLPFGDGPRNCIGARFAVYQTKVGLITILRNHKVEVCEKTIIPYEFD

PGAFLLSPKGGIYLKITKI

>EGI67003.1

MEPFEILCGIAVAVLALYYFLTSTFDFWKLRGVPGPRPIPGLGNFKDVMLNNISAGDYLK

EMYNTYKNEPLIGIFARKTPILIIKDLDLIKDILIKDFTKFADRGFPVLEKADPLSQHLF

FLEPKRWRPLRMKLSPAFTSGKLKEMFALMSECADHLIQYMDKITNSGNPVECRELTAKY

TTDVIGNCVFGIEMNALSDENSEFRKMGREAFNPSWLNILRIRLRQIFPWFYNILFGYFL

PQTNVTKFFTRVIVENMDYRETNNIIRNDFIDILRELKNHPDKLDDIDPTDSLIASQAFV

FFLAGFETSSTTISNVLYELALNQEIQDKLREEIDEVYAKHDGNLIYDNIKKMNYLDIVF

KETLRKYPPGTIIMRQSTSSYTFDGTKVSIPKDQKVWIPIYAIHRDPNIYPKPDVFDPER

FNNEAVHSRHSMAYLPFGDGPRNCIGARFAIYQSKLGLTKILRNYKIETCEKTPIPYVND

PKAFILAPKDGLHLKMVKINRI

>383855465

MATYLEILCGLIAICLAFYYYFLSVLDFWKIRGVPGPKPLPLLGNIKDVMLMKMSMSDYL

RKIYHEYKNEPMVGIFARKTPILVLQDPDLIKDVLIKDFSKFANRGLPVLEKIEPLSPHL

FNLEAERWRPLRTRLSPVFTSGKIREMFPLILECADHLEQYLEKIVARGEPIECRELTAK

YTTDVIGSCAFGIEINAISDDESEFRRVGKSVFATNFLQLVRFRARQMFPTFYNLLGYVL

PSSEVTTFFTKVIVDTMRYREENNVTRPDFVNMLIELKKHPDKLENVKLTDSLLTAQAFV

FFIAGFETSSTAISNALYELALNPEVQDKLRKEIKEMYDKNNGELQYEKIKDMEYLDLVF

KETLRKYPPGPLLLRRSICKYTFEGTKITIPAKQFIWIPIYAIQRDPDIYPNPDAFIPER

FTEDAVAARHPMHYLPFGDGPRNCIGARFAIYQSKVGLIKILRNYKIDVCEKTMIPYVID

PAAFLLAPKGGIYLKITKIES

>ABB36783.1

MASSFEILCGIAVLFLALYYYLTSTFDFWKSRGVVGPKPVPFFGTTKDLILVKKSTAHFV

KDIYEKYKNEPMVGLYATRSPFLLLNDPELIKDILIRDFSKFANRGLGVFERTEPLSPHL

LNLEVERWRPLRSRLSPIFTSGKLKEMFYLIIECSLNLETYLDKLIEKNEPIECRELTAR

FTTDVIGSCAFGIDMSSMTNENSEFRRMGREVFAVNFMNVMRMKLKQFMPRLYDLLGYVM

PDRTFAPFFTRVVTDTIKYRNDNNIVRPDFINMLMELQKNPQKLENIKLTDSLIAAQAFV

FFLAGFETSSTTMSNALYELALNQDVQKKLREEINTFCPKNNKELKYDDIKEMEYLDKVF

KETLRMYPPASILMRKAISDYTFNDTKITIPKEMKIWIPAFAIHRDSAIYPNPDSFDPER

FDQDAMASRHPMHYLPFGDGPRNCIGARFAVYQTKVGLITILRNHKVEVCEKTIIPYEFD

PGAFLLSPKDGIYLKITKI

>289177173

MGVGILEILAGIGLLFLACYYYFTSTFNYWQSRGIPGPKPAPIYGNLKDIMFGKMSVGDF

VKQEYDKFKDKRMFGIYAIRTPVLIVNDPELIKDILIRNFSSFANRGVRLFEKVEPLSAN

LVNLEAKRWRPLRAKQITMFTFSKLKEMFYIVSECADNLEKILAGMIEKNDVVECCEVSA

RYTTDVIGACVFGIDMKALDDENSQFRRVGRKFIHADKWRAFKLRFKQIFPSLYNLLCPI

MYDHEINDFFINTMIATMKHRKENNVRRGDFVDLLNDLKDNPGKLDGIELTDTLLTAQAF

AFFFAGFDTSATTMSHALYELALNKSIQNKLREEIKEDLAENNGKLLLESLKNMKYLDKV

FNETLRKYPVTTILTRQAMESYTFDGTDLTIPKDTKVWIPVYGIHRDPKIYPNPDTFDPE

RFDEEKVQERHPSYYLPFGAGPRNCLGARFGNYQTKVGLITILKNYSVDICDQTPIPYVS

NRRRFLLGPAAGIRLKFTNLS

>380021413

MTSLEIIFGIILLFLAIYYYFTSTFDFWKVRGVPGPKPIPIFGNIKNVMLLRTSMCHYLK

KLCEEYKHESMIGIFTRKTPILIIQDPDLIKDVLIKDFSKFANRGIPIHEKAEPLSSHLF

NLEVERWRPLRTRLSPVFTSGKLKEMFPLILDCAKHLEQYLDKVVVREESIECRELTAKY

TTDVIGSCAFGIEMNALSDEESEFRRIGRKVFATSFWQILRFRFAQIFPRIYNLLAFVLP

PMEITKFLTNIIVSTMKYRQENNIVRPDFVNMLIELKKHPDKLENIKLTDTLLTAQAFVF

FIAGFETSSSAISNALYELALNPEVQDKLRQEIKEYFNKHNELKYEYIKDMTYLDLVFRE

TLRKYPPGPIILRKSITNYTFNNTKISIPEKSFVWIPLYAIHHDPKIYPNPDAFIPERFN

DDAIAIRHPMHYLPFGDGPRNCIGARFAVYQSKIGLITILRNYKVEVCNKTMIPYEINPA

AFLLTPKGGIYLKFTKIKNDEEILN

>340723483

MATYFEIICGIIALLLAFYYYFTSTFDFWKVRGVPGPKPVPMFGNIKDVMLLRTSMCTYL

KKLWEEYKDEPMIGIFTRKTPILILQDPELIKDVLIRDFSVFADRGIPVHEKAEPLSPHL

FNLESQRWRPLRTRLSPVFTSGKLKEMFPLILECSKHLEQYLDSLVAKGEPIECRELTAK

YTTDVIGSCAFGIEMNALSDEDSEFRRMGKQVFATSFGQILRFRFRQIFPYLYNLLGSII

APLEITTFFTKIIVDTMKYRDDNNIVRPDFVNMLMELKRHPDKLENIKLTDTLLTAQAFV

FFIAGFETSSSAISNVLYELALNPEIQDKLRQEIREHFEENNGELKYDQIKDLTYLDLVF

RETLRKYPAGPLILRKSVSSYTFNNTKVSIPEKTFIWIPLYAIHHNPDIYPNPDAFIPER

FNDDAVASRDSMHYLPFGDGPRNCIGARFAVYQSKVGLITILRNHKVEVCEKTMIPYVID

PAAFLLAPKGGIYLKLIKVES

>340723503

MDYFQLLLGIAVILLAIYYYYTSVYDTWKNRGVPGPKPSMFFGNFVDILLKRRAVATIVK

DLYNEYKSEPVFGIYEGTSPILVINDLDLIKDVLIRDFSLFVDRGFKVLEKIEPLSQHLF

LLEAKRWRPLRAKLSPIFTSGKLKEMFPLIVECAGNLEKYLDKVVEKSVPIVECRDLAAK

FTTDVIGSCAFGISTNALEDENSEFRRMGKQIFAPNLKQTIRESCRRLAPFLFKIVGYFL

RTTEVNNFFINLVRDTMEYRKTNNITRPDFIYLLMQLKEHPEKMENVELTDSLIAAQAFV

FFIAGFETSSSTIAHALYELAQNQEMQDKLRQEIRDAYNKDGGTLTYEGIKGMKYLDKVF

KETLRKYPILTVLTRQAMENYTFKGTKITIPKETIVWIPIYGIQHDPNIYSDPEKFDPER

FNEDAVAARHPMSYLSFGDGPRNCIGARFAHYQSKVGLATILHNHKVDVCEKTKIPYEAD

KEAFLLSLSGGVNLKITKA

>350426726

MSGFEILCGLAALLLAFYYYSTSTFDFWKSRGVRGPKPVFLFGNTIDLMFARISMVAYQQ

NLYKVYKNEPMIGLYMRRSPVLILKDPELIKDVMIRDFSRFADRGFAVHERTEPLSMHLF

NLEPKRWRPLRSKLTPMFTSGKLKDMFGLILECADHFEKYLDKLTAKEEPIDFREVTAKF

TTDVIGSCAFGIEMSSLSDEDSEFREIGRQIFALNLENVIRLKLRLYMPKLYDLLGYIVP

DKRHAPFFTKLVTDTMKYRKEHNIYRPDFIHMLMELKEHPEKLGDIKLTDSLLTAQAFVF

FAAGFETSSSAMSNALYELALNQEIQDKLRQEIREHLAKHNGELQYEHVKDMEYLEKVFK

ETLRKYPPGSILPRRSTSAYTFKNTKVSIPKGLMIWIPTYSLHHDADIYPNPDVFNPENF

NEDAIEARHPMTYLPFGDGPRNCIGARFAIYQTKVGLITILRNYKVDVCDKTMIPYEFTK

TSFLLTPKGGIYLKLTKLET

>EGI61667.1

MESFEILCGIAAVVLAIYYFLTSTFDFWKSRGVPGPRPIPGLGNFKDVILNKKFVGDYVK

EIYNTYKNEPLIGAFARKTPILIVKDLDLIKNILIKDFSNFADRGFPNLKKVEPLSQHLF

TLEPKRWRPLRMRLSPVFTSGKLKEMFSLISECADHLILYMEKITVECRELMAKYATDVI

GTCAFGIDMNALSNEDSEFRKMGRKILIPTWTNILRLRLRESFPRLYEMLGYILPQTEIT

KFFTRVIVETINYREVNNITRNDFIDMLRELKKHPDKLGDNFDLTDSLLTAQAFVFFVAG

FETSSSTISHALYELALNEKIQNSLRKEINEVYAKHGDNLTYDNVKKMDYLDKVFRETLR

KYPPVTFLQRQSMSSYTFDGTKVSIPKNQQVWIPIYAIQRDSDIYPKPDVFDPERFCDDA

VQSRHPMTYLPFGDGPRNCIGARFGVYQSKIGLIKALRNYKFEICEKTSIPYVNNPKTLL

LTPKDGLHLRIIKIN

>380021333

MIHIEILCGIAIVSFLVFYHYFISAFNFWKNRGIPGPKPEFLFGNIKDIMLSKISTPMFI

KNIYDTYTNEAMVGLYIGRNPILFLKDPELIKDVLIRDFSKFADRGFNVHEKVEPLSQHL

FNLEPKRWRPLRSKLSPMFTSKKLKEMFHLILECGHHFENYLDQLAAQQEPVDFYEVTAK

YTTDVIGSCAFGINMNAMSNEESEFRVAGRKIFEPTWNAIIRLKFKITMPSLYDFLGPLI

PEREVTPFFIKIVTDAMKYRKENNVSRPDFIDMLMKLRDDSKSLSDIELTDAFLTAQVYV

FFAAGFETGASTISNALYELAQNQKIQDKLREEIREHYDKYNGELIYENIKEMEYFDKVF

TETLRKYPPGTLIPRRSISDYTFKDTNVTIPKGTMIWIPAFSIHRDANIYPNPDDFNPEN

FTEDAINNRHPMNYLPFSNGPRNCIGARFANYQVKIGLIMILRNYKVEVCEKTMIPYQFD

PNLFLLGPKGGIHLKITKVES

>EFN65184.1

MGSFEILCGIAAVLLVVYYYFTSNYDFWKSRGIRGPKPIPIFGNFKDVMLGKKLPGIYLM

EIYNEYKNESMIGIFNRKTPILIVKNPDLIKDILIKDFSKFADRGFPRGSEKVDPLSQHL

FGLEPKRWRPLRTCLSPVFTSGKLKEMFSLISECADHLEQYIEKLVSKNEPIEFREIMAK

YTTDVIGTCAFGIEMNALSDEDSELRRMGRMVFTPTWKSILRATIKQSMPRFSEILNYII

SLQTQASQFFMRVVIEMMNYRETNNIIRHDFIDILRELKKNPDKMGDIEMTDSLIASQAF

VFFLAGFETSSLTMSHALYELALNEQIQDKLREEIDQEYTNHDSNLTYEKIKKMNYLDKV

FKETLRKYPVVTILIRQSTSSYIFDGARLDIPKDQKVWIPVLAIHRDPDIYPKPDVFDPK

RFNDEAVQARHPMHYLPFGDGPRNCIGARFAIYQTRIGLVKILRNYKVEVCDKKQKPYII

NPKATMLAPKHGIHLRITKINR

>340723497

MSGFEILCGLAALLLALYYYSTSTFDFWKSRGVQGPKPVFLFGNTIDLMLARISMVAYQQ

NLYKVYKNEPMVGLYMRRSPVLVLKDPELIKDVMIRDFSKFADRGFAVHERTEPLSMHLF

NLEPKRWRPLRSKLTPMFTSGKLKDMFGLILECADHFEKYLDKLTAEEEPIDFREVTAKF

TTDVIGSCAFGIEMSSLSDEDSEFRKIGRQIFALNFENVIRLKLRLYMSKLYDLLGYIIP

DRRLAPFFTKLVTDTMKYRKEHNIYRPDFIHMLMELKEHPEKLGDIKLTDSLLTAQAFVF

FAAGFETSSSAMSNALYELALNQEIQDKLRQEIKEHLAKNNGELQYEHVKDMEYLEKVFK

ETLRKYPPGSLLPRRSTSAYTFRNTKVSIPEGLIIWIPIYSLHHDADIYPNPDVFNPENF

NEDAIEARHPMTYLPFGDGPRNCIGARFAIYQTKVGLITILRNYKVDVCDKTMIPYEFSK

TSFLLTPKGGIYLKLTKLKT

>340723499

MYVGFEILCGLAALLLALYYYSTSTFDFWKSRGVQGPKPVFLFGNTIDLMLARISMVAYQ

QNLYKVYKNEPMVGLYMRRSPVLVLKDPELIKDVMIRDFSKFADRGFAVHERTEPLSMHL

FNLEPKRWRPLRSKLTPMFTSGKLKDMFGLILECADHFEKYLDKLTAEEEPIDFREVTAK

FTTDVIGSCAFGIEMSSLSDEDSEFRKIGRQIFALNFENVIRLKLRLYMSKLYDLLGYII

PDRRLAPFFTKLVTDTMKYRKEHNIYRPDFIHMLMELKEHPEKLGDIKLTDSLLTAQAFV

FFAAGFETSSSAMSNALYELALNQEIQDKLRQEIKEHLAKNNGELQYEHVKDMEYLEKVF

KETLRKYPPGSLLPRRSTSAYTFRNTKVSIPEGLIIWIPIYSLHHDADIYPNPDVFNPEN

FNEDAIEARHPMTYLPFGDGPRNCIGARFAIYQTKVGLITILRNYKVDVCDKTMIPYEFS

KTSFLLTPKGGIYLKLTKLKT

>EFN81691.1

MTEYLTEIYHYYKNETMIGIFARRTPILIVKDPDLIKDILIKDFSNFADRGLTTHKKAEP

LSQHLFLLEPKRWRPLRIRLSPVFTSGKLKDMFPLILECSSHLERYVETLANKNESVECR

ELTAKYTTDVIGSCAFGIDTNSLSEEDSEFRKMGREIFAPSWHNMIRMRMRQSLPWLYDK

LGYILPQTRTTKFFTRIIVENIDYREKNNIVRHDVVDMLREIKKHPEKLNIELTDTLLTS

QAFVFFIAGFETSSTTMSNALYELALNQEIQDKLREEIHQKYMNINGDLTYDNIKKMSYL

DKVFKETLRKYPPLTFIMRKATSSYTFARSKITIPEGQRIWIPAYAIQRDPNIYPEPDVF

DPERFSEEIMQTRHAMTYLPFGDGPRNCIGARFAVYQTKVGLIKILRYYKVEACEKTQIP

YVIDPNTFLLAPKDGIHLKITKID

>350422652

MATYFEIICGIIALFFAFYFYFTSTFDFWKVRGVPGPKPVPVFGNIKDVMLLRTSMCAYL

KKLWEEYKDEPMVGIFTRKTPILILQDPELIKDVLIRDFSIFADRGIPVHEKAEPLSPHL

FNLESQRWRPLRTRLSPVFTSGKLKEMFPLILECSKHLEQYLDNLVAKGEPIECRELTAK

YTTDVIGSCAFGIEMNALSDEDSEFRRMGKQVFATSFRQILRFRLRQIFPHLYNLLGSIM

APLEITTFLTKIIVDTMKYRDDNNIVRPDFVNMLMELKKHPDKLENIKLTDTLLTAQAFV

FFVAGFETSSSAISNALYELALNPEIQDKLRQEIKEHFEKNDGELKYDQIKDLTYLDLVF

RETLRKYPAGPLILRKSVSSYTFNNTKVSIPEKTFVWIPLYAIHHNPDIYPNPDAFIPER

FNDDAVASRDSMHYLPFGDGPRNCIGARFAVYQSKVGLITILRNHKVEVCEKTMIPYVID

PAAFLLAPKGGIYLKLIKVES

>EFN78989.1

MSDYLMEIYNSYKDEPVIGIFARRSPILIVKDPNLIKDVLIKDFSNFADRGLTIHEKTEP

LSQHLVALEPKRWRPLRTKLSPVFTSGKLKDMFPLILECSEHLEQYVEKLASKNEPVECR

ELTAKYTTDVIGSCAFGIDTNSLSDVDSEFRRMGREVFATKWQNVMRLRIKQAAPWFYNI

LGYILPQTNFTKFFTRILMENLDYREKNNIVRHDFVDMLLELKRHPDKIDIELTDSVIAS

QAFVFFLAGFETSSTTMSHALYELAQNHKIQDKLREEINHKYAKNDGILTYNNIKEMDYL

DKVFKETLRKYPPATFLMRKSTSDYTFDGTEINISKGQTVWIPVYAIQRDPNIYPDPEVF

DPERFNEEAMQTRNAMFYLPFGDGPRNCIGARFAVYQTKIGLVKILRNYIVDICEKTQIP

YINDPKAFLLAPTGGIYLRIIKINQT

>110765952

MAYIEILCGIIVSMLVFYYYFTSVFNFWKIRGIPGPKPKFLFGNIRDIILSRISTPTFIK

NIYDTYTNEPMVGLYMGRNPILLLKDPELIKDVLIRDFSKFADRGFNVHEKVEPLSQHLF

NLEPKRWRPLRSKLSPMFTSKKLKEMFGLILECGHHFEKYVDGLAARRQPVDFCEVAAKY

TTDVIGSCAFGINMNAMSSEGSEFREAGRKIFEPTWNSIIRLKFKITMPTLYDLLGPLVP

EREVTPFFIKVVTDAMKYRKERNVFRPDFIDTLMKLRDDPESLSDIELTDAFLTAQAYVF

FAAGFETGASTISNTLYELAQNQGMQDRLREEIREHCDKYGGELMYENIKEMEYLDKVFK

ETLRKYPPGTLIPRRSVSEYTFKNTNVTIPKGTMIWIPAFPIHRDPNIYPNPDDFNPENF

TEDAINNRHPMNYLAFSNGPRNCIGARFANYQVKIGLIMILRNYKVEVCEKTVIPYQFDP

NLFLLGPKGGIYLRVTKVE

>EFN70316.1

MIAKEQNGRAQKEEEKAKSFGFHRTSLYRSWRNIDIDLTLCETIDQHLNYKVMESFEIIC

GIAVVFLGAYYYFTLNFGFWKSRGIRGPQPTPGFGNIKDIMLMKKSLPDFLMEIYNDYKD

EPMVGIFANRTPTLIVKDPDLIKDILIKDFTNFADRGFLFHEKLDPLSQHLFALEPKRWR

PLRTRLSPVFTSGKLKEMFSLITQCADRFERYIEKLASKNEPIECRELTAKYTTDVIGTC

AFGIEMNALSDEDSEFRNMGRLVFAPTWTNIVQRRMRQLLPKRFCDILSYILPIKEVTKF

FTRVVVETMYYRDTNNIVRHDFIDTLRDLKKHPDKVSNIDLTENLIAAQAFVFFAAGFET

SSTAMSNALYELALNQEIQKKLREEINEECIRHNGNFKYENVKKMNYLDKVFKETLRKYP

PLVQLTRKATSSYTFHNINVNIPKGQDIWIPIYAIQRDPDIYPKPDVFDPERFSDKVAQI

KHSMFYLPFGDGPRNCIGARFAVHQTKIGLIKILRNYKVEICEKTQIPYINDPRSSVILT

PLGGIYLKMIKINRL

>340723493

MTNYFEILCGMAALLLALYYYATLTFNFWKNRGVIGPQPLPFLGNTNELMFARKSIPHYL

MEIYDTYKNEPMVGLYMRRSPLLIVQDPEFIKDILIRDFPKFADRGFHVYERTEPLSQSI

FNLESERWRPLRTMFSPIFTSGKLREMFYLIIECSQHLEKYLDKRVEKGEPIECCEVTAK

FTTDVIGTCVFGIEMSAMADEDSEFRKMGREVFAVNVENVIRQKMKLFMPKLYHLLGYII

PDRKLAPFFIKIVTDTIKYRKENNIVRPDFINMLMEVQKHPEKFENIELTDSLLTAQAFI

FFAAGFETSSMTMSNALYELALHQDIQDKLREEIKEHCCENDEELKFQDIKGMQYLEKVF

KETLRMYPPGAFIPRRTKSTYTFNNTKVTIPQGTLIWIPVFAIHRDPDIYPNPDLFDPER

FNEDAVAARHPMHYLPFGDGPRNCIGARFANYETKVGLITILRNYKVEVCDKTVIPYEFD

PGAFVTAPKGGIYLKITKI

>383857845

MEYFEILCGIAAVLFLLYYYFTYNYDFWKKQGVVGPKPIPFFGNTIEIMLAIKSVAEYSE

ELYQKYKNERMIGLYALNIPVLLLKDPELIKDVLIRDFSSFTDRGFNVNDKAEPLSKNLL

NLEPRRWRPLRSKLSPIFTSGKLKEMFGLILDCAQHFDQYLDKVVAKGEPVDIRELTAKF

TTDAIGTCAFGIDMNALSEEDSEFRKMGRDIFVPNFLNVFRQKSNIFFPKLYGIVGRFLP

DKFTPFFTKMVTESMAYRKKNNIYRPDFINMLMDLKSHPMQVGDIELTDELLTAQAFVFF

AAGFETSSTTMSNALYELAQNHDIQDKLRDEIREHDEANNGDLKYEHIKQMEYLDKVFKE

TLRKYPPGSLVPRRATADHTFEGTNVTIHKSMRVWVPIYAIHRDPDIYPNPEVFDPERFN

EDAVQARHPMNYLPFSDGPRNCIGARFAVYQTKVGLITILRKYKVDVCEKTVIPYTLSAG

AFVLAPKEAIYLKLTKLTK

>383857571

MADYFQLFSAFVVVFLALYYYFTSTFDFWKNRGVPGPRPIPVFGNAKDVVLGRETLQIYI

TRLYRKYKDEPMFGIFVRGSPNLVLCDLDLIKDVLIKDFTMFEDRGITVVERVEPLSTNL

FNLEPERWRPIRKSLTPVFTSGKLKEMFPLILECSNQLEGCLEKIIEQGGLLDCREVAAR

FTTDAIGSCAFGINMNALSDEESEFRRIGKEIFKSDFQALMKLTFREALPQFYNMLGFVL

PQNEITTFLTAIVSETIKYRKENNIVRPDFINLLMELKENPHKLENIDMTDTLLTAQAFV

FFAAGFETSSTTIGHALYEMALNPDIQDKVRNEIREHYTKNNGKWTYDDVKDMKYLDKIF

KETLRKYPPGQLLRRRCNSNYTFNGTKVTIPKDTMVLIPLYAIQTDPAIYPNPDVFDPER

FNEDVESTRHPMTFLPFGDGPRNCIGARFAIFQTKLGLIQTLRNYKVDVCDQTMVPYVRH

PIARVFTPKQRIILKISKV

>380021415

MDYFQLLCAIGALLFAIYYYFTLTFDTWKSRGIPGPKPMIFFGNFQEVIFKKISLAEKTK

QLYQEYKNELVFGIFQGRTPVLVINDLDTIKDVLIRDFSLFADRGIHINPKVEPIFQTLF

ALEPKTWRPLRTKLSPVFTSGKLKDMFPLILDCAKNLEEYVEKVTESGEAVDCRDMAAKF

TTDVIGSCAFGVCMNSLSPEGSEFRRMGEQLGKFNFKRLARDFTRLYMPFLFDIIGGYLQ

SHEINNFFINLIRDSIKYRQENNVFRPDFVNTLKELKEHPEKLENVELTDALLTSQALVF

FLAGFETSSTTISNALYELAQNPEMQDKLREEIKEVYQNNGGALSYTDMKEMKYLDKVFK

ETLRKYPVLPVISRKAMENYTFKNTKITISKGTRVWIPAYGIQHDPNIYPEPQVFDPERF

EDDAFASRHPMSYLPFGDGPRNCIGARFAHYQSKVGLITILRDHKVEVCAETLIPYESEP

RNILMIPKGGKVELKITKV

>350422583

MTNYFEILCGMAALLLALYYYATLTFNFWKNRGVVGPQPLPFLGNTNELMFARKSMPHYL

KEIYNTYKNEPMVGLYMRRSPLLVVQDAEFIKDILIRDFPKFADRGFHVYERTEPLSQSI

FNLESERWRPLRTMFSPIFTSGKLREMFYLIIECSQHLEKYLDKCVEKGEPIECCEVMAK

FTTDVIGTCAFGIEMSAMANEDSEFRKMGREVFAVNLENVIRQKMKLFMPKLYHLLGYVI

PDRKLAPFFTKIVTDTIKYRKENNIVRPDFINMLMEVKKHPEKFGNIELTDSLLTAQAFI

FFAAGFETSSMTMSNALYELALNQDIQDKLREEIREHCCKSNEGLRFEDIKGMQYLEKIF

KETLRMYPPGAFIPRRTKSSYTFNNTKVTIPQGTLIWIPVFAIHRDLDIYPNPDSFDPEK

FNEEAVAARHPMHYLPFGDGPRNCIGARFANYVTKVGLITILRNYKVEVCDKTIIPYEFE

PGAFVSAPKGGIYLKITKIRSEC

>340723501

MAYYLIIICAAVAVLLAYYYILVFNFWKKRNISGPKPVFFFGNVIDIMFSGIALPQYIKK

LYETYTGRMIGLYMRTTPVLVLKDPELIKDVLIRDFSKFTDRGFNVHERVEPLSQHLFNL

EAKRWRPLRSKLTVYTSGKLKNMFDLIIECANYFEKYLDKMVAQGEPVDFREITAKFTTD

VIGSCAFGINMGALSDEESEFRKIGKQIFEPSLEAIIRMKFKLMMPKLYDLLGYIVPDRR

LAPFLTKVVVDTMKYRKENNVYKPDFIHTLMQLKEDPEKLHDLELTDSLITAQAYVFFAA

GFETGSTTMSNALYELALNPKIQEKLREEINEHYKKHNGELNYEHIKDMEYLDKVFKETL

RMYPPGTLIPRKSNSEYIFRDSQISIPKGVMMWIPVYAIHRDPEIYPNPDVFNPENFTRD

AIDARHPMHYLPFGSGPRNCIGARFAVYQTKVGIITILRKYKVDVCEKTMIPYQFDPSAF

LLAPKGGICLKITKI

>66565910

MDYFQILCAISIVILTIYYYYSLKYAFWKDRGISGPKPIIFFGNFGNSIIKKKSLSETVK

KWYDDYKHESVFGIFEGTIPVLVINDLDMIKDILIRDFSLFVDRGFHIFPKIEPLTQHLF

LLEAERWRPMRMKLSPIFTSGKLKEMFSLIVESAGNLEKYLDEVIKKNEMVECRDLAAKF

TTDVIGSCAFGINTNSLLEEDSEFRRMGKKIFSPSLKLMIGNTCKVFFPSLYEVIGNIFT

MKDVDEFFINLVSDTMKYRKDNDIVRSDFINMLMQLKEHPEKMDNIELTDTLLTAQAVVF

FIAGFETSSSTIAFGLYELAQNQEIQDKLREEIRKMHEKNKGILTYTDIKEMKYLDKVFK

ETLRKYPILSTLSRKAMENYTFKGTKITIPKGTKVWVPVYGIQHDPNIYPKPEVFDPERF

EDDAFASRHPMSYLPFGDGPRNCIGARFAHYQSKVGLITILRNHKVNVCEKTTIPFKADE

RSFLLALKGGVHLKITKI

>383857849

MIEYFQILCGIAIVFLSLYYYNISTYDFWKIRKVPGPTPQAFFGNFKEIFYKQASMSEHI

KKLYDDYKNEPMFGIFEGRTPTLMVNDTNEIKNVLVRDFNVFADRGIPYYKRVEPLSEHL

FFLESERWKPVRAKLSPVFTSGKMKEMFPLIVDCAVKLEQYLDKLVNKGKPIECRELAAK

FTTDVIGSCVFGIETKALSDENSEFREMGRRFFEPCIRTTVRTAIRQFLPGLYNIIGDYL

QAVGVSDFFIKVVIDTMKYRKAHNIYRPDFINALIDLRDHPEKVPTAELTDTFLTSQAFV

FFVAGFETSSTTIAHTLYELAQNHDVQNKLREEIVRSNAIHGKELTYDIVKAMKYLDKVF

KETLRKYPILPMIPREALADYTFESTKVSIPKGTKVWIPASGIHADPNFYPNPDKFDPER

FSEEAVASRNPMSYLPFGDGPRICIGARFAYYQAKVGIISIIRNYKVDVCEESIIPYKHE

PQALLLSPEGGVTLKVTKLSQLNGHMQSAK

>340723507

MDYFQLVCAIGMVLLAMYYYYTSTFDYWKIRGIPGPQPTVLIGNLKDILLRKTSLGDKLK

DMYEEYKKEPMFGIFEGMTPILVINDLELIKDVLIKDFPLFVNRGFRLFKKAEPLGEHLF

ALEAERWRPMRAKLSPVFTSGKLKEMFPLVIECSKNLEKYLDGVSQTGKPVECRDLAGKF

TTDVIGSCAFGINMNALSDENSEFREVGKKMFTQTMRMQIRDICRQFLPNIYEIFGHLLQ

IPGVDQFLIDVVRDTIKYRRENKIVRPDFINTLMELQDHPEKLEDIELTDSLLTSQAFVF

FAAGFETSSTTISHALYELALNQHIQDKLRKEIREVCDKHQGALTYEAIKEMKYLDKFFK

EVLRMYPLIPFVMREASENYTFKGTKVTIEKGTKLWVPAYGIQRDANIYPEPEKFDPERF

NDDAVAARHPMAYLPFGDGPRNCIGSRFAQYQSKVGIIAIIRNHKVDVCEKTKIPYESDP

RSFMLALKGGVHLKITKVKN

>350422635

MVYYLIIICTVVAVLLVYYYYILVFNFWKKRKISGPKPVFFFGNFIDIMFSEIALPQYIK

NLYETYTGRMIGLYMRSTPVLVLKDPELIKDVLIRDFSKFADRGFNVHERVEPLSQHLFN

LEAKRWRPLRSKLTVYTSGKLKNMFDLIIECANYFEEYLDKMVAQGEPVDFREITAKFTT

DVIGSCAFGINMGALSDEESEFRKIGKQIFEPSLEAIIRMKFKLMMPKLYDLLGYIVPDR

RLAPFLTKLVTDTMKCRKQNNVYRPDFIHTLMQLKEDSKKLHDLELTDSFITAQAYVFFA

AGFETGSTTMSNALYELALNPKIQEKLREEINEHYKKHNGELNYEYIKDMEYLDKVFKET

LRMYPPGTLLPRKSNSEYIFRDPQISIPKGVMIWIPVYAIHRDPEIYPNPDVFNPENFTR

NAIDARHPMHYLPFGTGPRNCIGARFAVYQTKVGIITILRKYKVDVCEKTMIPYQFDPNA

FLLAPKGGICLKITKI

>350426550

MDFQLLLGIAAILLAIYYYYSSVYDTWKSRGVPGPKPSIFVGNFVDLLLKRQAVATIVKN

FYNEYKSEPVFGIYEGTSPILVINDLDLIKDVLIRDFSLFVDRGFKVLEKIEPLSQHLFL

LEAKRWRPLRTKLSPIFTSGKLKEMFPLITECAGNLEKYLNNIVEKDVPVVECRDLAAKF

TTDVIGSCAFGISMNALEDENSEFRRMGKQIFTPNIKQTIRESCRRLTPFLFSVVGYFLR

MTEMNNFFINLVRDTMEYRKTNNIVRPDFIYQLMQLKEHPEKMENVGEXGIXKYILNEIA

AQAFVFFIAGFETSSSTIAHALYELAQNQEIQDKLREEIRDAYDKDGGTLTYEGIKGMKY

LDKVFKETLRKYPILTILSRQAMENYTFKGTKITIPKGTIVWIPVYGIQHDSNIYSDPEK

FDPERFNEDPVAARHPMSYLPFGDGPRNCIGARFANYQSKVGLATILHNHKVDVCEKTKI

PYEPDKQAFLLTLAGGINLKITKAQC

>289177051

MELGVVEILIGMALLFLGFYYYLTSNFDFWKKRQVLGPKPIPIFGNFKDVILGNINPALL

VQKFYDEFKNEQFIGLFSRSSPSLMIKDPDLIRDVLIKDFNVFADRAVASIPKNDPFSEN

LLNLEHERWRPLRNRLSPIFTSGKLKDMFYLMVDCAQNLEEYVNELVKKDQPVECRELSA

KFTTDSIGACAFGLNTNALHDENSEFRRFGRDLFASTLKNKFRRLLRELFPKLYAISGQW

LQNDATLFFIRSIKETTEYRKKNKVRRNDFVDLLMDLQNQPEKLSTFEFTDSLLTAQALV

FFIAGFETSSTTISNALYELAFHQDEQDKLREEIISELKKNENKLTYESIKSMKYLDKVV

KESLRKYPPGSILRRTSLAPYTFYGSKVTIPKHTPVLIPVWAIHRDPEIYPNPGEFDPER

FSEENEKSRHPMHYLPFGDGPHNCIGARFAKYQTKIGIVAIINKFKVDVCDKTCRTYFIE

NRSIIPTPVGGIHLKITKL

>350426301

MAAFEILCGFIVFFLAFYYYLVKPYEYWEKRGIPGPKPQLLFGNFFPVIFGRISIGDRLM

SFYKQYKHEPVFGLFVRTQRILAINDPDLIKTVLIKDFSKFSNRGLAVNEVAEPLSQHLF

SLEPERWRPLRTRLSPTFTSGKLKDMFSLIDECSNTLEKYLESLISTKSYIEVRELAARF

TTDVIGSCAFGVDMNAMSDMQCKFRDIGREFFGPGLKQTLKNRLRENLPTLYTLLGYILP

QDETTIFFTNAVLDMIKHRRKNKIVRPDFINTLMDLQDHPEKLSIKLTEPLLVAQAFLFF

VAGFETSSSTIANALYELAQNQDVQDKLRAEIKEHHELNDGKWQYENIKKMPILDAVFKE

TLRKYPPVTVIMRKNTEKYTFEDIKLTIPKDTRIFISAYGIHRDPEIYPNPDVFDIDRFK

EDAVAARHPMHYLPFGDGPRNCVGARFAIFQTKIGLIKILRTYKVDVCERTQIPYINEPR

TFTLAPKHGLDLKITKVES

>340723873

MAAFEILCGFIVLFLAFYYYLVKPYEYWEKRGIPGPKPQLLFGNFFPVIFGRISIGDRVM

SYYKQYKHEPVFGLFVRTQRILAINDPDLIKTVLIKDFSKFSNRGLAMNEVAEPLSQHLF

SLEVERWRPLRTRLSPTFTSGKLKDMFSLIVECSNTLEKYLESLVSTKSCIEVRELAARF

TTDVIGSCAFGVDMNAMSDTQCKFRDIGREFFGPGLKQILKNRLRENLPTFYTLLGYILP

RDETTIFFTNAVLDMIKHRRKNKIVRPDFINTLMDLQDHPEKLSIKLTEPLLVAQAFLFF

VAGFETSSSTIGNALYELAQNQDIQDKLRAEIKEHHELYDGKWQYENIKKMPILDAVFKE

TLRKYPPVTVIMRKSTEKYTFEDIKLTIPKDTRIFIPSYGIHRDPEIYPNPDVFDIDRFN

EDAVAARHPMHYLPFGDGPRNCVGARFAIFQTKIGLIKILRTYKVDVCERTQIPYINEPR

TFILAPKHGIDLKITKVESYRGVPGPKPTFLFGNTMKSMFGMESFPQLLTRNYNDFKSEP

LVGIFLRTVPVLMVKDPDLIKDILIKDFSKFANRGFLKSEPAVPLSNHLFSLEAKRWRPL

RTQLSPVFTSGKLRGTFSLILECSNHLESYLNAMIEKGDPIDVRELAARFTTDVIGSCAF

GIEMNSLSEKESDFRRIGKQIFSTDFWKILNMRIRQILPPLYAFFARILPETIEYREKNN

IVRPDFMNILMELKRHPERVADITAQTFIFFAAGFETSSTTISNALYELALNHDIQEKLR

EEIKQFAAKNNGEWKYETIKQMQYLDKVFQETLRKYPALPFLSRKSLEDYTFENAKVTIP

KETLIWVPVFPIHRDPEIYPDPEKFDPERFSEDKIEERNPMYYLPFGHGPRNCVGARFAI

YQTKIGLIKILHKNKVGVCSKTLIPYKYNPFSFVLAPTTGLYLTITRVED

>328785290

MYIGFEIIYGIVIVFIAFYYYLTINNNFWKHRGISGPKPVLGFGNMKKIILGEESMSQYL

TKLYHKYKNESMIGIFRLRTPALIIKDPDLIKIVLIKDFSKFMNRGLLPIISGEPISQHL

FNIEAERWRPLRIHLTPVFTANKLRGMFSLILECSMHFVSYVDSLVKKGEPVNVREVAAR

FTTDVVGSCGFGVEMNSLSEKESEFRRVGKSVFATNYARIIKHRIREFMPRLYNYILYLW

PTDEMAEKIIKLTRETLEYREKNNLFRPDFMNILLDLKKHPEKIGLDVTNEFLAAQAFIF

FVAGFETSSSTISNALYELALNPDVQDKLRKEIKEFAAKNDGEWRYETIKEMEYLGKVFQ

ETLRKYPSLPFLTRELIEDYTFESNKVTIPKGLKIWIPTYAIHNDPDIYPDPDKFDPERF

SDDKIKQRHPMHFLPFGHGPRNCIGARFAIYQTKIGLINILRNFKLDVCDKTLIPYKHHP

RGLLLMPLTDLYLKITRLTN

>110765954

MDYFQLLCVIGALLFAIYYYLTLTFDTWKNRGIPGPKPTIFFGNFQEVILKKISLAEKTK

QLYQEYKNELVFGIFQGRTPILVINDLEMIKDVLIRDFSVFPDRGIHVNPKVEPIFQTLF

SLKSKTWRPLRMKLSPVFTSGKLKDMFPLILDCAKNLEEFVEKVRNSGEPVDCRDMAAKF

TTDVIGSCAFGVCMNSLSPEGSEFRRMGEQLGKFSFKKLARDFTRLYMPFLFDIIGGYLQ

SHEVNNFFINLIRDSIKYRQENNVYRPDFVNTLKELKEHPEKLENIELTDALLTSQALVF

FLAGFETSSTTISNALYELAQNPEMQDKLRKEIKEVYENNGGALSYTDVKEMKYLDKVFK

ETLRKYPVLAALSRQATENYTFKDTKIKISKGTRIWIPVYGIQHDPNIYPEPEVFDPERF

EDDAFASRHPMTYLPFGDGPRNCIGARFAHYQSKVGLITILRNNKVEVCAKTLIPYKSEP

RNILMIPKGGKVELRITKV

>383857841

MVCIELLTSLVVVFLALYYYFTSTFNFWKTRDVPGPKPVPVLGNLKNIINGHSAIDDYIT

KLYYEYKNEPMIGIFVKRTPNLILTDLDLIKDVLIKDFSTFNDRGITLFPRAEPLSLNIF

SLEAERWRPLRMRLSPIFTSGKLKDMFPLILECSNALEGCLEKIIKEEKLLDCREVAARF

TTDVIGSCIFGISMNALADEGSEFRQLGKRMFEVGNKARLRGILREVAPWLYCTLALLLP

SHKVTTFLTKMVSETIRYRKENSVVRPDFINVLMELKYNPHKLENIEVTDTLLTAQAAIF

FAAGFETSSSTIGHALYEMALNPEVQEKLRKEIKEFYEKNKGNWTYENIKEMSYLDKVFK

ETLRKYPPVTVLKRRCNSNYTFSGTKVSIPKDTVVHIPVYAIHTDPDHYKNPEVFDPERF

NEDAEASRHPMCLLPFGDGPRNCIGARFAVYQTKIGLIQMLKNYRVDVSDETMIPYEKHP

FSHLLAPKRSLLLKISKVQSQTSDSNNVHIGE

>383857573

MPDYFQILIAIVAVLFSVYLYVSYTAITQFWRKLNVPGPKPNVVFGNLKGFITQKKSVSD

IIKELYEAYKHEPVFGIFQARTPILVINDLDLAKDVLIKDFSLFVNRGIPYFEKVEPLGQ

HLFLLEAERWRPLRVNLSPVFTSGKLKEMFPLIVDCSKHLEKYLDTVASKGEPIECRELA

AKFTTDVIGNCAFGIDMNALEDDDSEFRKMGRKIFVPSFKTTMRNLLRQTSPSLYKIIGH

KLQPEGVDNFFTKVVVDTIKYRKSNNVSRPDFINMLMELKEHPEKLHNVDLTDSLLTSQA

FVFFVAGFETSSTTISHALYELALNHEIQDKLRAEIKESDEKHGETLTYDRVKQMKYLDK

VFKETLRKYPVLPMLNRQALEDYTFRDIKVTIPKGTQIWIPVIGIQSDPNIYPNPETFDP

ERFEEEAVAARHQMTFLPFGDGPRNCIGARFAVFQTKVGIITVLRNYKVDVCEKTTVPYK

ADPRAFLLTLQGGVNLKIVKV

>340723487

MADYFQILSAIVAVFLAVYYYLTSTFDFWKDRGVVGPRPIPGFGNIKDVMLRKISIGDYI

ARLYEKYKNEPMIGIFVRGKPSLILCDLDLIKDVLIKDFSTFNNRGLNVSERTDPLATNL

FNVEARRWRPLRTKLSPVFTSGKLREMFPLILECAENLEQCLEKIVEKGGFVDCREITAR

FSTDVIGSCVFGINMNALSNEESEFRRIGRKIFDQNLKMILRITFRDTFPRLYNFLWFLI

PQSEVSIFITKLVADTMKYREENNIVRPDFINMLMELKKHPEKLADIELTDTILAAQAFV

FFAAGFETSSTTISHTLYEMALNPDIQDKLRREIIEFHVKSNGNLKYEQVKEMKYLDKIF

KETLRKYSAGTLLRRQCNANYTFSGTKVTIPKGTEVFIPVYAIQRDSNIYPDPEKFDPER

FNEDAVAARHPMCYLPFGDGPRNCIGARFAIYQTKMGLIKILHKFKVDVCEKTITTYVHN

PNSLTLSPKGGIHLKISKVES

>66523006

MYISLEIFCGIVVALIALYYYLTVNNNFWKNRGIAGPEPVLGFGNMKKVLLGKESMSQFL

TKIYHEYKNEPIIGIFTTRTPQLIIKDPDLIKTILIKDFSKIMNRGLLPMVSGEPISQHL

FNIEAERWRPLRIHLTPVFTANKLRGMFSLILECSMHFVSYVDSLVKKGEPVNVREVAAR

FTTDVVGSCGFGVEMNSLSEKESEFRRVGKSVFATNYARIIKHRIREFMPRLYNYILYLW

PTDEMAEKIIKLTRETLEYREKNNLFRPDFMNILLDLKKHPEKIGLDVTNEFLAAQAFIF

FVAGFETSSSTISNALYELALNPDVQDKLRKEIKEFAAKNDGEWRYETIKEMEYLGKVFQ

ETLRKYPSLPFLTRELIEDYTFESNKVTIPKGLKIWIPTYAIHNDPDIYPDPDKFDPERF

SDDKIKQRHPMHFLPFGHGPRNCIGARFAIYQTKIGLINILRNFKLDVCDKTLIPYKHHP

RGLLLMPLTDLYLKITRLTN

>66522973

MLLKTSMCHYLKKLCEEYKHEPMIGIFTRKTPILIIQDPDLIKDVLIRDFSKFANRGIPI

HEKAEPLSPHLFNLEVERWRPLRTRLSPVFTSGKLKEMFPLILDCAKHLEQYLDKLVLRE

EFIECRELTAKYTTDVIGSCAFGIEMNALSDEESEFRRIGRKVFSNSFGQILRFRFRQIF

PRIYNLLGFVLPPMEVTKFLTNIIVSTMKYRQENNIVRPDFVNMLIELKKHPDKLENIKL

TDTLLTAQAFVFFIAGFETSSSAISNALYELALNPEVQNKLRQEIKEYFNKHNELKYEYI

KNMIYLDLVFRETLRKYPPGPLILRKSITNYTFNNTKVSIPEESFVWIPLYAIHHDPKIY

PNPDAFIPERFNDDAIATRHPMHYLPFGDGPRNCIGARFAVYQSKIGLITILWNYKVEVC

DKTMIPYEINPAAFLLTPKGGIYLKFTKIKNNEEILN

>350422675

MADYFQILSVIVAVFLAVYYYLTCTFDFWKDRGVVGPRPIPGFGNIKDVMLRKISIGDYI

ARLYEKYKNEPMIGIFVRGSPSLILCDLDLIKDVLIKDFSTFDNRGLNVPERTDALATNL

FNVEARRWRPLRTKLSPVFTSGKLREMFPLILECAENLEQCLEKVIEKGGFMDCREITAR

FSTDVIGSCVFGINMNALSDEESEFRRIGRKIFDQNLKTILRITFRDTFPRLYNFLWFLI

PQSEVTTFITKLVADTMKYREENNIVRPDFINMLMELKKHPEKLADIELTDTILAAQAFV

FFAAGFETSSTTISHALYEMALNPDIQDKLRREIIEFHVKTNGNLKYEQVKEMKYLDKIF

KETLRKYSAGTLLRRQSNANYTFSGTKVTIPKGTGVFIPVYAIQRDSNIYPDPEKFDPER

FNEDAVAARHPMSYLPFGDGPRNCIGARFAIYQTKMGLIKILHKFKVDVCEKTITTYVHN

PNSLTLSPKGGIHLKISKVES

>48135592

MDYFQILCAISIVILTIYYYYSSKYTFWKKRGISGPKPIIFFGNFVDSIIQKRSTSEAVK

KWYDDYKHESVFGIFGGTTPLLVINDLDMIKDVLIRDFSLFVDRGFHIFPKIEPLSEHLF

LLEAERWRPMRMKLSPIFTSGKLKEMFFLIMESAGNLEKYLDEVIKKDEMVECRELAAKF

MTDVIGSCAFGINTNSLLEEDSEFRRMGKKISTPNLKVMLGNICKEFFPPLYEIVGSIFT

LKDVNEFFINLVSDTMKYRKDNNIIRSDFINMLMQLKEHPEKMENIELTNTLLTAQAVVF

FVAGFETSSSTMAFSLYELAQNQEIQDKLREEIRNMHEKNKGVLTYTDVKEMKYLDKVFK

ETLRKYPILPMLFRQAMENYTFKDTKITIPKGMKLWVPVHGIHHDPNIYPEPEVFDPERF

EDDAFASRHPMSYLPFGDGPRNCIGARFAHYQSKVGLITILRHHKVNVCEKTTIPFKADE

RSFLLTLKGGVHLKITKI

>350426723

MAFEFLCYVTVVFIALYYYFTAKFNFWKDRSVRGPKPIPVFGNIFGPMIGKISIAEYLQK

LHNEYKDEPMIGLFVRRTPHLVIQDPEIIKDVLIKDFSKFANRGFLKQEIAEPLSQHLFS

LEVERWRPLRMQLSPVFTSGKLKGIFSLIFECAKRIESYLDILIAKGEPIEVRELAARYT

TDVIGSCAFGIEVNSLSDEENEFRRMGKEIFATHWKAIIKFRMKECMGELYDLLGYILPP

DKVNVFIERITIETLNYRKEHNIVRHDFMNTLLELRNNPEKVKDIKLTDTLLAAQAFVFF

AAGFETSSMTISNALYELALNQEIQKKLREEIKEFDAKNAGDWKYETIKQMSYLDKVFKE

TLRKYPPLAFISRETTDNYTFSNTKVSLQKGMRVWIPVFSIHRDPNIYSDPDKFDPERFS

KEAVDARHPMHYLPFGHGPRNCIGARFAIYQTKIGLIKILQNYEVHVCEKTPIPYELNPY

AFIICPKGGLYLNIIKSKN

>328785304

MLHHFHILTAFVAIFLALYYYLTSKFDFWKNRGVSGPRPVPFFGNAKDVLLRKIGIGSFI

AELYKRYDNEAMFGIFIGRSPNLVLRDLDLIKDVLIKDFSIFDNRGLNIPERAEPFSVNL

FSVDATRWRPLRMRLSPVFTSGKLKEMFPLILECAEHLEQCLEDAVKRGGPVDCFEIPAR

YTTDVIGSCAFGINMNALSDERSEFRKMGRNMFDQNMIKFTRNLLRDFFPRFYNLLGFVL

PYTESTVFMTKLIKGTIKYREENDVVRPDFVNLLMELKKHPEKLKNIEITDTLLAAQASV

FFAAGFETSSTTMAHALYEMALNPDIQDKLRNEMKEFHAKNNGNLKYEDIKEMKYLDKVF

RETLRKYPPGMLLRRKCNSNYTFHGTKVSIPAGTSVIIPLYAIQIDPKFYENPDVFDPER

FNEDAVAARHPMTYLPFGDGPRNCVGARFAVYQTKVGLIKILQNFRVDVCEKTMIPYVKK

INSITLAPRDGIFLKIEKITD

>383857851

MTGFELLCGIVLILLALYYYMVQDHDFWKQRGIPGPKPVPLFGNFMDMLLGRMSIGDQMV

KFYHQYKNEPVIGLFIREKRLLAVKDLDLIKTILIKDFSKFAHRGIAINEVAEPLSQHLF

SLEPKRWRPLRAKLSPTFTSGKLKEMFTLILECSLTFEKYLAKMVSEGKPVDCRELAARF

TTDVIGSCAFGIETNAMTQKESEFRDAGKEFFSSGLKSTIRLRLREGFPRLYTLLGYIIP

YNKMTKFFTKAVSDMIEYRKKNNVVRHDFINTLIELQAHPEKLGIELTESLLSAQAFLFF

AAGFETSASTVTHALYELAQNQDVQDRVREEIKEHYALNNGKWLYDDIKEMPILDAVFKE

TLRKYPPLTVIMREAVEDYTFEDLKLSIPKGTRIFIPAYAIHRDPDIYPNPEVYDINRFQ

EDEVAKRHPMSYLPFGDGPRNCIGARFAIFQTKIALIKVIIKYKVDVCEETHVPFENDPR

SVFLVPNHPIVLKITELPIFLVNIRRHPSYLTTDINELSS

>350426697

MDYFQLVCVIGAVLLAFYYYYTSTFDYWKIRGILGPQPTVFIGNFKDILLRRVSLADKLR

EFYEEYKKEPMFGIFEGTTPILVVKDLELVKDVLIKDFPLFVNRGLRLFPKAEPLGEHLF

ALEEERWRPMRAKLSPVFTSGKLREMFPLVIECSKDLEKYLDSVVETGKPVECRDLAGKF

TTDVIGSCAFGINMNALSDENSEFREVGKKIFTQTMRMQIRDICRQFLPNIYEIFGHLLQ

IPRVDQFLIDVVRDTIKYRRENKIVRPDFINTLMELRDHPEKLENIELTDSLLTSQAFVF

FAAGFETSSTTISHALYELALNQHIQDRLRKEIREVYDKHQGVLTYDAVKEMKYLDKFFK

EVLRMYPLIPFIMREVSENYTFKGTKISIEKGTKVWVPTYGIQRDADVYPEPEKFDPERF

NDDAVAARHPMAYLPFGDGPRNCIGSRFAQYQSRVGIIAVIRNHKVDVCEKTTIPYESEP

LSFMLTLKGGVHLKITKVEN

>340723511

MAFEILCYVTVVFIALYYYFTAKFNFWKDRSVRGPKPIPVFGNIFWPMIGKISIAEYLQK

LHNEYKDEPMIGLFVRRTPHLVIQDPELIKDVLIKDFSKFANRGFLKQEIAEPLSQHLFS

LEVERWRPLRMQLSPVFTSGKLKGIFSLIFECAKRIESYLDILIAKGEPIEVRELAAKYT

TDVIGSCAFGIEMNSLSDKESEFRRMGKEIFATHWKAILKFRMKECMGELYDLLGYILPA

DKVNVFIERITIETLNYRKEHNIVRQDFMNTLLELRNNPEKIKDIKLTDTLLAAQAFVFF

AAGFETSSMTISNALYELALNQELQKKLREEIKEFDTKNSGDWKYETIKQMTYLDKVFKE

TLRKYPPLAFISRETTDNYTFSNTKVSLEKCMRVWIPVFSIHRDPNIYSDPDKFDPERFS

KEAVDARHPMHYLPFGHGPRNCIGARFAIYQTKIGLIKILHNYEVHVCEKTPIPYELNPF

AFIICPKGGLYLKIIKSEN

>350426266

MFITLEILCGLALALIVLYCYLTIRYNFWTSRGVPGPKPTLLFGNIMKSMFGKESLPQLL

TRNYNDFKNEPMVGIFLRTVPVLMVKDPDLIKDILIKDFSKFANRGFIKSEPAIPLSNHL

FSLEAKRWRPLRTQLSPVFTSGKLRGTFSLILECSNHLESYLNTMIEKGDPIDVRELAAR

FTTDVIGSCAFGIEMNSLSENESDFRRIGKQIFSTDFWKILRIRIRQILPPLHVLLARIL

PYDEETKMIMGITRETIEYREKNKIVRPDFMNILMELKRHPERVADIKLSDDLLAAQTFI

FFAAGFETSSTTISNALYELALNHDIQDKLREEIEHFAAKNDGEWKYETIKQMQYLDKVF

QETLRKYPALPFLSRESVEDYTFENTKVTIPKETLIWVPVFPIHRDPEIYPDPEKFDPER

FSEDKMKERNPMYYLPFGHGPRNCVGARFAIYQTKIGLIKILHKNKVGVCSKTPIPYEYN

PLSFVLTPTTGLYLTITKVED

>345486212

MEVGTLEIIIGLSVLSFIFHYYLTYHFDFWKKQNVVGPKPIPFFGNVKEILFGEIHAGLL

MKRYYDQYKSEPMIGIYGNRTPSLILKDPSLIKDVMIRDFNVFPDRGFYVNAKADPVNQN

LANLEHARWRPLRTKLTPIFTSGKLKEMFYLLVECANSFEKYVEKLIEKDEPVEVRELTS

KFTIQAIGVCVLGLDTKALEENSQFRTYGRALFVPTIVNVGRILLQQLAPWIYESLGPFK

TSEPVRFFAKSTKEIVEYRRKNKIRRNDFVGLLMDLQDQPNTLSNIDFSDEFLAGQILAF

FAAGFETSSTTVSNALYELAFNQNIQDKLRNEIREEIERNNGQLTYDSIKRMKYLDKIYK

ETLRKYPPGSIIQRRSNAQYTFTGTKVTIPANTTLIIPVWAIHRDPDLYPDPDIFDPERF

NEDNESSRHPMNYLPFGDGPHNCIAVRYSNYQTKVGLIAAINKFKVNVCDKTCISYQVEP

RAIFTAPVGGIYLKISKVL

>110776794

MDFFQIFCAICIMLLAIYYYYTSIYNYWKVRGIPGPEPTIIIGNFMEVFLKKISINDKLR

FLYNKYKNEPMFGIFEGSSPILVLNDLDLIKDVLIKDFSIFSNRGFRIFPKAEPLGEHLF

ALETERWRPMRAKLSPIFTSGKLKEMFPLIIECSKNMEPYLDKIAERGKYIECRDLAAKF

TTDVIGSCAFGIDMNSISDKDSEFRIIGRKLFTPTFKTIVRDVCRQFLPGLYDVIGHKLQ

IEEVNEFLTNLIKDTINYRKENKIVRPDFVNTLIELKDHPEKLETIKLTDSMIASQAFVF

FVAGFETSSSTISHALYELAQNQEIQDKLREEIREVYEKHGELTYDVIKNMKYLDKVLKE

TLRKYPIMAMLTREAQENYTFKGTKVTIEKGIKVWILPYGIQNDPDIFPNPDIFDPERFD

EEAVAARHPMSYLPFGDGPRNCIGARFAQFQSKIGIITIVRNHKIDVCEQTKIPYESDPF

QFLLALKGGINLKISKI

>EFN64016.1

MMGSLEMLCGIAVIFLAIYYYLTSIYDFWKSRDIRGPKPILIFGNYKDVILNKKCEADYF

KDIYDEYKDESVIGIFTGVTPALIVKDLDLIKDILIKDFSKFGNKGPIVSNNTDLLSHNL

AGLEHKRWRPLRIRLSPAFTSSKIKEMFPLISECADYLEQYIEKLVSKNEPIDCRELTAK

YTTDVIGSCGFGIEMNALLDQDSEFRKIGKMIFLPTWSNILRYRIKQIFPWLDKMLSYII

PNIIQTEVNRFFVRFVTEIMNYRETNNVIRHDFIDILREIKKNSDKVGDIKVSENFIAAQ

AFIFFFAGFETSSTTMSNTLYELALNQQIQDKLREEINQAYTKHGSNLSYENVKEMNYLD

KVFKETLRKYPTVSYLIRQSTTSYTFGGSTLDIPKNLQVFIPVFGIHRDPDIYPKPDVFD

PERFSDEAIQTRHPMHYLPFGDGPRNCIGARFAIHQTKIGLIKILRNYKVEICDKTEIPY

INDPKSFILLTPKNGIHLRIIKIKL

>110762387

MAAFEILCGFIIFIFAFYYYLIKPQEYWKNRGVPGPKPIPIFGNFFRLTFARISIGDLMT

KFYKEYKHEPVFGLYMRNVRVLAINNPDLIKTVLIKDFSKFAHRGLALNEVTEPLSQHLF

VLEPKRWRPLRTKLSPIFTSGKLKDMFSLIIECSNTLENYVEHLISKNDRVEVRDLAAKF

TTDVIGSCGFGVDMNAMSDVQCKFRDIGREFFGPSFKQILKIRLRENLPRLYTFLGYILP

RDETTTFFTNVVLDMIKYRKTNDIYRPDFINALINIQNHPEKLDIELTEPLLVAQAFLFF

VAGFETSSLTIATALYELAQNQDIQDKLRDEITEHHKLNNGEWQYENIKNMPYLDAVFKE

TLRKYVPLTVLMRQSLEDYTFESINLTIPKDTRIFIPIYAIHRDPDIYPNPEVFDINRFS

KEAEATRHPMHYLPFGDGPRNCIGARFAIFQTKIGLIKILRTYKVDVCNETQIPFINEPR

TFTLAPKHDLTLKITKIEN

>380021398

MDYFQILCALGVVILTIYYYYSSIYSFWKNRGISGPKPTILFGNFINSAIRKLSISETIK

NWYDEYKHESVFGIFEGMTPILIINDLDMIKDVLIRDFSLFVDRGFQIFPKIEPLTQHLF

LLEAERWRPMRMKLSPIFTSGKLKEMFPLIMESAGNLEKYLDEVIKKNEMVECRDLAAKF

TTDVIGSCAFGINTNSLLDEDSDFRRMGKKIFSPTLKLMLGNICKGIFPSLYNVIGNMFT

MKDVDEFFINLVSDTMKYRKDNDIVRADFINMLMELKEHPEKMDNIELTDILLTAQAVVF

FVAGFETSSSTMAFSLYELAQNQEIQDKLRQEIKKVYEKSNGVLTYTDIKEMKYLDKVFK

ETLRKYPILSMLSRRVMENYTFKGTKITIPKGTKIWVPVCGIQHDPNIYPEPQVFDPERF

EDDAFASRHPMSYLPFGDGPRNCIGARFAHYQSKVGLITILRNHKVNVCEKTTIPFKSEE

RSFLLTLKGGVYLKITKI

>383857577

MASYLEIILGLAAACLALYYYFIWNFNFWKSRGIPGPKPIPMFGNVLAPMIGTVSLAQFL

TDLYMKYRNEPMIGIFARRTPILIVQDPDIIKDVLIKDFSKFANRGFMKHEIAEPLSQHL

FSLEAERWRPLRTQLSPVFTSGKLKGTFTLILDCATHLEKYLNTLVEKGEPIEIRELAAQ

YTTDVIGSCAFGIDMNSLSEKESKFREVGRHIFATNLTALVKFRIKECLPLLYDLFGYVM

TMDKAMTFIKDVTIDTIEYRKKNNIVRPDFMNTLMELRNHPERVSDIQLTDTILAAQAYV

FFAAGFETSSTTIANTLYELALNQDIQDKLREEIREFDAKNNGEWKYEAVKQMTYLDKVF

RETLRKYPPLPFLSREATENYTFENPKLTVRKHDKVWIPLFSIHRDPAIYPNPEKFDPER

FSEEAVRSRHPMNYIPFGDGPRNCIGARFAVYQTKIGLITIIRNFKCDVSEKTEIPFEFD

PLAFIMCPKNGLYIKITKIDS

>350426531

MDYFQLLCGISILLLAIYYHYSSCYNFWKSRNIPGPKPIIFFGNFLDVLLKRESMADWMK

RLYDQYKNEPAFGIYIGTSPFLMPNNLDMIKDILIKDFSLFADRGFKIYDEKIEPIQQNL

IMLEAERWRPLRAKLSPVFTSGKLKEMFPLITECAEKLEKHLEKLAQKDEPVECRELTAK

FGIDSIGSCVFGLNMNALEDKNSEFLRMSKQIFAVNAKQIIRDFCREFTPLLYKIIGSYL

QPKEVNDFFVNLFIDTVKYRMDNNIIRPDFVHLLMELKKHPDRVNNIELTDALLASQTFA

FFVGGFETTSSTMSHALYELAQNLEIQDKLREEIRDVYDKSNGVLTYADIKRMKYLDKVL

KETLRKYPPLPMLNRQAMENYTFRDTNISIPKGTDIFISIYAIQNDSNVYPDPEKFDPER

FNEDAVAARHPMSYLSFGDGPRNCIGARFAQYQSKVGLATILRNHRVDVCEKTKIPYEND

IHAFILTLKGGVYLKIAKA

>380021417

MLDHFQILTAFVAAFFALYYYFTSKFDFWKNRGVIGPRPIPFFGNAKDVLLRKIGIGSFI

TELYKRYDNEAMFGIFFGGSPNLVLRDLDLIKDVLIKDFSIFDNRGLNTPERVEPFSVNL

FSVDAKRWRPLRTRLSPVFTSGKLKEMFPLILECAEHLEQCLEDVVKRGGPVDCFEIPAR

YTTDVIGSCAFGINMNALSDERSEFRRMGKKIFEQNAIKFMRNFLRDFFPRFYNLLGFIL

PYPESTVFVTKVITGTIKYREENNVVRPDFVNLLMELKRHPEKLKSIEITDTLLAAQASV

FFAAGFETSSTTMAHALYEMALNPNVQDKLRKEMKEFHGKNNGDLKYEDIKEMKYLDKVF

RETLRKYPPGMLLRRQCNSNYTFHGTKVSIPAETSILIPLYAIQTDPKFYENPDVFDPER

FNEDAVAARHPMTYLPFGDGPRNCIGARFAVYQTKVGLIKMLQNFRVDVCEKTMIPYVKK

INSLTLAPRDGIFLKIEKIID

>380021374

MAVFEILCGFIIFILVFYHYLLKPQEYWKNLGVPGPKPIPVFGNFFRLTIATMSIGDLMT

KFYKEYKHEPVFGLYIRNVRVLAINDPDLIKTVLIKDFSKFAHRGLALNEVMEPLSQHLF

VLEPRRWRPLRTRLSPIFTSGKLKDMFSLIIECSNTLEKYVEHLISKNDRVEIRELAAKF

TTDVIGSCGFGVDMNAMSDVQCKFRDIGREFFGPSFKQILKIRLRENLPRLYTFLGHILP

RDETTTFFTDVVLDMLKHRRKNNIYRPDFINTLMNIQDHPEKLDIELTEPLLVAQAFLFF

VAGFETSSLTIATALYELAQNQDIQDKLRDEIREHHKLNNGEWQYENIKSMPCLDAVFKE

TLRKYVPLTVLMRQSLEDYTFENINLTIPKNTRIFIPIYAIHRDPDIYPNPEVFDINRFS

KEAEATRHPMHYLPFGDGPRNCIGARFAIFQTKIGLIKILRTYKVDVCNETQIPFINEPR

TFTLAPKHDLILKITKIEN

>383864143

MSWSLLETVGFFSAIILLLYYYSTSMLEYWQKRGVKGPKPIPFLGNFKNVFLGKTSVNDC

FVKAYYEFKDEPLIGVFSGHDPILIVKDPDLMKDVLIKDFVNFADRMRDPDAEVEPFSLH

LFRLEAKRWKPLRNRFSPVFTSGKLKEMLHLILECANHFEKYLEKIAEKGKHIDCREISA

KFTTDVIGSCAFGIEANALAAEDSEFRKMGRQVFQSSFKTVIRDRLREYPLLFKIFGRFV

VDYEIVDFFTKITKESIDYRLKHNVRRHDFVDVLVELKQNPGKIGFSDVNDVLLTAQAFV

FFAAGFETSSVTITNALYELALNPPIQENLRAEIKDVLQRNNGEITYDSIMEMKYLDAVL

KETLRKYPVVLWLSRKAMANYTFSGTKVTIPKGQFVVLPVYAIQHDPDIFPQPEVFNPNR

FLSENTEIGHPMYFLPFGDGPRNCIGARFAKIQSKVALMKIVSNFKIDVCKETVTTYQID

KKPLFLLQPSHEVNLKLTKVYT

>345492590

MAVGIVEVLLGMGTILLLLYYFMIMGFDFWEKRGIPGPRPIPLFGNFKDVILAKMHQADY

LKSIYENYPKEPAVGVFMWNRPVLVVKDLDMIKDVLIKSFSDFSDRGIFVIHEDIDPLSK

HLVFLEPKRWKPLRKKLTPAFSSGKLREMFYLINECATHYDQYMQTIVDKKEPVDCRDLA

AKFTTDVIGVCAFGLNMNALLNEESEFRKMGRLIFADSWHNRARRLTRLLPHWSARFLKP

IARDEKIIDFFVTTLRETMEYRKKNNVRKHDIVDVLMDIKENPEQFNDEEITELFLTAQA

FVFFIAGFDTTSNAMSHAMYELALNPDIQEKLRQEVNATYSENNGKFSYDSVRNMKYLDK

VFKETLRKYPPAFTLSRKSMNNHSFSGTKITIPKGTSLMIPTYAIHHDPSYYPDPDKFDP

ERFDEGNANNRIHMTFLPFGDGPRNCIGERFANNQFKVGIAMTVRKFKLAVCERTCKNYV

KHARLSTLSPTDGLYLKFKSI

>350426701

MYIITFYYYYTSTFDYWKIRGILGPQPTVFIGNFKDILLRRVSLADKLREFYEEYKKEPM

FGIFEGTTPILVVKDLELVKDVLIKDFPLFVNRGLRLFPKAEPLGEHLFALEEERWRPMR

AKLSPVFTSGKLREMFPLVIECSKDLEKYLDSVVETGKPVECRDLAGKFTTDVIGSCAFG

INMNALSDENSEFREVGKKIFTQTMRMQIRDICRQFLPNIYEIFGHLLQIPRVDQFLIDV

VRDTIKYRRENKIVRPDFINTLMELRDHPEKLENIELTDSLLTSQAFVFFAAGFETSSTT

ISHALYELALNQHIQDRLRKEIREVYDKHQGVLTYDAVKEMKYLDKFFKEVLRMYPLIPF

IMREVSENYTFKGTKISIEKGTKVWVPTYGIQRDADVYPEPEKFDPERFNDDAVAARHPM

AYLPFGDGPRNCIGSRFAQYQSRVGIIAVIRNHKVDVCEKTTIPYESEPLSFMLTLKGGV

HLKITKVEN

>380029373

MIYKKKMYISLEILCGIIVALIMFYYYLTINNNFWKNREISGPQPVIGFGNMLKVILGKE

SISQFLTRIYNEYKNEPMIGIFSKNNPALVIKNPDLIKTVLIKDFHKFANRGLFPINSRE

PLSQNLFSIEAERWRPLRINFTPIFTTSKLRGICSLILECSEQLEKYMDTLIRKGEPLDV

REITARFTTDVIGSCAFGIEMNSLSEKESEFRRLGKGVFTPTFRRIVKTRIRNLTPWLYN

FFLRILPWDEITKKIVKLTSETIEYREKNNIVRSDFINVLLDLKRHPEKIAEIELTDDLL

SAQTFVFFGAGFETSSTSISNALYELALNHDIQHKLREEIKEFEKKNDGKWTYESIKEMQ

YLEKIFQETLRKYPVVPFLNRELIDDYTFENSKITIPKGLKIWIPVYGIHHDPNIYPNPE

KFDPERFSEDKIKERHPMHYLPFGHGPRNCIGARFGTYQTKIGLVKIIRKYKVDICDKTL

IPYKFHKFANFLMPLTGIYLMITELEN

>110762372

MYINLEIFCAIVIAFIAFYYYLTINNNFWKHRGISGPKPVLGFGNMKKIILGEESMSQYL

TKLYHKYKNESMIGIFRLRTPALIIKDPDLIKIVLIKDFSKFMNRGLLPIISTEPISHHL

FALEAERWHPLRKHLTSGFTSNKLKGMFCMIHECSKHLVNYLDILVRKEEPVNVREVAAR

FTTDVVGSCGFGVEMNSLSEQESEFRRLGKSIFNTNVQKIIKDRIRELTPQVYNFLLYIL

PLDGISPKILKLMKETIKYRKKYDIFRPDFMNIILELKKHPEKINIDITNELLAAQIFIF

FAAGFETSSTLISNALYELALNPNIQDKLREEIKKFESQNDEEWKYETIKKMDYLEKVIQ

ETLRKYPPVPFLNRELIDDYTFESNKVTIPKGLKIWIPTYAIHNDPDIYPDPDKFDPERF

SEDNIKQRHPMHFLPFGHGPRNCIGIRFAEYQTKIGLINILRNFKLDVCDKTLIPYKLHP

RGLILIPLTDLYLKITRLTN

>380021331

MFINLETLCGFVIVFITFYYYLTSNNNFWKNRGIPGPKPIIGFGNMRTVMFGKESFSQFL

TTVYNEYKDESMIGIFFRRRPVLLLKDFDLIKDVMIKDFSKFANRGFLKTNPKVPLTNHL

FALEVKRWRPLRSHLSPVFTSGKLKGTFAQILNCSNDLVTYMDTLLKIEESINVREVAAK

FTTDVIGSCVFGIKMNSLSEKESEFRRIGRNIFATNFTNILKVRLLQSMPLLHSLLCNIL

SNEETEMFMRITKETIEYREKNNIVRPDFMNILLELKKHPERITDFELTDDLLAAQAYIF

FAAGFETSSTTISNALYELALNHDMQDKLRKEIKEFEAKNNGEWRYETIKEMQYLGKIFQ

ETLRKYPALPFLSRELIINDYTFQNTKLTISKGTLVWIPVFPIHHDPNIYPDPEKFDPER

FTEDKIKERNLMHYFPFGHGPRNCIGARFAIYQTKIGLIKILRNYKVDVCSKTLIPYKYD

PFSFILVPLGGLYLKMTKIQD

>380021345

MYINLEIFCGIVITLIAFYCYLTSNNNFWKNRGISGPKPMLGFGNMKKVIFGEESLVQFL

TKMYHEYKNESMIGIFKLKTPALIIKDPDLIKTVLIKDFSKFMNRGLLPIISTEPISQHL

FALEAKRWQPLRKHLTSGFTSNKLKGMFYLIHECSQQLVNYVDILIRKEESINVREVAAR

FTTDVVGSCGFGVDMNSLSEKESEFRRLGKSIFDTNVYKIIIDRIRELTPQFYNLLLYIL

PLDKISPNILKLTKETIEYRIKNDIFRPDFINILLELKKHPEKIDLEITDELLAAQVFIF

FTAGFETSSTMLSNALYELALNPDVQNKLREEIKEFESKNDEEWKHETVKKMNYLEKIFQ

ETLRKYPSVPFLNREIIEDYTFESNKVTVPKGLKIWIPTYAIHNDPDIYPDPEKFDPERF

SEDNIKQRHPMYFLPFGHGPRNCIGIRFAVYQVKIGLINIIRNFKLDVCDKTLIPYKLHP

RGLILIPLTGLYLKITRYVTNEFLAAQAFIFFIAGFETSSTTISNALYELALNPDIQDKL

RKEIKEFEEKNDGDWKYETIKEMEYLEKIFQETLRKYPSLPFLNREIIDDYTFESNKVTL

PKGLKIWIPIYAIHHDPDIYPDPEKFDPERFSEDKIKQRHPMHFSPFGHGPRNCIGARFA

VYQVKIGLIKILRNFKLDVCDKTLIPYKFHPRALLLSPFTDIYLKITRLTD

>383852226

MAFPILEVIALAAVILFLLYYYFTSALQFWEKRGVKGPKPILFLGNFKDVFLGKVSINDC

LVKVYNDFKDEPMVGIFGGHIPHLVLRDPDLIKDVLIKDFPNFVDRLYRHEKVERLSEHL

FSLEAKRWRPLRACLSPIFTSGKLKEMFHLLVECGNHFEQYLIKLVKKGEDIPCREICSK

YTTDVIGSCAFGIEMNALAAEDSEFRKMGRQIFQTNFKSVIKDRLREYPFLFNLIGPFFA

DREVEDFFTRITNESINYRMQHNFKRHDFIDTLTELKKDSRKVSDEPLSEVFLAAQAFVF

FAAGFETSSITVTHALYELAFNQSVQDKLRAEVKEVLQRHNGQITYEMLKEMKYLDAVMN

ETLRKYPVVLWLSRTAMNDYTFSNTKVTIPKGQHVIVPVEAIQMDPDIYPEPEVFDPNRF

LDENVKDRHAMFYLPFGDGPRNCIGARFAKIQSKVAMIKIFSNFKVDVCDKTMKYNRDVK

AFFLLQPTHEIYLKMTKI

>380021421

MYINLEILCGIVITLIALYCYLTFDNNFWKNRGVIGPKPVLGFGNVKDVIFGKESLSQFL

TRMYNEYKNEPMIGIFSKRTPILIIKDPDLIKTILIKDFPKFANRGLFPIFSRDPLTHHL

FNLEVERWKPLRTQFTPLFTASKLKEMFSLILECADQLENYMDTLIKKGEPIDVREVSAR

FTTDVVGSCAFGIEMNSLSEKESIFRRLGKLIFATTLRKILSLRMQDMFPWLYNLFLSIF

PRDEKTRIITKLMIDTMKYREENNVFRPDFINMLLDLKKHPEKIDIELTNDLLSAQIFIF

FAAGFETSSSTISNALYELALNPDIQDKLRKEIKKFEVENNGEWKYXDHKRMEYLEKVFQ

ETLRKYPALPILSRKLINDYTFENSKITVPKDLKIWIPIYGIHHDPDIYLNPEKFDPERF

SKEEIKKRHPMHFLSFGHGPRNCIGARFAVYQTKIGLIKILRNFEVHVCNKTLIPYKVNP

YTALLVPITGLYLNVVKLKN

>350426729

MIGLYMRRSPVLILKDPELIKDVMIRDFSRFADRGFAVHERTEPLSMHLFNLEPKRWRPL

RSKLTPMFTSGKLKDMFGLILECADHFEKYLDKLTAKEEPIDFREVTAKFTTDVIGSCAF

GIEMSSLSDEDSEFREIGRQIFALNLENVIRLKLRLYMPKLYDLLGYIVPDKRHAPFFTK

LVTDTMKYRKEHNIYRPDFIHMLMELKERYFAELTDSLLTAQAFVFFAAGFETSSSAMSN

ALYELALNQEIQDKLRQEIREHLAKHNGELQYEHVKDMEYLEKVFKETLRKYPPGSILPR

RSTSAYTFKNTKVSIPKGLMIWIPTYSLHHDADIYPNPDVFNPENFNEDAIEARHPMTYL

PFGDGPRNCIGARFAIYQTKVGLITILRNYKVDVCDKTMIPYEFTKTSFLLTPKGGIYLK

LTKLET

>66558893

MNISLEILCGIIVALIVFYYYLIINNNFWKNRKISGPKPVIGFGNMLSIILGKESTSQFL

TRIYNEYKNEPMIGIFSKNNPALVIKNPDLIKTVLIKDFHKFANRGLFPVNSREPLSQNL

FGLEVERWRPLRIHFSPIFTTNKLKGLCSLILECSEQLEKYMDILIRKGEPLDIREIAAR

FTTDVIGSCAFGIEMNSLSENESEFRRLGKGVFNTTFRRIVKTRIRNLMPWLYNFFLRIL

PWDEITKKIVKLTTETIEYRNKNNIVRSDFINVLLNLKKHPEKIAEIELTNDLLSAQTFV

FFGAGFETSSTTISNALYELALNHDIQYKLREEIKEFEKKNDGKWTYESIKEMQYLNKIF

QETLRKYPVVPFLNRELISDYTFENSKITIPKGLKIWIPVYGIHHDPDIYPNPEKFDPER

FSEDKIKERHSMHYLPFGHGPRNCIGSRFGTYQTKIGLVKIIRKYKVEICDKTLIPYKFN

SFANFLMPSTGLYLMITDVEN

>340723495

MVGLYMRRSPLLIVQDPEFIKDILIRDFPKFADRGFHVYERTEPLSQSIFNLESERWRPL

RTMFSPIFTSGKLREMFYLIIECSQHLEKYLDKRVEKGEPIECCEVTAKFTTDVIGTCVF

GIEMSAMADEDSEFRKMGREVFAVNVENVIRQKMKLFMPKLYHLLGYIIPDRKLAPFFIK

IVTDTIKYRKENNIVRPDFINMLMEVQKHPEKFENIAQAFIFFAAGFETSSMTMSNALYE

LALHQDIQDKLREEIKEHCCENDEELKFQDIKGMQYLEKVFKETLRMYPPGAFIPRRTKS

TYTFNNTKVTIPQGTLIWIPVFAIHRDPDIYPNPDLFDPERFNEDAVAARHPMHYLPFGD

GPRNCIGARFANYETKVGLITILRNYKVEVCDKTVIPYEFDPGAFVTAPKGGIYLKITKI

>383857847

MIDYFQILCGLLVLVLSFYYYYTATFDFWKNRNVRGPKPVVFFGNLKETAFKKVSMTEHV

KNMYDQFKHEPVFGIFQGKTPTIVVTDLELIKNVLIRDFSQFSQRGLGYSRRVEPLEEHL

FFLDTPRWRPLRPKFSTIFSSGNLKEMFNLILKCSNDLEKYMDKVIGNGGLVECRELAGK

YTTDVIANCMFGLDISTLPNEDSEFREMGRRVFKPSKTMVVRDTLKQFVPGLYNWIGHAV

QPEGVINFFTRAMLDTIKYREEKKIFRPDCINVLMDIHQNPEKAGSFKPTEQLLATQGFV

FFLAGFENTSLTISHALYELAQNHEIQDKLRAEIQEDYAENGETLTYDRIRDLKYLDMVF

KETLRKYPVLTTLFRENIEDYTFKGTDVTIPKGTKVWIPVYAIQNDENHYPNPEKFDPER

FTKEAEAARHPMSYLPFGDGPRNCIGARFSQNQVKLGILTVIRKYKVDVCQQTTVPYQHE

KRSLMLMLKGGVNLKLSKVDY

>380021401

MIANLEIFCGIIVILIAFYYYITARNNFWKIRGIPGPEPLPGFGNVLMIVLGKEAPFQFL

TRVYNEFKNEALVGVFMKTHPALVVKDPDLIKDIMIKDFCKFPTRGFPQSDPADPLTQHL

FLVEEDKWRPLRTQLSPVFSTGKLKGTFTQILHCSDHLVTYMDKLVEKGEPIDVRDVTAK

FTTDVIGSCVFGIKMNSLSEKESEFRRFGRQIFATSFIKILRLRIKQFLPMIHYLLVRIL

PPDEETKIMLKLTRDTFKFRETHNIVRPDFMNILMELKKHPEKVPTLDLTDGLIAAQAYI

FFAAGFETSSTTIANTLYELALNPDIQEKLREEIKEFEANNNGEWKYETIQEMKYLDKTF

KETLRKYPVLPYLSRRALEDYTFEGTKVSIPKGTLICIPVFPIHHDSSIYPNPEKFDPER

FSEDEVKKRHPMHYFPFGHGPRNCIGLRFAIYQSKIGLIKILSNYKIEVCDKTLIPYKYN

PFSFISVPLTGIFLKISKLEN

>383857843

MADYFQLLSAFALVFLALYYYFTSTFDFWKKRGVAGPRPVPIFGNIKDVLLEKCSLNECI

VKFYQEYKNERLVGIFERRKPNILLTDLDLVKDVLIKDFSIFNDRGHLVFEKTEPLSLNI

FSLEAKRWRPLRAKLTPVFTSGKLKEMFPLILECSNQLEGCLEKIIEQGGFIDCREVAAR

FTTDAIGSCAFGINMNALSDEGSEFRRIGKKIFKPDIKKRAIATMKETVPWLYRLLGFVL

PQNEVTMFLTNIVTETIKYRKENNIVRPDFINVLMELRDNPHTLENIDKVTDVMLAAQAA

VFFAAGFETSSTTIGHALYEMALNPDMQDKLRKEIKEFYNKNKGNWTYDDVREMSYLDKV

FKETLRKYPPGGLLRRRCNIDYTFNGTKDPSIYPKPDVFDPERFNDDVAASRHPMAYMPF

GDGPRNCIGARFAVYQTKVGIIQMLNNYKVEVCDKTLIPYIRHPTARVLSPIRSIILKLS

KVQS

>EFN88978.1

MESLGILYGIVAVILALYYYFTSTFGFWKSRGIHGPQPIPLFGNVKDVMFARKAMCNYLM

EICNVYKDEPMIGIFVRRTPVLIVKDPDLIKDILIKDFSNFPERGFTTHERADPLSQHLV

ALEEKRWRPLRTKLSPVFTSGKLREMFLLILKCSEHLERYVEKLVSRSEIIECCALTAKY

TTDVISSCAFGIDTNSLSDVESEFLKMGREVFDPKWYNLIRLKIKQNAPQLFDILGYVLP

QTKVTKFFTRIIMENIEYRERNNIIRHDFVDVLRELKRHPDKIDIELTDSVIASQAFIFF

LAGFESSSSAMTHALYELAQNHKIQDTLREEIEQECAKDEDVLTYDNVKKMNYLDKVLRE

TLRKYPPATFLMRQSTSSYTFDSVKTNIPKGQPVWIPVYAIQRDPNIYPNPDIFDPERFK

GEVMQNRNTMYYLPFGSGPRNCIGVVE

>328780005

MSWLMIETVGLIATVFFLLYYYSMSKLDYWRKRGVKGPKPLPFLGNFKDVLLAKESTMDC

FERAYKEFKDEPMVGMYGSHEPLLILRDLDLIKDVLIKDFNKFAQRTQGAIREVEPLSEQ

LFRLDAERWRPLRLKLSSFFSSGKLKEMFHLFVECSDNFEKYLEKMVEKGGLVECRDAAA

KFSTDVIGACAFSIHTNALTDENSQFRKMGKQALATNLQQFLNDRLREYPFLFKIFGRFF

VDHEVTNFFANSIKDAMDYRIQNNVHLRDVIDILADIRENPTKCGLKEADNLFLTSQAVL

FFLAGFENASLTISNALYELAWKPEIQEKARAEIVNVLQKYDGKITYDGLEEMKYLEACI

FETLRMYPVLQWLSREAMETYTFTGTKVTIPKGQQVFLPIYAIQRDPDIYPNPDNFDPER

FTDDKIKTRHSMTHLPFGDGPRHCSGIRLAKKQLKVGLVTVLSKFKVEVCEKTRKIYQKD

KKPLFLLQPVDGIHLKISKVSV

>380026113

MSWLMLETVGLIATIFFLLYYYSMSRMDYWRKRGVKGPKPLPFLGNFKDVLLAKESTMDC

FERAYKKFKDEPVIGIYGGHEPLLILRDLDLIKDVLIKDFNKFAQRTHGAIREVEPLSEQ

LFRLDAERWRPLRLKLSSFFSSGKLKEMFHLFVECSNNFEKYLEKMVEKGEPVECRDAAA

KFTTDVIGACAFSIHTNALTDENSQFRKMGKQALATNLQQFLNDRLREYPFLFRIFGRFF

VDHEVTNFFVNAIKDAMDYRIQNNVYLRDVIDILADVRENPTNCSLKEADNLFLTSQAVL

FFLAGFENSSLTISNALYELAWKPEIQEKARTEIVNVLQKYDGKITYDGLEEMKYLEACI

FETLRMYPVLQWLSREAMETYTFTGTKVTIPKGQQVFLPVYAIQRDPDIYPNPDNFDPER

FTDDKIKTRHSMTLLPFGDGPRHCSGIRLAKKQLKVGLATVLSKFKVEVCEKTHKIYQKD

KKPLFLLQPVDGIHLKISKVSV

>EFN75490.1

MVLAILFVCVTILLSFYYCLANFDYWKSRGVKGPQPIPFCGTIAKHLFKKICLGAYLREI

YEQYPDEAMVGVFFREQPALVLRDPDLIKHVLIKDFSVFPEREIAVFDKAEPLSLHLFRI

DGARWRPLRTQLSPVFTSGKLKDMFHLLLECAEHLERHLNEIVSKDLIIECRELTSKYTI

DVIGSCAFGIEMNALKQKDSEFQKLGVKIFRTDMKTYIRKLFRDVAPKLYNRIGIILDDH

DVTNIMTNMIRNTIDYRKKNNFHRHDFIDALITLKNHPEKLGLDHVPDIYIAAQLFVFFA

AGFETSSTTMSNVLFELAQHLTIQKKVQAEIRQVLDNNNGAISYDSIKKMTYLEQVLKET

LRKYPPVMFLARNAQQKYTFEHTNVSIDKNLKVYIPVFGIHYDPNIYPDPEVFDPERFND

QNMQSRNPMHYLPFGNGARFAAYQAKVGLIKILLNYDISVCEKTQIPLILNRQQQMMFQT

DHGMYVKFTKLS

>340714730

MSWLMIETIGLIVTVFVLLYYYSMIKLNYWRKRGVKGPKPLPFLGNFKDVLLGKDSTMDC

FQKAYNEFRDEPMVGVYGGHEPLLVLRDLDLIKDVLIKDFNKFAQRTHGAVREVEPLSEQ

IFRLEAERWRPLRIKLSPFFSSGKLKEMFHLMTDCANSYDKYLEKLAEKGEPIEFRDTAA

KFTIDVIAACAFSIQTNAITDENCQFRKMGKKALDTNIEQFLTDRLREYPFLFRIFGRLF

TDHEVINFFETATREAMDYRIQNNVHTNDVIDILADLRQHPEKINLKEADSLFLASQAQL

FFLAGFGNSSLTISNALYELAWNQTVQDKLREEIQSILKKYNGEITYDGICEMKYLNACL

LETLRKYPVIQWLSRTAMETYTFSGTKVTVPKGQQIFLPVYAIQKDAEIYPNPEVFDPDR

FSDENIKTRHAMAHLPFGDGPRHCSGIRLAKKQLEVGMVTILSKFKVEVCEKTRKVYKPD

KRPLFLLQPADGIYLKMSKVAAA

>340723505

MADWMKRLYDQYKNEPAFGIYIGTSPFLMPNNLDMIKDILIKDFSLFADRGFKLFDEKIE

PINQNLIMLEAERWRPLRAKLSPVFTSGKLKEMFPLIVECAGKLEKYLEEIAQKDEPVEC

RELTAKFGIDSIGSCVFGLNMNALEDKNSEFLRMSKKIFAPNAKQIIRDFCREFIPSLYK

IIGSYLQPKEVNDFFLNLFIDTVKYRMDNNIIRPDFVHLLMELKKHPDKVNNIELTNSLL

ASQTFAFFVGGFETSSSTMSHALYELAQNLEIQDKLREEIRNVYDQNNGVLTYADIKRMK

YLDKVLKETLRKYPPLPMLNRQAMENYTFKDTNISIPKGTDICISIYAIQNDSNVYPDPE

KFDPERFNEDAVAARHPMSYLSFGDGPRNCIGARFAQYQSKVGLATILRNHRVDVCEKTK

IPFENDIHAFISTLKGGVYLKISKA

>328788701

MIANLEIFCGIIVIVIAFYYYITARNNFWKIRGIPGPEPLPGFGNVLMIVLGKEAPFQFL

TRVYNEFKNEALIGVFMKTYPALVVKDPDLIKDIMIKDFYKFPNRGFPKSDSADPLTKHL

FLVEEEKWRPLRTXMSPVFSTGKLRGTFTQILDCSNHLVTYMDKLVEIGEPIDVREVTAK

FTTDVIGSCVFGIKMNSLSGKESEFRRFGRQIFAMNFLKILRLRIKQFLPMLHYLLVRIL

PPDEETKIMLKLTRDTFKFREAHNIVRPDFMNILMELKKHPEKVPSLDLTDGLLAAQAFI

FFAAGFETSSTTVANTLYELALNPDIQEKLRQEIKEFEANNDGEWKYETIQEMKYLDKTF

KETLRKYPVLPYLSRRSIEDYTFEGTKVSIPKNTLICIPVYPIHHDSSIYPNPEKFDPER

FSEDEVKKRHSMHYFPFGHGPRNCIGLRFAIYQSKIALIKILSNYKIEICDKTLIPYKYD

PFSFISLPLTGIFLKITKLQN

>350405964

MSWLMIETIGFIVTVFVLLYYYSMIKLNYWRKRGVKGPKPLPFLGNFKNVLLGKDSTTDC

FQKAYNEFRDEPMVGVYGGHEPLLVLRDLDLIKDVLIKDFNKFAQRTHGAVRQVEPLSEQ

IFRLEAERWRPLRIKLSPFFSTGKLKEMFHLMTDCANTYDKYLEKLAEKGEPIEFRDTAA

KFTIDVIAACAFSIQTNAITDENCQFRKMGKRALAANTGQFLTDRLREYPFLFRIFGRLF

TDHDVINFFETATREAMDYRIQNNVHTKDVIDILADLRQHPEKINLKEADSLFLASQAQL

FFLAGFGNSSLTISNALYELAWNQTVQDKLREEIQSVLKKYNGEITYDGICEMKYLNACL

LETLRKYPVIQWLSRTAMETYTFSGTKVTVPKGQQIFLPVYAIQKDPEIYPNPEVFDPDR

FSDENIKTRHAMAHLPFGDGPRHCSGIRLAMKQLHVGMVTIVSKFKVEVCEKTRKVYKPE

KRQLFLLQPADGIYLKMSKVAAA

>328785269

MFDYFQILIAFVASFLALYYYLTSNFDFWKNRNVVAPKPIPFFGNTKDVVLKKIEISNFI

AELYKKYENEAMFGIFFGGSPNLILRDLDLIKDVLIKDFSTFDERGFKISERADPLNANL

FNMDVTRWRPLRIKLSPVFTSGKLKEMFPLILKCAERLEQCLEDAVKRGGPVDCFEISAR

YTTDVIGSCAFGINMNALSDERSEFRRIGKRIFDLDKNILRSFLRQFFPRFYNLLGFVIP

YSETSKFVTKFISEMIKYREENNVVKADFVNLLMELKKHPEKLQNIKITDNLLAAQAFVF

FAAGFETSSTTMAHALYEMALNPNIQDKLRKEIKEFYANNNFTYEEVKKMKYLDKVFKET

LRKYPPGVFLKRKCNSNYTFKDPKFYENPDVFDPERFNEDAVAARHPMTYLPFGDGPRKC

IGIRFGVYQTKVGLIKMLKNFKVNVCEKTMIPYIKKINSFTLAPKDGIFLKIEKNN

>328785255

MFDYFQILIAFVASFLALYYYLTSNFDFWKNRNVVAPKPIPFFGNTKDVVLKKIEISNFI

AELYKKYENEAMFGIFFGGSPNLILRDLDLIKDVLIKDFSTFDERGFKISERADPLNANL

FNLDVTRWRSLRIKLSPVFTSGKLKEIFPLILKCAERLEQCLEDAVKRGGSVDCFEISAR

YTTDVIGSCAFGINMNALSDERSEFRRIGKRIFDLDKNILRSFLRQFFPRFYNLLGFVIP

YSETSKFVTKFISEMIKYREENNVVKADFVNLLMELKKHPEKLQNIKITDNLLAAQAFVF

FAAGFETSSTTMAHALYEMALNPNIQDKLRKEIKEFYANNNFTYEEVKKMKYLDKVFKET

LRKYPPGVFLKRKCNSNYTFKDPKFYENPDVFDPERFNEDAVAARHPMTYLPFGDGPRKC

IGIRFGVYQTKVGLIKMLKNFKVNVCEKTMIPYIKKINSFTLAPKDGIFLKVEKNN

>380021335

MFDYFQILIAFIAISLALYYYLTSNFDFFKNRGVVAPKPKPFFGNTKDVAMFGLFFGESP

NLVLRDLDLIKDVLIQDFSTFDEXGFNISERDPLNANLFNIDVTRWRPLRTRLSSVFTSG

KLXKEMFPLILKCAELEQCLEDVVKRGGPVDCXNINMNALSDERSEFRRIGKKIFELDKN

ILRSFLRQFFPRFYNLLGFVIPYSETSIFVMKFISEIIKYREENNVVRADFINLLMELKK

HPEKLKNIKITNSLLAAQVFVFFAAGFETSSTTMKHVLYEMALNPNIQDKLRKEMKQFHT

KNNKNLTYEEVKKMKYLDKVFKETLRKYPPGVLLKRKCNSNYTFNDTKVSIPAETSIIIP

VYAIHTDPKFYENPDVFDPERFNEDAVAASHPMTYLLFGDGPRNCIGARFAVYQTKVGLI

KMLQNYKVDVCKRTMIPYVKKINSFTLAPRDGIFLKVEKITDYGKVQNK

>380013473

MWNIIRELLEQFLLPGLFLGILYCFLTSTFNFWKNRGVPFRKPTVLFGNFAPMLLFRKSL

PEGIKEMYEWFKNEKYFGAFRVRSPVLIVRDPDLVKNICVKNFTCFSNRGIPVNSQDPLS

AHLFNLEGKKWKSLRSKLTPAFSSGKLKRMFYLLAECSEEFQKLIDTSSETDRPYEIREL

AAKFTIDVIGTCAFGIQINALTDEESEFHRAAKKLSKPSYKATLWRMLRTAMPRLYKFLG

VQVIDPEVTEFFKDVVSQMIKQREEYGIKRHDFMDLLIELKNKGTLDESGSGLALLFIEL

DENSIAAQAFVFFAAGYETSSNTIAFCLHELALNTEIQEKTRRDIQDAIDSRNGKLTYDA

VQDMKYLDMVIAETLRKYPPASMLSRRCEYQYQIPNSKVELPAGIRVIIPIYGLHHDPDY

YPSPAMFNPERFTEENKRTRHPYAYLPFGEGPRNCIGMRFALLQIKVGIISFLRNHRVET

CQKTITPIKFSRRSLVTTSEKGFWLRIK

>380013471

MWNIIRELLEQFLLPGLFLGILYCFLTSTFNFWKNRGVPFRKPTVLFGNFAPMLLFRKSL

PEGIKEMYEWFKNEKYFGAFRVRSPVLIVRDPDLVKNICVKNFTCFSNRGIPVNSQDPLS

AHLFNLEGKKWKSLRSKLTPAFSSGKLKRMFYLLAECSEEFQKLIDTSSETDRPYEIREL

AAKFTIDVIGTCAFGIQINALTDEESEFHRAAKKLSKPSYKATLWRMLRTAMPRLYKFLG

VQVIDPEVTEFFKDVVSQMIKQREEYGIKRHDFMDLLIELKNKGTLDESGSGLVCNDEDA

ETADEIELDENSIAAQAFVFFAAGYETSSNTIAFCLHELALNTEIQEKTRRDIQDAIDSR

NGKLTYDAVQDMKYLDMVIAETLRKYPPASMLSRRCEYQYQIPNSKVELPAGIRVIIPIY

GLHHDPDYYPSPAMFNPERFTEENKRTRHPYAYLPFGEGPRNCIGMRFALLQIKVGIISF

LRNHRVETCQKTITPIKFSRRSLVTTSEKGFWLRIK

>380013475

MWNIIRELLEQFLLPGLFLGILYCFLTSTFNFWKNRGVPFRKPTVLFGNFAPMLLFRKSL

PEGIKEMYEWFKNEKYFGAFRVRSPVLIVRDPDLVKNICVKNFTCFSNRGIPVNSQDPLS

AHLFNLEGKKWKSLRSKLTPAFSSGKLKRMFYLLAECSEEFQKLIDTSSETDRPYEIREL

AAKFTIDVIGTCAFGIQINALTDEESEFHRAAKKLSKPSYKATLWRMLRTAMPRLYKFLG

VQVIDPEVTEFFKDVVSQMIKQREEYGIKRHDFMDLLIELKNKGTLDESGKLDENSIAAQ

AFVFFAAGYETSSNTIAFCLHELALNTEIQEKTRRDIQDAIDSRNGKLTYDAVQDMKYLD

MVIAETLRKYPPASMLSRRCEYQYQIPNSKVELPAGIRVIIPIYGLHHDPDYYPSPAMFN

PERFTEENKRTRHPYAYLPFGEGPRNCIGMRFALLQIKVGIISFLRNHRVETCQKTITPI

KFSRRSLVTTSEKGFWLRIK

>380013477

MWNIIRELLEQFLLPGLFLGILYCFLTSTFNFWKNRGVPFRKPTVLFGNFAPMLLFRKSL

PEGIKEMYEWFKNEKYFGAFRVRSPVLIVRDPDLVKNICVKNFTCFSNRGIPVNSQDPLS

AHLFNLEGKKWKSLRSKLTPAFSSGKLKRMFYLLAECSEEFQKLIDTSSETDRPYEIREL

AAKFTIDVIGTCAFGIQINALTDEESEFHRAAKKLSKPSYKATLWRMLRTAMPRLYKFLG

VQVIDPEVTEFFKDVVSQMIKQREEYGIKRHDFMDLLIELKNKGTLDESGSGLVCNDEDA

LLFIELDENSIAAQAFVFFAAGYETSSNTIAFCLHELALNTEIQEKTRRDIQDAIDSRNG

KLTYDAVQDMKYLDMVIAETLRKYPPASMLSRRCEYQYQIPNSKVELPAGIRVIIPIYGL

HHDPDYYPSPAMFNPERFTEENKRTRHPYAYLPFGEGPRNCIGMRFALLQIKVGIISFLR

NHRVETCQKTITPIKFSRRSLVTTSEKGFWLRIK

>350404797

MWNILEQFLLLGLLLGIIYCFFTSTFDFWKNRGVPFRKPIVLFGNFASLLLFQKSLPEGI

KDMYEWFKDERYFGAFRVMSPVLILRDPDLVKDICVKSFACFTNRGIPVNSQNSLSAHLF

NLEGKRWKSLRSKLTPAFSSGKLKRMFYLLAECGEEFQKLIDASSETHRPFEIRELAAKF

TIDVIGSCAFGIQINALTDEESEFHRTAKKLSKPSYTATLWRMLRTAMPKLYKFLDVQVI

DPEVSKFFKNVVSQMINEREEHGIKRHDFMDLLIELKNKGSLDECGSGQVCNDEDEKITE

EIELDENSIAAQAFVFFAAGYETSSNTIAFCLHELALNTEIQEKTRRDIQDAIDSRNGKL

SYDAIQDMKYLDMVIAETLRKYPPAPMLSRRCEHQYQIPGSKVELPVGMRVIIPIYGFHH

DPDYYPDPATFDPERFTEENKRTRHPYTYLPFGEGPRNCIGMRFALLQIKVGVISFLRKH

RVETCERTITPIKFSRRSLVTTSEKGFWLKIIQYS

>380013479

MWNIIRELLEQFLLPGLFLGILYCFLTSTFNFWKNRGVPFRKPTVLFGNFAPMLLFRKSL

PEGIKEMYEWFKNEKYFGAFRVRSPVLIVRDPDLVKNICVKNFTCFSNRGIPVNSQDPLS

AHLFNLEGKKWKSLRSKLTPAFSSGKLKRMFYLLAECSEEFQKLIDTSSETDRPYEIREL

AAKFTIDVIGTCAFGIQINALTDEESEFHRAAKKLSKPSYKATLWRMLRTAMPRLYKFLG

VQVIDPEVTEFFKDVVSQMIKQREEYGIKRHDFMDLLIELKNKGTLDDIAAQAFVFFAAG

YETSSNTIAFCLHELALNTEIQEKTRRDIQDAIDSRNGKLTYDAVQDMKYLDMVIAETLR

KYPPASMLSRRCEYQYQIPNSKVELPAGIRVIIPIYGLHHDPDYYPSPAMFNPERFTEEN

KRTRHPYAYLPFGEGPRNCIGMRFALLQIKVGIISFLRNHRVETCQKTITPIKFSRRSLV

TTSEKGFWLRIK

>383855398

MWNVIREFLEQFLLLGLFLSLLYCFLTSTFNFWEIRGVPFRKPIVLFGNYASLLLFRKSL

PEGIKEMYDWFKDERYFGTFRVKSPILILRDPDLIKNICVKNFACFSNRGIPVNGQDSLS

AHLFNLEGKKWKSLRCKLTPAFSSGKLKRMFYLLAECGEEFQKLIDSSSDRPFEIRELAA

KFTIDVIGSCAFGIQINALTDEESEFHRAAKKLSRPSYKATLWRMLRTAMPRLYKLLGVQ

VIDPEVTRFFKNVVSQMLKQREEQAVKRHDFMDLLIELKDKGTLENESGNGLIGNEEDAD

TAEEIELDENSIAAQAFVFFAAGYETSSNTIAFCLHELALNAEIQEKTRRDIHDAIDSRG

GKLTYDAVQDMKYLDMVIAETLRKYPPASLLSRRCEYQYQIPGSKVELPPGMRVIIPIYG

LHHDPDYYPNPSMFDPERFTEENKRTRHPYTYLPFGEGPRNCIGMRFALLQIKVGIISFL

RNHRVEVCEKTVTPIKFSRRSLVTTSEKGFWLKIVQY

>EFN89993.1

MLDVATELLDRPFLLIGLLFLGALYLYFTATFNFWECRGVPFRKPTVLVGNFSSSLLFRK

SVPESVEEMYQWFKDERFFGAFRVRSPVLIIRDPDLVRSVCVKDFACFSNRGIPINNFQD

PLSGHLFNLEGRKWKGLRSKLTPAFSSGKLKRMFYLLVECSEELQKLIDTSCNVDRTVVE

VRELAAKFTIDVIGSCAFGIQINALTNEESEFHRAAKRLSKPSYKATLWRMLRTAVPRLY

RWLGVQVIDPSVTRFFKNVVSQMISQREENSTRRHDFMDLLVELKNRGTLENEAGSTQFY

SEEDAQAAKEIELDENTIAAQAFVFFAAGYETSSNTIAFCLHELASNQEIQERARRDIRD

AIEARGGKLTYEAVQEMKYLDMVILETLRKYPPAPLLSRKCEYKYQIPNSKVELPAGMRV

VIPIYGLHHDPNYYPDPATFDPERFTEENKRTRHPYTYLPFGEGPRNCIGMRFALLQIKM

GMISFLKNHRVEICDKSIVPIKFSRRSLVTSSEAGFWLSVTQSS

>340721277

MWSILEKFLLPGLLLGILYCFLTSTFDFWKNRGVPFRKPTVLFGNFASLLLFQKSLPEGI

KDMYEWFRDERYFGAFRVTSPVLILRDPDLVKDICVKSFACFTNRGIPVNSQDSLSAHLF

NLEGKRWKSLRSKLTPAFSSGKLKRMFYLLAECGEEFQKLIDASSETHRPFEIRELAAKF

TIDVIGSCAFGIQINALTDEESEFHRAAKKLSKPSYTATLWRMLRTAMPKLYKFLDVQVI

DPEVSKFFKNVVSQMINEREEHGIKRHDFMDLLIELKNKGTLDECGNGQVCNDEDEEITE

EIELDENSIAAQAFVFFAAGYETSSNTIAFCLHELALNTEIQEKTRRDIQDAIDSRNGKL

SYDAIQDMKYLDMVIAETLRKYPPASMLSRRCEHQYQIPGSKVELPVGIRVIIPIYGFHH

DPDYYPDPATFDPERFTEENKRTRHPYTYLPFGEGPRNCIGMRFALLQIKVGIISFLRKH

RVETCERTIAPIKFSRRSLVTTSEKGFWLKIIQYS

>EFN69191.1

MLKITVAESLQMMYKQFEGYPFVGIYELTTPVLLLRDPKLIKCILVKDFTCFQSRGIVIN

EHADPLSANLININGQRWRKLRMKLASVFTTSKTRHVFELITEVSETFKQFIDQCADNEE

PVEFQNLAAKFTSEIIATCFFGLKTNTIQNGDSEFRRMCKKMMDPSLEIAMKRYVRDYMP

RIYKWLSVCLTPRDVTVYFMRIVKEIIIHREMNNMRRNDIMQALIDLRYKETKMKDAQSQ

NGTSESNDIVIDDKLITAQAFIFLLAGFETSSTAMSFAMYELAANPKIQEKLYDEIQTIY

EKHGWFFFFTISEMKYLDCIVHETLRKYPPVGATQRICEKSYKIPDSDVVLEKGTKVLVP

IYAIHHDSLYYKNPNAFDPDRFIDENKKLHDNNTYLPFGDGPRICIGMKFAYIQIKVGLV

TLMANYKVELSEKTPIPLQFEPTGITLTMLLDPFLLIGLLLGSLYFYLSATFDFWQCRGV

PFKKPTVLVGNFGPLLLFRKSQAEGVKEMYQWFKDERFFGAFRVRSPVLILRDPDLVKSV

FVKDFAYFTNRGIPINNAQDPLSGHLFNLEGRKWKNLRSKLTPAFSSGKMKRMFYLLVEC

CEELQRLIDASCSGDRSTIEVREFAAKFTIDVIGSCAFGIQINALTDEESEFHQAAKRLS

RPSYKTTLWRMLRTAMPGLYRLLGVQVIDPTVTTFFKSVVSQMIGQRERNEGRRHDFMDL

LIELKNKGVLENESGAQIYDDENAQAAKEIELDEDAIAAQAFVFFAAGYETSSNTIAFCL

HELALNQEIQERTRREICNALGMRDNKLTYDAVQDMKYLDMVILETLRKYPPAPLISRKC

EYNYQIPNSKVELPSGMRVIIPIYGFHHDPNYYPDPMKFNPERFTEENKRTRHPYTYLPF

GEGPRNCIGMRFALLQIKMGIISFLKNHRVEICERSIVPIKFSRRSLVTTSERGFWLAIT

PSS

>EGI63690.1

MLDVVAILLDPFLLIGLVLCSLYFYLTATFDFWKCRGVPFKKPTVLVGNFGPLLLFRTSQ

PEGVKEMYQWFKDERFFGAFRVRSPVLILRDPDLVRSVCVKDFACFVNRGIPINNDQDPL

SGHLFNLEGRKWKGLRSKLTPAFSSGKLKRMFYLLMECCEELQRLINEASNFGDQTVEVR

ELAAKFIIDVIGSCAFGIQINALTDEESEFHRAVKKLSKPSYKATLWRMLRTAMPRLYRL

MGVQLVDPSVTQFFTHVVSQMISQRESNGTIRHDFMDLLIELKNKGTLESEATNPRICSD

EDAQAAKEIELDENTIAAQAFGFFAAGYETSSNTIAFCLYELALNQEIQDKTRREVHNAI

GTRDGKLTYDAVQEMKYLDMVILETLRKYPPAPLFARRCEYKYRIPDSNVELPAGLRVVI

PIYGLHHDPNYYPDPARFDPERFTEENKRTRHSYTYLPFGEGPRNCIGMRFALLQIKMGI

IAFLKDHRVEICEKSPVPIKFSRRSLVTASETGYWLTITSSS

>EGI59272.1

MVLAELIGVFIFALSILYVYYKFVIFNFWHKRGIFYVKPVVPTGNLTDLITGKVSLGELF

QNAYMKYKDHRAFGMYTLFKPNLVIADPDLIRTVLTKEFGSFHDRGMYFNEKVDPLSGHL

FLMPGKKWRNLRVKLTPTFTSGKIKQMFPIMKEYSEKLAKYLKEKAQTKDSIEMKEMLAR

YTTDVIMSTAFGIESDCIEDPNNQYRGYQKKILEINPIWIALSMFAPKIMDFFSIPFTDR

NVISFYMNMFRETVEYRQSHNIVRYDFVNLLLQLMKRNYVDPDDDKKITNVSSTVNKLTM

TEATAQSYVFFVAGFETSSTTATYALYELAQHQDIQDKVRKEIDEILEKHGELSYDAMNE

MTYLHKVVNETMRKYPPVPVLNRICTKEITLPTNIHVPEGTSVTIPVLGLHRDPSIYPNP

DKFDPERFNADKIEERHAYTYLPFGEGPRNCIGSRFGYTQTKVGLVSLLSRYIFKLHSQT

PIPLIFSENSFILTAKSGVHFIIEPRK

>156555149

MNMMESLAPMLHTKLFWLAAVVTLIWVHFKFVIYTYWKRKGIPHDEPVVPFGSTLPIFLG

KSSMGNLVKQKYQKSKKYPLYGIYMFHQPMLLINDPDLVRIILIKEFNKFRDRGLYLNEK

VDPLSGHLFLLPGERWRKLRAKLTPTFTSGKLKQMFPLLVEIGDELIKVCEKIIQTDSVI

EFKDLNARYTVDSISSIAFGFNCKSLDNPNNEFKRYGSMVFDQSPIRNALGVFAPVVLDT

LRIPLIRRVIIDFFSQTFKDMVDHRHSNKIVRKDFINLLMQLMDKGVLEEDEKSQKSNDH

TKAGLIDNEKISMVEAQAQAFVFFLGGFETTSSTITYCLYELALNPHIQEKLQAEIDEHL

AKPGGMTYDRIVNELEYLHMVFSETLRKHPSVPILNRLCIEDCDLPNTNFRIKKGTGVMI

SVSGMQRDPNIYPDPDKFDPLRFTKENIASRSPYVYLPFGDGPRVCIGTRFGILQSKIAL

IALLAKYKFSVCDKTSIPINYSKRSFTQSPEGGVYLRMEKRIK

>328785604

MWNIIRELLEQFLLPGLFLGILYCFLTSTFDFWKNRGVPFRKPTVLFGNFAPMLLFRKSL

PEGIKEMYEWFKDERYFGAFRVRSPVLILRDPDLVKNICVKNFTSFSNRGIPVNSQDPLS

AHLFNLEGKKWKSLRSKLTPAFSSGKLKRMFYLLAECGEEFEKLIDISSETDRPYEIREL

AAKFTIDVIGTCAFGIQINALTDEESEFHRAAKKLSKPSYKATLWRMLRTAMPRLYKFLG

VQVIDPGVTKFFKDVVSQMIKQRGEYGIKRHDFMDLLIELKNKGTLDEFGSGLVCNDEDA

ETADEIELDENSIAAQAFVFFAAGYETSSNTIAFCLHELALNTEIQEKTRRDIQDAIDSR

NGNLTYDAVQDMKYLDMVIAGIRVIIPIYGLHHDPDYYPSPAIFNPERFTEENKRTRHPY

AYLPFGEGPRNCIGMRFALLQIKVGIISFLRNHRVETCQKTITPIKFSRRSLVTTSEKGF

WLRIK

>328712333

MFTDNWWIYVITPCTIIVTIVYYFCVSTFKKWENLNVPYIKPVPLFGNFLNVALGKEHHI

DFYNKFYHKFAGHKYAGVFQMRLPILMIIDPEIINDVLIKDFSSFPNRGFSVDFKANPLS

NNLFLMENPQWKIIRNKLTPAFTSGKLKVMYDQIKECGEELMKNIDIDLKKSGDEIEVRD

IMGKYSTDVIGTCAFGLKLDAINDDESPFRKHGKSIFAPSLRQLFREMCMLISPVLVKVV

RVKDFPKDATDFFHAAFKETMKYRHENKIVRNDLVHCLMQARNDLVLNTDLPKHEKFTES

QIVANAFIMFAAGFETVSSAISYCLYELALNKSIQDRVREEIQLKLSKNDGQINHEFLME

LHYLDMVLAETLRKYPPLVFLMRKALQTYRLPNDSLTIEKDQKVIIPVYAIHHDSKYYPE

PENFIPERFSTEEKAKRPNGTYMPFGDGPRICIGKRFAEVEMKLAMVEMLTKFEVFPCEK

TEVPLKYSHKTITLMPKHGIWLKFKKIN

>289177177

MDSITPILHKKLFWAFTLIIVIYAYFKFVIYDYWKRRGIPHDTPIIPVGNAFPLISGSIG

NFIKQKYNKSKKYPVYGIYIFHMPLLLINDPNVIQLILIKEFNKFRDRGLYFNEKVNPLS

VHLFLLPGERWRQLRAKLTPTFTSSKLKHMYPLLLKIGDNLIRVCDQIIKKDNIVEFKDL

IGRYTTDSISSIAFGVDCQSLDNGSSEFQKHGSMAFDLPPIKNTLGTFVPKLLDIFRVPL

YNKAMTQFFTKTFKDVLEERRRNKIARKDFLDLLMQLLDKGSLDEDKDLKQGDNSANGSA

LGKISLSEAIGQAFIFYLAGFETTSSTITYCLYELALNSEIQEKVQAEIDEFSKRDGGIT

YEIMSNDMKYLHMVFLETLRKHPSIPFLNRECIEDCDIPNTNFRIEKGTKLLICMNAMHR

DPDIFPDPEKFDPLRFTKENIASRQPYTYLPFGDGPRACIGIRFGMLQTKIALIGLLSNY

NFSVCEKTSIPVMYARRSFTQTPEYGIYLKTEKRNKSTFLYKNNKGQ

>ABQ42340.1

MLTAYLLYVFASIVVLIYFYLNNKYKYWKNKNIAGPEPEFLFGNLKESFHRRKHIATVFK

EIYDQYPDEKVVGMFRMTSPTLIVRDLDIVKQIMIKDYAKFNERGIKFSKEGLGDNLFHA

DADAWKAVRSHLTPMFSSGKLKNMVRVLSQTGDRFVDHVTNGVRLRPEQELVSLFLKYNI

ATIMACSFSMETDINDMQVYEMLNVAVFENSYISELDMVFPGILRKTNRSIFGPTVNNFC

YEIVEFIKNERNGKPANRNDMMDMLLGVNCDKLKMKNGSEDFVEITEHVMAGQVFVFFAA

GYVNNTITLTFSLYHLAKDQSIQERLMREIETVLENHNNVLTLEAINEMSYLEMIFLETL

RMHPTTNTLQRSALEDYTIPGTDIEIEKGTLVLIPPLAFHHDEKIYPEPEKFDPERFSVE

NHKSRHACAFLSFGIGPRTCIGIRLAKLQYKVCMVKFLTKYRVAPSTRTKDAYKIDPLRT

ALEPEGGVYVDILPRS

>AAK69504.1

MLTIFIVTATLFAILYLYFTRNFNYWKDRNVVGPEPTVFFGNIMESVIRRKHLIMIYKDI

YEAFPKEKVVGIYRMTTPCLLLRDLDVIKHVMIKDFDLFNDRGVEFSEEGLGLNIFHADG

DRWRVLRQAFTPLFTSGKLKNMLNLMSDRGDKFIKMVEKICDKEPEQQIIPLVRKFTMAS

ITTCAFGMELDEEMIETLDKLDSLIFTTSYGNEIDMMYPGILKKLNSSIFSKMIAPFFDN

LTKTIIEQRGGKPTNRKDLMDLILELRQKKAIEPMKKTQDEQVTTLELTDSVIAAQTFIF

YAAGYETSASTMSFLLFELAENPDIQEKVIAEVDETLKRHNGEITYDTLSEMTYLTQVFH

ETLRKYPVADILLRNAKADYAVPGTNVTLKKGQTVVVSGFGIHYDPKYYPDPEKFDPERF

SPENVKNRHPCAYIPFGAGQRKCLGMRFGQWQVQVCIIKLLSKFRLEPSTKTKSQFIYDP

MRLFVYPKSGIFLNILPRNY

>AAK69478.1

MLTIFIVTATLFAILYLYFTRNFNYWKDRNVVGPEPTVFFGNIMESVIRRKHLIMIYKDI

YEAFPKEKVVGIYRMTTPCLLLRDLDVIKHVMIKDFDLFNDRGVEFSEEGLGLNIFHADG

DRWRVLRQAFTPLFTSGKLKNMLNLMSDRGDKFIKMVEKICDKEPEQQIIPLVRKFTMAS

ITTCAFGMELDEEMIETLDKLDSLIFTTSYGNEIDMMYPGILKKLNSSIFSKMIGPFFDN

LTKTIIEQRGGKPTNRKDLMDLILELRQKKAIEPMKKTHDEPVTTFELTDSVIAAQTFVF

YAAGYETSATTMSFLLFELAENPDIQEKVIAEVDETLKRHNGEITYDTLSEMTYLTQVFH

QTLRKYPVADILLRKANADYAVPGTNVTLKKGQTVLVSGFGIHYDPKYYPDPEKFDPERF

GPENVKNRHPCAYIPFGAGQRKCLGMRFGQWQVQVCIIKLLSKFRLEPSTKTKSQFNYDP

MRLFVYPKSGIFLNILPRNY

>AAK69503.1

MLTIFIVTATLFAILYLYFTRNFNYWKDRNVVGPEPTVFFGNIMESVIRRKHLIMIYKDI

YEAFPKEKVVGIYRMTTPCLLLRDLDVIKHVMIKDFDLFNDRGVEFSEEGLGLNIFHADG

DRWRVLRQAFTPLFTSGKLKNMLNLMSDRGDKFIKMVEKICDKEPEQQIIPLVRKFTMAS

ITTCAFGMELDEEMIETLDKLDSLIFTTSYGNEIDMMYPGILKKLNGSIFSKMIAPFFDN

LTKTIIEQRGGKPTNRKDLMDLILELRQKKAIEPMKKTQDGQVTTLELTDSVIAAQTFIF

YAAGYETSAPTMSFLLFELAENPDIQEKVIAEVDETLKRHNGEITYDTLSEMTYLTQVFH

ETLRKYPVADILLRNAKADYAVPGTNVTLKKGQTVVVSGFGIHYDPKYYPDPEKFDPERF

SPENVKNRHPCAYIPFGAGQRKCLGMRFGQWQVQVCIIKLLSKFRFEPSTKTMSEFSYDP

TRLLVYPKSGIFLNILPRNY

>AAK69500.1

MLTIFIVTATLFAILYLYFTRNFNYWKDRNVVGPKPTVFSGNIMESVIRRKHLIMIYKDI

YEAFPKEKVVGIYRMTTPCLLLRDLDVIKHVMIKDFDLFSDRGIEFSEEGLGLNIFHADG

DRWRVLRQAFTPLFTSGKLKNMLNLMSDRGDKFIKMVEKICDKEPEQQIILLVRKFTMAS

IAACAFGMELGEDMIETLDKLDSLIFTNSYVNEVDMMYPGILKKLNSSIFSKMIGPFFDN

LTKTIIEQRGGKPTNRKDLMDLILELRQKKAIEPMKKTHDEPVTKFELTDSVIAAQTFVF

YAAGYETSATTMSFLLFELAENPDIQEKVIAEVDETLKRHNGEITYDTLSEMTYLTQVFH

ETLRKYPVADILLRKANADYAVPGTNVTLKKGQTVLVSGFGIHYDPKYYPDPEKFDPERF

SPENVKNRHPCAYIPFGAGQRKCLGMRFGQWQVQVCIIKLLSKFRLEPSTKTKSQFIYDP

MRLFVYPKSGIFLNILPRNY

>ADE05578.1

MVLLYLAVGVVLTTALALYAYFTRTFNYWKSKNVVGPEPVAFFGNLKESTLRRKNIGVVF

QEFYKQFPNEKVVGVYQMTTPCLLLRDLDIIKHIMIKDFDVFIERGIEFSKEGLGANLFH

ADADTWRSLRNRFTPLFTSGKLKNMLHLITERADKFRDYVYHLCNDNPEQEIHSLFQMYT

MGTISACAFGVDIDTLHDKLETFHEMDKLVLTPSYASELDMMFPGILKWFNSSIFSVKIK

DFFYDLVKSVISQRGGLPTKRNDFMDLILELRQQGRIETTKRHDETQKLILELSDDIIAA

QAFIFYVGGYETTATTVSFLLYQLAMNPDIQDKLLAEIDQSLEDNNGVVNYDTIQSMRYL

DKVLNETLRMYPIVEPLKRIAKVDYKIPGTDVTIEKGTTVIVSPIGIHHDEKYYPNPHIF

DPERFSPENSGQRHPCAYIPFGTGPRNCIGMRFAKLQGSVGLVKLLSKVRVEPSKNTPKI

LDIEPNRIIIGPKGGVRLNIIQRKPVNA

>AAK69499.1

MLTIFIVTATLFAILYLYFTRNFNYWKDRNVVGPEPTVFFGNIMESVIRRKHLIMIYKDI

YEAFPKEKVVGIYRMTTPCLLLRDLDVIKHVMIKDFDLFNDRGVEFSEEGLGLNIFHADG

DRWRVLRQAFTPLFTSGKLKNMLNLMSDRGDKFIKMVEKTCDKEPEQQIIPLVRKFTMAS

ITTCAFGMDLDEEMIETLDKLDSLIFTTSYGNEIDMMYPGILKKLNSSIFSKMIAPFFDN

LTKTIIEQRGGKPTNRKDLMDLILELRQKKAIEPMKKTQDEQVTTLELTDSVIAAQTFIF

YAAGYETSASTMSFLLFELAENPDIQEKVIAEVDETLKRHNGEITYDTLSEMTYLTQVFH

ETLRKYPVADILLRNAKADYAVPGTNVTLKKGQTVVVSGFGIHYDPKYYPDPEKFDPERF

SPENVRNRHPCAYIPFGAGQRKCLGMRFGQWQVQVCIIKLLSKFRFEPSTKTMSEFSYDP

TRLLVYPKSGIFLNILPRNY

>AAK69497.1

MLTIFIVTATLFAILYLYFTRNFNYWKDRNVVGPEPTVFFGNIMESVIRRKHLIMIYKDI

YEAFPKEKVVGIYRMTTPCLLLRDLDVIKHVMIKDFDLFNDRGVEFSEEGLGLNIFHADG

DRWRVLRQCFTPLFTSGKLKNMLNLMSDRGDKFIKMVEKICDKEPEQQIIPLVRKFTMAS

ITTCAFGMELDEEMIETLDKLDSLIFTTSYGNEIDMMYPGILKKLNSSIFSKMIAPFFDN

LTKTIIEKRGGKPTNRKDLMDLILELRQKKAIEPMKKTQDEQVTTLELTDSVIAAQTFIF

YAAGYETSASTMSFLLFELAENPDIQEKVIAEVDETLKRHNGEITYDTLSEMTYLTQVFH

ETLRKYPVADILLRNAKADYAVPGTNVTLKKGQTVVVSGFGIHYDPKYYPDPEKFDPERF

SPENIRNRHPCAYIPFGAGQRKCLGMRFGQWQVQVCIIKLLSKFRFEPSTKTKSEFNYDP

TRLLVYPKSGICLNILPRNY

>AAK69495.1

MLTIFIVTATLFAILYLYFTRNFNYWKDRNVVGPEPTVFFGNIMESVIRRKHLIMIYKDI

YEAFPKEKVVGIYRMTTPCLLLRDLDVIKHVMIKDFDLFNDRGVEFSEEGLGLNIFHADG

DRWRVLRQAFTPLFTSGKLKNMLNLMSDRGDKFIKMVEKICDKEPEQQIIPLVRKFTMAS

ITTCAFGMELDEEMIETLDKLDSLIFTTSYGNEIDMMYPGILKKLNSSIFSKMIAPFFDN

LTKTIIEQRGGKPTNRKDLMDLILELRQKKAIEPMKKTHDEQVTTLELTDSVIAAQTFIF

YAAGYETSASTMSFLLFELAENPDIQEKVIAEVDETLKRHNGEITYDTLSEMTYLTQVFH

ETLRKYPVADILLRNAKADYAVPGTNVTLKKGQTVVVSGFGIHYDPKYYPDPEKFDPERF

SPENVRNRHPCAYIPFGAGQRKCLGMRFGQWQVQVCIIKLLSKFRFEPSTKTMSEFSYDP

TRLLVYPKSGIFLNILPRNY

>Q27902.1

MLTIFIVTATLFAILYFYFTRNFNYWKDRNVVGPEPTVFFGNIMESVIRRKHLIMIYKDI

YEAFPKEKVVGIYRMTTPCLLLRDLDVIKHVMIKDFDLFNDRGVEFSEEGLGLNIFHADG

DRWRVLRQCFTPLFTSGKLKNMLNLMSDRGDKFIKMVEKFCDKEPEQQIIPLVRKFTMAS

ITTCAFGMELDEEMIETLDKLDSLIFTTSYGNEIDMMYPGILKKLNSSIFSKMIAPFFDN

LTKTIIEQRGGKPTNRKDLMDLILELRQKKAIEPMKKTHDEQVTTLELTDSVIAAQTFIF

YAAGYETSASTMSFLLFELAENPDIQEKVIAEVDETLKRHNGEITYDTLSEMTYLTQVFH

ETLRKYPVADILLRNAKADYAVPGTNVTLKKGQTVVVSGFGIHYDPKYYPDPEKFDPERF

SPENVRNRHPCAYIPFGAGQRKCLGMRFGQWQVQVCIIKLLSKFRFEPSTKTMSEFNYDP

KRLLVYPKSGIFLNIIPRNY

>AAK69477.1

MLTIFIVTATLFAILYLYFTRNFNYWKDRNVAGPEPTVFFGNIMESVIRRKHLIMIYKDI

YEAFPKEKVVGIYRMTTPCLLLRDLDAIKHVMIKDFDLFNDRGVEFSEEGLGLNIFHADG

DRWRVLRQAFTPLFTSGKLKNMLNLMSDRGDKFIKMVEKICDKEPEQQIIPLVRKFTMAS

ITTCAFGMELDEEMIETLDKLDSLIFTTSYGNEIDMMYPGILKKLNSSIFSKMIAPFFDN

LTKTIIEQRGGKPTNRKDLMDLILELRQKKAIEPMKKTQDEQVTTLELTDSVIAAQTFIF

YAAGYGTSASTMSFLLFELAENPDIQEKVIAEVDETLKRHNGEITYDTLSEMTHLTQVFH

ETLRKYPVADILLRNAKADYAVPGTNVTLKKGQTVVVSGFGIHYDPKYYPDPEKFDPERF

SPENVKNRHPCAYIPFGAGQRKCLGMRFGQWQVQVCIIKLLSKFRFEPSTKTMSEFNYDP

TRLLVYPKSGIFLNIIPRNY

>AAB06742.1

MLTIFIVTATLFAILYLYFTRNFNYWKDRNVVGPEPTVFFGNIMESVIRRKHLIMIYKDI

YEAFPKEKVVGIYRMTTPCLLLRDLDVIKHVMIKDFDLFNDRGVEFSEEGLGLNIFHADG

DRWRVLRQCFTPLFTSGKLKNMLNLMSDRGDKFIKMVEKICDKEPEQQIIPLVRKFTMAS

ITTCAFGMELDEEMIETLDKLDSLIFTTSYGNEIDMMYPGILKKLNSSLFSKMIAPFFDN

LTKTIIEQRGGKPTNRKDLMDLILELRQKKAIEPMKKTHDEQVTTFELTDSVIAAQTFIF

YAAGYETSASTMSFLLFELAENPDIQEKVIAEVDETLKRHNGEITYDTLSEMTYLTQVFH

ETLRKYPVADILLRNAKADYAVPGTNVTLKKGQTVVVSGFGIHYDPKYYPDPEKFDPERF

SPENVRNRHPCAYIPFGAGQRKCLGMRFGQWQVQVCIIKLLSKFRFEPSTKTMSEFSYDP

TRLLVYPKSGIFLNILPRNY

>AAK69479.1

MLTIFIVTATLLAILYLYFTRNFNYWKDRNVVGPEPTVFFGNIMESVIRRKHLIMIYKDI

YEAFPKEKVVGIYRMTTPCLLLRDLDVIKHVMIKDFDLFNDRGVEFSEEGLGLNIFHADG

DRWRVLRQFFTPLFTSGKLKNMLNLMSDRGDKFIKMVAKICDKEPEQQIIPLVRKFTMAS

ITTCAFGMELDEEMFESLDKLDSLIFTTSYGNEIDMMYPGILKKLNSSLFSKMIAPFFDN

LTKTIIEQRGGKPTNRKDLMDLILELRQKKAIEPMKKTHDEQVTTLELTDSVIAAQTFIF

YAAGYETSASTMSFLLFELAENPDIQEKVIAEVDETLKRHNGEITYDTLSEMTYLTQVFH

ETLRKYPVADILLRNAKADYAVPGTNVTLKKGQTVVVSGFGIHYDPKYYPDPEKFDPERF

SPENVKNRHPCAYIPFGAGQRKCLGMRFGQWQVQVCIIKLLSKFRFEPSTKTRSEFSYEP

TRLLVYPKSGICLNILPRNY

>AAK69480.1

MLTIFIVTATLFAILYLYFTRNFNYWKDRNVVGPKPTVFFGNVMESVIRRKHLIMIYKDI

YEAFPKEKVVGIYRMTTPCLLLRDLDVIKHVMIKDFDLFSDRGIEFSEQGLGLNIFHADG

DRWRVLRQAFTPLFTSGKLKNMLNLMSDRGDKFIKMVEKICDKEPEQQIILLVRKFTMAS

ITACAFGMELGEDMIETLDKLDSLIFTNSYVNEVDMMYPGILKKLNSSIFSKMIGPFFDN

LTKTIIEQRGGKPTNRKDLMDLILELRQKKAIEPMKKTHDEPVTTFELTDSVIAAQTFVF

YAAGYETSATTMSFLLFELAENPDIQEKVIAEVDETLKRHNGEITYDTLSEMTYLTQVFH

ETLRKYPVADILLRKANADYAVPGTNVTLKKGQTVLVSGFGIHYDPKYYPDPEKFDPERF

SPENVKNRHPCAYIPFGAGQRKCLGMRFGQWQVQVCIIKLLSKFRLEPSTKTKSQFNYDP

MRLFVYPKSGVF

>ADE05579.1

MLLLYLALGIVTAAVALYAYFTRTFNYWKSRNVAGPEPVALFGNLKESTLRSKNIGIVFQ

ELYNQFPNEKVVGIYRMTTPCLLLRDLDVIKHIMIKDFDVFSDRGVEFSKEGLGVNLFHA

DGDTWRTLRNRFTPIFTSGKLKNMLYLITERADKFSNYVEKLCYDQPEQEVHSLIQKYTM

GTIAACAFGVDIDTLYDKLDTLLLIDKLILQGTYATELDMMFPGILKKLNQSLFPQTVKD

FFYDLVKSVMTQRGGVPSNRKDFMDLLLELRQQGHIETTKRNDERERLSLAISEDVIAAQ

AFVFYVAGYETTATTVSFLLYELAMNPDIQNKLLAEIDDTLAANNGVVDYDTIKSMSYLD

RVFDETLRKYPIVEPLQRNAKADYKIPGTDVVVKKGMTVLISPMGIQNDEKYFPKPHIFD

PERFTPENAGKRHPCAYIPFGTGPRNCIGMRFARLQGATGLVKLLSKVRVEPSKNTPPIM

EIEPKRVIVGPKNGIQLKLIPRKPVKS

>AFP20587.1

MSALYFLAAVPVLAYAIYYYFTRTFNYWKSRNVAGPEPTAFFGNIKESVVRTKNIGIVMH

DIYNAFPNEKVVGMFRMTSPCLLIRDLDIVKHVMIKDFEAFSDRGVEFSKEGLGQNLFHA

DGETWTALRNRFTPIFTTGKLKNMFYLINEGGDSFIEYINTECQRKQEFDIQPLLQTYTL

STISACAFGITYDSLDDKFETLKLVDKIFSAPSYANELDMMYPGLLKALNLSLFPSSIQK

FFENLVKTVIEQRNGKPSGRNDFMDLILALREIGEVTNAKYDSAKPVEITPEVMAAQAFV

FYAGGYETSATTMTFMLYQLAMNPDIQNKLTAEIDEVLQANNGQVTYDSIKEMKYLNKVF

DETLRMYSIVEPLQRKAVRDYQVPGTNLTLEKNTIVLISPRGIHYDEKYYDNPEQFNPDR

FDPEEVGKRHPCAYLPFGFGQRNCIGMRFARLQSQLCIVKLLSKFQVEPSKNTARKLEIE

PCRFIIGPKGGVRLNIVPRKLKA

>AAK69494.1

MLTIFIVTATLFAILYLYFTRNFNYWKDRNVVGPEPTVFFGNIMESVIRRKHLIMIYKDI

YEAFPKEKVVGIYRMTTPCLLLRDLDVIKHVMIKDFDLFNDRGVEFSEEGLGLNIFHADG

DRWRVLRQAFTPLFTSGKLKNMLNLMSDRGDKSIKMVEKICDKEPEQQIIPLVRKFTMAS

ITTCAFGMELDEEMIETLDKLDSLIFTTSYGNEIDMMYPGILKKLNSSIFSKMIAPFFDN

LTKTIIEQRGGKPTNRKDLMDLILELRQKKAIEPMKKTHDEQVTTLELTDSVIAAQTFIF

YAAGYETSASTMSFLLFELAENPDIQEKVIAEVDETLKRHNGEITYDTLSEMTYLTQVFH

ETLRKYPVADILLRNAKADYAVPGTNVTLKKGQTVVVSGFGIHYDPKYYPDPEKFDPERF

SPENVRNRHPCAYIPFGAGQRKCLGMRFGQWQVQVCIIKLLSKFRFEPSTKTMSEFSYDP

TRLLVYPKSGICLNILPRNY

>ACV88721.2

MSALYFLAAVPVLAYAIYYYFTRTFNYWKSRNVPGPEPTAFFGNIKESVVRTKNIGIVMH

DIYKAFPNEKVVGMFRMTSPCLLIRDLDIVKHVMIKDFEAFSDRGVEFSKEGLGQNLFHA

DGETWTALRNRFTPIFTTGKLKNMFYLLNEGGDSFIEYVSTECQKKQEFDIQPLLQTYTV

STISACAFGISYDSLDDKLETLKLVDKVFSSPSYAFELDMMYPGLLKALNLSLFPSSIQK

FFDNLVKTVIAQRNGKPSGRHDFMDLILALREIGEVTNAKHDSAKPVEITPEVMAAQAFV

FYAGGYETSATTMTFMLYQLAMNPDIQNKLRAEIDEVIQANNGQVTYESIKEMKYLNKVF

DETLRMYSIVEPLQRKVARDYQVPGTNLTLEKDTIVLISPRGIHYDEKYYDNPEQFNPDR

FNPEEVGKRHPCAYLPFGLGQRNCIGLRFGKLQSQLCIVKFLSKFQVEPSKNTARKLEFE

PCRTIIGPKGGIRLNIVPRKLKA

>ACY41036.1

MSALYFLAAVPVLAYAIYYYFTRTFNYWKSRNVPGPEPTAFFGNIKESVVRTKNIGIVMH

DIYKAFPNEKVVGMFRMTSPCLLIRDLDIVKHVMIKDFEAFSDRGVEFSKEGLGQNLFHA

DGETWTALRNRFTPIFTTGKLKNMFYLLNEGGDSFIEYVSTECQKKQEFDIQPLLQTYTV

STISACAFGISYDSLDDKLETLKLVDKVFSSPSYAFELDMMYPGLLKALNLSLFPSSIQK

FFDNLVKTVIAQRNGKPSGRHDFMDLILALREIGEVTNAKHDSAKPVEITPEVMAAQAFV

FYAGGYETSATTMTFMLYQLAMNPDIQNKLRAEIDEVIQANNGQVTYESIKEMKYLNKVF

DETLRMYSIVEPLQRKVARDYQVPGTNLTLEKDTIVLISPRGIHYDEKYYDNPEQFNPDR

FNPEEVGKRHPCAYLPFGLGQRNCIGLRFGKLQSQLCIVKFLSKFQVEPSKNTARKLEFE

PCRTIIGPKGGIRLNIAPRKLKA

>BAM18141.1

MYFLLAVTTLLLSLYYYFTRTFDYWKKRNVVGPKPLPFFGNIIDSVLRREHLIMTLKKIY

PAYPNEKVVGLYRMTTPCLMLRDLDIIKHVLIKDFDLFVDRGISFSEEGLGQNMFHADGD

KWRVLRHRFTPLFTSGKLKNMLYLMTDRGDKVIDYIGQIYDKQTELPMHLLLQKFTIGSI

TACAFGVDLDDEMMQTLENFDKIMFVTNYGVELDMMYPGILKKFNSSLFPKNIRTFFYDL

TKSVIKQRGGMPTNRKDFMDLILELRQNKTIESYKRNDNEDVKTLEITDSIIAAQTFVFY

AAGYETSASTMTHLFYELAKNPDVQDKLITEIEEVLKRHNGEITYESLNDMTYLHQVFDE

TLRKYPIADVILRKAQVDYKLPGTDITLEKGTSVVLSTWGIHHDPKYYPNPEKFDPENFS

PENEKKRHPCAYLPFGTGPRNCIGMRFAKLQSQVFIVKFLSKYRVEPSKSTPSEIKYDPM

RVLLYSQGGLYLKVVRRDY

>AAK69496.1

MLTIFIVTATLFAILYLYLTRNFNYWKDRNVVGPEPTVFFGNIMESVIRRKHLIMIYKDI

YEAFPKEKVVGIYRMTTPCLLLRDLDVIKHVMIKDFDLFNDRGVEFSEEGLGLNIFHADG

DRWRVLRQCFTPLFTSGKLKNMLNLMSDRGDKFIKMVEKICDKEPEQQIILLVRKFTMAS

IAACAFGMELDEEMFESLDKLDSLIFTTSYGNEIDMMYPGILKKLNSSLFSKMIAPFFDN

LTKTIIEQRGGKPTNRKDLMDLILELRQKKAIEPMKKTHDEQVTTLELTDSVIAAQTFIF

YAAGYETSASTMSFLLFELAENPDIQEKVIAEVDETLKRHYGEITYDTLSEMTYLTQVFH

ETLRKYPVADILLRNAKADYAVPGTNVTLKKGQTVVASGFGIHYDPKYYPDPEKFDPERF

SPENVRNRHPCAYIPFGAGQRKCLGMRFGQWQVQVCIIKLLSKFRFEPSTKTRSEFSYEP

TRLLVYPKSGICLNILPRNY

>AAK69498.1

MLTIFIVTATLFAILYLYFTRNFNYWKDRNVVGPEPTVFFGNIMESVIRRKHLIMIYKDI

YEAFPKAKVVGIYRMTTPCLLLRDLDVIKHVMIKDFDLFNDRGVEFSEEGLGLNIFHADG

DRWRVLRQCFTPLFTSGKLKNMLNLMSDRGDKFIKMVEKICDKEPEQQIIPLVRKFTMAS

ITTCAFGMELDEEMFESLDKLDSLIFTTSYGNEIDMMYPGILKKLNSSLFSKMIAPFFDN

LTKTIIEQRGGKPTNRKDLMDLILELRQKKAIEPMKKTHDEQVTTLELTDSVIAAQTFIF

YAAGYETSASTMSFLLFELAENPDIQEKVIAEVDETLKRHNGEITYDTLSEMTYLTQVFH

ETLRKYPVADILLRNAKADYVVPGTNVTLKKGQTVVVSGFGIHYDPKYYPDPEKFDPERF

SPENVRNRHPCAYIPFGAGQRKCLGMRFGQWQVQVCIFKLLSKFRFDPSTKTMSEFNYDP

TRLLVYPKSGIFLNILPRNY

>ADA68173.1

MSALYFLAAIPVLAYAVYYYFTRTFNYWKSRNVAGPEPTAFFGNIKESVVRTKNIGIVMH

DIYKAFPNEKVVGMFRMTSPCLLIRDLDIVKHVMIKDFEAFSDRGVEFSKEGLGQNLFHA

DGETWTALRNRFTPIFTTGKLKNMFYLINEGGDTFIEYINTECQKKQEFDIQPLLQTYTM

STISACAFGISYDSLDDKLETLKLVDKIFSAPSYANELDMMYPGLLKALNLSLFPSSIQK

FFENLVKTVIAQRNGKPSGRHDFMDLILALREIGEVSNAKHDSAKPVEITPEVMAAQAFV

FYAAGYETSATTMTFMLYQLAMNPDIQNKLRAEIDEVIQANNGQVTYESIKEMKYLNKVF

DETLRMYSIVEPLQRKVVREYQVPGTNLTLEKNTIVLISPRGIHYDEKYYDNPEQFNPDR

FDPEEVGKRHPCAYLPFGLGQRNCIGMRFGRLQSQLCIVKLLSKFQVEPSKNTARKMEFE

PCRFIIGPKGGIRLNIVPRKLKA

>AAL89656.1

MWVLYLPAVLSVLIVTLYLYFTRTFNYWKKRNVRGPEPTVFFGNLKDSTLRKKNIGIVME

EIYNQFPDEKVVGMYRMTTPCLLVRDLDVIKHIMIKDFEAFRDRGVEFSKEGLGQNLFHA

DGETWRALRNRFTPIFTSGKLKNMFYLMHEGADNFIDHVSKECEKKQEFEVHSLLQTYTM

STISSCAFGVSYNSISDKVQTLEIVDKIISEPSYAIELDYMYPKLLAKLNLSIIPTPVQH

FFKSLVDSIISQRNGKPAGRNDFMDLILELRQMGEVTSNKYLDGVTSLEITDEVICAQAF

VFYVAGYETSAITMSYLIYQLSLNQDVQNKLIAEVDEAIKASDGKVTYDTVKEMKYLNKV

FDETLRMYSIVEPLQRKATRDYQIPGTDVVIEKDTMVLISPRGIHYDPKYYDNPKQFNPD

RFDAEEVGKRHPCAYLPFGLGQRNCIGMRFGRLQSLLCITKILSKFRIEPSKNTDRNLQV

EPRRVIIGPKGGIRVNIVPRKIVS

>AAF06102.1

MWVLYLPAVLSVLIVTLYLYFTRTFNYWKKRNVRGPEPTVFFGNLKDSTLRKKNIGIVME

EIYNQFPDEKVVGMYRMTTPCLLVRDLDVIKHIMIKDFEAFRDRGVEFSKEGLGQNLFHA

DGETWRALRNRFTPIFTSGKLKNMFYLMHEGADNFIDHVSKECEKKQEFEVHSLLQTYTM

STISSCAFGVSYNSISDKVQTLEIVDKIISEPSYAIELDYMYPKLLAKLNLSIIPTPVQH

FFKSLVDSIISQRNGKPAGRNDFMDLILELRQMGEVTSNKYLDGVTSLEITDEVICAQAF

VFYVAGYETSATTMSYLIYQLSLNQDVQNKLIAEVDEAIKASDGKVTYDTVKEMKYLNKV

FDETLRMYSIVEPLQRKATRDYQIPGTDVVIEKDTMVLISPRGIHYDPKYYDNPKQFNPD

RFDAEEVGKRHPCAYLPFGLGQRNCIGMRFGRLQSLLCITKILSKFRIEPSKNTDRNLQV

EPRRVIIGPKGGIRVNIVPRKIVS

>AAT36350.1

MWVLYLPAVLSVLIVTLYLYFTRTFNYWKKRNVRGPEPTVFFGNLKDSTLRKKNIGIVME

EIYNQFPDEKVVGMYRMTTPCLLVRDLDVIKHIMIKDFEAFRDRGVEFSKEGLGQNLFHA

DGETWRALRNRFTPIFTSGKLKNMFYLMHEGADNFIDHVSKECEKKQEFEVHSLLQTYTM

STISSCAFGVSYNSISDKVQTLEIVDKIISEPSYAIELDYMYPKLLAKLNLSIIPTPVQH

FFKSLVDNIISQRNGKPAGRNDFMDLILELRQMGEVTSNKYLDGVTSLEITDEVICAQAF

VFYVAGYETSATTMSYLIYQLSLNQDVQNKLIAEVDEAIKASDGKVTYDTVKEMKYLNKV

FDETLRMYSIVEPLQRKATRDYQIPGTDVVIEKDTMVLISPRGIHYDPKYYDNPKQFNPD

RFDAEEVGKRHPCAYLPFGLGQRNCIGMRFGRLQSLLCITKILSKFRIEPSKNTDRNLQV

EPRRVIIGPKGGIRVNIVPRKIVS

>AAM90317.1

MWVLYLPAVLSVLIVTLYLYFTRTFNYWKKRNVRGPEPTVFFGNLKDSTLRKKNIGIVME

EIYNQFPDEKVVGMYRMTTPCLLVRDLDVIKHIMIKDFEAFRDRGVEFSKEGLGQNLFHA

DGETWRALRNRFTPIFTSGKLKNMFYLMHEGADNFIDHVSKECEKKQEFEVHSLLQTYTM

STISSCAFGVSYNSISDKVQTLEIVDKIISEPSYAIELDYMYPKLLAKLNLSIIPTPVQH

FFKSLVDSIISQRNGKPAGRNDFMDLILELRQMGEVTSNKYLDGVTSLEITDEVICAQAF

VFYVAGYETSATTMSYLIYQLSLNQDVQNKLIAEVDEAIKASDGKVTYDTVKEMKYLNKV

FDETLRMYSIVEPLQRKASRDYQIPGTDVVIEKDTMVLISPRGIHYDPKYYDNPKQFNPD

RFDAEEVGKRHPCAYLPFGLGQRNCIGMRFGRLQSLLCITKILSKFRIEPSKNTDRNLQV

EPRRVIIGPKGGIRVNIVPRKIVS

>AAT01211.1

MWVLYLPAVLSVLIVTLYLYFTRTFNYWKKRNVRGPEPTVFFGNLKDSTLRKKNIGIVME

EIYNQFPDEKVVGMYRMTTPCLLVRDLDVIKHIMIKDFEAFRDRGVEFSKEGLGQNLFHA

DGETWRALRNRFTPIFTSGKLKNMFYLMHEGADNFIDHVSKECEKKQEFEVHSLLQTYTM

STISSCAFGVSYNSISDKVQTLEIVDKIISEPSYATELDYMYPKLLAKLNLSIIPTPVQH

FFKSLVDNIISQRNGKPAGRNDFMDLILELRQMGEVTSNKYLDGVTSLEITDEVICAQAF

VFYVAGYETSATTMSYLIYQLSLNQDVQNKLIAEVDEAIKASDGKVTYDTVKEMKYLNKV

FDETLRMYSIVEPLQRKVTRDYQIPGTDVVIEKDTMVLISPRGIHYDPKYYDNPKQFNPD

RFDAEEVGKRHPCAYLPFGLGQRNCIGMRFGRLQSLLCITKILSKFRIEPSKNTDRNLQV

EPRRVIIGPKGGIRVNIVPRKIVS

>AAY21921.1

MWILYFPAVISVLIVTLYFYFTRTFNYWKKRNVRGPEPTVFFGNLKDSALRKKNMGVVME

ELYNMFPEEKVIGIYRMTSPCLLVRDLEVIKHIMIKDFEVFSDRGVEFSKEGLGSNLFHA

DGETWRAWRNRFTPIFTSGKLKNMFYLMHEGADNFIDHVSAECEKNQEFEVHSLLQTYTM

STIAACAFGISYDSIGDKVKALDIVDKIISEPSYAIELDMMYPGLLSKLNLSIFPTAVKN

FFKSLVDNIVAQRNGKPSGRNDFMDLILELRQLGEVTSNKYGSSASSLEITDEVICAQAF

VFYIAGYETSATTMAYMIYQLALNPDIQNKLIAEVDEVLEANDGKVTYDTVKEMKYLNKV

FDETLRMYSIVEPLQRKATRDYKIPGTDVVIEKDTIVLISPRGIHYDPKYYDNPKQFNPD

RFDAEEVGERHPCAYLPFGLGQRNCIGMRFGRLQSLLCITKILSKFRIEPSKNTDRNLQV

EPHRGLIGPKGGIRVNVVPRKLVS

>AAY21920.1

MWILYFPAVISVLIVTLYFYFTRTFNYWKKRNVRGPEPTVFFGNLKDSALRKKNMGVVME

ELYNMFPEEKVIGIYRMTSPCLLVRDLEVIKHIMIKDFEVFSDRGVEFSKEGLGSNLFHA

DGETWRALRNRFTPIFTSGKLKNMFYLMHEGADNFIDHVSAECEKNQEFEVHSLLQTYTM

STIAACAFGISYDSIGDKVKALDIVDKIISEPSYAIELDMMYPGLLSKLNLSIFPTAVKN

FFKSLVDNIVAQRNGKPSGRNDFMDLILELRQLGEVTSNKYGSSASSLEITDEVICAQAF

VFYIAGYETSATTMAYMIYQLALNPDIQNKLIAEVDEVLKANDGKVTYDTVKEMKYLNKV

FDETLRMYSIVEPLQRKATRDYKIPGTDVVIEKDTIVLISPRGIHYDPKYYDNPKQFNPD

RFDAEEVGKRHPCAYLPFGLGQRNCIGMRFGRLQSLLCITKILSKFRIEPSKNTDRNLQV

EPHRGLIGPKGGIRVNVVPRKLVS

>Q04552.1

MLYLLALVTVLAGLLHYYFTRTFNYWKKRNVAGPKPVPFFGNLKDSVLRRKPQVMVYKSI

YDEFPNEKVVGIYRMTTPSVLLRDLDIIKHVLIKDFESFADRGVEFSLDGLGANIFHADG

DRWRSLRNRFTPLFTSGKLKSMLPLMSQVGDRFINSIDEVSQTQPEQSIHNLVQKFTMTN

IAACVFGLNLDEGMLKTLEDLDKHIFTVNYSAELDMMYPGILKKLNGSLFPKVVSKFFDN

LTKNVLEMRKGTPSYQKDMIDLIQELREKKTLELSRKHENEDVKALELTDGVISAQMFIF

YMAGYETSATTMTYLFYELAKNPDIQDKLIAEIDEVLSRHDGNITYECLSEMTYLSKVFD

ETLRKYPVADFTQRNAKTDYVFPGTDITIKKGQTIIVSTWGIQNDPKYYPNPEKFDPERF

NPENVKDRHPCAYLPFSAGPRNCLGMRFAKWQSEVCIMKVLSKYRVEPSMKSSGPFKFDP

MRLFALPKGGIYVNLVRR

>AAA29790.1

MLYLLALVTVLAGLLHYYFTRTFDYWKKRNVAGPKPVPFFGNLKDSVLRRKPQVMVYKSI

YDEFPNEKVVGIYRMTTPSVLLRDLDIIKHVLIKDFESFADRGVEFSLDGLGANIFHADG

DRWRSLRNRFTPLFTSGKLKSMLPLMSQVGDRFIKCIDEVSQTQPEQSIHNLVQKFTMTN

IAACVFGLNLDEGMLKTLEDLDKHIFTVNYSAELDMMYPGILKKLNGSLFPKVVSKFFDN

LTRNVLEMRKGTPSYQKDMIDLIQELREKKTLELSRKHENEDVKSLELTDGVVSAQMFIF

YMAGYETSATTMTYLFYELAKNPDIQDKLIAEIDEVLSRHDGNITYECLSEMTYLSKVFD

ETLRKYPVADFTQRNAKTDYVFPGTDITIKKGQTIIVSTWGIQNDPKYYPNPEKFDPERF

NPENVKDRHPCAYLPFSAGPRNCLGMRFAKWQSEVCIVKVLSKYRVEPSMKSSGEFKFDP

MRLFALPKGGIYVNIVRR

>AAL93212.1

MWVLYLPAVLSVLIVTLYLYFTRTFNYWKKRNVRGPEPTVFFGNLKDSTLRKKNIGIVME

EIYNQFPDEKVVGMYRMTTPCLLVRDLDVIKHIMIKDFEAFRDRGVEFSKEGLGQNLFHA

DGETWRALRNRFTPIFTSGKLKNMFYLMHEGADNFIDHVSKECEKKQEFEVHSLLQTYTM

STISSCAFGVSYNSISDKVQTLEIVDKIISEPSYAIELDYMYPKLLAKLNLSIIPTPVQH

FFKSLVDSIISQRNGKPAGRNDFMDLILELRQMGEVTSNKYLGGVTSLEITDEVICAQAF

VFYVAGYETSATTMSYLIYQLSLNQDVQNKLIAEVDEAIKASDGKVTYDTVKEMKYLNKV

FDETLRVYSIVEPLQRKATRDYQIPGTGVVIEKDTMVLISPRGIHYDPKYYDNPKQFNPD

RFDAEEVGKRHPCAYLPFGLGQRNCIGMRFGRLQSLLCITKILSKFRIEPSKNTDRNLQV

EPRRVIIGPKGGIRVNIVPRKIVS

>AAA16154.1

MLYLLALVTVLAGLLHYYFTRTFDYWKKRNVAGPKPVPFFGNLKDSVLRRKPQVMVYKSI

YDEFPNEKVVGIYRMTTPSVLLRDLDIIKHVLIKDFESFADRGVEFSLDGLGANIFHADG

DRWRSLRNRFTPLFTSGKLKSMLPLMSQVGDRFIKCIDEVSQTQPEQSIHNLVQKFTMTN

IAACVFGLNLDEGMLKTLEDLDKHIFTVNYSAELDMMYPGILKKLNGSLFPKVVSKFFDN

LTKNVLEMRKGTPSYQKDMIDLIQELREKKTLELSRKHENEDVKALELTDGVISAQMFIF

YMAGYETSATTMTYLFYELAKNPDIQDKLIAEIDEVLSRHDGNITYECLSEMTYLSKVFD

ETLRKYPVADFTQRNAKTDYVFPGTDITIKKGQTIIVSTWGIQNDPKYYPNPEKFDPERF

NPENVKDRHPCAYLPFSAGPRNCLGMRFAKWQSEVCIMKVLSKYRVEPSMKSSGPFKFDP

MRLFALPKGGIYVNLVRR

>ADA68174.1

MSFLYIAAGLSALVYALYYYFTRTFNYWKSRNVPGPEPIPFFGNIKESVLRQKNIGIIIH

DIYKAYPNEKVVGLFRMTSPCLLIRDLDIVKHIMIKDFEVFTDRGVDFSKEGLGQNLFHA

DGETWTALRNRFSPIFTTGKLKNMFYLMNEGADSFVDYVTTECQTRQEFEVHLLLQSYTV

STISACAFGVSYDSLQDKMEALKIVDQVISSPSYIVELDFMYPGLLRSLNLSLFPKVVLH

FFANLVDNIISQRNGKPSGRNDFMDLILELRQKGEVESTKYGHSGKTLEITSDVIAAQAF

VFYVAGYETSATTMGYMLYQLALNPDIQKRLTEEIDESLKANNGQITYDTIKEMKYLNKV

FDETLRMYSIVEPLQRKAVREYKVPGTDLTIEKDTIVLVSPRGIHYDEKYYDNPEVFNPD

RFDPEVAGNRHPCAYLPFGIGQRNCIGMRFGRLQSQLCITKLLSKFQVEPSKNTARNLEV

EPHRGIIGPKGGIKLNVVPRKIKS

>AAM90316.1

MWVLYLPAVLSVLIVTLYLYFTRTFNYWKKRNVRGPEPTVFFGNLKDSTLRKKNIGIVME

EIYNQFPDEKVVGMYRMTTPCLLVRDLDVIKHIMIKDFEAFRDRGVEFSKEGLGQNLFHA

DGETWRALRNRFTPIFTSGKLKNMFYLMHEGADNFIDHVSKECEKKQEFEVHSLLQTYTM

STISSCAFGVSYNSISDKVQTLEIVDKIISEPSYAIELDYMYPKLSAKLNLSIIPTPVQH

FFKSLVDSIISQRNGKPAGRNDFMDLILELRQMGEVTSNKYLDGVTSLEITDEVICAQAF

VFYVAGYETSATTMSYLIYQLTLNQDVQNKLIAEVDEAIKASDGKVTYDTVKEMKYLNKV

FDETLRMYSIVEPLRRRATRDYQIPGTDVVIEKDTMVLISPRGIHYDPKYYDNPKQFNPD

RFDAEEVGKRHPCAYLPFGLGQRNCIGMRFGRLQSLLCITKILSKFRIEPSKNTDRNLQV

EPRRVIIGPKGGIRVNIVPRKIVS

>ABE60885.1

MWVFYLPVVISVLAVALYYYFTRTFNYWKNRNVRGPEPTVFFGNLKDSVLRRKNIGVVME

EMYNMFPDEKVIGIYRMTTPCVLVRDLDIVKHIMIKDFEVFSDRGLEFSKEGLGQNLFHA

DGDTWRTLRNRFTPIFTSGKLKNMFYLMNEGADNFIDHVSKECEKHQEFEIHTLLQTYTM

STISSCAFGVSYDTISDKLDTLAIVDKIISEPSYAIELDMMYPGLLPKLNLSIFPSVVHK

FFKNLVNTIVTQRNGKPSGRNDFMDLILELRQMGEITSNKYGNNMSTLEITESVMCAQAF

VFYIAGYETSATTMAYLTYQLALNPDIQNKLIAEIDEAIKANGGKVTYDTVKDMKYLNKV

FDETLRMYSIVEPLQRKAIRDYKLPGTDVVIEKDTVVLISPRGIHYDPKYYDNPKQFNPD

RFDAEEVGKRHPCAYLPFGLGQRNCIGMRFGRLQSLLCITKLLSKFRLEPSKNTDRNLQV

EPYRFIIGPKGGIRLNIVPRKDVS

>ABE60886.1

MWIFYFPAVISVLIVTLYLYFTRTFNYWKKRNVRGPEPVVFFGNLKDSALRKKNMGVVME

ELYNMFPEEKVIGIYRMTSPCLLVRDLEVIKHIMIKDFEVFSDRGVEFSKEGLGSNLFHA

DGETWRALRNRFTPIFTSGKLKNMFYLMHEGADNFIDHVSAECEKNQEFEVHSLLQTYTM

STIAACAFGISYDSIGDKVKALDIVDKIISEPSYAIELDMMYPGLLSKLNLSIFPTAVKN

FFKSLVDNIVAQRNGKPSGRNDFMDLILELRQLGEVTSNKYGSSASSLEITDEVICAQAF

VFYIAGYETSATTMAYMIYQLALNPDIQNKLIAEVDEVLKANDGKVTYDTVKEMKYLNKA

FDETLRMYSIVEPLQRKATRDYKIPGTDVVIEKDTIVLISPRGIHYDPKYYDNPKQFNPD

RFDAEEVGKRHPCAYLPFGLGQRNCIGMRFGRLQSLLCITKILSKFRIEPSKNTDRNLQV

EPHRGLIGPKGGIRVNVVPRKLVS

>ADH82407.1

MWVLYLPAVLSVLIVTLYLYFTRTFNYWKKRNVRGPEPTVFFGNLKDSTLRKKNIGIVME

EIYNQFPDEKVVGMYRMTTPCLLVRDLDVIKHIMIKDFEAFRDRGVEFSKEGLGQNLFHA

DGETWRALRNRFTPIFTSGKLKNVFYLMHEGADNFIDHVSKECEKKQEFEVHSLLQTYTM

STISSCAFGVSYNSISDKVQTLEIVDKIISEPSYAIELDYMYPKLLAKLNLSIIPTPVQH

FFKSLVDNIISQRNGEPAGRNDFMDLILELRQMGEVTSNKYLDGVTSLEITDEVICAQAF

VFYVAGYETSATTMSYLICQLSLNQDVQNKLIAEVDEAIKASDGKVTYDTVKEMKYLNKV

FDETLRMYSIVEPLQRKATRDYQIPGTDVVIEKDTMVLISPRGIHYDPKYYDNPKQFNPD

RFDAEEVGKRHPCAYLPFGLGQRNCIGMRFGRLQSLLCITKILSKFRIEPSKNTDRNLQV

EPRRVIIGPKGGIRVNIVPRKIVS

>ABC96862.1

MLYVITLVTVLAGLLHYYFTRNFDYWKKRNVAGPKPIPFFGNLKDSVLRRKHQVMVYKSI

YDEFPNEKVVGIYRMTTPSVLIRDLDIIKHVLIKDFESFADRGVEFSIDGLGANIFHADG

DRWRSLRNRFTPLFTSGKLKTMLPLMSQVGDKFIKYIDEVSQTKSEQSIHDLVQIFTITN

IAACAFGLNLDENMLKTLQDLDKYIFTVNYSGEFDMLYPGILKKFNGSIFPKVVKQFFDK

LTKDIFEMRKGTSSCQKDMIDSIQELRQQKTVDLWRKHDNEDVKPLELIDGVISAQMFIF

YAAGYETSATTMTYLFYELAKNPDIQDKLIAEIDEVLSRHDGNITYECLSEMTYLSKVFD

ETLRKYPVGDFTQRNAKTDYVFPGTDITIKKGQTVIISTWSIQNDPKYYPNPEKFDPERF

NPENIKNRHPCAYLPFSAGPRNCLGLRFAKWQIEVCVVKVLSKYRVEPSNKSSGEFKFDP

MRLFVLPKGGIFVNIVRR

>AAM90318.1

MWIFYFPAVISVLIVTLYFYFTRTFNYWKKRNVRGPEPVVFFGNLKDSALRKKNMGVVME

EMYNMFPEEKVIGIYRMTSPCLLVRDLDVIKHIMIKDFEVFSDRGVEFSKEGLGSNLFHA

DGETWRALRNRFTPIFTSGKLKNMFYLMHEGADNFIDHVSAECEKNQEFEVHSLLQTYTM

STIAACAFGISYDSIGDKVKALDIVDKIISEPSYAIELDMMYPGLLSKLNLSIFPTAVKN

FFKSLVDNIVAQRNGKPSGRNDFMDLILELRQLGEVTSNKYGSSASSLEITDEVICAQAF

VFYIAGYETSATTMAYMIYQLALNPDIQNKLIAEVDEVLKANDGKVTYDTVKEMKYLNKA

FDETLRMYSIVEPLQRKATRDYKIPVTDVVIEKDTIVLISPRGIHYDPKYYDNPKQFNPD

RFDAEEVGKRHPCAYLPFGLGQRNCIGMRFGRLQSLLCITKILSKFRIEPSKNTDRNLQV

EPHRGLIGPKGGIRVNVVPRKLVS

>AFP20590.1

MLLLYLPIVLTFFIYILYLYFTRTFKYWKSRKVRGPEPTVFFGNIKESALRQKNIGVIMQ

EIYNNYPNEKVVGVYRMTSPCLLIRDLEIVKHIMIKDFEVFGDRGVEFSKKGLGQNLFHA

DGDTWTALRNRFSPIFTTGKLKNMFYLMNEGANNFVEHISTECQKKQEFEIHSLLQTYTV

STISACAFGVGYDSLDDKLETLKLVDQIISAPTYANELDMMYPGLLTSLNLSIIPVPIQK

FFKNLVDNIIAQRNGKPTGRNDFMDLILELREMREVNYTKYGNFVKPLEITPEVMAAQAF

VFYVAGYETSATTMAYLMYQLALNQDIQNRLIAEIDEVIQRNTGEVTYDMIKEMKYLNKV

FDETLRMYSIVEPLQRKATRDYQVPGIDLVIEKDTIVLISPRGIHYDEKYYDNPDVFNPD

RFDPEEVGKRHPCAYLPFGLGQRNCVGMRFGRLQSQLCIVKVLSKFRVEPSKNTDRNLKV

TPHRAIIGPKGGIHLNIIPRKIKA

>O61387.1

MWVLYLPAVLSVLIVTLYLYFTRTFNYWKKRNVRGPEPTVFFGNLKDSTLRKKNIGIVME

EIYNQFPYEKVVGMYRMTTPCLLVRDFHVIKHIMIKDFEAFRDRGVEFSKEGLGQNLFHA

DGETWRALRNRFTPIFTSGKLKNMFYLMHEGADNFIDHVSKECEKKQEFEVHSLLQTYTM

STISSCAFGVSYNSISDKVQTLEIVDKIISEPSYAIELDYMYPKLLAKLNLSIIPTPVQH

FFKSLVDNIISQRNGKPAGRNDFMDLILELRQMGEVTSNKYLDGVTSLEITDEVICAQAF

VFYVAGYETSATTMSYLIYQLSLNQDVQNKLIAEVDEAIKASDGKVTYDTVKEMKYLNKV

FDETLRMYSIVEPLQRKATRDYQIPGTDVVIEKDTMVLISPRGIHYDPKYYDNPKQFNPD

RFDAEEVGKRHPCAYLPFGLGQRNCIGMRFGRLQSLLCITKILSKFRIEPSKNTDRNLQV

EPRRVTIGPKGGIRVNIVPRKIVS

>ACL77781.1

MSFLYITLGLSALVYAFYYYFTRTFNYWKSRNVPGPEPIPFFGNIKESVLRQKNIGVVIH

DIYKAFPNEKVVGLYRMTSPCLLVRDLEIIKHIMIKDFEVFTDRGVDFSKQGLGQNLFHA

DGETWTALRNRFSPIFTTGKLKNMFYLMNEGADSFVDYVSTECQSRQEFEVHLLLQSYTV

STISACAFGVSYDSLQDKMEALKLVDQVISSPSYIVELDMMYPGLLRSLNLSLFPKVVMK

FFENLVDNIITQRNGKPSGRNDFIDLILELREKKEVNSTKFGHSGKTLEITSDVIAAQAF

VFYVAGYETSATTMGYMLYQLAMNPDVQKKLTAEIDEVIQANNGQVTYETIKEMKYLNKV

FDETLRMYSIVEPLQRKAVREYKVPGTDLTIEKDTIVLVSPRGIHYDEKYYEDPEVFNPE

RFDPEVAGNRHPCAYLPFGIGQRNCIGMRFGRLQSQLCITKLLSKFQVEPSKNTARNLEV

EPHRGIIGPKGGIRLNVIPRKIKS

>ABC96866.1

MLYVITLVTVLAGLLHYYFTRNFDYWKKRNVAGPKPIPFFGNLKDSVLRRKHQVMVYKSI

YDEFPNEKVVGIYRMTTPSVLIRDLDIIKHVLIKDFESFADRGVEFSIDGLGANIFHADG

DRWRSLRNRFTPLFTSGKLKTMLPLMSQVGDKFIKYIDEVSQTKSEQSIHDLVQIFTITN

IAACAFGLNLDENMLKTLQDLDKYIFTVNYSGEFDMLYPGILKKFNGSIFPKVVKQFFDK

LTKDIFEMRKGTSSCQKDMIDSIQELRQQKTVDLWRKHDNEDVKPLELIDGVISAQMFIF

YAAGYETSATTMTYLFYELAKNPDIQDKLIAEIDEVLSRHDGNITYECLSEMTYLSKVFD

ETLRKYPVADFTQRNAKTDYVFPGTDITIKKGQTIIVSTWGIQNDPKYYPNPEKFDPERF

NPENVKDRHPCAYLPFSAGPRNCLGMRFAKWQSEVCIMKVLSKYRVEPSMKSSGPFKFDP

MRLFALPKGGIYVNIVRR

>AAB06741.1

MLYVITLVTVLAGLLHYYFTRNFDYWKKRNVAGPKPIPFFGNLKDSVLRRKHQVMVYKSI

YDEFPNEKVVGIYRMTTPSVLIRDLDIIKHVLIKDFESFADRGVEFSIDGLGANIFHADG

DRWRSLRNRFTPLFTSGKLKTMLPLMSQVGDKFINTSDEVSQTKSEQSIHDLVQIFTITN

IAACAFGLNLDENMLKTLQDLDKYIFTVNYSGEFDMLYPGILKKFNGSIFPKVVKQFFDK

LTKDIFEMRKGTSSCQKDMIDSIQELRQQKTVDLWRKHDNEDVKPLELIDGVISAQMFIF

YAAGYETSATTMTYLFYELAKNPDIQDKLIAEIDEVLSRHDGNITYECLSEMTYLSKVFD

ETLRKYPVGDFTQRNAKTDYVFPGTDITIKKGQTVIISTWSIQNDPKYYPNPEKFDPERF

NPENIKNRHPCAYLPFSAGPRNCLGLRFAKWQIEVCVVKVLSKYRVEPSNKSSGEFKFDP

MRLFVLPKGGIFVNIVRR

>Q95031.1

MWIFYFPAVISVLIVSLYFYFTRTFNYWKKRNVRGPEPTVFFGNLKDSALPRKNMGVVME

ELYNMFPEEKVIGIYRMTSPCLLVRDLEVIKHIMIKDFEVFSDRGVEFSKEGLGSNLFHA

DGETWRALRNRFTPIFTSGKPKNMFYLMHEGADNFIDHVSAECEKNQEFEVHSLLQTYTM

STIAACAFGISYDSIGDKVKALDIVDKIISEPSYAIELDMMYPGLLSKLNLSIFPTAVKN

FFKSLVDNIVAQRNGKPSGRNDFMDLILELRQLGEVTSNKYGSSASSLEITDEVICAQAF

VFYIAGYETSATTMAYMIYQLALSPDIQNKLIAEVDEVLKANDGKVTYDTVKEMKYMNKA

FDETLRMYSIVEPLQRKATRDYKIPGTDVVIEKDTIVLISPRGIHYDPKYYDNPKQFNPD

RFDAEEVGKRHPCAYLPFGLGQRNCIGMRFGRLQSLLCITKILSKFRIEPSKNTDRNLQV

EPHRGLIGPKGGIRVNVVPRKLVS

>ABC96865.1

MLYVITLVTVLAGLLHYYFTRNFDYWKKRNVAGPKPVPFFGNLKDSVLRRKPQVMVYKSI

YDEFPNEKVVGIYRMTTPSVLLRDLDIIKHVLIKDFESFADRGVEFSLDGLGANIFHADG

DRWRSLRNRFTPLFTSGKLKTMLPLMSQVGDKFIKYIDEVSQTKSEQSIHDLVQIFTITN

IAACAFGLNLDENMLKTLQDLDKYIFTVNYSGEFDMLYPGILKKFNGSIFPKVVKQFFDK

LTKDIFEMRKGTSSCQKDMIDSIQELRQQKTVDLWRKHDNEDVKPLELIDGVISAQMFIF

YAAGYETSATTMTYLFYELAKNPDIQDKLIAEIDEVLSRHDGNITYECLSEMTYLSKVFD

ETLRKYPVGDFTQRNAKTDYVFPGTDITIKKGQTVIISTWSIQNDPKYYPNPEKFDPERF

NPENIKNRHPCAYLPFSAGPRNCLGLRFAKWQIEVCVVKVLSKYRVEPSNKSSGEFKFDP

MRLFVLPKGGIFVNIVRR

>Q27664.1

MWIFYFPAVISVLIVTLYFYFTRTFNYWKKRNVRGPEPVVFFGNLKDSALRKKNMGVVME

ELYNMFPEEKVIGIYRMTSPCLLVRDLEVIKHIMIKDFEVFSDRGLEFSKEGLGQNLFHA

DGDTWRTLRNRFTPIFTSGKLKNMFYLMNEGADNFIDHVSKECEKHQEFEIHTLLQTYTM

STISSCAFGVSYDTISDKLDTLAIVDKIISEPSYAIELDMMYPGLLPKLNLSIFPSVVHK

FFKNLVNTIVTQRNGKPSGRNDFMDLILELRQMGEITSNKYGNNMSTLEITESVMCAQAF

VFYIAGYETSATTMAYLTYQLALNPDIQNKLIAEIDEAIKANGGKVTYDTVKDMKYLNKV

FDETLRMYSIVEPLQRKAIRDYKLPGTDVVIEKDTVVLISPRGIHYDPKYYDNPKQFNPE

RFFAEEVGKRHPCAYLPFGLGQRNCIGMRFGRLQSLLCITKLLSKFRLEPSKNTDRNLQV

EPYRFIIGPKGGIRLNIVPRKDVS

>ABB58823.1

MAIIYILLASVVLPLLLYLYFTRHFNYWKKRNVPGPKPVPLFGNLMESALRKKNIGIVFK

ELYENFPNEKVVGIYRMTTPCLLIRDLDVIKNIMIKDFDVFVDRGVELSKSGLGANLFHA

DGDTWRVLRNRFTPLFTSGKLKNMLHLMIERANKYIEHVEMLCDHQPEQDIHTLVQKYTM

ATIAACAFGLDIDTTDPNKDQLKTLEEIDRLSLTANFAFELDMMYPGVLKKLNSTLFPGF

VSRFFKDVVKTIIEQRNGKPTDRNDFMDLILALRQLGDIQATKRNSEDKEYSIELTDELI

EAQAFVFYIAGYETSATTMTFMLYQLALNPDLQDKVIAEIDQGLKESKGEVTYEMLQNLT

YFEKAFNETLRMYSIVEPLQRNAKIDYKIPDTDIVIEKGTTVLFSPLGIHHDEKYYPNPS

KFDPERFSPANISARHPCAHIPFGTGPRNCIGMRFAKIQSRVCMVKMFSKFRFELAKNTP

RNLDIDPTRLLLGPKGGIPLKIVRR

>Q27756.1

MLYVITLVTVLAGLLHYYFTRNFDYWKKRNVAGPKPIPFFGNLKDSALRRKPQVMVYKSI

YDEFPNEKVVGIYRMTTPSVLLRDLDIIKHVPTKDFEPFADRGVEFSLDGLGANIFHADG

DRWRSLRNRFTPLFTSGKLKTMLPLMSQVGDKFINTSDEVSQTKSEQSIHDLVQIFTITN

IAACAFGLNLDENMLKTLQDLDKYIFTVNYSGEFDMLYPGILKKFNGSIFPKVVKQFFDK

LTKDIFEMRKGTSSCQKDMIDSIQELRQQKTVDLWRKHDNEDVKPLELTDGVISAQMFIF

YAAGYETSATTMTYLFYELAKNPDIQDKLIAEIDEVLSRHDGNITYECLGEMTFLGRVFD

ETLRKYPVGDFTQRNAKTDYVFPGTDITIKKGQTIIVSTWGIQNDPKYYPNPEKFDPERF

NPENIKNRHPCAYLPFSAGPRNCLGLRFAKWQIEVCVVKVLSKYRVEPSNKSSGEFKFDP

MRLFVLPKGGIFVNIVRR

>ABB58822.1

MAIIYILLASVVLPLLLYLYFTRHFNYWKKRNVPGPKPVPLFGNLMESALRKKNIGIVFK

ELYENFPNEKVVGIYRMTTPCLLIRDQDVIKNIMIKDFDVFVDRGVELSKSGLGANLFHA

DGDTWRVLRNRFTPLFTSGKLKNMLHLMIERANKYIEHVEMLCDHQPEQDIHTLVQKYTM

ATIAACAFGLDIDTTDPNKDQLKTSEEIDRLSLTANFAFELDMMYPGVLKKLNSTLFPGF

VSRFFKDVVKTIIEQRNGKPTDWNDFMDLILALRQLGDIQATKRNSEDKEYSIELTDELI

EAQAFVFYIAGYETSATTMTFMLYQLALNPDIQDKVIAEIDQGLKESKGEVTYEMLQNLT

YFEKASNETLRMYSIVEPLQRNAKIDCKIPDTDIVIEKGTTVLFSPLGIHHDEKYYPNPS

KFDPERFSPANISARHPCAHIPFGTGPRNCIGMRFAKIQSRVCMVKMFSKFRFELAKNTP

RNLDIDPTRLLLGPKGGIPLKIVRR

>BAM18052.1

MYDAFPEEKVIGVYRMTTPCLLLRDLDVIKHIMIKDFDSFVDRGVEFSKSGLGMNLFHAD

GDTWRVLRNRFTPIFTSGKLKNMLYLMLGRGDIFVNYINEICNKRPTELETHSLVQKYTL

ATISACAFGIDLDSFTPENMKTLETVDKLIFTSSYAIELDYMYPGILKKLNSSLFPKKVT

DFFDGLVKTVVKLRDGMPTNRKDFMDLLLELKQQDKITGPKKLENEKEKIVELTESLIAA

QAFVFYAAGYETSATTMSFLLYELAKNPDVQEKAYEEIDTTLKKYNGEMTYEMLNELKYL

ENIVAETLRKYPLVEPLQRNAAIDYKIPGTEVTVKKGQTVLISPFGIHNDEKYYKNPEKF

DPERFNPENSKERHPCAYLPFGVGPRNCIGMRFAKLQSHVCIAKLLSNFRVTPSKKIYG

>AAD25107.1

MWILYLPIVIPVLLVTLYFYFTRTYWKKGNVRGPEPTVFFGNLKDSALRKKNAGIVMEEL

YNMFPDEKVIGIYRMTSPCLLVRDLDVIKHIMIKDFEVFSDRGVEFSKEGLGSNLFHADG

ETWRALRNRFTPIFTSGKLKNMFYLMHEGADNFIDHVSAECEKNQEFEVHSLLQTYTMST

IAACAFGISYDSIGDKMKALEIVDKIISAPSYAIELDMMYPGLLSKLNLSIFPNEVKNFF

KSLVDNIITQRNGKPSGRNDFMDLILELRQLGQVTSNKYGSSVSSLEITDEVICAQAFVF

YIAGYETSATTMAYLIYELALNPDIQNRLIAEIDEAVKANDGKVTYDTVKEMKYLNKVFD

ETLRMYSIVEPLQRKAIRDYKIPGTDVVIEKDTIVLISPRGIHYDPKYYDNPKQFNPDIF

DAEEVGKRHPCAYLPFGVGQRNCIGKGLDIS

>328712339

MTMFTASWWINVITPCTIIVTITYYFCVSTFKKWEKLNVPYIKPIPLFGNFLNVALGKNH

PLEFYNKIYHEFAGQKYAGVFQMRTPYLMVRDPEIINDVMIKNFSSFPDRGIYSDFVAEP

LTNNLLLMENPQWKIIRNKLTPAFTAGKLKTMYDQIKECGDELMKNIDIDLNRTSNEIEV

KDIMGKYSTDVIGTCAFGLKLNAINDDESPFRKYGKLIFKPSLRVLMRELCVMITPALLK

VVRLKKFPTAATDFFHAAFNETMTYRLENNIVRNDFVHYLMQARNDLVLNTDLPKHEKFA

ESQIVANAFVLFAAGFETVSSAISYCLYELALNKSIQDRVRKEIQLQLSKNNGQINHELL

IDLNYLDMVIAETLRKYPPLVALFRKASQTYRVPNSSLIIEKGQKIIIPIYAIHYDNKYY

SDPEKFIPERFSAEEKAKRPSGVYLPFGDGPRICIGKRFAEMEIKLAFVEILTKFEVFPC

EKTEIPLKYSKKVLTLVSKHGIWLRFKRIN

>328712337

MFTANWWINVITPCTIIVTIAYYFCVSTFKKWEKLNVPYIKPIPLFGNFLNVAVGKDHPL

EFYGKIYNEFAAHKYGGLYQMRTPYLMVRDPEIINDMLIKDFSSFPDRGIYSDFVANPLS

NGLFFMENPQWKIIRNKLTPAFTSGKLKTMYDQIKECGDELMKTIDIELIKNGKEIEVRD

IMGKYSTDVIGTCAFGLKLNAINDDESPFRKHGKSIFTPSLRSLFRELCLMVTPALLKVV

RVKDFPTDATDFFHAVFKETITYRLENKIVRNDFVQCLIQARNDLVLNADLPNHEKFTES

QIVANAFGMFAAGFETVSSTISYCLYELALNKSIQDRLRKEIQLKLSKNDGQINPEFLMD

LNYLDMVIAETLRKYPPLVALFRKASQKYRLPNDSLIIEKGQKIIIPIYALHYDNKYFTD

PENFIPERFSAEEKAKRPNGIYLPFGDGPRICIGKRFAEMEMKLAFVEMLTKFEVFPCDK

TDIPLKYSNNVITLVPKHGIWLTFKRIN

>328712335

MTMFTANWWINFITPCTIIVTIAYYFCVSTFKKWEQLNVPYIKPIPLFGNLLNVALGKDH

PLDFYNKIYHKFAAHKYAGVFQMRTPYLMVRDPEIINDMLIKDFSSFPDRGIYSDFVAEP

FSNHLFFMENPQWKIIRNKLTPAFTSGKLKMMYDQIKECSDELMKTIDIELIKNDDEIEV

RDIIGKYSTDVIGTCAFGLKLNAIKDDESPFRKHGKTLFEPSLRALFKELCLMIAPALLK

VIKVKDFPTDATDFLHTVFKETITYRQKNKIVRNDIFQCLIQVRNDLVLNADLSKNEKFT

ETQIVANAFAMFAAGFETVSSAISYCLYELALNKSIQDRVREDIELKLSNNDGQINHELL

IDLNYLDMVIAETLRKYPPVVALFRKASQTYRVPNDSLIIEKGQKIIIPIYALHYDSKYY

TDPEKFIPERFSAEEKAKRPSGIHLPFGDGPRICIGKRFAEMEMKLAFVEILTKFEVFPC

EKTEIPLKYSNKVFTLMPKHGIWLRFKRIN

>328712342

MYPSSSSATDWWIYIVTPCLVAVTITYYFCISTFNKWEKLNVPYIKPIPLFGNFLKVALA

KDHPLEFYDKIYYKFSGLKYGGLFQMRTPYLMVRDPEIINNMLIKDFSSFPNRGIYSDLA

ANPLSDNLFFMENPRWKTIRSKLTPAFTSGKLKIMYDQIKECGDKLMKNIDNDLKGKNDE

IEVRDIMGKYSTDVIGTCAFGLKLNSISDDESPFRKYGKSIFIPSLRTLFRELCLMVSPA

LLKVVRVKDFPTDATAFFNAAFKETITYRLENKIVRNDFVNCLMQARNDLTLNTNLPKHE

RFSESQIVANAFVMFAAGFETTSTTLSYCLYELALNIHIQDKVRQEIQLKLSKSDGQIDN

EFLMGLNYLDMVIAETLRKYPPLIALFRKASQTYRLPDNLILEKGQKIVIPIYSIHFDSK

YFEDPLKFNPERFSSEERAKRPNCVYLPFGDGPRTCIGKRFAELEMKLALVEMLTKFEVL

PCGKTEVPLKYSNKALTLMPKHGIWLRFKKIV

>ADL59603.1

MNLSSSTTDWWIYIASACLVGVTITYYFCISTFSKWEKLNVPYIRPIPLFGNFVRVALSK

DHPLEFYNKIYYKFAGLKYGGLFQMRTPYLMIRDPEIINNVLIKDFSSFPDRGIYSDLAA

NPLSDNLFFMENPRWKTIRNKLTPAFTSGKLKTMYDQIKECGDVLMTNIDKCLRGGNEEI

EVRDIMGKYSTDVIGTCAFGLKLNSISDDESPFRKYGKSIFIPSLRTLFRELCLMVTPSL

LKVVRVKDFPTDATDFFHSAFKETIAYRLENKIVRNDFVNCLMQARNELTLNANLPKEEK

FSESQIVANAFVMFAAGFETTSTTLSYILYELALNTSIQDKVRQEFQLKLSNSDGQIDNE

FLMSLNYMDMVIAETLRKYPPLIALFRKASQTYRLPDNLILEKGQKIVIPIYSLHFDDKY

FEDPQKFDPERFSPENKDKRPNGVYLPFGDGPRMCIGKRFAEMEMRLALLEMLSKFEVLP

CEKTEVPLKYSNKVLTLMPKHGIWLKFQKIA

>328718669

MFSFIFIWWINIITPCLFIFTITYYFCTLTYSKWEKINVPYIQPIPLFGNFLDVALGMQH

PIDFYRKIYYELAGYKYGGLFQMRTPYLMIRDPEIINNVLIKDFSNFPNRGIYSDFSANP

LSNQLFFMENPQWKIIRKILSPAFTSGKLKLMYDQIKECGDELMKNIHKNLTKTDNKMEV

RDILGKYSTDVIGTCIFGLKLNAVSDDNSTFRKYGKSLFLPSLRTHLRELSLMITPALLN

ILRFKDFPADATEFFHSAFHETITYREKNNIVRNDFVQTLIQARNDLVLNKNIPQRERFL

ESQIVANAFVMFAAGFETVSTAISFCLYELSLKKHIQDKVREEINLKLSKNNGLINNDLL

IDLNYLDMVLAETLRKYPPTFALFRKASQTYHVPNDSLTIEKDQKVIIPIYSLHYDPKYF

ADPEVFDPERFSPEEKSKRISGTYLPFGDGPRICIGKRFAELEMKLALVEILTKFETEPC

ERTEVPIRFSKKALITMPENGIWLTFKKITNQ

>326439076

MLIFANFWMDFIILVTVLFSIIYYYCTSTFNVWKKLNVPYVRPIPLFGNYLKVALGIENP

METYKNIYYELAGFQYGGMFQMRTPYLMIRDPEIVNNILIKDFSYFTDRGIHVDFKAEPL

SEVLFLMENPRWKKLRSKLSPAFTSGKLKQMYSQIEKCGQDMIINIFAELKKNPNEIDIR

DILAKYSIDVIGSCAFGLALNVASDDTSLFRSYGKTAFGPSIFYFIREICVMISPAILKI

VRLPFFPNKTTAFFGSVFKETMMYREKNNVVRNDIVHALIQAHQANENSSKDEIFTESQI

LSNAFGFFAAGFDTTSTSLSYCLYELALKKTIQDRVRDEIKLTKSKYNGVIDNDFLNDLN

YLDMVIAETLRKYPLLFALFRVATKTYRVPNDSLIIEKGQKIIIPTFSLHFDPRYFSDPE

VFNPERFSTKEKAMRPNGVYLPFGDGPRLCIGKRFAEMEMKLALVEILSKFEVEPCEKTE

IPIQFSKLSVVVIPKDEKILLKLNPLSE

>328705783

MLIFANFWIDFIILITVLFSIIYYYCTSTFNVWKKLNVPYIRPIPLFGNYLRVALGIENP

METYRKIYCELAGFKYGGMFQMRTPYLMIRDPEIINNILIKDFSYFTDRGIYVDFKTEPL

SEVLFLMNNPRWKKFRSKLSPAFSSGKLKQMFNQIEKCGHDMINNIFAELKKNPNDIDMR

DVVAKYSMDVIGSCAFGLTLNVASDDTSLFRKYGKTAFGPSILYFIREICVMISPAILKI

LRLTFFPSKTTAFFGSVFKETKTYREQNNVLRNDIVHALIQAHQSDENSSKDETLMESQI

LSNAFGFFAAGFDTTSTSISYCLYELALKKNIQDRVREEIKLTKSKYNGVIDNEFLNDLH

YLDMVIAESLRKYPLMFALFRVATKTYRVPNDSLIIEKGQKIIIPTFSLHYDPKYFSDPE

VFNPERFSPKEKAMRPNGVYLPFGDGPRLCIGKRFAEMEMKLALVEILSKFEVEPSEKTM

IPVQFSKLSVVVIPRDEKILLKLNPLSE

>328705779

MLIFANFWIDFIILITVLFSIIYYYCTSTFNVWKKLNVPYIRPIPLFGNYLRVALGIENP

METYRKIYCELAGFKYGGMFQMRTPYLMIRDPEIINNILIKDFSYFTDRGIYVDFKTEPL

SEVLFLMNNPRWKKFRSKLSPAFSSGKLKQMFNQIEKCGHDMINNIFAELKKNPNDIDMR

DVVSKYSMDVIGSCAFGLTLNVASDDTSLFRKYGKTAFGPSILYFIREICVMISPAILKI

LRLTFFPSKTTAFFGSVFKETKTYREQNNVLRNDIVHALIQAHQSDENSSKDETLMESQI

LSNAFGFFAAGFDTTSTSISYCLYELALKKNIQDRVREEIKLTKSKYNGVIDNEFLNDLH

YLDMVIAESLRKYPLMFALFRVATKTYRVPNDSLIIEKGQKIIIPTFSLHYDPKYFSDPE

VFNPERFSPKEKAMRPNGVYLPFGDGPRHCIGKRFAEMEMKLALVEILSKFEVEPSEKTM

IPVQFSKLSVVVIPRDEKILLKLNPLSE

>328720616

MSASQLLVDLAAGWWTVAVLALLAATVYHFCTSTFGYWRDRGVPYVRPTVPLFGNIGGLA

LGVEHQARMFGRIYDGFRGQRYGGFFQMRTPHLMVCDPALVNRVLIGDFAHFTDHGMYTA

GPDENPLANGLFNMNGAQWKIMRQKLSPVFTAGKLRHMRGQVTECSEQLMRNVAADVPTG

GGQMEIRDVLGKYSTDVIGTCAFGLHLNAINDERSSFRKHGKAVFAPSFRVLLKELAWMV

TPALRRALRIGDMPPDAAQFFTAAFTDTMKYREEHGIVRDDFMQSLIQARTDLVVNKTEP

SVEFLETDIVANAFILFAAGFETVSTAMSFCLYELALKKPIQDKVREEMNTTKKKHNAEI

DNDFLKDLHYLEMVLAETLRKYPPLLTLFREATQDYQVPDDTFVIEKGTKVLIPAYAIHH

DYRYYPDPETFDPERFSPEEKAKRPNGTYMPFGDGPRLCIGKRFAEMEMKLALTELLTTY

EVEPCEKTDIPMRFSKRSLIITPENGIWLKFKPIHTSK

>193713791

MIDVISCSIIGLLSSVYILYATVFLSIAYYLCTSTHDKWRKLNVPYTKPLPLFGNSMNLV

LAREHPMDFFTGLYNRFPDEKLCGFYQMTTPFLMIRDPKLINNIMVRDFSYFTDHGFDTD

PSVNILANSLFMLNGDRWRTMRQKLSPGFTSGKLKDTHDQIKECTDQLINIVDDNLKVSD

HFEIRELVGNFSTDVIGMSAFGLKLDTIRNGNLDFRKFGKKIFQSDFKQLFVQAMMLFCP

KLVTILKLKQFPDDAADFYGSMFRDVLEYRDRNNVIRNDVTQTLIQAKKDLVTNNDGDDS

TSKNKWTEMDIVGNAILMFVAGAETVSITICFCLYQLALNKDIQDKLREEIVTTNAKHGG

QLNNDFLTNLHYMNMVLEEVSRMYSITMILFRQATKNYEVPGQSLVIEKGQKIIIPAYCI

HNDPKYYPNPGTFDPERFSTEEKAKRLNGTYIPFGDGPRLCIGKRFAELEMKLVLSKILL

KYEVLPCEKTEVPINIRGAGSIVNPKNGVWLSFKPIVAN

>193671582

MISCLIYVLFGTPAIAAVAVLAAILYYYTTNTYDKWLKLKVPHDPPWPLVGNTAKMMTLI

EHQLTTIDGIYKRFSGEKYCGFYQMKTPFLMIRDPELINNILIKDFSNFADRGFHKDPAL

NIIANGLFFMEGPKWKMMRQKLSPGFTSGKLKLAHNQIAECSDELMRFIAAKMKENDQIE

VKETMSKYSTDVIGTCAFGLKLDTVKNEGSDFRLYGRKILKLSFRFLLAEMVSPKILKLL

GVAEFPPDASAFYESAFKEVIRYREENGIVRHDVAQSLIEARKELVLDSTDENGFTEQHI

IANAILMFLAGFETVSSTLSFCLYHLALNQDVQEKIRDEMNSKLKQHGKINNDFLVNLHY

TDMVLAETERMYVVTNALFREAVKTYHVPGDTLVIEKGTKIMIPIYSIHHDPTYYPEPYI

FDPQRFSPEEKAKRQSSTYLPFGDGPRFCIGKRFAELEMKMVLSQIITTFRILPCEKTEV

PLKLQNGLPMMVAKNGIWLRFQSISE

>193587097

MISFMTDWLHDNVTCLSLIAVLASFYYYSTSTYGKWRILNIPYVPPVPLFGNTTRMMLRL

EHPIDMFERFYNSFPDVKLFGFYQMRDPVLLVRDPELINAILVKDFSYFTDHGIDLDSST

SVLANSLFFANGQKWRTMRQKLSPGFTSGKLKDTHGQINECSDEMVSGIVESIKKKTDQI

DVKTITGGFSTDVIGTCAFGMKLDTIKNDDSDFRRYVKIMFQSTPKQMIVQVLLMICPWV

IKVLKINMFSVEATNFFHNVFTDVFKYREEHNVIRNDLTQTLMQARKELVLKENSSIEDK

FTDADIIGNAILMFTAGSETISSMLSFCLYELALNIEIQDRLRSEICSMKAKHDGHLNND

YLMDLYYTNMVLEETARKYSIAFNLMRVATKTYTLPDESFVIEKGQKLIIPMFSIHRDPK

YYPDPLRFDPERFSTEQKSQRPNGIYMPFGDGPRLCIGKRFAESEMKLVLSNVLSKFEVL

PCEKTEIPVNIRSMSGFITPKNGIVLKFRPIVEH

>328723999

MISYLTNLLFDYIFLSLIIVCTFLYYYTTSTYDTWRKLNVPFAKPVPFFGNIFKMFTGLE

RQVDAFGRIYQQFPDEKFCGFYQMSTPFLMLRDPELINTVIIKDFSYFTDHGIDMNPSVN

VMARSLFFATGQKWKTMRQKLSPGFTSGKLKGTHEQIRECSDQLTNCIYEKSQKTDAIEV

YELVGNTATDVIGTCAFGMKLDTINNDNSSFRQNVKKVFKPSGKVIFAQILGVLFPKIVK

FLKLQTSPVDVDAVNFFHSVFGEVIEYRTKNDVVRNDLTQTLMKARQDLVVSSDYKGEEK

YCELDIIANAMLLFTAGSETVTATASFCFYELALNKVIQDRLRDEIISSKIKHGGQLNNE

FLEDLHYADMVLDETHRKYTIITALLRGATQNYNVPGESLLIEKGQKILIPIYSIHHDPK

YYPNPETFDPERFTAEEKSKRPNGTFLPFGDGPRHCIGKRFAELELKLILSKILTKFEIS

PCEKTEIPLQMNKERGITSPKNGIWLNFRPIVE

>193589574

MFEFVYELFDLKMLLVTAFLGAIYVYSTWTHSHWSKLGISSPSAPVPLFGHAMPSMLGQM

HFMDVLHNLYKELGDQRFGGIYTMRTPQLLVKDPELIGHILIKDFNNFTDRGLYAGTHTN

PLNNNIFFTRGERWKTMRQKLSPTFTANKLKYMNEQVKECSDGLLSTIGKNLDDDAGRIE

IREMMAKYSTDVIGSCAFGLKLDAINDPDSEFRKHGKTVFQPSLRSKIRVAVIFMQPSLL

SIFRVHHYSHRTIRFFHDAFQQTIEYREKHNEDRKDFVQHLMKAREDLVLNPNLKPEEKF

TEMDIVANAYILFIAGFETVSTSMSFCMYELALRKDVQDKVRKEILEVKSKYNGQMNSEC

LNELHYMGMVIKETLRKYPPLVTLNRVVTKPYVIPGTQIKLKIGTKIVVPVHAIHYDPKY

YSDPEAFEPDRFSDENIHNIQPNTYMPFGDGPRFCIGKRFAEFEMKMALSEVLTNYEVMA

CDKTQIPIKYVIGSFVNIPESVWLKFRKVNT

>193636641

MISWMFNCLIDSFTLICTTVIGLLFYYYSTSTYKKWRKANVPHTKPVPFFGNFFRSTLGF

ETINDTYHNIYKQFPDKKFCGFYQMRTPTLMIRDPELINNVLIKDFSHFTDHGLDMDPSV

NFLASSLFFTRGQKWKIMRQKMSAGFTSGKLKLMHSQIKDCSKEMIDYIDRKSKTTDQFD

MHDIMNKYATDVIGTCAFGLKLGSMKDEDNEFRKFTKLLFKPSFRLIFTNILSLISPKTS

NILKIKTSSPEVMEYFTTSFQNVIEYREKNNMDRNDVAQTLMRARKELKFTEMDIISNAI

LMYLAGAEPVSDTLGFCLHELAINKHVQDKLRKHINTKRKEHGGEFTNDYLMDLHYADMV

LTETLRKCNGTIVLFRKATKAYQVPDSSLVIEKGQQIIIPTYSIHHDPKYYTNPDVFDPE

RFSPEEKSKRPSSTELLFGDGPRFCIGKRLAELEMKLGLSEIISKFEILPCEKTENPVQL

ANAGGAIKPKNGIWLI

>328725226

MMYFLTDWLLDNFTYLSLIAVFTGFYYYSTSTYGKWQKLNIPYIPPVPLFGNAFRMVTKL

ECPMDMYDRLYKQFPDVKLLGFYQMTEPMLLIRDPELINAILIKDFPYFTDHGFVMDPST

TVMAKSLFFSNGQRWRKMRQKLSPGFTSGKLRDTYLAINECSNQMVSSIVEKLGKTDRLA

IRSIISGFSNDVIGMCAFGIQLDSMNNEDSDFRRYSERIFEKTTKQIIVQAVTTIFPFVI

NLFKIQMFSAEATNFFRKVFADVINYREKNNIVRNDLTQTLLQARKELVLKENSTAEDQF

TDDDIIGNAIVLFAAGAETISSIVSFCLYELALNKEIQDKMRAEICSMKAKHDGQFNNDF

LMDLRYTNMVLEETGRKYSIASILMREATKTYTLPDESFVIEKGQKLIIPMFSIHRDPKY

YPDPLIFDPERFSKEQKSQRPNGIYMPFGDGPRMCMGKRFAELEMKLVLSNVLSKFEVLP

CEETEIPLEITDETGVIAPKRDLVLKFRPIIED

>328701135

MISFLIDCLVNNVTCLSLIVIFTGSFYYYSTSTYNKWRKLKIPYVPPVPLFGNTFRMLAR

LEHPIDTFDKIYNHFPDFKLFGFYQMREPMLLVRDPELINMILVKDFLYFTDHGVDIDPS

MSTLAKSLFFANGQKWRTMRQKLSPGFTSGKLKGTYCQINECSDEMVSSIVEAIGKKTDR

IELKTITGRFSTDVIATCAFGLKLDSIKNGDSEFRRYVKILFQTTTKQAIILILSLICPR

VVKILRLQFFSLEATNFFSKVFADVIKYREDHNVSRNDITQTLIEARKELVLKEISTTED

KFTDDDIIGNAIFLFSAGSETISSLVCFCLYELALNKEIQDKLRAEIYSMKAKHNGKLNN

DYLVDLRYTNMVLEETGRKYSIAFNITRVATKTYTLPDESFVIEKGQKLIIPMFNIHRDP

KYYPDPLRFDPERFSMEQKSQRPNGTYIPFGDGPRLCIGKRFAEAEMKLVLSKVLSKFEV

QPCEQTEIPLDIRSGSGLLSPKNGLVLKFKPIIEH

>328725975

MLIFANFWIDFIILITVLFSIIYYYCTSTFNVWKKLNVPYIRPIPLFGNYLRVALGIENP

METYRKIYCELAGFKYGGMFQMRTPYLMIRDPEIINNILIKDFSYFTDRGIYVDFKTEPL

SEVLFLMNNPRWKKFRSKLSPAFSSGKLKQMFNQIEKCGHDMINNIFAELKKNPNDIDMR

DVVAKYSMDVIGSCAFGLTLNVASDDTSLFRKYGKTAFGPSILYFIREICVMISPAILKI

LRLTFFPSKTTAFFGSVFKETKTYREQNNVLRNDIVHALIQAHQSDENSSKDETLMESQI

LSNAFGFFAAGFDTTSTSISYCLYELALKKNIQDRVREEIKLTKSKYNGVIDNEFLNDLH

YLDMVIA

>CAH65681.2

MNAFGYEVSWFDIFFLVVSLVSIIYYFGINTFSHWEKKGIKFMKPVPFFGNTLDLAIGRQ

HITTCHQVIYDYFADDAYGGWLANRSPLLMVKDPELIQQILVKDFVHFYDRGTTVDMKLD

PLNANLLNMTGQRWKALRQKLTPAFSSGKLKLMSPQFNECVDDLCGLIDEKSNKKELIDV

QESMSKLATDVIGSCAFGLQFNSLKNPESEFRRMGRDILRPSWRFKVRTFMRVISDSLPS

LLGVKAFDKSKEDFFINLVNDTMKYREDNKVERNDFIQILMNLKKIDENMEIDPNNESHV

ILDDKLLAANTFIFFIAGFETTATTLTFSMYELAVNQEIQDKLRQEVQTTFEKYGAINYD

STKDMDYLDRVISETLRKYPIAGSLIRRCTKAWQVPGAKGKLEVGDRVVIPVYPIHHDPK

YYPDPQKFDPERFTPENKRSRPPCTYMPFGDGPRICIGARFALQELKTTLSSILLHYKLT

LNEKSKATLPLKMKPRRILMQSEQEIYINFTKIK

>193718347

MSGVWSIPFVQLCAAAVLLVTFLGYMYLTYHYGKWTGLGVPHAAPSPPFGSLRDVVMGRV

PLVDAIHSLYRRFDGQRYFGIYEGRQPLLVVCDPQLVHTIMVKDFRSFVDRNAGKVSFVH

DKLFDHLVNLRGEQWKAIRAKLSPTFSAAKLKSMLGDINVCTARLIDNLNGQITKNSGIV

DVSEASAQFTTDTIGSCAFGLDCNALSNPDSEFRRTGRAIFTPSLRSNLLNITRLVGFGR

LLDVFRIRGMSGNIYDFFDNLLDTTMEQHKSGENTRNDFIALLVKLKDEEKQKEHGQKLF

TDDILAANSFVFFVAGFETTASTISYCLYELAMNPEIQVKLRENIKKTLDANDGKLAYDT

LKDMKYLDMVINETFRLHPPVPVLNRVCTQKYTITDSNITLNVGDKLIIPTYSLHHDSKY

YSDPEIFDPERFTEENISSRPHGTFLPFGDGPRICIGLRFAMMEAKTGLAEILSKFEVSP

CKETQTPIKIKPRSILLTPNESIRLSFKSIDQ

>328724563

MICFSCWLEIVPIAAIASALLTYVYCTRYYGHWTALGVPHTKPAPLLGHFAGPTMGRESG

TITVDTLYRRFVGHRYFGVYQLRHPMLVVRDPVLVHAVLATEFGSFHDRVMSRTSFEHDG

LFNSLVNLRGDKWKAVRAKLSPTFTVAKLKAMFASLHVCTGQLTDKLLLLTSGGQGIVNV

TDVSSKFTIDTIGRCAFGINCNTLFDSNTEFQRAGQAVFTPTLKSSVLNFMRLIDLGWLV

DLFRLRSMPDLVYEFYLNLFQDTLELRKNEKEDRNDFVSILVKLRNDEKINNSRVELFTD

DVLASNAFIFFAAGFETTASAMSYCLYELALNQDIQVELRKQIQHTLNENGGILTYDVLK

DMKYLDMVLNETLRMHPPGPGLLRVCTKKFKIPDSDITLDTGMKVLIPTYSLHHDPAYYP

NPELFDPLRFTEDNKALRPNGTFLPFGDGPRICIGLRFALMEAKTGLAEIISKFEIFPCK

YTKIPIKLNPRSILLTPNEPISLLFKQIA

>383851580

MALLTASWAVDGILLLMPFTAFLYYLLTRNYKYWAKRGVMEIPRTPLLGNFGDCMLGRTS

PSEYLQGLYNKSKGLPYMGFYIFNKPYFLARDPNLVKHILVKDFNIFGDRHGTADDTHDR

LGYANLFLIKNPEWKMLRTKLTPIFTTGKLKRMFDLMLLIAKDLEQHLDARNLEGDGKTI

ELKDLCANFTTDMIASTAFGLRVKSLEDPKAQFREVGREIFGYDLRRSLEFIIIFFLPEY

IKYTRPKFFGKNASDFLRNVFWDVINERINSKQKRNDLIDLLIELRQKHGNDKDMEGFDF

NGDDLVAQAAVFFTGGFETSSTTMSFTLYELALQPDIQKTLRNEIHEALEESDGKITYEM

VMTLPYLDMVISETLRKYPPLAFLDRVTSEDYKVPNSDLVLEKGTPVYIPMMGIHRDPEY

YPDPDKYDPLRFTEENKQKRPNFTYFPFGEGPHICIGSRLGLMQSKLGIVQVIKDYEVMP

CDKTTVPMVLDPRGLTTTARDGLYVKFRKLTTAAE

>380027414

MTLLTPYWSLDILIVSSSLMIAAYLFMTRKFKYWSKKGVMEITPTPFLGNFGDCLLLKKS

ASEFIKELYEQSEGLPFMGFYIFDKPFLLVRDPELIKHILVKDFNIFADKFASTDSKNDR

IGYSNLFIMKNPAWKYLRTKLTPIFTSGKIKKMFDLMLMISKNLENHLESLNLDGNGKVI

EFKDLCANFTTDLIASTAFGVNLNSLKDPNADFRVYGRIIFDYNIKRAIDFLSIFFAPYL

TKYLSVKFFGKATNLLRNAFWNVINERIESNVKRNDLIDCLIELREKHKDEQIHDFKFDG

DDLVSQAGVFFTGGFETSSTTISFTLYELALNQEVQKTLRTEIHEALTQTDGKITYDMIT

NLPYLDMVISETLRKYPPLGFLDRVALQNYKIPNSDVTLEKGTPIYIPMIGLHYDPKYFP

NPEKYDPLRFSENVKKSRPNFVYMPFGEGPHICIGMRLGLLQSKLGIIEILKNYEITPCE

KTRIPMVLDPKGLTTTALGGLYLNIRKITIAGG

>340722611

MALLTPYWGLDGILILSSLMVCAYLFVTRKFNYWSKRGVKELAPTPFVGNFMDCILSRTS

ASEFVRDLYNYGEGLPFLGFYIFDKPYLLVRDPELVKHVLVKDFNYFADRYASADEKNDR

LGYANVFMMKNPEWKSLRAKLTPIFTSGKLKKMFELMQIVADDLGKHLDSLHLEGEGKVI

EFKDICANFTTDMIGSTAFGLKVDSLENPKAPFREYGRKIFDYDFYRNMEFLTIFFFPGL

VKYLKPKFFGKKATNFLRSLFWDVIEQRVGSGQKRNDLIDLLIEMREKYRNDESLKDYKF

EGDDLVSQAAIFFTGGFETSSTTMSFTLHELAMNQDVQKTLRAEIHDALAKTDGKITYDM

LMTLPYLDMVISETLRKYPPLAFLDRVTLADYKMPNSDLVLEKGTPIFISMMGSHYDPRY

FPNPEKYDPLRFTDEAKSARPNFVYFPFGEGPHICIGMRLGLMQSKLGVVQVLKDYEISP

CEKTKSPVILDAKALTTTALGGLYLNIRKTTTAAG

>328724565

MICFSCWLEIVPIAAIASALLTYVYCTRYYGHWTALGVPHTKPAPLLGHFAGPTMGRESG

TITVDTLYRRFVGHRYFGVYQLRHPMLVVRDPVLVHAVLATEFGSFHDRVMSRTSFEHDG

LFNSLVNLRGDKWKAVRAKLSPTFTVAKLKAMFASLHVCTGQLTDKLLLLTSGGQGIVNV

TDVSSKFTIDTIGRCAFGINCNTLFDSNTEFQRAGQAVFTPTLKSSVLNFMRLIDLGWLV

DLFRLRSMPDLVYEFYLNLFQDTLELRKNEKEDRNDFVSILVKLRNDEKINNSRVGNLQE

RHFIDVLASNAFIFFAAGFETTASAMSYCLYELALNQDIQVELRKQIQHTLNENGGILTY

DVLKDMKYLDMVLNETLRMHPPGPGLLRVCTKKFKIPDSDITLDTGMKVLIPTYSLHHDP

AYYPNPELFDPLRFTEDNKALRPNGTFLPFGDGPRICIGLRFALMEAKTGLAEIISKFEI

FPCKYTKIPIKLNPRSILLTPNEPISLLFKQIA

>350418637

MALLTPYWGLDGILILSSLMVCAYLFVTRKFNYWSKRGVKELAPTPFVGNFMDCILSRTS

ASEFVRDLYNYGEGLPFLGFYIFDKPYLLVRDPELVKHVLVRDFNYFADRYATADEKNDR

LGYANVFMMKNPEWKSLRTKLTPIFTSGKLKKMFELMSVVADDLGKHLDSLHLEGEGKVI

EFKDICANFTTDMIGSTAFGLKVDSLENPKAPFREYGRKIFDYDFYRNMEFLTIFFFPGL

VKYLKPKFFGKKATNFLRSLFWDVIEQRISSGQKRNDLIDLLIEMREKYRNDESLKDYKF

EGDDLVSQAAIFFTGGFETSSSTMSFTLHELAMNPDVQKTLRAEIRDALAKTDGKITYDM

VMTLPYLDMVISETLRKYPPLAFLDRVTLADYKMPNSDLVLEKGTPIFISMMGSHYDPRY

FPNPEKYDPLRFTEEAKNARPNFVYFPFGEGPHVCIGMRLGLMQSKLGVIQVLKDYEVSP

CEKTKTPVVLDPKALTTTALGGLYLNITKSTTAAG

>ADY62482.1

MNLLTPYWSLDILIVSSSLMIAVYLYASWKLKYWSRRGIMQITPSPLFGNFKKCILFQKS

VSEIIRELYGQNEGLPFMGFYIFYKPFFLVRDIELVKHILVKDFNTFANKHTSADSKNDR

IGYSNLFIIKNPAWKYLRGKLTSVFTSGKLKKMFDLMLIIGKNLEKHLELLNLDGNGKEV

ELKDLCANFTTDLIGTTAFGVNLNSLKDPNSDFRENGRLVFDYNLKRAFEFFSIFFFPNL

NKYFSVKFFGKATDYFRNSFWSVINQRIESNVKRNDLIDCLIELREKHKNDESFEGFRFD

GDDLVSQAAIFFTGGFETSSTTISFTLYELALNKDIQKTVRTEIHEALAQTDGKITYDMI

TNLPYLDMVVSETLRKYPPLGFLDRVALHDYKIPNSDVTIDKDTPVIIPMIAFHYDPKYF

PNPEKYDPLRFSEEVKKTRPSYVYMPFGEGPHICIGMRLGLLQSKLGIIEILKDYEVSPC

EKTKIPMVLDPKGLTTTALGGLYLNIRKITIAAG

>328703261

MFPAVVIIVACCTTVILFLYKYTTYTYKYWKSKSVTFATPVPLFGNIKDHVTLKMTQGEC

LKNIYNDFPREKFVGMYQLQTPTLLLRDPETIRLFLVKSFAHFTDRGFSYDGHREPLTKH

LVNLEGDTWKILRQKLTPTFSSGKIKSMLGLLQGCGVQLIEYMDATIESGKTEFEIRDLT

AKFTTDVIGTCAFGLECNSLKDSQSEFRRMGCAVLNSSASLALAKMVRVFFPKLFKALKL

RTFPAEVQQFFMGIVKQTIDFRNTNRVRRNDFIQLLLEIKNQNHNQENAIKSIELTEELI

AAQVFVFFLAGFETSSTTLSFCLHEMAVNQDIQNRVYDEINETANMYGLPFSYEAISSMN

YLEQCLKETMRKYPPVQALARVCTKQFRVPGTDLDLDVGTAVLIPVYAIHHDPQYYPEPD

TFNPDRFAKDGDGGGGDNGRPSGVFLPFGDGPRICIGMRFAMLEMKLALAQFLHRYLVTL

SDKSCTRIEFEPASFLSCPKGGIWLNVNKRKA

>350418640

MALLTPYWGLDGILILSSLMVAAYLLVTRKFNYWTKRGVKELPPTPFVGNFMDCLLLRKS

ASEFVRDLYNYGEGLPFLGFYIFDKPYLLVRDPELVKHVLVKDFNYFADRNATADEKNDR

LGYANVFLMKNPGWKSLRTKLTPFFTSGKLKKMFELMLVVADDLAKHLDSLHLEDEGKVV

EFKNICANFTTDMIGSTAFGLRVGSLENPKAPFREYGRKIFDYDFYRSMEFLSIFFFPGI

VKYFKPKFFGKKATKFFRSVFWDVIEQRISSGQKRNDLIDLLIEMREKYKDDESLKDYKF

EGDDLVSQAAIFFTGGFETSSSTMSFTLHELAMNPDVQKTLRAEIRDALAKTDGKITYDM

IMTLPYLDMVISETLRKYPPLAFLDRITLADYKMPNSDLVLEKGTPIFLSMMGSHYDPQY

FPNPEKYDPLRFSEEAKHSRPNCVYFPFGEGPHICIGLRLGLLQSKLGVVQVLKDYEVSP

CEKTKTPVVLDPKALTTTSLGGLYLNIRKITTAAG

>383851651

MALLTASLILDLLIVVASISAYIYYRLTRNVKYWEKRGVLEIPRSLLFGNFADCLLGKIA

PCEYLQDLYNKSKGLPYMGFYIFNKPYFLARDPELLKHILVKDFNVFADRNATADAERDR

LGHANLFMIKNPTWKPLRTKLTPIFTTGKLKKMFDLMLLIAKDLETCLNNMNLEGDGKIV

EVKDLCANFTTDMIGSTAFGLQVGSLLKPNVPFRDCGRKMFDFNLRRGLELTVVFFLPEY

VKYTRVKVFGEDTSKFLRDVFWQVINERVKSQEKRNDLIDVLIELRRKHEHDTDMAGFQF

NGDDLVSQALIFFTGGFETSSTTMSFTLYELALHPEIQQTLRSEILQALEESGGKITYEM

ITTLPYLDMVISETLRKYPPLAFLDRVTSQDYKVPNSDLVLEKGTPVFIPMMGIHRDPEY

YPDPDKYDPSRFTEENKQKRPNFTYFPFGEGPHICIGSRLGLMQSKLGIVQVIKDYEVMP

CDKTTVPVVLNPRGLTTTARDGLYVKLRKLTTAAG

>EFN71133.1

MAFITTYWGLDGMIALTVLIITAYLYTTRKFNYWKKRNVVEVSPMPFWGNFKECALLKIP

AGFLMRDLYTQAKGIPYIGFYIIDEPCLLIRDREMIKNVLVKDFSYFSDRNGSPSPNDRL

GLSNLFFLKNPAWKILRAKLTPIFTSGKLKKMFELMIECAKNLETYLESLKVNDAGKEID

MKDLSAKFTTDMIGSTAYGLNVNSLKNPNAEFRKYGKMIFDYGYVRGFEMLASFFFPNIT

RWANIKMFGKDSSNFLRKVFWETMNQRTESGVKRNDLIDILIDLKKNYGHQDIEGFRFDG

DDLLAQAVVFFTGGYETSSTTMAFTLYELALQPEIQDKLRKEILDALDKNDDTITYDMIL

SLPYLDMVVSETLRMYPTLPFLDRMTTETYKMPNSDLVLEKGTPIYISMLGMHFDPEYFP

DPDKFDPERFNEENKRNRPSCVYMPFGEGPRACIGTRLGLLQTKLGLIKILSKYEVTPSK

RTLIPMILNPKAPTTTPLNGGLYLNIRRINAN

>EDS32055.1

MLFYLALVAVSLTFFWVGRRFSYWKQLGVPYVEASFPFGNLKGMNSRHLGYIAQDLYVKL

KSTGKKFGGTFFFVSPTALILDLDFAKDVFVKDFQYFHDRGVYCNEKADPITAHLVTMSG

TKWKNLRTKLTPTFTSGKMKMMFPTIVGVAEEFRKGLLVEAEAGGEVEMKEFLARFTTDV

IGTCAFGLECNSLKDPDAEFRRMGKKALTLSTMDFLRQILTVTFKDVAMKFNVRLSNPDV

ANFFMNAVRETVEYPQSFVFFLAGFETSSTAMTFCLYELAQNQELQDKARKNVLDVLKEH

GSISYEAVHDMKYIEMCINESLRKYPPIANILREVTKDYPVQDMNVTLPKGHRVMLPIYA

IHHDPEYYPAPDQYDPERFTPAAVAARHQMAFVPFGEGPRVCIGQRFGMMQARVGLAYLL

KNFRFRLSEKTAVPLKILASSTVLATEGGLWLHIEKL

>EAT32869.1

MALLELYLAIGVTLVLATAGCVFLFLDKKRSFWKDRNFPCTGRAKMIYGDYKNMNQTEHM

QYINQRIYNEFKARKLPIGGTVLFLVPSTVVVDPDLIKAMLVKDFNFFHDRGVYNNPEVD

PLTGHLFSLEGQAWRQLRAKLSPTFTSGKMKMMFSTILNVADDLKEFLWEKTESGSTELE

MKNVLAGFTTDVIGSCAFGIECNSLRATHCRFREVSRKIFEQSVGQMLWMIVLMLFKGVA

TKLKLKATPAEVENFFTNMVQETIDHRERNNVQRSDFMNILIQMKNSTNLEEKLTLNEIT

AQSRIRDFLDHHGQLPVELAMIRDIQEKLRAEIFKVCGEGDLTYESVSSVEYLNMVIDET

LRKHPVVDSLLRTSTQPYNIPNTDLKIPKGTFVFIPVHALHHDPDYYPDPDRFDPERFNA

ENRASRHPFVYLPFGEGPRNCIGMRFGLMQTRVGLITVLRNFRVRPSSNTPERLVVNPKS

GIPAPLGGIPLLIERI

>AFM08403.1

MLIPAVLVACFGLVWLYIHFKRRYLFWLKRDVPYLEPTFPVGNVVDIFSPTIHFAHLIEK

LYRRLKGRGDYAGIYFFRDPVLLVLSPDFARTILVKDFNYFVDRGVYSNEEVDPLSANLF

FMDGNRWRKLRSKLAPTFTSGKLKAMFHTIVDVGNRLDQYLADRCIRLQRIDIKELLARF

MTDVIGSCAFGIECNSLDNPNSQFRLMGKRMINLPKLKALKVFFAMMFRKQAQLLGIRYN

DKDVSEFFMNVVRDTIRYREEKGVRRDDFMQLLIDMMKQDGTGAGEALTFEEIAAQAFVF

FFGGFETSTTTITCALHLLAKHPKAQRKARKCVRSVLAKHGNQLTYEAITEMDYIECIIN

ETLRLYPPVATIHRMTSQPYQLPNGKILPVGVGVIISNLAFHHDPTYFPDPHLFKPERFE

DKAFGKNNPSYLPFGDGPRMCIAMRFGQLQTRLGLAMLLRNYNFAIDDCDADRPLLIDPI

NLMHGPAGAVWLNVERTVEKNS

>EAA05452.4

MALPIALYLCVSVVCLLVLWIRRRRNYWSTRNVPTIPGHFLLGNFEGLRRQSVADVSNEL

YRQVDRRERFCGLSIMLQPTLMITDLDLIKSVLIKDFAHFTDHGVYHNERVDVLSCHLFC

LEGAKWKSIRSKLSPAFTTGRIRAMFPILRQVADNFAHFLRTEVDGKAELDMKDCCARMM

IDNIGGCAFGIECNSFHEPDSAFRRTGELVFDKPRHSQVVSSLLRLYPALGHLLGLKVNH

DEVVEFFTGMMRRTVAMRESSNTATPKRNDLIDMMLQLRAASNGSALSMDELMAQAFGFF

LAGYETSSSNVTFCLYELALNEHCQERARACVREALRKHGGLTYEALGEMEYLDRCINET

LRKYPPLPLLPRLTSKAYRIPGSDVILPAKMRIHLPVYAIQRDERYYPDPDRFDPDRFAP

ERVAERHFSAFLPFGEGPRICIGQRLGVLQSRIGLATVLANFRVRPGPRTPIPLVFAKDA

VTLQSAGEVYLQVEPLQEHE

>EDO64395.1

MALPIALYLCVSVVCLLVLWIRRQRNYWSTRNVPTIPGHFLLGNFEGLRRQSVADVSNEL

YRQVDRRERFCGLSIMLQPTLMITDLDLIKSVLIKDFAHFTDHGVYHNERVDVLSCHLFC

LEGAKWKSIRSKLSPAFTTGRIRAMFPILRQVADNFAHFLRTEVDGKAELDMKDCCARMM

IDNIGGCAFGIECNSFHEPDSAFRRTGELVFDKPRHSQVVSSLLRLYPALGHLLGLKVNH

DEVVEFFTGMMRRTVAMRESSNTATPKRNDLIDMMLQLRAASNGSALSMDELMAQAFGFF

LAGYETSSSNVTFCLYELALNEHCQERARACVREALRKHGGLTYEALGEMEYLDRCINET

LRKYPPLPLLPRLTSKAYRIPGSDVILPAKMRIHLPVYAIQRDERYYPDPDRFDPDRFAP

ERVAERHFSAFLPFGEGPRICIGQRLGVLQSRIGLATVLANFRVRPGPRTPIPLVFAKDA

VTLQSAGEVYLQVEPLQEHE

>EAA12405.2

MLIVAVFVAFFGIFWIYFHFRQRYLFWLNRDVPYLEPTFPFGNMIDIFNPNIHFAHLIQK

LYRQLKDRGDYVGLFFFRDPVLLVLSPEFARTVMVKDFNYFVDRGVYSNEEFDPLSANLF

FMEGAKWRKLRSKLTPTFTSGKLKAMFHTIVDVANRLDQHLGERCVRMQRIDIKELLARF

MTDVIGSCAFGIECNTLENPNSQFRLMGKRMINLPKLKSLKVFFAMMFRKQAQALGIRYN

DKDVADFFMNVVRDTIRYRAENGVRRDDFMQLLIDMMQENGAGAGESLTFEEIAAQAFVF

FFGGFETSTTTLTFALHLLAQHPKAQRKARKCVRSTLAKHGNEMTYEAIKDMYYLECVIH

ETLRLYPPVASIHRMTSQPYQLPNGEVIPEGVGVIISNLAFQHDPTLFPDPLAFKPERFE

DKTFAKTNPSYLAFGDGPRMCIAMRFGKLQTCLGLAMLLKSYTFSLEDCDVDRPLLIDPI

NLMYGPAGPVWLNVERYVEGSKSS

>AAK32958.2

MFVQFLLVVSLGWLWIYLHFNQRYRFWVERQVPFLEPSFPAGNVPDTLKPTIHFAYIIEK

LYKRLKSKGDYVGIYFFRDPVLLVLSPEFARTVLVKDFNYFVDRGVYSNEEVDPLSANLF

FLEGNRWGKLRSKLAPTFTSGKLKAMFHTIVDVGNRLDQHLAEKCQQVKRIDVRQLMACF

LTDVIGSCAFGIDCNSLENPNSQFRVMGKRMINLPKLKALKVFFAMMFRKQARALGIRVQ

TMKDVSDFFMNVVRDTIRYREEHSERRDDFMQLLIDMMKQDGAAEEDPLKFEEVAAQAFV

FFFAGFETSATTLTFVLHLLAKHPEVQLEGRECVRDVLAKHDNKLSYDAVMEMEYLGWIV

NETLRLYPPVATLHRITTQPYQLPNGAILPEGVGVILPNLAFHYDPDYFPDPYDFKPERF

AVKNDFKNNFSYLPFGEGPRICIGMRFGLLQTRLGLAMLLRNYHFTIDPSDAARPLRIDP

INLTHGPEGQVWLNVERIA

>EDS44710.1

MWLVLLTWLAIALPLGAFAWFKKRFRFWSDRGVEFIEPELPNKLSPTFTSGRIKMTFPLV

VEVCKQFCDHLEEVVQGVNEVEMHDLLSRYTIDVIGTCAFGIECNSFRDPDNEFRRYGKI

AFDKLPHSPLVVYLMKAFRKYANALGMKMLHEDVSSFFSNVVRDTIDYREKNNVVRNDFM

DLLLKLKNTGRLEEAGEEIGKLSFDEIAAQAFIFFNAGFDTSSTAMTYTLYELAMNREAQ

EKARKCVLDIFAANGGQLTYESVANMGYLDQCISETLRKHPPVAILERNADKDYRIPDTD

LVIAKGRKIMVPIFAMHHDEEHFPDPEAYKPERFSPEEVAQRDPYCYLPFGEGPRICIGM

RFGQIQARVGLANLLRRFRFSVCDRTQIPVKYSRTNFILGPANGVWLKVEKL

>AFM08402.1

MFVHLLLVASLALLWIYLHFKQRYRFWADQNVPYMEPAFPAGNVADTLKPTIHFAHIIEK

LYKKLKSRGDYVGIYFFRDPVLLVLSPDFARTILVKDFNYFVDRGVYSNEEVDPLSANLF

FMEGNRWRKLRSKLAPTFTSGKLKAMFHTIVDVGNRLDQYLAERCNQEQRLDIKELLARF

LTDVIGSCAFGIDCNSIDNPNSQFRVMGKRMINLPKLKALKVFFAMMFRKQARALGIRFN

DKDVSDFFLTVVRDTIRYREENNVRRDDFMQLLIDMMKQDGVGAGECLTFEEIAAQAFVF

FFAGFETSTTTITCALHLLAKHPEVQNEARKCIRETLIKHNNLLSYDAVMEMEYIGWIIN

ETLRMYPPVATLHRITTQPYQLPNGAVLPEGIGVILPNLAFQNDPDYFPNPRDFKPERFS

IKNDFKNNFSYLPFGEGPRICIGMRFGLLQTRLGLAMLVRNYNFTIDPSDAERPLRIDPI

NLLHGPAGEVWLNVKRIVDM

>EFA05730.1

MNTTIEILTIIILTIFCFYKWKFSYWKSRKIPYLDPKFPFGNVSNPFFMKEKVGMTLARF

YNDMKQRGWKYGGFYFGVSPICLIVDPDLIKNIVTRDFEHFVDRGVYFNEKDDPLSGHLA

NLDGQKWKNMRRKLNPTFSASKTKNMFPIVKFCVENMMEKVKSCYENAHKCCNINEVFPA

FSINVICSCAFGLQEENALFKAYADECVSWTKYKYLVQILATFFDKTAKRMGITTVPRNA

AKFFMKVVTDSVKYREENDYVRSDFMQLLIEMKKQNQLTIEEIAAQCFVFFIAGFETSSA

TLTFALYELAKNQNLQNVARDEILANCPNEITYDSLADLKFLDQVVDETLRKYPPLLYVT

RQCVKNYKIPDEDVVIEKGTLVAIPISALHSDEEFYPKPETFEPERFSKTEKSLRHPYTF

LPFGEGPRMCIGKRFGLLEVKLALTCLLKNYKFSVNCKTKEPLAMRVDTVVLAPKDEIWL

DLEKI

>91094085

MILLVLSVVLLVALRIFFFWRFQYWEKLQIPYLRPYCPFGCLPNPLINKEKGGIILKRLY

RNVRDKGWKHAGIFVFVTPVYLPTNLDYIKNILTKDFEHFDDRGLYYNEKDDPLSGHLIN

LGGPKGVTMRKKLNSLFTPSSLKMMFQTLNTSIEDLLQQIHSRNTIDIHKLFTCFTIHVI

ASCTLGLECNIFKEEHLLFESYANKFLVWSRGKWCLQLLAAMYIDTARKLKVRTVPKETE

DFFVKIVTKNIKYREENDYFRQDMMQFFIEQKNANTMSINEIIAQCFIFFIAAYETSTTT

LTFALYELAQNLDIQDRVRREITSVLLKHQKYSYEAIAEMKYLNQIVDETMRKYPPFLYV

VRRCTKDYTIPGEDVTIKKGIFALISILGIHHDEEIYPNPQKFDPDRFSKENKKMRHPYA

YLPFGEGPRICIGKRFALMQIKFGLAALLEKYKFTLNEKTKVPLEIRTDTLVVAPKGDVL

LDIQKIN

>383849312

MQGREIFSHYTMLLTMSVTTWLIPEILSAVIIGTIGLYVYFKLFIFNFWRKRGVFYVEPT

FPTGNILPLILGTRSQAKFTKDIYDDTKKHRVTGIFMFNKPFLVVNDLDLIRNVLTKNFM

SFHDRGIFCNEKTDPLSGNLLQLPGKKWRYLRVKLTPTFTSGKLKSIFPVLQESGEKLSK

YLDDKASVRGIIDVKDVFAMYSVDIIMSAAFGITCDSFKNPNNEFNYWGKKVLEPKPFWL

AVGIWAPQVLDLLSLPYTERGVTNFFLKMFEDIVTYRENNHIVRKDFLNLLIQLMKNGYV

DSDEKSDEPNVMKTTETKLTITEAAAQAFVFYGAGFETSSTTVTFCLYELAMHQDIQDRV

REEIRTVLKKHSELSYNAVNDMPHLHKVVSETLRKYPPVVFLNHICTKDIQIETTDFHIP

TGTSILIPVFGIHRDPDIYPDPDKFDPERFSEENVKARHPYAYLPFGEGPRICIGLRFGL

IQTKVGIIRSLFKNRFKLAPNTPTSLEFKEGSLILMAKRGVNLRIESVE

>AAL15173.1

MIYFILALAIIWAFVKLYTHVFNYWEQRGFPYVEGKFPLGSDPCLSRPSKFLGLEVQEHY

RKLSGHPLGGIYVGRRPDLIVRDPKIIKNIMVKDFAHFRNRGVEIPSKDSPLTQHLFALE

GTKWRALRVKLTPTFTSGKLKLMYSLFVECAQRLERKLNEDSMKNEGVVDIKDTIARFTT

DIIGSCAFGLEIDSLNNPDEPFRKIGMRLFQRNLKGRLIELIYSLAPNLRNYLKLSRTSK

ETEKMFMSGIGQTIEYREKNNVRRNDFLDLLIELKNQGHLYVDRQKDSNIENDHSQDGEA

QEKIEMDLGMITAQCFVFFIAGFETSSSVQSYALYELAYHQVIQDKVRREIEEVTSKFGG

LNYQALHEMPYLDQVINETIRKYPTLHTLTRVCTKPYAVPGTKYVIEEGIRVLIPVYSLH

HDRQYFPDPERFDPDRFSDTNKSSITPFTFLPFGEGPRNCIGMRFGLLQTKIGLVTLLKS

KRVLPTSQTKSPLVFSSSLSVISQNEGSIVLKLENI

>AAL15174.1

MIYFILALAIIWAFVKLYTHVFNYWEQRGFPYVEGKFPLGSDPCLSRPSKFLGLEVQEHY

RKLSGHPLGGIYVGRRPDLIVRDPKIIKNIMVKDFAHFRNRGVEIPSKDSPLTQHLFALE

GTKWRALRVKLTPTFTSGKLKLMYSLFVECAQRLERKLNEDSMKNEGVVDIKDTIARFTT

DIIGSCAFGLEIDSLNNPDEPFRKIGMRLFQRNLKGRLIELIYSLAPNLRNYLKLSRTSK

ETEKMFMSGIGQTIEYREKNNVRRNDFLDLLIELKNQGHLYVDRQKDSNIENDHSQDGEA

QEKIEMDLGMITAQCFVFFIAGFETSSSVQSYALYELAYHQVIQDKVRREIEEVTSKFGG

LNYQALHEMPYLDQVINETIRKYPTLHTLTRVCTKPYAVPGTKYVIEEGIRVLIPVYSLH

HDRQYFPDPERFDPDRFSDTNKSSITPFTFLPFGEGPRNCIGMRFGLLQTKIGLVTLLKS

KRVLPTAQTKSPLVFSSSLSVISQNEGSIVLKLENI

>AFI45024.1

MAFGLAYVVIGVISFIIYVKWRQSYWKRKGVPSSDADFLLGDNTKIFTNQESLGEISVNR

YNKFKAQGKKHAGFYNFLQPVYFPLDTDIIKHIMQQDANYFASHATYNNPDDVLSKHLFA

LEGERWKDLRAKLTPAFTSGKLKMMFETLTNKTVGLENAVAKMVDKKEAFVLKEFLCNFT

TDVIASCGFGIESDCLGDPNDAFRRAGKKIFEPRPFKLALLSLFNWDFLAKLGYKHFPNE

LTVFFTNVVTSTIRHRENNQIFRKDFMHLLLQLKNQGVLSDDDKVTGSGNGKGIISESDI

IAQCFVFFIAGFETSSSTMTFTMLELAQHQDIQDKLRKEILEVLAKHDGKITYEAVMEMP

YLDKVISEALRKFPPLPVIPRRCTKTYKVPGTDLVLERGTDVQIPVWAIQNDPEYYENPE

VFDPERFSEENKAARPEYAFFPFGAGPRVCIGLRLGKLETKVGLITLLRKFKFTINKKTP

FPIQMEKRGFISTIQGDLWVDAVRI

>EEB15938.1

MDAIAVNSWWFIYFGTFIGTLLALMYYYVTSNNDFWDKLNVPYIKPEFVYGNMKEIAKCV

KSQNEAVWEFYQYFKKLGHKYGGIFQLRDPVMIICHPQLVKDVLVKNFPHFHDRHIAHDE

HFDPLSAHLIALEGSKWKYIRSKLNPAFSTSKLKGMFQSMTNICNELIKNLNGAAEEGKM

LDARDFLGRYTVDIIGNVAFGIDVNAMKEDSEFLHMGQKFFEPTQFERVGQIIANTNYWM

YKIFKLRMVSKKVTDFFINVVNNTMTYRRENNVERNDFLHFLMKINETKGTDLKDMDEMK

GVKNYNMAVSENSIGVIAAQAFVFFVAGYETSSSAMSACLFELAHHPEIQEKLYEEVTQV

LKTNDDVTYDSVQQLTYTEQVLEETLRLYPPVGILLRKCTQPYEIPETSIILPKGCQLFI

PVYAFHHDPEYFPDPEEFNPERFSSENRKNIPPYAYMPFGHGPRVCIGFRLAMMEMKLTL

ALIIKNFKILKCEKTKPFKMCPSSFFLVPKDGFWIKVIKRNENLLI

>AFI45036.1

MAVVSTGFIIFDLCLIVGAAFLAVLIYFKHSYRYWKVKRVPYLEPTFPLGNDEYFFTKSA

TFAGEAKSWYDQLKAGGHKFGGVWSFSRKALVLVDPDYIRDVLTQDFHHFANRDIFVNEK

FDPQGSHLFNVRNAHWKPLRQKLTPTFTSGKMRMMFESVMRCCDHLVDHLKECAETGENI

DSRETLSCFTTDVIGSVAFGVDCNSFSKEESDFRKYGKLFFRPNLLDYIRLLFLKSFPDL

ARKLRLKLGNTRRTKFFSDMVKHTIDHRTQFKVTRPDFLQLLINMNLENDGKETLSFDQI

VANTILFFVAGFDTSSTAMNFALFELARNPDLQEKARQEVLQVMARNDGKITYQGLQEMT

YVKQVLDESMRMYPPGLTLSRVCTKDYKLRDTDIVIEKGTSVVISTLVLGRDPEYFPDPQ

RFDPDRFSAEEKAKRHPYVHIPFGEGPRNCIGLRFGVMQSKIGLARILSNFRLTVSPKTK

LPITLDKKLFMLKANETIYWKAEKL

>AFI45026.1

MALSLAYVVIGVISFIIYVKWRQSYWKRKGVPSSDADFLLGDNTKIFTNKESLGEISVKR

YKKFKAQGQKHAGFYNFLQPVYFPIDTDIIKHIMQQDANYFASHATYSNPDDVLSKHLFA

LEGERWKDLRAKLTPAFTSGKLKMMFETLTNKTVGLENAVAKMVDKKEAFVLKEFLCNFT

TDVIASCGFGIESDCLGDPNDAFRRAGKKIFEPRPFKLALLSLFNWDFLAKLGYKHFPNE

LTVFFTNVVTSTIRHRENNQIFRKDFMHLLLQLKNQGVLTDDDKVIGSGSEKGILSESDI

VAQCFVFFIAGFETSSSTMTFAMLELAQHQDIQDKLRKEIVEVLAKHDGKITYEAVMEMP

YLDKVISEALRKFPPLPIIPRRCTKTYKVPGTDLVLERGTDVQIPVWAIQNDPEYYENPE

VFDPERFSEENKAGRPEYAFFPFGAGPRVCIGLRLGKLETKVGLITLLRKFKFTINKKTP

FPIQMEKRGFISTIQGDLWVDAVRI

>AEL88548.1

MGLSLAYIVIGVISFIIYVKWRQSYWKRKGVPSSDADFLLGDNTKIFTNKESLGEISVER

YKKFKPQGLKHAGFYNFLQPVYFPIDIDIIKHIMQQDANYFASHATYSNPDDVLSKHLFA

LEGERWKDLRAKLTPAFTSGKLKMMFETITHKTVGLEKAVATMVDKKEAFVLKEFLCNFT

TDVIASCGFGIESDCLGDPNDAFRRAGKKIFEPRPFRFAILSLFGWDLLPKLGYKHFSSE

LTNFFTNVVTSTIKHREENQIFRKDFMHLLLQLKNQGVLTDDDKVIGSGKGNGILSESDI

IAQCFVFFIAGFETSSSTMTFAMLELAQHPEIQDKLRDEILEVLAKHDGKITYEAVMEMP

YLEKVICEALRKFPPLPIIPRRCTKTYKVPGTDLVLERGTDVQIPVWAIQNDPEYYENPE

VFDPERFSEENKKGRPEYAYLPFGAGPRACIGLRLGKLETKVGLITLLRKFKFTVNKKTP

FPIEMDKRGFISTIQGDLWVDAVRV

>AFM78645.1

MKTWVGEHYASIYFNTNGYKFVGFYQFQKPKLMLRDLNIIKDVFTKEFSTFPNRGIVFDD

KLEPLTGNLLTLEGHKWKVLRNKLTPAFTIGKIKNMIDLIDGRAQEMVRILEAPAVIDEQ

VEFKELLARFSTDVISIVAFGFETNSLANPDAEFRKVGRMLFSTSIESIIRNALNALAPS

LIGLLKVRSIKKEYADFFYNVVNDTVRYREENNIQRNDFLDLLMKIKRGQNLASEEESKL

VLNENEGKEDFKFTMDVLAAQCFVWFIGGYETSSITLTFTFFELAQNPEVQMRAQDEIDS

VLIKHNGKLTYEILQEMTYLDMIVSEALRKYPPVPNLTRKAVKPYKLPNSDFTLNKGLQV

VIPVYAIHNDPEYWPEPEKFIPERFTEDEKHNRPQYAYLPFGAGPRLCIGMRFGMMQVKV

ALFRILSTYNISLSKNMKLPIKLNPKTIPANPDGGMFLHVTKRKN

>EDS26133.1

MELVVSIFGFFIGLSVVVYCYLRRQQSHLHRAGLPVASKPHLVYGNVKGIAQTEHACKVA

QRLYQEFRERGLPLGGFMNFFIPSVLVTDPELVKCVLVRNFDVFHDRGLFADTESDPISG

SLFSLDGTHWRAMRQKLSPAFTSGKMKHMFGLMVDVAEQMRRFMDTNCHRADRLEMKDAL

QRYSIDVIGNVAFGVECNSMENPDSEFRRMGTKAFQFDTVQRLKFFFGGRYKALMKGLGL

KVFPGDVSGFFRKLTASTVEYREKSGVRRNDFINLMLEIRNKNGEEGLTMDELSAQCFIF

FTAGFETSSATMNFCLYELALNPFIQNRLRHEIEQVIGKDNDGFTYEKMLSMDYLDRVVK

ETLRKYPPAESLFRISAAPFAVPDTKYVIPANTFIQIPVYALHHDPHHYPHPDRFDPDRF

LEEAVQKRHPYSYLPFGEGPRNCVGMRFGLMQVKVGLVMLLRHFSFAPNAQTPLKIEFDP

EFVILAPTKGMYFDIEPVSRNKLVQA

>EAA07729.4

MVAFTWVLVLCTIVLTGVAVRMFLRRRQTFWQRHGIPAARHPHPLYGNVGGLVTKHHTAT

VLQRLYREFRERKLPAGGFNLYFSPVLLVVDRQLIERVLVNDFAQFQNRGLYASATVNPL

SSETLFSVAGERWHRMRQQLRPALGSSSVRTMFATTARIAQTLVVYIGAQSRRRDLEWTD

LMARYATDVIGSCAFGIDCRTIRDPGSEFRAMGHRAFRCSLRRMWKLRFGYACRRVADAM

RLCVSEPSVEQFFLQLCQSTVLHRESYQVVKRDFLQQLLEMKANGALDMRQVAGQCYSFF

IAGFETSASLLSFCLYELAKHGAVQDRLRGSIVAALDETDGQLSYDMVMALGYLDQVVKE

TLRMYPPVDFLFRVASNDYPIDGFGTIPQGTLLVVPVHALHRDPAYYPQPDVYNPDRFAA

SSKLSGASKNRPPFMPFGLGPRHCIGDTFGLMLVKVGLVAMVRSFRFTLDVDRTPENVTF

KPRSLVLAAGSGMYLNVERV

>AFI45025.1

MVVAILVIGVITFLIYVKWRQTYWTRRGLPTAKTEFFFGDMRKAITGVEPLAVRTKNRYN

EIKALGVKHAGFYMFWRQIYFPVDLQIIKNIMQQDADYFQSHGGYYHSDDVLSTHLFTLE

NQRWKELRAKLTPAFTSGKIKMMFETLTEKTELLIDAVGKLADETEGFAIKNVCGNFTTD

VIASCGFGIECDSLNEPDNAFRNFGKKLLVPNLLRFALTNIVNWNLLVKLGFTGFGSKTS

QFFIDLVKDTIKHREDNEVFRKDFMHLLLQLKNQGIISDKTNVNRTKSEAKGILSENDII

GQCFVFFVAGFETSSSTMTFTMLELAQNQDIQDKLRKEIKEVLAKHDGKLTYEAAMEMPY

LDKVVNEALRKFPPLPFIPRKCTRNYKIPGTDVVLEKGTGVHIPVWGIHNDPEYFPNPDV

FDPERFSEKNKASRPEYSYLPFGAGPRICIGLRFGKLQAKVGLIALLNNFKFTLNEKTPM

PIQIDRGFIITVKGNVWVDAVRL

>EDS26135.1

MFATLPAVALLVVALFGLYIRRILHLRRAHWLSRRIPFVGWPHILFGNVRGIWSVEHSST

MQQQIYQELKERRLAAGGFNVFFSPSIMVVDPELIKQILVTDANVFDDRGLYRDKLCDPL

NYTLFGSTGEVWQGLHENAMRLLTAESVKGMFGLVAGVADELRLQILKTIDRQRLDIHDL

AVRYSTHVIGTSVFGIRCRTIQVGCTEFREMGRRANEFTRLVLFKQWLGVSFPRFARILG

LKMIDSPVEEFFIGLCTATVLHRESYGVEKDDFLQSYIHLRRDKRLSMEELTAQCYSFVK

NGLETCSSVMTFCLYELASAPAIQDRLRNEINHSLEDTDGQLTYEVINSMTYLDQVINET

LRKYPPVDYLMRRSKTTYNHIPDGTLFIVPTYALHHDPDHYPEPEKFDPERFAPSAVRKR

HPYSFLPFGAGPRNCIGVRFGTMLVKTGIVTMVRSFRFTRRGGDDGLRFKPSSLVLSPAE

GSMGCVDVKRI

>157107783

MGFEQCKADNCLYRKRLRSAPTSDKPSCRQRCPRFAQTIGTLPGVSLLQMISGTVCIVLV

LANVAFLVLFVRGVLQSRQVYWVRRRIPFVAWPHLLFGNVRRLWRHEHSSTIGQRLYRDL

KARRLAAGGFNLLVSPSILVADPDLAEEVLVGNVRRFPDRGLHVDAEVDPLSETLFALSG

NSWQDKRNQLTPVFSEETLKPVFRMIASFADELRKEISKNLDRRLQDVQEWVSRYVTQVM

GKSVFGMRCRMMQDPNTDFRRYGRISTELSWLLLLKNWIGVTMPWIARKVGLRITDATVE

KFYVDLCRSNVLVRESYKVKENDILQLFMRLREARQLTMEELTTACYSFVKHGMEPCTSV

MTFCLYELAKNVSIQKRLRDEISHYLEDTDGQLTYDVIMSMNYLDQVVNETMRKYPPVDF

IYRRSSQSRDNIPQGTLFVIPVYAFHHDPDHFPAPEKFDPERFTAKQARTRHPYCYLPFG

AGPRECLGARFGLLVVKAGLVTLLRRFRFAMPEELVHEKLQFKPNASVLSPVEGSVRLRV

EAI

>EAT32871.2

MISGTVCIVLVLANVAFLVLFVRGVLQSRQVYWVRRRIPFVAWPHLLFGNVRRLWRHEHS

STIGQRLYRDLKARRLAAGGFNLLVSPSILVADPDLAEEVLVGNVRRFPDRGLHVDAEVD

PLSETLFALSGNSWQDKRNQLTPVFSEETLKPVFRMIASFADELRKEISKNLDRRLQDVQ

EWVSRYVTQVMGKSVFGMRCRMMQDPNTDFRRYGRISTELSWLLLLKNWIGVTMPWIARK

VGLRITDATVEKFYVDLCRSNVLVRESYKVKENDILQLFMRLREARQLTMEELTTACYSF

VKHGMEPCTSVMTFCLYELAKNVSIQKRLRDEISHYLEDTDGQLTYDVIMSMNYLDQVVN

ETMRKYPPVDFIYRRSSQSRDNIPQGTLFVIPVYAFHHDPDHFPAPEKFDPERFTAKQAR

TRHPYCYLPFGAGPRECLGARFGLLVVKAGLVTLLRRFRFAMPEELVHEKLQFKPNASVL

SPVEGSVRLRVEAI

>AFI45028.1

MHSASYLIYFLIVFVGLLGYLFRKAKKTYNYWTEHGVPSENNTHLLYGNLKESILLKKSD

AEVYGEFYQKFKKRGLKHGGVYFYSRPTWILVDLDLIKTVLISDFQYFAAHNPMYLNTIF

FNNLFHMQGEQWKDMRRKLAPTFTSGKMKMMFNILLEKTHGLADLVADMASTGEATEIKE

LVARFTTDVIGSCGFGIECDSLNDVNSTFFQHGQNIFKKLTPKAAPKFYKLILLSLGLMK

RFNINLDETEAFFRKLVEDTVKYREENKIVRKDFLHLMIQIKNNGTITDILNDSDIFIKS

RDEAGLSMEEMVGECLLFFMAGFETSSTTLSFVFLELSRNQSIQEKLRKEIHTVLEKYNG

TLSYDSLQEMTYCECVINETLRKYPPVASLPRMCTKDYAVPGSDALIEKNTMVVIPLLAL

HNDPDYFPEPEKFIPERFSAKNEKIPFIYMPFGEGPRQCLGLRFGVMQVKAAIATLLNRY

NFTLDKKTPYPPKFTPGSPVLSVEEGVWVRATKVK

>AFI45027.1

MVVAILVIGVITFLIYVKWRQTYWTRRGLPTAKTEFFFGDVRKAITGVEPLAITTKNRYN

EIKALGVKHAGFYMFWRQIYFPVDLQIIKNIMQQDADHFQSHGGYYHSDDVLSTHLFILE

NQRWKELRAKLTPAFTSGKIKIMFETLTEKTELLIAAVGKLADETEGFAIKNVCGNFTTD

VIASCGFGIECDSLNEPDNAFRNFGKKLLVPNLLRFALTNIVNWNLLVKLGFTGFGRKTS

QFFIDLVKDTIKHREDNEVFIKDFMHLLLQLKNQGVISDKTNVNRTKSAKGILSENDIIG

QCFVFFIAGFETSSSTMTFTMLELAQNQDIQDKLRKEIKEVLAKHDGKLTYEAAMEMPYL

DKVVNEALRKFPPLPFIPRKCTRNYKIPGTDVVLEKGIGVHIPVWGIHNDPEYFPNPDVF

DPERFSEENKASRPEYSYSPFGAGPRICIGLRFGKLQAKVGLTALLNNFKFTLNEKTPMP

IQIDRGFIITVKGNVWVDAVRL

>AEE62280.1

MTVLGLLPQLLLYISATLVSLFLCVYVYFSYSFTYWKRKGAPYIKPKFPLGNNDCFGTES

FSYGLETMDWYKEAKQKGHKFIGAWNYSNPVLLVVDPDYIKSVLVKDFNHFTDRDFFHNP

KHDPKNESIFVAENEEWRQLRQRLTPVFSTAKMRIMFELVNKCADPMMQLFQTNSRSGDP

MDIKEVVAMFTTDVIGCVAFGLDAGSFTSEDAKFRVIGKKLFESSLKTKMHLLVGRINVK

LAQFLGMRVIPESVTEFFSEVITENARFRQENNVRKIDLMQLLLDLYESSKGQDDGFSFD

DFVGNVIAFFIAGFETSSTTMHNVLYELARNPDVQEKTREEINNVLKKHGGVLTYEAVQD

MTYFRQVIDETLRMYPPVQNVARFCVKPYTFKDSNVTVEKGVSVVVPLVALGRDPDNYPN

PERFDPDRFSPQSKESINKSVYIPFGDGPRNCIGKRFGLMQTSIGLMQVVRDYKITINSK

TPMPLKLKRGVFLMETTNKLYLNVTKV

>345486271

MNFDLLSVLSYIVLLALSAFTLFYMYAKQKQTYWSKRGVYSPPCHWFYGHFRDAFLMKKS

FPQAFGEMHNLVDSSIPVVGVYVMQKPFLLLRNPEVIKQVLIKDFNVFCNRYFAAQRKTD

VIGSENLFSIKYAQWKYIRSKLSPAFSSGKLKRYFQLMLESAANTRNYLKSQIPDGEKVT

IECRKLSNKYTTDVISSFSFGIKTNSYVEPEPEFYLRSRKIFQLGLVRTLQILTTFFFPK

ISDYLMGSMLGDSNDFFRNAFWESMNERKESGIKRGDIIDFLVELSNEKQSQEFKFEGDH

LVAQSAILLVAGVETSAVTIGFTLLELAKHPEIQRRVREEIREKLCKDGQLAYEDVAVMT

YLSQVISETLRLYPPAPLIDRLASEDYKLPGTDIVIEKGTAVYAALKAGLHEDPHYHPNP

TKFDPDRFSEERKNEIQPCTYMPFGEGPRVCVGMRIGQLETMVGLITILSEYEVSLNPQH

ITELNVRALFLAPADGIKLYFQRIKTCQ

>380029591

MAVLNSLFMPIVISIAFLVIILILYIKYNRTYWQRRGVPTVPGHWLFGNVKKMLDLEKPP

AYVIGDICQKSSENDGILGIYVFFKPFLLIKDPAIIKQILIKDFNYFPNRSFTIRSFGDK

IGNTSLFSLKNPQWKYLRTKLSPVFSSAKMKKLFHLMVETANSMNKYLDDQFLNGTKTKT

IMMKDVTLKYTTDIISSIAFGIQVNSFNPETVQFYEEAQKGLKMTFFRNIQLVISFFFPK

LSLLLNARMLGTSTNFFRKVFWNSMDTREITKSKREDLIDFLIELKNDKQDEDFKFEGDA

LLSQSAIFFVAGRESSVSTICFTLYELAKHPEIQKRTREEINEKLKEHGMTYEGVQNMKY

LHQVVSEVLRLYPPAPLIDRIAVSDYKIPGTDIVIEKGTSVFIALTALHYDPKYHLDPLR

FDPDRFSDENKENIKQCTYMPFGEGPRVCIGVRMGQLQSVIGLITIIKNYEISLNSKCKG

DLDIRNIFVSPINDFSLNLTKI

>383848552

MAVLSSFLLNLGFTVVALLTILVLYVKYKRSYWQRRGVSTLPGHWLFGNIKDAALQKKSF

AQVFGELYEQAADTDDVLGIYVLHKPFLLIRNPEVIKQILIKDFNVFPNRYFSVRSLHDD

IGHRNLFSIENPPWKYLRTKLSPVFTGVKLKKLFHLIVQNANSMSKYMESQFLNGTKTKN

MRVRDIALKYTTDIISNIAFGIEVNSFDPEKVEFFNKVQEGLQFSFKRGLEFSMMFFFPT

IAKYIGTQMLGSSTDYFRKVFWDSMDTRESNKIKREDLIDLLLELKNEKQENSEFKFEGD

TLLSQSAIFFVAGRESSVSTICFTLAELAKHPEIQKRTRAEILEKLEQYGMTYEAVQNMK

YLHQVISETLRLYPPAPILDRVPVEDYKIPGTDIVLEKGTPVYISLTGLHRDPRYYRDPL

SYNPDRYTDENKNDIPPSTYIPFGEGPRVCIGTRLGQLQSAIGILTILKDYEVSYDSTCK

CDIEKRNVFLSPVEDFRLNVTKI

>EFN68268.1

MAMLTDCIFLDTLLIFFSLFATLFLWLKWKHSYWQRRGVRTLPAHWFFGHFKDAMLMRKP

AGVVFGELHRQAADEDDVIGIYILHEPFLLVRNPELIKQIMIRDFHVFPNHHFDGRSKSD

TLGNWSLFTIKNPEWKQLRTKMSPVFTSGKLKSLFLLMQESGEMMREYLQGQFVDSNIAS

IPIKDIFYKYTTIVIASMAFGIRANCFDTPPPEFFINSRKAFQPTLSRTLRFFFMFFLPK

IGKYLGLKMLGDYTDYFRKVFWDSMDNRSITKTKRGDLIDSLLQLKNENADDTDFRFEGD

ILLAQAAIFFVAGRETSITTMTYALCELAKYSEMQRRAREEILEKIQTANGVTYEAVNNM

KYLHQVINETLRLYPSAPILDRTPIEDYTLPNTNITIEKGTPIYVALYGIQRDLRFYEDP

KRFDPERFSDERKNEIVPCTFLPFGEGPRNCIGMRLGILQTAVGLIAILRDYEVSLNPSW

MSIVDTRNVFTTPPAGFLLNLKKI

>350402326

MAILNSCFFNIFLTIATLVTFLLLYVKYRRTYWQRRGVPTLPAHWFFGNVKDVIQFKKSP

SIVIGDLHSEASDNDDVLGIYIFHKPFLLLRNAELIKQILIKDFDYFPDRYFTAQSIRDK

IGSSNLFTMHNPEWRQMRTKISPVFSSGKMKKLFHLIGETAGSMIKYLEQQFSHDAKTKT

IFVRDIALRYTTDIISSVAFGIKVNSFDPKTVQFFEKAQEGTRITFLRAIQFSVMFFFPQ

FSKWIAGQMLGSSTNYFRKIFWNSMDTRNITKAKRGDLIDSLIDLKNENDLKLEGDELVS

QAAIFFVAGRESSVTTICLTLYELAKHPEIQKRTREEIHEKLKEHGMTYEAFQSMKYLNQ

VISETLRIYPPAPLIDRICVKDYKIPGTETVIEKGTPVYVALTGLHHDPRYFSNPQHFDP

DRFSDKNKDNIKQCTYMPFGDGPRVCVGVRLGLLQSSMALIAILKDYEISLDPTCNKDGI

DVRNVFLTVTEDFRLNFTKL

>328784477

MAMQNSLFMPIIFSIAFFVIILILYLKYTRTYWLRRGVPTVPGHWLFGNIKKILDLKKPP

AYVISDIYQKCSENDDILGIYIFFKPFLLIKDPAIIKQILIKDFNYFPDRNFTIQSFYDE

IGNKSLFTLKNPQWKYLRTKLSPIFSSAKVKKLFHLMVEAANSMNKYLDDEFSNDTKTKT

IMIKDVTLKYTTNVISSVAFGIQVNSFNPKTIQFYEEAQKGLKTTFSRSMQLCISFFFPK

LSPYLNTRMLGSSTNFFRKVFWNSMDNREITKTKREDLIDSLMELKNAKQDKDFKFEGDA

LLSQSAIFFIAGRETSISIICLTLYELAKHPEIQKRTREEINEKLKEHGMTYEGVQSMKY

LHQVVSEILRIYPPTPIIDRVAVADYKIPGTDIVIEKGTSVFIVLTALHNDPKYHPDPLR

FNPDRFSDENKENIKPFTYIPFGEGPRICIGARIGQLQSIIGLITIIKNYEISFISKCKG

DLDVRNIFITPINDFSDLDVRNIFITPINDFSLNLTKI

>EFN84331.1

MWNVLLIFSALLAALYLWMKWKHSYWQRRGIPTLPGHWFFGHFKDPILMRKSPGMVAGNL

HKQAADNDDVLGIYILHKPFLLVRNHELIKQILIKDFHIFPNHHFTGQSIADKVGSSSLF

GITSSQWKYLRVKMTPVFTSGKLKNLFLLMMESAELTRVHLREQFQGDSKVASFTVKDTF

YKYTTNVIASMAFGVRSNCFDPATSSEFYKYSTEAFQMTFLRAIQFFFLFFFPNLGKYLG

GSMLNTSTDYFRKVFWDSMDNRTVTKMKRGDLIDSLLQLKTENVSDEIFQFEGDALFAQA

AIFFVAGRETTITVITYTLCELAKKPEIQKRLRQEILEKIEVANGITYEAVQDMKYLQQV

IYEAMRLYPPVPILDRVPLEDYTFPGTNITVEKGMPIYIGVYGLHTDPKYYSNPMTFDPD

RFSDERKGEILPCTYLPFGEGPRNCIGSRLGYLQTAVGLITVLRDHEIALNPFWQCTVDE

KNVFTSPPSGFKLDMKKI

>EFN68266.1

MLFLWLKWKHTYWQRRGVRTLPAHWFFGHIKDAILMRKSIGVILGELHRQAADEDDVIGI

YILHKPFLLVRNPEVIKQIMIKDFHVFPNHYFDGKSKSDVLTNWILFTIKNPEWKHLRTK

MSPVFTSGKLKSLFLLMQESGEMMREYLCGEFTDSNIVSIPIKDIFDKYTTIVIASLAFG

IRANCFDIPPPEFFVNSHKASQRTLLRNLRFFFMFFLPEIGKYVGAQLLGDYTDYFRKVF

WDSMDNRSITKTKRGDLIDSLLQLKNENADDSHFRFEGDILLAQAAVFFVAGRETSIPTM

TYALCELAKYPEMQRRATEEILEKIQTANGITYEAVNNMKYLHQVINETLRFYPIVPILD

RAPIEDYTLPNTNITIEKGTPIYVALYGIQRDPRFYEDPKRFDPERFSDERKNEIVPCTF

LPFGEGPRNCIGMRLGILQTAIGLIAILRDYEVSLNPSWMSVTDTRRVFTSSPAGFLLDL

KKI

>EFN68267.1

MAILTDCVLLDTLLVFSLLFVTLFLWLKWKHSYWQRRGVRTLPAHWFFGHLKDAMLLRKS

AGVVIGELHRQAADEDNVIGIYILYKPFLLVRNPELIKQIMIKDFHLFPNRHFDGKSNSD

TLGNWSLFSVKNPEWRQLRTKMSPVFTSGKLKSLFLLMQESGEMMREYLQGQFADSNIVS

ISVKDLFSKYTTIVIASMAFGIRANCFDTPPPEFFVNYRKKQSHIVMHNPFCFLTYTAQK

AFLRNLSVNFRFFFLFFLPEIGKYLGLKTLGDYTDYFRKVFWDSMDNRSITKTKRGDLID

SLLQLKNENADDTDFRFEGDILLAQAALFFGAGRETSITTMTYDLCELAKYPEIQRRARE

EILEEIQTANGVTYEAVNKMKYLHQVINETLRLYPSAPVLDRISIEDYMLPNTNITIEKG

TPIYVALYGIQRDPRFYKDPERFDPERFSDERKNEIVPCTFLPFGEGPRNCIGMRLGILQ

TAIGLIAILRDYEVSLNPSWMSVTDTRRVFTSSPAGFLLDLKKI

>340711383

MLILNCFLNILLTVAILVTFLLLYVKNKRTYWQRRGVPTLPAHWFFGNVKDVIQFKKSPS

IVIGDLHNEASDNDDVLGIYIFHKPFLLLRNAELIKQILIKDFDYFPDRYFTAQSIRDKI

GSSNLFTMHNPEWRQTRTKISPVFSSGKIKKLFHLIGETAGSMIKYLEQQFSHDAKTKTI

FIRDVALRYTTDIISSVAFGIKVNSFDPETVQFFEKAQEGTQMTFVRAIQFGFMFFFPQF

SKWIAGQMLGSSTNYFRKVFWNSMDTRNMTKAKRGDLIDSLIDLKNKDDLKLEGDELHPE

IQKRTREEIHEKLKEHGMTYEAFQSMKYLNQVMSETLRMYPPAPLIDRICVKDYKIPGTE

IVIEKGTPVYVALTGLQSDPRYFSDPQHFDPDRFNDKNKDNIKQCTYMPFGDGPRVCIGV

RLGLLQSAMALIAILKDYEISLDPTCNKDIIDVRNVFLTVAEDYKLNFTKL

>EFN84330.1

MLATLVLLATAVIFFCFYARYKLNYWKRRGVESIPADYIFGNFKDAILFRSAPGFHLGEL

HNVAKSDAPFVGFYIFHKPCLLLRDPEIIKHFMIRDFDNFSDRHFAGSNQTDSIGMKNLF

GLKNPAWKYLRTKITPTLTRGKLKQMFPLMMETGEPMMKFLKNQPADMNNTKIVDAQDLS

YKYATDLIASVALGIKVDSFHYPDTEFTTAVAEFFHGFKRMIALVTVFFMPELVELFGTK

LLFNASFIKKVFWDVMENREKSGQKRGDFIDSLLQLKYGEQNPDYKFEGDNLLFQSGTFF

SGFESSSACLSFTLMELANHTEYQELAREDINNAIEKYGWTYEAFHNMKYLDQVIAEGLR

MHPPVSTIDRYTRQDYTIPGTDVIIEKGTPIYISLYGLHRDPRFFDESHIFNPDRFAEGN

QINDAYIPFGTGPRMCIGSKAGTLHAKVVLSMLLREYKIWQSSDNKTSLDTRSTFTAAAD

GIKLHFKKVVR

>Q964R0.1

MVTITGCALCDALVILATLIVAAYLYYAVRFTYWKRKGVVNPKPLPVFGNFLPSVLQKRS

PGQILWDIYKAAEAPFVGFYIFARPAILIKDPNIIKHVLVKDFNAFSDRHASAAESDTLG

SQNLFTLNGAPWKYLRVKLSPTFTSGRMKKMYPLVESCAKQLQDYLKENCNTKAIEVKET

TAKYATDVISTCAFGIESNSLKDPNAEFREFGRKIFEFTRYRTFEVMALFFSPGLVKFLN

GNFFTKETTEFLRKVFWDTINFRESNKISRDDFMDLLIQLKNKGTIDNEDGEVTEKVDKI

DKDSHLFEFTGDNLVSQPALFFTAGFETNATTLSFTLYELSLQPDLQNRLRSEIAGVMKT

SNGKPTYEDVFGMPYLHMVVSETLRKYPPLPLLDRVCLQDYKVPGTDLIIERDTPVFIAL

LGLHRDPQYYPNPERYDPERFSEENKRQRKAYTYLPFGEGPHNCIGLRFGYMAVKTALVH

MLAEFEVKPCKDTPIPLELSTRSSVLATTSGIPLTFVKSTAQVS

>156554006

MPGLLVSLALLGLVLLAFRFYARYKLDYWKRRGVEQLPQSNVVFGNFKDAVLFQTAPGWH

LGVLHKSAVNQDAPFLGFYIFHKPCLLLRDPEIIKQIMIRDFDNFSDRHFAGSQQKDSIG

MKNLFGLKNPAWKYLRSKISPTLARGKLKQMLPLMMQTGKPMMEYLEAQGIDANNEKMID

AQELNYKYATDLIANVALGTPMDSFHRPNEEFSKAVYQFFHGFKRMIALVTVFFVPELVE

LIGPSLLFNSKFVRKVFWNAVNSREKNGEKRGDFIDSLIQLKNGEQNPVYKFEGENLLYQ

SGTFFSGFESSSTTTAFTLMELAAHPEIQERARQDIERAIEEHGMTYEAFNDMKYLDQCL

AEGVRLHPPVSTIDRYTRQDYTIPGTDIVIEKGTAIYISLYGLQEDPRFFTRPSVFDPER

FADDRKVSDAFIPFGIGPRMCVGMKAGQLHAKVVLAMILREYTVTQRPENKLVLDPRSTF

TAAADGINLHFRKNIK

>289177164

MALLTHHWGLDGLILLSSLLVGIYLYFTRNFKYWQKRGIKQVPPTALFGNIGAPLLGQKP

FIKLLSDMYKAGEDEPYIGYYVLDKPFLLLRDPELIKYVLIKDFNNFPNRVAASRKTDVV

GSMNLFVVNNPEWKYIRQKLSPIFTSGRLKKMFDLILEVDKDLDVHMNSLKLEGSGRDIE

VKDICSKFTTDIIGLTAYGMKFNSLTDPDAMIRKVGKDIFGRSYRRYLEFFAVLQVPTLV

NVFGFEVFGKAATKFLRYIFWNAINERIQSGAKRADLIDLLVGLKKEQESDSNQNSFKLE

GDTLVAQAAVFFTGGFETSSSTMSFGLYELAKNPTIQDKLRKEIRDTLDANNKKVTYDMI

MSSLPYLDMVISEVLRMYPILPYLDRNTEADYQLPGTKLVLKAGTPVVIPMQAMHMDSRY

FPKPEIFDPERFSPENKKNILPNTYFPFGDGPRICIGMRLGLMQTKLGLIHLISKYEFSP

CKDTLIPMEFDKYSVFTTSRNDIILNVRKLRD

>345481896

MTFECLNCITNAALLFVFIITILYLYCKYRLSYWNRHGVKSLPTHLFFGNFKDTFTFKKP

PGQVIQEIYEKAAFNEPYIGFYIFHEPKLLLRDLYLMKQLMVKDFDVFPNRCFGGESQKD

SVGLVNLLGIHQPRWKYLRQKLTPSVTGFKLRAMIPLIKECGAPLLKFVENAKSQSDGWK

ELELKDISSRYTTDVIASLAFGISTNSFDDNDVSFWEAGQKILSGAKRGIILIILFFLPG

LIKFIENFMVGPAKYFRLIFWDSMNTREKTGIKRGDMIDHFINLKNGEQISEYKFEGDNL

LSQAVSFYVAGFEASSTAIAFSLYELSLHREYEERLYKEIKDHLTGKELTIDLINEMTFL

DQVVNEALRMYPPLPIVDRVAVRDYKLPGSNVTIQKGTPIYISVTGTNRDPKYFEKPNEF

IPTRPRLETADLPSSSLAFGMGPRSCVGQRLGLIMTKMALITILSEYTLANDKKNDKALL

KPITVFTAAADGIHVKLKKRN

>ADE05586.1

MEILTNALSQNGIIYFGLPVVCLLIYLYCCYKLNYWKRRGVPQLKDQNLLFGDFKDGILF

RTAPGYHLGEIYRRAPANAPYVGFYIFHKPCLLLKDPELIKQILTRDFDNFSNRHFAGPQ

QKDSIGMQNLFGLKNPAWRYLRKKISPTLSTRAKLRQMLPLMMETGEPMMGYLKEKVATN

NESKVIDAQDVNYKYTADLIANVALGTKTDSFTYPDSDYSKCLMNFFHSFKRMVALVTVF

FMPELVDMIGSPILFDSSFVREIFWKAVESREKDGKKRGDYIDSIISLKNGEQDPLYRFE

GDNLFYQSGTFFSGFESSSTVSAFTLMELAKNKAYQDRARADIKRAIDKYGWTIEAFNDM

KYLDQCIAEGVRLHPPVSTIDRYTHRDYKIPNTDVVIEKGTPIYISLYGLHGDHDHFDQP

EVYDPERFTDDRVVSEAYVPFGLGPRKCVGMRVGQLHVKVVISMILSEYEVYQKQAEVAK

LDTRSTFTAAADGIHMKFMKLEK

>EGI65437.1

MAFITAYWSLDGMIVLMTLIITVHLYMTHKFNYWKKRNVLHAQPISILGNFKNSLLLKRS

PGYLLKDFYDEGKGMPYIGFYILDKPFLLVRDRELIKNFLIKDFDYFIDRHATPNTTDRL

AYFSLFFIKNPAWKILRTKLSPTFTSGKLKKMFELMLECGDNLDTYLESLKLEDKDIELD

MKDLSSKFFIDIVGTTAYGLNVNSLNNPHNEFPKYSKQIFEVSILHAFEFLMILFLPNVI

RFINARLFNKKVTAFLRNVFWQTIIQRIKSGKKRNDFIDALIELKGTHRDEDSGREFTFD

GDDLLAQAAIFFIASYETSSTTMIFTLYELAKHPEVQNRLRKEILNALDEIDGKITYDMV

MSLPYLDMVVSEILRMYPPLPFLDRITKETYKVPNSDLVLEKGTPIYISLLGVHYDPEYY

PNPDKFDPERFTKENKRNRSPYVYLPFGDGPRMCIGSRMGLLQSKLGIIVILRKYEVKPC

KKTLIPMNSSKMALITAYWGLDGMIILMTLMITAYLYMTRKFKYWKKRGILEITPMPFFG

NFKECLFQKKAPAYFLKDIYDEMKGLPYVGFYVLDKPFLLVRDRELVKNILVKNFNYFSD

RYNMADPIDRIGYANLFFIKNPAWKIIRTKLTPFFTSGKMKKMFDLMLTCVKNLDEYLDA

LELEGNGKTIEVRELTAKFATDIIGSTAYGLDVNSFKNPNAEFRKYGKMIFHYDTYRSFE

MLAIFFLPTIVRLTRIKMFGKEPTDFMRKVFWETLTQRMKSGLKRNDLIDILLELKNNNN

NDEDLKDFTFDGDDLLAQAASFFSAGFETSSTTTTFALYELAMQLEIQNTLRQEIFEALK

KSNGKITYDMVWSLPYLDMVMSETLRMYPPLGYLNRMPNQTYKVPEFNLVIEKGTPVYIS

MLGLHYDPEYFPNPNKFDPERFNEENKRKRPPCVYFPFGEGPHACIGNRFGLLQTKLTLL

KILSKCEVTLCKETLVPVVIDPRGAMTVPLNGILHLNFRKINTNAI

>EFN69386.1

MAYLTLCLASLVIFIGLFYIYAKYKLSYWSRRGVKTPPMNLFFGNFKDCITLKKAPGEVL

REIYNSADPDDPYIGFYIFHKPMLLLRNHDLIKQMLITDFDVFPNRRFGACNQRDPIGLD

SILSMKQPRWRYLRSKLSSPLTGQKLKNMLPLMEECGKPMLKFIEKLQEDTFGHHKCEIK

DFSSCYTNDVLASIAFGINTNSFDEEENAFWKNGSKVFRGFMSGITFIILFFIPAWGPLV

KPFIQGPANYFREVFWDSMNTREKIGFKRGDVIDSLLSLKNGEQNPIYKFEGDNLVAQSS

SLYIAGIDTSSTTIAFALHNLSRNPEHQTTLYNEIQTHVLGKKLTIDLINEMSFLDCVVN

ESLRLHPPLPVIDRIATRDYKLPGFELIIEKDVSVYVSVNAINQDSKYFLNPHDFIPSRI

KTEQKKFCDSLAFGIGPRTCVGQRFGILLVKVALIMILSNYIVCYDKNEKPILKDKNEIH

VFAYAADGLYLQFKKRQQ

>350418633

MVLLTLHWSLDGILILSFLMVAAYLFVTRKFNYWSKRGVKELAPTPFVGNFMDCLLSKKS

APQFLKDLYNYGRDLPLLGFYVFDKPYLLIRDPELAKHVLMKDFDYFADRYSTADEKNDR

LGYANLFMMKNPGWKSLRPKLTPFFTPGKLKKMFDIMLPVADDFAKHLDSLHLEGEGKVI

EFKDICANFTTDIIGRTAFGLKVSSLENPKAPFRECGRKIFDYDFHRSMEFLAIFFFPGL

VKYLKPKFFGKEATKFFRSIFWDVIEQRVGSGQKRNDLIDMLIEMRETYKNDESLKDYKF

DGDDLVSQAAIFYIGGSETSASTMSFTLHELALNPDVQKTLRTEIQDALEKTGGKITYDM

ITTLPYLDMVISETLRKYPSIAYLDRVTLADYKVPNYDLVLEKDTPIFISTMGSHYDPRY

FPNPEKYDPLRFTEEAKRARPSFAYLPFGEGPRVCIGMRLGLMQSKLGVVQVLKDYEVSP

CEKTKTPIILDPKALTTTALGGIHLNIRKTTTAAG

>EFA12639.1

MAFISLTILAILLLLAVTLFYKWKFQYWKTRQVPFLPPKIPFGNIPNPFTLKEKPGIFIK

QFYQEMKRNGWKHGGFYFFACPVYMVVDPDYIKQVLIKDFQYFIDRGIYSNEKDDPISHN

LFSANETKWKYLRTLLNPSFTSKKIKAMFTILLACEKFLTQNVENNQVIDANHIFANFTI

DVITTCAFGLDCNGNQNLALKHFGEKTINYSKLKMFVLMLASIFPKLAKKFGCVIMHKNT

QEFFVQFVADIIKFREENNYTRDDFMQFLIKLKNKSAKQVFTLEEVASQCVLFFIAGFET

SHATMSFALYELAKHQDIQQKVREEFWAVVDSQESEITYEAVQKLKYLDQVLNETLRKYP

VVMYLTRRCIKDYKVPEDGAVIEKGTTVIIPTVALNYDENYYPNPKKFDPQRFSEENKKL

RHPCIHLPFGLGPRFCIGRRFALIQVKVGLVLLLKNYSFTVNPNTREPIKLKIHSFISAP

EDKILLDVHKL

>189238354

MKEKKWKHGGFYQQLSPVYVICDLNYVKNIMTRDFNYFPYRGLYYNETSDPLSAHIFNTS

GAKWKRMRRKLDQIFTSSKLKTIFPLLPNCGEKLAGKCEPVDIKKVLDDFISDVIELCVF

GSECKVFKQLGRKILNRSRLRRLKGIIINNFPKLAKILDIVLIPRDASTFFKKVVAEIVE

ERKKGQTGRNDFLQVLVEMEKKFEITIDEIAAQCFIFLIAGMSTSPTAMTFALYELARHQ

DIQEKVRQDIAKFGKVTYDSLQEMKYLGQVFDETLRMYPPAAYLNRKCERNYEIPDGSGI

IVEKGTIVAIPVLGIHYDRDYYPDPEKFDPEHFSEENKRLRPNFAFLPFGEGPRICIGMR

FGLMQSKLGLVSLLKRYKFTVSEKTREPLKMKPNSLVLSAEGEIWLDVTEIKI

>AFI45032.1

MVLIIALGVLLSTILLYCAYSKWKFQYWARQGVPQLNPRIFFGDTLPLVRGKPVQDFHLD

IYQKFKPTGAKCVGMYNSHIPELVAIDINLLKDIIVKDYSSFASHGLFYHEKNVLTPHVF

NIEGQIWKERRTKLTPLFTSAKMKQMFATVAGKSPGLIRLVGLSVDANKPADIKEILSRF

TTDVISEAAFGLDCKSLDEPDNMFRSIGREAFKPNSIKLYIENLFPSQFLGNIGYQAFSS

RIVSYFSKVVNETIQYREKNNVQRNDFLQLMLQLKQHGSLVKEDGTVDVKETGAYITDKE

ILSESFVIFLAGHETSSSASTFALFALAQNPDIQDKLRTEINEVLKRHDGKFGYDAIIEM

EYLDKVVRETLRKYPVAPVIPRRCTKDYKIRDTNIVIEKGTRIYIPVIGVHLDPEYYPDP

DRFDPERFSPENKAIRPDIAWMPFGDGPRQCLGMRFGLLQTKVALASLLQEFKFTLNKAM

KAPYTADTGTFVYMFKQDVLLDATRVN

>AEL88546.1

MAVLGLFPQLLLYISATLVSLFLCVYVYFSYSFTYWKRKGAPYIKPKFPLGNNDCFGTES

FSYGLETMDWYKEAKQKGHKLLGAWNYSNPVLLVVDPDYIKSVLVKDFNHFTDRDFFHNP

KHDPKNESIFVAENEDWRKLRQRLSPVFSTAKMRIMFELVNKCAEPMMQLFESYCQSGDP

MDIKEVVAMFTTDVIGCVAFGLDAGSFTSQDAKFRVIGKKLFESSLKTKMHLLMGRINVK

IAQFLGMRVIPESVTEFFAEVIAENARFRQENNVRKTDLMQLLLDLYESSKGQDDGFSFD

DFVGNVIVFFIAGFETSSTTMHNALYELARNPDVQERARVEIKTILKKHGGELTYEALQD

MTYFRQVIDETLRMYPPVQNVARFCVKPYTFKNSNVTVEKGFSVVIPLVALSRDPDNYPD

PERFDPDRFSPQNRDSINKSVYIPFGDGPRNCLGKRFGLMQTSIGLMQIVRDYKISINSK

TPMPLKLKRGVFLMETTHKLYLNVTKV

>AFI45031.1

MALIIAVGLLLSIILLYCVYSKWKFQHWTRKGVPQLNPRLFFGDTLPLVRGQALKDFHLG

LYQKFKSTGAKCVGIYNAHMPELVPIDIILLKDIMVKDYSYFSSHGVFYHEKNVLTSHLF

NIEGQSWKERRTKLTPVFTSAKMKQMFDTVASKSPGLVRLVGLSADANKPADIKEILSRF

TTDVISGAAFGLECKSLYEPDNMFRAIGKEAFEPNALKLYIEYLFPRQLLGSIGYQTFSS

RIVSYFSKVVNETIQYREKNNVQRNDFMQLMLQLKQHGSLVKEDGSVDVKKTETQITDRE

ILSESFLMFLAGHETSSSTSTFALFALSQNQDIQEKVRNEINEVLERHNGKLGYDALMEM

EYLDKVIRETLRKYPTVPVIPRRCTKDYQIKNTNTVIDKGTRLYIPVIGVHLDPEYYPDP

ERFDPERFSPENKAKRPDIAWMPFGEGPRQCLGMRFGLLQSKVALASLLPTFRFTINKAM

KPPYIADAGTLVYMFKQDVLLDATRIN

>AFI45041.1

MAVLGLLSQLLLYISAALVTLFLCVYVYFSYSFTYWKRKGAPYIKPKFPLGNNDCFGAEA

LSYGLETVDWYKEAKRKGHTIVGTWNYSNPVLLLIDPEVIKNVLVKDFNHFTDRDFFHNP

KHDPKNESIFVAENEEWRQLRQRLSPVFSTAKMRVMFELVNKCAEPMMQLFETYSQSGDP

MDIKDIVAMFATDVIGCVAFGLDAGSFNPHEAKFRLIGRKFFESNLKTKIHLIIGRINVK

IAQFLGLRVIPDFLTNFFTEVIAENARFRQENNVRKADLMQLLLDLYESSKGQNDAFTFD

DFVGNVIVFFIAGFDTSSTTMHYALYELARNPDLQEKTRVEIETVLKKHGGHLTYESFQD

MTYLRQVIDETLRLYPPVQNVARFCVKPYTFKDSNVTVEKGVSVVVPLVALGRDPDHYPD

PELFDPDRFSSQNKDSINKFTYIPFGEGPRNCIGKRFGLMQSSIGLIKILKTYKITINSK

TKMPLKLKRGVFLMQPVDKLYVTATKI

>AEL88547.1

MTVLGIFPQLLLYISATLVSLFLCTYVYFSYSFTYWKRKGAPYLKPKFPLGNNDCFGAES

LSYGLETVDWYKEAKEKGHKIVGTWNYSNPVLLLIDPEVIKNVLVKDFNHFTDRDFFHNP

KHDPKNESIFVAENEEWRQLRQRLSPVFSTAKMRVMFELVNKCAAPMMQLFESYSQSVDP

MDIKDVVAMFTTDVISCVAFGLDAGSFNPHEAKFRLIGRKFFESNLKTKMHLIIGRINVK

VAQFLGMRVIPDFLTNFFTEVIAENARFRQENNVRKADLMQLLLDLYESSKGQDDPFTFD

NFVGNVIVFFLAGFDTSSTTMQYALYELARNPDLQEKTRVEIETVLKKHGGHLTYESFQD

MTYLRQVIDETLRLYPPVQNVARFCVKPYTFKGTNVTVEKGVSIVIPLVALGRDPDHYPD

PERFDPDRFSSQNKDSINKFAYIPFGEGPRNCIGKRFGLMQASIGLIEILKTFKITISSK

TKMPLKLKRGVFLMQPVDKLYVNATKI

>AEL88549.1

MAVVSTGFIVFDLCLIIGAAFLAALVYFKHSYRYWKVKRVPYLEPTFPLGNDEYFFTKSA

TFAGEAKSWYDQLKARGHKFGGAWSFYRKVLVLVDPDYIRDVLTRDFHHFANRDMFANEK

FDLQGCHLFNVRNSHWKPLRQKLTPTFTSGKMKMMFESVMQCCDHLVEHLEECAETGENI

DSRKTLSSFTIDVIGSVAFGVDCNSFSKEESDFRKYANLFFRPNFLDYIRLLCLKLFPDL

ARKLRLKIGNTRRTKFFRDMVKQTIDHRIQFKVTRPDFLQLLINMNVERDGKEVLSFDQI

VANTILFFVAGFDTPSTAMNFALFELARNPDLQEKARQEVRKVMGRNDGQTTYEGLREMT

YVKQVLDESMRMYPPLLTLSRVCTKDYELRNTDIVIEKGTSVVISTLGLGRDPEYFPDPE

RFDPDRFSAEEKAKRHPYVHIPFGEGPRNCIGLRFGVMQSKIGLARILSNFRLSVSLKTK

LRINLDKKLFLLKENEPIYLKAEKL

>AAZ94270.1

MNYLLELLIFLVTLSISVTIFVHYRYGYWKRRNVPYLKPIFPMGNNTSIFPKGISLGIIS

KYFYDEFTKMGCKIGGVFFGIDPQLVIVDPDVAKDILTSDFGYFVARGVYKTSSDPTTVN

LFTQDGFEWRTARAKSTSLFTSAKMRKFLETMSKCADEMAASLEQNAIENSDVKMLEVMA

CFTTDVIGSAVLGLNCNSFKDPNAEFRNIGRQLFDEFTLMDTIKIFLTISFPKLCEKIGV

SNIQPEVSEFFCRTVKDLVKYREDNNLIRSDFLQMLIDMKNSTNQLTMDEIIGQVFIFFS

AGFETSSTTATLTLFELSQKPEIQNKLRGEIEECMEKNDNRITYDALMGMPYMNAVVEET

LRKWPSVTTLTRVCTKDYTFRDSGITIQKNTNVIIPVLGFHRDSNYHPKPMEFDPERFMN

KNSSYPGYLPFGEGPRMCIGMRFGQMQVKLGLLTILRKYQVYPSPKTSLPLEIDPDTFVL

HTKQPIYLKLKKL

>AFI45033.1

MALLIALGLLLSIILLYCVYSKWMFQHWARKGVPQLNPRLFFGDTLPLVRGQALRDFHLG

LYQNFKSTGAKCVGIYNTHKPELVPIDINLLKDILVKDYSSFSSHGVFYHENNVLTSHVF

NMEGQPWKDRRTKLTPIFTSAKMKQMFDTVASKSPGLVRLVGLSADANKPADIKEILSRF

TTDVISGAAFGLECKSLYEPDNMLRSIGKEAFEPNALKLYIEYLFHRKFLGTIGYQTFSS

RIVSYFTKVVNETIQYREKNNVQRNDFMQLMLQLKQHGSLMKEDGSVDVKKTETYLTDQE

ILSECFVIFLAGHETSSSTSTFTLFALSQHQDIQEKVRNEINEVLERHDGKLCYDAVMEM

EYLDKVIRESLRKYPTVPVIPRRCTKDYKIKNTNTVIDKGTRLYIPVIGVHLDPEYYPDP

ERFDPERFSPENKATRPDIAWMPFGEGPRQCLGMRFGMLQSKVALASLLHKFRFTLSKAM

KPPFIADAGTLVYMFKQDVLLDATRVN

>AFI45030.1

MLPVPLPPSIIMIVILVLLILSAAIGFWKFTFTYWKKRKCNFLEPTIPFGNAQNFFLGKR

GFGELFGDWYLEMKAKGWDMGGAYFTRRPVFIPINNQLIKRILVTDFAYFQNHGLYINEK

IDPLSGHLFNLENRKWKNLRSKMPAAFSSSKLKNYTVIMANFTEVLVNRLRNMSQSKLPI

EIKEVLSKFNVDAISACLFGIDTKSLDNENVELMKHGRAFFDIQFCRVVNTLVLLIPRPI

LTFFKFRVFPTHVTKYFMNFFSDIKAQRAGENIKRNDLTDILINLCDKTKSVSDIGGDGV

MEPLTSTEFVAQMHLFFEAGFETTGSTQTFALYELATNPDIQNRLRVEINTVLNKFNGIV

GYDAITEMKYLDQVINETLRKYPVFPILPRLCENDYRIPNSNVTIEKGTLVMVTNLGIHY

DPEYYPDPMRFDPERFTSENKAKRPFCSFMPFGEGQRSCVGKLLGILQSKVGLISILRNF

KVTLSEKTKMPFSFDKSGVILNTNGKLWISLETIE

>EFA04605.1

MLLLTAIELSSVFIISLLFAYYLYYRYCFTYWKKRGAPQLTPTFPVGDLSTAAWGKESVY

LRLQTLYHKFKDAGHKFGGIYFLNGPIYFPVHPDLIKRLLVTDFEHFVDRGMYIDEEKLP

LTAHIFSMKGEQWKNVRMKFTPTFTSGKMKSMYNILVQVSQHLIKTLDPVAEQGLEVDIK

ELLMRYTTDIIGSTAFGVDCNSLDNPNTEFNRMVAMIFNNPSWKLYKMALEEGLQNPGNI

IKIAHDNKVVQKYFNDLVRDTIEYRDKNNIVRDDFMNILMQMRSTGMEFKEIVAQAFLFF

TAGFETSSSTMSNCIHELAHNQEIQDKLREEIHENLGRESSKYTYDDVLALPYLDKCVKE

TLRKYPPVAMLNRICVKPYKIPDTETVVEVNTPVIVSVLGLHRDPEYFPEPLEFDPERFS

NSNKIAPFTYLPFGDGPRNCIGLRFGLMQTKLGLATLLNDFKFYPSPKSPRHLAVDPSTT

TLVFSVLNGVHTKIVKV

>EEB11225.1

MGFIMYVGLFTLIALFGFYKKNFNYWKNKNVKMTNPWFPLGDISRAGFYKNIGIVMKEIY

DKFPGEKFNGCWVFYRPHLMIRDPELIKTIFVKDFNYFRDRGLYFNEKTDPLSANLFFMG

GNKWRSLRTKLTSNFSSGKMKKMFENMHEKSLELQKSVKDFAEKDKIIEPKYFTVRYTHD

VAATCLLGFQAKCLTNNDSILPYVGHKVFEDTWTNIIKIAVSVFVPDIGTLFRIKITPKD

VTNFFFNTVKDVMEFRESQNKDGGFNDFMQTLVKLKKEGYTEYEDVKDYALAQAFIFWMG

AFESSASTMQFALYEMSLNNLIQEKARQEVKSVLKKFNGQLTYESLPYMEYLGRVVDETL

RKYPPVSVLTRVCLKKYKIPESDVVIEKGTPLIITVLGLHMDPNYYPNPEKFDPERFTEE

EKRKRHHYAYIPFGGGLHQCMGQRYGLMQIKIGLANLLVNYKFDISFKTPQRAKICRKNV

LYTVRNGIPLDFNKVNLKYHRTGERSHSSVKIDYNLAR

>EEB20095.1

MDYYYVTLLIIIISALTWFIYALANRNCNYWKNRNVPYVSPVPFFGNLKSLLFLEKNVGE

FLGDLYNSEKLPDHGFYGIYLTNNDPALIIRDPELIRNICIKDFQHFSNRNATSDEKNDP

LGYNNLFTLTGSKWKFIRTKLTPTFTSGKMKQMFTLVKETSDELIRYLNENSKEKNYTIM

ETKRILAKFTVDVISTCAFGIKSNCLRDEKSTFGFFSDMIFETTVKRAYEWRAFFFVHWM

IKGLGGVFLRKESTEYFRKVFDETMKERESQKIFRNDFVDNLIKLKNEQEEKLKKEENGK

KTFDKDEIFELKGDLLLAQSVAFFTAGFETTSTTMTYSLYELALNPEVQEKLREEILRSM

EKHDGCLTYDGIMEMNYLSMVVSETLRKYPPLPFLDRICTMDYKIPGTDVVVKKGTPIYI

PLFNGLHYDSKYYPEPEKYIPERFTEENKKTRHPYSYLPFGEGPRTCIGARFGSMVSKIG

LCAILSNFKVETCSKTAVPLEQNHRSLLFCTKDGCFLKYTKL

>AFI45035.1

MEFLLLLVLVGVVFLLLLLLAKRQLTYWARNGVAQLNPELFYGDLRDMLSGQVPIGSAFV

NFYNTFKQKNLRFGGIYWAFTPMFVPVDVQLIKAILVKDFAYFQDRGTYHHENDVLSQNL

FRIGGEPWRTLRQKLSPTFTSSRMRMMFETMQQKIQQLEPILDRCIQEGRINVTDVSACL

TTDVIGSCAFGIECNALQDPDCQLRQQGRRISIPMPLKQWVENTIPRKLLGYTGFKAFPG

QEGFFRKLVLDTMAYREKNSVYRKDFMHLLLQLRNKGVLTDDGNFSREQSHKGILTDDEI

IAQCFIFFTAGFDTSSSTMAFAMRELALQPDLQARVREEVLQVFSKYGGNITYEGTKELL

LTGRVIAETLRKYPVLPNIPRICTKDYKIPDSNVVLRKGTQIQLPVWGLHMDPDYYPDPH

KFDPERFSEAAKATRPEFAYFPFGEGPRICIGARFAQLEAKLGLAALLRRGKFSVLPHAP

TTLTFKPSMVLLHATEDIQLKVEQA

>EFA04674.1

MFLFDLQVSVIQLLSLFGVSILSGYYFYYRYCYTYWKKKGVPTSKPRFPFGDLSSSAWGK

QSVYSRLETLYHQFKAKGYKHAGIYFFNGPIYFPIDPDLVKRVLVTDFEYFVDRGMYGNG

DELPLSCHIFSMKGEEWKNIRTKMSPSFTAGKMKSIYNIVVHNCENLVKVFQPLAETRTV

IDIKDILMRFTADVIGSTSFGIDCNSLDHPDTEFSRMVNRISNHSGWKLLRVALEEGLQN

PGNIEKIAYNDKVVEDFFTNLVKETMEYRDKNNIIRNDFLHMLMQLRESAGMSLKEIVSQ

SFLFFIAGFKTSALTMCYCIHELAHSQDLQDKVREEIHKNLGRDVSKYAYEELIALPTLD

KVVKETMRKYPPLPMLNRICVKSYQVPGTDVVIDKDTPVIIPVLGLQRDPEFYPEPLKFD

PERFSNDNNIVPYTYLPFGDGPRNCIGLRFGMVKTKLAVAALLNNFRFLASSKTRRNLAI

DPSTTSILFNVSEGIYTKIIKV

>EFA04677.1

MFVLLCLVISLSLVSWYLYYVHCFNYWKKKQVPTHPPTFPTGNILKSVLGKENIFITIEN

YYKEFKRKGHKHAGLYFFNGPVYLPIDPEIVKSIMTTDFEHFVDRGVFVDEKNFPLSAHL

FSIKGSKWRNLRTKLSPTFSSGKLKTMYEIFMKMTQNFIETLEPKAGRKEDINIKYIAAN

FTADIIFSTAFGLEGNTLQNPDSKLGNVVQNLFNPSTWIVIKSLALEGLKNPGGIVRAFI

SDKALEEFFVNLVKNTVKHRDENKIVRNDFLSLLLEIRDKEGLSFNEIVAQCFLFFLAGY

DTSAQTISYCIHSLAYNEDIQDKLRKEILDNLGSDYTKYTYDNVLKLPYLEQVLNETLRL

YPVLGFFNRICVKPYKVPGTNVVLDVGTPVLIPTLGLQRDPEYFPDPLKFDPDRFRKDNS

LVPFTFMPFGEGPRFCIGMRYGKLQTKLGVASLLSQFKFKPSPETPELIKPENFSKSLSL

VPVKGVTVKIEKL

>EFA04676.1

MFKFDVFNTGVFELVCLFISLSIVSWYLYYIHCFNYWKKKQVPTHPPQFPAGNILKPVLG

RENLFISVENYYKEFKRKGYKHAGLYFLNGPVYLPIDPEIVKSILTTDFDHFIDRGVFVD

EQNFPLSAHLFSIKGSKWRSLRTKLSPTFSSGKLKTMYEIFMKMTQNFIETLEPKAGRKE

DINIKYIAANFTADIIFSTAFGLEGNTLQYPDSKLGNLAKNLFNPSTWIVIKSLALEGLQ

NPGSILRAFISNKSIEEFFVDLVKKTVKHRDENKVVRKDFLSLLLEIRDREGLSFNEIVA

QCFLFFLAGFETSSQTISYTIHSLAYNQDVQDKLRKEIVDNLGSDFTKYTYDDVLKLPYL

DKVFNETLRLYPVLGFLNRICVKPYKVPGTDVVLDVGTPVLISTLGLQRDPEYFPNPFKF

DPERFSEDNSQVPFTFMPFGEGPRFCIGMRYGKLQTKLGIASLVSQFRFKPSPETPEFIE

FDKFSKSLAMSPVKGITVKIEKL

>EFA04678.1

MFKFDMNNTTVFELLCTFITISLLSWYLYYGYCFNYWKKKGVPTTPPKFPAGNILKPVLG

KENLFVTVEDYYREFKREGHKHAGLYFFNGPVYLPIDPEIVKSILTTDFDHFVDRGVFYD

EQNFPLSTHLFSMRGTEWKSLRMKLSPTFTSGKLKTMYEIFIKMTENFINVVKPKAETGE

EINIKYTSANFTSDIIFNAAFGMEGHTLENPDNELSKVVLNVFNPSNWLFFKSILIEGLQ

NPGSLLRVVLSNKSMEKFFMDLVRTTVTHRDENKIVRKDFLSLLLEIRDKEGLSFNEIAA

QSFLFFLAGAETSSQTISYCIHQLAYHQDLQDKLRKEILDNLGTDFTKYTYDDIFKLPYL

DKVFNETLRLYPVLGFLNRICVKPYKVPGTNVVIDKGTPVLISLLGLQRDPEYFPEPLKF

DPERFSEGLEQVPFTFLPFGDGPRFCIGMRYGKLQTKLGIAALISQFRFLPSPKTPKFVE

YDRPSKSLAMVPAKGIIVKVEKL

>AFI45029.1

MLPVPAALMIIIIILLLIIFSIVITFSTYTFSYWKKRKFNFLEPTIPFGNAQSFFLGKKG

LGELYSDWYLEMKAKGWDMGGAYFGSKPVFIPIDNKLIKTILVKDFSNFQNHGFYINEKI

DPLSGHIYNLESSKWKNLRSKILPAFSSSKLRNHIVVMDSLTKVLVNRLRNMAQSQLPID

IKSSLDRFTLDVTSASLLGIETECLKDKNAELMKQTRAFFDIQLCRLFNTLVLLIPRNIL

IFFNFKVYPTQVTNYFINFFGNLKAQRSVEKIRRNDLTDILIDLCDKTKIVSGDSGNGLT

EPLTIKEFAAQMHLFLDTYETSSATETFALCELAAYPDMQTRLRNEINTVLSRFNGVVEY

DAITEMNYLDQVVNETLRKYPVLPVLPRVCESDYPIPDSKLTIEKGTLVMVTNMGIHYDP

EYYPDPMRFDPERFTSENIAKRPFSSFVPFGEGPRSCVGKRLGMLQAKVGLIAILRNFKI

TLSEKTKMPIQFEKSGLFLNPEGKIWINLETIE

>EFN89626.1

MYTFFKPNLMIADLDLIRTVLTKEFGSFHDRGMFCNEKIDPLSGHLFLLPGKKWRNLRVK

LTPTFTSGKIKQMFMVLKECGEEFTKSLESKARTKDVIEIKDIFARYSTDIIMSTAFGIK

ANCLVEPNNEFRRWGKKTFEVNAFWNALMIFAPQVLNFFSIPNTDRGVSKFFIKIFKDNV

EYRKANNVVRHDFMNLLIQLMDKGYVEPDVDKENDKVNETVNTNKLTMLEATAQAFVFFL

AGFESSSTTATFCLYELALHQDMQDKVRQEIDETLEKHGELTYDAISEMTYLHKVVQGKC

NTHSAHQSLRKYPPVPILNRVCTKEIVVPTTNVHVPEGTLITISVLGVQRDPSIYPNPDK

FDPGRFDADQVTTRHPYAFLPFGEGPRACIGTRFGYIQTKVGIISLLSKFKFKLHPQTPV

PLLFSESPIPLSPKGGIHLFIESR

>EDV54893.1

MWTILLAILAVGLLHRYVRRHYTHWQRLGVEEEPAKIPFGVMDTVMKKERSLGMALGDIY

ARHEGKIVGIYMMNKRSILIRDAQLARQIMTSDFASFHDRGVYVDEKRDPLSANLFNLRG

ASWRNLRQKLTPSFSSGKIKGMFGTIDDVGDKLVRHLEGALDQSDEVEIKDVMTTYAVDI

IGSVIFGLEIDSFSNPQNEFREISSSSSRDESLLLKIHNMSMFICPPIAKLMNRLGYESR

ILTSLRDMMKRTIEFREKHNVVRKDMLQLLIRLRNTGKIGEDDDQVWDMETAQWQLKSMS

IEKIAAQAFLFYVAGSESTAAASAFTLYELSMYPELLKEAREEVDAVLVKHKLKPKDRFT

YEAVQDLRFLDLCIMETIRKYPGLPFLNRECTEDYPVPGTDHIISKGTPILISLFGIQRD

PGYFPNPNGYDPHRFDADTMNYDQAAYMPFGEGPRHCIALRMGKVNSKVAVAKVLANFDL

IQSPRKEVEFRFDAAPVLVTKEPLKLRLTKRM

>EDX08232.1

MWAILLTILSVGLLYRYVRRHYTHWQRLGVDEEPAKIPFGVMDTVMKQERSLGMALGDIY

ARHEGKIVGIYMMNKRSILIRDAQLARQIMTSDFASFHDRGVYVDEDKDPLSANLFNLRG

ASWRNLRQKLTPSFSSGKIKGMFGTIDDVGDKLVQHLQGALDESDEVEIKDVMTTYAVDI

IGSVIFGLEIDSFSNPKNEFREISSSTSTDESLLIAKLMNRLCYESRILTSLRDMMKRTI

EFREEHNVVRKDMLQLLIRLRNTGKIGEDDDQVWDMETAQKQLKSMSIEKIAAQAFLFYV

AGSESTAAASAFTLYELSMYPELLKEAREEVDAVLMKHNLKPKDRFTYEAVQDLKFLDTC

IMETIRKYPGLPFLNRECTEDYPVPGTNHIIAKGTPILISLLGIQRDPVYFPNPNGYDPH

RFDSDTMNYDQAAYMPFGEGPRHCIALRMGKVNSKVAVAKILANFDLIQSPRKEVEFRFD

AAPVLVTKEPLKLRMTKRN

>EDW31899.1

MWAILTLLTLALGLLYQFVRRHYSSWQRLGVDEETPKIPMGILDTVMKQEMSLGMVLSDI

YKRHQGKIVGIYMMNKRSILVRDAQLARQIMTSDFASFHDRGVYVDEKNDPLSANLFNLR

GASWRNLRKKMTPSFSSGKIKGMFGTIDDVGDKMVQHLKGVLERAGADNEIEIKEMMTTY

AVDIIGSVIFGLEIDSFTNPNNEFRAISNEEARSKSLLLKIHNMAMFICPPIAKLMNRLG

FESAILTALRDMMKRTIEFRESNNVVRKDLLQLLIRLRNTGRIGEDDDEVWDMETVQEEL

KAMSIEKIAAQAFLFYVAGSESTAAAAAFTLYELAMYPELLKEARDELDTVLQRHNLEGT

GKLTYEAVQDLKFLDLCLKETLRKYPGLPFLNRECTEDYLVPGTRHTITKGTPILISLFA

IHRDPVYFPNPEGYDPHRFDADNMNYDQAAYMPFGEGPRHCIALRMGEVNTKVAVAKILA

NFDLVQAPHKEVEFRFDAAPVMVPKEGLKLRLNKRK

>EAL25058.1

MWAILTLLTLALGLLYQFVRRHYSSWQRLGVDEETPKIPMGILDTVMKQEMSLGMVLSDI

YKRHKGKIVGIYMMNKRSILVRDAQLARQIMTSDFASFHDRGVYVDEKNDPLSANLFNLR

GASWRNLRKKMTPSFSSGKIKGMFGTIDDVGDKMVQHLKGVLERAGADNEIDIKEMMTTY

AVDIIGSVIFGLEIDSFANPNNEFRAISNEEARSKSLLLKIHNMAMFICPPIAKLMNRLG

FESAILTALRDMMKRTIEFRESNNVVRKDLLQLLIRLRNTGRIGEDDDEVWDMETVQEEL

KAMSIEKIAAQAFLFYVAGSESTAAAAAFTLYELAMYPELLKEARDELDTVLQRHNLEGT

GKLTYEAVHDLKFLDLCLKETLRKYPGLPFLNRECTEDYLVPGTRHTITKGTPILISLFG

IHRDPVYFPNPEGYDPHRFDADKMNYDQAAYMPFGEGPRHCIALRMGEVNTKVAVAKILA

NFDLVQAPHKEVEFRFDAAPVMVPKEGLKLRLNKRK

>EDW91818.1

MNKRSILIRDAQLARQIMTSDFASFHDRGVYVDENKDPLSANLFNLRGASWRNLRQKLTP

SFSSGKIKGMFGTIDDVGVKLVQHLEGALDQSDEVEIKDVMTTYAVDIIGSVIFGLEIDS

FSNPKNEFREISSSTSRDESLLLKIHNMSMFICPPIAKLMNRLGYESRVLTSLRDMMRRT

IEFREQHNVVRKDMLQLLIRLRNTGKIGEDDDQVWDMETAQEQLKSMSIEKIAAQAFLFY

VAGSESTAAASAFTLYELSMYPGLLQEARDEVDAVLVKHKLEPKDRFTYEAVQDLKFLDL

CIMETIRKYPGLPFLNRECTEDYPVPGTDHIITKGTPILISLFGIQRDPAYFPNPNGYDP

HRFDADTMNYDQAAYMPFGEGPRHCIALRMGKVNSKVAVAKVLANFDLIQSPRKEVEFRF

DAAPVLVTKEPLKLRLIKRK

>EDW74325.1

MWLILFTTILAGLIYQYVKRHYTFWYREGIDEEPAKFPFGILNPVIKQERSFGLVLSDIY

QRYSGDIVGIYMINKQTLLVRDAQLARQIMTSDFNSLHDRGLYVDEERDPLSANLFNLPG

ASWRNLRQKLAPSFTSGKIKAMFETVNDVGDKLVQHLLKVIDETPGNDIEIKDKLTTYAV

DIIGSVIFGLELDSFTDPDNEFRKVSKTVLKSETIWMKLHNMATIMCPPVAKLMNLLGIE

NEVFVSLRNIMKETIEFREKHNVIRKDLLQLLIRLRNTSKIGDDDDQVWDIETTREELKD

MSIDKIAAQAFLFYIAGSETTAATSAFTLYELAMYPDLLKEVRDELDSVLQKHNLKRGDK

LTYDAIQDLKFLDQCIMETVRKYPGLPFLNRQCTEDYEVPKTKYVIRKRTPILIPLFGIH

RDPAYFPNPVDYDPHRFDVDNMNYDQAAYMPFGEGPRHCIALRMGKVNSKVAVAKILANF

DVVPTPRMEVEFGFDPTPVLVPIGGLKLRLNKRT

>EDW61417.1

MYLVLLLTILLALLVQYLRRIYSYWQREGVAEEPAKFPFGVLDKLVKRQRGFGLIISDVY

ERHSSKIVGIYMMHKPTLLVRDAQLARQIMTSDFSSFHDRGIYVDEKHDPLSANLFNLQG

ASWRNLRNKLTPSFSSGKIKGMFTTIDDVGDRLVQHLMGVIEKAPNEAIEIKEKLTTYAV

DIIGSVIFGLEIDSFTNPKNEFRAVSDSLLVEDELVLKIHNMASVIFPPLAKLMNRLGYE

NKVLAKLRDIMQHTIEFREKHNVVRKDLLQLLIRLRNTGKIGDDDDEVWDIETAQEELKA

MSIEKIAAQAFLFYIAGSETTAAASSFTLYELAMYPELLKEARDELDAVMKRHNLERGDK

FTYEAVQELKFLDHCILETIRKYPGLPFLNRECTQDFTVPDIDYTIKKGTPILISLFGLQ

RDPSYFPNPEGYDPHRFDADVMNYDQTAYMPFGEGPRHCIALRMGKVNAKVAVAKILANF

NLIEAPRKEVKFRFDPAPVLLPEGGLHVRLVKRP

>EDW02654.1

MYLILLVTISLALLVNYLRRIYTHWQREGIEEEPAKFPFGILDALVKRQRSFGIIVSDVY

EQHKSQIVGIYMMHKPTILVRDAQLARQVMTSEFNSFHDRGVYVDEKRDPLSANLFNLQG

AAWRNLRNKLTPSFSSGKIKGMFSTIDDVGDRLVQHLMGLIEKAPNDPIEIKQKLTTYAI

DIIGSVIFGLEIDSFTNPKNEFRAVSDSLLVEDGILIKMHNMASVIFPPVAKLMNRLGFE

NKVMIKLRDIMQHTIEFRERHNVVRKDLLQLLIRLRNTGKIGDDDDEVWDIETAQEELKA

MSIDKIAAQAFLFYIAGSETTAAASAFTLYELAMYPELLKEARDELDVVMERHNLKPGDK

FTYEAVQDLKFLELCILETIRKYPALPFLNRECTKDFHVDDMNYTIKKGTPILISLLGLH

RDPAYFPNPMDYDPHRYDADNMNYDPTAYMPFGEGPRHCIALRMGKVNAKVAVAKVLANF

NLIPAPRKEIKFRFDPTPVLMPEGGLSVRLSKRA

>EDW09055.1

MYLVLLVTILLILLMQYIRRIYSYWQREGVDEEPATFPFGSMLKLLKKQRGFGLILSDIY

ERHNSKIVGLYVMHKPVLLVRDPHLARQIMTSDFASFHDRGMYVDEEHDPLSANLFNLQG

ASWRNLRQKLTPSFSSGKIKGMFDTIDDVGDRLVQHLQGIVDKAPNESIEMKAILTTFAV

DIIGSVIFGLDIDSFTDPKNEFRAISDSLLSEIGILLRIHNLATIIFPPLAKLMNRMGFE

NKVLGKLRDIMKRTVEFREKNNVVRKDLLQLLIRLRNTGKIGDDDDKVWDVETAREELKA

MSIDKITAQGFLFYIAGSETTAATSAFTLYELSMYPELLKEARDELDAVMERHQLKRGDK

FTYEAVQDLKFLDMCIMETIRKYPGLPFLNRECTQDFLVPDLNYTIKRGTGILISLFGLH

RDAKYFPNPEDYGPHRFDADNMNYNQTAYMPFGEGPRHCIAIRMGKVNAKVAVAKVLLNF

DLVHAPRKEVKFRFDPAPVLLPEGGLHVRLVKRA

>AAA81513.1

MLLLLLLIVVTTLYIFAKLHYTKWERLGFESDKATIPLGSMAKVFHKERPFGLVMSDIYD

KCHEKVVGIYLFFKPALLVRDAELARQILTTDFNSFHDRGLYVDEKNDPMSANLFVMEGQ

SWRTLRMKLAPSFSSGKLKGMFETVDDVADKLINHLNERLKDGQTHVLEIKSILTTYAVD

IIGSVIFGLEIDSFTHPDNEFRVLSDRLFNPKKSTMLERIRNLSTFMCPPLAKLLSRLGA

KDPITYRLRDIVKRTIEFREEKGVVRKDLLQLFIQLRNTGKISDDNDKLWHDVESTAENL

KAMSIDMIASNSFLFYIAGSETTAATTSFTIYELAMYPEILKKAQSEVDECLQRHGLKPQ

GRLTYEAIQDMKYLDLCVMETTRKYPGLPFLNRKCTQDFQVPDTKLTIPKETGIIISLLG

IHRDPQYFPQPEDYRPERFADESKDYDPAAYMPFGEGPRHCIAQRMGVINSKVALAKILA

NFNIQPMPRQEVEFKFHSAPVLVPVNGLNVGLSKRW

>AAC46931.1

MLLLLLLIVVTTLYIFAKLHYTKWERLGFESDKATIPLGSMAKVFHKERPFGLVMSDIYD

KCHEKVVGIYLFFKPALLVRDAELARQILTTDFNSFHDRGLYVDEKNDPMSANLFVMEGQ

SWRTLRMKLAPSFSSGKLKGMFETVDDVAAKLLNHLNERLKDGQSHVLEIKSILTTYAVD

IIGSVIFGLEIDSFTHPDNEFRVLSDRLFNPKKSTMLQRFRNLSNFICPPLAKLLSRLGA

KDPITYRLRDIVKRTIEFREEKGVVRKDLLQLFIQLRNTGKISDDNDKLWHDVESTAENL

KAMSIDMIASNSFLFYIAGSETTAATTSFTIYELAMYPEILKKAQSEVDECLQRHGLKPQ

GRLTYEAIQDMKYLDLCVMETTRKYPGLPFLNRKCTQDFQVPDTKLTIPKETGIIISLLG

IHRDPQYFPQPEDYRPERFADESKDYDPAAYMPFGEGPRHCIAQRMGVINSKVALAKILA

NFNIQPMPRQEVEFKFHSAPVLVPVNGLNVGLSKRW

>Q27698.1

MLLLLLLIVVTTLYIFAKLHYTKWERLGFESDKATIPLGSMAKVFHKERPFGLVMSDIYD

KCHEKVVGIYLFFKPALLVRDAELARQILTTDFNSFHDRGLYVDEKNDPMSANLFVMEGQ

SWRTLRMKLAPSFSSGKLKGMFETVDDVAAKLLNHLNERLKDGQSHVLEIKSILTTYAVD

IIGSVIFGLEIDSFTHPDNEFRVLSDRLFNPKKSTMLQRFRNLSNFICPPLAKLLSRLGA

KDPITYRLRDIVKRTIEFREEKGVVRKDLLQLFIQLRNTGKISDDNDKLWHDVESTAENL

KAMSIDMIASNSFLFYIAGSETTAATTSFTIYELAMYPEILKKAQSEVDECLQRHGLKPQ

GRLTYEAIQDMKYLDLCVMETTRKYPGLPFLNRKCTQDFQVPDTKLTIPKETGIIISLLG

IHRDPQYFPQPEDYRPERFADESKDYDPAAYMPFGEGPRHCIAQRMGVMNSKVALAKILA

NFNIQPMPRQEVEFKFHSAPVLVPVNGLNVGLSKRW

>BAB41135.1

MLLYLLITLVSAFFIFAKRHYTQWQRLGLASDEAVIPFGSLAKVFRKERPFGLVMYDLYE

KFQEQVVGIYLFFQPALLIRDAELARQILTSDFNSFHDRGIYVDEKNDPMSANLFALEGQ

SWRTLRMKLTPSFSSGKLKGMFETVDDVGNKLLEYLNNQLKDGQTHVLDIKSILTTYAID

IIGSVIFGLEIDSFTNPDNEFRVLSDRSFDNESRSFLSKLRNLTNFVCPPIAKLLTRLGT

KEPVVYRLRDIVKRTIEFREENNVVRKDLLHLLIQLRNTGKISDDNDNLWNKVESTATNL

KAMSIDMIASNSFLFYIAGSETTASSTSFTIYELAMNPEALKKAQNEVDECLKKHGIKPD

GRITYEAIQDMKYLDLCVKETTRKYPGLPFLNRQCTQDFKVPNSKFTIKKDTNVIISLLG

LHRDAKYFPEPLAYKPERFADETKDYDAAAYMPFGEGPRHCIAQRMGVMNTKVALAKILA

NFNIEPMPHKEAEFQFNTAPVLVPVNGLRVGLSKRSFN

>AAD32478.1

MLLLLLLIVVTTLYIFAKLHYTKWERLGFESDKATIPLGSMAKVFHKERPFGLVLSDIYD

KCHEKVVGIYLFFKPALLVRDAELARQILTTDFNSFHDRGLYVDEKNDPMSANLFVMEGQ

SWRTLRMKLAPSFSSGKLKGMFETVDDVAAKLLNHLNERLKDGQSHVLEIKSILTTYAVD

INGSVIFGLEIDSFTHPDNEFRVLSDRLFNPKKSTMLERFRNLSTFMCPPLAKLLSRLGA

KDPITYRLRDIVKRTIEFREETGVVRKDLLQLFIQLRNTGKISDDNDKLWHDVESTAENL

KAMSIDMIASNSFLFYIAGSETTAATTSFTIYELAMYPEILKKAQSEVDECLQRHGLKPQ

GRLTYEAIQDMKYLDLCVMETTRKYPGLPFLNRKCTQDFQVPDTKLTIPKETGIIISLLG

IHRDPQYFPQPEDYRPERFADESKDYNPAAYMPFGEGPRHCIAQRMGVINSKVALAKILA

NFNIQPMPRQEVEFKFHSAPVLVPVNGLNVGLSKRW

>Q964Q7.1

MLLFLLITLLSAIFIFAKRHYTQWQRLGLVSDEAVIPFGSLAKVFRKERPFGLVMYDLYE

KFQEQVVGIYLFFKPALLIRDAELARQILTSDFNSFHDRGIYVDEKNDPMSANLFALEGQ

SWRTLRMKLTPSFSSGKLKGMFETVDDVGNKLLEYLNNQLKDGQTHVLDIKSILTTYAID

IIGSVIFGLEIDSFTNPDNEFRVLSDRSFDNESRSFLSKLQNLTNFVCPPIAKLLTRLGT

KEPVVYRLRDIVKRTIEFREENNVVRKDLLHLLIQLRNTGKISDDNDNLWNKVESTATNL

KAMSIDMIASNSFLFYIAGSETTASSTSFTIYELAMNPEALKKAQNEVDECLKKHGIKPD

GRITYEAIQDMKYLDLCVKETTRKYPGLPFLNRQCTQDFKVPNSKFTIKKDTNVIISLLG

LHRDAKYFPEPLAYKPERFADETKDYDAAAYMPFGEGPRHCIAQRMGVMNTKVALAKILA

NFNIEPMPHKEAEFQFNTAPVLVPVNGLRVGLSKRSFNRR

>AAC46930.1

MLLLLLIVVTTLYIFAKLHYTKWERLGFESDKATIPLGSMAKVFHKERPFGLVLSDIYDK

CHEKVVGIYLFFKPALLVRDAELARQILTTDFNSFHDRGLYVDEKNDPMSANLFVMEGQS

WRTLRMKLAPSFSSGKLKGMFETVDDVAAKLLNHLNERLKDGQSHVLEIKSILTTYAVDI

IGSVIFGLEIDSFTHPDNEFRVLSDRLFNPKKSTMLQRFRNLSNFICPPLAKLLSRLGAK

DPITYRLRDIVKRTIEFREEKGVVPKDLLQLFIQLRNTGKISDDNDKLWHDVESTAENLK

AMSIDMIASNSFLFYIAGSETTAATTSFTIYELAMYPEILKKAQSEVDECLQRHGLKPQG

RLTYEAIQDMKYLDLCVMETTRKYPGLPFLNRKCTQDFQVPDTKLTIPKETGIIISLLGI

HRDPQYFPQPEDYRPERFADESKDYDPAAYMPFGEGPRHCIAQRMGVMNSKVALAKILAN

FNIQPMPRQEVEFKFHSAPVLVPVNGLNVGLSKRW

>AAK69552.1

MLLFLLITLLSAFFIFAKRHYTQWQRLGLVSDEAVIPFGSLAKVFRKERPFGLVMYDLYE

KFQEQVVGIYLFFKPALLIRDAELARQILTSDFNSFHDRGIYVDEKNDPMSANLFALEGQ

SWRTLRMKLTPSFSSGKLKGMFETVDDVGNKLLEYLNNQLKDGQTHVLDIKSILTTYAID

IIGSVIFGLEIDSFTNPDNEFRVLSDRSFDNESRSFLSKLRNLTNFVCPPIAKLLTRLGT

KEPVVYRLRDIVKRTIEFREENNVVRKDLLHLLIQLRNTGKISDDNDNLWNKVESTATNL

KAMSIDMIASNSFLFYIAGSETTASSTSFTIYELAMNPEALKKAQNEVDECLKKHGIKPD

GRITYEAIQDMKYLDLCVKETTRKYPGLPFLNRQCTQDFKVPNSKFTIKKDTNVIISLLG

LHRDAKYFPEPLAYKPERFADETKDYDAAAYMPFGEGPRHCIAQRMGVMNTKVALAKILA

NFNIEPMPHKEAEFQFNTAPVLVPVNGLRVGLSKRSFN

>AAG28563.1

MLLFLLITLLSAFFIFAKRHYTQWQRLGLVSDEAVIPFGSLAKVFRKERPFGLVMYDLYE

KFQEQVVGIYLFFKPALLIRDAELARQILTSDFNSFHDRGIYVDEKNDPMSANLFALEGQ

SWRTLRMKLTPSFSSGKLKGMFETVDDVGNKLLEYLNNQLKDGQTHVLDIKSILTTYAID

IIGSVIFGLDIDSFTNPDNEFRVLSDRSFDNESRSFLSKLRNLTNFVCPPIAKLLTRLGT

KEPVVYRLRDIVKRTIEFREENNVVRKDLLHLLIQLRNTGKISDDNDNLWNKVESTATNL

KAMSIDMIASNSFLFYIAGSETTASSTSFTIYELAMNPDALKKVQNEVDECLKKHGIKPD

GRLTYEAIQDMKYLDLCVKETTRKYPGLPFLNRQCTQDFKVPNSKFTIKKDTNVIISLLG

LHRDAKYFPEPLAYKPERFADETKDYDAAAYMPFGEGPRHCIAQRMGVMNTKVALAKILA

NFNIEPMPHKEAEFQFNTAPVLVPVNGLRVGLSKRSFN

>EDV54106.1

MFSLVLLAISLLTLAWFYLKRHYEYWERRGFPVDKHSGIPFGCLDSVWRQEKSMGLAIYD

VYVKSKERVLGIYLLFRPAVLVRDADLARRVLAQDFASFHDRGVYVDEERDPLSANIFSL

RGQSWRSMRHMLSPCFTSGKLKSMFSTSEDIGDKMVAHLQKELPEEGFKEVDMKKVMQNY

AIDIIASTIFGLDVNSFENPDNKFRKLVSLARANNRFNALFGMMIFLVPSIAQFLFRIGF

KNPVGLAMLQIVKETVEYREKHGIVRKDLLQLLIQLRNTGKIDENDEKSFSIQKTPDGHI

KTISLEAITAQAFIFYIAGQETTGSTAAFTIYELAQYPELLKRLQDEVDETLAKNDGRIT

YDSLHKMEFLDLCVQETIRKYPGLPILNRECTQDYIVPDTNHVIPKGTPVVISLYGIHHD

AEYFPDPETYDPDRFSEQSRNFNPTAFMPFGEGPRICIAQRMGRINSKLAIIKILQNFKV

EVMSRSEIEFENSGIALIPKHGVRVRLSKRVPKLP

>Q9VCW1.1

MFSLILLAVTLLTLAWFYLKRHYEYWERRGFPFEKHSGIPFGCLDSVWRQEKSMGLAIYD

VYVKSKERVLGIYLLFRPAVLIRDADLARRVLAQDFASFHDRGVYVDEERDPLSANIFSL

RGQSWRSMRHMLSPCFTSGKLKSMFSTSEDIGDKMVAHLQKELPEEGFKEVDIKKVMQNY

AIDIIASTIFGLDVNSFENPDNKFRKLVSLARANNRFNAMFGMMIFLVPSIAQFLFRIGF

KNPVGLAMLQIVKETVEYREKHGIVRKDLLQLLIQLRNTGKIDENDEKSFSIQKTPDGHI

KTISLEAITAQAFIFYIAGQETTGSTAAFTIYELAQYPELLKRLQDEVDETLAKNDGKIT

YDSLNKMEFLDLCVQETIRKYPGLPILNRECTQDYTVPDTNHVIPKGTPVVISLYGIHHD

AEYFPDPETYDPERFSEESRNYNPTAFMPFGEGPRICIAQRMGRINSKLAIIKILQNFNV

EVMSRSEIEFENSGIALIPKHGVRVRLSKRVPKLS

>EDW98043.1

MFSLVLLAIHCSLLTLAWFYLKRHYEYWERRGFPFDKHFGIPFGCLDSVWRQEKSMGLAI

YDVYVKSKERVLGIYLLFRPAVLVRDADLARRVLAQDFASFHDRGVYVDEERDPLSANIF

SLRGQSWRSMRHMLSPCFTSGKLKSMFSTSEDIGDKMVAHLQKELPEEGFKEVDVKKVMQ

NYAIDIIASTIFGLDVNSFENPDNKFRKLVSLARANNRFNALFGMMIFLVPSIAQFLFRI

GFKNPVGLAMLQIVKETVEYREKHGIVRKDLLQLLIQLRNTGKIDENDEKSFSIQKTPDG

HIKTISLEAITAQAFIFYIAGQETTGSTAAFTIYELAQYPELLKRLQDEVDETLAKNDGK

ITYDSLHKMEFLDLCVQETIRKYPGLPILNRECTQDYTVPDTNHVIPKGTPVVISLYGIH

HDAEYFPDPETYDPDRFSEESRNYNPTAFMPFGEGPRICIAQRMGRINSKLAIIKILQNF

NVEVMSKSEIEFENSGIALIPKHGVRVRLSKRVPKLQ

>EDX13971.1

MFSLILLAVTLLTLAWFYLKRHYEYWERRGFPFEKHSGIPFGCLDSVWRQEKSMGLAIYD

VYVKSKERVLGIYLLFRPAVLVRDADLARRVLAQDFASFHDRGVYVDEERDPLSASIFSL

RGQSWRSMRHMLSPCFTSGKLKSMFSTSEDIGDKMVAHLQKELPEEGFKEVDMKKVMQNY

AIDIIASTIFGLDVNSFENPDNKFRKLVSLARANNRFNALFGMMIFLVPSIAQFLFRIGF

KNPVGLAMLQIVKETVEYREKHGIVRKDLLQLLIQLRNTGKIDENDEKSFSIQKTPDGHI

KTISLEAITAQAFIFYIAGQETTGSTAAFTIYELAQYPELLKRLQDEVDETLAKNDGKIT

YDSLNKMEFLDLCVQETIRKYPGLPILNRECTQDYTVPDTNHVIPKGTPVVISLYGIHHD

AEYFPDPETYDPERFSEESRNYNPTAFMPFGEGPRICIAQRMGRINSKLAIIKILQNFNV

EVMSRSEIEFENSGIALIPKHGVRVRLSKRVPKLP

>EDV93023.1

MLSLGLLIITLLVLGWYYLKHQYSYWERRGFPYDKDAGIPFGCLASVWRMERGMGMAIYD

VYMKSKERFLGTYLLYRPAVLVRDAELARRVLTQDFASFHDRGVYVEEEKNPLSASIFSL

RGQSWRSMRHKLSPCFTSGKLKSMYSTSEDIGNKMVAHLQKELPEQGAKEIDLKEVTQTY

AIDIIASVIFGLDVNSFEQPDNKFKKVVSLIRRNNRFAAFIGMIIVLIPSIAKFMVRMGL

RNPVAVALLAIVKDTIEYREKHGIVRKDMLQLLMQLRNKGSIDDDENKNWNIQTSEDGEI

KSISLETITAQAFIFYVAGQETTASTTAFTLYELAQYPEHLKRLQAEVDETLKQNDGKIT

YDALNKMEFLELCLQESLRKYPGLPILNRECTEDYTIPDTNHVIKKGTPVVISLHGIHRD

PEYFPDPDKYDPYRFAEETKNYNPIAYMPFGEGPRICIAQRMGRVNVKLAIVKILQNFNI

EVMSKRELEFEISGIVLTPKNGVKVRLSKRVLN

>EDV43083.1

MFSILLLVASLLTLAWFYLKHHYDYWQRRGMPVYEPSGIPFGCLDSVWRQERSMGLAIHD

VYLRTKEKVVGIYLLFRPGVLIRDAALVRRVLTQDFASFHDRGVYVDETNDPLSANLFSL

QGQSWRSMRHMLSPCFTSGKLKAMYSTSEDIGDKMVSHLQKVLPEEGSQEVDLKKVMQNY

AVDIIASTIFGLDVNSFDHPDNKFNNLVAIARKNNRFTALFGMMIFLVPSIAQFLFKVGL

KNPVGLAMLEIVKETIEHREKHGIVRKDMLQLLMNFRNTGKIEENDEKCFSIQKSPEGHI

KAISLEAITAQAFIFYIAGQETTGSTAAFTIYELAQYPEHLKRLQAEVDETLAKNDGKIT

YDALHKMEFLELCVQETLRKYPGLPMLNRECTQDYTVPETNHVIPKGTPIVISLYGLHRD

EEFFPNPETYDPNRFTEESHNYNPTAFMPFGEGPRICIAQRMGRMNSKLAIIKILQNFNV

EVMSKRELEFETHGIALMPKHGVKVRLSKRIPKMA

>EDV93021.1

MLSLGLLIITLLVLGWYYLKHQYSYWERRGFPYDKDAGIPFGCLASVWRMERGMGMAIYD

VYMKSKERFLGTYLLYRPAVLVRDAELARRVLTQDFASFHDRGVYVEEEKNPLSASIFSL

RGQSWRSMRHKLSPCFTSGKLKSMYSTSEDIGNKMVAHLQKELPEQGAKEIDLKEVTQTY

AIDIIASVIFGLDVNSFEQPDNKFKKVVSLIRRNNRFAALIGMMIFLIPAIAKFMFRMGL

RNPVAVALLAIVKDTIEYREKHGIVRKDMLQLLMQLRNKGSIDDDENKNCNIQTSEDGEI

KSISLETITAQAFIFYVAGQETTGSTTAFTLYELAQYPEHLKRLQAEVDETLKQNDGKIT

YDALNKMEFLELCLQESLRKYPGLPMLNRECTEDYTIPDTNHVIKKGTPVVISLHGIHRD

PEYFPDPDKYDPYRFAEETKNYNPIAYMPFGEGPRICIAQRMGRVNVKLAIVKILQNFNI

EVMSKRELEFEISGIVLTPKNGVKVRLSKRVLN

>EDW67808.1

MLSIFLLGLTLLALCWYYLKHHYSYWERRGFPYDKNAGIPFGCLASVWRMEKAMGMAVYD

VYKNSKERFLGTYLLFRPAVLVRDAELARRVLAQDFASFHDRGVYVDEDKDPLSASIFAL

RGQSWRSMRHKLSPCFTSGKLKGMYSTSEDIGIKMIAHLQKELPEQGSKEVDLKAVLQTY

AIDIIASVIFGLDVNSYEQPDNKFKKLVTIIRRNTRFSALFGMMIFLVPSVAKFLFRIGF

KNPVGLAMLAIVKDTIEYREQHGIVRKDMLQLLMQLRNKGSIDDDDSKSWSLQTNDDGQL

QSISLEKITAQAFIFYVAGQETTGSTAAFTIFELAQYPEHLKRLQGEVDETLKQNDGKIT

YDVLNKMEFLELCLQETMRKYPGLPMLNRECTQDYTVPDTNHVIKKGTPVVISLHGIHHD

AEYFPDPDKYDPYRFAEDTKNYNPIAYMPFGEGPRICIAQRMGRVNAKLAIVKILQNFNI

EVMSKRELEFETSGIALLPKDGIKVRLSKRMPN

>EDW23747.1

MFSLIVLAGTLLALGWFYLKHHYQYWERRGFPFDRHSTIPFGCLDSVWRREKGMGMAIYD

AYQKSKERFLGIYLLFRPAVLVRDAALARRVLAQDFASFHDRGVYVDEVNDPLSGSIFAL

RGQSWRSMRHKLSPCFTSGKLKGMFSTSEEIANNMVNHLQKTLPEQGSQEVDLKTVMQTY

AIDIIASTIFGLEMNSYLTPDNKFRNLVTIARRNTRFTTMFGMMIFLVPSVAKFLFSLGF

KNQVALAMLEIVKETIEYREKHGIVRKDLLQLLMELRNTGAVDESDENIWKIQKSATDQI

KCISLEAITAQAFIFYIAGQETTGSTAAFVLFELAQYPELLERLQTEVDETLAKNDGHIT

YDALQKMEFLDLCVQESLRKYPGLPMLNRECTQDYTVPDTDHVIPKGTPVVISLYGIHHD

AEYFPEPETYDPDRYSEENRNFSPTAFMPFGEGPRICIAQRMGLVNAKLAIINVLKNFNV

EVMSKRQLEFENSGIALLPKNGVKVRLSKRLPK

>EAL27266.1

MFSLIVLAGTLLALGWFYLKHHYQYWERRGFPFDRQSTIPFGCLDSVWRREKGMGMAIYD

AYQKSKERFLGIYLLFRPAVLVRDAALARRVLAQDFASFHDRGVYVDEVNDPLSGSIFAL

RGQSWRSMRHKLSPCFTSGKLKGMFSTSEEIANNMVNHLQKTLPEQGSQEVDLKTVMQTY

AIDIIASTIFGLEMNSYLTPDNKFRNLVTIARRNTRFTTMFGMMIFLVPSVAKFLFSLGF

KNQVALAMLEIVKETIEYREKHGIVRKDLLQLLMELRNTGAVDESDENIWKIQKSATDQI

KCISLESITAQAFIFYIAGQETTGSTAAFVLFELAQYPELLERLQTEVDETLAKNDGHIT

YDALQKMEFLDLCVQESLRKYPGLPMLNRECTQDYTVPDTDHVIPKGTPVVISLYGIHHD

AEYFPEPETYDPDRYSEENRNFSPTAFMPFGEGPRICIAQRMGLLNAKLAIINVLKNFNV

EVMSKRQLEFENSGIALLPKNGVKVRLSKRLPK

>EDW80627.1

MLTFIVLVGTLLGLGWFYLKHIYSYWERIGFPYDKNSAIPFGCLTPVWDMKQSMGLAIHE

VYKNSKERFLGVYLLFRPAVLVRDAELARRVMAQDFSSFHDRGIYVDEEKNPLSGSIFAL

RGQNWRSMRHKLSPCFTSGKLKAMFHVSEDIADKMVSHLDTLMPQEGSVEVDVKSVMQTY

AIDIIGSVIFGLDINSYEHPDNKFRTLVTLARSNTRFTALFGMMIFLVPSIAKFLFALGF

QNKVGQAMMEIVKETIENREKKGIVRKDMLQLLMQLRNTGQIEEDDEKSFNIQTTETGQL

QALSLETITAQAFIFYIAGQETTGSTVAFTLFELAQYPELLKRVQTEVDETLKQNDGKIT

YDALQKMEFLDHCVQEITRLYPGLPLLNRECTQDYAIPDTNHVIRKGTPVVISLYGIHHD

PEYFPNPEVFDPDRFSEENRNYNPTAFMPFGEGPRICIAQRMGRVNAKLAIVKVLQHFNV

EVMSKRKIEFEASGIALLPKSGVKVRLSKRTKN

>EDW14614.1

MLYLFLFALTLLNLGWYYLKHHYLYWERHGFPYDENISIPFGCLGSVWRREKAMGMALYD

VYRNSKERFLGTFLLFRPAILVRDAELARRILTQDFASFHDRGVYVDEKHNPLSASIFAL

RGQRWRSMRHMLSPCFTSGKLKAMYSTSEDIGNKMVAHLQKELPENGAKEVDLKQVMQIY

AVDIIASVIFGLEVNSYDQPDNKFRKVVTVARRNSRFNALFGMMLFLVPPILKFLFRIGL

KNPVGMAMLAIVKDTIEYREQNGIVRKDLLQLLMQLRNKGSIEEDDSKSWNIQTSEDGHL

KGISLEAITAQAFIFYIAGQETTASTAAFTIFEVAQYPEHLERLQAEVDETLKQNGGNIS

YDVLSKMEFLELCVLETMRKYPGLPMLNRECTQDYKVPDSNHIIKKGTPVVISLHGIHHD

AEYFPDPEKYDPNRFADESKNYNPIAYMPFGEGPRICIAQRMGRVNVKLAIVKILQHFDI

EVMSRRELEFENAGIGLLPKDGVRVRLSKKKTNSTEL

>EDW83125.1

MAWFPLLIVAIGLVYVYLKWTFSFWERKGFPYAAVNIPFGVLDSVRRGKRSFGMAIYDLY

VASKEPVVGMYLSLRPALLVRDPQLVHDMLLKDFASFHDRGIYVDEKNDPMSAGLFQMEG

SSWKTMRTKLTPSFTSGKLKAMFGTAEAIGDKMIAHLNERLAKESGGAPLEIEMKSLMAT

YAIDIIASTIFGLDVDSFADPKNEFATISKMVNRPIAKDILRGTTGFLYPGLEKLFNRLG

WKQEGQERMRELVHRTVDYREKNNVVRRDFLQLLLQLRNTGKISTDDGIWSAETAANNLK

SMSTDLIAGQLFLFFVAGYETSASTAAFTLYELTQNPALLAKAQEDVRSALERHEGKLSY

DAIQDMKYLELCIMETARKYPALPLLNRICTQDYPIPNSKLVIEKGTPIVISLIGMHRDE

ENFPEPLTYQPERYTEEHKNYNPNAYMPFGEGPRHCIAARLGKLNVKIALAKILSTFNIE

TRTELREIEFSVHGIPLMPEGGVPVRILKKI

>EDW83124.1

MIFIFLVITATSLLYVYLKWTFSYWERNGFPSTGTSIPFGTLDAVCRGKRSFGMAIYDMY

LSCKDPLVGIYLSLRPALLVRDAQLAHDMLVKDFASFHDRGVYVDEKKDPMSAGLFALKG

TDWKTMRHKLSPSFTTGKLKAMFSTSEAVGDKLVAHLNAKLPQEGSMEIDIKKLMSIYAI

DIIGSTIFGLDVDSFADPKNEFYSISKTVNRPIFKDIIRGTSSFLYPKLEKFFVAIGWKQ

ESLEDMKDLVNRTIVFREQNNVIRRDMLQLLLQLRNTGSISSDDNMWSAEKNQSNYLKTL

SNDLIAGQLFLFFVAGYETTASTAAFTLYEMAQNPDVLAKAQENIQRTLEKHDGKLTYDA

IQDMQYLELCLKETARKYPALPLLNRECTKDYEVAGGKLIIKQGTPIIISLIGLHRDEEY

FPDPLRYFPERFTEEHMNHNPAAYMPFGEGPRQCIAARMGKVNAKIALVKILINYNLETL

PEKREIEFSNHGVPLIAKGGVPVKLSQKVGKFA

>ACR19218.1

MFLYIIIAVCSAILFYFKWCYSYWQRHGYPYIPAQVPFGVLDPVVRTWKKSVGMAIYDIY

EQTREHKVVGMYLINRPALLVRDAQLARDMLTKDFASFHDRGIYVDEIHDPMSGGIFFLK

GQQWKTLRTKLAPSFTSGKLRGMFDTIHDVSLRMVDHLNGQLPEREFKQIEVKHLFVTYA

IDIIASSIFGLDVDSFSDPQNEFFKLSRNVNENSYRGVFRGTCQFMYPGLEKIFQRLGWT

EPAPDYMKKIVRQTIEKREKHNIVRKDMLQLLLQLRNTGKINGDEDEGWSAVKTTDALAS

MSIELIAAQLFLFYVAGFETSAATAAFTIYELARNAELLEKAQEDVEQALAKHGSLSYDS

LKDMKFLDLCVMETTRKYPGLPILNRECTKDYPLPDSELVVKKGTPIIISLFGIHRDEEY

FPDALRYDPYRFEKENYNAAAYMPFGEGPRHCIAQRMGKLNVKVGVAQILQHFEVKVDPS

APEIEFDNFGLPIMPKGGVPVTLAKKQKTNVIKS

>EDV44045.1

MIGIYLLIAVATLIYVYLKWTFSYWDRKGFRSAGSSIPFGVLEAVSKGKRSFGMAINDLY

NSTKGPVVGMYLTLRPALLIRDAQLAHDVLVKDFTSFHDRGVYVDEENDPISAGLFSLEG

ASWKTMRTKLSPSFTSGKLKAMFETSDAVGDKLVAHLNSLLPAKGSKEVELKALMATYAV

DIIASTIFGLDVDSFADPNNVFLGISKTINRNTFTDVVRGTTSFLYPGLEKLFVRLGWKQ

DAIEGMRELASQTIDLREKNNIVRKDLLQLLLQLRNQGEISTDDSVWTAENSKQGLKSMS

KDLIAGQLFLFYAAGFETTASTASFTLFELSQNPEAMARAQEDVRSAIASHGGKLTYDAI

QDMKYLEACVLETARKYPALPILNRICTQDYPVPDSKLVIRKGTPIVISLIGMHRDADNF

PDPLSYQPERYLEESKSFNQAAYMPFGEGPRHCIGARMGKVNVKLALAKVLSNFNVDISP

EKREIEFGPHGITLMAEGGVPVRISRKQ

>Q9VFP1.1

MIGIYLLIAAVTLLYVYLKWTFSYWDRKGFPSTGVSIPFGALESVTKGKRSFGMAIYDMY

KSTKEPVIGLYLTLRPALLVRDAQLAHDVLVKDFASFHDRGVYVDEKNDPMSASLFQMEG

ASWRALRNKLTPSFTSGKLKAMFETSDSVGDKLVDSIRKQLPANGAKELELKKLMATYAI

DIIATTIFGLDVDSFADPNNEFQIISKKVNRNNIEDIIRGTSSFLYPGLEKFFVKIGWKQ

EATERMRELSNRTVDLREQNNIVRKDLLQLLLQLRNQGKINTDDNIWSAESTKNGVKSMS

KDLIAGQLFLFYVAGYETTASTTSFTLYELTQNPEVMEKAKEDVRSAIEKHGGKLTYDAI

SDMKYLEACILETARKYPALPLLNRICTKDYPVPDSKLVIQKGTPIIISLIGMHRDEEYF

PDPLAYKPERYLENGKDYTQAAYLPFGEGPRMCIGARMGKVNVKIAIAKVLSNFDLEIRK

EKCEIEFGVYGIPLMPKSGVPVRLSLKK

>ACL89495.1

MIGIYLLIAAVTLLYVYLKWTFSYWDRKGFPSTGVSIPFGALESVTKGKRSFGMAIYDMY

KSTKEPVIGLYLTLRPALLVRDAQLAHDVLVKDFASFHDRGVYVDEKNDPMSASLFQMEG

ASWRALRNKLTPSFTSGKLKAMFETSDSVGDKLVDSIRKQLPANGAKELELKKLMATYAI

DIIATTIFGLDVDSFADPNNEFQIISKKVNRNNIEDIIRGTSSFLYPGLEKFFVKIGWKQ

EATERMRELSNRTVDLREQNNIVRKDLLQLLLQLRNQGKINTDDNIWSAESTKNGVKSMS

KDLIAGQLFLFYVAGYETTASTTSFTLYELTQNPEVMEKAKEDVRSAIEKHGGKLTYDAI

SDMKYLEACILETARKYPALPLLNRICTKDYPVPDSKLVIQKGTPIIISLIGMHRDEEYF

PDPLAYKPERYLENGKDYTQAAYLPFGEGPRMCIGARMGKVNVKIAIAKVLSNFDLEIRK

EKCEIEFGVYGIPLMPKSGVPVRLSLKK

>EDW97636.1

MIGIYLLIGAVTLLYVYLKWTFSYWDRKGFPSTGASIPFGALESVTKGKRSFGMAIYDMY

NSTKEPVIGMYLTLRPALLIRDAQLAHDVLVKDFASFHDRGVYVDEKNDPMSASLFQMEG

ASWRALRNKLTPSFTSGKLKAMFETSDSVGDKLVDCIKKQLPATGAKELELKKLMATYAI

DIIATTIFGLDVDSFADPNNEFQVISRKVNRNNFEDIIRGASSFLYPGLEKFFVQIGWKQ

EATERMRELSNRTVDLREQNNIVRKDLLQLLLQLRNQGQVNTDDNVWSAESSKNGEKSMS

KDLIAGQLFLFYVAGYETTASTTSFTLYELTQNPEVMEKAKEDVRSAIEKHGGLTYDAIS

DMKYLEACVLETARKYPALPLLNRICTKDYPVPDSKLVIRKGTPIIISLIGIHRDEEYFP

DPLQYRPERYLEDAKDFNQAAFLPFGEGPRMCIGARMGKVNVKIAIAKILSNFDLEIRKE

KREIEFGVYGIPLMPKSGVPVRLSLKK

>EDW04984.1

MIVLLILLALAALLFVYLRWTFSFWERKGFPYIEASIPLGALDSVRQNKRSFGLAMYDMY

LASSKPFVGIYLTLRPALLVRDAQIAHDVLTTDFAHFHDRGVYVDEKHDPLSANLFSMEG

SSWKSMRNKLSPSFTSGKLKGMFGTAEDVADKMMAHLNGKIEQTGTVEVDMKNLMSSFAI

DIIASTIFGLDVDSFLKPNNELRHISNEINKPTAKNILRGTTTFLYPRLEKIFNRLGWQF

SGQKEMRDLVYHTISVREKNNIERRDILQLLLQLRNTGQINKDDGVWSAQVAGHSFKSLS

KANIAAQMFIFFSAGYETTASTAAFTLYELAQNPDVLAKLMEDINQTLAKHNGELNYDCI

QAMKYLDLCIMETTRKYPALPLLNRMCNKDYPLPNSKLVLEKGTEIIISVMGLHRDAEYF

PDPLRYQPERFTEEHKNYTPAAYMPFGIGPRQCIAARMGKMNVKIALTKILTNYTIEMRK

EVRELEFTVNGITLLPKGGVPVKLSKRM

>EDV95032.1

MIVLLILLALAALLFVYLRWTFSFWERKGFPYIEASIPLGALDSVRQNKRSFGLAMYDMY

LASSKPFVGIYLTLRPALLVRDAQIAHDVLTTDFAHFHDRGVYVDEKHDPLSANLFSMEG

TSWKSMRNKLSPSFTSGKLKGMFGTAEDVADKMMAHLNGKIEQTGTVEVDMKNLMSSFAI

DIIASTIFGLDVDSFLKPNNELRHISNEINKPTAKNILRGTTTFLYPRLEKIFNRLGWQF

SGQKEMRDLVYHTISVREKNNIERRDILQLLLQLRNTGQINKDDGVWSAQVAGHSFKSLS

KANIAAQMFIFFSAGYETTASTAAFTLYELAQNPDVLAKLMEDINQTLAKHNGELNYDCI

QAMKYLDLCIMETTRKYPALPLLNRMCNKDYPLPNSKLVLEKGTEIIISVMGLHRDAEYF

PDPLRYQPERFTEEHKNYTPAAYMPFGIGPRQCIAARMGKMNVKIALTKILTNYTIEMRK

EVRELEFTVNGITLLPKGGVPVKLSKRM

>EDW24590.1

MIGVLLLVAAVSLLYVYLKWTFSYWDRQGFPSAGVTIPFGALDSVRRGKRSFGMAIYDMY

NATKEPVMGMYLTVRPALLVRDPQLAHDILVKDFASFHDRGIYVDEKNDPMSATIFAMEG

SNWRNLRAKLTPSFTSGKLKAMFSTSEAIGDKMVAHLNGKLPKEGSQEIEMKELLLTYAV

DIIASTIFGLDVDSFADPNNEFLSITKTINQNTFTDILRGTTSFLFPGLEKIFVRLGWSQ

EGPERIRELSNRTVDLREEKNIERRDLLQLLLQLRNTGKISNDDNMWSAQNANKGLKSVS

KDLIAGQLFLFFVAGYETTASTAAYTIYELTQNPEVMARALEDVRSTLEKHDGKLSYDAI

QDMKYLEACVLETARKYPALPLLNRVCTKEYQIPNSKLIVKKGTPIIISLIGMHRDAENF

PDPLAYKPERYLDENKNFNPTAYMPFGEGPHNCIGLRMGKVNVKIAVAKILSNFNLEMSK

EKREIEFSVHGIPLMPKGGVPVRISRKK

>EDW15278.1

MSAAIYLCVIGVLLYTYLKWTFSFWERKGFPCNRGSIPFGALDSVRRKKRSFGMAIYDMY

LSTKKPFIGIYLTLRPALLVRDAQLAYDMLVKDFASFHDRGIYVDEKHDALSANLFSMEG

KSWKTMRTKMSPSFTSGKLKAMFSTSEDVADKMVAHLNKNLQQSGATEIDMKNLMACYGI

DIIASTILGLDVDSFTQPENELRRISKMVTDNKAENILRGTTLFLYQGKANVCIIECLTN

IPLNYSLEKLFVRLGWRPPGMKEMQSLVHHTMTLREKTNVERRDLMQLLLQLRNTGQINN

DDGIWSAQATVNSVKTLSKDNVVAQMFLFFIAGYETTASATAFTLYELAQNPDVLAKLLD

DINQTLAKHNGELNYDSIQDMKYLELCVMETTRKYPGLPILNRMCNQDYPLPNSNLVLKK

GTEIIISLLGMQRDEEYFPDPLRYQPERFTEEHRNYTPAAYMPFGVGPRQCIAARMGKLN

VKIALTKILTNFKVESQEKREIEFTVHGVPLLPKGGVPVRLSKKQ

>EAL27841.1

MIGILLLVAAVSLLYVYLKWTFSYWDRQGFPSAGVTIPFGALDSVRRGKRSFGMAIYDMY

NATKEPVMGMYLTLRPALLVRDPQLAHDILVKDFASFHDRGIYVDEKNDPMSATIFAMEG

SNWRNLRAKLTPSFTSGKLKAMFSTSEAIGDKMVAHLNGKLPKEGSQEIEMKELLLTYAV

DIIASTIFGLDVDSFADPNNEFLSITKTINQNTFTDILRGTTSFLFPGLEKIFVRLGWSQ

EGPERIRELSNRTVDLREEKNIERRDLLQLLLQLRNTGKISNDDNMWSAQNDNKGLKSVS

KDLIAGQLFLFFVAGYETTASTAAYTIYELTQNPEVMARALEDVRSTLEKHDGKLSYDAI

QDMKYLEACVLETARKYPALPLLNRVCTKEYQIPNSKLIVKKGTPIIISLIGMHRDAENF

PDPLAYKPERYLDENKNFNPTAYMPFGEGPHNCIGLRMGKVNVKIAVAKILSNFSLEMSK

EKREIEFSVHGIPLMPKGGVPVRISRKK

>ADO24531.2

MFLLLLIAVCTFSFFYVKWIYTFWKRKGFPYLEPKIPFAVLDPVFKREAALGTAIPDVYR

RTKDKVLGIYLITRPVLLIRDAQLARDILTKHFESFHDRGIHVNDEDDPQFSGGLFNLKG

QKWKSLRVKMTPLLTSGKLKAMFETVEEVGDRMVNYVNAQLSDVGSKELELRHVMSTFAI

DIIASVFLGLEVNSFEEPNNPILKIRDKLNEPTFGSVVRGTAQMLFPSLEMLFIRLGWRE

KAPEMMREIVKRTIEMREERNITRKDMLQLLMQLRNKGQVNDNDNDWSAKATDKDSAKHM

SIETIASNLVLFYVAGYETTASTISFTAFELAQYPEALEKAQRDVRDAIERHDNKFTCES

VQDMKYLDLCIMETTRKYETTRKYPGLPILNRQCTQDYQLPDSDLVIKNGTPVIIPLLGF

HRDEKYFPDAMRWDPDRILNKNYDPVAFMPFGEGPRHCIAQRMGRVNAKVALAKLLLNFN

IEFNPVRKEIEIANFGVPIMAKGGINVRLSKRNRETQ

>EAT39048.1

MLFIYSVALLCIAVTLALKYVYSYWDRHGLPSVKPHIPFGNLKTVVKKTESFGIAINQLY

WQTKGQLAGIYLFFRPAILVRDAHLAQQIMTTDFNHFHDRGIYCNEEGDPFSANLFALPG

KRWRNLRNKLTPLFTGGQLRGMMPTILEVGEKLQKHLEPVAERQEVVEIRDIVSRFVLEI

IATVFFGFEANCIEDRDDSFSKVLREAQGERLSAVLRAAAMFVCPGLLRYTGISSLEPQV

IAFVSEIVTKQIEHREKNSVTRKDFIQQLIEIRRGSGENQVPAMSIEQCAANVFLFYAAG

SETSTGTIAFSMHELSHHADVMKKLQDEIDDALAKSNGAITYESVMQMQYLDLCVKETLR

KYPGLPFLNRECTMDYKVPDSDLVIRKGTQLVLPIYGFSMDEQYFPEPECYIPERFEEAS

KNYDEKAYYPFGDGPRNCIAYRMGVLITKIGLILLLSKFTFEATQGPKMMFSSASVPLLP

KDGISLKISNRKR

>AEB77682.1

MLIIYTVALVSIAVTLGLKYVYSYWDRHGLHNIKPHIPFGNLKTVVNKTESFGVAINQLY

WQTKGQLVGIYLFFRPAILIRDAHLAQQIMTTDFNHFHDRGVYCNEEHDPFSASLFSLPG

KRWRNLRNKLTPLFTGGQLRGMMPTILEVGDKLQKYLEPAAEREEVLEIRDIVSRFVLEI

IATVFFGFEANCIKDRDDAFSRVLREAQRQKFATNFRAAAVFVCPGLLKYTGISSLEPQV

KDFVSGIVTKQIEHREKSGVTRKDFIQQLIDLRREDGQDQAPKLSIEQCAANVFLFYVAG

SETSTGTITFTMHELSHHADVMQKLQAEIDDALVKSNGQITYDIVNQMQYLDLCVKETLR

KYPGLPILNRECTLDYRVPDSDRVIRKGTQVVIPLYGISMDERYFPEPERYKPERFEEVS

KDYDEKAYYPFGEGPRNCIAFRMGVLVTKIGLILLLSKFNFEATQGPKIVFSPASVPLSP

KGGISLKISNRKK

>EDS41752.1

MILLSLALLGLILFLGLRYIYSYWERHELPHVKPEIPYGNLRPVAERRECIGVAISELYK

KSKEQLLGIYLFFQPAILVRDARLAKQIMTSDFASFHDRGVFVDEKNDPFSANLFALPGK

KWRTLRNKLTPTFTSGKLRNMLPTILDESKRFQDYLTRVVGDKGDTVEMRDLASRYIVDI

IASVFFGFEADSMNDPEHPFRVVGREAVADSFSNNIRSAATFVCPGLLKLIQIPSTPPKI

SGFMIDLVKQQMEYREKNNVSRKDFFQQLVDLRKEDLLKGEDTMSIEECAAQVFLFYIAG

SETTSVTITYTLHELSHNSKAMEQLVAEIDETLAKSNGEINYDIVKGMRYLDVCVKETLR

KYPGLPILNRECTQDFQVPQSKVVIKKGTQIIIPISAYGMDERYFPDPDSYIPERFFEES

KNYDDNAYQPFGDGPRNCIGIRMGVFVAKAALIMLLSNFTFEATQGPKIEFAASSVTLLP

KSGIKFKVGQRKVKAA

>EAA12450.2

MFVYTLALVAAVIFLVLRYIYSHWERHGLPHLKPEIPYGNIRTVAEKKESFGTAINNLYH

KSSDRLLGIYLFFRPAILIRDPHLAKRIMVNDFQNFHDRGVYCNEEHDPFSANLFALPGQ

RWKNLRAKLTPTFTSGQLRNMLPTLLDVGNKLIDRMNKVADEKAIVDMRDIASRFVLDTI

ASVFFGFEANCIHNSEDPFLSTLRRANRGRNFIDNFRSSGVFICPGLLKLTRMTSLQPEL

IKFVMEIITHQIDHREKNQITRKDFVQLLIDLRREADKGSEEALTIEQCAANVFLFYIAG

AETSTATISFTLHELSHNPEAMAKLQQEIDEMMERYNGEITYENIKEMKYLDLCVKETLR

KYPGLPILNRECTIDYKVPDSDVVIRKGTQVIIPLLSISMNEKYFPDPELYSPERFDEAT

KNYDADAYYPFGAGPRNCIGLRQGVLVSKIGLVFLLSKFKFQATTPAKVKFAAATVGLTP

DAGFPMRIDHRK

>ABV80276.1

MFVYTLALVAAVIFLVLRYIYSHWERHGLPHLKPEIPYGNIRTVAEKKESFGTAINNLYH

KSSDRLLGIYLFFRPAILIRDPHLAKRIMVNDFQNFHDRGVYCNEEHDPFSANLFALPGQ

RWKNLRAKLTPTFTSGQLRNMLPTLLDVGNKLIDRMNKVADEKAIVDMRDIASRFVLDTI

ASVFFGFEANCIHNSEDPFLTTLRRANRGRNFIDNFRSSGVFICPGLLKLTRMTSLQPEL

IKFVMEIITHQIDHREKNQITRKDFVQLLIDLRREADKGSEEALTIEQCAANVFLFYIAG

AETSTATISFTLHELSHNPEAMAKLQQEIDEMMERYNGEITYENIKEMKYLDLCVKETLR

KYPGLPILNRECTIDYKVPDSDVVIRKGTQVIIPLLSISMNEKYFPDPELYSPERFDEAT

KNYDADAYYPFGAGPRNCIGLRQGVLVSKIGLVFLLSKFKFQATTPAKVKFAAATVGLTP

DAGFPMRIDHRK

>EAL39621.1

MFVYTLALVATVIFLVLRYIYSHWERHELPHLKPEIPYGNIRTVAEKKESFGIAINNLYH

KSSDRLLGIYLFFRPAILIRDPHLAKRIMVNDFQNFHDRGVYCNEEHDPFSANLFALPGQ

RWKNLRAKLTPTFTSGQLRNMLPTLLDVGNKLIDRMNKVADEKAIVDMRDIASRFVLDTI

ASVFFGFEANCIHNSEDPFLSTLQRLTKSRKFMDNFRTSGVFICPGLLKLTRITSLPPEL

ISFVMEIITHQIDHREKNQITRKDFVQLLIDLRREAENGSEKALSIEQCAANVFLFYIAG

AETSTATISFTLHELSHNPEAMAKLQQEIDEMMERYNGEITYENIKEMKYLDLCVKETLR

KYPGLPILNRECTIDYKVPDSDVVIRKGTQVIIPLLSISMNEKYFPDPELYSPERFDEAT

KNYDADAYYPFGAGPRNCIGLRQGVFVSKIGLVLLLSKYNFQATTPAKVKFAVATVVVTP

EDGFPMRVEHRC

>BAI77924.1

MILLSLALLGLILFLGLRYIYSYWERHELPHVKPEIPYGNLRPVAERRECFGVAISELYK

KSTEQLLGIYLFFAPAIMVRDARLAKQIMTSDFASFHDRGVFVDERNDPFSANLFALPGK

KWRTLRNKLTPTFTSGKLRNMLPTILDESKRFQDYLTRVVGDKGDTVEMRDLASRYIVDI

IASVFFGFEADSMNDPEHPFRVVGREAVADSFSNNIRSAATFVCPGLLKLIQIPSTPPKI

SGFMIDLVKQQMEYREKNNVSRKDFFQQLVDLRKEDLLKGEDTMSIEECAAQVFLFYIAG

SETTSVTITYTLHELSHNSKAMEQLVAEIDETLAKSNGEINYDIVKGMRYLDVCVKETLR

KYPGLPILNRECTQDFQVPQSKVVIKKGTQIIIPISAYGMDERYFPDPDSYIPERFFEES

KNYDDNAYQPFGDGPRNCIGIRMGVFVAKAALIMLLSNFTFEATQGPKIEFAASSVTLLP

KGGIKFKVGQRKVKAA

>EDS41753.1

MILLSLALLGLILFLGLRYIYSYWERHELPHVKPEIPYGNLRPVAERRECFGVAISELYK

KSTEQLLGIYLFFAPAIMVRDARLAKQIMTSDFASFHDRGVFVDERNDPFSANLFALPGK

KWRTLRNKLTPTFTSGKLRNMLPTILDESKRFQDYLTRVVGDKGDTVEMRDLASRYIVDI

IASVFFGFEADSMNDPEHPFRVVGREAVADSFSNNIRSAATFVCPGLLKLIQIPSTPPKI

SGFMIDLVQQQMEYREKNNVSRKDFFQQLVDLRKEDLLKGEDTMSIEECAAQVFLFYIAG

SETTSVTITYTLHELSHNSKAMEQLVAEIDETLAKSNGEINYDIVKGMRYLDVCVKETLR

KYPGLPILNRECTQDFQVPQSKVVIKKGTQIIIPISAYGMDERYFPDPDSYIPERFFEES

KNYDDNAYQPFGDGPRNCIGIRMGVFVAKAALIMLLSNFTFEATQGPKIEFAASSVTLLP

KGGIKFKVGQRKVKAA

>EDV43080.1

MLSPCFTSGKLKAMYSTSEDIGDKMVAHLQKVLPVQEFHEVDIKRVMQNYAIDIIGSTIF

GLEVNSFENPNNKFRNLVTLAQPKNRFGAMFFMMLFLLPSVAKMLFRIGLKNPVGLAMLE

IVKETIEHREKHGIIRKDMLQLLMNLRNTGKIEENDEKCFSIQKTPEGQIKAISLEAITA

QSFFFYIAGQETTAATAAFTIFELAQYPEHLKRLQSEIDETLSKNDGRISYDSLQKMEFL

ELCLQETLRKYPGLPMMNRECTQDYPVPESNHVIPKGTPVVISLYGIHRDPEFFPNPETY

DPYRFTEESRNYSPTAFMPFGEGPRICIAQRMGRMNSKLAIVKILQNFNVGELKKQELEF

DSSGITLMPKNGVKVHLSRRVFNES

>AAL96667.1

MFVYTLALVAAVIFLVLRYIYSHWERHGLPHLKPEIPYGNIRTVAEKKESFGTAINNLYH

KSSDRLLGIYLFFRPAILIRDPHLAKRIMVNDFQNFHDRGVYCNEEHDPFSANLFALPGQ

RWKNLRAKLTPTFTSGQLRNMLPTLLDVGNKLIDRMNKVADEKAIVDMRDIASRFVLDTI

ASVFFGFEANCIHNSEDPFLSTLRRANRGRNFIDNFRSSGVFICPGLLKLTRMTSLQPEL

IKFVMEIITHQIDHREKNQITRKDFVQLLIDLRREADKGSEEALTIEQCAANVFLFYIAG

AETSTATISFTLHELSHNPEAMAKLQQEIDEMMERYNGEITYENIKEMKYLDLCVKETLR

KYPGLPILNRECTIDYKVPDSDVVIRKGTQVIIPLLSISMNEKYFPDPELYSPERFDEAT

KNYDADAYYPFGAGPRNCIGQGVLVSKIGLVFLLSKFNFQATTPAKVKFAAATVGLTPDA

GFPMRIDHRK

>AFM08407.1

MFVYTLALFPIVIFLLLRYIYSHWERYGLPHVKPEIPYGNLRTVVEKKESFGIAINNLYH

RSSDRLLGVYLFFRPAILIRDPQLAKRIMVNDFQHFHDRGVYCNEKGDPFSANLFALSGD

RWKNLRAKLTPTFTSGQLRNMLPTLIDVGSKLNDRMNTLADEKTVVDMRDIASRFVLDTI

ASVFFGFEANCIHNSDDPFLKTLLQVNKRRSFVDNIRTSGVFICPGLLKLTGLTSLPTEL

INFVMEIITHQIDHREKNQISRKDFVQLLIDLRRDASSQGEQALSIEQCAANVFLFYIAG

SETSTAAISFTLHELSHNPDALAKLQQEIDEMMERHNGEITYENINELKYLDLCVKETLR

KYPGLPILNRECTIEYAVPDSDVVIRKGTQVIIPLLSLSMNEKYFPNPELYSPERFDEAT

KNYDPDAYYPFGVGPRNCIGNRSSSRCTGVQNWSCFIAFQVQLPRHNSCKGEVCSHQRRT

YAGGRFTHADRAQKVSGVTIDCDV

>Q9V674.1

MVLTEVLFVVVAALVALYTWFQRNHSYWQRKGIPYIPPTPIIGNTKVVFKMENSFGMHLS

EIYNDPRLKDEAVVGIYSMNKPGLIIRDIELIKSILIKDFNRFHNRYARCDPHGDPLGYN

NLFFVRDAHWKGIRTKLTPVFTSGKVKQMYTLMQEIGKDLELALQRRGEKNSGSFITEIK

EICAQFSTDSIATIAFGIRANSLENPNAEFRNYGRKMFTFTVARAKDFFVAFFLPKLVSL

MRIQFFTADFSHFMRSTIGHVMEERERSGLLRNDLIDVLVSLRKEAAAEPSKPHYAKNQD

FLVAQAGVFFTAGFETSSSTMSFALYEMAKHPEMQKRLRDEINEALVEGGGSLSYEKIQS

LEYLAMVVDEVLRMYPVLPFLDREYESVEGQPDLSLKPFYDYTLENGTPVFIPIYALHHD

PKYWTNPSQFDPERFSPANRKNIVAMAYQPFGSGPHNCIGSRIGLLQSKLGLVSLLKNHS

VRNCEATMKDMKFDPKGFVLQADGGIHLEIVNDRLYDQSAPSLQ

>ABG02643.1

MVLTEVLFVVVAALVALYTWFQRNHSYWQRKGIPYIPPTPIIGNTKVVFKMENSFGMHLS

EIYNDPRLKDEAVVGIYSMNKPGLIIRDIELIKSILIKDFNRFHNRYARCDPHGDPLGYN

NLFFVRDAHWKGIRTKLTPVFTSGKVKQMYTLMQEIGKDLELALQRRGEKNSGSFITEIK

EICAQFSTDSIATIAFGIRANSLENPNAEFRNYGRKMFTFTVARAKDFFVAFFLPKLVSL

MRIQFFTADFSHFMRSTIGHVMEERERSGLLRNDLIDVLVSLRKEAAAEPSKPHYAKNQD

FLVAQAGVFFTAGFESSSSTMSFALYEMAKHPEMQKRLRDEINEALVEGGGSLSYEKIQS

LEYLAMVVDEVLRMYPVLPFLDREYESVEGQPDLSLKPFYDYTLENGTPVFIPIYALHHD

PKYWTNPSQFDPERFSPANRKNIVAMAYQPFGSGPHNCIGSRIGLLQSKLGLVSLLKNHS

VRNCEATMKDMKFDPKGFVLQADGGIHLEIVNDRLYDQSAPSLQ

>ABA81842.1

MVLTEVLFVVVAALVALYTWFQRNHSYWQRKGIPYIPPTPIIGNTKVVFKMENSFGMHLS

EIYNDPRLKDEAVVGIYSMNKPGLIIRDIELIKSILIKDFNRFHNRYARCDPHGDPLGYN

NLFFVRDAHWKGIRTKLTPVFTSGKVKQMYTLMQEIGKDLELALQRRGEKNSGSFITEIK

EICAQFSTDSIATIAFGIRANSLENPNAEFRNYGRKMFTFTVARAKDFFVAFFLPKLVSL

MRIQFFTADFSHFMRSTIGHVMEERERSGLLRNDLIDVLVSLRKEAAAEPSKPHYAKNQD

FLVAQAGVFFTAGFETSSSTMSFALYEMAKHPEMQKRLRDEINEALVEGGGSLSYEKIQS

LEYLAMVVDEVLRMYPVLPFLDREYESVEGQPDLSLKPFYDYTLENGTPVFIPIYALHHD

PKYWTNPSQFDPERFSPANRKNIVAMAYQPFGSGPHNCIGSRIGLLQSKLGLVILLKNHS

VRNCEATMKDMKFDPKGFVLQADGGIHLEIVNDRLYDQSAPSLQ

>AAW28870.1

MVLTEVLFVVVAALVALYTWFQRNHSYWQRKGIPYIPPTPIIGNTKVVFKMENSFGMHLS

EIYNDPRLKDEAVVGIYSMNKPGLIIRDIELIKSILIKDFNRFHNRYARCDPHGDPLGYN

NLVFVRDAHWKGIRTKLTPVFTSGKVKQMYTLMQEIGKDLELALQRRGEKNSGSFITEIK

EICAQFSTDSIATIAFGIRANSLENPNAEFRNYGRKMFTFTVARAKDFFVAFFLPKLVSL

MRIQFFTADFSHFMRSTIGHVMEERERSGLLRNDLIDVLVSLRKEAAAEPSKPHYAKNQD

FLVAQAGVFFTAGFESSSSTMSFALYEMAKHPEMQKRLRDEINEALVEGGGSLSYEKIQS

LEYLAMVVDEVLRMYPVLPFLDREYESVEGQPDLSLKPFYDYTLENGTPVFIPIYALHHD

PKYWTNPSQFDPERFSPANRKNIVAMAYQPFGSGPHNCIGSRIGLLQSKLGLVSLLKNHS

VRNCEATMKDMKFDPKGFVLQADGGIHLEIVNDRLYDQSAPSLQ

>AAS15782.1

MVLTEVLFVVVAALVALYTWFQRNHSYWQRKGIPYIPPTPIIGNTKVVFKMENSFGMHLS

EIYNDPRLKDEAVVGIYSMNKPGLIIRDIELIKSILIKDFNRFHNRYARCDPHGDPLGYN

NLFFVRDAHWKGIRTKLTPVFTSGKVKQMYTLMQEIGKDLELALQRRGEKNSGSFITEIK

EICAQFSTDSIATIAFGIRANSLENPNAEFRNYGRKMFTFTVARAKDFFVAFFLPKLVSL

MRIQFFTADFSHFMRSTIGHVMEERERSGLLRNDLIDVLVSLRKEAAAEPSKPHYAKNQD

FLVAQAGVFFTAGFETSSSTMSFALYELAKHPEMQQRLREEINEALVEGGGSLSYEKIQS

LEYLAMVVDEVLRMYPVLPFLDREYESVQGQPDLSLKPFYDYTLENGTPVFIPIYALHHD

PKYWTNPRQFDPERFSPANRKNIVAMAYQPFGSGPHNCIGSRIGLLQSKLGLVSLLKNHS

VRSCEATMKDMKFDPKGFVLQADGGIHLEVVNDRLYEQSAPSIQ

>AAS15780.1

MVFTEVLFVVVAALVALYTWFQRNHSYWQRKGIPYIPPTPIIGNTKVVFKMENSFGMHLS

EIYNDPRLKDEAVVGIYSMNKPGLVIRDIELIKSILIKDFNRFHNRYARCDPHGDPLGYN

NLFFVRDTHWKDIRTKLTPVFTSGKVKQMYTLMQEIGNDLELALQRHGEKNSGNFITEIK

EICAQFSTDSIATIAFGIRANSLQNPNAEFRNYGRKLFTFTVGRAKDFFVAFFLPKLVSL

MRIQFFTPDFSHFMRSTIGHVMEERERSGLLRNDLIDVLVTLRKEAAAEPSKPHYAKNQD

FLVAQAGVFFTAGFETSSSTMSFALYELAKHPEMQQRLREEINEALVEGGGSLSYEKIQS

LEYLAMVVDEVLRMYPVLPFLDREYESVEGQPDLSLKPFYDYTLENGTPVFIPIYALHHD

PKYWTNPSQFDPERFSPANRKNIVAMAYQPFGSGPHNCIGSRIGLLQSKLGLVSLLKNHS

VRNCEATMKDMKFDPKGLVLQADGGIHLEIVNDRLYDQSAPSLQ

>AAS15772.1

MVLTEVLFVVVAALVALYTWFQRNHSYWQRKGIPYIPPTPIIGNTKVVFKMENSFGMHLS

EIYNDPRLKDEAVVGIYSMNKPGLVIRDIELIKSILIKDFNRFHNRYARCDPHGDPLGYN

NLFFVRDTHWKDIRTKLTPVFTSGKVKQMYTLMQEIGNDLELALQRHGEKNSGNFITEIK

EICAQFSTDSIATIAFGIRANSLQNPNAEFRNYGRKLFTFTVGRAKDFFVAFFLPKLVSL

MRIQFFTPDFSHFMRSTIGHVMEERERSGLLRNDLIDVLVSLRKEAAAEPSKPHYAKNQD

FLVAQAGVFFTAGFETSSSTMSFALYELAKHPEMQQRLREEINEALVEGGGSLSYEKIQS

LEYLAMVVDEVLRMYPVLPFLDREYESVQGQPDLSLKPFYDYTLENGTPVFIPIYALHHD

PKYWTNPRQFDPERFSPANRKNIVAMAYQPFGSGPHNCIGSRIGLLQSKLGLVSLLKNHS

VRSCEATMKDMKFDPKGFVLQADGGIHLEVVNDRLYEQSAPSIQ

>AAS15790.1

MVLTEVLFVVVAALVALYTWFQRNHSYWQRKGIPYIPPTPIIGNTKVVFKMENSFGMHLS

EIYNDPRLKDEAVVGIYSMNKPGLVIRDIELIKSILIKDFNRFHNRYARCDPHGDPLGYN

NLFFVRDTHWKDIRTKLTPVFTSGKVKQMYTLMQEIGNDLELALQRHGEKNSGSFITEIK

EICAQFSTDSIATIAFGIRANSLQNPNAEFRNYGRKLFTFTVARAKDFFVAFFLPKLVSL

MRIQFFTPDFSHFMRSTIGHVMEERERSGLLRNDLIDVLVSLRKEADAEPSKPHYAKNQD

FLVAQAGVFFTAGFETSSSTMSFALYELAKHPEMQQRLREEINEALVEGGGPLSYEKIQS

LEYLAMVVDEVLRMYPVLPFLDREYESVQGQPDLSLKPFYDYTLENGTPVFIPIYALHHD

PKYWTNPRQFDPERFSPANRKNIVAMAYQPFGSGPHNCIGSRIGLLQSKLGLVSLLKNHS

VRSCEATMKDMKFDPKGFVLQADGGIHLEVVNDRLYEQSAPSIQ

>AAS15779.1

MVLTEVLFVVVAALVALYTWFQRNHSYWQRKGIPYIPPTPIIGNTKVVFKMENSFGMHLS

EIYNDPRLKDEAVVGIYSMNKPGLVIRDIELIKSILIKDFNRFHNRYARCDPHGDPLGYN

NLFFVRDTHWKDIRTKLTPVFTSGKVKQMYTLMQEIGNDLELALQRHGEKNSGNFITEIK

EICAQFSTDSIATIAFGIRANSLQNPNAEFRNYGRKLFTFTVGRAKDFFVAFFLPKLVSL

MRIQFFTPDFSHFMRSTIGHVMEERERSGLLRNDLIDVLVSLRKEAAAEPSKPHYAKNQD

FLVAQAGVFFTAGFESSSSTMSFALYELAKHPEMQQRLREEINEALVEGGGSLSYEKIQS

LEYLAMVVDEVLRMYPVLPFLDREYESVQGQPDLSLKPFYDYTLENGTPVFIPIYALHHD

PKYWTNPRQFDPERFSPANRKNIVAMAYQPFGSGPHNCIGSRIGLLQSKLGLVSLLKNHS

VRSCEATMKDMKFDPKGFVLQADGGIHLEVVNDRLYEQSAPSIQ

>AAS15798.1

MVLTEVLFVVVAALVALYTWFQRNHSYWQRKGIPYIPPTPIIGNTKVVFKMENSFGMHLS

EIYNDPRLKDEAVVGIYSMNKPGLVIRDIELIKSILIKDFNRFHNRYARCDPHGDPLGYN

NLFFVRDTHWKDIRTKLTPVFTSGKVKQMYTLMQEIGNDLELALQRHGEKNSGSFITEIK

EICAQFSTDSIATIAFGIRANSLQNPNAEFRNYGRKLFTFTVARAKDFFVAFFLPKLVSL

MRIQFFTPDFSHFMRSTIGHVMEERERSGLLRNDLIDVLVTLRKEAAAEPSKPHYAKNQD

FLVAQAGVFFTAGFETFSSTMSFALYELAKHPEMQQRLREEINEALVEGGGSLSYEKIQS

LEYLAMVVDEVLRMYPVLPFLDREYESVQGQPDLSLKPFYDYTLENGTPVFIPIYALHHD

PKYWTNPRQFDPERFSPANRKNIVAMAYQPFGSGPHNCIGSRIGLLQSKLGLVSLLKNHS

VRSCDATMKDMKFDPKGFVLQADGGIHLEVVNDRLYEQSAPSIQ

>AAS15773.1

MVLTEVLFVVVAALVALYTWFQRNHSYWQRKGIPYIPPTPIIGNTKVVFKMENSFGMHLS

EIYNDPRLKDEAVVGIYSMNKPGLVIRDIELIKSILIKDFNRFHNRYARCDPHGDPLGYN

NLFFVRDTHWKDIRTKLTPVFTSGKVKQMYTLMQEIGNDLELALQRHGEKNSGNFITEIK

EICAQFSTDSIATIAFGIRANSLQNPNAEFRNYGRKLFTFTVGRAKDFFVAFFLPKLVSL

MRIQFFTPDFSHFMRSTIGHVMEERERSGLLRNDLIDVLVSLRKEAAAEPSKPHYAKNQD

FLVAQAGVFFTAGFETSSSTMSFALYELAKHPEMQQRLREEINEALVEGGGSLSYEKIQS

LEYLAMVVDEVLRMYPVLPFLDREYXSVQGQPDLSLKPFYDYTLENGTPVFIPIYALHHD

PKYWTNPRQFDPERFSPANRKNIVAMAYQPFGSGPHNCIGSRIGLLQSKLGLVSLLKNHS

VRSCEATMKDMKFDPKGFVLQADGGIHLEVVNDRLYEQSAPSIQ

>AAS15792.1

MVLTEVLFVVVAALVALYTWFQRNHSYWQRKGIPYIPPTPIIGNTKVVFKMENSFGMHLS

EIYNDPRLKDEAVVGIYSMNKPGLVIRDIELIKSILIKDFNRFHNRYARCDPHGDPLGYN

NLFFVRDTHWKDIRTKLTPVFTSGKVKQMYTLMQEIGNDLELALQRHGEKNSGNFITEIK

EICAQFSTDSIATIAFGIRANSLQNPNAEFRNYGRKLFTFTVGRAKDFFVAFFLPKLVSL

MRIQFFTPDFSHFMRSTIGHVMEERERSGLLRNDLIDVLVSLRKEAAAEPSKPHYAKNQD

FLVAQAGVFFTAGFETSSSTMSFALYELAKHPEMQQRLREEINEALVEGGGSLSYEKIQS

LEYLAMVVDEVLRMYPVLPFLDREYESVQGQPDLSLKPFYDYTLENGTPVFIPIYALHHD

PKYWTNPRQFDPERFSPANRKNIVAMAYQPFGSGPHNCIGSRIGLLQSKLGLVSLLKNHS

VRSCEATMKDTKFDPKGFVLQADGGIHLEVVNDRLYEQSAPSIQ

>AAS15786.1

MVLTEVLFVVVAALVALYTWFQRNHSYWQRKGIPYIPPTPIIGNTKVVFKMENSFGMHLS

EIYNDPRLKDEAVVGIYSMNKPGLVIRDIELIKSILIKDFNRFHNRYARCDPHGDPLGYN

NLFFVRDTHWKDIRTKLTPVFTSGKVKQMYTLMQEIGNDLELALQRHGEKNSGNFITEIK

EICAQFSTDSIATIAFGIRAYSLQNPNAEFRNYGRKLFTFTVGRAKDFFVAFFLPKLVSL

MRIQFFTPDFSHFMRSTIGHVMEERERSGLLRNDLIDVLVSLRKEAAAEPSKPHYAKNQD

FLVAQAGVFFTAGFETSSSTMSFALYELAKHPEMQQRLREEINEALVEGGGSLSYEKIQS

LEYLAMVVDEVLRMYPVLPFLDREYESVQGQPDLSLKPFYDYTLENGTPVFIPIYALHHD

PKYWTNPRQFDPERFSPANRKNIVAMAYQPFGSGPHNCIGSRIGLLQSKLGLVSLLKNHS

VRSCEATMKDMKFDPKGFVLQADGGIHLEVVNDRLYEQSAPSIQ

>AAS15794.1

MVLTEVLFVVVAALVALYTWFQRNHSYWQRKGIPYIPPTPIIGNTKVVFKMENSFGMHLS

EIYNDPRLKDEAVVGIYSMNKPGLVIRDIELIKSILIKDFNRFHNRYARCDPHGDPLGYN

NLFFVRDTHWKDIRTKLTPVFTSGKVKQMYTLMQEIGNDLELALQRHGEKNSGNFITEIK

EICAQFSTDSIATIAFGIRANSLQNPNAEFRNYGRKLFTFTVGRAKDFFVAFFLPKLVSL

MRIQFFTPDFSHFMRSTIGHVMEERERSGLLRNDLIDVLVSLRKEAAAEPSKPHYAKNQD

FLVAQAGVFFTAGFETSSSTMSFALYELAKHPEMQQRLREEINEALVEGGGSLSYEKIQS

LEYLAMVVDEVLRMYPVLPFLDREYESVQGQPDLSLKPFYDYTLENGTPVFIPIYALHHD

PKYWTNPRQFDPERFSLANRKNIVAMAYQPFGSGPHNCIGSRIGLLQSKLGLVSLLKNHS

VRSCEATMKDMKFDPKGFVLQADGGIHLEVVNDRLYEQSAPSIQ

>EDV56368.1

MVMTEVLFLVVAALVAIYSWFQRNHSYWQRKGIPYVPPTPIIGNTKVVFKMENSFGMHIS

EIYNDPRLKDEAVVGIYSLNKPGLIIRDIELIKSILIKDFNRFHNRYGRADPHRDPLGYN

NLFFVRDAHWKEIRTKLTPVFTSGKVKQMYTLMQEIGNDLELALKRHSEKSSGKFITEIK

EICAQFSTDSIATIAFGIRANSLENPNAEFRNYGRELFAFTISRAKDFFVVFFLPKLVSL

LRIQIFTQDFSHFLRSTIGHVMKEREQTGELRNDLIDVLVALRKEAAAEPSKPHYARNQD

FLVAQAGVFFSAGFETSSSTMSFALYELAKHPEMQHRLRKEINEALLEGGGSLTYEKIQS

LEYLAMVVDEVLRMYPVLPFLDREYESVQGQPDLNLKPFYDYTFENGTPVFIPIYALHHD

PKYWTNPSQFDPERFSAANRKSIVAMAYQPFGSGPHNCIGSRIGLLQSKLGLVSLLKNHS

VRNCGATMKHMKFDSKGFVLQADGGIHLEIVNDRLYEESAPSVP

>AAS15796.1

MVFTEVLFVVVAALVALYTWFQRNHSYWQRKGIPYIPPTPIIGNTKVVFKMENSFGMHLS

EIYNDPRLKDEAVVGIYSMNKPGLVIRDIELIKSILIKDFNRFHNRYARCDPHGDPLGYN

NLFFVGDTHWKDIRTKLTPVFTSGKVKQMYTLMQEIGNDLELALQRHGEKNPGNFITEIK

EICAQFSTDSIATIAFGIRANSLQNPNAEFRNYGRKLFTFTVGRAKDFFVAFFLPKLVSL

MRIQFFTPDFSHFMRSTIGHVMEERERSGLLRNDLIDVLVSLRKEAAAEPSKPHYAKNQD

FLVAQAGVFFTAGFETSSSTMSFALYELAKHPEMQQRLREEINEALVEGGGSLSYEKIQS

LEYLAMVVDEVLRMYPVLPFLDREYESVQGQPDLSLKPFYDYTLENGTPVFIPIYALHHD

PKYWTNPRQFDPERFSPANRKNIVAMAYQPFGSGPHNCIGSRIGLLQSKLGLVSLLKNHS

VRSCEATMKDMKFDPKGFVLQADGGIHLEVVNDRLYEQSAPSIQ

>EDW90823.1

MVLTEVLFLVVAVLVALYTWFQRNHSYWQRKGIPYVPPTPIIGNTKVVFKMENSFGMHLS

EIYNDPRLTDEAVVGIYSMNKPGLIIRDIELIKSILIKDFNRFHNRYARCDPHRDPLGYN

NLFFVRDAHWKDIRTKLTPVFTSGKVKQMYTLMQEIGNDLELALKRHGEKSSGKFITEIK

EICAQFSTDSIATIAFGIRANSLENPNAEFRNYGRKLFTFNISRAKDFFVAFFLPKLVSL

LRIQFFTPDFSHFLRSTIGHVMKEREQSGLLRNDLIDVLVALRKEAAAEPSKPHYARNQD

FLVAQAGVFFTAGFETSSSTMSFALYELAKHPEMQHRLREEINGALLEGGGSLTYEKIQS

LEYLSMVVDEVLRMYPVLPFLDREYESVQGQPDLSLKPYYDYTLENGTPVFIPIYALHHD

PKYWTNPSQFDPERFSPENRKSIVAMAYQPFGSGPHNCIGSRIGLLQSKLGLVSLLKNHS

VRDCEATMKQMKFDPKGFVLQADGGIHLEVVNDRLYEESAQSVQ

>AAS13452.1

MVLTEVLFLVVAVLVALYTWFQRNHSYWQRKGIPYVPPTPIIGNTKVVFKMENSFGMHLS

EIYNDPRLTDEAVVGIYSMNKPGLIIRDIELIKSILIKDFNRFHNRYARCDPHRDPLGYN

NLFFVRDAHWKDIRTKLTPVFTSGKVKQMYTLMQEIGNDLELALKRHGEKSSGKFITEIK

EICAQFSTDSIATIAFGIRANSLENPNAEFRNYGRKLFTFNISRAKDFFVAFFLPKLVSL

LRIQFFTPDFSHFLRSTIGHVMKEREQSGLLRNDLIDVLVALRKEAAAEPSKPHYARNQD

FLVAQAGVFFTAGFETSSSTMSFALYELAKHPEMQHRLREEINGALLEGGGSLTYEKIQS

LEYLSMVVDEVLRMYPVLPFLDREYESVQGQPDLSLKPYYDYTLENGTPVFIPIYALHHD

PKYWTNPSQFDPERFSPENRKSIVAMAYQPFGSGPHNCIGSRIGLLQSKLGLVSLLKNHS

VRDCEATMKQMKFDPKGFVLQADGGIHLEVVNDRIYEESAQSVQ

>AAC33298.1

MVLTEVLFVVVAALVALYTWFQRNHSYWQRRHTYIPPRDHCNTKVVFKWTLGDLCNTKVV

FKMENSFGMHPSEIYNDPRLKDEAVVGIYSMNKPGLIIRDIELIKSILIKDFNRFHNRYG

RLRIPWRSIGLYNLFFVRDAIGREFRTKLTPVFTSGKVKQMYTLLQEIGKDLELALQRRG

EKNSGSFITEIKEICAQFSTDSIATIAFGIRANSLENPNAEFRNYGRKMFTFTVARAKDF

FVAFFLPKLVSLMRIQFFTADFSHFMRSTIGHVMEERERSGLLRNSLIDVLVSLRKEAAA

EPSKPHYAKNQDFLVVSAGVFFTAGFETSSSTMSFALYEMAKHPEMQKRLRDEINEALVE

GGGSLSYEKIQSLEYLAMVVDEVLRMYPVLPFLDREYESVEGQPDLSLKPFYDYTLENGT

PVFIPIYALHHDPKYWTNPSQFDPERFSPANRKNIVAMAYQPFGSGPHNCIGSRIGLLQS

KLGLVSLLKNHSVRNCEATMKDMKFDPKGFVHQADGGIHLEIVNDRLYDQSAPSLQ

>EDV36183.1

MVLTEALFLVVAALVAVYLWFQRNHTYWQRKGIPYIPPTPIIGNTKPAFKLETSFGLYLS

DIYNDPRAKDEAVVGIYALNKPGLIIRDVDLIKSVLIKDFNRFHNRYAQCDPHGDPLGYN

NLFFVRNNHWKDIRTKLTPVFTSGKVKQMYPLMQEIGRDLEAALKRHGEESSGKYITEIK

EICAQFSTDSIATIAYGIRANSLENANAEFRNHGRKLFTFNVSRAKDFFVAFFLPKLVSL

LRIRFFTPEFSYFMRSTIGHVLEDRERTGFVRNDLIDVLVALRKEAAAEPSKPHYARNQD

FLVAQAGVFFTAGFETSSSTMSFALYELAQHPEMQHRLRQEIHEALLEGGGSLSYEKIQS

LEYLSMVVDEVLRLYPVLPFLDREYESVQGQPDLSLKPFYDFSYENGTPVFIPVFGLQRD

PKYWPNPNQFDPERFSPENRKNIQAMAYLPFGAGPHNCIGSRIGLLQSKLGLVHFLKNHA

VRNCADTMKQMKFDPKGFVLQADGGIHLEVVNDSLYDVGARDSNQSV

>EDW31718.1

MALTEALFLIVGALSAVYIWFQRNHSYWQRKGIPYIPPTPIIGNTEPAFKLKNSFGLHLS

EIYSDPKMQDEAVVGVYALNKPCLIIRDPELIKSVMVKDFNRFHNRYGRCDPHGDPLGSN

NLFFVRDTHWKDIRTKLTPVFTSGKVKQMYSLMLEIAHDLEEHLKKHVQNNSNSGKYITE

IKEVCAQFSTDSIATIAFGIRANSLQNPNAEFRNHGRKLFTFTLSRAKDFFVAFFLPKLV

SLLRIQFFTQEFAHFLRSTISHVMEERERTGFVRNDLIDVLVGLRKEAAEEPSKPHYARN

FDFLVAQAGVFFTAGFETSSSTMSFALYELAKNSHLQTRLRQEINEALVEGGGNLSYEKI

QSLEYLAMVVDEVLRMYPVLPFLDREYNSIKDQPDLSLKPYYDFTFEHGTPVFIPIYGLQ

HDPKYWPKPEQFDPERFSPENRKSIVAMAYQPFGSGPHNCIGSRIGLLQTKLGLVHILKN

HSVKMCADTIKEMKFDPKGFVLQSLTGIYLEIVNDRLYEESAARVH

>EAL24935.2

MALTEALFLIVGALSAVYIWFQRNHSYWQRKGIPYIPPTPIIGNTEPAFKLKNSFGLHLS

EIYSDPKMQDEAVVGVYALNKPCLIIRDPELIKSVMVKDFNRFHNRYGRCDPHGDPLGSN

NLFFVRDTHWKDIRTKLTPVFTSGKVKQMYTLMLEIAHDLEEHLKKHVQNNSNSGKYITE

IKEVCAQFSTDSIATIAFGIRANSLQNPNAEFRNHGRKLFSFTLSRAKDFFVAFFLPKLV

SLLRIQFFTQEFAHFLRSTISHVMEERERTGFVRNDLIDVLVGLRKEAAEEPSKPHYARN

FDFLVAQAGVFFTAGFETSSSTMSFALYELAKNSHLQTRLRQEINEALVEGGGNLSYEKI

QSLEYLAMVVDEVLRMYPVLPFLDREYNSIKDQPDLSLKPYYDFTFEHGTPVFIPIYGLQ

HDPKYWPKPEQFDPERFSPENRKSIEAMAYQPFGSGPHNCIGSRIGLLQTKLGLVHILKN

HSVKMCADTIKEMKFDPKGFVLQSLTGIYLEIVNDRLYEESAARVH

>EDW77075.1

MALTEALLLIAAGLGAIYLWFQRNHSYWQRKGLPYIPPTPIIGNTKAVFKLENSFGLHLS

EIYNDPRMKNEAVVGMYSINKPGLVIRDPELVKSILIKDFNRFHNRYGQCDPHGDPLASN

NLFFVRNNAWKEMRTKLTPVFTSGKIKQMYSLMLEIADDLELHLSKYPNKFITEIKEVCA

QFTTDTIASTAFGIRAHSLHNPDAEFRYHGRKLFTFTLSRAKDFFVAFFVPKLVGLFRIK

FFAPEFSHFMRSTISHVIEDRERTGTVRNDLIDVFVGLRKEAAQEPNKPHYARNKDFLVA

QAGLFFTAGFETSSSTMSFTLYEMAKHPELQQRLRDEIVGALVEEGGKLSYERINSLEYL

SMVVDEVLRLYPVLPFLDREYKSVKGEPDWSLKPYYDFSLENGTPVFIPIYGLQRDPKYW

PNPNQFDPERFAPENRKSIDTRIYQPFGSGPHNCIGSRIGLLQTKLGLVHFLKNHYVRTC

ADTIKEMKFDPKGFVLQSLTGIHLETVNDCLYDEKVKQ

>EDW60585.1

MALTEALFVIVSVLSAIYLWFQRNHSYWQRKGLPFMPPSPIIGNTASVFKQESSFGMYIS

QVYSEPRMQDEAVVGIYVINKPALVIREPELIKSVLIKDFNRFSNRYARCDPHGDPLGYN

NLFFVRNPHWKQIRSKLTPVFTSGKVKQMYPLMVEIAEDLESHLARHRPAQGTEYITEVK

DICARFTTDSIATIAFGVRANSLANPEAEFRNQGRKVLGASLSRSKDYFVAFFMPKLVSL

LRIKFFTEEYSNFIRNTINHVMSEREGTATVRNDLIDVLVGLRKEAALDPSKPHLANNND

FLVAQAGVFFTAGFETSSSTMAFGLLELAKQPELQERLRQEINEALVEEGGKLSYEKINS

LEYLAMVVDEVLRLYPVLPFLDREYQSIQGLPDLSLKPYYDYKMENGTPVFIPVYGLQRD

PKYWPNPNHFDPERFSPENRKSIEPMAYQPFGLGPHNCIGSRIGLLQSKLGLVHILKNHY

VRCCSQTPKDIQFDAKTIVLQLDKEMFLEIVNDRLYDEKAV

>EDW60584.1

MALTEALFVIVSVLSAIYLWFQRNHSYWQRKGLPFMPPSPIIGNTASVFKQESSFGMYIS

QVYSEPRMQDEAVVGIYVINKPALVIREPELIKSVLIKDFNRFSNRYARCDPHGDPLGYN

NLFFVRNPHWKQIRSKLTPVFTSGKVKQMYPLMVEIAEDLESHLARHRPAQGTEYITEVK

DICARFTTDSIATIAFGVRVNSLENPEAEFRSQGRTVVEATLLRSKDYFVAFFLPKLVSL

FRVKFYTEKYSNFLRKTINHVMSERERTATVRNDLIDVLVGLRKEAALDPSKPHLANNND

FLVAQAGVFFTAGFETSSSTMAFGLLELAKQPELQERLRQEINEALVEEGGKLSYEKINS

LEYLAMVVDEVLRLYPVLPFLDREYQSIQGLPDLSLKPYYDYKMENGTPVFIPVYGLQRD

PKYWPNPNHFDPERFSPENRKSIEPMAYQPFGLGPHNCIGSRIGLLQAKLGLVHILKNHY

VRCCSQTPKDIQFDAKTIVLQLDKEMFLDIVNDRLYDEKAI

>EDW60586.1

MALTEALFLIVGALSAIYLWFQRNHSYWHRKGLPFMPPSPIIGNTASVFKQESSFGMYIS

KVYSEPRMQDEAVVGIYVINKPALVIREPELIKSVLIKDFNRFSNRYGRCDPHGDALGSN

NLFFVRNPQWKEIRTKLTPVFTSGKVKQMYPLMLEIAADLESHLARHRPAQGTEYITEVK

DICARFTTDSIATIAFGVRANSLANPEAEFRNQGRKVLGASLSRSKDYFVAFFMPKLVSL

LRIKFFTEEYSNFIRNTINHVMSEREGTATVRNDLIDVLVGLRKEAALDPSKPHLANNND

FLVAQAGVFFTAGFETSSSTMAFGLLELAKQPELQERLRQEINEALVEEGGKLSYEKINS

LEYLAMVVDEVLRLYPVLPFLDREYQSIQGLPDLSLKPYYDYKMENGTPVFIPVYGLQRD

PKYWPNPNHFDPERFSPENRKSIEPMAYQPFGLGPHNCIGSRIGLLQAKLGLVHILKNHY

VRCCSQTSKDIQFDAKTFVLQLDKEMLLEIVNDRLYDEKAV

>EDV98209.1

MALTEGLFLIVGALSAIYLWFQRSHSYWQRKGIPYIQPTPIIGNTSPVFTQKNSFGLHIS

EVYNDPKMQDEAVVGIYVLNKPALVIREPELIKSVMIKDFQRFSNRYARCDPHGDALGNN

NLFFVRNPQWKEIRSKLTPVFTSGKLKQMYPLMQEIADDLEAHLAQQKTGQDASNIVEIK

DTCSRFSTDSIATIAFGVRANSLTNPDAEFRTEGRKVLEGTVSRSKDFFTVFFLPKLVSL

LRIKFFTKEYSNFIRKTIGHVISERERTGTMRNDLIDVLVGLRKEAAQQPNKPHLANIPD

FLVAQAGVFFTAGFETSSSTMAFGLLELAKQPELQDRLRREINNAFLEEGSGKLSYEKIN

SLEYLSMVVDEVLRLYPVLPFLDREYQGIKGQPDLSLKPHYDFQLENGTPVFIPIYGLQR

DPKYWPNPNVFDPERFSPENRKTIEAMAYQPFGLGPHNCIGSRIGLLQTKLGLVHILKNH

YVRCCSQTPKDITFDPKTFVLQLKSGMFLEIVNDRFYDDRAAQAL

>EDW09596.1

MVLTAALFVIIGALSAIYVWFQRNHSYWQRKGIPYMQPTPIIGNTGPVFTQSNSFGLHIS

AVYSDPRMANEAAVGIYVINKPGLVIREPELIKSVLIKDFNRFTNRYGRTDPHYDALGSN

NLFFVRNPQWKEIRTKLTPVFTSGKMKQMYPLMQEIAADLESMLAKERPANGDKYVTDIK

DVCARFTTDSIATIAYGVRANSLSNPDAEFRRQGRKIFAPTLARSKDFFVAFFLPKLVSL

LRIKVFPDDFSKFLRDTIGHVMTERERTATVRNDLIDVLVGLRKEAALEPGKPHLANNND

FLVAQAGVFFTAGFETSSSAMSFALLELARQPELQKRLRQEIHEALLEEKDGKLSYDKIH

SLEYLSMVVDEVLRMYPVLPFLDREYQSIKGEADLSLKPYYDHKLENGTPIFIPVYALQR

DPKYWTNPNLFNPERFSAENRKSIEPMAYQPFGLGPHNCIGSRIGVLQTKLGLVHFFKNH

FVRSCPETVKEIEFDPKSFILQLKSGMFLEIVNDSLYDANAL

>EDV98210.1

MQDEAVVGIYVLNKPALVIREPELIKSVMIKDFQRFSNRYARCDPHGDALGNNNLFFVRN

PQWKEIRSKLTPVFTSGKLKQMYPLMQEIADDLEAHLAQQKTGQDASNIVEIKDTCSRFS

TDSIATIAFGVRANSLTNPDAEFRKQGRRVLEATVARSIDFFTVFFLPKLVSLFRVKFFT

KEYSNFIRKTIGHVISERERMGTMRNDLIDVLVGLRKEAAQQPNKPHLANIPDFLVAQAG

VFFTAGFETSSSTMAFGLLELAKQPELQDRLRREINNAFLEEGSGKLSYEKINSLEYLSM

VVDEVLRLYPVLPFLDREYQGIKGQPDFSLKPYYDFQLENGTPVFIPIYGLQRDPKYWPN

PNVFDPERFSPENRKTIEAMAYQPFGLGPHNCIGSRIGLLQTKLGLVHILKNHYVRCCSQ

TPKDIEFDPKTVVLQLNSGMFLEIVNDRLYVNMAE

>ABK91492.1

MIASSILIFITCILGLVYYWSKHTYSYWQRKGIPYIKPTPIIGNTKEAFQLKTSFGLHLS

DIYNDPKVADEAVVGIYTFNQPGLIVREPELLKSILIKDFHYFNNRFSKCDPHGDALGNS

NLFFARDSYWKDLRTKISPVFTSGKIKQMYPLMMEVSSQLEHHLAQKGERFQAEVKEICA

LFTTDLIATIAFGINANSLKNPDGEFRTHCRKIFLFNIPRAIEFSIAFFLPKLVSLFRVK

LFTPDFSNFLRSTIKYVMQERENSGKLRNDLIDILVAIKKEVATGNINEAIAKNPDVLVA

QAAVFLTAGFETSSSTMTFTLFELAKRPDLQERLREEINEALLKENGNLSYETITTLEYL

NMVVEEVLRLYPILPFLDRQHNRPAGEKQGFSLKPYYDYTLPDDMPVYIPIFGIQRDPKH

WPNPNTFDPERFSPENKKQHNSMTYMPFGTGPHNCIGMRIGLLQTKIGLVHILKNHKVEL

CAETPTKSIFDPKAFLLQYENGVPLNVIRDNLYDKNILVK

>ACR19217.1

MIASSVLLAITFLLSALYFWCRQTYRYWQRNGIPYSKPTMFIGNTKEAFSLKTSFGLHLS

NIYNEPKVRDEPVVGIYIFNQPGLVVRDPELIKSVLIKDFHLFTNRYGQCDPHGDVLGNN

NMFFARNAYWKELRTKISPVFTSGKVKQMYPLMLEVASQLENYLSQKGDHFTTEVKEICA

LFTTDLISTIAFGINANSIKNPDGEFRSQCRKFFLFTLPRAIDFSVAFFLPKFVSLLRVK

IFPKDFSKFLRSTITHVMTERERTGIPRNDLIDILVALKKEAAKNNHSFMQNHDYLVAQA

AVFLTAGFETSSSTMAFALFEMSKKPDLQDRLRREICNALLSEKNGTLTYEQINSLEYLN

MVVEETLRLYPVLPFLDRQHQKPAGVSKGFTLKPYYDYTLPDGMPIYIPIYALQRDPKYW

PNPNEFDPERFTSENKKSQVSMTYMPFGTGPHNCIGSRIGLLQSKLGLVHFLKNHRVETC

NKTPTCEQFDPKALLLQFEQGITLNVVRDQLYENARKQN

>ACO83223.1

MIASNILLAITILLTAVYLWCRRTYSYWQRHGIPFAKPTMLIGNTKEAFSLKTSIGLHLS

QLYNLPEMQDEPVVGIYMFHQPALVIREPELIKSILIKDFNLFTNRYGKCDPHGDVLGNS

NLFFARNNYWKELRTKISPVFTSGKVKQMYPLMLEVATGLEEHLYLKGNNFTTEVKEICA

SFTTDLISTIAFGINANSLTNPNGEFRSQCRNMFLFTLPRAFDFSVAFFLPKLVSLLRVK

IFSPEFSKFLRSTINHVMRERERTQIPRNDLIDILVALKKDASLEGNKINPKDYQDCLVA

QAAVFLTAGFETSSSTMAFTLFEMAKKPELQERLRSEIVNALALEKSGHLSYENIHSLNY

LNMVVEETLRLYPVLPFLDRQHQRPAGIHKGFSLKPYYEYTLPDEMPVYIPIFAIQRDPK

YWPNPNEFDPERFSPENKKLNNNSTTYMPFGTGPHNCIGSRIGLLQTKLGLVHFLKNYRV

QTCSKTPIQEDFDPKALVLQFENGVTLNVVRDNLYEKNSKSK

>AAL28318.1

MFTFTVARAKDFFVAFFLPKLVSLMRIQFFTADFSHFMRSTIGHVMEERERSGLLRNDLI

DVLVSLRKEAAAEPSKPHYAKNQDFLVAQAGVFFTAGFETSSSTMSFALYEMAKHPEMQK

RLRDEINEALVEGGGSLSYEKIQSLEYLAMVVDEVLRMYPVLPFLDREYESVEGQPDLSL

KPFYDYTLENGTPVFIPIYALHHDPKYWTNPSQFDPERFSPANRKNIVAMAYQPFGSGPH

NCIGSRIGLLQSKLGLVSLLKNHSVRNCEATMKDMKFDPKGFVLQADGGIHLEIVNDRLY

DQSAPSLQ

>EDW74578.1

MFSEILLLLTTLVAVFYFWQKWNYGFWERHGVKHSKPVPIFGNLSRLLTGKTPFYMELSD

LHNKPEFEKEALIGMYLPTRPALLIRDLELIKTVMIKKFNYFSNRAMSTDPHNDPLGYNN

LFFVRNPDWKDLRAKISPVFTSGKIKQMYPLMVEIAKDLETNLSKRSESSVLAIKDLCAR

FTTDLIATIAFGLKANSLNQLDSEFFKSNQAIFAPNLGRAFDFTIIFTLPALATLARVKV

FSKKTSEFIRNSINYVLSEREKSGARRNDLIDILLAMKKEAAAHPDKPNRAKEMDYLVAQ

AAIFQTAGYETSSSTMTLTLYELAKNEELQDRLRAEITDYFGDEDHVDYDKIQEMPYLTK

VVNETLRKYPIVGYAERECTQPAEGERFSLKSYGYDMEVPNGMPVYVAVLAIQRDPQYWP

DPDKFDPERFDPANRHNLNMDAYMPFGIGPHNCIGMRLGLLQTKLGLVHLLRNHRVLTCD

KTVKNIVFAPKCPVILSKDEINLQLQRV

>EDW38679.1

MSTELLFLIATVAIVFYVWHKRRHSFWKRHGVKEIGPLPILGDTVGFLTGRLPFFDQIRK

FHEAPGLENEPIIGVYLAYRPALVIRDLDLVKTVMIKKFSYFTNRKLQTDPHDDALGYNN

LFFVRSPEWKELRNKISPVFTTGKIKQMYPLMVKIGKNLEANVGREPKDSIVTIKSLCAR

FTTDLIATIAFGLDANALQDPKSEFFRHNQAIFAPSVSRAIDFAIIFMLPALTTLAKVKV

FSKSASQFIRSSINYVMAERERSGLRRNDLVDVLLALKREAAASPDKNNKAKNMDYLVAQ

AAVFQTAGYETSSSTMTLALYEVAKNEEIQNRLRQEIEEYFGDDDHISYERIQEMPYLSK

VVNETLRLYPIVGYAERECAQPSQGERFTLAPHHDMELPDGTPVYVSAVAIQRDPKYWPE

PEKFDPERFDASNRDKLNMDAYMPFGVGPRNCIGMRLGLLQSKLGLVHLLRNHRVLWCDK

TVKTIEFAPFCGVLASKDEIYLRVERVSS

>EDV95133.1

MVYSTNILLCIVTILTAVFIWSRRTYVYWQRRRVKFVKPTHLLGNLKRVLKLEESFAMQM

RRFYFNERFRNEPVVGIYLFHQPALLIRDLQLVRTVLVEDFVSFSNRFAQCDLRRDKMGS

LNLFFARQPEWRDIRTRLSPVFTGAKIKQMFSLMEEIGCDLEWYLKRLTRDMRRGDADRG

VVVSIKDVCDLYNTDMIASIAFGLRSYSLRNTQSEIGSHCREIFRPNVRRMVDFFVIFFL

PKLAPLLRCKLFTEPHSEFLRRVIQLVIEERERGGDLRNDLIEMLLTLKKEADLQQDKSH

FTHNQDFLAAQAASFEVAGIATCSATLSFALYELAKQPLIQERLRREIRDAFNAGPGLSY

ERIAGMHYLEMVVEETLRMYPIVPILERECTPINKKRFYSLRPHAECYARRGMPVFISNL

AIQHDPQYWPEPDRFDPERFSAENKPKQTPMSYLPFGAGPHNCIGLHIGQLMIKLGLIYF

LRQHRVEFCDKTVERISFDAKFALLASEHCIYLKIADCI

>EAL26532.2

MSTELLFLIATVAIVFYVWHKRRHSFWKRHGVKEIGPLPILGDTVGFLTGRLPFFDQIRK

FHEAPGLENEPIIGVYLAYRPALVIRDLDLVKTVMIKKFSYFTNRKLQTDPHDDALGYNN

LFFVRSPEWKELRNKISPVFTTGKIKQMYPLMVKIGKNLEANVGREPKDSIVTIKSLCAR

FTTDLIATIAFGLDANALQDPKSEFFRHNQAIFAPSVSRAIDFAIIFMLPALTTLAKVKV

FSKSTSQFIRSSINYVMAERERSGLRRNDLVDVLLALKREAAASPDKNNKAKNMDYLVAQ

AAVFQTAGYETSSSTMTLALYEVAKNEEIQNRLRQEIEEYFGDDDHISYERIQEMPYLSK

VVNETLRLYPIVGYAERECAQPSQGERFTLAPHHDMELPDGTPVYVSAVAIQRDPKHWPE

PEKFDPERFDASNRDKLNMDAYMPFGVGPRNCIGMRLGLLQSKLGLVHLLRNHRVLWCDK

TVKTIEFAPFCGVLASKDEIYLRVERVSS

>EDV36184.1

MALGLLILVVILLTTACWLLQQHYSYWRRRGVPEIKPRWIVGNLMGLLNMRTSPPEFIAQ

LYNHPEAENEPVVGIHLFHKPGLLLRDPGLIRKIMVKDFAKFSNRYSNSDHKGDPLGSQN

IFFLKNPAWKEVRLKLSPFFTSNRLKNMFPLIEEVGASLDAHLRQKPLHNERLRCFDIEA

KELCALFTTDVISTVAFGVQANSFTDPNCEFRRHGRSVFEFTLLRAAEFTLVFFLPHWVP

FFGFKVVPAESTRFLRKTINYVLDERERSGQIRNDLIDILIEFRRNTQQAKAKGVKDQFV

FEGDILVAQAVLFFTAGFESSSSTMAFAMYELAKDADVQQRLREEIKEALVASGGQVTMK

MIESLEYMQMILMEVLRMYPPLPFLDRECTSDDLYSLEPFHNFQVPHGMPVYIPCYALHM

DPQHFPQPRKFQPERFSATNRKLHTPYTYMPFGLGPHGCIGERFGFLQAKVGLVSLLRNH

LVTTSERTQRRMQLNPKAMITQAKGGIHLRLVRDPLGI

>EDW74579.1

MFAETLILLATLAIVFYIWQKRNYSYWKRHGIKYIKPLPLLGNMRSSLTGKAPFYEQLSE

LHNEQGFEKEPLIGIYRAHRPSLLIRDLELIKTVMIKKFNYFSDRALQTDPHNDTLGYNN

LFFIRNPDWRELRAKLSPVFTSGKMKQMYPLMVEVAKDLETNLSSRQNGSELSIKDLCAR

FTTDLIATIAFGVKANSLTTSTSEFYAANQAIFELSLGRAFDFAIILVLPALATLARTKV

FSKKTTKFISESINYVLAERERTGVKRNDLIDTLLAMKREAALHPDKPNRARDIDYLVAQ

AAVFQLAGYETSSSTMTMAFYELSLNEEIQDRLRQEISDYFGEEDLVEYEKIQEMPYLTK

VINETIRKYPVVPYVERECTQPAQGERFNLKPHYDLEVPNGMPVYVSLLAIHRDPQYWPQ

PEIFDPERFDPANRHNLNMDAYMPFGIGPHNCIGMRLGLLQSKLGLVHLLRNHRIVRSDK

TLKDIAFNPLTPIMSSKEDILLRLERL

>EDW65417.1

MVYSTNILLGIVTILTAVFIWSRRTYVYWQRRRVKFVQPTHLLGNLKKVLKLEESFALQL

RRFYFDERFRNEPVVGIYLFHQPALLIRDLQLVRTVLVEDFVSFSNRFAKCDVRHDKMGS

LNLFFARQPEWREIRTRLSPVFTGAKIKQMFSLMEEIGCDLEWYLKRLTRDLRRGDAARG

VVVSIKDVCDLYNTDMIASIAFGLRSYSLRNTQSEIGSHCQDIFRPNVRRIIDFFVIFFL

PKLVPLLRSKLFTEPHSEFLRRVIQLVIEERERGGDLRNDLIEMLLTLKKEADLQQDKTH

FTHHQDFLAAQAASFEVAGIETCSSTMSFALYELAKQPLMQERLRREIRHAFALAAAHGS

NGAGPGLSYELIGSMKYLEMVVEETLRKYPIVPLLERECSPVNKKRFYSLRPHAECYARR

GMPVFISNLAIHHDPQYWPEPERFDPERFNAENKSKQTPMSYLPFGAGPRNCIGLPICQL

QIKLGLIYFLRQHRVEFCDKTVERITFDAKFALLASEHSIYLKVVDCL

>Q9V675.1

MELVLLILVASLIGIAFLALQQHYSYWRRMGVREIRPKWIVGNLMGLLNMRMSPAEFISQ

LYNHPDAENEPFVGIHVFHKPALLLRDPEMVRNILVKDFAGFSNRYSSSDPKGDPLGSQN

IFFLKNPAWKEVRLKLSPFFTGNRLKQMFPLIEEVGASLDAHLRQQPLHNERMRCFDLEA

KELCALYTTDVIATVAYGVSANSFTDPKCEFRRHGRSVFEFNLLRAAEFTLVFFLPHLVP

FVRFKVVPAEATRFLRKTINYVMSEREKSGQKRNDLIDILIEFRRSTQLAKASGIKDQFV

FEGDILVAQAVLFFTAGFESSSSTMAFAMYELAKDTDVQQRLREEIKDALVESGGQVTLK

MIESLEFMQMILLEVLRMYPPLPFLDRECTSGRDYSLAPFHKKFVVPKGMPVYIPCYALH

MDPQYFPQPRKFLPERFSPENRKLHTPYTYMPFGLGPHGCIGERFGYLQAKVGLVNLLRN

HMITTSERTPHRMQLDPKAIITQAKGGIHLRLVRDALGV

>EDW09597.1

MTLGTVLLLLFCILICGYYYLRHHYAYWIVRGVPELRPRLFVGNLGGLLSMRSSPADFIG

EMYNHPDAQDKPFVGIHVFYKPALLLRDPELIKRIMIKDFAKFSNRYSNSDYRGDPLGSQ

NIFFLKNPAWKEVRLKLSPFFTSNRLKNMFPLIDEVGRNLDAYLRQQPLHNERMRCFDLE

AKELCALFTTDVIATVAYGIQANSFTHPNGEFRRHGRAVFQFSLKRAAEFTLVFFLPQLV

PYFGFKVVPAESAKFLRDTISYVMAEREKSQKTRNDLIDLLIDFKRSTQQSKFVFDGDIL

VAQAVLFFTAGFESSSSTMAFAMYELAKHPEVQQRLRAEIKQALIASGGCVNQQLIESLE

YMQMILQEVLRMYPPLPFLDRECTSDEGYSLAPFHKFCVPKGMPVYIPAYALHMDPQYYP

QPRQFQPERFSSSNRKQHIPYTYMPFGLGPHGCIGERYGYLQAKVGLVYVLRNHLVTTSE

RTPRRMKLDAKAIILQAEGGINLRLVRDPLGV

>AAR88134.1

MELVLLILVASLIGIAFLALQQHYSYWRRMGVREIRPKWIVGNLMGLLNMRMSPAEFISQ

LYNHPDAENEPFVGIHVFHKPALLLRDPEMVRNILVKDFAGFSNRYSSSDPKGDPLGSQN

IFFLKNPAWKEVRLKLSPFFTGNRLKQMFPLIEEVGASLDAHLRQQPLHNERMRCFDLEA

KELCALYTTDVIATVAYGVSANSFTDPKCEFRRHGRSVFEFNLLRAAEFTLVFFLPHLVP

FVRFKVVPAEATRFLRKTINYVMSEREKSGQKRNDLIDILIEFRRSTQLAKASGIKDQFV

FEGDILVAQAVLFFTAGFESSSSTMAFAMYELAKDTDVQQRLREEIKDALVESGGQVTLK

MIESLEFMQMILLEVLRMYPPLPFLDRECTSGRDYSLAPFHKKFVVPKGMPVYIPCYALH

MDPQYFPQPRKFLPERFSPENHKLHTPYTYMPFGLGPHGCIGERFGYLQAKVGLVNLLRN

HMITTSERTPHRMQLDPKAIITQAKGGIHLRLVRDALGV

>EDV30042.1

MAYSTNILLAIVTILTGIFIWSRRTYVYWQRRRVKFVQPTHILGNLSRVLRLEESFALQL

RRFYFDERFRNEPVVGIYLFHQPALLIRDLNLARTVLIEDFVSFSNRFAKCDARSDKMGA

LNLFLAKQPEWREIRTRLDPAFAGAKLKQMFSLMEEIGCDLEWYLKRLTRDLRRGDAERG

AVVSIKDVCDLYNTDMIASIAFGLRSYSLRNTQSEIGSHCQDLFKPNVWRIIDLFVIFYL

PKFVPLLRPKLFTEPHSEFLRRVIQLVIEERERGGDLRNDLIELLLTLKKEADLQQDKSH

FTHHRDFLAAQAASFEVAGIETCSSSMSFALYELARHPLMQSRLRREICTAFGESPGGKL

TYEAITGMEFLDMVVKETLRKYPIVPLLERECTPINKKRFYSLRPHAECYARRGMPAFIS

VLAIHHDPKYWPDPERFDPERFAPESRSQHTPMSYLPFGAGPHNCIGMGLGLLQIKLGLT

YFLHQHRVELCEETCDQIRFDAKFALLASEQRIYLKVDCL

>EDV47553.1

MVYSTNILLAIVTILTGVFIWSRRTYVYWQRRRVKFVQPTHLLGNLSRVLRLEESFALQL

RRFYFDERFRNEPVVGIYLFHQPALLIRDLQLVRTVLVEDFVSFSNRFAKCDGRSDKMGA

LSLFLAKQPEWREIRTRLAPAFAGAKLKQMFSLMEEIGCDLEWYLKRLTRDLRRGDAERG

AIVSIKDVCDLYNTDMIASIAFGLRSYSLRNTQSEIGSHCQDLFRPNVRRVIDLFVIFYL

PKLVPLLRPKLFTEPHATFLRRVIQLVIEERERGGDLRNDLIEMLLTLKKEADLQQDKSH

FTHHRDFLAAQAASFEVAGIETCSASMSFALYELAKQPLMQSRLRREIREAFADNPNGRL

TYEAVARMEFLDMVVEETLRKYPIVPLLERECTPINKKRFYSLRPHAECYARRGMPVFIS

NLAIHHDPKYWPDPDRFDPERFSAANKALQAPMSYMPFGAGPHNCIGMQIGLLQIKLGLV

YFLHRHRVEICDRTVERIQFDAKFALLASEKRIYLKVDCL

>EAL24937.1

MVLGLFLLVVIFLATICFLLQQHYAYWRRRGVRELRPHWILGNLAGLLSMRDSPADFMGQ

LYNHPDALNQPFVGIHVFHRPALLLRDPDLIKRILIKDFGKFSNRYSNSDYKGDPLGSQN

IFFLKNPAWKEVRFKLSPFFTSNKLKSMFPLIVEVGSNLDAYLREQPLHNERTRCFDLEA

KELCALFTTDVIASVAYGVQANSFTQPNGEFRRHGRAVFEFSLKRAAEFTLVFFLPHLVP

LFGFKVVPSEATRFLRNTINYVMAERERSQQTRNDLIDILIEFRRSTQQARAAGDHGQFV

FEGDILVAQAVLFFTAGFESSSSTMAFAMYELAKDADIQLRVRHEIKEALIASGGQVTLQ

LIDSLEYMQMVLLEVLRMYPPLPFLDRECTAEDEDYSLTPYHGFRVPKGMPIYIPCYALH

MDPQYFPQPRKFQPERFSPTNRKLQTPYTYMPFGLGPHGCIGERFGLLQAKVGLVNLMRN

HQVTTSERTPRRMKLDPKAIILQAQGGIHLRLVRDPLGI

>EDW60583.1

MTLGTMLVILVLLLVSGYYYLRQHYAYWTRRGVPELRPSLLLGNLGGLLSMRSSPADFIG

ELYNHPDAQDKPFVGIHVFHKPALLLRDPTLIKRIMVKDFPKFANRYSNSDYKGDPLGSQ

NLFFLKNPAWKEVRLKLSPFFTSNRLKHMFPLIDEVGCNLDAYLRQQPLHNERMRCFDLE

AKELCALFTTDVIATVAYGVQANSFNHPNSEFRRHGRAVFKFSLKRAAEFTLVFFLPQLV

PYFGFKVVPAESTEFLRKTINYVMAEREKSKQPRNDLIDLLIDFKRSTQQAQAEGNQFVF

EGDILVAQAVIFFTAGFESSSSTMAFAMYELARHPEVQQRLRNEIKNALTASGGRVTQQL

IESLEYMQMIIQEVLRMYPPLPFLDRECTSEDGYDLAPFHKFCVPKGMPIYIPAYALHMD

PQYYPQPKQFQPERFSSSNRKLHTPYTYMPFGLGPHGCIGERYSFLQAKVGLVYVLRNHL

VTTSERTLRRMKLDPKAIILQADGGINLRLVRDPLGV

>EDX03029.1

MVYSTNILLAIVTILTGVFIWSRRTYVYWQRRRVKFVQPTHLLGNLSRVLRLEESFALQL

RRFYFDERFRNEPVVGIYLFHQPALLIRDLQLVRTVLVEDFVSFSNRFAKCDGRSDKMGA

LSLFLAKQPEWREIRTRLAPAFAGAKLKQMFSLMEEIGCDLDWYLKRLTRDLRRGDAERG

AIVSIKDVCDLYNTDMIASIAFGLRSYSLRNTQSEIGSHCQDLFRPNVRRVIDLFVIFYL

PKLVPLLRPKLFTEPHAEFLRRVIQLVIEERERGGDLRNDLIEMLLTLKKEADLQQDKTH

FTHHRDFLAAQAASFEVAGIETCSASMSFALYELAKQPLMQSRLRREIREAFADNPNGRL

TYEAVARMEFLDMVVEETLRKYPIVPLLERECTPINKKRFYSLRPHAECYARRGMPVFIS

NLAIHHDPKYWPDPDRFDPERFSAANKALQAPMSYMPFGAGPHNCIGMQIGLLQIKLGLV

YFLHRHRVEICDRTVERIQFDAKFALLASEQRIYLKVDCL

>EDW75215.1

MAYSTNILLGIVTILTAVFIWSRRTYVYWQRRRVKFVQPIPLLGNLKKVLKLEESFALQL

RRFYFDRRFQREPVIGIYLFHQPALLIRDLELVRTVLIEDFVSFSNRFAKCDLRHDKMGA

LNLFFAKQAEWREIRTRLSPAFTEAKIKQMFSLMEEIGCDLEWYLKRLMRSLRRDDSTSD

RSGAIVSIKDICDLYNTDMIASIAFGLRSYSLRNTQSEIGSHCQDIFRPNMRRIIDFFVI

FYLPKLVPLLRSKLFTESHSEFLRRVIQLVIEERERGGILRNDLIEMLLTLKKEADLQQD

KSHFTHHQDFLAAQAASFEVAGIETCSSSMSFALFELAKQPLMQARLRREIREAFRLQSD

DGSESLSYERIASMEYLRMVVEETLRKYPIVPLLERECTPTSKKRFYSLRPHAECYARRG

MPIYISNLAIHHDPKYWPDPERFDPERFTALNKSHQVPMSYLPYGAGPHNCIGMQIGQLQ

IKLGLIYFLRQHQVELYIGEDDEGRNTGPLTVEERNEANYEKRIKANRLWEAEQKQQAKQ

QAKQQAKQQAKRQAKQQPHKQLDEQPYKQPHKQPQQYHRHNEQHQQRNAQHRQRQDHQQH

HHQLHHRKKDERPQRCQLRRRSSSHMRPEFHYPAYTPTNAHSDNADVQAQRQRTERRRRN

SRENSVIALHRARAAKLDNLQTHLGSAKESAVNRIRNEQKLMQTPHNPHSSQIKYRARRN

RAF

>EDX18476.1

MVYSTNILLAIVTILTGVFIWSRRTYVYWQRRRVKFVQPTHLLGNLSRVLRLEESFALQL

RRFYFDERFRNEPVVGIYLFHQPALLIRDLQLVRTVLVEDFVSFSNRFAKCDGRSDKMGA

LSLFLAKQPEWREIRTRLAPAFAGAKLKQMFSLMEEIGCDLEWYLKRLTRDLRRGDAERG

AIVSIKDVCDLYNTDMIASIAFGLRSYSLRNTQSEIGSHCQDLFRPNVRRVIDLFVIFYL

PKLVPLLRPKLFTEPHAEFLRRVIQLVIEERERGGDLRNDLIEMLLTLKKEADLQQDKSH

FTHHRDFWAAQAASFEVAGIETCSASMSFALYELAKQPLMQSRLRREIREAFAGNPNGRL

TYEAVARMEFLDMVVEETLRKYPIVPLLERECTPINKKRFYSLRPHAECYARRGMPVFIS

NLAIHHDPKYWPDPDRFDPERFSAANKALQAPMSYMPFGAGPHNCIGMQIGLLQIKLGLV

YFLHRHRVEICDRTVERIQFDAKFALLASEQRIYLKVDCL

>EAL32098.1

MVYSTNILLAIVTILTAVFVWSRRTYVYWQRRRVKFVQPAHLLGNLSKVLRLEESFALQL

RRFYFDERFRNEPIVGIYLFHQPALLIRDLSLVRTVLVEDFVSFSNRFAKCDLRHDKMGS

LNLFFAKQPEWREIRTRLSPVFTGAKIKQMFSLMEEIGCDLEYYLKRLTRDMRRSDASAR

GVIVSIKDVCDLYNTDMIASIAFGLRSYSLRSTQSEIGSHCQDIFRPNVRRIIDFFVIFY

LPKLVPLLRPRLFTEPHAEFLRRVIQLVIEERERGGDLRNDLIEMLLTLKKEADLQKDKS

HFTHHRDFLAAQAASFEVAGIETCSSSLSFALYELAKQPLMQARLRREIRETFEESPAGS

LSYELIGRMQYLEMIVEETHRKYPIVPLLERECTPINKKRFYSLRPHAECYARRGMPIYI

SNLAIHHDPKYWPDPERFDPERFSAENKSHQAPMSYLPFGAGPKHCIGMQIGQLQIKLGL

IYFLRRHRVELCEKTVERISFDAKFALLASEHRIYLKVADCL

>EDW29975.1

MVYSTNILLAIVTILTAVFVWSRRTYVYWQRRRVKFVQPAHLLGNLSKVLRLEESFALQL

RRFYFDERFRNEPIVGIYLFHQPALLIRDLSLVRTVLVEDFVSFSNRFAKCDLRHDKMGS

LNLFFAKQPEWREIRTRLSPVFTGAKIKQMFSLMEEIGCDLEYYLKRLTRDMRRSDASAR

GVIVSIKDVCDLYNTDMIASIAFGLRSYSLRSTQSEIGSHCQDIFRPNVRRIIDFFVIFY

LPKLVPLLRPRLFTEPHAEFLRRVIQLVIEERERGGDLRNDLIEMLLTLKKEADLQKDKS

HFTHHRDFLAAQAASFEVAGIETCSSSLSFALYELAKQPLMQARLRREIRETFEESPAGS

LSYELIGRMQYLEMIVEETHRKYPIVPLLERECTPINKKRFYSLRPHAECYARRGMPIYI

SNLAIHHDPKYWPDPERFDPERFSAENKSHQAPMSYLPFGAGPKHCIGMQIGQLQIKLGL

IYFLRRHRVELCEKTVERISFDAKFALLASEHRIYLKVADCL

>EDW77077.1

MELGIFLPSILIVVISIYFFLQRHYGYWLRQGVRELRPHWLVGNLSGLLSMKSSPADFIS

DLYNHPDAVDQPFVGIHVFHKPAILLREPDLIKKIMIKDFCQFGNRYSNSDYKGDPLGSQ

NIFFLKQPQWKEVRLKLSPFFTSTKLKHMFPLIDEVGRNLDAHLRQQPLHNERMRSFDVE

AKELCALFTTDVIATVAYGVQANSFSVPDSEFRRHGRAVFEFSLKRAAEFTLVFFLPHLV

PLFGFKVVPKEPTEFLRKTINYVMAERERSKQTRNDLIDILIEFKRSTQQAKDTGKNGNQ

FVFEGDILVAQAVLFFTAGFESSSSTMSFALYELAKNLEIQERLRDEIKKALIESKGQVT

QQMIESLEYLQMILYEVLRMYPPLPFLDRECTSDEAYSLAPYHSFRVPRGMPIYIPAYAL

HMDPQYYPQPRKFLPERFSPTNRKLNTPYTYMPFGLGPHGCIGERFGFLQAKVGLVYLLR

NHLVTTSERTQGRMKLDTKAIILQAQGGIHLKLVRNPLGV

>EDW56308.1

MDNNTAVSALDEFCAKTNNTPPTYHFINGEDGGFVCKVQLLEIEALGNDDQNKSEEVTTA

GTHAPQNNLRSKIGLSKALYAYPCTIQPPLDPTSDPASEMVYSTNILLAIVTILTGVFIW

SRRTYVYWQRRRVKFVQPTHLLGNLSRVLRLEESFALQLRRFYFDERFRNEPVVGIYLFH

QPALLIRDLQLVRTVLVEDFVSFSNRFAKCDGRSDKMGALTLFLAEQPEWREIRTRLAPA

FAGAKLKQMFSLMEEIGCDLEWYLKRLTRDLRRGDAERGAIVSIKDVCDLYNTDMIASIA

FGLRSYSLRNTQSEIGSHCQDLFRPNVRRVIDLFLIFYLPKLVPLLRPKLFTEPHAEFLR

RVIQLVIEERERGGDLRNDLIEMLLTLKKEADLQQDKSHFTHHRDFLAAQAASFEVAGIE

TCSASMSFALYELAKQPLMQSRLRREIREAFAGNPNGRLTYEAVARMQFLDMVVEETLRK

YPIVPLLERECTPINKKRFYSLRPHAECYARRGMPVFISNLAIHHDPKYWPDPDRFDPER

FSAANKALQAPMSYMPFGAGPHNCIGMQIGLLQIKLGLVYFLHRHRVEICDRTVERIQFD

AKFAVLASEQRIYLKVDCL

>EDX15543.1

MELVLLILVGSLIGIAFLALQQHYSYWRRMGVREIRPKWIVGNLMGLLNMRMSPAEFICQ

LYNHPDAEKEPFVGIHVFHKPALLLRDPEMVRNILVKDFAGFSNRYSNSDPKSDPLGSQN

IFFLKNPAWKEVRLKLSPFFTGNRLKQMFPLIEEVGASLDAHLRQQPLHNERMRCFDLEA

KELCALYTTDVIATVAYGVSANSFTDPKCEFRRHGRSVFEFNLRRAAEITLVFFLPHLVP

LLRFKVVPAEATLFLRKTINYVMSEREKSGQKRNDLIDILIEFRRSTQMAKESGIKDQFV

FEGDILVAQAVLFFTAGFESSSSTMAFAMYELAKDADVQQRLREEIKDALVESGGQVTLK

MIESLEFMQLILLEVLRMYPPLPFLDRECTSGKDYSLAPFHNKFVVPKGMPVYIPCYALH

MDPQYFPQPRKFLPERFSPENRKLHTPYTYMPFGLGPHGCIGERFGYLQAKVGLVYLLRN

HMITTSERTPHRMQLDPKAIITQAKGGIHLRLVRDPLGV

>EDW47513.1

MELVLLILVGSLIGIAFLALQQHYSYWRRMEVREISPKWIVGNLMGLLNMRMSPAEFICQ

LYNHPDAEKEPFVGIHVFHKPALLLRDPEMVRNILVKDFAGFSNRYSNSDPKGDPLGSQN

IFFLKNPAWKEVRLKLSPFFTGNRLKQMFPLIEEVGASLDAHLRQQPLHNERMRCFDLEA

KELCALYTTDVIATVAYGVSANSFTDPKCEFRRHGRSVFEFNLRRAAEITLVFFLPHLVP

LLRFKVVPAEATLFLRKTINYVMSEREKSGQKRNDLIDILIEFRRSTQMAKESGIKDQFL

FEGDILVAQAVLFFTAGFESSSSTMAFAMYELAKDADVQQRLRDEIKDALVESGGQVTLK

MIESLEFMHMILLEVLRMYPPLPFLDRECTSGKDYSLAPFHNKFVVPKGMPVYIPCYALH

MDPQYFPQPRKFLPERFSPENRKLHTPYTYMPFGLGPHGCIGERFGYLQAKVGLAYLLRN

HMITTSERTPHRMQLDPKAIITQAKGGIQIRLVRDPLGV

>EDV56367.1

MELVLLILLVSLIGIAYAALQQHYSYWRRKGVREIKPKWIVGNLMGLLNMQNSPAEFISQ

LYNHPDAENEPFVGIHVFHKPALLLRDPEMVRNILAKDFTGFSNRYSNSDHKADPLGSQN

IFFLKNPAWKEVRYKLSPFFTGHRLKQMFPLIEEVGASLDAHLRQQPLHNERMRCFDVEA

KELCALYTTDVIATVAYGVSANSFTDPKGEFRRHGRSVFEFSLLRAAEFTLVFFLPDLVP

FFGFKVVPVEATRFLRKTINYVMSERERTGQKRNDLIDILIEFRNSTQQAKASGVKDQFL

FEGDILVAQAVLFFTAGFETSSSTMAFAMYELAKDADVQQRLREEIKDALVESGGQVTLN

MIESLEFMQMILLEVLRMYPPLPFLDRECTSGKNYSLAPFHKFVVPKGMPVYIPCYALHM

DPQYFPQPRKFLPERFSQENRKLHTPYTYMPFGLGPHGCIGERFAYLQAKVGLVYLLRNH

LVTTSERTPHRMQLDPKAIITQAKDGIHLRLVRDPLGV

>EDW90822.1

MELLLLILLVSLIGIAYVALQQHYSYWRRMGVREIKPKWIVGNLMGLLNMQKSPAEFISQ

LYNHPDAENEPFVGIHVFHKPALLLRDPEMVRNILVKDFGGFSNRYSNSDHKADPLGSQN

IFFLKNPAWKEVRLKLSPFFTGNRLKQMFPLIEEVGASLDAHLRQQPLHNERMRCFDLEA

KELCALYTTDVIATVAYGVRANSFTDPKGEFRRHGRSVFEFSLLRAAEFTLVFFLPHLVP

FFGFKVVPAGATSFLRKTINYVMSERERSGEKRNDLIDILIEFRKSTQQAKKAGLKDQFV

FEGDILVAQAVLFFTAGFESSSSTMAFAMYELAKDADVQQRLREEIKDALVESRGQVTLK

MIESLEFMQMIILEVLRMYPPLPFLDRECTSGKDYALAPFHKFVVPKGMPVYIPCYALHM

DSQYFPQPRKFLPERFSQENRKLHTPYTYMPFGLGPHGCIGERFGILQAKVGLVYLLRNH

FVTTSERTPHRMQLEPKAIITQAKGGVHLRLVRDPLGV

>EDW89797.1

MLYELVLLLGTLTLVFYIWQRSTLSFWKRHGVKYISPLPIVGCTRDFLTARVPFFEQIQK

FHEAPGFENEPFVGVYMTHRPALVIRDLELIKTVMIKKFQYFNNRALQTDPHNDALGYNN

LFFARSPEWRQLRTKISPVFTTGKIKQMYPLMVKIGKHLEDSVERLGSGTKVQVKDLCAR

FTTDLIATIAFGVEANALQDTKSEFFYHNKAIFSMTLSRAIDFAIIFMLPSLASLARVKL

FSKETTKFIRSSINFVLTERERTGEKRNDLVDILLAIKHEAAANPKKLSKEIDLDYLVAQ

AAIFQTAGFETSSSTMTLSLYELARNEALQERLREEIVDFFGDDDHISYERIQEMPYLSQ

VINETLRKYPIVGYIERECSQPAEGERFNLKPFHNMELPHGMSIYMSTLAIHRDPQYWPD

PEKFDPERFSPGNRENLNMDAYMPFGVGPRNCIGLRLGLLQSKLGLVHLLRNHKFHACDQ

TVKKIEWAPVSPVIASKHDIVLRLEKVSRNYVFGERLPTK

>EDV38145.1

MSTELLLLVATLAIVFYIWQRRAFSFWQRHGVKYIRPLPFLGCTREFLTARVPFFEQIKK

FHEAPGFEKEPFVGVYLAYKPALVVRDLDLIKTVMIKKFQYFHNRVLQTDPHNDALGFNN

LFFARSPEWKELRHKISPVFTSGKIKQMYPLMVKIGKDLESSAERLAGASPVPIKDLCAR

FTTDLIATIAFGVEANALKDAKSEFFYHNQAIFALTWSRAIDFTIIFMLPALVSIARVKL

FSKETTKFIRNTINYVLSERERTGVKRNDLVDILLALKREAEANPDQLQKYKDLDYLVAQ

AAVFQTAGFETSSSTMTLTLYHLAQNESLQDRLCQEITEYLGDEDHVSYDRIQQMPYLSQ

VVNETLRMYPIVGYAERQCAQPAEGEKFTLEPHSSMELPHGMAVYVSAIAIHHDPQYWPE

PEKFDPDRFDPANRSNLNMDAYMPFGVGPRNCIGMRLGLLQSKLGLVHLLRHHRVHATDQ

TPKKIEWAATSPLIASKHEIYLRLEKVSS

>EDX05859.1

MLLLLLLGSLTIVFYIWQRRTLSFWARHGVKYIRPLPILGCTREFLTARVPFFEQIQKFH

EAPGFEKEPFVGVYMTHRPALVIRDLDLIKTVMIKKFQYFNNRALQTDPHNDALGYNNLF

FARSPAWKELRTKISPAFTSGKIKQMYPLMVKIGKNLEDSAERLGSGSEVQVKDLCARFT

TDLIATIAFGVEANALQDAKSEFFYHNRAIFSLTLSRGIDFAIIFMIPALASLARVKLFS

RETTKFIRSSINFVLKERERTGEKRNDLIDILLALKREAAANPEKLSKVVDLDYLVAQAA

IFQTAGFETSSSTMTLTLYELAKNEALQDRLRQEIVDFFGDEDHISYERIQEMPYLSQVV

NETLRKYPIVGYIERECSQPAEGERFNLEPFHNMELPHGMSIYMSTLAVHRDPQYWPDPE

KFDPERFSSSNRENLNMDAYMPFGVGPRNCIGMRLGLLQSKLGLVHMLRNHRIHMCDKTI

RKIEWAATSPVIASKHDIVLRLEKVSGNNDVGQKLSMK

>ACL90681.1

MLLLLLLGSLTIVFYIWQRRTLSFWERHGVKYIRPFPVVGCTREFLTAKVPFFEQIQKFH

EAPGFENEPFVGVYMTHRPALVIRDLELIKTVMIKKFQYFNNRVLQTDPHNDALGYKNLF

FARSPGWRELRTKISPVFTSGKIKQMYPLMVKIGKNLQDSAERLGSGTEVQVKDLCSRFT

TDLIATIAFGVEANALQDAKSEFFYHNRAIFSLTLSRGIDFAIIFMIPALASLARVKLFS

RETTKFIRSSVNYVLKERERTGEKRNDLIDILLALKREAAANPGKMSKEVDLDYLVAQAA

VFQTAGFETSASTMTMTLYELAKNEALQDRLRQEIVDFFGDEDHISYERIQEMPYLSQVV

NETLRKYPIVGYIERECSQPAEGERFTLEPFHNMELPHGMSIYMSTVAVHRDPQYWPDPE

KYDPERFNSSNRDNLNMDAYMPFGVGPRNCIGMRLGLLQSKLGLVHILRNHRFHTCDKTI

KKIEWAPTSPVMASKRDIILRVEKVSGKKDFGQK

>EDV98208.1

MAFGTLLLLLIIILVSGYYFLRQHYDYWIRQDVPQLEPSLFLGNLGGLLSMQSNPADFIA

QLYNHPDAQDKPFVGIHVFHKPALLLRDPALIKRIMVKDFPKFANRYSNSDYKGDPLGSQ

NIFFLKNPAWKEVRLKLSPFFTSTRLKHMFPLINEVGCNLDAYLRQQPLHNERARCFDLE

AKELCALYTTDVIATVAYGVQANSFTQPNSEFRRHGRAIFEFSWKRAAEFTLVFFLPQLV

PYLGFKVVPAEPTAFMRKTINYVIAEREKSKQPRNDLIDLLIDFKRSSKNADNQFVFEGD

ILVAQAVLFFTAGFESSSSTMAFAMYELARNPSVQQRLREEIKTALIASGGCVTQQLIES

LEYMQMTLQEVLRMYPPLPFLDRECTSDNGYSLAPFHKFRVPKGMPIYIPAYGLHMDPQY

YPEPRQFQPERFSARNRKEHTPYTYMPFGLGQHGCIGERYGFLLAKVGLVYVLRNHLVTT

SERTQRRMKLDPKAIILQAAGGIHLRLVRDSLGV

>EDW31717.1

MVLGLFLLVVIFLATICFLLQQHYAYWRRRGVRELRPHWILGNLAGLLSMRDSPADFMGQ

LYNHPDALNQPFVGIHVFHRPALLLRDPDLIKRILIKDFGKFSNRYSNSDYKGDPLGSQN

IFFLKNPAWKEVRFKLSPFFTSNKLKSMFPLIVEVGSNLDAYLREQPLHNERTRCFDLEA

KELCALFTTDVIASVAYGVQANSFTQPNGEFRRHGRAVFEFSLKRAAEFTLVFFLPHLVP

LLGFKMVPSEATRFLRNTINYVMAERERSQQTRNDLIDILIEFRRSTQQARAAGDHGQFV

FEGDILVAQAVLFFTAGFESSSSTMAFAMYELAKDADIQLRVRHEIKEALIASGGQVLRM

YPPLPFLDRECTAEDEDYSLTPYHGFRVPKGMPIYIPCYALHMDPQYFPQPRKFQPERFS

PTNRKLQTPYTYMPFGLGPHGCIGERFGLLQAKVGLVNLMRNHQVTTSERTPRRMKLDPK

AIILQAQGGIHLRLVRDPLGI

>EDW06986.1

MVYSTNILLGIVTILTAVFIWSRRTYVYWQRRRVKFVQPTHLLGNLKKVLKLEESFPLQL

RRFYFDERFRNEPVVGIYLFHQPALLIRDLQLVRTVLIEDFVSFSNRFAKCDMRHDKMGS

LNLFFARQPEWREIRTRLSPVFTGAKIRQMFSLMEEIGCDLEWYLKRLTRDLRRGEASRG

VIVSIKDVCDLYNTDMIASIAFGLRSYSLRNTQSEIGSHCQDIFKPNLFTEMHSEFLRRV

IQLVIEERERGGDLRNDLIEMLLTLKKEADLQQDKTHFTHHQDFLAAQAATFEVAGIETC

SSTMSFALYELAKQPLMQERLRREIRHTFALAAAHGDGPGLSYELIGGMRYLEMVVEETL

RKYPIVPVLERECSPINKKRFYSLRPHAECYARRGMPVFISNLAIHHDPQYWPEPERFDP

ERFSAENKPKQTPMSYLPFGAGPHNCIGMHIGMLQIKLGLIYFLRQHRVEFCDRTVDRIS

FDAKFALLASEHRIYLRVVDCL

>EDW84524.1

MFTETILLIATLALVLFVWQKRTNNFWDRVGVKHVPTKFIVGNAREFLTGKVPFYEQIYK

LHTAPGFEKEPFIGVNMLHRPGLIIRDLELIKTVMIKKFNCFTNRVIQADPHVDVLGANN

LFFARNPSWRELRTKITPVFTSGKIKQMYPLLVEVGKELESYLDRLPKEAELRVKELAGR

FTTDLIATSAMGVKANSLKDVNSEFFLNNRNFFKFTWGRVFDSFFIFLIPSLAPIVRARV

FPKEVTKFFQRIVGHVMEEREKSGVHRNDLIDILLAMQKEALADPDQKNKAKDVNFLVAQ

AAGFQTGGYETSASTMTFTLFELANNLEIQNRLHQEIQEYYGEGKSISYEQLHEMPYLSK

VINETLRKYPIAGLAERECTQPSEGERFNLKPYNNFELPNGMPVYVSSLGIHRDPQYWPE

PEKYDPERFDPSNRHNINMDAYMPFGIGPHNCIGMRLGLLQAKLGIIHMLRNHRVTPSPS

TIKRIEFNPLSVVLTSKDEILLNIERI

>EDW60383.1

MSTELLLLIATLAIVFYIWQRRAYSYWLRSGVKQIEPSPIFGNARSILTGRVAFFEQIRM

LHTAKGFENEPFVGVYLLHRPALIIRDLDLIKTVMIKKFSYFANRALQTDPHRDPLGNNN

LFFVRNPDWKELRNKISPVFTSGKIKQMYPLMLEIGKNLEANLAELPADSLISIKSICAR

FTTDLIATTAFGLKANALGDIKSEFYKYNKSIFDINFRRGIDFTIIFMLPMLASLARVTV

FSKQASNFMRRSISYVLAEREKSGVRRNDLIDILLTMKREAAAQPDKKDNRAKELDYLVA

QAAIFQTAGYETSASTMTMTLFEFAHHPEIQERLRREINDYFGSEDHIDYDRIQEMPYLT

QVVNETLRKYPIVGYAERECTQPATGERFTLAPYSDLELPSGLPIYISSIGIHRDEQYWP

EPEKYNPDRFAPENRDSLNMDAYMPFGIGPRNCIGMRLGLLQAKLGLVHLLRNYRVVKCD

KTPKTIRFANFSAVITPKDEIFLKLQRA

>EDV38144.1

MSTELLLLLATLAIVFYIWQRRAFSFWQRHGVKYIRPLPFLGCTREFLTARVPFFEQLKK

FHEAPGFEKEPFVGVYLAYKPALVVRDLDLIKTVMIKKFQYFHNRVLATDPHNDALGFNN

LFFARSPEWKELRHKISPVFTSGKIKQMYPLMIKIGKDLENSADGLAGRSPVPIKSLCGR

FTTDMIATIAFGVEANALKDAKSEFFHHNQAIFAVTWSRAIDFTIIFMLPALASIARVKV

FSKETTKFIRNTINYVLSERERTGVKRNDLVDILLALKREAEANPDQLQKCKDLDYLVAQ

AAIFQTGGFETSASTMTLTLFELARNESIQDRLCQEITEYFGDEDHVSYERIQEMPYLSQ

VVNETLRMYPIVADAERQCSQPAEGEQFTLEPHSLMELPHGMPIYVSILAIHHDLQYWPE

PEKFDPDRFDPANRSNLNMDAYMPFGVGPRNCIGMRLGLLQTKLGLVHLLRHHRVHTTDQ

TPKKIEWAATSPLIASKHEIYLRLEKVSS

>EDW49567.1

MLLLFLLGSLTIVFYIWQRRTLSFWARHGVKYIRPLPILGCTREFLTARVPFFEQIQKFH

EAPGFEKEPFVGVYMTHRPALVIRDLDLIKTVMIKKFQYFNNRALQTDPHNDALGYNNLF

FARSPAWRELRTKISPVFTSGKIKQMYPLMVKIGKNLEDSAERLGSGSEVQVKDLCARFT

TDLIATIAFGVEANALRDAKSEFFYHNRAIFSLTLSRGIDFAIIFMIPALASLARVKLFS

RETTKFIRSSINFVLKERERTGEKRNDLIDILLALKREAAANPEKLSKVVDLDYLVAQAA

IFQTAGFETSSSTMTLTLYELAKNEALQDRLRQEIVDFFGDEDHISYERIQEMTYLSSGC

QRDTPQVPDRGLCRTRDSQPAEGERYNLEPFHNMELPHGMSIYMSTLAVHRDPQYWPDPE

KFDPERFSSSNRENLNMDAYMPFGVGPRNCIGMRLGLLQSKLGLVHILRNHRIHMCDKTI

RKIEWAATSPVIASKHDIVLRLEKVSGNNDIGQKLSMK

>EDW00573.1

MSTEVLLLIASLAIIFYVWQRRAFNFWQRLGVKHIPPSPIIGNLNSLLTGRASFLEQISL

LHNTKGFENEPVVGIYLFNRPGLLVKDLDLVKTIMIKKFNYFVNRAMKTDHHHDVLGTNN

IFFLHGQPWRDLRYKLSPFFTSGKMKQMYPLMVEIGKDLEKYLDKLPKGSMLKIKDVCGR

FTTDLIATTAFGLKANALGSAKNEFYENNKAIFQVSLRRGIDFAIIFALPMLVSLARTKL

FPKQTADFVRRIIKYALDEREKSGQRRNDLIDILLAMKHEAKSDKPLDFDYLVAQAALFQ

TAGYETSASTMTMALFELVHHPEMQERLRTEIREYFGDKQSIDFDGIHQMPYLNQVVNET

LRKYPIVGYAERECVQPATGEKFNLAPYYDLELPHGTPVYVATLAIHRDPQYWPDPEKFD

PDRFAAENRDNLNMDAYMPFGIGPRNCVGMRLGLLQAKLGLVHLLRHHRVIKDEKTTSLA

DFKLCNFSAVIAPKDEICLQLQRT

>383851647

MALLTASWAVDGILLLMPFTAFLYYLLTRNYKYWAKRGVMEIPRTPLLGNFGDCMLGRTS

PSEHLHGLYNKSKGLPYMGFYIFNKPYFLARDPDLVKHILVKDFHVFADRHVTADEIHDR

LGYANPFLIKNPEWKMLRTKLTTIFTTGKLKKMFDLMLLIAKDLEQHLNALNLEGNGKAI

ELKDLSANFTTDMIASTSFGLSVKSLENPKAPFREAGREIFGYSLRRSLEFMIIFFLPEY

IKYTRPKFLGRNASEFLRNVFWDVINERIKSKQKRNDLIDLLIELREKHGNDKDMKGFDF

NGDDLVAQASGFFVGGFETSSSTMSFTLYELALHPEIQKTLRNEIHEALEESGGKITYEM

VMTLPYLDMVISETLRKYPPLGFLDRVTCQDYKVPNSDLVLEKGTPVYIPMMGIHHDPEY

YPDPDKYDPLRFTEENKRKRPNMTYFPFGDGPHNCFGSRLGLMQSKLGVVQVIKDYEVTP

CDKTTVPMVLDPRGLTTTAQGGLYVKFRKLTTAAG

>EDV31883.1

MTLNIIAIVVAIGLPVLLAINGRWVLLFIAAIVWLWQKHHFNFWQRLGVPYIPATPFVGN

VWNLLRGGCCFGDQFKEMYQSSAAAGHPYVGIHVLHNHALLLRDPALIKRIMVEDFSKFS

SRFETTDPIHDTMGSQNLFFCKYETWREIHKIFAPFFAVGKVRQMYGLLERVGQQLEDHM

DVQLGGKKSMELEVKQLCALFTTDIIASLAFGIEAHSLQNPQAEFRQMCIEVNDPRPKRL

IHLFTMFFFPRLSGRIRTHLYSEEYERFMRQSMDFILAEREGSGVKRHDLIDIFLQLKGS

ESADSIIHRPDFFVAQAAFLLLAGFDTSSSTITFGLYELAKHPVIQTRLREELREVLQSS

QNKQLSCDIINGMTYLRQVVDEVLRMYPPTAFLDRCCNAPEGYDLSEWNCGTPFKLRPGT

PVYISVLGLHHDPQFWPNPEVFDPERFAAGQRQQHHPMTYLPFGAGPRGCIGTLLGQLEI

KVGLLHILKNFTVELCDKTLPEMKFDPKAFVLTAKGGTYLRFVENRL

>345486241

MLNNKCEKSGDSLYKHYSLRKHATLAQADIRGKLRGRAVSAPPTVADAAVIPAYYSLAMA

VLTSDWCLEAAICASALLLGLCLHARRKHDYWRKRGVKYAEPRFLFGNVAACLLGKKSPH

DLIRETYEKGAGEKVIGLYVFDKPYLILRDPELIKYVFIKDFGNFSNKVLCGKHTDVMSA

ANLFLVNNPPWKYIRTKISPIFTSGKLKKMYELMMEICVDLQDHLDKFDIDEKVGKPLEI

KEICSRFTTDLIGATAYGLQLNSLKDPDAIFRKNGRQVFRSSFKRYLQLLALFFIPSLRP

YTNAKFFDEKATEFLRNTFGDVINRRMESGDKRADLVDMLIEIKKKQDRDPNAHYKLEGD

ALIAQAAVFFTGGFETSSTTMSFALYALARHPEIQAKLRTEILNALETPGGQMSYEKLMS

LPYLNMVVNEALRLYPVLPWIDRIPETDYTFPGTNITVEKGVPLILPMRTLHLDPDYFPD

PDKFIPERFSEENKKNIVPCTFFPFGEGPRNCIGLRLGIIQSKLGIVNFLSRYELSICKE

TDFKIDHMHVLTQAKGGICLNFRKLPA

>340710545

MALLTPYWSLDGILIVSSLMVAAYLFVTRKFNYWSKRGVKELPPTPFVGNFMDCLLSRVS

AAEFIKDLYKYGEGLPFLGFYIFDKPYLLIRDPELVKHVLVSDFNYFADRYACADEKNDR

LGYANLFMMKNPGWKSLRSKLTPIFTVGKLKKMFELMLVVADDLAKYLDSLHLDGNGKVI

EFKDICGNFTTDIIGSTAYGLKVGSLENPKATFRVYGRTILNYDFYRNIDLLTIFFFPGF

VKYLKPKFFGKQATNFLRSIFWDVIEQRINSGQKRNDVIDLLIEMREKYKNDENLKDYKF

DGDDLVSQASIFFAAGFETSSSTISFTLYELALNPDVQKTLRAELQDALAKTDGKITYDM

IMTLPYLDMVISETLRKYPVLAFLDRITLADYKVPNSDLMIEKGTPIFISMTGSHYDPRY

FPNPEKYDPLRFTEEAKRARQNFVYFPFGEGPHSCIGMRLGLMQSKLGVVQILKDHEVSP

CGKTKIPMVLDPNGLSTTALGGIYLNIQKIATTAG

>289177160

MALITSDWSLDAWIFISAVLLGLYLYFRRNLDYWKKRGVKYVEPTLFFGNTVPCLLARKT

PDVLIREIYEKGAGEQFIGFHVFNKPYLVVRDPELIKNVFIKDFNNFTNKVLCGNHTDVM

SSTNLFVASNPPWKSIRTKLSPIFTSGKLRKMYELMEEICDDLQVYLDKLDIDEKVGKPI

EIKEICAKFTTDLIGTTAFGMRLNSLTDPNAEFRKNGRRVFRSSFKRYLQLLALFFIPPL

RPYTNAKFFDEKATDFLRTEFWNVINERIKSGFKRDDLLDMLIEIKNNQDNGTDNTFRLE

GDALVAQAAIFFTGGFETSSTTMSFALYELARNPESQTKLRNEILNALETTGGKVTYEMM

TTLPYLHMVTLEALRLYPVIAWLDRIPETDYTFPGTNVTVEKGVPVVLPLRALQLSPQYF

PNPNQWDPERFSEENKKNIVPFTYFPFGEGPRSCIGIRLGYIQTKLGILNFISKYELTVS

KESANLQPEPLNIITQAKGGIYLNLRKLTA

>EFA12857.1

MLLTPYLPLDTVVLLSVLALLLYKYFSRNFDHWEKKNVFYFKPIPFFGNFVDISLFRTTI

GEHLAKLYNQTTEPFFGIFVFDKPHLIIKSPELVKTILVRDFNNFDDRCIASPHHDPLVK

NMLFLNKNPEWKNVRVKMTPVFTTGKLKGMIPLINDVGETMTKYIAQKIPNFSLEAKEIC

AKFSTDVIAKCAFGINANSFKNEDAEFRKIGRRIFDFRWSTAIQQTSYFFLPGLVNLLKF

RMLDKDASDFLRETFWHTIKLREEKNLKANDLIDAIIALKDNQEFCKNMNFEGDKVVAQA

AQFFVAGFETTSSTMAFTLYELCLQPQFQRRVRAEIATCLKEHNGLTYEALQSMKYLNMC

VCETLRKYPVLPFLDRTCKEDYKLPNSNVVIEKGTPVFIPMFGLHYDPQYFPNPQKYDPE

RFSDENMQNITPFSYIPFGEGPRNCIGERFGLIGTKLGLIHILSEFEVEKSSDTPVPLEF

EPKSFVLASKVGLPMKFKKVMTSAA

>350427327

MALLTSYWSLDGILIVWSLMIAAYLYMTRNFNYWTKRGVKQMPPTPFVGNFMDCLLSRVP

AAIFVKNLYNYGEGLPFLGFYIFDKPYLLIRDPELVKHVLVSDFNYFANRYATADEKDDR

LGYANLFMMKNPGWKSLRSKLTPMFTTGKIKKMFELMLVVADDLAKYLDSLHLEGNGKVI

EFTDMCGNFTTDIIGSIAFGLKVGSLQNPKAAFREYGKAVFDFNFYRNLDLLTIFFCPWL

VKYLKPKFFGKEANNFFRSVFWNAIEERIKSGQKRNDVTDLLIEMREKYKNDENLKDYKF

DGDDLVSQASIFFIAGFETSSTTISYTLQELALNPDIQKTLRAELQDALAKTDGKITYDM

VMTLPYLDMVISETLRKYPPLTFLDRITLADYKVPNFDLVIEKGTPIYISNVGAHHDPRY

FPNPEKYDPLRFTEEAKRARQNFVYFPFGEGPHSCVGMRLGLMQSKLGVVQIMKDHEVFP

CEETIIPTVHTPTGFTTTPLGGINLNIRKTATTAG

>340723208

MALITPYWGLDGILIVSSLMAAAYLFVTRKFNYWSKRGVKELPPTPFVGNFMDCLLLKKS

SAQFLSDAYNYGEGFPLLGFYIFHKPCLLVRDPEFAKHVLVKDFEYFADRYATADENNDR

LGYANMILMKNPAWKSLRPKMTPIFTPGKLKKMFVLMAMVADDLGKYLDSFHLEGDGKAM

EFKDICASFATDIISGIAFGLKVGSLENPEAPFRECGRRIFDSNSERNMDFLTITFMPQL

SKFLKPKFFGKKATNFLRSVFWDMIEQRDNSGQKRNDLIDVLTEIRETYKNDERLKNYRF

DGEDLVAQAAIFFAAGFETSAATMSFTLYELALNTDIQKTLRAEIHDALEENDGKITYNM

ITTLPYLDMVISETLRKYPSVPNLNRVTLADYKVPDSDLVIQKGTPIFISIVALHYDSRY

FPNPEKYDPLRFTEEAKSNRPSSAYLPFGAGPRICLGMRLGLMQAKLGVVQILKDYEVSP

CEKTKIPIVLDPNAVISTALGGVHLNIRKTTTAIYFISESTKFRPNFDGDDLLAQAAILF

AAGFETSSTTMSFTLYELALNPDVQKKLRAEIQDALEETGGKITYDMITTLPYLDMVVSE

SLRKYPAVAYMDRISLADYKVPNSDLVIEKGTPIFISIMGLHYDSRYFPNPEKYDPLRFT

EEAKSNRPSFAYLPFGGGPRGCIGMRLGLMQSKLGVVQILKDYEVSPCEKTKIPIVLDPQ

GIITTALGGVHLNIRKITTAAG

>289177162

MAVITSNWSLDAWIFISAVLLGLYLYFARKFSYWKKRGVKYVEPTFLFGNAAPCLIAAKK

PDDLIREFYEEGAGEKVIGFYVFDKPYLVVRDPELIRHVFVKDFSNFSNKLLCGKNTDVM

GSTNLFQVMNPPWKSIRNKLSPFFTSGKLKKMYELMEEICTDLQVYLDKLEIDEKVGKPI

EIKEICAKFTTDLIGTTAYGMRLNSLTDPNAEFRKSGREMFRSSFMRYLQMLAIFFIPPL

RPYINAKFFDVRPTEFLRKAFWHVINERIATGIKRPDLLDMLIEIKNNQDNGTDDTFKLE

GDALVAQAAIFFTGGFETSSTTMSFALYELARNPETQTKLRNEILSALETTGGKVTYDMI

TTLPYLNMVTLEALRLYPVISWLDRLPEADYTFPGTNITVEKDVPVILPMRTLHLDPQYF

PNPDQWDPERFSEENKKNIVPFTYFPFGEGPRSCIGTRLGYIQTKLGILNFISKYEVTTL

KETNVKIDKLNIFTQAEDGIYLNLRKLSA

>EFN78001.1

MTLITNHWGLDGIILLTTLMVAAYLYMTRNFKYWKRRGVLEVSPVPFIGNFTECMLMQKN

PGYFMKDLYNHARGLPYIGFYVFDKPSLLVCDRELVKNVLVKDFNHFSDRYGTPDTNDRL

GYANLFFIKNPAWKILRTKLTPIFTSGKLKKMFGLMLECGNNLDIYINSLKLEGSSKMLD

VKELSAKFTTDVIASTAYGLNVNSLNNPDAEFRKYGKKMFHYDFFRGLEILAVFFLPNLV

RLTGVKMFGKESTDFLRKAFWETMLQRMESGEKRNDLIDILIELKKTHNNQDIGGFKFDG

DDLVAQAAVFFAAGFETSSTVTAFTLYELAVQPEIQTRLRKEIIEALDETNGEITYDMTL

SLPYLDMVVCETLRKYPTLPFLDRTTVETYKMPNSDLVIEKGTPIYISMLGMHYDPEYFP

DPEKYDPERFNEENKRNIPSCAYFPFGEGPRACIGTRFGLLTTKLALIKIFSKCEVTPCE

KTMIPIILDPKASFASPLGGKIYLNIRKVSEPTESLDRTEIVSSALYKS

>340722609

MALLTLHWSLDGIIILSSLIIAAYLFVTRKFNYWSKRGVKELAPTPFVGNFMDCLLSKKS

APQFLKDLYNYGRDLSLLGFYIFDKPYLLIRDPELIKHVLVKDFDYFPDRYSCADEKDDR

LGYANVFMMKNPRWKSLRQKLTPFFTPGKLKKMFDIMLAVTDDLGKHLGSLHLEGEGKVI

EFKDICGNFTTDMIGSTAFGLKVGSLENPKAPFRECGRKIFDYDFHRSMEFLIIFFIPGL

VKYLKPKFFGKKATNFLRSVFWDVIEQRVDSDQKRNDLIDLLIEMREKYKNDESLKDYKF

DGDDLVSQAAIFYIGGSETSATTMSFTLYELALNLDVQKTLRAEIHDALVKTDGKITYDM

ITTLPYLNMVISETLRKYPSIAYLDRVTLADYKVPNSDLVLEKGTPIFISIMGSHYDPRY

FPNPEKYDPLRFTEEAKRARPSFVYLPFGEGPRVCIGMRLGLMQSKLGVVQVLKDYEVSP

CEKTETPVTLDPKAIVTTALGGLYLNVRKTTTAAG

>350418756

MALLTLYWSLCGIFIFSSLLVAAYLFVIRKYNYWSRRGIIQLKPTPFVGNFMDCILSKRS

GAQFLSDAYNYDKDMPLLGFYIFHKPCLLVRDPELAKHILVKDFEYFADRYATADEKNDR

LGYANMILMKNPAWKSLRPKMTPIFTPGKLKKMFMLMAMIADDLGKYLDSLHLEGDGKVI

EFKDICASFATDMISGIAFGLKLNSLENPKTPFREYGRRIFDTNYQRNMEFLTIIFMPQL

SKYLKPKLFGKIATKFLQSVFWDVIEQRVVSGQKRHDLIDVLTEIRETYKSDERLKNYNF

EGDDLVAQAAIFFAAGFETSAATMSFTLYELARNSDVQQTLRKEILDALEKTDGEITYDM

ITTLPYLDMVLFETLRKYPSVPNLNRVTLADYKVPNSDLVIEKGTPIFISIMGLHYDSRY

FPNPEKYDPLRFTEEAKSTRPNFAYLPFGAGPRICLGMRLGLMQAKLGIVQIELGVVQIL

KDYEISPCEKTKIPIILDPKGIITTALGGVHLNIRKTTAAV

>EDW80387.1

MIIVLLLLLLIAIFGLLILCFLAISGSGFFLLLLVWLWHRRHFGYWKRLGVPYIPVTPFL

GNVWRLLRGACCFGDQFKELYGSRAARGHAYVGMHVLHNHAILLREPALIKRILVEDFAL

FSSRFETTDPIGDTMGAQNLFFAKHETWRETHKIFAPFFSVSKVRQMYGLLSQIGITLET

YMETQMGGDTSMELEVKQMCALFTTDIIASLAFGIEAHSLKQPQAEFRRMCIEVNDPRPK

RLIHLFTMFFFPRFSRTLRTHLYSDEYERFMRRSMEFVLAQREASGELRHDLIDIFLQLK

RTHPPESIVQAQAAFLLLAGFDTSSSSITFALYELSKHPSIQQRLRDELKRALHNSQASR

ITYDCISGLPYLRQVVDEVLRLYPPTAFLDRCCNAESGYDLSSWQCGAPLSLPIGTPVYI

SVLGLHRDAQYWPNPDVFDPERFSPEQREQHTPMTYLPFGAGPRGCIGTLLGLLEIKVGL

FHILKNYRVEECTRTLPEMKFDPKSFVLTAAGGTYLRFVKDHL

>340723206

MIVAYLYMTRKFNHWSKRGITELAPTPFVGNFMDCLLSRKSGSEFLKDVYEYGKNLPLLG

FYVFDKPHLLIRDPELVKHVLVKDFDYFPDRYACADEKDDRLGYANVVMMKNPGWKSLRP

KLTPIFTPGKLRTMFDLMLVVADELGMYLDSLHLEGDGKVVEFKDICASFATDTIGSVAF

GLKLGSLENPKTPFREYGRKIFYSNFYRNVELLIISFSPQLIKYMKPKFFGRKATNFLRS

VFWDAIDQRVDSAQKRNDLIDVLLEIRKTYMNDENLKDYNFDGEDLLAQAAILFAAGFET

SSSTMSFTLHELAVNPDVQKTLRAEIQDALEKTGGKITYDMIMTLPYLDMVISESLRKYP

VVGYTDRNTLADYKVPNSDLVLEKGTPIYISIMGLHYDSRYFPNPNKYDPHRFTEEAKST

RPSFAYLPFGAGPRICLGMRLGLMQSKLGVVQILKDYEVLPCEKTKIPIVLDPKGIMTTA

LGGVHLNIRKITTAYNLHVPEFDGDDLVAQAIMFFISGIETSSIAISFTLHELAVNPDLQ

KKLRAEIHDALEKSGGKITYDMITTLPYLDMVMSETLRKYPSVGHLDRVTLADYKLPNSD

LVLEKGTPIFISIMGLHYDSRYFPNPKKYDPLRFTEEAKCTRPSFVYLPFGGGPRGCIGI

RLGLMQSKLGVVQILKDYEVSPCDKTKISVILDPKTIITTALGGVQLNIRKITAAAD

>EDY70529.1

MILVIGVLFIAATVAAIGLLFFVVFPLVCAAGLIIFGIIWLWQRHHFCHWLRLGVPYIRA

TPFVGNVWKLLRGSCCFGDQFRQLYESKEAAGHAYVGIHVLHNHALLLRDPALIKRILVE

DFSQFSSRFETTDPIGDTMGAQNLFFSKHETWRETHKIFAPFFAVSKVRQMYGLLEHIGQ

QLEEHMEDQLNGKSEIELEVKQLCALFTTDIIASLAFGLEAHSLQKPDAEFRRMCVEVND

PRPKRLLHLFTMFFFPRLSRMVRTHLYSDEYERFMRQSMDFVLAQREKSGEKRHDLIDIF

LQLKRTEPPTSIIHRPDFFVAQASFLLLAGFDTSSSTITFALYELAKAPAIQKRLRDELS

DALRTNRHQPLNFDSVNGLSYLRQVVDEVLRLYPPTAFLDRCCNDENGYDLSAWNCGRAL

KLPAGAPVYISVLGLHRDPVHWPNPNQFDPEHFSPEQREQHHPMTYLPFGAGPRGCIGTL

LGQLEIKVGLLHILKNFRVELCERTLPEMVFDPKAFVLTAKGGTYLRFVKDTL

>EDW25553.1

MILVIGVLFIAAAVAAIGLLFFVVFPLVLAAGLIIFGIIWLWQRHHFCHWLRLGVPYIRA

TPFVGNVWKLLRGSCCFGDQFRQLYESKEAAGHAYVGIHVLHNHALLLRDPALIKRILVE

DFSQFSSRFETTDPIGDTMGAQNLFFSKHETWRETHKIFAPFFAVSKVRQMYGLLEHIGQ

QLEEHMEDQLNGKSEIELEVKQLCALFTTDIIASLAFGLEAHSLQKPDAEFRRMCVEVND

PRPKRLLHLFTMFFFPRLSRMVRTHLYSDEYERFMRQSMDFVLAQREKSGEKRHDLIDIF

LQLKRTEPPTSIIHRPDFFVAQASFLLLAGFDTSSSTITFALYELAKAPAIQKRLRDELS

DALRTNRHQPLNFDSVNGLSYLRQVVDEVLRLYPPTAFLDRCCNDENGYDLSAWNCGRAL

KLPAGAPVYISVLGLHRDPVHWPNPNQFDPEHFSPEQREQHHPMTYLPFGAGPRGCIGTL

LGQLEIKVGLLHILKNFRVELCERTLPEMVFDPKAFVLTAKGGTYLRFVKDTL

>157108137

MIYVNSDALTGYLTLVLVCLAGFAVWFLSTSGTRYWQFFRVPYIEGRPLVGNFLDAFLFR

KSTFELMDALYQNPKVEGTKLFGVSIMMQPALVIRDPELVKQVLIKDATYFSNRYMCTDR

IHDAIGYYNLLMIKNPHWKQLRAYLTPSLSLNKIKRMYSLVDQIGNNMLAHLDGVQQKPT

KLRETEFKELCARFTTDVIASTFFGIQANCLSDEESEFRYYGRKIFEYGPKRALNMAAFF

FMPELVPYLGFKLFPRDTERFLKTIIEQEIARRETSGENRGDFIDSMIALKNNEATIGVE

EKIPLKGDILVAQAATFYMASFETTSSVLSFTLYELTKNPEIQQRLREEIHNCIKKYGRD

LSYECLVNEMPYLGMVISEAARLYPVLPFIERQCSLPAGATGYKLDPFHNFVVPNKMPVL

VPIYAIHRDPKYFPDPLRFDPDRFSKDNADNIVPCSYMPFGVGPRTCLGSHFGTLQVKVA

ITRLLSKYRILRSESSPETLTYRKNAFTLHSNEGLYADLELDELC

>EFA12856.1

MLLTSYLPLDTFLLLSVFTLLLYKYFSRNYDHWEKKNVFFFKPTPFFGNILDISLFRTTI

GEHLAKLYNQTTEPFFGIFVFDKPHLIIKSPELVKTILVRDFNNFDDRGVASPHHDPLVK

NMLFFNKNPEWKNVRVKMTPVFTTGKLKGMIPLINDIGETLTKYIAQKTSNLSLEAKEIS

AKYTTDVIAKCAFGINANSLKNEDAEFRNLGRRFFDFRWSNAIQQTSYFLLPGLVNVLKL

RVMDKKDSNFLRETFWQTIKLRQENNSKAKDLIDAIIAMKENKEFCKNFNFEGDKVVAQA

AQFFIAGFETTSATMAFTLYELCLQPQIQSKVRTEIMTCVKEHNGLTYEALQDMKYLNMC

VCETLRKYPVLPFLDRTCKEDYKLPNSNVVIEKGTPVFIPMFGLHYDPQYFPNPQKYDPE

RFSDENMQNITPFSYIPFGEGPRNCIGERFGLISTKLGLIHVLSNFEVERSSDTPVPLEF

EPKSFVLASKVGLPMKFKKVMTSAA

>EDW11976.1

MFILGLLLLLLLAWIWQRWHFGYWQRLGVPQTPATPFVGNVWRLLRGCCCFGDQFRELYE

SAAAKGEAYVGIHVLHNHALLLREPALIKRILVEDFAQFSSRFETTDAIGDTMGSQNLFF

SKYEMWRETHKIFAPFFAAGKVRNMFGLLQQIGQQLEQHMARQLAASGSLATAFELEVKQ

LSALFSTDIIASLAFGLDANCLQQPDAEFRRMCIEVNEPRPKRLLHLFTMFFFPRCSRQL

RTHLYSEEYERFMRKSMNWVMEQRAASGEQRHDLIDIFLQLQRNQPPESVVHRRDFYVAQ

AAFLLLAGFDTSSSTITFALYELARQPAIQQRLRQELAAALLGCVSGHLDYETLSGLPYL

RQVVDEVLRLYPPTAFLDRCCNAKAGYDLSAWGRGKPLRLPPGTPVYISVLGLHRDEQYW

PQPLKFDPERFAPEQRAQHQPMTYLPFGAGPRGCLGTMLGLLEIKVGLFYILKNYRVESC

PRTLPEMKFDAKSFVLTAAGGTYLRFIHEPL

>EDV54444.1

MVIVVLLFFSAALAVGSLVLLPLVISGGWLFALITVWLWQRRHFCLWRRLGVPYVPAAPF

VGNVWNLLCGACCFGDQFRELYESKEAAGRAFVGIHVLHNQALLIRDPALIKRILVEDFA

QFSSRFETTDAIRDTMGSQNLFFSKYETWRETHKIFAPFFAVGKVRQMYGLLQKIGQNLE

AHMEKNLNGAESVELEVKQLCALFTTDIIASLAFGIEAHSLQNPEAEFRRMCIEVNDPRP

KRLLHLFTMFFFPRLSHRVRTHLYSVEYERFMRKSMNYALAQRAENGEKRNDLIDIFLQL

QGTEPAESIVHRPDFFVAQAAFLLLAGFDTSSSTITFALYELAKNPTIQHRLRAELRAAF

QSSHNQQLSYDTVTGLVYLRQVVDEVLRLYPPTAFLDRCCNSREGYDLSSWNCGSPFTLR

AGTPIYISVLGIQRNSQYWPNPEIFDPDRFSPEQRQQHHPMTYLPFGAGPRGCIGTLLGQ

LEIKVGLLHTLKHFRVELCERTSPEMRFDPKAFVLTAHNGTYLRFVRSSL

>EDV99440.1

MFIAFLVGILLLIWLWQRWHFGHWHRRGVPYIPATPFVGNVWRLLRSCCCFGDQFRELYE

SPCAKGHAYLGIHVLHNHALLLREPALIKRILVEDFAQFSSRFETTDAIGDTMGSQNLFF

SKYEVWRETHKIFAPFFAAGKVRHMYGLLQKIGQQLEQHMARQLAASDTDGHTLELEVKQ

LSALFSTDIIASLAFGLDANCLRQPDAEFRRLCVEVNEPRPKRLLHLFTMFFFPRCSRQL

RTHLYSEEYERFMRQSMNWAMEQRAASGEQRHDLIDIFLQLQQTQPPDSVVHRRDFFVAQ

AAFLLLAGFDTSSSTITFGLYELAKQPAIQQRLRQELKEAIAQSDTVDQLGYETLSSLPY

LRQVVDEVLRLYPPTAFLDRCCNSKAGYDLSAWSCGLPMRLSPGTPVYISVLGLHRDEQY

WPEPLKFDPERFSPEQRVQHQPMTYLPFGAGPRGCLGTLLGLLEIKVGLFYILKNYRVET

CTKTLSEMKFDPRAFVLTAAGGTYLRFVKDQL

>289177166

MAFITHCWTLDILITLVCALMVAYVYLTKSHSYWKKRGVKSVPEPNIFGNFSDCLFARKP

LRDFLSNVYFVGEGEKFIGFYVFDKPFLILRDPEAIKNVLVRDFKNFSNRCVAPNHSDYI

GNVNIFSLRNPAWKVLRQKLSPVFTSSKLKNMLDLIVETGVDLDTHLQNIISNDEWNVVE

VKELCEKFTTDVIGSTMFGLKLNALNNPKAEFHNEGKKHFQNSYGRYFSLLSMFFMPQLA

KLFGITFFDPDVPNFFKRVIPEAMNERQKDGIKRNDLLDVFMEMKESFEDEKNDLHYYFQ

VNGLVATAIVFFTGGFEPSASMMYLVLYELSKQSEIQDKLRKEILDALEETEGKITYDMM

MNLPYLDMVVSEGLRKYPLLHFLDREAEHDYTFPDTDVTIDKGTPIIIPMSAVHMDPKHF

PDPHVFDPQRFSPENKRKIAPFTYFPFGEGPRNCIGMRIGLAQSKIGLMQILSKYEVAPC

KETPIPLEFDSYTLFTKSRHNMKLNVRRVRSI

>350418747

MALLTLYWSLDGILILSTLMVAAYLYMTRKFNYWSKRGITQLAPTPFVGNIMDCLLSRKS

ASEFLKDVYDNGKGLPLMGFYAFDNPHLLIRDPELAKHVLVKDFDYFPDRYATADEKDDR

IGYANMILMRNPGWKSFRPKMTPIFTPGKQKIMFDLILAVADDLGMYLDSLHLEGDGKVI

EFKDICGNFITDMIGSTAFGLKVGAIKNPKTPFREYGRKIFHSNFYRNIESHIISFSPQL

VKCLKPKLLSKNVTNFFESVFWDVIEQRVDSGQKKNDLVDVLIEIRKTYKNDEHLKDYKF

DGDDLVAQAIMFLIAGTETSSTAISFTLHELAMNPDIQKTLRAEIRDALAKTDGKITYDM

IMTLPYLDMVISETLRKYPSAAHLDRVTVADYKVPNSDLVIEKGTGIFISIMGLHYDPRY

FPNPEKYDPLRFTEEAKSTRPSFAYLPFGGGPRGCIGMRLGLMQSKLGIVQILKDYEVSP

CEKTKTSVVLDPKGVITKALGGVHLNIRKITTAAD

>EAT43627.2

MPLVAWLAAIALICYLAWKRNNFWNRHGVPYVLEIPAVGNFSSVALQMHSMFDYVARIYD

HVRTRDADFFGINIFFRKALVIRNPDMVKKMLVADSRYFINRQMCTDREGDHFGYYNLMM

IKEPLWKDLRGYLSPSVTSSRLRRMFSLIDEIGNNMLAHLDGVQQKPTKLRETEFKELCA

RFTTDVIASTFFGIQANCLSDEESEFRYYGRKIFEYGPKRALNMAAFFFMPELVPYLGFK

LFPRDTERFLKTIIEQEIARRETSGENRGDFIDSMIALKNNEATIGVEEKIPLKGDILVA

QAATFYMASFETTSSVLSFTLYELTKNPEIQQRLREEIHNCIKKYGRDLSYECLVNEMPY

LGMVISEAARLYPVLPFIERQCSLPAGATGYKLDPFHNFVVPNKMPVLVPIYAIHRDPKY

FPDPLRFDPDRFSKDNADNIVPCSYMPFGVGPRTCLGSHFGTLQVKVAITRLLSKYRILR

SESSPETLTYRKNAFTLHSNEGLYADLELDELC

>EDX03109.1

MIIVLFSLIAAALAAGFLLVLLPVVVSSGCVLVLVIVWLWQLRHFGHWRRLGVPFVPAAP

FVGNVWNLLRGACCFGDQFRELYESKEAAGRAFVGIHVLHNHALLLRDPALIKRIMVEDF

AQFSSRFETTDPTCDTMGSQNLFFSKYETWRETHKIFAPFFAAGKVRQMYGLLENIGQKL

EEHMEQKLSGRDSMELEVKQLCALFTTDIIASLAFGIEAHSLQNPEAEFRRMCIEVNDPR

PKRLLHLFTMFFFPRLSHRVRTHLYSEEYERFMRKSMDYVLSQRAESGEKRHDLIDIFLQ

LKRSESAESVIHRPDFFAAQAAFLLLAGFDTSSSTITFGLYELAKNTTIQNRLRAELRAA

LQSSQDRQLSCDTVTGLAYLRQVVDEVLRLYPPTAFLDRCCNSRTGYDLAPWNGGSPFKL

RAGTPVYISVLGLHRDAQYWPNPELFDPERFSAEQRQQHHPMTYLPFGAGPRGCIGTLLG

QLEIKVGLLHILKHFRVELCERSLPEMRFDPKAFVLTAHNGAYLRFVKSPL

>EDS30708.1

MIVTISLLVSAALAVYVYLTWNFSYWKQRNVPGPDPRPLLGNFPSLLLRHRTAMDEMDQM

YSDYRAKFNFVGVFNIRQPRIFVTSPALYRDIITKHFRSFSDNEFAELTDVETDPLMGRN

PFLLTGEEWQAKRAEISPAFTASRIKALFAIVEELASRMTKYIEQNQHTALEAKDLAAKL

TIDVVSSCIFDTDAQSFSEDHGEIPEMARKMLEPSFSSLLIMILRSLCPKIAKALNIGMI

PKSVEVFFTNLMKEAIRYRETNSIKRVDYLEHLISLRNKKEITELDMAAHGVTFFFDGFE

ASSLALSFTLYELGRHPAIQTRLRTELLEATDAKGSIDYDTLLELPFLDQVVNESLRLWP

AGPFLSKRCTQPIDLDLTPTQQVRIEPGVCAMIPVWAIHRDPECYTDPDTFNPDRFSPET

GGTNPYREMGCFLPFNEGPRQCLGMRFAKMLVKRGLYEVVKNFEFSVDSKTEEPLRMNPK

VVLTAVVGGIWLNFEPIITK

>EDS26034.1

MIVTISLLVSAALAVYVYLTWNFSYWKQRNVPGPDPRPLLGNFPSLLLRHRTAMDEMDQM

YSDYRAKFNFVGVFNIRQPRIFVTSPALYRDIITKHFRSFSDNEFAELTDVETDPLMGRN

PFLLTGEEWQAKRAEISPAFTASRIKALFAIVEALASRMTKYIEQNQHTALEAKDLAAKL

TIDVVSSCIFDTDAQSFSEDHGEIPEMARKMLEPSFSSLLIMILRSLCPKIAKALNIGMI

PKSVEVFFTNLMKEAIRYRETNSIKRVDYLEHLISLRNKKEITELDMAAHGVTFFFDGFE

ASSLALSFTLYELGRHPAIQTRLRTELLEATNAKGSIDYDTLLELPFLDQVVNESLRLWP

AGPFLSKRCTQPIDLDLSPTQQVRIEPGVCAMIPVWAIHRDPECYTDPDTFNPDRFSPET

GGTNPYREMGCFLPFNEGPRQCLGMRFAKMLVKRGLYEVVKNFEFSVDSKTEEPLRMNPK

VVLTAVVGGIWLNFEPIITK

>EDS30928.1

MIVTISLLVSAALAVYVYLIWNFSYWKQRNVPGPDPRPLLGNFPSLLLRHRTIMDEMGQM

YSDYRAKFNFVGVFNIRQPRLFVTSPVLCRDIITKHFRSFSDNEFAELTDVDTDPLMGRN

PFMLTGEEWKAKRSEVTPAFTASRMKTLFAIVEALASRMTKYIEQNQHTALEGKDLAAKL

TIDVVSSCIFDTDAQSFSDDHGEIQEMGRKLVEPSFGSLLTVILRSLSPRIAKALNIAMI

PKSIEIFFTNLMKQAIRYREANSIKRADYLEHLISLRNKKEITELDMAAHGVTFFFDGFE

ASSSTISFTLYELGRHPAIQARLRTELMEATNDKGSIDYDTLFELPFLDQVVNESLRLWP

GGPFLSKRCTEPIDLDLTPTQQVRIEPGVCAMIPFWAIHRDPECYTDPDTFNPDRFSPET

GGVNPYREMGCFFPFSEGPRQCLGMRFARMLVKRGVYEVVKNFEFSVDSKTEEPLRMNPK

LFLTAVIGGIWLNFKPIVNE

>EAT32234.1

MLVTVGLLLTAFAALYLYLTWHFDYWRKRNVPGPEPLPLVGNFPAFFRRNRPVMEEKYQI

YKDYCSKYNFVGIFTNRSPQIFITSPALARDILVKYFKNFHDNEIGLITNKELDPLFGRN

PFVLNGAAWKAKRAEITPAFTASRIKALYVSVENVCAQMTKYVKEHCESPIEMKELGDKF

TTDVVSSCIFGADAQSFIHQDAEIRDMGSKLMDSSLSFALKMAVMTVLPSVAKIANMSLV

SKPREKFFIKLMAEAIRHREESSEKYLDFLDYLSMLKKEKNITELDMAAHGVTFFLDGNE

TSSATLSLNLYELAKQPEIQKRLREELMNATNDDGTISYETLSELPFLEQVFSEGLRLWP

PVTFMSKVCTDPIELDLTSTRKVPIERGTCAIISNWSLHRDPNFYEDPLKFNPDRFAPEK

GGIFPYKEKGCYMPFGDGPRQCLGMRFGRMQVKRGIYEVIRNFEISVASRTSDPLKIVSS

PAISLGLSGIWLSFKPIRS

>EAT44803.1

MLVTVGLLLTAFAALYLYLTWHFDYWRKRNVPGPEPLPLVGNFPAFFRRNRPVMEEKYQI

YKDYCSKYNFVGIFTNRSPQIFITSPALARDILVKYFKNFHDNEIGLITNKELDPLFGRN

PFVLNGAAWKAKRAEITPAFTASRIKALYVSVENVCAQMTKYVKEHCESPIEMKELGDKF

TTDVVSSCIFGADAQSFIHQDAEIRDMGSKLMDSSLSFALKMAVMTVLPSVAKIANMSLV

SKPREKFFIKLMAEAIRHREESNEKYLDFLDYLSMLKKEKNITELDMAAHGVTFFLDGNE

TSSATLSLNLYELAKQPEIQKRLREELMNATNDDGTISYETLSELPFLEQVFSEGLRLWP

PVTFMSKVCTDPIELDLTSTRKVPIERGTCAIISNWSLHRDPNFYEDPLKFNPDRFAPEK

GGIFPYKEKGCYMPFGDGPRQCLGMRFGRMQVKRGIYEVIRNFEISVASCTSDPLKIVSS

PAISLGLSGIWLSFKPIRS

>EAT41340.2

MWLIVISILVTIVSLVYHYLTWNFNYWKYRGVPGPLPKPFLGTFSSTFTQKEHPIEENNR

IYSLFREYRKDVPFIGGFSFRSPQLFALSPTLVKDILVKYHKHFRANEVGGTFDSKADPL

LARNPFFLDGEEWRSKRAQITPAFTNSRLKALLPIMDNICNNMVSYIDRHIPNGPIESKE

LSAKYTTDVVSSCIFGAEGGSLTSDRSEIREMGNALFQQTFMFIVLAVISSIAPILKRFV

KLSLIPKSIENYFVGLMTEAVRKRKASGTKQVDYLDHLINLQEQKEISILDMAAHGVTFF

IDGFETTSEVLGFSLLELSIDKEIQNRLRQEIHSAEDGQLTFETIMELPYLDQIVNETLR

KWPPAYALSKRCTEEITFRLKDNHEVLIEKGITAILPIWAIHLDKEFYPDPNRFNPDRFS

EEDGGHSVRYYQEKGVFLPFGDGPRACIGRRIGLLQVKRALVEIVKNYDFTVNSKTVLPI

KIDPKNIAVTPLGGIWIDYRKL

>EDS36417.1

MWLAVVASILISIATLLYLYLTWNFNYWKDRGVSGPRPKLFLGTFSSGFTQKEHPVEENN

RIYNHYKNDATFIGGFSFRSPQLFILSSTLARDILLKYHKHFQANDVSIDVSADPLLARH

PFFLQGEDWRVQRAQVTPAFTSNRLKSLFPLMDNVCSKMDDHIELEAKLGPIESKELAAK

YTTDVVASCIFGIEGGSFTAEKSEIREISKALLNQTFMFVVLTVVQSIAPFVRRFVKLSL

IPKRIEDFFVGLMGEAIRTRKANGTRQSDYLDYLIGLQEKKEISVLDMAAHGVTFFIDGF

ETTSEVLAFALFEIAMNRDVQKRLRQEILDTENREGSLLFETIMEMSFLEQVVNETLRKW

PPAFALTKVCTEDINLELANGKSVMIERGISAILPIWAIQLDEHHFPDPERFDPGRFSEA

EGGHSAKYYQERGVFLPFGDGPRACIGRRIGLLQVKRALVEIVKKYEITISSKTILPLRI

DPKQFVVAPLGGIWIDYNKI

>EAT41343.1

MWWTVVGVLGGILSAIYLFLSWNFNCWKKDGIKGPKPRLLFGNLPNVLTQKKHIFYEYEK

IYNDFKTEPVVGYFSVRTPQLMIREPELIKEVLIKGFRYFSANEFSDVVDEKSDPLFARN

PFSLSGEKWKTRRGEITPAFTNNRIKALSTLMDEVCDRMTDHVKKQKESALETKELMSKY

TTDVVSNCVFAIDAQSFSKDKPEIREMGRRIMDFNFAAQIILMVTTFLPSVKKFYKFTFV

PREVEQFFIRIMKDAIRHRKENNIVRNDYLDHLLSLQEKKQISEIDMAGHGVSFFADGFE

TSSLVMTYCLFDLASHPEIQTRLREEIRNVQATKGGINYDNIGEMTYLDQVLNETLRIHP

IIPVLAKRCTESTVLVGPKDQKIPVSAGTTVVIPYFVQLDSQYYQEPNKYNPERFSPENG

GTKPYRERGVYFPFGEGPRMCLGMRFAIAQVKRGIIEIIDKFEISVNSKTQVPLKYEPKM

FMLYPVGGIWLNYKPIK

>AAG36958.1

MWWTVVGVLGGILSAIYLFLSWNFDCWKKDGIKGPKPRLLFGNLPNVLTQKKHIFYEYEK

IYNDFKTEPVVGYFSVRTPQLMIREPELIKEVLIKGFRYFSANEFSDVVDEKSDPLFARN

PFSLSGEKWKTRRGEITPAFTNNRIKALSTLMDEVCDRMTDHVKKQKESALETKELMSKY

TTDVVSNCVFAIDAQSFSKDKPEIREMGRRIMDFNFAAQIILMVTTFLPSVKKFYKFTFV

PREVEQFFIRIMKDAIRHRKENNIVRNDYLDHLLSLQEKKQISEIDMAGHGVSFFADGFE

TSSLVMTYCLFDLASHPEIQTRLREEIRNVQATKGGINYDNIGEMTYLDQVLNETLRIHP

IIPVLAKRCTESTVLVGPKDQKIPVSAGTTVVIPYFVQLDSQYYQEPNKYNPERFSPENG

GTKPYRERGVYFPFGEGPRMCLGMRFAIAQVKRGIIEIIDKFEISVNSKTQLPLKYEPKM

FMLYPVGGIWLDYKPIK

>EAT41345.1

MWWTVVGVLGGILSAIYLFLSWNFDCWKKDGIKGPKPRLLFGNLPNVLKQKKHIFYEYEK

IYNDFKTEPVVGYFSVRTPQLMIREPELIKEVLIKGFRYFSANEFSDAVDEKSDPLFARN

PFSLSGEKWKTRRGEITPAFTNNRIKALSTLMDEVCDRMTDHVKKQKEPAVDTKELMSKY

TTDVVSNCVFAIDAQSFSKDKPEIREMGRRIMDFNFRAQIILMITTFLPSVKKFYKFTFL

PREVEQFFIRIMKDAIRHRKENNIVRNDYLDHLLSLQEKKQISEIDMAGHGVSFFADGFE

TSSTVMTNCLFDLASHPEIQTRLREEIRNVQATKGGINYDNIGEMTYLDQVLNETLRIHP

IIPVLRKRCTESTVLVGPKDQKIPVSAGTTVVIPYFVQLDSQYYQEPNKYNPERFSPENG

GTKPYRERGVYFPFGEGPRMCLGMRFAIAQVKRGIIEIIDKFEISVNSKTQVPLKYEPKM

FMLYPVGGIWLDYKSIK

>EGK97220.1

MLLILFGTVVAFVALVYIFLTWEFGFWEKYGVKGPKPKILLGNIPYLLTKKRHTVYNYDQ

LYNDFKNEPAVGFFSIRSPQLMVRDPELIKEVLSKGFQNFAENDFSDTVDEKSDPLLARN

PFSLSGERWKTRRAEITPGFTNNRIKAMAHLMDEVCEHMTDYLRTQAKSSDGVATLDAKE

VMAKYTTDVVASCIFAIDAQSFVKENPEIRLMGKRIMNFNFVVQLALLVTTFFPSDAIRY

RKENKINRTDFLDHLLQLQDRKQITDIDIAGHGVSFFADGFETSSIVLTYCLYDLAKYPT

IQQELRDEIAKAKQVGGGTMSYEQLGEMALLEQIICESMRIHPLMAVMAKRCTTDTTLVG

PKDRKIFVKKGTTVVLPYYSICFDAEHFADPYKYDPKRFAAENGGSKAYRERGVYFPFGD

GPRMCLGMRFAVTQVRRAIVEVVERFSISVSSKTIEPFEMDPDQFMLMPKGSIWLDFKPI

A

>AAK32957.1

MLIVAWLLSAVLSLCFVLCVIHIRKKYSFWSERGVPFVPARFPLGNIQHASHLMLDLYRE

LKGKHPFGGIFQFTEPVAMITDPEMIRNVLVRDFRHFYDRGGYINRQHDPLSGHMLNSGS

ERWSVLRHASSPIFSTGRLRAFLPEMVQMIDQFQAYLDAKLAAEGEMIELKGVLERHNTD

IAMRFLLGREGNNLLAPEGGLHEAVMRGGFLLPSVWKLFLMTSYRSVARKLRLKVCSREL

TETVERVAAEAINSKLHEHRPEASRRVDLIDQLLKAPGFDGKSSLTLSEIAAQVFLFVAA

YETNAITTFYCLYELAQRPELQQRARACVCEALEKHGGITYEAIAQMPYLDQCINETLRK

HPLAINLIRVVTEDYPVPDSTGIVLPKGLNIIVPVYAIHYDPQHYPEPERFDPDRFTPEG

CRQRAPYTFLPFGAGPKICIGYRQGKLQLRTMLAVLLSSYEFATCAKSTPGALSNAHTVI

KPQGDLWLKVKKLNRVVAA

>EAA12451.2

MLIVAWLLSAVLSLCFVLCVIHIRKKYSFWSERGVPFVPARFPLGNIQHASHLMLDLYRA

LKGKHPFGGIFQFTEPVAMITDPEMIRNVLVRDFRHFYNRGGYINRQHDPLSGHMLNSGS

ERWSVLRHASSPIFSTGRLRAFLPDMVQMIDQFQAYLDAKLAAEGEMIELKGVLERHNTD

IAMRFLLGREGNNLLAPEGGLHEAVMRGGFLLPSVWKLFLMTSYRAVARKLRLKVCSREL

RETVERAVEEAINSKLHEHRPEASRRVDLIDQLVKAPGFDGKSSLTLSEIAAQVFLFVAA

YETNAITTFYCLYELAQRPELQQRARACVCEALEKHGGITYEAIAQMPYLDQCINETLRK

HPLAINLIRVVTEDYPVPDSTGIVLPKGLNIIVPVYAIHYDPQHYPEPERFDPDRFTPEA

CRQRAPYTFLPFGAGPKICIGYRQGKLQLRTMLAVLLSSYEFATCANTTPGALTNAHTVI

KPQGDLWLKVKKLNRVVAA

>AFM08401.1

MLPVLWLLLTVSSFLTFLMCVIHIRKKYSFWRELGVPYIPASFPLGNIQHTSHLMVELYQ

ELKGKHPFGGLFQFTEPVALITDPEMVKNVLVRDCRFFYDRGGYTDGKHDPLTGHMLNSE

SERWCALRHAASPIFSTGRIRAFLPVMLEKLNHFREYLNGKVNTEGTKVELKEMIGKLNT

DIAMSFVLGIEGNNLIAPDGDLHEALMREAFLLPNVAKLFLMTCYRSMAKKMRLKMFSNY

ITELFQQVVRETIEHRKDISYLNRRTDLIDQLLAMPGFDGKSTLTLSQMAAQVFLFLGAY

DATGITTFFCLYELAQRPAVQERARACVLEALEKHDGITYDALADMPYIDQCLNETLRMH

PLAINLVRVVTKNYPVPDASGVVLPKGLNIVVPVYAIHYDPQYYPEPERFDPDRFLPEAC

RQRTPYTFLPFGVGPKSCIGYRQGKIQLRAMMAVLLSSYEFTKCPNITQGSRSKAHTVLK

LDGDLWLNVKPINRTNDD

>EFR25766.1

MLLSISLSLSVLVTLTVILCVYFLRKRAHFWVDQGVPSVAVKFPFGNIQHSSHLMLDLYR

KLKGRAPFGGIFQFVVPVAFVSDPEMIQNVLVRHYRHFADRGGYSNAKHEPLSGHMLNAE

AHRWTLLRRAAIPAFSSGRLKAFYPAMVQLVDQLMEGLHKKLSPSGECIIECQEELGRLT

TDVIASFLLGIDGKSLSQPDGGLYDRVNGKTFLIPPVWKLFLMTSCRTIAQALRVKIFSH

DITVLFQRMARDVIAARANSNEQRKDLLQRFLQLSKENGKPLLTEDEIAAEVLLFFAAGH

ESTSLMVCYCLDQLARNPELQERARDCALEAFAKHGDLNYEALQDMQYIDQCLAGINAIR

VVTEDYQVPGSSIVLRKGQNIIIPVYAIHYDPEYYPDPERYDPDRFTPEACRARIPFTYM

PFGEGPKMCIAYRLAKLQMRIVLAKLLCSYRFYPCDEAAESRQSATYTVLKSSKGLYLKM

SRIVK

>289177175

MYIRLMLLCGLYNCTYDMSLEIINIMDSIAPILHTKLFLVTALITVIYMYFKFVIYGYWK

RRGIPHAPPIIPVGNAFPLISGKVSIGNLMRQKYNEGKKYPVYGIYMFHKPILLINDPNV

IQLILIKEFNKFCNRGLYFNDKVDTLSLNLFFLPGERWRQLRTKLTPTFTSGKLKQMYPL

LLEIGDNLIRVCDQIIKKDNIVEFKDLIGRYTTDSISSIAFGVDCQSLDNGSSEFQKHGI

MVFDLPPSKIASAVFAPIILDIFRIPVFNNTITQFFVKTFNDVLEERRRNKITRKDFLNS

LMQLLDKGSLDEDKDLKQKDNNNSANGSALGKISLSEAIGQAFIFYLAGFETTSSTITYC

LYELALNPEIQEKVQAEIDEFSKRDGGITYEIMSNDMKYLHMVFLETLRKHPSIPFLNRE

CIEDCDIPNTNFRIEKGTKLLICMNAMHRDPDIFPDPEKFDPFRFTKENIASRQPYTYLP

FGDGPRACIGIRFGMLQTKIALIGLLSNYNFSVCEKTSIPVMYARRSFTQTPEYGIYLKT

EKRNKSTFLYKNRQ

>AED99066.1

MFVATVLAAVAALLAAFWYYFSRNFGYWTERGVVSCNPFSLTGDRGGGIPFGKPFYELLT

PIYNKYKGKERVVGTFQARRPILVALDPEVTKAVLTKDFSHFHGRGQPTDESDPFSQNLF

FLEGARWKNLRMKLSPTFTSGKLKGMFPLMAGITDQLNDAVREKAAHGDKQVEFKDILLR

YSTDVIGSVAFGIDCNTVKGGNDDFLNMSRELFRRNFIFLLRFFLISVHPVFVKLLPFKR

IFKDMTEFFLKLMSDTVNYREKNKVERNDFVQIMMQLREEDRNRSTLDRASHVELNNDTM

AAQAFLFFVAGLDSVANTIGFALHELAMNHALQRRAVAEIQENIRKHGSLTYDAVRDMEL

IERIVRESLRKYSPVGILTRQPSEPIKIPDSDVVIDPSVMVWIPVWQAMHDPDIFPEPER

FDPDRFTEEAKKTRHQYNYLPFGEGPRFCVAERFAILEMKLCIAGLLQQHVFDAGSKTDV

KLKLDPKTSSPTPQNGFWLQVTDRPTSAL

>157132677

MLPLLLAVVTILLTAAALYIRSRHRFWSDRGIPCAPNPEFLFGHVRGQVTNKHAAYVNRE

LYQQFKARGEGFGGYSFFAVPAVIIVDPELVKTILVRDFAVFHDRGIYNNPKDDPLSGQL

FLLEGLQWKILRQMLTPTFTSGRMKAMFGTIMDVAEEFRQFLVDSRKRESVIEMKEILAS

FTTDVIGTCAFGIECNTLKNPDSDFLKYGKKVFEQRVSTLMKFIFASLFKDLARKLRIKI

TDAGVEKFFLGLVRETVEFREKNNVLRNDFMNLLLQLKNKGRLVDQLDEADEVAARGLTM

EELAAQCFVFFIAGYETSSTTMNFCLYELAKNPDIQEKLREDIEEAVASNSGRVTYDLVM

GLWHPMMCE

>EFR21007.1

MVALWTLVLSAFGSASLLLCFVFLRRRRTFWLRHGIPTTSEPAHLLYGNVRGISTDHHTA

IILQRLYREFRSRNLPAGGFNLFFSPVLLTIDSDLIERVLVSDFSKFRDRGVYANATVNP

LTSGTLFSLAGDQWQHLRQVLAPAHATVNIRTLFTDTVRNAQGLVSFVGTSIGQGLDSLE

WTDLTARYTTDVIGSCIGIDCRAIREPHRAEFRTMGHRAFRFSVRRMWKLRFGYTFRRLA

DALRLCINESIVERFFLQLCRSTGLHRESYRTVKRDFLQIMLEMKRRGLLDMTQVAAQCY

QFFIAGFETSATLLNFALYELARDASVQKRLRSAIQRALDETDGQLSYDMVMSLDYLDQI

VKETLRMYPPVDFLFRVSNVDYTIRDVGTVPRGTLFVVPVHAIHHDAQYYPQPERFNPDR

FSATQRQPAKNAFLPFGLGPRHCIGEEFALMLVKVGLVALVRAFRFSLDTNRMPASGEPI

VFKPRSLVLAPTGGMYLRVERI

>ABV80275.1

MILYTIGLIVAFVFLALKYVYSYWDRQGLPNLRPEIPYGNLRILAQKKESFNVAINDLYD

RSSERLVGVYLFFRPAILVRDAHLAKRIMVNDFQHFHDRGVYCNEHNDPMSANLFALPGQ

RWKNLRAKLTPTFTSGQLRHMLPTFLAVGSKLEQYLERLANEKQIVDMRDIVSRYVLDVV

ASVFFGFEANCLHDPDDAFRVALRDLNNPDSFMNNIRTAGVFLCPGLLKFTGINSLSPPM

KKFTTEVISSHLHQRETGQVMRKDFIQMLTDLRRKAGSSGEETLTDAQCAANVFLFYGAG

ADTSTGTITFTLHELTHNAEAMAKLQQEVDEMMERHHGEITYDNINGMKYLDLCVKETLR

IYPALAVLNRECTIDYKVPDSDTVIRKGTQMIIPLLGISMNEKYFPEPELYSPERFDEAT

KNYDADAYYPFGAGPRNCIGLRQGLLLSKIALVMMLSRFNFSATIPRKIKFEPVSITLAP

KGGLPMRIENRAKH

>EAA12490.2

MILYTIGLIVTFVFLALKYVYSYWDRQGLPNLRPEIPYGNLRILAQKKESFNVAINDLYD

RSSERLVGVYLFFRPAILVRDAHLAKRIMVNDFQHFHDRGVYCNEHSDPMSANLFALPGQ

RWKNLRAKLTPTFTSGQLRHMLPTFLAVGSKLEQYLERLANEKQIVDMRDIVSRYVLDVV

ASVFFGFEANCLHDPDDAFRVALRDLNNPDSFINNIRTAGVFLCPGLLKFTGINSLSPPM

KKFTTEVISSHLHQRETGQVTRKDFIQMLTDLRRKAGSSGEETLTDAQCAANVFLFYGAG

ADTSTGTITFTLHELTHNAEAMAKLQQEVDEMMERHHGEITYDNINGMKYLDLCVKETLR

IYPALAVLNRECTIDYKVPDSDTVIRKGTQMIIPLLGISMNEKYFPEPELYSPERFDEAT

KNYDADAYYPFGAGPRNCIGLRQGLLLSKIALVMMLSRFNFSATIPRKIKFEPVSITLAP

KGGLPMRIENRAKH

>AAL93296.1

MILYTIGLIVAFVFLALKYVYSYWDRQGLPNLRPEIPYGNLRILAQKKESFNVAINDLYD

RSSERLVGVYLFFRPAILVRDAHLAKRIMVNDFQHFHDRGVYCNEHSDPMSANLFALPGQ

RWKNLRAKLTPTFTSGQLRHMLPTFLAVGSKLEQYLERLANEKQIVDMRDIVSRYVLDVV

ASVFFGFEANCLHDPDDAFRVALRDLNNPDSFMNNIRTAGVFLCPGLLKFTGINSLSPPM

KKFTTEVISSHLHQRETGQVMRKDFIQMLTDLRRKAGSSGEETLTDAQCAANVFLFYGAG

ADTSTGTITFTLHELTHNAEAMAKLQREVDEMMERHHGEITYDNITGMKYLDLCVKETLR

IYPALAVLNRECTIDYKVPDSDTVIRKGTQMIIPLLGISMNEKYFPEPELYSPERFDEAT

KNYDADAYYPFGAGPRNCIGLRQGLLLSKIALVMMLSRFNFSATIPRKIKFEPVSITLAP

KGGLPMRIENRVKH

>AFM08406.1

MILYAIAVLPVIVFLALKYVYSYWDRHGIPNLKPEIPYGNLRILAEKKESLNIAVNNLYT

RSTDRLVGVYLFFRPAILIRDAHLAKRIMVNDFQHFHDRGVYCNEHGDPMSANLFALPGQ

RWKNLRGKLTPTFTSGQLRNMLPTFLDVGKKLQQFLDNLAMDHQIVNMRDIVSRYVLDVV

ASVFFGFEANCLHDPDDPFRVALRDLTNPESFLNNIRSAGVFLCPGLLKFTGISALAPAM

KKFTMEVISSHLHQRETGQVTRRDFIQMLTDLRRNAGNNAEDSLSEAQCAANVFLFYAAG

ADTSTAAISFTLHELTHNPEVMGKVQREIDEMMNRYNGEITYDNITELKYLDMCIKETLR

MYPALAVLNRECTIDYPVPDSDIIIRKGTQVIIPLLGISMNEKYFPNPELYSPERFDEAT

KNYDADAYYPFGVGPRNCIGKIALVLMLTKFVFSATIPRKLCFEPVAVTLAPKGGFPMRI

ERRV

>EAA07605.4

MFVYTLALFAVVLYLALRYVYSYWARQGFPHLRPEIPYGNLRALAEKRESFGVAVNELYT

RSTERLLGVYLFFRPAILVRDAHLAKRILVADFQHFHDRGVFCDEHRDPMSANLFALPGA

RWKRLRAKLTPTFTSGQLRQMMPTFLDVSEKLLAELEPLAAEGRVVDMRDISSRYVLDVI

ATVFFGFETHCLRDPNDPFVEPLRDLNNPNSFVNNIRSAGVFVCPGLLKLTGLSSLPPGM

RQFATEVVTHQIEHRERHPQQRRKDFIQLLIDLRREANGQDALTIAQCAANVFLFYVAGA

DTSTGVISFTLHELTHNGEAMAKARQEIDHVLERHGGVISYESLQELAYLDLCVKETLRK

YPGLPILNRVCTEDYPVPGAADVVIRAGTQVIIPLLGISMDEKYFPEPEVYMPQRFDEQA

PNYDPDAYYPFGLGPRNCIGLRQGIMLSKIGLVLMLSRFDFEATVPRKVRFEPANVTLTP

DGGLPMKITVRGDRAKVQ

>EAT39045.1

MFIYTFALFWLALVLVLRYIYSYWDRNGLASIKPQIPYGNLKSVAQKTQSFGVATCELYW

KSQERLAGIYLFFRPAVLIRDAHLAQRIMTTDFSYFHDRGVYCNEEIDPFSANLFALPGK

RWRNLRHRFTPLFTSGQLRCMMPTILDVGHKLQKFLEPAAERQEVVDIREIVSRGVLELI

ASLFFGFEADCINDPDDAFSKTLREFQLGGFMNNFRTACTFVCPELLQVTRISSLSPQMI

KFATDVVTKQIEHREKNNVSRKDFIQLLIDLRREEANNNEVALSFEQCAANVFLFYVAGS

DTSTSAITFTLHELTQNPEVMDKLQSEIDEMLVQTNGELTYTAIKELPYLDLCVKETLRK

YPGLAILNRKCTKSYAVPESSVVIQEGTQIMIPLLAYGMDEKYFPEPERYYPERFNKQSK

NYDEKAYYPFGEGPRNCIAYRMGVMVSKIGLILLLSKFKFEATQGPKIVFSAATVPLVPK

GGIPVKISNR

>AFM73688.1

MFIYTFALFWLAVAFAIRYIYSYWDRNGLPSIKPHIPYGNLKTVANRTESFGVATCDLYW

KSKDRLVGIYLFFRPAVLIRDAHLAQQIMTTDFSHFHDRGVFCNEEVDPFSANLFALAGK

RWRNLRNKFTPLFTGGQLRCMMPTILNVGHKLQNVLEPAAKKQEVLEIRELVSRCVLDII

ASVFFGFEANCINDPNDAFIQNLRELQYDGFFNNLRAAATFICPELLKLTRISSLSPEMI

RFVTDIVTKQIEHREKNNVTRKDFIQLLIDLRREDANNNEATLGLEECAANVFLFYVAGS

DTSTSAVAFTLHELTQNVETMEKLQTEIDEMLVKTSGELTYDGIKEMSYLDLCVKETLRK

YPGLAILNRECTKSYAVPNSNIVLKKGTQVVIPLLAYGMDEKYFPEPDRYLPERFDKSTK

NYDEKAFYPFGEGPRNCIAFRMGVMVSKICLVLLLSRFNFEATRGPKINFTPSTVALLPK

GGIPVKISIR

>EAT39047.1

MFIYTFALFWLAVAFAIRYIYSYWDRNGLPSIKPHIPYGNLKAVANRTESFGVATCDLYW

KSKDRLVGIYLFFRPAVLIRDAHLAQQIMTTDFSHFHDRGVFCNEEVDPFSANLFALAGK

RWRNLRNKFTPLFTAGQLRCMMPIILSVGHKLQNVLEPAAKKQEVLEIRELVSRCVLDII

ASVFFGFEANCINDPNDAFIQNLRELQYDGFFNNLRAAASFICPELLKLTRISSLSPEMI

RFVTDIVTKQIEHREKNKVTRKDFIQLLIDLRREDTNNNEAALGFEECAANVFLFYVAGS

DTSTSAVAFTLHELTQNAETMGKLQTEIDEMLVKTSGELTYDGIKEMSYLDLCVKETLRK

YPGLAILNRECTKSYAVPNSDILLKKGTQVVIPLLAYGMDEKYFPEPDRYLPERFDKSTK

NYDEKAFYPFGEGPRNCIAFRMGVMVSKICLVLLLSRFNFEATRGPKIDFTPSTVALLPK

GGIPVKISIR

>EDS30845.1

MIIYSLILICSSIYLILRYIYSYWDRHDLPNLKPDIPFGNIRTVALKQESFGVALNALHA

KTTGQLVGIYLLFRPAILIRDAHLAHRIITSDFNYFHDRGVHCDESSDPFSAHLFALPGK

RWRSLRNKLTPTFTAGQLRGMLPTILTVGRKIQAFLEPKAKRGQVIEARDLISRFVLEIV

ASVFFGYEINSIHDPEDSFRTVLRSFREDNHVTNLRTVGAFLCPALLKVSRVKTVPEVLD

NFVNKIVREQIEYREKNNVTRKDFIQLLIDLRQEKSDFGLSLEQCAANVFLFYAAGADTT

TDAITYTIHELTHRPDLMQKVQVEIDDALEKSNGEINYDVVHEMKLLDNCVKETLRKYPF

PILNRECTQDYQVPDSKLIIRKGTPVIIPLQAYGMSEEYFPEPNRYLPERFDPSTKNYDE

KAYFPFGDGPRNCIGSRMGSSVSKIGIIMLLSRFNFEATQGAQIDFARAQIALAPENGIS

LKISNRISNLM

>EDS31518.1

MFLLSIVLLGLVTFLGLYYIFSYWSRYGLPHLKPEVPYGNLRAVAERRQSFGVAISELYR

KSTEQLLGIYLFFQPAILVRDASLAKQIMTSDFGSFHDRGIYHDEKSFSSNIFSASLKPW

KQVRHKLTPNFSTGKLRNMVPTVLDVGKKLESYLAEHADRGEIVELREICTRYVIDIIAL

VLFGFEMDSINDPDHPFYVVGRELVRNSFSNNLRTAATFICPGILKLTKISSVSPKVSDF

MVELVKKQLELREKDCVTRKDFFQSLIDLRHEDRDREALSIVECASNVNLFYIAGSETTG

GTIIFTLHELTHNPAVMKQLLAEIDEALERSGGQIDYDVVKGMRYLDVCIKETLRKYPGL

PILNRKCTQDYRVPNSKMTIRKGTQLIIPLLAYAMDERYFPEPEKYMPERFFEETKNYDD

DAYAPFGDGPRQCIATQMGITVAKVTLVMLLSKFSFEATHGQRIEFAPSSVPLGPKDGIK

LKISRRNI

>EDS42257.1

MFLLTIGLLGLVLYLGLRYIFSHWDRHQLPSIKPEVPYGNLRDVAKRRQSFGVLISDLYW

KSTEQLLGIYLFFQPAILVRDAHLAKQIMVTDFGSFHDRGVYNDEKTFSSNVFSLPLKPW

KPLRQKLAPNFSTGRLRSIIPTILDAGKKLEHYLDRSADKEEVVELREVVTRYVLDIIGL

VMFDLKVDTLNNPEHPFCLVGQEILRNSFSNNLRTAATFVCPGIYKITNISSISPIVETF

MIDLFTRQKEIREKSSLNKKDFIQSLIDLHSEDRDQNREALNTLECGSNMFIFYIAGSET

SSGTTIFTLHELTHHPEVMAKLVKEIDETLERSKGLIDYDVIKGMQYLDVCVKETLRKYP

GLPILNRKCTQDYQVPNSKTVIRKGTQLIIPLLGFAMDERYFPEPEKYSPERFLEASKNY

VDDAYAPFGDGPRQCIATQMGITVVKVTLVMLLSKLNFEATQGPKIKFAAASVPLRPDGG

IKLKISRRIVAAK

>EDS30844.1

MLLLSVALLAIVLLLGLQYIFSYWHRHGLPHLKPEIPYGNLRSVAKRQQSLGVAITELYR

RSTEPLVGIYLFFTPAILVRDATLAKRILTVDFGSFQDRGVLYDKKRDLFSCNMFAAPLS

SWRPVRSKLTAAFSLSQLRGMLPTVLDVGTKLEDRLGVAAESGDAVDLNGICMRYVVDVI

AEVLFGFKADSIGNPEHPFYTIGEYCDEQRSYIRNVHLSSISRKVSNFMISLVRQQMKYR

AKNDLARKDFLQLLTDLHQEDLSVEECAANVNLFYTAGSETTKATVIYTLHELAHHPEVM

GRLVEEVDECLKQSGGEISYDLVKSMSYLDVCRKETLRKYPGLFFLNRKCTHDYQVPNSW

LVIKKGTQLVIPLKAYGMDERYFPNPESYIPERFLEDTKNYDENAYAPFGNGPRKCIAPR

MGIFVAKVTLVRLLSKFRFEATQELKIEFAPSVVPLVPKDGVRMKIHRRCI

>EDS42256.1

MFLLPIALLVLVSFFERRQCIGVAISELYRQSAEQLVGVYLFFKPAILVRDAGLANQIMT

SDFASFHDRGTWNNPRSSNVFSLPLRPWKQVRHVLSPCFTPGKLRLMIPTVLDVGKKLED

YLSESAERGEIVELRDICTRYVLDIIALTMLGFEADTLNHPDHPFYTIGRELVRNSFLNN

IRTAATFLFPEFYKLTKIPSISPKLSRYLVSLFNEQSDFRERNNVSRKDFFQSLLDLRQE

DREHNRKALSVQQCASNINVFYIAGSETTGGTVIFTLHELTHHPEVMEKLVNEIDETLER

SEGLIDYDVIKGMRYLDVCVKETLRKYPGLPILNRKCTQDYQVPNSKVIIRKGTQLIIPL

LAYAKDERYFPEPDKYSPERFLEESKNFVDDAYTPFGDGPRQCIATQMGIVIAKITLVML

LSKFRFEATQGPTLEFAVSQVPLGPKHGIKLRIFRRNVNTAENTGENLTWVGMFELTTFG

LRVQPPPAIPPE

>AAX35341.1

MFVLYLLILFGSVCYLWMKFVYSYWSRTKFPYLKPNIPFGNVSEAAMGKKSMGINLYELH

KSSNEPIIGIYLLFRPALLVRDADLAKDMLTTDFANFYDRGIYHNPEDPVASHMLMLPGQ

EWKQIRSKLTPTFTSGKLKGMMPAILNIAEILKQKLLPSSQKGEIVEMRDLLMRFSLDVI

GTVGFGLDVNTLDDPNHSFREIEKCVNNGELINRIRLIGAFFCPKILEVLHMSSLPKEFK

EFMMNIVQKTMEFREKNQVQQKDFIQLLMELRKPGKLNDNDGTDTIPNDKHSKQDGLTIE

QCAAQVALFYLAGFDTTSSAVSFCLFELSRQPDLMTRAQCEIDDIMNKYNNTITYESLNE

MPFLDACVRESVGKYPVFPFLNGMCTKDYKIPNMDLTITNETPIIISLLGIMRDPKNFPH

PEQFLPDRYDEKDPKYNPTAFIPFGDYYCYSAGIRMGKMVAKIALIFLLHNYSFICTENR

ELEFDIIL

>EDX16386.1

MIVVFALIAATLAVGFLVLLPVVVSGGCLLVVAIVWLWQLLHFGHWLRLGVPFVPAAPFV

GNVWNLLRGACCFGDQFRELYESKEAAGRAFVGIHVLHNHALLLRDPALIKRIMVEDFAQ

FSSRFETTDPTCDTMGSQNLFFSKYETWRETHKIFAPFFAAGKVRQMYGLLENIGQKLEE

HMEQKLSGRDSMELEVKQLCALFTTDIIASLAFGIEAHSLQNPEAEFRRMCIEVNDPRPK

RLLHLFTMFFFPRLAHRVRTHLYSEEYERFMRKSMDYVLSQRAESGENRHDLIDIFLQLR

RSESAESIIHRPDFFAAQAAFLLLAGFDTSSSTITFALYELAKNTTIQNRLRTELRAALQ

SSQDRQLSCDAVTGLAYLRQVVDEVLRLYPPTAFLDRCCNSRTGYDLSPWNGGSPFKLRA

GTPVYISVLGLHRDAQYWPNPEVFDPERFSAEQRQQHHPMTYLPFGAGPRGCIGTLLGQL

EIKVGLLHILKHFRVELCGRTLPEMRFDPKAFVLTAHNGAYLRFVRDSL

>EDW56395.1

MIAVFALIAATLAVGFLVLLPVVVSGGCLLVVAIVWLWQLLHFGHWRRLGVPFVPAAPFV

GNVWNLLRGACCFGDQFRELYESKEAAGRAFVGIHVLHNHALLLRDPALIKRIMVEDFAQ

FSSRFETTDPTCDTMGSQNLFFSKYETWRETHKIFAPFFAAGKVRHMYGLLENIGQKLEE

HMEQKLSRRDSMELEVKQLCALFTTDIIASLAFGIEAHSLQNPEAEFRRMCIEVNDPRPK

RLLHLFTMFFFPRLAHRVRTHLYSEEYERFMRKSMDYLKRSESAESIIHRPDFFAAQAAF

MLLAGFDTSSSTITFALYELAKNTTIQNRLRTELRAALQSSQDRQLSCDAVTGLVYLRQV

VDEVLRLYPPTAFLDRCCNSRTGYDLSPWNGGSPFKLRAGTPVYISVLGLHRDAQYWPNP

EVFDPERFSAEQRQQHHPMTYLPFGAGPRGCIGTLLGQLEIKVGLLHILKHFRVELCGRT

LPEMRFDPKAFVLTAHNGPFLRFVRDSL

>EDV47631.1

MIIVVFALFAAALGVGCLLAVTAVGGGCLLVLGIAWLWQHRHFGHWRRLGVPCVPAAPFV

GNVWRLLRGACCFGDQFRELYESKEAAGRAFVGIHVLHNHALLLRDPALIKRVMVEDFAQ

FSSRFETTDSTRDTMGSQNLFFSKYETWRQTHKLFAPFFAAGKVRQMYGLLEHIGQRLEE

HMERQLGGRDSMELEVKQLCALFTTDIIASLAFGIEARSLQNPEAEFRRMCIEVNDPRPK

RLLHLFTMFFFPRLAHRVRTHLYSEEYERFMRKSMDYVLTQRATSGEKRHDLIDIFLQLQ

RTESADSIIHRPDFFAAQAAFLLLAGFDTSSSTITFALYELAKNATIQHRLRTELRSALH

SSQDRQLGCDSVSGLPYLRQVVDEVLRLYPPTAFLDRCCNSSAGYDLSPWNGGSPFTLRA

GTPVYISVLGLHRDAQYWPSPEVFDPERFSAEQRQQHHPMTYLPFGAGPRGCIGTLLGQL

EIKVGLLHILRHFRVELCERSLPQMRFDPKSFVLTAHNGTYLRFVRSPL

>EDW57969.1

MITFGLLAVLLLAWLWQRWHFGHWQRLGVPHAPAAPFVGNVWRLLRGCCCFGDQFRELYE

CPSAKGQAYLGIHVLHNHALLLREPALIKRILVEDFGQFSSRFETTDAIGDTMGAQNLFF

SKYEMWRETHKIFAPFFAAGKVRHMFGLLQQIGQQLEQHMARQLTASGTKEGTLELEVKQ

LSALFSTDIIASLAFGLEANCLQQPDAEFRRMCIEVNEPRPKRLLHLFTMFFFPRCSRQL

RTHLYSEEYERFMRQSMNWVMEQRAASGEQRHDLIDIFLQLQRTQPPESVVHRRDFFVAQ

AAFLLLAGFDTSSSTITFALYELAKQPAIQQRLRQELAAALLGSGATAGQLDYETLSGLP

YLRQVVDEVLRLYPPTAFLDRCCNAKAGYDLSAWSCGSALRLPPGTPVYISVLGLHRDEQ

YWPQPLKFDPERFSPEQRVQHQPMTYLPFGAGPRERPKPLC

>EDW74580.1

MLAETLILLATLATIFYIWQKWNYSYWERHGVKYIKPVPILGNMVNLFKGKVPFYLQVSE

LHKEPGFEKEPLVGIYLAHRPALLIRDLELIKTVMIKKFNYFANRALSTDPHNDQLGYNN

LFFVRNPDWKDLRAKLSPVFTSGKMKQMYPLMIEIAKDLETNLSERSTGSVLPIKNICAR

FTTDLIATIAFALATLARVKVFSKKTSEFIRHSINYVLAEREKSGVKRNDLIDILLAMKK

EAAENPDKPNRAHDIDYLVAQAAVFQTAGYETSSSTMALALYELARNEEAQNRLRAEISD

YFGDEDHISYERIQEMPYLSKVVNETLRLYPIVGYAERECIQPAQGERFNLKPHYDLEVP

NGMPVYIGVLAIQRDPQYWPDPDKFDPERFDPTNRHNLNMDAYMPFGIGPRNCIGMRLGL

LQSKLGLVHLLRHYRVQTCDRTVKSIVYAPLSPALQSKDEIYLKLERV

>289177118

MLEFTYSKLFWITVVLAAVYAYLKYYLYNYWNRYNVPNETPSIPLGNVSIDYLRGKIHFA

GFIDSIYNKFKHHRFYGVYVFQRPILCINDPELIKLILVKDFDLFADRGWFYDGKVDPMS

SHLFLLPSEKWKRLRIKFSPIFTSGKLRRMYPYLVEISKHMIKTCDAELKTTDIVDVNDM

LARYTTDIISSIVYGLDSKSLDNPKSEFQTKGEKALNFGRFNLLMAMFAPEMMSWATVPL

FQENVSKFFLNIFIEAVTYRRKEKIQKKDFLDLIMQLIDYGKVEDDDQIMQTNGKNSEHF

NKLTIEEAAANAFVFFLGGYETSSSTTSFCLYELAQNPEIQEKLQAEIDEVVKSPTGLTY

ESIAEMEYLDMVLSETLRKHPSLPILNRIAKEDYPLPTTDFVIKKDMRIMISLSGIQNNP

EYYPDPEKFDPLRFTKEKAVARNKYINIPFGDGERMCIGKRFAVMQVKIALAAMLHNYQF

SISEKTQVPPQYETMNFTQTVRNGVKLRVKRRDLEKILRKRS

>289177188

MLLNIWQILCIKTLWISIAIAGVYLYLKFVLFNYWSRKKVPHVPPNIVLGNINKNFLLDK

ITIGELVRQHYDKFRKEKYFGIYIFHNPILVINDLELIRLILVKDFPYFYDRGIHSNVDV

DPMSFNLFRLPGERWKNLRTKLTPIFTSGKLKQLYPLLYEVSHEFVDICDKILEVTDVID

MKDLVERFITDCISNVAFGFNCNSLKDPNNEFRNNGKSGTDLGKYSATLSIFGSNILNFL

RLRVFSSNVNKFFIRIFEDVVNHRVKSKIVRNDFINLLMDLTDHKQGSDKAPIVNKSDPA

EMKNRKLNMLEAAAQVFVFFIAGFETTSTTVTYCLHELAKYPEVQEKLQEEIDEVANSPE

GFNYDNIMNMKYLDMTFNETLRKHPPVPFVNRLCTKDYQIPGTDLRIPKGMRLAISVSGL

QRDPDIYPEPEKFEPLRFSKENLAQRSAYCFLPFGEGPRICIAKRIALMQGKLALATLLS

KYTFSICDETPVPLEYSNRTFLTVAEKDLYLKLTKR

>345482007

MDFYEDLVYSKLFWATTVLFGVYLYLKYIMYNCWSRNGVPHDSPSIPLGNVPFSYFTENV

PHGEIIKSSYEKYKNHRYHGLYILNEPILVVNDPELIRLILVKNFTNFCNRGTYYNESTD

PLSCALPRLVDDKWKRLRAKLSPTFTSGSLKKMYPMFKEICDKLVKTFDTALDESDVLEM

KDIASRFTMDCISSIVLGFNVNSLQNPEHEFRKYGKIVNDFGKVFVLLSFYVPDMIDFYS

FIPKKRKKVHAFFVYLFQKTVAYRRAKKIVRNDFLNMLMQLMDRGYVENEFEASSTNVSD

DDRLSMTEAVAQSVIFFAAGQETTSSALCCCLYELALHQDVQKKLQWEIDQAFASPEGLT

YEKIFEMPYLDMVLCETLRKHPGAPVLNRISLADLNVPDSKFRIPKGMRLVIPVNGIHSD

PNVYPDPDRFDPSRFTPENRAKRHPLVYLPFGEGPRHCIGKRLGVLKPKIALCYLLKNYS

FFSCDKTQPRLSYMSNSFVQVPAGGVYLRVERRRQERTEASP

>345481151

MEPLSAILYTKTFWITSLFLTIYLYLKYVVYNVWANRGVPHDPPSIPLGNIPVKYFQGKT

THGAIVKESYDRYKTNPFYGMYMLYMPILVVKDPELIQLILVKHFNSFYNRGTLYNESKD

PLSTGLVRLYDEKWKRLRVKLSPTFTSGKLKLMYPLVKEICDELLRVYEDVLDKSDVVEI

KDITNRFTIDCISSIAFGFNCDSLHQPDNEFLKYGKISNDLGRVFLIFSFFVPRLVSLFP

IPEKRKKVSEFFYDLFEKMVSHRRKDKTVRNDFLNMLMQLMDHGKVEEDDDAPSKSNSLT

KITDADKITMVEAVGQSMFFFAAGQETTASAITYCLYELSFNQDIQEELYNEISQVAHSP

EGLTYEKLFSMPYLDMVFRETLRKHPGAPMLNRQASEDFVVPGSHFVIKKGTRIVIPIKA

LHADPDIYPDPDKFDPTRFTSENKAKRHAFTYIPFGEGPRHCIGKRLGILKPKIALFYLL

LNYKFSVCDKTPIPLRYMTDYFVQVPAGQVYLKVEKRNGTLSDKASRPWNSAN

>345482200

MTKGSVLNSKLFWISAIIFVFYSYFKLIIYSYWKRHATPHDKPTVPFGNVGMDLITXKVS

IGAKMKSSYDKYKKHPFHGLYMFHNPTLMVNDPEVTKYVLVKEFAHFHDRGMYFNDKIDP

ISGHLFFMTGDRXRALRIKLSPSLTSGKLKNMYPFVQEIADEMVNIANENLEKTDVLEIK

ELVASLEQMLFFHRYTTDTIFSLAFGFNANHLRNPDSEFRHYGDLSIKNVPVINAMAAFT

PYILEFFKILFTNAKATAFFIKLFKDMVVQRKMXKKDFLNLLIELMDHGQIRDQEGENGT

DEPAVNLTKLELLEAAAQAFVFFVAGFETSSSIVTHTLXELALNPDEQEKLHQEIDELLQ

RDGGFNYENLMHLEYLEMAFYETIRKHPSVPFLNRICAKDCKLPNSDFVVPEGMPILISI

SGLHRDSNIYPDPDKFDPTRFSKENIAARHPYAYLAFGKAPKICIGKRLGIMMSMVALAT

LLSKFKFSVCDRTPIPNRYSLNSILQMPRDGIHLGMEKRKK

>ACD84797.1

MELLEIVKSAMDTHSVLLFLSVMVYLLYVYRDKFHYWSKRGVPCQSPAQSIVRTFRLVLR

MDSFTDNFYRVYKAFDGHPYVGSLELTKPILVVRDPELARIVLVKSFSSFSGRLKSPDTT

LDPLSNHLFTLNGEKWRQVRHKTATAFSTAKLKNMFHSLKDCAREMDAYMERAIGDKGDV

EFDAFKVMSNYTLEVIGACAMGIKCDSIHDEETEFKRLSRDFFRFDARRMIFTLLDLLHP

KLPVLLKWKAVRPEVENFFREAIKEAASLKESEAAARTDFLQILIDFQKSEKASKTDAGN

DTELVFTDNIIGGVIGSFFSAGYEPTAAALTFCLYELARHPQVQAKLHEEILAVKEKLGD

DIEYENLKEFKYANQVIDETLRLYPASGILVRTCTEPFKLPDSDVVIEKGTKVFVSSYGL

QTDPRYFPEPEKFDPERFSEENKEKILPGTYLPFGDGPRLCIAMRLALMDVKMMMVRLVS

KYEIHTTPKTPKKITFDTNSFTVQPAEKVWLRFRRRASTP

>ACS92724.1

MELLEIVKSAMDTHSVLLFLSVMVYLLYVYRDKFHYWSKRGVPCQSPAQSIVRTFRLVLR

MDSFTDNFYRVYKAFDGHPYVGSLELTKPILVVRDPELARIVLVKSFSSFSGRLKSPDTT

LDPLSNHLFTLNGEKWRQVRHKTATAFSTAKLKNMFHSLKDCAREMDAYMERAIGDKGDV

EFDAFKVMSNYTLEVIGACAMGIKCDSIHDEETEFKRLSRDFFRFDARRMIFTLLDLLHP

KLPVLLKWKAVRPEVENFFREAIKEAASLKESEAAARTDFLQILIDFQKSEKASKTDAGN

DTELVFTDNIIGGVIGSFFSAGYEPTAAALTFCLYELARHPQVQAKLHEGILAVKEKLGD

DIEYENLKEFKYANQVIDETLRLYPASGILVRTCTEPFKLPDSDVVIEKGTKVFVSSYGL

QTDPRYFPEPEKFDPERFSEENKEKILPGTYLTFGDGPRLCIAMRLALMDVKMMMVRLVS

KYEIHTTPKTPKKITFDTNSFTVQPAEKVWLRFRRRASTP

>AEK21827.1

MGSLVELLSSFGSPSLNSVLIVASVGLLAWYYLEDKVAYWSKRGVPNVPASKCIAQTLTF

LAGRKTMNEIFKEQYQLLEGQKYGGTFQGLYPSILVRDPAMIKEWLVKSFSSFHDRAPEP

DENVDKLAGNLFQLTGDRWRTVRHKMSPAFSSAKLKIMYETLKDCAEECNAHLEARCASK

GETEIDIKDFVSNYTMDVIGACAMGIKCNAVRDPENCEMKKVVKEMFAVGWRSILFQLLE

MIHPKLPELLRIVPRQPAVEQFLMTITKNAMEMKTKAGQSTRKDFLQILMNISSSESDVT

RDSNEDLSVLEGGVVHEGPIFTENIIAGVVTSFLFAGLEPVSAITTFCLYELVRHPEMQE

RLFQEIQSARKESCGEIKYEDLKKLRYLDQVVNETFRKYPVASILARNCTENVQISGESV

VVEKGVKVFISTYALHRDPTFFPDPDKFDPERFSEENVDKIIPGSYLPFGDGPRFCIAQR

LALMDVKTMVITLVSGYTLHTCSKTVDHIEMDPKAFTLNPKGSVWLRLKKRI

>ACT68012.1

MELLTSILSLPVNSVLVFTGAALFLWYYLQDQLSYWRKRGVPYISPIKCFSFTMAQTLRT

KSVLETTKEMYELLEGHSYGGIYRLFRPAVILRDPGMVKEWLVKGFPSFHDRNSPPDKKA

RNLSGNLFTLTGDRWHTVRTKLAPAFSSAKLKVMYQTLKGCASDVNAHLQGLCSAKGEVE

IDIKDLVSRYAMDVIGACALGIQCNAIKDPENCQMRKAVQQIFRINWRFSLQILLSAIHP

KLALLFSLSRGTSEVTKFLTSVTKEAMDVKKKAGSSRKDFLQIMMSAGDTEIEKRKLKSA

EDEDFVFTENVITSMIGLFLSAGLDPVATTVTFCLYELAYHPEIQDRLFQEIQPVRNECG

DEIGFEDLKKLQYLEQVVNETLRKYPPAGVLSRKCTESFQIPDSSVVIEKGLGLMIPAAC

FHHDPKYFPDPEKFNPERFSKENIGNIVPGSYIPFGEGPRFCIANRLALMDVKTMLITLV

SDYALHPCARTKNHFEFDRETFTLNPKGEVWLRLKKRN

>ACT78507.2

MELLTSILSLPVNSVLVFTGAALFLWYYLQDQLSYWRKRGVPYISPIKCFSFTMAQTLRT

KSVLETTKEMYELLEGHSYGGIYRLFRPAVILRDPGMVKEWLVKGFPSFHDRNSPPDKKA

RNLSGNLFTLTGDRWHTVRTKLAPAFSSAKLKVMYQTLKGCASDVNAHLQGLCSAKGEVE

IDIKDLVSRYAMDVIGACALGIQCNAIKDPENCQMRKAVQQIFRINWRFSLQILLSAIHP

KLALLFSLSRGTSEVTKFLTSVTKEAMDVKKKAGSSRKDFLQIMMSAGDTEIEKRKLKST

EDEDFVFTENVITSMIGLFLSAGLDPVATTVTFCLYELAYHPEIQDRLFQEIQTVRNECG

DEIGFEDLKKLQYLEQVVNETLRKYPPAGVLSRKCTESFQIPDSSVVIEKGLGLMIPAAC

FHHDPKYFPDPEKFNPERFSKENIGNIVPGSYIPFGEGPRFCIANRLALMDVKTMLITLV

SDYALRPCARTKNHFEFDRETFILNPKGEVWLRLKKRN

>AEK21825.1

MELLQGLLSMPSNSTLVCIGISLFVYYYLQDKVNYWAKRGVAYVPASKCLSLFIPQMLQN

RTMLEVVSEQYDLLEGHSYGGVYQLTRPTVILRDPAMTKEWLVKGFTSFHDRGPEVEEGQ

GNLSENLFSLTGDRWHTVRTKLSPAFSSTKLKVMYQTLKGCAEELNAHLQGLCEKRDEAE

IDIKDLVGRYAMDAIGACAMGIKCNAIKDPDNCEMKKAVKAIFTINWRFTLMQLCMLIHP

KLVKIFGLAGPPKDLTKFLTSVTKQAMDMKTKSGQPRKDFLQIMMSASESETQNGKEHSQ

NGDQTVKSEDNIFTENVITGVISTFLSAGLDPVAATVTFGLYELAFHPEIQERLFEEIQA

ARMKSGGEIEYDDIKDMQYLEQVVNETLRLYPAAGFLFRFCREPFQIQDSSVVIEKSVNL

LVPQMCIQRDPKYFPEPMKFDPERFSKENVDKILPGSYLPFGEGPRFCIANRFALMNAKT

MMITLVSDYTLHPCAKTKSRMTFDKKTFTLSPEGEVWLKLKKRN

>EDV37696.1

MWVIPLLLLCLVLLYKYVRHHYTSWQRLGVDEEPAQIPFGIMTSVMKQEKSLGVVLRDIY

DRHTEKIVGIYMMNKRSILIRDAVLARQIMTTDFHSFHDRGVYVDEEKDPLSGNLFNLRG

ASWRNLRQKLTPSFSSGKIKGMFGTIDDVGDKLVQHLEGTLEQNEVVEIKDVMTTYAVDI

IGSVIFGLEIDSFKNPQNEFREISSSTAKDKSLLLKIHNMSMFICPPIAKLMNRLGYESR

ILTSLRDMMKRTIEFREKNNVVRKDLLQLLIRLRNTGKIGEDDDEVWDVETAQDQLKSMS

IEKIAAQAFVFYVAGSESTAAASAFTLYELSMYPELLKQAREEVDAVLKKHNLKPKDKFT

YEAVQDMKFLELCIMETIRKYPGLPFLNRECTEDYPVPGTNHVITKGTPILISLFGINRD

PIYFPNPNDYDPHRFDADIMNYNPAAYMPFGEGPRHCIALRMGKINSKVAVAKVLANFNV

IQSPRKEVEFAFDAAPVLVTKEGLKLHLTKRN

**CYP7**

>CAB57878.1

MMTISLIWGIAVLVSCCIWFIVGIRRRKAGEPPLENGLIPYLGCALKFGSNPLEFLRANQ

RKHGHVFTCKLMGKYVHFITNSLSYHKVLCHGKYFDWKKFHYTTSAKAFGHRSIDPNDGN

TTENINNTFTKTLQGDALCSLSEAMMQNLQSVMRPPGLXKSKSNAWVTEGMYAFCYRVMF

EAGYLTLFGRDISKTDTQKALILNNLDNFKQFDQVFPALVAGLPIHLFKTAHKAREKLAE

GLKHKNLCVRDQVSELIRLRMFLNDTLSTFDDMEKAKTHLAILWASQANTIPATFWSLFQ

MIRSPEAMKAASEEVSGALQSAGQELSSGGSAIYLDQVQLNDLPVLDSIIKEALRLSSAS

LNIRTAKEDFTLHLEDGSYNIRKDDMIALYPQLMHLDPEIYPDPLTFKYDRYLDESGKAK

TTFYSNGNKLKCFYMPFGSGATICPGRLFAVQEIKQFLILMLSCFELEFVESQVKCPPLD

QSRAGLGILPPLHDIEFKYKLKH

>AAC95426.1

MAGEVSAATGRFSLERLGLPGLALAAALLLLALCLLVRRTRRPGEPPLIKGWLPYLGVVL

NLRKDPLRFMKTLQKQHGDTFTVLLGGKYITFILDPFQYQLVIKNHKQLSFRVFSNKLLE

KAFSISQLQKNHDMNDELHLCYQFLQGKSLDILLESMMQNLKQVFEPQLLKTTSWDTAEL

YPFCSSIIFEITFTTIYGKVIVCDNNKFISELRDDFLKFDDKFAYLVSNIPIELLGNVKS

IREKIIKCFSSEKLAKMQGWSEVFQSRQDVLEKYYVHEDLEIGAHHLGFLWASVANTIPT

MFWAMYYLLRHPEAMAAVRDEIDHLLQSTGQKKGSGFPIHLTREQLDSLICLESSIFEAL

RLSSYSTTIRFVEEDLTLSSETGDYCVRKGDLVAIFPPVLHGDPEIFEAPEEFRYDRFIE

DGKKKTTFFKRGKKLKCYLMPFGTGTSKCPGRFFALMEIKQLLVILLTYFDLEIIDDKPI

GLNYSRLLFGIQYPDSDVLFRYKVKS

>P18125.1

MMTISLIWGIAVLVSCCIWFIVGIRRRKAGEPPLENGLIPYLGCALKFGSNPLEFLRANQ

RKHGHVFTCKLMGKYVHFITNSLSYHKVLCHGKYFDWKKFHYTTSAKAFGHRSIDPNDGN

TTENINNTFTKTLQGDALCSLSEAMMQNLQSVMRPPGLPKSKSNAWVTEGMYAFCYRVMF

EAGYLTLFGRDISKTDTQKALILNNLDNFKQFDQVFPALVAGLPIHLFKTAHKAREKLAE

GLKHKNLCVRDQVSELIRLRMFLNDTLSTFDDMEKAKTHLAILWASQANTIPATFWSLFQ

MIRSPEAMKAASEEVSGALQSAGQELSSGGSAIYLDQVQLNDLPVLDSIIKEALRLSSAS

LNIRTAKEDFTLHLEDGSYNIRKDDMIALYPQLMHLDPEIYPDPLTFKYDRYLDESGKAK

TTFYSNGNKLKCFYMPFGSGATICPGRLFAVQEIKQFLILMLSCFELEFVESQVKCPPLD

QSRAGLGILPPLHDIEFKYKLKH

>AAA41042.1

MMTISLIWGIAVLVSCCIWFIVGIRRRKAGEPPLENGLIPYLGCALKFGSNPLEFLRANQ

RKHGHVFTCKLMGKYVHFITNSLSYHKVLCHGKYFDWKKFHYTTSAKAFGHRSIDPNDGN

TTENINNTFTKTLQGDALCSLSEAMMQNLQSVMRPPGLPKSKSNAWVTEGMYAFCYRVMF

EAGYLTLFGRDISKTDTQKALILNNLDNFKQFDQVFPALVAGLPIHLFKTAHKAREKLAE

GLKHKNLCVRDQVSELIRLRMFLNDTLSTFDDMEKAKTHLAILWASQANTIPATFWSLFQ

MIRSPEAMKAASEEVSGALQSAGQELSSGGSAIYLDQVQLNDLPVLDSIIKEALRLSSAS

LNIRTAKEDFSLHLEDGSYNIRKDDMIALYPQLMHLDPEIYPDPLTFKYDRYLDESGKAK

TTFYSNGNKLKCFYMPFGSGATICPGRLFAVQEIKQFLILMLSCFELEFVESQVKCPPLD

QSRAGLGILPPLHDIEFKYKLKH

>EDL05690.1

MMSISLIWGIAVVVSCCIWFIIGIRRRKVGEPPLDNGLIPYLGCALKFGSNPLEFLRAKQ

RKHGHVFTCKLMGKYVHFITNSLSYHKVLCHGKYFDWKKFHYTTSAKAFGHRSIDPSDGN

TTENINKTFNKTLQGDALCSLSEAMMQNLQSVMRPPGLPKSKSAVWVTEGMYAFCYRVMF

EAGYLTLFGKDISKTDTQRAFIQNNLDSFKQFDQVFPALVAGVPIHLFKTAHKARERLAE

SLKHKNLYMRDQVSELIRLRMFLNDTLSTFDDMEKAKTHLVILWASQANTIPATFWSLFQ

MIRSPEAMKAASEEVNGALQSAGQELSSGGNAIYLDQEQLNNLPVLDSIIKEALRLSSAS

LNIRTAKEDFTLHLEDGSYNIRKDDIIALYPQLMHLDPEIYPDPLTFKYDRYLDESGKAK

TTFYRNGNKLKYFYMPFGSGATICPGRLFAVQEIKQFLILMLSYFELELVESHTKCPPLD

QSRAGLGILPPLNDIEFKYKLKH

>AAI30262.1

MMSISLIWGIAVVVSCCIWFIIGIRRRKVGEPPLDNGLIPYLGCALKFGSNPLEFLRAKQ

RKHGHVFTCKLMGKYVHFITNSLSYHKVLCHGKYFDWKKFHYTTSAKAFGHRSIDPSDGN

TTENINKTFNKTLQGDALCSLSEAMMQNLQSVMRPPGLPKSKSAVWVTEGMYAFCYRVMF

EAGYLTLFGKDISKTDTQRAFIQNNLDSFKQFDQVFPALVAGVPIHLFKTAHKARERLAE

SLKHKNLYMRDQVSELIRLRMFLNDTLSTFDDMEKAKTHLVILWASQANTIPATFWSLFQ

MIRSPEAMKAASEEVNGSLQSAGQELSSGGNAIYLDQEQLNNLPVLDSIIKEALRLSSAS

LNIRTAKEDFTLHLEDGSYNIRKDDIIALYPQLMHLDPEIYPDPLTFKYDRYLDESGKAK

TTFYRNGNKLKYFYMPFGSGATICPGRLFAVQEIKQFLILMLSYFELELVESHTKCPPLD

QSRAGLGILPPLNDIEFKYKLKH

>Q64505.2

MMSISLIWGIAVVVSCCIWFIIGIRRRKVGEPPLDNGLIPYLGCALKFGSNPLEFLRAKQ

RKHGHVFTCKLMGKYVHFITNSLSYHKVLCHGKYFDWKKFHYTTSAKAFGHRSIDPSDGN

TTENINKTFNKTLQGDALCSLSEAMMQNLQSVMRPPGLPKSKSAVWVTEGMYAFCYRVMF

EAGYLTLFGKDISKTDSQRAFIQNNLDSFKQFDQVFPALVAGVPIHLFKTAHKARERLAE

SLKHKNLYMRDQVSELIRLRMFLNDTLSTFDDMEKAKTHLVILWASQANTIPATFWSLFQ

MIRSPEAMKAASEEVNGALQSAGQELSSGGNAIYLDQEQLNNLPVLDSIIKEALRLSSAS

LNIRTAKEDFTLHLEDGSYNIRKDDIIALYPQLMHLDPEIYPDPLTFKYDRYLDESGKAK

TTFYRNGNKLKYFYMPFGSGATICPGRLFAVQEIKQFLILMLSYFELELVESHTKCPPLD

QSRAGLGILPPLNDIEFKYKLKH

>AAA68867.1

MMSISLIWGIAVVVSCCIWFIIGIRRRKVGEPPLDNGLIPYLGCALKFGSNPLEFLRAKQ

RKHGHVFTCKLMGKYVHFITNSLSYHKVLCHGKYFDWKKFHYTTSAKAFGHRSIDPSDGN

TTENINKTFNKTLQGDALCSLSEAMMQNLQSVMRPPGLPKSKSAVWVTEGMYAFCYRVMF

EAGYLTLFGKDISKTDTQRAFIQNNLDSFKQFDQVFPALVAGVPIHLLKTAHKARERLAE

SLKHKNLYMRDQVSELIRLRMFLNDTLSTFDDMEKAKTHLVILWASQANTIPATFWSLFQ

MIRSPEAMKAASEEVNGSLQSAGQELSSGGNAIYLDQEQLNNLPVLDSIIKEALRLSSAS

LNIRTAKEDFTLHLEDGSYNIRKDDIIALYPQLMHLDPEIYPDPLTFKYDRYLDESGKAK

TTFYRNGNKLKYFYMPFGSGATICPGRLFAVQEIKQFLILMLSYFELELVESHTKCPPLD

QSRAGLGILPPLNDIEFKYKLKH

>P46634.1

MMTISLIWGIAMVVCCCIWVIFDRRRRKAGEPPLENGLIPYLGCALKFGSNPLEFLRANQ

RKHGHVFTCKLMGKYVHFITNSLSYHKVLCHGKYFDWKKFHYTTSAKAFGHRSIDPNDGN

TTENINNTFTKTLQGDALHSLSEAMMQNLQFVLRPPDLPKSKSDAWVTEGMYAFCYRVMF

EAGYLTLFGRDTSKPDTQRVLILNNLNSFKQFDQVFPALVAGLPIHLFKAAHKAREQLAE

GLKHENLSVRDQVSELIRLRMFLNDTLSTFDDMEKAKTHLAILWASQANTIPATFWSLFQ

MIRSPDALRAASEEVNGALQSAGQKLSSEGNAIYLDQIQLNNLPVLDSIIKEALRLSSAS

LNIRTAKEDFTLHLEDGSYNIRKDDIIALYPQLMHLDPAIYPDPLTFKYDRYLDENKKAK

TSFYSNGNKLKYFYMPFGSGATICPGRLFAVQEIKQFLILMLSYFELELVESHVKCPPLD

QSRAGLGILPPLNDIEFKYKLKHL

>332213879

MMTISLIWGIAIAACCCLWLILGIRRRQTGEPPLENGLIPYLGCALQFGANPLEFLRANQ

RKHGHVFTCKLMGKYVHFITNPLSYHKVLCHGKYFDWKKFHFATSAKAFGHRSIDPRDGN

TTENINDTFIKTLQGNALNSLTESMMENLQRIMRPPVSSNSKTAAWVTEGMYSFCYRVMF

EAGYLTIFGRDLTRQDTQKAHILNNLDNFKQFDKVFPALVAGLPIHMFRTAHNAREKLAE

SLRHENLQKRESISELIRLRMFLNDTLSTFDDLEKAKTHLVVLWASQANTIPATFWSLFQ

MIRNPEAMKAATEEVKRTLENAGQKVSLEGNPICLSQTELNDLPVLDSIIKESLRLSSAS

LNIRTAKEDFTLHLEDGSYNIRKDDIIALYPQLMHLDPEIYPDPLTFKYDRYLDENGKTK

TTFYCNGLKLKYYYMPFGSGATICPGRLFAIHEIKQFLILMLSCFELELVEGQAKCPPLD

QSRAGLGILPPLNDIEFKYKFKHL

>EHH28524.1

MMTISLIWGIAIAACCCLWLILGIRRRQTGEPPLENGLIPYLGCALQFGANPLEFLRANQ

RKHGHVFTCKLMGKYVHFITNPLSYHKVLCHGKYFDWKKFHFATSAKAFGHRSIDPKDGN

TTENINNTFIKTLQGNALNSLTESMMENLQRIMRPPVFSNSKTAAWVTEGMYSFCYRVMF

EAGYLTIFGRDLTRQDTQKAHILNNLDNFKQFDKVFPALVAGLPIHMFRTAHSAREKLAE

SLRHENLQKRESVSELIRLRMFLNDTLSTFDDLEKAKTHLVVLWASQANTIPATFWSLFQ

MIRNPEAMKAATEEVKRTLENAGQKVSLEGNPICLSQTQLNDLPVLESIIKESLRLSSAS

LNIRTAKEDFTLHLEDGSYNIRKDDIIALYPQLMHLDPEIYPDPLIFKYDRYLDENGKTK

TTFYCNGLKLKYYYMPFGSGATICPGRVFAIHEIKQFLVLMLSYFELELVEGQDKCPPLD

QSRAGLGILPPLYDIEFKYKFKHL

>EHH64186.1

MMTISLIWGIAIAACCCLWLILGIRRRQTGEPPLENGLIPYLGCALQFGANPLEFLRANQ

RKHGHVFTCKLMGKYVHFITNPLSYHKVLCHGKYFDWKKFHFATSAKAFGHRSIDPKDGN

TTENINNTFIKTLQGNALNSLTESMMENLQRIMRPPVFSNSKTAAWVTEGMYSFCYRVMF

EAGYLTIFGRDLTRQDTQKAHILNNLDNFKQFDKVFPALVAGLPIHMFRTAHSAREKLAE

SLRHENLQKRESVSELIRLRMFLNDTLSTFDDLEKAKTHLVVLWASQANTIPATFWSLFQ

MIRNPEAMKAATEEVKRTLENAGQKVSLEGNPICLSQTQLNDLPVLESIIKESLRLSSAS

LNIRTAKEDFTLHLEDGSYNIRKDDIIALYPQLMHLDPEIYPDPLIFKYDRYLDENGKTK

TTFYCNGLKLKYYYMPFGSGATICPGRVFAIHEIKQFLVLMLSYFELELVEGQDKCPPLD

QSRAGLGILPPLYDIEFKYKFKHL

>402878301

MMTISLIWGIAIAACCFLWLILGIRRRQTGEPPLENGLIPYLGCALQFGANPLEFLRANQ

RKHGHVFTCKLMGKYVHFITNPLSYHKVLCHGKYFDWKKFHFATSAKAFGHRSIDPKDGN

TTENINNTFIKTLQGNALNSLTESMMENLQRIMRPPVFSNSKTAAWVTEGMYSFCYRVMF

EAGYLTIFGRDLTRQDTQKAHILNNLDNFKQFDKVFPALVAGLPIHMFRTAHSAREKLAE

SLRHENLQKRESVSELIRLRMFLNDTLSTFDDLEKAKTHLVVLWASQANTIPATFWSLFQ

MIRNPEAMKAATEEVKRTLENAGQKVSLEGNPICLSQTQLNDLPVLESIIKESLRLSSAS

LNIRTAKEDFTLHLEDGSYNIRKDDIIALYPQLMHLDPEIYPDPLTFKYDRYLDENGKTK

TTFYCNGLKLKYYYMPFGSGATICPGRVFAIHEIKQFLVLMLSYFELELVEGQAKCPPLD

QSRAGLGILPPLYDIEFKYKFKHL

>302563835

MMTISLIWGIAIAACCCLWLILGIRRRQTGEPPLENGLIPYLGCALQFGANPLEFLRANQ

RKHGHVFTCKLMGKYVHFITNPLSYHKVLCHGKYFDWKKFHFATSAKAFGHRSIDPKDGN

TTENINNTFIKTLQGNALNSLTESMMENLQRIMRPPVFSNSKTAAWVTEGMYSFCYRVMF

EAGYLTIFGRDLTRQDTQKAHILNNLDNFKQFDKVFPALVAGLPIHMFRTAHSAREKLAE

SLRHENLQKRESVSELIRLRMFLNDTLSTFDDLEKAKTHLVVLWASQANTIPATFWSLFQ

MIRNPEAMKAATEEVKRTLENAGQKVSLEGNPICLSQTQLNDLPVLESIIKESLRLSSAS

LNIRTAKEDFTLHLEEGSYNIRKDDIIALYPQLMHLDPEIYPDPLSFKYDRYLDENGKTK

TTFYCNGLKLKYYYMPFGSGATICPGRVFAIHEIKQFLVLMLSYFELELVEGQDKCPPLD

QSRAGLGILPPLYDIEFKYKFKHL

>297682932

MMTISLIWGIAIAACCCLWLILGIRRRQTGEPPLENGLIPYLGCALQFGANPLEFLRANQ

RKHGHVFTCKLMGKYVHFITNPLSYHKVLCHGKYFDWKKFHFATSAKAFGHRSIDPRDGN

TTENINDTFIKTLQGNALNSLTESMMENLQRIMRPPVSSNSKTAAWVTEGMYSFCYRVMF

EAGYLTIFGRDLTRQDTQKAHILNNLDNFKQFDKVFPALVAGLPIHMFRTAHNAREKLAE

SLRHENLQKRESISELISLRMFLNDTLSTFDDLEKAKTHLVVLWASQANTIPATFWSLFQ

MIRNPEAMKAATEEVKRTLENAGQKVTLEGNPICLSQTELNDLPVLDSIIKESLRLSSAS

LNIRTAKEDFTLHLEDGSYNIRKDDIIALYPQLMHLDPEIYPDPLTFKYDRYLDENGKTK

TTFYCNGLKLKYYYMPFGSGATICPGRLFAIHEIKQFLILMLSYFELELVEGQAKCPPLD

QSRAGLGILPPLNDIEFKYKFKHL

>114620228

MMTTSLIWGIAIAACCCLWLILGIRRRQTGEPPLENGLIPYLGCALQFGANPLEFLRANQ

RKHGHVFTCKLMGKYVHFITNPLSYHKVLCHGKYFDWKKFHFATSAKAFGHRSIDPMDGN

TTENINDTFIKTLQGHALNSLTESMMENLQRIMRPPVSSNSKTAAWVTEGMYSFCYRVMF

EAGYLTIFGRDLTRRDTQKAHILNNLDNFKQFDKVFPALVAGLPIHMFRTAHNAREKLAE

SLRHENLQKRESVSELISLRMFLNDTLSTFDDLEKAKTHLVVLWASQANTIPATFWSLFQ

MIRNPEAMKAATEEVKRTLENAGQKVSLEGNPICLSQAELNDLPVLDSIIKESLRLSSAS

LNIRTAKEDFTLHLEDGSYNIRKDDIIALYPQLMHLDPEIYPDPLTFKYDRYLDENGKTK

TTFYCNGLKLKYYYMPFGSGATICPGRLFAIHEIKQFLILMLSYFELELIEGQAKCPPLD

QSRAGLGILPPLNDIEFKYKFKHL

>397502783

MMTTSLIWGIAIAACCCLWLILGIRRRQTGEPPLENGLIPYLGCALQFGANPLEFLRANQ

RKHGHVFTCKLMGKYVHFITNPLSYHKVLCHGKYFDWKKFHFATSAKAFGHRSIDPMDGN

TTENINDTFIKTLQGHALNSLMESMMENLQRIMRPPVSSNSKTAAWVTEGMYSFCYRVMF

EAGYLTIFGRDLTRRDTQKAHILNNLDNFKQFDKVFPALVAGLPIHMFRTAHNAREKLAE

SLRHENLQKRESISELISLRMFLNDTLSTFDDLEKAKTHLVVLWASQANTIPATFWSLFQ

MIRNPEAMKAATEEVKRTLENAGQKVSLEGNPICLSQAELNDLPVLDSIIKESLRLSSAS

LNIRTAKEDFTLHLEDGSYNIRKDDIIALYPQLMHLDPEIYPDPLTFKYDRYLDENGKTK

TTFYCNGLKLKYYYMPFGSGATICPGRLFAIHEIKQFLILMLSYFELELIEGQVKCPPLD

QSRAGLGILPPLNDIEFKYKFKHL

>P22680.2

MMTTSLIWGIAIAACCCLWLILGIRRRQTGEPPLENGLIPYLGCALQFGANPLEFLRANQ

RKHGHVFTCKLMGKYVHFITNPLSYHKVLCHGKYFDWKKFHFATSAKAFGHRSIDPMDGN

TTENINDTFIKTLQGHALNSLTESMMENLQRIMRPPVSSNSKTAAWVTEGMYSFCYRVMF

EAGYLTIFGRDLTRRDTQKAHILNNLDNFKQFDKVFPALVAGLPIHMFRTAHNAREKLAE

SLRHENLQKRESISELISLRMFLNDTLSTFDDLEKAKTHLVVLWASQANTIPATFWSLFQ

MIRNPEAMKAATEEVKRTLENAGQKVSLEGNPICLSQAELNDLPVLDSIIKESLRLSSAS

LNIRTAKEDFTLHLEDGSYNIRKDDIIALYPQLMHLDPEIYPDPLTFKYDRYLDENGKTK

TTFYCNGLKLKYYYMPFGSGATICPGRLFAIHEIKQFLILMLSYFELELIEGQAKCPPLD

QSRAGLGILPPLNDIEFKYKFKHL

>403288777

MLTISLIWGIAIAACCCLWLILGIRRRQTGEPPLENGLIPYLGCALQFGANPLEFLRANQ

RKHGHVFTCKLMGKYVHFITNPLSYHKVLCHGKYFDWKKFHFATSAKAFGHGSIDPRDGN

TTENINDTFIKTLQGSALNSLTESMMENLQRILRPPVFSNSKTSAWVTEGMYSFCYRVMF

EAGYLTLFGRDLTSQDTQRARILNNLDNFKQFDKVFPALVAGLPIHMFRTAHNAREKLAE

SLRHENLKKRGNVSELVSLRMFLNDTLSTFDDLEKAKTHLVILWASQANTIPATFWSLFQ

MIRNPEAMKAATEEVKRTLENAGQKVSLEGNPICLSQTQMNDLPVLDSIIKESLRLSSAS

LNIRTAKEDFTLLLEDGSYNIRKDDIIALYPQLMHLDPEIYPDPSTFKYDRYLDENGKTK

TTFYRNGLKLKCYYMPFGSGATICPGRLFAIHEIKQFLILMLSYFELELVEGQAKCPPLD

QSRAGLGILPPLNDIEFKYKFKHL

>CAA39568.1

MMTTSLIWGIAIAACCCLWLILGIRRRQTGEPPLENGLIPYLGCALQFGANPLEFLRANQ

RKHGHVFTCKLMGKYVHFITNPLSYHKVLCHGKYFDWKKFHFATSAKAFGHRSIDPMDGN

TTENINDTFIKTLQGHALNSLTESMMENLQRIMRPPVSSNSKTAAWVTEGMYSFCYRVMF

EAGYLTIFGRDLTRRDTQKAHILNNLDNFKQFDKVFPALVAGLPIHMFRTAHNAREKLAE

SLRHENLQKRESISELISLRMFLNDTLSTFDDLEKAKTHLVVLWASQANTIPATFWSLFQ

MIRNPEAMKAATEEVKRTLENAGQKVSLEGNPICLSQAELNDLPVLNSIIKESLRLSSAS

LNIRTAKEDFTLHLEDGSYNIRKDSIIALYPQLMHLDPEIYPDPLTFKYDRYLDENGKTK

TTFYCNGLKLKYYYMPFGSGATICPGRLFAIHEIKQFLILMLSYFELELIEGQAKCPPLD

QSRAGLGILPPLNDIEFKYKFKHL

>296226538

MVTISLIWGIAIAACCCLWLILGIRRRQTGEPPLENGLIPYLGCALQFGANPLEFLRANQ

RKHGHVFTCKLMGKYVHFITNPLSYHKVLCHGKYFDWKKFHFATSAKAFGHGSIDPRDGN

TTENINNTFIKTLQGNALNSLTESMMENLQRIMRPPVFSNSKTSAWVTEGMYSFCYRVMF

EAGYLTLFGRDLTSQDTQRAHILNNLDNFKQFDKVFPALVAGLPIHMFRTAHNAREKLAE

SLRHENLKKRGNVSELVSLRMFLNDTLSTFNDLEKAKTHLVILWASQANTIPATFWSLFH

MIRNPEAMKAATEEVKRTLENAGQKVSLEGNPICLSQTQMNDLPVLDSIIKESLRLSSAS

LNIRTAKEDFTLLLEDGSYNIRKDDIIALYPQLMHLDPEIYPDPSSFKYDRYLDENGKTK

TTFYCNGLKLKCHYMPFGSGATICPGRLFAIHEIKQFLILMLSYFELELVEGQAKCPPLD

QSRAGLGILPPLNDIEFKYKFKHL

>149721382

MLTVSLIWGIVIAVGCCLWLILGRRRRQMGEPPLENGLIPYLGCALQFGANPLEFLRANQ

RKHGHVFTCKLMGNYVHFITNPLSYHKVLCHGKYLDWKKFHFTTSAKAFGHSSIDPSDGN

TTENISKTFIKTLQGDALNSLTEAMMENLQLVIRAPVVPKSQMPAWVTEGMYSFCYRVMF

EAGYLTLFGRDLTGQDTQKALILNNLDNFKQFDKIFPALVAGLPIHVFKTAHHAREKLAE

GLRHQTLGKRDHISELVSFMNDTLSTLDDMEKAKTHLAVLWASQANTIPATFWSLFQMIR

SPEAMKAATEEVNKTLENAGQKVSFEGGPICLNQMQLNDMPVLDSIIKESLRLSSASLNI

RTAKEDFTLHLQDGSYHIRKDDIIALYPQLMHLDPEIYPDPLAFKYDRYLDENGKTKTTF

YSNGLKLKYYYMPFGSGATICPGRLFAVQEIKQFLILMLSYFELELVESHVKCPPLDQSR

AGLGILPPLNDIEFKYKFKHL

>57107891

MMTISLIWGIVVAVCCCLWLIFGMRRRQMGEPPLENGLIPYLGCALKFGANPLEFLRANQ

RKYGHVFTCRLMGNYVHFITNPLSYQKVLCHGKYLDWKKFHFTTSAKAFGHRSIDPSDGN

TTENINKTFIKTLQGDALNSLTEAMMENLQLVMRCPLVSKSKTPAWVTEGMYSFCYRVMF

EAGYLTLFGKDLTGQDAQKGLILNNLDHFKEFDKIFPALVAGLPIHVFKTAHHAREKLAE

GLRHENLGKRDHISELVRFLNDMLSTLDDMDKAKTHLAILWASQANTIPATFWSLFQMIR

SPEAMKAATEEVNKTLENAGQKISLDGSPICLNQMQLNDMPVLDSLIKESLRLSSASLNI

RTATEDFTLHLQDSSYNIRKDDIIAFYPQLVHLDPEIYPDPLTFKYDRYLDENGKTKTTF

YSNGIKLKYYYMPFGSGATMCPGRLFAVQEIKQFLILMLSYFELELVESQVKCPPLDQSR

AGLGILPPLNDIEFKYKFKHL

>348557311

MPIYVVWGIALAVCCCLWIIFGVRRRKIGEPPLENGLIPYLGCALKFGANPLEFLRANQR

KHGHVFTCKLMGKYVHFITNSLSYHKVLCHGKYFDWKKFHYTTSAKAFGHRSIDPSDGNT

TENIQSTFSKTLQGDALAALTQAMMENLQVTLRPQGAGPLTAAWVTEGMYAFCYRVMFEA

GYLTLFGRDLARQDVQRGLIRSSLDHFKEFDRVFPALVAGLPIRAFKTAHRAREKLAEGL

GHERLRRRARVSELISLRMHLNDTLSTFDDAEKARTHLALLWASQANTIPATFWSLFQMI

RSPEAMEAASEEVQKALEKAGQQLSFEGKPVHLSQMQLNDLPVLDSIIKEALRLSSASLN

IRTAKEDFTLHLEDGSYNIRKDDIIALYPQLMHLDPEIYPDPLTFKYDRYLDENRKTKTT

FYSNGIKLKYYYMPFGSGATICPGRLFAVQEIKQFLILMLLYFELELVESHVKCPPLDQS

RAGLGILPPLHDIEFKYKFKYVSTCG

>395841807

MMTMSLIWGIVVAVCCCLWLILGIRRRRKGEPPLENGWIPYLGCALRFGANPLEFLRTNQ

RKHGHVFTCRLMGKYVHFITNPLSYHKVLCHGKYFDWKKFHFTTSAKAFGHRSIDPSDGH

TSENINDTFVRTLQGDALNSLTEAMMENLQLIMRPPAPSESQLAAWVTEGMYSFCYRVMF

EAGYLTLFGRDLTRQDSRAHMLNNLDNFKQFDKVFPALVAGLPIHLFQMAHRAREELATS

LKHEKLRERDRVSELVSLRMLLNDTLSTFNDLEKGKTHLAILWASQANTIPATFWSLFQM

IRNPEAMKAATEEVNRTLEKAGQKISLQGKPICLNQMQLNDLPVLDSIIKESLRLSSASL

NIRTAKEDFILSIEDGSYNIRKDDIIALYPQLMHLDPDIYPDPLTFKYDRYLDENGKTKT

TFYSNGLKLKYYYMPFGSGATICPGRLFAVYEIKQFLILMLSYFELELVESHVKCPPLDQ

SRAGLGILPPLNDIKFKYKFKHL

>P51542.1

MITIFWIWGICLSVCCCLWLILGLRRRRMGEPPLEKGWIPYLGCALQFGANPLDFLRANQ

RKYGHVFTCKLMGKYVHFITNSLSYHKVLCHGKYFDWKKFHFTTSAKAFGHRSIDPRDGN

TTENINNTFNKTLQGDALISLTDAMMENLQLTLRRPEPKSRAWVTEGMYSFCYRVMFEAG

YLTLFGRELTRQDAQRAFILNSLEDFKQFDKVFPALVAGLPIHIFMTAHNAREKLAEGLK

HDNLRTRDHISELIRLRMFLNDTLSTFDAMEKAKTHLAILWASQANTIPATFWSLFHMMR

SSEALKAATEEVNKALEDADQQINFEGKPIHLNQTQLNDMPVLDSIIKESLRLSSASLNI

RTAKEDFTLHLEDGSYNIRKDDIIALYPQLMHLDPEIYPDPMTFKYDRYLDENRKTKTTF

YSKGLKLKYYYMPFGSGATICPGRLFAIQEIKQFLILMLSYFELEFVDSHVKCPPLDQSR

AGLGILPPLNDIEFKYKFKHL

>301765926

MMTISLIWGIVVAVCCCLWLILGMRRRQMGEPPLENGLIPYLGCALKFGANPLEFLRANQ

RKHGHVFTCRLMGNYVHFITNPLSYQKVLCHGKYLDWKKFHFATSAKAFGHRSIDPSDGN

TTDNISKTFVKTLQGDGLNSLTEAMMENLQLVMRRPLVSKSKVPAWVTEGMYSFCYRVMF

EAGYLTLFGKDLTGQDAQKRHILNNLDHFKEFDKIFPALVAGLPIHVFKTAHHAREKLAE

GLRHENLGKRDHISELVRFLNDMLSTLDDMDKAKTHLAILWASQANTIPATFWSLFQMIR

SPEAMKAATEEVNKTLENAGQKISFDGSPICLNQMQLNAMPVLDSIIKESLRLSSASLNI

RTAKEDFTLHLQDSSYNIRKDDIIALYPQLMHLDPEIYPDPLTFKYDRYLDENRKTKTTF

YSNGIKLKYYYMPFGSGATICPGRIFAVQEIKQFLILMLSYFELELVESQVKCPPLNQSR

AGLGILPPLNDIEFKYKFKHL

>3DAXA

MAKKTSSRRRQTGEPPLENGLIPYLGCALQFGANPLEFLRANQRKHGHVFTCKLMGKYVH

FITNPLSYHKVLCHGKYFDWKKFHFATSAKAFGHRSIDPMDGNTTENINDTFIKTLQGHA

LNSLTESMMENLQRIMRPPVSSNSKTAAWVTEGMYSFCYRVMFEAGYLTIFGRDLTRRDT

QKAHILNNLDNFKQFDKVFPALVAGLPIHMFRTAHNAREKLAESLRHENLQKRESISELI

SLRMFLNDTLSTFDDLEKAKTHLVVLWASQANTIPATFWSLFQMIRNPEAMKAATEEVKR

TLENAGQKVSLEGNPICLSQAELNDLPVLDSIIKESLRLSSASLNIRTAKEDFTLHLEDG

SYNIRKDDIIALYPQLMHLDPEIYPDPLTFKYDRYLDENGKTKTTFYCNGLKLKYYYMPF

GSGATICPGRLFAIHEIKQFLILMLSYFELELIEGQAKCPPLDQSRAGLGILPPLNDIEF

KYKFKHHHHHH

>3SN5A

MAKKTSSRRRQTGEPPLENGLIPYLGCALQFGANPLEFLRANQRKHGHVFTCKLMGKYVH

FITNPLSYHKVLCHGKYFDWKKFHFALSAKAFGHRSIDPMDGNTTENINDTFIKTLQGHA

LNSLTESMMENLQRIMRPPVSSNSKTAAWVTEGMYSFCYRVMFEAGYLTIFGRDLTRRDT

QKAHILNNLDNFKQFDKVFPALVAGLPIHMFRTAHNAREKLAESLRHENLQKRESISELI

SLRMFLNDTLSTFDDLEKAKTHLVVLWASQANTIPATFWSLFQMIRNPEAMKAATEEVKR

TLENAGQKVSLEGNPICLSQAELNDLPVLDSIIKESLRLSSASLNIRTAKEDFTLHLEDG

SYNIRKDDIIALYPQLMHLDPEIYPDPLTFKYDRYLDENGKTKTTFYCNGLKLKYYYMPF

GSGATICPGRLFAIHEIKQFLILMLSYFELELIEGQAKCPPLDQSRAGLGILPPLNDIEF

KYKFKHHHHHH

>O46491.1

MMSISLLGGIVTAVCCCLWLLLGMRRRQTGEPPLENGIIPYLGCALQFGANPLEFLRANQ

RKHGHIFTCQLMGNYVHFITNPLSYHKVLCHGKYLDWKKFHFTASAKAFGHRSIDPSDGN

TTDNINKTIIKTLQGDALNLLAAAMMENLQLVLRPQVAPQPEKPAWVTEGMYSFCYRVMF

EAGYVTLFGKDPIGHDAQKALILNNLDNFKQFDKIFPALVAGFPIHVFKTGHYAREKLAE

GLRLQKLRKRDHISELVRFLNDTLSTLDDAEKAKSLLAVLWASQANTIPATFWCLFQTIR

SPEAMKAASEEVNKTLEKAGQKISLDDKPIYLNQIELDSMPVLDSIIKESLRLSSASLNI

RTAKEDFTLHLQDGSYNIRKDDIIALYPQLMHLDPEIYPDPLTFKYDRYLDENGKTKTTF

YSHGLKLKYYYMPFGSGATICPGRLFAVQEIKQFLILMLSYFDLELVESHVKCPPLDQSR

AGLGILPPSNDIEFRYKLKHL

>DAA22744.1

MMSLSLIWGIVIAVCCCLYLLGMRRRQMGEPPLENGLIPYLGCALQFGANPLEFLRANQR

KHGHVFTCRLMGNYVHFITNPLSYHKVLCHGKYFDWKKFHFTASAKAFGHRSIDPSDGNT

TDTISKTIIKTLQGDALSSLTEAMMGNLQLVLRPQGPPQPPTPTWVTEGMYSFCYRVMFE

AGYLTLFGRDLAGQDAQKALILNSLDNFKQFDKIFPALVAGFPIHVFKTGHYAREKLTEG

LRLQKFRERDHISELVRFLNDTFATLDDTERAKSLLAVLWASQANTIPATFWSLFQMIRN

PEAMKAATEEVNKTLENAGQKVSFEDSPIHLNQTQLDNMPVLDSIIKESLRLSSASLNIR

TAKEDFTLHLQDGSYNIRKDDIIALYPQLMHLDPEIYPDPLTFKYDRYLDENGKTKTTFY

SNGLKLKYYYMPFGSGVTICPGRLFAVQEIKQFLILMLSYFELELVESCVKCPPLDQSRA

GLGILPPLYDTEFRYKFKHS

>344296700

MTISLLWGIVVAVCCCLWLILGIRRRQMGEPPLENGLIPYLGCALQFGANPLEFLRANQR

KHGHVFTCKLMGKYVHFITNPLSYHKVLCHGKHFDWRKFHFNTSAKVFGHRSVDPGDGHT

TENINKTFIKTLQGDALNALAEAMMENLLLVLRPSVLPKPKMAGWVKEGMYSFCYRVMFE

AGYLTLFGKDLTRQDSQRALMLSNLDNFKQFDKIFPALAAGLPIRMFKSAHRAREKLAEG

LRHENLRRRDGVSDLVSLRMLLNDQLSTFDDTEKAKTHLVLLWASQANTIPATFWSLFQT

IRSPEAMKAATEEVNRTLENAGQKIIPEGSPICLNQMQLNDMPVLDSIIKESLRLSSASL

SIRAAKEDFTLHIQDGSFNIRKDDFIAFYPQLIHLDPGIYPDPLTFKYDRYLDENGKAKT

NFYCNGLKLNYYYMPFGSGATICPGRLFAVHEIKQFLVLMLSYFELELVERHVKCPPLDQ

SRAGLGILPPLNDVEFKYKFKYL

>126321090

MLTISLIWGIVVAICCCLWLITGIRRRRMGEPPLENGLIPYVGCALQFGANPLEFLRINK

RKYGHIFTCKLMGKYVHFITNPFSYTTVMRHGKYFDSKKFNLAASAKAFGHRSIDPSDGN

TTENVHETFIRTLQGDALNSLSEAMMENLQYVMRPPGLSKTNPDSWVTEGMYSFCNKVMF

EAGYLTLFGKDLTRQEAQRTSILNNLDNFKQFDKIFPALVAGLPIHVFKNAHNAREKLAE

DLKHENLQKRDNISELITTRMFLNDTLSTFDDMEKAKTHLALLWAAQANTLPATFWCLFH

TIRSSEAMKMATEEVRKTLEKSGQKISFEGKPISLSQMQLNDMPVLDSIIKEALRLSSAS

LNIRPAKEDFTLHLEEGSYNIRKDDIIAIYPQLLHLDPEIYPDPLTFKYDRYLDENGKPK

NSFYCNGIKLKYYYMPFGWGLTMCPGRLFAVSEIKQFLILMLSYFEMQLVDSQVKCPPLD

QSRVGLGILPPTNDIEFKYRLKYL

>395511109

MLTISISLIWGFVVAVCCCLWLIIGIRRRRLGEPPLDNGLIPYVGCALQFGANPLEFLRT

KKRKYGHIFTCKLMGKYVHFITNPFSYNTVLRHGKYFDWKKINYATSAKAFGHRSIDPSD

GNTTENVHETLIKTLQGDALNSLTEAMMENLQYVMKPSVLSKTNPDSWVTEGMCSFCYRV

MFEAGYLTLFGKDLTRQEVQRTFILNSLNNFKQFDKIFPALVAGLPIHVFKNAHNAREKL

AETLRHENLQKRDNISELITTRMFLNDTLSTFDDMEKAKTHLALLWAAQANTLPATFWCL

FHTIRSPEAMKTATEEVRKTLENSGQKISFEGKPISLSQMQLNDMPVLDSIIKEALRLCS

ASLNIRAAKEDFTLHLEEGSYSIRKDDIIAFYPQLLHFDPEIYPDPLVFKYDRYLDENGK

PKTNFYYNGIKLKYYYMPFGSGLSLCPGRLFAVHEIKQFLILMLSYFEMKLVDSQVKYPP

LDQSRLGLGILPPTNDIDFKYKLKHL

>149411112

MMTMSLIWGVLVAVCCCLWLITGIRRRHPGEPPLDNGPLPYLGCALQFGANPLKFLRAKQ

KKVGNIFTCKIVGKYVHFLTNPFSFHTVMRHGKHFDWKKFHFTTAAKVFGHRSIDPNDGN

TTENMHQTFIKTLQGDALHSLTENMMENLQHVLKPSVPPQENPAPWVTDGLYSFCYRVMF

EAGYLTLFGKELNSNKDLQKQKAQRAIYLENFKEFDKIFPALVAGLPIHVFKSAYNAREK

LAEALLHKNLQKRDKISELVTLRMFLNDTQSTFDDMEKAKTHLAVLWAAQANTIPATFWS

LFQTIRSPEALKAAVEEVHNVLKNAGQKVSFDGKPIVLTQAQLNDMPVLDSIIKESIRLS

SASMNVRAAKEDFILHLEDGSYSIRKDDVIALYPPLVHLDPEIYPDPLTFKYNRYLDENG

KTKTNFYCNGLKLKYYYLPFGSGLTKCPGRIFAIYEIKEFLILMLSYFELELVDRQPRCP

PLDQSRAGLGILPPTNDIDFRYRLKCL

>ACO25495.1

MFISSWIWGIVIILCCTFWFLLGRRRRQKGEPPLENGFLPYLGCALQFGANPLEFLREKQ

KKHGHIFTCRVAGKYIHFLTDPFSYHSLIRHGKHLDWKKFHFATSAKAFGHGSIDPAEGN

TTENFHQTFIRTLQGNALDDLIKAMMENLQDVMLQSRTSKLQFNAWITEGLYTFCCQVMF

ESGFLTLFGKEFNSNHDKNLSKQETERAHILNALENFKEFDRIFPALVAGLPIHLFKSAH

SAREKLGEALLHKNLLKRNNLSELVTLRMFLNDTLSTFDDMEKAKTHVAVLWASQANTIP

ATFWSLFYLLKNPEAMRAATKEVQSILESAGEKISLDGKHISLNRKQLDNMPVLDSIIKE

AMRLSSASMTFRVAKEDFTLHLENDFYNIRKDDIVALYPQLLHFDPEIYADPLTFKYDRY

LNENGEEKTDFYRNGRKLKYYYMPFGTGIAKCPGRLFAVHEIKQFLVLVFSYFEIELVDN

NVQCPSLDQSRAGLGILQPSNDIDFRYRLKCL

>AFN07713.1

MFIAPWIWGIVIILCCSFWFLLGRRRRQKGEPPLENGFLPYLGCALQFGANPLEFLKEKQ

KKHGHIFTCRVAGKYIHFLTDPFSYHSLIRHGKHLDWKKFHFATSAKAFGHGSIDSAEGN

TTENFHQTFIRTLQGNALDDLIKAMMENLQDVMLQSRTSEPQFNAWTTEGLYKFCCQVMF

ESGFLTLFGKEFNSNHEKNLSKQETERAHILNALENFKEFDRIFPALVAGLPIHLFKSAH

SAREKLGEALLHKNLLKRNNLSELVTLRMFLNDTLSTFDDMEKAKTHVAVLWASQANTIP

ATFWSLFYLLKNPEAMRAAIKEVQSILESAGEKISLDGKHISLNRKQLDNMPVLDSIIKE

AMRLSSASMTFRVAKEDFTLHLENDFYNIRKDDIVALYPQLLHFDPEIYADPLTFKYDRY

LNENGEEKTDFYRNGRKLKYYFMPFGTGIAKCPGRLFAVHEIKQFLVLIFSYFEIELVDN

NVQCPSLDQSRAGLGILQPSNDIDFRYRLKCL

>AAW33560.1

MITTSWIWGTVIIVCCSFWFLFGRRRRRRQGEPPLENGFLPYLGCALQFGANPLKFLREK

QKKHGHIFTCQVAGKYIHFLTDPFSYHSLIRQGKYLDWKKFHFATSAKAFGHGSIDPAEG

NTTENFHHTFIRTLQGNALDALIKAMMENLQYVMLQSRASKFQPNTWVTEGLYTFCCQVM

FESGFLTLFGKEFNSNHDKNLSKRETERARILNALENFKEFDKIFPALVAGLPIHLFKSA

HSAREKLGEALLHKNLLKRDNLSELVMLRMFLNDTLSTFDDMEKAKTHVAVLWASQANTI

PATFWSLFTFLRNPEAMRAATKEVQSVLESAGEKISLDGNYISLNRKQLDNMPVLDSIIK

EAMRLSSASMTFRVAKEDFTLHLENSFYNIRKDDIVALYPQLLHFDPEIYADPLTFKYDR

YLNENKEEKTDFYRNGRKLKYYYMPFGAGIAKCPGRLFAVHEIKQFLVLIFSYFEIDLVD

SNVQCPSLDQSRAGLGILQPSNDIDFRYRLKCL

>326917662

MFITPWIWGTVIIVCCTFWFLFGRRRRRRQGEPPLENGFLPYLGCALQFGANPLEFLREK

QKKHGHIFTCQVAGKYIHFLTDPFSYNSLMRQGKHLDWKKFHFATSAKAFGHGSIDPAEG

NTTENFHHTFIRTLQGNALDALIEAMMENLQYVMLQSRASKFQPNTWVTEGLYTFCCQVM

FESGFLTLFGKEFNPNHDKNLSKRETERARILNALENFKEFDKIFPALVAGLPIHLFKSA

HSAREKLGEALLHKNLLKRDNLSELVMLRMFLNDTLSTFDDMEKAKTHVAVLWASQANTI

PATFWSLFYLLKNPEAMRAATKEVQSILESAGEEIRLDGNYISLNRKQLDNMPVLDSIIK

EAMRLSSASMTFRVAKEDFTLHLENSFYNIRKDDIVALYPQLLHFDPEIYADPLTFKYDR

YLNENKEEKTDFYRNGRKLKYYYMPFGAGIAKCPGRLFAVHEIKQFLVLIFSYFEIDLVD

SNVQCPSLDQSRAGLGILQPSNDIDFRYRLKCL

>224046284

MFLASWIWGMAVILCCVFWFLLGRRRRERQGEPPLENGFLPYLGCALKFGANPLEFLKEK

QKKHGHIFTCHVAGKYIHFLTDPFSYHALMRQGKHLDWKKFHFATSAKAFGHGSIDPAEG

NTTENFHQTFIRTLQGNALDALIEAMMENLQYVMLQSRAPKLQSNTWVTEGLYTFCCQVM

FESGFLTLFGKEFNSNNDKNPSSKQETERAHILNALENFKEFDKIFPALVAGLPIHLFKS

AHSAREKLGEALLHKNLLKRDNLSELVTLRMFLNDTLSTFDDMEKAKTHVAVLWASQANT

IPATFWTLFYLLKSPEAMKAATKEVQSVLETDEESIGLDGKHISLNRKQLDNMPILDSII

KEAMRLSSASMTFRVAKEDFTLHLENDFYNIRKDDIVALYPQLLHFDPEIYADPLTFKYD

RFLNEKGEEKTDFYRNGRKLKYYYMPFGTGIAKCPGRLFAVHEIKQFLALIFSYFEIELV

DSNVKCPSLDQSRAGLGILQPSNDIDFRYRMKCL

>BAD01520.1

MFIAPWIWGTVIIVCCSFWFLFGRRRRRRQGEPPLENGFLPYLGCALQFGANPLKFLREK

QKKHGHIFTCQVAGKYIHFLTDPFSYHSLIRQGKHLDWKKFHFATSAKAFGHGSIDPAEG

NTTENFHHTFIRTLQGNALDALIKAMMENLQYVMLQSRASKFQPNTWVTEGLYTFCCQVM

FESGFLTLFGKEFNSNHDKNLSKRETERARILNALENFKEFDKIFPALVAGLPIHLFKSA

HSAREKLGEALLHKNLLKRDNLSELVMLRMFLNDTLSTFDDMEKAKTHVAVLWASQANTI

PATFWSLFYLLKNPEAMRAATKEVQSVLESAGEKISLDGNYISLNRKQLDNMPVLDSIIK

EAMRLSSASMTFRVAKEDFTLHLENSFYNIRKDDIVALYPQLLHFDPEIYADPLTLKYDR

YLNENKEEKTDFYRNGRKLKYYYMPFGAGIAKCPGRLFAVHEIKQFLVLIFSYFEIDLVD

SNVQCPSLDQSRAGLGILQPSNDIDFRYRLKCL

>AAH54571.1

MILTISFIWAIVVGLCCCLWLITGIRRRHPAEPPLENGWIPFLGCALQFGANPLEFLRSR

QKKHGHIFTCKIAGQYVHFLCDPFSYHAVIRQGRHLDWKKFHFDASAKAFGHESMDPSQG

YTTENLHQTFLKTLQGDALSSLIETMMENLQGTMLQSGMLKATTSEWQSDGIYAFCYKVM

FEAGYLTLFGKELDGDQSIARQQAQKALVLNALDNFKEFDKIFPALIAGLPIHVFKSAYS

AREKLAKTMLHENLSRRANVSDLISLRMLLNDTLSTFNELSKARTHVAILWASQANTLPA

TFWTLFHMIRCPAAMKAASEEVRQTFESSNQKVDPTNSRLVLTREQLDNMPVLDSIIKEA

MRLSSASLNVRMAKSDFLLQLDNKESYHIRKDDVIAMYPPMIHFDPEIYDDPLEFKYDRY

IDEKGQEKTAFYRNGRKLRYYYMPFGSGVTKCPGRFFAVHEIKQFLSLLLSYFEMELLDS

DVKEPPLDQSRAGLGVLQPTYDVDFRYRLKSL

>EHB05065.1

MLLFMAYFWNKEKAFGHRSIDPSDGNTTENINSTFNKTLQGKALDALTEAMMENLQFILR

PPVPPTSKSAAWVTEGMYSFCYRVMFEAGYLTLFGRDLTMQDTQKALILNNLDNFKQFDK

VFPALAAGLPIRAFKTAHKAREKLAEDLRHDRLRVRASVSELIRLRMHLNDTLSTFDDAE

KAKTHLAVLWASQANTIPATFWSLFQMIRSPEAMKAAAEEVNRALESAGHQLSVEGNPIH

LNQMQLNDLPVLDSIIKEALRLSSASLNIRTAKEDFTLHLEDGSYNIRKDDIIALYPQLM

HLDPEIYPDPLTFKYDRYLDENKKTKTTFYSNGIKLKYYYMPFGSGATICPGRLFAVQEI

KQFLILMLLYFELELVESQAKCPPLDQSRVGLGILPPLNDIEFKYKLKHVSMCG

>301613314

MLTVSLIWGLVVALCCFFWLIVGIRRRQPGEPPLENGLIPYLGCALQFGANPLEFLRVRQ

NKFGNVFTCKIAGQFVHFVTDPFSFNSVMRHGRHFDWQKFHFATSAKAFGHSNIDSSDSE

VTQNVHDSFLKTLQGDALDPLISNMMENLQHTMLQNSSYKVNSKDWVTEGLYAFCYRVMF

EAGYLTLFGKEFNSPEDKNLARQEAQRALILNAIENFKEFDKIFPALVAGLPIHVFKSAY

SARENLAKDLLHENLRKRNNISELISLRMFLNESMTSLNDMEKAKTHLALLWASQANTLP

ATFWSVFYLLRCPHAMKASTEEVQRVLEKASQKVNCDGRYIFLNRHELDDMPVLDSIIKE

AMRLSSASLNIRVAKENFVLHIDDKQAFNIRKDDIVALYPQMVHLNPDIYEDPNNFKYDR

YLGEDGKEKTSFFLNGRKLKYYYMPFGSGKTKCPGRQFAVHEIKQMLTLIICYFDMELVD

KNIRSPPLDQSRAGLGILQPTHDVDFRYKLKAH

>327269709

MLALLFLWGAVVMLCCSCCFFLRRRRRPNEPPIENGILPYLGCALQFGANPFQFLRAREK

KYGPIFTCRLAGKYVHFLTDSFSYHAVMQHGRHLDWKKFHFATSAKAFGHGSIDPSDGNT

TENMQQTFTRTLQGNALTYLSEAMMENLHYVMVKSREAKVSSNDWVTDKLYEFCCRVMFE

SGFLTLFGTEFNSNYGKILSSIQENQRASILHALENFKAFDQIFPALVAGLPIHLFKSAH

SAREKLAETLLHENLKKRENISELISLRMFLNDTLSTFNDKEKAKTHLAVLWASQANTIP

ATFWTLFHLLRNPKALEAATEEVKRILQDVSGNIGSSGNPIHLNRKQLDDMPVLDSIIKE

AIRLSSASMTVRVAKEDFTLQLGSSSYNIRKDDVVALYPQLLHFDPEIYSDPMTFKYDRY

LNENGEEKTVFYRNGRRLKYYYMPFGTGLAKCPGRLFAVHEIKQFLTLLLSYFEVELTNS

NVTCPSLDQSRAGLGILQPTSDIDFKYRFKVL

>EGV95812.1

MMQNLQFVLRPPDLPKSKRDAWVTEGMYTFCYRVMFEAGYLTLFGRDTSKPDTQRALILN

NLDNFKQFDQIFPALVAGLPIHLFKTAHKAREQLAEGLKHENLSVRDQVSELIRLRMFLN

DTLSTFDDMEKAKTHLAILWASQANTIPATFWSLFQMIRSPEALRAASEEVNGALQSAGQ

KLSSEGNAIYLDQIQLNSLPVLDSIIKEALRLSSASLNIRTAKEDFTLHLEDGSYNIRKD

DIIALYPQLMHLDPEIYPDPLTFKYDRYLDENKKAKTSFYSNGNKLKYFYMPFGSGATIC

PGRLFAVQEIKQFLILMLSYFELGLVESHVKCPPLDQSRAGLGILPPLNDIEFKYKLKHL

>348538597

MIISIALIWAVVVGFCCLLWLAVGIRHRQPGEPAVENGLIPYLGCALQFGANPLQFLRSR

QKKYGHIFTCKIAGKYIHFLCDPFSYHSVIRQGRHLDWRKFHFATSVKAFGHDSFDPRHG

HTTENLHQTFLKTLQGEALPSLIETMMGHLQDVMLKSDTLSPSRDHWQVDGIFAFCYKVM

FESGYLTLFGKELGEDKCQARQEAQKALVLNALENFKEFDKIFPALVAGLPIHVFKSAYS

ARENLAKTMHAEHLSKRENVSDLISMRMILNDSLSTFNDISKARTHVALLWASQANTLPA

TFWSLFYMIRSPDAMKAAHEEVQKILGDSGKTVDPSDPSLKLTRDQLDNMPVLDSIIKEA

MRLSSASMNVRVAKEDFLLHLDNQEAYHIRKDDVIALYPPLLHYDPEIYEDPYEYKFDRF

LDNKGQEKTTFYRSGRRLRYFYMPFGSGVTKCPGRFFAVYEIKQFLTLVLSYFDMELLDP

GIKAPPLDQSRAGLGILQPTYDIDFRYKLKSSQ

>125822555

MSTFLFTVAAILLTLFIVWICGHTRKRKPGEPPLVTGWLPFIGVTKDYVSNPLGFLTETQ

KKHGNVFTCLIAGKYFTFVTDPFSFSSVVRQGKNLDFQKFAINFSQRVFGHADFNSPQLS

GNYREIHSIFRQTLQGPSLQHLTQSMLCNLQTVLERCLPHEHDWLEEGLQSFTNRIMFEA

GFLTLFGKEDIGFLQDHGMSGNRISMPKATQDFLVFDHAFPLLAAGVPIGLCTKAWRARE

ALAEELLHDELHRRKCISDLIQRRMDIFDRMLLDETGKARTHVCMLWASQANTLPAAFWS

LYYTLKSVDAFTAARTEVNRVLLGQSLNNKDQTVYLSKDQLDSMTVLESIIEEALRLSSA

SMMIRVASDDFTLTLESDQTAAIRKGDYIALYPQLLHLDPEIYPNPTEFKYNRFLDENDQ

PKTNFFKSGRRLRNFLMPFGSGASECPGRFFAVYEIKLFLALTLWHYDLQLRDPDVPVVQ

DSARAGLGIMPPSQDVLLRFRKKQRTPGNEHKDTK

>O75881.2

MAGEVSAATGRFSLERLGLPGLALAAALLLLALCLLVRRTRRPGEPPLIKGWLPYLGVVL

NLRKDPLRFMKTLQKQHGDTFTVLLGGKYITFILDPFQYQLVIKNHKQLSFRVFSNKLLE

KAFSISQLQKNHDMNDELHLCYQFLQGKSLDILLESMMQNLKQVFEPQLLKTTSWDTAEL

YPFCSSIIFEITFTTIYGKVIVCDNNKFISELRDDFLKFDDKFAYLVSNIPIELLGNVKS

IREKIIKCFSSEKLAKMQGWSEVFQSRQDVLEKYYVHEDLEIGAHHLGFLWASVANTIPT

MFWAMYYLLRHPEAMAAVRDEIDRLLQSTGQKKGSGFPIHLTREQLDSLICLESSIFEAL

RLSSYSTTIRFVEEDLTLSSETGDYCVRKGDLVAIFPPVLHGDPEIFEAPEEFRYDRFIE

DGKKKTTFFKRGKKLKCYLMPFGTGTSKCPGRFFALMEIKQLLVILLTYFDLEIIDDKPI

GLNYSRLLFGIQYPDSDVLFRYKVKS

>114620308

MAGEVSAATGRFSLERLGLPGLALAAALLLLALCLLVRRTRRPGEPPLIKGWLPYLGVVL

NLRKDPLRFMKTLQKQHGDTFTVLLGGKYITFILDPFQYQLVIKNHKQLSFRVFSNKLLE

KAFSISQLQKNHDMNDELHLCYQFLQGKSLDILLESMMQNLKQVFEPQLLKTTSWDMAQL

YPFCSSIIFEITFTTIYGKVIVCDNNKFISELRDDFLKFDDKFAYLVSNIPIELLGNVKS

IREKIIKCFSSEKLAKMQGWSEVFQSRQDVLEKYYVHEDLEIGAHHLGFLWASVANTIPT

MFWAMYYLLRHPEAMAAVRDEIDRLLQSTGQKKGSGFPIHLTREQLDSLICLESSIFEAL

RLSSYSTTIRFVEEDLTLSSETGDYCVRKGDLVAIFPPILHGDPEIFEAPEEFRYDRFIE

DGKKKTTFFKRGKKLKCYLMPFGTGTSKCPGRFFALMEIKQLLVILLTYFDLEIIDDKPI

GLNYSRLLFGIQYPDSDVLFRYKVKS

>397522769

MAGEVSAATGRFSLERLGLPGLALAAALLLLALCLLVRRTRRPGEPPLIKGWLPYLGVVL

NLRKDPLRFMKTLQKQHGDTFTVLLGGKYITFILDPFQYQLVIKNHKQLSFRVFSNKLLE

KAFSISQLQKNHDMNDELHLCYQFLQGKSLDILLESMMQNLKQVFEPQLLKTTSWDMAQL

YPFCSSIIFEITFTTIYGKVIVCDNNKFISELRDDFLKFDDKFAYLVSNIPIELLGNVKS

IREKIIKCFSSEKLAKMQGWSEVFQSRQDVLEKYYVHEDLEIGAHHLGFLWASVANTIPT

MFWAMYYLLRHPEAMAAVRDEIDRLLQSTGQKKGSGFPIHLTREQLDSLICLESSIFEAL

RLSSYSTTIRFVEEDLTLSSETGDYCVRKGDLVAIFPPILHGDPEIFEAPEEFRYDRFIE

DGKKKTTFFKRGKKLKCYLMPFGTGTSKCPGRFFALMEIKQLLVILLTYFDLEIIDDKPI

GLNYSRLLFGIQYPDSDVLFRYKVKS

>332238429

MAGEVSAATGRFSLEQLGLPGPALAAALLLLALCLLVRRTRRPGEPPLIKGWLPYLGVAL

NLRKDPLSFMKTLQKQHGDTFTVLLGGKYITFILDPFQYQLVIKNHKQLSFRVFSNKLLE

KAFSISQLQKNHDMNDELHLCYQFLQGKSLDILLESMMQNLKQVFESQLLKTTSWDTAQL

YPFCSSIIFEITFTTIYGKVIVCDSNKFISELRDDFLKFDDKFAYLVSNIPIELLGNVKS

IRKKIIKCFSSENLAKMQGWSEVFQSRQDVLEKYYVHEDLEIGAHHLGFLWASVANTIPT

MFWAMYYLLRHPEAMAAVRDEIDRLLQSTGQKKGSGFPIHLTREQLDSLICLESTIFEAL

RLSSYSTTMRFVEEDLTLSAETGDYRVRKGDLVAIFPPILHGDPEIFEAPEEFRYDRFIE

DGKKKTTFFKRGKKLKCYLMPFGTGTSKCPGRFFALMEIKQLLVILLTYFDLEIIDDKPI

GLNYSRLLFGIQYPDSDVLFRYKVKS

>AFE80926.1

MAGEVSAAMGRFSLEWLVLPGLALAAALLLLALCLLDRRTRRPGEPPLIKGWLPYLGVVL

KLRKDPLSFMKTLQKQHGDTFTVLLGGKYITFILDPFQYQLVIKNHKQLSFRLFSNKLLE

KAFSISQLQKNHDMNDELHLCYQFLQGKSLDILLESMMQNLKQVFESQLLKTTSWDTAQL

YPFCSSIIFEITFTTIYGKVLVCDNKFISELRDDFLKFDDKFAYLVSNIPIELLGNVKSI

RKKIIKCLSSENLAKMQGWSEVFQSRQDVLEKYYVHEDLEIGAHHLGFLWASVANTIPTM

FWAMYYLLRHPEAMAAVRDEIDRLLQSTGQKKGSGFPIHLTREQLDSLICLESTIFEALR

LSSYSTTIRFVEEDLTLSAETGDYCVRKGDLVAIFPPILHGDPEIFEAPEEFRYDRFIED

GKKKTTFFKRGKKLKCYLMPFGTGTSKCPGRFFALMEIKQLLVILLTYFDLEIIDDKPIG

LNYNRLLFGIQYPDSDVLFRYKVKS

>EHH64198.1

MAGEVSAAMGRFSLEWLGLPGLALAAALLLLALCLLDRRTRRPGEPPLIKGWLPYLGVVL

KLRKDPLSFMKTLQKQHGDTFTVLLGGKYITFILDPFHYQLVIKNHKQLSFRLFSNKLLE

KAFSISQLQKNHDMNDELHLCYQFLQGKSLDILLESMMQNLKQVFESQLLKTTSWDTAQL

YPFCSSIIFEITFTTIYGKVLVCDNKFISELRDDFLKFDDKFAYLVSNIPIELLGNVKSI

RKKIIKCLSSENLAKMQGWSEVFQSRQDVLEKYYVHEDLEIGAHHLGFLWASVANTIPTM

FWAMYYLLRHPEAMAAVRDEIDRLLQSTGQKKGSGFPIHLTREQLDSLICLESTIFEALR

LSSYSTTIRFVEEDLTLSAETGDYCVRKGDLVAIFPPILHGDPEIFEAPEEFRYDRFIED

GKKKTTFFKRGKKLKCYLMPFGTGTSKCPGRFFALMEIKQLLVILLTYFDLEIIDDKPIG

LNYNRLLFGIQYPDSDVLFRYKVKS

>297682972

MKRAFRPGQLLERRPGEPPLIKGWLPYLGVVLNLRKDPLRFMKTLQKQHGDTFTVLLGGK

YITFILDPFQYQLVIKNHKQLSFRLFSNKLLEKAFSISQLQKNHDMNDELHLCYQFLQGK

SLDILLESMMQNLKQVFEPQLLKTTSWDTAQLYPFCSSIIFEITFTTIYGKVIVCDNNKF

ISELRDDFLKFDDKFAYLVSNIPIDLLGNVKSVREKIIKCFSSEKLAKLQGWSEVFQSRQ

DVLEKYYVHEDLEIGAHHLGFLWASVANTIPTMFWAMYYLLRHPEAMAAVRDEIDRLLQS

TGQKKGSGFPIHLTREQLDSLICLESTIFEALRLSSYSTTIRFVEEDLTLSAETGDYCVR

KGDLVAIFPPILHGDPEIFEAPEEFRYDRFIEDGKKKTTFFKRGKKLKCYLMPFGTGTSK

CPGRFFALMEIKQLLVILLTYFDLEIIDDKPIGLNYSRLLFGIQYPDSEVLFRYKVKS

>297299497

MELDSELSLQQEINEKLMLNSEKIQQRPGEPPLIKGWLPYLGVVLKLRKDPLSFMKTLQK

QHGDTFTVLLGGKYITFILDPFQYQLVIKNHKQLSFRLFSNKLLEKAFSISQLQKNHDMN

DELHLCYQFLQGKSLDILLESMMQNLKQVFESQLLKTTSWDTAQLYPFCSSIIFEITFTT

IYGKVLVCDNKFISELRDDFLKFDDKFAYLVSNIPIELLGNVKSIRKKIIKCLSSENLAK

MQGWSEVFQSRQDVLEKYYVHEDLEIGAHHLGFLWASVANTIPTMFWAMYYLLRHPEAMA

AVRDEIDRLLQSTGQKKGSGFPIHLTREQLDSLICLESTIFEALRLSSYSTTIRFVEEDL

TLSAETGDYCVRKGDLVAIFPPILHGDPEIFEAPEEFRYDRFIEDGKKKTTFFKRGKKLK

CYLMPFGTGTSKCPGRFFALMEIKQLLVILLTYFDLEIIDDKPIGLNYNRLLFGIQYPDS

DVLFRYKVKS

>296226585

MAELSAAIGRFSLEQFGVWGLAFAAALLLLALCLLGGRTRRPGEPPLIKGWLPYFGVVLE

LRKDPLRFMKTLQKQHGDTFTVLLGGKYITFILDPFQYQLVIKNQKQLSFRVFSDELVRK

AFSISQLQKNHDMNDELHLCYQFLQGKSLDILLESMMQNLKQVFESQLLKTTSWDTVQLY

AFCSSIIFDITFTTIYGKLLVCDNNKFINELRDDFLKFDDNFTYLVSDIPIELLGNIKSI

RKNIIKCLSSENLDKMQGWSEILQRRQDVLEKYYVHEDLEIGAHHXGFLWASVANTIPAM

FWAMYYLLRHPEAMAAVRDEIDRLLQSTGQKKGSGFPIYLTREQLDSLICLESTVFEALR

LSSYSTTIRFVEEDLTLSAETGDFCVRKGDLLAVFPPIFHGDPEIFEAPEEFRYDRFVED

GKKKTTFFKRGKKLKCYLMPFGTGTTKCPGRFFAIMEIKQLLVILLTYFDLEIIDDKPIG

LDYRRMLFGIQCPNSDVLFRYKVKSYRMLISVWSEISPIRNASTKKKKKEMQRKVERRKK

ENSDTHILVYLLILQ

>EHH28538.1

MAGEVSAAMGRFSLEWLGLPGLALAAALLLLALCLLDRRTRRPGEPPLIKGWLPYLGVVL

KLRKDPLSFMKTLQKQHGDTFTVLLGXXXXXXXXXXXXXXXXXXXXXXXXXXXXNKLLEK

AFSISQLQKNHDMNDELHLCYQFLQGKSLDILLESMMQNLKQVFESQLLKTTSWDTAQLY

PFCSSIIFEITFTTIYGKVLVCDNKFISELRDDFLKFDDKFAYLVSNIPIELLGNVKSIR

KKIIKCLSSENLAKMQGWSEVFQSRQDVLEKYYVHEDLEIGAHHLGFLWASVANTIPTMF

WAMYYLLRHPEAMAAVRDEIDRLLQSTGQKKGSGFPIHLTREQLDSLICLESTIFEALRL

SSYSTTIRFVEEDLTLSAETGDYCVRKGDLVAIFPPILHGDPEIFEAPEEFRYDRFIEDG

KKKTTFFKRGKKLKCYLMPFGTGTSKCPGRFFALMEIKQLLVILLTYFDLEIIDDKPIGL

NYNRLLFGIQYPDSDVLFRYKVKS

>403288816

MDRFSLEQSGPWGLALAAALLLLALCLLGGRTRRPGEPPLIKGWLPYFGVVLELRKDPLR

FMKTLQKQHGDTFTVLLGGKYITFILDPLQYQLVIKNQKQLSFRVFSDELVGKAFSVSQL

QKNHNMNDELHLCYQFLQGKSLDILLESMMQNLKQVFESQLLKTTSWDTVQLYAFCSSII

FDITFTTIYGKLVCDNNKFVNELRDDFLKFDDNFTYLVSDIPIELLGNIKSIRKKIIKCL

SSENLAKMQGWSEILQSRQDVLEKYYVHEDLEIGAHHLGFLWASVANTIPAMFWAMYYLL

RHPEAMAAVRDEIDRLLQSTGQKKGSGFPIYLTREQLDSLICLESTIFETLRLSSYSTTI

RFVEEDLTLSAETGDFRVRKGDLLAVFPPIFHGDPEIFEAPEEFRYDRFVEDGKKKTTFF

KRGKKVKCYLMPFGTGTTKCPGRFFAIMEIKQLLVILLTYFDLEIIDDKPIGLDYRRMLF

GIQYPDSNVLFRYKVKS

>301761978

MAISDLEILQYNHRRPGEPPLVKGWLPYLGEALKLQKDPLGFMKTLQKQHGDTFTVLLGG

KYITFILDPFQYQSVMKSHKLSFRIFTNKLLRKAFCIEKLAANDDLNDDLHGCYHLLRGT

SLDTLTENMMQNLKQVFELQLLKTTNWDTANLLTFCSSVIFEVTFTTIYGKSLAGNRKKF

ITELRDDFLKFDDKFPYLISDIPVELLGNVKSIRKKLITCLTSEHLAKTQEWSEVVQMRQ

DILEKYYTLEDFEIGAHHLGFLWASVTNTIPTMFWAMYYLLRHPEAMAVLRDEIDHLLQS

TGQKKGSGFPIHLTREQLDSLVYLESTVLEVLRLCSFSSIIRFVQEDLTLQSETQDYCLR

KGDLVAIFPPAIHHDPEIFEAPEEFRFDRFVEDGKKKTAFFKNGKKLKYYLLPFGFGASK

CPGRFLAIVEVKQLLVVLLTYFDLEIIDGKPMEANYSRLLFGIQHPASDVLFKYRVKS

>338728273

MGLLLSRDGREAPRHVALTDGLEGKVFQAQLKIEESWRPGEPPLIKGWLPYLGESLKLQK

DALGFLKTLQKQHGDIFTILLAGKYITFILDPFQFQLVMKNHKLSFRIFSNKITVKAFSI

KKLVTNDDLNNELHVCYQCLRGKSLDVLMENMMQNLKQVFEPQLLKTTNWEMACLLTFCS

SVIFEITFTTIHGKLLAGDRKKFITELREDMLKFDDKFPYLASDIPIELLGNVKSIRKKL

IKLLTSENLAKIRGWSEVVQMRQDILEKYYMPKDFEIGAHHFGFLWASVTNTIPTMFWAM

YYLLRHPEAMAVLRDEIDHLLQSTGQKKGSGFSIHLTREQLDSLVYLESTILEVLRLCSF

SVIMRFVEEDLTLPSETGDYCLRKGDSIAIFPAALHHDPEIYEAPEEFRFDRFVEDGKKK

TTFFKGGKKLKYYLLPFGFGASKCPGRFLAIMEIKQLLVVLLTYFDLEIMDQKPIELNRS

RLLFGIQHPDSDVSFRYKVKT

>395849396

MDGTFPGGVAAGPPCGPSESDVCSAVVLPGGSTAQAFPRARRPGEPPLIKGWLPYLGVAL

QFQKNPLNYLKALQKQHGDIFTVFLGGRYITFILDPFQYQLLIKNHNKLSFKVFCHKFFK

AFSLTKLLEHGELNDDIHTCYQFLQGKSLDILLEDLTKNLKQVFEPHLLRATNWNVAQLC

TFCSSILFEVTFTTIYGHLPADNRKELINELRDDFFKFDGKFYYLLSDIPLDLLGNVRSL

RKKLIQCLSSEKLAKMQGQSEVFQKRQNTLEKYYAREDFEIGAHHLAFLWASVTNSIPTM

MWATYYLMRHPEAMSAVRDEIDHLLQSTGQKKGSGFSIHLTREQLDSMICLESTVLEVLR

LCSLSGTIRFVQEDLTLDSELKDSCLRKGDFVAIFPAFLHNDPEIFEAPQEFRYDRFIED

GKKKTTFYKRGKKLKSYLMPFGTGTTKCPGRFFAVLEIKIVLIVLLTYFDLELIDDKPVK

PNYNRLLFGIEYPDSEVLFRYRAKS

>345793247

MARTLGVLESEWTKKGESAAKRQKVTELQVRCEYNVNGDRAEMSAPPPSGGSTWAAIQWM

GPSSTKEERGCDEQISPRRPGEPPLIKGWLPYLGEALKLQKDPLGFLRTLQKQHGDTFTL

FAGGKYITFILDPFQYQLIMKTHKLSFRIFSNKLSRKVFSIKKLETTDDLSNDLHGCYHL

LQGKSLDVLTETMMQNLKQVFELHLLKTTNWDMAHLLTFCSSIIFEITLKTIYGKSLAGN

GEKFITELRDNFLKFDDKFPYLISDIPIELLGNIKSIRKKLIKHLASEHLGKTQGWSEIV

QMRQDVLEKYYTLEDFEVGAHHLGFLWASVTNTIPTMFWAMYYLLQHPEAMAVLRDEIDH

LLQSTGQKKGSGFPMHLTREQLDNLVYLESTVLEVLRIRSFSSIIRFVQEDLTLHSETQD

YCLRKGDLVALFPPAIHYDPEIFEAPEEFRFDRFVEDGKKKTTFFKKGKRLKYYLLPFGI

GTSKCPGRFLAVVEIKQLLVVLLTYFDLELIDDKPVVANRSRLLLGVQHPASDVFFRYKV

KT

>350596279

MVSSDSVEKLSISIARVLDLTFPRMQRLNEIWRSSFPFLSVDLVMPVAQVFMLVVFGFPG

VPPGKGLGSGPPPLHPFLKHPSWLRPGEPPLVKSWLPFIGEALKLQRDPIGFMTTLQKQY

GDIFTVLLGGKYITFILDPFQYQSVIKNQKLSFRGFSNKVLLRVFSIKKLVEDDAMNDEL

HTCYQLLQGRHLDMLMENTMQSLKQVFEPQLLKTTSWHTESLFTFCNSVIFEMTLTTVYG

SILLGHRKTFITELKDDFFKFDGKFTQLASGMPIELLGNIKSIRLKLIKLLTTENLAKFQ

GWSEVVQRRQDILEKYYLPQDIEIGAHHLGLLWASVANTAPTMSWTMYYLLRNPEAMAVL

RDEIDHLLQSTGQKKGPGFSIHLTREQLDSLVYLESTILESLRLSSFSSTFRFVQEDLTL

HLEAQDCCLRKGDFVGVFPPILHYDPEIFEAPEEFRFDRFVENGKKKTTFFKRGKKLKYY

LMPFGFGISKCPGRFLAVVEIKQLLVILLTYFDLEIIDDKPVKLNFSRSLFGIQHPDSDV

SFRYKVKS

>DAA22734.1

MNAGMGGFWPEPWLTRSLGYPGLALAAALLLLVLCLNARRTRRPGEPPLIKGWLPYFGQA

LKLQKDPLGFMTSLQKQYGDIFTLLLGGKYITFILDPFHYTSVAKNQKLSFQIFTNKFLK

RVFSIKKMLTDSDLIDEIHSTYQFLQGKHLDILMESTMQNLKQVFEPQLLKTTSWSTEYL

LPFCNSVIFEMTFTTIYGNILADDKKTFITELKDDFLKFDEKFTRLASGIPIELLGNIKS

VRTKLIKDLTIESLAKLQGMSEVVQRRNDILEKYYTPKDTEIGAHHLGLLWASVTNTVPT

MFWAMYYLLRNPKAMAVLRDEIDHLLQSTGQKKGPGFSIYLTREQLDSLVYLESTILEVL

RLCSFSGIFRFVQEDLTLHLESQDCCLRKGDFVVIFPPILHHDPEIFEAPDEFRFDRFTE

NGKKKTTFFKRGKKLKYYHLPFGLGVSKCPGRFLAMVEIKQLLVVLLTYFDLEIIDNKPL

ELNYSRFLFGIPYPDSDVLFRYKIKS

>344272871

MCQDSATPSAHSSVALGSPICSPAMRLPLPSILTDTPILNYPLLGRPGEPPLIKGWLPYL

GQGLNFQKAPSAFLKTLQKQHGDIFTVLLGGKYITFILDPLQFQLVTKNPKHLSFQIFST

KLISKVFSIEMMLTNQELNHDIHVCYQYLQGKSLDILFETMMQNLKHVFESKLLKTRNWD

TERMLTFCTSVVFEITFTTLYGKLPDADRKKFISELRDDFIKFDDKFPYLVSGIPIKLLG

NALSLRKKLTECFTQENLAKMQGWSEVIQMRQDTLEKYYEPDNSEIGAHHLALLWASVAN

TIPTVFWAMYYLLRHPEAMAALRDEIDRLLQSTGQKKGSEFFIDFTREQLDSLVYLESIV

LEVLRLCSFSTTIRFVEEDFALPVESGECLLRKGDLVAIFPPALHKDPEVFEAPEEFRYD

RFVEDGKKKTTFFKSGKKLKYCVMPFGSGVSKCPGRFFAACEMKQLLVALVTYFDLEIID

DKPVELSYSRMLLGIPHPTTDVSFRYRVKS

>291388042

MAMLSLLPFERLDVPQELSVDEKFTTIHEKRAGEPPLIKGWIPYLGVTLKFQKDPLGFLK

SLQKQHGDIFTILLGGKYITFILDPFQYQLVMKNQKQLSFRKFGNKLSEKTFSIKQMIHN

NDMDDELHVVYQLLQGKSLDILLEDLMQNIKQMFEVQLLKTTNWKMAQLLTFCGTMIFDI

TFTTLHGKFLAGDSTKYISELRENFFKFDDKFSLLASEIPIELLGNIKSVQQKLIKCLTS

EKLAEFKGSSEIVQKRQEILEKYYVREDFEIGAHHLAFLWASVANTFPAMFWAMYYLLRH

PEAMAAVRDEIDRLLQSTGQKQGSGFSIYLTREQLDSLVYLESVIFETFRLCSFSGTIRV

IEEDLTLSSETGNFCLRKGDMLCVLPAVTHTDPEIFEAPEEFRFDRFTEDGKKKTTFFKG

ERKIKYYLSPFGFGASKCPGRFFAVSEIKQLLVLLLTYLDLELIDDKPIGLDHSRLMFGI

QHPNSDVLFRYKAKS

>348588480

MSPAGCITHLFCSQRPALLSMRFGTTSALTEAVGASSILTVNHTFRLLLLLWDLPPVALQ

RPGEPPLIKGWLPYFGMTMKFQKDPLGLIRTLQKQYGDIFTILLGGKYITFILDPFQYQL

LNKYHKKLSFQRLTRKLSVRVFSIKILENYEDLDHELHGAYQFLQGKSLDIILEDMLQNI

KQTFESQLLKTTNWNKTQMLTFCCTMVFEITFTTMYGKVPEDDRKTFISELRDNILKFDD

KFPYLMSDIPIVFLRNVKSIREKLIKCMTPEELAKIPGSSEVVQKRQDILEKYYGLEDLD

IGAHHFALLWASIANSIPVMFWAMYYVLRHPEAAEALRDEIDHLLQSTGQKKGSGFSLHL

TREQLDSLVCLESIIFEALRLSSYSSTIRFVEEDFILSSDTGDCSLRKGDLVAIFPPAIH

GDPEVFEAPEEFRFDRFMEDGKKKTTFYKRGKRLKFYLVPFGFGANKCPGRFLAVMEIKL

LLLVLLTYFDLELVENKSIKLNYTRLFFGIQYPNSDILFRYKVKS

>EDM01054.1

MEGATTPDAASPGPLSLLGLLFAVTLLLPVLFLLTRRTRRPCEPPLIKGWIPYLGMALKF

WKDPLAFLQTLQRQYGDTFTVLLGGKYITFVLNPFQYQYVMKNPKQLSFEKFSRRLSAKA

FSVKKLLTDDDLSNDIHRGYLLLQGKSLDGLLETMIQEVKEIFESRLLKLTDWNTARVFD

FCSSLVFEITFTTIYGKILAANKKQIISELRDDFLKFDDHFPYLVSDIPIQLLRNAEFMQ

KKIIKCLTPEKVAQMQRRSEIVQERQEMLKKYYGHEEFEIGAHHLGLLWASLANTIPAMF

WAMYYLLQHPEAMEVLRDEIDSFLQSTGQKKGPGISVHFTREQLDSLVCLESAILEVLRL

CSYSSIIREVQEDMDFSSESRSYRLRKGDFVAVFPPMIHNDPEVFDAPKDFRFDRFVEDG

KKKTTFFKGGKKLKSYIIPFGLGTSKCPGRYFAINEMKLLVIILLTYFDLEVIDTKPIGL

NHSRMFLGIQHPDSDISFRYKAKSWRS

>158186667

MEGATTPDAASPGPLSLLGLLFAVTLLLPVLFLLTRRTRRPCEPPLIKGWIPYLGMALKF

WKDPLAFLQTLQRQCGDTFTVLLGGKYITFVLNPFQYQYVMKNPKQLSFEKFSRRLSAKA

FSVKKLLTNDDLSNDIHRGYLLLQGKSLDGLLETMIQEVKEIFESRLLKLTDWNTARVFD

FCSSLVFEITFTTIYGKILAANKKQIISELRDDFLKFDDHFPYLVSDIPIQLLRNAEFMQ

KKIIKCLTPEKVAQMQRRSEIVQERQEMLKKYYGHEEFEIGAHHLGLLWASLANTIPAMF

WAMYYLLQHPEAMEVLRDEIDSFLQSTGQKKGPGISVHFTREQLDSLVCLESAILEVLRL

CSYSSIIREVQEDMDFSSESRSYRLRKGDFVAVFPPMIHNDPEVFDAPKDFRFDRFVEDG

KKKTTFFKGGKKLKSYIIPFGLGTSKCPGRYFAINEMKLLVIILLTYFDLEVIDTKPIGL

NHSRMFLGIQHPDSDISFRYKAKSWRS

>BAE24236.1

MQGATTLDAASPGPLALLGLLFAATLLLSALFLLTRRTRRPCEPPLIKGWLPYLGMALKF

FKDPLTFLKTLQRQHGDTFTVFLVGKYITFVLNPFQYQYVTKNPKQLSFQKFSSRLSAKA

FSVKKLLTDDDLNEDVHRAYLLLQGKPWDALLETMIQEVKELFESQLLKITDWNTERIFA

FCGSLVFEITFATLYGKILAGNKKQIISELRDDFFKFDDMFPYLVSDIPIQLLRNEESMQ

KKIIKCLTSEKVAQMQGQSKIVQERQDLLKRYYRHDDPEIGAHHLGFLWASLANTIPAMF

WAMYYILRHPEAMEALRDEIDSFLQSTGQKKGPGISVHFTREQLDSLVCLESTILEVLRL

CSYSSIIREVQEDMNLSLESKSFSLRKGDFVALFPPLIHNDPEIFDAPKEFRFDRFIEDG

KKKSTFFKGGKKLKTYVMPFGLGTSKCPGRYFAVNEMKLLLIMLLTYFDLEIIDRKPIGL

NHSRMFLGIQHPDSAVSFRYKAKSWRS

>354498264

MRSCTQILCTFAKLLVAVIACSSTLKMGRRKWWRPDEPPLIKGWLPYLGIALSLQKNPLA

FMKILQRKHGDTFTVLLAGRYTTFILDPFQYHVIMKNPKQLNFWKFSRQLSAKAFGIKKF

ATDDDLNEDLHRHYLLLQGKPLDGLLECANQQLKEIFEFQLLKTTDWNTASLFGFCASLI

FETTFSVIYGKTLAGNRKKLISELRDDFLKYDEMFPYLISDLPIPLLKHAKAAQMKVIKH

LMPEKLAQMQGWSEIIKERHSVLEKQYLPEDFEIGGHHLGFLWASMGNTIPAMFWAMYYL

LRHPECMEALREEIDSLLQSTGQKKGPGFSIHFNREQLDSLVGLESTIFEVMRLCSYSTI

IREVEEDMTLNLETSSYHVRKGDFVALFPPILHMDPEIFEAPEEFRFDRFIEDGKKKNTF

FKGGKKLKYSVMPFGLGASKCPGRYFAINELKLLIALLLTYFDIEITDNKTIELNYSRLL

FGVQHPNSDIQFRYKVKSWRS

>Q60991.2

MQGATTLDAASPGPLALLGLLFAATLLLSALFLLTRRTRRPREPPLIKGWLPYLGMALKF

FKDPLTFLKTLQRQHGDTFTVFLVGKYITFVLNPFQYQYVTKNPKQLSFQKFSSRLSAKA

FSVKKLLTDDDLNEDVHRAYLLLQGKPLDALLETMIQEVKELFESQLLKITDWNTERIFA

FCGSLVFEITFATLYGKILAGNKKQIISELRDDFFKFDDMFPYLVSDIPIQLLRNEESMQ

KKIIKCLTSEKVAQMQGQSKIVQERQDLLKRYYRHDDPEIGAHHLGFLWASLANTIPAMF

WAMYYILRHPEAMEALRDEIDSFLQSTGQKKGPGISVHFTREQLDSLVCLESTILEVLRL

CSYSSIIREVQEDMNLSLESKSFSLRKGDFVALFPPLIHNDPEIFDAPKEFRFDRFIEDG

KKKSTFFKGGKKLKTYVMPFGLGTSKCPGRYFAVNEMKLLLIMLLTYFDLEIIDRKPIGL

NHSRMFLGIQHPDSAVSFRYKAKSWRS

>AAH38810.1

MQGATTLDAASPGPLALLGLLFAATLLLSALFLLTRRTRRPREPPLIKGWLPYLGMALKF

FKDPLTFLKTLQRQHGDTFTVFLVGKYITFVLNPFQYQYVTKNPKQLSFQKFSSRLSAKA

FSVKKLLTDDDLNEDVHRAYLLLQGKPLDALLETMIQEVKELFESQLLKITDWNTERIFA

FCGSLVFEITFATLYGKILAGNKKQIISELRDDFFKFDDMFPYLVSDIPIQLLRNEESMQ

KKIIKCLTSEKVAQMQGQSKIVQERQDLLKRYYRHDDPEIGAHHLGFLWASLANTIPAMF

WAMYYILRHPEAMEALRDEIDSFLQSTGQKKGPGISVHFTREQLDSLVCLESTILEVLRL

CSYSSIIREVQEDMNLSLESKSFSLRKGDFVALFPPLIHNDPEIFDAPKEFRFDRFIEDG

KKKSTFFKGGKRLKTYVMPFGLGTSKCPGRYFAVNEMKLLLIMLLTYFDLEIIDRKPIGL

NHSRMFLGIQHPDSAVSFRYKAKSWRS

>AAA92615.1

MQGATTLDAASPGPLALLGLLFAATLLLSALFLLTRRTRRPREPPLIKGWLPYLGMALKF

FKDPLTFLKTLQRQHGDTFTVFLVGKYITFVLNPFQYQYVTKNPKQLSFQKFSSRLSAKA

FSVKKLLTDDDLNEDVHRAYLLLQGKPLDALLETMIQEVKELFESQLLKITDWNTERIFA

FCGSLVFEITFATLYGKILAGNKKQIISELRDDFFKFDDMFPYLVSDIPIQLLRNEESMQ

KKIIKCLTSEKVAQMQGQSKIVQESQDLLKRYYRHDDSEIGAHHLGFLWASLANTIPAMF

WAMYYILRHPEAMEALRDEIDSFLQSTGQKKGPGISVHFTREQLDSLVCLESTILEVLRL

CSYSSIIREVQEDMNLSLESKSFSLRKGDFVALFPPLIHNDPEIFDAPKEFRFDRFIEDG

KKKSTFFKGGKRLKTYVMPFGLGTSKCPGRYFAVNEMKLLLIELLTYFDLEIIDRKPIGL

NHSRMFLGIQHPDSAVSFRYKAKSWRS

>224046311

MTPLWQKTFFSLACGFFSAFGGSCCEKRSAELALPCRVTCSCRASSAVLCAVKAALTPDA

NHTPPVDLQDYRTGKKDEGYLFCVVCRRPRKTGEPPLINGWIPYLGKALIFRKDAYKFLL

DQQKKFGDIFTVHIAGRYITFIMDPFQYVYVIRNSKQLEFHEFANKMASKTFDYPALSKG

KFPELKENLHRIYQYLQGKPLDIISDHMMKNLQDIFEWKCSQATDWETEKMYKFCCSVMF

EASFVTLYGRVPAADGHKVISEIRDKFIKFDASFPYLAANIPIELLGATKKVRKELIHHF

LLQNMTKWLGGSKVVQARQDIFEKYELLGDYDKAAHHFAFLWASVGNTIPATFWAMYYLL

RHPEALAAVRDEIDHLLQSTGQKRGPTYNIHLTREQLDNLVYLESALNESLRMCSSSMNI

RISQEDFVLKLEGNQEVVLRKGDWIALYPQILHMDPEVYEDPKEYKFDRYIENGKKKTAF

YKAGRKLKYFLMPFGSGISMCPGRFLAMNEMKMFLFILLSHFDVELAENKAVRLDNSRMG

LGILLPDVDIAFRYKLRSLRN

>BAF95940.1

MGPEALPLGPYSGAAAAALLLWAVCVLCRRKRKTGEPPLINGWIPYLGKALIFRKDAFKF

LLDQQKKLGDIFTVHIAGRYITFIMDPFQYVYVIRNSKQLEFHEFADKMASKTFDYPALS

NVKFPDLKENLHRIYQYLQGKPLEIISDHMMKNLQDIFEWKCSQATDWETEKMYKFCCSV

MFEASFVTLYGRVPAADGRKVISEIRDKFIKFDASFPYLAANIPIELLGATKKVRKELIH

HFLLQNMTKWLGGSKVVQARQDIFEKYELLGDYDKAAHHFAFLWASVGNTIPATFWAMYY

LLRHPEALAAVRDEIDHLLQSTGQKRGPTYNIHLTREQLDNLVYLESALNESLRMCSSSM

NIRISQEDFVLKLEGDQEVSLRKGDWIALYPQILHMDPEVYEDPKEYKFDRYIENGKKKT

AFYKAGRKLKYFLMPFGSGISMCPGRFLAMNEMKMFLFLLLAHFDVELGENKDVRLDNGR

MGLGILLPDTDIAFRYKLRSLRN

>326917688

MVALDLRAMDLRKSKIHWTEEAATKCKKYNRFGTPSSEETKSEVEDLKCSMKIKGRREGK

FSDDKDRLLGETGNSITEYLQEQHGKTGEPPLINGWIPYLGKALLFRKDAFKFLLDQQKK

LGDIFTVHIAGRYITFIMDPFQYVYVIRNSKQLEFHEFADKMASKTFDYPALSNVKFPDL

KENLHRIYQYLQGKPLEIISDHMMKNLQDIFEWKCSQATDWETEKMYKFCCSVMFEASFV

TLYGRVPAADGRKVISEIRDKFIKFDASFPYLAANIPIELLGATKKVRKELIHHFLLQNM

TKWLGGSKVVQARQDIFEKYELLGDYDKAAHHFAFLWASVGNTIPATFWAMYYLLRHPEA

LAAVRDEIDHLLQSTGQKRGPTYNIHLTREQLDNLVYLESALNESLRMCSSSMNIRISQE

DFVLKLEGDQEVSLRKGDWIALYPQILHMDPEVYEDPKEYKFDRYIENGKKKTTFYKAGR

KLKYFLMPFGSGISMCPGRFLAMNEMKMFLFLLLAHFDVELGENKAVRLDNSRMGLGILL

PDTDIAFRYKLRSLRN

>363730966

MEMKTHFCEQPAVGRKLKQCCISLRSTRSPAVVFNGAKLIAVELNAAEANDDPHVLAIAK

CLPDVIPQTWSIKLIFPWISELRPLYPAESWKTGEPPLINGWIPYLGKALLFRKDAFKFL

LDQQKKLGDIFTVHIAGRYITFIMDPFQYVYVIRNSKQLEFHEFADKMASKTFDYPALSN

VKFPDLKENLHRIYQYLQGKPLEIISDHMMKNLQDIFEWKCSQATDWETEKMYKFCCSVM

FEASFVTLYGRVPAADGRKVISEIRDKFIKFDASFPYLAANIPIELLGATKKVRKELIHH

FLLQNMTKWLGGSKVVQARQDIFEKYELLGDYDKAAHHFAFLWASVGNTIPATFWAMYYL

LRHPEALAAVRDEIDHLLQSTGQKRGPTYNIHLTREQLDNLVYLESALNESLRMCSSSMN

IRISQEDFVLKLEGDQEVSLRKGDWIALYPQILHMDPEVYEDPKKYKFDRYIENGKKKTT

FYKAGRKLKYFLMPFGSGISMCPGRFLAMNEMKMFLFLLLAHFDVELGENKAVRLDNSRM

GLGILLPDTDIAFRYKLRSLRN

>334325514

MDLDLKGLSEIIQFNYFSQIRKSRRPGEPPLINGWIPFFGVAFQMKTDPFKKLKEYQKKY

GDFFTLYINGKYFTFVLDPFQYPLVLKNHKQFDFFKFSNEIIHKVFTVSYRAQVMEKMKE

NYKYLQSKLLEKTVYNLMQNLQHVIKKQLLQTKNWEMSQLFKFCKSIMFEATYVTLYGKD

PSPNSDEVIPELLEHFDKFDNEFLNFLFPIPTLSLRAAKISREKLIQNFSSEHMYKMLET

SEIIRSRRNCLEKFTELPDHEKGAHHLVFLWASVANTIPTTFWTVYYLLQHPEAVAVLRE

EINHLLQSTGQKKEPDFNVQFTREQLDSLVYLDSIIFEALRMISASANLRVNEEDFTLST

DKGEINLRKGDSIIIYPPMLHFDPEVFEQPEEFKFDRFVKDGKKKTTFFKRGQELPYCLM

SFGSGITKCPGRFFAVFEIKQLLILLFMFFDMELIEKEPLQFRKSRMFFGIQCPQSDVSF

RYKLRC

>345306802

MALRPKEPPLVPSWIPFLGRALQFKNDPYHFLKAQQKIHGDVFTVLFVGKYITFILDPFQ

FPTITRTSKHFDFKGFANHLGQKIFNYQSLYTIPNLSKDIHESFRYLQGKPLDKLVSQVM

HSLQCIFKEKFSRMADWETEQMYTFCHAVIFEATFTTFYGKYPTADGHKVINEIRETFAK

FDANFPKLAANIPIELLGSTKKNREFLIRQFTPELMVKRQPLAEIIQFRREFLEKYKLLQ

DYSKAAHHFGFLWASVGNTIPASFWALYYLLRHPEALAVVRDEIDHLLQSTGQANRPGSF

IYLTREQLDNLVYLDSLILESFRLNSASTNFRIIQEDFTLQLEDGRDFNLRKGDWLALFP

QTMHRDPEIFEDPEVFKFDRFVENGKKKTTFFKRGRKLKYFLMPFGSGVSMCPGRFFAVS

EIKQFLILLLVHLEAEIIEEKNLVSNNERLGFGILYPNSDISFRYRWKGCGSSLVC

>327269725

MNSLIKGKFDIVALQETHIAQNHVSYLKNPKLGREFFSADARKKRGVVLYIKEKIEAELK

FKDSEGRFVGVEISLGNRKILICNVYVPNGPKTQFIKELRDQITKSEAEHIMIFGDFNGV

LDPIQDKTRKTKRPKKDDCRRLPGNMLLMKEEFHLQDIWRYHNPTKRDYTFYSNRHDSWS

RIDMIWASSSLLTNSIDIKILPRDKSDHCPITMCLNNYPVYHKWRLDDNLIKSEEDINRY

KDITKEFFKFNTQKDTSLQTIWDAYKAVLRGHLIQQKSRKNKQKFQKINELKREIDIIEK

QLKQNSQDKQAHQKLKELKKEKERNELEDTAKKLKFIKQYNFENANKPEAGPGLSGVPLE

AAQSSQPPMREPSGFGGKLPRALLEAGKGKVKGEGWEFPRAWSNFPRKEEEEERKEDGEA

TGARKSDGMGLVDLLPLPPLGLALATLLALGALWHFCRRRRRPGEPPLEAGWIPFLGEAL

NFRKDATSFLLSLQKKHGDVFTIYLAGKYITFILDPLQFPSVIKNGKHLEFSEIADEISS

RAFDHPPVLKEIYSNLSENVHRGYQYLIGKSLDELSDSMMKNLQSLFKAKFSHTADWEKE

TVNKFCFSMIFEASFRTLYGRDPDADGYSVTDAIGDKFLKFDASFAYLAANIPAELLGET

KGIRKELINFFKPANMSKWLGCSEVVQDRKNVFEMYEALGDYDKSAHHFAFLWASVGNTI

PATFWALYYLLRHPEALSVVRDEIDHLLQSTGQERGPGFDIHVTREQLDNLVYLESAINE

SLRLCSSSMNLRLTKERFILKLEGNLEVSLRKGDYIAIYPPTLHMDPEVYQDPEQYKFDR

YIENKKNKTAFYKQGKKLKYFLMPFGSGVSKCPGRFFAINEIKLCLVLLLSYFDMELLEQ

KPIGMDKSRFGLGIYLPDSDVLFRYKLKS

>AAI61410.1

MLDTLLYTVTGLVLGGLLLLLLLPRRQRREGEPPLENGWIPFIGLAYEFHKNALEFLISR

QQKYGDIFTVHVAGKYITFIMDSTQFQYVIKHGKQLDFHEFANSLSSRTFDHPRLTEAKF

PHLNDKLHRIYKIMQGRALDKLTDSMMGNLQRVFKWKFSQATDWKAEKMYQFCCCIMFEA

SFMTLYGRDPIADGHKVISEIREKFTKFDAKFPYLVINIPIALLGATKKIREELIHFFFP

NKMEKRSEISEVVQERKNVLEQYELQDYDRAAHHFAFLWASVGNTIPATFWAMYYLVRHP

EALAAVRDEIDHLLQSTGQKKGPEYDIHITREQLDSMVLLGSAIKESFRLCAASMNIRLV

QEDFDLELEGNQTIRLRKDDFIALYPPALHMDPEIYEDPERYKYDRFVENGKEKILFYKK

GKKLKEYLMPFGSGTSKCPGRFFAMNEIKQFLAVLLIYVDMELVEHKALGHDNRRSGLGI

LLPNSDIMFRFKPRTLDL

>BAE42849.1

MQGATTLDAASPGPLALLGLLFAATLLLSALFLLTRRTRRPREPPLIKGWLPYLGMALKF

FKDPLTFLKTLQRQHGDTFTVFLVGKYITFVLNPFQYQYVTKNPKQLSFQKFSSRLSAKA

FSVKKLLTDDDLNEDVHRAYLLLQGKPLDVLLETMIQEVKELFESQLLKITDWNTERIFA

FCGSLVFEITFATLYGKILAGNKKQIISELRDDFFKFDDMFPYLVSDIPIQLLRNEESMQ

KKIIKCLTSEKVAQMQGQSKIVQERQDLLKRYYRHDDPEIGAHHLGFLWASLANTIPAMF

WAMYYILRHPEAMEALRDEIDSFLQSTGQKKGPGISVHFTREQLDSLVCLESTILEVLRL

CSYSSIIREVQEDMNLSLESKSFSLRKGDFVALFPPLIHNDPEIFDAPKEFRFDRFIEDG

K

>BAG72175.1

MLFSGFSGEEKALQWLFSVYTLTLGAAALLAYLCFSGSRRRRRAGEPPLETGWIPYLGAL

QFHKNPSNFLKSLQKKHGDIFTVQLLGKYMSFIMDPSQYQYVTKRGKQLDFTEFAGQMAS

RTFDYPQMSEAKFPQLHDNMHKGYKILQGEGLNELTDSMMRNLQHIFKWKFSQATDWRTG

KLYEFCCSVMFEASFMTLYGKEPTDGHKVISEIRDKFSKFDAKFPYLFTSIPIALLGATK

KTREALIHIFFPDIMSKRLTVSEVVQYRSSVLDKYEQLRDYDKAAHHFAFMWASVGNTIP

ATFWAMYYLVQHPEALAVVRDEIDHLLKSTGQTRSPEYNIHFTKDQLDALVYLDSAVHES

FRMSTASMNIRVVQQDFLLKLEGNQEWSLRKGDMLALFPPTLHNDPEVYSDPEVFQFDRF

VENGAKKTSFYKRGKKLKYYLMPFGSGVSKCPGRFFAVNEIKQFLAMMVTYLDMEFIENK

KVGLDNSRSGLGVLLPDSDIQFRCKPRR

>395511081

MTERGGRPGEPPLINGYIPFFGVLFQIKNNPYKSIQDYQKKYGNIFTLKINGNYFTFVLD

PFQYPLVLKNHKNFTFENFKDEFIKKIFLISIKPHMKKEFKDSQKYMQSKLLDLSYTLMQ

NMKHIMKERFLQAKNWETSQLYKFCISLIFEVTYIGFYGKDPGTNSGEVICELLNNFVKF

DNEFLKLLLPLPTLSLRTLKNVREKLIQNFLSEHISNMQETSEIIKARKILLDKFTDLQD

HEKAAHHFAFLWASVANTIPVTFWTVYYLLQHPEAVAVLRDEIDHLLQSTGQKKEPDFNI

QFTREQLDSLVYLESIINESFRMTSISSNVRYSEEDITLNLGNEDVNLRKGDIIIIYPPF

LHYDPEIFEQPEKFKFDRFVEDGKKKTTFFKRGKEVPYFLLPFGSGISKCPGRFLAMLEM

KQFLILLFMYFDLELIEFKPLEFNNSRILFGIQCPESDISFRYKLRC

>ACI33395.1

MLEFVLPLFLGFLALYLLSVRFGRTRRDGEPPLINGWIPFLGKAMEFGKNAHGFLAAHKE

KHGDVFTVLIAGKYMTFIMNPLLYPYVIKHQKQLDFHEFSDQVAPLTFGYPPVRSGKFPG

MEEHIQRSFRLLQGDNLNNLTESMMGNLMFVFRQDYLTGESEWRTESVYQLCNSIMFEAT

FLTLFGKPAHTSRHNGMVTLREDFVKFDTMFPLLIARIPISLLGGTKAIRDKLINYFHPQ

RMSPWSNTSGFIKERAALFEQYDSMRDVDKAAHHFATLWASVGNTVPATFWAMYYLVTHP

EALAVVREEIHGVLLVSGIETDHNRDIAFTREQLDSLLYLESSINESLRLSSASMNIRVA

QEDFSLRLEGERSIGVRKGDIVSLYPQSMHMDPGIYKNPEIYKFDRYIENGKEKTDFYKD

GQKLKYYRMSFGSGSTKCPGRYFAVNEIKQFLSLLLLYFDVEVLEGQEPCTLDPSRAGLG

ILLPASDVQIRYRLR

>125806840

MLEYLLTILLGFISVLLLYSFCGRRRKDGEPPLIKGWIPFLGKALEFRKNSQEFLTRLQK

EHGDDFTVLIAGRYITFIMNPLQYPSVIKHGKQLDFHTFSDSAASRTFDYPPIRSGHFPG

LSDQIQRSYTLLQGPALDPLTFSMMGNLQKVLRQLFLSGDEADDHWQQAELYKFCKSAMF

QSTFKTLYGHSPHLDLDQLREDFEKFDDMFPLLMARIPIGLLGKTKEIREKLIHFFYPQR

MMEWTAPCEFVQTRTALFQDYDTLQDRDKAAHHFAMLWASVGNTIPAAFWCLYHLISCPE

ALAAVREEITNVLGEKNMQLIFKEDIAITRQQLEQMVYLESSVNESLRLYSVSMNIRVVQ

EDFCLRLDGQRSINMRKDDIIALYPQSTHLDPEIYKEPKRFQFDRFVKDGKEKTDFYKSG

QKVRYYRMPFGSGATQCPGRFFAMNELKQFVCVTLLMCEMQLLDGQQEASMDNSRAGLGI

LPPANPIPFKYKTRKTLDCTDREE

>348541431

MSPLLVLVLGLLLPLLLLVLRGRTRSAWFSVLGQGSSSGPPGGQQHTTHSLYYTFITHFL

SGCVPFGLSSAPAQYGGVLLQVGQEEIPLSVGHKDWKCRGTPSAAQEKTHSQNLGFVKGM

MERFQPLRMNAAQREDEPPLIKGWIPFIGKALEFGKDAHKFLKEHKRKFGDVFTVQIAGK

YMTFIMDPLLYPNIIKQGRQLDFHEFSNRVAPFAFGYPPVTSKFPGLREQIKRSFILLQG

DNLTPLTESMMGNLMLVFRQDYLEQESSWKTAYMYKFCNSIMFEATFLTMYGKPASASRH

SGMSVLEKDFIKFDNMFPLLVAQIPIWLLGRTKAIRQRLINYFLPHQMSCWSHSSEFIRT

RFEMFEQYDTLRDFDKAAHHFAILWASLGNTVPATFWAMYYLVSHPEALQFVRQELEDVL

RLSGVEFSRDQDVTLTPAHLEKLLYMESAIKESFRLCSSSMNIRVAQEDFSLRLDAERSA

AVRKGDVIALYPQALHLDPEIYEDPETFRFDRYVQNGQKKTDFYKDGQKLKHYLMPFGSG

ATKCPGRFFAINEIKQFLSVILLYLDLELEEGQSKATLDLSRAGLGVMLPAKDVRFRYRL

RPV

**CYP8**

>Q16647.1

MAWAALLGLLAALLLLLLLSRRRTRRPGEPPLDLGSIPWLGYALDFGKDAASFLTRMKEK

HGDIFTILVGGRYVTVLLDPHSYDAVVWEPRTRLDFHAYAIFLMERIFDVQLPHYSPSDE

KARMKLTLLHRELQALTEAMYTNLHAVLLGDATEAGSGWHEMGLLDFSYSFLLRAGYLTL

YGIEALPRTHESQAQDRVHSADVFHTFRQLDRLLPKLARGSLSVGDKDHMCSVKSRLWKL

LSPARLARRAHRSKWLESYLLHLEEMGVSEEMQARALVLQLWATQGNMGPAAFWLLLFLL

KNPEALAAVRGELESILWQAEQPVSQTTTLPQKVLDSTPVLDSVLSESLRLTAAPFITRE

VVVDLAMPMADGREFNLRRGDRLLLFPFLSPQRDPEIYTDPEVFKYNRFLNPDGSEKKDF

YKDGKRLKNYNMPWGAGHNHCLGRSYAVNSIKQFVFLVLVHLDLELINADVEIPEFDLSR

YGFGLMQPEHDVPVRYRIRP

>AAD19877.1

MVLWGPVLGALLVVIAGYLCLPGMLRQRRPWEPPLDKGTVPWLGHAMAFRKNMFEFLKRM

RTKHGDVFTVQLGGQYFTFVMDPLSFGPILKDTQRKLDFGQYAKKLVLKVFGYRSVQGDH

EMIHSASTKHLRGDGLKDLNETMLDSLSFVMLTSKGWSLDASCWHEDSLFRFCYYILFTA

GYLSLFGYTKDKEQDLLQAGELFMEFRKFDLLFPRFVYSLLWPREWLEVGRLQRLFHKML

SVSHSQEKEGISNWLGNMLQFLREQGVPSAMQDKFNFMMLWASQGNTGPTSFWALLYLLK

HPEAIRAVREEATQVLGEARLETKQSFAFKLGALQHTPVLDSVVEETLRLRAAPTLLRLV

HEDYTLKMSSGQEYLFRHGDILALFPYLSVHMDPDIHPEPTVFKYDRFLNPNGSRKVDFF

KTGKKIHHYTMPWGSGVSICPGRFFALSEVKLFILLMVTHFDLELVDPDTPLPHVDPQRW

GFGTMQPSHDVRFRYRLHPTE

>BAA11910.1

MAWAALLGLLVALLLLLLLSRRRTRRPGEPPLDLGSIPWLGYALDFGKDAASFLTRMKEK

HGDIFTILVGGRYVTVLLDPHSYDAVVWEPRTRLDFHAYAIFLMERIFDVQLPHYSPSDE

KARMKLTLLHRELQALTEAMYTNLHAVLLGDATEAGSGWHEMGLLDFSYSFLLRAGYLTL

YGIEALPRTHESQAQDRVHSADVFHTFRQLDRLLPKLARGSLSVGDKDHMCSVKSRLWKL

LSPARLARRAHRSKWLESYLLHLEEMGVSEEMQARALVLQLWATQGNMGPAAFWLLLFLL

KNPEALAAVRGELESILWQAEQPVSQTTTLPQKVLDSTPVLDSVLSESLRLTAAPFITRE

VVVDLAMPMADGREFNLRRGDRLLLFPFLSPQRDPEIYTDPEVFKYNRFLNPDGSEKKDF

YKDGKRLKNYNMPWGAGHNHCLGRSYAVNSIKQFVFLVLVHLDLELINADVEIPEFDLSR

YGFGLMQPEHDVPVRYRIRP

>AAG31785.1

MAWAALLGLLAALLLLLLLSRRRTRRPGEPPLDLGSIPWLGYALDFGKDAASFLTRMKEK

HGDIFTILVGGRYVTVLLDPHSYDAVVWEPRTRLDFHAYAIFLMERIFDVQLPHYSPSDE

KARMKLTLLHRELQALTEAMYTNLHAVLLGDATEAGSGWHEMGLLDFSYSFLLRAGYLTL

YGIEALPRTHESQAQDRVHSADVFHTFRQLDRLLPKLARGSLSVGDKDHMCSVKSRLWKL

LSPARLARRAHRSKWLESYLLHLEEMGVSEEMQARALVLQLWATQGNMGPAAFWLLLFLL

KNPEALAAVRGELESILWQAEQPVSQTTTLPQKVLDSTPVLDSVLSESLRLTAAPFITRE

VVVDLAMPMADGREFNLRSGDRLLLFPFLSPQRDPEIYTDPEVFKYNRFLNPDGSEKKDF

YKDGKRLKNYNMPWGAGHNHCLGRSYAVNSIKQFVFLVLVHLDLELINADVEIPEFDLSR

YGFGLMQPEHDVPVRYRIRP

>AAG31783.1

MAWAALLGLLAALLLLLLLSRRRTRRPGEPPLDLGSILWLGYALDFGKDAASFLTRMKEK

HGDIFTILVGGRYVTVLLDPHSYDAVVWEPRTRLDFHAYAIFLMERIFDVQLPHYSPSDE

KARMKLTLLHRELQALTEAMYTNLHAVLLGDATEAGSGWHEMGLLDFSYSFLLRAGYLTL

YGIEALPRTHESQAQDRVHSADVFHTFRQLDRLLPKLARGSLSVGDKDHMCSVKSRLWKL

LSPARLARRAHRSKWLESYLLHLEEMGVSEEMQARALVLQLWATQGNMGPAAFWLLLFLL

KNPEALAAVRGELESILWQAEQPVSQTTTLPQKVLDSTPVLDSVLSESLRLTAAPFITRE

VVVDLAMPMADGREFNLRRGDRLLLFPFLSPQRDPEIYTDPEVFKYNRFLNPDGSEKKDF

YKDGKRLKNYNMPWGAGHNHCLGRSYAVNSIKQFVFLVLVHLDLELINADVEIPEFDLSR

YGFGLMQPEHDVPVRYRIRP

>AAG31784.1

MAWAALLGLLAALLLLLLLSRRRTRRPGEPPLDLGSIPWLGYALDFGKDAASFLTRMKEK

HGDIFTILVGGRYVTVLLDPHSYDAVVWEPRTRLDFHAYAIFLMERIFDVQLPHYSPRDE

KARMKLTLLHRELQALTEAMYTNLHAVLLGDATEAGSGWHEMGLLDFSYSFLLRAGYLTL

YGIEALPRTHESQAQDRVHSADVFHTFRQLDRLLPKLARGSLSVGDKDHMCSVKSRLWKL

LSPARLARRAHRSKWLESYLLHLEEMGVSEEMQARALVLQLWATQGNMGPAAFWLLLFLL

KNPEALAAVRGELESILWQAEQPVSQTTTLPQKVLDSTPVLDSVLSESLRLTAAPFITRE

VVVDLAMPMADGREFNLRRGDRLLLFPFLSPQRDPEIYTDPEVFKYNRFLNPDGSEKKDF

YKDGKRLKNYNMPWGAGHNHCLGRSYAVNSIKQFVFLVLVHLDLELINADVEIPEFDLSR

YGFGLMQPEHDVPVRYRIRP

>3B6HA

MAKKTSSLLSRRRTRRPGEPPLDLGSIPWLGYALDFGKDAASFLTRMKEKHGDIFTILVG

GRYVTVLLDPHSYDAVVWEPRTRLDFHAYAIFLMERIFDVQLPHYSPSDEKARMKLTLLH

RELQALTEAMYTNLHAVLLGDATEAGSGWHEMGLLDFSYSFLLRAGYLTLYGIEALPRTH

ESQAQDRVHSADVFHTFRQLDRLLPKLARGSLSVGDKDHMCSVKSRLWKLLSPARLARRA

HRSKWLESYLLHLEEMGVSEEMQARALVLQLWATQGNMGPAAFWLLLFLLKNPEALAAVR

GELESILWQAEQPVSQTTTLPQKVLDSTPVLDSVLSESLRLTAAPFITREVVVDLAMPMA

DGREFNLRRGDRLLLFPFLSPQRDPEIYTDPEVFKYNRFLNPDGSEKKDFYKDGKRLKNY

NMPWGAGHNHCLGRSYAVNSIKQFVFLVLVHLDLELINADVEIPEFDLSRYGFGLMQPEH

DVPVRYRIRPHHHHHHHH

>2IAGA

RTRRPGEPPLDLGSIPWLGYALDFGKDAASFLTRMKEKHGDIFTILVGGRYVTVLLDPHS

YDAVVWEPRTRLDFHAYAIFLMERIFDVQLPHYSPSDEKARMKLTLLHRELQALTEAMYT

NLHAVLLGDATEAGSGWHEMGLLDFSYSFLLRAGYLTLYGIEALPRTHESQAQDRVHSAD

VFHTFRQLDRLLPKLARGSLSVGDKDHMCSVKSRLWKLLSPARLARRAHRSKWLESYLLH

LEEMGVSEEMQARALVLQLWATQGNMGPAAFWLLLFLLKNPEALAAVRGELESILWQAEQ

PVSQTTTLPQKVLDSTPVLDSVLSESLRLTAAPFITREVVVDLAMPMADGREFNLRRGDR

LLLFPFLSPQRDPEIYTDPEVFKYNRFLNPDGSEKKDFYKDGKRLKNYNMPWGAGHNHCL

GRSYAVNSIKQFVFLVLVHLDLELINADVEIPEFDLSRYGFGLMQPEHDVPVRYRIRPHH

HH

>397475824

MPFLALSNGDKCHGFCRRPGEPPLDLGSIPWLGYALDFGKDAASFLTRMKEKHGDIFTIL

VGGRYVTVLLDPHSYDAVVWEPRNRLDFHAYAIFLMERIFDVQLPHYSPSDEKARMKPTL

LHRELQALTEAMYTNLHTVLLGDATEAGSGWHEMGLLDFSYSFLLRAGYLTLYGIEALPR

THESQAQDRVHSADVFHTFRQLDRLLPKLARGSLSVGDKDHMCSVKSRLWKLLSPARLAT

RAHRSKWLESYLLHLEEMGVSEEMQARALVLQLWATQGNMGPAAFWLLLFLLKNPEALAA

VRGELESILWQAEQPVSQMTTLPQKVLDSTPVLDSVLSESLRLTAAPFITREVVVDLAMP

MADGREFNLRRGDRLLLFPFLSPQRDPEIYADPEVFKYNRFLNPDGSEKKDFYKDGKRLK

NYNMPWGAGHNHCLGRSYAVNSIKQFVFLVLVHLDLELINADVEIPEFDLSRYGFGLMQP

EHDVPVRYRIRP

>332858747

MPAASQSRSRDGLGALLGLWRTVAAAATEPRRTRRPGEPPLDLGSIPWLGYALDFGKDAA

SFLTRMKEKHGDIFTILVGGRYITVLLDPHSYDAVVWEPRTRLDFHAYAIFLMERIFDVQ

LPHYSPSDEKARMKPTLLHRELQALTEAMYTNLHTVLLGDATEAGSGWHEMGLLDFSYSF

LLRAGYLTLYGIEALPRTHESQAQDRVHSADVFHTFRQLDRLLPKLARGSLSVGDKDHMC

SVKSRLWKLLSPARLATRAHRSKWLESYLLHLEEMGVSEEMQARALVLQLWATQGNMGPA

AFWLLLFLLKNPEALAAVRGELESILWQAEQPVSQTTTLPQKVLDSTPVLDSVLSESLRL

TAAPFITREVVVDLAMPMADGREFNLRRGDRLLLFPFLSPQRDPEIYADPEVFKYNRFLN

PDGSEKKDFYKDGKRLKNYNMPWGAGHNHCLGRSYAVNSIKQFVFLVLVHLDLELINADV

EIPEFDLSRYGFGLMQPEHDVPVRYRIRP

>332207767

MAWAALLGLLAALLLLLLLSRRRTRRPGEPPLDLGSIPWLGHALDFGKDAASFLTRMKEK

HGDIFTILVGGRYVTVLLDPHSYDAVVWEPRTRLDFHAYAVFLMERIFDVQLPHYSPSDE

KARMKPTLLHRELQALTEAMYTNLHTVLLGDAMEAGSGWHEMGLLDFSYSFLLRAGYLTL

YGIEALPRTHQSQAQDRVHSADVFHTFRQLDQLLPKLARGSLSVGDKDHMCSVKSRLWKL

LSPARLATRAHRSKWLESYLLHLEEMGVSEEMQARALVLQLWATQGNMGPAAFWLLLFLL

KNPEALAAVRGELESILWQAEQPVSQMTTLPQKVLDSTPVLDSVLSESLRLTAAPFITRE

VVVDLAMPMADGREFNLRRGDRLLLFPFLSPQRDPEIYTDPEVFKYNRFLNPDGSEKKDF

YKDGKRLKNYNMPWGAGHNHCLGRSYAINSIKQFVFLVLVHLDLELINADVEIPEFDLSR

YGFGLMQPEHDVPIRYRIRP

>109091639

MTWAALLGLLAALLLLLLLSRRRTRRPGEPPLDLGSIPWLGYALDFGKDAASFLTRMKEK

HGDIFTILVGGRYVTVLLDPHSYDAVVWESRTRLDFHAYAVFLMERIFDVQLPHYSPSDE

KARMKPTLLHRELQALTEAMYTNLHTVLLGDATEAGSGWHEMGLLDFSYSSLLRAGYLTL

YGIEALPRTHQSQAQDRVHSADVFHTFRQLDRLLPKLARGSLSVGDKDHMCSVKSRLWKL

LSPARLATRAHRSKWLESYLLHLEEMGVSEEMQARALVLQLWATQGNMGPAAFWLLLFLL

KNPEALAAVRGELESILWEAEQPVSQMTTLPQKVLDGTPVLDSVLSESLRLTAAPFITRE

VVVDLAMPMADGREFNLRRGDRLLLFPFLSPQRDPEIYTDPEVFKYNRFLNPDGSEKKDF

YKDGKRLKNYNMPWGAGHNHCLGKSYAVNSIKQFVFLVLVHLDLELINADVEIPEFDLSR

YGFGLMQPEHDVPIRYRIRP

>EHH19608.1

MPFLASSNGDKCHGFCRRPGEPPLDLGSIPWLGYALDFGKDAASFLTRMKEKHGDIFTIL

VGGRYVTVLLDPHSYDAVVWESRTRLDFHAYAVFLMERIFDVQLPHYSPSDEKARMKPTL

LHRELQALTEAMYTNLHTVLLGDATEAGSGWHEMGLLDFSYSSLLRAGYLTLYGIEALPR

THQSQAQDRVHSADVFHTFRQLDRLLPKLARGSLSVGDKDHMCSVKSRLWKLLSPARLAT

RAHRSKWLESYLLHLEEMGVSEEMQARALVLQLWATQGNMGPAAFWLLLFLLKNPEALAA

VRGELESILWEAEQPVSQMTTLPQKVLDGTPVLDSVLSESLRLTAAPFITREVVVDLAMP

MADGREFNLRRGDRLLLFPFLSPQRDPEIYTDPEVFKYNRFLNPDGSEKKDFYKDGKRLK

NYNMPWGAGHNHCLGKSYAVNSIKQFVFLVLVHLDLELINADVEIPEFDLSRYGFGLMQP

EHDVPIRYRIRP

>EHH65254.1

MPFLASSNGDKCHGFCRRPGEPPLDLGSIPWLGYALDFGKDAASFLTRMKEKHGDIFTIL

VGGRYVTVLLDPHSYDAVVWESRTRLDFHAYAVFLMERIFDVQLPHYSPSDEKARMKPTL

LHRELQALTEAMYTNLHTVLLGDATEAGSGWHEMGLLDFSYSSLLRAGYLTLYGIEALPR

THQSQAQDRVHSADVFHTFRQLDRLLPKLARGSLSVGDKDHMCSVKSRLWKLLSPARLAT

RAHRSKWLESYLLHLEEMGVSEEMQARALVLQLWATQGNMGPAAFWLLLFLLKNPEALAA

VRGELESILWEAEQPVSQMTTLPQKVLDGTPVLDSVLSESLRLTAAPFITREVVVDLAMP

MADGREFNLRRGDRLLLFPFLSPQRDPEIYTDPEVFKYNRFLNPDGSEKKDFYKDGKRLK

NYNMPWGAGHNHCLGKSYAVNSIKQFVFLVLVHLDLELINADVEIPEFDLSRYGFGLMQP

EHDVPIRYRIRP

>402882289

MAPKAGEKANMKETHFSRSLQQISGRATGTGGWHPKHQVAATMATTSGGPNMHQPRQPRQ

PRQPRQPRDDLGPRLLGLLAALLLLLLLSRRRTRRPGEPPLDLGSIPWLGYALDFGKDAA

SFLTRMKEKHGDIFTILVGGRYVTVLLDPHSYDAVVWESRTRLDFHAYAVFLMERIFDVQ

LPHYSPSDEKARMKPTLLHRELQALTEAMYTNLHTVLLGDATEAGSGWHEMGLLDFSYSS

LLRAGYLTLYGIEALPRTHQSQAQDRIHSADVFHTFRQLDRLLPKLARGSLSVGDKDHMC

SVKSRLWKLLSPARLATRAHRSKWLESYLLHLEEMGVSEEMQARALVLQLWATQGNMGPA

AFWLLLFLLKNPEALAAVRGELESILWEAEQPVSQMTTLPQKVLDGTPVLDSVLSESLRL

TAAPFITREVVVDLAMPMADGREFNLRRGDRLLLFPFLSPQRDPEIYTDPEVFKYNRFLN

PDGSEKKDFYKDGKRLKNYNMPWGAGHNHCLGKSYAVNSIKQFVFLVLVHLDLELINADV

EIPEFDLSRYGFGLMQPEHDVPIRYRIRP

>296237163

MAFRVEPDHMVLGKHLKGGFKALMTSIKQLSSEELEQFQKTGKKIETKPDHRQWRGAELV

GAGTGGWHPKHQVAVTRATTSGGPNMHQPRQPGQHGDVWAALLGLLAALLLLLLLLSRRR

TRRPGEPPLDLGSIPWLGYALDFGKDAASFLTRMKEKHGDIFTILVGGRYITVLLDPHSY

DAVVWEPHTRFDFHAYAVFLMERIFDVQLPHYSPSDEKARMKPTLLHRELQALTEAMYAN

LCTVLLDDATEVGSGWHEMGLFDFSYSFLLRAGYLTLYGIEALPRTHQSQAEDRIHSADV

FHTFRQLDRLLPKLARGSLSVGDKDHMSNVKSHLWKLLSPARLATRAHRSKWLESYLLHL

EEMDVSEEMQARALVLQLWATQGNMGPAAFWLLLFLLKNPEALAAVRGELESILWPAEKP

FLQMTTLPQKVLDSTPVLDSVLSESLRLTAAPFITREVVVDLAMPMADGREFNLRRGDRL

LLFPFLSPQRDPEIYTDPEVFKYNRFLNPDGSEKKDFYKDGKRLKNYNMPWGAGHNHCLG

RSYAVNSIKQFVFLVLVHLDLELINADVEIPEFDLSRYGFGLMQPEHDVPIRYRIRP

>403282624

MPQHVPVLRDSQQQGLDGRKEVISSNPLPLIQTCFLFPGSGTGDWHPKHQVAVTRATTSG

GPNMHQPPPHAVSAPLRPASLDKGGGSRPGCSPGVSEPAAGLARVQPQYPKQGETLGGGS

PGAPHPGEGARPCVTPAKNAQGGRCGSRCVPRGGSPTGTNPGLALSDGGDLRGGFRRPGE

PPLDLGSIPWLGYALDFGKDAAGFLTRMKEKHGDIFTILVGGRYVTILLDPHSYDAVVWE

PRTRFDFHAYAVFLMERIFDVQLPHYSPSDEKARMKPTLLHRELQALTEAMYANLCTVLL

DDATEAGSGWHEMGLFDFSYNFLLRAGYLTLYGIEALPRTHQSRAEDRIHSADVFHTFRQ

LDQLLPKLARGSLSVGDKDHMSSVKSHLWKLLSPARLATRAHRSKWLESYLLHLEEMGVS

EEMQARALVLQLWATQGNMGPAAFWLLLFLLKNPEALAAVRGELESILWPAEQPVSQMTT

LPQKVLDSTPVLDSVLSESLRLTAAPFITREVVVDLAMPMADGREFNLRRGDRLLLFPFL

SPQRDPEIYTDPEVFKYNRFLNPDGSEKKDFYKDGKRLKNYNMPWGAGHNHCLGRSYAVN

SIKQFVFLVLVHLDLELINRDVEIPEFDLSRYGFGLMQPEQDVPIRYRIRP

>395829171

MSWAALLGLLAALLLLLLLTRRRTRRPGEPPLDLGSIPWLGYALDFGKDAASFLTRMKER

HGDIFTILVGGRYVTILLDPHSYDTVVWEPRTRLDFHGYAVFLMERMFDVQLPHYNPSDE

KARLKPTLLHRDLQVLTDAMYTNLRTVLLGDATEADSGWHEMGLLDFSYSVLLRAGYLTL

YGIEALPRTHESQAQDRVHSAEVFHTFRQLDLLLPKLARGSLSAGDKDRACSIKSRLWKL

LSPGRLATRAHRSKWLESYLLHLEEMGVSEEMKARALVLQLWATQGNMGPAAFWLLLFLL

KNPEALAAVREELQDILWQAEQPVSQMATLPQKVLDSTPVLDSVLSESLRLTAAPFITRE

VVVDLTMVMADGREFNLRRGDRLLLFPFLSPQRDPEIYTDPEVFKYNRFLNPDGSEKKDF

YKDGKRLKNYNMPWGAGHNQCLGRTYATTSIKQFVVLVLMQLDLELMDAGVEVPKFDLSR

YGFGLLQPEHDVPIRYRVRP

>359322687

MNNRRPGEPPLDLGSIPWLGHALEFGKDAASFLTRMKKKHGDIFTVLVGGRYVTVLLDPH

SYDAVVWEPHSKLDFHAYAVFLMERIFDVQLPHYSPSDEKAKMKPTLLHKDLQALTDSMY

ANLRTVLLGDTTEAGSGWHEISLLDFSYSCLLRAGYLTLYGVEALPRTHESQAQDRAHSA

DVFHTFRQLDLLLPKLARGSLSAGDKDQACRVKGRLWKLLSPARLATRAHRSNWLESYLL

YLEEMGVSADMQARALVLQLWATQGNMGPAAFWLLIFLLKNPEALAAVRGEFEQLLSRAE

QPISQMTTLPQKLLDNMPVLDSVLSESLRLTAAPFITREVMVDLAMPMADGREFSLRRGD

RLLLFPFLSPQKDPEIYTDPEVFKYNRFLNSDGSEKKDFYKDGKRLKNYSMPWGAGHNQC

LGKAYAISSIKQFVFLVLVHLDLELINPDMEIPEFDLSRYGFGLMQPEHDVPVRYRTRP

>301754395

MSPKLCDLRGVFRLRLLLSLQILVLISRRKLRRPGEPPLDLGSIPWLGHALEFGKDAASF

LTRMKKKHGDIFTVLVGGRYVTVLLDPHSYDVVVWEPRAKLDFHAYAVFLMERIFDVQLP

HYSPSEEKAKMKPTLLHRDLQALMDAMYTNLRTVLLGDTTGAASGWQEMGLLDFSYSCLL

RAGYLTLYGVEALPRTHESQAQDRTHSADVFHTFRQLDLLLPKLARGSLSVGDKDHACRV

KGRLWKLLSPARLAARAQRSSWLESYLLHLQETGVSEDMQARAMVLQLWATQGNMGPAAF

WLLLFLLKNPEALAAVRGELEQLLSRAEQPISQMTTLPQKVLDSMPVLDSVLSESLRLTA

APFITREAVVDLALPMADGREFSLRRGDRLLLFPFLSPQRDPEIYTDPEVFKYNRFLNSD

GSEKKDFYKDGKRLKNYSMPWGAGHNQCLGKAYAISSIKQFVFLVLVHFDLELVNPDVEI

PEFDLSRYGFGLMQPEHDVPIRYSARP

>Q29626.1

MSWAVVFGLLAALLLLLLLTRRRTRRPGEPPLDLGSIPWLGHALEFGKDAAGFLTRMKEK

HGDIFTVLVGGRHVTVLLDPHSYDAVVWEPRSRLDFHAYAVFLMERIFDVQLPHYNPGDE

KSKMKPTLLHKELQALTDAMYTNLRTVLLGDTVEAGSGWHEMGLLEFSYGFLLRAGYLTQ

YGVEAPPHTQESQAQDRVHSADVFHTFRQLDLLLPKLARGSLSAGDKDRVGKVKGRLWKL

LSPTRLASRAHRSRWLESYLLHLEEMGVSEEMQARALVLQLWATQGNMGPAAFWLLLFLL

KNPEALAAVRGELETVLLGAEQPISQMTTLPQKVLDSMPVLDSVLSESLRLTAAPFITRE

VVADLALPMADGREFSLRRGDRLLLFPFLSPQKDPEIYTDPEVFKYNRFLNPDGSEKKDF

YKDGKRLKNYSLPWGAGHNQCLGKGYAVNSIKQFVFLVLTQFDLELITPDVDIPEFDLSR

YGFGLMQPEHDVPVRYRIRP

>194224516

MSWAVLLGLLAALLLLLLLSRRRARRPGEPPLDLGSIPWLGYALEFGKDAASFLTRMKEK

HGDIFTVLVGGRYVTVLLDPHSYETVVWEPRSKLDFHAYAVFLMERIFDVQLPHYNPSDE

KAKMKPTLLHRDLQALTEAMYTNLCTVLLGDTTEAGSGWQEMGLLDFSYSFMLRAGYLTL

YGVEALPRTPESQAQDRIHSADVFHTFRQLDLLLPKLARGSLSVGDKDQVCKVKGHLWKL

LSPARLACRAHRSSWLDSYLQHLEEMGASEEMQARALALQLWATQGNMGPAAFWLLLFLL

KNPEALAAVRGELEQILLQAEQPVSQMTTLPQKVLDSMPVLDSVLSETLRLTAAPFITRE

VMVDLALPMADGREFTLRRGDRLLLFPFLSPQKDPEVYTDPEVFQYNRFLNPDGTEKKDF

YKDGKRLKNYSVPWGAGHNQCLGRAYATSSIKQFVFLVLTRCDLELTSPDAEIPEFDLSR

YGFGLMQPERDVPIRYRLRP

>350595035

MATIFRAPAALQARVAVEADPQLPALGRGSPVAGPAMSWAVVFGLLAALLLLLLLLLLTR

RRTRTGSPAAPGEPFRGGSAAPRPEGFPRCSRPRGAGPAERQLPRGHKVGRRSWEALSNG

NKCCGFRRRPGEPPLDVGSIPWLGYALEFGKDAASFLTRMKEKHGDIFTVLVGGRYVTVL

LDPHSYDSVVWEPRTKLDFHAYAVFLMERIFDVQLPHYNPGDEKSKMKPTLLHRELQLLT

EAMYTNLRTVLLGDTMETGSGWHEMGLFDFSYSFLLRAGYLTLYGVETPPWTQESQAQDR

VHSADVFHTFRQLDLLLPKLARGSLSVGDKDRVCRVKGRLWKLLSPARLATRAHRSKWLE

SYLQHLEEMGVSEQMQARALVLQLWATQGNMGPAAFWLLLFLLKNPEALAAVRGELEPIL

SRAEQPISQMTTLPQKVLDSTPVLDSVLSESLRLTAAPFITREVVADLALPMADGREFTL

RRGDRLLLFPFLSPQKDPAIYTDPEVFKYNRFLNPDGLEKRDFYKDGKRLKNYSLPWGAG

HNQCLGRAYAVSSIKQFVFLVLAHFDLELSSPDVEMPEFDLSRYGFGLMQPERDVSIRYR

LRA

>AAA53674.1

MSWAVVFGLLAALLLLLLLTRRRTRRPGEPPLDLGSIPWLGHALEFGKDAAGFLTRMKEK

HGDIFTVLVGGRHVTVLLDPHSYDAVVWEPRSRLDFHAYAVFLMERIFDVQLPHYNPGDE

KSKMKPTLLHKELQALTDAMYTNLRTVLLGDTVEAGSGWHEMGLLEFSYGFLLRAGYLTQ

YGVEAPPHTQENQAQDRVHSADVFHTFRQLDLLLPKLARGSLSAGDKDRVGKVKGRLWKL

LSPTRLASRAHRSRWLESYLLHLEEMGVSEEMQARALVLQLWATQGNMGPAAFWLLLFLL

KNPEALAAVRGELETVLLGAEQPISQMTTLPQKVLDSMPVLDSVLSESLRLTPAPFITRE

VVADLALPMADGREFSLRRGDRLLLFPFLSPQKDPEIYTDPEVFKYTRFLNPDGSEKKDF

YKDGKRLKNYSLPWGAGHNQCLGKGYAVNSIKQFVFLVLTQFDLELITPDVDIPEFDLSR

YGFGLMQPEHDVPVRYRIRP

>O35074.1

MSWAALLGLLAVLLLLLLLLSRRRARRPGEPPLDLGSIPWLGHALEFGRDAASFLTRMKE

KHGDIFTVLVGGRYVTVLLDPHSYDTVVWELRTRLDFHPYAIFLMERIFDLQLPNFNPSE

EKARMKPTLMHRDLQALTEAMYTNLRTVLLGDSTEAGSGWQETGLLEFSYNALLSAGYLT

LYGVEASPRTHESQAQDRVHSADVFHTFRQLDLLLPKLARGSLSAGDKDHACSVKNRLWK

LLSPARLASRADRSSWLESYLRHLEEMGVSEEMQARALVLQLWATQGNMGPTAFWLLLFL

LKNPEALAAVRAELKHTVWQAEQPVSQMTTLPQKILDSMPVLDSVLNETLRLTAAPFITR

EVMADLALPMADGREFSLRRGDRLLLFPFLSPQKDPEIYTEPEVFKYNRFLNPDGSEKKD

FYKDGKRLKNYNMPWGAGHNQCLGKSYAINSIKQFVVLLLTHFDLELGSEDTEVPEFDLS

RYGFGLMQPEEDVPIRYRARL

>EGW05508.1

MSWAALLGLLAALLLLLLLLSRRRARRPGEPPLDLGSIPWLGHALEFGRDAASFLTRMKE

KHGDIFTVLVGGRYVTVLLDPHSYDTVVWEPRTRLDFHPYAIFLMERIFDLQLPNFNPSE

EKAKMKPMLMHRDLQALTEAMFTNLRTVLLGDSTEAAGGWQETGLLDFAYSSLLRAGYLT

LYGVEALPRTHESQAQDRVHSADVFTTFRQLDLLLPKLARGSLSTGDKDHACSVKGRLWK

LLSPARLATRADRSSWLESYLKHLEEMGLSEEMQARALVLQLWATQGNMGPTAFWLLLFL

LKNPEALEAVHEELKRTVWKAEQPVSQMITLPQKTLDNMPVLDSVLNETLRLTAAPFITR

EVMADLALPMADGREFSLRRGDRLLLFPFLSPQKDPEIYTEPEVFKYNRFLNPDGSEKKD

FYKDGKRLKNYTLPWGAGHNQCLGKNYAINSIKQFVVLLLTHFDLELGSKDTEVPEFDLS

RYGFGLMQPEEDVPIRYRSRL

>Q62969.1

MSWAALLGLLAVLLLLLLLLSRRRARRPGEPPLDLGSIPWLGHALEFGKDAASFLTRMKE

KHGDIFTVLVGGRYVTVLLDPHSYDTVVWDLRTRLDFHPYAIFLMERIFDLQLPNFNPSE

EKARMKPTLMHKDLQALTEAMYTNLRTVLLGDSTEGGSGWQEKGLLEFSYSSLLSAGYLT

LYGVEASPRTHESQALDRDHSADVFRTFRQLDLMLPKLARGSLSVGDKDHACSVKSRLWK

LLSPAGLASRADRSSWLESYLRHLEEMGVSEDMQARALVLQLWATQGNMGPTAFWLLLFL

LKNPEALDAVHAELKRIVWQAEKPVLQMTALPQKILDSMPVLDSVLNETLRLTAAPFITR

EVMADLALPMADRREFSLRRGDRLLLFPFLSPQKDPEIYTEPEVFKYNRFLNPDGSEKKD

FYKDGKRLKNYNMPWGAGHNQCLGKSYAINSIKQFVVLLLTHFDLELVSEDTEVPEFDLS

RYGFGLMQPEEDVPIRYRTRL

>EDL96416.1

MSWAALLGLLAVLLLLLLLLSRRRARRPGEPPLDLGSIPWMGHALEFGKDAASFLTRMKE

KHGDIFTVLVGGRYVTVLLDPHSYDTVVWDLRTRLDFHPYAIFLMERIFDLQLPNFNPSE

EKARMKPTLMHKDLQALTEAMYTNLRTVLLGDSTEGGSGWQEKGLLEFSYSSLLSAGYLT

LYGVEASPRTHESQALDRDHSADVFRTFRQLDLMLPKLARGSLSVGDKDHACSVKSRLWK

LLSPAGLASRADRSSWLESYLRHLEEMGVSEDMQARALVLQLWATQGNMGPTAFWLLLFL

LKNPEALDAVHAELKRIVWQAEKPVLQMTALPQKILDSMPVLDSVLNETLRLTAAPFITR

EVMADLALPMADRREFSLRRGDRLLLFPFLSPQKDPEIYTEPEVFKYNRFLNPDGSEKKD

FYKDGKRLKNYNMPWGAGHNQCLGKSYAINSIKQFVVLLLTHFDLELVSEDTEVPEFDLS

RYGFGLMQPEEDVPIRYRTRL

>348564132

MAPPASRCPQPSPRRGPGLRLESGGPGAAPRASSRGTPGASGETEAPKASPALTAGTRQN

RACLSQPRGPPNFGTLKRSQIMVLAGDKDKIMTRPGEPPLDLGNIPWLGHALEFGRDSAS

FLTRMKEKHGDIFTVLVKGRYVTVLLDPHSYDTVTWESRTKLDFHGYAVILMEKIFNVRL

PHYDPSDEKAKMKLTLQHRDLQALTDAMYSNLRTVLLGEPAEAAGDWHKTGLFEFSYSAL

LRAGFLTLYGVEASPGTPESQAQDRTHSAEVFHTFRQLDLLFPKLARGSLSAGEKDSACS

VRGRLWKLLSPASLAPRALRSSWLQSYLAHLEELGVSPEMQARALVLQLWATQGNMGPAA

FWLLVFLLKNPEALAAVCEELERILLQTEQPVSPMGALPQKALDNTPVLDSVLSESLRLT

AAPFITRDVVVDMALPLADGREFALRRGDRLLLFPFLSPQRDPEIYTDPEVFKYNRFLNP

DGSEKRDFYKGGKRLKNYNMPWGAGHNQCLGKSYAIYSIKQFVFLMLTHFDLELVSTDEE

IPKFDLSRYGFGLMQPEHDVAVQYRVRP

>EDL06503.1

MSWAALLGLLAVLLLLLLLLSRRRARRPGEPPLDLGSIPWLGHALEFGRDAASFLTRMKE

KHGDIFTVLVGGRYVTVLLDPHSYDTVVWELRTRLDFHPYAIFLMERIFDLQLPNFNPSE

EKARMKPTLMHRDLQALTEAMYTNLRTVLLGDSTEAGSGWQETGLLEFSYNALLSAGYLT

LYGVEASPRTHESQAQDRVHSADVFHTFRQLDLLLPKLARGSLSAGDKDHACSVKNRLWK

LLSPARLASRADRSSWLESYLRHLEEMGVSEEMQARALVLQLWATQGNMGPTAFWLLLFL

LKNPEALAAVRAELKHTVWQAEQPVSQMTTLPQKILDSMPVLDSVLNETLRLTAAPFITR

EVMADLALPMADGREFSLRRGDRLLLFPFLSPQKDPEIYTEPEVFKYNRFLNPDGSEKKD

FYKDGKRLKNYNMPWGAGHNQCLGKSYAINSIKHGGIVFVVIVIIIVQRFKQEARPGQQG

QVVFLLPHYLIWSPTSRCYPHSGQSSYIN

>344279746

MRVLVGGRYVTVLLDPHCYDTVVWESRTKLDFHAYAVFLMERIFDVQLPHYNPSDEKARM

KPTLLHRDLQALTEAMYTNLRTVLLDDATEAGGGWHETGLLDFSYSSLLRAGYLTLYGVE

ALPRTHQSQTQDRAHSADVFHTFRQLDLLLPKLARGSLSVGDKDHVSKAKGRLWKLLSPA

RLATRAHRSTWLESYLLHLEEMGVSEEMQARALVLQLWATQGNMGPAAFWLLLFLLKHPE

ALTAVRGELERALCQVEEPVSQMTTLPQKVLDSMSVLDSVLSESLRLTAAPFITREVVVD

MAIPMADGREFSLRRGDRLLLFPFLSPQRDSEIYTDPEKFKYDRFLNADGSEKKDFYKDG

KRLKNYNMPWGAGHNQCLGRSYAINSIKQFVFLMLVHFDLELTNPDVEIPEFDLSRYGFG

LMQPEHDVPIRYRFRP

>EHB04133.1

MKDKHGDIFTVLLGGRYVTVLLDPHSYDKVMWEPRTRLDFHAYAVLLMERIFSVQLPHYD

PSNEKAKMKPTLEHSNLQALTDAMYANLHTVLRGDPAEAAGDWHESGLFEFCYSGLLRAG

YLTLFGVEASPGTLESQAQDRTHSANIFHTFRQLDLLLPKLARGSLSAGEKDQVRSVRGH

LWKLLSPARVATRALRSSWLESYLLHLEEMGASVEMQGRALVLQLWATQGNMGPAAFWLL

LFLLKNPEALAAVRRELQCILCQPGQPVSQMSALPPKALDSTPVLDSVLSESLRLTAAPF

ITREVVVDMALPLADGREFSLRRGDRLLLFPFLSPQRDPEIYTDPEAFKYNRFLNPEGTE

KKDFYKGGKQLKNYNLPWGAGHNQCLGKNFAIYSLKQFVFLALMHFDLKLVRADEEIPEF

DLSRYGFGLMQPKNDVLVRYRAQL

>334312362

MPWFFVAALLAALLLLLLLSRRRMRRPGEPPLDLGLIPWLGHASEFGKDAARFLSRMEGK

HGDIFTVLVAGRFVTVLLDPHSYDTVVWESSSKLDFHKYAIFLMEKIFDVQLANYNPSME

KAKIKPTLLHKNLHALTEAMFNNLCNILLRDAGDIGKDWKETGLFQFSYNVMLRAGYLTL

YGTEGDDPVSQAQDRTHSEEVFQSFRQLDQLLMKLARSSLSAEEKKEASHIKARLWKLLS

PQGLMGRAHRSAWLDSYHQYLQDLGAAEEKQARAMLLQLWSTQGNAGPAAFWLLTFLLKT

PTALAAVLGELESTLQKEGQVLTQMASIPQGVLDNMPIFDSVLSESLRLTAAPFITREVL

EDLVLPLADGREYSLRQGDRLLLFPFLSPQKDPEIYEDPEVFKYNRFLNQDGSEKRDFYK

GGKRLKNYNLPWGAGTNMCLGRSHAISSIKQFVIILLMQFDLKLKNPEEKIPSFDTSRYG

FGLMQPEHDITICYRFKPRETPGSTGLSFPAPDGITEA

>297707306

MCECPRRTPDPLSASPRAGYLTLYGIEALPRTHQSQAQDRVHSADVFHTFRQLDRLLPKL

ARGSLSVGDKDHMCSVKSRLWKLLSPARLATRAHRSKWLESYLLHLEEMGVSEEMQARAL

VLQLWATQGNMGPAAFWLLLFLLKNPEALAAVRGELESILWQAEQPVSQMTTLPQKVLDS

TPVLDSVLSESLRLTAAPFITREVVVDLAMPMADGREFNLRRGDRLLLFPFLSPQRDPEI

YTDPEVFKYNRFLNPDGSEKKDFYKDGKRLKNYNMPWGAGHNHCLGRSYAVNSIKQFVFL

VLVHLDLELISADVEIPEFDLSRYGFGLMQPEHDVPVRYRIRP

>395506875

MPWFFLAALLAALLLLLFLSRRRMRKPGEPPLDQGLIPWLGHASEFGKDAAGFLSRMEKK

HGDIFTVLVAGRFITVLLDPHSYDTVVWESSSKLDFHKYAIFLMEKIFDVQLTNYDPGLE

KAKVKPTLLHKNLQALTGAMFNNLCNILKDAGDMSTDWQETGLFQFSYNVMLRAGYLTLF

GTEGDDPVSQARDRAHSAKVFQGFYQLDQLLMKLARNSLSGEEKKKASLIKAHLWKLLSP

QSLVGRAHRSAWLDSYQQYLEELGAGEEMQARAMLLQLWATQGNAGPAAFWLLLFLLKTP

TALAAVIGEIESTLQKEGQLLTEMPSIPQEVLDSMPIFDSVLSESLRLTAAPFITREVLV

DLVLPLADGREYSLRQGDRLLLFPFLSPQRDAEIYEDPEEFKYNRFLNQDGSEKQDFYKA

GKRLKNYSLPWGAGTNMCLGRSHAISSIKQFVIVLLMQFDLKLKNPEEKIPSFDTSRYGF

GLMQPEHDITICYRPKSRGTASSTELPFPAPGGTTET

>327271842

MTHRRPDEPPLDRGLIPWLGHALEFGKDAVKFLSEMKKKHGDIFTVQAAGKFITILLDPL

SYDVVLWESRSKLDFNPYARILMDRMFDVRLPEYDPNEEKAMLRMTLQKPNLSSLTKAMS

FNLRTVLLYDPSIASRQWKEEDLFHLCYSTMLRAGYLTLYGNEMKDYEDVASQTKDQAHS

LEVYNEFYKLDRLLMKAARSSLSAGEKKKFDSIKKDLWELLSVKRLDGRANRSVWLDSYK

QHLVDLGVDEDMQTRAMLLQLWSTQSNAGPAVFWLLLFLLKHPSAMAAVRGEMEKTLSRK

QTTGQVHEISQEKLDSTPIFDSALNETLRLTAAPFITREVLQDMCLRLHDCREYRLRKGD

RLCLFPYISPQMDPEIYEQPEIFKYDRFLNVDGTEKTDFYKGGEKLKYYTMPWGAGINVC

IGKFFAVNSIKQCIFLFLTYFDLELKDPKAKIPDFDKERYGFGMLQPEIDVIVRYKLKEQ

>AFJ51025.1

MGAFLLSASWLGFFLLLLLLLLLVTSLSGRTRRHDEPPLDKGHIPWLGHALAFGKDAAKF

LSEMKEKHGDIFTVKVAGKFVTVLLDPHSYDVVLWESCAKLDFGQYARILMKRMFDVTLP

SYDSNEEKAMLRPTLKDKNLDSLTKAMCLNLQNILLPGPDCAGRQWKEEGLFQLSYSTMF

RAGFLTLYGNESKNYEDVNSQDKDRTHSMEVYNAYYKLDRFLMKAARSTLSAGEKLKFYN

IRKDLWNFFSMERLDSRANQSVWLNSYKEHLVDLGVNEDMQTRAMVLQLWSTQSNAGPAV

FWLLLFLLKHPPAMAAVQAEIEKLFRNRRLTTRPICEIISQDVLDSTPIFDSALNETLRL

TAAPFISREVLQDMALRLAAGREYHLRKGDRLCLFPFISPQMDPEIYQEPEKFVYDRFLN

SDGTEKKDFYKGGKRLKYYNMPWGAGVNACIGKFFAVNSIKLCVFHLLNCFDLELIDSAK

KIPGFDKERHGFGMLQPEMDVRIRYKLKAQ

>348507565

MIWTILLLVLLIFILTNRWRSKGEPPLDKGAIPWLGHALEFGKDASKFINRMKMKHGDVF

TVRVAGRYVTMLLDPHSYDTVLGDSDSLDFTRYAQVLMERIFRLHLPYSQQAKSKEIMKR

HFVGMNLATLNSAMSRNLQALMKAKTTQNQKDWKEEGLFNFSYSLLFRAGYLMLFGGEQN

NNPIDPSSVYEEYKKFDGLLVKMARGTLTQEEKKTAQSVQNKLWELLAPAGLTDDSGSSP

WLHAYRQFLQEDGTDVEMQNRAILMQLWATQGNVGPAAFWLLGFLLTNPEALMAVKREFS

QNTQMETTTGTPLMDSQVNTPVFDSALEEALRLTAAPFITREVVREKTLRMADGRQYLLR

KGDRVCLFPYVSPQMDPEIYHEPQKYKYDRFLREDGSVKKDFYKGGKRLKYYTMPWGAGT

NGCVGKQFAINTIRQFVYMVLTNYDLELCDPNAQMPEINASRYGFGMLQPEGDLPVRYKP

RSTH

>ABX51998.1

MMWTALLLVGLSILVIVLYGRRTRRRNEPPLDKGMIPWLGHALEFGKDAAKFLTRMKEKH

GDIFTVRAAGLYITVLLDSNCYDAVLSDVASLDQTSYAQVLMKRIFNMILPSHNPESEKK

RAEMHFQGASLTQLSNSMQNNLRLLMTPSEMGLKTSEWKKDGLFNLCYSLLFKTGYLTVF

GAENNNSAALTQIYEEFRRFDKLLPKLARTTVNKEEKQIASAAREKLWKWLTPSGLDRKP

REQSWLGSYVKQLQDEGIDAEMQRRAMLLQLWVTQGNAGPAAFWVMGYLLTHPEALRAVR

EEIQGGKHLRLEERQKNTPVFDSVLWETLRLTAAALITRDVTQDKKICLSNGQEYHLRRG

DRLCVFPFISPQMDPQIHQQPEMFQFDRFLNADRTEKKDFFKNGARVKYPSVPWGTEDNL

CPGRHFAVHAIKELVFTILTRFDVELCDKNATVPLVDPSRYGFGILQPAGDLEIRYRIRF

>53986561

MTFWAVFLCTLAVSLLGGLYVLGTFRRRRPNEPPLDKGIIPWLGYALDFRQDSSGFLSKM

QRKHGDIFTVLIGGHYFTFVMDPFCFGAIVKESRSKLDFLTFAAKLVLRVFGYKSNKDNN

SALHSFNTKHLMGEGLSLLTQATMDNFQKLMLFKLSSGEEKQPWQEAGLFHYCYNIVFRA

GYLALYGTESYQGTGNKEKAHEQDLIHSNQLFTEFRKYDRLFPRLAYAVLPPKDKVEAER

LKRFFWDALSVSKDSQKENVSRWISEQDQYLAESGVPDYMRDRFKFMMLWASQGNTGPTA

FWLLLYLMKDPEAMKAVKGEVEKVLREHGQEVKPGSPPVNITRDMLDQTPFLDSAVEETL

RLVAAPILIRAVLQDVNLKMSSGREYTLRKGDRVALFPHLSVQMNPEIHPEPHKFKYDRF

VNPDGTKKDFYKNGKRLKYFNMPWGAGVSTCPGRFFATAEMKLFVFLMLTHYDLELVNDK

EEIPAIDSSRWGFGTMQPVHDVRFRYRPRV

>3B98A

MAKKTSSVLYGRRTRRRNEPPLDKGMIPWLGHALEFGKDAAKFLTRMKEKHGDIFTVRAA

GLYITVLLDSNCYDAVLSDVASLDQTSYAQVLMKRIFNMILPSHNPESEKKRAEMHFQGA

SLTQLSNSMQNNLRLLMTPSEMGLKTSEWKKDGLFNLCYSLLFKTGYLTVFGAENNNSAA

LTQIYEEFRRFDKLLPKLARTTVNKEEKQIASAAREKLWKWLTPSGLDRKPREQSWLGSY

VKQLQDEGIDAEMQRRAMLLQLWVTQGNAGPAAFWVMGYLLTHPEALRAVREEIQGGKHL

RLEERQKNTPVFDSVLWETLRLTAAALITRDVTQDKKICLSNGQEYHLRRGDRLCVFPFI

SPQMDPQIHQQPEMFQFDRFLNADRTEKKDFFKNGARVKYPSVPWGTEDNLCPGRHFAVH

AIKELVFTILTRFDVELCDKNATVPLVDPSRYGFGILQPAGDLEIRYRIRFHHHH

>ABC87983.1

MMWTALLLLGVVVLFLYARRTRRRNEPPLDKGMIPWLGHALEFGKDAAKFLTRMKQKHGD

IFTVRAAGKYITVLLDSNCYDEVLADVITLDQTAYAHLLMKRIFNLNLPNHNPESEKKRA

ETHFQGPALTQLRSSMKNNLLRLMDSLEMDLKMSEWKEDGLFNFCYSLLFKTGYLTVFGV

ENNNSAALTEVYEEFRRFDKLLPKLARTTVNKEEKQIANSAREKLWKFLTSQCLDRTKEQ

SWLGKYVEQLQDEGLDTETQRRAMLLQLWVTQGNAGPAAFWVLGCLLTHPEALRAVREEI

QGGKHLRPDETQKNTPVFDSVLKETLRLTAAALITREAIQDKKIRLSNGQEYQLRRGDRL

CVFPFISPQMDPQIHQQPEMFQFDRFLNADGTEKKEFFKEGVRVKHPSVPWGTQDNLCPG

RYFAVSAIKELVFTILDRFDRELCNKNATLPLVDPSRYGFGILQPAGDLEIRYRIRS

>348521870

MWWTLLSVITGVLILVVRFSTRRRRRNEPPLDKGWIPWLGHAIEFGKDAAKFLARMKEKH

GDIFTVRVAGLRVTVLLDPHSFDSVLNDRDSFDFTRIRNQVIQKVFSLQLTNIEPTSERR

WMEQHFEGSSLSKLSSSMNTHLHTLLLHCHNGSEWKQEGLFDLCYSLLFRAGYLTFFQRT

DNAAAAYEEFRKFDSLLTKLARGSLRREERRAVSYSQERLWELLSPHGLRGGSESSTWQK

SYCDFLQQAGIDADMQKKALLLQLWTTQCNAGPAAFWLLGFLLTHPEAMEAVKSEIRGLS

TRRDTSFQQPLNNLLEAHSTPVFDSVLSETLRLTAAVMIRREVVQDKILDMANGQRYHLR

QGDRVCLFPFLSPQMDPQIHQEPQTFKYDRFLNEDMTVKNEFYKDGKKLKYYSLPWGAGK

NSCVGKEFAVSTIKRFVFLALTHLDLELCDPEAKLPPVNPSRYGFGMLQPDGDLQVRYKL

KRT

>326921359

MAFWTVFLCTLVASLLGGLYLLGVFRRRRPNEPPLDKGIIPWLGYALDFRKDSSGFLSKM

QRKHGDIFTVLIGGYYFTFVMDPSCFGAIVKESRSKLDFLTFSAKLVLQVFGYKSDKNIN

SALHAFNTKHLMGEGLSLLTQATMDNFQKLMLFKLSSGEEKRPWQEAGLFHFCYNIVFRA

GYLALYGTESHQGTGNKEKAHEQDLIHSNQLYTEFRKYDRLFPRLAYAVLPPKDKVEAER

LKRFFWNALSVTKSSQKENVSRWISEQDQFLAESGVPDYMRDRFKFMMLWASQGNTGPTS

FWLLLYLMKNPEAMKAVKGEIEKVLREHGQEVKPGSPPINITRDMLAQTPFLDSAVEETL

RLVAAPILIRAVLQDVSLKMSGGTEYTLRKGDRLALFPHLSVQMNPEIHPEPHEFKYDRF

VNPDGTKKDFYKNGKRLKYFNMPWGAGVSICPGRFFATAEIKLFVSLMLTHYDLELVNDK

EEIPAIDSSRWGFGTMQPVHDVQFRYRQRV

>301619961

MALFLPIILALLVSVIGGLYLLGMFRKRRPDEPPLDKGTIPWLGYALDFRKNTSTFLQKM

HKKHGDIFTVQIAGYYFTFVMDPLSFGPIIKESKGNLDFEEFAKDLVLRVFGYQSFTNDH

KMLEKSSTKHLMGDGLIVMTQAMMENLQNLMVHNIGSGKGEREWQQDGLFNYSYNIVFRA

GYLALYGNEPAKNKGSKEKAKEFDRKHSDELFYEFRKYDQLFPRLAYAVLPPKDKIEAER

LKRLFWNMLSVKKTLQKENISGWIGEQHQQRAEQGLPEYMQDRFMFLLLWASQGNTGPAS

FWFLLYLLKHPEALKAVREEVEAVLKETGQEVKPGGPLINLTRDMLMKTPVMDSAVEETL

RLTAAPVLIRAVKQDMKIKMASGKDFSMRKGDRVALFPYIAVQMDPEIHAEPEKFKYNRF

LNEDGTKKTEFFKNGKKVKYYTMPWGAGSTICPGRFFATNELKQFAFLMLTYFEFELVNP

NEEIPSIDPNRWGFGTMQPTRDVQFRYRLRY

>348501158

MELLLPILLGFLACLVGGLYLLRVFRQRRPGEPPLDKGLIPWLGHVLEFRRNTLKFLERM

EKKHGDIFTVQLGGFYITFVQDPFSFGGFVKESRDKLDARKVALHLAHRVFGYKAVEGEH

SILRISSNKHLKGEGLEEVTQAFMSNLQNMMLHNIGSVAEDRTWKEGGLFRYSYNVVFRA

GYLALFGNVPHKSEQSEEKAKEKDRAESEALFHEFRKYDQLFPRLAYGVLPPMEKLAAER

LKAYFWDVLSILKMKTKDNISGWVWDIQQGKEEMGIDMSMINRYMFVLLWASQGNIGPSS

FWLLLFLMKHPEAMKAVKEEVDKVLKESGQEVRPGGPMVNLTREMLLKTPILDSAVEENL

RLTAAPLLTRAVIKDMTFKMADGREYLLRKGDRIAIFPFSSVHHDPEIHPEPHSFKYDRF

LNPDGTKKTDFYKAGKRVKYYTMPWGSGVSMCPGRFFATSELKQFVFLMLVYFELELMNP

DEKIPETDVSRYGFGTMQPVRDVQFRYRLRY

>224044552

MVLWVALLCTLVSSLLGGLYVLGVFRRQRPSEPPLDKGTIPWLGYLLEFRKDSSEFLKRM

QSKHGDVFTVLIGGYYFTFVMDPFCFGTIVKESRSKLDFRTSATQLVVQVFGYKPTEATH

SVVHAASTKHLMGDGLTALTQATMESFRKLLLCSLSSGEKRVWQEENLFHYCYNIIFRAG

YLALYGSEPPRGAGNKEEAEEQDRLHSNQLFQEFRKYDRLFPHLAFALLPPKDKREAERL

KRHFWNVLSVKKVCQKDNISGWISDQEQLLAENGVPEYMRDRFMFMLLWASQGNTGPSAF

WLLLYLLKHPEALKAVRDEVDKVLRESGQEVKAGSPPVTVTRDMLNQTPLLDSALEETLR

LVAAPMLVRAVLEDTSLRTSSGTEFTLRRGDRVALFPHISVQMNPEIHQEPHKFKYDRFV

NPDGTRKDFYRNGKKLKFVSMPWGAGVSTCPGRFFATAEMKLFVFLMLSHFDLELVNEEE

ETPAIDSSRWGFGTMQPVRDIRFRYRPRF

>CAF98429.1

MAPLLLILLTLLVALLGGLYLLGVFRQRRPGEPPLDKGLIPWLGHVLEFRRNTWKFLDRM

KQKHGDVFTIQLAGFYITFIQDPMSFGAFVKESRDKLDFRKFAAHLVYRVFGYITVEGDH

ESLNTSSNRHLKGDGLEVMTQAMMSSLQNLMLHNVGPCSDHMTWREDKLFAYCYNIVFRA

GYLSLYGNEPFNSNISEEKAKEKDRAESEALYHEFRKYDQLFPRLAYGVLPPKKQREATK

LLEYFWNVLSIQKVKSRDNISRWIWDVQESKDNAGVKEDMLSRYMFVLLWASQGNTGPSS

FWLLLFLMKHPEALRAVKEEINKVVRESGQEVKRGGPLVNLSREMLMKTPILDSAVEETL

RLSAAPLLTRAVLQDMTINMADGKKYFIRQGDRVALFPYSAVQMDPEIHPDPHSFKYDRF

LNPDGSEKTDFYKAGNKVKYYTMPWGSGISMCPGRFFATNELKQFAFLMLLYFELELINP

DEEIPEMDQSRYGFGTMQPDRDVQFRYRLRY

>AAH77160.1

MALVQILLALLISVIGALYLLGSFRRRRTGEPPLEKGPIPWLGHVLEFRKDTAKFLNRMK

AKHGDIFTVQLGGFYFTFITDPLSFGAVVKEARAKLDFTKFAEQLVQRVFGYHSIQSEHK

VLQASSTKHLMGDGLVVMTQAMMYNLQNLMLHSVGSGNGKVWQESGLFAYSYNIVFRAGY

LSLFGNESPKGTGKESVEKAKEIDRQESNDLFWEFRKYDQLFPNLAYGVLGPSEKMEAER

LKRLFWSTLSVQKMRARDNISGWVSDQQQVRAEHGMQEFMQDRYMFLLLWASQGNTGPSA

FWLLLYLMKHPEAMSAVRKEVEEILKEAGQEVKPGGPLIDLSRDMLLKTPILDSAVEETL

RLTAAPILTRAVMQDMTIIMANGQEYKIREGDRVAVFPYVVHVDPEVHPDPLTFKYDRFL

NADGSRKTDFYKGGKKLKYYSMPWGAGTTMCPGRFFATNELKQFVFLMLSYFDFELTNPN

EQIPGIDIRRWGFGSMQSDRDIQFRYRPRI

>AAI54282.1

MESLVQILLALLLSVIGALYLLGGFRRRRTGEPPLEKGLIPWLGCVLEFRKDTVKFLQRM

KAKHGDIFTVQLGGFYFTFITDPQSFGAVVKESRAKLDFTNFADQLVERVFGYGAVEIKH

KFFKNLQTTSTKHLMGDGLVVMTQAMMYNLQNLMLHSVGSGNGKVWQESGLFAYSYNIVF

RAGYLSLFGNESPKGTGKESVEKAKEIDLQHSNDLFWEFRKYDQLFPNLAYGVLGPRGKM

EAERLKRLFWNMLSVQKMKARDNVSGWVSDQQQVRAEHGMQEFMQDRFMFLLLWASQGNT

GPSAFWLLLYLMKHPEAMSAVRKEVEEILKEAGQEVKPGGPLIDLSRDMLLKTPILDSAV

EETLRLTTAPLLTRGVMEDMTIIMANGQEYKIREGDRVSVFPYLVHIDPEVHPDPLTFKY

DRFLNADGSRKTDFYKDGKKLKYYSMPWGAGTTMCPGRFFATNELKQFVFLMLSYFDLEL

KNPNEEIPSIETRRWGFGSMHPDRDIQFRYRPRI

>CAF98424.1

MAPLLLILLALLVALLGGLYLLGVFRQRRPGEPPLDKGLIPWLGHVLEFRRNTWKFLDRM

KQKHGDVFTIQLAGFYITFIQDPMSFGAFVKESRNKLDFRKFSAHLVYRVFGYITVEGDH

EGLNTSSNRHLKGDGLEVMTQAMMSSLQNLMLHNVGPCSDHMTWREDKLFAYCYNIVFRA

GYLSLYGNEPFNSNISEEKAKEKDRAESEALYHEFRKYDQLFPRLAYGVLPPKKQREATK

LLEYFWNVLSIQKVKSRDNISRWIWDVQESKDNAGVKEDMLSRYMFVLLWASQGNTGPSS

FWLLLFLMKHPEALRAVKEEINKVVRKSGQEVKRGGPLVNLSREMLMKTPILDSAVEETL

RLSAAPLLTRAVLQDMTINMADGKKYFIRQGDRVALFPYSAVQMDPEIHPDPHSFKYDRF

LNPDGSKKTDFYKAGNKVKYYTMPWGSGISMCPGRFFATNELKQFAFLMLLYFELELINP

DEEIPEMDQSRYGFGTMQPDRDVQFRYRLRY

>334333694

MALWVFVFIILVSSVLVVLYALGVLRQRRAREPPLDKGPIPWFGYTWHLSKDASSFLKKM

HRRYGNIFTILLGGKYVTFVTDPLSFDAILKENRSKLDFSNFASQLVFKIFEFQGPKQYQ

KVLKSHSTKYLMGSSLAVMTQAMMDNLQVLLLELDADMKPGLQEWKQDGLLEHCYNIVFR

AGYLALFGSLPTSGKSGHIQDLLRSQKVFEEFRKFDRLFPRAFLSALLPNEKQEMKRLRR

FFWDILAPQKILEKDNVSPWIYFSQQQLADSGVSDIGQSQNNLILLWASQGNTGIVSFWA

LLFLLKNPGALKAVREEAEGILRKMGKERRPGGVPIQVEYSMLQQTPVLDSVMEETLRLV

VAPFLTRSVMQDMILKMDDGREYALRKGDEVCVFPYLFLHMDPEIYPDPTTFKYDRFLNL

DGSQKVDFYKQGRRIKSYMLPWGAGPSLCPGRFFALNEMKMFVFLMVTQYDIELVDQEVE

IPPINVKRWGIGAMQPIYDVQFRYRMRF

>395516267

MAFWASVLIVLMTTVLVVLFRLGAFRQRRFNEPPLDKGLIPWLGHAMDMSRNASSFLKKM

QERHGEIFTVHIAGSYITFVTDPFSFDVILKESKSRLDFSKFASELVFKIFEFQMPKNHR

GMVINNSSKHLMSIGLVGLTQAMMDSLQILMLQPDADKRLGPQEWKQEGLFQYCYNTVFR

AGYLALFGNLPTNGKAGHAQDLLQSQKVFEEFRKFDILFPRMFHSVILPKEKQEIKRLRY

FFWDILSARQMLEKDNVSGWISSIQRELADLGVPELGQSQYNLILLWASQGNTGVVSFWA

LLFLLKNPEALKAVREEAEGVLRKMGKEMRPGGAPIIVEYSMLQQTPVLDSVMEETLRLR

VAPLLTRAVKQDVILKMDNGKEYALRQGDRVCMFPYLFLQMDPEIYPDPTTFKYDRFLNP

DGSRKVAFYKQGKKLKSHMLPWGGGPSICPGRFFALNEMKMFVFLMVAQYDIELVDQDVK

IPPINVKRFGIGAMQPIQDVQFRYRIRF

>334333692

MALWASVLIVLVAIVLIGLYKLGALRQRKAREPPLDKGPIPWLGYSKEFSKDASIFLKKM

QERHGDIFTVQLCGRYVTFVTDPFTYDAILKESKDKLGFSNLVSEKVIKIFDIQYEEGHQ

KISSNIHTKHLSASSLVVITQAVMDNLQTLLLEPDGGIRPASQEWKQDGLLEYCYSVIFR

AGYLALFGSFPTSGKTGRIQDLLHSQKVFQEFRKFDRLFPRAFYSILHPKEKQEMKRLRH

FFWDILSSQQILEKDNVSSWIHSIQQEMIENGVSDKGQSQNNLVLLWASQGSTTGVNFWT

LFFLLKNPEALKAVREEAEGILRKMGKEKRPGGVPINFDYNMLKQTPVLDSVMEETLRLV

VAPFLARSVMQDMTLKMDDGREYALRKGDEVCVFPYLFLHMDPEIHSDPTTFKYDRFLTL

DGSQKVVFYKQGRRIKSHMLPWGAGTTLCPGRFLALNQIKMFIFLMVTQYDMELVDQEAG

IPPINVKRWGMGPLQPMYDVQFRYRLRF

>327274367

MDFWLMVLCALLAAILGGLYLVGAFRKRRANEPPVDKGLIPWLGHGLNFRNDTLSFLQRM

HKKHGDVFTVLIAGHYFTFLIDPLSFGAVVKEGRAKLDFITFASELVSNVFAFRPSEKTH

KVIETSSVKHLKGDGLVVMTQAMMDSLQKVMLHDLGSGESPKPWNQDGLFHFCYNMVFRA

GYFALYGNKPGKSEKEKESALKQDLKESEEIFKEFRKYDQLFPRLSYSMLSFGEKKEVES

LWSYFWNILSVKNVYQKDNISGWINDQQQQLAETGMPEHMRDRFMFLLLWAAQGNTGPAS

FWLLAHLLKNPQAMEEVRKEVTAVVRDSGQELKPGNEVLNVTREMLGQTPVLDSAVKEAL

RMGAAPMLIRAVLEDMQLKMNDGRAYALRKGDKVAIFPYLTHMDPEIHPEPLVFKHDRFL

TTDGNKKEFYKNGEKVKYATMPWGAGVSICPGRFFAVNEIKLFAFLMVTCFEMELVNPED

KIPPIDKRRFGFGVMQPTHDIQFRYRLRY

>327274337

MNFWETVLSAFLASLLAIILGGLYTIGAFRKRRPKEPPLDKGFIPWLGYAIQFKKTPAEF

LERMRKKHGDIFTVLVGGNYINFVSDPHTYEPLMKEPKEKLDFEKFSSMVVHNVFGFQPT

QVHHHIVETISKKYLNGKHLSELNQVMMEKLQVVMLNGRSSGDGQKSWQQTGLFHFSYKT

IFQAGFLSLFGTKPGKDEGSKEKLVQTNLAQCAETFELFQKFDHYFPQLVVKLLNPAEQA

ETQKLQSTLWDILSIDKLYQRDDINSWVVENDQEMVNNGMTEKMRTQFQLLLLWASQANT

GPATFWIILYLLKHPEAIKAVREELGRVLKETGQEVKPGSPFLSLALDKIKTPLLDSAIQ

EMLRLKGSVFIVRGVMEDMDIKMGDGREYTLRKGDSLFLFPYIGLHMDPEIYPEPQTFKY

DRFINLDGTKKEFYKNGKLLKHYIMPFGGGSTMCPGRYFAVSEMKMFVILMLVYFDMELV

NAEEDMPPTDEARCGFGAASPSRDIQFRYRLRH

>327274365

MNFWETVLSAFLASLLAIILGGLYTIGAFRKRRPKEPPLDKGFIPWLGYAIQFRKSPAEF

LERMRKKHGDIFTVLVGGNYINFVADPHTHEPLMKEPKEKLDFEKFSSTVVHNAFGFQPT

QVHHHAVQTISKKFLNGKHLSELNQVMMEKLQISMLKGHGPGNGQRTWHQAGLFHFSYKT

IFQAAFLSLFGTKSDKESGSKDSSVETNLAQCEKIFEIFHKFDHYFPQLAVKLLDPIGQA

ETQKLQSILWGILSVDKLYQRDDINSWVVENDREMVNIGMTEKMRTQFLLLLLWASQANT

GPATFWIILYLLKHPEAMKAVREELGRVLKETGQEVKPGSPFLSLALDKIKTPLLDSAIQ

ETLRLKGSVFIIRGVMEDIDIKMGDGREYILRKGDTLFLFPYIGLHMDPEIYPDPQTFKY

DRFINPDGTKKEFYKNGKLLKHYIMPFGGGSTMCPGRYFAVSEMKMFVILMLVYFDVELV

NAEEDMPPTDEARCGFGAASPSHDIQFRYRLRCSD

>327274339

MDFWLMVLCALLAAVLGGLYLVGAFRKRGANEPPVDKGLIPWLGHGLNFRNDTLSFLQQM

QKKHGDVFTVLIAGHFYTFLIDPLSFEAVVKEGRAKLDFYPIGYELVGNVFAFRPSEKMH

KVIETSSVKHLSGDGLEVMTKAMMDSLQKVMLHKLGSEESPKPWKQDGFFHFCYNKVFRA

GYFALYGNKPGKGKKEKESALKQDLKESEEIFKQFRKYDQLFPRFSYSMLTFGEKKEVES

LWSYFWNILSVKNVYQKDNISGWIHDQQQQLAETGMPEHMRDRFMFLLLWAAQSNTGPAS

FWLLAHLLKNPQAMEEVRKEVTAVVRDSGQELKPGNAVLNVTREMLSQTPVLDSAVKETL

RMAVAPMLIRAVLEDMQLKMNDGRAYALRKGDRVAIFPYLTHMDPEIHPEPLVFKHDRFL

TTDGKKKKFYKNGEKVKYATMPWGAGVSVCRGRFFAINEIKLFAFLMVTCFEMELVNPEV

KIPPIDKRRFGFGVMQPSHDIQFRYRLRY

>EHH16466.1

MVLWGPVLGALLVVIAGYLCLPGMLRQRRPREPPLDKGTVPWLGYAMAFRKNMFEFLKRM

RSKHGDVFTVQLGGQYFTFVMDPLSFGPILKDTQRKLDFGQYAKKLVLKVFGYRSVQGDH

EMIHSASTKHLRGDGLKDLNETMLDSLSFVMLKSKGWSLDASCWHEDSLFHFCYYILFTA

GYLSLFGYTKDKEQDLLQAGELFVEFRKFDLLFPRFVYSLLWPQEWLEVGRLQRLFHKML

SVSHSQEKEGISNWLCNMLQFLREQGVPSAMQDKFNFMMLWASQGNTGPTSFWALLFLLK

HPEAIRAVRQETTQVLGEARLETKQSFAFKLSALQHTPVLDSVVEETLRLRAAPTLLRLV

HEDYTLKMASGQEYLFRRGDILALFPYLSVHVDPDIHPEPTIFKYDRFLNPNGSRKVDFF

KAGKKIHHYTMPWGSGVSICPGRFFALSEVKLFILLMVTHFDLELVDPDTPLPHVDPQRW

GFGTMQPSHDVRFRYRLRPTV

>296224949

MVLWGPVLGALLVVIVGYLCLPGILRQRRPWEPPLDKGTVPWLGHATAFRKNMFEFLKRM

RTKHGDVFTVQLGGQYFTFVMDPLSFGPILKDARRKLDFVEYAKKLVLKVFGYHSVQGDN

EMIHSASTKHLMGDGLKDLNEVMLDSLSFVMLKSKGRSLDASCWHEDSLFEFCYYILFTA

GYLSLFGYTKDKERDLLQAGKLFKGFRKFDLLFPRFVYSLLWPQERLEVGRLQRFFHKML

SVRHTQEKDGISNWLCNMLQFLREQGVPSAMQDKFSFMMLWASQGNTGPASFWTLFFLLK

YPEAIQAVREEATQVLGEARLETKQPFAFKLSALQHTTVLDSVVEETLRLRAAPTLLRMV

HEDYTLKMASGQEYLIRHGDILALFPYLSVHLDPDIHPEPTVFKYNRFLNPDGSRKVDFF

KAGKKTRHYTMPWGSGVSICPGRFFALSEVKLFVLLMVTHFDLELVDPDAPVPHVDPKRW

GFGMMQPSHDVRFRYRLRPTA

>402860458

MVLWGPVLGALLVVIAGYLCLPGMLRQRRPREPPLDKGTVPWLGYAMAFRKNMFEFLKRM

RSKHGDVFTVQLGGQYFTFVMDPLSFGPILKDTQRKLDFGQYAKKLVLKVFGYRSVQGDH

EMIHSASTKHLRGDGLKDLNETMLDSLSFVMLKSKGWSLDASCWHEDSLFHFCYYVLFTA

GYLSLFGYTKDKEQDLLQAGELFVEFRKFDLLFPRFVYSLLWPQEWLEVGRLQRLFHKML

SVSHSQEKEGISNWLCNMLQFLREQGVPSAMQDKFNFMMLWASQGNTGPTSFWALLFLLK

HPEAIRAVREEATQVLGEARLETKQSFAFKLSALQHTPVLDSVVEETLRLRAAPTLLRLV

HEDYTLKMASGQEYLFRRGDILALFPYLSVHVDPDIHPEPTVFKYDRFLNPNGSRKVDFF

KAGKKIHHYTMPWGSGVSICPGRFFALSEVKLFILLMVTHFDLELVDPDTPLPHVDPQRW

GFGTMQPSHDVHFRYRLRPIV

>57103812

MVFWGLVLGALLVVTTGCLYLLGVLRQRRPQEPPLDKGSIPWLGHAMAFRKNMFEFLKHM

WAKHGDVFTVQLGGQYFTFVMDPLSFGPILKDAQRKLDFVKYAEKLVLKVFGYHSGQGDY

RMIHSASTKYLMGDGLEDLNKTMLDSLSLVMLGPTGQSLDSSCWHEDGLFHFCYNVLFKA

GYLSLFGYTKDQEQDLRQAEELFVQFRKFDRLFPRFVYSLLGPREWREVGRLQRLFHKAL

SVKRNLEKNGISRWISYMLQYLRERGVAPAMQDKFNFMMLWASQGNTGPTSFWALLFLLK

HPEALRAVREEATQVLGEARLKARQSFDFKVSTLHHTPVLDSVMEETLRLGAAPTLLRVV

HNDHILKMASGQEYLLRSGDIVALFPYLSVHMDPDIHPEPTTFKYDRFLNPNGSRKVDFY

KAGKKIHHYTMPWGSGVSICPGRFLATNEMKLFVLLMVTYFDLELVDPNTPVPPVDPQRW

GFGTTQPSHEVRFRYRLRPTS

>EHH51382.1

MVLWGPVLGALLVVIAGYLCLPGMLRQRRPREPPLDKGTVPWLGYAMAFRKNMFEFLKRM

RSKHGDVFTVQLGGQYFTFVMDPLSFGPILKDTQRKLDFGQYAKKLVLKVFGYRSVQGDH

EMIHSASTKHLRGDGLKDLNETMLDSLSFVMLKSKGWSLDASCWHEDSLFHFCYYILFTA

GYLSLFGYTKDKEQDLLQAGELFVEFRKFDLLFPRFVYSLLWPQEWLEVGRLQRLFHKML

SVSHSQEKEGISNWLCNMLQFLREQGVPSAMQDKFNFMMLWASQGNTGPTSFWALLFLLK

HPEAIRAVRQEATQVLGEAKLETKQSFAFKLSALQHTPVLDSVVEETLRLRAAPTLLRLV

HEDYTLKMASGQEYLFRRGDILALFPYLSVHVDPDIHPEPTIFKYDRFLNPNGSRKVDFF

KAGKKIHHYTMPWGSGVSICPGRFFALSEVKLFILLMVTHFDLELVDPDTPLPHVDPQRW

GFGTMQPSHDVRFRYRLCPIV

>403268386

MVLWGPVLGALLVVIVVYLCLPGILRQRRPWEPPLDKGTVPWLGHAMAFRKNMFEFLKRM

RTKHGDVFTVQLGGQYFTFVMDPLSFGPILKDARRKLDFVEYAKKLVLKVFGYRSVQGDN

EMIHSASTKHLMGDGLKDLNEVMLDSLSFVMLKSKGRSLDASCWHEDSLFDFCYYILFTA

GYLSLFGYTKDKEQDLLQAGRLFKEFRKFDLLFPRFVYSLLWPREWLEVGRLQRLFHKML

SVKHSQEKDGISNWLCNMLQFLREQGVPSAMQDKFNFMMLWASQGNTGPASFWTLLFLLK

HQEAIQAVREEAIRVLGEARLETKQPFAFKLSALQHTPVLDSVVEETLRLRAAPTLLRMV

HEDYTLKMASGQEYLIRHGDTLALFPYLSVHLDPDIHPEPTVFKYDRFLNPDGSRKVDFF

KAGKKTHHYTMPWGSGVSICPGRFFALSEVKLFILLMVTHFDLELVDPDAPVPQVDPRRW

GFGMMQPSHDVRFRHRLHPTA

>327274343

MDFCLTVLSALLAAVLGGLYLVGAFRKRRANEPPVDKGLIPWLGHGLNFKNDTLSLLQQM

HKKHGDIFTVLIAGHFYTFLIDPLSFGAVVKEGRAKLDFYPIGYELVSNVFAFRPSQNMR

KIIETSSVKHLSGDGLEVMTQAMMDSLQKVMLHDLGSGESPKPWNQDGLFHFCYNMVFRA

GYFALYGNKPGKGKKEKESALKQDLKESEEIFKEFLKYDQLVPRLSYSMLSFGEKKEVES

LWSYFWNILSVKNVFQKDNISGWIIEQQQQLAETGMPEHMRDRFMFCLLWAAQSNTGPAS

FWLLAHLLKNPQAMEEVRKEVTAVVRDSGQELKPGNAVLNVTREMLSQTPVLDSAVKETL

RMAVAPMVTRAVLEDMQLKMNDGRVYALRKGDRVAIFPYLTHMDPEIHPEPLVFKHDRFL

TTDGNKKEFYKNGEKVKYATMPWGAGISVCRGRFLAVNEIKLFAFFVVTCFEMELMNPED

RIPPTDNRRYGFGVMHPTHDIQFRYRLHY

>395843563

MVLWSLVLGLLLVVIVGYLFLPGLLRQRRPQEPPLDKGTVPWLGHALAFRKNMFEFLKRM

QAKHGEVFTVLLGGQYFHFVMDPFSFGPILKDSRRKLDFVQYAQELVLKVFGYSPMQGDN

NMVHSASTKYLMGDGLEELNEVMLDSLSLVMLRPKGQSLDAGCWYEDGLFHFCYNILFKA

GYLSLFGYTKDKEQDLLQAEDLFTQFRKYDLLFPRFVYSLLGPREWLEVGRLQSFFHKML

SVKHTQEKDGLSNWVAYMLQSLREQGVTPAMQDKFNFMMLWASQGNTGPTSFWALLFLLK

HPEAMRAVKEEATQVLGKVRLEAEQPFPFKLSALQHTPVLDSVMEETLRLRAAPTLIRAV

CKDYILKMASGKEYLFRQGDMVALFPYLSVHMDPEIHPEPTAFKYNRFLNPNGSRKVDFY

KAGKKIHHYTMPWGSGVSICPGRFLALSEMKLFILLMVTYFDLELVDPGTPVPPVDPQRW

GFGTTQPSHDVHFRYRLQPAQ

>O02766.3

MVLWGLLGALLMVMVGWLCLPGLLRQRRPQEPPLDKGSIPWLGHAMTFRKNMLEFLKHMR

SKHGDVFTVQLGGQYFTFVMDPVSFGPILKDGQRKLDFVEYAKGLVLKVFGYQSIEGDHR

MIHLASTKHLMGHGLEELNKAMLDSLSLVMLGPEGRSPDASRWHEDGLFHFCYGVMFKAG

YLSLFGHTSDKRQDLLQAEEIFIKFRRFDLLFPRFVYSLLGPREWREVGRLQQLFHELLS

VKHNPEKDGMSNWIGHMLQYLSEQGVAPAMQDKFNFMMLWASQGNTGPASFWALIYLLKH

PEAMRAVKEEATRVLGEPRLEAKQSFTVQLSALQHIPVLDSVMEETLRLGAAPTLYRVVQ

KDILLKMASGQECLLRQGDIVTLFPYLSVHMDPDIHPEPTTFKYDRFLNPNGSRKVDFYK

AGQKIHHYTMPWGSGVSICPGRFFALSEMKLFVLLMVQYFDLELVDPNTPVPPIDPRRWG

FGTMQPTHDVRIRYRLKPLE

>397475876

MVLWGPVLGALLVVIAGYLCLPGMLRQRRPWEPPLDKGTVPWLGHAMAFRKNMFEFLKRM

RTKHGDVFTVQLGGQYFTFVMDPLSFGPILKDTQRKLDFGQYAKKLVLKVFGYRSVQGDH

EMIHSASTKHLRGDGLKDLNETMLDSLSFVMLTSKGSSLDASCWHEDSLFRFCYYILFTA

GYLSLFGYTKDKEQDLLQAGELFMEFRKFDLLFPRFVYSLLWPREWLEVGRLQRLFHKML

SVSHSQEKEGISNWLGNMLQFLREQGVPSAMQDKFNFMMLWASQGNTGPTSFWALLFLLK

HPEAIRAVREEATQVLGEARLETKQSFAFKLGALQHTPVLDSVVEETLRLRAAPTLLRLV

HEDYTLKMSSGQEYLFRHGDILALFPYLSVHMDPDIHPEPTVFKYDRFLNPNGSRKVDFF

KAGKKIHHYTMPWGSGVSICPGRFFALSEVKLFILLMVTHFDLELVDPDTPLPHVDPQRW

GFGTMQPSHDVRFRYRLHPTE

>Q9UNU6.2

MVLWGPVLGALLVVIAGYLCLPGMLRQRRPWEPPLDKGTVPWLGHAMAFRKNMFEFLKRM

RTKHGDVFTVQLGGQYFTFVMDPLSFGSILKDTQRKLDFGQYAKKLVLKVFGYRSVQGDH

EMIHSASTKHLRGDGLKDLNETMLDSLSFVMLTSKGWSLDASCWHEDSLFRFCYYILFTA

GYLSLFGYTKDKEQDLLQAGELFMEFRKFDLLFPRFVYSLLWPREWLEVGRLQRLFHKML

SVSHSQEKEGISNWLGNMLQFLREQGVPSAMQDKFNFMMLWASQGNTGPTSFWALLYLLK

HPEAIRAVREEATQVLGEARLETKQSFAFKLGALQHTPVLDSVVEETLRLRAAPTLLRLV

HEDYTLKMSSGQEYLFRHGDILALFPYLSVHMDPDIHPEPTVFKYDRFLNPNGSRKVDFF

KTGKKIHHYTMPWGSGVSICPGRFFALSEVKLFILLMVTHFDLELVDPDTPLPHVDPQRW

GFGTMQPSHDVRFRYRLHPTE

>114586374

MVLWGPVLGALLVVIAGYLCLPGMLRQRRPWEPPLDKGTVPWLGHAMAFRKNMFEFLKRM

RTKHGDVFTVQLGGQYFTFVMDPLSFGPILKDTQRKLDFGQYAKKLVLKVFGYRSVQGDH

EMIHSASTKHLRGDGLKDLNETMLDSLSFVMLTSKGSSLDASCWHEDSLFRFCYYILFTA

GYLSLFGYTKDKEQDLLQAGELFMEFRKFDLLFPRFVYSLLWPREWLEVGRLQRLFHKML

SVSHSQEKEGISNWLGNMLQFLREQGVPSAMQDKFNFMMLWASQGNTGPTSFWALLFLLK

HPEAIRAVREEATQVLGEARLETKQSFAFKLGALQHTPVLDSEVEETLRLKAAPTLLRLV

HEDYTLKMSSGQEYLFRHGDILALFPYLSVHMDPDIHPEPTVFKYDRFLNPNGSRKVDFF

KAGKKIHHYTMPWGSGVSICPGRFFALSEVKLFILLMVTHFDLELVDPDTPLPHVDPQRW

GFGTMQPSHDVRFRYRLHPTE

>297671598

MVLWGPVLGALLVVIAGYLCLPGMLRQRRPREPPLDKGTVPWLGHAMAFRKNMFEFLKRM

RTKHGDVFTVQLGGQYFTFVMDPLSFGPILKDTQRKLDFGQYAKKLVLKVFGYRSVQGDH

EMIHSASTKHLRGDGLKDLNETMLDSLSFVMLMSKGWSLHASCWHEDSLFRFCYYILFTA

GYLSLFGYTKDKEQDLLQAGELFVEFRKFDLLFPRFVYSLLWPQEWLEVGRLQRLFHKML

SVSHSQEKEGISNWLGNMLQFLREQGVPSAMQDKFNFMMLWASQGNTGPTSFWALFFLLK

HPEAIRAVREEATQVLGEARLETKQSFAFKLSALQHTPVLDSVVEETLRLRAAPTLLRLV

HEDYTLKMSSGQEYLFRHGDILALFPYLSVHMDPDIHPEPTVFKYDRFLNPNGSRKVDFF

KAGKKIHHYTMPWGSGVSICPGRFFALSEVKLFILLMVTHFDIELVDPDIPLPHVDPQRW

GFGTMQPSHDVRFRYRLHPTE

>BAG37730.1

MVLWGPVLGALLVVIAGYLCLPGILRQRRPWEPPLDKGTVPWLGHAMAFRKNMFEFLKRM

RTKHGDVFTVQLGGQYFTFVMDPLSFGPILKDTQRKLDFGQYAKKLVLKVFGYRSVQGDH

EMIHSASTKHLRGDGLKDLNETMLDSLSFVMLTSKGWSLDASCWHEDSLFRFCYYILFTA

GYLSLFGYTKDKEQDLLQAGELFMEFRKFDLLFPRFVYSLLWPREWLEVGRLQRLFHKML

SVSHSQEKEGISNWLGNMLQFLREQGVPSAMQDKFNFMMLWASQGNTGPTSFWALLYLLK

HPEAIRAVREEATQVLGEARLETKQSFAFKLGALQHTPVLDSVVEETLRLRAAPTLLRLV

HEDYTLKMSSGQEYLFRHGDILALFPYLSVHMDPDIHPEPTVFKYDRFLNPNGSRKVDFF

KTGKKIHHYTMPWGSGVSICPGRFFALSEVKLFILLMVTHFDLELVDPDTPLPHVDPQRW

GFGTMQPSHDVRFRYRLHPTE

>AAH67434.1

MVLWGPVLGALLVVIAGYLCLPGMLRQRRPWEPPLDKGTVPWLGHAMAFRKNMFEFLKRM

RTKHGDVFTVQLGGQYFTFVMDPLSFGPILKDTQRKLDFGQYAKKLVLKVFGYRSVQGDH

EMIHSASTKHLRGDGLKDLNETMLDSLSFVMLTSKGWSLDASCWHEDSLFRFCYYILFTA

GYLSLFGYTKDKEQDLLQAGELFMEFRKFDLLFPRFVYSLLWPREWLEVGRLQRLFHKML

SVSHSQEKEGISNWLGNMLQFLREQGVPSAMQDKFNFMMLWASQGNTGPTSFWALLYLLK

HPEAIRAVREEATQVLGEARLETKQSFAFKLGALQHTPVLDSVVEETLRLRAAPTLLRLV

HEDYTLKMSSGQEYLFRHGDILALFPYLSVHMDPDIHPEPTVFKYDRFLNPNGSRKVDFF

KTGKKIHHYTMPWGSGVSICPGRFFALSEVKLFTLLMVTHFDLELVDPDTPLPHVDPQRW

GFGTMQPSHDVRFRYRLHPTE

>EGW04094.1

MVLWGPVLGALLTVIVGCLCLSTLLRHRRPQEPPLDKGFVPWLGHAMAFRKNMFEFLKGM

WTKHGDVFTVQLGGQYFTFVMDPLSFGPILKDTQKTLDFIKYAQELVLKVFGYQSMDGDH

HMVHSASTKHLMGQGLEDLNKTMLDSLSLVMLGPEGPSLGASSWHEDGLFRFCYSILFKA

GFLGLFGYTKDRTQDLQEAEELFTKFRRFDLLFPRFVYSLLGPREWVEVSQLQRLFHQKL

SVEQNLEKDGLSNWLGCMLQSLREQGIDSSMQDKFNFMMLWASQGNTGPTCFWVLLFLLK

HQDAMKAVKEEATRVLGEVRLEDKKSFVFRLSALKCTPVLDSVMEETLRLRATPTLLRVV

QKDFILTMASGQEYQIRRGDKVALFPYLSVHMDPDIHPEPKTFKYNRFLNPDGTRKMDFY

KSGKKIHHYNMPWGSGVSICPGRFFALSEMKIFVLLIVMYYDLELVDPDMPVPPIDPRRW

GFGTSQPSHEVCFRYRLKPMQ

>AAH67444.1

MVLWGPVLGALLVVIAGYLCLPGMLRQRRPWEPPLDKGTVPWLGHAMAFRKNMFEFLKRV

RTKHGDVFTVQLGGQYFTFVMDPLSFGPILKDTQRKLDFGQYAKKLVLKVFGYRSVQGDH

EMIHSASTKHLRGDGLKDLNETMLDSLSFVMLTSKGWSLDASCWHEDSLFRFCYYILFTA

GYLSLFGYTKDKEQDLLQAGELFMEFRKFDLLFPRFVYSLLWPREWLEVGRLQRLFHKML

SVSHSQEKEGISNWLGNMLQFLREQGVPSAMQDKFNFMMLWASQGNTGPTSFWALLYLLK

HPEAIRAVREEATQVLGEARLETKQSFAFKLGALQHTPVLDSVVEETLRLRAAPTLLRLV

HEDYTLKMSSGQEYLFRHGDILALFPYLSVHMDPDIHPEPTVFKYDRFLNPNGSRKVDFF

KTGKKIHHYTMPWGSGVSICPGRFFALSEVKLFILLMVTHFDLELVDLDTPLPHVDPQRW

GFGTMQPSHDVRFRYRLHPTE

>194221445

MVLWVPVLGVLLVAIVGYLCVLGLLRQRRPQEPPLDKGSIPWLGHAVAFRKNMFEFLKHM

RAKHGDVFTVQLGGQYFTFVMDPLSFGPILKDAQRKLDFVEYAEKLVLKVFGYHSVQGDH

RMIHSASTKHLMGDGLEDLNKVMLDSLSLVILGPTGRSPDASCWREDGLFHFCYDILFKA

GYLSLFGYTKDKEQDLLQAEELFIHFRKFDCLFPRFVYSLLWPREWLEVGRLQRLFHKTL

SVKHNLEKDGISNWISYMLQFLREQEVAPAMQDKFNFMMLWASQGNTGPTSFWALLFLLK

HPEAMRAVRKEATRVLGEARLEARQSFNFKVSALHHTPVLDSVMEETLRLGAAPTLLRVV

HGDHTLKMASGQEYRLRSGDIVALFPYLSVHMDPDIHPDPTAFKYDRFLNPNGSRKVAFY

KAGKKIHHYTMPWGSGVSICPGRFLALSEMKLFILLMVTHFDLELVDPDTPVPPVDPKRW

GFGTTQPSHDVRFRYRLQPTE

>109041324

MVLWGPVLGALLVVIAGYLCLPGMLRQRRPREPPLDKGTVPWLGYAMAFRKNMFEFLKRM

RSKHGDVFTVQLGGQYFTFVMDPLSFGPILKDTQRKLDFGQYAKKLVLKVFGYRSVQGDH

EMIHSASTKHLRGDGLKDLNETMLDSLSFVMLKSKGWSLDASCWHEDSLFHFCYYILFTA

GYLSLFGYTKDKEQDLLQAGELLGEFRKFDLLFPRFVYSLLCMMLWASQGNTGPTSFWAL

LFLLKHPEAIRAVRQETTQVLGEARLETKQSFAFKLSALQHTPVLDSVVEETLRLRAAPT

LLRLVHEDYTLKMASGQEYLFRRGDILALFPYLSVHVDPDIHPEPTIFKYDRFLNPNGSR

KVDFFKAGKKIHHYTMPWGSGVSICPGRFFALSEVKLFILLMVTHFDLELVDPDTPLPHV

DPQRWGFGTMQPSHDVRFRYRLRPIV

>301780458

MVLWGLVLGALLVVTMGCLCLLGLLRQRRPQEPPLDKGSIPWLGHAVAFRKNMFEFLKHM

RAKHGDVFTVQLGGQYFTFVMDPLSFGPILKDAQRKLDFVEYAEKLVLKVFGYQSVQGDY

RMIHSASTKYLMGDGLEDLNKTMLDSLALVMLGPTGRSLDSSCWHEDGLFHFCYNVLFKA

GYLSLFGYTKDQEQDLRQAEELFAQFRKFDRLFPRFVYSLLGPREWLEVGRLQSLFHKTL

SVRHNLEKNGISNWISYMLHYLREQGVDPAMQDKFNFMMLWASQGNTGPASFWALLFLLK

HPEAMRAVREEATQVLGEARLKARLSFDFDVSALHHTPVLDSVMEETLRLGAAPTLLRVV

HSDHVLKMANGQEYLLRSKDIVALFPYISVHMDPDIHPQPTAFKYDRFLNPNGSRKVDFY

KAGKKIHHYTMPWGSGVSICPGRFLAISEMKLFVLLMVTYFDLELVDPATPVPPVDPHRW

GFGTTQPSHEVRFRYRLRPTN

>Q7YRB2.1

MVLWGPVLGVLLVAIVGYLCLQGLLRQRRPEEPPLDKGSVPWLGHAMTFRKNMLEFLKHM

WARHGDIFTVQLGGQYFTFVMDPLSFGPILKDAKRKLDFVEYAEKLVLKVFGYRSMQGDH

RMIHSASTKHLMGDGLEELNKAMLDNLSLVMLGPKGPSPDASCWREDGLFHFCYDILFKA

GYLSLFGRTEDKEQDLLQAEELFMQFRKFDRMFPRFVYSLLGPREWLEVGRLQCLFHKML

SVEHSLERHGISSWITDMLQVLREQGVAPAMQDKFNFMMLWASQGNTGPTTFWALLFLLK

HPEAMRAVREEATRVLGEARLEDKQSFDVEVSALNHMPVLDSVMEETLRLGAAPTLLRVV

NSDQILKMASGQEYRLRHGDILALFPYLSVHMDPDIHPEPTTFKYDRFLTPSGSRKVNFY

KAGKKIHHYTMPWGSGISICPGRFFALTEMKLFVLLMVTHFDLELVDPDTPVPPVDPQRW

GFGTMQPSYEVRFRYRLRPTE

>BAA82169.1

MLWGSVLGALLMAVGCLCLSLLPRHRRPWEPPLDKGFVPWLGHTMAFRKNMFEFLKGMRA

KHGDVFTLQLGGQYFTFVMDPLSFGPIIKSTQKVLDFVTYARELVFKVFGYQSMDEDHQM

LHVASTKHLMGQGLEDLNRAMLDSLSLVMLGPKGRSLGARSWCEDGLFHFCYSILFKAGF

LSLFGCTKDKEQDLDEADELFRKFRRFDLLFPRFVYSLLGPLEWVEVSQLQRLFHQRLSV

EQNLEKDGISNWLGFMLRFLRERGMASSMQDKFNFMMLWASQGNTGPTCFWALLFLLKHQ

DAMKAVREEATRVLGEARLEAETSFAFTLSALKCTPVLDSVMEETLRLCATPTLLGVVQE

DYVLKMASGQEYQIRRGDKVALFPYLSVHMDPDIHPEPTTFKYNRFLNPDGTRKVDFYKS

GKKIHHYNMPWGSGVSICPGRFFAPSEMKTFVLLMVMYFDFELVDPDMPVPPIDPRRWGF

GTSQPSHEVRFRYRLKPMQ

>BAA76602.1

MLWGSVLGALLMAVGCLCLSLLPRHRSPWEPPLDKGFVPWLGHTMAFRKNMFEFLKGMRA

KHGDVFTLQLGGQYFTFVMDPLSFGPIIKSTQKVLDFVTYPRELVFKVFGVQSMDVGHQM

LHVASTKHLMGQGLEDLKRAMLDSLSLVMLGPKGRSLGARSWCEDGLFHFCYSILFKAGF

LSLFGCTKDKEQDLDEADELFRKFRRFDLLFPRFVYSLLGPLEWVEVSQLQRLFHQRLSV

EQNLEKDGISNWLGFMLRFLRERGMASSMQDKFNFMMLWASQGNTGPTCFWALLFLLKHQ

DAMKAVREEATRVLGEARLEAETSFAFTLSALKCTPVLDSVMEETLRLCATPTLLRVVQE

DYVLKMASGQEYQIRRGDKVALFPYLSVHMDPDIHPEPTTFKYNRFLNPDGTRKVDFYKS

GKKIHHYNMPWGSGVSICPGRFFALSEMKTFVLLMVMYFDFELVDPDMPVPPIDPRRWGF

GTSQPSHEVRFRYRLKPMQ

>O88962.1

MTLWCTVLGALLTVVGCLCLSLLLRHRRPWEPPLDKGFVPWLGHSMAFRKNMFEFLKGMR

AKHGDVFTVQLGGQYFTFVMDPLSFGPIIKNTEKALDFQSYAKELVLKVFGYQSVDGDHR

MIHLASTKHLMGQGLEELNQAMLDSLSLVMLGPKGSSLGASSWCEDGLFHFCYRILFKAG

FLSLFGYTKDKQQDLDEADELFRKFRRFDFLFPRFVYSLLGPREWVEVSQLQRLFHQRLS

VEQNLEKDGISCWLGYMLQFLREQGIASSMQDKFNFMMLWASQGNTGPTCFWVLLFLLKH

QDAMKAVREEATRVMGKARLEAKKSFTFTPSALKHTPVLDSVMEESLRLCATPTLLRVVQ

EDYVLKMASGQEYQIRRGDKVALFPYLSVHMDPDIHPEPTAFKYDRFLNPDGTRKVDFYK

SGKKIHHYSMPWGSGVSKCPGRFFALSEMKTFVLLMIMYFDFKLVDPDIPVPPIDPRRWG

FGTSQPSHEVRFLYRLKPVQ

>AAD19876.1

MTLWCTVLGALLTVVGCLCLSLLLRHRRPWEPPLDKGFVPWLGHSMAFRKNMFEFLKGMR

AKHGDVFTVQLGGQYFTFVMDPLSFGPIIKNTEKALDFQSYAKELVLKVFGYQSVDGDHR

MIHLASTKHLMGQGLEELNQAMLDSLSLVMLGPKGSSLGASSWCEDGLFHFCYRILFKAG

FLSLFGYTKDKQQDLDEADELFRKFRRFDFLFPRFVYSLLGPREWVEVSQLQRLFHQRLS

VKQNLEKDGISCWLGYMLQFLREQGIASSMQDKFNFMMLWASQGNTGPTCFWVLLFLLKH

QDAMKAVREEATRVMGKARLEAKKSFTFTPSALKHTPVLDSVMEESLRLCATPTLLRVVQ

EDYVLKMASGQEYQIRRGDKVALFPYLSVHMDPDIHPEPTAFKYDRFLNPDGTRKVDFYK

SGKKIHHYSMPWGSGVSKCPGRFFALSEMKTFVLLMIMYFDFKLVDPDIPVPPIDPRRWG

FGTSQPSHEVRFLYRLKPVQ

>EDL76812.1

MLWGSVLGALLMAVGCLCLSLLPRHRRPWEPPLDKGFVPWLGHTMAFRKNMFEFLKGMRA

KHGDVFTLQLGGQYFTFVMDPLSFGPIIKSTQKVLDFVTYARELVFKVFGYQSMDEDHQM

LHIASTKHLMGQGLEDLNRAMLDSLSLVMLGPKGRSLGARSWCEDGLFHFCYSILFKAGF

LSLFGCTKDKEQDLDEADELFRKFRRFDLLFPRFVYSLLGPREWVEVSQLQRLFHQRLSV

EQNLEKDGISNWLGFMLRFLRERGMASSMQDKFNFMMLWASQGNTGPTCFWALLFLLKHQ

DAMKAVREEATRVLGEARLEAETSFAFTLSALKCTPVLDSVMEETLRLCATPTLLRVVQE

DYVLKMASGQEYQIRRGDKVALFPYLSVHMDPDIHPEPTTFKYNRFLNPDGTRKVDFYKS

GKKIHHYNMPWGSGVSICPGRFFALSEMKTFVLLMVMYFDFELVDPDVPVPPIDPRRWGF

GTSQPSHEVRFRYRLKPMQ

>348582586

MVMWGLVLAAVLVATLGYLCLPGLLRQRRPQEPALDKGLIPGVGHAIAIRKNMLEFLKAM

RAKHGDVFTVQLGSHFFTFVMDPLSFGPIIKNTNKNLDFMEHAKELVRKVFGYQPLKSCH

QLAHSASTKYLMGDGLEDMNKVMLDNLCLMLLGPRGPEQDASCWREDGLFHFCYNVLFKA

GYLSLFGCTKDKEQDLLQAEELFRNFRRFDILFPRFVYSLLGPREWLEVRQLQRVFHKTL

SVSHNSQKDGISNWLSYMLCYLTEWGTSPEMLGKFNFTMLWASQGNTGPSSFWVLLFLLK

HPEAMQAVKKEATQVLQGTKLEAKQPFAFQVSALQHTPILNSVMEETLRLVAVPTLIRAV

QEDCTLKMASGCEYLLRRGDRVALFPYLSVHMDPDIHPDPCTFKYDRFLNPDGSRKVDFF

KAGKKIHHYLMPWGSGMSMCPGRFFALSEMKLFVLLMVTYFDLELVDPNTPVPPLEPRRW

GFGISQPSYEVRFRYHLKPMD

>348582584

MVMWGLVLAAVLVATLGYLYLPTLLRQRRPQEPPLDKGPIPGVGHAIAIRKNMLEFLKAM

RTKHGDVFTVQLGSHFFTFVMDPLSFGPIIKNTNKNLDFRQFAQELVQKGFGYQHVKDDR

RLVPFASTKFLMGEGLEDMNKVMLESLSLVLLGPGGPRQNASCWREDGLFHFCYNVLFKA

GYLSLFGCTKDKEQDLLQAEELFRNFRRFDVLFPRFVYSLLGPREWLEVRQLQRVFHKTL

SVSHNSQKDGISNWLSYMLCYLTEWGTSPEMLGKFNFTMLWASQGNTGPSSFWVLLFLLK

HPEAMQAVKKEAAQVLQGTKLEAKQTFAFQVSALQHTPVLNSVMEETLRLVAVPTLIRAV

QEDCTLKMASGCEYLLRRGDRVALFPYLSVHMDPDIHPDPCTFKYDRFLNPDGSRKVDFF

KAGKKIHHYLMPWGSGVSMCPGRFFALSEMKLFVLLMVTYFDLELVDPNTPVPPLEPSRW

GFGISQPSYEVRFRYHLKPMD

>301619959

MTFSILILATVFIISGLYLLGMLRMRRPGEPPLIKGAIPWLGNVLEFKNNGHAFLQEMLK

NHGDVFTVQLAGHYFTFVMDPLSFGPVIREATSVLDFNEFARESAIKLFGYHSTLNDLHM

LKESSTKHLMGDGLVVLAQAMMENLQTLMLYNMGRVKGDRKWLQGGLFNFTYSIIFKAGY

CSLFGNEPVKSKGSEKRAQDCDWERSEKLFQEFVKYDQFVPRLIYSALSPKDKIEAERLK

RFFWHRLSVKQTLQKENINGWITEQIRKRTEMGVPEHMQDRFLFSLLWVSQSNTGPACFW

FLYYLMKYPEAMKAVRAEVEGVLKGTGQDIKPGGPLINLTRDMLRKVPIMDSAMEETLRL

KAAPVLTRVAKENVKFKMASGMDVSIRREDRVVLFPYVSVQMNPEIHTDPQKFKYDRFLN

EDGTKKTAFFKNGEKVKYYTMPWGAGGSICPGRFFAVNELKQFVFLMLSYFELELVNPNE

EIPSIDPNRWGFGIMQPNNDVQFRYRLRF

>327274341

MDFCLTVLCALLAAVLGGLYLVGAFRKRRANEPPVDKGLIPWLGHGLNFRNNIFSFLQQM

HKKHGDVFTVLIAGHYYTFLIDPLSFGAVVKEVRAKLDYTPIAFELVSNVFALQSSETMH

QAIETISVKHLSGDGLEVMTQAMMDSFQKVMLHDLGSREAPTPWKQDGLFHFCYNMVFRA

GYFAFYGNKPGKSEKEKESALKQDLKESEEILKEFRKYDQLFFRLSYSLLSFGEKKEVES

LWSYFWNTLSVKNVYQKDNISGWINDHQQLLAEIGMPEHMRDRFMFCLLSAVQGNTGPAS

FWLLAHLLKNPQAMEEVRKEVTAVVRDSGQEPGNAVLNVTREMLSQTPVLDSTVKEVLRM

RAAPMVTRAVLEDMQLKMNDGRVYALRKGDRVAIFPYLTHMDPEIHPQPLVFKHDRFLTT

DGNKKEFFKNGKKVKYATMPWGAGVSVCRGRFLAVNEIKLFAFLMVTCFEMELVNPEDKI

PPIEKRRYGFSIMHPTHDIQFRYRLRY

>327274309

MNFWETVLCASLASLLAIILGGLHSMGAFRKRRPKEPPLDKGFIPWLGHGITFKKSPLTF

LEGMQKKHGDIFTCLVGGNYLHFVLDPFIYEALLKESEEKLDFSKSASMIAFNLFGVQPT

QDQHKVAHMNMKHLRGKTLEILNQRLMEKLQHIMLDGKHLRDGERSWQQDGIFHFSYRII

FQATFLTLFGDETGRAALNKEADKESQPMSYGEMFEKFLKFDHLAPQMVIGMLDPQRKEE

TDHLRNIFWDILSVQKISLNENISDWISEQDRQLAEIGMDETMRTKVMFLFFWVSQVNTG

PAIFWILTYLLKHPEAMEAVRDEVDRALKGTGEEVKPGSPFIKATLATIKTPLLDSAILE

TLRLRASPFMFRTVVQDTDIRMADGREYLVRKGDALLLFPFLAIHMDPEIHPDPHAFKFD

RFVSPNGTRKEFYKDGKTLKHSILPFGAGHTKCPGRFLAVNEMKIFVFLMLIYFDIELVN

PEEGIPSVEGSSYGFGTVKPSHDIQFKYRLRF

>327274311

MNFWETVLCASLASLLAIILGGLYAIGAFRKRKPKEPPLDKGLIPWLGHGITFKKTPFAF

LTEMRKKHGDIFTCLVGGKYLHFVLDPCTYGPLTRESENKLDFHAFATIISGNIFDFQAS

KALDKVAKMNSKYLKGKELGALDHRLMEMLQTMMLRCQQHKEGEKSWQEDTLFHFSFKTI

FQAAFLTLFGTEPQSQENIKERELKQCGEIFEMFLEFDRFVPQMAFGMLDPSGKEKAERL

RNIFWDLLDVEKVYEKENISTWIVKQDQNLAEIGESDGIRTRIMFLFLWVSQVNTGPAVF

WILAFLLKHPEAMKAVRDEVDRILREVGQEVKPGSPSINVSLDMMKTPLLDSAIQESLRL

RVSPFLFRGVMQDTEVRMANGRVYILRKGDALVLSPFIALHMDPEIYPDPHAFKYDRFVN

PDGTRKEFSKNGKTLRHSILPFGAGHSMCPGRYFAVNEMKIFVILMLTYFDMELVNPEEE

IPPVKESSYGFGTLKPSCDMRFKYRLRF

>AAH81623.1

MEIFLQCSSEAFTSLSSQIRCLSAQWSKSQEQSWTSQNFSDQLVERVFGYHPMENEHKFL

QTASTKHLMGDGLVVMTQAMMYNLQNLMLHSVGSGNGKVWQESGLFAYCYNIVFRAGYLS

LFGNESPKGTGKESVEKAKEIDLQHSNDLFCEFRKYDQLFPNLAYGVLGPRGKMEAERLK

RLFWSTLSVQKMRVKDNISGWISDQQQVRAELDMQEFMQDRFMFLLLWASQGNTGPSAFW

LLLYLMKHPEAMSAVRKEVEEILKEAGQEVKPGGPLIDLSRDMLLKTPILDSAVEETLRL

TTVPLLTRGVVQDMTIIMANGQEYKIREGDRVSVFPYLVHIDPEVHPDPLTFKYDRFLNA

DGSRKTDFYKDGKKLKYYSMPWGAGTTMCPGRFFATNELKQFVFLMLSYFDFELTNPNEQ

IPDIDIRRWGFGSMHPDRDIQFRYRPRI

**CYP9**

>ACJ04711.1

MIIIIWTLAIGLALLLYLKQIYCYFSKHEIKSITPLPILGNMGKIVFKINHFVDDISQLY

NKFPEERFVGRYEFVNPVIYIRDIEIVKRITIKDFEHFLDHRTIVNEETDPIFGRNLFSL

KGQEWKDMRSTLSPAFTSSKMKLMMPLIVEVGEQMINALKKNIKNSGVGYVDIDTKDLTT

RYANDVIASCAFGLKVDSLTEENNQFYAMGKAASNFSGKPILLLLGFISFPKLMKMTKFT

LFSEETSGFFKELIMGTMKDREMRKIIRPDMIHLLMEAKKGKLVHDDKSSKDTDAGFATV

EESAVGKKQIDRVWTDDDIIAQAVLFFVAGFETVSSAMTFLLHELALNPEVQDKLVEEIK

ENKERNNGKFDYNSIQNMVYLDMVVSELLRLWPPGVSMDRICVQDYNLGKPNDKAKRDFT

LRKGTGVAIPVWAFHRNPEFFPDPQKFDPERFSEENKHNIKPFAYLPFGVGPRNCIGSRF

ALCEVKVMAYQLLQHTEISPCEKTCIPSKLSKETFNLRLEGGHWVRLKIRD

>EFA09148.1

MWWFFVLLLGAPIVFFIFLYRTQKHWERRGLKQKNMGVTIPDNWAETKYPITFPDYVQKF

YEMFPNRRYVGYYTISNPSLFIRDLDLIKQIIVKDFEYFTDHQHFIWEENDPLWSQNLFS

LKGKKWREMRTTLSPTFTSSKIKYMFTLVQKNGEQFARYFLDKNENFMVFEMKDIFTRFT

NDVIASSAFGVECDSLREPQNEFYLMSKAVTDFTGLWKIVAGTFYFLMPSVAKFLNISLF

PKKGSKFFSNLVKSNIKSRLQHGIVRPDMINIMLEARKHGLKHEETQPSQDTGFATIEES

ELGKSGKVKKEITDDDITSQALVFIFAGFETVSSMMCFLSYELALNPDIQKRLIQEIDQT

LDACGGSITYESLMGMKYLDMVLTESLRKWPISAGLDRVCTKEYVIEPKLPGENPVRVDK

GTTILVPVYALLRDPKYFPNPEKFDPERFSDENKGKIVPYTFLPFGAGPRSCIGTRFAIM

ETKVLFFSILRHFEIVRVEKTAVPIRYIPQQITLSSADGFWLGFKRRVKV

>EFA01242.1

MFILVAFLIPLLGITGTIYLGIFCAIFGYFYYYVKRQQNVWHQKGIKYCKPKFLLGSLEI

IRSKSLAQIIQELYNAFPEERYHGFYQFLLPTLIIKDPDLIKQITIKDFDHFVNHRRFVN

ADDDPFWSRNLRGRDEKWRDMRATLSPSFTSSKMRIMFELMDECAREFVQHFKSQNSDTV

ELEMKDTLRRFTNDVIASTAFGLKINSLKDRKNEFLLMGAAATDFGYIRTMKFLLYTLSP

KLCQLLKMKIIPTKVADFFTVIIKDTIKKRKEKNIIRPDMIHLLLEAQKGRQHPEEHLDI

DEGFSSVSESKLTKPNEKLEITTEDIISQALIFFLAGFDTVSTLLNFLFYELAVNPDVQT

RLRTEIQSVDEKITYETLLKLKYLDMVMSEALRKWPPAIATDREVAKNYTIEPTKPDEKP

LLLEEGMLCSIPICAIHRDEKYYPEPEKFDPERFNDENKHKINPLTFIPFGAGPRNCIGS

RFALLESKLLVFHVVKNFEIVPIDKTLIPLVMDKYQINWSVKGGTWLGLKNI

>EEZ99187.1

MLLLLLGTLIVILVLYLVNLSTKNFDHWEKRRVKYVKPFPFVGNLLPTVLKTKSTAELIQ

HLYKAFPNERFVGIFQFNTPILLIRDPELIKSIAIKNFDNFVDHYGFANDDIDPLWAKNL

FASQGERWRDLRQTLSPVFTSSKMRTMFVLMDECVKQLTEYFKDQKQDVIDIELKDIFSR

YTTDVIATTAFGIKVDSLRNRNNDFVVSGREFTDFSGLRGLAFFINGSYPRLAKFLNIKI

VSDRLGNFFRTIIKETLTLREEKKIVRPDLIHSLMLARKGKLKYEELMELPEAGFAAVEE

SNLTKSNNNVNFLTDEDITAQALVFFLGGFDTTSSLMCFAGYELAINPHIQKRLKDEVLA

TDRECNGQITYEKLLNMKYLDMVVSETLRKWNQAVWLDRKCTKEIEIESETSGEPSVTLK

VGDIIWMPAYAIHHDPKYYPNPELFDPERFSDENKDKIRTGTYLPFGVGPRNCIGSRFAL

LETKLLLYNLLLNFDLVTNHKTENPIKLRRDTPLLMPQNGFQVSLRKNDAFSEK

>AAC33297.1

MDLILLLSIVGLIYFVYKWATARHNEFELRGLPFEKPLPIFGNNAAVVTGRASFQKSSPS

SIARTRQHKMVGFFNFRTPMIQLNDPEIIKKITVKDFEYFPNHQLFFTTEERLINDMLSV

MKDQRWKHMRNTLTPVFTSAKMRSMFSLMNESFAECMDHLDQMSKTAVKPGGSFELELKE

VCNRLSNDLIATTAFGLKVSSYKKPDNDFYEIGKSIVFFRGKALYKLFACPTTLPAVFKL

LGFKIFDAQKTDFFIRLVVDAMKYREENNIVRPDMIQLLMEAKKESTEHWSDDELVAQCF

IFFFAAFENNASLICTTAYELLNNPDVQQRLYEEVQETYDALKGEMLTYDAVTKMKYMDL

VASESLRKWTLAASTDRECAKDYTLYDDDASKLFEFKAGDRINIPIVGLHLDDKFFPEPH

KFIPERFSDGNKDQIVPYTYLPFGAGPRNCIGNRYALMQAKAMLYNLVLKYKIERSPKTV

KDLLSDSRGFQLTPQSGYWVHLVPRK

>AAX62142.1

MWPFVVQKKHITEIIADIYRQFPTDRYVGYYHFMTPTLLLRDVELIKQIGVKEFDSFHDH

VSVSNTNTEPLLSEALTNLPGEKWKEMRATLSPAFTSSKIKNMYSLISECAENFVDFFQG

REQDVEIKEVFSRFSNDVIATAAFGIRVNSVQEPNNEFYAMGRSLTTFSAFQILKIFLAH

LVPKIAEFFEYRVLPRKVTDFFEGLILDNMQKREAEKIVRPDLIHLLMEARKGKLKHDNS

NEGGEGFATVEESEIGKNTKQVELTDEKIVAQALLFFFAGFETVSTGVSFMAYELATNPH

VQKKLQKEIDLTLQENHGKISYNVLQSMKYLDQVVCESLRLWPPAPQTDRLCNKNFVIEA

SKPHERTFTVEKDTMVMISMFAIHRDPQYFPDPEKFDPERFSDENKAKIVPGTYMPFGVG

PRNCIAKFDIVPNGKTVIPIKISPSTANMVPQEGIHLTFKPRL

>AAR97606.1

MMSSLASFWVWALTINLIIMLLYIIGTWTHKHFSKKNVPCLKPLPVFGNMGPLAFGTKSF

PEFIADVYNKLKGHKYGGFYEFLKPVVVLRDPELIKMVTIKDFEHFLDHRNLITEDSEPL

FGKALFNLQGPRWRHMRSTLSPAFTSSKMKNMFTLVSECGDQMGDFLHRCINDKTMKIES

CKIQRADRRLEIEMKDLFTRYTNDVITTTALGIGCDSLKNPQNEFYTMGKKITTMGTLQR

LVFLGYSLSPKLMKALKLRFISDKLSDYFRSLIQKTMDTRENKGIIRPDMIHLLMQAKKG

TLKNEEDDDANGTPSIKPKLEDDDIISQAFIFFFAGFETVSTLMCFASHLLAVHPDIQTR

LQKEIDQTLQENDGKVTYESVHSMKYLDMVLSETLRLYPPAVFMDRKCVKTYTLPTEPSY

TLQPGEGIWIPVHGIHHDPEYFPDPEKFDPERFSDENKDKIKPFTYLPFGSGPRNCIGNR

FALMESKIVLVHMLSRFNFKVVSRTPIPIKITRRGLNITVDGGFWLGLEERI

>Q9VG82.1

MLWEFFALFAIAAALFYRWASANNDFFKDRGIAYEKPVLYFGNMAGMFLRKRAMFDIVCD

LYTKGGSKKFFGIFEQRQPLLMVRDPDLIKQITIKDFDHFINHRNVFATSSDDDPHDMSN

LFGSSLFSMRDARWKDMRSTLSPAFTGSKMRQMFQLMNQVAKEAVDCLKQDDSRVQENEL

DMKDYCTRFTNDVIASTAFGLQVNSFKDRENTFYQMGKKLTTFTFLQSMKFMLFFALKGL

NKILKVELFDRKSTQYFVRLVLDAMKYRQEHNIVRPDMINMLMEARGIIQTEKTKASAVR

EWSDRDIVAQCFVFFFAGFETSAVLMCFTAHELMENQDVQQRLYEEVQQVDQDLEGKELT

YEAIMGMKYLDQVVNEVLRKWPAAIAVDRECNKDITFDVDGQKVEVKKGDVIWLPTCGFH

RDPKYFENPMKFDPERFSDENKESIQPFTYFPFGLGQRNCIGSRFALLEAKAVIYYLLKD

YRFAPAKKSCIPLELITSGFQLSPKGGFWIKLVQRN

>ABZ81070.1

MLVEVIVFLITTLVAYYLYVYKKIHYFYDARGVKYQPGIPVLGNILKSSLGTGHFWEDID

KIYKAFPGERYIGYIEGTTPILMIKDPEIIKNITVRDFDHFVNHKEFFPVEIDALFGGSL

FMMKDDKWRDMRTTLSPAFTGSKMRLMLPFMIDISKNIVEYLKGHQLEDVDVDDLMRRYT

NDVIASAGFGLQVNSLVDKDNEFYECGQAMFSTSWPQRFKMILAAQFPTLAKKIGIKVFP

QKVTRFFREIVTSTMDYRLKNNVERPDMIQLLMDAYKGTLKNESNESDEKNVGFAMTEEM

LKPKGNVRKWTQDELTAQVFIFFLAGFESSANGLTLCIHELALNPEAQEKLYEAIIKFKE

EKGPLTYDNIGELKYLDCVLNETSRKWSAAIIVDRVCSKPYELPPPREGGKPYKLQPGDI

VYNSVNSIQMDPEHHPDPEKFDPDRFLDENKHKIKPFTFLPFGAGPRNCIGSRFALLELK

VLIYYIVLNFKIIKTEKTLSPIKLQPGEFNIKVLGGTWTQFEARE

>157167198

MKTDTGSSVVLLFSFNNHFSLLGLLRKNMVEVNLFSALAVGAVILLLYHYIAKKYHYFLT

KPIPCIKPTFLLGIFDMVVLKRVELVFGSKLLYNSYPDAKIIGYYELTKPTYMVRDPEMI

KKIAIKDFDSFTDRTPVFGDAVPADSLFFNSLFSLRGQKWRDMRSTLSPAFTGSRMRHMA

ELVAKCATSMTDFIHSEAKAGRRLEFNMKDTFSRFVCDAIASVAFGIEVDSFRDPENEFY

KKGNESQKIHTFKSLATFVTLRFVPFLQKVFNFDFVDANVAGYFKKLILDNMDQRKKQGI

VRNDLVNMLMETKKGALKYEEPDMQVSEGYATVEESHVGKSTHSRIWTDNELISQCFFFF

FAAFDNVSSILAFLSYELTVNQDIQRRLYEEIAVTESTLNGQPITYEALQKMAYLDMVVS

EALRKYPTATLTDRYANKDYVFDDEEGLRFVIEKGKTIWIPMLALHHDPKYFPEPERFDP

ERFSEDNRSKIVPGTYLPFGAGPRSCIGPRLALLEVKMALYHLVKDFNLQASEKTQIPLR

LSKSAFTMQAENGVWLELKARPKA

>AAL93294.1

MLGTLASLLFSLEGVLLVALLALLYYYVVVRSTRFFADRGVPYLPPVPLLGNGADFITRK

RLLTDVLSDMYHRFASHRFFGYFDFLSPIYVVRDLELVKQICIKDFDHFVNHRIQLDENH

DPLFGRALFAMRDTRWRNMRTILSPAFTGSKMRLMFGLITSYCDGAVRTIRSELGADGTA

ELEMKELFRRFGNDIVATCAFGIEINSFRDRANAFFTLGKELTNLDGVQGLKFLAFSSFP

RVMRALRLRLFSAKMTSFFRHVVMDTITQREQRGIVRHDMINLLMQARKQELRFDENENI

ETNGGGSQKRSVDWTDDDLTAQCTVFFFGGYDTVSTLSAFMAHELAVNPDVQERLRDEID

RVRSTIGSADKLNYETLQSMRYLDMVANETLRKWTPAPFLDRTCTKPYMLEDYDGRRVQL

QPGDGLWIPAAAIMRDPQLFPEPDRFWPERFEPESAGAPVDSSAMLAFGLGPRNCIGSRF

ALMETKAVFFFLLTHFLLEAGPRTQHPIRLKKSSLGPCAEKGSWIRFRLRK

>AAL96668.1

MEINLMYVIGIVSVLVALYVYLTHNNDFFKKYPIPCLPVEPLFGSSRQFLLKKISFSEFV

RSNYERFPNAKMYGMFEMFTPMFVIRDPELIKQITVKDFDHFINHRPLMKADNSSNSTAM

FSKILFNLTGQRWRNVRTTLSPTFTGSKMRQMFAMILECSDNMVQALAHPTGRECEVKDL

FIRFTNDVIASCAFGVHVNSFRDKDNVFFRYGKDLSNFSRLKVALKIMGYQVFPKLMAQL

QMDIFDSTHVQFFTEMFRQSVQEREEHGIVRPDLIHLLIQARKGQLRYQPQESEETDGFA

TAKESNEQKILPEDMVKLSENEMIAQCLLFFLAGFDTIATSMTFVLYEVTLAPEIQQRLY

EEIQQVSETLDGKALTYDALQGMRYLDMVVSETLRKWSPSPGTDRMCNQDYTIPGDPDIV

IPKGATVFIPIAGLHYDPRFYPDPDRFDPERFNDENKHKIPLGAYLPFGIGPRNCIASRF

ALMEVKAIVYHILLNYELKRSERTSVPVKLAKGFSPLKPENGMYLKFNPRMKN

>156546809

MTSIMLLAAYGLAALLFYASLLVLHQFTYWKRRKVLHLELVPIIGNMAPIMFRTSSFPIH

SQNMYKKFPGARYYGFFDLQKPSVIIKDPDLIREIFVKSFDHFTDHDAFVTEKMDPIVGR

NIFSLRGQRWKEVRNTLTPSYTAARIKMLFALVAECSKDFVQYFVDHPELAENFEAKDAI

TRYTNDVVASAAFGVKVDSMKDQDNEFYLHGKEVTNFSALAALKFTILKNFPNIMKFFGT

TFLPNATDKFFKKLITNTVTTRIEKGITRQDMMQLLIQAMKKDDGIKVTMDDIIGQAFFF

FLAGFESTSSAMCFALQELAANPDIQDRLRQEIDDEIEQHGGELTYESLVNLKYLDMVMS

ETLRKYPPVAITNRLCNKEYTFPPLMEGYPEYQMEVGTSILISMFGLHRDPKYFPDPEKF

DPERFNDENKHKINPYTYMPFGIGPRQCIANRFALMEAKIVIIDILRKFVVKFTEKSHHP

VTLSKKTFILSAEEGYWFKFEGRKKVD

>ABF06554.1

RPLNHFTKMGVKQTNTALPIFGDRWGVELRLDKSYFDLIKRVYFSCDKDDRFVGLYNFTR

PILFIRDPQLIKELGIKHFDSFNHRIPHIDPDSDPLWAANLTQIKGERWKEMRQSLSGSF

TSSKMKFIFELLNKSCSQFAEHYAAANGPTEVDMNDVSAMLTTDSIASSAYGIEVNSFKD

PDNLFFMMSKNILNLTTLRSQIKVLLTTICPFLLRIFKVGLFDKSVTDNISKIVEDTIAV

REKTGFVRPDMINVLLETRKVAKTNTAEDNTMETGYATAKESTALDKQKVKKRPLTNFEI

ASQAFVFFFAGQSSNSTSITFTFYELAVNPDIQERLRADIKETHKKNGNKATYESVLGIK

YLDMVVSESLRKWSPIVNFDRVCTKDFTIEPVRPGEKPIHLKRGDCIGIVPACIQRDPKY

FPNPDVFDPERFSEENIHKIVPYTYIPFGLGPRNCIGSRYALLQTKLAVYHILLNCKIVP

SSRTPVPMKTGFNWFLLHPENGLHLALEPLKE

>EFA09242.1

MLWILVSIAIIALSYLFLIRPFKYWTNLGVEQGNPVPVFGHSWGTIVHTQSFADLVQMVY

NIAPGARYSGMYQFFTPTLVLKDPDLIKQIAVKEFDSFLNHRSFIPEDVDPLWSKNLFSL

TNQKWRDMRSTLSPAFTSSKMKYMFVLISESAEQFIGHFLKQKEDVITIEMKDTFTRFTN

DVIANTAFGVKIDSLEERNNEFYLMGKEATDFKGFWRNLKFLILLSCPTLFKYLGINFFP

KAVSSFFRRLVKDNINNREKHGIVRPDMINLLLEARKNGLKNEEQEPILDTGFATVEESD

IGKNGAIKQKITDDDITAQALLFFFGGFDSVSSLMCFMSYELGINPDVQEKLHQEIDETL

EACKGKLTYEALMKMKYMDMVISETLRLWPTAVASDRLCNKPYTIEPKDPTEKPIHLEKN

SVVWIPIFAIHRDPQYFPEPNRFDPERFSDENKNNINPYAYMPFGVGPRNCIGSRFALLE

TKALFFHILSKFEIVPIEKTEIPLQLNRKSFSMTAENGFWFGLKRRESNK

>Q9W130.1

MVFVELSIFVAFIGLLLYKWSVYTFGYFSKRGVAHEKPIPLLGNIPWSVLMGKESYIKHS

IDLHLRLKQHKVYGVFNLRDPLYYLSDPELIRQVGIKNFDTFTNHRKGITEGFNDTSVIS

KSLLSLRDRRWKQMRSTLTPTFTSLKIRQMFELIHFCNVEAVDFVQRQLDAGTSELELKD

FFTRYTNDVIATAAFGIQVNSFKDPNNEFFSIGQRISEFTFWGGLKVMLYILMPKLMKAL

RVPVMDMNNVDYFKKLVFGAMKYRKEQSIVRPDMIHLLMEAQRQFKAEQEGSAESAAQQD

KAEFNDDDLLAQCLLFFSAGFETVATCLSFTSYELMMNPEVQEKLLAEILAVKEQLGEKP

LDYDTLMGMKYLNCVVSESLRKWPPAFIVDRMCGSDFQLKDEEGEVVVNLREDDLVHINV

GALHHDPDNFPEPEQFRPERFDEEHKHEIRQFTYLPFGVGQRSCIGNRLALMEVKSLIFQ

LVLRYHLKPTDRTPADMMSSISGFRLLPRELFWCKLESRGPA

>Q9V6H1.1

MDQSMIALALFIILLVLLYKWSVAKYDVFSERGVSHEKPWPLIGNIPLKAMIGGMPVLKK

MIELHTKHTGSPVYGIYALRDAVFFVRDPELIKLIGIKEFDHFVNHNSMHNNIQESILSK

SLISLRDGRWKEMRNILTPAFTGSKMRIMYDLIQSCSEEGVIHIQEQLELSQDASIELEM

KDYFTRFANDVIATVAFGISINSFRRKDNEFFRIGQAMSRISAWSVVKAMLYALFPRLMK

VLRIQVLDTKNIDYFSSLVTAAMRYRQEHKVVRPDMIHLLMEAKQQRLADLSDKSKDELY

YSEFTADDLLAQCLLFFFAGFEIISSSLCFLTHELCLNPTVQDRLYEEIISVHEELKGQP

LTYDKLTKMKYLDMVVLEALRKWPPSISTDRECRQDIDLFDENGQKLFSARKGDVLQIPI

FSLHHDPENFEDPEFFNPERFADGHALESRVYMPFGVGPRNCIGNRMALMELKSIVYQLL

LNFKLLPAKRTSRDLLNDIRGHGLKPKNGFWLKFEARQ

>156538028

MDLLTFALIVVACYTIYYYFKNNLDYFKNRDIPYYPAWPLLGNMATPFFRRKHISDLVRD

MYNYNPDAKYIGGFDFVRPVIVLRDLELIKSVAIKNFDSFPDHKSFVDDSLDPLFGGNLF

NIAGDRWKEARNLLSPAFTSSKMKGMFELIAQCAENFVKYLEERPASENKMIATKDLFTR

YTNDAIATCAFGIAVDSMKDPKNQFYVLGREATNLEGLMTVKFFIARAFPQILKFFRITF

VRDEIVKFFKDIVGATVAMRDEQGISRPDMIQLMMDARGKESKHLKLDLTEMTAQAFIFF

FGGFDTTSTQMCIIAHLLAIHPNVQRKLQEEIDEVMRKHQGKPTYEDINGMAYLDAVFNE

SMRRHTQVGFIDRLCTKKFELPPALPGGKGFTVLPGMNIWIPAIGIHMDPKYYDDPETFD

PERYYEKKVSINDVTNLGFGIGPRACIGNRFAIMETKLMLFFLLSKFNLVANEKTAKPFV

YSKHTFSIVAKGGYWLGIEKRN

>156553042

MDYWIWSLLVVTLTYAITSIYRQLSYFSRWGIPHPREMPIVGAIYALFFPRRHINDFIKY

IYDFNPDAKYVGAHIFTKPTIFIRDLDLVKTVLVKHFDHFADRKTFVDEKADPLFGRNLN

FLNGDRWKEIRTILSPAFTSSKMKTMYVLMSKCAENFTDEFLKKYGDAEAVDMKGAIGKY

TNDTIASCAFGIEVDSMKDPNNEFYLQAKEINFKGVTLLKIQLEMIFPTLTRKFGMRIVS

KKVTDFFVNAIATTMSVREEQGIFRPDMLQLMMEARSKMDKEAAERFDLLEMTAQAFLFF

LAGFDASSNQFCIIAHELAANPDIQSRLQAEVDDLMQKCDNKPTYDALNHMPYMDAVISE

SLRRHPIAFLNRLCSKEFELPPAVPDSKPYTMKPGDGLMIPVAGIHMDPNLYPNPERFDP

DRFIDKKIAISDVTNLGFGLGPRMCIGNRFAILSMKSLLVQLLSRCNLVPCSKTCIPLEY

CKNIFAPTPKNGFWLRIEPRTESR

>ABQ18318.1

MIIIIWTLAIGLAFLLYLKQIYCYFSKHEIKSITPLPILGNMGKIVFKINHFVDDISQLY

NKFPEERFVGRYEFVNPVIYIRDIEIVKRITIKDFEHFLDHRTIVNEETDPIFGRNLFSL

KGQEWKDMRSTLSPAFTSSKMKLMMPLIVEVGEQMIYALKKNIKNSGVGYVDIDTKDLTT

RYANDVIASCAFGLKVDSLTEENNQFYAMGKAASNFSFKQILLLLGFISFPKMMKMTKFT

LFSEETSGFFKELIMGTMKDREMRKIIRPDMIHLLMEAKKGKLVHDDKSSKDTDAGFATV

EESAVGKKQIDRVWTDDDIIAQAVLFFVAGFETVSSAMTFLLHELALNPEVQDKLVEEIK

ENKERNNGKFDYNSIQNMVYLDMVVSELLRLWPPGVSMDRICVQDYNLGKPNDKAKRDFI

LRKGTGVAIPVWAFHRNPEFFPDPLKFDPERFSEENKHNIKPFAYLPFGVGPRNCIGSRF

ALCEVKVMAYQLLQHMEISPCEKTCIPSKLSKETFNLRLEGGHWVRLKIRD

>ABQ08710.1

MIIIIWTLVIGLALLLYLKQTYCYFSKHEIKSITPLPILGNMAKIVFKINHFVDDISQLY

SKFPEERFVGRYEFVNPVIYIRDIEIVKRITIKDFEHFLDHRTIVNEETDPIFGRNLFSL

KGQEWKDMRSTLSPAFTSSKMKLMMPLIVEVGEQMINALKKNIKNSGVGYVDIDTKDLTT

RYANDVIASCAFGLKVDSLTEENNQFYAMGKAASNFSFKQILLLLGFISFPKLMKMTKFT

LFSEETSGFFKELIMGTMKDREMRKIIRPDMIHLLMEAKKGKLVHDDKSSKDTDAGFATV

EESAVGKKQIDRVWTDDDIIAQAVLFFVAGFETVSSAMTFLLHELALNPEVQDKLVEEIK

ENKERNNGKFDYNSIQNMVYLDMVVSEILRLWPPAVSMDRICVQDYNLGKPNDKAKRDFI

LRKGTGVAIPVWAFHRNPEFFPDPQKFDPERFSEENKHNIKPFAYLPFGVGPRNCIGSRF

ALCEVKVMAYQLLQHMEISPCEKTCIPSKLSKETFNLRLEGGHWVRLKIRD

>ABN71369.1

MIIIIWTLAIGLAFLLYLKQIYCYFSKHEIESITPLPILGNMGKIVFKINHFVDDISQLY

NKFPEERFVGRYEFVNPVIYIRDIEIVKRITIKDFEHFLDHRTIVNEETDPIFGRNLFSL

KGQEWKDMRSTLSPAFTSSKMKLMMPLIVEVGEQMINALKKNIKNSGVGYVDIDTKDLTT

RYANDVIASCAFGLKVDSLTEENNQFYAMGRAASNFSFKQILLLLGFISFPKMMKMTKFT

LFSEETSGFFKELIMGTMKDREMRKIIRPDMIHLLMEAKKGKLVHDDKSSKDTDAGYATV

EESAVGKKQIDRVWTDDDIIAQAVLFFVAGFETVSSAMTFLLHELALNPEVQDKLVEEIM

ENKERNNGKFDYNSIQNMVYLDMVVSEPLRLWPPGVSMDRICVQDYNLGKPNDKAKRDFI

LRKGTGVAIPVWAFHRNPEFFPDPLKFDPERFSEENKHNIKPFAYLPFGVGPRNCIGSRF

ALCEVKVMAYQLLQHMEISPCEKTCIPSKLSKETFNLRLEGGHWVRLKIRD

>ABP02071.1

MIIIIWTLVIGLALLLYLKQTYSYFSKHEIKSVTPLPILGNMGKIVFKFNHLVDDISQLY

NNFPEERFVGRYEFVNPVIYIRDIEIVKRITIKDFEHFLDHRTIVNEDSDPMFGRNLFSL

KGQEWKDMRSTLSPAFTSSKMKLMMPLIVEVGEQMINAIKENIKNSGVGYVDIDTKDLTT

RYANDVIASCAFGLKVDSLTEENNRFYAMGKAATNFSFKQILMLLGFISFPKLMKMTKFR

LFSEETSGFFKELIMGTMKDREMRKIIRPDMIHLLMEAKKGKLVHDNKSSKDTDAGFATV

EESAVGKKQIDRVWTDDDIIAQAVLFFIAGFETVSSAMTFLLHELALNPEVQEKLVEEIK

ENKERNNGKFDYNSIQNMAYLDMVVSELLRLWPPAVSMDRICVQDYNLGKPNDKAKRDFI

LRKGTGVAIPVWAFHRNPEFFPDPQKFDPERFSEENKHNIKPFTYLPFGVGPRNCIGSRF

ALCEVKVMAYQLLQHMEISPCEKTCIPSKLSKEIFNLRLEGGHWVRLKIRD

>ABQ08709.1

MIIIIWTLVIGLALLLYLKQTYSYFSKHEIKSVTPLPILGNMGKIVFKFNHLVDDISQLY

NNFPDERFVGRYEFVNPVIYIRDIEIVKRITIKDFEHFLDHRTIVNEDSDPMFGRNLFSL

KGQEWKDMRSTLSPAFTSSKMKLMMPLIVEVGEQMINAIKKNIKNSDGGYVDIDTRDLTT

RYANDVIASCAFGLKVGSLTEENNKFYAMGKAASNFSFKQILLLLGFISFPNLMKMTKFR

LFSEETSGFFKELIMGTMKDREMRKIIRPDMIHLLMEAKKGKLVHDNKSSKDTDAGFATV

EESAVGKKQINRVWTDDDIIAQAVLFFIAGFETVSSAMTFLLHELALNPEVQEKLVVEIK

ENKERNNGKFDYNSIQNMPYLDMVVSELLRLWPPAVSMDRICVQDYNLGKPNDKAKRDFI

LRKGTGVAIPVWAFHRNPELFPDPLKFDPERFSEENKHNIKPFTYLPFGVGPRNCIGSRF

ALCEVKVMAYQLLQHMEISPCEKTCIPTKLSKEIFNLRLEGGHWVRLKIRD

>ABO07439.1

MILLIWAVVLIAAFVLFYKQAYSLFSKHGVKGFTPLPFFGNMGRIVIKMDHFSDHIQSLY

DSFPEERFVGRYEFLNPMVIIRDIELLKKITVKDFEHFLDHRTIINKDTDPFFGRSLFFL

RDQDWKDMRSTLSPAFTSSKMKLMMPFIVEVGEQMNKALKQRIQEAGVGYVDIDSKDLTT

RYANDVIASCAFGLKVDSITEENNQFYAMGKAASTFNFRQLLIFFGLASVPKLVKILRIT

LFQKEIKTFFRELILGTMKNREAQNIIRPDMIHLLMEAKKGKLRHDEKSTKDSDAGFATV

EESSVGKKDINRVWTDDDLVAQAVLFFVAGFETVSSAMTFLLHELALNPEVQEKLVEEIR

ENEKNNNGKFDYNSIQNMVYLDMVVSEVLRLWPPVIALDRMCVKDYNLGKPNDKSKEDFI

IRKDVAVGIPVWGLHRDPEFFPNPLKFDPERFSEENKHNIKPFSYMPFGLGPRNCIGSRF

ALCEVKVMTYQLLQHMEISPCEKTCIPSKLSKETFNLRLEGGHWIRLKIRN

>BAI47532.1

MILLIWAVVLIAAFVLFYKQAYSLFSKHGVKGFTPLPFFGNMGRTVIKMDHFSDHIQSLY

DSFPEERFVGRYEFLNPMVIIRDIELLKKITVKDFEHFLDHRTIINKDTDPFFGRSLFFL

RDQDWKDMRSTLSPAFTSSKMKLMMPFIVEVGEQMNKALKQRIQEAGVGYVDIDSKDLTT

RYANDVIASCAFGLKVDSITEENNQFYAMGKAASTFNFRQLLIFFGLASVPKLVKILRIT

LFQKEIKTFFRELILGTMKNREAQNIIRPDMIHLLMEAKKGKLRHDEKSTKDSDAGFATV

EESSVGKKDINRVWTDDDLVAQAVLFFVAGFETVSSAMTFLLHELALNPEVQEKLVEEIR

ENEKNNNGKFDYNSIQNMVYLDMVVSEVLRLWPPVIALDRMCVKDYNLGKPNDKSKEDFI

IRKDVAVGIPVWGLHRDPEFFPNPLKFDPERFSEENKHNIKPFSYMPFGLGPRNCIGSRF

ALCEVKVMTYQLLQHMEISPCEKTCIPSKLSKETFNLRLEGGHWIRLKIRN

>ACJ05915.1

MILLIWAVVLIAAFVLFYKQAYSLFSKQGVKGFTPLPFFGNMGRIVIKMDHFSDHIQSLY

DSFPEERFVGRYEFLNPMVIIRDIELLKKITVKDFEHFLDHRTIINKDTDPFFGRSLFFL

RDQDWKDMRSTLSPAFTSSKMKLMMPFIVEVGQQMNKALKQRIQEAGVGYVDIDSKDLTT

RYANDVIASCAFGLKVDSITEENNQFYAMGKAASTFNFRQLLIFFGLASVPKLVKILRIT

LFQKKIKTFFRELILGTMKNREAQNIIRPDMIHLLMEAKKGKLRHDEKSTRDSDAGFATV

EESSVGKKDINRVWTDDDLVAQAVLFFVAGFETVSSAMTFLLHELALNPEVQEKLVQEIR

ENEKNNNGKFDYNSIQNMVYLDMVVSEVLRLWPPVIALDRICVKDYNLGKPNDKSKEDFI

IRKDVAVGIPVWGLHRDPEFFPNPLKFDPERFSEENKHNIKPFSYMPFGLGPRNCIGSRF

ALCEVKVMTYQLLQHMEISPCEKTCIPSKLSKETFNLRLEGGHWIRLKIRN

>ABF14737.1

MILLIWAVVLIAAFVLFYKQAYSLFSKHGVKGFTPLPFFGNMGRIVIKMDHFSDHIQSLY

DSFPEERFVGRYEFLNPMVIIRDIELLKKITVKDFEHFLDHRTIINKDTDPFFGRSLFFL

RDQDWKDMRSTLSPAFTSSKMKLMMPLIVEVGQQMNKALKQRIQEAGVGYVDIDSNDLTT

RYANDVIASCAFGLKVDSITEENNQFYAMGKAASTFNFRQLHIFFGLASVPKLCEDLRIT

LFQKEIKTFFRELILGTMKNREAQNIIRPDMIHLLMEAKKGKLRHDEKSTKDSDAGFATV

EESSVGKKDINRVWTDDDLVAQAVLFFVAGFETVSSAMTFLLHELALNPEVQEKLVEEIQ

ENEKNNNGKFDYNSIQNMVYLDMVVSEVLRLWPPVIALDRICVKDYNLGKPNDKSKEDFI

IRKDVAVGIPVWGLHRDPEFFPNPLKFNPERFSEENKHNIKPFSYMPFGLGPRNCIGSRF

ALCEVKVMTYQLLQHMEISPCEKTCIPSKLSKETFNLRLEGGHWIRLKIRN

>AAR26518.1

MIILLIWVAVLIAVLVLYLRQVYSRSSRYGVKHFKPVPLLGDMTGILFRVNHFTDDIVNL

YDSFPEEKFVGRFEFLNEMVMIRDIELVKKITVKDFEHFLDHRSIFSTSDSFFSRNLFSL

KGQEWKDMRSTLSPAFTSSKMRLMVPFMVEVGDQMMSSLQKKIKESESGYIDIDCKDLTT

RYANDVIASCAFGLKVDSHNDVDNEFYAMGKVSSTFNFRQMLMFFLIVNAPKLAKVLKLD

FLSEASKIFFRKLVLETMANREMKNVIRPDMIHLLMEAKKGKLTHEDIKSSDEAAGFATV

EESSVGQKEINRVWTDDDLIAQAVLFFIAGFETVSAGMSFLLYELALNPDVQERLAQEIK

ETDAKNGGKFDFNSIQNMPYMDMVVSELLRLWPPGIALDRICTKDYNLGKPNEKAEKDFI

VRKGTGISIPAYAFHRDPQFFPNPDKFDPERFSEENKHNIQMFAYMPFGVGPRNCIGSRF

ALCEMKVMAYQILQHLEVSPCEKTTIPAKLDKQTFNIRLEGGHWLRFRARK

>CAZ65619.1

MLMYVWLAAMAAAVWLYCRQIYSRFSKAGVKHFKPLPIVGNMLKILLRMDHFTDNIESLY

FAYPDEKFVGRYEFINPMVMIKDVDLLKKITVKDFEHFIDHRTLVNEKTDPFFGRNLFSL

KGDEWKDMRSTLSPAFTSSKMKLMLPFMVEVGNQMVDSLKKKIKESNATHIEIDAKDLTT

RYANDVIASCAFGLKVDSQTDTENHFYVMGKNTTAFNFRQILTFFALANFPKIMDKFKVT

LFTENTRQFFQHLVMDTMNERELKKIIRPDMIHLLMEAKKGRLTHDDKASHDPDAGFATV

EESNIGKKNINREWSDDDLTAQAFLFFFAGFETVSSAMSFALYELAVNPDVQEKFLEEIK

EADAKNGGKFDYNSIQNMTYMDMVVSEVLRLWPPGIAMDRICVKDYNLGKPNDKATSDYI

IRKGESIMIPAWAIHHDPKFFPNPMKFDPERFSEENKHNMDVTAYMPFGVGPRNCIGSRF

ALCEVKVMLYQLLLHMEISPSPRTSIPAQLSKETFNVRIKGGHWLNFRSRT

>AAY21809.1

MILVLVWVAVLIAVAVLYLRQVYSRFSRYGVKQFRPVPILGNMTRILLKQDHFVDDTMRY

YNSFPEERFVGKFEFIKELVVIRDIELVKKIAVKDFEHFLDHRSMFSSSDSFFSRNLFSL

KGQEWKDMRSTLSPAFTSSKMRMMVPFMVEVGDQMMAAIKNKIKESGNGYIDIECKDLTT

RYANDVIASCAFGLKVDSHNETDNEFYTMGKLSSTFNFRQMLVFFFIANAPTVAKILKLD

FLSEAAKKFFRNLVLDTMKNRELNHIIRPDMIHLLMEAKKGKLTHEEIKSNDVTAGFATV

EESAVGQKEITRVWTDEDLIAQAVLFFIAGFETVSSGMSFLLYELAVNPDVQERLAQEIK

ENDAKNGGKFDFNSIQNLQYMDMVVSELLRLWPPGAALDRICTKDYNLGKPNDKAKHDFI

VRKGTGISIPAFAFHRDPQFFPNPEKFDPERFSEENKHNIQSFAYMPFGIGPRNCIGSRF

ALCEMKVMAYQILQHMEVSPCERTCIPAKLDTETFNMRLKGGHWLRFRPRQ

>AAV28704.1

MILALVWVAVLIAVAVLYLRQVYSRFSRYGVKQFRPVPILGNMTRILLKQDHFVDDTMRY

YNSFPEERFVGKFEFIKELVVIRDIELAKKIAVKDFEHFLDHRSMFSSSDSFFSRNLFSL

KGQEWKDMRSTLSPAFTSSKMRMMVPFMVEVGDQMMAAIKNKIKESGNGYIDIECKDLTT

RYANDVIASCAFGLKVDSHNETDNEFYTMGKLSSTFNFRQMLVFFFIANAPTVAKILKLD

FLSEAAKKFFRNLVLDTMKNRELNHIIRPDMIHLLMEAKKGKLTHEEIKSNDVTAGFATV

EESAVGQKEITRVWTDEDLIAQAVLFFIAGFETVSSGMSFLLYELAVNPDVQERLAQEIK

ENDAKNGGKFDFNSIQNLQYMDMVVSELLRLWPPGAALDRICTKDYNLGKPNDKAKHDFI

VRKGTGISIPAFAFHRDPQFFPNPEKFDPERFSEENKHNIQSFAYMPFGIGPRNCIGSRF

ALCEMKVMAYQILQHMEVSPCERTCIPAKLDTETFNMRLKGGHWLRFRLRM

>ACJ37388.1

MILVLVWVAVLIAVAVLYLRQVYSRFSRYGVKQFRPVPILGNMTRILLKQDHFVDDTMRY

YNSFPEERFVGKFEFIKELVVIRDIELVKKIAVKDFEHFLDHRSMFSSSDSFFSRNLFSL

KGQEWKDMRSTLSPAFTSSKMRMMVPFMVEVGDQMMAAIKNKIKESGNGYIDIECKDLTT

RYANDVIASCAFGLKVDSHNETDNEFYTMGKLSSTFNFRQMLVFFFIANAPTVAKILKLD

FLSEAAKKFFRNLVLDTMKNRELNHIIRPDMIHLLMEAKKGKLTHEEIKSNAVTAGFATV

EESAVGQKEITRVWTDEDLIAQAVLFFIAGFETVSSGMSFLLYELAVNPDVQERLAQEIK

ENDAKNGGKFDFNSIQNLQYMDMVVSELLRLWPPGAALDRICTKDYNLGKPNDKAKHDFI

VRKGTGISIPAFAFHRDPQFFPNPEKFDPERFSEENKHNIQSFAYMPFGIGPRNCIGSRF

ALCEMKVMAYQILQHMEVSPCERTCIPAKLDTETFNMRLKGGHWLRFRPRQ

>ACB30272.1

MILALVWVAVLIAVAVLYLRQVYSRFSRYGVKQFRPVPILGNMTRILLKQDHFVDDTMRY

YNSFPEERFVGKFEFIKELVVIRDIELAKKIAVKDFEHFLDHRSMFSSSDSFFSRNLFSL

KGQEWKDMRSTLSPAFTSSKMRMMVPFMVEVGDQMMAAIKNKIKESGNGYIDIECKDLTT

RYANDVIASCAFGLKVDSHNETDNEFYTMGKLSSTFNFRQMVVFFFIANAPTVAKILKLD

FLSEAAKKFFRNLVLDTMKNRELNHIIRPDMIHLLMEAKKGKLTHEEIKSNDVTAGFATV

EESAVGQKEITRVWTDEDLIAQAVLFFIAGFETVSSGMSFLLYELAVNPDVQERLAQEIK

ENDAKNGGKFDFNSIQNLQYMDMVVSELLRLWPPGAALDRICTKDYNLGKPNDKAKHDFI

VRKGTGISIPAFAFHRDPQFFPNPEKFDPERFSEENKHNIQSFAYMPFGIGPRNCIGSRF

ALCEMKVMAYQILQHMEVSPCERTCIPAKLDTETFNMRLKGGHWLRFRPRQ

>ACB30273.2

MILVLVWVAVLITVAVLYLRQIYSRFSRYGVKHFRPVPLLGNMTRMVLKQDHLIEDILRY

YNSFPGERFVGRFEFISEMVVIRDLELVKKIAVKDFEHFLDHRSIFSSSDSFFSRNLFSL

KGQEWKDMRSTLSPAFTSSKMRMMVPFMVEVGDQMMDAIKKKIKESGNGYIDLECKDLTT

RYANDVIASCAFGLKVDSHNETDNEFYTMGKLSSTFRFREMIMFFVIANAPTVAKILKLD

FLSEAAKKFFRNLVLDTMKNRELNHIIRPDMIHLLMEAKKGKLTHEEIKSNDVTAGFATV

EESAVGQKEITRVWTDEDLIAQAVLFFIAGFETVSSGMSFLLYELAVNPDVQERLAQEIK

ENDAKNGGKFDFNSIQNLQYMDMVVSELLRLWPPGAALDRICTKDYNLGKPNDKAKHDFI

VRKGTGISIPAFAFHRDPQFFPNPEKFDPERFSEENKHNIQSFAYMPFGIGPRNCIGSRF

ALCEMKVMAYQILQHMEVSPCERTCIPAKLDTETFNMRLKGGHWLRFRPRQ

>ABY47595.1

MILVLVWVAVLITVAVLYLRQIYSRFSRYGVKHFRPVPLLGNMTRMVLKQDHLIEDILRY

YNSFPGERFVGRFEFISEMVVIRDLELVKKIAVKDFEHFLDHRSIFSSSDSFFSRNLFSL

KGQEWKDMRSTLSPAFTSSKMRMMVPFMVEVGDQMMDAIKKKIKESGNGYIDLECKDLTT

RYANDVIASCAFGLKVDSHNETDNEFYTMGKLSSTFRFREMIMFFVIANAPTVAKILKLD

FLSEAAKKFFRNLVLDTMKNRELNHIIRPDMIHLLMEAKKGKLTHEEIKSNDVTAGFATV

EESAVGQKEITRVWTDEDLIAQAVLFFIAGFETVSSGMSFLLYELAVNPDVQERLAQEIK

ENDARDGGKFDFNSIQNLQYMDMVVSELLRLWPPGAALDRICTKDYNLGKPNDKAKHDFI

VRKGTGISIPAFAFHRDPQFFPNPEKFDPERFSEENKHNIQSFAYMPFGIGPRNCIGSRF

ALCEMKVMAYQILQHMEVSPCERTCIPAKLDTETFNMRLKGGHWLRFRPRQ

>ABH09252.1

MILVLVWVAVLIAVAVLYLRQIYSRFSRYGVKHFRPVPLLGNMTRMVLKQDHLIDDILRY

YNSFPGERFVGRFEFINEMVVIRDLELVKKIAVKDFEHFLDHRSVFSSSDSFFSRNLFSL

KGQEWKDMRSTLSPAFTSSKMRMMVPFMVEVGDQMMDAIRKKIKESGNGYIDLECKDLTT

RYANDVIASCAFGLKVDSHNETDNEFYTMGKLSSTFKFRDMLMFFVIANAPTVAKILRLD

FLSEAAKKFFRNLVLDTMKNRELNHIIRPDMIHLLMEAKKGKLTRDEIKSNDVAAGFATV

EESAVGQKEITRVWTDEDLIAQAVLFFIAGFETVSSGMSFLLYELAVNPDVQERLAQEIK

ENDAKNGGKFDFNSIQNLQYMDMVVSELLRLWPPGAALDRICTKDYNLGKPNDKAKHDFI

VRKGTGISIPAFAFHRDPQFFPNPEKFDPERFSEENKHNIQSFAYMPFGIGPRNCIGSGF

ALCEMKVMAYQILQHMEVSPCERTCIPAKLDTETFGMRLKGGHWLRFRPRQ

>AAQ73544.1

MILVLVWVAVLITVAVLYLRQIYSRFSRYGVKHFRPVPLLGNMTRMVLKQDHLIEDILRY

YNSFPGERFVGRFEFISEMVVIRDLELVKKIAVKDFEHFLDHRSIFSSSDSFFSRNLFSL

KGQEWKDMRSTLSPAFTSSKMRMMVPFMVEVGDQMMDTIKKKIKESGNGYIDLECKDLTT

RYANDVIASCAFGLKVDSHNETDNEFYTMGKLSSTFRFREMIMFFVIANAPTVAKILKLD

FLSEAAKKFFRNFVLDTMKNRELNHIIRPDMIHLLMEAKKGKLTHEEIKSNDVTAGFATV

EESAVGQKEITRVWTEEDLIAQAVLFFIAGFETVSSGMSFLLYGLAVNPDVQERLAQEIK

ENDAKNGGKFDFNSIQNLQYMDMVVSELLRLWPPGAALDRICTKDYSLGKPNDKAKHDFI

VRKGTGISIPAFAFHRDPQFFPNPEKFDPERFSEENKHNIQSFAYMPFGIGPRNCIGSRF

ALCEMKVMAYQILQHMEVSPCERTCIPAKLDTETFNMRLKGGHWLRFRPRQ

>ACZ97417.2

MIFLIWGLLLSAALWLYLRQVYSRFSKYGVRHLKPIPLLGNNAKILFRMQHMTENVEENY

NAFPDDRFVGSYEFVNPMTIIRDVDLIKKITVKDFEYFLNHRVILNEDTDPFFGRSLFSL

QDQKWKDMRSTLSPAFTSSKIRLMVPFMEEVGDQMIKSLKINIKQSGNDFIDVDSKDLLA

RYANDVIASCAFGLKVDSHLEKDNQFYKMGKLATTFKFMETLKIFGYIIAPLIMKKLKIA

VINKESTNFLRDLVMNTMKDREIKNIKRNDMIHLLMEAKKGKLTHEGQAAGDTDVGFATV

EESDVGKKTVQRVWTDDDLTAQATLFFFAGFETVSTVMTFLLYELAVNPDIQDKLVKEIK

ETHQKNNGKIDYSSIQHMKYMDMVVSEGLRLWPPAPATDRKCVKDYNLGKANNYATDYII

RKGESIAIPIWAIHRSAEYFPDPLKFDPERFSEENKHNIKPFTYMPFGLGPRNCIASRFA

LCEVKVMLYQLLLHMVVSPAAKTCIPVKLSLESFNLRMKGGDWIRLSIRI

>BAG71410.1

MIILWIWLVVLVAVITLYFRRVYSSFDRHGIKHIKPTPMLGNMTKVILRAEHMADALKKV

YNDFPEERFVGRYEFIKELIVVRDLELVKKITVKDFEHFLDHRPLLGNSDSYFSRNLFSL

RGQEWKDMRSTLSPAFTSSKMRLMVPFMVEVGDQMMRSLHKIIKESKNGSVDLDCKDLTT

RYTNDVIASCAFGLKVDSHNDKNNSFYALGKETSTFDFSQIIKAFLFANLPEVAKFLKLD

FLSKSSKNAFKRLVLYTMENREMKNIIRPDMIHLLMEAKKGKLTHDDIKSNDLAAGFATV

TESAVGKKKVNRVWSDEDLVAQAVLFFIAGFEAVSSGTSFLLHELAMNPDVQERLAQEIK

EHDAKNGGKFDFNSIQNMVYMDMVVSEVLRLWPAAGALDRICTKDYNMGKPNAKAEKDYI

IRKGTGVWIPVFSIHRDPQYFPNPLKFDPERFSEENRHNINPLAYMPFGVGPRNCIGSRF

ALCEVKVMTYQIIREMVVSPCERTCIPAKLCPETFNMRLKGGHWLNFRPRTEQIRM

>AAP80766.1

MIILLIWSAVLVAVITLYFRRLYSSFSRHGIKHIKPVPILGNMAKVVLRAEHVSDGLTKV

YNDFPEERVVGRYEFTKELIVVRDLELVKKITVKDFEHFLDHRPLLGNGESYFSRNLFSL

RGQEWKDMRSTLSPAFTSSKMRLMVPFMVEVGDQMMRSLHRTIKESKNGSVDVDCKDLTA

RYANDVIASCAFGLKVDSHNDKNNNFYELGKETSTFNFRQVIKAFLFANLPEVAKLLKLD

FLSESSKNAFKRLVLDTMENRELKNIIRPDMIHLLMEAKKGQLTHEDIKSKDQAVGFATV

TESAVGKIKVNREWTDEDLVAQAVLFFIAGFEAVSSSMSFLLYELAVNPDVQERLAQEIK

ETDAKNGGKFDFNSIQNMVYMDMVVSEVLRLWPTAGALDRICTKDYNMGKPNLKAEKDYI

IRKGTGMWIPVFTIHRDPDYFPNPLKFDPERFSEENRHTINPLAYMPFGVGPRNCIGSRF

ALCEVKVMTYQILREMEVSPCEKTCIPAKLCNETFNLRLRGGHWLKFRART

>ABQ08706.1

MITLIWLGLLLVTLTLHLRKVYSRFKDYGVNHFTPIPVLGNAGPITVRLRHVAEDFDMVY

KAFPEDRFTGRFDLLRPTVIIKDLDLIKQITIKDFEHFLDHRALVDDTADPFFGRNLFSL

RGQEWKDMRSTLSPAFTSSKMRGMVPFMVEVNNQMIDMIKKKIVANADGYLDCEGKDLTT

RYANDVIASCAFGVKVDSHTNEENQFYLMGRDMADFGFRKIMVFLGYSSFPKLMKKFNAK

LLSDETGHFFTDLVLRTMEDREVKEIVRPDMIHLLMEAKQGKLSYDEKSTKEADTGFATV

EESDVGKKTINRIWSNTDLIAQATLFFVAGFETISSAMSFALHELALNPEIQDRLVQEIK

ENYAKTGGKFDFNCIQDLTYMDMFVSEVLRLWTPVVGMDRLCVKDYNLGRANKNATKDFI

LRKGEGLSIPTWSIHHNPEYYPEPYKFDPERFSEENKRNIKPFTYLPFGTGPRNCIGSRF

ALCEVKVMLYQLLQQIEVLPSDKTKVRAKLAKDTFNVKIEGGHWIRLKLRD

>ABQ08708.2

MITLIWLGLLLVTLTLHLRKVYSRFKDYGVNHFTPIPVLGNAGPITVRLRHVAEDFDMVY

KAFPEDRFTGRFDLLRPTVIIKDLDLIKQITIKDFEHFLDHRALVDDTADPFFGRNLFSL

RGQEWKDMRSTLSPAFTSSKMRGMVPFMVEVNNQMIDMIKKKIVANADGYLDCEGKDLTT

RYANDVIASCAFGVKVDSHTNEENQFYLMGRDMADFGFRKIMVFLGYSSFPKLMKKFNAK

LLSDETGHFFTDLVLRTMEDREVKEIVRPDMIHLLMEAKQGKLSYDEKSTKEADTGFATV

EESDVGKKTINRIWSNTDLIAQATLFFVAGFETISSAMSFALHELALNPEIQDRLVQEIK

ENYAKTGGKFDFNCIQDLTYMDMFVSEVLRLWTPVVGMDRLCVKDYNLGRANKNATKDFI

LRKGEGLSIPTWSIHHNPEYYPEPYKFDPERFSEENKRNIKPFTYLPFGTGPRNCIGSRF

ALCEVKVMLYQLLQQIEVLPSDKTKVRAKLAKDTFNVKIEGGHWIRLKLRD

>ABP65279.1

MITLIWLGVLLVTLTLHLRKVYSRFKDYGVNHFTPIPVLGNAGPITVRLRHVAEDFDMVY

KAFPEDRFTGRFDLLRPTVIIKDLDLIKQITIKDFEHFLDHRALVDDTADPFFGRNLFSL

RGQEWKDMRSTLSPAFTSSKMRGMVPFMVEVNNQMIDMIKKKIVANADGYLDCEGKDLTT

RYANDVIASCAFGVKVDSHTNEENQFYLMGRDMADFGFRKIMVFLGYSSFPKLMKKFNAK

LLSDETGHFFTDLVLRTMEDREVKEIVRPDMIHLLMEAKQGKLSYDEKSTKEADTGFATV

EESDVGKKTINRIWSNTDLIAQATLFFVAGFETISSAMSFALHELALNPEIQDRLVQEIK

ENYAKTGGKFDFNCIQDLTYMDMFVSEVLRLWTPVVGMDRLCVKDYNLGRANKNATKDFI

LRKGEGLSIPTWSIHHNPEYYPEPYKFDPERFSEENKRNIKPFTYLPFGTGPRNCIGSRF

ALCEVKVMLYQLLQQIEVLPSDKTKVRAKLAKDTFNVKIEGGHWIRLKLRD

>ACV88722.1

MIIPLIWLAVLIGVAALYFRRVYSSFSRQGIKHFKPVPVFGNLAKMLMRTEHFCDHIVNL

YQSFPEGRFVGRYEFVKQIIFVRDLELIKKMTVKDFEHFLDHQSILSTSDSYFSRNLFSL

QGQEWKDMRSTLSPAFTSSKMRLMLPFMVEVGDQMMQMLHRQIRESKNGSITVDCKDLTT

RYANDVIATCAFGLKVDSQTDKTNSFYMLGKESSEFGFRKLMSFLIVSNAPALARLLKLD

FLSEACKQGFKKIVLSTMQTREMKNIIRPDMIHLLMEAKKGKLTHDDVKTNDVAAGFATV

EESAVGKKSADRVWSDEDLVAQAVLFFIAGFETVSTGMIFLLYELAVNPDVQERLAQEIK

EVDAKNGGKFDFNSIQNMVYMDMVVSETLRLWPPAVTLDRICTKDYNMGKPNPKAEKDVI

LRKGTGVWIPTYAFHRDPQYFPNPDKFDPERFSEENRHTINPFAYMPFGVGPRNCIGSRF

ALCEIKVMVYQILKEMVLSPSETTCIPAKLSKDTFSMRLEGGHWLKFSPRN

>ABH09253.1

MIALLWLVVLVAALTLYLRQVYSRFSRFGVKHFEPVPLVGNLSTVLMRKAHASEDFNNLY

QAFPGERFVGRYEFLRNIVMIRDLELVKSITVKDFEHFIDHRMLADADVEPLFGRNLFSL

RGHEWKEMRSTLSPAFTSSKMKAMVPFMMEVSEQMINFLKMQIKESGGKHADIECKDLMT

RYANDVIASCAFGLKVDSHNDRENEFYSIGTETANFDFKKMLVIFGYACFPAIMKKFNGK

MFSELIVNFFKNIVIGTMRNRQKNNILRPDMIHLLMEAKKGKLTHEEKVAEANTGFATVE

ESDIGKVTLKKEWTEDDLTAQAVLFFVAGYETISSAMAFLIYELAVHPEVQEKLAKEIKE

HDAKNGGKFDFNSIQNMPYLDMVISEVLRLWPPAVGLDRECSKDYNLGKPNDKAEKDYIL

RKGEALVIPVWSIHHDPEYFPDPYKFDPERFSEENKHKIKPFSYMPFGLGPRNCIGSRFA

LCEVKVMAYQLIQQMELSPCEKTSIPAVLAKDTFNLKVEGGHYIRVKLRQ

>ABY47596.1

MIALLWLAVLVAALTLYLRQVYSRFSRFGVKHFEPVPLVGNLSTVLMRKAHASEDFNNLY

QAFPGERFVGRYEFLRNIVMIRDLELVKSITVKDFEHFIDHRMLADADVEPLFGRNLFSL

RGHEWKEMRSTLSPAFTSSKMKAMVPFMMEVSEQMINFLKMQIKESGGKHADIECKDLVT

RYANDVIASCAFGLKVDSHNDRENEFYSIGTETANFDFKKMLVIFGYACFPAVMKKFNVK

MFSELIVNFFKNIVIGTMRNRQKNNILRPDMIHLLMEAKKGKLTHEEKAAEANTGFATVE

ESDIGKVTVKKEWSEDDLTAQAVLFFVAGYETISSAMAFLIYELAVHPEVQEKLAKEIRE

HDAKNGGKFDFNSIQNMPYLDMVISEVLRLWPPAVGLDRECSKDYNLGKPNDKAEKDYIL

RKGEALVIPVWSIHHDPEYFPDPYKFDPERFSEENKHKIKPFSYMPFGLGPRNCIGSRFA

LCEVKVMAYQLIQQMELSPCEKTSIPAVLAKDTFNLKVEGGHYIRVKLRQ

>BAI49180.1

MITLIWLGVLLVTLTLHLRKVYSRFKDYGVNHFTPIPVLGNAGPITVRLRHVAEDFDMVY

KAFPEDRFTGRFDLLRPTVIIKDLDLIKQITIKDFEHFLDHRALVDDTADPFFGRNLFSL

RGQEWKDMRSTLSPAFTSSKMRGMVPFMVEVNNQMIDMIKKKIVANADGYLDCEGKDLTT

RYANDVIASCAFGVKVDSHTNEENQFYLMGRDMADFGFRKIMVFLGYSSFPKLMKKFNAK

LLSDETGHFFTDLVLRTMEDREVKEIVRPDMIHLLMEAKQGKLSYDEKSTKEADTGFATV

EESDVGKKTINRIWSNTDLIAQATLFFVAGFETISSAMPFALHELALNPEIQDRLVQEIK

ENYAKTGGKFDFNCIQDLTYMDMFVSEVLRLWTPVVGMDRLCVKDYNLGRANKNATKDFI

LRKGEGLSIPIWSIHHNPEYYPEPYKFDPERFSEENKRNIKPFTYLPFGTGPRNCIGSRF

ALCEVKVMLYQLLQQIEVLPSDKTKVRAKLAKDTFNVKIEGGHWIRLKLRD

>ABQ08707.1

MITLIWLGLLLVTLTLHLRKVYSRFKDYGVNHFTPIPVLGNAGPTTVRLRHVAEDFDMVY

KAFPEDRFTGRFDLLRPTVIIKDLDLIKQITIKDFEHFLDHRALVDDTADPFFGRNLFSL

RGQEWKDMRSTLSPAFTSSKMRGMVPFMVEVNNQMIDMIKKKIVANADGYLDCEGKDLTT

RYANDVIASCAFGVKVDSHTNEEDQFYLMGRDMADFGFRKIMVFLGYSSFPKLMKKFNAK

LLSDETGHFFTDLVLRTMEDREVKEIVRPDMIHLLMEAKKGKLSYDEKSTKEADTGFATV

EESDVGKKTINRIWSDTDLIAQATLLFVAGFETISSAMSFALHELALNPEIQDRLVQEIK

ENYAKTGGKFDFNCIQDLTYMDMFVSEVLRLWTPVVGMDRLCVKDYNLGRANKNATKDFI

LRKGEGLSIPTWSIHHNPEYYPEPYKFDPERFSEENKRNIKPFTYLPFGTGPRNCIGSRF

ALCEVKVMLYQLLQQIEVLPSDKTKIRAKLAKDTFNVKIEGGHWIRLKLRD

>AAR37015.1

MIALLWLAVLVAALTLYLRQVYSRFSRFGVKHFEPVPLVGNLSTVLMRKAHASEDFNNLY

QAFPGERFVGRYEFLRNIVMIRDLELVKSITVKDFEHFIDHRMLADADVEPLFGRNLFSL

RGHEWKEMRSTLSPAFTSSKMKAMVPFMMEVSEQLINFLKMQIKESGGKHADIECKDLMT

RYANDVIASCAFGLKVDSHNDRENEFYSIGTETANFDFKKMLVIFGYACFPAIMKKFNVK

MFSELIVNFFKNIVIGTMRNRQKNNILRPDMIHLLMVAKKGKLTHEEKVAEANTGFATVE

ESDIGKVTVKKEWTEDDLTAQAVLFFVAGYETISSAMAFLIYELAVHPEVQEKLAKEIRE

HDAKNGGKFDFNSIQNMPYLDMVISEVLRLWPPAVGLDRECSKDYNLGKPNDKAEKDYIL

RKGEALVIPVWSIHHDPEYFPDPYKFDPERFSEENKHKIQPFSYMPFGLGPRNCIGSRFA

LCEVKVMAYQLIQQMELSPCEKTSIPAVLAKDTFNLKVEGGHYIRVKLRQ

>ACZ97407.2

MILITICSLVLLATLWLYLRQVYSRFSKYGVKHLPPVPLLGNHAKFTLGLEDLGTLTVKT

YNSFSEERFVGSFAFLNPVVFIRDLELLRKITVKDFEHFLNHRPLVDENVDPFMGKILFS

LNDQKWKDMRSTLSPAFTNSKIRLMVPFMVEVGDQMVRSLNKKIEKSAGGYIDIDVKDLM

TRYTNDVIASCAFGLKVDSHTDEHNQFYKLGRFSTNYTLKQILTFSAYNAFPNLMKTLNA

HVFREKTNKFFKDLVLNTMKDREAKKIIRNDMIHLLMEARKGNLKHDDKVTKDWGGFAAV

EESDVGKKQVNAVWTDEELIAQAVMFFIAGFETVSTAMVFCLHELAMNPDIQDTLVREIQ

ENHEKSDGEIDYNSIQQMAYMDMVVSETLRLWSPVFTTDRMCIKDYNIGKPNKYATEDFI

VRKGEEINVPIWAFHRSPEYFPDPLKFDPERFSEENKHKIKPFSYIPFGIGPRNCLGSRF

ALCEIKVMLYQLLLAMEVSVADKTCIPAEVAKGTGFHLRMKGGHWVRMKTRN

>ADP55210.1

MIIFFIWLVVLIAALALHLRQMHSAFSKHGIKYLNPAPLLGNMINTIFLREHFAYDVLRL

YGSFPKDRFVGRYEFTNKAIMLRDLDLVKKITVKDFEVFLDHRDFGGDTFFGRTLILMRG

QEWKDMRSTLSPAFTSSKIRLMVPFMVEVGDQMIQSLNKEIEKSSDGYIDVDCKDLTTRY

ANDVIASCAFGLKVNSHAEKNNEFYIMGRTAANFGLREMFMFIFMVIAPKIVALLKWNLV

PDKMKNYFTNLVLDTMKDRELRHIFRPDMIHLLMEAKKGQLTHEDAKSNNDTTGFATVEE

STVGLKKTTNRVWNDNDLSAQAFLFFIAGFETVSTSMSFLLYELAVNPDVQERLAQEIKE

HDVKHGGKFDFNSIQNMKYMDMVVSELLRLWPPLTVMDRECNRDYNMGKPNEDFDKDYIL

PKGTTVFIPTFAFHRDPQYFPDPEKFDPERFSEENRHKLNLNAYMPFGVGPRNCIGSRFA

LCELKVLTYQILLHMELSPSEKTQIPARLAPDNINVRLQGGHWLRFRQRK

>ABB69055.1

MTILLLIWALVLAVIVLRDLRRLYSTFSKDGIKHFKPVPLLGNMSSVIFRRNHYADNILK

FYKSFPDEKFVGRFEFVNQSIVILDLELVKKITIKDFEHFPDHRNFGAESFFSRTLFLLA

GQEWKDMRSTLTPAFTSSKIRLMVPFMVEVGDQMILSLKKKLEESKDDYIEVDCKDLTTR

YANDVIASCAFGLKVDSHMDRDNRFYAMGGSLFEFGFRTMIMFFVLLNAPKVAKFFKWDI

VPASVRKFFTNLVLDTMKDREMRHIIRPEMIHLLMEANKGKLTHEDIMPNDNAVGFATVE

ESAIGLNSSKRVWTDEDIVAQAFVFFAAGFETVSATMAFLLYELAVHPEVQERLAQEIRE

YDDKNDGKFDFNSIQDMKYMDMVVSEVLRKWPAPLVLDRICTKDYNLGKPNNAAEKDFII

RKGSGVQIPVYAFHNDPRYFPNPEKFDPERFSPDNKHKFNANAYMPFGVGPRNCIGSRFA

LCEVKVITYQILRHMEVSPCKSTCIPAKLATNNLNLRLKGGHSLRFKLRK

>EHJ65260.1

MIVFLLWVVVLVAALALYYRQIYSRFSKFGVKQNNIVPFFGNMLKITLGMEHFAENIDSM

YKNFPEERFIGRFEFSKPGVLIRDLELIKKVTVKDFEHFLDHRGFVDDKVEPLFARNLFS

LKGQEWKDMRSTLSPAFTSSKIKQMVPFMEEVGEMMVRAVKKNIENSKVGQIEIDVKDLT

SRYSNDVIASCAFGLKVDSHTTEDNEFYEMGKKASTFKFKQMLLFFMNSALPFVARLFKL

KLFSRETTDFFVDLVQDTMKEREDKKIIRPDMIHLLMEAKKGKLTYDVANGKADSNVGFS

TVEESDLGQKPVTRVWSDSDLIAQAVMFFITAFETISTVASFTLYELAVNPDVQDKLFQE

IKEHNAKFGGKLDFNSIQNLTYLDMVVSEVLRLWPPAIVLDRVCNKDYNLGKPNSKATED

FIVRKGELVGVPVMSIHRDPQYFQNPNKFDPERFSEENRHLIDLMTYMPFGYGPRNCIGS

RFALCEVKVLTYQIILNFEISPAEKTCIPAKLSTGNLNLRLKEGHGLLFKLR

>EHJ65261.1

MSIRNRICVLRLQMILEIWIVVLFVAAVLYYRQVYSRFTKYGVKQQTPFPLFGNMWRVTL

RLEHFTDTMNSMYKEFPEERFIGRFEFIKPCLFVTDIELIKKITVKDFEHFLDRSGFSVE

VDPLFSRNLFSLKGQEWKDMRSTLSPAFTSSKIKQMVPFMEEVGEMMIQAVKNKIKNSGS

EWIEIDCKDLTTRYTNDVIASCAFGIKVDSHTNVDNDFYKIGKIASTFKTRDVILFQLYS

AAPFLGKFFKLELLSTKTRNFFIDLVLSTMKERETHNIIRPDMIHLLMEAKKGRLHHEKD

VINADDGFATVEESEVGKNNKHKSWSDTDLIAQAVLFFIAAFEVVSTAMTFTLTELALHP

DVKERLALEIKEHNEKYGGKIDLHSIQTLTYLDMVISEVLRLWVPTILIERMCVKDYNMG

KSNGEAKEDFILRKGESLHIPTWCIHRDPQFFPNPNKFDPERFSKENRHLIHPIAYMPFG

LGPRNCIGSRFALCELKLLVYQILLNFDLSPTKKTCIPARLNKSSFHLQLEGGHALKFRS

RYNLIL

>AAD25104.1

MILLLTWLVVIITAVLLYFRSVYSQLSKQGVNHLPTIPVFGNLMWMVMKQEHFVDTLGRC

VKAFPDDKIVGHYDMVSPILVVLDVDTVKRITVKDFEHFVDRRSFTSSFDPIFGRGLLLL

HGDEWKAMRSTMSPAFTSSKMRLMVPFMEEIALEMIRVLRGKIKDSGKPYIDVEAKSVMT

MYANDVIASCAFGLNVNSQASDHEFYVNSQAITKFKFSAFLKVLFFQCLPSVAQKLKMSL

VPRECSDYFSNVVLTTMKDREKNKVVRNDLINILMEVKKGQLTHEKDDAEADAGFATVEE

SHIGRKQHNYEWTDSDLIAQAALFLFAGFDTVSTSMSFLLYELAVNPDVQDRLLQEIREY

DEKNHGKIDYNVVQSMTYLDMVVSEGLRLWPPAAVVDRVCVKDYNIGRPNEQATKDLIIH

TGQAVAISPWLFHRNPKFFPEPAKFDPERFSPENRHKIQPFTYFPFGLGPRNCIGSRFAL

CEIKVILYLLIREMEVYPFEKTIYPPQLSKDRFNMHLEGGAWVRLRVRPEKS

>AAC25787.1

MILLLTWLVVIITAVLLYFRSVYSQLSKQGVNHLPTIPVFGNLMWMVMKQEHFVDTLGRC

VKAFPDDKIVGHYDMVSPILVVLDVDTVKRITVKDFEHFVDRRSFTSSFDPIFGRGLLLL

HGDEWKAMRSTMSPAFTSSKMRLMVPFMEEIALEMIRVLRGKIKDSGKPYIDVEAKSMMT

RYANDVIASCAFGLKVNSQASDHEFYVNSQAITKFKFSAFLKVLFFRCLPSVAQKLKMSL

VPRECSDYFSNVVLTTMKDREKNKVVRNDLINILMEVKKGQLTHEKDDADADAGFATVEE

SHIGRKQHNYEWTDSDLIAQAALFLFAGFDTVSTSMSFLLYELAVNPDVQDRLLQEIREY

DEKNHGKIDYNVVQSMTYLDMVVSEGLRLWPPAAVVDRVCVKDYNIGRPNKKATKDLIIH

TGQAVAISPWLFHRNPKFFPEPAKFDPERFSPENRHKILPFTYFSFCLGPRNCIGSRFAL

CEIKVILYLLIREMEVYPFEKTIYPPQLSKDRFNMHLEGGAWVRLRVRPEKS

>AEL87781.1

MLLLLTWVVVIVTALVLYLRQAYGSLRRDGINHLPTVPFFGNIFWVMFQKEHFVDLIMKI

TNAFPDDKIVGNYDMMTPILIIRDLEMLKRITVKDFEHFIDRRSFTTELDPMFGRGLLLL

HGDEWKAMRSTMSPAFTSSKIRLMVPFMEEVCLEMVKVLKQRIKDSGTSYYDMECKEVMT

RYANDVIASCAFGLKVESLTRDSEFYVHSKSITSKSITKFTFWRFMKFMFYRLLPSVAGM

LQLSLVSQKTTDYFTNVVLGTMKDREKNKIVRNDMINILMEVKKGQLTHEKEGKDADAGF

ATVEESHVGKKQHNYEWTDTDLVAQAALFLFAGFDTISTAMSFILYELAINPDVQDRLVQ

EIREYDAKNNGKIDYNSIQSMTYLDMVVSEGMRLWPPAPFMDRICVNDYNIGRPNKEATK

DLIIRKGQCVVIPAYTLHRNPEYFPNPSKFDPERFSHENRDKIVPFTYLPFGLGPRNCIG

SRFALCEVKVMLYLLLRDLKLTTCDKPASLRG

>Q964T2.1

MMSLESLWLWTFAISFIVLIAYLIGTWNHDFFSKRNIPSLKTVPFLGNMGPLVLRKASFA

EHSQNIYNRLKGYKYGGMFEFMNPVLVLRDPELIKMVTVKDFEYFLDHRAPISEEAEPMF

GKNLFNLRGHRWKEMRSTLSPAFTSSKMKNMFVLVSECGKQLGEFLMECSRDKNKKTEGC

KIEREGDLLTVELKDLYTRYTNDVIATSAFGIGCDSLKNPKNEFFQMGKDVTNFGGIRQF

IFLGYLFSPRLMKYLNLKFLSSKATEFFRFLVHNTMDTRKTKGIIRPDMIHLLMQAKEGT

LKSEENGETNGKIASKPKWDDDDLTAQAVLFFFAGFDTASTLLCFMSHLLATNPDVQNRL

QDEIDQSLEENDGKLTYEAIHSMKYLDMVVSESLRLYPPAIFTDRKCVKNYRLPMEPSYT

LEPGDAVWIPIYAIHHDPKYYPNPEKFDPERFSDENKDNIKPFTYLPFGSGPRNCIGNRF

ALMESKIALVHLLCRFNLKVVSKTPIPIKITKKGFNMTVDGGFWLGLEERTNQ

>EFA09141.1

MLTIILSVVLGALIYYKLIKPASFWDERNITHASAWPLIGSMWESVIQKKNFNEISSDFY

KKYPNDRYVGYMQFSNPVLLIRDLDLIKQIGVKVFDNFHDHLAITGIKTDPLFSKSLIVL

RGEEWKQMRATLSPAFTSSKMKNMYHLIYECAENFAKHFQDEKKNVEVKDIFSKFTNDVI

ATVAFGIQINSLKEPNNEFYSMGKLLTTFKGFELMKFFFALALPQIFELTKLSLFNQKVT

NFFRNIIIDNMHKREKDGIVRPDLIHLLMEARKGKLKHDNSNEGGDGFATVEESEIGKNS

KKMELTDDLMVAQALLFFFAGFETISTGFSFMAYELATNPDVQKKLQKEIDLTLQENHGK

ISYNVLQSMKYLDQVVCESLRLWPPAPQTDRFCNTDFVLEPTKPHERRFTIERGVTIIIP

IYGIHRDPEYFPNPDKFDPERFSDENKAKIVPGTYMPFGVGPRNCIGSRLALLELKTLFF

HLLSKCDIVTNSKTVIPLKISPNNGNITPKEGIHLTFKPRNVA

>EFA09240.1

MFWILIVIAAIIAISYHQLRRYHKYWSEKGVQQGDPLPLFGHCLGTTLQKQSLSEFIQMS

YNVAPDARYCGIYQFYSPILIVRDPELIKQITVKDFDHFVNRMAFIPEDCDPLWGKNLVA

LTDQRWRDMRSTLSPAFTSSKMKYMFSLISESAEQFAQYFANQDQDLITIEMKDIFTRFT

NDVIANTAFGVKIDSLEERNNEFYLMGIEATDFRGFWRNMKIVIYFIFPTLFKSLGWRFF

SKNVADFFVKLVRDNIAKREKHGIVRPDLINLFLEARKNGLKYEEQEHALDTGFATAEES

EIGKNGMVRQTLTDVDIAAQALLFFFGGFDSVSSMMCFMSYELALNPDVQAKLLREVDET

FESCDGKLTYEALIKMKYLDMVVSETLRLWPTNVSADRVCTKPYTIEPKNPNEKALHLEK

NTNVIVPIFGIHRDAKNFPEPNRFDPERFSDENKANIRPYTYLPFGAGPRNCIGSRFALL

ETKALFFHLLSKFEITPVEQTQIPIQLNRKSFNMTAENGFWLGLKRRKNDN

>EDS34508.1

MYYQCNLVLSDKTPDTQKYDFFEERNIPFIKPVFLLGNTAPVLLKRQDLLQNVKEMYNAF

PSSKVVGLFDFIKPVLMIRDLDMLKQIGVKDFDHFSDHTPIFTRADVEDVGGDSLFGNSL

FALRGQKWRDMRSTLSPAFTGSKMRHMFELVSQSAQSTVEFFRAEAKAGRTLEYEMKDVF

SRFSNDVIASVAFGIEVDSFRERENSFYVKGKAMLNFQNVKTIVKMLLLRTIPNLMIRLK

VDIQSKESTEYFKSMITDNMRQRDVHNIVRNDMIHMLMQVRKGALKHQKDEQETKDAGFA

TVEESNVGKVTHSRVWTDNELVSQCFLFFLAGFDTVSTALTFLSYELLVNPDIQQRLYEE

ILSVEETLNGKSLSYEVLQRMQYMDMVVSETLRKWPPAPFVDRFCVKDYQYDDGEGLRLQ

ISKNQILWIPIVALHHDPKYYPNPEKFDPERFSEANRASINPAAYVPFGVGPRNCIGSRF

ALMEVKALVYNLVKSFTFERSAKTKVPLELVKSIVGVAIDGGMWLELKARS

>EAT41568.1

MEVDLFAAIAVGALILAVYHHLLKRYQYFLTKPVSCVKPSFPMGSSGVMLTRKRDIFSHI

QMMYNTYPDAKIMGFYDFTKPVYMIRDPEVIKRITVKDFDHFIDHTPSMTGQGEEPGENS

LLGNTLFALRGQKWRDMRSTMSPAFTGSKMRHMFELVAESGQSTAKFLLAEAKARKRLEF

EMKDTFTRFGNDVIATVAFGIKVDSMRDRDNEFYMKGKQLLNFQTFLLKIKFIMMRAMPT

LAEKLGVDLLDAEAVKYFKGMILENMKQRKAHGIIRNDMIHMLMEVRKGALKHEKDEQDT

KDAGFATVEESQVGKTTHSRIWKDNELVAQCFIFFVAGFDTVSTGLTFLAYELALNPEIQ

QRLYEEIIETETTLEGKSLTYEVLQKMKYLDMVVSEGLRKWPAGILGDRYCTKDYQYKDA

AGSFVIEKGTSLWIPTIAIHNDPQYYPNPEKFDPERFSDENKSKINPFAYMPFGVGPRNC

IGSRLALMEMKLIMYYLLREFSFEPTEKTQIPLKLVMSGFALQGEKDVWLEFKPRAL

>EAT41567.1

MLEVNLFAAIAVGALILAVYHHISKRYQYFLSKPVPCMKPTFLVGNSGPMLTRKKDIASH

IRTMYDTYPDAKMIGFYDLTKPVYLVRDPEVVKTMTVKDFEHFTDHTPTMTGTGEEVSEK

SLFGNSLFALRGQKWRDMRSTLSPAFTGSKMRHMFELVVECGQSMAEFLISEAKAGKRLE

FEMKDTFTRFGNDVIATVAFGIKVDSMRDRENEFYMKGKQLLNFQRFTLMIKFLLMRAMP

ALAEKLGADFVDAEAGKYFTGVIMENMKQRKAHGIVRNDMIHMLMEVRKGALKHEKGEQE

TKDAGFATVEESQVGKTTHSRIWKDNELVAQCFIFFLAGFDTLSTGLTFLTYELALNPEI

QQRLYEEVMETESNLDGKPLTYEVLQQMKYMDMVISESLRKWPPGIVADRYCTKDYQFKD

GPGSFLIEKGTSLWIPTIAIHNDPRYYPNPDKFDPERFSDENKSKINPAAYIPFGVGPRN

CIGSRLALMEMKSVVYYLLREFSFEPTEKTQIPLKLTMSGFTLQGEKGVWLEFKPRSI

>157167200

MVEVDLYVAVAIGAIILLLYHYGSKKYEYFLTKPIPALKPTFLLGNTGAMMFRRRDVSAH

VKLLYNSFEGYKVAGFYDLMKPIYMLRDPEVIKQIAVKDFDYFMDHTPTMTNSKADDEVG

GDSLFGNSLFALRGQKWRDMRATLSPAFTGSKMRHMFELVADCAKSMAEFFKSEAAAGKK

LEYEMKDTFSRFGNDVIATVAFGIKVDSLRDRDNEFYMKGKNMLNFQSVSVLFKFLLLRA

FPKLSQKIGVDFVDSNLTEYFKGMIVDNMKQRDAHNIFRNDMIQMLMEVRKGSLKHQKDE

KETKDAGFATVEESNVGKSTINRVWTENELIAQCFLFFLAGFDTVSTCMTFLTYELMLNP

DIQQRLFDEVMETEESLNGKPLTYEVLQRMEYMDMVVSEALRKWPPAVVSDRFCVKNYMY

DDGKGTRFPIEKGQTMWIPTIAIHSDPRYYENPEKFDPERFNEENRSKIDTGAYLPFGVG

PRNCIGSRLALMEVKVIIYNLLKEFSLEASEKTQIPLKIAKNFFALQAENGVWLELKPRK

H

>EDS34505.1

MVQVDLFTAGAVFAIILLAYHYLSKRYEYFLTKPIPCLKPTFLVGNTGAMMFRQRDIAAH

VKTLYYAFPDFKITGFYDLMNPIFMLRDPDVIKKIGVKDFDVFMDHTPTMAGATEAEVSG

EQLFANSLFALRGSKWRDMRATLSPAFTGSKMRHMFELVAECAVSMSDFFKAEAKAGKKL

EFEMKDTFSRFGNDVIATVAFGIKVDSMRERENDFFVKGKQMLNFQSLIVLLKFFIFRAL

PSLAAKLELDFIDKDLARYFKGMIIDNMKQRDAHGIIRNDMIHMLMEVQKGALKHQKDEK

DTKDAGFATVEESTVGKSTHSRVWTENELIAQCFLFFLAGFDTVSTCMTFLTYELALNPE

IQNRLYEEIMETERSLNGSPLNYETLQKMVYMDMVVSEALRKWPPAVVSDRYSNRDYTYD

DGAGSRFLIEKGRTIWIPTIAIHNDPKYYPNPDKFDPERFNEQNRPNINLGAYLPFGIGP

RNCIGSRLALMEVKSIIYCLLKDFCLEPSEKTEIPLKMSKNLFALQAENGVWLEFKPRKM

>EAL38705.3

MEISLMVLVPLVAILGFIYHYITKGNKYFHDKPIPSLAAEPLFGSTRRLMMKQTSFHDFV

ISIYNKYPLVKVFGMIDTMLPMFVVRDPELLKRIAVKDFEYFMNHRPVFGNNDDPHNEAL

FGKTLIAITDQKWRDMRATLSPAFTGSKMRQMFELIVECSSNMAQHYKEQIRSNGAGSRE

HEMKDVFTRYANDVIATCAFGIKVDSLKHAENDFFVNGKKMMAFNRPIVMLRMMGFRLLP

KLMNFFNLDLFDREQRVFFTEIIKDTVRTRDANGIVRPDMVHLLMQARKGALKHQRDTEE

REEVKGFATVEESEVGQTQASKGMQMTELEMIAQCLIFFLAGFDTVSTCLTFLAYELTVN

RDVQDKLYEEILQTDKALSGGPLTYDAVQGMQYMDMVVSEGLRMWAPAPATDRLCVRDYV

VDDGDRLKFTIDKGTVVFIPIAGLHHDPQYYPNPSKFDPERFSVENRDKINPNTYLPFGI

GPRNCIGSRFALMEVKAIIYYMLREFSFERTPNTQVPLRLAKGFAGMRSEKGVFVEFRPR

KQAAN

>EAT33137.1

MVEVDLYVALAVGAIVVLLYHYAAKKYEYFLTKPIPALKPTMLFGNTGPMMFRQRDVSSH

LKMLYNTYEGSKMIGFYDLMKPIYMLRDPEVIKQIAVKDFDYFMDHTPTMTNSNPEDEVG

GDSLFGNSLFALRGQKWRDMRATLSPAFTGSKMRHMFELVADCAKSMAEFFKAEAAAGKT

LEYEMKDTFSRFGNDVIATVAFGIKVDSLRDRDNEFYLKGKAMLNFQSLSVLLKVLFLRA

FPKLSHKLGLDFVDSTLTEYFKQMIVDNMKQRAAHGIMRNDMIQMLMEVRKGSLRHQKDE

KETKDAGFATVEESNVGKSNINRVWTENELISQCFLFFVAGFDTVSTCMTFLTYELMLNQ

NIQERLYDEVMETEKSLNGKPLTYEVLQKMEYMDMVVSEALRKWPPAVVSDRFCVKNYMY

DDGQGTRFLVEKGQTMWIPTIAIHSDPKYYENPEKFDPERFNEENRSKIDTGAYLPFGVG

PRNCIGSRLALMEVKVIIYNLLKDFSLESSEKTQIPLKMSKNFFVLQAENGVWLELKPRK

R

>EFA09142.1

MLTIILSVILGALIYYKLIKPASFWEERNITHASAWPLVGSMWESVIQKKNVNEVSTDIY

NKYPNDRYVGYMQFTDPVLLIRDLDLIKQIGVKVFDNFRDHHAIAGFNAEPLFTKSLIFL
[truncated: 7,569,757 more chars]
